# Diet during pregnancy and infancy and risk of allergic or autoimmune disease: A systematic review and meta-analysis

**DOI:** 10.1371/journal.pmed.1002507

**Published:** 2018-02-28

**Authors:** Vanessa Garcia-Larsen, Despo Ierodiakonou, Katharine Jarrold, Sergio Cunha, Jennifer Chivinge, Zoe Robinson, Natalie Geoghegan, Alisha Ruparelia, Pooja Devani, Marialena Trivella, Jo Leonardi-Bee, Robert J. Boyle

**Affiliations:** 1 Department of International Health, Johns Hopkins School of Public Health, Baltimore, Maryland, United States of America; 2 Respiratory Epidemiology, Occupational Medicine and Public Health, National Heart and Lung Institute, Imperial College London, London, United Kingdom; 3 Section of Paediatrics, Department of Medicine, Imperial College London, London, United Kingdom; 4 Centre for Statistics in Medicine, University of Oxford, Oxford, United Kingdom; 5 Division of Epidemiology and Public Health, University of Nottingham, Nottingham, United Kingdom; 6 Centre of Evidence Based Dermatology, University of Nottingham, Nottingham, United Kingdom; Stanford University, UNITED STATES

## Abstract

**Background:**

There is uncertainty about the influence of diet during pregnancy and infancy on a child’s immune development. We assessed whether variations in maternal or infant diet can influence risk of allergic or autoimmune disease.

**Methods and findings:**

Two authors selected studies, extracted data, and assessed risk of bias. Grading of Recommendations Assessment, Development and Evaluation (GRADE) was used to assess certainty of findings. We searched Medical Literature Analysis and Retrieval System Online (MEDLINE), Excerpta Medica dataBASE (EMBASE), Web of Science, Central Register of Controlled Trials (CENTRAL), and Literatura Latino Americana em Ciências da Saúde (LILACS) between January 1946 and July 2013 for observational studies and until December 2017 for intervention studies that evaluated the relationship between diet during pregnancy, lactation, or the first year of life and future risk of allergic or autoimmune disease. We identified 260 original studies (964,143 participants) of milk feeding, including 1 intervention trial of breastfeeding promotion, and 173 original studies (542,672 participants) of other maternal or infant dietary exposures, including 80 trials of maternal (*n* = 26), infant (*n* = 32), or combined (*n* = 22) interventions. Risk of bias was high in 125 (48%) milk feeding studies and 44 (25%) studies of other dietary exposures. Evidence from 19 intervention trials suggests that oral supplementation with nonpathogenic micro-organisms (probiotics) during late pregnancy and lactation may reduce risk of eczema (Risk Ratio [RR] 0.78; 95% CI 0.68–0.90; I^2^ = 61%; Absolute Risk Reduction 44 cases per 1,000; 95% CI 20–64), and 6 trials suggest that fish oil supplementation during pregnancy and lactation may reduce risk of allergic sensitisation to egg (RR 0.69, 95% CI 0.53–0.90; I^2^ = 15%; Absolute Risk Reduction 31 cases per 1,000; 95% CI 10–47). GRADE certainty of these findings was moderate. We found weaker support for the hypotheses that breastfeeding promotion reduces risk of eczema during infancy (1 intervention trial), that longer exclusive breastfeeding is associated with reduced type 1 diabetes mellitus (28 observational studies), and that probiotics reduce risk of allergic sensitisation to cow’s milk (9 intervention trials), where GRADE certainty of findings was low. We did not find that other dietary exposures—including prebiotic supplements, maternal allergenic food avoidance, and vitamin, mineral, fruit, and vegetable intake—influence risk of allergic or autoimmune disease. For many dietary exposures, data were inconclusive or inconsistent, such that we were unable to exclude the possibility of important beneficial or harmful effects. In this comprehensive systematic review, we were not able to include more recent observational studies or verify data via direct contact with authors, and we did not evaluate measures of food diversity during infancy.

**Conclusions:**

Our findings support a relationship between maternal diet and risk of immune-mediated diseases in the child. Maternal probiotic and fish oil supplementation may reduce risk of eczema and allergic sensitisation to food, respectively.

## Abstract 2

Robert Boyle and colleagues reveal the importance of a pregnant woman's diet. Diets supplemented with probiotics and fish oils may reduce the likelihood of eczema and other autoimmune conditions in children.

## Author summary

**Why was this study done?:**

- There is uncertainty about the effects of specific dietary variations in early life on risk of immune-mediated disease.

**What did the researchers do and find?:**

- We undertook a systematic review of observational and intervention studies of maternal diet during pregnancy and lactation and infant diet during the first year of life to evaluate whether dietary exposures in early life influence risk of allergic or autoimmune disease.
- We found that supplementation with micro-organisms (probiotics) during late pregnancy and breastfeeding may reduce risk of eczema, and fish oil supplementation during pregnancy and breastfeeding may reduce risk of sensitisation to food allergens.
- We found more limited data that longer duration of breastfeeding may reduce risk of eczema and type 1 diabetes mellitus.

**What do these findings mean?:**

- A mother’s diet during pregnancy and lactation may influence her child’s risk of developing allergic disease.

## Introduction

Immune-mediated health conditions such as allergic and autoimmune diseases appear to have increased in prevalence in many countries and are leading causes of chronic illness in young people [1]. There is evidence that early dietary exposures may influence the development of these diseases, but a comprehensive analysis of the relationship between all dietary exposures during pregnancy, lactation, or the first year of life and risk of allergic or autoimmune disease has not been undertaken [1]. Relevant dietary exposures may include intake of individual foods or food groups, nutrients or nutrient groups, dietary supplements, avoidance of specific allergenic foods, timing of introduction of specific foods or food groups to the infant diet, overall dietary pattern, and duration of breastfeeding. A recent World Allergy Organization guideline recommended probiotic and prebiotic supplements for eczema prevention [2,3], but European, North American, and Australasian guidelines do not support this [4–6]. Several guidelines recommend exclusive breastfeeding for at least 4 to 6 months to reduce risk of eczema, food allergy, and wheezing [4,5,7], and a recent Australasian guideline recommends oily fish or omega-3 fatty acid supplements during pregnancy to reduce eczema [6]. Recent focused systematic reviews support a relationship between breastfeeding and reduced asthma risk [8,9] and between probiotics and prebiotics and reduced eczema risk [2,10,11]. In order to inform United Kingdom dietary recommendations for infants and their pregnant or lactating mothers, we undertook an updated and comprehensive systematic review of diet during pregnancy and infancy and risk of allergic sensitisation, allergic disease, or autoimmune disease.

## Methods

This review is reported in accordance with PRISMA guidance. The review is part of a series of systematic reviews commissioned by the UK Food Standards Agency in order to inform UK dietary recommendations for infants and their pregnant or lactating mothers, under the title ‘Review of scientific published literature on infant feeding and development of atopic and autoimmune disease'. The protocols for the systematic reviews were registered with the International Prospective Register of Systematic Reviews (PROSPERO CRD42013003802 ‘Review A:milk feeding’; CRD42013004239 ‘Review B:timing of allergenic food introduction’; CRD42013004252 ‘Review C:maternal and infant diet’) on 5 August 2013, prior to title screening or selecting any studies from the search results. This manuscript reports findings from the majority of the project, comprising Reviews A and C, ‘milk feeding’ and ‘maternal and infant diet’. Review B, regarding timing of introduction of allergenic foods, and one part of Review C, regarding use of hydrolysed infant formula, have been published separately [12,13]. Thus, this manuscript describes outcomes for maternal or infant intake of individual foods or food groups, nutrients or nutrient groups, dietary supplements, maternal allergenic food avoidance, timing of introduction of solid foods or nonallergenic foods to the infant diet, overall dietary pattern and duration of breastfeeding. This manuscript does not include timing of introduction of allergenic foods (milk, soya, egg, peanuts, tree nuts, fish, seafood, wheat) to the infant diet or use of hydrolysed formula in infants. Measures of dietary diversity and objective measures of nutritional status (with the exception of blood vitamin D level) were not included in these systematic reviews. These measures were considered to be too indirect as indicators of dietary intake to be used to inform public health guidance in the UK. As part of this project, we also searched for other systematic reviews covering the same topic published since 1 January 2011.

### Study interventions and comparators

Studies of total breastfeeding duration, exclusive breastfeeding duration [14], and timing of solid food introduction were included in Review A ‘milk feeding’. Studies of any other nutritional exposure, ranging from dietary pattern to specific micronutrient supplementation and from single to multifaceted interventions, were included in Review C ‘maternal and infant diet’. For observational studies, data on dietary exposures were acquired from interviews, health records, diaries, and questionnaires. With the exception of blood vitamin D level, we did not include assessments of nutrient status as an exposure, and we did not include measures of dietary diversity as an exposure.

### Study designs and populations

We included all intervention trials, and observational studies described as cohort, case control or cross-sectional analytic studies. Intervention trials were classified as randomised controlled trials (RCTs), where the method of treatment allocation was random, and as controlled clinical trials (CCTs), where treatment allocation was nonrandom and likely to lead to significant imbalance between treatment groups. CCTs were analysed separately from RCTs. Prospective and retrospective observational studies were analysed separately. We included studies of diet during pregnancy or lactation and of infant feeding between birth and 12 months of age. We excluded studies in which participants or their mothers were defined by the presence of a preexisting disease state, including very low birth weight or very premature infants.

### Study outcomes

Allergic and autoimmune outcomes were selected on the basis of their population prevalence in children and young adults [15]. We included diseases with a prevalence of at least 1 in 1,000 in children/adolescents or young adults (aged <40 years) but did not include rarer diseases. We did not include pernicious anaemia or adult-onset rheumatoid arthritis despite a high prevalence in middle-aged and elderly people because their prevalence in young people is lower than 1 in 1,000, and prospective studies of infant feeding in relation to diseases of older adults were thought unlikely to have been reported. Allergic outcomes that met our inclusion criteria were asthma or wheeze, eczema, allergic rhinitis and/or conjunctivitis, food allergy (defined by food challenge, medical diagnosis, or self/parent report), allergic sensitisation, i.e., skin prick or specific immunoglobulin E (sIgE) assessment, and total immunoglobin E (IgE) level. We categorised asthma as wheeze, recurrent wheeze, or atopic wheeze depending on the definition used in the original publication, and we included measures of lung function, specifically bronchial hyper-reactivity, forced vital capacity, peak expiratory flow rate, and forced expiratory volume in 1 second. Physician-diagnosed asthma was included within the category ‘recurrent wheeze’, unless defined as a single episode of wheeze. Autoimmune diseases that met our inclusion criteria were type 1 diabetes mellitus (TIDM) (doctor diagnosed or serological diagnosis), coeliac disease defined serologically or clinically (positive Immunoglobulin A tissue Transglutaminase antibodies on one or more visits or a positive small bowel biopsy), inflammatory bowel disease, autoimmune thyroid disease, juvenile rheumatoid arthritis, vitiligo, and psoriasis, all as doctor diagnosis. For clinical allergic outcomes, we grouped age at assessment as 0–4 years, 5–14 years, and ≥15 years. Allergic sensitisation and autoimmune diseases were not stratified by age at outcome assessment.

### Data sources

We searched The Cochrane Library (2013, Issue 7), Excerpta Medica dataBASE (EMBASE) (1947 to July 2013), Literatura Latino Americana em Ciências da Saúde (LILACS) (1982 to July 2013), Medical Literature Analysis and Retrieval System Online (MEDLINE) (1946 to July 2013), and Web of Science (1970 to July 2013) on 25 July 2013. We reran the searches for intervention trials and systematic reviews on 26 February 2017 and for intervention trials again on 15 December 2017. We included all studies published up to that date and studies in progress or completed but unpublished studies identified through http://apps.who.int/trialsearch/. Both peer-reviewed publications and abstract publications were included. We reviewed the bibliography of eligible studies for possible additional publications and included all eligible publications, regardless of the language. Study authors were not contacted for original data. The search strategies were extensively piloted and refined to optimise sensitivity, comparing search results with those of other systematic reviews. The search strategies are shown in S1 Text.

### Study selection

Title and abstract screening was undertaken by a team of 9 trained researchers (RJB, VGL, DI, NG, KJ, JC, ZR, AR, PD). Two researchers undertook title screening independently and met to agree upon included and excluded titles. Their screening was checked by a third person, and uncertainties were resolved at a weekly team meeting between February and April 2014, 2015, and 2017 (RB, SC, VGL). The full texts of all potentially eligible studies were reviewed.

### Risk of bias assessment

Risk of bias assessment was undertaken in duplicate, using modified versions of the Cochrane Collaboration Risk of Bias tool for intervention trials and the National Institute for Clinical Excellence methodological checklist for cohort and case–control studies [16]. Risk of bias domains for intervention trials were Selection bias (sequence generation and allocation concealment), Assessment bias (blinding of outcome assessors and validity of the outcome assessment tool), and Attrition bias (considered high when <70% of randomised participants had outcome data). Risk of bias domains for observational studies were Selection bias (low if cases and controls came from similar populations and participation rate ≥80%), Assessment bias (the validity of exposure and outcome assessment tools), and Confounding bias (whether study design and analysis accounted for most potential confounders). We separately included an assessment of Conflict of Interest for each study, judged as low where there was no evidence of industry involvement in study design, analysis, interpretation, or publication and no evidence that study authors received remuneration from relevant industry partners for other activities.

### Data extraction

Data extraction was undertaken in duplicate. Disagreements and uncertainties about data coding and risk of bias were discussed within the team. For foreign language studies, data were extracted by VGL together with a native speaker of the relevant language (see Acknowledgemnts section). We extracted all relevant data from included studies, including data not able to be meta-analysed. Data were extracted using either raw frequencies or crude or adjusted effect estimates. Random effects meta-analysis was undertaken, and where this was not possible, study results were summarised in a narrative table.

### Data selection for analysis

For intervention trials, we extracted outcome data that adhered to the intention-to-treat principle in preference to data based on per protocol analyses. Where studies included multiple intervention groups, we performed pairwise comparisons where we split the number of events and no events in the unexposed/control group to prevent double counting. Where studies reported data at multiple timepoints, we extracted the most complete dataset available beyond the intervention period (i.e., from 1 year of age onwards); this is the dataset with the largest denominator or, where the denominator is identical for multiple time points, the largest numerator (number of events). Where studies reported multiple assessments of the same outcome at the same timepoint, clinical assessments were selected in preference to serological assessments, and skin prick in preference to sIgE assessment of allergic sensitisation. For observational studies of breastfeeding or timing of solid or other food introduction to the infant diet, data were only included in meta-analysis where the reference group was complete, i.e., ‘less than’ a certain duration or, in some cases, ‘never’. Where more than one exposure group was compared with the reference group, we analysed data for the latest exposure group. For all observational studies, adjusted effect estimates were used in preference to unadjusted estimates for meta-analysis if both were reported.

### Data synthesis

Pooled results for binary outcomes were presented as Risk Ratio (RR) calculated using the Mantel–Haenszel method, with continuity correction of 0.5 for zero cell frequencies, and odds ratios (ORs) were pooled using the generic inverse variance method using R (version 3.1.0 2014-04-10, www.r-project.org). For continuous outcomes measured using similar scales, data were summarised as mean differences with 95% CI. Key findings are presented in a Summary of Findings table with Grading of Recommendations Assessment, Development and Evaluation (GRADE) assessment of certainty of findings [17]. Heterogeneity was quantified using I^2^ and classified as low (I^2^ < 25%), moderate (I^2^ 25%*–*50%), high (I^2^ 50%*–*75%) or extreme (I^2^ > 75%). Data were not pooled where I^2^ ≥ 80%. We assessed for publication bias using Funnel plots and Egger asymmetry tests where there were ≥ 10 studies in a meta-analysis. We undertook a priori defined subgroup analyses for meta-analyses with > 5 studies according to overall risk of bias; disease risk; study design; conflict of interest; for some analyses, by features of the intervention or exposure assessment; and method of outcome assessment. Data that could not be included in meta-analysis were reported narratively, and the outcomes of both meta-analyses and narrative reports were considered when interpreting data and making conclusions. Trial sequential analysis (TSA) was used to quantify the statistical reliability of moderate or high certainty findings using 2-sided 5% significance and 80% power to estimate optimum heterogeneity-adjusted information sizes needed to identify relative risk reductions of 20% and 30%. Control event rates were estimated using random effects meta-analyses of the pooled proportions from the largest studies included in the meta-analysis and compared with event rates from large population-based studies. TSA quantifies the statistical reliability of data in a cumulative meta-analysis in a similar way to an interim analysis in a single randomised clinical trial.

## Results

**Fig 1:**
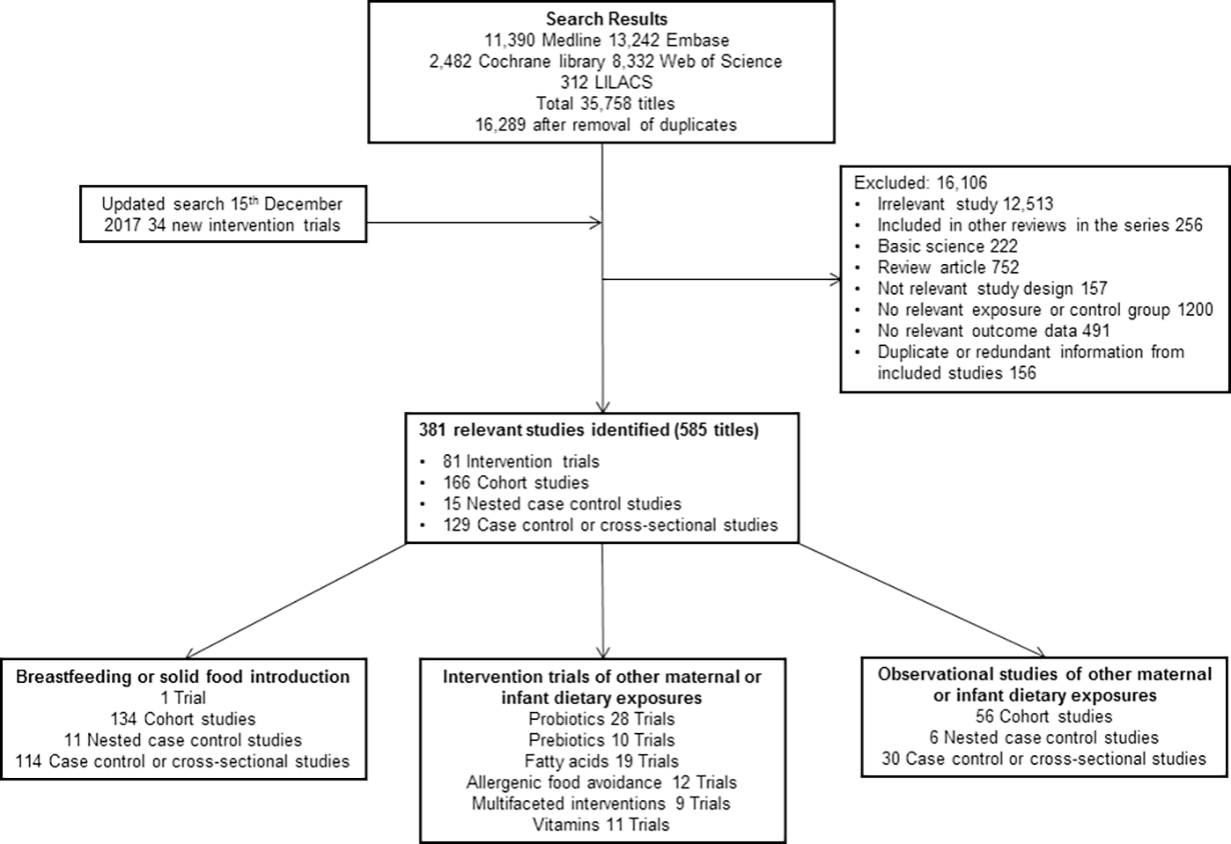
PRISMA flow chart. CENTRAL, Central Register of Controlled Trials; EMBASE, Excerpta Medica dataBASE; LILACS, Literatura Latino Americana em Ciências da Saúde; MEDLINE, Medical Literature Analysis and Retrieval System Online.

**Fig 2:**
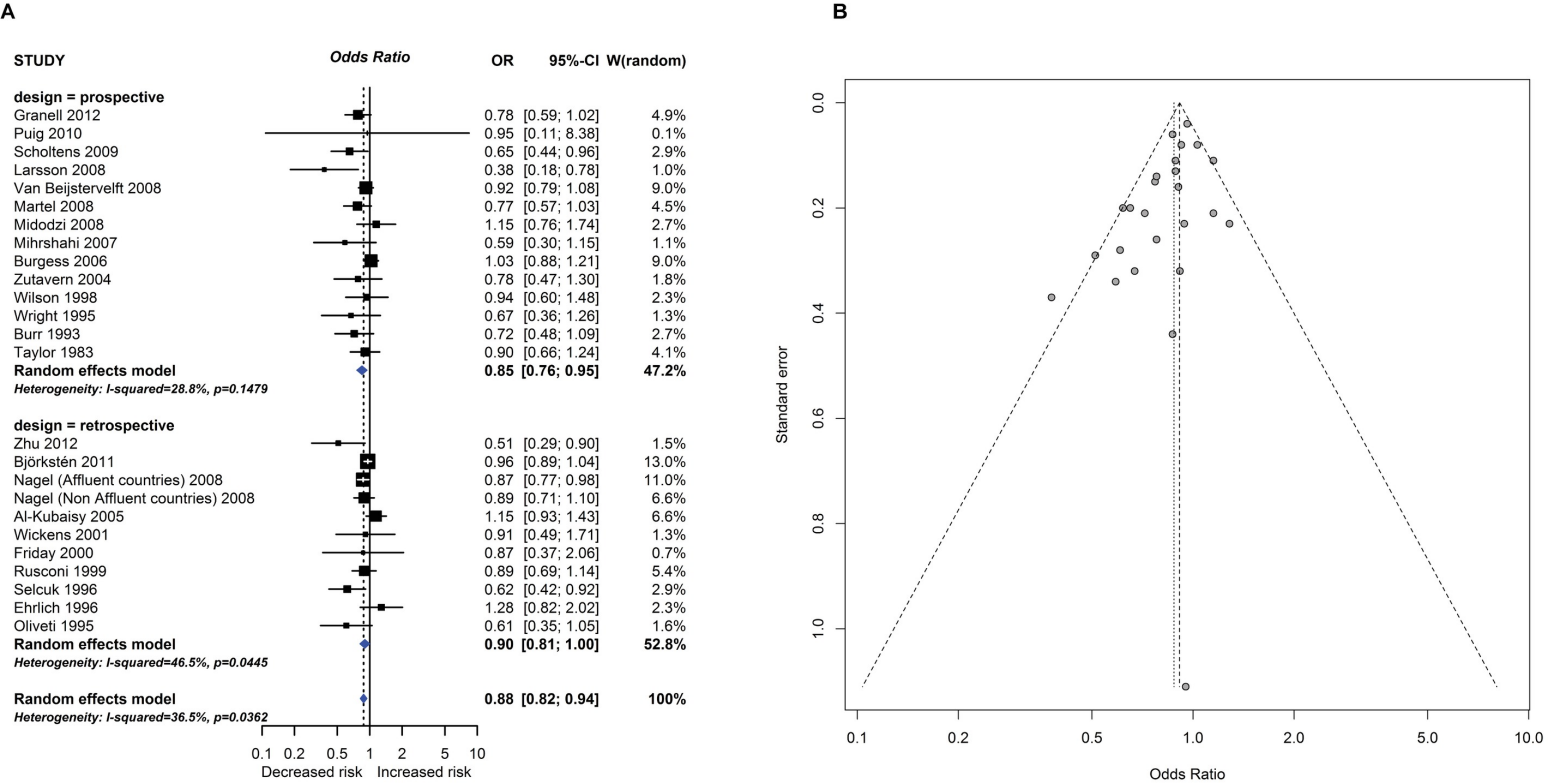
Observational study findings for a relationship between breastfeeding ever and recurrent wheeze at age 5–14 years (A) and a Funnel plot for this analysis showing evidence of publication bias (B). Egger test *P* = 0.012. CI, confidence interval; OR, odds ratio; W, weight.

**Fig 3:**
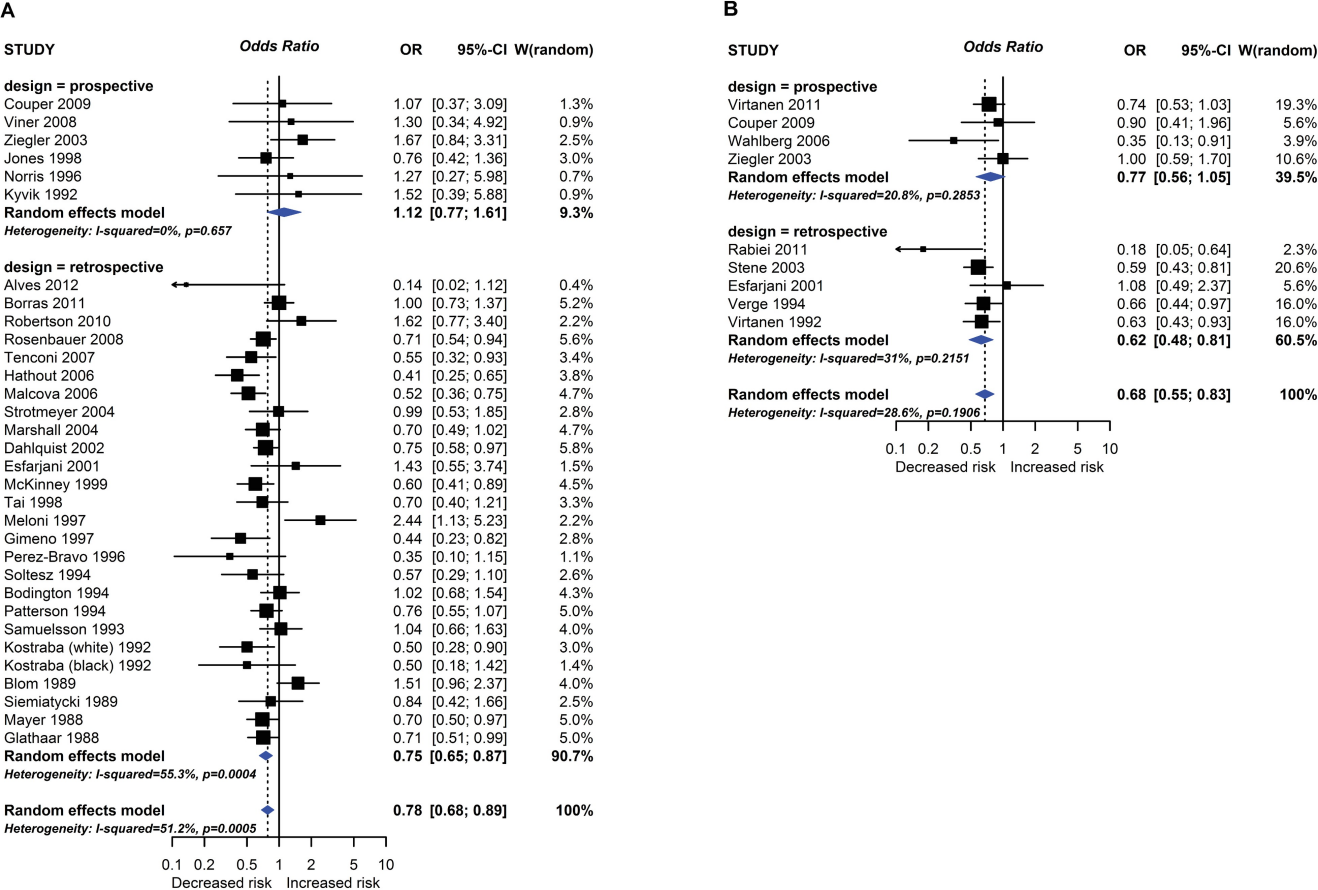
Observational study findings for a relationship between breastfeeding ever (A) or exclusive breastfeeding for ≥3–4 months (B) and type 1 diabetes mellitus. CI, confidence interval; OR, odds ratio; W, weight.

**Fig 4:**
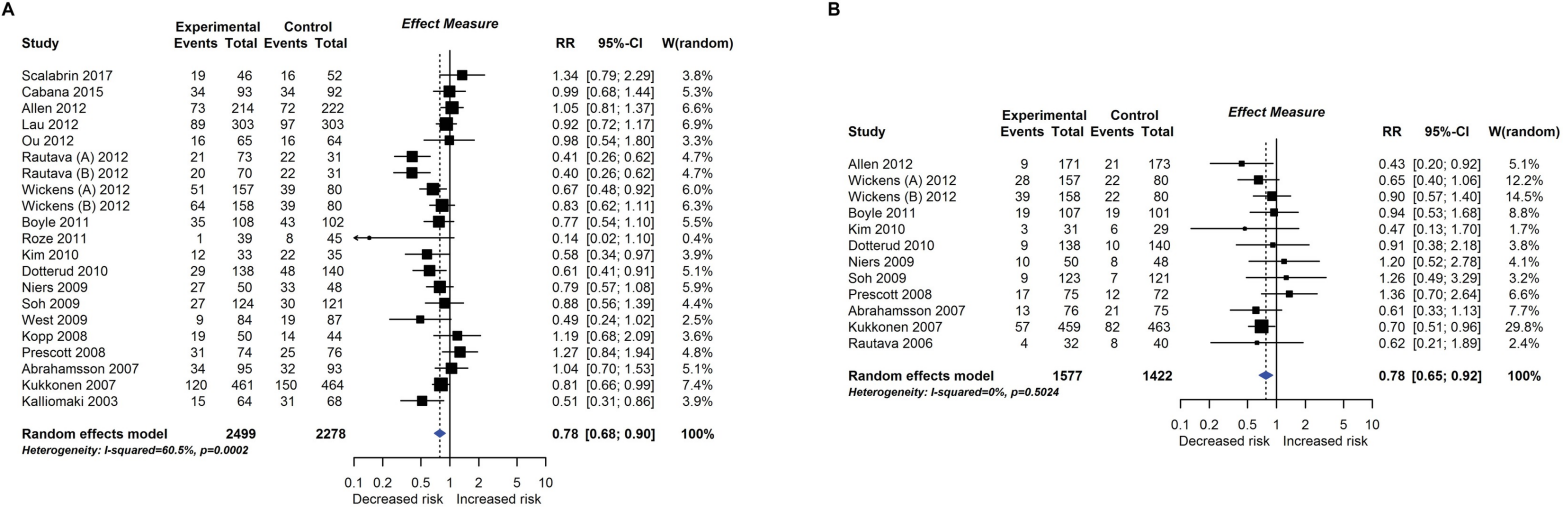
RCT findings for probiotic supplementation compared with no probiotics and risk of eczema (A) or atopic eczema (B) at age ≤4 years. CI, confidence interval; RCT, randomised controlled trial; RR, risk ratio; W, weight.

**Fig 5:**
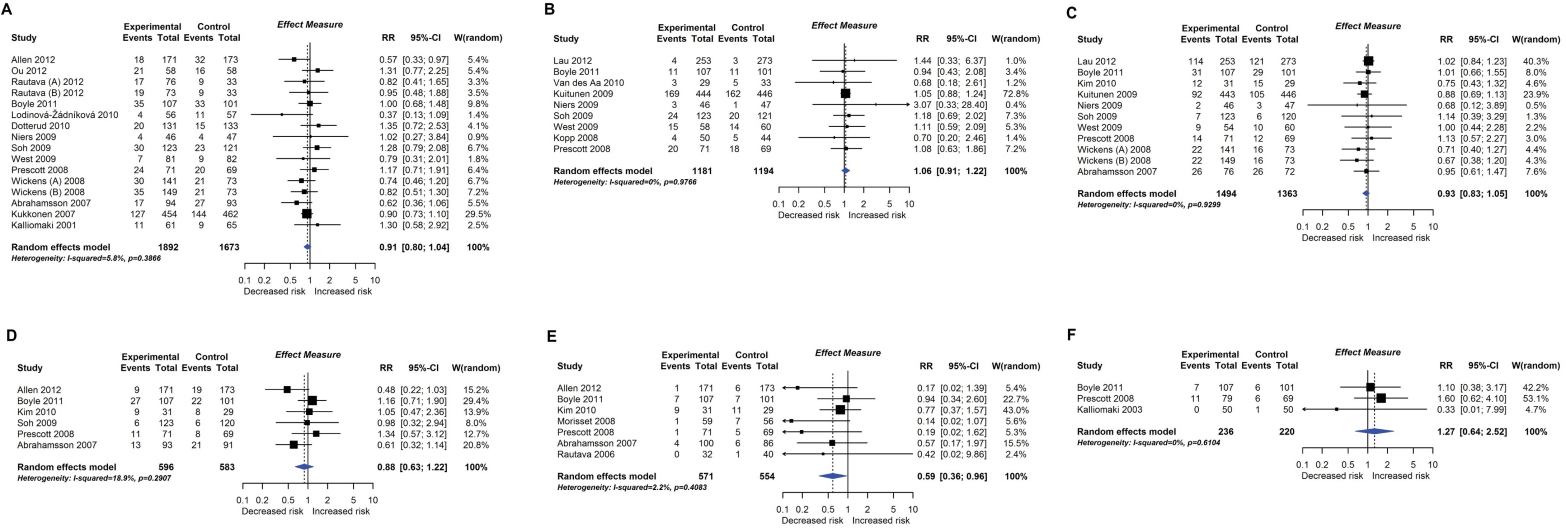
RCT findings for probiotic supplementation compared with no probiotics and risk of allergic sensitisation to any allergen (A), any inhalant allergen (B), any food allergen (C), egg (D), milk (E), or peanut (F). CI, confidence interval; RCT, randomised controlled trial; RR, risk ratio; W, weight.

**Fig 6:**
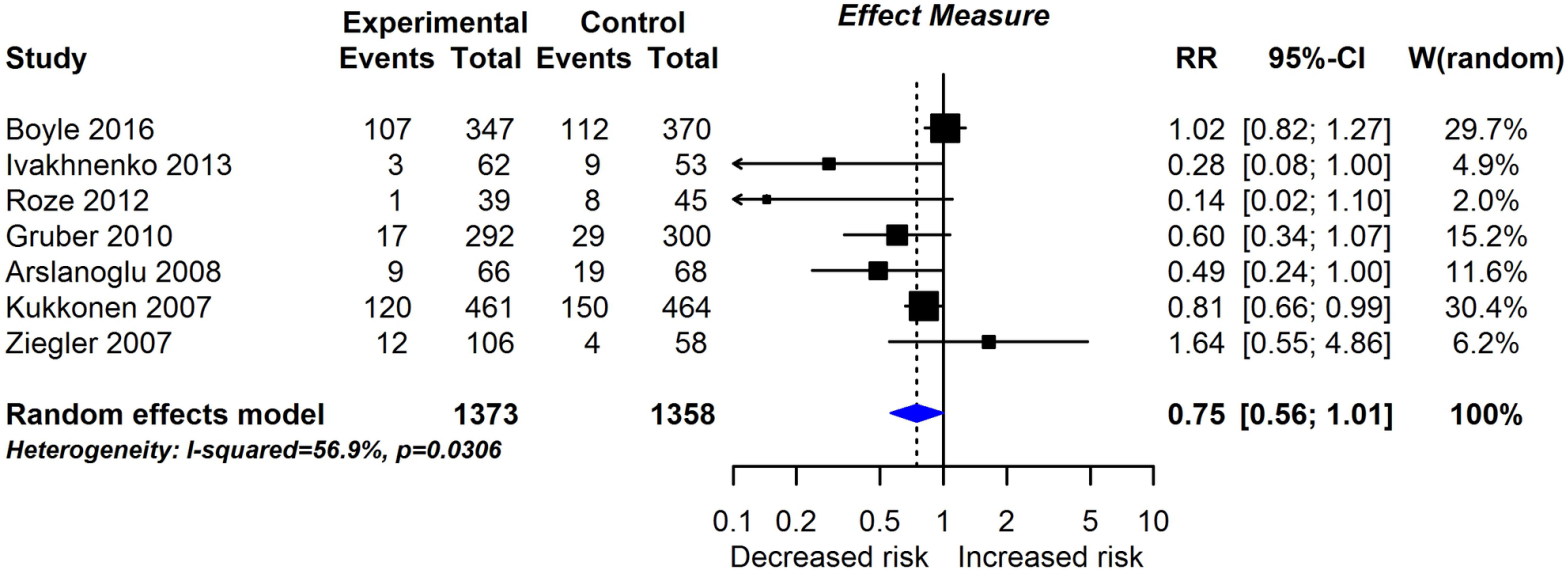
RCT findings for prebiotic supplementation compared with no prebiotics and risk of eczema at age ≤4 years. CI, confidence interval; RCT, randomised controlled trial; RR, risk ratio; W, weight.

**Fig 7:**
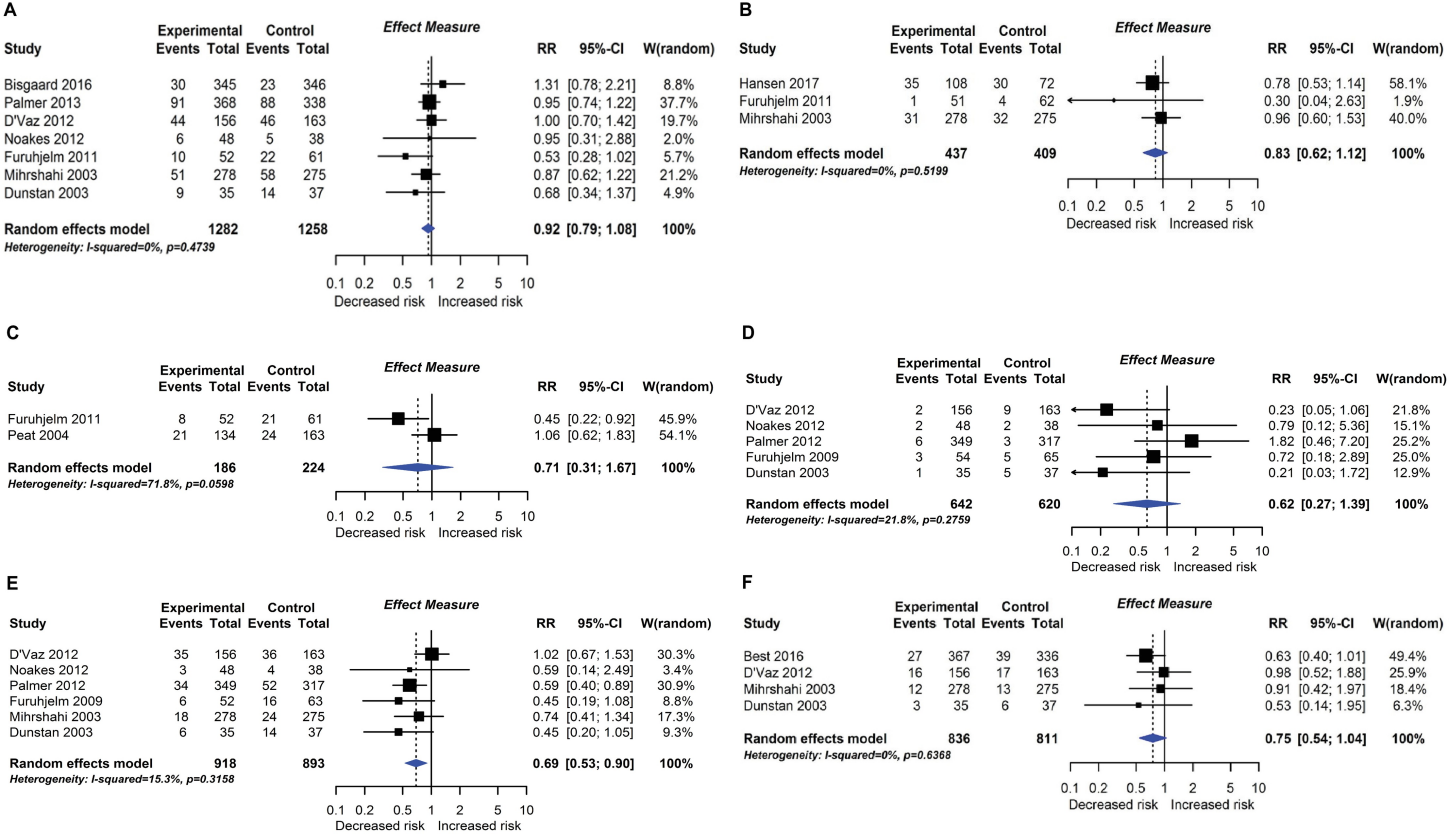
RCT findings for omega-3 polyunsaturated fatty acid supplementation compared with no polyunsaturated fatty acids and risk of allergic sensitisation to any allergen (A), any inhalant allergen (B), any food allergen (C), milk (D), egg (E), or peanut (F). CI, confidence interval; RCT, randomised controlled trial; RR, risk ratio; W, weight.

**Fig 8:**
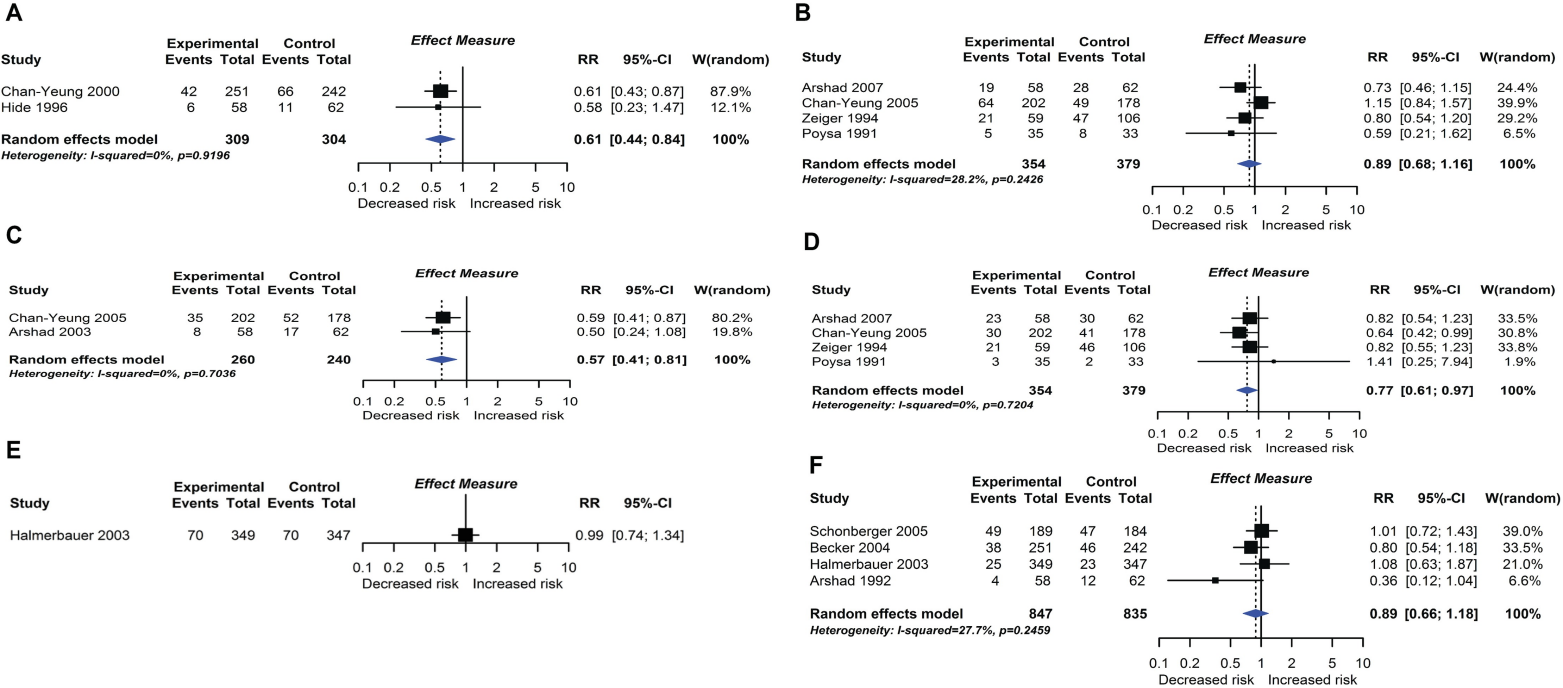
Randomised controlled trial findings for multifaceted dietary interventions compared with no multifaceted intervention and risk of allergic rhinitis at age ≤4 years (A) or 5–14 years (B), wheeze (C) or recurrent wheeze (D) at age 5–14 years, and wheeze (E) or recurrent wheeze (F) at age ≤4 years. CI, confidence interval; RCT, randomised controlled trial; RR, risk ratio; W, weight.

**Fig 9:**
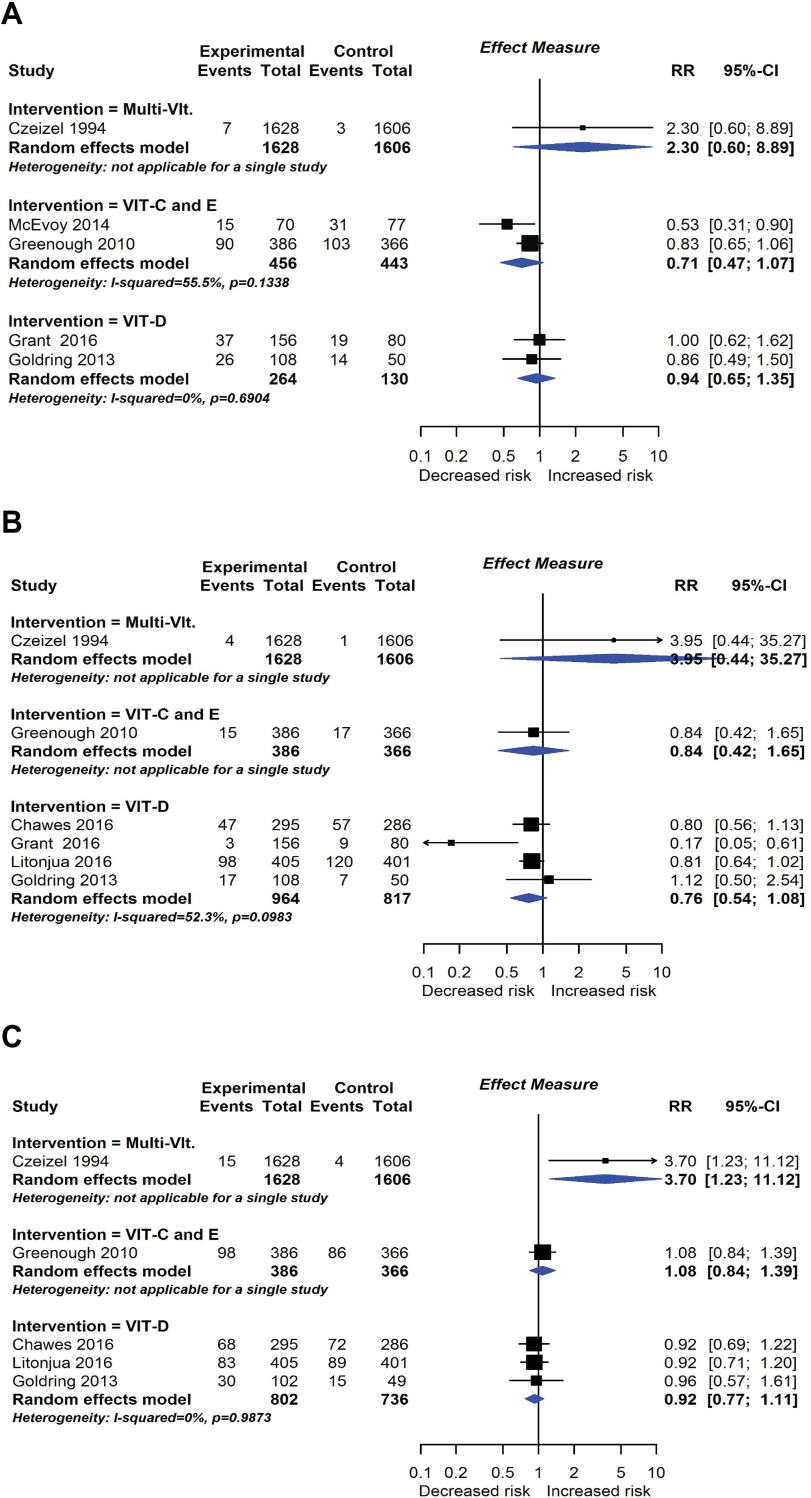
RCT findings for vitamin supplementation compared with no vitamin supplementation and risk of wheeze (A), recurrent wheeze (B), or eczema (C) at age ≤4 years. CI, confidence interval; RCT, randomised controlled trial; RR, risk ratio; W, weight.

Our original search identified 16,289 original titles. Title, abstract, and full text screening yielded a total of 343 titles (260 original studies; 964,143 participants) of milk feeding and 281 titles (173 original studies; 542,672 participants) of maternal and infant diet, including 80 trials of maternal (*n* = 26), infant (*n* = 32), or combined (*n* = 22) interventions. Full details of the search results are shown in Fig 1 (PRISMA flow chart), and characteristics of included studies and risk of bias are summarised in S1 Appendix. Risk of bias was high in 125 (48%) studies of milk feeding and 44 (25%) studies of maternal and infant diet (most commonly due to attrition bias in intervention studies and confounding bias in observational studies). Key findings are shown in Table 1, which includes a GRADE assessment of certainty for each finding [18], and in Figs 2–9. The full report, with a detailed description of the methodology used and all review findings, is available on the Food Standards Agency website (https://www.food.gov.uk/science/research/allergy-research/fs305005ac). Summary reports to the Food Standards Agency for studies of milk feeding and studies of maternal and infant diet are in S2 Text and S3 Text, respectively. Detailed appraisal of findings underlying the 2 reports are in S1 Data and S2 Data, respectively, and all data used for meta-analyses are in S3 Data. An associated statement by the Committee on Toxicity is available on the Food Standards Agency website (https://cot.food.gov.uk/cotstatements/cotstatementsyrs/cot-statements-2017/statementonrevsaandc).

**Table 1:** GRADE assessment and summary of key findings.

GRADE of evidence assessment							Summary of findings		Absolute Risk Reduction*	
No of studies and design	Risk of bias	Inconsistency	Indirectness	Imprecision	Publication bias	Other considerations	Relative risk	GRADE of evidence	Control Risk	Risk Difference
									Cases per 1,000 population	Cases per 1,000 population
**Intervention: Breastfeeding promotion intervention vs no intervention** **Outcome: Eczema at age 0–4** **Study design: RCT**										
1 RCT	Not Serious Unblinded outcome assessment	Serious No effect seen at age 6–7 years, and no clear evidence for an effect seen at age 16 years. No association seen in observational studies.	No	Serious Wide CIs	No	No association found between exclusive breastfeeding duration and eczema in an observational analysis of this trial	**OR 0.54 95% CI 0.31–0.95**	**⨂⨂○○** **Low**	**200** **(low risk)**	**81 cases** **95% CI** **8–128**
Eczema									**300** **(high risk)**	**112 cases** **95% CI** **11–183**
									**400** **(very high risk)**	**135 cases** **95% CI** **12–229**
**Exposure: Total breastfeeding duration—Breastfed ever versus never** **Outcome: Recurrent wheeze at age 5–14 and Type 1 Diabetes Mellitus** **Study design: Prospective or retrospective studies**										
14 prospective (13 PC, 1 NCC all in meta-analysis) 13 retrospective (2 RC, 4 CC, 7 CS, 1 RC, and 1 CS not in meta-analysis) Recurrent wheeze	Not serious 4 prospective studies, and 7 retrospective studies, at high risk of bias, but no subgroup difference according to risk of bias	Not serious I^2^ = 29% prospective, 47% retrospective. Study estimates vary from 0.38 to 1.15 prospective; 0.51 to 1.28 retrospective. Similar findings not consistently seen for wheeze at age 5–14 years, nor for wheeze or recurrent wheeze at ages 0–4 or 15+ years.	Not serious Mixed study populations, but several studies included large numbers of participants from representative populations	No Large numbers of participants and events, with relatively narrow CIs	Serious Asymmetrical Funnel plot for all studies combined Egger *P* = 0.012	Analysis of other cut-offs for duration of total breastfeeding showed a similar association between longer BF duration and reduced risk of recurrent wheeze at age 5–14, but with evidence of publication bias; no evidence of subgroup differences by adjustment of analysis; by disease risk; by risk of bias; or by prospective versus retrospective study design	**ProspectiveOR = 0.85** **(0.76, 0.95)** **Retrospective** **OR = 0.90** **(0.81, 1.00)** **Pooled effect** **OR = 0.88** **(0.82, 0.94)**	**⨂○○○** **Very Low**	**50** **(low risk)**	**6 cases** **95% CI** **3–9**
									**100** **(high risk)**	**11 cases** **95% CI** **5–16**
									**150** **(very high risk)**	**16 cases** **95% CI** **8–24**
7 prospective (4 PC, 3 in meta-analysis; 3 NCC, all in meta-analysis) 28 retrospective (27 CC, 24 in meta-analysis; 1 CS in meta-analysis) TIDM	Not serious 4 prospective studies, and 11 retrospective studies, at high risk of bias	Serious I^2^ = 0% prospective, 55% retrospective. Study estimates vary from 0.76 to 1.67 prospective; 0.14 to 2.44 retrospective. Significant difference between prospective studies, with no association seen, and retrospective studies, where breastfeeding was associated with reduced TIDM.	Not serious 3 studies used serological diagnosis in populations at high inherited risk of TIDM, others used clinical diagnosis in more representative populations	No Large numbers of studies and events	No Symmetrical funnel plot for all studies combined Egger *P* = 0.81	Analysis of other cut-offs for duration of total breastfeeding showed a similar pattern of a significant association between longer BF duration and reduced odds of TIDM in retrospective studies, where clinical TIDM was used for outcome assessment; but no consistent evidence of a relationship seen in prospective studies, where serological TIDM was usually used for outcome assessment	**ProspectiveOR = 1.12** **(0.77, 1.61)** **Retrospective** **OR = 0.75** **(0.65, 0.87)** **Pooled effect** **OR = 0.78 (0.68, 0.89)**	**⨂○○○** **Very Low**	**1** **(low risk)**	**Prospective0.2 cases** **95% CI** **0.1–0.3**
									**10** **(high risk)**	**2 cases** **95% CI** **1–3**
									**100** **(very high risk)**	**20 cases** **95% CI** **10–30**
**Exposure: Exclusive breastfeeding duration greater than 3–4 months versus less than 3–4 months** **Outcome: Type 1 Diabetes Mellitus** **Study design: Prospective or retrospective studies**										
5 prospective (5 PC, 4 in meta-analysis) 5 retrospective (5 CC, all in meta-analysis) TIDM	Not serious 2 prospective studies, and 3 retrospective studies, at high risk of bias; no subgroup difference according to risk of bias	Not serious I^2^ = 21% prospective, 31% retrospective. Study estimates vary from 0.35 to 1.00 prospective; 0.18 to 1.08 retrospective. Nonsignificant difference between prospective studies, with no association seen, and retrospective studies, where longer exclusive breastfeeding was associated with reduced TIDM.	Not serious Prospective studies used serological diagnosis in populations at high inherited odds of TIDM, retrospective studies used clinical diagnosis in more representative populations	Not serious Reasonable numbers of studies and events.	Not tested (*n* < 10)	Analysis of other cut-offs for duration of exclusive breastfeeding showed a similar pattern of a significant association between longer duration and reduced odds of TIDM in retrospective studies, where clinical TIDM was used for outcome assessment; but no consistent evidence of a relationship seen in prospective studies, where serological TIDM was used for outcome assessment	**ProspectiveOR = 0.77** **(0.56, 1.05)** **Retrospective** **OR = 0.62** **(0.48, 0.81)** **Pooled effect** **OR = 0.68 (0.55, 0.83)**	**⨂⨂○○** **Low**	**1** **(low risk)** **10** **(high risk)** **100** **(very high risk)**	**0.3 cases** **95% CI** **0.2 to 0.4** **3 cases** **95% CI** **2 to 4** **30 cases** **95% CI** **16 to 42**
**Intervention: Maternal or infant probiotic supplement vs no probiotic supplement** **Outcome: Eczema, atopic eczema or cow’s milk sensitisation at age 0–4** **Study design: RCT**										
23 RCT (19 in meta-analysis) 1 CCT Eczema	Not Serious <20% of studies had a high risk of bias	Serious I^2^ = 61% Eczema Study estimates vary from 0.14 to 1.34; subgroup analysis suggests greatest effect if postnatal component includes maternal supplementation	No	No	No	Most RCTs were undertaken in populations at high risk of eczema due to family history of allergic disease. No evidence of an effect on eczema beyond age 0–4.	**RR = 0.78** **(0.68, 0.90)**	**⨂⨂⨂○** **Moderate**	**200** **(low risk)**	**44 cases** **95% CI** **20–64**
									**300** **(high risk)**	**66 cases** **95% CI** **30–96**
									**400** **(very high risk)**	**88 cases** **95% CI** **40–128**
11 RCT Atopic Eczema	Not Serious <20% of studies had a high risk of bias	Serious I^2^ = 0% Study estimates vary from 0.47 to 1.36. But no beneficial effect seen at age 5–14 years RR 0.99 (0.80, 1.22; I^2^ = 0%).	No	No	No Egger *P* = 0.70	Most RCTs were undertaken in populations at high risk of eczema due to family history of allergic disease.	**RR = 0.78 (0.65, 0.92)**	**⨂⨂⨂○** **Moderate**	**100** **(low risk)** **150** **(high risk)** **200** **(very high risk)**	**22 cases** **95% CI** **8–35** **33 cases** **95% CI** **12–53** **44 cases** **95% CI** **16–70**
97 RCT (8 in meta-analysis) Cow’s milk sensitisation	Not Serious <20% of studies had a high risk of bias	Not Serious I^2^ = 0% Study estimates vary from 0.14 to 0.98. No beneficial effect seen for allergic sensitisation to other allergens.	Serious Milk sensitisation is a surrogate measure of clinical food allergy. No effect of the intervention was seen on clinical food allergy.	Serious Low event numbers and borderline statistical significance	Not tested (*n* < 10) No evidence of publication bias in Funnel plots for other forms of allergic sensitisation	Most RCTs were undertaken in populations at high risk of eczema due to family history of allergic disease	**RR = 0.60 (0.37, 0.96)**	**⨂⨂○○** **Low**	**30** **(low risk)** **100** **(high risk)** **300** **(very high risk)**	**12 cases** **95% CI** **1–19** **40 cases** **95% CI** **4–63** **120 cases** **95% CI** **12–189**
**Intervention: Infant prebiotic supplement versus no prebiotic supplement** **Outcome: Eczema at age 0–4** **Study design: RCT**										
9 RCT (7 in meta-analysis) Eczema	Serious 4 studies with high risk of attrition bias, 5 with high risk of conflict of interest	Serious I^2^ = 57% Study estimates vary from 0.14 to 1.64	Serious 6 studies restricted inclusion to infants fully formula fed from birth, 3 days, < 2 weeks, <6 weeks (n = 2) and <8 weeks; 3 other studies used co-interventions–hydrolysed formula and probiotics	Serious Wide CIs, borderline statistical significance	Not tested (n<10)	3 RCTs were undertaken in populations at high risk due to family history of allergic disease, 4 at normal risk, and 1 at low risk due to absence of family history of allergic disease. All studies used galactooligosaccharides, usually combined with other oligosaccharides; 2 studies combined the intervention with probiotic(s).	**RR = 0.75** **(0.56, 1.01)**	**○○○○** **No evidence**	**-**	**-**
**Intervention: Maternal fish oil (n-3 PUFA) vs no fish oil** **Outcome: Allergic sensitisation to egg** **Study design: RCT**										
6 RCT Allergic sensitisation to egg	Not Serious 2 studies with unclear overall risk of bias and 2 studies with unclear risk of conflict of interest	Not Serious I^2^ = 15% Study estimates vary from 0.45 to 1.02. No significant effect seen for allergic sensitisation to other allergens, but direction of effect is inconsistent with that seen for egg.	Serious Egg sensitisation is a surrogate measure of clinical food allergy. No effect of the intervention was seen on clinical food allergy.	Not serious Limits of 95% CI are all important effects at a population level.	Not tested (*n* < 10)	All RCTs were undertaken in populations at high risk of allergy due to family history of allergic disease. Subgroup analysis suggests effect is greatest if treatment is given during pregnancy.	**RR = 0.69** **(0.53, 0.90)**	**⨂⨂⨂○** **Moderate**	**100** **(low risk)** **150** **(high risk)** **200** **(very high risk)**	**31 cases** **95% CI** **10–47** **46 cases** **95% CI** **15–71** **62 cases** **95% CI** **20–94**
**Intervention: Multifaceted interventions vs control** **Outcome: Allergic rhinitis at age 0–4** **Study design: RCT**										
3 RCT (2 in meta-analysis) Allergic rhinitis	Not serious No study with high risk of bias or high risk of conflict of interest	Not serious I^2^ = 0% Study estimates vary from 0.58 to 0.61	Serious Both studies in meta-analysis included environmental control measures such as dust mite and tobacco smoke avoidance	Serious Sparse data, inconsistent with data for ages 5–14 years, where no effect was seen. Study not included in meta-analysis found no effect.	Not tested (*n* < 10)	All RCTs were undertaken in populations at high risk due to family history of allergic disease. All studies included delayed introduction of allergenic foods and maternal allergenic food avoidance	**RR = 0.61** **(0.44, 0.84)**	**⨂⨂○○** **Low**	**50** **(low risk)** **100** **(high risk)** **150** **(very high risk)**	**20 cases** **95% CI** **8–28** **39 cases** **95% CI** **16–56** **59 cases** **95% CI** **24–84**
**Intervention: Multifaceted interventions vs control** **Outcome: Wheeze or recurrent wheeze at age 5–14** **Study design: RCT**										
2 RCT Wheeze	Not serious No study with high risk of attrition bias or high risk of conflict of interest	Not serious I^2^ = 0% Study estimates vary from 0.50 to 0.59	Serious Both studies included environmental control measures such as dust mite and tobacco smoke avoidance, which are known to reduce respiratory symptoms	Serious Sparse data, inconsistent with data for age 0–4 and lung function data, where no effect was seen	Not tested (*n* < 10)	All RCTs were undertaken in populations at high risk due to family history of allergic disease. Both studies included delayed introduction of allergenic foods and maternal allergenic food avoidance	**RR = 0.57** **(0.41, 0.81)**	**⨂⨂○○** **Low**	**100** **(low risk)** **150** **(high risk)** **200** **(very high risk)**	**43 cases** **95% CI** **19–59** **65 cases** **95% CI** **29–89** **86 cases** **95% CI** **38–118**
4 RCT Recurrent wheeze	Not serious 1 study high risk of attrition bias, and no study with high risk of conflict of interest	Not serious I^2^ = 0% Study estimates vary from 0.64 to 1.41	Serious 3 studies included environmental control measures such as dust mite and tobacco smoke avoidance, which are known to reduce respiratory symptoms	Serious Sparse data, inconsistent with data for age 0–4 and lung function data, where no effect was seen	Not tested (*n* < 10)	All RCTs were undertaken in populations at high risk due to family history of allergic disease. All studies included delayed introduction of allergenic foods; three also included maternal allergenic food avoidance.	**RR = 0.77** **(0.61, 0.97)**	**⨂⨂○○** **Low**	**50** **(low risk)** **100** **(high risk)** **150** **(very high risk)**	**12 cases** **95% CI** **2–20** **23 cases** **95% CI** **3–39** **35 cases** **95% CI** **5–59**
**Intervention: Maternal vitamin A vs no vitamin A** **Outcome: Lung function at age 11 years** **Study design: RCT**										
1 RCT Lung function	Not serious Low risk of bias	Very serious Supplementation with the vitamin A precursor beta carotene in the same trial had no effect on lung function. Neither vtamin A nor beta carotene had an effect on risk of wheeze. Separate trial with similar design but lower vitamin A dose as part of a multivitamin intervention found no effect on FEV1 or FVC at age 8.	Serious Study population was a poor rural Nepalese population at risk of vitamin A deficiency	Serious Single study, with wide CIs	Not tested (*n* < 10)	Dose response relationship between maternal postpartum serum retinol levels and lung function outcomes	**Mean Difference** **FEV**_**1**_ **46mls (6, 86)** **FVC 46mls (8, 84)**	**○○○○** **No evidence**	**-**	**-**

* Absolute risk reduction is expressed per 1,000 people treated. Control risks were derived from control event rates in included studies or, where data were sparse or unrepresentative from included studies, from large international surveys such as the ISAAC survey [19]. Abbreviations: BF, breastfeeding; CC case control; CS, cross-sectional; NCC, nested case control; OR, odds ratio; PC, prospective cohort; RC, retrospective cohort; RCT, randomised controlled trial; RR, Risk Ratio; TIDM, type 1 diabetes mellitus

### Breastfeeding duration and timing of solid food introduction

One intervention trial (17,046 participants) and 259 observational studies (947,097 participants) reported the association between total breastfeeding, exclusive breastfeeding or timing of solid food introduction, and allergic or autoimmune diseases. The intervention trial found reduced eczema in the first year of life in centres allocated to deliver a breastfeeding promotion intervention. Meta-analyses of 24 observational studies showed an association between total breastfeeding duration and reduced recurrent wheeze at 5 to 14 years (e.g., Fig 2), but we also found evidence of publication bias. Meta-analyses of 31 observational studies showed an association between total and exclusive breastfeeding duration and reduced TIDM (e.g. Fig 3). Associations were statistically significant for retrospective studies but not for prospective studies. The GRADE certainty of these breastfeeding findings was low to very low. There was no association found for other allergic outcomes or for the other autoimmune diseases studied (coeliac disease, 12 studies; inflammatory bowel disease, 13 studies; juvenile rheumatoid arthritis, 3 studies; autoimmune thyroid disease, 1 study).

### Intervention trials of other maternal or infant dietary exposures

#### Probiotic and prebiotic supplementation

Twenty-eight intervention trials (6,705 participants) evaluated probiotic supplements. These were either single or multiple organisms, given as capsules, powder, or part of a drink or infant formula milk, at a dose of 1 to 10 billion colony-forming units per day. Meta-analysis showed an association between probiotic supplementation and reduced eczema (Fig 4; 19 studies; RR 0.78; 95% CI 0.68–0.90; I^*2*^ = 61%) or ‘atopic’ eczema (Fig 4; 11 studies; RR 0.78; 95% CI 0.65–0.92; I^*2*^ = 0%) at age ≤ 4 years and reduced allergic sensitisation to cow’s milk at age 1 to 2 years (Fig 5; 97 studies). No significant association was seen with eczema at age 5 to 14 years, nor with allergic sensitisation to other allergens. The GRADE certainty of these findings was moderate for eczema and low for cow’s milk sensitisation. Subgroup analysis for eczema showed a significant difference between supplementing mothers during the postnatal period (9 interventions, RR 0.64; 95% CI 0.51–0.80; I^*2*^ = 59%) and studies that just supplemented infants during the postnatal period (11 interventions, RR 0.93; 95% CI 0.81–1.06; I^*2*^ = 31%; test for subgroup difference *P* = 0.016). Ten intervention trials (4,242 participants) evaluated prebiotic supplements (always a galacto-oligosaccharide) either alone or combined with other prebiotics or with probiotics. Nine studies involved comparison of an infant formula milk with versus without prebiotic. Meta-analysis showed no clear evidence that prebiotic supplementation reduces eczema at age ≤ 4 years (Fig 6; 7 studies; RR 0.75; 95% CI 0.56–1.01; I^*2*^ = 57%) and no association at age 5 to 14 years. There was no association between probiotics or prebiotics and other allergic outcomes. In our updated search on 15 December 2017, we identified one publication reporting no effect of a perinatal probiotic supplement on risk of coeliac disease at age 13 years from the trial of Kukkonen [20]. No other studies reported autoimmune outcomes.

#### Polyunsaturated fatty acid supplementation

Nineteen intervention trials (14,479 participants) evaluated fatty acid supplements. These were given as fish oil supplements in 12 trials, advice to eat oily fish in 1 trial, supplementation of infant formula milk with omega-3 fatty acids in 3 trials, and other oils in 3 trials. Comparators were corn, soya, canola, olive, sunflower, or vegetable oil, or no supplement. Meta-analysis showed an association between omega-3 polyunsaturated fatty acid supplementation using fish oil during pregnancy and lactation and reduced allergic sensitisation to egg at 1 year (Fig 7; 6 studies; RR 0.69 95% CI 0.53–0.90; I^*2*^ = 15%). No significant association was seen with allergic sensitisation to other allergens. The GRADE certainty of this finding was moderate. Subgroup analysis showed a significant difference between studies which supplemented mothers during pregnancy (4 studies; RR 0.55 95% CI 0.40–0.76; I^*2*^ = 0%) and studies which supplemented during lactation only (2 studies; RR 0.92 95% CI 0.65–1.28; I^*2*^ = 0%; test for subgroup difference *P* = 0.032). Similarly, for allergic sensitisation to peanut, there was reduced risk in the subgroup of studies which supplemented mothers during pregnancy (2 studies; RR 0.62 95% CI 0.40–0.96 I^*2*^ = 0%). There was no association between omega-3 polyunsaturated fatty acid supplementation and other allergic outcomes. Data were sparse and inconclusive for food allergy and for omega-6 supplementation and allergic outcomes. No autoimmune outcomes were reported, and no new trials were identified in our update search on 15 December 2017.

#### Maternal allergenic food avoidance and multifaceted interventions

Twelve intervention trials (1,945 participants) evaluated maternal allergenic food avoidance during pregnancy (2 trials), lactation (6 trials), or both (4 trials). The foods excluded were milk alone (2 trials), egg alone (1 trial), milk and egg (3 trials), or multiple foods (6 trials). We did not find that maternal allergenic food avoidance during pregnancy or lactation reduces risk of allergic disease or TIDM development. Nine trials (2,317 participants) evaluated multifaceted interventions—defined as an intervention with more than one category of dietary intervention. Six trials included promotion of prolonged breastfeeding, avoidance of allergenic foods for mother or infant, and delayed solid food introduction; 6 included environmental control measures to reduce dust mite, pet, or tobacco smoke exposure; 7 used an alternative formula milk as part of the intervention. Meta-analysis shown in Fig 8 found an association between multifaceted interventions and reduced allergic rhinitis at age ≤ 4 years (2 studies; RR 0.61 95% CI 0.44–0.84; I^*2*^ = 0%), but not at age 5 to 14 years (4 studies). We also found an association between multifaceted interventions and reduced wheeze (2 studies; RR 0.57 95% CI 0.41–0.81; I^*2*^ = 0%) and recurrent wheeze (4 studies; RR 0.77 95% CI 0.61–0.97; I^*2*^ = 0%) at age 5–14 years. There was no association at age ≤ 4 years (4 studies), nor with measures of lung function. The GRADE certainty of findings for multifaceted interventions and wheeze or rhinitis was low, there were no autoimmune outcomes reported, and no new trials were identified in our update search on 15 December 2017.

#### Vitamin and mineral supplementation

Eleven intervention trials (15,753 participants) evaluated vitamin and mineral supplements. Two trials used vitamin A in infants; 1 used vitamin A or beta carotene in pregnancy; 2 used vitamin C or E during pregnancy; 3 used vitamin D during pregnancy; 1 used vitamin D during pregnancy and infancy; and 2 used multivitamins during pregnancy. Meta-analysis shown in Fig 9 found no association between vitamin supplementation and risk of wheeze (5 studies), recurrent wheeze (6 studies), or eczema (5 studies) at age 0 to 4 years, nor for these outcomes at other ages or other allergic outcomes. One trial reported an association between vitamin A (but not beta carotene) supplementation during pregnancy and increased lung function in childhood [21]. This was not consistent with findings from another similar trial [22]. No autoimmune outcomes were reported, and no new trials were identified in our update search on 15 December 2017.

### Observational studies of other maternal or infant dietary exposures

Ninety-two observational studies (485,733 participants) evaluated other maternal or infant dietary exposures, including fruit and vegetable intake (35 studies), classified as fruits, vegetables, citrus fruits, nuts, or sometimes as individual foods or food groups; fat and fatty acid intake (26 studies), classified as total fat intake, omega 3 or omega 6 poly-unsaturated fatty acids or other types of fatty acids, and specific fatty acids (we also included fish intake and fish oil supplementation in this group); vitamin and mineral intake (50 studies), including antioxidant vitamins A, C, E, and their precursors, vitamin D and folic acid, mineral intake, and blood vitamin D levels; and a wide range of ‘other dietary exposures’ (47 studies), including dietary pattern, maternal allergenic food avoidance, alcohol, tea or coffee intake, and maternal or infant meat, cereal, milk, or egg intake. Opportunities for meta-analysis of these observational studies were very limited due to heterogeneity of exposure assessment methods and reporting—all analyses are described in the full Food Standards Agency report (https://www.food.gov.uk/science/research/allergy-research/fs305005ac). Overall, there was no consistent association between these dietary exposures and risk of allergic outcomes or those autoimmune diseases studied (TIDM, 23 studies; coeliac disease, 2 studies; inflammatory bowel disease, 2 studies; juvenile idiopathic arthritis, 1 study). There was insufficient evidence to either support or refute the intervention trial findings for fish oil supplementation and allergic sensitization to foods, in the observational studies of fatty acid intake during pregnancy.

### Trial sequential analysis of moderate or high certainty findings

**Fig 10:**
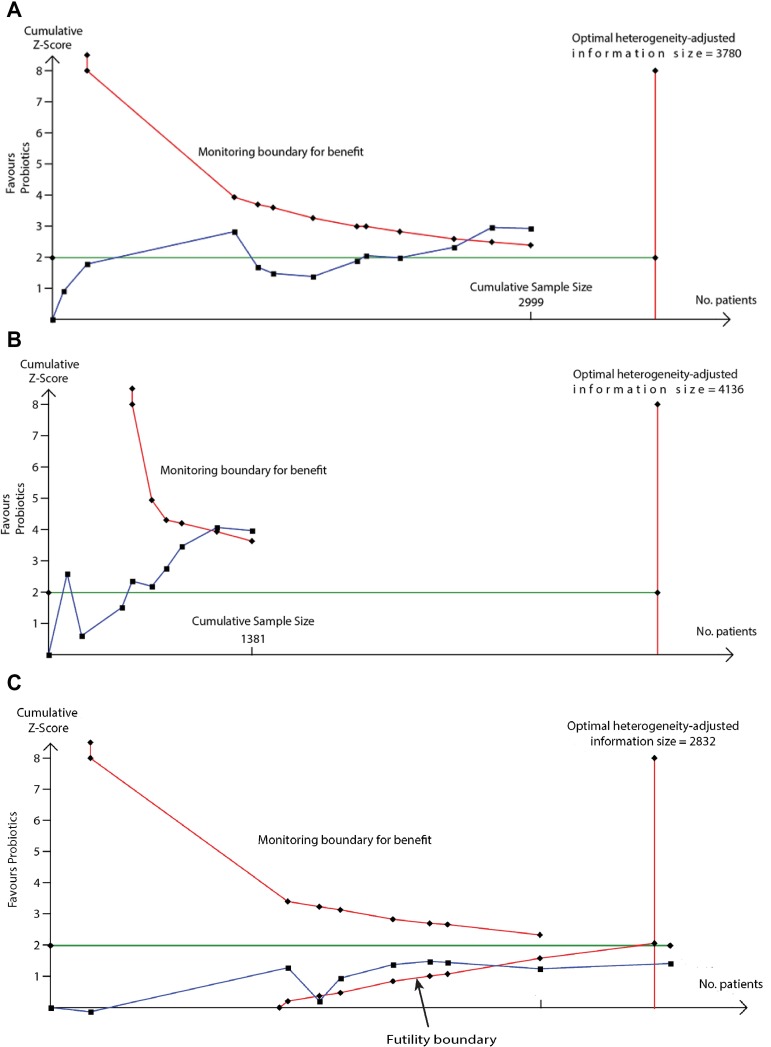
TSA of intervention trials evaluating the effect of probiotics on risk of eczema (A) and the subgroups of studies that did (B) or did not (C) supplement lactating mothers during the postnatal period. The vertical red line is the optimal information size, i.e., the cumulative sample size required to establish with 80% power and 5% 2-sided significance whether the intervention reduces risk of the outcome by ≥20%, allowing for repeatedly meta-analysing the accumulating studies. The horizontal green line is a z score of +1.96, equal to two-sided *P* = 0.05. The cumulative Z-statistic (blue line) does not reach the optimal information size in analysis of all studies (A) or maternal supplementation studies (B) but does cross the trial sequential monitoring boundary (curved red line), showing evidence for ≥20% relative risk reduction. The cumulative Z-statistic (blue line) for studies without maternal supplementation (C) crosses the futility boundary and reaches the optimal information size without crossing ±1.96, indicating evidence of futility such that further trials of this intervention are not required. Findings were similar for ≥30% relative risk reduction and for eczema associated with allergic sensitisation. No., number; TSA, trial sequential analysis.

**Fig 11:**
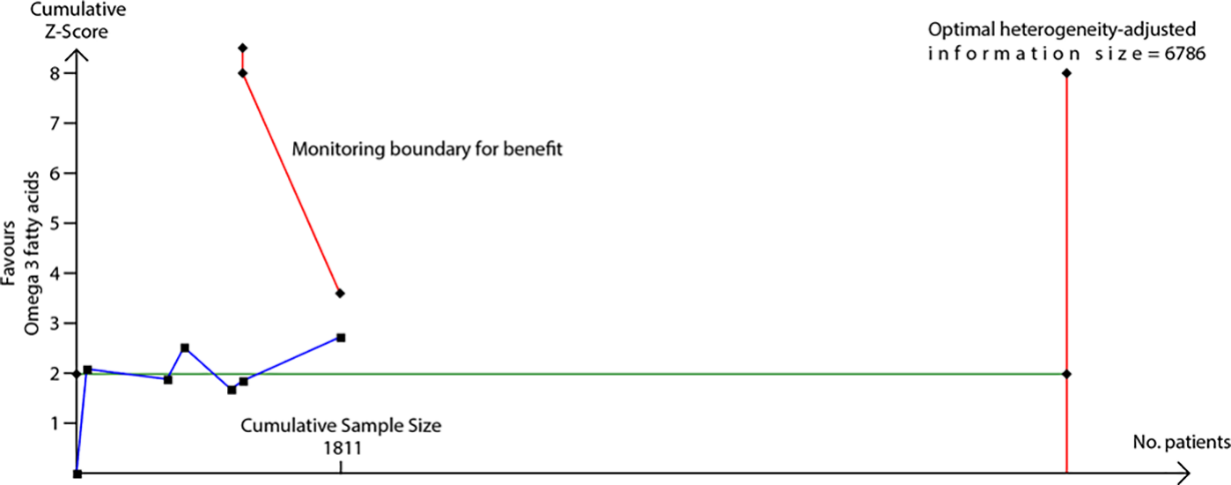
TSA of intervention trials evaluating the effect of fish oil supplementation on risk of allergic sensitisation to egg. The vertical red line is the optimal information size, i.e., the cumulative sample size required to establish with 80% power and 5% 2-sided significance whether the intervention reduces risk of the outcome by ≥20%, allowing for repeatedly meta-analysing the accumulating studies. The horizontal green line is a z score of +1.96, equal to two-sided *P* = 0.05. The cumulative Z-statistic (blue line) does not reach the optimal information size and does not cross the trial sequential monitoring boundary (curved red line), indicating no clear evidence for ≥20% relative risk reduction. Findings were similar for ≥30% relative risk reduction. No., number; TSA, trial sequential analysis.

We used TSA to evaluate whether probiotics reduce risk of eczema by ≥20% or ≥30%. The heterogeneity-adjusted optimal information size for detection of a 20% relative risk reduction for eczema was 3,780 study participants, and TSA for this outcome is shown in Fig 10. The optimal information size has not been reached, but the monitoring boundary has been crossed, suggesting that this treatment reduces eczema risk by ≥20%. Findings were similar for ≥30% risk reduction, for atopic eczema, and for the subgroup of studies that supplemented lactating mothers during the postnatal period (Fig 10). TSA for studies that didn’t supplement lactating mothers showed evidence of futility (Fig 10). We also used TSA to evaluate whether maternal fish oil supplementation reduces risk of allergic sensitisation to egg by ≥20% or ≥30% (Fig 11). The optimal information size for detection of a 20% relative risk reduction was 6,786 participants, which has not been reached, and the monitoring boundary has not been crossed, suggesting that further studies are required to confirm the effect.

## Discussion

### Principal findings

In this systematic review of diet during pregnancy and infancy and risk of allergic or autoimmune disease, we found a relationship between maternal diet during pregnancy and lactation and eczema or allergic sensitisation to food during childhood. Positive health effects were found for probiotic and fish oil supplements. We found weaker support for the hypotheses that multifaceted interventions reduce risk of allergic rhinitis and wheeze; that longer duration of breastfeeding is associated with reduced eczema, TIDM, and wheezing; and that longer exclusive breastfeeding is associated with reduced TIDM. There was no association between other dietary exposures and risk of allergic or autoimmune diseases, including timing of solid food introduction, prebiotic supplementation, maternal allergenic food avoidance, and vitamin, mineral, fruit, or vegetable intake. Previously, we reported that hydrolysed formula does not appear to influence risk of allergic or autoimmune diseases and that early peanut or egg introduction to the infant diet reduces risk of peanut or egg allergy [12,13]. Taken together, our findings suggest that while infant diet may influence immune development through allergen-specific mechanisms, maternal diet during prenatal life and lactation may have broader effects on the developing immune system. Future efforts to promote immune health through the life course should consider maternal dietary interventions during pregnancy and lactation as an important area of investigation. Our finding that probiotic supplements such as *Lactobacillus rhamnosus* at a dose of 1 to 10 billion colony-forming units per day may reduce risk of eczema is consistent with a recent World Allergy Organization (WAO) systematic review and guideline, although we rated the certainty of findings as stronger than the WAO group, who downgraded the certainty to very low due to risk of selection and attrition bias in the trials, variation in probiotic(s) used, and variation in the timing of the probiotic supplement intervention [3,10]. Our subgroup analysis and TSA findings provide new evidence that maternal (rather than infant) supplementation may be important for this intervention. In contrast, we did not find support for the WAO group’s recommendation to use prebiotic supplements such as galacto-oligosaccharides for preventing eczema due to our serious concerns about risk of bias, inconsistency, imprecision, and indirectness, some of which were shared by the WAO group [2,23]. Although microbial exposures have long been postulated as influencing risk of allergic disease, a clear mechanism through which probiotics might reduce risk of eczema is currently lacking. Our finding that fish oil supplementation during pregnancy may reduce allergic sensitisation to the most common food allergens affecting young children, hen’s egg and peanut, is new. In contrast to our findings for probiotics, those for fish oil have a plausible mechanistic basis, since fish oil has known anti-inflammatory effects [24].Further work is required to clarify whether the effect of fish oil on allergic sensitisation leads to reduced risk of clinical food allergy. We did not find support for recent Australasian guidelines or other systematic reviews suggesting that fish or fish oil supplementation may reduce eczema risk [6,25,26]. Other findings were of low or very low GRADE certainty and are not likely to impact infant feeding guidance at present. The findings for multifaceted interventions and reduced risk of rhinitis or wheezing are unlikely to be related to dietary components of the interventions, and the findings that longer breastfeeding or exclusive breastfeeding duration may reduce risk of TIDM, eczema, and wheezing are not likely to change current breastfeeding recommendations [14]. Others have reported stronger support for an association between breastfeeding duration and reduced risk of wheezing or other allergic outcomes, but only by pooling quite heterogeneous data [8,9]. Our meta-analyses of observational studies of breastfeeding duration and wheezing found new evidence of publication bias, which also suggests that previous reports of links between breastfeeding and wheezing may be misleading. We did not find evidence to support a role for vitamin D supplementation in preventing wheezing or other outcomes. This is consistent with a WAO systematic review [27] that was undertaken prior to the publication of two key vitamin D trials. Two other recent analyses concluded that prenatal vitamin D supplementation may reduce risk of recurrent wheeze. A systematic review of prenatal vitamins and allergic outcomes did not identify one of our included trials and made a data entry error in another trial (Goldring et al. control and active data switched) [28]. A meta-analysis of the two largest trials, undertaken by their authors, also had positive findings but did not include the other two published studies which we identified [29]. Overall, we found relatively few opportunities for meaningful meta-analysis in the observational studies, due mainly to variations in exposure definition and assessment. Even when observational study meta-analysis is possible, differences between populations studied or methods of exposure assessment can lead to increased variability of findings and thereby reduce statistical power compared with a single large, well-conducted study. This may partly explain the stronger findings in relation to intervention trials of dietary supplements than for observational studies of dietary pattern or specific nutritional intake.

### Interpretation

Our key findings suggest that a mother’s diet during pregnancy and lactation may influence the risk that her child develops allergic disease, and this has implications for future research in this field. There are two specific implications for pregnant women. First, a daily probiotic supplement such as *L*. *rhamnosus*, taken from around 36 to 38 weeks gestation through the first 3 to 6 months of lactation, may reduce risk of eczema in the child. Although probiotics are generally considered safe, their pro-inflammatory effects may have potential adverse consequences when used earlier in pregnancy, and serious adverse effects in people with intestinal disorders or immune deficiency have been documented [30][31]. Second, a fish oil supplement, taken from around 20 weeks gestation through the first 3 to 4 months of lactation, may reduce risk of allergic sensitisation to egg or peanut in the child. In one included trial, the fish oil intervention was administered via advice to consume at least 2 servings per week of low mercury oily fish (farmed salmon), which is consistent with current United States Food and Drug Administration guidance [32]. It was not possible to reliably assess whether the findings in this small trial differed significantly from the findings in higher dose fish oil supplementation trials.

### Strengths and limitations of the study

This comprehensive and wide-ranging systematic review has highlighted key dietary interventions that may reduce risk of allergic outcomes, information which will be useful for pregnant women and their healthcare providers. However, for many dietary exposures, data were inconclusive or inconsistent, such that we are unable to exclude the possibility of beneficial or harmful effects. One limitation of this study is the 2013 search date for observational studies, such that review findings that are derived from these studies may benefit from an update in due course. We included abstract publications, since their exclusion can lead to publication bias; however, their inclusion carries a risk of methodological bias due to the lack of peer review. While the list of dietary exposures included was comprehensive, we did not evaluate food diversity during infancy, which has been suggested as an important factor in relation to allergic outcomes, and we did not evaluate objective markers of nutritional status, with the exception of blood vitamin D level [33]. This means that we may have missed important relationships for food diversity or nutritional markers. Given the volume of titles included in this systematic review, we were unable to contact the corresponding authors of included studies for data verification, as has been recommended by some authors [34]. Finally, some outcome measures were reported using a wide variety of assessment tools—for example, wheeze and recurrent wheeze were defined in several different ways—and this may have contributed to some of the statistical heterogeneity seen in meta-analyses. There is a need for more well-powered intervention trials of dietary interventions, given the positive findings with some specific dietary supplements identified here.

### Conclusions and policy implications

These findings, together with our previous findings regarding hydrolysed formula and allergenic food introduction to the infant diet, suggest that current infant feeding guidance needs revision. Guideline committees will need to carefully consider the key findings together with an evaluation of the safety, acceptability, and cost implications of advising probiotic or fish oil supplementation for pregnant and lactating women.

## Supporting information

S1 PRISMA Checklist PRISMA Checklist. (DOC)

S1 Text Systematic review search strategies. (DOCX)

S2 Text Review A (milk feeding) over-arching report. (DOC)

S3 Text Review C (maternal and infant diet) over-arching report. (DOC)

S1 Appendix Characteristics of included studies and risk of bias tables. (DOCX)

S1 Data Review A (milk feeding) report files. (ZIP)

S2 Data Review C (maternal and infant diet) report files. (ZIP)

S3 Data Data used for meta-analyses. (XLSX)

## Abbreviations

CCT: controlled clinical trial
CENTRAL: Central Register of Controlled Trials
COT: Committee on Toxicity of Chemicals in Food, Consumer Products and the Environment
EMBASE: Excerpta Medica dataBASE
GRADE: Grading of Recommendations Assessment, Development and Evaluation
LILACS: Literatura Latino Americana em Ciências da Saúde
MEDLINE: Medical Literature Analysis and Retrieval System Online
OR: odds ratio
RCT: randomised controlled trial
RR: Risk Ratio
SACN: Scientific Advisory Committee on Nutrition
sIgE: specific immunoglobulin E
TIDM: type 1 diabetes mellitus
TSA: trial sequential analysis
WAO: World Allergy Organization
