# Supplementary material for: Diet during pregnancy and infancy and risk of allergic or autoimmune disease: A systematic review and meta-analysis
Source: PLoS Med. 2018 Feb 28;15(2):e1002507. doi: 10.1371/journal.pmed.1002507 (PMC5830033; doi:10.1371/journal.pmed.1002507)
Supplement: S1 Text — (DOCX) [file pmed.1002507.s002.docx]

**Diet during pregnancy and infancy, and risk of allergic or autoimmune disease: a systematic review and meta-analysis**

Vanessa Garcia-Larsen* assistant professor in human nutrition^1, 2^, Despo Ierodiakonou* post-doctoral research fellow^2, 3^, Katharine Jarrold medical student^3^, Sergio Cunha research fellow^2^, Jennifer Chivinge medical student^3^, Zoe Robinson medical student^3^, Natalie Geoghegan medical student^3^, Alisha Ruparelia medical student^3^, Pooja Devani medical student^3^, Tim Reeves librarian^2^, Marialena Trivella senior medical statistician^4^, Jo Leonardi-Bee professor of medical statistics and epidemiology^5^, Robert J Boyle clinical reader in paediatric allergy^3, 6^

^*^ Joint first authors

^1^Department of International Health, Johns Hopkins School of Public Health, N Wolfe Street, Baltimore, MD 21205, USA ^2^Respiratory Epidemiology, Occupational Medicine and Public Health, National Heart and Lung Institute, Imperial College London, Manresa Road, London SW3 6LR, UK ^3^Section of Paediatrics, Department of Medicine, Imperial College London, Norfolk Place, London W2 1PG, UK ^4^Centre for Statistics in Medicine, University of Oxford, Windmill Road, Oxford OX3 7LD, UK ^5^Division of Epidemiology and Public Health, University of Nottingham, Hucknall Road, Nottingham NG5 1PB, UK ^6^Centre of Evidence Based Dermatology, University of Nottingham, Lenton Lane, Nottingham NG7 2NR, UK

Address for Correspondence:

Robert Boyle, Wright Fleming Building, Norfolk Place, London W2 1PG

Tel: +44 207 594 3990 Fax: +44 207 594 3984. Email: r.boyle@imperial.ac.uk

Table of Contents

[**Appendix 1 Search Strategies for other systematic reviews** 3](#_Toc503440715)

[**Medline** 3](#_Toc503440716)

[**Embase** 17](#_Toc503440717)

[**COCHRANE Reviews and DARE** 31](#_Toc503440718)

[**Appendix 2 Search Strategies for original articles on milk feeding** 45](#_Toc503440719)

[**Medline** 45](#_Toc503440720)

[**Embase** 50](#_Toc503440721)

[**LILACS** 55](#_Toc503440722)

[**COCHRANE Library** 56](#_Toc503440723)

[**Web of Science** 61](#_Toc503440724)

[**Appendix 3 Search Strategies for original articles on other maternal or infant dietary exposures** 62](#_Toc503440725)

[**Medline** 62](#_Toc503440726)

[**Embase** 74](#_Toc503440727)

[**LILACS** 86](#_Toc503440728)

[**COCHRANE Library** 87](#_Toc503440729)

[**Web of Science** 99](#_Toc503440730)

**Appendix 1 Search Strategies for other systematic reviews**

**Medline**

1. breast feeding.ab,ti.

2. breastfeeding.ab,ti.

3. breast fed.ab,ti.

4. breastfed.ab,ti.

5. Breast Feeding/

6. Milk, Human/

7. formula?.ab,ti.

8. hydrolysed.ab,ti.

9. bottlefed.ab,ti.

10. bottle fed.ab,ti.

11. (bottle adj3 feed$).ab,ti.

12. Infant Formula/

13. Bottle Feeding/

14. wean$.ab,ti.

15. Weaning/

16. 1 or 2 or 3 or 4 or 5 or 6 or 7 or 8 or 9 or 10 or 11 or 12 or 13 or 14 or 15

17. complementary food?.ab,ti.

18. (introduc$ adj2 food?).ab,ti.

19. wean$.ab,ti.

20. Weaning/

21. solid?.ab,ti.

22. semi-solid?.ab,ti.

23. baby food?.ab,ti.

24. Infant Food/

25. Infant Nutritional Physiological Phenomena/

26. breast feeding.ab,ti.

27. breastfeeding.ab,ti.

28. breast fed.ab,ti.

29. breastfed.ab,ti.

30. Breast Feeding/

31. Milk, Human/

32. formula?.ab,ti.

33. hydrolysed.ab,ti.

34. bottlefed.ab,ti.

35. bottle fed.ab,ti.

36. (bottle adj3 feed$).ab,ti.

37. Infant Formula/

38. Bottle Feeding/

39. liquid?.ab,ti.

40. milk.ab,ti.

41. Milk/

42. egg?.ab,ti.

43. Egg Proteins/

44. Egg Proteins, Dietary/

45. nut?.ab,ti.

46. peanut?.ab,ti.

47. almond?.ab,ti.

48. (brazil? adj5 nut?).ab,ti.

49. walnut?.ab,ti.

50. pecan?.ab,ti.

51. pistachio?.ab,ti.

52. cashew?.ab,ti.

53. hazelnut?.ab,ti.

54. macadamia?.ab,ti.

55. Nuts/

56. Arachis hypogaea/

57. Prunus/

58. Bertholletia/

59. Juglans/

60. Carya/

61. Pistacia/

62. Anacardium/

63. Corylus/

64. Macadamia/

65. wheat.ab,ti.

66. Triticum/

67. soya.ab,ti.

68. Soybeans/

69. gluten$.ab,ti.

70. Glutens/

71. fish.ab,ti.

72. Fishes/

73. 17 or 18 or 19 or 20 or 21 or 22 or 23 or 24 or 25 or 26 or 27 or 28 or 29 or 30 or 31 or 32 or 33 or 34 or 35 or 36 or 37 or 38 or 39 or 40 or 41 or 42 or 43 or 44 or 45 or 46 or 47 or 48 or 49 or 50 or 51 or 52 or 53 or 54 or 55 or 56 or 57 or 58 or 59 or 60 or 61 or 62 or 63 or 64 or 65 or 66 or 67 or 68 or 69 or 70 or 71 or 72

74. Diet/

75. Diet Therapy/

76. Nutritional Sciences/

77. Child Nutrition Sciences/

78. diet.ab,ti.

79. diets.ab,ti.

80. Diet, Mediterranean/

81. mediterranean diet$.ab,ti.

82. dietetic.ab,ti.

83. dietary.ab,ti.

84. eat.ab,ti.

85. eating.ab,ti.

86. intake.ab,ti.

87. nutrient?.ab,ti.

88. nutrition.ab,ti.

89. Diet, Vegetarian/

90. vegetarian?.ab,ti.

91. vegan$.ab,ti.

92. Diet, Macrobiotic/

93. macrobiotic?.ab,ti.

94. Food/

95. food$.ab,ti.

96. feed.ab,ti.

97. feeding.ab,ti.

98. cereal$.ab,ti.

99. grain$.ab,ti.

100. granary.ab,ti.

101. wholegrain.ab,ti.

102. wholewheat.ab,ti.

103. whole wheat.ab,ti.

104. wheat.ab,ti.

105. wheatgerm.ab,ti.

106. rye.ab,ti.

107. barley.ab,ti.

108. oat?.ab,ti.

109. exp Cereals/

110. root?.ab,ti.

111. tuber?.ab,ti.

112. exp Vegetables/

113. vegetable$.ab,ti.

114. onion$.ab,ti.

115. spinach.ab,ti.

116. chard.ab,ti.

117. tomato$.ab,ti.

118. pepper$.ab,ti.

119. carrot$.ab,ti.

120. beetroot.ab,ti.

121. asparagus.ab,ti.

122. garlic.ab,ti.

123. pumpkin.ab,ti.

124. sprouts.ab,ti.

125. broccoli.ab,ti.

126. cabbage$.ab,ti.

127. celery.ab,ti.

128. ginger.ab,ti.

129. potato$.ab,ti.

130. crisps.ab,ti.

131. fries.ab,ti.

132. syrup.ab,ti.

133. honey.ab,ti.

134. Honey/

135. Fruit/

136. fruit$.ab,ti.

137. apple?.ab,ti.

138. pear?.ab,ti.

139. banana?.ab,ti.

140. orange?.ab,ti.

141. grape?.ab,ti.

142. kiwi?.ab,ti.

143. citrus.ab,ti.

144. grapefruit?.ab,ti.

145. pulses.ab,ti.

146. beans.ab,ti.

147. lentil?.ab,ti.

148. chickpea?.ab,ti.

149. legume?.ab,ti.

150. lupin?.ab,ti.

151. soy.ab,ti.

152. soya.ab,ti.

153. nut?.ab,ti.

154. almond?.ab,ti.

155. peanut?.ab,ti.

156. groundnut?.ab,ti.

157. Nuts/

158. seed?.ab,ti.

159. sesame.ab,ti.

160. mustard.ab,ti.

161. Seeds/

162. exp Meat/

163. meat.ab,ti.

164. beef.ab,ti.

165. pork.ab,ti.

166. lamb.ab,ti.

167. poultry.ab,ti.

168. chicken.ab,ti.

169. turkey.ab,ti.

170. duck.ab,ti.

171. fish.ab,ti.

172. Fatty Acids/

173. exp Fatty Acids, Omega-3/

174. exp Fatty Acids, Omega-6/

175. omega-3.ab,ti.

176. omega-6.ab,ti.

177. PUFA.ab,ti.

178. fat.ab,ti.

179. fats.ab,ti.

180. fatty.ab,ti.

181. egg.ab,ti.

182. eggs.ab,ti.

183. exp Eggs/

184. Bread/

185. bread.ab,ti.

186. oil.ab,ti.

187. oils.ab,ti.

188. oily.ab,ti.

189. omega.ab,ti.

190. exp Seafood/

191. seafood.ab,ti.

192. shellfish.ab,ti.

193. crustacean?.ab,ti.

194. mollusc?.ab,ti.

195. Shellfish/

196. Dairy Products/

197. dairy.ab,ti.

198. exp Milk/

199. milk.ab,ti.

200. Infant Formula/

201. formula?.ab,ti.

202. hydrolysed.ab,ti.

203. Infant Food/

204. yoghurt.ab,ti.

205. probiotic.ab,ti.

206. prebiotic?.ab,ti.

207. butter.ab,ti.

208. herb?.ab,ti.

209. spice?.ab,ti.

210. chilli$.ab,ti.

211. condiment?.ab,ti.

212. exp Condiments/

213. Beverages/

214. beverage?.ab,ti.

215. fluid intake.ab,ti.

216. water.ab,ti.

217. drink$.ab,ti.

218. exp Food Preservation/

219. pickled.ab,ti.

220. bottled.ab,ti.

221. canned.ab,ti.

222. canning.ab,ti.

223. smoked.ab,ti.

224. preserved.ab,ti.

225. preservatives.ab,ti.

226. nitrosamine.ab,ti.

227. hydrogenation.ab,ti.

228. fortified.ab,ti.

229. nitrates.ab,ti.

230. nitrites.ab,ti.

231. ferment$.ab,ti.

232. processed.ab,ti.

233. antioxidant$.ab,ti.

234. genetic modif$.ab,ti.

235. genetically modif$.ab,ti.

236. Cooking/

237. cooking.ab,ti.

238. cooked.ab,ti.

239. grill.ab,ti.

240. grilled.ab,ti.

241. fried.ab,ti.

242. fry.ab,ti.

243. roast.ab,ti.

244. bake.ab,ti.

245. baked.ab,ti.

246. stewing.ab,ti.

247. stewed.ab,ti.

248. casserol$.ab,ti.

249. broil.ab,ti.

250. broiled.ab,ti.

251. boiled.ab,ti.

252. poach.ab,ti.

253. poached.ab,ti.

254. steamed.ab,ti.

255. barbecue$.ab,ti.

256. chargrill$.ab,ti.

257. salt.ab,ti.

258. salting.ab,ti.

259. salted.ab,ti.

260. fiber.ab,ti.

261. fibre.ab,ti.

262. polysaccharide$.ab,ti.

263. starch.ab,ti.

264. starchy.ab,ti.

265. carbohydrate$.ab,ti.

266. lipid$.ab,ti.

267. linoleic acid$.ab,ti.

268. sugar$.ab,ti.

269. sweetener$.ab,ti.

270. saccharin$.ab,ti.

271. aspartame.ab,ti.

272. sucrose.ab,ti.

273. xylitol.ab,ti.

274. cholesterol.ab,ti.

275. hydrogenated lard.ab,ti.

276. dietary protein.ab,ti.

277. dietary proteins.ab,ti.

278. protein intake.ab,ti.

279. animal protein$.ab,ti.

280. total protein$.ab,ti.

281. vegetable protein$.ab,ti.

282. plant protein$.ab,ti.

283. exp Dietary Carbohydrates/

284. exp Dietary Fats/

285. exp Dietary Fiber/

286. exp Dietary Proteins/

287. exp Dietary Supplements/

288. exp Food Additives/

289. exp Vitamins/

290. supplements.ab,ti.

291. supplement.ab,ti.

292. vitamin$.ab,ti.

293. retinol.ab,ti.

294. carotenoid$.ab,ti.

295. tocopherol.ab,ti.

296. folate$.ab,ti.

297. folic acid.ab,ti.

298. methionine.ab,ti.

299. riboflavin.ab,ti.

300. thiamine.ab,ti.

301. niacin.ab,ti.

302. pyridoxine.ab,ti.

303. cobalamin.ab,ti.

304. mineral$.ab,ti.

305. sodium.ab,ti.

306. iron.ab,ti.

307. calcium.ab,ti.

308. selenium.ab,ti.

309. iodine.ab,ti.

310. magnesium.ab,ti.

311. potassium.ab,ti.

312. zinc.ab,ti.

313. copper.ab,ti.

314. phosphorus.ab,ti.

315. manganese.ab,ti.

316. chromium.ab,ti.

317. phytochemical.ab,ti.

318. polyphenol$.ab,ti.

319. phytoestrogen$.ab,ti.

320. genistein.ab,ti.

321. saponin$.ab,ti.

322. coumarin$.ab,ti.

323. flavonoid$.ab,ti.

324. polyphenol$.ab,ti.

325. flavonol$.ab,ti.

326. flavone$.ab,ti.

327. isoflavone$.ab,ti.

328. catechin$.ab,ti.

329. ascorbic acid$.ab,ti.

330. hydroxy cholecalciferol$.ab,ti.

331. hydroxycholecalciferol$.ab,ti.

332. tocotrienol$.ab,ti.

333. carotene$.ab,ti.

334. cryptoxanthin$.ab,ti.

335. lycopene$.ab,ti.

336. lutein$.ab,ti.

337. zeaxanthin$.ab,ti.

338. selenium$.ab,ti.

339. organic diet?.ab,ti.

340. Food, Organic/

341. 74 or 75 or 76 or 77 or 78 or 79 or 80 or 81 or 82 or 83 or 84 or 85 or 86 or 87 or 88 or 89 or 90 or 91 or 92 or 93 or 94 or 95 or 96 or 97 or 98 or 99 or 100 or 101 or 102 or 103 or 104 or 105 or 106 or 107 or 108 or 109 or 110 or 111 or 112 or 113 or 114 or 115 or 116 or 117 or 118 or 119 or 120 or 121 or 122 or 123 or 124 or 125 or 126 or 127 or 128 or 129 or 130 or 131 or 132 or 133 or 134 or 135 or 136 or 137 or 138 or 139 or 140 or 141 or 142 or 143 or 144 or 145 or 146 or 147 or 148 or 149 or 150 or 151 or 152 or 153 or 154 or 155 or 156 or 157 or 158 or 159 or 160 or 161 or 162 or 163 or 164 or 165 or 166 or 167 or 168 or 169 or 170 or 171 or 172 or 173 or 174 or 175 or 176 or 177 or 178 or 179 or 180 or 181 or 182 or 183 or 184 or 185 or 186 or 187 or 188 or 189 or 190 or 191 or 192 or 193 or 194 or 195 or 196 or 197 or 198 or 199 or 200 or 201 or 202 or 203 or 204 or 205 or 206 or 207 or 208 or 209 or 210 or 211 or 212 or 213 or 214 or 215 or 216 or 217 or 218 or 219 or 220 or 221 or 222 or 223 or 224 or 225 or 226 or 227 or 228 or 229 or 230 or 231 or 232 or 233 or 234 or 235 or 236 or 237 or 238 or 239 or 240 or 241 or 242 or 243 or 244 or 245 or 246 or 247 or 248 or 249 or 250 or 251 or 252 or 253 or 254 or 255 or 256 or 257 or 258 or 259 or 260 or 261 or 262 or 263 or 264 or 265 or 266 or 267 or 268 or 269 or 270 or 271 or 272 or 273 or 274 or 275 or 276 or 277 or 278 or 279 or 280 or 281 or 282 or 283 or 284 or 285 or 286 or 287 or 288 or 289 or 290 or 291 or 292 or 293 or 294 or 295 or 296 or 297 or 298 or 299 or 300 or 301 or 302 or 303 or 304 or 305 or 306 or 307 or 308 or 309 or 310 or 311 or 312 or 313 or 314 or 315 or 316 or 317 or 318 or 319 or 320 or 321 or 322 or 323 or 324 or 325 or 326 or 327 or 328 or 329 or 330 or 331 or 332 or 333 or 334 or 335 or 336 or 337 or 338 or 339 or 340

342. allerg$.ab,ti.

343. asthma$.ab,ti.

344. wheeze.ab,ti.

345. wheezing.ab,ti.

346. bronchial hyperresponsiveness.ab,ti.

347. bronchial hyperreactivity.ab,ti.

348. Forced expiratory volume.ab,ti.

349. FEV1.ab,ti.

350. "FEV 1".ab,ti.

351. "FEV0.5".ab,ti.

352. "FEV 0.5".ab,ti.

353. Forced vital capacity.ab,ti.

354. FVC.ab,ti.

355. Peak expiratory flow rate.ab,ti.

356. PEFR.ab,ti.

357. AD.ab,ti.

358. neurodermatitis.ab,ti.

359. rhinitis.ab,ti.

360. besniers prurigo.ab,ti.

361. rhinoconjunctivitis.ab,ti.

362. hayfever.ab,ti.

363. (hay adj fever).ab,ti.

364. poll?nosis.ab,ti.

365. SAR.ab,ti.

366. (pollen adj allergy).ab,ti.

367. conjunctivitis.ab,ti.

368. immunoglobulin e.ab,ti.

369. Total IgE.ab,ti.

370. autoimmune disease?.ab,ti.

371. diabetes.ab,ti.

372. diabetic.ab,ti.

373. type 1.ab,ti.

374. c?eliac disease.ab,ti.

375. crohn$ disease.ab,ti.

376. Inflammatory Bowel Disease?.ab,ti.

377. Ulcerative colitis.ab,ti.

378. (Lympho$ adj3 thyroiditi$).ab,ti.

379. (Thyroiditi$ adj3 autoimmune).ab,ti.

380. (Hashimoto$ adj3 (syndrome? or thyroiditi$ or disease?)).ab,ti.

381. (Thyroiditi$ adj3 (post-partum or postpartum)).ab,ti.

382. Graves? disease.ab,ti.

383. Basedow$ disease.ab,ti.

384. exophthalmic goiter?.ab,ti.

385. (Still? Disease adj3 (juvenile or onset)).ab,ti.

386. (Juvenile adj3 arthriti$).ab,ti.

387. vitiligo.ab,ti.

388. Psorias?s.ab,ti.

389. (Arthriti? adj3 Psoria$).ab,ti.

390. atopic disease.ab,ti.

391. atopic dermatitis.ab,ti.

392. (food? adj3 sensiti$).ab,ti.

393. (food? adj3 toleran$).ab,ti.

394. (food? adj3 intoleran$).ab,ti.

395. ((aero or air$) adj3 allergen?).ab,ti.

396. (aeroallergen? adj3 sensiti$).ab,ti.

397. (allergen? adj3 sensiti$).ab,ti.

398. skin prick test$.ab,ti.

399. atopy.ab,ti.

400. hypersensitiv$.ab,ti.

401. Hypersensitivity/

402. exp Food Hypersensitivity/

403. Respiratory Hypersensitivity/

404. Asthma/

405. Bronchial Hyperreactivity/

406. Forced Expiratory Volume/

407. Vital Capacity/

408. Peak Expiratory Flow Rate/

409. AD/

410. Neurodermatitis/

411. Rhinitis/

412. Rhinitis, Allergic, Perennial/

413. Rhinitis, Allergic, Seasonal/

414. Conjunctivitis/

415. Immunoglobulin E/

416. Autoimmune Diseases/

417. Diabetes Mellitus, Type 1/

418. Celiac Disease/

419. Crohn Disease/

420. Inflammatory Bowel Diseases/

421. Colitis, Ulcerative/

422. Thyroiditis, Autoimmune/

423. Hashimoto Disease/

424. Postpartum Thyroiditis/

425. Graves Disease/

426. Arthritis, Juvenile Rheumatoid/

427. Vitiligo/

428. Psoriasis/

429. Arthritis, Psoriatic/

430. Dermatitis, Atopic/

431. Hypersensitivity, Immediate/

432. 342 or 343 or 344 or 345 or 346 or 347 or 348 or 349 or 350 or 351 or 352 or 353 or 354 or 355 or 356 or 357 or 358 or 359 or 360 or 361 or 362 or 363 or 364 or 365 or 366 or 367 or 368 or 369 or 370 or 371 or 372 or 373 or 374 or 375 or 376 or 377 or 378 or 379 or 380 or 381 or 382 or 383 or 384 or 385 or 386 or 387 or 388 or 389 or 390 or 391 or 392 or 393 or 394 or 395 or 396 or 397 or 398 or 399 or 400 or 401 or 402 or 403 or 404 or 405 or 406 or 407 or 408 or 409 or 410 or 411 or 412 or 413 or 414 or 415 or 416 or 417 or 418 or 419 or 420 or 421 or 422 or 423 or 424 or 425 or 426 or 427 or 428 or 429 or 430 or 431

433. infant?.ab,ti.

434. ((one or two or three or four or five or six or seven or eight or nine or ten or eleven or twelve or thirteen or fourteen or fifteen or sixteen or seventeen or eighteen or nineteen or twenty or "twenty one" or "twenty two" or "twenty three" or "twenty four" or "twenty five" or "twenty six") adj week?).ab,ti.

435. ((one or two or three or four or five or six or seven or eight or nine or ten or eleven or twelve or thirteen or fourteen or fifteen or sixteen or seventeen or eighteen or nineteen or twenty or "twenty one" or "twenty two" or "twenty three" or "twenty four") adj month?).ab,ti.

436. 434 or 435

437. (old or age?).ab,ti.

438. 436 and 437

439. (("one year?" or "two year?") adj3 (old or age?)).ab,ti.

440. ((first or second or two) adj3 "year? of life").ab,ti.

441. Infant/

442. Infant, Newborn/

443. (maternal or pregnan$).ab,ti.

444. (lactat$).ab,ti.

445. (mother?).ab,ti.

446. 433 or 438 or 439 or 440 or 441 or 442 or 443 or 444 or 445

447. MEDLINE.tw.

448. systematic review.tw.

449. meta-analysis.pt.

450. intervention$.ti.

451. 447 or 448 or 449 or 450

452. 16 or 73 or 341

453. 432 and 446 and 451 and 452

454. limit 453 to yr="2011 -Current"

**Embase**

1. breast feeding.ab,ti.

2. breastfeeding.ab,ti.

3. breast fed.ab,ti.

4. breastfed.ab,ti.

5. breast feeding/

6. breast milk/

7. formula?.ab,ti.

8. hydrolysed.ab,ti.

9. bottlefed.ab,ti.

10. bottle fed.ab,ti.

11. (bottle adj3 feed$).ab,ti.

12. artificial milk/

13. bottle feeding/

14. wean$.ti,ab.

15. weaning/

16. 1 or 2 or 3 or 4 or 5 or 6 or 7 or 8 or 9 or 10 or 11 or 12 or 13 or 14 or 15

17. complementary food?.ab,ti.

18. (introduc$ adj2 food?).ab,ti.

19. wean$.ab,ti.

20. weaning/

21. solid?.ab,ti.

22. semi-solid?.ab,ti.

23. baby food?.ab,ti.

24. baby food/

25. infant nutrition/

26. breast feeding.ab,ti.

27. breastfeeding.ab,ti.

28. breast fed.ab,ti.

29. breastfed.ab,ti.

30. breast feeding/

31. breast milk/

32. formula?.ab,ti.

33. hydrolysed.ab,ti.

34. bottlefed.ab,ti.

35. bottle fed.ab,ti.

36. (bottle adj3 feed$).ab,ti.

37. artificial milk/

38. bottle feeding/

39. liquid?.ti,ab.

40. milk.ti,ab.

41. milk/

42. egg?.ti,ab.

43. egg/

44. egg protein/

45. nut?.ab,ti.

46. peanut?.ab,ti.

47. almond?.ab,ti.

48. (brazil? adj5 nut?).ab,ti.

49. walnut?.ab,ti.

50. pecan?.ab,ti.

51. pistachio?.ab,ti.

52. cashew?.ab,ti.

53. hazelnut?.ab,ti.

54. macadamia?.ab,ti.

55. nut/

56. peanut/

57. almond/

58. Brazil nut/

59. exp walnut/

60. pecan/

61. pistachio/

62. cashew nut/

63. hazelnut/

64. Corylus avellana/

65. Macadamia/

66. wheat.ti,ab.

67. exp wheat/

68. soya.ti,ab.

69. soybean/

70. gluten$.ti,ab.

71. gluten/

72. fish$.ti,ab.

73. fish/

74. 17 or 18 or 19 or 20 or 21 or 22 or 23 or 24 or 25 or 26 or 27 or 28 or 29 or 30 or 31 or 32 or 33 or 34 or 35 or 36 or 37 or 38 or 39 or 40 or 41 or 42 or 43 or 44 or 45 or 46 or 47 or 48 or 49 or 50 or 51 or 52 or 53 or 54 or 55 or 56 or 57 or 58 or 59 or 60 or 61 or 62 or 63 or 64 or 65 or 66 or 67 or 68 or 69 or 70 or 71 or 72 or 73

75. diet/

76. diet therapy/

77. nutritional science/

78. diet.ti,ab.

79. diets.ti,ab.

80. Mediterranean diet/

81. mediterranean diet$.ab,ti.

82. dietetic.ab,ti.

83. dietary.ab,ti.

84. eat.ab,ti.

85. eating.ab,ti.

86. intake.ab,ti.

87. nutrient?.ab,ti.

88. nutrition.ab,ti.

89. vegetarian diet/

90. vegetarian?.ti,ab.

91. vegan$.ti,ab.

92. macrobiotic diet/

93. macrobiotic?.ti,ab.

94. food/

95. food$.ab,ti.

96. feed.ab,ti.

97. feeding.ab,ti.

98. cereal$.ab,ti.

99. grain$.ab,ti.

100. granary.ab,ti.

101. wholegrain.ab,ti.

102. wholewheat.ab,ti.

103. whole wheat.ab,ti.

104. wheat.ab,ti.

105. wheatgerm.ab,ti.

106. rye.ab,ti.

107. barley.ab,ti.

108. oat?.ab,ti.

109. exp cereal/

110. root?.ti,ab.

111. tuber?.ti,ab.

112. exp vegetable/

113. vegetable$.ab,ti.

114. onion$.ab,ti.

115. spinach.ab,ti.

116. chard.ab,ti.

117. tomato$.ab,ti.

118. pepper$.ab,ti.

119. carrot$.ab,ti.

120. beetroot.ab,ti.

121. asparagus.ab,ti.

122. garlic.ab,ti.

123. pumpkin.ab,ti.

124. sprouts.ab,ti.

125. broccoli.ab,ti.

126. cabbage$.ab,ti.

127. celery.ab,ti.

128. ginger.ab,ti.

129. potato$.ab,ti.

130. crisps.ab,ti.

131. fries.ab,ti.

132. syrup.ab,ti.

133. honey.ab,ti.

134. honey/

135. fruit/

136. fruit$.ab,ti.

137. apple?.ab,ti.

138. pear?.ab,ti.

139. banana?.ab,ti.

140. orange?.ab,ti.

141. grape?.ab,ti.

142. kiwi?.ab,ti.

143. citrus.ab,ti.

144. grapefruit?.ab,ti.

145. pulses.ab,ti.

146. beans.ab,ti.

147. lentil?.ab,ti.

148. chickpea?.ab,ti.

149. legume?.ab,ti.

150. lupin?.ab,ti.

151. soy.ab,ti.

152. soya.ab,ti.

153. nut?.ab,ti.

154. almond?.ab,ti.

155. peanut?.ab,ti.

156. groundnut?.ab,ti.

157. exp nut/

158. seed?.ti,ab.

159. sesame.ti,ab.

160. mustard.ti,ab.

161. plant seed/

162. meat/

163. meat.ab,ti.

164. beef.ab,ti.

165. pork.ab,ti.

166. lamb.ab,ti.

167. poultry.ab,ti.

168. chicken.ab,ti.

169. turkey.ab,ti.

170. duck.ab,ti.

171. fish.ab,ti.

172. fatty acid/

173. omega 3 fatty acid/

174. omega 6 fatty acid/

175. omega-3.ab,ti.

176. omega-6.ab,ti.

177. PUFA.ab,ti.

178. fat.ab,ti.

179. fats.ab,ti.

180. fatty.ab,ti.

181. egg.ab,ti.

182. eggs.ab,ti.

183. exp egg/

184. bread/

185. bread.ti,ab.

186. oil.ti,ab.

187. oils.ti,ab.

188. oily.ti,ab.

189. omega.ti,ab.

190. sea food/

191. seafood.ti,ab.

192. shellfish.ti,ab.

193. crustacean?.ti,ab.

194. mollusc?.ti,ab.

195. shellfish/

196. exp dairy product/

197. dairy.ti,ab.

198. milk/

199. milk.ti,ab.

200. artificial milk/

201. formula?.ti,ab.

202. hydrolysed.ti,ab.

203. baby food/

204. yoghurt.ab,ti.

205. probiotic.ab,ti.

206. prebiotic?.ab,ti.

207. butter.ab,ti.

208. herb?.ab,ti.

209. spice?.ab,ti.

210. chilli$.ab,ti.

211. condiment?.ab,ti.

212. exp condiment/

213. beverage/

214. beverage?.ti,ab.

215. fluid intake.ti,ab.

216. water.ti,ab.

217. drink$.ti,ab.

218. exp food preservation/

219. pickled.ab,ti.

220. bottled.ab,ti.

221. canned.ab,ti.

222. canning.ab,ti.

223. smoked.ab,ti.

224. preserved.ab,ti.

225. preservatives.ab,ti.

226. nitrosamine.ab,ti.

227. hydrogenation.ab,ti.

228. fortified.ab,ti.

229. nitrates.ab,ti.

230. nitrites.ab,ti.

231. ferment$.ab,ti.

232. processed.ab,ti.

233. antioxidant$.ab,ti.

234. genetic modif$.ab,ti.

235. genetically modif$.ab,ti.

236. cooking/

237. cooking.ab,ti.

238. cooked.ab,ti.

239. grill.ab,ti.

240. grilled.ab,ti.

241. fried.ab,ti.

242. fry.ab,ti.

243. roast.ab,ti.

244. bake.ab,ti.

245. baked.ab,ti.

246. stewing.ab,ti.

247. stewed.ab,ti.

248. casserol$.ab,ti.

249. broil.ab,ti.

250. broiled.ab,ti.

251. boiled.ab,ti.

252. poach.ab,ti.

253. poached.ab,ti.

254. steamed.ab,ti.

255. barbecue$.ab,ti.

256. chargrill$.ab,ti.

257. salt.ab,ti.

258. salting.ab,ti.

259. salted.ab,ti.

260. fiber.ab,ti.

261. fibre.ab,ti.

262. polysaccharide$.ab,ti.

263. starch.ab,ti.

264. starchy.ab,ti.

265. carbohydrate$.ab,ti.

266. lipid$.ab,ti.

267. linoleic acid$.ab,ti.

268. sugar$.ab,ti.

269. sweetener$.ab,ti.

270. saccharin$.ab,ti.

271. aspartame.ab,ti.

272. sucrose.ab,ti.

273. xylitol.ab,ti.

274. cholesterol.ab,ti.

275. hydrogenated lard.ab,ti.

276. dietary protein.ab,ti.

277. dietary proteins.ab,ti.

278. protein intake.ab,ti.

279. animal protein$.ab,ti.

280. total protein$.ab,ti.

281. vegetable protein$.ab,ti.

282. plant protein$.ab,ti.

283. carbohydrate diet/

284. carbohydrate intake/

285. fat intake/

286. dietary fiber/

287. protein intake/

288. diet supplementation/

289. food additive/

290. exp vitamin/

291. supplements.ab,ti.

292. supplement.ab,ti.

293. vitamin$.ab,ti.

294. retinol.ab,ti.

295. carotenoid$.ab,ti.

296. tocopherol.ab,ti.

297. folate$.ab,ti.

298. folic acid.ab,ti.

299. methionine.ab,ti.

300. riboflavin.ab,ti.

301. thiamine.ab,ti.

302. niacin.ab,ti.

303. pyridoxine.ab,ti.

304. cobalamin.ab,ti.

305. mineral$.ab,ti.

306. sodium.ab,ti.

307. iron.ab,ti.

308. calcium.ab,ti.

309. selenium.ab,ti.

310. iodine.ab,ti.

311. magnesium.ab,ti.

312. potassium.ab,ti.

313. zinc.ab,ti.

314. copper.ab,ti.

315. phosphorus.ab,ti.

316. manganese.ab,ti.

317. chromium.ab,ti.

318. phytochemical.ab,ti.

319. polyphenol$.ab,ti.

320. phytoestrogen$.ab,ti.

321. genistein.ab,ti.

322. saponin$.ab,ti.

323. coumarin$.ab,ti.

324. flavonoid$.ab,ti.

325. polyphenol$.ab,ti.

326. flavonol$.ab,ti.

327. flavone$.ab,ti.

328. isoflavone$.ab,ti.

329. catechin$.ab,ti.

330. ascorbic acid$.ab,ti.

331. hydroxy cholecalciferol$.ab,ti.

332. hydroxycholecalciferol$.ab,ti.

333. tocotrienol$.ab,ti.

334. carotene$.ab,ti.

335. cryptoxanthin$.ab,ti.

336. lycopene$.ab,ti.

337. lutein$.ab,ti.

338. zeaxanthin$.ab,ti.

339. selenium$.ab,ti.

340. organic diet?.ab,ti.

341. organic food/

342. 75 or 76 or 77 or 78 or 79 or 80 or 81 or 82 or 83 or 84 or 85 or 86 or 87 or 88 or 89 or 90 or 91 or 92 or 93 or 94 or 95 or 96 or 97 or 98 or 99 or 100 or 101 or 102 or 103 or 104 or 105 or 106 or 107 or 108 or 109 or 110 or 111 or 112 or 113 or 114 or 115 or 116 or 117 or 118 or 119 or 120 or 121 or 122 or 123 or 124 or 125 or 126 or 127 or 128 or 129 or 130 or 131 or 132 or 133 or 134 or 135 or 136 or 137 or 138 or 139 or 140 or 141 or 142 or 143 or 144 or 145 or 146 or 147 or 148 or 149 or 150 or 151 or 152 or 153 or 154 or 155 or 156 or 157 or 158 or 159 or 160 or 161 or 162 or 163 or 164 or 165 or 166 or 167 or 168 or 169 or 170 or 171 or 172 or 173 or 174 or 175 or 176 or 177 or 178 or 179 or 180 or 181 or 182 or 183 or 184 or 185 or 186 or 187 or 188 or 189 or 190 or 191 or 192 or 193 or 194 or 195 or 196 or 197 or 198 or 199 or 200 or 201 or 202 or 203 or 204 or 205 or 206 or 207 or 208 or 209 or 210 or 211 or 212 or 213 or 214 or 215 or 216 or 217 or 218 or 219 or 220 or 221 or 222 or 223 or 224 or 225 or 226 or 227 or 228 or 229 or 230 or 231 or 232 or 233 or 234 or 235 or 236 or 237 or 238 or 239 or 240 or 241 or 242 or 243 or 244 or 245 or 246 or 247 or 248 or 249 or 250 or 251 or 252 or 253 or 254 or 255 or 256 or 257 or 258 or 259 or 260 or 261 or 262 or 263 or 264 or 265 or 266 or 267 or 268 or 269 or 270 or 271 or 272 or 273 or 274 or 275 or 276 or 277 or 278 or 279 or 280 or 281 or 282 or 283 or 284 or 285 or 286 or 287 or 288 or 289 or 290 or 291 or 292 or 293 or 294 or 295 or 296 or 297 or 298 or 299 or 300 or 301 or 302 or 303 or 304 or 305 or 306 or 307 or 308 or 309 or 310 or 311 or 312 or 313 or 314 or 315 or 316 or 317 or 318 or 319 or 320 or 321 or 322 or 323 or 324 or 325 or 326 or 327 or 328 or 329 or 330 or 331 or 332 or 333 or 334 or 335 or 336 or 337 or 338 or 339 or 340 or 341

343. allerg$.ab,ti.

344. asthma$.ab,ti.

345. wheeze.ab,ti.

346. wheezing.ab,ti.

347. bronchial hyperresponsiveness.ab,ti.

348. bronchial hyperreactivity.ab,ti.

349. Forced expiratory volume.ab,ti.

350. FEV1.ab,ti.

351. "FEV 1".ab,ti.

352. "FEV0.5".ab,ti.

353. "FEV 0.5".ab,ti.

354. Forced vital capacity.ab,ti.

355. FVC.ab,ti.

356. Peak expiratory flow rate.ab,ti.

357. PEFR.ab,ti.

358. AD.ab,ti.

359. neurodermatitis.ab,ti.

360. rhinitis.ab,ti.

361. besniers prurigo.ab,ti.

362. rhinoconjunctivitis.ab,ti.

363. hayfever.ab,ti.

364. (hay adj fever).ab,ti.

365. poll?nosis.ab,ti.

366. SAR.ab,ti.

367. (pollen adj allergy).ab,ti.

368. conjunctivitis.ab,ti.

369. immunoglobulin e.ab,ti.

370. Total IgE.ab,ti.

371. autoimmune disease?.ab,ti.

372. diabetes.ab,ti.

373. diabetic.ab,ti.

374. type 1.ab,ti.

375. c?eliac disease.ab,ti.

376. crohn$ disease.ab,ti.

377. Inflammatory Bowel Disease?.ab,ti.

378. Ulcerative colitis.ab,ti.

379. (Lympho$ adj3 thyroiditi$).ab,ti.

380. (Thyroiditi$ adj3 autoimmune).ab,ti.

381. (Hashimoto$ adj3 (syndrome? or thyroiditi$ or disease?)).ab,ti.

382. (Thyroiditi$ adj3 (post-partum or postpartum)).ab,ti.

383. Graves? disease.ab,ti.

384. Basedow$ disease.ab,ti.

385. exophthalmic goiter?.ab,ti.

386. (Still? Disease adj3 (juvenile or onset)).ab,ti.

387. (Juvenile adj3 arthriti$).ab,ti.

388. vitiligo.ab,ti.

389. Psorias?s.ab,ti.

390. (Arthriti? adj3 Psoria$).ab,ti.

391. atopic disease.ab,ti.

392. atopic dermatitis.ab,ti.

393. (food? adj3 sensiti$).ab,ti.

394. (food? adj3 toleran$).ab,ti.

395. (food? adj3 intoleran$).ab,ti.

396. ((aero or air$) adj3 allergen?).ab,ti.

397. (aeroallergen? adj3 sensiti$).ab,ti.

398. (allergen? adj3 sensiti$).ab,ti.

399. skin prick test$.ab,ti.

400. atopy.ab,ti.

401. hypersensitiv$.ab,ti.

402. exp hypersensitivity/

403. respiratory tract allergy/

404. asthma/

405. wheezing/

406. bronchus hyperreactivity/

407. forced expiratory volume/

408. forced vital capacity/

409. peak expiratory flow/

410. AD/

411. neurodermatitis/

412. rhinitis/

413. rhinoconjunctivitis/

414. hay fever/

415. pollen allergy/

416. perennial rhinitis/

417. conjunctivitis/

418. immunoglobulin E/

419. autoimmune disease/

420. diabetes mellitus/

421. insulin dependent diabetes mellitus/

422. celiac disease/

423. Crohn disease/

424. enteritis/

425. ulcerative colitis/

426. autoimmune thyroiditis/

427. Hashimoto disease/

428. postpartum thyroiditis/

429. Graves disease/

430. juvenile rheumatoid arthritis/

431. vitiligo/

432. psoriasis/

433. psoriatic arthritis/

434. atopic dermatitis/

435. nutritional intolerance/

436. 343 or 344 or 345 or 346 or 347 or 348 or 349 or 350 or 351 or 352 or 353 or 354 or 355 or 356 or 357 or 358 or 359 or 360 or 361 or 362 or 363 or 364 or 365 or 366 or 367 or 368 or 369 or 370 or 371 or 372 or 373 or 374 or 375 or 376 or 377 or 378 or 379 or 380 or 381 or 382 or 383 or 384 or 385 or 386 or 387 or 388 or 389 or 390 or 391 or 392 or 393 or 394 or 395 or 396 or 397 or 398 or 399 or 400 or 401 or 402 or 403 or 404 or 405 or 406 or 407 or 408 or 409 or 410 or 411 or 412 or 413 or 414 or 415 or 416 or 417 or 418 or 419 or 420 or 421 or 422 or 423 or 424 or 425 or 426 or 427 or 428 or 429 or 430 or 431 or 432 or 433 or 434 or 435

437. infant?.ab,ti.

438. ((one or two or three or four or five or six or seven or eight or nine or ten or eleven or twelve or thirteen or fourteen or fifteen or sixteen or seventeen or eighteen or nineteen or twenty or "twenty one" or "twenty two" or "twenty three" or "twenty four" or "twenty five" or "twenty six") adj week?).ab,ti.

439. ((one or two or three or four or five or six or seven or eight or nine or ten or eleven or twelve or thirteen or fourteen or fifteen or sixteen or seventeen or eighteen or nineteen or twenty or "twenty one" or "twenty two" or "twenty three" or "twenty four") adj month?).ab,ti.

440. 438 or 439

441. (old or age?).ab,ti.

442. 440 and 441

443. (("one year?" or "two year?") adj3 (old or age?)).ab,ti.

444. ((first or second or two) adj3 "year? of life").ab,ti.

445. infant/

446. newborn/

447. (maternal or pregnan$).ti,ab.

448. (lactat$).ti,ab.

449. (mother?).ti,ab.

450. 437 or 442 or 443 or 444 or 445 or 446 or 447 or 448 or 449

451. MEDLINE.tw.

452. exp systematic review/

453. systematic review.tw.

454. meta analysis/

455. intervention$.ti.

456. 451 or 452 or 453 or 454 or 455

457. 16 or 74 or 342

458. 436 and 450 and 456 and 457

459. limit 458 to yr="2011 -Current"

**COCHRANE Reviews and DARE**

1. “breast feeding”:ab,ti

2. breastfeeding:ab,ti

3. “breast fed”:ab,ti

4. breastfed:ab,ti

5. MeSH descriptor [Breast Feeding] this term only

6. MeSH descriptor [Milk, Human] this term only

7. formula*:ab,ti

8. hydrolysed:ab,ti

9. bottlefed:ab,ti

10. “bottle fed”:ab,ti

11. (bottle NEAR/3 feed*):ab,ti

12. MeSH descriptor [Infant Formula] this term only

13. MeSH descriptor [Bottle Feeding] this term only

14. wean*:ab,ti

15. MeSH descriptor [Weaning] this term only

16. 1 or 2 or 3 or 4 or 5 or 6 or 7 or 8 or 9 or 10 or 11 or 12 or 13 or 14 or 15

17. “complementary food*”:ab,ti

18. (introduc* NEAR/2 food*):ab,ti

19. wean*:ab,ti

20. MeSH descriptor [Weaning] this term only

21. solid*:ab,ti

22. semi-solid*:ab,ti

23. “baby food*”:ab,ti

24. MeSH descriptor [Infant Food] this term only

25. MeSH descriptor [Infant Nutritional Physiological Phenomena] this term only

26. “breast feeding”:ab,ti

27. breastfeeding:ab,ti

28. “breast fed”:ab,ti

29. breastfed:ab,ti

30. MeSH descriptor [Breast Feeding] this term only

31. MeSH descriptor [Milk, Human] this term only

32. formula*:ab,ti

33. hydrolysed:ab,ti

34. bottlefed:ab,ti

35. “bottle fed”:ab,ti

36. (bottle NEAR/3 feed*):ab,ti

37. MeSH descriptor [Infant Formula] this term only

38. MeSH descriptor [Bottle Feeding] this term only

39. liquid*:ab,ti

40. milk:ab,ti

41. MeSH descriptor [Milk] this term only

42. egg*:ab,ti

43. MeSH descriptor [Egg Proteins] this term only

44. MeSH descriptor [Egg Proteins, Dietary] this term only

45. nut*:ab,ti

46. peanut*:ab,ti

47. almond*:ab,ti

48. (brazil* NEAR/5 nut*):ab,ti

49. walnut*:ab,ti

50. pecan*:ab,ti

51. pistachio*:ab,ti

52. cashew*:ab,ti

53. hazelnut*:ab,ti

54. macadamia*:ab,ti

55. Nuts] this term only

56. MeSH descriptor [Arachis hypogaea] this term only

57. MeSH descriptor [Prunus] this term only

58. MeSH descriptor [Bertholletia] this term only

59. MeSH descriptor [Juglans] this term only

60. MeSH descriptor [Carya] this term only

61. MeSH descriptor [Pistacia] this term only

62. MeSH descriptor [Anacardium] this term only

63. MeSH descriptor [Corylus] this term only

64. MeSH descriptor [Macadamia] this term only

65. wheat:ab,ti

66. MeSH descriptor [Triticum] this term only

67. soya:ab,ti

68. MeSH descriptor [Soybeans] this term only

69. gluten*:ab,ti

70. MeSH descriptor [Glutens] this term only

71. fish:ab,ti

72. MeSH descriptor [Fishes] this term only

73. 17 or 18 or 19 or 20 or 21 or 22 or 23 or 24 or 25 or 26 or 27 or 28 or 29 or 30 or 31 or 32 or 33 or 34 or 35 or 36 or 37 or 38 or 39 or 40 or 41 or 42 or 43 or 44 or 45 or 46 or 47 or 48 or 49 or 50 or 51 or 52 or 53 or 54 or 55 or 56 or 57 or 58 or 59 or 60 or 61 or 62 or 63 or 64 or 65 or 66 or 67 or 68 or 69 or 70 or 71 or 72

74. MeSH descriptor [Diet] this term only

75. MeSH descriptor [Diet Therapy] this term only

76. MeSH descriptor [Nutritional Sciences] this term only

77. MeSH descriptor [Child Nutrition Sciences] this term only

78. diet:ab,ti

79. diets:ab,ti

80. MeSH descriptor [Diet, Mediterranean] this term only

81. “mediterranean diet*”:ab,ti

82. dietetic:ab,ti

83. dietary:ab,ti

84. eat:ab,ti

85. eating:ab,ti

86. intake:ab,ti

87. nutrient*:ab,ti

88. nutrition:ab,ti

89. MeSH descriptor [Diet, Vegetarian] this term only

90. vegetarian*:ab,ti

91. vegan*:ab,ti

92. MeSH descriptor [Diet, Macrobiotic] this term only

93. macrobiotic*:ab,ti

94. MeSH descriptor [Food] this term only

95. food*:ab,ti

96. feed:ab,ti

97. feeding:ab,ti

98. cereal*:ab,ti

99. grain*:ab,ti

100. granary:ab,ti

101. wholegrain:ab,ti

102. wholewheat:ab,ti

103. “whole wheat”:ab,ti

104. wheat:ab,ti

105. wheatgerm:ab,ti

106. rye:ab,ti

107. barley:ab,ti

108. oat*:ab,ti

109. MeSH descriptor [Cereals] explode all trees

110. root*:ab,ti

111. tuber*:ab,ti

112. MeSH descriptor [Vegetables] explode all trees

113. vegetable*:ab,ti

114. onion*:ab,ti

115. spinach:ab,ti

116. chard:ab,ti

117. tomato*:ab,ti

118. pepper*:ab,ti

119. carrot*:ab,ti

120. beetroot:ab,ti

121. asparagus:ab,ti

122. garlic:ab,ti

123. pumpkin:ab,ti

124. sprouts:ab,ti

125. broccoli:ab,ti

126. cabbage*:ab,ti

127. celery:ab,ti

128. ginger:ab,ti

129. potato*:ab,ti

130. crisps:ab,ti

131. fries:ab,ti

132. syrup:ab,ti

133. honey:ab,ti

134. MeSH descriptor [Honey] this term only

135. MeSH descriptor [Fruit] this term only

136. fruit*:ab,ti

137. apple*:ab,ti

138. pear*:ab,ti

139. banana*:ab,ti

140. orange*:ab,ti

141. grape*:ab,ti

142. kiwi*:ab,ti

143. citrus:ab,ti

144. grapefruit*:ab,ti

145. pulses:ab,ti

146. beans:ab,ti

147. lentil*:ab,ti

148. chickpea*:ab,ti

149. legume*:ab,ti

150. lupin*:ab,ti

151. soy:ab,ti

152. soya:ab,ti

153. nut*:ab,ti

154. almond*:ab,ti

155. peanut*:ab,ti

156. groundnut*:ab,ti

157. MeSH descriptor [Nuts] this term only

158. seed*:ab,ti

159. sesame:ab,ti

160. mustard:ab,ti

161. MeSH descriptor [Seeds] this term only

162. MeSH descriptor [Meat] explode all trees

163. meat:ab,ti

164. beef:ab,ti

165. pork:ab,ti

166. lamb:ab,ti

167. poultry:ab,ti

168. chicken:ab,ti

169. turkey:ab,ti

170. duck:ab,ti

171. fish:ab,ti

172. MeSH descriptor [Fatty Acids] this term only

173. MeSH descriptor [Fatty Acids, Omega-3] explode all trees

174. MeSH descriptor [Fatty Acids, Omega-6] explode all trees

175. omega-3:ab,ti

176. omega-6:ab,ti

177. PUFA:ab,ti

178. fat:ab,ti

179. fats:ab,ti

180. fatty:ab,ti

181. egg:ab,ti

182. eggs:ab,ti

183. MeSH descriptor [Eggs] explode all trees

184. MeSH descriptor [Bread] this term only

185. bread:ab,ti

186. oil:ab,ti

187. oils:ab,ti

188. oily:ab,ti

189. omega:ab,ti

190. MeSH descriptor [Seafood] explode all trees

191. seafood:ab,ti

192. shellfish:ab,ti

193. crustacean*:ab,ti

194. mollusc*:ab,ti

195. MeSH descriptor [Shellfish] this term only

196. MeSH descriptor [Dairy Products] this term only

197. dairy:ab,ti

198. MeSH descriptor [Milk] explode all trees

199. milk:ab,ti

200. MeSH descriptor [Infant Formula] this term only

201. formula*:ab,ti

202. hydrolysed:ab,ti

203. MeSH descriptor [Infant Food] this term only

204. yoghurt:ab,ti

205. probiotic:ab,ti

206. prebiotic*:ab,ti

207. butter:ab,ti

208. herb*:ab,ti

209. spice*:ab,ti

210. chilli*:ab,ti

211. condiment*:ab,ti

212. MeSH descriptor [Condiments] explode all trees

213. MeSH descriptor [Beverages] this term only

214. beverage*:ab,ti

215. “fluid intake”:ab,ti

216. water:ab,ti

217. drink*:ab,ti

218. MeSH descriptor [Food Preservation] explode all trees

219. pickled:ab,ti

220. bottled:ab,ti

221. canned:ab,ti

222. canning:ab,ti

223. smoked:ab,ti

224. preserved:ab,ti

225. preservatives:ab,ti

226. nitrosamine:ab,ti

227. hydrogenation:ab,ti

228. fortified:ab,ti

229. nitrates:ab,ti

230. nitrites:ab,ti

231. ferment*:ab,ti

232. processed:ab,ti

233. antioxidant*:ab,ti

234. “genetic modif*”:ab,ti

235. “genetically modif*”:ab,ti

236. MeSH descriptor [Cooking] this term only

237. cooking:ab,ti

238. cooked:ab,ti

239. grill:ab,ti

240. grilled:ab,ti

241. fried:ab,ti

242. fry:ab,ti

243. roast:ab,ti

244. bake:ab,ti

245. baked:ab,ti

246. stewing:ab,ti

247. stewed:ab,ti

248. casserol*:ab,ti

249. broil:ab,ti

250. broiled:ab,ti

251. boiled:ab,ti

252. poach:ab,ti

253. poached:ab,ti

254. steamed:ab,ti

255. barbecue*:ab,ti

256. chargrill*:ab,ti

257. salt:ab,ti

258. salting:ab,ti

259. salted:ab,ti

260. fiber:ab,ti

261. fibre:ab,ti

262. polysaccharide*:ab,ti

263. starch:ab,ti

264. starchy:ab,ti

265. carbohydrate*:ab,ti

266. lipid*:ab,ti

267. “linoleic acid*”:ab,ti

268. sugar*:ab,ti

269. sweetener*:ab,ti

270. saccharin*:ab,ti

271. aspartame:ab,ti

272. sucrose:ab,ti

273. xylitol:ab,ti

274. cholesterol:ab,ti

275. “hydrogenated lard”:ab,ti

276. “dietary protein”:ab,ti

277. “dietary proteins”:ab,ti

278. “protein intake”:ab,ti

279. “animal protein*”:ab,ti

280. “total protein*”:ab,ti

281. “vegetable protein*”:ab,ti

282. “plant protein*”:ab,ti

283. MeSH descriptor [Dietary Carbohydrates] explode all trees

284. MeSH descriptor [Dietary Fats] explode all trees

285. MeSH descriptor [Dietary Fiber] explode all trees

286. MeSH descriptor [Dietary Proteins] explode all trees

287. MeSH descriptor [Dietary Supplements] explode all trees

288. MeSH descriptor [Food Additives] explode all trees

289. MeSH descriptor [Vitamins] explode all trees

290. supplements:ab,ti

291. supplement:ab,ti

292. vitamin*:ab,ti

293. retinol:ab,ti

294. carotenoid*:ab,ti

295. tocopherol:ab,ti

296. folate*:ab,ti

297. “folic acid”:ab,ti

298. methionine:ab,ti

299. riboflavin:ab,ti

300. thiamine:ab,ti

301. niacin:ab,ti

302. pyridoxine:ab,ti

303. cobalamin:ab,ti

304. mineral*:ab,ti

305. sodium:ab,ti

306. iron:ab,ti

307. calcium:ab,ti

308. selenium:ab,ti

309. iodine:ab,ti

310. magnesium:ab,ti

311. potassium:ab,ti

312. zinc:ab,ti

313. copper:ab,ti

314. phosphorus:ab,ti

315. manganese:ab,ti

316. chromium:ab,ti

317. phytochemical:ab,ti

318. polyphenol*:ab,ti

319. phytoestrogen*:ab,ti

320. genistein:ab,ti

321. saponin*:ab,ti

322. coumarin*:ab,ti

323. flavonoid*:ab,ti

324. polyphenol*:ab,ti

325. flavonol*:ab,ti

326. flavone*:ab,ti

327. isoflavone*:ab,ti

328. catechin*:ab,ti

329. “ascorbic acid*”:ab,ti

330. “hydroxy cholecalciferol*”:ab,ti

331. hydroxycholecalciferol*:ab,ti

332. tocotrienol*:ab,ti

333. carotene*:ab,ti

334. cryptoxanthin*:ab,ti

335. lycopene*:ab,ti

336. lutein*:ab,ti

337. zeaxanthin*:ab,ti

338. selenium*:ab,ti

339. “organic diet*”:ab,ti

340. MeSH descriptor [Food, Organic] this term only

341. 74 or 75 or 76 or 77 or 78 or 79 or 80 or 81 or 82 or 83 or 84 or 85 or 86 or 87 or 88 or 89 or 90 or 91 or 92 or 93 or 94 or 95 or 96 or 97 or 98 or 99 or 100 or 101 or 102 or 103 or 104 or 105 or 106 or 107 or 108 or 109 or 110 or 111 or 112 or 113 or 114 or 115 or 116 or 117 or 118 or 119 or 120 or 121 or 122 or 123 or 124 or 125 or 126 or 127 or 128 or 129 or 130 or 131 or 132 or 133 or 134 or 135 or 136 or 137 or 138 or 139 or 140 or 141 or 142 or 143 or 144 or 145 or 146 or 147 or 148 or 149 or 150 or 151 or 152 or 153 or 154 or 155 or 156 or 157 or 158 or 159 or 160 or 161 or 162 or 163 or 164 or 165 or 166 or 167 or 168 or 169 or 170 or 171 or 172 or 173 or 174 or 175 or 176 or 177 or 178 or 179 or 180 or 181 or 182 or 183 or 184 or 185 or 186 or 187 or 188 or 189 or 190 or 191 or 192 or 193 or 194 or 195 or 196 or 197 or 198 or 199 or 200 or 201 or 202 or 203 or 204 or 205 or 206 or 207 or 208 or 209 or 210 or 211 or 212 or 213 or 214 or 215 or 216 or 217 or 218 or 219 or 220 or 221 or 222 or 223 or 224 or 225 or 226 or 227 or 228 or 229 or 230 or 231 or 232 or 233 or 234 or 235 or 236 or 237 or 238 or 239 or 240 or 241 or 242 or 243 or 244 or 245 or 246 or 247 or 248 or 249 or 250 or 251 or 252 or 253 or 254 or 255 or 256 or 257 or 258 or 259 or 260 or 261 or 262 or 263 or 264 or 265 or 266 or 267 or 268 or 269 or 270 or 271 or 272 or 273 or 274 or 275 or 276 or 277 or 278 or 279 or 280 or 281 or 282 or 283 or 284 or 285 or 286 or 287 or 288 or 289 or 290 or 291 or 292 or 293 or 294 or 295 or 296 or 297 or 298 or 299 or 300 or 301 or 302 or 303 or 304 or 305 or 306 or 307 or 308 or 309 or 310 or 311 or 312 or 313 or 314 or 315 or 316 or 317 or 318 or 319 or 320 or 321 or 322 or 323 or 324 or 325 or 326 or 327 or 328 or 329 or 330 or 331 or 332 or 333 or 334 or 335 or 336 or 337 or 338 or 339 or 340

342. allerg*:ab,ti

343. asthma*:ab,ti

344. wheeze:ab,ti

345. wheezing:ab,ti

346. “bronchial hyperresponsiveness”:ab,ti

347. “bronchial hyperreactivity”:ab,ti

348. “Forced expiratory volume”:ab,ti

349. “FEV1”:ab,ti

350. "FEV 1":ab,ti

351. "FEV0.5":ab,ti

352. "FEV 0.5":ab,ti

353. “Forced vital capacity”:ab,ti

354. FVC:ab,ti

355. “Peak expiratory flow rate”:ab,ti

356. PEFR:ab,ti

357. AD:ab,ti

358. neurodermatitis:ab,ti

359. rhinitis:ab,ti

360. “besniers prurigo”:ab,ti

361. rhinoconjunctivitis:ab,ti

362. hayfever:ab,ti

363. “hay fever”:ab,ti

364. poll*nosis:ab,ti

365. SAR:ab,ti

366. “pollen allergy”:ab,ti

367. conjunctivitis:ab,ti

368. immunoglobulin e:ab,ti

369. Total IgE:ab,ti

370. “autoimmune disease*”:ab,ti

371. diabetes:ab,ti

372. diabetic:ab,ti

373. “type 1”:ab,ti

374. “c*eliac disease”:ab,ti

375. “crohn* disease”:ab,ti

376. “Inflammatory Bowel Disease*”:ab,ti

377. “Ulcerative colitis”:ab,ti

378. (Lympho* NEAR/3 thyroiditi*):ab,ti

379. (Thyroiditi* NEAR/3 autoimmune):ab,ti

380. (Hashimoto* NEAR/3 (syndrome* or thyroiditi* or disease*)):ab,ti

381. (Thyroiditi* NEAR/3 (post-partum or postpartum)):ab,ti

382. “Graves* disease”:ab,ti

383. “Basedow* disease”:ab,ti

384. “exophthalmic goiter*”:ab,ti

385. (“Still* Disease” NEAR/3 (juvenile or onset)):ab,ti

386. (Juvenile NEAR/3 arthriti*):ab,ti

387. vitiligo:ab,ti

388. Psorias*s:ab,ti

389. (Arthriti* NEAR/3 Psoria*):ab,ti

390. “atopic disease”:ab,ti

391. “atopic dermatitis”:ab,ti

392. (food* NEAR/3 sensiti*):ab,ti

393. (food* NEAR/3 toleran*):ab,ti

394. (food* NEAR/3 intoleran*):ab,ti

395. ((aero or air*) NEAR/3 allergen*):ab,ti

396. (aeroallergen* NEAR/3 sensiti*):ab,ti

397. (allergen* NEAR/3 sensiti*):ab,ti

398. “skin prick test*”:ab,ti

399. atopy:ab,ti

400. hypersensitiv*:ab,ti

401. MeSH descriptor [Hypersensitivity] this term only

402. MeSH descriptor [Food Hypersensitivity] explode all trees

403. MeSH descriptor [Respiratory Hypersensitivity] this term only

404. MeSH descriptor [Asthma] this term only

405. MeSH descriptor [Bronchial Hyperreactivity] this term only

406. MeSH descriptor [Forced Expiratory Volume] this term only

407. MeSH descriptor [Vital Capacity] this term only

408. MeSH descriptor [Peak Expiratory Flow Rate] this term only

409. MeSH descriptor [AD] this term only

410. MeSH descriptor [Neurodermatitis] this term only

411. MeSH descriptor [Rhinitis] this term only

412. MeSH descriptor [Rhinitis, Allergic, Perennial] this term only

413. MeSH descriptor [Rhinitis, Allergic, Seasonal] this term only

414. MeSH descriptor [Conjunctivitis] this term only

415. MeSH descriptor [Immunoglobulin E] this term only

416. MeSH descriptor [Autoimmune Diseases] this term only

417. MeSH descriptor [Diabetes Mellitus, Type 1] this term only

418. MeSH descriptor [Celiac Disease] this term only

419. MeSH descriptor [Crohn Disease] this term only

420. MeSH descriptor [Inflammatory Bowel Diseases] this term only

421. MeSH descriptor [Colitis, Ulcerative] this term only

422. MeSH descriptor [Thyroiditis, Autoimmune] this term only

423. MeSH descriptor [Hashimoto Disease] this term only

424. MeSH descriptor [Postpartum Thyroiditis] this term only

425. MeSH descriptor [Graves Disease] this term only

426. MeSH descriptor [Arthritis, Juvenile Rheumatoid] this term only

427. MeSH descriptor [Vitiligo] this term only

428. MeSH descriptor [Psoriasis] this term only

429. MeSH descriptor [Arthritis, Psoriatic] this term only

430. MeSH descriptor [Dermatitis, Atopic] this term only

431. MeSH descriptor [Hypersensitivity, Immediate] this term only

432. 342 or 343 or 344 or 345 or 346 or 347 or 348 or 349 or 350 or 351 or 352 or 353 or 354 or 355 or 356 or 357 or 358 or 359 or 360 or 361 or 362 or 363 or 364 or 365 or 366 or 367 or 368 or 369 or 370 or 371 or 372 or 373 or 374 or 375 or 376 or 377 or 378 or 379 or 380 or 381 or 382 or 383 or 384 or 385 or 386 or 387 or 388 or 389 or 390 or 391 or 392 or 393 or 394 or 395 or 396 or 397 or 398 or 399 or 400 or 401 or 402 or 403 or 404 or 405 or 406 or 407 or 408 or 409 or 410 or 411 or 412 or 413 or 414 or 415 or 416 or 417 or 418 or 419 or 420 or 421 or 422 or 423 or 424 or 425 or 426 or 427 or 428 or 429 or 430 or 431

433. infant*:ab,ti

434. ((one or two or three or four or five or six or seven or eight or nine or ten or eleven or twelve or thirteen or fourteen or fifteen or sixteen or seventeen or eighteen or nineteen or twenty or "twenty one" or "twenty two" or "twenty three" or "twenty four" or "twenty five" or "twenty six") NEAR/1 week*):ab,ti

435. ((one or two or three or four or five or six or seven or eight or nine or ten or eleven or twelve or thirteen or fourteen or fifteen or sixteen or seventeen or eighteen or nineteen or twenty or "twenty one" or "twenty two" or "twenty three" or "twenty four") NEAR/1 month*):ab,ti

436. 434 or 435

437. (old or age*):ab,ti

438. 436 and 437

439. (("one year*" or "two year*") NEAR/3 (old or age*)):ab,ti

440. ((first or second or two) NEAR/3 "year* of life"):ab,ti

441. MeSH descriptor [Infant] this term only

442. MeSH descriptor [Infant, Newborn] this term only

443. (maternal or pregnan*):ab,ti

444. (lactat*):ab,ti

445. (mother*):ab,ti

446. 433 or 438 or 439 or 440 or 441 or 442 or 443 or 444 or 445

447. 16 or 73 or 341

448. 432 and 446 and 447

Publication date from 2011

**Appendix 2 Search Strategies for original articles on milk feeding**

**Medline**

1. breast feeding.ab,ti.

2. breastfeeding.ab,ti.

3. breast fed.ab,ti.

4. breastfed.ab,ti.

5. Breast Feeding/

6. Milk, Human/

7. formula?.ab,ti.

8. hydrolysed.ab,ti.

9. bottlefed.ab,ti.

10. bottle fed.ab,ti.

11. (bottle adj3 feed$).ab,ti.

12. Infant Formula/

13. Bottle Feeding/

14. wean$.ab,ti.

15. Weaning/

16. 1 or 2 or 3 or 4 or 5 or 6 or 7 or 8 or 9 or 10 or 11 or 12 or 13 or 14 or 15

17. allerg$.ab,ti.

18. asthma$.ab,ti.

19. wheeze.ab,ti.

20. wheezing.ab,ti.

21. bronchial hyperresponsiveness.ab,ti.

22. bronchial hyperreactivity.ab,ti.

23. Forced expiratory volume.ab,ti.

24. FEV1.ab,ti.

25. "FEV 1".ab,ti.

26. "FEV0.5".ab,ti.

27. "FEV 0.5".ab,ti.

28. Forced vital capacity.ab,ti.

29. FVC.ab,ti.

30. Peak expiratory flow rate.ab,ti.

31. PEFR.ab,ti.

32. AD.ab,ti.

33. neurodermatitis.ab,ti.

34. rhinitis.ab,ti.

35. besniers prurigo.ab,ti.

36. rhinoconjunctivitis.ab,ti.

37. hayfever.ab,ti.

38. (hay adj fever).ab,ti.

39. poll?nosis.ab,ti.

40. SAR.ab,ti.

41. (pollen adj allergy).ab,ti.

42. conjunctivitis.ab,ti.

43. immunoglobulin e.ab,ti.

44. Total IgE.ab,ti.

45. autoimmune disease?.ab,ti.

46. diabetes.ab,ti.

47. diabetic.ab,ti.

48. type 1.ab,ti.

49. c?eliac disease.ab,ti.

50. crohn$ disease.ab,ti.

51. Inflammatory Bowel Disease?.ab,ti.

52. Ulcerative colitis.ab,ti.

53. (Lympho$ adj3 thyroiditi$).ab,ti.

54. (Thyroiditi$ adj3 autoimmune).ab,ti.

55. (Hashimoto$ adj3 (syndrome? or thyroiditi$ or disease?)).ab,ti.

56. (Thyroiditi$ adj3 (post-partum or postpartum)).ab,ti.

57. Graves? disease.ab,ti.

58. Basedow$ disease.ab,ti.

59. exophthalmic goiter?.ab,ti.

60. (Still? Disease adj3 (juvenile or onset)).ab,ti.

61. (Juvenile adj3 arthriti$).ab,ti.

62. vitiligo.ab,ti.

63. Psorias?s.ab,ti.

64. (Arthriti? adj3 Psoria$).ab,ti.

65. atopic disease.ab,ti.

66. atopic dermatitis.ab,ti.

67. (food? adj3 sensiti$).ab,ti.

68. (food? adj3 toleran$).ab,ti.

69. (food? adj3 intoleran$).ab,ti.

70. ((aero or air$) adj3 allergen?).ab,ti.

71. (aeroallergen? adj3 sensiti$).ab,ti.

72. (allergen? adj3 sensiti$).ab,ti.

73. skin prick test$.ab,ti.

74. atopy.ab,ti.

75. hypersensitiv$.ab,ti.

76. Hypersensitivity/

77. exp Food Hypersensitivity/

78. Respiratory Hypersensitivity/

79. Asthma/

80. Bronchial Hyperreactivity/

81. Forced Expiratory Volume/

82. Vital Capacity/

83. Peak Expiratory Flow Rate/

84. AD/

85. Neurodermatitis/

86. Rhinitis/

87. Rhinitis, Allergic, Perennial/

88. Rhinitis, Allergic, Seasonal/

89. Conjunctivitis/

90. Immunoglobulin E/

91. Autoimmune Diseases/

92. Diabetes Mellitus, Type 1/

93. Celiac Disease/

94. Crohn Disease/

95. Inflammatory Bowel Diseases/

96. Colitis, Ulcerative/

97. Thyroiditis, Autoimmune/

98. Hashimoto Disease/

99. Postpartum Thyroiditis/

100. Graves Disease/

101. Arthritis, Juvenile Rheumatoid/

102. Vitiligo/

103. Psoriasis/

104. Arthritis, Psoriatic/

105. Dermatitis, Atopic/

106. Hypersensitivity, Immediate/

107. 17 or 18 or 19 or 20 or 21 or 22 or 23 or 24 or 25 or 26 or 27 or 28 or 29 or 30 or 31 or 32 or 33 or 34 or 35 or 36 or 37 or 38 or 39 or 40 or 41 or 42 or 43 or 44 or 45 or 46 or 47 or 48 or 49 or 50 or 51 or 52 or 53 or 54 or 55 or 56 or 57 or 58 or 59 or 60 or 61 or 62 or 63 or 64 or 65 or 66 or 67 or 68 or 69 or 70 or 71 or 72 or 73 or 74 or 75 or 76 or 77 or 78 or 79 or 80 or 81 or 82 or 83 or 84 or 85 or 86 or 87 or 88 or 89 or 90 or 91 or 92 or 93 or 94 or 95 or 96 or 97 or 98 or 99 or 100 or 101 or 102 or 103 or 104 or 105 or 106

108. infant?.ab,ti.

109. ((one or two or three or four or five or six or seven or eight or nine or ten or eleven or twelve or thirteen or fourteen or fifteen or sixteen or seventeen or eighteen or nineteen or twenty or "twenty one" or "twenty two" or "twenty three" or "twenty four" or "twenty five" or "twenty six") adj week?).ab,ti.

110. ((one or two or three or four or five or six or seven or eight or nine or ten or eleven or twelve or thirteen or fourteen or fifteen or sixteen or seventeen or eighteen or nineteen or twenty or "twenty one" or "twenty two" or "twenty three" or "twenty four") adj month?).ab,ti.

111. 109 or 110

112. (old or age?).ab,ti.

113. 111 and 112

114. (("one year?" or "two year?") adj3 (old or age?)).ab,ti.

115. ((first or second or two) adj3 "year? of life").ab,ti.

116. Infant/

117. Infant, Newborn/

118. 108 or 113 or 114 or 115 or 116 or 117

119. clinical trial?.mp.

120. random$.mp.

121. factorial$.mp.

122. crossover$.mp.

123. placebo$.mp.

124. (doubl$ adj blind$).mp.

125. (singl$ adj blind$).mp.

126. assign$.mp.

127. volunteer$.mp.

128. cohort stud$.mp.

129. longitudinal$.mp.

130. follow-up.mp.

131. prospectiv$.mp.

132. retrospectiv$.mp.

133. case control.mp.

134. case referent.mp.

135. exp clinical trial/

136. Cross-Over Studies/

137. Placebos/

138. Double-Blind Method/

139. Single-Blind Method/

140. exp Cohort Studies/

141. case-control studies/

142. 119 or 120 or 121 or 122 or 123 or 124 or 125 or 126 or 127 or 128 or 129 or 130 or 131 or 132 or 133 or 134 or 135 or 136 or 137 or 138 or 139 or 140 or 141

143. 16 and 107 and 118 and 142

**Embase**

1. breast feeding.ab,ti.

2. breastfeeding.ab,ti.

3. breast fed.ab,ti.

4. breastfed.ab,ti.

5. breast feeding/

6. breast milk/

7. formula?.ab,ti.

8. hydrolysed.ab,ti.

9. bottlefed.ab,ti.

10. bottle fed.ab,ti.

11. (bottle adj3 feed$).ab,ti.

12. artificial milk/

13. bottle feeding/

14. wean$.ti,ab.

15. weaning/

16. 1 or 2 or 3 or 4 or 5 or 6 or 7 or 8 or 9 or 10 or 11 or 12 or 13 or 14 or 15

17. allerg$.ab,ti.

18. asthma$.ab,ti.

19. wheeze.ab,ti.

20. wheezing.ab,ti.

21. bronchial hyperresponsiveness.ab,ti.

22. bronchial hyperreactivity.ab,ti.

23. Forced expiratory volume.ab,ti.

24. FEV1.ab,ti.

25. "FEV 1".ab,ti.

26. "FEV0.5".ab,ti.

27. "FEV 0.5".ab,ti.

28. Forced vital capacity.ab,ti.

29. FVC.ab,ti.

30. Peak expiratory flow rate.ab,ti.

31. PEFR.ab,ti.

32. AD.ab,ti.

33. neurodermatitis.ab,ti.

34. rhinitis.ab,ti.

35. besniers prurigo.ab,ti.

36. rhinoconjunctivitis.ab,ti.

37. hayfever.ab,ti.

38. (hay adj fever).ab,ti.

39. poll?nosis.ab,ti.

40. SAR.ab,ti.

41. (pollen adj allergy).ab,ti.

42. conjunctivitis.ab,ti.

43. immunoglobulin e.ab,ti.

44. Total IgE.ab,ti.

45. autoimmune disease?.ab,ti.

46. diabetes.ab,ti.

47. diabetic.ab,ti.

48. type 1.ab,ti.

49. c?eliac disease.ab,ti.

50. crohn$ disease.ab,ti.

51. Inflammatory Bowel Disease?.ab,ti.

52. Ulcerative colitis.ab,ti.

53. (Lympho$ adj3 thyroiditi$).ab,ti.

54. (Thyroiditi$ adj3 autoimmune).ab,ti.

55. (Hashimoto$ adj3 (syndrome? or thyroiditi$ or disease?)).ab,ti.

56. (Thyroiditi$ adj3 (post-partum or postpartum)).ab,ti.

57. Graves? disease.ab,ti.

58. Basedow$ disease.ab,ti.

59. exophthalmic goiter?.ab,ti.

60. (Still? Disease adj3 (juvenile or onset)).ab,ti.

61. (Juvenile adj3 arthriti$).ab,ti.

62. vitiligo.ab,ti.

63. Psorias?s.ab,ti.

64. (Arthriti? adj3 Psoria$).ab,ti.

65. atopic disease.ab,ti.

66. atopic dermatitis.ab,ti.

67. (food? adj3 sensiti$).ab,ti.

68. (food? adj3 toleran$).ab,ti.

69. (food? adj3 intoleran$).ab,ti.

70. ((aero or air$) adj3 allergen?).ab,ti.

71. (aeroallergen? adj3 sensiti$).ab,ti.

72. (allergen? adj3 sensiti$).ab,ti.

73. skin prick test$.ab,ti.

74. atopy.ab,ti.

75. hypersensitiv$.ab,ti.

76. exp hypersensitivity/

77. respiratory tract allergy/

78. asthma/

79. wheezing/

80. bronchus hyperreactivity/

81. forced expiratory volume/

82. forced vital capacity/

83. peak expiratory flow/

84. AD/

85. neurodermatitis/

86. rhinitis/

87. rhinoconjunctivitis/

88. hay fever/

89. pollen allergy/

90. perennial rhinitis/

91. conjunctivitis/

92. immunoglobulin E/

93. autoimmune disease/

94. diabetes mellitus/

95. insulin dependent diabetes mellitus/

96. celiac disease/

97. Crohn disease/

98. enteritis/

99. ulcerative colitis/

100. autoimmune thyroiditis/

101. Hashimoto disease/

102. postpartum thyroiditis/

103. Graves disease/

104. juvenile rheumatoid arthritis/

105. vitiligo/

106. psoriasis/

107. psoriatic arthritis/

108. atopic dermatitis/

109. nutritional intolerance/

110. 17 or 18 or 19 or 20 or 21 or 22 or 23 or 24 or 25 or 26 or 27 or 28 or 29 or 30 or 31 or 32 or 33 or 34 or 35 or 36 or 37 or 38 or 39 or 40 or 41 or 42 or 43 or 44 or 45 or 46 or 47 or 48 or 49 or 50 or 51 or 52 or 53 or 54 or 55 or 56 or 57 or 58 or 59 or 60 or 61 or 62 or 63 or 64 or 65 or 66 or 67 or 68 or 69 or 70 or 71 or 72 or 73 or 74 or 75 or 76 or 77 or 78 or 79 or 80 or 81 or 82 or 83 or 84 or 85 or 86 or 87 or 88 or 89 or 90 or 91 or 92 or 93 or 94 or 95 or 96 or 97 or 98 or 99 or 100 or 101 or 102 or 103 or 104 or 105 or 106 or 107 or 108 or 109

111. infant?.ab,ti.

112. ((one or two or three or four or five or six or seven or eight or nine or ten or eleven or twelve or thirteen or fourteen or fifteen or sixteen or seventeen or eighteen or nineteen or twenty or "twenty one" or "twenty two" or "twenty three" or "twenty four" or "twenty five" or "twenty six") adj week?).ab,ti.

113. ((one or two or three or four or five or six or seven or eight or nine or ten or eleven or twelve or thirteen or fourteen or fifteen or sixteen or seventeen or eighteen or nineteen or twenty or "twenty one" or "twenty two" or "twenty three" or "twenty four") adj month?).ab,ti.

114. 112 or 113

115. (old or age?).ab,ti.

116. 114 and 115

117. (("one year?" or "two year?") adj3 (old or age?)).ab,ti.

118. ((first or second or two) adj3 "year? of life").ab,ti.

119. infant/

120. newborn/

121. 111 or 116 or 117 or 118 or 119 or 120

122. clinical trial?.mp.

123. random$.mp.

124. factorial$.mp.

125. crossover$.mp.

126. placebo$.mp.

127. (doubl$ adj blind$).mp.

128. (singl$ adj blind$).mp.

129. assign$.mp.

130. volunteer$.mp.

131. cohort stud$.mp.

132. longitudinal$.mp.

133. follow-up.mp.

134. prospectiv$.mp.

135. retrospectiv$.mp.

136. case control.mp.

137. case referent.mp.

138. exp clinical trial/

139. crossover procedure/

140. placebo/

141. double blind procedure/

142. single blind procedure/

143. cohort analysis/

144. longitudinal study/

145. follow up/

146. prospective study/

147. retrospective study/

148. exp case control study/

149. 122 or 123 or 124 or 125 or 126 or 127 or 128 or 129 or 130 or 131 or 132 or 133 or 134 or 135 or 136 or 137 or 138 or 139 or 140 or 141 or 142 or 143 or 144 or 145 or 146 or 147 or 148

150. 16 and 110 and 121 and 149

**LILACS**

(tw:((breast feeding) or breastfeeding or (breast fed) or breastfed or formula* or hydrolysed or bottlefed or (bottle fed) or (bottle feed*) or wean*)

AND

(tw:(allerg* or asthma* or wheez* or (bronchial hyperresponsiveness) or (bronchial hyperreactivity) or (Forced expiratory volume) or FEV1 or (FEV 1) or FEV0.5 or (FEV 0.5) or (Forced vital capacity) or FVC or (Peak expiratory flow rate) or PEFR or AD or neurodermatitis or rhinitis or (besniers prurigo) or rhinoconjunctivitis or hayfever or (hay fever) or poll?nosis or SAR or (pollen allergy) or conjunctivitis or (immunoglobulin e) or (Total IgE) or (autoimmune disease*) or diabetes or diabetic or (type 1) or (c?eliac disease) or (crohn* disease) or (Inflammatory Bowel Disease*) or (Ulcerative colitis) or (Lympho* thyroiditi*) or (Thyroiditi* autoimmune) or (Hashimoto* syndrome*) or (Hashimoto* thyroiditis*) or (Hashimoto* disease*) or (Thyroiditi* post-partum) or (Thyroiditi* postpartum) or (Graves* Disease) or (Basedow* disease) or (exophthalmic goiter*) or (Still’s Disease) or (Stills disease) or (Juvenile arthriti*) or vitiligo or Psorias?s or (Arthriti* Psoria*) or (atopic disease) or (atopic dermatitis) or (food* sensiti*) or (food* toleran*) or (food* intoleran*) or (aero allergen*) or (air* allergen*) or (aeroallergen* sensiti*) or (allergen* sensiti*) or (skin prick test*) or atopy or hypersensitive*)

AND

db:(“LILACS”)

AND

type_of_study:(“clinical_trials” or “case_control” or “cohort” or “systematic_reviews”)

AND

limit:(“infant” or “newborn” or “preschool” or “child”)

**COCHRANE Library**

1. “breast feeding”:ab,ti

2. breastfeeding:ab,ti

3. “breast fed”:ab,ti

4. breastfed:ab,ti

5. MeSH descriptor [Breast Feeding] this term only

6. MeSH descriptor [Milk, Human] this term only

7. formula*:ab,ti

8. hydrolysed:ab,ti

9. bottlefed:ab,ti

10. “bottle fed”:ab,ti

11. (bottle NEAR/3 feed*):ab,ti

12. MeSH descriptor [Infant Formula] this term only

13. MeSH descriptor [Bottle Feeding] this term only

14. wean*:ab,ti

15. MeSH descriptor [Weaning] this term only

16. 1 or 2 or 3 or 4 or 5 or 6 or 7 or 8 or 9 or 10 or 11 or 12 or 13 or 14 or 15

17. allerg*:ab,ti

18. asthma*:ab,ti

19. wheeze:ab,ti

20. wheezing:ab,ti

21. “bronchial hyperresponsiveness”:ab,ti

22. “bronchial hyperreactivity”:ab,ti

23. “Forced expiratory volume”:ab,ti

24. “FEV1”:ab,ti

25. "FEV 1":ab,ti

26. "FEV0.5":ab,ti

27. "FEV 0.5":ab,ti

28. “Forced vital capacity”:ab,ti

29. FVC:ab,ti

30. “Peak expiratory flow rate”:ab,ti

31. PEFR:ab,ti

32. AD:ab,ti

33. neurodermatitis:ab,ti

34. rhinitis:ab,ti

35. “besniers prurigo”:ab,ti

36. rhinoconjunctivitis:ab,ti

37. hayfever:ab,ti

38. “hay fever”:ab,ti

39. poll*nosis:ab,ti

40. SAR:ab,ti

41. “pollen allergy”:ab,ti

42. conjunctivitis:ab,ti

43. “immunoglobulin e”:ab,ti

44. “Total IgE”:ab,ti

45. “autoimmune disease*”:ab,ti

46. diabetes:ab,ti

47. diabetic:ab,ti

48. “type 1”:ab,ti

49. “c*eliac disease”:ab,ti

50. “crohn* disease”:ab,ti

51. “Inflammatory Bowel Disease*”:ab,ti

52. “Ulcerative colitis”:ab,ti

53. (Lympho* NEAR/3 thyroiditi*):ab,ti

54. (Thyroiditi* NEAR/3 autoimmune):ab,ti

55. (Hashimoto* NEAR/3 (syndrome* or thyroiditi* or disease*)):ab,ti

56. (Thyroiditi* NEAR/3 (post-partum or postpartum)):ab,ti

57. “Graves* disease”:ab,ti

58. “Basedow* disease”:ab,ti

59. “exophthalmic goiter*”:ab,ti

60. (Still* Disease NEAR/3 (juvenile or onset)):ab,ti

61. (Juvenile NEAR/3 arthriti*):ab,ti

62. vitiligo:ab,ti

63. Psorias*s:ab,ti

64. (Arthriti* NEAR/3 Psoria*):ab,ti

65. “atopic disease”:ab,ti

66. “atopic dermatitis”:ab,ti

67. (food* NEAR/3 sensiti*):ab,ti

68. (food* NEAR/3 toleran*):ab,ti

69. (food* NEAR/3 intoleran*):ab,ti

70. ((aero or air*) NEAR/3 allergen*):ab,ti

71. (aeroallergen* NEAR/3 sensiti*):ab,ti

72. (allergen* NEAR/3 sensiti*):ab,ti

73. “skin prick test*”:ab,ti

74. atopy:ab,ti

75. hypersensitiv*:ab,ti

76. MeSH descriptor [Hypersensitivity] this term only

77. MeSH descriptor [Food Hypersensitivity] explode all trees

78. MeSH descriptor [Respiratory Hypersensitivity] this term only

79. MeSH descriptor [Asthma] this term only

80. MeSH descriptor [Bronchial Hyperreactivity] this term only

81. MeSH descriptor [Forced Expiratory Volume] this term only

82. MeSH descriptor [Vital Capacity] this term only

83. MeSH descriptor [Peak Expiratory Flow Rate] this term only

84. MeSH descriptor [AD] this term only

85. MeSH descriptor [Neurodermatitis] this term only

86. MeSH descriptor [Rhinitis] this term only

87. MeSH descriptor [Rhinitis, Allergic, Perennial] this term only

88. MeSH descriptor [Rhinitis, Allergic, Seasonal] this term only

89. MeSH descriptor [Conjunctivitis] this term only

90. MeSH descriptor [Immunoglobulin E] this term only

91. MeSH descriptor [Autoimmune Diseases] this term only

92. MeSH descriptor [Diabetes Mellitus, Type 1] this term only

93. MeSH descriptor [Celiac Disease] this term only

94. MeSH descriptor [Crohn Disease] this term only

95. MeSH descriptor [Inflammatory Bowel Diseases] this term only

96. MeSH descriptor [Colitis, Ulcerative] this term only

97. MeSH descriptor [Thyroiditis, Autoimmune] this term only

98. MeSH descriptor [Hashimoto Disease] this term only

99. MeSH descriptor [Postpartum Thyroiditis] this term only

100. MeSH descriptor [Graves Disease] this term only

101. MeSH descriptor [Arthritis, Juvenile Rheumatoid] this term only

102. MeSH descriptor [Vitiligo] this term only

103. MeSH descriptor [Psoriasis] this term only

104. MeSH descriptor [Arthritis, Psoriatic] this term only

105. MeSH descriptor [Dermatitis, Atopic] this term only

106. MeSH descriptor [Hypersensitivity, Immediate] this term only

107. 17 or 18 or 19 or 20 or 21 or 22 or 23 or 24 or 25 or 26 or 27 or 28 or 29 or 30 or 31 or 32 or 33 or 34 or 35 or 36 or 37 or 38 or 39 or 40 or 41 or 42 or 43 or 44 or 45 or 46 or 47 or 48 or 49 or 50 or 51 or 52 or 53 or 54 or 55 or 56 or 57 or 58 or 59 or 60 or 61 or 62 or 63 or 64 or 65 or 66 or 67 or 68 or 69 or 70 or 71 or 72 or 73 or 74 or 75 or 76 or 77 or 78 or 79 or 80 or 81 or 82 or 83 or 84 or 85 or 86 or 87 or 88 or 89 or 90 or 91 or 92 or 93 or 94 or 95 or 96 or 97 or 98 or 99 or 100 or 101 or 102 or 103 or 104 or 105 or 106

108. infant*:ab,ti

109. ((one or two or three or four or five or six or seven or eight or nine or ten or eleven or twelve or thirteen or fourteen or fifteen or sixteen or seventeen or eighteen or nineteen or twenty or "twenty one" or "twenty two" or "twenty three" or "twenty four" or "twenty five" or "twenty six") NEAR/1 week*):ab,ti

110. ((one or two or three or four or five or six or seven or eight or nine or ten or eleven or twelve or thirteen or fourteen or fifteen or sixteen or seventeen or eighteen or nineteen or twenty or "twenty one" or "twenty two" or "twenty three" or "twenty four") NEAR/1 month*):ab,ti

111. 109 or 110

112. (old or age*):ab,ti

113. 111 and 112

114. (("one year*" or "two year*") NEAR/3 (old or age*)):ab,ti

115. ((first or second or two) NEAR/3 "year* of life"):ab,ti

116. MeSH descriptor [Infant] this term only

117. MeSH descriptor [Infant, Newborn] this term only

118. 108 or 113 or 114 or 115 or 116 or 117

119. “clinical trial*”

120. random*

121. factorial*

122. crossover*

123. placebo*

124. “doubl* blind*”

125. “singl* blind*”

126. assign*

127. volunteer*

128. “cohort stud*”

129. longitudinal*

130. follow-up

131. prospectiv*

132. retrospectiv*

133. “case control”

134. “case referent”

135. MeSH descriptor [clinical trial] explode all trees

136. MeSH descriptor [Cross-Over Studies] this term only

137. MeSH descriptor [Placebos] this term only

138. MeSH descriptor [Double-Blind Method] this term only

139. MeSH descriptor [Single-Blind Method] this term only

140. MeSH descriptor [Cohort Studies] explode all trees

141. MeSH descriptor [case-control studies] this term only

142. 119 or 120 or 121 or 122 or 123 or 124 or 125 or 126 or 127 or 128 or 129 or 130 or 131 or 132 or 133 or 134 or 135 or 136 or 137 or 138 or 139 or 140 or 141

143. 16 and 107 and 118 and 142

**Web of Science**

1. TOPIC = (“breast feeding” or breastfeeding or “breast fed” or breastfed or formula$ or hydrolysed or bottlefed or “bottle fed” or (bottle NEAR/3 feed*) or wean*)

2. TOPIC = (allerg* or asthma* or wheeze or wheezing or “bronchial hyperresponsiveness” or “bronchial hyperreactivity” or “Forced expiratory volume” or “FEV1” or "FEV 1" or "FEV0.5" or "FEV 0.5" or “Forced vital capacity” or FVC or “Peak expiratory flow rate” or PEFR or AD or neurodermatitis or rhinitis or “besniers prurigo” or rhinoconjunctivitis or hayfever or “hay fever” or poll$nosis or SAR or “pollen allergy” or conjunctivitis or “immunoglobulin e” or “Total IgE” or “autoimmune disease$” or diabetes or diabetic or “type 1” or “c$eliac disease” or “crohn* disease” or “Inflammatory Bowel Disease$” or “Ulcerative colitis” or (Lympho* NEAR/3 thyroiditi*) or (Thyroiditi* NEAR/3 autoimmune) or (Hashimoto* NEAR/3 (syndrome$ or thyroiditis* or disease$)) or (Thyroiditi* NEAR/3 (post-partum or postpartum)) or “Graves$ Disease” or “Basedow* disease” or “exophthalmic goiter$” or (“Still$ Disease” NEAR/3 (juvenile or onset)) or (Juvenile NEAR/3 arthriti*) or vitiligo or Psorias$s or (Arthriti$ NEAR/3 Psoria*) or “atopic disease” or “atopic dermatitis” or (food$ NEAR/3 sensiti*) or (food$ NEAR/3 toleran*) or (food$ NEAR/3 intoleran*) or ((aero or air*) NEAR/3 allergen$) or (aeroallergen$ NEAR/3 sensiti*) or (allergen$ NEAR/3 sensiti*) or “skin prick test*” or atopy or hypersensitive*)

3. TOPIC = (infant$ or (("one year$" or "two year$") NEAR/3 (old or age$)) or ((first or second or two) NEAR/3 "year$ of life"))

4. TOPIC = ((one or two or three or four or five or six or seven or eight or nine or ten or eleven or twelve or thirteen or fourteen or fifteen or sixteen or seventeen or eighteen or nineteen or twenty or "twenty one" or "twenty two" or "twenty three" or "twenty four" or "twenty five" or "twenty six") NEAR/1 week$)

5. TOPIC = ((one or two or three or four or five or six or seven or eight or nine or ten or eleven or twelve or thirteen or fourteen or fifteen or sixteen or seventeen or eighteen or nineteen or twenty or "twenty one" or "twenty two" or "twenty three" or "twenty four") NEAR/1 month$)

6. 4 or 5

7. TOPIC = ((old or age$))

8. 7 and 6

9. 8 or 3

10. TOPIC = (“clinical trial$” or random* or factorial* or crossover* or placebo* or “doubl* blind*” or “singl* blind*” or assign* or volunteer* or “cohort stud*” or longitudinal* or follow-up or prospective* or retrospective* or “case control” or “case referent”)

11. 1 and 2 and 9 and 10

**Appendix 3 Search Strategies for original articles on other maternal or infant dietary exposures**

**Medline**

1. Diet/

2. Diet Therapy/

3. Nutritional Sciences/

4. Child Nutrition Sciences/

5. diet.ab,ti.

6. diets.ab,ti.

7. Diet, Mediterranean/

8. mediterranean diet$.ab,ti.

9. dietetic.ab,ti.

10. dietary.ab,ti.

11. eat.ab,ti.

12. eating.ab,ti.

13. intake.ab,ti.

14. nutrient?.ab,ti.

15. nutrition.ab,ti.

16. Diet, Vegetarian/

17. vegetarian?.ab,ti.

18. vegan$.ab,ti.

19. Diet, Macrobiotic/

20. macrobiotic?.ab,ti.

21. Food/

22. food$.ab,ti.

23. feed.ab,ti.

24. feeding.ab,ti.

25. cereal$.ab,ti.

26. grain$.ab,ti.

27. granary.ab,ti.

28. wholegrain.ab,ti.

29. wholewheat.ab,ti.

30. whole wheat.ab,ti.

31. wheat.ab,ti.

32. wheatgerm.ab,ti.

33. rye.ab,ti.

34. barley.ab,ti.

35. oat?.ab,ti.

36. exp Cereals/

37. root?.ab,ti.

38. tuber?.ab,ti.

39. exp Vegetables/

40. vegetable$.ab,ti.

41. onion$.ab,ti.

42. spinach.ab,ti.

43. chard.ab,ti.

44. tomato$.ab,ti.

45. pepper$.ab,ti.

46. carrot$.ab,ti.

47. beetroot.ab,ti.

48. asparagus.ab,ti.

49. garlic.ab,ti.

50. pumpkin.ab,ti.

51. sprouts.ab,ti.

52. broccoli.ab,ti.

53. cabbage$.ab,ti.

54. celery.ab,ti.

55. ginger.ab,ti.

56. potato$.ab,ti.

57. crisps.ab,ti.

58. fries.ab,ti.

59. syrup.ab,ti.

60. honey.ab,ti.

61. Honey/

62. Fruit/

63. fruit$.ab,ti.

64. apple?.ab,ti.

65. pear?.ab,ti.

66. banana?.ab,ti.

67. orange?.ab,ti.

68. grape?.ab,ti.

69. kiwi?.ab,ti.

70. citrus.ab,ti.

71. grapefruit?.ab,ti.

72. pulses.ab,ti.

73. beans.ab,ti.

74. lentil?.ab,ti.

75. chickpea?.ab,ti.

76. legume?.ab,ti.

77. lupin?.ab,ti.

78. soy.ab,ti.

79. soya.ab,ti.

80. nut?.ab,ti.

81. almond?.ab,ti.

82. peanut?.ab,ti.

83. groundnut?.ab,ti.

84. Nuts/

85. seed?.ab,ti.

86. sesame.ab,ti.

87. mustard.ab,ti.

88. Seeds/

89. exp Meat/

90. meat.ab,ti.

91. beef.ab,ti.

92. pork.ab,ti.

93. lamb.ab,ti.

94. poultry.ab,ti.

95. chicken.ab,ti.

96. turkey.ab,ti.

97. duck.ab,ti.

98. fish.ab,ti.

99. Fatty Acids/

100. exp Fatty Acids, Omega-3/

101. exp Fatty Acids, Omega-6/

102. omega-3.ab,ti.

103. omega-6.ab,ti.

104. PUFA.ab,ti.

105. fat.ab,ti.

106. fats.ab,ti.

107. fatty.ab,ti.

108. egg.ab,ti.

109. eggs.ab,ti.

110. exp Eggs/

111. Bread/

112. bread.ab,ti.

113. oil.ab,ti.

114. oils.ab,ti.

115. oily.ab,ti.

116. omega.ab,ti.

117. exp Seafood/

118. seafood.ab,ti.

119. shellfish.ab,ti.

120. crustacean?.ab,ti.

121. mollusc?.ab,ti.

122. Shellfish/

123. Dairy Products/

124. dairy.ab,ti.

125. exp Milk/

126. milk.ab,ti.

127. Infant Formula/

128. formula?.ab,ti.

129. hydrolysed.ab,ti.

130. Infant Food/

131. yoghurt.ab,ti.

132. probiotic.ab,ti.

133. prebiotic?.ab,ti.

134. butter.ab,ti.

135. herb?.ab,ti.

136. spice?.ab,ti.

137. chilli$.ab,ti.

138. condiment?.ab,ti.

139. exp Condiments/

140. Beverages/

141. beverage?.ab,ti.

142. fluid intake.ab,ti.

143. water.ab,ti.

144. drink$.ab,ti.

145. exp Food Preservation/

146. pickled.ab,ti.

147. bottled.ab,ti.

148. canned.ab,ti.

149. canning.ab,ti.

150. smoked.ab,ti.

151. preserved.ab,ti.

152. preservatives.ab,ti.

153. nitrosamine.ab,ti.

154. hydrogenation.ab,ti.

155. fortified.ab,ti.

156. nitrates.ab,ti.

157. nitrites.ab,ti.

158. ferment$.ab,ti.

159. processed.ab,ti.

160. antioxidant$.ab,ti.

161. genetic modif$.ab,ti.

162. genetically modif$.ab,ti.

163. Cooking/

164. cooking.ab,ti.

165. cooked.ab,ti.

166. grill.ab,ti.

167. grilled.ab,ti.

168. fried.ab,ti.

169. fry.ab,ti.

170. roast.ab,ti.

171. bake.ab,ti.

172. baked.ab,ti.

173. stewing.ab,ti.

174. stewed.ab,ti.

175. casserol$.ab,ti.

176. broil.ab,ti.

177. broiled.ab,ti.

178. boiled.ab,ti.

179. poach.ab,ti.

180. poached.ab,ti.

181. steamed.ab,ti.

182. barbecue$.ab,ti.

183. chargrill$.ab,ti.

184. salt.ab,ti.

185. salting.ab,ti.

186. salted.ab,ti.

187. fiber.ab,ti.

188. fibre.ab,ti.

189. polysaccharide$.ab,ti.

190. starch.ab,ti.

191. starchy.ab,ti.

192. carbohydrate$.ab,ti.

193. lipid$.ab,ti.

194. linoleic acid$.ab,ti.

195. sugar$.ab,ti.

196. sweetener$.ab,ti.

197. saccharin$.ab,ti.

198. aspartame.ab,ti.

199. sucrose.ab,ti.

200. xylitol.ab,ti.

201. cholesterol.ab,ti.

202. hydrogenated lard.ab,ti.

203. dietary protein.ab,ti.

204. dietary proteins.ab,ti.

205. protein intake.ab,ti.

206. animal protein$.ab,ti.

207. total protein$.ab,ti.

208. vegetable protein$.ab,ti.

209. plant protein$.ab,ti.

210. exp Dietary Carbohydrates/

211. exp Dietary Fats/

212. exp Dietary Fiber/

213. exp Dietary Proteins/

214. exp Dietary Supplements/

215. exp Food Additives/

216. exp Vitamins/

217. supplements.ab,ti.

218. supplement.ab,ti.

219. vitamin$.ab,ti.

220. retinol.ab,ti.

221. carotenoid$.ab,ti.

222. tocopherol.ab,ti.

223. folate$.ab,ti.

224. folic acid.ab,ti.

225. methionine.ab,ti.

226. riboflavin.ab,ti.

227. thiamine.ab,ti.

228. niacin.ab,ti.

229. pyridoxine.ab,ti.

230. cobalamin.ab,ti.

231. mineral$.ab,ti.

232. sodium.ab,ti.

233. iron.ab,ti.

234. calcium.ab,ti.

235. selenium.ab,ti.

236. iodine.ab,ti.

237. magnesium.ab,ti.

238. potassium.ab,ti.

239. zinc.ab,ti.

240. copper.ab,ti.

241. phosphorus.ab,ti.

242. manganese.ab,ti.

243. chromium.ab,ti.

244. phytochemical.ab,ti.

245. polyphenol$.ab,ti.

246. phytoestrogen$.ab,ti.

247. genistein.ab,ti.

248. saponin$.ab,ti.

249. coumarin$.ab,ti.

250. flavonoid$.ab,ti.

251. polyphenol$.ab,ti.

252. flavonol$.ab,ti.

253. flavone$.ab,ti.

254. isoflavone$.ab,ti.

255. catechin$.ab,ti.

256. ascorbic acid$.ab,ti.

257. hydroxy cholecalciferol$.ab,ti.

258. hydroxycholecalciferol$.ab,ti.

259. tocotrienol$.ab,ti.

260. carotene$.ab,ti.

261. cryptoxanthin$.ab,ti.

262. lycopene$.ab,ti.

263. lutein$.ab,ti.

264. zeaxanthin$.ab,ti.

265. selenium$.ab,ti.

266. organic diet?.ab,ti.

267. Food, Organic/

268. 1 or 2 or 3 or 4 or 5 or 6 or 7 or 8 or 9 or 10 or 11 or 12 or 13 or 14 or 15 or 16 or 17 or 18 or 19 or 20 or 21 or 22 or 23 or 24 or 25 or 26 or 27 or 28 or 29 or 30 or 31 or 32 or 33 or 34 or 35 or 36 or 37 or 38 or 39 or 40 or 41 or 42 or 43 or 44 or 45 or 46 or 47 or 48 or 49 or 50 or 51 or 52 or 53 or 54 or 55 or 56 or 57 or 58 or 59 or 60 or 61 or 62 or 63 or 64 or 65 or 66 or 67 or 68 or 69 or 70 or 71 or 72 or 73 or 74 or 75 or 76 or 77 or 78 or 79 or 80 or 81 or 82 or 83 or 84 or 85 or 86 or 87 or 88 or 89 or 90 or 91 or 92 or 93 or 94 or 95 or 96 or 97 or 98 or 99 or 100 or 101 or 102 or 103 or 104 or 105 or 106 or 107 or 108 or 109 or 110 or 111 or 112 or 113 or 114 or 115 or 116 or 117 or 118 or 119 or 120 or 121 or 122 or 123 or 124 or 125 or 126 or 127 or 128 or 129 or 130 or 131 or 132 or 133 or 134 or 135 or 136 or 137 or 138 or 139 or 140 or 141 or 142 or 143 or 144 or 145 or 146 or 147 or 148 or 149 or 150 or 151 or 152 or 153 or 154 or 155 or 156 or 157 or 158 or 159 or 160 or 161 or 162 or 163 or 164 or 165 or 166 or 167 or 168 or 169 or 170 or 171 or 172 or 173 or 174 or 175 or 176 or 177 or 178 or 179 or 180 or 181 or 182 or 183 or 184 or 185 or 186 or 187 or 188 or 189 or 190 or 191 or 192 or 193 or 194 or 195 or 196 or 197 or 198 or 199 or 200 or 201 or 202 or 203 or 204 or 205 or 206 or 207 or 208 or 209 or 210 or 211 or 212 or 213 or 214 or 215 or 216 or 217 or 218 or 219 or 220 or 221 or 222 or 223 or 224 or 225 or 226 or 227 or 228 or 229 or 230 or 231 or 232 or 233 or 234 or 235 or 236 or 237 or 238 or 239 or 240 or 241 or 242 or 243 or 244 or 245 or 246 or 247 or 248 or 249 or 250 or 251 or 252 or 253 or 254 or 255 or 256 or 257 or 258 or 259 or 260 or 261 or 262 or 263 or 264 or 265 or 266 or 267

269. allerg$.ab,ti.

270. asthma$.ab,ti.

271. wheeze.ab,ti.

272. wheezing.ab,ti.

273. bronchial hyperresponsiveness.ab,ti.

274. bronchial hyperreactivity.ab,ti.

275. Forced expiratory volume.ab,ti.

276. FEV1.ab,ti.

277. "FEV 1".ab,ti.

278. "FEV0.5".ab,ti.

279. "FEV 0.5".ab,ti.

280. Forced vital capacity.ab,ti.

281. FVC.ab,ti.

282. Peak expiratory flow rate.ab,ti.

283. PEFR.ab,ti.

284. AD.ab,ti.

285. neurodermatitis.ab,ti.

286. rhinitis.ab,ti.

287. besniers prurigo.ab,ti.

288. rhinoconjunctivitis.ab,ti.

289. hayfever.ab,ti.

290. (hay adj fever).ab,ti.

291. poll?nosis.ab,ti.

292. SAR.ab,ti.

293. (pollen adj allergy).ab,ti.

294. conjunctivitis.ab,ti.

295. immunoglobulin e.ab,ti.

296. Total IgE.ab,ti.

297. autoimmune disease?.ab,ti.

298. diabetes.ab,ti.

299. diabetic.ab,ti.

300. type 1.ab,ti.

301. c?eliac disease.ab,ti.

302. crohn$ disease.ab,ti.

303. Inflammatory Bowel Disease?.ab,ti.

304. Ulcerative colitis.ab,ti.

305. (Lympho$ adj3 thyroiditi$).ab,ti.

306. (Thyroiditi$ adj3 autoimmune).ab,ti.

307. (Hashimoto$ adj3 (syndrome? or thyroiditi$ or disease?)).ab,ti.

308. (Thyroiditi$ adj3 (post-partum or postpartum)).ab,ti.

309. Graves? disease.ab,ti.

310. Basedow$ disease.ab,ti.

311. exophthalmic goiter?.ab,ti.

312. (Still? Disease adj3 (juvenile or onset)).ab,ti.

313. (Juvenile adj3 arthriti$).ab,ti.

314. vitiligo.ab,ti.

315. Psorias?s.ab,ti.

316. (Arthriti? adj3 Psoria$).ab,ti.

317. atopic disease.ab,ti.

318. atopic dermatitis.ab,ti.

319. (food? adj3 sensiti$).ab,ti.

320. (food? adj3 toleran$).ab,ti.

321. (food? adj3 intoleran$).ab,ti.

322. ((aero or air$) adj3 allergen?).ab,ti.

323. (aeroallergen? adj3 sensiti$).ab,ti.

324. (allergen? adj3 sensiti$).ab,ti.

325. skin prick test$.ab,ti.

326. atopy.ab,ti.

327. hypersensitiv$.ab,ti.

328. Hypersensitivity/

329. exp Food Hypersensitivity/

330. Respiratory Hypersensitivity/

331. Asthma/

332. Bronchial Hyperreactivity/

333. Forced Expiratory Volume/

334. Vital Capacity/

335. Peak Expiratory Flow Rate/

336. AD/

337. Neurodermatitis/

338. Rhinitis/

339. Rhinitis, Allergic, Perennial/

340. Rhinitis, Allergic, Seasonal/

341. Conjunctivitis/

342. Immunoglobulin E/

343. Autoimmune Diseases/

344. Diabetes Mellitus, Type 1/

345. Celiac Disease/

346. Crohn Disease/

347. Inflammatory Bowel Diseases/

348. Colitis, Ulcerative/

349. Thyroiditis, Autoimmune/

350. Hashimoto Disease/

351. Postpartum Thyroiditis/

352. Graves Disease/

353. Arthritis, Juvenile Rheumatoid/

354. Vitiligo/

355. Psoriasis/

356. Arthritis, Psoriatic/

357. Dermatitis, Atopic/

358. Hypersensitivity, Immediate/

359. 269 or 270 or 271 or 272 or 273 or 274 or 275 or 276 or 277 or 278 or 279 or 280 or 281 or 282 or 283 or 284 or 285 or 286 or 287 or 288 or 289 or 290 or 291 or 292 or 293 or 294 or 295 or 296 or 297 or 298 or 299 or 300 or 301 or 302 or 303 or 304 or 305 or 306 or 307 or 308 or 309 or 310 or 311 or 312 or 313 or 314 or 315 or 316 or 317 or 318 or 319 or 320 or 321 or 322 or 323 or 324 or 325 or 326 or 327 or 328 or 329 or 330 or 331 or 332 or 333 or 334 or 335 or 336 or 337 or 338 or 339 or 340 or 341 or 342 or 343 or 344 or 345 or 346 or 347 or 348 or 349 or 350 or 351 or 352 or 353 or 354 or 355 or 356 or 357 or 358

360. infant?.ab,ti.

361. ((one or two or three or four or five or six or seven or eight or nine or ten or eleven or twelve or thirteen or fourteen or fifteen or sixteen or seventeen or eighteen or nineteen or twenty or "twenty one" or "twenty two" or "twenty three" or "twenty four" or "twenty five" or "twenty six") adj week?).ab,ti.

362. ((one or two or three or four or five or six or seven or eight or nine or ten or eleven or twelve or thirteen or fourteen or fifteen or sixteen or seventeen or eighteen or nineteen or twenty or "twenty one" or "twenty two" or "twenty three" or "twenty four") adj month?).ab,ti.

363. 361 or 362

364. (old or age?).ab,ti.

365. 363 and 364

366. (("one year?" or "two year?") adj3 (old or age?)).ab,ti.

367. ((first or second or two) adj3 "year? of life").ab,ti.

368. Infant/

369. Infant, Newborn/

370. (maternal or pregnan$).ab,ti.

371. (lactat$).ab,ti.

372. (mother?).ab,ti.

373. 360 or 365 or 366 or 367 or 368 or 369 or 370 or 371 or 372

374. clinical trial?.mp.

375. random$.mp.

376. factorial$.mp.

377. crossover$.mp.

378. placebo$.mp.

379. (doubl$ adj blind$).mp.

380. (singl$ adj blind$).mp.

381. assign$.mp.

382. volunteer$.mp.

383. cohort stud$.mp.

384. longitudinal$.mp.

385. follow-up.mp.

386. prospectiv$.mp.

387. retrospectiv$.mp.

388. case control.mp.

389. case referent.mp.

390. exp clinical trial/

391. Cross-Over Studies/

392. Placebos/

393. Double-Blind Method/

394. Single-Blind Method/

395. exp Cohort Studies/

396. case-control studies/

397. 374 or 375 or 376 or 377 or 378 or 379 or 380 or 381 or 382 or 383 or 384 or 385 or 386 or 387 or 388 or 389 or 390 or 391 or 392 or 393 or 394 or 395 or 396

398. 268 and 359 and 373 and 397

**Embase**

1. diet/

2. diet therapy/

3. nutritional science/

4. diet.ti,ab.

5. diets.ti,ab.

6. Mediterranean diet/

7. mediterranean diet$.ab,ti.

8. dietetic.ab,ti.

9. dietary.ab,ti.

10. eat.ab,ti.

11. eating.ab,ti.

12. intake.ab,ti.

13. nutrient?.ab,ti.

14. nutrition.ab,ti.

15. vegetarian diet/

16. vegetarian?.ti,ab.

17. vegan$.ti,ab.

18. macrobiotic diet/

19. macrobiotic?.ti,ab.

20. food/

21. food$.ab,ti.

22. feed.ab,ti.

23. feeding.ab,ti.

24. cereal$.ab,ti.

25. grain$.ab,ti.

26. granary.ab,ti.

27. wholegrain.ab,ti.

28. wholewheat.ab,ti.

29. whole wheat.ab,ti.

30. wheat.ab,ti.

31. wheatgerm.ab,ti.

32. rye.ab,ti.

33. barley.ab,ti.

34. oat?.ab,ti.

35. exp cereal/

36. root?.ti,ab.

37. tuber?.ti,ab.

38. exp vegetable/

39. vegetable$.ab,ti.

40. onion$.ab,ti.

41. spinach.ab,ti.

42. chard.ab,ti.

43. tomato$.ab,ti.

44. pepper$.ab,ti.

45. carrot$.ab,ti.

46. beetroot.ab,ti.

47. asparagus.ab,ti.

48. garlic.ab,ti.

49. pumpkin.ab,ti.

50. sprouts.ab,ti.

51. broccoli.ab,ti.

52. cabbage$.ab,ti.

53. celery.ab,ti.

54. ginger.ab,ti.

55. potato$.ab,ti.

56. crisps.ab,ti.

57. fries.ab,ti.

58. syrup.ab,ti.

59. honey.ab,ti.

60. honey/

61. fruit/

62. fruit$.ab,ti.

63. apple?.ab,ti.

64. pear?.ab,ti.

65. banana?.ab,ti.

66. orange?.ab,ti.

67. grape?.ab,ti.

68. kiwi?.ab,ti.

69. citrus.ab,ti.

70. grapefruit?.ab,ti.

71. pulses.ab,ti.

72. beans.ab,ti.

73. lentil?.ab,ti.

74. chickpea?.ab,ti.

75. legume?.ab,ti.

76. lupin?.ab,ti.

77. soy.ab,ti.

78. soya.ab,ti.

79. nut?.ab,ti.

80. almond?.ab,ti.

81. peanut?.ab,ti.

82. groundnut?.ab,ti.

83. exp nut/

84. seed?.ti,ab.

85. sesame.ti,ab.

86. mustard.ti,ab.

87. plant seed/

88. meat/

89. meat.ab,ti.

90. beef.ab,ti.

91. pork.ab,ti.

92. lamb.ab,ti.

93. poultry.ab,ti.

94. chicken.ab,ti.

95. turkey.ab,ti.

96. duck.ab,ti.

97. fish.ab,ti.

98. fatty acid/

99. omega 3 fatty acid/

100. omega 6 fatty acid/

101. omega-3.ab,ti.

102. omega-6.ab,ti.

103. PUFA.ab,ti.

104. fat.ab,ti.

105. fats.ab,ti.

106. fatty.ab,ti.

107. egg.ab,ti.

108. eggs.ab,ti.

109. exp egg/

110. bread/

111. bread.ti,ab.

112. oil.ti,ab.

113. oils.ti,ab.

114. oily.ti,ab.

115. omega.ti,ab.

116. sea food/

117. seafood.ti,ab.

118. shellfish.ti,ab.

119. crustacean?.ti,ab.

120. mollusc?.ti,ab.

121. shellfish/

122. exp dairy product/

123. dairy.ti,ab.

124. milk/

125. milk.ti,ab.

126. artificial milk/

127. formula?.ti,ab.

128. hydrolysed.ti,ab.

129. baby food/

130. yoghurt.ab,ti.

131. probiotic.ab,ti.

132. prebiotic?.ab,ti.

133. butter.ab,ti.

134. herb?.ab,ti.

135. spice?.ab,ti.

136. chilli$.ab,ti.

137. condiment?.ab,ti.

138. exp condiment/

139. beverage/

140. beverage?.ti,ab.

141. fluid intake.ti,ab.

142. water.ti,ab.

143. drink$.ti,ab.

144. exp food preservation/

145. pickled.ab,ti.

146. bottled.ab,ti.

147. canned.ab,ti.

148. canning.ab,ti.

149. smoked.ab,ti.

150. preserved.ab,ti.

151. preservatives.ab,ti.

152. nitrosamine.ab,ti.

153. hydrogenation.ab,ti.

154. fortified.ab,ti.

155. nitrates.ab,ti.

156. nitrites.ab,ti.

157. ferment$.ab,ti.

158. processed.ab,ti.

159. antioxidant$.ab,ti.

160. genetic modif$.ab,ti.

161. genetically modif$.ab,ti.

162. cooking/

163. cooking.ab,ti.

164. cooked.ab,ti.

165. grill.ab,ti.

166. grilled.ab,ti.

167. fried.ab,ti.

168. fry.ab,ti.

169. roast.ab,ti.

170. bake.ab,ti.

171. baked.ab,ti.

172. stewing.ab,ti.

173. stewed.ab,ti.

174. casserol$.ab,ti.

175. broil.ab,ti.

176. broiled.ab,ti.

177. boiled.ab,ti.

178. poach.ab,ti.

179. poached.ab,ti.

180. steamed.ab,ti.

181. barbecue$.ab,ti.

182. chargrill$.ab,ti.

183. salt.ab,ti.

184. salting.ab,ti.

185. salted.ab,ti.

186. fiber.ab,ti.

187. fibre.ab,ti.

188. polysaccharide$.ab,ti.

189. starch.ab,ti.

190. starchy.ab,ti.

191. carbohydrate$.ab,ti.

192. lipid$.ab,ti.

193. linoleic acid$.ab,ti.

194. sugar$.ab,ti.

195. sweetener$.ab,ti.

196. saccharin$.ab,ti.

197. aspartame.ab,ti.

198. sucrose.ab,ti.

199. xylitol.ab,ti.

200. cholesterol.ab,ti.

201. hydrogenated lard.ab,ti.

202. dietary protein.ab,ti.

203. dietary proteins.ab,ti.

204. protein intake.ab,ti.

205. animal protein$.ab,ti.

206. total protein$.ab,ti.

207. vegetable protein$.ab,ti.

208. plant protein$.ab,ti.

209. carbohydrate diet/

210. carbohydrate intake/

211. fat intake/

212. dietary fiber/

213. protein intake/

214. diet supplementation/

215. food additive/

216. exp vitamin/

217. supplements.ab,ti.

218. supplement.ab,ti.

219. vitamin$.ab,ti.

220. retinol.ab,ti.

221. carotenoid$.ab,ti.

222. tocopherol.ab,ti.

223. folate$.ab,ti.

224. folic acid.ab,ti.

225. methionine.ab,ti.

226. riboflavin.ab,ti.

227. thiamine.ab,ti.

228. niacin.ab,ti.

229. pyridoxine.ab,ti.

230. cobalamin.ab,ti.

231. mineral$.ab,ti.

232. sodium.ab,ti.

233. iron.ab,ti.

234. calcium.ab,ti.

235. selenium.ab,ti.

236. iodine.ab,ti.

237. magnesium.ab,ti.

238. potassium.ab,ti.

239. zinc.ab,ti.

240. copper.ab,ti.

241. phosphorus.ab,ti.

242. manganese.ab,ti.

243. chromium.ab,ti.

244. phytochemical.ab,ti.

245. polyphenol$.ab,ti.

246. phytoestrogen$.ab,ti.

247. genistein.ab,ti.

248. saponin$.ab,ti.

249. coumarin$.ab,ti.

250. flavonoid$.ab,ti.

251. polyphenol$.ab,ti.

252. flavonol$.ab,ti.

253. flavone$.ab,ti.

254. isoflavone$.ab,ti.

255. catechin$.ab,ti.

256. ascorbic acid$.ab,ti.

257. hydroxy cholecalciferol$.ab,ti.

258. hydroxycholecalciferol$.ab,ti.

259. tocotrienol$.ab,ti.

260. carotene$.ab,ti.

261. cryptoxanthin$.ab,ti.

262. lycopene$.ab,ti.

263. lutein$.ab,ti.

264. zeaxanthin$.ab,ti.

265. selenium$.ab,ti.

266. organic diet?.ab,ti.

267. organic food/

268. 1 or 2 or 3 or 4 or 5 or 6 or 7 or 8 or 9 or 10 or 11 or 12 or 13 or 14 or 15 or 16 or 17 or 18 or 19 or 20 or 21 or 22 or 23 or 24 or 25 or 26 or 27 or 28 or 29 or 30 or 31 or 32 or 33 or 34 or 35 or 36 or 37 or 38 or 39 or 40 or 41 or 42 or 43 or 44 or 45 or 46 or 47 or 48 or 49 or 50 or 51 or 52 or 53 or 54 or 55 or 56 or 57 or 58 or 59 or 60 or 61 or 62 or 63 or 64 or 65 or 66 or 67 or 68 or 69 or 70 or 71 or 72 or 73 or 74 or 75 or 76 or 77 or 78 or 79 or 80 or 81 or 82 or 83 or 84 or 85 or 86 or 87 or 88 or 89 or 90 or 91 or 92 or 93 or 94 or 95 or 96 or 97 or 98 or 99 or 100 or 101 or 102 or 103 or 104 or 105 or 106 or 107 or 108 or 109 or 110 or 111 or 112 or 113 or 114 or 115 or 116 or 117 or 118 or 119 or 120 or 121 or 122 or 123 or 124 or 125 or 126 or 127 or 128 or 129 or 130 or 131 or 132 or 133 or 134 or 135 or 136 or 137 or 138 or 139 or 140 or 141 or 142 or 143 or 144 or 145 or 146 or 147 or 148 or 149 or 150 or 151 or 152 or 153 or 154 or 155 or 156 or 157 or 158 or 159 or 160 or 161 or 162 or 163 or 164 or 165 or 166 or 167 or 168 or 169 or 170 or 171 or 172 or 173 or 174 or 175 or 176 or 177 or 178 or 179 or 180 or 181 or 182 or 183 or 184 or 185 or 186 or 187 or 188 or 189 or 190 or 191 or 192 or 193 or 194 or 195 or 196 or 197 or 198 or 199 or 200 or 201 or 202 or 203 or 204 or 205 or 206 or 207 or 208 or 209 or 210 or 211 or 212 or 213 or 214 or 215 or 216 or 217 or 218 or 219 or 220 or 221 or 222 or 223 or 224 or 225 or 226 or 227 or 228 or 229 or 230 or 231 or 232 or 233 or 234 or 235 or 236 or 237 or 238 or 239 or 240 or 241 or 242 or 243 or 244 or 245 or 246 or 247 or 248 or 249 or 250 or 251 or 252 or 253 or 254 or 255 or 256 or 257 or 258 or 259 or 260 or 261 or 262 or 263 or 264 or 265 or 266 or 267

269. allerg$.ab,ti.

270. asthma$.ab,ti.

271. wheeze.ab,ti.

272. wheezing.ab,ti.

273. bronchial hyperresponsiveness.ab,ti.

274. bronchial hyperreactivity.ab,ti.

275. Forced expiratory volume.ab,ti.

276. FEV1.ab,ti.

277. "FEV 1".ab,ti.

278. "FEV0.5".ab,ti.

279. "FEV 0.5".ab,ti.

280. Forced vital capacity.ab,ti.

281. FVC.ab,ti.

282. Peak expiratory flow rate.ab,ti.

283. PEFR.ab,ti.

284. AD.ab,ti.

285. neurodermatitis.ab,ti.

286. rhinitis.ab,ti.

287. besniers prurigo.ab,ti.

288. rhinoconjunctivitis.ab,ti.

289. hayfever.ab,ti.

290. (hay adj fever).ab,ti.

291. poll?nosis.ab,ti.

292. SAR.ab,ti.

293. (pollen adj allergy).ab,ti.

294. conjunctivitis.ab,ti.

295. immunoglobulin e.ab,ti.

296. Total IgE.ab,ti.

297. autoimmune disease?.ab,ti.

298. diabetes.ab,ti.

299. diabetic.ab,ti.

300. type 1.ab,ti.

301. c?eliac disease.ab,ti.

302. crohn$ disease.ab,ti.

303. Inflammatory Bowel Disease?.ab,ti.

304. Ulcerative colitis.ab,ti.

305. (Lympho$ adj3 thyroiditi$).ab,ti.

306. (Thyroiditi$ adj3 autoimmune).ab,ti.

307. (Hashimoto$ adj3 (syndrome? or thyroiditi$ or disease?)).ab,ti.

308. (Thyroiditi$ adj3 (post-partum or postpartum)).ab,ti.

309. Graves? disease.ab,ti.

310. Basedow$ disease.ab,ti.

311. exophthalmic goiter?.ab,ti.

312. (Still? Disease adj3 (juvenile or onset)).ab,ti.

313. (Juvenile adj3 arthriti$).ab,ti.

314. vitiligo.ab,ti.

315. Psorias?s.ab,ti.

316. (Arthriti? adj3 Psoria$).ab,ti.

317. atopic disease.ab,ti.

318. atopic dermatitis.ab,ti.

319. (food? adj3 sensiti$).ab,ti.

320. (food? adj3 toleran$).ab,ti.

321. (food? adj3 intoleran$).ab,ti.

322. ((aero or air$) adj3 allergen?).ab,ti.

323. (aeroallergen? adj3 sensiti$).ab,ti.

324. (allergen? adj3 sensiti$).ab,ti.

325. skin prick test$.ab,ti.

326. atopy.ab,ti.

327. hypersensitiv$.ab,ti.

328. exp hypersensitivity/

329. respiratory tract allergy/

330. asthma/

331. wheezing/

332. bronchus hyperreactivity/

333. forced expiratory volume/

334. forced vital capacity/

335. peak expiratory flow/

336. AD/

337. neurodermatitis/

338. rhinitis/

339. rhinoconjunctivitis/

340. hay fever/

341. pollen allergy/

342. perennial rhinitis/

343. conjunctivitis/

344. immunoglobulin E/

345. autoimmune disease/

346. diabetes mellitus/

347. insulin dependent diabetes mellitus/

348. celiac disease/

349. Crohn disease/

350. enteritis/

351. ulcerative colitis/

352. autoimmune thyroiditis/

353. Hashimoto disease/

354. postpartum thyroiditis/

355. Graves disease/

356. juvenile rheumatoid arthritis/

357. vitiligo/

358. psoriasis/

359. psoriatic arthritis/

360. atopic dermatitis/

361. nutritional intolerance/

362. 269 or 270 or 271 or 272 or 273 or 274 or 275 or 276 or 277 or 278 or 279 or 280 or 281 or 282 or 283 or 284 or 285 or 286 or 287 or 288 or 289 or 290 or 291 or 292 or 293 or 294 or 295 or 296 or 297 or 298 or 299 or 300 or 301 or 302 or 303 or 304 or 305 or 306 or 307 or 308 or 309 or 310 or 311 or 312 or 313 or 314 or 315 or 316 or 317 or 318 or 319 or 320 or 321 or 322 or 323 or 324 or 325 or 326 or 327 or 328 or 329 or 330 or 331 or 332 or 333 or 334 or 335 or 336 or 337 or 338 or 339 or 340 or 341 or 342 or 343 or 344 or 345 or 346 or 347 or 348 or 349 or 350 or 351 or 352 or 353 or 354 or 355 or 356 or 357 or 358 or 359 or 360 or 361

363. infant?.ab,ti.

364. ((one or two or three or four or five or six or seven or eight or nine or ten or eleven or twelve or thirteen or fourteen or fifteen or sixteen or seventeen or eighteen or nineteen or twenty or "twenty one" or "twenty two" or "twenty three" or "twenty four" or "twenty five" or "twenty six") adj week?).ab,ti.

365. ((one or two or three or four or five or six or seven or eight or nine or ten or eleven or twelve or thirteen or fourteen or fifteen or sixteen or seventeen or eighteen or nineteen or twenty or "twenty one" or "twenty two" or "twenty three" or "twenty four") adj month?).ab,ti.

366. 364 or 365

367. (old or age?).ab,ti.

368. 366 and 367

369. (("one year?" or "two year?") adj3 (old or age?)).ab,ti.

370. ((first or second or two) adj3 "year? of life").ab,ti.

371. infant/

372. newborn/

373. (maternal or pregnan$).ti,ab.

374. (lactat$).ti,ab.

375. (mother?).ti,ab.

376. 363 or 368 or 369 or 370 or 371 or 372 or 373 or 374 or 375

377. clinical trial?.mp.

378. random$.mp.

379. factorial$.mp.

380. crossover$.mp.

381. placebo$.mp.

382. (doubl$ adj blind$).mp.

383. (singl$ adj blind$).mp.

384. assign$.mp.

385. volunteer$.mp.

386. cohort stud$.mp.

387. longitudinal$.mp.

388. follow-up.mp.

389. prospectiv$.mp.

390. retrospectiv$.mp.

391. case control.mp.

392. case referent.mp.

393. exp clinical trial/

394. crossover procedure/

395. placebo/

396. double blind procedure/

397. single blind procedure/

398. cohort analysis/

399. longitudinal study/

400. follow up/

401. prospective study/

402. retrospective study/

403. exp case control study/

404. 377 or 378 or 379 or 380 or 381 or 382 or 383 or 384 or 385 or 386 or 387 or 388 or 389 or 390 or 391 or 392 or 393 or 394 or 395 or 396 or 397 or 398 or 399 or 400 or 401 or 402 or 403

405. 268 and 362 and 376 and 404

**LILACS**

(tw:((breast feeding) or breastfeeding or (breast fed) or breastfed or formula* or hydrolysed or bottlefed or (bottle fed) or (bottle feed*) or wean*)

AND

(tw:(allerg* or asthma* or wheez* or (bronchial hyperresponsiveness) or (bronchial hyperreactivity) or (Forced expiratory volume) or FEV1 or (FEV 1) or FEV0.5 or (FEV 0.5) or (Forced vital capacity) or FVC or (Peak expiratory flow rate) or PEFR or AD or neurodermatitis or rhinitis or (besniers prurigo) or rhinoconjunctivitis or hayfever or (hay fever) or poll?nosis or SAR or (pollen allergy) or conjunctivitis or (immunoglobulin e) or (Total IgE) or (autoimmune disease*) or diabetes or diabetic or (type 1) or (c?eliac disease) or (crohn* disease) or (Inflammatory Bowel Disease*) or (Ulcerative colitis) or (Lympho* thyroiditi*) or (Thyroiditi* autoimmune) or (Hashimoto* syndrome*) or (Hashimoto* thyroiditis*) or (Hashimoto* disease*) or (Thyroiditi* post-partum) or (Thyroiditi* postpartum) or (Graves* Disease) or (Basedow* disease) or (exophthalmic goiter*) or (Still’s Disease) or (Stills disease) or (Juvenile arthriti*) or vitiligo or Psorias?s or (Arthriti* Psoria*) or (atopic disease) or (atopic dermatitis) or (food* sensiti*) or (food* toleran*) or (food* intoleran*) or (aero allergen*) or (air* allergen*) or (aeroallergen* sensiti*) or (allergen* sensiti*) or (skin prick test*) or atopy or hypersensitive*)

AND

db:(“LILACS”)

AND

type_of_study:(“clinical_trials” or “case_control” or “cohort” or “systematic_reviews”)

AND

limit:(“infant” or “newborn” or “preschool” or “child”)

**COCHRANE Library**

1. MeSH descriptor: [Diet] this term only

2. MeSH descriptor: [Diet Therapy] this term only

3. MeSH descriptor: [Nutritional Sciences] this term only

4. MeSH descriptor: [Child Nutrition Sciences] this term only

5. diet:ab,ti

6. diets:ab,ti

7. MeSH descriptor: [Diet, Mediterranean] this term only

8. “mediterranean diet*”:ab,ti

9. dietetic:ab,ti

10. dietary:ab,ti

11. eat:ab,ti

12. eating:ab,ti

13. intake:ab,ti

14. nutrient*:ab,ti

15. nutrition:ab,ti

16. MeSH descriptor: [Diet, Vegetarian] this term only

17. vegetarian*:ab,ti

18. vegan*:ab,ti

19. MeSH descriptor: [Diet, Macrobiotic] this term only

20. macrobiotic*:ab,ti

21. MeSH descriptor: [Food] this term only

22. food*:ab,ti

23. feed:ab,ti

24. feeding:ab,ti

25. cereal*:ab,ti

26. grain*:ab,ti

27. granary:ab,ti

28. wholegrain:ab,ti

29. wholewheat:ab,ti

30. “whole wheat”:ab,ti

31. wheat:ab,ti

32. wheatgerm:ab,ti

33. rye:ab,ti

34. barley:ab,ti

35. oat*:ab,ti

36. MeSH descriptor: [Cereals] explode all trees

37. root*:ab,ti

38. tuber*:ab,ti

39. MeSH descriptor: [Vegetables] explode all trees

40. vegetable*:ab,ti

41. onion*:ab,ti

42. spinach:ab,ti

43. chard:ab,ti

44. tomato*:ab,ti

45. pepper*:ab,ti

46. carrot*:ab,ti

47. beetroot:ab,ti

48. asparagus:ab,ti

49. garlic:ab,ti

50. pumpkin:ab,ti

51. sprouts:ab,ti

52. broccoli:ab,ti

53. cabbage*:ab,ti

54. celery:ab,ti

55. ginger:ab,ti

56. potato*:ab,ti

57. crisps:ab,ti

58. fries:ab,ti

59. syrup:ab,ti

60. honey:ab,ti

61. MeSH descriptor: [Honey] this term only

62. MeSH descriptor: [Fruit] this term only

63. fruit*:ab,ti

64. apple*:ab,ti

65. pear*:ab,ti

66. banana*:ab,ti

67. orange*:ab,ti

68. grape*:ab,ti

69. kiwi*:ab,ti

70. citrus:ab,ti

71. grapefruit*:ab,ti

72. pulses:ab,ti

73. beans:ab,ti

74. lentil*:ab,ti

75. chickpea*:ab,ti

76. legume*:ab,ti

77. lupin*:ab,ti

78. soy:ab,ti

79. soya:ab,ti

80. nut*:ab,ti

81. almond*:ab,ti

82. peanut*:ab,ti

83. groundnut*:ab,ti

84. MeSH descriptor: [Nuts] this term only

85. seed*:ab,ti

86. sesame:ab,ti

87. mustard:ab,ti

88. MeSH descriptor: [Seeds] this term only

89. MeSH descriptor: [Meat] explode all trees

90. meat:ab,ti

91. beef:ab,ti

92. pork:ab,ti

93. lamb:ab,ti

94. poultry:ab,ti

95. chicken:ab,ti

96. turkey:ab,ti

97. duck:ab,ti

98. fish:ab,ti

99. MeSH descriptor: [Fatty Acids] this term only

100. MeSH descriptor: [Fatty Acids, Omega-3] explode all trees

101. MeSH descriptor: [Fatty Acids, Omega-6] explode all trees

102. omega-3:ab,ti

103. omega-6:ab,ti

104. PUFA:ab,ti

105. fat:ab,ti

106. fats:ab,ti

107. fatty:ab,ti

108. egg:ab,ti

109. eggs:ab,ti

110. MeSH descriptor: [Eggs] explode all trees

111. MeSH descriptor: [Bread] this term only

112. bread:ab,ti

113. oil:ab,ti

114. oils:ab,ti

115. oily:ab,ti

116. omega:ab,ti

117. MeSH descriptor: [Seafood] explode all trees

118. seafood:ab,ti

119. shellfish:ab,ti

120. crustacean*:ab,ti

121. mollusc*:ab,ti

122. MeSH descriptor: [Shellfish] this term only

123. MeSH descriptor: [Dairy Products] this term only

124. dairy:ab,ti

125. MeSH descriptor: [Milk] explode all trees

126. milk:ab,ti

127. MeSH descriptor: [Infant Formula] this term only

128. formula*:ab,ti

129. hydrolysed:ab,ti

130. MeSH descriptor: [Infant Food] this term only

131. yoghurt:ab,ti

132. probiotic:ab,ti

133. prebiotic*:ab,ti

134. butter:ab,ti

135. herb*:ab,ti

136. spice*:ab,ti

137. chilli*:ab,ti

138. condiment*:ab,ti

139. MeSH descriptor: [Condiments] explode all trees

140. MeSH descriptor: [Beverages] this term only

141. beverage*:ab,ti

142. “fluid intake”:ab,ti

143. water:ab,ti

144. drink*:ab,ti

145. MeSH descriptor: [Food Preservation] explode all trees

146. pickled:ab,ti

147. bottled:ab,ti

148. canned:ab,ti

149. canning:ab,ti

150. smoked:ab,ti

151. preserved:ab,ti

152. preservatives:ab,ti

153. nitrosamine:ab,ti

154. hydrogenation:ab,ti

155. fortified:ab,ti

156. nitrates:ab,ti

157. nitrites:ab,ti

158. ferment*:ab,ti

159. processed:ab,ti

160. antioxidant*:ab,ti

161. “genetic modif*”:ab,ti

162. “genetically modif*”:ab,ti

163. MeSH descriptor: [Cooking] this term only

164. cooking:ab,ti

165. cooked:ab,ti

166. grill:ab,ti

167. grilled:ab,ti

168. fried:ab,ti

169. fry:ab,ti

170. roast:ab,ti

171. bake:ab,ti

172. baked:ab,ti

173. stewing:ab,ti

174. stewed:ab,ti

175. casserol*:ab,ti

176. broil:ab,ti

177. broiled:ab,ti

178. boiled:ab,ti

179. poach:ab,ti

180. poached:ab,ti

181. steamed:ab,ti

182. barbecue*:ab,ti

183. chargrill*:ab,ti

184. salt:ab,ti

185. salting:ab,ti

186. salted:ab,ti

187. fiber:ab,ti

188. fibre:ab,ti

189. polysaccharide*:ab,ti

190. starch:ab,ti

191. starchy:ab,ti

192. carbohydrate*:ab,ti

193. lipid*:ab,ti

194. “linoleic acid*”:ab,ti

195. sugar*:ab,ti

196. sweetener*:ab,ti

197. saccharin*:ab,ti

198. aspartame:ab,ti

199. sucrose:ab,ti

200. xylitol:ab,ti

201. cholesterol:ab,ti

202. “hydrogenated lard”:ab,ti

203. “dietary protein”:ab,ti

204. “dietary proteins”:ab,ti

205. “protein intake”:ab,ti

206. “animal protein*”:ab,ti

207. “total protein*”:ab,ti

208. “vegetable protein*”:ab,ti

209. “plant protein*”:ab,ti

210. MeSH descriptor: [Dietary Carbohydrates] explode all trees

211. MeSH descriptor: [Dietary Fats] explode all trees

212. MeSH descriptor: [Dietary Fiber] explode all trees

213. MeSH descriptor: [Dietary Proteins] explode all trees

214. MeSH descriptor: [Dietary Supplements] explode all trees

215. MeSH descriptor: [Food Additives] explode all trees

216. MeSH descriptor: [Vitamins] explode all trees

217. supplements:ab,ti

218. supplement:ab,ti

219. vitamin*:ab,ti

220. retinol:ab,ti

221. carotenoid*:ab,ti

222. tocopherol:ab,ti

223. folate*:ab,ti

224. “folic acid”:ab,ti

225. methionine:ab,ti

226. riboflavin:ab,ti

227. thiamine:ab,ti

228. niacin:ab,ti

229. pyridoxine:ab,ti

230. cobalamin:ab,ti

231. mineral*:ab,ti

232. sodium:ab,ti

233. iron:ab,ti

234. calcium:ab,ti

235. selenium:ab,ti

236. iodine:ab,ti

237. magnesium:ab,ti

238. potassium:ab,ti

239. zinc:ab,ti

240. copper:ab,ti

241. phosphorus:ab,ti

242. manganese:ab,ti

243. chromium:ab,ti

244. phytochemical:ab,ti

245. polyphenol*:ab,ti

246. phytoestrogen*:ab,ti

247. genistein:ab,ti

248. saponin*:ab,ti

249. coumarin*:ab,ti

250. flavonoid*:ab,ti

251. polyphenol*:ab,ti

252. flavonol*:ab,ti

253. flavone*:ab,ti

254. isoflavone*:ab,ti

255. catechin*:ab,ti

256. “ascorbic acid*”:ab,ti

257. “hydroxy cholecalciferol*”:ab,ti

258. hydroxycholecalciferol*:ab,ti

259. tocotrienol*:ab,ti

260. carotene*:ab,ti

261. cryptoxanthin*:ab,ti

262. lycopene*:ab,ti

263. lutein*:ab,ti

264. zeaxanthin*:ab,ti

265. selenium*:ab,ti

266. “organic diet*”:ab,ti

267. MeSH descriptor: [Food, Organic] this term only

268. 1 or 2 or 3 or 4 or 5 or 6 or 7 or 8 or 9 or 10 or 11 or 12 or 13 or 14 or 15 or 16 or 17 or 18 or 19 or 20 or 21 or 22 or 23 or 24 or 25 or 26 or 27 or 28 or 29 or 30 or 31 or 32 or 33 or 34 or 35 or 36 or 37 or 38 or 39 or 40 or 41 or 42 or 43 or 44 or 45 or 46 or 47 or 48 or 49 or 50 or 51 or 52 or 53 or 54 or 55 or 56 or 57 or 58 or 59 or 60 or 61 or 62 or 63 or 64 or 65 or 66 or 67 or 68 or 69 or 70 or 71 or 72 or 73 or 74 or 75 or 76 or 77 or 78 or 79 or 80 or 81 or 82 or 83 or 84 or 85 or 86 or 87 or 88 or 89 or 90 or 91 or 92 or 93 or 94 or 95 or 96 or 97 or 98 or 99 or 100 or 101 or 102 or 103 or 104 or 105 or 106 or 107 or 108 or 109 or 110 or 111 or 112 or 113 or 114 or 115 or 116 or 117 or 118 or 119 or 120 or 121 or 122 or 123 or 124 or 125 or 126 or 127 or 128 or 129 or 130 or 131 or 132 or 133 or 134 or 135 or 136 or 137 or 138 or 139 or 140 or 141 or 142 or 143 or 144 or 145 or 146 or 147 or 148 or 149 or 150 or 151 or 152 or 153 or 154 or 155 or 156 or 157 or 158 or 159 or 160 or 161 or 162 or 163 or 164 or 165 or 166 or 167 or 168 or 169 or 170 or 171 or 172 or 173 or 174 or 175 or 176 or 177 or 178 or 179 or 180 or 181 or 182 or 183 or 184 or 185 or 186 or 187 or 188 or 189 or 190 or 191 or 192 or 193 or 194 or 195 or 196 or 197 or 198 or 199 or 200 or 201 or 202 or 203 or 204 or 205 or 206 or 207 or 208 or 209 or 210 or 211 or 212 or 213 or 214 or 215 or 216 or 217 or 218 or 219 or 220 or 221 or 222 or 223 or 224 or 225 or 226 or 227 or 228 or 229 or 230 or 231 or 232 or 233 or 234 or 235 or 236 or 237 or 238 or 239 or 240 or 241 or 242 or 243 or 244 or 245 or 246 or 247 or 248 or 249 or 250 or 251 or 252 or 253 or 254 or 255 or 256 or 257 or 258 or 259 or 260 or 261 or 262 or 263 or 264 or 265 or 266 or 267

269. allerg*:ab,ti

270. asthma*:ab,ti

271. wheeze:ab,ti

272. wheezing:ab,ti

273. “bronchial hyperresponsiveness”:ab,ti

274. “bronchial hyperreactivity”:ab,ti

275. “Forced expiratory volume”:ab,ti

276. “FEV1”:ab,ti

277. "FEV 1":ab,ti

278. "FEV0.5":ab,ti

279. "FEV 0.5":ab,ti

280. “Forced vital capacity”:ab,ti

281. FVC:ab,ti

282. “Peak expiratory flow rate”:ab,ti

283. PEFR:ab,ti

284. AD:ab,ti

285. neurodermatitis:ab,ti

286. rhinitis:ab,ti

287. “besniers prurigo”:ab,ti

288. rhinoconjunctivitis:ab,ti

289. hayfever:ab,ti

290. “hay fever”:ab,ti

291. poll*nosis:ab,ti

292. SAR:ab,ti

293. “pollen allergy”:ab,ti

294. conjunctivitis:ab,ti

295. “immunoglobulin e”:ab,ti

296. “Total IgE”:ab,ti

297. “autoimmune disease*”:ab,ti

298. diabetes:ab,ti

299. diabetic:ab,ti

300. “type 1”:ab,ti

301. “c*eliac disease”:ab,ti

302. “crohn* disease”:ab,ti

303. “Inflammatory Bowel Disease*”:ab,ti

304. “Ulcerative colitis”:ab,ti

305. (Lympho* NEAR/3 thyroiditi*):ab,ti

306. (Thyroiditi* NEAR/3 autoimmune):ab,ti

307. (Hashimoto* NEAR/3 (syndrome* or thyroiditi* or disease*)):ab,ti

308. (Thyroiditi* NEAR/3 (post-partum or postpartum)):ab,ti

309. “Graves* disease”:ab,ti

310. “Basedow* disease”:ab,ti

311. “exophthalmic goiter*”:ab,ti

312. (Still* Disease NEAR/3 (juvenile or onset)):ab,ti

313. (Juvenile NEAR/3 arthriti*):ab,ti

314. vitiligo:ab,ti

315. Psorias*s:ab,ti

316. (Arthriti* NEAR/3 Psoria*):ab,ti

317. “atopic disease”:ab,ti

318. “atopic dermatitis”:ab,ti

319. (food* NEAR/3 sensiti*):ab,ti

320. (food* NEAR/3 toleran*):ab,ti

321. (food* NEAR/3 intoleran*):ab,ti

322. ((aero or air*) NEAR/3 allergen*):ab,ti

323. (aeroallergen* NEAR/3 sensiti*):ab,ti

324. (allergen* NEAR/3 sensiti*):ab,ti

325. “skin prick test*”:ab,ti

326. atopy:ab,ti

327. hypersensitiv*:ab,ti

328. MeSH descriptor: [Hypersensitivity] this term only

329. MeSH descriptor: [Food Hypersensitivity] explode all trees

330. MeSH descriptor: [Respiratory Hypersensitivity] this term only

331. MeSH descriptor: [Asthma] this term only

332. MeSH descriptor: [Bronchial Hyperreactivity] this term only

333. MeSH descriptor: [Forced Expiratory Volume] this term only

334. MeSH descriptor: [Vital Capacity] this term only

335. MeSH descriptor: [Peak Expiratory Flow Rate] this term only

336. MeSH descriptor: [AD] this term only

337. MeSH descriptor: [Neurodermatitis] this term only

338. MeSH descriptor: [Rhinitis] this term only

339. MeSH descriptor: [Rhinitis, Allergic, Perennial] this term only

340. MeSH descriptor: [Rhinitis, Allergic, Seasonal] this term only

341. MeSH descriptor: [Conjunctivitis] this term only

342. MeSH descriptor: [Immunoglobulin E] this term only

343. MeSH descriptor: [Autoimmune Diseases] this term only

344. MeSH descriptor: [Diabetes Mellitus, Type 1] this term only

345. MeSH descriptor: [Celiac Disease] this term only

346. MeSH descriptor: [Crohn Disease] this term only

347. MeSH descriptor: [Inflammatory Bowel Diseases] this term only

348. MeSH descriptor: [Colitis, Ulcerative] this term only

349. MeSH descriptor: [Thyroiditis, Autoimmune] this term only

350. MeSH descriptor: [Hashimoto Disease] this term only

351. MeSH descriptor: [Postpartum Thyroiditis] this term only

352. MeSH descriptor: [Graves Disease] this term only

353. MeSH descriptor: [Arthritis, Juvenile Rheumatoid] this term only

354. MeSH descriptor: [Vitiligo] this term only

355. MeSH descriptor: [Psoriasis] this term only

356. MeSH descriptor: [Arthritis, Psoriatic] this term only

357. MeSH descriptor: [Dermatitis, Atopic] this term only

358. MeSH descriptor: [Hypersensitivity, Immediate] this term only

359. 269 or 270 or 271 or 272 or 273 or 274 or 275 or 276 or 277 or 278 or 279 or 280 or 281 or 282 or 283 or 284 or 285 or 286 or 287 or 288 or 289 or 290 or 291 or 292 or 293 or 294 or 295 or 296 or 297 or 298 or 299 or 300 or 301 or 302 or 303 or 304 or 305 or 306 or 307 or 308 or 309 or 310 or 311 or 312 or 313 or 314 or 315 or 316 or 317 or 318 or 319 or 320 or 321 or 322 or 323 or 324 or 325 or 326 or 327 or 328 or 329 or 330 or 331 or 332 or 333 or 334 or 335 or 336 or 337 or 338 or 339 or 340 or 341 or 342 or 343 or 344 or 345 or 346 or 347 or 348 or 349 or 350 or 351 or 352 or 353 or 354 or 355 or 356 or 357 or 358

360. infant*:ab,ti

361. ((one or two or three or four or five or six or seven or eight or nine or ten or eleven or twelve or thirteen or fourteen or fifteen or sixteen or seventeen or eighteen or nineteen or twenty or "twenty one" or "twenty two" or "twenty three" or "twenty four" or "twenty five" or "twenty six") NEAR/1 week*):ab,ti

362. ((one or two or three or four or five or six or seven or eight or nine or ten or eleven or twelve or thirteen or fourteen or fifteen or sixteen or seventeen or eighteen or nineteen or twenty or "twenty one" or "twenty two" or "twenty three" or "twenty four") NEAR/1 month*):ab,ti

363. 361 or 362

364. (old or age*):ab,ti

365. 363 and 364

366. (("one year*" or "two year*") NEAR/3 (old or age*)):ab,ti

367. ((first or second or two) NEAR/3 "year* of life"):ab,ti

368. MeSH descriptor: [Infant] this term only

369. MeSH descriptor: [Infant, Newborn] this term only

370. (maternal or pregnan*):ab,ti

371. (lactat*):ab,ti

372. (mother*):ab,ti

373. 360 or 365 or 366 or 367 or 368 or 369 or 370 or 371 or 372

374. “clinical trial*”

375. random*

376. factorial*

377. crossover*

378. placebo*

379. “doubl* blind*”

380. “singl* blind*”

381. assign*

382. volunteer*

383. “cohort stud*”

384. longitudinal*

385. follow-up

386. prospectiv*

387. retrospectiv*

388. “case control”

389. “case referent”

390. MeSH descriptor: [clinical trial] explode all trees

391. MeSH descriptor: [Cross-Over Studies] this term only

392. MeSH descriptor: [Placebos] this term only

393. MeSH descriptor: [Double-Blind Method] this term only

394. MeSH descriptor: [Single-Blind Method] this term only

395. MeSH descriptor: [Cohort Studies] explode all trees

396. MeSH descriptor: [case-control studies] this term only

397. 374 or 375 or 376 or 377 or 378 or 379 or 380 or 381 or 382 or 383 or 384 or 385 or 386 or 387 or 388 or 389 or 390 or 391 or 392 or 393 or 394 or 395 or 396

398. 268 and 359 and 373 and 397

**Web of Science**

1. TOPIC = (diet$ or “mediterranean diet*” or dietetic or dietary or eat or eating or intake or nutrient$ or nutrition or vegetarian$ or vegan$ or macrobiotic$ or food$ or feed or feeding or cereal$ or grain$ or granary or wholegrain or wholewheat or “whole wheat” or wheat or wheatgerm or rye or barley or oat$ or root$ or tuber$ or vegetable$ or onion$ or spinach or chard or tomato* or pepper$ or carrot$ or beetroot or asparagus or garlic or pumpkin or sprouts or broccoli or cabbage$ or celery or ginger or potato* or crisps or fries or syrup or honey or fruit$ or apple$ or pear$ or banana$ or orange$ or grape$ or kiwi$ or citrus or grapefruit$ or pulses or bean$ or lentil$ or chickpea$ or legume$ or lupin$ or soy or soya or nut$ or almond$ or peanut$ or groundnut$ or seed$ or sesame or mustard or meat$ or beef or pork or lamb or poultry or chicken or turkey or duck or fish* or omega-3 or omega-6 or PUFA or fat$ or fatty or egg$ or bread or oil$ or omega or seafood or shellfish or crustacean$ or mollusc$ or dairy or milk or formula$ or hydrolysed or yoghurt or probiotic$ or prebiotic$ or butter or herb$ or spice$ or chilli* or condiment$ or beverage$ or “fluid intake” or water or drink* or pickled or bottled or canned or canning or smoked or preserved or preservative$ or nitrosamine or hydrogenation or fortified or nitrates or nitrites or ferment* or processed or antioxidant$ or “genetic modif*” or “genetically modif*” or cooking or cooked or grill or grilled or fried or fry or roast or bake or baked or stewing or stewed or casserole* or broil or broiled or boiled or poach or poached or steamed or barbecue$ or chargrill* or salt or salting or salted or fiber or fibre or polysaccharide$ or starch or starchy or carbohydrate$ or lipid$ or “linoleic acid$” or sugar$ or sweetener$ or saccharin$ or aspartame or sucrose or xylitol or cholesterol or “hydrogenated lard” or “dietary protein$” or “protein intake” or “animal protein$” or “total protein$” or “vegetable protein$” or “plant protein$” or supplement$ or vitamin$ or retinol or carotenoid$ or tocopherol or folate$ or “folic acid” or methionine or riboflavin or thiamine or niacin or pyridoxine or cobalamin or mineral$ or sodium or iron or calcium or selenium or iodine or magnesium or potassium or zinc or copper or phosphorus or manganese or chromium or phytochemical or polyphenol$ or phytoestrogen$ or genistein or saponin$ or coumarin$ or flavonoid$ or polyphenol$ or flavonol$ or flavone$ or isoflavone$ or catechin$ or “ascorbic acid$” or “hydroxy cholecalciferol$” or “hydroxycholecalciferol$” or tocotrienol$ or carotene$ or cryptoxanthin$ or lycopene$ or lutein$ or zeaxanthin$ or selenium$ or “organic diet$”)

2. TOPIC = (allerg* or asthma* or wheeze or wheezing or “bronchial hyperresponsiveness” or “bronchial hyperreactivity” or “Forced expiratory volume” or “FEV1” or "FEV 1" or "FEV0.5" or "FEV 0.5" or “Forced vital capacity” or FVC or “Peak expiratory flow rate” or PEFR or AD or neurodermatitis or rhinitis or “besniers prurigo” or rhinoconjunctivitis or hayfever or “hay fever” or poll$nosis or SAR or “pollen allergy” or conjunctivitis or “immunoglobulin e” or “Total IgE” or “autoimmune disease$” or diabetes or diabetic or “type 1” or “c$eliac disease” or “crohn* disease” or “Inflammatory Bowel Disease$” or “Ulcerative colitis” or (Lympho* NEAR/3 thyroiditi*) or (Thyroiditi* NEAR/3 autoimmune) or (Hashimoto* NEAR/3 (syndrome$ or thyroiditis* or disease$)) or (Thyroiditi* NEAR/3 (post-partum or postpartum)) or “Graves$ Disease” or “Basedow* disease” or “exophthalmic goiter$” or (“Still$ Disease” NEAR/3 (juvenile or onset)) or (Juvenile NEAR/3 arthriti*) or vitiligo or Psorias$s or (Arthriti$ NEAR/3 Psoria*) or “atopic disease” or “atopic dermatitis” or (food$ NEAR/3 sensiti*) or (food$ NEAR/3 toleran*) or (food$ NEAR/3 intoleran*) or ((aero or air*) NEAR/3 allergen$) or (aeroallergen$ NEAR/3 sensiti*) or (allergen$ NEAR/3 sensiti*) or “skin prick test*” or atopy or hypersensitive*)

3. TOPIC = (infant$ or (("one year$" or "two year$") NEAR/3 (old or age$)) or ((first or second or two) NEAR/3 "year$ of life") or (maternal) or (pregnan*) or (lactat*) or (mother$))

4. TOPIC = ((one or two or three or four or five or six or seven or eight or nine or ten or eleven or twelve or thirteen or fourteen or fifteen or sixteen or seventeen or eighteen or nineteen or twenty or "twenty one" or "twenty two" or "twenty three" or "twenty four" or "twenty five" or "twenty six") NEAR/1 week$)

5. TOPIC = ((one or two or three or four or five or six or seven or eight or nine or ten or eleven or twelve or thirteen or fourteen or fifteen or sixteen or seventeen or eighteen or nineteen or twenty or "twenty one" or "twenty two" or "twenty three" or "twenty four") NEAR/1 month$)

6. 4 or 5

7. TOPIC = ((old or age$))

8. 7 and 6

9. 8 or 3

10. TOPIC = (“clinical trial$” or random* or factorial* or crossover* or placebo* or “doubl* blind*” or “singl* blind*” or assign* or volunteer* or “cohort stud*” or longitudinal* or follow-up or prospective* or retrospective* or “case control” or “case referent”)

11. 1 and 2 and 9 and 10
