# Supplementary material for: Diet during pregnancy and infancy and risk of allergic or autoimmune disease: A systematic review and meta-analysis
Source: PLoS Med. 2018 Feb 28;15(2):e1002507. doi: 10.1371/journal.pmed.1002507 (PMC5830033; doi:10.1371/journal.pmed.1002507)
Supplement: S2 Text — (DOC) [file pmed.1002507.s003.doc]

**Review of scientific published literature on infant feeding and development of atopic and autoimmune disease**

**Review A: duration of total and exclusive breastfeeding, and timing of solid food introduction**

Robert J Boyle^1^, Vanessa Garcia-Larsen^2^, Despo Ierodiakonou^3^, Jo Leonardi-Bee^4^, Tim Reeves^5^, Jennifer Chivinge^6^, Zoe Robinson^6^, Natalie Geoghegan^6^, Katharine Jarrold^6^, Alisha Ruparelia^6^, Pooja Devani^6^Evangelia Andreou^7^, Nara Tagiyeva-Milne^8^, Ulugbek Nurmatov^9^, Sergio Cunha^10^

**^1^**Clinical Senior Lecturer, Section of Paediatrics, Imperial College London; **^2^**Post-Doctoral Research Associate, Respiratory Epidemiology and Public Health, National Heart and Lung Institute, Imperial College London; **^3^**Post-Doctoral Research Associate, Departments of Paediatric and Respiratory Epidemiology and Public Health Group, Imperial College London. **^4^**Associate Professor of Community Health Sciences, University of Nottingham; **^5^**Research Support Librarian, Faculty of Medicine, Imperial College London; **^6^**Undergraduate medical students, Imperial College London; **^7^**Research Associate, Imperial Consultants; **^8^**Research Fellow, University of Aberdeen; ^9^Research Fellow, University of Edinburgh; **^10^**Research Associate, Respiratory Epidemiology and Public Health, National Heart and Lung Institute, Imperial College London

Imperial Consultants,

58 Princes Gate,

Exhibition Road,

London SW7 2PG

**TABLE OF CONTENTS**

[1. Introduction 4](#__RefHeading___Toc419878098)

[1.1. REVIEW A: Duration of total and exclusive breastfeeding, and timing of solid food introduction 6](#__RefHeading___Toc419878099)

[1.2. Specific review questions addressed in Review A 7](#__RefHeading___Toc419878100)

[1.3. Glossary of key terms 7](#__RefHeading___Toc419878101)

[2. Executive Summary 9](#__RefHeading___Toc419878102)

[2.1 Duration of TBF and EBF, or timing of solid food introduction, and Wheeze 11](#__RefHeading___Toc419878103)

[2.2 Duration of TBF and EBF, or timing of solid food introduction, and Eczema 12](#__RefHeading___Toc419878104)

[2.3 Duration of TBF and EBF, or timing of solid food introduction, and Rhino-conjunctivitis 14](#__RefHeading___Toc419878105)

[2.4 Duration of TBF and EBF, or timing of solid food introduction, and Allergic Sensitisation 15](#__RefHeading___Toc419878106)

[2.5 Duration of TBF and EBF, or timing of solid food introduction, and Food Allergy 16](#__RefHeading___Toc419878107)

[2.6 Duration of TBF and EBF, or timing of solid food introduction, and Type 1 Diabetes Mellitus 17](#__RefHeading___Toc419878108)

[2.7 Duration of TBF and EBF, or timing of solid food introduction, and Other Autoimmune Diseases 18](#__RefHeading___Toc419878109)

[3. Methods 19](#__RefHeading___Toc419878110)

[3.1. Inclusion Criteria 19](#__RefHeading___Toc419878111)

[3.1.1. Types of study included 19](#__RefHeading___Toc419878112)

[3.1.2. Participants/population 20](#__RefHeading___Toc419878113)

[3.1.3. Interventions/ exposures 20](#__RefHeading___Toc419878114)

[3.2. Search strategy 22](#__RefHeading___Toc419878115)

[3.3. Study Outcomes 24](#__RefHeading___Toc419878116)

[3.3.1. Atopic outcomes: 25](#__RefHeading___Toc419878117)

[3.3.2. Autoimmune outcomes: 26](#__RefHeading___Toc419878118)

[3.4. Study selection and data extraction 27](#__RefHeading___Toc419878119)

[3.4.1. Study selection 27](#__RefHeading___Toc419878120)

[3.4.2. Data extraction 28](#__RefHeading___Toc419878121)

[3.4.3. Data cleaning and coding 28](#__RefHeading___Toc419878122)

[3.5. Risk of bias (quality) assessment 31](#__RefHeading___Toc419878123)

[3.5.1. Review level bias 31](#__RefHeading___Toc419878124)

[3.5.2. Study level bias 31](#__RefHeading___Toc419878125)

[3.6. Strategy for data synthesis 32](#__RefHeading___Toc419878126)

[3.6.1. Data extraction 33](#__RefHeading___Toc419878127)

[3.6.2. Heterogeneity 33](#__RefHeading___Toc419878128)

[3.7. Data analysis 33](#__RefHeading___Toc419878129)

[3.7.1. Planned subgroup analyses 34](#__RefHeading___Toc419878130)

[3.7.2. Graphical exploration of heterogeneity 35](#__RefHeading___Toc419878131)

[3.8. Review registration 35](#__RefHeading___Toc419878132)

[3.9. Differences between the protocol and the review 36](#__RefHeading___Toc419878133)

[4. Results 37](#__RefHeading___Toc419878134)

[4.1. Overview of recent high quality systematic reviews 37](#__RefHeading___Toc419878135)

[4.2. Systematic review of original studies 38](#__RefHeading___Toc419878136)

[5. Discussion 39](#__RefHeading___Toc419878137)

[6. Conclusions 40](#__RefHeading___Toc419878138)

[7. Acknowledgements 42](#__RefHeading___Toc419878139)

[References 43](#__RefHeading___Toc419878140)

[Appendix 1 Search Strategies for other systematic reviews 51](#__RefHeading___Toc419878141)

[1.1. Medline 51](#__RefHeading___Toc419878142)

[1.2. Embase 62](#__RefHeading___Toc419878143)

[1.3. COCHRANE Reviews and DARE 73](#__RefHeading___Toc419878144)

[Appendix 2 Search Strategies for original articles (Review A) 83](#__RefHeading___Toc419878145)

[2.1. Medline 83](#__RefHeading___Toc419878146)

[2.2. Embase 87](#__RefHeading___Toc419878147)

[2.3. LILACS 91](#__RefHeading___Toc419878148)

[2.4. COCHRANE Library 92](#__RefHeading___Toc419878149)

[2.5. Web of Science 96](#__RefHeading___Toc419878150)

# Introduction

This is one of 4 reports resulting from a comprehensive review of the scientific literature on infant feeding and the development of atopic and autoimmune diseases, commissioned by the UK Food Standards Agency. Atopic conditions such as asthma, eczema, rhinoconjunctivitis and food allergy appear to have increased in prevalence in recent decades in many countries, and are now the leading causes of chronic illness in children and young adults living in the UK [^3^](#_ENREF_3) [^4^](#_ENREF_4) [^5^](#_ENREF_5). The apparently rapid changes in disease prevalence, combined with data from migration studies, suggest that early-life environmental factors may be important modulators of atopic disease risk. Similar findings apply to the autoimmune diseases type I diabetes mellitus and Crohn’s disease, which appear to have increased in prevalence in some countries [**^6^**](#_ENREF_6). Significant attention has focussed on early-life dietary exposures in relation to these atopic and autoimmune diseases due to the rapid changes in the human diet in recent decades, and the potential effects of such changes on the intestinal and systemic immune development [^7^](#_ENREF_7). The gut associated lymphoid tissue is our largest collection of immune tissue, and our most mature immune organ at the time of birth [**^8^**](#_ENREF_8). Hence enteral exposures in infancy are likely to be especially potent modulators of immune development and risk of immune-mediated disease. Although there are a large number of observational studies, some intervention trials and several systematic reviews in this area, they tend to focus on one specific area of diet and a limited number of immune outcomes. The purpose of this project is to assess comprehensively and systematically the existing literature regarding the relationship between dietary exposures during pregnancy, lactation and infancy, and a child’s risk of developing any of the common atopic and/or autoimmune diseases.

This project consists of a series of systematic reviews which together have very broad inclusion criteria and were registered as 3 separate review protocols on the International Prospective Register of Systematic Reviews (PROSPERO references CRD42013003802 – REVIEW A; CRD42013004239 – REVIEW B; CRD42013004252 – REVIEW C; [www.crd.york.ac.uk/Prospero](http://www.crd.york.ac.uk/Prospero)) on the 5^th^ August 2013. The overall purpose of the work is to inform UK Government feeding guidance for mothers and their infants. The outcomes of this project will be summarised in 4 separate reports, with a distinct set of dietary exposures examined in each report:

1. REVIEW A: DURATION OF TOTAL AND EXCLUSIVE BREASTFEEDING, AND TIMING OF SOLID FOOD INTRODUCTION
2. REVIEW B: TIMING OF INTRODUCTION OF ALLERGENIC FOODS INTO THE INFANT DIET
3. REVIEW C PART I: HYDROLYSED FORMULA IN PLACE OF STANDARD UNHYDROLYSED COW’S MILK BASED INFANT FORMULA, OR BREAST MILK
4. REVIEW C PART II: OTHER MATERNAL AND INFANT DIETARY EXPOSURES

The specific outcomes of interest for all of these reviews, chosen due to their high prevalence in the UK population, and described in more detail below, are:

**Atopic disorders**: Food allergy, Eczema, Asthma, Allergic rhinitis, Allergic conjunctivitis, Allergic sensitisation

**Autoimmune disorders**: Type 1 diabetes mellitus, Coeliac disease, Inflammatory bowel disease, Autoimmune thyroid disease, Juvenile rheumatoid arthritis, Vitiligo, Psoriasis.

- 1. REVIEW A: Duration of total and exclusive breastfeeding, and timing of solid food introduction

This first review focuses on the relationship between breastfeeding and risk of atopic/autoimmune diseases. Human breast milk is the main nutritional exposure during infancy, and usually the sole exposure during the critical early months of immune maturation. Cow’s milk based infant formula has a very different composition to human breast milk, and is deficient in the high concentrations of IgG and IgA antibodies and growth factors, cellular components, cytokines and other immune active factors that influence infant immunity [^9^](#_ENREF_9). The introduction of non-human formula milk, and the introduction of non-milk feeds (here called ‘solid food’) are both associated with significant changes in the intestinal microbiome and gastrointestinal physiology, which in turn influence infant immune development [^10^](#_ENREF_10). Through their impact on immune development, the exposures assessed in this report may in theory be able to influence a developing child’s risk of atopic or autoimmune disorders both during feeding and through the life course. Thus this first report addresses the question whether duration of total or exclusive breastfeeding, and/or timing of solid food introduction influence the development of atopic or autoimmune disorders.

- 1. Specific review questions addressed in Review A

A1. Does the total duration of breastfeeding up to 2 years, influence children’s future risk of atopic disease, allergic sensitisation or autoimmune disease?

A2. Does the duration of exclusive/predominant breastfeeding during the first year of life, influence children’s future risk of atopic disease, allergic sensitisation or autoimmune disease?

A3. Does the timing of introduction of solid food during the first year of life, influence children’s future risk of atopic disease, allergic sensitisation or autoimmune disease?

- 1. Glossary of key terms

**Allergic sensitisation**: production of specific IgE antibodies directed against harmless environmental antigens such as pollens, mites, milk, egg or peanut; or production of increased serum total IgE levels. Allergic sensitisation is strongly associated with Atopic disease.

**Atopic disease**: chronic health conditions associated with (but not always directly caused by) the production of IgE antibodies to harmless environmental antigens.

**Exclusive breastfeeding duration (EBF)**: age at which another food other than human breast milk (but excluding oral rehydration solution, vitamins, minerals or medications) is introduced into the infant diet.

**Predominant breastfeeding duration**: age at which another food other than human breast milk (but excluding oral rehydration solution, vitamins, minerals, medications, water, water-based drinks or fruit juice) is introduced into the infant diet.

**Solid food introduction**: time at which the first non-milk food is introduced into the infant diet eg rice cereal, pureed vegetables or fruits.

**Total breastfeeding duration (TBF)**: time until all breastfeeding has ceased.

**GRADE evaluation of evidence**: grade of evidence in this report is assigned using the GRADE system, which has 4 categories HIGH, MODERATE, LOW or VERY LOW. Evidence is initially assigned as HIGH if coming from a randomised trial; LOW from observational studies; VERY LOW from other evidence. The grade of evidence is then reduced if there are serious (-1) or very serious (-2) limitations to study quality or uncertainties about directness of association; important inconsistency (-1), imprecise or sparse data (-1) or a high probability of reporting bias (-1). Grade of evidence is increased if strong evidence of association is seen (eg RR >2 or <0.5) from ≥2 observational studies with no plausible confounders (+1) or very strong direct evidence (RR >5 or <0.2) with no major threats to validity (+2); if there is evidence of a dose-response gradient (+1) or if all plausible confounders would have reduced the effect/association seen (+1). The interpretation of GRADE evidence assessments is that for HIGH level evidence further research is very unlikely to change our confidence in the estimate of effect; for MODERATE evidence further research is likely to have an important impact on our confidence in the estimate of effect and may change the estimate; for LOW level evidence further research is very likely to have an important impact on our confidence in the estimate of effect and is likely to change the estimate; and for VERY LOW level evidence any estimate of effect is very uncertain. Further detailed explanation of GRADE can be found at:

<http://www.gradeworkinggroup.org/publications/Grading_evidence_and_recommendations_BMJ.pdf>

# Executive Summary

The key findings of this review are summarised below, in relation to each of 7 outcome domains of interest – wheeze (including asthma and lung function), eczema, rhino-conjunctivitis, allergic sensitisation, food allergy, type 1 diabetes mellitus, and all other autoimmune diseases. We used the GRADE approach to assessing the strength of evidence, and specify in the text of the Executive Summary where judgements have been made that result in lower or higher grade evidence [^11^](#_ENREF_11).

This is followed by a summary of the Methods used to identify studies, extract, analyse and appraise data, and a Discussion of the main findings in the context of existing literature. The 7 separate systematic reviews which provide the basis for this Executive Summary and constitute the substance of REVIEW A, can be accessed from Table 1 below.

**Table 1 Individual study reports in relation to 7 outcome domains of interest.**

| **Outcome** | **Report** |
| --- | --- |
| Wheeze |  |
| Eczema |  |
| Rhino-conjunctivitis |  |
| Food Allergy |  |
| Allergic Sensitisation |  |
| Type 1 Diabetes Mellitus |  |
| Other Autoimmune Diseases |  |

# Duration of TBF and EBF, or timing of solid food introduction, and Wheeze

Data from over 500,000 participants in 100 studies contributed to this report. In the single randomised controlled trial (RCT) of a breastfeeding promotion intervention, there was no evidence for an effect on wheeze at age 1 year or 6.5 years. In the observational studies, there was an association between increased TBF duration and reduced risk of wheeze (TBF ever vs never at age 5-14, and TBF ≥3-4 months at age 15+), and recurrent wheeze (TBF ≥3-4 months at age 0-4; TBF ever vs never, ≥3-4, ≥18 months at age 5-14). There was no evidence for reduced risk at other cut-offs and ages. There was evidence of publication bias for TBF and wheeze/recurrent wheeze at aged 5-14 (GRADE -1; reporting bias). EBF ≥0-2 months was associated with reduced wheeze at ages 0-4 and 5-14, but not recurrent wheeze, and no consistent relationships were seen with other EBF durations. Some EBF analyses had high statistical heterogeneity, and some evidence of publication bias (GRADE -2; inconsistency, reporting bias). We did not find evidence for an association between timing of solid food introduction and asthma/wheeze. There was no consistent evidence that increased TBF and EBF duration are associated with bronchial hyper-reactivity or measures of lung function.

**Conclusion**:

Increased TBF duration may be associated with reduced risk of recurrent wheeze at age 5-14.

*Grade of evidence: VERY LOW*.

There is no evidence for an association between TBF or EBF duration, or timing of solid food introduction, and other measures of wheezing, bronchial hyper-reactivity or lung function.

# Duration of TBF and EBF, or timing of solid food introduction, and Eczema

Data from over 250,000 participants in 90 studies contributed to this report. Some analyses were limited by small numbers of studies reporting relevant data, especially for EBF and timing of solid food introduction. One RCT of a breastfeeding promotion intervention found reduced eczema at age 1 year (OR 0.54 95% CI 0.31, 0.95; GRADE -2; imprecision, inconsistency) but not at age 6.5 years. . From the observational studies, we found no consistent evidence that duration of TBF or EBF, or timing of solid food introduction, are associated with risk of eczema. Some analyses suggested an association in the opposite direction i.e. increased duration of TBF and EBF were associated with increased eczema risk at age 0-4 (TBF ever vs never), at age 5-14 (TBF ≥1-2, ≥3-4, ≥5-7 and ≥8-12 months; EBF ≥0-2 months) and at age 15+ (TBF ≥3-4 months; EBF ≥0-2 months). Several of these analyses were dominated by one study, albeit one with low risk of bias. Other analyses showed either no evidence of association, or reduced eczema risk with increased breastfeeding duration (for EBF≥3-4 months only, and eczema at age 0-4). These associations were not seen in studies which couldn’t be included in meta-analysis, and subgroup analysis for TBF found no association in studies with low risk of bias (GRADE -2; inconsistency, study quality). Finally, very few studies failed to make allowance in their analyses for the possibility of reverse causation ie prolonged TBF/EBF when infants develop eczema, due to concerns about associated food allergy. We found no association between timing of solid food introduction and eczema risk.

**Conclusion**:

A breastfeeding promotion intervention may reduce risk of eczema in the first year.

*Grade of evidence: LOW*.

There is no consistent evidence from observational studies, for an association between TBF and EBF duration, or timing of solid food introduction, and risk of developing eczema.

# Duration of TBF and EBF, or timing of solid food introduction, and Rhino-conjunctivitis

Data from over 200,000 participants in 33 studies contributed to this report. Overall we found no evidence to support an association between duration of TBF or EBF and rhinoconjunctivitis (RC) risk at age 0-4, 5-14 or 15+. In the single RCT of a breastfeeding promotion intervention, there was no evidence for an effect on RC at age 6.5 years.. One meta-analysis of 5 observational studies (18,000 participants) found reduced RC risk at age 0-4 years associated with EBF ≥ 3-4 months (OR 0.77 95% CI 0.60, 0.99), with no statistical heterogeneity. However statistical significance was borderline, and we were unable to confirm this association using other cut-offs for EBF duration or at ages 5-14 or 15 years and older (GRADE -2 imprecision; -1 inconsistency). Data for timing of solid food introduction and disease risk were limited, but do not support an association.

**Conclusion**:

There is no consistent evidence for an association between TBF duration, EBF duration or timing of solid food introduction and RC.

# Duration of TBF and EBF, or timing of solid food introduction, and Allergic Sensitisation

Data from over 30,000 participants in over 40 studies contributed to this report. Overall we found no clear evidence to support an association between TBF, EBF or timing of solid food introduction and allergic sensitisation. In the single RCT of a breastfeeding promotion intervention, there was no evidence for an effect on RC at age 6.5 years. From the observational studies, one meta-analysis of 3 prospective cohort studies (2000 participants) found increased risk of food AS associated with EBF ≥0-2 months with no statistical heterogeneity, and one study from that meta-analysis also reported increased risk of food AS with TBF ever vs never. However, there was no association seen in analysis of other age cut-offs for TBF or EBF duration and food AS, which included similar numbers of participants than the positive analyses. There was also no association between TBF/EBF and other forms of allergic sensitisation (i.e. milk, egg or peanut individually, to aeroallergens or to ‘any allergen’), or in studies which could not be included in meta-analysis (GRADE -2; inconsistency, imprecision). The possibility of reverse causality was explored in one of the 3 positive studies, and one negative study, with no evidence found that the outcome was influencing the exposure however reverse causality cannot be excluded in the other studies.

**Conclusion**:

There is no evidence for an association between TBF and EBF duration, or timing of solid food introduction, and risk of developing allergic sensitisation.

# Duration of TBF and EBF, or timing of solid food introduction, and Food Allergy

Data from over 70,000 participants in 29 studies contributed to this report. Food allergy was defined using medical assessments with allergy testing and clinical history in most cases, using oral food challenges in some, and parental report of food allergic reaction(s) alone in others. No intervention trial was identified, which evaluated food allergy as an outcome. From the observational studies we found some evidence to support an association between TBF ever vs never and increased risk of food allergy, but the data were mainly unadjusted, associations were not seen in other analyses of TBF, and this finding is vulnerable to reverse causation, since the 2 included studies did not undertake restricted analysis excluding cases of food allergy which arose during the breastfeeding period (GRADE -2; inconsistency, study quality).

The available data did not suggest any association between EBF duration or timing of solid food introduction and food allergy.

**Conclusion**:

There is no evidence for an association between TBF and EBF duration, or timing of solid food introduction, and risk of developing food allergy.

The evidence is limited by small numbers of studies and participants, often without appropriate statistical adjustments, so that important associations cannot be confidently excluded for this outcome.

# Duration of TBF and EBF, or timing of solid food introduction, and Type 1 Diabetes Mellitus

Data from over 15,000 participants in over 60 studies contributed to this report. Most studies were case-control studies, some of which were nested in prospective cohorts. Overall we found evidence that longer duration of TBF at all ages from birth to 1 year, and longer duration of EBF at all ages from birth to 4 months, is associated with reduced diabetes risk. For TBF duration there was moderate/high statistical heterogeneity, and for both TBF and EBF a stronger association was seen between breastfeeding duration and TIDM risk in the retrospective studies (GRADE -1; inconsistency). Prospective studies often used surrogate serological diagnosis of TIDM at an early age, but recall bias in the retrospective studies must also be considered as an explanation for the difference in findings between prospective and retrospective studies. We found no evidence for differences between adjusted and unadjusted data, and plausible confounders such as socioeconomic status would be expected to lead to an association between longer duration of breastfeeding and *increased* TIDM risk, since both are associated with higher socioeconomic status in many settings (GRADE +1; confounding). So we consider that the risk of the association between TIDM and breastfeeding duration being due to hidden confounding is low. We found no association between timing of solid food introduction and diabetes risk.

**Conclusion**:

Total and exclusive breastfeeding duration are associated with reduced TIDM risk.

*Grade of evidence: LOW*.

There is no evidence for that timing of solid food introduction influences TIDM risk.

# Duration of TBF and EBF, or timing of solid food introduction, and Other Autoimmune Diseases

Data from over 250,000 participants in 12 studies contributed to assessment of coeliac disease; 13,000 participants/ 13 studies inflammatory bowel disease; 1200 participants/ 3 studies juvenile rheumatoid arthritis; 189 participants/ 1 study thyroid disease. We did not identify studies for vitiligo/psoriasis. Most included studies were case-control design. We found shorter TBF or EBF duration in those with coeliac disease, but data were often unadjusted, and there was high statistical heterogeneity due to differences between the 2 prospective studies (100 cases) with no association, and 6 retrospective studies (1100 cases) which found reduced coeliac disease with longer TBF or EBF (GRADE -2; inconsistency, study quality). A further 1 prospective (50 cases) and 3 retrospective studies (220 cases) found no association but could not be included in meta-analyses due to the way the data were reported.

Two retrospective studies (570 cases) reporting unadjusted data found reduced risk of Crohn’s disease with TBF ≥5-7 months and ≥3-4 months. There was no association at ≥1-2 months, or ever vs never; nor with EBF or solid food introduction, and no association between Ulcerative colitis and TBF/EBF or solid food introduction. Data for arthritis and thyroid disease were non-significant, but limited by low study/participant numbers.

**Conclusion**:

There is no evidence for an association between TBF and EBF duration, or timing of solid food introduction, and risk of autoimmune diseases.

# Methods

## 3.1. Inclusion Criteria

### 3.1.1. Types of study included

We included recent high quality systematic reviews published from 2011 until the search date (25^th^ July 2013; updated on 26^th^ February 2017). Older systematic reviews were not included, due to the likelihood of being out of date. We quality assessed eligible systematic reviews using the revised AMSTAR criteria [^12^](#_ENREF_12) and extracted data from systematic reviews with revised AMSTAR score ≥32. A summary of the identified reviews is included at the end of this report, and relevant review findings are included in each of the 7 individual systematic reviews that comprise Review A, as appropriate.

We included other research studies published at any time prior to the search date (25^th^ July 2013; updated on 26^th^ February 2017for intervention trials only). Original studies eligible for inclusion were randomised controlled trials (RCT), quasi RCT (RCT where the allocation sequence was predictable but not thought likely to lead to imbalance), controlled clinical trials (CCT where the allocation sequence was predictable, and thought likely to lead to significant imbalance between groups in important risk factors for the outcome(s) of interest), prospective cohort or longitudinal studies, retrospective cohort studies, nested case-control studies, other case control studies and cross-sectional surveys. We took a hierarchical approach to study design, such that where data were absent or limited from systematic reviews or intervention trials, we included observational study data. Where a large number of intervention trials were identified, we did not analyse data from observations studies that assessed the same intervention/exposure. We did not include non-comparative studies, or non-human studies.

### 3.1.2. Participants/population

*Inclusion criteria*: Infants between birth and the end of their 12^th^ post-partum month (questions A2 and A3) or birth and the end of the 24^th^ post-partum month (question A1). The reason for this is that the primary focus of the review is the first year of life, which encompasses food introduction and the end of exclusive breastfeeding in most circumstances, but total breastfeeding duration is recommended to be at least 2 years by the World Health Organisation (WHO). If infants were characterised as high or normal/low risk for atopic or autoimmune disease based on family history or genotype, this information was be recorded so that it could be used for the planned subgroup analysis by disease risk.

*Exclusion criteria*: We excluded studies in which participants were defined by a disease state - e.g. pregnant women with specific nutritional deficiencies, infants born prematurely (<31 weeks gestation) or other groups clearly representing <5% of the UK population, since the results of this review should apply to the general UK population. We did not exclude studies on the basis of including specific ethnic groups, studies of high risk infants, since this applies to many UK-born infants for allergic disease, and it is difficult to undertake studies of autoimmune disease prevention in the general population without stratifying by genetic/family risk due to the relatively low prevalence of autoimmune diseases.

### 3.1.3. Interventions/ exposures

*3.1.3.1. Review question A1*

Timing of the total duration of breastfeeding (i.e. time to complete cessation of breastfeeding). We extracted data for total breastfeeding duration when reported either as a continuous variable, and when reported categorically.

*3.1.3.2. Review question A2*

Timing of the transition from exclusive/predominant breastfeeding (as defined by WHO), to partial or no breastfeeding (i.e. introduction of a complementary food including infant formula). We extracted data for exclusive breastfeeding duration reported either as a continuous or categorical variable. Where possible, we planned to characterise studies as defining exclusive/predominant breastfeeding according to the WHO definition, or not, so that this information could be used for the planned subgroup analysis by breastfeeding definition.

*Exclusive breastfeeding* is defined by WHO as ‘no other food or drink, not even water, except breast milk (including milk expressed or from a wet nurse) other than oral rehydration solution, and drops/syrups (vitamins, minerals and medicines)’.

*Predominant breastfeeding* is defined by WHO as meaning ‘the infant's predominant source of nourishment is breast milk (as defined above), but they may also receive liquids (water and water-based drinks, fruit juice) and ritual fluids in addition to those permitted under exclusive breastfeeding.’

The vast majority of studies define exclusive breastfeeding as the period where the main source of feeding is breast milk without other milk feed or complementary food. Thus, in this report the term exclusive breastfeeding is the equivalent of predominant breastfeeding as defined by WHO. Where possible, we planned to characterise studies as defining predominant breastfeeding according to the WHO definition, or not, so that this information could be used for the planned subgroup analysis by breastfeeding.

*3.1.3.3. Review question A3*

Timing of the transition from liquid infant milk feed (i.e. breast milk and/or infant formula) to the introduction of other complementary foods. We extracted data for this exposure, termed ‘solid food’ when reported either as a continuous variable, or categorically.

## 3.2. Search strategy

The search strategies included both text terms and subject heading terms where appropriate. The search strategies were initially developed for use on the MEDLINE database and then adapted for use on other databases. We searched the following databases, with no specified start date:

- The Cochrane Library (2013, Issue 7)
- EMBASE (1947 to July 2013)
- LILACS (1982 to July 2013)
- MEDLINE (1946 to July 2013)
- Web of Science (1970 to July 2013)

The search was run on 25^th^ July 2013 and included all studies published up to that date, and was updated on 26^th^ February 2017for intervention trials and systematic reviews. We included peer reviewed publications, and abstract publications if they contained data that had not subsequently been published as a peer reviewed publication. We reviewed the bibliography of eligible studies for possible additional publications, and included all eligible publications, regardless of the language. We did not contact the authors of eligible or potentially eligible studies to request original data. The search strategies were extensively piloted and refined to optimise sensitivity, comparing search results with those of other high quality published systematic reviews. The final search strategies for review A are listed at the end of this document, as *Appendices*.

The search for existing systematic reviews which cover any of the same exposure(s)/outcome(s) as the original studies was limited to publications from 1^st^ January 2011 to 25^th^ July 2013 in the original search, and to 26^th^ February 2017in the update. The search strategy was partly based on the search strategies used for Review A, Review B and Review C but included a search filter for retrieving systematic reviews (Lee et al., 2012). Open Grey was searched using the terms ‘(breast OR lactation OR formula) AND (allergy OR autoimmune OR asthma OR eczema OR rhinitis OR conjunctivitis OR food allergy OR vitiligo OR psoriasis OR arthritisi OR thyroiditis OR atopy OR IgE OR diabetes OR coeliac OR inflammatory bowel disease)’ for studies relevant to Review A; the terms ‘(wean OR peanut OR egg OR milk OR soya OR nut OR fish OR wheat) AND (allergy OR autoimmune OR asthma OR eczema OR rhinitis OR conjunctivitis OR food allergy OR vitiligo OR psoriasis OR arthritisi OR thyroiditis OR atopy OR IgE OR diabetes OR coeliac OR inflammatory bowel disease)’ for studies relevant to review B; the terms ‘(lactation OR pregnancy OR infant OR mother) AND (allergy OR autoimmune OR asthma OR eczema OR rhinitis OR conjunctivitis OR food allergy OR vitiligo OR psoriasis OR arthritisi OR thyroiditis OR atopy OR IgE OR diabetes OR coeliac OR inflammatory bowel disease)’ for studies relevant to review C.

The International Prospective Register of Systematic Reviews (PROSPERO) database was also searched for relevant systematic reviews. Due to the limited functionality of this resource individual keywords with date limits were used to search PROSPERO: we searched for titles containing ‘breast OR infant OR lactation OR wean OR infant’ for studies relevant to review A; ‘nut OR wheat OR egg OR food OR diet’ for studies relevant to review B; ‘pregnant OR infant OR diet OR nutrition OR supplement’ for studies relevant to review C.

The citations identified in searches were imported into Endnote libraries for de-duplication and title screening.

## 3.3. Study Outcomes

We selected atopic and autoimmune outcomes on the basis of their population prevalence in children and young adults in the UK. We included diseases with a prevalence of at least 1 in 1000, in children/adolescents or young adults (aged <40 years), but did not include rarer diseases [^13^](#_ENREF_13). We did not include pernicious anaemia or adult-onset rheumatoid arthritis despite a high prevalence in middle aged or elderly people, because their prevalence in young people is lower than 1 in 1000, and prospective studies of infant feeding in relation to diseases of older adults are unlikely to have been undertaken. We did not specifically exclude rare manifestations of food allergy such as eosinophilic oesophagitis, if they were reported as part of a food allergy definition, but did exclude them if they were reported as a unique outcome measure since their prevalence is less than 1 in 1000. For atopic outcomes, age at assessment was grouped as 1-4 years, 5-14 years, 15-24 years, 25-44 years, 45-64 years and ≥65 years. Due to a paucity of studies in adults, we pooled all age groups ≥ 15 years for almost all reports. For autoimmune outcomes, we did not stratify analyses by age at outcome assessment. Where studies reported the same outcome at different timepoints within one of these frames, we used the timepoint with the most complete dataset ie lowest percentage of missing data, as the primary assessment point for inclusion in meta-analysis. Where possible we chose a timepoint for outcome assessment that did not fall within the relevant exposure period i.e. first 1 year for A2 and A3, first 2 years for A1. For each outcome measure in this review, there is more than one possible method of assessment. We therefore included our preferred method of assessment for each outcome, which is the *a priori*  ‘primary outcome measure’, assessed at the optimal age as defined above.

### 3.3.1. Atopic outcomes:

*1. Asthma/Wheeze* - defined as either ‘asthma’, ‘infantile wheeze’ or similar, using parent/self-report, doctor diagnosis, a validated questionnaire, scoring system or objective measure such as bronchial hyper-reactivity, forced vital capacity, peak expiratory flow rate or reversible airways obstruction using forced expiratory volume in 1 second. We included data for ‘atopic’ asthma/wheeze i.e. wheeze associated with allergic sensitisation, and for recurrent wheezing and atopic recurrent wheezing. We did not include different wheezing entities based on the timing of onset/resolution of the disease such as ‘early transient wheeze’ or ‘persistent wheeze’ due to heterogeneity in definition between studies. We did not include outcomes such as ‘bronchitis’ or ‘bronchiolitis’ which included some subjects with wheezing but others without wheezing.

*2. Eczema* – defined using parent/self-report, doctor diagnosis, a validated questionnaire, scoring system or objective measure. We included data for ‘atopic’ eczema i.e. eczema associated with allergic sensitisation. We did not include reports of rashes which were likely to have included other cutaneous problems, such as nappy rash, contact dermatitis ‘rash’, ‘skin problem’ etc, but did include reports of ‘recurrent itchy rash in infancy’ or similar descriptions which were likely to represent eczema.

*3. Allergic Rhinoconjunctivitis* – defined using parent/self-report, doctor diagnosis, a validated questionnaire, scoring system or objective measure. We included data for ‘atopic’ rhinoconjunctivitis i.e. rhinoconjunctivitis associated with allergic sensitisation. We included data for ‘allergic rhinitis’, ‘allergic conjunctivitis’ or ‘allergic rhinoconjunctivitis’ and planned to analyse ‘allergic conjunctivitis’ separately where data were reported separately.

*4. Food allergy* - defined by double blind placebo controlled food challenge, by open food challenge, by medical diagnosis or by self/parent report. We included reports of ‘any food allergy’, and specific food allergies to cow’s milk, egg or peanut. We did not include reports of ‘food intolerance’ that we judged were unlikely to meet current definitions of food allergy [^14^](#_ENREF_14).

*6. Allergic sensitisation* – to an inhalant, an ingestant, or both – defined as positive skin prick test and/or specific IgE test to the relevant allergen using recognised methodologies and scoring criteria [^15^](#_ENREF_15). We combined data for skin prick and specific IgE testing due to limited numbers of studies available for each meta-analysis, and assessed ‘any allergic sensitisation’, ‘food allergic sensitisation’, ‘aeroallergen sensitisation’, ‘cow’s milk sensitisation’, ‘egg sensitisation’ and ‘peanut sensitisation’ separately. We included Total IgE data when measured using a recognised technology such as ImmunoCAP (ThermoFisher, Massachusets).

### 3.3.2. Autoimmune outcomes:

*1. Type I diabetes mellitus* – defined as a medical diagnosis e.g. using the 1999 WHO recommendations for diagnosis and classification of diabetes mellitus [^16^](#_ENREF_16), or a surrogate marker such as autoantibodies against insulin, GAD65, IA-2 or the ZnT8 transporter in the first 3 years of life. We did not include reports where the outcome was stated as ‘diabetes’ and thought likely to include some cases of type II diabetes mellitus or other disease entities.

*2. Coeliac disease* – defined by characteristic histological features (intraepithelial lymphocytes, crypt hyperplasia and villous atrophy) with improvement in symptoms and histology after institution of a gluten free diet, a medical diagnosis, or a surrogate marker such as IgA tissue transglutaminase or IgA endomysial antibodies.

*3. Inflammatory bowel disease* (Crohn's disease or Ulcerative colitis) – defined as a medical diagnosis.

*4. Autoimmune thyroid disease* (Graves' disease or Hashimoto's thyroiditis) - defined as a medical diagnosis.

*5. Juvenile rheumatoid arthritis* – defined as a medical diagnosis eg using the 2001 revised International League of Associations for Rheumatology (ILAR) classification criteria [^17^](#_ENREF_17).

*6. Vitiligo* - defined as a medical diagnosis.

Primary assessment: medical diagnosis using the Vitiligo European Task Force 2007 criteria or similar [^18^](#_ENREF_18).

*7. Psoriasis* - defined as a medical diagnosis.

## 3.4. Study selection and data extraction

### 3.4.1. Study selection

Title and abstract screening was undertaken in duplicate by a team of 7 researchers (RB, VGL, DI, NG, KJ, JC, ZR). Two researchers undertook title screening independently, and met to agree included and excluded titles. Their screening was checked by a third member of the team, and uncertainties were brought to a full team meeting for discussion. This procedure took place between February and April 2014, with weekly team meetings to discuss uncertainties about study eligibility, and again in April 2017. The full text of all potentially eligible studies was reviewed, and where electronic copies were not available, hard copies of articles were ordered from the British Library.

### 3.4.2. Data extraction

An Excel data extraction form was developed, piloted and refined by DI, VGL, RB and JL-B – separate forms were used for intervention studies, cohort studies and case control studies. Data extraction was undertaken in duplicate by a team of 8 researchers (DI, RB, UN, SC, VGL, NT-M, NG, EA). Disagreements and uncertainties about data coding were discussed within the team with leads as follows - RB (clinical queries), VGLA (dietetic queries), DI (analysis and coding queries) and JL-B (study design and statistics queries). For foreign language studies, data were extracted by VGL together with a native speaker of the relevant language (see Acknowledgements section). We extracted all relevant data from included studies, including data that could not (not appropriately reported) or would not (see ‘data cleaning below) be included in meta-analysis, text information such as ‘no significant association found’, and information that adjusted or unadjusted analyses were performed but not reported.

### 3.4.3. Data cleaning and coding

Data were extensively cleaned and coded for analysis with further data checks to identify publications related to the same parent study, and to identify the most appropriate output for inclusion in meta-analysis from studies reporting multiple assessments of closely related exposures/outcomes at the same age in the same population. Data cleaning was undertaken by DI, NG and RJB. Data on duration of breastfeeding and timing of solid food introduction were extracted independent of exposure cut-offs used in the studies. However, in order to have homogeneous exposure reference group(s), data were only included in meta-analysis where the reference group (cut-off) of breastfeeding duration (or timing of solid food introduction) was complete i.e. ‘less than’ a certain duration, or in some cases ‘never breast fed’. Studies evaluating timing of cow’s milk or other specific food introduction into the infant diet, or comparing different types of infant formula milk, are included in separate reports – REVIEWS B, REVIEW C PART I, AND REVIEW C PART II.

Where more than one exposure group was compared with the reference group (≤ than a specific cut-off) in relation to the same outcome at the same age, we chose the exposure furthest from the cut-off point. For example, a study reporting the relationship between total breastfeeding duration and wheeze at age 2 years, with data for ≤4 versus 5-7 and ≤4 versus >7 months duration, we would include the comparison ≤4 versus >7 months. This would be grouped for meta-analysis with studies comparing ≤4 versus >4 months duration. We used the following exposure cut-offs for total duration of breastfeeding, which were selected based on the distribution of the data presented in published reports so as to maximise our ability to undertake meta-analysis: never vs. any (and where possible a dose response of never vs. short (≥1-3 months)/ never vs. intermediate (≥4-6 months) / never vs. long (≥7-12 months) duration); ≤ 1-2 vs. >1-2; ≤3-4 vs >3-4; ≤5-7 vs.>5-7 ; ≤8-12 vs. >8-12 and ≤ 12-24 vs. >12-24 months duration. For duration of exclusive breastfeeding and timing of solid food introduction we again grouped studies according to the distribution of data presented in study reports - using groupings of ≤0-2 vs. >0-2; ≤3-4 vs >3-4 and ≤5 vs >5 months duration for exclusive breastfeeding, and ≤3-4 vs >3-4 months for timing of solid food introduction. Data that could not fit in any group were not included in meta-analysis, but were reported narratively. The outcomes of both meta-analysed and narratively reported studies were considered together when interpreting data and making conclusions.

From individual studies reporting more than one measure for the same outcome, we selected data for analysis reporting time to event (hazard ratio) in preference to cumulative incidence or lifetime prevalence i.e. ‘disease ever’, in turn in preference to point prevalence data i.e. ‘disease in the last 12 months’ for all binary outcomes with the exception of the non-clinical outcomes allergic sensitisation and lung function, where point prevalence was analysed in preference to cumulative measures.

For allergic outcomes we grouped studies reporting outcome at ages 0-4, 5-15 and 15+ years. If a study reported associations (within or between publications) at more than one age within the same age group (i.e. age 1 and 3 years), we selected data for analysis within specific age groups that were most complete i.e. had the largest number of participants assessed. We also included data from longitudinal models (e.g. generalised estimating equation), and they were grouped according to the last age included in the model. Where appropriate we also considered the outcomes reported at other ages which were not included in meta-analysis, in our interpretation of the data. Age groups were not used for autoimmune diseases. Where different methods of outcome assessment were used within a study we prioritised validated and patient-centred outcomes – for example we prioritised clinical diagnosis of diabetes over diabetes-associated autoantibody detection; we prioritised patient or parent-reported wheeze using a validated instrument such as the ISAAC questionnaire, over doctor diagnosis of wheeze or study physician assessment. Again, where appropriate the impact of these decisions was taken into account in our interpretation of findings.

For included studies which did not report numerical data in a form that could be included in meta-analysis, for example medians, or means without a standard deviation or standard error, or ‘no significant difference’ statements, we summarised the findings in a ‘narrative table’. Each of the 7 systematic reviews contains a single narrative table for each of the 3 exposures of interest – A1. Total breastfeeding duration; A2. Exclusive (predominant) breastfeeding duration; A3. Solid food introduction.

## 3.5. Risk of bias (quality) assessment

### 3.5.1. Review level bias

Publication bias was assessed using funnel plots and Egger's test, for those meta-analyses with ≥10 studies included. Possible causes for asymmetry other than publication bias (e.g. between study heterogeneity, small study effects) were also considered. We also took into consideration both the outcomes of meta-analyses and the findings of studies not included in meta-analysis, when interpreting systematic review outcomes.

### 3.5.2. Study level bias

The risk of bias in included intervention studies was assessed using a modified version of the Cochrane Collaboration Risk of Bias tool, which assessed sequence generation and allocation concealment (Selection Bias), blinding of outcome assessors and validity of outcome assessment tool (Assessment Bias), incomplete outcome data (Attrition Bias – considered high where <70% of randomised participants had outcome data available) [^19^](#_ENREF_19). RCTs were considered at low overall risk of bias where the risk of bias was judged to be low for all 3 key domains selection, assessment and attrition bias. The risk of bias in included cohort and case control studies was assessed using a modified version of the National Institute for Clinical Excellence methodological checklist for cohort and case-control studies respectively [^20^](#_ENREF_20). Key domains were Selection Bias (low if cases and controls were selected from similar populations, if the participation rate was ≥80%, or <80% but investigators explored and adjusted for characteristic differences between participants and non-participants), Assessment Bias (low if validated and reliable tools were used to assess exposure and/or outcome), and Confounding Bias (low if most likely confounders are identified and taken into account in study design and analysis). Observational studies were considered at low overall risk of bias where the risk of bias was judged to be low for all 3 key domains selection, assessment and confounding bias. For assessment of Confounding Bias, factors that we expected to be adjusted for within studies of allergic outcomes were: siblings (parity or birth order or family size); gender; age at outcome assessment; disease risk based on family history; maternal or household smoking (asthma/wheeze outcomes); maternal age; maternal education or socioeconomic status; mode of delivery. For studies on autoimmune outcomes we expected matching and/or adjusting for gender, age, address, socioeconomic status, smoking and disease risk. For all studies we also assessed possible Conflict of Interest, judged as low where there was no evidence of industry involvement in study design, analysis, interpretation or publication, and no evidence that study authors receive remuneration from relevant industry partners for other activities. For all study reports, we created a summary Table of Study Characteristics with key study features, and a separate summary Risk of Bias Figure showing the risk of bias for all included studies – whether included in meta-analyses or reported in the narrative table.

## 3.6. Strategy for data synthesis

Meta-analysis was undertaken where ≥2 studies reported the same outcome for a given exposure. Where meta-analysis was deemed inappropriate due to differences in population, exposure/intervention or outcome; or where meta-analysis was not possible due to the nature of the data reported - individual study results were summarised in a narrative table at the end of each report. Separate analyses were undertaken for each disease outcome, for each (age) group of similar outcome assessment methods for any given disease, and for each intervention/exposure (group). In general our approach to meta-analysis was inclusive, with data pooled for maximum statistical power, but explored for important sources of statistical or clinical heterogeneity. Results for randomised or quasi-randomised controlled trials were pooled separately from controlled clinical trials, and observational studies were pooled with a planned subgroup analysis of those with prospective (cohort or nested case control) or retrospective (other case control or cross-sectional) design.

### 3.6.1. Data extraction

Data were extracted either using raw frequencies, crude estimates of effect (including odds ratios, risk ratios, incidence rate ratios, hazard ratios, mean differences) or as adjusted estimates of effect. Adjusted estimates of effect were used in preference for primary analyses, where available. Random effect meta-analyses were performed to allow for heterogeneity between studies.

### 3.6.2. Heterogeneity

Heterogeneity was quantified using I^2^. We explored reasons for heterogeneity using subgroup analyses based on study level factors. We classified heterogeneity as low (I^2^<25%), moderate (I^2^ 25-50%), high (I^2^ 50-75%) or extreme (I^2^>75%). For single study analyses, and where I^2^ exceeded 80% we did not pool data in meta-analysis but presented studies in a forest plot without a pooled effect shown. Individual patient data analysis was not undertaken in this review, and study authors were not contacted to clarify data queries or request further participant data.

## 3.7. Data analysis

Pooled results for binary outcomes from intervention studies are presented as Risk ratios calculated from the frequencies given in the study; for observational studies as odds ratios – both with 95% confidence intervals as the vast majority of cohorts, case controls and cross sectional studies reported this measure of effect. Data from individual studies were pooled using the generic inverse variance method for pooled OR and Mantel-Haenszel method (with continuity correction of 0.5 in studies with zero cell frequencies) or inverse variance method for single studies calculation of pooled RR in the statistical programme R version 3.1.0 (2014-04-10). Pooled results for continuous outcomes measured using similar scales are presented as mean differences with 95% confidence intervals. Where different scales are pooled across studies, we planned to report results using standardised mean differences. Where the only information given in the study was mean (SD) exposure in diseased and non diseased children, those were used for calculating pooled mean differences between cases and controls. We planned to present key findings from intervention trials in Summary of Findings tables similar to those used by the Cochrane Collaboration [^21^](#_ENREF_21).

### 3.7.1. Planned subgroup analyses

We planned certain subgroup and stratified analyses prior to running our search. Subgroup analysis was undertaken for all meta-analyses with ≥ 6 studies included. We planned and undertook stratified analysis according to *type of data* - unadjusted versus adjusted data. Adjusted data were used preferentially in primary analyses. Stratified analysis was undertaken of all unadjusted data available and all adjusted data available separately to help understand the potential influence of confounding on analysis results. We also undertook planned subgroup analyses according to:

*1. Risk of bias* – studies with low, versus unclear/high overall risk of bias based on the criteria described above.

*2. Disease risk* - studies of populations at increased risk for atopic or autoimmune disease, versus those at normal or low risk of disease.

*3. Clear definition of breastfeeding status* - studies which used definitions of exclusive/predominant breastfeeding which conform to WHO definitions versus those which did not report having used such definitions.

*4. Exclusive breastfeeding* – we planned to separately analyse data for ‘exclusive’ versus ‘predominant’ breastfeeding duration as defined by WHO. However, we did not identify relevant data for this analysis since studies very rarely used WHO definitions of exclusive or predominant breastfeeding.

*5*. *Study design* – due to controversy about the value of combining case-control and cohort studies in the same meta-analysis, we also included a subgroup analysis for prospective versus retrospective study design in all reports.

For some reports, further specific subgroup analyses were undertaken appropriate to the outcome of interest – for example in type 1 diabetes (TIDM) meta-analyses we planned a subgroup analysis of serological versus clinical TIDM as an outcome measure, and in the Allergic Sensitisation meta-analyses we planned a subgroup of specific IgE versus Skin Prick Test as outcome measure.

.

### 3.7.2. Graphical exploration of heterogeneity

Studies were ordered by year of publication in forest plots, in order to be able to assess any cohort effect, since human breast milk composition may have changed over time due to changing diet, and therefore have different effects on child health at different times. Due to insufficient information in included studies, it was not possible to order forest plots by year of birth for the study population or year of assessment.

## 3.8. Review registration

The protocol for this systematic review was registered with the International Prospective Register of Systematic Reviews (PROSPERO CRD42013003802; CRD42013004239; CRD42013004252; [www.crd.york.ac.uk/Prospero](http://www.crd.york.ac.uk/Prospero)) on the 5^th^ August 2013, prior to title screening or selecting any studies from the search results. The protocol was revised following detailed review by the UK Food Standards Agency, the UK Scientific Advisory Committee on Nutrition, independent experts Professor Graham Devereux and Dr Carina Venter, and the Lancet peer review service, prior to being registered on PROSPERO.

## 3.9. Differences between the protocol and the review

Following external statistical review of preliminary reports, a decision was made to not undertake pooled meta-analysis where statistical heterogeneity was ≥80%. Due to insufficient data in included studies, we did not order forest plots by participant year of birth or year of outcome assessment. Instead we ordered by year of publication. New authors joined the review team due to the high workload of title screening and data extraction – NT-M, SC, UN, NG, ZR, JC, KJ, EA.

# Results

## 4.1. Overview of recent high quality systematic reviews

Our search results are summarised in the PRISMA flow chart Figure 1.

We identified 469 titles after removing duplicates, of which 33 were considered to be a report from a relevant or potentially relevant systematic review after title and abstract screening. All these studies underwent full text review and revised AMSTAR scoring by 2 authors independently (VGL, RJB) – Table 1 summarises the AMSTAR scores. Two studies were only available in Chinese, and these were scored by a native Chinese speaker Dr Sze-Chin Tan, Consultant Allergist from Tan Tock Seng Hospital Singapore, who was trained in the revised AMSTAR scoring procedure prior to undertaking this task. Of the 33 scored studies, and a further 10 review identified in the update search in February 2017, 11 were eligible for inclusion in the project overall, of which 3 are relevant to Review A. Fifteen studies were excluded due to low AMSTAR score. One study was not a systematic review. Six studies were excluded because they were either protocols for a systematic review (n=2) or were abstract publications with insufficient detail to establish the AMSTAR score or sufficient detail regarding study outcomes (n=4). One of these has subsequently been published, that is relevant to Review A, but R-AMSTAR scoring excluded the review from our report based on the quality of the systematic review [^5^](#_ENREF_5). Eight systematic reviews were excluded because they were only relevant to REVIEWS B or C. The updated search on 26^th^ February 2017 identified a total of 3 systematic reviews relevant to Review A, but AMSTAR scores were below 32. A summary of R-AMSTAR scores for existing systematic reviews is shown in Table 2, and a summary of the 3 included reviews is shown in Table 3. The 3 included reviews are reviews of observational data on breastfeeding and atopic outcomes (n=2) and breastfeeding and coeliac disease (n=1). Data from these prior systematic reviews are included in the relevant sections of this report (Table 1).

## 4.2. Systematic review of original studies

Our search results are summarised in the PRISMA flow chart Figure 2.

We identified 1 relevant intervention trial, 134 cohort studies, 11 nested case control studies and 114 case control or cross-sectional studies for inclusion in Review A. No further eligible intervention trials were identified from the updated search on 26^th^ February 2017. Detailed findings, analyses and discussion for the 7 separate systematic reviews are shown in the attached reports (Table 1), and summarised in the Executive Summary.

1. Discussion

In these systematic reviews of mainly observational data regarding breastfeeding duration, timing of solid food introduction and atopic and autoimmune diseases, we did not find strong and consistent evidence of a relationship between the exposures and atopic outcomes, however for most analyses we were unable to confidently exclude clinically important effects due to wide confidence intervals.

We identified a single RCT of a breastfeeding promotion intervention, which resulted in increased TBF, increased EBF and delayed SF introduction. We classified the RCT under TBF, as this was arguably the major intervention of the trial – however the trial initiative was the widely-used UNICEF Baby Friendly Initiative which advises EBF to 6 months and continued TBF to at least 2 years, consistent with WHO guidance [^22^](#_ENREF_22). This trial found LOW certainty evidence that a breastfeeding promotion intervention reduces odds of eczema during the first year of life. We did also identify the Enquiring About Tolerance (EAT) trial, which randomised exclusively breastfed infants to SF introduction at 3 months versus 6 months – however results from this trial were included in Review B regarding timing of allergenic food introduction, since the solid foods were specific allergenic foods in this trial [^23^](#_ENREF_23). EAT did not identify a clear effect of SF introduction on allergic outcomes other than food allergy and food sensitisation, which are discussed in detail in Review B.

From the observational studies we found VERY LOW certainty evidence that longer TBF duration is associated with reduced risk of recurrent wheezing at age 5-14.

A recent systematic review found that increased duration of breastfeeding may be associated with reduced risk of wheeze/asthma[^24^](#_ENREF_24). We found a similar association, but there was significant evidence of publication bias, leading to our conclusion that there is only VERY LOW evidence for an association between longer total or exclusive breastfeeding duration and reduced risk of wheeze or recurrent wheeze at age 5-14. The intervention trial did not support an effect of breastfeeding promotion on risk of wheeze/asthma. In contrast with a recent separate systematic review, we found no consistent evidence for an association between longer total or exclusive breastfeeding and increased lung function [^25^](#_ENREF_25).

We found LOW and VERY LOW grade evidence that longer EBF and TBF duration respectively are associated with reduced Type 1 Diabetes Mellitus risk. One hypothesis for this association is that bovine insulin, which is present in cow’s milk formula, leads to immune sensitisation in the formula fed infant, which in turn increases the risk of an autoimmune response directed against human insulin and associated antibodies, the cause of TIDM. However, a recent large randomised controlled trial comparing cow’s milk with, and without (extensively hydrolysed) bovine insulin failed to confirm any difference in risk of serological TIDM [^26^](#_ENREF_26). Our systematic reviews of timing of cow’s milk introduction to the infant diet (REVIEW B), and of hydrolysed infant formula (REVIEW C Part I), also failed to support a relationship between cow’s milk or bovine insulin exposure and risk of TIDM. An alternative hypothesis is that TIDM is triggered by enterovirus infection, and the association between longer EBF/TBF and reduced TIDM risk may therefore be explained by the reduction in gastrointestinal infection seen with longer breastfeeding duration, in part attributable to the high levels of antibodies present in human milk.[^27^](#_ENREF_27) [^9^](#_ENREF_9)

There was more mixed evidence for an association between breastfeeding duration and reduced risk of other autoimmune diseases, with no clear conclusions – but this is an area which requires further study.

1. Conclusions

These findings are not of sufficient strength to make new recommendations about the timing of solid food introduction in infancy, but they do support existing recommendations that breastfeeding is the optimal mode of infant feeding, and should be continued for at least 2 years since we found VERY LOW evidence that increased TBF is associated with reduced TIDM, and this association continued through to the second year of life. We found LOW certainty evidence that EBF duration of <3-4 months compared with ≥3-4 months may increase risk of Type 1 Diabetes Mellitus, but we did not find evidence of a relationship between longer EBF duration than this and TIDM risk – for EBF duration ≥ 5-9 months, 1 retrospective study found reduced TIDM risk, and one prospective study found no association. Duration of exclusive breastfeeding is largely determined by timing of cow’s milk formula introduction, and to a lesser extent timing of solid food introduction, in the UK. The available evidence for timing of solid food introduction and TIDM risk was inconclusive due to extreme heterogeneity, and data for timing of cow’s milk formula introduction and risk of TIDM, reported in REVIEW B, found no consistent evidence for an association. Further work is needed to understand possible effects of longer breastfeeding duration on risk of TIDM, and risk of childhood eczema and asthma.

# Acknowledgements

We are grateful to the independent expert peer reviewers Professor Graham Devereux and Dr Carina Venter, and to members of the UK Food Standards Agency, the Committee on Toxicity of Chemicals in Food, Consumer Products and the Environment (COT), and the UK Scientific Advisory Committee on Nutrition for their comments on the study protocol and previous versions of this report. We are grateful to Dr Marialena Trivella and Professor Doug Altman for advice on statistical analysis. We are grateful to Dr Yujie Zhao, Mr Szymon Mikolajewski, Dr Andre Amaral, Dr Mari Kihara, Dr Christian Nielsen, Mr Radoslav Latinovic, Dr Stephanie MacNeill, Dr Andreas Forsters, Dr Daniel Munblit, Dr Sze-Chin Tan, and Dr Claudia Gore for their assistance with translation of foreign language reports. We are grateful to Jackie Cousins of the St. Mary’s library at Imperial College London for assistance with literature search training.

# References

**Table 2** Quality assessment of recent or ongoing overlapping systematic reviews using R-AMSTAR scoring.

| **Author/Reference** | **VGL Score** | **BB Score** | **CONSENSUS** |
| --- | --- | --- | --- |
| Antico [^28^](#_ENREF_28) | 19 | 14 | Exclude |
| Braeger [^29^](#_ENREF_29) | 30 | 29 | Exclude |
| Brew [^30^](#_ENREF_30) | **35** | **37** | Include – Review A |
| Cardwell [^31^](#_ENREF_31) | 31 | 25 | Exclude |
| Christesen [^32^](#_ENREF_32) | 24 | 25 | Exclude |
| De Silva [^5^](#_ENREF_5) | 26 | 29 | Exclude |
| Dick [^33^](#_ENREF_33) | 22 | 19 | Exclude |
| Doege [^34^](#_ENREF_34) | 28 | 21 | Exclude |
| Flohr [^35^](#_ENREF_35) | 19 | 19 | Exclude |
| Foolad [^36^](#_ENREF_36) | 18 | 22 | Exclude |
| Gunaratne | Protocol and Abstract publication only | | |
| Henricksson [^39^](#_ENREF_39) | 18 | 24 | Exclude |
| Kheirkhah [^40^](#_ENREF_40) | Abstract publication only | | |
| Klemens [^41^](#_ENREF_41) | **36** | **35** | Exclude – Review C |
| Kramer [^27^](#_ENREF_27) | **38** | **40** | Include – Review A |
| Kramer [^42^](#_ENREF_42) | **37** | **40** | Exclude – Review C |
| Kremmyda [^43^](#_ENREF_43) | 21 | 20 | Exclude |
| Middleton [^44^](#_ENREF_44) | Abstract publication only | | |
| Osborne [^45^](#_ENREF_45) | Abstract publication only | | |
| Osborne [^46^](#_ENREF_46) | **38** | **40** | Exclude – Review C |
| Pelucci [^47^](#_ENREF_47) | **33** | **36** | Exclude – Review C |
| Patelarou [^48^](#_ENREF_48) | 23 | 24 | Exclude |
| Schindler [^49^](#_ENREF_49) | Protocol only | | |
| Schmitt [^50^](#_ENREF_50) | Not a systematic review | | |
| Szajeweska [^51^](#_ENREF_51) | **36** | **35** | Include – Reviews A and B |
| Tang [^52^](#_ENREF_52) | **34*** | | Exclude – Review C |
| Waidyatillake [^25^](#_ENREF_25) | 24 | 25 | Exclude |
| Wang [^53^](#_ENREF_53) | 31* | | Exclude |

A score of ≥32 was required for inclusion; * Chinese language, scored by Dr Sze-Chin Tan

**Table 3** Characteristics of included systematic reviews

| **Study**  Relevant SR | **Databases searched** | **No. studies**  **No. (range) participants** | **Study designs included** | **Population** | **Intervention/ Exposure(s)**  **Comparator(s)** | **Outcomes relevant to the project** | **Subgroup analyses relevant to project** |
| --- | --- | --- | --- | --- | --- | --- | --- |
| **Brew** [^30^](#_ENREF_30)  Review A | MEDLINE, EMBASE  01/2000 - 06/2010 | 31 studies  417,880 (180 – 168,330) participants | 16 Birth cohort  6 Case-control  9 Cross-sectional | - Birth cohort, cross-sectional, case-control study - Age 5-18 years at follow-up | Exclusive BF for ≥3-4 months vs. less | ‘Current wheezing illness’, defined in various ways, but usually including wheezing in last 12 months | • Study design  • Definition of asthma/wheeze  • Adjustment for relevant confounders  • Study participation/ retention rate  •Age at assessment |
| **Kramer** [^27^](#_ENREF_27)  Review A | MEDLINE EMBASE CENTRAL 2011, Issue 6 CINAHL BIOSIS African Index Medicus IMEMR LILACS  06/2011 | 23 studies  11,299 (30 – 3,483) participants  Relevant outcomes reported in 3 studies with 4128 (135 – 3483) participants | 1 Prospective cohort  2 Prospective cohorts nested within a RCT | Lactating mothers and their healthy, term, singleton infants exclusively or predominantly breast fed for at least 3 months | Continued exclusive/ predominant breast feeding to 6-7 months, versus introduction of other liquids or complementary foods at 3-4 months | - Wheezing/asthma - Eczema - Rhinoconjunctivitis (hayfever) - Allergic sensitisation - Food allergy | Nil |
| **Szajeweska** [^51^](#_ENREF_51)  Review A  Review B | CENTRAL, MEDLINE, EMBASE (up to July 2012) | 12 studies  266,728 participants | 3 cohort studies  8 case control studies  1 RCT  Data | Infants at population risk or increased risk of developing Coeliac disease (defined by HLA status, first-degree relative with celiac disease or type 1 diabetes mellitus) | - Duration of breastfeeding | - Coeliac disease - Coeliac autoantibodies (anti-TG2 or EMA) | Nil |

RCT: Randomised controlled trial, BF: Breast feeding

# Appendix 1 Search Strategies for other systematic reviews

These search strategies were used to identify recent SRs relevant to Reviews A, B or C

## 1.1. Medline

1. breast feeding.ab,ti.

2. breastfeeding.ab,ti.

3. breast fed.ab,ti.

4. breastfed.ab,ti.

5. Breast Feeding/

6. Milk, Human/

7. formula?.ab,ti.

8. hydrolysed.ab,ti.

9. bottlefed.ab,ti.

10. bottle fed.ab,ti.

11. (bottle adj3 feed$).ab,ti.

12. Infant Formula/

13. Bottle Feeding/

14. wean$.ab,ti.

15. Weaning/

16. 1 or 2 or 3 or 4 or 5 or 6 or 7 or 8 or 9 or 10 or 11 or 12 or 13 or 14 or 15

17. complementary food?.ab,ti.

18. (introduc$ adj2 food?).ab,ti.

19. wean$.ab,ti.

20. Weaning/

21. solid?.ab,ti.

22. semi-solid?.ab,ti.

23. baby food?.ab,ti.

24. Infant Food/

25. Infant Nutritional Physiological Phenomena/

26. breast feeding.ab,ti.

27. breastfeeding.ab,ti.

28. breast fed.ab,ti.

29. breastfed.ab,ti.

30. Breast Feeding/

31. Milk, Human/

32. formula?.ab,ti.

33. hydrolysed.ab,ti.

34. bottlefed.ab,ti.

35. bottle fed.ab,ti.

36. (bottle adj3 feed$).ab,ti.

37. Infant Formula/

38. Bottle Feeding/

39. liquid?.ab,ti.

40. milk.ab,ti.

41. Milk/

42. egg?.ab,ti.

43. Egg Proteins/

44. Egg Proteins, Dietary/

45. nut?.ab,ti.

46. peanut?.ab,ti.

47. almond?.ab,ti.

48. (brazil? adj5 nut?).ab,ti.

49. walnut?.ab,ti.

50. pecan?.ab,ti.

51. pistachio?.ab,ti.

52. cashew?.ab,ti.

53. hazelnut?.ab,ti.

54. macadamia?.ab,ti.

55. Nuts/

56. Arachis hypogaea/

57. Prunus/

58. Bertholletia/

59. Juglans/

60. Carya/

61. Pistacia/

62. Anacardium/

63. Corylus/

64. Macadamia/

65. wheat.ab,ti.

66. Triticum/

67. soya.ab,ti.

68. Soybeans/

69. gluten$.ab,ti.

70. Glutens/

71. fish.ab,ti.

72. Fishes/

73. 17 or 18 or 19 or 20 or 21 or 22 or 23 or 24 or 25 or 26 or 27 or 28 or 29 or 30 or 31 or 32 or 33 or 34 or 35 or 36 or 37 or 38 or 39 or 40 or 41 or 42 or 43 or 44 or 45 or 46 or 47 or 48 or 49 or 50 or 51 or 52 or 53 or 54 or 55 or 56 or 57 or 58 or 59 or 60 or 61 or 62 or 63 or 64 or 65 or 66 or 67 or 68 or 69 or 70 or 71 or 72

74. Diet/

75. Diet Therapy/

76. Nutritional Sciences/

77. Child Nutrition Sciences/

78. diet.ab,ti.

79. diets.ab,ti.

80. Diet, Mediterranean/

81. mediterranean diet$.ab,ti.

82. dietetic.ab,ti.

83. dietary.ab,ti.

84. eat.ab,ti.

85. eating.ab,ti.

86. intake.ab,ti.

87. nutrient?.ab,ti.

88. nutrition.ab,ti.

89. Diet, Vegetarian/

90. vegetarian?.ab,ti.

91. vegan$.ab,ti.

92. Diet, Macrobiotic/

93. macrobiotic?.ab,ti.

94. Food/

95. food$.ab,ti.

96. feed.ab,ti.

97. feeding.ab,ti.

98. cereal$.ab,ti.

99. grain$.ab,ti.

100. granary.ab,ti.

101. wholegrain.ab,ti.

102. wholewheat.ab,ti.

103. whole wheat.ab,ti.

104. wheat.ab,ti.

105. wheatgerm.ab,ti.

106. rye.ab,ti.

107. barley.ab,ti.

108. oat?.ab,ti.

109. exp Cereals/

110. root?.ab,ti.

111. tuber?.ab,ti.

112. exp Vegetables/

113. vegetable$.ab,ti.

114. onion$.ab,ti.

115. spinach.ab,ti.

116. chard.ab,ti.

117. tomato$.ab,ti.

118. pepper$.ab,ti.

119. carrot$.ab,ti.

120. beetroot.ab,ti.

121. asparagus.ab,ti.

122. garlic.ab,ti.

123. pumpkin.ab,ti.

124. sprouts.ab,ti.

125. broccoli.ab,ti.

126. cabbage$.ab,ti.

127. celery.ab,ti.

128. ginger.ab,ti.

129. potato$.ab,ti.

130. crisps.ab,ti.

131. fries.ab,ti.

132. syrup.ab,ti.

133. honey.ab,ti.

134. Honey/

135. Fruit/

136. fruit$.ab,ti.

137. apple?.ab,ti.

138. pear?.ab,ti.

139. banana?.ab,ti.

140. orange?.ab,ti.

141. grape?.ab,ti.

142. kiwi?.ab,ti.

143. citrus.ab,ti.

144. grapefruit?.ab,ti.

145. pulses.ab,ti.

146. beans.ab,ti.

147. lentil?.ab,ti.

148. chickpea?.ab,ti.

149. legume?.ab,ti.

150. lupin?.ab,ti.

151. soy.ab,ti.

152. soya.ab,ti.

153. nut?.ab,ti.

154. almond?.ab,ti.

155. peanut?.ab,ti.

156. groundnut?.ab,ti.

157. Nuts/

158. seed?.ab,ti.

159. sesame.ab,ti.

160. mustard.ab,ti.

161. Seeds/

162. exp Meat/

163. meat.ab,ti.

164. beef.ab,ti.

165. pork.ab,ti.

166. lamb.ab,ti.

167. poultry.ab,ti.

168. chicken.ab,ti.

169. turkey.ab,ti.

170. duck.ab,ti.

171. fish.ab,ti.

172. Fatty Acids/

173. exp Fatty Acids, Omega-3/

174. exp Fatty Acids, Omega-6/

175. omega-3.ab,ti.

176. omega-6.ab,ti.

177. PUFA.ab,ti.

178. fat.ab,ti.

179. fats.ab,ti.

180. fatty.ab,ti.

181. egg.ab,ti.

182. eggs.ab,ti.

183. exp Eggs/

184. Bread/

185. bread.ab,ti.

186. oil.ab,ti.

187. oils.ab,ti.

188. oily.ab,ti.

189. omega.ab,ti.

190. exp Seafood/

191. seafood.ab,ti.

192. shellfish.ab,ti.

193. crustacean?.ab,ti.

194. mollusc?.ab,ti.

195. Shellfish/

196. Dairy Products/

197. dairy.ab,ti.

198. exp Milk/

199. milk.ab,ti.

200. Infant Formula/

201. formula?.ab,ti.

202. hydrolysed.ab,ti.

203. Infant Food/

204. yoghurt.ab,ti.

205. probiotic.ab,ti.

206. prebiotic?.ab,ti.

207. butter.ab,ti.

208. herb?.ab,ti.

209. spice?.ab,ti.

210. chilli$.ab,ti.

211. condiment?.ab,ti.

212. exp Condiments/

213. Beverages/

214. beverage?.ab,ti.

215. fluid intake.ab,ti.

216. water.ab,ti.

217. drink$.ab,ti.

218. exp Food Preservation/

219. pickled.ab,ti.

220. bottled.ab,ti.

221. canned.ab,ti.

222. canning.ab,ti.

223. smoked.ab,ti.

224. preserved.ab,ti.

225. preservatives.ab,ti.

226. nitrosamine.ab,ti.

227. hydrogenation.ab,ti.

228. fortified.ab,ti.

229. nitrates.ab,ti.

230. nitrites.ab,ti.

231. ferment$.ab,ti.

232. processed.ab,ti.

233. antioxidant$.ab,ti.

234. genetic modif$.ab,ti.

235. genetically modif$.ab,ti.

236. Cooking/

237. cooking.ab,ti.

238. cooked.ab,ti.

239. grill.ab,ti.

240. grilled.ab,ti.

241. fried.ab,ti.

242. fry.ab,ti.

243. roast.ab,ti.

244. bake.ab,ti.

245. baked.ab,ti.

246. stewing.ab,ti.

247. stewed.ab,ti.

248. casserol$.ab,ti.

249. broil.ab,ti.

250. broiled.ab,ti.

251. boiled.ab,ti.

252. poach.ab,ti.

253. poached.ab,ti.

254. steamed.ab,ti.

255. barbecue$.ab,ti.

256. chargrill$.ab,ti.

257. salt.ab,ti.

258. salting.ab,ti.

259. salted.ab,ti.

260. fiber.ab,ti.

261. fibre.ab,ti.

262. polysaccharide$.ab,ti.

263. starch.ab,ti.

264. starchy.ab,ti.

265. carbohydrate$.ab,ti.

266. lipid$.ab,ti.

267. linoleic acid$.ab,ti.

268. sugar$.ab,ti.

269. sweetener$.ab,ti.

270. saccharin$.ab,ti.

271. aspartame.ab,ti.

272. sucrose.ab,ti.

273. xylitol.ab,ti.

274. cholesterol.ab,ti.

275. hydrogenated lard.ab,ti.

276. dietary protein.ab,ti.

277. dietary proteins.ab,ti.

278. protein intake.ab,ti.

279. animal protein$.ab,ti.

280. total protein$.ab,ti.

281. vegetable protein$.ab,ti.

282. plant protein$.ab,ti.

283. exp Dietary Carbohydrates/

284. exp Dietary Fats/

285. exp Dietary Fiber/

286. exp Dietary Proteins/

287. exp Dietary Supplements/

288. exp Food Additives/

289. exp Vitamins/

290. supplements.ab,ti.

291. supplement.ab,ti.

292. vitamin$.ab,ti.

293. retinol.ab,ti.

294. carotenoid$.ab,ti.

295. tocopherol.ab,ti.

296. folate$.ab,ti.

297. folic acid.ab,ti.

298. methionine.ab,ti.

299. riboflavin.ab,ti.

300. thiamine.ab,ti.

301. niacin.ab,ti.

302. pyridoxine.ab,ti.

303. cobalamin.ab,ti.

304. mineral$.ab,ti.

305. sodium.ab,ti.

306. iron.ab,ti.

307. calcium.ab,ti.

308. selenium.ab,ti.

309. iodine.ab,ti.

310. magnesium.ab,ti.

311. potassium.ab,ti.

312. zinc.ab,ti.

313. copper.ab,ti.

314. phosphorus.ab,ti.

315. manganese.ab,ti.

316. chromium.ab,ti.

317. phytochemical.ab,ti.

318. polyphenol$.ab,ti.

319. phytoestrogen$.ab,ti.

320. genistein.ab,ti.

321. saponin$.ab,ti.

322. coumarin$.ab,ti.

323. flavonoid$.ab,ti.

324. polyphenol$.ab,ti.

325. flavonol$.ab,ti.

326. flavone$.ab,ti.

327. isoflavone$.ab,ti.

328. catechin$.ab,ti.

329. ascorbic acid$.ab,ti.

330. hydroxy cholecalciferol$.ab,ti.

331. hydroxycholecalciferol$.ab,ti.

332. tocotrienol$.ab,ti.

333. carotene$.ab,ti.

334. cryptoxanthin$.ab,ti.

335. lycopene$.ab,ti.

336. lutein$.ab,ti.

337. zeaxanthin$.ab,ti.

338. selenium$.ab,ti.

339. organic diet?.ab,ti.

340. Food, Organic/

341. 74 or 75 or 76 or 77 or 78 or 79 or 80 or 81 or 82 or 83 or 84 or 85 or 86 or 87 or 88 or 89 or 90 or 91 or 92 or 93 or 94 or 95 or 96 or 97 or 98 or 99 or 100 or 101 or 102 or 103 or 104 or 105 or 106 or 107 or 108 or 109 or 110 or 111 or 112 or 113 or 114 or 115 or 116 or 117 or 118 or 119 or 120 or 121 or 122 or 123 or 124 or 125 or 126 or 127 or 128 or 129 or 130 or 131 or 132 or 133 or 134 or 135 or 136 or 137 or 138 or 139 or 140 or 141 or 142 or 143 or 144 or 145 or 146 or 147 or 148 or 149 or 150 or 151 or 152 or 153 or 154 or 155 or 156 or 157 or 158 or 159 or 160 or 161 or 162 or 163 or 164 or 165 or 166 or 167 or 168 or 169 or 170 or 171 or 172 or 173 or 174 or 175 or 176 or 177 or 178 or 179 or 180 or 181 or 182 or 183 or 184 or 185 or 186 or 187 or 188 or 189 or 190 or 191 or 192 or 193 or 194 or 195 or 196 or 197 or 198 or 199 or 200 or 201 or 202 or 203 or 204 or 205 or 206 or 207 or 208 or 209 or 210 or 211 or 212 or 213 or 214 or 215 or 216 or 217 or 218 or 219 or 220 or 221 or 222 or 223 or 224 or 225 or 226 or 227 or 228 or 229 or 230 or 231 or 232 or 233 or 234 or 235 or 236 or 237 or 238 or 239 or 240 or 241 or 242 or 243 or 244 or 245 or 246 or 247 or 248 or 249 or 250 or 251 or 252 or 253 or 254 or 255 or 256 or 257 or 258 or 259 or 260 or 261 or 262 or 263 or 264 or 265 or 266 or 267 or 268 or 269 or 270 or 271 or 272 or 273 or 274 or 275 or 276 or 277 or 278 or 279 or 280 or 281 or 282 or 283 or 284 or 285 or 286 or 287 or 288 or 289 or 290 or 291 or 292 or 293 or 294 or 295 or 296 or 297 or 298 or 299 or 300 or 301 or 302 or 303 or 304 or 305 or 306 or 307 or 308 or 309 or 310 or 311 or 312 or 313 or 314 or 315 or 316 or 317 or 318 or 319 or 320 or 321 or 322 or 323 or 324 or 325 or 326 or 327 or 328 or 329 or 330 or 331 or 332 or 333 or 334 or 335 or 336 or 337 or 338 or 339 or 340

342. allerg$.ab,ti.

343. asthma$.ab,ti.

344. wheeze.ab,ti.

345. wheezing.ab,ti.

346. bronchial hyperresponsiveness.ab,ti.

347. bronchial hyperreactivity.ab,ti.

348. Forced expiratory volume.ab,ti.

349. FEV1.ab,ti.

350. "FEV 1".ab,ti.

351. "FEV0.5".ab,ti.

352. "FEV 0.5".ab,ti.

353. Forced vital capacity.ab,ti.

354. FVC.ab,ti.

355. Peak expiratory flow rate.ab,ti.

356. PEFR.ab,ti.

357. eczema.ab,ti.

358. neurodermatitis.ab,ti.

359. rhinitis.ab,ti.

360. besniers prurigo.ab,ti.

361. rhinoconjunctivitis.ab,ti.

362. hayfever.ab,ti.

363. (hay adj fever).ab,ti.

364. poll?nosis.ab,ti.

365. SAR.ab,ti.

366. (pollen adj allergy).ab,ti.

367. conjunctivitis.ab,ti.

368. immunoglobulin e.ab,ti.

369. Total IgE.ab,ti.

370. autoimmune disease?.ab,ti.

371. diabetes.ab,ti.

372. diabetic.ab,ti.

373. type 1.ab,ti.

374. c?eliac disease.ab,ti.

375. crohn$ disease.ab,ti.

376. Inflammatory Bowel Disease?.ab,ti.

377. Ulcerative colitis.ab,ti.

378. (Lympho$ adj3 thyroiditi$).ab,ti.

379. (Thyroiditi$ adj3 autoimmune).ab,ti.

380. (Hashimoto$ adj3 (syndrome? or thyroiditi$ or disease?)).ab,ti.

381. (Thyroiditi$ adj3 (post-partum or postpartum)).ab,ti.

382. Graves? disease.ab,ti.

383. Basedow$ disease.ab,ti.

384. exophthalmic goiter?.ab,ti.

385. (Still? Disease adj3 (juvenile or onset)).ab,ti.

386. (Juvenile adj3 arthriti$).ab,ti.

387. vitiligo.ab,ti.

388. Psorias?s.ab,ti.

389. (Arthriti? adj3 Psoria$).ab,ti.

390. atopic disease.ab,ti.

391. atopic dermatitis.ab,ti.

392. (food? adj3 sensiti$).ab,ti.

393. (food? adj3 toleran$).ab,ti.

394. (food? adj3 intoleran$).ab,ti.

395. ((aero or air$) adj3 allergen?).ab,ti.

396. (aeroallergen? adj3 sensiti$).ab,ti.

397. (allergen? adj3 sensiti$).ab,ti.

398. skin prick test$.ab,ti.

399. atopy.ab,ti.

400. hypersensitiv$.ab,ti.

401. Hypersensitivity/

402. exp Food Hypersensitivity/

403. Respiratory Hypersensitivity/

404. Asthma/

405. Bronchial Hyperreactivity/

406. Forced Expiratory Volume/

407. Vital Capacity/

408. Peak Expiratory Flow Rate/

409. Eczema/

410. Neurodermatitis/

411. Rhinitis/

412. Rhinitis, Allergic, Perennial/

413. Rhinitis, Allergic, Seasonal/

414. Conjunctivitis/

415. Immunoglobulin E/

416. Autoimmune Diseases/

417. Diabetes Mellitus, Type 1/

418. Celiac Disease/

419. Crohn Disease/

420. Inflammatory Bowel Diseases/

421. Colitis, Ulcerative/

422. Thyroiditis, Autoimmune/

423. Hashimoto Disease/

424. Postpartum Thyroiditis/

425. Graves Disease/

426. Arthritis, Juvenile Rheumatoid/

427. Vitiligo/

428. Psoriasis/

429. Arthritis, Psoriatic/

430. Dermatitis, Atopic/

431. Hypersensitivity, Immediate/

432. 342 or 343 or 344 or 345 or 346 or 347 or 348 or 349 or 350 or 351 or 352 or 353 or 354 or 355 or 356 or 357 or 358 or 359 or 360 or 361 or 362 or 363 or 364 or 365 or 366 or 367 or 368 or 369 or 370 or 371 or 372 or 373 or 374 or 375 or 376 or 377 or 378 or 379 or 380 or 381 or 382 or 383 or 384 or 385 or 386 or 387 or 388 or 389 or 390 or 391 or 392 or 393 or 394 or 395 or 396 or 397 or 398 or 399 or 400 or 401 or 402 or 403 or 404 or 405 or 406 or 407 or 408 or 409 or 410 or 411 or 412 or 413 or 414 or 415 or 416 or 417 or 418 or 419 or 420 or 421 or 422 or 423 or 424 or 425 or 426 or 427 or 428 or 429 or 430 or 431

433. infant?.ab,ti.

434. ((one or two or three or four or five or six or seven or eight or nine or ten or eleven or twelve or thirteen or fourteen or fifteen or sixteen or seventeen or eighteen or nineteen or twenty or "twenty one" or "twenty two" or "twenty three" or "twenty four" or "twenty five" or "twenty six") adj week?).ab,ti.

435. ((one or two or three or four or five or six or seven or eight or nine or ten or eleven or twelve or thirteen or fourteen or fifteen or sixteen or seventeen or eighteen or nineteen or twenty or "twenty one" or "twenty two" or "twenty three" or "twenty four") adj month?).ab,ti.

436. 434 or 435

437. (old or age?).ab,ti.

438. 436 and 437

439. (("one year?" or "two year?") adj3 (old or age?)).ab,ti.

440. ((first or second or two) adj3 "year? of life").ab,ti.

441. Infant/

442. Infant, Newborn/

443. (maternal adj7 pregnan$).ab,ti.

444. (maternal adj7 lactat$).ab,ti.

445. (mother? adj7 pregnan$).ab,ti.

446. 433 or 438 or 439 or 440 or 441 or 442 or 443 or 444 or 445

447. MEDLINE.tw.

448. systematic review.tw.

449. meta-analysis.pt.

450. intervention$.ti.

451. 447 or 448 or 449 or 450

452. 16 or 73 or 341

453. 432 and 446 and 451 and 452

454. limit 453 to yr="2011 -Current"

## 1.2. Embase

1. breast feeding.ab,ti.

2. breastfeeding.ab,ti.

3. breast fed.ab,ti.

4. breastfed.ab,ti.

5. breast feeding/

6. breast milk/

7. formula?.ab,ti.

8. hydrolysed.ab,ti.

9. bottlefed.ab,ti.

10. bottle fed.ab,ti.

11. (bottle adj3 feed$).ab,ti.

12. artificial milk/

13. bottle feeding/

14. wean$.ti,ab.

15. weaning/

16. 1 or 2 or 3 or 4 or 5 or 6 or 7 or 8 or 9 or 10 or 11 or 12 or 13 or 14 or 15

17. complementary food?.ab,ti.

18. (introduc$ adj2 food?).ab,ti.

19. wean$.ab,ti.

20. weaning/

21. solid?.ab,ti.

22. semi-solid?.ab,ti.

23. baby food?.ab,ti.

24. baby food/

25. infant nutrition/

26. breast feeding.ab,ti.

27. breastfeeding.ab,ti.

28. breast fed.ab,ti.

29. breastfed.ab,ti.

30. breast feeding/

31. breast milk/

32. formula?.ab,ti.

33. hydrolysed.ab,ti.

34. bottlefed.ab,ti.

35. bottle fed.ab,ti.

36. (bottle adj3 feed$).ab,ti.

37. artificial milk/

38. bottle feeding/

39. liquid?.ti,ab.

40. milk.ti,ab.

41. milk/

42. egg?.ti,ab.

43. egg/

44. egg protein/

45. nut?.ab,ti.

46. peanut?.ab,ti.

47. almond?.ab,ti.

48. (brazil? adj5 nut?).ab,ti.

49. walnut?.ab,ti.

50. pecan?.ab,ti.

51. pistachio?.ab,ti.

52. cashew?.ab,ti.

53. hazelnut?.ab,ti.

54. macadamia?.ab,ti.

55. nut/

56. peanut/

57. almond/

58. Brazil nut/

59. exp walnut/

60. pecan/

61. pistachio/

62. cashew nut/

63. hazelnut/

64. Corylus avellana/

65. Macadamia/

66. wheat.ti,ab.

67. exp wheat/

68. soya.ti,ab.

69. soybean/

70. gluten$.ti,ab.

71. gluten/

72. fish$.ti,ab.

73. fish/

74. 17 or 18 or 19 or 20 or 21 or 22 or 23 or 24 or 25 or 26 or 27 or 28 or 29 or 30 or 31 or 32 or 33 or 34 or 35 or 36 or 37 or 38 or 39 or 40 or 41 or 42 or 43 or 44 or 45 or 46 or 47 or 48 or 49 or 50 or 51 or 52 or 53 or 54 or 55 or 56 or 57 or 58 or 59 or 60 or 61 or 62 or 63 or 64 or 65 or 66 or 67 or 68 or 69 or 70 or 71 or 72 or 73

75. diet/

76. diet therapy/

77. nutritional science/

78. diet.ti,ab.

79. diets.ti,ab.

80. Mediterranean diet/

81. mediterranean diet$.ab,ti.

82. dietetic.ab,ti.

83. dietary.ab,ti.

84. eat.ab,ti.

85. eating.ab,ti.

86. intake.ab,ti.

87. nutrient?.ab,ti.

88. nutrition.ab,ti.

89. vegetarian diet/

90. vegetarian?.ti,ab.

91. vegan$.ti,ab.

92. macrobiotic diet/

93. macrobiotic?.ti,ab.

94. food/

95. food$.ab,ti.

96. feed.ab,ti.

97. feeding.ab,ti.

98. cereal$.ab,ti.

99. grain$.ab,ti.

100. granary.ab,ti.

101. wholegrain.ab,ti.

102. wholewheat.ab,ti.

103. whole wheat.ab,ti.

104. wheat.ab,ti.

105. wheatgerm.ab,ti.

106. rye.ab,ti.

107. barley.ab,ti.

108. oat?.ab,ti.

109. exp cereal/

110. root?.ti,ab.

111. tuber?.ti,ab.

112. exp vegetable/

113. vegetable$.ab,ti.

114. onion$.ab,ti.

115. spinach.ab,ti.

116. chard.ab,ti.

117. tomato$.ab,ti.

118. pepper$.ab,ti.

119. carrot$.ab,ti.

120. beetroot.ab,ti.

121. asparagus.ab,ti.

122. garlic.ab,ti.

123. pumpkin.ab,ti.

124. sprouts.ab,ti.

125. broccoli.ab,ti.

126. cabbage$.ab,ti.

127. celery.ab,ti.

128. ginger.ab,ti.

129. potato$.ab,ti.

130. crisps.ab,ti.

131. fries.ab,ti.

132. syrup.ab,ti.

133. honey.ab,ti.

134. honey/

135. fruit/

136. fruit$.ab,ti.

137. apple?.ab,ti.

138. pear?.ab,ti.

139. banana?.ab,ti.

140. orange?.ab,ti.

141. grape?.ab,ti.

142. kiwi?.ab,ti.

143. citrus.ab,ti.

144. grapefruit?.ab,ti.

145. pulses.ab,ti.

146. beans.ab,ti.

147. lentil?.ab,ti.

148. chickpea?.ab,ti.

149. legume?.ab,ti.

150. lupin?.ab,ti.

151. soy.ab,ti.

152. soya.ab,ti.

153. nut?.ab,ti.

154. almond?.ab,ti.

155. peanut?.ab,ti.

156. groundnut?.ab,ti.

157. exp nut/

158. seed?.ti,ab.

159. sesame.ti,ab.

160. mustard.ti,ab.

161. plant seed/

162. meat/

163. meat.ab,ti.

164. beef.ab,ti.

165. pork.ab,ti.

166. lamb.ab,ti.

167. poultry.ab,ti.

168. chicken.ab,ti.

169. turkey.ab,ti.

170. duck.ab,ti.

171. fish.ab,ti.

172. fatty acid/

173. omega 3 fatty acid/

174. omega 6 fatty acid/

175. omega-3.ab,ti.

176. omega-6.ab,ti.

177. PUFA.ab,ti.

178. fat.ab,ti.

179. fats.ab,ti.

180. fatty.ab,ti.

181. egg.ab,ti.

182. eggs.ab,ti.

183. exp egg/

184. bread/

185. bread.ti,ab.

186. oil.ti,ab.

187. oils.ti,ab.

188. oily.ti,ab.

189. omega.ti,ab.

190. sea food/

191. seafood.ti,ab.

192. shellfish.ti,ab.

193. crustacean?.ti,ab.

194. mollusc?.ti,ab.

195. shellfish/

196. exp dairy product/

197. dairy.ti,ab.

198. milk/

199. milk.ti,ab.

200. artificial milk/

201. formula?.ti,ab.

202. hydrolysed.ti,ab.

203. baby food/

204. yoghurt.ab,ti.

205. probiotic.ab,ti.

206. prebiotic?.ab,ti.

207. butter.ab,ti.

208. herb?.ab,ti.

209. spice?.ab,ti.

210. chilli$.ab,ti.

211. condiment?.ab,ti.

212. exp condiment/

213. beverage/

214. beverage?.ti,ab.

215. fluid intake.ti,ab.

216. water.ti,ab.

217. drink$.ti,ab.

218. exp food preservation/

219. pickled.ab,ti.

220. bottled.ab,ti.

221. canned.ab,ti.

222. canning.ab,ti.

223. smoked.ab,ti.

224. preserved.ab,ti.

225. preservatives.ab,ti.

226. nitrosamine.ab,ti.

227. hydrogenation.ab,ti.

228. fortified.ab,ti.

229. nitrates.ab,ti.

230. nitrites.ab,ti.

231. ferment$.ab,ti.

232. processed.ab,ti.

233. antioxidant$.ab,ti.

234. genetic modif$.ab,ti.

235. genetically modif$.ab,ti.

236. cooking/

237. cooking.ab,ti.

238. cooked.ab,ti.

239. grill.ab,ti.

240. grilled.ab,ti.

241. fried.ab,ti.

242. fry.ab,ti.

243. roast.ab,ti.

244. bake.ab,ti.

245. baked.ab,ti.

246. stewing.ab,ti.

247. stewed.ab,ti.

248. casserol$.ab,ti.

249. broil.ab,ti.

250. broiled.ab,ti.

251. boiled.ab,ti.

252. poach.ab,ti.

253. poached.ab,ti.

254. steamed.ab,ti.

255. barbecue$.ab,ti.

256. chargrill$.ab,ti.

257. salt.ab,ti.

258. salting.ab,ti.

259. salted.ab,ti.

260. fiber.ab,ti.

261. fibre.ab,ti.

262. polysaccharide$.ab,ti.

263. starch.ab,ti.

264. starchy.ab,ti.

265. carbohydrate$.ab,ti.

266. lipid$.ab,ti.

267. linoleic acid$.ab,ti.

268. sugar$.ab,ti.

269. sweetener$.ab,ti.

270. saccharin$.ab,ti.

271. aspartame.ab,ti.

272. sucrose.ab,ti.

273. xylitol.ab,ti.

274. cholesterol.ab,ti.

275. hydrogenated lard.ab,ti.

276. dietary protein.ab,ti.

277. dietary proteins.ab,ti.

278. protein intake.ab,ti.

279. animal protein$.ab,ti.

280. total protein$.ab,ti.

281. vegetable protein$.ab,ti.

282. plant protein$.ab,ti.

283. carbohydrate diet/

284. carbohydrate intake/

285. fat intake/

286. dietary fiber/

287. protein intake/

288. diet supplementation/

289. food additive/

290. exp vitamin/

291. supplements.ab,ti.

292. supplement.ab,ti.

293. vitamin$.ab,ti.

294. retinol.ab,ti.

295. carotenoid$.ab,ti.

296. tocopherol.ab,ti.

297. folate$.ab,ti.

298. folic acid.ab,ti.

299. methionine.ab,ti.

300. riboflavin.ab,ti.

301. thiamine.ab,ti.

302. niacin.ab,ti.

303. pyridoxine.ab,ti.

304. cobalamin.ab,ti.

305. mineral$.ab,ti.

306. sodium.ab,ti.

307. iron.ab,ti.

308. calcium.ab,ti.

309. selenium.ab,ti.

310. iodine.ab,ti.

311. magnesium.ab,ti.

312. potassium.ab,ti.

313. zinc.ab,ti.

314. copper.ab,ti.

315. phosphorus.ab,ti.

316. manganese.ab,ti.

317. chromium.ab,ti.

318. phytochemical.ab,ti.

319. polyphenol$.ab,ti.

320. phytoestrogen$.ab,ti.

321. genistein.ab,ti.

322. saponin$.ab,ti.

323. coumarin$.ab,ti.

324. flavonoid$.ab,ti.

325. polyphenol$.ab,ti.

326. flavonol$.ab,ti.

327. flavone$.ab,ti.

328. isoflavone$.ab,ti.

329. catechin$.ab,ti.

330. ascorbic acid$.ab,ti.

331. hydroxy cholecalciferol$.ab,ti.

332. hydroxycholecalciferol$.ab,ti.

333. tocotrienol$.ab,ti.

334. carotene$.ab,ti.

335. cryptoxanthin$.ab,ti.

336. lycopene$.ab,ti.

337. lutein$.ab,ti.

338. zeaxanthin$.ab,ti.

339. selenium$.ab,ti.

340. organic diet?.ab,ti.

341. organic food/

342. 75 or 76 or 77 or 78 or 79 or 80 or 81 or 82 or 83 or 84 or 85 or 86 or 87 or 88 or 89 or 90 or 91 or 92 or 93 or 94 or 95 or 96 or 97 or 98 or 99 or 100 or 101 or 102 or 103 or 104 or 105 or 106 or 107 or 108 or 109 or 110 or 111 or 112 or 113 or 114 or 115 or 116 or 117 or 118 or 119 or 120 or 121 or 122 or 123 or 124 or 125 or 126 or 127 or 128 or 129 or 130 or 131 or 132 or 133 or 134 or 135 or 136 or 137 or 138 or 139 or 140 or 141 or 142 or 143 or 144 or 145 or 146 or 147 or 148 or 149 or 150 or 151 or 152 or 153 or 154 or 155 or 156 or 157 or 158 or 159 or 160 or 161 or 162 or 163 or 164 or 165 or 166 or 167 or 168 or 169 or 170 or 171 or 172 or 173 or 174 or 175 or 176 or 177 or 178 or 179 or 180 or 181 or 182 or 183 or 184 or 185 or 186 or 187 or 188 or 189 or 190 or 191 or 192 or 193 or 194 or 195 or 196 or 197 or 198 or 199 or 200 or 201 or 202 or 203 or 204 or 205 or 206 or 207 or 208 or 209 or 210 or 211 or 212 or 213 or 214 or 215 or 216 or 217 or 218 or 219 or 220 or 221 or 222 or 223 or 224 or 225 or 226 or 227 or 228 or 229 or 230 or 231 or 232 or 233 or 234 or 235 or 236 or 237 or 238 or 239 or 240 or 241 or 242 or 243 or 244 or 245 or 246 or 247 or 248 or 249 or 250 or 251 or 252 or 253 or 254 or 255 or 256 or 257 or 258 or 259 or 260 or 261 or 262 or 263 or 264 or 265 or 266 or 267 or 268 or 269 or 270 or 271 or 272 or 273 or 274 or 275 or 276 or 277 or 278 or 279 or 280 or 281 or 282 or 283 or 284 or 285 or 286 or 287 or 288 or 289 or 290 or 291 or 292 or 293 or 294 or 295 or 296 or 297 or 298 or 299 or 300 or 301 or 302 or 303 or 304 or 305 or 306 or 307 or 308 or 309 or 310 or 311 or 312 or 313 or 314 or 315 or 316 or 317 or 318 or 319 or 320 or 321 or 322 or 323 or 324 or 325 or 326 or 327 or 328 or 329 or 330 or 331 or 332 or 333 or 334 or 335 or 336 or 337 or 338 or 339 or 340 or 341

343. allerg$.ab,ti.

344. asthma$.ab,ti.

345. wheeze.ab,ti.

346. wheezing.ab,ti.

347. bronchial hyperresponsiveness.ab,ti.

348. bronchial hyperreactivity.ab,ti.

349. Forced expiratory volume.ab,ti.

350. FEV1.ab,ti.

351. "FEV 1".ab,ti.

352. "FEV0.5".ab,ti.

353. "FEV 0.5".ab,ti.

354. Forced vital capacity.ab,ti.

355. FVC.ab,ti.

356. Peak expiratory flow rate.ab,ti.

357. PEFR.ab,ti.

358. eczema.ab,ti.

359. neurodermatitis.ab,ti.

360. rhinitis.ab,ti.

361. besniers prurigo.ab,ti.

362. rhinoconjunctivitis.ab,ti.

363. hayfever.ab,ti.

364. (hay adj fever).ab,ti.

365. poll?nosis.ab,ti.

366. SAR.ab,ti.

367. (pollen adj allergy).ab,ti.

368. conjunctivitis.ab,ti.

369. immunoglobulin e.ab,ti.

370. Total IgE.ab,ti.

371. autoimmune disease?.ab,ti.

372. diabetes.ab,ti.

373. diabetic.ab,ti.

374. type 1.ab,ti.

375. c?eliac disease.ab,ti.

376. crohn$ disease.ab,ti.

377. Inflammatory Bowel Disease?.ab,ti.

378. Ulcerative colitis.ab,ti.

379. (Lympho$ adj3 thyroiditi$).ab,ti.

380. (Thyroiditi$ adj3 autoimmune).ab,ti.

381. (Hashimoto$ adj3 (syndrome? or thyroiditi$ or disease?)).ab,ti.

382. (Thyroiditi$ adj3 (post-partum or postpartum)).ab,ti.

383. Graves? disease.ab,ti.

384. Basedow$ disease.ab,ti.

385. exophthalmic goiter?.ab,ti.

386. (Still? Disease adj3 (juvenile or onset)).ab,ti.

387. (Juvenile adj3 arthriti$).ab,ti.

388. vitiligo.ab,ti.

389. Psorias?s.ab,ti.

390. (Arthriti? adj3 Psoria$).ab,ti.

391. atopic disease.ab,ti.

392. atopic dermatitis.ab,ti.

393. (food? adj3 sensiti$).ab,ti.

394. (food? adj3 toleran$).ab,ti.

395. (food? adj3 intoleran$).ab,ti.

396. ((aero or air$) adj3 allergen?).ab,ti.

397. (aeroallergen? adj3 sensiti$).ab,ti.

398. (allergen? adj3 sensiti$).ab,ti.

399. skin prick test$.ab,ti.

400. atopy.ab,ti.

401. hypersensitiv$.ab,ti.

402. exp hypersensitivity/

403. respiratory tract allergy/

404. asthma/

405. wheezing/

406. bronchus hyperreactivity/

407. forced expiratory volume/

408. forced vital capacity/

409. peak expiratory flow/

410. eczema/

411. neurodermatitis/

412. rhinitis/

413. rhinoconjunctivitis/

414. hay fever/

415. pollen allergy/

416. perennial rhinitis/

417. conjunctivitis/

418. immunoglobulin E/

419. autoimmune disease/

420. diabetes mellitus/

421. insulin dependent diabetes mellitus/

422. celiac disease/

423. Crohn disease/

424. enteritis/

425. ulcerative colitis/

426. autoimmune thyroiditis/

427. Hashimoto disease/

428. postpartum thyroiditis/

429. Graves disease/

430. juvenile rheumatoid arthritis/

431. vitiligo/

432. psoriasis/

433. psoriatic arthritis/

434. atopic dermatitis/

435. nutritional intolerance/

436. 343 or 344 or 345 or 346 or 347 or 348 or 349 or 350 or 351 or 352 or 353 or 354 or 355 or 356 or 357 or 358 or 359 or 360 or 361 or 362 or 363 or 364 or 365 or 366 or 367 or 368 or 369 or 370 or 371 or 372 or 373 or 374 or 375 or 376 or 377 or 378 or 379 or 380 or 381 or 382 or 383 or 384 or 385 or 386 or 387 or 388 or 389 or 390 or 391 or 392 or 393 or 394 or 395 or 396 or 397 or 398 or 399 or 400 or 401 or 402 or 403 or 404 or 405 or 406 or 407 or 408 or 409 or 410 or 411 or 412 or 413 or 414 or 415 or 416 or 417 or 418 or 419 or 420 or 421 or 422 or 423 or 424 or 425 or 426 or 427 or 428 or 429 or 430 or 431 or 432 or 433 or 434 or 435

437. infant?.ab,ti.

438. ((one or two or three or four or five or six or seven or eight or nine or ten or eleven or twelve or thirteen or fourteen or fifteen or sixteen or seventeen or eighteen or nineteen or twenty or "twenty one" or "twenty two" or "twenty three" or "twenty four" or "twenty five" or "twenty six") adj week?).ab,ti.

439. ((one or two or three or four or five or six or seven or eight or nine or ten or eleven or twelve or thirteen or fourteen or fifteen or sixteen or seventeen or eighteen or nineteen or twenty or "twenty one" or "twenty two" or "twenty three" or "twenty four") adj month?).ab,ti.

440. 438 or 439

441. (old or age?).ab,ti.

442. 440 and 441

443. (("one year?" or "two year?") adj3 (old or age?)).ab,ti.

444. ((first or second or two) adj3 "year? of life").ab,ti.

445. infant/

446. newborn/

447. (maternal adj7 pregnan$).ti,ab.

448. (maternal adj7 lactat$).ti,ab.

449. (mother? adj7 pregnan$).ti,ab.

450. 437 or 442 or 443 or 444 or 445 or 446 or 447 or 448 or 449

451. MEDLINE.tw.

452. exp systematic review/

453. systematic review.tw.

454. meta analysis/

455. intervention$.ti.

456. 451 or 452 or 453 or 454 or 455

457. 16 or 74 or 342

458. 436 and 450 and 456 and 457

459. limit 458 to yr="2011 -Current"

## 1.3. COCHRANE Reviews and DARE

1. “breast feeding”:ab,ti

2. breastfeeding:ab,ti

3. “breast fed”:ab,ti

4. breastfed:ab,ti

5. MeSH descriptor [Breast Feeding] this term only

6. MeSH descriptor [Milk, Human] this term only

7. formula*:ab,ti

8. hydrolysed:ab,ti

9. bottlefed:ab,ti

10. “bottle fed”:ab,ti

11. (bottle NEAR/3 feed*):ab,ti

12. MeSH descriptor [Infant Formula] this term only

13. MeSH descriptor [Bottle Feeding] this term only

14. wean*:ab,ti

15. MeSH descriptor [Weaning] this term only

16. 1 or 2 or 3 or 4 or 5 or 6 or 7 or 8 or 9 or 10 or 11 or 12 or 13 or 14 or 15

17. “complementary food*”:ab,ti

18. (introduc* NEAR/2 food*):ab,ti

19. wean*:ab,ti

20. MeSH descriptor [Weaning] this term only

21. solid*:ab,ti

22. semi-solid*:ab,ti

23. “baby food*”:ab,ti

24. MeSH descriptor [Infant Food] this term only

25. MeSH descriptor [Infant Nutritional Physiological Phenomena] this term only

26. “breast feeding”:ab,ti

27. breastfeeding:ab,ti

28. “breast fed”:ab,ti

29. breastfed:ab,ti

30. MeSH descriptor [Breast Feeding] this term only

31. MeSH descriptor [Milk, Human] this term only

32. formula*:ab,ti

33. hydrolysed:ab,ti

34. bottlefed:ab,ti

35. “bottle fed”:ab,ti

36. (bottle NEAR/3 feed*):ab,ti

37. MeSH descriptor [Infant Formula] this term only

38. MeSH descriptor [Bottle Feeding] this term only

39. liquid*:ab,ti

40. milk:ab,ti

41. MeSH descriptor [Milk] this term only

42. egg*:ab,ti

43. MeSH descriptor [Egg Proteins] this term only

44. MeSH descriptor [Egg Proteins, Dietary] this term only

45. nut*:ab,ti

46. peanut*:ab,ti

47. almond*:ab,ti

48. (brazil* NEAR/5 nut*):ab,ti

49. walnut*:ab,ti

50. pecan*:ab,ti

51. pistachio*:ab,ti

52. cashew*:ab,ti

53. hazelnut*:ab,ti

54. macadamia*:ab,ti

55. Nuts] this term only

56. MeSH descriptor [Arachis hypogaea] this term only

57. MeSH descriptor [Prunus] this term only

58. MeSH descriptor [Bertholletia] this term only

59. MeSH descriptor [Juglans] this term only

60. MeSH descriptor [Carya] this term only

61. MeSH descriptor [Pistacia] this term only

62. MeSH descriptor [Anacardium] this term only

63. MeSH descriptor [Corylus] this term only

64. MeSH descriptor [Macadamia] this term only

65. wheat:ab,ti

66. MeSH descriptor [Triticum] this term only

67. soya:ab,ti

68. MeSH descriptor [Soybeans] this term only

69. gluten*:ab,ti

70. MeSH descriptor [Glutens] this term only

71. fish:ab,ti

72. MeSH descriptor [Fishes] this term only

73. 17 or 18 or 19 or 20 or 21 or 22 or 23 or 24 or 25 or 26 or 27 or 28 or 29 or 30 or 31 or 32 or 33 or 34 or 35 or 36 or 37 or 38 or 39 or 40 or 41 or 42 or 43 or 44 or 45 or 46 or 47 or 48 or 49 or 50 or 51 or 52 or 53 or 54 or 55 or 56 or 57 or 58 or 59 or 60 or 61 or 62 or 63 or 64 or 65 or 66 or 67 or 68 or 69 or 70 or 71 or 72

74. MeSH descriptor [Diet] this term only

75. MeSH descriptor [Diet Therapy] this term only

76. MeSH descriptor [Nutritional Sciences] this term only

77. MeSH descriptor [Child Nutrition Sciences] this term only

78. diet:ab,ti

79. diets:ab,ti

80. MeSH descriptor [Diet, Mediterranean] this term only

81. “mediterranean diet*”:ab,ti

82. dietetic:ab,ti

83. dietary:ab,ti

84. eat:ab,ti

85. eating:ab,ti

86. intake:ab,ti

87. nutrient*:ab,ti

88. nutrition:ab,ti

89. MeSH descriptor [Diet, Vegetarian] this term only

90. vegetarian*:ab,ti

91. vegan*:ab,ti

92. MeSH descriptor [Diet, Macrobiotic] this term only

93. macrobiotic*:ab,ti

94. MeSH descriptor [Food] this term only

95. food*:ab,ti

96. feed:ab,ti

97. feeding:ab,ti

98. cereal*:ab,ti

99. grain*:ab,ti

100. granary:ab,ti

101. wholegrain:ab,ti

102. wholewheat:ab,ti

103. “whole wheat”:ab,ti

104. wheat:ab,ti

105. wheatgerm:ab,ti

106. rye:ab,ti

107. barley:ab,ti

108. oat*:ab,ti

109. MeSH descriptor [Cereals] explode all trees

110. root*:ab,ti

111. tuber*:ab,ti

112. MeSH descriptor [Vegetables] explode all trees

113. vegetable*:ab,ti

114. onion*:ab,ti

115. spinach:ab,ti

116. chard:ab,ti

117. tomato*:ab,ti

118. pepper*:ab,ti

119. carrot*:ab,ti

120. beetroot:ab,ti

121. asparagus:ab,ti

122. garlic:ab,ti

123. pumpkin:ab,ti

124. sprouts:ab,ti

125. broccoli:ab,ti

126. cabbage*:ab,ti

127. celery:ab,ti

128. ginger:ab,ti

129. potato*:ab,ti

130. crisps:ab,ti

131. fries:ab,ti

132. syrup:ab,ti

133. honey:ab,ti

134. MeSH descriptor [Honey] this term only

135. MeSH descriptor [Fruit] this term only

136. fruit*:ab,ti

137. apple*:ab,ti

138. pear*:ab,ti

139. banana*:ab,ti

140. orange*:ab,ti

141. grape*:ab,ti

142. kiwi*:ab,ti

143. citrus:ab,ti

144. grapefruit*:ab,ti

145. pulses:ab,ti

146. beans:ab,ti

147. lentil*:ab,ti

148. chickpea*:ab,ti

149. legume*:ab,ti

150. lupin*:ab,ti

151. soy:ab,ti

152. soya:ab,ti

153. nut*:ab,ti

154. almond*:ab,ti

155. peanut*:ab,ti

156. groundnut*:ab,ti

157. MeSH descriptor [Nuts] this term only

158. seed*:ab,ti

159. sesame:ab,ti

160. mustard:ab,ti

161. MeSH descriptor [Seeds] this term only

162. MeSH descriptor [Meat] explode all trees

163. meat:ab,ti

164. beef:ab,ti

165. pork:ab,ti

166. lamb:ab,ti

167. poultry:ab,ti

168. chicken:ab,ti

169. turkey:ab,ti

170. duck:ab,ti

171. fish:ab,ti

172. MeSH descriptor [Fatty Acids] this term only

173. MeSH descriptor [Fatty Acids, Omega-3] explode all trees

174. MeSH descriptor [Fatty Acids, Omega-6] explode all trees

175. omega-3:ab,ti

176. omega-6:ab,ti

177. PUFA:ab,ti

178. fat:ab,ti

179. fats:ab,ti

180. fatty:ab,ti

181. egg:ab,ti

182. eggs:ab,ti

183. MeSH descriptor [Eggs] explode all trees

184. MeSH descriptor [Bread] this term only

185. bread:ab,ti

186. oil:ab,ti

187. oils:ab,ti

188. oily:ab,ti

189. omega:ab,ti

190. MeSH descriptor [Seafood] explode all trees

191. seafood:ab,ti

192. shellfish:ab,ti

193. crustacean*:ab,ti

194. mollusc*:ab,ti

195. MeSH descriptor [Shellfish] this term only

196. MeSH descriptor [Dairy Products] this term only

197. dairy:ab,ti

198. MeSH descriptor [Milk] explode all trees

199. milk:ab,ti

200. MeSH descriptor [Infant Formula] this term only

201. formula*:ab,ti

202. hydrolysed:ab,ti

203. MeSH descriptor [Infant Food] this term only

204. yoghurt:ab,ti

205. probiotic:ab,ti

206. prebiotic*:ab,ti

207. butter:ab,ti

208. herb*:ab,ti

209. spice*:ab,ti

210. chilli*:ab,ti

211. condiment*:ab,ti

212. MeSH descriptor [Condiments] explode all trees

213. MeSH descriptor [Beverages] this term only

214. beverage*:ab,ti

215. “fluid intake”:ab,ti

216. water:ab,ti

217. drink*:ab,ti

218. MeSH descriptor [Food Preservation] explode all trees

219. pickled:ab,ti

220. bottled:ab,ti

221. canned:ab,ti

222. canning:ab,ti

223. smoked:ab,ti

224. preserved:ab,ti

225. preservatives:ab,ti

226. nitrosamine:ab,ti

227. hydrogenation:ab,ti

228. fortified:ab,ti

229. nitrates:ab,ti

230. nitrites:ab,ti

231. ferment*:ab,ti

232. processed:ab,ti

233. antioxidant*:ab,ti

234. “genetic modif*”:ab,ti

235. “genetically modif*”:ab,ti

236. MeSH descriptor [Cooking] this term only

237. cooking:ab,ti

238. cooked:ab,ti

239. grill:ab,ti

240. grilled:ab,ti

241. fried:ab,ti

242. fry:ab,ti

243. roast:ab,ti

244. bake:ab,ti

245. baked:ab,ti

246. stewing:ab,ti

247. stewed:ab,ti

248. casserol*:ab,ti

249. broil:ab,ti

250. broiled:ab,ti

251. boiled:ab,ti

252. poach:ab,ti

253. poached:ab,ti

254. steamed:ab,ti

255. barbecue*:ab,ti

256. chargrill*:ab,ti

257. salt:ab,ti

258. salting:ab,ti

259. salted:ab,ti

260. fiber:ab,ti

261. fibre:ab,ti

262. polysaccharide*:ab,ti

263. starch:ab,ti

264. starchy:ab,ti

265. carbohydrate*:ab,ti

266. lipid*:ab,ti

267. “linoleic acid*”:ab,ti

268. sugar*:ab,ti

269. sweetener*:ab,ti

270. saccharin*:ab,ti

271. aspartame:ab,ti

272. sucrose:ab,ti

273. xylitol:ab,ti

274. cholesterol:ab,ti

275. “hydrogenated lard”:ab,ti

276. “dietary protein”:ab,ti

277. “dietary proteins”:ab,ti

278. “protein intake”:ab,ti

279. “animal protein*”:ab,ti

280. “total protein*”:ab,ti

281. “vegetable protein*”:ab,ti

282. “plant protein*”:ab,ti

283. MeSH descriptor [Dietary Carbohydrates] explode all trees

284. MeSH descriptor [Dietary Fats] explode all trees

285. MeSH descriptor [Dietary Fiber] explode all trees

286. MeSH descriptor [Dietary Proteins] explode all trees

287. MeSH descriptor [Dietary Supplements] explode all trees

288. MeSH descriptor [Food Additives] explode all trees

289. MeSH descriptor [Vitamins] explode all trees

290. supplements:ab,ti

291. supplement:ab,ti

292. vitamin*:ab,ti

293. retinol:ab,ti

294. carotenoid*:ab,ti

295. tocopherol:ab,ti

296. folate*:ab,ti

297. “folic acid”:ab,ti

298. methionine:ab,ti

299. riboflavin:ab,ti

300. thiamine:ab,ti

301. niacin:ab,ti

302. pyridoxine:ab,ti

303. cobalamin:ab,ti

304. mineral*:ab,ti

305. sodium:ab,ti

306. iron:ab,ti

307. calcium:ab,ti

308. selenium:ab,ti

309. iodine:ab,ti

310. magnesium:ab,ti

311. potassium:ab,ti

312. zinc:ab,ti

313. copper:ab,ti

314. phosphorus:ab,ti

315. manganese:ab,ti

316. chromium:ab,ti

317. phytochemical:ab,ti

318. polyphenol*:ab,ti

319. phytoestrogen*:ab,ti

320. genistein:ab,ti

321. saponin*:ab,ti

322. coumarin*:ab,ti

323. flavonoid*:ab,ti

324. polyphenol*:ab,ti

325. flavonol*:ab,ti

326. flavone*:ab,ti

327. isoflavone*:ab,ti

328. catechin*:ab,ti

329. “ascorbic acid*”:ab,ti

330. “hydroxy cholecalciferol*”:ab,ti

331. hydroxycholecalciferol*:ab,ti

332. tocotrienol*:ab,ti

333. carotene*:ab,ti

334. cryptoxanthin*:ab,ti

335. lycopene*:ab,ti

336. lutein*:ab,ti

337. zeaxanthin*:ab,ti

338. selenium*:ab,ti

339. “organic diet*”:ab,ti

340. MeSH descriptor [Food, Organic] this term only

341. 74 or 75 or 76 or 77 or 78 or 79 or 80 or 81 or 82 or 83 or 84 or 85 or 86 or 87 or 88 or 89 or 90 or 91 or 92 or 93 or 94 or 95 or 96 or 97 or 98 or 99 or 100 or 101 or 102 or 103 or 104 or 105 or 106 or 107 or 108 or 109 or 110 or 111 or 112 or 113 or 114 or 115 or 116 or 117 or 118 or 119 or 120 or 121 or 122 or 123 or 124 or 125 or 126 or 127 or 128 or 129 or 130 or 131 or 132 or 133 or 134 or 135 or 136 or 137 or 138 or 139 or 140 or 141 or 142 or 143 or 144 or 145 or 146 or 147 or 148 or 149 or 150 or 151 or 152 or 153 or 154 or 155 or 156 or 157 or 158 or 159 or 160 or 161 or 162 or 163 or 164 or 165 or 166 or 167 or 168 or 169 or 170 or 171 or 172 or 173 or 174 or 175 or 176 or 177 or 178 or 179 or 180 or 181 or 182 or 183 or 184 or 185 or 186 or 187 or 188 or 189 or 190 or 191 or 192 or 193 or 194 or 195 or 196 or 197 or 198 or 199 or 200 or 201 or 202 or 203 or 204 or 205 or 206 or 207 or 208 or 209 or 210 or 211 or 212 or 213 or 214 or 215 or 216 or 217 or 218 or 219 or 220 or 221 or 222 or 223 or 224 or 225 or 226 or 227 or 228 or 229 or 230 or 231 or 232 or 233 or 234 or 235 or 236 or 237 or 238 or 239 or 240 or 241 or 242 or 243 or 244 or 245 or 246 or 247 or 248 or 249 or 250 or 251 or 252 or 253 or 254 or 255 or 256 or 257 or 258 or 259 or 260 or 261 or 262 or 263 or 264 or 265 or 266 or 267 or 268 or 269 or 270 or 271 or 272 or 273 or 274 or 275 or 276 or 277 or 278 or 279 or 280 or 281 or 282 or 283 or 284 or 285 or 286 or 287 or 288 or 289 or 290 or 291 or 292 or 293 or 294 or 295 or 296 or 297 or 298 or 299 or 300 or 301 or 302 or 303 or 304 or 305 or 306 or 307 or 308 or 309 or 310 or 311 or 312 or 313 or 314 or 315 or 316 or 317 or 318 or 319 or 320 or 321 or 322 or 323 or 324 or 325 or 326 or 327 or 328 or 329 or 330 or 331 or 332 or 333 or 334 or 335 or 336 or 337 or 338 or 339 or 340

342. allerg*:ab,ti

343. asthma*:ab,ti

344. wheeze:ab,ti

345. wheezing:ab,ti

346. “bronchial hyperresponsiveness”:ab,ti

347. “bronchial hyperreactivity”:ab,ti

348. “Forced expiratory volume”:ab,ti

349. “FEV1”:ab,ti

350. "FEV 1":ab,ti

351. "FEV0.5":ab,ti

352. "FEV 0.5":ab,ti

353. “Forced vital capacity”:ab,ti

354. FVC:ab,ti

355. “Peak expiratory flow rate”:ab,ti

356. PEFR:ab,ti

357. eczema:ab,ti

358. neurodermatitis:ab,ti

359. rhinitis:ab,ti

360. “besniers prurigo”:ab,ti

361. rhinoconjunctivitis:ab,ti

362. hayfever:ab,ti

363. “hay fever”:ab,ti

364. poll*nosis:ab,ti

365. SAR:ab,ti

366. “pollen allergy”:ab,ti

367. conjunctivitis:ab,ti

368. immunoglobulin e:ab,ti

369. Total IgE:ab,ti

370. “autoimmune disease*”:ab,ti

371. diabetes:ab,ti

372. diabetic:ab,ti

373. “type 1”:ab,ti

374. “c*eliac disease”:ab,ti

375. “crohn* disease”:ab,ti

376. “Inflammatory Bowel Disease*”:ab,ti

377. “Ulcerative colitis”:ab,ti

378. (Lympho* NEAR/3 thyroiditi*):ab,ti

379. (Thyroiditi* NEAR/3 autoimmune):ab,ti

380. (Hashimoto* NEAR/3 (syndrome* or thyroiditi* or disease*)):ab,ti

381. (Thyroiditi* NEAR/3 (post-partum or postpartum)):ab,ti

382. “Graves* disease”:ab,ti

383. “Basedow* disease”:ab,ti

384. “exophthalmic goiter*”:ab,ti

385. (“Still* Disease” NEAR/3 (juvenile or onset)):ab,ti

386. (Juvenile NEAR/3 arthriti*):ab,ti

387. vitiligo:ab,ti

388. Psorias*s:ab,ti

389. (Arthriti* NEAR/3 Psoria*):ab,ti

390. “atopic disease”:ab,ti

391. “atopic dermatitis”:ab,ti

392. (food* NEAR/3 sensiti*):ab,ti

393. (food* NEAR/3 toleran*):ab,ti

394. (food* NEAR/3 intoleran*):ab,ti

395. ((aero or air*) NEAR/3 allergen*):ab,ti

396. (aeroallergen* NEAR/3 sensiti*):ab,ti

397. (allergen* NEAR/3 sensiti*):ab,ti

398. “skin prick test*”:ab,ti

399. atopy:ab,ti

400. hypersensitiv*:ab,ti

401. MeSH descriptor [Hypersensitivity] this term only

402. MeSH descriptor [Food Hypersensitivity] explode all trees

403. MeSH descriptor [Respiratory Hypersensitivity] this term only

404. MeSH descriptor [Asthma] this term only

405. MeSH descriptor [Bronchial Hyperreactivity] this term only

406. MeSH descriptor [Forced Expiratory Volume] this term only

407. MeSH descriptor [Vital Capacity] this term only

408. MeSH descriptor [Peak Expiratory Flow Rate] this term only

409. MeSH descriptor [Eczema] this term only

410. MeSH descriptor [Neurodermatitis] this term only

411. MeSH descriptor [Rhinitis] this term only

412. MeSH descriptor [Rhinitis, Allergic, Perennial] this term only

413. MeSH descriptor [Rhinitis, Allergic, Seasonal] this term only

414. MeSH descriptor [Conjunctivitis] this term only

415. MeSH descriptor [Immunoglobulin E] this term only

416. MeSH descriptor [Autoimmune Diseases] this term only

417. MeSH descriptor [Diabetes Mellitus, Type 1] this term only

418. MeSH descriptor [Celiac Disease] this term only

419. MeSH descriptor [Crohn Disease] this term only

420. MeSH descriptor [Inflammatory Bowel Diseases] this term only

421. MeSH descriptor [Colitis, Ulcerative] this term only

422. MeSH descriptor [Thyroiditis, Autoimmune] this term only

423. MeSH descriptor [Hashimoto Disease] this term only

424. MeSH descriptor [Postpartum Thyroiditis] this term only

425. MeSH descriptor [Graves Disease] this term only

426. MeSH descriptor [Arthritis, Juvenile Rheumatoid] this term only

427. MeSH descriptor [Vitiligo] this term only

428. MeSH descriptor [Psoriasis] this term only

429. MeSH descriptor [Arthritis, Psoriatic] this term only

430. MeSH descriptor [Dermatitis, Atopic] this term only

431. MeSH descriptor [Hypersensitivity, Immediate] this term only

432. 342 or 343 or 344 or 345 or 346 or 347 or 348 or 349 or 350 or 351 or 352 or 353 or 354 or 355 or 356 or 357 or 358 or 359 or 360 or 361 or 362 or 363 or 364 or 365 or 366 or 367 or 368 or 369 or 370 or 371 or 372 or 373 or 374 or 375 or 376 or 377 or 378 or 379 or 380 or 381 or 382 or 383 or 384 or 385 or 386 or 387 or 388 or 389 or 390 or 391 or 392 or 393 or 394 or 395 or 396 or 397 or 398 or 399 or 400 or 401 or 402 or 403 or 404 or 405 or 406 or 407 or 408 or 409 or 410 or 411 or 412 or 413 or 414 or 415 or 416 or 417 or 418 or 419 or 420 or 421 or 422 or 423 or 424 or 425 or 426 or 427 or 428 or 429 or 430 or 431

433. infant*:ab,ti

434. ((one or two or three or four or five or six or seven or eight or nine or ten or eleven or twelve or thirteen or fourteen or fifteen or sixteen or seventeen or eighteen or nineteen or twenty or "twenty one" or "twenty two" or "twenty three" or "twenty four" or "twenty five" or "twenty six") NEAR/1 week*):ab,ti

435. ((one or two or three or four or five or six or seven or eight or nine or ten or eleven or twelve or thirteen or fourteen or fifteen or sixteen or seventeen or eighteen or nineteen or twenty or "twenty one" or "twenty two" or "twenty three" or "twenty four") NEAR/1 month*):ab,ti

436. 434 or 435

437. (old or age*):ab,ti

438. 436 and 437

439. (("one year*" or "two year*") NEAR/3 (old or age*)):ab,ti

440. ((first or second or two) NEAR/3 "year* of life"):ab,ti

441. MeSH descriptor [Infant] this term only

442. MeSH descriptor [Infant, Newborn] this term only

443. (maternal NEAR/7 pregnan*):ab,ti

444. (maternal NEAR/7 lactat*):ab,ti

445. (mother* NEAR/7 pregnan*):ab,ti

446. 433 or 438 or 439 or 440 or 441 or 442 or 443 or 444 or 445

447. 16 or 73 or 341

448. 432 and 446 and 447

Publication date from 2011

# Appendix 2 Search Strategies for original articles (Review A)

## 2.1. Medline

1. breast feeding.ab,ti.

2. breastfeeding.ab,ti.

3. breast fed.ab,ti.

4. breastfed.ab,ti.

5. Breast Feeding/

6. Milk, Human/

7. formula?.ab,ti.

8. hydrolysed.ab,ti.

9. bottlefed.ab,ti.

10. bottle fed.ab,ti.

11. (bottle adj3 feed$).ab,ti.

12. Infant Formula/

13. Bottle Feeding/

14. wean$.ab,ti.

15. Weaning/

16. 1 or 2 or 3 or 4 or 5 or 6 or 7 or 8 or 9 or 10 or 11 or 12 or 13 or 14 or 15

17. allerg$.ab,ti.

18. asthma$.ab,ti.

19. wheeze.ab,ti.

20. wheezing.ab,ti.

21. bronchial hyperresponsiveness.ab,ti.

22. bronchial hyperreactivity.ab,ti.

23. Forced expiratory volume.ab,ti.

24. FEV1.ab,ti.

25. "FEV 1".ab,ti.

26. "FEV0.5".ab,ti.

27. "FEV 0.5".ab,ti.

28. Forced vital capacity.ab,ti.

29. FVC.ab,ti.

30. Peak expiratory flow rate.ab,ti.

31. PEFR.ab,ti.

32. eczema.ab,ti.

33. neurodermatitis.ab,ti.

34. rhinitis.ab,ti.

35. besniers prurigo.ab,ti.

36. rhinoconjunctivitis.ab,ti.

37. hayfever.ab,ti.

38. (hay adj fever).ab,ti.

39. poll?nosis.ab,ti.

40. SAR.ab,ti.

41. (pollen adj allergy).ab,ti.

42. conjunctivitis.ab,ti.

43. immunoglobulin e.ab,ti.

44. Total IgE.ab,ti.

45. autoimmune disease?.ab,ti.

46. diabetes.ab,ti.

47. diabetic.ab,ti.

48. type 1.ab,ti.

49. c?eliac disease.ab,ti.

50. crohn$ disease.ab,ti.

51. Inflammatory Bowel Disease?.ab,ti.

52. Ulcerative colitis.ab,ti.

53. (Lympho$ adj3 thyroiditi$).ab,ti.

54. (Thyroiditi$ adj3 autoimmune).ab,ti.

55. (Hashimoto$ adj3 (syndrome? or thyroiditi$ or disease?)).ab,ti.

56. (Thyroiditi$ adj3 (post-partum or postpartum)).ab,ti.

57. Graves? disease.ab,ti.

58. Basedow$ disease.ab,ti.

59. exophthalmic goiter?.ab,ti.

60. (Still? Disease adj3 (juvenile or onset)).ab,ti.

61. (Juvenile adj3 arthriti$).ab,ti.

62. vitiligo.ab,ti.

63. Psorias?s.ab,ti.

64. (Arthriti? adj3 Psoria$).ab,ti.

65. atopic disease.ab,ti.

66. atopic dermatitis.ab,ti.

67. (food? adj3 sensiti$).ab,ti.

68. (food? adj3 toleran$).ab,ti.

69. (food? adj3 intoleran$).ab,ti.

70. ((aero or air$) adj3 allergen?).ab,ti.

71. (aeroallergen? adj3 sensiti$).ab,ti.

72. (allergen? adj3 sensiti$).ab,ti.

73. skin prick test$.ab,ti.

74. atopy.ab,ti.

75. hypersensitiv$.ab,ti.

76. Hypersensitivity/

77. exp Food Hypersensitivity/

78. Respiratory Hypersensitivity/

79. Asthma/

80. Bronchial Hyperreactivity/

81. Forced Expiratory Volume/

82. Vital Capacity/

83. Peak Expiratory Flow Rate/

84. Eczema/

85. Neurodermatitis/

86. Rhinitis/

87. Rhinitis, Allergic, Perennial/

88. Rhinitis, Allergic, Seasonal/

89. Conjunctivitis/

90. Immunoglobulin E/

91. Autoimmune Diseases/

92. Diabetes Mellitus, Type 1/

93. Celiac Disease/

94. Crohn Disease/

95. Inflammatory Bowel Diseases/

96. Colitis, Ulcerative/

97. Thyroiditis, Autoimmune/

98. Hashimoto Disease/

99. Postpartum Thyroiditis/

100. Graves Disease/

101. Arthritis, Juvenile Rheumatoid/

102. Vitiligo/

103. Psoriasis/

104. Arthritis, Psoriatic/

105. Dermatitis, Atopic/

106. Hypersensitivity, Immediate/

107. 17 or 18 or 19 or 20 or 21 or 22 or 23 or 24 or 25 or 26 or 27 or 28 or 29 or 30 or 31 or 32 or 33 or 34 or 35 or 36 or 37 or 38 or 39 or 40 or 41 or 42 or 43 or 44 or 45 or 46 or 47 or 48 or 49 or 50 or 51 or 52 or 53 or 54 or 55 or 56 or 57 or 58 or 59 or 60 or 61 or 62 or 63 or 64 or 65 or 66 or 67 or 68 or 69 or 70 or 71 or 72 or 73 or 74 or 75 or 76 or 77 or 78 or 79 or 80 or 81 or 82 or 83 or 84 or 85 or 86 or 87 or 88 or 89 or 90 or 91 or 92 or 93 or 94 or 95 or 96 or 97 or 98 or 99 or 100 or 101 or 102 or 103 or 104 or 105 or 106

108. infant?.ab,ti.

109. ((one or two or three or four or five or six or seven or eight or nine or ten or eleven or twelve or thirteen or fourteen or fifteen or sixteen or seventeen or eighteen or nineteen or twenty or "twenty one" or "twenty two" or "twenty three" or "twenty four" or "twenty five" or "twenty six") adj week?).ab,ti.

110. ((one or two or three or four or five or six or seven or eight or nine or ten or eleven or twelve or thirteen or fourteen or fifteen or sixteen or seventeen or eighteen or nineteen or twenty or "twenty one" or "twenty two" or "twenty three" or "twenty four") adj month?).ab,ti.

111. 109 or 110

112. (old or age?).ab,ti.

113. 111 and 112

114. (("one year?" or "two year?") adj3 (old or age?)).ab,ti.

115. ((first or second or two) adj3 "year? of life").ab,ti.

116. Infant/

117. Infant, Newborn/

118. 108 or 113 or 114 or 115 or 116 or 117

119. clinical trial?.mp.

120. random$.mp.

121. factorial$.mp.

122. crossover$.mp.

123. placebo$.mp.

124. (doubl$ adj blind$).mp.

125. (singl$ adj blind$).mp.

126. assign$.mp.

127. volunteer$.mp.

128. cohort stud$.mp.

129. longitudinal$.mp.

130. follow-up.mp.

131. prospectiv$.mp.

132. retrospectiv$.mp.

133. case control.mp.

134. case referent.mp.

135. exp clinical trial/

136. Cross-Over Studies/

137. Placebos/

138. Double-Blind Method/

139. Single-Blind Method/

140. exp Cohort Studies/

141. case-control studies/

142. 119 or 120 or 121 or 122 or 123 or 124 or 125 or 126 or 127 or 128 or 129 or 130 or 131 or 132 or 133 or 134 or 135 or 136 or 137 or 138 or 139 or 140 or 141

143. 16 and 107 and 118 and 142

## 2.2. Embase

1. breast feeding.ab,ti.

2. breastfeeding.ab,ti.

3. breast fed.ab,ti.

4. breastfed.ab,ti.

5. breast feeding/

6. breast milk/

7. formula?.ab,ti.

8. hydrolysed.ab,ti.

9. bottlefed.ab,ti.

10. bottle fed.ab,ti.

11. (bottle adj3 feed$).ab,ti.

12. artificial milk/

13. bottle feeding/

14. wean$.ti,ab.

15. weaning/

16. 1 or 2 or 3 or 4 or 5 or 6 or 7 or 8 or 9 or 10 or 11 or 12 or 13 or 14 or 15

17. allerg$.ab,ti.

18. asthma$.ab,ti.

19. wheeze.ab,ti.

20. wheezing.ab,ti.

21. bronchial hyperresponsiveness.ab,ti.

22. bronchial hyperreactivity.ab,ti.

23. Forced expiratory volume.ab,ti.

24. FEV1.ab,ti.

25. "FEV 1".ab,ti.

26. "FEV0.5".ab,ti.

27. "FEV 0.5".ab,ti.

28. Forced vital capacity.ab,ti.

29. FVC.ab,ti.

30. Peak expiratory flow rate.ab,ti.

31. PEFR.ab,ti.

32. eczema.ab,ti.

33. neurodermatitis.ab,ti.

34. rhinitis.ab,ti.

35. besniers prurigo.ab,ti.

36. rhinoconjunctivitis.ab,ti.

37. hayfever.ab,ti.

38. (hay adj fever).ab,ti.

39. poll?nosis.ab,ti.

40. SAR.ab,ti.

41. (pollen adj allergy).ab,ti.

42. conjunctivitis.ab,ti.

43. immunoglobulin e.ab,ti.

44. Total IgE.ab,ti.

45. autoimmune disease?.ab,ti.

46. diabetes.ab,ti.

47. diabetic.ab,ti.

48. type 1.ab,ti.

49. c?eliac disease.ab,ti.

50. crohn$ disease.ab,ti.

51. Inflammatory Bowel Disease?.ab,ti.

52. Ulcerative colitis.ab,ti.

53. (Lympho$ adj3 thyroiditi$).ab,ti.

54. (Thyroiditi$ adj3 autoimmune).ab,ti.

55. (Hashimoto$ adj3 (syndrome? or thyroiditi$ or disease?)).ab,ti.

56. (Thyroiditi$ adj3 (post-partum or postpartum)).ab,ti.

57. Graves? disease.ab,ti.

58. Basedow$ disease.ab,ti.

59. exophthalmic goiter?.ab,ti.

60. (Still? Disease adj3 (juvenile or onset)).ab,ti.

61. (Juvenile adj3 arthriti$).ab,ti.

62. vitiligo.ab,ti.

63. Psorias?s.ab,ti.

64. (Arthriti? adj3 Psoria$).ab,ti.

65. atopic disease.ab,ti.

66. atopic dermatitis.ab,ti.

67. (food? adj3 sensiti$).ab,ti.

68. (food? adj3 toleran$).ab,ti.

69. (food? adj3 intoleran$).ab,ti.

70. ((aero or air$) adj3 allergen?).ab,ti.

71. (aeroallergen? adj3 sensiti$).ab,ti.

72. (allergen? adj3 sensiti$).ab,ti.

73. skin prick test$.ab,ti.

74. atopy.ab,ti.

75. hypersensitiv$.ab,ti.

76. exp hypersensitivity/

77. respiratory tract allergy/

78. asthma/

79. wheezing/

80. bronchus hyperreactivity/

81. forced expiratory volume/

82. forced vital capacity/

83. peak expiratory flow/

84. eczema/

85. neurodermatitis/

86. rhinitis/

87. rhinoconjunctivitis/

88. hay fever/

89. pollen allergy/

90. perennial rhinitis/

91. conjunctivitis/

92. immunoglobulin E/

93. autoimmune disease/

94. diabetes mellitus/

95. insulin dependent diabetes mellitus/

96. celiac disease/

97. Crohn disease/

98. enteritis/

99. ulcerative colitis/

100. autoimmune thyroiditis/

101. Hashimoto disease/

102. postpartum thyroiditis/

103. Graves disease/

104. juvenile rheumatoid arthritis/

105. vitiligo/

106. psoriasis/

107. psoriatic arthritis/

108. atopic dermatitis/

109. nutritional intolerance/

110. 17 or 18 or 19 or 20 or 21 or 22 or 23 or 24 or 25 or 26 or 27 or 28 or 29 or 30 or 31 or 32 or 33 or 34 or 35 or 36 or 37 or 38 or 39 or 40 or 41 or 42 or 43 or 44 or 45 or 46 or 47 or 48 or 49 or 50 or 51 or 52 or 53 or 54 or 55 or 56 or 57 or 58 or 59 or 60 or 61 or 62 or 63 or 64 or 65 or 66 or 67 or 68 or 69 or 70 or 71 or 72 or 73 or 74 or 75 or 76 or 77 or 78 or 79 or 80 or 81 or 82 or 83 or 84 or 85 or 86 or 87 or 88 or 89 or 90 or 91 or 92 or 93 or 94 or 95 or 96 or 97 or 98 or 99 or 100 or 101 or 102 or 103 or 104 or 105 or 106 or 107 or 108 or 109

111. infant?.ab,ti.

112. ((one or two or three or four or five or six or seven or eight or nine or ten or eleven or twelve or thirteen or fourteen or fifteen or sixteen or seventeen or eighteen or nineteen or twenty or "twenty one" or "twenty two" or "twenty three" or "twenty four" or "twenty five" or "twenty six") adj week?).ab,ti.

113. ((one or two or three or four or five or six or seven or eight or nine or ten or eleven or twelve or thirteen or fourteen or fifteen or sixteen or seventeen or eighteen or nineteen or twenty or "twenty one" or "twenty two" or "twenty three" or "twenty four") adj month?).ab,ti.

114. 112 or 113

115. (old or age?).ab,ti.

116. 114 and 115

117. (("one year?" or "two year?") adj3 (old or age?)).ab,ti.

118. ((first or second or two) adj3 "year? of life").ab,ti.

119. infant/

120. newborn/

121. 111 or 116 or 117 or 118 or 119 or 120

122. clinical trial?.mp.

123. random$.mp.

124. factorial$.mp.

125. crossover$.mp.

126. placebo$.mp.

127. (doubl$ adj blind$).mp.

128. (singl$ adj blind$).mp.

129. assign$.mp.

130. volunteer$.mp.

131. cohort stud$.mp.

132. longitudinal$.mp.

133. follow-up.mp.

134. prospectiv$.mp.

135. retrospectiv$.mp.

136. case control.mp.

137. case referent.mp.

138. exp clinical trial/

139. crossover procedure/

140. placebo/

141. double blind procedure/

142. single blind procedure/

143. cohort analysis/

144. longitudinal study/

145. follow up/

146. prospective study/

147. retrospective study/

148. exp case control study/

149. 122 or 123 or 124 or 125 or 126 or 127 or 128 or 129 or 130 or 131 or 132 or 133 or 134 or 135 or 136 or 137 or 138 or 139 or 140 or 141 or 142 or 143 or 144 or 145 or 146 or 147 or 148

150. 16 and 110 and 121 and 149

## 2.3. LILACS

(tw:((breast feeding) or breastfeeding or (breast fed) or breastfed or formula* or hydrolysed or bottlefed or (bottle fed) or (bottle feed*) or wean*)

AND

(tw:(allerg* or asthma* or wheez* or (bronchial hyperresponsiveness) or (bronchial hyperreactivity) or (Forced expiratory volume) or FEV1 or (FEV 1) or FEV0.5 or (FEV 0.5) or (Forced vital capacity) or FVC or (Peak expiratory flow rate) or PEFR or eczema or neurodermatitis or rhinitis or (besniers prurigo) or rhinoconjunctivitis or hayfever or (hay fever) or poll?nosis or SAR or (pollen allergy) or conjunctivitis or (immunoglobulin e) or (Total IgE) or (autoimmune disease*) or diabetes or diabetic or (type 1) or (c?eliac disease) or (crohn* disease) or (Inflammatory Bowel Disease*) or (Ulcerative colitis) or (Lympho* thyroiditi*) or (Thyroiditi* autoimmune) or (Hashimoto* syndrome*) or (Hashimoto* thyroiditis*) or (Hashimoto* disease*) or (Thyroiditi* post-partum) or (Thyroiditi* postpartum) or (Graves* Disease) or (Basedow* disease) or (exophthalmic goiter*) or (Still’s Disease) or (Stills disease) or (Juvenile arthriti*) or vitiligo or Psorias?s or (Arthriti* Psoria*) or (atopic disease) or (atopic dermatitis) or (food* sensiti*) or (food* toleran*) or (food* intoleran*) or (aero allergen*) or (air* allergen*) or (aeroallergen* sensiti*) or (allergen* sensiti*) or (skin prick test*) or atopy or hypersensitive*)

AND

db:(“LILACS”)

AND

type_of_study:(“clinical_trials” or “case_control” or “cohort” or “systematic_reviews”)

AND

limit:(“infant” or “newborn” or “preschool” or “child”)

## 2.4. COCHRANE Library

1. “breast feeding”:ab,ti

2. breastfeeding:ab,ti

3. “breast fed”:ab,ti

4. breastfed:ab,ti

5. MeSH descriptor [Breast Feeding] this term only

6. MeSH descriptor [Milk, Human] this term only

7. formula*:ab,ti

8. hydrolysed:ab,ti

9. bottlefed:ab,ti

10. “bottle fed”:ab,ti

11. (bottle NEAR/3 feed*):ab,ti

12. MeSH descriptor [Infant Formula] this term only

13. MeSH descriptor [Bottle Feeding] this term only

14. wean*:ab,ti

15. MeSH descriptor [Weaning] this term only

16. 1 or 2 or 3 or 4 or 5 or 6 or 7 or 8 or 9 or 10 or 11 or 12 or 13 or 14 or 15

17. allerg*:ab,ti

18. asthma*:ab,ti

19. wheeze:ab,ti

20. wheezing:ab,ti

21. “bronchial hyperresponsiveness”:ab,ti

22. “bronchial hyperreactivity”:ab,ti

23. “Forced expiratory volume”:ab,ti

24. “FEV1”:ab,ti

25. "FEV 1":ab,ti

26. "FEV0.5":ab,ti

27. "FEV 0.5":ab,ti

28. “Forced vital capacity”:ab,ti

29. FVC:ab,ti

30. “Peak expiratory flow rate”:ab,ti

31. PEFR:ab,ti

32. eczema:ab,ti

33. neurodermatitis:ab,ti

34. rhinitis:ab,ti

35. “besniers prurigo”:ab,ti

36. rhinoconjunctivitis:ab,ti

37. hayfever:ab,ti

38. “hay fever”:ab,ti

39. poll*nosis:ab,ti

40. SAR:ab,ti

41. “pollen allergy”:ab,ti

42. conjunctivitis:ab,ti

43. “immunoglobulin e”:ab,ti

44. “Total IgE”:ab,ti

45. “autoimmune disease*”:ab,ti

46. diabetes:ab,ti

47. diabetic:ab,ti

48. “type 1”:ab,ti

49. “c*eliac disease”:ab,ti

50. “crohn* disease”:ab,ti

51. “Inflammatory Bowel Disease*”:ab,ti

52. “Ulcerative colitis”:ab,ti

53. (Lympho* NEAR/3 thyroiditi*):ab,ti

54. (Thyroiditi* NEAR/3 autoimmune):ab,ti

55. (Hashimoto* NEAR/3 (syndrome* or thyroiditi* or disease*)):ab,ti

56. (Thyroiditi* NEAR/3 (post-partum or postpartum)):ab,ti

57. “Graves* disease”:ab,ti

58. “Basedow* disease”:ab,ti

59. “exophthalmic goiter*”:ab,ti

60. (Still* Disease NEAR/3 (juvenile or onset)):ab,ti

61. (Juvenile NEAR/3 arthriti*):ab,ti

62. vitiligo:ab,ti

63. Psorias*s:ab,ti

64. (Arthriti* NEAR/3 Psoria*):ab,ti

65. “atopic disease”:ab,ti

66. “atopic dermatitis”:ab,ti

67. (food* NEAR/3 sensiti*):ab,ti

68. (food* NEAR/3 toleran*):ab,ti

69. (food* NEAR/3 intoleran*):ab,ti

70. ((aero or air*) NEAR/3 allergen*):ab,ti

71. (aeroallergen* NEAR/3 sensiti*):ab,ti

72. (allergen* NEAR/3 sensiti*):ab,ti

73. “skin prick test*”:ab,ti

74. atopy:ab,ti

75. hypersensitiv*:ab,ti

76. MeSH descriptor [Hypersensitivity] this term only

77. MeSH descriptor [Food Hypersensitivity] explode all trees

78. MeSH descriptor [Respiratory Hypersensitivity] this term only

79. MeSH descriptor [Asthma] this term only

80. MeSH descriptor [Bronchial Hyperreactivity] this term only

81. MeSH descriptor [Forced Expiratory Volume] this term only

82. MeSH descriptor [Vital Capacity] this term only

83. MeSH descriptor [Peak Expiratory Flow Rate] this term only

84. MeSH descriptor [Eczema] this term only

85. MeSH descriptor [Neurodermatitis] this term only

86. MeSH descriptor [Rhinitis] this term only

87. MeSH descriptor [Rhinitis, Allergic, Perennial] this term only

88. MeSH descriptor [Rhinitis, Allergic, Seasonal] this term only

89. MeSH descriptor [Conjunctivitis] this term only

90. MeSH descriptor [Immunoglobulin E] this term only

91. MeSH descriptor [Autoimmune Diseases] this term only

92. MeSH descriptor [Diabetes Mellitus, Type 1] this term only

93. MeSH descriptor [Celiac Disease] this term only

94. MeSH descriptor [Crohn Disease] this term only

95. MeSH descriptor [Inflammatory Bowel Diseases] this term only

96. MeSH descriptor [Colitis, Ulcerative] this term only

97. MeSH descriptor [Thyroiditis, Autoimmune] this term only

98. MeSH descriptor [Hashimoto Disease] this term only

99. MeSH descriptor [Postpartum Thyroiditis] this term only

100. MeSH descriptor [Graves Disease] this term only

101. MeSH descriptor [Arthritis, Juvenile Rheumatoid] this term only

102. MeSH descriptor [Vitiligo] this term only

103. MeSH descriptor [Psoriasis] this term only

104. MeSH descriptor [Arthritis, Psoriatic] this term only

105. MeSH descriptor [Dermatitis, Atopic] this term only

106. MeSH descriptor [Hypersensitivity, Immediate] this term only

107. 17 or 18 or 19 or 20 or 21 or 22 or 23 or 24 or 25 or 26 or 27 or 28 or 29 or 30 or 31 or 32 or 33 or 34 or 35 or 36 or 37 or 38 or 39 or 40 or 41 or 42 or 43 or 44 or 45 or 46 or 47 or 48 or 49 or 50 or 51 or 52 or 53 or 54 or 55 or 56 or 57 or 58 or 59 or 60 or 61 or 62 or 63 or 64 or 65 or 66 or 67 or 68 or 69 or 70 or 71 or 72 or 73 or 74 or 75 or 76 or 77 or 78 or 79 or 80 or 81 or 82 or 83 or 84 or 85 or 86 or 87 or 88 or 89 or 90 or 91 or 92 or 93 or 94 or 95 or 96 or 97 or 98 or 99 or 100 or 101 or 102 or 103 or 104 or 105 or 106

108. infant*:ab,ti

109. ((one or two or three or four or five or six or seven or eight or nine or ten or eleven or twelve or thirteen or fourteen or fifteen or sixteen or seventeen or eighteen or nineteen or twenty or "twenty one" or "twenty two" or "twenty three" or "twenty four" or "twenty five" or "twenty six") NEAR/1 week*):ab,ti

110. ((one or two or three or four or five or six or seven or eight or nine or ten or eleven or twelve or thirteen or fourteen or fifteen or sixteen or seventeen or eighteen or nineteen or twenty or "twenty one" or "twenty two" or "twenty three" or "twenty four") NEAR/1 month*):ab,ti

111. 109 or 110

112. (old or age*):ab,ti

113. 111 and 112

114. (("one year*" or "two year*") NEAR/3 (old or age*)):ab,ti

115. ((first or second or two) NEAR/3 "year* of life"):ab,ti

116. MeSH descriptor [Infant] this term only

117. MeSH descriptor [Infant, Newborn] this term only

118. 108 or 113 or 114 or 115 or 116 or 117

119. “clinical trial*”

120. random*

121. factorial*

122. crossover*

123. placebo*

124. “doubl* blind*”

125. “singl* blind*”

126. assign*

127. volunteer*

128. “cohort stud*”

129. longitudinal*

130. follow-up

131. prospectiv*

132. retrospectiv*

133. “case control”

134. “case referent”

135. MeSH descriptor [clinical trial] explode all trees

136. MeSH descriptor [Cross-Over Studies] this term only

137. MeSH descriptor [Placebos] this term only

138. MeSH descriptor [Double-Blind Method] this term only

139. MeSH descriptor [Single-Blind Method] this term only

140. MeSH descriptor [Cohort Studies] explode all trees

141. MeSH descriptor [case-control studies] this term only

142. 119 or 120 or 121 or 122 or 123 or 124 or 125 or 126 or 127 or 128 or 129 or 130 or 131 or 132 or 133 or 134 or 135 or 136 or 137 or 138 or 139 or 140 or 141

143. 16 and 107 and 118 and 142

## 2.5. Web of Science

1. TOPIC = (“breast feeding” or breastfeeding or “breast fed” or breastfed or formula$ or hydrolysed or bottlefed or “bottle fed” or (bottle NEAR/3 feed*) or wean*)

2. TOPIC = (allerg* or asthma* or wheeze or wheezing or “bronchial hyperresponsiveness” or “bronchial hyperreactivity” or “Forced expiratory volume” or “FEV1” or "FEV 1" or "FEV0.5" or "FEV 0.5" or “Forced vital capacity” or FVC or “Peak expiratory flow rate” or PEFR or eczema or neurodermatitis or rhinitis or “besniers prurigo” or rhinoconjunctivitis or hayfever or “hay fever” or poll$nosis or SAR or “pollen allergy” or conjunctivitis or “immunoglobulin e” or “Total IgE” or “autoimmune disease$” or diabetes or diabetic or “type 1” or “c$eliac disease” or “crohn* disease” or “Inflammatory Bowel Disease$” or “Ulcerative colitis” or (Lympho* NEAR/3 thyroiditi*) or (Thyroiditi* NEAR/3 autoimmune) or (Hashimoto* NEAR/3 (syndrome$ or thyroiditis* or disease$)) or (Thyroiditi* NEAR/3 (post-partum or postpartum)) or “Graves$ Disease” or “Basedow* disease” or “exophthalmic goiter$” or (“Still$ Disease” NEAR/3 (juvenile or onset)) or (Juvenile NEAR/3 arthriti*) or vitiligo or Psorias$s or (Arthriti$ NEAR/3 Psoria*) or “atopic disease” or “atopic dermatitis” or (food$ NEAR/3 sensiti*) or (food$ NEAR/3 toleran*) or (food$ NEAR/3 intoleran*) or ((aero or air*) NEAR/3 allergen$) or (aeroallergen$ NEAR/3 sensiti*) or (allergen$ NEAR/3 sensiti*) or “skin prick test*” or atopy or hypersensitive*)

3. TOPIC = (infant$ or (("one year$" or "two year$") NEAR/3 (old or age$)) or ((first or second or two) NEAR/3 "year$ of life"))

4. TOPIC = ((one or two or three or four or five or six or seven or eight or nine or ten or eleven or twelve or thirteen or fourteen or fifteen or sixteen or seventeen or eighteen or nineteen or twenty or "twenty one" or "twenty two" or "twenty three" or "twenty four" or "twenty five" or "twenty six") NEAR/1 week$)

5. TOPIC = ((one or two or three or four or five or six or seven or eight or nine or ten or eleven or twelve or thirteen or fourteen or fifteen or sixteen or seventeen or eighteen or nineteen or twenty or "twenty one" or "twenty two" or "twenty three" or "twenty four") NEAR/1 month$)

6. 4 or 5

7. TOPIC = ((old or age$))

8. 7 and 6

9. 8 or 3

10. TOPIC = (“clinical trial$” or random* or factorial* or crossover* or placebo* or “doubl* blind*” or “singl* blind*” or assign* or volunteer* or “cohort stud*” or longitudinal* or follow-up or prospective* or retrospective* or “case control” or “case referent”)

11. 1 and 2 and 9 and 10
