# Supplementary material for: Diet during pregnancy and infancy and risk of allergic or autoimmune disease: A systematic review and meta-analysis
Source: PLoS Med. 2018 Feb 28;15(2):e1002507. doi: 10.1371/journal.pmed.1002507 (PMC5830033; doi:10.1371/journal.pmed.1002507)
Supplement: S3 Text — (DOC) [file pmed.1002507.s004.doc]

**Dietary Exposures during Pregnancy, Lactation and/or Infancy for Reducing Risk of Allergic or Autoimmune Outcomes: a systematic review and meta-analysis (Review C Part II).**

Robert J Boyle^1^, Vanessa Garcia-Larsen^2^, Despo Ierodiakonou^3^, Tasnia Khan^4^, Thalia Afxentiou^5^, Jo Leonardi-Bee^6^, Tim Reeves^7^, Jennifer Chivinge^4^, Zoe Robinson^4^, Natalie Geoghegan^4^, Katharine Jarrold^4^, Alisha Ruparelia^4^, Pooja Devani^4^, Evangelia Andreou^8^, Sergio Cunha^9^ , Marialena Trivella^10^

^1^Clinical Senior Lecturer, Section of Paediatrics, Imperial College London; ^2^Post-Doctoral Research Associate, Respiratory Epidemiology, Occupational Medicine, and Public Health Group, National Heart and Lung Institute, Imperial College London; ^3^Post-Doctoral Research Associate, Departments of Paediatric and Respiratory Epidemiology, Occupational Medicine, and Public Health Group, Imperial College London; ^4^Undergraduate medical student, Imperial College London; ^5^Specialist Registrar in Dermatology, Imperial College London; ^6^Associate Professor of Community Health Sciences, University of Nottingham; ^7^Research Support Librarian, Faculty of Medicine, Imperial College London; ^8^Research Associate, Imperial Consultants;  ^9^Research Associate, Respiratory Epidemiology, Occupational Medicine, and Public Health Group, National Heart and Lung Institute, Imperial College London; ^10^Centre for Statistics in Medicine, Oxford University

Imperial Consultants,

58 Princes Gate,

Exhibition Road,

London SW7 2PG

List of contents

[List of Tables 4](#__RefHeading___Toc439844717)

[I. Background 5](#__RefHeading___Toc439844718)

[1. Review C Part II: other maternal (pregnancy/ lactation) and/or infant dietary exposures 7](#__RefHeading___Toc439844719)

[1.1. Specific questions addressed in Review C 7](#__RefHeading___Toc439844720)

[1.2. Glossary of key terms 8](#__RefHeading___Toc439844721)

[1.3. Executive summary 10](#__RefHeading___Toc439844722)

[1.3.1. Probiotic supplementation 12](#__RefHeading___Toc439844723)

[1.3.2. Prebiotic supplementation 13](#__RefHeading___Toc439844724)

[1.3.3. PUFA supplementation 14](#__RefHeading___Toc439844725)

[1.3.4. Maternal allergenic food avoidance 15](#__RefHeading___Toc439844726)

[1.3.5. Multifaceted interventions 16](#__RefHeading___Toc439844727)

[1.3.6. Fruits and vegetables – observational studies 17](#__RefHeading___Toc439844728)

[1.3.7. Fats and fatty acids – observational studies 19](#__RefHeading___Toc439844729)

[1.3.8. Vitamins and minerals 17](#__RefHeading___Toc439844730)

[1.3.9. ‘Other dietary exposures’ – observational studies 20](#__RefHeading___Toc439844731)

[2. Methodology 21](#__RefHeading___Toc439844732)

[2.1. Inclusion Criteria 21](#__RefHeading___Toc439844733)

[2.1.1. Types of study included 21](#__RefHeading___Toc439844734)

[2.1.2. Participants/population 22](#__RefHeading___Toc439844735)

[2.1.3. Interventions/Exposures 22](#__RefHeading___Toc439844736)

[2.1.4. Study outcomes 22](#__RefHeading___Toc439844737)

[2.1.5. Atopic outcomes: 23](#__RefHeading___Toc439844738)

[2.2. Search strategy 25](#__RefHeading___Toc439844739)

[2.3. Study selection, data extraction and analysis 26](#__RefHeading___Toc439844740)

[2.3.1. Study selection 26](#__RefHeading___Toc439844741)

[2.3.2. Data extraction 27](#__RefHeading___Toc439844742)

[2.3.3. Data cleaning and coding 27](#__RefHeading___Toc439844743)

[2.3.4. Risk of bias assessment 28](#__RefHeading___Toc439844744)

[2.3.5. Strategy for data synthesis 29](#__RefHeading___Toc439844745)

[2.4. Review registration 31](#__RefHeading___Toc439844746)

[2.5. Differences between the protocol and review 32](#__RefHeading___Toc439844747)

[3. Results of the Searches 33](#__RefHeading___Toc439844748)

[3.1. Systematic reviews identified 33](#__RefHeading___Toc439844749)

[3.2. Original studies identified 33](#__RefHeading___Toc439844750)

[4. General Conclusions 34](#__RefHeading___Toc439844751)

[Acknowlegements 35](#__RefHeading___Toc439844752)

[References 36](#__RefHeading___Toc439844753)

[Appendix 1 Tables 39](#__RefHeading___Toc439844754)

[Appendix 2 Search Strategies for other systematic reviews 47](#__RefHeading___Toc439844755)

[Appendix 3 Search Strategies for original articles (Review C) 77](#__RefHeading___Toc439844756)

# List of Tables

[Table 1 Individual study reports in relation to 5 intervention groups of interest 11](#__RefHeading___Toc427216080)

[Table 2 Quality assessment of recent SRs using R-AMSTAR scoring 42](#__RefHeading___Toc427216081)

[Table 3 Characteristics of included systematic reviews 43](#__RefHeading___Toc427216082)

[Table 4 Summary of findings 45](#__RefHeading___Toc427216083)

# Background

Review C Part II is one of 4 reports resulting from a comprehensive review of the scientific literature on infant feeding and the development of atopic and autoimmune diseases, commissioned by the UK Food Standards Agency. Atopic conditions such as asthma, eczema, rhinoconjunctivitis and food allergy appear to have increased in prevalence in recent decades in many countries, and are some of the commonest causes of chronic illness in children and young adults living in the UK . An apparent increase in disease prevalence, combined with data from migration studies, suggests that early-life environmental factors may be important modulators of atopic disease risk. Similarly the autoimmune diseases type I diabetes mellitus and Crohn’s disease appear to have increased in some countries . Significant attention has focussed on early-life dietary exposures in relation to these atopic and autoimmune diseases, due to recent changes in the human diet, and the potential effects of such changes on intestinal and systemic immune development . The gut associated lymphoid tissue is the largest collection of immune tissue in humans, and is the most mature immune organ at the time of birth . So enteral exposures in infancy are likely to be especially potent modulators of immune development and risk of immune-mediated disease. Although there is a large number of observational studies, some intervention trials and several systematic reviews in this area, they tend to focus on one specific area of diet and a limited number of immune outcomes. The purpose of this project is to assess comprehensively and systematically the existing literature regarding the relationship between dietary exposures during pregnancy, lactation and infancy, and a child’s risk of developing any of the common atopic and autoimmune diseases.

This project consists of a series of systematic reviews which together have very broad inclusion criteria and were registered as 3 separate review protocols on the International Prospective Register of Systematic Reviews (PROSPERO references CRD42013003802 – REVIEW A; CRD42013004239 – REVIEW B; CRD42013004252 – REVIEW C; [www.crd.york.ac.uk/Prospero](http://www.crd.york.ac.uk/Prospero)) on the 5^th^ August 2013. The overall purpose of the work is to inform UK Government feeding guidance for mothers and their infants.

The outcomes of this project will be summarised in 4 separate reports, with a distinct set of dietary exposures examined in each report:

1. REVIEW A: DURATION OF TOTAL AND EXCLUSIVE BREASTFEEDING, AND TIMING OF SOLID FOOD INTRODUCTION
2. REVIEW B: TIMING OF INTRODUCTION OF ALLERGENIC FOODS INTO THE INFANT DIET
3. REVIEW C PART I: HYDROLYSED FORMULA IN PLACE OF STANDARD UNHYDROLYSED COW’S MILK BASED INFANT FORMULA, OR BREAST MILK
4. REVIEW C PART II: OTHER MATERNAL AND INFANT DIETARY EXPOSURES (INTERVENTION TRIALS AND OBSERVATIONAL STUDIES)

The specific outcomes of interest for all of these reviews, chosen due to their high prevalence in the UK population, and described in more detail below, are:

**Atopic disorders**: Food allergy, Eczema, Asthma/Wheeze, Allergic rhinitis, Allergic conjunctivitis, Allergic sensitisation

**Autoimmune diseases**: Type 1 diabetes mellitus, Coeliac disease, Inflammatory bowel disease, Autoimmune thyroid disease, Juvenile rheumatoid arthritis, Vitiligo, Psoriasis.

# Review C Part II: other maternal (pregnancy/ lactation) and/or infant dietary exposures

Review C includes all dietary interventions and exposures not captured elsewhere in the review, ie dietary patterns, specific food or food group intake, dietary supplements and micro/macronutrient intake. The review includes supplements given directly to infants, either alone, as part of a more complex intervention, or given to infants indirectly via maternal supplementation during pregnancy and/or lactation. In this report we present data from intervention studies as well as from observational studies.

## Specific questions addressed in Review C

Review C addresses 2 key research questions, to help understand the potential role of dietary exposures during pregnancy/ lactation and infancy for reducing an infant’s risk of atopic or autoimmune disease:

1. does exposure to specific dietary patterns, food groups or nutrients during the first year of life, influence a child’s future risk of developing atopic disease, allergic sensitisation or autoimmune disease.
2. does maternal exposure to specific dietary patterns, food groups or nutrients during pregnancy or lactation, influence a child’s future risk of developing atopic disease, allergic sensitisation or autoimmune disease.

## Glossary of key terms

**Allergic sensitisation**: production of specific IgE antibodies directed against harmless environmental antigens such as pollens, mites, milk, egg or peanut; and sometimes associated with increased serum total IgE levels. Allergic sensitisation is strongly associated with atopic disease. Although there are also other forms of allergic sensitisation which do not involve IgE, these are poorly characterised in the context of food allergy and have not been included in this review.

**Atopic disease**: chronic health conditions associated with the production of IgE antibodies to harmless environmental antigens.

**Atopy:** a predisposition to mount IgE antibody responses

**Case control study:** a retrospective study that compares people with an outcome of interest (cases) with people without the outcome (controls), in relation to specific risk factor(s).

**Controlled clinical trial:** An intervention trial which used a predicatable allocation sequence, thought likely to lead to unbalanced treatment groups in relation to an important risk factor for the outcome(s) of interest.

**Extensively hydrolysed formula**: cow’s milk formula described in original publication as extensively hydrolysed.

**GRADE evaluation of evidence**: grade of evidence in this report is assigned using the GRADE system, which has 4 categories HIGH, MODERATE, LOW or VERY LOW. Evidence is initially assigned as HIGH if coming from a randomised trial; LOW from observational studies; VERY LOW from other evidence. The grade of evidence is then reduced if there are serious (-1) or very serious (-2) limitations to study quality or uncertainties about directness of association; important inconsistency (-1), imprecise or sparse data (-1) or a high probability of reporting bias (-1). Grade of evidence is increased if strong evidence of association is seen (eg RR >2 or <0.5) from ≥2 observational studies with no plausible confounders (+1) or very strong direct evidence (RR >5 or <0.2) with no major threats to validity (+2); if there is evidence of a dose-response gradient (+1) or if all plausible confounders would have reduced the effect/association seen (+1). The interpretation of GRADE evidence assessments is that for HIGH level evidence further research is very unlikely to change our confidence in the estimate of effect; for MODERATE evidence further research is likely to have an important impact on our confidence in the estimate of effect and may change the estimate; for LOW level evidence further research is very likely to have an important impact on our confidence in the estimate of effect and is likely to change the estimate; and for VERY LOW level evidence any estimate of effect is very uncertain. Further detailed explanation of GRADE can be found at :

<http://www.gradeworkinggroup.org/publications/Grading_evidence_and_recommendations_BMJ.pdf>

**Maternal allergenic food avoidance:** avoidance of one or more of the following foods during any part of pregnancy and/or lactation - cow’s milk, soya, egg, peanut, tree nuts, wheat, fish, seafood, which are the ‘major food allergens’ as defined by the US Food Allergen Labeling and consumer Protection Act of 2004.

**Multifaceted interventions:** an intervention which includes more than one category of dietary intervention that was eligible for inclusion in this FSA/COT systematic review project. Many such interventions also include environmental control measures such as house dust mite reduction and tobacco smoke avoidance.

**Nested case control study:** a case control study in which cases and controls are identified from within a larger prospective cohort study

**Prospective cohort study:** a study in which people are followed over time to determine whether they develop an outcome of interest, and exposure data are collected prior to outcome data.

**Prebiotic:** selectively fermented ingredient (typically oligosaccharides), that allows specific changes in the composition and/or activity of the gastrointestinal microflora, and thereby confer a health benefit.

**Probiotic:** live microorganisms which, when administered in adequate amounts, confer a health benefit on the host.

**PUFA**: polyunsaturated fatty acids – n-3 or n-6 fatty acids, including essential and also some non-essential fatty acids.

**RCT**: Randomised controlled trial. An intervention trial which reported that a random method of treatment allocation was used.

**Retrospective cohort study:** a special type of cohort study in which the outcome of interest has already occurred at the time when participants are categorised by exposure status.

**Synbiotic**: a combination of probiotic and prebiotic administered to the same individual.

## Executive summary

The key findings of this review are summarised below, in relation to each of 9 reports – intervention trials of probiotics, prebiotics, polyunsaturated fatty acids (PUFAs), maternal allergenic food avoidance, and multifaceted interventions; observational studies of fruits and vegetables, fats and fatty acids and ‘other dietary exposures’; and a report on vitamin and mineral exposures which includes both intervention and observational studies. We used the GRADE approach to assessing the strength of evidence, and specify in the text of the Executive Summary section of each individual report where judgements have been made that result in lower or higher grade evidence .

This is followed by a summary of the Methods used to identify studies, extract, analyse and appraise data, and a Discussion of the main findings in the context of existing literature. The 9 separate systematic reviews which provide the basis for this Executive Summary and constitute the substance of REVIEW C, can be accessed from Table 1 below.

Table 1 Individual study reports in relation to 9 exposure groups of interest

| **Dietary exposure** | **Report** |
| --- | --- |
| Probiotic supplementation intervention trials |  |
| Prebiotic supplementation intervention trials |  |
| PUFA supplementation  intervention trials |  |
| Maternal allergenic food avoidance intervention trials |  |
| Multifaceted interventions intervention trials |  |
| Vitamins and minerals  intervention and observational studies |  |
| Fruits and vegetables  Observational studies |  |
| Dietary sources of fatty acids  observational studies |  |
| Other foods  observational studies |  |

##

## Probiotic supplementation

Data from over 6000 participants in 28 intervention trials contributed to this report. We found MODERATE level evidence (-1 inconsistency) that probiotics can prevent atopic AD ie AD with associated positive skin prick test or specific IgE test (11 studies, 12 interventions; 3000 participants RR 0.78; 95% CI 0.65 to 0.92; I^2^=0%). We found MODERATE level evidence (-1 inconsistency) that probiotics can prevent *all* atopic dermatitis (AD) (19 studies; 4500 participants; RR 0.78; 95% CI 0.68 to 0.90; I^2^=61%) in children ≤ 4 years old. The majority of the evidence in these positive meta-analyses came from studies of high risk infants ie those with a positive family history of atopic disease (10 of 12 interventions for atopic AD; 16 of 19 trials for *all* AD). In subgroup analyses we found some evidence that probiotic supplementation during pregnancy and lactation, rather than just given directly to infants, is important for efficacy. Approximately half of the trials evaluating AD as an outcome used *Lactobacillus rhamnosus* strains either alone or in combination with others. We found no evidence that probiotics reduce risk of AD at age 5-14 (11 studies; 2500 participants; RR 0.87; 95% CI 0.74, 1.02; I^2^=39%). We found LOW level evidence

(-1 indirect outcome; -1 inconsistency) that probiotics can prevent allergic sensitisation to cow’s milk in high risk infants, but no evidence that probiotics prevent allergic sensitisation to other allergens or prevent clinical cow’s milk allergy. We found no evidence that probiotics prevent wheezing, allergic rhinitis or food allergy, but we found no studies reporting data for allergic conjunctivitis or autoimmune outcomes.

**Conclusion**:

Probiotics during pregnancy/lactation/infancy reduce risk of AD in high risk infants at age ≤4 years.

*Grade of evidence: MODERATE*.

Probiotics during pregnancy/lactation/infancy reduce risk of allergic sensitisation to cow’s milk in high risk infants.

*Grade of evidence: LOW.*

There is no evidence that probiotics influence risk of wheeze, allergic rhinitis, food allergy, or other forms of allergic sensitisation. Further research is needed to understand whether probiotics influence risk of autoimmune diseases.

## Prebiotic supplementation

Data from almost 4000 participants in 10 intervention trials contributed to this report. For all analyses data were either sparse or inconsistent, and carried a significant risk of conflict of interest, and of overall bias, especially attrition bias. The findings have largely resulted from industry-sponsored studies undertaken in populations of infants with very limited or no breastfeeding, and often with significant post-randomisation losses/exclusions. Given the very high levels of prebiotics naturally present in human breast milk, the role of prebiotics in infant nutrition is likely to be as an additive to formula milk in formula-fed infants, rather than as a supplement for all infants.

We found no evidence that probiotics reduce risk of wheezing, allergic sensitisation, AD, food allergy or allergic rhinitis.

**Conclusion**:

There is no evidence that prebiotics influence risk of wheeze, AD, allergic rhinitis, allergic sensitisation or food allergy. Further research is needed to confirm these findings, and we did not identify any studies reporting prebiotic effects on allergic conjunctivitis or autoimmune diseases.

## PUFA supplementation

Data from over 14,000 participants in 19 intervention trials contributed to this report. Overall the quality of evidence was moderate - there was a low or unclear risk of bias and risk of conflict of interest in most studies. Half of the trials studied infants at high risk of allergic outcomes due to family history of allergic disease, half studied ‘normal risk’ populations.

We found MODERATE level evidence (-1 indirectness of outcome measure) that n-3 supplementation using fish oil during pregnancy +/- lactation, reduces allergic sensitisation to egg in high risk infants (6 studies; 1800 participants; RR 0.69; 95% CI 0.53, 0.90; I^2^=15%). No effect was seen for allergic sensitisation to other allergens, and no studies evaluated this outcome in a ‘normal risk’ population.

There was no evidence for any effect of n-3 supplementation on the other allergic outcomes reported – AD, AR, wheeze or food allergy, but data were sparse and inconclusive for n-3 supplementation and risk of food allergy, suggesting a need for further research. Data were also sparse and inconclusive for n-6 supplementation and allergic outcomes. We did not find any studies reporting effects of PUFA supplementation on autoimmune outcomes.

**Conclusion**:

Fish oil supplementation during pregnancy +/- lactation in high risk infants reduces risk of allergic sensitisation to eggs.

*Grade of evidence:* MODERATE.

There is no evidence that fish oil or other n-3 polyunsaturated fatty acid sources influence risk of wheezing, AD or allergic rhinitis, and further work is needed to assess whether the effect on egg sensitisation leads to an effect on food allergy. We did not identify any reports of PUFA supplementation on risk of autoimmune diseases.

## Maternal allergenic food avoidance

Data from over 1900 participants in 12 intervention trials (9 RCT, 3 CCT) contributed to this report. Overall the quality of evidence was low - there was a high or unclear risk of bias in all studies, and data were sparse for all outcomes, especially TIDM, food allergy and allergic rhinitis. No reports of other autoimmune outcomes were identified. All studies evaluated participants at high risk of allergic outcomes. There was a low risk of conflict of interest in most studies.

Two studies intervened in pregnancy only, 6 during lactation and 4 during both periods of time. Seven studies were single intervention (AFA), 5 were multifaceted (typically both maternal and infant dietary interventions, with environmental control measures to reduce allergen/ irritant exposure). The foods excluded were: milk alone (2), egg alone (1), milk and egg (3), or multiple other food groups (6), the latter usually including milk and egg as well.

We found no evidence that maternal allergenic food avoidance during pregnancy and/or lactation reduces risk of allergic disease or TIDM development.

**Conclusion**:

There is no evidence that maternal allergenic food avoidance reduces risk of allergic or autoimmune outcomes. Data are sparse, and high quality adequately-powered studies are needed to confidently exclude a beneficial effect. Data are lacking for ‘normal risk’ populations, and for autoimmune diseases other than TIDM.

## Multifaceted interventions

Data from over 2000 participants in 9 intervention trials contributed to this report. Overall the quality of evidence was mixed. There was an unclear risk of bias in most studies, largely due to insufficient details about the randomisation process (selection bias). All the trials studied infants at high risk of allergic outcomes due to family history of allergic disease. Data were generally sparse, such that clinically significant effects could not be confidently excluded.

We found LOW level evidence that multifaceted interventions reduce risk of AR at age ≤ 4 years (-1 indirect intervention; -1 imprecision). Two studies, 613 participants, RR 0.61 95% CI 0.44, 0.84; I^2^=0%. We found no evidence for an effect on AC, or on AR at age 5-14.

We found LOW level evidence (-1 indirect intervention; -1 imprecision) that multifaceted interventions reduce risk of wheeze (2 studies, 500 participants, RR 0.57 95% CI 0.41, 0.81; I^2^=0%) or recurrent wheeze (4 studies, 730 participants, RR 0.77 95% CI 0.61, 0.97; I^2^=0%) at age 5-14 years. We found no evidence for an effect on lung function, or on wheeze at age ≤ 4 years.

**Conclusion**:

Multifaceted interventions reduce risk of AR in high risk infants, at age ≤4.

*Grade of evidence: LOW*.

Multifaceted interventions prevent wheeze/recurrent wheeze in high risk children at age 5-14.

*Grade of evidence: LOW*.

There is no evidence that multifaceted interventions reduce risk of food allergy, allergic sensitisation, allergic conjunctivitis or AD, but data were sparse such that clinically significant effects cannot be confidently excluded. We found no data for autoimmune outcomes, nor for ‘normal risk’ populations.

## Vitamins and minerals

*Intervention trials*: Data from over 15,000 participants in 11 intervention trials contributed to this report. Five of the 11 studies were at high risk of bias due to high attrition bias. Opportunities for meta-analysis were limited due to differing interventions and outcome measures. Two studies used vitamin A in infants; one vitamin A/beta carotene in pregnancy; 2 studies used vitamin C +/- E during pregnancy; 3 used vitamin D during pregnancy, one vitamin D during pregnancy and infancy and 2 multivitamins during pregnancy. One trial found evidence that vitamin A supplementation during pregnancy increases lung function in offspring; but this was downgraded to no evidence, due to inconsistency, indirectness of the study population and imprecision. We found no evidence for other effects, and no reports of autoimmune outcomes.

*Observational studies*: We identified 50 observational studies which reported the association between infant, maternal or combined intake of vitamins and minerals and allergic or autoimmune outcomes. Of these, 32 were prospective cohorts, 4 were nested case-control studies, and 14 were case-control studies. The main dietary exposures studied were antioxidant vitamins A, C, E, and their precursors, vitamin D and folic acid.

We found no consistent evidence that vitamin or mineral exposures influence allergic or autoimmune outcomes.

**Conclusion**:

There is no evidence that vitamin or mineral supplementation reduces risk of allergic outcomes, but data were sparse and heterogeneous such that clinically significant effects cannot be confidently excluded. We found no data for autoimmune outcomes.

## Fruits and vegetables – observational studies

A total of 35 observational studies reported the association between infant and/or maternal intake of fruit and vegetable and allergic or autoimmune diseases. Of these studies, 27 were prospective cohort studies, 6 case-controls, and 2 nested case control studies. Nineteen studies investigated the association between risk of AD and fruit or vegetable intake, nine studies investigated food allergy (FA), 1 study investigated lung function (LF), 19 studied wheeze, seven studied rhino-conjunctivitis (RC), 15 studied allergenic sensitisation, nine studied type 1 diabetes mellitus (T1DM) and one investigated inflammatory bowel disease (IBD).

Risk of bias was generally low, and opportunities for meta-analysis were very limited due to heterogeneity in reporting of associations.

Overall, we found no consistent evidence to suggest that intake of fruits and vegetables early in life influences risk of other allergic or autoimmune diseases.

**Conclusion**:

There is no evidence that maternal or infant fruit or vegetable intake or introduction is associated with risk of allergic or autoimmune outcomes.

## Fats and fatty acids – observational studies

A total of 26 studies investigated intake of fats and risk of allergic or autoimmune diseases. Twenty were prospective cohort studies, 1 nested case-control, 1 retrospective cohort, 3 case-control and 1 cross-sectional study. Outcomes studied were atopic dermatitis (AD) (n=13), food allergy (FA) (n=3), wheeze (n=13), lung function (LF) (n=1), allergic sensitisation (AS) (n=6), type 1 diabetes mellitus (T1DM) (n=3) and juvenile idiopathic arthritis (JIA) (n=1).

Risk of bias was generally low, and opportunities for meta-analysis were very limited due to heterogeneity in reporting of associations.

We found inconsistent evidence regarding infant fish intake and risk of later AD or RC, with one study finding increased infant fish intake associated with reduced AD or RC, but other studies failing to support this association.

In our report of intervention trials for fish/fish oil supplementation we identified studies of fish oil supplementation during infancy, which did not show a protective effect on allergic outcomes – however we did not identify any intervention trials of fish supplementation in infancy.

We found no evidence for an association between other measures of maternal or infant fat and fatty acid intake, and risk of allergic outcomes or autoimmune disease.

**Conclusion:**

We found no evidence for associations between infant fish, fat or fatty acid intake, or maternal fish, fat or fatty acid intake during pregnancy or lactation, and risk of allergic or autoimmune outcomes.

## ‘Other dietary exposures’ – observational studies

We identified a total of 47 observational studies that investigated the association between other infant or maternal dietary exposures, and allergic or autoimmune diseases. 27 studies were prospective cohorts (PC), 4 were nested case-controls (NCC), 12 were case-controls (CC), and 4 cross-sectional studies (CS). 22 studies included atopic dermatitis (AD) as outcome; 2 studied food allergy (FA); 2 lung function (LF); 21 wheeze; 7 rhino-conjunctivitis (RC); 10 allergic sensitisation (AS); 10 type 1 diabetes mellitus (T1DM); one studied juvenile idiopathic arthritis (JIA), and one studied inflammatory bowel disease (IBD). ).

Risk of bias was generally low, and opportunities for meta-analysis were very limited due to heterogeneity in reporting of associations. We found no consistent evidence for associations between other maternal or infant dietary exposures and allergic or autoimmune outcomes.

**Conclusion**:

Overall we did not find consistent evidence for associations between other maternal or infant dietary exposures, and allergic or autoimmune outcomes in this report.

# Methodology

The systematic review was undertaken according to PRISMA guidelines for intervention trial evidence.The systematic review protocol was registered on PROSPERO. Studies included in the review were those relevant to maternal dietary exposures during pregnancy and/or lactation, and allergic and autoimmune outcomes as described in more detail below.

## Inclusion Criteria

## Types of study included

We included recent high quality systematic reviews published from 2011 until the search date (25^th^ July 2013; updated on 26^th^ February 2017). Older systematic reviews were not included, due to the likelihood of being out of date. We quality assessed eligible systematic reviews using the revised AMSTAR criteria and extracted data from systematic reviews with revised AMSTAR score ≥32. A summary of the identified reviews and data is included in the General Conclusions section at the end of this report.

We included original research studies published at any time prior to the search date (25^th^ July 2013; updated on 26^th^ February 2017for intervention trials only). Original studies eligible for inclusion were randomised controlled trials (RCT), quasi RCT (RCT where the allocation sequence was predictable but not thought likely to lead to imbalance), controlled clinical trials (CCT where the allocation sequence was predictable, and thought likely to lead to significant imbalance between groups in important risk factors for the outcome(s) of interest), prospective cohort or longitudinal studies, retrospective cohort studies, nested case-control studies, other case control studies and cross-sectional surveys. We took a hierarchical approach to study design, such that where data were absent or limited from systematic reviews or intervention trials, we included observational study data. Where a significant number of intervention trials were identified, we did not analyse data from observational studies that assessed the same intervention/exposure. We did not include non-comparative studies, or non-human studies.

## Participants/population

*Inclusion criteria*: Infants between birth and the end of their 12^th^ post-partum month, and their mothers during pregnancy and lactation up to the end of the 24^th^ month postnatally. If infants were characterised as high or normal/low risk for atopic or autoimmune disease based on family history or genotype, this information was recorded so that it could be used for the planned subgroup analysis by disease risk.

*Exclusion criteria*: We excluded studies in which participants were defined by a disease state - eg pregnant women with specific nutritional deficiencies, infants born prematurely (<31 weeks gestation) or other groups clearly representing <5% of the UK population, since the results of this review should apply to the general UK population. We did not exclude studies on the basis of including specific ethnic groups, studies of high risk infants, since this applies to many UK-born infants for allergic disease, and it is difficult to undertake studies of autoimmune disease prevention in the general population without stratifying by genetic/family risk due to the relatively low prevalence of autoimmune diseases.

## Interventions/Exposures

*Dietary Interventions and Exposures* of interest in this report were any nutritional intervention, from dietary pattern to specific micronutrient supplementation.

*Comparators* of interest were no intervention/exposure, or any control intervention or control level of exposure.

## Study outcomes

We selected atopic and autoimmune outcomes on the basis of their population prevalence in children and young adults in the UK. We included diseases with a prevalence of at least 1 in 1000, in children/adolescents or young adults (aged <40 years), but did not include rarer diseases . We did not include pernicious anaemia or adult-onset rheumatoid arthritis despite a high prevalence in middle aged or elderly people, because their prevalence in young people is lower than 1 in 1000, and prospective studies of infant feeding in relation to diseases of older adults are unlikely to have been undertaken. We did not specifically exclude rare manifestations of food allergy such as eosinophilic oesophagitis, if they were reported as part of a food allergy definition, but did exclude them if they were reported as a unique outcome measure since their prevalence is less than 1 in 1000. For atopic outcomes, age at assessment was grouped as 1-4 years, 5-14 years, 15-24 years, 25-44 years, 45-64 years and ≥65 years. Due to a paucity of studies in adults, we pooled all age groups ≥ 15 years for almost all reports. For autoimmune outcomes, we did not stratify analyses by age at outcome assessment. Where studies reported the same outcome at different timepoints within one of these frames, we used the timepoint with the most complete dataset ie lowest percentage of missing data, as the primary assessment point for inclusion in meta-analysis. Where possible we chose a timepoint for outcome assessment that did not fall within the relevant exposure period ie the first year of life. For each outcome measure in this review, there is more than one possible method of assessment. We therefore included our preferred methods of assessment for each outcome as below *a priori*. In general where multiple measures of the same outcome were reported we selected outcomes that included the most complete data, used a published or validated assessment tool, and were meaningful for patients eg patient/parent-reported measures.

## Atopic outcomes:

*1. Asthma/Wheeze* - defined as either ‘asthma’, ‘infantile wheeze’ or similar, using parent/self-report, doctor diagnosis, a validated questionnaire, scoring system or objective measure such as bronchial hyper-reactivity, forced vital capacity, peak expiratory flow rate or reversible airways obstruction using forced expiratory volume in 1 second. We included data for ‘atopic’ asthma/wheeze ie wheeze associated with allergic sensitisation, and for recurrent wheezing. For all asthma/wheeze outcomes, data were analysed as ‘wheeze’, ‘atopic wheeze’ or ‘recurrent wheeze’ depending on the definition of wheeze/asthma used in the original publication. We did not include different wheezing entities based on the timing of onset/resolution of the disease such as ‘early transient wheeze’ or ‘persistent wheeze’ due to heterogeneity in definition between studies. We did not include outcomes such as ‘bronchitis’ or ‘bronchiolitis’ which included some subjects with wheezing but others without wheezing.

*2. Eczema* – defined using parent/self-report, doctor diagnosis, a validated questionnaire, scoring system or objective measure. We also included data for ‘atopic’ eczema ie eczema associated with allergic sensitisation. We did not include reports of rashes which were likely to have included other cutaneous problems, such as nappy rash, contact dermatitis, ‘rash’, ‘skin problem’ etc, but did include reports of ‘recurrent itchy rash in infancy’ or similar descriptions which were likely to represent eczema.

*3. Allergic Rhinoconjunctivitis* – defined using parent/self-report, doctor diagnosis, a validated questionnaire, scoring system or objective measure. We included data for ‘atopic’ rhinoconjunctivitis ie rhinoconjunctivitis associated with allergic sensitisation. We included data for ‘allergic rhinitis’, ‘allergic conjunctivitis’ or ‘allergic rhinoconjunctivitis’ and planned to analyse ‘allergic conjunctivitis’ separately where data were reported separately.

*4. Food allergy* - defined by double blind placebo controlled food challenge, by open food challenge, by medical diagnosis or by self/parent report. We included reports of ‘any food allergy’, and specific food allergies to cow’s milk, egg or peanut. We did not include reports of ‘food intolerance’ that we judged were unlikely to meet current definitions of food allergy .

*6. Allergic sensitisation* – to an inhalant, an ingestant, or both – defined as positive skin prick test and/or specific IgE test to the relevant allergen using recognised methodologies and scoring criteria . We combined data for skin prick and specific IgE testing (using SPT where both were reported in the same study) due to limited numbers of studies available for each meta-analysis, and assessed ‘any allergic sensitisation’, ‘food allergic sensitisation’, ‘aeroallergen sensitisation’, ‘cow’s milk sensitisation’, ‘egg sensitisation’ and ‘peanut sensitisation’ separately. We included Total IgE data when measured using a recognised technology such as ImmunoCAP (ThermoFisher, Massachusets).

2.1.6. Autoimmune outcomes:

*1. Type I diabetes mellitus* – defined as a medical diagnosis eg using the 1999 WHO recommendations for diagnosis and classification of diabetes mellitus , or a surrogate marker such as autoantibodies against insulin, GAD65, IA-2 or the ZnT8 transporter in the first 3 years of life. We did not include reports where the outcome was stated as ‘diabetes’ and thought likely to include some cases of type II diabetes mellitus or other disease entities.

*2. Coeliac disease* – defined by characteristic histological features (intraepithelial lymphocytes, crypt hyperplasia and villous atrophy) with improvement in symptoms and histology after institution of a gluten free diet, a medical diagnosis, or a surrogate marker such as IgA tissue transglutaminase or IgA endomysial antibodies.

*3. Inflammatory bowel disease* (Crohn's disease or ulcerative colitis) – defined as a medical diagnosis.

*4. Autoimmune thyroid disease* (Graves' disease or Hashimoto's thyroiditis) - defined as a medical diagnosis.

*5. Juvenile rheumatoid arthritis* – defined as a medical diagnosis eg using the 2001 revised International League of Associations for Rheumatology (ILAR) classification criteria .

*6. Vitiligo* - defined as a medical diagnosis.

Primary assessment: medical diagnosis using the Vitiligo European Task Force 2007 criteria or similar .

*7. Psoriasis* - defined as a medical diagnosis.

## Search strategy

The search strategies included both text terms and subject heading terms where appropriate. The search strategies were initially developed for use on the MEDLINE database and then adapted for use on other databases. We searched the following databases, with no specified start date:

- The Cochrane Library (2013, Issue 7)
- EMBASE (1947 to July 2013)
- LILACS (1982 to July 2013)
- MEDLINE (1946 to July 2013)
- Web of Science (1970 to July 2013)

The search was run on 25^th^ July 2013 and included all studies published up to that date, and was updated on 26^th^ February 2017 for intervention trials and systematic reviews. We included peer reviewed publications, and abstract publications if they contained data that had not subsequently been published as a peer reviewed publication. We reviewed the bibliography of eligible studies for possible additional publications, and included all eligible publications, regardless of the language. We did not contact the authors of eligible or potentially eligible studies to request original data. The search strategies were extensively piloted and refined to optimise sensitivity, comparing search results with those of other high quality published systematic reviews. The final search strategies for review C are included at the end of this report as *Appendices*.

The search for existing systematic reviews which cover any of the same exposure(s)/outcome(s) as the original studies was limited to publications from 1^st^ January 2011 to 25^th^ July 2013 in the original search, and to 26th February 2017 in the update. The search strategy was partly based on the search strategies used for Review A, Review B and Review C but included a search filter for retrieving systematic reviews . Open Grey was searched using the terms ‘(breast OR lactation OR formula) AND (allergy OR autoimmune OR asthma OR eczema OR rhinitis OR conjunctivitis OR food allergy OR vitiligo OR psoriasis OR arthritisi OR thyroiditis OR atopy OR IgE OR diabetes OR coeliac OR inflammatory bowel disease)’ for studies relevant to Review A; the terms ‘(wean OR peanut OR egg OR milk OR soya OR nut OR fish OR wheat) AND (allergy OR autoimmune OR asthma OR eczema OR rhinitis OR conjunctivitis OR food allergy OR vitiligo OR psoriasis OR arthritisi OR thyroiditis OR atopy OR IgE OR diabetes OR coeliac OR inflammatory bowel disease)’ for studies relevant to review B; the terms ‘(lactation OR pregnancy OR infant OR mother) AND (allergy OR autoimmune OR asthma OR eczema OR rhinitis OR conjunctivitis OR food allergy OR vitiligo OR psoriasis OR arthritisi OR thyroiditis OR atopy OR IgE OR diabetes OR coeliac OR inflammatory bowel disease)’ for studies relevant to review C.

The International Prospective Register of Systematic Reviews (PROSPERO) database was also searched for relevant systematic reviews. Due to the limited functionality of this resource individual keywords with date limits were used to search PROSPERO: we searched for titles containing ‘breast OR infant OR lactation OR wean OR infant’ for studies relevant to review A; ‘nut OR wheat OR egg OR food OR diet’ for studies relevant to review B; ‘pregnant OR infant OR diet OR nutrition OR supplement’ for studies relevant to review C.

The citations identified in searches were imported into Endnote libraries for de-duplication and title screening.

## Study selection, data extraction and analysis

## Study selection

Title and abstract screening was undertaken in duplicate by a team of 7 researchers (RB, VGL, DI, NG, KJ, JC, ZR). Two researchers undertook title screening independently, and met to agree included and excluded titles. Their screening was checked by a third member of the team, and uncertainties were brought to a full team meeting for discussion. This procedure took place between February and April 2014, with weekly team meetings to discuss uncertainties about study eligibility, and again in April 2017. The full text of all potentially eligible studies was reviewed, and where electronic copies were not available, hard copies of articles were ordered from the British Library.

## Data extraction

An Excel data extraction form was developed, piloted and refined by DI, VGL, RB and JL-B – separate forms were used for intervention studies, cohort studies and case control studies. Data extraction was undertaken in duplicate by a team of 10 researchers (DI, RB, UN, SC, VGL, NT-M, NG, EA, AR, PD). Disagreements and uncertainties about data coding were discussed within the team with leads as follows - RB (clinical queries), VGLA (dietetic queries), DI (analysis and coding queries) and JL-B (study design and statistics queries). For foreign language studies, data were extracted by VGL together with a native speaker of the relevant language (see Acknowledgements section). We extracted all relevant data from included studies, including data that could not (not appropriately reported) or would not (see ‘data cleaning’ below) be included in meta-analysis, text information such as ‘no significant association found’, and information that adjusted or unadjusted analyses were performed but not reported.

## Data cleaning and coding

Data were extensively cleaned and coded for analysis with further data checks to identify publications related to the same parent study, and to identify the most appropriate output for inclusion in meta-analysis from studies reporting multiple assessments of closely related exposures/outcomes at the same age in the same population. In general from individual studies reporting more than one measure for the same outcome, we selected data for analysis reporting time to event (hazard ratio) in preference to cumulative incidence or lifetime prevalence ie ‘disease ever’, in turn in preference to point prevalence data ie ‘disease in the last 12 months’ for all binary outcomes with the exception of the non-clinical outcomes allergic sensitisation and lung function, where point prevalence was analysed in preference to cumulative measures. Data cleaning was undertaken by DI, VGL and RJB. The outcomes of both meta-analysed and narratively reported studies were considered together when interpreting data and making conclusions.

For allergic outcomes we grouped studies reporting outcome at ages 0-4, 5-15 and 15+ years. If a study reported associations (within or between publications) at more than one age within the same age group (eg age 1 and 3 years), we selected data for analysis within specific age groups that were most complete ie had the largest number of participants assessed. We also included data from longitudinal models (eg generalised estimating equation), and they were grouped according to the last age included in the model. Where appropriate we also considered the outcomes reported at other ages which were not included in meta-analysis, in our interpretation of the data. Age groups were not used for autoimmune diseases. Where different methods of outcome assessment were used within a study we prioritised validated and patient-centred outcomes – for example we prioritised clinical diagnosis of diabetes over diabetes-associated autoantibody detection; we prioritised patient or parent-reported wheeze using a validated instrument such as the ISAAC questionnaire, over doctor diagnosis of wheeze or study physician assessment. Again where appropriate the impact of these decisions was taken into account in our interpretation of findings.

For included studies which did not report numerical data in a form that could be included in meta-analysis, for example medians, or means without a standard deviation or standard error, or ‘no significant difference’ statements, we summarised the findings narratively in the text or tables of the review.

## Risk of bias assessment

1. *Review level bias*

Publication bias was assessed using funnel plots and Egger's test, for those meta-analyses with ≥10 studies included. Possible causes for asymmetry other than publication bias (eg between study heterogeneity, small study effects) were also considered. We also took into consideration both the outcomes of meta-analyses and the findings of studies not included in meta-analysis, when interpreting systematic review outcomes.

1. *Study level bias*

The risk of bias in included intervention studies was assessed using a modified version of the Cochrane Collaboration Risk of Bias tool, which assessed sequence generation and allocation concealment (Selection Bias), blinding of outcome assessors and validity of outcome assessment tool (Assessment Bias), incomplete outcome data (Attrition Bias – considered high where <70% of randomised participants had outcome data available). RCTs were considered at low overall risk of bias where the risk of bias was judged to be low for all 3 key domains selection, assessment and attrition bias. We assessed risk of Conflict of Interest as low where there was no evidence of industry involvement in study design, analysis, interpretation or publication, and no evidence that study authors receive remuneration from relevant industry partners for other activities. The summary Risk of Bias Figures show the risk of bias for all studies reporting the relevant outcome, whether or not their data could be included in meta-analyses.

The risk of bias in included cohort and case control studies was assessed using a modified version of the National Institute for Clinical Excellence methodological checklist for cohort and case-control studies respectively [^25^](#_ENREF_25). Key domains were Selection Bias (low if cases and controls were selected from similar populations, if the participation rate was ≥80%, or <80% but investigators explored and adjusted for characteristic differences between participants and non-participants), Assessment Bias (low if validated and reliable tools were used to assess exposure and/or outcome), and Confounding Bias (low if most likely confounders are identified and taken into account in study design and analysis). Observational studies were considered at low overall risk of bias where the risk of bias was judged to be low for all 3 key domains selection, assessment and confounding bias. For assessment of Confounding Bias, factors that we expected to be adjusted for within studies of allergic outcomes were: siblings (parity or birth order or family size); gender; age at outcome assessment; disease risk based on family history; maternal or household smoking (asthma/wheeze outcomes); maternal age; maternal education or socioeconomic status; mode of delivery. For studies on autoimmune outcomes we expected matching and/or adjusting for gender, age, address, socioeconomic status, smoking and disease risk. For all studies we also assessed possible Conflict of Interest, judged as low where there was no evidence of industry involvement in study design, analysis, interpretation or publication, and no evidence that study authors receive remuneration from relevant industry partners for other activities. For all study reports, we created a summary Table of Study Characteristics with key study features, and a separate summary Risk of Bias Figure showing the risk of bias for all included studies – whether included in meta-analyses or reported in the narrative table.

## Strategy for data synthesis

Meta-analysis was undertaken where ≥2 studies reported the same outcome for a given exposure. Where meta-analysis was deemed inappropriate due to differences in population, exposure/intervention or outcome; or where meta-analysis was not possible due to the nature of the data reported - individual study results were summarised in a narrative table at the end of each report. Separate analyses were undertaken for each disease outcome, for each (age) group of similar outcome assessment methods for any given disease, and for each intervention/exposure (group). In general our approach to meta-analysis was inclusive, with data pooled for maximum statistical power, but explored for important sources of statistical or clinical heterogeneity. Results for randomised or quasi-randomised controlled trials were pooled separately from controlled clinical trials.

1. *Data extraction*

Where studies reported data at multiple timepoints within one of our pre-defined age groupings, we extracted the most complete dataset available, beyond the intervention period (ie from 1 year of age onwards). This is the dataset with the largest denominator, or where the denominator is identical for multiple time points then the largest numerator (number of events) is used. We planned to undertake subgroup/stratified analyses for meta-analyses which contained a total of >5 studies, and to assess publication bias using Funnel plots and Egger’s test where there were ≥10 studies in a meta-analysis.

1. *Heterogeneity*

Heterogeneity was quantified using I^2^. We explored reasons for heterogeneity using subgroup analyses based on study level factors. We classified heterogeneity as low (I^2^<25%), moderate (I^2^ 25-50%), high (I^2^ 50-75%) or extreme (I^2^>75%). For single study analyses, and where I^2^ exceeded 80% we did not pool data in meta-analysis but presented studies in a forest plot without a pooled effect shown. Individual patient data analysis was not undertaken in this review, and study authors were not contacted to clarify data queries or request further participant data.

1. *Data analysis*

Pooled results for binary outcomes from intervention studies are presented as RR calculated from the frequencies given in the study. Data from individual studies were pooled using the generic inverse variance method for pooled RR and Mantel-Haenszel method (with continuity correction of 0.5 in studies with zero cell frequencies) or inverse variance method for single studies calculation of pooled RR in the statistical programme R version 3.1.0 (www.r-project.org). Pooled results for continuous outcomes measured using similar scales are presented as mean differences with 95% confidence intervals.

1. *Planned subgroup analyses*

We planned certain subgroup and stratified analyses prior to running our search. Subgroup analysis was undertaken for all meta-analyses with >5 studies included, since subgroup analysis was unlikely to yield clear subgroup differences where the total number of studies was ≤5. We undertook planned subgroup analyses according to:

*1. Risk of bias* – studies with low, versus unclear/high overall risk of bias based on the criteria described above.

*2. Disease risk* - studies of populations at increased risk for atopic or autoimmune disease, versus those at normal or low risk of disease.

*3.*  *Study design* – we also included a subgroup analysis for qRCT versus RCT.

In the Allergic Sensitisation meta-analyses we planned a subgroup analysis of specific IgE (sIgE) versus Skin Prick Test (SPT) as outcome measure, and in type 1 diabetes mellitus meta-analyses for clinical versus serological diabetes. For all analyses we planned subgroup analyses according to timing of the intervention used (ie pre/postnatal, and postnatal to infant alone or mother +/- infant). For probiotics and prebiotics we planned a subgroup analysis for those studies which used a symbiotic, versus those which used just probiotic or prebiotic alone; and for probiotics we planned a subgroup analysis for the specific species used (*L. rhamnosus* vs others).

.

1. *Graphical exploration of heterogeneity*

Studies were ordered by year of publication in forest plots, in order to be able to assess any cohort effect, since the composition of infant formula and the prevalence of allergic and autoimmune diseases appear to have changed over time. Due to insufficient information in included studies, it was not possible to order forest plots by year of birth for the study population or year of assessment.

## Review registration

The protocol for this systematic review was registered with the International Prospective Register of Systematic Reviews (PROSPERO CRD42013003802; CRD42013004239; CRD42013004252; [www.crd.york.ac.uk/Prospero](http://www.crd.york.ac.uk/Prospero)) on the 5^th^ August 2013, prior to title screening or selecting any studies from the search results. The protocol was revised following detailed review by the UK Food Standards Agency, the UK Scientific Advisory Committee on Nutrition, independent experts Professor Graham Devereux and Dr Carina Venter, and the Lancet peer review service, prior to being registered on PROSPERO.

## Differences between the protocol and review

Following external statistical review of preliminary reports, a decision was made to not undertake pooled meta-analysis where statistical heterogeneity was ≥80%. Due to insufficient data in included studies, we did not order forest plots by participant year of birth or year of outcome assessment. Instead we ordered by year of publication. New authors joined the review team due to the high workload of title screening and data extraction – TA, TK, SC, NG, ZR, JC, KJ, EA, AR, PD.

# Results of the Searches

## Systematic reviews identified

Our search results are summarised in the PRISMA flow chart Figure 1.

We identified 469 titles after removing duplicates, of which 33 were considered to be a report from a relevant or potentially relevant systematic review after title and abstract screening. All these studies underwent full text review and revised AMSTAR scoring by 2 authors independently (VGL, RJB) – Table 1 summarises the AMSTAR scores. Two studies were only available in Chinese, and these were scored by a native Chinese speaker Dr Sze-Chin Tan, Consultant Allergist from Tan Tock Seng Hospital Singapore, who was trained in the revised AMSTAR scoring procedure prior to undertaking this task. Of the 33 scored studies, and a further 10 review identified in the update search in February 2017, 11 were eligible for inclusion in the project overall, of which 5 were relevant to Review C. Fifteen studies were excluded due to low AMSTAR score. One study was not a systematic review. Six studies were excluded because they were either protocols for a systematic review (n=2) or were abstract publications with insufficient detail to establish the AMSTAR score or sufficient detail regarding study outcomes (n=4). Six high-quality systematic reviews were excluded because they were only relevant to REVIEWS A or B. A summary of R-AMSTAR scores for existing systematic reviews identified in the original search is shown in Table 2, and a summary of the 5 included reviews from the original search is shown in Table 3. The 11 systematic reviews are reviews of probiotics (n=5), prebiotics (n=1), maternal allergenic food avoidance (n=1), vitamin D (n=2), dietary patterns (n=1) and maternal fatty acid supplementation (n=1). Data from these prior systematic reviews are included in the relevant reports where appropriate (Table 1).

## Original studies identified

Our search results are summarised in the PRISMA flow chart Figure 2.

We identified 80 relevant intervention trials for inclusion in Review C part II. A small number of identified trials were included in more than one report, due to use of a synbiotic intervention (included in both probiotic and prebiotic reports) or due to use of a multifaceted intervention which included maternal allergenic food avoidance (included in both multifaceted, and maternal allergenic food avoidance reports). We identified 92 observational studies - several observational studies were included in more than one report, due to multiple exposures assessed in the same study population.

Detailed findings, analyses and discussion for the 9 separate systematic reviews are shown in the attached reports (Table 1), and summarised in the Executive Summary.

# General Conclusions

In these systematic reviews of intervention trial data regarding nutritional exposures during pregnancy, lactation and infancy, we found evidence that nutritional exposures can influence allergic disease development, at least in populations at high risk of allergic disease based on family history. We did not find evidence for effects on risk of autoimmune diseases, but data for these outcomes were either lacking or very sparse in all cases.

Specifically we found MODERATE evidence that probiotics can reduce risk of AD; LOW evidence that multifaceted interventions can reduce risk of wheezing and allergic rhinitis; MODERATE evidence that n-3 PUFA can reduce risk of allergic sensitisation to egg; and LOW level evidence that probiotics can reduce risk of allergic sensitisation to cow’s milk..

From the observational studies we found no consistent evidence for associations between maternal or infant dietary exposures and allergic or autoimmune outcome. While single case-control studies reported significant associations in some cases, we did not consider such findings to be significant evidence, in the absence of replication.

Taken together our analyses support recent international recommendations to use probiotics in people at high risk of developing AD, and suggest the need for further trials of maternal n-3 PUFA supplementation.

# Acknowlegements

We are grateful to the independent expert peer reviewers Professor Graham Devereux and Dr Carina Venter, and to members of the UK Food Standards Agency, the Committee on Toxicity of Chemicals in Food, Consumer Products and the Environment (COT), and the UK Scientific Advisory Committee on Nutrition for their comments on the study protocol and previous versions of this report. We are grateful to Dr Yujie Zhao, Mr Szymon Mikolajewski, Dr Andre Amaral, Dr Mari Kihara, Dr Christian Nielsen, Mr Radoslav Latinovic, Dr Stephanie MacNeill, Dr Andreas Forsters, Dr Daniel Munblit, Dr Sze-Chin Tan, and Dr Claudia Gore for their assistance with translation of foreign language reports. We are grateful to Jackie Cousins of the St. Mary’s library at Imperial College London for assistance with literature search training.

# References

# Appendix 1 Tables

Table 2 Quality assessment of recent SRs using R-AMSTAR scoring

| **Author/Reference** | **VGL Score** | **BB Score** | **CONSENSUS** |
| --- | --- | --- | --- |
| Antico | 19 | 14 | Exclude |
| Braeger | 30 | 29 | Exclude |
| Brew | **35** | **37** | Exclude – Review A |
| Cardwell | 31 | 25 | Exclude |
| Christesen | 24 | 25 | Exclude |
| De Silva | 26 | 29 | Exclude |
| Dick | 22 | 19 | Exclude |
| Doege | 28 | 21 | Exclude |
| Flohr | 19 | 19 | Exclude |
| Foolad | 18 | 22 | Exclude |
| Gunaratne | Protocol and Abstract publication only | | |
| Henricksson | 18 | 24 | Exclude |
| Kheirkhah | Abstract publication only | | |
| Klemens | **36** | **35** | Include – Review C |
| Kramer | **38** | **40** | Exclude – Review A |
| Kramer | **37** | **40** | Include – Review C |
| Kremmyda | 21 | 20 | Exclude |
| Middleton | Abstract publication only | | |
| Osborn | Abstract publication only | | |
| Osborn | **38** | **40** | Include – Review C |
| Pelucci | **33** | **36** | Include – Review C |
| Patelarou | 23 | 24 | Exclude |
| Schindler | Protocol only | | |
| Schmitt | Not a systematic review | | |
| Szajeweska | **36** | **35** | Exclude – Reviews A and B |
| Tang | **34*** | | Include – Review C |
| Waidyatillake | 24 | 25 | Exclude |
| Wang | 31* | | Exclude |

A score of ≥32 was required for inclusion; * Chinese language, scored by Dr Sze-Chin Tan

Table 3 Characteristics of included systematic reviews

| **Study**  Relevant SR | **Databases searched** | **No. studies**  **No. (range) participants** | **Study designs included** | **Population** | **Intervention/ Exposure(s)**  **Comparator(s)** | **Outcomes relevant to the project** | **Subgroup analyses relevant to project** |
| --- | --- | --- | --- | --- | --- | --- | --- |
| **Klemens**  Review C | MEDLINE, EBM reviews, ACP Journal Club, DARE, CCTR, CMR, HTA, NHSEED (1950 – 10/2010) | 5 studies  949 (98 -402) participants | All RCTs | - Pregnant or lactating women and their offspring - Two high risk and 3 normal risk studies | Supplementation of pregnant or lactating women with n-3 PUFA, versus olive oil (3), placebo not described (1) or soya oil (1) | - Asthma - Eczema - Food allergy in infancy or childhood - Egg sensitisation in infancy | Nil |
| **Kramer**  Review C | Cochrane Pregnancy and childbirth Group’s Trials Register*  07/2012 | 5 studies  925 (19 – 497) participants | All RCTs | Pregnant or lactating women at high risk for having an atopic child | Exclusion or reduced intake of potentially allergenic foods such as cow milk, egg, peanuts, fish, versus no exclusion | - Eczema - Wheezing - Rhinoconjunctivitis, - Positive skin prick test to egg, milk and peanut - Cord blood IgE | Nil |
| **Osborn**  Review C | CENTRAL 2012; Issue 8  MEDLINE (1948 -8/2012)  EMBASE (1974-8/ 2012) | 4 studies  1,428 (113-830) participants | All RCTs | Infants in the first six months of life without clinical evidence of allergy, both with and without risk factors for allergic disease | Prebiotics added to human milk or infant formula, compared to control (placebo or no treatment) or a different prebiotic | - Asthma/wheeze - Eczema - Rhinitis [no data] - Food allergy [no data] | - High risk of allergic disease based on family history - Breastfeeding status - Age – infant (under 2), child (2-10), adolescent (10-18), adult - Study quality [no data – all high risk of bias] |
| **Pelucci**  Review C | MEDLINE, EMBASE, the Cochrane Library (reviews only) 10/2011 | 14 studies  1,417 (12-368) participants | All RCTs | Pregnant women, lactating women and their infants | Any probiotic or combination of probiotics, compared with no probiotic | Eczema or IgE-associated eczema, up to age 12 | - High risk of allergic disease - Treatment in pregnancy/ lactation/ infancy - Method of diagnosis |
| **Tang** †  Review C | PUBMED, MEDLINE, SPRINGER LINK, HIGHWIRE, COCHRANE, VIP, WANFANG DATA, CKNI (09/2011) | 11 studies  1007 participants | All RCTs | Infants at high risk of atopic disease (first degree relative with any atopic disease including asthma, allergic rhinitis, atopic eczema, food allergy etc) and their mothers | Prenatal and/or postnatal probiotic use versus no probiotic use | - Eczema* - Asthma* - Food sensitisation (positive SPT and/or specific IgE to a food) | Nil |

SR: Systematic reviews, RCT: Randomised controlled trial, PUFA: Polyunsaturated fatty acid

Table 4 Summary of findings from intervention trials

| **GRADE of evidence assessment** | | | | | | | **Summary of findings** | | **Absolute Risk Reduction*** | |
| --- | --- | --- | --- | --- | --- | --- | --- | --- | --- | --- |
| **No of studies and design** | **Risk of bias** | **Inconsistency** | **Indirectness** | **Imprecision** | **Publication bias** | **Other considerations** | **Relative risk** | **GRADE of evidence** | **Control Risk**  **Cases per 1000 population** | **Risk Difference**  **Cases per 1000 population** |
| **Intervention: Maternal or infant probiotic vs no probiotic**  **Outcome: Eczema at age 0-4**  **Study design: RCT** | | | | | | | | | | |
| 1 RCT  Eczema | Not Serious  Unblinded outcome assessment | Serious  No effect seen at age 6-7 years. No association seen in observational studies | No | Serious  Wide confidence intervals | No | No association found between exclusive breastfeeding duration of and eczema in an observational analyslis of this trial | **OR 0.54 95% CI 0.31, 0.95** | **⮿⮿🌕🌕**  **Low** | **200**  **(low risk)**  **300**  **(high risk)**  **400**  **(very high risk)** | **81 cases**  **95% CI**  **8 to 128**  **112 cases**  **95% CI**  **11 to 183**  **135 cases**  **95% CI**  **12 to 229** |
| **Intervention: Maternal or infant probiotic vs no probiotic**  **Outcome: Eczema, atopic eczema or cow’s milk sensitisation at age 0-4**  **Study design: RCT** | | | | | | | | | | |
| 23 RCT  (19 in meta-analysis)  1 CCT  Eczema | Not Serious  <20% of studies had a high risk of bias | Serious  I^2^=61% Eczema  Study estimates vary from 0.14 to 1.34; subgroup analysis suggests greatest effect if postnatal component includes maternal supplementation | No | No | No | Most RCTs were undertaken in populations at high risk of eczema due to family history of allergic disease.  No evidence of an effect on eczema beyond age 0-4. | **RR = 0.78**  **(0.68, 0.90)** | **⮿⮿⮿🌕**  **Moderate** | **200**  **(low risk)**  **300**  **(high risk)**  **400**  **(very high risk)** | **44 cases**  **95% CI**  **20 to 64**  **66 cases**  **95% CI**  **30 to 96**  **88 cases**  **95% CI**  **40 to 128** |
| 11 RCT  Atopic Eczema | Not Serious  <20% of studies had a high risk of bias | Serious  I^2^=0% Study estimates from 0.47 to 1.36.  No effect at age 5-14 years RR 0.99 (0.80, 1.22; I^2^=0%) | No | No | No  Egger’s P=0.70 | Most RCTs were undertaken in populations at high risk of eczema due to family history of allergic disease. | **RR = 0.78 (0.65, 0.92)** | **⮿⮿⮿🌕**  **Moderate** | **100**  **(low risk)**  **150**  **(high risk)**  **200**  **(very high risk)** | **22 cases**  **95% CI**  **8 to 35**  **33 cases**  **95% CI**  **12 to 53**  **44 cases**  **95% CI**  **16 to 70** |
| 9 RCT  (8 in meta-analysis)  Cow’s milk sensitisation | Not Serious  <20% of studies had a high risk of bias | Not Serious  I^2^=0% Study estimates vary from 0.14 to 0.98.  No beneficial effect seen for allergic sensitisation to other allergens. | Serious  Sensitisation is a surrogate measure of clinical food allergy. No effect of intervention on food allergy | Serious  Low event numbers and borderline statistical significance | Not tested  (n<10)  No evidence of publication bias in Funnel plots for other forms of allergic sensitisation | Most RCTs were undertaken in populations at high risk of eczema due to family history of allergic disease | **RR = 0.60 (0.37, 0.96)** | **⮿⮿🌕🌕**  **Low** | **30**  **(low risk)**  **100**  **(high risk)**  **300**  **(very high risk)** | **12 cases**  **95% CI**  **1 to 19**  **40 cases**  **95% CI**  **4 to 63**  **120 cases**  **95% CI**  **12 to 189** |
| **Intervention: Infant prebiotic versus no prebiotic**  **Outcome: Eczema at age 0-4**  **Study design: RCT** | | | | | | | | | | |
| 9 RCT  (7 in meta-analysis)  Eczema | Serious  4 studies with high risk of attrition bias, 5 with high risk of conflict of interest | Serious  I^2^=57%  Study estimates vary from 0.14 to 1.64 | Serious  6 studies restricted inclusion to infants fully formula fed from birth, 3 days, < 2 weeks, <6 weeks (n=2) and <8 weeks; 3 other studies used co-interventions – hydrolysed formula and probiotics | Serious  Wide confidence intervals, borderline statistical significance | Not tested  (n<10) | 3 RCTs were undertaken in populations at high risk due to family history of allergic disease, 4 at normal risk, and 1 at low risk due to absence of family history of allergic disease.  All studies used galactooligosaccharides, usually combined with other oligosaccharides; 2 studies combined the intervention with probiotic(s) | **RR = 0.75**  **(0.56, 1.01)** | **🌕🌕🌕🌕**  **No evidence** | **-** | **-** |
| **Intervention: Maternal fish oil (n-3 PUFA) vs no fish oil**  **Outcome: Allergic sensitisation to egg**  **Study design: RCT** | | | | | | | | | | |
| 6 RCT  Allergic sensitisation to egg | Not Serious  2 studies with unclear overall risk of bias, and 2 studies with unclear risk of conflict of interest | Not Serious  I^2^=15%  Study estimates vary from 0.45 to 1.02.  No significant effect seen for allergic sensitisation to other allergens, but direction of effect is not inconsistent with that seen for egg | Serious  Egg sensitisation is a surrogate measure of clinical food allergy. No effect of the intervention was seen on clinical food allergy. | Not serious  Limits of 95% CI are all important effects at a population level. | Not tested  (n<10) | All RCTs were undertaken in populations at high risk of allergy due to family history of allergic disease.  Subgroup analysis suggests effect is greatest if treatment is given during pregnancy. | **RR = 0.69**  **(0.53, 0.90)** | **⮿⮿⮿🌕**  **Moderate** | **100**  **(low risk)**  **150**  **(high risk)**  **200**  **(very high risk)** | **31 cases**  **95% CI**  **10 to 47**  **46 cases**  **95% CI**  **15 to 71**  **62 cases**  **95% CI**  **20 to 94** |
| **Intervention: Multifaceted interventions vs control**  **Outcome: Allergic rhinitis at age 0-4**  **Study design: RCT** | | | | | | | | | | |
| 3 RCT  (2 in meta-analysis)  Allergic rhinitis | Not serious  No study with high risk of bias or high risk of conflict of interest | Not serious  I^2^=0%  Study estimates vary from 0.58 to 0.61 | Serious  Both studies in meta-analysis included environmental control measures such as dust mite and tobacco smoke avoidance | Serious  Sparse data, inconsistent with data for age 5-14, where no effect was seen. Study not included in meta-analysis found no effect | Not tested  (n<10) | All RCTs were undertaken in populations at high risk due to family history of allergic disease.  All studies included delayed introduction of allergenic foods and maternal allergenic food avoidance | **RR = 0.61**  **(0.44, 0.84)** | **⮿⮿🌕🌕**  **Low** | **50**  **(low risk)**  **100**  **(high risk)**  **150**  **(very high risk)** | **20 cases**  **95% CI**  **8 to 28**  **39 cases**  **95% CI**  **16 to 56**  **59 cases**  **95% CI**  **24 to 84** |
| **Intervention: Multifaceted interventions vs control**  **Outcome: Wheeze or recurrent wheeze at age 5-14**  **Study design: RCT** | | | | | | | | | | |
| 2 RCT  Wheeze | Not serious  No study with high risk of attrition bias or high risk of conflict of interest | Not serious  I^2^=0%  Study estimates vary from 0.50 to 0.59 | Serious  Both studies included environmental control measures such as dust mite and tobacco smoke avoidance, which are known to reduce respiratory symptoms | Serious  Sparse data, inconsistent with data for age 0-4 and lung function data, where no effect was seen | Not tested  (n<10) | All RCTs were undertaken in populations at high risk due to family history of allergic disease.  Both studies included delayed introduction of allergenic foods and maternal allergenic food avoidance | **RR = 0.57**  **(0.41, 0.81)** | **⮿⮿🌕🌕**  **Low** | **100**  **(low risk)**  **150**  **(high risk)**  **200**  **(very high risk)** | **43 cases**  **95% CI**  **19 to 59**  **65 cases**  **95% CI**  **29 to 89**  **86 cases**  **95% CI**  **38 to 118** |
| 4 RCT  Recurrent wheeze | Not serious  1 study high risk of attrition bias, and no study with high risk of conflict of interest | Not serious  I^2^=0%  Study estimates vary from 0.64 to 1.41 | Serious  3 studies included environmental control measures such as dust mite and tobacco smoke avoidance, which are known to reduce respiratory symptoms | Serious  Sparse data, inconsistent with data for age 0-4 and lung function data, where no effect was seen | Not tested  (n<10) | All RCTs were undertaken in populations at high risk due to family history of allergic disease.  All studies included delayed introduction of allergenic foods; three also included maternal allergenic food avoidance | **RR = 0.77**  **(0.61, 0.97)** | **⮿⮿🌕🌕**  **Low** | **50**  **(low risk)**  **100**  **(high risk)**  **150**  **(very high risk)** | **12 cases**  **95% CI**  **2 to 20**  **23 cases**  **95% CI**  **3 to 39**  **35 cases**  **95% CI**  **5 to 59** |
| **Intervention: Maternal vitamin A vs no vitamin A**  **Outcome: Lung function at age 11 years**  **Study design: RCT** | | | | | | | | | | |
| 1 RCT  Lung function | Not serious  Low risk of bias | Very serious  Supplementation with the vitamin A precursor beta carotene in the same trial had no effect on lung function. Neither vtamin A nor beta carotene had an effect on risk of wheeze. Separate trial with similar design but lower vitamin A dose as part of a multivitamin intervention found no effect on FEV1 or FVC at age 8 | Serious  Study population was a poor rural Nepalese population at risk of vitamin A deficiency | Serious  Single study, with wide confidence intervals | Not tested  (n<10) | Dose response relationship between maternal postpartum serum retinol levels and lung function outcomes | **Mean Difference**  **FEV_1_ 46mls (6, 86)**  **FVC 46mls (8, 84)** | **🌕🌕🌕🌕**  **No evidence** | **-** | **-** |

# Appendix 2 Search Strategies for other systematic reviews

These search strategies were used to identify recent SRs relevant to Reviews A, B or C

**Medline**

1. breast feeding.ab,ti.

2. breastfeeding.ab,ti.

3. breast fed.ab,ti.

4. breastfed.ab,ti.

5. Breast Feeding/

6. Milk, Human/

7. formula?.ab,ti.

8. hydrolysed.ab,ti.

9. bottlefed.ab,ti.

10. bottle fed.ab,ti.

11. (bottle adj3 feed$).ab,ti.

12. Infant Formula/

13. Bottle Feeding/

14. wean$.ab,ti.

15. Weaning/

16. 1 or 2 or 3 or 4 or 5 or 6 or 7 or 8 or 9 or 10 or 11 or 12 or 13 or 14 or 15

17. complementary food?.ab,ti.

18. (introduc$ adj2 food?).ab,ti.

19. wean$.ab,ti.

20. Weaning/

21. solid?.ab,ti.

22. semi-solid?.ab,ti.

23. baby food?.ab,ti.

24. Infant Food/

25. Infant Nutritional Physiological Phenomena/

26. breast feeding.ab,ti.

27. breastfeeding.ab,ti.

28. breast fed.ab,ti.

29. breastfed.ab,ti.

30. Breast Feeding/

31. Milk, Human/

32. formula?.ab,ti.

33. hydrolysed.ab,ti.

34. bottlefed.ab,ti.

35. bottle fed.ab,ti.

36. (bottle adj3 feed$).ab,ti.

37. Infant Formula/

38. Bottle Feeding/

39. liquid?.ab,ti.

40. milk.ab,ti.

41. Milk/

42. egg?.ab,ti.

43. Egg Proteins/

44. Egg Proteins, Dietary/

45. nut?.ab,ti.

46. peanut?.ab,ti.

47. almond?.ab,ti.

48. (brazil? adj5 nut?).ab,ti.

49. walnut?.ab,ti.

50. pecan?.ab,ti.

51. pistachio?.ab,ti.

52. cashew?.ab,ti.

53. hazelnut?.ab,ti.

54. macadamia?.ab,ti.

55. Nuts/

56. Arachis hypogaea/

57. Prunus/

58. Bertholletia/

59. Juglans/

60. Carya/

61. Pistacia/

62. Anacardium/

63. Corylus/

64. Macadamia/

65. wheat.ab,ti.

66. Triticum/

67. soya.ab,ti.

68. Soybeans/

69. gluten$.ab,ti.

70. Glutens/

71. fish.ab,ti.

72. Fishes/

73. 17 or 18 or 19 or 20 or 21 or 22 or 23 or 24 or 25 or 26 or 27 or 28 or 29 or 30 or 31 or 32 or 33 or 34 or 35 or 36 or 37 or 38 or 39 or 40 or 41 or 42 or 43 or 44 or 45 or 46 or 47 or 48 or 49 or 50 or 51 or 52 or 53 or 54 or 55 or 56 or 57 or 58 or 59 or 60 or 61 or 62 or 63 or 64 or 65 or 66 or 67 or 68 or 69 or 70 or 71 or 72

74. Diet/

75. Diet Therapy/

76. Nutritional Sciences/

77. Child Nutrition Sciences/

78. diet.ab,ti.

79. diets.ab,ti.

80. Diet, Mediterranean/

81. mediterranean diet$.ab,ti.

82. dietetic.ab,ti.

83. dietary.ab,ti.

84. eat.ab,ti.

85. eating.ab,ti.

86. intake.ab,ti.

87. nutrient?.ab,ti.

88. nutrition.ab,ti.

89. Diet, Vegetarian/

90. vegetarian?.ab,ti.

91. vegan$.ab,ti.

92. Diet, Macrobiotic/

93. macrobiotic?.ab,ti.

94. Food/

95. food$.ab,ti.

96. feed.ab,ti.

97. feeding.ab,ti.

98. cereal$.ab,ti.

99. grain$.ab,ti.

100. granary.ab,ti.

101. wholegrain.ab,ti.

102. wholewheat.ab,ti.

103. whole wheat.ab,ti.

104. wheat.ab,ti.

105. wheatgerm.ab,ti.

106. rye.ab,ti.

107. barley.ab,ti.

108. oat?.ab,ti.

109. exp Cereals/

110. root?.ab,ti.

111. tuber?.ab,ti.

112. exp Vegetables/

113. vegetable$.ab,ti.

114. onion$.ab,ti.

115. spinach.ab,ti.

116. chard.ab,ti.

117. tomato$.ab,ti.

118. pepper$.ab,ti.

119. carrot$.ab,ti.

120. beetroot.ab,ti.

121. asparagus.ab,ti.

122. garlic.ab,ti.

123. pumpkin.ab,ti.

124. sprouts.ab,ti.

125. broccoli.ab,ti.

126. cabbage$.ab,ti.

127. celery.ab,ti.

128. ginger.ab,ti.

129. potato$.ab,ti.

130. crisps.ab,ti.

131. fries.ab,ti.

132. syrup.ab,ti.

133. honey.ab,ti.

134. Honey/

135. Fruit/

136. fruit$.ab,ti.

137. apple?.ab,ti.

138. pear?.ab,ti.

139. banana?.ab,ti.

140. orange?.ab,ti.

141. grape?.ab,ti.

142. kiwi?.ab,ti.

143. citrus.ab,ti.

144. grapefruit?.ab,ti.

145. pulses.ab,ti.

146. beans.ab,ti.

147. lentil?.ab,ti.

148. chickpea?.ab,ti.

149. legume?.ab,ti.

150. lupin?.ab,ti.

151. soy.ab,ti.

152. soya.ab,ti.

153. nut?.ab,ti.

154. almond?.ab,ti.

155. peanut?.ab,ti.

156. groundnut?.ab,ti.

157. Nuts/

158. seed?.ab,ti.

159. sesame.ab,ti.

160. mustard.ab,ti.

161. Seeds/

162. exp Meat/

163. meat.ab,ti.

164. beef.ab,ti.

165. pork.ab,ti.

166. lamb.ab,ti.

167. poultry.ab,ti.

168. chicken.ab,ti.

169. turkey.ab,ti.

170. duck.ab,ti.

171. fish.ab,ti.

172. Fatty Acids/

173. exp Fatty Acids, Omega-3/

174. exp Fatty Acids, Omega-6/

175. omega-3.ab,ti.

176. omega-6.ab,ti.

177. PUFA.ab,ti.

178. fat.ab,ti.

179. fats.ab,ti.

180. fatty.ab,ti.

181. egg.ab,ti.

182. eggs.ab,ti.

183. exp Eggs/

184. Bread/

185. bread.ab,ti.

186. oil.ab,ti.

187. oils.ab,ti.

188. oily.ab,ti.

189. omega.ab,ti.

190. exp Seafood/

191. seafood.ab,ti.

192. shellfish.ab,ti.

193. crustacean?.ab,ti.

194. mollusc?.ab,ti.

195. Shellfish/

196. Dairy Products/

197. dairy.ab,ti.

198. exp Milk/

199. milk.ab,ti.

200. Infant Formula/

201. formula?.ab,ti.

202. hydrolysed.ab,ti.

203. Infant Food/

204. yoghurt.ab,ti.

205. probiotic.ab,ti.

206. prebiotic?.ab,ti.

207. butter.ab,ti.

208. herb?.ab,ti.

209. spice?.ab,ti.

210. chilli$.ab,ti.

211. condiment?.ab,ti.

212. exp Condiments/

213. Beverages/

214. beverage?.ab,ti.

215. fluid intake.ab,ti.

216. water.ab,ti.

217. drink$.ab,ti.

218. exp Food Preservation/

219. pickled.ab,ti.

220. bottled.ab,ti.

221. canned.ab,ti.

222. canning.ab,ti.

223. smoked.ab,ti.

224. preserved.ab,ti.

225. preservatives.ab,ti.

226. nitrosamine.ab,ti.

227. hydrogenation.ab,ti.

228. fortified.ab,ti.

229. nitrates.ab,ti.

230. nitrites.ab,ti.

231. ferment$.ab,ti.

232. processed.ab,ti.

233. antioxidant$.ab,ti.

234. genetic modif$.ab,ti.

235. genetically modif$.ab,ti.

236. Cooking/

237. cooking.ab,ti.

238. cooked.ab,ti.

239. grill.ab,ti.

240. grilled.ab,ti.

241. fried.ab,ti.

242. fry.ab,ti.

243. roast.ab,ti.

244. bake.ab,ti.

245. baked.ab,ti.

246. stewing.ab,ti.

247. stewed.ab,ti.

248. casserol$.ab,ti.

249. broil.ab,ti.

250. broiled.ab,ti.

251. boiled.ab,ti.

252. poach.ab,ti.

253. poached.ab,ti.

254. steamed.ab,ti.

255. barbecue$.ab,ti.

256. chargrill$.ab,ti.

257. salt.ab,ti.

258. salting.ab,ti.

259. salted.ab,ti.

260. fiber.ab,ti.

261. fibre.ab,ti.

262. polysaccharide$.ab,ti.

263. starch.ab,ti.

264. starchy.ab,ti.

265. carbohydrate$.ab,ti.

266. lipid$.ab,ti.

267. linoleic acid$.ab,ti.

268. sugar$.ab,ti.

269. sweetener$.ab,ti.

270. saccharin$.ab,ti.

271. aspartame.ab,ti.

272. sucrose.ab,ti.

273. xylitol.ab,ti.

274. cholesterol.ab,ti.

275. hydrogenated lard.ab,ti.

276. dietary protein.ab,ti.

277. dietary proteins.ab,ti.

278. protein intake.ab,ti.

279. animal protein$.ab,ti.

280. total protein$.ab,ti.

281. vegetable protein$.ab,ti.

282. plant protein$.ab,ti.

283. exp Dietary Carbohydrates/

284. exp Dietary Fats/

285. exp Dietary Fiber/

286. exp Dietary Proteins/

287. exp Dietary Supplements/

288. exp Food Additives/

289. exp Vitamins/

290. supplements.ab,ti.

291. supplement.ab,ti.

292. vitamin$.ab,ti.

293. retinol.ab,ti.

294. carotenoid$.ab,ti.

295. tocopherol.ab,ti.

296. folate$.ab,ti.

297. folic acid.ab,ti.

298. methionine.ab,ti.

299. riboflavin.ab,ti.

300. thiamine.ab,ti.

301. niacin.ab,ti.

302. pyridoxine.ab,ti.

303. cobalamin.ab,ti.

304. mineral$.ab,ti.

305. sodium.ab,ti.

306. iron.ab,ti.

307. calcium.ab,ti.

308. selenium.ab,ti.

309. iodine.ab,ti.

310. magnesium.ab,ti.

311. potassium.ab,ti.

312. zinc.ab,ti.

313. copper.ab,ti.

314. phosphorus.ab,ti.

315. manganese.ab,ti.

316. chromium.ab,ti.

317. phytochemical.ab,ti.

318. polyphenol$.ab,ti.

319. phytoestrogen$.ab,ti.

320. genistein.ab,ti.

321. saponin$.ab,ti.

322. coumarin$.ab,ti.

323. flavonoid$.ab,ti.

324. polyphenol$.ab,ti.

325. flavonol$.ab,ti.

326. flavone$.ab,ti.

327. isoflavone$.ab,ti.

328. catechin$.ab,ti.

329. ascorbic acid$.ab,ti.

330. hydroxy cholecalciferol$.ab,ti.

331. hydroxycholecalciferol$.ab,ti.

332. tocotrienol$.ab,ti.

333. carotene$.ab,ti.

334. cryptoxanthin$.ab,ti.

335. lycopene$.ab,ti.

336. lutein$.ab,ti.

337. zeaxanthin$.ab,ti.

338. selenium$.ab,ti.

339. organic diet?.ab,ti.

340. Food, Organic/

341. 74 or 75 or 76 or 77 or 78 or 79 or 80 or 81 or 82 or 83 or 84 or 85 or 86 or 87 or 88 or 89 or 90 or 91 or 92 or 93 or 94 or 95 or 96 or 97 or 98 or 99 or 100 or 101 or 102 or 103 or 104 or 105 or 106 or 107 or 108 or 109 or 110 or 111 or 112 or 113 or 114 or 115 or 116 or 117 or 118 or 119 or 120 or 121 or 122 or 123 or 124 or 125 or 126 or 127 or 128 or 129 or 130 or 131 or 132 or 133 or 134 or 135 or 136 or 137 or 138 or 139 or 140 or 141 or 142 or 143 or 144 or 145 or 146 or 147 or 148 or 149 or 150 or 151 or 152 or 153 or 154 or 155 or 156 or 157 or 158 or 159 or 160 or 161 or 162 or 163 or 164 or 165 or 166 or 167 or 168 or 169 or 170 or 171 or 172 or 173 or 174 or 175 or 176 or 177 or 178 or 179 or 180 or 181 or 182 or 183 or 184 or 185 or 186 or 187 or 188 or 189 or 190 or 191 or 192 or 193 or 194 or 195 or 196 or 197 or 198 or 199 or 200 or 201 or 202 or 203 or 204 or 205 or 206 or 207 or 208 or 209 or 210 or 211 or 212 or 213 or 214 or 215 or 216 or 217 or 218 or 219 or 220 or 221 or 222 or 223 or 224 or 225 or 226 or 227 or 228 or 229 or 230 or 231 or 232 or 233 or 234 or 235 or 236 or 237 or 238 or 239 or 240 or 241 or 242 or 243 or 244 or 245 or 246 or 247 or 248 or 249 or 250 or 251 or 252 or 253 or 254 or 255 or 256 or 257 or 258 or 259 or 260 or 261 or 262 or 263 or 264 or 265 or 266 or 267 or 268 or 269 or 270 or 271 or 272 or 273 or 274 or 275 or 276 or 277 or 278 or 279 or 280 or 281 or 282 or 283 or 284 or 285 or 286 or 287 or 288 or 289 or 290 or 291 or 292 or 293 or 294 or 295 or 296 or 297 or 298 or 299 or 300 or 301 or 302 or 303 or 304 or 305 or 306 or 307 or 308 or 309 or 310 or 311 or 312 or 313 or 314 or 315 or 316 or 317 or 318 or 319 or 320 or 321 or 322 or 323 or 324 or 325 or 326 or 327 or 328 or 329 or 330 or 331 or 332 or 333 or 334 or 335 or 336 or 337 or 338 or 339 or 340

342. allerg$.ab,ti.

343. asthma$.ab,ti.

344. wheeze.ab,ti.

345. wheezing.ab,ti.

346. bronchial hyperresponsiveness.ab,ti.

347. bronchial hyperreactivity.ab,ti.

348. Forced expiratory volume.ab,ti.

349. FEV1.ab,ti.

350. "FEV 1".ab,ti.

351. "FEV0.5".ab,ti.

352. "FEV 0.5".ab,ti.

353. Forced vital capacity.ab,ti.

354. FVC.ab,ti.

355. Peak expiratory flow rate.ab,ti.

356. PEFR.ab,ti.

357. eczema.ab,ti.

358. neurodermatitis.ab,ti.

359. rhinitis.ab,ti.

360. besniers prurigo.ab,ti.

361. rhinoconjunctivitis.ab,ti.

362. hayfever.ab,ti.

363. (hay adj fever).ab,ti.

364. poll?nosis.ab,ti.

365. SAR.ab,ti.

366. (pollen adj allergy).ab,ti.

367. conjunctivitis.ab,ti.

368. immunoglobulin e.ab,ti.

369. Total IgE.ab,ti.

370. autoimmune disease?.ab,ti.

371. diabetes.ab,ti.

372. diabetic.ab,ti.

373. type 1.ab,ti.

374. c?eliac disease.ab,ti.

375. crohn$ disease.ab,ti.

376. Inflammatory Bowel Disease?.ab,ti.

377. Ulcerative colitis.ab,ti.

378. (Lympho$ adj3 thyroiditi$).ab,ti.

379. (Thyroiditi$ adj3 autoimmune).ab,ti.

380. (Hashimoto$ adj3 (syndrome? or thyroiditi$ or disease?)).ab,ti.

381. (Thyroiditi$ adj3 (post-partum or postpartum)).ab,ti.

382. Graves? disease.ab,ti.

383. Basedow$ disease.ab,ti.

384. exophthalmic goiter?.ab,ti.

385. (Still? Disease adj3 (juvenile or onset)).ab,ti.

386. (Juvenile adj3 arthriti$).ab,ti.

387. vitiligo.ab,ti.

388. Psorias?s.ab,ti.

389. (Arthriti? adj3 Psoria$).ab,ti.

390. atopic disease.ab,ti.

391. atopic dermatitis.ab,ti.

392. (food? adj3 sensiti$).ab,ti.

393. (food? adj3 toleran$).ab,ti.

394. (food? adj3 intoleran$).ab,ti.

395. ((aero or air$) adj3 allergen?).ab,ti.

396. (aeroallergen? adj3 sensiti$).ab,ti.

397. (allergen? adj3 sensiti$).ab,ti.

398. skin prick test$.ab,ti.

399. atopy.ab,ti.

400. hypersensitiv$.ab,ti.

401. Hypersensitivity/

402. exp Food Hypersensitivity/

403. Respiratory Hypersensitivity/

404. Asthma/

405. Bronchial Hyperreactivity/

406. Forced Expiratory Volume/

407. Vital Capacity/

408. Peak Expiratory Flow Rate/

409. Eczema/

410. Neurodermatitis/

411. Rhinitis/

412. Rhinitis, Allergic, Perennial/

413. Rhinitis, Allergic, Seasonal/

414. Conjunctivitis/

415. Immunoglobulin E/

416. Autoimmune Diseases/

417. Diabetes Mellitus, Type 1/

418. Celiac Disease/

419. Crohn Disease/

420. Inflammatory Bowel Diseases/

421. Colitis, Ulcerative/

422. Thyroiditis, Autoimmune/

423. Hashimoto Disease/

424. Postpartum Thyroiditis/

425. Graves Disease/

426. Arthritis, Juvenile Rheumatoid/

427. Vitiligo/

428. Psoriasis/

429. Arthritis, Psoriatic/

430. Dermatitis, Atopic/

431. Hypersensitivity, Immediate/

432. 342 or 343 or 344 or 345 or 346 or 347 or 348 or 349 or 350 or 351 or 352 or 353 or 354 or 355 or 356 or 357 or 358 or 359 or 360 or 361 or 362 or 363 or 364 or 365 or 366 or 367 or 368 or 369 or 370 or 371 or 372 or 373 or 374 or 375 or 376 or 377 or 378 or 379 or 380 or 381 or 382 or 383 or 384 or 385 or 386 or 387 or 388 or 389 or 390 or 391 or 392 or 393 or 394 or 395 or 396 or 397 or 398 or 399 or 400 or 401 or 402 or 403 or 404 or 405 or 406 or 407 or 408 or 409 or 410 or 411 or 412 or 413 or 414 or 415 or 416 or 417 or 418 or 419 or 420 or 421 or 422 or 423 or 424 or 425 or 426 or 427 or 428 or 429 or 430 or 431

433. infant?.ab,ti.

434. ((one or two or three or four or five or six or seven or eight or nine or ten or eleven or twelve or thirteen or fourteen or fifteen or sixteen or seventeen or eighteen or nineteen or twenty or "twenty one" or "twenty two" or "twenty three" or "twenty four" or "twenty five" or "twenty six") adj week?).ab,ti.

435. ((one or two or three or four or five or six or seven or eight or nine or ten or eleven or twelve or thirteen or fourteen or fifteen or sixteen or seventeen or eighteen or nineteen or twenty or "twenty one" or "twenty two" or "twenty three" or "twenty four") adj month?).ab,ti.

436. 434 or 435

437. (old or age?).ab,ti.

438. 436 and 437

439. (("one year?" or "two year?") adj3 (old or age?)).ab,ti.

440. ((first or second or two) adj3 "year? of life").ab,ti.

441. Infant/

442. Infant, Newborn/

443. (maternal adj7 pregnan$).ab,ti.

444. (maternal adj7 lactat$).ab,ti.

445. (mother? adj7 pregnan$).ab,ti.

446. 433 or 438 or 439 or 440 or 441 or 442 or 443 or 444 or 445

447. MEDLINE.tw.

448. systematic review.tw.

449. meta-analysis.pt.

450. intervention$.ti.

451. 447 or 448 or 449 or 450

452. 16 or 73 or 341

453. 432 and 446 and 451 and 452

454. limit 453 to yr="2011 -Current"

**Embase**

1. breast feeding.ab,ti.

2. breastfeeding.ab,ti.

3. breast fed.ab,ti.

4. breastfed.ab,ti.

5. breast feeding/

6. breast milk/

7. formula?.ab,ti.

8. hydrolysed.ab,ti.

9. bottlefed.ab,ti.

10. bottle fed.ab,ti.

11. (bottle adj3 feed$).ab,ti.

12. artificial milk/

13. bottle feeding/

14. wean$.ti,ab.

15. weaning/

16. 1 or 2 or 3 or 4 or 5 or 6 or 7 or 8 or 9 or 10 or 11 or 12 or 13 or 14 or 15

17. complementary food?.ab,ti.

18. (introduc$ adj2 food?).ab,ti.

19. wean$.ab,ti.

20. weaning/

21. solid?.ab,ti.

22. semi-solid?.ab,ti.

23. baby food?.ab,ti.

24. baby food/

25. infant nutrition/

26. breast feeding.ab,ti.

27. breastfeeding.ab,ti.

28. breast fed.ab,ti.

29. breastfed.ab,ti.

30. breast feeding/

31. breast milk/

32. formula?.ab,ti.

33. hydrolysed.ab,ti.

34. bottlefed.ab,ti.

35. bottle fed.ab,ti.

36. (bottle adj3 feed$).ab,ti.

37. artificial milk/

38. bottle feeding/

39. liquid?.ti,ab.

40. milk.ti,ab.

41. milk/

42. egg?.ti,ab.

43. egg/

44. egg protein/

45. nut?.ab,ti.

46. peanut?.ab,ti.

47. almond?.ab,ti.

48. (brazil? adj5 nut?).ab,ti.

49. walnut?.ab,ti.

50. pecan?.ab,ti.

51. pistachio?.ab,ti.

52. cashew?.ab,ti.

53. hazelnut?.ab,ti.

54. macadamia?.ab,ti.

55. nut/

56. peanut/

57. almond/

58. Brazil nut/

59. exp walnut/

60. pecan/

61. pistachio/

62. cashew nut/

63. hazelnut/

64. Corylus avellana/

65. Macadamia/

66. wheat.ti,ab.

67. exp wheat/

68. soya.ti,ab.

69. soybean/

70. gluten$.ti,ab.

71. gluten/

72. fish$.ti,ab.

73. fish/

74. 17 or 18 or 19 or 20 or 21 or 22 or 23 or 24 or 25 or 26 or 27 or 28 or 29 or 30 or 31 or 32 or 33 or 34 or 35 or 36 or 37 or 38 or 39 or 40 or 41 or 42 or 43 or 44 or 45 or 46 or 47 or 48 or 49 or 50 or 51 or 52 or 53 or 54 or 55 or 56 or 57 or 58 or 59 or 60 or 61 or 62 or 63 or 64 or 65 or 66 or 67 or 68 or 69 or 70 or 71 or 72 or 73

75. diet/

76. diet therapy/

77. nutritional science/

78. diet.ti,ab.

79. diets.ti,ab.

80. Mediterranean diet/

81. mediterranean diet$.ab,ti.

82. dietetic.ab,ti.

83. dietary.ab,ti.

84. eat.ab,ti.

85. eating.ab,ti.

86. intake.ab,ti.

87. nutrient?.ab,ti.

88. nutrition.ab,ti.

89. vegetarian diet/

90. vegetarian?.ti,ab.

91. vegan$.ti,ab.

92. macrobiotic diet/

93. macrobiotic?.ti,ab.

94. food/

95. food$.ab,ti.

96. feed.ab,ti.

97. feeding.ab,ti.

98. cereal$.ab,ti.

99. grain$.ab,ti.

100. granary.ab,ti.

101. wholegrain.ab,ti.

102. wholewheat.ab,ti.

103. whole wheat.ab,ti.

104. wheat.ab,ti.

105. wheatgerm.ab,ti.

106. rye.ab,ti.

107. barley.ab,ti.

108. oat?.ab,ti.

109. exp cereal/

110. root?.ti,ab.

111. tuber?.ti,ab.

112. exp vegetable/

113. vegetable$.ab,ti.

114. onion$.ab,ti.

115. spinach.ab,ti.

116. chard.ab,ti.

117. tomato$.ab,ti.

118. pepper$.ab,ti.

119. carrot$.ab,ti.

120. beetroot.ab,ti.

121. asparagus.ab,ti.

122. garlic.ab,ti.

123. pumpkin.ab,ti.

124. sprouts.ab,ti.

125. broccoli.ab,ti.

126. cabbage$.ab,ti.

127. celery.ab,ti.

128. ginger.ab,ti.

129. potato$.ab,ti.

130. crisps.ab,ti.

131. fries.ab,ti.

132. syrup.ab,ti.

133. honey.ab,ti.

134. honey/

135. fruit/

136. fruit$.ab,ti.

137. apple?.ab,ti.

138. pear?.ab,ti.

139. banana?.ab,ti.

140. orange?.ab,ti.

141. grape?.ab,ti.

142. kiwi?.ab,ti.

143. citrus.ab,ti.

144. grapefruit?.ab,ti.

145. pulses.ab,ti.

146. beans.ab,ti.

147. lentil?.ab,ti.

148. chickpea?.ab,ti.

149. legume?.ab,ti.

150. lupin?.ab,ti.

151. soy.ab,ti.

152. soya.ab,ti.

153. nut?.ab,ti.

154. almond?.ab,ti.

155. peanut?.ab,ti.

156. groundnut?.ab,ti.

157. exp nut/

158. seed?.ti,ab.

159. sesame.ti,ab.

160. mustard.ti,ab.

161. plant seed/

162. meat/

163. meat.ab,ti.

164. beef.ab,ti.

165. pork.ab,ti.

166. lamb.ab,ti.

167. poultry.ab,ti.

168. chicken.ab,ti.

169. turkey.ab,ti.

170. duck.ab,ti.

171. fish.ab,ti.

172. fatty acid/

173. omega 3 fatty acid/

174. omega 6 fatty acid/

175. omega-3.ab,ti.

176. omega-6.ab,ti.

177. PUFA.ab,ti.

178. fat.ab,ti.

179. fats.ab,ti.

180. fatty.ab,ti.

181. egg.ab,ti.

182. eggs.ab,ti.

183. exp egg/

184. bread/

185. bread.ti,ab.

186. oil.ti,ab.

187. oils.ti,ab.

188. oily.ti,ab.

189. omega.ti,ab.

190. sea food/

191. seafood.ti,ab.

192. shellfish.ti,ab.

193. crustacean?.ti,ab.

194. mollusc?.ti,ab.

195. shellfish/

196. exp dairy product/

197. dairy.ti,ab.

198. milk/

199. milk.ti,ab.

200. artificial milk/

201. formula?.ti,ab.

202. hydrolysed.ti,ab.

203. baby food/

204. yoghurt.ab,ti.

205. probiotic.ab,ti.

206. prebiotic?.ab,ti.

207. butter.ab,ti.

208. herb?.ab,ti.

209. spice?.ab,ti.

210. chilli$.ab,ti.

211. condiment?.ab,ti.

212. exp condiment/

213. beverage/

214. beverage?.ti,ab.

215. fluid intake.ti,ab.

216. water.ti,ab.

217. drink$.ti,ab.

218. exp food preservation/

219. pickled.ab,ti.

220. bottled.ab,ti.

221. canned.ab,ti.

222. canning.ab,ti.

223. smoked.ab,ti.

224. preserved.ab,ti.

225. preservatives.ab,ti.

226. nitrosamine.ab,ti.

227. hydrogenation.ab,ti.

228. fortified.ab,ti.

229. nitrates.ab,ti.

230. nitrites.ab,ti.

231. ferment$.ab,ti.

232. processed.ab,ti.

233. antioxidant$.ab,ti.

234. genetic modif$.ab,ti.

235. genetically modif$.ab,ti.

236. cooking/

237. cooking.ab,ti.

238. cooked.ab,ti.

239. grill.ab,ti.

240. grilled.ab,ti.

241. fried.ab,ti.

242. fry.ab,ti.

243. roast.ab,ti.

244. bake.ab,ti.

245. baked.ab,ti.

246. stewing.ab,ti.

247. stewed.ab,ti.

248. casserol$.ab,ti.

249. broil.ab,ti.

250. broiled.ab,ti.

251. boiled.ab,ti.

252. poach.ab,ti.

253. poached.ab,ti.

254. steamed.ab,ti.

255. barbecue$.ab,ti.

256. chargrill$.ab,ti.

257. salt.ab,ti.

258. salting.ab,ti.

259. salted.ab,ti.

260. fiber.ab,ti.

261. fibre.ab,ti.

262. polysaccharide$.ab,ti.

263. starch.ab,ti.

264. starchy.ab,ti.

265. carbohydrate$.ab,ti.

266. lipid$.ab,ti.

267. linoleic acid$.ab,ti.

268. sugar$.ab,ti.

269. sweetener$.ab,ti.

270. saccharin$.ab,ti.

271. aspartame.ab,ti.

272. sucrose.ab,ti.

273. xylitol.ab,ti.

274. cholesterol.ab,ti.

275. hydrogenated lard.ab,ti.

276. dietary protein.ab,ti.

277. dietary proteins.ab,ti.

278. protein intake.ab,ti.

279. animal protein$.ab,ti.

280. total protein$.ab,ti.

281. vegetable protein$.ab,ti.

282. plant protein$.ab,ti.

283. carbohydrate diet/

284. carbohydrate intake/

285. fat intake/

286. dietary fiber/

287. protein intake/

288. diet supplementation/

289. food additive/

290. exp vitamin/

291. supplements.ab,ti.

292. supplement.ab,ti.

293. vitamin$.ab,ti.

294. retinol.ab,ti.

295. carotenoid$.ab,ti.

296. tocopherol.ab,ti.

297. folate$.ab,ti.

298. folic acid.ab,ti.

299. methionine.ab,ti.

300. riboflavin.ab,ti.

301. thiamine.ab,ti.

302. niacin.ab,ti.

303. pyridoxine.ab,ti.

304. cobalamin.ab,ti.

305. mineral$.ab,ti.

306. sodium.ab,ti.

307. iron.ab,ti.

308. calcium.ab,ti.

309. selenium.ab,ti.

310. iodine.ab,ti.

311. magnesium.ab,ti.

312. potassium.ab,ti.

313. zinc.ab,ti.

314. copper.ab,ti.

315. phosphorus.ab,ti.

316. manganese.ab,ti.

317. chromium.ab,ti.

318. phytochemical.ab,ti.

319. polyphenol$.ab,ti.

320. phytoestrogen$.ab,ti.

321. genistein.ab,ti.

322. saponin$.ab,ti.

323. coumarin$.ab,ti.

324. flavonoid$.ab,ti.

325. polyphenol$.ab,ti.

326. flavonol$.ab,ti.

327. flavone$.ab,ti.

328. isoflavone$.ab,ti.

329. catechin$.ab,ti.

330. ascorbic acid$.ab,ti.

331. hydroxy cholecalciferol$.ab,ti.

332. hydroxycholecalciferol$.ab,ti.

333. tocotrienol$.ab,ti.

334. carotene$.ab,ti.

335. cryptoxanthin$.ab,ti.

336. lycopene$.ab,ti.

337. lutein$.ab,ti.

338. zeaxanthin$.ab,ti.

339. selenium$.ab,ti.

340. organic diet?.ab,ti.

341. organic food/

342. 75 or 76 or 77 or 78 or 79 or 80 or 81 or 82 or 83 or 84 or 85 or 86 or 87 or 88 or 89 or 90 or 91 or 92 or 93 or 94 or 95 or 96 or 97 or 98 or 99 or 100 or 101 or 102 or 103 or 104 or 105 or 106 or 107 or 108 or 109 or 110 or 111 or 112 or 113 or 114 or 115 or 116 or 117 or 118 or 119 or 120 or 121 or 122 or 123 or 124 or 125 or 126 or 127 or 128 or 129 or 130 or 131 or 132 or 133 or 134 or 135 or 136 or 137 or 138 or 139 or 140 or 141 or 142 or 143 or 144 or 145 or 146 or 147 or 148 or 149 or 150 or 151 or 152 or 153 or 154 or 155 or 156 or 157 or 158 or 159 or 160 or 161 or 162 or 163 or 164 or 165 or 166 or 167 or 168 or 169 or 170 or 171 or 172 or 173 or 174 or 175 or 176 or 177 or 178 or 179 or 180 or 181 or 182 or 183 or 184 or 185 or 186 or 187 or 188 or 189 or 190 or 191 or 192 or 193 or 194 or 195 or 196 or 197 or 198 or 199 or 200 or 201 or 202 or 203 or 204 or 205 or 206 or 207 or 208 or 209 or 210 or 211 or 212 or 213 or 214 or 215 or 216 or 217 or 218 or 219 or 220 or 221 or 222 or 223 or 224 or 225 or 226 or 227 or 228 or 229 or 230 or 231 or 232 or 233 or 234 or 235 or 236 or 237 or 238 or 239 or 240 or 241 or 242 or 243 or 244 or 245 or 246 or 247 or 248 or 249 or 250 or 251 or 252 or 253 or 254 or 255 or 256 or 257 or 258 or 259 or 260 or 261 or 262 or 263 or 264 or 265 or 266 or 267 or 268 or 269 or 270 or 271 or 272 or 273 or 274 or 275 or 276 or 277 or 278 or 279 or 280 or 281 or 282 or 283 or 284 or 285 or 286 or 287 or 288 or 289 or 290 or 291 or 292 or 293 or 294 or 295 or 296 or 297 or 298 or 299 or 300 or 301 or 302 or 303 or 304 or 305 or 306 or 307 or 308 or 309 or 310 or 311 or 312 or 313 or 314 or 315 or 316 or 317 or 318 or 319 or 320 or 321 or 322 or 323 or 324 or 325 or 326 or 327 or 328 or 329 or 330 or 331 or 332 or 333 or 334 or 335 or 336 or 337 or 338 or 339 or 340 or 341

343. allerg$.ab,ti.

344. asthma$.ab,ti.

345. wheeze.ab,ti.

346. wheezing.ab,ti.

347. bronchial hyperresponsiveness.ab,ti.

348. bronchial hyperreactivity.ab,ti.

349. Forced expiratory volume.ab,ti.

350. FEV1.ab,ti.

351. "FEV 1".ab,ti.

352. "FEV0.5".ab,ti.

353. "FEV 0.5".ab,ti.

354. Forced vital capacity.ab,ti.

355. FVC.ab,ti.

356. Peak expiratory flow rate.ab,ti.

357. PEFR.ab,ti.

358. eczema.ab,ti.

359. neurodermatitis.ab,ti.

360. rhinitis.ab,ti.

361. besniers prurigo.ab,ti.

362. rhinoconjunctivitis.ab,ti.

363. hayfever.ab,ti.

364. (hay adj fever).ab,ti.

365. poll?nosis.ab,ti.

366. SAR.ab,ti.

367. (pollen adj allergy).ab,ti.

368. conjunctivitis.ab,ti.

369. immunoglobulin e.ab,ti.

370. Total IgE.ab,ti.

371. autoimmune disease?.ab,ti.

372. diabetes.ab,ti.

373. diabetic.ab,ti.

374. type 1.ab,ti.

375. c?eliac disease.ab,ti.

376. crohn$ disease.ab,ti.

377. Inflammatory Bowel Disease?.ab,ti.

378. Ulcerative colitis.ab,ti.

379. (Lympho$ adj3 thyroiditi$).ab,ti.

380. (Thyroiditi$ adj3 autoimmune).ab,ti.

381. (Hashimoto$ adj3 (syndrome? or thyroiditi$ or disease?)).ab,ti.

382. (Thyroiditi$ adj3 (post-partum or postpartum)).ab,ti.

383. Graves? disease.ab,ti.

384. Basedow$ disease.ab,ti.

385. exophthalmic goiter?.ab,ti.

386. (Still? Disease adj3 (juvenile or onset)).ab,ti.

387. (Juvenile adj3 arthriti$).ab,ti.

388. vitiligo.ab,ti.

389. Psorias?s.ab,ti.

390. (Arthriti? adj3 Psoria$).ab,ti.

391. atopic disease.ab,ti.

392. atopic dermatitis.ab,ti.

393. (food? adj3 sensiti$).ab,ti.

394. (food? adj3 toleran$).ab,ti.

395. (food? adj3 intoleran$).ab,ti.

396. ((aero or air$) adj3 allergen?).ab,ti.

397. (aeroallergen? adj3 sensiti$).ab,ti.

398. (allergen? adj3 sensiti$).ab,ti.

399. skin prick test$.ab,ti.

400. atopy.ab,ti.

401. hypersensitiv$.ab,ti.

402. exp hypersensitivity/

403. respiratory tract allergy/

404. asthma/

405. wheezing/

406. bronchus hyperreactivity/

407. forced expiratory volume/

408. forced vital capacity/

409. peak expiratory flow/

410. eczema/

411. neurodermatitis/

412. rhinitis/

413. rhinoconjunctivitis/

414. hay fever/

415. pollen allergy/

416. perennial rhinitis/

417. conjunctivitis/

418. immunoglobulin E/

419. autoimmune disease/

420. diabetes mellitus/

421. insulin dependent diabetes mellitus/

422. celiac disease/

423. Crohn disease/

424. enteritis/

425. ulcerative colitis/

426. autoimmune thyroiditis/

427. Hashimoto disease/

428. postpartum thyroiditis/

429. Graves disease/

430. juvenile rheumatoid arthritis/

431. vitiligo/

432. psoriasis/

433. psoriatic arthritis/

434. atopic dermatitis/

435. nutritional intolerance/

436. 343 or 344 or 345 or 346 or 347 or 348 or 349 or 350 or 351 or 352 or 353 or 354 or 355 or 356 or 357 or 358 or 359 or 360 or 361 or 362 or 363 or 364 or 365 or 366 or 367 or 368 or 369 or 370 or 371 or 372 or 373 or 374 or 375 or 376 or 377 or 378 or 379 or 380 or 381 or 382 or 383 or 384 or 385 or 386 or 387 or 388 or 389 or 390 or 391 or 392 or 393 or 394 or 395 or 396 or 397 or 398 or 399 or 400 or 401 or 402 or 403 or 404 or 405 or 406 or 407 or 408 or 409 or 410 or 411 or 412 or 413 or 414 or 415 or 416 or 417 or 418 or 419 or 420 or 421 or 422 or 423 or 424 or 425 or 426 or 427 or 428 or 429 or 430 or 431 or 432 or 433 or 434 or 435

437. infant?.ab,ti.

438. ((one or two or three or four or five or six or seven or eight or nine or ten or eleven or twelve or thirteen or fourteen or fifteen or sixteen or seventeen or eighteen or nineteen or twenty or "twenty one" or "twenty two" or "twenty three" or "twenty four" or "twenty five" or "twenty six") adj week?).ab,ti.

439. ((one or two or three or four or five or six or seven or eight or nine or ten or eleven or twelve or thirteen or fourteen or fifteen or sixteen or seventeen or eighteen or nineteen or twenty or "twenty one" or "twenty two" or "twenty three" or "twenty four") adj month?).ab,ti.

440. 438 or 439

441. (old or age?).ab,ti.

442. 440 and 441

443. (("one year?" or "two year?") adj3 (old or age?)).ab,ti.

444. ((first or second or two) adj3 "year? of life").ab,ti.

445. infant/

446. newborn/

447. (maternal adj7 pregnan$).ti,ab.

448. (maternal adj7 lactat$).ti,ab.

449. (mother? adj7 pregnan$).ti,ab.

450. 437 or 442 or 443 or 444 or 445 or 446 or 447 or 448 or 449

451. MEDLINE.tw.

452. exp systematic review/

453. systematic review.tw.

454. meta analysis/

455. intervention$.ti.

456. 451 or 452 or 453 or 454 or 455

457. 16 or 74 or 342

458. 436 and 450 and 456 and 457

459. limit 458 to yr="2011 -Current"

**COCHRANE Reviews and DARE**

1. “breast feeding”:ab,ti

2. breastfeeding:ab,ti

3. “breast fed”:ab,ti

4. breastfed:ab,ti

5. MeSH descriptor [Breast Feeding] this term only

6. MeSH descriptor [Milk, Human] this term only

7. formula*:ab,ti

8. hydrolysed:ab,ti

9. bottlefed:ab,ti

10. “bottle fed”:ab,ti

11. (bottle NEAR/3 feed*):ab,ti

12. MeSH descriptor [Infant Formula] this term only

13. MeSH descriptor [Bottle Feeding] this term only

14. wean*:ab,ti

15. MeSH descriptor [Weaning] this term only

16. 1 or 2 or 3 or 4 or 5 or 6 or 7 or 8 or 9 or 10 or 11 or 12 or 13 or 14 or 15

17. “complementary food*”:ab,ti

18. (introduc* NEAR/2 food*):ab,ti

19. wean*:ab,ti

20. MeSH descriptor [Weaning] this term only

21. solid*:ab,ti

22. semi-solid*:ab,ti

23. “baby food*”:ab,ti

24. MeSH descriptor [Infant Food] this term only

25. MeSH descriptor [Infant Nutritional Physiological Phenomena] this term only

26. “breast feeding”:ab,ti

27. breastfeeding:ab,ti

28. “breast fed”:ab,ti

29. breastfed:ab,ti

30. MeSH descriptor [Breast Feeding] this term only

31. MeSH descriptor [Milk, Human] this term only

32. formula*:ab,ti

33. hydrolysed:ab,ti

34. bottlefed:ab,ti

35. “bottle fed”:ab,ti

36. (bottle NEAR/3 feed*):ab,ti

37. MeSH descriptor [Infant Formula] this term only

38. MeSH descriptor [Bottle Feeding] this term only

39. liquid*:ab,ti

40. milk:ab,ti

41. MeSH descriptor [Milk] this term only

42. egg*:ab,ti

43. MeSH descriptor [Egg Proteins] this term only

44. MeSH descriptor [Egg Proteins, Dietary] this term only

45. nut*:ab,ti

46. peanut*:ab,ti

47. almond*:ab,ti

48. (brazil* NEAR/5 nut*):ab,ti

49. walnut*:ab,ti

50. pecan*:ab,ti

51. pistachio*:ab,ti

52. cashew*:ab,ti

53. hazelnut*:ab,ti

54. macadamia*:ab,ti

55. Nuts] this term only

56. MeSH descriptor [Arachis hypogaea] this term only

57. MeSH descriptor [Prunus] this term only

58. MeSH descriptor [Bertholletia] this term only

59. MeSH descriptor [Juglans] this term only

60. MeSH descriptor [Carya] this term only

61. MeSH descriptor [Pistacia] this term only

62. MeSH descriptor [Anacardium] this term only

63. MeSH descriptor [Corylus] this term only

64. MeSH descriptor [Macadamia] this term only

65. wheat:ab,ti

66. MeSH descriptor [Triticum] this term only

67. soya:ab,ti

68. MeSH descriptor [Soybeans] this term only

69. gluten*:ab,ti

70. MeSH descriptor [Glutens] this term only

71. fish:ab,ti

72. MeSH descriptor [Fishes] this term only

73. 17 or 18 or 19 or 20 or 21 or 22 or 23 or 24 or 25 or 26 or 27 or 28 or 29 or 30 or 31 or 32 or 33 or 34 or 35 or 36 or 37 or 38 or 39 or 40 or 41 or 42 or 43 or 44 or 45 or 46 or 47 or 48 or 49 or 50 or 51 or 52 or 53 or 54 or 55 or 56 or 57 or 58 or 59 or 60 or 61 or 62 or 63 or 64 or 65 or 66 or 67 or 68 or 69 or 70 or 71 or 72

74. MeSH descriptor [Diet] this term only

75. MeSH descriptor [Diet Therapy] this term only

76. MeSH descriptor [Nutritional Sciences] this term only

77. MeSH descriptor [Child Nutrition Sciences] this term only

78. diet:ab,ti

79. diets:ab,ti

80. MeSH descriptor [Diet, Mediterranean] this term only

81. “mediterranean diet*”:ab,ti

82. dietetic:ab,ti

83. dietary:ab,ti

84. eat:ab,ti

85. eating:ab,ti

86. intake:ab,ti

87. nutrient*:ab,ti

88. nutrition:ab,ti

89. MeSH descriptor [Diet, Vegetarian] this term only

90. vegetarian*:ab,ti

91. vegan*:ab,ti

92. MeSH descriptor [Diet, Macrobiotic] this term only

93. macrobiotic*:ab,ti

94. MeSH descriptor [Food] this term only

95. food*:ab,ti

96. feed:ab,ti

97. feeding:ab,ti

98. cereal*:ab,ti

99. grain*:ab,ti

100. granary:ab,ti

101. wholegrain:ab,ti

102. wholewheat:ab,ti

103. “whole wheat”:ab,ti

104. wheat:ab,ti

105. wheatgerm:ab,ti

106. rye:ab,ti

107. barley:ab,ti

108. oat*:ab,ti

109. MeSH descriptor [Cereals] explode all trees

110. root*:ab,ti

111. tuber*:ab,ti

112. MeSH descriptor [Vegetables] explode all trees

113. vegetable*:ab,ti

114. onion*:ab,ti

115. spinach:ab,ti

116. chard:ab,ti

117. tomato*:ab,ti

118. pepper*:ab,ti

119. carrot*:ab,ti

120. beetroot:ab,ti

121. asparagus:ab,ti

122. garlic:ab,ti

123. pumpkin:ab,ti

124. sprouts:ab,ti

125. broccoli:ab,ti

126. cabbage*:ab,ti

127. celery:ab,ti

128. ginger:ab,ti

129. potato*:ab,ti

130. crisps:ab,ti

131. fries:ab,ti

132. syrup:ab,ti

133. honey:ab,ti

134. MeSH descriptor [Honey] this term only

135. MeSH descriptor [Fruit] this term only

136. fruit*:ab,ti

137. apple*:ab,ti

138. pear*:ab,ti

139. banana*:ab,ti

140. orange*:ab,ti

141. grape*:ab,ti

142. kiwi*:ab,ti

143. citrus:ab,ti

144. grapefruit*:ab,ti

145. pulses:ab,ti

146. beans:ab,ti

147. lentil*:ab,ti

148. chickpea*:ab,ti

149. legume*:ab,ti

150. lupin*:ab,ti

151. soy:ab,ti

152. soya:ab,ti

153. nut*:ab,ti

154. almond*:ab,ti

155. peanut*:ab,ti

156. groundnut*:ab,ti

157. MeSH descriptor [Nuts] this term only

158. seed*:ab,ti

159. sesame:ab,ti

160. mustard:ab,ti

161. MeSH descriptor [Seeds] this term only

162. MeSH descriptor [Meat] explode all trees

163. meat:ab,ti

164. beef:ab,ti

165. pork:ab,ti

166. lamb:ab,ti

167. poultry:ab,ti

168. chicken:ab,ti

169. turkey:ab,ti

170. duck:ab,ti

171. fish:ab,ti

172. MeSH descriptor [Fatty Acids] this term only

173. MeSH descriptor [Fatty Acids, Omega-3] explode all trees

174. MeSH descriptor [Fatty Acids, Omega-6] explode all trees

175. omega-3:ab,ti

176. omega-6:ab,ti

177. PUFA:ab,ti

178. fat:ab,ti

179. fats:ab,ti

180. fatty:ab,ti

181. egg:ab,ti

182. eggs:ab,ti

183. MeSH descriptor [Eggs] explode all trees

184. MeSH descriptor [Bread] this term only

185. bread:ab,ti

186. oil:ab,ti

187. oils:ab,ti

188. oily:ab,ti

189. omega:ab,ti

190. MeSH descriptor [Seafood] explode all trees

191. seafood:ab,ti

192. shellfish:ab,ti

193. crustacean*:ab,ti

194. mollusc*:ab,ti

195. MeSH descriptor [Shellfish] this term only

196. MeSH descriptor [Dairy Products] this term only

197. dairy:ab,ti

198. MeSH descriptor [Milk] explode all trees

199. milk:ab,ti

200. MeSH descriptor [Infant Formula] this term only

201. formula*:ab,ti

202. hydrolysed:ab,ti

203. MeSH descriptor [Infant Food] this term only

204. yoghurt:ab,ti

205. probiotic:ab,ti

206. prebiotic*:ab,ti

207. butter:ab,ti

208. herb*:ab,ti

209. spice*:ab,ti

210. chilli*:ab,ti

211. condiment*:ab,ti

212. MeSH descriptor [Condiments] explode all trees

213. MeSH descriptor [Beverages] this term only

214. beverage*:ab,ti

215. “fluid intake”:ab,ti

216. water:ab,ti

217. drink*:ab,ti

218. MeSH descriptor [Food Preservation] explode all trees

219. pickled:ab,ti

220. bottled:ab,ti

221. canned:ab,ti

222. canning:ab,ti

223. smoked:ab,ti

224. preserved:ab,ti

225. preservatives:ab,ti

226. nitrosamine:ab,ti

227. hydrogenation:ab,ti

228. fortified:ab,ti

229. nitrates:ab,ti

230. nitrites:ab,ti

231. ferment*:ab,ti

232. processed:ab,ti

233. antioxidant*:ab,ti

234. “genetic modif*”:ab,ti

235. “genetically modif*”:ab,ti

236. MeSH descriptor [Cooking] this term only

237. cooking:ab,ti

238. cooked:ab,ti

239. grill:ab,ti

240. grilled:ab,ti

241. fried:ab,ti

242. fry:ab,ti

243. roast:ab,ti

244. bake:ab,ti

245. baked:ab,ti

246. stewing:ab,ti

247. stewed:ab,ti

248. casserol*:ab,ti

249. broil:ab,ti

250. broiled:ab,ti

251. boiled:ab,ti

252. poach:ab,ti

253. poached:ab,ti

254. steamed:ab,ti

255. barbecue*:ab,ti

256. chargrill*:ab,ti

257. salt:ab,ti

258. salting:ab,ti

259. salted:ab,ti

260. fiber:ab,ti

261. fibre:ab,ti

262. polysaccharide*:ab,ti

263. starch:ab,ti

264. starchy:ab,ti

265. carbohydrate*:ab,ti

266. lipid*:ab,ti

267. “linoleic acid*”:ab,ti

268. sugar*:ab,ti

269. sweetener*:ab,ti

270. saccharin*:ab,ti

271. aspartame:ab,ti

272. sucrose:ab,ti

273. xylitol:ab,ti

274. cholesterol:ab,ti

275. “hydrogenated lard”:ab,ti

276. “dietary protein”:ab,ti

277. “dietary proteins”:ab,ti

278. “protein intake”:ab,ti

279. “animal protein*”:ab,ti

280. “total protein*”:ab,ti

281. “vegetable protein*”:ab,ti

282. “plant protein*”:ab,ti

283. MeSH descriptor [Dietary Carbohydrates] explode all trees

284. MeSH descriptor [Dietary Fats] explode all trees

285. MeSH descriptor [Dietary Fiber] explode all trees

286. MeSH descriptor [Dietary Proteins] explode all trees

287. MeSH descriptor [Dietary Supplements] explode all trees

288. MeSH descriptor [Food Additives] explode all trees

289. MeSH descriptor [Vitamins] explode all trees

290. supplements:ab,ti

291. supplement:ab,ti

292. vitamin*:ab,ti

293. retinol:ab,ti

294. carotenoid*:ab,ti

295. tocopherol:ab,ti

296. folate*:ab,ti

297. “folic acid”:ab,ti

298. methionine:ab,ti

299. riboflavin:ab,ti

300. thiamine:ab,ti

301. niacin:ab,ti

302. pyridoxine:ab,ti

303. cobalamin:ab,ti

304. mineral*:ab,ti

305. sodium:ab,ti

306. iron:ab,ti

307. calcium:ab,ti

308. selenium:ab,ti

309. iodine:ab,ti

310. magnesium:ab,ti

311. potassium:ab,ti

312. zinc:ab,ti

313. copper:ab,ti

314. phosphorus:ab,ti

315. manganese:ab,ti

316. chromium:ab,ti

317. phytochemical:ab,ti

318. polyphenol*:ab,ti

319. phytoestrogen*:ab,ti

320. genistein:ab,ti

321. saponin*:ab,ti

322. coumarin*:ab,ti

323. flavonoid*:ab,ti

324. polyphenol*:ab,ti

325. flavonol*:ab,ti

326. flavone*:ab,ti

327. isoflavone*:ab,ti

328. catechin*:ab,ti

329. “ascorbic acid*”:ab,ti

330. “hydroxy cholecalciferol*”:ab,ti

331. hydroxycholecalciferol*:ab,ti

332. tocotrienol*:ab,ti

333. carotene*:ab,ti

334. cryptoxanthin*:ab,ti

335. lycopene*:ab,ti

336. lutein*:ab,ti

337. zeaxanthin*:ab,ti

338. selenium*:ab,ti

339. “organic diet*”:ab,ti

340. MeSH descriptor [Food, Organic] this term only

341. 74 or 75 or 76 or 77 or 78 or 79 or 80 or 81 or 82 or 83 or 84 or 85 or 86 or 87 or 88 or 89 or 90 or 91 or 92 or 93 or 94 or 95 or 96 or 97 or 98 or 99 or 100 or 101 or 102 or 103 or 104 or 105 or 106 or 107 or 108 or 109 or 110 or 111 or 112 or 113 or 114 or 115 or 116 or 117 or 118 or 119 or 120 or 121 or 122 or 123 or 124 or 125 or 126 or 127 or 128 or 129 or 130 or 131 or 132 or 133 or 134 or 135 or 136 or 137 or 138 or 139 or 140 or 141 or 142 or 143 or 144 or 145 or 146 or 147 or 148 or 149 or 150 or 151 or 152 or 153 or 154 or 155 or 156 or 157 or 158 or 159 or 160 or 161 or 162 or 163 or 164 or 165 or 166 or 167 or 168 or 169 or 170 or 171 or 172 or 173 or 174 or 175 or 176 or 177 or 178 or 179 or 180 or 181 or 182 or 183 or 184 or 185 or 186 or 187 or 188 or 189 or 190 or 191 or 192 or 193 or 194 or 195 or 196 or 197 or 198 or 199 or 200 or 201 or 202 or 203 or 204 or 205 or 206 or 207 or 208 or 209 or 210 or 211 or 212 or 213 or 214 or 215 or 216 or 217 or 218 or 219 or 220 or 221 or 222 or 223 or 224 or 225 or 226 or 227 or 228 or 229 or 230 or 231 or 232 or 233 or 234 or 235 or 236 or 237 or 238 or 239 or 240 or 241 or 242 or 243 or 244 or 245 or 246 or 247 or 248 or 249 or 250 or 251 or 252 or 253 or 254 or 255 or 256 or 257 or 258 or 259 or 260 or 261 or 262 or 263 or 264 or 265 or 266 or 267 or 268 or 269 or 270 or 271 or 272 or 273 or 274 or 275 or 276 or 277 or 278 or 279 or 280 or 281 or 282 or 283 or 284 or 285 or 286 or 287 or 288 or 289 or 290 or 291 or 292 or 293 or 294 or 295 or 296 or 297 or 298 or 299 or 300 or 301 or 302 or 303 or 304 or 305 or 306 or 307 or 308 or 309 or 310 or 311 or 312 or 313 or 314 or 315 or 316 or 317 or 318 or 319 or 320 or 321 or 322 or 323 or 324 or 325 or 326 or 327 or 328 or 329 or 330 or 331 or 332 or 333 or 334 or 335 or 336 or 337 or 338 or 339 or 340

342. allerg*:ab,ti

343. asthma*:ab,ti

344. wheeze:ab,ti

345. wheezing:ab,ti

346. “bronchial hyperresponsiveness”:ab,ti

347. “bronchial hyperreactivity”:ab,ti

348. “Forced expiratory volume”:ab,ti

349. “FEV1”:ab,ti

350. "FEV 1":ab,ti

351. "FEV0.5":ab,ti

352. "FEV 0.5":ab,ti

353. “Forced vital capacity”:ab,ti

354. FVC:ab,ti

355. “Peak expiratory flow rate”:ab,ti

356. PEFR:ab,ti

357. eczema:ab,ti

358. neurodermatitis:ab,ti

359. rhinitis:ab,ti

360. “besniers prurigo”:ab,ti

361. rhinoconjunctivitis:ab,ti

362. hayfever:ab,ti

363. “hay fever”:ab,ti

364. poll*nosis:ab,ti

365. SAR:ab,ti

366. “pollen allergy”:ab,ti

367. conjunctivitis:ab,ti

368. immunoglobulin e:ab,ti

369. Total IgE:ab,ti

370. “autoimmune disease*”:ab,ti

371. diabetes:ab,ti

372. diabetic:ab,ti

373. “type 1”:ab,ti

374. “c*eliac disease”:ab,ti

375. “crohn* disease”:ab,ti

376. “Inflammatory Bowel Disease*”:ab,ti

377. “Ulcerative colitis”:ab,ti

378. (Lympho* NEAR/3 thyroiditi*):ab,ti

379. (Thyroiditi* NEAR/3 autoimmune):ab,ti

380. (Hashimoto* NEAR/3 (syndrome* or thyroiditi* or disease*)):ab,ti

381. (Thyroiditi* NEAR/3 (post-partum or postpartum)):ab,ti

382. “Graves* disease”:ab,ti

383. “Basedow* disease”:ab,ti

384. “exophthalmic goiter*”:ab,ti

385. (“Still* Disease” NEAR/3 (juvenile or onset)):ab,ti

386. (Juvenile NEAR/3 arthriti*):ab,ti

387. vitiligo:ab,ti

388. Psorias*s:ab,ti

389. (Arthriti* NEAR/3 Psoria*):ab,ti

390. “atopic disease”:ab,ti

391. “atopic dermatitis”:ab,ti

392. (food* NEAR/3 sensiti*):ab,ti

393. (food* NEAR/3 toleran*):ab,ti

394. (food* NEAR/3 intoleran*):ab,ti

395. ((aero or air*) NEAR/3 allergen*):ab,ti

396. (aeroallergen* NEAR/3 sensiti*):ab,ti

397. (allergen* NEAR/3 sensiti*):ab,ti

398. “skin prick test*”:ab,ti

399. atopy:ab,ti

400. hypersensitiv*:ab,ti

401. MeSH descriptor [Hypersensitivity] this term only

402. MeSH descriptor [Food Hypersensitivity] explode all trees

403. MeSH descriptor [Respiratory Hypersensitivity] this term only

404. MeSH descriptor [Asthma] this term only

405. MeSH descriptor [Bronchial Hyperreactivity] this term only

406. MeSH descriptor [Forced Expiratory Volume] this term only

407. MeSH descriptor [Vital Capacity] this term only

408. MeSH descriptor [Peak Expiratory Flow Rate] this term only

409. MeSH descriptor [Eczema] this term only

410. MeSH descriptor [Neurodermatitis] this term only

411. MeSH descriptor [Rhinitis] this term only

412. MeSH descriptor [Rhinitis, Allergic, Perennial] this term only

413. MeSH descriptor [Rhinitis, Allergic, Seasonal] this term only

414. MeSH descriptor [Conjunctivitis] this term only

415. MeSH descriptor [Immunoglobulin E] this term only

416. MeSH descriptor [Autoimmune Diseases] this term only

417. MeSH descriptor [Diabetes Mellitus, Type 1] this term only

418. MeSH descriptor [Celiac Disease] this term only

419. MeSH descriptor [Crohn Disease] this term only

420. MeSH descriptor [Inflammatory Bowel Diseases] this term only

421. MeSH descriptor [Colitis, Ulcerative] this term only

422. MeSH descriptor [Thyroiditis, Autoimmune] this term only

423. MeSH descriptor [Hashimoto Disease] this term only

424. MeSH descriptor [Postpartum Thyroiditis] this term only

425. MeSH descriptor [Graves Disease] this term only

426. MeSH descriptor [Arthritis, Juvenile Rheumatoid] this term only

427. MeSH descriptor [Vitiligo] this term only

428. MeSH descriptor [Psoriasis] this term only

429. MeSH descriptor [Arthritis, Psoriatic] this term only

430. MeSH descriptor [Dermatitis, Atopic] this term only

431. MeSH descriptor [Hypersensitivity, Immediate] this term only

432. 342 or 343 or 344 or 345 or 346 or 347 or 348 or 349 or 350 or 351 or 352 or 353 or 354 or 355 or 356 or 357 or 358 or 359 or 360 or 361 or 362 or 363 or 364 or 365 or 366 or 367 or 368 or 369 or 370 or 371 or 372 or 373 or 374 or 375 or 376 or 377 or 378 or 379 or 380 or 381 or 382 or 383 or 384 or 385 or 386 or 387 or 388 or 389 or 390 or 391 or 392 or 393 or 394 or 395 or 396 or 397 or 398 or 399 or 400 or 401 or 402 or 403 or 404 or 405 or 406 or 407 or 408 or 409 or 410 or 411 or 412 or 413 or 414 or 415 or 416 or 417 or 418 or 419 or 420 or 421 or 422 or 423 or 424 or 425 or 426 or 427 or 428 or 429 or 430 or 431

433. infant*:ab,ti

434. ((one or two or three or four or five or six or seven or eight or nine or ten or eleven or twelve or thirteen or fourteen or fifteen or sixteen or seventeen or eighteen or nineteen or twenty or "twenty one" or "twenty two" or "twenty three" or "twenty four" or "twenty five" or "twenty six") NEAR/1 week*):ab,ti

435. ((one or two or three or four or five or six or seven or eight or nine or ten or eleven or twelve or thirteen or fourteen or fifteen or sixteen or seventeen or eighteen or nineteen or twenty or "twenty one" or "twenty two" or "twenty three" or "twenty four") NEAR/1 month*):ab,ti

436. 434 or 435

437. (old or age*):ab,ti

438. 436 and 437

439. (("one year*" or "two year*") NEAR/3 (old or age*)):ab,ti

440. ((first or second or two) NEAR/3 "year* of life"):ab,ti

441. MeSH descriptor [Infant] this term only

442. MeSH descriptor [Infant, Newborn] this term only

443. (maternal NEAR/7 pregnan*):ab,ti

444. (maternal NEAR/7 lactat*):ab,ti

445. (mother* NEAR/7 pregnan*):ab,ti

446. 433 or 438 or 439 or 440 or 441 or 442 or 443 or 444 or 445

447. 16 or 73 or 341

448. 432 and 446 and 447

Publication date from 2011

# Appendix 3 Search Strategies for original articles (Review C)

**Medline**

1. Diet/

2. Diet Therapy/

3. Nutritional Sciences/

4. Child Nutrition Sciences/

5. diet.ab,ti.

6. diets.ab,ti.

7. Diet, Mediterranean/

8. mediterranean diet$.ab,ti.

9. dietetic.ab,ti.

10. dietary.ab,ti.

11. eat.ab,ti.

12. eating.ab,ti.

13. intake.ab,ti.

14. nutrient?.ab,ti.

15. nutrition.ab,ti.

16. Diet, Vegetarian/

17. vegetarian?.ab,ti.

18. vegan$.ab,ti.

19. Diet, Macrobiotic/

20. macrobiotic?.ab,ti.

21. Food/

22. food$.ab,ti.

23. feed.ab,ti.

24. feeding.ab,ti.

25. cereal$.ab,ti.

26. grain$.ab,ti.

27. granary.ab,ti.

28. wholegrain.ab,ti.

29. wholewheat.ab,ti.

30. whole wheat.ab,ti.

31. wheat.ab,ti.

32. wheatgerm.ab,ti.

33. rye.ab,ti.

34. barley.ab,ti.

35. oat?.ab,ti.

36. exp Cereals/

37. root?.ab,ti.

38. tuber?.ab,ti.

39. exp Vegetables/

40. vegetable$.ab,ti.

41. onion$.ab,ti.

42. spinach.ab,ti.

43. chard.ab,ti.

44. tomato$.ab,ti.

45. pepper$.ab,ti.

46. carrot$.ab,ti.

47. beetroot.ab,ti.

48. asparagus.ab,ti.

49. garlic.ab,ti.

50. pumpkin.ab,ti.

51. sprouts.ab,ti.

52. broccoli.ab,ti.

53. cabbage$.ab,ti.

54. celery.ab,ti.

55. ginger.ab,ti.

56. potato$.ab,ti.

57. crisps.ab,ti.

58. fries.ab,ti.

59. syrup.ab,ti.

60. honey.ab,ti.

61. Honey/

62. Fruit/

63. fruit$.ab,ti.

64. apple?.ab,ti.

65. pear?.ab,ti.

66. banana?.ab,ti.

67. orange?.ab,ti.

68. grape?.ab,ti.

69. kiwi?.ab,ti.

70. citrus.ab,ti.

71. grapefruit?.ab,ti.

72. pulses.ab,ti.

73. beans.ab,ti.

74. lentil?.ab,ti.

75. chickpea?.ab,ti.

76. legume?.ab,ti.

77. lupin?.ab,ti.

78. soy.ab,ti.

79. soya.ab,ti.

80. nut?.ab,ti.

81. almond?.ab,ti.

82. peanut?.ab,ti.

83. groundnut?.ab,ti.

84. Nuts/

85. seed?.ab,ti.

86. sesame.ab,ti.

87. mustard.ab,ti.

88. Seeds/

89. exp Meat/

90. meat.ab,ti.

91. beef.ab,ti.

92. pork.ab,ti.

93. lamb.ab,ti.

94. poultry.ab,ti.

95. chicken.ab,ti.

96. turkey.ab,ti.

97. duck.ab,ti.

98. fish.ab,ti.

99. Fatty Acids/

100. exp Fatty Acids, Omega-3/

101. exp Fatty Acids, Omega-6/

102. omega-3.ab,ti.

103. omega-6.ab,ti.

104. PUFA.ab,ti.

105. fat.ab,ti.

106. fats.ab,ti.

107. fatty.ab,ti.

108. egg.ab,ti.

109. eggs.ab,ti.

110. exp Eggs/

111. Bread/

112. bread.ab,ti.

113. oil.ab,ti.

114. oils.ab,ti.

115. oily.ab,ti.

116. omega.ab,ti.

117. exp Seafood/

118. seafood.ab,ti.

119. shellfish.ab,ti.

120. crustacean?.ab,ti.

121. mollusc?.ab,ti.

122. Shellfish/

123. Dairy Products/

124. dairy.ab,ti.

125. exp Milk/

126. milk.ab,ti.

127. Infant Formula/

128. formula?.ab,ti.

129. hydrolysed.ab,ti.

130. Infant Food/

131. yoghurt.ab,ti.

132. probiotic.ab,ti.

133. prebiotic?.ab,ti.

134. butter.ab,ti.

135. herb?.ab,ti.

136. spice?.ab,ti.

137. chilli$.ab,ti.

138. condiment?.ab,ti.

139. exp Condiments/

140. Beverages/

141. beverage?.ab,ti.

142. fluid intake.ab,ti.

143. water.ab,ti.

144. drink$.ab,ti.

145. exp Food Preservation/

146. pickled.ab,ti.

147. bottled.ab,ti.

148. canned.ab,ti.

149. canning.ab,ti.

150. smoked.ab,ti.

151. preserved.ab,ti.

152. preservatives.ab,ti.

153. nitrosamine.ab,ti.

154. hydrogenation.ab,ti.

155. fortified.ab,ti.

156. nitrates.ab,ti.

157. nitrites.ab,ti.

158. ferment$.ab,ti.

159. processed.ab,ti.

160. antioxidant$.ab,ti.

161. genetic modif$.ab,ti.

162. genetically modif$.ab,ti.

163. Cooking/

164. cooking.ab,ti.

165. cooked.ab,ti.

166. grill.ab,ti.

167. grilled.ab,ti.

168. fried.ab,ti.

169. fry.ab,ti.

170. roast.ab,ti.

171. bake.ab,ti.

172. baked.ab,ti.

173. stewing.ab,ti.

174. stewed.ab,ti.

175. casserol$.ab,ti.

176. broil.ab,ti.

177. broiled.ab,ti.

178. boiled.ab,ti.

179. poach.ab,ti.

180. poached.ab,ti.

181. steamed.ab,ti.

182. barbecue$.ab,ti.

183. chargrill$.ab,ti.

184. salt.ab,ti.

185. salting.ab,ti.

186. salted.ab,ti.

187. fiber.ab,ti.

188. fibre.ab,ti.

189. polysaccharide$.ab,ti.

190. starch.ab,ti.

191. starchy.ab,ti.

192. carbohydrate$.ab,ti.

193. lipid$.ab,ti.

194. linoleic acid$.ab,ti.

195. sugar$.ab,ti.

196. sweetener$.ab,ti.

197. saccharin$.ab,ti.

198. aspartame.ab,ti.

199. sucrose.ab,ti.

200. xylitol.ab,ti.

201. cholesterol.ab,ti.

202. hydrogenated lard.ab,ti.

203. dietary protein.ab,ti.

204. dietary proteins.ab,ti.

205. protein intake.ab,ti.

206. animal protein$.ab,ti.

207. total protein$.ab,ti.

208. vegetable protein$.ab,ti.

209. plant protein$.ab,ti.

210. exp Dietary Carbohydrates/

211. exp Dietary Fats/

212. exp Dietary Fiber/

213. exp Dietary Proteins/

214. exp Dietary Supplements/

215. exp Food Additives/

216. exp Vitamins/

217. supplements.ab,ti.

218. supplement.ab,ti.

219. vitamin$.ab,ti.

220. retinol.ab,ti.

221. carotenoid$.ab,ti.

222. tocopherol.ab,ti.

223. folate$.ab,ti.

224. folic acid.ab,ti.

225. methionine.ab,ti.

226. riboflavin.ab,ti.

227. thiamine.ab,ti.

228. niacin.ab,ti.

229. pyridoxine.ab,ti.

230. cobalamin.ab,ti.

231. mineral$.ab,ti.

232. sodium.ab,ti.

233. iron.ab,ti.

234. calcium.ab,ti.

235. selenium.ab,ti.

236. iodine.ab,ti.

237. magnesium.ab,ti.

238. potassium.ab,ti.

239. zinc.ab,ti.

240. copper.ab,ti.

241. phosphorus.ab,ti.

242. manganese.ab,ti.

243. chromium.ab,ti.

244. phytochemical.ab,ti.

245. polyphenol$.ab,ti.

246. phytoestrogen$.ab,ti.

247. genistein.ab,ti.

248. saponin$.ab,ti.

249. coumarin$.ab,ti.

250. flavonoid$.ab,ti.

251. polyphenol$.ab,ti.

252. flavonol$.ab,ti.

253. flavone$.ab,ti.

254. isoflavone$.ab,ti.

255. catechin$.ab,ti.

256. ascorbic acid$.ab,ti.

257. hydroxy cholecalciferol$.ab,ti.

258. hydroxycholecalciferol$.ab,ti.

259. tocotrienol$.ab,ti.

260. carotene$.ab,ti.

261. cryptoxanthin$.ab,ti.

262. lycopene$.ab,ti.

263. lutein$.ab,ti.

264. zeaxanthin$.ab,ti.

265. selenium$.ab,ti.

266. organic diet?.ab,ti.

267. Food, Organic/

268. 1 or 2 or 3 or 4 or 5 or 6 or 7 or 8 or 9 or 10 or 11 or 12 or 13 or 14 or 15 or 16 or 17 or 18 or 19 or 20 or 21 or 22 or 23 or 24 or 25 or 26 or 27 or 28 or 29 or 30 or 31 or 32 or 33 or 34 or 35 or 36 or 37 or 38 or 39 or 40 or 41 or 42 or 43 or 44 or 45 or 46 or 47 or 48 or 49 or 50 or 51 or 52 or 53 or 54 or 55 or 56 or 57 or 58 or 59 or 60 or 61 or 62 or 63 or 64 or 65 or 66 or 67 or 68 or 69 or 70 or 71 or 72 or 73 or 74 or 75 or 76 or 77 or 78 or 79 or 80 or 81 or 82 or 83 or 84 or 85 or 86 or 87 or 88 or 89 or 90 or 91 or 92 or 93 or 94 or 95 or 96 or 97 or 98 or 99 or 100 or 101 or 102 or 103 or 104 or 105 or 106 or 107 or 108 or 109 or 110 or 111 or 112 or 113 or 114 or 115 or 116 or 117 or 118 or 119 or 120 or 121 or 122 or 123 or 124 or 125 or 126 or 127 or 128 or 129 or 130 or 131 or 132 or 133 or 134 or 135 or 136 or 137 or 138 or 139 or 140 or 141 or 142 or 143 or 144 or 145 or 146 or 147 or 148 or 149 or 150 or 151 or 152 or 153 or 154 or 155 or 156 or 157 or 158 or 159 or 160 or 161 or 162 or 163 or 164 or 165 or 166 or 167 or 168 or 169 or 170 or 171 or 172 or 173 or 174 or 175 or 176 or 177 or 178 or 179 or 180 or 181 or 182 or 183 or 184 or 185 or 186 or 187 or 188 or 189 or 190 or 191 or 192 or 193 or 194 or 195 or 196 or 197 or 198 or 199 or 200 or 201 or 202 or 203 or 204 or 205 or 206 or 207 or 208 or 209 or 210 or 211 or 212 or 213 or 214 or 215 or 216 or 217 or 218 or 219 or 220 or 221 or 222 or 223 or 224 or 225 or 226 or 227 or 228 or 229 or 230 or 231 or 232 or 233 or 234 or 235 or 236 or 237 or 238 or 239 or 240 or 241 or 242 or 243 or 244 or 245 or 246 or 247 or 248 or 249 or 250 or 251 or 252 or 253 or 254 or 255 or 256 or 257 or 258 or 259 or 260 or 261 or 262 or 263 or 264 or 265 or 266 or 267

269. allerg$.ab,ti.

270. asthma$.ab,ti.

271. wheeze.ab,ti.

272. wheezing.ab,ti.

273. bronchial hyperresponsiveness.ab,ti.

274. bronchial hyperreactivity.ab,ti.

275. Forced expiratory volume.ab,ti.

276. FEV1.ab,ti.

277. "FEV 1".ab,ti.

278. "FEV0.5".ab,ti.

279. "FEV 0.5".ab,ti.

280. Forced vital capacity.ab,ti.

281. FVC.ab,ti.

282. Peak expiratory flow rate.ab,ti.

283. PEFR.ab,ti.

284. eczema.ab,ti.

285. neurodermatitis.ab,ti.

286. rhinitis.ab,ti.

287. besniers prurigo.ab,ti.

288. rhinoconjunctivitis.ab,ti.

289. hayfever.ab,ti.

290. (hay adj fever).ab,ti.

291. poll?nosis.ab,ti.

292. SAR.ab,ti.

293. (pollen adj allergy).ab,ti.

294. conjunctivitis.ab,ti.

295. immunoglobulin e.ab,ti.

296. Total IgE.ab,ti.

297. autoimmune disease?.ab,ti.

298. diabetes.ab,ti.

299. diabetic.ab,ti.

300. type 1.ab,ti.

301. c?eliac disease.ab,ti.

302. crohn$ disease.ab,ti.

303. Inflammatory Bowel Disease?.ab,ti.

304. Ulcerative colitis.ab,ti.

305. (Lympho$ adj3 thyroiditi$).ab,ti.

306. (Thyroiditi$ adj3 autoimmune).ab,ti.

307. (Hashimoto$ adj3 (syndrome? or thyroiditi$ or disease?)).ab,ti.

308. (Thyroiditi$ adj3 (post-partum or postpartum)).ab,ti.

309. Graves? disease.ab,ti.

310. Basedow$ disease.ab,ti.

311. exophthalmic goiter?.ab,ti.

312. (Still? Disease adj3 (juvenile or onset)).ab,ti.

313. (Juvenile adj3 arthriti$).ab,ti.

314. vitiligo.ab,ti.

315. Psorias?s.ab,ti.

316. (Arthriti? adj3 Psoria$).ab,ti.

317. atopic disease.ab,ti.

318. atopic dermatitis.ab,ti.

319. (food? adj3 sensiti$).ab,ti.

320. (food? adj3 toleran$).ab,ti.

321. (food? adj3 intoleran$).ab,ti.

322. ((aero or air$) adj3 allergen?).ab,ti.

323. (aeroallergen? adj3 sensiti$).ab,ti.

324. (allergen? adj3 sensiti$).ab,ti.

325. skin prick test$.ab,ti.

326. atopy.ab,ti.

327. hypersensitiv$.ab,ti.

328. Hypersensitivity/

329. exp Food Hypersensitivity/

330. Respiratory Hypersensitivity/

331. Asthma/

332. Bronchial Hyperreactivity/

333. Forced Expiratory Volume/

334. Vital Capacity/

335. Peak Expiratory Flow Rate/

336. Eczema/

337. Neurodermatitis/

338. Rhinitis/

339. Rhinitis, Allergic, Perennial/

340. Rhinitis, Allergic, Seasonal/

341. Conjunctivitis/

342. Immunoglobulin E/

343. Autoimmune Diseases/

344. Diabetes Mellitus, Type 1/

345. Celiac Disease/

346. Crohn Disease/

347. Inflammatory Bowel Diseases/

348. Colitis, Ulcerative/

349. Thyroiditis, Autoimmune/

350. Hashimoto Disease/

351. Postpartum Thyroiditis/

352. Graves Disease/

353. Arthritis, Juvenile Rheumatoid/

354. Vitiligo/

355. Psoriasis/

356. Arthritis, Psoriatic/

357. Dermatitis, Atopic/

358. Hypersensitivity, Immediate/

359. 269 or 270 or 271 or 272 or 273 or 274 or 275 or 276 or 277 or 278 or 279 or 280 or 281 or 282 or 283 or 284 or 285 or 286 or 287 or 288 or 289 or 290 or 291 or 292 or 293 or 294 or 295 or 296 or 297 or 298 or 299 or 300 or 301 or 302 or 303 or 304 or 305 or 306 or 307 or 308 or 309 or 310 or 311 or 312 or 313 or 314 or 315 or 316 or 317 or 318 or 319 or 320 or 321 or 322 or 323 or 324 or 325 or 326 or 327 or 328 or 329 or 330 or 331 or 332 or 333 or 334 or 335 or 336 or 337 or 338 or 339 or 340 or 341 or 342 or 343 or 344 or 345 or 346 or 347 or 348 or 349 or 350 or 351 or 352 or 353 or 354 or 355 or 356 or 357 or 358

360. infant?.ab,ti.

361. ((one or two or three or four or five or six or seven or eight or nine or ten or eleven or twelve or thirteen or fourteen or fifteen or sixteen or seventeen or eighteen or nineteen or twenty or "twenty one" or "twenty two" or "twenty three" or "twenty four" or "twenty five" or "twenty six") adj week?).ab,ti.

362. ((one or two or three or four or five or six or seven or eight or nine or ten or eleven or twelve or thirteen or fourteen or fifteen or sixteen or seventeen or eighteen or nineteen or twenty or "twenty one" or "twenty two" or "twenty three" or "twenty four") adj month?).ab,ti.

363. 361 or 362

364. (old or age?).ab,ti.

365. 363 and 364

366. (("one year?" or "two year?") adj3 (old or age?)).ab,ti.

367. ((first or second or two) adj3 "year? of life").ab,ti.

368. Infant/

369. Infant, Newborn/

370. (maternal adj7 pregnan$).ab,ti.

371. (maternal adj7 lactat$).ab,ti.

372. (mother? adj7 pregnan$).ab,ti.

373. 360 or 365 or 366 or 367 or 368 or 369 or 370 or 371 or 372

374. clinical trial?.mp.

375. random$.mp.

376. factorial$.mp.

377. crossover$.mp.

378. placebo$.mp.

379. (doubl$ adj blind$).mp.

380. (singl$ adj blind$).mp.

381. assign$.mp.

382. volunteer$.mp.

383. cohort stud$.mp.

384. longitudinal$.mp.

385. follow-up.mp.

386. prospectiv$.mp.

387. retrospectiv$.mp.

388. case control.mp.

389. case referent.mp.

390. exp clinical trial/

391. Cross-Over Studies/

392. Placebos/

393. Double-Blind Method/

394. Single-Blind Method/

395. exp Cohort Studies/

396. case-control studies/

397. 374 or 375 or 376 or 377 or 378 or 379 or 380 or 381 or 382 or 383 or 384 or 385 or 386 or 387 or 388 or 389 or 390 or 391 or 392 or 393 or 394 or 395 or 396

398. 268 and 359 and 373 and 397

***Embase***

1. diet/

2. diet therapy/

3. nutritional science/

4. diet.ti,ab.

5. diets.ti,ab.

6. Mediterranean diet/

7. mediterranean diet$.ab,ti.

8. dietetic.ab,ti.

9. dietary.ab,ti.

10. eat.ab,ti.

11. eating.ab,ti.

12. intake.ab,ti.

13. nutrient?.ab,ti.

14. nutrition.ab,ti.

15. vegetarian diet/

16. vegetarian?.ti,ab.

17. vegan$.ti,ab.

18. macrobiotic diet/

19. macrobiotic?.ti,ab.

20. food/

21. food$.ab,ti.

22. feed.ab,ti.

23. feeding.ab,ti.

24. cereal$.ab,ti.

25. grain$.ab,ti.

26. granary.ab,ti.

27. wholegrain.ab,ti.

28. wholewheat.ab,ti.

29. whole wheat.ab,ti.

30. wheat.ab,ti.

31. wheatgerm.ab,ti.

32. rye.ab,ti.

33. barley.ab,ti.

34. oat?.ab,ti.

35. exp cereal/

36. root?.ti,ab.

37. tuber?.ti,ab.

38. exp vegetable/

39. vegetable$.ab,ti.

40. onion$.ab,ti.

41. spinach.ab,ti.

42. chard.ab,ti.

43. tomato$.ab,ti.

44. pepper$.ab,ti.

45. carrot$.ab,ti.

46. beetroot.ab,ti.

47. asparagus.ab,ti.

48. garlic.ab,ti.

49. pumpkin.ab,ti.

50. sprouts.ab,ti.

51. broccoli.ab,ti.

52. cabbage$.ab,ti.

53. celery.ab,ti.

54. ginger.ab,ti.

55. potato$.ab,ti.

56. crisps.ab,ti.

57. fries.ab,ti.

58. syrup.ab,ti.

59. honey.ab,ti.

60. honey/

61. fruit/

62. fruit$.ab,ti.

63. apple?.ab,ti.

64. pear?.ab,ti.

65. banana?.ab,ti.

66. orange?.ab,ti.

67. grape?.ab,ti.

68. kiwi?.ab,ti.

69. citrus.ab,ti.

70. grapefruit?.ab,ti.

71. pulses.ab,ti.

72. beans.ab,ti.

73. lentil?.ab,ti.

74. chickpea?.ab,ti.

75. legume?.ab,ti.

76. lupin?.ab,ti.

77. soy.ab,ti.

78. soya.ab,ti.

79. nut?.ab,ti.

80. almond?.ab,ti.

81. peanut?.ab,ti.

82. groundnut?.ab,ti.

83. exp nut/

84. seed?.ti,ab.

85. sesame.ti,ab.

86. mustard.ti,ab.

87. plant seed/

88. meat/

89. meat.ab,ti.

90. beef.ab,ti.

91. pork.ab,ti.

92. lamb.ab,ti.

93. poultry.ab,ti.

94. chicken.ab,ti.

95. turkey.ab,ti.

96. duck.ab,ti.

97. fish.ab,ti.

98. fatty acid/

99. omega 3 fatty acid/

100. omega 6 fatty acid/

101. omega-3.ab,ti.

102. omega-6.ab,ti.

103. PUFA.ab,ti.

104. fat.ab,ti.

105. fats.ab,ti.

106. fatty.ab,ti.

107. egg.ab,ti.

108. eggs.ab,ti.

109. exp egg/

110. bread/

111. bread.ti,ab.

112. oil.ti,ab.

113. oils.ti,ab.

114. oily.ti,ab.

115. omega.ti,ab.

116. sea food/

117. seafood.ti,ab.

118. shellfish.ti,ab.

119. crustacean?.ti,ab.

120. mollusc?.ti,ab.

121. shellfish/

122. exp dairy product/

123. dairy.ti,ab.

124. milk/

125. milk.ti,ab.

126. artificial milk/

127. formula?.ti,ab.

128. hydrolysed.ti,ab.

129. baby food/

130. yoghurt.ab,ti.

131. probiotic.ab,ti.

132. prebiotic?.ab,ti.

133. butter.ab,ti.

134. herb?.ab,ti.

135. spice?.ab,ti.

136. chilli$.ab,ti.

137. condiment?.ab,ti.

138. exp condiment/

139. beverage/

140. beverage?.ti,ab.

141. fluid intake.ti,ab.

142. water.ti,ab.

143. drink$.ti,ab.

144. exp food preservation/

145. pickled.ab,ti.

146. bottled.ab,ti.

147. canned.ab,ti.

148. canning.ab,ti.

149. smoked.ab,ti.

150. preserved.ab,ti.

151. preservatives.ab,ti.

152. nitrosamine.ab,ti.

153. hydrogenation.ab,ti.

154. fortified.ab,ti.

155. nitrates.ab,ti.

156. nitrites.ab,ti.

157. ferment$.ab,ti.

158. processed.ab,ti.

159. antioxidant$.ab,ti.

160. genetic modif$.ab,ti.

161. genetically modif$.ab,ti.

162. cooking/

163. cooking.ab,ti.

164. cooked.ab,ti.

165. grill.ab,ti.

166. grilled.ab,ti.

167. fried.ab,ti.

168. fry.ab,ti.

169. roast.ab,ti.

170. bake.ab,ti.

171. baked.ab,ti.

172. stewing.ab,ti.

173. stewed.ab,ti.

174. casserol$.ab,ti.

175. broil.ab,ti.

176. broiled.ab,ti.

177. boiled.ab,ti.

178. poach.ab,ti.

179. poached.ab,ti.

180. steamed.ab,ti.

181. barbecue$.ab,ti.

182. chargrill$.ab,ti.

183. salt.ab,ti.

184. salting.ab,ti.

185. salted.ab,ti.

186. fiber.ab,ti.

187. fibre.ab,ti.

188. polysaccharide$.ab,ti.

189. starch.ab,ti.

190. starchy.ab,ti.

191. carbohydrate$.ab,ti.

192. lipid$.ab,ti.

193. linoleic acid$.ab,ti.

194. sugar$.ab,ti.

195. sweetener$.ab,ti.

196. saccharin$.ab,ti.

197. aspartame.ab,ti.

198. sucrose.ab,ti.

199. xylitol.ab,ti.

200. cholesterol.ab,ti.

201. hydrogenated lard.ab,ti.

202. dietary protein.ab,ti.

203. dietary proteins.ab,ti.

204. protein intake.ab,ti.

205. animal protein$.ab,ti.

206. total protein$.ab,ti.

207. vegetable protein$.ab,ti.

208. plant protein$.ab,ti.

209. carbohydrate diet/

210. carbohydrate intake/

211. fat intake/

212. dietary fiber/

213. protein intake/

214. diet supplementation/

215. food additive/

216. exp vitamin/

217. supplements.ab,ti.

218. supplement.ab,ti.

219. vitamin$.ab,ti.

220. retinol.ab,ti.

221. carotenoid$.ab,ti.

222. tocopherol.ab,ti.

223. folate$.ab,ti.

224. folic acid.ab,ti.

225. methionine.ab,ti.

226. riboflavin.ab,ti.

227. thiamine.ab,ti.

228. niacin.ab,ti.

229. pyridoxine.ab,ti.

230. cobalamin.ab,ti.

231. mineral$.ab,ti.

232. sodium.ab,ti.

233. iron.ab,ti.

234. calcium.ab,ti.

235. selenium.ab,ti.

236. iodine.ab,ti.

237. magnesium.ab,ti.

238. potassium.ab,ti.

239. zinc.ab,ti.

240. copper.ab,ti.

241. phosphorus.ab,ti.

242. manganese.ab,ti.

243. chromium.ab,ti.

244. phytochemical.ab,ti.

245. polyphenol$.ab,ti.

246. phytoestrogen$.ab,ti.

247. genistein.ab,ti.

248. saponin$.ab,ti.

249. coumarin$.ab,ti.

250. flavonoid$.ab,ti.

251. polyphenol$.ab,ti.

252. flavonol$.ab,ti.

253. flavone$.ab,ti.

254. isoflavone$.ab,ti.

255. catechin$.ab,ti.

256. ascorbic acid$.ab,ti.

257. hydroxy cholecalciferol$.ab,ti.

258. hydroxycholecalciferol$.ab,ti.

259. tocotrienol$.ab,ti.

260. carotene$.ab,ti.

261. cryptoxanthin$.ab,ti.

262. lycopene$.ab,ti.

263. lutein$.ab,ti.

264. zeaxanthin$.ab,ti.

265. selenium$.ab,ti.

266. organic diet?.ab,ti.

267. organic food/

268. 1 or 2 or 3 or 4 or 5 or 6 or 7 or 8 or 9 or 10 or 11 or 12 or 13 or 14 or 15 or 16 or 17 or 18 or 19 or 20 or 21 or 22 or 23 or 24 or 25 or 26 or 27 or 28 or 29 or 30 or 31 or 32 or 33 or 34 or 35 or 36 or 37 or 38 or 39 or 40 or 41 or 42 or 43 or 44 or 45 or 46 or 47 or 48 or 49 or 50 or 51 or 52 or 53 or 54 or 55 or 56 or 57 or 58 or 59 or 60 or 61 or 62 or 63 or 64 or 65 or 66 or 67 or 68 or 69 or 70 or 71 or 72 or 73 or 74 or 75 or 76 or 77 or 78 or 79 or 80 or 81 or 82 or 83 or 84 or 85 or 86 or 87 or 88 or 89 or 90 or 91 or 92 or 93 or 94 or 95 or 96 or 97 or 98 or 99 or 100 or 101 or 102 or 103 or 104 or 105 or 106 or 107 or 108 or 109 or 110 or 111 or 112 or 113 or 114 or 115 or 116 or 117 or 118 or 119 or 120 or 121 or 122 or 123 or 124 or 125 or 126 or 127 or 128 or 129 or 130 or 131 or 132 or 133 or 134 or 135 or 136 or 137 or 138 or 139 or 140 or 141 or 142 or 143 or 144 or 145 or 146 or 147 or 148 or 149 or 150 or 151 or 152 or 153 or 154 or 155 or 156 or 157 or 158 or 159 or 160 or 161 or 162 or 163 or 164 or 165 or 166 or 167 or 168 or 169 or 170 or 171 or 172 or 173 or 174 or 175 or 176 or 177 or 178 or 179 or 180 or 181 or 182 or 183 or 184 or 185 or 186 or 187 or 188 or 189 or 190 or 191 or 192 or 193 or 194 or 195 or 196 or 197 or 198 or 199 or 200 or 201 or 202 or 203 or 204 or 205 or 206 or 207 or 208 or 209 or 210 or 211 or 212 or 213 or 214 or 215 or 216 or 217 or 218 or 219 or 220 or 221 or 222 or 223 or 224 or 225 or 226 or 227 or 228 or 229 or 230 or 231 or 232 or 233 or 234 or 235 or 236 or 237 or 238 or 239 or 240 or 241 or 242 or 243 or 244 or 245 or 246 or 247 or 248 or 249 or 250 or 251 or 252 or 253 or 254 or 255 or 256 or 257 or 258 or 259 or 260 or 261 or 262 or 263 or 264 or 265 or 266 or 267

269. allerg$.ab,ti.

270. asthma$.ab,ti.

271. wheeze.ab,ti.

272. wheezing.ab,ti.

273. bronchial hyperresponsiveness.ab,ti.

274. bronchial hyperreactivity.ab,ti.

275. Forced expiratory volume.ab,ti.

276. FEV1.ab,ti.

277. "FEV 1".ab,ti.

278. "FEV0.5".ab,ti.

279. "FEV 0.5".ab,ti.

280. Forced vital capacity.ab,ti.

281. FVC.ab,ti.

282. Peak expiratory flow rate.ab,ti.

283. PEFR.ab,ti.

284. eczema.ab,ti.

285. neurodermatitis.ab,ti.

286. rhinitis.ab,ti.

287. besniers prurigo.ab,ti.

288. rhinoconjunctivitis.ab,ti.

289. hayfever.ab,ti.

290. (hay adj fever).ab,ti.

291. poll?nosis.ab,ti.

292. SAR.ab,ti.

293. (pollen adj allergy).ab,ti.

294. conjunctivitis.ab,ti.

295. immunoglobulin e.ab,ti.

296. Total IgE.ab,ti.

297. autoimmune disease?.ab,ti.

298. diabetes.ab,ti.

299. diabetic.ab,ti.

300. type 1.ab,ti.

301. c?eliac disease.ab,ti.

302. crohn$ disease.ab,ti.

303. Inflammatory Bowel Disease?.ab,ti.

304. Ulcerative colitis.ab,ti.

305. (Lympho$ adj3 thyroiditi$).ab,ti.

306. (Thyroiditi$ adj3 autoimmune).ab,ti.

307. (Hashimoto$ adj3 (syndrome? or thyroiditi$ or disease?)).ab,ti.

308. (Thyroiditi$ adj3 (post-partum or postpartum)).ab,ti.

309. Graves? disease.ab,ti.

310. Basedow$ disease.ab,ti.

311. exophthalmic goiter?.ab,ti.

312. (Still? Disease adj3 (juvenile or onset)).ab,ti.

313. (Juvenile adj3 arthriti$).ab,ti.

314. vitiligo.ab,ti.

315. Psorias?s.ab,ti.

316. (Arthriti? adj3 Psoria$).ab,ti.

317. atopic disease.ab,ti.

318. atopic dermatitis.ab,ti.

319. (food? adj3 sensiti$).ab,ti.

320. (food? adj3 toleran$).ab,ti.

321. (food? adj3 intoleran$).ab,ti.

322. ((aero or air$) adj3 allergen?).ab,ti.

323. (aeroallergen? adj3 sensiti$).ab,ti.

324. (allergen? adj3 sensiti$).ab,ti.

325. skin prick test$.ab,ti.

326. atopy.ab,ti.

327. hypersensitiv$.ab,ti.

328. exp hypersensitivity/

329. respiratory tract allergy/

330. asthma/

331. wheezing/

332. bronchus hyperreactivity/

333. forced expiratory volume/

334. forced vital capacity/

335. peak expiratory flow/

336. eczema/

337. neurodermatitis/

338. rhinitis/

339. rhinoconjunctivitis/

340. hay fever/

341. pollen allergy/

342. perennial rhinitis/

343. conjunctivitis/

344. immunoglobulin E/

345. autoimmune disease/

346. diabetes mellitus/

347. insulin dependent diabetes mellitus/

348. celiac disease/

349. Crohn disease/

350. enteritis/

351. ulcerative colitis/

352. autoimmune thyroiditis/

353. Hashimoto disease/

354. postpartum thyroiditis/

355. Graves disease/

356. juvenile rheumatoid arthritis/

357. vitiligo/

358. psoriasis/

359. psoriatic arthritis/

360. atopic dermatitis/

361. nutritional intolerance/

362. 269 or 270 or 271 or 272 or 273 or 274 or 275 or 276 or 277 or 278 or 279 or 280 or 281 or 282 or 283 or 284 or 285 or 286 or 287 or 288 or 289 or 290 or 291 or 292 or 293 or 294 or 295 or 296 or 297 or 298 or 299 or 300 or 301 or 302 or 303 or 304 or 305 or 306 or 307 or 308 or 309 or 310 or 311 or 312 or 313 or 314 or 315 or 316 or 317 or 318 or 319 or 320 or 321 or 322 or 323 or 324 or 325 or 326 or 327 or 328 or 329 or 330 or 331 or 332 or 333 or 334 or 335 or 336 or 337 or 338 or 339 or 340 or 341 or 342 or 343 or 344 or 345 or 346 or 347 or 348 or 349 or 350 or 351 or 352 or 353 or 354 or 355 or 356 or 357 or 358 or 359 or 360 or 361

363. infant?.ab,ti.

364. ((one or two or three or four or five or six or seven or eight or nine or ten or eleven or twelve or thirteen or fourteen or fifteen or sixteen or seventeen or eighteen or nineteen or twenty or "twenty one" or "twenty two" or "twenty three" or "twenty four" or "twenty five" or "twenty six") adj week?).ab,ti.

365. ((one or two or three or four or five or six or seven or eight or nine or ten or eleven or twelve or thirteen or fourteen or fifteen or sixteen or seventeen or eighteen or nineteen or twenty or "twenty one" or "twenty two" or "twenty three" or "twenty four") adj month?).ab,ti.

366. 364 or 365

367. (old or age?).ab,ti.

368. 366 and 367

369. (("one year?" or "two year?") adj3 (old or age?)).ab,ti.

370. ((first or second or two) adj3 "year? of life").ab,ti.

371. infant/

372. newborn/

373. (maternal adj7 pregnan$).ti,ab.

374. (maternal adj7 lactat$).ti,ab.

375. (mother? adj7 pregnan$).ti,ab.

376. 363 or 368 or 369 or 370 or 371 or 372 or 373 or 374 or 375

377. clinical trial?.mp.

378. random$.mp.

379. factorial$.mp.

380. crossover$.mp.

381. placebo$.mp.

382. (doubl$ adj blind$).mp.

383. (singl$ adj blind$).mp.

384. assign$.mp.

385. volunteer$.mp.

386. cohort stud$.mp.

387. longitudinal$.mp.

388. follow-up.mp.

389. prospectiv$.mp.

390. retrospectiv$.mp.

391. case control.mp.

392. case referent.mp.

393. exp clinical trial/

394. crossover procedure/

395. placebo/

396. double blind procedure/

397. single blind procedure/

398. cohort analysis/

399. longitudinal study/

400. follow up/

401. prospective study/

402. retrospective study/

403. exp case control study/

404. 377 or 378 or 379 or 380 or 381 or 382 or 383 or 384 or 385 or 386 or 387 or 388 or 389 or 390 or 391 or 392 or 393 or 394 or 395 or 396 or 397 or 398 or 399 or 400 or 401 or 402 or 403

405. 268 and 362 and 376 and 404

*2.3. LILACS*

(tw:((breast feeding) or breastfeeding or (breast fed) or breastfed or formula* or hydrolysed or bottlefed or (bottle fed) or (bottle feed*) or wean*)

AND

(tw:(allerg* or asthma* or wheez* or (bronchial hyperresponsiveness) or (bronchial hyperreactivity) or (Forced expiratory volume) or FEV1 or (FEV 1) or FEV0.5 or (FEV 0.5) or (Forced vital capacity) or FVC or (Peak expiratory flow rate) or PEFR or eczema or neurodermatitis or rhinitis or (besniers prurigo) or rhinoconjunctivitis or hayfever or (hay fever) or poll?nosis or SAR or (pollen allergy) or conjunctivitis or (immunoglobulin e) or (Total IgE) or (autoimmune disease*) or diabetes or diabetic or (type 1) or (c?eliac disease) or (crohn* disease) or (Inflammatory Bowel Disease*) or (Ulcerative colitis) or (Lympho* thyroiditi*) or (Thyroiditi* autoimmune) or (Hashimoto* syndrome*) or (Hashimoto* thyroiditis*) or (Hashimoto* disease*) or (Thyroiditi* post-partum) or (Thyroiditi* postpartum) or (Graves* Disease) or (Basedow* disease) or (exophthalmic goiter*) or (Still’s Disease) or (Stills disease) or (Juvenile arthriti*) or vitiligo or Psorias?s or (Arthriti* Psoria*) or (atopic disease) or (atopic dermatitis) or (food* sensiti*) or (food* toleran*) or (food* intoleran*) or (aero allergen*) or (air* allergen*) or (aeroallergen* sensiti*) or (allergen* sensiti*) or (skin prick test*) or atopy or hypersensitive*)

AND

db:(“LILACS”)

AND

type_of_study:(“clinical_trials” or “case_control” or “cohort” or “systematic_reviews”)

AND

limit:(“infant” or “newborn” or “preschool” or “child”)

**COCHRANE Library**

1. MeSH descriptor: [Diet] this term only

2. MeSH descriptor: [Diet Therapy] this term only

3. MeSH descriptor: [Nutritional Sciences] this term only

4. MeSH descriptor: [Child Nutrition Sciences] this term only

5. diet:ab,ti

6. diets:ab,ti

7. MeSH descriptor: [Diet, Mediterranean] this term only

8. “mediterranean diet*”:ab,ti

9. dietetic:ab,ti

10. dietary:ab,ti

11. eat:ab,ti

12. eating:ab,ti

13. intake:ab,ti

14. nutrient*:ab,ti

15. nutrition:ab,ti

16. MeSH descriptor: [Diet, Vegetarian] this term only

17. vegetarian*:ab,ti

18. vegan*:ab,ti

19. MeSH descriptor: [Diet, Macrobiotic] this term only

20. macrobiotic*:ab,ti

21. MeSH descriptor: [Food] this term only

22. food*:ab,ti

23. feed:ab,ti

24. feeding:ab,ti

25. cereal*:ab,ti

26. grain*:ab,ti

27. granary:ab,ti

28. wholegrain:ab,ti

29. wholewheat:ab,ti

30. “whole wheat”:ab,ti

31. wheat:ab,ti

32. wheatgerm:ab,ti

33. rye:ab,ti

34. barley:ab,ti

35. oat*:ab,ti

36. MeSH descriptor: [Cereals] explode all trees

37. root*:ab,ti

38. tuber*:ab,ti

39. MeSH descriptor: [Vegetables] explode all trees

40. vegetable*:ab,ti

41. onion*:ab,ti

42. spinach:ab,ti

43. chard:ab,ti

44. tomato*:ab,ti

45. pepper*:ab,ti

46. carrot*:ab,ti

47. beetroot:ab,ti

48. asparagus:ab,ti

49. garlic:ab,ti

50. pumpkin:ab,ti

51. sprouts:ab,ti

52. broccoli:ab,ti

53. cabbage*:ab,ti

54. celery:ab,ti

55. ginger:ab,ti

56. potato*:ab,ti

57. crisps:ab,ti

58. fries:ab,ti

59. syrup:ab,ti

60. honey:ab,ti

61. MeSH descriptor: [Honey] this term only

62. MeSH descriptor: [Fruit] this term only

63. fruit*:ab,ti

64. apple*:ab,ti

65. pear*:ab,ti

66. banana*:ab,ti

67. orange*:ab,ti

68. grape*:ab,ti

69. kiwi*:ab,ti

70. citrus:ab,ti

71. grapefruit*:ab,ti

72. pulses:ab,ti

73. beans:ab,ti

74. lentil*:ab,ti

75. chickpea*:ab,ti

76. legume*:ab,ti

77. lupin*:ab,ti

78. soy:ab,ti

79. soya:ab,ti

80. nut*:ab,ti

81. almond*:ab,ti

82. peanut*:ab,ti

83. groundnut*:ab,ti

84. MeSH descriptor: [Nuts] this term only

85. seed*:ab,ti

86. sesame:ab,ti

87. mustard:ab,ti

88. MeSH descriptor: [Seeds] this term only

89. MeSH descriptor: [Meat] explode all trees

90. meat:ab,ti

91. beef:ab,ti

92. pork:ab,ti

93. lamb:ab,ti

94. poultry:ab,ti

95. chicken:ab,ti

96. turkey:ab,ti

97. duck:ab,ti

98. fish:ab,ti

99. MeSH descriptor: [Fatty Acids] this term only

100. MeSH descriptor: [Fatty Acids, Omega-3] explode all trees

101. MeSH descriptor: [Fatty Acids, Omega-6] explode all trees

102. omega-3:ab,ti

103. omega-6:ab,ti

104. PUFA:ab,ti

105. fat:ab,ti

106. fats:ab,ti

107. fatty:ab,ti

108. egg:ab,ti

109. eggs:ab,ti

110. MeSH descriptor: [Eggs] explode all trees

111. MeSH descriptor: [Bread] this term only

112. bread:ab,ti

113. oil:ab,ti

114. oils:ab,ti

115. oily:ab,ti

116. omega:ab,ti

117. MeSH descriptor: [Seafood] explode all trees

118. seafood:ab,ti

119. shellfish:ab,ti

120. crustacean*:ab,ti

121. mollusc*:ab,ti

122. MeSH descriptor: [Shellfish] this term only

123. MeSH descriptor: [Dairy Products] this term only

124. dairy:ab,ti

125. MeSH descriptor: [Milk] explode all trees

126. milk:ab,ti

127. MeSH descriptor: [Infant Formula] this term only

128. formula*:ab,ti

129. hydrolysed:ab,ti

130. MeSH descriptor: [Infant Food] this term only

131. yoghurt:ab,ti

132. probiotic:ab,ti

133. prebiotic*:ab,ti

134. butter:ab,ti

135. herb*:ab,ti

136. spice*:ab,ti

137. chilli*:ab,ti

138. condiment*:ab,ti

139. MeSH descriptor: [Condiments] explode all trees

140. MeSH descriptor: [Beverages] this term only

141. beverage*:ab,ti

142. “fluid intake”:ab,ti

143. water:ab,ti

144. drink*:ab,ti

145. MeSH descriptor: [Food Preservation] explode all trees

146. pickled:ab,ti

147. bottled:ab,ti

148. canned:ab,ti

149. canning:ab,ti

150. smoked:ab,ti

151. preserved:ab,ti

152. preservatives:ab,ti

153. nitrosamine:ab,ti

154. hydrogenation:ab,ti

155. fortified:ab,ti

156. nitrates:ab,ti

157. nitrites:ab,ti

158. ferment*:ab,ti

159. processed:ab,ti

160. antioxidant*:ab,ti

161. “genetic modif*”:ab,ti

162. “genetically modif*”:ab,ti

163. MeSH descriptor: [Cooking] this term only

164. cooking:ab,ti

165. cooked:ab,ti

166. grill:ab,ti

167. grilled:ab,ti

168. fried:ab,ti

169. fry:ab,ti

170. roast:ab,ti

171. bake:ab,ti

172. baked:ab,ti

173. stewing:ab,ti

174. stewed:ab,ti

175. casserol*:ab,ti

176. broil:ab,ti

177. broiled:ab,ti

178. boiled:ab,ti

179. poach:ab,ti

180. poached:ab,ti

181. steamed:ab,ti

182. barbecue*:ab,ti

183. chargrill*:ab,ti

184. salt:ab,ti

185. salting:ab,ti

186. salted:ab,ti

187. fiber:ab,ti

188. fibre:ab,ti

189. polysaccharide*:ab,ti

190. starch:ab,ti

191. starchy:ab,ti

192. carbohydrate*:ab,ti

193. lipid*:ab,ti

194. “linoleic acid*”:ab,ti

195. sugar*:ab,ti

196. sweetener*:ab,ti

197. saccharin*:ab,ti

198. aspartame:ab,ti

199. sucrose:ab,ti

200. xylitol:ab,ti

201. cholesterol:ab,ti

202. “hydrogenated lard”:ab,ti

203. “dietary protein”:ab,ti

204. “dietary proteins”:ab,ti

205. “protein intake”:ab,ti

206. “animal protein*”:ab,ti

207. “total protein*”:ab,ti

208. “vegetable protein*”:ab,ti

209. “plant protein*”:ab,ti

210. MeSH descriptor: [Dietary Carbohydrates] explode all trees

211. MeSH descriptor: [Dietary Fats] explode all trees

212. MeSH descriptor: [Dietary Fiber] explode all trees

213. MeSH descriptor: [Dietary Proteins] explode all trees

214. MeSH descriptor: [Dietary Supplements] explode all trees

215. MeSH descriptor: [Food Additives] explode all trees

216. MeSH descriptor: [Vitamins] explode all trees

217. supplements:ab,ti

218. supplement:ab,ti

219. vitamin*:ab,ti

220. retinol:ab,ti

221. carotenoid*:ab,ti

222. tocopherol:ab,ti

223. folate*:ab,ti

224. “folic acid”:ab,ti

225. methionine:ab,ti

226. riboflavin:ab,ti

227. thiamine:ab,ti

228. niacin:ab,ti

229. pyridoxine:ab,ti

230. cobalamin:ab,ti

231. mineral*:ab,ti

232. sodium:ab,ti

233. iron:ab,ti

234. calcium:ab,ti

235. selenium:ab,ti

236. iodine:ab,ti

237. magnesium:ab,ti

238. potassium:ab,ti

239. zinc:ab,ti

240. copper:ab,ti

241. phosphorus:ab,ti

242. manganese:ab,ti

243. chromium:ab,ti

244. phytochemical:ab,ti

245. polyphenol*:ab,ti

246. phytoestrogen*:ab,ti

247. genistein:ab,ti

248. saponin*:ab,ti

249. coumarin*:ab,ti

250. flavonoid*:ab,ti

251. polyphenol*:ab,ti

252. flavonol*:ab,ti

253. flavone*:ab,ti

254. isoflavone*:ab,ti

255. catechin*:ab,ti

256. “ascorbic acid*”:ab,ti

257. “hydroxy cholecalciferol*”:ab,ti

258. hydroxycholecalciferol*:ab,ti

259. tocotrienol*:ab,ti

260. carotene*:ab,ti

261. cryptoxanthin*:ab,ti

262. lycopene*:ab,ti

263. lutein*:ab,ti

264. zeaxanthin*:ab,ti

265. selenium*:ab,ti

266. “organic diet*”:ab,ti

267. MeSH descriptor: [Food, Organic] this term only

268. 1 or 2 or 3 or 4 or 5 or 6 or 7 or 8 or 9 or 10 or 11 or 12 or 13 or 14 or 15 or 16 or 17 or 18 or 19 or 20 or 21 or 22 or 23 or 24 or 25 or 26 or 27 or 28 or 29 or 30 or 31 or 32 or 33 or 34 or 35 or 36 or 37 or 38 or 39 or 40 or 41 or 42 or 43 or 44 or 45 or 46 or 47 or 48 or 49 or 50 or 51 or 52 or 53 or 54 or 55 or 56 or 57 or 58 or 59 or 60 or 61 or 62 or 63 or 64 or 65 or 66 or 67 or 68 or 69 or 70 or 71 or 72 or 73 or 74 or 75 or 76 or 77 or 78 or 79 or 80 or 81 or 82 or 83 or 84 or 85 or 86 or 87 or 88 or 89 or 90 or 91 or 92 or 93 or 94 or 95 or 96 or 97 or 98 or 99 or 100 or 101 or 102 or 103 or 104 or 105 or 106 or 107 or 108 or 109 or 110 or 111 or 112 or 113 or 114 or 115 or 116 or 117 or 118 or 119 or 120 or 121 or 122 or 123 or 124 or 125 or 126 or 127 or 128 or 129 or 130 or 131 or 132 or 133 or 134 or 135 or 136 or 137 or 138 or 139 or 140 or 141 or 142 or 143 or 144 or 145 or 146 or 147 or 148 or 149 or 150 or 151 or 152 or 153 or 154 or 155 or 156 or 157 or 158 or 159 or 160 or 161 or 162 or 163 or 164 or 165 or 166 or 167 or 168 or 169 or 170 or 171 or 172 or 173 or 174 or 175 or 176 or 177 or 178 or 179 or 180 or 181 or 182 or 183 or 184 or 185 or 186 or 187 or 188 or 189 or 190 or 191 or 192 or 193 or 194 or 195 or 196 or 197 or 198 or 199 or 200 or 201 or 202 or 203 or 204 or 205 or 206 or 207 or 208 or 209 or 210 or 211 or 212 or 213 or 214 or 215 or 216 or 217 or 218 or 219 or 220 or 221 or 222 or 223 or 224 or 225 or 226 or 227 or 228 or 229 or 230 or 231 or 232 or 233 or 234 or 235 or 236 or 237 or 238 or 239 or 240 or 241 or 242 or 243 or 244 or 245 or 246 or 247 or 248 or 249 or 250 or 251 or 252 or 253 or 254 or 255 or 256 or 257 or 258 or 259 or 260 or 261 or 262 or 263 or 264 or 265 or 266 or 267

269. allerg*:ab,ti

270. asthma*:ab,ti

271. wheeze:ab,ti

272. wheezing:ab,ti

273. “bronchial hyperresponsiveness”:ab,ti

274. “bronchial hyperreactivity”:ab,ti

275. “Forced expiratory volume”:ab,ti

276. “FEV1”:ab,ti

277. "FEV 1":ab,ti

278. "FEV0.5":ab,ti

279. "FEV 0.5":ab,ti

280. “Forced vital capacity”:ab,ti

281. FVC:ab,ti

282. “Peak expiratory flow rate”:ab,ti

283. PEFR:ab,ti

284. eczema:ab,ti

285. neurodermatitis:ab,ti

286. rhinitis:ab,ti

287. “besniers prurigo”:ab,ti

288. rhinoconjunctivitis:ab,ti

289. hayfever:ab,ti

290. “hay fever”:ab,ti

291. poll*nosis:ab,ti

292. SAR:ab,ti

293. “pollen allergy”:ab,ti

294. conjunctivitis:ab,ti

295. “immunoglobulin e”:ab,ti

296. “Total IgE”:ab,ti

297. “autoimmune disease*”:ab,ti

298. diabetes:ab,ti

299. diabetic:ab,ti

300. “type 1”:ab,ti

301. “c*eliac disease”:ab,ti

302. “crohn* disease”:ab,ti

303. “Inflammatory Bowel Disease*”:ab,ti

304. “Ulcerative colitis”:ab,ti

305. (Lympho* NEAR/3 thyroiditi*):ab,ti

306. (Thyroiditi* NEAR/3 autoimmune):ab,ti

307. (Hashimoto* NEAR/3 (syndrome* or thyroiditi* or disease*)):ab,ti

308. (Thyroiditi* NEAR/3 (post-partum or postpartum)):ab,ti

309. “Graves* disease”:ab,ti

310. “Basedow* disease”:ab,ti

311. “exophthalmic goiter*”:ab,ti

312. (Still* Disease NEAR/3 (juvenile or onset)):ab,ti

313. (Juvenile NEAR/3 arthriti*):ab,ti

314. vitiligo:ab,ti

315. Psorias*s:ab,ti

316. (Arthriti* NEAR/3 Psoria*):ab,ti

317. “atopic disease”:ab,ti

318. “atopic dermatitis”:ab,ti

319. (food* NEAR/3 sensiti*):ab,ti

320. (food* NEAR/3 toleran*):ab,ti

321. (food* NEAR/3 intoleran*):ab,ti

322. ((aero or air*) NEAR/3 allergen*):ab,ti

323. (aeroallergen* NEAR/3 sensiti*):ab,ti

324. (allergen* NEAR/3 sensiti*):ab,ti

325. “skin prick test*”:ab,ti

326. atopy:ab,ti

327. hypersensitiv*:ab,ti

328. MeSH descriptor: [Hypersensitivity] this term only

329. MeSH descriptor: [Food Hypersensitivity] explode all trees

330. MeSH descriptor: [Respiratory Hypersensitivity] this term only

331. MeSH descriptor: [Asthma] this term only

332. MeSH descriptor: [Bronchial Hyperreactivity] this term only

333. MeSH descriptor: [Forced Expiratory Volume] this term only

334. MeSH descriptor: [Vital Capacity] this term only

335. MeSH descriptor: [Peak Expiratory Flow Rate] this term only

336. MeSH descriptor: [Eczema] this term only

337. MeSH descriptor: [Neurodermatitis] this term only

338. MeSH descriptor: [Rhinitis] this term only

339. MeSH descriptor: [Rhinitis, Allergic, Perennial] this term only

340. MeSH descriptor: [Rhinitis, Allergic, Seasonal] this term only

341. MeSH descriptor: [Conjunctivitis] this term only

342. MeSH descriptor: [Immunoglobulin E] this term only

343. MeSH descriptor: [Autoimmune Diseases] this term only

344. MeSH descriptor: [Diabetes Mellitus, Type 1] this term only

345. MeSH descriptor: [Celiac Disease] this term only

346. MeSH descriptor: [Crohn Disease] this term only

347. MeSH descriptor: [Inflammatory Bowel Diseases] this term only

348. MeSH descriptor: [Colitis, Ulcerative] this term only

349. MeSH descriptor: [Thyroiditis, Autoimmune] this term only

350. MeSH descriptor: [Hashimoto Disease] this term only

351. MeSH descriptor: [Postpartum Thyroiditis] this term only

352. MeSH descriptor: [Graves Disease] this term only

353. MeSH descriptor: [Arthritis, Juvenile Rheumatoid] this term only

354. MeSH descriptor: [Vitiligo] this term only

355. MeSH descriptor: [Psoriasis] this term only

356. MeSH descriptor: [Arthritis, Psoriatic] this term only

357. MeSH descriptor: [Dermatitis, Atopic] this term only

358. MeSH descriptor: [Hypersensitivity, Immediate] this term only

359. 269 or 270 or 271 or 272 or 273 or 274 or 275 or 276 or 277 or 278 or 279 or 280 or 281 or 282 or 283 or 284 or 285 or 286 or 287 or 288 or 289 or 290 or 291 or 292 or 293 or 294 or 295 or 296 or 297 or 298 or 299 or 300 or 301 or 302 or 303 or 304 or 305 or 306 or 307 or 308 or 309 or 310 or 311 or 312 or 313 or 314 or 315 or 316 or 317 or 318 or 319 or 320 or 321 or 322 or 323 or 324 or 325 or 326 or 327 or 328 or 329 or 330 or 331 or 332 or 333 or 334 or 335 or 336 or 337 or 338 or 339 or 340 or 341 or 342 or 343 or 344 or 345 or 346 or 347 or 348 or 349 or 350 or 351 or 352 or 353 or 354 or 355 or 356 or 357 or 358

360. infant*:ab,ti

361. ((one or two or three or four or five or six or seven or eight or nine or ten or eleven or twelve or thirteen or fourteen or fifteen or sixteen or seventeen or eighteen or nineteen or twenty or "twenty one" or "twenty two" or "twenty three" or "twenty four" or "twenty five" or "twenty six") NEAR/1 week*):ab,ti

362. ((one or two or three or four or five or six or seven or eight or nine or ten or eleven or twelve or thirteen or fourteen or fifteen or sixteen or seventeen or eighteen or nineteen or twenty or "twenty one" or "twenty two" or "twenty three" or "twenty four") NEAR/1 month*):ab,ti

363. 361 or 362

364. (old or age*):ab,ti

365. 363 and 364

366. (("one year*" or "two year*") NEAR/3 (old or age*)):ab,ti

367. ((first or second or two) NEAR/3 "year* of life"):ab,ti

368. MeSH descriptor: [Infant] this term only

369. MeSH descriptor: [Infant, Newborn] this term only

370. (maternal NEAR/7 pregnan*):ab,ti

371. (maternal NEAR/7 lactat*):ab,ti

372. (mother* NEAR/7 pregnan*):ab,ti

373. 360 or 365 or 366 or 367 or 368 or 369 or 370 or 371 or 372

374. “clinical trial*”

375. random*

376. factorial*

377. crossover*

378. placebo*

379. “doubl* blind*”

380. “singl* blind*”

381. assign*

382. volunteer*

383. “cohort stud*”

384. longitudinal*

385. follow-up

386. prospectiv*

387. retrospectiv*

388. “case control”

389. “case referent”

390. MeSH descriptor: [clinical trial] explode all trees

391. MeSH descriptor: [Cross-Over Studies] this term only

392. MeSH descriptor: [Placebos] this term only

393. MeSH descriptor: [Double-Blind Method] this term only

394. MeSH descriptor: [Single-Blind Method] this term only

395. MeSH descriptor: [Cohort Studies] explode all trees

396. MeSH descriptor: [case-control studies] this term only

397. 374 or 375 or 376 or 377 or 378 or 379 or 380 or 381 or 382 or 383 or 384 or 385 or 386 or 387 or 388 or 389 or 390 or 391 or 392 or 393 or 394 or 395 or 396

398. 268 and 359 and 373 and 397

***Web of Science***

1. TOPIC = (diet$ or “mediterranean diet*” or dietetic or dietary or eat or eating or intake or nutrient$ or nutrition or vegetarian$ or vegan$ or macrobiotic$ or food$ or feed or feeding or cereal$ or grain$ or granary or wholegrain or wholewheat or “whole wheat” or wheat or wheatgerm or rye or barley or oat$ or root$ or tuber$ or vegetable$ or onion$ or spinach or chard or tomato* or pepper$ or carrot$ or beetroot or asparagus or garlic or pumpkin or sprouts or broccoli or cabbage$ or celery or ginger or potato* or crisps or fries or syrup or honey or fruit$ or apple$ or pear$ or banana$ or orange$ or grape$ or kiwi$ or citrus or grapefruit$ or pulses or bean$ or lentil$ or chickpea$ or legume$ or lupin$ or soy or soya or nut$ or almond$ or peanut$ or groundnut$ or seed$ or sesame or mustard or meat$ or beef or pork or lamb or poultry or chicken or turkey or duck or fish* or omega-3 or omega-6 or PUFA or fat$ or fatty or egg$ or bread or oil$ or omega or seafood or shellfish or crustacean$ or mollusc$ or dairy or milk or formula$ or hydrolysed or yoghurt or probiotic$ or prebiotic$ or butter or herb$ or spice$ or chilli* or condiment$ or beverage$ or “fluid intake” or water or drink* or pickled or bottled or canned or canning or smoked or preserved or preservative$ or nitrosamine or hydrogenation or fortified or nitrates or nitrites or ferment* or processed or antioxidant$ or “genetic modif*” or “genetically modif*” or cooking or cooked or grill or grilled or fried or fry or roast or bake or baked or stewing or stewed or casserole* or broil or broiled or boiled or poach or poached or steamed or barbecue$ or chargrill* or salt or salting or salted or fiber or fibre or polysaccharide$ or starch or starchy or carbohydrate$ or lipid$ or “linoleic acid$” or sugar$ or sweetener$ or saccharin$ or aspartame or sucrose or xylitol or cholesterol or “hydrogenated lard” or “dietary protein$” or “protein intake” or “animal protein$” or “total protein$” or “vegetable protein$” or “plant protein$” or supplement$ or vitamin$ or retinol or carotenoid$ or tocopherol or folate$ or “folic acid” or methionine or riboflavin or thiamine or niacin or pyridoxine or cobalamin or mineral$ or sodium or iron or calcium or selenium or iodine or magnesium or potassium or zinc or copper or phosphorus or manganese or chromium or phytochemical or polyphenol$ or phytoestrogen$ or genistein or saponin$ or coumarin$ or flavonoid$ or polyphenol$ or flavonol$ or flavone$ or isoflavone$ or catechin$ or “ascorbic acid$” or “hydroxy cholecalciferol$” or “hydroxycholecalciferol$” or tocotrienol$ or carotene$ or cryptoxanthin$ or lycopene$ or lutein$ or zeaxanthin$ or selenium$ or “organic diet$”)

2. TOPIC = (allerg* or asthma* or wheeze or wheezing or “bronchial hyperresponsiveness” or “bronchial hyperreactivity” or “Forced expiratory volume” or “FEV1” or "FEV 1" or "FEV0.5" or "FEV 0.5" or “Forced vital capacity” or FVC or “Peak expiratory flow rate” or PEFR or eczema or neurodermatitis or rhinitis or “besniers prurigo” or rhinoconjunctivitis or hayfever or “hay fever” or poll$nosis or SAR or “pollen allergy” or conjunctivitis or “immunoglobulin e” or “Total IgE” or “autoimmune disease$” or diabetes or diabetic or “type 1” or “c$eliac disease” or “crohn* disease” or “Inflammatory Bowel Disease$” or “Ulcerative colitis” or (Lympho* NEAR/3 thyroiditi*) or (Thyroiditi* NEAR/3 autoimmune) or (Hashimoto* NEAR/3 (syndrome$ or thyroiditis* or disease$)) or (Thyroiditi* NEAR/3 (post-partum or postpartum)) or “Graves$ Disease” or “Basedow* disease” or “exophthalmic goiter$” or (“Still$ Disease” NEAR/3 (juvenile or onset)) or (Juvenile NEAR/3 arthriti*) or vitiligo or Psorias$s or (Arthriti$ NEAR/3 Psoria*) or “atopic disease” or “atopic dermatitis” or (food$ NEAR/3 sensiti*) or (food$ NEAR/3 toleran*) or (food$ NEAR/3 intoleran*) or ((aero or air*) NEAR/3 allergen$) or (aeroallergen$ NEAR/3 sensiti*) or (allergen$ NEAR/3 sensiti*) or “skin prick test*” or atopy or hypersensitive*)

3. TOPIC = (infant$ or (("one year$" or "two year$") NEAR/3 (old or age$)) or ((first or second or two) NEAR/3 "year$ of life") or (maternal NEAR/7 pregnan*) or (maternal NEAR/7 lactat*) or (mother$ NEAR/7 pregnan*))

4. TOPIC = ((one or two or three or four or five or six or seven or eight or nine or ten or eleven or twelve or thirteen or fourteen or fifteen or sixteen or seventeen or eighteen or nineteen or twenty or "twenty one" or "twenty two" or "twenty three" or "twenty four" or "twenty five" or "twenty six") NEAR/1 week$)

5. TOPIC = ((one or two or three or four or five or six or seven or eight or nine or ten or eleven or twelve or thirteen or fourteen or fifteen or sixteen or seventeen or eighteen or nineteen or twenty or "twenty one" or "twenty two" or "twenty three" or "twenty four") NEAR/1 month$)

6. 4 or 5

7. TOPIC = ((old or age$))

8. 7 and 6

9. 8 or 3

10. TOPIC = (“clinical trial$” or random* or factorial* or crossover* or placebo* or “doubl* blind*” or “singl* blind*” or assign* or volunteer* or “cohort stud*” or longitudinal* or follow-up or prospective* or retrospective* or “case control” or “case referent”)

11. 1 and 2 and 9 and 10
