# Supplementary material for: Diet during pregnancy and infancy and risk of allergic or autoimmune disease: A systematic review and meta-analysis
Source: PLoS Med. 2018 Feb 28;15(2):e1002507. doi: 10.1371/journal.pmed.1002507 (PMC5830033; doi:10.1371/journal.pmed.1002507)
Supplement: S1 Appendix — (DOCX) [file pmed.1002507.s005.docx]

**Diet during pregnancy and infancy, and risk of allergic or autoimmune disease: a systematic review and meta-analysis**

Vanessa Garcia-Larsen* assistant professor in human nutrition^1, 2^, Despo Ierodiakonou* post-doctoral research fellow^2, 3^, Katharine Jarrold medical student^3^, Sergio Cunha research fellow^2^, Jennifer Chivinge medical student^3^, Zoe Robinson medical student^3^, Natalie Geoghegan medical student^3^, Alisha Ruparelia medical student^3^, Pooja Devani medical student^3^, Tim Reeves librarian^2^, Marialena Trivella senior medical statistician^4^, Jo Leonardi-Bee professor of medical statistics and epidemiology^5^, Robert J Boyle clinical reader in paediatric allergy^3, 6^

^*^ Joint first authors

^1^Department of International Health, Johns Hopkins School of Public Health, N Wolfe Street, Baltimore, MD 21205, USA ^2^Respiratory Epidemiology, Occupational Medicine and Public Health, National Heart and Lung Institute, Imperial College London, Manresa Road, London SW3 6LR, UK ^3^Section of Paediatrics, Department of Medicine, Imperial College London, Norfolk Place, London W2 1PG, UK ^4^Centre for Statistics in Medicine, University of Oxford, Windmill Road, Oxford OX3 7LD, UK ^5^Division of Epidemiology and Public Health, University of Nottingham, Hucknall Road, Nottingham NG5 1PB, UK ^6^Centre of Evidence Based Dermatology, University of Nottingham, Lenton Lane, Nottingham NG7 2NR, UK

Address for Correspondence:

Robert Boyle, Wright Fleming Building, Norfolk Place, London W2 1PG

Tel: +44 207 594 3990 Fax: +44 207 594 3984. Email: r.boyle@imperial.ac.uk

Table of Contents

[Table S1 Characteristics of included intervention trials of breastfeeding duration or solid food introduction and allergic outcomes 3](#_Toc503440843)

[Table S2 Characteristics of included observational studies of breastfeeding or solid food introduction, and risk of allergic outcomes 4](#_Toc503440844)

[Table S3 Characteristics of included observational studies of breastfeeding or solid food introduction, and risk of autoimmune diseases 30](#_Toc503440845)

[Table S4 Characteristics of included intervention trials of other maternal or infant dietary exposures and allergic outcomes 40](#_Toc503440846)

[Table S5 Characteristics of included observational studies of other maternal or infant dietary exposures and risk of allergic outcomes 58](#_Toc503440847)

[Table S6 Characteristics of included observational studies of other maternal or infant dietary exposures and risk of autoimmune disease 71](#_Toc503440848)

[Table S7 Risk of bias in intervention trials of breastfeeding promotion or solid food introduction and allergic outcomes 76](#_Toc503440849)

[Table S8 Risk of bias in observational studies of breastfeeding or solid food introduction, and risk of allergic outcomes 77](#_Toc503440850)

[Table S9 Risk of bias in observational studies of breastfeeding or solid food introduction, and risk of autoimmune diseases 92](#_Toc503440851)

[Table S10 Risk of bias in intervention trials of other interventions and allergic or autoimmune outcomes 99](#_Toc503440852)

[Table S11 Risk of bias in observational studies of other maternal or infant dietary exposures and risk of allergic outcomes 106](#_Toc503440853)

[Table S12 Risk of bias in observational studies of other maternal or infant dietary exposures and risk of autoimmune disease 112](#_Toc503440854)

[References 115](#_Toc503440855)

# Table S1 Characteristics of included intervention trials of breastfeeding duration or solid food introduction and allergic outcomes

| **Study** | **Design** | **N Intervention/ Control** | **Country** | **Intervention** | **Disease risk** | **Age at outcome (years)** | **Outcomes reported**  **(method of assessment)** |
| --- | --- | --- | --- | --- | --- | --- | --- |
| Kramer, 2001 [[1](#_ENREF_1)]; Kramer, 2007 [[2](#_ENREF_2)] | cluster RCT | 8865/8181 | Belarus | TBF. Breastfeeding promotion program based on the WHO/UNICEF baby friendly hospital initiative, versus standard local breastfeeding policies | Normal | 1, 6.5 | AD (Hanifin and Rajka, ISAAC); Wheeze (ISAAC); AR (ISAAC); AS (SPT) |

AD: atopic dermatitis; AR: allergic rhinitis; AS: allergic sensitisation; DD doctor diagnosis; EBF: exclusive breastfeeding; FA: food allergy; ISAAC: International Study of Asthma and Allergies in Children; OFC: oral food challenge; RCT: randomised controlled trial; SPT: skin prick test

# Table S2 Characteristics of included observational studies of breastfeeding or solid food introduction, and risk of allergic outcomes

| **Study** | **Design** | **N/n cases** | **Country** | **Population** | **Exposures and method of assessment** | **Age at outcome (years)** | **Outcomes reported**  **(method of assessment)** |
| --- | --- | --- | --- | --- | --- | --- | --- |
| Alho, 1990 [[3](#_ENREF_3)] | PC | 2,130 | Finland | Birth cohort, population representative sample born 1985-1986 (normal risk of disease) | TBF, Q | 2 | Wheeze (DD Wheeze) |
| Allen, 2009 [[4](#_ENREF_4)]; Koplin, 2010 [[5](#_ENREF_5)] | PC | 310/50 | Australia | Part of the **HealthNUTS study**. Representative cohort of infants recruited from routine immunisation clinics 2008-2010 | TBF, Q | 1 | SPT to food; FA (OFC) |
| Alm, 2008[[6](#_ENREF_6)]; Goksor, 2009/11  [[7](#_ENREF_7),[8](#_ENREF_8)] | PC | 4,987 | Sweden | Infants of Western Sweden. Population birth cohort of infants born in 2003 | TBF, SFI, Q | 1, 1.4, 4.5 | Wheeze (ever; ≥3 episodes of wheeze in past year); FA (DD); AD (parent reported) |
| Bacopoulou, 2009 [[9](#_ENREF_9)] | PC | 6,643 | Greece | Population based sample of neonates born in 1983 | EBF, Q | 7 | Wheeze (DD) |
| Benn, 2004 [[10](#_ENREF_10)]; Linneberg 2006 [[11](#_ENREF_11)] | PC | 34,793 | Denmark | **DNBC study**. Population based birth cohort of children born between 1997-2002 | EBF, Q/I | 1.5 | AD (Physician assessment and parent report) |
| Bergmann, 2000 [[12](#_ENREF_12)]; Bergmann, 2002 [[13](#_ENREF_13)] Kulig, 2000 [[14](#_ENREF_14)] | PC | 1,314 | Germany | **MAS study**. Atopic risk enriched cohort of infants born in 1990 in 5 German cities | TBF, Q/I | 6, 7 | Wheeze (Physician assessment); RC (Physician assessment, Parent reported RC ever) |
| Berth-Jones, 1997 [[15](#_ENREF_15)] | PC | 413 | UK | Infants born in 1992 and registered with the two major Leicester obstetric units | TBF, Q | 1 | AD (Physician assessment) |
| Besednjak-Kocijancic, 2010 [[16](#_ENREF_16)] | PC | 408/24 | Slovenia | Infants with a positive history of parental allergy | EBF, Unclear | 1, 5 | FA (Infants with a positive history of parental allergy) |
| Bisgaard, 2009 [[17](#_ENREF_17)]; Giwercman, 2010 [[18](#_ENREF_18)] | PC | 354 | Denmark | **COPSAC study**. Infants of mothers with a history of doctor-diagnosed asthma, recruited from August 1998 to December 2001 | TBF, EBF, Q/I | 2, 3 | AD (Hanifin and Rajka criteria); Wheeze (Parent report) |
| Burr, 1989; Burr, 1993; Burr, 1993 (b) [[19-21](#_ENREF_19)]; Burr, 1997 [[22](#_ENREF_22)] | PC | 483 | UK | Infants with family history of allergic diseases in South Wales | TBF, D, Q | 1, 6.7, 7 | Wheeze (Parent reported wheeze; Wheeze ever, DD); AD (physician assessment); sIgE aero (any); RC (Parent reported) |
| Burgess, 2006 [[23](#_ENREF_23)] | PC | 4,964 | Australia | **Mater-University of Queensland Study of Pregnancy**. Population based birth cohort of infants born 1981-1984 | TBF, Q | 14 | Wheeze (Parent reported asthma) |
| Businco, 1987 [[24](#_ENREF_24)]; Bruno, 1995 [[25](#_ENREF_25)] | PC | 244 | Italy | Infants of atopic parents recruited from hospital and born in 1985-1988 | TBF, I | 0-2, 2, 8 | Wheeze DD asthma (≥3 episodes of wheeze); AD (physician assessment); RC (Physician assessment); sIgE to any, Total IgE |
| Cano Garcinuno, 2003 [[26](#_ENREF_26)] | PC | 234 | Spain | Children born in 1998-2002 attending primary health centre | EBF, Q | 3 | Wheeze (DD) |
| Caudri, 2013; Scholtens, 2009 [[27](#_ENREF_27),[28](#_ENREF_28)]; Kerkhof, 2003 [[29](#_ENREF_29)] | PC | 3,115 | Netherlands | **PIAMA**. Population-based study of children born in 1996-1997 (normal risk of disease) | EBF, TBF, Q | 1, 8 | Wheeze (Current wheeze; Parent reported current or ever wheeze (+/- sIgE); ISAAC; BHR: methacholine PC_20_); sIgE aero; AD (UK Working Party Criteria) |
| Cogswell, 1987 [[30](#_ENREF_30)] | PC | 73/32 | UK | Birth cohort of infants with family history of hay fever or asthma | EBF, D | 5 | SPT to any |
| Chuang, 2011[[31](#_ENREF_31)] | PC | 18,773 | Taiwan | **Taiwan Birth Cohort Study**. Population representative sample born in 1995 (normal risk of disease) | TBF, SFI, R/I | 1.5 | AD (Physician assessment) |
| da Costa Lima, 2003; Menezes, [[32](#_ENREF_32),[33](#_ENREF_33)] | PC | 4,297 | Brazil | **Pelotas Birth Cohort**. Population based birth cohort of infants born in 1982 in the city of Pelotas | EBF, TBF, Q/I | 18, 22 | Wheeze (ISAAC) |
| Dell, 2001; Midodzi, 2008 [[34](#_ENREF_34),[35](#_ENREF_35)]; Midodzi, 2010 [[36](#_ENREF_36)] | PC; CS | 2,711 | Canada | **National Longitudinal Survey of Children and Youth (NLSCY**) and the **Canadian Early Childhood Development Cohort Study** (part of NLSCY). First of longitudinal surveys of 12-24 months old children representative of the Canadian population | TBF, Q | 2, 5, 9 | Wheeze (≥2 episodes of wheeze; Preschool wheeze: <5 years but not beyond 6 years; Parent reported wheeze; DD) |
| Devereux, 2006 [[37](#_ENREF_37)] | PC | 1,704 | Scotland, UK | Population based birth cohort of infants born in 1998 | TBF, Q | 5 | Wheeze ; AD; RC (ISAAC); SPT aero |
| Dogaru, 2012 [[38](#_ENREF_38)] | PC | 1,458 | UK | Population based sample of children of white and south Asian ethnic origin born between 1993 and 1997, part of the Leicestershire cohorts | TBF, Q | 12 | Wheeze (Spirometry) |
| Elliott, 2008; Granell, 2012; Sherriff, 2001  [[39-41](#_ENREF_39)]; Abd, 2012 [[42](#_ENREF_42)]; Lack, 2003 [[43](#_ENREF_43)] | PC | 9,100 | UK | **ALSPAC study**. Population based cohort of children born 1991-1992 | EBF, TBF, Q | 3, 3.5, 7, 7.5 | Wheeze (DD asthma plus current wheeze; Parent reported wheeze; BHR: (methacholine PC_20_); SPT to any food; Visible flexural dermatitis; Parent reported AD; Parent report of food reaction (DBPCFC) |
| Eneli, 2006 [[44](#_ENREF_44)] | PC | 536 | Germany | **The Child Health and Environment Cohort Study**. community based in urban area, born 1994 (normal risk of disease) | TBF, Q | 10 | Wheeze (BHR hypertonic saline PC_15_) |
| Farooqi, 1998 [[45](#_ENREF_45)] | PC | 1,453 | UK | Representative sample of general practice born in 1975-84 | TBF, R | 16 | Wheeze (DD of recurrent episodes of wheeze after the age of two years); AD (DD); RC (DD) |
| Fergusson, 1983; Horwood, 1995  [[46](#_ENREF_46),[47](#_ENREF_47)] | PC | 1,110 | New Zealand | Christchurch Child Development Study. Population based cohort of infants born in 1977 in the Christchurch urban region | EBF, R/I |  | Wheeze (Parental report) |
| Fredriksson, 2007 [[48](#_ENREF_48)] | PC | 1,933 | Finland | Population-based study of children born between 1984 and 1989 | TBF, SFI, Q | 15 | Wheeze (ATS questionnaire: wheezing apart from colds or wheezing most days or nights during the past year; DD) |
| Forster, 1990[[49](#_ENREF_49)] | PC | 145 | Germany | Babies hospitalised in 1985 | TBF, Q | 1.5 | AD (Parental report) |
| Galbally, 2013 [[50](#_ENREF_50)] | PC | 4,507 | Australia | **Longitudinal Study of Australian Children**. Population based study of infants born between March 2003 and February 2004 and were enrolled in the Australian Medicare database | TBF, I | 1 | Wheeze (Parent reported wheezing ≥4 nights per week) |
| Gruber 2010 [[51](#_ENREF_51)] | PC | 167/15 | Central Europe | **MIPS-1 study**. Infants born in 2006 without family history of allergy, randomised to a prebiotic formula intervention if fully formula fed < 8 weeks | EBF, Q | 1 | sIgE CM, or egg; Total IgE |
| Gruskay 1982 [[52](#_ENREF_52)] | PC | 328 FH+/ 580 FH- | USA | Children born in 1961-1966 in a private paediatric practice | TBF, Unclear/Q | 3, 5, 15 | Wheeze (Physician assessment of recurrent wheezing); AD (Physician assessment); RC (parent reported) |
| Guida, 2009 [[53](#_ENREF_53)] | PC | 3,041 | France | Population based birth cohort of infants born in 2003 | TBF, Q | 1 | Wheeze (Parent reported) |
| Gustafsson, 2000 [[54](#_ENREF_54)] | PC | 94 | Sweden | Children with atopic dermatitis attending allergic clinic or referred by child welfare clinics | TBF, Q | 7, 8 | Wheeze (≥3 episodes of physician diagnosed wheezing); FA (Parent report of food reaction); SPT any |
| Halken, 1991[[55](#_ENREF_55)] | PC | 276 | Denmark | Population based birth cohort of children born in 1985 | TBF, Q | 1.5 | Wheeze (≥2 episodes of physician diagnosed wheeze) |
| Harris, 2001[[56](#_ENREF_56)]; Zutavern, 2004 [[57](#_ENREF_57)] | PC | 622 | UK | Population based birth cohort of children born between 1993 and 1995 in three general practices in Ashford | TBF, SFI, I | 2 | AD (UK Working Party Criteria) |
| Hagendorens, 2005 [[58](#_ENREF_58)]; Sariachvili, 2007 [[59](#_ENREF_59)]; Sariachvili 2010 [[60](#_ENREF_60)] | PC | 693 | Belgium | **PIPO study**. Recruited from university service, born 1997-2001 (normal risk of disease) | TBF, Q | 1 4 | Wheeze (Parent reported current wheeze); AD (Parent reported); sIgE any |
| Hesselmar, 2010 [[61](#_ENREF_61)] | PC | 184 | Sweden | **ALLERGYFLORA study**. Population based study of babies selected from antenatal clinics between 1998 and 2003 - mainly high risk of allergic disease | EBF, TBF, SFI, I | 0.5, 1.5 | Wheeze ( DD asthma; ≥3 episodes of wheeze); FA (Physician assessment: history +/- investigations); AD (UK Working Party Criteria); sIgE to food |
| Hetzner, 2009 [[62](#_ENREF_62)] | PC | 7,900 | USA | **Early Child Longitudinal Study Birth Cohort**. Nationally representative sample of children born during 2001 | EBF, I | 2 | Wheeze (DD) |
| Hide, 1981[[63](#_ENREF_63)]; Arshad, 1992 [[64](#_ENREF_64)] | PC | 843 | UK | **The Isle of Wight study**. Born in 1977-1978 (normal risk of disease) | EBF, TBF, SFI, D/Q | 1 | Wheeze (Wheeze ever; Parent reported wheeze); RC (Parent reported rhinitis symptoms); AD (DD); RC (Physician assessment), SPT to any, CM or egg |
| Hikino, 2001[[65](#_ENREF_65)] | PC | 21,766/2,381 | Japan | All children born in 1993-1995 attending well-baby check-ups funded by Fukuoka City | TBF, Q | 1.5 | FA (DD); AD (Physician assessment) |
| Hong, 2011 [[66](#_ENREF_66)] | PC | 970/361 | USA | **Boston Birth Cohort**. Predominantly African-American mother-infant pairs. | EBF, TBF, Q/I | 2.5 | sIgE to food |
| Hoppu, 2002 [[67](#_ENREF_67)] | PC | 114/27 | Finland | Birth cohort of infants with a family history of atopy | EBF, TBF, I | 1 | SPT to any |
| Host, 1991 [[68](#_ENREF_68)] | PC | 315/16 | Denmark | Population birth cohort of infants from the municipality of Odense born in 1985 | EBF, R/Q/I | 1 | FA (OFC) |
| Howie, 1990 [[69](#_ENREF_69)] | PC | 618 | UK | Population based birth cohort of infants born between 1983 and 1986 in Dundee | TBF, R/D | 1 | AD (Physician assessment) |
| Huang, 2013 [[70](#_ENREF_70)] | PC | 684 | China | Mother-infant pairs registered in Putuo District, Changzheng Town Community Health Service Center Child Health Clinic within the period from January to December, 2008 | EBF, Q | 2 | Wheeze (ISAAC) |
| Huurre, 2008 [[71](#_ENREF_71)] | PC | 98/29 | Finland | Cohort of infants whose mother participated in a nutritional intervention trial during pregnancy | EBF, TBF, I | 1 | SPT any |
| Joseph, 2011 [[72](#_ENREF_72)] | PC | 594/178 | USA | **WHEALS STUDY.** Recruited from hospital prenatal care and born in 2005 | SFI, I | 3 | sIgE CM, egg, PN |
| Juto, 1980 [[73](#_ENREF_73)] | PC | 56/NA | Sweden | Population based birth cohort of infants born in 1977 | TBF, Q | 0.25 | Total IgE |
| Kajosaari, 1991[[74](#_ENREF_74)] | PC | 135 | Finland | Exclusively breastfed infants with solid food introduction at 6 months versus 3 months | EBF, SFI, Unclear | 1, 5 | AD (Hanifin and Rajka) , FA (parental report), asthma (DD) |
| Karmaus, 2008 [[75](#_ENREF_75)]; Ogbuanu, 2009 [[76](#_ENREF_76)]; Soto-Ramırez, 2012 [[77](#_ENREF_77)] | PC | 1,336 | UK | **Isle of Wight Prevention Study**. Population based birth cohort of infants born in semi-rural areas between 1989 and 1990 | TBF, Q | 10, 18 | Wheeze (ISAAC and DD asthma, spirometry) |
| Kaufman, 1976 [[78](#_ENREF_78)] | PC | 94 | USA | Birth cohort of infants from allergic mothers | TBF, Unclear | 2 | Wheeze (DD) |
| Kellberger, 2012 [[79](#_ENREF_79)] | PC | 3,785/314 | Germany | **SOLAR.** Community based random sample of all pupils aged 9-11 years in 1995-1996 as part of ISAAC study, with follow-up survey 2002-2003 | EBF, Q | 9-18 | Ever AR (ISAAC) |
| Kemeny, 1991[[80](#_ENREF_80)] | PC | 180 | UK | Population based birth cohort of infants born at Dulwich and King’s College Hospitals in London | EBF, TBF, Unclear | 1 | Wheeze (≥2 episodes); FA (Parent report of ≥2 food reactions); AD (unclear); SPT and sIgE to CM, egg; Total IgE |
| Kerr, 1981[[81](#_ENREF_81)] | PC | 269 | New Zealand | Birth cohort hospital based, born 1977-1978 (normal risk of disease) | TBF, I | 0.5 | Wheeze (Parent reported) |
| Kim, 2011 [[82](#_ENREF_82)] | PC | 1177/61 | Korea | Population based birth cohort of infants born in Seoul between July 2006 and August 2007 | EBF, SFI, Q/I | 1 | FA (Parent report of ≥ 2 food reactions) |
| Kitz, 2006 [[83](#_ENREF_83)] | PC | 131 | Germany | Birth cohort of infants at increased risk for atopy participating in RCT on infant feeding intervention | EBF, D | 1 | AD (Hanifin and Rajka criteria) |
| Klinnert, 2001 [[84](#_ENREF_84)] | PC | 145 | USA | Birth cohort of infants at increased risk for atopy born between 1985 and 1987 | TBF, Q | 8 | Wheeze (DD) |
| Kramer, 2003; Kramer, 2009; Kramer, 2009 (b)  [[85-87](#_ENREF_85)] | PC | 13,889/455 | Belarus | **PROBIT**. Observational analysis of data from a cluster RCT of the UNICEF baby friendly initiative intervention. Infants born 1996-1997 | EBF, I | 6.5 | Current AR (ISAAC); SPT aero |
| Kull, 2002 [[88](#_ENREF_88)] | PC | 3,790 | Sweden | **BAMSE study**. Population based cohort of children born between 1994-1996 | EBF, TBF, Q | 2 | Wheeze (Self-reported wheeze; ≥3 episodes of wheeze OR inhaled corticosteroids); FA (DD); Parent reported OR DD AD; RC (Parent reported symptoms) |
| Kusel, 2005 [[89](#_ENREF_89)] | PC | 263/107 | Australia | Birth cohort of children at high risk of developing atopic disease born 1996-8 | TBF, Unclear | 5 | SPT any |
| Larsson, 2008 [[90](#_ENREF_90)] | PC | 4779 | Sweden | **DBH study**. Preschool children aged 1–6 years surveyed in 2000 and 2005 | TBF, SFI, Q | 9 | Wheeze (DD), AD (ISAAC); Current RC (DD, ISAAC) |
| Marini, 1996 [[91](#_ENREF_91)] | PC | Unclear | Italy | Infants with family history of allergy whose mothers were invited to participate in an allergy prevention program | EBF, TBF, SFI, Q | 3 | Wheeze (Physician assessment; ≥3 episodes of wheeze); AD and RC (Physician assessment) |
| Matheson, 2007 [[92](#_ENREF_92)] | PC | 5,729/2,610 | Australia | **Tasmanian Asthma Study.** Population based cohort born in 1961 | EBF, Q | 44 | RC (Physician assessment) |
| Midwinter, 1987 [[93](#_ENREF_93)] | PC | 453 | UK | Children born to parents with a family history of atopy in 1979-1981 | EBF, Unclear | 5 | Wheeze (DD asthma) |
| Mihrshahi, 2007 [[94](#_ENREF_94)] | PC | 516 | Australia | **CAPS study**. Observational analysis of participants from a RCT of omega-3 fatty acid supplementation. Infants born in 1997-1999 with family history of asthma or wheezing | EBF, TBF, SFI, I | 5 | Wheeze (DD plus current wheeze); AD (Visible flexural dermatitis or DD AD); SPT to any |
| Milner, 2004 [[95](#_ENREF_95)] | PC | 8,071 | USA | National Maternal and Infant Health Survey and Longitudinal Follow Up. Representative US population born in 1988. Black, low socioeconomic status, and premature infants intentionally overrepresented | TBF, Q | 3 | Wheeze (DD) |
| Miskelly, 1988 [[96](#_ENREF_96)] | PC | 482 | UK | Infants from antenatal clinics with family history of allergy randomised into a dietary intervention program | TBF, D | 1 | AD (Physician assessment) |
| Miyake, 2008 [[97](#_ENREF_97)]; Miyake, 2009 [[98](#_ENREF_98)] | PC | 763 | Japan | **OMCHS study**. Population based birth cohort of infants born in 2002-2003 | EBF, TBF, Q | 2 | Wheeze (ISAAC); AD (Physician assessment) |
| Morgan, 2004; Morgan, 2004 (b) [[99](#_ENREF_99),[100](#_ENREF_100)] | PC | 257 | UK | Infants from five prospective randomised dietary trials conducted in the UK between 1993 and 1997. One LBW, 2 premature and 2 appropriate weight/gestation cohorts | TBF, I | 0.25 | Parent reported AD; Physician assessment |
| Moore, 1985 [[101](#_ENREF_101)] | PC | 475 | UK | Infants born in a hospital in 1979-1980 with family history of AD or asthma, participating in a dietary intervention program | EBF, SFI, D/I | 0.25 | AD (Physician assessment) |
| Morales, 2012 [[102](#_ENREF_102)] | PC | 467 | Spain | **INMA project**. Population based birth cohort of infants born 2004-2006 | TBF, Q/I | 1 | Wheeze; AD (Parent reported) |
| Muiño, 2008 [[103](#_ENREF_103)] | PC | 897 | Brazil | Population based cohort of infants born in 1993 | TBF, Q | 12 | Wheeze (Parent reported current wheeze; Persistent wheeze: parent reported wheeze at 1, 4 and 8-10 year assessments; Early transient wheeze: parent reported) |
| Nielsen, 2013 [[104](#_ENREF_104)] | PC | 5429 | Demark | Population based birth cohort of infants born in 1995 | EBF, | 0.5 | Wheeze (unclear) |
| Nwaru, 2010 [[105](#_ENREF_105)]; Erkkola, 2012; Nwaru, 2013  [[106](#_ENREF_106),[107](#_ENREF_107)]; Virtanen, 2010 [[108](#_ENREF_108)] | PC | 3,675 | Finland | **DIPP study**. Infants at high risk (HLA) for TIDM born between 1996-2004 invited to the allergy study between 1998 and 2000 | EBF, TBF, D | 0.5, 5 | Wheeze (DD plus ISAAC; DD plus ISAAC (+/- sIgE)); AD (ISAAC); sIgE to aero, CM, egg or food; RC (Modified ISAAC questionnaire) |
| Oddy, 2003 [[109](#_ENREF_109)] | PC | 243 | USA | Birth cohort of infants participating in the Infant Immune Study in Tucson, Arizona. | EBF, TBF, Q | 1 | Wheeze (Parent reported wheeze ever) |
| Oddy, 1999; Oddy, 2003; Oddy, 2004  [[110-112](#_ENREF_110)] | PC, NCC | 2,456 | Australia | **Western Australia Pregnancy Cohort**. Recruited from antenatal clinics born in 1989-1992 (normal risk of disease) | EBF, TBF, D/I/Q | 1, 6, 8 | Wheeze (DD; parent reported current wheeze; DD asthma and ≥3 episodes of wheeze); SPT aero |
| Odelram, 1996 [[113](#_ENREF_113)] | PC | 70/23 | Sweden and Finland | Birth cohort of infant with family history and high cord blood IgE born in 1989-1990 | EBF, D | 1.5 | sIgE to CM, SPT to any, total IgE |
| Perez Tarazona, 2010 [[114](#_ENREF_114)] | PC | 620 | Spain | Population based birth cohort of infants born in 2007-2008 in Valencia | TBF, Q | 1 | Wheeze (Parent reported wheeze) |
| Pesonen, 2006 [[115](#_ENREF_115)] | PC | 160/19 | Finland | Population based birth cohort of infants born in 1981 | EBF, I | 5 | FA (Parent report of ≥ 2 food reactions) |
| Porch, 1998 [[116](#_ENREF_116)] | PC | 130 | USA | Infants recruited from prenatal services with family history of allergy (high risk of disease) | TBF, D | 1 | ISAAC - current AD |
| Poysa, 1990; Poysa, 1992 [[117](#_ENREF_117),[118](#_ENREF_118)] | PC | 120/41 | Finland | High risk of disease, born 1979-1980 | EBF, I | 5, 10 | Total IgE; SPT aero |
| Pratt, 1984 [[119](#_ENREF_119)] | PC | 198 | UK | Recruited in antenatal clinics, normal risk of disease | EBF, TBF, D/I | 4.5, 5 | AD (DD; Physician assessment) |
| Puig, 2010 [[120](#_ENREF_120)] | PC | 368 | Spain | **Part of AMICS**. Population based cohort of infants born in 1996-1998 in Barcelona | TBF, Q | 6 | Wheeze (DD) |
| Purvis, 2005 [[121](#_ENREF_121)] | PC | 550 | New Zealand | **Auckland Birth weight Collaborative study.** Birth cohort with representative sample of babies born in 1996-1997 | EBF, TBF, Q | 3.5 | AD (UK Working Party Criteria) |
| Rhodes, 2001[[122](#_ENREF_122)] | PC | 63 | UK | Hospital based, born 1976-1977, family history of allergy (high risk of disease) | TBF, Q | 22 | Wheeze (Current wheeze and BHR) |
| Rothenbacher, 2005 [[123](#_ENREF_123)] | PC | 803 | Germany | Recruited from university service, born in 2000-2001 (normal risk of disease) | EBF, TBF, Q/I | 2 | Wheeze (DD) |
| Rowntree, 1985 [[124](#_ENREF_124)] | PC | 80/20 | UK | Recruited from hospital and family history of atopy | TBF, I | 5 | sIgE to CM, egg |
| Rullo, 2007; Rullo, 2009; Rullo, 2009 (b); Rullo, 2010  [[125-128](#_ENREF_125)] | PC | 101 | Brazil | Recruited from hospital (high risk of asthma) | EBF, Q/I | 1.5, 2.5, 4, 5 | Wheeze (≥3 episodes of wheeze in past year; Persistent wheeze: ever wheezing treated with inhaled corticosteroids and beta-2 agonists in the past year) |
| Ruiz 1992 [[129](#_ENREF_129)] | PC | 39 | UK | Recruited from hospital and family history of atopy (high risk of disease) | TBF, SFI, Unclear | 1 | AD (Hanifin and Rajka criteria) |
| Saarinen, 1995 [[130](#_ENREF_130)] Saarinen, 1979 [[131](#_ENREF_131)] | PC | Unclear | Finland | Recruited from hospital and born in 1975 (normal risk of disease) | EBF, TBF, R | 0-1, 1, 5, 17 | Wheeze (Physician assessment); FA (Parent report of food reaction); AD (Parent reported or DD); sIgE to CM; Total IgE |
| Sears, 2002 [[132](#_ENREF_132)]; Mandhane 2007 [[133](#_ENREF_133)] | PC | 1,037 | New Zealand | **Dunedin Multidisciplinary Health and Development Research Study**. Population based cohort of infants born between 1972-1973 | TBF, EBF, R/I | 9, 26 | SPT aero; wheeze (Current wheeze plus BHR (methacholine PC_20_) |
| Shaheen, 1996 [[134](#_ENREF_134)] | PC | 395/44 | Guinea-Bissau | Young adults part of a cohort recruited for a survey of aged 0-6 year children in 1978-80 and living in a semi-rural district of Bissau | TBF, I | 21 | SPT aero |
| Shohet, 1985 [[135](#_ENREF_135)] | PC | 368 | Israel | Cohort born in 1980 | EBF, I | 0.5 | AD (Hanifin and Lobitz criteria) |
| Schoetzau, 2002 [[136](#_ENREF_136)] | PC | 829/92 | Germany | **GINI study**. Term newborn infants born 1995-8 from 2 regions of Germany who participated in an intervention program according to risk of allergy | EBF, D | 1 | sIgE CM |
| Schonberger, 2005 [[137](#_ENREF_137)] | PC | 443 | Netherlands | **PREVASC study**. Cohort analysis of participants in an intervention trial, born in 1997-2000 with a family history of asthma | TBF, D/Q | 2 | Wheeze (ISAAC) |
| Sicherer, 2010 [[138](#_ENREF_138)] | PC | 503/140 | USA | **The Consortium of Food Allergy Research.** Atopic children recruited from clinical services | TBF, SFI, I | 1 | sIgE to PN |
| Siltanen, 2003 [[139](#_ENREF_139)] | PC | 285/53 | Finland | Infants recruited from maternal hospital born in 1994-1995 | EBF, Q | 4 | RC (Physician assessment); sIgE any; SPT any, Total IgE |
| Silva, 2005 [[140](#_ENREF_140)] | PC | 73 | Brazil | Recruited from health services in urban area, born in 1998 (high risk of disease) | EBF, Q | 4 | Wheeze (Physician assessment of ≥3 episodes of wheeze) |
| Silvers, 2009; Silvers, 2011 [[141](#_ENREF_141),[142](#_ENREF_142)] | PC | 889/249 | New Zealand | **New Zealand Asthma and Allergy Cohort Study**. Population based birth cohort of infants born 1997-2001 | EBF, TBF, Q | 1, 5 | Wheeze (DD plus current wheeze; Parent reported wheeze); FA (DD); AD (parent reported); SPT any |
| Simon, 2008 [[143](#_ENREF_143)] ; Wang, 2007[[144](#_ENREF_144)]; Wegienka, 2006 [[145](#_ENREF_145)]; Salam, 2003 [[146](#_ENREF_146)] | PC | 372 | USA | **CAS study**. Middle class mother-infant pairs enrolled in a health maintenance organisation in 1987-89 | EBF, TBF, R/I | 0.5, 6, 7 | Wheeze (current DD; Transient wheezing: wheezing in the last 12 months at ages of 1,2 and/or 4 years but not at age of 6 years); SPT aero |
| Snijders, 2007; Snijders, 2008 [[147](#_ENREF_147),[148](#_ENREF_148)] | PC | 2,505 | Netherlands | **KOALA study**. Population based birth cohort of infants born between 2000-2002 (consisting of cohorts with conventional and alternative lifestyle) | TBF, SFI, Q | 2 | Wheeze (≥4 episodes); sIgE-to any, aero, CM, egg, PN; Total IgE; AD (parent reported) |
| Soto-Ramirez, 2013 [[149](#_ENREF_149)] | PC | 2,833 | USA | Population-based birth cohort selected from nationally distributed consumer opinion panel of 500,000 households (2005-2007) | TBF, Q | 1 | Wheeze (Parent reported current wheeze) |
| Strachan, 1997 [[150](#_ENREF_150)]; Strachan, 1996 [[151](#_ENREF_151)]; Lewis, 1995; Lewis, 1996 [[152](#_ENREF_152),[153](#_ENREF_153)]; Butland, 1997 [[154](#_ENREF_154)] | PC | 12,835 | UK | **British Cohort Studies**. Infants born in England, Wales, and Scotland in 1958 and 1970 | EBF, TBF, I | 5, 16, 35 | Wheeze (Parent reported); SPT aero; RC (DD); current AR (parent reported) |
| Strassburger, 2010 [[155](#_ENREF_155)] | PC | 325/94 | Brazil | Recruited from hospital, born 2001-2002 | EBF, I | 3.5 | SPT aero |
| Sunyer, 2006 [[156](#_ENREF_156)] | PC | 462 | Spain | Population representative sample born in 1997-1998 (normal risk of disease) | TBF, Q | 6.5 | Wheeze (DD) |
| Sunyer, 2001 [[157](#_ENREF_157)] | PC | 596 | Tanzania | Cohort born in 1995-1996 in urban area (normal risk of disease) | TBF, Q | 4 | Wheeze (ISAAC) |
| Taylor, 1983 [[158](#_ENREF_158)] ; Taylor, 1984 [[159](#_ENREF_159)] | PC | 12,608 | UK | **CHES study**. Population based cohort of children born in England, Scotland, and Wales in 1970 | TBF, I | 5, 6, 7 | Wheeze (Parent reported DD); AD and RC (Parent reported) |
| Tennant, 2008 [[160](#_ENREF_160)]  Tennant, 2010 [[161](#_ENREF_161)] | PC | 392 | UK | **Newcastle Thousand Families Study**. Population based sample of subject born in 1947 who were either traced through the National Health Service Central Register or contacted the study team in response to media publicity in the mid 1990 | TBF, I/Q | 14, 50, 51 | Wheeze (Spirometry) |
| Tian, 2009 [[162](#_ENREF_162)] | PC | 472 | China | Infants from urban areas born in 2004-2006 (normal risk of disease) | TBF, I | 2 | Wheeze (Physician assessment) |
| Van Asperen, 1983 [[163](#_ENREF_163)] | PC | 79/44 | Australia | Cohort recruited from medical service, born in 1980-1981 with family history of atopy | EBF, SFI, I | 1, 1.3 | RC (Physician assessment); SPT food |
| Van Beijstervelft, 2008 [[164](#_ENREF_164)] | PC | 24,018 | Netherlands | Netherlands Twin Register: born in 1987-2000 (normal risk of disease) | TBF, Q | 5 | Wheeze (DD) |
| van der Voort, 2012 [[165](#_ENREF_165)] | PC | 5,368 | Netherlands | **Generation R study**. Population-based multicultural birth cohort of infants born between 2002 and 2009 | EBF, TBF, Q | 1, 4 | Wheeze (ISAAC) |
| Vandenplas, 1988 [[166](#_ENREF_166)] | PC | 75/9 | Belgium | Infants with family history of atopy | EBF, Unclear | 0.33 | sIgE CM; SPT CM; Total IgE |
| van Merode, 2007 [[167](#_ENREF_167)] | PC | 222 | Netherlands | **PREVASC study**: cohort born in 2005 with family history of asthma (high risk of disease) | EBF, Q | 1 | Wheeze (ISAAC) |
| Venter, 2009 [[168](#_ENREF_168)] | PC | 891/58 | UK | Population based cohort recruited from antenatal clinics and born in 2001-2002 | EBF, TBF, SFI, Q | 1, 3 | FA (OFC); SPT to food |
| Watson,2013 [[169](#_ENREF_169)] | PC | 369 | New Zealand | Recruited from Polynesian women, non-random sample (high risk of disease) | EBF, Q/I | 1.5 | Wheeze (ISAAC) |
| Wetzig, 2000 [[170](#_ENREF_170)] | PC | 475 | Germany | **LARS study**. High allergy risk or low birth weight children born within one year in the City and District of Leipzig | TBF, Q | 1 | AD (Physician assessment) |
| Wilson, 1998 [[171](#_ENREF_171)] | PC | 545 | UK | **Dundee infant feeding study.** Population based cohort of infants born between 1983-1986 | EBF, TBF, Q | 7 | Wheeze (Self-reported wheeze; DD) |
| Wright, 2002 [[172](#_ENREF_172)] | PC | 499 | USA | Part of a metropolitan Boston prospective birth cohort study of infants born between 1994-1996 with family history of asthma or recruited from a Boston hospital | TBF, Q/I | 0-1 | Wheeze (Parent reported) |
| Wright, 1989; Wright, 1995 [[173](#_ENREF_173),[174](#_ENREF_174)]; Wright, 1999 [[175](#_ENREF_175)]; Wright, 1994 [[176](#_ENREF_176)] | PC | 988 | USA | **Tucson Children's Respiratory Study**. Healthy newborn infants recruited from local health maintenance organisation born in 1980-1984 (normal risk of disease) | TBF, SFI, Q/I | 1, 6 | Wheeze (DD; ≥4 episodes of wheeze in past year); Total IgE; RC (Parent reported) |
| Yamamoto, 2011 [[177](#_ENREF_177)] | PC | 1344 | Japan | **Tokyo Children's Health Illness and Development study (T-CHILD)** | TBF, Q | 3 | Wheeze (ISAAC) |
| Zutavern, 2006; Zutavern, 2008 [[178](#_ENREF_178),[179](#_ENREF_179)] | PC | 606 | UK | Cohort recruited from general practices and born in 1993-1995 (normal risk of disease) | EBF, TBF, SFI, Q/I | 2, 5.5, 6 | Wheeze (Parent reported current wheeze); AD (DD); SPT aero; sIgE any, food, aero, CM, egg, PN |
| Friday, 2000 [[180](#_ENREF_180)] | RC | 94 | Unclear | Unclear | TBF, Unclear | 10? | Wheeze (Physician assessed asthma); sIgE CM |
| McConnochie, 1986 [[181](#_ENREF_181)] | RC | 223 | USA | Historical cohort with subjects were drawn from the patient population of a five-paediatrician group practice in a suburb of Rochester, New York | TBF, R/I | 8 | Wheeze (ATS guideline: wheezing with and without colds or most days or nights; DD) |
| Monego, 1989 [[182](#_ENREF_182)] | RC | 144 | Italy | 144 Italian infants retrospectively followed to examine association between breastfeeding and allergic diseases | TBF, D | 4 | AD (DD) |
| Rona, 2005 [[183](#_ENREF_183)] | RC | 1,213 | Chile | Infants born in a hospital in 1974-1978 | TBF, R | 27 | Wheeze (ECRHS questionnaire, BHR (methacholine PC_20_); SPT aero |
| Mai, 2007 [[184](#_ENREF_184)] | NCC | 723 | Canada | Cases and controls selected from records of the Manitoba Health Services Insurance  Plan and all were born 1995 | EBF, Q | 10 | Wheeze (Canadian Asthma Consensus Guidelines: symptoms plus variable airway obstruction) |
| Martel, 2008 [[185](#_ENREF_185)] | NCC | 1,578 | Canada | Data originating from 3 interlinked administrative health databases on children health in the first 10 years of life | TBF, Q | <10 | Wheeze (DD plus asthma medication) |
| Maskell, 2010 [[186](#_ENREF_186)]; Oliver, 2010 [[187](#_ENREF_187)]; Munro, 2011 [[188](#_ENREF_188)] | NCC | 117/31 | UK | **EuroPrevall UK.** Cases and age-matched controls selected from a population based birth cohort of infants born in 2008 | EBF, SFI, D/R | 1 | Wheeze (Parent reported); FA (DBPCFC) |
| Ronmark, 1999 [[189](#_ENREF_189)] | NCC | 258 | Sweden | **Obstructive Lung Disease in Northern Sweden Study**. 7-8 years old children enrolled in school in 1996 in northern Sweden (born 1988-1989) | TBF, Q | 8 | Wheeze (DD plus ISAAC; Physician assessment (+/-sensitisation)) |
| Camara, 2003 [[190](#_ENREF_190)] | CC | 91 | Brazil | Cases and control were children who sought ED care | TBF, Q | 12 | Wheeze (wheezing that required therapy with inhaled β2-agonists as judged by the attending physician) |
| DesRoches, 2010 [[191](#_ENREF_191)] | CC | 403/202 | Canada | Hospital based cases and controls recruited 1998-2004 | TBF, SFI, Q | <1.5 | FA (Physician assessment: history +/- investigations) |
| Djenouhat, 2011 [[192](#_ENREF_192)] | CC | 450/150 | Algeria | Hospital based cases and family controls, born 1999-2005 | TBF, Unclear | 0.3 | FA (Physician assessment: history +/- investigations) |
| Fox, 2009 [[193](#_ENREF_193)] | CC | 283/133 | UK | Cases and controls were children referred for suspected FA to a large paediatric service | TBF, Q | <4 | FA (Physician assessment: history +/- investigations) |
| Ghaderi, 2014 [[194](#_ENREF_194)] | CC | 200 | Iran | Unclear source of population. Sex and age matched controls | TBF, I | 5 | AD (DD) |
| Haileamlak, 2005 [[195](#_ENREF_195)] | CC | 732 | Ethiopia | Children age 1- 5 years. Cases were defined according to the ISAAC criteria for AD and confirmed by clinical examination | TBF, SFI, I | 5 | AD (ISAAC) |
| Infante-Rivard, 1993 [[196](#_ENREF_196)] | CC | 914 | Canada | Cases were 3- and 4-year-old children with a first-time diagnosis of asthma made by a paediatrician 1988-90. Age and area matched controls chosen from computerized family allowance files for the target region | TBF, I | 3.5 | Wheeze (DD) |
| Juca, 2012 [[197](#_ENREF_197)] | CC | 590 | Brazil | Adolescents 13 to 14 years of age from Cuiabá, Mato Grosso State taking part in ISAAC | EBF, TBF, Q | 14 | Wheeze (ISAAC) |
| Karunasekera, 2001 [[198](#_ENREF_198)] | CC | 582 | Sri Lanka | Hospital-based cases aged 1-10 years old with age matched controls from inpatient clinics | TBF, SFI, Q | 10 | Wheeze (Physician assessment) |
| Kramer, 1981 [[199](#_ENREF_199)] | CC | 470 | Canada | Cases and controls were1 month to 20 years old children attending dermatology clinics visits | EBF, TBF, I | <20 | AD (Hanifin and Lobitz criteria) |
| Mavale-Manuel, 2003 [[200](#_ENREF_200)] | CC | 199 | Mozambique | Children aged 18 months to 8 years attending paediatrics clinic with history of asthma with age-match controls attending the clinic immediately after selection of the index case | TBF, Q/I | 8 | Wheeze (DD plus asthma medication) |
| Oliveti, 1995 [[201](#_ENREF_201)] | CC | 262 | USA | Cases and age matched controls were identified using rosters of patients followed during the previous year. he majority of children from each group were insured by Medicaid (low income) | TBF, Q | 9 | Wheeze (DD plus asthma medication) |
| Porro, 1993 [[202](#_ENREF_202)] | CC | 465 | Italy | Hospital based study with matched controls (normal risk of disease) | TBF, Q | <1.6 | Wheeze (Parent reported) |
| Ratageri, 2000 [[203](#_ENREF_203)] | CC | 180 | India | Hospital-based study (normal risk of disease) | EBF, Unclear | 9 | Wheeze (DD asthma using International Paediatric Consensus Group Criteria) |
| Rosas Vargas 2002 [[204](#_ENREF_204)] | CC | 148 | Mexico | Hospital-based study, cases born in 2000 (normal risk of disease) | TBF, Q | 3 | Wheeze (DD) |
| Rylander 1993 [[205](#_ENREF_205)] | CC | 550 | Sweden | Cases from a health service and control from population (normal risk of disease) | EBF, I | 4 | Wheeze (Physician assessment) |
| Ventura, 1988 [[206](#_ENREF_206)] | CC | 339/148 | Italy | Italian Working Group on CMPA. Cases selected from 15 paediatric centres and age and area matched controls from well-baby clinics | TBF, I | <1 | FA (Physician assessment: history +/- investigations) |
| Whu, 2007 [[207](#_ENREF_207)] | CC | 261 | USA | Hospital-based study cases born in 2000 | EBF, I | 2, 5.5 | Wheeze (Parent report, current) |
| Wickens, 2001 [[208](#_ENREF_208)] | CC | 474 | New Zealand | Population-based study | TBF, I | 6.5 | Wheeze (ISAAC) |
| Zhu, 2012 [[209](#_ENREF_209)] | CC | 542 | China | Population-based study | TBF, Q | 14 | Wheeze (DD) |
| Alper, 2006 [[210](#_ENREF_210)] | CS | 858 | Turkey | 7 years old children randomly selected from seven primary schools in Bursa in 1999 | TBF, Q | 7 | Wheeze (Parent reported wheeze classified using Martinez criteria) |
| Al-Kubaisy, 2005 [[211](#_ENREF_211)] | CS | 2,262 | Iraq | Primary school urban and rural children | TBF, Q | 12 | Wheeze (DD plus ISAAC) |
| Awasthi, 2004 [[212](#_ENREF_212)] Björkstén, 2011 [[213](#_ENREF_213)] ; Flohr, 2011 [[214](#_ENREF_214)] Nagel, 2009 [[215](#_ENREF_215)]; Kuyucu, 2004  [[216](#_ENREF_216)] | CS | 103,716 | Worldwide | **ISAAC Phase 2**. Schoolchildren aged 8–12 years from 27 centres in 21 affluent and non-affluent countries  **ISAAC Phase 3.** Schoolchildren aged 6-7 years from different countries and geographic regions  **ISAAC Phase 3-India.** Schoolchildren aged 6-7 and 13-14 years old from India | TBF, Q | 7, 12, 14 | Wheeze (Parent reported current wheeze; Parent reported asthma (+/- SPT); Spirometry; BHR hypertonic saline PC15); AD ever; RC (ISAAC) |
| Berjon, 1987 [[217](#_ENREF_217)] | CS | 2,690/148 | Spain | Patients attending a paediatric allergy clinic in Valladolid, Spain between 1970 and 1984, with and without diagnosed food allergy | TBF, Unclear | 14 | FA (DD) |
| Castro-Rodriguez, 2010 [[218](#_ENREF_218)]; Chong Neto, 2007 [[219](#_ENREF_219)] | CS | 3,003 | Brazil, Spain | **EISL study**. One-year old infants from urban and rural primary care health clinics born in 2004-2006 | EBF, Q | 1, 1.4 | Wheeze (ISAAC, parent reported wheeze ever) |
| Civelek, 2001[[220](#_ENREF_220)] | CS | 1,533 | Turkey | Representative sample of schoolchildren in 5 cities | EBF, TBF, Q | 11 | AD (ISAAC) |
| Ehlayel, 2008 [[221](#_ENREF_221)] | CS | 1,278 | Qatar | Children 0-5 years old attending primary healthcare centres for routine immunisation | EBF, Q | 5 | AR ever (ISAAC); FA (DD) |
| Ehrlich, 1996 [[222](#_ENREF_222)] | CS | 620 | South Africa | Second year black elementary school (7- 8 years) children. | TBF, Q/I | 9 | Wheeze (ISAAC) |
| Evenhouse, 2005 [[223](#_ENREF_223)] | CS | 16,903 | USA | **National Longitudinal Study of Adolescent Health (Add Health).** Nationally representative samples of adolescents from 80 school districts, 1994 | TBF, Q | 12-18 | Wheeze (Unclear) |
| Girolomoni, 2003 [[224](#_ENREF_224)] | CS | 1369 | Italy | Representative sample of children attending the fourth grade at elementary school | TBF, Q | 9 | AD (UK Working Party Criteria) |
| Han, 2009 [[225](#_ENREF_225)] | CS | 21,371 | Taiwan | Elementary and middle school children aged 6-15 years old in 2004 | TBF, Q | 15 | Wheeze (ISAAC) |
| Karino, 2008 [[226](#_ENREF_226)] | CS | 9615 | Japan | University students aged 18–19 years enrolled from 2003 through 2005. | TBF, Q | 18 | Wheeze (Self-reported asthma); AD (Self-reported); RC (DD) |
| Kucukosmanoglu, 2008 [[227](#_ENREF_227)] | CS | 1015/20 | Turkey | Hospital based selection of 8-18 months old Infants born in 2001-2002 | TBF, SFI, Q/I | 1 | SPT to egg |
| Kuehr, 1992 [[228](#_ENREF_228)] | CS | 1470/201 | Germany | Schoolchildren between 6 and 8 years of age | TBF, Q | 8 | SPT aero |
| Kurt, 2008 [[229](#_ENREF_229)]  Kurt, 2007 [[230](#_ENREF_230)] | CS | 25,843 | Turkey | **PARFAIT**. Prevalence and Risk Factors of Allergies in Turkey. Population representative sample of children aged 9-15 years old | TBF, Q | 15 | Wheeze; AD (Current; Parent reported); current RC (parent reported) |
| Liu 2012 [[231](#_ENREF_231)] | CS | 8733/397 | China | Sample of children from kindergarten and elementary schools in Shenyang | EBF, Q | 8 | RC (ATS questionnaire) |
| Miyake, 2003 [[232](#_ENREF_232)] | CS | 6845 | Japan | 12-15 years old children from all public junior high schools in Suita, Japan. | (EBF, TBF) Q | 15 | Wheeze; current RC (ISAAC) |
| Nakamura, 1999 [[233](#_ENREF_233)] | CS | 3850 | Japan | All 3 year old children participating in a health check-up in 1997 | TBF, Q | 3 | AD (DD) |
| Paton, 2012 [[234](#_ENREF_234)] | CS | 15,142/592 | Australia | Representative primary school entrants 2006-2009 | EBF, TBF, Q | 7 | FA (Parent-reported food reaction to any nut) |
| Prietsch, 2006 [[235](#_ENREF_235)] | CS | 685 | Brazil | Population representative sample of infants aged 12-15 months | EBF, Q | 13 | Wheeze (unclear) |
| Rusconi, 1999; Rusconi, 2005  [[236](#_ENREF_236),[237](#_ENREF_237)] | CS | 16933 | Italy | **SIDRIA** survey of a representative sample of children aged 6-7 years old | TBF, Q | 7 | Wheeze (Parent reported persistent wheezing≥1 in first 2 years, and in past 12 months); Transient early wheeze (wheeze in first 2 years but not past 12 months; ISAAC) |
| Rust, 2001 [[238](#_ENREF_238)] | CS | 6783 | USA | **NHANES III survey**. Children ages 2 months to 5 years from non-institutionalized U.S. population | TBF, I | <6 | Wheeze (DD; DD asthma ever) |
| Salem, 2002 [[239](#_ENREF_239)] | CS | 424 | Iraq | Population representative sample of children aged 0.16-2 years old | EBF, Q | 5 | Wheeze (Wheeze ever) |
| Selcuk, 1997 [[240](#_ENREF_240)] | CS | 5412 | Turkey | Children 7-12 years of 18 primary schools | TBF, Q | 12 | Wheeze (Parent reported wheeze ever; Parent reported current asthma); AD (parent reported current); r RC (Parent reported ever) |
| Suwanpromma, 2012 [[241](#_ENREF_241)] | CS | 215 | Thailand | Schoolchildren aged 6-18 years | TBF, Q | 18 | Wheeze (Spirometry; BHR hypertonic saline PC_15_) |
| Takemura, 2002 [[242](#_ENREF_242)] | CS | 23828 | Japan | **The Tokorozawa Childhood Asthma and Pollinosis Study.** Representative sample of children in public elementary schools (normal risk of disease) | EBF, TBF, Q | 15 | Wheeze (ATS questionnaire: DD asthma plus ≥2 episodes of wheeze) |
| Tanaka, 2009 [[243](#_ENREF_243)] | CS | 1957 | Japan | **Fukuoka Child Health Study.** All 3-year old children who had the examination at public health centres in Fukuoka city | EBF, TBF, Q | 3 | Wheeze (ISAAC); AD (ISAAC; current dermatitis) |
| Visser, 2010 [[244](#_ENREF_244)] | CS | 1115 | Netherlands | **EISL study**. One-year old infants from urban and rural primary care health clinics born in 2004-2006 | TBF, Q | 1 | Wheeze (Parent reported wheeze ever; ≥3 episodes of wheeze) |
| Wang, 2006 [[245](#_ENREF_245)] | CS | 8733 | China | Population representative sample of children in elementary schools and nurseries (normal risk of disease) | EBF, TBF, Q | 10 | Wheeze (ISAAC) |

Any: a panel of aero and food allergens; AD: atopic dermatitis; ATS: American Thoracic Society; BHR: bronchial hyper-responsiveness; CM: cow’s milk; CC: Case Control; CS: Cross-sectional; D: diary; DBPCFC: double blind placebo controlled food challenge; DD: doctor diagnosed; EBF: exclusive breastfeeding; ECRHS: European Community Respiratory Health Survey; FA: food allergy; I: interview; ISAAC: International Study of Asthma and Allergies in Children; OFC: open food challenge; PC: prospective cohort; PC_15_: provocative concentration to reduce by 15% a measure of lung function (usually forced expiratory volume in 1 second [FEV_1_]); PN: peanut; aero: aeroallergen; Q: questionnaire; R: medical records, Re-C: retrospective cohort; RC: rhino-conjunctivitis; SFI: solid food introduction; SPT: allergen skin prick test; sIgE: allergen-specific IgE; TBF: total breastfeeding.

# Table S3 Characteristics of included observational studies of breastfeeding or solid food introduction, and risk of autoimmune diseases

| **Study** | **Design** | **N/n cases** | **Country** | | **Population** | **Exposures and method of assessment** | **Age at outcome (years)** | **Outcomes reported**  **(method of assessment)** |
| --- | --- | --- | --- | --- | --- | --- | --- | --- |
| Couper, 1999 [[246](#_ENREF_246)]; Couper, 2009 [[247](#_ENREF_247)] | PC | 548/~30 | Australia | | **BABYDIAB study.** First degree relatives of diabetic children | TBF, EBF, D/Q/I | 2 | TIDM (serology) |
| Frederikson, 2012 (abstract) [[248](#_ENREF_248)]; Fronczak, 2003 [[249](#_ENREF_249)]; Lamb, 2013 [[250](#_ENREF_250)]; Norris, 2003 [[251](#_ENREF_251)]; Lamb 2008 [[252](#_ENREF_252)] | PC, NCC | 1,698 | USA | | **DAISY study**. High risk children via HLA screening, or Colorado register | TBF, EBF, SFI, Q/I | 4, <7, 9, 13 | TIDM (serology; DD) |
| Holmberg, 2007 [[253](#_ENREF_253)]; Karlen, 2012 [[254](#_ENREF_254)]; Wahlberg, 2006 [[255](#_ENREF_255)] | PC | 3,788/~51 | Sweden | | **ABIS study.** General population in Southeast Sweden | TBF, EBF, Q | 1, 2, 6 | TIDM (serology) |
| Ludvigsson, 2003 [[256](#_ENREF_256)] | PC | 205 | Sweden | | Relatives of diabetics | TBF, Q | 2 | TIDM (serology) |
| Viner, 2008 [[257](#_ENREF_257)] | PC | 11,211/61 | UK | | Unclear | TBF, Q | >10 | TIDM (DD) |
| Virtanen, 1992 [[258](#_ENREF_258)]; Virtanen, 1998 [[259](#_ENREF_259)]; Hypponen, 1999 [[260](#_ENREF_260)]; Virtanen, 2000 [[261](#_ENREF_261)] | PC, NCC | 697/43 | Finland | | **Childhood Diabetes in Finland Study**. Siblings of diabetic children | TBF, Q | 7, <25 | TIDM (serology; DD) |
| Virtanen, 2011 [[262](#_ENREF_262)] | PC | ~4,000/ ~160 | Finland | | Odu and Tampere University Hospitals | TBF, EBF, Q | 5 | TIDM (serology; DD) |
| Jones, 1998 [[263](#_ENREF_263)] | NCC | 518/60 | UK | | Hospital admission | TBF, R | 5.0-9 | TIDM (DD) |
| Kimpimaki, 2001 [[264](#_ENREF_264)] | NCC | 455/65 | Finland | | Turku, Oulu and Tampere Hospital births | TBF, EBF, Q | <4 | TIDM (DD) |
| Kyvik, 1992 [[265](#_ENREF_265)] | NCC | 228/76 | Denmark | | National Service Conscript records | TBF, R | <20 | TIDM (DD) |
| Norris, 1996 [[266](#_ENREF_266)]; | NCC | 171/18 | USA | | Siblings or offspring of Barbara Davies Centre Diabetics | TBF, Q/R | <7 | TIDM (DD) |
| Robertson, 2010 [[267](#_ENREF_267)] | NCC | 1444/361 | UK | | AMND and SSG register, hospital births | TBF, R | <15 | TIDM (DD) |
| Savilahti, 2009 [[268](#_ENREF_268)] | NCC | 6209/45 | Finland | | NHI database | TBF, SFI, R | 12 | TIDM (DD) |
| Ahadi, 2011 [[269](#_ENREF_269)] | CC | 202/101 | Iran | | Hospital admission | TBF, Q/I | 7 | TIDM (DD) |
| Alves, 2012 [[270](#_ENREF_270)] | CC | 246/123 | Brazil | | Siblings | TBF, EBF, SFI, I | 7 | TIDM (DD) |
| Ashraf, 2010 [[271](#_ENREF_271)] | CC | 195/128 | USA | | Children's hospital | TBF, Q | <10 | TIDM (DD) |
| Baruah, 2011 [[272](#_ENREF_272)] | CC | 86/43 | India | | Endocrinology ward | TBF, EBF, I | <18 | TIDM (DD) |
| Bener, 2009 [[273](#_ENREF_273)] | CC | 340 | Qatar | | Endocrinology clinic and community | TBF, I | <16 | TIDM (DD) |
| Blom, 1989 [[274](#_ENREF_274)] | CC | 867/339 | Sweden | | Paediatric referral and population register | TBF, Q | 7 | TIDM (DD) |
| Bodington, 1994 [[275](#_ENREF_275)] | CC | 393/209 | UK | | Independent sources and population register | TBF, Q | <15 | TIDM (DD) |
| Borras, 2011 [[276](#_ENREF_276)] | CC | 1,530/306 | Spain | | Diabetes register and Catalonia birth register | TBF, R | Not reported | TIDM (DD) |
| Dahlquist, 2002 [[277](#_ENREF_277)] | CC | 2,226/610 | Austria, Latvia, Lithuania, Luxembourg and UK | | **EURODIAB**. Diabetes register and population register | TBF, SFI, Q/I | <15 | TIDM (DD) |
| Esfarjani, 2001 [[278](#_ENREF_278)] | CC | 104/52 | Iran | | Endocrine clinic and paediatric OPD attendance | TBF, EBF, SFI, Q | <14 | TIDM (DD) |
| Gimeno, 1997 [[279](#_ENREF_279)] | CC | 626/313 | Brazil | | Juvenile Diabetes Association or hospital records | TBF, EBF, Q | <18 | TIDM (DD) |
| Hathout, 2006 [[280](#_ENREF_280)] | CC | 402/102 | USA | | Diabetes hospital care and Hospital Well Child clinics | TBF, Q/I | 7 | TIDM (DD) |
| Kostraba, 1992 [[281](#_ENREF_281)]; Kostraba, 1993 [[282](#_ENREF_282)] | CC | 264/132-white  108/54-black | USA | | Alleghany Hospital diabetes register Colorado IDDM Registry and motor vehicle driver register | TBF, EBF, Q/I | 10, <18 | TIDM (DD) |
| Liese, 2012 [[283](#_ENREF_283)] | CC | 709/505 | USA | | SEARCH surveillance (Colorado and South Carolina research centres) | EBF, SFI, I | <20 | TIDM (DD) |
| Majeed, 2011 [[284](#_ENREF_284)] | CC | 310/96 | Iraq | | Hospital admission or OPD | TBF, SFI, Q | <17 | TIDM (DD) |
| Malcova, 2006 [[285](#_ENREF_285)] | CC | 2,334/868 | Czech Republic | | Czech Childhood Diabetes Register and diabetes clinic | TBF, Q | <15 | TIDM (DD) |
| Marshall, 2004 [[286](#_ENREF_286)] | CC | 577/196 | UK | | Paediatric clinic and Local Health Authority Register | TBF, I | <16 | TIDM (DD) |
| Mayer, 1988 [[287](#_ENREF_287)] | CC | 747/268 | USA | | Colorado IDDM Registry or Barbara Davies Centre | TBF, Q/I | <18 | TIDM (DD) |
| McKinney, 1999 [[288](#_ENREF_288)] | CC | 521/196 | UK | | Yorkshire Childhood Diabetes Register and Family Health Service Authority Register | TBF, I | <16 | TIDM (DD) |
| Meloni, 1997 [[289](#_ENREF_289)] | CC | 200/100 | Italy | | Paediatric clinic or hospital admission | TBF, SFI, Q | <17 | TIDM (DD) |
| Patterson, 1994 [[290](#_ENREF_290)] | CC | 1,548/258 | UK | | Diabetes register, hospital discharge, Health Service records | TBF, EBF, R | <15 | TIDM (DD) |
| Perez-Bravo, 1996 [[291](#_ENREF_291)] | CC | 165/80 | Chile | | Santiago de Chile registry | TBF, SFI, Q/I | <15 | TIDM (DD) |
| Perez-Bravo, 2003 [[292](#_ENREF_292)] | CC | 250/143 | Chile | | School volunteers | TBF, Q | 8 | TIDM (DD) |
| Rabiei 2011 [[293](#_ENREF_293)] | CC | 300/100 | Iran | | Diabetes register | EBF, SFI, Q | 11 | TIDM (DD) |
| Rami, 1999 [[294](#_ENREF_294)] | CC | 609/114 | Austria | | Austrian diabetes register | TBF, EBF, Q | <15 | TIDM (DD) |
| Rosenbauer, 2008 [[295](#_ENREF_295)] | CC | 2631/760 | Germany | | Hospital based surveillance system ESPD and local registration office records | TBF, SFI, Q/I | <5 | TIDM (DD) |
| Sadauskaite-Kuehne, 2004 [[296](#_ENREF_296)]; Skrodeniene, 2010 [[297](#_ENREF_297)] | CC | 1,944/803 | Sweden/ Lithuania | | Population register and outpatients | TBF, EBF, Q | 7, 9 | TIDM (DD) |
| Samuelsson, 1993 [[298](#_ENREF_298)] | CC | 1,089/297 | Sweden | | Paediatric department and population register | TBF, EBF, Q/R | <15 | TIDM (DD) |
| Siemiatycki, 1989 [[299](#_ENREF_299)] | CC | 482/161 | Canada | | Hospital admission | TBF, I | <17 | TIDM (DD) |
| Sipetic, 2005 [[300](#_ENREF_300)] | CC | 315/105 | Serbia | | Hospital admission | TBF, I | <16 | TIDM (DD) |
| Soltesz, 1994 [[301](#_ENREF_301)] | CC | 305/130 | Hungary | | Incidence register | TBF, EBF, Q | <14 | TIDM (DD) |
| Stene, 2000 [[302](#_ENREF_302)] | CC | 1156/85 | Norway | | National Childhood Diabetes register | EBF, Q | <15 | TIDM (DD) |
| Stene, 2003 [[303](#_ENREF_303)] | CC | 2213/545 | Norway | | Diabetes register | EBF, SFI, Q | 9 | TIDM (DD) |
| Strotmeyer, 2004 [[304](#_ENREF_304)] | CC | 485/247 | China | | Diabetes register and population register | TBF, SFI, Q | 10 | TIDM (DD) |
| Tai, 1998 [[305](#_ENREF_305)] | CC | 310/117 | Taiwan | | Taipei City | TBF, I | 8 | TIDM (DD) |
| Telahun, 1994 [[306](#_ENREF_306)] | CC | 129/55 | Ethiopia | | Ethio-Swedish Children's Hospital Diabetic Clinic | TBF, Q | <15 | TIDM (DD) |
| Tenconi, 2007 [[307](#_ENREF_307)] | CC | 477/159 | Italy | | Diabetes register or paediatric admissions | TBF, R/I/Q | 16 | TIDM (DD) |
| Thorsdottir, 2000 [[308](#_ENREF_308)] | CC | 220/55 | Iceland | | Statistical Bureau of Iceland | TBF, EBF, I | 12 | TIDM (DD) |
| Verge, 1994 [[309](#_ENREF_309)] | CC | 475/217 | Australia | | New South Wales diabetes register and school records | TBF, Q | <15 | TIDM (DD) |
| Virtanen, 1993 [[310](#_ENREF_310)] | CC | 1,380/690 | Finland | | Finnish National Population Registry | TBF, EBF, Q | 14 | TIDM (DD) |
| Visalli, 2003 [[311](#_ENREF_311)] | CC | 900/150 | Italy | | **EURODIAB study** register and school records | TBF, SFI, Q | 6-18 | TIDM (DD) |
| Wadsworth, 1997 [[312](#_ENREF_312)] | CC | 639/276 | UK | | **BPASU** reporting system and District Health Authority Immunisation Register | TBF, Q | <5 | TIDM (DD) |
| Glatthaar, 1988 [[313](#_ENREF_313)] | CS | 946/~200 | Australia | | School register | TBF, Q | <18 | TIDM (DD) |
| Hummel, 2000 [[314](#_ENREF_314)]; Hummel, 2007 [[315](#_ENREF_315)]; Ziegler, 2003 [[316](#_ENREF_316)] | PC | 1460/~68 | Germany | | **German BABYDIAB study**. Offspring of mothers and/or fathers with TIDM born in Germany between 1989 and 2000 | EBF, TBF, SFI, Q | 2, 5, 8 | TIDM (serology), CD (IgA-tTG ) |
| Norris, 2005 [[317](#_ENREF_317)] | PC | 1560 | USA | | **DAISY study**. Children at increased risk for TIDM were enrolled at birth from 1993 to 2006 and/or identified by newborn screening for HLA genotype | TBF, Q/I | <5 | CD (IgA-tTG) |
| Welander, 2010 [[318](#_ENREF_318)] | PC | 9,414/ ~29 | Sweden | | **ABIS study**. Population based study of babies born between Oct 1997 and Oct 1999. | TBF, D/I | 8.4 | CD (IgA-tTG and biopsy) |
| Ascher, 1997 [[319](#_ENREF_319)] | CC | 81/8 | Sweden | | Cases of coeliac disease were compared with siblings at high genetic risk (DQA1*0501-DQB1*02), in whom the diagnosis was excluded | TBF, I | <18 | CD (ESPGHAN criteria) |
| Auricchio, 1983 [[320](#_ENREF_320)] | CC | 437/190 | Italy | | Source of cases unknown, controls unaffected siblings | TBF, R/I | <18 | CD (ESPGHAN criteria) |
| Baron, 2005 [[321](#_ENREF_321)] | CC | 444/222 | France | | Cases from EPIMAD registry (1988-97) with community-based sex, age, region matched controls | TBF, EBF, I | <17 | IBD (DD) |
| Bergstrand, 1983 [[322](#_ENREF_322)] | CC | 616/308 | Sweden | | Cases were residents of Stockholm County diagnosed with Crohn's disease between 1955 and 1974 with sex, age, residence matched controls from population registry in Stockholm County | TBF, Q/I | 20 | IBD (Unclear) |
| Castiglione, 2011 [[323](#_ENREF_323)] | CC | 1,030/ 468 | Italy | | Cases from gastroenterology units; controls comprised from physicians, nurses, and support services professionals from the participating sites | TBF, Q | 16-66 | IBD (ECCO guideline) |
| Corrao, 1997 [[324](#_ENREF_324)] | CC | 1,252/ 626 | Italy | | Cases identified in clinics with controls sex and age matched hospital-based control | TBF, I | 18-65 | IBD (DD including histology) |
| Decker 2010 [[325](#_ENREF_325)] | CC | 866/123 | Germany | | Cases from paediatric gastroenterology clinics; controls from ophthalmology and dental clinics | TBF, Q | <18 | CD (DD); IBD (DD) |
| Ellis, 2012 [[326](#_ENREF_326)] | CC | 655/246 | Australia | | **CLARITY**. Cases from paediatric rheumatology clinic; controls from paediatric surgery unit born in the same area | TBF; SFI, Q/I | 18 | JRA (DD) |
| Falth-Magnusson, 1996 [[327](#_ENREF_327)] | CC | 336/72 | Sweden | | Cases from paediatric department records, born in 1987-1989. Reference children were age matched from same county. | TBF, EBF, R/Q | <2 | CD (ESPGHAN criteria) |
| Fort, 1990 [[328](#_ENREF_328)] | CC | 189/59 | USA | | Cases being followed up in clinics with sibling or other controls | TBF, SFI, I | 15 | Autoimmune thyroid Disease (DD) |
| Gearry, 2010 [[329](#_ENREF_329)] | CC | 1,253/ 653 | | New Zealand | Canterbury Inflammatory Bowel Disease Project. Cases selected from patient advertising, letters to patients from their doctor, patient support groups; Community based (Electoral Roll) controls | TBF, R/Q | >20 | IBD (DD) |
| Gilat, 1987 [[330](#_ENREF_330)] | CC | 1,497/ 499 | 9 countries: USA, Canada, UK, Sweden, Denmark, Holland, France, Italy, Israel | | The International IBD Study Group: Cases and matched controls from several health centres (normal risk of disease) | TBF, Q/I | <25 | IBD (DD) |
| Greco, 1988 [[331](#_ENREF_331)] | CC | 2,150/ 201 | Italy | | Hospital-based cases born in 1976-1983 with age and area matched controls | EBF, R/Q | 2 | CD (ESPGHAN criteria) |
| Gruber, 1996 [[332](#_ENREF_332)] | CC | 144/54 | USA | | Children diagnosed with Crohn's disease with mothers who were volunteers from the Western New York Chapter of the Crohn's and Colitis Foundation of America, Inc. with age matched unrelated controls | EBF, TBF, Q | <22 | IBD (Unclear) |
| Hansen, 2011[[333](#_ENREF_333)] | CC | 534/  267 | Denmark | | All patients diagnosed with IBD in Copenhagen City and County (private and public sector) in 2003-4 with age, sex, ethnicity and area matched control with orthopaedic problems | TBF, Q | 38 | IBD (Copenhagen Diagnostic Criteria) |
| Ivarsson, 2002 [[334](#_ENREF_334)] | CC | 1,272/ 392 | Sweden | | Cases selected from CD Register born in 1992-1996 and sex age and area matched controls from the national population register | TBF, SFI, Q | 2, 15 | CD (ESPGHAN criteria) |
| Koletzko, 1991 [[335](#_ENREF_335)] | CC | 231/93 | Unclear | | Source of cases unclear. Sibling controls | EBF, TBF, SFI, Q | 15 | IBD (DD including histology) |
| Mason, 1995 [[336](#_ENREF_336)] | CC | 133/54 | USA | | Children seen at the outpatient paediatric rheumatology clinics with playmates matched for age and race as controls | TBF, SFI, I | 6 | JRA (DD) |
| Pacilio, 2010 [[337](#_ENREF_337)] | CC | 278/139 | Unclear | | Unclear source of cases and controls. Cases aged 0.5-2 years old with age matched healthy controls | TBF, Unclear | 2 | CD (Unclear) |
| Peters, 2001 [[338](#_ENREF_338)] | CC | 270/133 | Germany | | All newly diagnosed patients aged <10 years old were identified from paediatricians and a biannual meeting of the German Coeliac Disease Society in 1985–1995. Sex and aged matched control selected from population registry | TBF, Q | < 10 | CD (ESPGHAN criteria) |
| Roberts 2009 [[339](#_ENREF_339)] | CC | 248,521/ 90 | UK | | Cases identified from hospital admission codes, controls the rest of the population with linked record data | TBF, R | <24 | CD (ICD codes 269.0 (ICD-8) or 579.0 (ICD-9) or K90.0 (ICD-10)) |
| Rosenberg, 1996 [[340](#_ENREF_340)] | CC | 468/ 137 | Canada | | Cases from a health service and matched control from population | TBF, SFI, Q | <18 | JRA (American College of Rheumatology criteria) |
| Sonntag, 2007 [[341](#_ENREF_341)] | CC | 1,974/ 1,096 | Germany | | Cases identified from different sources and controls from partners (normal risk of disease) | TBF, Q | 40 | IBD (DD including histology) |
| Thompson, 1999 [[342](#_ENREF_342)] | NCC | 243/27 | UK | | Cases and matched for gender and social class controls were selected from the 1946 National Survey of Health & Development (NSHD) and the 1958 National Child Development Study (NCDS), two on-going, longitudinal birth cohort studies in UK. | TBF, R/I | 33-43 | IBD (DD) |
| Wang, 2013 [[343](#_ENREF_343)] | CC | 2,616/ 1,308 | China | | Cases from several health centres and matched controls from friends or neighbours (normal risk of disease) | TBF, I | <70 | IBD (Chinese diagnostic guideline including histology) |

CC: case control; CD: coeliac disease; CS: Cross-sectional; DD: doctor diagnosis; EBF: exclusive breastfeeding; IA: Islet autoantibodies; IBD: Inflammatory bowel disease; I: interview ; NCC: nested case control; PC: prospective cohort; Q: questionnaire; R: medical records; SFI: solid food introduction; TBF: total breastfeeding

# Table S4 Characteristics of included intervention trials of other maternal or infant dietary exposures and allergic outcomes

| Study | Design | N Intervention/ Control | Country | Intervention | Study Details | Disease risk | Age at outcome (years) | Outcomes reported  (method of assessment) |
| --- | --- | --- | --- | --- | --- | --- | --- | --- |
| Falth-Magnusson, [1987](#_bookmark21) [[344](#_ENREF_344)]  Falth-Magnusson[, 1992](#_bookmark22) [[345](#_ENREF_345)]  Ludvigsson, 2003 [[346](#_ENREF_346)] | RCT | 108/104 | Sweden | **Allergenic food avoidance** (maternal) | Pregnant women. Cow's milk and egg exclusion from 28 weeks gestation to delivery. | High | 1.5, 5 | AR  (physician assessment); Asthma  (≥3 episodes of  wheeze); AD (Hanifin and Lobitz); AS (SPT); FA (history); Total IgE; TIDM (serology) |
| Jirapinyo, 2013 [[347](#_ENREF_347)] | RCT | 30/32 | Thailand | **Allergenic food avoidance** (maternal) | Lactating women.  Cow's milk exclusion during lactation up to 4  months postpartum | High | <0.5 | AD (unclear) |
| Lilja, 1989 [[348](#_ENREF_348)] | RCT | 84/87 | Sweden | **Allergenic food avoidance** (maternal) | Pregnant women.  Milk and egg  exclusion during third trimester, versus 1 litre milk and 1 egg per day | High | 1.5 | Asthma (physician  assessment); AS (SPT); Allergic rhinitis (physician assessment); AD (physician assessment); FA (history) |
| Hattevig, 1990 [[349](#_ENREF_349)]; Paronen, 2000 [[350](#_ENREF_350)]; Hattevig, 1999 [[351](#_ENREF_351)]; Hattevig, 1989 [[352](#_ENREF_352)]; Sigurs, 1992 [[353](#_ENREF_353)] | CCT | 54/67 | Sweden | **Allergenic food avoidance** (maternal) | Lactating women. Cow's milk, egg and fish exclusion during first 3 months post- partum | High | 0.25,  1.5, 4,  10 | AS (SPT);  TIDM (serology), AD, Asthma, Wheeze; AR; FA (history), Total IgE |
| Herrmann, 1996 [[354](#_ENREF_354)] | CCT | 50/50 | Germany | **Allergenic food avoidance** (maternal) | Pregnant/lactating women. Cow's milk and egg exclusion from 28 weeks gestation through lactation, versus 1 litre cow's milk and 1 egg/day | High | 1 | AD (DD); AS (sIgE) |
| Kilburn, 1998 [[355](#_ENREF_355)] | CCT | 15/96 | UK | **Allergenic food avoidance** (maternal) | Lactating women.  Milk, egg, fish and nuts exclusion throughout lactation | High | 1.5 | AD (Hanifin and Rajka), AS (SPT) |
| Metcalfe, 2016 [[356](#_ENREF_356)] | RCT | 40, 44, 36 | Australia | **Allergenic food avoidance** (maternal) | First 6 weeks of lactation. Randomised to high-egg diet (>4 eggs per week), low-egg diet (1-3 eggs per week) and egg-free diet | High | 0.33 | AS (sIgE egg) |
| Becker, 2004 [[357](#_ENREF_357)]  Chan-  Yeung, 2000 [[358](#_ENREF_358)] & 2005 [[359](#_ENREF_359)];Wong, 2013 [[360](#_ENREF_360)] Protudjer, 2011 [[361](#_ENREF_361)]; Carlsten, 2013 [[362](#_ENREF_362)] | RCT | 281/268 | Canada | **Multifaceted including maternal allergenic food avoidance** | **CAPPS Study.** Pregnant/lactating women. BF encouraged for 4 months, allergenic food exclusion during pregnancy/lactation, delayed solid (6 months) and allergenic (12 months) food, whey pHF if necessary, environmental control | High | 1, 7, 15 | Asthma; Recurrent wheeze (ISAAC and modified ECRHS); AS (SPT); AR (DD); AD (DD); BHR (PC_20_); Lung function (FEV_1_) |
| Hide, 1994 [[363](#_ENREF_363)]  Hide, 1996,[[364](#_ENREF_364)]  Arshad, 1992 [[365](#_ENREF_365)]  Arshad, 2003 [[366](#_ENREF_366)]  Arshad, 2007 [[367](#_ENREF_367)]  Scott, 2012 [[368](#_ENREF_368)] | RCT | 71/68 | UK | **Multifaceted including maternal allergenic food avoidance** | **Isle of Wight Study.** Lactating women. Allergenic food exclusion during lactation, delayed allergenic (9-11 months) food, soya hydrolysate if necessary, environmental control. | High | 1, 2, 4, 8, 18 | Recurrent wheeze (≥3 episodes); BHR (PC_20_); AD (Physician assessment); FA (OFC; Physician assessment); AS (SPT, total IgE); AR (physician assessment); Total IgE |
| Lovegrove, 1994 [[369](#_ENREF_369)] | RCT | 12/14 | UK | **Multifaceted including maternal allergenic food avoidance** | CM exclusion during pregnancy and lactation with hydrolysed milk if necessary. BF encouraged for 6 months and EHF if needed | High | 1 | AD (DD) |
| Shao, 2006 [[370](#_ENREF_370)] | RCT | 23/23 | China | **Multifaceted including maternal allergenic food avoidance** | Lactating women. EBF encouraged for 4 months, allergenic food exclusion during lactation, delayed solid (4 months) and allergenic (6-12 months) food, pHF if necessary | High | 1.5 | AD (Wolkerstorfer score); AS (SPT) |
| Zeiger, 1992 [[371](#_ENREF_371)]  Zeiger, 1989, [[372](#_ENREF_372)] Zeiger 1994 [[373](#_ENREF_373)] | RCT | 103/185 [seen at 4 months] | USA | **Multifaceted including maternal allergenic food avoidance** | Pregnant/lactating women. Allergenic food exclusion during pregnancy/lactation, delayed allergenic (1-3 years) food, casein eHF if necessary | High | 1, 4, 7 | AD (Hanifin and Rajka Criteria); AR (DD); FA - Any (DD), Wheeze ( ≥2 physician diagnosed episodes); AS (SPT) |
| Halmerbauer, 2002, 2003 [[374](#_ENREF_374),[375](#_ENREF_375)] | RCT | 349/347 | UK, Germany, Austria | **Multifaceted** | **SPACE Study.** EBF encouraged beyond 3 months, delayed solid (6 months) and allergenic (1-3 years) food, environmental control | High | 1 | AD (DD); AS (SPT, sIgE); Wheeze (Parent reported wheeze ever), Asthma (≥3 episodes of wheeze); FA (DD) |
| Matthew, 1977 [[376](#_ENREF_376)] | RCT | 27/35 | UK | **Multifaceted** | BF encouraged for 6 months, delayed solid food (3 months) and allergenic food (6 months), soy milk if necessary, environmental control | High | 1 | AD (Physician assessment); AS (Total IgE) |
| Poysa, 1991 [[377](#_ENREF_377)]  Poysa 1989 [[378](#_ENREF_378)]  Kuikka 1985 [[379](#_ENREF_379)] | RCT | Unclear - outcome reported in 35/33 | Finland | **Multifaceted** | EBF encouraged for 3 months, solid food and cow's milk formula after 3 months plus environmental control | High | 5, 10 | Asthma (≥3 episodes of wheeze); AR (Physician assessment); AS (SPT, total IgE); FA (oral food challenge); AD (Hanifin and Rajka) |
| Schonberger,  2005 [[137](#_ENREF_137)] | RCT | 222/221 | The Netherlands | **Multifaceted** | **PREVASC study.** BF encouraged to ≥6 months, delayed solid and allergenic (6 months) food, eHF if formula introduced, environmental control | High | 2 | AD (ICHPPC); Wheeze (Dutch Guideline ‘‘Asthma in Children’’ and ISAAC); AS (sIgE) |
| Boyle 2015 [[380](#_ENREF_380)] [[381](#_ENREF_381)]  Boyle 2016 [[382](#_ENREF_382)] | RCT | 432/431 | Australia, Singapore, England and Ireland | **Prebiotic**  Immunofortis  GOS/FOS + AOS | **PATCH Study**. Term infants with  ≥one parent with allergic disease, and formula introduction <18 weeks. Intervention until 6 months. Co- intervention with partially hydrolysed formula | High | 1 | AD (Hanifin and Rajka)  AS (total IgE, sIgE milk, sIgE egg) |
| Gruber 2010 [[383](#_ENREF_383)]  Gruber 2015 [[384](#_ENREF_384)] | RCT | 414/ 416 | Netherlands,  Austria, Switzerland, Italy, Germany | **Prebiotic** Immunofortis GOS/FOS + AOS | **MIPS-1 Study.** Full formula feeds  <8 weeks | Low | 1, 5 | AS (sIgE to CM or egg; total IgE); AD (UKWPC), AD (DD); Wheeze (DD); AR (DD); FA (DD) |
| Ivakhnenko 2013 [[385](#_ENREF_385)] | RCT | 80/ 80 | Ukraine | **Prebiotic** GOS/FOS | Fully formula fed – no breastfeeding at all. Intervention until 2 months. | Normal | 1.5 | AD (Harrigan and Rabinowitz); FA (unclear) |
| Moro 2006 [[386](#_ENREF_386)], van Hoffen 2009 [[387](#_ENREF_387)], Arslanoglu 2008 [[388](#_ENREF_388)] Arslanoglu 2012 [[389](#_ENREF_389)] | RCT | 129/ 130 | Italy | **Prebiotic** Immunofortis | Formula commenced <2 weeks, and full formula feeds  <6 weeks | High | 0.5, 2, 5 | Allergic sensitisation (sIgE to CM; total IgE), AD (Harrigan and Rabinowitz), Wheeze (≥3 episodes), Allergic rhinoconjunctivitis (physician assessment) |
| Sierra, 2015 [[390](#_ENREF_390)] | RCT | 188/ 177 | Spain | **Prebiotic** Nutradefense. GOS | Infants aged <2 months, exclusively formula fed for ≥15 days. Intervention until 1 year. | Normal | 1 | AD (physician assessment); Wheeze (physician assessment); AS (SPT); FA (unclear) |
| Ziegler 2007[[391](#_ENREF_391)] | RCT | 150/ 76 | USA | **Prebiotic** GOS/Polydextrose +/- lactulose | Full formula feeds  <2 weeks | Normal | 0.3 | AD (unclear) |
| Chien, 2016 [[392](#_ENREF_392)] | RCT | Unclear – outcome reported in 45 (synbiotic), 39 (prebiotic), 45 (control) | Singapore | **Prebiotic & Synbiotic**  B. breve  scGOS/lcGOS | Mixed fed infants born by elective Caesarean. Infant formula supplemented with scGOS/lcFOS (0.8g/100ml) and B. breve M-16V (7.5x108CFU/100ml), or formula with scGOS/lcFOS (0.8g/100ml), or control formula from birth until 4 months. | Unclear | 0.4 | AD (unclear) |
| Kukkonen 2007 [[393](#_ENREF_393)] Kuitunen 2009 [[394](#_ENREF_394)] Kukkonen 2011[[395](#_ENREF_395)] | RCT | 610/ 613 | Finland | **Synbiotic**  L. rhamnosus, B. breve, P. freudenreichii; GOS | Pregnant women (2-4 weeks before delivery) and infants up to 6 months. Representative population | High | 2, 5 | AD (UK Working party criteria), Wheeze (≥2 episodes + interval symptoms), ARC (symptoms + sensitisation), Allergic sensitisation (Total IgE and SPT/sIgE to common allergens) |
| Roze 2012 [[396](#_ENREF_396)] | RCT | 48/ 49 | France | **Synbiotic**  L. rhamnosus; GOS/FOS | Infants (first 6 months). Full formula feeds until randomised (up to day 3) | Normal | 0.5 | AD (UK Working party criteria) |
| Van der Aa 2010 [[397](#_ENREF_397)] | RCT | 46/ 44 | Netherlands | **Synbiotic**  B. breve; GOS/FOS | Whey-based formula combined with Synbiotic, for 3 months. Infants who already had AD, with SCORAD >15 | High | 1 | Wheeze (≥3 episodes),  Allergic sensitisation (Total IgE) |
| Abrahamsson 2007 [[398](#_ENREF_398)]  Abrahamsson 2013 [[399](#_ENREF_399)] | RCT | 117/ 115 | Sweden | **Probiotic**  L.reuteri | Pregnant women (week 36) and infants (for 12 months) | High | 2, 7 | AD (Seymour), Wheeze (single, or ≥2 episodes), ARC (watery discharge ≥2 times with same allergen), Allergic sensitisation (SPT common allergens) |
| Allen 2012 [[400](#_ENREF_400)]  Allen 2014 [[401](#_ENREF_401)] | RCT | 220/ 234 | UK | **Probiotic**  L. salivarius, L. paracasei, B. animalis, B. bifidum | Pregnant women (week 36) and infants (for 6 months) | High | 2 | AD (DD), Allergic sensitisation (SPT common allergens, CM, Egg), Wheeze (unclear), ARC (unclear), Food Allergy (parent report) |
| Boyle 2011 [[402](#_ENREF_402)] | RCT | 125/ 125 | Australia | **Probiotic**  L. rhamnosus | Pregnant women (week 36) | High | 1 | AD (UK Working party criteria), Wheeze (wheeze + loose API), Allergic sensitisation (SPT common allergens) |
| Cabana, 2015 [[403](#_ENREF_403)] | RCT | 93/ 92 | USA | **Probiotic**  Lactobacillus GG | Birth to 6 months to infants daily | High | 2 | Wheeze (unclear); AD (unclear) |
| De Leon 2007 [[404](#_ENREF_404)] Simon 2007 [[405](#_ENREF_405)] | RCT | Total = 33 | Philippines | **Probiotic** Lactobacillus Bifidobacterium | Birth to 4 months to infants, or to their breastfeeding mothers | High | 0.5 | AD (unclear)  Allergic sensitisation (Total IgE) |
| Dotterud 2010 [[406](#_ENREF_406)]  Simpson, 2015 [[407](#_ENREF_407)] | RCT | 211/ 204 | Norway | **Probiotic**  L. rhamnosus, B. animalis, L. acidophilus | Mothers only (36 weeks gestation to 3 months after birth) | Normal | 2, 6 | AD (UKWPC), Wheeze (≥3 episodes + ICS, parent report), ARC (DD, parent report), Allergic sensitisation (SPT common allergens, sIgE) |
| Enomoto 2014 [[408](#_ENREF_408)] | CCT | 130/36 | Japan | **Probiotic**  B. longum, B. breve | Pregnant women (week 36) and infants (for 6 months) | Normal | 1.5 | AD (Hanifin and Rajka), Wheeze (physician assessment), ARC (physician assessment) |
| Huurre 2008 [[409](#_ENREF_409)] | RCT | 72/ 68 | Finland | **Probiotic**  L. rhamnosus, B. animalis | Infants (for 6 months) | High | 1 | AD (Hanifin and Rajka),  Allergic sensitisation (SPT common allergens) |
| Kalliomaki 2001 [[410](#_ENREF_410)] Kalliomaki 2003 [[411](#_ENREF_411)] Kalliomaki 2007 [[412](#_ENREF_412)]  Rautava 2002 [[413](#_ENREF_413)] | RCT | 77/ 82 | Finland | **Probiotic**  L. rhamnosus | Mothers and infants; from 2–4 weeks before expected delivery and 6 months of age | High | 2 | AD (relapsing itchy lesions with typical location), Wheeze (symptoms + ICS), ARC (symptoms with allergen exposure), Food Allergy (CMA by DBPCFC), Allergic sensitisation (Total IgE and SPT/sIgE to common allergens) |
| Kim 2010 [[414](#_ENREF_414)] | RCT | 57/ 55 | Korea | **Probiotic**  B. bifidum, B. animalis, L. acidophilus | Pregnant women and infants from 4 to 6 months | High | 1 | AD (Hanifin and Rajka),  Allergic sensitisation (Total IgE and sIgE to common allergens) |
| Kopp 2008 [[415](#_ENREF_415)] | RCT | 54/ 51 | Germany | **Probiotic**  L. rhamnosus | Mothers (2-4 weeks before birth until 3 months post birth) and infants (months 4-6) | High | 2 | AD (UK Working party criteria), Wheeze (≥5 episodes),  Allergic sensitisation (Total IgE and sIgE to common allergens) |
| Lau 2012 [[416](#_ENREF_416)] | RCT | 303/ 303 | Germany | **Probiotic**  E. coli, E. faecalis | Infant from 5 weeks up to 7 months age | High | 2 | AD (Hanifin and Rajka), Allergic sensitisation (Total IgE and sIgE) |
| Lodinová-Žádníková 2010 [[417](#_ENREF_417)] | RCT | 56/ 57 | Czech Republic | **Probiotic**  E. coli | Infants - birth to age 4 weeks | High | 1 | AD (unclear), Food allergy (unclear), Wheeze (unclear)  Allergic sensitisation (Total IgE and sIgE to common allergens) |
| Lundelin, 2016 [[418](#_ENREF_418)]  Luoto, 2014 [[419](#_ENREF_419)] | RCT | 31, 31, 32 | Finland | **Probiotic**  L. rhamnosus GG, B. lactis, L. paracasei ST11, B.longum BL999 | Perinatal administration of probiotic to preterm infants (32-36 weeks gestation) | Normal | 1 | Wheeze (ISAAC); ARC ISAAC); AD (ISAAC); FA (unclear) |
| Morisset 2008 [[420](#_ENREF_420)] | RCT | 59/ 56 | France | **Probiotic**  Fermented formula without live bacteria | Infant – from birth or weaning, to age 1 year | High | 1 | Food allergy (CMA by physician assessment)  Allergic sensitisation (sIgE to cow’s milk) |
| Niers 2009 [[421](#_ENREF_421)]  Gorissen, 2014 [[422](#_ENREF_422)] | RCT | 78/ 78 | Netherlands | **Probiotic**  B. bifidum, B. animalis, Lc. lactis | **PANDA study.** Pregnant women during the last 6 weeks of pregnancy and their infants until age 1 year | High | 2, 6 | AD (modified ECRHS, UKWPC), Wheeze (physician assessment),  AS (Total IgE and SPT/sIgE to common allergens), Food allergy (physician assessment); ARC (ISAAC); Lung function (FEV1) |
| Ou 2012 [[423](#_ENREF_423)] | RCT | 95/ 96 | Taiwan | **Probiotic**  L. rhamnosus | Pregnant women (third trimester) and then breastfeeding mothers, or directly to infants, until age 6 months | High | 3 | AD (ISAAC), Wheeze (ISAAC), ARC (ISAAC), Allergic sensitisation (sIgE to common allergens) |
| Taylor 2007 [[424](#_ENREF_424)], Prescott 2008 [[425](#_ENREF_425)] Jensen 2012 [[426](#_ENREF_426)] | RCT | 115/ 111 | Australia | **Probiotic**  L. acidophilus | Infants until 6 months | High | 1, 2.5, 5 | AD (DD), Food allergy (any food - physician assessment), Wheeze (DD), ARC (symptoms + sensitisation), Allergic sensitisation (SPT common allergens) |
| Rautava 2006 [[427](#_ENREF_427)] | RCT | 38/ 43 | Finland | **Probiotic**  B. animalis, L. rhamnosus | Infant formula; infants until age 1 year | Normal | 1 | AD (Hanifin and Rajka), Food allergy (CMA by DBPCFC)  Allergic sensitisation (SPT food allergens) |
| Rautava 2012 [[428](#_ENREF_428)] | RCT | 82/ 78 | Finland | **Probiotic**  B. longum, L. rhamnosus | Infants fed daily until age 1 year | High | 2 | AD (Hanifin and Rajka),  Allergic sensitisation (SPT common allergens) |
| Scalabrin 2009 [[429](#_ENREF_429)]  Scalabrin 2014 [[430](#_ENREF_430)]  Scalabrin 2017 [[431](#_ENREF_431)] | RCT | 95/ 95 | USA | **Probiotic**  L. rhamnosus | Infants (first 12 months) fed extensively hydrolysed formula from 14 days | Normal | 0.4, 3, 5 | Allergic sensitisation (sIgE to common allergens), AD (unclear), Wheeze (unclear), ARC (unclear), Food Allergy (unclear) |
| Soh 2009 [[432](#_ENREF_432)]  Loo 2014 [[433](#_ENREF_433)] | RCT | 127/ 126 | Singapore | **Probiotic**  L. rhamnosus, B. longum | Cow’s milk-based infant formula supplemented with Probiotic; infants up to 6 months of age | High | 1 | AD (Seymour),  Allergic sensitisation (Total IgE and SPT/sIgE to common allergens) Food allergy (physician assessment), ARC (physician assessment) |
| West 2009 [[434](#_ENREF_434)]  West 2013 [[435](#_ENREF_435)] | RCT | 89/ 90 | Sweden | **Probiotic**  L paracasei | To infant from 4 until 13 months | Normal | 1, 8-9 | AD (itchy rash with typical distribution, or DD), Wheeze (DD), ARC (DD), Allergic sensitisation (Total IgE and sIgE to common allergens) Food allergy (physician assessment) |
| Wickens 2008 [[436](#_ENREF_436)]  Wickens 2012 [[437](#_ENREF_437)]  Wickens 2013 [[438](#_ENREF_438)] | RCT | 341/ 171 | New Zealand | **Probiotic**  L. rhamnosus, B. animalis | To pregnant women (35 weeks gestation to end up of BF or 6 months post-partum) and infants from 2-16 days of birth to 2 years | High | 2, 4, 6 | AD (UKWPC); Wheeze (ISAAC); ARC (ISAAC); Allergic sensitisation (SPT common allergens) |
| Berman, 2015 [[439](#_ENREF_439)] | RCT | Unclear – total 114 | USA | **Fatty acids**  Ω-3 | DHA (Ω -3) or EPA (Ω -3) rich fish oil prenatal supplementation, or soy oil | Unclear | 3 | Wheeze (parent report); AD (parent report) |
| Birch, 2010 [[440](#_ENREF_440)]  Foiles, 2015 [[441](#_ENREF_441)] | RCT | 88/ 90 | USA | **Fatty acids**  Ω-3  and Ω-6 | **DIAMOND study.** DHA (Ω-3) and arachidonic acid Ω -6) supplemented formula for first year | Normal | 3, 4 | Wheeze (DD); AD (DD); ARC (DD); FA (DD) |
| Bisgaard, 2016 [[442](#_ENREF_442)] | RCT | 365/371 | Denmark | **Fatty acids**  Ω-3 | **COPSAC trial.** Fish oil (2.4g/d Ω -3 LCPUFA), or olive oil to pregnant women 24 weeks gestation until 1 week post-partum | Normal | 5 | Wheeze (physician assessment); AD (Hanifin and Rajka); AS (SPT, sIgE); ARC (DD);  Lung function (FEV1) |
| Harslof, 2014 [[443](#_ENREF_443)] | RCT | 61/ 60 | The  Netherlands | **Fatty acids**  Ω-6 | **EFATOP trial.** Borage oil (100 mg GLA) daily from 1-2 weeks to 6 months age, or sunflower oil | High | 1 | AD (UKWPC); AS (sIgE); Total IgE |
| Lucas, 1999 [[444](#_ENREF_444)] | RCT | 75/ 79 | Denmark | **Fatty acids**  Ω-3 | Fish oil (1.2g/d Ω-3 PUFA), or sunflower oil to infants from 9 to 18 months | Normal | 1.5 | AS (total IgE) |
| van Gool, 2003 [[445](#_ENREF_445)] | RCT | 55/ 76 | Germany | **Fatty acids**  Ω-6 | 100mg/day GLA supplementation of mothers during lactation, or infant formula (160mg/day) in first 5 months | High | 1 | AD (Hanifin); Total IgE |
| Kitz, 2006 [[83](#_ENREF_83)] | RCT | 154/155 | UK | **Fatty acids**  Ω-3  and Ω-6 | 0.3% arachidonic acid (Ω -6), 0.32% DHA (Ω -3) supplemented formula from birth to 6 months | Normal | 0.75 | AD (parent report); Wheeze/Asthma (parent report, DD) |
| Linnamaa, 2010 [[446](#_ENREF_446)] | RCT | 151/162 | Finland | **Fatty acids**  Ω-6 | 3g/day blackcurrant seed oil (essential fatty acid) during pregnancy and exclusive breastfeeding, 1ml/day to infant to age 2, or olive oil | Normal | 1 | AD (H&R); AS (SPT); Total IgE |
| Mihrshahi, 2003 [[447](#_ENREF_447)]  Peat, 2004 [[448](#_ENREF_448)]  Marks, 2006 [[449](#_ENREF_449)] | RCT | 312/304 | Australia | **Fatty acids**  Ω-3 | **CAPS trial.** 500mg fish oil (=184mg n-3 PUFA) daily from 6 months, or in formula if introduced before then, plus canola oil (high in n-3 PUFA) for family, plus environmental control, until 5 y age | High | 1.5,  3, 5 | Asthma (ISAAC/ parent report), FEV_1_; AD (ISAAC, UKWPC);  AR (ISAAC); AS (SPT); Total IgE |
| Damsgaard, 2007 [[450](#_ENREF_450)] | RCT | 45/ 49 | Denmark | **Fatty acids**  Ω-3 | Fish oil ~3.4mls/day (571mg  EPA, 381mg DHA) from 9 to 12 months | Normal | 1 | Total IgE |
| Palmer, 2012  & 2013 [[451](#_ENREF_451),[452](#_ENREF_452)]  Best, 2015 & 2016 [[453](#_ENREF_453),[454](#_ENREF_454)] | RCT | 368/338 | Australia | **Fatty acids**  Ω-3 | **DOMINO trial.** Fish oil capsules with 800mg DHA, 100mg EPA daily from 21 weeks gestation to delivery, or vegetable oil | High | 1, 3, 6 | AD (physician assessment, ISAAC); Wheeze (physician assessment, ISAAC), FA (physician assessment); AR (physician assessment, ISAAC); AS (SPT) |
| Dunstan, 2003 [[455](#_ENREF_455)] | RCT | 52/ 46 | Australia | **Fatty acids**  Ω-3 | Fish oil 4g daily  from 20 weeks gestation to delivery, containing 2.07g DHA, 1.02g EPA, from 20 week of gestation until delivery or olive oil | High | 1 | FA (history), Asthma (≥2 episodes of wheeze), AD (DD); AS (SPT) |
| D'Vaz, 2012 [[456](#_ENREF_456)] | RCT | 218/202 | Australia | **Fatty acids**  Ω-3 | Daily fish oil with 280mg DHA and 110mg EPA (changed to 250/60 part way through trial) from birth to 6 months, or 650mg olive oil | High | 1 | AD (´typical skin lesions´); AS (SPT); FA (parent report + SPT); Wheeze (unclear) |
| Furuhjelm, 2009 [[457](#_ENREF_457)]  Furuhjelm, 2011 [[458](#_ENREF_458)] | RCT | 70/ 75 | Sweden | **Fatty acids**  Ω-3 | Daily fish oil (1.6 g EPA, 1.1 g DHA) from 25 weeks gestation to end of lactation (or through 3.5 months of breastfeeding), or soy oil | High | 1, 2 | AS (SPT and sIgE);  FA (parent report + SPT/sIgE); AR (seasonal symptoms); Wheeze (DD, $\geq$ 3 episodes of wheezing), AD (parent report, Seymour criteria) |
| Lauritzen, 2005 [[459](#_ENREF_459)] | RCT | 62/ 60 | Denmark | **Fatty acids**  Ω-3 | **Lactation**. 4.5 g fish oil daily (=~1.5 g/d of n-3 LCPUFA) from 1-2 week to 4 months, or olive oil | Normal | 2.5 | Wheeze (parent report), FA  (parent report); AD (DD); Total IgE |
| Olsen, 2008 [[460](#_ENREF_460)]  Hansen, 2017 [[461](#_ENREF_461)] | RCT | 266/267 | Denmark | **Fatty acids**  Ω-3 | **Pregnant women**. Fish oil 4g/day (1.28g EPA, 920mg DHA) from 30 weeks of gestation to delivery | Normal | 16, 24 | Asthma (DD, ISAAC, physician assessment); AS (sIgE); AR (DD, ISAAC); Lung function (FEV1); |
| Dotterud, 2013 [[462](#_ENREF_462)] | CCT | 2,860/5,743 | Norway | **Fatty acids**  Ω-3 | **PACT trial.**  Oily fish twice/week and 5ml cod liver oil (1.2 g n-3 PUFA) daily through pregnancy and to infant from 4–6 weeks with oily fish twice/ week from 6 months | Normal | 2 | Wheeze/Asthma (parent report; DD); AD (Parent report) |
| Imhoff- Kunsch, 2011 [[463](#_ENREF_463)] | RCT | 547/547 | Mexico | **Fatty acids**  Ω-3 | 400 mg DHA daily from ~20 weeks gestation to delivery, or corn/soy oil | Normal | 0.5 | Wheeze  (parent report) |
| Noakes, 2012 [[464](#_ENREF_464)] | RCT | 62/ 61 | USA | **Fatty acids**  Ω-3 | **SiPS trial**. 2 x 150g portions/week of farmed salmon (~163mg EPA, 331mg DHA per day) from 20 weeks gestation to delivery | High | 0.5 | Wheeze (study assessment); AD (Physician assessment); AS (SPT) |
| Aage, 2015 [[465](#_ENREF_465)] | RCT | 2145/ 2200 | Guinea-Bissau | **Vitamins** | Neonates supplemented with Vitamin A 50,000 IU plus 10IU vitamin E, or 10IU vitamin E alone, at time of BCG vaccination. Population at high risk of vitamin A deficiency. | Normal | 10 | AS (SPT); Wheeze (ISAAC); AD (ISAAC) |
| Chawes, 2016 [[442](#_ENREF_442)] | RCT | 315/308 | Denmark | **Vitamins** | **COPSAC** Vitamin D3 (2400 IU/d) to pregnant women 24 weeks gestation to 1 week post-partum, or placebo. All women also took 400 IU/day of vitamin D3. | Normal | 3 | Wheeze (physician assessment); AD (Hanifin and Rajka); AS (SPT, sIgE) |
| Checkley, 2010 [[466](#_ENREF_466)]  Checkley, 2011 [[467](#_ENREF_467)] | cluster RCT | 803, 885, 771 | Nepal | **Vitamins** | **NIPPS 2 study.** Vitamin A (23,300 IU) or Beta Carotene (42mg) versus peanut oil placebo weekly from preconception through lactation, in married women at high risk of vitamin A deficiency | Normal | 11 | Wheeze (ISAAC, DD); LF (FEV_1_; FVC; PEF) |
| Czeizel, 1994 [[468](#_ENREF_468)]; Dobo, 1998 [[469](#_ENREF_469)] | RCT | 2090/2032 | Hungary | **Vitamins** | **Hungarian Optimal Family Planning Programme**. Multivitamins from preconception to second missed period, in women who wanted to become pregnant | Normal | 1, 6 | Wheeze (physician assessment); Recurrent wheeze (physician assessment); AD (physician assessment); FA (unclear) |
| Devakumar, 2015 [[470](#_ENREF_470)] | RCT | 600/600 | Nepal | **Vitamins** | Micronutrient daily supplement (800$\mu$g Vit A, 10mg Vit E, 5$\mu$g Vit D, 1.4mg Vit B1, 1.4mg Vit B2, 18mg Niacin, 1.9mg Vit B6, 2.6$\mu$g Vit B12, 400$\mu$g Folic acid, 70mg Vit C, 30mg Iron, 15mg Zinc, 2mg Copper, Selenium 65$\mu$g, Iodine 150$\mu$g) versus iron 60mg and folic acid 400$\mu$g, to pregnant women in the second and third trimester. Population at risk of micronutrient deficiencies. | Normal | 8 | Lung function (FEV1, FVC); Wheeze (ISAAC); AR (ISAAC) |
| Grant, 2016 [[471](#_ENREF_471)] | RCT | 87, 86, 87 | New Zealand | **Vitamins** | Vitamin D daily from 27 weeks gestation to birth (pregnant women) and birth to 6 months (infants) at 1000 IU/ 400 IU, or 2000 IU/ 800 IU, versus placebo | Normal | 1.5 | Wheeze (DD); AS (SPT, sIgE) |
| Greenough, 2010 [[472](#_ENREF_472)] | RCT | 1199/1205 | UK | **Vitamins** | **Vitamins in Pre-eclampsia trial.** Vitamin C (100mg) and Vitamin E (400IU) from 14-21 weeks to delivery, in women with clinical risk factors for pre-eclampsia. | Normal | 1 | Wheeze (parent-reported); Recurrent wheeze (parent-reported); AD (parent-reported) |
| Goldring, 2013 [[473](#_ENREF_473)] | RCT | 120/60 | UK | **Vitamins** | Vitamin D 200,000 IU bolus at 27 weeks or 800IU daily from 27 weeks to delivery, versus no treatment Ethnically stratified as Asian, Middle Eastern, Black, White. | Normal | 3 | Wheeze (ISAAC); Recurrent wheeze (≥episodes); AR (ISAAC); AS (SPT); AD (ISAAC); FA (physician assessment); total IgE |
| Kiraly, 2013 [[474](#_ENREF_474)] | RCT | 227/235 | Guinea-Bissau | **Vitamins** | Vitamin A 100,000-200,000 IU at 6-9 months age, versus no supplement, in infants with no history of measles or vitamin A supplementation. Population at risk of vitamin A deficiency. | Normal | 7 | AS (SPT) |
| Litonjua, 2016 [[475](#_ENREF_475)] | RCT | Unclear – 881 total | USA | **Vitamins** | Vitamin D (4000 IU/d) versus placebo from 10-18 weeks gestation to delivery. All women also received 400 IU/d vitamin D as part of a multivitamin supplement. | High | 3 | Wheeze (physician assessment); AD (DD); AS (total IgE, sIgE) |
| McEvoy, 2014[[476](#_ENREF_476)] | RCT | 89/90 | USA | **Vitamins** | **Vitamin C** 500mg daily from ≤22 weeks gestation to delivery versus corn starch, in women aged ≥15 years old who reported being current smokers (≥1 cigarette per day) randomised at 22 weeks of gestation | Normal | 1 | Wheeze (unclear); LF (spirometry) |

AD: atopic dermatitis; AOS acidic oligosaccharides; API Asthma Predictive Index; AR: Allergic Rhinitis; ARC: Allergic Rhinoconjunctivis; AS: allergic sensitisation; B. Bifidobacterium; BF: breastfeeding; BHR: bronchial hyper-responsiveness; CM: cow’s milk; CCT controlled clinical trial; DBPCFC: double blind placebo-controlled food challenge; DD doctor’s diagnosis in the community (Physician assessment refers to assessment by a study physician); DHA: docosahexanoic acid; E. Escherichia; ECRHS European Community Respiratory Health Survey; eHF extensively hydrolysed formula; EPA: eicosapentaenoic acid; FA: food allergy; FEV_1_ forced expiratory volume in one second; FOS long-chain fructo-oligosaccharides; FVC; forced vital capacity; GLA gamma linolenic acid; GOS galacto-oligosaccharides; ICHPPC: International Classification of Health Problems in Primary Care; ICS inhaled corticosteroids; ISAAC International Study of Asthma and Allergies in Childhood; IU International Units; L. Lactobacillus; Lc. Lactococcus; LCPUFA: long chain polyunsaturated fatty acid; LF: lung function; OFCs oral food challenges; PEF: peak expiratory flow; pHF: partially hydrolysed formula; RCT randomised controlled trial; SPT skin prick test; sIgE allergen-specific Immunoglobulin E sensitisation; TIDM: type 1 diabetes mellitus UKWPC: the UK Working Party Criteria (to define AD; Seymour criteria and UKWPC are both modifications of the Hanifin and Rajka criteria).

# Table S5 Characteristics of included observational studies of other maternal or infant dietary exposures and risk of allergic outcomes

| **Study** | **Design** | **N/n cases** | **Country** | **Population** | **Exposures and method of assessment** | **Age at outcome (years)** | **Outcomes reported**  **(method of assessment)** |
| --- | --- | --- | --- | --- | --- | --- | --- |
| Alm, 2009 [[6](#_ENREF_6)]; Goksor, 2011; [[477](#_ENREF_477)] Alm, 2012;[[478](#_ENREF_478)] | PC | 4,941 | Sweden | **Infants of Western Sweden**: Population based birth cohort of infants born in the region in 2003 | Infant vitamin intake [any]; infant fish, fat intake; Dietary Pattern; vegetarianism in the family, gluten, type of fat on bread, lactic acid (yoghurt, fermented vegetables); vegetables; alcohol, Q | 1 | AD (parent reported); FA (DD) |
| Andreasyan, 2007; [[479](#_ENREF_479)] | PC | 498 | Australia | **CARHS.** Some of the participants at high risk for sudden infant death syndrome born in 1988-1989 were identified in the northern region of Tasmania through school records | Fruit syrup; fruit juice (orange); honey, Q | 9 | AD, wheeze and RC (ISAAC Q); SPT any |
| Back, 2009 [[480](#_ENREF_480)] | PC | 123 | Sweden | The first 206 babies born in 1998 recruited at University Hospital of Umea. All children were prescribed vitamin A&D supplements from 6 weeks to 24 months of age | Infant vitamin  intake [any], Q | 6 | AD, wheeze and rec wheeze (ISAAC Q) |
| Baiz, 2013[[481](#_ENREF_481)] | PC | 239 | France | **EDEN.** Population based birth cohort with pregnant women recruited from prenatal clinics in Nancy and Poitiers,  < 24th weeks of gestation in 2003 | Vitamin D cord blood, S | 1, 2, 3, 5 | AD (DD); RC (ISAAC Q); Rec wheeze (DD asthma plus medication and/or current symptoms) |
| Bekkers, 2012 [[482](#_ENREF_482)]; Willers, 2008 [[483](#_ENREF_483)] | PC | 3,786 | The Netherlands | **PIAMA.** Population based birth cohort of children born in 1996/97 recruited prenatally, Netherlands. The children were allocated to an intervention study or natural history study depending on their family risk for allergy | Maternal vitamin supplement [any] and Vitamin D cord blood, Q | 2, 8 | AD (DD); LF (BHR, spirometry); sIgE any; Wheeze (ISAAC); Rec wheeze (DD asthma plus ≥4 episodes of wheeze); Total IgE; sIgE-aero; sIgE to food |
| ;Bertelsen, 2013 [[484](#_ENREF_484)] | PC | 54,740 | Norway | **MoBa study.** Population birth cohort with pregnant women recruited at ~17 weeks of pregnancy with children born between 2000 and 2005 in Denmark | Probiotics (milk based), Q | 0.5, 3 | AD (parent reported); Rec wheeze (parent reported asthma) |
| Bisgaard, 2009 [[17](#_ENREF_17)] | PC | 354 | Denmark | **COPSAC study.** Healthy newborns of mothers with a history of doctor-diagnosed asthma recruited in Copenhagen, Denmark between 1998 and 2001 | Alcohol, Q | 3 | Rec wheeze (physician assessment) |
| Carmargo, 2010 [[485](#_ENREF_485)] | PC | 823 | New Zealand | **The New Zealand Asthma and Allergy Cohort study.** Population based birth cohort with pregnant women recruited at maternity care centres in Wellington and Christchurch 1997-2001 | Vitamin D cord blood, S | 1.25, 3, 5 | Wheeze (parent reported wheeze); Rec wheeze (DD asthma plus medication and/or current symptoms) |
| Romieu, 2007; [[486](#_ENREF_486)]  Chatzi, 2008 [[487](#_ENREF_487)] | PC | 468 | Spain | **Menorca birth cohort.** Population based birth cohort with women recruited from antenatal care at all general practices in Menorca between 1997-1998 | Fruits, legumes, nuts, vegetables; Dietary Pattern-adherence to Mediterranean diet; cereal; dairy; meat; white meat; maternal fish intake, Q | 4.6; 6.5 | SPT-aero; Atopic wheeze (parent reported wheeze plus positive SPT); Rec wheeze (parent reported ≥1 episodes of wheeze) AD (DD, parent reported); AS (any, food, aeroallergen by sIgE) |
| de Jong, 2012 [[488](#_ENREF_488)]; De Jong, 2012 [[489](#_ENREF_489)]; Leermakers, 2013; [[490](#_ENREF_490)] | PC | 8742 | The Netherlands | **GENERATION R.** Population based birth cohort , with pregnant women recruited < 25 weeks gestation in Rotterdam | Maternal vitamin supplement [any]; maternal fish intake, Q | 4 | AD and wheeze (ISAAC Q) |
| Dubakiene, 2012 [[491](#_ENREF_491)]; Butiene, 2011[[492](#_ENREF_492)];  Oliver, 2010; [[187](#_ENREF_187)] Grimshaw, 2012 [[493](#_ENREF_493)] | PC  NCC | 1158  123/41 | Lithuania;  UK | **EuroPrevall birth cohort.** Over 12,000 newborns in 9 European countries 2005–2009 using a standardised approach across 9 European countries.  Lithuanian birth cohort compared cases with age-matched controls; UK birth cohort compared each case with two controls | Maternal allergenic food avoidance to milk or egg;  Infant mineral intake; infant fat intake; maternal fish oil supplement; Dietary carbohydrates; Wheat; dietary energy; dietary protein, Q | 1, 2 | AD (parent reported)  SPT to food;  FA any (DD with DBPCFC) |
| Dunlop, 2006 [[494](#_ENREF_494)] | PC | 1326 | Slovakia | **Slovak birth cohort.** The 1st 250 pregnant women delivering at maternity hospitals in the selected study sites were recruited between 1997 and 1999 | Citrus fruits, Q | 1 | AD (physician assessment) |
| Fergusson, 1990 [[495](#_ENREF_495)] | PC | 1,067 | New Zealand | **Christchurch child development study.** A cohort of children born in the Christchurch urban region New Zealand during mid1977 | Fruits, vegetables, cereal, meat, R/D | 10 | AD (Physician assessment; DD , duration of > 3years and use of regular medication) |
| Fitzsimon, 2007 [[496](#_ENREF_496)] | PC | 631 | Ireland | **LIFE-WAYS.** cohort of children born in 2002 whose mother had completed FFQ during pregnancy were followed up from birth through general practice records | Maternal fat, fruit and vegetable intakes, Q | 3 | Rec wheeze (physician assessment of asthma) |
| Gale, 2008 [[497](#_ENREF_497)] | PC | 440 | UK | Population based birth cohort with pregnant women recruited on their first visit to midwives' antenatal booking clinic in 1991 | Maternal plasma Vitamin D, S | 0.75, 9 | AD (UKWPC, visible AD on examination, parent reported); Rec wheeze (Parent reported asthma) |
| Harris, 2001; [[56](#_ENREF_56)] Zutavern, 2004 [[57](#_ENREF_57)] | PC | 604/ 622 | UK | Population based birth cohort of newly pregnant women who presented at one of three general practices in Ashford, Kent UK between 1993 and 1995 | Rice; cereal; meat | 2, 5.5 | AD ( DD; visible AD on examination); SPT aero; Rec wheeze (Parent reported) |
| Hesselmar, 2010 [[61](#_ENREF_61)] | PC | 184 | Sweden | **ALLERGYFLORA.** Birth cohort in Sweden enriched with children with family history of allergies | Fruits, I/Q | 0.5/1.5 | AD (physician assessment, and full-filling Williams’ criteria); FA CM (physician assessment plus OFC); sIgE any |
| Hypponen, 2004; [[498](#_ENREF_498)]  Hypponen, 2001 [[499](#_ENREF_499)] | PC | 10,366/81 | Finland | **Northern Finland Birth Cohort.**  Population based birth cohort with women recruited between the 24th and 28th week of gestation in Oulu and Lapland in 1995-1966 | Infant vitamin intake [any], Q | 31, 2 | SPT aero; rec wheeze (self-reported asthma plus medication and/or symptoms) |
| Hoppu, 2000 [[67](#_ENREF_67)] | PC | 115 | Finland | Birth cohort of infants of breastfeeding mothers (for at least 3 months) with a positive family history of atopic disease | Maternal intake of vitamins, fats, R | 1 | AS (SPT to any) |
| Jedrychowski, 2008 [[500](#_ENREF_500)]; Jedrychowski 2011 [[501](#_ENREF_501)] | PC | 469 | USA and Poland | Prospective birth cohort of infants from non-smoking healthy mothers who gave birth at 29 - 43 weeks of gestation between 2001 and 2004 | Maternal fish intake, Q | 1, 2 | AD (physician assessment); Wheeze (parent reported) |
| Kemp, 2011 [[502](#_ENREF_502)] | PC | 310 | Tasmania | **THIS.** Cohort of infants at high risk for sudden infant death syndrome born in 1988-1989 were identified in the northern region of Tasmania through school records | Maternal allergenic food avoidance (peanut), Q | 3.5 | sIgE to peanut |
| Kull, 2006; [[503](#_ENREF_503)] Magnusson, 2013 [[504](#_ENREF_504)]; | PC | 3230 | Sweden | **BAMSE.**  Prospective birth cohort of newborns in a predefined area of Stockholm, between 1994 and 1997 | Infant fish intake, infant vitamin intake, Q | 4, 12 | Wheeze (Self-reported wheeze; ≥3 episodes of wheeze OR inhaled corticosteroids); FA (DD); Parent reported OR DD AD; RC (Parent reported symptoms); AS (sIgE) |
| Martindale, 2005; [[505](#_ENREF_505)] Devereux, 2006; [[37](#_ENREF_37)] Devereux, 2007 [[506](#_ENREF_506)]; Willers, 2007[[507](#_ENREF_507)] | PC | 3230 | Scotland | **Aberdeen birth cohort**. Population based birth cohort with pregnant women recruited 1997-99 while attending a hospital antenatal clinic at ~12weeks gestation | Infant fish intake; maternal vitamin intake and mineral intake; Fruits; citrus fruits; fruit juice; vegetables; green leafy vegetables; apples; Maternal fat intake from different sources; whole grain products, Q | 2, 4, 5, 12 | AR (DD); AS-any (sIgE); Rec wheeze (≥ 3 episodes in past year); AD (ISAAC, UKWPC); LF (Spirometry); RC and wheeze (ISAAC Q) |
| Lack, 2003 [[43](#_ENREF_43)];  Shaheen, 2009 [[508](#_ENREF_508)]; Wills, 2013[[509](#_ENREF_509)]; Granell, 2008 [[510](#_ENREF_510)] | PC; NCC | 11,352 | UK | **ALSPAC.** The study enrolled pregnant women living in the Avon Health Authority are, UK, expected to delivery between 1991 and 1992. Cases and controls, who did and did not develop food allergy respectively, were not matched | Allergenic food avoidance (peanuts/nuts); allergenic food avoidance (soybean meat); peanut; Dietary Patterns: 'Traditional'; 'Health Conscious'; 'Processed'; 'Confectionery'; 'Vegetarian'; 'Processed'; 'Vegetarian'; maternal plasma vitamin and vitamin supplement [any], Q | 0.5, 2.5, 3.5, 7, 7.5, 8, 8.7 | FA peanut (DD); Rec wheeze (parent reported persistent wheeze, DD asthma); AD (parent reported); LF (BHR slope, FEV_1_); RC (parent reported); SPT aero; Total IgE |
| Laitinen, 2005 [[511](#_ENREF_511)] | PC | 95 | Finland | Children with a family history of AD (mother, father and/or older sibling with AD, AR or asthma), who participated in a prospective allergy prevention study (probiotic intervention trial) | Infant fat, mineral, and vitamin intake [any]; Dietary carbohydrates; dietary energy; dietary protein, D | 0.5, 1 | AD (physician assessment); FA cow’s milk (physician assessment) |
| Lange, 2010 [[512](#_ENREF_512)];  Litonjua, 2006; [[513](#_ENREF_513)] Camargo, 2007 [[514](#_ENREF_514)] | PC | 1376 | USA | **Project Viva study**. Population based birth cohort with pregnant women at <22 weeks of gestation recruited from 8 obstetric offices of a large multispecialty suburban/urban group practice in eastern Massachusetts US between 1999 and 2002 | Dietary Pattern derived from Principal Component Analysis -Alternate Healthy Eating Index for pregnancy (AHEI-P); Adherence to intake of Mediterranean foods; maternal vitamin and mineral intake; infant vitamin and vitamin supplement [any]; fruits and vegetables, Q | 2, 3 | AD (DD); Wheeze (parent reported); Rec wheeze (parent reported in at least one questionnaire at 1,2,3 years old, ≥2 episodes of wheeze plus medication and/or current symptoms) |
| Liu, 2011 [[515](#_ENREF_515)] | PC | 649 | USA | **Boston Birth Cohort.** Mother-infant pairs recruited at birth at Boston Medical Centre | Vitamin D cord blood, S | 2 | sIgE cow’s milk; sIgE egg; sIgE food; sIgE peanut |
| Marini, 1996 [[91](#_ENREF_91)] | PC | 68 | Italy | Infants with family history of allergy born in maternity wards of 3 hospitals from 1989 whose mothers were refused to participate in an allergy prevention intervention program | Citrus fruit; meat, Q | 1, 3 | AD (DD; parent reported); RC (Physician assessment + parent reported); Rec wheeze (physician assessment > 3 episodes of wheeze); sIgE to food; sIgE aero; sIgE any |
| Mommers, 2009; [[516](#_ENREF_516)] Magdelijn, 2011; [[517](#_ENREF_517)] Cremers, 2011[[518](#_ENREF_518)] | PC | 2465 | The Netherlands | **KOALA.** Population based birth cohort with healthy pregnant women recruited in week 10 -14 of their pregnancy from an ongoing PC study on pregnancy-related pelvic girdle pain and through posters in organic food shops, anthroposophical, physician offices, and midwives | Maternal plasma vitamin D and vitamin supplement [any], S, Q | 2, 5, 6.5 , 7 | AD (UKWPC, (ISAAC); LF (spirometry); sIgE any; Total IgE; Wheeze (ISAAC Q); Rec wheeze (DD asthma plus medication) |
| Maslova, 2012 [[519](#_ENREF_519)]; Maslova, 2013 [[520](#_ENREF_520)] [[521](#_ENREF_521)]; Linneberg, 2004; [[522](#_ENREF_522)] | PC | 28,758 | Denmark | **DNBC.** Population based birth cohort with pregnant women recruited between 1996 and 2002 at ~12weeks gestation | Maternal vitamin intake; maternal intake of peanut and pistachio; tree nuts alcohol; artificially-sweetened non-carbonated soft drinks, Q | 1.5, 7 | AD (parent reported + DD); ARC ( DD + Medication registry); RC (DD); Rec wheeze (DD asthma plus ≥1 episode of wheeze in the last year) |
| Magnus, 2013 [[523](#_ENREF_523)] Haberg, 2009 [[524](#_ENREF_524)] | NCC, PC | 32,077 | Norway | Cases with asthma & non asthmatic controls were recruited from the participants of the Norwegian Mother and Child Cohort Study | Maternal plasma vitamin d and vitamin supplement [any] S, R | 1.5, 3 | Rec wheeze (DD or asthma medication); Wheeze (parent reported) |
| Milner, 2004 [[95](#_ENREF_95)] | PC | 8,073 | USA | **NMIH.** Survey of mothers who gave birth in 1988 with a follow-up survey conducted in 1991. Blacks, individuals with low socioeconomic status, and premature infants were intentionally overrepresented in the survey sample | Infant vitamin intake [any], Q | 3 | FA any (DD); Rec wheeze (DD asthma) |
| Miskelly, 1988 [[96](#_ENREF_96)] | PC | 482 | UK | Infant recruited through two antenatal clinics in South Wales born to mothers with positive allergy history in at least one member of family, whose mothers were asked to participate in allergy preventive program | Fruits; meat; meat other than beef, D | 1 | AD (Physician assessment and parent reported); Wheeze (parent reported) |
| Miyake, 2009 [[525](#_ENREF_525)] and 2010 [[526-528](#_ENREF_526)] and 2011 [[529](#_ENREF_529),[530](#_ENREF_530)]; Satio, 2010 [[526](#_ENREF_526)] | PC | 763 | Japan | **OMCHS.** Population birth cohort with pregnant women between the 5-39th week of pregnancy recruited from a university hospital and three obstetric hospitals in municipalities of Osaka between 2001 and 2003 | Maternal mineral, fat, fish, and vitamin intake; fruits; apples; citrus fruits; vegetables; green and yellow vegetables; Dietary patterns derived from Factor Analysis; dairy; cheese; milk; egg; meat; yoghurt, Q | 0.33, 2 | AD and wheeze (parent reported DD, ISAAC) |
| Morales, 2012 [[531](#_ENREF_531)] | PC | 1,724 | Spain | **INMA Project**. Population based birth cohort study with pregnant women attending their first routine specialized antenatal care visit in 4 study areas: Menorca (1997-98), Valencia (2003-05), Sabadell (2004-06), Gipuzkoa (2006-08) | Maternal plasma Vitamin D, S | 1, 4, 5 | Wheeze (parent reported); Rec wheeze (DD asthma plus medication and/or current symptoms) |
| Morgan, 2004 [[100](#_ENREF_100)] | PC | 257 | UK | Healthy preterm births (<37 weeks gestational) from 3 hospitals in southeast England | Fruits, vegetables; Cereal; rice; rusks; desserts; meat; meat with vegetables, I | 1 | AD (Physician assessment and parent reported) |
| Narita, 2011; [[532](#_ENREF_532)] Ohya, 2011 [[533](#_ENREF_533)] | PC | 1,463 | Japan | **T-CHILD.** Population based birth cohort of Japanese mother-infant pairs with women recruited ~ second trimester in Tokyo | Maternal vitamin, mineral, fish intake; total energy intake, Q | 0.6, 1.5, 3 | AD (parent reported ISAAC Q); Wheeze (parent reported ISAAC Q); Rec wheeze (DD asthma) |
| Nwaru, 2010, 2011, 2012 and 2013 [[105](#_ENREF_105),[106](#_ENREF_106),[534](#_ENREF_534),[535](#_ENREF_535)] [[536](#_ENREF_536),[537](#_ENREF_537)];  Niinisto, 2012 [[538](#_ENREF_538)]; Lumia, 2011; [[539](#_ENREF_539)] Erkkola, 2012; [[107](#_ENREF_107)]; Maijaliisa, 2011; [[540](#_ENREF_540)]; Uusitalo 2008 [[541](#_ENREF_541)]  Erkkola, 2009; [[542](#_ENREF_542)] Marjamaki, 2010; [[543](#_ENREF_543)] | PC | 5,619 | Finland | **DIPP**. Prospective birth cohort of children at high risk of TIDM (HLA genotype conferred susceptibility) born between 1997 and 2004 in Oulu and Tampere University Hospital Finland | Maternal vitamin D, maternal fish and margarine intake; infant fish intake; malaceous fruits; green leafy vegetables; berries; citrus fruits; fruit juice; fruits; vegetables and roots; nuts and pulses; potatoes; fruits and berries; root vegetables; fruits and vegetables; vegetables; cabbage; carrots; Various cereals, dairy products, meat, egg, alcohol, rice, tea, coffee, chocolate, sweets, Q | 0.5, 5, <10 | AD and RC (DD, parent reported ISAAC); sIgE aero; sIgE cm; sIgE egg; sIgE food; Rec atopic wheeze (DD asthma + positive IgE); Rec wheeze (DD asthma); Wheeze (ISAAC); Rec wheeze (DD asthma plus medication and/or current symptoms) |
| Oien, 2010 [[544](#_ENREF_544)] | PC | 3,067 | Norway | **PACT**. A controlled primary intervention study on allergic diseases conducted in the central part of Norway in the city of Trondheim. Inclusion in the control cohort began in September 2000, and the interventional programme started in a separate cohort in July 2002 | Maternal fish/ fish oil; vegetable intake, Q | 2 | AD (ISAAC); Rec wheeze (DD asthma) |
| Pike, 2012 [[545](#_ENREF_545)] | PC | 739 | UK | **SWS**. Population birth cohort with 20-34 year old women recruited pre-conception from general practitioners in Southampton between 1998 and 2002 and subsequently become pregnant | Maternal plasma vitamin D and vitamin intake, S, Q | 1, 3, 6 | LF (EV_1_, FVC, BHR slope); SPT any; Wheeze (parent reported ISAAC Q); Rec wheeze (DD asthma) |
| Roduit, 2012 [[546](#_ENREF_546)] | PC | 1,041 | Austria, Finland, France, Germany, and Switzerland | **PASTURE**. Population based birth cohort with women recruited in third trimester of pregnancy from rural areas in 5 European countries (Austria, Finland, France, Germany, and Switzerland) and divided into 2 groups: those who lived or worked on family run farms and those not living on a farm from the same area | Infant fat source introduction; fruits and vegetables; Cereal; bread; chocolate; cake; Farm milk' - either boiled or not boiled; meat, D | 1, 4 | AD (DD) |
| Rothers, 2011 [[547](#_ENREF_547)] | PC | 208 | US | **IIS.** Prospective birth cohort study of healthy children born to pregnant women who planned to obtain care for their new-borns from collaborating paediatricians | Vitamin D cord blood, S | 1, 2, 3, 5 | RC (DD); sIgE aero; SPT aero: Total IgE; Rec wheeze (DD asthma) |
| Sausenthaler, 2007 [[548](#_ENREF_548)]; Zutavern, 2006; [[178](#_ENREF_178)] Zutavern, 2008; [[179](#_ENREF_179)] | PC | 2,540 | Germany | **LISA.** Population based cohort study of newborns recruited between 1997 and1999 from 4 German cities: Munich, Leipzig, Wesel, and Bad Honnef | Maternal fat intake; maternal fish intake; fruits; vegetables; apples; bananas; strawberries; citrus fruits; fruit juice; cabbage; salad; celery; sweet peppers; spinach; nuts; carrots; seeds; tomatoes; cereal, milk, cheese; yoghurt, Q | 1, 2, 6 | AD (DD, parent reported), AS (any, food, aeroallergen by sIgE) |
| Sicherer, 2010 [[138](#_ENREF_138)] | PC | 503 | USA | **The Consortium of Food Allergy Research** enrolled infants at 3 to 15 months of age with likely egg or milk allergy but without previously known peanut allergy | Peanut, Q | 1 | sIgE to peanut |
| Strassburger, 2010 [[155](#_ENREF_155)] | PC | 338 | Brazil | Birth cohort study nested in a dietary intervention randomized field trial in the city of São Leopoldo, southern Brazil in 2002 | Salty pureed food; fruit juice, R | 3.5 | SPT aero; Wheeze (parent reported) |
| Venter, 2009; [[168](#_ENREF_168)] Dean, 2007 [[549](#_ENREF_549)] | PC | 937 | UK | **The Isle of Wight cohort.** Population based birth cohort recruited through antenatal clinics and included all babies born on the Isle of Wight UK between 2001 and 2002 | Maternal allergenic food avoidance, Q | 2 | AS peanut (SPT) |
| Wang, 2007 [[144](#_ENREF_144)] | PC | 1,760 | Taiwan | **Taiwan National Birth Cohort Study** (Pilot study). Representative samples recruited post-natally using the national birth registration data in 2003 | Maternal mineral supplement; maternal seafood intake/ fish oil supplementation ; gingseng, Q | 0.5 | AD (DD) |
| Weisse, 2012 [[550](#_ENREF_550)] | PC | 272 | Germany | **LINA.** Population based birth cohort with mother–child pairs recruited between 2006 and 2008 in Leipzig | Maternal plasma Vitamin D and Vitamin D cord blood, S | 1, 2 | AD (DD, parent reported); FA any (DD, Total IgE; sIgE) |
| West, 2012 [[551](#_ENREF_551)] | PC | 319 | Australia | Mother-infant pairs from a pregnancy cohort, recruited in Perth, Western Australia from 2005 to 2008. Pregnant women with a family history of allergic diseases recruited to participate in a postnatal infant dietary intervention study | Maternal mineral and vitamin intake, Q | 1 | AD (DD); FA any (history of reaction to food plus positive SPT-food; SPT any) Wheeze (DD) |
| Whitrow, 2009 [[552](#_ENREF_552)] | PC | 490 | Australia | **Generation 1.** Population based birth cohort of women and their children recruited in the first 16 weeks of pregnancy between 1998 and 2000 from 4 antenatal clinics in Adelaide | Maternal vitamin intake, Q | 3.5, 5.5 | Rec wheeze (DD asthma) |
| Calvani, 2006 [[553](#_ENREF_553)] | RC | 988 | Italy | **APAL**. Children attending outpatients allergy clinic in Rome between 2001-2002 | Maternal fat intake, Q |  | AS aero, food, cow’s milk, egg (SPT) |
| Jones, 2012 [[554](#_ENREF_554)] | RC | 231 | Western Australia | Children with family history of atopy derived from a larger birth cohort in 2002-2009 | Maternal vitamin supplement [any] and Vit D cord blood, S, Q | 1 | AD (DD or evidence of typical skin lesions) ; FA any (history of reaction to food plus positive SPT-food); Rec wheeze (unclear) |
| Allen, 2013 [[555](#_ENREF_555)] | NCC | 2,758/240 | Australia | **HealthNUTS.** Cases & controls recruited from population-based sampling from governmental immunisation clinics in Melbourne | Infant plasma Vitamin D, S, Q | 1 | AD (DD); FA any, egg, peanut (DD with OFCs) |
| Sariachvili, 2010 [[60](#_ENREF_60)] | NCC | 557/ 252 | Belgium | **PIPO Cohort.** cases and controls with data regarding development of AD and timing of introduction of solid foods were identified from this PC | Fruits, fruit juice, vegetables; cereal meat, Q | 4 | AD (parent reported, ISAAC Q) |
| Binkley, 2011 [[556](#_ENREF_556)] | CC | 1,413/1,300 | Canada | Cases & controls were anaphylaxis registry's previous survey respondents, all having had previous anaphylactic food allergy reactions, although only cases to peanuts | Maternal vitamin supplement [any]; maternal allergenic food avoidance, Q | <18 | FA peanut (DD) |
| Dai, 1993 [[557](#_ENREF_557)] | CC | 70 | China | Children from the community and they had to reside there the last year | Maternal allergenic food avoidance [more than one food group]: any, Q | 0.5 | Rec wheeze (unclear) |
| DesRoches, 2010 [[191](#_ENREF_191)] | CC | 401/ 202 | Canada | Cases and controls were recruited from the Paediatric University Centre between 1998-2004 | Allergenic food avoidance (nut); allergenic food avoidance (soya); allergenic food avoidance (peanut), Q | <1.5 | FA peanut (history of a clinical reaction within 60 minutes of exposure to peanuts, combined with positive IgE and/or SPT to peanut) |
| Fox, 2009 [[193](#_ENREF_193)] | CC | 293/ 133 | UK | Cases and controls from specialist food allergy clinics, with cases sensitised to peanut and controls sensitised to egg but not peanut | Peanut, Q | <4 | FA peanut (DD DBPCFC) |
| Lopez Campos, 2001 [[558](#_ENREF_558)] | CC | 75/58 | Mexico | Asthmatic patients were recruited from Allergy clinics and control patients from familial medicine clinics of Hospital de Especialidades, Mexico | Chocolate; mango, Q | 6-10 | Rec wheeze (DDA asthma) |
| Mullins, 2012 [[559](#_ENREF_559)] | CC | 115/ 115 | Australia | Cases were peanut allergic patients born in Australian Capital Territory and population matched-controls: Australia | Infant plasma vitamin D, S | 6 | FA peanut (history of acute systemic allergic reaction within 2 hours of known food exposure, combined with a SPT to the relevant food) |
| Oliveti, 1995 [[201](#_ENREF_201)] | CC | 263/ 131 | USA | Cases & controls were identified from rosters of patients followed up at the Rainbow Babies & Children's Hospital continuity care clinic, Ohio | Alcohol, R | 4-9 | Rec wheeze (DD asthma) |
| Salam, 2005 [[560](#_ENREF_560)] | CC | 691/ 279 | USA | Children's Health Study: cases and controls selected from school-children who participated in a population-based study: USA | Maternal fat intake, Q | 9-16 | Re wheeze (DD asthma) |
| Castro-Rodriguez, 2010 [[218](#_ENREF_218)] | CS | 1,409/  594 | Spain | **EISL**: Spanish population attending primary healthcare clinics, cases with wheeze in first year of life and controls without: Spain | Maternal fat intake; Mediterranean diet; ‘industrial food’, Q | 1.4 | Wheeze (SAAC) |
| de Batlle, 2008 [[561](#_ENREF_561)] | CS | 1,476/402 | Mexico | Participants were recruited from a random sample of children in primary school in the Mexicali province, Mexico | Dietary pattern intake in pregnant mothers, Q | 6-7 | Wheeze and rec wheeze (parent reported); RC (parent reported) |
| Dela Bianca, 2012 [[562](#_ENREF_562)] | CS | 467 | Brazil | Participants were infants aged from 12 to 15 months who attended 9 selected health centres for routine immunisations. | Processed food, Q | 1 | Rec wheeze (parent reported ≥1 episode of wheezing in the past year) |
| Riedler, 2001 [[563](#_ENREF_563)] | CS | 812/ unclear | Austria, Germany, and Switzerland | **ALEX STUDY TEAM**: cases were children of farming families and controls of non-farming families from the study schools: Austria, Germany, & Switzerland | Farm milk/environment, I | 9 | AD (DD); RC (parent reported); sIgE aero; Rec wheeze ( DD asthma; asthma ≥1 episode of wheezing in the past year) |

AD: atopic dermatitis; AS: allergic sensitisation; AR: allergic rhinitis; ARC: allergic rhinoconjunctivitis; BHR bronchial hyper-responsiveness; CC: case-control study; CS: cross-sectional study; DBPCFC: double blind placebo-controlled food challenge; DD: Doctor diagnosis (community); FA: food allergy; FEV_1_: forced expiratory volume in 1 second; ISAAC International Study of Allergy and Asthma in Children; LF lung function; OFCs oral food challenges; PC: Prospective cohort study; PEF: peak expiratory flow; Physician assessment is assessment by study physician; Q: questionnaire; SPT: skin prick test, sIgE specific IgE.

# Table S6 Characteristics of included observational studies of other maternal or infant dietary exposures and risk of autoimmune disease

| **Study** | **Design** | **N/n cases** | **Country** | **Population** | **Exposures and method of assessment** | **Age at outcome (years)** | **Outcomes reported**  **(method of assessment)** |
| --- | --- | --- | --- | --- | --- | --- | --- |
| Brekke, 2007 [[564](#_ENREF_564)]  Brekke, 2010 [[565](#_ENREF_565)]; Wahlberg, 2006; [[566](#_ENREF_566)] | PC | 8,694 | Sweden | **ABIS:** Population based birth cohort of children born in Southeast Sweden between 1997 and 1999 | Infant vitamin intake [any] and maternal vitamin supplement [any]; potatoes and root vegetables; other vegetables; coffee, Q | 1, 2.5, 8.4, 17 | TIDM (serology); CD (DD + IgA-tTG +symptoms) |
| Fronczak, 2003 [[249](#_ENREF_249)]; Lamb 2008 [[252](#_ENREF_252)]; Simpson, 2011[[567](#_ENREF_567)]; Norris, 1996 [[568](#_ENREF_568)]; Frederikson, 2012 [[248](#_ENREF_248)]; Lamb, 2013; [[250](#_ENREF_250)] | PC; NCC | 222,222/  16  1698/49 | USA | **DAISY**: Prospective birth cohort of children at increased risk for TIDM recruited 1993 to 2004 in Denver, Colorado. Cases & their non-diabetic siblings, as controls, taken from diabetes care clinics & Colorado IDDM registry | Maternal vitamin intake; maternal fish intake; infant plasma vitamin D;  fruits; vegetables; potatoes; root vegetables, Q | 4, 9, 15 | CD (positive IgA-tTG on 2 visits or a positive small bowel biopsy and one tTG-positive); TIDM (serology) |
| Hypponen, 2004 [[498](#_ENREF_498)]  Hypponen, 2001 [[499](#_ENREF_499)] | PC | 10,366/81 | Finland | **Northern Finland Birth Cohort:** Population based birth cohort with women recruited between the 24th and 28th week of gestation in Oulu and Lapland in 1995-1966 | Infant vitamin intake [any], Q | 31, 2 | TIDM (DD) |
| Nwaru, 2010, 2011, 2012 and 2013 [[105](#_ENREF_105),[535](#_ENREF_535),[537](#_ENREF_537)] [[534](#_ENREF_534)] [[106](#_ENREF_106)]; Niinisto, 2012 [[538](#_ENREF_538)]; Lumia, 2011 [[539](#_ENREF_539)] ; Erkkola, 2012 [[107](#_ENREF_107)]; Virtanen, 2006 and 2011 [[569](#_ENREF_569),[570](#_ENREF_570)]; Uusitalo, 2008; [[541](#_ENREF_541)] | PC | 3,730 | Finland | **DIPP**: Prospective birth cohort of children at high risk of TIDM (HLA genotype conferred susceptibility) born between 1997 and 2004 in Oulu and Tampere University Hospital Finland | Maternal fish and margarine intake; infant fish intake; Wheat , Rye, Oats, Barley, Other cereals (maize, rice, millet, and buckwheat); cereal; Other cereals (maize, rice, millet, and buckwheat); milk; milk and dairy; cheese; meat; egg; alcohol; rice; tea; coffee; chocolate; chocolate and  sweets; dairy, Q | 0.5, 5, <10 | TIDM (serology; DD WHO criteria)) |
| Harsunen, 2012 [[571](#_ENREF_571)] | NCC | 33/33 | Germany | **BABYDIET study**: cases were study children who developed islet autoantibodies and TIDM during BABYDIET study follow-up and controls were children from the same original study cohort who did not: Germany | Dietary energy intake, D | <18 | TIDM (DD) |
| Miettinen, 2012 [[572](#_ENREF_572)] | NCC | 686/343 | Finland | All selected from the Finnish Maternity Cohort, with cases identified from the Finnish Diabetes Register | Maternal plasma Vitamin D, I | 3.4 | TIDM (DD) |
| Savilahti, 2009 [[268](#_ENREF_268)] | NCC | 6,209/45 | Finland | Cases and controls taken from the NHI database, Finland | Vegetables, R/D | 11.5 | TIDM (DD) |
| Sørensen, 2012 [[573](#_ENREF_573)] | NCC | 328/109 | Norway | Cases and controls selected from a population birth cohort linked to Norwegian Childhood Diabetes Registry | Maternal plasma Vitamin D, S | <15 | TIDM (DD) |
| Ahadi, 2011 [[269](#_ENREF_269)] | CC | 202/101 | Iran | Cases were diagnoses of TIDM referred to Children's Medical Centre Hospital and matched controls | Infant vitamin intake [any], Q | 6.7 | TIDM (DD) |
| Ashraf, 2010 [[271](#_ENREF_271)] | CC | 195 | USA | Cases and controls selected from electronic medical records | Infant mineral intake, Q | 10 | TIDM (DD) |
| Baron, 2005 [[321](#_ENREF_321)] | CC | 444/222 | France | Cases with Crohn's disease were identified from the **EPIMAD** registry with matched controls from the same area identified by random digit dialling: France; Cases with ulcerative colitis were identified from the EPIMAD registry with matched controls from the same area | Vegetables ; flour meat, I | <17 | IBD-CR (DD); IBD-UC (DD) |
| Ellis, 2012 [[326](#_ENREF_326)] | CC | 655/246 | Australia | **CLARITY:** cases were recruited during a clinic visit to Royal Children's Hospital, with diagnosed JIA using ILAR criteria: controls were patients in for elective surgery, also at the Royal Children's Hospital Day Surgery Unit | Maternal iron, multivitamin, folate, calcium, fish oil, vitamin D supplement; alcohol; coffee, Q | 0-18 | JIA (DD; ILAR criteria) |
| Bener, 2009 [[273](#_ENREF_273)] | CC | 340/170 | Qatar | Cases were insulin dependent or had a venous blood glucose >6.7mmol/L on 2 occasions with matched healthy controls selected from the community | Infant vitamin intake [any], I/Q | <16 | TIDM (DD) |
| EURODIAB substudy 2 study group, 1999 [[574](#_ENREF_574)] | CC | 2,934/746 | Luxembourg, N. Ireland, Romania, Lithuania, Bulgaria and Australia | **EURODIAB**: cases were <15years at TIDM diagnosis and matched with population-based controls. | Infant vitamin intake [any], I/Q | <15 | TIDM (DD; WHO criteria) |
| Gilat, 1987 [[330](#_ENREF_330)] | CC | 504/167 | 9 countries: USA, Canada, UK, Sweden, Denmark, Holland, France, Italy, Israel | **The International IBD Study Group**: cases were patients with proven Crohn's Disease in 14 centres across 9 countries with 2 controls per case, one with a different minor GI disease and the other with minor non-GI disease taken from hospitals or clinics. | Maternal vitamin supplement [any], Q | <25 | IBD-CR (DD); IBD-UC (DD) |
| Majeed, 2011 [[284](#_ENREF_284)] | CC | 395/96 | Iraq | Cases were TIDM patients admitted to hospitals or primary health centres in Basrah and controls were attendees to outpatients of the same institutions for non-diabetic complaints | Coffee; tea, Q | 15-17 | TIDM (DD) |
| Malcova, 2005 [[285](#_ENREF_285)] | CC | 2,334/868 | Czech Republic | Cases were identified from the Czech Childhood Diabetes Register, with unrelated aged-match controls selected from among the schoolmates of cases | Infant vitamin intake [any], Q | 7 | TIDM (DD) |
| Rosenbauer, 2007 and 2008 [[295](#_ENREF_295),[575](#_ENREF_575)] | CC | 2,631/760 | Germany | German study of newly diagnosed TIDM cases selected from a hospital-based surveillance system ESPED and controls from local registration office records | Coffee, Q | <5 | TIDM (DD) |
| Sipetic, 2003 [[576](#_ENREF_576)]; Sipetic, 2005 [[577](#_ENREF_577)] | CC | 315/105 | Serbia | Cases and controls were children admitted to hospital due to allergic conditions 1994-97 | Alcohol; coca cola, nitrosamine-rich foods, coffee, Q | 1-16 | TIDM (maternal report; DD WHO criteria) |
| Stene, 2003 [[578](#_ENREF_578)], Stene, 2008 [[579](#_ENREF_579)] | CC | 2213/545 | Norway | **Norwegian Childhood Diabetes Study Group**: cases were all children on the diabetes registry diagnosed 1997-2000 and controls were matched from the national population registry | Infant vitamin intake [any]; infant oil supplement; maternal vitamin/fish supplement [any], Q | 8.8 | TIDM (DD; WHO criteria) |
| Stene, 2000 [[302](#_ENREF_302)] | CC | 1131/84 | Norway | Cases were TIDM patients in Vest-Agder & on the National Childhood Diabetes Register 1982-98 and controls were selected randomly from the population register for the same age and period | Maternal vitamin supplement [any] ; maternal fish oil supplement, Q | <15 | TIDM (DD) |
| Svensson, 2005 [[580](#_ENREF_580)] | CC | 1152/475 | Denmark | Cases identified from the Danish National Register of incident cases diagnosed 1996-99 and matched controls from the Danish Population Register | Infant vitamin intake [any], Q | 8.4 | TIDM (DD) |
| Strotmeyer, 2004; [[304](#_ENREF_304)] | CC | 688/ 247 | China | **DiaMond:** WHO Multinational Project; cases selected from TIDM incidence registries 1985-98 and matched controls from local population | Vegetables; fruits; steamed bread; rice; noodles; meat, Q | 9.7 | TIDM (DD; WHO criteria) |
| Tenconi, 2007 [[307](#_ENREF_307)] | CC | 429/131 | Italy | Cases identified from TIDM population registry 1988-2000 and matched controls selected from hospitalised patients, not affected by metabolic disease or cancer | Maternal vitamin supplement [any], Q | 15.5 | TIDM (DD) |
| Virtanen, 1994 [[581](#_ENREF_581)] | CC | 1136/600 | Finland | **Childhood Diabetes in Finland**: cases were newly diagnosed TIDM with matched controls selected from the general population | Coffee, Q |  | TIDM (DD) |
| Visalli, 2003 [[311](#_ENREF_311)] | CC | 900/150 | Italy | **EURODIAB Italy**: Cases with TIDM selected from within the EURODIAB study, born 1977-89, with controls selected from school records for the same period | Infant vitamin intake [any]; fruits, vegetables; meat; tea; coffee, Q | 12 | TIDM (DD; WHO criteria) |

AR: allergic rhinitis; CC: case-control study; CD Coeliac disease; CR Crohn’s disease; DD: Doctor diagnosis (community); FA: food allergy; GADA: Glutamic acid decarboxylase antibodies; GI: gastro-intestinal; IA2A: tyrosine phosphatase IA2 antibodies; IAA: insulin autoantibodies; ILAR: International League of Associations for Rheumatology; IBD: inflammatory bowel disease; JIA: juvenile idiopathic arthritis; NCC: nested case-control study; PC: Prospective cohort study; Physician assessment is assessment by study physician; Q: questionnaire; RC: retrospective cohort; SPT: skin prick test; sIgE specific IgE; TIDM: type 1 diabetes mellitus; tTG: Antibodies to tissue transglutaminase; UC: ulcerative colitis ; WHO: World Health Organisation

# Table S7 Risk of bias in intervention trials of breastfeeding promotion or solid food introduction and allergic outcomes

| **Study** | **Design** | **N Intervention/ Control** | **Assessment** | **Selection** | **Attrition** | **Overall Bias** | **Conflict of Interest** |
| --- | --- | --- | --- | --- | --- | --- | --- |
| Kramer, 2001 [[1](#_ENREF_1)]; Kramer, 2007 [[2](#_ENREF_2)] | cluster RCT | 8865/8181 | Low | Low | Low | Low | Low |

RCT randomised controlled trial

# Table S8 Risk of bias in observational studies of breastfeeding or solid food introduction, and risk of allergic outcomes

| **Study** | **Design** | **N/n cases** | **Assessment** | **Selection** | **Confounding** | **Overall Bias** | **Conflict of interest** |
| --- | --- | --- | --- | --- | --- | --- | --- |
| Alho, 1990 [[3](#_ENREF_3)] | PC | 2,130 | Unclear | Low | High | High | Low |
| Allen, 2009 [[4](#_ENREF_4)]; Koplin, 2010 [[5](#_ENREF_5)] | PC | 310/50 | Low | Low | Low | Low | Low |
| Alm, 2008[[6](#_ENREF_6)]; Goksor, 2009/11  [[7](#_ENREF_7),[8](#_ENREF_8)] | PC | 4,987 | Unclear | Unclear | High | High | Low |
| Bacopoulou, 2009 [[9](#_ENREF_9)] | PC | 6,643 | Unclear | Unclear | High | High | Unclear |
| Benn, 2004 [[10](#_ENREF_10)]; Linneberg 2006 [[11](#_ENREF_11)] | PC | 34,793 | Low | Low | Low | Low | Low |
| Bergmann, 2000 [[12](#_ENREF_12)]; Bergmann, 2002 [[13](#_ENREF_13)] Kulig, 2000 [[14](#_ENREF_14)] | PC | 1,314 | Low | High | Low | High | Low |
| Berth-Jones, 1997 [[15](#_ENREF_15)] | PC | 413 | Unclear | Low | High | High | Low |
| Besednjak-Kocijancic, 2010 [[16](#_ENREF_16)] | PC | 408/24 | Unclear | Unclear | High | High | Unclear |
| Bisgaard, 2009 [[17](#_ENREF_17)]; Giwercman, 2010 [[18](#_ENREF_18)] | PC | 354 | Low | Low | Low | Low | Low |
| Burr, 1989; Burr, 1993; Burr, 1993 (b) [[19-21](#_ENREF_19)]; Burr, 1997 [[22](#_ENREF_22)] | PC | 483 | Low | Low | Low | Low | Low |
| Burgess, 2006 [[23](#_ENREF_23)] | PC | 4,964 | Low | Low | Low | Low | Low |
| Businco, 1987 [[24](#_ENREF_24)]; Bruno, 1995 [[25](#_ENREF_25)] | PC | 244 | Low | Low | Low | Low | Low |
| Cano Garcinuno, 2003 [[26](#_ENREF_26)] | PC | 234 | Unclear | Low | Low | Unclear | Low |
| Caudri, 2013; Scholtens, 2009 [[27](#_ENREF_27),[28](#_ENREF_28)]; Kerkhof, 2003 [[29](#_ENREF_29)] | PC | 3,115 | Low | Low | Low | Low | Low |
| Cogswell, 1987 [[30](#_ENREF_30)] | PC | 73/32 | Unclear | Low | High | High | Low |
| Chuang, 2011[[31](#_ENREF_31)] | PC | 18,773 | Low | Low | Low | Low | Low |
| da Costa Lima, 2003; Menezes, [[32](#_ENREF_32),[33](#_ENREF_33)] | PC | 4,297 | Low | High | Low | High | Low |
| Dell, 2001; Midodzi, 2008 [[34](#_ENREF_34),[35](#_ENREF_35)]; Midodzi, 2010 [[36](#_ENREF_36)] | PC; CS | 2,711 | Unclear | Low | Low | Unclear | Low |
| Devereux, 2006 [[37](#_ENREF_37)] | PC | 1,704 | Low | Low | Low | Low | Unclear |
| Dogaru, 2012 [[38](#_ENREF_38)] | PC | 1,458 | Low | Low | Low | Low | Low |
| Elliott, 2008; Granell, 2012; Sherriff, 2001  [[39-41](#_ENREF_39)]; Abd, 2012 [[42](#_ENREF_42)]; Lack, 2003 [[43](#_ENREF_43)] | PC | 9,100 | Low | Low | Low | Low | Low |
| Eneli, 2006 [[44](#_ENREF_44)] | PC | 536 | Low | Low | Low | Low | Unclear |
| Farooqi, 1998 [[45](#_ENREF_45)] | PC | 1,453 | Low | High | Low | High | Low |
| Fergusson, 1983; Horwood, 1995  [[46](#_ENREF_46),[47](#_ENREF_47)] | PC | 1,110 | Low | Low | Low | Low | Low |
| Fredriksson, 2007 [[48](#_ENREF_48)] | PC | 1,933 | Low | Low | Low | Unclear | Low |
| Forster, 1990[[49](#_ENREF_49)] | PC | 145 | Unclear | Low | Unclear | Unclear | Low |
| Galbally, 2013 [[50](#_ENREF_50)] | PC | 4,507 | Low | Low | Low | Low | Low |
| Gruber 2010 [[51](#_ENREF_51)] | PC | 167/15 | Low | Low | High | High | High |
| Gruskay 1982 [[52](#_ENREF_52)] | PC | 328 FH+/ 580 FH- | Unclear | High | High | High | Low |
| Guida, 2009 [[53](#_ENREF_53)] | PC | 3,041 | Unclear | Unclear | Low | Unclear | Low |
| Gustafsson, 2000 [[54](#_ENREF_54)] | PC | 94 | Unclear | Low | Low | Unclear | Low |
| Halken, 1991[[55](#_ENREF_55)] | PC | 276 | Low | Low | Low | Low | Low |
| Harris, 2001[[56](#_ENREF_56)]; Zutavern, 2004 [[57](#_ENREF_57)] | PC | 622 | Low | Low | Low | Low | Low |
| Hagendorens, 2005 [[58](#_ENREF_58)]; Sariachvili, 2007 [[59](#_ENREF_59)]; Sariachvili 2010 [[60](#_ENREF_60)] | PC | 693 | Unclear | Low | Low | Unclear | Low |
| Hesselmar, 2010 [[61](#_ENREF_61)] | PC | 184 | Low | Low | High | High | Low |
| Hetzner, 2009 [[62](#_ENREF_62)] | PC | 7,900 | Unclear | Low | High | High | Low |
| Hide, 1981[[63](#_ENREF_63)]; Arshad, 1992 [[64](#_ENREF_64)] | PC | 843 | Low | Low | Low | Low | Low |
| Hikino, 2001[[65](#_ENREF_65)] | PC | 21,766/2,381 | Unclear | Low | Low | Unclear | Low |
| Hong, 2011 [[66](#_ENREF_66)] | PC | 970/361 | Low | Unclear | Low | Unclear | Low |
| Hoppu, 2002 [[67](#_ENREF_67)] | PC | 114/27 | Low | Low | High | High | Low |
| Host, 1991 [[68](#_ENREF_68)] | PC | 315/16 | Unclear | Low | High | High | Unclear |
| Howie, 1990 [[69](#_ENREF_69)] | PC | 618 | Low | Low | Low | Low | Low |
| Huang, 2013 [[70](#_ENREF_70)] | PC | 684 | Unclear | Low | High | High | Low |
| Huurre, 2008 [[71](#_ENREF_71)] | PC | 98/29 | Low | Low | Low | Low | Unclear |
| Joseph, 2011 [[72](#_ENREF_72)] | PC | 594/178 | Unclear | Low | Low | Unclear | Low |
| Juto, 1980 [[73](#_ENREF_73)] | PC | 56/NA | Unclear | Low | High | High | Unclear |
| Kajosaari, 1991[[74](#_ENREF_74)] | PC | 135 | Low | Unclear | High | High | Low |
| Karmaus, 2008 [[75](#_ENREF_75)]; Ogbuanu, 2009 [[76](#_ENREF_76)]; Soto-Ramırez, 2012 [[77](#_ENREF_77)] | PC | 1,336 | Low | Low | Low | Low | Low |
| Kaufman, 1976 [[78](#_ENREF_78)] | PC | 94 | Unclear | Low | High | High | Low |
| Kellberger, 2012 [[79](#_ENREF_79)] | PC | 594/178 | Unclear | Low | Low | Unclear | High |
| Kemeny, 1991[[80](#_ENREF_80)] | PC | 180 | Unclear | Low | High | High | Unclear |
| Kerr, 1981[[81](#_ENREF_81)] | PC | 269 | Unclear | Low | High | High | Low |
| Kim, 2011 [[82](#_ENREF_82)] | PC | 1,177/61 | Unclear | Low | Low | Unclear | Low |
| Kitz, 2006 [[83](#_ENREF_83)] | PC | 131 | Unclear | Low | High | High | Unclear |
| Klinnert, 2001 [[84](#_ENREF_84)] | PC | 145 | Low | Low | Low | Low | Low |
| Kramer, 2003; Kramer, 2009; Kramer, 2009 (b)  [[85-87](#_ENREF_85)] | PC | 13,889/455 | Unclear | Low | Low | Unclear | Low |
| Kull, 2002 [[88](#_ENREF_88)] | PC | 3790 | Low | Low | Low | Low | Low |
| Kusel, 2005 [[89](#_ENREF_89)] | PC | 263/107 | Unclear | Low | Low | Unclear | Unclear |
| Larsson, 2008 [[90](#_ENREF_90)] | PC | 4779 | Low | Low | Low | Low | Low |
| Marini, 1996 [[91](#_ENREF_91)] | PC | Unclear | Unclear | Low | Low | Unclear | Unclear |
| Matheson, 2007 [[92](#_ENREF_92)] | PC | 5,729/2,610 | Unclear | Low | Low | Unclear | High |
| Midwinter, 1987 [[93](#_ENREF_93)] | PC | 453 | Unclear | Low | Low | Unclear | Unclear |
| Mihrshahi, 2007 [[94](#_ENREF_94)] | PC | 516 | Low | Low | Low | Low | Low |
| Milner, 2004 [[95](#_ENREF_95)] | PC | 8,071 | Low | Unclear | Low | Unclear | Low |
| Miskelly, 1988 [[96](#_ENREF_96)] | PC | 482 | Low | Low | High | High | Low |
| Miyake, 2008 [[97](#_ENREF_97)]; Miyake, 2009 [[98](#_ENREF_98)] | PC | 763 | Low | High | Low | High | Low |
| Morgan, 2004; Morgan, 2004 (b) [[99](#_ENREF_99),[100](#_ENREF_100)] | PC | 257 | Low | Low | Low | Low | Low |
| Moore, 1985 [[101](#_ENREF_101)] | PC | 475 | Low | Low | Low | Low | Low |
| Morales, 2012 [[102](#_ENREF_102)] | PC | 467 | Unclear | Low | Low | Unclear | Low |
| Muiño, 2008 [[103](#_ENREF_103)] | PC | 897 | Low | Low | High | High | Low |
| Nielsen, 2013 [[104](#_ENREF_104)] | PC | 5,429 | Low | Low | Low | Low | Low |
| Nwaru, 2010 [[105](#_ENREF_105)]; Erkkola, 2012; Nwaru, 2013  [[106](#_ENREF_106),[107](#_ENREF_107)]; Virtanen, 2010 [[108](#_ENREF_108)] | PC | 3,675 | Low | Low | Low | Low | Low |
| Oddy, 2003 [[109](#_ENREF_109)] | PC | 243 | Low | Unclear | High | High | Low |
| Oddy, 1999; Oddy, 2003; Oddy, 2004  [[110-112](#_ENREF_110)] | PC, NCC | 2,456 | Low | Low | Low | Low | Low |
| Odelram, 1996 [[113](#_ENREF_113)] | PC | 70/23 | Unclear | Low | High | High | Unclear |
| Perez Tarazona, 2010 [[114](#_ENREF_114)] | PC | 620 | Unclear | Low | High | High | Low |
| Pesonen, 2006 [[115](#_ENREF_115)] | PC | 160 | Low | Low | Low | Low | Low |
| Porch, 1998 [[116](#_ENREF_116)] | PC | 130 | Low | Low | High | High | Low |
| Poysa, 1990; Poysa, 1992 [[117](#_ENREF_117),[118](#_ENREF_118)] | PC | 68 | Unclear | Low | High | High | Unclear |
| Pratt, 1984 [[119](#_ENREF_119)] | PC | 198 | Low | Low | High | High | Low |
| Puig, 2010 [[120](#_ENREF_120)] | PC | 368 | Low | Low | Low | Low | Low |
| Purvis, 2005 [[121](#_ENREF_121)] | PC | 550 | Unclear | High | Low | High | Low |
| Rhodes, 2001[[122](#_ENREF_122)] | PC | 63 | Unclear | High | High | High | Low |
| Rothenbacher, 2005 [[123](#_ENREF_123)] | PC | 803 | Low | Low | Low | Low | Low |
| Rowntree, 1985 [[124](#_ENREF_124)] | PC | 80/20 | Unclear | Low | High | High | Unclear |
| Rullo, 2007; Rullo, 2009; Rullo, 2009 (b); Rullo, 2010  [[125-128](#_ENREF_125)] | PC | 101 | Low | Low | Low | Low | Low |
| Ruiz 1992 [[129](#_ENREF_129)] | PC | 39 | Unclear | High | High | High | Low |
| Saarinen, 1995 [[130](#_ENREF_130)] Saarinen, 1979 [[131](#_ENREF_131)] | PC | Unclear | Unclear | Low | High | High | Unclear |
| Sears, 2002 [[132](#_ENREF_132)]; Mandhane 2007 [[133](#_ENREF_133)] | PC | 1,037 | Low | Low | High | High | Low |
| Shaheen, 1996 [[134](#_ENREF_134)] | PC | 395/44 | Unclear | Unclear | Low | Unclear | Low |
| Shohet, 1985 [[135](#_ENREF_135)] | PC | 368 | Unclear | Low | High | High | Unclear |
| Schoetzau, 2002 [[136](#_ENREF_136)] | PC | 829 | Low | High | Low | High | Low |
| Schonberger, 2005 [[137](#_ENREF_137)] | PC | 443 | Low | Low | Low | Low | Low |
| Sicherer, 2010 [[138](#_ENREF_138)] | PC | 503/140 | Unclear | Low | High | High | Low |
| Siltanen, 2003 [[139](#_ENREF_139)] | PC | 285/53 | Unclear | Low | High | High | High |
| Silva, 2005 [[140](#_ENREF_140)] | PC | 73 | Unclear | Unclear | High | High | Unclear |
| Silvers, 2009; Silvers, 2011 [[141](#_ENREF_141),[142](#_ENREF_142)] | PC | 889/249 | Low | Low | Low | Low | Low |
| Simon, 2008 [[143](#_ENREF_143)] ; Wang, 2007[[144](#_ENREF_144)]; Wegienka, 2006 [[145](#_ENREF_145)]; Salam, 2003 [[146](#_ENREF_146)] | PC | 372 | Low | Low | Low | Low | Low |
| Snijders, 2007; Snijders, 2008 [[147](#_ENREF_147),[148](#_ENREF_148)] | PC | 2,505 | Low | Low | Low | Low | Low |
| Soto-Ramirez, 2013 [[149](#_ENREF_149)] | PC | 2,833 | Low | High | Low | High | Low |
| Strachan, 1997 [[150](#_ENREF_150)]; Strachan, 1996 [[151](#_ENREF_151)]; Lewis, 1995; Lewis, 1996 [[152](#_ENREF_152),[153](#_ENREF_153)]; Butland, 1997 [[154](#_ENREF_154)] | PC | 1,369/730 | Low | Low | Low | Low | Unclear |
| Strassburger, 2010 [[155](#_ENREF_155)] | PC | 325/94 | Unclear | Low | Low | Unclear | Low |
| Sunyer, 2006 [[156](#_ENREF_156)] | PC | 462 | Low | Low | Low | Low | Low |
| Sunyer, 2001 [[157](#_ENREF_157)] | PC | 596 | Low | Unclear | Low | Unclear | Low |
| Taylor, 1983 [[158](#_ENREF_158)] ; Taylor, 1984 [[159](#_ENREF_159)] | PC | 12,608 | Low | Low | Low | Low | Low |
| Tennant, 2008 [[160](#_ENREF_160)]  Tennant, 2010 [[161](#_ENREF_161)] | PC | 392 | Low | Low | Low | Low | Low |
| Tian, 2009 [[162](#_ENREF_162)] | PC | 472 | Unclear | Low | Low | Unclear | Low |
| Van Asperen, 1983 [[163](#_ENREF_163)] | PC | 79/44 | Unclear | Low | High | High | Low |
| Van Beijstervelft, 2008 [[164](#_ENREF_164)] | PC | 24,018 | Low | Low | Low | Low | Low |
| van der Voort, 2012 [[165](#_ENREF_165)] | PC | 5,368 | Low | Low | Low | Low | Low |
| Vandenplas, 1988 [[166](#_ENREF_166)] | PC | 75 | Unclear | Low | High | High | Unclear |
| van Merode, 2007 [[167](#_ENREF_167)] | PC | 222 | Low | Low | Low | Low | Low |
| Venter, 2009 [[168](#_ENREF_168)] | PC | 891/58 | Unclear | Low | High | High | Low |
| Watson,2013 [[169](#_ENREF_169)] | PC | 369 | Low | Low | High | High | Low |
| Wetzig, 2000 [[170](#_ENREF_170)] | PC | 475 | Low | Low | High | High | Low |
| Wilson, 1998 [[171](#_ENREF_171)] | PC | 545 | Low | Low | High | High | Low |
| Wright, 2002 [[172](#_ENREF_172)] | PC | 499 | Low | Low | Low | Low | Low |
| Wright, 1989; Wright, 1995 [[173](#_ENREF_173),[174](#_ENREF_174)]; Wright, 1999 [[175](#_ENREF_175)]; Wright, 1994 [[176](#_ENREF_176)] | PC | 988 | Unclear | Low | Low | Unclear | Low |
| Yamamoto, 2011 [[177](#_ENREF_177)] | PC | 1,344 | Unclear | Low | High | High | Low |
| Zutavern, 2006; Zutavern, 2008 [[178](#_ENREF_178),[179](#_ENREF_179)] | PC | 606 | Low | Low | Low | Low | Low |
| Friday, 2000 [[180](#_ENREF_180)] | RC | 94 | Unclear | Low | High | High | Low |
| McConnochie, 1986 [[181](#_ENREF_181)] | RC | 223 | Low | Low | Low | Low | Low |
| Monego, 1989 [[182](#_ENREF_182)] | RC | 144 | Low | Low | High | High | Low |
| Rona, 2005 [[183](#_ENREF_183)] | RC | 1,213 | Unclear | Low | Low | Unclear | Low |
| Mai, 2007 [[184](#_ENREF_184)] | NCC | 723 | Low | Low | Low | Low | Low |
| Martel, 2008 [[185](#_ENREF_185)] | NCC | 1,578 | Unclear | High | Low | High | Low |
| Maskell, 2010 [[186](#_ENREF_186)]; Oliver, 2010 [[187](#_ENREF_187)]; Munro, 2011 [[188](#_ENREF_188)] | PC; NCC | 700 | Low | Unclear | Unclear | Unclear | Low |
| Ronmark, 1999 [[189](#_ENREF_189)] | NCC | 258 | Unclear | Low | Low | Unclear | Low |
| Camara, 2003 [[190](#_ENREF_190)] | CC | 91 | Unclear | Low | High | High | Low |
| DesRoches, 2010 [[191](#_ENREF_191)] | CC | 403/202 | Unclear | Low | Unclear | Unclear | Low |
| Djenouhat, 2011 [[192](#_ENREF_192)] | CC | 450 | Unclear | Unclear | Unclear | Unclear | Low |
| Fox, 2009 [[193](#_ENREF_193)] | CC | 283/133 | Low | Unclear | High | High | Low |
| Ghaderi, 2014 [[194](#_ENREF_194)] | CC | 200 | Low | Unclear | High | High | Low |
| Haileamlak, 2005 [[195](#_ENREF_195)] | CC | 732 | Unclear | High | Low | High | Low |
| Infante-Rivard, 1993 [[196](#_ENREF_196)] | CC | 914 | Unclear | Low | Low | Unclear | Low |
| Juca, 2012 [[197](#_ENREF_197)] | CC | 590 | Low | Low | High | High | Low |
| Karunasekera, 2001 [[198](#_ENREF_198)] | CC | 582 | Unclear | Unclear | Low | Unclear | Low |
| Kramer, 1981 [[199](#_ENREF_199)] | CC | 470 | Low | Low | High | High | Low |
| Mavale-Manuel, 2003 [[200](#_ENREF_200)] | CC | 199 | High | Unclear | High | High | Low |
| Oliveti, 1995 [[201](#_ENREF_201)] | CC | 262 | Low | Unclear | High | High | Low |
| Porro, 1993 [[202](#_ENREF_202)] | CC | 465 | Unclear | Low | High | High | Low |
| Ratageri, 2000 [[203](#_ENREF_203)] | CC | 180 | Low | Low | Low | Low | Low |
| Rosas Vargas 2002 [[204](#_ENREF_204)] | CC | 148 | Unclear | Low | High | High | Low |
| Rylander 1993 [[205](#_ENREF_205)] | CC | 550 | Unclear | Low | Low | Unclear | Unclear |
| Ventura, 1988 [[206](#_ENREF_206)] | CC | 339/148 | Unclear | Unclear | High | High | Low |
| Whu, 2007 [[207](#_ENREF_207)] | CC | 261 | Low | Low | Low | Low | Unclear |
| Wickens, 2001 [[208](#_ENREF_208)] | CC | 474 | Low | Low | Low | Low | Low |
| Zhu, 2012 [[209](#_ENREF_209)] | CC | 542 | Unclear | High | High | High | Low |
| Alper, 2006 [[210](#_ENREF_210)] | CS | 858 | Unclear | Low | Low | Unclear | Low |
| Al-Kubaisy, 2005 [[211](#_ENREF_211)] | CS | 2,262 | Unclear | Low | High | High | Unclear |
| Awasthi, 2004 [[212](#_ENREF_212)] Björkstén, 2011 [[213](#_ENREF_213)] ; Flohr, 2011 [[214](#_ENREF_214)] Nagel, 2009 [[215](#_ENREF_215)]; Kuyucu, 2004  [[216](#_ENREF_216)] | CS | 2,471 | Unclear | Unclear | Low | Unclear | Low |
| Berjon, 1987 [[217](#_ENREF_217)] | CS | 2,690/148 | Unclear | Unclear | High | High | Low |
| Castro-Rodriguez, 2010 [[218](#_ENREF_218)]; Chong Neto, 2007 [[219](#_ENREF_219)] | CS | 3,003 | Low | Low | Low | Low | Low |
| Civelek, 2001[[220](#_ENREF_220)] | CS | 1533 | Unclear | Low | Low | Unclear | High |
| Ehlayel, 2008 [[221](#_ENREF_221)] | CS | 1,278 | Low | Low | Low | Low | Low |
| Ehrlich, 1996 [[222](#_ENREF_222)] | CS | 620 | Unclear | Low | Low | Unclear | Low |
| Evenhouse, 2005 [[223](#_ENREF_223)] | CS | 16,903 | Unclear | Low | Low | Unclear | Low |
| Girolomoni, 2003 [[224](#_ENREF_224)] | CS | 1,369 | Unclear | High | High | High | Low |
| Han, 2009 [[225](#_ENREF_225)] | CS | 21,371 | Unclear | High | Low | High | Low |
| Karino, 2008 [[226](#_ENREF_226)] | CS | 9,615 | Unclear | Low | Low | Unclear | Low |
| Kucukosmanoglu, 2008 [[227](#_ENREF_227)] | CS | 1,015/20 | Unclear | High | High | High | Low |
| Kuehr, 1992 [[228](#_ENREF_228)] | CS | 1,470/201 | Unclear | High | High | High | Unclear |
| Kurt, 2008 [[229](#_ENREF_229)]  Kurt, 2007 [[230](#_ENREF_230)] | CS | 25,843 | Low | Low | Low | Low | Low |
| Liu 2012 [[231](#_ENREF_231)] | CS | 8733 | Unclear | Unclear | High | High | Unclear |
| Miyake, 2003 [[232](#_ENREF_232)] | CS | 6,845 | Unclear | High | Low | High | Low |
| Nakamura, 1999 [[233](#_ENREF_233)] | CS | 3,850 | Unclear | Low | Low | Unclear | Low |
| Paton, 2012 [[234](#_ENREF_234)] | CS | 15,142/592 | Low | Unclear | High | High | Low |
| Prietsch, 2006 [[235](#_ENREF_235)] | CS | 685 | Unclear | Unclear | High | High | Unclear |
| Rusconi, 1999; Rusconi, 2005  [[236](#_ENREF_236),[237](#_ENREF_237)] | CS | 16,933 | Unclear | Low | High | High | Low |
| Rust, 2001 [[238](#_ENREF_238)] | CS | 6,783 | Unclear | Low | Low | Unclear | Low |
| Salem, 2002 [[239](#_ENREF_239)] | CS | 424 | Low | High | High | High | Low |
| Selcuk, 1997 [[240](#_ENREF_240)] | CS | 5,412 | Low | Low | Low | Low | Low |
| Suwanpromma, 2012 [[241](#_ENREF_241)] | CS | 215 | Unclear | Low | High | High | Low |
| Takemura, 2002 [[242](#_ENREF_242)] | CS | 23828 | Unclear | Low | Low | Unclear | Low |
| Tanaka, 2009 [[243](#_ENREF_243)] | CS | 1957 | Unclear | Unclear | Unclear | Unclear | Unclear |
| Visser, 2010 [[244](#_ENREF_244)] | CS | 1115 | Unclear | Low | Low | Unclear | Low |
| Wang, 2006 [[245](#_ENREF_245)] | CS | 8733 | Low | Low | High | High | Low |

CC Case Control study; CS Cross-sectional study; NCC Nested Case Control study; PC Prospective Cohort; RC Retrospective Cohort

# Table S9 Risk of bias in observational studies of breastfeeding or solid food introduction, and risk of autoimmune diseases

| **Study** | **Design** | **N/n cases** | **Assessment** | **Selection** | **Confounding** | **Overall Bias** | **Conflict of interest** |
| --- | --- | --- | --- | --- | --- | --- | --- |
| Couper, 1999 [[246](#_ENREF_246)]; Couper, 2009 [[247](#_ENREF_247)] | PC | 317/70 | Unclear | Unclear | High | High | Low |
| Frederikson, 2012 (abstract) [[248](#_ENREF_248)]; Fronczak, 2003 [[249](#_ENREF_249)]; Lamb, 2013 [[250](#_ENREF_250)]; Norris, 2003 [[251](#_ENREF_251)]; Lamb 2008 [[252](#_ENREF_252)] | PC | 1,698 | Low | Low | Low | Low | Low |
| Holmberg, 2007 [[253](#_ENREF_253)]; Karlen, 2012 [[254](#_ENREF_254)]; Wahlberg, 2006 [[255](#_ENREF_255)] | PC | 3788/~51 | Low | Low | Low | Low | Low |
| Ludvigsson, 2003 [[256](#_ENREF_256)] | PC | 205 | Unclear | Low | Low | Unclear | Unclear |
| Viner, 2008 [[257](#_ENREF_257)] | PC | 11211/61 | Unclear | High | Low | High | Low |
| Virtanen, 1992 [[258](#_ENREF_258)]; Virtanen, 1998 [[259](#_ENREF_259)]; Hypponen, 1999 [[260](#_ENREF_260)]; Virtanen, 2000 [[261](#_ENREF_261)] | PC, NCC | 697/43 | Low | Low | Low | Low | Low |
| Virtanen, 2011 [[262](#_ENREF_262)] | PC | ~4000/~160 | Unclear | Low | High | High | Low |
| Jones, 1998 [[263](#_ENREF_263)] | NCC | 518/60 | Low | High | High | High | Low |
| Kimpimaki, 2001 [[264](#_ENREF_264)] | NCC | 455/65 | Unclear | High | Low | High | Low |
| Kyvik, 1992 [[265](#_ENREF_265)] | NCC | 228/76 | Low | Unclear | High | High | Low |
| Norris, 1996 [[266](#_ENREF_266)]; | NCC | 171/18 | Low | Low | Low | Low | Low |
| Robertson, 2010 [[267](#_ENREF_267)] | NCC | 1444/361 | Low | Unclear | Low | Unclear | Low |
| Savilahti, 2009 [[268](#_ENREF_268)] | NCC | 6209/45 | Low | High | High | High | Low |
| Ahadi, 2011 [[269](#_ENREF_269)] | CC | 202/101 | Unclear | Unclear | Low | Unclear | Low |
| Alves, 2012 [[270](#_ENREF_270)] | CC | 246/123 | Unclear | Unclear | Low | Unclear | Low |
| Ashraf, 2010 [[271](#_ENREF_271)] | CC | 195/128 | Unclear | High | High | High | Low |
| Baruah, 2011 [[272](#_ENREF_272)] | CC | 86/43 | Unclear | Unclear | High | High | Low |
| Bener, 2009 [[273](#_ENREF_273)] | CC | 340 | Unclear | Unclear | High | High | Low |
| Blom, 1989 [[274](#_ENREF_274)] | CC | 867/339 | Unclear | Low | High | High | Low |
| Bodington, 1994 [[275](#_ENREF_275)] | CC | 393/209 | Unclear | High | High | High | Low |
| Borras, 2011 [[276](#_ENREF_276)] | CC | 1530/306 | Unclear | Unclear | High | High | Low |
| Dahlquist, 2002 [[277](#_ENREF_277)] | CC | 2226/610 | Unclear | Low | Low | Unclear | Low |
| Esfarjani, 2001 [[278](#_ENREF_278)] | CC | 104/52 | Low | Unclear | High | High | Low |
| Gimeno, 1997 [[279](#_ENREF_279)] | CC | 626/313 | Low | Low | Low | Low | Low |
| Hathout, 2006 [[280](#_ENREF_280)] | CC | 402/102 | Unclear | Unclear | Unclear | Unclear | Low |
| Kostraba, 1992 [[281](#_ENREF_281)]; Kostraba, 1993 [[282](#_ENREF_282)] | CC | 264/132- white; 108/54-black | Unclear | Low | High | High | Low |
| Liese, 2012 [[283](#_ENREF_283)] | CC | 709/505 | Unclear | Low | High | High | Low |
| Majeed, 2011 [[284](#_ENREF_284)] | CC | 310/96 | Low | Unclear | Low | Unclear | Low |
| Malcova, 2006 [[285](#_ENREF_285)] | CC | 2334/868 | Unclear | High | Low | High | Low |
| Marshall, 2004 [[286](#_ENREF_286)] | CC | 577/196 | Unclear | Low | High | High | Low |
| Mayer, 1988 [[287](#_ENREF_287)] | CC | 747/268 | Unclear | High | Low | High | Low |
| McKinney, 1999 [[288](#_ENREF_288)] | CC | 521/196 | Unclear | Low | Low | Unclear | Low |
| Meloni, 1997 [[289](#_ENREF_289)] | CC | 200/100 | Unclear | Unclear | Low | Unclear | Low |
| Patterson, 1994 [[290](#_ENREF_290)] | CC | 1548/258 | Low | Unclear | Low | Unclear | Low |
| Perez-Bravo, 1996 [[291](#_ENREF_291)] | CC | 165/80 | Unclear | Unclear | High | High | Low |
| Perez-Bravo, 2003 [[292](#_ENREF_292)] | CC | 250/143 | Unclear | Unclear | High | High | Low |
| Rabiei 2011 [[293](#_ENREF_293)] | CC | 300/100 | Low | Unclear | Low | Unclear | Low |
| Rami, 1999 [[294](#_ENREF_294)] | CC | 609/114 | Unclear | Unclear | High | High | Low |
| Rosenbauer, 2008 [[295](#_ENREF_295)] | CC | 2631/760 | Unclear | High | Low | High | Low |
| Sadauskaite-Kuehne, 2004 [[296](#_ENREF_296)]; Skrodeniene, 2010 [[297](#_ENREF_297)] | CC | 1944/803 | Low | Unclear | Low | Unclear | Low |
| Samuelsson, 1993 [[298](#_ENREF_298)] | CC | 1089/297 | Unclear | Low | Low | Unclear | Low |
| Siemiatycki, 1989 [[299](#_ENREF_299)] | CC | 482/161 | Unclear | Low | Low | Unclear | Low |
| Sipetic, 2005 [[300](#_ENREF_300)] | CC | 315/105 | Unclear | Low | Low | Unclear | Low |
| Soltesz, 1994 [[301](#_ENREF_301)] | CC | 305/130 | Unclear | Low | Low | Unclear | Low |
| Stene, 2000 [[302](#_ENREF_302)] | CC | 1156/85 | Low | High | Low | High | Low |
| Stene, 2003 [[303](#_ENREF_303)] | CC | 2213/545 | Unclear | Low | Low | Unclear | Low |
| Strotmeyer, 2004 [[304](#_ENREF_304)] | CC | 485/247 | Unclear | Unclear | Low | Unclear | Low |
| Tai, 1998 [[305](#_ENREF_305)] | CC | 310/117 | Unclear | Low | Low | Unclear | Low |
| Telahun, 1994 [[306](#_ENREF_306)] | CC | 129/55 | Low | Unclear | High | High | Low |
| Tenconi, 2007 [[307](#_ENREF_307)] | CC | 477/159 | Unclear | Unclear | Low | Unclear | Low |
| Thorsdottir, 2000 [[308](#_ENREF_308)] | CC | 220/55 | Low | Low | Low | Low | Low |
| Verge, 1994 [[309](#_ENREF_309)] | CC | 475/217 | Unclear | Unclear | High | High | Low |
| Virtanen, 1993 [[310](#_ENREF_310)] | CC | 1380/690 | Low | Low | Low | Low | Low |
| Visalli, 2003 [[311](#_ENREF_311)] | CC | 900/150 | Unclear | Low | Low | Unclear | Low |
| Wadsworth, 1997 [[312](#_ENREF_312)] | CC | 639/276 | Unclear | Low | Low | Unclear | Low |
| Glatthaar, 1988 [[313](#_ENREF_313)] | CS | 946/~200 | Unclear | Unclear | Low | Unclear | Low |
| Hummel, 2000 [[314](#_ENREF_314)]; Hummel, 2007 [[315](#_ENREF_315)]; Ziegler, 2003 [[316](#_ENREF_316)] | PC | 1460/~68 | Low | Low | Low | Low | Low |
| Norris, 2005 [[317](#_ENREF_317)] | PC | 1560 | Low | High | High | High | Low |
| Welander, 2010 [[318](#_ENREF_318)] | PC | 9414/~29 | Unclear | High | High | High | Low |
| Ascher, 1997 [[319](#_ENREF_319)] | CC | 81/8 | Unclear | Low | High | High | Low |
| Auricchio, 1983 [[320](#_ENREF_320)] | CC | 437/190 | Low | Unclear | High | High | Low |
| Baron, 2005 [[321](#_ENREF_321)] | CC | 444/222 | Unclear | Low | Low | Unclear | Low |
| Bergstrand, 1983 [[322](#_ENREF_322)] | CC | 616/308 | Unclear | High | High | High | Low |
| Castiglione, 2011 [[323](#_ENREF_323)] | CC | 1030/468 | Unclear | Unclear | High | High | Low |
| Corrao, 1997 [[324](#_ENREF_324)] | CC | 1252/626 | Unclear | Unclear | Low | Unclear | Low |
| Decker 2010 [[325](#_ENREF_325)] | CC | 866/123 | Unclear | Low | Low | Unclear | Low |
| Ellis, 2012 [[326](#_ENREF_326)] | CC | 655/246 | Unclear | High | Low | Unclear | Low |
| Falth-Magnusson, 1996 [[327](#_ENREF_327)] | CC | 336/72 | Low | Low | High | High | Unclear |
| Fort, 1990 [[328](#_ENREF_328)] | CC | 189/59 | Unclear | Unclear | High | High | Low |
| Gearry, 2010 [[329](#_ENREF_329)] | CC | 1253/653 | Low | High | Low | High | Low |
| Gilat, 1987 [[330](#_ENREF_330)] | CC | 1497/499 | Unclear | Unclear | Unclear | Unclear | Low |
| Greco, 1988 [[331](#_ENREF_331)] | CC | 2150/201 | Low | Low | High | High | High |
| Gruber, 1996 [[332](#_ENREF_332)] | CC | 144/54 | Low | Unclear | Low | Unclear | High |
| Hansen, 2011[[333](#_ENREF_333)] | CC | 534/267 | Unclear | High | Low | High | Low |
| Ivarsson, 2002 [[334](#_ENREF_334)] | CC | 1272/392 | Low | High | High | High | Low |
| Koletzko, 1991 [[335](#_ENREF_335)] | CC | 231/93 | Low | Low | Low | Low | Low |
| Mason, 1995 [[336](#_ENREF_336)] | CC | 133/54 | Unclear | Low | High | High | Low |
| Pacilio, 2010 [[337](#_ENREF_337)] | CC | 278/139 | Unclear | Unclear | High | High | Low |
| Peters, 2001 [[338](#_ENREF_338)] | CC | 270/133 | Low | High | Low | High | Low |
| Roberts 2009 [[339](#_ENREF_339)] | CC | 248521/ 90 | High | Unclear | High | High | Low |
| Rosenberg, 1996 [[340](#_ENREF_340)] | CC | 468/137 | Unclear | Unclear | High | High | Low |
| Sonntag, 2007 [[341](#_ENREF_341)] | CC | 1974/1096 | Low | High | High | High | Low |
| Thompson, 1999 [[342](#_ENREF_342)] | NCC | 243/27 | Low | Unclear | High | High | Low |
| Wang, 2013 [[343](#_ENREF_343)] | CC | 2616/1308 | Unclear | Unclear | High | High | Low |

CC Case Control study; CS Cross-sectional study; NCC Nested Case Control study; PC Prospective Cohort

# Table S10 Risk of bias in intervention trials of other interventions and allergic or autoimmune outcomes

| **Study** | **Design** | **N Intervention/ Control** | **Assessment** | **Selection** | **Attrition** | **Overall** | **Conflict of interest** |
| --- | --- | --- | --- | --- | --- | --- | --- |
| Falth-Magnusson, [1987](#_bookmark21) [[344](#_ENREF_344)]  Falth-Magnusson[, 1992](#_bookmark22) [[345](#_ENREF_345)]  Ludvigsson, 2003 [[346](#_ENREF_346)] | RCT | 108/104 | High | Unclear | Low | High | Low |
| Jirapinyo, 2013 [[347](#_ENREF_347)] | RCT | 30/32 | Unclear | Unclear | Low | Unclear | Low |
| Lilja, 1989 [[348](#_ENREF_348)] | RCT | 84/87 | Low | Unclear | Low | Unclear | Low |
| Hattevig, 1990 [[349](#_ENREF_349)]; Paronen, 2000 [[350](#_ENREF_350)]; Hattevig, 1999 [[351](#_ENREF_351)]; Hattevig, 1989 [[352](#_ENREF_352)]; Sigurs, 1992 [[353](#_ENREF_353)] | CCT | 54/67 | Unclear | Unclear | Low | Unclear | Unclear |
| Herrmann, 1996 [[354](#_ENREF_354)] | CCT | 50/50 | Unclear | High | Unclear | High | Low |
| Kilburn, 1998 [[355](#_ENREF_355)] | CCT | 15/96 | Unclear | High | Low | High | Low |
| Metcalfe, 2016 [[582](#_ENREF_582)] | RCT | 40, 44, 36 | Low | Low | High | High | Low |
| Becker, 2004 [[357](#_ENREF_357)]  Chan-  Yeung, 2000 [[358](#_ENREF_358)] & 2005 [[359](#_ENREF_359)];Wong, 2013 [[360](#_ENREF_360)] Protudjer, 2011 [[361](#_ENREF_361)]; Carlsten, 2013 [[362](#_ENREF_362)] | RCT | 281/268 | Low | Low | Low | Low | Low |
| Hide, 1994 [[363](#_ENREF_363)]  Hide, 1996,[[364](#_ENREF_364)]  Arshad, 1992 [[365](#_ENREF_365)]  Arshad, 2003 [[366](#_ENREF_366)]  Arshad, 2007 [[367](#_ENREF_367)]  Scott, 2012 [[368](#_ENREF_368)] | RCT | 71/68 | Low | Low | Low | Low | Low |
| Lovegrove, 1994 [[369](#_ENREF_369)] | RCT | 12/14 | Unclear | Unclear | Low | Unclear | Low |
| Shao, 2006 [[370](#_ENREF_370)] | RCT | 23/23 | Low | Unclear | Low | Unclear | Low |
| Zeiger, 1992 [[371](#_ENREF_371)]  Zeiger, 1989, [[372](#_ENREF_372)] Zeiger 1994 [[373](#_ENREF_373)] | RCT | 103/185 | Low | Low | High | High | Low |
| Halmerbauer, 2002, 2003 [[374](#_ENREF_374),[375](#_ENREF_375)] | RCT | 349/347 | Low | Unclear | Low | Unclear | Low |
| Matthew, 1977 [[376](#_ENREF_376)] | RCT | 27/35 | Low | Unclear | Low | Unclear | High |
| Poysa, 1991 [[377](#_ENREF_377)]  Poysa 1989 [[378](#_ENREF_378)]  Kuikka 1985 [[379](#_ENREF_379)] | RCT | 35/33 | Low | Unclear | Low | Unclear | Low |
| Schonberger,  2005 [[137](#_ENREF_137)] | RCT | 222/221 | Unclear | Unclear | Low | Unclear | Unclear |
| Boyle 2015 [[380](#_ENREF_380)] [[381](#_ENREF_381)]  Boyle 2016 [[382](#_ENREF_382)] | RCT | 432/431 | Low | Low | Low | Low | High |
| Gruber 2010 [[383](#_ENREF_383)]  Gruber 2015 [[384](#_ENREF_384)] | RCT | 414/ 416 | Low | Low | Unclear | Unclear | High |
| Ivakhnenko 2013 [[385](#_ENREF_385)] | RCT | 129/ 130 | Low | Low | High | High | High |
| Moro 2006 [[386](#_ENREF_386)], van Hoffen 2009 [[387](#_ENREF_387)], Arslanoglu 2008 [[388](#_ENREF_388)] Arslanoglu 2012 [[389](#_ENREF_389)] | RCT | 80/80 | Unclear | Low | High | High | Unclear |
| Sierra, 2015 [[390](#_ENREF_390)] | RCT | 188/ 177 | Low | Unclear | High | High | High |
| Ziegler 2007[[391](#_ENREF_391)] | RCT | 150/ 76 | Unclear | Unclear | Low | Unclear | High |
| Chien, 2016 [[392](#_ENREF_392)] | RCT | Unclear – outcome reported in 45 (synbiotic), 39 (prebiotic) / 45 (control) | Unclear | Unclear | Unclear | Unclear | Unclear |
| Kukkonen 2007 [[393](#_ENREF_393)] Kuitunen 2009 [[394](#_ENREF_394)] Kukkonen 2011[[395](#_ENREF_395)] | RCT | 610/ 613 | Low | Unclear | Low | Unclear | Unclear |
| Roze 2012 [[396](#_ENREF_396)] | RCT | 48/ 49 | Low | Low | Low | Low | High |
| Van der Aa 2010 [[397](#_ENREF_397)] | RCT | 46/ 44 | Low | Low | Unclear | Unclear | High |
| Abrahamsson 2007 [[398](#_ENREF_398)]  Abrahamsson 2013 [[399](#_ENREF_399)] | RCT | 117/ 115 | Unclear | Low | Low | Unclear | High |
| Allen 2012 [[400](#_ENREF_400)]  Allen 2014 [[401](#_ENREF_401)] | RCT | 220/ 234 | Unclear | Unclear | Low | Unclear | Unclear |
| Boyle 2011 [[402](#_ENREF_402)] | RCT | 125/ 125 | Low | Low | Low | Low | Low |
| Cabana, 2015 [[403](#_ENREF_403)] | RCT | 93/92 | Unclear | Unclear | Low | Unclear | Unclear |
| De Leon 2007 [[404](#_ENREF_404)] Simon 2007 [[405](#_ENREF_405)] | RCT | Total = 33 | Low | Unclear | Unclear | Unclear | Unclear |
| Dotterud 2010 [[406](#_ENREF_406)]  Simpson, 2015 [[407](#_ENREF_407)] | RCT | 211/ 204 | Low | Low | High | High | Unclear |
| Enomoto 2014 [[408](#_ENREF_408)] | CCT | 130/36 | Unclear | High | High | High | High |
| Huurre 2008 [[409](#_ENREF_409)] | RCT | 72/ 68 | Low | Unclear | Unclear | Unclear | Low |
| Kalliomaki 2001 [[410](#_ENREF_410)] Kalliomaki 2003 [[411](#_ENREF_411)] Kalliomaki 2007 [[412](#_ENREF_412)]  Rautava 2002 [[413](#_ENREF_413)] | RCT | 77/ 82 | Low | Low | Unclear | Unclear | Unclear |
| Kim 2010 [[414](#_ENREF_414)] | RCT | 57/ 55 | Low | Low | Low | Low | Unclear |
| Kopp 2008 [[415](#_ENREF_415)] | RCT | 54/ 51 | Low | Low | Low | Low | Unclear |
| Lau 2012 [[416](#_ENREF_416)] | RCT | 303/ 303 | Low | Low | Low | Low | High |
| Lodinová-Žádníková 2010 [[417](#_ENREF_417)] | RCT | 56/57 | Unclear | Unclear | Low | Unclear | Unclear |
| Lundelin, 2016 [[418](#_ENREF_418)]  Luoto, 2014 [[419](#_ENREF_419)] | RCT | 31 (prebiotic), 31 (probiotic)/ 32 (placebo) | Low | Low | Unclear | Unclear | High |
| Morisset 2008 [[420](#_ENREF_420)] | RCT | 59/ 56 | Unclear | Low | Low | Unclear | Unclear |
| Niers 2009 [[421](#_ENREF_421)]  Gorissen, 2014 [[422](#_ENREF_422)] | RCT | 78/ 78 | Low | Unclear | High | High | High |
| Ou 2012 [[423](#_ENREF_423)] | RCT | 95/ 96 | Low | Unclear | Low | Unclear | Unclear |
| Taylor 2007 [[424](#_ENREF_424)], Prescott 2008 [[425](#_ENREF_425)] Jensen 2012 [[426](#_ENREF_426)] | RCT | 115/ 111 | Low | Low | Unclear | Unclear | Low |
| Rautava 2006 [[427](#_ENREF_427)] | RCT | 38/ 43 | Unclear | Unclear | Low | Unclear | Low |
| Rautava 2012 [[428](#_ENREF_428)] | RCT | 82/ 78 | Low | Low | Low | Low | Low |
| Scalabrin 2009 [[429](#_ENREF_429)]  Scalabrin 2014 [[430](#_ENREF_430)]  Scalabrin 2017 [[431](#_ENREF_431)] | RCT | 95/ 95 | Low | Low | High | High | High |
| Soh 2009 [[432](#_ENREF_432)]  Loo 2014 [[433](#_ENREF_433)] | RCT | 127/ 126 | Low | Unclear | Low | Unclear | Unclear |
| West 2009 [[434](#_ENREF_434)]  West 2013 [[435](#_ENREF_435)] | RCT | 89/ 90 | Low | Unclear | Low | Unclear | Low |
| Wickens 2008 [[436](#_ENREF_436)]  Wickens 2012 [[437](#_ENREF_437)]  Wickens 2013 [[438](#_ENREF_438)] | RCT | 341/ 171 | Low | Low | Low | Low | Low |
| Berman, 2015 [[583](#_ENREF_583)] | RCT | Unclear – total 114 | Unclear | Unclear | Unclear | Unclear | Unclear |
| Birch, 2010 [[440](#_ENREF_440)]  Foiles, 2015 [[441](#_ENREF_441)] | RCT | 88/ 90 | Low | Unclear | High | High | High |
| Bisgaard, 2016 [[584](#_ENREF_584)] | RCT | 365/371 | Low | Low | Low | Low | Low |
| Harslof, 2014 [[443](#_ENREF_443)] | RCT | 75/ 79 | Low | Low | High | High | Low |
| Lucas, 1999 [[444](#_ENREF_444)] | RCT | 154/155 | Unclear | Low | Low | Unclear | High |
| van Gool, 2003 [[445](#_ENREF_445)] | RCT | 61/ 60 | Low | Unclear | Low | Unclear | High |
| Kitz, 2006 [[83](#_ENREF_83)] | RCT | 55/ 76 | Low | Unclear | Low | Unclear | Unclear |
| Linnamaa, 2010 [[446](#_ENREF_446)] | RCT | 151/162 | Unclear | Low | Low | Unclear | Low |
| Mihrshahi, 2003 [[447](#_ENREF_447)]  Peat, 2004 [[448](#_ENREF_448)]  Marks, 2006 [[449](#_ENREF_449)] | RCT | 312/304 | Low | Low | Low | Low | Low |
| Damsgaard, 2007 [[450](#_ENREF_450)] | RCT | 45/ 49 | Low | Low | High | High | Low |
| Palmer, 2012  & 2013 [[451](#_ENREF_451),[452](#_ENREF_452)]  Best, 2015 & 2016 [[453](#_ENREF_453),[454](#_ENREF_454)] | RCT | 368/338 | Low | Low | Low | Low | Low |
| Dunstan, 2003 [[455](#_ENREF_455)] | RCT | 52/ 46 | Low | Low | Low | Low | Low |
| D'Vaz, 2012 [[456](#_ENREF_456)] | RCT | 218/202 | High | Low | Unclear | High | Low |
| Furuhjelm, 2009 [[457](#_ENREF_457)]  Furuhjelm, 2011 [[458](#_ENREF_458)] | RCT | 70/ 75 | Low | Low | Low | Low | Unclear |
| Lauritzen, 2005 [[459](#_ENREF_459)] | RCT | 62/ 60 | Unclear | Unclear | High | High | Low |
| Olsen, 2008 [[460](#_ENREF_460)]  Hansen, 2017 [[461](#_ENREF_461)] | RCT | 266/267 | Unclear | Unclear | Low | Unclear | Low |
| Dotterud, 2013 [[462](#_ENREF_462)] | RCT | 2,860/5,743 | Low | High | Unclear | High | Low |
| Imhoff- Kunsch, 2011 [[463](#_ENREF_463)] | RCT | 547/547 | Low | Low | Low | Low | Low |
| Noakes, 2012 [[464](#_ENREF_464)] | RCT | 62/61 | Low | Low | Unclear | Unclear | Low |
| Aage, 2015 [[465](#_ENREF_465)] | RCT | 2145/ 2200 | Low | Low | High | High | Low |
| Chawes, 2016 [[442](#_ENREF_442)] | RCT | 315/308 | Low | Low | Low | Low | Low |
| Checkley, 2010 [[466](#_ENREF_466)]  Checkley, 2011 [[467](#_ENREF_467)] | cluster  RCT | 803, 885, 771 | Low | Unclear | High | High | Low |
| Czeizel, 1994 [[468](#_ENREF_468)]; Dobo, 1998 [[469](#_ENREF_469)] | RCT | 2090/2032 | Low | Unclear | Low | Unclear | Low |
| Devakumar, 2015 [[470](#_ENREF_470)] | RCT | 600/600 | Low | Low | High | High | Unclear |
| Grant, 2016 [[471](#_ENREF_471)] | RCT | 173/ 87 | Low | Low | Low | Low | Low |
| Greenough, 2010 [[472](#_ENREF_472)] | RCT | 1199/1205 | Unclear | Unclear | High | High | Low |
| Goldring, 2013 [[473](#_ENREF_473)] | RCT | 120/60 | Low | Low | Low | Low | Low |
| Kiraly, 2013 [[474](#_ENREF_474)] | RCT | 227/235 | Unclear | Low | High | High | Low |
| Litonjua, 2016 [[475](#_ENREF_475)] | RCT | Unclear – 881 total | Low | Low | Low | Low | Low |
| McEvoy, 2014[[476](#_ENREF_476)] | RCT | 89/90 | Low | Low | Low | Low | Low |

RCT Randomised Controlled Trial; CCT Controlled Clinical Trial

# Table S11 Risk of bias in observational studies of other maternal or infant dietary exposures and risk of allergic outcomes

| **Study** | **Design** | **N/n cases** | **Assessment** | **Selection** | **Confounding** | **Overall Bias** | **Conflict of interest** |
| --- | --- | --- | --- | --- | --- | --- | --- |
| Alm, 2009 [[6](#_ENREF_6)]; Goksor, 2011; [[477](#_ENREF_477)] Alm, 2012;[[478](#_ENREF_478)] | PC | 4941 | Low | High | Low | High | Low |
| Andreasyan, 2007; [[479](#_ENREF_479)] | PC | 498 | Unclear | Low | Unclear | Unclear | Low |
| Back, 2009 [[480](#_ENREF_480)] | PC | 123 | Low | High | Low | High | Unclear |
| Baiz, 2013[[481](#_ENREF_481)] | PC | 239 | Low | Low | Low | Low | Unclear |
| Bekkers, 2012 [[482](#_ENREF_482)]; Willers, 2008 [[483](#_ENREF_483)] | PC | 3786 | Unclear | Low | Low | Unclear | Low |
| ;Bertelsen, 2013 [[484](#_ENREF_484)] | PC | 54,740 | Unclear | Unclear | Low | Unclear | Unclear |
| Bisgaard, 2009 [[17](#_ENREF_17)] | PC | 354 | Low | Low | Low | Low | Low |
| Carmargo, 2010 [[485](#_ENREF_485)] | PC | 823 | Low | Low | Low | Low | Low |
| Romieu, 2007; [[486](#_ENREF_486)]  Chatzi, 2008 [[487](#_ENREF_487)] | PC | 468 | Low | Low | Low | Low | Low |
| de Jong, 2012 [[488](#_ENREF_488)]; De Jong, 2012 [[489](#_ENREF_489)]; Leermakers, 2013; [[490](#_ENREF_490)] | PC | 7,210 | Low | Low | Low | Low | Low |
| Dubakiene, 2012 [[491](#_ENREF_491)]; Butiene, 2011[[492](#_ENREF_492)];  Oliver, 2010; [[187](#_ENREF_187)] Grimshaw, 2012 [[493](#_ENREF_493)] | PC, NCC | 128 | Low | Low | Unclear | Unclear | Low |
| Dunlop, 2006 [[494](#_ENREF_494)] | PC | 1326 | Low | High | Low | High | Low |
| Fergusson, 1990 [[495](#_ENREF_495)] | PC | 1,067 | Low | Low | High | High | Low |
| Fitzsimon, 2007 [[496](#_ENREF_496)] | PC | 631 | Low | Low | Low | Low | Low |
| Gale, 2008 [[497](#_ENREF_497)] | PC | 440 | Low | Low | Unclear | Unclear | Low |
| Harris, 2001; [[56](#_ENREF_56)] Zutavern, 2004 [[57](#_ENREF_57)] | PC | 604,622 | Low | Low | Low | Low | Low |
| Hesselmar, 2010 [[61](#_ENREF_61)] | PC | 184 | Low | Low | High | High | Low |
| Hypponen, 2004; [[498](#_ENREF_498)]  Hypponen, 2001 [[499](#_ENREF_499)] | PC | 10,366/81 | Unclear | Unclear | Low | Unclear | Low |
| Hoppu, 2000 [[67](#_ENREF_67)] | PC | 115 | Low | Low | Unclear | Unclear | Low |
| Jedrychowski, 2008 [[500](#_ENREF_500)]; Jedrychowski 2011 [[501](#_ENREF_501)] | PC | 469 | Low | Low | Low | Low | Low |
| Kemp, 2011 [[502](#_ENREF_502)] | PC | 310 | Unclear | High | Low | High | Low |
| Kull, 2006; [[503](#_ENREF_503)] Magnusson, 2013 [[504](#_ENREF_504)]; | PC | 3,230 | Low | Low | Low | Low | Low |
| Martindale, 2005; [[505](#_ENREF_505)] Devereux, 2006; [[37](#_ENREF_37)] Devereux, 2007 [[506](#_ENREF_506)]; Willers, 2007[[507](#_ENREF_507)] | PC | 3,230 | Low | Low | Low | Low | Low |
| Lack, 2003 [[43](#_ENREF_43)];  Shaheen, 2009 [[508](#_ENREF_508)]; Wills, 2013[[509](#_ENREF_509)]; Granell, 2008 [[510](#_ENREF_510)] | PC; NCC | 11,352 | Low | Low | Low | Low | Low |
| Laitinen, 2005 [[511](#_ENREF_511)] | PC | 95 | Unclear | Unclear | Low | Unclear | Low |
| Lange, 2010 [[512](#_ENREF_512)];  Litonjua, 2006; [[513](#_ENREF_513)] Camargo, 2007 [[514](#_ENREF_514)] | PC | 1,376 | Low | Low | Low | Low | Low |
| Liu, 2011 [[515](#_ENREF_515)] | PC | 649 | Low | Unclear | Low | Unclear | Low |
| Marini, 1996 [[91](#_ENREF_91)] | PC | 68 | Unclear | Low | Low | Unclear | Unclear |
| Mommers, 2009; [[516](#_ENREF_516)] Magdelijn, 2011; [[517](#_ENREF_517)] Cremers, 2011[[518](#_ENREF_518)] | PC | 2,465 | Low | Low | Low | Low | Low |
| Maslova, 2012 [[519](#_ENREF_519)]; Maslova, 2013 [[520](#_ENREF_520)] [[521](#_ENREF_521)]; Linneberg, 2004; [[522](#_ENREF_522)] | PC | 28,758 | Low | Low | Low | Low | Low |
| Magnus, 2013 [[523](#_ENREF_523)] Haberg, 2009 [[524](#_ENREF_524)] | NCC, PC | 32,077 | Low | Unclear | Low | Unclear | Low |
| Milner, 2004 [[95](#_ENREF_95)] | PC | 8,073 | Low | Unclear | Low | Unclear | Low |
| Miskelly, 1988 [[96](#_ENREF_96)] | PC | 482 | Low | Low | High | High | Unclear |
| Miyake, 2009 [[525](#_ENREF_525)] and 2010 [[526-528](#_ENREF_526)] and 2011 [[529](#_ENREF_529),[530](#_ENREF_530)]; Satio, 2010 [[526](#_ENREF_526)] | PC | 763 | Low | Low | Low | Low | Low |
| Morales, 2012 [[531](#_ENREF_531)] | PC | 1,724 | Unclear | Low | Low | Unclear | Low |
| Morgan, 2004 [[100](#_ENREF_100)] | PC | 257 | Unclear | Low | Low | Unclear | Unclear |
| Narita, 2011; [[532](#_ENREF_532)] Ohya, 2011 [[533](#_ENREF_533)] | PC | 1,463 | Unclear | Unclear | Unclear | Unclear | Low |
| Nwaru, 2010, 2011, 2012 and 2013 [[105](#_ENREF_105),[106](#_ENREF_106),[534](#_ENREF_534),[535](#_ENREF_535)] [[536](#_ENREF_536),[537](#_ENREF_537)];  Niinisto, 2012 [[538](#_ENREF_538)]; Lumia, 2011; [[539](#_ENREF_539)] Erkkola, 2012; [[107](#_ENREF_107)]; Maijaliisa, 2011; [[540](#_ENREF_540)]; Uusitalo 2008 [[541](#_ENREF_541)]  Erkkola, 2009; [[542](#_ENREF_542)] Marjamaki, 2010; [[543](#_ENREF_543)] | PC | 5,619 | Low | Low | Low | Low | Low |
| Oien, 2010 [[544](#_ENREF_544)] | PC | 3,067 | Low | Low | Low | Low | Low |
| Pike, 2012 [[545](#_ENREF_545)] | PC | 739 | Low | Low | Low | Low | Low |
| Roduit, 2012 [[546](#_ENREF_546)] | PC | 1,041 | Low | Low | Low | Low | Unclear |
| Rothers, 2011 [[547](#_ENREF_547)] | PC | 208 | Low | Low | Low | Low | Low |
| Sausenthaler, 2007 [[548](#_ENREF_548)]; Zutavern, 2006; [[178](#_ENREF_178)] Zutavern, 2008; [[179](#_ENREF_179)] | PC | 2,540 | Low | Low | Low | Low | Low |
| Sicherer, 2010 [[138](#_ENREF_138)] | PC | 503 | Low | Low | Low | Low | Unclear |
| Strassburger, 2010 [[155](#_ENREF_155)] | PC | 338 | Low | Low | Low | Low | Low |
| Venter, 2009; [[168](#_ENREF_168)] Dean, 2007 [[549](#_ENREF_549)] | PC | 937 | Low | Low | High | High | Low |
| Wang, 2007 [[144](#_ENREF_144)] | PC | 1,760 | Low | Low | Low | Low | Low |
| Weisse, 2012 [[550](#_ENREF_550)] | PC | 272 | Low | High | Low | High | Low |
| West, 2012 [[551](#_ENREF_551)] | PC | 319 | Low | High | Low | High | Low |
| Whitrow, 2009 [[552](#_ENREF_552)] | PC | 490 | Low | Low | Low | Low | Low |
| Calvani, 2006 [[553](#_ENREF_553)] | RC | 988 | Unclear | Low | Low | Unclear | Low |
| Jones, 2012 [[554](#_ENREF_554)] | RC | 231 | Unclear | Low | Low | Unclear | Low |
| Allen, 2013 [[555](#_ENREF_555)] | NCC | 2,758/240 | Low | Low | Low | Low | Low |
| Sariachvili, 2010 [[60](#_ENREF_60)] | NCC | 557/252 | Low | Low | Low | Low | Unclear |
| Binkley, 2011 [[556](#_ENREF_556)] | CC | 1,413/1,300 | Unclear | Unclear | High | High | Low |
| Dai, 1993 [[557](#_ENREF_557)] | CC | 70 | Unclear | Unclear | High | High | Unclear |
| DesRoches, 2010 [[191](#_ENREF_191)] | CC | 401/202 | Low | Low | Low | Low | Low |
| Fox, 2009 [[193](#_ENREF_193)] | CC | 293/133 | Low | Unclear | Unclear | Unclear | Unclear |
| Lopez Campos, 2001 [[558](#_ENREF_558)] | CC | 75/58 | Unclear | Unclear | Unclear | Unclear | Unclear |
| Mullins, 2012 [[559](#_ENREF_559)] | CC | 115/115 | Low | Unclear | Low | Unclear | Low |
| Oliveti, 1995 [[201](#_ENREF_201)] | CC | 263/131 | Low | Unclear | Low | Unclear | Low |
| Salam, 2005 [[560](#_ENREF_560)] | CC | 691/ 279 | Unclear | Unclear | Low | Unclear | Low |
| Castro-Rodriguez, 2010 [[218](#_ENREF_218)] | CS | 1,409/ 594 | Unclear | High | Low | High | Low |
| de Batlle, 2008 [[561](#_ENREF_561)] | CS | 1,476/402 | Unclear | High | Low | High | Low |
| Dela Bianca, 2012 [[562](#_ENREF_562)] | CS | 467 | Low | Unclear | Low | Unclear | Low |
| Riedler, 2001 [[563](#_ENREF_563)] | CS | 812 | Unclear | High | Low | High | Low |

CC Case Control study; CS Cross-sectional study; NCC Nested Case Control study; PC Prospective Cohort; RC Retrospective Cohort

# Table S12 Risk of bias in observational studies of other maternal or infant dietary exposures and risk of autoimmune disease

| **Study** | **Design** | **N/n cases** | **Assessment** | **Selection** | **Confounding** | **Overall Bias** | **Conflict of interest** |
| --- | --- | --- | --- | --- | --- | --- | --- |
| Brekke, 2007 [[564](#_ENREF_564)]  Brekke, 2010 [[565](#_ENREF_565)]; Wahlberg, 2006; [[566](#_ENREF_566)] | PC | 8694 | Low | Low | Low | Low | Low |
| Fronczak, 2003 [[249](#_ENREF_249)]; Lamb 2008 [[252](#_ENREF_252)]; Simpson, 2011[[567](#_ENREF_567)]; Norris, 1996 [[568](#_ENREF_568)]; Frederikson, 2012 [[248](#_ENREF_248)]; Lamb, 2013; [[250](#_ENREF_250)] | PC; NCC | 222,222/  16  1698/49 | Low | Low | Low | Low | Low |
| Hypponen, 2004 [[498](#_ENREF_498)]  Hypponen, 2001 [[499](#_ENREF_499)] | PC | 10,366/81 | Unclear | Low | Low | Unclear | Low |
| Nwaru, 2010, 2011, 2012 and 2013 [[105](#_ENREF_105),[535](#_ENREF_535),[537](#_ENREF_537)] [[534](#_ENREF_534)] [[106](#_ENREF_106)]; Niinisto, 2012 [[538](#_ENREF_538)]; Lumia, 2011 [[539](#_ENREF_539)] ; Erkkola, 2012 [[107](#_ENREF_107)]; Virtanen, 2006 and 2011 [[569](#_ENREF_569),[570](#_ENREF_570)]; Uusitalo, 2008; [[541](#_ENREF_541)] | PC | 3730 | Low | Low | Low | Low | Low |
| Harsunen, 2012 [[571](#_ENREF_571)] | NCC | 33/33 | Unclear | Unclear | Low | Unclear | Low |
| Miettinen, 2012 [[572](#_ENREF_572)] | NCC | 686/343 | Low | Unclear | Low | Unclear | Low |
| Savilahti, 2009 [[268](#_ENREF_268)] | NCC | 6209/45 | Low | Unclear | High | High | Low |
| Sørensen, 2012 [[573](#_ENREF_573)] | NCC | 328/109 | Low | Low | Low | Low | Low |
| Ahadi, 2011 [[269](#_ENREF_269)] | CC | 202/101 | Unclear | Unclear | Low | Unclear | Low |
| Ashraf, 2010 [[271](#_ENREF_271)] | CC | 195 | Unclear | High | Unclear | High | Low |
| Baron, 2005 [[321](#_ENREF_321)] | CC | 444/222 | Unclear | Low | Low | Unclear | Low |
| Ellis, 2012 [[326](#_ENREF_326)] | CC | 655/246 | Unclear | Unclear | Low | Unclear | Low |
| Bener, 2009 [[273](#_ENREF_273)] | CC | 340/170 | Unclear | Low | Low | Unclear | Low |
| EURODIAB substudy 2 study group, 1999 [[574](#_ENREF_574)] | CC | 2934/746 | Low | Low | Low | Low | Low |
| Gilat, 1987 [[330](#_ENREF_330)] | CC | 504/167 | Low | Unclear | Low | Unclear | Low |
| Majeed, 2011 [[284](#_ENREF_284)] | CC | 395/96 | Unclear | Unclear | Unclear | Unclear | Unclear |
| Malcova, 2005 [[285](#_ENREF_285)] | CC | 2334/868 | Unclear | Low | Unclear | Unclear | Low |
| Rosenbauer, 2007 and 2008 [[295](#_ENREF_295),[575](#_ENREF_575)] | CC | 2631/760 | Low | Unclear | Low | Unclear | Low |
| Sipetic, 2003 [[576](#_ENREF_576)]; Sipetic, 2005 [[577](#_ENREF_577)] | CC | 315/105 | Low | Unclear | High | High | Low |
| Stene, 2003 [[578](#_ENREF_578)], Stene, 2008 [[579](#_ENREF_579)] | CC | 2213/545 | Unclear | High | Low | High | Unclear |
| Stene, 2000 [[302](#_ENREF_302)] | CC | 1131/84 | Unclear | High | Low | High | Low |
| Svensson, 2005 [[580](#_ENREF_580)] | CC | 1152/475 | Low | Low | Low | Low | Low |
| Strotmeyer, 2004; [[304](#_ENREF_304)] | CC | 688/247 | Unclear | Unclear | Low | Unclear | Low |
| Tenconi, 2007 [[307](#_ENREF_307)] | CC | 429/131 | Unclear | Unclear | Low | Unclear | Unclear |
| Virtanen, 1994 [[581](#_ENREF_581)] | CC | 1136/600 | Unclear | Low | High | High | Low |
| Visalli, 2003 [[311](#_ENREF_311)] | CC | 900/150 | Unclear | Low | Low | Unclear | Low |

CC Case Control study; NCC Nested Case Control study; PC Prospective Cohort; RC Retrospective Cohort

# References

1. Kramer MS, Chalmers B, Hodnett ED, Sevkovskaya Z, Dzikovich I, et al. (2001) Promotion of breastfeeding intervation trial (PROBIT): A randomized trial in the Republic of Belarus. Journal of the American Medical Association. pp. 413-420.

2. Kramer MS, Matush L, Vanilovich I, Platt R, Bogdanovich N, et al. (2007) Effect of prolonged and exclusive breast feeding on risk of allergy and asthma: cluster randomised trial. BMJ (Clinical research ed) 335: 815.

3. Alho OP, Koivu M, Sorri M, Rantakallio P (1990) Risk factors for recurrent acute otitis media and respiratory infection in infancy. International Journal of Pediatric Otorhinolaryngology 19: 151-161.

4. Allen KJ, Koplin J, Gurrin L, Gibson M, Thiele L, et al. (2009) Prevalence and environmental predictors of food allergy in infants. Journal of Allergy and Clinical Immunology 1): S108.

5. Koplin J, Osborne N, Martin P, Gurrin L, Robinson M, et al. (2010) Does age of introduction of foods affect the risk of having egg allergy? A population-based study of an infant cohort. Allergy: European Journal of Allergy and Clinical Immunology 65: 312.

6. Alm B, Aberg N, Erdes L, Mollborg P, Pettersson R, et al. (2009) Early introduction of fish decreases the risk of eczema in infants. Archives of Disease in Childhood 94: 11-15.

7. Goksor E, Alm B, Thengilsdottir H, Erdes L, Mollborg P, et al. (2009) Neonatal antibiotic treatment is a risk factor for multiple trigger wheeze at age 41/2 years. Pediatric Allergy and Immunology 20: 17.

8. Alm B, Erdes L, Mollborg P, Pettersson R, Norvenius SG, et al. (2008) Neonatal antibiotic treatment is a risk factor for early wheezing. Pediatrics 121: 697-702.

9. Bacopoulou F, Veltsista A, Vassi I, Gika A, Lekea V, et al. (2009) Can we be optimistic about asthma in childhood? A Greek cohort study. Journal of Asthma 46: 171-174.

10. Benn CS, Wohlfahrt J, Aaby P, Westergaard T, Benfeldt E, et al. (2004) Breastfeeding and risk of atopic dermatitis, by parental history of allergy, during the first 18 months of life. American Journal of Epidemiology 160: 217-223.

11. Linneberg A, Simonsen JB, Petersen J, Stensballe LG, Benn CS (2006) Differential effects of risk factors on infant wheeze and atopic dermatitis emphasize a different etiology. Journal of Allergy & Clinical Immunology 117: 184-189.

12. Bergmann RL, Edenharter G, Bergmann KE, Lau S, Wahn U (2000) Socioeconomic status is a risk factor for allergy in parents but not in their children. Clinical & Experimental Allergy 30: 1740-1745.

13. Bergmann RL, Diepgen TL, Kuss O, Bergmann KE, Kujat J, et al. (2002) Breastfeeding duration is a risk factor for atopic eczema. Clinical and Experimental Allergy 32: 205-209.

14. Kulig M, Klettke U, Wahn V, Forster J, Bauer CP, et al. (2000) Development of seasonal allergic rhinitis during the first 7 years of life. Journal of Allergy & Clinical Immunology 106: 832-839.

15. Berth-Jones J, George S, Graham-Brown RA (1997) Predictors of atopic dermatitis in Leicester children. British Journal of Dermatology 136: 498-501.

16. Besednjak-Kocijancic L (2010) Is longer exclusive breastfeeding associated with lover prevalence of asthma, atopic dermatitis and atopic sensitisation in 1 and 5-year-old Slovene children? Allergy: European Journal of Allergy and Clinical Immunology 65: 311-312.

17. Bisgaard H, Halkjaer LB, Hinge R, Giwercman C, Palmer C, et al. (2009) Risk analysis of early childhood eczema. Journal of Allergy & Clinical Immunology 123: 1355-1360.e1355.

18. Giwercman C, Halkjaer LB, Jensen SM, Bonnelykke K, Lauritzen L, et al. (2010) Increased risk of eczema but reduced risk of early wheezy disorder from exclusive breast-feeding in high-risk infants. Journal of Allergy & Clinical Immunology 125: 866-871.

19. Burr ML, Miskelly FG, Butland BK, Merrett TG, Vaughan-Williams E (1989) Environmental factors and symptoms in infants at high risk of allergy. Journal of Epidemiology & Community Health 43: 125-132.

20. Burr ML, Limb ES, Maguire MJ, Amarah L, Eldridge BA, et al. (1993) INFANT-FEEDING, WHEEZING, AND ALLERGY - A PROSPECTIVE-STUDY. Archives of Disease in Childhood 68: 724-728.

21. Burr ML, Limb ES, Maguire MJ, Amarah L, Eldridge BA, et al. (1993) Infant feeding, wheezing, and allergy: a prospective study. Archives of Disease in Childhood 68: 724-728.

22. Burr ML, Merrett TG, Dunstan FDJ, Maguire MJ (1997) The development of allergy in high-risk children. Clinical and Experimental Allergy 27: 1247-1253.

23. Burgess SW, Dakin CJ, O'Callaghan MJ (2006) Breastfeeding does not increase the risk of asthma at 14 years. Pediatrics 117: e787-792.

24. Businco L, Cantani A, Meglio P, Bruno G (1987) Prevention of atopy: Results of a long-term (7 months to 8 years) follow-up. Annals of Allergy 59: 183-186.

25. Bruno G, Cantani A, Ragno V, Milita O, Ziruolo G, et al. (1995) Natural history of IgE antibodies in children at risk for atopy. Annals of Allergy, Asthma and Immunology 74: 431-436.

26. Cano Garcinuno A, Perez Garcia I, Garcia Puertas J, Casas Rodriguez P (2003) [Tobacco, infant feeding, and wheezing in the first three years of life]. Anales de Pediatria 59: 541-547.

27. Scholtens S, Wijga AH, Brunekreef B, Kerkhof M, Hoekstra MO, et al. (2009) Breast feeding, parental allergy and asthma in children followed for 8 years. The PIAMA birth cohort study. Thorax 64: 604-609.

28. Caudri D, Savenije O, Smit HA, Postma DS, Koppelman G, et al. (2010) The relation between perinatal factors and phenotypes of wheeze in the first 8 years of life. American Journal of Respiratory and Critical Care Medicine 181 (1 MeetingAbstracts).

29. Kerkhof M, Koopman LP, van Strien RT, Wijga A, Smit HA, et al. (2003) Risk factors for atopic dermatitis in infants at high risk of allergy: the PIAMA study. Clinical and Experimental Allergy 33: 1336-1341.

30. Cogswell JJ, Mitchell EB, Alexander J (1987) Parental smoking, breast feeding, and respiratory infection in development of allergic diseases. Archives of Disease in Childhood 62: 338-344.

31. Chuang CH, Hsieh WS, Chen YC, Chang PJ, Hurng BS, et al. (2011) Infant feeding practices and physician diagnosed atopic dermatitis: a prospective cohort study in Taiwan. Pediatric Allergy & Immunology 22: 43-49.

32. Menezes AM, Lima RC, Minten GC, Hallal PC, Victora CG, et al. (2008) [Prevalence of wheezing in the chest among adults from the 1982 Pelotas birth cohort, Southern Brazil]. Revista de Saude Publica 42 Suppl 2: 101-107.

33. da Costa Lima R, Victora CG, Menezes AM, Barros FC (2003) Do risk factors for childhood infections and malnutrition protect against asthma? A study of Brazilian male adolescents. American Journal of Public Health 93: 1858-1864.

34. Midodzi WK, Rowe BH, Majaesic CM, Saunders LD, Senthilselvan A (2008) Predictors for wheezing phenotypes in the first decade of life. Respirology 13: 537-545.

35. Dell S, To T (2001) Breastfeeding and asthma in young children: findings from a population-based study. Archives of Pediatrics & Adolescent Medicine 155: 1261-1265.

36. Midodzi WK, Rowe BH, Majaesic CM, Saunders LD, Senthilselvan A (2010) Early life factors associated with incidence of physician-diagnosed asthma in preschool children: results from the Canadian Early Childhood Development cohort study. Journal of Asthma 47: 7-13.

37. Devereux G, Turner SW, Craig LC, McNeill G, Martindale S, et al. (2006) Low maternal vitamin E intake during pregnancy is associated with asthma in 5-year-old children. American Journal of Respiratory & Critical Care Medicine 174: 499-507.

38. Dogaru CM, Strippoli MP, Spycher BD, Frey U, Beardsmore CS, et al. (2012) Breastfeeding and lung function at school age: does maternal asthma modify the effect? American Journal of Respiratory & Critical Care Medicine 185: 874-880.

39. Elliott L, Henderson J, Northstone K, Chiu GY, Dunson D, et al. (2008) Prospective study of breast-feeding in relation to wheeze, atopy, and bronchial hyperresponsiveness in the Avon Longitudinal Study of Parents and Children (ALSPAC). Journal of Allergy & Clinical Immunology 122: 49-54, 54.e41-43.

40. Granell R, Sterne JAC, Henderson J (2012) Associations of Different Phenotypes of Wheezing Illness in Early Childhood with Environmental Variables Implicated in the Aetiology of Asthma. PLoS ONE 7.

41. Sherriff A, Peters TJ, Henderson J, Strachan D, Team AS (2001) Risk factor associations with wheezing patterns in children followed longitudinally from birth to 3 1/2 years. International Journal of Epidemiology 30: 1473-1484.

42. Abd A, Henderson J, Northstone K, Seddon P, Palmer C, et al. (2012) Breastfeeding duration and eczema risk in the avon longitudinal study of parents and children. Archives of Disease in Childhood 97: A43.

43. Lack G, Fox D, Northstone K, Golding J, Avon Longitudinal Study of P, et al. (2003) Factors associated with the development of peanut allergy in childhood. New England Journal of Medicine 348: 977-985.

44. Eneli IU, Karmaus WK, Davis S, Kuehr J (2006) Airway hyperresponsiveness and body mass index: the Child Health and Environment Cohort Study in Hesse, Germany. Pediatric Pulmonology 41: 530-537.

45. Farooqi IS, Hopkin JM (1998) Early childhood infection and atopic disorder. Thorax 53: 927-932.

46. Fergusson DM, Horwood LJ, Shannon FT (1983) Asthma and infant diet. Archives of Disease in Childhood 58: 48-51.

47. Horwood LJ, Fergusson DM, Shannon FT (1985) Social and familial factors in the development of early childhood asthma. Pediatrics 75: 859-868.

48. Fredriksson P, Jaakkola N, Jaakkola JJ (2007) Breastfeeding and childhood asthma: a six-year population-based cohort study. BMC Pediatrics 7: 39.

49. Forster J, Dungs M, Wais U, Urbanek R (1990) [Atopy-suggesting symptoms in the first 2 years of life. Effect of gestational age, nutrition and social class]. Klinische Padiatrie 202: 136-140.

50. Galbally M, Lewis AJ, McEgan K, Scalzo K, Islam FA (2013) Breastfeeding and infant sleep patterns: an Australian population study. Journal of Paediatrics & Child Health 49: E147-152.

51. Gruber C, van Stuijvenberg M, Mosca F, Moro G, Chirico G, et al. (2010) Reduced occurrence of early atopic dermatitis because of immunoactive prebiotics among low-atopy-risk infants. Journal of Allergy & Clinical Immunology 126: 791-797.

52. Gruskay FL (1982) Comparison of breast, cow, and soy feedings in the prevention of onset of allergic disease: a 15-year prospective study. Clinical Pediatrics 21: 486-491.

53. Guida F, Clarisse B, Momas I (2009) Risk factors of wheezing onset during infancy in the Paris birth cohort: Benefits of the cox model. Allergy: European Journal of Allergy and Clinical Immunology 64: 202.

54. Gustafsson D, Sjoberg O, Foucard T (2000) Development of allergies and asthma in infants and young children with atopic dermatitis--a prospective follow-up to 7 years of age. Allergy 55: 240-245.

55. Halken S, Host A, Husby S, Hansen LG, Osterballe O, et al. (1991) Recurrent wheezing in relation to environmental risk factors in infancy. A prospective study of 276 infants. Allergy 46: 507-514.

56. Harris JM, Cullinan P, Williams HC, Mills P, Moffat S, et al. (2001) Environmental associations with eczema in early life. British Journal of Dermatology 144: 795-802.

57. Zutavern A, von Mutius E, Harris J, Mills P, Moffatt S, et al. (2004) The introduction of solids in relation to asthma and eczema. Archives of Disease in Childhood 89: 303-308.

58. Hagendorens MM, Bridts CH, Lauwers K, van Nuijs S, Ebo DG, et al. (2005) Perinatal risk factors for sensitization, atopic dermatitis and wheezing during the first year of life (PIPO study). Clinical and Experimental Allergy 35: 733-740.

59. Sariachvili M, Droste J, Dom S, Wieringa M, Vellinga A, et al. (2007) Is breast feeding a risk factor for eczema during the first year of life? Pediatric Allergy & Immunology 18: 410-417.

60. Sariachvili M, Droste J, Dom S, Wieringa M, Hagendorens M, et al. (2010) Early exposure to solid foods and the development of eczema in children up to 4 years of age. Pediatric Allergy & Immunology 21: 74-81.

61. Hesselmar B, Saalman R, Rudin A, Adlerberth I, Wold A (2010) Early fish introduction is associated with less eczema, but not sensitization, in infants. Acta Paediatrica 99: 1861-1867.

62. Hetzner NMP, Razza RA, Malone LM, Brooks-Gunn J (2009) Associations among feeding behaviors during infancy and child illness at two years. Maternal and Child Health Journal 13: 795-805.

63. Hide DW, Guyer BM (1981) Clinical manifestations of allergy related to breast and cows' milk feeding. Archives of Disease in Childhood 56: 172-175.

64. Arshad SH, Hide DW (1992) Effect of environmental factors on the development of allergic disorders in infancy. Journal of Allergy & Clinical Immunology 90: 235-241.

65. Hikino S, Nakayama H, Yamamoto J, Kinukawa N, Sakamoto M, et al. (2001) Food allergy and atopic dermatitis in low birthweight infants during early childhood. Acta Paediatrica 90: 850-855.

66. Hong X, Wang G, Liu X, Kumar R, Tsai HJ, et al. (2011) Gene polymorphisms, breast-feeding, and development of food sensitization in early childhood. Journal of Allergy & Clinical Immunology 128: 374-381.e372.

67. Hoppu U, Kalliomaki M, Isolauri E (2000) Maternal diet rich in saturated fat during breastfeeding is associated with atopic sensitization of the infant. European Journal of Clinical Nutrition 54: 702-705.

68. Host A (1991) Importance of the first meal on the development of cow's milk allergy and intolerance. Allergy Proceedings 12: 227-232.

69. Howie PW, Forsyth JS, Ogston SA, Clark A, Florey CD (1990) Protective effect of breast feeding against infection. BMJ 300: 11-16.

70. Huang H, Zhang FY, Hang JQ, Zhu J, Wang R, et al. (2013) [Cohort study of 684 pairs of mother-and-child allergic diseases]. Zhonghua Erke Zazhi 51: 168-171.

71. Huurre A, Laitinen K, Rautava S, Korkeamäki M, Isolauri E (2008) Impact of maternal atopy and probiotic supplementation during pregnancy on infant sensitization: a double-blind placebo-controlled study. Clinical and experimental allergy : journal of the British Society for Allergy and Clinical Immunology. pp. 1342-1348.

72. Joseph CL, Ownby DR, Havstad SL, Woodcroft KJ, Wegienka G, et al. (2011) Early complementary feeding and risk of food sensitization in a birth cohort. Journal of Allergy & Clinical Immunology 127: 1203-1210.e1205.

73. Juto P, Bjorksten B (1980) Serum IgE in infants and influence of type of feeding. Clinical Allergy 10: 593-600.

74. Kajosaari M (1991) Atopy prophylaxis in high-risk infants. Prospective 5-year follow-up study of children with six months exclusive breastfeeding and solid food elimination. Advances in Experimental Medicine & Biology 310: 453-458.

75. Karmaus W, Dobai AL, Ogbuanu I, Arshard SH, Matthews S, et al. (2008) Long-term effects of breastfeeding, maternal smoking during pregnancy, and recurrent lower respiratory tract infections on asthma in children. Journal of Asthma 45: 688-695.

76. Ogbuanu IU, Karmaus W, Arshad SH, Kurukulaaratchy RJ, Ewart S (2009) Effect of breastfeeding duration on lung function at age 10 years: a prospective birth cohort study. Thorax 64: 62-66.

77. Soto-Ramirez N, Alexander M, Karmaus W, Yousefi M, Zhang H, et al. (2012) Breastfeeding is associated with increased lung function at 18 years of age: a cohort study. European Respiratory Journal 39: 985-991.

78. Kaufman HS, Frick OL (1976) The development of allergy in infants of allergic parents: a prospective study concerning the role of heredity. Annals of Allergy 37: 410-415.

79. Kellberger J, Dressel H, Vogelberg C, Leupold W, Windstetter D, et al. (2012) Prediction of the incidence and persistence of allergic rhinitis in adolescence: a prospective cohort study. Journal of Allergy & Clinical Immunology 129: 397-402, 402.e391-393.

80. Kemeny DM, Price JF, Richardson V, Richards D, Lessof MH (1991) THE IGE AND IGG SUBCLASS ANTIBODY-RESPONSE TO FOODS IN BABIES DURING THE 1ST YEAR OF LIFE AND THEIR RELATIONSHIP TO FEEDING REGIMEN AND THE DEVELOPMENT OF FOOD ALLERGY. Journal of Allergy and Clinical Immunology 87: 920-929.

81. Kerr AA (1981) Lower respiratory tract illness in Polynesian infants. New Zealand Medical Journal 93: 333-335.

82. Kim J, Chang E, Han Y, Ahn K, Lee SI (2011) The incidence and risk factors of immediate type food allergy during the first year of life in Korean infants: a birth cohort study. Pediatric Allergy & Immunology 22: 715-719.

83. Kitz R, Rose MA, Schonborn H, Zielen S, Bohles HJ (2006) Impact of early dietary gamma-linolenic acid supplementation on atopic eczema in infancy. Pediatric Allergy & Immunology 17: 112-117.

84. Klinnert MD, Nelson HS, Price MR, Adinoff AD, Leung DYM, et al. (2001) Onset and persistence of childhood asthma: Predictors from infancy. Pediatrics 108: art. no.-e69.

85. Kramer MS, Guo T, Platt RW, Sevkovskaya Z, Dzikovich I, et al. (2003) Infant growth and health outcomes associated with 3 compared with 6 mo of exclusive breastfeeding. American Journal of Clinical Nutrition 78: 291-295.

86. Kramer MS, Matush L, Bogdanovich N, Aboud F, Mazer B, et al. (2009) Health and development outcomes in 6.5-y-old children breastfed exclusively for 3 or 6 mo. American Journal of Clinical Nutrition 90: 1070-1074.

87. Kramer MS, Matush L, Bogdanovich N, Dahhou M, Platt RW, et al. (2009) The low prevalence of allergic disease in Eastern Europe: are risk factors consistent with the hygiene hypothesis? Clinical & Experimental Allergy 39: 708-716.

88. Kull I, Wickman M, Lilja G, Nordvall SL, Pershagen G (2002) Breast feeding and allergic diseases in infants-a prospective birth cohort study. Archives of Disease in Childhood 87: 478-481.

89. Kusel MM, Holt PG, de Klerk N, Sly PD (2005) Support for 2 variants of eczema. Journal of Allergy & Clinical Immunology 116: 1067-1072.

90. Larsson M, Hagerhed-Engman L, Sigsgaard T, Janson S, Sundell J, et al. (2008) Incidence rates of asthma, rhinitis and eczema symptoms and influential factors in young children in Sweden. Acta Paediatrica 97: 1210-1215.

91. Marini A, Agosti M, Motta G, Mosca F (1996) Effects of a dietary and environmental prevention programme on the incidence of allergic symptoms in high atopic risk infants: three years' follow-up. Acta Paediatrica Supplement 414: 1-21.

92. Matheson MC, Erbas B, Balasuriya A, Jenkins MA, Wharton CL, et al. (2007) Breast-feeding and atopic disease: a cohort study from childhood to middle age. Journal of Allergy & Clinical Immunology 120: 1051-1057.

93. Midwinter RE, Moore WJ, Soothill JF, Turner MW, Colley JR (1982) Infant feeding and atopy. Lancet 1: 339.

94. Mihrshahi S, Ampon R, Webb K, Almqvist C, Kemp AS, et al. (2007) The association between infant feeding practices and subsequent atopy among children with a family history of asthma. Clinical & Experimental Allergy 37: 671-679.

95. Milner JD, Stein DM, McCarter R, Moon RY (2004) Early infant multivitamin supplementation is associated with increased risk for food allergy and asthma. Pediatrics 114: 27-32.

96. Miskelly FG, Burr ML, Vaughan-Williams E, Fehily AM, Butland BK, et al. (1988) Infant feeding and allergy. Archives of Disease in Childhood 63: 388-393.

97. Miyake Y, Tanaka K, Sasaki S, Kiyohara C, Ohya Y, et al. (2008) Breastfeeding and the risk of wheeze and asthma in Japanese infants: the Osaka Maternal and Child Health Study. Pediatric Allergy & Immunology 19: 490-496.

98. Miyake Y, Tanaka K, Sasaki S, Kiyohara C, Ohya Y, et al. (2009) Breastfeeding and atopic eczema in Japanese infants: The Osaka Maternal and Child Health Study. Pediatric Allergy & Immunology 20: 234-241.

99. Morgan JB, Lucas A, Fewtrell MS (2004) Does weaning influence growth and health up to 18 months? Archives of Disease in Childhood 89: 728-733.

100. Morgan J, Williams P, Norris F, Williams CM, Larkin M, et al. (2004) Eczema and early solid feeding in preterm infants. Archives of Disease in Childhood 89: 309-314.

101. Moore WJ, Midwinter RE, Morris AF, Colley JR, Soothill JF (1985) Infant feeding and subsequent risk of atopic eczema. Archives of Disease in Childhood 60: 722-726.

102. Morales E, Garcia-Esteban R, Guxens M, Guerra S, Mendez M, et al. (2012) Effects of prolonged breastfeeding and colostrum fatty acids on allergic manifestations and infections in infancy. Clinical and Experimental Allergy 42: 918-928.

103. Muiño A, Menezes AMB, Reichert FF, Duquia RP, Chatkin M (2008) Padrões de sibilância respiratória do nascimento até o início da adolescência: coorte de Pelotas (RS) Brasil, 1993-2004

Wheezing phenotypes from birth to adolescence: a cohort study in Pelotas, Brazil, 1993-2004. J Bras Pneumol 34: 347-355.

104. Nielsen AM, Rasmussen S, Christoffersen MN (2002) [Morbidity of Danish infants during their first months of life. Incidence and risk factors]. Ugeskrift for Laeger 164: 5644-5648.

105. Nwaru BI, Erkkola M, Ahonen S, Kaila M, Haapala AM, et al. (2010) Age at the introduction of solid foods during the first year and allergic sensitization at age 5 years. Pediatrics 125: 50-59.

106. Nwaru BI, Takkinen HM, Niemela O, Kaila M, Erkkola M, et al. (2013) Timing of infant feeding in relation to childhood asthma and allergic diseases. Journal of Allergy and Clinical Immunology 131: 78-86.

107. Erkkola M, Nwaru BI, Kaila M, Kronberg-Kippila C, Ilonen J, et al. (2012) Risk of asthma and allergic outcomes in the offspring in relation to maternal food consumption during pregnancy: a Finnish birth cohort study. Pediatric Allergy & Immunology 23: 186-194.

108. Virtanen SM, Kaila M, Pekkanen J, Kenward MG, Uusitalo U, et al. (2010) Early introduction of oats associated with decreased risk of persistent asthma and early introduction of fish with decreased risk of allergic rhinitis. British Journal of Nutrition 103: 266-273.

109. Oddy WH, Halonen M, Martinez FD, Lohman IC, Stern DA, et al. (2003) TGF-beta in human milk is associated with wheeze in infancy. Journal of Allergy & Clinical Immunology 112: 723-728.

110. Oddy WH, Sly PD, de Klerk NH, Landau LI, Kendall GE, et al. (2003) Breast feeding and respiratory morbidity in infancy: a birth cohort study. Archives of Disease in Childhood 88: 224-228.

111. Oddy WH, de Klerk NH, Kendall GE, Mihrshahi S, Peat JK (2004) Ratio of omega-6 to omega-3 fatty acids and childhood asthma. Journal of Asthma 41: 319-326.

112. Oddy WH, Holt PG, Sly PD, Read AW, Landau LI, et al. (1999) Association between breast feeding and asthma in 6 year old children: findings of a prospective birth cohort study. BMJ 319: 815-819.

113. Odelram H, Vanto T, Jacobsen L, Kjellman NIM (1996) Whey hydrolysate compared with cow's milk-based formula for weaning at about 6 months of age in high allergy-risk infants: Effects on atopic disease and sensitization. Allergy: European Journal of Allergy and Clinical Immunology 51: 192-195.

114. Perez Tarazona S, Alfonso Diego J, Amat Madramany A, Chofre Escrihuela L, Lucas Saez E, et al. (2010) [Incidence of wheezing and associated risk factors in the first 6 months of life of a cohort in Valencia (Spain)]. Anales de Pediatria 72: 19-29.

115. Pesonen M, Kallio MJ, Ranki A, Siimes MA (2006) Prolonged exclusive breastfeeding is associated with increased atopic dermatitis: a prospective follow-up study of unselected healthy newborns from birth to age 20 years. Clinical & Experimental Allergy 36: 1011-1018.

116. Porch MC, Shahane AD, Leiva LE, Elston RC, Sorensen RU (1998) Influence of breast milk, soy or two hydrolyzed formulas on the development of allergic manifestations in infants at risk. Nutrition Research 18: 1413-1424.

117. Poysa L, Korppi M, Remes K, Juntunen-Backman K (1990) Predictive value of IgE levels in infancy. Acta Paediatrica Scandinavica 79: 970-972.

118. Poysa L, Pulkkinen A, Korppi M, Remes K, Juntunen-Backman K (1992) Diet in infancy and bronchial hyperreactivity later in childhood. Pediatric Pulmonology 13: 215-221.

119. Pratt HF (1984) Breastfeeding and eczema. Early Human Development 9: 283-290.

120. Puig C, Friguls B, Gomez M, Garcia-Algar O, Sunyer J, et al. (2010) Relationship between lower respiratory tract infections in the first year of life and the development of asthma and wheezing in children. [Spanish]

Relacion entre las infecciones respiratorias de vias bajas durante el primer ano de vida y el desarrollo de asma y sibilancias en ninos. Archivos de Bronconeumologia 46: 514-521.

121. Purvis DJ, Thompson JM, Clark PM, Robinson E, Black PN, et al. (2005) Risk factors for atopic dermatitis in New Zealand children at 3.5 years of age. British Journal of Dermatology 152: 742-749.

122. Rhodes HL, Sporik R, Thomas P, Holgate ST, Cogswell JJ (2001) Early life risk factors for adult asthma: a birth cohort study of subjects at risk. Journal of Allergy & Clinical Immunology 108: 720-725.

123. Rothenbacher D, Weyermann M, Beermann C, Brenner H (2005) Breastfeeding, soluble CD14 concentration in breast milk and risk of atopic dermatitis and asthma in early childhood: birth cohort study. Clinical & Experimental Allergy 35: 1014-1021.

124. Rowntree S, Cogswell JJ, Platts-Mills TA, Mitchell EB (1985) Development of IgE and IgG antibodies to food and inhalant allergens in children at risk of allergic disease. Archives of Disease in Childhood 60: 727-735.

125. Rullo VE, Arruda LK, Cardoso MR, Valente V, Zampolo AS, et al. (2009) Respiratory infection, exposure to mouse allergen and breastfeeding: role in recurrent wheezing in early life. International Archives of Allergy & Immunology 150: 172-178.

126. Rullo V, Arruda K, Valente V, Zampolo A, Cardoso M, et al. (2009) Allergen and endotoxin exposure, infection, and breastfeeding in early life, and recurrent wheezing in children: 48-month follow-up cohort study. Allergy: European Journal of Allergy and Clinical Immunology 64: 22.

127. Rullo VEV, Arruda LK, Valente V, Zampolo AS, Cardoso MR, et al. (2010) Allergen and endotoxin exposure, infection, and breastfeeding in early life, and persistent wheezing in children: 60-month follow-up of a cohort study. Journal of Allergy and Clinical Immunology 1): AB56.

128. Rider NL, Morton D, Strauss KA (2007) Allergen and endotoxin exposure, infection, and breastfeeding in early life, and recurrent wheezing in infants: 18-month follow-up of a cohort study. Journal of Allergy and Clinical Immunology 119: S70-S71.

129. Ruiz RGG, Kemeny DM, Price JF (1992) Higher risk of infantile atopic dermatitis from maternal atopy than from paternal atopy. Clinical and Experimental Allergy 22: 762-766.

130. Saarinen UM, Kajosaari M (1995) Breastfeeding as prophylaxis against atopic disease: prospective follow-up study until 17 years old. Lancet 346: 1065-1069.

131. Saarinen UM, Bjorksten F, Knekt P, Siimes MA (1979) Serum IgE in healthy infants fed breast milk or cow's milk-based formulas. Clinical Allergy 9: 339-335.

132. Sears MR, Greene JM, Willan AR, Taylor DR, Flannery EM, et al. (2002) Long-term relation between breastfeeding and development of atopy and asthma in children and young adults: a longitudinal study. Lancet 360: 901-907.

133. Mandhane PJ, Greene JM, Sears MR (2007) Interactions between breast-feeding, specific parental atopy, and sex on development of asthma and atopy. Journal of Allergy & Clinical Immunology 119: 1359-1366.

134. Shaheen SO, Aaby P, Hall AJ, Barker DJ, Heyes CB, et al. (1996) Measles and atopy in Guinea-Bissau. Lancet 347: 1792-1796.

135. Shohet L, Shahar E, Davidson S (1985) Breast feeding as prophylaxis for atopic eczema: a controlled study of 368 cases. Acta Paediatrica Hungarica 26: 35-39.

136. Schoetzau A, Filipiak-Pittroff B, Franke K, Koletzko S, Von Berg A, et al. (2002) Effect of exclusive breast-feeding and early solid food avoidance on the incidence of atopic dermatitis in high-risk infants at 1 year of age. Pediatric Allergy & Immunology 13: 234-242.

137. Schonberger HJ, Dompeling E, Knottnerus JA, Maas T, Muris JW, et al. (2005) The PREVASC study: the clinical effect of a multifaceted educational intervention to prevent childhood asthma. European Respiratory Journal 25: 660-670.

138. Sicherer SH, Wood RA, Stablein D, Lindblad R, Burks AW, et al. (2010) Maternal consumption of peanut during pregnancy is associated with peanut sensitization in atopic infants. Journal of Allergy & Clinical Immunology 126: 1191-1197.

139. Siltanen M, Kajosaari M, Poussa T, Saarinen KM, Savilahti E (2003) A dual long-term effect of breastfeeding on atopy in relation to heredity in children at 4 years of age. Allergy 58: 524-530.

140. Silva JM, Camara AA, Tobias KR, Macedo IS, Cardoso MR, et al. (2005) A prospective study of wheezing in young children: the independent effects of cockroach exposure, breast-feeding and allergic sensitization. Pediatric Allergy & Immunology 16: 393-401.

141. Silvers KM, Frampton CM, Wickens K, Pattemore PK, Ingham T, et al. (2012) Breastfeeding protects against current asthma up to 6 years of age. Journal of Pediatrics 160: 991-996.e991.

142. Silvers KM, Frampton CM, Wickens K, Epton MJ, Pattemore PK, et al. (2009) Breastfeeding protects against adverse respiratory outcomes at 15 months of age. Maternal & Child Nutrition 5: 243-250.

143. Simon MR, Havstad SL, Wegienka GR, Ownby DR, Johnson CC (2008) Risk factors associated with transient wheezing in young children. Allergy & Asthma Proceedings 29: 161-165.

144. Wang IJ, Guo YL, Weng HJ, Hsieh WS, Chuang YL, et al. (2007) Environmental risk factors for early infantile atopic dermatitis. Pediatric Allergy & Immunology 18: 441-447.

145. Wegienka G, Ownby DR, Havstad S, Williams LK, Johnson CC (2006) Breastfeeding history and childhood allergic status in a prospective birth cohort. Annals of Allergy, Asthma, & Immunology 97: 78-83.

146. Salam MT, Li YF, Langholz B, Gilliland FD, Children's Health S (2004) Early-life environmental risk factors for asthma: findings from the Children's Health Study. Environmental Health Perspectives 112: 760-765.

147. Snijders BE, Thijs C, van Ree R, van den Brandt PA (2008) Age at first introduction of cow milk products and other food products in relation to infant atopic manifestations in the first 2 years of life: the KOALA Birth Cohort Study. Pediatrics 122: e115-122.

148. Snijders BE, Thijs C, Dagnelie PC, Stelma FF, Mommers M, et al. (2007) Breast-feeding duration and infant atopic manifestations, by maternal allergic status, in the first 2 years of life (KOALA study). Journal of Pediatrics 151: 347-351, 351.e341-342.

149. Soto-Ramirez N, Karmaus W, Zhang H, Davis S, Agarwal S, et al. (2013) Modes of infant feeding and the occurrence of coughing/wheezing in the first year of life. Journal of Human Lactation 29: 71-80.

150. Strachan DP, Harkins LS, Johnston IDA, Anderson HR (1997) Childhood antecedents of allergic sensitization in young British adults. Journal of Allergy and Clinical Immunology 99: 6-12.

151. Strachan DP, Taylor EM, Carpenter RG (1996) Family structure, neonatal infection, and hay fever in adolescence. Archives of Disease in Childhood 74: 422-426.

152. Lewis S, Richards D, Bynner J, Butler N, Britton J (1995) PROSPECTIVE-STUDY OF RISK-FACTORS FOR EARLY AND PERSISTENT WHEEZING IN CHILDHOOD. European Respiratory Journal 8: 349-356.

153. Lewis S, Butland B, Strachan D, Bynner J, Richards D, et al. (1996) Study of the aetiology of wheezing illness at age 16 in two national British birth cohorts. Thorax 51: 670-676.

154. Butland BK, Strachan DP, Lewis S, Bynner J, Butler N, et al. (1997) Investigation into the increase in hay fever and eczema at age 16 observed between the 1958 and 1970 British birth cohorts. BMJ 315: 717-721.

155. Strassburger SZ, Vitolo MR, Bortolini GA, Pitrez PM, Jones MH, et al. (2010) Nutritional errors in the first months of life and their association with asthma and atopy in preschool children. Jornal de Pediatria 86: 391-399.

156. Sunyer J, Torrent M, Garcia-Esteban R, Ribas-Fito N, Carrizo D, et al. (2006) Early exposure to dichlorodiphenyldichloroethylene, breastfeeding and asthma at age six. Clinical & Experimental Allergy 36: 1236-1241.

157. Sunyer J, Mendendez C, Ventura PJ, Aponte JJ, Schellenberg D, et al. (2001) Prenatal risk factors of wheezing at the age of four years in Tanzania. Thorax 56: 290-295.

158. Taylor B, Wadsworth J, Golding J, Butler N (1983) Breast feeding, eczema, asthma, and hayfever. Journal of Epidemiology and Community Health 37: 95-99.

159. Taylor B, Wadsworth J, Wadsworth M, Peckham C (1984) Changes in the reported prevalence of childhood eczema since the 1939-45 war. Lancet 2: 1255-1257.

160. Tennant PW, Gibson GJ, Pearce MS (2008) Lifecourse predictors of adult respiratory function: results from the Newcastle Thousand Families Study. Thorax 63: 823-830.

161. Tennant PW, Gibson GJ, Parker L, Pearce MS (2010) Childhood respiratory illness and lung function at ages 14 and 50 years: childhood respiratory illness and lung function. Chest 137: 146-155.

162. Tian M, Zhao DY, Wen GY, Shi SY (2009) [The correlation factor about respiratory syncytial virus bronchiolitis and post-bronchiolitis wheezing in infant]. Chinese Journal of Experimental & Clinical Virology 23: 371-374.

163. Van Asperen PP, Kemp AS, Mellis CM (1984) Relationship of diet in the development of atopy in infancy. Clinical Allergy 14: 525-532.

164. van Beijsterveldt TC, Boomsma DI (2008) An exploration of gene-environment interaction and asthma in a large sample of 5-year-old Dutch twins. Twin Research & Human Genetics: the Official Journal of the International Society for Twin Studies 11: 143-149.

165. Sonnenschein-van der Voort AM, Jaddoe VW, van der Valk RJ, Willemsen SP, Hofman A, et al. (2012) Duration and exclusiveness of breastfeeding and childhood asthma-related symptoms. European Respiratory Journal 39: 81-89.

166. Vandenplas Y, Deneyer M, Sacre L, Loeb H (1988) Preliminary data on a field study with a new hypo-allergic formula. European Journal of Pediatrics 148: 274-277.

167. van Merode T, Maas T, Twellaar M, Kester A, van Schayck CP (2007) Gender-specific differences in the prevention of asthma-like symptoms in high-risk infants. Pediatric Allergy & Immunology 18: 196-200.

168. Venter C, Pereira B, Voigt K, Grundy J, Clayton CB, et al. (2009) Factors associated with maternal dietary intake, feeding and weaning practices, and the development of food hypersensitivity in the infant. Pediatric Allergy and Immunology 20: 320-327.

169. Watson PE, McDonald BW (2013) Subcutaneous body fat in pregnant new zealand women: association with wheeze in their infants at 18months. Maternal & Child Health Journal 17: 959-967.

170. Wetzig H, Schulz R, Diez U, Herbarth O, Viehweg B, et al. (2000) Associations between duration of breast-feeding, sensitization to hens' eggs and eczema infantum in one and two year old children at high risk of atopy. International Journal of Hygiene & Environmental Health 203: 17-21.

171. Wilson AC, Forsyth JS, Greene SA, Irvine L, Hau C, et al. (1998) Relation of infant diet to childhood health: seven year follow up of cohort of children in Dundee infant feeding study. BMJ 316: 21-25.

172. Wright RJ, Cohen S, Carey V, Weiss ST, Gold DR (2002) Parental stress as a predictor of wheezing in infancy: a prospective birth-cohort study. American Journal of Respiratory & Critical Care Medicine 165: 358-365.

173. Wright AL, Holberg CJ, Martinez FD, Morgan WJ, Taussig LM (1989) Breast feeding and lower respiratory tract illness in the first year of life. Group Health Medical Associates. BMJ 299: 946-949.

174. Wright AL, Holberg CJ, Taussig LM, Martinez FD (1995) Relationship of infant feeding to recurrent wheezing at age 6 years. Archives of Pediatrics & Adolescent Medicine 149: 758-763.

175. Wright AL, Sherrill D, Holberg CJ, Halonen M, Martinez FD (1999) Breast-feeding, maternal IgE, and total serum IgE in childhood. Journal of Allergy & Clinical Immunology 104: 589-594.

176. Wright AL, Holberg CJ, Martinez FD, Halonen M, Morgan W, et al. (1994) Epidemiology of physician-diagnosed allergic rhinitis in childhood. Pediatrics 94: 895-901.

177. Yamamoto K, Shoda T, Futamura M, Narita M, Sakamoto N, et al. (2011) Associated factors of wheezing at 3 years of age in Japan: Prospective birth cohort study. Allergy: European Journal of Allergy and Clinical Immunology 66: 549-550.

178. Zutavern A, Brockow I, Schaaf B, Bolte G, von Berg A, et al. (2006) Timing of solid food introduction in relation to atopic dermatitis and atopic sensitization: results from a prospective birth cohort study. Pediatrics 117: 401-411.

179. Zutavern A, Brockow I, Schaaf B, von Berg A, Diez U, et al. (2008) Timing of solid food introduction in relation to eczema, asthma, allergic rhinitis, and food and inhalant sensitization at the age of 6 years: results from the prospective birth cohort study LISA. Pediatrics 121: e44-52.

180. Friday GA, Jr., Smith J (2000) Breast-feeding and atopic dermatitis. Pediatric Asthma, Allergy and Immunology 14: 205-209.

181. McConnochie KM, Roghmann KJ (1986) Breast feeding and maternal smoking as predictors of wheezing in children age 6 to 10 years. Pediatric Pulmonology 2: 260-268.

182. Monego A, Infortuna M, Santisi G, Barberio G (1989) [Can breast feeding prevent atopic dermatitis? Epidemiologic study of 144 cases]. Minerva Pediatrica 41: 223-227.

183. Rona RJ, Smeeton NC, Bustos P, Amigo H, Diaz PV (2005) The early origins hypothesis with an emphasis on growth rate in the first year of life and asthma: a prospective study in Chile. Thorax 60: 549-554.

184. Mai XM, Becker AB, Sellers EAC, Liem JJ, Kozyrskyj AL (2007) The relationship of breast-feeding, overweight, and asthma in preadolescents. Journal of Allergy and Clinical Immunology 120: 551-556.

185. Martel MJ, Rey E, Malo JL, Perreault S, Beauchesne MF, et al. (2009) Determinants of the incidence of childhood asthma: a two-stage case-control study. American Journal of Epidemiology 169: 195-205.

186. Maskell J, Grimshaw K, King R, Oliver E, Gudgeon L, et al. (2010) Dietary patterns in the first year of life and food allergy risk. Allergy: European Journal of Allergy and Clinical Immunology 65: 46.

187. Oliver E, Grimshaw K, Scally K, Garland J, Gudgeon L, et al. (2010) Does nutritional intake in infancy effect later development of food allergy? Allergy: European Journal of Allergy and Clinical Immunology 65: 313.

188. Munro A, Grimshaw K, Oliver E, Foote K, Roberts G (2011) Risk factors associated with wheezing in the first 2 years of life: Europrevall cohort. Clinical and Experimental Allergy 41 (12): 1856.

189. Ronmark E, Jonsson E, Platts-Mills T, Lundback B (1999) Different pattern of risk factors for atopic and nonatopic asthma among children--report from the Obstructive Lung Disease in Northern Sweden Study. Allergy 54: 926-935.

190. Camara AA, Silva JM, Ferriani VPL, Tobias KRC, Macedo IS, et al. (2004) Risk factors for wheezing in a subtropical environment: Role of respiratory viruses and allergen sensitization. Journal of Allergy and Clinical Immunology 113: 551-557.

191. DesRoches A, Infante-Rivard C, Paradis L, Paradis J, Haddad E (2010) Peanut allergy: is maternal transmission of antigens during pregnancy and breastfeeding a risk factor? Journal of Investigational Allergology & Clinical Immunology 20: 289-294.

192. Djenouhat K, Ibsaine O, Abbadi M, Berrah H (2011) Cow's milk allergy and risk factors. Allergy: European Journal of Allergy and Clinical Immunology 66: 388.

193. Fox AT, Sasieni P, du Toit G, Syed H, Lack G (2009) Household peanut consumption as a risk factor for the development of peanut allergy. Journal of Allergy and Clinical Immunology 123: 417-423.

194. Ghaderi R, Makhmalbaf Z (2005) Effect of breast-feeding on the development of atopic dermatitis.[Retraction in Ghaderi R, Makhmalbaf Z. Iran J Allergy Asthma Immunol. 2007 Dec;6(4):225; PMID: 18357658]. Iranian Journal of Allergy Asthma & Immunology 4: 129-132.

195. Haileamlak A, Dagoye D, Williams H, Venn AJ, Hubbard R, et al. (2005) Early life risk factors for atopic dermatitis in Ethiopian children. Journal of Allergy & Clinical Immunology 115: 370-376.

196. Infante-Rivard C (1993) Childhood asthma and indoor environmental risk factors. Am J Epidemiol 137: 834-844.

197. Juca SC, Takano OA, Moraes LS, Guimaraes LV (2012) [Asthma prevalence and risk factors in adolescents 13 to 14 years of age in Cuiaba, Mato Grosso State, Brazil]. Cadernos de Saude Publica 28: 689-697.

198. Karunasekera KA, Jayasinghe JA, Alwis LW (2001) Risk factors of childhood asthma: a Sri Lankan study. Journal of Tropical Pediatrics 47: 142-145.

199. Kramer MS, Moroz B (1981) Do breast-feeding and delayed introduction of solid foods protect against subsequent atopic eczema? Journal of Pediatrics 98: 546-550.

200. Mavale-Manuel S, Alexandre F, Duarte N, Albuquerque O, Scheinmann P, et al. (2004) Risk factors for asthma among children in Maputo (Mozambique). Allergy 59: 388-393.

201. Oliveti JF, Kercsmar CM, Redline S (1996) Pre- and perinatal risk factors for asthma in inner city African-American children. American Journal of Epidemiology 143: 570-577.

202. Porro E, Indinnimeo L, Antognoni G, Midulla F, Criscione S (1993) Early wheezing and breast feeding. Journal of Asthma 30: 23-28.

203. Ratageri VH, Kabra SK, Dwivedi SN, Seth V (2000) Factors associated with severe asthma. Indian Pediatrics 37: 1072-1082.

204. Rosas Vargas MA, Gonzalez Reyes M, del Rio Navarro BE, Avila Castanon L, Velazquez Armenta Y, et al. (2002) [Allergen sensitization and asthma in children from 1 to 3 years of age]. Revista Alergia Mexico 49: 171-175.

205. Rylander E, Pershagen G, Eriksson M, Nordvall L (1993) Parental smoking and other risk factors for wheezing bronchitis in children. European Journal of Epidemiology 9: 517-526.

206. Anonymous (1988) Cow's milk allergy in the first year of life. An Italian Collaborative Study. Acta Paediatrica Scandinavica - Supplement 348: 1-14.

207. Whu R, Cirilo G, Wong J, Finkel ML, Mendez HA, et al. (2007) Risk factors for pediatric asthma in the South Bronx. Journal of Asthma 44: 855-859.

208. Wickens K, Crane J, Kemp T, Lewis S, D'Souza W, et al. (2001) A case-control study of risk factors for asthma in New Zealand children. Aust N Z J Public Health 25: 44-49.

209. Zhu CH, Liu JX, Zhao XH (2012) [Risk factors of asthma among children aged 0 - 14 in Suzhou city]. Chung-Hua Yu Fang i Hsueh Tsa Chih [Chinese Journal of Preventive Medicine] 46: 456-459.

210. Alper Z, Sapan N, Ercan I, Canitez Y, Bilgel N (2006) Risk factors for wheezing in primary school children in Bursa, Turkey. American Journal of Rhinology 20: 53-63.

211. Al-Kubaisy W, Ali SH, Al-Thamiri D (2005) Risk factors for asthma among primary school children in Baghdad, Iraq. Saudi Medical Journal 26: 460-466.

212. Awasthi S, Kalra E, Roy S, Awasthi S (2004) Prevalence and risk factors of asthma and wheeze in school-going children in Lucknow, North India. Indian Pediatrics 41: 1205-1210.

213. Innes Asher M, Robertson C, Ait-Khaled N, Anderson HR, Beasley R, et al. (2011) Global analysis of breast feeding and risk of symptoms of asthma, rhinoconjunctivitis and eczema in 6-7 year old children: ISAAC Phase Three. Allergologia et Immunopathologia 39: 318-325.

214. Flohr C, Nagel G, Weinmayr G, Kleiner A, Strachan DP, et al. (2011) Lack of evidence for a protective effect of prolonged breastfeeding on childhood eczema: lessons from the International Study of Asthma and Allergies in Childhood (ISAAC) Phase Two. British Journal of Dermatology 165: 1280-1289.

215. Nagel G, Buchele G, Weinmayr G, Bjorksten B, Chen YZ, et al. (2009) Effect of breastfeeding on asthma, lung function and bronchial hyperreactivity in ISAAC Phase II. European Respiratory Journal 33: 993-1002.

216. Kuyucu S, Saraclar Y, Tuncer A, Sackesen C, Adalioglu G, et al. (2004) Determinants of atopic sensitization in Turkish school children: effects of pre- and post-natal events and maternal atopy. Pediatric Allergy & Immunology 15: 62-71.

217. Bruno G, Giampietro PG, Businco L (1996) [Results of a multicentric study for the prevention of atopic allergy. 48 months of follow up]. Minerva Pediatrica 48: 413-419.

218. Castro-Rodriguez JA, Garcia-Marcos L, Sanchez-Solis M, Perez-Fernandez V, Martinez-Torres A, et al. (2010) Olive oil during pregnancy is associated with reduced wheezing during the first year of life of the offspring. Pediatric Pulmonology 45: 395-402.

219. Chong Neto HJ, Rosário NA, Solé D, Mallol J (2007) Prevalência de sibilância recorrente em lactentes

Prevalence of recurrent wheezing in infants. J Pediatr (Rio J) 83: 357-362.

220. Civelek E, Sahiner UM, Yuksel H, Boz AB, Orhan F, et al. (2011) Prevalence, burden, and risk factors of atopic eczema in schoolchildren aged 10-11 years: a national multicenter study. Journal of Investigational Allergology & Clinical Immunology 21: 270-277.

221. Ehlayel MS, Bener A (2008) Duration of breast-feeding and the risk of childhood allergic diseases in a developing country. Allergy & Asthma Proceedings 29: 386-391.

222. Ehrlich RI, Du Toit D, Jordaan E, Zwarenstein M, Potter P, et al. (1996) Risk factors for childhood asthma and wheezing. Importance of maternal and household smoking. American Journal of Respiratory & Critical Care Medicine 154: 681-688.

223. Evenhouse E, Reilly S (2005) Improved estimates of the benefits of breastfeeding using sibling comparisons to reduce selection bias. Health Services Research 40: 1781-1802.

224. Girolomoni G, Abeni D, Masini C, Sera F, Ayala F, et al. (2003) The epidemiology of atopic dermatitis in Italian schoolchildren. Allergy 58: 420-425.

225. Han YY, Lee YL, Guo YL (2009) Indoor environmental risk factors and seasonal variation of childhood asthma. Pediatr Allergy Immunol 20: 748-756.

226. Karino S, Okuda T, Uehara Y, Toyo-oka T (2008) Breastfeeding and prevalence of allergic diseases in Japanese university students. Annals of Allergy Asthma & Immunology 101: 153-159.

227. Kucukosmanoglu E, Yazi D, Yesil O, Akkoc T, Gezer M, et al. (2008) Prevalence of egg sensitization in Turkish infants based on skin prick test. Allergologia Et Immunopathologia 36: 141-144.

228. Kuehr J, Frischer T, Karmaus W, Meinert R, Barth R, et al. (1992) EARLY-CHILDHOOD RISK-FACTORS FOR SENSITIZATION AT SCHOOL AGE. Journal of Allergy and Clinical Immunology 90: 358-363.

229. Kurt S, Kisacik B, Kaplan Y, Yildirim B, Etikan I, et al. (2008) Obesity and carpal tunnel syndrome: Is there a causal relationship? European Neurology 59: 253-257.

230. Kurt E, Metintas S, Basyigit I, Bulut I, Coskun E, et al. (2007) Prevalence and risk factors of allergies in Turkey: Results of a multicentric cross-sectional study in children. Pediatric Allergy & Immunology 18: 566-574.

231. Liu P, Woo JMP, Parsa MF, Amarilyo G, McCurdy DK, et al. (2012) Ethnic differences in pediatric SLE early disease severity: A comparison between Hispanic-Americans and European-Americans. Pediatric Rheumatology 10.

232. Miyake Y, Yura A, Iki M (2003) Breastfeeding and the prevalence of symptoms of allergic disorders in Japanese adolescents. Clinical and Experimental Allergy 33: 312-316.

233. Nakamura Y, Oki I, Tanihara S, Ojima T, Ito Y, et al. (2000) Relationship between breast milk feeding and atopic dermatitis in children. Journal of epidemiology / Japan Epidemiological Association 10: 74-78.

234. Paton J, Kljakovic M, Ciszek K, Ding P (2012) Infant Feeding Practices and Nut Allergy over Time in Australian School Entrant Children. International Journal of Pediatrics 2012: 675724.

235. Prietsch SO, Fischer GB, Cesar JA, Cervo PV, Sangaletti LL, et al. (2006) [Risk factors for recurrent wheezing in children under 13 years old in the South of Brazil]. Pan American Journal of Public Health 20: 331-337.

236. Rusconi F, Galassi C, Bellasio M, Piffer S, Lombardi E, et al. (2005) [Risk factors in the pre-, perinatal and early life (first year) for wheezing in young children]. Epidemiologia e Prevenzione 29: 47-51.

237. Rusconi F, Galassi C, Corbo GM, Forastiere F, Biggeri A, et al. (1999) Risk factors for early, persistent, and late-onset wheezing in young children. SIDRIA Collaborative Group. American Journal of Respiratory & Critical Care Medicine 160: 1617-1622.

238. Rust GS, Thompson CJ, Minor P, Davis-Mitchell W, Holloway K, et al. (2001) Does breastfeeding protect children from asthma? Analysis of NHANES III survey data. Journal of the National Medical Association 93: 139-148.

239. Salem MB, Al-Sadoon IO, Hassan MK (2002) Prevalence of wheeze among preschool children in Basra governonate, southern Iraq. Eastern Mediterranean Health Journal 8: 503-508.

240. Selcuk ZT, Caglar T, Enunlu T, Topal T (1997) The prevalence of allergic diseases in primary school children in Edirne, Turkey. Clinical & Experimental Allergy 27: 262-269.

241. Suwanpromma S, Boonlarbtaweechoke C, Udomsubpayakul U, Preutthipan A (2012) Spirometric airflow obstruction in Bangkok school children: prevalence and risk factors. Journal of the Medical Association of Thailand 95: 1411-1417.

242. Takemura Y, Sakurai Y, Honjo S, Kusakari A, Hara T, et al. (2001) Relation between breastfeeding and the prevalence of asthma : the Tokorozawa Childhood Asthma and Pollinosis Study. American Journal of Epidemiology 154: 115-119.

243. Tanaka K, Miyake Y, Sasaki S (2010) Association between breastfeeding and allergic disorders in Japanese children. International Journal of Tuberculosis and Lung Disease 14: 513-518.

244. Visser CAN, Garcia-Marcos L, Eggink J, Brand PLP (2010) Prevalence and Risk Factors of Wheeze in Dutch Infants in Their First Year of Life. Pediatric Pulmonology 45: 149-156.

245. Wang HY, Chen YZ, Ma Y, Wong GW, Lai CK, et al. (2006) [Disparity of asthma prevalence in Chinese schoolchildren is due to differences in lifestyle factors]. Zhonghua Erke Zazhi 44: 41-45.

246. Couper JJ, Steele C, Beresford S, Powell T, McCaul K, et al. (1999) Lack of association between duration of breast-feeding or introduction of cow's milk and development of islet autoimmunity. Diabetes 48: 2145-2149.

247. Couper JJ, Beresford S, Hirte C, Baghurst PA, Pollard A, et al. (2009) Weight Gain in Early Life Predicts Risk of Islet Autoimmuity in Children With a First-Degree Relative With Type 1 Diabetes. Diabetes Care 32: 94-99.

248. Frederiksen B, Kroehl M, Lamb M, Seifert J, Barriga K, et al. (2012) Infant exposures and development of type 1 diabetes-the diabetes autoimmunity study in the young (DAISY). Diabetes 61: A352.

249. Fronczak CM, Baron AE, Chase HP, Ross C, Brady HL, et al. (2003) In utero dietary exposures and risk of islet autoimmunity in children. Diabetes Care 26: 3237-3242.

250. Lamb MM, Simpson MD, Seifert J, Scott FW, Rewers M, et al. (2013) The association between IgG4 antibodies to dietary factors, islet autoimmunity and type 1 diabetes: the Diabetes Autoimmunity Study in the Young. PLoS ONE [Electronic Resource] 8: e57936.

251. Norris JM, Barriga K, Klingensmith G, Hoffman M, Eisenbarth GS, et al. (2003) Timing of initial cereal exposure in infancy and risk of islet autoimmunity. JAMA 290: 1713-1720.

252. Lamb MM, Myers MA, Barriga K, Zimmet PZ, Rewers M, et al. (2008) Maternal diet during pregnancy and islet autoimmunity in offspring. Pediatric Diabetes 9: 135-141.

253. Holmberg H, Wahlberg J, Vaarala O, Ludvigsson J (2007) Short duration of breast-feeding as a risk-factor for beta-cell autoantibodies in 5-year-old children from the general population. The British journal of nutrition 97: 111-116.

254. Karlen J, Faresjo T, Ludvigsson J (2012) Could the social environment trigger the induction of diabetes related autoantibodies in young children? Scandinavian Journal of Public Health 40: 177-182.

255. Wahlberg J, Vaarala O, Ludvigsson J, group AB-s (2006) Dietary risk factors for the emergence of type 1 diabetes-related autoantibodies in 21/2 year-old Swedish children. Br J Nutr 95: 603-608.

256. Ludvigsson JF, Ludvigsson J (2003) Stressful life events, social support and confidence in the pregnant woman and risk of coeliac disease in the offspring. Scandinavian Journal of Gastroenterology 38: 516-521.

257. Viner RM, Hindmarsh PC, Taylor B, Cole TJ (2008) Childhood body mass index (BMI), breastfeeding and risk of Type 1 diabetes: findings from a longitudinal national birth cohort. Diabetic Medicine 25: 1056-1061.

258. Virtanen SM, Rasanen L, Aro A, Ylonen K, Lounamaa R, et al. (1992) Feeding in infancy and the risk of type 1 diabetes mellitus in Finnish children. The 'Childhood Diabetes in Finland' Study Group. Diabetic Medicine 9: 815-819.

259. Virtanen SM, Hypponen E, Laara E, Vahasalo P, Kulmala P, et al. (1998) Cow's milk consumption, disease-associated autoantibodies and type 1 diabetes mellitus: a follow-up study in siblings of diabetic children. Childhood Diabetes in Finland Study Group. Diabetic Medicine 15: 730-738.

260. Hypponen E, Kenward MG, Virtanen SM, Piitulainen A, Virta-Autio P, et al. (1999) Infant feeding, early weight gain, and risk of type I diabetes. Diabetes Care 22: 1961-1965.

261. Virtanen SM, Laara E, Hypponen E, Reijonen H, Rasanen L, et al. (2000) Cow's milk consumption, HLA-DQB1 genotype, and type 1 diabetes: a nested case-control study of siblings of children with diabetes. Childhood diabetes in Finland study group.[Erratum appears in Diabetes 2000 Sep;49(9):1617]. Diabetes 49: 912-917.

262. Virtanen SM, Takkinen HM, Nevalainen J, Kronberg-Kippila C, Salmenhaara M, et al. (2011) Early introduction of root vegetables in infancy associated with advanced beta-cell autoimmunity in young children with human leukocyte antigen-conferred susceptibility to Type 1 diabetes. Diabetic Medicine 28: 965-971.

263. Jones ME, Swerdlow AJ, Gill LE, Goldacre MJ (1998) Pre-natal and early life risk factors for childhood onset diabetes mellitus: a record linkage study. International Journal of Epidemiology 27: 444-449.

264. Kimpimaki T, Erkkola M, Korhonen S, Kupila A, Virtanen SM, et al. (2001) Short-term exclusive breastfeeding predisposes young children with increased genetic risk of Type I diabetes to progressive beta-cell autoimmunity. Diabetologia 44: 63-69.

265. Kyvik KO, Green A, Svendsen A, Mortensen K (1992) Breast feeding and the development of type 1 diabetes mellitus. Diabetic Medicine 9: 233-235.

266. Norris JM, Beaty B, Klingensmith G, Yu LP, Hoffman M, et al. (1996) Lack of association between early exposure to cow's milk protein and beta-cell autoimmunity - Diabetes autoimmunity study in the young (DAISY). Jama-Journal of the American Medical Association 276: 609-614.

267. Robertson L, Harrild K (2010) Maternal and neonatal risk factors for childhood type 1 diabetes: a matched case-control study. BMC Public Health 10: 281.

268. Savilahti E, Saarinen KM (2009) Early infant feeding and type 1 diabetes. European Journal of Nutrition 48: 243-249.

269. Ahadi M, Tabatabaeiyan M, Moazzami K (2011) Association between environmental factors and risk of type 1 diabetes - a case-control study. Endokrynologia Polska 62: 134-137.

270. Alves JGB, Figueiroa JN, Meneses J, Alves GV (2012) Breastfeeding protects against type 1 diabetes mellitus: A case-sibling study. Breastfeeding Medicine 7: 25-28.

271. Ashraf AP, Eason NB, Kabagambe EK, Haritha J, Meleth S, et al. (2010) Dietary iron intake in the first 4 months of infancy and the development of type 1 diabetes: A pilot study. Diabetology and Metabolic Syndrome 2.

272. Baruah MP, Ammini AC, Khurana ML (2011) Demographic, breast-feeding, and nutritional trends among children with type 1 diabetes mellitus. Indian J Endocrinol Metab 15: 38-42.

273. Bener A, Alsaied A, Al-Ali M, Al-Kubaisi A, Basha B, et al. (2009) High prevalence of vitamin D deficiency in type 1 diabetes mellitus and healthy children. Acta Diabetologica 46: 183-189.

274. Blom L, Dahlquist G, Nystrom L, Sandstrom A, Wall S (1989) The Swedish childhood diabetes study - social and perinatal determinants for diabetes in childhood. Diabetologia 32: 7-13.

275. Bodington MJ, McNally PG, Burden AC (1994) Cow's milk and type 1 childhood diabetes: no increase in risk. Diabetic Medicine 11: 663-665.

276. Borras V, Freitas A, Castell C, Gispert R, Jane M (2011) Type 1 diabetes and perinatal factors in Catalonia (Spain). Pediatric Diabetes 12: 419-423.

277. Group ESS (2002) Rapid early growth is associated with increased risk of childhood type 1 diabetes in various European populations. Diabetes Care 25: 1755-1760.

278. Esfarjani F, Azar MR, Gafarpour M (2001) IDDM and early exposure of infant to cow's milk and solid food. Indian Journal of Pediatrics 68: 107-110.

279. Gimeno SG, de Souza JM (1997) IDDM and milk consumption. A case-control study in Sao Paulo, Brazil. Diabetes Care 20: 1256-1260.

280. Hathout EH, Beeson WL, Ischander M, Rao R, Mace JW (2006) Air pollution and type 1 diabetes in children. Pediatric Diabetes 7: 81-87.

281. Kostraba JN, Dorman JS, LaPorte RE, Scott FW, Steenkiste AR, et al. (1992) Early infant diet and risk of IDDM in blacks and whites. A matched case-control study. Diabetes Care 15: 626-631.

282. Kostraba JN, Cruickshanks KJ, Lawler-Heavner J, Jobim LF, Rewers MJ, et al. (1993) Early exposure to cow's milk and solid foods in infancy, genetic predisposition, and risk of IDDM. Diabetes 42: 288-295.

283. Liese AD, Puett RC, Lamichhane AP, Nichols MD, Dabelea D, et al. (2012) Neighborhood level risk factors for type 1 diabetes in youth: the SEARCH case-control study. International Journal of Health Geographics [Electronic Resource] 11: 1.

284. Majeed AA, Mea, Hassan K (2011) Risk Factors for Type 1 Diabetes Mellitus among Children and Adolescents in Basrah. Oman Medical Journal 26: 189-195.

285. Malcova H, Sumnik Z, Drevinek P, Venhacova J, Lebl J, et al. (2006) Absence of breast-feeding is associated with the risk of type 1 diabetes: a case-control study in a population with rapidly increasing incidence. European Journal of Pediatrics 165: 114-119.

286. Marshall AL, Chetwynd A, Morris JA, Placzek M, Smith C, et al. (2004) Type 1 diabetes mellitus in childhood: a matched case control study in Lancashire and Cumbria, UK. Diabetic Medicine 21: 1035-1040.

287. Mayer EJ, Hamman RF, Gay EC, Lezotte DC, Savitz DA, et al. (1988) Reduced risk of IDDM among breast-fed children. The Colorado IDDM Registry. Diabetes 37: 1625-1632.

288. McKinney PA, Parslow R, Gurney KA, Law GR, Bodansky HJ, et al. (1999) Perinatal and neonatal determinants of childhood type 1 diabetes. A case-control study in Yorkshire, U.K. Diabetes Care 22: 928-932.

289. Meloni T, Marinaro AM, Mannazzu MC, Ogana A, La Vecchia C, et al. (1997) IDDM and early infant feeding. Sardinian case-control study. Diabetes Care 20: 340-342.

290. Patterson CC, Carson DJ, Hadden DR, Waugh NR, Cole SK (1994) A case-control investigation of perinatal risk factors for childhood IDDM in Northern Ireland and Scotland. Diabetes Care 17: 376-381.

291. Perez-Bravo F, Carrasco E, Gutierrez-Lopez MD, Martinez MT, Lopez G, et al. (1996) Genetic predisposition and environmental factors leading to the development of insulin-dependent diabetes mellitus in Chilean children. J Mol Med (Berl) 74: 105-109.

292. Perez-Bravo F, Oyarzun A, Carrasco E, Albala C, Dorman JS, et al. (2003) Duration of breast feeding and bovine serum albumin antibody levels in type 1 diabetes: a case-control study. Pediatric Diabetes 4: 157-161.

293. Rabiei S (2011) The association of nutrition style through the first 2 years of life with type 1 diabetes mellitus and some of the other effective factors in 2-15 years old children. [Persian]. Iranian Journal of Endocrinology and Metabolism 13: 113.

294. Rami B, Schneider U, Imhof A, Waldhor T, Schober E (1999) Risk factors for type I diabetes mellitus in children in Austria. European Journal of Pediatrics 158: 362-366.

295. Rosenbauer J, Herzig P, Giani G (2008) Early infant feeding and risk of type 1 diabetes mellitus-a nationwide population-based case-control study in pre-school children. Diabetes/Metabolism Research Reviews 24: 211-222.

296. Sadauskaite-Kuehne V, Ludvigsson J, Padaiga Z, Jasinskiene E, Samuelsson U (2004) Longer breastfeeding is an independent protective factor against development of type 1 diabetes mellitus in childhood. Diabetes/Metabolism Research Reviews 20: 150-157.

297. Skrodeniene E, Marciulionyte D, Padaiga Z, Jasinskiene E, Sadauskaite-Kuehne V, et al. (2010) Associations between HLA class II haplotypes, environmental factors and type 1 diabetes mellitus in Lithuanian children with type 1 diabetes and controls. Polish Annals of Medicine 17: 7-15.

298. Samuelsson U, Johansson C, Ludvigsson J (1993) Breast-feeding seems to play a marginal role in the prevention of insulin-dependent diabetes mellitus. Diabetes Research & Clinical Practice 19: 203-210.

299. Siemiatycki J, Colle E, Campbell S, Dewar RA, Belmonte MM (1989) Case-control study of IDDM. Diabetes Care 12: 209-216.

300. Sipetic S, Vlajinac H, Kocev N, Bjekic M, Sajic S (2005) Early infant diet and risk of type 1 diabetes mellitus in Belgrade children. Nutrition 21: 474-479.

301. Soltesz G, Jeges S, Dahlquist G (1994) Non-genetic risk determinants for type 1 (insulin-dependent) diabetes mellitus in childhood. Hungarian Childhood Diabetes Epidemiology Study Group. Acta Paediatrica 83: 730-735.

302. Stene LC, Ulriksen J, Magnus P, Joner G (2000) Use of cod liver oil during pregnancy associated with lower risk of Type I diabetes in the offspring.[Erratum appears in Diabetologia 2000 Nov;43(11):1451]. Diabetologia 43: 1093-1098.

303. Stene LC, Joner G (2003) Use of cod liver oil during the first year of life is associated with lower risk of childhood-onset type 1 diabetes: a large, population-based, case-control study. The American journal of clinical nutrition 78: 1128-1134.

304. Strotmeyer ES, Yang Z, LaPorte RE, Chang YF, Steenkiste AR, et al. (2004) Infant diet and type 1 diabetes in China. Diabetes Research & Clinical Practice 65: 283-292.

305. Tai TY, Wang CY, Lin LL, Lee LT, Tsai ST, et al. (1998) A case-control study on risk factors for Type 1 diabetes in Taipei City. Diabetes Res Clin Pract 42: 197-203.

306. Telahun M, Abdulkadir J, Kebede E (1994) The relation of early nutrition, infections and socio-economic factors to the development of childhood diabetes. Ethiopian Medical Journal 32: 239-244.

307. Tenconi MT, Devoti G, Comelli M, Pinon M, Capocchiano A, et al. (2007) Major childhood infectious diseases and other determinants associated with type 1 diabetes: a case-control study. Acta Diabetologica 44: 14-19.

308. Thorsdottir I, Birgisdottir BE, Johannsdottir IM, Harris DP, Hill J, et al. (2000) Different beta-casein fractions in Icelandic versus Scandinavian cow's milk may influence diabetogenicity of cow's milk in infancy and explain low incidence of insulin-dependent diabetes mellitus in Iceland. Pediatrics 106: 719-724.

309. Verge CF, Howard NJ, Irwig L, Simpson JM, Mackerras D, et al. (1994) Environmental factors in childhood IDDM. A population-based, case-control study. Diabetes Care 17: 1381-1389.

310. Virtanen SM, Rasanen L, Ylonen K, Aro A, Clayton D, et al. (1993) Early introduction of dairy products associated with increased risk of IDDM in Finnish children. Diabetes 42: 1786-1790.

311. Visalli N, Sebastiani L, Adorisio E, Conte A, De Cicco AL, et al. (2003) Environmental risk factors for type 1 diabetes in Rome and province. Archives of Disease in Childhood 88: 695-698.

312. Wadsworth EJ, Shield JP, Hunt LP, Baum JD (1997) A case-control study of environmental factors associated with diabetes in the under 5s. Diabetic Medicine 14: 390-396.

313. Glatthaar C, Whittall DE, Welborn TA, Gibson MJ, Brooks BH, et al. (1988) Diabetes in Western Australian children: Descriptive epidemiology. Medical Journal of Australia 148: 117-123.

314. Hummel M, Fuchtenbusch M, Schenker M, Ziegler AG (2000) No major association of breast-feeding, vaccinations, and childhood viral diseases with early islet autoimmunity in the German BABYDIAB Study. Diabetes Care 23: 969-974.

315. Hummel S, Hummel M, Banholzer J, Hanak D, Mollenhauer U, et al. (2007) Development of autoimmunity to transglutaminase C in children of patients with type 1 diabetes: relationship to islet autoantibodies and infant feeding. Diabetologia 50: 390-394.

316. Ziegler AG, Schmid S, Huber D, Hummel M, Bonifacio E (2003) Early infant feeding and risk of developing type 1 diabetes-associated autoantibodies. JAMA 290: 1721-1728.

317. Norris JM, Barriga K, Hoffenberg EJ, Taki I, Miao D, et al. (2005) Risk of celiac disease autoimmunity and timing of gluten introduction in the diet of infants at increased risk of disease. Journal of the American Medical Association 293: 2343-2351.

318. Welander A, Tjernberg AR, Montgomery SM, Ludvigsson J, Ludvigsson JF (2010) Infectious disease and risk of later celiac disease in childhood. Pediatrics 125: e530-536.

319. Ascher H, Krantz I, Rydberg L, Nordin P, Kristiansson B (1997) Influence of infant feeding and gluten intake on coeliac disease. Archives of Disease in Childhood 76: 113-117.

320. Auricchio S, Follo D, de Ritis G, Giunta A, Marzorati D, et al. (1983) Does breast feeding protect against the development of clinical symptoms of celiac disease in children? Journal of Pediatric Gastroenterology & Nutrition 2: 428-433.

321. Baron S, Turck D, Leplat C, Merle V, Gower-Rousseau C, et al. (2005) Environmental risk factors in paediatric inflammatory bowel diseases: a population based case control study. Gut 54: 357-363.

322. Bergstrand O, Hellers G (1983) Breast-feeding during infancy in patients who later develop Crohn's disease. Scandinavian Journal of Gastroenterology 18: 903-906.

323. Castiglione F, Diaferia M, Morace F, Labianca O, Meucci C, et al. (2012) Risk factors for inflammatory bowel diseases according to the "hygiene hypothesis": a case-control, multi-centre, prospective study in Southern Italy. Journal of Crohn's & colitis 6: 324-329.

324. Corrao G, Tragnone A, Caprilli R, Trallori G, Papi C, et al. (1998) Risk of inflammatory bowel disease attributable to smoking, oral contraception and breastfeeding in Italy: a nationwide case-control study. Cooperative Investigators of the Italian Group for the Study of the Colon and the Rectum (GISC). International Journal of Epidemiology 27: 397-404.

325. Decker E, Engelmann G, Findeisen A, Gerner P, Laass M, et al. (2010) Cesarean delivery is associated with celiac disease but not inflammatory bowel disease in children. Pediatrics 125: e1433-1440.

326. Ellis JA, Ponsonby AL, Pezic A, Chavez RA, Allen RC, et al. (2012) CLARITY - ChiLdhood Arthritis Risk factor Identification sTudY. Pediatric Rheumatology Online Journal 10: 37.

327. Falth-Magnusson K, Franzen L, Jansson G, Laurin P, Stenhammar L (1996) Infant feeding history shows distinct differences between Swedish celiac and reference children. Pediatric Allergy & Immunology 7: 1-5.

328. Fort P, Moses N, Fasano M, Goldberg T, Lifshitz F (1990) Breast and soy-formula feedings in early infancy and the prevalence of autoimmune thyroid disease in children. Journal of the American College of Nutrition 9: 164-167.

329. Gearry RB, Richardson AK, Frampton CM, Dodgshun AJ, Barclay ML (2010) Population-based cases control study of inflammatory bowel disease risk factors. Journal of Gastroenterology & Hepatology 25: 325-333.

330. Gilat T, Hacohen D, Lilos P, Langman MJ (1987) Childhood factors in ulcerative colitis and Crohn's disease. An international cooperative study. Scandinavian Journal of Gastroenterology 22: 1009-1024.

331. Greco L, Auricchio S, Mayer M, Grimaldi M (1988) Case control study on nutritional risk factors in celiac disease. Journal of Pediatric Gastroenterology & Nutrition 7: 395-399.

332. Gruber M, Marshall JR, Zielezny M, Lance P (1996) A case-control study to examine the influence of maternal perinatal behaviors on the incidence of Crohn's disease. Gastroenterology Nursing 19: 53-59.

333. Hansen TS, Jess T, Vind I, Elkjaer M, Nielsen MF, et al. (2011) Environmental factors in inflammatory bowel disease: a case-control study based on a Danish inception cohort. Journal of Crohn's & colitis 5: 577-584.

334. Ivarsson A, Hernell O, Stenlund H, Persson LA (2002) Breast-feeding protects against celiac disease. American Journal of Clinical Nutrition 75: 914-921.

335. Koletzko S, Griffiths A, Corey M, Smith C, Sherman P (1991) Infant feeding practices and ulcerative colitis in childhood. BMJ 302: 1580-1581.

336. Mason T, Rabinovich CE, Fredrickson DD, Amoroso K, Reed AM, et al. (1995) Breast feeding and the development of juvenile rheumatoid arthritis. Journal of Rheumatology 22: 1166-1170.

337. Pacilio A, Piccolo E, Scala MG, Auricchio R (2010) The natural history of celiac disease. Digestive and Liver Disease 42: S357.

338. Peters U, Schneeweiss S, Trautwein EA, Erbersdobler HF (2001) A case-control study of the effect of infant feeding on celiac disease. Annals of Nutrition & Metabolism 45: 135-142.

339. Roberts SE, Williams JG, Meddings D, Davidson R, Goldacre MJ (2009) Perinatal risk factors and coeliac disease in children and young adults: a record linkage study. Aliment Pharmacol Ther 29: 222-231.

340. Rosenberg AM (1996) Evaluation of associations between breast feeding and subsequent development of juvenile rheumatoid arthritis. Journal of Rheumatology 23: 1080-1082.

341. Sonntag B, Stolze B, Heinecke A, Luegering A, Heidemann J, et al. (2007) Preterm birth but not mode of delivery is associated with an increased risk of developing inflammatory bowel disease later in life. Inflammatory Bowel Diseases 13: 1385-1390.

342. Thompson NP, Montgomery SM, Wadsworth MEJ, Pounder RE, Wakefield AJ (2000) Early determinants of inflammatory bowel disease: use of two national longitudinal birth cohorts. European Journal of Gastroenterology & Hepatology 12: 25-30.

343. Wang YF, Ou-Yang Q, Xia B, Liu LN, Gu F, et al. (2013) Multicenter case-control study of the risk factors for ulcerative colitis in China. World Journal of Gastroenterology 19: 1827-1833.

344. Falth-Magnusson K, Kjellman NI (1987) Development of atopic disease in babies whose mothers were receiving exclusion diet during pregnancy--a randomized study. Journal of Allergy & Clinical Immunology 80: 868-875.

345. Falth-Magnusson K, Kjellman NI (1992) Allergy prevention by maternal elimination diet during late pregnancy--a 5-year follow-up of a randomized study. Journal of Allergy & Clinical Immunology 89: 709-713.

346. Ludvigsson J (2003) Cow-milk-free diet during last trimester of pregnancy does not influence diabetes-related autoantibodies in nondiabetic children. Annals of the New York Academy of Sciences 1005: 275-278.

347. Jirapinyo P, Densupsoontorn N, Kangwanpornsiri C, Limlikhit T (2013) Lower prevalence of a topic dermatitis in breast-fed infants whose allergic mothers restrict dairy products. Journal of the Medical Association of Thailand 96: 192-195.

348. Lilja G, Dannaeus A, Foucard T, Graff-Lonnevig V, Johansson SG, et al. (1989) Effects of maternal diet during late pregnancy and lactation on the development of atopic diseases in infants up to 18 months of age--in-vivo results. Clinical & Experimental Allergy 19: 473-479.

349. Hattevig G, Kjellman B, Sigurs N, Grodzinsky E, Hed J, et al. (1990) The effect of maternal avoidance of eggs, cow's milk, and fish during lactation on the development of IgE, IgG, and IgA antibodies in infants. Journal of Allergy & Clinical Immunology 85: 108-115.

350. Paronen J, Bjorksten B, Hattevig G, Akerblom HK, Vaarala O (2000) Effect of maternal diet during lactation on development of bovine insulin-binding antibodies in children at risk for allergy. Journal of Allergy & Clinical Immunology 106: 302-306.

351. Hattevig G, Sigurs N, Kjellman B (1999) Effects of maternal dietary avoidance during lactation on allergy in children at 10 years of age. Acta Paediatrica 88: 7-12.

352. Hattevig G, Kjellman B, Sigurs N, Bjorksten B, Kjellman NI (1989) Effect of maternal avoidance of eggs, cow's milk and fish during lactation upon allergic manifestations in infants. Clin Exp Allergy 19: 27-32.

353. Sigurs N, Hattevig G, Kjellman B (1992) Maternal avoidance of eggs, cow's milk, and fish during lactation: effect on allergic manifestations, skin-prick tests, and specific IgE antibodies in children at age 4 years. Pediatrics 89: 735-739.

354. Herrmann ME, Dannemann A, Gruters A, Radisch B, Dudenhausen JW, et al. (1996) Prospective study of the atopy preventive effect of maternal avoidance of milk and eggs during pregnancy and lactation. European Journal of Pediatrics 155: 770-774.

355. Kilburn SA, Pollard C, Bevin S, Hourihane JO, Warner JO, et al. (1998) Allergens in mother's milk: Tolerisation or sensitization. Nutrition Research 18: 1351-1361.

356. Metcalfe JR, D'Vaz N, Makrides M, Gold MS, Quinn P, et al. (2016) Elevated IL-5 and IL-13 responses to egg proteins predate the introduction of egg in solid foods in infants with eczema. Clinical and experimental allergy : journal of the British Society for Allergy and Clinical Immunology 46: 308-316.

357. Becker A, Watson W, Ferguson A, Dimich-Ward H, Chan-Yeung M (2004) The Canadian asthma primary prevention study: outcomes at 2 years of age. Journal of Allergy & Clinical Immunology 113: 650-656.

358. Chan-Yeung M, Manfreda J, Dimich-Ward H, Ferguson A, Watson W, et al. (2000) A randomized controlled study on the effectiveness of a multifaceted intervention program in the primary prevention of asthma in high-risk infants. Archives of Pediatrics & Adolescent Medicine 154: 657-663.

359. Chan-Yeung M, Ferguson A, Watson W, Dimich-Ward H, Rousseau R, et al. (2005) The Canadian Childhood Asthma Primary Prevention Study: outcomes at 7 years of age. Journal of Allergy & Clinical Immunology 116: 49-55.

360. Wong T, Chan-Yeung M, Rousseau R, Dybuncio A, Kozyrskyj AL, et al. (2013) Delayed introduction of food and effect on incidence of food allergy in a population at high risk for atopy: The Canadian asthma primary prevention study (CAPPS). Journal of Allergy and Clinical Immunology 1): AB96.

361. Protudjer JLP, Robertson L, Kozyrskyj A, Ramsey CD, Chan-Yeung M, et al. (2011) Non-atopic asthma and atopic asthma are associated with reduced pulmonary function by age 7 years. American Journal of Respiratory and Critical Care Medicine 183 (1 MeetingAbstracts).

362. Carlsten C, Dimich-Ward H, Ferguson A, Watson W, Rousseau R, et al. (2013) Atopic dermatitis in a high-risk cohort: Natural history, associated allergic outcomes, and risk factors. Annals of Allergy, Asthma and Immunology 110: 24-28.

363. Hide DW (1994) The Isle of Wight study, an approach to allergy prevention. Pediatric Allergy & Immunology 5: 61-64.

364. Hide DW, Matthews S, Tariq S, Arshad SH (1996) Allergen avoidance in infancy and allergy at 4 years of age. Allergy 51: 89-93.

365. Arshad SH, Matthews S, Gant C, Hide DW (1992) Effect of allergen avoidance on development of allergic disorders in infancy. Lancet 339: 1493-1497.

366. Arshad SH, Bateman B, Matthews SM (2003) Primary prevention of asthma and atopy during childhood by allergen avoidance in infancy: a randomised controlled study. Thorax 58: 489-493.

367. Arshad SH, Bateman B, Sadeghnejad A, Gant C, Matthews SM (2007) Prevention of allergic disease during childhood by allergen avoidance: the Isle of Wight prevention study. Journal of Allergy & Clinical Immunology 119: 307-313.

368. Scott M, Roberts G, Kurukulaaratchy RJ, Matthews S, Nove A, et al. (2012) Multifaceted allergen avoidance during infancy reduces asthma during childhood with the effect persisting until age 18 years. Thorax 67: 1046-1051.

369. Lovegrove JA, Hampton SM, Morgan JB (1994) The immunological and long-term atopic outcome of infants born to women following a milk-free diet during late pregnancy and lactation: a pilot study. British Journal of Nutrition 71: 223-238.

370. Shao J, Sheng J, Dong W, Li YZ, Yu SC (2006) [Effects of feeding intervention on development of eczema in atopy high-risk infants: an 18-month follow-up study]. Zhonghua Erke Zazhi 44: 684-687.

371. Zeiger RS, Heller S, Mellon MH, Halsey JF, Hamburger RN, et al. (1992) Genetic and environmental factors affecting the development of atopy through age 4 in children of atopic parents: A prospective randomized study of food allergen avoidance. Pediatric Allergy and Immunology 3: 110-127.

372. Zeiger RS, Heller S, Mellon MH, Forsythe AB, O'Connor RD, et al. (1989) Effect of combined maternal and infant food-allergen avoidance on development of atopy in early infancy: a randomized study J Allergy Clin Immunol 1989 Nov;84(5 Pt 1):677. Journal of Allergy and Clinical Immunology. pp. 72-89.

373. Zeiger RS (1994) Dietary manipulations in infants and their mothers and the natural course of atopic disease. Pediatric Allergy & Immunology 5: 33-43.

374. Halmerbauer G, Gartner C, Schierl M, Arshad H, Dean T, et al. (2003) Study on the Prevention of Allergy in Children in Europe (SPACE): Allergic sensitization at 1 year of age in a controlled trial of allergen avoidance from birth. Pediatric Allergy and Immunology 14: 10-17.

375. Halmerbauer G, Gartner C, Schierl M, Arshad H, Dean T, et al. (2002) Study on the Prevention of Allergy in Children in Europe (SPACE): Allergic sensitization in children at 1 year of age in a controlled trial of allergen avoidance from birth. Pediatric Allergy and Immunology, Supplement 13: 47-54.

376. Matthew DJ, Taylor B, Norman AP, Turner MW (1977) Prevention of eczema. Lancet 1: 321-324.

377. Poysa L, Korppi M, Remes K, Juntunen-Backman K (1991) Atopy in childhood and diet in infancy. A nine-year follow-up study. I. Clinical manifestations. Allergy Proceedings 12: 107-111.

378. Poysa L, Remes K, Korppi M, Juntunen-Backman K (1989) Atopy in children with and without a family history of atopy. I. Clinical manifestations, with special reference to diet in infancy. Acta Paediatrica Scandinavica 78: 896-901.

379. Kuikka L, Korppi M, Remes K (1995) Atopy and skin reactivity at school age in children followed up from birth, with special reference to atopy prevention in infancy and atopic findings at preschool age. Allergy Proceedings 16: 313-317.

380. Boyle RJ, Brown N, Chiang WC, Chien CM, Gold M, et al. (2015) Partially hydrolysed prebiotic supplemented whey formula for the prevention of allergic manifestations in high risk infants: a multicentre double blind randomised controlled trial. Clin Trans Allergy 5: P30.

381. Boyle RJ BN, Chiang WC, Chien CM, Gold M, Hourihane J (2015) Partially hydrolysed prebiotic supplemented whey formula for the prevention of allergic manifestations in high risk infants: a multicentre double blind randomised controlled trial. Clinical Translational Allergy 15: 30.

382. Boyle RJ, Tang MLK, Chiang WC, Chua MC, Ismail I, et al. (2016) Prebiotic-supplemented partially hydrolysed cow's milk formula for the prevention of eczema in high-risk infants: a randomized controlled trial. Allergy 71: 701-710.

383. Gruber C, Van Stuijvenberg M, Mosca F, Moro G, Chirico G, et al. (2010) Reduced occurrence of early atopic dermatitis because of immunoactive prebiotics among low-atopy-risk infants. Journal of Allergy and Clinical Immunology 126: 791-797.

384. Gruber C, Margriet VS, Mosca F, Moro G, Chirico G, et al. (2015) Prevention of early atopic dermatitis among low-atopy-risk infants by immunoactive prebiotics is not sustained after the first year of life. Blackwell Publishing Ltd. pp. 286-287.

385. Ivakhnenko O, Niankovskyy S (2013) [Clinical effectiveness of probiotics in complex treatment of infants with cow's milk allergy]. Georgian medical news. pp. 39-45.

386. Moro G, Arslanoglu S, Stahl B, Jelinek J, Wahn U, et al. (2006) A mixture of prebiotic oligosaccharides reduces the incidence of atopic dermatitis during the first six months of age. Archives of Disease in Childhood 91: 814-819.

387. van Hoffen E, Ruiter B, Faber J, M'Rabet L, Knol EF, et al. (2009) A specific mixture of short-chain galacto-oligosaccharides and long-chain fructo-oligosaccharides induces a beneficial immunoglobulin profile in infants at high risk for allergy. Allergy 64: 484-487.

388. Arslanoglu S, Moro GE, Schmitt J, Tandoi L, Rizzardi S, et al. (2008) Early dietary intervention with a mixture of prebiotic oligosaccharides reduces the incidence of allergic manifestations and infections during the first two years of life. Journal of Nutrition 138: 1091-1095.

389. Arslanoglu S, Moro GE, Boehm G, Wienz F, Stahl B, et al. (2012) Early neutral prebiotic oligosaccharide supplementation reduces the incidence of some allergic manifestations in the first 5 years of life. Journal of Biological Regulators & Homeostatic Agents 26: 49-59.

390. Sierra C, Bernal MJ, Blasco J, Martinez R, Dalmau J, et al. (2015) Prebiotic effect during the first year of life in healthy infants fed formula containing GOS as the only prebiotic: a multicentre, randomised, double-blind and placebo-controlled trial. European Journal of Nutrition 54: 89-99.

391. Ziegler E, Vanderhoof JA, Petschow B, Mitmesser SH, Stolz SI, et al. (2007) Term infants fed formula supplemented with selected blends of prebiotics grow normally and have soft stools similar to those reported for breast-fed infants. Journal of Pediatric Gastroenterology & Nutrition 44: 359-364.

392. Chien CM, Anne GEN, Chin CW, Rao R, Charmaine C, et al. (2016) A synbiotic mixture of scGOS/lcFOS and Bifidobacterium breve M-16V is able to restore the delayed colonization of bifidobacterium observed in C-section Delivered Infants. Lippincott Williams and Wilkins. pp. 681.

393. Kukkonen K, Savilahti E, Haahtela T, Juntunen-Backman K, Korpela R, et al. (2007) Probiotics and prebiotic galacto-oligosaccharides in the prevention of allergic diseases: a randomized, double-blind, placebo-controlled trial. Journal of Allergy & Clinical Immunology 119: 192-198.

394. Kuitunen M, Kukkonen K, Juntunen-Backman K, Korpela R, Poussa T, et al. (2009) Probiotics prevent IgE-associated allergy until age 5 years in cesarean-delivered children but not in the total cohort. Journal of Allergy & Clinical Immunology 123: 335-341.

395. Kukkonen AK, Kuitunen M, Savilahti E, Pelkonen A, Malmberg P, et al. (2011) Airway inflammation in probiotic-treated children at 5 years. Pediatric Allergy & Immunology 22: 249-251.

396. Roze JC, Barbarot S, Butel MJ, Kapel N, Waligora-Dupriet AJ, et al. (2012) An alpha-lactalbumin-enriched and symbiotic-supplemented v. a standard infant formula: A multicentre, double-blind, randomised trial. British Journal of Nutrition 107: 1616-1622.

397. Van Der Aa L, Heymans H, Van Aalderen W, Sillevis Smitt H, Nauta A, et al. (2010) Specific synbiotic mixture prevents asthma-like symptoms in infants with atopic dermatitis. Allergy: European Journal of Allergy and Clinical Immunology 65: 313.

398. Abrahamsson TR, Jakobsson T, Bottcher MF, Fredrikson M, Jenmalm MC, et al. (2007) Probiotics in prevention of IgE-associated eczema: a double-blind, randomized, placebo-controlled trial. Journal of Allergy & Clinical Immunology 119: 1174-1180.

399. Abrahamsson TR, Jakobsson T, Bjorksten B, Oldaeus G, Jenmalm MC (2013) No effect of probiotics on respiratory allergies: a seven-year follow-up of a randomized controlled trial in infancy. Pediatric Allergy & Immunology 24: 556-561.

400. Allen SJ, Jordan S, Storey M, Thornton CA, Gravenor MB, et al. (2012) Probiotics and atopic ECZEMA: A double-blind randomised controlled trial. Archives of Disease in Childhood 97: A2.

401. Allen SJ, Jordan S, Storey M, Thornton CA, Gravenor MB, et al. (2014) Probiotics in the prevention of eczema: a randomised controlled trial. Archives of Disease in Childhood 99: 1014-1019.

402. Boyle RJ, Ismail IH, Kivivuori S, Licciardi PV, Robins-Browne RM, et al. (2011) Lactobacillus GG treatment during pregnancy for the prevention of eczema: a randomized controlled trial. Allergy 66: 509-516.

403. Cabana M, McKean M, Caughey A, Leong R, Wong A, et al. (2015) A randomized controlled trial of early probiotic supplementation to prevent early markers of asthma for high-risk infants. European Respiratory Journal ( varpagings).

404. De Leon J, Sumpaico MW, Recto MT, Tan RA (2007) A preliminary study on the role of probiotics (lactobacillus acidophilus/bifidobacterium) in the prevention of atopic dermatitis in high-risk infants (0-2 weeks old): A randomized placebo-controlled trial. Annals of Allergy Asthma & Immunology 98: A84-A84.

405. Simon AL, Sumpaico MW, Recto MT, Castor MR, Tan RA (2007) The effects of probiotics on total ige levels of infants at risk for the development of atopic disease: A randomized triple blind placebo controlled clinical trial. Annals of Allergy Asthma & Immunology 98: A94-A95.

406. Dotterud CK, Storro O, Johnsen R, Oien T (2010) Probiotics in pregnant women to prevent allergic disease: a randomized, double-blind trial. British Journal of Dermatology 163: 616-623.

407. Simpson MR, Brede G, Johansen J, Johnsen R, Storro O, et al. (2015) Human Breast Milk miRNA, Maternal Probiotic Supplementation and Atopic Dermatitis in Offspring. PloS one 10: e0143496.

408. Enomoto T, Sowa M, Nishimori K, Shimazu S, Yoshida A, et al. (2014) Effects of bifidobacterial supplementation to pregnant women and infants in the prevention of allergy development in infants and on fecal microbiota. Allergology International 63: 575-585.

409. Huurre A, Laitinen K, Rautava S, Korkeamaki M, Isolauri E (2008) Impact of maternal atopy and probiotic supplementation during pregnancy on infant sensitization: a double-blind placebo-controlled study. Clinical & Experimental Allergy 38: 1342-1348.

410. Kalliomaki M, Salminen S, Arvilommi H, Kero P, Koskinen P, et al. (2001) Probiotics in primary prevention of atopic disease: a randomised placebo-controlled trial. Lancet 357: 1076-1079.

411. Kalliomaki M, Salminen S, Poussa T, Arvilommi H, Isolauri E (2003) Probiotics and prevention of atopic disease: 4-year follow-up of a randomised placebo-controlled trial. Lancet 361: 1869-1871.

412. Kalliomaki M, Salminen S, Poussa T, Isolauri E (2007) Probiotics during the first 7 years of life: a cumulative risk reduction of eczema in a randomized, placebo-controlled trial. Journal of Allergy & Clinical Immunology 119: 1019-1021.

413. Rautava S, Kalliomäki M, Isolauri E (2002) Probiotics during pregnancy and breast-feeding might confer immunomodulatory protection against atopic disease in the infant. The Journal of allergy and clinical immunology. pp. 119-121.

414. Kim JY, Kwon JH, Ahn SH, Lee SI, Han YS, et al. (2010) Effect of probiotic mix (Bifidobacterium bifidum, Bifidobacterium lactis, Lactobacillus acidophilus) in the primary prevention of eczema: a double-blind, randomized, placebo-controlled trial. Pediatric Allergy & Immunology 21: e386-393.

415. Kopp MV, Hennemuth I, Heinzmann A, Urbanek R (2008) Randomized, double-blind, placebo-controlled trial of probiotics for primary prevention: no clinical effects of Lactobacillus GG supplementation. Pediatrics 121: e850-856.

416. Lau S, Gerhold K, Zimmermann K, Ockeloen CW, Rossberg S, et al. (2012) Oral application of bacterial lysate in infancy decreases the risk of atopic dermatitis in children with 1 atopic parent in a randomized, placebo-controlled trial. Journal of Allergy & Clinical Immunology 129: 1040-1047.

417. Lodinová-Zádníková R, Prokesová L, Kocourková I, Hrdý J, Zizka J (2010) Prevention of allergy in infants of allergic mothers by probiotic Escherichia coli. International archives of allergy and immunology. pp. 201-206.

418. Lundelin K, Poussa T, Salminen S, Isolauri E (2016) Long-term safety and efficacy of perinatal probiotic intervention: Evidence from a follow-up study of four randomized, double-blind, placebo-controlled trials. United Kingdom: Blackwell Publishing Ltd (E-mail: customerservices@oxonblackwellpublishing.com). pp. no pagination.

419. Luoto R, Kinnunen TI, Aittasalo M, Kolu P, Raitanen J, et al. (2017) Primary prevention of gestational diabetes mellitus and large-for-gestational-age newborns by lifestyle counseling: a cluster-randomized controlled trial. Plos medicine. pp. e1001036.

420. Morisset M, Soulaines P, Aubert-Jacquin C, Codreanu F, Maamri N, et al. (2008) Double blind test of a fermented infantile formula in cow's milk allergy prevention [Abstract]. Journal of Allergy and Clinical Immunology. pp. S244 [941].

421. Niers L, Martin R, Rijkers G, Sengers F, Timmerman H, et al. (2009) The effects of selected probiotic strains on the development of eczema (the PandA study). Allergy 64: 1349-1358.

422. Gorissen DM, Rutten NB, Oostermeijer CM, Niers LE, Hoekstra MO, et al. (2014) Preventive effects of selected probiotic strains on the development of asthma and allergic rhinitis in childhood. The Panda study. Clin Exp Allergy 44: 1431-1433.

423. Ou CY, Kuo HC, Wang L, Hsu TY, Chuang H, et al. (2012) Prenatal and postnatal probiotics reduces maternal but not childhood allergic diseases: a randomized, double-blind, placebo-controlled trial. Clinical & Experimental Allergy 42: 1386-1396.

424. Taylor AL, Dunstan JA, Prescott SL (2007) Probiotic supplementation for the first 6 months of life fails to reduce the risk of atopic dermatitis and increases the risk of allergen sensitization in high-risk children: a randomized controlled trial. Journal of Allergy & Clinical Immunology 119: 184-191.

425. Taylor SN, Wagner CL, Hollis BW (2008) Vitamin D supplementation during lactation to support infant and mother. Journal of the American College of Nutrition 27: 690-701.

426. Jensen MP, Meldrum S, Taylor AL, Dunstan JA, Prescott SL (2012) Early probiotic supplementation for allergy prevention: long-term outcomes. Journal of Allergy & Clinical Immunology 130: 1209-1211.e1205.

427. Rautava S, Arvilommi H, Isolauri E (2006) Specific probiotics in enhancing maturation of IgA responses in formula-fed infants. Pediatric Research 60: 221-224.

428. Rautava S, Kainonen E, Salminen S, Isolauri E (2012) Maternal probiotic supplementation during pregnancy and breast-feeding reduces the risk of eczema in the infant. Journal of Allergy & Clinical Immunology 130: 1355-1360.

429. Scalabrin DM, Johnston WH, Hoffman DR, P'Pool VL, Harris CL, et al. (2009) Growth and tolerance of healthy term infants receiving hydrolyzed infant formulas supplemented with Lactobacillus rhamnosus GG: randomized, double-blind, controlled trial. Clinical Pediatrics 48: 734-744.

430. Scalabrin DMF, Harris C, Strong PV, Liu B, Berseth CL (2014) Infant supplementation with Lactobacillus rhamnosus GG (LGG) and long-term growth and health: A 5-year follow-up. Allergy: European Journal of Allergy and Clinical Immunology 69: 196-197.

431. Scalabrin D, Harris C, Johnston W, Berseth C (2017) Long-term safety assessment in children who received hydrolyzed protein formulas with Lactobacillus rhamnosus GG: a 5-year follow-up. 2 ed. Germany: Springer Verlag (E-mail: service@springer.de). pp. 217-224.

432. Soh SE, Aw M, Gerez I, Chong YS, Rauff M, et al. (2009) Probiotic supplementation in the first 6 months of life in at risk Asian infants--effects on eczema and atopic sensitization at the age of 1 year. Clinical & Experimental Allergy 39: 571-578.

433. Loo EX, Llanora GV, Lu Q, Aw MM, Lee BW, et al. (2014) Supplementation with probiotics in the first 6 months of life did not protect against eczema and allergy in at-risk Asian infants: a 5-year follow-up. International Archives of Allergy & Immunology 163: 25-28.

434. West CE, Hammarstrom ML, Hernell O (2009) Probiotics during weaning reduce the incidence of eczema. Pediatric Allergy & Immunology 20: 430-437.

435. West CE, Hammarstrom ML, Hernell O (2013) Probiotics in primary prevention of allergic disease--follow-up at 8-9 years of age. Allergy 68: 1015-1020.

436. Wickens K, Black PN, Stanley TV, Mitchell E, Fitzharris P, et al. (2008) A differential effect of 2 probiotics in the prevention of eczema and atopy: a double-blind, randomized, placebo-controlled trial. Journal of Allergy & Clinical Immunology 122: 788-794.

437. Wickens K, Black P, Stanley TV, Mitchell E, Barthow C, et al. (2012) A protective effect of Lactobacillus rhamnosus HN001 against eczema in the first 2 years of life persists to age 4 years. Clinical & Experimental Allergy 42: 1071-1079.

438. Wickens K, Stanley TV, Mitchell EA, Barthow C, Fitzharris P, et al. (2013) Early supplementation with Lactobacillus rhamnosus HN001 reduces eczema prevalence to 6 years: does it also reduce atopic sensitization? Clinical & Experimental Allergy 43: 1048-1057.

439. Biron-Shental T, Sukenik-Halevy R, Naboani H, Liberman M, Kats R, et al. (2015) Telomeres are shorter in placentas from pregnancies with uncontrolled diabetes. Placenta 36: 199-203.

440. Birch EE, Khoury JC, Berseth CL, Castaneda YS, Couch JM, et al. (2010) The impact of early nutrition on incidence of allergic manifestations and common respiratory illnesses in children. Journal of Pediatrics 156: 902-906, 906.e901.

441. Foiles AM, Kerling EH, Wick JA, Scalabrin DMF, Colombo J, et al. (2016) Formula with long-chain polyunsaturated fatty acids reduces incidence of allergy in early childhood. 2 ed. United Kingdom: Blackwell Publishing Ltd (E-mail: customerservices@oxonblackwellpublishing.com). pp. 156-161.

442. Chawes BL, Bonnelykke K, Stokholm J, Vissing NH, Bjarnadottir E, et al. (2016) Effect of Vitamin D3 Supplementation During Pregnancy on Risk of Persistent Wheeze in the Offspring: A Randomized Clinical Trial. JAMA 315: 353-361.

443. Harslof LBS, Damsgaard CT, Andersen AD, Aakjaer DL, Michaelsen KF, et al. (2015) Reduced ex vivo stimulated IL-6 response in infants randomized to fish oil from 9 to 18 months, especially among PPARG2 and COX2 wild types. Prostaglandins Leukotrienes and Essential Fatty Acids 94: 21-27.

444. Lucas A, Stafford M, Morley R, Abbott R, Stephenson T, et al. (1999) Efficacy and safety of long-chain polyunsaturated fatty acid supplementation of infant-formula milk: a randomised trial. Lancet 354: 1948-1954.

445. van Gool CJ, Thijs C, Henquet CJ, van Houwelingen AC, Dagnelie PC, et al. (2003) Gamma-linolenic acid supplementation for prophylaxis of atopic dermatitis--a randomized controlled trial in infants at high familial risk. American Journal of Clinical Nutrition 77: 943-951.

446. Linnamaa P, Savolainen J, Koulu L, Tuomasjukka S, Kallio H, et al. (2010) Blackcurrant seed oil for prevention of atopic dermatitis in newborns: a randomized, double-blind, placebo-controlled trial. Clinical & Experimental Allergy 40: 1247-1255.

447. Mihrshahi S, Peat JK, Marks GB, Mellis CM, Tovey ER, et al. (2003) Eighteen-month outcomes of house dust mite avoidance and dietary fatty acid modification in the Childhood Asthma Prevention Study (CAPS) J Allergy Clin Immunol. 2003 Apr;111(4):735. Journal of Allergy and Clinical Immunology. pp. 162-168.

448. Peat JK, Mihrshahi S, Kemp AS, Marks GB, Tovey ER, et al. (2004) Three-year outcomes of dietary fatty acid modification and house dust mite reduction in the Childhood Asthma Prevention Study. Journal of Allergy & Clinical Immunology 114: 807-813.

449. Marks GB, Mihrshahi S, Kemp AS, Tovey ER, Webb K, et al. (2006) Prevention of asthma during the first 5 years of life: a randomized controlled trial. Journal of Allergy & Clinical Immunology 118: 53-61.

450. Damsgaard CT, Lauritzen L, Kjaer TMR, Holm PMI, Fruekilde MB, et al. (2007) Fish oil supplementation modulates immune function in healthy infants. Journal of Nutrition 137: 1031-1036.

451. Palmer DJ, Sullivan T, Gold MS, Prescott SL, Heddle R, et al. (2012) Effect of n-3 long chain polyunsaturated fatty acid supplementation in pregnancy on infants' allergies in first year of life: randomised controlled trial. BMJ 344: e184.

452. Palmer DJ, Sullivan T, Gold MS, Prescott SL, Heddle R, et al. (2013) Effect of n-3 polyunsaturated fatty acid supplementation in pregnancy on early childhood allergic disease: Randomized controlled trial. Journal of Paediatrics and Child Health 49: 56.

453. Best K, Sullivan T, Gold M, Kennedy D, Martin J, et al. (2015) Six-year follow up of children at high hereditary risk of allergy, born to mothers supplemented with docosahexaenoic acid (DHA) in the domino trial. Journal of Paediatrics and Child Health. pp. 58.

454. Best KP, Sullivan T, Palmer D, Gold M, Kennedy DJ, et al. (2016) Prenatal fish oil supplementation and allergy: 6-Year follow-up of a randomized controlled trial. Pediatrics.

455. Dunstan JA, Mori TA, Barden A, Beilin LJ, Taylor AL, et al. (2003) Fish oil supplementation in pregnancy modifies neonatal allergen-specific immune responses and clinical outcomes in infants at high risk of atopy: a randomized, controlled trial. Journal of Allergy & Clinical Immunology 112: 1178-1184.

456. D'Vaz N, Meldrum SJ, Dunstan JA, Martino D, McCarthy S, et al. (2012) Postnatal fish oil supplementation in high-risk infants to prevent allergy: randomized controlled trial. Pediatrics 130: 674-682.

457. Furuhjelm C, Warstedt K, Larsson J, Fredriksson M, Bottcher MF, et al. (2009) Fish oil supplementation in pregnancy and lactation may decrease the risk of infant allergy. Acta Paediatrica 98: 1461-1467.

458. Furuhjelm C, Warstedt K, Fagerås M, Fälth-Magnusson K, Larsson J, et al. (2011) Allergic disease in infants up to 2 years of age in relation to plasma omega-3 fatty acids and maternal fish oil supplementation in pregnancy and lactation. Pediatric allergy and immunology : official publication of the European Society of Pediatric Allergy and Immunology. pp. 505-514.

459. Lauritzen L, Kjaer TM, Fruekilde MB, Michaelsen KF, Frokiaer H (2005) Fish oil supplementation of lactating mothers affects cytokine production in 2 1/2-year-old children. Lipids 40: 669-676.

460. Olsen SF, Osterdal ML, Salvig JD, Mortensen LM, Rytter D, et al. (2008) Fish oil intake compared with olive oil intake in late pregnancy and asthma in the offspring: 16 y of registry-based follow-up from a randomized controlled trial. American Journal of Clinical Nutrition 88: 167-175.

461. Hansen S, Strom M, Maslova E, Dahl R, Hoffmann HJ, et al. (2017) Fish oil supplementation during pregnancy and allergic respiratory disease in the adult offspring. Journal of Allergy and Clinical Immunology 139: 104.

462. Dotterud CK, Storro O, Simpson MR, Johnsen R, Oien T (2013) The impact of pre- and postnatal exposures on allergy related diseases in childhood: a controlled multicentre intervention study in primary health care. BMC Public Health 13: 123.

463. Imhoff-Kunsch B, Stein AD, Martorell R, Parra-Cabrera S, Romieu I, et al. (2011) Prenatal docosahexaenoic acid supplementation and infant morbidity: randomized controlled trial. Pediatrics 128: e505-512.

464. Noakes PS, Vlachava M, Kremmyda LS, Diaper ND, Miles EA, et al. (2012) Increased intake of oily fish in pregnancy: effects on neonatal immune responses and on clinical outcomes in infants at 6 mo. American Journal of Clinical Nutrition 95: 395-404.

465. Aage S, Kiraly N, Da Costa K, Byberg S, Bjerregaard-Andersen M, et al. (2015) Neonatal vitamin A supplementation associated with increased atopy in girls. 8 ed. United Kingdom: Blackwell Publishing Ltd (E-mail: customerservices@oxonblackwellpublishing.com). pp. 985-994.

466. Checkley W, West KP, Wise RA, Baldwin MR, Wu L, et al. (2010) Maternal Vitamin A Supplementation and Lung Function in Offspring. New England Journal of Medicine 362: 1784-1794.

467. Checkley W, West KP, Jr., Wise RA, Wu L, LeClerq SC, et al. (2011) Supplementation with vitamin A early in life and subsequent risk of asthma. European Respiratory Journal 38: 1310-1319.

468. Czeizel AE, Dobo M (1994) Postnatal somatic and mental development after periconceptional multivitamin supplementation. Archives of Disease in Childhood 70: 229-233.

469. Dobo M, Czeizel AE (1998) Long-term somatic and mental development of children after periconceptional multivitamin supplementation. European Journal of Pediatrics 157: 719-723.

470. Devakumar D, Stocks J, Ayres JG, Kirkby J, Yadav SK, et al. (2015) Effects of antenatal multiple micronutrient supplementation on lung function in mid-childhood: Follow-up of a double-blind randomised controlled trial in Nepal. European Respiratory Journal 45: 1566-1575.

471. Grant C, Knight J, Milne T, Sinclair J, Camargo C (2016) Vitamin D supplementation during pregnancy and infancy reduces food allergen sensitisation and parental-reported food allergy: A randomised controlled trial. 11 ed. Netherlands: Springer Verlag. pp. 1437-1438.

472. Greenough A, Shaheen SO, Shennan A, Seed PT, Poston L (2010) Respiratory outcomes in early childhood following antenatal vitamin C and E supplementation. Thorax 65: 998-1003.

473. Goldring ST, Griffiths CJ, Martineau AR, Robinson S, Yu C, et al. (2013) Prenatal vitamin d supplementation and child respiratory health: a randomised controlled trial. PLoS ONE [Electronic Resource] 8: e66627.

474. Kiraly N, Balde A, Lisse IM, Eriksen HB, Aaby P, et al. (2013) Vitamin A supplementation and risk of atopy: long-term follow-up of a randomized trial of vitamin A supplementation at six and nine months of age. BMC Pediatr 13: 190.

475. Litonjua AA, Carey VJ, Laranjo N, Harshfield BJ, McElrath TF, et al. (2016) Effect of Prenatal Supplementation With Vitamin D on Asthma or Recurrent Wheezing in Offspring by Age 3 Years: The VDAART Randomized Clinical Trial. JAMA 315: 362-370.

476. McEvoy CT, Schilling D, Clay N, Jackson K, Go MD, et al. (2014) Vitamin C supplementation for pregnant smoking women and pulmonary function in their newborn infants: a randomized clinical trial. JAMA 311: 2074-2082.

477. Goksor E, Alm B, Thengilsdottir H, Mollborg P, Pettersson R, et al. (2011) Characteristics and risk factors for doctor-diagnosed food allergy at preschool age. Allergy: European Journal of Allergy and Clinical Immunology 66: 219.

478. Alm B, Goksor E, Thengilsdottir H, Pettersson R, Mollborg P, et al. (2011) Early protective and risk factors for allergic rhinitis at age 41/2 yr. Pediatric Allergy & Immunology 22: 398-404.

479. Andreasyan K, Ponsonby AL, Dwyer T, Dear K, Cochrane J (2007) Infant feeding and childhood atopy: does early introduction of non-milk fluids matter? Pediatric Allergy & Immunology 18: 250-257.

480. Back O, Blomquist HK, Hernell O, Stenberg B (2009) Does vitamin D intake during infancy promote the development of atopic allergy? Acta Dermato-Venereologica 89: 28-32.

481. Baiz N, Dargent-Molina P, Wark JD, Souberbielle JC, Annesi-Maesano I, et al. (2014) Cord serum 25-hydroxyvitamin D and risk of early childhood transient wheezing and atopic dermatitis. J Allergy Clin Immunol 133: 147-153.

482. Bekkers MB, Elstgeest LE, Scholtens S, Haveman-Nies A, de Jongste JC, et al. (2012) Maternal use of folic acid supplements during pregnancy, and childhood respiratory health and atopy. European Respiratory Journal 39: 1468-1474.

483. Willers SM, Wijga AH, Brunekreef B, Kerkhof M, Gerritsen J, et al. (2008) Maternal food consumption during pregnancy and the longitudinal development of childhood asthma. American Journal of Respiratory & Critical Care Medicine 178: 124-131.

484. Bertelsen RJ, Brantsaeter AL, Haugen M, Meltzer HM, London SJ (2013) Maternal probiotic intake and respiratory and allergy outcomes in early childhood. Journal of Allergy and Clinical Immunology 1): AB129.

485. Camargo CA, Jr., Ingham T, Wickens K, Thadhani R, Silvers KM, et al. (2011) Cord-blood 25-hydroxyvitamin D levels and risk of respiratory infection, wheezing, and asthma. Pediatrics 127: e180-187.

486. Romieu I, Torrent M, Garcia-Esteban R, Ferrer C, Ribas-Fito N, et al. (2007) Maternal fish intake during pregnancy and atopy and asthma in infancy. Clinical & Experimental Allergy 37: 518-525.

487. Chatzi L, Torrent M, Romieu I, Garcia-Esteban R, Ferrer C, et al. (2008) Mediterranean diet in pregnancy is protective for wheeze and atopy in childhood. Thorax 63: 507-513.

488. Kiefte-de Jong JC, Timmermans S, Jaddoe VW, Hofman A, Tiemeier H, et al. (2012) High circulating folate and vitamin B-12 concentrations in women during pregnancy are associated with increased prevalence of atopic dermatitis in their offspring. Journal of Nutrition 142: 731-738.

489. Kiefte-de Jong JC, de Vries JH, Franco OH, Jaddoe VW, Hofman A, et al. (2012) Fish consumption in infancy and asthma-like symptoms at preschool age. Pediatrics 130: 1060-1068.

490. Leermakers ET, Sonnenschein-van der Voort AM, Heppe DH, de Jongste JC, Moll HA, et al. (2013) Maternal fish consumption during pregnancy and risks of wheezing and eczema in childhood: the Generation R Study. European Journal of Clinical Nutrition 67: 353-359.

491. Dubakiene R, Rudzeviciene O, Butiene I, Sezaite I, Petronyte M, et al. (2012) Studies on early allergic sensitization in the Lithuanian birth cohort. Thescientificworldjournal 2012: 909524.

492. Butiene I, Dubakiene R, Vaicekauskaite D, Drasutiene G (2011) Prevalence of food sensitisation in young children from EuroPrevall Lithuanian birth cohort. Allergy: European Journal of Allergy and Clinical Immunology 66: 233.

493. Grimshaw KEC, Oliver EM, Kemp T, Mills ENC, Beyer K, et al. (2012) Maternal dietary intake and subsequent allergy development. Journal of Allergy and Clinical Immunology 1): AB173.

494. Dunlop AL, Reichrtova E, Palcovicova L, Ciznar P, Adamcakova-Dodd A, et al. (2006) Environmental and dietary risk factors for infantile atopic eczema among a Slovak birth cohort. Pediatric Allergy & Immunology 17: 103-111.

495. Fergusson DM, Horwood LJ, Shannon FT (1990) Early solid feeding and recurrent childhood eczema: a 10-year longitudinal study. Pediatrics 86: 541-546.

496. Fitzsimon N, Fallon U, O'Mahony D, Loftus BG, Bury G, et al. (2007) Mothers' dietary patterns during pregnancy and risk of asthma symptoms in children at 3 years. Irish Medical Journal 100: suppl 27-32.

497. Gale CR, Robinson SM, Harvey NC, Javaid MK, Jiang B, et al. (2008) Maternal vitamin D status during pregnancy and child outcomes. European Journal of Clinical Nutrition 62: 68-77.

498. Hypponen E, Sovio U, Wjst M, Patel S, Pekkanen J, et al. (2004) Infant vitamin d supplementation and allergic conditions in adulthood: northern Finland birth cohort 1966. Annals of the New York Academy of Sciences 1037: 84-95.

499. Hypponen E, Laara E, Reunanen A, Jarvelin MR, Virtanen SM (2001) Intake of vitamin D and risk of type 1 diabetes: a birth-cohort study. Lancet 358: 1500-1503.

500. Jedrychowski W, Flak E, Mroz E, Pac A, Jacek R, et al. (2008) Modulating effects of maternal fish consumption on the occurrence of respiratory symptoms in early infancy attributed to prenatal exposure to fine particles. Annals of Nutrition & Metabolism 52: 8-16.

501. Jedrychowski W, Perera F, Maugeri U, Mrozek-Budzyn D, Miller RL, et al. (2011) Effects of prenatal and perinatal exposure to fine air pollutants and maternal fish consumption on the occurrence of infantile eczema. International Archives of Allergy & Immunology 155: 275-281.

502. Kemp AS, Ponsonby AL, Dwyer T, Cochrane JA, Pezic A, et al. (2011) Maternal antenatal peanut consumption and peanut and rye sensitization in the offspring at adolescence. Clinical & Experimental Allergy 41: 224-231.

503. Kull I, Bergstrom A, Lilja G, Pershagen G, Wickman M (2006) Fish consumption during the first year of life and development of allergic diseases during childhood. Allergy 61: 1009-1015.

504. Magnusson J, Kull I, Rosenlund H, Hakansson N, Wolk A, et al. (2013) Fish consumption in infancy and development of allergic disease up to age 12 y. American Journal of Clinical Nutrition 97: 1324-1330.

505. Martindale S, McNeill G, Devereux G, Campbell D, Russell G, et al. (2005) Antioxidant intake in pregnancy in relation to wheeze and eczema in the first two years of life. American Journal of Respiratory & Critical Care Medicine 171: 121-128.

506. Devereux G, Litonjua AA, Turner SW, Craig LC, McNeill G, et al. (2007) Maternal vitamin D intake during pregnancy and early childhood wheezing. American Journal of Clinical Nutrition 85: 853-859.

507. Willers SM, Devereux G, Craig LC, McNeill G, Wijga AH, et al. (2007) Maternal food consumption during pregnancy and asthma, respiratory and atopic symptoms in 5-year-old children. Thorax 62: 773-779.

508. Shaheen SO, Northstone K, Newson RB, Emmett PM, Sherriff A, et al. (2009) Dietary patterns in pregnancy and respiratory and atopic outcomes in childhood. Thorax 64: 411-417.

509. Wills AK, Shaheen SO, Granell R, Henderson AJ, Fraser WD, et al. (2013) Maternal 25-hydroxyvitamin D and its association with childhood atopic outcomes and lung function. Clin Exp Allergy 43: 1180-1188.

510. Granell R, Heron J, Lewis S, Davey Smith G, Sterne JA, et al. (2008) The association between mother and child MTHFR C677T polymorphisms, dietary folate intake and childhood atopy in a population-based, longitudinal birth cohort.[Erratum appears in Clin Exp Allergy. 2008 Apr;38(4):699 Note: Smith, G D [corrected to Davey Smith, G]]. Clinical & Experimental Allergy 38: 320-328.

511. Laitinen K, Kalliomaki M, Poussa T, Lagstrom H, Isolauri E (2005) Evaluation of diet and growth in children with and without atopic eczema: follow-up study from birth to 4 years. British Journal of Nutrition 94: 565-574.

512. Lange NE, Rifas-Shiman SL, Camargo CA, Jr., Gold DR, Gillman MW, et al. (2010) Maternal dietary pattern during pregnancy is not associated with recurrent wheeze in children. Journal of Allergy & Clinical Immunology 126: 250-255, 255.e251-254.

513. Litonjua AA, Rifas-Shiman SL, Ly NP, Tantisira KG, Rich-Edwards JW, et al. (2006) Maternal antioxidant intake in pregnancy and wheezing illnesses in children at 2 y of age. American Journal of Clinical Nutrition 84: 903-911.

514. Camargo CA, Jr., Rifas-Shiman SL, Litonjua AA, Rich-Edwards JW, Weiss ST, et al. (2007) Maternal intake of vitamin D during pregnancy and risk of recurrent wheeze in children at 3 y of age. American Journal of Clinical Nutrition 85: 788-795.

515. Liu X, Wang G, Hong X, Wang D, Tsai HJ, et al. (2011) Gene-vitamin D interactions on food sensitization: a prospective birth cohort study. Allergy 66: 1442-1448.

516. Mommers M, Bronsveld A, Van Ree R, Jansen E, Thijs C (2009) Vitamin D status during pregnancy and early life and atopic outcomes in childhood. Allergy: European Journal of Allergy and Clinical Immunology 64: 21.

517. Magdelijns FJ, Mommers M, Penders J, Smits L, Thijs C (2011) Folic acid use in pregnancy and the development of atopy, asthma, and lung function in childhood. Pediatrics 128: e135-144.

518. Cremers E, Thijs C, Penders J, Jansen E, Mommers M (2011) Maternal and child's vitamin D supplement use and vitamin D level in relation to childhood lung function: the KOALA Birth Cohort Study. Thorax 66: 474-480.

519. Maslova E, Granstrom C, Hansen S, Petersen SB, Strom M, et al. (2012) Peanut and tree nut consumption during pregnancy and allergic disease in children-should mothers decrease their intake? Longitudinal evidence from the Danish National Birth Cohort. Journal of Allergy & Clinical Immunology 130: 724-732.

520. Maslova E, Hansen S, Strom M, Jensen CB, Thorne-Lyman AL, et al. (2013) Maternal 25(OH) vitamin D prediction scores in pregnancy and child asthma and allergic rhinitis: a prospective study from the Danish National Birth Cohort. Acta Obstetricia Et Gynecologica Scandinavica 92: 24-25.

521. Maslova E, Strom M, Olsen SF, Halldorsson TI (2013) Consumption of Artificially-Sweetened Soft Drinks in Pregnancy and Risk of Child Asthma and Allergic Rhinitis. PLoS ONE 8.

522. Linneberg A, Petersen J, Gronbaek M, Benn CS (2004) Alcohol during pregnancy and atopic dermatitis in the offspring. Clinical & Experimental Allergy 34: 1678-1683.

523. Magnus MC, Nafstad P, Stene LC, Haberg SE, London SJ, et al. (2013) Maternal vitamin D status during pregnancy and asthma in the offspring among participants in the Norwegian Mother and Child Cohort Study. Journal of Allergy and Clinical Immunology 1): AB128.

524. Haberg SE, London SJ, Stigum H, Nafstad P, Nystad W (2009) Folic acid supplements in pregnancy and early childhood respiratory health.[Erratum appears in Arch Dis Child. 2009 Jun;94(6):485]. Archives of Disease in Childhood 94: 180-184.

525. Miyake Y, Sasaki S, Tanaka K, Ohfuji S, Hirota Y (2009) Maternal fat consumption during pregnancy and risk of wheeze and eczema in Japanese infants aged 16-24 months: the Osaka Maternal and Child Health Study. Thorax 64: 815-821.

526. Saito K, Yokoyama T, Miyake Y, Sasaki S, Tanaka K, et al. (2010) Maternal meat and fat consumption during pregnancy and suspected atopic eczema in Japanese infants aged 3-4 months: the Osaka Maternal and Child Health Study. Pediatric Allergy & Immunology 21: 38-46.

527. Miyake Y, Sasaki S, Tanaka K, Hirota Y (2010) Consumption of vegetables, fruit, and antioxidants during pregnancy and wheeze and eczema in infants. Allergy 65: 758-765.

528. Miyake Y, Sasaki S, Tanaka K, Hirota Y (2010) Dairy food, calcium and vitamin D intake in pregnancy, and wheeze and eczema in infants. European Respiratory Journal 35: 1228-1234.

529. Miyake Y, Sasaki S, Tanaka K, Hirota Y (2011) Maternal B vitamin intake during pregnancy and wheeze and eczema in Japanese infants aged 16-24 months: the Osaka Maternal and Child Health Study. Pediatric Allergy & Immunology 22: 69-74.

530. Miyake Y, Okubo H, Sasaki S, Tanaka K, Hirota Y (2011) Maternal dietary patterns during pregnancy and risk of wheeze and eczema in Japanese infants aged 16-24 months: the Osaka Maternal and Child Health Study. Pediatric Allergy & Immunology 22: 734-741.

531. Morales E, Romieu I, Guerra S, Ballester F, Rebagliato M, et al. (2012) Maternal vitamin D status in pregnancy and risk of lower respiratory tract infections, wheezing, and asthma in offspring. Epidemiology 23: 64-71.

532. Narita M, Qiu D, Hamaguchi M, Doi M, Futamura M, et al. (2011) Maternal diet during pregnancy and wheeze and eczema in infants; the Japanese birth cohort (T-CHILD) study. Journal of Allergy and Clinical Immunology 1): AB174.

533. Ohya Y, Yonemoto J, Ogata T, Sone H, Sakamoto N, et al. (2011) Influence of environmental chemicals and drugs taken before and during pregnancy on onset of childhood asthma and eczema. Allergy: European Journal of Allergy and Clinical Immunology 66: 554.

534. Nwaru BI, Ahonen S, Kaila M, Erkkola M, Haapala AM, et al. (2010) Maternal diet during pregnancy and allergic sensitization in the offspring by 5 yrs of age: a prospective cohort study. Pediatric Allergy & Immunology 21: 29-37.

535. Nwaru BI, Erkkola M, Ahonen S, Kaila M, Lumia M, et al. (2011) Maternal diet during lactation and allergic sensitization in the offspring at age of 5. Pediatric Allergy & Immunology 22: 334-341.

536. Nwaru BI, Erkkola M, Ahonen S, Kaila M, Kronberg-Kippila C, et al. (2011) Intake of antioxidants during pregnancy and the risk of allergies and asthma in the offspring. European Journal of Clinical Nutrition 65: 937-943.

537. Nwaru BI, Erkkola M, Lumia M, Kronberg-Kippila C, Ahonen S, et al. (2012) Maternal intake of fatty acids during pregnancy and allergies in the offspring. British Journal of Nutrition 108: 720-732.

538. Niinisto S, Takkinen HM, Uusitalo L, Nevalainen J, Kenward M, et al. (2012) Maternal fatty acid composition of diet during lactation and risk of preclinical and clinical type 1 diabetes. Diabetologia 55: S141-S142.

539. Lumia M, Luukkainen P, Tapanainen H, Kaila M, Erkkola M, et al. (2011) Dietary fatty acid composition during pregnancy and the risk of asthma in the offspring. Pediatric Allergy & Immunology 22: 827-835.

540. Maijaliisa E, Bright IN, Minna K, Kronberg-Kippila C, Jorma I, et al. (2011) Maternal food consumption during pregnancy and risk of asthma and allergic outcomes in the offspring. Annals of Nutrition and Metabolism 58: 369.

541. Uusitalo L, Kenward MG, Virtanen SM, Uusitalo U, Nevalainen J, et al. (2008) Intake of antioxidant vitamins and trace elements during pregnancy and risk of advanced beta cell autoimmunity in the child. American Journal of Clinical Nutrition 88: 458-464.

542. Erkkola M, Kaila M, Nwaru BI, Kronberg-Kippila C, Ahonen S, et al. (2009) Maternal vitamin D intake during pregnancy is inversely associated with asthma and allergic rhinitis in 5-year-old children. Clinical & Experimental Allergy 39: 875-882.

543. Marjamaki L, Niinisto S, Kenward MG, Uusitalo L, Uusitalo U, et al. (2010) Maternal intake of vitamin D during pregnancy and risk of advanced beta cell autoimmunity and type 1 diabetes in offspring. Diabetologia 53: 1599-1607.

544. Oien T, Storro O, Johnsen R (2010) Do early intake of fish and fish oil protect against eczema and doctor-diagnosed asthma at 2 years of age? A cohort study. Journal of Epidemiology & Community Health 64: 124-129.

545. Pike KC, Inskip HM, Robinson S, Lucas JS, Cooper C, et al. (2012) Maternal late-pregnancy serum 25-hydroxyvitamin D in relation to childhood wheeze and atopic outcomes. Thorax 67: 950-956.

546. Roduit C, Frei R, Loss G, Buchele G, Weber J, et al. (2012) Development of atopic dermatitis according to age of onset and association with early-life exposures. Journal of Allergy & Clinical Immunology 130: 130-136.e135.

547. Rothers J, Wright AL, Stern DA, Halonen M, Camargo CA, Jr. (2011) Cord blood 25-hydroxyvitamin D levels are associated with aeroallergen sensitization in children from Tucson, Arizona. Journal of Allergy & Clinical Immunology 128: 1093-1099.e1091-1095.

548. Sausenthaler S, Koletzko S, Schaaf B, Lehmann I, Borte M, et al. (2007) Maternal diet during pregnancy in relation to eczema and allergic sensitization in the offspring at 2 y of age. American Journal of Clinical Nutrition 85: 530-537.

549. Dean T, Venter C, Pereira B, Grundy J, Clayton CB, et al. (2007) Government advice on peanut avoidance during pregnancy--is it followed correctly and what is the impact on sensitization? Journal of Human Nutrition & Dietetics 20: 95-99.

550. Weisse K, Winkler S, Hirche F, Herberth G, Hinz D, et al. (2013) Maternal and newborn vitamin D status and its impact on food allergy development in the German LINA cohort study. Allergy 68: 220-228.

551. West CE, Dunstan J, McCarthy S, Metcalfe J, D'Vaz N, et al. (2012) Associations between maternal antioxidant intakes in pregnancy and infant allergic outcomes. Nutrients 4: 1747-1758.

552. Whitrow MJ, Moore VM, Rumbold AR, Davies MJ (2009) Effect of supplemental folic acid in pregnancy on childhood asthma: a prospective birth cohort study. American Journal of Epidemiology 170: 1486-1493.

553. Calvani M, Alessandri C, Sopo SM, Panetta V, Pingitore G, et al. (2006) Consumption of fish, butter and margarine during pregnancy and development of allergic sensitizations in the offspring: role of maternal atopy. Pediatric Allergy and Immunology 17: 94-102.

554. Jones AP, Palmer D, Zhang G, Prescott SL (2012) Cord blood 25-hydroxyvitamin D3 and allergic disease during infancy. Pediatrics 130: e1128-1135.

555. Allen KJ, Koplin JJ, Ponsonby AL, Gurrin LC, Wake M, et al. (2013) Vitamin D insufficiency is associated with challenge-proven food allergy in infants. Journal of Allergy and Clinical Immunology 131: 1109-1116.e1106.

556. Binkley KE, Leaver C, Ray JG (2011) Antenatal risk factors for peanut allergy in children. Allergy, Asthma, & Clinical Immunology : Official Journal of the Canadian Society of Allergy & Clinical Immunology 7: 17.

557. Dai L (1993) [An analysis of the risk factors of child bronchial asthma]. Chung-Hua Liu Hsing Ping Hsueh Tsa Chih Chinese Journal of Epidemiology 14: 131-135.

558. Lopez Campos C, Munoz Hernandez MA, Lopez Campos JE, Carrillo Lucero J, Rincon Castaneda CB, et al. (2001) [Asthma risk factors]. Revista Alergia Mexico 48: 103-106.

559. Mullins RJ, Clark S, Wiley V, Eyles D, Camargo Jr CA (2012) Neonatal vitamin D status and childhood peanut allergy: A pilot study. Annals of Allergy, Asthma and Immunology 109: 324-328.

560. Salam MT, Li YF, Langholz B, Gilliland FD (2005) Maternal fish consumption during pregnancy and risk of early childhood asthma. Journal of Asthma 42: 513-518.

561. de Batlle J, Garcia-Aymerich J, Barraza-Villarreal A, Anto JM, Romieu I (2008) Mediterranean diet is associated with reduced asthma and rhinitis in Mexican children. Allergy 63: 1310-1316.

562. Dela Bianca A, Wandalsen G, Mallol J, Sole D (2012) Risk factors for wheezing disorders in infants in the first year of life living in Sao Paulo, Brazil. Journal of Tropical Pediatrics 58: 501-504.

563. Riedler J, Braun-Fahrlander C, Eder W, Schreuer M, Waser M, et al. (2001) Exposure to farming in early life and development of asthma and allergy: a cross-sectional survey. Lancet 358: 1129-1133.

564. Brekke HK, Ludvigsson J (2007) Vitamin D supplementation and diabetes-related autoimmunity in the ABIS study. Pediatric Diabetes 8: 11-14.

565. Brekke HK, Ludvigsson J (2010) Daily vegetable intake during pregnancy negatively associated to islet autoimmunity in the offspring--the ABIS study. Pediatric Diabetes 11: 244-250.

566. Wahlberg J, Fredriksson J, Nikolic E, Vaarala O, Ludvigsson J, et al. (2005) Environmental factors related to the induction of beta-cell autoantibodies in 1-yr-old healthy children. Pediatric Diabetes 6: 199-205.

567. Simpson M, Brady H, Yin X, Seifert J, Barriga K, et al. (2011) No association of vitamin D intake or 25-hydroxyvitamin D levels in childhood with risk of islet autoimmunity and type 1 diabetes: the Diabetes Autoimmunity Study in the Young (DAISY). Diabetologia 54: 2779-2788.

568. Norris JM, Beaty B, Klingensmith G, Yu L, Hoffman M, et al. (1996) Lack of association between early exposure to cow's milk protein and beta-cell autoimmunity. Diabetes Autoimmunity Study in the Young (DAISY). JAMA 276: 609-614.

569. Virtanen SM, Uusitalo L, Kenward MG, Nevalainen J, Uusitalo U, et al. (2011) Maternal food consumption during pregnancy and risk of advanced beta-cell autoimmunity in the offspring. Pediatric Diabetes 12: 95-99.

570. Virtanen SM, Kenward MG, Erkkola M, Kautiainen S, Kronberg-Kippila C, et al. (2006) Age at introduction of new foods and advanced beta cell autoimmunity in young children with HLA-conferred susceptibility to type 1 diabetes. Diabetologia 49: 1512-1521.

571. Harsunen MH, Hummel S, Beyerlein A, Joslowski G, Von Meyer A, et al. (2012) Short chain fatty acids and neutrophils in the pathogenesis of type 1 diabetes. Diabetologia 55: S141.

572. Miettinen ME, Reinert L, Kinnunen L, Harjutsalo V, Koskela P, et al. (2012) Serum 25-hydroxyvitamin D level during early pregnancy and type 1 diabetes risk in the offspring. Diabetologia 55: 1291-1294.

573. Sorensen IM, Joner G, Jenum PA, Eskild A, Torjesen PA, et al. (2012) Maternal serum levels of 25-hydroxy-vitamin D during pregnancy and risk of type 1 diabetes in the offspring. Diabetes 61: 175-178.

574. Anonymous (1999) Vitamin D supplement in early childhood and risk for Type I (insulin-dependent) diabetes mellitus. The EURODIAB Substudy 2 Study Group. Diabetologia 42: 51-54.

575. Rosenbauer J, Herzig P, Kaiser P, Giani G (2007) Early nutrition and risk of Type 1 diabetes mellitus--a nationwide case-control study in preschool children. Experimental & Clinical Endocrinology & Diabetes 115: 502-508.

576. Sipetic S, Vlajinac H, Kocev N, Radmanovic S (2003) Characteristics and habits of parents of children with insulin-dependent diabetes mellitus. [Croatian]

Neke karakteristike i navike roditelja dece sa insulin-zavisnim dijabetes melitusom. Srpski arhiv za celokupno lekarstvo 131: 238-243.

577. Sipetic SB, Vlajinac HD, Kocev NI, Marinkovic JM, Radmanovic SZ, et al. (2005) The Belgrade childhood diabetes study: a multivariate analysis of risk determinants for diabetes. European Journal of Public Health 15: 117-122.

578. Stene LC, Joner G, Norwegian Childhood Diabetes Study G (2003) Use of cod liver oil during the first year of life is associated with lower risk of childhood-onset type 1 diabetes: a large, population-based, case-control study. American Journal of Clinical Nutrition 78: 1128-1134.

579. Stene LC, Thorsby PM, Berg JP, Ronningen KS, Joner G, et al. (2008) Peroxisome proliferator-activated receptor-2 Pro12Ala polymorphism, cod liver oil and risk of type 1 diabetes. Pediatric Diabetes 9: 40-45.

580. Svensson J, Carstensen B, Mortensen HB, Borch-Johnsen K, Danish Study Group of Childhood D (2005) Early childhood risk factors associated with type 1 diabetes--is gender important? European Journal of Epidemiology 20: 429-434.

581. Virtanen SM, Rasanen L, Aro A, Ylonen K, Lounamaa R, et al. (1994) Is children's or parents' coffee or tea consumption associated with the risk for type 1 diabetes mellitus in children? European Journal of Clinical Nutrition 48: 279-285.

582. Metcalfe JR, Marsh JA, D'Vaz N, Geddes DT, Lai CT, et al. (2016) Effects of maternal dietary egg intake during early lactation on human milk ovalbumin concentration: a randomized controlled trial. 12 ed. United Kingdom: Blackwell Publishing Ltd (E-mail: customerservices@oxonblackwellpublishing.com). pp. 1605-1613.

583. Berman D, Limb R, Somers E, Clinton C, Romero V, et al. (2015) Prenatal omega-3 supplementation and risk of eczema among offspring at age 36 months: Long-term follow-up of the mothers, omega-3, & mental health trial. American journal of obstetrics and gynecology. pp. S162.

584. Bisgaard H, Stokholm J, Chawes BL, Vissing NH, Bjarnadottir E, et al. (2016) Fish oil-derived fatty acids in pregnancy and wheeze and asthma in offspring. New England Journal of Medicine 375: 2530-2539.

1. Kajosaari M. Atopy prophylaxis in high-risk infants. Prospective 5-year follow-up study of children with six months exclusive breastfeeding and solid food elimination. Advances in Experimental Medicine & Biology 1991;**310**:453-8

2. Kramer MS, Chalmers B, Hodnett ED, et al. Promotion of breastfeeding intervation trial (PROBIT): A randomized trial in the Republic of Belarus. Journal of the American Medical Association 2001; 285(4). <http://onlinelibrary.wiley.com/o/cochrane/clcentral/articles/706/CN-00441706/frame.html>.

3. Kramer MS, Matush L, Vanilovich I, et al. Effect of prolonged and exclusive breast feeding on risk of allergy and asthma: cluster randomised trial. BMJ (Clinical research ed) 2007;**335**(7624):815

4. Alho OP, Koivu M, Sorri M, et al. Risk factors for recurrent acute otitis media and respiratory infection in infancy. International Journal of Pediatric Otorhinolaryngology 1990;**19**(2):151-61

5. Allen KJ, Koplin J, Gurrin L, et al. Prevalence and environmental predictors of food allergy in infants. Journal of Allergy and Clinical Immunology 2009;**1)**:S108

6. Koplin J, Osborne N, Martin P, et al. Does age of introduction of foods affect the risk of having egg allergy? A population-based study of an infant cohort. Allergy: European Journal of Allergy and Clinical Immunology 2010;**65**:312

7. Alm B, Aberg N, Erdes L, et al. Early introduction of fish decreases the risk of AD in infants. Archives of Disease in Childhood 2009;**94**(1):11-5

8. Goksor E, Alm B, Thengilsdottir H, et al. Neonatal antibiotic treatment is a risk factor for multiple trigger wheeze at age 41/2 years. Pediatric Allergy and Immunology 2009;**20**:17

9. Alm B, Erdes L, Mollborg P, et al. Neonatal antibiotic treatment is a risk factor for early wheezing. Pediatrics 2008;**121**(4):697-702

10. Bacopoulou F, Veltsista A, Vassi I, et al. Can we be optimistic about asthma in childhood? A Greek cohort study. Journal of Asthma 2009;**46**(2):171-4

11. Benn CS, Wohlfahrt J, Aaby P, et al. Breastfeeding and risk of atopic dermatitis, by parental history of allergy, during the first 18 months of life. American Journal of Epidemiology 2004;**160**(3):217-23

12. Linneberg A, Simonsen JB, Petersen J, et al. Differential effects of risk factors on infant wheeze and atopic dermatitis emphasize a different etiology. Journal of Allergy & Clinical Immunology 2006;**117**(1):184-9

13. Bergmann RL, Edenharter G, Bergmann KE, et al. Socioeconomic status is a risk factor for allergy in parents but not in their children. Clinical & Experimental Allergy 2000;**30**(12):1740-5

14. Bergmann RL, Diepgen TL, Kuss O, et al. Breastfeeding duration is a risk factor for atopic AD. Clin Exp Allergy 2002;**32**(2):205-09

15. Kulig M, Klettke U, Wahn V, et al. Development of seasonal allergic rhinitis during the first 7 years of life. Journal of Allergy & Clinical Immunology 2000;**106**(5):832-9

16. Berth-Jones J, George S, Graham-Brown RA. Predictors of atopic dermatitis in Leicester children. British Journal of Dermatology 1997;**136**(4):498-501

17. Besednjak-Kocijancic L. Is longer exclusive breastfeeding associated with lover prevalence of asthma, atopic dermatitis and atopic sensitisation in 1 and 5-year-old Slovene children? Allergy: European Journal of Allergy and Clinical Immunology 2010;**65**:311-12

18. Bisgaard H, Halkjaer LB, Hinge R, et al. Risk analysis of early childhood AD. Journal of Allergy & Clinical Immunology 2009;**123**(6):1355-60.e5

19. Giwercman C, Halkjaer LB, Jensen SM, et al. Increased risk of AD but reduced risk of early wheezy disorder from exclusive breast-feeding in high-risk infants. Journal of Allergy & Clinical Immunology 2010;**125**(4):866-71

20. Burr ML, Miskelly FG, Butland BK, et al. Environmental factors and symptoms in infants at high risk of allergy. Journal of Epidemiology & Community Health 1989;**43**(2):125-32

21. Burr ML, Limb ES, Maguire MJ, et al. INFANT-FEEDING, WHEEZING, AND ALLERGY - A PROSPECTIVE-STUDY. Archives of Disease in Childhood 1993;**68**(6):724-28

22. Burr ML, Limb ES, Maguire MJ, et al. Infant feeding, wheezing, and allergy: a prospective study. Archives of Disease in Childhood 1993;**68**(6):724-8

23. Burr ML, Merrett TG, Dunstan FDJ, et al. The development of allergy in high-risk children. Clinical and Experimental Allergy 1997;**27**(11):1247-53

24. Burgess SW, Dakin CJ, O'Callaghan MJ. Breastfeeding does not increase the risk of asthma at 14 years. Pediatrics 2006;**117**(4):e787-92

25. Businco L, Cantani A, Meglio P, et al. Prevention of atopy: Results of a long-term (7 months to 8 years) follow-up. Annals of Allergy 1987;**59**(5 PART II):183-86

26. Bruno G, Cantani A, Ragno V, et al. Natural history of IgE antibodies in children at risk for atopy. Annals of Allergy, Asthma and Immunology 1995;**74**(5):431-36

27. Cano Garcinuno A, Perez Garcia I, Garcia Puertas J, et al. [Tobacco, infant feeding, and wheezing in the first three years of life]. Anales de Pediatria 2003;**59**(6):541-7

28. Scholtens S, Wijga AH, Brunekreef B, et al. Breast feeding, parental allergy and asthma in children followed for 8 years. The PIAMA birth cohort study. Thorax 2009;**64**(7):604-9

29. Caudri D, Savenije O, Smit HA, et al. The relation between perinatal factors and phenotypes of wheeze in the first 8 years of life. American Journal of Respiratory and Critical Care Medicine 2010;**181 (1 MeetingAbstracts)**

30. Kerkhof M, Koopman LP, van Strien RT, et al. Risk factors for atopic dermatitis in infants at high risk of allergy: the PIAMA study. Clin Exp Allergy 2003;**33**(10):1336-41 doi: 10.1046/j.1365-2222.2003.01751.x[published Online First: Epub Date]|.

31. Cogswell JJ, Mitchell EB, Alexander J. Parental smoking, breast feeding, and respiratory infection in development of allergic diseases. Archives of Disease in Childhood 1987;**62**(4):338-44

32. Chuang CH, Hsieh WS, Chen YC, et al. Infant feeding practices and physician diagnosed atopic dermatitis: a prospective cohort study in Taiwan. Pediatric Allergy & Immunology 2011;**22**(1 Pt 1):43-9

33. Menezes AM, Lima RC, Minten GC, et al. [Prevalence of wheezing in the chest among adults from the 1982 Pelotas birth cohort, Southern Brazil]. Revista de Saude Publica 2008;**42 Suppl 2**:101-7

34. da Costa Lima R, Victora CG, Menezes AM, et al. Do risk factors for childhood infections and malnutrition protect against asthma? A study of Brazilian male adolescents. American Journal of Public Health 2003;**93**(11):1858-64

35. Midodzi WK, Rowe BH, Majaesic CM, et al. Predictors for wheezing phenotypes in the first decade of life. Respirology 2008;**13**(4):537-45

36. Dell S, To T. Breastfeeding and asthma in young children: findings from a population-based study. Archives of Pediatrics & Adolescent Medicine 2001;**155**(11):1261-5

37. Midodzi WK, Rowe BH, Majaesic CM, et al. Early life factors associated with incidence of physician-diagnosed asthma in preschool children: results from the Canadian Early Childhood Development cohort study. Journal of Asthma 2010;**47**(1):7-13

38. Devereux G, Turner SW, Craig LC, et al. Low maternal vitamin E intake during pregnancy is associated with asthma in 5-year-old children. American Journal of Respiratory & Critical Care Medicine 2006;**174**(5):499-507

39. Dogaru CM, Strippoli MP, Spycher BD, et al. Breastfeeding and lung function at school age: does maternal asthma modify the effect? American Journal of Respiratory & Critical Care Medicine 2012;**185**(8):874-80

40. Elliott L, Henderson J, Northstone K, et al. Prospective study of breast-feeding in relation to wheeze, atopy, and bronchial hyperresponsiveness in the Avon Longitudinal Study of Parents and Children (ALSPAC). Journal of Allergy & Clinical Immunology 2008;**122**(1):49-54, 54.e1-3

41. Granell R, Sterne JAC, Henderson J. Associations of Different Phenotypes of Wheezing Illness in Early Childhood with Environmental Variables Implicated in the Aetiology of Asthma. PLoS ONE 2012;**7**(10)

42. Sherriff A, Peters TJ, Henderson J, et al. Risk factor associations with wheezing patterns in children followed longitudinally from birth to 3 1/2 years. Int J Epidemiol 2001;**30**(6):1473-84 doi: 10.1093/ije/30.6.1473[published Online First: Epub Date]|.

43. Abd A, Henderson J, Northstone K, et al. Breastfeeding duration and AD risk in the avon longitudinal study of parents and children. Archives of Disease in Childhood 2012;**97**:A43

44. Lack G, Fox D, Northstone K, et al. Factors associated with the development of peanut allergy in childhood. New England Journal of Medicine 2003;**348**(11):977-85

45. Eneli IU, Karmaus WK, Davis S, et al. Airway hyperresponsiveness and body mass index: the Child Health and Environment Cohort Study in Hesse, Germany. Pediatric Pulmonology 2006;**41**(6):530-7

46. Farooqi IS, Hopkin JM. Early childhood infection and atopic disorder. Thorax 1998;**53**(11):927-32

47. Fergusson DM, Horwood LJ, Shannon FT. Asthma and infant diet. Archives of Disease in Childhood 1983;**58**(1):48-51

48. Horwood LJ, Fergusson DM, Shannon FT. Social and familial factors in the development of early childhood asthma. Pediatrics 1985;**75**(5):859-68

49. Fredriksson P, Jaakkola N, Jaakkola JJ. Breastfeeding and childhood asthma: a six-year population-based cohort study. BMC Pediatrics 2007;**7**:39

50. Forster J, Dungs M, Wais U, et al. [Atopy-suggesting symptoms in the first 2 years of life. Effect of gestational age, nutrition and social class]. Klinische Padiatrie 1990;**202**(3):136-40

51. Galbally M, Lewis AJ, McEgan K, et al. Breastfeeding and infant sleep patterns: an Australian population study. Journal of Paediatrics & Child Health 2013;**49**(2):E147-52

52. Gruber C, van Stuijvenberg M, Mosca F, et al. Reduced occurrence of early atopic dermatitis because of immunoactive prebiotics among low-atopy-risk infants. Journal of Allergy & Clinical Immunology 2010;**126**(4):791-7

53. Gruskay FL. Comparison of breast, cow, and soy feedings in the prevention of onset of allergic disease: a 15-year prospective study. Clinical Pediatrics 1982;**21**(8):486-91

54. Guida F, Clarisse B, Momas I. Risk factors of wheezing onset during infancy in the Paris birth cohort: Benefits of the cox model. Allergy: European Journal of Allergy and Clinical Immunology 2009;**64**:202

55. Gustafsson D, Sjoberg O, Foucard T. Development of allergies and asthma in infants and young children with atopic dermatitis--a prospective follow-up to 7 years of age. Allergy 2000;**55**(3):240-5

56. Halken S, Host A, Husby S, et al. Recurrent wheezing in relation to environmental risk factors in infancy. A prospective study of 276 infants. Allergy 1991;**46**(7):507-14

57. Harris JM, Cullinan P, Williams HC, et al. Environmental associations with AD in early life. British Journal of Dermatology 2001;**144**(4):795-802

58. Zutavern A, von Mutius E, Harris J, et al. The introduction of solids in relation to asthma and AD. Archives of Disease in Childhood 2004;**89**(4):303-8

59. Hagendorens MM, Bridts CH, Lauwers K, et al. Perinatal risk factors for sensitization, atopic dermatitis and wheezing during the first year of life (PIPO study). Clinical and Experimental Allergy 2005;**35**(6):733-40 doi: 10.1111/j.1365-2222.2005.02254.x[published Online First: Epub Date]|.

60. Sariachvili M, Droste J, Dom S, et al. Is breast feeding a risk factor for AD during the first year of life? Pediatric Allergy & Immunology 2007;**18**(5):410-7

61. Sariachvili M, Droste J, Dom S, et al. Early exposure to solid foods and the development of AD in children up to 4 years of age. Pediatric Allergy & Immunology 2010;**21**(1 Pt 1):74-81

62. Hesselmar B, Saalman R, Rudin A, et al. Early fish introduction is associated with less AD, but not sensitization, in infants. Acta Paediatrica 2010;**99**(12):1861-7

63. Hetzner NMP, Razza RA, Malone LM, et al. Associations among feeding behaviors during infancy and child illness at two years. Maternal and Child Health Journal 2009;**13**(6):795-805

64. Hide DW, Guyer BM. Clinical manifestations of allergy related to breast and cows' milk feeding. Archives of Disease in Childhood 1981;**56**(3):172-5

65. Arshad SH, Hide DW. Effect of environmental factors on the development of allergic disorders in infancy. Journal of Allergy & Clinical Immunology 1992;**90**(2):235-41

66. Hikino S, Nakayama H, Yamamoto J, et al. Food allergy and atopic dermatitis in low birthweight infants during early childhood. Acta Paediatrica 2001;**90**(8):850-55

67. Hong X, Wang G, Liu X, et al. Gene polymorphisms, breast-feeding, and development of food sensitization in early childhood. Journal of Allergy & Clinical Immunology 2011;**128**(2):374-81.e2

68. Hoppu U, Kalliomaki M, Isolauri E. Maternal diet rich in saturated fat during breastfeeding is associated with atopic sensitization of the infant. European Journal of Clinical Nutrition 2000;**54**(9):702-5

69. Host A. Importance of the first meal on the development of cow's milk allergy and intolerance. Allergy Proceedings 1991;**12**(4):227-32

70. Howie PW, Forsyth JS, Ogston SA, et al. Protective effect of breast feeding against infection. Bmj 1990;**300**(6716):11-6

71. Huang H, Zhang FY, Hang JQ, et al. [Cohort study of 684 pairs of mother-and-child allergic diseases]. Zhonghua Erke Zazhi 2013;**51**(3):168-71

72. Huurre A, Laitinen K, Rautava S, et al. Impact of maternal atopy and probiotic supplementation during pregnancy on infant sensitization: a double-blind placebo-controlled study. Clinical and experimental allergy : journal of the British Society for Allergy and Clinical Immunology 2008; 38(8). <http://onlinelibrary.wiley.com/o/cochrane/clcentral/articles/465/CN-00648465/frame.html>

<http://onlinelibrary.wiley.com/store/10.1111/j.1365-2222.2008.03008.x/asset/j.1365-2222.2008.03008.x.pdf?v=1&t=hsx7b6h9&s=f1e2d199628aed5ec3d2a4a0ce155b613e11a1d9>.

73. Joseph CL, Ownby DR, Havstad SL, et al. Early complementary feeding and risk of food sensitization in a birth cohort. Journal of Allergy & Clinical Immunology 2011;**127**(5):1203-10.e5

74. Juto P, Bjorksten B. Serum IgE in infants and influence of type of feeding. Clinical Allergy 1980;**10**(5):593-600

75. Karmaus W, Dobai AL, Ogbuanu I, et al. Long-term effects of breastfeeding, maternal smoking during pregnancy, and recurrent lower respiratory tract infections on asthma in children. Journal of Asthma 2008;**45**(8):688-95

76. Ogbuanu IU, Karmaus W, Arshad SH, et al. Effect of breastfeeding duration on lung function at age 10 years: a prospective birth cohort study. Thorax 2009;**64**(1):62-6

77. Soto-Ramirez N, Alexander M, Karmaus W, et al. Breastfeeding is associated with increased lung function at 18 years of age: a cohort study. European Respiratory Journal 2012;**39**(4):985-91

78. Kaufman HS, Frick OL. The development of allergy in infants of allergic parents: a prospective study concerning the role of heredity. Annals of Allergy 1976;**37**(6):410-5

79. Kellberger J, Dressel H, Vogelberg C, et al. Prediction of the incidence and persistence of allergic rhinitis in adolescence: a prospective cohort study. Journal of Allergy & Clinical Immunology 2012;**129**(2):397-402, 02.e1-3

80. Kemeny DM, Price JF, Richardson V, et al. THE IGE AND IGG SUBCLASS ANTIBODY-RESPONSE TO FOODS IN BABIES DURING THE 1ST YEAR OF LIFE AND THEIR RELATIONSHIP TO FEEDING REGIMEN AND THE DEVELOPMENT OF FOOD ALLERGY. Journal of Allergy and Clinical Immunology 1991;**87**(5):920-29 doi: 10.1016/0091-6749(91)90413-i[published Online First: Epub Date]|.

81. Kerr AA. Lower respiratory tract illness in Polynesian infants. New Zealand Medical Journal 1981;**93**(684):333-5

82. Kim J, Chang E, Han Y, et al. The incidence and risk factors of immediate type food allergy during the first year of life in Korean infants: a birth cohort study. Pediatric Allergy & Immunology 2011;**22**(7):715-9

83. Kitz R, Rose MA, Schonborn H, et al. Impact of early dietary gamma-linolenic acid supplementation on atopic AD in infancy. Pediatric Allergy & Immunology 2006;**17**(2):112-7

84. Klinnert MD, Nelson HS, Price MR, et al. Onset and persistence of childhood asthma: Predictors from infancy. Pediatrics 2001;**108**(4):art. no.-e69 doi: 10.1542/peds.108.4.e69[published Online First: Epub Date]|.

85. Kramer MS, Guo T, Platt RW, et al. Infant growth and health outcomes associated with 3 compared with 6 mo of exclusive breastfeeding. American Journal of Clinical Nutrition 2003;**78**(2):291-5

86. Kramer MS, Matush L, Bogdanovich N, et al. Health and development outcomes in 6.5-y-old children breastfed exclusively for 3 or 6 mo. American Journal of Clinical Nutrition 2009;**90**(4):1070-4

87. Kramer MS, Matush L, Bogdanovich N, et al. The low prevalence of allergic disease in Eastern Europe: are risk factors consistent with the hygiene hypothesis? Clinical & Experimental Allergy 2009;**39**(5):708-16

88. Kull I, Wickman M, Lilja G, et al. Breast feeding and allergic diseases in infants-a prospective birth cohort study. Archives of Disease in Childhood 2002;**87**(6):478-81

89. Kusel MM, Holt PG, de Klerk N, et al. Support for 2 variants of AD. Journal of Allergy & Clinical Immunology 2005;**116**(5):1067-72

90. Larsson M, Hagerhed-Engman L, Sigsgaard T, et al. Incidence rates of asthma, rhinitis and AD symptoms and influential factors in young children in Sweden. Acta Paediatrica 2008;**97**(9):1210-5

91. Marini A, Agosti M, Motta G, et al. Effects of a dietary and environmental prevention programme on the incidence of allergic symptoms in high atopic risk infants: three years' follow-up. Acta Paediatrica Supplement 1996;**414**:1-21

92. Matheson MC, Erbas B, Balasuriya A, et al. Breast-feeding and atopic disease: a cohort study from childhood to middle age. Journal of Allergy & Clinical Immunology 2007;**120**(5):1051-7

93. Midwinter RE, Moore WJ, Soothill JF, et al. Infant feeding and atopy. Lancet 1982;**1**(8267):339

94. Mihrshahi S, Ampon R, Webb K, et al. The association between infant feeding practices and subsequent atopy among children with a family history of asthma. Clinical & Experimental Allergy 2007;**37**(5):671-9

95. Milner JD, Stein DM, McCarter R, et al. Early infant multivitamin supplementation is associated with increased risk for food allergy and asthma. Pediatrics 2004;**114**(1):27-32

96. Miskelly FG, Burr ML, Vaughan-Williams E, et al. Infant feeding and allergy. Archives of Disease in Childhood 1988;**63**(4):388-93

97. Miyake Y, Tanaka K, Sasaki S, et al. Breastfeeding and the risk of wheeze and asthma in Japanese infants: the Osaka Maternal and Child Health Study. Pediatric Allergy & Immunology 2008;**19**(6):490-6

98. Miyake Y, Tanaka K, Sasaki S, et al. Breastfeeding and atopic AD in Japanese infants: The Osaka Maternal and Child Health Study. Pediatric Allergy & Immunology 2009;**20**(3):234-41

99. Morgan JB, Lucas A, Fewtrell MS. Does weaning influence growth and health up to 18 months? Archives of Disease in Childhood 2004;**89**(8):728-33

100. Morgan J, Williams P, Norris F, et al. AD and early solid feeding in preterm infants. Archives of Disease in Childhood 2004;**89**(4):309-14 doi: 10.1136/adc.2002.020065[published Online First: Epub Date]|.

101. Moore WJ, Midwinter RE, Morris AF, et al. Infant feeding and subsequent risk of atopic AD. Archives of Disease in Childhood 1985;**60**(8):722-6

102. Morales E, Garcia-Esteban R, Guxens M, et al. Effects of prolonged breastfeeding and colostrum fatty acids on allergic manifestations and infections in infancy. Clinical and Experimental Allergy 2012;**42**(6):918-28 doi: 10.1111/j.1365-2222.2012.03969.x[published Online First: Epub Date]|.

103. Muiño A, Menezes AMB, Reichert FF, et al. Padrões de sibilância respiratória do nascimento até o início da adolescência: coorte de Pelotas (RS) Brasil, 1993-2004

Wheezing phenotypes from birth to adolescence: a cohort study in Pelotas, Brazil, 1993-2004. J Bras Pneumol 2008;**34**(6):347-55

104. Nielsen AM, Rasmussen S, Christoffersen MN. [Morbidity of Danish infants during their first months of life. Incidence and risk factors]. Ugeskrift for Laeger 2002;**164**(48):5644-8

105. Nwaru BI, Erkkola M, Ahonen S, et al. Age at the introduction of solid foods during the first year and allergic sensitization at age 5 years. Pediatrics 2010;**125**(1):50-9

106. Nwaru BI, Takkinen HM, Niemela O, et al. Timing of infant feeding in relation to childhood asthma and allergic diseases. Journal of Allergy and Clinical Immunology 2013;**131**(1):78-86

107. Erkkola M, Nwaru BI, Kaila M, et al. Risk of asthma and allergic outcomes in the offspring in relation to maternal food consumption during pregnancy: a Finnish birth cohort study. Pediatric Allergy & Immunology 2012;**23**(2):186-94

108. Virtanen SM, Kaila M, Pekkanen J, et al. Early introduction of oats associated with decreased risk of persistent asthma and early introduction of fish with decreased risk of allergic rhinitis. British Journal of Nutrition 2010;**103**(2):266-73

109. Oddy WH, Halonen M, Martinez FD, et al. TGF-beta in human milk is associated with wheeze in infancy. Journal of Allergy & Clinical Immunology 2003;**112**(4):723-8

110. Oddy WH, Sly PD, de Klerk NH, et al. Breast feeding and respiratory morbidity in infancy: a birth cohort study. Archives of Disease in Childhood 2003;**88**(3):224-8

111. Oddy WH, de Klerk NH, Kendall GE, et al. Ratio of omega-6 to omega-3 fatty acids and childhood asthma. Journal of Asthma 2004;**41**(3):319-26

112. Oddy WH, Holt PG, Sly PD, et al. Association between breast feeding and asthma in 6 year old children: findings of a prospective birth cohort study. Bmj 1999;**319**(7213):815-9

113. Odelram H, Vanto T, Jacobsen L, et al. Whey hydrolysate compared with cow's milk-based formula for weaning at about 6 months of age in high allergy-risk infants: Effects on atopic disease and sensitization. Allergy: European Journal of Allergy and Clinical Immunology 1996;**51**(3):192-95

114. Perez Tarazona S, Alfonso Diego J, Amat Madramany A, et al. [Incidence of wheezing and associated risk factors in the first 6 months of life of a cohort in Valencia (Spain)]. Anales de Pediatria 2010;**72**(1):19-29

115. Pesonen M, Kallio MJ, Ranki A, et al. Prolonged exclusive breastfeeding is associated with increased atopic dermatitis: a prospective follow-up study of unselected healthy newborns from birth to age 20 years. Clinical & Experimental Allergy 2006;**36**(8):1011-8

116. Porch MC, Shahane AD, Leiva LE, et al. Influence of breast milk, soy or two hydrolyzed formulas on the development of allergic manifestations in infants at risk. Nutrition Research 1998;**18**(8):1413-24

117. Poysa L, Korppi M, Remes K, et al. Predictive value of IgE levels in infancy. Acta Paediatrica Scandinavica 1990;**79**(10):970-2

118. Poysa L, Pulkkinen A, Korppi M, et al. Diet in infancy and bronchial hyperreactivity later in childhood. Pediatric Pulmonology 1992;**13**(4):215-21

119. Pratt HF. Breastfeeding and AD. Early Human Development 1984;**9**(3):283-90

120. Puig C, Friguls B, Gomez M, et al. Relationship between lower respiratory tract infections in the first year of life and the development of asthma and wheezing in children. [Spanish]

Relacion entre las infecciones respiratorias de vias bajas durante el primer ano de vida y el desarrollo de asma y sibilancias en ninos. Archivos de Bronconeumologia 2010;**46**(10):514-21

121. Purvis DJ, Thompson JM, Clark PM, et al. Risk factors for atopic dermatitis in New Zealand children at 3.5 years of age. British Journal of Dermatology 2005;**152**(4):742-9

122. Rhodes HL, Sporik R, Thomas P, et al. Early life risk factors for adult asthma: a birth cohort study of subjects at risk. Journal of Allergy & Clinical Immunology 2001;**108**(5):720-5

123. Rothenbacher D, Weyermann M, Beermann C, et al. Breastfeeding, soluble CD14 concentration in breast milk and risk of atopic dermatitis and asthma in early childhood: birth cohort study. Clinical & Experimental Allergy 2005;**35**(8):1014-21

124. Rowntree S, Cogswell JJ, Platts-Mills TA, et al. Development of IgE and IgG antibodies to food and inhalant allergens in children at risk of allergic disease. Archives of Disease in Childhood 1985;**60**(8):727-35

125. Rullo VE, Arruda LK, Cardoso MR, et al. Respiratory infection, exposure to mouse allergen and breastfeeding: role in recurrent wheezing in early life. International Archives of Allergy & Immunology 2009;**150**(2):172-8

126. Rullo V, Arruda K, Valente V, et al. Allergen and endotoxin exposure, infection, and breastfeeding in early life, and recurrent wheezing in children: 48-month follow-up cohort study. Allergy: European Journal of Allergy and Clinical Immunology 2009;**64**:22

127. Rullo VEV, Arruda LK, Valente V, et al. Allergen and endotoxin exposure, infection, and breastfeeding in early life, and persistent wheezing in children: 60-month follow-up of a cohort study. Journal of Allergy and Clinical Immunology 2010;**1)**:AB56

128. Rider NL, Morton D, Strauss KA. Allergen and endotoxin exposure, infection, and breastfeeding in early life, and recurrent wheezing in infants: 18-month follow-up of a cohort study. Journal of Allergy and Clinical Immunology 2007;**119**(1):S70-S71 doi: 10.1016/j.jaci.2006.11.300[published Online First: Epub Date]|.

129. Ruiz RGG, Kemeny DM, Price JF. Higher risk of infantile atopic dermatitis from maternal atopy than from paternal atopy. Clin Exp Allergy 1992;**22**(8):762-66

130. Saarinen UM, Kajosaari M. Breastfeeding as prophylaxis against atopic disease: prospective follow-up study until 17 years old. Lancet 1995;**346**(8982):1065-9

131. Saarinen UM, Bjorksten F, Knekt P, et al. Serum IgE in healthy infants fed breast milk or cow's milk-based formulas. Clinical Allergy 1979;**9**(4):339-5

132. Sears MR, Greene JM, Willan AR, et al. Long-term relation between breastfeeding and development of atopy and asthma in children and young adults: a longitudinal study. Lancet 2002;**360**(9337):901-7

133. Mandhane PJ, Greene JM, Sears MR. Interactions between breast-feeding, specific parental atopy, and sex on development of asthma and atopy. Journal of Allergy & Clinical Immunology 2007;**119**(6):1359-66

134. Shaheen SO, Aaby P, Hall AJ, et al. Measles and atopy in Guinea-Bissau. Lancet 1996;**347**(9018):1792-6

135. Shohet L, Shahar E, Davidson S. Breast feeding as prophylaxis for atopic AD: a controlled study of 368 cases. Acta Paediatrica Hungarica 1985;**26**(1):35-9

136. Schoetzau A, Filipiak-Pittroff B, Franke K, et al. Effect of exclusive breast-feeding and early solid food avoidance on the incidence of atopic dermatitis in high-risk infants at 1 year of age. Pediatric Allergy & Immunology 2002;**13**(4):234-42

137. Schonberger HJ, Dompeling E, Knottnerus JA, et al. The PREVASC study: the clinical effect of a multifaceted educational intervention to prevent childhood asthma. European Respiratory Journal 2005;**25**(4):660-70

138. Sicherer SH, Wood RA, Stablein D, et al. Maternal consumption of peanut during pregnancy is associated with peanut sensitization in atopic infants. Journal of Allergy & Clinical Immunology 2010;**126**(6):1191-7

139. Siltanen M, Kajosaari M, Poussa T, et al. A dual long-term effect of breastfeeding on atopy in relation to heredity in children at 4 years of age. Allergy 2003;**58**(6):524-30

140. Silva JM, Camara AA, Tobias KR, et al. A prospective study of wheezing in young children: the independent effects of cockroach exposure, breast-feeding and allergic sensitization. Pediatric Allergy & Immunology 2005;**16**(5):393-401

141. Silvers KM, Frampton CM, Wickens K, et al. Breastfeeding protects against current asthma up to 6 years of age. Journal of Pediatrics 2012;**160**(6):991-6.e1

142. Silvers KM, Frampton CM, Wickens K, et al. Breastfeeding protects against adverse respiratory outcomes at 15 months of age. Maternal & Child Nutrition 2009;**5**(3):243-50

143. Simon MR, Havstad SL, Wegienka GR, et al. Risk factors associated with transient wheezing in young children. Allergy & Asthma Proceedings 2008;**29**(2):161-5

144. Wang IJ, Guo YL, Weng HJ, et al. Environmental risk factors for early infantile atopic dermatitis. Pediatric Allergy & Immunology 2007;**18**(5):441-7

145. Wegienka G, Ownby DR, Havstad S, et al. Breastfeeding history and childhood allergic status in a prospective birth cohort. Annals of Allergy, Asthma, & Immunology 2006;**97**(1):78-83

146. Salam MT, Li YF, Langholz B, et al. Early-life environmental risk factors for asthma: findings from the Children's Health Study. Environmental Health Perspectives 2004;**112**(6):760-5

147. Snijders BE, Thijs C, van Ree R, et al. Age at first introduction of cow milk products and other food products in relation to infant atopic manifestations in the first 2 years of life: the KOALA Birth Cohort Study. Pediatrics 2008;**122**(1):e115-22

148. Snijders BE, Thijs C, Dagnelie PC, et al. Breast-feeding duration and infant atopic manifestations, by maternal allergic status, in the first 2 years of life (KOALA study). Journal of Pediatrics 2007;**151**(4):347-51, 51.e1-2

149. Soto-Ramirez N, Karmaus W, Zhang H, et al. Modes of infant feeding and the occurrence of coughing/wheezing in the first year of life. Journal of Human Lactation 2013;**29**(1):71-80

150. Strachan DP, Harkins LS, Johnston IDA, et al. Childhood antecedents of allergic sensitization in young British adults. Journal of Allergy and Clinical Immunology 1997;**99**(1):6-12 doi: 10.1016/s0091-6749(97)81038-x[published Online First: Epub Date]|.

151. Strachan DP, Taylor EM, Carpenter RG. Family structure, neonatal infection, and hay fever in adolescence. Archives of Disease in Childhood 1996;**74**(5):422-6

152. Lewis S, Richards D, Bynner J, et al. PROSPECTIVE-STUDY OF RISK-FACTORS FOR EARLY AND PERSISTENT WHEEZING IN CHILDHOOD. European Respiratory Journal 1995;**8**(3):349-56 doi: 10.1183/09031936.95.08030349[published Online First: Epub Date]|.

153. Lewis S, Butland B, Strachan D, et al. Study of the aetiology of wheezing illness at age 16 in two national British birth cohorts. Thorax 1996;**51**(7):670-6

154. Butland BK, Strachan DP, Lewis S, et al. Investigation into the increase in hay fever and AD at age 16 observed between the 1958 and 1970 British birth cohorts. Bmj 1997;**315**(7110):717-21

155. Strassburger SZ, Vitolo MR, Bortolini GA, et al. Nutritional errors in the first months of life and their association with asthma and atopy in preschool children. Jornal de Pediatria 2010;**86**(5):391-9

156. Sunyer J, Torrent M, Garcia-Esteban R, et al. Early exposure to dichlorodiphenyldichloroethylene, breastfeeding and asthma at age six. Clinical & Experimental Allergy 2006;**36**(10):1236-41

157. Sunyer J, Mendendez C, Ventura PJ, et al. Prenatal risk factors of wheezing at the age of four years in Tanzania. Thorax 2001;**56**(4):290-5

158. Taylor B, Wadsworth J, Golding J, et al. Breast feeding, AD, asthma, and hayfever. Journal of Epidemiology and Community Health 1983;**37**:95-99

159. Taylor B, Wadsworth J, Wadsworth M, et al. Changes in the reported prevalence of childhood AD since the 1939-45 war. Lancet 1984;**2**(8414):1255-7

160. Tennant PW, Gibson GJ, Pearce MS. Lifecourse predictors of adult respiratory function: results from the Newcastle Thousand Families Study. Thorax 2008;**63**(9):823-30

161. Tennant PW, Gibson GJ, Parker L, et al. Childhood respiratory illness and lung function at ages 14 and 50 years: childhood respiratory illness and lung function. Chest 2010;**137**(1):146-55

162. Tian M, Zhao DY, Wen GY, et al. [The correlation factor about respiratory syncytial virus bronchiolitis and post-bronchiolitis wheezing in infant]. Chinese Journal of Experimental & Clinical Virology 2009;**23**(5):371-4

163. Van Asperen PP, Kemp AS, Mellis CM. Relationship of diet in the development of atopy in infancy. Clinical Allergy 1984;**14**(6):525-32

164. van Beijsterveldt TC, Boomsma DI. An exploration of gene-environment interaction and asthma in a large sample of 5-year-old Dutch twins. Twin Research & Human Genetics: the Official Journal of the International Society for Twin Studies 2008;**11**(2):143-9

165. Sonnenschein-van der Voort AM, Jaddoe VW, van der Valk RJ, et al. Duration and exclusiveness of breastfeeding and childhood asthma-related symptoms. European Respiratory Journal 2012;**39**(1):81-9

166. Vandenplas Y, Deneyer M, Sacre L, et al. Preliminary data on a field study with a new hypo-allergic formula. European Journal of Pediatrics 1988;**148**(3):274-7

167. van Merode T, Maas T, Twellaar M, et al. Gender-specific differences in the prevention of asthma-like symptoms in high-risk infants. Pediatric Allergy & Immunology 2007;**18**(3):196-200

168. Venter C, Pereira B, Voigt K, et al. Factors associated with maternal dietary intake, feeding and weaning practices, and the development of food hypersensitivity in the infant. Pediatric Allergy and Immunology 2009;**20**(4):320-27 doi: 10.1111/j.1399-3038.2008.00832.x[published Online First: Epub Date]|.

169. Watson PE, McDonald BW. Subcutaneous body fat in pregnant new zealand women: association with wheeze in their infants at 18months. Maternal & Child Health Journal 2013;**17**(5):959-67

170. Wetzig H, Schulz R, Diez U, et al. Associations between duration of breast-feeding, sensitization to hens' eggs and AD infantum in one and two year old children at high risk of atopy. International Journal of Hygiene & Environmental Health 2000;**203**(1):17-21

171. Wilson AC, Forsyth JS, Greene SA, et al. Relation of infant diet to childhood health: seven year follow up of cohort of children in Dundee infant feeding study. Bmj 1998;**316**(7124):21-5

172. Wright RJ, Cohen S, Carey V, et al. Parental stress as a predictor of wheezing in infancy: a prospective birth-cohort study. American Journal of Respiratory & Critical Care Medicine 2002;**165**(3):358-65

173. Wright AL, Holberg CJ, Martinez FD, et al. Breast feeding and lower respiratory tract illness in the first year of life. Group Health Medical Associates. Bmj 1989;**299**(6705):946-9

174. Wright AL, Holberg CJ, Taussig LM, et al. Relationship of infant feeding to recurrent wheezing at age 6 years. Archives of Pediatrics & Adolescent Medicine 1995;**149**(7):758-63

175. Wright AL, Sherrill D, Holberg CJ, et al. Breast-feeding, maternal IgE, and total serum IgE in childhood. Journal of Allergy & Clinical Immunology 1999;**104**(3 Pt 1):589-94

176. Wright AL, Holberg CJ, Martinez FD, et al. Epidemiology of physician-diagnosed allergic rhinitis in childhood. Pediatrics 1994;**94**(6 Pt 1):895-901

177. Yamamoto K, Shoda T, Futamura M, et al. Associated factors of wheezing at 3 years of age in Japan: Prospective birth cohort study. Allergy: European Journal of Allergy and Clinical Immunology 2011;**66**:549-50

178. Zutavern A, Brockow I, Schaaf B, et al. Timing of solid food introduction in relation to atopic dermatitis and atopic sensitization: results from a prospective birth cohort study. Pediatrics 2006;**117**(2):401-11

179. Zutavern A, Brockow I, Schaaf B, et al. Timing of solid food introduction in relation to AD, asthma, allergic rhinitis, and food and inhalant sensitization at the age of 6 years: results from the prospective birth cohort study LISA. Pediatrics 2008;**121**(1):e44-52

180. Friday GA, Jr., Smith J. Breast-feeding and atopic dermatitis. Pediatric Asthma, Allergy and Immunology 2000;**14**(3):205-09

181. McConnochie KM, Roghmann KJ. Breast feeding and maternal smoking as predictors of wheezing in children age 6 to 10 years. Pediatric Pulmonology 1986;**2**(5):260-8

182. Monego A, Infortuna M, Santisi G, et al. [Can breast feeding prevent atopic dermatitis? Epidemiologic study of 144 cases]. Minerva Pediatrica 1989;**41**(5):223-7

183. Rona RJ, Smeeton NC, Bustos P, et al. The early origins hypothesis with an emphasis on growth rate in the first year of life and asthma: a prospective study in Chile. Thorax 2005;**60**(7):549-54

184. Mai XM, Becker AB, Sellers EAC, et al. The relationship of breast-feeding, overweight, and asthma in preadolescents. Journal of Allergy and Clinical Immunology 2007;**120**(3):551-56 doi: 10.1016/j.jaci.2007.05.004[published Online First: Epub Date]|.

185. Martel MJ, Rey E, Malo JL, et al. Determinants of the incidence of childhood asthma: a two-stage case-control study. American Journal of Epidemiology 2009;**169**(2):195-205

186. Maskell J, Grimshaw K, King R, et al. Dietary patterns in the first year of life and food allergy risk. Allergy: European Journal of Allergy and Clinical Immunology 2010;**65**:46

187. Oliver E, Grimshaw K, Scally K, et al. Does nutritional intake in infancy effect later development of food allergy? Allergy: European Journal of Allergy and Clinical Immunology 2010;**65**:313

188. Munro A, Grimshaw K, Oliver E, et al. Risk factors associated with wheezing in the first 2 years of life: Europrevall cohort. Clinical and Experimental Allergy 2011;**41 (12)**:1856

189. Ronmark E, Jonsson E, Platts-Mills T, et al. Different pattern of risk factors for atopic and nonatopic asthma among children--report from the Obstructive Lung Disease in Northern Sweden Study. Allergy 1999;**54**(9):926-35

190. Camara AA, Silva JM, Ferriani VPL, et al. Risk factors for wheezing in a subtropical environment: Role of respiratory viruses and allergen sensitization. J Allergy Clin Immunol 2004;**113**(3):551-57

191. DesRoches A, Infante-Rivard C, Paradis L, et al. Peanut allergy: is maternal transmission of antigens during pregnancy and breastfeeding a risk factor? Journal of Investigational Allergology & Clinical Immunology 2010;**20**(4):289-94

192. Djenouhat K, Ibsaine O, Abbadi M, et al. Cow's milk allergy and risk factors. Allergy: European Journal of Allergy and Clinical Immunology 2011;**66**:388

193. Fox AT, Sasieni P, du Toit G, et al. Household peanut consumption as a risk factor for the development of peanut allergy. Journal of Allergy and Clinical Immunology 2009;**123**(2):417-23 doi: 10.1016/j.jaci.2008.12.014[published Online First: Epub Date]|.

194. Ghaderi R, Makhmalbaf Z. Effect of breast-feeding on the development of atopic dermatitis.[Retraction in Ghaderi R, Makhmalbaf Z. Iran J Allergy Asthma Immunol. 2007 Dec;6(4):225; PMID: 18357658]. Iranian Journal of Allergy Asthma & Immunology 2005;**4**(3):129-32

195. Haileamlak A, Dagoye D, Williams H, et al. Early life risk factors for atopic dermatitis in Ethiopian children. Journal of Allergy & Clinical Immunology 2005;**115**(2):370-6

196. Infante-Rivard C. Childhood asthma and indoor environmental risk factors. Am J Epidemiol 1993;**137**(8):834-44

197. Juca SC, Takano OA, Moraes LS, et al. [Asthma prevalence and risk factors in adolescents 13 to 14 years of age in Cuiaba, Mato Grosso State, Brazil]. Cadernos de Saude Publica 2012;**28**(4):689-97

198. Karunasekera KA, Jayasinghe JA, Alwis LW. Risk factors of childhood asthma: a Sri Lankan study. Journal of Tropical Pediatrics 2001;**47**(3):142-5

199. Kramer MS, Moroz B. Do breast-feeding and delayed introduction of solid foods protect against subsequent atopic AD? Journal of Pediatrics 1981;**98**(4):546-50

200. Mavale-Manuel S, Alexandre F, Duarte N, et al. Risk factors for asthma among children in Maputo (Mozambique). Allergy 2004;**59**(4):388-93

201. Oliveti JF, Kercsmar CM, Redline S. Pre- and perinatal risk factors for asthma in inner city African-American children. American Journal of Epidemiology 1996;**143**(6):570-7

202. Porro E, Indinnimeo L, Antognoni G, et al. Early wheezing and breast feeding. Journal of Asthma 1993;**30**(1):23-8

203. Ratageri VH, Kabra SK, Dwivedi SN, et al. Factors associated with severe asthma. Indian Pediatrics 2000;**37**(10):1072-82

204. Rosas Vargas MA, Gonzalez Reyes M, del Rio Navarro BE, et al. [Allergen sensitization and asthma in children from 1 to 3 years of age]. Revista Alergia Mexico 2002;**49**(6):171-5

205. Rylander E, Pershagen G, Eriksson M, et al. Parental smoking and other risk factors for wheezing bronchitis in children. European Journal of Epidemiology 1993;**9**(5):517-26

206. Anonymous. Cow's milk allergy in the first year of life. An Italian Collaborative Study. Acta Paediatrica Scandinavica - Supplement 1988;**348**:1-14

207. Whu R, Cirilo G, Wong J, et al. Risk factors for pediatric asthma in the South Bronx. Journal of Asthma 2007;**44**(10):855-9

208. Wickens K, Crane J, Kemp T, et al. A case-control study of risk factors for asthma in New Zealand children. Aust N Z J Public Health 2001;**25**(1):44-9

209. Zhu CH, Liu JX, Zhao XH. [Risk factors of asthma among children aged 0 - 14 in Suzhou city]. Chung-Hua Yu Fang i Hsueh Tsa Chih [Chinese Journal of Preventive Medicine] 2012;**46**(5):456-9

210. Alper Z, Sapan N, Ercan I, et al. Risk factors for wheezing in primary school children in Bursa, Turkey. American Journal of Rhinology 2006;**20**(1):53-63

211. Al-Kubaisy W, Ali SH, Al-Thamiri D. Risk factors for asthma among primary school children in Baghdad, Iraq. Saudi Medical Journal 2005;**26**(3):460-6

212. Awasthi S, Kalra E, Roy S, et al. Prevalence and risk factors of asthma and wheeze in school-going children in Lucknow, North India. Indian Pediatrics 2004;**41**(12):1205-10

213. Innes Asher M, Robertson C, Ait-Khaled N, et al. Global analysis of breast feeding and risk of symptoms of asthma, rhinoconjunctivitis and AD in 6-7 year old children: ISAAC Phase Three. Allergologia et Immunopathologia 2011;**39**(6):318-25

214. Flohr C, Nagel G, Weinmayr G, et al. Lack of evidence for a protective effect of prolonged breastfeeding on childhood AD: lessons from the International Study of Asthma and Allergies in Childhood (ISAAC) Phase Two. British Journal of Dermatology 2011;**165**(6):1280-89 doi: 10.1111/j.1365-2133.2011.10588.x[published Online First: Epub Date]|.

215. Nagel G, Buchele G, Weinmayr G, et al. Effect of breastfeeding on asthma, lung function and bronchial hyperreactivity in ISAAC Phase II. European Respiratory Journal 2009;**33**(5):993-1002

216. Kuyucu S, Saraclar Y, Tuncer A, et al. Determinants of atopic sensitization in Turkish school children: effects of pre- and post-natal events and maternal atopy. Pediatric Allergy & Immunology 2004;**15**(1):62-71

217. Bruno G, Giampietro PG, Businco L. [Results of a multicentric study for the prevention of atopic allergy. 48 months of follow up]. Minerva Pediatrica 1996;**48**(10):413-9

218. Castro-Rodriguez JA, Garcia-Marcos L, Sanchez-Solis M, et al. Olive oil during pregnancy is associated with reduced wheezing during the first year of life of the offspring. Pediatric Pulmonology 2010;**45**(4):395-402

219. Chong Neto HJ, Rosário NA, Solé D, et al. Prevalência de sibilância recorrente em lactentes

Prevalence of recurrent wheezing in infants. J Pediatr (Rio J) 2007;**83**(4):357-62

220. Civelek E, Sahiner UM, Yuksel H, et al. Prevalence, burden, and risk factors of atopic AD in schoolchildren aged 10-11 years: a national multicenter study. Journal of Investigational Allergology & Clinical Immunology 2011;**21**(4):270-7

221. Ehlayel MS, Bener A. Duration of breast-feeding and the risk of childhood allergic diseases in a developing country. Allergy & Asthma Proceedings 2008;**29**(4):386-91

222. Ehrlich RI, Du Toit D, Jordaan E, et al. Risk factors for childhood asthma and wheezing. Importance of maternal and household smoking. American Journal of Respiratory & Critical Care Medicine 1996;**154**(3 Pt 1):681-8

223. Evenhouse E, Reilly S. Improved estimates of the benefits of breastfeeding using sibling comparisons to reduce selection bias. Health Services Research 2005;**40**(6):1781-802 doi: 10.1111/j.1475-6773.2005.00453.x[published Online First: Epub Date]|.

224. Girolomoni G, Abeni D, Masini C, et al. The epidemiology of atopic dermatitis in Italian schoolchildren. Allergy 2003;**58**(5):420-5

225. Han YY, Lee YL, Guo YL. Indoor environmental risk factors and seasonal variation of childhood asthma. Pediatr Allergy Immunol 2009;**20**(8):748-56 doi: 10.1111/j.1399-3038.2009.00871.x[published Online First: Epub Date]|.

226. Karino S, Okuda T, Uehara Y, et al. Breastfeeding and prevalence of allergic diseases in Japanese university students. Annals of Allergy Asthma & Immunology 2008;**101**(2):153-59

227. Kucukosmanoglu E, Yazi D, Yesil O, et al. Prevalence of egg sensitization in Turkish infants based on skin prick test. Allergologia Et Immunopathologia 2008;**36**(3):141-44 doi: 10.1016/s0301-0546(08)72538-2[published Online First: Epub Date]|.

228. Kuehr J, Frischer T, Karmaus W, et al. EARLY-CHILDHOOD RISK-FACTORS FOR SENSITIZATION AT SCHOOL AGE. Journal of Allergy and Clinical Immunology 1992;**90**(3):358-63 doi: 10.1016/s0091-6749(05)80015-6[published Online First: Epub Date]|.

229. Kurt S, Kisacik B, Kaplan Y, et al. Obesity and carpal tunnel syndrome: Is there a causal relationship? European Neurology 2008;**59**(5):253-57 doi: 10.1159/000115639[published Online First: Epub Date]|.

230. Kurt E, Metintas S, Basyigit I, et al. Prevalence and risk factors of allergies in Turkey: Results of a multicentric cross-sectional study in children. Pediatric Allergy & Immunology 2007;**18**(7):566-74

231. Liu P, Woo JMP, Parsa MF, et al. Ethnic differences in pediatric SLE early disease severity: A comparison between Hispanic-Americans and European-Americans. Pediatric Rheumatology 2012;**10**

232. Miyake Y, Yura A, Iki M. Breastfeeding and the prevalence of symptoms of allergic disorders in Japanese adolescents. Clinical and Experimental Allergy 2003;**33**(3):312-16 doi: 10.1046/j.1365-2222.2003.t01-1-01607.x[published Online First: Epub Date]|.

233. Nakamura Y, Oki I, Tanihara S, et al. Relationship between breast milk feeding and atopic dermatitis in children. Journal of epidemiology / Japan Epidemiological Association 2000;**10**(2):74-78

234. Paton J, Kljakovic M, Ciszek K, et al. Infant Feeding Practices and Nut Allergy over Time in Australian School Entrant Children. International Journal of Pediatrics 2012;**2012**:675724

235. Prietsch SO, Fischer GB, Cesar JA, et al. [Risk factors for recurrent wheezing in children under 13 years old in the South of Brazil]. Pan American Journal of Public Health 2006;**20**(5):331-7

236. Rusconi F, Galassi C, Bellasio M, et al. [Risk factors in the pre-, perinatal and early life (first year) for wheezing in young children]. Epidemiologia e Prevenzione 2005;**29**(2 Suppl):47-51

237. Rusconi F, Galassi C, Corbo GM, et al. Risk factors for early, persistent, and late-onset wheezing in young children. SIDRIA Collaborative Group. American Journal of Respiratory & Critical Care Medicine 1999;**160**(5 Pt 1):1617-22

238. Rust GS, Thompson CJ, Minor P, et al. Does breastfeeding protect children from asthma? Analysis of NHANES III survey data. Journal of the National Medical Association 2001;**93**(4):139-48

239. Salem MB, Al-Sadoon IO, Hassan MK. Prevalence of wheeze among preschool children in Basra governonate, southern Iraq. Eastern Mediterranean Health Journal 2002;**8**(4-5):503-8

240. Selcuk ZT, Caglar T, Enunlu T, et al. The prevalence of allergic diseases in primary school children in Edirne, Turkey. Clinical & Experimental Allergy 1997;**27**(3):262-9

241. Suwanpromma S, Boonlarbtaweechoke C, Udomsubpayakul U, et al. Spirometric airflow obstruction in Bangkok school children: prevalence and risk factors. Journal of the Medical Association of Thailand 2012;**95**(11):1411-7

242. Takemura Y, Sakurai Y, Honjo S, et al. Relation between breastfeeding and the prevalence of asthma : the Tokorozawa Childhood Asthma and Pollinosis Study. American Journal of Epidemiology 2001;**154**(2):115-9

243. Tanaka K, Miyake Y, Sasaki S. Association between breastfeeding and allergic disorders in Japanese children. International Journal of Tuberculosis and Lung Disease 2010;**14**(4):513-18

244. Visser CAN, Garcia-Marcos L, Eggink J, et al. Prevalence and Risk Factors of Wheeze in Dutch Infants in Their First Year of Life. Pediatric Pulmonology 2010;**45**(2):149-56 doi: 10.1002/ppul.21161[published Online First: Epub Date]|.

245. Wang HY, Chen YZ, Ma Y, et al. [Disparity of asthma prevalence in Chinese schoolchildren is due to differences in lifestyle factors]. Zhonghua Erke Zazhi 2006;**44**(1):41-5

246. Couper JJ, Steele C, Beresford S, et al. Lack of association between duration of breast-feeding or introduction of cow's milk and development of islet autoimmunity. Diabetes 1999;**48**(11):2145-9

247. Couper JJ, Beresford S, Hirte C, et al. Weight Gain in Early Life Predicts Risk of Islet Autoimmuity in Children With a First-Degree Relative With Type 1 Diabetes. Diabetes Care 2009;**32**(1):94-99 doi: 10.2337/dc08-0821[published Online First: Epub Date]|.

248. Frederiksen B, Kroehl M, Lamb M, et al. Infant exposures and development of type 1 diabetes-the diabetes autoimmunity study in the young (DAISY). Diabetes 2012;**61**:A352

249. Fronczak CM, Baron AE, Chase HP, et al. In utero dietary exposures and risk of islet autoimmunity in children. Diabetes Care 2003;**26**(12):3237-42

250. Lamb MM, Simpson MD, Seifert J, et al. The association between IgG4 antibodies to dietary factors, islet autoimmunity and type 1 diabetes: the Diabetes Autoimmunity Study in the Young. PLoS ONE [Electronic Resource] 2013;**8**(2):e57936

251. Norris JM, Barriga K, Klingensmith G, et al. Timing of initial cereal exposure in infancy and risk of islet autoimmunity. JAMA 2003;**290**(13):1713-20

252. Lamb MM, Myers MA, Barriga K, et al. Maternal diet during pregnancy and islet autoimmunity in offspring. Pediatric Diabetes 2008;**9**(2):135-41

253. Holmberg H, Wahlberg J, Vaarala O, et al. Short duration of breast-feeding as a risk-factor for beta-cell autoantibodies in 5-year-old children from the general population. The British journal of nutrition 2007;**97**(1):111-16

254. Karlen J, Faresjo T, Ludvigsson J. Could the social environment trigger the induction of diabetes related autoantibodies in young children? Scandinavian Journal of Public Health 2012;**40**(2):177-82

255. Wahlberg J, Vaarala O, Ludvigsson J, et al. Dietary risk factors for the emergence of type 1 diabetes-related autoantibodies in 21/2 year-old Swedish children. Br J Nutr 2006;**95**(3):603-8

256. Ludvigsson JF, Ludvigsson J. Stressful life events, social support and confidence in the pregnant woman and risk of coeliac disease in the offspring. Scandinavian Journal of Gastroenterology 2003;**38**(5):516-21

257. Viner RM, Hindmarsh PC, Taylor B, et al. Childhood body mass index (BMI), breastfeeding and risk of Type 1 diabetes: findings from a longitudinal national birth cohort. Diabetic Medicine 2008;**25**(9):1056-61

258. Virtanen SM, Rasanen L, Aro A, et al. Feeding in infancy and the risk of type 1 diabetes mellitus in Finnish children. The 'Childhood Diabetes in Finland' Study Group. Diabetic Medicine 1992;**9**(9):815-9

259. Virtanen SM, Hypponen E, Laara E, et al. Cow's milk consumption, disease-associated autoantibodies and type 1 diabetes mellitus: a follow-up study in siblings of diabetic children. Childhood Diabetes in Finland Study Group. Diabetic Medicine 1998;**15**(9):730-8

260. Hypponen E, Kenward MG, Virtanen SM, et al. Infant feeding, early weight gain, and risk of type I diabetes. Diabetes Care 1999;**22**(12):1961-65

261. Virtanen SM, Laara E, Hypponen E, et al. Cow's milk consumption, HLA-DQB1 genotype, and type 1 diabetes: a nested case-control study of siblings of children with diabetes. Childhood diabetes in Finland study group.[Erratum appears in Diabetes 2000 Sep;49(9):1617]. Diabetes 2000;**49**(6):912-7

262. Virtanen SM, Takkinen HM, Nevalainen J, et al. Early introduction of root vegetables in infancy associated with advanced beta-cell autoimmunity in young children with human leukocyte antigen-conferred susceptibility to Type 1 diabetes. Diabetic Medicine 2011;**28**(8):965-71 doi: 10.1111/j.1464-5491.2011.03294.x[published Online First: Epub Date]|.

263. Jones ME, Swerdlow AJ, Gill LE, et al. Pre-natal and early life risk factors for childhood onset diabetes mellitus: a record linkage study. International Journal of Epidemiology 1998;**27**(3):444-9

264. Kimpimaki T, Erkkola M, Korhonen S, et al. Short-term exclusive breastfeeding predisposes young children with increased genetic risk of Type I diabetes to progressive beta-cell autoimmunity. Diabetologia 2001;**44**(1):63-9

265. Kyvik KO, Green A, Svendsen A, et al. Breast feeding and the development of type 1 diabetes mellitus. Diabetic Medicine 1992;**9**(3):233-5

266. Norris JM, Beaty B, Klingensmith G, et al. Lack of association between early exposure to cow's milk protein and beta-cell autoimmunity - Diabetes autoimmunity study in the young (DAISY). Jama-Journal of the American Medical Association 1996;**276**(8):609-14 doi: 10.1001/jama.276.8.609[published Online First: Epub Date]|.

267. Robertson L, Harrild K. Maternal and neonatal risk factors for childhood type 1 diabetes: a matched case-control study. BMC Public Health 2010;**10**:281

268. Savilahti E, Saarinen KM. Early infant feeding and type 1 diabetes. European Journal of Nutrition 2009;**48**(4):243-9

269. Ahadi M, Tabatabaeiyan M, Moazzami K. Association between environmental factors and risk of type 1 diabetes - a case-control study. Endokrynologia Polska 2011;**62**(2):134-7

270. Alves JGB, Figueiroa JN, Meneses J, et al. Breastfeeding protects against type 1 diabetes mellitus: A case-sibling study. Breastfeeding Medicine 2012;**7**(1):25-28

271. Ashraf AP, Eason NB, Kabagambe EK, et al. Dietary iron intake in the first 4 months of infancy and the development of type 1 diabetes: A pilot study. Diabetology and Metabolic Syndrome 2010;**2**(1)

272. Baruah MP, Ammini AC, Khurana ML. Demographic, breast-feeding, and nutritional trends among children with type 1 diabetes mellitus. Indian journal of endocrinology and metabolism 2011;**15**(1):38-42 doi: 10.4103/2230-8210.77583[published Online First: Epub Date]|.

273. Bener A, Alsaied A, Al-Ali M, et al. High prevalence of vitamin D deficiency in type 1 diabetes mellitus and healthy children. Acta Diabetologica 2009;**46**(3):183-9

274. Blom L, Dahlquist G, Nystrom L, et al. The Swedish childhood diabetes study - social and perinatal determinants for diabetes in childhood. Diabetologia 1989;**32**(1):7-13

275. Bodington MJ, McNally PG, Burden AC. Cow's milk and type 1 childhood diabetes: no increase in risk. Diabetic Medicine 1994;**11**(7):663-5

276. Borras V, Freitas A, Castell C, et al. Type 1 diabetes and perinatal factors in Catalonia (Spain). Pediatric Diabetes 2011;**12**(4 Pt 2):419-23

277. Group ESS. Rapid early growth is associated with increased risk of childhood type 1 diabetes in various European populations. Diabetes Care 2002;**25**(10):1755-60

278. Esfarjani F, Azar MR, Gafarpour M. IDDM and early exposure of infant to cow's milk and solid food. Indian Journal of Pediatrics 2001;**68**(2):107-10

279. Gimeno SG, de Souza JM. IDDM and milk consumption. A case-control study in Sao Paulo, Brazil. Diabetes Care 1997;**20**(8):1256-60

280. Hathout EH, Beeson WL, Ischander M, et al. Air pollution and type 1 diabetes in children. Pediatric Diabetes 2006;**7**(2):81-7

281. Kostraba JN, Dorman JS, LaPorte RE, et al. Early infant diet and risk of IDDM in blacks and whites. A matched case-control study. Diabetes Care 1992;**15**(5):626-31

282. Kostraba JN, Cruickshanks KJ, Lawler-Heavner J, et al. Early exposure to cow's milk and solid foods in infancy, genetic predisposition, and risk of IDDM. Diabetes 1993;**42**(2):288-95

283. Liese AD, Puett RC, Lamichhane AP, et al. Neighborhood level risk factors for type 1 diabetes in youth: the SEARCH case-control study. International Journal of Health Geographics [Electronic Resource] 2012;**11**:1

284. Majeed AA, Mea, Hassan K. Risk Factors for Type 1 Diabetes Mellitus among Children and Adolescents in Basrah. Oman Medical Journal 2011;**26**(3):189-95

285. Malcova H, Sumnik Z, Drevinek P, et al. Absence of breast-feeding is associated with the risk of type 1 diabetes: a case-control study in a population with rapidly increasing incidence. European Journal of Pediatrics 2006;**165**(2):114-9

286. Marshall AL, Chetwynd A, Morris JA, et al. Type 1 diabetes mellitus in childhood: a matched case control study in Lancashire and Cumbria, UK. Diabetic Medicine 2004;**21**(9):1035-40

287. Mayer EJ, Hamman RF, Gay EC, et al. Reduced risk of IDDM among breast-fed children. The Colorado IDDM Registry. Diabetes 1988;**37**(12):1625-32

288. McKinney PA, Parslow R, Gurney KA, et al. Perinatal and neonatal determinants of childhood type 1 diabetes. A case-control study in Yorkshire, U.K. Diabetes Care 1999;**22**(6):928-32

289. Meloni T, Marinaro AM, Mannazzu MC, et al. IDDM and early infant feeding. Sardinian case-control study. Diabetes Care 1997;**20**(3):340-2

290. Patterson CC, Carson DJ, Hadden DR, et al. A case-control investigation of perinatal risk factors for childhood IDDM in Northern Ireland and Scotland. Diabetes Care 1994;**17**(5):376-81

291. Perez-Bravo F, Carrasco E, Gutierrez-Lopez MD, et al. Genetic predisposition and environmental factors leading to the development of insulin-dependent diabetes mellitus in Chilean children. Journal of molecular medicine 1996;**74**(2):105-9

292. Perez-Bravo F, Oyarzun A, Carrasco E, et al. Duration of breast feeding and bovine serum albumin antibody levels in type 1 diabetes: a case-control study. Pediatric Diabetes 2003;**4**(4):157-61

293. Rabiei S. The association of nutrition style through the first 2 years of life with type 1 diabetes mellitus and some of the other effective factors in 2-15 years old children. [Persian]. Iranian Journal of Endocrinology and Metabolism 2011;**13**(1):113

294. Rami B, Schneider U, Imhof A, et al. Risk factors for type I diabetes mellitus in children in Austria. European Journal of Pediatrics 1999;**158**(5):362-6

295. Rosenbauer J, Herzig P, Giani G. Early infant feeding and risk of type 1 diabetes mellitus-a nationwide population-based case-control study in pre-school children. Diabetes/Metabolism Research Reviews 2008;**24**(3):211-22

296. Sadauskaite-Kuehne V, Ludvigsson J, Padaiga Z, et al. Longer breastfeeding is an independent protective factor against development of type 1 diabetes mellitus in childhood. Diabetes/Metabolism Research Reviews 2004;**20**(2):150-7

297. Skrodeniene E, Marciulionyte D, Padaiga Z, et al. Associations between HLA class II haplotypes, environmental factors and type 1 diabetes mellitus in Lithuanian children with type 1 diabetes and controls. Polish Annals of Medicine 2010;**17**(1):7-15

298. Samuelsson U, Johansson C, Ludvigsson J. Breast-feeding seems to play a marginal role in the prevention of insulin-dependent diabetes mellitus. Diabetes Research & Clinical Practice 1993;**19**(3):203-10

299. Siemiatycki J, Colle E, Campbell S, et al. Case-control study of IDDM. Diabetes Care 1989;**12**(3):209-16

300. Sipetic S, Vlajinac H, Kocev N, et al. Early infant diet and risk of type 1 diabetes mellitus in Belgrade children. Nutrition 2005;**21**(4):474-9

301. Soltesz G, Jeges S, Dahlquist G. Non-genetic risk determinants for type 1 (insulin-dependent) diabetes mellitus in childhood. Hungarian Childhood Diabetes Epidemiology Study Group. Acta Paediatrica 1994;**83**(7):730-5

302. Stene LC, Ulriksen J, Magnus P, et al. Use of cod liver oil during pregnancy associated with lower risk of Type I diabetes in the offspring.[Erratum appears in Diabetologia 2000 Nov;43(11):1451]. Diabetologia 2000;**43**(9):1093-8

303. Stene LC, Joner G. Use of cod liver oil during the first year of life is associated with lower risk of childhood-onset type 1 diabetes: a large, population-based, case-control study. The American journal of clinical nutrition 2003;**78**(6):1128-34

304. Strotmeyer ES, Yang Z, LaPorte RE, et al. Infant diet and type 1 diabetes in China. Diabetes Research & Clinical Practice 2004;**65**(3):283-92

305. Tai TY, Wang CY, Lin LL, et al. A case-control study on risk factors for Type 1 diabetes in Taipei City. Diabetes research and clinical practice 1998;**42**(3):197-203

306. Telahun M, Abdulkadir J, Kebede E. The relation of early nutrition, infections and socio-economic factors to the development of childhood diabetes. Ethiopian Medical Journal 1994;**32**(4):239-44

307. Tenconi MT, Devoti G, Comelli M, et al. Major childhood infectious diseases and other determinants associated with type 1 diabetes: a case-control study. Acta Diabetologica 2007;**44**(1):14-9

308. Thorsdottir I, Birgisdottir BE, Johannsdottir IM, et al. Different beta-casein fractions in Icelandic versus Scandinavian cow's milk may influence diabetogenicity of cow's milk in infancy and explain low incidence of insulin-dependent diabetes mellitus in Iceland. Pediatrics 2000;**106**(4):719-24

309. Verge CF, Howard NJ, Irwig L, et al. Environmental factors in childhood IDDM. A population-based, case-control study. Diabetes Care 1994;**17**(12):1381-9

310. Virtanen SM, Rasanen L, Ylonen K, et al. Early introduction of dairy products associated with increased risk of IDDM in Finnish children. Diabetes 1993;**42**(12):1786-90

311. Visalli N, Sebastiani L, Adorisio E, et al. Environmental risk factors for type 1 diabetes in Rome and province. Archives of Disease in Childhood 2003;**88**(8):695-8

312. Wadsworth EJ, Shield JP, Hunt LP, et al. A case-control study of environmental factors associated with diabetes in the under 5s. Diabetic Medicine 1997;**14**(5):390-6

313. Glatthaar C, Whittall DE, Welborn TA, et al. Diabetes in Western Australian children: Descriptive epidemiology. Medical Journal of Australia 1988;**148**(3):117-23

314. Hummel M, Fuchtenbusch M, Schenker M, et al. No major association of breast-feeding, vaccinations, and childhood viral diseases with early islet autoimmunity in the German BABYDIAB Study. Diabetes Care 2000;**23**(7):969-74

315. Hummel S, Hummel M, Banholzer J, et al. Development of autoimmunity to transglutaminase C in children of patients with type 1 diabetes: relationship to islet autoantibodies and infant feeding. Diabetologia 2007;**50**(2):390-4

316. Ziegler AG, Schmid S, Huber D, et al. Early infant feeding and risk of developing type 1 diabetes-associated autoantibodies. JAMA 2003;**290**(13):1721-8

317. Norris JM, Barriga K, Hoffenberg EJ, et al. Risk of celiac disease autoimmunity and timing of gluten introduction in the diet of infants at increased risk of disease. Journal of the American Medical Association 2005;**293**(19):2343-51

318. Welander A, Tjernberg AR, Montgomery SM, et al. Infectious disease and risk of later celiac disease in childhood. Pediatrics 2010;**125**(3):e530-6

319. Ascher H, Krantz I, Rydberg L, et al. Influence of infant feeding and gluten intake on coeliac disease. Archives of Disease in Childhood 1997;**76**(2):113-7

320. Auricchio S, Follo D, de Ritis G, et al. Does breast feeding protect against the development of clinical symptoms of celiac disease in children? Journal of Pediatric Gastroenterology & Nutrition 1983;**2**(3):428-33

321. Baron S, Turck D, Leplat C, et al. Environmental risk factors in paediatric inflammatory bowel diseases: a population based case control study. Gut 2005;**54**(3):357-63

322. Bergstrand O, Hellers G. Breast-feeding during infancy in patients who later develop Crohn's disease. Scandinavian Journal of Gastroenterology 1983;**18**(7):903-6

323. Castiglione F, Diaferia M, Morace F, et al. Risk factors for inflammatory bowel diseases according to the "hygiene hypothesis": a case-control, multi-centre, prospective study in Southern Italy. Journal of Crohn's & colitis 2012;**6**(3):324-9

324. Corrao G, Tragnone A, Caprilli R, et al. Risk of inflammatory bowel disease attributable to smoking, oral contraception and breastfeeding in Italy: a nationwide case-control study. Cooperative Investigators of the Italian Group for the Study of the Colon and the Rectum (GISC). International Journal of Epidemiology 1998;**27**(3):397-404

325. Decker E, Engelmann G, Findeisen A, et al. Cesarean delivery is associated with celiac disease but not inflammatory bowel disease in children. Pediatrics 2010;**125**(6):e1433-40 doi: 10.1542/peds.2009-2260[published Online First: Epub Date]|.

326. Ellis JA, Ponsonby AL, Pezic A, et al. CLARITY - ChiLdhood Arthritis Risk factor Identification sTudY. Pediatric Rheumatology Online Journal 2012;**10**(1):37

327. Falth-Magnusson K, Franzen L, Jansson G, et al. Infant feeding history shows distinct differences between Swedish celiac and reference children. Pediatric Allergy & Immunology 1996;**7**(1):1-5

328. Fort P, Moses N, Fasano M, et al. Breast and soy-formula feedings in early infancy and the prevalence of autoimmune thyroid disease in children. Journal of the American College of Nutrition 1990;**9**(2):164-67

329. Gearry RB, Richardson AK, Frampton CM, et al. Population-based cases control study of inflammatory bowel disease risk factors. Journal of Gastroenterology & Hepatology 2010;**25**(2):325-33

330. Gilat T, Hacohen D, Lilos P, et al. Childhood factors in ulcerative colitis and Crohn's disease. An international cooperative study. Scandinavian Journal of Gastroenterology 1987;**22**(8):1009-24

331. Greco L, Auricchio S, Mayer M, et al. Case control study on nutritional risk factors in celiac disease. Journal of Pediatric Gastroenterology & Nutrition 1988;**7**(3):395-9

332. Gruber M, Marshall JR, Zielezny M, et al. A case-control study to examine the influence of maternal perinatal behaviors on the incidence of Crohn's disease. Gastroenterology Nursing 1996;**19**(2):53-9

333. Hansen TS, Jess T, Vind I, et al. Environmental factors in inflammatory bowel disease: a case-control study based on a Danish inception cohort. Journal of Crohn's & colitis 2011;**5**(6):577-84

334. Ivarsson A, Hernell O, Stenlund H, et al. Breast-feeding protects against celiac disease. American Journal of Clinical Nutrition 2002;**75**(5):914-21

335. Koletzko S, Griffiths A, Corey M, et al. Infant feeding practices and ulcerative colitis in childhood. Bmj 1991;**302**(6792):1580-1

336. Mason T, Rabinovich CE, Fredrickson DD, et al. Breast feeding and the development of juvenile rheumatoid arthritis. Journal of Rheumatology 1995;**22**(6):1166-70

337. Pacilio A, Piccolo E, Scala MG, et al. The natural history of celiac disease. Digestive and Liver Disease 2010;**42**:S357

338. Peters U, Schneeweiss S, Trautwein EA, et al. A case-control study of the effect of infant feeding on celiac disease. Annals of Nutrition & Metabolism 2001;**45**(4):135-42

339. Roberts SE, Williams JG, Meddings D, et al. Perinatal risk factors and coeliac disease in children and young adults: a record linkage study. Aliment Pharmacol Ther 2009;**29**(2):222-31 doi: 10.1111/j.1365-2036.2008.03871.x[published Online First: Epub Date]|.

340. Rosenberg AM. Evaluation of associations between breast feeding and subsequent development of juvenile rheumatoid arthritis. Journal of Rheumatology 1996;**23**(6):1080-2

341. Sonntag B, Stolze B, Heinecke A, et al. Preterm birth but not mode of delivery is associated with an increased risk of developing inflammatory bowel disease later in life. Inflammatory Bowel Diseases 2007;**13**(11):1385-90

342. Thompson NP, Montgomery SM, Wadsworth MEJ, et al. Early determinants of inflammatory bowel disease: use of two national longitudinal birth cohorts. European Journal of Gastroenterology & Hepatology 2000;**12**(1):25-30 doi: 10.1097/00042737-200012010-00006[published Online First: Epub Date]|.

343. Wang YF, Ou-Yang Q, Xia B, et al. Multicenter case-control study of the risk factors for ulcerative colitis in China. World Journal of Gastroenterology 2013;**19**(11):1827-33

344. Falth-Magnusson K, Kjellman NI. Development of atopic disease in babies whose mothers were receiving exclusion diet during pregnancy--a randomized study. Journal of Allergy & Clinical Immunology 1987;**80**(6):868-75

345. Falth-Magnusson K, Kjellman NI. Allergy prevention by maternal elimination diet during late pregnancy--a 5-year follow-up of a randomized study. Journal of Allergy & Clinical Immunology 1992;**89**(3):709-13

346. Ludvigsson J. Cow-milk-free diet during last trimester of pregnancy does not influence diabetes-related autoantibodies in nondiabetic children. Annals of the New York Academy of Sciences 2003;**1005**:275-8

347. Jirapinyo P, Densupsoontorn N, Kangwanpornsiri C, et al. Lower prevalence of a topic dermatitis in breast-fed infants whose allergic mothers restrict dairy products. Journal of the Medical Association of Thailand 2013;**96**(2):192-95

348. Lilja G, Dannaeus A, Foucard T, et al. Effects of maternal diet during late pregnancy and lactation on the development of atopic diseases in infants up to 18 months of age--in-vivo results. Clinical & Experimental Allergy 1989;**19**(4):473-9

349. Hattevig G, Kjellman B, Sigurs N, et al. The effect of maternal avoidance of eggs, cow's milk, and fish during lactation on the development of IgE, IgG, and IgA antibodies in infants. Journal of Allergy & Clinical Immunology 1990;**85**(1 Pt 1):108-15

350. Paronen J, Bjorksten B, Hattevig G, et al. Effect of maternal diet during lactation on development of bovine insulin-binding antibodies in children at risk for allergy. Journal of Allergy & Clinical Immunology 2000;**106**(2):302-6

351. Hattevig G, Sigurs N, Kjellman B. Effects of maternal dietary avoidance during lactation on allergy in children at 10 years of age. Acta Paediatrica 1999;**88**(1):7-12

352. Hattevig G, Kjellman B, Sigurs N, et al. Effect of maternal avoidance of eggs, cow's milk and fish during lactation upon allergic manifestations in infants. Clin Exp Allergy 1989;**19**(1):27-32

353. Sigurs N, Hattevig G, Kjellman B. Maternal avoidance of eggs, cow's milk, and fish during lactation: effect on allergic manifestations, skin-prick tests, and specific IgE antibodies in children at age 4 years. Pediatrics 1992;**89**(4 Pt 2):735-9

354. Herrmann ME, Dannemann A, Gruters A, et al. Prospective study of the atopy preventive effect of maternal avoidance of milk and eggs during pregnancy and lactation. European Journal of Pediatrics 1996;**155**(9):770-4

355. Kilburn SA, Pollard C, Bevin S, et al. Allergens in mother's milk: Tolerisation or sensitization. Nutrition Research 1998;**18**(8):1351-61 doi: 10.1016/s0271-5317(98)00114-6[published Online First: Epub Date]|.

356. Becker A, Watson W, Ferguson A, et al. The Canadian asthma primary prevention study: outcomes at 2 years of age. Journal of Allergy & Clinical Immunology 2004;**113**(4):650-6

357. Chan-Yeung M, Manfreda J, Dimich-Ward H, et al. A randomized controlled study on the effectiveness of a multifaceted intervention program in the primary prevention of asthma in high-risk infants. Archives of Pediatrics & Adolescent Medicine 2000;**154**(7):657-63

358. Chan-Yeung M, Ferguson A, Watson W, et al. The Canadian Childhood Asthma Primary Prevention Study: outcomes at 7 years of age. Journal of Allergy & Clinical Immunology 2005;**116**(1):49-55

359. Wong T, Chan-Yeung M, Rousseau R, et al. Delayed introduction of food and effect on incidence of food allergy in a population at high risk for atopy: The Canadian asthma primary prevention study (CAPPS). Journal of Allergy and Clinical Immunology 2013;**1)**:AB96

360. Protudjer JLP, Robertson L, Kozyrskyj A, et al. Non-atopic asthma and atopic asthma are associated with reduced pulmonary function by age 7 years. American Journal of Respiratory and Critical Care Medicine 2011;**183 (1 MeetingAbstracts)**

361. Carlsten C, Dimich-Ward H, Ferguson A, et al. Atopic dermatitis in a high-risk cohort: Natural history, associated allergic outcomes, and risk factors. Annals of Allergy, Asthma and Immunology 2013;**110**(1):24-28

362. Hide DW. The Isle of Wight study, an approach to allergy prevention. Pediatric Allergy & Immunology 1994;**5**(6 Suppl):61-4

363. Hide DW, Matthews S, Tariq S, et al. Allergen avoidance in infancy and allergy at 4 years of age. Allergy 1996;**51**(2):89-93

364. Arshad SH, Matthews S, Gant C, et al. Effect of allergen avoidance on development of allergic disorders in infancy. Lancet 1992;**339**(8808):1493-7

365. Arshad SH, Bateman B, Matthews SM. Primary prevention of asthma and atopy during childhood by allergen avoidance in infancy: a randomised controlled study. Thorax 2003;**58**(6):489-93

366. Arshad SH, Bateman B, Sadeghnejad A, et al. Prevention of allergic disease during childhood by allergen avoidance: the Isle of Wight prevention study. Journal of Allergy & Clinical Immunology 2007;**119**(2):307-13

367. Scott M, Roberts G, Kurukulaaratchy RJ, et al. Multifaceted allergen avoidance during infancy reduces asthma during childhood with the effect persisting until age 18 years. Thorax 2012;**67**(12):1046-51

368. Lovegrove JA, Hampton SM, Morgan JB. The immunological and long-term atopic outcome of infants born to women following a milk-free diet during late pregnancy and lactation: a pilot study. British Journal of Nutrition 1994;**71**(2):223-38

369. Shao J, Sheng J, Dong W, et al. [Effects of feeding intervention on development of AD in atopy high-risk infants: an 18-month follow-up study]. Zhonghua Erke Zazhi 2006;**44**(9):684-7

370. Zeiger RS, Heller S, Mellon MH, et al. Genetic and environmental factors affecting the development of atopy through age 4 in children of atopic parents: A prospective randomized study of food allergen avoidance. Pediatric Allergy and Immunology 1992;**3**(3):110-27

371. Zeiger RS, Heller S, Mellon MH, et al. Effect of combined maternal and infant food-allergen avoidance on development of atopy in early infancy: a randomized study J Allergy Clin Immunol 1989 Nov;84(5 Pt 1):677. Journal of Allergy and Clinical Immunology 1989; 84(1). <http://onlinelibrary.wiley.com/o/cochrane/clcentral/articles/438/CN-00678438/frame.html>

<http://ac.els-cdn.com/0091674989901814/1-s2.0-0091674989901814-main.pdf?_tid=eeeb9810-ae8e-11e3-b839-00000aacb35d&acdnat=1395141646_2e5789458ac8f08bfa4dde5ca1eaa46d>.

372. Zeiger RS. Dietary manipulations in infants and their mothers and the natural course of atopic disease. Pediatric Allergy & Immunology 1994;**5**(6 Suppl):33-43

373. Halmerbauer G, Gartner C, Schierl M, et al. Study on the Prevention of Allergy in Children in Europe (SPACE): Allergic sensitization at 1 year of age in a controlled trial of allergen avoidance from birth. Pediatr Allergy Immunol 2003;**14**(1):10-17

374. Halmerbauer G, Gartner C, Schierl M, et al. Study on the Prevention of Allergy in Children in Europe (SPACE): Allergic sensitization in children at 1 year of age in a controlled trial of allergen avoidance from birth. Pediatric Allergy and Immunology, Supplement 2002;**13**(15):47-54

375. Matthew DJ, Taylor B, Norman AP, et al. Prevention of AD. Lancet 1977;**1**(8007):321-4

376. Poysa L, Korppi M, Remes K, et al. Atopy in childhood and diet in infancy. A nine-year follow-up study. I. Clinical manifestations. Allergy Proceedings 1991;**12**(2):107-11

377. Poysa L, Remes K, Korppi M, et al. Atopy in children with and without a family history of atopy. I. Clinical manifestations, with special reference to diet in infancy. Acta Paediatrica Scandinavica 1989;**78**(6):896-901

378. Kuikka L, Korppi M, Remes K. Atopy and skin reactivity at school age in children followed up from birth, with special reference to atopy prevention in infancy and atopic findings at preschool age. Allergy Proceedings 1995;**16**(6):313-7

379. Boyle RJ BN, Chiang WC, Chien CM, Gold M, Hourihane J. Partially hydrolysed prebiotic supplemented whey formula for the prevention of allergic manifestations in high risk infants: a multicentre double blind randomised controlled trial. Clinical Translational Allergy 2015;**15**(Suppl 3):30

380. Gruber C, Van Stuijvenberg M, Mosca F, et al. Reduced occurrence of early atopic dermatitis because of immunoactive prebiotics among low-atopy-risk infants. J Allergy Clin Immunol 2010;**126**(4):791-97

381. Ivakhnenko O, Niankovskyy S. [Clinical effectiveness of probiotics in complex treatment of infants with cow's milk allergy]. Georgian medical news 2013; (216). <http://onlinelibrary.wiley.com/o/cochrane/clcentral/articles/797/CN-01123797/frame.html>.

382. Sierra C, Bernal MJ, Blasco J, et al. Prebiotic effect during the first year of life in healthy infants fed formula containing GOS as the only prebiotic: a multicentre, randomised, double-blind and placebo-controlled trial. European Journal of Nutrition 2015;**54**(1):89-99 doi: [http://dx.doi.org/10.1007/s00394-014-0689-9[published](http://dx.doi.org/10.1007/s00394-014-0689-9%5bpublished) Online First: Epub Date]|.

383. Ziegler E, Vanderhoof JA, Petschow B, et al. Term infants fed formula supplemented with selected blends of prebiotics grow normally and have soft stools similar to those reported for breast-fed infants. Journal of Pediatric Gastroenterology & Nutrition 2007;**44**(3):359-64

384. Kukkonen K, Savilahti E, Haahtela T, et al. Probiotics and prebiotic galacto-oligosaccharides in the prevention of allergic diseases: a randomized, double-blind, placebo-controlled trial. Journal of Allergy & Clinical Immunology 2007;**119**(1):192-8

385. Kuitunen M, Kukkonen K, Juntunen-Backman K, et al. Probiotics prevent IgE-associated allergy until age 5 years in cesarean-delivered children but not in the total cohort. Journal of Allergy & Clinical Immunology 2009;**123**(2):335-41

386. Kukkonen AK, Kuitunen M, Savilahti E, et al. Airway inflammation in probiotic-treated children at 5 years. Pediatric Allergy & Immunology 2011;**22**(2):249-51

387. Roze JC, Barbarot S, Butel MJ, et al. An alpha-lactalbumin-enriched and symbiotic-supplemented v. a standard infant formula: A multicentre, double-blind, randomised trial. British Journal of Nutrition 2012;**107**(11):1616-22

388. Van Der Aa L, Heymans H, Van Aalderen W, et al. Specific synbiotic mixture prevents asthma-like symptoms in infants with atopic dermatitis. Allergy: European Journal of Allergy and Clinical Immunology 2010;**65**:313

389. Abrahamsson TR, Jakobsson T, Bottcher MF, et al. Probiotics in prevention of IgE-associated AD: a double-blind, randomized, placebo-controlled trial. Journal of Allergy & Clinical Immunology 2007;**119**(5):1174-80

390. Abrahamsson TR, Jakobsson T, Bjorksten B, et al. No effect of probiotics on respiratory allergies: a seven-year follow-up of a randomized controlled trial in infancy. Pediatric Allergy & Immunology 2013;**24**(6):556-61 doi: [http://dx.doi.org/10.1111/pai.12104[published](http://dx.doi.org/10.1111/pai.12104%5bpublished) Online First: Epub Date]|.

391. Allen SJ, Jordan S, Storey M, et al. Probiotics and atopic AD: A double-blind randomised controlled trial. Archives of Disease in Childhood 2012;**97**:A2

392. Allen SJ, Jordan S, Storey M, et al. Probiotics in the prevention of AD: a randomised controlled trial. Archives of Disease in Childhood 2014;**99**(11):1014-9 doi: [http://dx.doi.org/10.1136/archdischild-2013-305799[published](http://dx.doi.org/10.1136/archdischild-2013-305799%5bpublished) Online First: Epub Date]|.

393. Boyle RJ, Ismail IH, Kivivuori S, et al. Lactobacillus GG treatment during pregnancy for the prevention of AD: a randomized controlled trial. Allergy 2011;**66**(4):509-16

394. De Leon J, Sumpaico MW, Recto MT, et al. A preliminary study on the role of probiotics (lactobacillus acidophilus/bifidobacterium) in the prevention of atopic dermatitis in high-risk infants (0-2 weeks old): A randomized placebo-controlled trial. Annals of Allergy Asthma & Immunology 2007;**98**(1):A84-A84

395. Simon AL, Sumpaico MW, Recto MT, et al. The effects of probiotics on total ige levels of infants at risk for the development of atopic disease: A randomized triple blind placebo controlled clinical trial. Annals of Allergy Asthma & Immunology 2007;**98**(1):A94-A95

396. Dotterud CK, Storro O, Johnsen R, et al. Probiotics in pregnant women to prevent allergic disease: a randomized, double-blind trial. British Journal of Dermatology 2010;**163**(3):616-23

397. Enomoto T, Sowa M, Nishimori K, et al. Effects of bifidobacterial supplementation to pregnant women and infants in the prevention of allergy development in infants and on fecal microbiota. Allergology International 2014;**63**(4):575-85 doi: [http://dx.doi.org/10.2332/allergolint.13-OA-0683[published](http://dx.doi.org/10.2332/allergolint.13-OA-0683%5bpublished) Online First: Epub Date]|.

398. Huurre A, Laitinen K, Rautava S, et al. Impact of maternal atopy and probiotic supplementation during pregnancy on infant sensitization: a double-blind placebo-controlled study. Clinical & Experimental Allergy 2008;**38**(8):1342-8

399. Kalliomaki M, Salminen S, Arvilommi H, et al. Probiotics in primary prevention of atopic disease: a randomised placebo-controlled trial. Lancet 2001;**357**(9262):1076-9

400. Kalliomaki M, Salminen S, Poussa T, et al. Probiotics and prevention of atopic disease: 4-year follow-up of a randomised placebo-controlled trial. Lancet 2003;**361**(9372):1869-71

401. Kalliomaki M, Salminen S, Poussa T, et al. Probiotics during the first 7 years of life: a cumulative risk reduction of AD in a randomized, placebo-controlled trial. Journal of Allergy & Clinical Immunology 2007;**119**(4):1019-21

402. Rautava S, Kalliomäki M, Isolauri E. Probiotics during pregnancy and breast-feeding might confer immunomodulatory protection against atopic disease in the infant. The Journal of allergy and clinical immunology 2002; 109(1). <http://onlinelibrary.wiley.com/o/cochrane/clcentral/articles/174/CN-00377174/frame.html>

<http://ac.els-cdn.com/S0091674902251945/1-s2.0-S0091674902251945-main.pdf?_tid=59bab422-ae9f-11e3-807d-00000aacb35d&acdnat=1395148698_7296ee208e68e2f49afe70de62143737>.

403. Kim JY, Kwon JH, Ahn SH, et al. Effect of probiotic mix (Bifidobacterium bifidum, Bifidobacterium lactis, Lactobacillus acidophilus) in the primary prevention of AD: a double-blind, randomized, placebo-controlled trial. Pediatric Allergy & Immunology 2010;**21**(2 Pt 2):e386-93

404. Kopp MV, Hennemuth I, Heinzmann A, et al. Randomized, double-blind, placebo-controlled trial of probiotics for primary prevention: no clinical effects of Lactobacillus GG supplementation. Pediatrics 2008;**121**(4):e850-6

405. Lau S, Gerhold K, Zimmermann K, et al. Oral application of bacterial lysate in infancy decreases the risk of atopic dermatitis in children with 1 atopic parent in a randomized, placebo-controlled trial. Journal of Allergy & Clinical Immunology 2012;**129**(4):1040-7

406. Lodinová-Zádníková R, Prokesová L, Kocourková I, et al. Prevention of allergy in infants of allergic mothers by probiotic Escherichia coli. International archives of allergy and immunology 2010; 153(2). <http://onlinelibrary.wiley.com/o/cochrane/clcentral/articles/180/CN-00762180/frame.html>

<http://www.karger.com/Article/Pdf/312638>.

407. Morisset M, Soulaines P, Aubert-Jacquin C, et al. Double blind test of a fermented infantile formula in cow's milk allergy prevention [Abstract]. Journal of Allergy and Clinical Immunology 2008; 21(2 Suppl 1). <http://onlinelibrary.wiley.com/o/cochrane/clcentral/articles/516/CN-00679516/frame.html>.

408. Niers L, Martin R, Rijkers G, et al. The effects of selected probiotic strains on the development of AD (the PandA study). Allergy 2009;**64**(9):1349-58

409. Ou CY, Kuo HC, Wang L, et al. Prenatal and postnatal probiotics reduces maternal but not childhood allergic diseases: a randomized, double-blind, placebo-controlled trial. Clinical & Experimental Allergy 2012;**42**(9):1386-96

410. Taylor AL, Dunstan JA, Prescott SL. Probiotic supplementation for the first 6 months of life fails to reduce the risk of atopic dermatitis and increases the risk of allergen sensitization in high-risk children: a randomized controlled trial. Journal of Allergy & Clinical Immunology 2007;**119**(1):184-91

411. Taylor SN, Wagner CL, Hollis BW. Vitamin D supplementation during lactation to support infant and mother. Journal of the American College of Nutrition 2008;**27**(6):690-701

412. Jensen MP, Meldrum S, Taylor AL, et al. Early probiotic supplementation for allergy prevention: long-term outcomes. Journal of Allergy & Clinical Immunology 2012;**130**(5):1209-11.e5

413. Rautava S, Arvilommi H, Isolauri E. Specific probiotics in enhancing maturation of IgA responses in formula-fed infants. Pediatric Research 2006;**60**(2):221-24 doi: 10.1203/01.pdr.0000228317.72933.db[published Online First: Epub Date]|.

414. Rautava S, Kainonen E, Salminen S, et al. Maternal probiotic supplementation during pregnancy and breast-feeding reduces the risk of AD in the infant. Journal of Allergy & Clinical Immunology 2012;**130**(6):1355-60

415. Scalabrin DM, Johnston WH, Hoffman DR, et al. Growth and tolerance of healthy term infants receiving hydrolyzed infant formulas supplemented with Lactobacillus rhamnosus GG: randomized, double-blind, controlled trial. Clinical Pediatrics 2009;**48**(7):734-44

416. Scalabrin DMF, Harris C, Strong PV, et al. Infant supplementation with Lactobacillus rhamnosus GG (LGG) and long-term growth and health: A 5-year follow-up. Allergy: European Journal of Allergy and Clinical Immunology 2014;**69**:196-97 doi: [http://dx.doi.org/10.1111/all.12493[published](http://dx.doi.org/10.1111/all.12493%5bpublished) Online First: Epub Date]|.

417. Soh SE, Aw M, Gerez I, et al. Probiotic supplementation in the first 6 months of life in at risk Asian infants--effects on AD and atopic sensitization at the age of 1 year. Clinical & Experimental Allergy 2009;**39**(4):571-8

418. Loo EX, Llanora GV, Lu Q, et al. Supplementation with probiotics in the first 6 months of life did not protect against AD and allergy in at-risk Asian infants: a 5-year follow-up. International Archives of Allergy & Immunology 2014;**163**(1):25-8 doi: [http://dx.doi.org/10.1159/000356338[published](http://dx.doi.org/10.1159/000356338%5bpublished) Online First: Epub Date]|.

419. West CE, Hammarstrom ML, Hernell O. Probiotics during weaning reduce the incidence of AD. Pediatric Allergy & Immunology 2009;**20**(5):430-7

420. West CE, Hammarstrom ML, Hernell O. Probiotics in primary prevention of allergic disease--follow-up at 8-9 years of age. Allergy 2013;**68**(8):1015-20 doi: [http://dx.doi.org/10.1111/all.12191[published](http://dx.doi.org/10.1111/all.12191%5bpublished) Online First: Epub Date]|.

421. Wickens K, Black PN, Stanley TV, et al. A differential effect of 2 probiotics in the prevention of AD and atopy: a double-blind, randomized, placebo-controlled trial. Journal of Allergy & Clinical Immunology 2008;**122**(4):788-94

422. Wickens K, Black P, Stanley TV, et al. A protective effect of Lactobacillus rhamnosus HN001 against AD in the first 2 years of life persists to age 4 years. Clinical & Experimental Allergy 2012;**42**(7):1071-9

423. Wickens K, Stanley TV, Mitchell EA, et al. Early supplementation with Lactobacillus rhamnosus HN001 reduces AD prevalence to 6 years: does it also reduce atopic sensitization? Clinical & Experimental Allergy 2013;**43**(9):1048-57 doi: [http://dx.doi.org/10.1111/cea.12154[published](http://dx.doi.org/10.1111/cea.12154%5bpublished) Online First: Epub Date]|.

424. Birch EE, Khoury JC, Berseth CL, et al. The impact of early nutrition on incidence of allergic manifestations and common respiratory illnesses in children. Journal of Pediatrics 2010;**156**(6):902-6, 06.e1

425. Lucas A, Stafford M, Morley R, et al. Efficacy and safety of long-chain polyunsaturated fatty acid supplementation of infant-formula milk: a randomised trial. Lancet 1999;**354**(9194):1948-54

426. van Gool CJ, Thijs C, Henquet CJ, et al. Gamma-linolenic acid supplementation for prophylaxis of atopic dermatitis--a randomized controlled trial in infants at high familial risk. American Journal of Clinical Nutrition 2003;**77**(4):943-51

427. Linnamaa P, Savolainen J, Koulu L, et al. Blackcurrant seed oil for prevention of atopic dermatitis in newborns: a randomized, double-blind, placebo-controlled trial. Clinical & Experimental Allergy 2010;**40**(8):1247-55

428. Mihrshahi S, Peat JK, Marks GB, et al. Eighteen-month outcomes of house dust mite avoidance and dietary fatty acid modification in the Childhood Asthma Prevention Study (CAPS) J Allergy Clin Immunol. 2003 Apr;111(4):735. Journal of Allergy and Clinical Immunology 2003; 111(1). <http://onlinelibrary.wiley.com/o/cochrane/clcentral/articles/174/CN-00676174/frame.html>

<http://ac.els-cdn.com/S0091674902912984/1-s2.0-S0091674902912984-main.pdf?_tid=32cb6320-ae9f-11e3-95b0-00000aacb35e&acdnat=1395148632_3591a57752a3749b0fcd9acf89c00bd3>.

429. Peat JK, Mihrshahi S, Kemp AS, et al. Three-year outcomes of dietary fatty acid modification and house dust mite reduction in the Childhood Asthma Prevention Study. Journal of Allergy & Clinical Immunology 2004;**114**(4):807-13

430. Marks GB, Mihrshahi S, Kemp AS, et al. Prevention of asthma during the first 5 years of life: a randomized controlled trial. Journal of Allergy & Clinical Immunology 2006;**118**(1):53-61

431. Damsgaard CT, Lauritzen L, Kjaer TMR, et al. Fish oil supplementation modulates immune function in healthy infants. Journal of Nutrition 2007;**137**(4):1031-36

432. Palmer DJ, Sullivan T, Gold MS, et al. Effect of n-3 long chain polyunsaturated fatty acid supplementation in pregnancy on infants' allergies in first year of life: randomised controlled trial. Bmj 2012;**344**:e184

433. Palmer DJ, Sullivan T, Gold MS, et al. Effect of n-3 polyunsaturated fatty acid supplementation in pregnancy on early childhood allergic disease: Randomized controlled trial. Journal of Paediatrics and Child Health 2013;**49**:56

434. Dunstan JA, Mori TA, Barden A, et al. Fish oil supplementation in pregnancy modifies neonatal allergen-specific immune responses and clinical outcomes in infants at high risk of atopy: a randomized, controlled trial. Journal of Allergy & Clinical Immunology 2003;**112**(6):1178-84

435. D'Vaz N, Meldrum SJ, Dunstan JA, et al. Postnatal fish oil supplementation in high-risk infants to prevent allergy: randomized controlled trial. Pediatrics 2012;**130**(4):674-82

436. Furuhjelm C, Warstedt K, Larsson J, et al. Fish oil supplementation in pregnancy and lactation may decrease the risk of infant allergy. Acta Paediatrica 2009;**98**(9):1461-7

437. Furuhjelm C, Warstedt K, Fagerås M, et al. Allergic disease in infants up to 2 years of age in relation to plasma omega-3 fatty acids and maternal fish oil supplementation in pregnancy and lactation. Pediatric allergy and immunology : official publication of the European Society of Pediatric Allergy and Immunology 2011; 22(5). <http://onlinelibrary.wiley.com/o/cochrane/clcentral/articles/572/CN-00812572/frame.html>

<http://onlinelibrary.wiley.com/store/10.1111/j.1399-3038.2010.01096.x/asset/j.1399-3038.2010.01096.x.pdf?v=1&t=hsx79hch&s=55fa750a8ca2de44b6754fa121f69e7234ddc103>.

438. Lauritzen L, Kjaer TM, Fruekilde MB, et al. Fish oil supplementation of lactating mothers affects cytokine production in 2 1/2-year-old children. Lipids 2005;**40**(7):669-76

439. Olsen SF, Osterdal ML, Salvig JD, et al. Fish oil intake compared with olive oil intake in late pregnancy and asthma in the offspring: 16 y of registry-based follow-up from a randomized controlled trial. American Journal of Clinical Nutrition 2008;**88**(1):167-75

440. Dotterud CK, Storro O, Simpson MR, et al. The impact of pre- and postnatal exposures on allergy related diseases in childhood: a controlled multicentre intervention study in primary health care. BMC Public Health 2013;**13**:123

441. Imhoff-Kunsch B, Stein AD, Martorell R, et al. Prenatal docosahexaenoic acid supplementation and infant morbidity: randomized controlled trial. Pediatrics 2011;**128**(3):e505-12

442. Noakes PS, Vlachava M, Kremmyda LS, et al. Increased intake of oily fish in pregnancy: effects on neonatal immune responses and on clinical outcomes in infants at 6 mo. American Journal of Clinical Nutrition 2012;**95**(2):395-404

443. Checkley W, West KP, Wise RA, et al. Maternal Vitamin A Supplementation and Lung Function in Offspring. N Engl J Med 2010;**362**(19):1784-94 doi: 10.1056/NEJMoa0907441[published Online First: Epub Date]|.

444. Czeizel AE, Dobo M. Postnatal somatic and mental development after periconceptional multivitamin supplementation. Archives of Disease in Childhood 1994;**70**(3):229-33

445. Dobo M, Czeizel AE. Long-term somatic and mental development of children after periconceptional multivitamin supplementation. European Journal of Pediatrics 1998;**157**(9):719-23

446. Greenough A, Shaheen SO, Shennan A, et al. Respiratory outcomes in early childhood following antenatal vitamin C and E supplementation. Thorax 2010;**65**(11):998-1003

447. Goldring ST, Griffiths CJ, Martineau AR, et al. Prenatal vitamin d supplementation and child respiratory health: a randomised controlled trial. PLoS ONE [Electronic Resource] 2013;**8**(6):e66627

448. Kiraly N, Balde A, Lisse IM, et al. Vitamin A supplementation and risk of atopy: long-term follow-up of a randomized trial of vitamin A supplementation at six and nine months of age. BMC pediatrics 2013;**13**:190 doi: 10.1186/1471-2431-13-190[published Online First: Epub Date]|.

449. McEvoy CT, Schilling D, Clay N, et al. Vitamin C supplementation for pregnant smoking women and pulmonary function in their newborn infants: a randomized clinical trial. JAMA 2014;**311**(20):2074-82 doi: [http://dx.doi.org/10.1001/jama.2014.5217[published](http://dx.doi.org/10.1001/jama.2014.5217%5bpublished) Online First: Epub Date]|.

450. Goksor E, Alm B, Thengilsdottir H, et al. Characteristics and risk factors for doctor-diagnosed food allergy at preschool age. Allergy: European Journal of Allergy and Clinical Immunology 2011;**66**:219

451. Alm B, Goksor E, Thengilsdottir H, et al. Early protective and risk factors for allergic rhinitis at age 41/2 yr. Pediatric Allergy & Immunology 2011;**22**(4):398-404

452. Andreasyan K, Ponsonby AL, Dwyer T, et al. Infant feeding and childhood atopy: does early introduction of non-milk fluids matter? Pediatric Allergy & Immunology 2007;**18**(3):250-7

453. Back O, Blomquist HK, Hernell O, et al. Does vitamin D intake during infancy promote the development of atopic allergy? Acta dermato-venereologica 2009;**89**(1):28-32

454. Baiz N, Dargent-Molina P, Wark JD, et al. Cord serum 25-hydroxyvitamin D and risk of early childhood transient wheezing and atopic dermatitis. The Journal of allergy and clinical immunology 2014;**133**(1):147-53 doi: 10.1016/j.jaci.2013.05.017[published Online First: Epub Date]|.

455. Bekkers MB, Elstgeest LE, Scholtens S, et al. Maternal use of folic acid supplements during pregnancy, and childhood respiratory health and atopy. European Respiratory Journal 2012;**39**(6):1468-74

456. Willers SM, Wijga AH, Brunekreef B, et al. Maternal food consumption during pregnancy and the longitudinal development of childhood asthma. American Journal of Respiratory & Critical Care Medicine 2008;**178**(2):124-31

457. Bertelsen RJ, Brantsaeter AL, Haugen M, et al. Maternal probiotic intake and respiratory and allergy outcomes in early childhood. Journal of Allergy and Clinical Immunology 2013;**1)**:AB129

458. Camargo CA, Jr., Ingham T, Wickens K, et al. Cord-blood 25-hydroxyvitamin D levels and risk of respiratory infection, wheezing, and asthma. Pediatrics 2011;**127**(1):e180-7

459. Romieu I, Torrent M, Garcia-Esteban R, et al. Maternal fish intake during pregnancy and atopy and asthma in infancy. Clinical & Experimental Allergy 2007;**37**(4):518-25

460. Chatzi L, Torrent M, Romieu I, et al. Mediterranean diet in pregnancy is protective for wheeze and atopy in childhood. Thorax 2008;**63**(6):507-13

461. Kiefte-de Jong JC, Timmermans S, Jaddoe VW, et al. High circulating folate and vitamin B-12 concentrations in women during pregnancy are associated with increased prevalence of atopic dermatitis in their offspring. Journal of Nutrition 2012;**142**(4):731-8

462. Kiefte-de Jong JC, de Vries JH, Franco OH, et al. Fish consumption in infancy and asthma-like symptoms at preschool age. Pediatrics 2012;**130**(6):1060-8

463. Leermakers ET, Sonnenschein-van der Voort AM, Heppe DH, et al. Maternal fish consumption during pregnancy and risks of wheezing and AD in childhood: the Generation R Study. European Journal of Clinical Nutrition 2013;**67**(4):353-9

464. Dubakiene R, Rudzeviciene O, Butiene I, et al. Studies on early allergic sensitization in the Lithuanian birth cohort. Thescientificworldjournal 2012;**2012**:909524

465. Butiene I, Dubakiene R, Vaicekauskaite D, et al. Prevalence of food sensitisation in young children from EuroPrevall Lithuanian birth cohort. Allergy: European Journal of Allergy and Clinical Immunology 2011;**66**:233

466. Grimshaw KEC, Oliver EM, Kemp T, et al. Maternal dietary intake and subsequent allergy development. Journal of Allergy and Clinical Immunology 2012;**1)**:AB173

467. Dunlop AL, Reichrtova E, Palcovicova L, et al. Environmental and dietary risk factors for infantile atopic AD among a Slovak birth cohort. Pediatric Allergy & Immunology 2006;**17**(2):103-11

468. Fergusson DM, Horwood LJ, Shannon FT. Early solid feeding and recurrent childhood AD: a 10-year longitudinal study. Pediatrics 1990;**86**(4):541-6

469. Fitzsimon N, Fallon U, O'Mahony D, et al. Mothers' dietary patterns during pregnancy and risk of asthma symptoms in children at 3 years. Irish Medical Journal 2007;**100**(8):suppl 27-32

470. Gale CR, Robinson SM, Harvey NC, et al. Maternal vitamin D status during pregnancy and child outcomes. European Journal of Clinical Nutrition 2008;**62**(1):68-77

471. Hypponen E, Sovio U, Wjst M, et al. Infant vitamin d supplementation and allergic conditions in adulthood: northern Finland birth cohort 1966. Annals of the New York Academy of Sciences 2004;**1037**:84-95

472. Hypponen E, Laara E, Reunanen A, et al. Intake of vitamin D and risk of type 1 diabetes: a birth-cohort study. Lancet 2001;**358**(9292):1500-3
[truncated: 19,347 more chars]
