# Supplementary material for: Diet during pregnancy and infancy and risk of allergic or autoimmune disease: A systematic review and meta-analysis
Source: PLoS Med. 2018 Feb 28;15(2):e1002507. doi: 10.1371/journal.pmed.1002507 (PMC5830033; doi:10.1371/journal.pmed.1002507)
Supplement: S1 Data — (ZIP) [file pmed.1002507.s006.zip › Review_A_reports/ALLERGIC SENSITISATION.docx]

**Breastfeeding, Solid Food Introduction and Allergic Sensitisation**

Robert J Boyle^1^, Vanessa Garcia-Larsen^2^, Despo Ierodiakonou^3^, Jo Leonardi-Bee^4^, Tim Reeves^5^, Jennifer Chivinge^6^, Zoe Robinson^6^, Natalie Geoghegan^6^, Katharine Jarrold^6^, Andrew Logan^6^, Annabel Groome^6^ , Evangelia Andreou^7^, Nara Tagiyeva-Milne^8^, Ulugbek Nurmatov^9^, Sergio Cunha^10^

^1^ Clinical Senior Lecturer, Section of Paediatrics; ^2^ Post-Doctoral Research Associate & Honorary Research Fellow, Royal Brompton and Harefield NHS Foundation Trust, Respiratory Epidemiology and Public Health Group, National Heart and Lung Institute; ^3^ Post-Doctoral Research Associate, Departments of Paediatric and Respiratory Epidemiology and Public Health Group, Imperial College London; ^4^Associate Professor of Community Health Sciences, University of Nottingham; ^5^ Research Support Librarian, Faculty of Medicine, Imperial College London; ^6^ Undergraduate medical students, Imperial College London; ^7^Research Associate, Imperial Consultants; ^8^Research Fellow, University of Aberdeen; ^9^Research Fellow, University of Edinburgh; ^10^Research Associate, Respiratory Epidemiology and Public Health Group, National Heart and Lung Institute, Imperial College London

Imperial Consultants,

58 Princes Gate,

Exhibition Road,

London SW7 2PG

Contents

[**List of Figures 3**](#_Toc416357166)

[**List of Tables 5**](#_Toc416357167)

[**1 Total breastfeeding duration and Allergic Sensitisation** 6](#_Toc416357168)

[1.1 Overall characteristics of studies, risk of bias and summary of results 6](#_Toc416357169)

[1.2 Total breastfeeding duration and allergic sensitisation 13](#_Toc416357170)

[1.2.1 Allergic Sensitisation to Any Allergen 14](#_Toc416357171)

[1.2.2 Allergic sensitisation to Food 15](#_Toc416357172)

[1.2.3 Allergic sensitisation to Cow’s Milk (CM-AS) 16](#_Toc416357173)

[1.2.4 Allergic Sensitisation to Egg (EGG-AS) 18](#_Toc416357174)

[1.2.5 Allergic Sensitisation to Peanut (PN-AS) 19](#_Toc416357175)

[1.2.6 Allergic Sensitisation to any Aeroallergen (AERO-AS) 20](#_Toc416357176)

[1.2.7 Total IgE 24](#_Toc416357177)

[1.3 Data for TBF duration and AS that were not suitable for meta-analysis 24](#_Toc416357178)

[2 **Exclusive breastfeeding duration and Allergic Sensitisation** 28](#_Toc416357179)

[2.1.1 Overall characteristics of studies, risk of bias and summary of results 28](#_Toc416357180)

[2.2 Exclusive breastfeeding and allergic sensitisation 34](#_Toc416357181)

[2.2.1 Allergic sensitisation to any allergen 34](#_Toc416357182)

[2.2.2 EBF and allergic sensitisation to Food 37](#_Toc416357183)

[2.2.3 EBF and allergic sensitisation Cow’s Milk 38](#_Toc416357184)

[2.2.4 EBF and allergic sensitisation to Egg 40](#_Toc416357185)

[2.2.5 EBF and allergic sensitisation to Peanut 41](#_Toc416357186)

[2.2.6 EBF and allergic sensitisation to aeroallergen 41](#_Toc416357187)

[2.2.7 Total IgE 43](#_Toc416357188)

[2.3 Data for EBF duration and AS that were not suitable for meta-analysis 44](#_Toc416357189)

[**3 Age at solid food introduction and allergic sensitisation** 47](#_Toc416357190)

[3.1.1 Overall characteristics of studies, risk of bias and summary of results 47](#_Toc416357191)

[3.2 Solid food introduction and allergic sensitisation 50](#_Toc416357192)

[3.2.1 SF and allergic sensitisation to any allergen 50](#_Toc416357193)

[3.2.2 SF and allergic sensitisation to Food 51](#_Toc416357194)

[3.2.3 SF and allergic sensitisation to Cow’s Milk 51](#_Toc416357195)

[3.2.4 SF and allergic sensitisation to Egg 52](#_Toc416357196)

[3.2.5 SF and allergic sensitisation to Peanut 52](#_Toc416357197)

[3.2.6 SF and allergic sensitisation to Aeroallergen 53](#_Toc416357198)

[3.3 Data for SF and AS that were not suitable for meta-analysis 54](#_Toc416357199)

[**4 Conclusion** 56](#_Toc416357200)

[**5 References** 57](#_Toc416357201)

# List of Figures

[Figure 1 Risk of bias in studies of TBF duration and allergic sensitisation 13](#_Toc416357945)

[Figure 2 TBF Ever vs. Never and risk of any allergic sensitisation 14](#_Toc416357946)

[Figure 3 TBF ≥5-7 months vs. <5-7 months and risk of allergic sensitisation 14](#_Toc416357947)

[1.2.2.2 Figure 4 TBF Ever vs. Never and risk of allergic sensitisation to food 15](#_Toc416357948)

[Figure 5 TBF ≥5-7 months vs. <5-7 months and risk of allergic sensitisation 16](#_Toc416357949)

[Figure 6 TBF Ever vs. Never and risk of allergic sensitisation to cow’s milk 16](#_Toc416357950)

[Figure 7 TBF ≥3-4 months vs. <3-4 months and risk of allergic sensitisation to cow’s milk 18](#_Toc416357951)

[Figure 8 TBF ≥5-7 months vs. <5-7 months and risk of allergic sensitisation to cow’s milk 18](#_Toc416357952)

[Figure 9 TBF Ever vs. Never and risk of allergic sensitisation to Egg 19](#_Toc416357953)

[Figure 10 TBF medium duration (4-6months) vs. Never and risk of allergic sensitisation to egg 19](#_Toc416357954)

[Figure 11 TBF long duration (7-12months) vs. Never and risk of allergic sensitisation to egg 19](#_Toc416357955)

[Figure 12 TBF Ever vs. Never and risk of allergic sensitisation to PN 20](#_Toc416357956)

[Figure 13 TBF Ever vs. Never and risk of allergic sensitisation to aeroallergen 21](#_Toc416357957)

[Figure 14 TBF medium duration (4-6months) vs. Never and risk of allergic sensitisation to aeroallergen 21](#_Toc416357958)

[Figure 15 TBF long duration (7-12months) vs. Never and risk of allergic sensitisation to aeroallergen 21](#_Toc416357959)

[Figure 16 TBF ≥1-2 months vs. <1-2 months and risk of allergic sensitisation to aeroallergen 22](#_Toc416357960)

[Figure 17 TBF ≥3-4 months vs. <3-4 months and risk of allergic sensitisation to aeroallergen 22](#_Toc416357961)

[Figure 18 TBF ≥5-7 months vs. <5-7 months and risk of allergic sensitisation to aeroallergen 23](#_Toc416357962)

[Figure 19 TBF ≥8-12 months vs. <8-12 months and risk of allergic sensitisation to aeroallergen 23](#_Toc416357963)

[Figure 20 TBF Ever vs. Never and risk of raised Total IgE 24](#_Toc416357964)

[Figure 57 Risk of bias in studies of EBF duration and allergic sensitisation 34](#_Toc416357965)

[Figure 22 EBF ≥0-2 months vs. <0-2 months and risk of allergic sensitisation in children 35](#_Toc416357966)

[Figure 23 EBF ≥3-4 months vs. <3-4 months and risk of allergic sensitisation 36](#_Toc416357967)

[Figure 24 EBF ≥5-9 months vs. <5-9 months and risk of allergic sensitisation 37](#_Toc416357968)

[Figure 25 EBF ≥0-2 months vs. <0-2 months and risk of allergic sensitisation to food 37](#_Toc416357969)

[Figure 26 EBF ≥3-4 months vs. <3-4 months and risk of allergic sensitisation to food in children 38](#_Toc416357970)

[Figure 27 EBF ≥0-2 months vs. <0-2 months and risk of allergic sensitisation in children 38](#_Toc416357971)

[Figure 28 EBF ≥3-4 months vs. <3-4 months and risk of allergic sensitisation 39](#_Toc416357972)

[Figure 29 EBF ≥5-9 months vs. <5-9 months and risk of allergic sensitisation 40](#_Toc416357973)

[Figure 30 EBF ≥0-2 months vs. <0-2 months and risk of allergic sensitisation to Egg 40](#_Toc416357974)

[Figure 31 EBF ≥3-4 months vs. <3-4 months and risk of allergic sensitisation to Egg 41](#_Toc416357975)

[Figure 32 Exclusive breastfeeding ≥3-4 months vs. <3-4 months and risk of allergic sensitisation to PN 41](#_Toc416357976)

[Figure 33 EBF ≥0-2 months vs. <0-2 months and risk of allergic sensitisation to aeroallergen 42](#_Toc416357977)

[Figure 34 EBF ≥3-4 months vs. <3-4 months and risk of allergic sensitisation to aeroallergen 42](#_Toc416357978)

[Figure 35 EBF ≥5-7 months vs. <5-7 months and risk of allergic sensitisation to aeroallergen 43](#_Toc416357979)

[Figure 36 EBF ≥3-4 months vs. <3-4 months and risk of raised Total IgE 43](#_Toc416357980)

[Figure 37 EBF ≥5-9 months vs. <5-9 months and risk of raised Total IgE 44](#_Toc416357981)

[Figure 38 Risk of bias in studies of SF introduction and allergic sensitisation 50](#_Toc416357982)

[Figure 39 SF ≥3-4 months vs. <3-4 months and risk of allergic sensitisation 50](#_Toc416357983)

[Figure 40 SF ≥3-4 months vs. <3-4 months and risk of allergic sensitisation to food 51](#_Toc416357984)

[Figure 41 SF ≥3-4 months vs. <3-4 months and risk of allergic sensitisation to cow’s milk 52](#_Toc416357985)

[Figure 42 SF ≥3-4 months vs. <3-4 months and risk of allergic sensitisation to Egg 52](#_Toc416357986)

[Figure 43 SF ≥3-4 months vs. <3-4 months and risk of allergic sensitisation to Peanut 53](#_Toc416357987)

[Figure 44 SF ≥3-4 months vs. <3-4 months and risk of allergic sensitisation to aeroallegen 54](#_Toc416357988)

# List of Tables

[Table 1 Characteristics of included studies evaluating TBF duration and allergic sensitisation 8](#_Toc416358862)

[Table 2 Studies investigating the association between TBF duration and allergic sensitisation which were not eligible for meta-analysis 25](#_Toc416358863)

[Table 3 Characteristics of included studies evaluating EBF duration and allergic sensitisation 30](#_Toc416358864)

[Table 4 EBF for 6 months versus 3 months and risk of RC 35](#_Toc416358865)

[Table 4 Studies investigating the association between EBF and allergic sensitisation which were not eligible for meta-analysis 45](#_Toc416358866)

[Table 5 Characteristics of included studies evaluating SF introduction and allergic sensitisation 48](#_Toc416358867)

[Table 6 Studies investigating the association between SF introduction and allergic sensitisation which were not eligible for meta-analysis 55](#_Toc416358868)

# Total breastfeeding duration and Allergic Sensitisation

## Overall characteristics of studies, risk of bias and summary of results

Table 1 describes the main characteristics of the studies that assessed total breastfeeding duration (TBF) in relation to food and aero-allergen allergic sensitisation (AS) risk. A total of 1 cluster randomised controlled trial and 35 observational studies, reported the association between TBF and AS. Of these, 30 were prospective cohort studies, 2 retrospective cohort studies and 3 cross-sectional studies. The majority of studies (n=23) are from Europe – others are from North America (n=4) and Australasia (n=5), Africa (n=1), South America (n=1) and unclear (n=1). Overall, valid data on TBF duration in the first 2 years of life and AS risk were available from over 31,000 subjects. Information on AS was obtained from skin prick testing using standard techniques in 19 studies, and by blood specific IgE testing in 16 studies. With regards to time of outcome diagnosis, 16 studies explored the association between TBF duration and AS at age 0-4, 16 at ages 5-14, and 3 at age 15 years and over. For assessment of TBF duration, 11 studies used an interview, one parent diaries, and 2 a medical records review; in 3 studies the method of exposure assessment was unclear and in all others a questionnaire method was used.

Risk of bias was assessed using the NICE Methodological checklists for cohort and case-control studies. Figure 1 illustrates the distribution of bias across the five main methodological areas of the studies. Over 40% of studies had a high risk of bias, most commonly due to lack of adjustment for confounding bias i.e. no adjusted data presented, and a similar number of studies had unclear risk of assessment bias, usually due to unclear method for determining TBF duration. Risk of conflict of interest was generally assessed as low (over 70% of studies) or unclear.

Where data were available, five levels of comparison were used to assess the risk of sensitization to allergen (food and aero-allergens) according to TBF duration, namely ‘any (including ever) vs. never’, ‘≥1-2 months vs. <1-2 months’, ‘≥3-4 months vs. <3-4 months’, ‘≥5-7 months vs. <5-7 months’, and ‘≥8-12 months vs. <8-12 months’.

*Main Findings*

In the single intervention trial there was no evidence of a relationship between breastfeeding promotion and risk of allergic sensitisation. For observational studies, across all cut-offs where data were available, there was no consistent evidence of a relationship between risk of AS and initiation or prolongation of TBF. One study found food sensitisation to be increased in children who were breastfed ever, but other studies did not find a relationship between longer TBF and food sensitisation, or initiation/duration of TBF and sensitisation to specific foods cow’s milk (CM), Egg and Peanut (PN). In general there were only small numbers of studies with data available for food sensitisation. The studies of aeroallergen sensitisation, or ‘any’ sensitisation, consistently showed no evidence of a relationship with initiation of breastfeeding. Studies of Total IgE showed no consistent relationship with TBF initiation or duration.

Studies which could not be included in meta-analysis generally found no evidence of a relationship between TBF duration and AS. Certainly there was no support for a protective effect of TBF duration against AS. In one study (Wright 1999 [[1](#_ENREF_1)]) the authors’ analysis suggested that increased TBF duration may be associated with increased Total IgE in the child when mother is atopic, but with reduced Total IgE in the child where mother is non-atopic.

**Table 1 Characteristics of included studies evaluating TBF duration and allergic sensitisation**

| **Study** | **N/n cases** | **Design** | **Country** | **Exposure assessment** | **Outcome** | **Age at outcome (years)** | **Population characteristics/ Intervention** |
| --- | --- | --- | --- | --- | --- | --- | --- |
| Kramer, 2001 [[2](#_ENREF_2)]; Kramer, 2007 [[3](#_ENREF_3)] | 8865/8181 | Cluster RCT | Belarus | - | SPT-Aero | 6.5 | Breastfeeding promotion program based on the WHO/UNICEF baby friendly hospital initiative, versus standard local breastfeeding policies |
| Hesselmar, 2010 [[2](#_ENREF_2)] | 169/25 | PC | Sweden | I | sIgE - FOOD | 1.5 | ALLERGYFLORA study. Population based study of babies selected from antenatal clinics between 1998 and 2003 |
| Elliott, 2008 [[3](#_ENREF_3)] | 5779/ 1235 | PC | UK | Q | SPT-ANY | 7 | ALSPAC study. Population based cohort of children born 1991-1992 |
| Juto, 1980 [[4](#_ENREF_4)] | 56/NA | PC | Sweden | Q | Total IgE | 0.25 | Population based birth cohort of infants born in 1977 |
| Hong, 2011 [[5](#_ENREF_5)] | 970/ 361 | PC | USA | Q/I | sIgE-FOOD | 2.5 | Boston Birth Cohort. Predominantly African-American mother-infant pairs. |
| Burr, 1997 [[6](#_ENREF_6)] | 437/ 109 | PC | UK | Unclear | sIgE-AERO/ANY | 7 | Infants with family history of allergic diseases in South Wales |
| Bruno, 1995 [[7](#_ENREF_7)] | 174 | PC | Italy | I | sIgE-ANY, Total IgE | 0-2, 2 | Infants of atopic parents recruited from hospital and born in 1985-1988 |
| Mihrshahi, 2007 [[8](#_ENREF_8)] | 516/ 217 | PC | Australia | I | SPT-ANY | 5 | CAPS study. Infants born in 1997-1999 with family history of asthma or wheezing |
| Wegienka, 2006 [[9](#_ENREF_9)] | 403/ 127 | PC | USA | I | SPT-AERO | 7 | CAS study. Middle class mother-infant pairs enrolled in a health maintenance organisation in 1987-89 |
| Devereux, 2006 [[10](#_ENREF_10)] | 700/ 54 | PC | UK | Q | SPT-AERO | 5 | Population based birth cohort of infants born in 1998 |
| Nwaru, 2010 [[11](#_ENREF_11)] | 994/ 200 | PC | Finland | D | sIgE-AERO/CM/EGG/FOOD | 5 | DIPP study. Infants at high risk (HLA) for TIDM born between 1996-2004 invited to the allergy study between 1998 and 2000 |
| Rowntree, 1985 [[12](#_ENREF_12)] | 80/ 20 | PC | UK | I | sIgE-CM/EGG | 5 | Recruited from hospital and family history of atopy |
| Saarinen, 1979 [[13](#_ENREF_13)] | 95/NA | PC | Finland | R | sIgE-CM; Total IgE | 0-1, 1 | Recruited from hospital and born in 1975 |
| Sicherer, 2010 [[14](#_ENREF_14)] | 503/ 140 | PC | USA | I | sIgE-PN | 1 | The Consortium of Food Allergy Research: atopic children recruited from clinical services |
| Wright, 1999 [[1](#_ENREF_1)] | 982/ NA | PC | USA | Q/I | Total IgE | 1, 6 | Tuscon Children's Respiratory Study: Healthy newborn infants recruited from local health maintenance organisation born in 1980-1984 |
| Venter, 2009 [[15](#_ENREF_15)] | 891/27 | PC | UK | Q | SPT-FOOD | 1, 3 | Recruited from antenatal clinics and born in 2001-2002 |
| Oddy, 1999 [[16](#_ENREF_16)] | 1595/ 651 | PC | Australia | D/Q | SPT-AERO | 6 | Western Australia Pregnancy Cohort: Recruited from antenatal clinics born in 1989-1992 |
| Zutavern 2004 [[17](#_ENREF_17)] | 623/ 103 | PC | UK | I | SPT-AERO | 5.5 | Cohort recruited from general practices and born in 1993-1995 |
| Shaheen, 1996 [[18](#_ENREF_18)] | 395/44 | PC | Guinea-Bissau | I | SPT-AERO | 21 | Young adults part of a cohort recruited for a survey of aged 0-6 year children in 1978-80 and living in a semi-rural district of Bissau |
| Gustafsson, 1999 [[19](#_ENREF_19)] | 94/57 | PC | Sweden | Q | SPT-ANY | 8 | Children with atopic dermatitis attending allergic clinic or referred by child welfare clinics |
| Allen, 2009 [[20](#_ENREF_20)] | 310/50 | PC | Australia | Q | SPT-FOOD | 1 | Part of the HealthNUTS study. Representative cohort of infants recruited from routine immunisation clinics 2008-2010 |
| Hoppu, 2002 [[21](#_ENREF_21)] | 114/27 | PC | Finland | I | SPT-ANY | 1 | Birth cohort of infants with a family history of atopy |
| Huurre, 2008 [[22](#_ENREF_22)] | 98/29 | PC | Finland | I | SPT-ANY | 1 | Cohort of infants whose mother participated in a nutritional intervention trial during pregnancy |
| Kemeny, 1991 [[23](#_ENREF_23)] | 189/11 | PC | UK | Unclear | SPT and sIgE-CM/EGG; Total IgE | 1 | Population based birth cohort of infants born at Dulwich and King’s College Hospitals in London |
| Snijders, 2007; Snijders, 2008  [[24](#_ENREF_24), [25](#_ENREF_25)] | 776/ 218 | PC | Netherlands | Q | sIgE-ANY/AERO/CM/EGG/PN; Total IgE. | 2 | KOALA study. Population based birth cohort of infants born between 2000-2002 |
| Kusel, 2005 [[26](#_ENREF_26)] | 263/ 107 | PC | Australia | Unclear | SPT-ANY | 5 | Birth cohort of children at high risk of developing atopic disease born 1996-8 |
| Wetzig, 2000 [[27](#_ENREF_27)] | 475/ 43 | PC | Germany | Q | sIgE-CM/EGG | 1 | LARS study. High allergy risk or low birth weight children born within one year in Leipzig |
| Strachan, 1997 (BCS58 and BCS70) [[28](#_ENREF_28)] | 1369/ 730 | PC | UK | I | SPT-AERO | 35 | British Cohort Study: infants born in England, Wales, and Scotland in 1958 and 1970 |
| Silvers, 2009 [[29](#_ENREF_29)] | 889/ 249 | PC | New Zealand | Q | SPT-ANY | 1 | New Zealand Asthma and Allergy Cohort Study. Population based birth cohort of infants born 1997-2001 |
| Sears, 2002 | 714/ 319 | PC | New Zealand | R/I | SPT-AEROALLERGEN | 13 | Dunedin Multidisciplinary Health and Development Research Study. Population based cohort of infants born between 1972-1973 |
| Scholtens, 2009 [[30](#_ENREF_30)] | 1553/ 427 | PC | Netherlands | Q | sIgE-AERO | 8 | PIAMA. Population-based cohort born in 1996-1997 |
| Hagendorens, 2005 [[31](#_ENREF_31)] | 810/ 107 | PC | Belgium | Q | sIgE-ANY | 1 | PIPO study. Recruited from university service, born 1997-2001 |
| Friday, 2000 [[32](#_ENREF_32)] | 94/18 | RC | Unclear | Unclear | sIgE-CM | ~10 | Unclear |
| Rona, 2005 [[33](#_ENREF_33)] | 1213/ 345 | RC | Chile | R | SPT-AERO | 27 | Infants born in a hospital in 1974-1978 |
| Kuyucu, 2004  [[34](#_ENREF_34)] | 2774/ 570 | CS | Turkey | Q | SPT-AERO | 11 | ISAAC Phase 2. Schoolchildren aged 8–12 years |
| Kucukosmanoglu, 2008 [[35](#_ENREF_35)] | 1015/ 20 | CS | Turkey | Q/I | SPT-EGG | 1 | Hospital based selection of 8-18 months old Infants born in 2001-2002 |
| Kuehr, 1992 [[36](#_ENREF_36)] | 1470/ 201 | CS | Germany | Q | SPT-AERO | 8 | Schoolchildren between 6 and 8 years of age |

Q: questionnaire, I: interview, R: medical records, D: diary, PC: prospective cohort, RC: retrospective cohort, CS: Cross-sectional, SPT: allergen skin prick test, sIgE: allergen-specific IgE; ANY: a panel of aero and food allergens; CM: cow’s milk, PN: peanut, AERO: aeroallergen.

Figure 1 Risk of bias in observational studies of TBF duration and allergic sensitisation to foods or aero-allergens

## Total breastfeeding duration and allergic sensitisation to food or aero-allergens

The single intervention trial of a breastfeeding promotion intervention was rated as having a low risk of bias on all domains, and a low risk of conflict of interest. Kramer found no significant difference in odds of allergic sensitisation to at least one aeroallergen at the age of 6.5 years – cluster adjusted odds ratio 1.2 (95% CI 0.5, 2.6). A sensitivity analysis excluding sites with unusually high rates of allergic sensitisation showed increased odds of allergic sensitisation in the breastfeeding promotion group – cluster adjusted odds ratio 2.0 (95% CI 1.1, 3.4). All other evidence was derived from observational studies. For assessment of TBF and AS using our pre-defined analysis strategy, most exposure/outcome meta-analyses contained either one or zero eligible studies. We therefore pooled data for studies reporting sIgE and SPT together, and pooled data from different age groups 0-4 years, 5-14 years and 15+ years. We did however take into consideration differences between study populations and AS assessment methods when interpreting the outputs from individual meta-analyses.

### Allergic Sensitisation to Any Allergen

#### TBF Ever vs. Never

Figure 2 shows the outcomes of 3 eligible observational studies evaluating TBF any vs never and risk of AS to any allergen. The data show no significant relationship, with no statistical heterogeneity (I^2^=0%). All three studies are prospective cohort studies at low or unclear risk of bias, reporting prospectively collected breastfeeding information and adjusted data. Elliott in a normal risk population at aged 7 years using SPT; Mihrshahi in a high risk population at age 5 using SPT; Snijders in a normal risk population at age 2 using sIgE. The Snijders study included a population within the cohort, with an anthroposophic lifestyle. There were insufficient data for a meaningful dose-response analysis.

Figure 2 TBF Ever vs. Never and risk of any allergic sensitisation


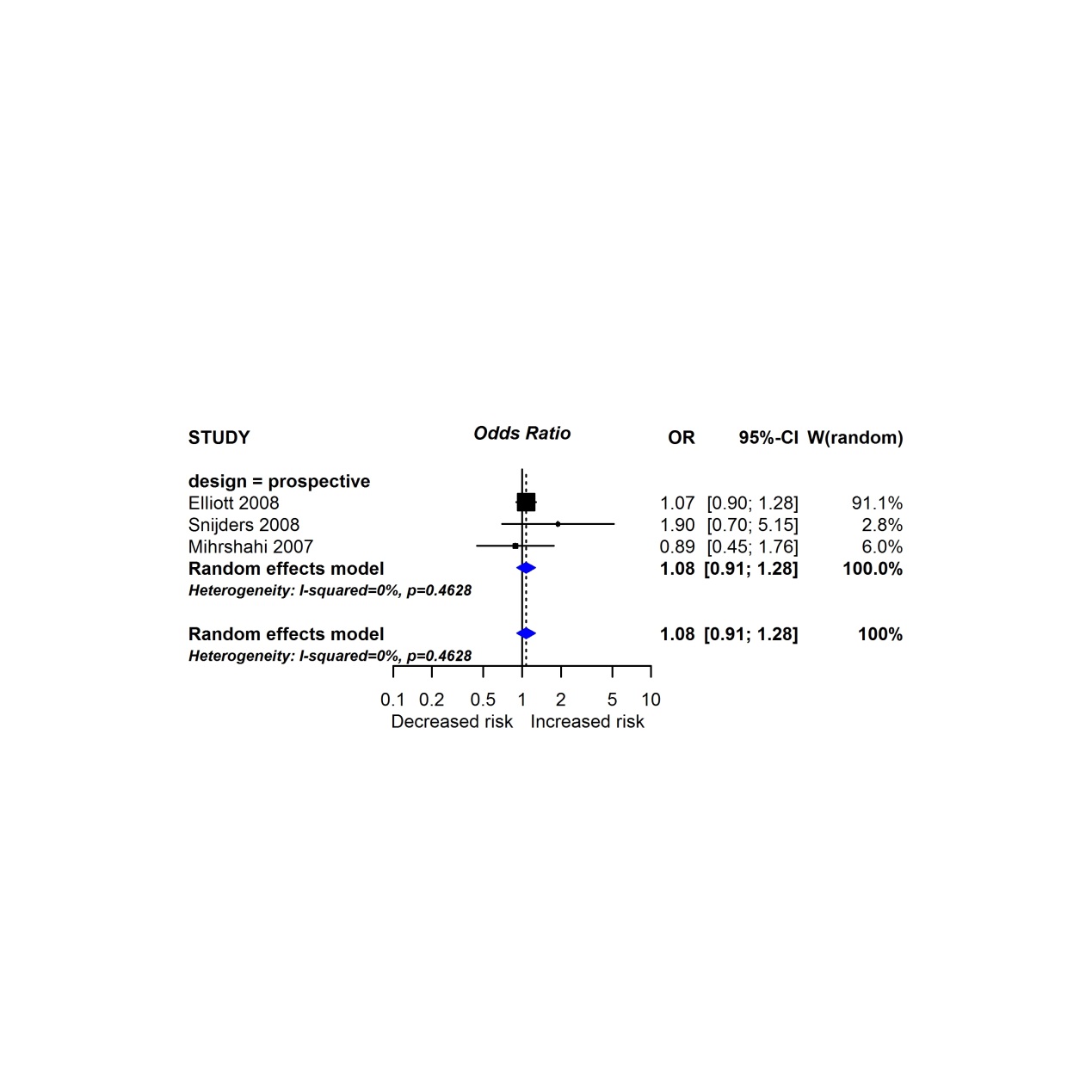


#### TBF ≥5-7 months vs <5-7 months

Figure 3 shows the outcomes of 2 eligible observational studies evaluating TBF duration of ≥5-7 months vs <5-7 months and risk of AS to any allergen. The data show no significant relationship, with high statistical heterogeneity (I^2^=70%). Mihrshahi reported adjusted data in a high risk population at age 5 years using SPT; Huurre reported adjusted data in a high risk population at age 1 using SPT. Both studies had a low risk of bias on all parameters, and the reason for the high statistical heterogeneity is not clear.

Figure 3 TBF ≥5-7 months vs. <5-7 months and risk of allergic sensitisation to any allergens


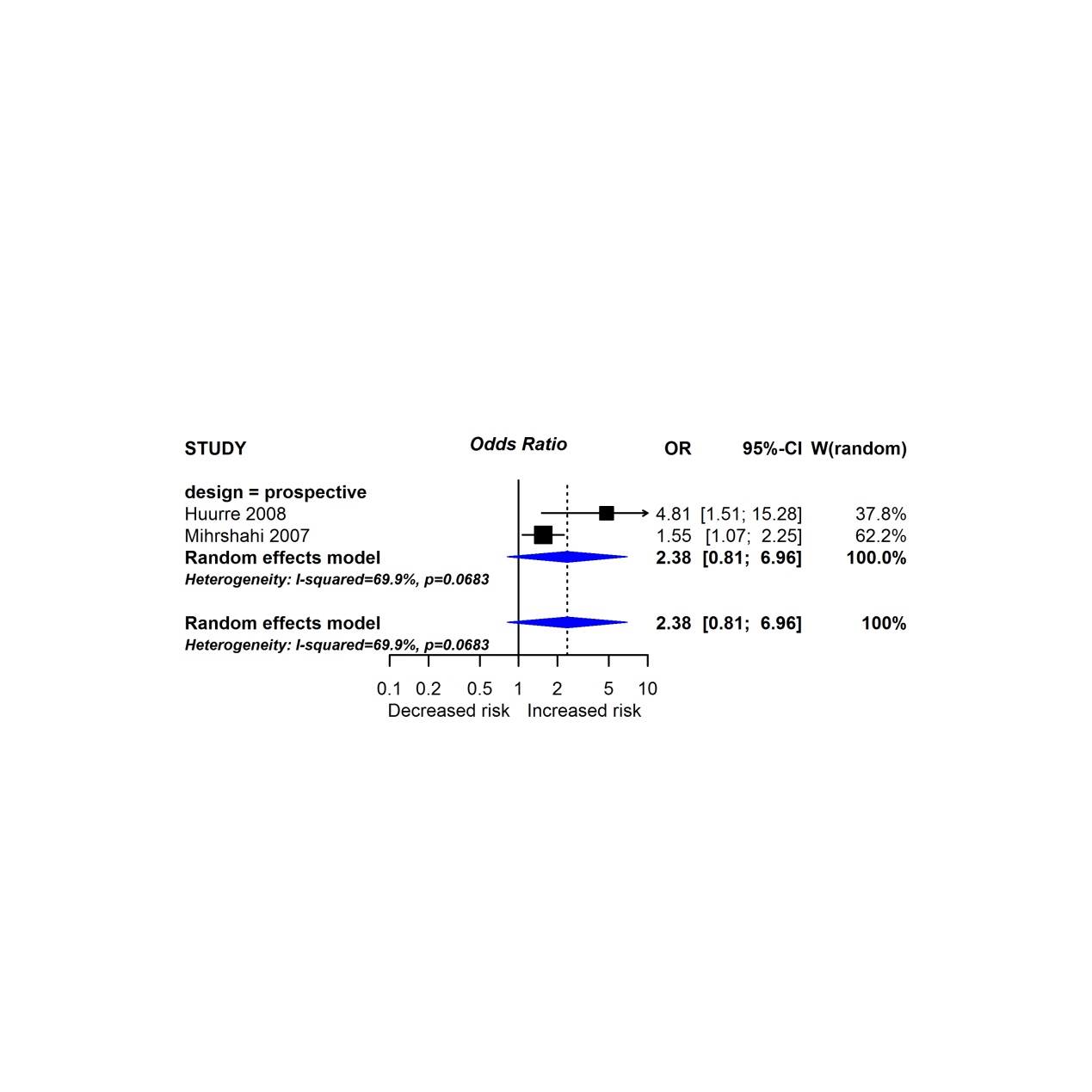


### Allergic sensitisation to Food

#### Ever vs. Never

Figure 4 shows the outcome of 1 eligible observational study evaluating TBF duration of any vs never and risk of AS to any food allergen. The data show increased food sensitisation in breastfed infants. Hong reported adjusted data in a high risk population at age 5 years using SPT. The authors did not find this effect varied by family history of allergy, but it was significantly modified by certain genetic polymorphisms related to cytokines. No data were available for dose response analysis.

#### Figure 4 TBF Ever vs. Never and risk of allergic sensitisation to food

####


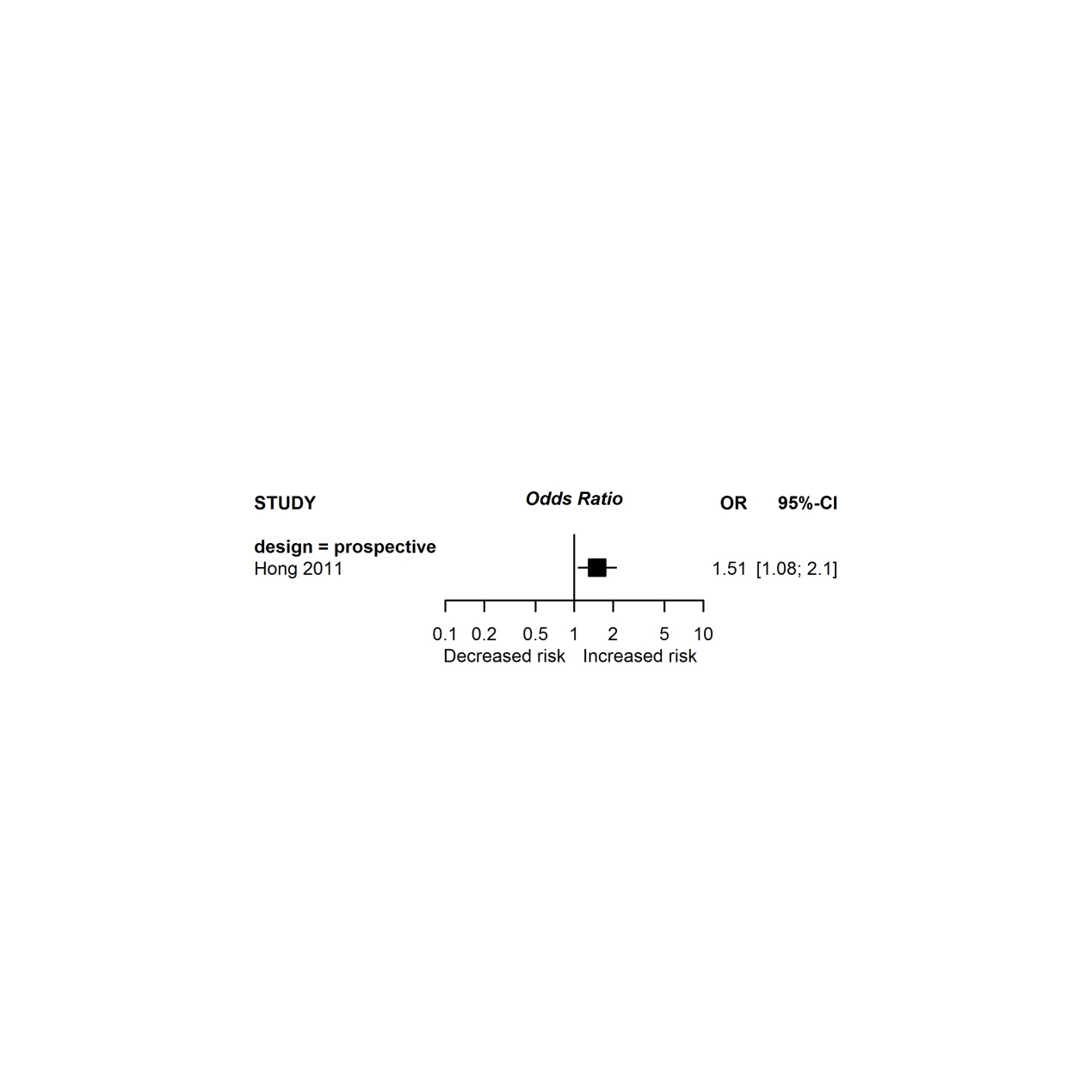


#### TBF ≥5-7 months vs <5-7 months

Figure 5 shows the outcome of 1 eligible observational study evaluating TBF duration of ≥5-7 months vs. <5-7 months and risk of AS to any food allergen. The data show no association between TBF and AS to any food allergen. Nwaru reported adjusted data in a normal risk population at age 5 using sIgE. The authors also reported no significant association between TBF duration and CM-AS (P=0.15) or Egg-AD (P=0.83) using sIgE at age 5 years.

Figure 5 TBF ≥5-7 months vs. <5-7 months and risk of allergic sensitisation to any food


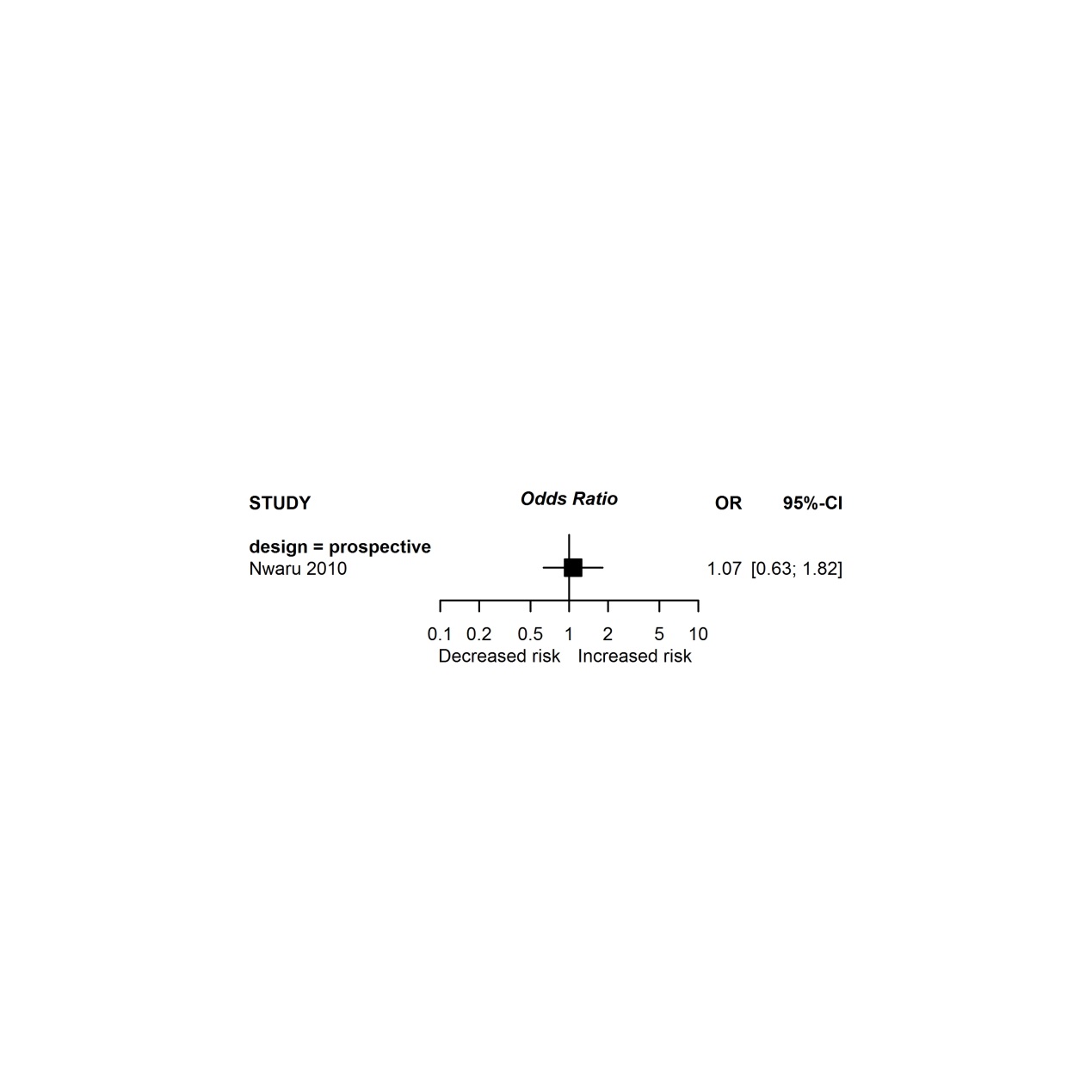


### Allergic sensitisation to Cow’s Milk (CM-AS)

#### TBF Ever vs. Never

Figure 6 shows the outcome of 2 eligible observational studies evaluating TBF duration of any vs never and risk of CM-AS. The data show no significant relationship, with no statistical heterogeneity. Snijders reported adjusted data in a prospective cohort study of a normal risk population at age 2 years using sIgE. The population included a population with anthroposophic lifestyle. Friday reported unadjusted data in a retrospective cohort study of a high risk population at age 10, with a consequent high risk of bias. Data from the Kemeny and Rowntree studies for CM-AS could not be meta-analysed, but show no significant association with TBF duration using different cutoffs for SPT or sIgE to CM. No data were available for meaningful dose response analysis.

**Figure 6 TBF Ever vs. Never and risk of allergic sensitisation to cow’s milk**


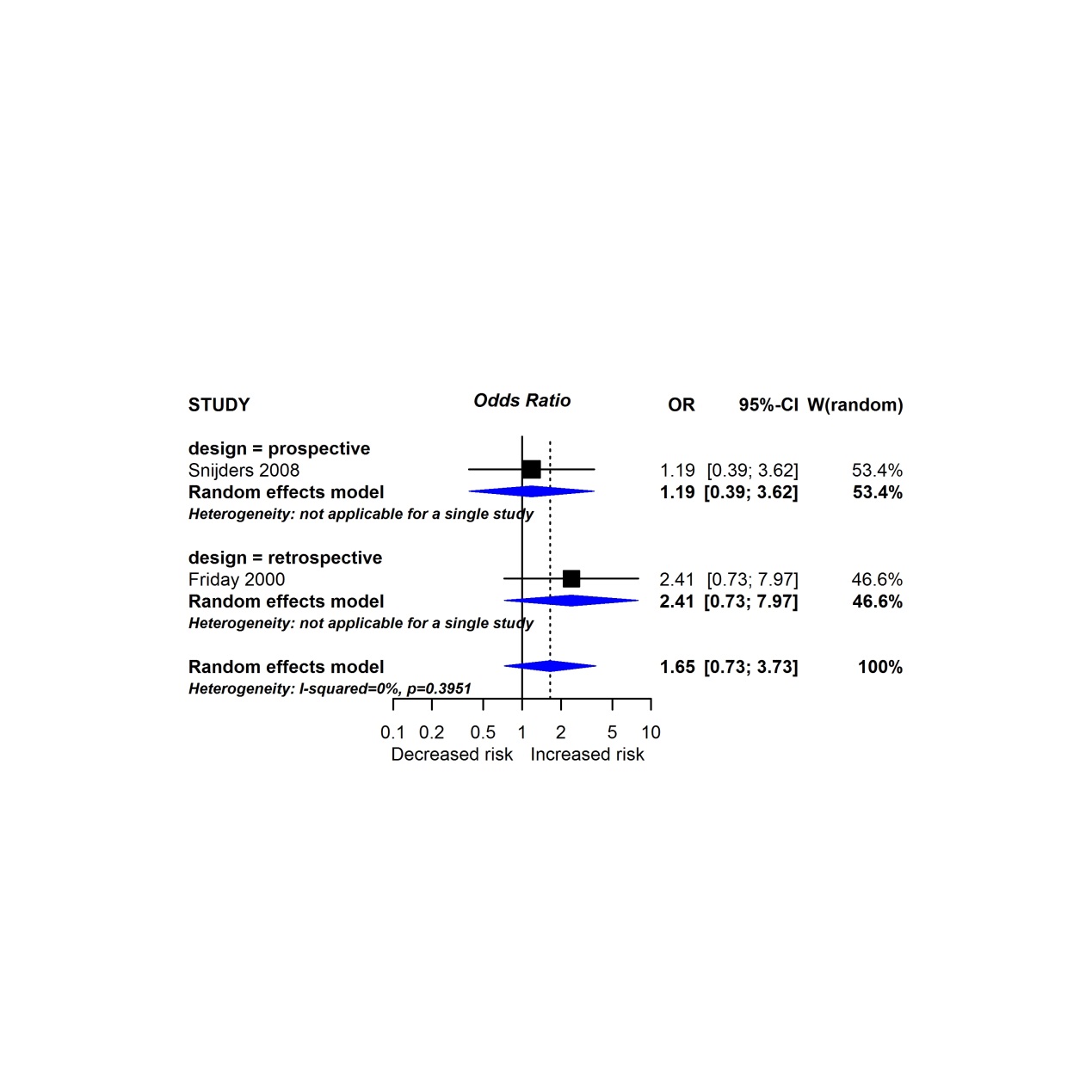


#### CM-AS and longer TBF durations

Friday also reported CM-AS risk with TBF ≥3 months vs. <3 months (Figure 7) where there was no evidence of an association; and the same study showed data for CM-AS risk and TBF ≥6 months vs. <6 months (Figure 8) where they reported an association between prolonged TBF and increased CM-AS risk. However these unadjusted data were judged to have a high risk of confounding bias.

Figure 7 TBF ≥3-4 months vs. <3-4 months and risk of allergic sensitisation to cow’s milk


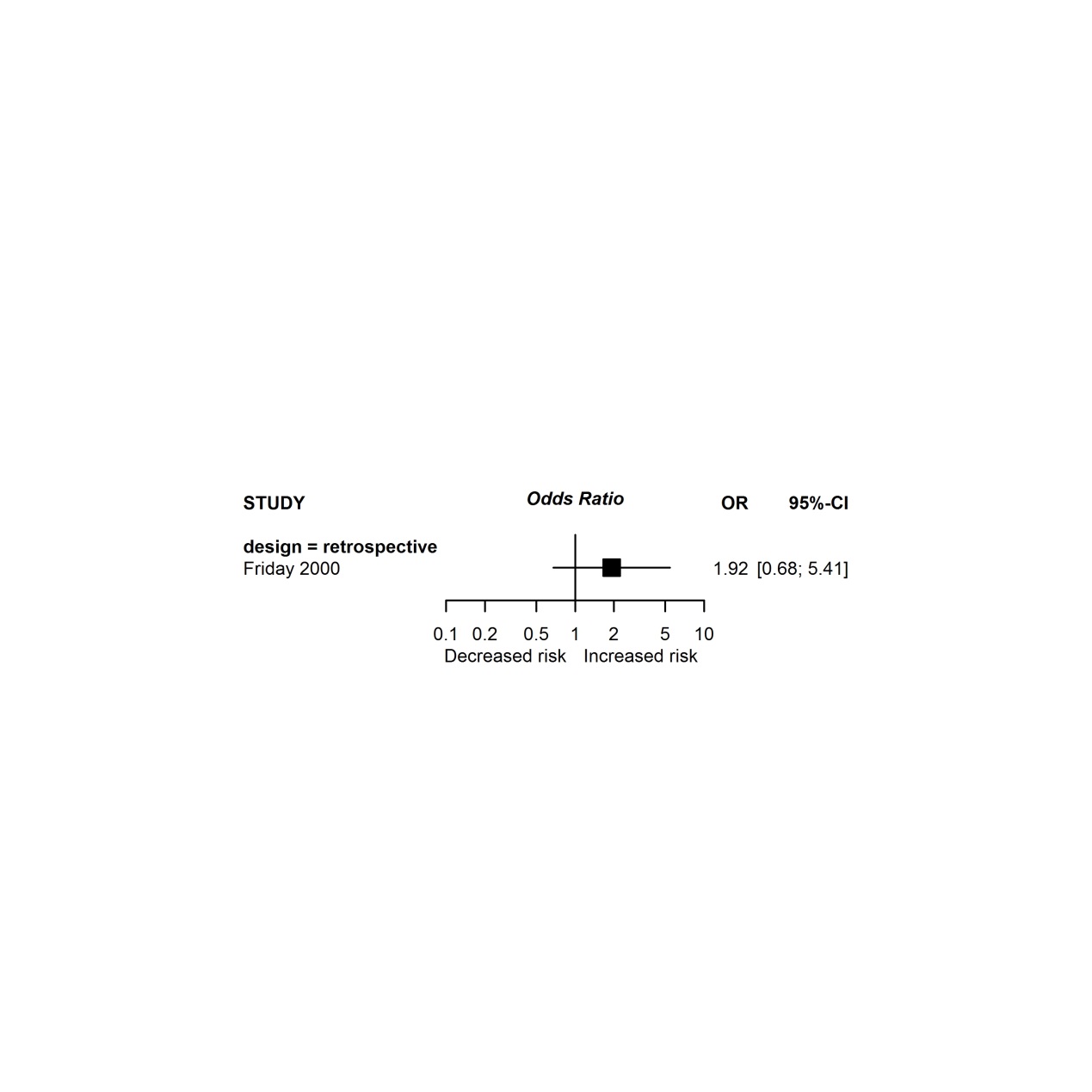


Figure 8 TBF ≥5-7 months vs. <5-7 months and risk of allergic sensitisation to cow’s milk


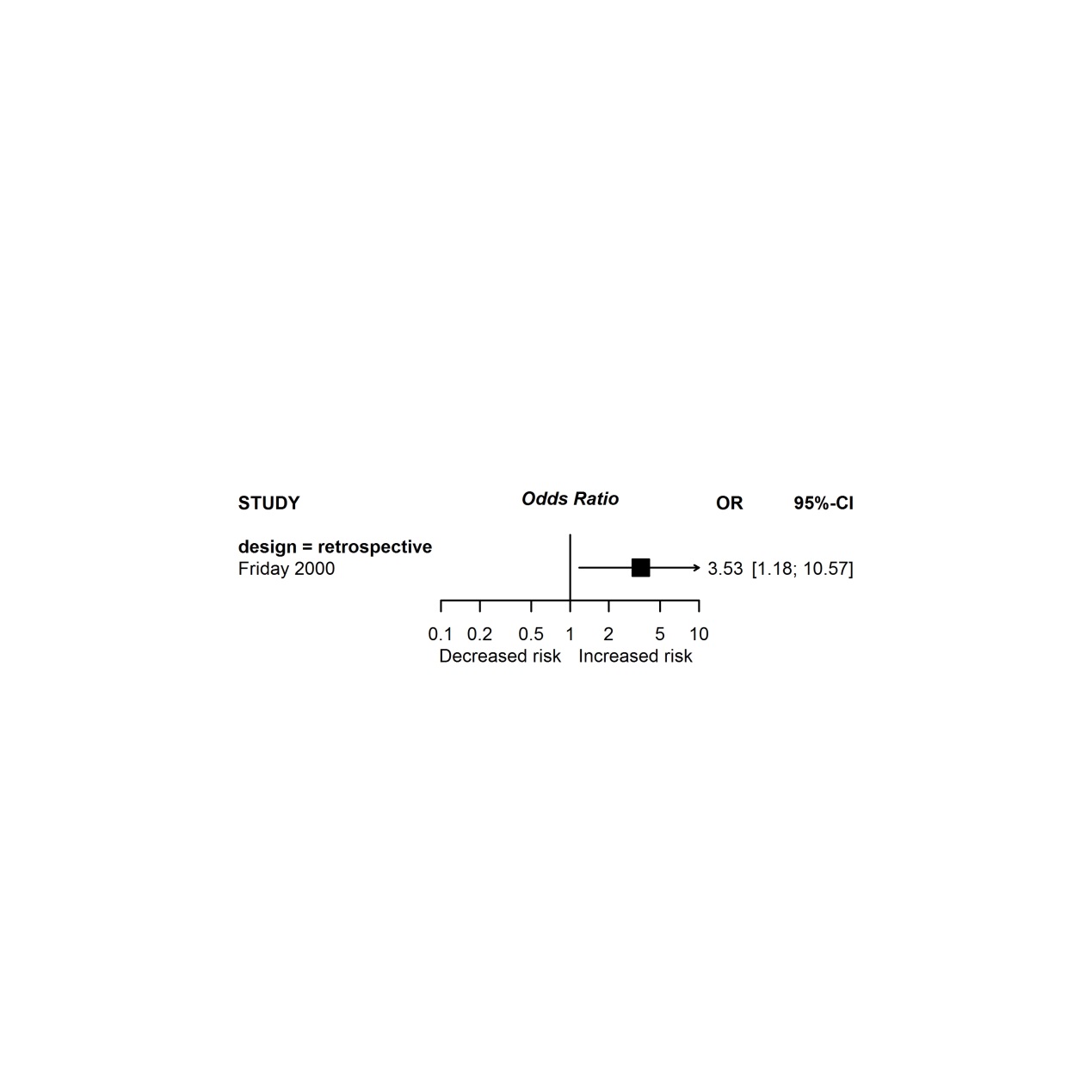


### Allergic Sensitisation to Egg (EGG-AS)

#### TBF Ever vs. Never

Figure 9 shows the outcome of 3 eligible observational studies evaluating TBF duration of ever vs never and risk of EGG-AS. The data show no significant relationship, with no statistical heterogeneity. Snijders reported adjusted data in a prospective cohort study of a normal risk population at age 2 years using sIgE. The population included a population with anthroposophic lifestyle. Kemeny and Rowntree reported unadjusted data in prospective cohort studies of normal and high risk populations, at ages 1 year and 5 years,respectively, and used both SPT and sIgE to assess AS. Both studies were judged as at high risk of confounding bias. Kemeny and Rowntree also found no difference when EGG-AS was measured as skin prick test rather than sIgE (sIgE in Figure). Dose response analysis did not show evidence of a dose response relationship (Figures 10-11).

**Figure 9 TBF Ever vs. Never and risk of allergic sensitisation to Egg**


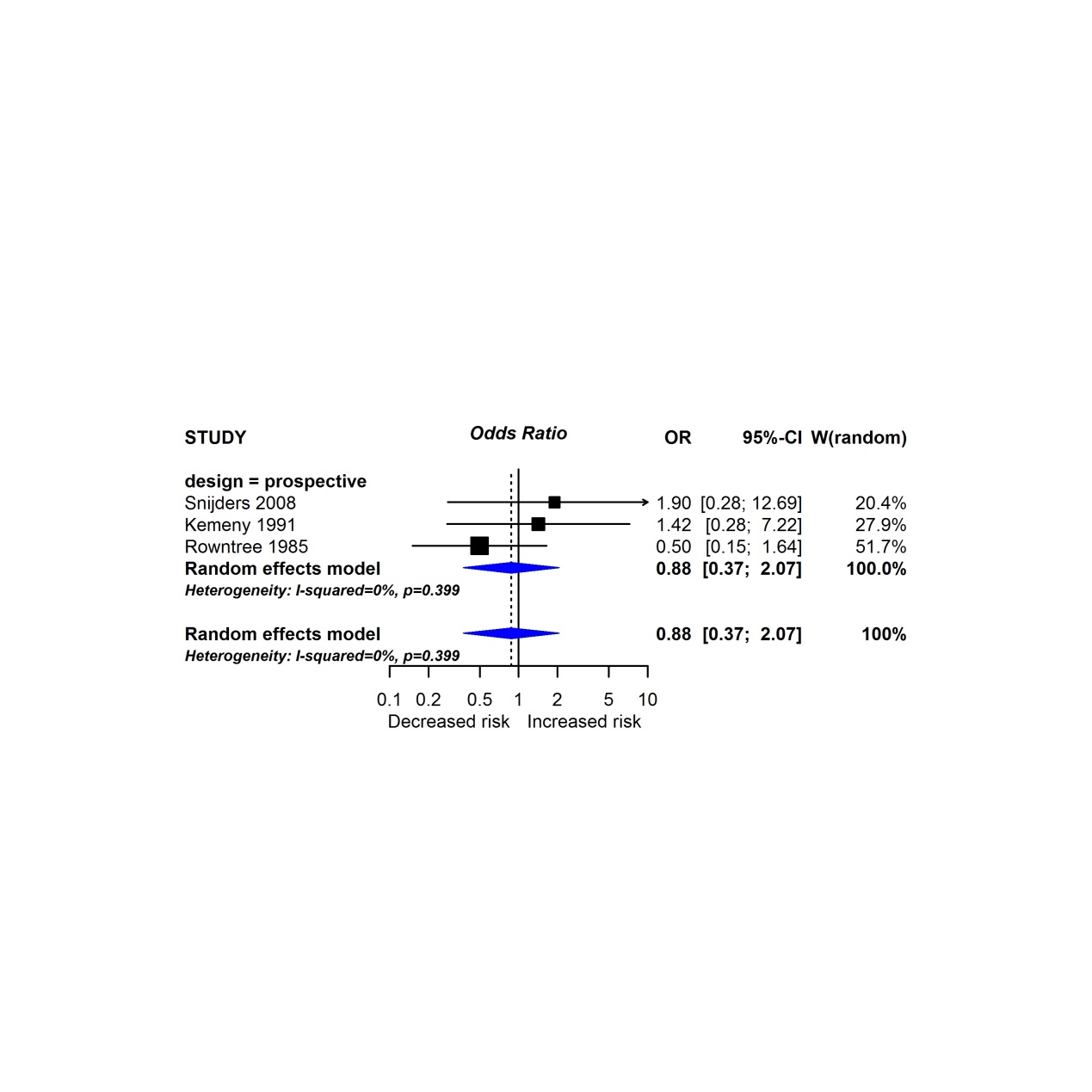


Figure 10 TBF medium duration (4-6months) vs. Never and risk of allergic sensitisation to egg


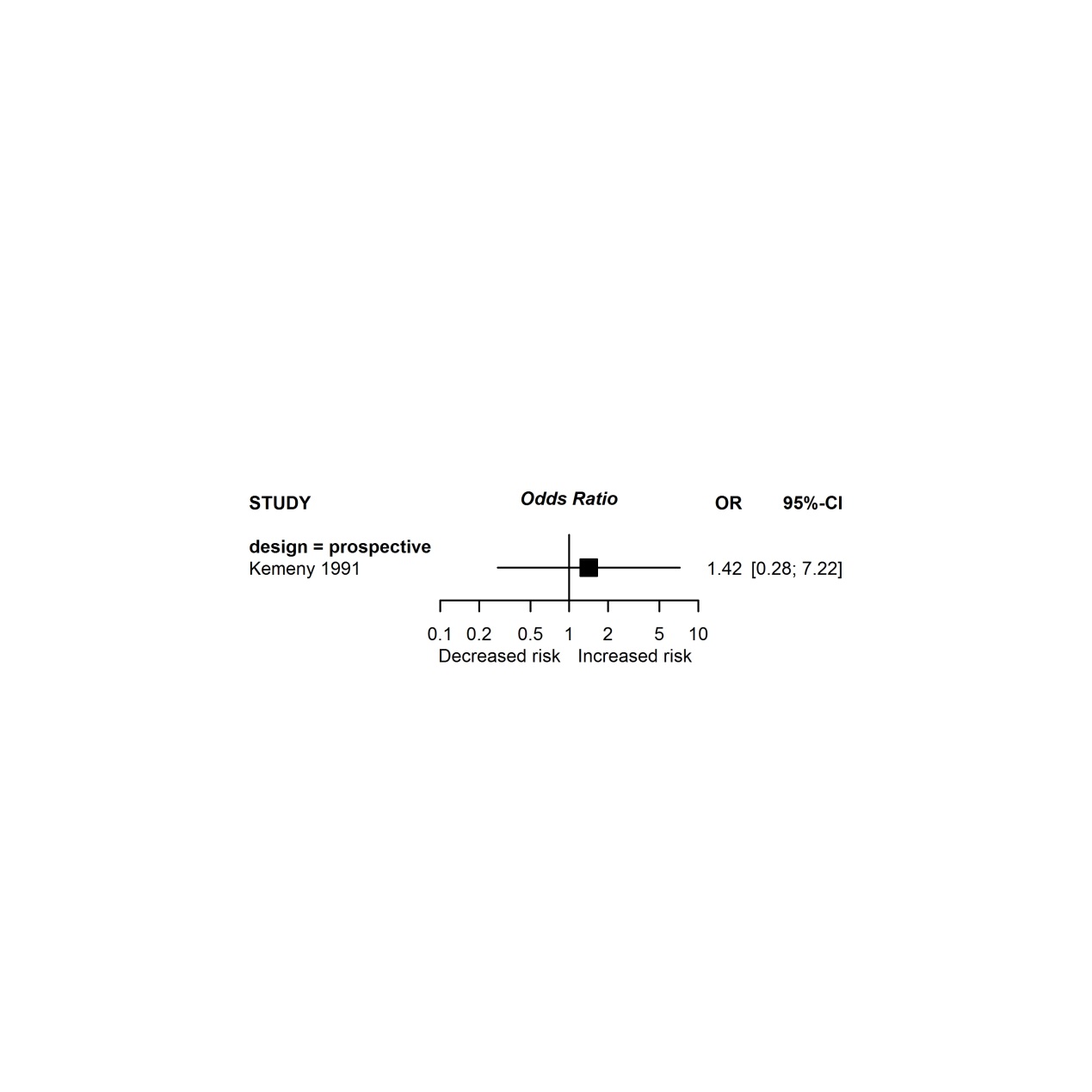


Figure 11 TBF long duration (7-12months) vs. Never and risk of allergic sensitisation to egg


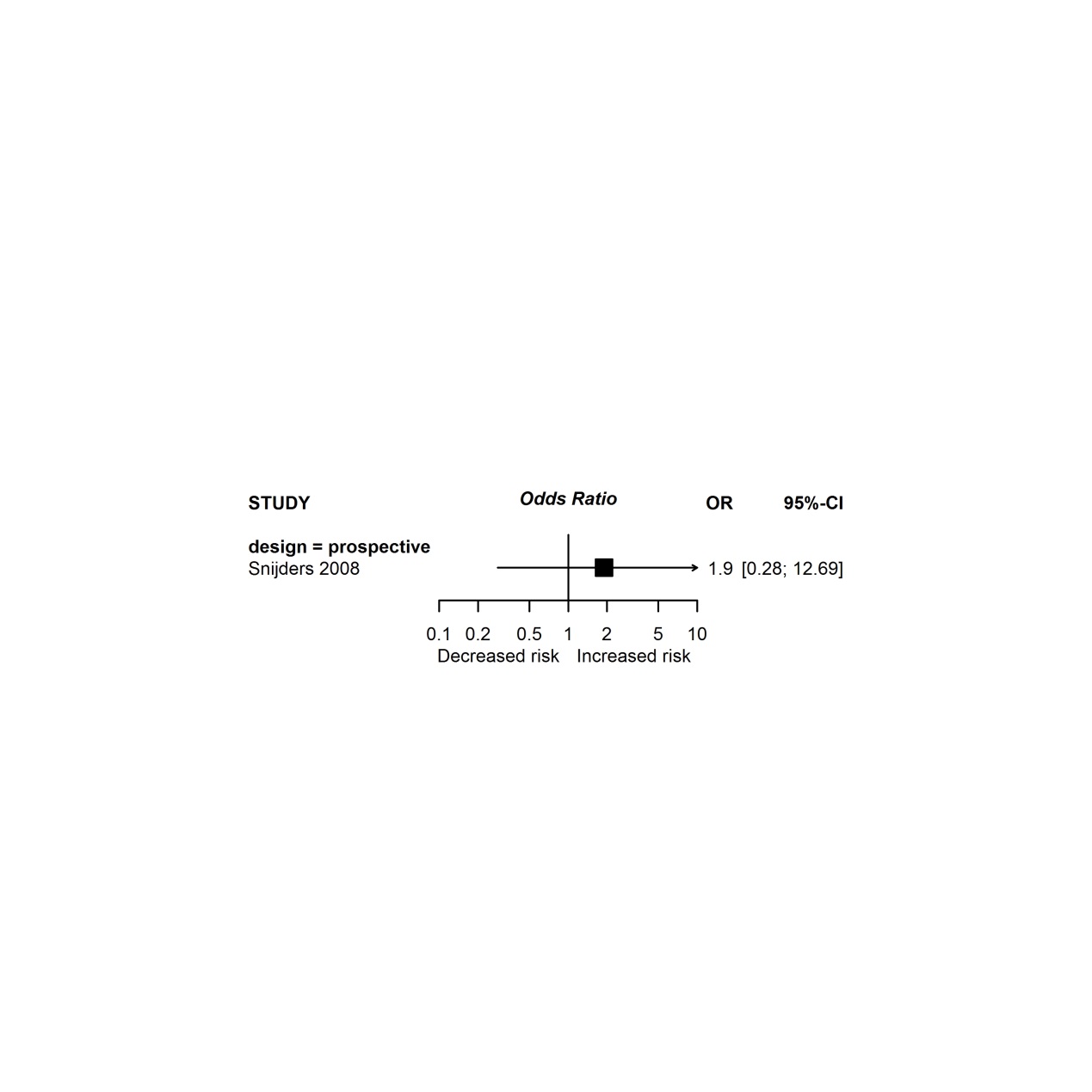


### Allergic Sensitisation to Peanut (PN-AS)

#### Ever vs. Never

Figure 12 shows the outcome of 2 eligible observational studies evaluating TBF duration of any vs never and risk of PN-AS. The data show no significant relationship, with no statistical heterogeneity. Snijders reported adjusted data in a prospective cohort study of a normal risk population at age 2 using sIgE. The study included a population with anthroposophic lifestyle. Sicherer reported unadjusted data in a prospective cohort study of a high risk population at age 5 using sIgE. Adjusted analyses in the same study showed no evidence of a relationship between BF any vs never and PN-AS. There were insufficient data for meaningful dose response analysis.

#### Figure 12 TBF Ever vs. Never and risk of allergic sensitisation to PN

**
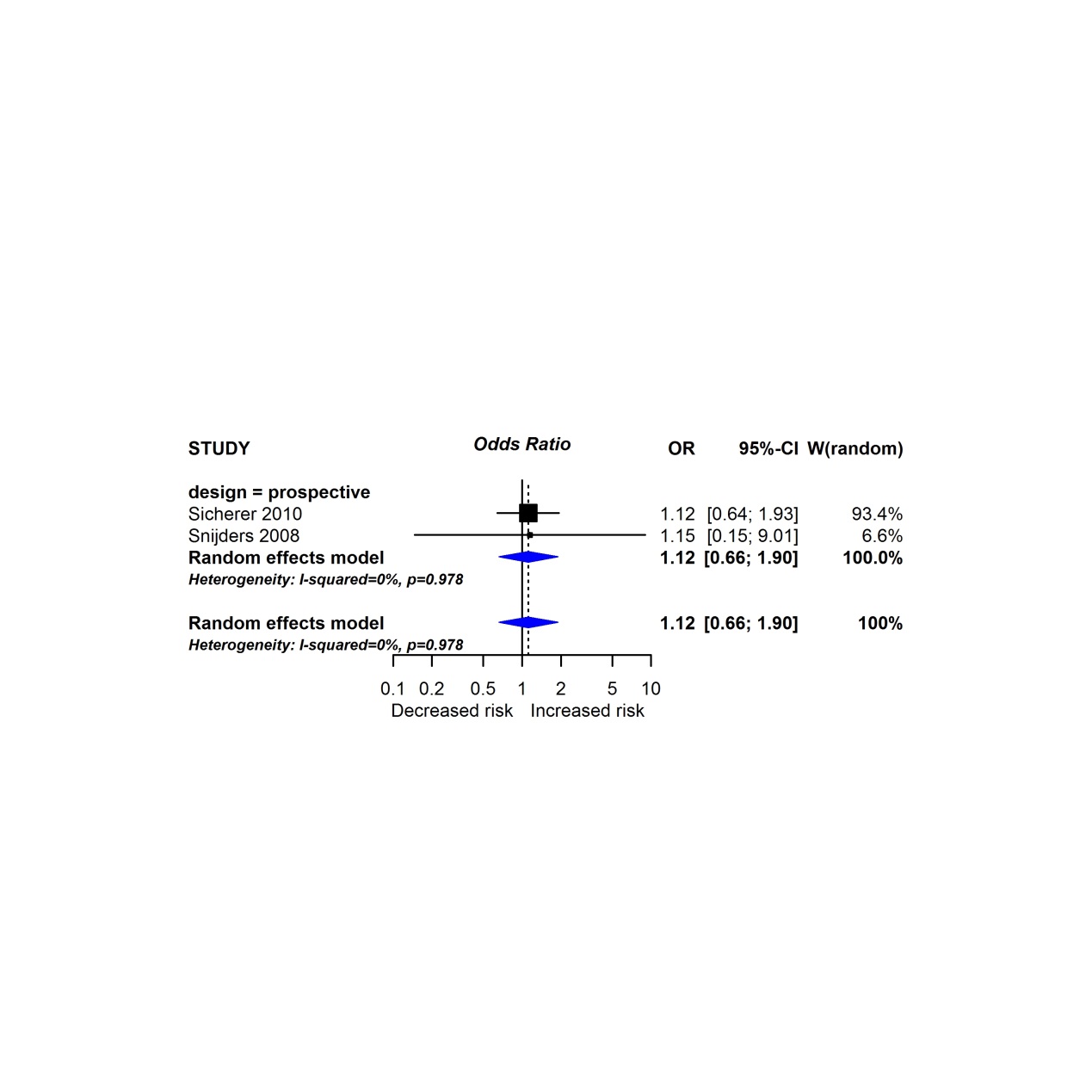
**

### Allergic Sensitisation to any Aeroallergen (AERO-AS)

#### Ever vs. Never

Figure 13 shows the outcome of 5 eligible observational studies evaluating TBF duration of any vs never and risk of AERO-AS. The data show no significant relationship, with low statistical heterogeneity. All studies reported adjusted data in a prospective cohort study of a normal risk population - Snijders at age 2 years using sIgE. The study included a population with anthroposophic lifestyle. Scholtens at age 8 years using sIgE; Wegienka, Strachan and Zutavern using SPT at age 7, 35 and 5.5 years respectively. These studies were generally judged to be at low risk of bias on most parameters. Dose response analysis (Figure 14 and Figure 15) did not show clear evidence of a dose response relationship between increasing duration of TBF and risk of AERO-AS when compared with never TBF.

#### Figure 13 TBF Ever vs. Never and risk of allergic sensitisation to aeroallergen


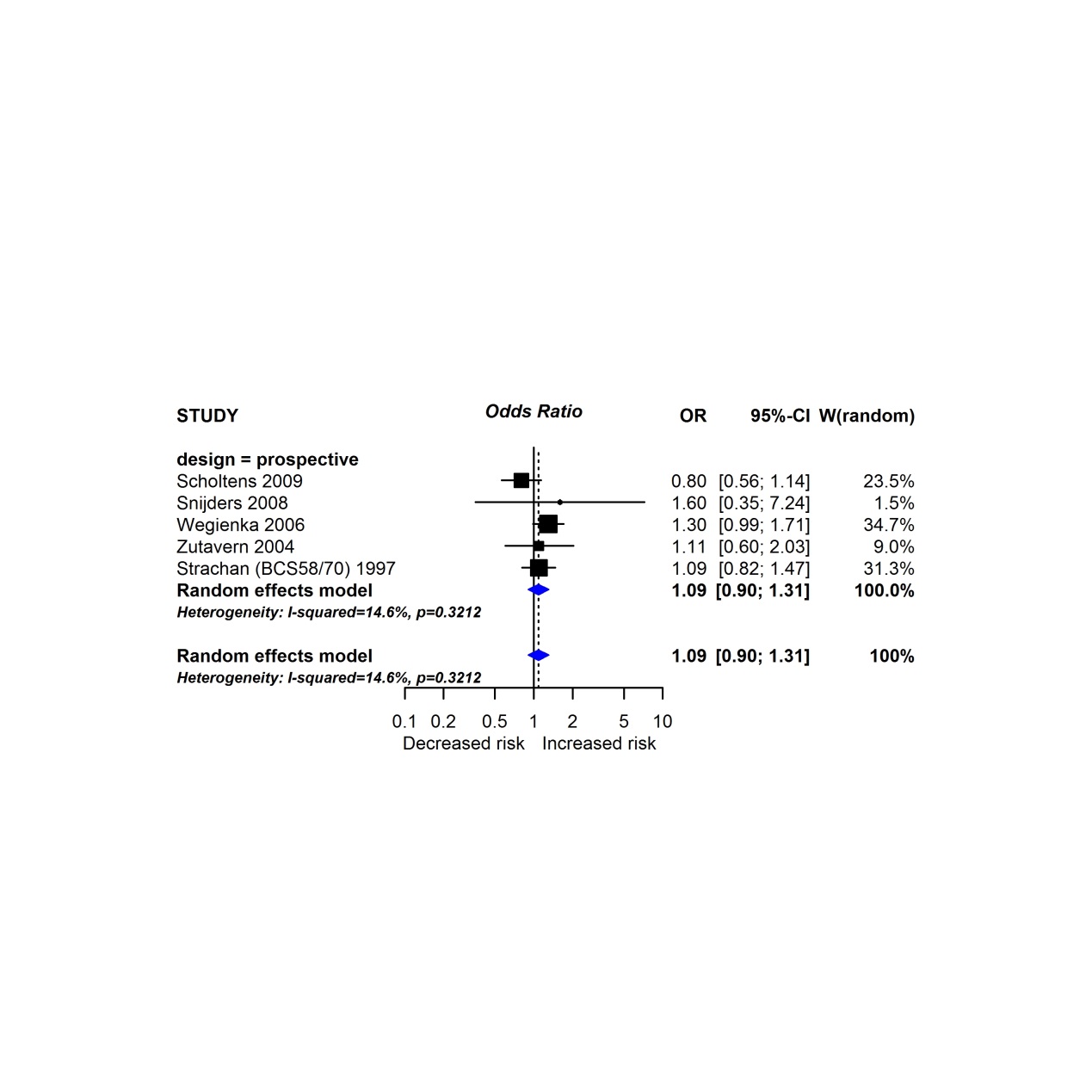


Figure 14 TBF medium duration (4-6months) vs. Never and risk of allergic sensitisation to aeroallergen


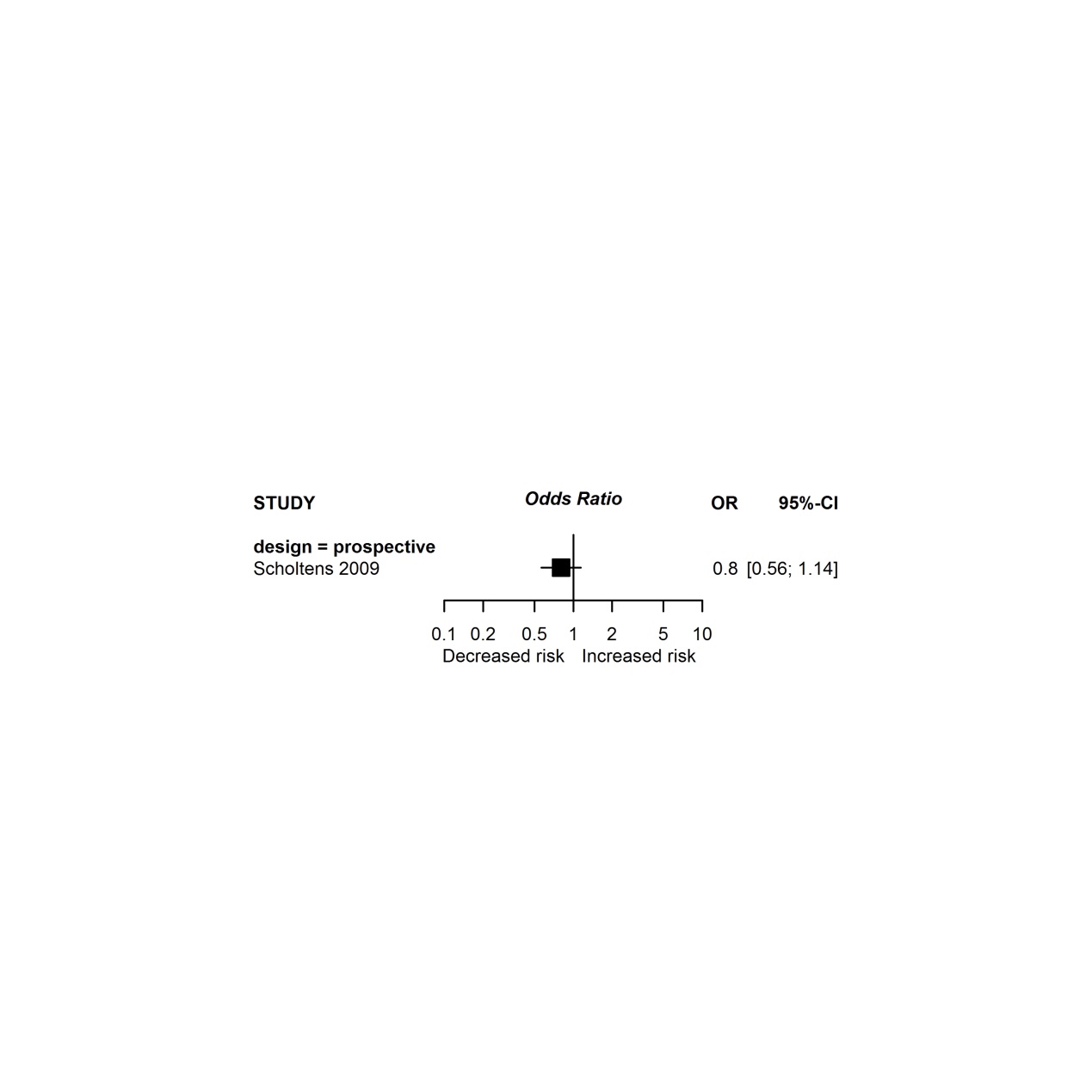


Figure 15 TBF long duration (7-12months) vs. Never and risk of allergic sensitisation to aeroallergen


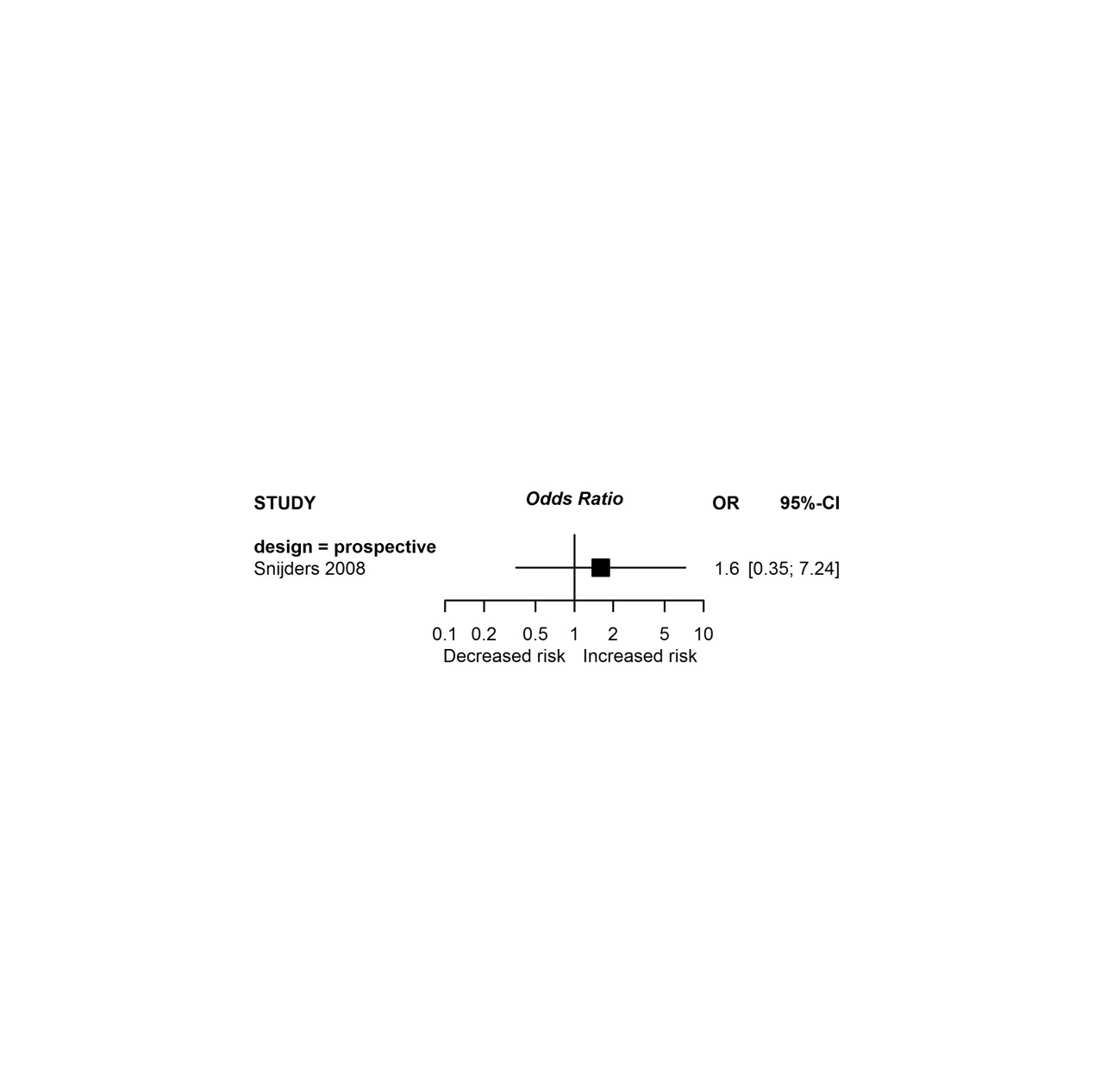


#### TBF ≥1-2 months vs. <1-2 months 1-2 months

Figure 16 shows the outcome of 3 eligible observational studies evaluating TBF duration of ≥1-2 months vs. <1-2 months and risk of AERO-AS. The data show no significant relationship, with extreme statistical heterogeneity (I^2^ = 75.6%). The study of Kuehr was a cross sectional study which reported unadjusted data in normal risk population at age 8 using SPT. The study of Rona was a retrospective cohort study which reported adjusted data in a normal risk population at age 27 years using SPT. The study of Sears is a prospective cohort with SPT assessed at 13 years reporting adjusted estimates. Given the nature of the cross-sectional and retrospective cohort studies, it is possible that some recall bias occurred, which might partly explain the statistical heterogeneity observed.

Figure 16 TBF ≥1-2 months vs. <1-2 months and risk of allergic sensitisation to aeroallergen


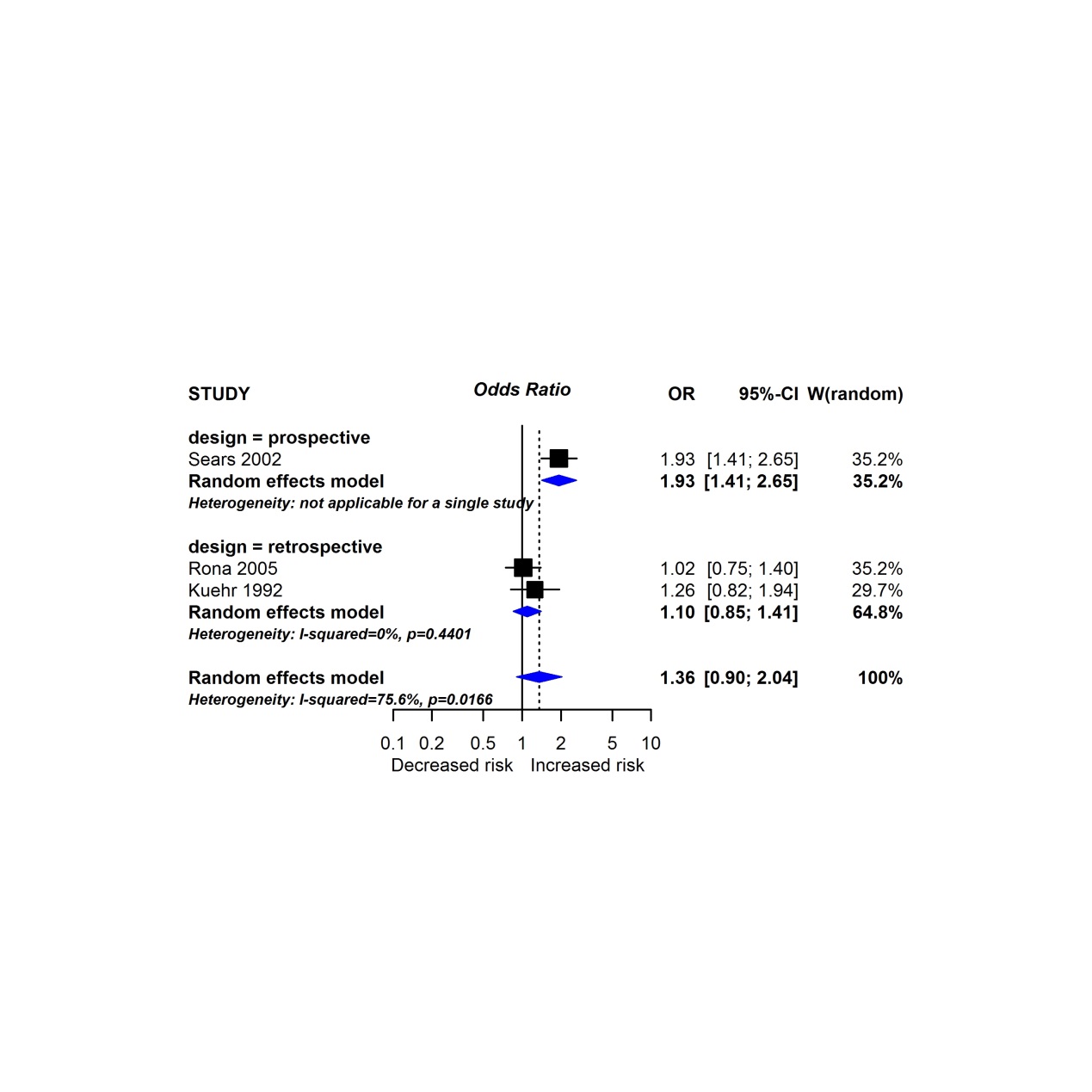


#### TBF ≥3-4 months vs. <3-4 months

Figure 17 shows the outcome of 1 eligible observational study evaluating TBF duration of ≥3-4 months vs. <3-4 months and risk of AERO-AS. The data show no significant relationship. The study of Oddy was a prospective cohort study which reported adjusted data in a normal risk population at age 6 years using SPT.

Figure 17 TBF ≥3-4 months vs. <3-4 months and risk of allergic sensitisation to aeroallergen


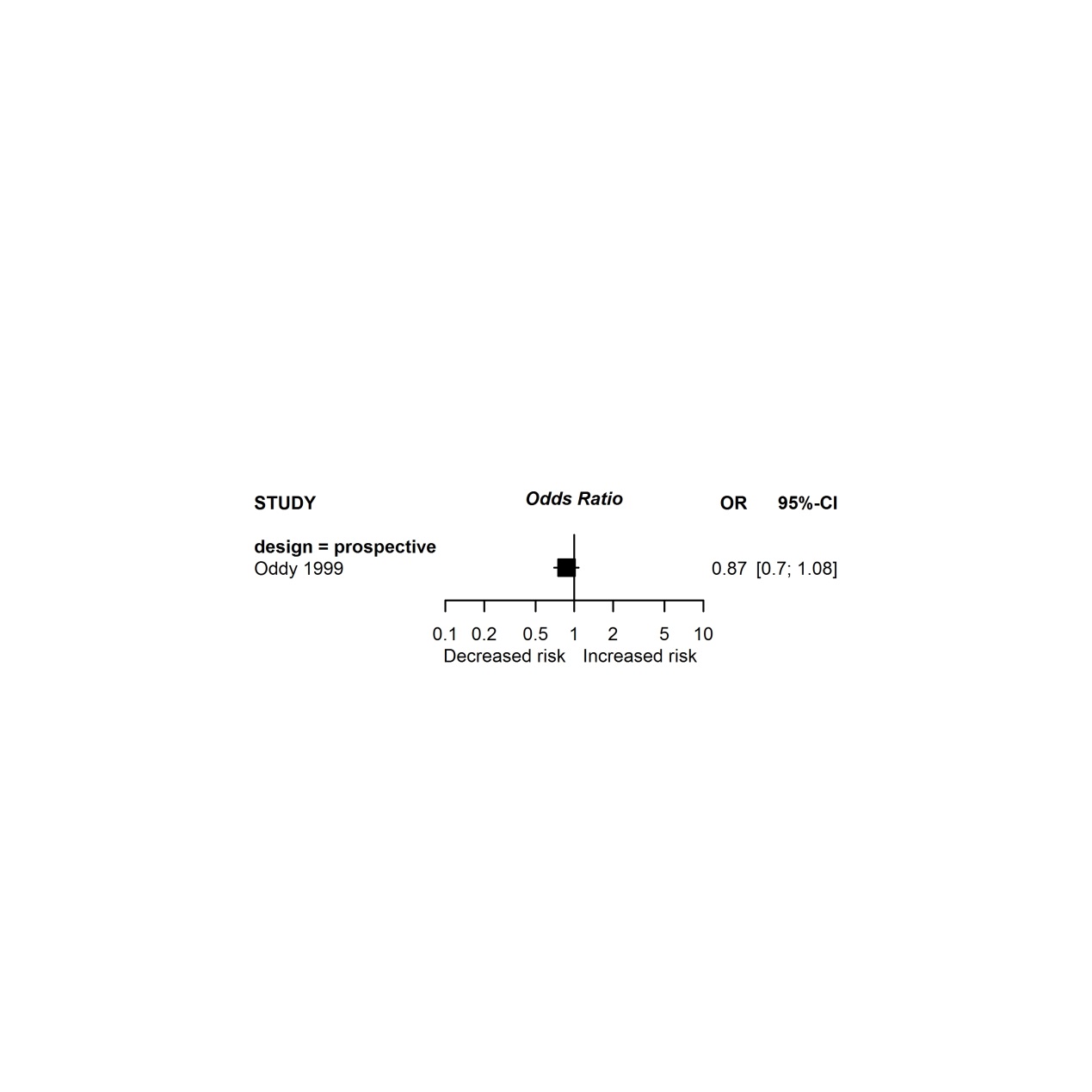


#### TBF ≥5-7 months vs. <5-7 months

Figure 18 shows the outcome of 3 eligible observational studies evaluating TBF duration of ≥5-7 months vs. <5-7 months and risk of AERO-AS. The data show reduced risk of sensitisation with prolonged TBF duration, with moderate statistical heterogeneity and borderline statistical significance. The study of Oddy was a prospective cohort study which reported adjusted data in a normal risk population at age 6 years using SPT. Nwaru reported adjusted data in a normal risk population at age 5 using sIgE. Kuyucu reported adjusted data in a normal risk population at age 11 years in a cross-sectional study using SPT. Kuyucu also reported an interaction with maternal atopic status, such that children of atopic mothers with TBF ≥6 months vs. <6 months had reduced risk of a positive SPT to aeroallergen, whereas this was not the case for children of non-atopic mothers.

Figure 18 TBF ≥5-7 months vs. <5-7 months and risk of allergic sensitisation to aeroallergen


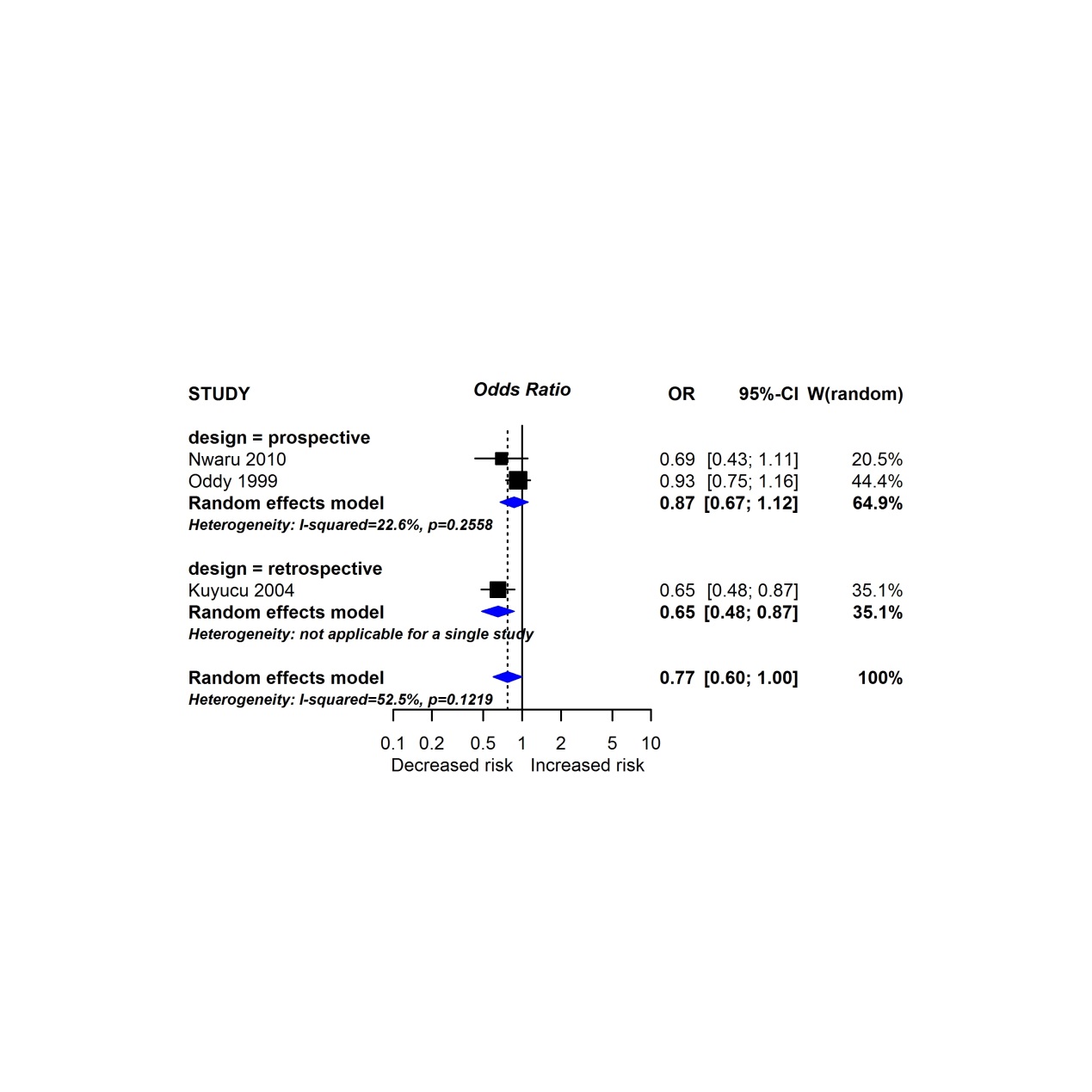


#### TBF ≥8-12 months vs. <8-12 months

Figure 19 shows the outcome of 1 eligible observational study evaluating TBF duration of ≥8-12 months vs. <8-12 months and risk of AERO-AS. The data show no significant relationship. The study of Shaheen was a prospective cohort study which reported adjusted data in a normal risk population in Guinea-Bissau at age 21 years using SPT.

Figure 19 TBF ≥8-12 months vs. <8-12 months and risk of allergic sensitisation to aeroallergen


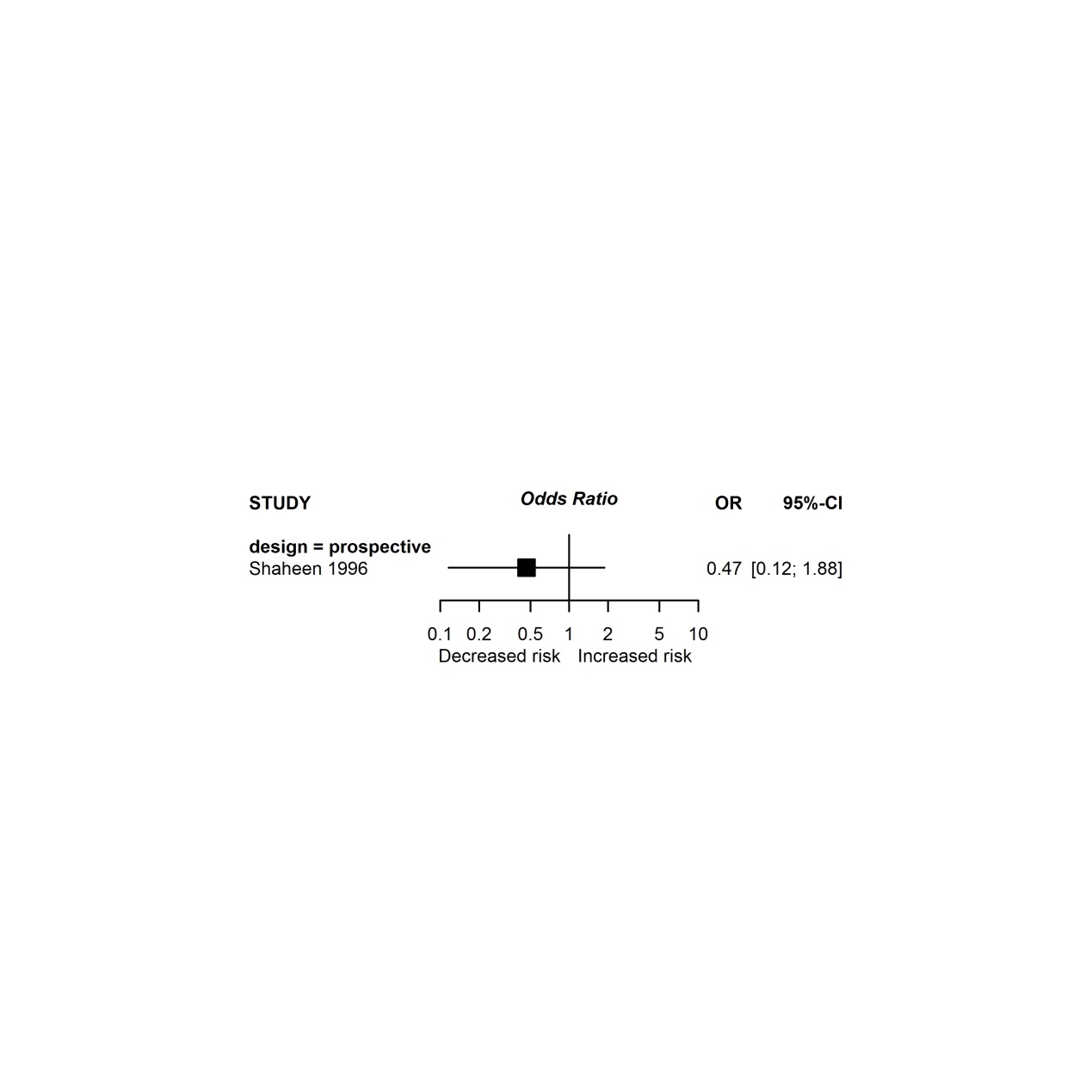


### Total IgE

#### TBF any vs never and Total IgE

One study reported OR for raised total IgE (>10IU/mL) in breastfed versus not breastfed infants, and found increased total IgE in breastfed infants at the age of 2 years, with borderline statistical significance. The sample population in this study was not representative of the general population as it only included children from families who had an anthroposofic lifestyle (Figure 20).

Figure 20 TBF Ever vs. Never and risk of raised Total IgE


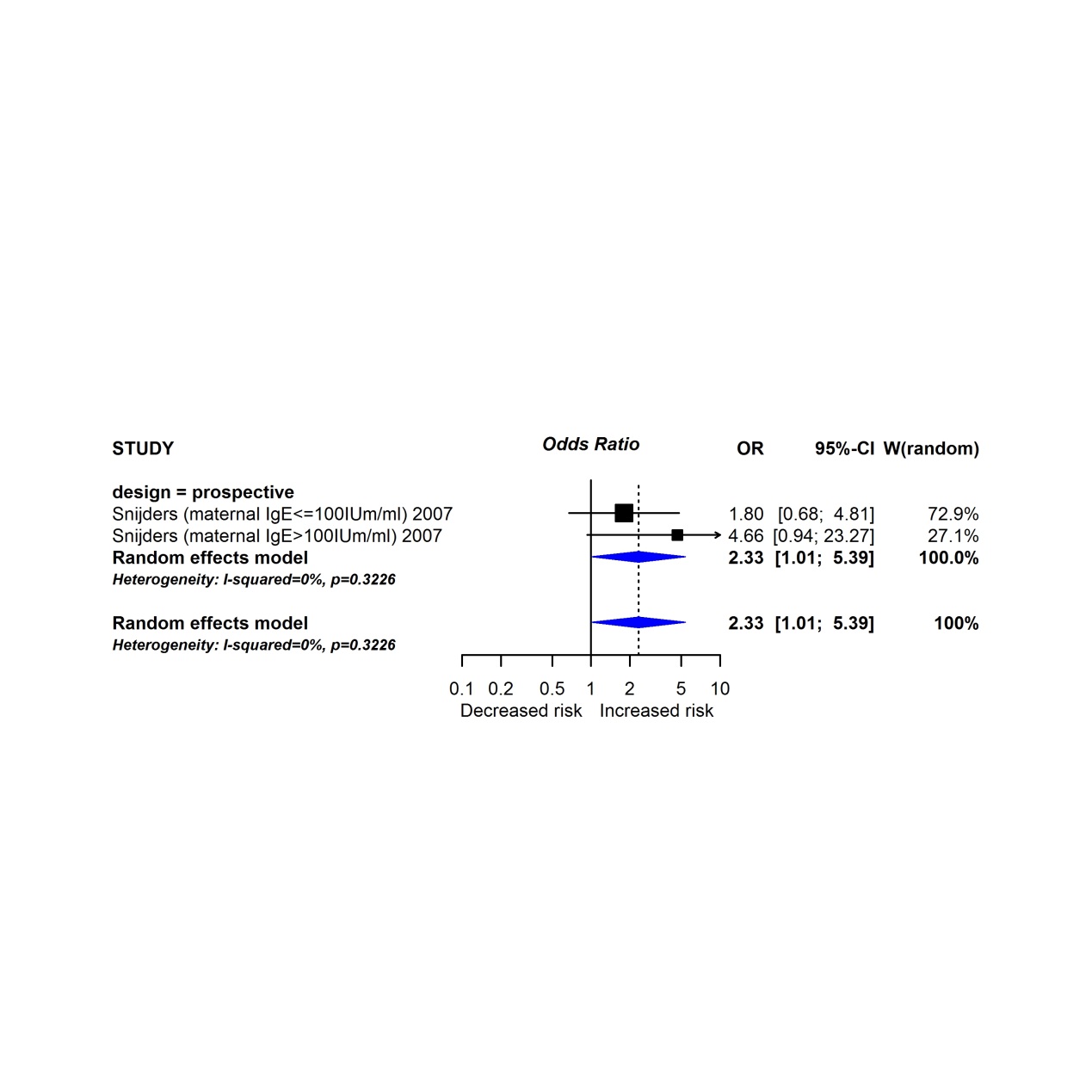


- 1. **Data for TBF duration and AS that were not suitable for meta-analysis**

Almost half of all included studies (17 of 35) did not report data that could be included in a meta-analysis, and are summarised below in Table 2. In 2 studies there was a significant positive association between TBF initiation or duration and specific AS risk; in 2 further studies there was evidence of such a relationship in some analyses, but not in the most complete analyses; in 1 study there was evidence of reduced specific AS risk associated with TBF; in 8 other studies there was no relationship between specific AS and TBF. Total IgE was reported by 5 of the narrative studies in relation to TBF. Here 4 studies (~950 participants) found no relationship between TBF initiation or duration and Total IgE; 1 study (95 participants) found increased Total IgE with TBF initiation or prolongation. In one of these studies the authors found a possible interaction between TBF and Total IgE, in relation to maternal atopic status.

Table 2 Studies investigating the association between TBF duration and allergic sensitisation which were not eligible for meta-analysis

| **Study** | **Design** | **Outcome** | **Age** | **N/n cases** | **Data** | **Measure** | **TBF in AS** | **TBF in No AS** | **P** |
| --- | --- | --- | --- | --- | --- | --- | --- | --- | --- |
| Hoppu, 2002 [[21](#_ENREF_21)] | PC | SPT-ANY | 1 | 114/27 | Continuous | Mean (95%CI) | 7.5 (6.9, 8.2) | 8.1 (6.4, 9.8) | NS |
| Hesselmar 2010 [[2](#_ENREF_2)] | PC | sIgE-FOOD | 1.5 | 169/25 | Continuous | Median (IQR) | 7 (4.7, 10) | 7 (4, 9) | NS |
| Silvers, 2009 [[29](#_ENREF_29)] | PC | SPT-ANY | 1 | 889/249 | Continuous | Average | Adjusted OR for AS, for each month increase in TBF duration was 1.03 (1, 1.06) (P=0.051) | | |
| Hagendorens, 2005 [[31](#_ENREF_31)] | PC | sIgE-ANY | 1 | 810/107 | Categorical | Odds ratio | Unadjusted OR for AS in ever versus never breastfed, is >1 and not statistically significant | | |
| Bruno, 1995 [[7](#_ENREF_7)] | PC | sIgE-ANY, Total IgE | 2 | 174 | Categorical/ Continuous |  | No significant difference in Total IgE according to feeding type. sIgE-ANY was significantly less common in ever versus never breastfed. | | |
| Kemeny, 1991 [[23](#_ENREF_23)] | PC | Total IgE | 1 | 189/NA | Continuous |  | No significant difference in Total IgE according to TBF≥3 months versus <3 months versus never | | |
| Saarinen, 1979 [[13](#_ENREF_13)] | PC | Total IgE; sIgE-CM | 1 | 95/NA | Continuous/Categorical | Adjusted geometric mean Total IgE significantly higher in ever vs. never breastfed at up to 4 months; CM-IgE not detected in never BF, rarely detected in ever BF up to 1 year | | | |
| Juto, 1980 [[4](#_ENREF_4)] | PC | Total IgE | 1 | 56/NA | Continuous | No significant difference | | | |
| Wetzig, 2000 [[27](#_ENREF_27)] | PC | sIgE-CM, sIgE-Egg | 1 | 475/43 | Categorical | Infants TBF >5 months had more Egg-AS than <5 months (significant in a subgroup with high cord IgE), but no significant difference for CM-AS | | | |
| Kucukosmanoglu 2008 [[35](#_ENREF_35)] | CS | SPT-EGG | 1 | 1015/20 | Categorical | No significant difference in Egg-AS between ever and never BF. P=0.32 | | | |
| Allen, 2009 [[20](#_ENREF_20)] | PC | SPT-FOOD | 1 | 310/50 | Categorical | Sensitised infants significantly more frequently BF at 12 months; no significant difference in ever BF (2.5% AS, 10.5% Not AS) | | | |
| Venter, 2009 [[15](#_ENREF_15)] | PC | SPT-FOOD | 1, 3 | 891/27 | Categorical | No significant relationship between Food-AS and TBF ever/never, >/<6 months or >/<9 months at age 1 or 3 | | | |
| Burr, 1997 [[6](#_ENREF_6)] | PC | sIgE-AERO/ SPT-AERO | 7 | 338 | Categorical |  | AS to AEROs by SPT or sIgE not associated with TBF duration in adjusted analysis | | |
| Devereux 2006 [[10](#_ENREF_10)] | PC | SPT-AERO | 5 | 700/54 | Categorical |  | No significant relationship between BF ever and AS | | |
| Kusel, 2005 [[26](#_ENREF_26)] | PC | SPT-ANY | 5 | 263/107 | Continuous |  | Analysis of children with eczema – those with AS had longer TBF than no AS, but this was not statistically significant in adjusted analyses | | |
| Gustafsson, 1999 [[19](#_ENREF_19)] | PC | SPT-ANY | 8 | 94/57 | Categorical |  | No association between TBF >6 months, or any other duration and AS | | |
| Wright, 1999 (All) [[1](#_ENREF_1)] | PC | Total IgE | 6 | 534/NA | Categorical |  | No association overall between TBF Never, <4 months or ≥4 months and Total IgE at 0.75, 6 or 11 years | | |
| Wright, 1999 (low maternal IgE) [[1](#_ENREF_1)] | PC | Total IgE | 6 | 534/NA | Continuous |  | Significantly reduced Total IgE in children of mothers with low Total IgE for ever versus never. P<0.02 | | |
| Wright, 1999 (high maternal IgE) [[1](#_ENREF_1)] | PC | Total IgE | 6 | 534/NA | Continuous |  | Significantly increased Total IgE in children of mothers with high Total IgE for ≥4 vs. <4 months. P<0.005 | | |

# Exclusive breastfeeding duration and Allergic Sensitisation

### Overall characteristics of studies, risk of bias and summary of results

Table 3 describes the main characteristics of the studies analysed in this report. A total of 1 systematic review which included 1 RCT analysed as a cohort study, and 1 other prospective cohort study; and 25 prospective cohort studies, reported the association between duration of exclusive breastfeeding (EBF) and risk of AS. The majority of studies were undertaken in Europe – others in Australasia (n=4), North America (n=1) and South America (n=1). Overall, valid data on EBF duration and AS risk were available from over 31,000 subjects. Information on AS was obtained using skin prick testing in 16 studies, and by blood IgE testing in 12 studies. With regards to time of outcome diagnosis, 15 studies explored the association between EBF duration and AS at age 0-4 years, and 12 at age 5-14 years. Eight studies used a questionnaire to assess the exposure (EBF), 10 used an interview, three studies used a diary, in three the method was unclear, one used medical records.

Risk of bias in both the studies included in the systematic review was assessed as ‘low’ on all parameters. Risk of bias for the observational studies is shown in Figure 21. Over half of studies had a high risk of bias, due to lack of adjustment for confounding bias i.e. no adjusted data presented. A similar number of studies had unclear risk of assessment bias, due to inadequate information about the method for classifying EBF duration. Risk of conflict of interest was generally assessed as low.

Where data were available, three levels of comparison were used to assess the risk of AS according to EBF duration, namely ≥0-2 months vs. <0-2 months; ≥3-4 months vs. <3-4 months; ≥5-9 months vs. <5-9 months. Across most cut-offs there was no consistent evidence of a lower risk of AS if EBF was prolonged. In general there were only small numbers of studies with available data, and several analyses were restricted to single studies with high risk of bias. We did find some evidence from one meta-analysis of 3 observational studies (Figure 25) that EBF ≥0-2 months may be associated with increased risk of AS to food OR 1.61 (1.17, 2.22) with no statistical heterogeneity (I^2^=0%). This was not reflected in data for AS to specific foods, to aeroallergens or any allergen, or at different EBF cut-offs. The studies included in this analysis did not take measures to account for the possibility that mothers of infants with symptoms associated with food sensitisation might extend the duration of EBF due to concern about symptoms. In general there was significant unexplained statistical heterogeneity in several analyses, even when restricted to studies with low risk of bias reporting adjusted data. We found evidence from one study of a gene-environment interaction affecting the relationship between EBF and Food AS, which may be relevant to some of the unexplained statistical heterogeneity between individual studies [[5](#_ENREF_5)].

We found no evidence of a relationship between EBF duration and total IgE levels.

**Table 3** **Characteristics of included studies evaluating EBF duration and allergic sensitisation**

| **Study** | **N/n cases** | **Design** | **Country** | **Exposure assessment** | **Outcome** | **Age at outcome (years)** | **Population characteristics** |
| --- | --- | --- | --- | --- | --- | --- | --- |
| Kramer [[37](#_ENREF_37)] | 2651 | SR of PC | Finland/Belarus | - | SPT-ANY | 5-7 | Study #1: Population based cohort study |
| Hesselmar, 2010 [[2](#_ENREF_2)] | 169/  25 | PC | Sweden | I | sIgE-FOOD | 1.5 | ALLERGYFLORA study. Population based study of babies selected from antenatal clinics between 1998 and 2003 - mainly high risk of allergic disease |
| Elliott, 2008 [[3](#_ENREF_3)] | 5095/1235 | PC | UK | Q | SPT-ANY | 7 | ALSPAC study. Population based cohort of children born 1991-1992. |
| Kull, 2004; Kull, 2010 [[38](#_ENREF_38), [39](#_ENREF_39)] | 2370/654 | PC | Sweden | Q | sIgE-AERO/ANY/CM/EGG/FOOD/PN | 4, 8 | BAMSE study. Population based cohort of children born between 1994-1996 |
| Hong, 2011 [[5](#_ENREF_5)] | 970/  361 | PC | USA | Q/I | sIgE-FOOD | 2.5 | Boston Birth Cohort. Predominantly African-American mother-infant pairs. |
| Bruno, 1995 [[40](#_ENREF_40)] | 107 | PC | Italy | Unclear | Total IgE | 5 | Infants of atopic parents recruited from hospital and born in 1985-1988 |
| Mihrshahi, 2007 [[8](#_ENREF_8)] | 516/  217 | PC | Australia | I | SPT-ANY | 5 | CAPS study. Infants born in 1997-1999 with family history of asthma or wheezing |
| Cogswell, 1987 [[41](#_ENREF_41)] | 73/32 | PC | UK | D | SPT-ANY | 5 | Birth cohort of infants with family history of hay fever or asthma |
| Nwaru, 2010 [[11](#_ENREF_11)] | 994/  200 | PC | Finland | D | sIgE-AERO/CM/EGG/FOOD | 5 | DIPP study. Infants at high risk (HLA) for TIDM born between 1996-2004 invited to the allergy study between 1998 and 2000 |
| Schoetzau, 2002 [[42](#_ENREF_42)] | 829/92 | PC | Germany | D | sIgE-CM | 1 | GINI study. Term newborn infants born  1995-8 from 2 regions of Germany who participated in an intervention program according to risk of allergy |
| Hoppu, 2000 [[21](#_ENREF_21)] | 114/27 | PC | Finland | I | SPT-ANY | 1 | Birth cohort of infants with a family history of atopy |
| Huurre, 2008 [[22](#_ENREF_22)] | 98/29 | PC | Finland | I | SPT-ANY | 1 | Infants born to mothers who participated in a nutritional intervention trial during pregnancy |
| Arshad, 1992[[43](#_ENREF_43)] | 344/123 | PC | UK | D/Q | SPT-ANY/CM/EGG | 1 | Isle of Wight Study. Population based birth cohort of infants born in semi-rural areas between 1989 and 1990 |
| Kemeny, 1991 [[23](#_ENREF_23)] | 189/11 | PC | UK | Unclear | SPT and sIgE-CM/EGG; SPT-CM; Total IgE | 1 | Population based birth cohort of infants born at Dulwich and King’s College Hospitals in London |
| Gruber 2010 [[44](#_ENREF_44)] | 167/15 | PC | Central Europe | Q | sIgE-CM/EGG; Total IgE | 1 | MIPS-1 study. Infants born in 2006 without family history of allergy, randomised to a prebiotic formula intervention if fully formula fed < 8 weeks |
| Silvers, 2009 [[29](#_ENREF_29)] | 889/249 | PC | New Zealand | Q | SPT-ANY | 1 | New Zealand Asthma and Allergy Cohort Study. Population based birth cohort of infants born between 1997 and 2001 |
| Odelram, 1996 [[45](#_ENREF_45)] | 70/23 | PC | Sweden and Finland | D | sIgE-CM; SPT-ANY; Total IgE | 1.5 | Birth cohort of infant with family history and high cord blood IgE born in 1989-1990 |
| Poysa, 1990; Poysa, 1992  [[46](#_ENREF_46), [47](#_ENREF_47)] | 120/41 | PC | Finland | I | Total IgE; SPT-AERO | 5,10 | High risk of disease, born 1979-1980 |
| Kramer, 2009 [[48](#_ENREF_48)] | 138889/3135 | PC | Belarus | I | SPT-AERO | 6.5 | PROBIT study: recruited in hospitals , born 1996-1997 |
| Siltanen,2003 [[49](#_ENREF_49)] | 285/75 | PC | Finland | Q | sIgE-ANY; SPT-ANY; Total IgE | 4 | Infants recruited from maternal hospital born in 1994-5 |
| Strassburger, 2010 [[50](#_ENREF_50)] | 325/94 | PC | Brazil | I | SPT-AERO | 3.5 | Recruited from hospital, born 2001-2002 |
| Van Asperen, 1983 [[51](#_ENREF_51)] | 79/13 | PC | Australia | I | SPT-FOOD | 1 | Cohort recruited from medical service, born in 1980-1981 with family history of atopy |
| Vandenplas, 1988 [[52](#_ENREF_52)] | 75/9 | PC | Belgium | Unclear | sIgE-CM; SPT-CM; Total IgE | 0.33 | Infants with family history of atopy |
| Venter, 2009 [[15](#_ENREF_15)] | 891/27 | PC | UK | Q | SPT-FOOD | 3 | Recruited from antenatal clinics and born in 2001-2002 |
| Oddy, 2004; Oddy, 1999 [[16](#_ENREF_16), [53](#_ENREF_53)] | 1595/651 | PC | Australia | D/Q | SPT-AERO | 6 | Western Australia Pregnancy Cohort: Recruited from antenatal clinics born in 1989-1992 |
| Zutavern, 2004 [[17](#_ENREF_17)] | 623/103 | PC | UK | I | SPT-AERO | 5.5 | Cohort recruited from general practices and born in 1993-1995 |

Q: questionnaire, I: interview, R: medical records, D: diary, PC: prospective cohort, SPT: allergen skin prick test, sIgE: allergen-specific IgE; ANY: a panel of aero and food allergens; CM: cow’s milk.

Figure 21 Risk of bias in studies of EBF duration and allergic sensitisation

## Exclusive breastfeeding and allergic sensitisation

For assessment of EBF and AS most exposure/outcome meta-analyses contained either one or zero eligible studies. We therefore pooled data for studies reporting sIgE and SPT together, and pooled data from different age groups 0-4 years, 5-14 years and 15+ years. We did however take into consideration differences between study populations and AS assessment methods when interpreting the outputs from individual meta-analyses.

### Allergic sensitisation to any allergen

#### EBF ≥0-2 months vs. <0-2 months

Three prospective cohort studies reported data that could be pooled to calculate OR for any AS, in infants with EBF for ≥0-2 months vs. <0-2 months duration and are shown in Figure 22. They show no association between EBF duration and AS risk, with high statistical heterogeneity. All three studies used skin prick testing as an outcome measure. The studies of Elliott and Huurre reported adjusted data, and the study of Cogswell reported unadjusted data. We were not able to confidently explain the heterogeneity between Huurre and Elliott results, but the data of Huurre are based on a subgroup of infants born to allergic mothers whereas Elliott reported on a large representative population-based cohort and it is possible that EBF duration has different effects depending on the allergic status of the mother.

Figure 22 EBF ≥0-2 months vs. <0-2 months and risk of allergic sensitisation in children


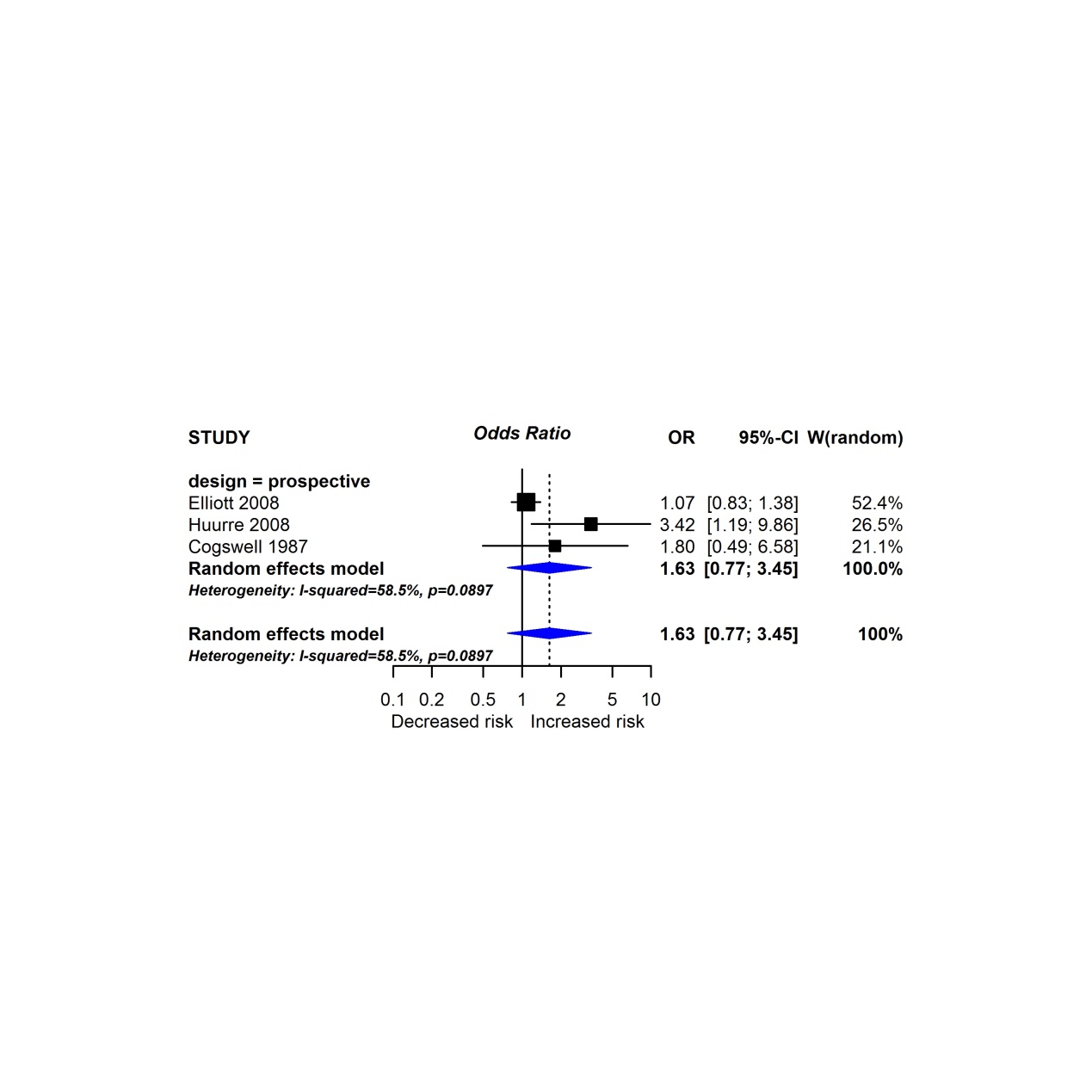


#### EBF ≥3-4 vs. <3-4 Months

##### Systematic reviews and intervention trials

As shown in Table 4, the single systematic review found no evidence that longer exclusive/predominant breastfeeding (for 6 months vs 3 months in the systematic review) influenced risk of AS at 6-7 years. Kramer’s intervention trial of breastfeeding promotion, which we have included under TBF which aimed to increase EBF duration, also found no effect on AS.

Table 4 Promotion of increased EBF duration and risk of AS

| **Study** | **Outcome measure** | **No. participants (studies)** | **Outcome (95% CI)** |
| --- | --- | --- | --- |
| **Kramer 2012** [[37](#_ENREF_37)]  Oddy, 1999 [[16](#_ENREF_16), [53](#_ENREF_53)] | Positive skin prick test at 6 years | 331 (1) | RR 0.99 (0.73, 1.35) |

##### Observational studies

Four prospective cohort studies reported data that could be pooled to calculate OR for any AS, in infants with EBF for ≥3-4 months vs. <3-4 months duration and are shown in Figure 23. They show no association between AS and EBF duration, with high statistical heterogeneity. The data from Arshad and Siltanen are unadjusted data using skin prick testing, with high risk of confounding bias. The data from Kull and Mihrshahi are adjusted data using specific IgE and skin prick testing respectively. The studies of Siltanen and Kull included normal risk infants, the studies of Arshad and Mihrshahi included high risk infants.

Figure 23 EBF ≥3-4 months vs. <3-4 months and risk of allergic sensitisation


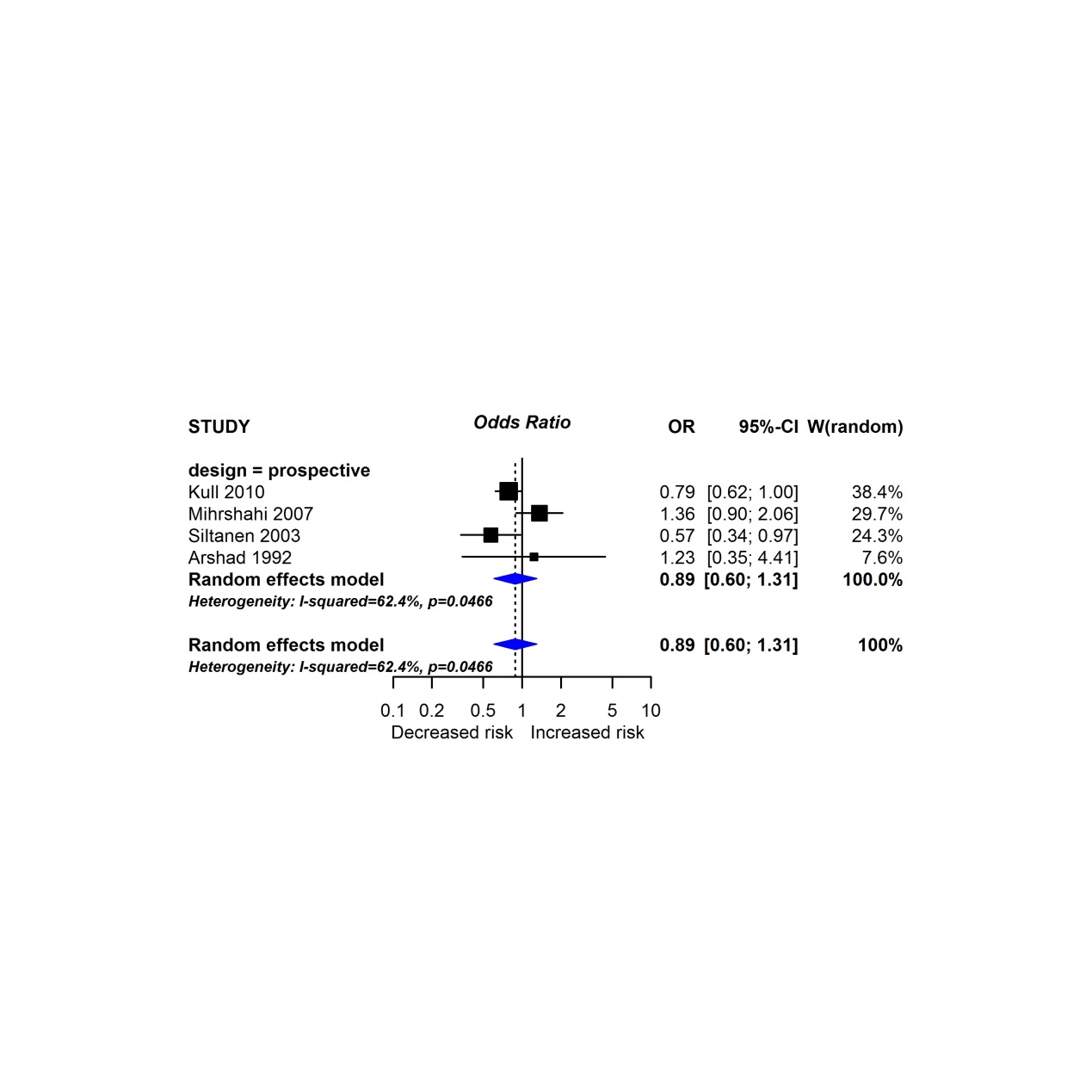


#### EBF ≥5 months vs. <5 months

A single study reported unadjusted data regarding risk of any AS in infants EBF for at least 9 months compared with less than 9 months, and found no significant association (Figure 24).

Figure 24 EBF ≥5-9 months vs. <5-9 months and risk of allergic sensitisation


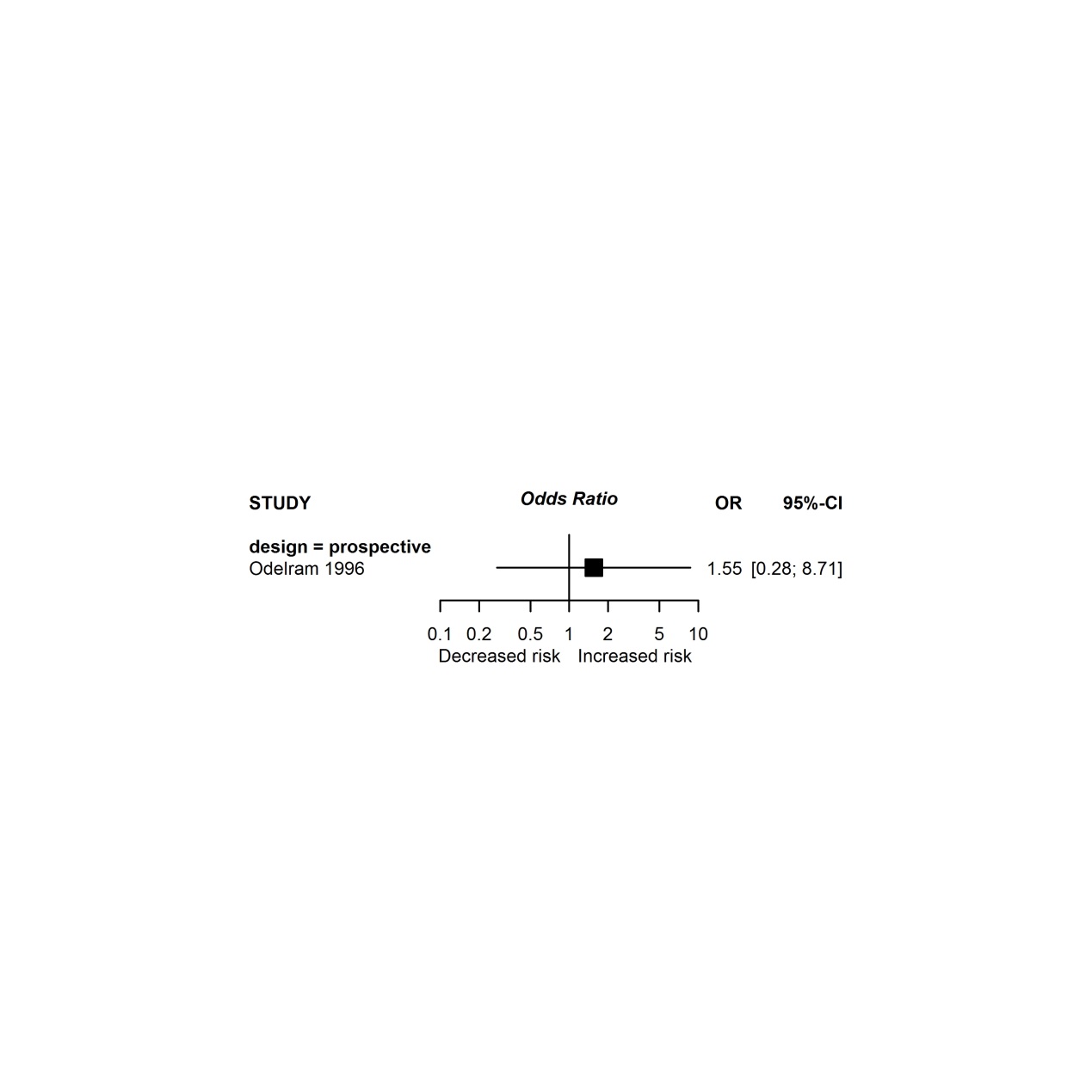


### EBF and allergic sensitisation to Food

#### EBF ≥0-2 months vs. <0-2 months

Three prospective cohort studies reported data that could be pooled to calculate OR for Food AS, in infants with EBF for ≥0-2 months vs. <0-2 months duration and are shown in Figure 25. They show an association between increased EBF duration and increased risk of food sensitisation OR1.61 (1.17, 2.22), with no statistical heterogeneity. The study of Van Asperen reported unadjusted data using skin prick testing at 1 year as an outcome measure in a high risk population, the other studies reported adjusted data used specific IgE at 2.5 and 5 years in normal risk populations. The study of Nwaru separately reported no association between EBF duration and Egg allergy (P=0.30) or CMA (P=0.71). The study of Hong also excluded the occurrence of allergic symptoms during the first 4 months of infancy, and found this did not alter the study findings suggesting that reverse causality was not relevant.

Figure 25 EBF ≥0-2 months vs. <0-2 months and risk of allergic sensitisation to food


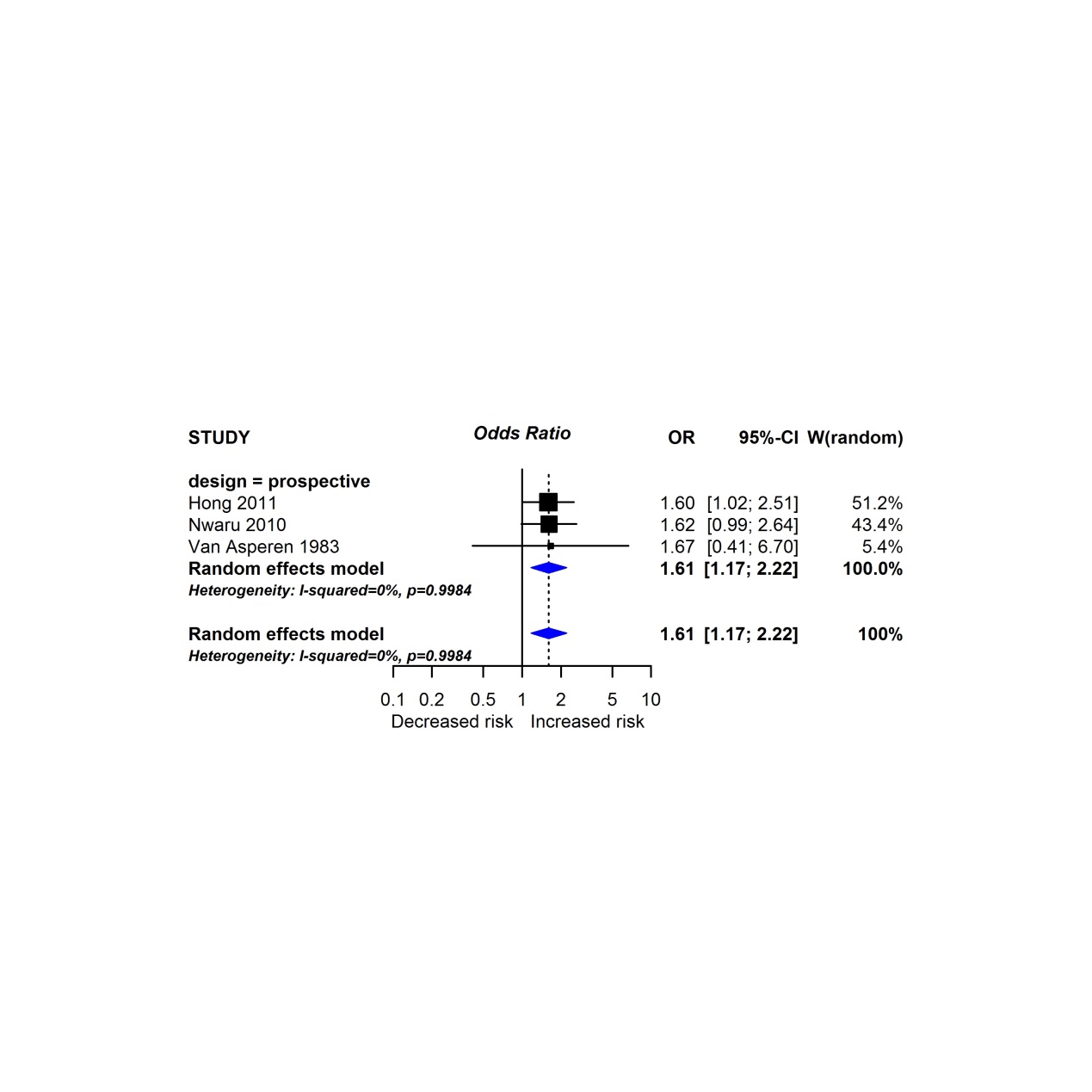


#### EBF ≥3-4 months vs. <3-4 months

Two prospective cohort studies reported data that could be pooled to calculate OR for Food AS, in infants with EBF for ≥3-4 months vs. <3-4 months duration and are shown in Figure 26. They show no association between EBF duration and risk of food sensitisation, with no statistical heterogeneity. The study of Van Asperen reported unadjusted data using skin prick testing at 1 year as an outcome measure in a high risk population, the study of Kull reported adjusted data using specific IgE in a normal risk population at age 8.

Figure 26 EBF ≥3-4 months vs. <3-4 months and risk of allergic sensitisation to food in children

**
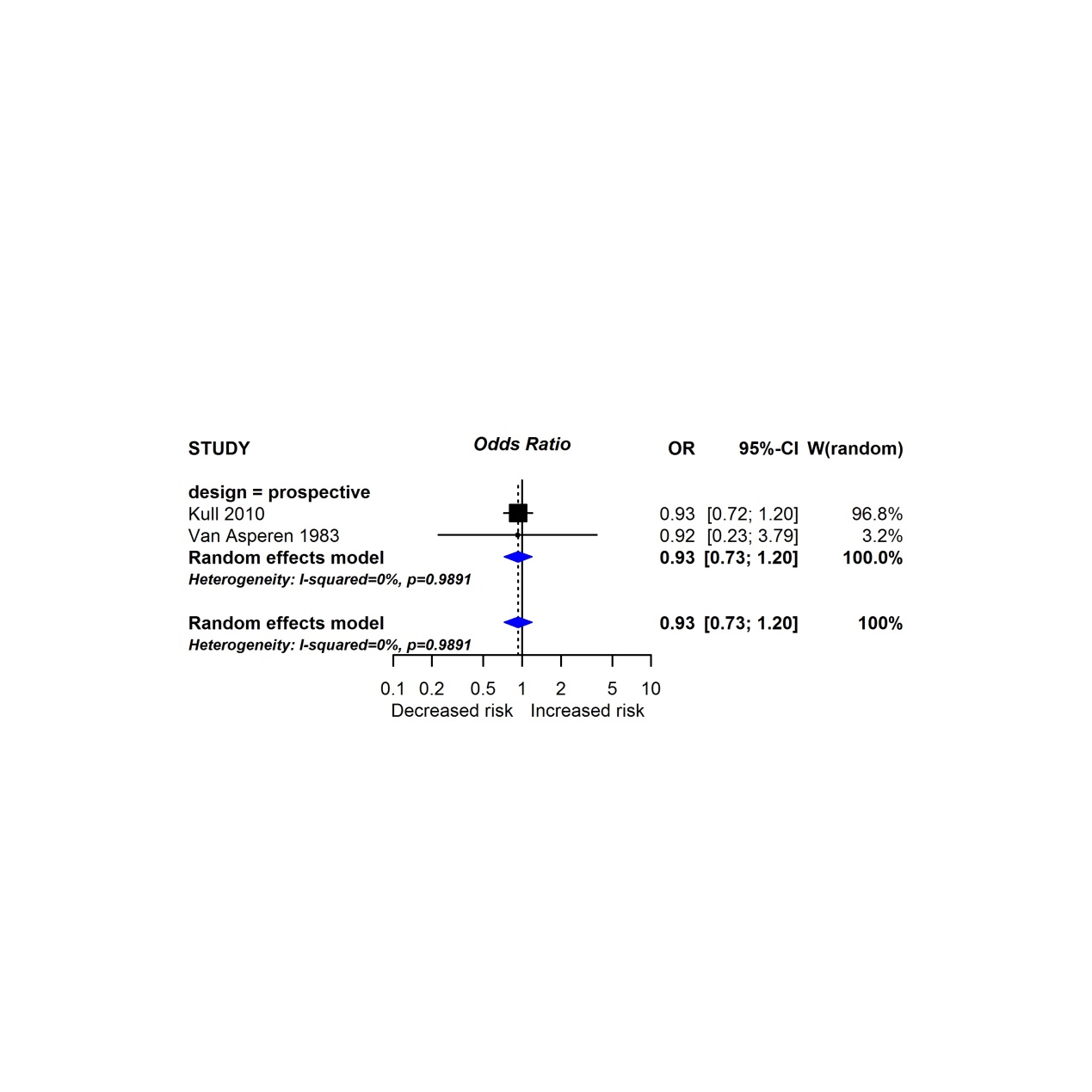
**

### EBF and allergic sensitisation Cow’s Milk

#### EBF ≥0-2 months vs. <0-2 months 0-2

One prospective cohort study reported OR for CM AS, in infants with EBF for ≥0-2 months vs. <0-2 months duration and is shown in Figure 27. There was no association found, with wide confidence intervals due to low study power, and data were unadjusted so carry a high risk of confounding bias.

Figure 27 EBF ≥0-2 months vs. <0-2 months and risk of allergic sensitisation in children to cow’s milk


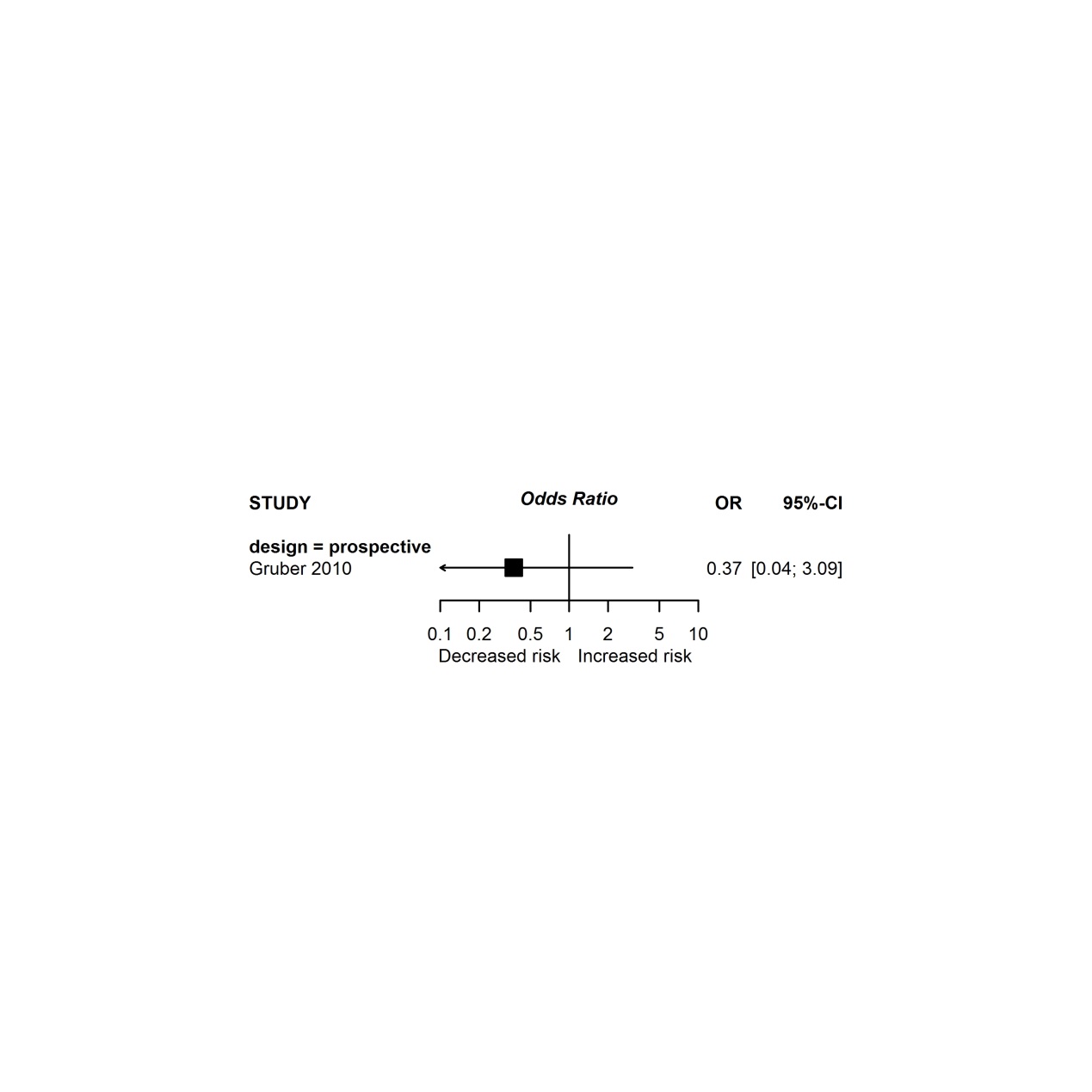


#### EBF ≥3-4 months vs. <3-4 months 3-4

Four prospective cohort studies reported OR for CM AS, in infants with EBF for ≥3-4 months vs. <3-4 months duration and are shown in Figure 28. There was no association found, moderate statistical heterogeneity. All studies other than that of Arshad used specific IgE for assessment; all studies other than Kull assessed at 1 year age; Kull and Schoetzau reported adjusted data, Kemeny and Arshad unadjusted data. In the study of Kemeny there were no participants with positive SPT to cow’s milk (sIgE data presented in Figure 28).

Figure 28 EBF ≥3-4 months vs. <3-4 months and risk of allergic sensitisation to cow’s milk


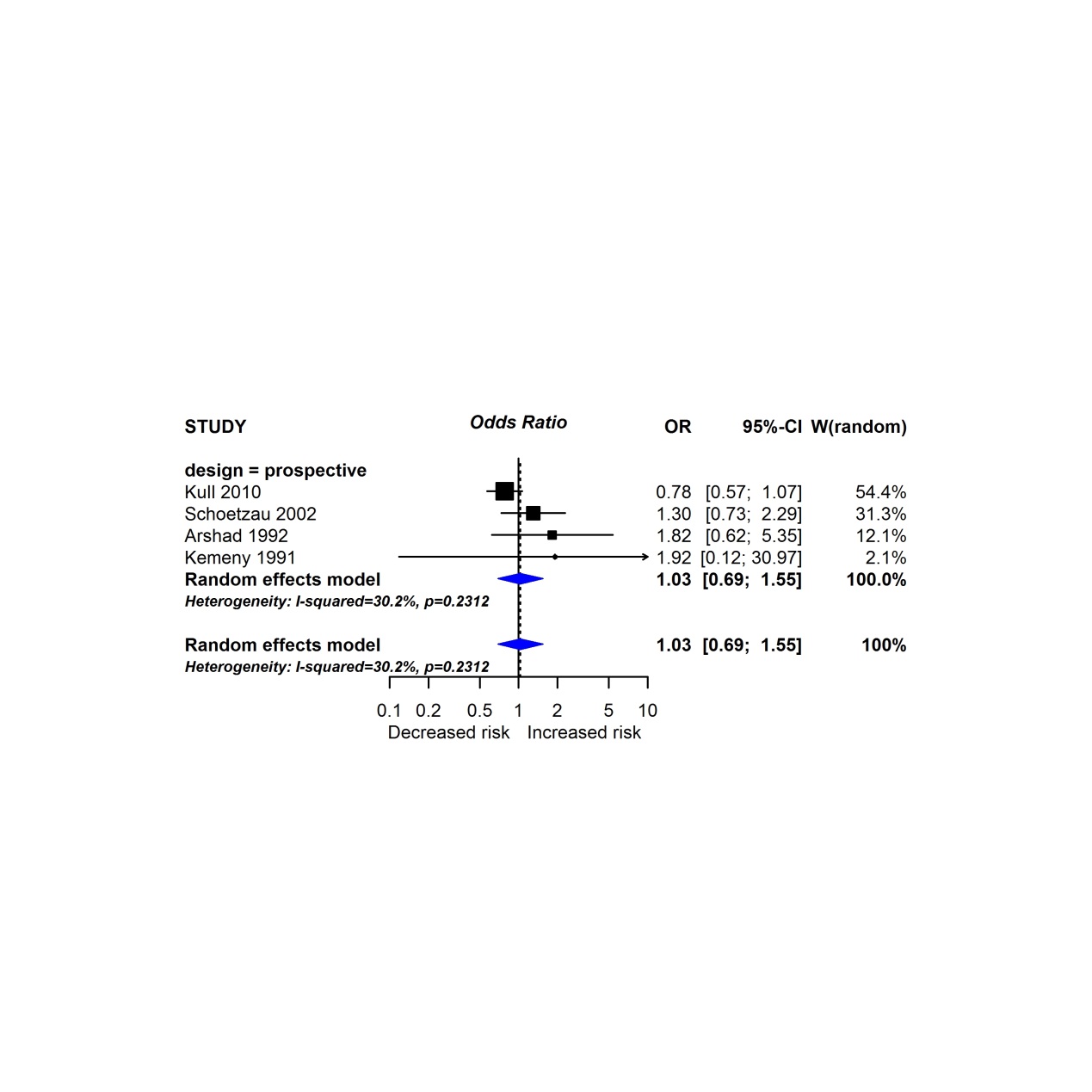


#### EBF for ≥5-9 months vs. <5-9 months

One prospective cohort study reported OR for CM AS, in infants with EBF for ≥5-9 months vs. <5-9 months duration and is shown in Figure 29. There was no association found, with wide confidence intervals due to low study power, and data were unadjusted so carry a high risk of confounding bias.

Figure 29 EBF ≥5-9 months vs. <5-9 months and risk of allergic sensitisation to cow’s milk


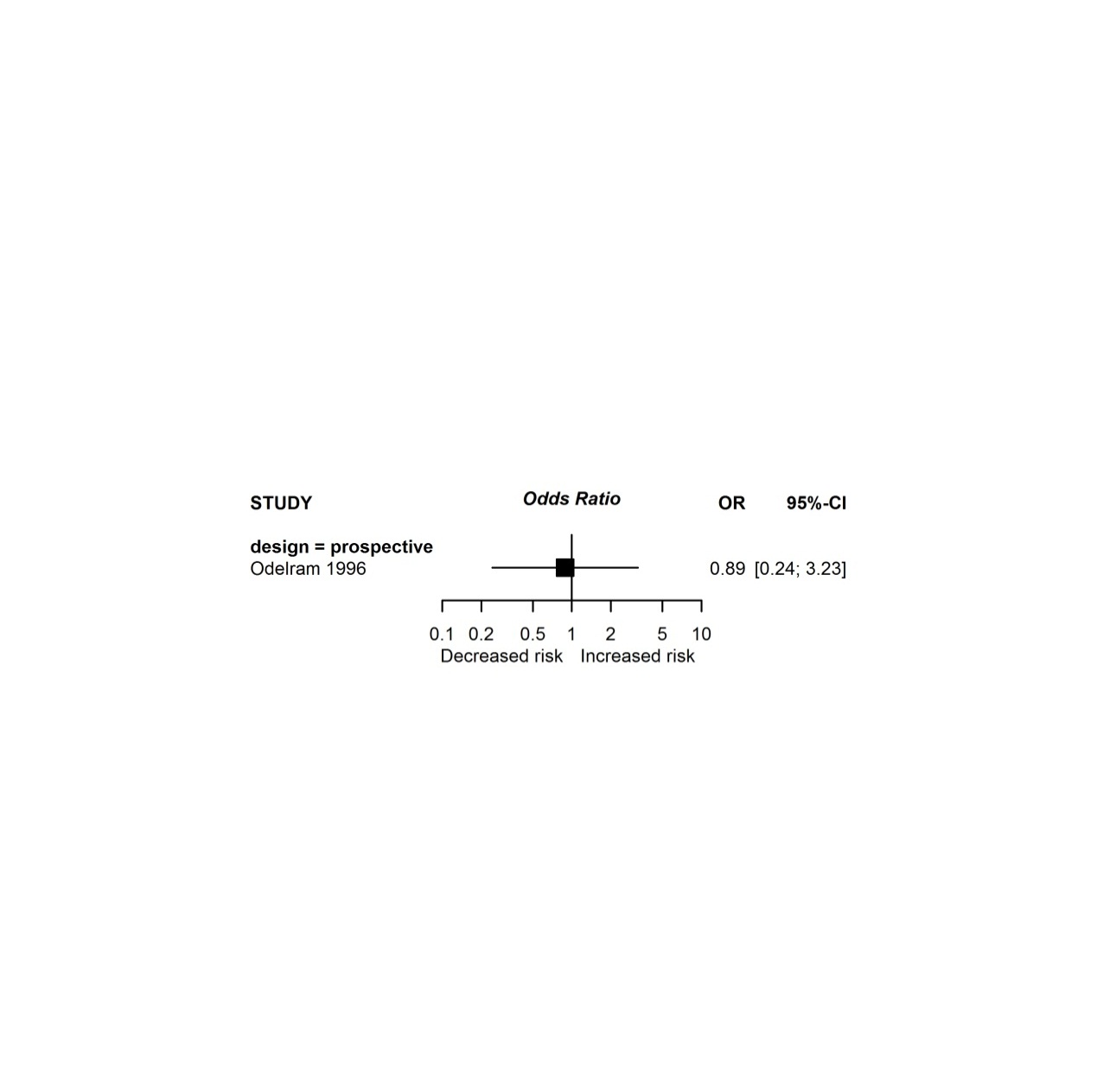


### EBF and allergic sensitisation to Egg

#### EBF ≥0-2 months vs. <0-2 months

One prospective cohort study reported OR for Egg AS, in infants with EBF for ≥0-2 months vs. <0-2 months duration and is shown in Figure 30. There was no association found, with wide confidence intervals due to low study power, and data were unadjusted so carry a high risk of confounding bias

Figure 30 EBF ≥0-2 months vs. <0-2 months and risk of allergic sensitisation to Egg


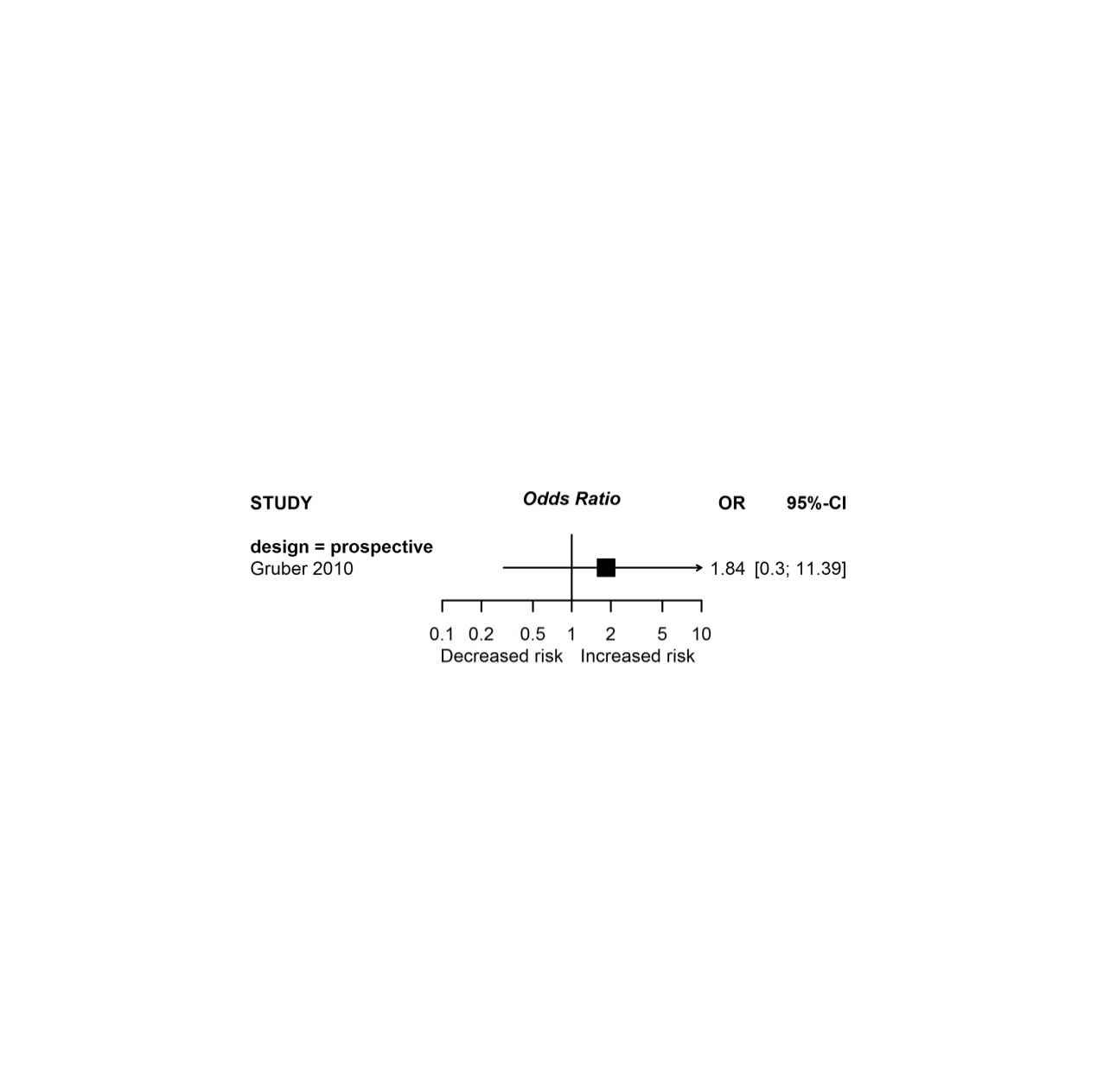


#### EBF ≥3-4 months vs. <3-4 months

Three prospective cohort studies reported OR for Egg AS, in infants with EBF for ≥3-4 months vs. <3-4 months duration and are shown in Figure 31. There was no association found, with no statistical heterogeneity. All studies other than that of Arshad used specific IgE for assessment; all studies other than Kull assessed at 1 year age; Kull reported adjusted data, Kemeny and Arshad unadjusted data. In the study of Kemeny skin prick testing data showed very similar findings (sIgE data presented in Figure 31).

Figure 31 EBF ≥3-4 months vs. <3-4 months and risk of allergic sensitisation to Egg


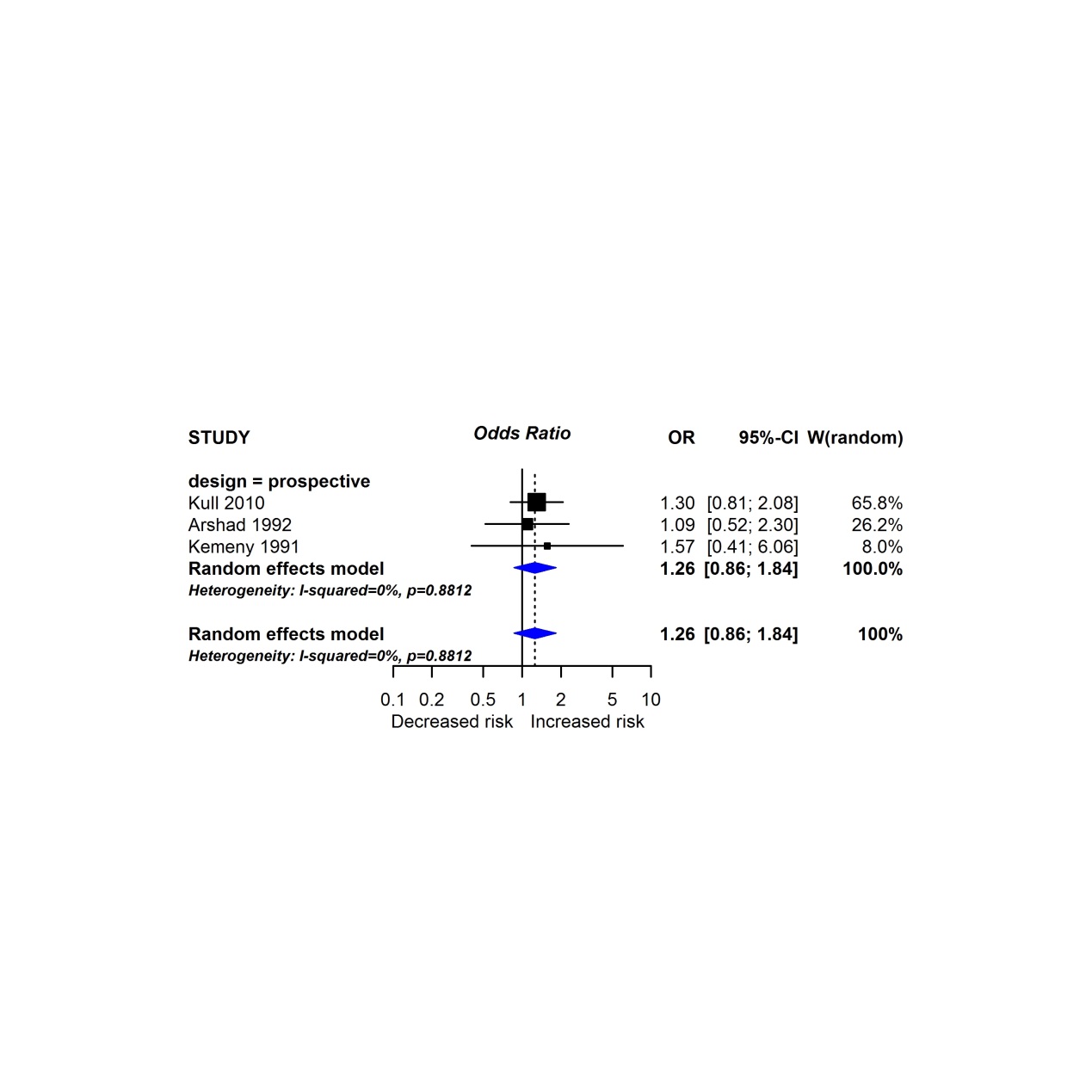


### EBF and allergic sensitisation to Peanut

#### EBF ≥3-4 months vs. <3-4 months

One prospective cohort study reported OR for PN AS, in infants with EBF for ≥3-4 months vs. <3-4 months duration and is shown in Figure 32. There was no association found. This was a study with low risk of bias on all parameters, which reported adjusted data for PN sensitisation at age 8, measured using specific IgE.

Figure 32 Exclusive breastfeeding ≥3-4 months vs. <3-4 months and risk of allergic sensitisation to PN


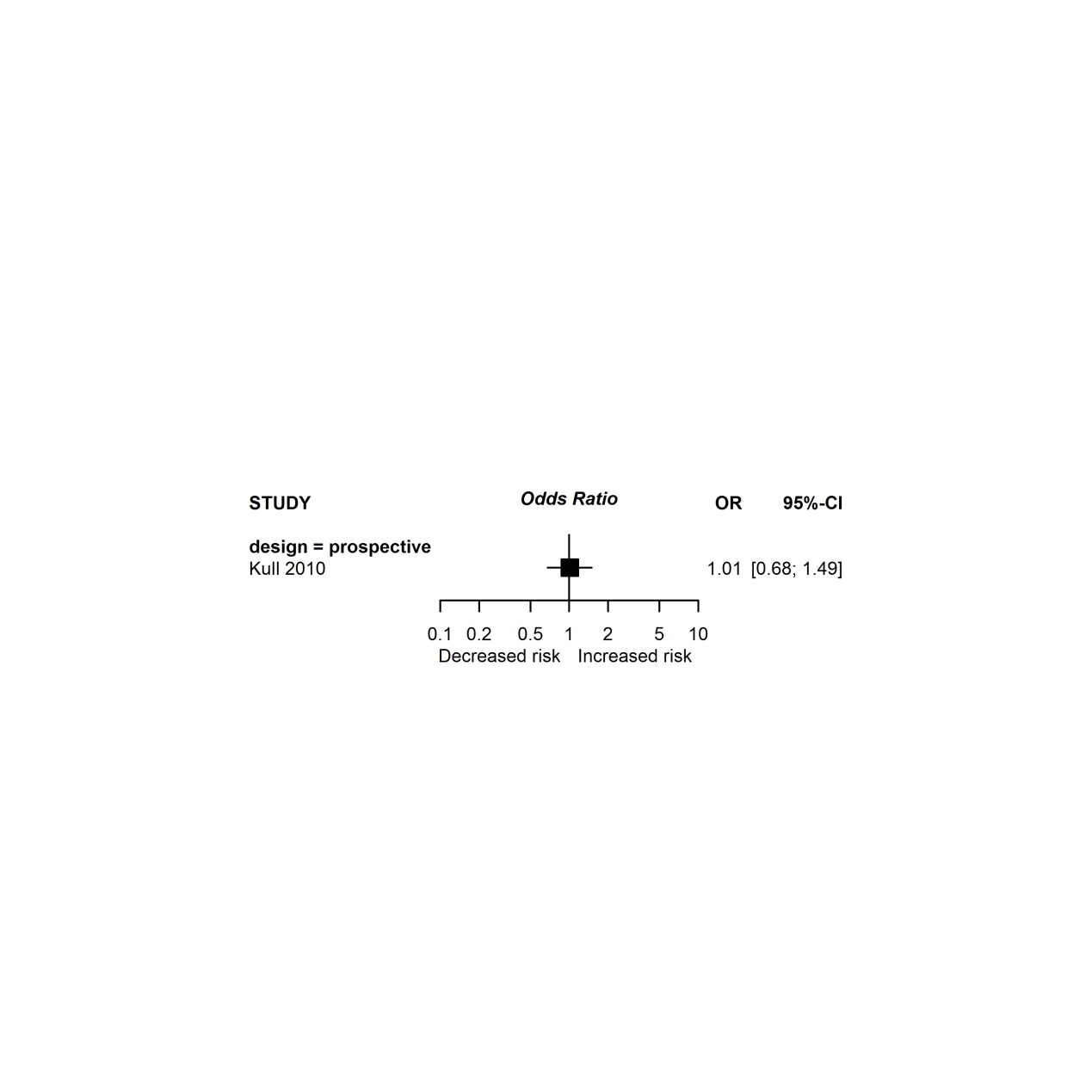


### EBF and allergic sensitisation to aeroallergen

#### EBF ≥0-2 months vs. <0-2 months

Two prospective cohort studies reported OR for Aeroallergen AS, in infants with EBF for ≥0-2 months vs. <0-2 months duration and are shown in Figure 33. There was no association found, with moderate statistical heterogeneity (I^2^=40%). Both studies reported adjusted data on sIgE in normal risk populations, and were considered at low risk of bias.

Figure 33 EBF ≥0-2 months vs. <0-2 months and risk of allergic sensitisation to aeroallergen


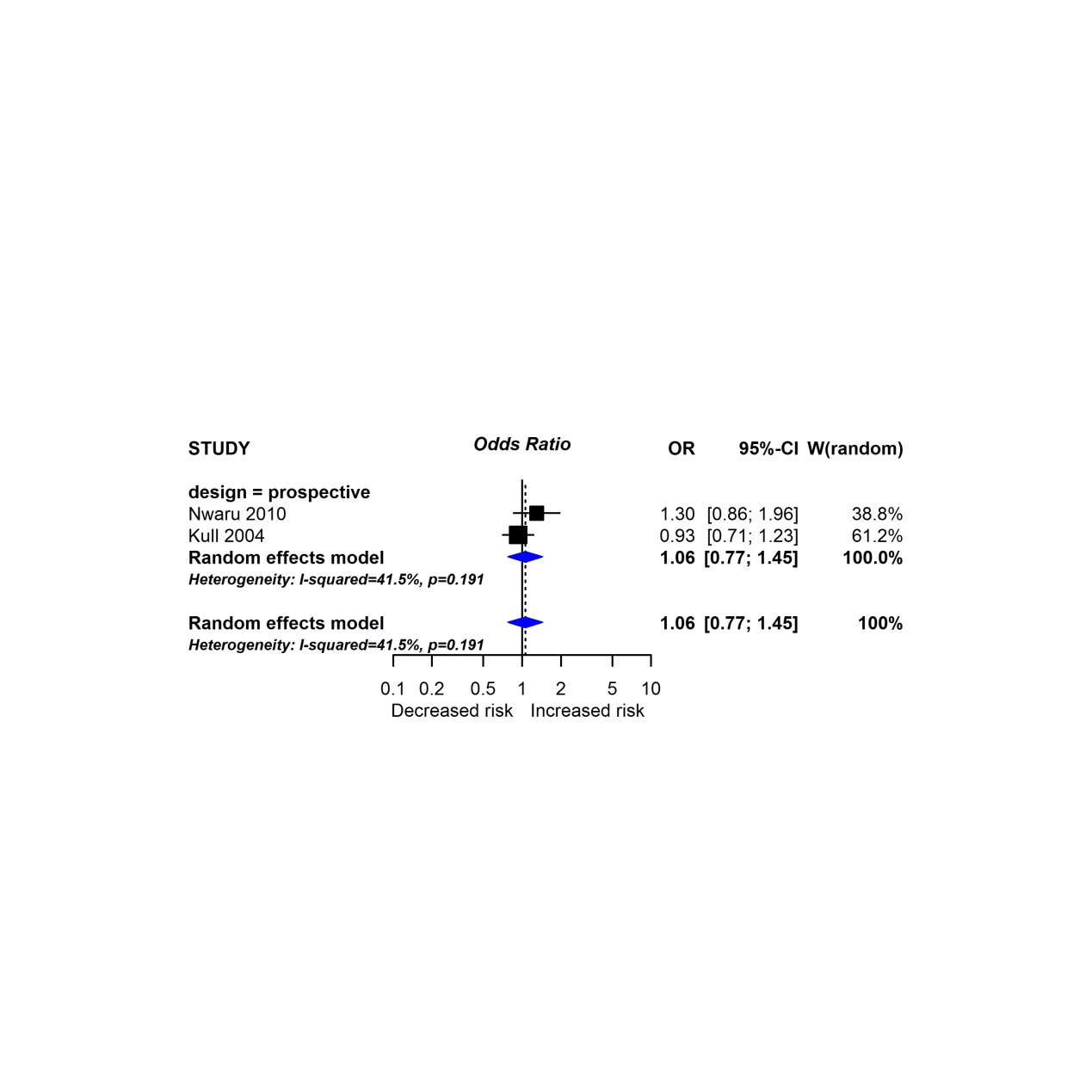


#### EBF ≥3-4 months vs. <3-4 months

Five prospective cohort studies reported OR for Aeroallergen AS, in infants with EBF for ≥3-4 months vs. <3-4 months duration and are shown in Figure 34. Data were not pooled due to extreme statistical heterogeneity (I^2^=83%), largely driven by the study of Sears. Two of the studies (Oddy and Kramer) were reported in the included systematic review. All studies are prospective cohorts reporting adjusted data in normal risk populations, other than Poysa which reports unadjusted data in a high risk population. All studies used SPT other than Kull. Ages at outcome assessment ranged from 6 to 21 years.

Figure 34 EBF ≥3-4 months vs. <3-4 months and risk of allergic sensitisation to aeroallergen


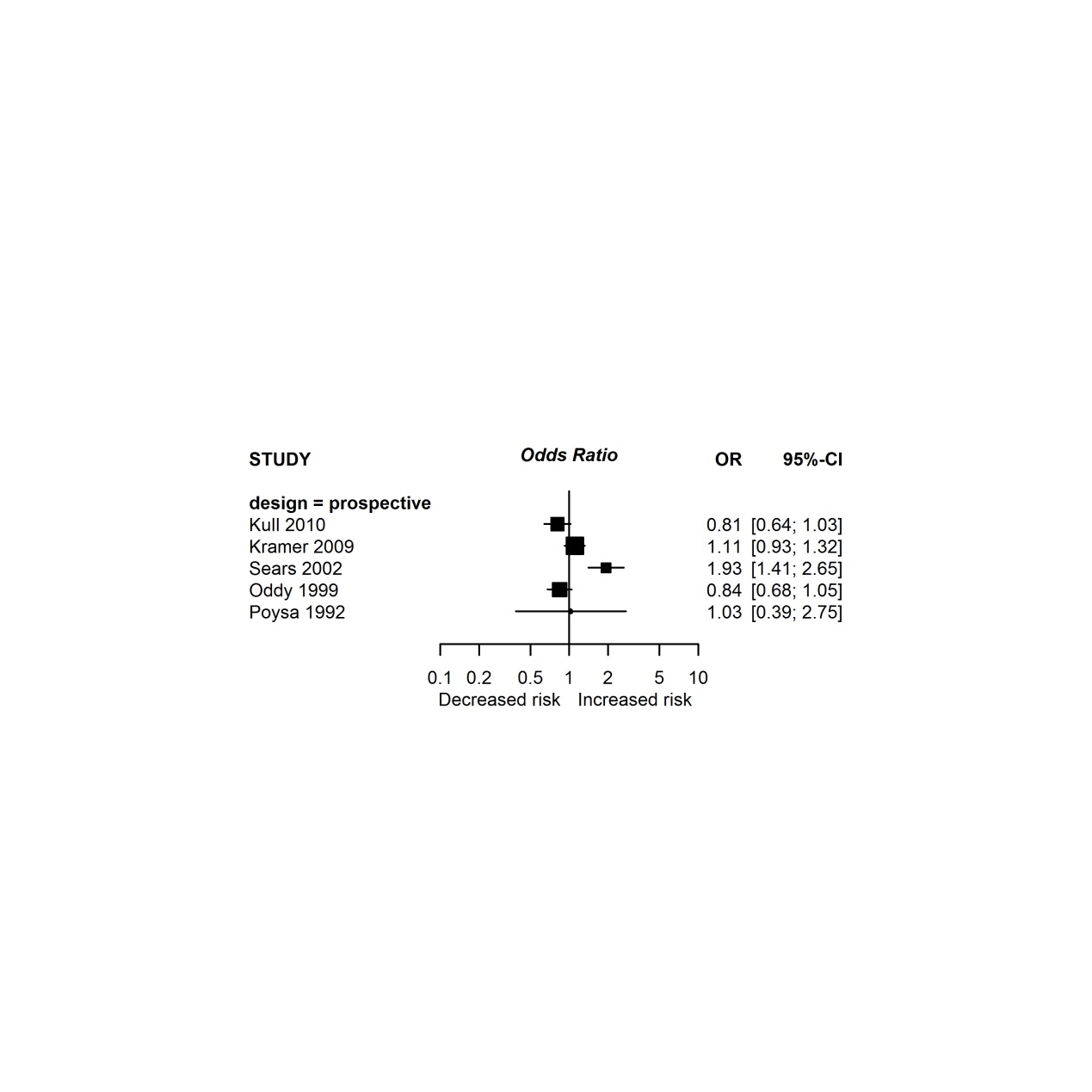


#### EBF ≥5-9 months vs. <5-9 months

Two prospective cohort studies reported OR for Aeroallergen AS, in infants with EBF for ≥5-7 months vs. <5-7 months duration and are shown in Figure 35. There was no association found, with moderate statistical heterogeneity. Both studies reported adjusted data in normal risk populations, and were considered at low risk of bias on all parameters and used SPT for assessment of AS at 3.5-6 years. We were unable to explain the statistical heterogeneity.

Figure 35 EBF ≥5-7 months vs. <5-7 months and risk of allergic sensitisation to aeroallergen


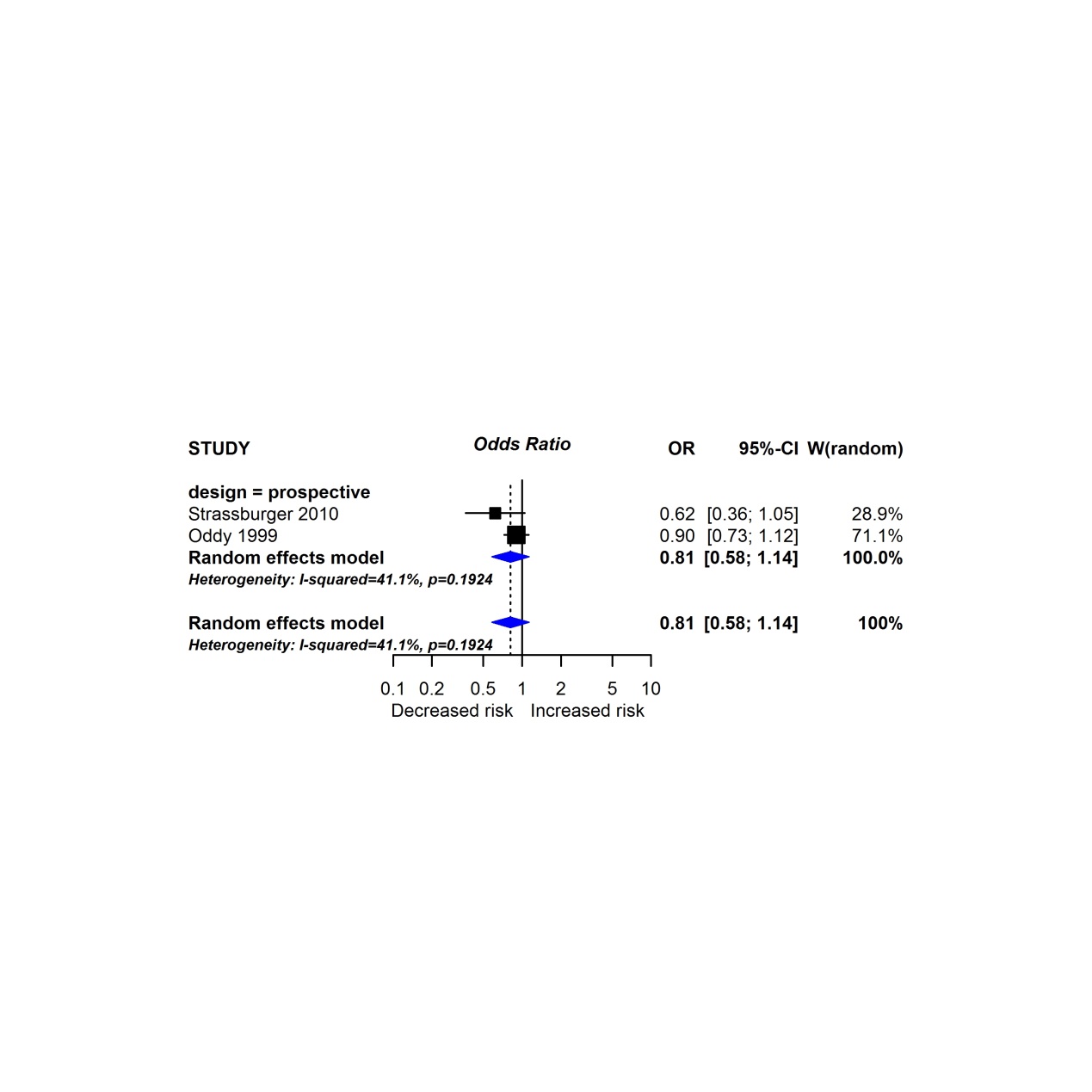


### Total IgE

#### EBF ≥3-4 months vs. <3-4 months and elevated Total IgE

Two studies reported the relationship between EBF duration ≥3-4 months and risk of raised Total IgE, and are shown in Figure 36. Pooled data show no evidence of association, with high statistical heterogeneity for unexplained reasons.

Figure 36 EBF ≥3-4 months vs. <3-4 months and risk of raised Total IgE


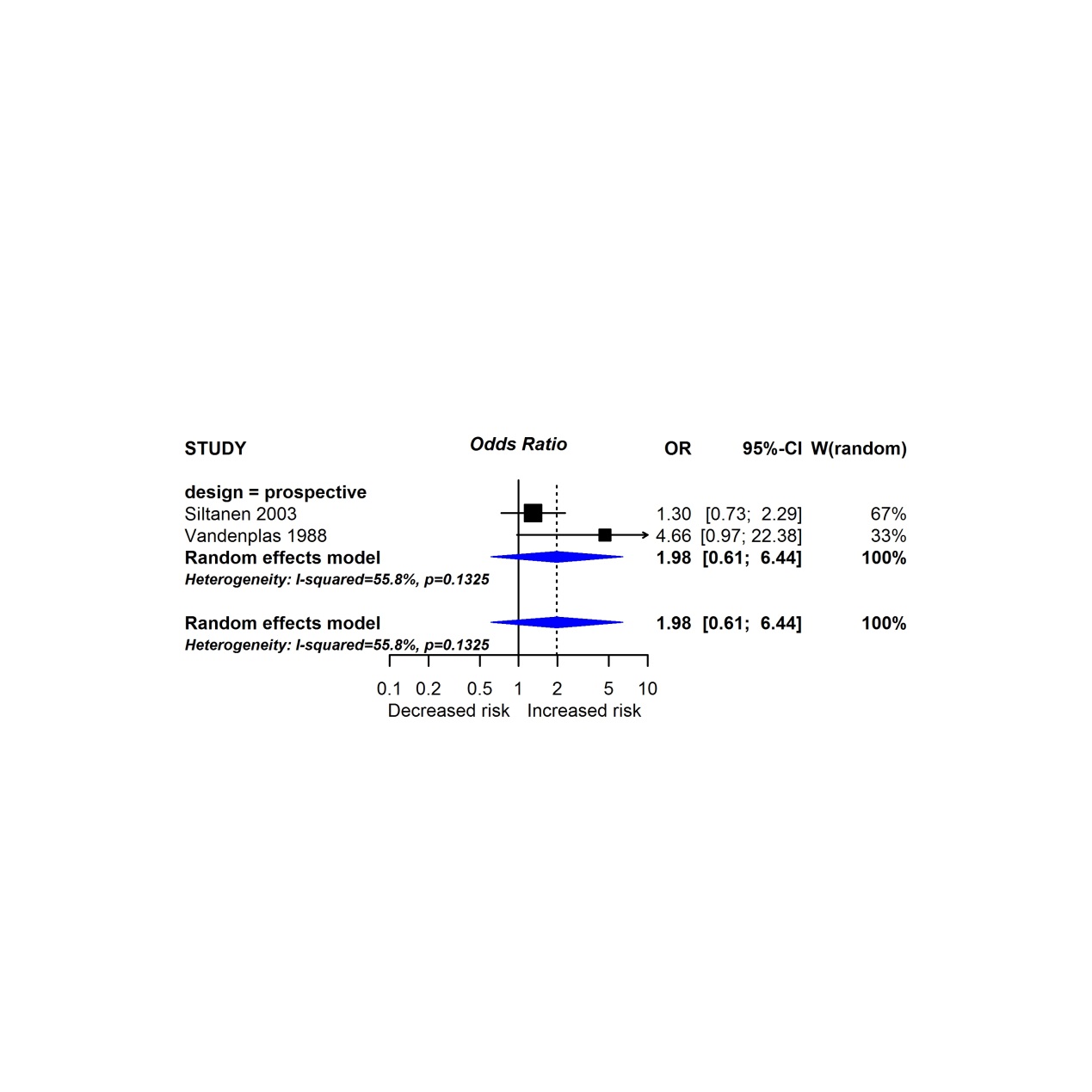


#### EBF ≥5-9 months vs. <5-9 months and elevated Total IgE

One study reported the relationship between EBF duration ≥5-9 months and risk of raised Total IgE, and is shown in Figure 37. There was no evidence of association.

Figure 37 EBF ≥5-9 months vs. <5-9 months and risk of raised Total IgE


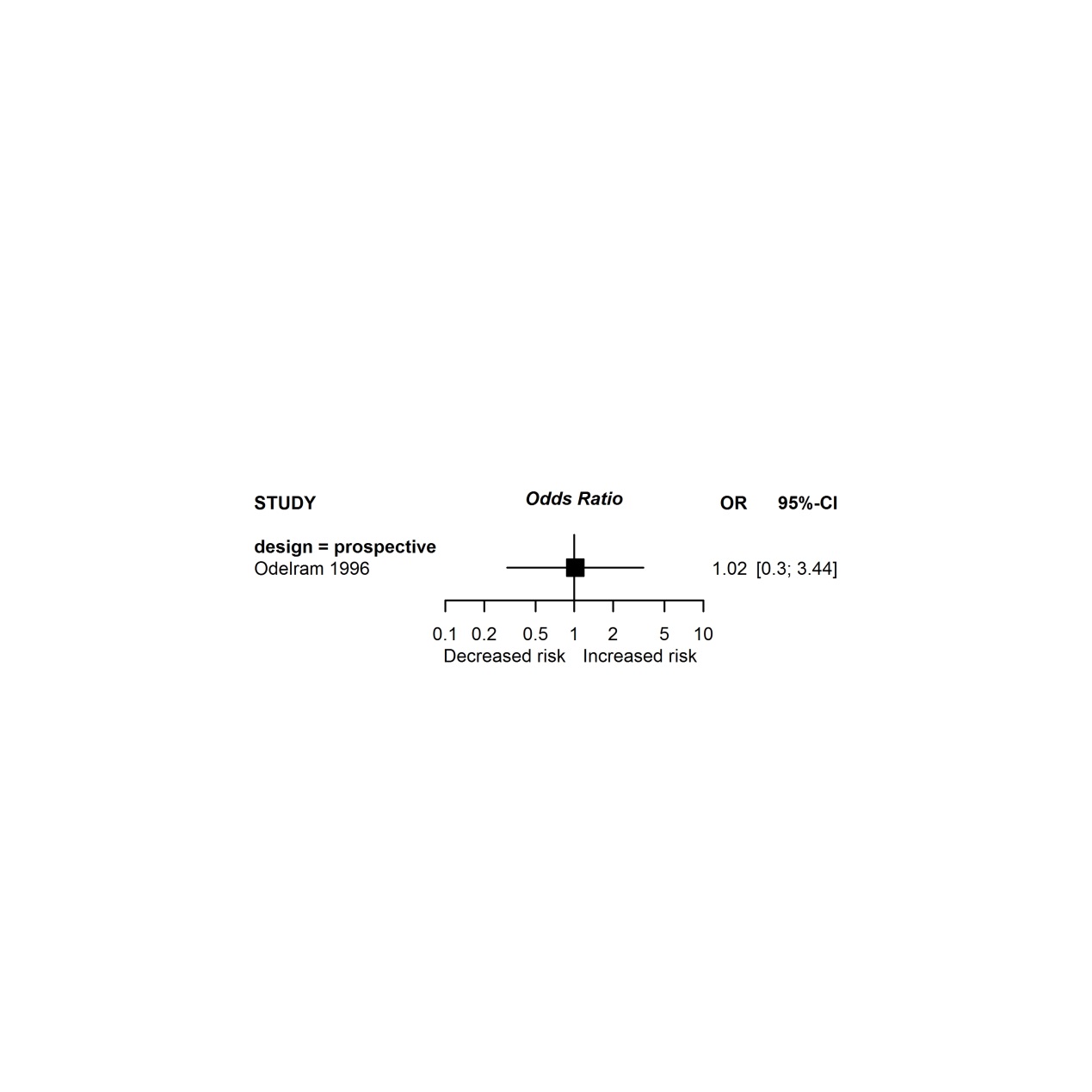


- 1. **Data for EBF duration and AS that were not suitable for meta-analysis**

Nine of the 25 included studies did not report data that could be included in a meta-analysis, and are summarised below in Table 4. In 6 studies reporting EBF duration and specific AS risk there was evidence of a positive relationship in just one small study reporting unadjusted data. In all others there was no evidence of association. In 3 studies reporting EBF duration and Total IgE there was no evidence of association.

Table 5 Studies investigating the association between EBF and allergic sensitisation which were not eligible for meta-analysis

| **Study** | **Design** | **Outcome** | **Age** | **N/n** | **Data** | **Measure** | **EBF in no AS** | **EBF in AS** | **P-value** |
| --- | --- | --- | --- | --- | --- | --- | --- | --- | --- |
| Vandenplas 1988 [[52](#_ENREF_52)] | PC | sIgE-CM, SPT-CM, Total IgE | 0.33 | 75/9 | Categorical |  | Increased AS in those with longer EBF. 3/60 vs 0/15 (sIgE-CM), 7/60 vs 0/15 (SPT-CM). Unadjusted data. | | |
| Venter, 2009 [[15](#_ENREF_15)] | PC | SPT-FOOD | 3 | 891/27 | Categorical |  | No relationship between EBF duration of >3 or <3 months, and food sensitisation at 1 or 3 years | | |
| Silvers, 2009 [[29](#_ENREF_29)] | PC | SPT-ANY | 1 | 889/249 | Continuous |  | Adjusted OR for AS, for each month increase in EBF duration was  1.03 (0.96, 1.1) P=0.40 | | |
| Hoppu, 2000 [[21](#_ENREF_21)] | PC | SPT-ANY | 1 | 114/27 | Continuous | Mean (95%C) | 3.3 (3, 3.7) | 3.2 (2.6, 3.8) | NS |
| Hesselmar 2010 [[2](#_ENREF_2)] | PC | sIgE-FOOD | 1.5 | 169/25 | Continuous | Median (IQR) | 4 (4, 4.5) | 4 (2.5, 4.5) | 0.58 |
| Zutavern 2004 [[17](#_ENREF_17)] | PC | SPT-AERO | 5.5 | 623/103 | Categorical | No significant relationship between EBF duration of >8 or <8 weeks, and food sensitisation | | | |
| Bruno, 1995 [[40](#_ENREF_40)] | PC | Total IgE | 5 | 107 | Continuous |  | Geometric mean Total IgE was 8.5 in those EBF for 6 months, and 14 for shorter EBF duration. The difference was no statistically significant | | |
| Gruber 2010 [[44](#_ENREF_44)] | PC | Total IgE | 1 | 167/15 | Continuous |  | Least squares mean (kU/L) for those EBF for >2 months 10.9 (8.2, 14.5) versus <2 months 11.9 (10.2, 13.8). Not statistically significant | | |
| Poysa, 1990 [[46](#_ENREF_46)] | PC | Total IgE | 5 | 91 | Continuous |  | Total IgE not significantly different between those EBF for ≥3 versus <3 months | | |

# Age at solid food introduction and allergic sensitisation

### Overall characteristics of studies, risk of bias and summary of results

Table 5 describes the main characteristics of the studies analysed in this report. A total of 9 prospective cohort studies and one cross-sectional study, reported the association between timing of solid food introduction (SF) and risk of AS. The studies were from Europe (n=6), USA (n=2) and Australia (n=2). Overall, valid data on SF and AS risk were available from over 7000 subjects. Information on AS was obtained from a skin prick test in 5 studies, and blood IgE measurement in 5 studies. With regards to time of outcome diagnosis, 7 studies explored the association between SF and AS in the first 4 years of life, 3 studies assessed at age 5-14 years. Four studies used a questionnaire and/or interview to assess the exposure (SF), 6 used an interview alone.

Risk of bias is summarised in Figure 38. Just under half of studies had a high risk of bias, due to lack of adjustment for confounding bias i.e. no adjusted data presented. One third of studies had unclear risk of assessment bias. Risk of conflict of interest was generally assessed as low.

The risk of AS according to SF was categorised as ≥3-4 months vs. <3-4 months. Overall the evidence base was limited due to small numbers of included studies. We found no evidence that the timing of solid food introduction is associated with risk of AS.

**Table 6** **Characteristics of included studies evaluating SF introduction and allergic sensitisation**

| **First Author & Publication Year** | **N/n cases** | **Design** | **Country** | **Exposure assessment** | **Method of outcome assessment** | **Age at outcome (years)** | **Population characteristics** |
| --- | --- | --- | --- | --- | --- | --- | --- |
| Hesselmar, 2010 [[2](#_ENREF_2)] | 169/25 | PC | Sweden | I | sIgE-FOOD | 1.5 | ALLERGYFLORA study. Population based study of babies selected from antenatal clinics between 1998 and 2003 - mainly high risk of allergic disease |
| Mihrshahi, 2007 [[8](#_ENREF_8)] | 516/217 | PC | Australia | I | SPT-ANY | 5 | CAPS study. Infants born in 1997-1999 with family history of asthma or wheezing |
| Snijders, 2008 [[24](#_ENREF_24)] | 776/218 | PC | Netherlands | Q | sIgE-AERO/ANY/CM/EGG/PN | 2 | KOALA study. Population based birth cohort of infants born between 2000-2002 (consisting of cohorts with conventional and alternative lifestyle) |
| Zutavern, 2006; Zutavern, 2008  [[55](#_ENREF_55), [56](#_ENREF_56)] | 2612/258; 1123/301 | PC | Germany | Q/I | sIgE-ANY/FOOD/AERO/CM/EGG/PN | 2, 6 | LISA study. Population based birth cohort of infants born 1997-9 at selected maternity hospitals in 4 German cities |
| Sicherer, 2010 [[14](#_ENREF_14)] | 503/140 | PC | USA | I | sIgE-PN | 1 | The Consortium of Food Allergy Research: atopic children recruited from services |
| Van Asperen, 1983 [[51](#_ENREF_51)] | 79/13 | PC | Australia | I | SPT-FOOD | 1.3 | Cohort recruited from medical service, born in 1980-1981 with family history of atopy |
| Venter, 2009 [[15](#_ENREF_15)] | 757/27 | PC | UK | Q | SPT-FOOD | 1, 3 | Recruited from antenatal clinics and born in 2001-2002 |
| Joseph, 2011 [[57](#_ENREF_57)] | 594/178 | PC | USA | I | sIgE-CM/EGG/PN | 3 | WHEALS STUDY: Recruited from hospital prenatal care and born in 2005 |
| Zutavern, 2004 [[17](#_ENREF_17)] | 623/103 | PC | UK | I | SPT-AERO | 5.5 | Cohort recruited from general practices and born in 1993-5 |
| Kucukosmanoglu, 2008 [[35](#_ENREF_35)] | 1015/20 | CS | Turkey | Q/I | SPT-EGG | 1 | Hospital based selection of 8-18 months old Infants born in 2001-2002 |

Figure 38 Risk of bias in studies of SF introduction and allergic sensitisation

## Solid food introduction and allergic sensitisation

### SF and allergic sensitisation to any allergen

Three prospective cohort studies reported OR for AS to any allergen, in infants with SF ≥3-4 months vs. <3-4 months and are shown in Figure 39. There was no significant association found, with extreme statistical heterogeneity. All studies reported adjusted data, and were considered at low risk of bias on all domains. The study of Mihrshahi used skin prick testing for assessment at age 5, and the other studies used blood specific IgE testing for similar combined food/aeroallergen profiles at age 2 years. We were unable to confidently explain the heterogeneity between the findings of Snijders and Zutavern, however the study of Snijders included very few participants with SF introduction prior to 4 months (n=50), and included participants with an anthroposophic lifestyle (~20% of participants).

Figure 39 SF ≥3-4 months vs. <3-4 months and risk of allergic sensitisation to any allergen


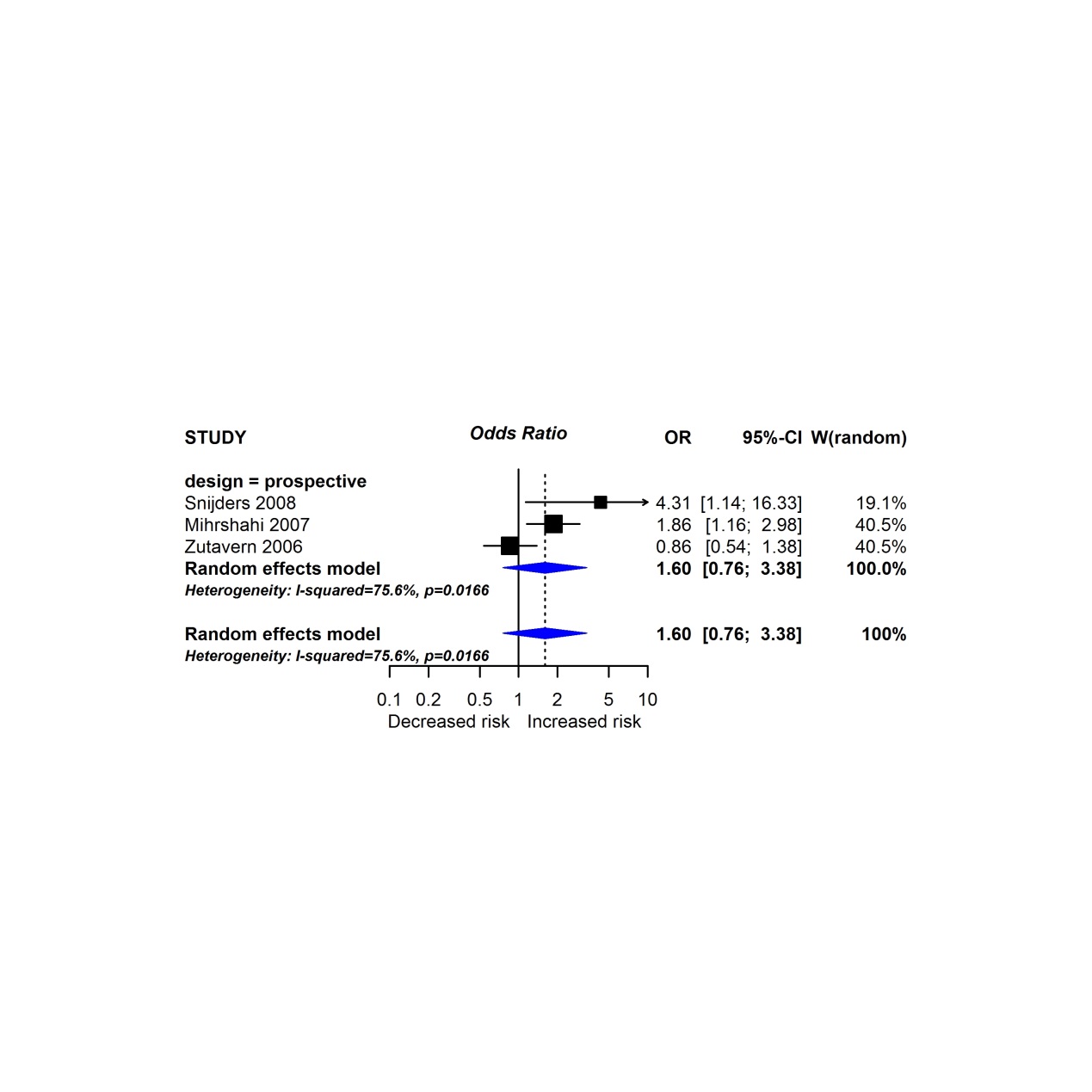


### SF and allergic sensitisation to Food

Two prospective cohort studies reported OR for AS to any food allergen at age 0-4 years, in infants with SF ≥3-4 months vs. <3-4 months and are shown in Figure 40. There was no significant association found, with extreme statistical heterogeneity. The study of Zutavern reported adjusted data using specific IgE blood testing at aged 2, and was considered at low risk of bias on all domains. The study of Venter reported unadjusted data using skin prick testing at age 1, and was therefore considered at high risk of bias. This difference in adjustment for potential confounding variables may explain the statistical heterogeneity.

Figure 40 SF ≥3-4 months vs. <3-4 months and risk of allergic sensitisation to food


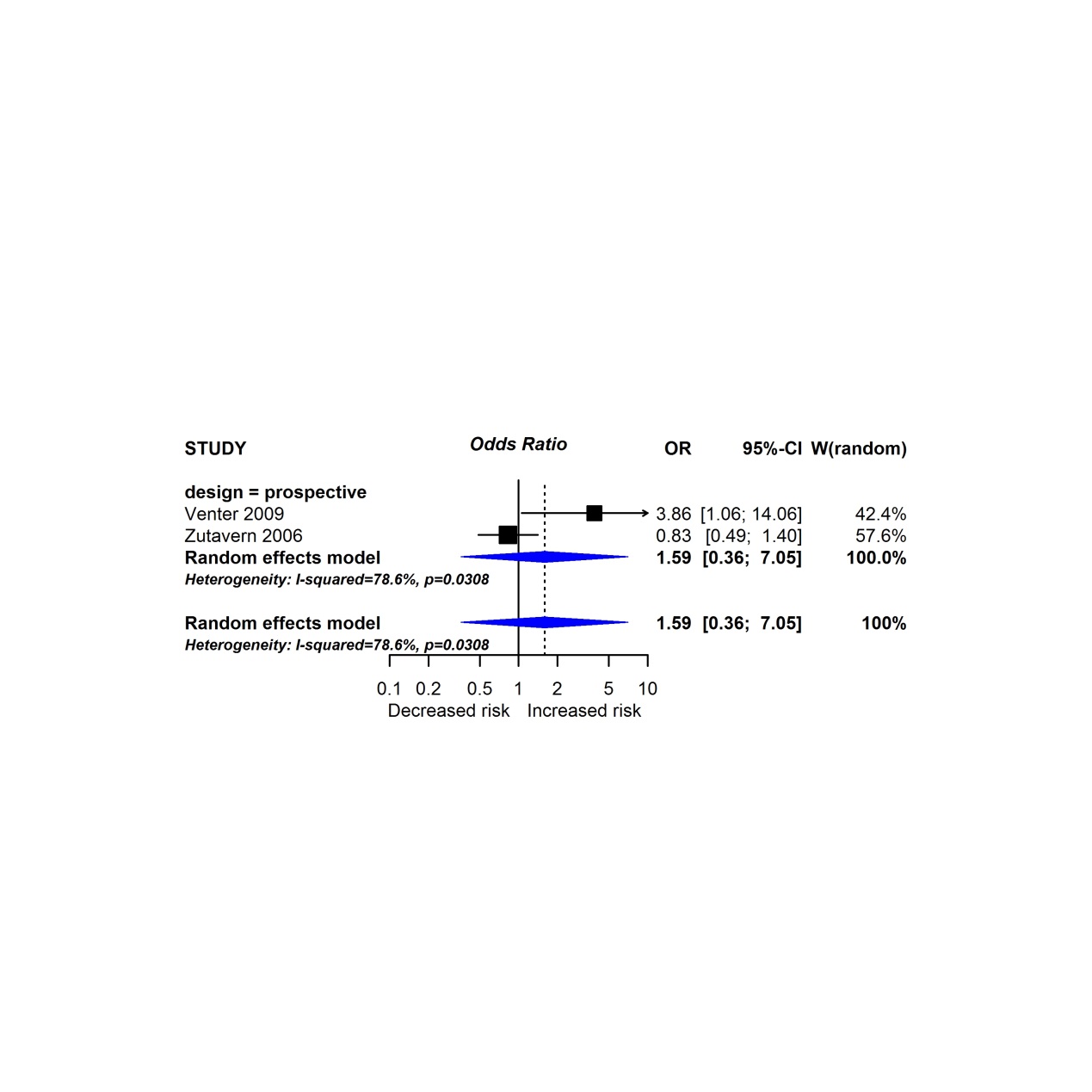


### SF and allergic sensitisation to Cow’s Milk

Two prospective cohort studies reported OR for AS to CM at age 0-4 years, in infants with SF ≥3-4 months vs. <3-4 months and are shown in Figure 41. There was no significant association found, with no statistical heterogeneity. Both studies assessed sensitisation using specific IgE blood testing at aged 2 years. The study of Snijders reported adjusted data and was considered at low risk of bias on all domains. The study of Joseph reported unadjusted data, and was therefore considered at high risk of bias.

Figure 41 SF ≥3-4 months vs. <3-4 months and risk of allergic sensitisation to cow’s milk


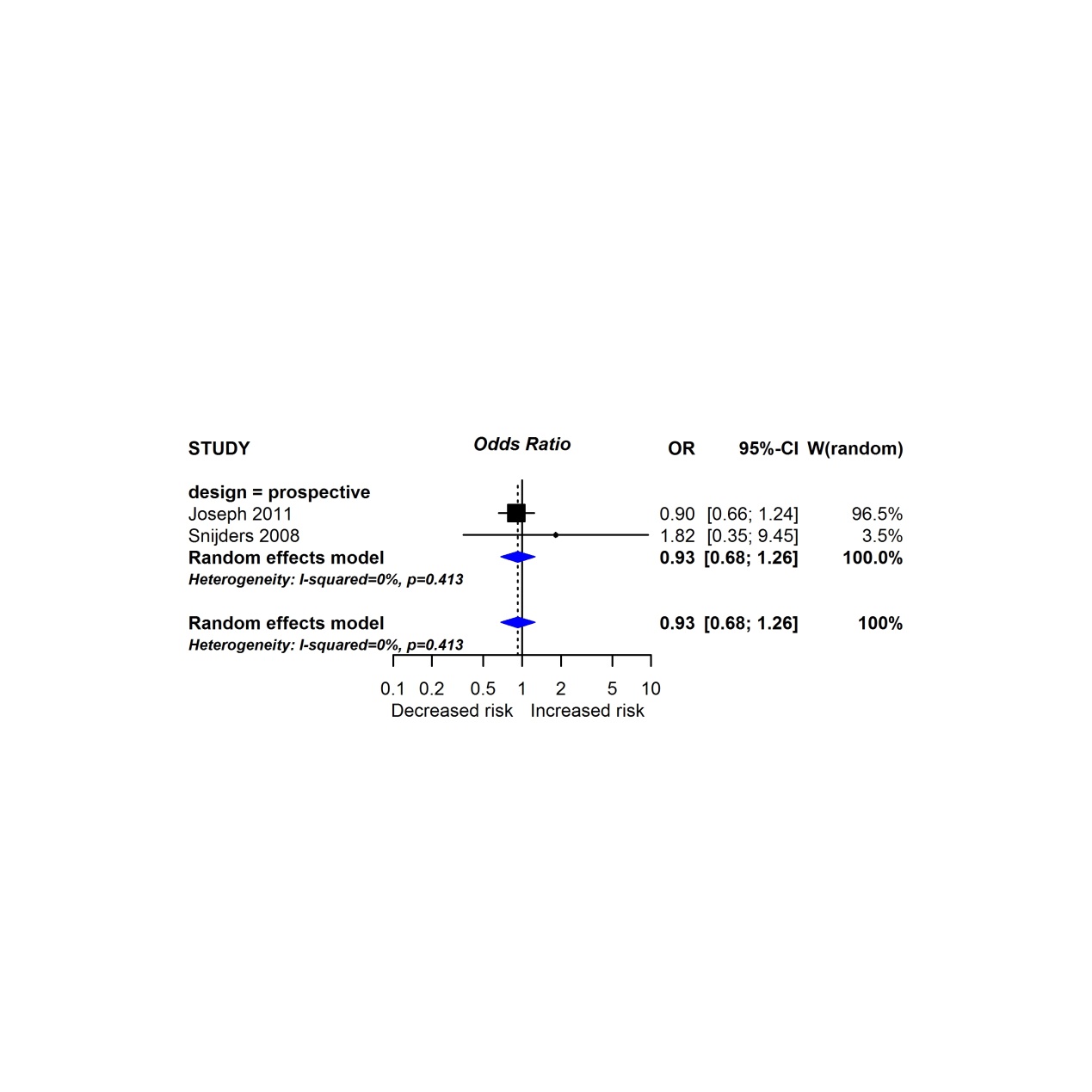


### SF and allergic sensitisation to Egg

Two prospective cohort studies reported OR for AS to Egg at age 0-4, in infants with SF ≥3-4 months vs. <3-4 months and are shown in Figure 42. There was no significant association found, with moderate statistical heterogeneity. Both studies assessed sensitisation using specific IgE blood testing at aged 2 years. The study of Snijders reported adjusted data and was considered at low risk of bias on all domains. The study of Joseph reported unadjusted data, and was therefore considered at high risk of bias.

Figure 42 SF ≥3-4 months vs. <3-4 months and risk of allergic sensitisation to Egg


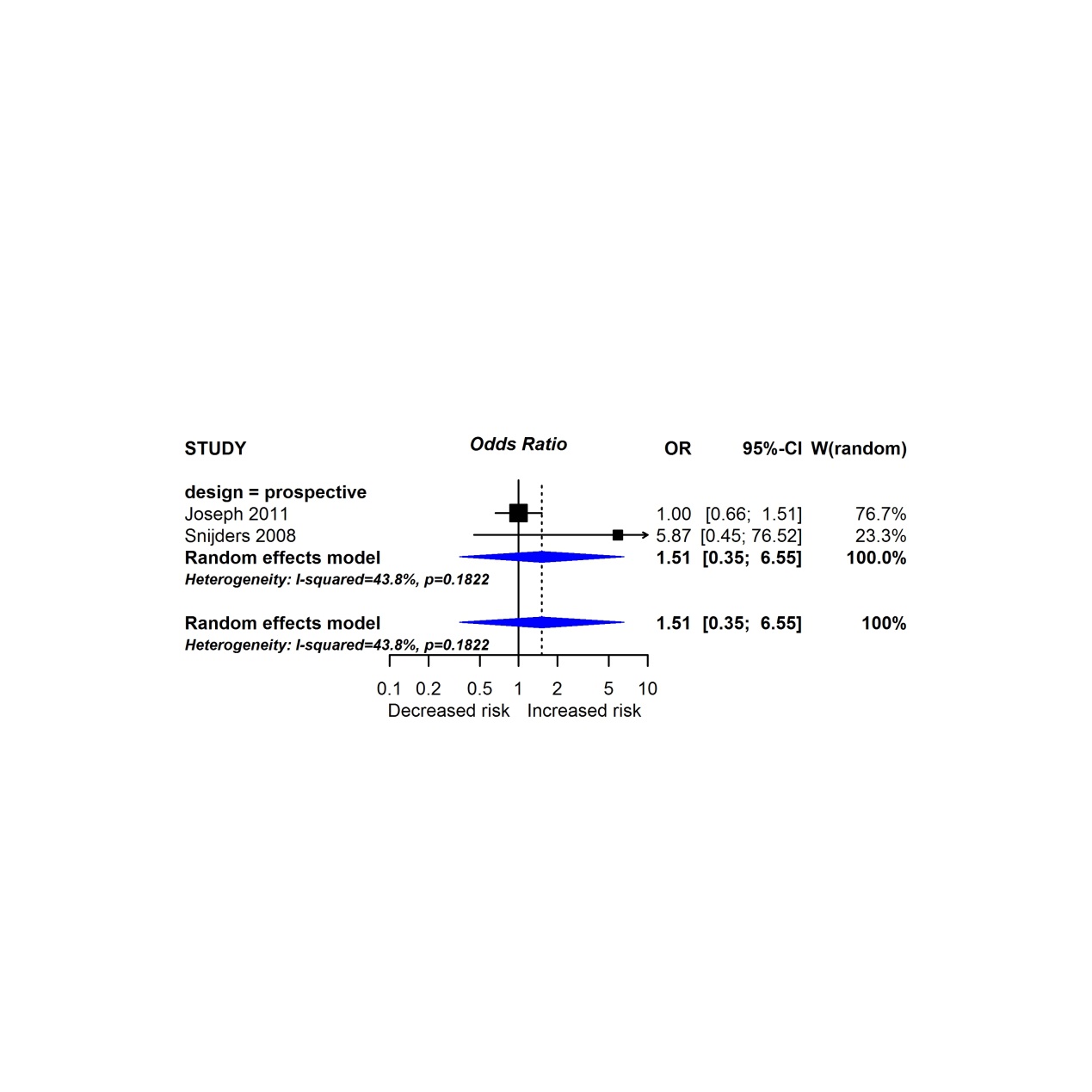


### SF and allergic sensitisation to Peanut

Two prospective cohort studies reported OR for AS to PN at age 0-4 years, in infants with SF ≥3-4 months vs. <3-4 months and are shown in Figure 43. There was no significant association found, with low statistical heterogeneity. Both studies assessed sensitisation using specific IgE blood testing at aged 2 years. The study of Snijders reported adjusted data and was considered at low risk of bias on all domains. The study of Joseph reported unadjusted data, and was therefore considered at high risk of bias. A third study Zutavern 2008 could only be reported in narrative form, and found increased PN sensitisation associated with later SF. A fourth study Sicherer 2010 could only be reported in narrative form, and found no association between SF and PN sensitisation.

#### Figure 43 SF ≥3-4 months vs. <3-4 months and risk of allergic sensitisation to Peanut


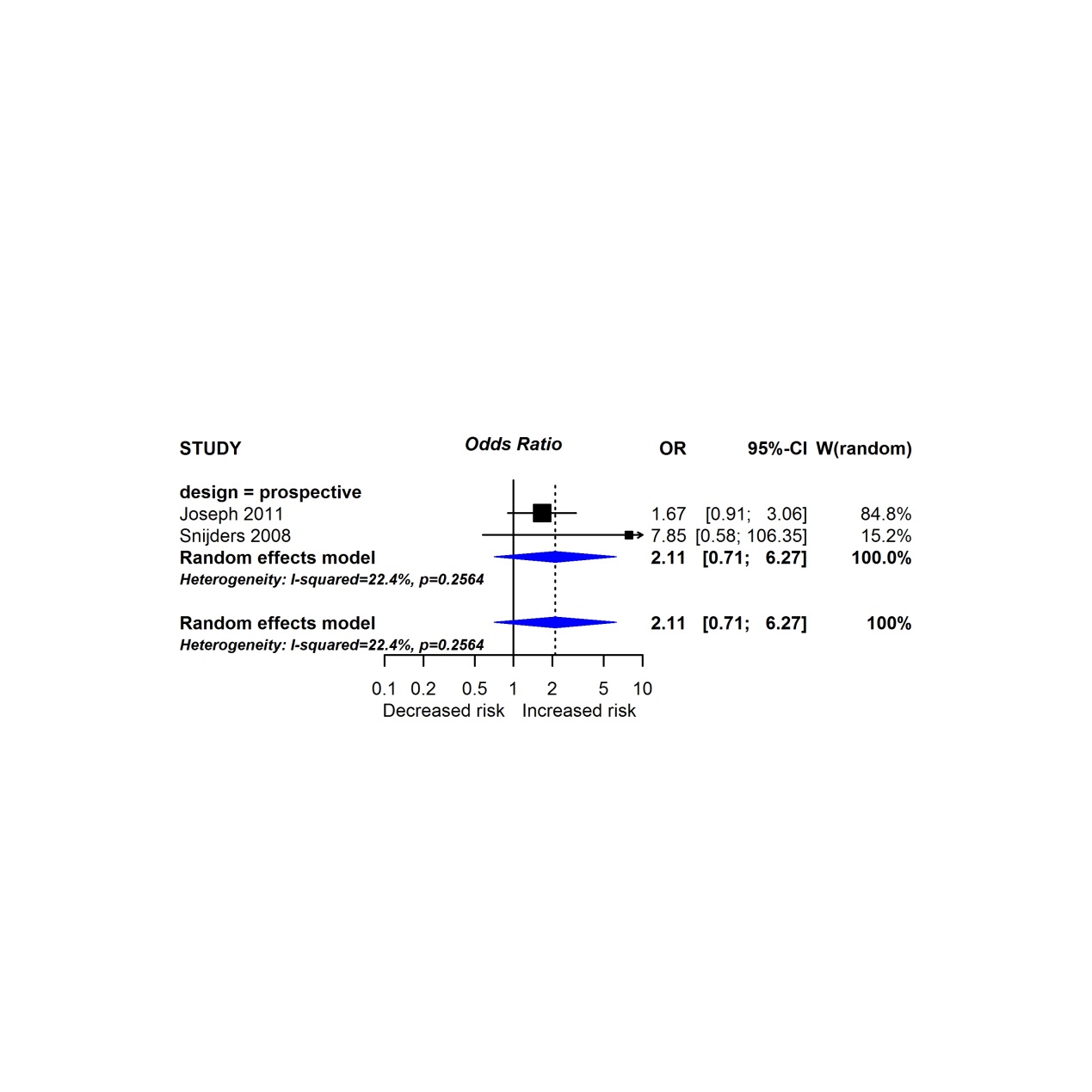


### SF and allergic sensitisation to Aeroallergen

Two prospective cohort studies reported OR for AS to any aerollergen at age 0-4 years, in infants with SF ≥3-4 months vs. <3-4 months and are shown in Figure 44. The data were not pooled due to extreme statistical heterogeneity. Both studies reported adjusted data using specific IgE testing at age 2 years, and were considered at low risk of bias on all domains. The study of Zutavern found no association; the study of Snijders found increased odds of aeroallergen sensitisation with later SF introduction. We were unable to confidently explain the heterogeneity between the findings of Snijders and Zutavern, however the study of Snijders included very few participants with SF introduction prior to 4 months (n=50), and included participants with an anthroposophic lifestyle (~20% of participants).

Figure 44 SF ≥3-4 months vs. <3-4 months and risk of allergic sensitisation to aeroallegen


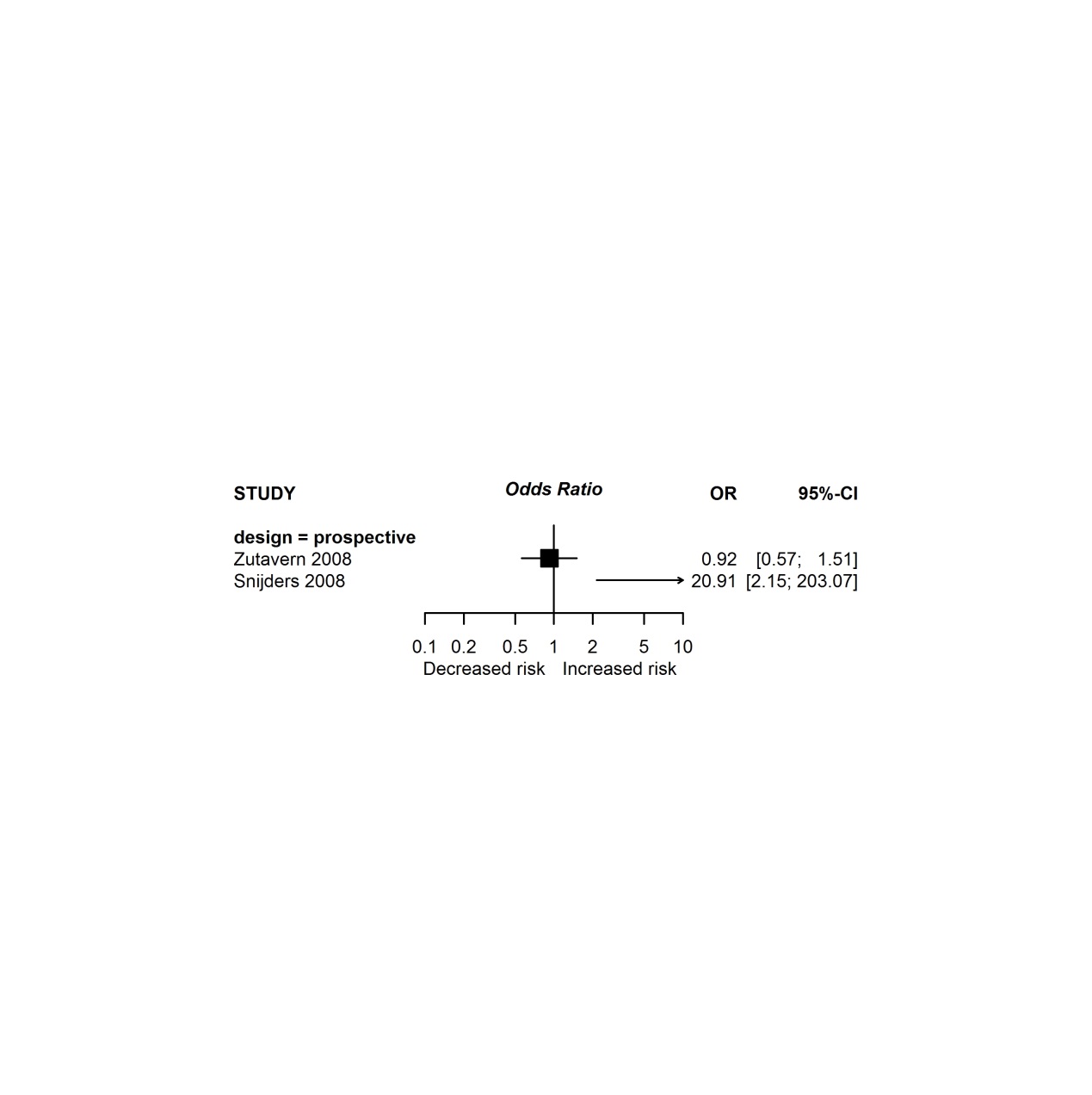


## Data for SF and AS that were not suitable for meta-analysis

No studies were identified for SF and Total IgE.

Six of the 10 included studies did not report data that could be included in a meta-analysis, and are summarised below in Table 6. In one study there was a significant association between delayed SF introduction and increase PN-AS (but not CM or Egg AS). In all other studies there was no association seen.

Table 7 Studies investigating the association between SF introduction and allergic sensitisation which were not eligible for meta-analysis

| **Study** | **Design** | **Outcome** | **Age** | **N/n** | **Data** | **Measure** | **SF in no AS** | **SF in AS** | **P** |
| --- | --- | --- | --- | --- | --- | --- | --- | --- | --- |
| Hesselmar 2010  [[2](#_ENREF_2)] | PC | sIgE-FOOD | 1.5 | 169/25 | Continuous | Median (IQR) | 4 (4, 5) | 4 (4, 5) | 0.84 |
| Kucukosmanoglu 2008  [[35](#_ENREF_35)] | CS | SPT-EGG | 1 | 1015/20 | Continuous | Mean (SD) | 4.11 (1.91) | 4.26 (1.91) | 0.77 |
| Van Asperen, 1983  [[51](#_ENREF_51)] | PC | SPT-FOOD | 1.3 | 79/13 | Categorical | No significant difference in food sensitisation in those with SF before and after 6 months | | | |
| Sicherer, 2010  [[14](#_ENREF_14)] | PC | sIgE-PN | 1 | 503/140 | Categorical | No significant association between SF timing and PN sensitisation. One analysis gave OR 1.03 (0.89, 1.18) P=0.71. | | | |
| Zutavern, 2008  [[56](#_ENREF_56)] | PC | sIgE-CM/EGG/PN | 6 | 1123/301 | Categorical | Significant association between SF >4 months and increased PN sensitisation (P<0.05), but not milk or Egg sensitisation | | | |
| Zutavern 2004 [[17](#_ENREF_17)] | PC | SPT-AERO | 5.5 | 623/103 | Categorical | No significant relationship between SF timing and AERO sensitisation | | | |

1. **Conclusion**

This report summarises the results of over 40 studies investigation the association between total and exclusive breastfeeding duration, timing of solid food introduction and risk of allergic sensitisation. The majority of studies were prospective cohort studies. We identified one systematic review and one intervention trial which found no evidence for an association between EBF duration or TBF duration respectively, and risk of AS. From the observational studies, we overall found no clear evidence to support an association between TBF or EBF duration, or SF introduction and AS. One meta-analysis found increased risk of food AS associated with EBF ≥0-2 months vs <0-2 months with no statistical heterogeneity, and one study from that meta-analysis also reported increased risk of food AS with TBF ever vs never. However there was no association seen in analysis of other cut-offs for TBF or EBF duration and food AS, no association between these exposures and other forms of allergic sensitisation (i.e. AS to milk, egg or peanut individually, AS to aeroallergens or to any allergen), or in studies which could not be included in meta-analysis. The possibility of reverse causality in those 3 studies should be considered, and was adjusted for in some studies, where they did not find evidence that early symptoms associated with food sensitisation is associated with extension of TBF or EBF duration in infancy. Overall this report has not identified evidence of a relationship between TBF, EBF, SF introduction and AS, and we did not identify any other detailed systematic review of these outcomes with which to compare our findings.

# References

1. Wright AL, Sherrill D, Holberg CJ, Halonen M, Martinez FD. Breast-feeding, maternal IgE, and total serum IgE in childhood. Journal of Allergy & Clinical Immunology 1999;104: 589-594.

2. Hesselmar B, Saalman R, Rudin A, Adlerberth I, Wold A. Early fish introduction is associated with less eczema, but not sensitization, in infants. Acta Paediatrica 2010;99: 1861-1867.

3. Elliott L, Henderson J, Northstone K, Chiu GY, Dunson D, London SJ. Prospective study of breast-feeding in relation to wheeze, atopy, and bronchial hyperresponsiveness in the Avon Longitudinal Study of Parents and Children (ALSPAC). Journal of Allergy & Clinical Immunology 2008;122: 49-54, 54.e41-43.

4. Juto P, Bjorksten B. Serum IgE in infants and influence of type of feeding. Clinical Allergy 1980;10: 593-600.

5. Hong X, Wang G, Liu X, Kumar R, Tsai HJ, Arguelles L, et al. Gene polymorphisms, breast-feeding, and development of food sensitization in early childhood. Journal of Allergy & Clinical Immunology 2011;128: 374-381.e372.

6. Burr ML, Merrett TG, Dunstan FDJ, Maguire MJ. The development of allergy in high-risk children. Clinical and Experimental Allergy 1997;27: 1247-1253.

7. Bruno G, Cantani A, Ragno V, Milita O, Ziruolo G, Businco L. Natural history of IgE antibodies in children at risk for atopy. Annals of Allergy, Asthma and Immunology 1995;74: 431-436.

8. Mihrshahi S, Ampon R, Webb K, Almqvist C, Kemp AS, Hector D, et al. The association between infant feeding practices and subsequent atopy among children with a family history of asthma. Clinical & Experimental Allergy 2007;37: 671-679.

9. Wegienka G, Ownby DR, Havstad S, Williams LK, Johnson CC. Breastfeeding history and childhood allergic status in a prospective birth cohort. Annals of Allergy, Asthma, & Immunology 2006;97: 78-83.

10. Devereux G, Turner SW, Craig LC, McNeill G, Martindale S, Harbour PJ, et al. Low maternal vitamin E intake during pregnancy is associated with asthma in 5-year-old children. American Journal of Respiratory & Critical Care Medicine 2006;174: 499-507.

11. Nwaru BI, Erkkola M, Ahonen S, Kaila M, Haapala AM, Kronberg-Kippila C, et al. Age at the introduction of solid foods during the first year and allergic sensitization at age 5 years. Pediatrics 2010;125: 50-59.

12. Rowntree S, Cogswell JJ, Platts-Mills TA, Mitchell EB. Development of IgE and IgG antibodies to food and inhalant allergens in children at risk of allergic disease. Archives of Disease in Childhood 1985;60: 727-735.

13. Saarinen UM, Bjorksten F, Knekt P, Siimes MA. Serum IgE in healthy infants fed breast milk or cow's milk-based formulas. Clinical Allergy 1979;9: 339-335.

14. Sicherer SH, Wood RA, Stablein D, Lindblad R, Burks AW, Liu AH, et al. Maternal consumption of peanut during pregnancy is associated with peanut sensitization in atopic infants. Journal of Allergy & Clinical Immunology 2010;126: 1191-1197.

15. Venter C, Pereira B, Voigt K, Grundy J, Clayton CB, Higgins B, et al. Factors associated with maternal dietary intake, feeding and weaning practices, and the development of food hypersensitivity in the infant. Pediatric Allergy and Immunology 2009;20: 320-327.

16. Oddy WH, Holt PG, Sly PD, Read AW, Landau LI, Stanley FJ, et al. Association between breast feeding and asthma in 6 year old children: findings of a prospective birth cohort study. BMJ 1999;319: 815-819.

17. Zutavern A, von Mutius E, Harris J, Mills P, Moffatt S, White C, et al. The introduction of solids in relation to asthma and eczema. Archives of Disease in Childhood 2004;89: 303-308.

18. Shaheen SO, Aaby P, Hall AJ, Barker DJ, Heyes CB, Shiell AW, et al. Measles and atopy in Guinea-Bissau. Lancet 1996;347: 1792-1796.

19. Gustafsson D, Sjoberg O, Foucard T. Development of allergies and asthma in infants and young children with atopic dermatitis--a prospective follow-up to 7 years of age. Allergy 2000;55: 240-245.

20. Allen KJ, Koplin J, Gurrin L, Gibson M, Thiele L, Miles L, et al. Prevalence and environmental predictors of food allergy in infants. Journal of Allergy and Clinical Immunology 2009;1: S108.

21. Hoppu U, Kalliomaki M, Isolauri E. Maternal diet rich in saturated fat during breastfeeding is associated with atopic sensitization of the infant. European Journal of Clinical Nutrition 2000;54: 702-705.

22. Huurre A, Laitinen K, Rautava S, Korkeamäki M, Isolauri E. Impact of maternal atopy and probiotic supplementation during pregnancy on infant sensitization: a double-blind placebo-controlled study. Clinical and experimental allergy : journal of the British Society for Allergy and Clinical Immunology [Internet]. 2008; 38(8):[1342-1348 pp.]. Available from: <http://onlinelibrary.wiley.com/o/cochrane/clcentral/articles/465/CN-00648465/frame.html>

23. Kemeny DM, Price JF, Richardson V, Richards D, Lessof MH. The IgE and IgG subclass antibody-response to foods in babies during the 1^st^ year of life and their relationship to feeding regimen and the development of food allergy. Journal of Allergy and Clinical Immunology 1991;87: 920-929.

24. Snijders BE, Thijs C, van Ree R, van den Brandt PA. Age at first introduction of cow milk products and other food products in relation to infant atopic manifestations in the first 2 years of life: the KOALA Birth Cohort Study. Pediatrics 2008;122: e115-122.

25. Snijders BE, Thijs C, Dagnelie PC, Stelma FF, Mommers M, Kummeling I, et al. Breast-feeding duration and infant atopic manifestations, by maternal allergic status, in the first 2 years of life (KOALA study). Journal of Pediatrics 2007;151: 347-351, 351.e341-342.

26. Kusel MM, Holt PG, de Klerk N, Sly PD. Support for 2 variants of eczema. Journal of Allergy & Clinical Immunology 2005;116: 1067-1072.

27. Wetzig H, Schulz R, Diez U, Herbarth O, Viehweg B, Borte M. Associations between duration of breast-feeding, sensitization to hens' eggs and eczema infantum in one and two year old children at high risk of atopy. International Journal of Hygiene & Environmental Health 2000;203: 17-21.

28. Strachan DP, Harkins LS, Johnston IDA, Anderson HR. Childhood antecedents of allergic sensitization in young British adults. Journal of Allergy and Clinical Immunology 1997;99: 6-12.

29. Silvers KM, Frampton CM, Wickens K, Epton MJ, Pattemore PK, Ingham T, et al. Breastfeeding protects against adverse respiratory outcomes at 15 months of age. Maternal & Child Nutrition 2009;5: 243-250.

30. Scholtens S, Wijga AH, Brunekreef B, Kerkhof M, Hoekstra MO, Gerritsen J, et al. Breast feeding, parental allergy and asthma in children followed for 8 years. The PIAMA birth cohort study. Thorax 2009;64: 604-609.

31. Hagendorens MM, Bridts CH, Lauwers K, van Nuijs S, Ebo DG, Vellinga A, et al. Perinatal risk factors for sensitization, atopic dermatitis and wheezing during the first year of life (PIPO study). Clinical and Experimental Allergy 2005;35: 733-740.

32. Friday GA, Jr., Smith J. Breast-feeding and atopic dermatitis. Pediatric Asthma, Allergy and Immunology 2000;14: 205-209.

33. Rona RJ, Smeeton NC, Bustos P, Amigo H, Diaz PV. The early origins hypothesis with an emphasis on growth rate in the first year of life and asthma: a prospective study in Chile. Thorax 2005;60: 549-554.

34. Kuyucu S, Saraclar Y, Tuncer A, Sackesen C, Adalioglu G, Sumbuloglu V, et al. Determinants of atopic sensitization in Turkish school children: effects of pre- and post-natal events and maternal atopy. Pediatric Allergy & Immunology 2004;15: 62-71.

35. Kucukosmanoglu E, Yazi D, Yesil O, Akkoc T, Gezer M, Bakirci N, et al. Prevalence of egg sensitization in Turkish infants based on skin prick test. Allergologia Et Immunopathologia 2008;36: 141-144.

36. Kuehr J, Frischer T, Karmaus W, Meinert R, Barth R, Herrmankunz E, et al. EARLY-CHILDHOOD RISK-FACTORS FOR SENSITIZATION AT SCHOOL AGE. Journal of Allergy and Clinical Immunology 1992;90: 358-363.

37. Kramer MS, Kakuma R. Optimal duration of exclusive breastfeeding. Cochrane Database of Systematic Reviews 2012;8: CD003517.

38. Kull I, Almqvist C, Lilja G, Pershagen G, Wickman M. Breast-feeding reduces the risk of asthma during the first 4 years of life. Journal of Allergy & Clinical Immunology 2004;114: 755-760.

39. Kull I, Melen E, Alm J, Hallberg J, Svartengren M, van Hage M, et al. Breast-feeding in relation to asthma, lung function, and sensitization in young schoolchildren. Journal of Allergy & Clinical Immunology 2010;125: 1013-1019.

40. Businco L, Bruno G, Grandolfo ME, Novello F, Fiove L, Amato C. Soy formula feeding and immunological response in babies of atopic families. Lancet 1989;2: 625-626.

41. Cogswell JJ, Mitchell EB, Alexander J. Parental smoking, breast feeding, and respiratory infection in development of allergic diseases. Archives of Disease in Childhood 1987;62: 338-344.

42. Schoetzau A, Filipiak-Pittroff B, Franke K, Koletzko S, Von Berg A, Gruebl A, et al. Effect of exclusive breast-feeding and early solid food avoidance on the incidence of atopic dermatitis in high-risk infants at 1 year of age. Pediatric Allergy & Immunology 2002;13: 234-242.

43. Arshad SH, Hide DW. Effect of environmental factors on the development of allergic disorders in infancy. Journal of Allergy & Clinical Immunology 1992;90: 235-241.

44. Gruber C, van Stuijvenberg M, Mosca F, Moro G, Chirico G, Braegger CP, et al. Reduced occurrence of early atopic dermatitis because of immunoactive prebiotics among low-atopy-risk infants. Journal of Allergy & Clinical Immunology 2010;126: 791-797.

45. Odelram H, Vanto T, Jacobsen L, Kjellman NIM. Whey hydrolysate compared with cow's milk-based formula for weaning at about 6 months of age in high allergy-risk infants: Effects on atopic disease and sensitization. Allergy: European Journal of Allergy and Clinical Immunology 1996;51: 192-195.

46. Poysa L, Korppi M, Remes K, Juntunen-Backman K. Predictive value of IgE levels in infancy. Acta Paediatrica Scandinavica 1990;79: 970-972.

47. Poysa L, Pulkkinen A, Korppi M, Remes K, Juntunen-Backman K. Diet in infancy and bronchial hyperreactivity later in childhood. Pediatric Pulmonology 1992;13: 215-221.

48. Kramer MS, Matush L, Bogdanovich N, Dahhou M, Platt RW, Mazer B. The low prevalence of allergic disease in Eastern Europe: are risk factors consistent with the hygiene hypothesis? Clinical & Experimental Allergy 2009;39: 708-716.

49. Siltanen M, Kajosaari M, Poussa T, Saarinen KM, Savilahti E. A dual long-term effect of breastfeeding on atopy in relation to heredity in children at 4 years of age. Allergy 2003;58: 524-530.

50. Strassburger SZ, Vitolo MR, Bortolini GA, Pitrez PM, Jones MH, Stein RT. Nutritional errors in the first months of life and their association with asthma and atopy in preschool children. Jornal de Pediatria 2010;86: 391-399.

51. Van Asperen PP, Kemp AS, Mellis CM. Relationship of diet in the development of atopy in infancy. Clinical Allergy 1984;14: 525-532.

52. Vandenplas Y, Deneyer M, Sacre L, Loeb H. Preliminary data on a field study with a new hypo-allergic formula. European Journal of Pediatrics 1988;148: 274-277.

53. Oddy WH, Sherriff JL, de Klerk NH, Kendall GE. Breastfeeding, body mass index, and asthma and atopy in children. Advances in Experimental Medicine & Biology 2004;554: 387-390.

54. Kramer MS, Matush L, Vanilovich I, Platt R, Bogdanovich N, Sevkovskaya Z, et al. Effect of prolonged and exclusive breast feeding on risk of allergy and asthma: cluster randomised trial. BMJ (Clinical research ed.) 2007;335: 815.

55. Zutavern A, Brockow I, Schaaf B, Bolte G, von Berg A, Diez U, et al. Timing of solid food introduction in relation to atopic dermatitis and atopic sensitization: results from a prospective birth cohort study. Pediatrics 2006;117: 401-411.

56. Zutavern A, Brockow I, Schaaf B, von Berg A, Diez U, Borte M, et al. Timing of solid food introduction in relation to eczema, asthma, allergic rhinitis, and food and inhalant sensitization at the age of 6 years: results from the prospective birth cohort study LISA. Pediatrics 2008;121: e44-52.

57. Joseph CL, Ownby DR, Havstad SL, Woodcroft KJ, Wegienka G, MacKechnie H, et al. Early complementary feeding and risk of food sensitization in a birth cohort. Journal of Allergy & Clinical Immunology 2011;127: 1203-1210.e1205.
