# Supplementary material for: Diet during pregnancy and infancy and risk of allergic or autoimmune disease: A systematic review and meta-analysis
Source: PLoS Med. 2018 Feb 28;15(2):e1002507. doi: 10.1371/journal.pmed.1002507 (PMC5830033; doi:10.1371/journal.pmed.1002507)
Supplement: S1 Data — (ZIP) [file pmed.1002507.s006.zip › Review_A_reports/AUTOIMMUNE DISEASE.docx]

**Breastfeeding, Solid Food Introduction and Autoimmune Diseases**

Natalie Geoghegan^1^, Despo Ierodiakonou^2^, Vanessa Garcia-Larsen^3^, Jo Leonardi-Bee^4^, Tim Reeves^5^, Jennifer Chivinge^1^, Zoe Robinson^1^, Katharine Jarrold^1^, Evangelia Andreou^6^, Nara Tagiyeva-Milne^7^, Ulugbek Nurmatov^8^, Sergio Cunha^9^, Robert J Boyle^10^

^1^ Undergraduate medical students, Imperial College London; ^2^ Post-Doctoral Research Associate, Departments of Paediatric and Respiratory Epidemiology and Public Health Group, Imperial College London, ^3^ Post-Doctoral Research Associate & Honorary Research Fellow, Royal Brompton Hospital and Harefield NHS Foundation Trust, Respiratory Epidemiology and Public Health, National Heart and Lung Institute, Imperial College London; ^4^Associate Professor of Community Health Sciences, University of Nottingham; ^5^ Research Support Librarian, Faculty of Medicine, Imperial College London; ^6^ Research Associate, Imperial Consultants; ^7^ Research Fellow, University of Aberdeen; ^8^ Research Fellow, University of Edinburgh; ^9^ Research Associate, Respiratory Epidemiology and Public Health, National Heart and Lung Institute, Imperial College London; ^10^ Clinical Senior Lecturer, Section of Paediatrics, Imperial College London

Imperial Consultants,

58 Princes Gate,

Exhibition Road,

London SW7 2PG

Contents

[**List of Figures** 4](#_Toc416350424)

[**List of Tables** 5](#_Toc416350425)

[**1 Total breastfeeding duration and Autoimmune Diseases** 7](#_Toc416350426)

[1.1 Total breastfeeding duration and Coeliac Disease 7](#_Toc416350427)

[1.1.1 Overall characteristics of studies, risk of bias and summary of results 7](#_Toc416350428)

[1.1.2 Total Breastfeeding duration and Coeliac Disease 12](#_Toc416350429)

[1.2 Total breastfeeding duration and inflammatory bowel disease 19](#_Toc416350430)

[1.2.1 Overall characteristics of studies, risk of bias and summary of results 19](#_Toc416350431)

[1.2.2 Total Breastfeeding duration and Inflammatory Bowel Disease 24](#_Toc416350432)

[1.3 Total breastfeeding duration and Thyroid disease 33](#_Toc416350433)

[1.3.1 Overall characteristics of studies, risk of bias and summary of results 33](#_Toc416350434)

[1.3.2 Data for TBF and thyroid disease which were not suitable for meta-analysis 35](#_Toc416350435)

[1.4 Total breastfeeding duration and juvenile rheumatoid arthritis 36](#_Toc416350436)

[1.4.1 Overall characteristics of studies, risk of bias and summary of results 36](#_Toc416350437)

[1.4.2 Total Breastfeeding duration and juvenile rheumatoid arthritis 38](#_Toc416350438)

[1.4.3 Total Breastfeeding dose-response and juvenile rheumatoid arthritis 39](#_Toc416350439)

[**2 Exclusive breastfeeding duration and Autoimmune Diseases** 41](#_Toc416350440)

[2.1 Exclusive breastfeeding duration and Coeliac Disease 41](#_Toc416350441)

[2.1.1 Overall characteristics of studies, risk of bias and summary of results 41](#_Toc416350442)

[2.1.2 Exclusive breastfeeding duration and Coeliac Disease 44](#_Toc416350443)

[2.2 Exclusive breastfeeding duration and inflammatory bowel disease 47](#_Toc416350444)

[2.2.1 Overall characteristics of studies, risk of bias and summary of results 47](#_Toc416350445)

[2.2.2 Data for EBF duration and IBD which were not suitable for meta-analysis 49](#_Toc416350446)

[**3 Solid food introduction and Autoimmune Disease** 50](#_Toc416350447)

[3.1 Solid food introduction and Coeliac Disease 50](#_Toc416350448)

[3.1.1 Overall characteristics of studies, risk of bias and summary of results 50](#_Toc416350449)

[3.1.2 Solid food introduction and Coeliac Disease 52](#_Toc416350450)

[3.2 Solid food introduction and inflammatory bowel disease 52](#_Toc416350451)

[3.2.1 Overall characteristics of studies, risk of bias and summary of results 52](#_Toc416350452)

[3.2.2 Data for solid food introduction and IBD which were not suitable for meta-analysis 54](#_Toc416350453)

[3.3 Solid food introduction and juvenile rheumatoid arthritis 54](#_Toc416350454)

[3.3.1 Overall characteristics of studies, risk of bias and summary of results 54](#_Toc416350455)

[3.3.2 Data for solid food introduction and juvenile rheumatoid arthritis which were not suitable for meta-analysis 55](#_Toc416350456)

[3.4 Solid food introduction and Thyroid disease 56](#_Toc416350457)

[3.4.1 Overall characteristics of studies, risk of bias and summary of results 56](#_Toc416350458)

[3.4.2 Data for solid food introduction and thyroid disease which were not suitable for meta-analysis 58](#_Toc416350459)

[**4 Conclusion** 59](#_Toc416350460)

[**5 References** 61](#_Toc416350461)

# List of Figures

[Figure 1 Risk of bias in studies of TBF duration and Coeliac Disease 11](#_Toc416349766)

[Figure 2 TBF any vs. never and risk of Coeliac Disease 14](#_Toc416349767)

[Figure 3 TBF ≥1-2 months vs. <1-2 months and risk of Coeliac Disease 15](#_Toc416349768)

[Figure 4 TBF ≥3-4 months vs. <3-4 months and risk of Coeliac Disease 15](#_Toc416349769)

[Figure 5 TBF short vs. never and risk of Coeliac Disease 16](#_Toc416349770)

[Figure 6 TBF medium vs. never and risk of Coeliac Disease 16](#_Toc416349771)

[Figure 7 TBF long vs. never and risk of Coeliac Disease in children 16](#_Toc416349772)

[Figure 8 Risk of bias in studies of TBF duration and inflammatory bowel disease 24](#_Toc416349773)

[Figure 9 TBF any vs. never and risk of IBD 24](#_Toc416349774)

[Figure 10 TBF ≥5-7 months vs. <5-7 months and risk of IBD 25](#_Toc416349775)

[Figure 11 TBF any vs. never and risk and risk of Crohn’s Disease 26](#_Toc416349776)

[Figure 12 TBF ≥1-2 months vs. <1-2 months and risk of Crohn’s Disease 28](#_Toc416349777)

[Figure 13 TBF ≥3-4 months vs. <3-4 months and risk of Crohn’s Disease 28](#_Toc416349778)

[Figure 14 TBF ≥5-7 months vs. <5-7 months and risk of Crohn’s Disease 29](#_Toc416349779)

[Figure 15 TBF any vs. never and risk and risk of ulcerative colitis 29](#_Toc416349780)

[Figure 16 TBF ≥5-7 months vs. <5-7 months and risk of ulcerative colitis 31](#_Toc416349781)

[Figure 17 Risk of bias in studies of TBF duration and Thyroid disease 34](#_Toc416349782)

[Figure 18 Risk of bias in studies of TBF duration and juvenile rheumatoid arthritis 38](#_Toc416349783)

[Figure 19 TBF any vs. never and risk of juvenile rheumatoid arthritis 38](#_Toc416349784)

[Figure 20 TBF any vs. never and risk of pauciarticular juvenile rheumatoid arthritis 39](#_Toc416349785)

[Figure 21 TBF any vs. never and risk of polyarticular juvenile rheumatoid arthritis 39](#_Toc416349786)

[Figure 22 TBF short vs never and risk of juvenile rheumatoid arthritis 40](#_Toc416349787)

[Figure 23 TBF medium vs never and risk of juvenile rheumatoid arthritis 40](#_Toc416349788)

[Figure 24 Risk of bias in studies of EBF duration and coeliac disease 44](#_Toc416349789)

[Figure 25 EBF ≥0-2 months vs <0-2 months and risk of Coeliac Disease 44](#_Toc416349790)

[Figure 26 EBF ≥3-4 months vs <3-4 months and risk of Coeliac Disease 45](#_Toc416349791)

[Figure 27 Risk of bias in studies of EBF duration and inflammatory bowel disease 48](#_Toc416349792)

[Figure 28 Risk of bias in studies of solid food introduction and Coeliac Disease 51](#_Toc416349793)

[Figure 29 SF ≥3-4 months vs. <3-4 months and risk of Coeliac Disease 52](#_Toc416349794)

[Figure 30 Risk of bias in studies of solid food introduction and inflammatory bowel disease 53](#_Toc416349795)

[Figure 31 Risk of bias in studies of solid food introduction and Juvenile rheumatoid arthritis 55](#_Toc416349796)

[Figure 32 Risk of bias in studies of solid food introduction and Thyroid disease 57](#_Toc416349797)

# List of Tables

[Table 1 Characteristics of included studies evaluating TBF duration and Coeliac Disease 9](#_Toc416350746)

[Table 2 Other studies evaluating TBF duration and coeliac disease which were not suitable for meta-analysis 18](#_Toc416350747)

[Table 3 Characteristics of included studies evaluating TBF duration and inflammatory bowel disease 21](#_Toc416350748)

[Table 4 Subgroup Analyses of risk of TBF any vs. never and the risk of Crohn’s disease 27](#_Toc416350749)

[Table 5 Subgroup Analyses of risk of TBF any vs. never and the risk of ulcerative colitis 30](#_Toc416350750)

[Table 6 Studies which could not be included in meta-analysis of TBF and IBD 32](#_Toc416350751)

[Table 7 Characteristics of included studies evaluating TBF duration and Thyroid disease 34](#_Toc416350752)

[Table 8 Study evaluating TBF duration and thyroid disease which were not suitable for meta-analysis 35](#_Toc416350753)

[Table 9 Characteristics of included studies evaluating TBF duration and juvenile rheumatoid arthritis 37](#_Toc416350754)

[Table 10 Characteristics of included studies evaluating EBF duration and Coeliac Disease 43](#_Toc416350755)

[Table 11 Other studies reporting data on EBF duration and coeliac disease which couldn’t be meta-analysis 46](#_Toc416350756)

[Table 12 Characteristics of included studies evaluating EBF duration and inflammatory bowel disease 48](#_Toc416350757)

[Table 13 Studies reporting data on EBF duration and IBD which were not suitable for meta-analysis 49](#_Toc416350758)

[Table 14 Characteristics of included studies evaluating solid food introduction and Coeliac Disease 51](#_Toc416350759)

[Table 15 Characteristics of included studies evaluating solid food introduction and inflammatory bowel disease 53](#_Toc416350760)

[Table 16 Studies reporting solid food introduction and IBD which were not suitable for meta-analysis 54](#_Toc416350761)

[Table 17 Characteristics of included studies evaluating solid food introduction and juvenile rheumatoid arthritis 55](#_Toc416350762)

[Table 18 Studies reporting solid food introduction and juvenile rheumatoid arthritis which were not suitable for meta-analysis 55](#_Toc416350763)

[Table 19 Characteristics of included studies evaluating solid food introduction and Thyroid disease 57](#_Toc416350764)

[Table 20 Data for solid food introduction and thyroid disease 58](#_Toc416350765)

# Total breastfeeding duration and Autoimmune Diseases

## Total breastfeeding duration and Coeliac Disease

### Overall characteristics of studies, risk of bias and summary of results

Table 1 describes the main characteristics of the studies analysed in this report. A total of 1 systematic review including 11 observational studies, and 9 further observational studies reported the association between duration of breastfeeding (namely ‘any vs. never’, ‘≥1-2 months vs. <1-2 months’ and ‘≥3-4 months vs. <3-4 months’) and risk of coeliac disease. Of the original studies, 3 were prospective cohort studies and 6 were case-control studies. Most of the studies (n=7) were from Europe, with one from North America and one with origin unknown. Overall, relevant data on total breastfeeding duration in the first year of life (TBF) and coeliac disease was available from 15,108 subjects in the original studies, and over 250,000 subjects in the systematic review. Information on coeliac disease was obtained from serology (autoantibodies to transglutaminase, here termed IgA-tTG) in the 3 prospective studies and via medical diagnosis using ESPGHAN criteria in the remaining (case control) studies; method of outcome assessment was unclear in one study. With regards to time of outcome diagnosis, 5 studies explored the association between exposure to breastfeeding and coeliac disease in the first 5 years of life and others evaluated coeliac disease in older children or young adults. All studies used interview or questionnaire to assess the exposure (TBF), with 3 studies combining this with diary or medical record information.

Risk of bias in original studies was assessed using the NICE Methodological checklists for cohort and case-control studies. Figure 1 illustrates the distribution of bias across the five main methodological areas of the studies. Over 80% of studies had a high risk of bias, most commonly due to lack of adjustment for confounding bias i.e. no adjusted data presented.

Risk of coeliac disease was measured in relation to each of the three cut-offs for breastfeeding duration mentioned above. Dose response relationships were assessed by analysing risk of coeliac disease according to total breastfeeding duration ‘ever vs never’, ‘short duration vs never’, ‘medium duration vs never’ and ‘long duration vs never’.

*Main Findings*

Meta-analyses in this review were characterised by significant clinical and statistical heterogeneity, and the majority of cases and studies could not be included in meta-analysis. While some meta-analyses found an association between increased TBF duration and reduced coeliac disease risk, those studies which could not be included in meta-analysis more often reported increased TBF duration associated with increased coeliac disease risk. Thus these data must be interpreted as inconclusive, requiring further investigation. Certainly the available data have not reported a consistent relationship between TBF duration and coeliac disease, but many studies only reported unadjusted data and therefore carry a high risk of bias. So a significant association between TBF and coeliac disease cannot be confidently excluded based on the available data.

Table 1 Characteristics of included studies evaluating TBF duration and Coeliac Disease

| **Study** | **Design** | **N/n cases** | **Exposure assessment** | **Method of outcome assessment** | **Age at outcome (years)** | **Country** | **Population characteristics** |
| --- | --- | --- | --- | --- | --- | --- | --- |
| Welander, 2010 [[1](#_ENREF_1)] | PC | 9414/~29 | D/I | IgA-tTG and biopsy | 8.4 | Sweden | ABIS study. Population based study of babies born between Oct 1997 and Oct 1999. |
| Ascher, 1997 [[2](#_ENREF_2)] | CC | 81/8 | I | ESPGHAN criteria | <18 | Sweden | Cases of coeliac disease were compared with the siblings at high genetic risk (DQA1*0501-DQB1*02), in whom the diagnosis was excluded |
| Auricchio, 1983 [[3](#_ENREF_3)] | CC | 437/190 | R/I | ESPGHAN criteria | <18 | Italy | Source of cases unknown, controls unaffected siblings |
| Roberts 2009 [[4](#_ENREF_4)] | CC | 248521/ 90 | R | ICD codes 269.0 (ICD-8) or 579.0 (ICD-9) or K90.0 (ICD-10 | <24 | UK | Cases identified from hospital admission codes, controls the rest of the population with linked record data |
| Decker 2010 [[5](#_ENREF_5)] | CC | 866/123 | Q | Medical diagnosis | <18 | Germany | Cases from paediatric gastroenterology clinics; controls from ophthalmology and dental clinics |
| Norris, 2005 [[6](#_ENREF_6)] | PC | 1560 | Q/I | IgA-tTG | <5 | USA | DAISY study. Children at increased risk for T1DM were enrolled at birth from 1993 to 2006 and/or identified by newborn screening for HLA genotype |
| Falth-Magnusson, 1996 [[7](#_ENREF_7)] | CC | 336/72 | R/Q | ESPGHAN criteria | <2 | Sweden | Cases from paediatric department records, born in 1987-1989. Reference children were age matched from same county. |
| Ziegler, 2003 [[8](#_ENREF_8)] | PC | 1460/81 | Q | IgA-tTG | 5 | Germany | German BABYDIAB study. Offspring of mothers and/or fathers with T1DM born in Germany between 1989 and 2000 |
| Ivarsson, 2002 [[9](#_ENREF_9)] | CC | 1272/392 | Q | ESPGHAN criteria | 2, 15 | Sweden | Cases selected from CD Register born in 1992-1996 and sex age and area matched controls from the national population register |
| Pacilio, 2010 [[10](#_ENREF_10)] | CC | 278/139 | Unclear | Unclear | 2 | Not known | Unclear source of cases and controls. Cases aged 0.5-2 years old with age matched healthy controls |
| Peters, 2001 [[11](#_ENREF_11)] | CC | 270/133 | Q | ESPGHAN criteria | < 10 | Germany | All newly diagnosed patients aged <10 years old were identified from paediatricians and a biannual meeting of the German Coeliac Disease Society in 1985–1995. Sex and aged matched control selected from population registry |

Q: questionnaire, I: interview, R: medical records, PC: prospective cohort, NCC: nested case control, CS: Cross-sectional, CC: case control

Figure 1 Risk of bias in studies of TBF duration and Coeliac Disease

###

### Total Breastfeeding duration and Coeliac Disease

#### Evidence from prior systematic reviews

Overall there was no evidence for an association between duration of exclusive/predominant breastfeeding and coeliac disease.

Szajewska et al identified 11 studies evaluating this relationship, 6 of which were previously included in a systematic review by Akobeng et al which had concluded that a short duration of breastfeeding predisposed to coeliac disease [[12](#_ENREF_12)]. The 11 studies and their findings are summarised below – the conclusion of Szajewska is that there is no evidence of a relationship between duration of breastfeeding and risk of coeliac disease. Meta-analysis was not undertaken in either review, due to heterogeneity of studies.

All studies identified by Szajewska were identified in our review, under TBF or EBF as the exposure, and four of them could be included in at least one meta-analysis in our review, together with a more recent study which we identified in our own systematic review of original studies.

**Table 4. Relationship between duration of breastfeeding and autoimmune outcomes (coeliac disease) -** data from Szajewska et al 2012 [[12](#_ENREF_12)]

| **Study ID** | **Study design** | **No. participants** | **Exposure/Comparison** | **Outcome** |
| --- | --- | --- | --- | --- |
| Auricchio 1983 | Case control | 505 | Breastfed for >30 days versus less | OR 4.05 [2.20, 7.27] for CD with short duration |
| Ascher 1997 | Case control | 81 | Duration of BF in CD versus controls | No association |
| Falth-Magnusson 1996 | Case control | 336 | Duration of BF in CD versus controls | Median 2.5 months CD; 4 months controls P=0.003 |
| Greco 1988* | Case control | 2150 | BF for >90 days versus less | OR 4.97 [3.5, 6.9] for CD with short duration |
| Ivarsson 2002 | Case control | 1272 | Duration of BF in CD versus controls | Children <2 median 5 months CD; 7 months controls  P <0.001  Children >2: No significant difference |
| Peters 2001 | Case control | 280 | BF for >2months versus less | OR 0.37 [0.21, 0.64] for CD with long duration |
| Decker 2010 | Case control | 866 | Duration of BF in CD versus controls | No difference |
| Norris 2005 | Cohort | 1560 | Duration of BF in CD serology positive versus negative | Mean 8.3 months CD, 6.7 months controls NS |
| Roberts 2008 | Cohort | 248,521 | Duration of BF in CD versus controls | No association |
| Welander 2010 | Case control | 9364 | Duration of BF in CD versus controls | No association |
| Ziegler 2003 | Cohort | 1610 | Duration of BF in CD serology positive versus negative | No association |

BF breastfeeding; CD coeliac disease

*This study is included in EBF in our analysis, because BF exposure was judged to represent EBF rather than TBF exposure

#### Evidence from original studies and new meta-analyses

##### TBF Any vs. Never

Figure 2 shows the combined effect of all studies investigating the association between any duration of breastfeeding versus never breastfeeding, and coeliac disease risk. Overall there was a significant reduction in risk of disease in infants who were breast fed (OR 0.40, 95% CI 0.16, 0.98) with high heterogeneity between studies (I^2^=57.3%). Subgroup and stratified analyses was not performed due to the small number of studies included. Pacilio presented unadjusted data in a case control study comparing TBF ever versus never; Ziegler presented adjusted HR in a prospective cohort study of high risk participants (family history of autoimmune disease) comparing TBF 3-6 months versus never; Peters presented adjusted OR in a case-control study comparing TBF ≥7 months versus never. The high statistical heterogeneity may be explained by this heterogeneity in study design and analysis.

Figure 2 TBF any vs. never and risk of Coeliac Disease


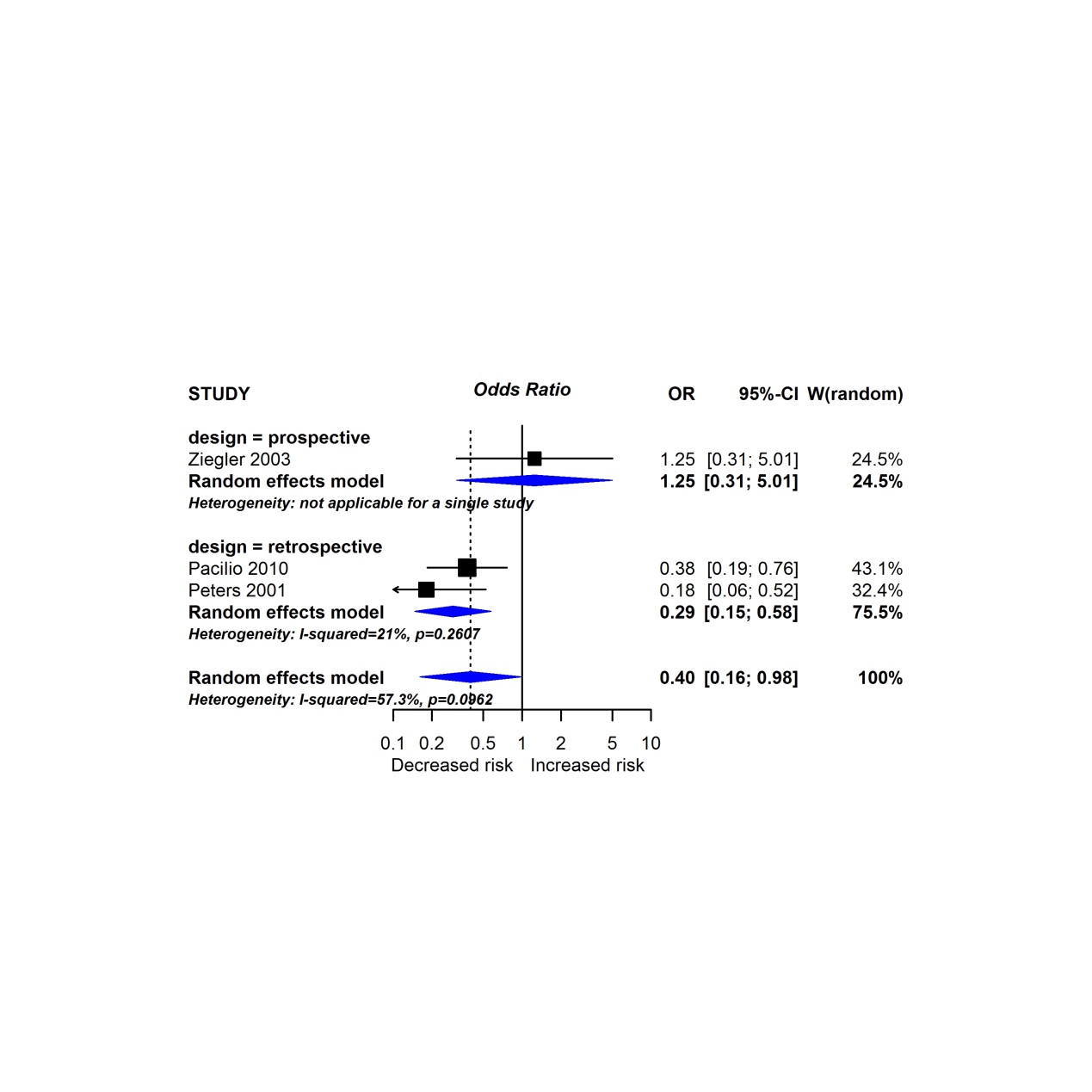


##### TBF ≥1-2 months vs. <1-2 months

Three studies examined the risk of coeliac disease in infants who were breast fed for over 1-2 months compared to less than this duration. Figure 3 shows that the combined risk of T1DM is significantly lower if infants were breastfed for at least 1-2 months, (OR 0.48, 95% CI 0.31, 074) with moderate heterogeneity between studies (I^2^=31.7%). Welander presented unadjusted HR from a prospective cohort study comparing TBF ≥11-12 vs 0-2 months; Peters presented adjusted OR from a case control study comparing TBF ≥2 vs <2 months; Auricchio presented unadjusted OR from a case control study comparing TBF ≥1 vs <1 month. The moderate statistical heterogeneity may be explained by this heterogeneity in study design and analysis.

Figure 3 TBF ≥1-2 months vs. <1-2 months and risk of Coeliac Disease


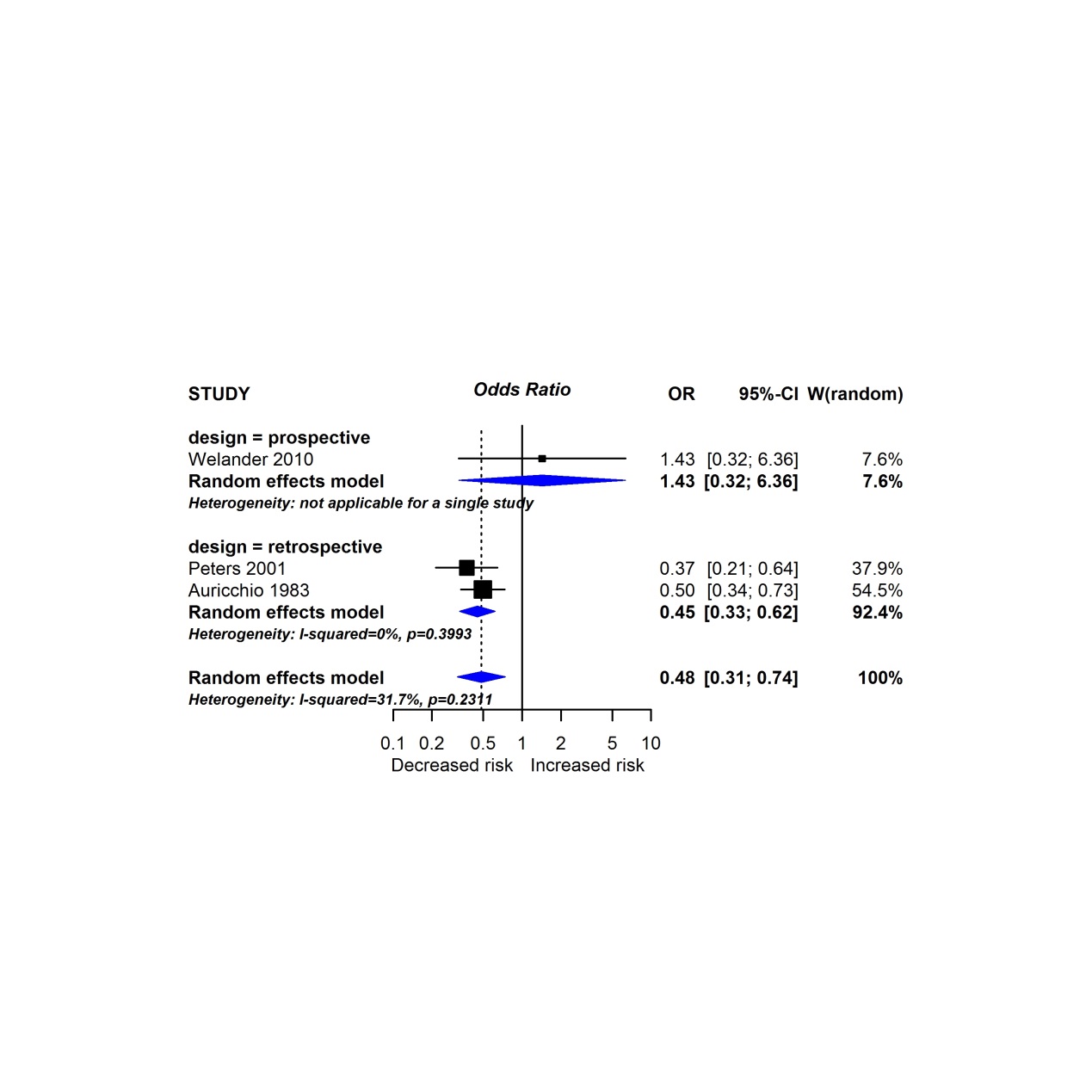


##### TBF ≥3-4 months vs. <3-4 months

The association between breastfeeding for at least 3-4months versus less than this duration, and risk of coeliac disease, was examined in only one study (Figure 4). The study found no evidence of a relationship between TBF over 3-4 months and coeliac disease risk (OR 0.84, 95% CI 0.28, 2.5).

Figure 4 TBF ≥3-4 months vs. <3-4 months and risk of Coeliac Disease


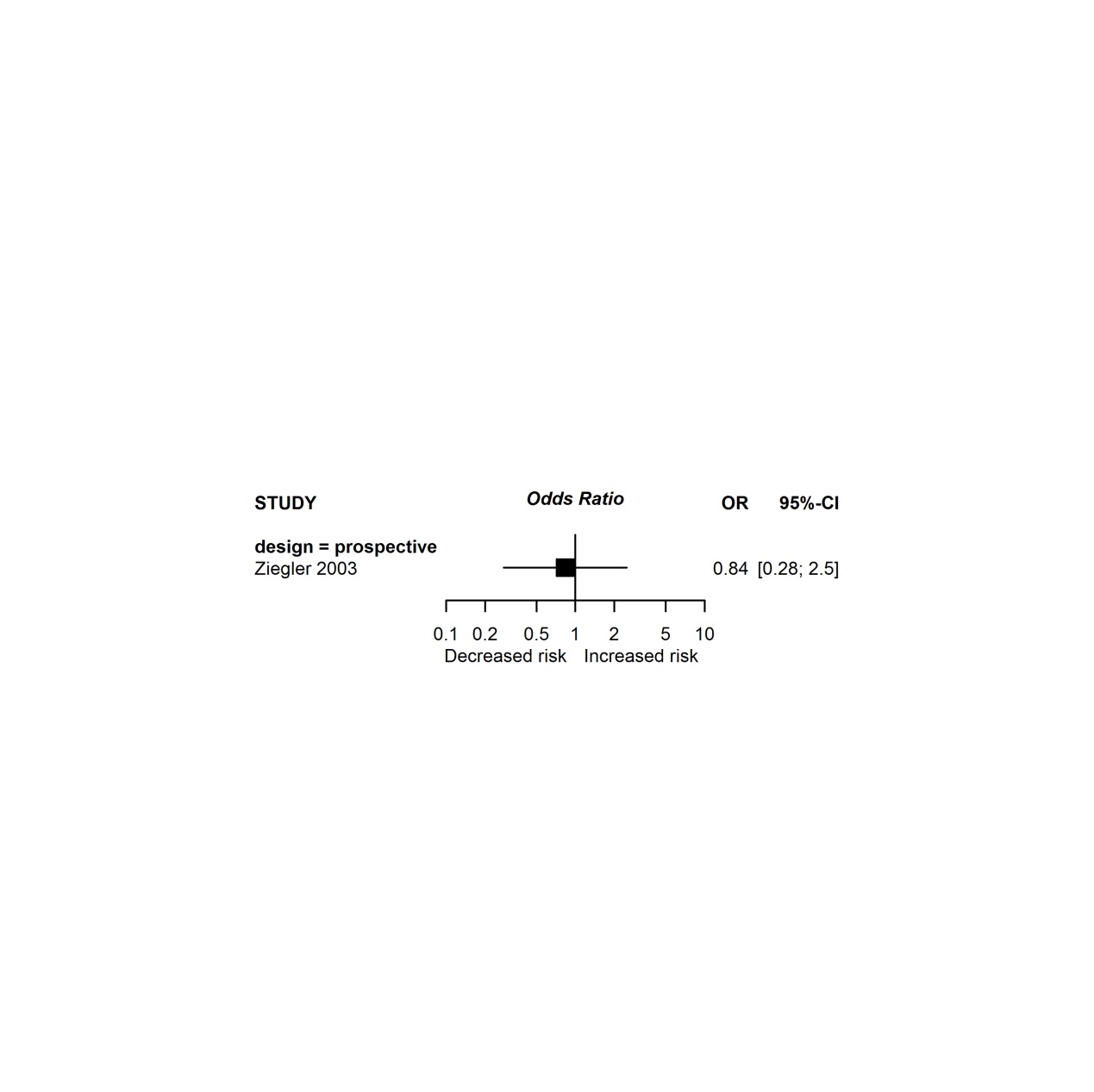


#### Dose response analysis of TBF duration and Coeliac Disease

We also analysed TBF duration by grouping studies according to exposure duration – short (1-3 months), medium (4-6 months) and long ((≥ 6 months) in comparison with never breastfed. These results are shown in Figures 5, 6 and 7. The very small number of studies reporting relevant data limited the power of these analyses. Of note the study of Peters [[11](#_ENREF_11)] did report a lower OR for coeliac disease with increased TBF duration.

Figure 5 TBF short vs. never and risk of Coeliac Disease


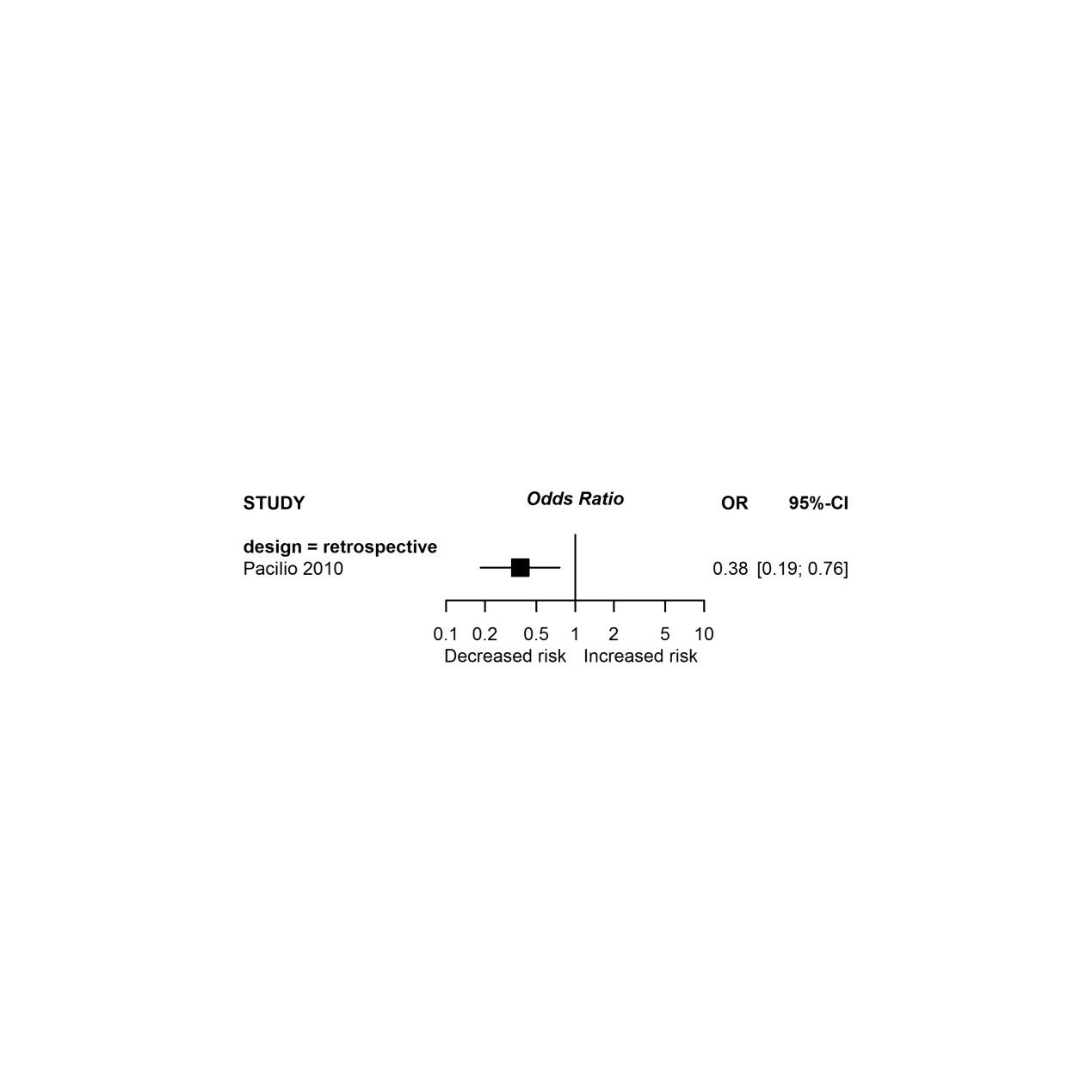


Figure 6 TBF medium vs. never and risk of Coeliac Disease


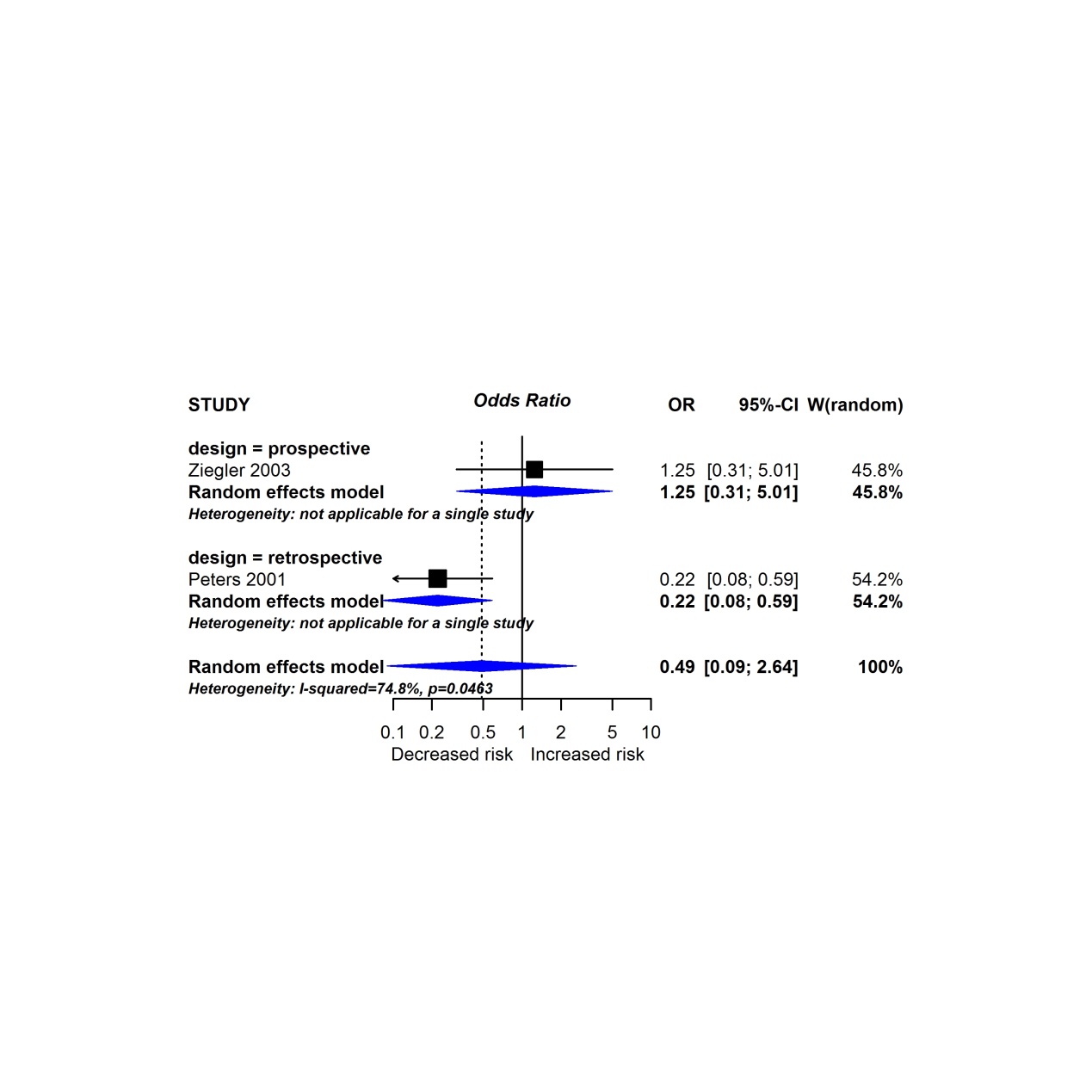


Figure 7 TBF long vs. never and risk of Coeliac Disease in children


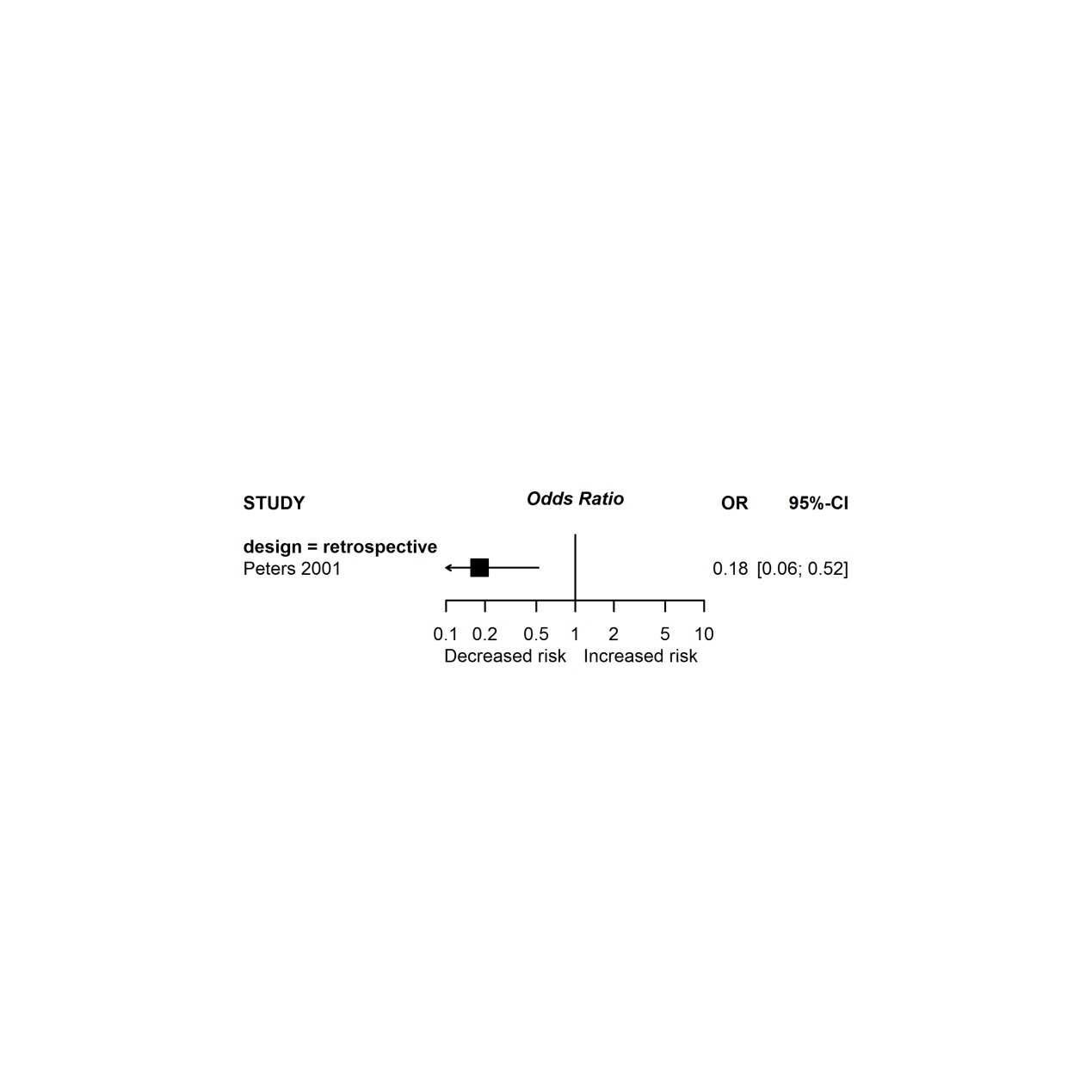


#### Data for TBF duration and Coeliac Disease which were not suitable for meta-analysis

Meta-analyses included 5 studies, reporting data on at least 491 participants with coeliac disease. A further 5 case control studies and one prospective cohort study reported relevant data which could not be reported in meta-analysis, in relation to at least 850 participants with coeliac disease. The reason for exclusion from meta-analysis was the type of effect measure used, which in five of these studies was median or mean, or as in 2 studies, risk effects that could not be combined. These studies are summarised in Table 2. TBF duration was shorter in people with coeliac disease in two studies (one significantly so), longer in three studies (not significant), and unclear in the other.

| **First Author and year of publication** | **Design** | **N/n cases** | **TBF duration (months)** | **Descriptive measure** | **TBF in Unaffected** | **TBF in Affected** | **P-value** |
| --- | --- | --- | --- | --- | --- | --- | --- |
| Ascher, 1997 [[2](#_ENREF_2)] | CC | 81  /8 | continuous | Median (range) | 5 (0-14) | 6.5 (1.5-9) | NS |
| Falth-Magnusson 1996 [[7](#_ENREF_7)] | CC | 336  /72 | continuous | Mean (range) | 5.3 (0-20) | 3.9 (0-9) | **<0.05** |
| Roberts 2009 [[4](#_ENREF_4)] | CC | 248521  /90 | categorical | Unadjusted cumulative incidence rate per 100,000 births 43.2 (27.1, 65.4) no BF, 32.4 (22.9, 44.5) BF. P=0.28 | | | |
| Decker 2010 [[5](#_ENREF_5)] | CC | 866  /123 | categorical | Increased TBF in cases vs controls. OR 1.99 (1.12, 3.51) which was not significant in adjusted analyses | | | |
| Norris 2005 [[6](#_ENREF_6)] | PC | 1560  /51 | continuous | Mean (SD) | 6.7 (6.8) | 8.3 (8.8) | NS |
|  |  |  |  | HR of 1.02 (95% CI 0.99-1.05) for each month increase in breastfeeding | | | |
| Ivarsson 2002 (0-2 y.o) [[9](#_ENREF_9)] | CC | 1018  /392 | continuous | Median (IQR) | 7 (4, 9) | 5 (3, 7) | **<0.001** |
| Ivarsson 2002 (2-14 y.o) [[9](#_ENREF_9)] |  | 254  /99 | continuous | Median (IQR) | 6 (3, 9) | 6 (4, 8) | NS |

Table 2 Other studies evaluating TBF duration and coeliac disease which were not suitable for meta-analysis

## Total breastfeeding duration and inflammatory bowel disease

### Overall characteristics of studies, risk of bias and summary of results

Table 3 describes the main characteristics of the studies analysed in this report. A total of 13 observational studies, and no intervention studies, reported the association between duration of total breastfeeding (TBF) and risk of inflammatory bowel disease (IBD). Of these, 1 was a nested case-control study, and 12 were case-control studies. Over half of the studies (n=8) were from Europe – others are from North America (n=1), and Asia/Pacific (n=2). One study involved subjects from a variety of 9 countries and one did not report location. Overall, valid data on TBF duration and IBD risk were available from over 13,000 subjects. Information on IBD was obtained via medical diagnosis, using diagnostic criteria or histology. With regards to time of outcome diagnosis, no studies explored the association between duration of TBF and IBD in the first 5 years of life; studies evaluated the outcome in older children and adults up to 70 years old. All studies used interview or questionnaire to assess TBF duration, and 2 studies combined this information with data from medical records.

Risk of bias was assessed using the NICE Methodological checklist for case-control studies. Figure 8 illustrates the distribution of bias across the five main methodological areas of the studies. Almost half of the studies had a high risk, most commonly due to lack of adjustment for confounding bias i.e., no adjusted data presented. A third of the studies had an ‘unclear’ overall risk of bias, most commonly due to insufficient information to assess selection and assessment bias.

Where data were available, five levels of comparison were used to assess the risk of FA according to TBF duration, namely ‘any (including ever) vs. never’, ‘≥1-2 months vs. <1-2 months’, ‘≥3-4 months vs. <3-4 months’, ‘≥5-7 months vs. <5-7 months’, and ‘≥8-12 months vs. <8-12 months’.

*Main Findings*

Overall the data show significant statistical heterogeneity, especially for Crohn’s disease analysis. The heterogeneity remained unexplained after subgroup analysis, but careful review of the included studies suggests that it may be partly related to varied methods for acquisition of exposure data, often decades following cessation of breastfeeding, with methods often carrying an unclear and variable risk of assessment bias. Based on the available data, we found no evidence for an association between TBF and IBD risk – this was most clearly the case for UC, where large protective effects of TBF seem unlikely, whereas the evidence base for Crohn’s disease was more mixed, with individual studies finding an association in either direction, but inconclusive overall.

Table 3 Characteristics of included studies evaluating TBF duration and inflammatory bowel disease

| **First Author & Publication Year** | **Design** | **N/n cases** | **Exposure assessment** | **Method of outcome assessment** | **Age at outcome (years)** | **Country** | **Population characteristics** |
| --- | --- | --- | --- | --- | --- | --- | --- |
| Baron, 2005 [[13](#_ENREF_13)] | CC | 444/222 | I | DD | <17 | France | Cases from EPIMAD registry (1988-97) with community-based sex, age, region matched controls |
| Gearry, 2010 [[14](#_ENREF_14)] | CC | 1253/653 | R/Q | DD | >20 | New Zealand | Canterbury Inflammatory Bowel Disease Project. Cases selected from patient advertising, letters to patients from their doctor, patient support groups; Community based (Electoral Roll) controls |
| Castiglione, 2011 [[15](#_ENREF_15)] | CC | 1030/468 | Q | ECCO guideline | 16-66 | Italy | Cases from gastroenterology units; controls comprised from physicians, nurses, and support services professionals from the participating sites |
| Corrao, 1997 [[16](#_ENREF_16)] | CC | 1252/626 | I | DD including histology | 18-65 | Italy | Cases identified in clinics with controls sex and age matched hospital-based control |
| Decker 2010 [[5](#_ENREF_5)] | CC | 1286/374Crohn, 169 UC |  | Medical diagnosis | <18 | Germany | Cases from paediatric gastroenterology clinics; controls from ophthalmology and dental clinics |
| Gruber, 1996 [[17](#_ENREF_17)] | CC | 144/54 | Q | Unclear | <22 | USA | Children diagnosed with Crohn's disease with mothers who were volunteers from the Western New York Chapter of the Crohn's and Colitis Foundation of America, Inc. with age matched unrelated controls |
| Hansen, 2011[[18](#_ENREF_18)] | CC | 534/267 | Q | Copenhagen Diagnostic Criteria | 38 | Denmark | All patients diagnosed with IBD in Copenhagen City and County (private and public sector) in 2003-4 with age, sex, ethnicity and area matched control with orthopaedic problems |
| Bergstrand, 1983 [[19](#_ENREF_19)] | CC | 616/308 | Q/I | Unclear | 20 | Sweden | Cases were residents of Stockholm County diagnosed with Crohn's disease between 1955 and 1974 with sex, age, residence matched controls from population registry in Stockholm County |
| Koletzko, 1991 [[20](#_ENREF_20)] | CC | 231/93 | Q | DD including histology | 15 | Not Available | Source of cases unclear. Sibling controls |
| Thompson, 1999 [[21](#_ENREF_21)] | NCC | 243/27 | R/I | DD | 33-43 | UK | Cases and matched for gender and social class controls were selected from the 1946 National Survey of Health & Development (NSHD) and the 1958 National Child Development Study (NCDS), two on-going, longitudinal birth cohort studies in UK. |
| Sonntag, 2007 [[22](#_ENREF_22)] | CC | 1974/1096 | Q | DD including histology | 40 | Germany | Cases identified from different sources and controls from partners (normal risk of disease) |
| Gilat, 1987 [[23](#_ENREF_23)] | CC | 1497/499 | Q/I | DD | <25 | 9 countries: USA, Canada, UK, Sweden, Denmark, Holland, France, Italy, Israel | The International IBD Study Group: Cases and matched controls from several health centres (normal risk of disease) |
| Wang, 2013 [[24](#_ENREF_24)] | CC | 2616/1308 | I | Chinese diagnostic guideline including histology | <70 | China | Cases from several health centres and matched controls from friends or neighbours (normal risk of disease) |

Q: questionnaire, I: interview, R: medical records, PC: prospective cohort, NCC: nested case control, CS: Cross-sectional, CC: case control

Figure 8 Risk of bias in studies of TBF duration and inflammatory bowel disease

### Total Breastfeeding duration and Inflammatory Bowel Disease

#### TBF duration and any IBD

##### TBF Any vs. Never

One study reported the relationship between any breastfeeding duration compared to never being breastfed, and risk of IBD (Figure 9). There was no association found in adjusted analysis (OR 1.07, 95% CI 0.53, 2.17).

##### Figure 9 TBF any vs. never and risk of IBD


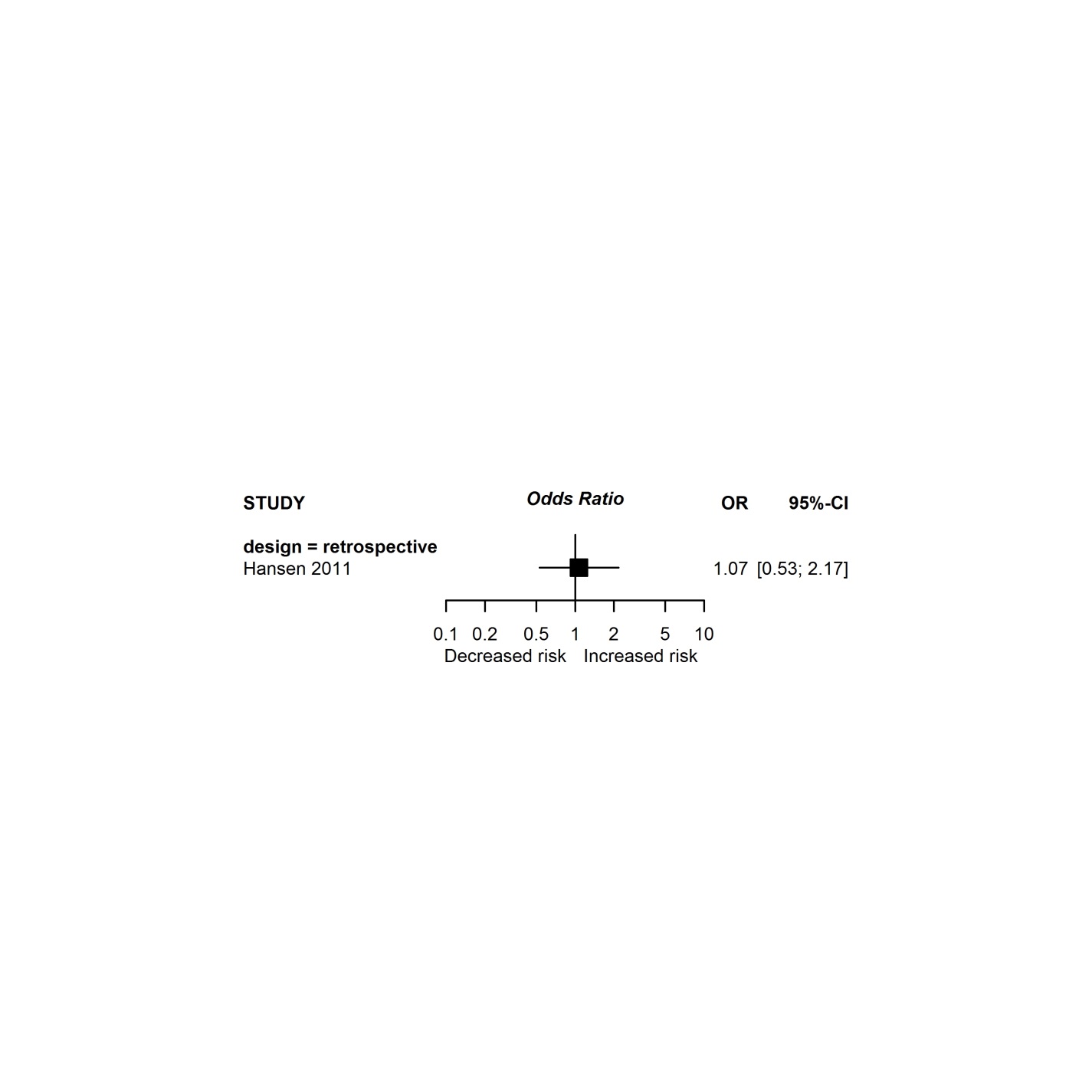


##### TBF ≥5-7 months vs. <5-7

Figure 10 illustrates comparison of TBF ≥6 months vs <6 months in the same study. The study reported a reduced risk of IBD with longer breastfeeding duration but this result failed to reach statistical significance (OR 0.50, 95% CI 0.22, 1.12).

Figure 10 TBF ≥5-7 months vs. <5-7 months and risk of IBD


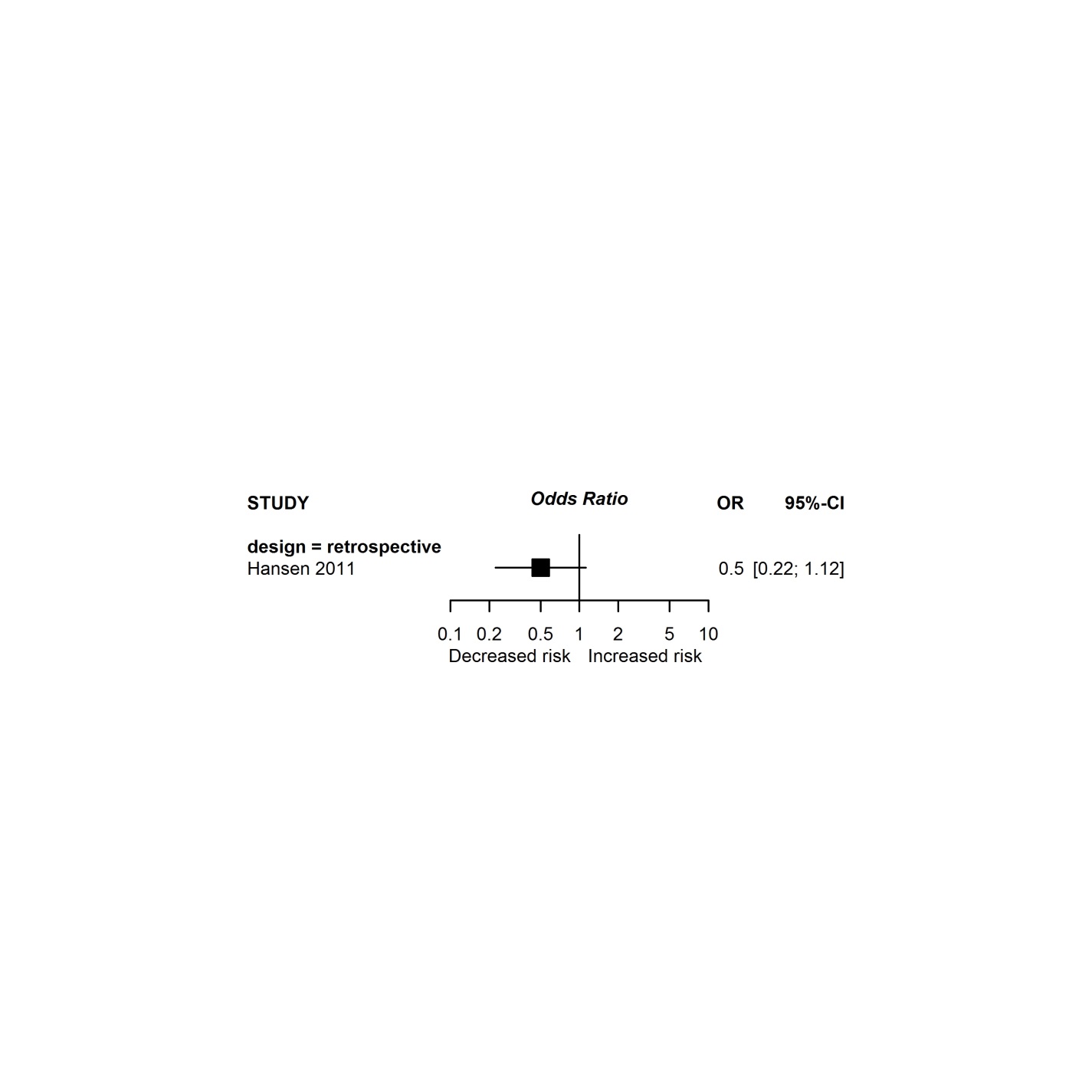


#### Total Breastfeeding duration and Crohn’s Disease

##### TBF Any vs. Never

Nine studies which reported the association between TBF any vs never and risk of Crohn’s disease are shown in Figure 11. Crohn’s disease was not association with breastfeeding initiation (OR 0.79, 95%CI 0.58, 1.08), however there was high statistical heterogeneity between studies (I^2^=76.1%). Bergstrand compared short vs never and the rest of the studies compared ever vs never and risk of Crohn’s disease. Thompson was the only prospective study contributing to the analysis. All of the studies were assessed as at high or unclear overall risk of bias. Excluding each study individually did not materially impact on the statistical heterogeneity. Stratified and subgroup analyses were limited by the number of studies, but showed similar findings in adjusted and unadjusted analyses. Studies used quite varied methods for assessment of breastfeeding duration, often with an unclear risk of assessment bias, and this may account for some of the statistical heterogeneity between studies.

Figure 11 TBF any vs. never and risk of Crohn’s Disease


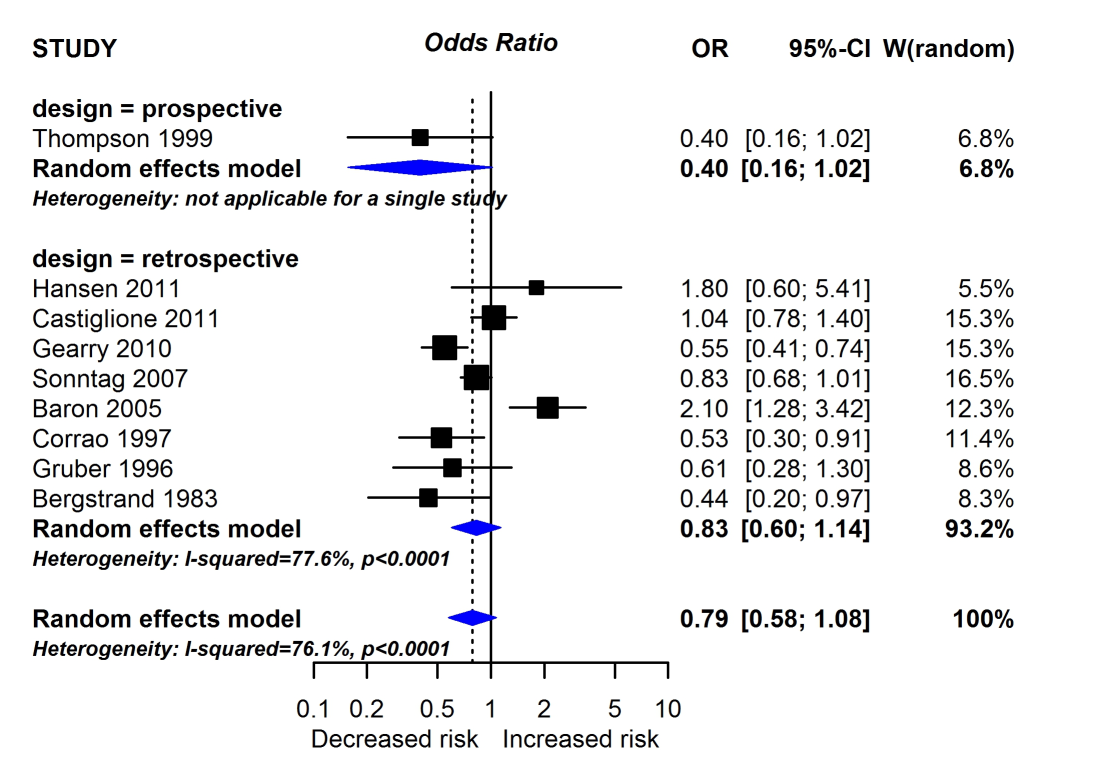


Table 4 Subgroup Analyses of risk of TBF any vs. never and the risk of Crohn’s disease

|  | **Number of studies** | **OR [95% CI]** | **I^2^ (%)** | **P-value for between groups difference** |
| --- | --- | --- | --- | --- |
| **Overall (if adjusted NA, unadjusted value used)**  **Adjusted**  **Unadjusted** | 9 | 0.79 [0.58; 1.10] | 76.1 |  |
|  | 4 | 0.97 [0.45; 2.06] | 88.1 | Not tested |
|  | 8 | 0.86 [0.64; 1.14] | 63.1 |  |
| Study Design – Prospective  Study Design – Retrospective | 1  8 | 0.40 [0.16; 1.02]  0.83 [0.60; 1.14] | -  77.6 | 0.14 |
| Risk of disease – High  Risk of disease – Normal | 0  9 | 0.79 [0.58; 1.08] | 77.6 | - |
| Risk of bias – Low  Risk of bias – High/Unclear | 0  9 | 0.79 [0.58; 1.08] | 77.6 | - |

##### TBF ≥1-2 months vs. <1-2 months

Only one study reported unadjusted data using 1-2 months as a cut-off for Crohn’s disease (Figure 12). The study by Bergstrand showed a non-significant reduction in risk with increased TBF duration (OR 0.67, 95% CI 0.41, 1.09).

Figure 12 TBF ≥1-2 months vs. <1-2 months and risk of Crohn’s Disease


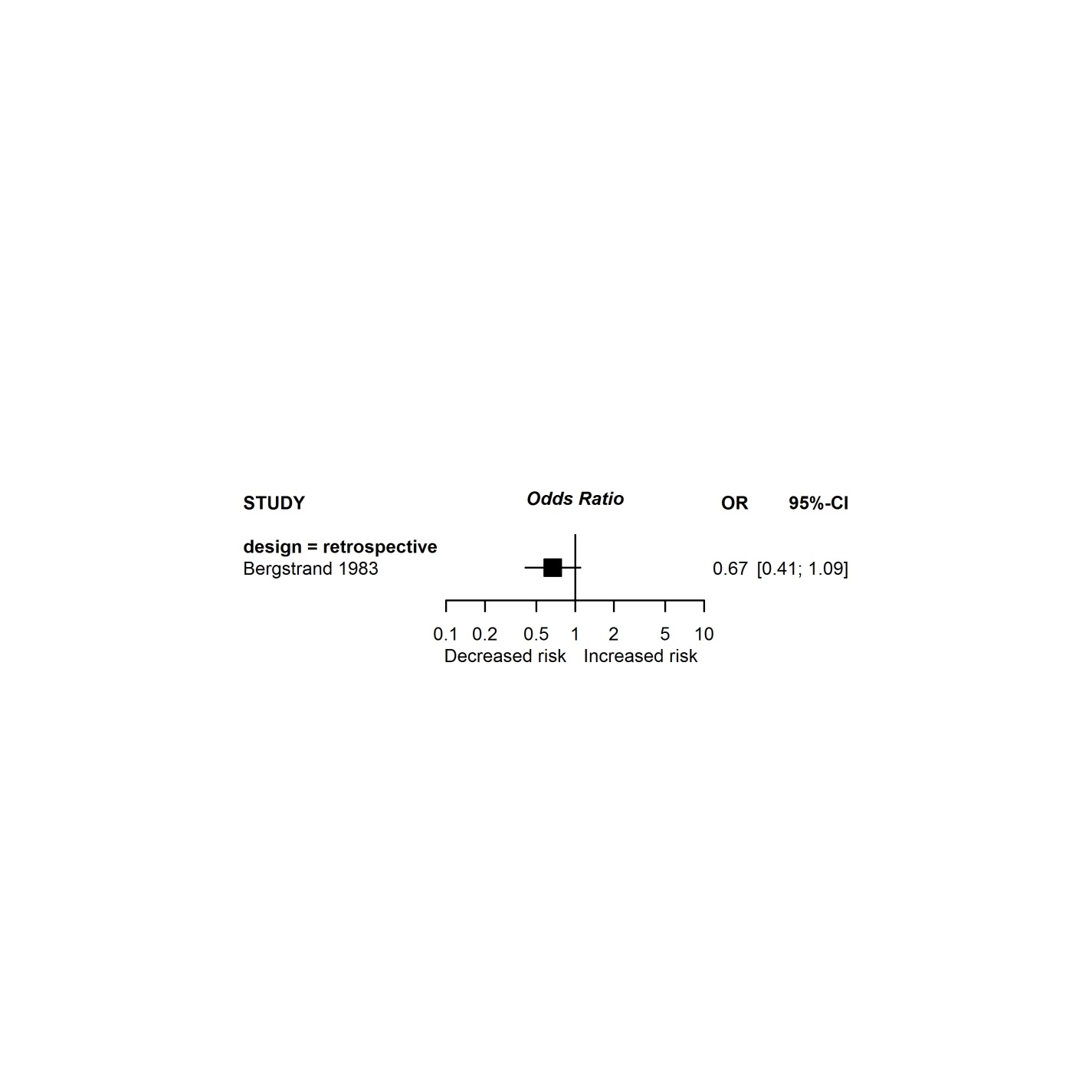


##### TBF ≥3-4 months vs. <3-4 months

The same study by Bergstrand also reported the association between risk of Crohn’s disease and exposure to TBF for ≥3-4 months (Figure 13). Longer duration of breastfeeding was associated with a statistically significant reduction in risk of disease in unadjusted analysis (OR 0.58, 95% CI 0.39, 0.85).

Figure 13 TBF ≥3-4 months vs. <3-4 months and risk of Crohn’s Disease


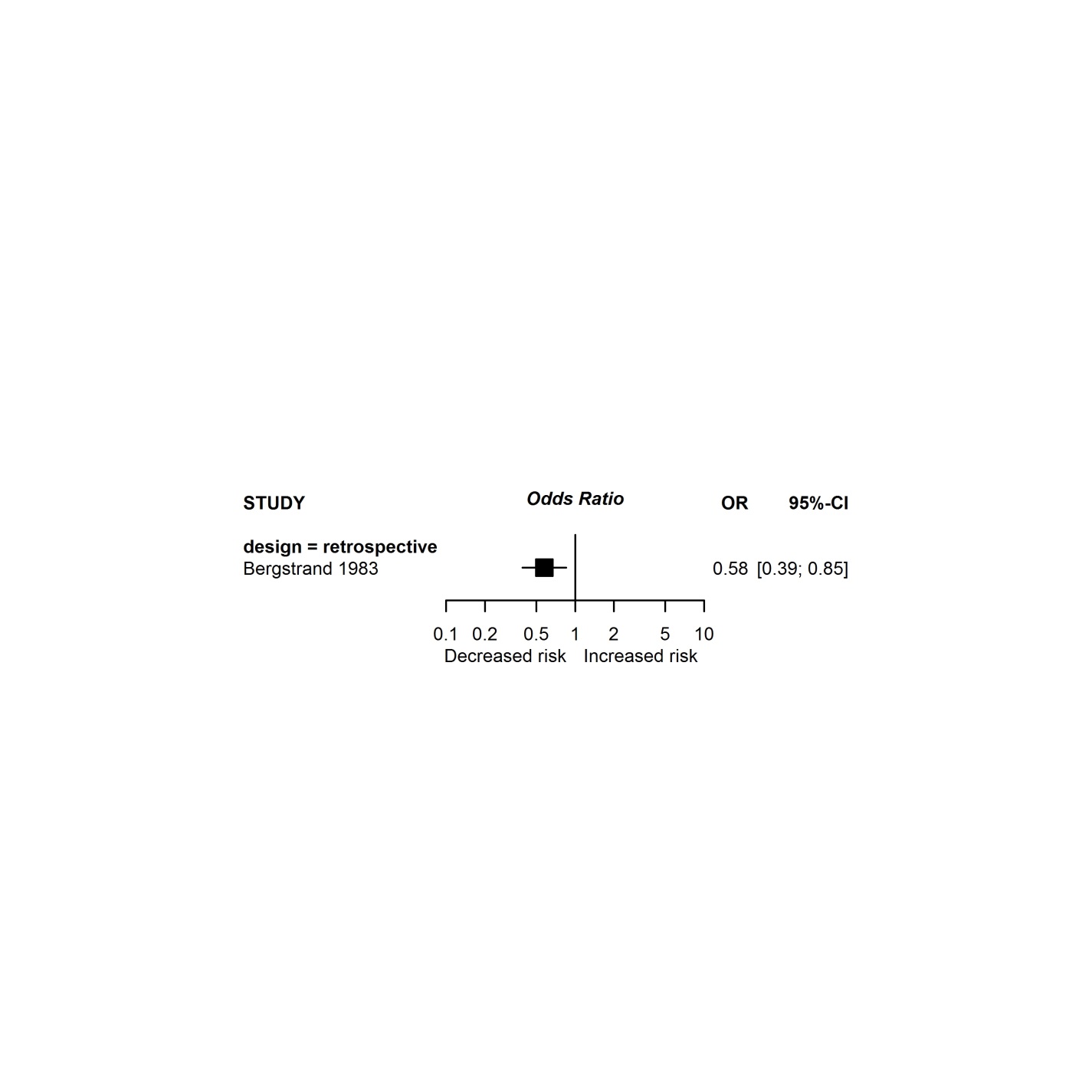


##### TBF ≥5-7 months vs. <5-7 months

Two studies assessed the effect of breastfeeding for more than 5-7 months compared to shorter durations on risk of Crohn’s disease (Figure 14). There was a significantly reduced risk of disease associated with longer breastfeeding (OR 0.50, 95% CI 0.36, 0.70) with no heterogeneity (I^2^=0%). The study by Bergstrand reported an stronger association with reduced Crohn’s risk, with increased duration of breastfeeding across the three cut-offs analysed. However these unadjusted data from a case control study carry a high risk of recall and confounding bias.

Figure 14 TBF ≥5-7 months vs. <5-7 months and risk of Crohn’s Disease


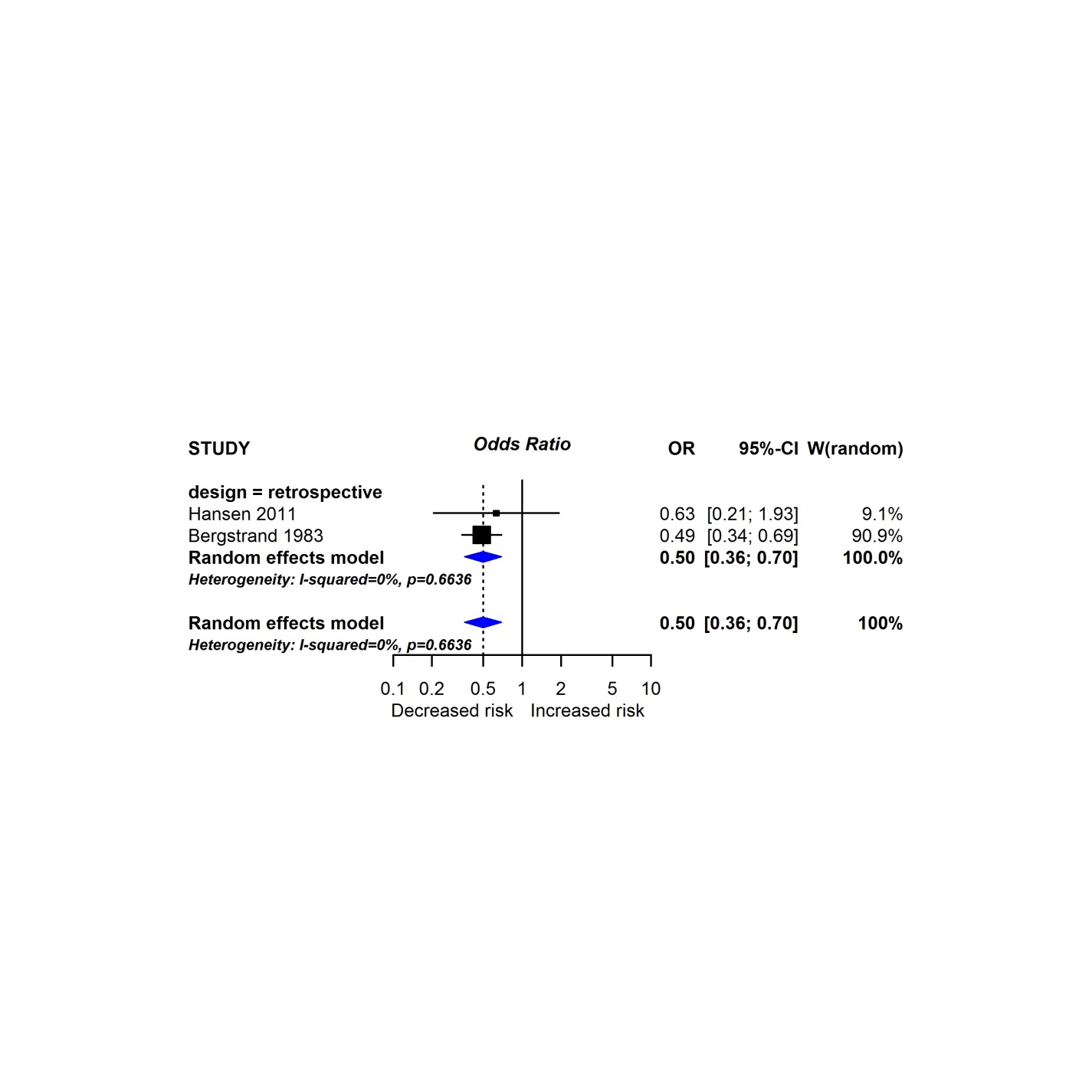


#### Total Breastfeeding duration and Ulcerative Colitis

##### TBF Any vs. Never

Eight studies reported data which could be meta-analysed for risk of ulcerative colitis in relation to TBF any vs never (Figure 15). Overall, there was no association shown between exposure and outcome (OR 0.98, 95% CI 0.78, 1.23), with high heterogeneity between studies (I^2^=61.63%). Stratified and subgroup analyses are shown in table 5. The highest ORs were seen in the only prospective study Thompson, and the only case control study using sibling controls Koletzko.

Figure 15 TBF any vs. never and risk and risk of ulcerative colitis


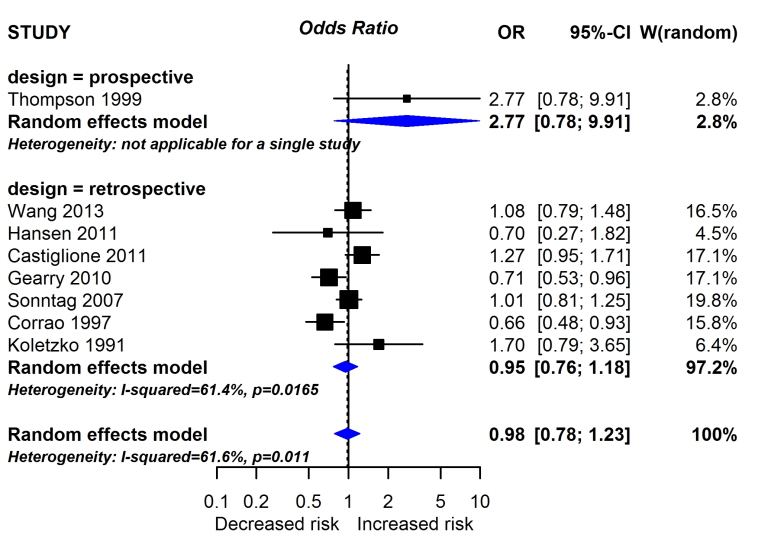


Table 5 Subgroup Analyses of risk of TBF any vs. never and the risk of ulcerative colitis

|  | **Number of studies** | **OR [95% CI]** | **I^2^ (%)** | **P-value for between groups difference** |
| --- | --- | --- | --- | --- |
| **Overall (if adjusted NA, unadjusted value used)**  **Adjusted**  **Unadjusted** | 8 | 0.98 [0.78; 1.23] | 61.6 |  |
|  | 4 | 0.77 [0.57; 1.06] | 40.4 | Not tested |
|  | 6 | 1.00 [0.80; 1.27] | 56.4 |  |
| Study Design – Prospective  Study Design – Retrospective | 1  7 | 2.77 [0.77; 9.91]  0.95 [0.76; 1.18] | -  61.4 | 0.104 |
| Risk of disease – High  Risk of disease – Normal | 0  8 | 0.98 [0.78; 1.23] | 61.6 | - |
| Risk of bias – Low  Risk of bias – High/Unclear | 1  7 | 1.70 [0.79; 3.65]  0.94 [0.75; 1.18] | -  62.1 | 0.148 |

##### TBF ≥5-7 months vs. <5-7 months

Only one study reported data on the association between TBF for at least 5-7 months compared to less than this, in association with ulcerative colitis (Figure 16). The study found that increased duration of TBF was associated with a reduction in disease (OR 0.4) but this result was not statistically significant in adjusted analysis (95% CI 0.13, 1.27).

Figure 16 TBF ≥5-7 months vs. <5-7 months and risk of ulcerative colitis


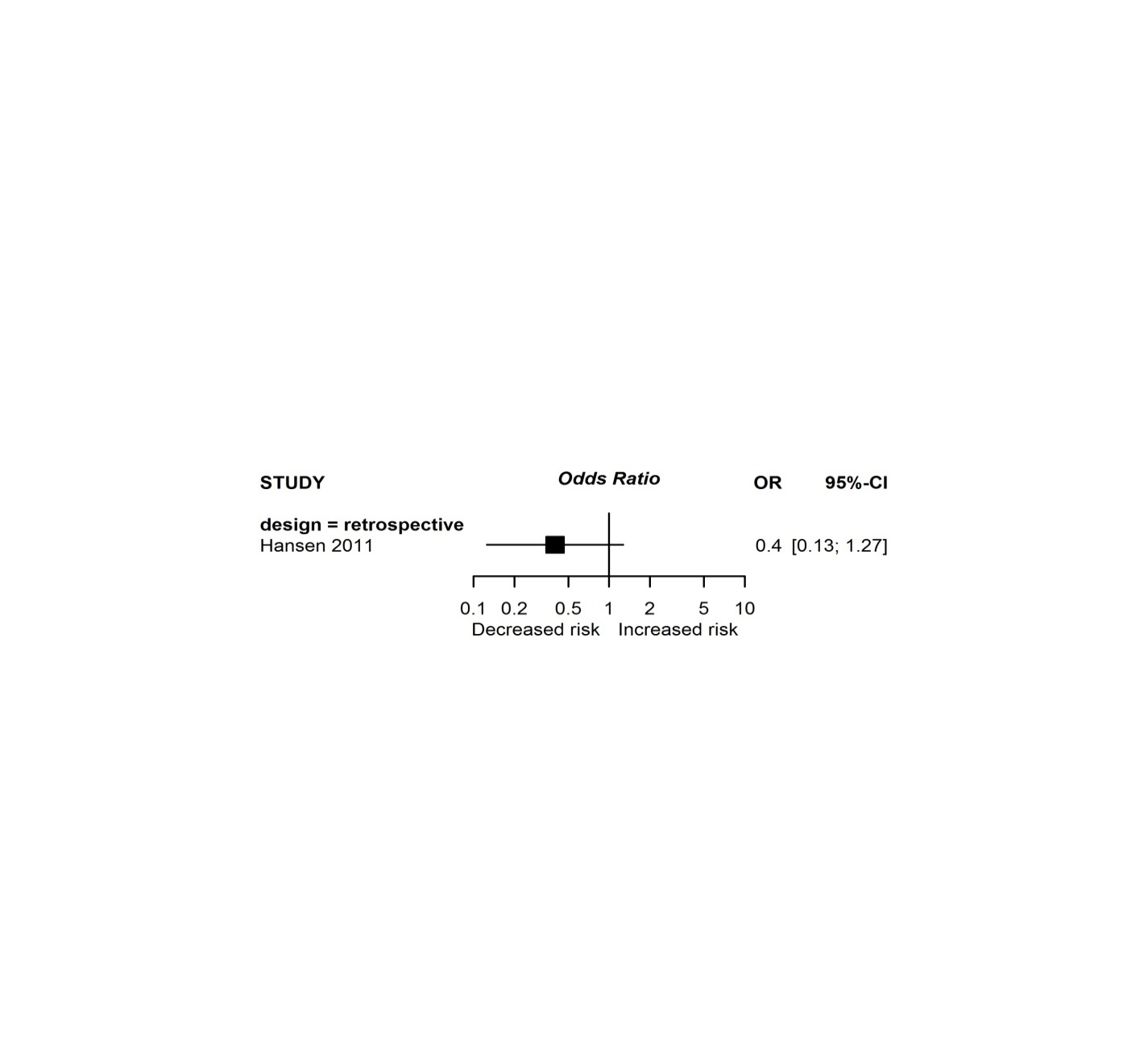


#### Data for TBF duration and IBD which were not suitable for meta-analysis

Meta-analyses included 11 studies, including at least 5,122 participants with IBD. Two other studies were identified which could not be included in meta-analysis (Table 6), which contained information regarding 1042 participants with disease. These studies used different definitions for breastfeeding duration and one of them did not provide figures for the effects estimates. The data are shown below. In one study increased TBF was associated with reduced UC (but not Crohn’s) risk. In the other study no association was found.

| **First Author and year of publication** | **Design** | **N/n cases** | **TBF duration (months)** | **Descriptive measure** | **TBF in Unaffected** | **Exposure in Affected** | **P-value** |
| --- | --- | --- | --- | --- | --- | --- | --- |
| Decker 2010 [[5](#_ENREF_5)] | CC | 1286/  374 Crohn, 169 UC | continuous | Adjusted analyses showed reduced UC risk with longer TBF duration OR 0.93 (0.89, 0.98) but not for Crohn’s (OR 0.99 (0.96, 1.01) | | | |
| Gilat, 1987 [[23](#_ENREF_23)] | CC | 1497/499 | continuous | No significant difference in breastfeeding duration between diseased and healthy subjects | | | |

**Table 6 Studies which could not be included in meta-analysis of TBF and IBD**

## Total breastfeeding duration and Thyroid disease

### Overall characteristics of studies, risk of bias and summary of results

Table 7 shows the characteristics of the only study reporting data on TBF and risk of thyroid autoimmune disease. This was an American case control study reporting data on 189 subjects. Exposure data were collected by interview and outcome data included medical diagnosis of disease and auto-antibody results. The study reported narrative data, shown in Table 7.

A summary of the risk of bias of this study is shown in Figure 17. The study was categorised as having a high risk of overall bias due to reliance on unadjusted data, hence high risk of confounding bias.

*Main Findings*

The authors found reduced TBF duration in cases compared with controls, but this was not statistically significant and overall the study was underpowered to identify an effect.

Table 7 Characteristics of included studies evaluating TBF duration and Thyroid disease

| **Study** | **Design** | **N/n cases** | **Exposure assessment** | **Method of outcome assessment** | **Age at outcome (years)** | **Country** | **Population characteristics** |
| --- | --- | --- | --- | --- | --- | --- | --- |
| Fort, 1990 [[25](#_ENREF_25)] | CC | 189/59 | I | DD including autoantibody testing - Hashimoto and Graves | 15 | USA | Cases being followed up in clinics with sibling or other controls |

I: interview, CC: case control

Figure 17 Risk of bias in studies of TBF duration and Thyroid disease

| **First Author and year of publication** | **Design** | **N/n cases** | **Measure** | **TBF in Unaffected** | **TBF in Affected** | **P-value** |
| --- | --- | --- | --- | --- | --- | --- |
| Fort, 1990 (Controls – siblings) [[25](#_ENREF_25)] | CC | 189/  59 | Mean (SD) | 5.6 (3.2) | 5.2 (3.7) | NS |
| Fort, 1990 (Controls – healthy controls) [[25](#_ENREF_25)] |  |  | Mean (SD) | 8.7 (4.5) | 5.2 (3.7) |  |

### Data for TBF and thyroid disease which were not suitable for meta-analysis

The case control study by Fort et al (regarding 59 cases of thyroid disease) reported no significant difference in mean breast feeding duration between affected and unaffected subjects (Table 8).

Table 8 Study evaluating TBF duration and thyroid disease which were not suitable for meta-analysis

## Total breastfeeding duration and juvenile rheumatoid arthritis

### Overall characteristics of studies, risk of bias and summary of results

General characteristics of included studies are summarised in Table 9. No intervention trials were identified. Data were available from a total of 3 case-control studies evaluating total breastfeeding duration (‘never vs ever’, ‘short duration vs never’ and ‘medium duration vs never’) and juvenile rheumatoid arthritis risk (JRA). The studies were North American (n=2) and Australian (n=1).

All studies evaluated JRA risk in children over 5 years old, and obtained information on duration of breastfeeding based on questionnaire or interview data.

Based on the distribution of data reported in included studies, meta-analysis compared JRA risk and each of the breastfeeding definitions indicated above. Separate analysis of pauciarticular and polyarticular JRA was also undertaken.

A summary of the risk of bias in included studies is shown in Figure 18. All the studies had a high overall risk of bias, due either to reliance on unadjusted data (hence high risk of confounding bias), or high/unreported inclusion rates (leading to high risk of selection bias).

*Main Findings*

Overall the data do not provide evidence for a relationship between TBF duration and JRA risk. Interpretation of the strength of the evidence is limited by the small number of studies and the high risk of bias observed in all of them.

Table 9 Characteristics of included studies evaluating TBF duration and juvenile rheumatoid arthritis

| **First Author & Publication Year** | **Design** | **N/n cases** | **Exposure assessment** | **Specific outcome/Method of outcome assessment** | **Age at outcome (years)** | **Country** | **Population characteristics** |
| --- | --- | --- | --- | --- | --- | --- | --- |
| Ellis, 2012 [[26](#_ENREF_26)] | CC | 655/246 | Q/I | DD | <18 | Australia | CLARITY study. Cases from paediatric rheumatology clinic; controls from pediatric surgery unit and born in the same area |
| Mason, 1995 [[27](#_ENREF_27)] | CC | 133/54 | I | DD | 6 | USA | Children seen at the outpatient paediatric rheumatology clinics with playmates matched for age and race as controls |
| Rosenberg, 1996 [[28](#_ENREF_28)] | CC | 468/137 | Q | American College of Rheumatology criteria | <18 | Canada | Cases from a health service and matched control from population |

Q: questionnaire, I: interview, R: medical records, PC: prospective cohort, NCC: nested case control, CS: Cross-sectional, CC: case control

Figure 18 Risk of bias in studies of TBF duration and juvenile rheumatoid arthritis

### Total Breastfeeding duration and juvenile rheumatoid arthritis

##### TBF Any vs. Never

Figure 19 shows the results of meta-analysis of the association between any breastfeeding never vs ever and risk of JRA. Overall, there was no significant association (OR 0.79, 95% CI 0.38, 1.65), but with extreme statistical heterogeneity (I^2^=75%). The study of Ellis reported adjusted data, and the other two studies unadjusted, which may account for some of the statistical heterogeneity.

Figure 19 TBF any vs. never and risk of juvenile rheumatoid arthritis


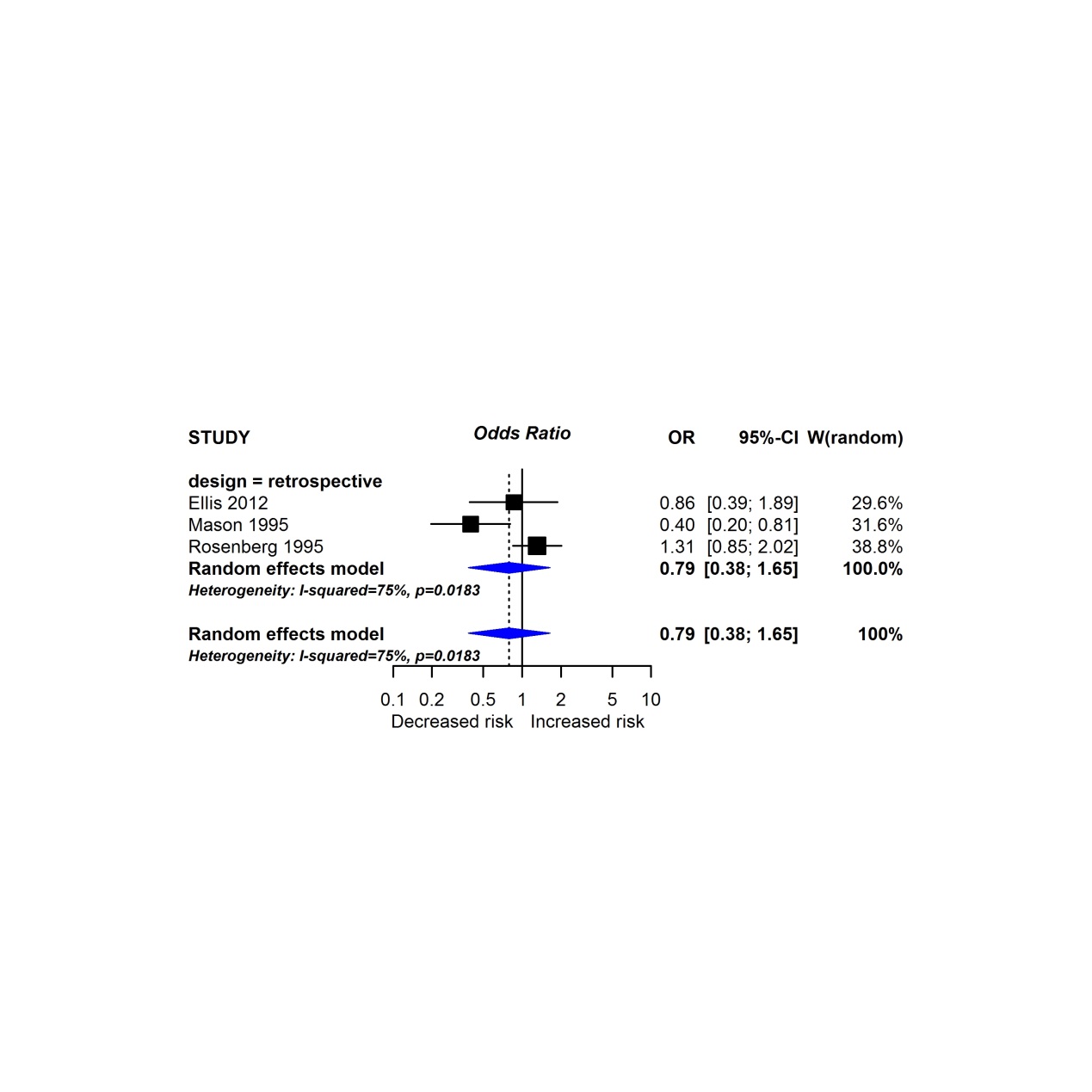


##### Any vs. never and pauciarticular and polyarticular JRA

Two studies reported the effect of any vs never breastfeeding and risk of both pauciarticular JRA and polyarticular JRA (Figure 20 and Figure 21 respectively). Studies reporting association between ever breastfeeding and pauciartiular JRA were extremely heterogeneous (I^2^=88.8%) and could not be meta-analysed. There was a reduction in risk of polyarticular JRA with nil heterogeneity but this association did not reach statistical significance (OR 0.68 95% CI 0.40, 1.41).

Figure 20 TBF any vs. never and risk of pauciarticular juvenile rheumatoid arthritis


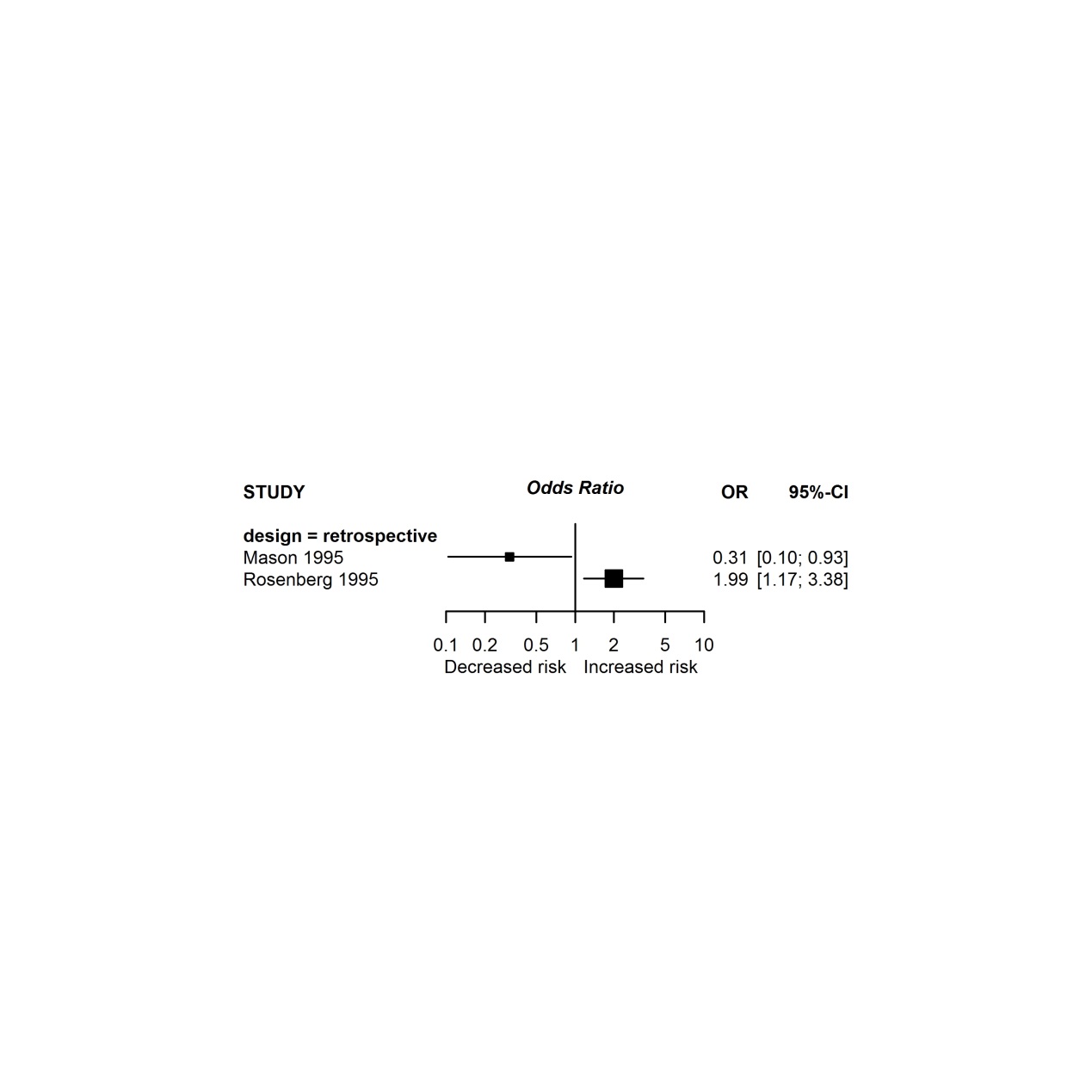


Figure 21 TBF any vs. never and risk of polyarticular juvenile rheumatoid arthritis


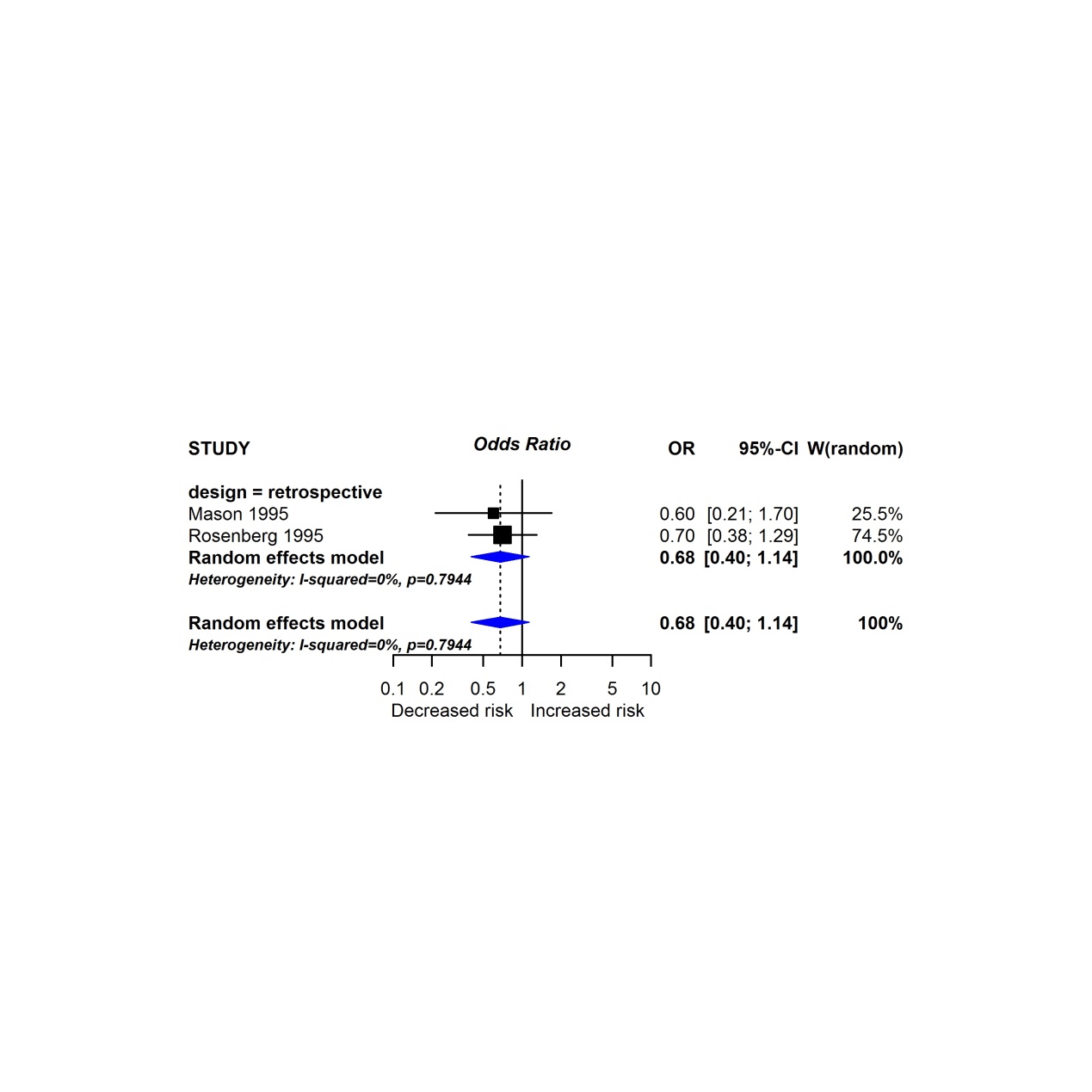


### Total Breastfeeding dose-response and juvenile rheumatoid arthritis

We also analysed the dose response relationship between breastfeeding duration and JRA (Figure 22 and Figure 23). The single included study by Mason et al [[27](#_ENREF_27)] shows an increasing protective effect with continuation of breastfeeding from short duration (0-3 months) (OR 0.56, 95% 0.28, 1.13) to medium duration (>3 months) (OR 0.28, 95% 0.12, 0.68) although these were unadjusted analyses.

Figure 22 TBF short vs never and risk of juvenile rheumatoid arthritis


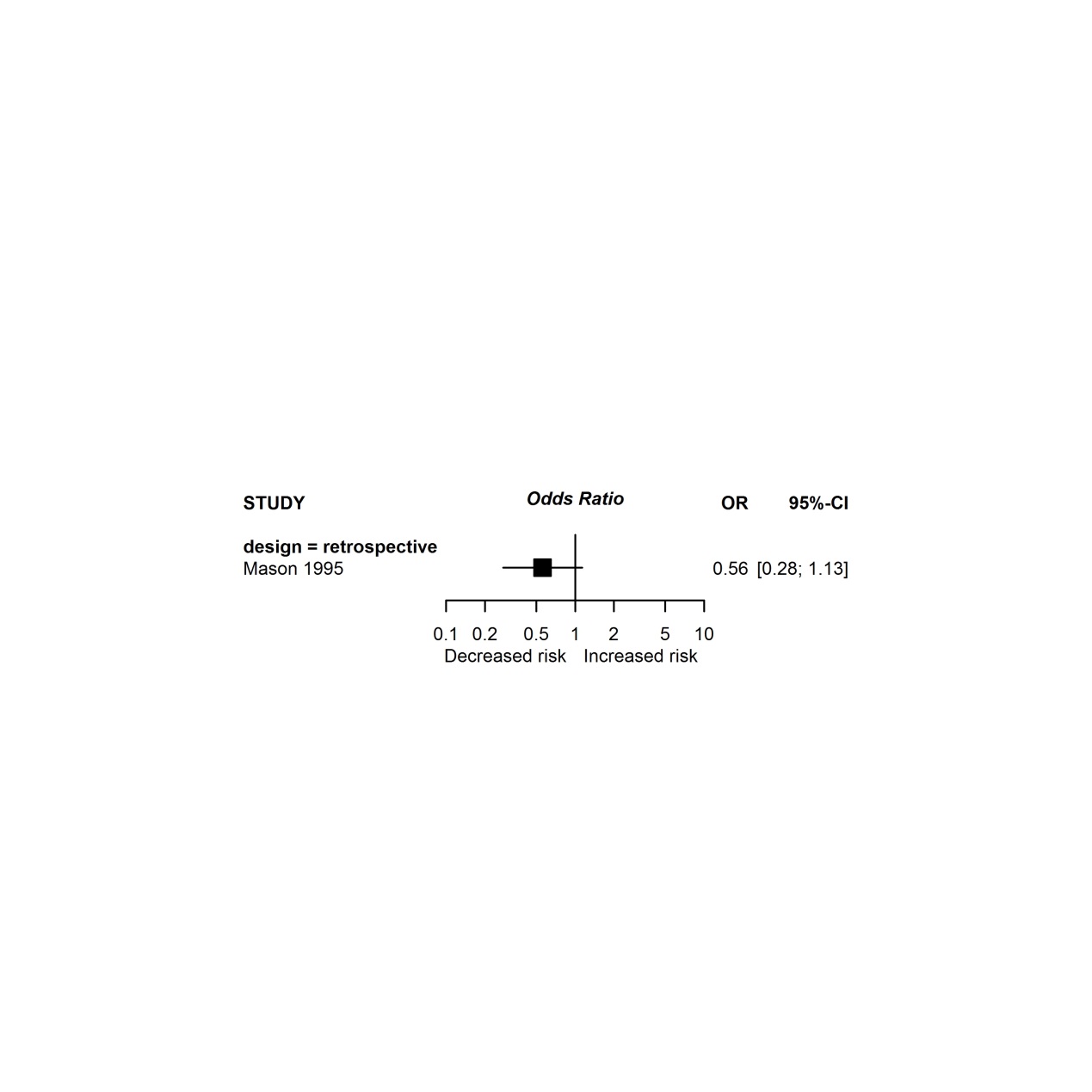


Figure 23 TBF medium vs never and risk of juvenile rheumatoid arthritis


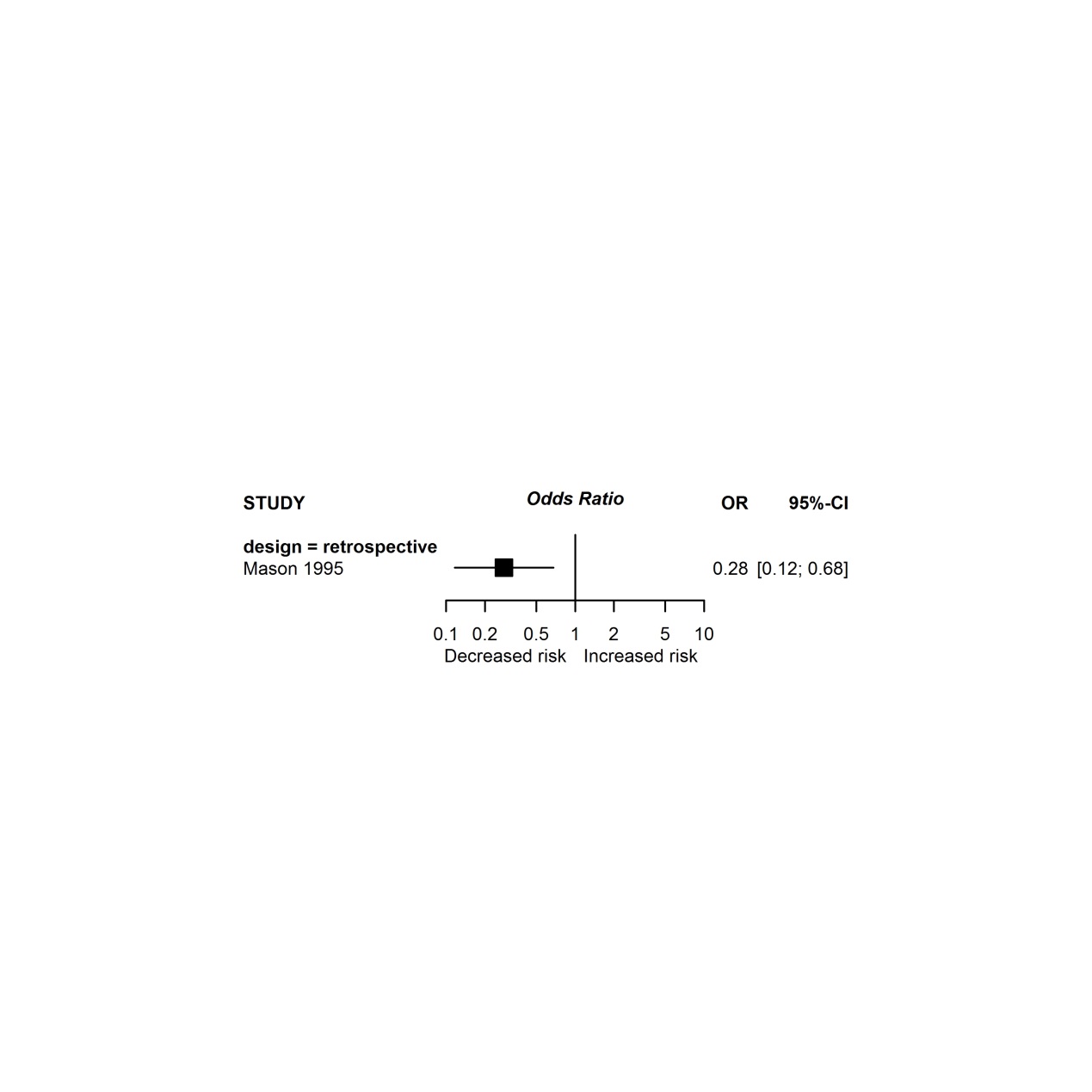


# Exclusive breastfeeding duration and Autoimmune Diseases

## Exclusive breastfeeding duration and Coeliac Disease

### Overall characteristics of studies, risk of bias and summary of results

Table 10 describes the main characteristics of the studies analysed in relation to exclusive breastfeeding duration and coeliac disease. A total of 4 observational studies were identified; of these, 1 was a prospective cohort studies and the remaining 3 were case-control studies. All of the studies originated in Europe. Overall, valid data on exclusive breastfeeding duration and coeliac disease risk were available from 4,216 subjects – this information was obtained by questionnaires in all studies, with two studies combining this with medical record information. Information on coeliac disease was obtained mainly from medical diagnosis using ESPGHAN criteria, although one study used serological transglutaminase auto-antibodies. With regards to time of outcome diagnosis, 3 studies explored the association between exposure to exclusive breastfeeding and coeliac disease in the first 5 years of life and one evaluated coeliac disease in older children.

Risk of bias was assessed using the NICE Methodological checklists for cohort and case-control studies. Figure 24 illustrates the distribution of bias across the five main methodological areas of the studies. Over half of studies had a high risk of bias, most commonly due to lack of adjustment for confounding bias.

Two levels of comparison were used to assess the risk of coeliac disease according to exclusive breastfeeding duration, namely ‘≥0-2 months vs. <0-2 months’ and ‘≥3-4 months vs. <3-4 months’. Stratified and subgroup analyses were not performed due to the small number of studies included in analysis.

*Main Findings*

In total 3 case control studies found that EBF duration was associated with reduced risk of coeliac disease, and 1 prospective cohort study in a population with a paternal or maternal history of TIDM failed to confirm this relationship. This is a similar pattern to that seen with TBF and coeliac disease, and TBF/EBF and TIDM, where retrospective and prospective studies have discrepant findings, with retrospective studies showing a relationship but prospective studies – often with smaller numbers of cases in specific high risk populations, using surrogate outcomes for clinical disease – not finding a relationship between BF and disease.

Table 10 Characteristics of included studies evaluating EBF duration and Coeliac Disease

| **First Author & Publication Year** | **Design** | **N/n cases** | **Exposure assessment** | **Method of outcome assessment** | **Age at outcome (years)** | **Country** | **Population characteristics** |
| --- | --- | --- | --- | --- | --- | --- | --- |
| Falth-Magnusson, 1996 [[7](#_ENREF_7)] | CC | 336/72 | R/Q | ESPGHAN criteria | <2 | Sweden | Cases from paediatric department records, born in 1987-1989. Reference children were age matched from same county. |
| Ziegler, 2003 [[8](#_ENREF_8)] | PC | 1460/27 | Q | IgA-tTG | 5 | Germany | German BABYDIAB study. Offspring of mothers and/or fathers with T1DM born in Germany between 1989 and 2000 |
| Greco, 1988 [[29](#_ENREF_29)] | CC | 2150/201 | R/Q | ESPGHAN criteria | 2 | Italy | Hospital-based cases born in 1976-1983 with age and area matched controls |
| Peters, 2001 [[11](#_ENREF_11)] | CC | 270/133 | Q | ESPGHAN criteria | < 10 | Germany | Cases born in 1985-1995 identified from registries of several hospitals and matched control from population registry (normal risk of disease) |

Q: questionnaire, I: interview, R: medical records, PC: prospective cohort, NCC: nested case control, CS: Cross-sectional, CC: case control

Figure 24 Risk of bias in studies of EBF duration and coeliac disease

### Exclusive breastfeeding duration and Coeliac Disease

#### EBF ≥0-2 months vs <0-2 months

Figure 25 shows the one study which reported the association between exclusive breastfeeding for 0-2 months or more, versus shorter durations. There was no difference in risk of disease between these exposures (OR 1.0, 95% CI 0.40, 2.51).

**Figure 25 EBF ≥0-2 months vs <0-2 months and risk of Coeliac Disease**


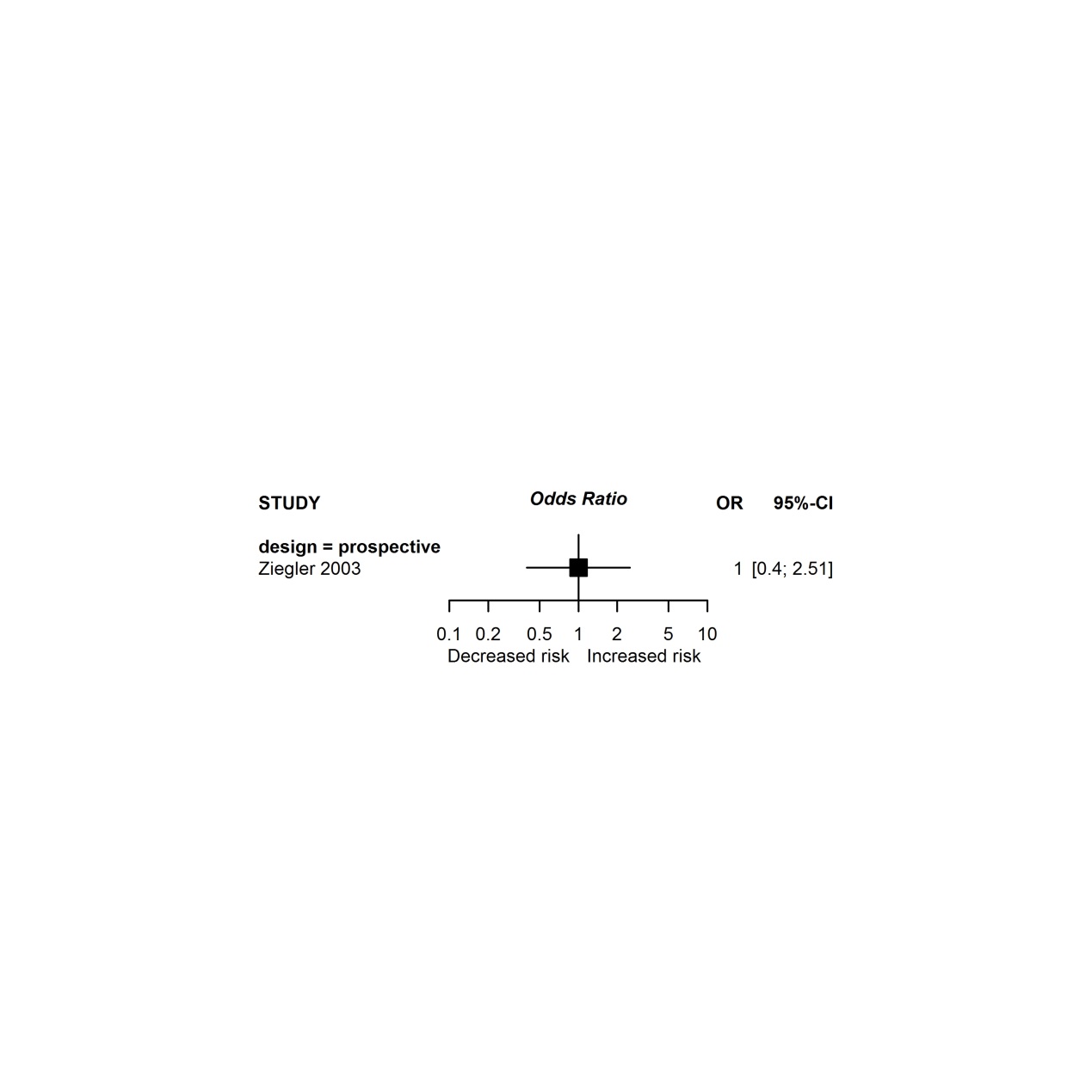


#### EBF ≥3-4 months vs <3-4 months

Two studies reported the association between exclusive breastfeeding for more than vs less than 3-4 months. Data could not be pooled due to extreme statistical heterogeneity (I^2^=82.8%) between the case control study of Greco reporting unadjusted OR, and the prospective cohort study of Ziegler reporting adjusted HR.

**Figure 26 EBF ≥3-4 months vs <3-4 months and risk of Coeliac Disease**


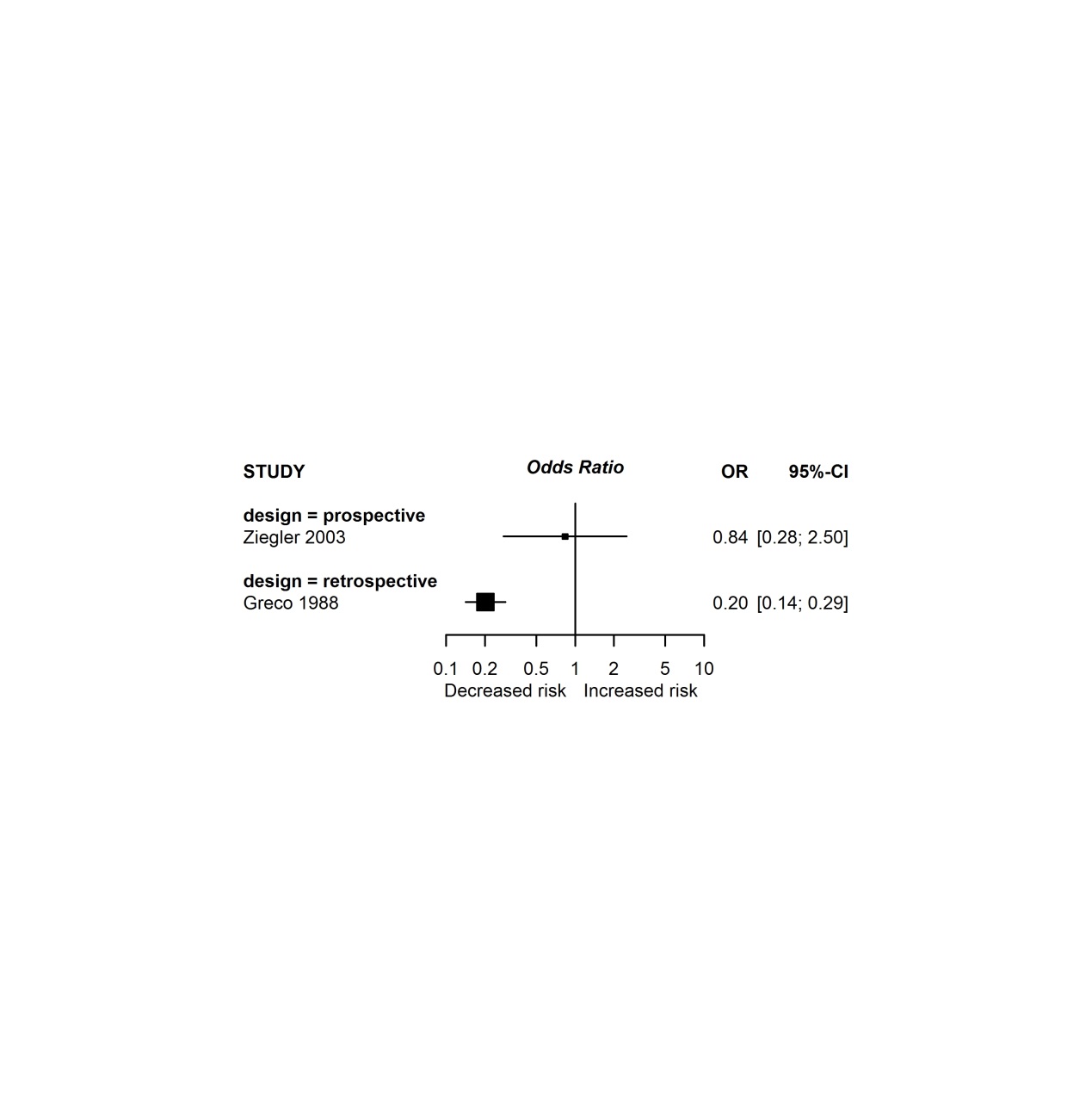


#### Data for EBF duration and coeliac disease which were not suitable for meta-analysis

Meta-analysis involved two studies, containing information on 228 subjects with coeliac disease. A further two studies, with 205 subjects with coeliac disease, could not be meta-analysed. The reasons for exclusion from meta-analysis was the type of measurement used to report data (mean, median, or percentage), which made comparisons of effect size not possible. Details of these studies are shown in Table 11.

Both studies showed that individuals with coeliac disease had been exclusively breastfed for a significantly shorter duration than unaffected controls. The study by Peters et al [[11](#_ENREF_11)] also reported that risk of coeliac disease was reduced by 12% for each month of exclusive breastfeeding (OR 0.88, 95% CI 0.79,0.98).

| **First Author and year of publication** | **Design** | **N/n cases** | **EBF duration (months)** | **Descriptive measure** | **EBF in Unaffected** | **Exposure in Affected** | **P-value** |
| --- | --- | --- | --- | --- | --- | --- | --- |
| Peters, 2001 [[11](#_ENREF_11)] | CC | 270/133 | Continuous | Mean (SD) | 2.7 (2.3) | 2.1 (2.5) | **<0.05** |
|  |  |  |  | Coeliac disease was reduced by 12% for each month of exclusive breastfeeding in adjusted analysis (OR 0.88, 95% CI 0.79,0.98) | | | |
| Falth-Magnusson 1996 [[7](#_ENREF_7)] | CC | 336/72 | Continuous | Median (range) | 4 (0-10) | 2 (0-6) | **<0.05** |

Table 11 Other studies reporting data on EBF duration and coeliac disease which couldn’t be meta-analysis

## Exclusive breastfeeding duration and inflammatory bowel disease

### Overall characteristics of studies, risk of bias and summary of results

Table 12 shows the characteristics of the studies included in analysis of exclusive breastfeeding duration and IBD. Two studies were identified: both were case control studies and included a total of 675 subjects. One study was from Europe while the origin of the second study was unknown. Exposure information was obtained by questionnaire or interview and outcome assessment used medical diagnosis. Both studies assessed children older than 5 years old.

Risk of bias was assessed using the NICE Methodological checklists for case-control studies (Figure 27). One study had a high overall risk of bias due to lack of adjustment for confounding bias whereas the other study was considered to have low risk of bias. Meta-analysis was not performed due to only narrative data being reported. The limited data show no evidence of a relationship between EBF duration and risk of IBD.

Table 12 Characteristics of included studies evaluating EBF duration and inflammatory bowel disease

| **Study** | **Design** | **N/ cases** | **Exposure assessment** | **Outcome assessment** | **Age (yrs)** | **Country** | **Population characteristics** |
| --- | --- | --- | --- | --- | --- | --- | --- |
| Baron, 2005 [[13](#_ENREF_13)] | CC | 444/222 | I | DD | <17 | France | Cases from EPIMAD registry (1988-97) with community-based sex, age, region matched controls |
| Koletzko, 1991[[20](#_ENREF_20)] | CC | 231/93 | Q | DD including histology | 15 | Unclear | Source of cases unclear. Sibling controls |

Q: questionnaire, I: interview, R: medical records, PC: prospective cohort, NCC: nested case control, CS: Cross-sectional, CC: case control

Figure 27 Risk of bias in studies of EBF duration and inflammatory bowel disease

### Data for EBF duration and IBD which were not suitable for meta-analysis

There was no significant difference in EBF duration between cases and controls (Table 13), using either continuous analysis (mean) or categorical analysis (OR).

Table 13 Studies reporting data on EBF duration and IBD which were not suitable for meta-analysis

| **Study** | **Design** | **N/n cases** | **EBF duration (months)** | **Descriptive measure** | **EBF in Unaffected** | **Exposure in Affected** | **P** |
| --- | --- | --- | --- | --- | --- | --- | --- |
| Koletzko, 1991 [[20](#_ENREF_20)] | CC | 231/93 | >0 vs 0 months | Adj.OR |  |  | NS |
| Baron, 2005 [[13](#_ENREF_13)] | CC | 444/222 | continuous | Mean | 2 | 2.5 | NS |

# Solid food introduction and Autoimmune Disease

## Solid food introduction and Coeliac Disease

### Overall characteristics of studies, risk of bias and summary of results

Table 14 Characteristics of included studies evaluating solid food introduction and Coeliac Disease shows the characteristics of the only study included in analysis of timing of solid food introduction and risk of coeliac disease. This was a European prospective cohort study containing relevant information form 1219 subjects. Exposure data were collected using a questionnaire and transglutaminase auto-antibody serology was used as the outcome measure. The authors reported that neither breastfeeding or its duration nor the age of first exposure to gluten was associated with the risk of developing transglutaminase antibodies.

Risk of bias assessment is reported in Figure 28. The study was categorised as having a high overall risk of bias due to a lack of adjustment for confounders. Due to the information reported in the paper, the cut-off of solid food introduction ‘≥3-4 months vs <3-4 months’ was chosen.

*Main Findings*

We found no evidence to support a relationship between timing of SF introduction and risk of Coeliac disease.

Table 14 Characteristics of included studies evaluating solid food introduction and Coeliac Disease

| **Study** | **Design** | **N/n cases** | **Exposure** | **Outcome** | **Age (yrs)** | **Country** | **Population characteristics** |
| --- | --- | --- | --- | --- | --- | --- | --- |
| Hummel, 2007 [[30](#_ENREF_30)] | PC | 1219  /27 | Q | IgA-tTG | 8 | Germany | German BABYDIAB. Offspring of mothers and/or fathers with T1DM born in Germany between 1989 and 2000 |

Q: questionnaire, I: interview, R: medical records, PC: prospective cohort, NCC: nested case control, CS: Cross-sectional, CC: case control

Figure 28 Risk of bias in studies of solid food introduction and Coeliac Disease

### Solid food introduction and Coeliac Disease

#### SF ≥3-4 months vs. <3-4 months

There was a non-significant reduction in risk of coeliac disease associated with delaying solid food introduction until after 3-4 months (Figure 29, OR 0.55, 95% CI 0.17, 1.83).

**Figure 29 SF ≥3-4 months vs. <3-4 months and risk of Coeliac Disease**


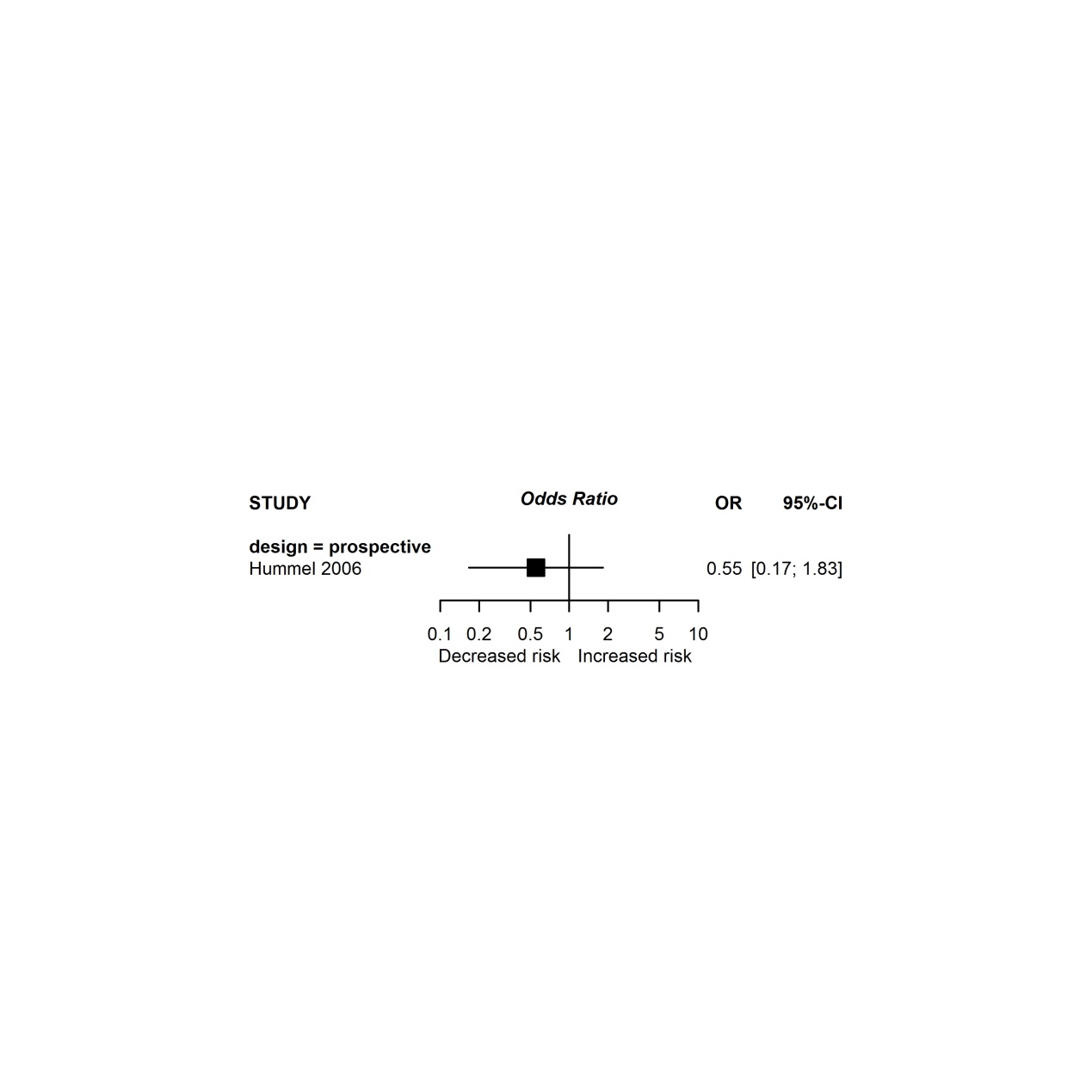


## Solid food introduction and inflammatory bowel disease

### Overall characteristics of studies, risk of bias and summary of results

One study reported the association between timing of solid food introduction and risk of inflammatory bowel disease (Ulcerative colitis; Table 15). This was a case control study of unknown location reporting relevant data for 231 subjects. Data on solid food introduction was collected by questionnaire and medical diagnosis of IBD (including histology) was used as the outcome measure of interest. The authors reported that age of solid food introduction was not different in children with ulcerative colitis (no estimates provided).

Figure 30 demonstrates the results of risk of bias assessment using the NICE Methodological guideline for case control studies. The study was considered to have a low overall risk of bias, scoring well in all domains.

*Main Findings*

We found no evidence to support a relationship between timing of SF introduction and risk of inflammatory bowel disease. Only narrative data were reported on the lack of association between exposure and outcome, so meta-analysis was not undertaken.

Table 15 Characteristics of included studies evaluating solid food introduction and inflammatory bowel disease

| **Study** | **Design** | **N/**  **cases** | **Exposure** | **Outcome** | **Age (yrs)** | **Country** | **Population** |
| --- | --- | --- | --- | --- | --- | --- | --- |
| Koletzko, 1991 [[20](#_ENREF_20)] | CC | 231/93 | Q | DD including histology | 15 | Not Available | Source of cases unclear. Sibling controls |

Q: questionnaire, I: interview, R: medical records, PC: prospective cohort, NCC: nested case control, CS: Cross-sectional, CC: case control

Figure 30 Risk of bias in studies of solid food introduction and inflammatory bowel disease

### Data for solid food introduction and IBD which were not suitable for meta-analysis

The study by Koletzko et al found that age at solid food introduction did not affect risk of inflammatory bowel disease (Table 16), with a p-value >0.05.

Table 16 Studies reporting solid food introduction and IBD which were not suitable for meta-analysis

| **Study** | **Design** | **N/**  **n cases** | **Age at SF introduction (months)** | **Descriptive measure** | **Effect** | **P-value** |
| --- | --- | --- | --- | --- | --- | --- |
| Koletzko, 1991 [[20](#_ENREF_20)] | CC | 231/93 | >0 vs 0 months | Adj.OR |  | NS |

## Solid food introduction and juvenile rheumatoid arthritis

### Overall characteristics of studies, risk of bias and summary of results

One study reported data regarding timing of solid food introduction and risk of JRA (Table 17). This was an Australian case control study with relevant data regarding 655 subjects. Exposure data was collected via questionnaire and medical diagnosis of JRA was used as the outcome measure.

Risk of bias was assessed and reported in Figure 31 Risk of bias in studies of solid food introduction and Juvenile rheumatoid arthritis. The study had a high overall risk of bias, due to a high dropout rate and therefore high selection bias.

*Main Findings*

We found no evidence to support a relationship between timing of SF introduction and risk of JRA. Only narrative data were reported, which are shown below.

Table 17 Characteristics of included studies evaluating solid food introduction and juvenile rheumatoid arthritis

| **Study** | **Design** | **N/n cases** | **Exposure** | **Outcome** | **Age (yrs)** | **Country** | **Population characteristics** |
| --- | --- | --- | --- | --- | --- | --- | --- |
| Ellis, 2012 [[26](#_ENREF_26)] | CC | 655/246 | Q/I | DD | 18 | Australia | CLARITY. Cases from paediatric rheumatology clinic; controls from paediatric surgery unit born in the same area |

Q: questionnaire, I: interview, R: medical records, PC: prospective cohort, NCC: nested case control, CS: Cross-sectional, CC: case control

Figure 31 Risk of bias in studies of solid food introduction and Juvenile rheumatoid arthritis

### Data for solid food introduction and juvenile rheumatoid arthritis which were not suitable for meta-analysis

The study by Ellis et al showed no significant difference in mean age at solid food introduction between cases and controls (Table 18).

Table 18 Studies reporting solid food introduction and juvenile rheumatoid arthritis which were not suitable for meta-analysis

| **Study** | **Design** | **N/n cases** | **Age (yrs)** | **Measure** | **TBF in No JRA** | **TBF in JRA** | **P** |
| --- | --- | --- | --- | --- | --- | --- | --- |
| Ellis, 2012 [[26](#_ENREF_26)] | CC | 655/246 | continuous | Mean (SD) | 5.6 (2.2) | 5.5 (3.8) | NS |

## Solid food introduction and Thyroid disease

### Overall characteristics of studies, risk of bias and summary of results

The only study reporting data on solid food introduction timing and risk of thyroid disease is shown in Table 19. This was an American case control study with relevant data about 189 subjects. Outcome assessment included medical diagnosis and autoantibody testing, reporting data on Hashimoto and Grave’s disease. Interviews were used to collect information on timing of solid food introduction.

The study was assessed as having a high overall risk of bias (Figure 32), due to lack of adjustment for confounders.

*Main Findings*

We found no evidence to support a relationship between timing of SF introduction and risk of thyroid disease. The paper reported only narrative data, which is shown below.

Table 19 Characteristics of included studies evaluating solid food introduction and Thyroid disease

| **Study** | **Design** | **N/n cases** | **Exposure assessment** | **Method of outcome assessment** | **Age at outcome (years)** | **Country** | **Population characteristics** |
| --- | --- | --- | --- | --- | --- | --- | --- |
| Fort, 1990 [[25](#_ENREF_25)] | CC | 189/59 | I | DD including autoantibody testing - Hashimoto (52) and Graves (7) | 15 | USA | Cases being followed up in clinics with sibling or other controls |

Q: questionnaire, I: interview, R: medical records, PC: prospective cohort, NCC: nested case control, CS: Cross-sectional, CC: case control

Figure 32 Risk of bias in studies of solid food introduction and Thyroid disease

| **Study** | **Design** | **N/n cases** | **Measure** | **TBF in no thyroid disease** | **TBF in thyroid disease** | **P-value** |
| --- | --- | --- | --- | --- | --- | --- |
| Fort, 1990 [[25](#_ENREF_25)] | CC | 189/59 | Mean (SD) | 3.3 (3) | 3.7 (3.3) | NS |

### Data for solid food introduction and thyroid disease which were not suitable for meta-analysis

Table 20 shows the results of the study by Ford et al, who found there was no significant difference in mean age at introduction of solid food between cases and controls.

Table 20 Data for solid food introduction and thyroid disease

# Conclusion

This report summarises the results of 30 studies investigating the association between total and exclusive breastfeeding duration, timing of solid food introduction and risk of autoimmune disease. The majority of studies were retrospective case-control studies. Overall, we found some evidence to support an association between longer duration of total and exclusive breastfeeding and reduced risk of coeliac disease. However, there were high levels of statistical heterogeneity in all relevant analyses, attributed to negative findings in 2 prospective studies but positive findings of an association in the retrospective studies. This is similar to the pattern seen with TIDM, where there are a greater number of studies and events included in meta-analyses.

The current recommendations from the Committee on Nutrition of the European Society for Paediatric Gastroenterology, Hepathology and Nutrition (ESPGHAN) suggest to avoid both early (< 4 months) and late (7 or more months) introduction of gluten. The American Academy of Pediatrics (AAP) recommends that complementary foods can be introduced between 4 and 6 months of age; gluten-containing foods should be introduced while the infant is receiving only breast milk and not infant formula or other bovine milk products. Both AAP and ESPGHAN recommend that gluten is introduced whilst the infant is being breastfed, but do not make specific recommendations about breastfeeding duration in relation to coeliac disease or other autoimmune diseases as an outcome. We did not identify other systematic reviews of these exposures and outcomes with which to compare our findings. Our data would suggest that there is currently no consistent evidence to support a relationship between breastfeeding duration and risk of coeliac disease.

Our data suggest no association between UC and TBF, but the data were extremely heterogeneous for Crohn’s and TBF, such that it is not possible to exclude a significant association – part of this heterogeneity may relate to varied and unreliable methods of exposure assessment.

Analyses of JRA, thyroid disease, and timing of solid food introduction, were limited by small numbers of included subjects and studies, and cannot be taken as evidence for or against an association between the relevant exposure and outcome. For other autoimmune diseases vitiligo and psoriasis, we found no eligible studies. Given the inconsistent signal seen with coeliac disease and TIDM, further study of the relationship between TBF, EBF and other autoimmune diseases such as Crohn’s disease, JRA, thyroid disease, vitiligo and psoriasis seems warranted.

# References

1. Welander A, Tjernberg AR, Montgomery SM, Ludvigsson J, Ludvigsson JF. Infectious disease and risk of later celiac disease in childhood. Pediatrics 2010;125: e530-536.

2. Ascher H, Krantz I, Rydberg L, Nordin P, Kristiansson B. Influence of infant feeding and gluten intake on coeliac disease. Archives of Disease in Childhood 1997;76: 113-117.

3. Auricchio S, Follo D, de Ritis G, Giunta A, Marzorati D, Prampolini L, et al. Does breast feeding protect against the development of clinical symptoms of celiac disease in children? Journal of Pediatric Gastroenterology & Nutrition 1983;2: 428-433.

4. Roberts SE, Williams JG, Meddings D, Davidson R, Goldacre MJ. Perinatal risk factors and coeliac disease in children and young adults: a record linkage study. Aliment Pharmacol Ther 2009;29: 222-231.

5. Decker E, Engelmann G, Findeisen A, Gerner P, Laass M, Ney D, et al. Cesarean delivery is associated with celiac disease but not inflammatory bowel disease in children. Pediatrics 2010;125: e1433-1440.

6. Norris JM, Barriga K, Hoffenberg EJ, Taki I, Miao D, Haas JE, et al. Risk of celiac disease autoimmunity and timing of gluten introduction in the diet of infants at increased risk of disease. Journal of the American Medical Association 2005;293: 2343-2351.

7. Falth-Magnusson K, Franzen L, Jansson G, Laurin P, Stenhammar L. Infant feeding history shows distinct differences between Swedish celiac and reference children. Pediatric Allergy & Immunology 1996;7: 1-5.

8. Ziegler AG, Schmid S, Huber D, Hummel M, Bonifacio E. Early infant feeding and risk of developing type 1 diabetes-associated autoantibodies. JAMA 2003;290: 1721-1728.

9. Ivarsson A, Hernell O, Stenlund H, Persson LA. Breast-feeding protects against celiac disease. American Journal of Clinical Nutrition 2002;75: 914-921.

10. Pacilio A, Piccolo E, Scala MG, Auricchio R. The natural history of celiac disease. Digestive and Liver Disease 2010;42: S357.

11. Peters U, Schneeweiss S, Trautwein EA, Erbersdobler HF. A case-control study of the effect of infant feeding on celiac disease. Annals of Nutrition & Metabolism 2001;45: 135-142.

12. Szajewska H, Chmielewska A, Piescik-Lech M, Ivarsson A, Kolacek S, Koletzko S, et al. Systematic review: Early infant feeding and the prevention of coeliac disease. Alimentary Pharmacology and Therapeutics 2012;36: 607-618.

13. Baron S, Turck D, Leplat C, Merle V, Gower-Rousseau C, Marti R, et al. Environmental risk factors in paediatric inflammatory bowel diseases: a population based case control study. Gut 2005;54: 357-363.

14. Gearry RB, Richardson AK, Frampton CM, Dodgshun AJ, Barclay ML. Population-based cases control study of inflammatory bowel disease risk factors. Journal of Gastroenterology & Hepatology 2010;25: 325-333.

15. Castiglione F, Diaferia M, Morace F, Labianca O, Meucci C, Cuomo A, et al. Risk factors for inflammatory bowel diseases according to the "hygiene hypothesis": a case-control, multi-centre, prospective study in Southern Italy. Journal of Crohn's & colitis 2012;6: 324-329.

16. Corrao G, Tragnone A, Caprilli R, Trallori G, Papi C, Andreoli A, et al. Risk of inflammatory bowel disease attributable to smoking, oral contraception and breastfeeding in Italy: a nationwide case-control study. Cooperative Investigators of the Italian Group for the Study of the Colon and the Rectum (GISC). International Journal of Epidemiology 1998;27: 397-404.

17. Gruber M, Marshall JR, Zielezny M, Lance P. A case-control study to examine the influence of maternal perinatal behaviors on the incidence of Crohn's disease. Gastroenterology Nursing 1996;19: 53-59.

18. Hansen TS, Jess T, Vind I, Elkjaer M, Nielsen MF, Gamborg M, et al. Environmental factors in inflammatory bowel disease: a case-control study based on a Danish inception cohort. Journal of Crohn's & colitis 2011;5: 577-584.

19. Bergstrand O, Hellers G. Breast-feeding during infancy in patients who later develop Crohn's disease. Scandinavian Journal of Gastroenterology 1983;18: 903-906.

20. Koletzko S, Griffiths A, Corey M, Smith C, Sherman P. Infant feeding practices and ulcerative colitis in childhood. BMJ 1991;302: 1580-1581.

21. Thompson NP, Montgomery SM, Wadsworth MEJ, Pounder RE, Wakefield AJ. Early determinants of inflammatory bowel disease: use of two national longitudinal birth cohorts. European Journal of Gastroenterology & Hepatology 2000;12: 25-30.

22. Sonntag B, Stolze B, Heinecke A, Luegering A, Heidemann J, Lebiedz P, et al. Preterm birth but not mode of delivery is associated with an increased risk of developing inflammatory bowel disease later in life. Inflammatory Bowel Diseases 2007;13: 1385-1390.

23. Gilat T, Hacohen D, Lilos P, Langman MJ. Childhood factors in ulcerative colitis and Crohn's disease. An international cooperative study. Scandinavian Journal of Gastroenterology 1987;22: 1009-1024.

24. Wang YF, Ou-Yang Q, Xia B, Liu LN, Gu F, Zhou KF, et al. Multicenter case-control study of the risk factors for ulcerative colitis in China. World Journal of Gastroenterology 2013;19: 1827-1833.

25. Fort P, Moses N, Fasano M, Goldberg T, Lifshitz F. Breast and soy-formula feedings in early infancy and the prevalence of autoimmune thyroid disease in children. Journal of the American College of Nutrition 1990;9: 164-167.

26. Ellis JA, Ponsonby AL, Pezic A, Chavez RA, Allen RC, Akikusa JD, et al. CLARITY - ChiLdhood Arthritis Risk factor Identification sTudY. Pediatric Rheumatology Online Journal 2012;10: 37.

27. Mason T, Rabinovich CE, Fredrickson DD, Amoroso K, Reed AM, Stein LD, et al. Breast feeding and the development of juvenile rheumatoid arthritis. Journal of Rheumatology 1995;22: 1166-1170.

28. Rosenberg AM. Evaluation of associations between breast feeding and subsequent development of juvenile rheumatoid arthritis. Journal of Rheumatology 1996;23: 1080-1082.

29. Greco L, Auricchio S, Mayer M, Grimaldi M. Case control study on nutritional risk factors in celiac disease. Journal of Pediatric Gastroenterology & Nutrition 1988;7: 395-399.

30. Hummel S, Hummel M, Banholzer J, Hanak D, Mollenhauer U, Bonifacio E, et al. Development of autoimmunity to transglutaminase C in children of patients with type 1 diabetes: relationship to islet autoantibodies and infant feeding. Diabetologia 2007;50: 390-394.
