# Supplementary material for: Diet during pregnancy and infancy and risk of allergic or autoimmune disease: A systematic review and meta-analysis
Source: PLoS Med. 2018 Feb 28;15(2):e1002507. doi: 10.1371/journal.pmed.1002507 (PMC5830033; doi:10.1371/journal.pmed.1002507)
Supplement: S1 Data — (ZIP) [file pmed.1002507.s006.zip › Review_A_reports/ECZEMA.docx]

**Breastfeeding, Solid Food Introduction and Risk of Eczema**

Robert J Boyle^1^, Vanessa Garcia-Larsen^2^, Despo Ierodiakonou^3^, Jo Leonardi-Bee^4^, Tim Reeves^5^, Jennifer Chivinge^6^, Zoe Robinson^6^, Natalie Geoghegan^6^, Katharine Jarrold^6^, Andrew Logan^6^, Annabel Groome^6^ , Evangelia Andreou^7^, Nara Tagiyeva-Milne^8^, Ulugbek Nurmatov^9^, Sergio Cunha^10^

^1^Clinical Senior Lecturer, Section of Paediatrics, Imperial College London; ^2^Post-Doctoral Research Associate & Honorary Research Fellow Royal Brompton and Harefield NHS Foundation Trust, Respiratory Epidemiology, Occupational Medicine, and Public Health Group, National Heart and Lung Institute, Imperial College London; ^3^Post-Doctoral Research Associate, Departments of Paediatric and Respiratory Epidemiology and Public Health Group, Imperial College London. ^4^Associate Professor of Community Health Sciences, University of Nottingham; ^5^Research Support Librarian, Faculty of Medicine, Imperial College London; ^6^Undergraduate medical students, Imperial College London; ^7^Research Associate, Imperial Consultants; ^8^Research Fellow, University of Aberdeen; ^9^Research Fellow, University of Edinburgh; ^10^Research Associate, Respiratory Epidemiology and Public Health, National Heart and Lung Institute, Imperial College London

Imperial Consultants,

58 Princes Gate,

Exhibition Road,

London SW7 2PG

CONTENTS

[**List of figures** 3](#_Toc416340840)

[**List of tables** 5](#_Toc416340841)

[**1** **Total breastfeeding duration and eczema** 6](#_Toc416340842)

[1.1 Overall characteristics of studies, risk of bias and summary of results 6](#_Toc416340843)

[1.2 Total breastfeeding duration and eczema 16](#_Toc416340844)

[1.2.1 TBF and risk of eczema in children aged 0-4 years 16](#_Toc416340845)

[1.2.2 TBF and risk of eczema in children aged 5-14 years 22](#_Toc416340846)

[1.2.3 TBF and risk of eczema in children aged 15+ years 28](#_Toc416340847)

[1.3 Data for TBF and risk of eczema that couldn’t be analysed 29](#_Toc416340848)

[**2 Exclusive Breastfeeding Duration and Eczema** 33](#_Toc416340849)

[2.1 Overall characteristics of studies, risk of bias and summary of results 33](#_Toc416340850)

[2.2 Exclusive breastfeeding duration and eczema 42](#_Toc416340851)

[2.2.1 EBF duration and risk of eczema in children aged 0-4 years 42](#_Toc416340852)

[2.2.2 EBF duration and the risk of eczema in children aged 5-14 years 48](#_Toc416340853)

[2.2.3 EBF duration and risk of eczema in children aged 15+ years 50](#_Toc416340854)

[2.3 Exclusive breastfeeding duration and atopic eczema 52](#_Toc416340855)

[2.3.1 EBF and atopic eczema in children aged 0-4 years 52](#_Toc416340856)

[2.3.2 EBF and atopic eczema in children aged 5-14 years 52](#_Toc416340857)

[2.4 Data for TBF and risk of eczema that couldn’t be analysed 53](#_Toc416340858)

[**3 Solid food introduction and atopic eczema** 56](#_Toc416340859)

[3.1 Overall characteristics of studies, risk of bias and summary of results 56](#_Toc416340860)

[3.1.1 SF introduction and risk of eczema in children aged 0-4 years 61](#_Toc416340861)

[3.1.2 SF introduction and risk of eczema in children aged 5-14 years 64](#_Toc416340862)

[3.2 Data for SF and risk of eczema that couldn’t be analysed 64](#_Toc416340863)

[**4 Conclusions** 66](#_Toc416340864)

[**5 References** 68](#_Toc416340865)

# List of figures

[Figure 1 Risk of bias in studies of TBF duration and eczema 16](#_Toc497230439)

[Figure 2 TBF Ever vs. Never and risk of eczema at age 0-4 years 17](#_Toc497230440)

[Figure 3 Risk of publication bias in studies investigating TBF Ever vs. Never and risk of eczema in children aged 0-4 years 18](#_Toc497230441)

[Figure 4 TBF short duration (1-3 months) vs. never and risk of eczema at age 0-4 years 20](#_Toc497230442)

[Figure 5 TBF medium duration (4-6 months) and risk of eczema at age 0-4 years 20](#_Toc497230443)

[Figure 6 TBF long duration (≥7-12 months) and risk of eczema at age 0-4 years 20](#_Toc497230444)

[**Figure 7 TBF ≥3-4 months vs. <3-4 months and risk of eczema at age 0-4 years** 21](#_Toc497230445)

[**Figure 8 TBF ≥5-7 months vs. <5-7 months and risk of eczema at age 0-4 years** 21](#_Toc497230446)

[**Figure 9 TBF ≥8-12 months vs. <8-12 months and risk of eczema at age 0-4 years** 22](#_Toc497230447)

[Figure 10 TBF Ever vs. Never and risk of eczema at age 5-14 years 23](#_Toc497230448)

[Figure 11 Risk of publication bias in studies investigating TBF and risk of eczema in children aged 5-14 years 23](#_Toc497230449)

[Figure 12 TBF short duration (1-3 months) vs. never and risk of eczema at age 5-14 years 25](#_Toc497230450)

[Figure 13 TBF medium duration (4-6 months) vs. never and risk of eczema at age 5-14 years 25](#_Toc497230451)

[Figure 14 TBF long duration (7-12 months) vs. never and risk of eczema at age 5-14 years 25](#_Toc497230452)

[**Figure 15 TBF ≥1-2 months vs. <1-2 months and risk of eczema at age 5-14 years** 26](#_Toc497230453)

[**Figure 16 TBF ≥3-4 months vs. <3-4 months and risk of eczema at age 5-14 years** 26](#_Toc497230454)

[**Figure 17 TBF for ≥5-7 months vs. <5-7 months and risk of eczema at age 5-14 years** 27](#_Toc497230455)

[**Figure 18 TBF for ≥8-12 months vs. <8-12 months and risk of eczema at age 5-14 years** 28](#_Toc497230456)

[**Figure 19 TBF Never vs. Any and risk of eczema at age 15+ years** 29](#_Toc497230457)

[**Figure 20 TBF ≥3-4 months vs. <3-4 months and risk of eczema at age 15+ years** 29](#_Toc497230458)

[Figure 21 Risk of bias in studies of exclusive breastfeeding duration and eczema 42](#_Toc497230459)

[**Figure 22 EBF ≥0-2 months vs. <0-2 months and risk of eczema at age 0-4 years** 42](#_Toc497230460)

[**Figure 23 EBF for ≥3-4 months vs. <3-4 months and risk of eczema at age 0-4 years** 45](#_Toc497230461)

[**Figure 24 Risk of publication bias in studies investigating EBF duration for ≥3-4 months vs. <3-4 months and risk of eczema in children aged 0-4 years** 46](#_Toc497230462)

[**Figure 25 EBF ≥5 -9months vs. <5-9 months and risk of eczema at age 0-4 years** 48](#_Toc497230463)

[**Figure 26 EBF ≥0-2 months vs. <0-2 months and risk of eczema at age 5-14 years** 49](#_Toc497230464)

[**Figure 27 EBF for ≥3-4 months vs. <3-4 months and risk of eczema at age 5-14 years** 49](#_Toc497230465)

[**Figure 28 EBF for ≥5 months vs. <5 months and risk of eczema at age 5-14 years** 50](#_Toc497230466)

[**Figure 29 EBF for ≥0-2 months vs. <0-2 months and risk of eczema at age 15+ years** 51](#_Toc497230467)

[**Figure 30 EBF ≥3-4 months vs. <3-4 months and risk of eczema at age 15+ years** 51](#_Toc497230468)

[**Figure 31 EBF ≥5 months vs. <5 months and risk of eczema at age 15+ years** 51](#_Toc497230469)

[**Figure 32 EBF ≥3-4 months vs. <3-4 months and risk of atopic eczema at age 0-4 years** 52](#_Toc497230470)

[**Figure 33 EBF ≥0-2 months vs. <0-2 months and risk of atopic eczema at age 5-14 years** 52](#_Toc497230471)

[**Figure 34 EBF for ≥3-4 months vs. <3-4 months and risk of atopic eczema at age 5-14 years** 53](#_Toc497230472)

[Figure 35 Risk of bias in studies of solid food introduction and eczema 61](#_Toc497230473)

[Figure 36 SF ≥3-4 months vs. <3-4 months and risk of atopic eczema at age 0-4 years 62](#_Toc497230474)

[Figure 37 Risk of publication bias in studies investigating SF introduction ≥3-4 months vs. <3-4 months and risk of eczema in children aged 0-4 years 62](#_Toc497230475)

[Figure 38 SF ≥3-4 months vs. <3-4 months and risk of eczema at age 5-14 years 64](#_Toc497230476)

#

# List of tables

[Table 1 Characteristics of included studies evaluating TBF duration and eczema 8](#_Toc497230477)

[Table 2 Subgroup Analyses of risk of atopic dermatitis and TBF Ever vs. Never in children aged 0-4 years 19](#_Toc497230478)

[Table 3 Subgroup analyses of risk of eczema and TBF Ever vs. Never in children aged 5-14 years 24](#_Toc497230479)

[Table 4 Studies investigating the association between TBF duration and eczema which were not eligible for meta-analysis 30](#_Toc497230480)

[Table 5 Characteristics of included studies evaluating EBF duration and eczema 35](#_Toc497230481)

[Table 6 Subgroup Analyses EBF duration ≥0-2 vs. <0-2 months and risk of AD at 0-4 years 43](#_Toc497230482)

[Table 7 EBF for 6 months versus 3 months and risk of RC 44](#_Toc497230483)

[Table 8 Subgroup Analyses of risk of atopic dermatitis and EBF duration ≥3-4 months vs. <3-4 months in children aged 0-4 years 47](#_Toc497230484)

[Table 9 Studies investigating the association between EBF duration and eczema which were not eligible for meta-analysis 54](#_Toc497230485)

[Table 10 Characteristics of included studies evaluating solid food introduction and eczema 57](#_Toc497230486)

[Table 11 Subgroup Analyses of risk of atopic dermatitis and SF ≥3-4 months vs. <3-4 months in children aged 0-4 years 63](#_Toc497230487)

[Table 12 Studies investigating the association between solid food introduction and eczema which were not eligible for meta-analysis 65](#_Toc497230488)

# Total breastfeeding duration and eczema

## Overall characteristics of studies, risk of bias and summary of results

Table 1 describes the main characteristics of the studies that assessed total breastfeeding duration (TBF) in relation to eczema/atopic dermatitis (AD) risk. In this report AD or eczema refers to any eczema/atopic dermatitis, and ‘atopic AD’ refers to eczema in association with evidence of IgE sensitisation to dietary or inhalant allergens. A total of 1 cluster randomised controlled trial and 57 observational studies, reported the association between TBF and AD. Of these, 43 were prospective cohort studies, 1 retrospective cohort study, 1 nested case-control study, 3 case control studies, and 9 cross-sectional studies. The majority of studies (n=40) are from Europe – others are from Asia (n=7), North America (n=3) and Australasia (n=3), Africa (n=1), Middle East (n=1) and worldwide (n=2). Overall, valid data on TBF duration in the first 2 years of life and AD risk were available from over 250,000 subjects. Information on AD was obtained from a medical assessment (n=23), parental report (n=15), Hanifin and Rajka criteria or modifications of these (n=8), the ISAAC questionnaire (n=9), self-report (n=1) and unclear methodology (n=1). With regards to time of outcome diagnosis, 33 studies explored the association between TBF duration and AD at age 0-4, 18 at ages 5-14, and six at age 15 and over. For assessment of TBF duration, 10 studies used an interview, 8 parent diaries, and 4 a medical records review; in one study the method of exposure assessment was unclear and in all others (n=24) a questionnaire method was used.

Risk of bias was assessed using the NICE Methodological checklists for cohort and case-control studies. Figure 1 illustrates the distribution of bias across the five main methodological areas of the studies. Approximately 30% of studies had a high risk of bias due to lack of adjustment for confounding bias i.e. no adjusted data presented, and a further 10-15% had a high risk of selection bias. Risk of conflict of interest was generally assessed as low.

Where data were available, five levels of comparison were used to assess the risk of AD according to TBF duration, namely ‘ever vs. never’, ‘≥1-2 months vs. <1-2 months’, ‘≥3-4 months vs. <3-4 months’, ‘≥5-7 months vs. <5-7 months’, and ‘≥8-12 months vs. <8-12 months’.

*Main Findings*

In the single intervention trial there was LOW certainty evidence (-1 imprecision; -1 inconsistency) of a relationship between breastfeeding promotion and reduced risk of AD in the first year of life; OR 0.54 (95% CI 0.31, 0.95). This effect was not seen at later follow up at age 6-7 years. For observational studies, across all cut-offs where data were available, there was no consistent evidence of a relationship between risk of AD and initiation or prolongation of BF. There was weak evidence that ever BF may be associated with increased AD risk at age 0-4 years; OR 1.15 (1.00, 1.32; I^2^=57%). This was not the case in the subgroup of studies with low risk of bias, prospective study design, or adjusted data analysis. Studies which could not be included in meta-analysis, which represented over 16,000 participants in 16 studies, almost universally found no evidence of a relationship between TBF duration and AD.

Table 1 Characteristics of included studies evaluating TBF duration and eczema

| **Study** | **N/n cases** | **Design** | **Country** | **Exposure assessment** | **Method of outcome assessment** | **Age at outcome (years)** | **Population characteristics** |
| --- | --- | --- | --- | --- | --- | --- | --- |
| Kramer, 2001 ([1](#_ENREF_1)); Kramer, 2007 ([2](#_ENREF_2)) | 8865/8181 | Cluster RCT | Belarus | - | SPT-Aero | 6.5 | Breastfeeding promotion program based on the WHO/UNICEF baby friendly hospital initiative, versus standard local breastfeeding policies |
| Hesselmar, 2010 ([3](#_ENREF_3)) | 184 | PC | Sweden | I | UK Working Party Criteria | 1.5 | ALLERGYFLORA study. Population based study of babies selected from antenatal clinics between 1998 and 2003 - mainly high risk of allergic disease |
| Abd, 2012 ([4](#_ENREF_4)) | Unclear | PC | UK | Q | Visible flexural dermatitis; Parent reported eczema | 3.5, 7.5 | ALSPAC study. Population based cohort of children born 1991-1992. |
| Purvis, 2005([5](#_ENREF_5)) | 550 | PC | New Zealand | Q | UK Working Party Criteria | 3.5 | Auckland Birthweight Collaborative study. Birth cohort with representative sample of babies born in 1996-1997 |
| Kull, 2002([6](#_ENREF_6)) | 3790 | PC | Sweden | Q | Parent reported OR DD eczema | 2 | BAMSE study. Population based cohort of children born between 1994-1996 |
| Berth-Jones, 1997 ([7](#_ENREF_7)) | 413 | PC | UK | Q | Physician assessment | 1 | Infants born in 1992 and registered with the two major Leicester obstetric units |
| Taylor, 1984 ([8](#_ENREF_8)) | 14498 | PC | UK | I/Q | Parent reported eczema | 5, 6, 7 | British Cohort Studies of children born in England and Wales in 1946; England, Scotland and Wales in 1958 and 1970 |
| Taylor, 1983 ([9](#_ENREF_9)) | 10886 | PC | UK | I | Parent reported eczema | 5 | CHES study. Population based cohort of children born in England, Scotland, and Wales in 1970 |
| Burr, 1989; Burr, 1993; Burr, 1993 (b)  ([10-12](#_ENREF_10)) | 483 | PC | UK | D, Q | Physician assessment | 1, 7 | Infants with family history of allergic diseases in South Wales |
| Businco, 1987([13](#_ENREF_13)) | 244 | PC | Italy | I | Physician assessment | 8 | Infants of atopic parents recruited from hospital and born in 1985-1988 |
| Mihrshahi, 2007 ([14](#_ENREF_14)) | 516 | PC | Australia | I | Visible flexural dermatitis OR DD eczema | 5 | CAPS study. Infants born in 1997-1999 with family history of asthma or wheezing |
| Bisgaard, 2009 ([15](#_ENREF_15)) | 354 | PC | Denmark | Q | Hanifin and Rajka criteria | 3 | COPSAC study. Infants of mothers with a history of doctor-diagnosed asthma, recruited from August 1998 to December 2001. |
| Larsson, 2008 ([16](#_ENREF_16)) | 4779 | PC | Sweden | Q | ISAAC | 9 | DBH study. Preschool children aged 1–6 years surveyed in 2000 and 2005. |
| Devereux, 2006 ([17](#_ENREF_17)) | 1704 | PC | UK | Q | ISAAC | 5 | Population based birth cohort of infants born in 1998 |
| Nwaru, 2013([18](#_ENREF_18)) | 3109 | PC | Finland | D | ISAAC | 0.5 | DIPP study. Infants at high risk (HLA) for TIDM born between 1996-2004 invited to the allergy study between 1998 and 2000 |
| Farooqi, 1998 ([19](#_ENREF_19)) | 1453 | PC | UK | R | DD | 16 | Representative sample of general practice born in 1975-84 |
| Forster, 1990([20](#_ENREF_20)) | 145 | PC | Germany | Q | Parental report | 1.5 | Babies hospitalised in 1985 |
| Harris, 2001([21](#_ENREF_21)) | 622 | PC | UK | I | UK Working Party Criteria | 2 | Population based birth cohort of children born between 1993 and 1995 in three general practices in Ashford |
| Hikino, 2001([22](#_ENREF_22)) | 21766 | PC | Japan | Q | Physician assessment | 1.5 | All children born in 1993-1995 attending Well-baby check-ups funded by Fukuoka City |
| Howie, 1990([23](#_ENREF_23)) | 618 | PC | UK | R/D | Physician assessment | 1 | Population based birth cohort of infants born between 1983 and 1986 in Dundee |
| Gruskay, 1982 ([24](#_ENREF_24)) | 908 | PC | USA | Q | Physician assessment | 3, 5, 15 | Children born in 1961-1966 in a private paediatric practice |
| Alm, 2008 ([25](#_ENREF_25)) | 4941 | PC | Sweden | Q | Parent reported eczema | 1 | Infants of Western Sweden. Population birth cohort of infants born in 2003 |
| Morales, 2012 ([26](#_ENREF_26)) | 467 | PC | Spain | Q/I | Parent reported eczema | 1 | INMA project. Population based birth cohort of infants born 2004-2006 |
| Kemeny, 1991([27](#_ENREF_27)) | 180 | PC | UK | Unclear | Unclear | 1 | Population based birth cohort of infants born at Dulwich and King’s College Hospitals in London |
| Snijders, 2007; Snijders, 2008([28](#_ENREF_28), [29](#_ENREF_29)) | 2510/ 783 | PC | Netherlands | Q | Parent reported eczema | 2 | KOALA study. Population based birth cohort of infants born between 2000-2002 (conventional and anthroposophic lifestyle) |
| Wetzig, 2000([30](#_ENREF_30)) | 475 | PC | Germany | Q | Physician assessment | 1 | LARS study. High allergy risk or low birth weight children born within one year in the City and District of Leipzig |
| Marini, 1996 ([31](#_ENREF_31)) | 279 | PC | Italy | Q | Physician assessment | 1 | Infants with family history of allergy whose mother were proposed to participate in an allergy prevention program |
| Miskelly, 1988([32](#_ENREF_32)) | 482 | PC | UK | D | Physician assessment | 1 | Infants from antenatal clinics with family history of allergy randomised into a dietary intervention program |
| Moore, 1985([33](#_ENREF_33)) | 475 | PC | UK | D/I | Physician assessment | 0.25 | Infants born in a hospital in 1979-1980 with family history of eczema or asthma, participating in a dietary intervention program |
| Morgan, 2004; Morgan, 2004 (b) ([34](#_ENREF_34), [35](#_ENREF_35)) | 257 | PC | UK | I | Parent reported eczema; Physician assessment | 1, 1.5 | Infants from five prospective randomised dietary trials conducted in the UK between 1993 and 1997. One LBW, 2 premature and 2 appropriate weight/gestation cohorts |
| Bergmann, 2000; Bergmann, 2002([36](#_ENREF_36), [37](#_ENREF_37)) | 1314 | PC | Germany | Q/I | Physician assessment; Hanifin and Rajka criteria | 6, 7 | MAS study. Atopic risk enriched cohort of infants born in 1990 in 5 German cities |
| Silvers, 2009 ([38](#_ENREF_38)) | 987 | PC | New Zealand | Q | Parent reported eczema | 1 | New Zealand Asthma and Allergy Cohort Study. Population based birth cohort of infants born between 1997 and 2001 |
| Miyake, 2009([39](#_ENREF_39)) | 763 | PC | Japan | Q | Physician assessment | 2 | OMCHS study. Population based birth cohort of infants born in 2002-2003 |
| Kerkhof, 2003([40](#_ENREF_40)) | 304 | PC | Netherlands | Q | UK Working Party Criteria | 1 | PIAMA: population-based born in 1996-1997 (normal risk of disease) |
| Porch, 1998([41](#_ENREF_41)) | 130 | PC | USA | D | ISAAC - current AD | 1 | Infants recruited from prenatal services with family history of allergy (high risk of disease) |
| Pratt, 1984([42](#_ENREF_42)) | 198 | PC | UK | D/I | DD; Physician assessment | 4.5, 5 | Recruited in antenatal clinics, normal risk of disease |
| Rothenbacher, 2005([43](#_ENREF_43)) | 803 | PC | Germany | Q/I | DD | 2 | Recruited from university service, born in 2000-2001 (normal risk of disease) |
| Ruiz 1992([44](#_ENREF_44)) | 39 | PC | UK | Unclear | Hanifin and Rajka criteria | 1 | Recruited from hospital and family history of atopy (high risk of disease) |
| Saarinen, 1995([45](#_ENREF_45)) | 150 | PC | Finland | R | Parent reported OR DD eczema | 5, 17 | Recruited from hospital and born in 1975 (normal risk of disease) |
| Chuang, 2011([46](#_ENREF_46)) | 18773 | PC | Taiwan | R/I | Physician assessment | 1.5 | Taiwan Birth Cohort Study: population representative sample born in 1995 (normal risk of disease) |
| Hide, 1981([47](#_ENREF_47)) | 843 | PC | UK | D/Q | DD | 1 | The Isle of Wight study: born in 1977-1978 (normal risk of disease) |
| Wang, 2007([48](#_ENREF_48)) | 1760 | PC | Taiwan | Q | DD | 0.5 | Tiawan National Birth Cohort Study: population representative sample born in 2003 (normal risk of disease) |
| Zutavern, 2004([48](#_ENREF_48)) | 604 | PC | UK | I | DD | 5.5 | Cohort recruited from general practices and born in 1993-1995 (normal risk of disease) |
| Hagendorens, 2005; Sariachvili, 2007([49](#_ENREF_49), [50](#_ENREF_50)) | 976 | PC | Belgium | Q | Parent reported eczema | 1 | PIPO study: recruited from university service, born 1997-2001 (normal risk of disease) |
| Sariachvili 2010 ([51](#_ENREF_51)) | 557 | NCC | Belgium | Q | ISAAC | 4 | PIPO: nested case control study within the PIPO cohort |
| Monego, 1989([52](#_ENREF_52)) | 144 | RC | Italy | D | DD | 4 | 144 Italian infants retrospectively followed to examine association between breastfeeding and allergic diseases |
| Kramer, 1981 ([53](#_ENREF_53)) | 470 | CC | Canada | I | Hanifin and Lobitz criteria | <20 | Cases and controls were1 month to 20 years old children attending dermatology clinics visits |
| Ghaderi, 2014 ([54](#_ENREF_54)) | 200 | CC | Iran | I | DD | 5 | Unclear source of population. Sex and age matched controls |
| Haileamlak, 2005 ([55](#_ENREF_55)) | 732 | CC | Ethiopia | I | ISAAC | 5 | Children age 1- 5 years. Cases were defined according to the ISAAC criteria for AD and confirmed by clinical examination |
| Flohr, 2011 ([56](#_ENREF_56)) | 51,  119 | CS | Worldwide | Q | Parent reported eczema | 12 | ISAAC Phase 2. Schoolchildren aged 8–12 years from 27 centres in 21 affluent and nonaffluent countries |
| Civelek, 2001([57](#_ENREF_57)) | 1533 | CS | Turkey | Q | ISAAC | 11 | Representative sample of schoolchildren in 5 cities |
| Nakamura, 1999([58](#_ENREF_58)) | 3850 | CS | Japan | Q | DD | 3 | All 3 year old children participating in a health check-up in 1997 |
| Tanaka, 2009([59](#_ENREF_59)) | 1957 | CS | Japan | Q | ISAAC - current AD | 3 | Fukuoka Child Health Study. All 3-year old children examined at public public health centre’s in Fukuoka city |
| Girolomoni,2003([60](#_ENREF_60)) | 1369 | CS | Italy | Q | UK Working Party Criteria | 9 | Representative sample of children attending the fourth grade at elementary school |
| Björkstén, 2011([61](#_ENREF_61)) | 103716 | CS | Worldwide | Q | ISAAC - AD ever | 7 | ISAAC Phase 3: Schoolchildren aged 6-7 years from different countries and geographic regions |
| Karino, 2008([62](#_ENREF_62)) | 9615 | CS | Japan | Q | Self-reported AD | 18 | University students aged 18–19 years enrolled from 2003 through 2005. |
| Kurt, 2007 ([63](#_ENREF_63)) | 25843 | CS | Turkey | Q | Parent reported current eczema | 15 | Prevalence and Risk Factors of Allergies in Turkey (PARFAIT): population representative sample of children aged 9-15 years old (normal risk of disease0 |
| Selcuk, 1997 ([64](#_ENREF_64)) | 5412 | CS | Turkey | Q | Parent reported current eczema | 12 | Children 7-12 yrs. from 18 primary schools (normal risk of disease) |

Q: questionnaire, I: interview, R: medical records, D: diary, PC: prospective cohort, RC: retrospective cohort, NCC: nested case-control, CC: case-control, CS: Cross-sectional, AD: eczema, DD: doctor diagnosis of AD, in contrast to ‘Physician assessment’ where AD diagnosis was always made by a study physician as part of the study protocol.

Figure 1 Risk of bias in studies of TBF duration and eczema

## Total breastfeeding duration and eczema

The single intervention trial of a breastfeeding promotion intervention was rated as having a low risk of bias on all domains, and a low risk of conflict of interest. Kramer found reduced odds of AD at age 1 year – cluster adjusted odds ratio 0.54 (95% CI 0.31, 0.95). This outcome was an unblinded assessment of AD by study paediatricians, and the authors reported poor agreement between sites in the outcome measure. The effect on AD was not seen at later follow-up in the same trial at age 6.5 years (OR 1.0 95% CI 0.50, 1.80). Certainty of evidence was downgraded for imprecision (wide confidence intervals) and for inconsistency with later outcome data and with the findings of observational studies. All other evidence was derived from observational studies.

### TBF and risk of eczema in children aged 0-4 years

#### TBF Ever vs. Never

Figure 2 shows the outcomes of 20 eligible observational studies reporting OR for TBF never vs ever and AD. The pooled data show increased risk of AD in breastfed infants, with borderline statistical significance and high heterogeneity (I^2^=57%). The outlier study of Purvis had very few participants in the reference group of never breastfed (n=26, including just 3 cases of AD), which may account for the extreme OR and wide confidence intervals.

A Funnel plot to explore publication bias is shown in Figure 3, and shows no clear evidence of publication bias.

Subgroup analyses are shown in Table 2. Here we did not find clear subgroup differences, but there was a trend to different outcome according to risk of allergic disease. Those children with no increased risk of allergic disease (i.e. not selected for family history of allergic problems) had the strongest association between TBF initiation and increased AD. Heterogeneity was high (I^2^=53%).

Analysis of dose-response relationship, using studies with short, medium or long duration/cut-off for the exposed group, with ‘never BF’ as reference group, showed some evidence of a dose-response relationship between TBF and AD although with moderate to high heterogeneity in each analysis (Figures 4 and 5).

Figure 2 TBF Ever vs. Never and risk of eczema at age 0-4 years


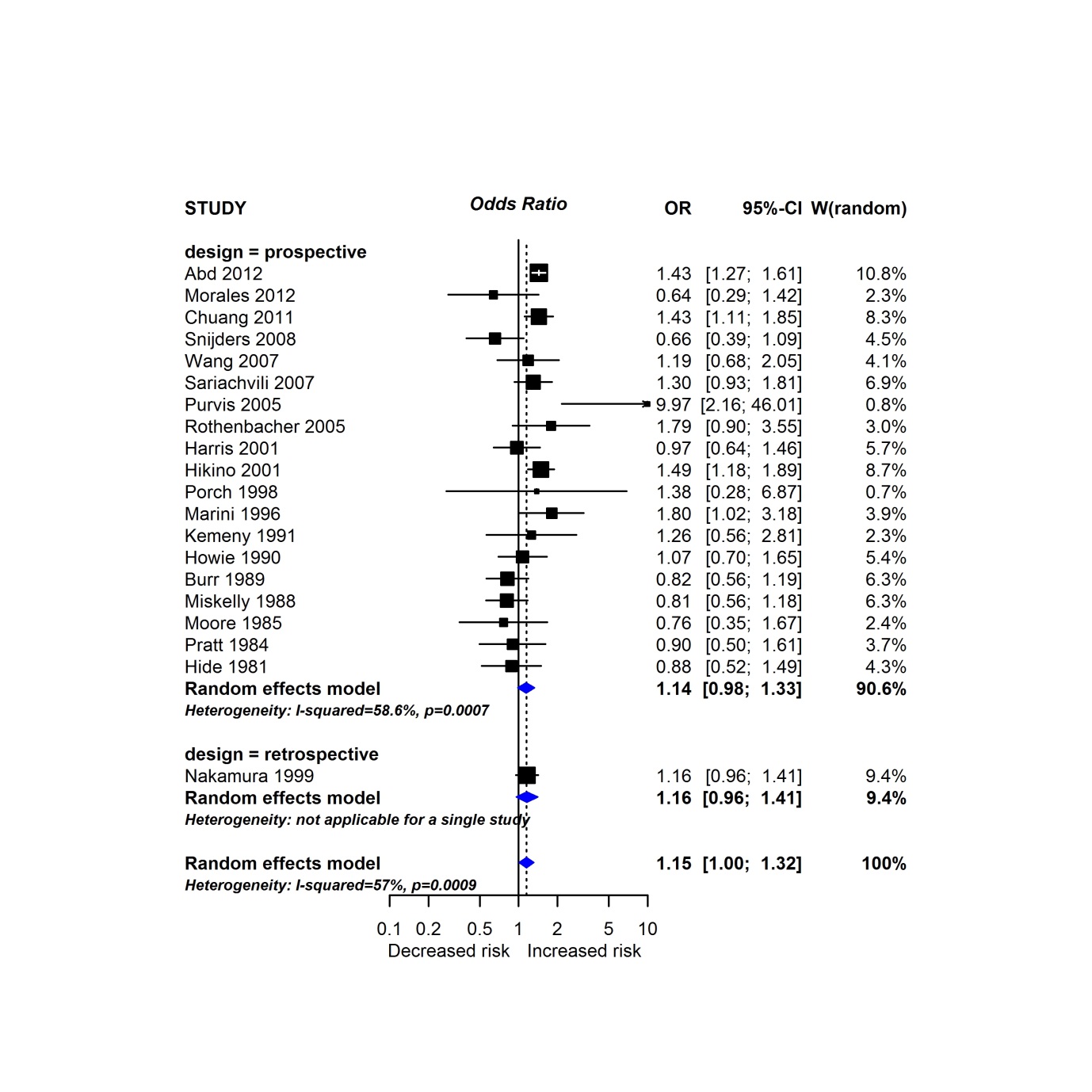


Figure 3 Risk of publication bias in studies investigating TBF Ever vs. Never and risk of eczema in children aged 0-4 years


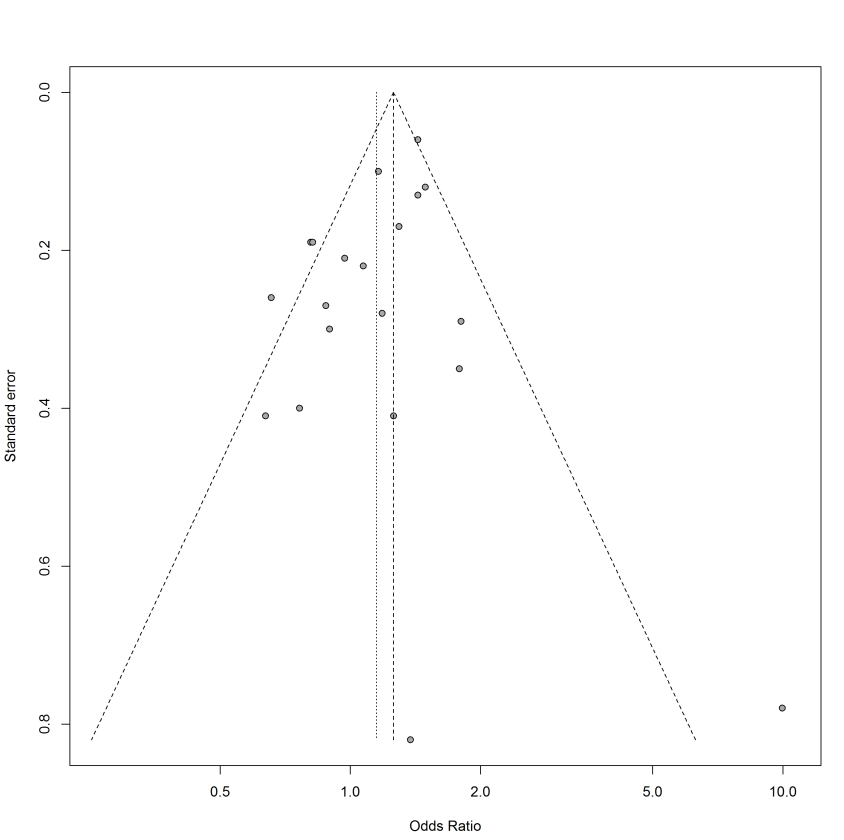


Egger’s test p-value = 0.116

Table 2 Subgroup Analyses of risk of atopic dermatitis and TBF Ever vs. Never in children aged 0-4 years

|  | **Number of studies** | **OR [95% CI]** | **I^2^ (%)** | **P-value for between group difference** |
| --- | --- | --- | --- | --- |
| **Overall**  **Adjusted**  **Unadjusted** | 20 | 1.15 [1.00; 1.32] | 57.0 |  |
|  | 7 | 1.17 [0.87; 1.57] | 70.1 | Not tested |
|  | 19 | 1.78 [1.03; 1.35] | 58.3 |  |
| Study Design – Prospective  Study Design – Retrospective | 19 | 1.14 [0.98; 1.33] | 58.6 | 0.90 |
|  | 1 | 1.16 [0.96; 1.41] | - |  |
| Risk of disease – High  Risk of disease – Normal | 5 | 0.97 [0.70; 1.34] | 40.0 | 0.22 |
|  | 15 | 1.21 [1.05; 1.39] | 53.0 |  |
| Risk of bias – Low  Risk of bias – High/Unclear | 7 | 1.02 [0.80; 1.32] | 55.8 | 0.23 |
|  | 13 | 1.22 [1.04; 1.44] | 54.3 |  |

Figure 4 TBF short duration (1-3 months) vs. never and risk of eczema at age 0-4 years


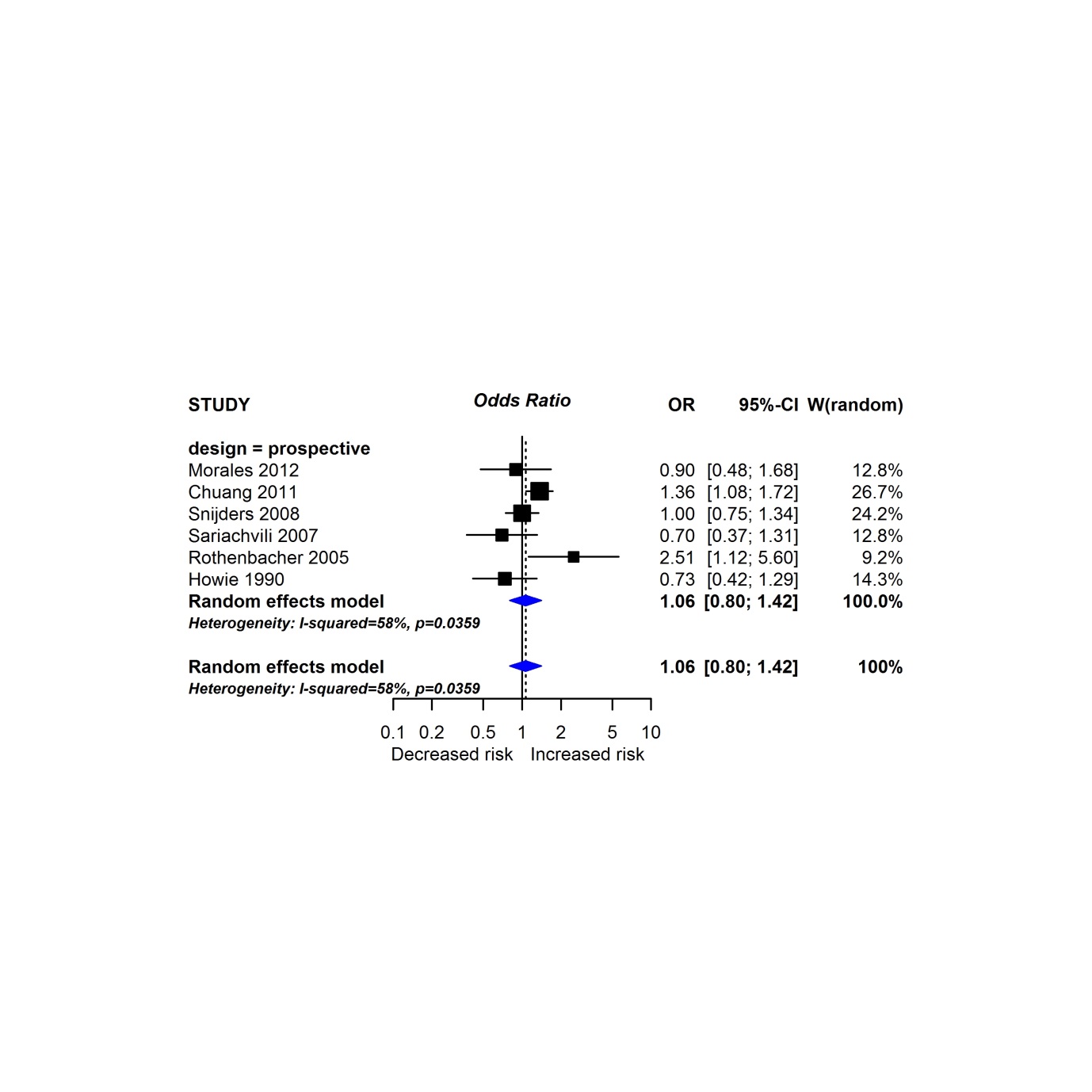


Figure 5 TBF medium duration (4-6 months) and risk of eczema at age 0-4 years


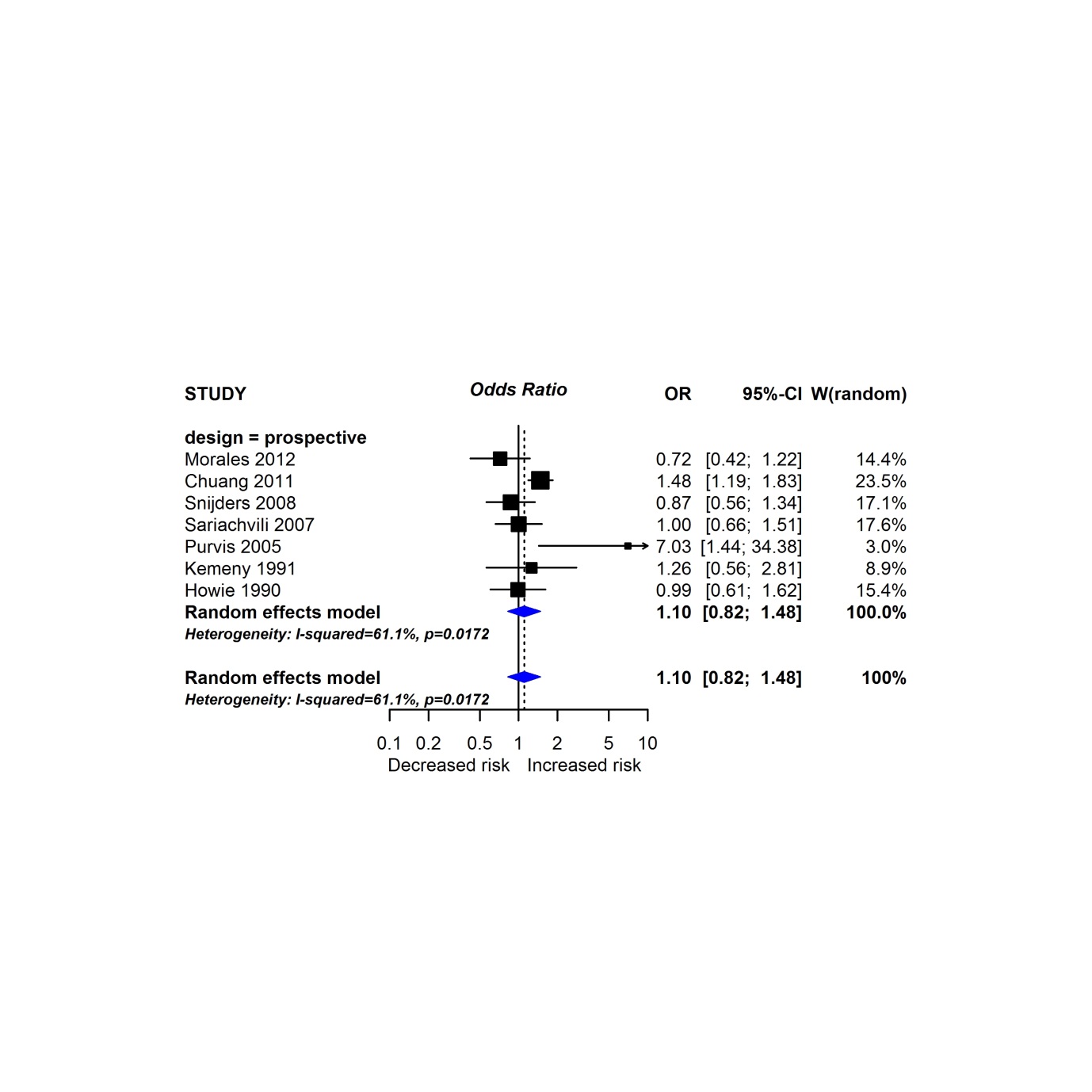


Figure 6 TBF long duration (≥7-12 months) and risk of eczema at age 0-4 years


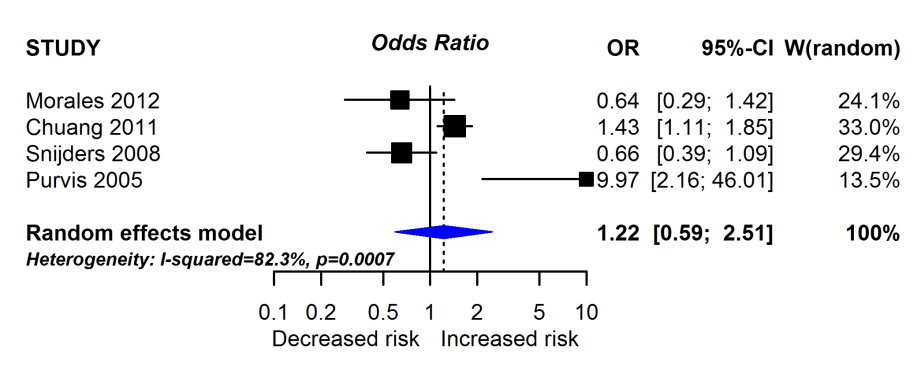


#### TBF ≥3-4 months vs. <3-4 months

One nested case control study and two prospective cohort studies reported data that could be pooled to calculate OR for AD, in infants with TBF for ≥3-4 months vs. <3-4 months duration and are shown in Figure 7. They show no evidence of association between TBF and AD risk, with low statistical heterogeneity. All studies report unadjusted data, so carry a high risk of confounding bias.

**Figure 7 TBF ≥3-4 months vs. <3-4 months and risk of eczema at age 0-4 years**


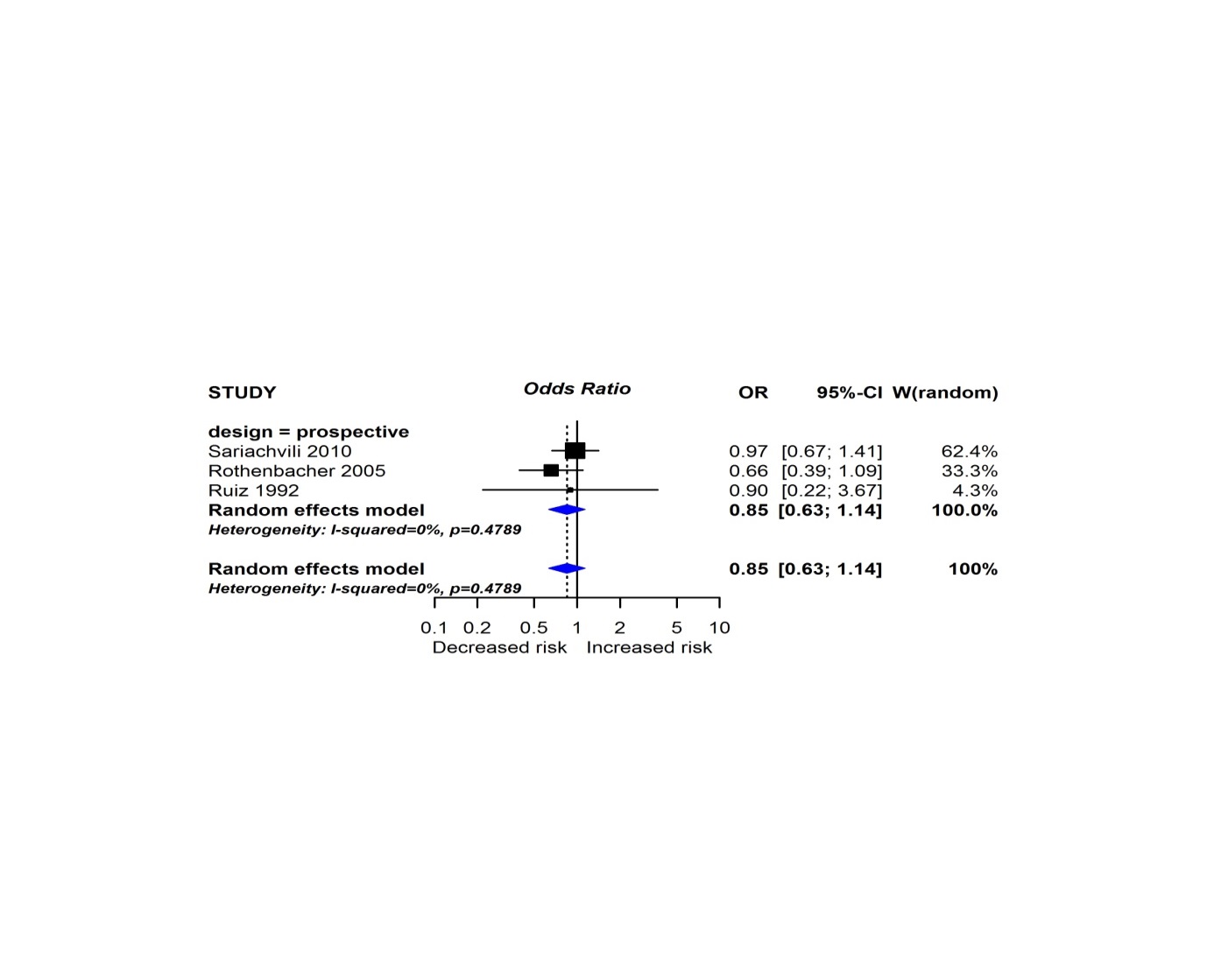


#### TBF ≥5-7 months vs. <5-7 months

One cross-sectional and three prospective cohort studies reported data that could be pooled to calculate OR for AD, in infants with TBF for ≥5-7 months vs. <5-7 months duration and are shown in Figure 8. They show no evidence of association between TBF and AD risk, with low statistical heterogeneity. Three of the four studies report adjusted data, and the study of Hide reported unadjusted data.

**Figure 8 TBF ≥5-7 months vs. <5-7 months and risk of eczema at age 0-4 years**


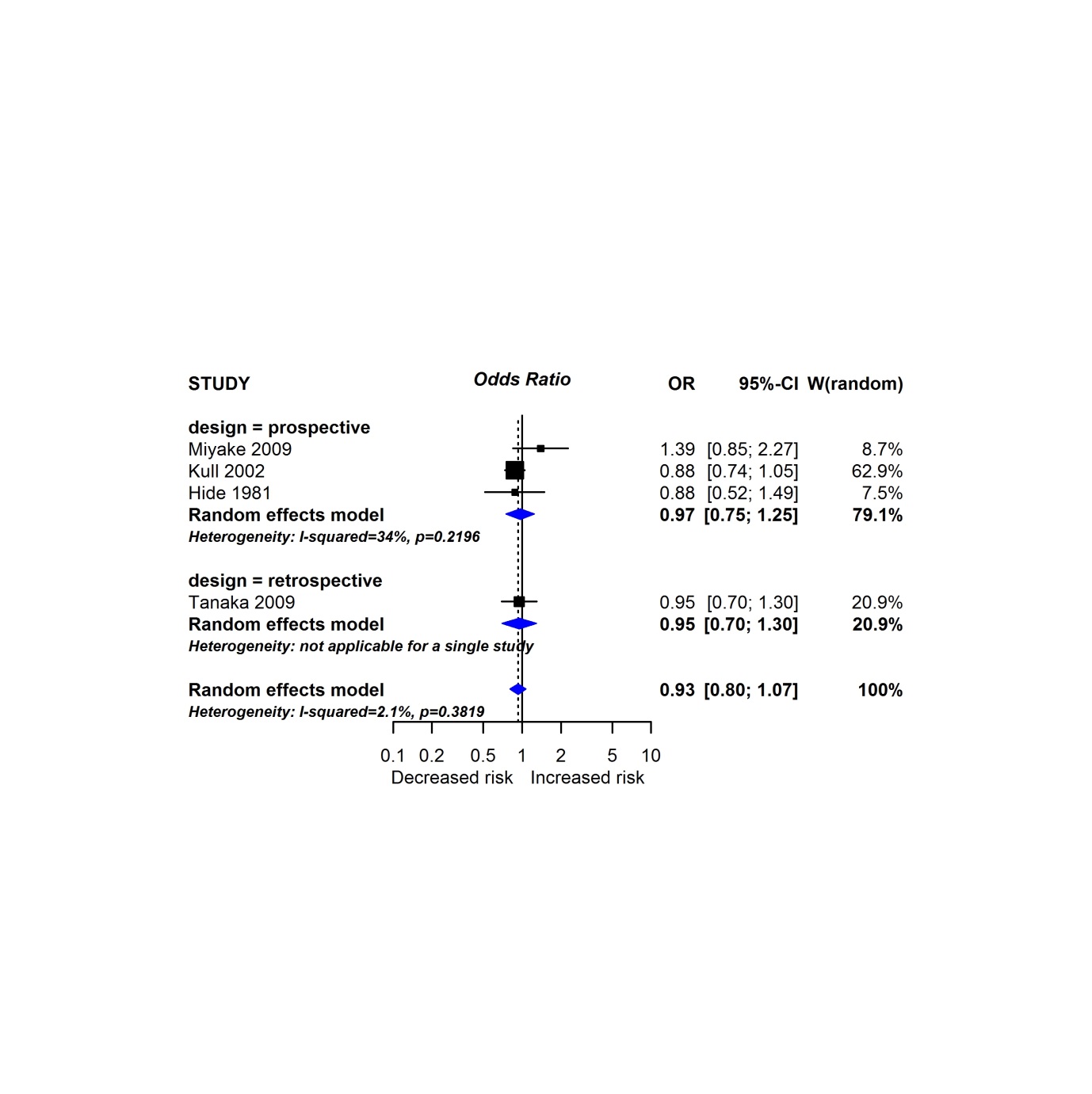


#### TBF ≥8-12 months vs. <8-12 months

Two case-control studies reported data that could be pooled to calculate OR for AD, in infants with TBF for ≥12 months vs. <12 months duration and are shown in Figure 9. Due to extreme statistical heterogeneity (I^2^= 84.6%) we did not undertake pooled analysis. The study of Haileamlak reported adjusted data, and the study of Ghaderi reported unadjusted data.

**Figure 9 TBF ≥8-12 months vs. <8-12 months and risk of eczema at age 0-4 years**


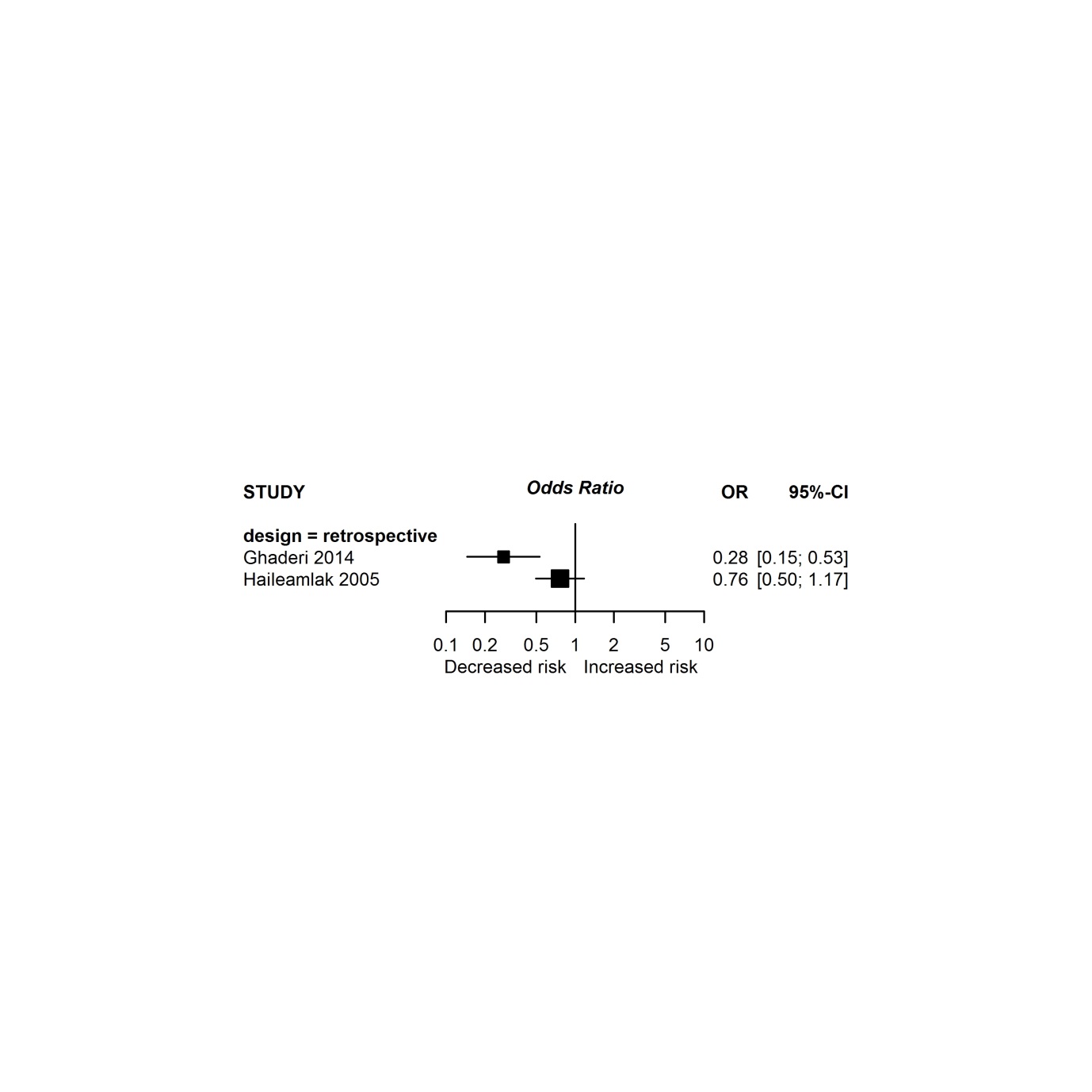


### TBF and risk of eczema in children aged 5-14 years

#### TBF Ever vs. Never

Figure 10 shows the outcomes of 13 eligible observational studies reporting OR for TBF never vs ever and AD at age 5-14 years. The pooled data show association between breastfeeding and risk of AD, but with extreme statistical heterogeneity (I^2^=79%). The analysis was dominated by the ISAAC phase II and III surveys published in 2011, and the British Birth Cohorts published in 1984. A Funnel plot to explore publication bias is shown in Figure 11. There is no asymmetry, and Egger’s test does not suggest clear evidence of publication bias.

Subgroup analyses are shown in Table 3. Here we did not find clear subgroup differences, but of note the studies at low overall risk of bias, using prospective study design and reporting adjusted data found least evidence of an association between TBF any vs never and AD risk, compared with studies at high risk of bias, with retrospective design or reporting unadjusted data. However all subgroup analyses had moderate, high or extreme levels of statistical heterogeneity.

Analysis of dose response, with forest plots for never versus short, medium and long duration/cut-off of TBF showed no evidence of a dose-response relationship between TBF duration and risk of AD at age 5-14 years (Figure 12, Figure 13, and Figure 14).

#### Figure 10 TBF Ever vs. Never and risk of eczema at age 5-14 years


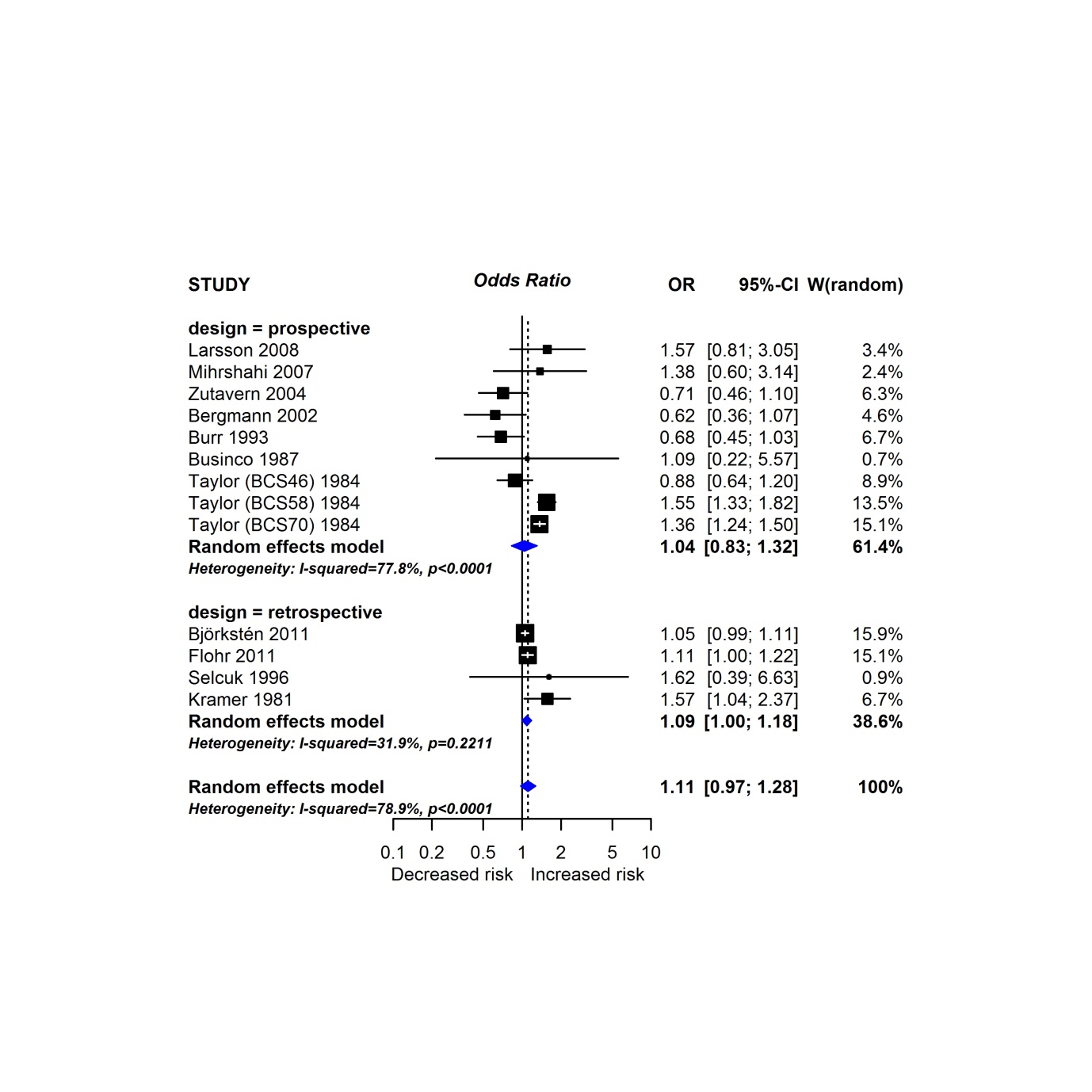


Figure 11 Risk of publication bias in studies investigating TBF and risk of eczema in children aged 5-14 years


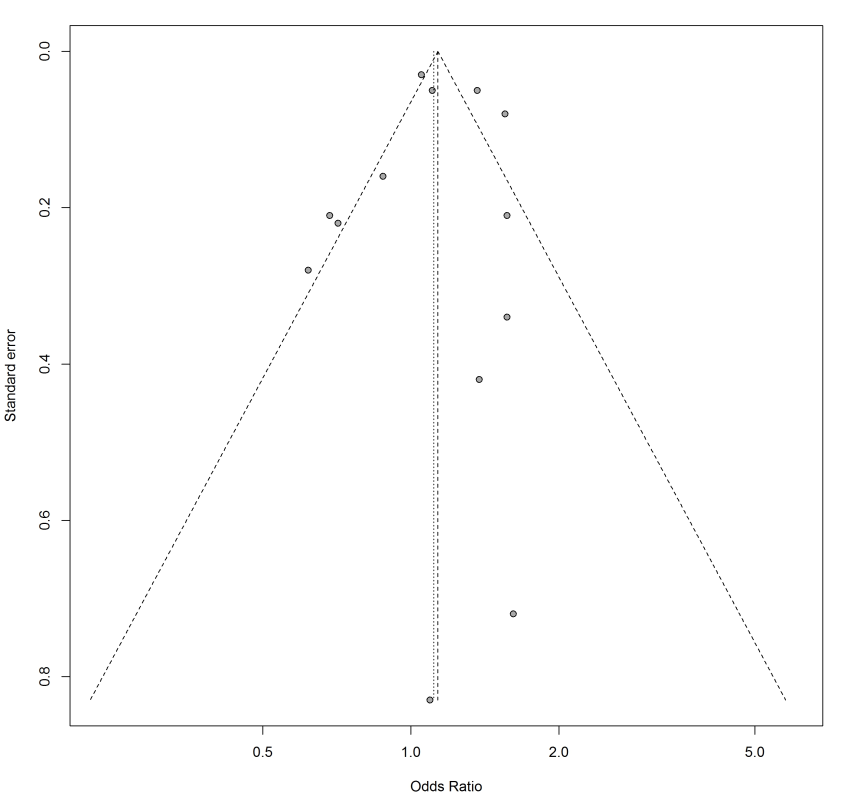


Egger’s test p-value = 0.139

Table 3 Subgroup analyses of risk of eczema and TBF Ever vs. Never in children aged 5-14 years

|  | **Number of studies** | **OR [95% CI]*** | **I^2^ (%)** | **P-value for between groups difference** |
| --- | --- | --- | --- | --- |
| **Overall (if adjusted NA, unadjusted used)**  **Adjusted**  **Unadjusted** | 13 | 1.11 [0.97; 1.28] | 78.9 |  |
|  | 6 | 1.04 [0.93; 1.15] | 40.9 | Not tested |
|  | 9 | 1.17 [0.94; 1.44] | 75.6 |  |
| Study Design – Prospective  Study Design – Retrospective | 9 | 1.04 [0.83; 1.31] | 77.8 | 0.72 |
|  | 4 | 1.09 [1.00; 1.18] | 31.9 |  |
| Risk of disease – High  Risk of disease – Normal | 3 | 0.84 [0.54; 1.31] | 15.7 | 0.20 |
|  | 10 | 1.14 [1.00; 1.32] | 82.3 |  |
| Risk of bias – Low  Risk of bias – High/Unclear | 7 | 0.95 [0.79; 1.17] | 39.6 | 0.057 |
|  | 6 | 1.24 [1.03; 1.50] | 80.6 |  |

Figure 12 TBF short duration (1-3 months) vs. never and risk of eczema at age 5-14 years


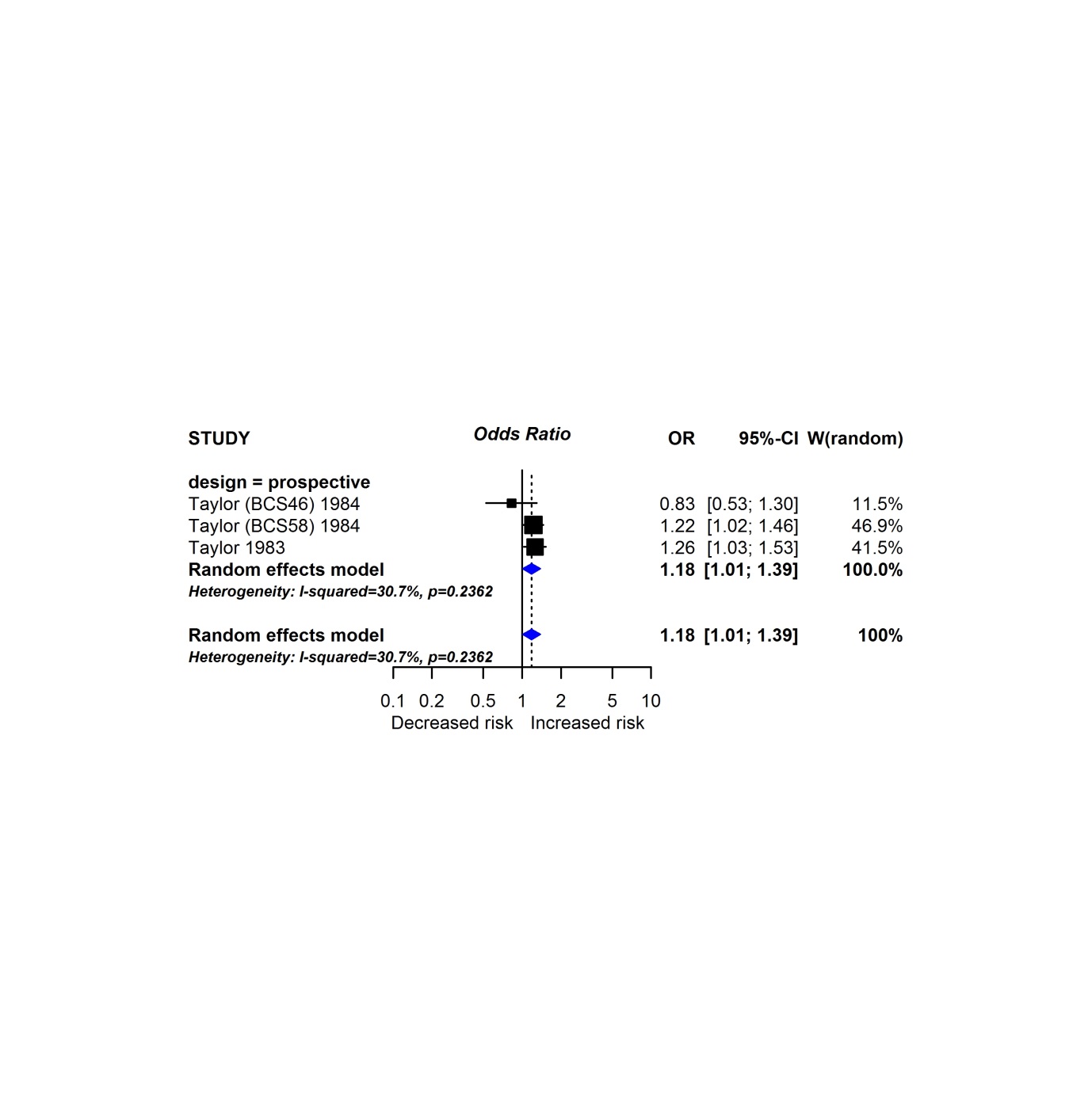


Figure 13 TBF medium duration (4-6 months) vs. never and risk of eczema at age 5-14 years


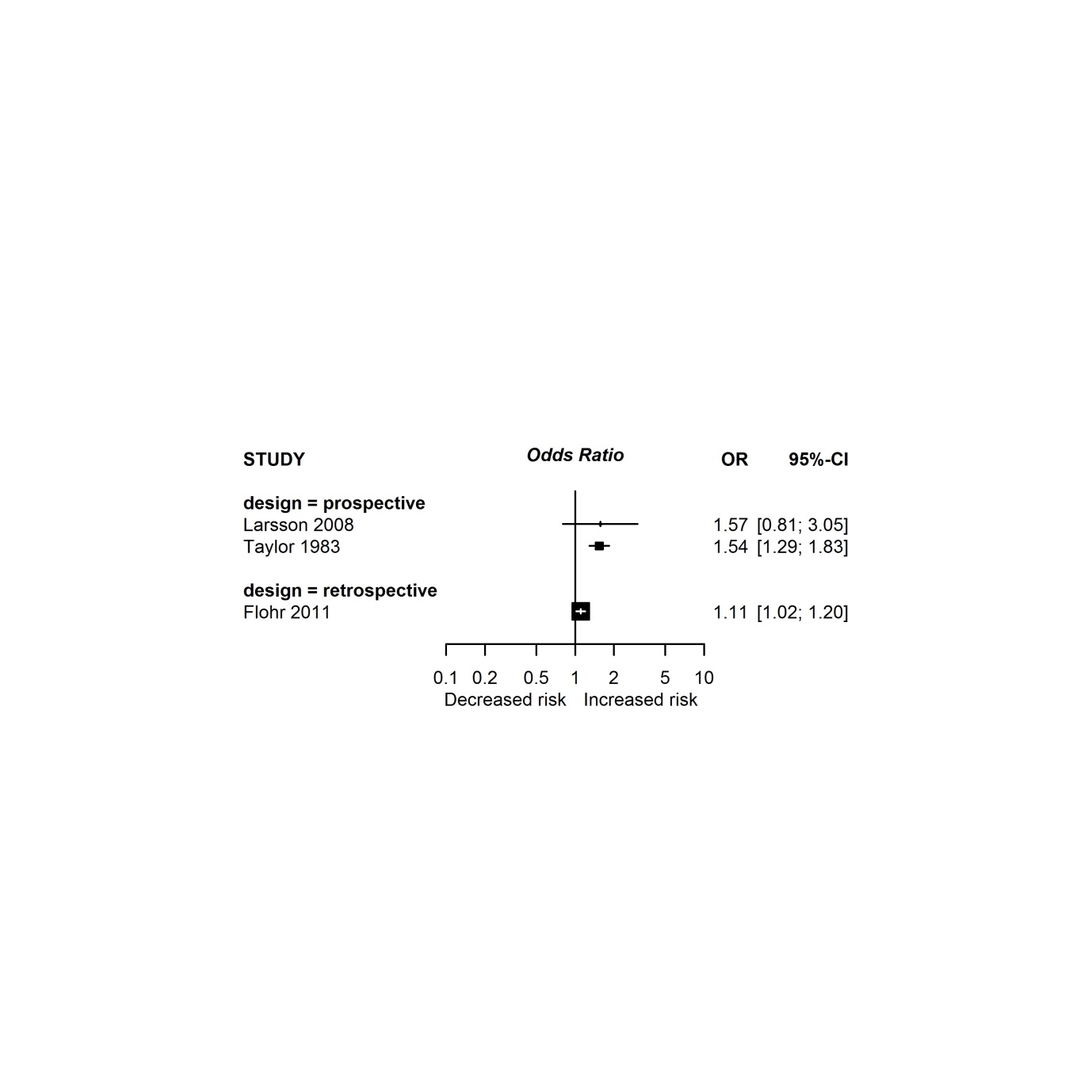


Figure 14 TBF long duration (7-12 months) vs. never and risk of eczema at age 5-14 years


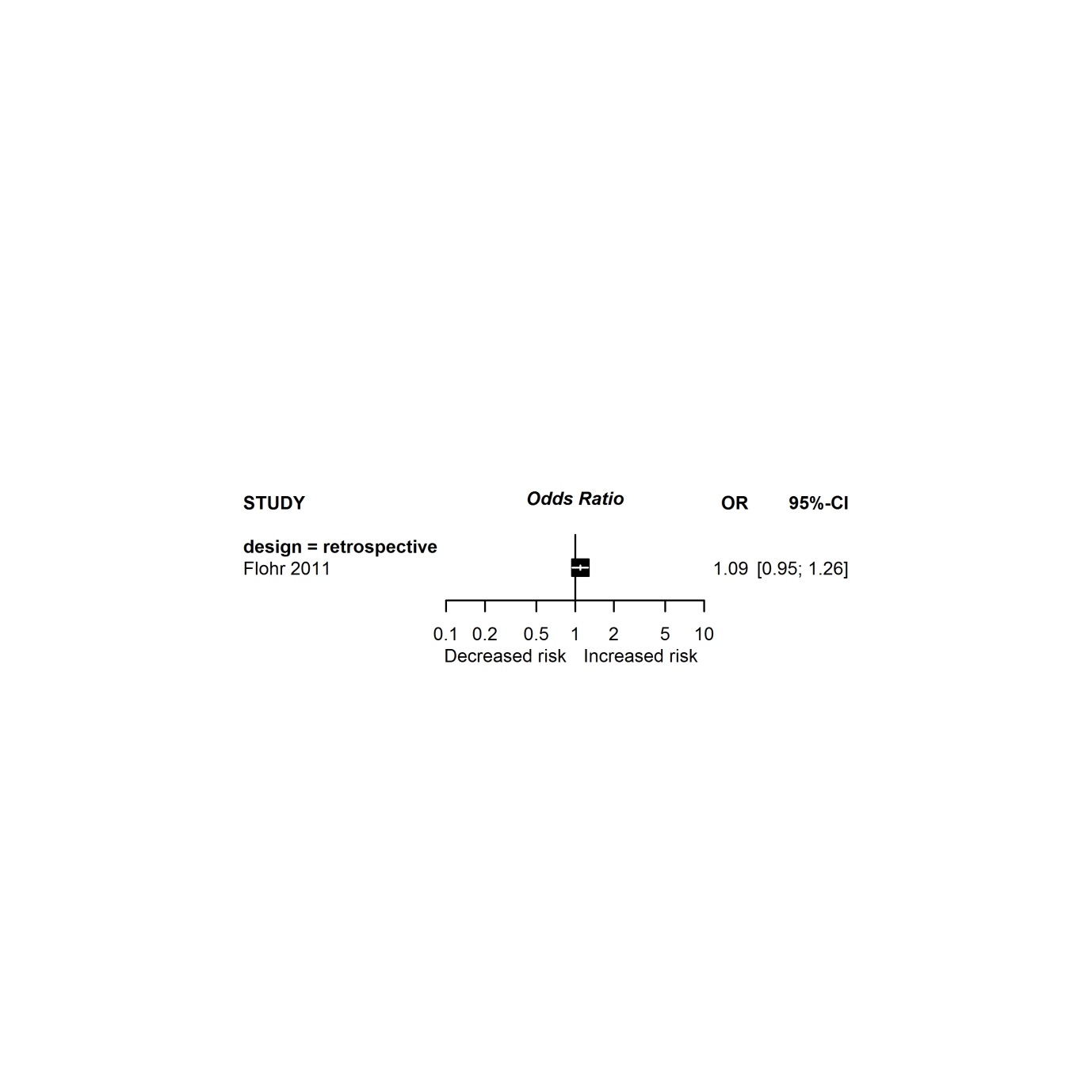


#### TBF ≥1-2 months vs. <1-2 months

One study reported data for TBF ≥1-2 months vs. <1-2 months duration and risk of AD at age 5-14 years, and is shown in Figure 15. The study suggests increased AD risk with increased TBF duration. This is a prospective cohort study with low risk of bias in all domains, which reported adjusted data for several TBF cut-offs and found an overall significant association between increased TBF duration and increased AD risk - OR 1.03 (1.00, 1.06) for each additional month TBF P=0.034.

**Figure 15 TBF ≥1-2 months vs. <1-2 months and risk of eczema at age 5-14 years**


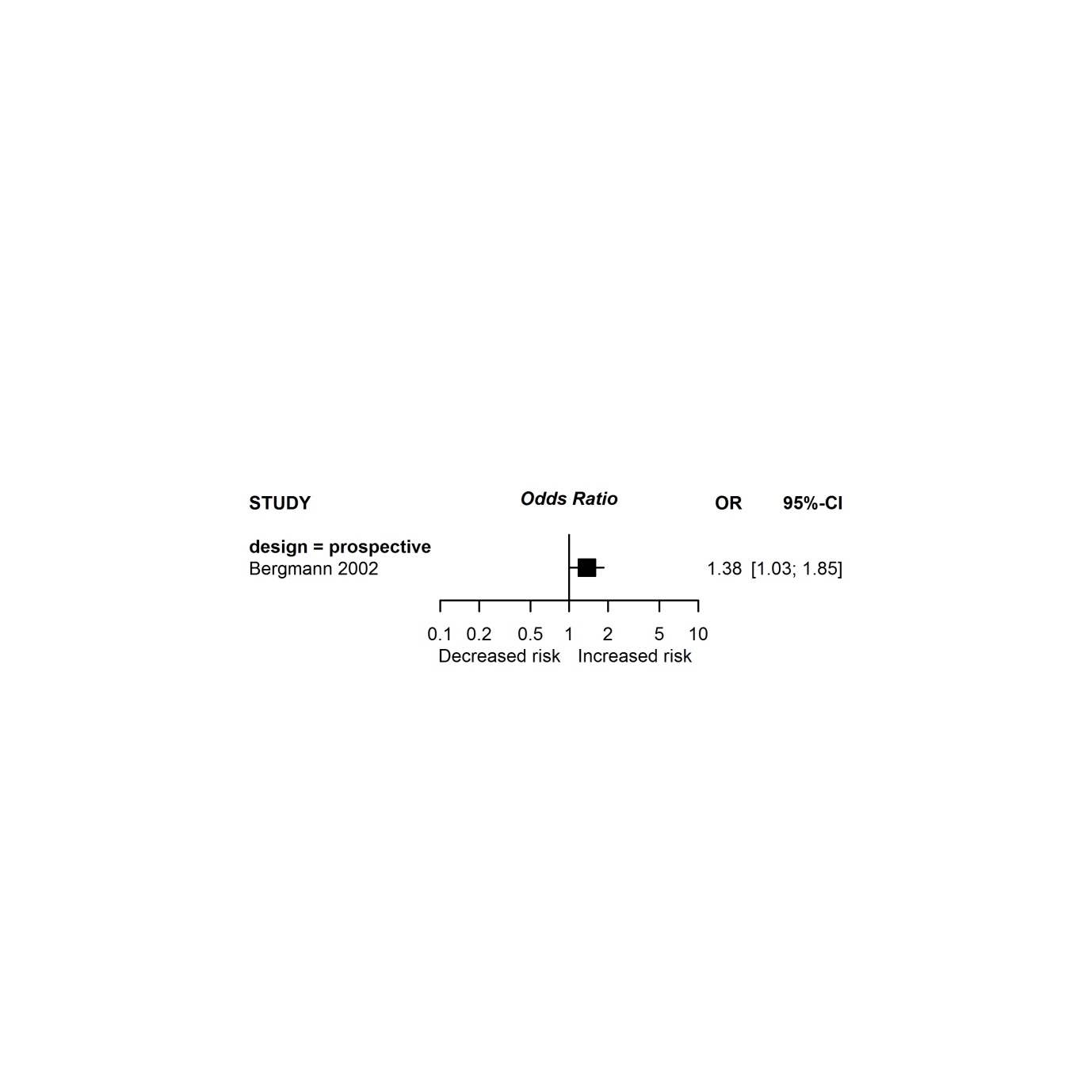


#### TBF ≥3-4 months vs. <3-4 months

Two studies reported data for TBF ≥3-4 months vs. <3-4 months duration and risk of AD at age 5-14 years, and are shown in Figure 16. They show borderline significant increase risk of AD with prolonged breastfeeding (≥3-4 months), without statistical heterogeneity. Both studies are prospective cohort studies in a normal risk population, with low risk of bias in all domains, reporting adjusted OR.

**Figure 16 TBF ≥3-4 months vs. <3-4 months and risk of eczema at age 5-14 years**


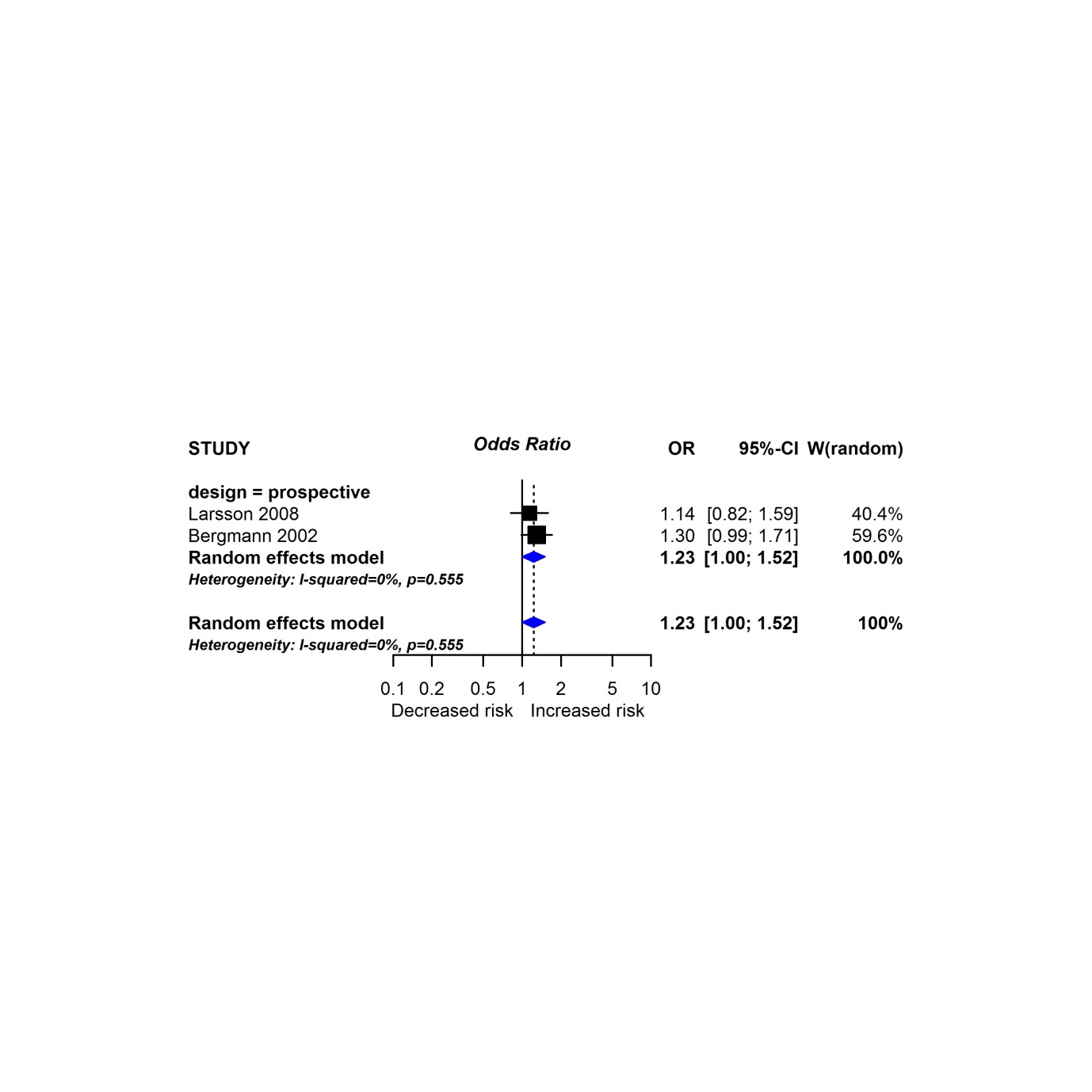


#### TBF ≥5-7 months vs. <5-7 months

Two studies reported data for TBF ≥5-7 months vs. <5-7 months duration and risk of AD at age 5-14 years and are shown in Figure 17. They show increased risk of AD with longer TBF, with no statistical heterogeneity. Both studies are prospective cohort studies with low risk of bias in all domains, reporting adjusted OR.

**Figure 17 TBF for ≥5-7 months vs. <5-7 months and risk of eczema at age 5-14 years**

**
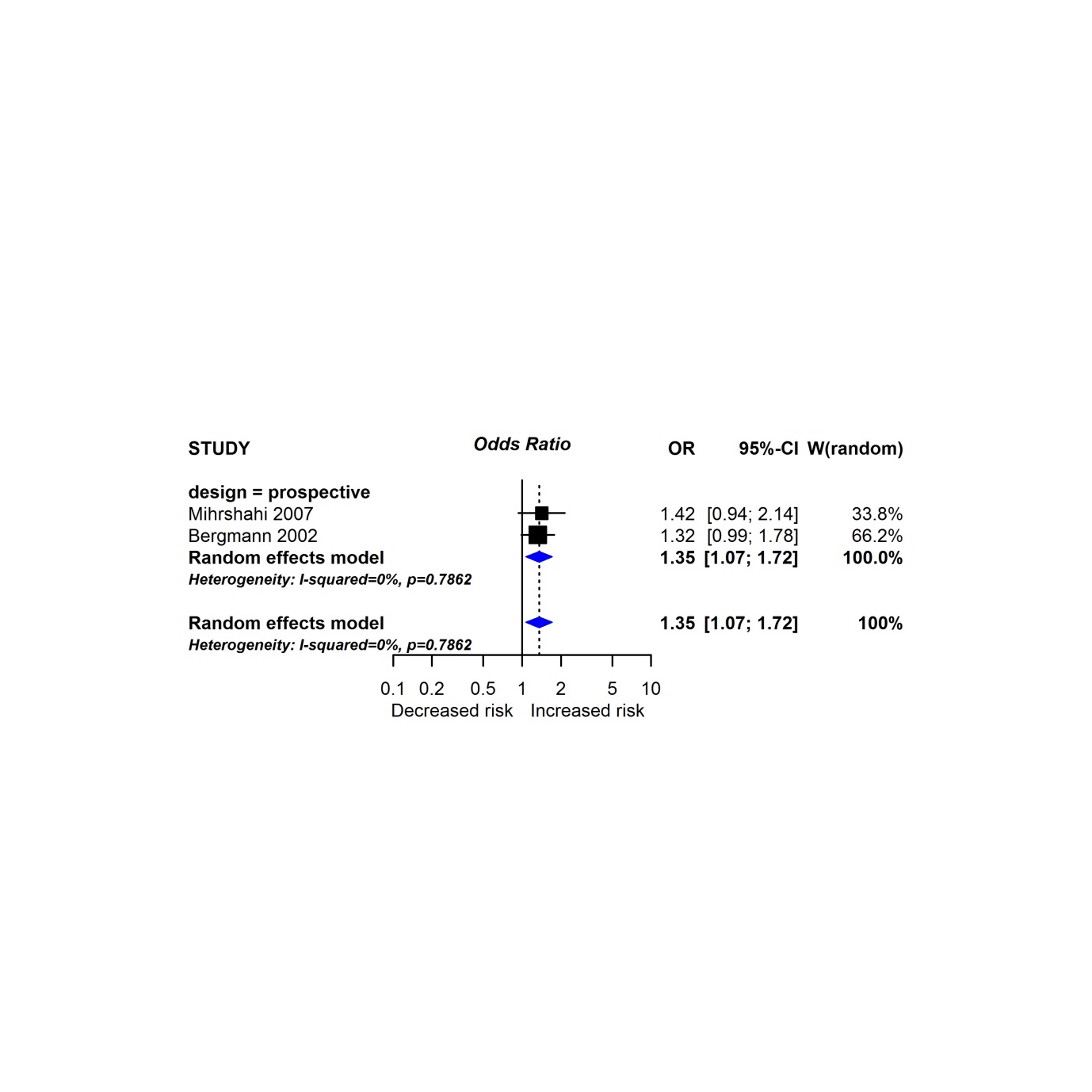
**

#### TBF ≥8-12 months vs. <8-12 months

One study reported data for TBF ≥8-12 months vs. <8-12 months duration and risk of AD at age 5-14 years, and is shown in Figure 18. The study found no significant association using this cut-off. As mentioned above, the authors found an overall significant association between increased TBF duration and increased AD risk in this study.

**Figure 18 TBF for ≥8-12 months vs. <8-12 months and risk of eczema at age 5-14 years**

**
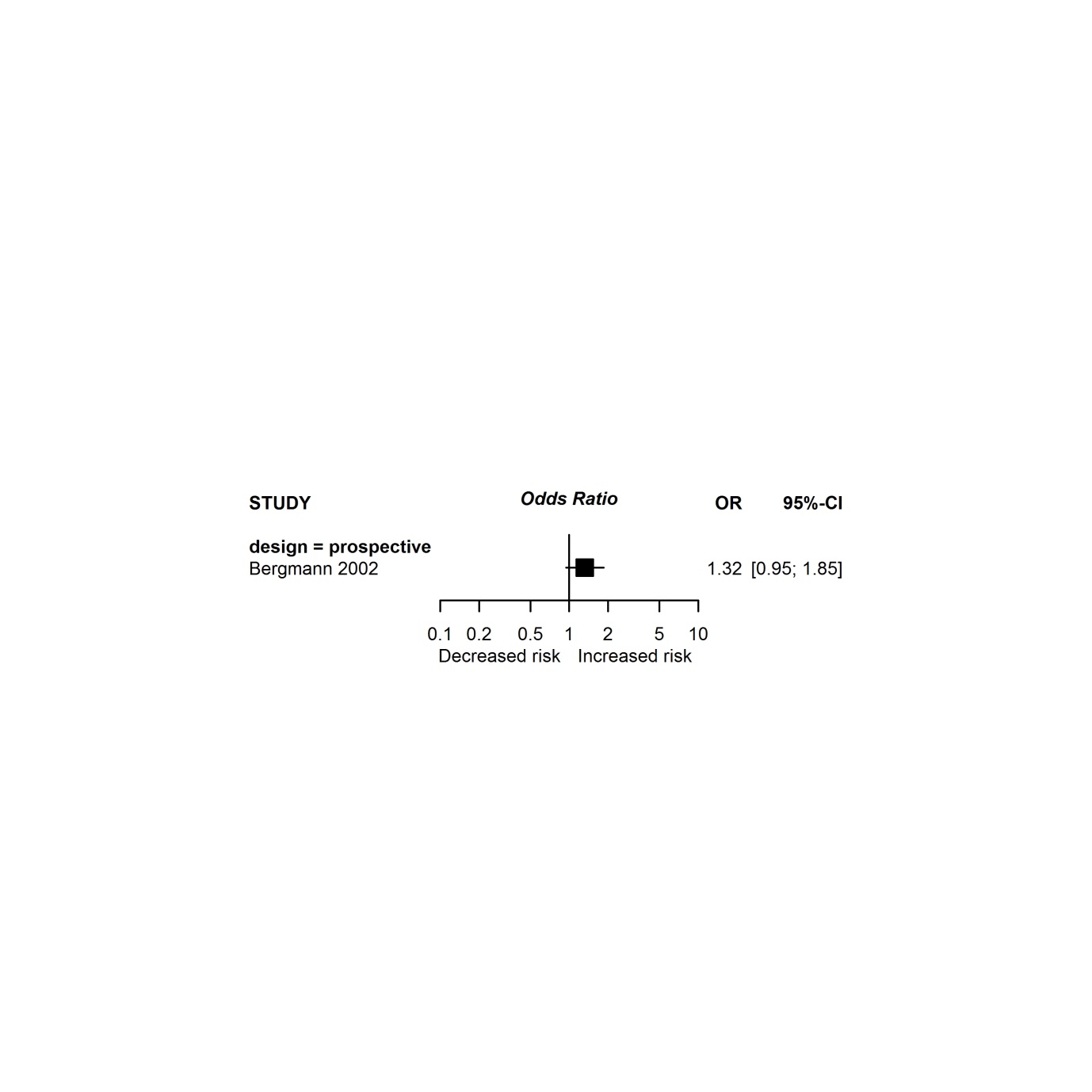
**

### TBF and risk of eczema in children aged 15+ years

#### TBF Never vs. Any

Figure 19 shows the outcomes of 3 eligible observational studies reporting OR for TBF never vs ever and AD at age 15+ years. The pooled data show no association between breastfeeding and risk of AD, but with moderate statistical heterogeneity (I^2^=38%). The studies of Miyake and Kurt report adjusted data from cross-sectional surveys; the study of Farooqi reports unadjusted data from a prospective cohort study – they do state in the manuscript that adjusted analysis showed no significant association between TBF and AD. The study of Kurt was assessed as at low risk of bias on all domains, the other two studies as at high risk of bias overall. These differences may be relevant to the statistical heterogeneity seen in the meta-analysis.

**Figure 19 TBF Never vs. Any and risk of eczema at age 15+ years**


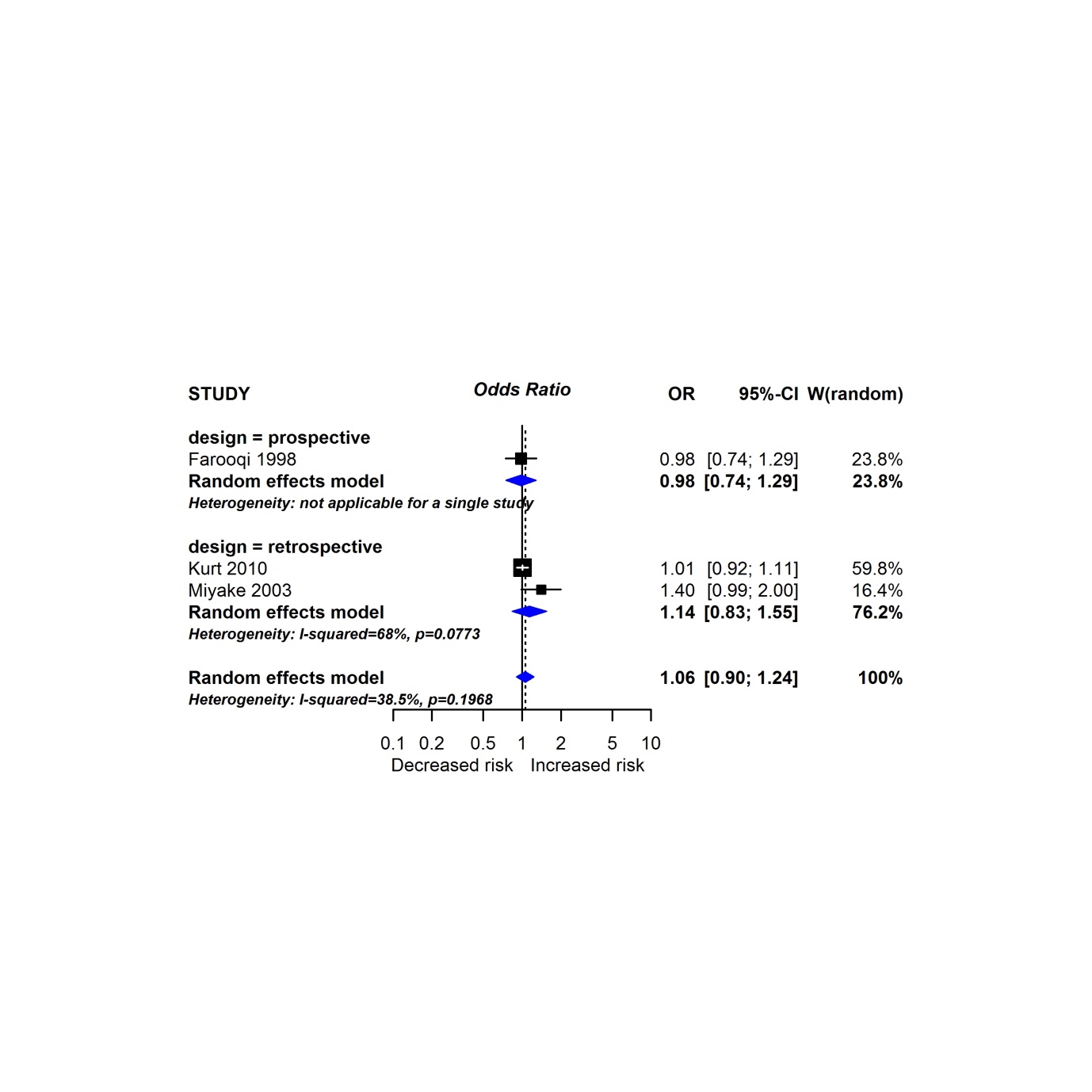


#### TBF ≥3-4 months vs. <3-4 months

One study reported data for TBF ≥3-4 months vs. <3-4 months duration and risk of AD by age 18 years in a cross sectional survey reporting adjusted data. As shown in Figure 20, there was an association between longer TBF duration and increased risk of AD, with borderline statistical significance.

**Figure 20 TBF ≥3-4 months vs. <3-4 months and risk of eczema at age 15+ years**


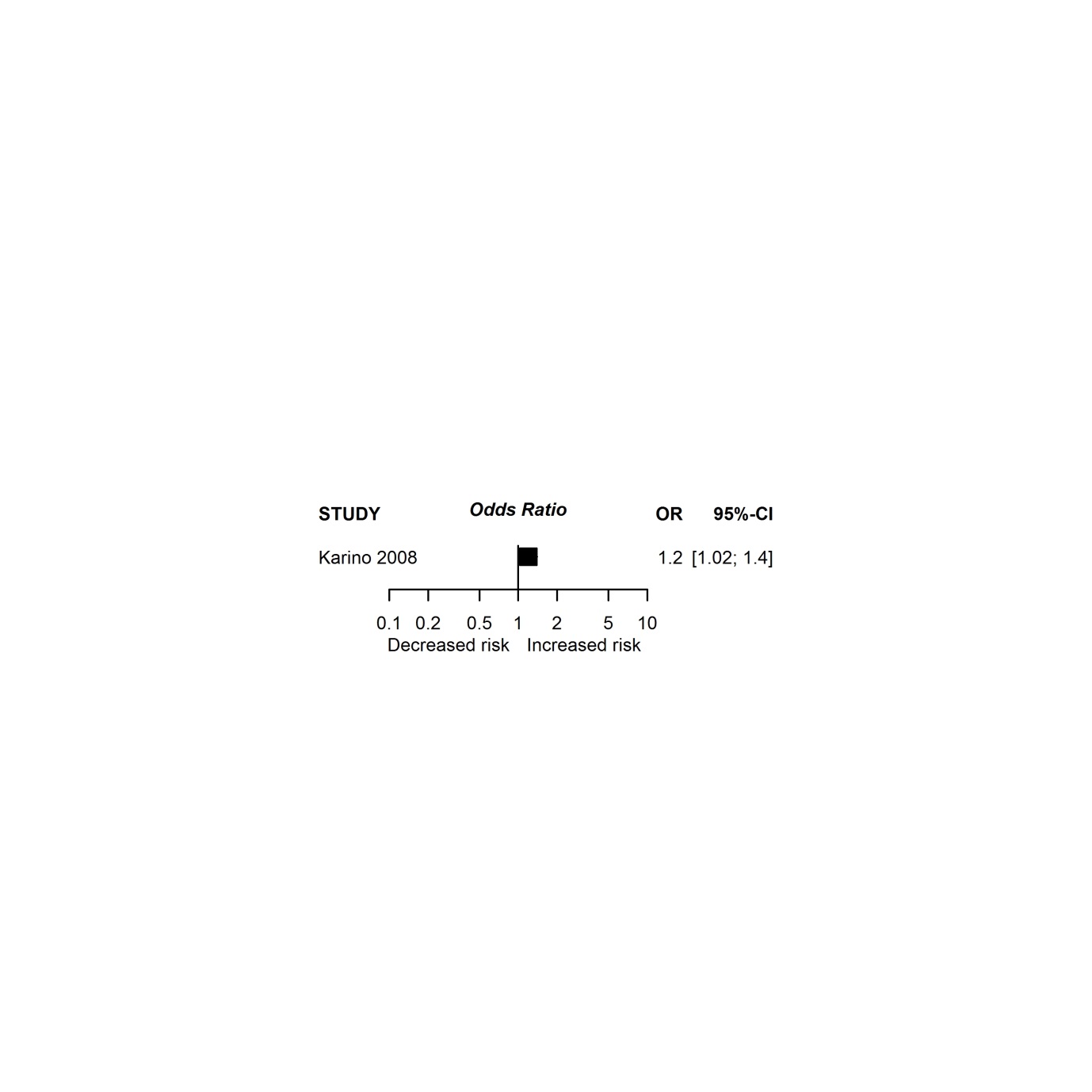


## Data for TBF and risk of eczema that couldn’t be analysed

A further 14 prospective cohort studies and 2 cross-sectional studies reported relevant data from over 16000 study participants, which could not be included in meta-analysis. These studies are summarised in Table 4. In one small prospective cohort study there was reduced AD associated with TBF ≥5 months. In all other studies and analyses reported there was no association found between TBF and AD.

Table 4 Studies investigating the association between TBF duration and eczema which were not eligible for meta-analysis

| **Study** | **Design** | **Outcome** | **Age** | **N/n** | **Data** | **Measure** | **TBF in AD** | **TBF in No AD** | **P** |
| --- | --- | --- | --- | --- | --- | --- | --- | --- | --- |
| Berth-Jones, 1997 ([7](#_ENREF_7)) | PC | AD | 1 | 413 | Continuous | Average | 6.2 | 5.7 | NS |
| Hesselmar, 2010 ([3](#_ENREF_3)) | PC | AD | 1.5 | 184 | Continuous | Median (IQR) | 6.7 (6, 9) | 7.2 (4, 10) | 0.82 |
| Forster, 1990 ([20](#_ENREF_20)) | PC | AD | 1.5 | 145 | Continuous | No significant association between TBF duration and AD risk | | | |
| Alm, 2008  ([25](#_ENREF_25)) | PC | AD | 1 | 4941 | Continuous | No significant association between TBF duration and AD risk | | | |
| Morgan, 2004 ([35](#_ENREF_35)) | PC | AD | 1 | 257 | Continuous/ Categorical | No significant association between TBF duration and AD risk | | | |
| Kerkhof, 2003 ([40](#_ENREF_40)) | PC | AD | 1 | 304 | Continuous | No significant association between TBF duration and AD risk | | | |
| Nwaru, 2013 ([18](#_ENREF_18)) | PC | AD | 5 | 3109/ 476 | Continuous | No significant association between TBF duration either as a categorical or continuous variable in adjusted analyses, and risk of AD up to age 5. Median TBF 7 (4, 11) AD; 7 (4, 11) no AD. P=0.57 | | | |
| Silvers, 2009 ([38](#_ENREF_38)) | PC | AD | 1 | 987 | Continuous | No significant association between TBF duration and AD.  Adjusted OR 1 (0.98-1.03) per month increase in TBF. P=0.79 | | | |
| Bisgaard, 2009 ([15](#_ENREF_15)) | PC | AD | 3 | 354 | Continuous | No significant association between TBF duration and AD.  Adjusted OR 2.8 (0.9-9.0) per year increase in TBF. P=0.10 | | | |
| Hagendorens, 2005 ([49](#_ENREF_49)) | PC | AD | 1 | 669 | Categorical | No significant association between TBF ever vs never, and AD. OR>1, data in graphical form. NS | | | |
| Monego 1989 ([52](#_ENREF_52)) | PC | AD | 4 | 144 | Categorical | No significant association between TBF duration >1 month and AD. | | | |
| Wetzig, 2000([30](#_ENREF_30)) | PC | AD | 1 | 323 | Categorical | Significantly reduced risk of AD with TBF >5 vs <5 months | | | |
| Civelek, 2001([57](#_ENREF_57)) | CS | AD | 11 | 1533/743 | Continuous | No significant association between TBF duration and AD risk.  Mean duration 11.0 months AD, 11.7 months No AD | | | |
| Devereux 2006 ([17](#_ENREF_17)) | PC | AD | 5 | 1704 | Categorical | No significant relationship between TBF ever vs never and AD risk | | | |
| Girolomoni,2003 ([60](#_ENREF_60)) | CS | AD | 9 | 1369 | Categorical | No significant relationship between TBF ever vs never and AD risk | | | |
| Saarinen, 1995([45](#_ENREF_45)) | PC | AD | 5 | 150 | >1 | No significant relationship between TBF <1 month, 1-6 months, >6 months and AD at 5 or 17 years | | | |

# Exclusive Breastfeeding Duration and Eczema

## Overall characteristics of studies, risk of bias and summary of results

Table 5 describes the main characteristics of the studies analysed in this report. A total of 1 systematic review, which included 1 RCT analysed as a cohort study and 1 other prospective cohort study, and 45 observational studies, reported the association between duration of exclusive breastfeeding (EBF) and risk of AD. Of these, 37 were prospective cohort studies, 3 case-control studies and 5 cross-sectional studies. Most studies (n=30) are from Europe – others are from Australasia (n=6), Asia (n=5), North America (n=3), Middle East (n=2) and worldwide (n=1). Overall, valid data on EBF duration and AD risk were available from over 100,000 subjects. Information on AD was obtained mainly from a medical assessment in 21 studies, using Hanifin and Rajka criteria or adaptations of these (n=12), via parental report (n=7), using the ISAAC questionnaire (n=5), and by unclear methodology in 2 studies. With regards to time of outcome diagnosis, 25 studies explored the association between EBF duration and AD at age 0-4 years, when most AD begins, 17 evaluated AD at age 5-14 years, and five at age 15-44 years. Most studies used a questionnaire to assess the exposure (EBF), 11 used an interview, 6 used a diary, one medical records and in 3 studies the method was unclear.

Risk of bias is shown in Figure 21. Over half of studies had a high risk of bias, most commonly due to lack of adjustment for confounding bias i.e. no adjusted data presented. Over half of studies had unclear risk of assessment bias, mainly due to unclear method for assessing/classifying EBF duration. Risk of conflict of interest was generally assessed as low.

Where data were available, three levels of comparison were used to assess the risk of AD according to EBF duration, namely ≥0-2 months vs. <0-2 months; ≥3-4 months vs. <3-4 months; ≥5-9 months vs. <5-9 months. Across all cut-offs there was no consistent evidence of a lower risk of AD if EBF was prolonged. In general, there were only small numbers of studies with available data in each analysis, and a significant number of studies and participants could only be analysed in narrative form due to the way the data were presented (Table 9). The relatively large number of observational studies and participants did not suggest that there is likely to be an association between EBF duration and AD risk. We did however find some evidence for gene-environment interaction in the relationship between EBF duration and AD risk.

Table 5 Characteristics of included studies evaluating EBF duration and eczema

| **Study** | **N/n cases** | **Design** | **Country** | **Exposure assessment** | **Method of outcome assessment** | **Age at outcome (years)** | **Population characteristics** |
| --- | --- | --- | --- | --- | --- | --- | --- |
| Kramer 2012 ([65](#_ENREF_65)) | 3618 | SR of PC | Finland/Belarus | - | #1 Hanifin and Rajka  #2 ISAAC | 1, 5-6.5 | Study #1: Infants of atopic parents  Study #2: Healthy, term, breastfed newborns participating in a breastfeeding promotion intervention trial |
| Kajosaari, 1991 ([66](#_ENREF_66)) | 135 | PC | Finland | Unclear | Hanifin and Rajka | 1, 5 | Solid food introduction at 6 months versus 3 months, in exclusively breastfed infants |
| Ludvigsson, 2005 ([67](#_ENREF_67)) | 8346 | PC | Sweden | Q | Parent reported eczema | 1 | ABIS study. Population based study of babies born between Oct 1997 and Oct 1999. |
| Hesselmar 2010 ([3](#_ENREF_3)) | 184 | PC | Sweden | I | UK Working Party Criteria | 1.5 | ALLERGYFLORA study. Population based study of babies selected from antenatal clinics between 1998 and 2003 - mainly high risk of allergic disease |
| Purvis, 2005 ([5](#_ENREF_5)) | 550 | PC | New Zealand | Q | UK Working Party Criteria | 3.5 | Auckland Birthweight Collaborative study. Birth cohort with representative sample of babies born in 1996-1997 |
| Kull, 2002; Kull, 2005; Kull, 2009 ([6](#_ENREF_6), [68](#_ENREF_68), [69](#_ENREF_69)) | 3791 | PC | Sweden | Q | Parent report OR DD eczema (+/- sIgE) | 2, 4, 8 | BAMSE study. Population based cohort of children born between 1994-1996 |
| Besednjak-Kocijancic, 2010 ([70](#_ENREF_70)) | 408 | PC | Slovenia | Unclear | Unclear | 1, 5 | Infants with a positive history of parental allergy |
| Peters, 1987 ([71](#_ENREF_71)) | 11920 | PC | UK | Q | Parent reported eczema | 5 | British Cohort Study: sample of all infants born in 1970 in Britain |
| Mihrshahi, 2007 ([14](#_ENREF_14)) | 516 | PC | Australia | I | Visible flexural dermatitis OR DD eczema | 5 | CAPS study. Infants born in 1997-1999 with family history of asthma or wheezing |
| Fergusson, 1982; Fergusson 1990 ([72](#_ENREF_72)) | 1175 | PC | New Zealand | R/D/I | DD eczema; DD eczema for >=3 years needing medication | 1, 10 | Christchurch Child Development Study. Population based cohort of infants born in 1977 in the Christchurch urban region |
| Cogswell, 1987 ([73](#_ENREF_73)) | 73 | PC | UK | D | Physician assessment (+ SPT) | 5 | Birth cohort of infants with family history of hay fever or asthma |
| Giwercman, 2010 ([74](#_ENREF_74)) | 306 | PC | Denmark | I | Hanifin and Rajka criteria | 2 | COPSAC study. Infants of mothers with a history of doctor-diagnosed asthma, recruited from August 1998 to December 2001. |
| Benn, 2004; Linneberg, 2006  ([75](#_ENREF_75), [76](#_ENREF_76)) | 34793 | PC | Denmark | Q/I | Physician assessment; Parent reported eczema | 1.5 | DNBC. Population based birth cohort of children born between 1997-2002 |
| Nwaru, 2013 ([18](#_ENREF_18)) | 3109 | PC | Finland | D | ISAAC | 5 | DIPP study. Infants at high risk (HLA) for TIDM born between 1996-2004 invited to the allergy study 1998 - 2000 |
| Laubereau, 2003 ([77](#_ENREF_77)) | 2251 | PC | Germany | I | DD | 3 | GINI study. Term newborn infants born 1995-8 were recruited from 2 regions of Germany to participate into an intervention program |
| Arshad, 1992 ([78](#_ENREF_78)) | 1167 | PC | UK | D/Q | Physician assessment | 1 | Isle of Wight Study. Population based birth cohort of infants born in semi-rural areas between 1989 and 1990 |
| Jedrychowski, 2011 ([79](#_ENREF_79)) | 469 | PC | USA and Poland | I | Physician assessment | 1 | Birth cohort of full-term infants born between 2001 and 2004 |
| Kemeny, 1991 ([27](#_ENREF_27)) | 180 | PC | UK | Unclear | Unclear | 1 | Population based birth cohort of infants born at Dulwich and King’s College Hospitals in London |
| Kitz, 2006 ([80](#_ENREF_80)) | 131 | PC | Germany | D | Hanifin and Rajka criteria | 1 | Birth cohort of infants at increased risk for atopy participate in RCT on infant feeding intervention |
| Marini, 1996 ([31](#_ENREF_31)) | 359 | PC | Italy | Q | Physician assessment | 1 | Infants with family history of allergy whose mother were proposed to participate in an allergy prevention program |
| Midwinter, 1987 ([81](#_ENREF_81)) | 455 | PC | UK | Unclear | DD | 5 | Children born to parents with a family history of atopy in 1979-1981 |
| Gruber 2010 ([82](#_ENREF_82)) | 300 | PC | Netherlands, Austria, Switzerland, Italy, Germany | Q | Hanifin and Rajka criteria | 1 | MIPS-1 study. Infants born in 2006 without family history of allergy, randomised to intervention if fully formula fed < 8 weeks |
| Moore, 1985 ([33](#_ENREF_33)) | 475 | PC | UK | D/I | Physician assessment | 0.25 | Infants born in a hospital in 1979-1980 with family history of eczema or asthma (high risk of disease) |
| Nentwich, 2009 ([83](#_ENREF_83)) | 121 | PC | Germany | Q/I | DD | 0.5 | Newborns with a hereditary risk for atopy |
| Silvers, 2009 ([38](#_ENREF_38)) | 987 | PC | New Zealand | Q | Parent reported eczema | 1 | New Zealand Asthma and Allergy Cohort Study. Population based birth cohort of infants born between 1997 and 2001 |
| Miyake, 2009 ([39](#_ENREF_39)) | 763 | PC | Japan | Q | Physician assessment | 2 | OMCHS study. Population based birth cohort of infants born in 2002-2003 |
| Pesonen, 2006 ([84](#_ENREF_84)) | 160 | PC | Finland | I | Physician assessment | 5, 20 | Population based birth cohort of infants born in 1981 |
| Kerkhof, 2003 ([40](#_ENREF_40)) | 304 | PC | Netherlands | Q | UK Working Party Criteria | 1 | PIAMA: population-based born in 1996-1997 (normal risk of disease) |
| Pratt, 1984 ([42](#_ENREF_42)) | 198 | PC | UK | D/I | Physician assessment | 5 | Recruited in antenatal clinics, normal risk of disease |
| Kramer, 2003; Kramer, 2009 ([85](#_ENREF_85), [86](#_ENREF_86)) | 2951 | PC | Belarus | I | Physician assessment// ISAAC | 1, 6.5 | PROBIT study: recruited in hospitals , born 1996-1997 (normal risk of disease) |
| Moore, 2004 ([87](#_ENREF_87)) | 1005 | PC | USA | Q | DD | 0.5, 6.5 | PROJECT VIVA: hospital based and born 1998-2002 (normal risk of disease) |
| Saarinen, 1979 ([88](#_ENREF_88)) | 177 | PC | Finland | Q | Physician assessment | 1 | Recruited from hospital and born in 1975 (normal risk of disease) |
| Siltanen,2003 ([89](#_ENREF_89)) | 285 | PC | Finland | Q | Hanifin and Rajka criteria | 4 | Infants recruited from maternal hospital born in 1994-1995 (normal risk of disease) |
| Shohet, 1985 ([90](#_ENREF_90)) | 368 | PC | Israel | I | Hanifin and Lobitz criteria | 0.5 | Cohort born in 1980 (normal risk of disease) |
| Dunlop, 2006 ([91](#_ENREF_91)) | 1326 | PC | Slovakia | Q | Physician assessment | 1 | Recruited from hospital, born 1997-1999 (normal risk of disease) |
| Matheson, 2007 ([92](#_ENREF_92)) | 5729 | PC | Tasmania | Q | Parent reported eczema | 44 | Tasmanian ASTHMA Study: population based born in 1961 (normal risk of disease) |
| Wang, 2007 ([48](#_ENREF_48)) | 1760 | PC | Taiwan | Q | DD | 0.5 | Tiawan National Birth Cohort Study: population representative sample born in 2003 (normal risk of disease) |
| Van Asperen, 1983 ([48](#_ENREF_48)) | 79 | PC | Australia | I | Physician assessment | 1 | Cohort recruited from medical service, born in 1980-1981 with family history of atopy (high risk of disease) |
| Zutavern 2004 ([93](#_ENREF_93)) | 604 | PC | UK | I | DD | 5.5 | Cohort recruited from general practices and born in 1993-1995 (normal risk of disease) |
| Amri 2012 ([94](#_ENREF_94)) | 343 | CC | Saudi Arabia | Q | Hanifin and Rajka criteria | <15 | Cases from hospital, source of control unknown |
| Sahakyan, 2006 ([95](#_ENREF_95)) | 240 | CC | Armenia | Q | UK Working Party Criteria | 7 | Hospital-based study with matched controls (low risk of disease) |
| Kramer, 1981 ([31](#_ENREF_31)) | 142 | CC | Canada | I | Hanifin and Lobitz criteria | <20 | Cases and controls were1 month to 20 years old children attending dermatology clinics visits |
| Tanaka, 2009 ([59](#_ENREF_59)) | 1957 | CS | Japan | Q | ISAAC - current AD | 3 | Fukuoka Child Health Study. All 3-year old children examined at public health centres in Fukuoka city |
| Civelek, 2001 ([57](#_ENREF_57)) | 1533 | CS | Turkey | Q | ISAAC | 11 | Representative sample of schoolchildren in 5 cities |
| Ehlayel, 2008 ([96](#_ENREF_96)) | 1278 | CS | Qatar | Q | Parent reported eczema | 5 | Children 0-5 years old attending primary healthcare centres for routine immunisation |
| Flohr,2011([56](#_ENREF_56)) | 51 119 | CS | Worldwide | Q | Parent reported eczema | 12 | ISAAC Phase 2. Schoolchildren aged 8–12 years from 27 centres in 21 affluent and nonaffluent countries |
| Miyake, 2003 ([97](#_ENREF_97)) | 6845 | CS | Japan | Q | ISAAC - current AD | 15 | 12-15 years old children from all public junior high schools in Suita, Japan. |

Q: questionnaire, I: interview, R: medical records, D: diary, PC: prospective cohort, RC: retrospective cohort, NCC: nested case-control, CC: case-control, CS: Cross-sectional, AD: eczema, DD: doctor diagnosis of AD, in contrast to ‘Physician assessment’ where AD diagnosis was always made by a study physician as part of the study protocol.

Figure 21 Risk of bias in studies of exclusive breastfeeding duration and eczema

## Exclusive breastfeeding duration and eczema

### EBF duration and risk of eczema in children aged 0-4 years

#### EBF ≥0-2 months vs. <0-2

Seven prospective cohort studies reported data that could be pooled to calculate OR for AD, in infants with EBF for ≥0-2 months vs. <0-2 months duration and are shown in Figure 22. They show no association between EBF duration and AD risk, with high statistical heterogeneity. Most studies in this meta-analysis were at high risk of bias due to reporting unadjusted data, including those with most extreme OR, which may account for some of the statistical heterogeneity. Subgroup analysis (Table 6) did not identify significant differences by subgroup in this outcome.

**Figure 22 EBF ≥0-2 months vs. <0-2 months and risk of eczema at age 0-4 years**


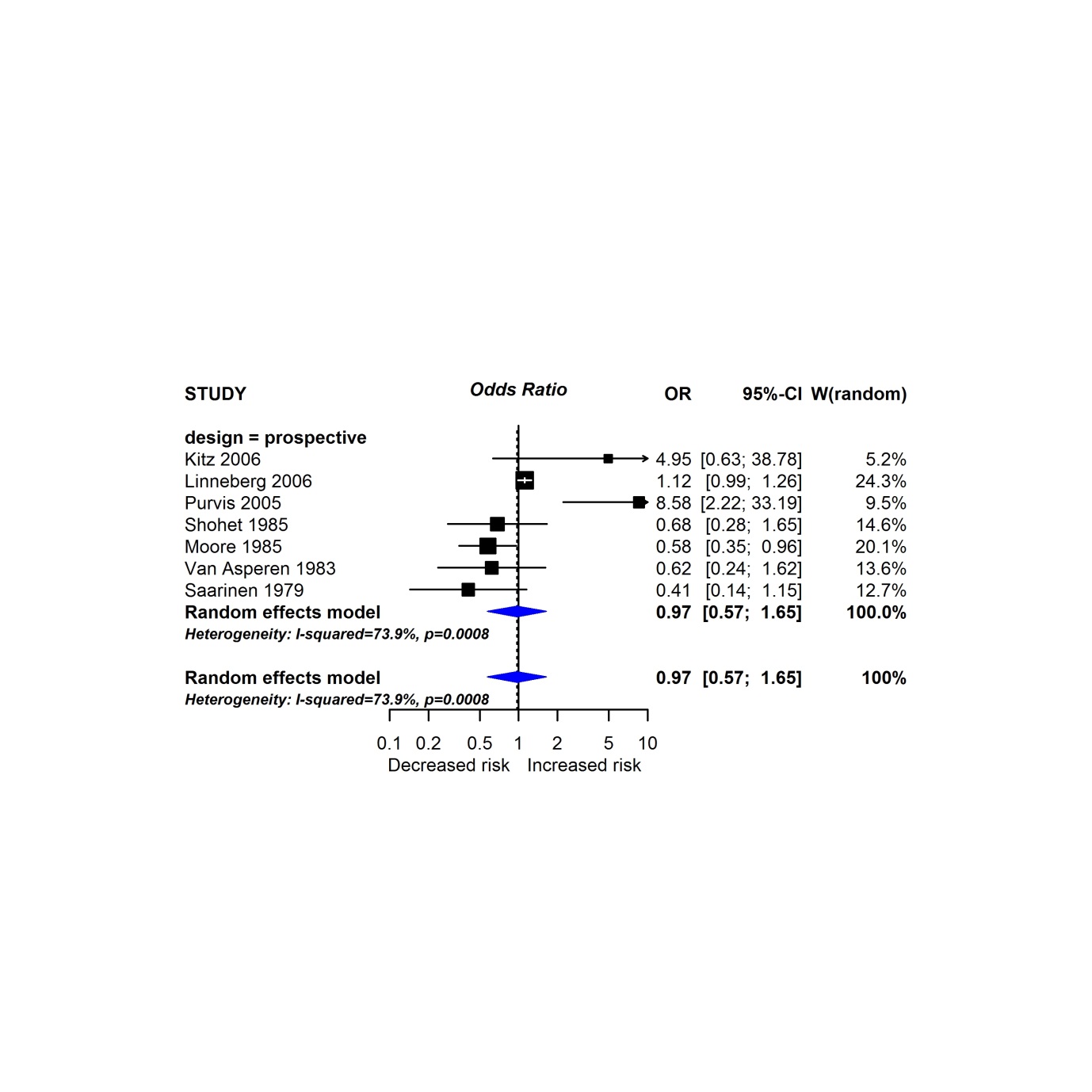


Table 6 Subgroup Analyses EBF duration ≥0-2 vs. <0-2 months and risk of AD at 0-4 years

|  | **Number of studies** | **OR [95% CI]** | **I^2^ (%)** | **P-value for between groups difference** |
| --- | --- | --- | --- | --- |
| **Overall (if adjusted NA, unadjusted used)** | 7 | 0.97 [0.57; 1.65] | 73.9 |  |
| **Adjusted** | 1 | 1.12 [0.99; 1.26] | -- | Not tested |
| **Unadjusted** | 6 | 1.01 [0.47; 2.19] | 72.6 |  |
| Study Design – Prospective | 7 | 0.97 [0.57; 1.65] | 73.9 | 1.00 |
| Study Design – Retrospective | 0 | - | - |  |
| Risk of disease – High | 3 | 0.77 [0.35; 1.72] | 49.5 | 0.51 |
| Risk of disease – Normal | 4 | 1.14 [0.50; 2.60] | 77.9 |  |
| Risk of bias – Low | 1 | 0.58 [0.35; 0.96] | -- | 0.11 |
| Risk of bias – High/Unclear | 6 | 1.13 [0.59; 2.17] | 70.6 |  |

#### EBF ≥3-4 months vs. <3-4 months

#### Systematic reviews and intervention trials

As shown in Table 7, the single systematic review found no evidence that exclusive/predominant breastfeeding for 6 months, compared with exclusive/predominant breastfeeding for 3 months, influenced risk of AD at 1 or 5 years. There was evidence of reduced AD at 1 year with prolonged EBF, from the study of Kajosaari, but the study of Kramer found no relationship, yielding significant statistical heterogeneity between the 2 studies. Of note, the study of Kramer separately reported reduced AD at 12 months in centres randomised to a breastfeeding promotion programme (OR 0.54 95% CI 0.30, 0.95), as discussed under ‘TBF’ in this report. The study of Kajosaari reported no effect on AD at age 5 years (Table 7), and the study of Kramer separately reported no effect on AD at age 6.5 years (OR 1.0 95% CI 0.50, 1.80).

Table 7 EBF for 6 months versus 3 months and risk of RC

| **Study** | **Outcome measure** | **No. participants (studies)** | **Outcome (95% CI)** |
| --- | --- | --- | --- |
| Kramer 2012 ([98](#_ENREF_98))  Kajosaari ([66](#_ENREF_66)) Kramer 2007 ([2](#_ENREF_2)) | AD at 1 year | 3618 (2) | RR 0.73 (0.49, 1.08) I^2^=78% |
| Kramer 2012 ([98](#_ENREF_98))  Kajosaari ([66](#_ENREF_66)) | AD at 5 years | 3584 (2) | RR 0.97 (0.50, 1.89) |

Data shown are from analyses of the trials of Kramer and Kajosaari in the 2012 Cochrane review of Kramer ([98](#_ENREF_98)).

#### Observational studies

Eighteen observational studies reported data that could be pooled to calculate OR for AD, in infants with EBF for ≥3-4 months vs. <3-4 months duration and are shown in Figure 23. They show reduced risk of AD with increased EBF duration, of borderline statistical significance, with moderate statistical heterogeneity. The Funnel plot (Figure 24) was not symmetrical, but Egger’s test was >0.05 suggesting no clear evidence of publication bias. Subgroup analyses (Table 8) suggested that the statistical heterogeneity may be explained by family disease risk – children at high risk of allergic disease had less AD where EBF ≥3-4 months, whereas for children without high risk of allergic disease there was no association. It is notable that two other large observational studies reported effect modification by family history, for EBF and AD, whereby children from non-allergic families had increased AD with increased EBF duration, but this was not seen from children from allergic families ([75](#_ENREF_75), [76](#_ENREF_76), [97](#_ENREF_97)). Other studies have suggested important gene environment interactions between EBF duration and allergic sensitisation, at least to foods ([99](#_ENREF_99)).

**Figure 23 EBF for ≥3-4 months vs. <3-4 months and risk of eczema at age 0-4 years**

**
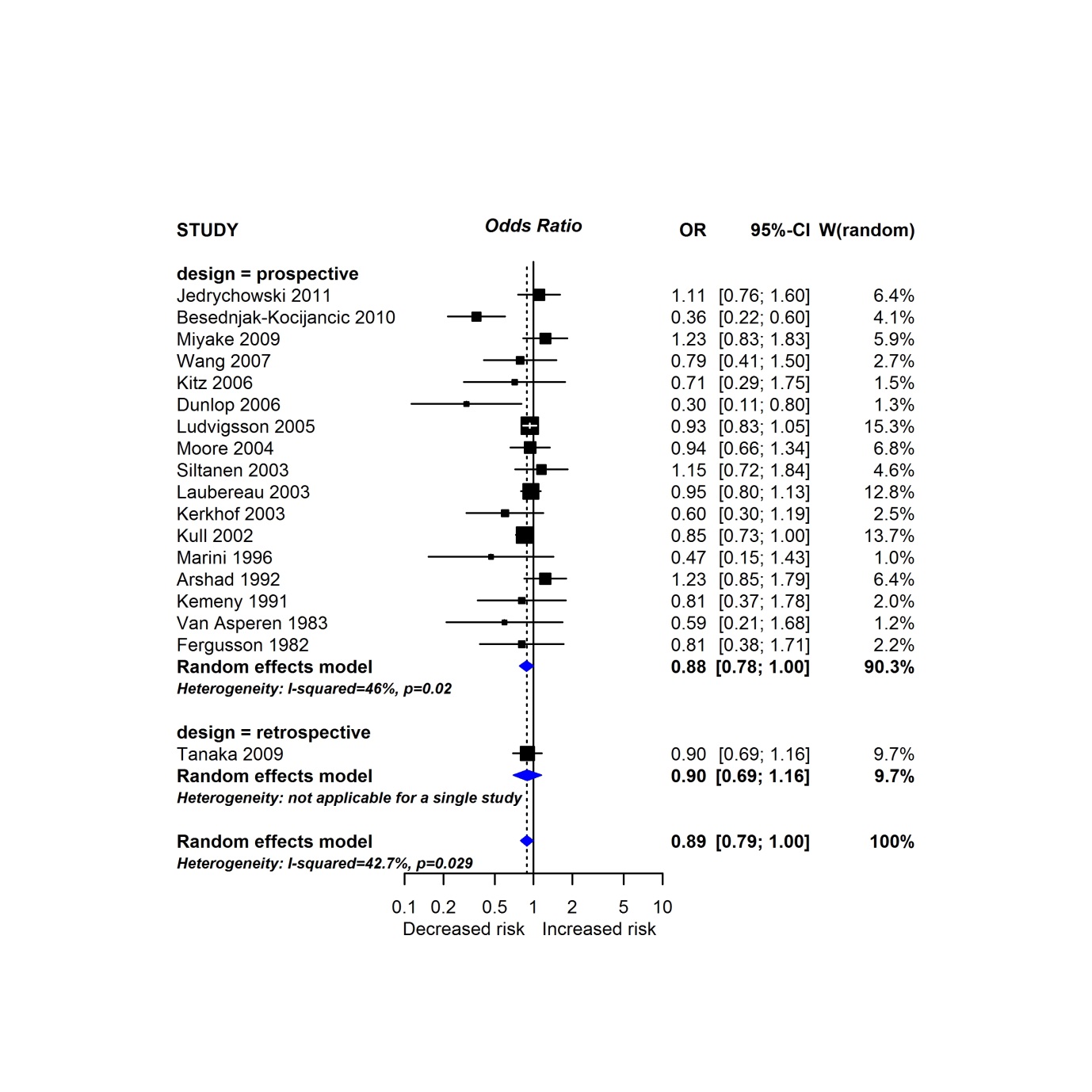
**

**Figure 24 Risk of publication bias in studies investigating EBF duration for ≥3-4 months vs. <3-4 months and risk of eczema in children aged 0-4 years**

**
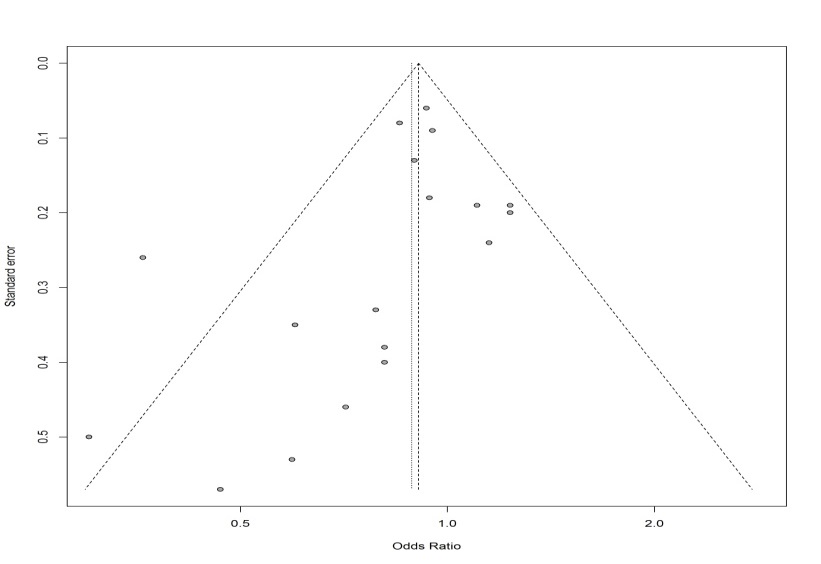
**

**Egger’s test p-value = 0.141**

Table 8 Subgroup Analyses of risk of atopic dermatitis and EBF duration ≥3-4 months vs. <3-4 months in children aged 0-4 years

|  | **Number of studies** | **OR [95% CI]*** | **I^2^ (%)** | **P-value for between groups difference** |
| --- | --- | --- | --- | --- |
| **Overall (if adjusted NA, unadjusted used)** | 18 | 0.89 [0.79; 1.00] | 42.7 |  |
| **Adjusted** | 6 | 0.92 [0.85; 0.99] | 0 | Not tested |
| **Unadjusted** | 16 | 0.89 [0.78; 1.03] | 47.8 |  |
| Study Design – Prospective | 17 | 0.88 [0.78; 1.00] | 46 | 0.92 |
| Study Design – Retrospective | 1 | 0.90 [0.69; 1.16] | -- |  |
| Risk of disease – High | 4 | 0.45 [0.31; 0.66] | 0 | 0.00 |
| Risk of disease – Normal | 14 | 0.93 [0.86; 1.01] | 9.2 |  |
| Risk of bias – Low | 1 | 0.95 [0.80; 1.14] | -- | 0.45 |
| Risk of bias – High/Unclear | 17 | 0.87 [0.76; 1.00] | 45.5 |  |

#### EBF for ≥5-9 months vs. <5-9 months

Three prospective cohort studies reported data that could be pooled to calculate OR for AD, in infants with EBF for ≥5-9 months vs. <5-9 months duration and are shown in Figure 25. They show no association between EBF duration and AD risk, with no statistical heterogeneity.

**Figure 25 EBF ≥5 -9months vs. <5-9 months and risk of eczema at age 0-4 years**


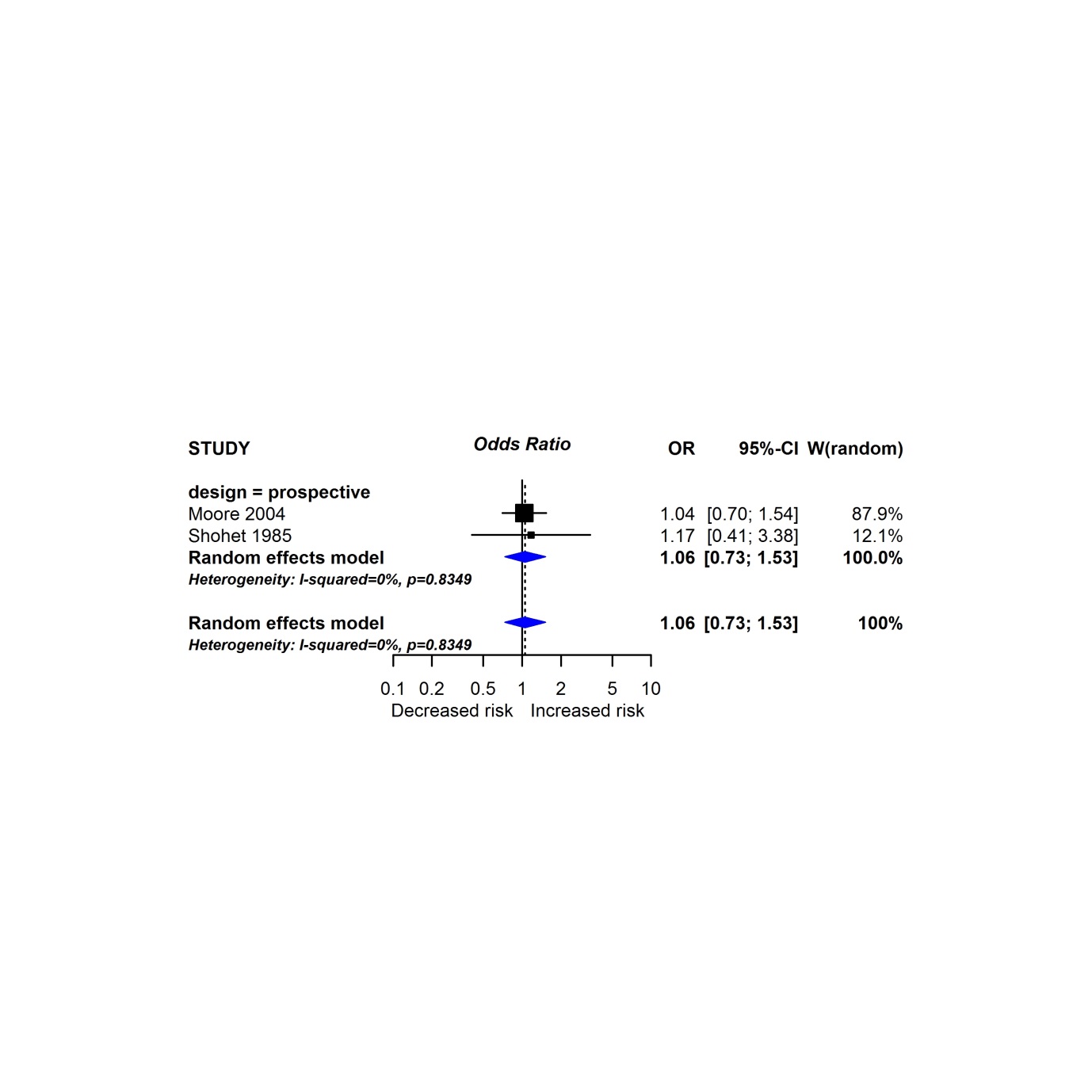


### EBF duration and the risk of eczema in children aged 5-14 years

#### EBF ≥0-2 months vs. <0-2 months

Four prospective cohort studies and one cross-sectional study reported data that could be pooled to calculate OR for AD, in infants with EBF for ≥0-2 months vs. <0-2 months duration and are shown in Figure 26. They show increased AD with increasing duration of EBF, with borderline statistical significance but no statistical heterogeneity (I^2^=0%). Two of the studies presented adjusted data including the cross-sectional study of Flohr, and three presented unadjusted data including the significant study of Peters.

**Figure 26 EBF ≥0-2 months vs. <0-2 months and risk of eczema at age 5-14 years**

**
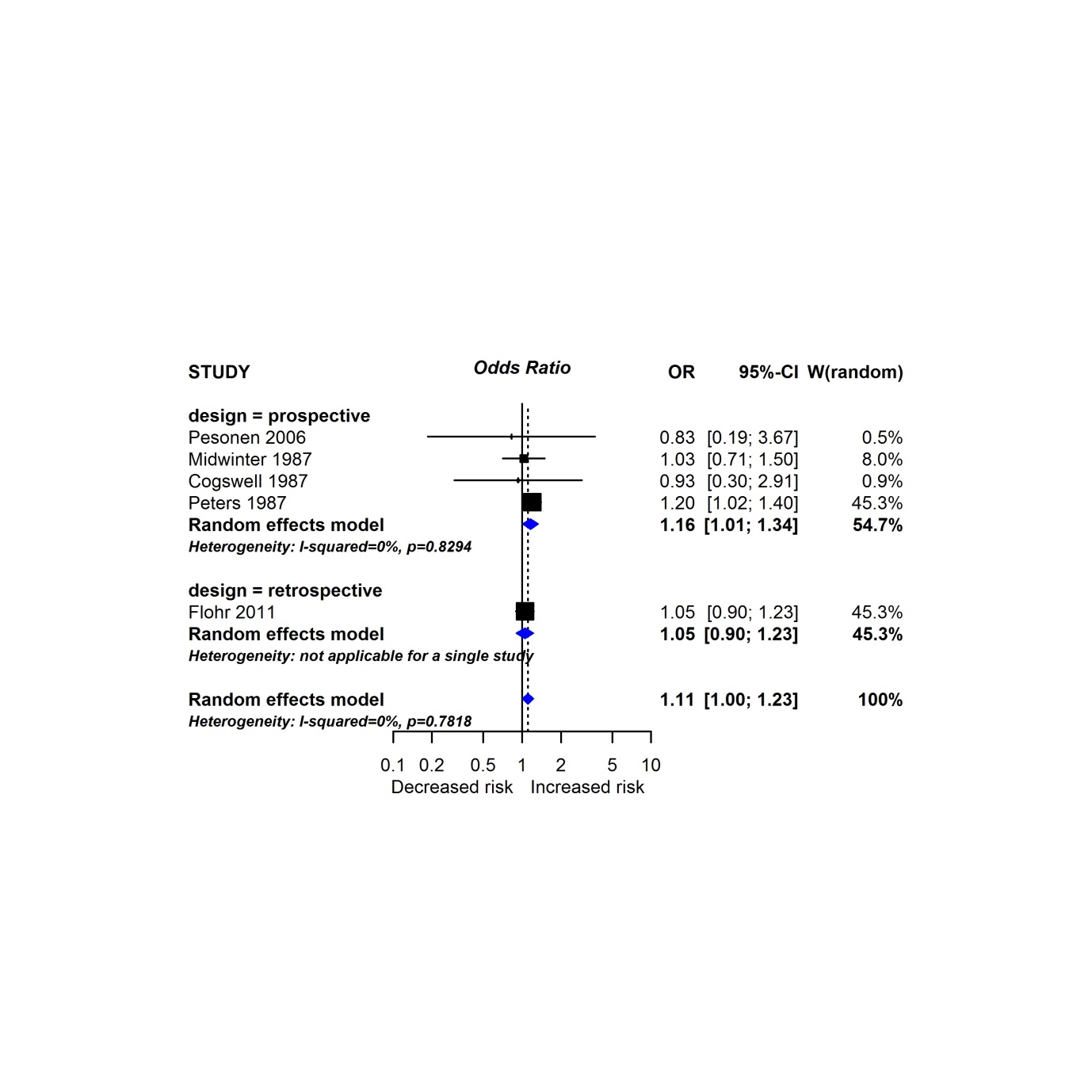
**

#### EBF ≥3-4 months vs. <3-4 months

Four prospective cohort studies reported OR for AD, in infants with EBF for ≥0-2 months vs. <0-2 months duration and are shown in Figure 27. The studies could not be pooled due to extreme statistical heterogeneity (I^2^=83%). The studies of Mihrshahi and Besednjak-Kocijancic were in populations at high risk of allergic outcomes, the latter study reported unadjusted data; the former adjusted. The studies of Kramer and Kull were in normal risk populations and reported adjusted data. Overall the data do not support an association between EBF ≥0-2 months vs. <0-2 months duration and altered AD risk.

**Figure 27 EBF for ≥3-4 months vs. <3-4 months and risk of eczema at age 5-14 years**

**
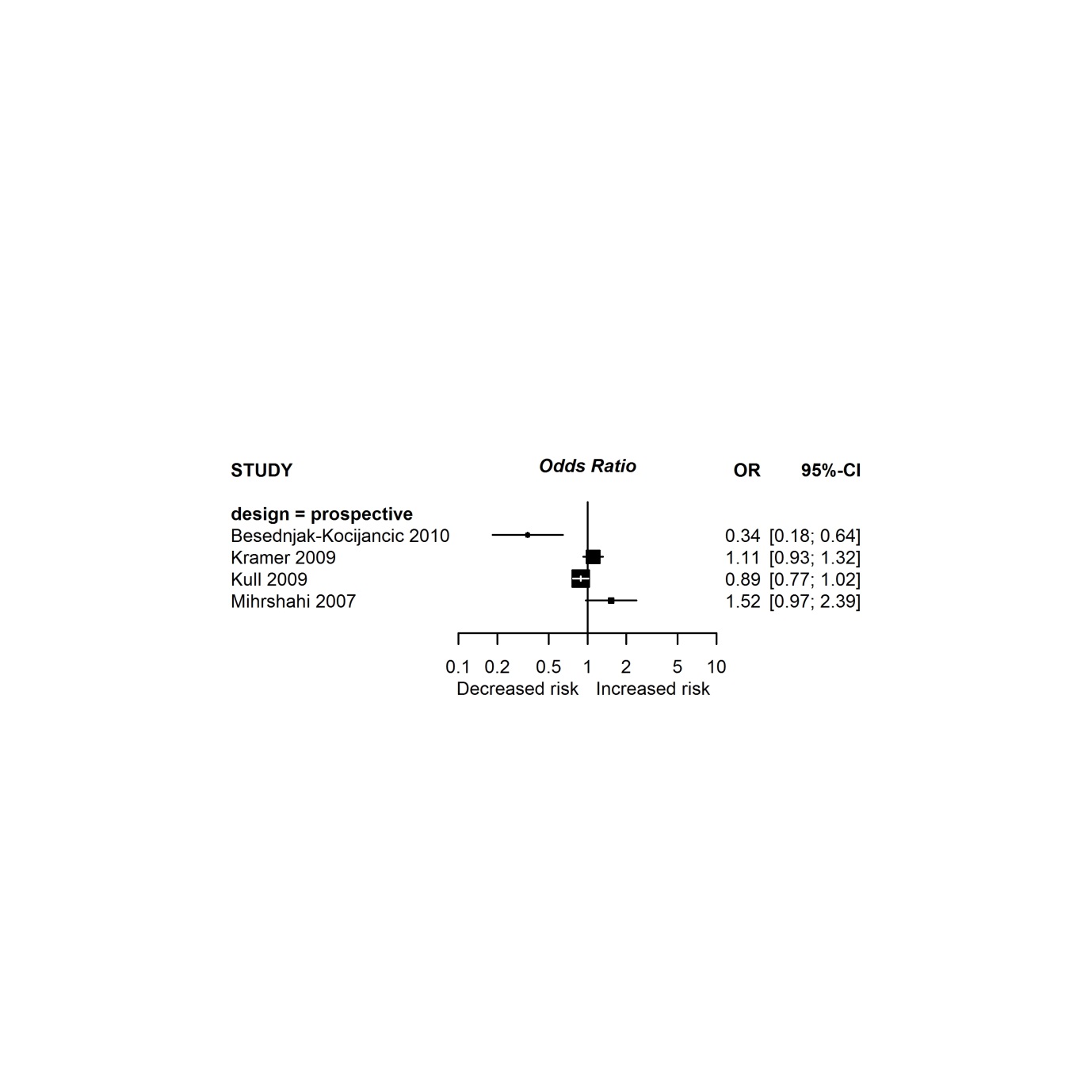
**

#### EBF ≥5-9 months vs. <5-9 months

Four observational studies reported OR for AD, in infants with EBF for ≥5-9 months vs. <5-9 months duration and are shown in Figure 28. The pooled OR show no association, with high statistical heterogeneity (I^2^=67%). The studies of Amri and Ehlayel were retrospective studies reporting unadjusted data and thus carry a high risk of confounding bias. The study of Pesonen compared EBF duration ≥9 vs <9 months which is an unusual duration of EBF and this analysis included relatively small numbers hence the wide confidence intervals. The study of Sahakya is a case-control study which reported adjusted OR and was judged as low or unclear risk of bias in all domains.

**Figure 28 EBF for ≥5 months vs. <5 months and risk of eczema at age 5-14 years**

**
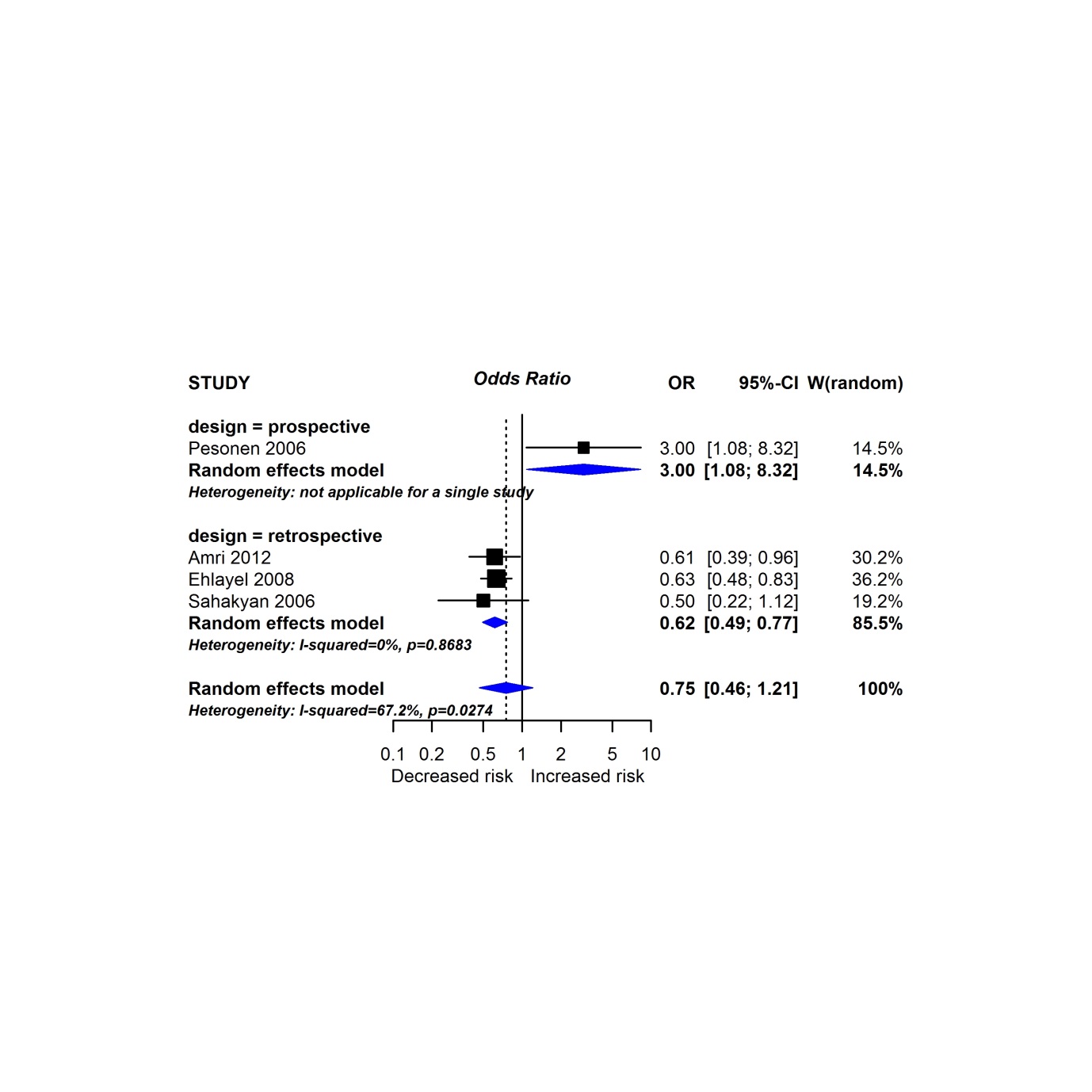
**

### EBF duration and risk of eczema in children aged 15+ years

#### EBF ≥0-2 months vs. <0-2 months

Two observational studies reported data that could be pooled to calculate OR for AD, in people aged 15+ years with EBF for ≥0-2 months vs. <0-2 months duration and are shown in Figure 29. They show increased AD with increasing duration of EBF, with no statistical heterogeneity (I^2^=0%). The study of Pesonen compared EBF duration ≥9 vs <9 months which is an unusual duration of EBF and this analysis included relatively small numbers hence the wide confidence intervals. The study of Miyake is a cross-sectional survey using the ISAAC questionnaire, which reported adjusted data. The same authors found a similar effect for partial BF in the first 3 months, as for EBF.

**Figure 29 EBF for ≥0-2 months vs. <0-2 months and risk of eczema at age 15+ years**

**
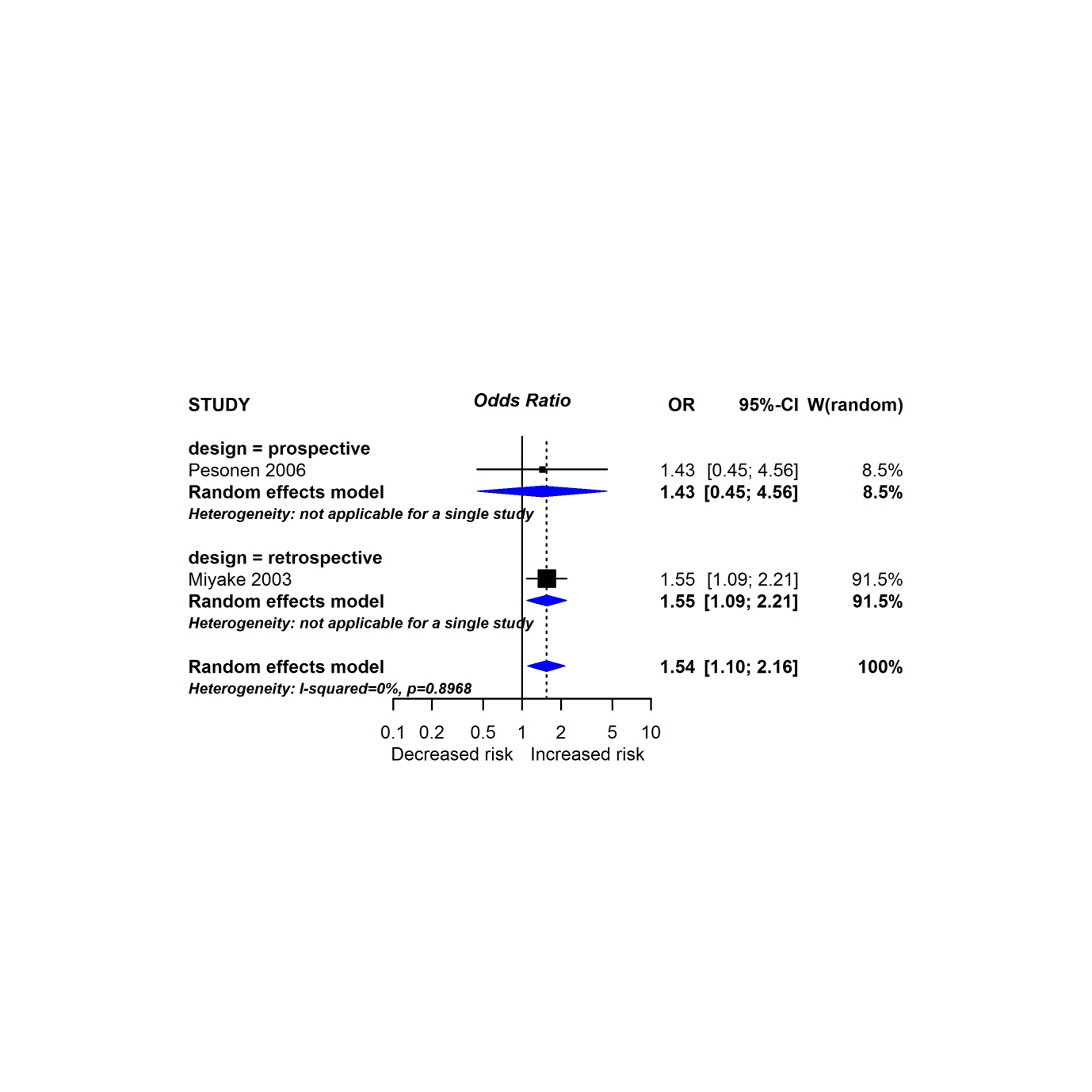
**

#### EBF ≥3-4 months vs. <3-4 months

One prospective cohort study reported OR for AD, in adults aged 44 years with EBF for ≥3-4 months vs. <3-4 months duration and is shown in Figure 30. There was no evidence of an association.

**Figure 30 EBF ≥3-4 months vs. <3-4 months and risk of eczema at age 15+ years**

**
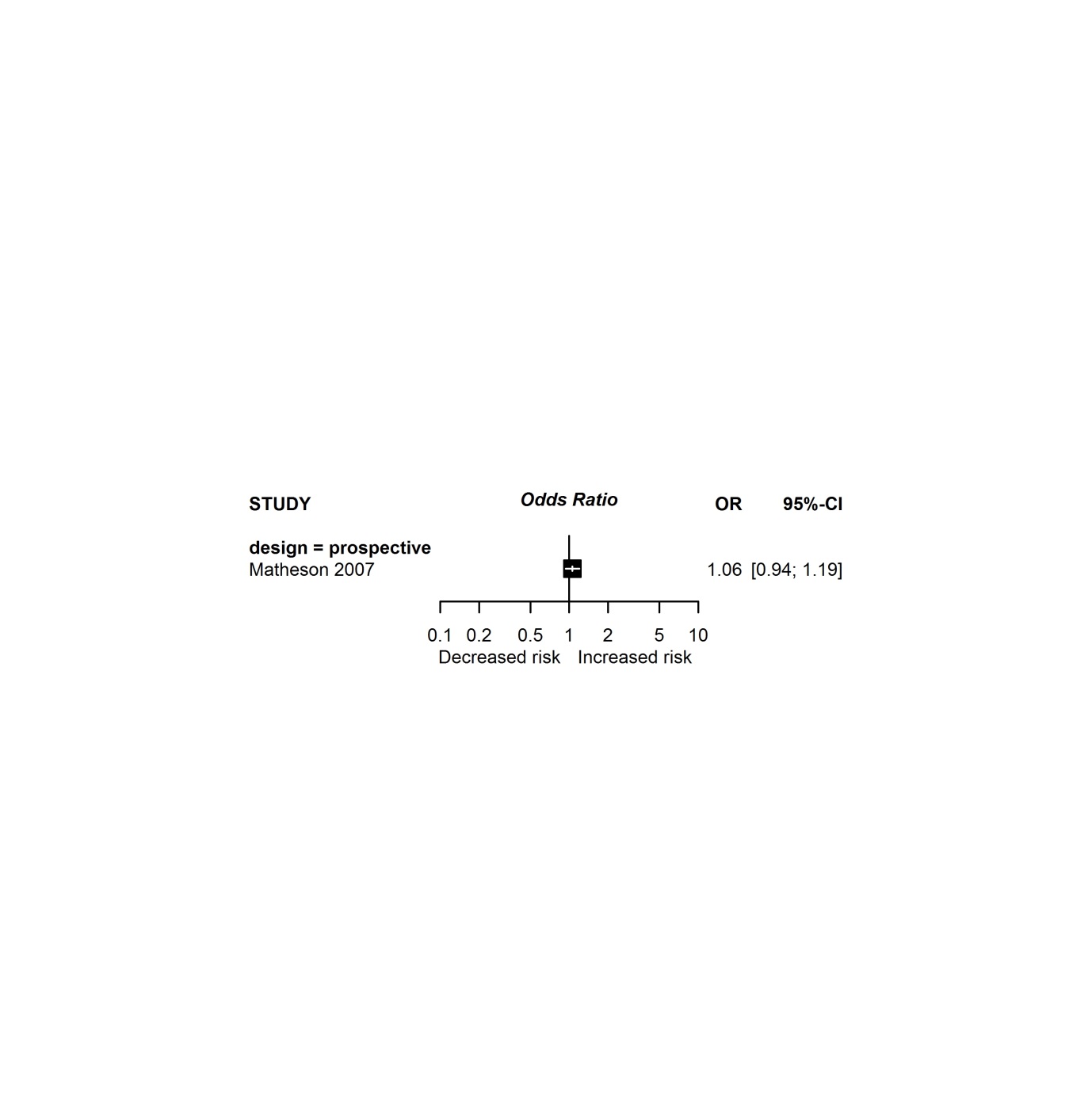
**

#### EBF 5-9 months vs. <5-9 months

One prospective cohort study reported OR for AD, in adults aged 44 years with EBF for ≥5-9 months vs. <5-9 months duration and is shown in Figure 31. There was no evidence of an association.

**Figure 31 EBF ≥5 months vs. <5 months and risk of eczema at age 15+ years**


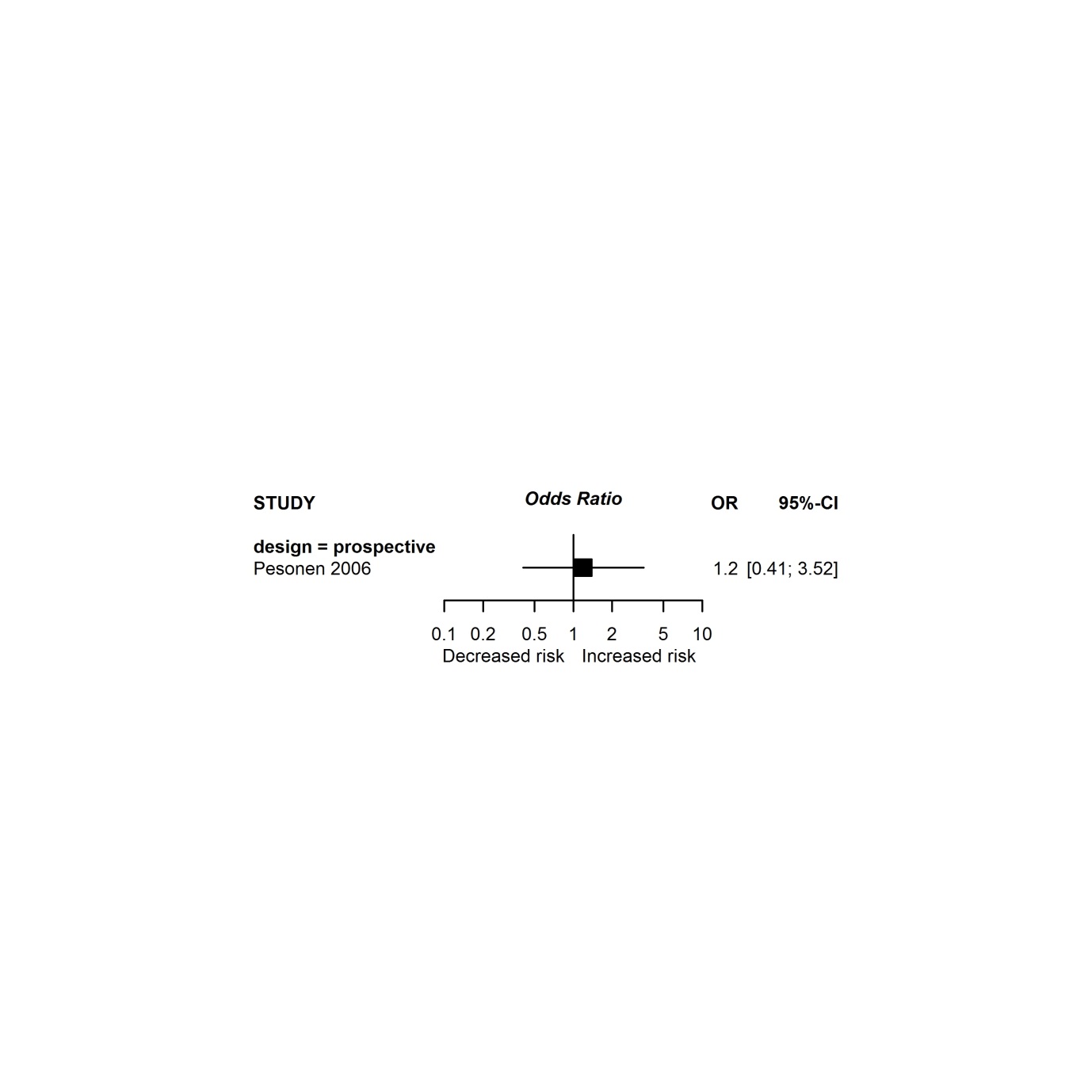


## Exclusive breastfeeding duration and atopic eczema

We also evaluated associations between EBF duration and eczema associated with atopy (i.e. a positive SPT or sIgE test). Relatively few included studies reported this outcome.

### EBF and atopic eczema in children aged 0-4 years

#### EBF ≥3-4 months vs. <3-4 months

One prospective cohort study reported OR for atopic-AD, in infants with EBF for ≥3-4 months vs. <3-4 months duration and is shown in Figure 32. There was no evidence of an association.

**Figure 32 EBF ≥3-4 months vs. <3-4 months and risk of atopic eczema at age 0-4 years**


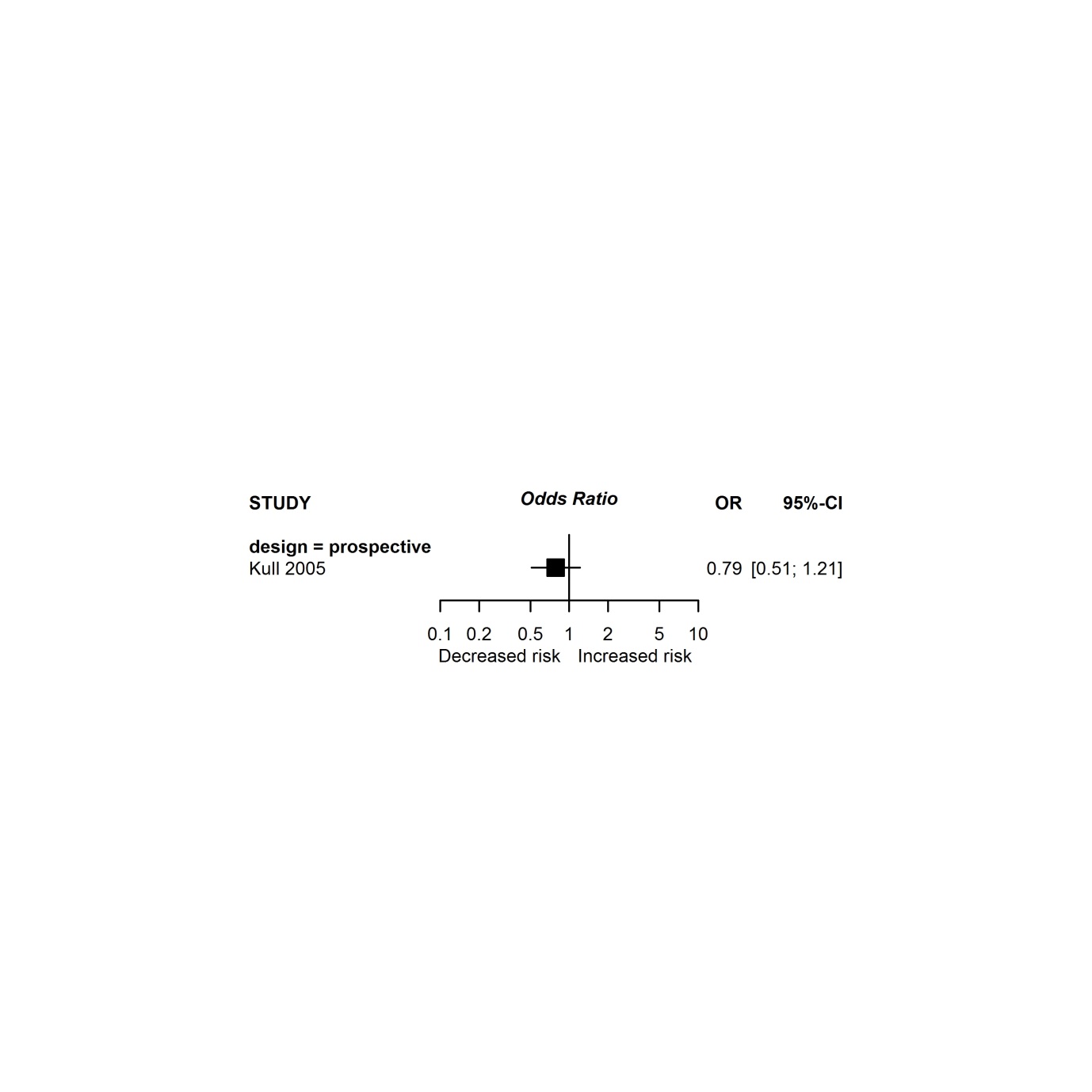


### EBF and atopic eczema in children aged 5-14 years

#### EBF ≥0-2 months vs. <0-2 months

One large cross-sectional study reported separately for affluent and non-affluent countries, calculated OR for atopic-AD, in children with EBF for ≥0-2 months vs. <0-2 months duration and is shown in Figure 33. There was no evidence of an association, with low statistical heterogeneity.

**Figure 33 EBF ≥0-2 months vs. <0-2 months and risk of atopic eczema at age 5-14 years**


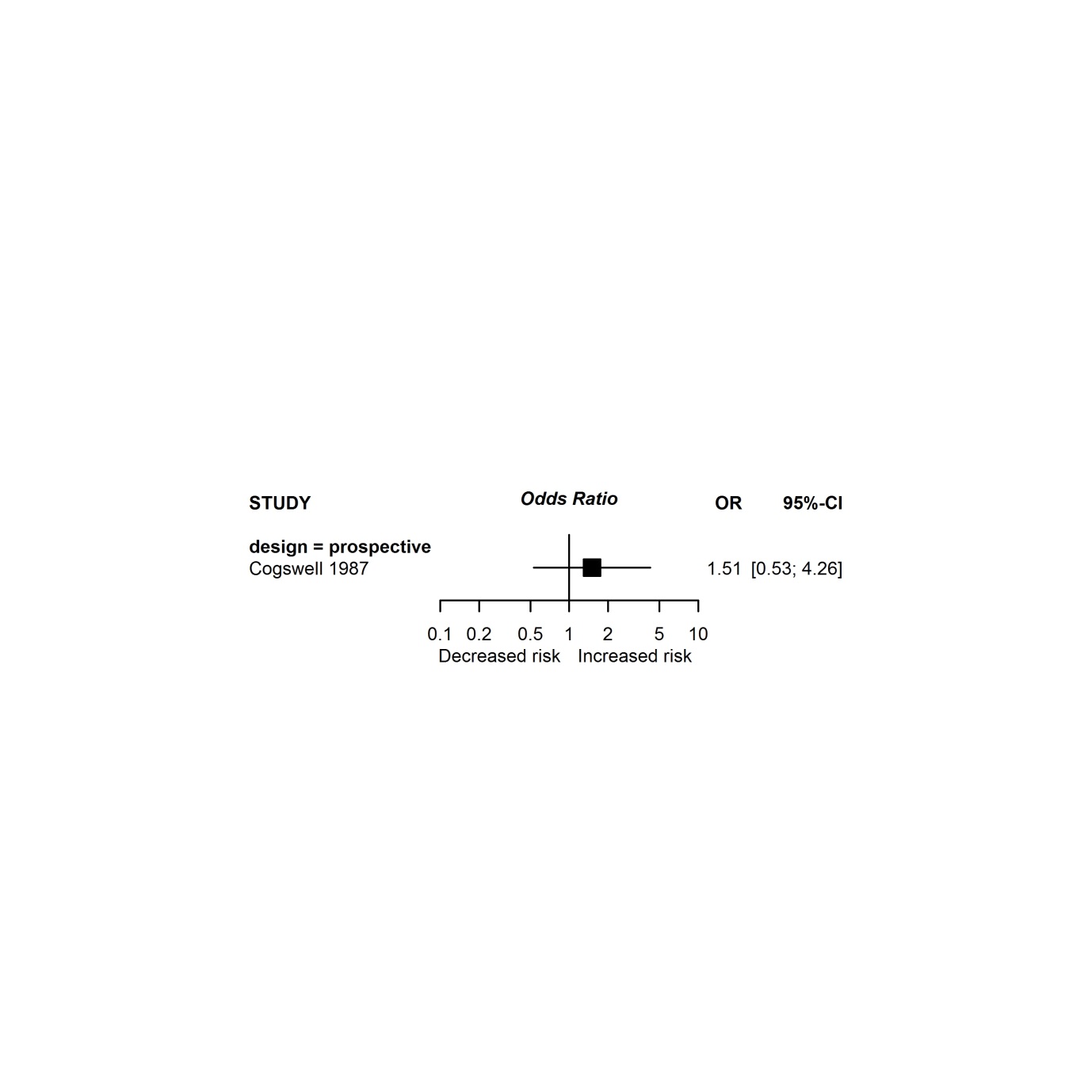


#### EBF ≥3-4 months vs. <3-4 months

One prospective cohort study reported OR for atopic-AD, in children with EBF for ≥3-4 months vs. <3-4 months duration and is shown in Figure 34. There was no evidence of an association.

**Figure 34 EBF for ≥3-4 months vs. <3-4 months and risk of atopic eczema at age 5-14 years**


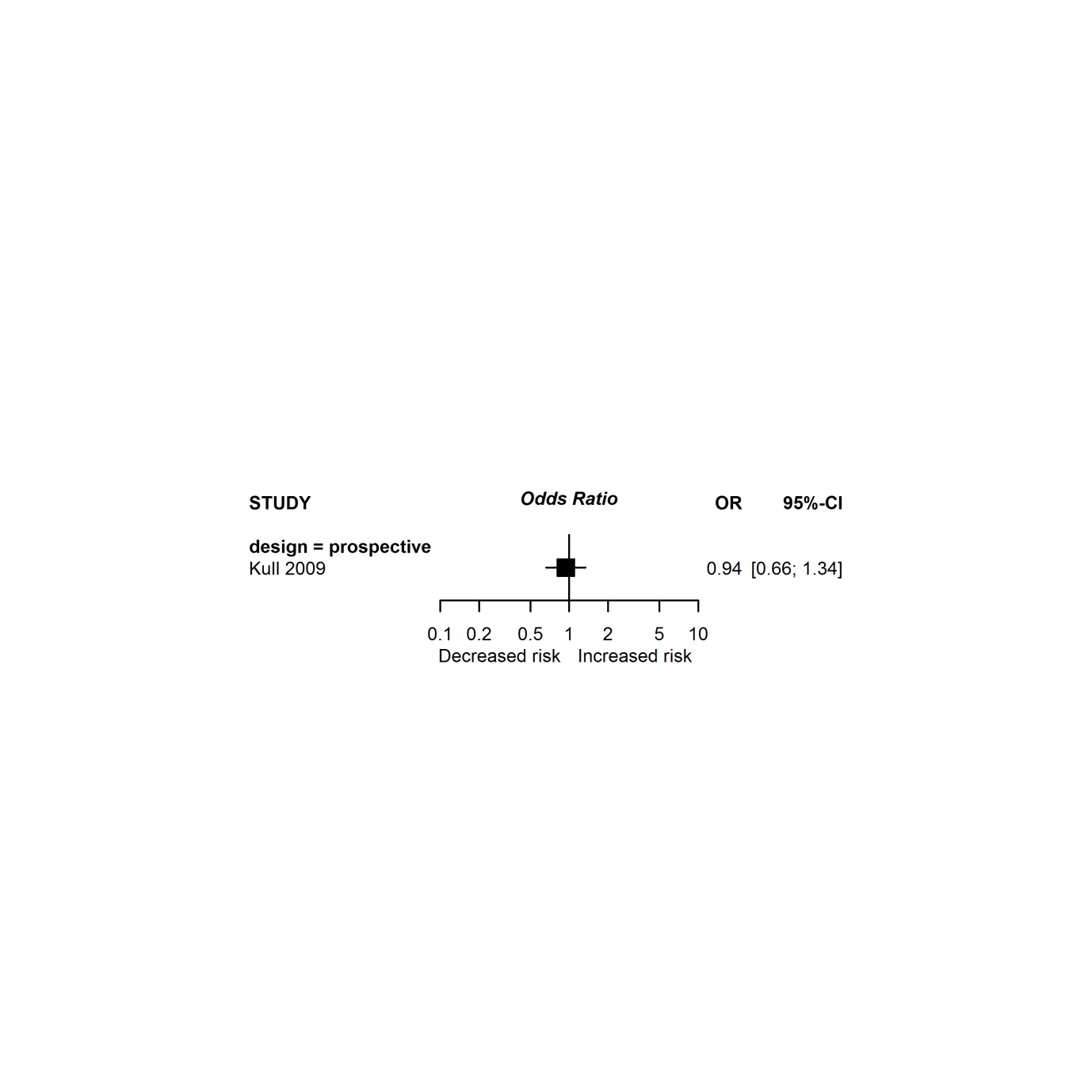


## Data for TBF and risk of eczema that couldn’t be analysed

A further 11 prospective cohort studies, 1 case-control and 1 cross-sectional study reported relevant data from over 35000 study participants, which could not be included in meta-analysis. These studies are summarised in Table 9. In one prospective cohort study there was increased AD associated with increased EBF duration. In all other studies reported there was no association found between EBF and AD.

Table 9 Studies investigating the association between EBF duration and eczema which were not eligible for meta-analysis

| **Study** | **Design** | **Outcome** | **Age** | **N/n cases** | **Data** | **Measure** | **EBF in AD** | **EBF in no AD** | **P** |
| --- | --- | --- | --- | --- | --- | --- | --- | --- | --- |
| Hesselmar, 2010 ([3](#_ENREF_3)) | PC | AD | 1.5 | 184 | Continuous | Median (IQR) | 4 (3.2, 4.5) | 4 (3.9, 4.5) | 0.63 |
| Giwercman, 2010 ([74](#_ENREF_74)) | PC | AD | 2 | 306 | Categorical | Risk of AD increased with increasing duration of EBF in analyses adjusted for allergy risk factors and filaggrin genotype HR 2.1 (1.2-3.8) P=0.016 | | | |
| Gruber, 2010 ([82](#_ENREF_82)) | PC | AD | 1 | 543/47 | Categorical | Cumulative incidence 7.3% (SE1.6%) with EBF>2 months vs 9.7% (SE1.5%) with EBF <2 months. Not adjusted. No statistical comparison. | | | |
| Ludvigsson, 2005 ([67](#_ENREF_67)) | PC | AD | 1 | 8346/ unclear | Categorical | Cumulative incidence of AD to 1 year in EBF ≥4 vs <4 months was not significantly different. Adjusted HR 1.0 (0.95, 1.06) | | | |
| Kramer, 2003 ([86](#_ENREF_86)) | PC | AD | 1 | 3483/96 | Categorical | Cumulative incidence of AD to 1 year in EBF ≥6 vs 3-5 months was not significantly different. Adjusted OR 1.1 (0.65, 2.0) | | | |
| Benn, 2004 ([75](#_ENREF_75)) | PC | AD | 1.5 | 15430 | Categorical | Cumulative incidence of AD to 1.5 years in EBF ≥4 vs <4 months was not significantly different. Adjusted HR 1.1 (0.98-1.28)  NB The authors found significantly increased AD with EBF ≥4 months where there was no parental allergy, but not if parents were allergic | | | |
| Nentwich, ([83](#_ENREF_83)) | PC | AD | 2 | 106 | Categorical | At 2 and 6 months of age there were more atopic symptoms in EBF group, but differences disappeared after 6 months | | | |
| Silvers, 2009 ([38](#_ENREF_38)) | PC | AD | 1 | 987 | Categorical | No association between duration of EBF and AD to 1 year.  Adjusted OR 0.96 (0.9-1.03) | | | |
| Nwaru, 2013 ([18](#_ENREF_18)) | PC | AD | 5 | 3109 | Categorical; Continuous | No significant association between EBF duration either as a categorical or continuous variable in adjusted analyses, and risk of AD up to age 5. Median EBF 1.8 (0.2, 3.5) AD; 1.5 (0.2, 3.5) no AD | | | |
| Kramer, 1981 ([53](#_ENREF_53)) | CC | AD | <20 | 142/ unclear | Categorical | No significant association between EBF >2 months and AD risk | | | |
| Zutavern, 2004 ([93](#_ENREF_93)) | PC | AD | 5.5 | 604 | Categorical | No significant association between EBF ≥2 months and AD risk, in adjusted analysis | | | |
| Fergusson, 1990 ([72](#_ENREF_72)) | PC | AD | 10 | 1067 | Categorical | No significant association between EBF ≥4 months and AD risk, in adjusted analysis. P>0.80 | | | |
| Civelek, 2001 ([57](#_ENREF_57)) | CS | AD | 11 | 1533/743 | Continuous | No significant association between EBF duration and AD risk, in adjusted analysis | | | |

## Solid food introduction and atopic eczema

## Overall characteristics of studies, risk of bias and summary of results

Table 10 describes the main characteristics of the studies analysed in this section of the report. A total of 25 observational studies including 21 prospective cohort studies, 1 retrospective cohort study, 1 nested case-control study and 2 case-control studies, reported the association between timing of solid food introduction (SF) and risk of AD. The studies were mainly from Europe (n=17), but also Australasia (n=3), Asia (n=3), USA (n=1) and Africa (n=1). Overall, valid data on SF and AD risk were available from over 45,000 subjects. Information on AD was obtained from a medical assessment in 11 studies, via parental report in 6 studies, using Hanifin and Rajka criteria or modified versions of the criteria in 4 studies, and ISAAC questionnaire in 4 studies. With regards to time of outcome diagnosis, 19 studies explored the association between SF and AD in the first 4 years of life, and 6 studies assessed at age 5-14 years. Eleven studies used a questionnaire to assess the exposure (SF), 3 diary, 10 interview and 1 used medical record review.

Risk of bias is summarised in Figure 35. Just over 40% of studies had a high risk of bias, most commonly due to lack of adjustment for confounding bias i.e. no adjusted data presented. Risk of conflict of interest was generally assessed as low.

The risk of AD according to SF was categorised as ≥3-4 months vs. <3-4 months. Overall the evidence base was limited by some unexplained statistical heterogeneity in both meta-analyses, but the available data do not suggest any relationship between timing of solid food introduction and AD risk.

Table 10 Characteristics of included studies evaluating solid food introduction and eczema

| **Study** | **N/n cases** | **Design** | **Country** | **Exposure assessment** | **Method of outcome assessment** | **Age at outcome (years)** | **Population characteristics** |
| --- | --- | --- | --- | --- | --- | --- | --- |
| Hesselmar, 2010 ([3](#_ENREF_3)) | 184 | PC | Sweden | I | UK Working Party Criteria | 1.5 | ALLERGYFLORA study. Population based study of babies selected from antenatal clinics between 1998 and 2003 - mainly high risk of allergic disease |
| Mihrshahi, 2007 ([14](#_ENREF_14)) | 516 | PC | Australia | I | Visible flexural dermatitis OR DD eczema | 5 | CAPS study. Infants born in 1997-1999 with family history of asthma or wheezing |
| Fergusson, 1981 ([100](#_ENREF_100)); Fergusson, 1982 ([100](#_ENREF_100)) | 1175 | PC | New Zealand | R/I | DD | 1, 2 | Christchurch Child Development Study. Population based cohort of infants born in 1977 in the Christchurch urban region |
| Larsson, 2008 ([16](#_ENREF_16)) | 4779 | PC | Sweden | Q | ISAAC | 9 | DBH study. Preschool children aged 1–6 years surveyed in 2000 and 2005. |
| Forsyth, 1993 ([101](#_ENREF_101)) | 455 | PC | UK | D/I | Parent reported eczema | 2 | Dundee infant feeding study. Population based cohort of infants born between 1983-1986 |
| Filipiak, 2007; Schoetzau, 2002  ([102-104](#_ENREF_102)) | 4753 | PC | Germany | Q, D | DD; Physician assessment | 1, 4 | GINI study. Term newborn infants born 1995-8 in 2 regions of Germany referred to an intervention program according to risk of allergy |
| Harris, 2001 ([21](#_ENREF_21)) | 622 | PC | UK | I | UK Working Party Criteria | 2 | Population based birth cohort of children born between 1993 and 1995 in three general practices in Ashford |
| Huang, 2013 ([105](#_ENREF_105)) | 684 | PC | China | Q | ISAAC | 2 | Mother-infant pairs registered in Putuo District, Changzheng Town Community Health Service Center Child Health Clinic, 2008 |
| Alm, 2008 ([25](#_ENREF_25)) | 4941 | PC | Sweden | Q | Parent reported eczema | 1 | Infants of Western Sweden. Population birth cohort of infants born in 2003 |
| Snijders, 2008 ([28](#_ENREF_28)) | 822 | PC | Netherlands | Q | Parent reported eczema | 2 | KOALA study. Population based birth cohort of infants born 2000-2002 (including a cohort with anthroposophic lifestyle) |
| Brockow, 2008;  Zutavern 2006 ; Zutavern, 2008  ([106-108](#_ENREF_106)) | 2474 | PC | Germany | I, Q | DD; Parent reported eczema | 2, 6 | LISA study. Population based birth cohort of infants born between 1997-9 at selected maternity hospitals in 4 German cities |
| Marini, 1996 ([31](#_ENREF_31)) | 359 | PC | Italy | Q | Physician assessment | 1 | Infants with family history of allergy whose mother were proposed to participate in an allergy prevention program |
| Moore, 1985 ([33](#_ENREF_33)) | 470 | PC | UK | D/I | Physician assessment | 1.0 | Infants born in a hospital in 1979-1980 with family history of eczema or asthma (high risk of disease) |
| Morgan, 2004; Morgan, 2004 (b) ([34](#_ENREF_34), [35](#_ENREF_35)) | 257 | PC | UK | I | Parent reported eczema; Physician assessment | 1, 1.5 | Infants from five prospective randomised dietary trials conducted in the UK 1993-7. Two term infants, one LBW infants, two premature infants |
| Ruiz, 1992 ([44](#_ENREF_44)) | 39 | PC | UK | I | Hanifin and Rajka criteria | 1 | Recruited from hospital and family history of atopy (high risk of disease) |
| Dunlop, 2006 ([91](#_ENREF_91)) | 1326 | PC | Slovakia | Q | Physician assessment | 1 | Recruited from hospital, born 1997-1999 (normal risk of disease) |
| Chuang, 2011 ([46](#_ENREF_46)) | 18773/  1050 | PC | Taiwan | R/I | Physician assessment | 1.5 | TaiwanBirth Cohort Study: population representative sample born in 1995 (normal risk of disease) |
| Joseph, 2011 ([109](#_ENREF_109)) | 594 | PC | USA | I | DD | 3 | WHEALS STUDY: Recruited from hospital prenatal care and born in 2005 (normal risk of disease) |
| Zutavern, 2004 ([93](#_ENREF_93)) | 604 | PC | UK | I | DD | 5.5 | Cohort recruited from general practices and born in 1993-1995 (normal risk of disease) |
| Hide, 1981 ([47](#_ENREF_47)) | 843 | PC | UK | D/Q | DD | 1 | The Isle of Wight study: born in 1977-1978 (normal risk of disease) |
| Van Asperen, 1983 ([110](#_ENREF_110)) | 79 | PC | Australia | I | Physician assessment | 1.3 | Cohort recruited from medical service, born in 1980-1981 with family history of atopy (high risk of disease) |
| Sariachvili, 2010 ([51](#_ENREF_51)) | 557 | NCC | Belgium | Q | ISAAC | 4 | PIPO COHORT: population representative sample, participants born in 1997-2001 (normal risk of disease) |
| von Geburstsreif, 1990 ([20](#_ENREF_20)) | 145 | RC | Germany | Q | Parental report | 1.5 | Incident cases from diabetes registry and controls from schools (normal risk of disease) |
| Haileamlak, 2005  ([55](#_ENREF_55)) | 732 | CC | Ethiopia | I | ISAAC | 5 | Children age 1- 5 years. Cases were defined according to the ISAAC criteria for AD and confirmed by clinical examination |
| Sahakyan, 2006 ([95](#_ENREF_95)) | 240 | CC | Armenia | Q | UK Working Party Criteria | 7 | Hospital-based study with matched controls (normal risk of disease) |

Q: questionnaire, I: interview, R: medical records, D: diary, PC: prospective cohort, RC: retrospective cohort, NCC: nested case-control, CC: case-control, CS: Cross-sectional, AD: eczema, DD: doctor diagnosis of AD, in contrast to ‘Physician assessment’ where AD diagnosis was always made by a study physician as part of the study protocol.

Figure 35 Risk of bias in studies of solid food introduction and eczema

### SF introduction and risk of eczema in children aged 0-4 years

#### SF ≥3-4 months vs. <3-4 months

Figure 36 shows the outcomes of 14 eligible observational studies evaluating SF ≥3-4 months vs. <3-4 months and risk of AD at age 0-4 years. The data show no significant relationship, with high statistical heterogeneity (I^2^=70%). Chuang also reported no relationship between SF introduction at >6 months and AD, in their prospective cohort study with low overall risk of bias in a normal risk population evaluated at age 1.5 years aOR 1.08 (0.74-1.57). In an analysis of a subset of the GINI dataset reported by Filipiak, Schoetzao also found no association between SF introduction at >6 months and risk of AD in the first year – unadjusted OR 1.8 (0.8-1.7). Funnel plot is shown in Figure 37, and does not suggest obvious publication bias. In the study of Snijders a cutoff of >7 versus 3 months was used.

Subgroup analyses are shown in Table 11 and do not identify important subgroup differences or explanations for the statistical heterogeneity.

Figure 36 SF ≥3-4 months vs. <3-4 months and risk of atopic eczema at age 0-4 years


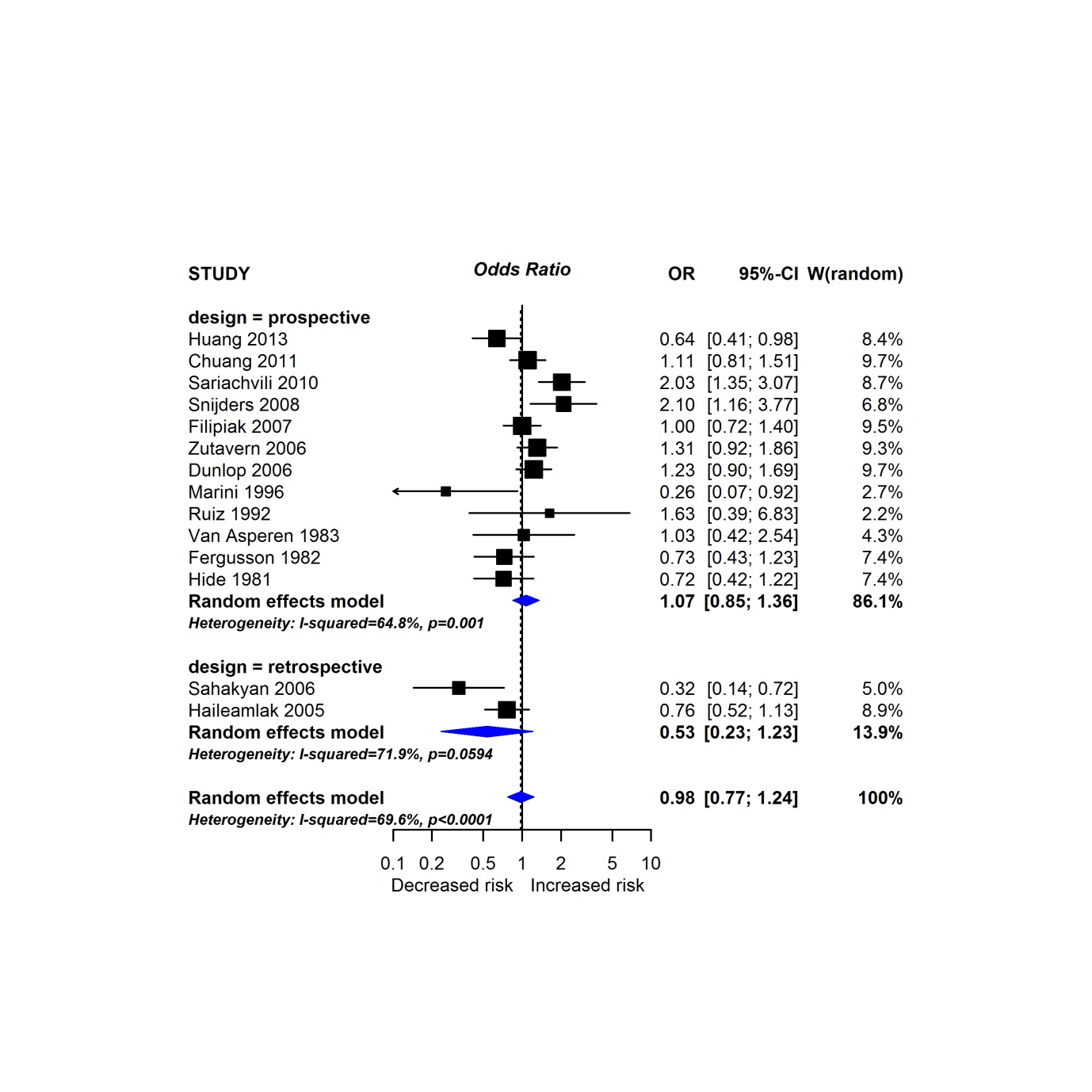


Figure 37 Risk of publication bias in studies investigating SF introduction ≥3-4 months vs. <3-4 months and risk of eczema in children aged 0-4 years


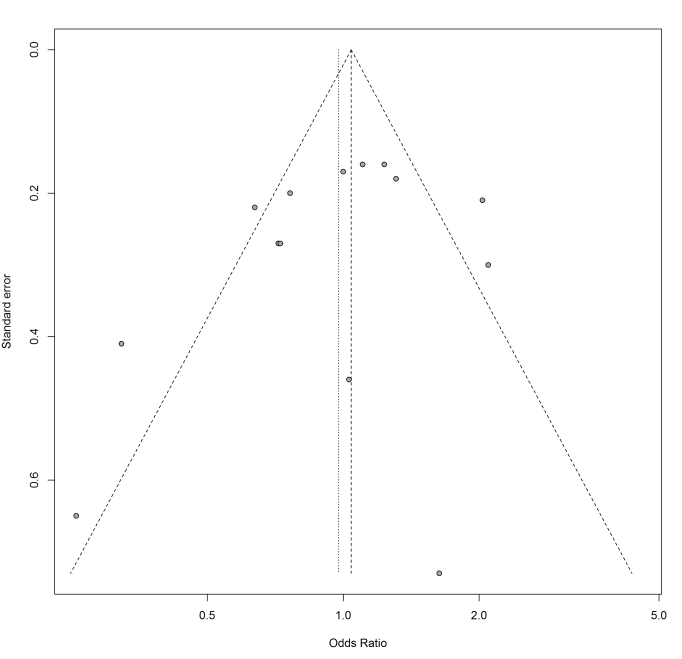


Egger’s test p-value = 0.241

Table 11 Subgroup Analyses of risk of atopic dermatitis and SF ≥3-4 months vs. <3-4 months in children aged 0-4 years

|  | **Number of studies** | **OR [95% CI]** | **I^2^ (%)** | **P-value for between groups difference** |
| --- | --- | --- | --- | --- |
| **Overall (if adjusted NA, unadjusted used)** | 14 | 0.98 [0.77; 1.24] | 69.6 |  |
| **Adjusted** | 8 | 1.03 [0.73; 1.46] | 77.9 | Not tested |
| **Unadjusted** | 9 | 1.10 [0.84; 1.43] | 63.3 |  |
| Study Design – Prospective | 12 | 1.07 [0.85; 1.36] | 64.8 | 0.11 |
| Study Design – Retrospective | 2 | 0.54 [0.23; 1.23] | 71.9 |  |
| Risk of disease – High | 3 | 0.76 [0.28; 2.07] | 53.6 | 0.60 |
| Risk of disease – Normal | 11 | 1.00 [0.78; 1.28] | 73.5 |  |
| Risk of bias – Low | 6 | 1.20 [0.84; 1.71] | 76.8 | 0.09 |
| Risk of bias – High/Unclear | 8 | 0.81 [0.62; 1.06] | 44.0 |  |

### SF introduction and risk of eczema in children aged 5-14 years

#### SF ≥3-4 months vs <3-4 months

Three observational studies reported OR for SF at ≥3-4 months vs <3-4 months and risk of AD at age 5-14 years. Data were not pooled due to extreme statistical heterogeneity (I^2^=83%). All 3 studies had a low risk of bias on all domains, and reported adjusted data – Zutavern at aged 6 in a normal risk population using parental report of eczema, Mihrshahi at aged 5 in a high risk population using doctor diagnosis, Larsson at aged 9 in a normal risk population using ISAAC. In the study of Larsson, there was no significant association between SF 3-6 months versus >6 months and AD OR 0.99 (0.77, 1.27). However, there was a significant association between SF >6 months vs <3 months and AD (data shown in Figure 38). We were unable to identify an explanation for the statistical heterogeneity seen between studies.

Figure 38 SF ≥3-4 months vs. <3-4 months and risk of eczema at age 5-14 years


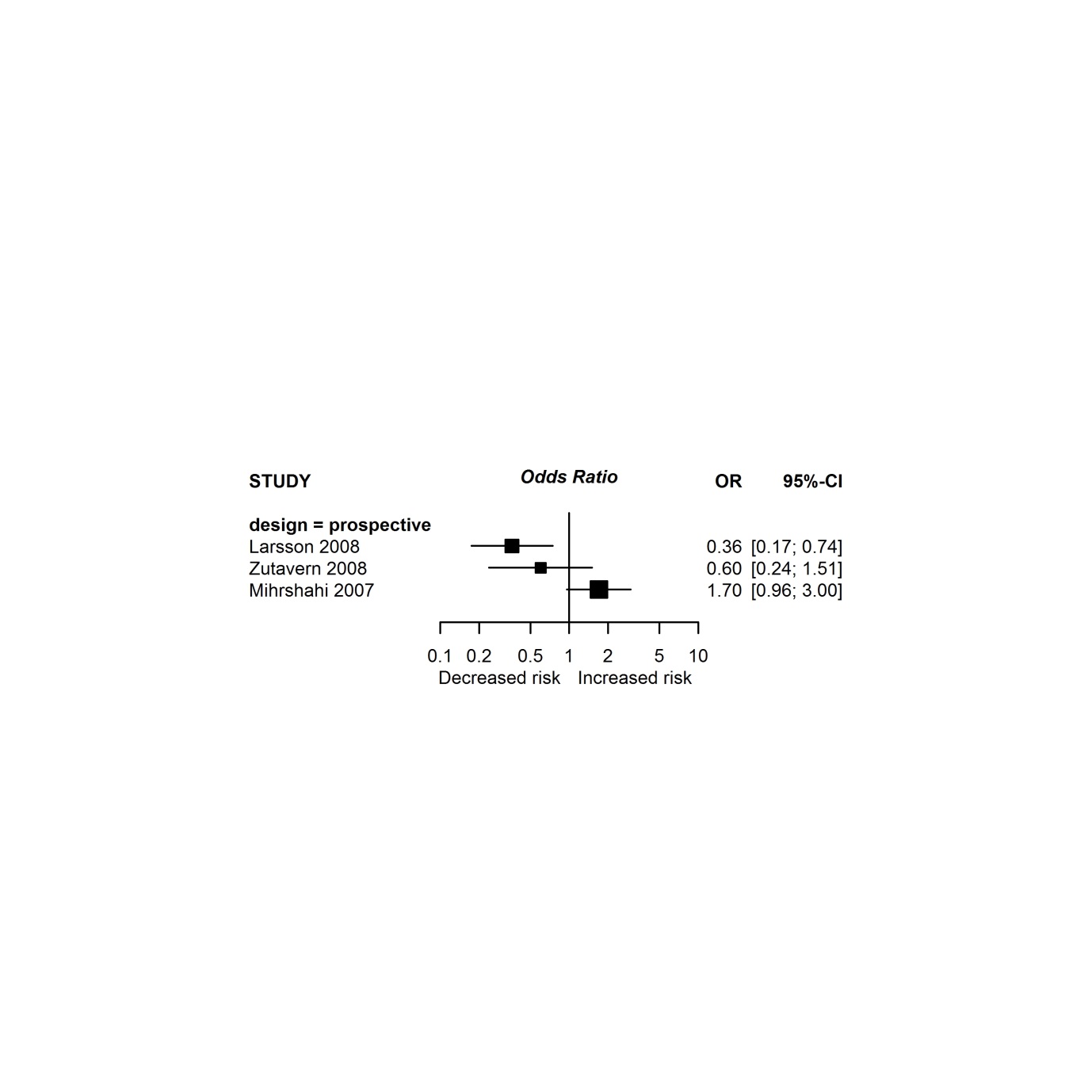


## Data for SF and risk of eczema that couldn’t be analysed

A further 7 prospective cohort studies reported relevant data from over 7500 study participants, which could not be included in meta-analysis. This was due to the way the studies presented the data, either as simply a narrative in the Results section, or with estimates that were not comparable. Their characteristics and results are summarised in Table 12. The studies show no evidence of an association between SF and AD.

Table 12 Studies investigating the association between solid food introduction and eczema which were not eligible for meta-analysis

| **Study** | **Design** | **Outcome** | **Age** | **N/n cases** | **Data** | **Measure** | **SF in AD** | **SF in no AD** | **P** |
| --- | --- | --- | --- | --- | --- | --- | --- | --- | --- |
| Hesselmar 2010 ([3](#_ENREF_3)) | PC | AD | 1.5 | 184/ unclear | Continuous | Median (IQR) | 4 (4, 5) | 4 (4, 4.6) | 0.97 |
| Moore, 1985 ([33](#_ENREF_33)) | PC | AD | 1 | 470 | Categorical |  | No significant association between SF >3 vs <3 and risk of AD (adjusted analysis) | | |
| Joseph 2011 ([109](#_ENREF_109)) | PC | AD | 3 | 594 | Categorical |  | No significant association between SF >4 vs <4 and risk of AD P=0.76 | | |
| Forsyth, 1993 ([101](#_ENREF_101)) | PC | AD | 2 | 455 | Categorical |  | SF >3 had less AD (8.3%) than SF 2-3 (17.0%) but more than SF <2 (5.4%) P<0.05. Adjusted analysis | | |
| Morgan, 2004 ([35](#_ENREF_35)) | PC | AD | 1.5 | 257 | Categorical |  | No significant association between SF >3 vs <3 and risk of AD (adjusted analysis) | | |
| Alm, 2008 ([25](#_ENREF_25)) | PC | AD | 1 | 4941/ unclear | Categorical |  | No significant association between SF and risk of AD | | |
| Harris, 2001 ([21](#_ENREF_21)) | PC | AD | 2 | 622/ 283 | Categorical |  | No significant association between SF and risk of AD | | |

## Conclusions

This report summarises the results of 57, 47, 25 studies of TBF, EBF and SF respectively, in relation to AD risk. Studies included over 250,000 people and AD is a common disease affecting 5-20% of most human populations. Thus we captured a significant dataset in which to explore the relationship between BF, SF and AD risk. Our search identified one high quality recent systematic review, which appraised 2 observational studies of EBF duration and AD and found no evidence for an association. One cluster RCT trial of a breastfeeding promotion intervention found reduced AD risk at 1 year (but not at 6.5 years) in centres randomised to the breastfeeding promotion intervention. Despite a large volume of data from observational studies, some analyses especially for EBF and solid food introduction were limited by small numbers of studies reporting relevant data. Overall we found no evidence from the observational studies that duration of TBF or EBF at any stage is associated with reduced risk of AD. Some analyses suggested an association in the opposite direction i.e. prolonged BF associated with increased AD – however these were not consistent between analyses, not robust to subgroup analysis, and not seen in studies which did not contribute to meta-analysis. The intervention trial evidence that breastfeeding promotion reduces risk of AD was graded at LOW certainty, due to imprecision and inconsistency with other sources of evidence. We found no association between timing of SF introduction and AD risk.

Our data suggest that breastfeeding promotion may reduce risk of AD, at least in the first year of life, but that the evidence for this is of LOW certainty. We did not find evidence that duration of EBF, or timing of SF introduction, influence AD risk. We did identify some evidence of gene-environment effects which were also seen in the ‘Allergic Sensitisation’ report – suggesting that human breast milk may be able to have an effect on AD risk, but that its composition or the genetic make-up of the infant are important effect modifiers. This is an unproven hypothesis with respect to AD, but needs exploration in future studies since our understanding of eczema pathogenesis may be enhanced by understanding the relationship between breast milk composition, maternal and infant genotype, and clinical outcome.

A previous overview of systematic reviews undertaken by the Cochrane Collaboration found no evidence of a relationship between these exposures and risk of eczema, but did highlight the single intervention trial which we also found, that reported reduced eczema risk in infants born in centres randomised to a breastfeeding promotion intervention ([111](#_ENREF_111)). Our comprehensive analysis of the observational studies literature has failed to strengthen support for this finding..

## References

1. Kramer MS, Matush L, Vanilovich I, Platt R, Bogdanovich N, Sevkovskaya Z, et al. Effect of prolonged and exclusive breast feeding on risk of allergy and asthma: cluster randomised trial. Bmj. 2007;335(7624):815.

2. Kramer MS, Matush L, Vanilovich I, Platt R, Bogdanovich N, Sevkovskaya Z, et al. Effect of prolonged and exclusive breast feeding on risk of allergy and asthma: cluster randomised trial. BMJ (Clinical research ed). 2007;335(7624):815.

3. Hesselmar B, Saalman R, Rudin A, Adlerberth I, Wold A. Early fish introduction is associated with less eczema, but not sensitization, in infants. Acta Paediatrica. 2010;99(12):1861-7.

4. Abd A, Henderson J, Northstone K, Seddon P, Palmer C, Mukhopadhyay S, et al. Breastfeeding duration and eczema risk in the avon longitudinal study of parents and children. Archives of Disease in Childhood. 2012;97:A43.

5. Purvis DJ, Thompson JM, Clark PM, Robinson E, Black PN, Wild CJ, et al. Risk factors for atopic dermatitis in New Zealand children at 3.5 years of age. British Journal of Dermatology. 2005;152(4):742-9.

6. Kull I, Wickman M, Lilja G, Nordvall SL, Pershagen G. Breast feeding and allergic diseases in infants-a prospective birth cohort study. Archives of Disease in Childhood. 2002;87(6):478-81.

7. Berth-Jones J, George S, Graham-Brown RA. Predictors of atopic dermatitis in Leicester children. British Journal of Dermatology. 1997;136(4):498-501.

8. Taylor B, Wadsworth J, Wadsworth M, Peckham C. Changes in the reported prevalence of childhood eczema since the 1939-45 war. Lancet. 1984;2(8414):1255-7.

9. Taylor B, Wadsworth J, Golding J, Butler N. Breast feeding, eczema, asthma, and hayfever. Journal of Epidemiology and Community Health. 1983;37:95-9.

10. Burr ML, Miskelly FG, Butland BK, Merrett TG, Vaughan-Williams E. Environmental factors and symptoms in infants at high risk of allergy. Journal of Epidemiology & Community Health. 1989;43(2):125-32.

11. Burr ML, Limb ES, Maguire MJ, Amarah L, Eldridge BA, Layzell JCM, et al. INFANT-FEEDING, WHEEZING, AND ALLERGY - A PROSPECTIVE-STUDY. Archives of Disease in Childhood. 1993;68(6):724-8.

12. Burr ML, Limb ES, Maguire MJ, Amarah L, Eldridge BA, Layzell JC, et al. Infant feeding, wheezing, and allergy: a prospective study. Archives of Disease in Childhood. 1993;68(6):724-8.

13. Businco L, Cantani A, Meglio P, Bruno G. Prevention of atopy: Results of a long-term (7 months to 8 years) follow-up. Annals of Allergy. 1987;59(5 PART II):183-6.

14. Mihrshahi S, Ampon R, Webb K, Almqvist C, Kemp AS, Hector D, et al. The association between infant feeding practices and subsequent atopy among children with a family history of asthma. Clinical & Experimental Allergy. 2007;37(5):671-9.

15. Bisgaard H, Halkjaer LB, Hinge R, Giwercman C, Palmer C, Silveira L, et al. Risk analysis of early childhood eczema. Journal of Allergy & Clinical Immunology. 2009;123(6):1355-60.e5.

16. Larsson M, Hagerhed-Engman L, Sigsgaard T, Janson S, Sundell J, Bornehag CG. Incidence rates of asthma, rhinitis and eczema symptoms and influential factors in young children in Sweden. Acta Paediatrica. 2008;97(9):1210-5.

17. Devereux G, Turner SW, Craig LC, McNeill G, Martindale S, Harbour PJ, et al. Low maternal vitamin E intake during pregnancy is associated with asthma in 5-year-old children. American Journal of Respiratory & Critical Care Medicine. 2006;174(5):499-507.

18. Nwaru BI, Takkinen HM, Niemela O, Kaila M, Erkkola M, Ahonen S, et al. Timing of infant feeding in relation to childhood asthma and allergic diseases. Journal of Allergy and Clinical Immunology. 2013;131(1):78-86.

19. Farooqi IS, Hopkin JM. Early childhood infection and atopic disorder. Thorax. 1998;53(11):927-32.

20. Forster J, Dungs M, Wais U, Urbanek R. [Atopy-suggesting symptoms in the first 2 years of life. Effect of gestational age, nutrition and social class]. Klinische Padiatrie. 1990;202(3):136-40.

21. Harris JM, Cullinan P, Williams HC, Mills P, Moffat S, White C, et al. Environmental associations with eczema in early life. British Journal of Dermatology. 2001;144(4):795-802.

22. Hikino S, Nakayama H, Yamamoto J, Kinukawa N, Sakamoto M, Hara T. Food allergy and atopic dermatitis in low birthweight infants during early childhood. Acta Paediatrica. 2001;90(8):850-5.

23. Howie PW, Forsyth JS, Ogston SA, Clark A, Florey CD. Protective effect of breast feeding against infection. Bmj. 1990;300(6716):11-6.

24. Gruskay FL. Comparison of breast, cow, and soy feedings in the prevention of onset of allergic disease: a 15-year prospective study. Clinical Pediatrics. 1982;21(8):486-91.

25. Alm B, Aberg N, Erdes L, Mollborg P, Pettersson R, Norvenius SG, et al. Early introduction of fish decreases the risk of eczema in infants. Archives of Disease in Childhood. 2009;94(1):11-5.

26. Morales E, Garcia-Esteban R, Guxens M, Guerra S, Mendez M, Molto-Puigmarti C, et al. Effects of prolonged breastfeeding and colostrum fatty acids on allergic manifestations and infections in infancy. Clinical and Experimental Allergy. 2012;42(6):918-28.

27. Kemeny DM, Price JF, Richardson V, Richards D, Lessof MH. THE IGE AND IGG SUBCLASS ANTIBODY-RESPONSE TO FOODS IN BABIES DURING THE 1ST YEAR OF LIFE AND THEIR RELATIONSHIP TO FEEDING REGIMEN AND THE DEVELOPMENT OF FOOD ALLERGY. Journal of Allergy and Clinical Immunology. 1991;87(5):920-9.

28. Snijders BE, Thijs C, van Ree R, van den Brandt PA. Age at first introduction of cow milk products and other food products in relation to infant atopic manifestations in the first 2 years of life: the KOALA Birth Cohort Study. Pediatrics. 2008;122(1):e115-22.

29. Snijders BE, Thijs C, Dagnelie PC, Stelma FF, Mommers M, Kummeling I, et al. Breast-feeding duration and infant atopic manifestations, by maternal allergic status, in the first 2 years of life (KOALA study). Journal of Pediatrics. 2007;151(4):347-51, 51.e1-2.

30. Wetzig H, Schulz R, Diez U, Herbarth O, Viehweg B, Borte M. Associations between duration of breast-feeding, sensitization to hens' eggs and eczema infantum in one and two year old children at high risk of atopy. International Journal of Hygiene & Environmental Health. 2000;203(1):17-21.

31. Marini A, Agosti M, Motta G, Mosca F. Effects of a dietary and environmental prevention programme on the incidence of allergic symptoms in high atopic risk infants: three years' follow-up. Acta Paediatrica Supplement. 1996;414:1-21.

32. Miskelly FG, Burr ML, Vaughan-Williams E, Fehily AM, Butland BK, Merret TG. Infant feeding and allergy. Archives of Disease in Childhood. 1988;63(4):388-93.

33. Moore WJ, Midwinter RE, Morris AF, Colley JR, Soothill JF. Infant feeding and subsequent risk of atopic eczema. Archives of Disease in Childhood. 1985;60(8):722-6.

34. Morgan JB, Lucas A, Fewtrell MS. Does weaning influence growth and health up to 18 months? Archives of Disease in Childhood. 2004;89(8):728-33.

35. Morgan J, Williams P, Norris F, Williams CM, Larkin M, Hampton S. Eczema and early solid feeding in preterm infants. Archives of Disease in Childhood. 2004;89(4):309-14.

36. Bergmann RL, Edenharter G, Bergmann KE, Lau S, Wahn U. Socioeconomic status is a risk factor for allergy in parents but not in their children. Clinical & Experimental Allergy. 2000;30(12):1740-5.

37. Bergmann RL, Diepgen TL, Kuss O, Bergmann KE, Kujat J, Dudenhausen JW, et al. Breastfeeding duration is a risk factor for atopic eczema. Clinical & Experimental Allergy. 2002;32(2):205-9.

38. Silvers KM, Frampton CM, Wickens K, Epton MJ, Pattemore PK, Ingham T, et al. Breastfeeding protects against adverse respiratory outcomes at 15 months of age. Maternal & Child Nutrition. 2009;5(3):243-50.

39. Miyake Y, Tanaka K, Sasaki S, Kiyohara C, Ohya Y, Fukushima W, et al. Breastfeeding and atopic eczema in Japanese infants: The Osaka Maternal and Child Health Study. Pediatric Allergy & Immunology. 2009;20(3):234-41.

40. Kerkhof M, Koopman LP, van Strien RT, Wijga A, Smit HA, Aalberse RC, et al. Risk factors for atopic dermatitis in infants at high risk of allergy: the PIAMA study. Clinical and Experimental Allergy. 2003;33(10):1336-41.

41. Porch MC, Shahane AD, Leiva LE, Elston RC, Sorensen RU. Influence of breast milk, soy or two hydrolyzed formulas on the development of allergic manifestations in infants at risk. Nutrition Research. 1998;18(8):1413-24.

42. Pratt HF. Breastfeeding and eczema. Early Human Development. 1984;9(3):283-90.

43. Rothenbacher D, Weyermann M, Beermann C, Brenner H. Breastfeeding, soluble CD14 concentration in breast milk and risk of atopic dermatitis and asthma in early childhood: birth cohort study. Clinical & Experimental Allergy. 2005;35(8):1014-21.

44. Ruiz RGG, Kemeny DM, Price JF. Higher risk of infantile atopic dermatitis from maternal atopy than from paternal atopy. Clinical and Experimental Allergy. 1992;22(8):762-6.

45. Saarinen UM, Kajosaari M. Breastfeeding as prophylaxis against atopic disease: prospective follow-up study until 17 years old. Lancet. 1995;346(8982):1065-9.

46. Chuang CH, Hsieh WS, Chen YC, Chang PJ, Hurng BS, Lin SJ, et al. Infant feeding practices and physician diagnosed atopic dermatitis: a prospective cohort study in Taiwan. Pediatric Allergy & Immunology. 2011;22(1 Pt 1):43-9.

47. Hide DW, Guyer BM. Clinical manifestations of allergy related to breast and cows' milk feeding. Archives of Disease in Childhood. 1981;56(3):172-5.

48. Wang IJ, Guo YL, Weng HJ, Hsieh WS, Chuang YL, Lin SJ, et al. Environmental risk factors for early infantile atopic dermatitis. Pediatric Allergy & Immunology. 2007;18(5):441-7.

49. Hagendorens MM, Bridts CH, Lauwers K, van Nuijs S, Ebo DG, Vellinga A, et al. Perinatal risk factors for sensitization, atopic dermatitis and wheezing during the first year of life (PIPO study). Clinical and Experimental Allergy. 2005;35(6):733-40.

50. Sariachvili M, Droste J, Dom S, Wieringa M, Vellinga A, Hagendorens M, et al. Is breast feeding a risk factor for eczema during the first year of life? Pediatric Allergy & Immunology. 2007;18(5):410-7.

51. Sariachvili M, Droste J, Dom S, Wieringa M, Hagendorens M, Stevens W, et al. Early exposure to solid foods and the development of eczema in children up to 4 years of age. Pediatric Allergy & Immunology. 2010;21(1 Pt 1):74-81.

52. Monego A, Infortuna M, Santisi G, Barberio G. [Can breast feeding prevent atopic dermatitis? Epidemiologic study of 144 cases]. Minerva Pediatrica. 1989;41(5):223-7.

53. Kramer MS, Moroz B. Do breast-feeding and delayed introduction of solid foods protect against subsequent atopic eczema? Journal of Pediatrics. 1981;98(4):546-50.

54. Ghaderi R, Makhmalbaf Z. Effect of breast-feeding on the development of atopic dermatitis.[Retraction in Ghaderi R, Makhmalbaf Z. Iran J Allergy Asthma Immunol. 2007 Dec;6(4):225; PMID: 18357658]. Iranian Journal of Allergy Asthma & Immunology. 2005;4(3):129-32.

55. Haileamlak A, Dagoye D, Williams H, Venn AJ, Hubbard R, Britton J, et al. Early life risk factors for atopic dermatitis in Ethiopian children. Journal of Allergy & Clinical Immunology. 2005;115(2):370-6.

56. Flohr C, Nagel G, Weinmayr G, Kleiner A, Strachan DP, Williams HC, et al. Lack of evidence for a protective effect of prolonged breastfeeding on childhood eczema: lessons from the International Study of Asthma and Allergies in Childhood (ISAAC) Phase Two. British Journal of Dermatology. 2011;165(6):1280-9.

57. Civelek E, Sahiner UM, Yuksel H, Boz AB, Orhan F, Uner A, et al. Prevalence, burden, and risk factors of atopic eczema in schoolchildren aged 10-11 years: a national multicenter study. Journal of Investigational Allergology & Clinical Immunology. 2011;21(4):270-7.

58. Nakamura Y, Oki I, Tanihara S, Ojima T, Ito Y, Yamazaki O, et al. Relationship between breast milk feeding and atopic dermatitis in children. Journal of epidemiology / Japan Epidemiological Association. 2000;10(2):74-8.

59. Tanaka K, Miyake Y, Sasaki S. Association between breastfeeding and allergic disorders in Japanese children. International Journal of Tuberculosis and Lung Disease. 2010;14(4):513-8.

60. Girolomoni G, Abeni D, Masini C, Sera F, Ayala F, Belloni-Fortina A, et al. The epidemiology of atopic dermatitis in Italian schoolchildren. Allergy. 2003;58(5):420-5.

61. Innes Asher M, Robertson C, Ait-Khaled N, Anderson HR, Beasley R, Bjorksten B, et al. Global analysis of breast feeding and risk of symptoms of asthma, rhinoconjunctivitis and eczema in 6-7 year old children: ISAAC Phase Three. Allergologia et Immunopathologia. 2011;39(6):318-25.

62. Karino S, Okuda T, Uehara Y, Toyo-oka T. Breastfeeding and prevalence of allergic diseases in Japanese university students. Annals of Allergy Asthma & Immunology. 2008;101(2):153-9.

63. Kurt E, Metintas S, Basyigit I, Bulut I, Coskun E, Dabak S, et al. Prevalence and risk factors of allergies in Turkey: Results of a multicentric cross-sectional study in children. Pediatric Allergy & Immunology. 2007;18(7):566-74.

64. Selcuk ZT, Caglar T, Enunlu T, Topal T. The prevalence of allergic diseases in primary school children in Edirne, Turkey. Clinical & Experimental Allergy. 1997;27(3):262-9.

65. Kramer MS, Kakuma R. Maternal dietary antigen avoidance during pregnancy and/or lactation for preventing or treating atopic disease in the child. Cochrane Database of Systematic Reviews. 2003(4):CD000133.

66. Kajosaari M. Atopy prophylaxis in high-risk infants. Prospective 5-year follow-up study of children with six months exclusive breastfeeding and solid food elimination. Advances in Experimental Medicine & Biology. 1991;310:453-8.

67. Ludvigsson JF, Mostrom M, Ludvigsson J, Duchen K. Exclusive breastfeeding and risk of atopic dermatitis in some 8300 infants. Pediatric Allergy and Immunology. 2005;16(3):201-8.

68. Kull I, Bohme M, Wahlgren CF, Nordvall L, Pershagen G, Wickman M. Breast-feeding reduces the risk for childhood eczema. Journal of Allergy & Clinical Immunology. 2005;116(3):657-61.

69. Kull I, Bergstrom A, Alm J, Melen E, Bohme M, Wahlgren C, et al. Exclusive breastfeeding reduces the risk of childhood eczema. Allergy: European Journal of Allergy and Clinical Immunology. 2009;64:63.

70. Besednjak-Kocijancic L. Is longer exclusive breastfeeding associated with lover prevalence of asthma, atopic dermatitis and atopic sensitisation in 1 and 5-year-old Slovene children? Allergy: European Journal of Allergy and Clinical Immunology. 2010;65:311-2.

71. Peters TJ, Golding J. The epidemiology of childhood eczema: II. Statistical analyses to identify independent early predictors. Paediatric and Perinatal Epidemiology. 1987;1(1):80-94.

72. Fergusson DM, Horwood LJ, Shannon FT. Early solid feeding and recurrent childhood eczema: a 10-year longitudinal study. Pediatrics. 1990;86(4):541-6.

73. Cogswell JJ, Mitchell EB, Alexander J. Parental smoking, breast feeding, and respiratory infection in development of allergic diseases. Archives of Disease in Childhood. 1987;62(4):338-44.

74. Giwercman C, Halkjaer LB, Jensen SM, Bonnelykke K, Lauritzen L, Bisgaard H. Increased risk of eczema but reduced risk of early wheezy disorder from exclusive breast-feeding in high-risk infants. Journal of Allergy & Clinical Immunology. 2010;125(4):866-71.

75. Benn CS, Wohlfahrt J, Aaby P, Westergaard T, Benfeldt E, Michaelsen KF, et al. Breastfeeding and risk of atopic dermatitis, by parental history of allergy, during the first 18 months of life. American Journal of Epidemiology. 2004;160(3):217-23.

76. Linneberg A, Simonsen JB, Petersen J, Stensballe LG, Benn CS. Differential effects of risk factors on infant wheeze and atopic dermatitis emphasize a different etiology. Journal of Allergy & Clinical Immunology. 2006;117(1):184-9.

77. Laubereau B, Brockow I, Zirngibl A, Koletzko S, Gruebl A, von Berg A, et al. Effect of breast-feeding on the development of atopic dermatitis during the first 3 years of life--results from the GINI-birth cohort study. Journal of Pediatrics. 2004;144(5):602-7.

78. Arshad SH, Hide DW. Effect of environmental factors on the development of allergic disorders in infancy. Journal of Allergy & Clinical Immunology. 1992;90(2):235-41.

79. Jedrychowski W, Perera F, Maugeri U, Mrozek-Budzyn D, Miller RL, Flak E, et al. Effects of prenatal and perinatal exposure to fine air pollutants and maternal fish consumption on the occurrence of infantile eczema. International Archives of Allergy & Immunology. 2011;155(3):275-81.

80. Kitz R, Rose MA, Schonborn H, Zielen S, Bohles HJ. Impact of early dietary gamma-linolenic acid supplementation on atopic eczema in infancy. Pediatric Allergy & Immunology. 2006;17(2):112-7.

81. Midwinter RE, Moore WJ, Soothill JF, Turner MW, Colley JR. Infant feeding and atopy. Lancet. 1982;1(8267):339.

82. Gruber C, van Stuijvenberg M, Mosca F, Moro G, Chirico G, Braegger CP, et al. Reduced occurrence of early atopic dermatitis because of immunoactive prebiotics among low-atopy-risk infants. Journal of Allergy & Clinical Immunology. 2010;126(4):791-7.

83. Nentwich I, Pazdirkova A, Lokaj J, Szepfalusi Z, Hrstkova H. [Early feeding in infancy and atopic dermatitis - a prospective observational study]. Klinische Padiatrie. 2009;221(2):78-82.

84. Pesonen M, Kallio MJ, Ranki A, Siimes MA. Prolonged exclusive breastfeeding is associated with increased atopic dermatitis: a prospective follow-up study of unselected healthy newborns from birth to age 20 years. Clinical & Experimental Allergy. 2006;36(8):1011-8.

85. Kramer MS, Matush L, Bogdanovich N, Dahhou M, Platt RW, Mazer B. The low prevalence of allergic disease in Eastern Europe: are risk factors consistent with the hygiene hypothesis? Clinical & Experimental Allergy. 2009;39(5):708-16.

86. Kramer MS, Guo T, Platt RW, Sevkovskaya Z, Dzikovich I, Collet JP, et al. Infant growth and health outcomes associated with 3 compared with 6 mo of exclusive breastfeeding. American Journal of Clinical Nutrition. 2003;78(2):291-5.

87. Moore MM, Rifas-Shiman SL, Rich-Edwards JW, Kleinman KP, Camargo CA, Jr., Gold DR, et al. Perinatal predictors of atopic dermatitis occurring in the first six months of life. Pediatrics. 2004;113(3 Pt 1):468-74.

88. Saarinen UM, Kajosaari M, Backman A, Siimes MA. Prolonged breast-feeding as prophylaxis for atopic disease. Lancet. 1979;2(8135):163-6.

89. Siltanen M, Kajosaari M, Poussa T, Saarinen KM, Savilahti E. A dual long-term effect of breastfeeding on atopy in relation to heredity in children at 4 years of age. Allergy. 2003;58(6):524-30.

90. Shohet L, Shahar E, Davidson S. Breast feeding as prophylaxis for atopic eczema: a controlled study of 368 cases. Acta Paediatrica Hungarica. 1985;26(1):35-9.

91. Dunlop AL, Reichrtova E, Palcovicova L, Ciznar P, Adamcakova-Dodd A, Smith SJ, et al. Environmental and dietary risk factors for infantile atopic eczema among a Slovak birth cohort. Pediatric Allergy & Immunology. 2006;17(2):103-11.

92. Matheson MC, Erbas B, Balasuriya A, Jenkins MA, Wharton CL, Tang ML, et al. Breast-feeding and atopic disease: a cohort study from childhood to middle age. Journal of Allergy & Clinical Immunology. 2007;120(5):1051-7.

93. Zutavern A, von Mutius E, Harris J, Mills P, Moffatt S, White C, et al. The introduction of solids in relation to asthma and eczema. Archives of Disease in Childhood. 2004;89(4):303-8.

94. Amri M, Elhani I, Doussari S, Amir A. [Atopic dermatitis and prolonged exclusive breast-feeding]. Annales de Dermatologie et de Venereologie. 2012;139(4):257-60.

95. Sahakyan A, Armenian HK, Breitscheidel L, Thompson ME, Enokyan G. Feeding practices of babies and the development of atopic dermatitis in children after 12 months of age in Armenia: is there a signal? European Journal of Epidemiology. 2006;21(9):723-5.

96. Ehlayel MS, Bener A. Duration of breast-feeding and the risk of childhood allergic diseases in a developing country. Allergy & Asthma Proceedings. 2008;29(4):386-91.

97. Miyake Y, Yura A, Iki M. Breastfeeding and the prevalence of symptoms of allergic disorders in Japanese adolescents. Clinical and Experimental Allergy. 2003;33(3):312-6.

98. Kramer MS, Kakuma R. Optimal duration of exclusive breastfeeding. Cochrane Database of Systematic Reviews. 2012;8:CD003517.

99. Hong X, Wang G, Liu X, Kumar R, Tsai HJ, Arguelles L, et al. Gene polymorphisms, breast-feeding, and development of food sensitization in early childhood. Journal of Allergy & Clinical Immunology. 2011;128(2):374-81.e2.

100. Fergusson DM, Horwood LJ, Beautrais AL, Shannon FT, Taylor B. Eczema and infant diet. Clinical Allergy. 1981;11(4):325-31.

101. Forsyth JS, Ogston SA, Clark A, Florey CD, Howie PW. Relation between early introduction of solid food to infants and their weight and illnesses during the first two years of life. Bmj. 1993;306(6892):1572-6.

102. Filipiak B, Zutavern A, Koletzko S, Von Berg A, Brockow I, Grubl A, et al. Early solid food introduction and development of eczema in the first 4 years. Results from the GINI birth cohort. [German]

Fruhe beikosteinfuhrung und auftreten von ekzem innerhalb der ersten vier lebensjahre - Ergebnisse der GINI-geburtskohorte. Allergo Journal. 2008;17(1):82-3.

103. Filipiak B, Zutavern A, Koletzko S, von Berg A, Brockow I, Grubl A, et al. Solid food introduction in relation to eczema: results from a four-year prospective birth cohort study. Journal of Pediatrics. 2007;151(4):352-8.

104. Schoetzau A, Filipiak-Pittroff B, Franke K, Koletzko S, Von Berg A, Gruebl A, et al. Effect of exclusive breast-feeding and early solid food avoidance on the incidence of atopic dermatitis in high-risk infants at 1 year of age. Pediatric Allergy & Immunology. 2002;13(4):234-42.

105. Huang H, Zhang FY, Hang JQ, Zhu J, Wang R, Chen PF, et al. [Cohort study of 684 pairs of mother-and-child allergic diseases]. Zhonghua Erke Zazhi. 2013;51(3):168-71.

106. Zutavern A, Brockow I, Schaaf B, Bolte G, von Berg A, Diez U, et al. Timing of solid food introduction in relation to atopic dermatitis and atopic sensitization: results from a prospective birth cohort study. Pediatrics. 2006;117(2):401-11.

107. Zutavern A, Brockow I, Schaaf B, von Berg A, Diez U, Borte M, et al. Timing of solid food introduction in relation to eczema, asthma, allergic rhinitis, and food and inhalant sensitization at the age of 6 years: results from the prospective birth cohort study LISA. Pediatrics. 2008;121(1):e44-52.

108. Brockow I, Zutavern A, Franke K, Schaaf B, von Berg A, Kramer U, et al. Influences of lifestyle-related factors on the immune system and the development of allergies in childhood (LISA). Design and results to date of a prospective birth cohort study. Monatsschrift Kinderheilkunde. 2008;156(3):249-55.

109. Joseph CL, Ownby DR, Havstad SL, Woodcroft KJ, Wegienka G, MacKechnie H, et al. Early complementary feeding and risk of food sensitization in a birth cohort. Journal of Allergy & Clinical Immunology. 2011;127(5):1203-10.e5.

110. Van Asperen PP, Kemp AS, Mellis CM. Relationship of diet in the development of atopy in infancy. Clinical Allergy. 1984;14(6):525-32.

111. Foisy M, Boyle RJ, Chalmers JR, Simpson EL, Williams HC. Overview of Reviews The prevention of eczema in infants and children: an overview of Cochrane and non-Cochrane reviews. Evid Based Child Health. 2011;6(5):1322-39.
