# Supplementary material for: Diet during pregnancy and infancy and risk of allergic or autoimmune disease: A systematic review and meta-analysis
Source: PLoS Med. 2018 Feb 28;15(2):e1002507. doi: 10.1371/journal.pmed.1002507 (PMC5830033; doi:10.1371/journal.pmed.1002507)
Supplement: S1 Data — (ZIP) [file pmed.1002507.s006.zip › Review_A_reports/FOOD ALLERGY.docx]

**Breastfeeding Duration, Solid Food Introduction and risk of Food Allergy**

Robert J Boyle^1^, Vanessa Garcia-Larsen^2^, Despo Ierodiakonou^3^, Jo Leonardi-Bee^4^, Tim Reeves^5^, Jennifer Chivinge^6^, Zoe Robinson^6^, Natalie Geoghegan^6^, Katharine Jarrold^6^, Andrew Logan^6^, Annabel Groome^6^ , Evangelia Andreou^7^, Nara Tagiyeva-Milne^8^, Ulugbek Nurmatov^9^, Sergio Cunha^10^

^1^ Clinical Senior Lecturer, Section of Paediatrics, Imperial College London; ^2^ Post-Doctoral Research Associate & Honorary Research Fellow, Royal Brompton Hospital and Harefield NHS Foundation Trust, Respiratory Epidemiology and Public Health, National Heart and Lung Institute, Imperial College London; ^3^ Post-Doctoral Research Associate, Departments of Paediatric and Respiratory Epidemiology and Public Health Group, Imperial College London. ^4^Associate Professor of Community Health Sciences, University of Nottingham; ^5^ Research Support Librarian, Faculty of Medicine, Imperial College London; ^6^ Undergraduate medical students, Imperial College London; ^7^Research Associate, Imperial Consultants; ^8^Research Fellow, University of Aberdeen; ^9^Research Fellow, University of Edinburgh; ^10^Research Associate, Respiratory Epidemiology and Public Health, National Heart and Lung Institute, Imperial College London

Imperial Consultants,

58 Princes Gate,

Exhibition Road,

London SW7 2PG

CONTENTS

[**List of Figures** 3](#_Toc416346461)

[**List of Tables** 4](#_Toc416346462)

[**1 Total Breastfeeding Duration and Food Allergy** 5](#_Toc416346463)

[1.1 Overall characteristics of studies, risk of bias and summary of results 5](#_Toc416346464)

[1.1 TBF and FA in children aged 0-4 years 10](#_Toc416346465)

[1.1.1 Any Food Allergy in children aged 0-4 years 10](#_Toc416346466)

[1.1.1.1 TBF Ever vs. Never 10](#_Toc416346467)

[1.1.1.2 TBF ≥ 5-7 months vs. <5-7 months 11](#_Toc416346468)

[1.1.2 Cow’s Milk Allergy in children aged 0-4 years 11](#_Toc416346469)

[1.1.2.1 TBF Ever vs. Never 11](#_Toc416346470)

[1.1.3 Egg Allergy in children aged 0-4 years 12](#_Toc416346471)

[1.1.3.1 TBF Ever vs. Never 12](#_Toc416346472)

[1.1.3.2 TBF ≥1-2 months vs < 1-2 months 12](#_Toc416346473)

[1.1.4 Peanut Allergy in children aged 0-4 years 12](#_Toc416346474)

[1.1.4.1 TBF Ever vs. Never 12](#_Toc416346475)

[1.2 TBF and FA in children aged 5-14 years 13](#_Toc416346476)

[1.2.1 Peanut Allergy in children aged 5-14 years 13](#_Toc416346477)

[1.2.1.1 TBF Ever vs. Never 13](#_Toc416346478)

[1.3 Data for TBF duration and FA which were not suitable for meta-analysis 15](#_Toc416346479)

[**2 Exclusive Breastfeeding Duration and Food Allergy** 18](#_Toc416346480)

[2.1 Overall characteristics of studies, risk of bias and summary of results 18](#_Toc416346481)

[2.2 Exclusive breastfeeding duration and risk of food allergy 22](#_Toc416346482)

[2.2.1 Systematic reviews and intervention trials 22](#_Toc416346483)

[2.2.2 Observational studies of EBF duration and FA in children aged 0-4 years 23](#_Toc416346484)

[2.2.3 Observational studies of EBF duration and FA in children aged 5-14 years 26](#_Toc416346485)

[2.2.4 Observational studies of EBF duration and FA at age 15+ 27](#_Toc416346486)

[2.2.5 Data for TBF duration and FA which were not suitable for meta-analysis 28](#_Toc416346487)

[**3 Solid Food Introduction and Food Allergy** 30](#_Toc416346488)

[3.1 Overall characteristics of studies, risk of bias and summary of results 30](#_Toc416346489)

[3.2 Solid food introduction and FA in children aged 0-4 years 32](#_Toc416346490)

[3.2.1 SF ≥3-4 months vs. <3-4 months 32](#_Toc416346491)

[3.2.2 Data for SF and FA reported narratively 33](#_Toc416346492)

[**4 Conclusion** 35](#_Toc416346493)

[**5 References** 36](#_Toc416346494)

# List of Figures

[Figure 1 Risk of bias in studies of TBF duration and food allergy 10](#_Toc419481662)

[Figure 2 TBF Ever vs. Never and risk of any FA at age 0-4 years 10](#_Toc419481663)

[Figure 3 TBF ≥5-7 months vs. <5-7 months and risk of any FA at age 0-4 years 11](#_Toc419481664)

[Figure 4 TBF Ever vs. Never and risk of FA to milk at age 0-4 years 11](#_Toc419481665)

[Figure 5 TBF Ever vs. Never and risk of FA to egg at age 0-4 years 12](#_Toc419481666)

[Figure 6 TBF ≥1-2 months vs. <1-2 months and risk of FA to egg at age 0-4 years 12](#_Toc419481667)

[Figure 7 TBF Ever vs. Never and risk of peanut allergy at age 0-4 years 13](#_Toc419481668)

[Figure 8 TBF Ever vs. Never and risk of FA to nut-peanut at age 5-14 years 14](#_Toc419481669)

[Figure 9 TBF 0-3 months vs. never and risk of FA to peanut at age 5-14 years 14](#_Toc419481670)

[Figure 10 TBF 3-5 months vs. never and risk of FA to peanut at age 5-14 years 14](#_Toc419481671)

[Figure 11 TBF ≥6 months vs. never and risk of FA to peanut at age 5-14 years 14](#_Toc419481672)

[Figure 12 Risk of bias in studies of EBF duration and food allergy 22](#_Toc419481673)

[Figure 13 EBF ≥0-2 months vs. <0-2 months and risk of any FA at age 0-4 years 23](#_Toc419481674)

[Figure 14 EBF ≥3-4 months vs. <3-4 months and risk of any FA at age 0-4 years 23](#_Toc419481675)

[Figure 15 EBF ≥5 months vs. <5 months and risk of any FA at age 0-4 years 24](#_Toc419481676)

[Figure 16 EBF ≥0-2 months vs. <0-2 months and risk of FA to cow’s milk at age 0-4 years 24](#_Toc419481677)

[Figure 17 EBF ≥3-4 months vs. <3-4 months and risk of FA to cow’s milk at age 0-4 years 25](#_Toc419481678)

[Figure 18 EBF ≥3-4 months vs. <3-4 months and risk of FA to egg at age 0-4 years 25](#_Toc419481679)

[Figure 19 EBF ≥0-2 months vs. <0-2 months and risk of any FA at age 5-14 years 26](#_Toc419481680)

[Figure 20 EBF ≥5 months vs. <5 months and risk of any FA at age 5-14 years 26](#_Toc419481681)

[Figure 21 EBF ≥5 months vs. <5 months and risk of FA to nut-peanut at age 5-14 years 27](#_Toc419481682)

[Figure 22 EBF ≥3-4 months vs. <3-4 months and risk of any FA at age 15+ years 28](#_Toc419481683)

[Figure 23 Risk of bias in studies of SF introduction and food allergy 32](#_Toc419481684)

[Figure 24 SF ≥3-4 months vs. <3-4 months and risk of any FA at age 0-4 years 32](#_Toc419481685)

[Figure 25 SF ≥3-4 months vs. <3-4 months and risk of FA to egg at age 0-4 years 33](#_Toc419481686)

# List of Tables

[Table 1 Characteristics of included studies evaluating TBF duration and food allergy 7](#_Toc419747560)

[Table 2 Studies investigating the association between TBF duration and food allergy which were not eligible for meta-analysis 16](#_Toc419747561)

[Table 3 Characteristics of included studies evaluating EBF duration and food allergy 19](#_Toc419747562)

[Table 4 EBF for 6 months versus 3 months and risk of food allergy 22](#_Toc419747563)

[Table 5 Studies investigating the association between EBF duration and food allergy which were not eligible for meta-analysis 29](#_Toc419747564)

[Table 6 Characteristics of included studies evaluating SF introduction and food allergy 31](#_Toc419747565)

[Table 7 Studies investigating the association between SF introduction and food allergy which were not eligible for meta-analysis 34](#_Toc419747566)

# Total Breastfeeding Duration and Food Allergy

## Overall characteristics of studies, risk of bias and summary of results

Table 1 describes the main characteristics of the studies analysed in this report. A total of 18 observational studies, and no intervention studies, reported the association between duration of breastfeeding and risk of Food Allergy (FA). Of these, 11 were prospective cohort studies, 1 nested case-control, 1 cross-sectional study and 4 case-control studies. Over half of the studies (n=12) are from Europe – others are from North America (n=2), Australasia (n=3), and Africa (n=1). Overall, valid data on Total Breastfeeding Duration in the first 2 years of life (TBF) and FA risk were available from over 72,000 subjects. Information on FA was obtained mainly from a medical assessment in 10 studies, via parental report in 4 studies and supervised oral food challenge in 4 studies. With regards to time of outcome diagnosis, 14 studies explored the association between TBF duration and FA in the first 5 years of life, where FA and anaphylaxis are most common, and 4 evaluated FA in older children or adolescents. Twelve studies used interview or questionnaire to assess the exposure (TBF), 2 studies assessed medical records only and in 3 studies the method of exposure assessment was unclear.

Risk of bias was assessed using the NICE Methodological checklists for cohort and case-control studies. Figure 1 illustrates the distribution of bias across the five main methodological areas of the studies. Over half of studies had a high risk of bias, most commonly due to lack of adjustment for confounding bias i.e. no adjusted data presented. Over a quarter of studies had an ‘unclear’ overall risk of bias, for varied reasons. Risk of conflict of interest was generally assessed as low.

Where data were available, five levels of comparison were used to assess the risk of FA according to TBF duration, namely ‘any (including ever) vs. never’, ‘≥1-2 months vs. <1-2 months’, ‘≥3-4 months vs. <3-4 months’, ‘≥5-7 months vs. <5-7 months’, and ‘≥8-12 months vs. <8-12 months’.

*Main Findings*

In general there were only small numbers of studies with data available, and several analyses were restricted to studies with high risk of bias. Two large prospective studies found significantly increased risk of FA in breastfed compared to never breastfed children (Figure 2) OR 1.57 (1.28, 1.93), with high statistical heterogeneity (I^2^=63%) which may be explained by appropriate adjustment for confounders in one study and not the other. Two additional studies reported data that could be used to calculate OR for peanut and/or other nut allergy in infants breastfed versus not breastfed, and found increased risk of peanut/nut FA in breastfed infants in unadjusted analyses, but not in adjusted analyses. Other studies did not support a relationship between different durations of TBF and FA, or TBF ever/never and allergy to specific foods CM, Egg or PN. However numbers of studies and participants were low for such analyses. Stratified and subgroup analyses and Funnel plots were not undertaken due to small numbers of studies in individual meta-analyses.

In summary we found weak evidence that FA may be more common in breastfed children. To our knowledge neither of the relevant studies excluded FA occurring during breastfeeding, in order to reduce the possibility that breastfeeding is prolonged due to the initiation of FA symptoms. Given the type and quality of the studies, these data provide no consistent evidence (GRADE -2; inconsistency, study quality) for a causal relationship between TBF duration and FA, and further work is needed to understand the relationship between breastfeeding and FA.

Table 1 Characteristics of included studies evaluating TBF duration and food allergy

| **First Author & Publication Year** | **N/n cases** | **Design** | **Country** | **Exposure assessment** | **Method of outcome assessment** | **Age at outcome (years)** | **Population characteristics** |
| --- | --- | --- | --- | --- | --- | --- | --- |
| Hesselmar, 2010 ([1](#_ENREF_1)) | 184/14 | PC | Sweden | I | Physician assessment: history +/- investigations | 0.5, 1.5 | ALLERGYFLORA. Population based cohort of mainly high risk infants recruited from antenatal clinics 1998-2003 |
| Lack, 2003 ([2](#_ENREF_2)) | 11179/  45(21) | PC | UK | Q/I | Parent report of food reaction (DBPCFC) | 5.5 | ALSPAC. Population based cohort of children born 1991-1992 |
| Kull, 2002 ([3](#_ENREF_3)) | 3790/768 | PC | Sweden | Q | DD | 2 | BAMSE. Population based cohort of children born 1994-1996 |
| Gustafsson, 2000 ([4](#_ENREF_4)) | 94/56 | PC | Sweden | Q | Parent report of food reaction | 7 | Children with atopic dermatitis attending allergic clinic or referred by child welfare clinics |
| Koplin, 2010 ([5](#_ENREF_5)) | 2589/231 | PC | Australia | Q | Open food challenge | 1 | HEALTHNUTS. Population based cohort of children born 2008-2010 recruited at 1 year at routine immunization clinics |
| Hikino, 2001 ([6](#_ENREF_6)) | 21766/2381 | PC | Japan | Q | DD | 1.5 | All children born in 1993-1995 attending well-baby check-ups funded by Fukuoka City |
| Goksor, 2011 ([7](#_ENREF_7)) | 4496/202 | PC | Sweden | Unclear | DD | 4.5 | Infants of Western Sweden. Population birth cohort of infants born in 2003 |
| Kemeny, 1991 ([8](#_ENREF_8)) | 180/9 | PC | UK | Unclear | Parent report of ≥2 food reactions | 1 | Population based birth cohort of infants born at Dulwich and King’s College Hospitals in London |
| Milner, 2004 ([9](#_ENREF_9)) | 8073/396 | PC | USA | Q | DD | 3 | National Maternal and Infant Health Survey and Longitudinal Follow Up. Representative population-based cohort born in 1988. Blacks, individuals with low socioeconomic status, and premature infants intentionally overrepresented |
| Saarinen, 1995 ([10](#_ENREF_10)) | 236/61 and 18 | PC | Finland | R | Parent report of food reaction | 5 and 17 | Cohort recruited from hospital and born in 1975 |
| Venter, 2009 ([11](#_ENREF_11)) | 891/58 | PC | UK | Q | Open food challenge | 3 | Population based cohort recruited from antenatal clinics and born in 2001-2002 |
| Oliver, 2010 ([12](#_ENREF_12)) | 93/31 | NCC | UK | R | DBPCFC | 1 | EuroPrevall UK. Cases and age-matched controls selected from a population based birth cohort of infants born in 2008 |
| DesRoches, 2010 ([13](#_ENREF_13)) | 403/202 | CC | Canada | Q | Physician assessment: history +/- investigations | <1.5 | Hospital based cases and controls recruited 1998-2004. |
| Djenouhat, 2011 ([14](#_ENREF_14)) | 450/150 | CC | Algeria | Unclear | Physician assessment: history +/- investigations | 0.3 | Hospital based cases and family controls, born 1999-2005 |
| Fox, 2009 ([15](#_ENREF_15)) | 283/133 | CC | UK | Q | Physician assessment: history +/- investigations | <4 | Cases and controls were children referred for suspected FA to a large paediatric service |
| Ventura, 1988 ([16](#_ENREF_16)) | 339/148 | CC | Italy | I | Physician assessment: history +/- investigations | <1 | Italian Working Group on CMPA. Cases selected from 15 paediatric centres in Italy and age and area matched controls from well-baby clinics |
| Paton, 2012 ([17](#_ENREF_17)) | 15142/592 | CS | Australia | Q | Parent-reported food reaction (any nut) | 7 | Representative primary school entrants 2006-2009 |
| Berjon, 1987 ([18](#_ENREF_18)) | 2690/148 | CS | Spain | Unclear | DD | 14 | Patients attending a paediatric allergy clinic in Valladolid, Spain between 1970 and 1984, with and without diagnosed food allergy |

Q: questionnaire, I: interview, R: medical records, PC: prospective cohort, NCC: nested case control, CS: Cross-sectional, CC: case control, DBPCFC: double blind placebo controlled food challenge, DD: doctor diagnosis of FA, in contrast to ‘Physician assessment’ where FA diagnosis was always made by a study physician as part of the study protocol, NA: not available

Figure 1 Risk of bias in studies of TBF duration and food allergy

## TBF and FA in children aged 0-4 years

## Any Food Allergy in children aged 0-4 years

## TBF Ever vs. Never

Figure 2 shows the pooled estimates of 2 eligible observational studies including 2777 people with FA, suggesting increased risk of FA if breastfeeding was initiated, with high heterogeneity between the two studies (I^2^=62%) The prospective cohort study of Milner presents OR adjusted just for multivitamin intake, while the prospective cohort study of Hikino adjusted for birthweight, sex and gestational age. The unadjusted OR (1.44) of the latter was very similar to the OR in the study of Milner. In the study of Hikino data were also presented for FA at age 3 years, where there was high risk of selection bias due to greater loss to follow up, but there remained increased odds of FA in breastfed children - unadjusted OR 4.45 (3.42, 5.78).

Figure 2 TBF Ever vs. Never and risk of any FA at age 0-4 years


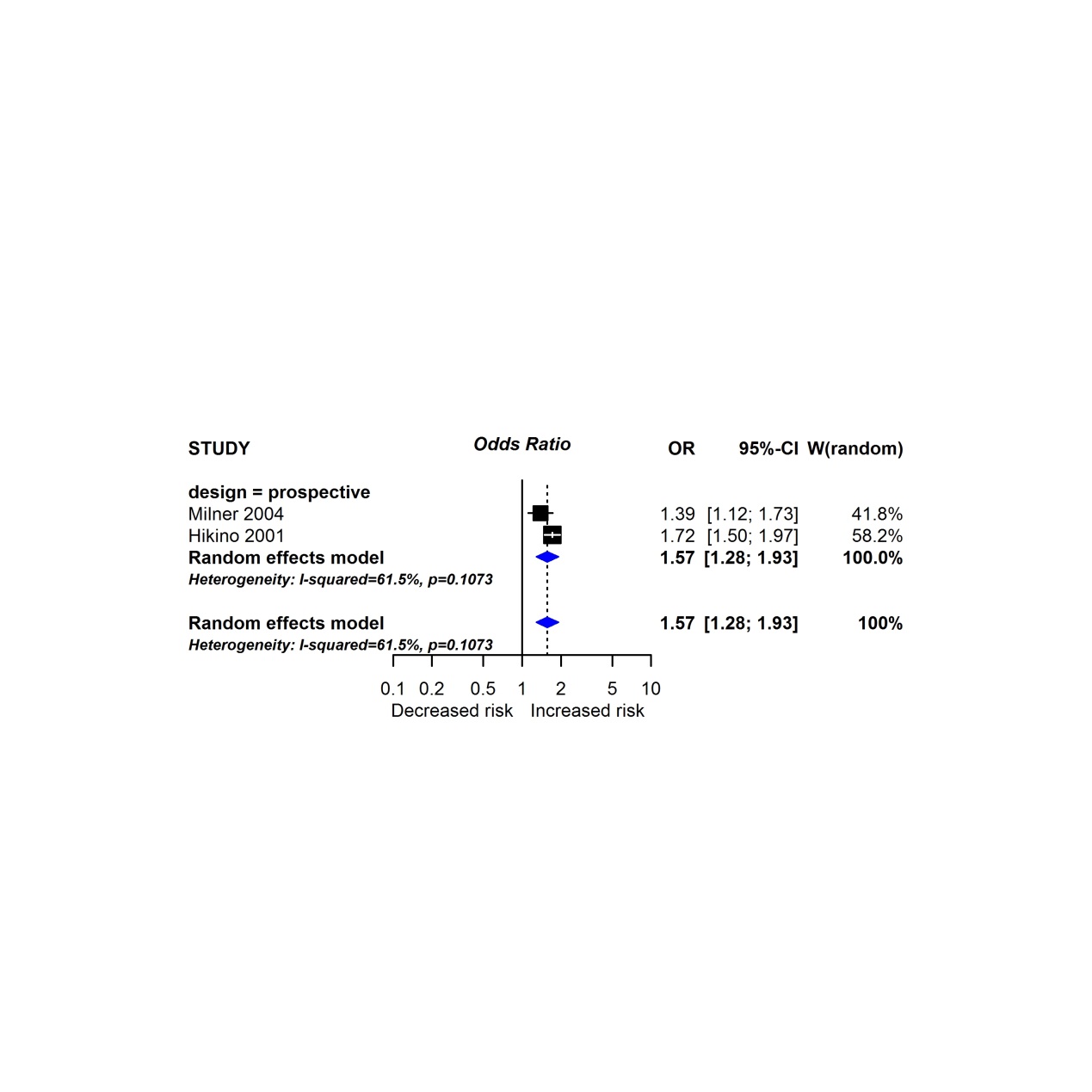


## TBF ≥ 5-7 months vs. <5-7 months

No studies reported data that could be used to calculate OR for FA with different TBF exposure/reference groups. One prospective cohort study including 768 cases of FA reported odds of diagnosed FA in children aged 2 who were breastfed for ≥ 6 months compared to <6 months and found no significant difference in adjusted analysis (Figure 3). The study risk of bias was judged as low in most domains.

Figure 3 TBF ≥5-7 months vs. <5-7 months and risk of any FA at age 0-4 years


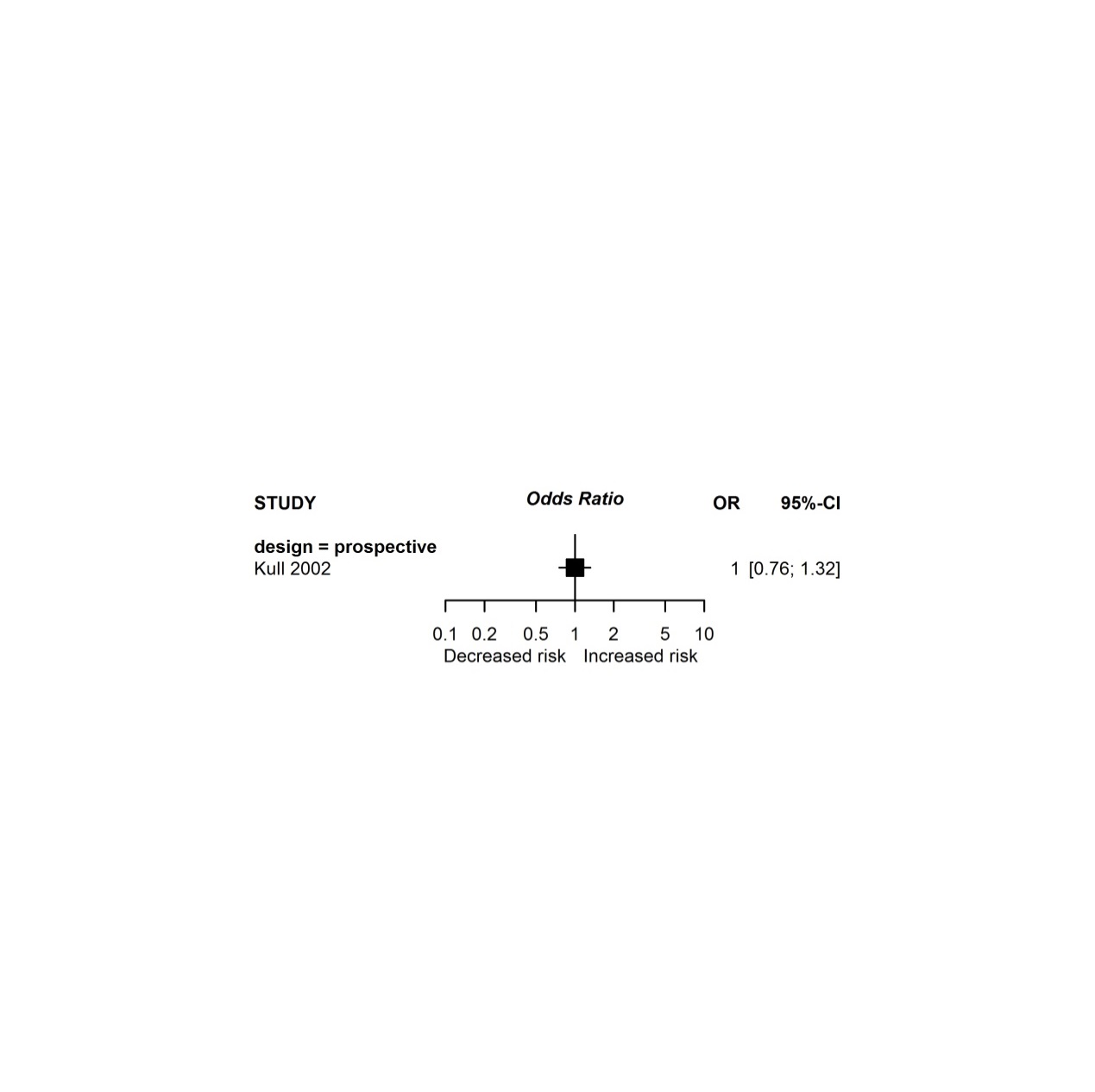


## Cow’s Milk Allergy in children aged 0-4 years

## TBF Ever vs. Never

One study reported data that could be used to calculate OR for Cow’s Milk Allergy (CMA) in infants breastfed versus not breastfed, and is shown in Figure 4. The data are unadjusted, and therefore carry a high risk of bias, and show no significant difference in OR for CMA in relation to breastfeeding status. In the same study, mean TBF duration was not significantly different between infants with (2.0 months) and without (1.6 months) CMA.

Figure 4 TBF Ever vs. Never and risk of FA to milk at age 0-4 years


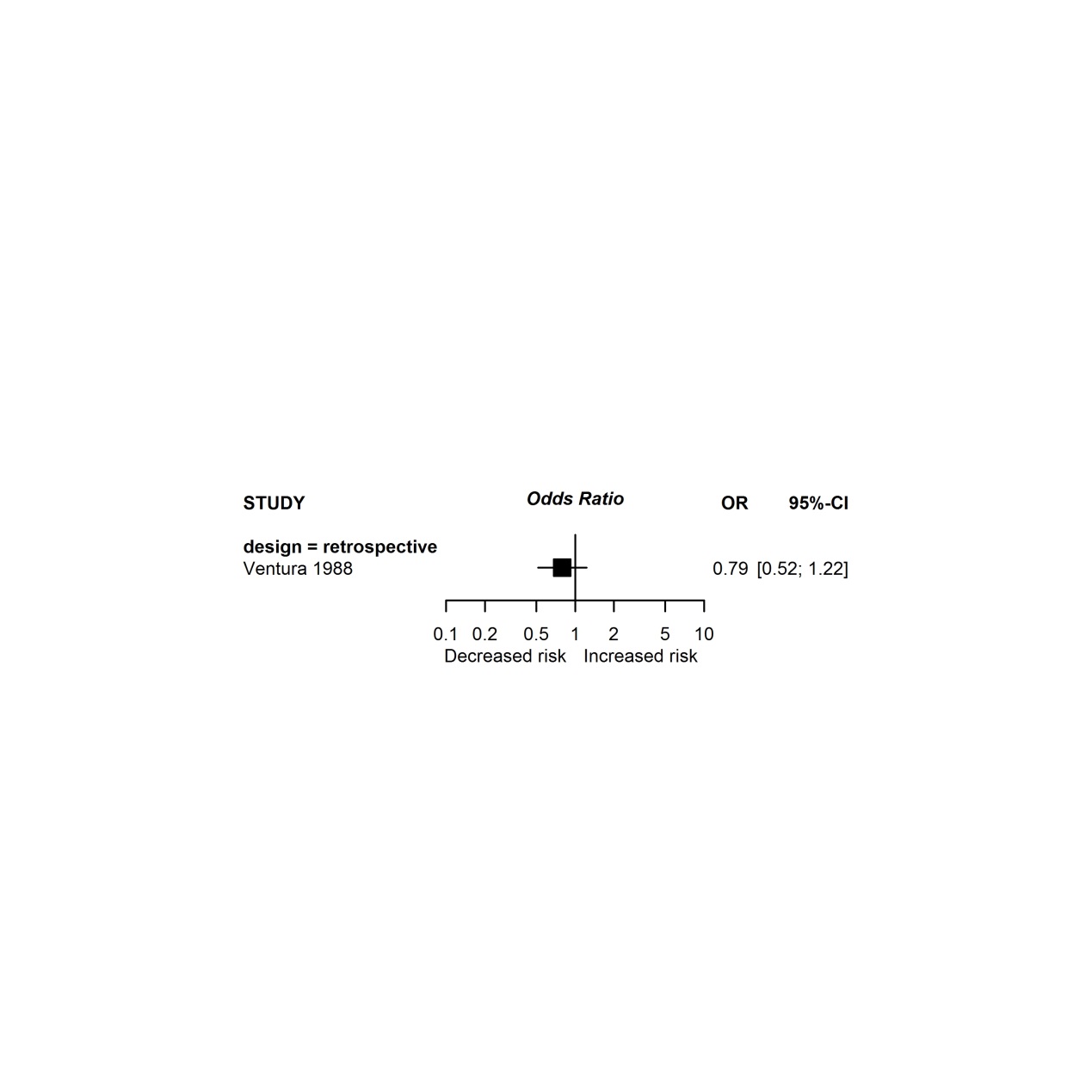


## Egg Allergy in children aged 0-4 years

## TBF Ever vs. Never

One study reported data that could be used to calculate OR for egg allergy in infants breastfed versus not breastfed, and is shown in Figure 5. The data are unadjusted and therefore carry a high risk of bias, and show no significant difference in OR for egg allergy in relation to breastfeeding status.

Figure 5 TBF Ever vs. Never and risk of FA to egg at age 0-4 years


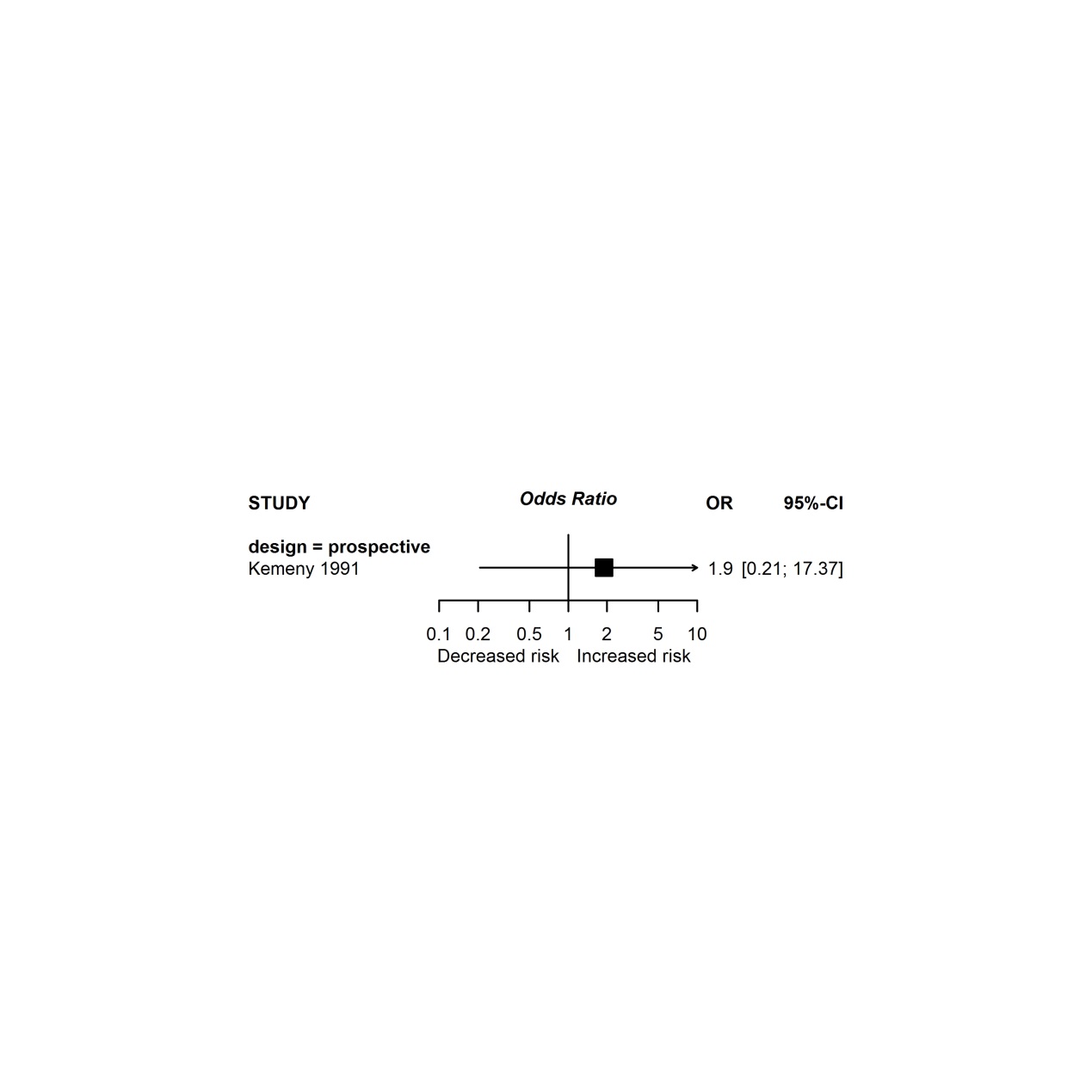


## TBF ≥1-2 months vs < 1-2 months

One study reported risk of egg allergy in infants breastfed longer than 1 month. The data are adjusted, the study had a low overall risk of bias, and they show no significant difference in OR for egg allergy in relation to breastfeeding status: OR 0.9 (95%CI 0.5, 2.2) for TBF 1-3 months; OR 1.1 (0.6, 2.3) 4-6 months; OR 0.9 (0.5, 1.9) 7-9 months; OR 0.9 (0.4, 1.8) 10-12 months; OR 0.7 (0.4, 1.4 for >12 months (shown in Figure 6).

Figure 6 TBF ≥1-2 months vs. <1-2 months and risk of FA to egg at age 0-4 years


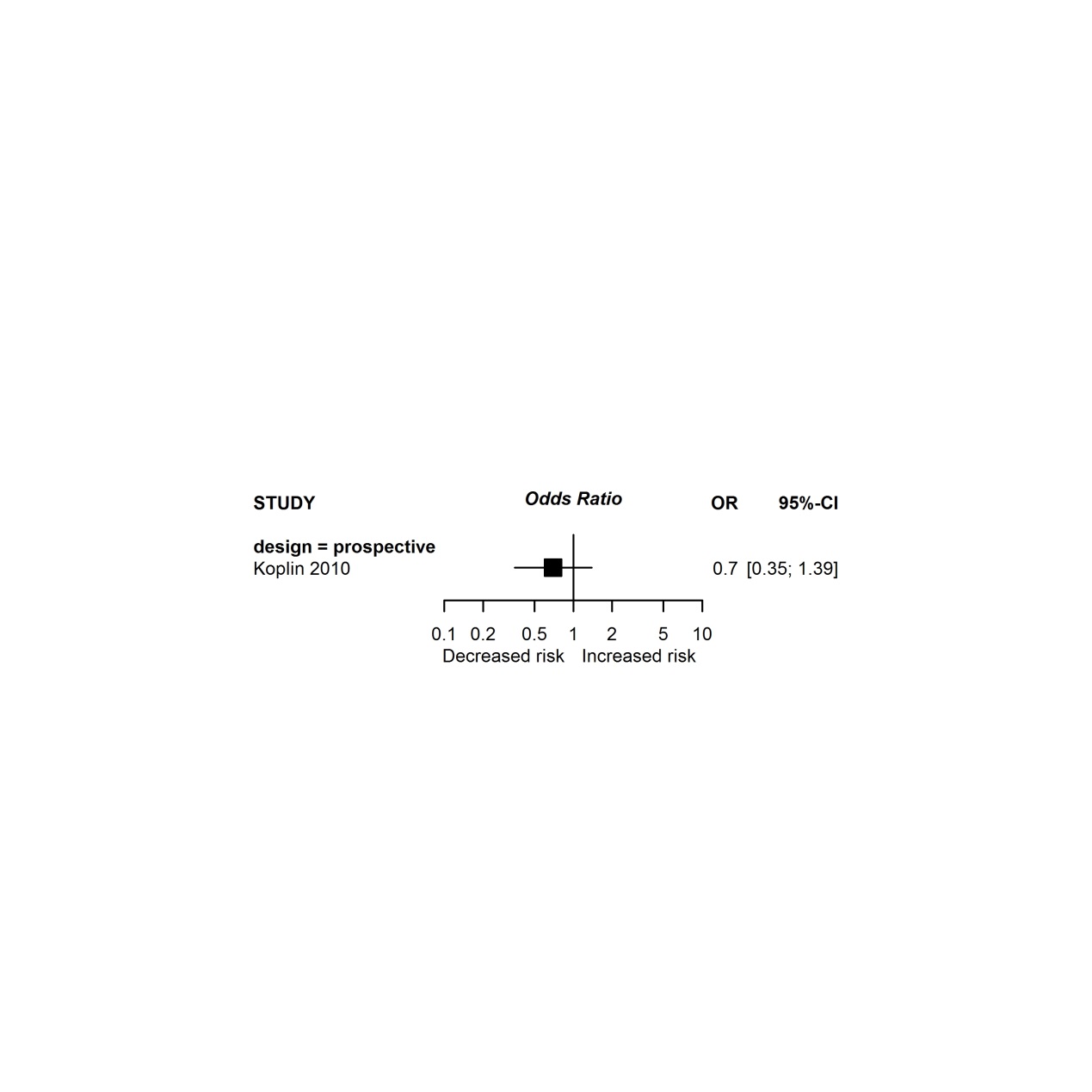


## Peanut Allergy in children aged 0-4 years

## TBF Ever vs. Never

One case control study reported data that could be used to calculate OR for peanut allergy in infants breastfed versus not breastfed, and is shown in Figure 7. The data are unadjusted and therefore carry a high risk of bias, and show no significant difference in OR for peanut allergy in relation to breastfeeding status. In the same study, mean TBF duration was not significantly different between infants with (6.3, sd 3.8) and without (5.7, sd 3.7) peanut allergy.

Figure 7 TBF Ever vs. Never and risk of peanut allergy at age 0-4 years


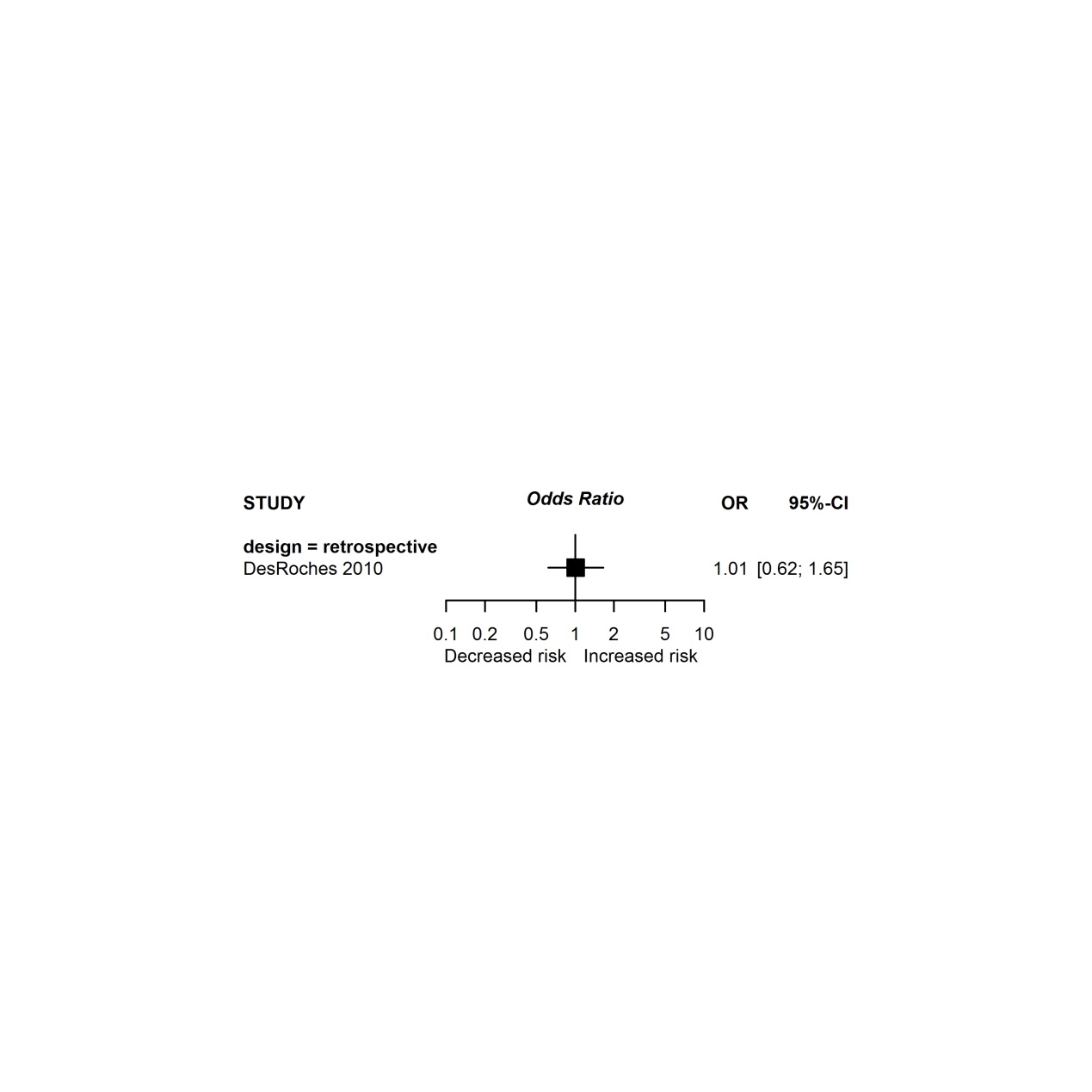


## TBF and FA in children aged 5-14 years

## Peanut Allergy in children aged 5-14 years

## TBF Ever vs. Never

Two studies reported data that could be used to calculate OR for peanut and/or other nut allergy in infants breastfed versus not breastfed, and are shown in Figure 8. The data on peanut allergy reported from the cohort study of Lack 2003 are unadjusted and therefore carry a high risk of bias, and Paton 2012 is a cross-sectional study which was judged as at high risk of confounding bias because data were only adjusted for age and sex and not for other factors such as family history of allergic disease and eczema which are associated with peanut and/or other nut allergy. Pooled analysis shows increased odds of peanut/nut allergy in breastfed infants OR 1.59 (1.17, 2.15) with no statistical heterogeneity. In the study of Lack 2003 unadjusted analysis showed increased odds of peanut allergy with breastfeeding duration of ≥6 months OR 2.60 (1.04, 6.53) and there was a trend to increased odds of peanut allergy with increased TBF duration (Figure 9, Figure 10, and Figure 11). Lack also reported the relationship between TBF and peanut allergy confirmed by DBPCFC in a subset of the population with reported peanut allergy. Here they found no significant association between TBF ever and peanut allergy – unadjusted OR 2.14 (0.91, 5.04). In adjusted analyses, the associations between TBF and peanut allergy were not significant.

Overall the data do not provide strong evidence for a relationship between TBF duration and peanut allergy.

Figure 8 TBF Ever vs. Never and risk of FA to nut-peanut at age 5-14 years


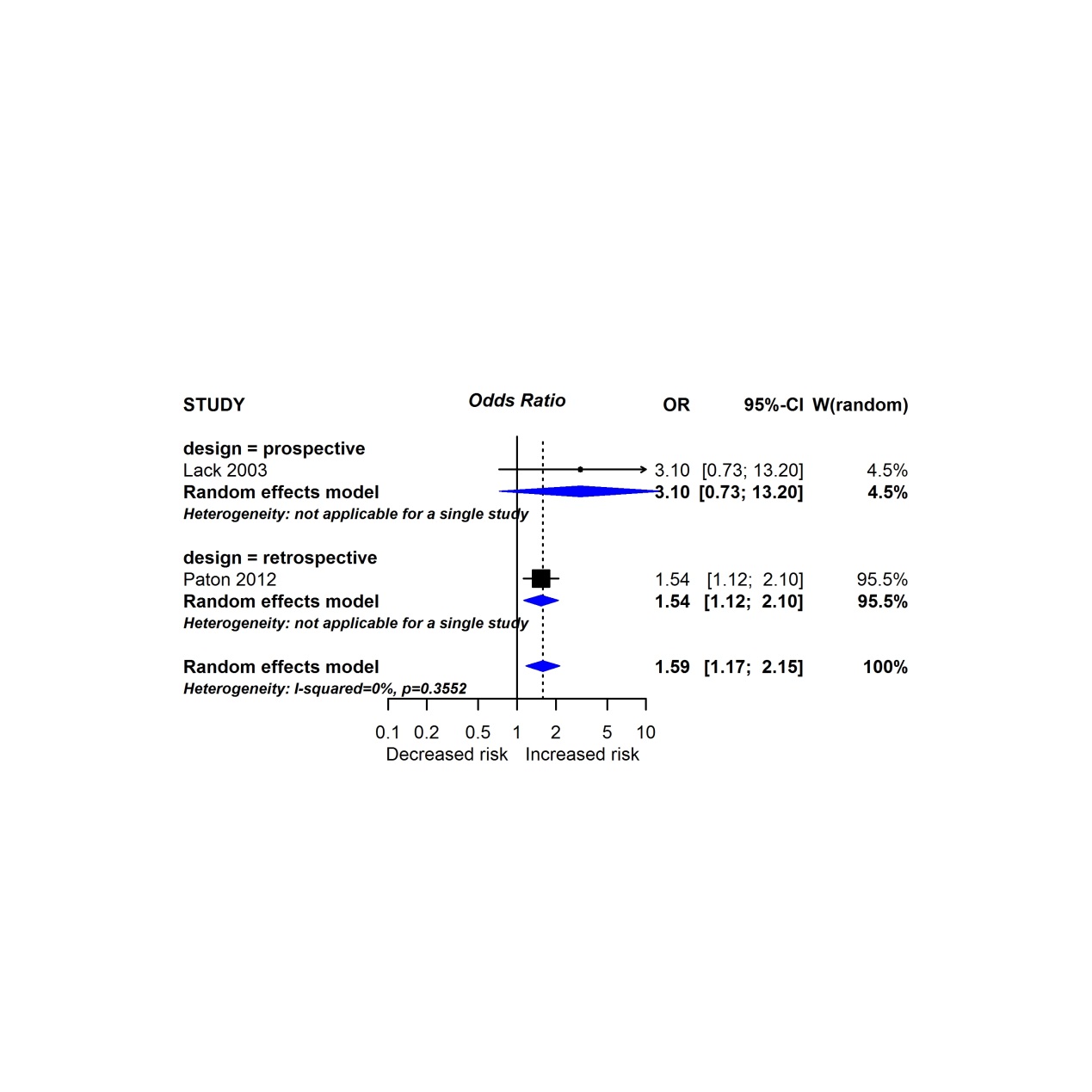


Figure 9 TBF 0-3 months vs. never and risk of FA to peanut at age 5-14 years


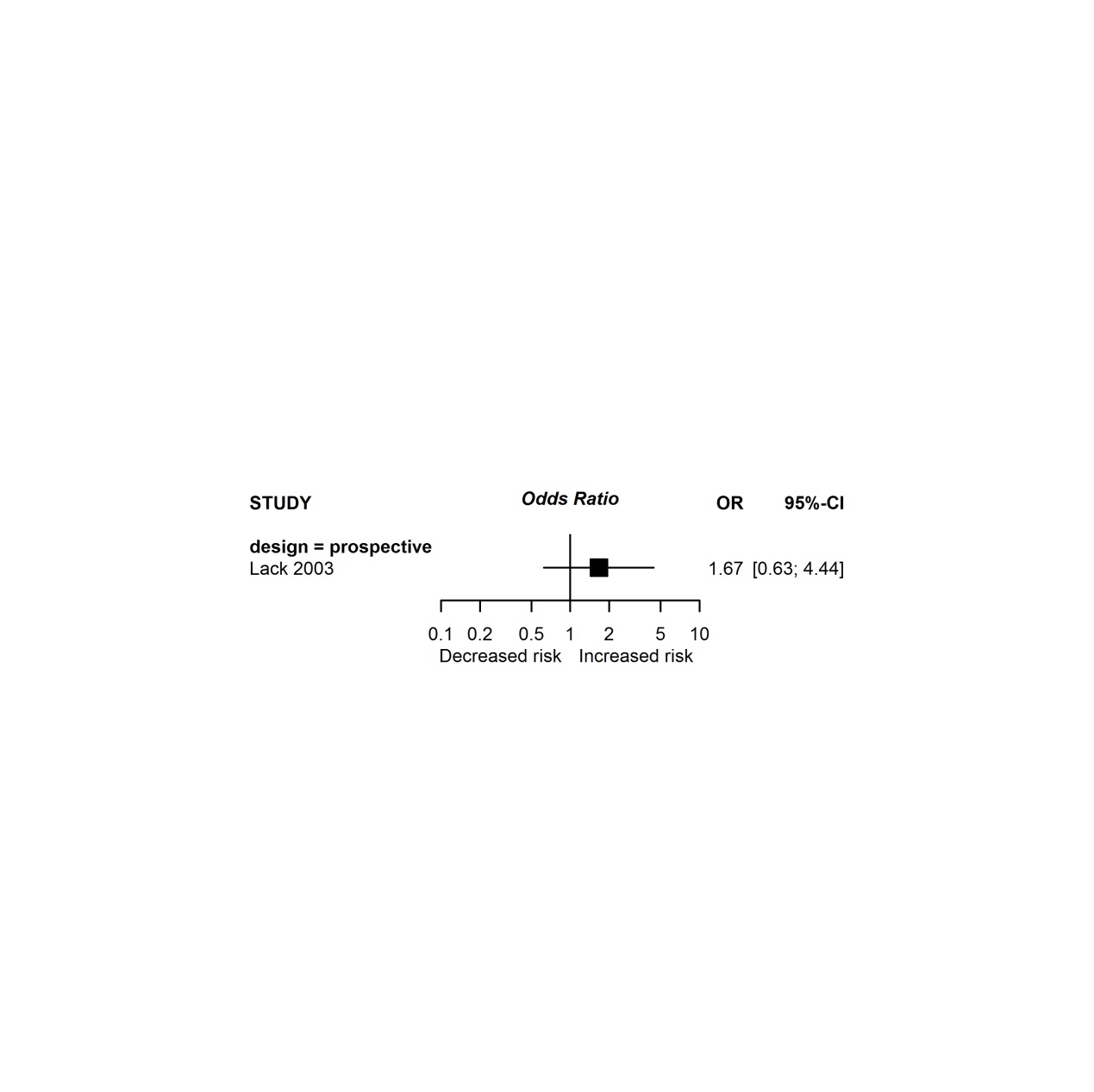


Figure 10 TBF 3-5 months vs. never and risk of FA to peanut at age 5-14 years


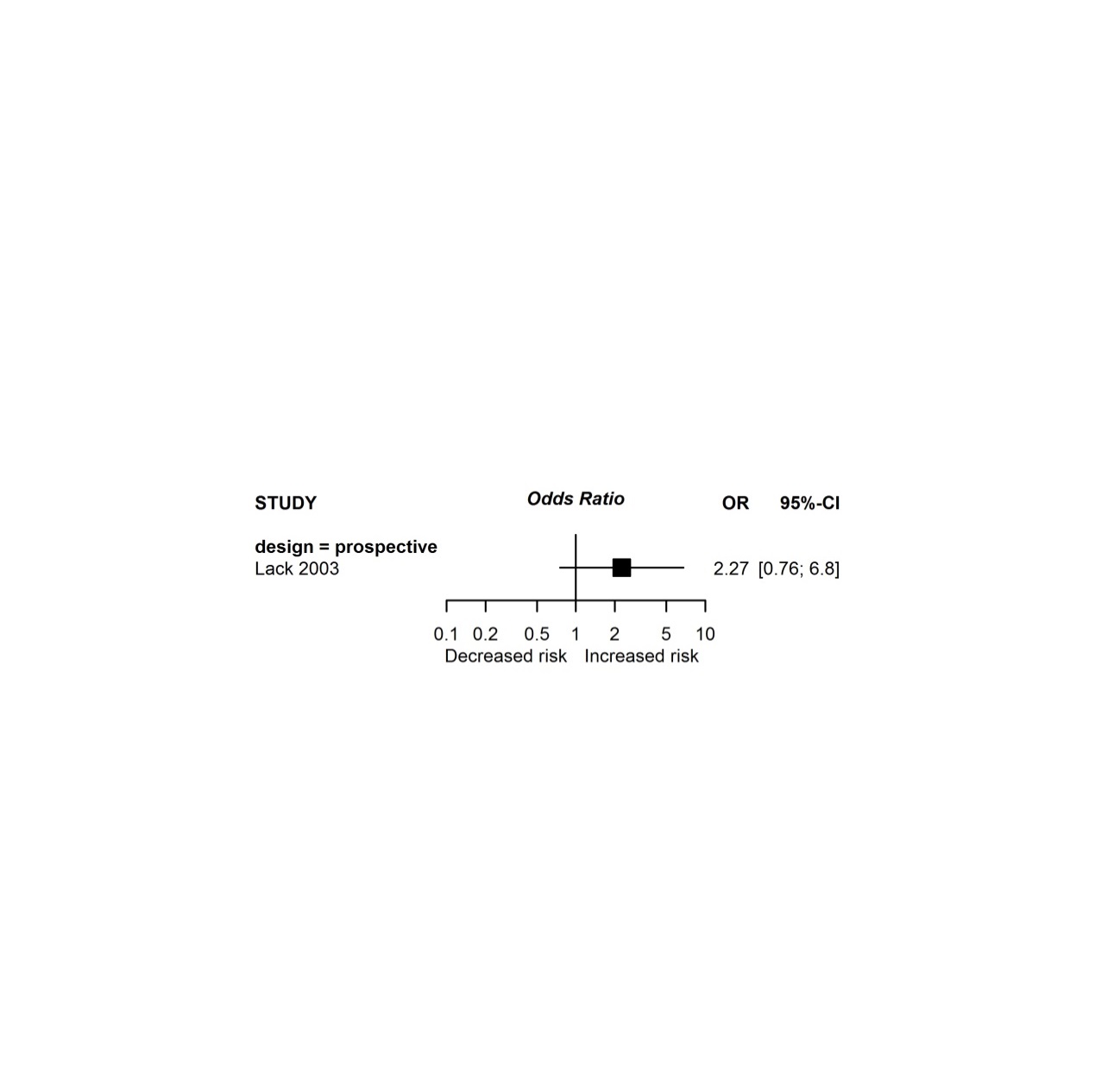


Figure 11 TBF ≥6 months vs. never and risk of FA to peanut at age 5-14 years


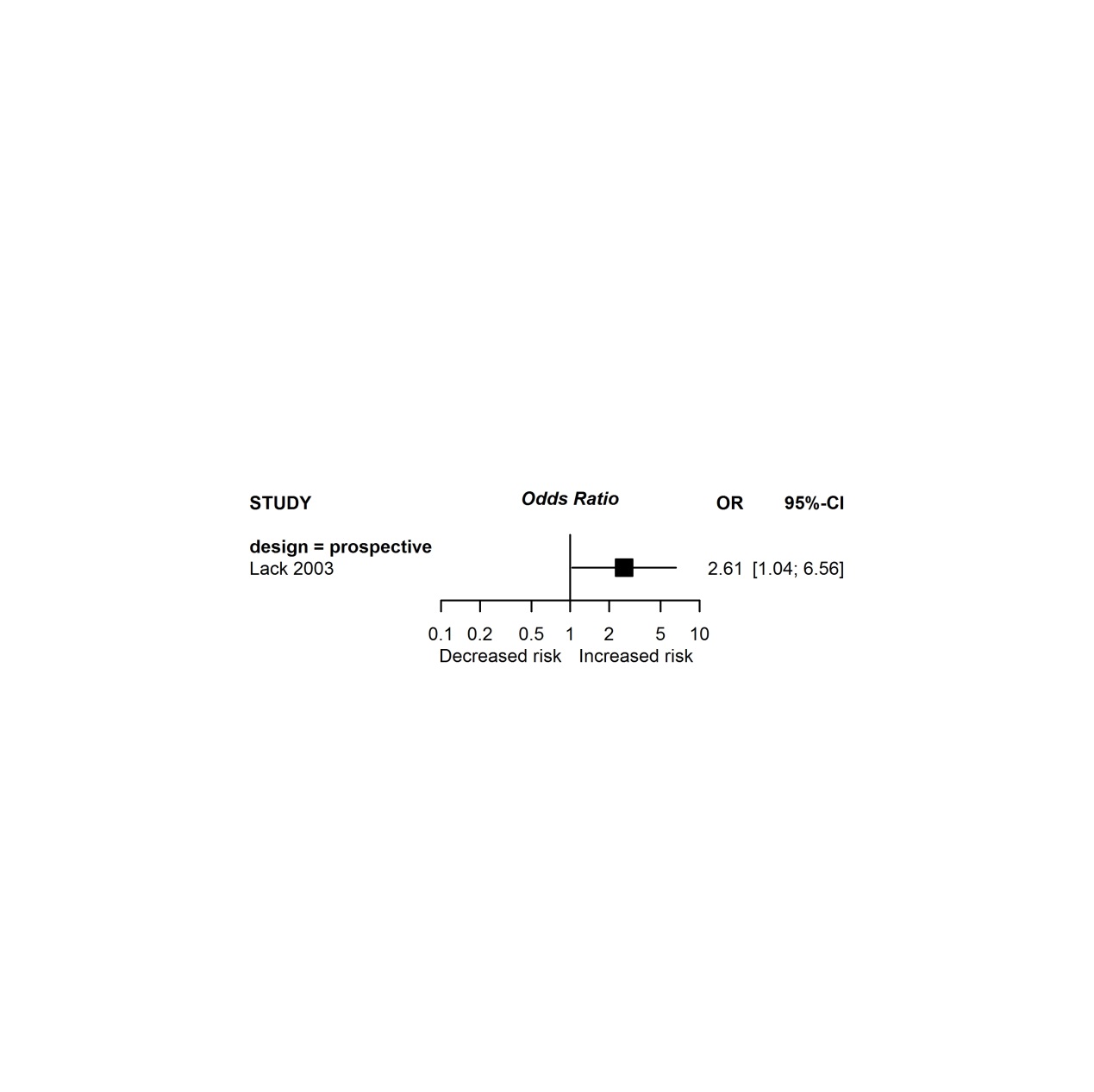


## Data for TBF duration and FA which were not suitable for meta-analysis

Meta-analyses included 9 studies, reporting data on at least 4200 participants with FA. A further 9 studies reported relevant data which could not be reported in meta-analysis, in relation to at least 800 participants with FA. Results in these studies did not allow creating effect estimates that could be combined in a meta-analysis. Four studies used mean or median, and six provided a narrative only describing the effect observed. These studies are summarised in Table 2. TBF duration was shorter in people with CMA in one study. In all other studies and analyses reported there was no association found.

Table 2 Studies investigating the association between TBF duration and food allergy which were not eligible for meta-analysis

| **Study** | **Design** | **Outcome** | **Age** | **N/n cases** | **Data** | **Measure** | **TBF in no FA** | **TBF in FA** | **P** |
| --- | --- | --- | --- | --- | --- | --- | --- | --- | --- |
| Hesselmar 2010 ([1](#_ENREF_1)) | PC | FA | 1.5 | 184/14 | Continuous | Median (IQR) | 7  (4.5) | 8.5  (5.5) | 0.25 |
|  |  | CMA | 0.5 | 184/unclear | Continuous | Median (IQR) | 7  (4.6) | 8  (4) | 0.35 |
| Oliver 2010 ([12](#_ENREF_12)) | NCC | FA | 1 | 93/31 | Continuous | Mean (weeks) | 22 | 15 | NS |
| Fox 2009 ([15](#_ENREF_15)) | CC | Peanut Allergy | <4 | 293/133 | Continuous | Mean (months) | 8.1 | 8.1 | NS |
| Berjon, 1987 ([18](#_ENREF_18)) | CS | FA | 14 | 2690/148 | Categorical | No significant difference between those with and without food allergy in TBF ever | | | |
| Djenouhat ([14](#_ENREF_14)) (2011) | CC | CMA | 0.3 | 450/150 | Categorical | Breastfeeding was associated with reduced CMA in adjusted analysis  (abstract publication) | | | |
| Venter, 2009 ([11](#_ENREF_11)) | PC | FA | 3 | 891/ 58 | Categorical | No significant difference between those with and without food allergy in TBF ever, >6 or >9 months (unadjusted) | | | |
| Goksor 2011 ([7](#_ENREF_7)) | PC | FA | 4.5 | 4496/202 | Categorical | No significant association between TBF >4 months and FA ? adjusted analysis | | | |
| Gustafsson, 2000 ([4](#_ENREF_4)) | PC | FA | 8 | 94/56 | Categorical | No significant association between TBF >1 or >6 months and FA | | | |
| Saarinen, 1995 ([10](#_ENREF_10)) | PC | FA | 5 and 17 | 256/61 and 18 | Categorical | No significant association between TBF <1, 1-6 or >6 months and FA | | | |

# Exclusive Breastfeeding Duration and Food Allergy

## Overall characteristics of studies, risk of bias and summary of results

Table 3 describes the main characteristics of the studies analysed in this report. A total of 1 systematic review describing 1 cohort study, and 14 observational studies, reported the association between duration of exclusive breastfeeding (EBF) and risk of Food Allergy (FA). Of these, 11 were prospective cohort studies, 1 nested case-control, 2 cross-sectional study and none case-control studies. Over half of the studies (n=10) are from Europe – others are from Australasia (n=3), and the Middle East (n=1). Overall, valid data on EBF duration and FA risk were available from over 30,000 subjects. Information on FA was obtained mainly from a medical assessment in 5 studies, via parental report in 6 studies, through supervised oral food challenge in 3 studies and not clear in 1 study. With regards to time of outcome diagnosis, 13 studies explored the association between EBF duration and FA in the first 5 years of life, where FA and anaphylaxis are most common, and 2 evaluated FA in older children or adults. Most studies used a questionnaire to assess the exposure (EBF), sometimes supplemented by a diary or medical record review, and in 2 studies the method of exposure assessment was unclear.

Risk of bias for the observational studies is shown in Figure 12. Over half of studies had a high risk of bias, due to lack of adjustment for confounding bias i.e. no adjusted data presented. The majority of studies also had ‘unclear’ risk of assessment bias due to inadequate definition of what was meant by EBF duration. Risk of conflict of interest was generally assessed as low.

Where data were available, three levels of comparison were used to assess the risk of FA according to EBF duration, namely ≥0-2 months vs. <0-2 months; ≥3-4 months vs. <3-4 months; ≥5-9 months vs. <5-9 months. Across all these cut-offs there was no consistent evidence of a lower risk of FA if EBF was prolonged. In general there were only small numbers of studies with data available, and several analyses were restricted to studies with high risk of bias. A single prospective RCT was underpowered and inconclusive, and the few prospective cohort studies reporting appropriately adjusted data yielded mixed and inconclusive findings.

Table 3 Characteristics of included studies evaluating EBF duration and food allergy

| **First Author & Publication Year** | **N/n cases** | **Design** | **Country** | **Exposure assessment** | **Method of outcome assessment** | **Age at outcome (years)** | **Population characteristics** |
| --- | --- | --- | --- | --- | --- | --- | --- |
| Kramer ([19](#_ENREF_19))  Kajosaari, 1991 ([20](#_ENREF_20)) | 135 | SR/PC | Finland | - | Parent reported FA and DBPCFC | 1, 5 | Infants of atopic parents |
| Hesselmar, 2010 ([1](#_ENREF_1)) | 184/14 | PC | Sweden | I | Physician assessment: history +/- investigations | 1.5 | ALLERGYFLORA. Population based cohort of mainly high risk infants recruited from antenatal clinics 1998-2003 |
| Kull, 2002 ([3](#_ENREF_3)) | 3791/768 | PC | Sweden | Q | DD | 2 | BAMSE. Population based cohort of children born 1994-1996 |
| Besednjak-Kocijancic, 2010 ([21](#_ENREF_21)) | 408/24 | PC | Slovenia | Unclear | Unclear | 1, 5 | Infants with a positive history of parental allergy |
| Arshad, 1992 ([22](#_ENREF_22)) | 1167/80 | PC | UK | D/Q | Parent report of ≥ 2 food reactions | 1 | Isle of Wight Study. Population based birth cohort of infants born in semi-rural areas between 1989 and 1990 |
| Kemeny, 1991 ([8](#_ENREF_8)) | 180/9 | PC | UK | Unclear | Parent report of ≥ 2 food reactions | 1 | Population based birth cohort of infants born at Dulwich and King’s College Hospitals in London |
| Kim, 2011 ([23](#_ENREF_23)) | 1177/61 | PC | Korea | Q/I | Parent report of ≥ 2 food reactions | 1 | Population based birth cohort of infants born in Seoul between July 2006 and August 2007 |
| Host, 1991 ([24](#_ENREF_24)) | 315/16 | PC | Denmark | R/Q/I | Open food challenge | 1 | Population birth cohort of infants from the municipality of Odense born in 1985 |
| Pesonen, 2006 ([25](#_ENREF_25)) | 160/19 | PC | Finland | I | Parent report of ≥ 2 food reactions | 5 | Population based birth cohort of infants born in 1981 |
| Saarinen, 1979 ([26](#_ENREF_26)) | 177/17 | PC | Finland | Q | Physician assessment: methodology unclear | 1 | Recruited from hospital and born in 1975 |
| Matheson, 2007 ([27](#_ENREF_27)) | 5729/568 | PC | Australia | Q | DD | 44 | Tasmanian Asthma Study: population based cohort born in 1961 |
| Venter, 2009 ([11](#_ENREF_11)) | 891/58 | PC | UK | Q | Open food challenge | 3 | Population based cohort recruited from antenatal clinics and born in 2001-2002 |
| Maskell, 2010 ([28](#_ENREF_28)) | 117/31 | NCC | UK | D | DBPCFC | 1 | EuroPrevall UK. Cases and age-matched controls selected from a population based birth cohort of infants born in 2008 |
| Paton, 2012 ([17](#_ENREF_17)) | 15142/592 | CS | Australia | Q | Parent-reported reaction to any nut | 7 | Representative primary school entrants 2006-2009. |
| Ehlayel, 2008 ([29](#_ENREF_29)) | 1278/ unclear | CS | Qatar | Q | DD | 5 | Children 0-5 years old attending primary healthcare centres for routine immunisation |

Q: questionnaire, I: interview, R: medical records, D: diary, PC: prospective cohort, NCC: nested case control, CS: Cross-sectional, CC: case control, DBPCFC: double blind placebo controlled food challenge, DD: doctor diagnosis of FA, in contrast to ‘Physician assessment’ where FA diagnosis was always made by a study physician as part of the study protocol.

Figure 12 Risk of bias in studies of EBF duration and food allergy

## Exclusive breastfeeding duration and risk of food allergy

### Systematic reviews and intervention trials

The single systematic review and included cohort study (Table 4) found no evidence that exclusive/predominant breastfeeding for 6 months, compared with exclusive/predominant breastfeeding for 3 months, influenced risk of challenge-proven food allergy at 1 year (RR 0.77 95%CI 0.25, 2.41) although parent reported food allergy at the same time-point in the same study was significantly reduced ([30](#_ENREF_30)). Food allergy at 5 years was not significantly different in the same study (RR 0.61 95% CI 0.12, 3.19).

Table 4 EBF for 6 months versus 3 months and risk of food allergy

| **Study** | **Outcome measure** | **No. participants (studies)** | **Outcome (95% CI)** |
| --- | --- | --- | --- |
| Kramer ([19](#_ENREF_19))  Kajosaari ([20](#_ENREF_20)) ([30](#_ENREF_30)) | Parent report - 1 year | 135 (1) | RR 0.19 [0.08, 0.48] |
|  | DBPCFC - 1 year | 135 (1) | RR 0.77 [0.25, 2.41] |
|  | FA diagnosis - 5 years | 113 (1) | RR 0.61 [0.12, 3.19] |

### Observational studies of EBF duration and FA in children aged 0-4 years

#### Any Food Allergy in children aged 0-4 years

##### EBF ≥0-2 months vs. <0-2 months

One prospective cohort study reported data that could be used to calculate OR for FA in the first year, in infants with EBF for 6 months vs. <2 months duration and is shown in Figure 13. The study found no significant association. The data are unadjusted, so carry a high risk of bias, and the authors reported similar findings comparing 2-6 months duration with <2 months, and using the same categorisations at age 3 years.

Figure 13 EBF ≥0-2 months vs. <0-2 months and risk of any FA at age 0-4 years


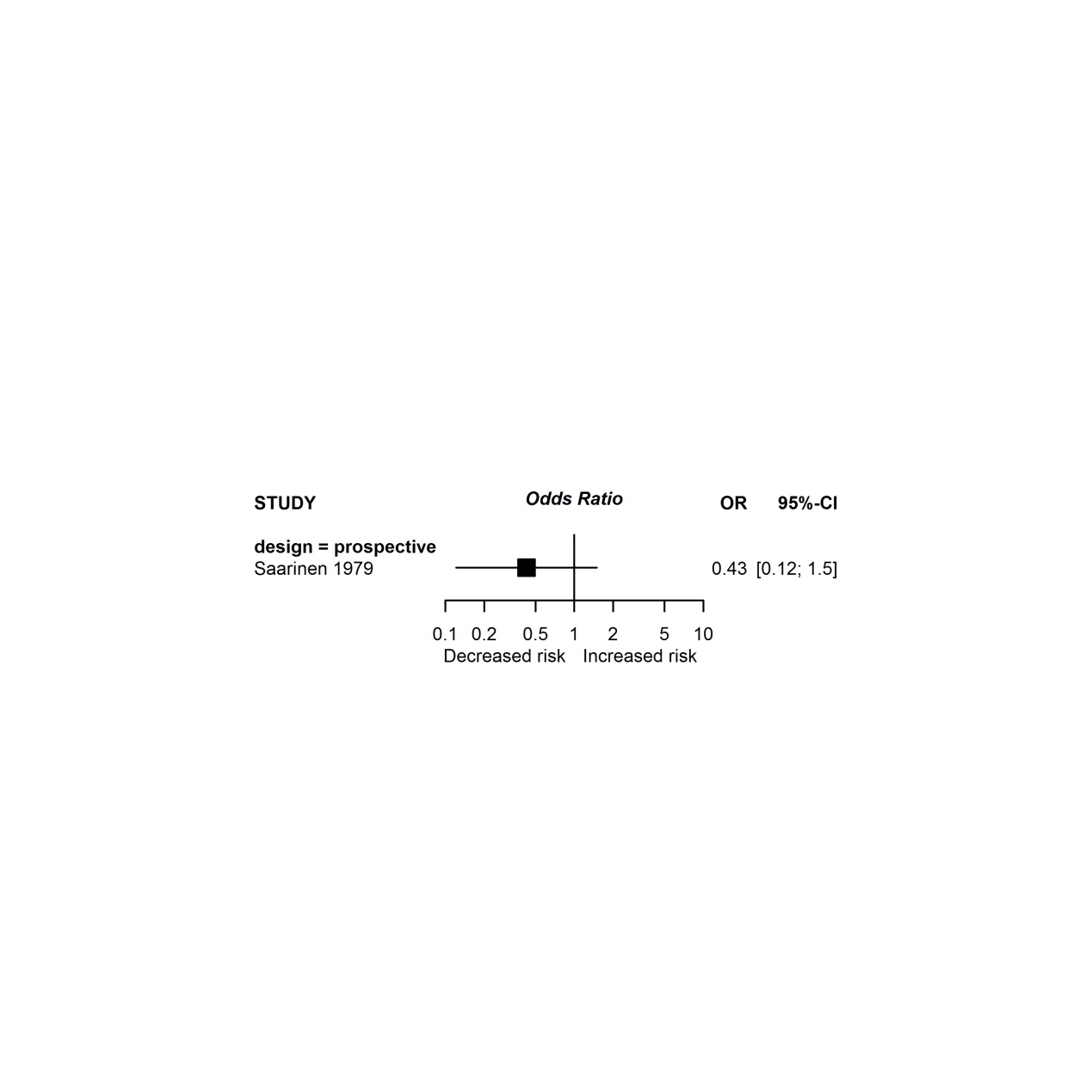


##### EBF ≥3-4 months vs. <3-4 months

Two prospective cohort studies reported data that could be used to calculate OR for FA at age 0-4 years, in infants with EBF for ≥3-4 months vs. <3-4 months duration and are shown in Figure 14. They show no significant association, with moderate heterogeneity. The data from Arshad 1992 are unadjusted, so carry a high risk of bias, but the data of Kull 2002 are adjusted. This may explain the statistical heterogeneity.

Figure 14 EBF ≥3-4 months vs. <3-4 months and risk of any FA at age 0-4 years


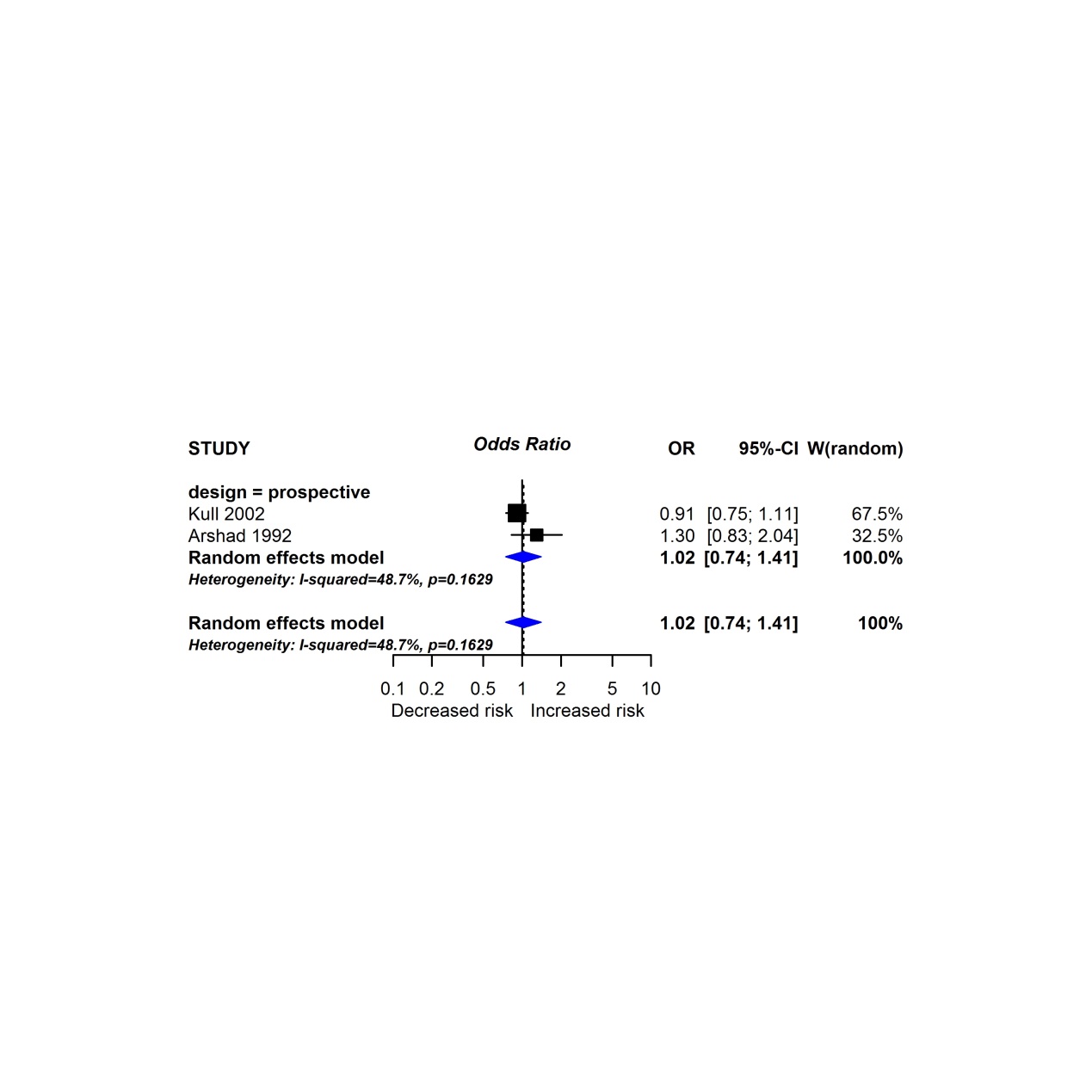


##### EBF ≥5-6 months vs. <5-6 months

One prospective cohort study reported data that could be used to calculate OR for FA in the first year in infants with EBF for ≥6 months vs. <6 months duration and is shown in Figure 15. The study reported adjusted data, and found no significant association.

Figure 15 EBF ≥5 months vs. <5 months and risk of any FA at age 0-4 years


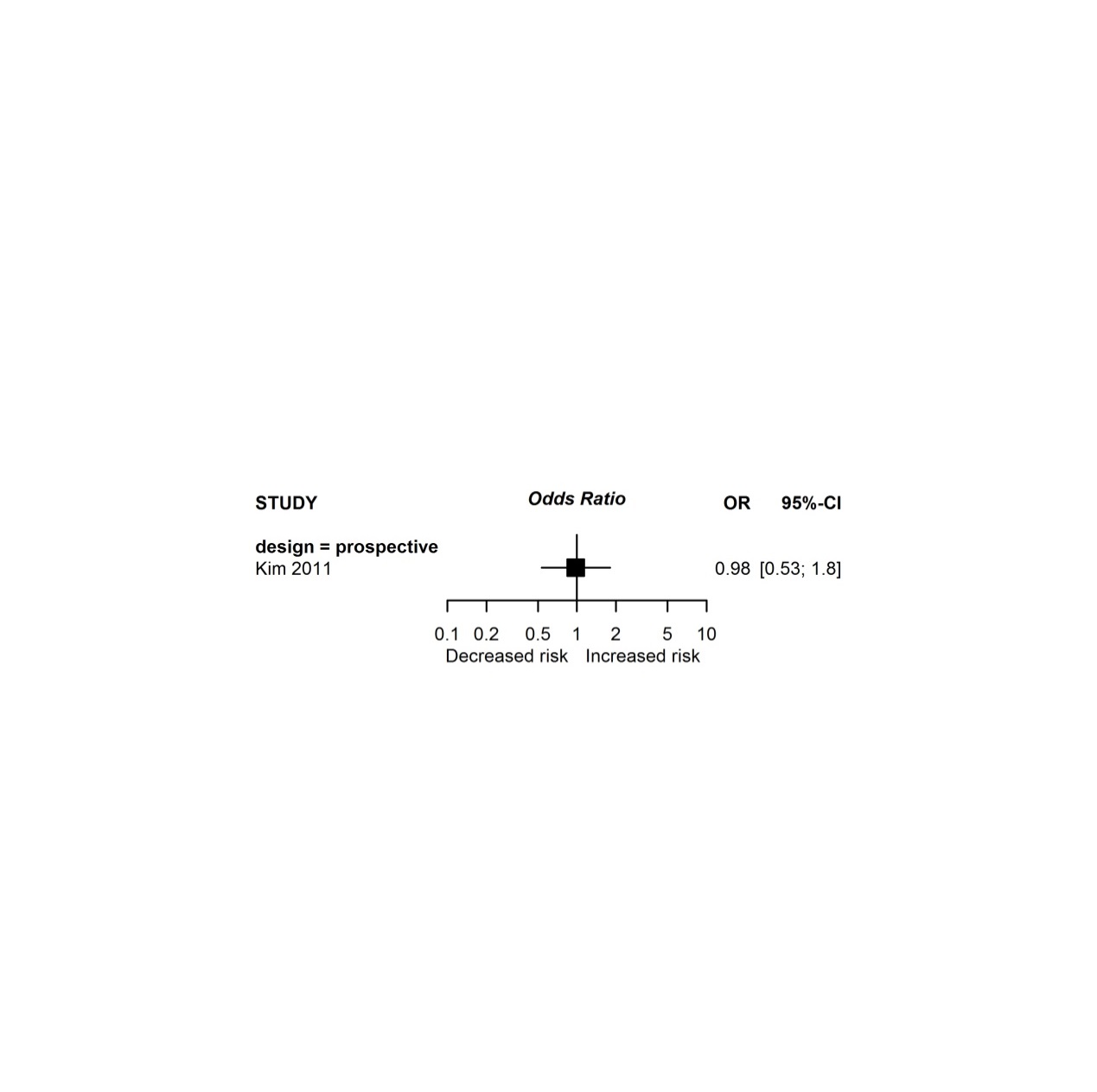


#### Cow’s Milk Allergy in children aged 0-4 years

##### EBF ≥0-2 months vs. <0-2 months

One prospective cohort study reported data that could be used to calculate OR for CMA in the first year, in infants with EBF for >1 month vs. <1 month duration and is shown in Figure 16. The study found no significant association. The data are unadjusted, so carry a high risk of bias, and the authors reported similar insignificant findings when assessing 1-3 months vs. <1 month, and >3 months vs. <1 month.

Figure 16 EBF ≥0-2 months vs. <0-2 months and risk of FA to cow’s milk at age 0-4 years


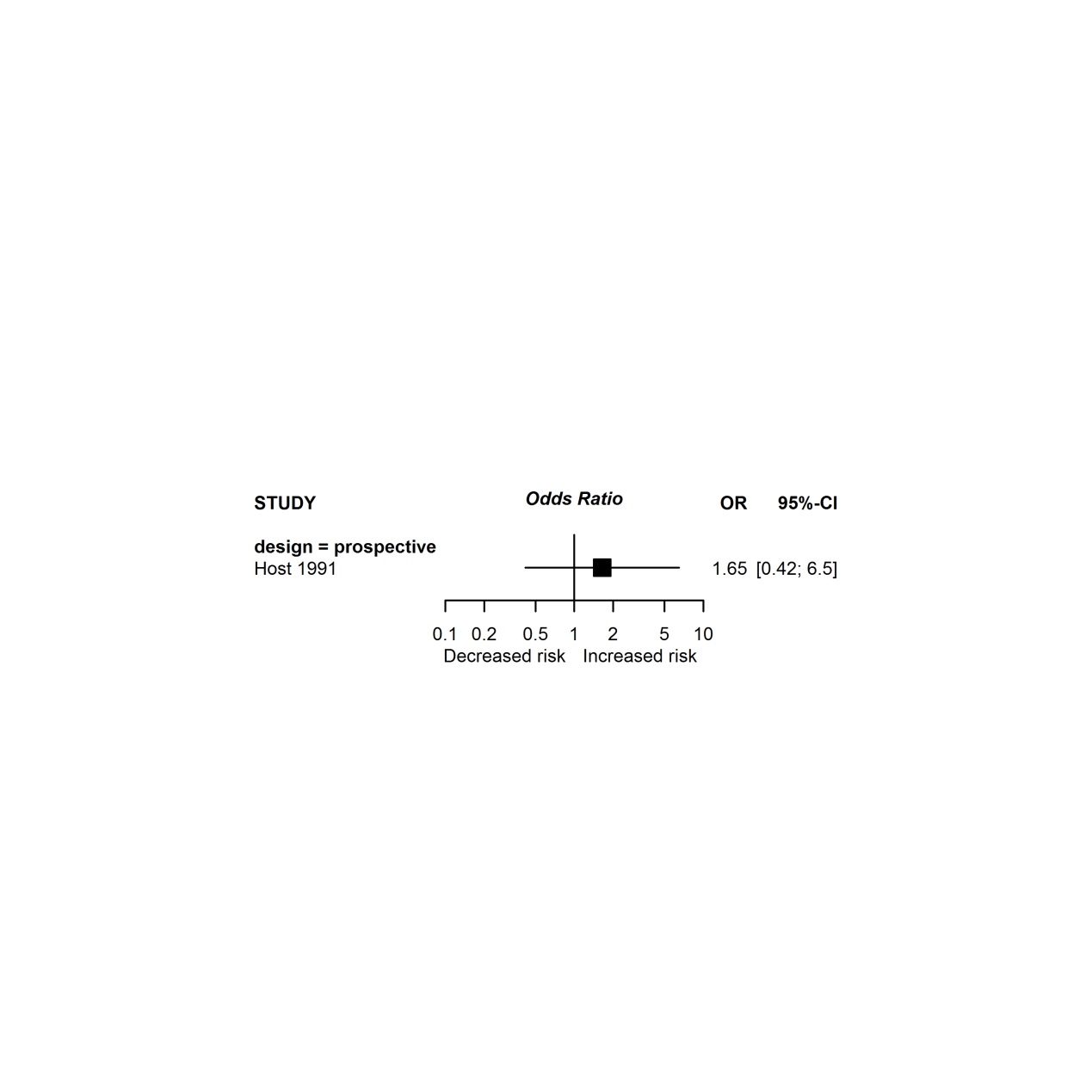


##### EBF ≥3-4 months vs. <3-4 months

One prospective cohort study reported data that could be used to calculate OR for CMA in the first year, in infants with EBF for ≥3-4 months vs. <3-4 months duration and is shown in Figure 17. The study found significantly reduced risk of CMA with longer EBF. The data are unadjusted, so carry a high risk of bias.

Figure 17 EBF ≥3-4 months vs. <3-4 months and risk of FA to cow’s milk at age 0-4 years


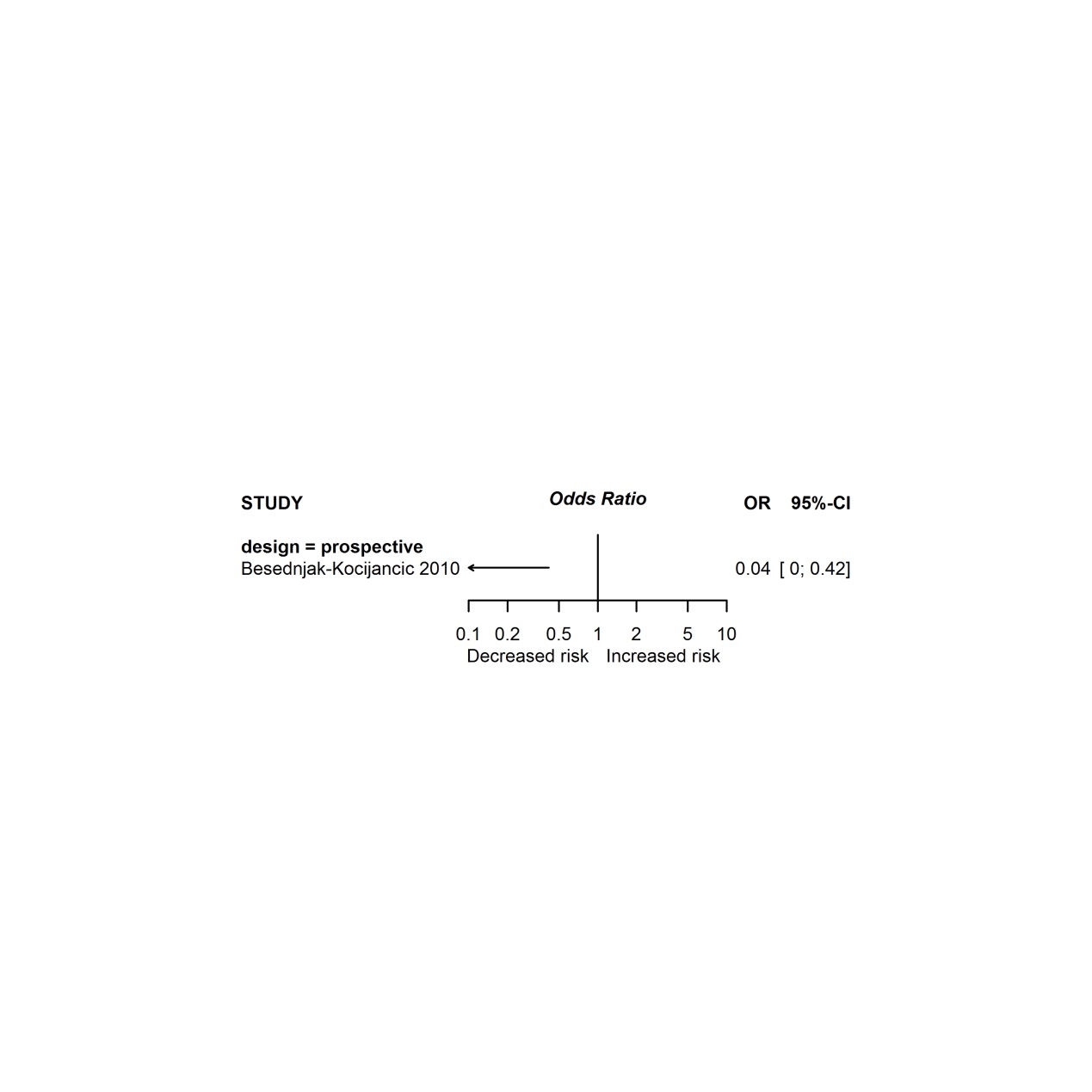


#### Egg Allergy in children aged 0-4 years

Two prospective cohort studies reported data that could be used to calculate OR for egg allergy at age 0-4 years, in infants with EBF for ≥3-4 months vs. <3-4 months duration and are shown in Figure 18. They show reduced odds of egg allergy with longer duration of EBF, with borderline statistical significance and no statistical heterogeneity. Both studies reported unadjusted data, and are therefore at high risk of bias.

Figure 18 EBF ≥3-4 months vs. <3-4 months and risk of FA to egg at age 0-4 years


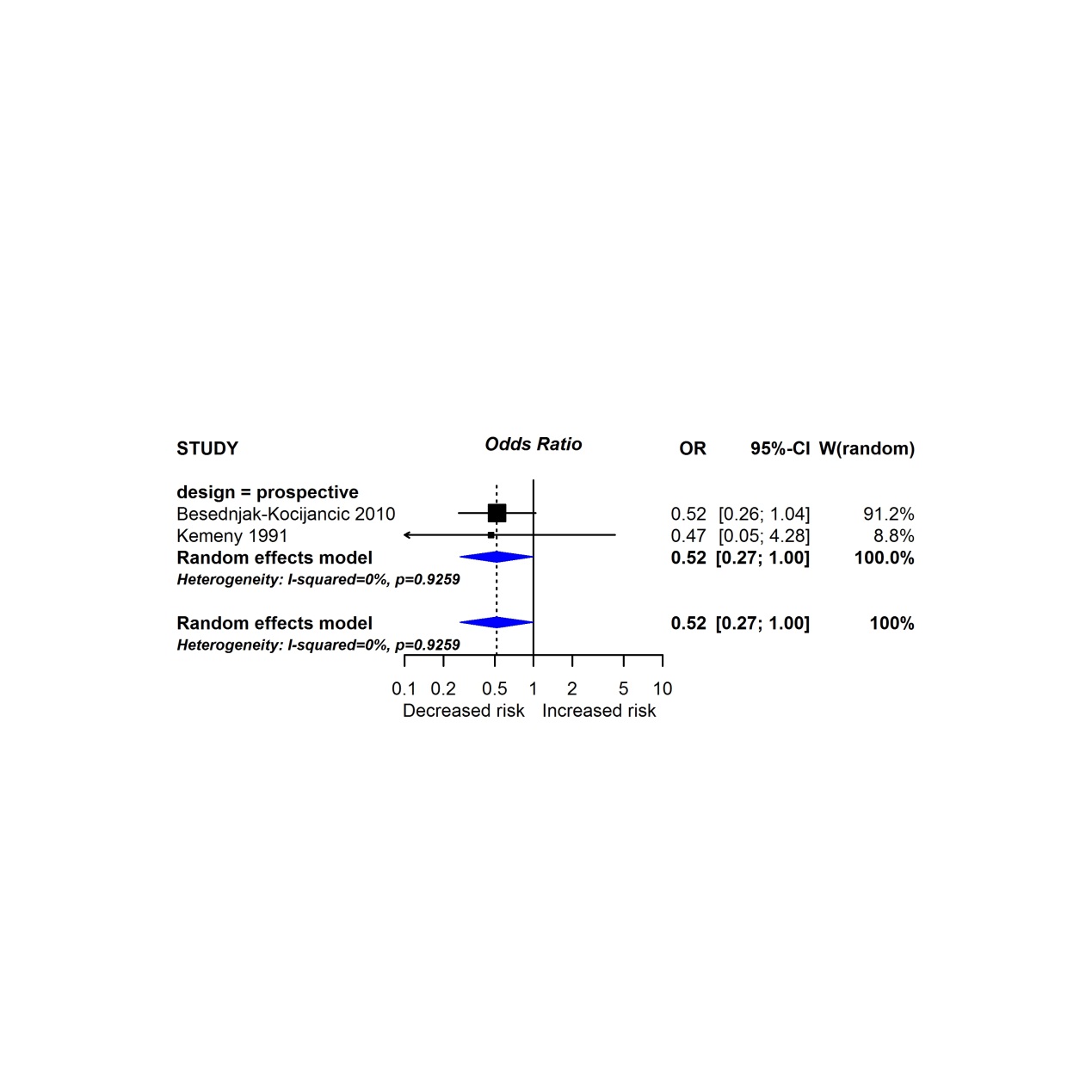


### Observational studies of EBF duration and FA in children aged 5-14 years

#### Any Food Allergy in children aged 5-14 years

##### EBF ≥0-2 months vs. <0-2 months

One prospective cohort study reported data that could be used to calculate OR for FA at age 5-14 years, in infants with EBF for ≥0-2 months vs. <0-2 months duration and is shown in Figure 19. The study found no significant association, and the data carry a high risk of bias because they are unadjusted.

Figure 19 EBF ≥0-2 months vs. <0-2 months and risk of any FA at age 5-14 years


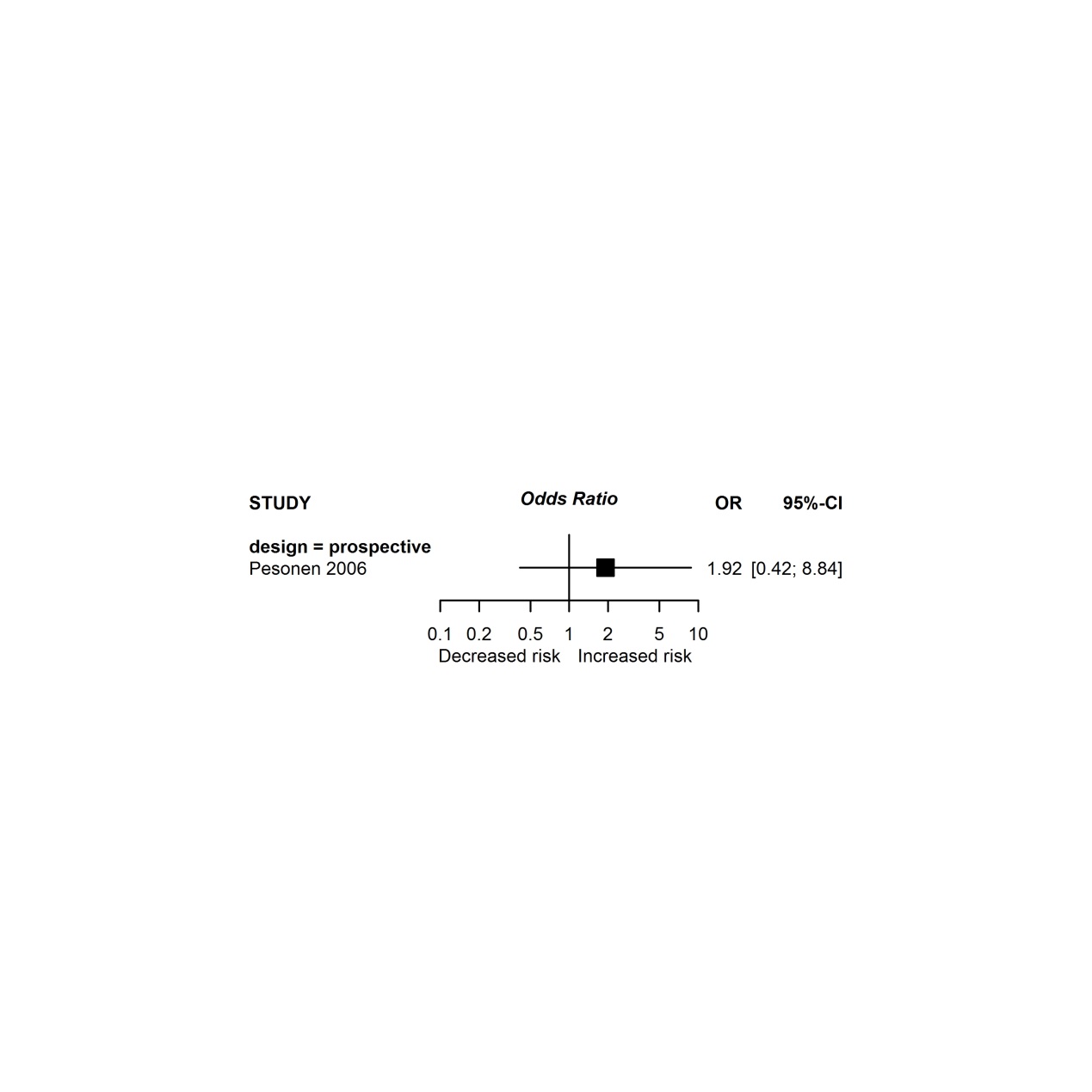


##### EBF ≥5 months vs. <5 months

Two prospective cohort studies reported data that could be used to calculate OR for FA at age 5-14 years, in infants with EBF for ≥5 months vs. <5 months duration and are shown in Figure 20. The data were not pooled due to extreme statistical heterogeneity. The cross-sectional study of Ethlayel 2008 reported unadjusted data, and therefore carries a high risk of bias. The prospective cohort study of Pesonen 2006 reported adjusted data, and had unclear risk of bias due to unclear method for defining EBF duration, which was categorised as ≥9 vs. <9 months, which is an unusually long duration of EBF in most human populations.

Figure 20 EBF ≥5 months vs. <5 months and risk of any FA at age 5-14 years


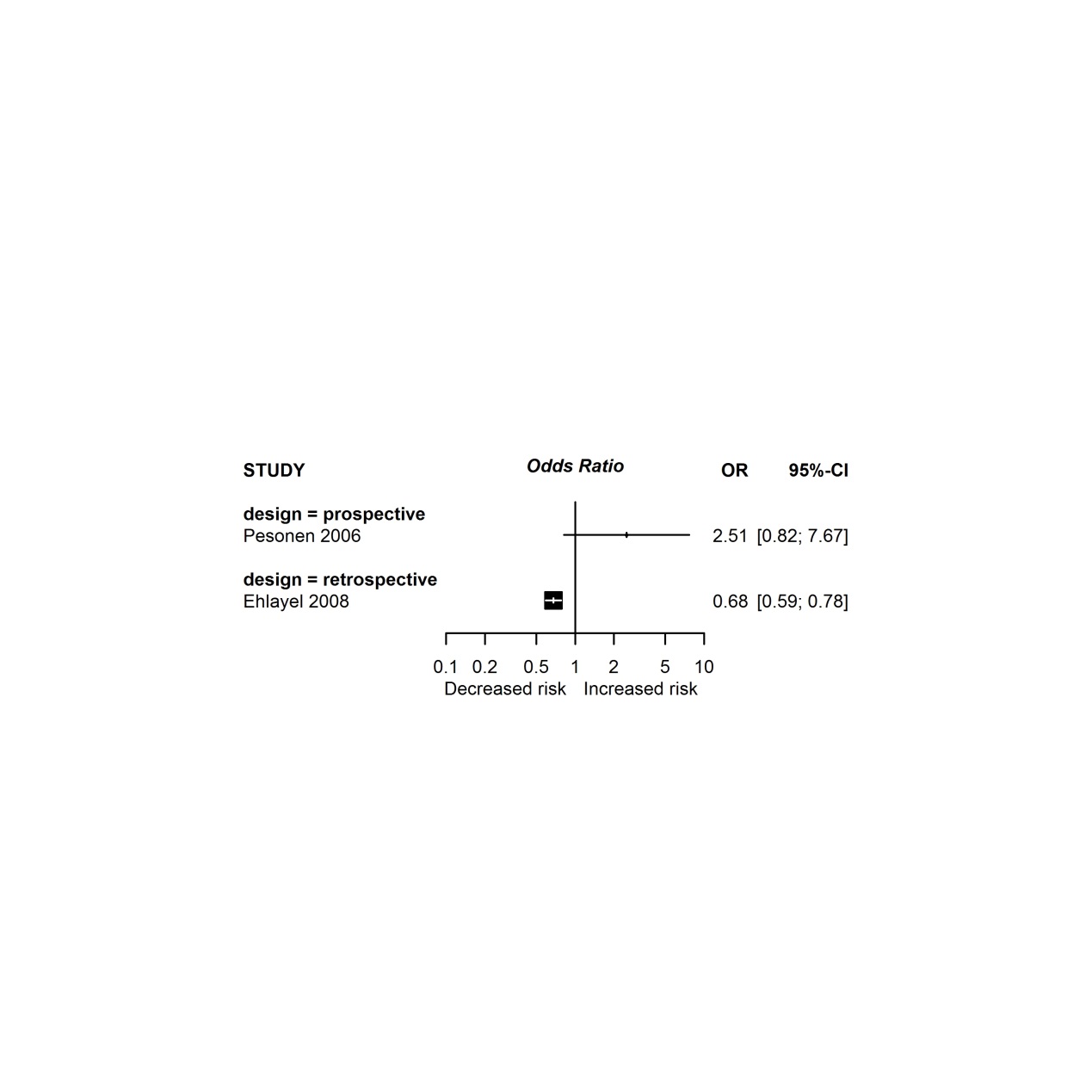


#### Peanut Allergy in children aged 5-14 years

##### EBF ≥5 months vs. <5 months

One cross-sectional study reported data that could be used to calculate OR for nut allergy (peanut or tree nut) at age 5-14 years, in infants with EBF for ≥5 months vs. <5 months duration and is shown in Figure 21. There were significantly increased odds of nut allergy in children who had longer duration of EBF. However the study was considered at high risk of bias due to inadequate adjustment for potential confounders – age and sex only were adjusted for.

Figure 21 EBF ≥5 months vs. <5 months and risk of FA to nut-peanut at age 5-14 years


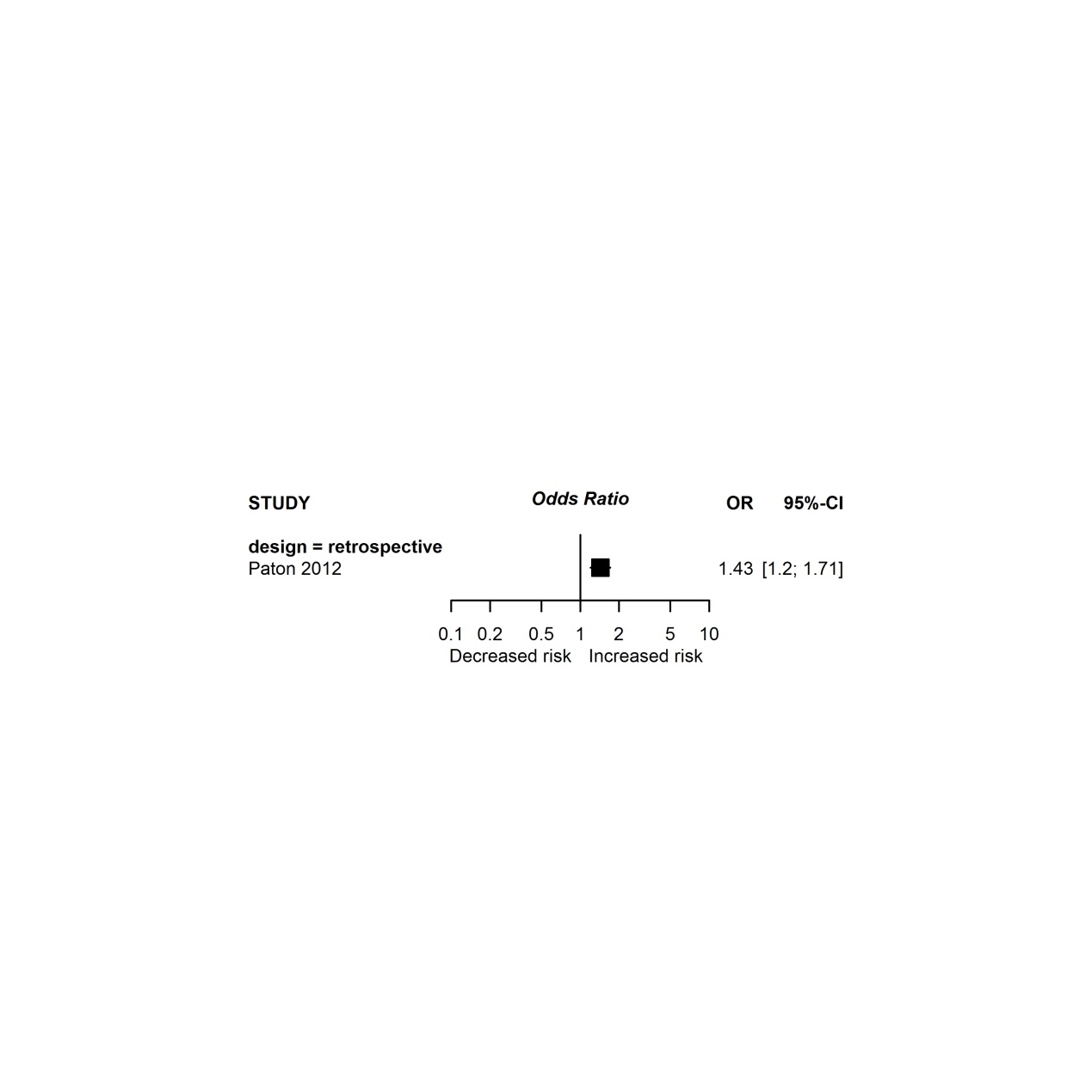


### Observational studies of EBF duration and FA at age 15+

#### Any Food Allergy in children aged 15+ years

One prospective cohort study reported data that could be used to calculate OR for any food allergy at age 44 years, in adults with a history of EBF ≥3-4 months vs. <3-4 months duration and is shown in Figure 22. There were significantly increased odds of self-reported FA in adults who had received longer duration of EBF. The data were appropriately adjusted, but the study was considered at unclear risk of bias due to inadequate definition of EBF, which was assessed retrospectively through parental report at aged 7 years. In the same study, reduced odds of parent-reported allergy to foods or medicines were found with EBF ≥3-4 months at age 7; aOR 0.75 (0.62, 0.92).

Figure 22 EBF ≥3-4 months vs. <3-4 months and risk of any FA at age 15+ years


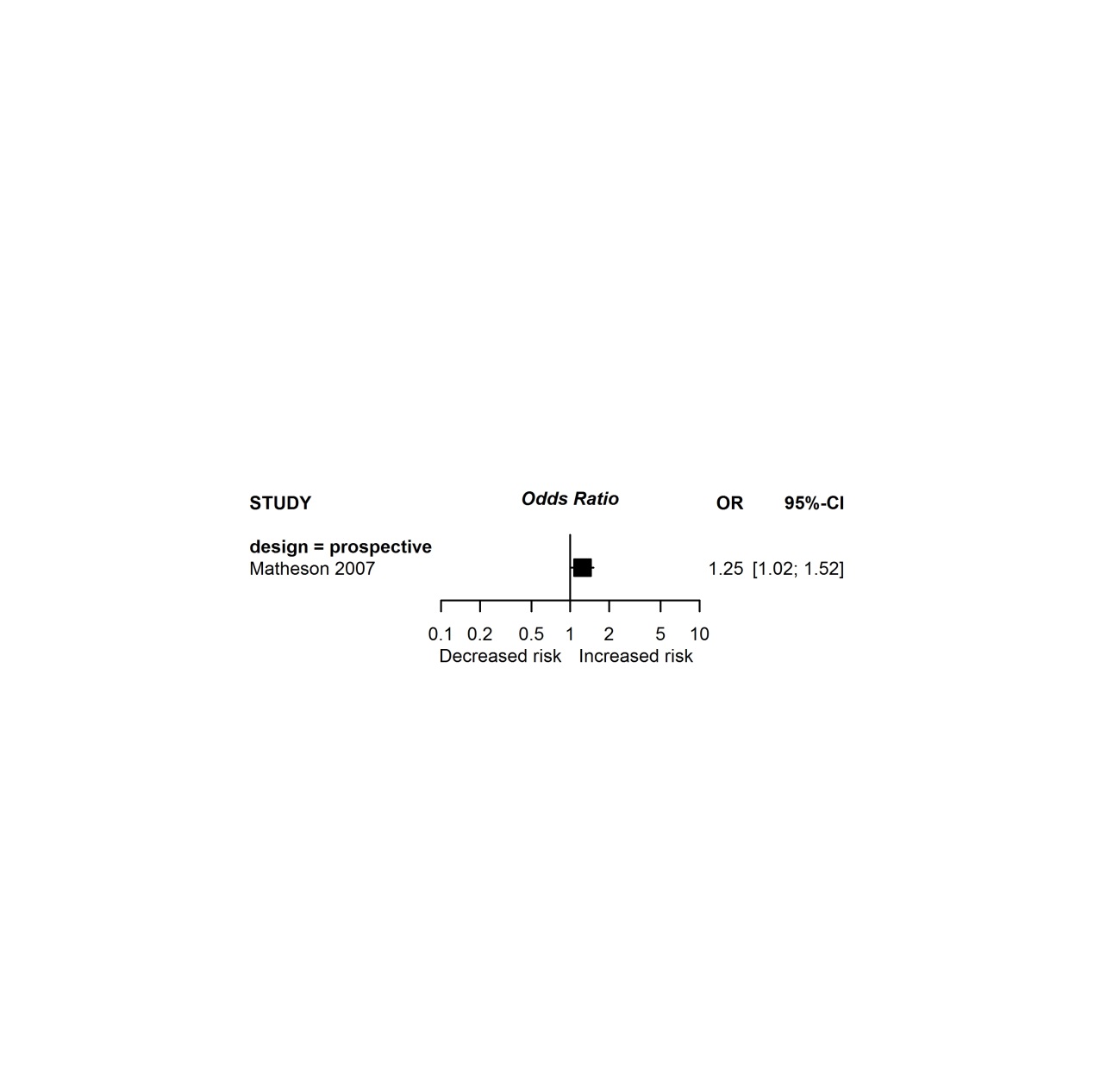


### Data for TBF duration and FA which were not suitable for meta-analysis

Meta-analyses included 12 studies, reporting data on at least 1800 participants with FA. A further 4 studies reported relevant data which could not be reported in meta-analysis, in relation to at least 100 participants with FA. These studies are summarised in Table 5. TBF duration was shorter in people with FA in one study. In all other studies and analyses reported there was no association found.

Table 5 Studies investigating the association between EBF duration and food allergy which were not eligible for meta-analysis

| **Study** | **Design** | **Outcome** | **Age** | **N/n cases** | **Data** | **Measure** | **EBF duration with no FA** | **EBF duration with FA** | **P** |
| --- | --- | --- | --- | --- | --- | --- | --- | --- | --- |
| Hesselmar 2010 ([1](#_ENREF_1)) | PC | FA | 1.5 | 184/14 | Continuous | Median (IQR) in months | 4  (4, 4.5) | 4  (3.3, 4) | NS |
| Maskell 2010 ([28](#_ENREF_28)) | NCC | FA | 1 | 117/39 | Continuous | Mean (weeks) | 5.4 | 3.2 | P=0.045 |
| Venter, 2009 ([11](#_ENREF_11)) | PC | FA | 3 | 891/58 | Categorical | No significant association between EBF duration of >3 months and food allergy at 1 or 3 years | | | |
| Kemeny, 1991 ([8](#_ENREF_8)) | PC | CMA | 1 | 180/0 | Categorical | No significant association between EBF duration of >3 months and food allergy at 1 year | | | |

# Solid Food Introduction and Food Allergy

## Overall characteristics of studies, risk of bias and summary of results

Table 6 describes the main characteristics of the studies analysed in this report. A total of 6 observational studies and no intervention studies, reported the association between timing of solid food introduction (SF) and risk of FA. Of these, 4 were prospective cohort studies, 1 nested case-control, 1 case-control study. Half of the studies (n=3) are from Europe, 2 from Australasia (n=3), and 1 North America. Overall, valid data on SF and FA risk were available from almost 3000 subjects. Information on FA was obtained mainly from a medical assessment in 2 studies, via parental report in 1 study, and through supervised oral food challenge in 3 studies. With regards to time of outcome diagnosis, all studies explored the association between SF and FA in the first 1.5 years of life. Four studies used a questionnaire to assess the exposure (SF), 1 used an interview and 1 a diary.

Risk of bias is summarised in Figure 23. Almost half of studies had a high risk of bias, due to lack of adjustment for confounding bias i.e. no adjusted data presented. In a further 25% of studies the risk of confounding bias was unclear. Risk of conflict of interest was generally assessed as low.

The risk of FA according to SF was categorised as ≥3-4 months vs. <3-4 months. Overall the evidence base was limited due to small numbers of studies and participants with food allergy included in analyses. We found no evidence that the timing of solid food introduction is associated with risk of FA, based on the available data.

Table 6 Characteristics of included studies evaluating SF introduction and food allergy

| **First Author & Publication Year** | **N/n cases** | **Design** | **Country** | **Exposure assessment** | **Method of outcome assessment** | **Age at outcome (years)** | **Population characteristics** |
| --- | --- | --- | --- | --- | --- | --- | --- |
| Hesselmar, 2010 ([1](#_ENREF_1)) | 184/14 | PC | Sweden | I | Physician assessment: history +/- investigations | 0.5, 1.5 | ALLERGYFLORA. Population based cohort of mainly high risk infants recruited from antenatal clinics 1998-2003 |
| Koplin, 2010 ([5](#_ENREF_5)) | 2589/231 | PC | Australia | Q | Open food challenge | 1 | HEALTHNUTS. Population based cohort of children born 2008-2010 recruited at 1 year at routine immunization clinics |
| Kim, 2011 ([23](#_ENREF_23)) | 1177/61 | PC | Korea | Q/I | Parent report of ≥2 food reactions | 1 | Population based birth cohort of infants born in Seoul between July 2006 and August 2007 |
| Venter, 2009 ([11](#_ENREF_11)) | 757/39 | PC | UK | Q | Open food challenge | 1 | Population based cohort recruited from antenatal clinics and born in 2001-2002 |
| Maskell, 2011; Oliver, 2010 ([12](#_ENREF_12), [28](#_ENREF_28)) | 117/31 | NCC | UK | D | DBPCFC | 1 | EuroPrevall UK. Cases and age-matched controls selected from a population based birth cohort of infants born in 2008 |
| DesRoches, 2010 ([13](#_ENREF_13)) | 403/202 | CC | Canada | Q | Physician assessment: history +/- investigations | 1.5 | Hospital based cases and controls recruited 1998-2004. |

Q: questionnaire, I: interview, D: diary, PC: prospective cohort, NCC: nested case control, CC: case control, DBPCFC: double blind placebo controlled food challenge.

Figure 23 Risk of bias in studies of SF introduction and food allergy

## Solid food introduction and FA in children aged 0-4 years

### SF ≥3-4 months vs. <3-4 months

#### Any Food Allergy in children aged 0-4 years

One prospective cohort study reported data that could be used to calculate OR for FA at age 0-4 years, in infants with SF ≥3-4 months vs. <3-4 months and is shown in Figure 24. There were significantly increased odds of FA in infants with delayed SF introduction. The data were unadjusted, so the study was considered at high risk of bias.

Figure 24 SF ≥3-4 months vs. <3-4 months and risk of any FA at age 0-4 years


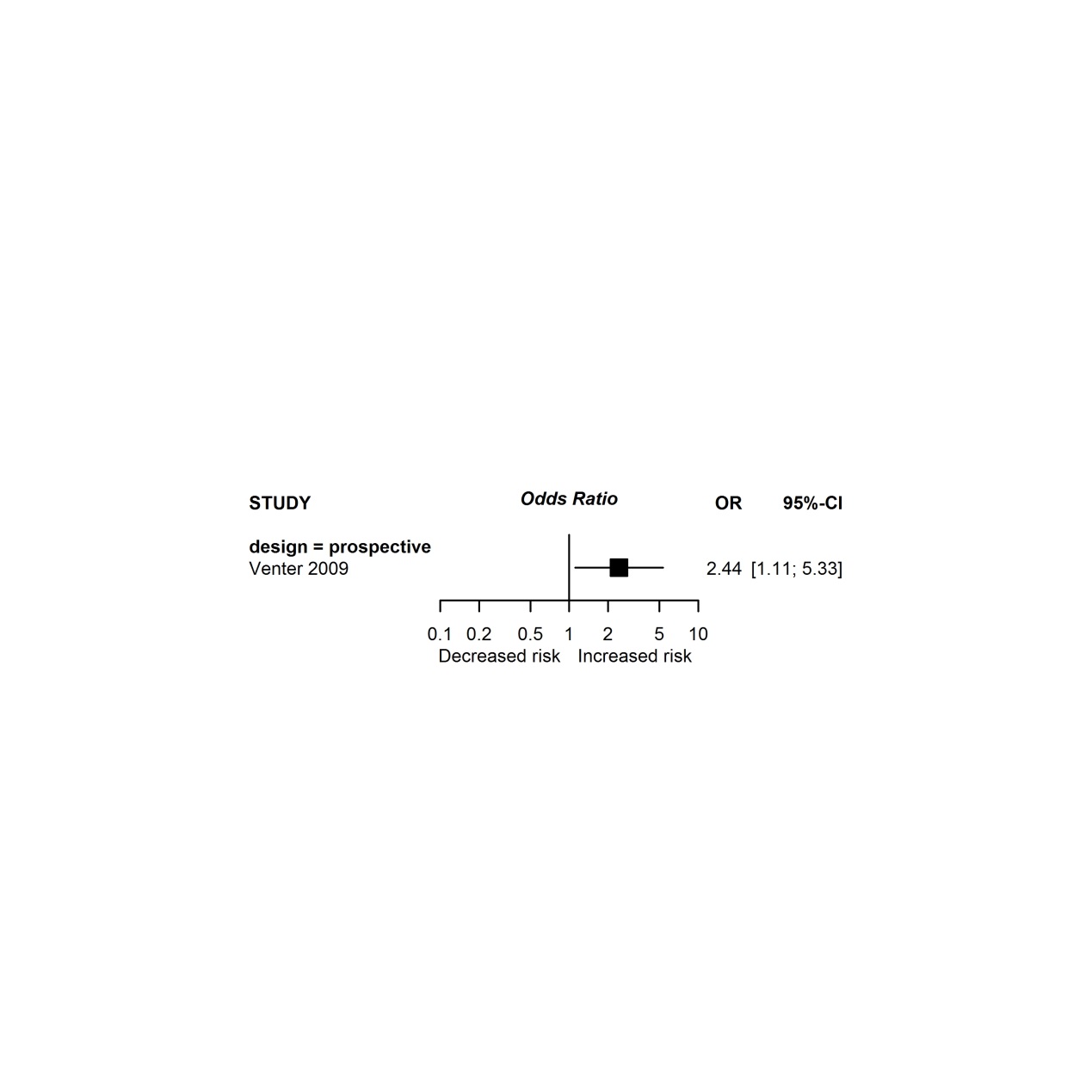


#### Egg Allergy in children aged 0-4 years

One prospective cohort study reported data that could be used to calculate OR for egg allergy at age 0-4 years, in infants with SF ≥3-4 months vs. <3-4 months and is shown in Figure 25. There were no association found in this analysis of SF≥6 vs <4 months. The same authors found no association between SF 4-5 vs <4 months – adjusted OR 2.1 (0.6, 7.0). The data were adjusted, and the study was considered at low overall risk of bias however confidence intervals were wide.

Figure 25 SF ≥3-4 months vs. <3-4 months and risk of FA to egg at age 0-4 years


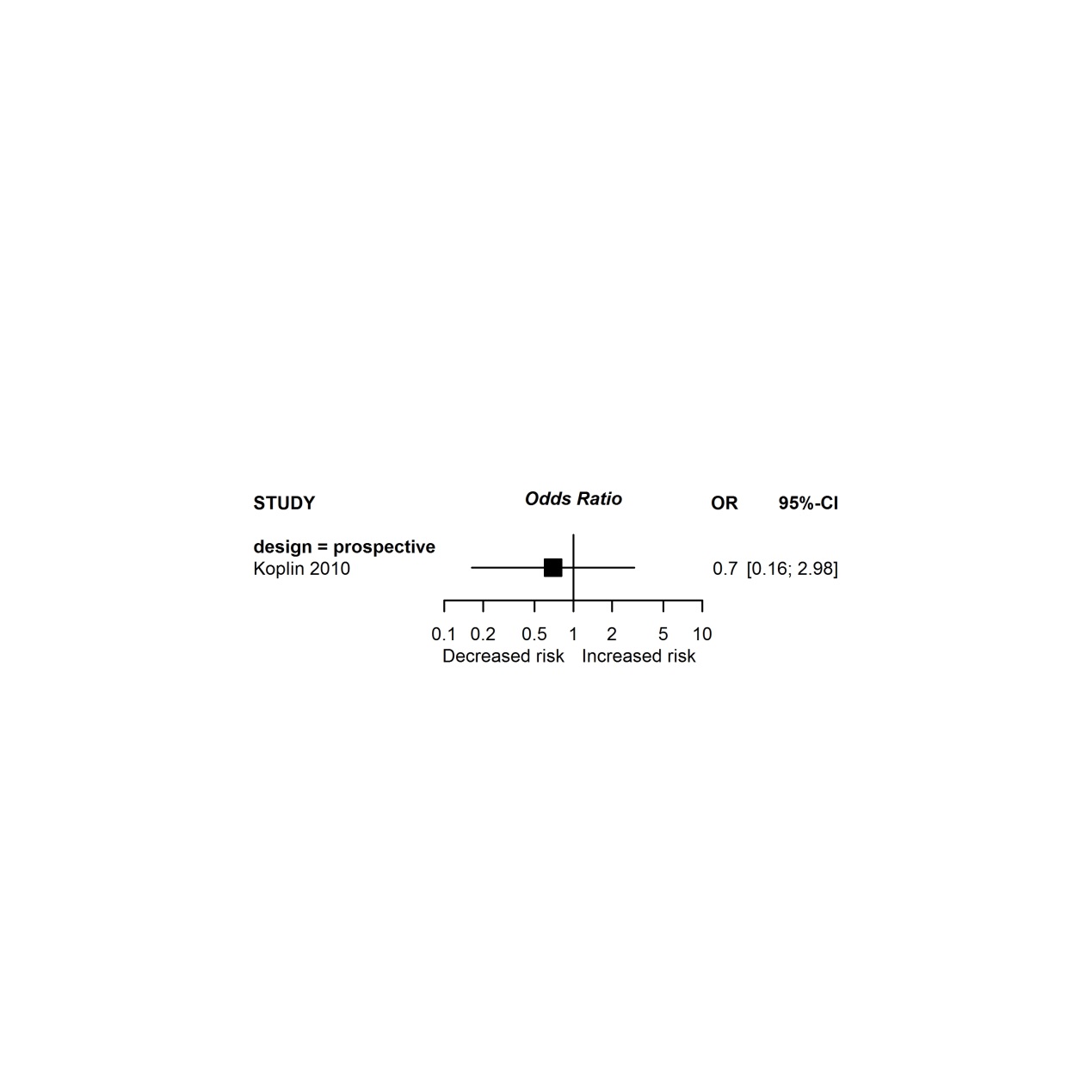


### Data for SF and FA reported narratively

Five other studies with 345 cases of FA reported data in a way that could not be included in the predefined meta-analyses. These studies are summarised in Table 7. No association was found between SF and FA in these studies.

Table 7 Studies investigating the association between SF introduction and food allergy which were not eligible for meta-analysis

| **Study** | **Design** | **Outcome** | | **Age** | | **N/n** | | **Data** | | **Measure** | | **SF timing with no FA** | **SF timing with FA** | | **P** |
| --- | --- | --- | --- | --- | --- | --- | --- | --- | --- | --- | --- | --- | --- | --- | --- |
| Kim, 2011 ([23](#_ENREF_23)) | PC | FA | | 1 | | 1177/61 | | Categorical (>6months) | | Adj.OR (95%CI) | | 1.4 (0.7-2.5) | | | NS |
| Hesselmar 2010 ([1](#_ENREF_1)) | PC | FA | | 1.5 | | 184/14 | | Continuous | | Median (IQR) in months | | 4 (4) | 4 (4) | | NS |
| Oliver 2010 ([12](#_ENREF_12)) | NCC | FA | | 1 | | 93/31 | | Continuous | | Mean (months) | | 4.2 | 4.2 | | NS |
| Maskell 2011 ([28](#_ENREF_28)) | NCC | FA | | 1 | | 117/39 | | Continuous | | No significant difference in timing of solid food introduction between cases and controls | | | | | |
| DesRoches, 2010 ([13](#_ENREF_13)) | CC | Peanut allergy | 1.5 | | 403/202 | | Continuous | | Mean (SD) in months | | 4.5 (1.6) | | | 4.3 (1.8) | NS |

# Conclusion

This report summarises the results of 29 studies investigation the association between total and exclusive breastfeeding duration, timing of solid food introduction and risk of food allergy. The majority of studies were prospective cohort studies. One systematic review of a single cohort study assessing EBF duration 6 months vs 3 months found evidence of reduced parent-reported food allergy at age 1 with longer EBF duration, however this effect was not seen in objective assessment of food allergy at the same age, nor in assessment of food allergy at age 5 years. From the observational studies we found weak evidence to support an association between longer TBF duration and increased risk of food allergy, but the data were not consistent. The quality and quantity of data was limited, especially for analysis of timing of solid food introduction in relation to FA. Few studies (2 in TBF, 2 in EBF and 2 in SFI) used an objective measure of food allergy (food challenge) which might contribute to the lack of evidence. Our conclusion is similar to that drawn by the EAACI Food Allergy Anaphylaxis Group, where the authors indicated that they found no evidence to suggest that breastfeeding could be protective against risk of food allergy in children ([31](#_ENREF_31)). The available data did not suggest an association between EBF or SF and FA, and the evidence for an association between TBF and FA was weak. There is a strong, dose-response association between severity of infant eczema and risk of food allergy, and we found a similar weak association between increased TBF duration and eczema in our eczema report. Both the association between increased TBF and FA, and between increased TBF and eczema, may be influenced by mother’s decision to prolong TBF when infants develop eczema or signs of food allergy. Only a minority of studies made appropriate adjustments to the analysis dataset to account for this possibility of reverse causation. Restricted analysis, excluding subjects with outcomes during breastfeeding should be undertaken in future studies of TBF/EBF and FA or eczema.

# References

1. Hesselmar B, Saalman R, Rudin A, Adlerberth I, Wold A. Early fish introduction is associated with less eczema, but not sensitization, in infants. Acta Paediatrica. 2010;99(12):1861-7.

2. Lack G, Fox D, Northstone K, Golding J, Avon Longitudinal Study of P, Children Study T. Factors associated with the development of peanut allergy in childhood. New England Journal of Medicine. 2003;348(11):977-85.

3. Kull I, Wickman M, Lilja G, Nordvall SL, Pershagen G. Breast feeding and allergic diseases in infants-a prospective birth cohort study. Archives of Disease in Childhood. 2002;87(6):478-81.

4. Gustafsson D, Sjoberg O, Foucard T. Development of allergies and asthma in infants and young children with atopic dermatitis--a prospective follow-up to 7 years of age. Allergy. 2000;55(3):240-5.

5. Koplin J, Osborne N, Martin P, Gurrin L, Robinson M, Slaa M, et al. Does age of introduction of foods affect the risk of having egg allergy? A population-based study of an infant cohort. Allergy: European Journal of Allergy and Clinical Immunology. 2010;65:312.

6. Hikino S, Nakayama H, Yamamoto J, Kinukawa N, Sakamoto M, Hara T. Food allergy and atopic dermatitis in low birthweight infants during early childhood. Acta Paediatrica. 2001;90(8):850-5.

7. Goksor E, Alm B, Thengilsdottir H, Mollborg P, Pettersson R, Erdes L, et al. Characteristics and risk factors for doctor-diagnosed food allergy at preschool age. Allergy: European Journal of Allergy and Clinical Immunology. 2011;66:219.

8. Kemeny DM, Price JF, Richardson V, Richards D, Lessof MH. The IgE and IgG subclass antibody-response to foods in babies during the 1^st^ year of life and their relationship to feeding regimen and the development of food allergy. J Allergy Clin Immunol. 1991;87(5):920-9.

9. Milner JD, Stein DM, McCarter R, Moon RY. Early infant multivitamin supplementation is associated with increased risk for food allergy and asthma. Pediatrics. 2004;114(1):27-32.

10. Saarinen UM, Kajosaari M. Breastfeeding as prophylaxis against atopic disease: prospective follow-up study until 17 years old. Lancet. 1995;346(8982):1065-9.

11. Venter C, Pereira B, Voigt K, Grundy J, Clayton CB, Higgins B, et al. Factors associated with maternal dietary intake, feeding and weaning practices, and the development of food hypersensitivity in the infant. Pediatric Allergy and Immunology. 2009;20(4):320-7.

12. Oliver E, Grimshaw K, Scally K, Garland J, Gudgeon L, Foote K, et al. Does nutritional intake in infancy effect later development of food allergy? Allergy: European Journal of Allergy and Clinical Immunology. 2010;65:313.

13. DesRoches A, Infante-Rivard C, Paradis L, Paradis J, Haddad E. Peanut allergy: is maternal transmission of antigens during pregnancy and breastfeeding a risk factor? Journal of Investigational Allergology & Clinical Immunology. 2010;20(4):289-94.

14. Djenouhat K, Ibsaine O, Abbadi M, Berrah H. Cow's milk allergy and risk factors. Allergy: European Journal of Allergy and Clinical Immunology. 2011;66:388.

15. Fox AT, Sasieni P, du Toit G, Syed H, Lack G. Household peanut consumption as a risk factor for the development of peanut allergy. J Allergy Clin Immunol. 2009;123(2):417-23.

16. Anonymous. Cow's milk allergy in the first year of life. An Italian Collaborative Study. Acta Paediatrica Scandinavica - Supplement. 1988;348:1-14.

17. Paton J, Kljakovic M, Ciszek K, Ding P. Infant Feeding Practices and Nut Allergy over Time in Australian School Entrant Children. International Journal of Pediatrics. 2012;2012:675724.

18. Bruno G, Giampietro PG, Businco L. [Results of a multicentric study for the prevention of atopic allergy. 48 months of follow up]. Minerva Pediatrica. 1996;48(10):413-9.

19. Kramer MS, Kakuma R. Optimal duration of exclusive breastfeeding. Cochrane Database Syst Rev. 2012;8:CD003517.

20. Kajosaari M. Atopy prophylaxis in high-risk infants. Prospective 5-year follow-up study of children with six months exclusive breastfeeding and solid food elimination. Advances in Experimental Medicine & Biology. 1991;310:453-8.

21. Besednjak-Kocijancic L. Is longer exclusive breastfeeding associated with lover prevalence of asthma, atopic dermatitis and atopic sensitisation in 1 and 5-year-old Slovene children? Allergy: European Journal of Allergy and Clinical Immunology. 2010;65:311-2.

22. Arshad SH, Hide DW. Effect of environmental factors on the development of allergic disorders in infancy. Journal of Allergy & Clinical Immunology. 1992;90(2):235-41.

23. Kim J, Chang E, Han Y, Ahn K, Lee SI. The incidence and risk factors of immediate type food allergy during the first year of life in Korean infants: a birth cohort study. Pediatric Allergy & Immunology. 2011;22(7):715-9.

24. Host A. Importance of the first meal on the development of cow's milk allergy and intolerance. Allergy Proceedings. 1991;12(4):227-32.

25. Pesonen M, Kallio MJ, Ranki A, Siimes MA. Prolonged exclusive breastfeeding is associated with increased atopic dermatitis: a prospective follow-up study of unselected healthy newborns from birth to age 20 years. Clinical & Experimental Allergy. 2006;36(8):1011-8.

26. Saarinen UM, Kajosaari M, Backman A, Siimes MA. Prolonged breast-feeding as prophylaxis for atopic disease. Lancet. 1979;2(8135):163-6.

27. Matheson MC, Erbas B, Balasuriya A, Jenkins MA, Wharton CL, Tang ML, et al. Breast-feeding and atopic disease: a cohort study from childhood to middle age. Journal of Allergy & Clinical Immunology. 2007;120(5):1051-7.

28. Maskell J, Grimshaw K, King R, Oliver E, Gudgeon L, Foote K, et al. Dietary patterns in the first year of life and food allergy risk. Allergy: European Journal of Allergy and Clinical Immunology. 2010;65:46.

29. Ehlayel MS, Bener A. Duration of breast-feeding and the risk of childhood allergic diseases in a developing country. Allergy & Asthma Proceedings. 2008;29(4):386-91.

30. Kajosaari M, Saarinen UM. Prophylaxis of atopic disease by six months' total solid food elimination. Evaluation of 135 exclusively breast-fed infants of atopic families. Acta paediatrica Scandinavica. 1983;72(3):411-4.

31. de Silva D, Geromi M, Halken S, Host A, Panesar SS, Muraro A, et al. Primary prevention of food allergy in children and adults: systematic review. Allergy. 2014;69(5):581-9.
