# Supplementary material for: Diet during pregnancy and infancy and risk of allergic or autoimmune disease: A systematic review and meta-analysis
Source: PLoS Med. 2018 Feb 28;15(2):e1002507. doi: 10.1371/journal.pmed.1002507 (PMC5830033; doi:10.1371/journal.pmed.1002507)
Supplement: S1 Data — (ZIP) [file pmed.1002507.s006.zip › Review_A_reports/RHINOCONJUNCTIVITIS.docx]

**Breastfeeding, Solid Food Introduction and Rhinoconjunctivitis**

Robert J Boyle^1^, Vanessa Garcia-Larsen^2^, Despo Ierodiakonou^3^, Jo Leonardi-Bee^4^, Tim Reeves^5^, Jennifer Chivinge^6^, Zoe Robinson^6^, Natalie Geoghegan^6^, Katharine Jarrold^6^, Andrew Logan^6^, Annabel Groome^6^ , Evangelia Andreou^7^, Nara Tagiyeva-Milne^8^, Ulugbek Nurmatov^9^, Sergio Cunha^10^

^1^ Clinical Senior Lecturer, Section of Paediatrics, Imperial College London; ^2^ Post-Doctoral Research Associate & Honorary Research Fellow Royal Brompton Hospital and Harefield NHS Foundation Trust, Respiratory Epidemiology and Public Health, National Heart and Lung Institute, Imperial College London; ^3^ Post-Doctoral Research Associate, Departments of Paediatric and Respiratory Epidemiology and Public Health Group, Imperial College London. ^4^Associate Professor of Community Health Sciences, University of Nottingham; ^5^ Research Support Librarian, Faculty of Medicine, Imperial College London; ^6^ Undergraduate medical students, Imperial College London; ^7^Research Associate, Imperial Consultants; ^8^Research Fellow, University of Aberdeen; ^9^Research Fellow, University of Edinburgh; ^10^Research Associate, Respiratory Epidemiology and Public Health, National Heart and Lung Institute, Imperial College London

Imperial Consultants,

58 Princes Gate,

Exhibition Road,

London SW7 2PG

Contents

[List of Figures 3](#_Toc416344090)

[List of Tables 4](#_Toc416344091)

[1 Total breastfeeding and rhinoconjunctivitis 5](#_Toc416344092)

[1.1 Overall characteristics of studies, risk of bias and summary of results 5](#_Toc416344093)

[1.2 TBF and rhinoconjunctivitis 11](#_Toc416344094)

[1.2.1 TBF duration and risk of RC in children aged 0-4 years 11](#_Toc416344095)

[1.2.2 TBF duration and risk of RC in children aged 5-14 years 12](#_Toc416344096)

[1.2.3 TBF duration and the risk of RC in children aged 15+ years 17](#_Toc416344097)

[1.3 TBF and Atopic RC 18](#_Toc416344098)

[1.4 Data for TBF duration and RC that couldn’t be meta-analysed 18](#_Toc416344099)

[2 Exclusive breastfeeding and rhinoconjunctivitis 20](#_Toc416344100)

[2.1 Overall characteristics of studies, risk of bias and summary of results 20](#_Toc416344101)

[2.2 EBF durarion and risk of rhinoconjunctivitis 25](#_Toc416344102)

[2.3 EBF duration and risk of RC in children aged 0-4 years 25](#_Toc416344103)

[2.3.2 EBF and risk of RC in children aged 5-14 years 26](#_Toc416344104)

[2.3.3 EBF and risk of RC in children aged 15+ years 28](#_Toc416344105)

[2.4 Data for EBF duration and RC that couldn’t be meta-analysed 29](#_Toc416344106)

[3 Solid Food Introduction and Rhinoconjunctivitis 30](#_Toc416344107)

[3.1 Overall characteristics of studies, risk of bias and summary of results 30](#_Toc416344108)

[3.2 SF introduction and risk of rhinoconjunctivitis 32](#_Toc416344109)

[3.2.1 SF introduction and risk of RC in children aged 0-4 years 32](#_Toc416344110)

[3.2.2 SF introduction and risk of RC in children aged 5-14 years 32](#_Toc416344111)

[3.3 Data for SF introduction and RC that couldn’t be meta-analysed 33](#_Toc416344112)

[4 Conclusion 34](#_Toc416344113)

[5 References 35](#_Toc416344114)

# List of Figures

[Figure 1 Risk of bias in studies of TBF duration and rhino conjunctitis 11](#_Toc497230671)

[Figure 2 TBF Ever vs. Never and RC at age 0-4 years 11](#_Toc497230672)

[Figure 3 TBF ≥5-7 months vs. <5-7 months and risk of RC at age 0-4 years 12](#_Toc497230673)

[Figure 4 TBF Ever vs. Never and risk of RC at age 5-14 years 14](#_Toc497230674)

[Figure 5 TBF short duration (≥1-3 months) vs. never and risk of RC at age 5-14 years 14](#_Toc497230675)

[Figure 6 TBF medium duration (≥4-6 months) vs. never and risk of RC at age 5-14 years 14](#_Toc497230676)

[Figure 7 TBF long duration (≥7-12 months) vs. never and risk of RC at age 5-14 years 14](#_Toc497230677)

[Figure 8 TBF ≥3-4 months vs. <3-4 months and RC risk at age 5-14 years 16](#_Toc497230678)

[Figure 9 TBF ≥5-7 months vs. <5-7 months and risk of RC at age 5-14 years 16](#_Toc497230679)

[Figure 10 TBF Ever vs. Never and risk of RC at age 15+ years 17](#_Toc497230680)

[Figure 11 TBF short duration (≥1-3 months) vs. never and risk of RC at age 15+ years 17](#_Toc497230681)

[Figure 12TBF medium duration (≥4-6 months) vs. never and risk of RC at age 15+ years 18](#_Toc497230682)

[Figure 13 Risk of bias in studies of EBF duration and rhinoconjunctivitis 25](#_Toc497230683)

[Figure 14 EBF ≥0-2 months vs. <0-2 months and RC risk at age 0-4 25](#_Toc497230684)

[Figure 15 EBF ≥3-4 months vs. <3-4 months and risk of RC at age 0-4 years 26](#_Toc497230685)

[Figure 16 EBF ≥0-2 months vs. <0-2 months and risk of RC at age 5-14 years 27](#_Toc497230686)

[Figure 17 EBF ≥3-4 months vs. <3-4 months and risk of RC at age 5-14 years 27](#_Toc497230687)

[Figure 18 EBF ≥5-7 months vs. <5-7 months and risk of RC at age 5-14 years 28](#_Toc497230688)

[Figure 19 EBF ≥0-2 months vs. <0-2 months and risk of RC in children aged 15+ years 28](#_Toc497230689)

[Figure 20 EBF ≥3-4 months vs. <3-4 months and risk of RC at age 15+ years 29](#_Toc497230690)

[Figure 21 Risk of bias in studies of SF introduction and rhinoconjunctivitis 32](#_Toc497230691)

[Figure 22 SF ≥3-4 months vs. <3-4 months and RC risk at age 0-4 32](#_Toc497230692)

[Figure 23 SF ≥3-4 months vs. <3-4 months and RC risk at age 5-14 years 33](#_Toc497230693)

# List of Tables

[Table 1 Characteristics of included studies evaluating TBF duration and rhinoconjunctivitis 7](#_Toc497230694)

[Table 2 Subgroup Analyses of risk of RC and TBF ever vs. never in children aged 5-14 years 15](#_Toc497230695)

[Table 3 Studies investigating the association between TBF and RC which were not eligible for meta-analysis 19](#_Toc497230696)

[Table 4 Characteristics of included studies evaluating EBF duration and rhinoconjunctivitis 22](#_Toc497230697)

[Table 5 Promotion of increased EBF duration and risk of RC 26](#_Toc497230698)

[Table 6 Characteristics of included studies evaluating SF introduction and rhinoconjunctivitis 31](#_Toc497230699)

[Table 7 Studies of SF introduction and RC which were not eligible for meta-analysis 33](#_Toc497230700)

# Total breastfeeding and rhinoconjunctivitis

## Overall characteristics of studies, risk of bias and summary of results

Table 1 describes the main characteristics of the studies that assessed total breastfeeding duration (TBF) in relation to rhinoconjunctivitis (RC) risk. A total of 1 cluster randomised controlled trial and 21 observational studies, reported the association between TBF and RC. Of these, 16 were prospective cohort studies, 5 cross-sectional studies. The majority of studies (n=17) are from Europe – others are from North America (n=2) and Japan (n=2). Overall, valid data on TBF duration in the first 2 years of life and RC risk were available from over 200,000 subjects. Information on RC was obtained solely from a medical assessment in 8 studies, and mainly via parental report in 13 studies. With regards to time of outcome diagnosis, 4 studies explored the association between TBF duration and RC at age 0-4, where allergic RC can be most difficult to distinguish from infectious RC, 10 at ages 5-14, and 7 at ages up to and beyond 15 years. Four studies used an interview, one parent diaries, and one a medical records review; in one study the method of exposure assessment was unclear and in all others a questionnaire method was used.

Risk of bias was assessed using the NICE Methodological checklists for cohort and case-control studies. Figure 1 illustrates the distribution of bias across the five main methodological areas of the studies. Over half of studies had a high risk of bias, most commonly due to lack of adjustment for confounding bias i.e. no adjusted data presented, but also in some cases due to high risk of selection bias. Risk of conflict of interest was generally assessed as low.

Where data were available, five levels of comparison were used to assess the risk of RC according to TBF duration, namely ‘ever vs. never’, ‘≥1-2 months vs. <1-2 months’, ‘≥3-4 months vs. <3-4 months’, ‘≥5-7 months vs. <5-7 months’, and ‘≥8-12 months vs. <8-12 months’.

*Main Findings*

In the single intervention trial there was no evidence of a relationship between breastfeeding promotion and risk of allergic rhinoconjunctivitis. For observational studies, across all cut-offs where data were available, there was no clear evidence of a relationship between risk of RC and initiation or prolongation of BF. In general there were only small numbers of studies with available data for individual meta-analyses, although the number of participants in some of the relevant studies was large. For RC at age 0-4 there was a trend to reduced risk with TBF ever and ≥5-7 months, but this was not statistically significant. For RC at older ages the large study sizes mean that we can be more confident of having excluded at least a large reduction in RC risk with increased initiation or duration of TBF.

Table 1 Characteristics of included studies evaluating TBF duration and rhinoconjunctivitis

| **First Author & Publication Year** | **N/n cases** | **Design** | **Country** | **Exposure assessment** | **Method of outcome assessment** | **Age at outcome (years)** | **Population characteristics** |
| --- | --- | --- | --- | --- | --- | --- | --- |
| Kramer, 2001 ([1](#_ENREF_1)); Kramer, 2007 ([2](#_ENREF_2)) | 8865/8181 | Cluster RCT | Belarus | - | SPT-Aero | 6.5 | Breastfeeding promotion program based on the WHO/UNICEF baby friendly hospital initiative, versus standard local breastfeeding policies |
| Kull, 2002 ([3](#_ENREF_3)) | 3790/262 | PC | Sweden | Q | Parent reported RC symptoms | 2 | BAMSE study. Population based cohort of children born between 1994-1996 |
| Butland, 1997 (BCS58 and BCS70) ([4](#_ENREF_4)) | 20582/3631 | PC | UK | I | Parent reported current AR | 16 | British Cohort Study (two samples): infants born in England, Wales, and Scotland in 1958 and 1970 |
| Taylor, 1983 (BSC70) ([5](#_ENREF_5)) | 10781/452 | PC | UK | I | Parent reported RC | 5 | CHES study. Population based cohort of children born in England, Scotland, and Wales in 1970 |
| Burr, 1993 ([6](#_ENREF_6)) | 453/117 | PC | UK | Q | Parent reported AR | 7 | Infants with family history of allergy born in 1982 |
| Businco, 1987 ([7](#_ENREF_7)) | 244/3 | PC | Italy | I | Physician assessment | 8 | Infants of atopic parents recruited from hospital and born in 1985-1988 |
| Larsson, 2008 ([8](#_ENREF_8)) | 4779/573  (in 2000), 975  (in 2005) | PC | Sweden | Q | DD; ISAAC - current AR | 9 | DBH study. Preschool children aged 1–6 years surveyed in 2000 and 2005 |
| Devereux, 2006 ([9](#_ENREF_9)) | 1253/54 | PC | UK | Q | ISAAC | 5 | Population based birth cohort of infants born in 1998 |
| Virtanen, 2010 ([10](#_ENREF_10)) | 1288/185 | PC | Finland | Q | Modified ISAAC questionnaire | 5 | DIPP study. Infants at high risk (HLA) for TIDM born between 1996-2004 invited to the allergy study 1998-2000 |
| Farooqi, 1998 ([11](#_ENREF_11)) | 1453 | PC | UK | R | DD | 16 | Representative sample of general practice born in 1975-84 |
| Gruskay 1982 ([12](#_ENREF_12)) | 908/22 | PC | USA | Unclear | Parent reported AR | 3, 5, 15 | Children born in 1961-1966 seen in a private pediatric practice |
| Marini, 1996 ([13](#_ENREF_13)) | 359/ | PC | Italy | Q | Physician assessment | 3 | Infants with family history of allergy who participated in an allergy prevention program |
| Miskelly, 1988 ([14](#_ENREF_14)) | 468/204 | PC | UK | D | Physician assessment | 1 | Infants from antenatal clinics with family history of allergy enrolled in a dietary intervention trial |
| Strachan, 1996 ([15](#_ENREF_15)) | 11765/1932 | PC | UK | I | DD | 16 | Population based cohort born in 1975 |
| Hide, 1981 ([16](#_ENREF_16)) | 843/198 | PC | UK | D/Q | Parent reported rhinitis symptoms | 1 | Isle of Wight study: infants born in 1977-1978 |
| Wright, 1994 ([17](#_ENREF_17)) | 747/313 | PC | USA | Q | Parent reported AR | 6 | Tuscon Children's Respiratory Study: recruited from local health maintenance organisation born in 1980-1984 |
| Bergmann, 2000; Kulig, 2000([18](#_ENREF_18), [19](#_ENREF_19)) | 587/88 | PC | Germany | Q/I | Physician assessment; Parent reported AR ever | 6, 7 | MAS study. Atopic risk enriched cohort of infants born in 1990 in 5 German cities |
| Björkstén, 2011 ([20](#_ENREF_20)) | 103716/unclear | CS | Worldwide | Q | ISAAC - AR ever | 7 | ISAAC Phase 3: Schoolchildren age 6-7 from different countries |
| Miyake, 2003 ([21](#_ENREF_21)) | 5614/1340 | CS | Japan | Q | ISAAC - current AR | 15 | 12-15 year old children from all public junior high schools in Suita, Japan |
| Karino, 2008 ([22](#_ENREF_22)) | 9615/4038 | CS | Japan | Q | DD | 18 | University students aged 18–19 years enrolled from 2003-2005 |
| Kurt, 2007 ([23](#_ENREF_23)) | 25843/4618 | CS | Turkey | Q | Parent reported current AR | 6-15 | Prevalence and Risk Factors of Allergies in Turkey. Representative sample of children aged 6-15 |
| Selcuk, 1997([24](#_ENREF_24)) | 5412/271 | CS | Turkey | Q | Parent reported RC ever | 7-12 | Children aged 7-12 at 18 primary schools |

Q: questionnaire, I: interview, R: medical records, D: diary, PC: prospective cohort, NCC: nested case control, CS: Cross-sectional, CC: case control, DD: doctor diagnosis of RC, in contrast to ‘Physician assessment’ where RC diagnosis was always made by a study physician as part of the study protocol.

Figure 1 Risk of bias in studies of TBF duration and rhino conjunctitis

## TBF and rhinoconjunctivitis

The single intervention trial of a breastfeeding promotion intervention was rated as having a low risk of bias on all domains, and a low risk of conflict of interest. Kramer found no significant difference in odds of ever having hayfever by the age of 6.5 years – cluster adjusted odds ratio 1.1 (95% CI 0.6, 1.9), or hayfever in the past 12 months at the same age OR 1.0 (0.6, 1.8). All other evidence was derived from observational studies.

### TBF duration and risk of RC in children aged 0-4 years

#### TBF Ever vs. Never

Figure 2 shows the outcomes of 3 eligible observational studies including over 600 children with RC. The data show reduced odds of RC in breastfed infants, but do not reach statistical significance. There is no statistical heterogeneity (I^2^=0%). All three studies are prospective cohort studies – the study of Marini reported adjusted data at age 3 and had overall unclear risk of bias due to unclear assessment bias; the other two studies reported unadjusted data at age 1, and are therefore at high risk of confounding bias. Thus the data do not show strong evidence of a relationship between breastfeeding ever and risk of RC.

Figure 2 TBF Ever vs. Never and RC at age 0-4 years


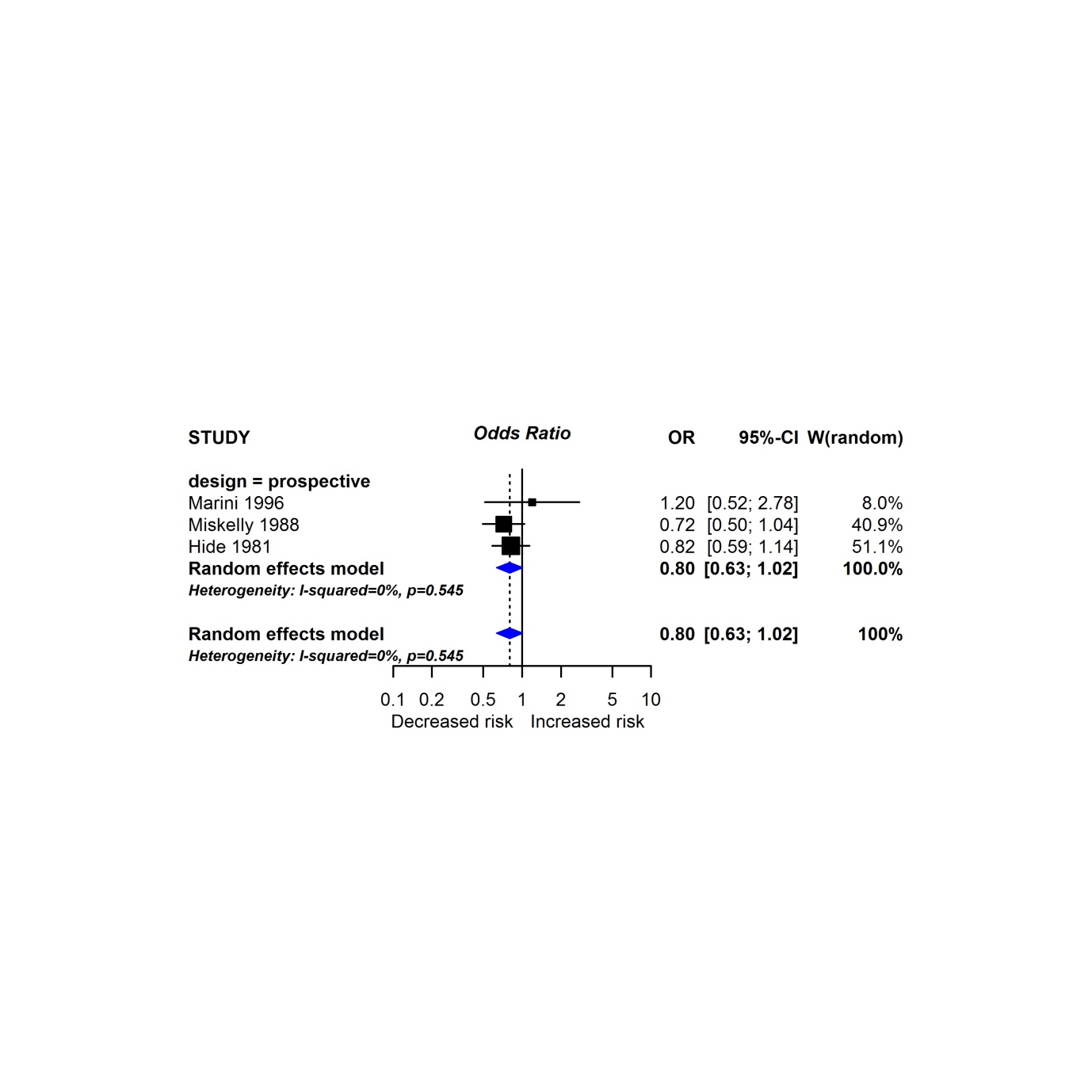


#### TBF ≥5-7 months vs. <5-7 months

Two prospective cohort studies reported data that could be used to calculate OR for RC at age 0-4 with TBF ≥ 6 months compared to <6 months and found reduced odds of RC with longer breastfeeding duration, which did not reach statistical significance. There was no statistical heterogeneity (I^2^=0%). The study of Hide assessed infants at 1 year and reported unadjusted data, so carries a high risk of bias. The study of Kull assessed infants at 2 years and reported adjusted data – there was unclear risk of assessment bias but low risk of bias on other parameters.

Figure 3 TBF ≥5-7 months vs. <5-7 months and risk of RC at age 0-4 years


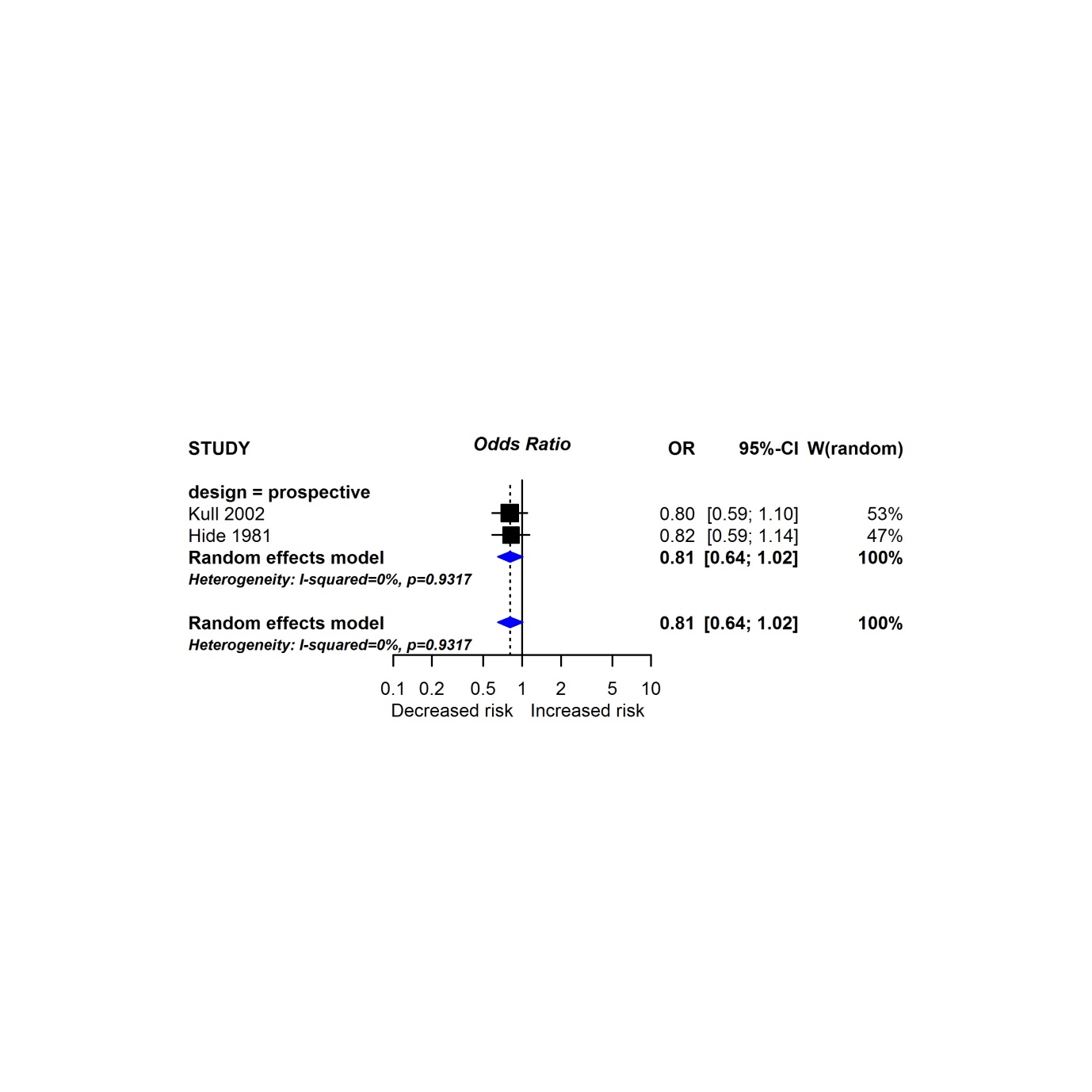


### TBF duration and risk of RC in children aged 5-14 years

#### TBF Ever vs. Never

Four prospective cohort studies and two cross-sectional surveys reported data that could be used to calculate OR for RC at age 5-14 with TBF ever vs. never and found no association between breastfeeding duration and odds of RC (Figure 4). There was high statistical heterogeneity (I^2^=72.2%). The studies of Selcuk and Bjorksten reported adjusted data and were judged to be at low overall risk of bias. Other studies reported unadjusted data and were therefore at high risk of confounding bias. Subgroup analyses (Table 2) did not identify a clear explanation for the statistical heterogeneity between studies, but there was no heterogeneity between the two studies reporting adjusted data.

Analysis of ‘dose response’ using a reference group of ‘never BF’ with comparison groups of short TBF (≥1-3; Figure 5), medium TBF (≥4-6; Figure 6) and long TBF (≥7-12; Figure 7) were limited by small numbers of eligible studies reporting only unadjusted data, and did not show evidence of a dose response relationship.

Figure 4 TBF Ever vs. Never and risk of RC at age 5-14 years


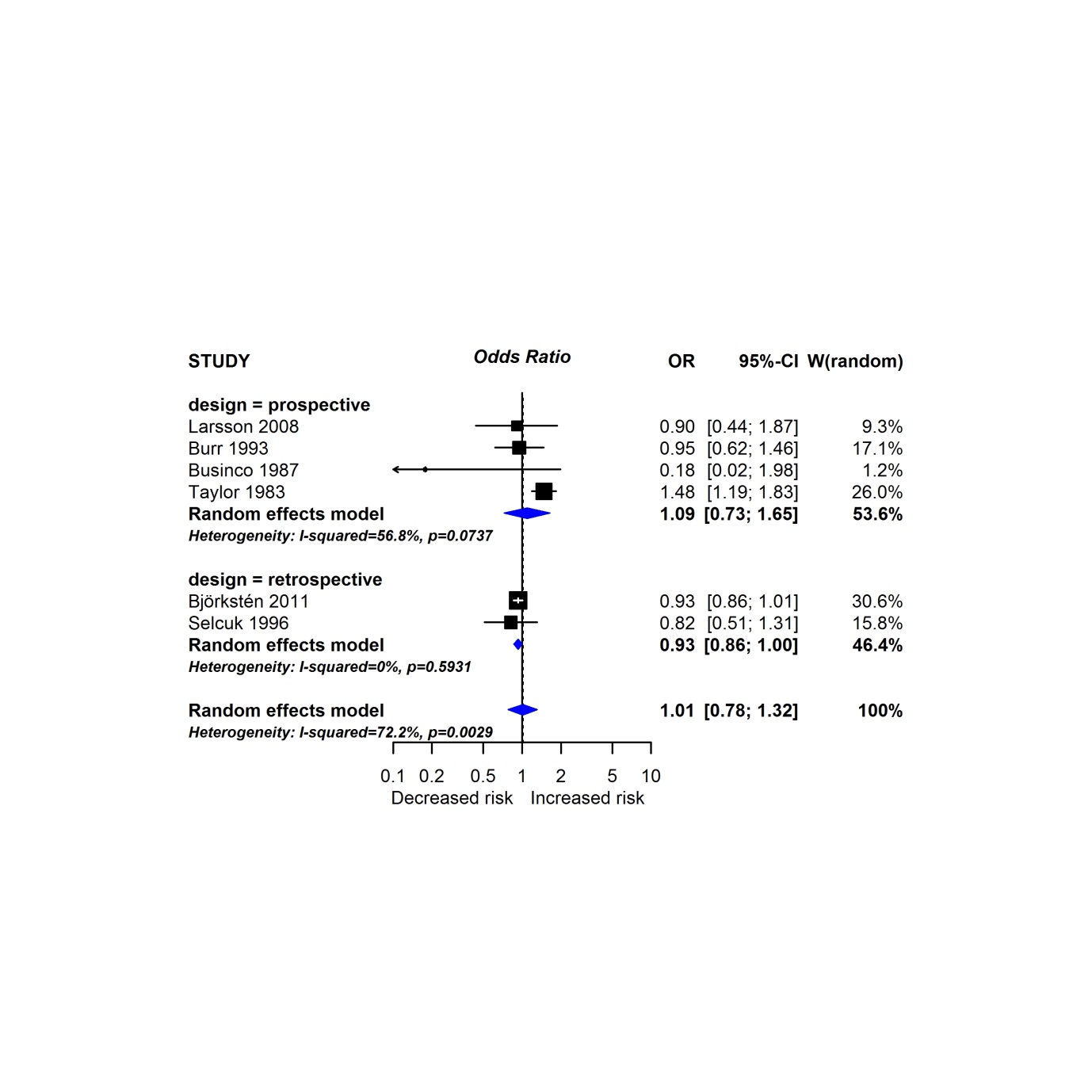


Figure 5 TBF short duration (≥1-3 months) vs. never and risk of RC at age 5-14 years


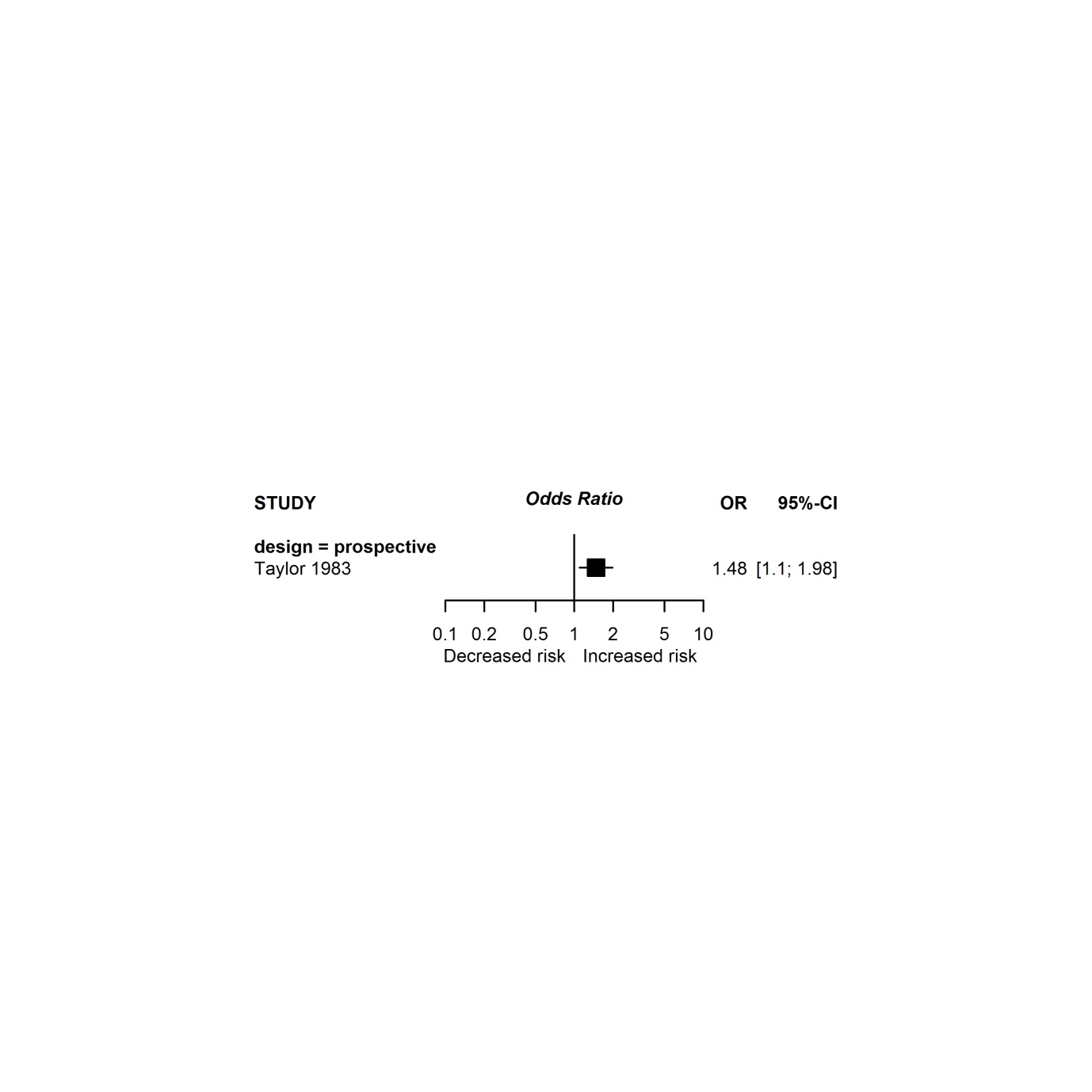


Figure 6 TBF medium duration (≥4-6 months) vs. never and risk of RC at age 5-14 years


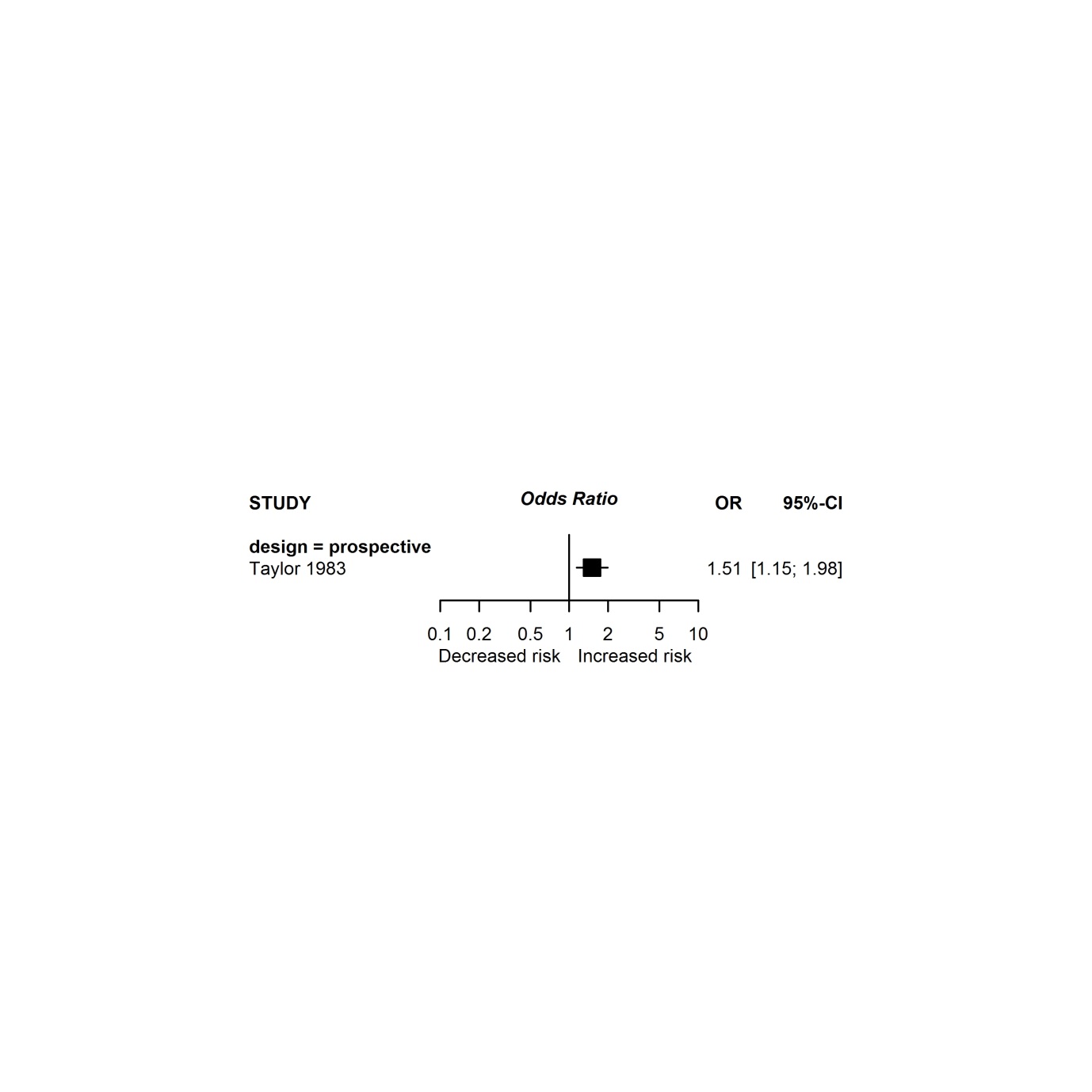


Figure 7 TBF long duration (≥7-12 months) vs. never and risk of RC at age 5-14 years


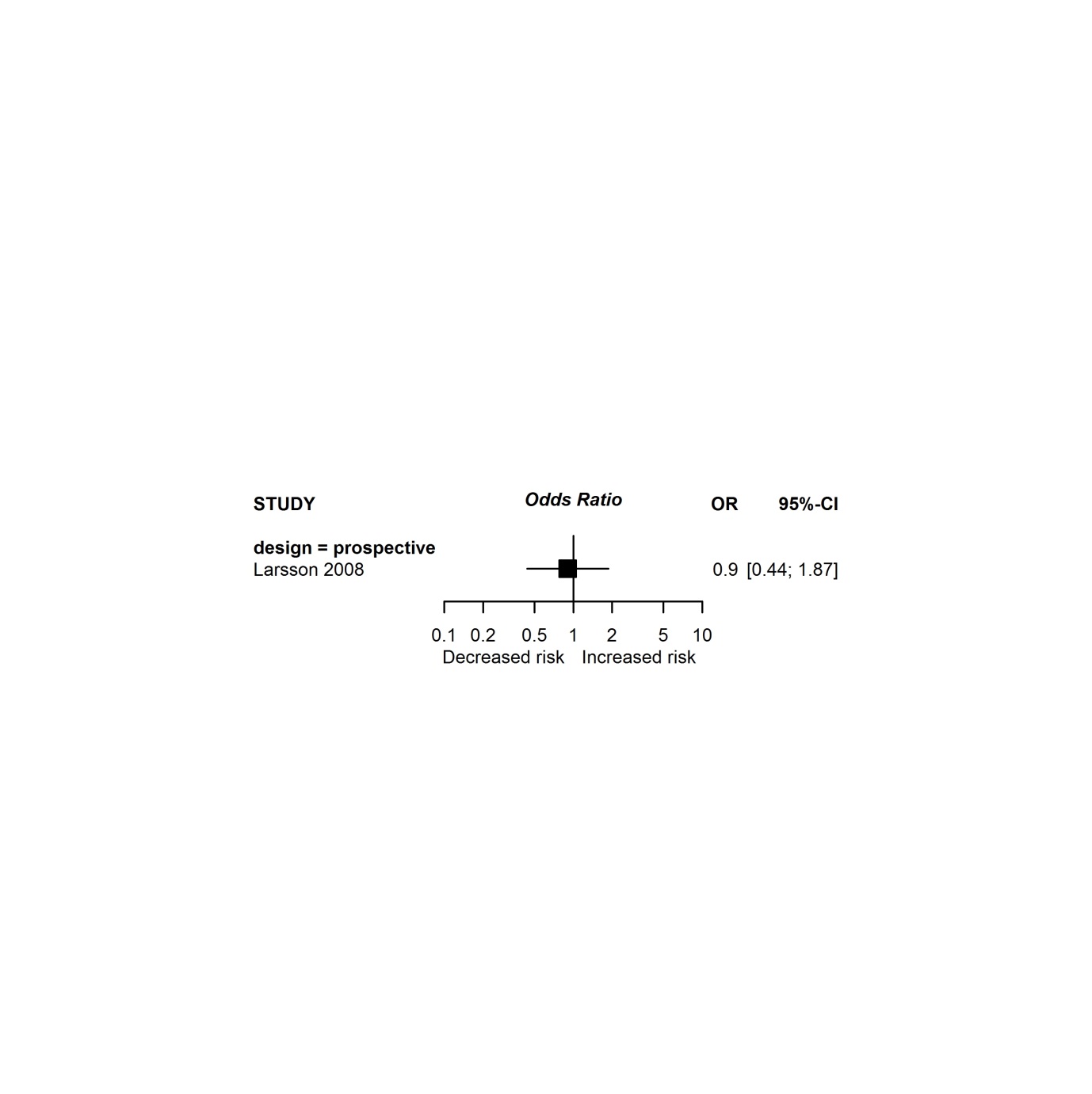


Table 2 Subgroup Analyses of risk of RC and TBF ever vs. never in children aged 5-14 years

|  | Number of studies | OR [95% CI] | I^2^ (%) | P-value for between groups difference |
| --- | --- | --- | --- | --- |
| Overall (if adjusted NA, unadjusted value used)  Adjusted  Unadjusted |  |  |  |  |
|  | 2 | 0.93 [0.86; 1.00] | 0.00 | Not tested |
|  | 4 | 1.10 [0.73; 1.65] | 56.8 |  |
| Study Design – Prospective  Study Design – Retrospective | 4  2 | 1.00 [0.62; 1.62]  0.93 [0.86; 1.00] | 58.6  0.00 | 0.44 |
| Risk of disease – High  Risk of disease – Normal | 2  4 | 0.64 [0.16; 2.59]  1.00 [0.76; 1.43] | 44.7  81.3 | 0.50 |
| Risk of bias – Low  Risk of bias – High/Unclear | 4  2 | 1.00 [0.76; 1.43]  0.64 [0.16; 2.59] | 81.3  44.7 | 0.50 |

#### TBF ≥3-4 Months vs. <3-4 months

One prospective cohort study reported data that could be used to calculate an OR for this comparison, shown in Figure 8. Unadjusted data, with a high risk of confounding bias, showed no significant association between TBF duration and risk of RC at age 5-14.

Figure 8 TBF ≥3-4 months vs. <3-4 months and RC risk at age 5-14 years


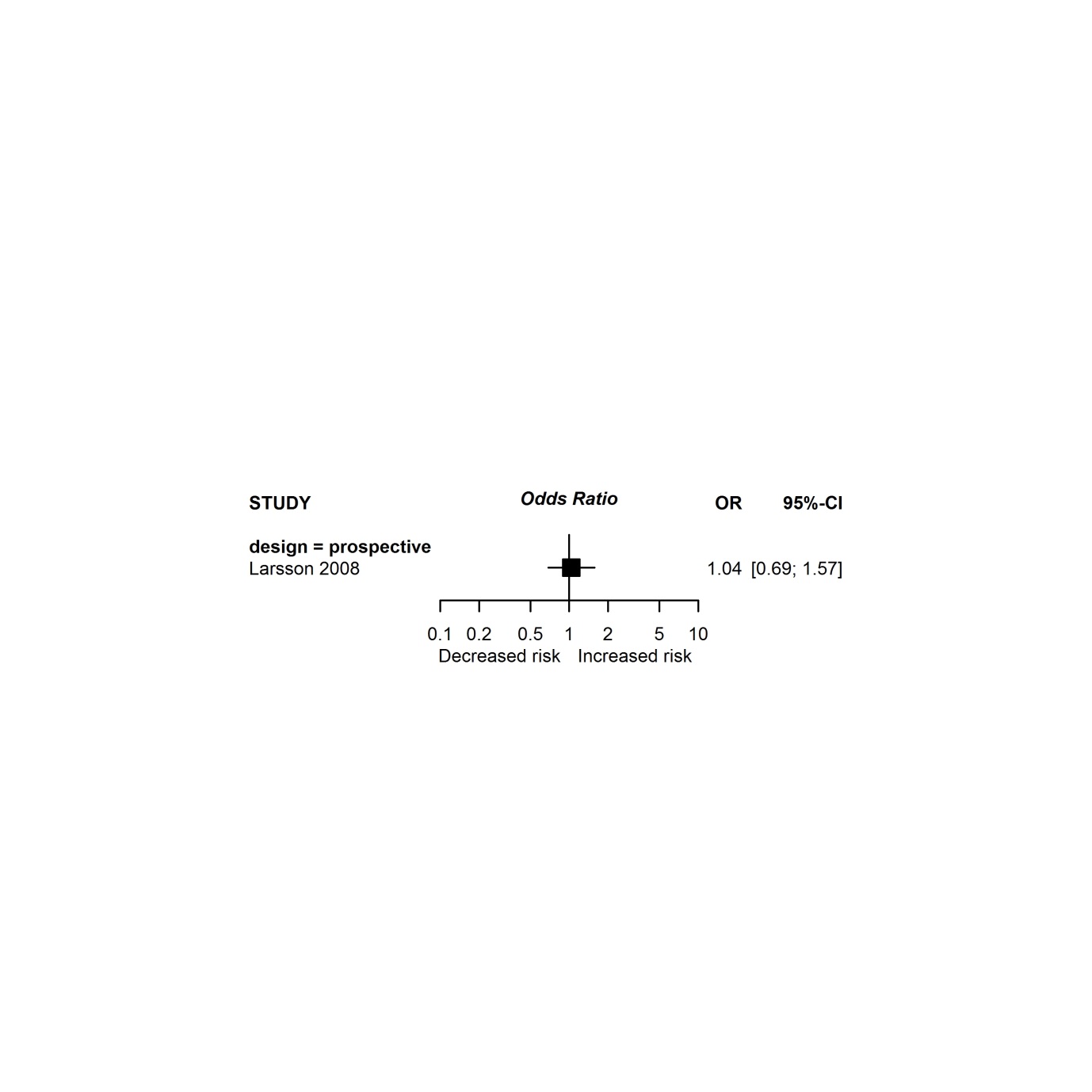


#### TBF ≥5-7 Months vs. <5-7 months

One prospective cohort study reported data that could be used to calculate an OR for this comparison, shown in Figure 9. Adjusted data showed no significant association between TBF duration and risk of RC at age 5-14. There was a high risk of selection bias due to loss to follow up of 33% of participants when assessed at age 6 years.

Figure 9 TBF ≥5-7 months vs. <5-7 months and risk of RC at age 5-14 years


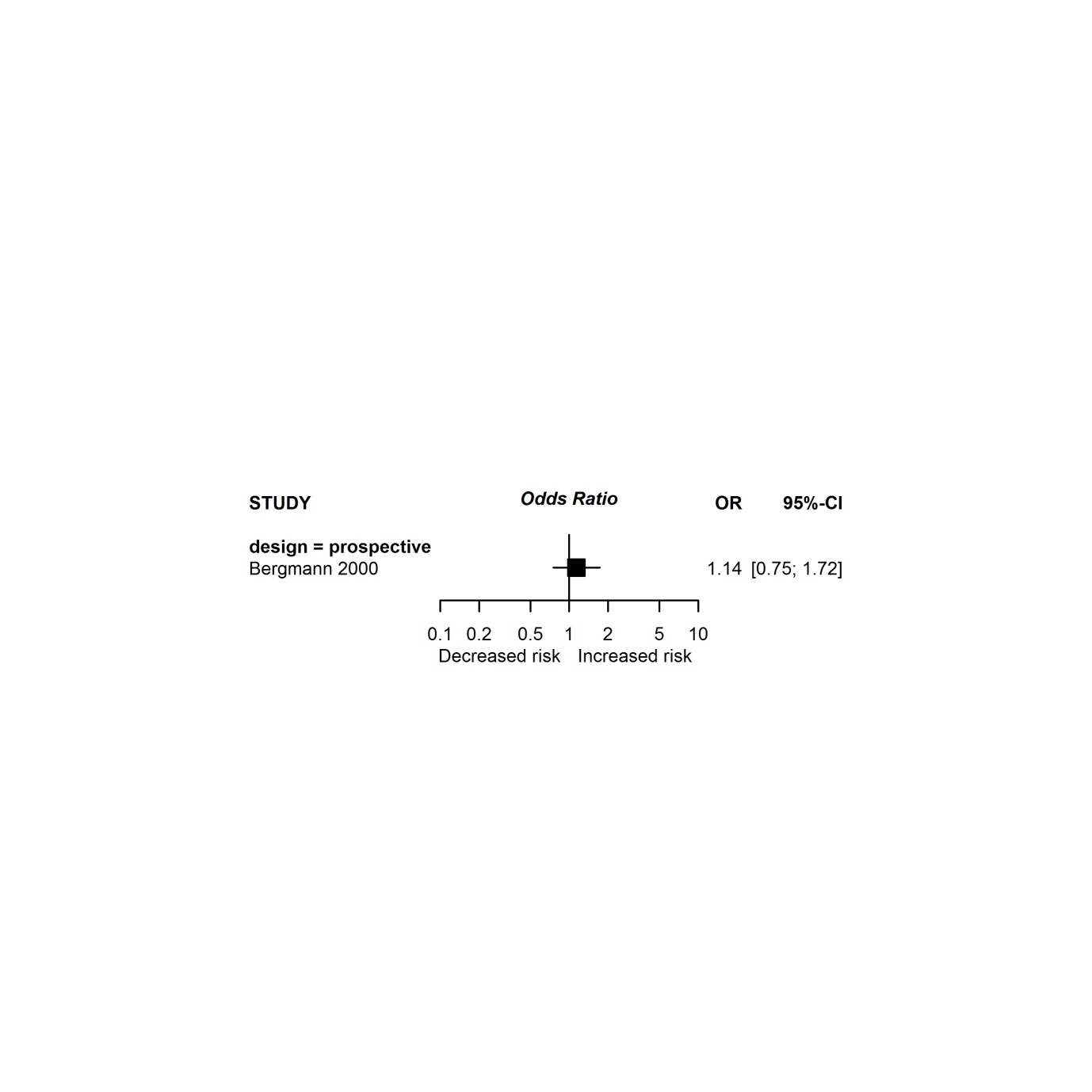


### TBF duration and the risk of RC in children aged 15+ years

#### TBF Ever vs. Never

Two cross-sectional and four prospective cohort studies reported data that could be used to calculate an OR for this comparison. Pooled data showed increased risk of RC in breastfed children at age 15+, but this did not reach statistical significance and there was high statistical heterogeneity (Figure 10). The cross-sectional studies of Miyake and Kurt, and the prospective study of Butland, reported adjusted data. The studies of Strachan and Farooqi reported unadjusted data and were therefore at high risk of confounding bias. It was not possible to identify a clear cause for the statistical heterogeneity seen in this analysis. Analysis of ‘dose response’ using a reference group of ‘never BF’ with comparison groups of short TBF (≥1-3; Figure 11) and medium TBF (≥4-6; Figure 12) were limited by small numbers of eligible studies and did not show evidence of a dose response relationship.

#### Figure 10 TBF Ever vs. Never and risk of RC at age 15+ years


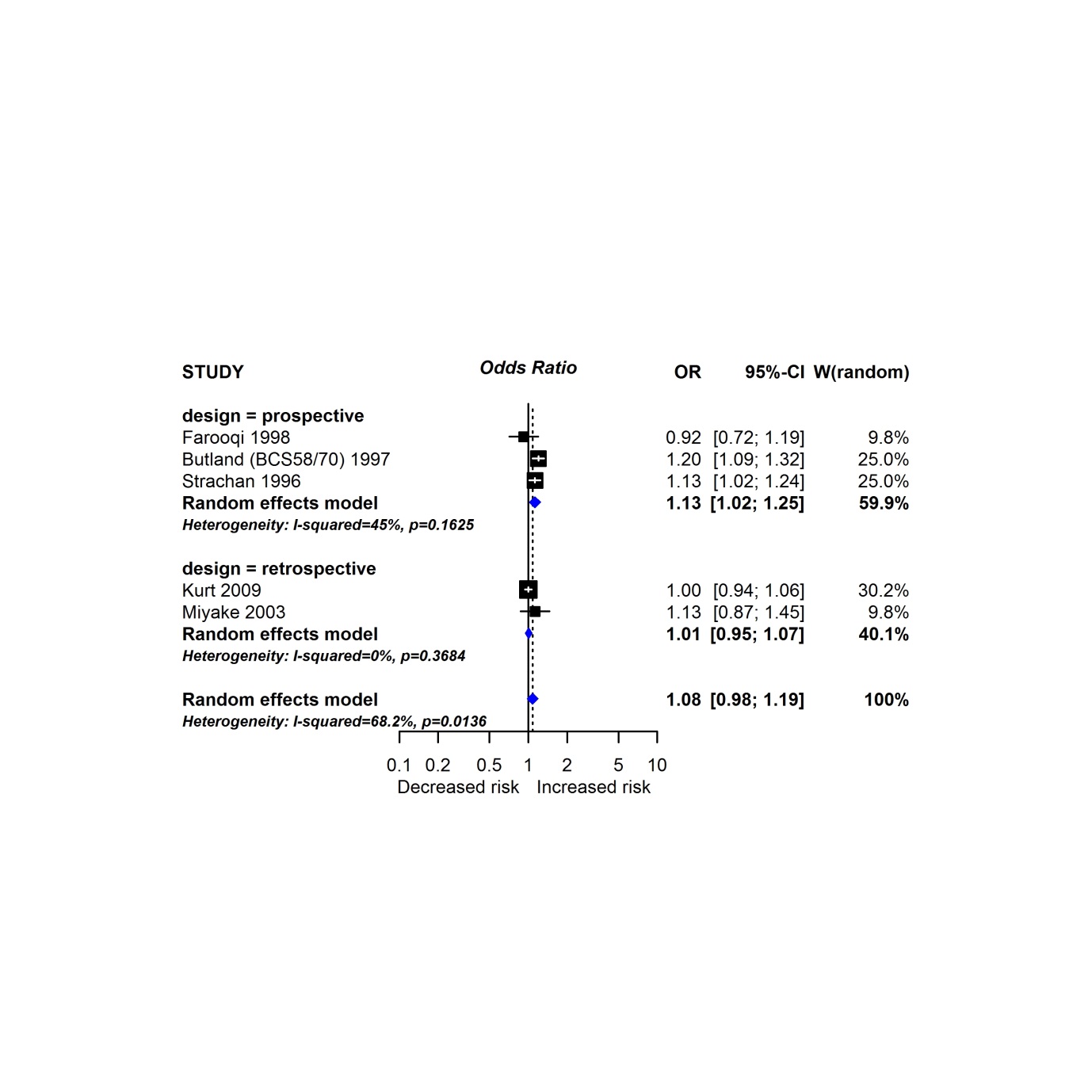


Figure 11 TBF short duration (≥1-3 months) vs. never and risk of RC at age 15+ years


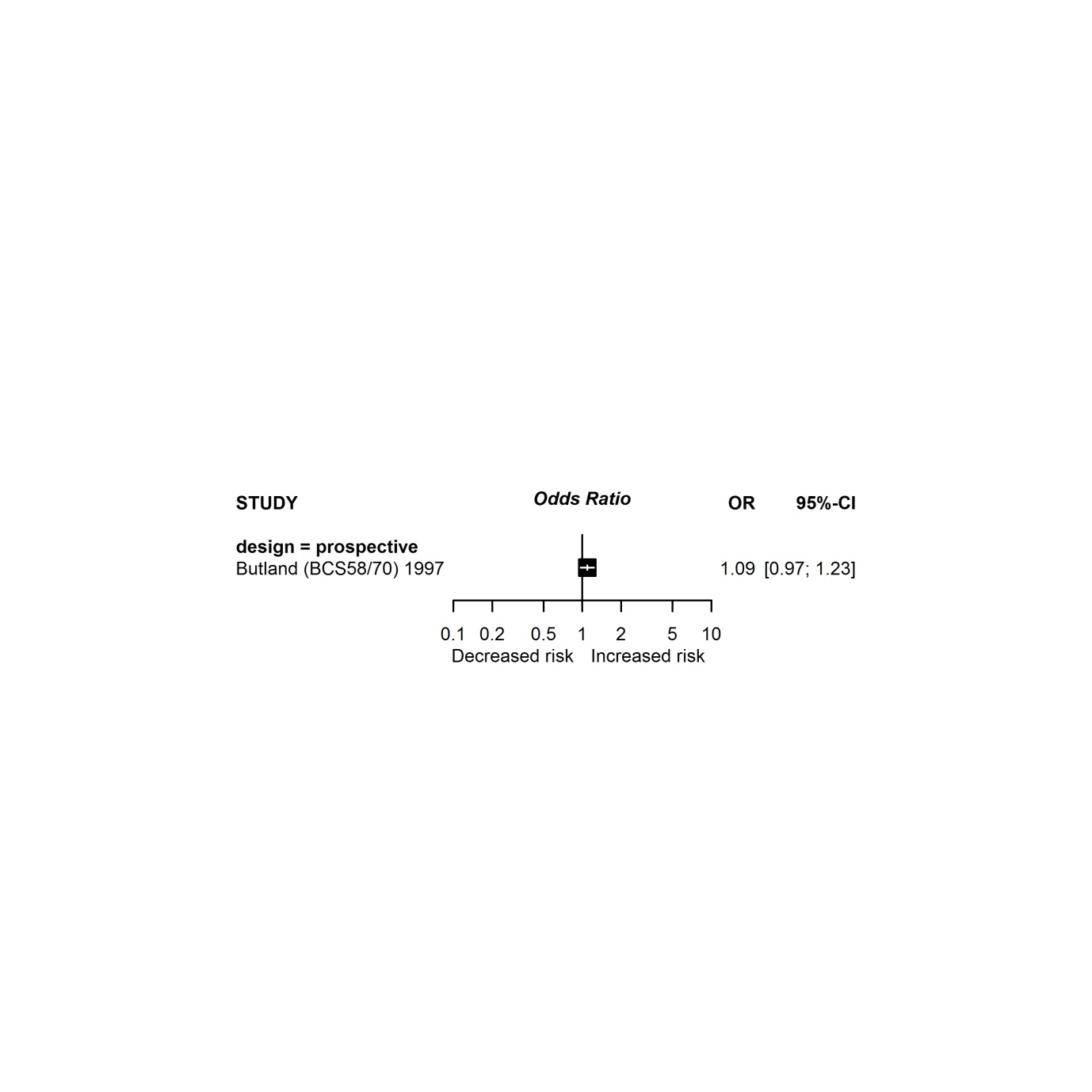


Figure 12TBF medium duration (≥4-6 months) vs. never and risk of RC at age 15+ years


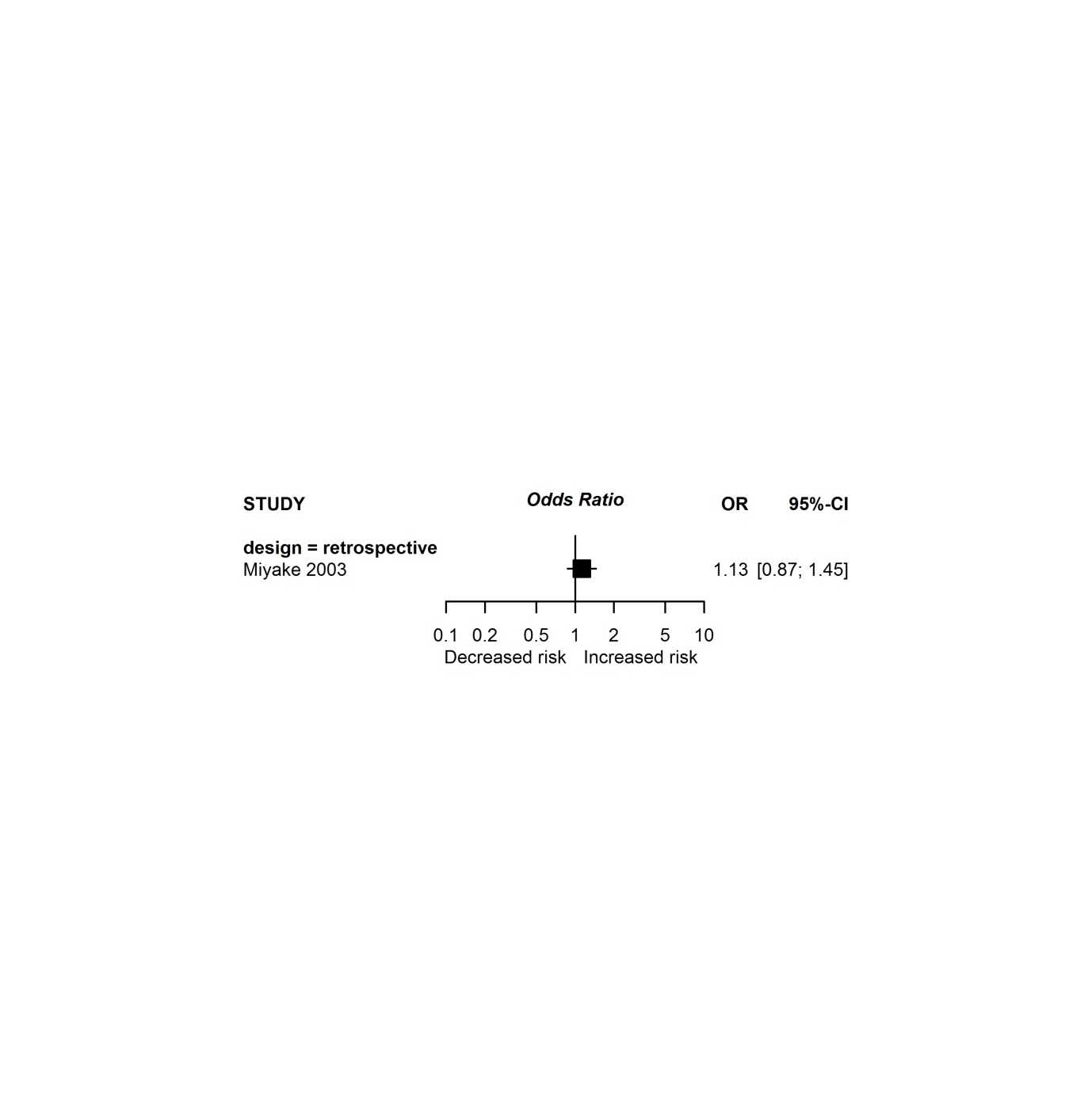


## TBF and Atopic RC

We also assessed the outcome ‘atopic RC’, where participants have the outcome if they are reported as having RC, and also have evidence of specific allergic sensitisation through skin prick or sIgE testing. This may be a more reliable measure of allergic rhinoconjunctivitis than RC alone, since infective rhinitis and conjunctivitis can be difficult to distinguish from allergic forms especially when patient or parent report is used for outcome assessment.

A single study was identified which reported TBF and atopic RC data that could be used to calculate ORs. In the study of Gruskay 6 of 782 formula fed children had RC at age 3, compared with none of 126 children who had been breastfed. At age five OR 3.51 (0.63, 19.61), at age 15 OR 1.71 (0.18, 15.80) there was no association found between TBF and atopic RC.

## Data for TBF duration and RC that couldn’t be meta-analysed

Meta-analyses included 10 studies, reporting data on at least 13,000 participants with RC. A further 5 studies reported relevant data which could not be reported in meta-analysis, in relation to at least 4500 participants with RC. These studies are summarised in Table 3. In all studies and analyses reported there was no association found.

Table 3 Studies investigating the association between TBF and RC which were not eligible for meta-analysis

| Study | Design | Age | N/n | Data | Measure | Outcome (s) | P |
| --- | --- | --- | --- | --- | --- | --- | --- |
| Wright, 1994 ([17](#_ENREF_17)) | PC | 6 | 747/313 | Continuous | No significant difference between groups in breastfeeding duration | | |
| Kulig, 2000 ([19](#_ENREF_19)) | PC | 7 | 587/88 | Continuous | No significant difference between groups in breastfeeding duration (P=0.91; adjusted) | | |
| Devereux, 2006 | PC | 5 | 1253/54 | Categorical | No significant relationship between BF ever and RC (adjusted) | | |
| Virtanen, 2010 ([10](#_ENREF_10)) | PC | 5 | 1288/185 | Categorical | aHR (95%CI) 1.1 (0.6-1.9) for TBF <5 vs. >9.5 months | | |
|  |  |  |  | Categorical | aHR (95%CI) 1.1 (0.6-1.6) for TBF 5-9 vs. >9.5 months | | |
| Karino, 2008 ([22](#_ENREF_22)) | CS | 18 | 9615/4038 | Categorical | BF duration not associated with RC when classified as never/ever; more/less than 1 month, 3 months or 6 months (unadjusted) | | |

# Exclusive breastfeeding and rhinoconjunctivitis

## Overall characteristics of studies, risk of bias and summary of results

Table 4 describes the main characteristics of the studies analysed in this report. A total of 1 systematic review which included 1 RCT analysed as a cohort study and 1 other prospective cohort study, and 13 observational studies, reported the association between duration of exclusive breastfeeding (EBF) and risk of RC. Of these, 10 were prospective cohort studies and 3 cross-sectional studies. Over half of the studies (n=9) are from Europe – others are from Australasia (n=4), and the Middle East (n=1). Overall, valid data on EBF duration and RC risk were available from over 29,000 subjects. Information on RC was obtained mainly from a medical assessment in 6 studies, via parental report in 8 studies. With regards to time of outcome diagnosis, 5 studies explored the association between EBF duration and RC at age 0-4, where allergic RC can be difficult to distinguish from infective RC, 5 evaluated RC at age 5-14, and 4 at age 15+. Most studies used a questionnaire to assess the exposure (EBF), one study used a diary and three used an interview.

Risk of bias for the observational studies is shown in Figure 13. Over half of studies had a high risk of bias, due to lack of adjustment for confounding bias i.e. no adjusted data presented. Risk of conflict of interest was generally assessed as low. All included studies reported data which could be analysed as odds ratios and presented using forest plots.

Where data were available, three levels of comparison were used to assess the risk of RC according to EBF duration, namely ≥0-2 months vs. <0-2 months; ≥3-4 months vs. <3-4 months; ≥5-9 months vs. <5-9 months.

*Main Findings*

There was no consistent evidence of an association between EBF and risk of RC. In general there were only small numbers of studies with available data for each analysis, and several analyses were restricted to studies with high risk of bias. Two prospective RCT showed inconsistent and non-significant findings. We did find some evidence from observational studies that EBF ≥3-4 months may reduce odds of RC at age 0-4 OR 0.77 (0.60, 0.99) with no statistical heterogeneity (I^2^=0%), but this was not supported by other data. One large cohort study suggested increased odds of hayfever by age 44 in those EBF ≥3 months however such an effect was not supported by shorter term observations in intervention studies or cohort studies.

Table 4 Characteristics of included studies evaluating EBF duration and rhinoconjunctivitis

| **Study** | **N/n cases** | **Design** | **Country** | **Exposure assessment** | **Method of outcome assessment** | **Age at outcome (years)** | **Population characteristics** |
| --- | --- | --- | --- | --- | --- | --- | --- |
| Kramer ([25](#_ENREF_25)) | 3584 | SR of PC nested in RCTs | Finland/Belarus | - | ISAAC – current AR | 5-7 | Study #1: Infants of atopic parents  Study #2: Healthy, term, breastfed newborns participating in a breastfeeding promotion trial. |
| Kajosaari, 1991([26](#_ENREF_26)) | 135 | PC | Finland |  | Parent report of pollen allergy | 5 | Solid food introduction at 6 months versus 3 months, in exclusively breastfed infants |
| Kellberger, 2012 ([27](#_ENREF_27)) | 3785/314 | PC | Germany | Q | ISAAC - ever AR | 9-18 | SOLAR. Community based random sample of all pupils aged 9-11 years in 1995-1996 as part of ISAAC study, with follow-up survey 2002-2003 |
| Kull, 2002 ([3](#_ENREF_3)) | 3791/263 | PC | Sweden | Q | Parent reported RC symptoms | 2 | BAMSE. Population based cohort of children born between 1994-1996 |
| Kramer, 2009 ([28](#_ENREF_28)) | 13889/455 | PC nested in RCT | Belarus | I | ISAAC - current AR | 6.5 | PROBIT. Born 1996-1997 |
| Siltanen,2003 ([29](#_ENREF_29)) | 285/53 | PC | Finland | Q | Physician assessment | 4 | Infants recruited from maternal hospital born in 1994-1995 |
| Strachan, 1996 ([15](#_ENREF_15)) | 11765/1932 | PC | UK | I | DD | 16 | Population representative sample born in 1975 |
| Matheson, 2007 ([30](#_ENREF_30)) | 5729/2610 | PC | Australia | Q | Physician assessment | 44 | Tasmanian Asthma Study: population based cohort born in 1961 |
| Van Asperen, 1983 ([31](#_ENREF_31)) | 79/44 | PC | Australia | I | Physician assessment | 1 | Cohort recruited from medical service, born in 1980-1981 with family history of atopy |
| Arshad, 1992 ([32](#_ENREF_32)) | 1167/38 | PC | UK | D/Q | Physician assessment | 1 | Isle of Wight Study. Population based birth cohort of infants born in semi-rural areas between 1989 and 1990 |
| Marini, 1996 ([13](#_ENREF_13)) | 359 | PC | Italy | Q | Physician assessment | 3 | Infants with family history of allergy whose mother agreed to participate in an allergy prevention program |
| Erkkola, 2012 ([33](#_ENREF_33)) | 2441/359 | PC | Finland | D | ISAAC - current AR | 5 | DIPP study. Infants at high risk (HLA) for TIDM born between 1996-2004 invited to the allergy study 1998-2000 |
| Ehlayel, 2008 ([34](#_ENREF_34)) | 1278 | CS | Qatar | Q | ISAAC - AR ever | 5 | Children 0-5 years old attending primary healthcare centers for routine immunisation |
| Liu 2012 ([35](#_ENREF_35)) | 8733/397 | CS | China | Q | ATS questionnaire | 8 | Sample of children from kindergarten and elementary schools in Shenyang |
| Miyake, 2003 ([21](#_ENREF_21)) | 6845/1340 | CS | Japan | Q | ISAAC - current AR | 15 | 12-15 year old children from all public junior high schools in Suita, Japan |

Q: questionnaire, I: interview, R: medical records, D: diary, PC: prospective cohort, NCC: nested case control, CS: Cross-sectional, CC: case control, DD: doctor diagnosis of RC, in contrast to ‘Physician assessment’ where RC diagnosis was always made by a study physician as part of the study protocol.

Figure 13 Risk of bias in studies of EBF duration and rhinoconjunctivitis

## EBF duration and risk of rhinoconjunctivitis

## EBF duration and risk of RC in children aged 0-4 years

#### EBF ≥0-2 months vs. <0-2 months

One prospective cohort study reported data that could be used to calculate OR for RC in the first year, in infants with EBF for ≥2 months vs. <2 months duration and is shown in Figure 14. The study found no significant association. The data are unadjusted, so carry a high risk of bias.

Figure 14 EBF ≥0-2 months vs. <0-2 months and RC risk at age 0-4


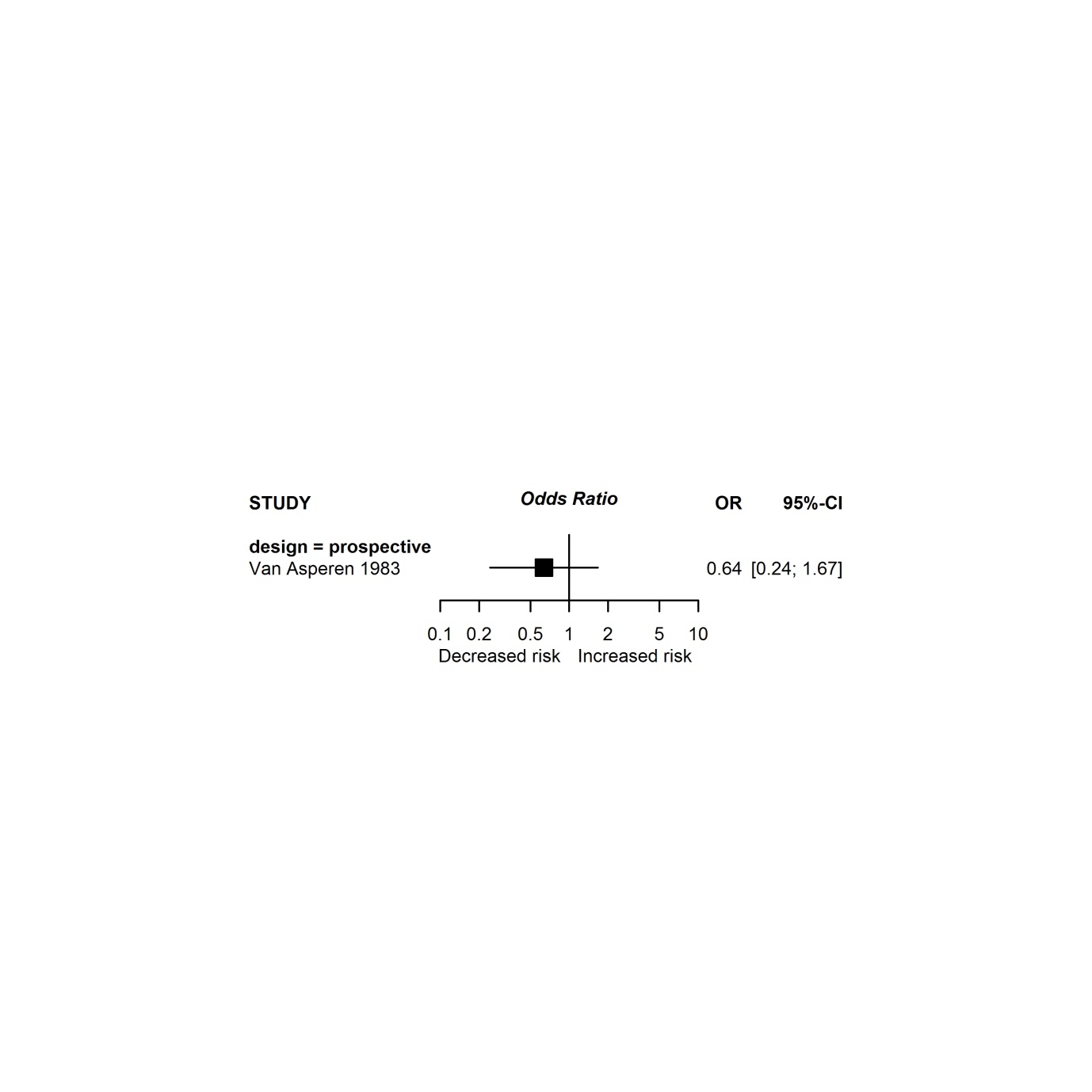


#### EBF ≥3-4 months vs. <3-4 months

Five prospective cohort studies reported data that could be used to calculate OR for RC at age 0-4, in infants with EBF for ≥3-4 months vs. <3-4 months duration and are shown in Figure 15. They show reduced odds of RC with longer EBF duration, with no statistical heterogeneity. The data from Arshad, Marini and Van Asperen are unadjusted, so carry a high risk of bias, but the data of Kull and Siltanen are adjusted.

Figure 15 EBF ≥3-4 months vs. <3-4 months and risk of RC at age 0-4 years


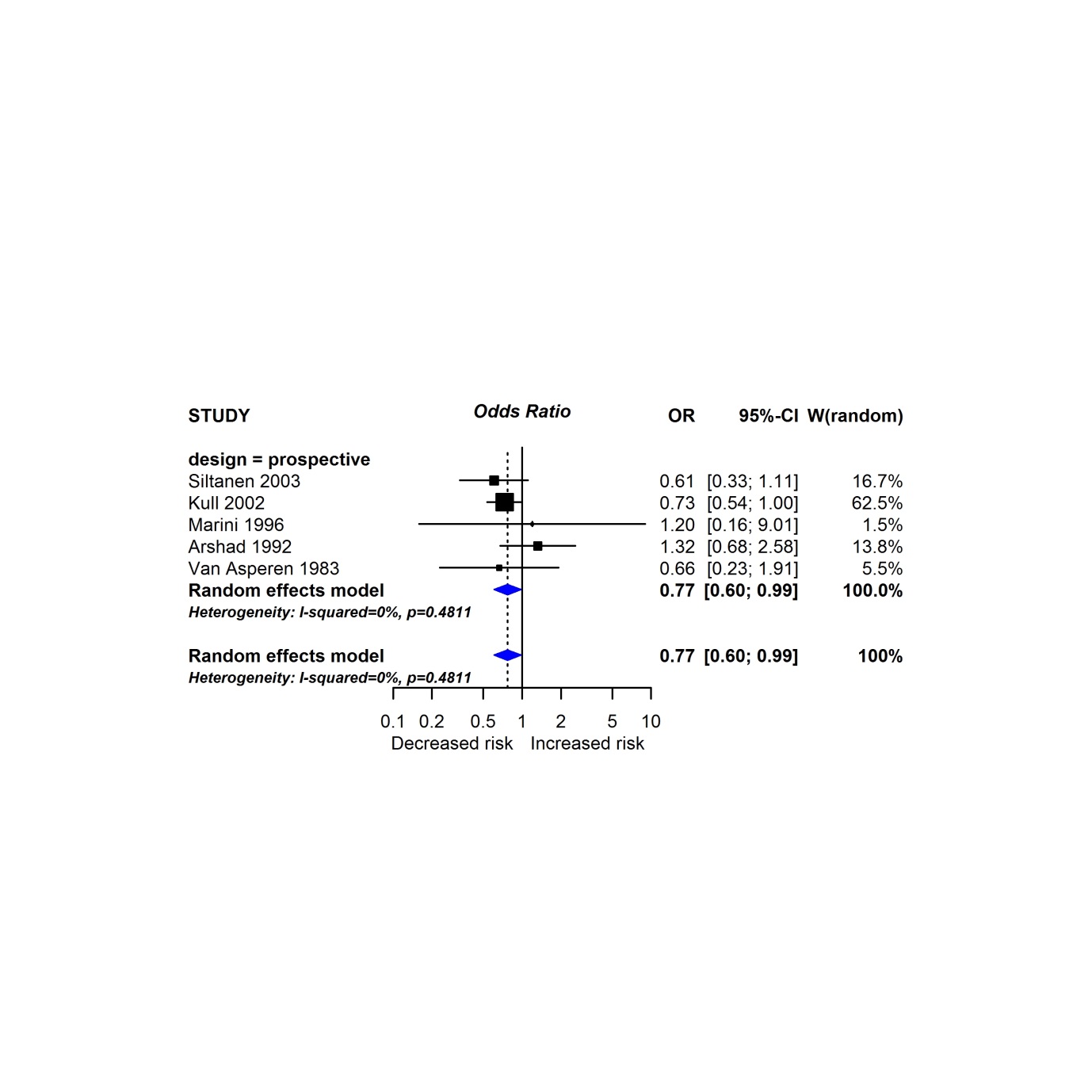


### EBF and risk of RC in children aged 5-14 years

#### Systematic reviews and intervention trials

As shown in Table 5, the single systematic review found no evidence that increased duration of exclusive/predominant breastfeeding influences risk of RC at 6.5 years, although there was heterogeneity in findings, with one small study finding borderline evidence for reduced risk of RC RR 0.53 (0.28, 1.01), and a larger study showing no evidence of association.

Table 5 Promotion of increased EBF duration and risk of RC

| **Study** | **Outcome measure** | **No. participants (studies)** | **Outcome (95% CI)** |
| --- | --- | --- | --- |
| Kajosaari 1991 ([26](#_ENREF_26)) | Pollen allergy at 5 years | 113 (1) | RR 0.5 (0.3, 1.0) |
| Kramer 2007 ([2](#_ENREF_2)) | Allergic rhinoconjunctivitis at age 6.5 years using ISAAC questionnaire | 17,046 (1) | RR 1.1 (0.6, 1.9) |

Data for Kajosaari 1991 are taken from the systematic review of Kramer 2012 ([25](#_ENREF_25)).

#### EBF ≥0-2 months vs. <0-2 months

One prospective cohort study reported data that could be used to calculate OR for RC at age 5-14, in infants with EBF for ≥0-2 months vs. <0-2 months duration and is shown in Figure 16. The study found reduced odds of RC with EBF duration of >3months vs. <1 month (Figure 18) and a trend to reduced RC with longer EBF when all 3 categories (<1 month, 1-3 months, >3 months) were considered (P=0.093). The data carry a high risk of bias because they are unadjusted.

Figure 16 EBF ≥0-2 months vs. <0-2 months and risk of RC at age 5-14 years


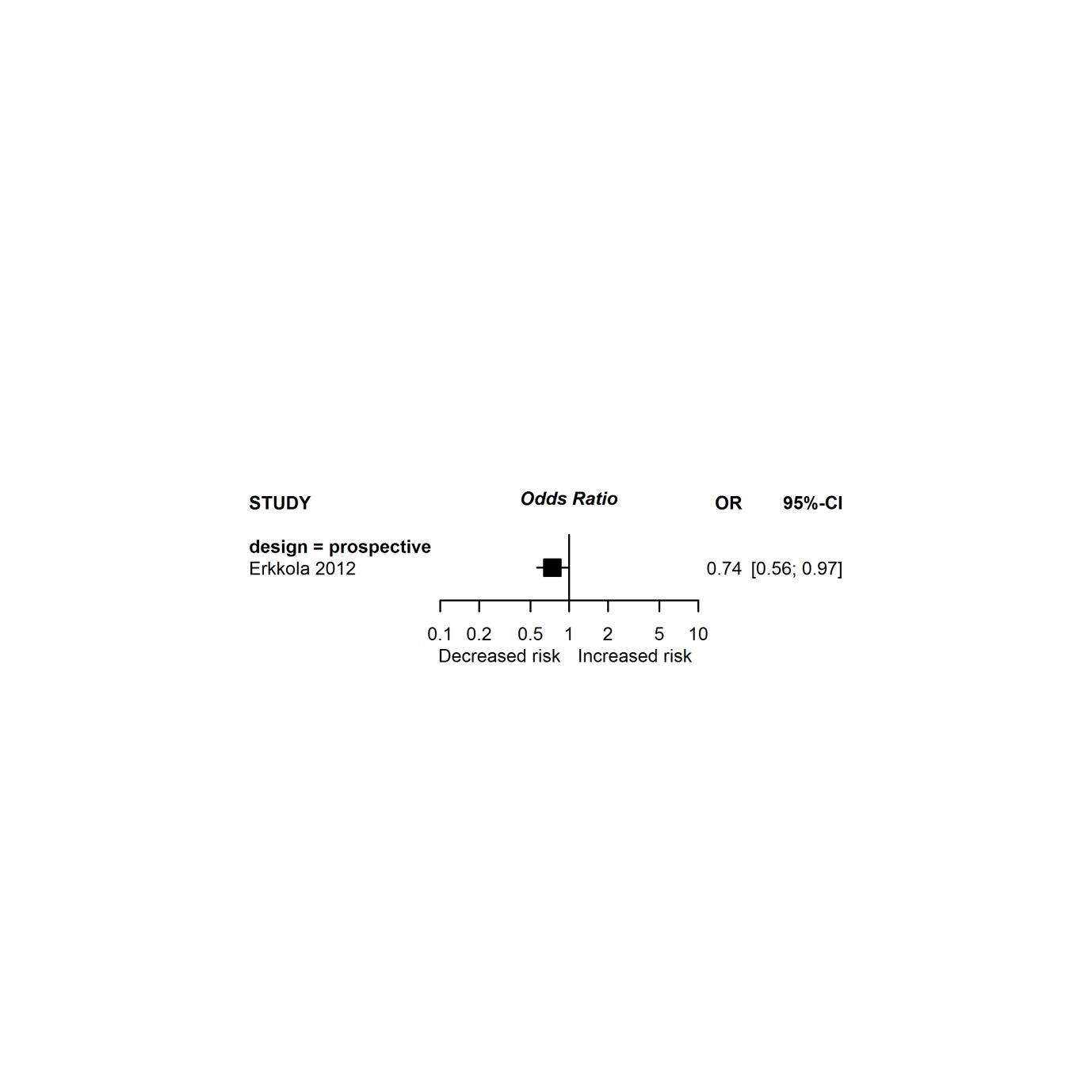


#### EBF ≥3-4 months vs. <3-4 months

Two observational studies reported data that could be used to calculate OR for RC at age 5-14, in infants with EBF for ≥3-4 months vs. <3-4 months duration are shown in Figure 17. Data were not pooled due to extreme statistical heterogeneity (I^2^ >80%). The cross-sectional study of Liu found reduced odds of RC with EBF duration of >3months vs. <3 months but data were unadjusted so carry a high risk of bias. The prospective cohort study of Kramer presented adjusted data showing no association when comparing EBF for >6 months with <3 months (data shown in Figure 17) but found increased odds of RC with EBF 3-6 months vs <3 months (OR 1.3 95%CI 1.1, 1.5).

Figure 17 EBF ≥3-4 months vs. <3-4 months and risk of RC at age 5-14 years


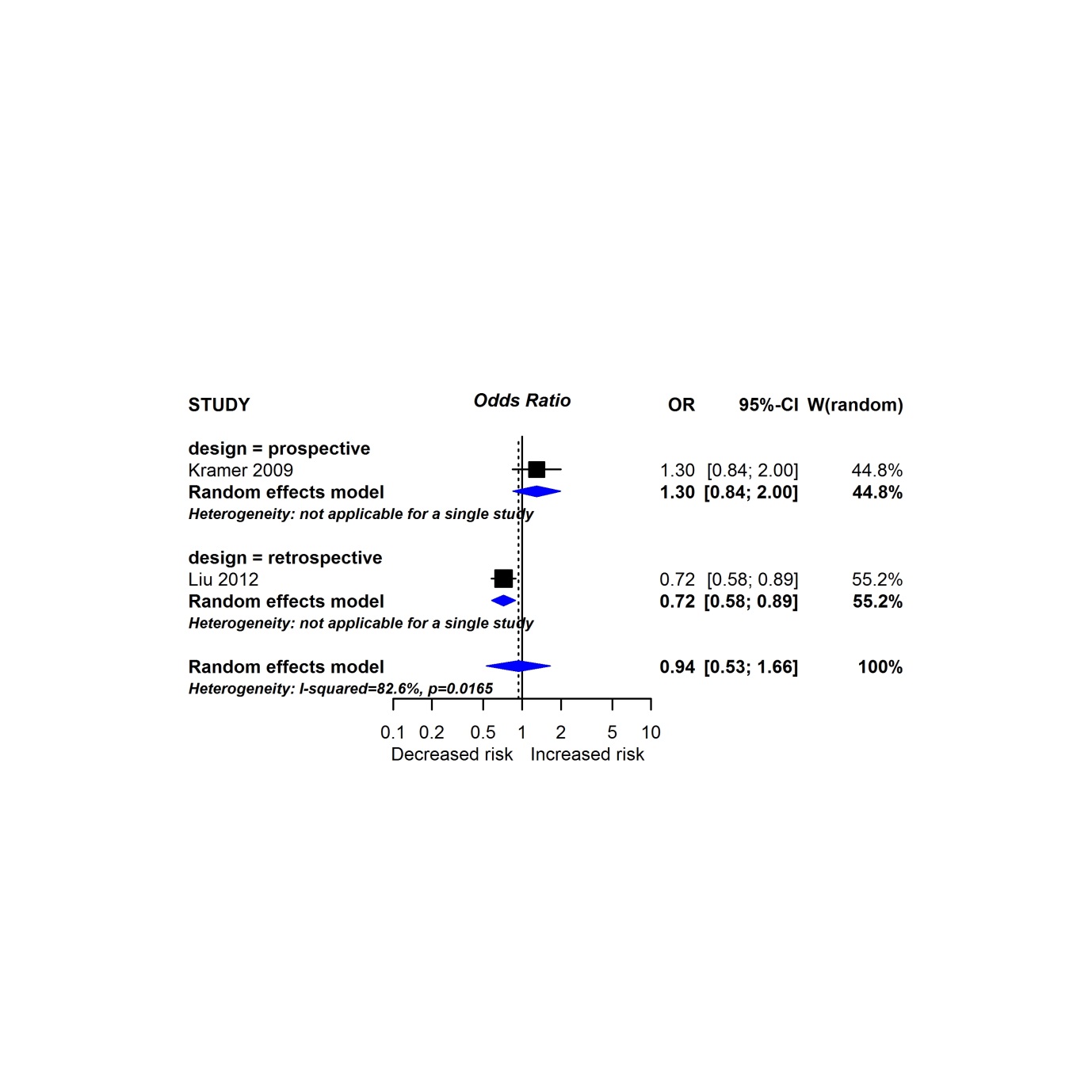


#### EBF ≥5-7 months vs. <5-7 months

One cross-sectional study reported OR for RC at age 5, in children EBF for ≥6 months vs. <6 months duration and is shown in Figure 18. There were reduced odds of RC with prolonged EBF duration, but this did not reach statistical significance. The study reported unadjusted data, and therefore carries a high risk of bias.

Figure 18 EBF ≥5-7 months vs. <5-7 months and risk of RC at age 5-14 years


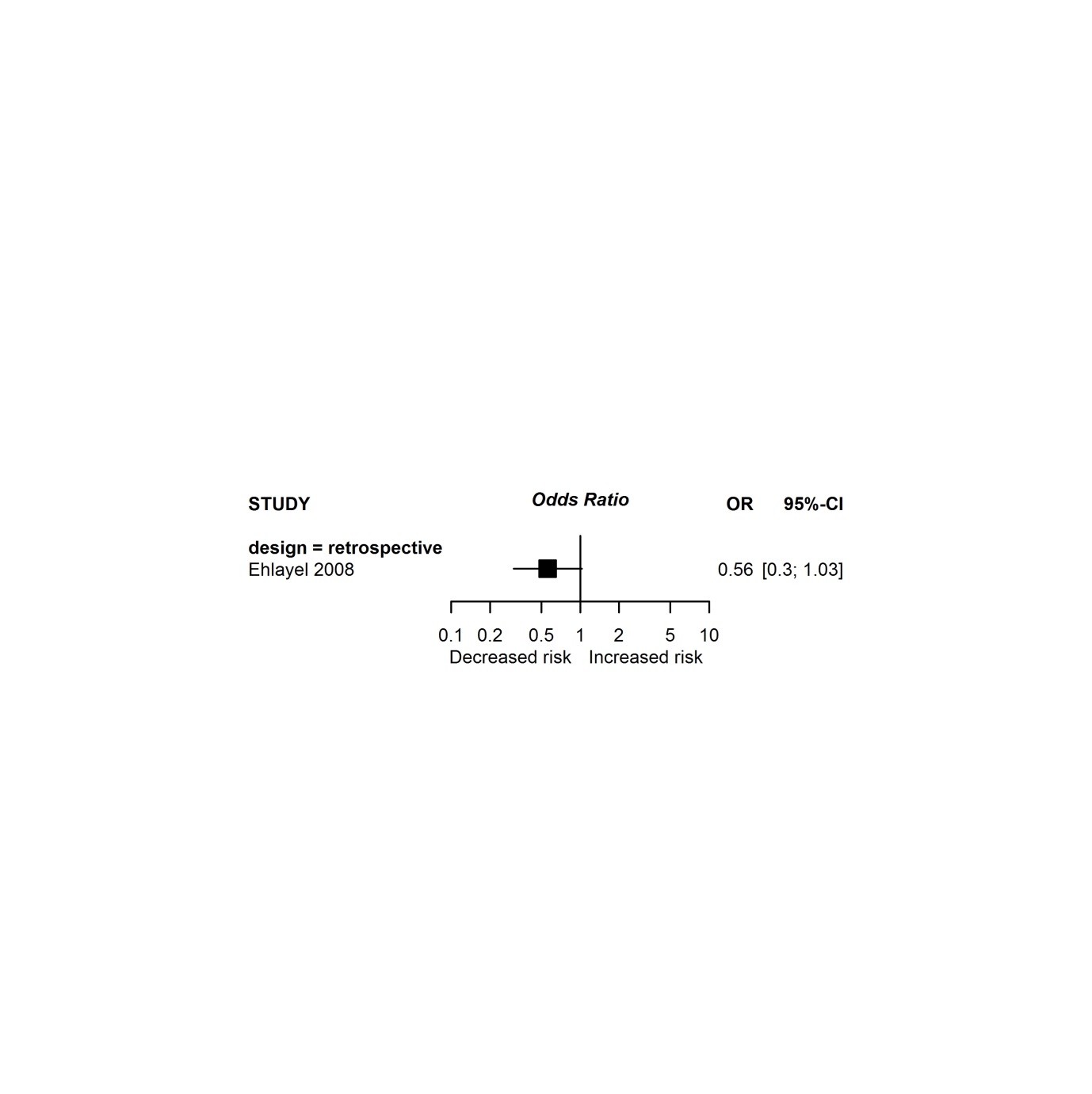


### EBF and risk of RC in children aged 15+ years

#### EBF ≥0-2 months vs. <0-2 months

Two prospective cohort studies (Strachan, Kellberger) and one cross-sectional study reported OR for RC at age 15+, in infants with EBF for ≥0-2 months vs. <0-2 months duration and are shown in Figure 19. Data were not pooled due to extreme heterogeneity (I^2^ >80%). The study of Kellberger reported adjusted data showing reduced odds of RC with prolonged EBF beyond 2 months; the studies of Miyake and Strachan reported adjusted and unadjusted data respectively, showing no association between EBF duration and RC risk.

Figure 19 EBF ≥0-2 months vs. <0-2 months and risk of RC in children aged 15+ years


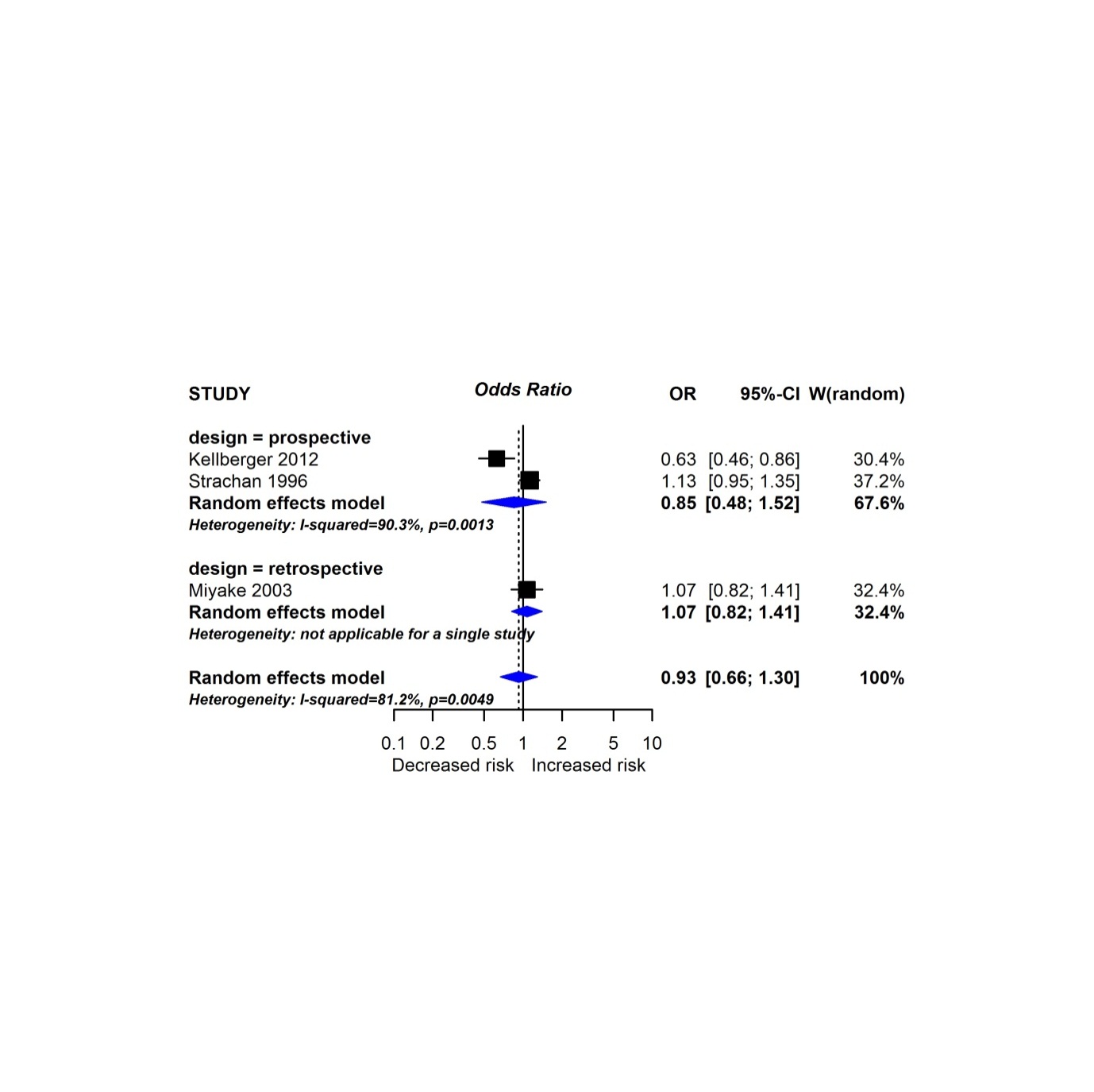


#### EBF ≥3-4 months vs. <3-4 months

One prospective cohort study reported data that could be used to calculate OR for RC at age 44, in infants with EBF ≥3-4 months vs. <3-4 months duration and is shown in Figure 20. There were significantly increased odds of self-reported hayfever in adults who had received longer duration of EBF. The data were appropriately adjusted, but the study was considered at unclear risk of bias due to inadequate definition of EBF, which was assessed retrospectively through parental report at aged 7 years.

Figure 20 EBF ≥3-4 months vs. <3-4 months and risk of RC at age 15+ years


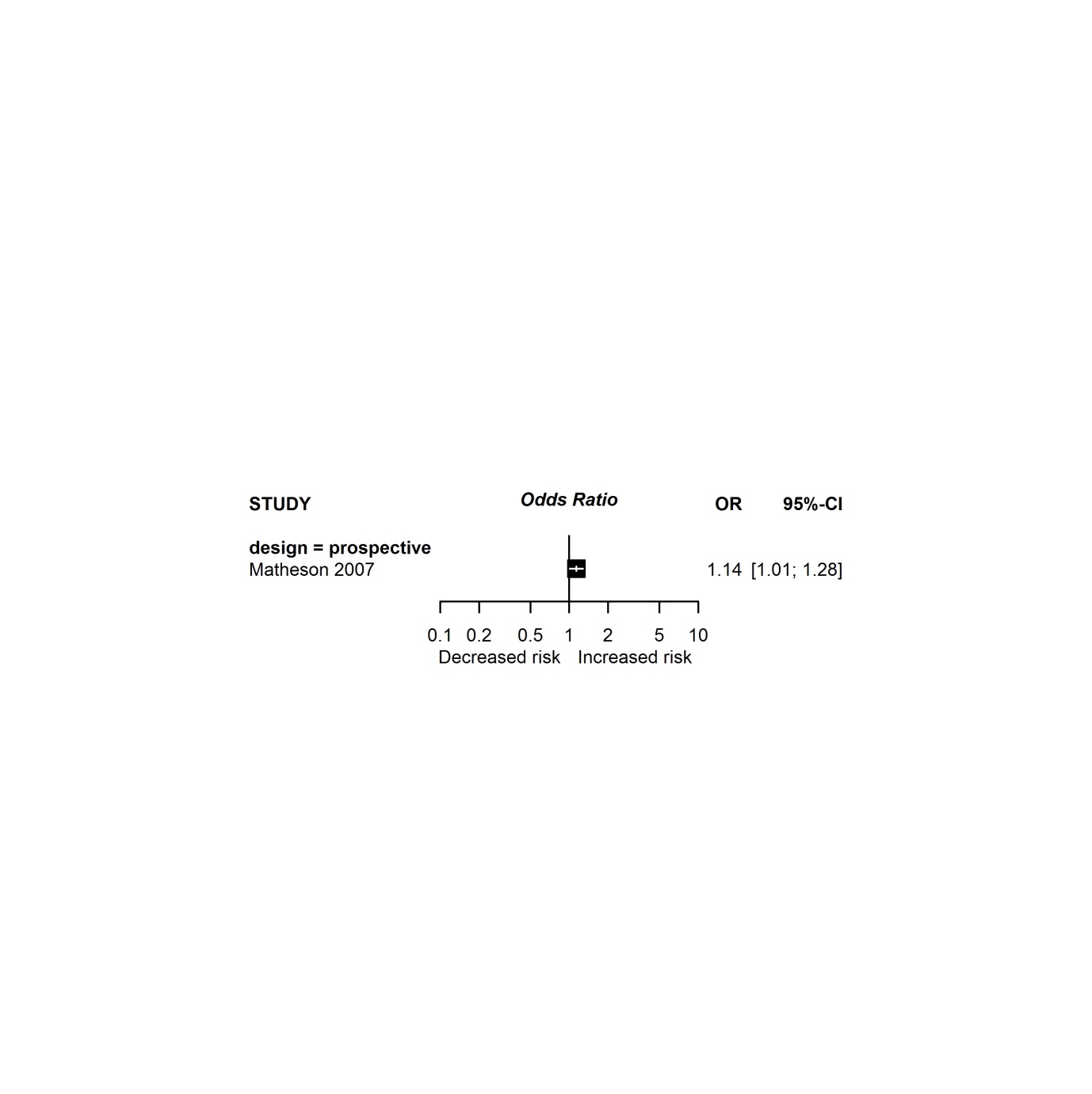


## Data for EBF duration and RC that couldn’t be meta-analysed

All included studies reported OR that have been presented as forest plots – there were no further data of note for EBF duration and RC risk.

# Solid Food Introduction and Rhinoconjunctivitis

## Overall characteristics of studies, risk of bias and summary of results

Table 6 describes the main characteristics of the studies analysed in this report. A total of 6 prospective cohort studies and no intervention studies, reported the association between timing of solid food introduction (SF) and risk of RC. The studies were from Europe (n=4), USA (n=1) and Australia (n=1). Overall, valid data on SF and RC risk were available from over 8000 subjects. Information on RC was obtained from a medical assessment in 3 studies, via parental report in 3 studies. With regards to time of outcome diagnosis, 3 studies explored the association between SF and RC in the first 4 years of life, when allergic RC can be difficult to distinguish from infectious RC, and 3 studies assessed at age 5-14. Five studies used a questionnaire +/- diary to assess the exposure (SF), 1 used an interview.

Risk of bias is summarised in Figure 21. Half of studies had a high risk of bias, due to lack of adjustment for confounding bias i.e. no adjusted data presented. Four of six studies had unclear risk of assessment bias. Risk of conflict of interest was generally assessed as low.

The risk of RC according to SF was categorised as ≥3-4 months vs. <3-4 months. Overall the evidence base was limited due to small numbers of included studies. We found no evidence that the timing of solid food introduction is associated with risk of RC, based on the available data.

Table 6 Characteristics of included studies evaluating SF introduction and rhinoconjunctivitis

| Study | N/n cases | Design | Country | Exposure assessment | Method of outcome assessment | Age at outcome (years) | Population characteristics |
| --- | --- | --- | --- | --- | --- | --- | --- |
| Larsson, 2008 ([8](#_ENREF_8)) | 4779/573 (in 2000), 975(in 2005) | PC | Sweden | Q | ISAAC - current AR | 9 | DBH study. Preschool children aged 1–6 years surveyed in 2000 and 2005. |
| Zutavern, 2008 ([36](#_ENREF_36)) | 2073/83 | PC | Germany | Q | DD | 6 | LISA study. Population based birth cohort of infants born 1997-1999 at selected maternity hospitals in 4 German cities |
| Marini, 1996 ([13](#_ENREF_13)) | 359 | PC | Italy | Q | Physician assessment | 3 | Infants with family history of allergy whose mother agreed to participate in an allergy prevention program |
| Hide, 1981 ([16](#_ENREF_16)) | 843/198 | PC | UK | D/Q | Parent reported rhinitis symptoms | 1 | The Isle of Wight study: born in 1977-1978 |
| Wright, 1994 ([17](#_ENREF_17)) | 747/313 | PC | USA | Q | Parent reported AR | 6 | Tuscon Children's Respiratory Study: Healthy newborn infants recruited from local health maintenance organisation born in 1980-1984 |
| Van Asperen, 1983 ([31](#_ENREF_31)) | 79/44 | PC | Australia | I | Physician assessment | 1.3 | Cohort recruited from medical service, born in 1980-1981 with family history of atopy |

Q: questionnaire, I: interview, R: medical records, D: diary, PC: prospective cohort, NCC: nested case control, CS: Cross-sectional, CC: case control, DD: doctor diagnosis of RC, in contrast to ‘Physician assessment’ where RC diagnosis was always made by a study physician as part of the study protocol.

Figure 21 Risk of bias in studies of SF introduction and rhinoconjunctivitis

## SF introduction and risk of rhinoconjunctivitis

### SF introduction and risk of RC in children aged 0-4 years

Three prospective cohort studies reported OR for RC at age 0-4, in infants with SF ≥3-4 months vs. <3-4 months and are shown in Figure 22. There was no significant association found. The study of Marini reported adjusted data at age 3 and had overall unclear risk of bias due to unclear assessment bias; the other two studies reported unadjusted data at age 1, and are therefore at high risk of confounding bias.

Figure 22 SF ≥3-4 months vs. <3-4 months and RC risk at age 0-4


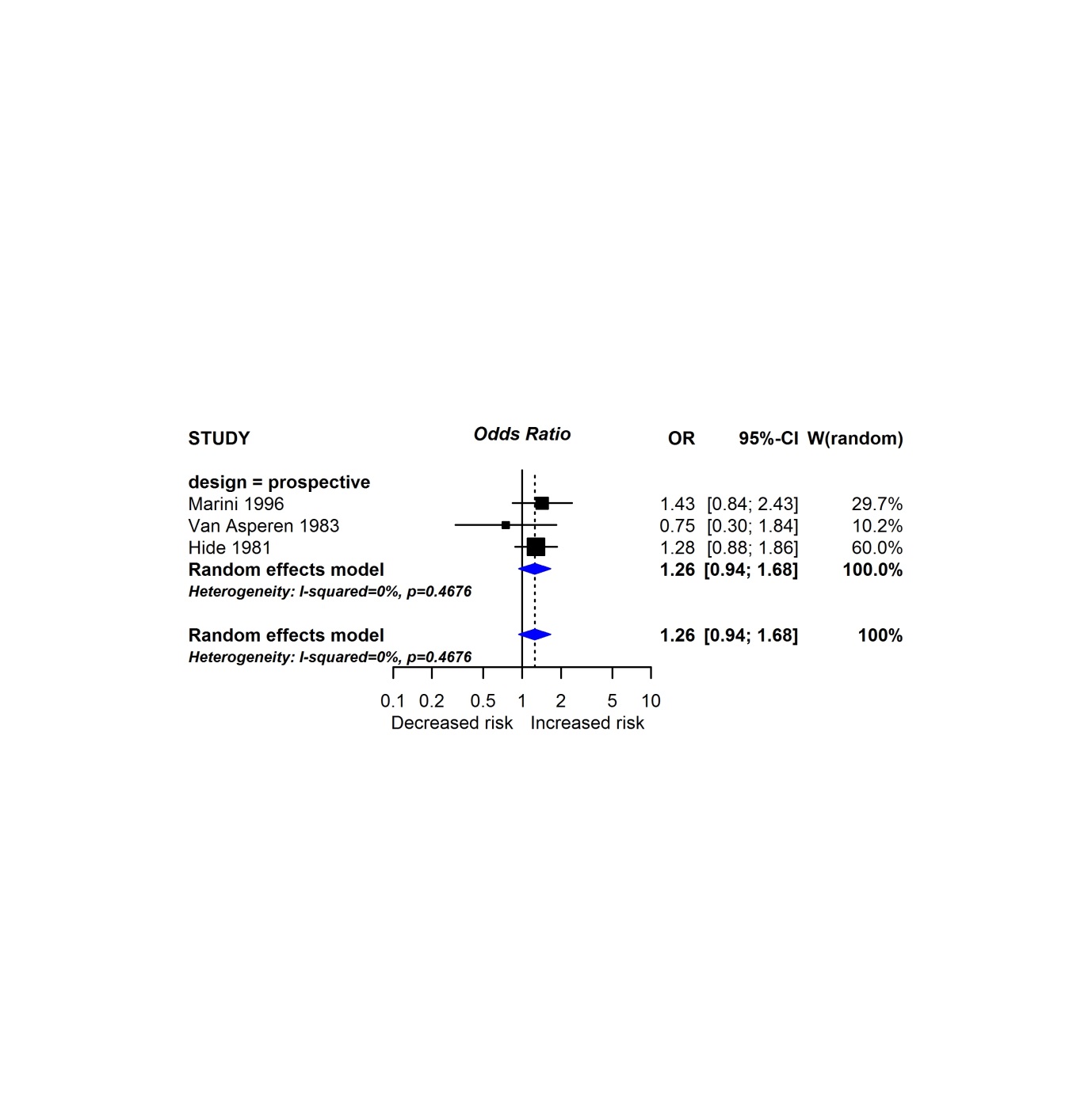


### SF introduction and risk of RC in children aged 5-14 years

Two prospective cohort studies reported OR for RC at age 5-14, in infants with SF ≥3-4 months vs. <3-4 months and is shown in Figure 23. There was no significant association found. The study of Zutavern reported adjusted data at age 6 and had overall low risk of bias; the study of Larsson reported unadjusted data but also stated that there was no significant relationship between SF introduction and RC in adjusted analyses, and had overall low risk of bias.

Figure 23 SF ≥3-4 months vs. <3-4 months and RC risk at age 5-14 years


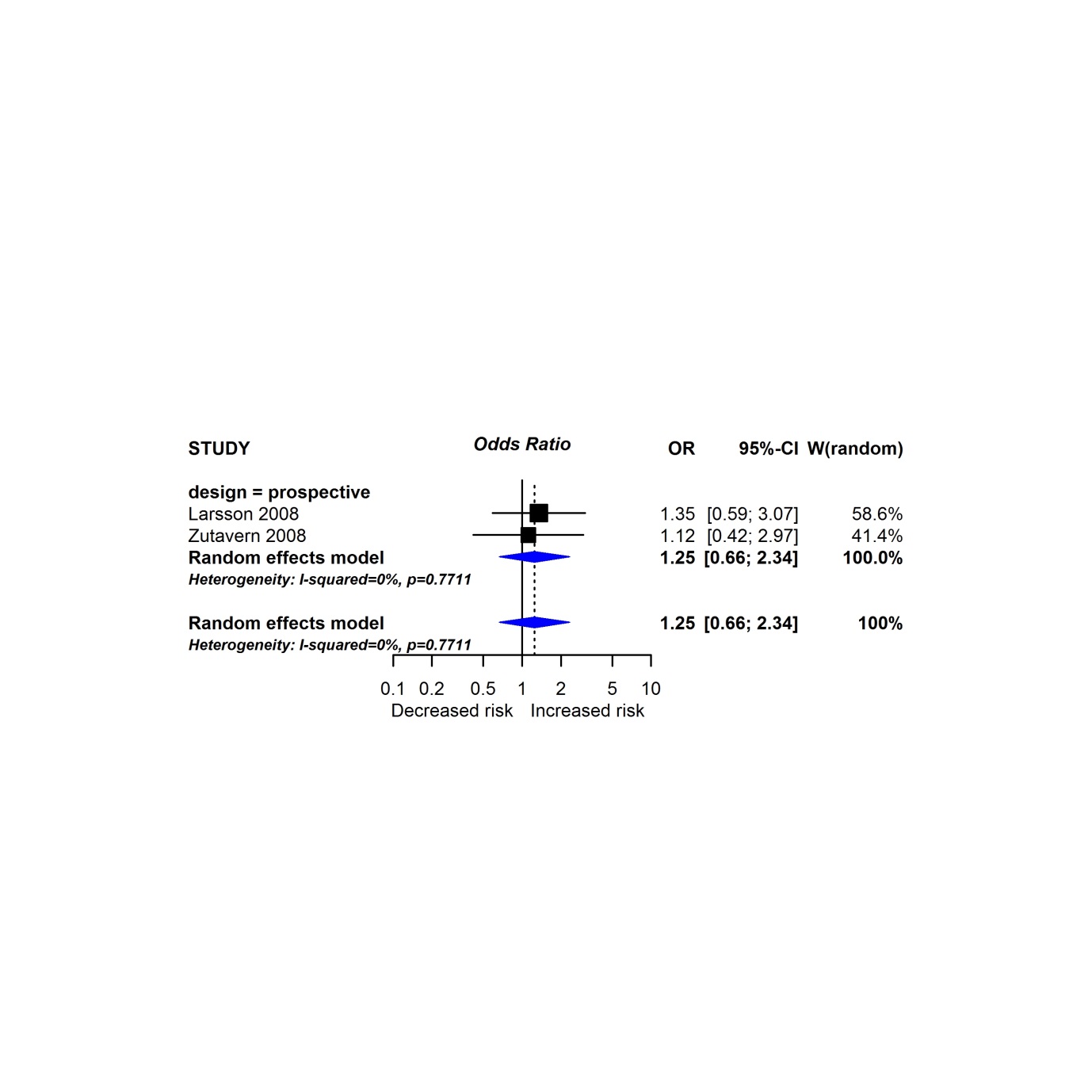


## Data for SF introduction and RC that couldn’t be meta-analysed

Meta-analyses included 5 studies, reporting data on ~ 900 participants with RC. One further study reporting RC in over 300 participants reported no relationship between timing of SF introduction and RC (Table 7).

Table 7 Studies of SF introduction and RC which were not eligible for meta-analysis

| Study | Design | Age | N/n cases | Data | Measure | TBF in no AR | TBF in AR | P |
| --- | --- | --- | --- | --- | --- | --- | --- | --- |
| Wright, 1994 ([17](#_ENREF_17)) | PC | 6 | 747/313 | Continuous | No relationship between timing of solid food introduction and RC | | | |

# Conclusion

This report summarises the results of 26 studies investigation the association between total and exclusive breastfeeding duration, timing of solid food introduction and risk of rhinoconjunctivitis. The majority of studies were prospective cohort studies, with one systematic review and one intervention trial. Overall we found no clear evidence to support an association between TBF duration, and RC risk at any age. The data from the intervention trial showed no evidence that promotion of longer TBF duration influences risk of RC – OR 1.1 (95% CI 0.6, 1.9),.One meta-analysis of observational studies found reduced risk of RC at ages 0-4 years associated with EBF ≥3-4 months vs <3-4 months with no statistical heterogeneity, based largely on 2 prospective cohort studies reporting adjusted data. However, statistical significance was borderline, and we were unable to confirm this association using other cutoffs for EBF duration. At other ages there was no association between EBF duration and RC risk. Data for timing of SF introduction and RC risk were limited, but the available data do not support an association between timing of SF introduction and RC.

The possible relationship between EBF duration and RC risk at age 0-4 years makes some physiological sense, and is consistent with findings from the systematic review of Kramer, who evaluated EBF duration in relation to a range of child health outcomes and found some evidence for reduced nasal symptoms with longer EBF duration ([25](#_ENREF_25)). Most RC in this age group is infective rather than allergic in origin and distinction of the two can be difficult in epidemiological studies. Breastfeeding duration is known to be associated with reduced risk of respiratory and gastrointestinal infections during infancy, so the possible association here may reflect a protective effect of human breast milk through the high immunoglobulin content which is thought to mediate the effects on other infections. The quality of data was limited, especially for analysis of timing of solid food introduction in relation to RC, and more research is needed to clarify whether there is an effect of different infant feeding regimens on upper airway and ocular health.

# References

1. Kramer MS, Matush L, Vanilovich I, Platt R, Bogdanovich N, Sevkovskaya Z, et al. Effect of prolonged and exclusive breast feeding on risk of allergy and asthma: cluster randomised trial. Bmj. 2007;335(7624):815.

2. Kramer MS, Matush L, Vanilovich I, Platt R, Bogdanovich N, Sevkovskaya Z, et al. Effect of prolonged and exclusive breast feeding on risk of allergy and asthma: cluster randomised trial. BMJ (Clinical research ed). 2007;335(7624):815.

3. Kull I, Wickman M, Lilja G, Nordvall SL, Pershagen G. Breast feeding and allergic diseases in infants-a prospective birth cohort study. Archives of Disease in Childhood. 2002;87(6):478-81.

4. Butland BK, Strachan DP, Lewis S, Bynner J, Butler N, Britton J. Investigation into the increase in hay fever and eczema at age 16 observed between the 1958 and 1970 British birth cohorts. Bmj. 1997;315(7110):717-21.

5. Taylor B, Wadsworth J, Golding J, Butler N. Breast feeding, eczema, asthma, and hayfever. Journal of Epidemiology and Community Health. 1983;37:95-9.

6. Burr ML, Limb ES, Maguire MJ, Amarah L, Eldridge BA, Layzell JC, et al. Infant feeding, wheezing, and allergy: a prospective study. Archives of Disease in Childhood. 1993;68(6):724-8.

7. Businco L, Cantani A, Meglio P, Bruno G. Prevention of atopy: Results of a long-term (7 months to 8 years) follow-up. Annals of Allergy. 1987;59(5 PART II):183-6.

8. Larsson M, Hagerhed-Engman L, Sigsgaard T, Janson S, Sundell J, Bornehag CG. Incidence rates of asthma, rhinitis and eczema symptoms and influential factors in young children in Sweden. Acta Paediatrica. 2008;97(9):1210-5.

9. Devereux G, Turner SW, Craig LC, McNeill G, Martindale S, Harbour PJ, et al. Low maternal vitamin E intake during pregnancy is associated with asthma in 5-year-old children. American Journal of Respiratory & Critical Care Medicine. 2006;174(5):499-507.

10. Virtanen SM, Kaila M, Pekkanen J, Kenward MG, Uusitalo U, Pietinen P, et al. Early introduction of oats associated with decreased risk of persistent asthma and early introduction of fish with decreased risk of allergic rhinitis. British Journal of Nutrition. 2010;103(2):266-73.

11. Farooqi IS, Hopkin JM. Early childhood infection and atopic disorder. Thorax. 1998;53(11):927-32.

12. Gruskay FL. Comparison of breast, cow, and soy feedings in the prevention of onset of allergic disease: a 15-year prospective study. Clinical Pediatrics. 1982;21(8):486-91.

13. Marini A, Agosti M, Motta G, Mosca F. Effects of a dietary and environmental prevention programme on the incidence of allergic symptoms in high atopic risk infants: three years' follow-up. Acta Paediatrica Supplement. 1996;414:1-21.

14. Miskelly FG, Burr ML, Vaughan-Williams E, Fehily AM, Butland BK, Merret TG. Infant feeding and allergy. Archives of Disease in Childhood. 1988;63(4):388-93.

15. Strachan DP, Taylor EM, Carpenter RG. Family structure, neonatal infection, and hay fever in adolescence. Archives of Disease in Childhood. 1996;74(5):422-6.

16. Hide DW, Guyer BM. Clinical manifestations of allergy related to breast and cows' milk feeding. Archives of Disease in Childhood. 1981;56(3):172-5.

17. Wright AL, Holberg CJ, Martinez FD, Halonen M, Morgan W, Taussig LM. Epidemiology of physician-diagnosed allergic rhinitis in childhood. Pediatrics. 1994;94(6 Pt 1):895-901.

18. Bergmann RL, Edenharter G, Bergmann KE, Lau S, Wahn U. Socioeconomic status is a risk factor for allergy in parents but not in their children. Clinical & Experimental Allergy. 2000;30(12):1740-5.

19. Kulig M, Klettke U, Wahn V, Forster J, Bauer CP, Wahn U. Development of seasonal allergic rhinitis during the first 7 years of life. Journal of Allergy & Clinical Immunology. 2000;106(5):832-9.

20. Innes Asher M, Robertson C, Ait-Khaled N, Anderson HR, Beasley R, Bjorksten B, et al. Global analysis of breast feeding and risk of symptoms of asthma, rhinoconjunctivitis and eczema in 6-7 year old children: ISAAC Phase Three. Allergologia et Immunopathologia. 2011;39(6):318-25.

21. Miyake Y, Yura A, Iki M. Breastfeeding and the prevalence of symptoms of allergic disorders in Japanese adolescents. Clinical and Experimental Allergy. 2003;33(3):312-6.

22. Karino S, Okuda T, Uehara Y, Toyo-oka T. Breastfeeding and prevalence of allergic diseases in Japanese university students. Annals of Allergy Asthma & Immunology. 2008;101(2):153-9.

23. Kurt E, Metintas S, Basyigit I, Bulut I, Coskun E, Dabak S, et al. Prevalence and risk factors of allergies in Turkey: Results of a multicentric cross-sectional study in children. Pediatric Allergy & Immunology. 2007;18(7):566-74.

24. Selcuk ZT, Caglar T, Enunlu T, Topal T. The prevalence of allergic diseases in primary school children in Edirne, Turkey. Clinical & Experimental Allergy. 1997;27(3):262-9.

25. Kramer MS, Kakuma R. Optimal duration of exclusive breastfeeding. Cochrane Database of Systematic Reviews. 2012;8:CD003517.

26. Kajosaari M. Atopy prophylaxis in high-risk infants. Prospective 5-year follow-up study of children with six months exclusive breastfeeding and solid food elimination. Advances in Experimental Medicine & Biology. 1991;310:453-8.

27. Kellberger J, Dressel H, Vogelberg C, Leupold W, Windstetter D, Weinmayr G, et al. Prediction of the incidence and persistence of allergic rhinitis in adolescence: a prospective cohort study. Journal of Allergy & Clinical Immunology. 2012;129(2):397-402, .e1-3.

28. Kramer MS, Matush L, Bogdanovich N, Dahhou M, Platt RW, Mazer B. The low prevalence of allergic disease in Eastern Europe: are risk factors consistent with the hygiene hypothesis? Clinical & Experimental Allergy. 2009;39(5):708-16.

29. Siltanen M, Kajosaari M, Poussa T, Saarinen KM, Savilahti E. A dual long-term effect of breastfeeding on atopy in relation to heredity in children at 4 years of age. Allergy. 2003;58(6):524-30.

30. Matheson MC, Erbas B, Balasuriya A, Jenkins MA, Wharton CL, Tang ML, et al. Breast-feeding and atopic disease: a cohort study from childhood to middle age. Journal of Allergy & Clinical Immunology. 2007;120(5):1051-7.

31. Van Asperen PP, Kemp AS, Mellis CM. Relationship of diet in the development of atopy in infancy. Clinical Allergy. 1984;14(6):525-32.

32. Arshad SH, Hide DW. Effect of environmental factors on the development of allergic disorders in infancy. Journal of Allergy & Clinical Immunology. 1992;90(2):235-41.

33. Erkkola M, Nwaru BI, Kaila M, Kronberg-Kippila C, Ilonen J, Simell O, et al. Risk of asthma and allergic outcomes in the offspring in relation to maternal food consumption during pregnancy: a Finnish birth cohort study. Pediatric Allergy & Immunology. 2012;23(2):186-94.

34. Ehlayel MS, Bener A. Duration of breast-feeding and the risk of childhood allergic diseases in a developing country. Allergy & Asthma Proceedings. 2008;29(4):386-91.

35. Liu P, Woo JMP, Parsa MF, Amarilyo G, McCurdy DK, Rullo OJ. Ethnic differences in pediatric SLE early disease severity: A comparison between Hispanic-Americans and European-Americans. Pediatric Rheumatology. 2012;10.

36. Zutavern A, Brockow I, Schaaf B, von Berg A, Diez U, Borte M, et al. Timing of solid food introduction in relation to eczema, asthma, allergic rhinitis, and food and inhalant sensitization at the age of 6 years: results from the prospective birth cohort study LISA. Pediatrics. 2008;121(1):e44-52.
