# Supplementary material for: Diet during pregnancy and infancy and risk of allergic or autoimmune disease: A systematic review and meta-analysis
Source: PLoS Med. 2018 Feb 28;15(2):e1002507. doi: 10.1371/journal.pmed.1002507 (PMC5830033; doi:10.1371/journal.pmed.1002507)
Supplement: S1 Data — (ZIP) [file pmed.1002507.s006.zip › Review_A_reports/TIDM.docx]

**Breastfeeding, Solid Food Introduction and Type I Diabetes Mellitus**

Natalie Geoghegan^1^, Despo Ierodiakonou^2^, Vanessa Garcia-Larsen^3^, Jo Leonardi-Bee^4^, Tim Reeves^5^, Jennifer Chivinge^1^, Zoe Robinson^1^, Katharine Jarrold^1^, Evangelia Andreou^6^, Nara Tagiyeva-Milne^7^, Ulugbek Nurmatov^8^, Sergio Cunha^9^, Robert J Boyle^10^

^1^ Undergraduate medical students, Imperial College London; ^2^ Post-Doctoral Research Associate, Departments of Paediatric and Respiratory Epidemiology and Public Health Group, Imperial College London; ^3^ Post-Doctoral Research Associate & Honorary Research Fellow, Royal Brompton Hospital and Harefield NHS Foundation Trust, Respiratory Epidemiology and Public Health, National Heart and Lung Institute, Imperial College London; ^4^Associate Professor of Community Health Sciences, University of Nottingham; ^5^ Research Support Librarian, Faculty of Medicine, Imperial College London; ^6^ Research Associate, Imperial Consultants; ^7^Research Fellow, University of Aberdeen; ^8^ Research Fellow, University of Edinburgh; ^9^ Research Associate, Respiratory Epidemiology and Public Health, National Heart and Lung Institute, Imperial College London; ^10^ Clinical Senior Lecturer, Section of Paediatrics, Imperial College London

Imperial Consultants,

58 Princes Gate,

Exhibition Road,

London SW7 2PG

**TABLE OF CONTENTS**

LIST OF FIGURES .3

1. Total breastfeeding duration and risk of TIDM 5

1.1. Overall characteristics of studies, risk of bias and summary of results 5

1.2. Outcomes from studies of total breastfeeding and TIDM 12

1.2.1. Ever vs. never breastfed 12

1.2.2. Total breastfeeding ≥1-2 months vs. < 1-2 months 15

1.2.3. Total breastfeeding duration ≥3-4 months vs. <3-4 months 16

1.2.4. Total duration of breastfeeding ≥5-7 months vs. < 5-7 months 19

1.2.5. Total duration of breastfeeding ≥8-12 months vs. <8-12 months 21

1.2.6. Dose response analysis of TBF and TIDM risk 22

1.2.7. Studies investigating TBF and TIDM as a continuous variable 23

1.2.8 Data for TBF and TIDM which couldn’t be meta-analysed 24

2. Exclusive breastfeeding duration and risk of TIDM 27

2.1 Overall characteristics of studies, risk of bias and summary of results 27

1.3. Outcomes from studies of exclusive breastfeeding and TIDM 32

2.2.1 Exclusive breastfeeding for ≥0-2 months vs. <0-2 months 32

2.2.2 Exclusive breastfeeding for ≥3-4 months vs < 3-4 months 34

2.2.3 Exclusive breastfeeding for ≥5-9 months vs. <5-9 months 36

2.2.4 Studies investigating EBF as a continuous variable and risk of TIDM 36

2.2.5 Other studies evaluating EBF and TIDM which couldn’t be meta-analysed 37

3 Age at introduction of solid food and risk of TIDM 40

3.1. Overall characteristics of studies, risk of bias and summary of results 40

3.2. Outcomes from studies of solid food introduction and TIDM 43

3.2.1 Solid food introduction at ≥ 3-4 months vs < 3-4 months, and risk of TIDM 43

3.2.2 Timing of solid food introduction as a continuous variable, and TIDM risk 45

3.2.3 Other studies solid food introduction and TIDM, which couldn’t be meta- analysed 45

4 Conclusions 47

5 References 50

# LIST OF FIGURES

[Figure 1 Risk of bias in observational studies of total breastfeeding duration and TIDM 12](#_Toc419734515)

[Figure 2 Breastfeeding ever vs. never and TIDM risk 13](#_Toc419734516)

[Figure 3 Risk of publication bias in observational studies investigating breastfed ever vs. never and TIDM risk 15](#_Toc419734517)

[Figure 4 Breastfeeding for ≥1-2 months vs. < 1-2 months and TIDM risk 16](#_Toc419734518)

[Figure 5 Breastfeeding for ≥3-4 months vs. <3-4 months and TIDM risk 17](#_Toc419734519)

[Figure 6 Risk of publication bias in observational studies investigating TBF ≥3-4 months vs. <3-4 months and TIDM risk 19](#_Toc419734520)

[Figure 7 Breastfeeding for ≥5-7 months vs. <5-7 months and TIDM risk 19](#_Toc419734521)

[Figure 8 Breastfeeding for ≥8-12 months vs. <8-12 months and TIDM risk 21](#_Toc419734522)

[Figure 9 Breastfeeding for short duration versus never 22](#_Toc419734523)

[Figure 10 Breastfeeding for medium duration versus never 23](#_Toc419734524)

[Figure 11 Breastfeeding for long duration versus never 23](#_Toc419734525)

[Figure 12 Risk of TIDM for each month increase in TBF duration 24](#_Toc419734526)

[Figure 13 Difference in TBF in people with TIDM versus unaffected subjects 24](#_Toc419734527)

[Figure 14 Risk of bias in observational studies of exclusive breastfeeding and TIDM 32](#_Toc419734528)

[Figure 15 Exclusive breastfeeding for ≥0-2 months vs. < 0-2 months and TIDM risk 32](#_Toc419734529)

[Figure 16 Exclusive BF for ≥ 3-4 months vs. < 3-4 months and TIDM risk 34](#_Toc419734530)

[Figure 17 Exclusive breastfeeding for ≥5-9 months vs. < 5-9 months and TIDM risk 36](#_Toc419734531)

[Figure 18 Difference in EBF in people with T1DM versus unaffected subjects 37](#_Toc419734532)

[Figure 19 Risk of bias in observational studies of solid food exposure and TIDM risk 43](#_Toc419734533)

[Figure 20 Introduction of solid food at age ≥3-4 months vs. <3-4 months and TIDM risk 43](#_Toc419734534)

[Figure 21 Timing of solid food introduction as a continuous variable, and TIDM risk 45](#_Toc419734535)

[Figure 22 Summary of Meta-Analysis findings for Duration of BF and TIDM risk 47](#_Toc419734536)

**LIST OF TABLES**

[Table 1 Description of observational studies on total breastfeeding duration and Risk of TIDM 7](#_Toc419734537)

[Table 2 Stratified and subgroup analyses of association between ever vs. never being breastfed and risk of TIDM 14](#_Toc419734538)

[Table 3 Stratified and subgroup analyses of breastfeeding ≥3-4 months vs. <3-4 months and TIDM risk 18](#_Toc419734539)

[Table 4 Stratified and subgroup analyses of breastfeeding ≥5-7 months vs. <5-7 months and TIDM risk 20](#_Toc419734540)

[Table 5 Other studies evaluating total breastfeeding and TIDM which couldn’t be meta-analysed 25](#_Toc419734541)

[Table 6 Characteristics of Included Studies for analysis of Exclusive Breast Feeding duration and TIDM Risk 29](#_Toc419734542)

[Table 7 Stratified and subgroup analyses of EBF duration ≥0-2 months vs. <0-2 months and TIDM risk 33](#_Toc419734543)

[Table 8 Stratified and subgroup analyses of EBF duration ≥3-4 months vs. <3-4 months and TIDM risk 35](#_Toc419734544)

[Table 9 Other studies evaluating exclusive breastfeeding and TIDM which couldn’t be meta-analysed 38](#_Toc419734545)

[Table 10 Characteristics of studies reporting timing of solid food and TIDM risk 41](#_Toc419734546)

[Table 11 Stratified and subgroup analyses of solid food introduction ≥3-4 months vs. <3-4 months and TIDM risk 44](#_Toc419734547)

[Table 12 Other studies evaluating timing of solid food introduction and TIDM which could not be meta-analysed 46](#_Toc419734548)

# Total breastfeeding duration and risk of TIDM

## Overall characteristics of studies, risk of bias and summary of results

Table 1 describes the main characteristics of the studies analysed in this report. A total of 65 observational studies, and no intervention studies, reported the association between duration of breastfeeding and risk of TIDM. Of these, 15 were prospective cohort studies, 7 nested case-controls, 1 cross-sectional study and 42 case-control studies. Over half of the studies (n=39) are from Europe – others are from North America (n=9), South America (n=4), Asia (n=8), the Middle East (n=4) and Africa (n=1). Overall, valid data on total breastfeeding duration in the first year of life (TBF) and TIDM risk were available from almost 50,000 subjects including over 12,000 with TIDM. Information on TIDM was obtained mainly from serology (Islet auto-antibodies) in 15 prospective studies and via medical diagnosis in 50 (mainly case control) studies. With regards to time of outcome diagnosis, 12 studies explored the association between exposure to breastfeeding and TIDM in the first 5 years of life, 2 didn’t report the age at outcome assessment, and others evaluated TIDM in older children or young adults. 58 studies used interview or questionnaire to assess the exposure (TBF), 7 studies assessed medical records only.

Risk of bias was assessed using the NICE Methodological checklists for cohort and case-control studies. Figure 1 illustrates the distribution of bias across the five main methodological areas of the studies. Over half of studies had a high risk of bias, most commonly due to lack of adjustment for confounding bias i.e. no adjusted data presented. Over a quarter of studies had an ‘unclear’ overall risk of bias, most commonly due to insufficient information to evaluate assessment bias. We undertook subgroup/stratified analyses for meta-analyses with >5 studies, and Funnel plots and Egger’s test where there were ≥10 studies in a meta-analysis.

Five levels of comparison were used to assess the risk of TIDM according to total breastfeeding duration, namely ‘ever vs. never’, ‘≥1-2 months vs. <1-2 months’, ‘≥3-4 months vs. <3-4 months’, ‘≥5-7 months vs. <5-7 months’, and ‘≥8-12 months vs. <8-12 months’.

*Main Findings*

Across all cut-offs for TBF duration, there was evidence of a lower risk of TIDM with longer duration of breastfeeding, however meta-analyses showed moderate to high statistical heterogeneity across studies. Stratified and subgroup analyses showed some evidence of risk difference when specific risk groups and study design characteristics were analysed. Prospective studies, which often used autoantibodies at a young age as a surrogate for TIDM, and sometimes reported HR rather than OR, tended to not show a significant association between TBF and TIDM risk. In contrast, retrospective studies using clinical TIDM as an outcome tended to report an association between longer TBF and reduced TIDM risk, sometimes with low statistical heterogeneity. It is possible that the difference observed between these two groups of study design lies in the type of outcome used to measure TIDM. In general adjusted and unadjusted analyses showed similar findings. We were not able to clearly confirm the relationship between TBF and TIDM in dose-response analysis, although data were limited for this analysis - and only a small number of the 24 studies (~1/3 of TIDM cases) which could not be included in any meta-analysis found the same association between TBF and reduced TIDM risk. Thus our data must be interpreted as VERY LOW certainty evidence (GRADE -1 inconsistency) that longer duration of TBF is associated with reduced TIDM risk.

**Table 1 Description of observational studies on total breastfeeding duration and Risk of TIDM**

| **First Author & Publication Year** | **N/n cases** | **Design** | **Country** | **Exposure assessment** | **Specific outcome** | **Age at outcome (years)** | **Population**  **characteristics** |
| --- | --- | --- | --- | --- | --- | --- | --- |
| Couper, 1999 ([1](#_ENREF_1)) | 317/70 | PC | Australia | D/Q | Islet autoantibodies | 2 | First degree relatives of diabetic children |
| Couper, 2009 ([2](#_ENREF_2)) | 548/~30 | PC | Australia | D/I | Islet autoantibodies | 2 | First degree relatives of diabetic children |
| Fronczak, 2003 ([3](#_ENREF_3)) | 222/~16 | PC | USA | Q | Islet autoantibodies | 4 | Newborn screening, or Colorado register |
| Holmberg, 2007 ([4](#_ENREF_4)) | 3788/~51 | PC | Sweden | Q | Islet autoantibodies | 6 | General population |
| Hummel, 2000 ([5](#_ENREF_5)) | 568 | PC | Germany | Q | Islet autoantibodies | 2 | Offspring of diabetic parents |
| Karlen, 2012 ([6](#_ENREF_6)) | 1409 | PC | Sweden | Q | Islet autoantibodies | 1 | General population |
| Lamb 2008 ([7](#_ENREF_7)) | 642 | PC | Australia | I | Islet autoantibodies | 13 | St. Joseph’s Hospital in Denver, Colorado |
| Lamb, 2013 ([8](#_ENREF_8)) | 260 | PC | America | I | Islet autoantibodies | Not reported | not reported |
| Ludvigsson, 2003 ([9](#_ENREF_9)) | 205 | PC | Sweden | Q | Islet autoantibodies | 2 | Relatives of diabetics |
| Norris, 2003 ([10](#_ENREF_10)) | 1183/~733 | PC | USA | I | Islet autoantibodies | 4 | St Joseph’s Hospital, Colorado |
| Viner, 2008 ([11](#_ENREF_11)) | 11211/61 | PC | UK | Q | Medical diagnosis | >10 | not reported |
| Virtanen, 1998 ([12](#_ENREF_12)) | 697/43 | PC | Finland | Q | Medical diagnosis, Islet autoantibodies | <25 | Siblings of diabetic children |
| Virtanen, 2011 ([13](#_ENREF_13)) | ~4000/~160 | PC | Finland | Q | Medical diagnosis or islet autoantibodies | 5 | Odu and Tampere University Hospitals |
| Wahlberg, 2006 ([14](#_ENREF_14)) | 8715/31 | PC | Sweden | Q | Islet autoantibodies | 2 | General population |
| Ziegler, 2003 ([15](#_ENREF_15)) | 1460/~68 | PC | Germany | Q | Islet autoantibodies | 5 | Newborn children |
| Jones, 1998 ([16](#_ENREF_16)) | 518/60 | NCC | UK | R | Medical diagnosis | 5.0-9 | Hospital admission |
| Kimpimaki, 2001 ([17](#_ENREF_17)) | 455/65 | NCC | Finland | Q | Medical diagnosis | <4 | Turku, Oulu and Tampere Hospital births |
| Kyvik, 1992 ([18](#_ENREF_18)) | 228/76 | NCC | Denmark | R | Medical diagnosis | <20 | National Service Conscript records |
| Norris, 1996 ([19](#_ENREF_19)) | 171/18 | NCC | USA | Q/R | Islet autoantibodies | <7 | Siblings or offspring of Barbara Davies Centre Diabetics |
| Robertson, 2010 ([20](#_ENREF_20)) | 1444/361 | NCC | UK | R | Medical diagnosis | <15 | AMND and SSG register, hospital births |
| Savilahti, 2009 ([21](#_ENREF_21)) | 6209/45 | NCC | Finland | R | Medical diagnosis | 12 | NHI database |
| Virtanen, 2000 ([22](#_ENREF_22)) | 287/33 | NCC – nested in Virtanen, 1998 ([12](#_ENREF_12)) | Finland | Q | Medical diagnosis | 7 | Siblings of previously diagnosed diabetic child |
| Glatthaar, 1988 ([23](#_ENREF_23)) | 946/~200 | CS | Australia | Q | Medical diagnosis | <18 | School register |
| Ahadi, 2011 ([24](#_ENREF_24)) | 202/101 | CC | Iran | Q/I | Medical diagnosis | 7 | Hospital admission |
| Alves, 2012 ([25](#_ENREF_25)) | 246/123 | CC | Brazil | I | Medical diagnosis | 7 | Siblings |
| Ashraf, 2010 ([26](#_ENREF_26)) | 195/128 | CC | USA | Q | Medical diagnosis | <10 | Children's hospital |
| Baruah, 2011 ([27](#_ENREF_27)) | 86/43 | CC | India | I | Medical diagnosis | <18 | Endocrinology ward |
| Bener, 2009 ([28](#_ENREF_28)) | 340 | CC | Qatar | I | Medical diagnosis | <16 | Endocrinology clinic and community |
| Blom, 1989 ([29](#_ENREF_29)) | 867/339 | CC | Sweden | Q | Medical diagnosis | 7 | Paediatric referral and population register |
| Bodington, 1994 ([30](#_ENREF_30)) | 393/209 | CC | UK | Q | Medical diagnosis | <15 | Independent sources and population register |
| Borras, 2011 ([31](#_ENREF_31)) | 1530/306 | CC | Spain | R | Medical diagnosis | not reported | Diabetes register and Catalonia birth register |
| Esfarjani, 2001 ([32](#_ENREF_32)) | 104/52 | CC | Iran | Q | Medical diagnosis | <14 | Endocrine clinic and paediatric OPD attendance |
| Dahlquist, 2002 ([33](#_ENREF_33)) | 2226/610 | CC | Austria, Latvia, Lithuania, Luxembourg and UK | Q/I | Medical diagnosis | <15 | Diabetes register and population register |
| Gimeno, 1997 ([34](#_ENREF_34)) | 626/313 | CC | Brazil | Q | Medical diagnosis | <18 | Juvenile Diabetes Association or hospital records |
| Hathout, 2006 ([35](#_ENREF_35)) | 402/102 | CC | USA | Q/I | Medical diagnosis | 7 | Diabetes hospital care and Hospital Well Child clinics |
| Hypponen, 1999 ([36](#_ENREF_36)) | 821/435 | CC | Finland | Q | Medical diagnosis | 8 | Finnish Population Registry |
| Kostraba, 1992 ([37](#_ENREF_37)) | 264/132-white | CC | USA | Q/I | Medical diagnosis | 10 | Alleghany Hospital diabetes register |
|  | 108/54-black |  |  |  |  |  |  |
| Kostraba, 1993 ([38](#_ENREF_38)) | 306/142 | CC | USA | Q | Medical diagnosis | <18 | Colorado IDDM Registry and otor vehicle driver register |
| Majeed, 2011 ([39](#_ENREF_39)) | 310/96 | CC | Iraq | Q | Medical diagnosis | <17 | Hospital admission or OPD |
| Malcova, 2006 ([40](#_ENREF_40)) | 2334/868 | CC | Czech Republic | Q | Medical diagnosis | <15 | Czech Childhood Diabetes Register and diabetes clinic |
| Marshall, 2004 ([41](#_ENREF_41)) | 577/196 | CC | UK | I | Medical diagnosis | <16 | Paediatric clinic and Local Health Authority Register |
| Mayer, 1988 ([42](#_ENREF_42)) | 747/268 | CC | USA | Q/I | Medical diagnosis | <18 | Colorado IDDM Registry or Barbara Davies Centre |
| McKinney, 1999 ([43](#_ENREF_43)) | 521/196 | CC | UK | I | Medical diagnosis | <16 | Yorkshire Childhood Diabetes Register and Family Health Service Authority Register |
| Meloni, 1997 ([44](#_ENREF_44)) | 200/100 | CC | Italy | Q | Medical diagnosis | <17 | Paediatric clinic or hospital admission |
| Patterson, 1994 ([45](#_ENREF_45)) | 1548/258 | CC | UK | R | Medical diagnosis | <15 | Diabetes register, hospital discharge, Health Service records |
| Perez-Bravo, 1996 ([46](#_ENREF_46)) | 165/80 | CC | Chile | Q/I | Medical diagnosis | <15 | Santiago de Chile registry |
| Perez-Bravo, 2003 ([47](#_ENREF_47)) | 250/143 | CC | Chile | Q | Medical diagnosis | 8 | School volunteers |
| Rami, 1999 ([48](#_ENREF_48)) | 609/114 | CC | Austria | Q | Medical diagnosis | <15 | Austrian diabetes register |
| Rosenbauer, 2008 ([49](#_ENREF_49)) | 2631/760 | CC | Germany | Q/I | Medical diagnosis | <5 | Hospital based surveillance system ESPD and local registration office records |
| Sadauskaite-Kuehne, 2004 ([50](#_ENREF_50)) | 1944/803 | CC | Sweden/Lithuania | Q | Medical diagnosis | 7 | Population register and outpatients |
| Samuelsson, 1993 ([51](#_ENREF_51)) | 1089/297 | CC | Sweden | Q/R | Medical diagnosis | <15 | Paediatric department and population register |
| Siemiatycki, 1989 ([52](#_ENREF_52)) | 482/161 | CC | Canada | I | Medical diagnosis | <17 | Hospital admission |
| Sipetic, 2005 ([53](#_ENREF_53)) | 315/105 | CC | Serbia | I | Medical diagnosis | <16 | Hospital admission |
| Skrodeniene, 2010 ([54](#_ENREF_54)) | 1099/286 | CC | Lithuania | Q | Medical diagnosis | 9 | Population register and outpatients |
| Soltesz, 1994 ([55](#_ENREF_55)) | 305/130 | CC | Hungary | Q | Medical diagnosis | <14 | Incidence register |
| Strotmeyer, 2004 ([56](#_ENREF_56)) | 485/247 | CC | China | Q | Medical diagnosis | 10 | Diabetes register and population register |
| Tai, 1998 ([57](#_ENREF_57)) | 310/117 | CC | Taiwan | I | Medical diagnosis | 8 | Taipei City |
| Telahun, 1994 ([58](#_ENREF_58)) | 129/55 | CC | Ethiopia | Q | Medical diagnosis | <15 | Ethio-Swedish Children's Hospital Diabetic Clinic |
| Tenconi, 2007 ([59](#_ENREF_59)) | 477/159 | CC | Italy | R/I/Q | Medical diagnosis | 16 | Diabetes register or paediatric admissions |
| Thorsdottir, 2000 ([60](#_ENREF_60)) | 220/55 | CC | Iceland | I | Medical diagnosis | 12 | Statistical Bureau of Iceland |
| Verge, 1994 ([61](#_ENREF_61)) | 475/217 | CC | Australia | Q | Medical diagnosis | <15 | New South Wales diabetes register and school records |
| Virtanen, 1992 (74) [(4](#_ENREF_46)6) (46) | 852/426 | CC | Finland | Q | Medical diagnosis | <14 | Hospital admissions |
| Virtanen, 1993 ([62](#_ENREF_62)) | 1380/690 | CC | Finland | Q | Medical diagnosis | 14 | Finnish National Population Registry |
| Visalli, 2003 ([63](#_ENREF_63)) | 900/150 | CC | Italy | Q | Medical diagnosis | 6-18 | EURODIAB study register and school records |
| Wadsworth, 1997 ([64](#_ENREF_64)) | 639/276 | CC | UK | Q | Medical diagnosis | <5 | BPASU reporting system and District Health Authority Immunisation Register |

Q: questionnaire, I: interview, R: medical records, PC: prospective cohort, NCC: nested case control, CS: Cross-sectional, CC: case control

Figure 1 Risk of bias in observational studies of total breastfeeding duration and TIDM

## Outcomes from studies of total breastfeeding and TIDM

## Ever vs. never breastfed

Figure 2 shows the combined effect of 32 eligible observational studies including over 6000 people with TIDM investigating the risk of TIDM according to whether infants were breastfed for any duration or never breastfed. Overall, there was a 22% (OR 0.78; 95% confidence interval [CI] 0.68, 0.89) reduction in the risk of having TIDM in infants who were ever breastfed, with high heterogeneity across studies (I^2^=51.2%). Subgroup analyses are shown in Table 2. Adjusted and unadjusted analyses yielded similar findings, with high heterogeneity in each. In the small number of studies with prospective design (total 313 cases), in those which used TIDM associated antibodies rather than disease as an outcome (and recruited high risk populations; total 116 cases) and in studies with low risk of bias (total 86 cases), there was no evidence of an association between BF and TIDM. Risk of bias was commonly considered unclear, due to lack of information about blinding of outcome assessors to exposure data. We found no evidence of publication bias (Egger’s test P-value=0.81) (Figure 3).

Figure 2 Breastfeeding ever vs. never and TIDM risk


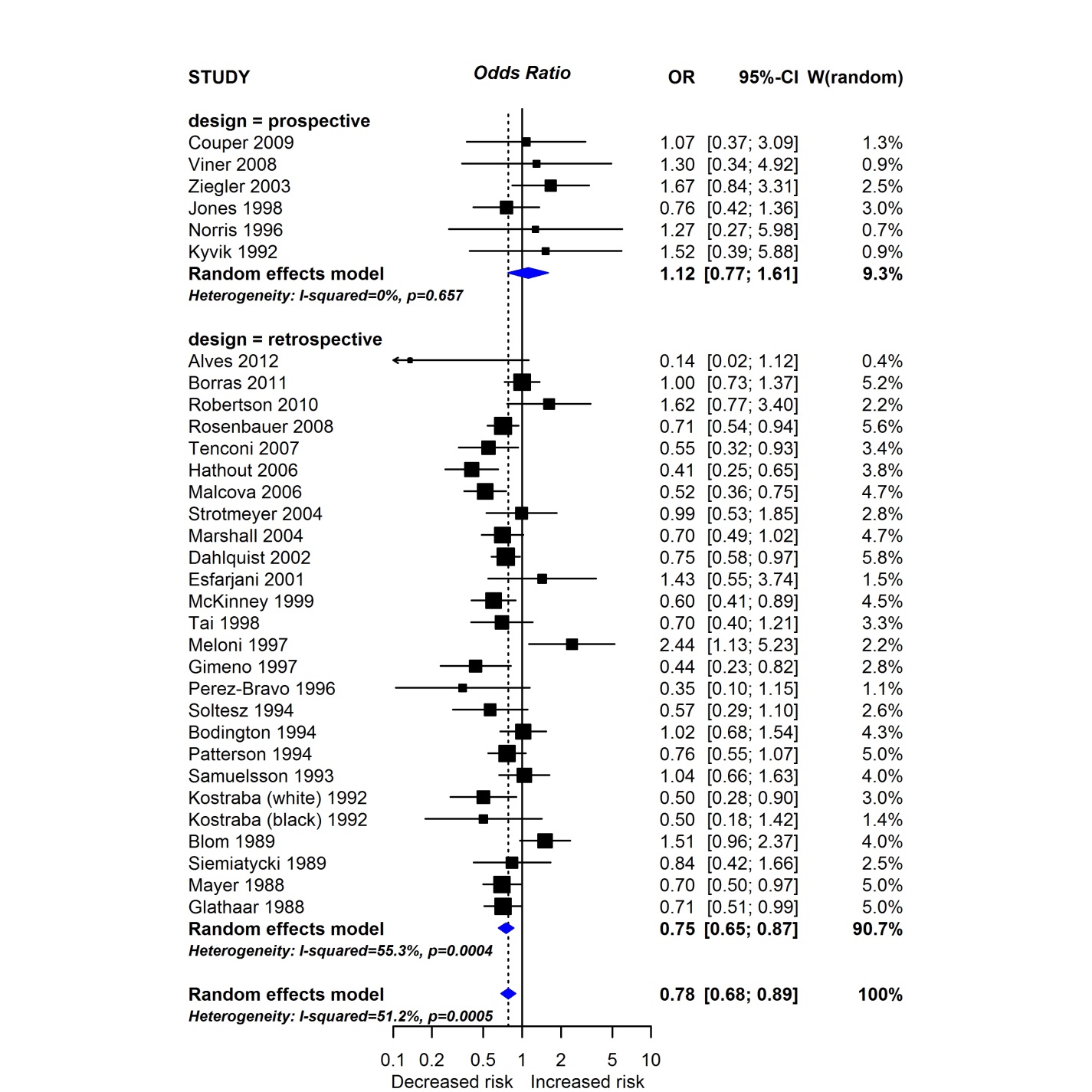


Table 2 Stratified and subgroup analyses of association between ever vs. never being breastfed and risk of TIDM

|  | **Number of studies** | **OR [95% CI]*** | **I^2^ (%)** | **P-value for between groups difference** |
| --- | --- | --- | --- | --- |
| **Overall (if adjusted NA, unadjusted value used)**  **Adjusted**  **Unadjusted** | 32  15  29 | 0.78 [0.68-0.89]  0.76 [0.64-0.91]  0.81 [0.70-0.94] | 51.2  51.8  54.0 | Not tested |
| Risk of disease – High  Risk of disease – Normal | 3  29 | 1.44 [0.84-2.47]  0.76 [0.67-0.87] | 0  51.4 | **0.025** |
| Risk of bias – Low  Risk of bias – High/Unclear | 2  30 | 1.59 [0.85-2.98]  0.76 [0.66-0.87] | 0  50.1 | **0.025** |
| Study Design – Prospective  Study Design - Retrospective | 6  26 | 1.15 [0.77-1.61]  0.75 [0.65-0.87] | 0  55.3 | **0.049** |
| Method of diagnosis – clinical  Method of diagnosis – serological (single or combination of antibodies) | 29  3 | 0.76 [0.66-0.87]  1.44 [0.84-2.47] | 51.4  0 | **0.025** |

*Pooled estimate [95%CI] of meta-analysis of studies reporting Odds or Relative risk or Risk ratios

Figure 3 Risk of publication bias in observational studies investigating breastfed ever vs. never and TIDM risk


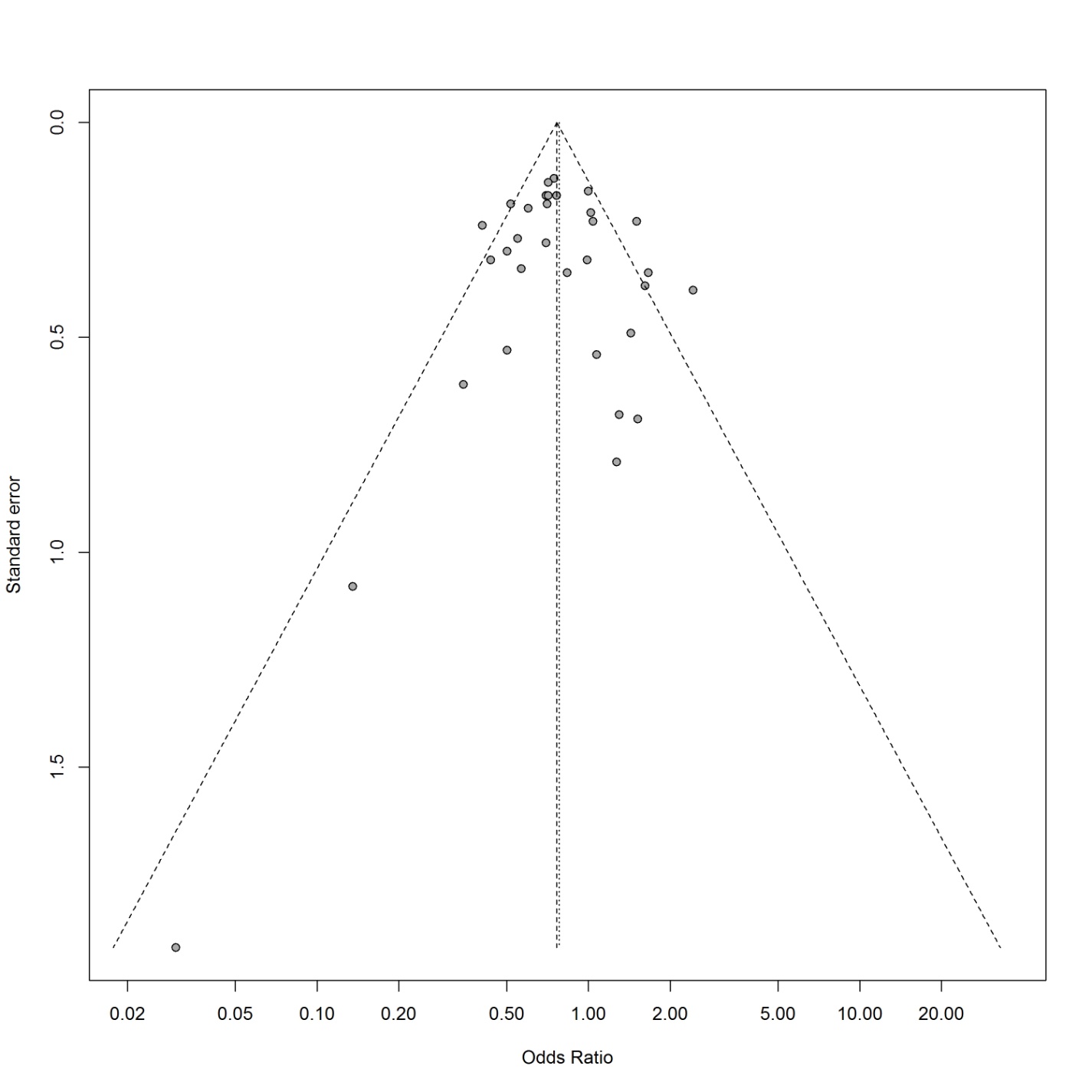


Egger’s test p=0.81

## Total breastfeeding ≥1-2 months vs. < 1-2 months

Four studies examined the risk of TIDM if infants were breastfed for over 1-2 months compared to less than this duration. Figure 4 shows that the combined risk of TIDM is 36% lower (Pooled OR 0.64; 95% CI 0.43, 0.96) if infants were breastfed for at least 1-2 months. There was moderate heterogeneity across studies (I^2^=45.3%). Stratified and subgroup analyses was not performed due to the small number of studies included. The study of Kyvik presented unadjusted data from a nested case control study, considered at high risk of bias, comparing ≥5 months with 0-1 months TBF; Virtanen and Verge presented adjusted data from case control studies; Wahlberg unadjusted data from a prospective cohort study using autoantibodies as an outcome. The reason for the statistical heterogeneity is not clear, but may relate to use of autoantibodies at age 2 as an outcome in Wahlberg, and presentation of unadjusted data in Kyvik.

**Figure 4 Breastfeeding for ≥1-2 months vs. < 1-2 months and TIDM risk**

**
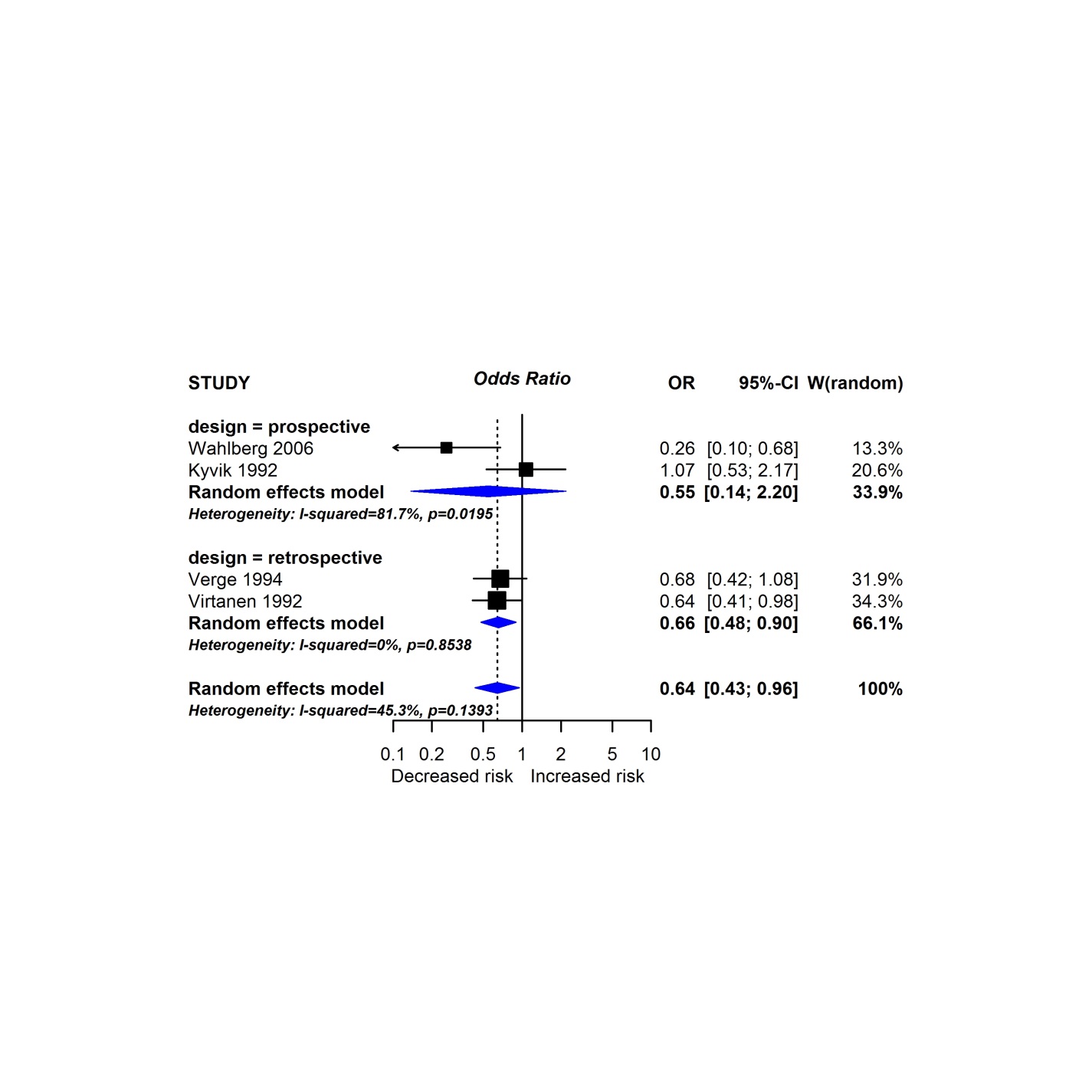
**

## Total breastfeeding duration ≥3-4 months vs. <3-4 months

The association between breastfeeding for at least 3-4 months vs. less than this duration and risk of TIDM was examined in 10 studies. Meta-analysis showed reduced risk of TIDM (OR 0.55; 95% CI 0.39, 0.77) (Figure 5) although there was high heterogeneity across studies (I^2^=61.2%). There was little evidence of different outcomes in subgroup or stratified analyses, other than a greater reduction in risk in individuals with high disease risk versus low/normal risk (Table 3). Ziegler, Fronczak and Couper presented HR from prospective studies using autoantibodies for TIDM diagnosis in young children. Holmberg also used autoantibodies in young children in a prospective study. In general heterogeneity was reduced in retrospective studies using clinical diagnosis of TIDM.

Figure 5 Breastfeeding for ≥3-4 months vs. <3-4 months and TIDM risk


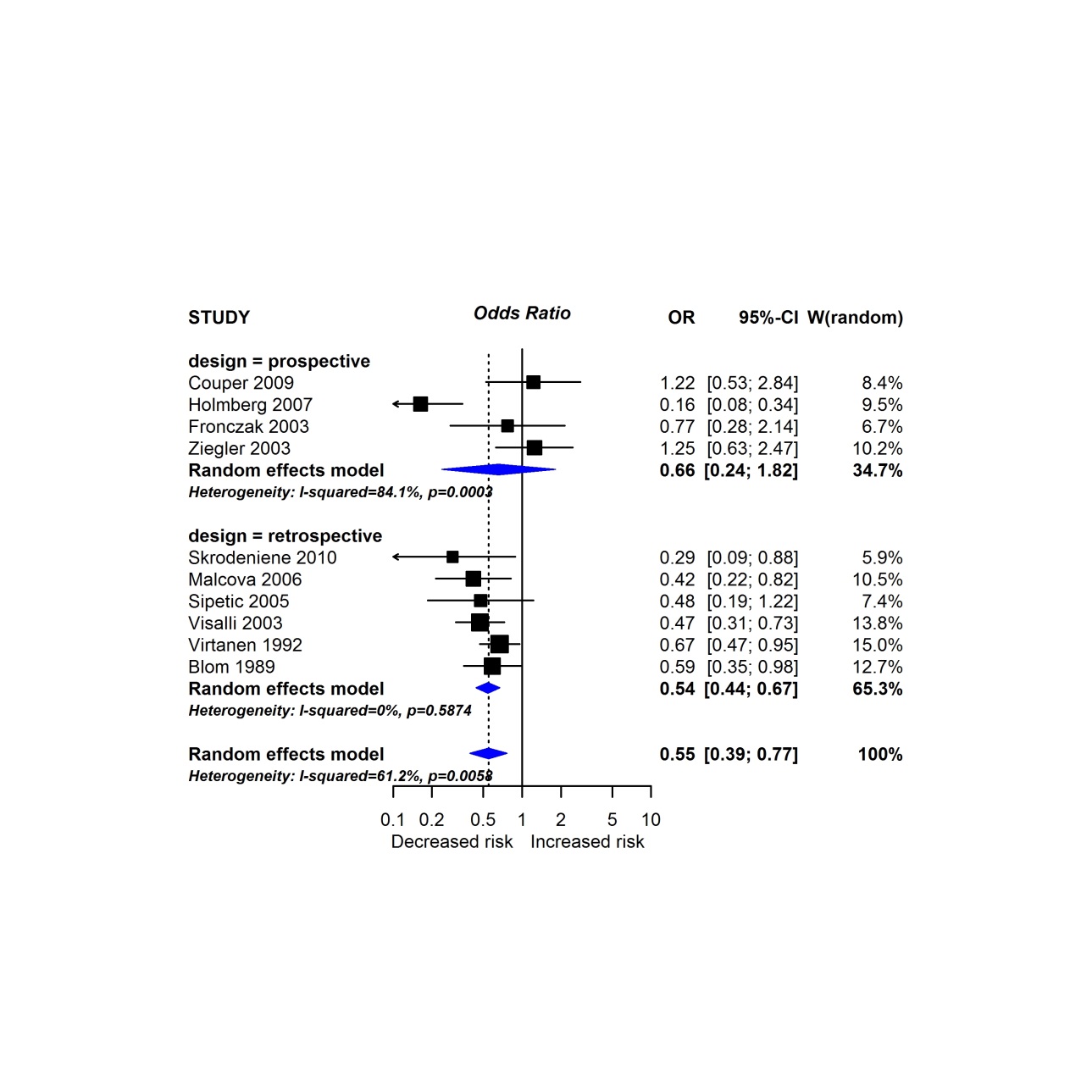


Table 3 Stratified and subgroup analyses of breastfeeding ≥3-4 months vs. <3-4 months and TIDM risk

|  | **Number of studies** | **OR [95% CI]*** | **I^2^ (%)** | **P-value for between groups difference** |
| --- | --- | --- | --- | --- |
| **Overall (if adjusted NA, unadjusted value used)**  **Adjusted**  **Unadjusted** | 10  5  7 | 0.55 [0.39-0.77]  0.58 [0.40-0.85]  0.63 [0.38-1.04] | 61.2  53.8  74.5 | Not tested |
| Risk of disease – High  Risk of disease – Normal | 3  7 | 1.12 [0.70-1.79]  0.45 [0.32-0.62] | 0  53.6 | **0.002** |
| Risk of bias – Low  Risk of bias – High/Unclear | 2  8 | 0.85 [0.47-1.53]  0.47 [0.32-0.69] | 59.7  53.7 | 0.099 |
| Study Design – Prospective  Study Design - Retrospective | 4  6 | 0.66 [0.24-1.82]  0.54 [0.44-0.67] | 84.1  0 | 0.719 |
| Method of diagnosis – clinical  Method of diagnosis – serological (single or combination of antibodies) | 6  4 | 0.54 [0.44-0.67]  0.66 [0.24-1.82] | 0  84.1 | 0.719 |

*Pooled estimate [95%CI] of meta-analysis of studies reporting Odds or Relative risk or Risk ratios

Figure 6 Risk of publication bias in observational studies investigating TBF ≥3-4 months vs. <3-4 months and TIDM risk


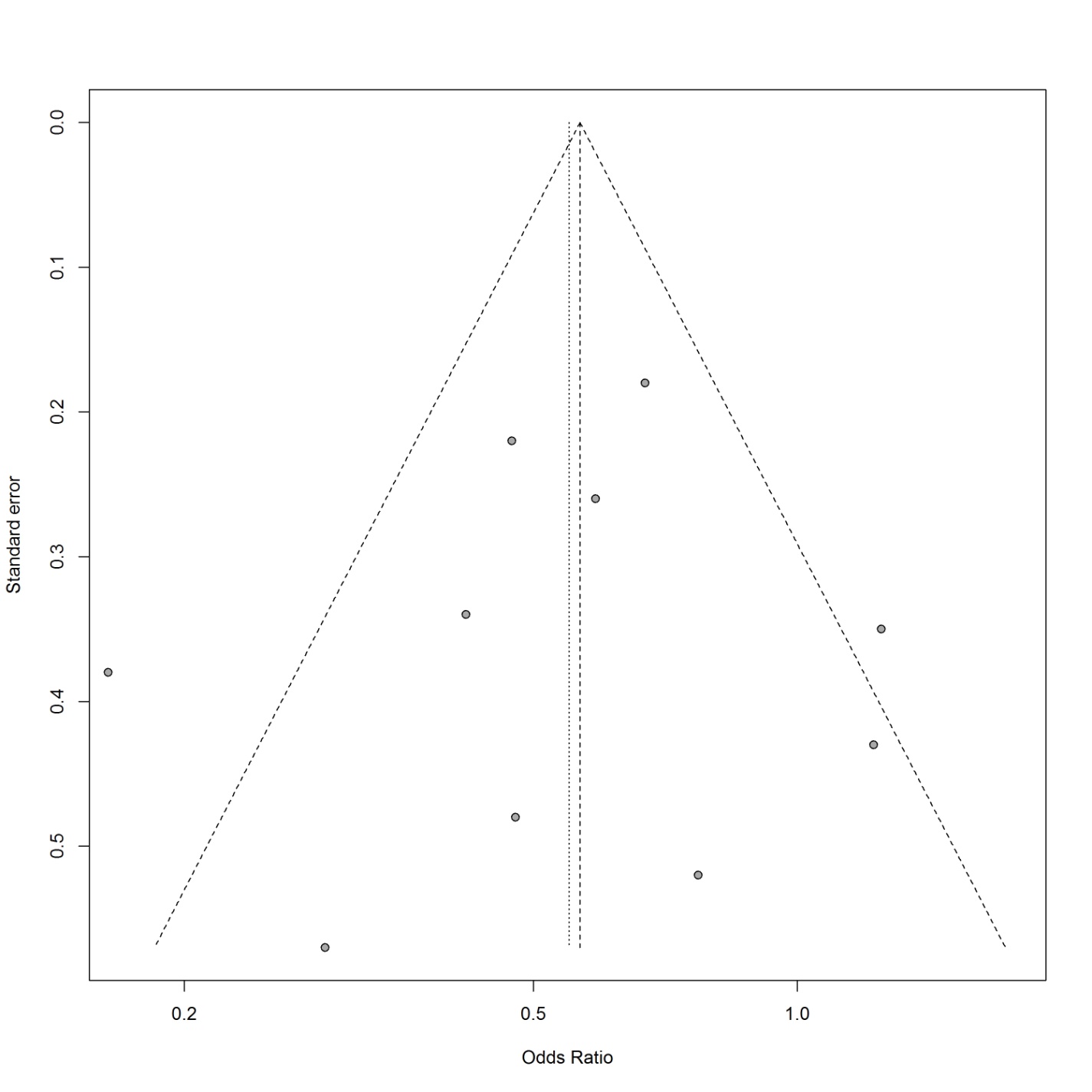


Egger’s test P=0.72

## Total duration of breastfeeding ≥5-7 months vs. < 5-7 months

Six studies contributed data to meta-analysis of TBF ≥5-7 vs. <5-7 months and TIDM risk (Figure 7). There was reduced risk associated with prolonged TBF (OR 0.59; 95% CI 0.37, 0.92) but there was extreme heterogeneity across studies (I^2^=76.6%). Table 4 shows subgroup and stratified analyses. There was reduced statistical heterogeneity in analysis of adjusted data. Studies of retrospective design and those with an unclear definition of breastfeeding showed a greater level of risk reduction for TIDM than prospective studies or those with clear TBF definition. Data from Majeed included in meta-analysis were unadjusted, and adjusted analysis in the same study was not statistically significant. Sensitivity analysis excluding the study by Majeed reduced heterogeneity (I^2^=54.2%, OR 0.72, 95% CI 0.53, 0.98).

Figure 7 Breastfeeding for ≥5-7 months vs. <5-7 months and TIDM risk


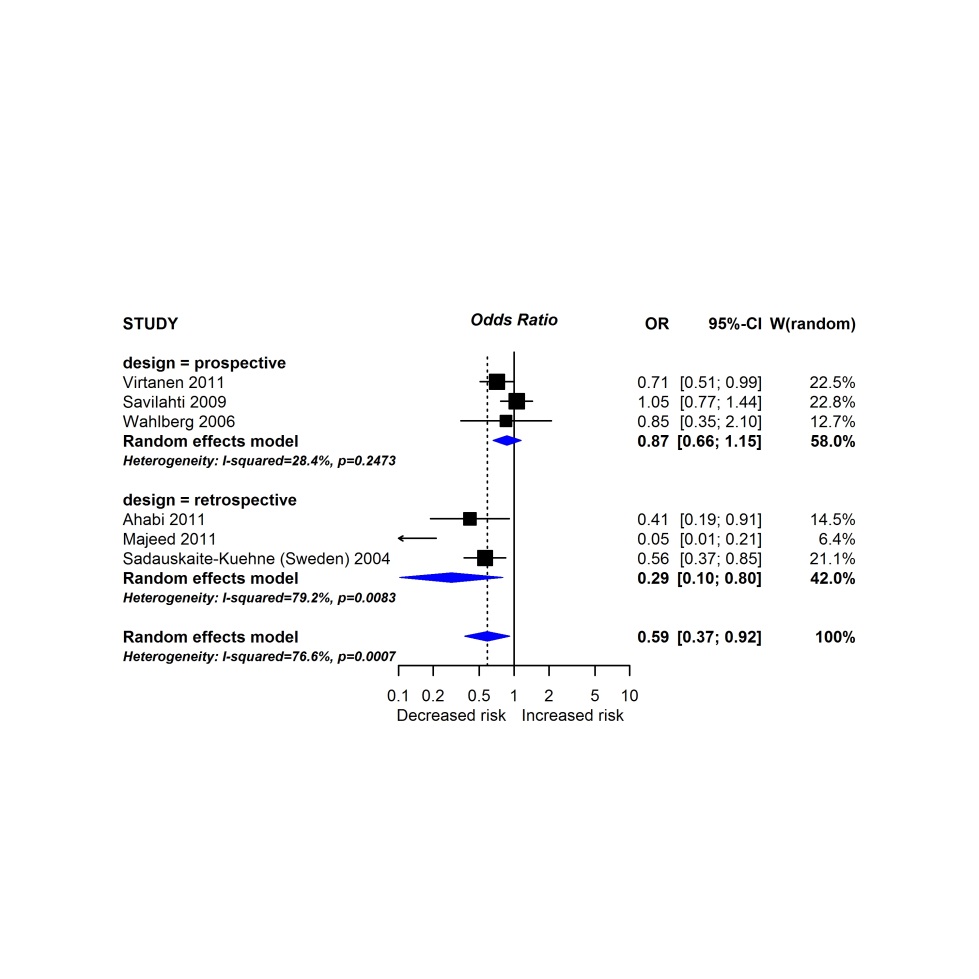


Table 4 Stratified and subgroup analyses of breastfeeding ≥5-7 months vs. <5-7 months and TIDM risk

|  | **Number of studies** | **OR [95% CI]*** | **I^2^ (%)** | **P-value for between groups difference** |
| --- | --- | --- | --- | --- |
| **Overall (if adjusted NA, unadjusted value used)**  **Adjusted**  **Unadjusted** | 6  2  5 | 0.59 [0.37-0.92]  0.65 [0.50-0.84]  0.61 [0.35-1.06] | 76.6  0  78.8 | Not tested |
| Risk of disease – High  Risk of disease – Normal | 1  5 | 0.71[0.51-0.99]  0.51 [0.27-0.97] | -  81.2 | 0.377 |
| Risk of bias – Low  Risk of bias – High/Unclear | 1  5 | 0.71 [0.51-0.99]  0.51 [0.27-0.97] | -  81.2 | 0.377 |
| Study Design – Prospective  Study Design - Retrospective | 3  3 | 0.87 [0.66-1.15]  0.29 [0.10-0.80] | 28.4  79.2 | **0.042** |
| Method of diagnosis – clinical  Method of diagnosis – serological (single or combination of antibodies) | 4  2 | 0.45 [0.21-0.96]  0.73 [0.53-0.99] | 85.9  0 | 0.244 |

*Pooled estimate [95%CI] of meta-analysis of studies reporting Odds or Relative risk or Risk ratios

## Total duration of breastfeeding ≥8-12 months vs. <8-12 months

Total breastfeeding duration for ≥8-12 months showed an overall reduced risk of TIDM compared to shorter TBF duration – 2 studies, pooled OR 0.55 (95% CI 0.38, 0.79) with no significant heterogeneity (I^2^=6.1%) (Figure 8). Both studies are case control studies using medical diagnosis of TIDM, one reporting adjusted and one (Ahabi) unadjusted data.

Figure 8 Breastfeeding for ≥8-12 months vs. <8-12 months and TIDM risk


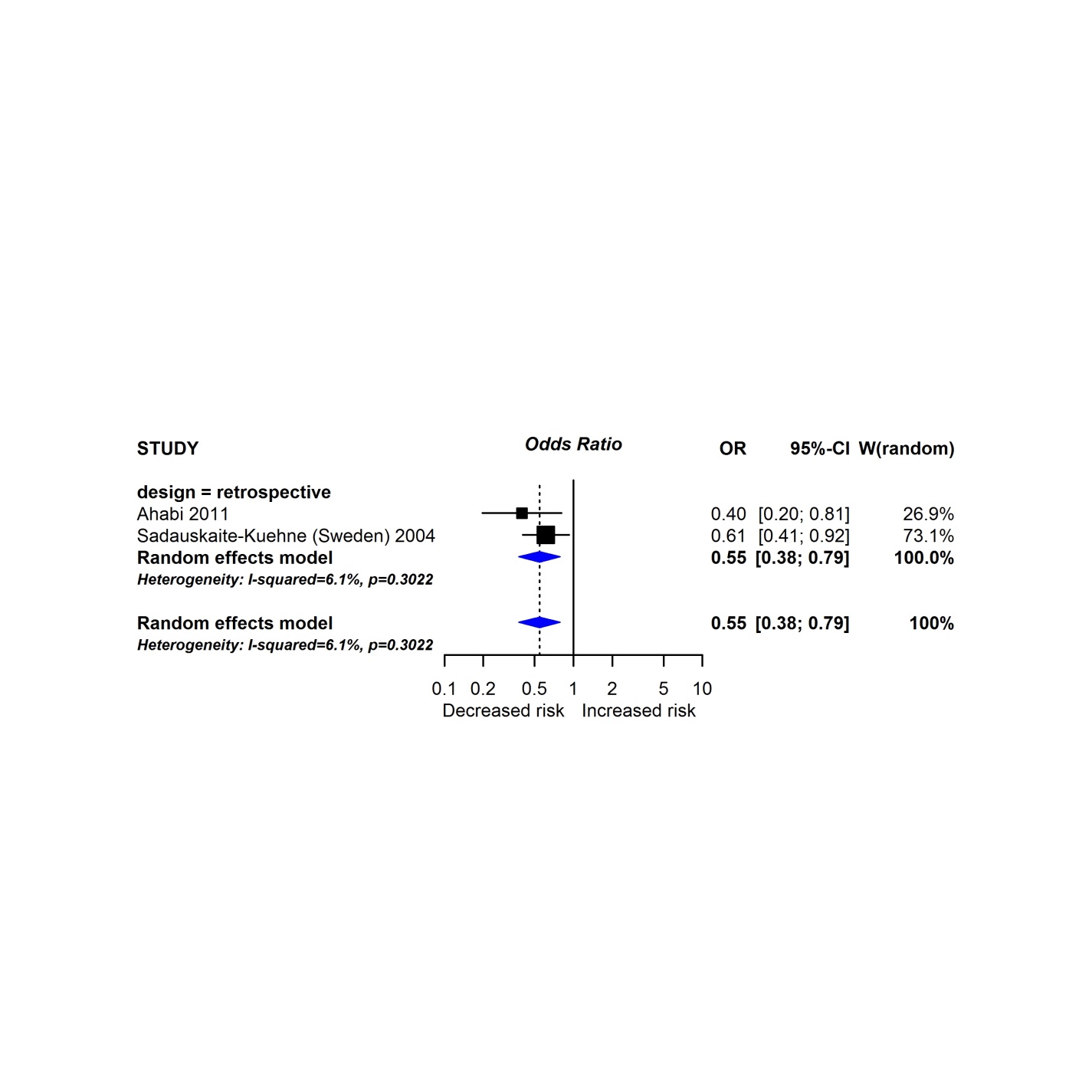


## Dose response analysis of TBF and TIDM risk

We also analysed TBF duration by grouping studies according to the exposure rather than the reference group – short (≥1-3 months), medium (≥4-6 months) and long (≥7-12 months); all compared to a reference group of never BF. These analyses are shown in Figures 9, 10 and 11. The data showed no significant difference between any time frame analysed versus never. There was low heterogeneity in the short and medium versus never analyses (I^2^=0 and I^2^=11.7% respectively). There was extreme heterogeneity in the long versus never breastfeeding meta-analysis (I^2^=84.3%) so pooled analysis was not reported; the reason for this heterogeneity is unclear. Sensitivity analysis excluding the study by Meloni reduced heterogeneity (I^2^=33.7%, OR 0.40, 95% CI 0.19, 0.84), although there appeared to be no major difference in method between studies to explain the difference in results. All 3 studies reported adjusted data from case control studies using clinical diagnosis of TIDM.

We were unable to identify a clear explanation for why dose response analysis did not mirror the positive association seen in analyses using reference groups to define the cut off. Four individual studies were included in more than one dose response analysis - they did not show a clear trend, but in general there tended to be a stronger association between TBF and reduced TIDM for longer TBF exposure ([11](#_ENREF_11), [42](#_ENREF_42), [49](#_ENREF_49), [57](#_ENREF_57)).

**Figure 9 Breastfeeding for short duration versus never**


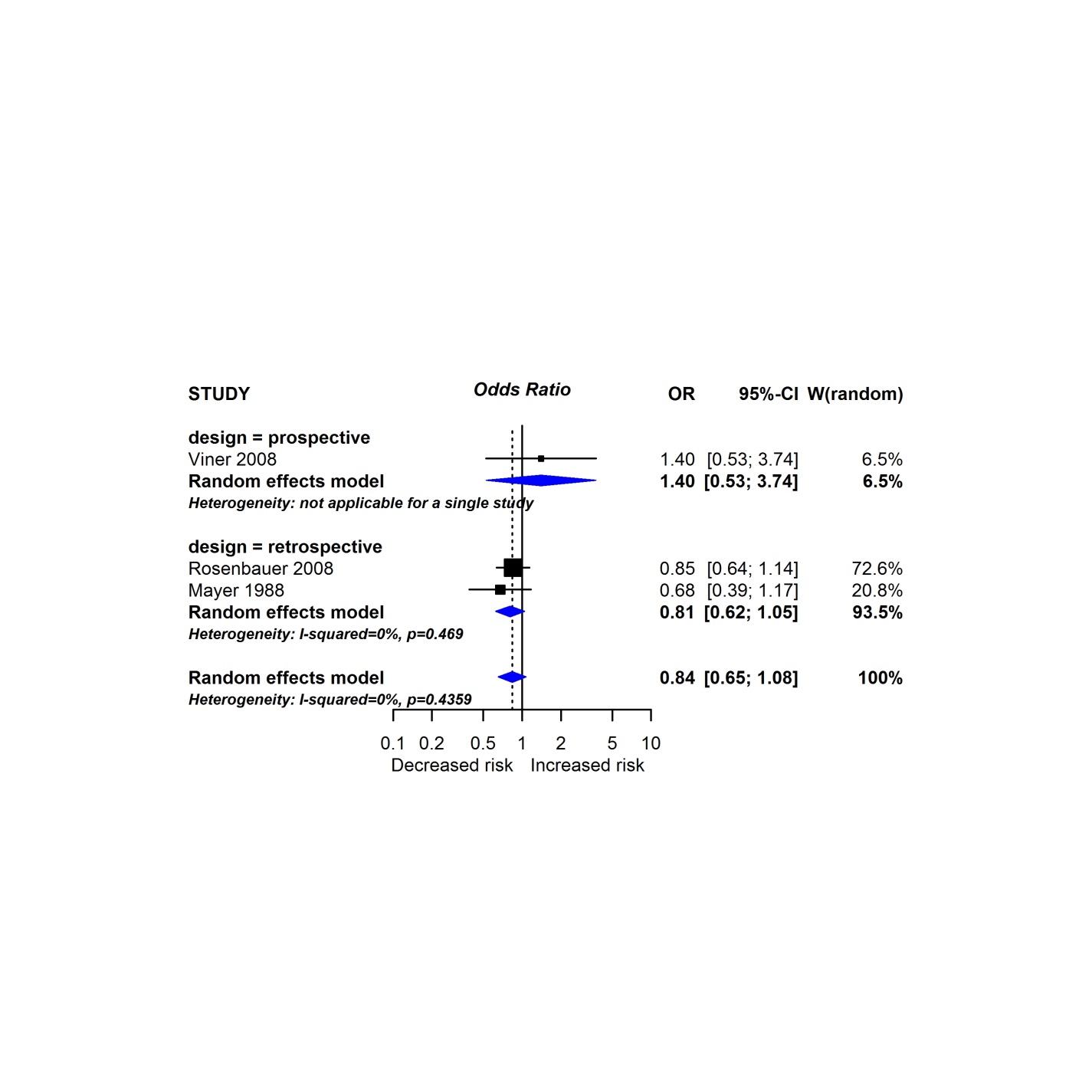


**Figure 10 Breastfeeding for medium duration versus never**


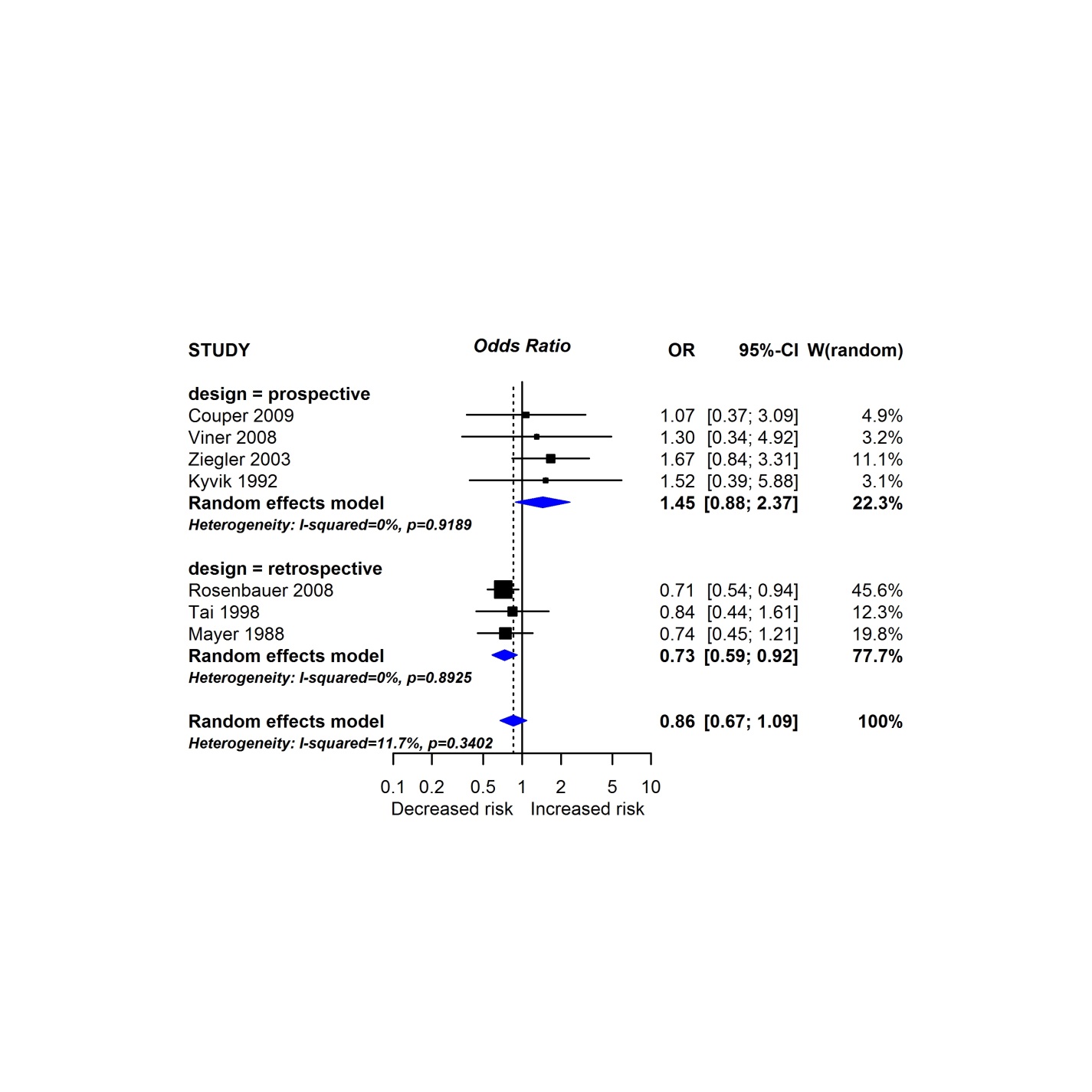


**Figure 11 Breastfeeding for long duration versus never**


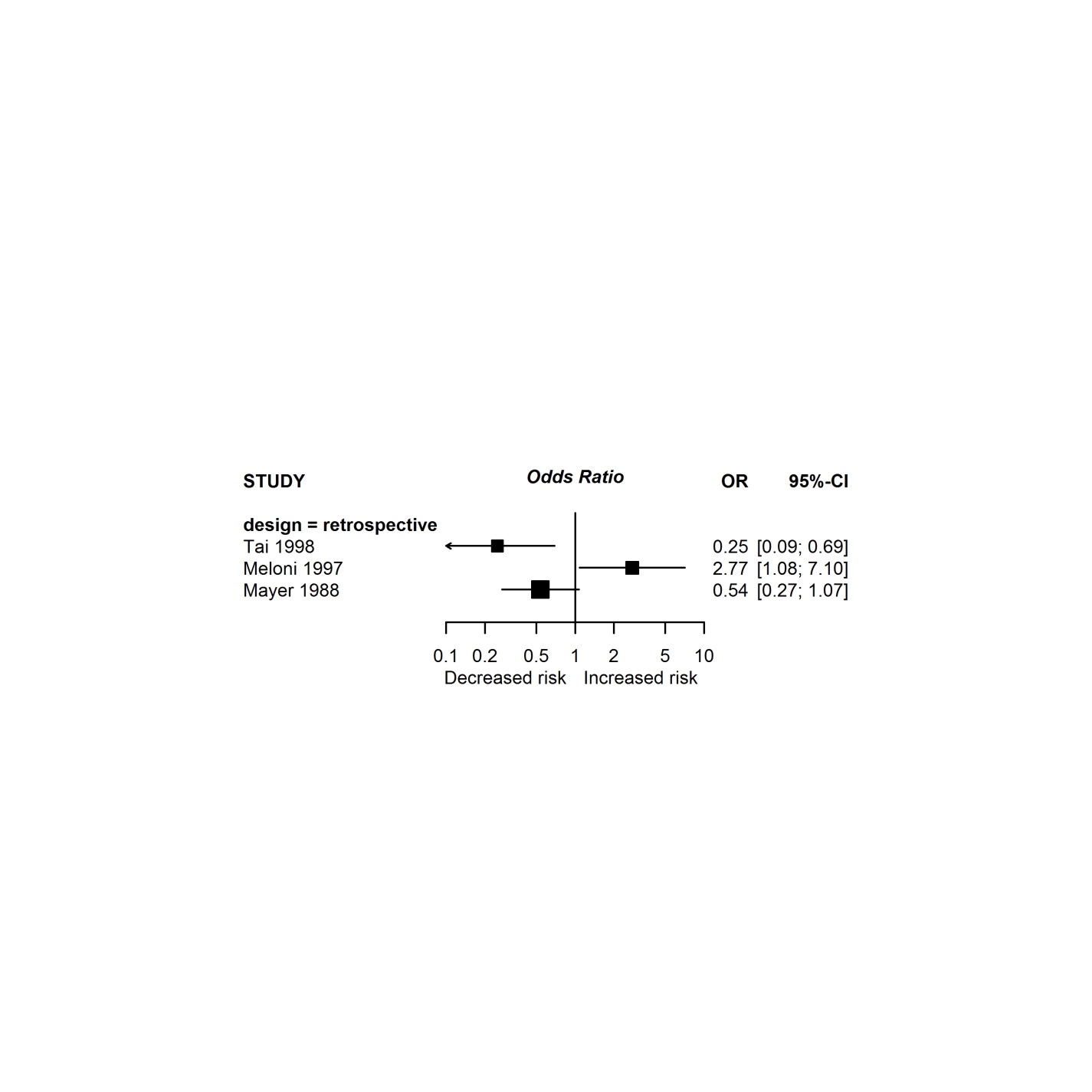


## Studies investigating TBF and TIDM as a continuous variable

Three studies reported OR of TIDM for each month increase in TBF duration. Meta-analysis of these data showed no significant association (pooled OR 1.01; 95 CI% 0.87, 1.18) but with extreme statistical heterogeneity (I^2^=76.8%; Figure 12). Three additional studies comparing the mean duration of TBF between TIDM and unaffected subjects were meta-analysed (Figure 13). Data could not be pooled to extreme statistical heterogeneity (I^2^=91.8%) which was present even when the study of Baruah (27) was excluded (I^2^=92.8%). This extreme heterogeneity may be related to the non-Gaussian distribution of TBF duration in the population.

Figure 12 Risk of TIDM for each month increase in TBF duration


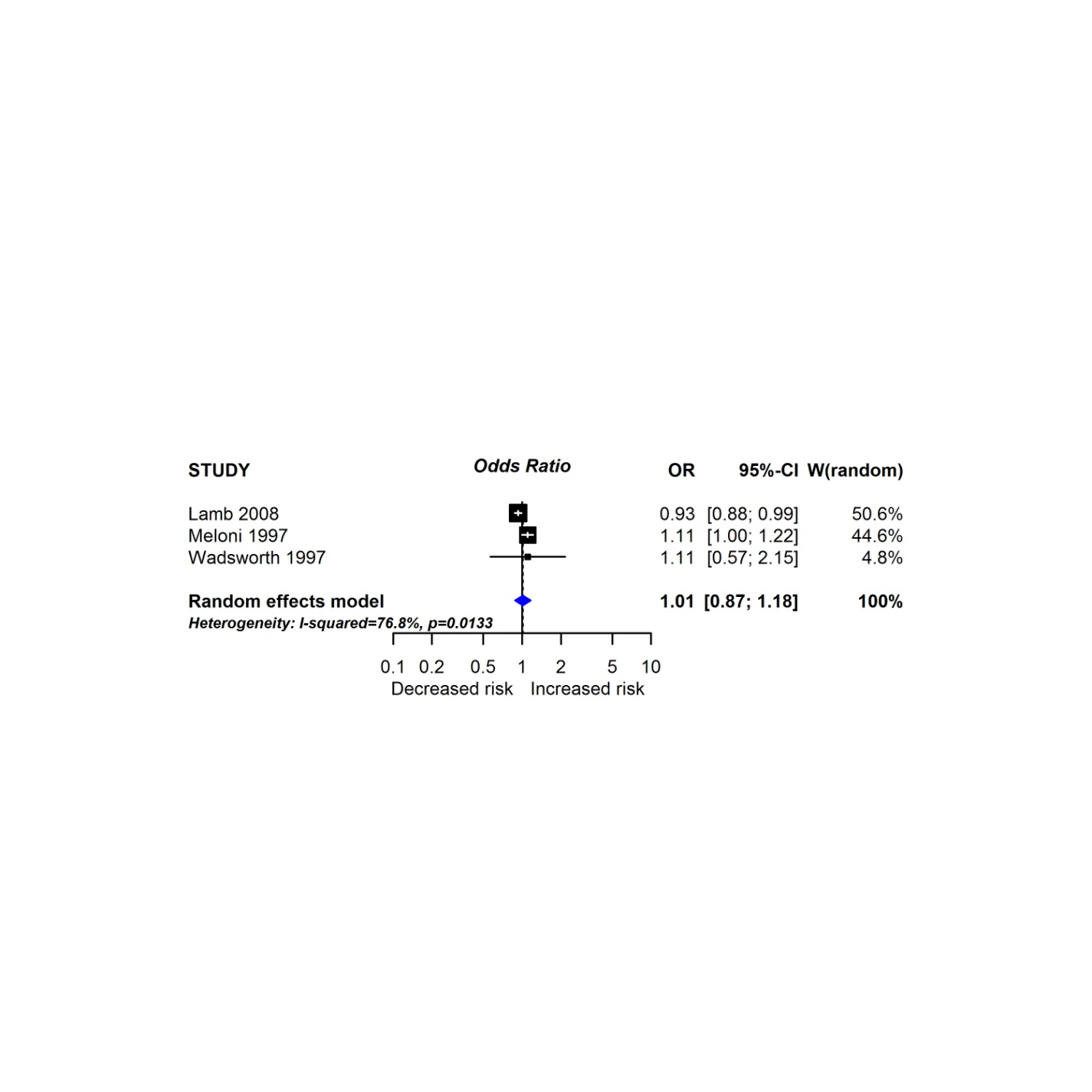


Figure 13 Difference in TBF in people with TIDM versus unaffected subjects


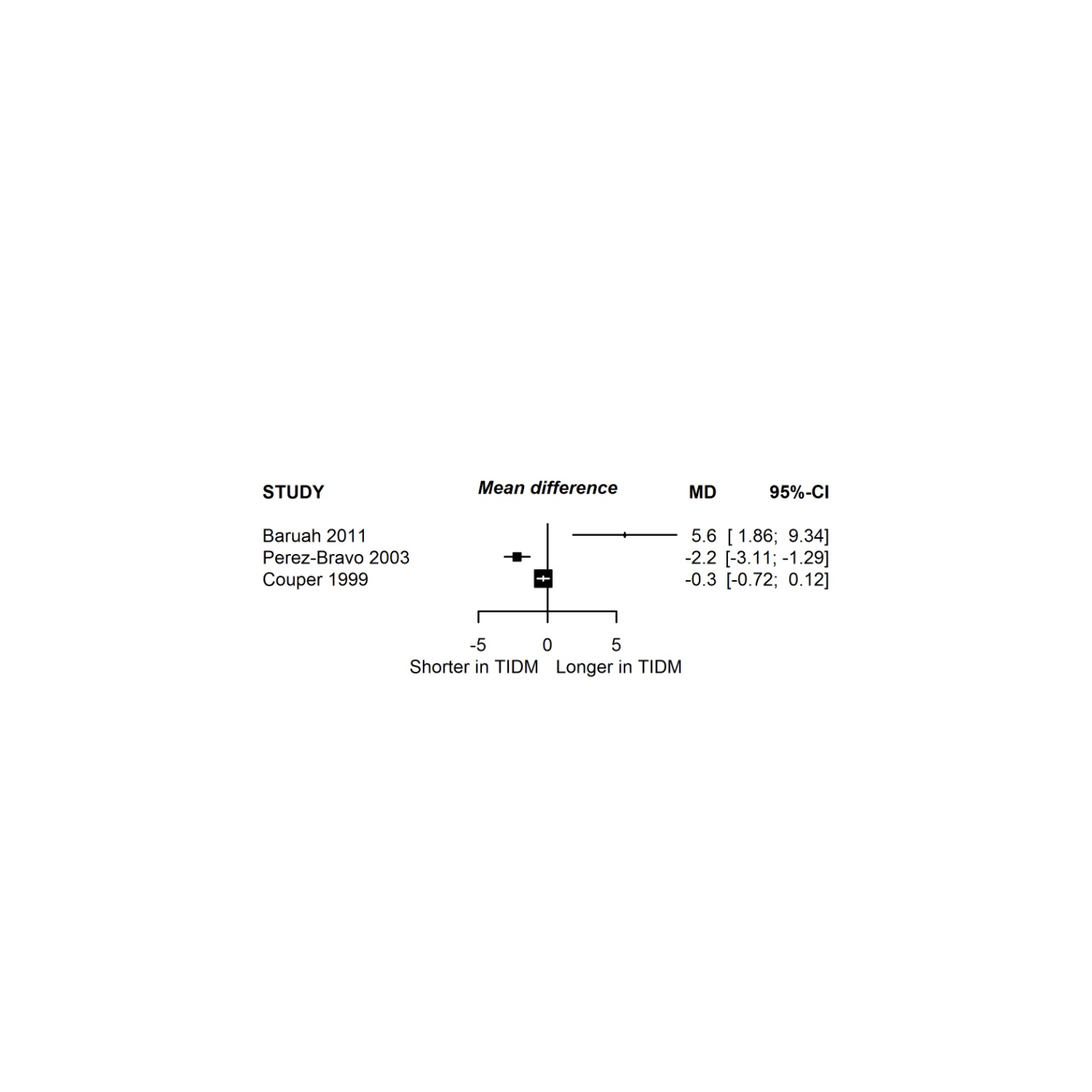


## 1.2.8 Data for TBF and TIDM which couldn’t be meta-analysed

Meta-analyses included 7 cohort, 4 nested case control, 1 cross sectional and 29 case control studies in total, including at least 8221 participants with TIDM. A further 7 cohort, 3 nested case control and 14 case control studies reported relevant data which could not be included in meta-analysis, in relation to at least 3909 participants with TIDM. These studies are summarised in Table 5. Two of the case control studies (Ashraf ([26](#_ENREF_26)), Perez-Bravo ([47](#_ENREF_47))) showed a significantly shorter TBF duration in TIDM compared to unaffected subjects. The other 22 studies showed no significant relationship, although TBF duration was shorter in people with TIDM in 9 of these 22 studies, longer in 4 and similar or unclear in 9. The study of Lamb 2008 (7) also reported adjusted HR 0.93 (95%CI 0.86, 0.99) for each additional month of breastfeeding.

**Table 5 Other studies evaluating total breastfeeding and TIDM which couldn’t be meta-analysed**

| **First Author and Year of Publication** | **Design** | **N/n cases** | **Total BF duration** | **Units** | **Descriptive measure** | **Unaffected** | | | **TIDM** | **P value** |
| --- | --- | --- | --- | --- | --- | --- | --- | --- | --- | --- |
| Baruah, 2011 ([27](#_ENREF_27)) | CC | 86/43 | continuous | Months | Mean | 13 | | | 18.6 | **-** |
| Kostraba, 1993 ([38](#_ENREF_38)) | CC | 306/142 | continuous | Months | Mean | 6.95 | | 6.2 | | - |
| Marshall, 2004 ([41](#_ENREF_41)) | CC | 577/196 | continuous | Months | Mean | 4.6 | | | 3.9 | - |
| Norris, 2003 ([10](#_ENREF_10)) | PC | 1183/~733 | continuous | Months | Mean | 6.1 | | | 5.9 | - |
| Perez-Bravo, 2003 ([47](#_ENREF_47)) | CC | 250/143 | continuous | Months | Mean | 7.6 | | | 5.4 | **<0.02** |
| Virtanen, 1993 ([62](#_ENREF_62)) | CC | 1380/690 | continuous | Months | Mean | 6.6 | | | 6.6 | NS |
| Ashraf, 2010 ([26](#_ENREF_26)) | CC | 195/128 | continuous | Months | Median(range) | 3 (0-4) | | | 1 (0-4) | **0.001** |
| Hummel, 2000 (mother with TIDM) ([5](#_ENREF_5)) | PC | 568 | continuous | Months | Median | 3 | | | 4 | NS |
| Hummel, 2000 (father with TIDM) |  |  | continuous | Months | Median | 4 | | | 2.50 | 0.41 |
| Kimpimaki, 2001 ([17](#_ENREF_17)) | NCC | 455/65 | continuous | Months | Median (IQR) | 6.5 (3-10) | | | 6.0 (2.9-10.5) | - |
| Norris, 1996 ([19](#_ENREF_19)) | NCC | 171/18 | continuous | Months | Median (IQR) | 8 | | | 10 | - |
| Rami, 1999 ([48](#_ENREF_48)) | CC | 609/114 | continuous | Months | Median (range) | 2 (0-72) | | | 2 (0-24) | 0.54 |
| Hypponen, 1999 ([36](#_ENREF_36)) | CC | 821/435 | continuous | Months | Average |  | | |  | NS |
| Lamb, 2013 ([8](#_ENREF_8)) | NCC | 260 | continuous | Months | Average |  | | |  | NS |
| Ludvigsson, 2003 ([9](#_ENREF_9)) | PC | 205 | continuous | Months | Average |  | | |  | NS |
| Telahun, 1994 ([58](#_ENREF_58)) | CC | 129/55 | continuous | Months | Average |  | | |  | NS |
| Lamb, 2008 ([7](#_ENREF_7)) | PC | 642 | continuous | Months | aHR(95%CI) | 0.93 (0.86, 0.99) per month TBF | | | | - |
| Virtanen, 1998 ([12](#_ENREF_12)) | PC | 697/~43 | categorical | >2 vs <2 months | aHR (95%CI) | 0.53 (0.2-1.6) | | | | - |
| Karlen, 2012 ([6](#_ENREF_6)) | PC | 1409 | categorical | >7 vs 0-6 months | uOR (95% CI) | 0.83 (0.52-1.32) | | | | - |
| Wadsworth, 1997 ([64](#_ENREF_64)) | CC | 639/276 | categorical | >2 vs <2 weeks | aOR (95% CI) | 1.1 (0.56-2.16) | | | | 0.7 |
| Bener, 2009 ([28](#_ENREF_28)) | CC | 340/170 | categorical | Breastfed (yes) | % | 97.2 | 95.1 | | | - |
| Couper, 1999 ([1](#_ENREF_1)) | PC | 317/70 | categorical | Breastfed (yes) | % | 75 | | | 61 | - |
|  |  |  | continuous | Months | Mean | 6.1 | | | 5.3 | NS |
| Sadauskaite-Kuehne, 2004 (Lithuania) ([50](#_ENREF_50)) | CC | 813/286 | categorical | Breastfed (yes) | % | 91.6 | | | 95 | NS |
| Strotmeyer, 2004 ([56](#_ENREF_56)) | CC | 485/247 | categorical | Breastfed (yes) | % | 91.1 | 91.0 | | | - |
| Thorsdottir, 2000 ([60](#_ENREF_60)) | CC | 220/55 | categorical | Breastfed (yes) | % |  | | |  | 0.10 |

# Exclusive breastfeeding duration and risk of TIDM

## Overall characteristics of studies, risk of bias and summary of results

Table 6 describes the main characteristics of the studies analysed in this report. A total of 28 observational studies, and no intervention studies, reported the association between duration of exclusive breastfeeding (EBF) and risk of TIDM. Of these, 8 were prospective cohort studies, 2 nested case-control, and 18 case-control studies. Over half of the studies (n=16) are from Europe – others are from North America (n=4), South America (n=2), Asia/Pacific (n=4), and the Middle East (n=2). Overall, valid data on EBF duration and TIDM risk were available from almost 35,000 subjects and over 5300 people with TIDM. Information on TIDM was obtained mainly from Islet auto-antibodies in 8 prospective studies and via medical diagnosis in 20 (mainly case control) studies. With regards to time of outcome diagnosis, 6 studies explored the association between duration of EBF and TIDM in the first 5 years of life and 22 studies evaluated the outcome in older children or adolescents. 26 studies used interview or questionnaire to assess EBF duration, 1 study assessed medical records and 1 did not report the method of exposure assessment.

Risk of bias was assessed using the NICE Methodological checklist for cohort and case-control studies. Figure 14 illustrates the distribution of bias across the five main methodological areas of the studies. Over half of the studies had a high risk, most commonly due to lack of adjustment for confounding bias i.e., no adjusted data presented. A third of the studies had an ‘unclear’ overall risk of bias, most commonly due to insufficient information to assess selection and assessment bias.

Three levels of comparison were used to meta-analyse binary data for TIDM risk and EBF duration, based on the distribution of data reported in included studies: EBF duration with a cut-off in the first 2 months (‘≥0-2 months vs. <0-2 months’); EBF duration ‘≥3-4 months vs. <3-4 months’; EBF duration ‘≥5-9 months vs. <5-9 months’.

*Main Findings*

Across all three EBF duration cut-offs there was some evidence of reduced TIDM risk with increased EBF duration. Stratified and subgroup analyses by specific risk groups and study characteristics showed little evidence of difference. Adjusted and unadjusted analyses gave similar findings. The evidence for an association between EBF and TIDM from the 14 studies (1588 TIDM cases) which could not be included in meta-analysis was weaker as only three studies showed statistically significant associations between increased EBF duration and reduced TIDM risk and the others were inconclusive. All but one study used mean or median to present results which prevented the use of the data in the meta-analysis, and one study used a definition of breastfeeding duration that was not possible to combine with the other studies (Table 9). Overall the data suggest there is LOW certainty evidence that longer duration of EBF is associated with reduced TIDM risk, with relatively low statistical heterogeneity within individual meta-analyses, and reasonable consistency between meta-analyses.

Table 6 Characteristics of Included Studies for analysis of Exclusive Breast Feeding duration and TIDM Risk

| **First Author & Publication Year** | **N/n cases** | **Design** | **Country** | **Exposure assessment** | **Specific outcome** | **Age at outcome (years)** | **Population**  **characteristics** |
| --- | --- | --- | --- | --- | --- | --- | --- |
| Couper, 1999 ([1](#_ENREF_1)) | 317/70 | PC | Australia | D/Q | Islet autoantibodies | 2 | First degree relatives of diabetic children |
| Couper, 2009 ([65](#_ENREF_65)) | 548/~30 | PC | Australia | D/I | Islet autoantibodies | 2 | First degree relatives of diabetic children |
| Frederikson, 2012 (abstract) ([66](#_ENREF_66)) | 1698 | PC | USA | - | Medical diagnosis | <7 | High risk children via HLA screening |
| Holmberg, 2007 ([67](#_ENREF_67)) | 3788/~51 | PC | Sweden | Q | Islet autoantibodies | 6 | General population |
| Hummel, 2000 ([5](#_ENREF_5)) | 568 | PC | Germany | Q | Islet autoantibodies | 2 | Offspring of diabetic parents |
| Virtanen, 2011 ([13](#_ENREF_13)) | ~4000/~160 | PC | Finland | Q | Medical diagnosis or islet autoantibodies | <18 | Odu and Tampere University Hospitals |
| Wahlberg, 2006 ([15](#_ENREF_15)) | 8715/31 | PC | Sweden | Q | Islet autoantibodies | 2 | General population |
| Ziegler, 2003 ([14](#_ENREF_14)) | 1460/~68 | PC | Germany | Q | Islet autoantibodies | 5 | Children of a mother/father withT1DM |
| Kimpimaki, 2001 ([17](#_ENREF_17)) | 455/65 | NCC | Finland | I | Islet autoantibodies | <4 | Turku, Oulu and Tampere Hospital births |
| Lamb, 2013 ([8](#_ENREF_8)) | 260 | NCC | USA | I | Islet autoantibodies | <7 | High risk children via HLA screening |
| Alves, 2012 ([25](#_ENREF_25)) | 246/123 | CC | Brazil | I | Medical diagnosis | 7 | Endocrine clinic attendance |
| Baruah, 2011 ([27](#_ENREF_27)) | 86/43 | CC | India | I | Medical diagnosis | <18 | Endocrinology ward |
| Esfarjani, 2001 ([32](#_ENREF_32)) | 104/52 | CC | Iran | Q | Medical diagnosis | <14 | Endocrine clinic attendance |
| Gimeno, 1997 ([45](#_ENREF_45)) | 626/313 | CC | Brazil | Q | Medical diagnosis | <18 | Juvenile Diabetes Association or hospital records |
| Kostraba, 1992 ([38](#_ENREF_38)) | 264/132-white  108/54-Black | CC | USA | Q/I | Medical diagnosis | 10 | Alleghany Hospital diabetes register |
| Liese, 2012 ([68](#_ENREF_68)) | 709/505 | CC | USA | I | Medical diagnosis | <20 | SEARCH surveillance (Colorado and South Carolina research centres) |
| Patterson, 1994 ([34](#_ENREF_34)) | 1548/258 | CC | UK | R | Medical diagnosis | <16 | Diabetes register, hospital discharge, Health Service records |
| Rabiei 2011 ([69](#_ENREF_69)) | 300/100 | CC | Iran | Q | Medical diagnosis | 11 | Diabetes register |
| Rami, 1999 ([48](#_ENREF_48)) | 609/114 | CC | Austria | Q | Medical diagnosis | <15 | Austrian diabetes register |
| Sadauskaite-Kuehne, 2004 ([50](#_ENREF_50)) | 1944/803 | CC | Sweden/Lithuania | Q | Medical diagnosis | 7 | Hospital admissions |
| Samuelsson, 1993 ([51](#_ENREF_51)) | 1026/297 | CC | Sweden | R/Q | Medical diagnosis | <17 | Paediatric department |
| Soltesz, 1994 ([55](#_ENREF_55)) | 305/130 | CC | Hungary | Q | Medical diagnosis | <14 | Incidence register |
| Stene, 2000 ([70](#_ENREF_70)) | 1156/85 | CC | Norway | Q | Medical diagnosis | <15 | National Childhood Diabetes register |
| Stene, 2003 ([71](#_ENREF_71)) | 2213/545 | CC | Norway | Q | Medical diagnosis | 9 | Diabetes register |
| Thorsdottir, 2000 ([60](#_ENREF_60)) | 220/55 | CC | Iceland | I | Medical diagnosis | 12 | General population |
| Verge, 1994 ([61](#_ENREF_61)) | 475/217 | CC | Australia | Q | Medical diagnosis | 9 | New South Wales diabetes register |
| Virtanen, 1992 (74) | 852/426 | CC | Finland | Q | Medical diagnosis | <14 | Hospital admissions |
| Virtanen, 1993 ([62](#_ENREF_62)) | 1380/690 | CC | Finland | Q | Medical diagnosis | <14 | Finnish National Population Registry |

PC = prospective cohort, NCC = nested case control, CC = case control, D = diary, I = interview, Q = questionnaire, R = medical records

Figure 14 Risk of bias in observational studies of exclusive breastfeeding and TIDM

## Outcomes from studies of exclusive breastfeeding and TIDM

## 2.2.1 Exclusive breastfeeding for ≥0-2 months vs. <0-2 months

Nine studies reported the association between EBF for a duration of at least 0-2 months, compared to less than this ([17](#_ENREF_17), [34](#_ENREF_34), [45](#_ENREF_45), [50](#_ENREF_50), [62](#_ENREF_62)). Meta-analysis showed significantly reduced TIDM risk with longer EBF duration (OR 0.74; 95% CI 0.63, 0.88) with low statistical heterogeneity (I^2^=10.2%; Figure 15). There was no evidence of differences in strength of association in the stratified or subgroup analyses (Table 7). Adjusted analyses and unadjusted analyses showed similar findings, but heterogeneity was lower in the retrospective case control studies reporting clinical TIDM as an outcome, than in the prospective studies reporting autoantibodies as an outcome.

Figure 15 Exclusive breastfeeding for ≥0-2 months vs. < 0-2 months and TIDM risk


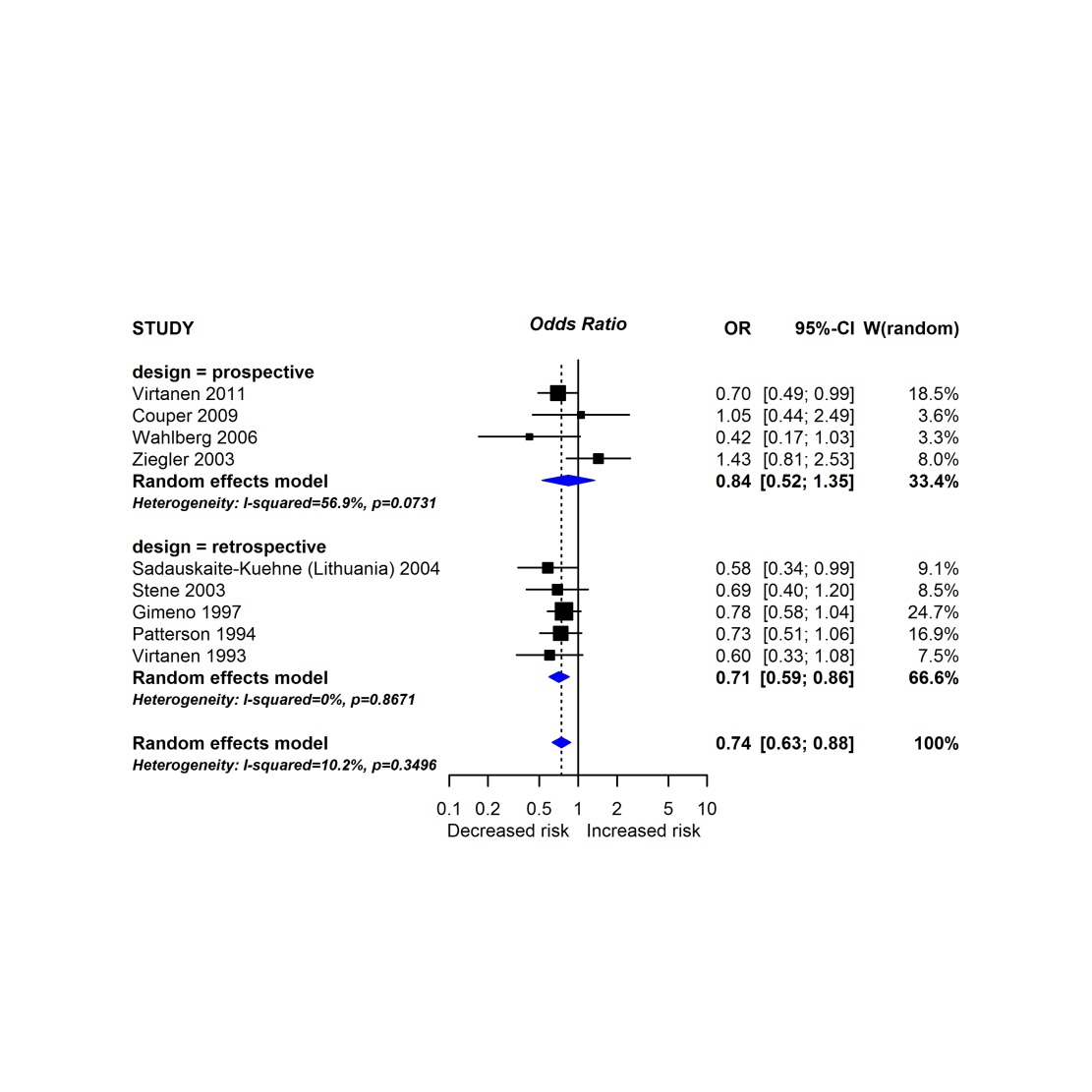


Table 7 Stratified and subgroup analyses of EBF duration ≥0-2 months vs. <0-2 months and TIDM risk

|  | **Number of studies** | **OR [95% CI]*** | **I^2^ (%)** | **P-value for between groups difference** |
| --- | --- | --- | --- | --- |
| **Overall (if adjusted NA, unadjusted used)**  **Adjusted**  **Unadjusted** | 9  6  7 | 0.74 [0.63-0.88]  0.76 [0.63-0.92]  0.77 [0.59-1.00] | 10.2  18.8  45.8 | Not tested |
| Risk of disease – High  Risk of disease – Normal/Low | 3  6 | 0.97 [0.59-1.59]  0.70 [0.58-0.84] | 57  0 | 0.221 |
| Overall risk of bias – Low  Overall risk of bias – High/Unclear | 2  7 | 0.96 [0.48-1.95]  0.71 [0.59-0.84] | 77.5  0 | 0.402 |
| Study Design – Prospective  Study Design – Retrospective | 4  5 | 0.84 [0.52-1.35]  0.71 [0.59-0.86] | 56.9  0 | 0.519 |
| Method of diagnosis – clinical  Method of diagnosis – serological | 5  4 | 0.71 [0.59-0.86]  0.84 [0.52-1.35] | 0  56.9 | 0.519 |
| Clear definition of exclusive breastfeeding  Unclear definition of exclusive breastfeeding | 4  5 | 0.78 [0.54-1.13]  0.72 [0.59-0.89] | 55.3  0 | 0.703 |

*Pooled estimate [95%CI] of meta-analysis of studies reporting Odds or Relative risk or Risk ratios

## Exclusive breastfeeding for ≥3-4 months vs < 3-4 months

Nine studies reported data which could be meta-analysed, for risk of TIDM in relation to EBF for more or less than 3-4 months (Figure 16). Pooled data showed significantly reduced risk of TIDM with longer EBF duration (OR 0.68; 95% CI 0.55, 0.83), with moderate statistical heterogeneity (I^2^ =28.6%). There was no evidence of differences in strength of association in the stratified or subgroup analyses (Table 8). Adjusted analyses and unadjusted analyses showed similar findings.

Figure 16 Exclusive BF for ≥ 3-4 months vs. < 3-4 months and TIDM risk


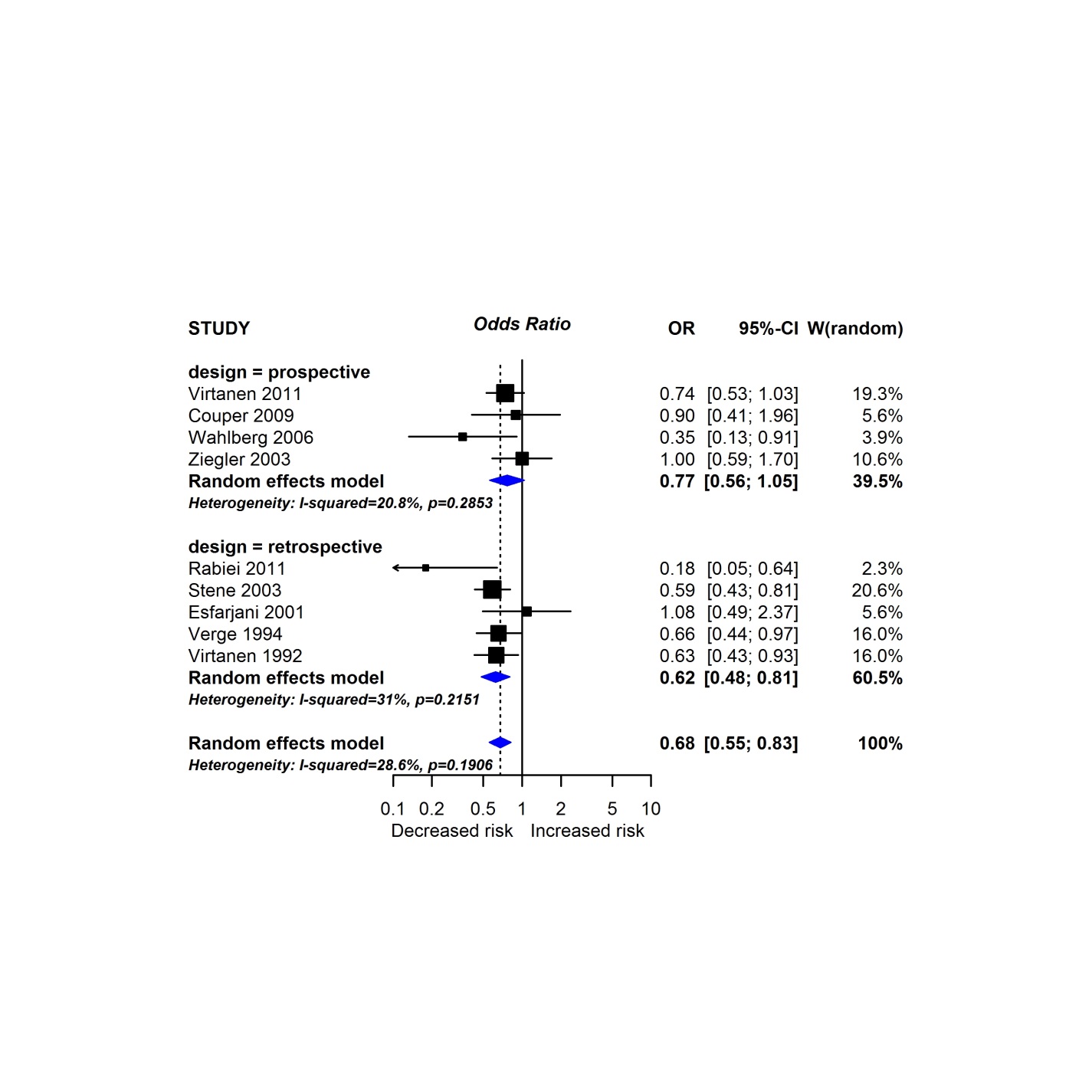


Table 8 Stratified and subgroup analyses of EBF duration ≥3-4 months vs. <3-4 months and TIDM risk

|  | **Number of studies** | **OR [95% CI]*** | **I^2^ (%)** | **P-value for between groups difference** |
| --- | --- | --- | --- | --- |
| **Overall (if adjusted NA, unadjusted used)**  **Adjusted**  **Unadjusted** | 9  6  8 | 0.68 [0.55-0.83]  0.66 [0.54-0.82]  0.71 [0.60-0.84] | 28.6  32.6  14.8 | Not tested |
| Risk of disease – High  Risk of disease – Normal/Low | 3  6 | 0.82 [0.63-1.06]  0.60 [0.47-0.78] | 0  30.2 | 0.104 |
| Risk of bias – Low  Risk of bias – High/Unclear | 3  6 | 0.74 [0.59-0.93]  0.62 [0.45-0.85] | 0  37.8 | 0.348 |
| Study Design – Prospective  Study Design - Retrospective | 4  5 | 0.77 [0.56-1.05]  0.62 [0.48-0.81] | 20.8  31.0 | 0.326 |
| Method of diagnosis – clinical  Method of diagnosis – serological | 5  4 | 0.62 [0.48-0.81]  0.77 [0.56-1.05] | 31.0  20.8 | 0.326 |
| Clear definition of exclusive breastfeeding  Unclear definition of exclusive breastfeeding | 4  5 | 0.71 [0.54-0.93]  0.65 [0.47-0.90] | 27.9  39.1 | 0.705 |

*Pooled estimate [95%CI] of meta-analysis of studies reporting Odds or Relative risk or Risk ratios

## Exclusive breastfeeding for ≥5-9 months vs. <5-9 months

Two studies reported the association between risk of TIDM and exposure to exclusive breastfeeding for ≥5-9 months. There was extreme statistical heterogeneity between studies (I^2^=81.2%). The study of Wahlberg reported unadjusted OR for diabetes associated antibodies in preschool children. The study of Sadauskaite-Kuehne reported adjusted OR for clinical TIDM in 7 year old children in a case-control study. These differences in study design and outcome assessment may explain the extreme heterogeneity. One study suggested a protective effect, and one study suggested no evidence of protection (Figure 17).

Figure 17 Exclusive breastfeeding for ≥5-9 months vs. < 5-9 months and TIDM risk


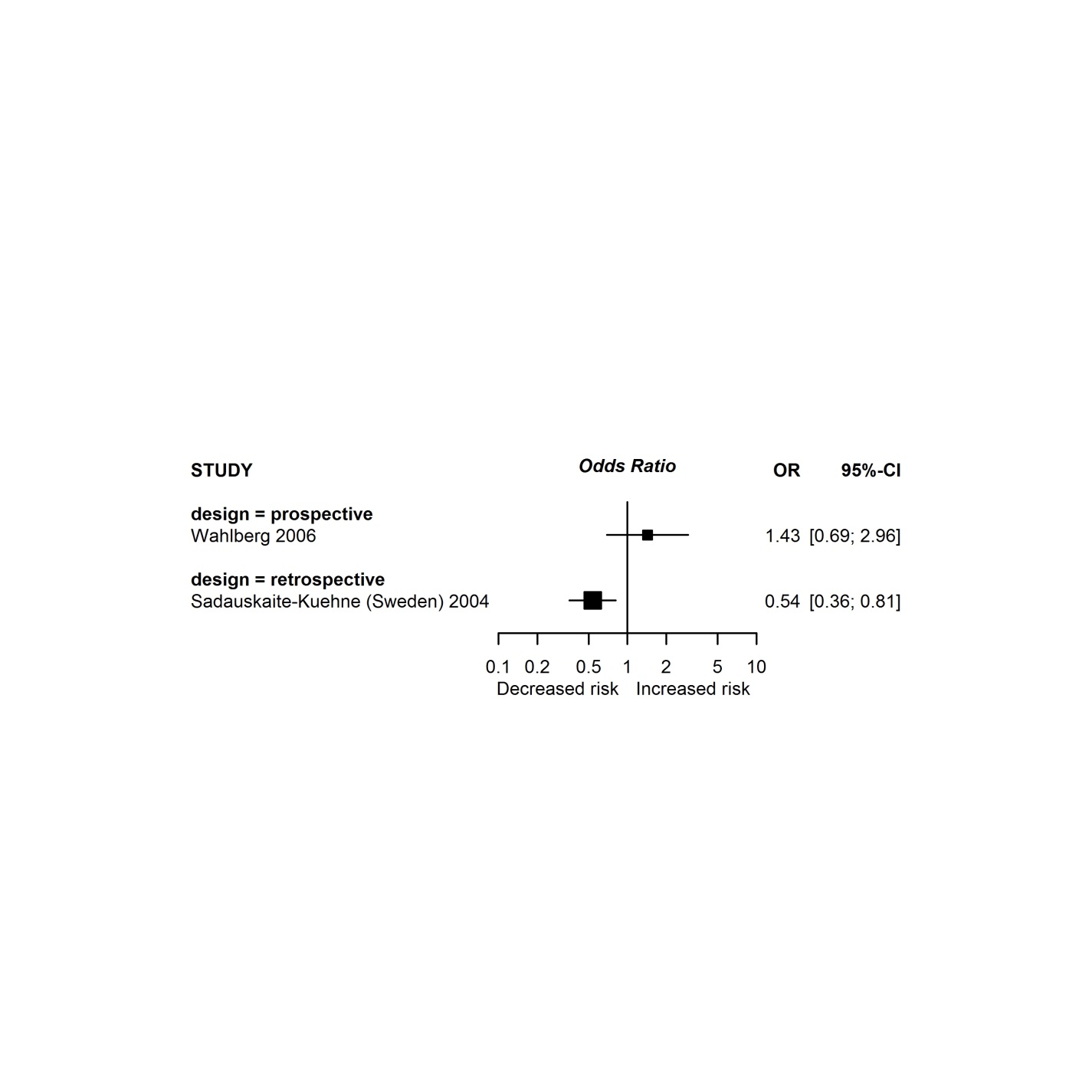


## 2.2.4 Studies investigating EBF as a continuous variable and risk of TIDM

Three studies reported the unadjusted relationship between EBF duration and TIDM risk, comparing the mean duration of EBF in TIDM versus unaffected subjects. There was extreme statistical heterogeneity (I^2^=92%) attributable to the study of Liese, so data were not pooled (Figure 18). There was also heterogeneity *within* the study of Liese, with shorter duration of EBF in controls than TIDM in South Carolina, but longer EBF in controls in Colorado. It is not clear that EBF is normally distributed in general, so one reason for the extreme heterogeneity may be inappropriate analysis of EBF duration as arithmetic mean (sd) in these studies.

**Figure 18 Difference in EBF in people with T1DM versus unaffected subjects**


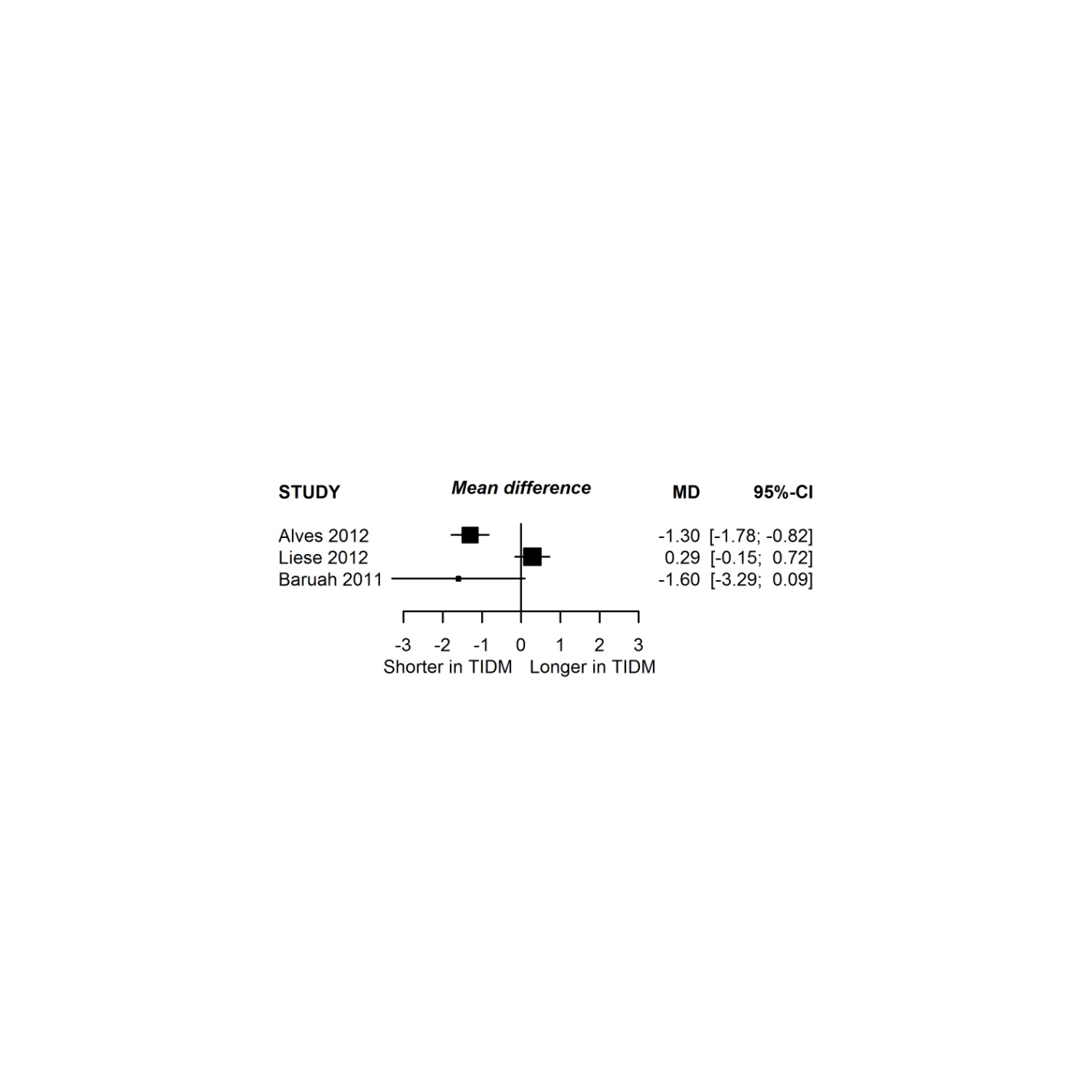


## 2.2.5 Other studies evaluating EBF and TIDM which couldn’t be meta-analysed

Meta-analyses included 4 cohort and 9 case control studies in total, including at least 3733 participants with TIDM. A further 4 cohort, 2 nested case control and 8 case control studies reported relevant data which could not be included in meta-analysis, in relation to at least 1588 participants with TIDM. All but one study used mean or median to present results which prevented the use of the data in the meta-analysis, and one study used a definition of breastfeeding duration that was not possible to combine with the other studies (Table 9). Two of the case control studies (Alves ([25](#_ENREF_25)), Baruah ([27](#_ENREF_27))) and one prospective cohort study (Holmberg ([67](#_ENREF_67))) showed a significantly shorter EBF duration in TIDM compared to unaffected subjects. The other 11 studies showed no significant relationship, although EBF duration was shorter in people with TIDM in 2 of these 11 studies, longer in 2 and similar or unclear in 7.

Table 9 Other studies evaluating exclusive breastfeeding and TIDM which couldn’t be meta-analysed

| **First Author and Year of Publication** | **Design** | **N/n cases** | **Exclusive BF duration** | **Units** | **Descriptive measure** | **Unaffected** | **TIDM** | **P value** |
| --- | --- | --- | --- | --- | --- | --- | --- | --- |
| Alves, 2009 ([25](#_ENREF_25)) | CC | 246/123 | Continuous | Months | Mean (SD) | 4.6 | 3.3 | **<0.001** |
| Baruah, 2011 ([27](#_ENREF_27)) | CC | 86/43 | Continuous | Months | Mean (SD) | 6.6 | 5 | **<0.05** |
| Couper, 1999 ([1](#_ENREF_1)) | PC | 317/70 | Continuous | Months | Mean (SD) | 4.5 | 3.4 | NS |
| Liese, 2012 ([68](#_ENREF_68)) | CC | 709/505 | Continuous | Months | Mean (SD) | 2.40 | 2.69 | 0.23 |
| Samuelsson, 1993 ([51](#_ENREF_51)) (<5y) | CC | 1089/297 | continuous | Months | Mean (SE) | 3.0 (0.5) | 4.5 (0.7) | 0.17 |
| Samuelsson, 1993 ([51](#_ENREF_51)) (5-9y) |  |  | continuous | Months | Mean (SE) | 3.6 (0.3) | 3.2 (0.3) | 0.34 |
| Samuelsson, 1993 ([51](#_ENREF_51)) (>10y) |  |  | continuous | Months | Mean (SE) | 2.4 (0.1) | 2.2 (0.2) | 0.31 |
| Hummel, 2000 (mother with TIDM) ([4](#_ENREF_4)) | PC | 568 | continuous | Months | Median | 1 | 2 | NS |
| Hummel, 2000 (father with TIDM) ([4](#_ENREF_4)) |  |  | continuous | Months | Median | 3 | 1 | 0.31 |
| Kimpimaki, 2001 ([17](#_ENREF_17)) | NCC | 455/65 | continuous | Months | Median (IQR) | 1.8 (0.5-3.9) | 2 (1-4) | - |
| Rami, 1999 ([48](#_ENREF_48)) | CC | 609/114 | continuous | Months | Median (range) | 2 (0-18) | 2 (0-7) | - |
| Soltesz, 1994 ([55](#_ENREF_55)) | CC | 305/130 | continuous | Months | Median | 2.5 | 2 | NS |
| Frederikson, 2012 ([66](#_ENREF_66)) | PC | 548 | continuous | Months | Average |  |  | NS |
| Kostraba, 1992 (white) ([38](#_ENREF_38)) | CC | 302/132  106/54 | continuous | Months | Average | 3.3 | 4.5 | 0.40 |
| Kostraba, 1992 (black) |  |  | continuous | Months | Average | 6.8 | 3.3 | 0.16 |
| Lamb, 2013 ([8](#_ENREF_8)) | NCC | 260 | continuous | Months | Average |  |  | NS |
| Thorsdottir, 2000 ([60](#_ENREF_60)) | CC | 220/55 | continuous | Months | Average |  |  | NS |
| Holmberg, 2007 ([67](#_ENREF_67)) | PC | 3788 | categorical | >4 vs 1-3 months | aOR (95% CI) | 0.50 (0.27-0.93) | | **0.028** |

# Age at introduction of solid food and risk of TIDM

## 3.1. Overall characteristics of studies, risk of bias and summary of results

General characteristics of included studies are summarised in Table 10. No intervention trials were identified. Data were available from a total of 17 studies with over 4000 people with TIDM. There were 2 prospective cohort studies, 1 nested case-control studies, and 14 case-control studies evaluating timing of solid food introduction and TIDM risk. The studies were European (n=8), North American (n=3), Asia-Pacific (n=1), South American (n=2) and Middle Eastern (n=3). This analysis addresses the first introduction of non-milk feed in any form (here termed ‘solid food’) into the infant diet.

Two studies evaluated TIDM risk only in young children (age ≤5), and 15 studies only in older children (up to 20 years old). Other than one study which didn’t describe the source of dietary exposure assessment, and another which used diary records, all studies obtained information on age of solid food introduction based on questionnaire or interview data.

Based on the distribution of data reported in included studies, meta-analysis of binary data compared TIDM risk and timing of solid food introduction ≥3-4 months vs. <3-4 months.

A summary of the risk of bias in included studies is shown in Figure 19. Just over half of studies had a high overall risk of bias, mainly due to reliance on unadjusted data, hence high risk of confounding bias. Meta-analyses showed extreme statistical heterogeneity, with no clear evidence that delaying introduction of solid food until after 3-4 months was associated with altered risk of TIDM. Studies which analysed EBF duration as a continuous variable, or could not be included in meta-analysis, also showed a mixed and unclear picture. We were not able to exclude a clinically important association, due to the small number of studies in meta-analysis and high statistical heterogeneity and confidence intervals.

Table 10 Characteristics of studies reporting timing of solid food and TIDM risk

| **First Author & Publication Year** | **N/n cases** | **Country** | **Design** | **Exposure assessment** | **Specific outcome** | **Age at outcome (years)** | **Population characteristics** |
| --- | --- | --- | --- | --- | --- | --- | --- |
| Frederiksen, 2012 ([66](#_ENREF_66)) | 1698 | USA | PC | - | Medical diagnosis | <7 | High risk children via HLA screening |
| Ziegler, 2003 ([15](#_ENREF_15)) | 1460/~68 | Germany | PC | Q | Islet autoantibodies | 5 | Newborn children |
| Savilahti, 2009 ([72](#_ENREF_72)) | 6209/45 | Finland | NCC | D | Medical diagnosis | 12 | NHI database |
| Alves, 2012 ([25](#_ENREF_25)) | 246/123 | Brazil | CC | I | Medical diagnosis | 9 | Endocrinology clinic |
| Dahlquist, 2002 ([33](#_ENREF_33)) | 2226/610 | Austria, Latvia, Lithuania, Luxembourg and UK | CC | Q/I | Medical diagnosis | <15 | Diabetes register |
| Esfarjani, 2001 ([32](#_ENREF_32)) | 104/52 | Iran | CC | Q | Medical diagnosis | <14 | Endocrine clinic attendance |
| Hypponen, 1999 ([36](#_ENREF_36)) | 821/435 | Finland | CC | Q | Medical diagnosis | 8 | Hospital admissions |
| Kostraba, 1993 ([38](#_ENREF_38)) | 309/142 | USA | CC | Q | Medical diagnosis | <18 | Colorado IDDM Registry |
| Liese, 2012 ([40](#_ENREF_40), [68](#_ENREF_68)) | 709/505 | USA | CC | I | Medical diagnosis | <20 | SEARCH surveillance (Colorado and South Carolina research centres) |
| Majeed, 2011 ([39](#_ENREF_39)) | 395/96 | Iraq | CC | Q | Medical diagnosis | <18 | Paediatric clinic |
| Meloni, 1997 ([44](#_ENREF_44)) | 200/100 | Italy | CC | Q/I | Medical diagnosis | <15 | Paediatric clinic |
| Perez-Bravo, 1996 ([73](#_ENREF_73)) | 165/80 | Chile | CC | Q | Medical diagnosis | <15 | Santiago de Chile registry |
| Rabiei, 2011 ([69](#_ENREF_69)) | 300/100 | Iran | CC | Q | Medical diagnosis | <15 | Diabetes register |
| Rosenbauer, 2008 ([49](#_ENREF_49)) | 2631/760 | Germany | CC | Q | Medical diagnosis | <5 | Hospital based surveillance system ESPD |
| Stene, 2003 ([71](#_ENREF_71)) | 2118/545 | Norway | CC | Q | Medical diagnosis | 9 | Diabetes register |
| Strotmeyer, 2004 ([56](#_ENREF_56)) | 690/247 | China | CC | Q | Medical diagnosis | 10 | Diabetes register |
| Visalli, 2003 ([63](#_ENREF_63)) | 900/150 | Italy | CC | Q | Medical diagnosis | 12 | EURODIAB register |

PC = prospective cohort, NCC = nested case control, CC = case control, D = diary, I = interview, Q = questionnaire, R = medical records

Figure 19 Risk of bias in observational studies of solid food exposure and TIDM risk

## 3.2. Outcomes from studies of solid food introduction and TIDM

## 3.2.1 Solid food introduction at ≥ 3-4 months vs < 3-4 months, and risk of TIDM

Six studies examined the association between delaying introduction of solid food for longer than 3-4 and risk of TIDM (Figure 20). There was extreme statistical heterogeneity (I^2^=83.5%) so data were not pooled for meta-analysis. This heterogeneity could not be attributed to a particular study. If the study of Ziegler was excluded, extreme heterogeneity (I^2^=86%) remained. Frederikson and Ziegler reported adjusted HR from prospective cohort studies, Ziegler using autoantibodies and Frederikson clinical TIDM for case definition. The other studies reported adjusted (Kostraba, Stene) or unadjusted OR from case control studies using clinical TIDM for case definition. Subgroup and stratified analyses did not show important differences in strength of association and extreme heterogeneity remained in all groups (Table 11).

Figure 20 Introduction of solid food at age ≥3-4 months vs. <3-4 months and TIDM risk


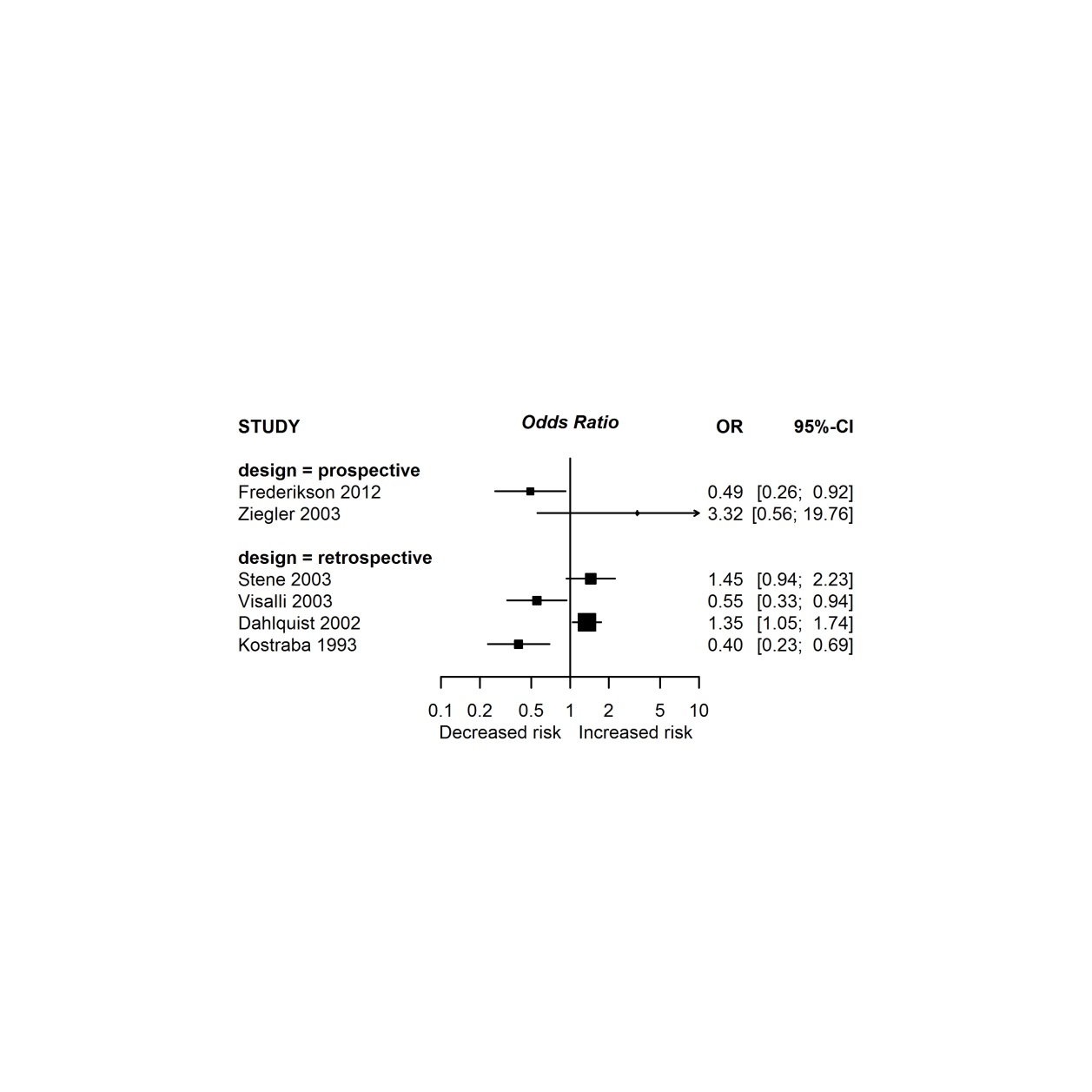


Table 11 Stratified and subgroup analyses of solid food introduction ≥3-4 months vs. <3-4 months and TIDM risk

|  | **Number of studies** | **OR [95% CI]*** | **I^2^ (%)** | **P-value for between groups difference** |
| --- | --- | --- | --- | --- |
| **Overall (if adjusted NA, unadjusted used)**  **Adjusted**  **Unadjusted** | 6  4  5 | 0.83 [0.49-1.40]  0.82 [0.36-1.87]  0.79 [0.48-1.29] | 83.5  83.5  81.0 | Not tested |
| Risk of disease – High  Risk of disease – Normal/Low | 2  4 | 1.06 [0.17-6.62]  0.84 [0.46-1.51] | 74.5  87.2 | 0.813 |
| Risk of bias – Low  Risk of bias – High/Unclear | 2  4 | 0.96 [0.12-7.48]  0.90 [0.54-1.50] | 79.8  82.2 | 0.947 |
| Study Design – Prospective  Study Design - Retrospective | 2  4 | 1.06 [0.17-6.62]  0.84 [0.46-1.51] | 74.5  87.2 | 0.813 |
| Method of diagnosis – clinical  Method of diagnosis – serological | 5  1 | 0.76 [0.44-1.30]  3.32 [0.56-19.76] | 86.0  - | 0.121 |

*Pooled estimate [95%CI] of meta-analysis of studies reporting Odds or Relative risk or Risk ratios or Hazard ratios

## Timing of solid food introduction as a continuous variable, and TIDM risk

Three studies compared the mean time of solid food introduction in subjects with T1DM versus unaffected subjects. Pooled data show no overall difference between cases and controls (MD -0.16; 95% CI -1.32, 0.99) with extreme statistical heterogeneity (I^2^=76.2%) (Figure 21). All 3 studies reported unadjusted data from case control studies. Heterogeneity was attributable to the study of Esfarjani ([32](#_ENREF_32)), for unclear reasons, but timing of SF introduction occurred later in the study of Esfarjani than the other 2 studies, and if timing of SF introduction is not normally distributed then analysis of arithmetic mean (sd) may not be appropriate and may have contributed to the heterogeneity.

Figure 21 Timing of solid food introduction as a continuous variable, and TIDM risk


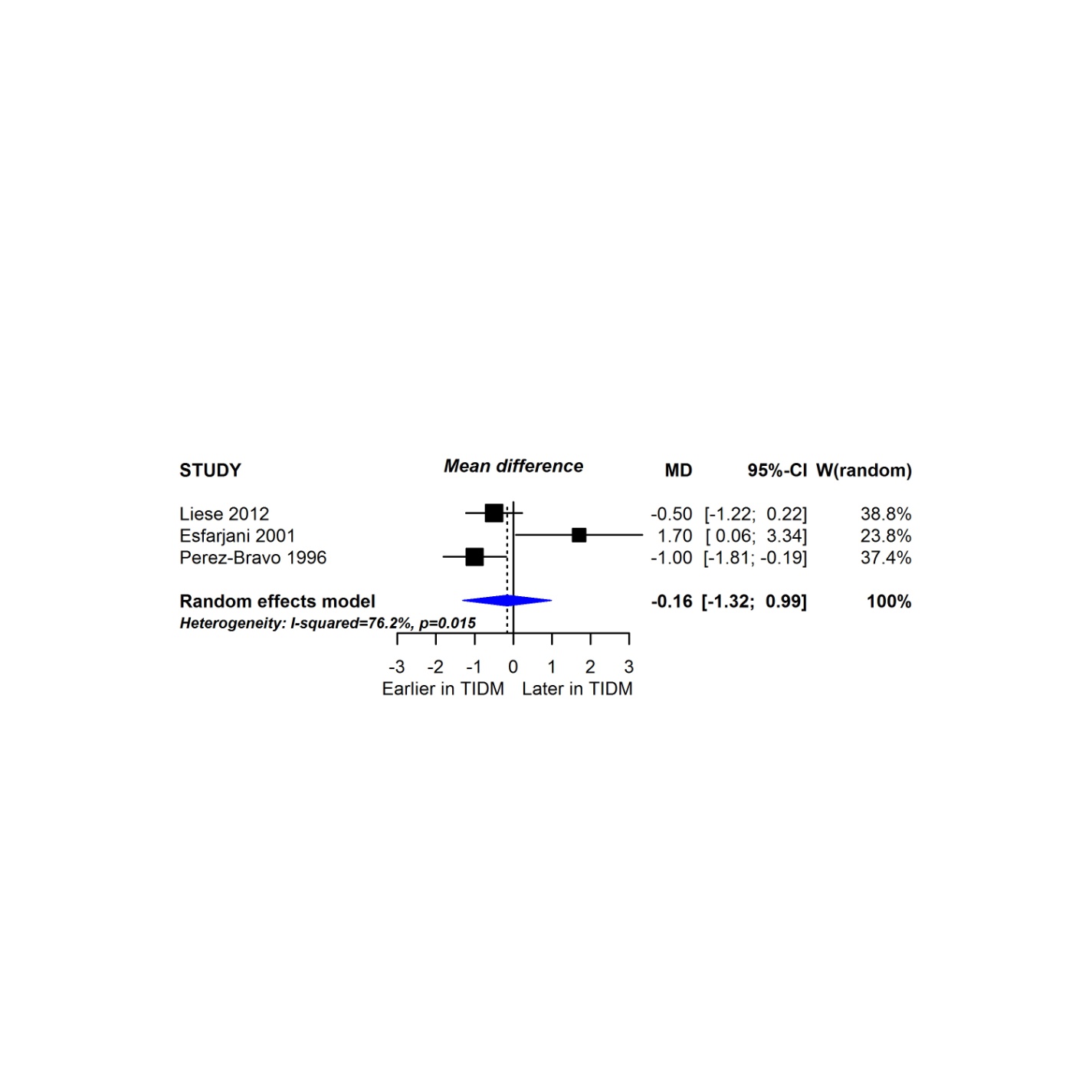


## 3.2.3 Other studies solid food introduction and TIDM, which couldn’t be meta-analysed

Meta-analyses included 1 cohort and 4 case control studies in total, including at least 2264 participants with TIDM. A further 1 nested case control and 7 case control studies reported relevant data which could not be included in meta-analysis, in relation to at least 1825 participants with TIDM. These studies are summarised in Table 12. Two of the case control studies (Kostraba ([38](#_ENREF_38), [49](#_ENREF_49)), Rosenbauer ([49](#_ENREF_49))) showed a significant relationship between early introduction of solid food in TIDM compared to unaffected subjects. 6 studies showed no significant relationship, although solid food introduction was earlier in people with TIDM in 3 of these 6 studies, and similar or unclear in 3.

Table 12 Other studies evaluating timing of solid food introduction and TIDM which couldn’t be meta-analysed

| **First Author and Year of Publication** | **Design** | **N/n cases** | **Age at first solid food introduction** | **Units** | **Descriptive measure** | | **Unaffected** | | **TIDM** | **P value** |
| --- | --- | --- | --- | --- | --- | --- | --- | --- | --- | --- |
| Kostraba, 1993 ([38](#_ENREF_38)) | CC | 309/142 | Continuous | Weeks | Mean | | 16.9 | 13.4 | | **0.01** |
| Savilahti, 2009 ([72](#_ENREF_72)) | NCC | 6209/45 | Continuous | Months | Mean | | 4 | 3.7 | | 0.09 |
| Hypponen, 1999 ([36](#_ENREF_36)) | CC | 821/435 | Continuous | Months | Average |  | |  | | NS |
| Majeed, 2011 ([39](#_ENREF_39)) | CC | 395/96 | Categorical | >6 vs <6 months |  | |  |  | | NS |
| Rabiei, 2011 ([69](#_ENREF_69)) | CC | 300/100 | Categorical | >6 vs <6 months | aOR (95% CI) | | 0.60 (0.27-1.32) | | | NS |
| Rosenbauer, 2008 ([49](#_ENREF_49)) | CC | 2631/760 | Categorical | >5 vs <5 months | aOR (95% CI) | | 0.82 (0.64-1.05) | | | NS |
|  |  |  |  |  | uOR (95% CI) | | 0.78 (0.65-0.93)* | | | **0.006** |
| Strotmeyer, 2004 ([56](#_ENREF_56)) | CC | 690/247 | 1-3 months | % |  | | 20 | 13 | | **<0.05** |
|  |  |  | 4-6 months | % |  | | 48 | 58 | | **<0.05** |
|  |  |  | 7-12 months | % |  | | 30 | 28 | | NS |

* Adjusted data from this study showed no statistically significant difference

# Conclusions

This report summarises the results of over 60 studies examining the association between total or exclusive breastfeeding duration, timing of solid food introduction and TIDM risk, including over 15,000 people with TIDM. The majority of the studies were case-control design, some of which were nested in cohort studies. Overall we found VERY LOW evidence that increased duration of TBF, and LOW evidence that increased duration of EBF, are associated with reduced risk of TIDM. We found no association between timing of SF introduction and TIDM risk.

Figure 22 Summary of Meta-Analysis findings for Duration of BF and TIDM risk

**A**

**B**

Figure 22. Pooled OR for TIDM in relation to TBF (A) and eBF (B). Bars are 95% CI; I^2^ is a measure of statistical heterogeneity.

In spite of the growing number of epidemiological studies examining the association between breastfeeding, solid food introduction and risk of TIDM, there are very few systematic reviews analysing this relationship. The current report probably provides the most comprehensive description of eligible studies on breastfeeding and TIDM to date. A recent literature review by Hornell and colleagues ([74](#_ENREF_74)) concluded that prolonged breastfeeding had a protective effect against the risk of TIDM. The authors based their conclusions on an earlier paper by Ip and colleagues ([75](#_ENREF_75)) and on a single prospective study, also included in our report ([2](#_ENREF_2)).

In our search for existing systematic reviews we identified two existing reviews of this area, by Patelarou et al ([76](#_ENREF_76)) and by Cardwell et al ([77](#_ENREF_77)). Both were scored below the recommended AMSTAR cut-off point for a high quality systematic review (24 and 31, respectively) and therefore were excluded from our Overview and we have not directly included their data in this report. Cardwell’s meta-analysis of individual patient data from 43 observational studies reported no clear association between TBF for more than 2 weeks (28 studies; OR = 0.93, 95% CI 0.81 to 1.07) or for more than 3 months (29 studies; OR = 0.88, 95% CI 0.78 to 1.00) and TIDM. They found a protective effect of EBF >2 weeks on risk of TIDM albeit with very high statistical heterogeneity (17 studies; aOR = 0.78, 95% CI 0.65, 0.93; I^2^=52%), but no such effect for EBF >3 months. Cardwell’s analysis differs from ours because they acquired individual patient data from the majority of the 43 included studies, including confounders, so that they could do adjusted analyses where possible. Due to their methods they were able to include larger numbers of studies in individual meta-analyses of specific cut-off durations. They did not however identify as many studies as our review, so had increased risk of publication bias from the methods used. They also only explored 2 time cut-offs i.e., 2 weeks and 3 months for their analyses. For example in their analysis of EBF >2 weeks versus shorter they included 13 distinct studies in meta-analysis – of these, 1 study was not included in our review ([78](#_ENREF_78)) because relevant data were only available through contact with the authors. In contrast we included 28 separate studies in our analysis of EBF duration, although a maximum of 6 studies could be included in meta-analysis of EBF duration and TIDM at any given time cut-off, due to lack of individual patient data. Our finding that TBF durations of ≥5-7 and 8-12 months are associated with reduced TIDM risk was not explored by Cardwell et al, and theirs was not a systematic review including all available data but rather a details analysis of studies well known to the authors. The review by Patelarou *et al* ([76](#_ENREF_76)) was methodologically weaker, only identifying 28 studies.

In the current report, the pooled data suggest reduced risk of TIDM is associated with increased duration of total or exclusive breastfeeding duration. Some meta-analyses showed significant statistical heterogeneity across studies, especially for TBF, which remained largely unexplained after subgroup and stratified analyses. There was no evidence for a difference between adjusted and unadjusted analyses, suggesting that confounding bias may not be a major issue for studies of TBF/EBF and TIDM. There was however reduced heterogeneity in some analyses when analysing retrospective and prospective studies separately, and in some analyses these 2 subgroups were significantly different i.e. retrospective studies showed more positive findings than prospective studies. The use of serology for outcome assessment in some of the prospective studies, often with shorter time between exposure and outcome assessment, may account for the reduced evidence for association between TBF/EBF and TIDM seen in prospective compared with retrospective studies. The relationship between serological diabetes and clinical diabetes is only moderately strong - approximately 60% of people with ≥2 diabetes-associated antibodies develop clinical TIDM over a 10 year follow up period ([79](#_ENREF_79)).

Evidence for an association was strongest for EBF and TIDM, where both meta-analysed and non meta-analysed data suggested prolonged EBF is associated with reduced TIDM risk, with low or moderate statistical heterogeneity and no significant difference between prospective and retrospective studies. Possible explanations for this association are reduced gastrointestinal infection, an established effect of prolonged EBF duration and a proposed risk factor for TIDM; or reduced cow’s milk exposure in infancy, also proposed as a risk factor for TIDM ([80](#_ENREF_80), [81](#_ENREF_81)). We found no evidence that timing of SF introduction is relevant.

Overall these data support an association between longer TBF and EBF duration and reduced risk of TIDM, and no association between delayed solid food introduction until after 3-4 months and risk of T1DM. The association seen with prolonged TIDM suggests a protective effect against infection as the most likely mechanism, rather than an adverse effect of early CM introduction, since other parts of this systematic review series have not identified a relationship between timing of CM introduction, or use of hydrolysed infant formula, and risk of TIDM.

# References

1. Couper JJ, Steele C, Beresford S, Powell T, McCaul K, Pollard A, et al. Lack of association between duration of breast-feeding or introduction of cow's milk and development of islet autoimmunity. Diabetes. 1999;48(11):2145-9.

2. Couper JJ, Beresford S, Hirte C, Baghurst PA, Pollard A, Tait BD, et al. Weight Gain in Early Life Predicts Risk of Islet Autoimmuity in Children With a First-Degree Relative With Type 1 Diabetes. Diabetes Care. 2009;32(1):94-9.

3. Fronczak CM, Baron AE, Chase HP, Ross C, Brady HL, Hoffman M, et al. In utero dietary exposures and risk of islet autoimmunity in children. Diabetes Care. 2003;26(12):3237-42.

4. Holmberg H, Wahlberg J, Vaarala O, Ludvigsson J. Short duration of breast-feeding as a risk-factor for beta-cell autoantibodies in 5-year-old children from the general population. The British journal of nutrition. 2007;97(1):111-6.

5. Hummel M, Fuchtenbusch M, Schenker M, Ziegler AG. No major association of breast-feeding, vaccinations, and childhood viral diseases with early islet autoimmunity in the German BABYDIAB Study. Diabetes Care. 2000;23(7):969-74.

6. Karlen J, Faresjo T, Ludvigsson J. Could the social environment trigger the induction of diabetes related autoantibodies in young children? Scandinavian Journal of Public Health. 2012;40(2):177-82.

7. Lamb MM, Myers MA, Barriga K, Zimmet PZ, Rewers M, Norris JM. Maternal diet during pregnancy and islet autoimmunity in offspring. Pediatric Diabetes. 2008;9(2):135-41.

8. Lamb MM, Simpson MD, Seifert J, Scott FW, Rewers M, Norris JM. The association between IgG4 antibodies to dietary factors, islet autoimmunity and type 1 diabetes: the Diabetes Autoimmunity Study in the Young. PLoS ONE [Electronic Resource]. 2013;8(2):e57936.

9. Ludvigsson JF, Ludvigsson J. Stressful life events, social support and confidence in the pregnant woman and risk of coeliac disease in the offspring. Scandinavian Journal of Gastroenterology. 2003;38(5):516-21.

10. Norris JM, Barriga K, Klingensmith G, Hoffman M, Eisenbarth GS, Erlich HA, et al. Timing of initial cereal exposure in infancy and risk of islet autoimmunity. JAMA. 2003;290(13):1713-20.

11. Viner RM, Hindmarsh PC, Taylor B, Cole TJ. Childhood body mass index (BMI), breastfeeding and risk of Type 1 diabetes: findings from a longitudinal national birth cohort. Diabetic Medicine. 2008;25(9):1056-61.

12. Virtanen SM, Hypponen E, Laara E, Vahasalo P, Kulmala P, Savola K, et al. Cow's milk consumption, disease-associated autoantibodies and type 1 diabetes mellitus: a follow-up study in siblings of diabetic children. Childhood Diabetes in Finland Study Group. Diabetic Medicine. 1998;15(9):730-8.

13. Virtanen SM, Takkinen HM, Nevalainen J, Kronberg-Kippila C, Salmenhaara M, Uusitalo L, et al. Early introduction of root vegetables in infancy associated with advanced beta-cell autoimmunity in young children with human leukocyte antigen-conferred susceptibility to Type 1 diabetes. Diabetic Medicine. 2011;28(8):965-71.

14. Wahlberg J, Vaarala O, Ludvigsson J, group AB-s. Dietary risk factors for the emergence of type 1 diabetes-related autoantibodies in 21/2 year-old Swedish children. Br J Nutr. 2006;95(3):603-8.

15. Ziegler AG, Schmid S, Huber D, Hummel M, Bonifacio E. Early infant feeding and risk of developing type 1 diabetes-associated autoantibodies. JAMA. 2003;290(13):1721-8.

16. Jones ME, Swerdlow AJ, Gill LE, Goldacre MJ. Pre-natal and early life risk factors for childhood onset diabetes mellitus: a record linkage study. International Journal of Epidemiology. 1998;27(3):444-9.

17. Kimpimaki T, Erkkola M, Korhonen S, Kupila A, Virtanen SM, Ilonen J, et al. Short-term exclusive breastfeeding predisposes young children with increased genetic risk of Type I diabetes to progressive beta-cell autoimmunity. Diabetologia. 2001;44(1):63-9.

18. Kyvik KO, Green A, Svendsen A, Mortensen K. Breast feeding and the development of type 1 diabetes mellitus. Diabetic Medicine. 1992;9(3):233-5.

19. Norris JM, Beaty B, Klingensmith G, Yu LP, Hoffman M, Chase HP, et al. Lack of association between early exposure to cow's milk protein and beta-cell autoimmunity - Diabetes autoimmunity study in the young (DAISY). Jama-Journal of the American Medical Association. 1996;276(8):609-14.

20. Robertson L, Harrild K. Maternal and neonatal risk factors for childhood type 1 diabetes: a matched case-control study. BMC Public Health. 2010;10:281.

21. Savilahti E, Saarinen KM. Early infant feeding and type 1 diabetes. European Journal of Nutrition. 2009;48(4):243-9.

22. Virtanen SM, Laara E, Hypponen E, Reijonen H, Rasanen L, Aro A, et al. Cow's milk consumption, HLA-DQB1 genotype, and type 1 diabetes: a nested case-control study of siblings of children with diabetes. Childhood diabetes in Finland study group.[Erratum appears in Diabetes 2000 Sep;49(9):1617]. Diabetes. 2000;49(6):912-7.

23. Glatthaar C, Whittall DE, Welborn TA, Gibson MJ, Brooks BH, Ryan P, et al. Diabetes in Western Australian children: Descriptive epidemiology. Medical Journal of Australia. 1988;148(3):117-23.

24. Ahadi M, Tabatabaeiyan M, Moazzami K. Association between environmental factors and risk of type 1 diabetes - a case-control study. Endokrynologia Polska. 2011;62(2):134-7.

25. Alves JGB, Figueiroa JN, Meneses J, Alves GV. Breastfeeding protects against type 1 diabetes mellitus: A case-sibling study. Breastfeeding Medicine. 2012;7(1):25-8.

26. Ashraf AP, Eason NB, Kabagambe EK, Haritha J, Meleth S, McCormick KL. Dietary iron intake in the first 4 months of infancy and the development of type 1 diabetes: A pilot study. Diabetology and Metabolic Syndrome. 2010;2(1).

27. Baruah MP, Ammini AC, Khurana ML. Demographic, breast-feeding, and nutritional trends among children with type 1 diabetes mellitus. Indian journal of endocrinology and metabolism. 2011;15(1):38-42.

28. Bener A, Alsaied A, Al-Ali M, Al-Kubaisi A, Basha B, Abraham A, et al. High prevalence of vitamin D deficiency in type 1 diabetes mellitus and healthy children. Acta Diabetologica. 2009;46(3):183-9.

29. Blom L, Dahlquist G, Nystrom L, Sandstrom A, Wall S. The Swedish childhood diabetes study - social and perinatal determinants for diabetes in childhood. Diabetologia. 1989;32(1):7-13.

30. Bodington MJ, McNally PG, Burden AC. Cow's milk and type 1 childhood diabetes: no increase in risk. Diabetic Medicine. 1994;11(7):663-5.

31. Borras V, Freitas A, Castell C, Gispert R, Jane M. Type 1 diabetes and perinatal factors in Catalonia (Spain). Pediatric Diabetes. 2011;12(4 Pt 2):419-23.

32. Esfarjani F, Azar MR, Gafarpour M. IDDM and early exposure of infant to cow's milk and solid food. Indian Journal of Pediatrics. 2001;68(2):107-10.

33. Group ESS. Rapid early growth is associated with increased risk of childhood type 1 diabetes in various European populations. Diabetes Care. 2002;25(10):1755-60.

34. Gimeno SG, de Souza JM. IDDM and milk consumption. A case-control study in Sao Paulo, Brazil. Diabetes Care. 1997;20(8):1256-60.

35. Hathout EH, Beeson WL, Ischander M, Rao R, Mace JW. Air pollution and type 1 diabetes in children. Pediatric Diabetes. 2006;7(2):81-7.

36. Hypponen E, Kenward MG, Virtanen SM, Piitulainen A, Virta-Autio P, Tuomilehto J, et al. Infant feeding, early weight gain, and risk of type I diabetes. Diabetes Care. 1999;22(12):1961-5.

37. Kostraba JN, Dorman JS, LaPorte RE, Scott FW, Steenkiste AR, Gloninger M, et al. Early infant diet and risk of IDDM in blacks and whites. A matched case-control study. Diabetes Care. 1992;15(5):626-31.

38. Kostraba JN, Cruickshanks KJ, Lawler-Heavner J, Jobim LF, Rewers MJ, Gay EC, et al. Early exposure to cow's milk and solid foods in infancy, genetic predisposition, and risk of IDDM. Diabetes. 1993;42(2):288-95.

39. Majeed AA, Mea, Hassan K. Risk Factors for Type 1 Diabetes Mellitus among Children and Adolescents in Basrah. Oman Medical Journal. 2011;26(3):189-95.

40. Malcova H, Sumnik Z, Drevinek P, Venhacova J, Lebl J, Cinek O. Absence of breast-feeding is associated with the risk of type 1 diabetes: a case-control study in a population with rapidly increasing incidence. European Journal of Pediatrics. 2006;165(2):114-9.

41. Marshall AL, Chetwynd A, Morris JA, Placzek M, Smith C, Olabi A, et al. Type 1 diabetes mellitus in childhood: a matched case control study in Lancashire and Cumbria, UK. Diabetic Medicine. 2004;21(9):1035-40.

42. Mayer EJ, Hamman RF, Gay EC, Lezotte DC, Savitz DA, Klingensmith GJ. Reduced risk of IDDM among breast-fed children. The Colorado IDDM Registry. Diabetes. 1988;37(12):1625-32.

43. McKinney PA, Parslow R, Gurney KA, Law GR, Bodansky HJ, Williams R. Perinatal and neonatal determinants of childhood type 1 diabetes. A case-control study in Yorkshire, U.K. Diabetes Care. 1999;22(6):928-32.

44. Meloni T, Marinaro AM, Mannazzu MC, Ogana A, La Vecchia C, Negri E, et al. IDDM and early infant feeding. Sardinian case-control study. Diabetes Care. 1997;20(3):340-2.

45. Patterson CC, Carson DJ, Hadden DR, Waugh NR, Cole SK. A case-control investigation of perinatal risk factors for childhood IDDM in Northern Ireland and Scotland. Diabetes Care. 1994;17(5):376-81.

46. Perez-Bravo F, Carrasco E, Gutierrez-Lopez MD, Martinez MT, Lopez G, de los Rios MG. Genetic predisposition and environmental factors leading to the development of insulin-dependent diabetes mellitus in Chilean children. Journal of molecular medicine. 1996;74(2):105-9.

47. Perez-Bravo F, Oyarzun A, Carrasco E, Albala C, Dorman JS, Santos JL. Duration of breast feeding and bovine serum albumin antibody levels in type 1 diabetes: a case-control study. Pediatric Diabetes. 2003;4(4):157-61.

48. Rami B, Schneider U, Imhof A, Waldhor T, Schober E. Risk factors for type I diabetes mellitus in children in Austria. European Journal of Pediatrics. 1999;158(5):362-6.

49. Rosenbauer J, Herzig P, Giani G. Early infant feeding and risk of type 1 diabetes mellitus-a nationwide population-based case-control study in pre-school children. Diabetes/Metabolism Research Reviews. 2008;24(3):211-22.

50. Sadauskaite-Kuehne V, Ludvigsson J, Padaiga Z, Jasinskiene E, Samuelsson U. Longer breastfeeding is an independent protective factor against development of type 1 diabetes mellitus in childhood. Diabetes/Metabolism Research Reviews. 2004;20(2):150-7.

51. Samuelsson U, Johansson C, Ludvigsson J. Breast-feeding seems to play a marginal role in the prevention of insulin-dependent diabetes mellitus. Diabetes Research & Clinical Practice. 1993;19(3):203-10.

52. Siemiatycki J, Colle E, Campbell S, Dewar RA, Belmonte MM. Case-control study of IDDM. Diabetes Care. 1989;12(3):209-16.

53. Sipetic S, Vlajinac H, Kocev N, Bjekic M, Sajic S. Early infant diet and risk of type 1 diabetes mellitus in Belgrade children. Nutrition. 2005;21(4):474-9.

54. Skrodeniene E, Marciulionyte D, Padaiga Z, Jasinskiene E, Sadauskaite-Kuehne V, Sanjeevi CB, et al. Associations between HLA class II haplotypes, environmental factors and type 1 diabetes mellitus in Lithuanian children with type 1 diabetes and controls. Polish Annals of Medicine. 2010;17(1):7-15.

55. Soltesz G, Jeges S, Dahlquist G. Non-genetic risk determinants for type 1 (insulin-dependent) diabetes mellitus in childhood. Hungarian Childhood Diabetes Epidemiology Study Group. Acta Paediatrica. 1994;83(7):730-5.

56. Strotmeyer ES, Yang Z, LaPorte RE, Chang YF, Steenkiste AR, Pietropaolo M, et al. Infant diet and type 1 diabetes in China. Diabetes Research & Clinical Practice. 2004;65(3):283-92.

57. Tai TY, Wang CY, Lin LL, Lee LT, Tsai ST, Chen CJ. A case-control study on risk factors for Type 1 diabetes in Taipei City. Diabetes research and clinical practice. 1998;42(3):197-203.

58. Telahun M, Abdulkadir J, Kebede E. The relation of early nutrition, infections and socio-economic factors to the development of childhood diabetes. Ethiopian Medical Journal. 1994;32(4):239-44.

59. Tenconi MT, Devoti G, Comelli M, Pinon M, Capocchiano A, Calcaterra V, et al. Major childhood infectious diseases and other determinants associated with type 1 diabetes: a case-control study. Acta Diabetologica. 2007;44(1):14-9.

60. Thorsdottir I, Birgisdottir BE, Johannsdottir IM, Harris DP, Hill J, Steingrimsdottir L, et al. Different beta-casein fractions in Icelandic versus Scandinavian cow's milk may influence diabetogenicity of cow's milk in infancy and explain low incidence of insulin-dependent diabetes mellitus in Iceland. Pediatrics. 2000;106(4):719-24.

61. Verge CF, Howard NJ, Irwig L, Simpson JM, Mackerras D, Silink M. Environmental factors in childhood IDDM. A population-based, case-control study. Diabetes Care. 1994;17(12):1381-9.

62. Virtanen SM, Rasanen L, Ylonen K, Aro A, Clayton D, Langholz B, et al. Early introduction of dairy products associated with increased risk of IDDM in Finnish children. Diabetes. 1993;42(12):1786-90.

63. Visalli N, Sebastiani L, Adorisio E, Conte A, De Cicco AL, D'Elia R, et al. Environmental risk factors for type 1 diabetes in Rome and province. Archives of Disease in Childhood. 2003;88(8):695-8.

64. Wadsworth EJ, Shield JP, Hunt LP, Baum JD. A case-control study of environmental factors associated with diabetes in the under 5s. Diabetic Medicine. 1997;14(5):390-6.

65. Couper JJ, Beresford S, Hirte C, Baghurst PA, Pollard A, Tait BD, et al. Weight gain in early life predicts risk of islet autoimmunity in children with a first-degree relative with type 1 diabetes. Diabetes Care. 2009;32(1):94-9.

66. Frederiksen B, Kroehl M, Lamb M, Seifert J, Barriga K, Rewers M, et al. Infant exposures and development of type 1 diabetes-the diabetes autoimmunity study in the young (DAISY). Diabetes. 2012;61:A352.

67. Holmberg H, Wahlberg J, Vaarala O, Ludvigsson J, Group AS. Short duration of breast-feeding as a risk-factor for beta-cell autoantibodies in 5-year-old children from the general population. British Journal of Nutrition. 2007;97(1):111-6.

68. Liese AD, Puett RC, Lamichhane AP, Nichols MD, Dabelea D, Lawson AB, et al. Neighborhood level risk factors for type 1 diabetes in youth: the SEARCH case-control study. International Journal of Health Geographics [Electronic Resource]. 2012;11:1.

69. Rabiei S. The association of nutrition style through the first 2 years of life with type 1 diabetes mellitus and some of the other effective factors in 2-15 years old children. [Persian]. Iranian Journal of Endocrinology and Metabolism. 2011;13(1):113.

70. Stene LC, Ulriksen J, Magnus P, Joner G. Use of cod liver oil during pregnancy associated with lower risk of Type I diabetes in the offspring.[Erratum appears in Diabetologia 2000 Nov;43(11):1451]. Diabetologia. 2000;43(9):1093-8.

71. Stene LC, Joner G. Use of cod liver oil during the first year of life is associated with lower risk of childhood-onset type 1 diabetes: a large, population-based, case-control study. The American journal of clinical nutrition. 2003;78(6):1128-34.

72. Savilahti E, Kukkonen K, Kuitunen M. Probiotics in the treatment and prevention of allergy in children. World Allergy Organization Journal. 2009;2(5):69-76.

73. Perez-Bravo F, Carrasco E, Gutierrez-Lopez MD, Martinez MT, Lopez G, de los Rios MG. Genetic predisposition and environmental factors leading to the development of insulin-dependent diabetes mellitus in Chilean children. Journal of Molecular Medicine. 1996;74(2):105-9.

74. Hornell A, Lagstrom H, Lande B, Thorsdottir I. Breastfeeding, introduction of other foods and effects on health: a systematic literature review for the 5th Nordic Nutrition Recommendations. Food & nutrition research. 2013;57.

75. Ip S, Chung M, Raman G, Chew P, Magula N, DeVine D, et al. Breastfeeding and maternal and infant health outcomes in developed countries. Evidence Report/Technology Assessment. 2007(153):1-186.

76. Patelarou E, Girvalaki C, Brokalaki H, Patelarou A, Androulaki Z, Vardavas C. Current evidence on the associations of breastfeeding, infant formula, and cow's milk introduction with type 1 diabetes mellitus: a systematic review. Nutrition Reviews. 2012;70(9):509-19.

77. Cardwell CR, Stene LC, Ludvigsson J, Rosenbauer J, Cinek O, Svensson J, et al. Breast-feeding and childhood-onset type 1 diabetes: a pooled analysis of individual participant data from 43 observational studies. Diabetes care. 2012;35(11):2215-25.

78. Ponsonby AL, Pezic A, Cochrane J, Cameron FJ, Pascoe M, Kemp A, et al. Infant anthropometry, early life infection, and subsequent risk of type 1 diabetes mellitus: a prospective birth cohort study. Pediatric Diabetes. 2011;12(4 Pt 1):313-21.

79. Ziegler AG, Rewers M, Simell O, Simell T, Lempainen J, Steck A, et al. Seroconversion to multiple islet autoantibodies and risk of progression to diabetes in children. JAMA. 2013;309(23):2473-9.

80. Kramer MS, Kakuma R. Optimal duration of exclusive breastfeeding. Cochrane Database of Systematic Reviews. 2012;8:CD003517.

81. Vaarala O, Knip M, Paronen J, Hämäläinen AM, Muona P, Väätäinen M, et al. Cow's milk formula feeding induces primary immunization to insulin in infants at genetic risk for type 1 diabetes. Diabetes [Internet]. 1999; 48(7):[1389-94 pp.]. Available from: <http://onlinelibrary.wiley.com/o/cochrane/clcentral/articles/791/CN-00164791/frame.html>.
