# Supplementary material for: Diet during pregnancy and infancy and risk of allergic or autoimmune disease: A systematic review and meta-analysis
Source: PLoS Med. 2018 Feb 28;15(2):e1002507. doi: 10.1371/journal.pmed.1002507 (PMC5830033; doi:10.1371/journal.pmed.1002507)
Supplement: S1 Data — (ZIP) [file pmed.1002507.s006.zip › Review_A_reports/WHEEZE.docx]

**Breastfeeding, Solid Food Introduction and Wheeze**

Vanessa Garcia-Larsen^1^, Despo Ierodiakonou^2^, Jo Leonardi-Bee^3^, Tim Reeves^4^, Jennifer Chivinge^5^, Zoe Robinson^5^, Natalie Geoghegan^5^, Katharine Jarrold^5^, Andrew Logan^5^, Annabel Groome^5^ , Evangelia Andreou^6^, Nara Tagiyeva-Milne^7^, Ulugbek Nurmatov^8^, Sergio Cunha^9^, Robert J Boyle^10^

^1^Post-Doctoral Research Associate, Respiratory Epidemiology and Public Health, National Heart and Lung Institute, Imperial College London; ^2^ Post-Doctoral Research Associate, Departments of Paediatric and Respiratory Epidemiology and Public Health Group, Imperial College London. ^3^Associate Professor of Community Health Sciences, University of Nottingham; ^4^ Research Support Librarian, Faculty of Medicine, Imperial College London; ^5^Undergraduate medical students, Imperial College London; ^6^Research Associate, Imperial Consultants; ^7^Research Fellow, University of Aberdeen; ^8^Research Fellow, University of Edinburgh; ^9^Research Associate, Respiratory Epidemiology and Public Health, National Heart and Lung Institute, Imperial College London ^10^ Clinical Senior Lecturer, Section of Paediatrics, Imperial College London

Imperial Consultants,

58 Princes Gate,

Exhibition Road,

London SW7 2PG

Contents

[List of figures 4](#_Toc495507908)

[List of Tables 9](#_Toc495507909)

[1 Total breastfeeding and wheeze 11](#_Toc495507910)

[1.1 Overall characteristics of studies, risk of bias and summary of results 11](#_Toc495507911)

[1.2 Total Breastfeeding and wheeze 30](#_Toc495507912)

[1.2.1 Age at outcome measurement 0-4 30](#_Toc495507913)

[1.2.2 Age at outcome measurement 5-14 38](#_Toc495507914)

[1.2.3 Age at outcome measurement 15+ 44](#_Toc495507915)

[1.3 Total Breastfeeding and atopic wheeze 46](#_Toc495507916)

[1.3.1 Age at outcome measurement 5-14 46](#_Toc495507917)

[1.4 Total Breastfeeding per month and Wheeze 47](#_Toc495507918)

[1.4.1 Age at outcome measurement 5-14 47](#_Toc495507919)

[1.5 Total Breastfeeding and Recurrent Wheeze 47](#_Toc495507920)

[1.5.1 Age at outcome measurement 0-4 47](#_Toc495507921)

[1.5.2 Age at outcome measurement 5-14 54](#_Toc495507922)

[1.5.3 Age at outcome measurement 15+ 66](#_Toc495507923)

[1.6 Data for TBF duration and lung function in children aged 5-14 years 74](#_Toc495507924)

[1.6.1 Outcome: FEV1(ml) 74](#_Toc495507925)

[1.6.2 Outcome: PEF (ml/sec) 74](#_Toc495507926)

[1.6.3 Outcome: FEV1% predicted 75](#_Toc495507927)

[1.6.4 Outcome: FVC (ml) 76](#_Toc495507928)

[1.6.5 Data for TBF duration and wheeze, BHR or lung function not included in meta-analysis 77](#_Toc495507929)

[1.6.6 Conclusion on association between TBF duration and lung function and bronchial hyper-responsiveness 83](#_Toc495507930)

[2 Exclusive breastfeeding and wheeze 84](#_Toc495507931)

[2.1 Overall characteristics of studies, risk of bias and summary of results 84](#_Toc495507932)

[2.2 Exclusive Breastfeeding and Wheeze 99](#_Toc495507933)

[2.2.1 Systematic reviews 99](#_Toc495507934)

[2.2.2 Age at outcome measurement 0-4 99](#_Toc495507935)

[2.2.3 Age at outcome measurement 5-14 105](#_Toc495507936)

[2.2.4 Age at outcome measurement 15+ 107](#_Toc495507937)

[2.3 Exclusive Breastfeeding per month and Wheeze 107](#_Toc495507938)

[2.3.1 Age at outcome measurement 0-4 107](#_Toc495507939)

[2.4 Exclusive Breastfeeding and Recurrent Wheeze 108](#_Toc495507940)

[2.4.1 Age at outcome measurement 0-4 108](#_Toc495507941)

[2.4.2 Age at outcome measurement 5-14 113](#_Toc495507942)

[2.4.3 Age at outcome measurement 15+ 118](#_Toc495507943)

[2.5 Exclusive Breastfeeding and Atopic Wheeze 119](#_Toc495507944)

[2.5.1 Age at outcome measurement 0-4 119](#_Toc495507945)

[2.5.2 Age at outcome measurement 5-14 120](#_Toc495507946)

[2.6 Exclusive Breastfeeding per month and recurrent wheeze 121](#_Toc495507947)

[2.6.1 Age at outcome measurement 0-4 121](#_Toc495507948)

[2.6.2 Age at outcome measurement 5-14 122](#_Toc495507949)

[2.7 Data for EBF duration and lung function (FEV1/FVC%) 123](#_Toc495507950)

[2.8 Data for EBF duration and Wheeze, BHR or lung function that were not suitable for meta-analysis 124](#_Toc495507952)

[3 Solid Food Introduction and Wheeze 130](#_Toc495507953)

[3.1 Overall characteristics of studies, risk of bias and summary of results 130](#_Toc495507954)

[3.2 Solid Food Introduction and Wheeze 135](#_Toc495507955)

[3.2.1 Age at outcome measurement 0-4 135](#_Toc495507956)

[3.2.2 Age at outcome measurement 5-14 135](#_Toc495507957)

[3.3 Solid Food Introduction and Recurrent Wheeze 136](#_Toc495507958)

[3.3.1 Age at outcome measurement 0-4 136](#_Toc495507959)

[3.3.2 Age at outcome measurement 5-14 137](#_Toc495507960)

[4 Conclusion 140](#_Toc495507961)

[5 References 142](#_Toc495507962)

# List of figures

[Figure 1 Risk of bias in studies of total breastfeeding and wheeze 30](#_Toc497227701)

[Figure 2 Total breastfeeding ever vs. never and risk of wheeze in children aged 0-4 years 32](#_Toc497227702)

[Figure 3 Risk of publication bias in studies investigating total breastfeeding ever vs. never and risk of wheeze in children aged 0-4 years 32](#_Toc497227703)

[Figure 4 Total breastfeeding (dose response) short vs. never and risk of wheeze in children aged 0-4 years 34](#_Toc497227704)

[Figure 5 Total breastfeeding (dose response) medium vs. never and risk of wheeze in children aged 0-4 years 34](#_Toc497227705)

[Figure 6 Total breastfeeding (dose response) long vs. never and risk of wheeze in children aged 0-4 years 34](#_Toc497227706)

[Figure 7 Total breastfeeding for ≥1-2 months vs. <1-2 months and risk of wheeze in children aged 0-4 years 35](#_Toc497227707)

[Figure 8 Total breastfeeding for ≥3-4 months vs. <3-4 months and risk of wheeze in children aged 0-4 years 36](#_Toc497227708)

[Figure 9 Total breastfeeding for ≥5-7 months vs. <5-7 months and risk of wheeze in children aged 0-4 years 36](#_Toc497227709)

[Figure 10 Total breastfeeding for ≥8-12 months vs. <8-12 months and risk of wheeze in children aged 0-4 years 38](#_Toc497227710)

[Figure 11 Total breastfeeding ever vs. never and risk of wheeze in children aged 5-14 year 39](#_Toc497227711)

[Figure 12 Risk of publication bias in studies investigating breastfeeding ever vs. never and risk of wheeze in children aged 5-14 years 40](#_Toc497227712)

[Figure 13 Total breastfeeding (dose response) short vs. never and risk of wheeze in children aged 5-14 years 42](#_Toc497227713)

[Figure 14 Total breastfeeding (dose response) medium vs. never and risk of wheeze in children aged 5-14years 42](#_Toc497227714)

[Figure 15 Total breastfeeding (dose response) long vs. never and risk of wheeze in children aged 5-14 years 42](#_Toc497227715)

[Figure 16 Total breastfeeding for ≥1-2 months vs. <1-2 months and risk of wheeze in children aged 5-14 years 43](#_Toc497227716)

[Figure 17 Total breastfeeding for ≥3-4 months vs. <3-4 months and risk of wheeze in children aged 5-14 years 43](#_Toc497227717)

[Figure 18 Total breastfeeding for ≥5-7 months vs. <5-7 months and risk of wheeze in children aged 5-14 years 44](#_Toc497227718)

[Figure 19 Total breastfeeding ever vs. never and risk of wheeze in children aged 15+ years 44](#_Toc497227719)

[Figure 20 Total breastfeeding for ≥1-2 months vs. <1-2 months and risk of wheeze in children aged 15+ years 45](#_Toc497227720)

[Figure 21 Total breastfeeding for ≥3-4 months vs. <3-4 months and risk of wheeze in children aged 15+ years 45](#_Toc497227721)

[Figure 22 Total breastfeeding (dose response) ever vs. never and risk of atopic wheeze in children aged 5-14 years 46](#_Toc497227722)

[Figure 23 Total breastfeeding (dose response) medium vs. never and risk of atopic wheeze in children aged 5-14 years 46](#_Toc497227723)

[Figure 24 Total breastfeeding (dose response) long vs. never and risk of atopic wheeze in children aged 5-14 years 46](#_Toc497227724)

[Figure 25 Total breastfeeding per month and risk of wheeze in children aged 5-14 years 47](#_Toc497227725)

[Figure 26 Total breastfeeding ever vs. never and risk of recurrent wheeze in children aged 0-4 years 48](#_Toc497227726)

[Figure 27 Risk of publication bias in studies investigating total breastfeeding ever vs. never and risk of recurrent wheeze in children aged 0-4 years 48](#_Toc497227727)

[Figure 28 Total breastfeeding short vs. never term and risk of recurrent wheeze in children aged 0-4 years 50](#_Toc497227728)

[Figure 29 Total breastfeeding medium vs. never term and risk of recurrent wheeze in children aged 0-4 years 50](#_Toc497227729)

[Figure 30Total breastfeeding long vs. never term and risk of recurrent wheeze in children aged 0-4 years 50](#_Toc497227730)

[Figure 31 Total breastfeeding for ≥1-2 months vs. <1-2 months and risk of recurrent wheeze in children aged 0-4 years 51](#_Toc497227731)

[Figure 32 Total breastfeeding for ≥3-4 months vs. <3-4 months and risk of recurrent wheeze in children aged 0-4 years 51](#_Toc497227732)

[Figure 33 Total breastfeeding for ≥5-7 months vs. <5-7 months and risk of recurrent wheeze in children aged 0-4 years 52](#_Toc497227733)

[Figure 34 Total breastfeeding for ≥8-12 months vs. <8-12 months and risk of recurrent wheeze in children aged 0-4 years 54](#_Toc497227734)

[Figure 35 Total Breastfeeding ever vs. never and risk of recurrent wheeze in children aged 5-14 years 55](#_Toc497227735)

[Figure 36 Risk of publication bias in studies investigating total breastfeeding ever vs. never and risk of recurrent wheeze in children aged 5-14 years 56](#_Toc497227736)

[Figure 37 Total breastfeeding (dose response) short vs. never term and risk of recurrent wheeze in children aged 5-14 years 58](#_Toc497227737)

[Figure 38 Total breastfeeding (dose response) medium vs. never term and risk of recurrent wheeze in children aged 5-14 years 58](#_Toc497227738)

[Figure 39 Total breastfeeding (dose response) long vs. never term and risk of recurrent wheeze in children aged 5-14 years 59](#_Toc497227739)

[Figure 40 Total breastfeeding for ≥1-2 months vs. <1-2 months and risk of recurrent wheeze in children aged 5-14 years 59](#_Toc497227740)

[Figure 41 Total breastfeeding for ≥3-4 months vs. <3-4 months and risk of recurrent wheeze in children aged 5-14 years 60](#_Toc497227741)

[Figure 42 Risk of publication bias in studies investigating total breast feeding and recurrent wheeze ≥3-4 months vs. <3-4 months in children aged 5-14 years 61](#_Toc497227742)

[Figure 43 Total breastfeeding for ≥5-7 months vs. <5-7 months and risk of recurrent wheeze in children aged 5-14 years 63](#_Toc497227743)

[Figure 44 Risk of publication bias in studies investigating total breast feeding and recurrent wheeze ≥5-7 months vs. <5-7 months in children aged 5-14 years 64](#_Toc497227744)

[Figure 45 Total breastfeeding for ≥18+ months vs. <18+ months and risk of recurrent wheeze in children aged 5-14 years 66](#_Toc497227745)

[Figure 46 Total breastfeeding ever vs. never and risk of recurrent wheeze in children aged 15+ years 67](#_Toc497227746)

[Figure 47 Total breastfeeding for ≥1-2 months vs. <1-2 months and risk of recurrent wheeze in children aged 15+ years 69](#_Toc497227747)

[Figure 48 Total breastfeeding for ≥3-4 months vs. <3-4 months and risk of recurrent wheeze in children aged 15+ years 69](#_Toc497227748)

[Figure 49 Total breastfeeding for ≥5-7 months vs. <5-7 months and risk of recurrent wheeze in children aged 15+ years 70](#_Toc497227749)

[Figure 50 Total Breastfeeding any duration vs. never and risk of bronchial hyper-responsiveness in children aged 5-14 years 71](#_Toc497227750)

[Figure 51 Total breastfeeding (dose response) never vs. ever and risk of bronchial hyper-responsiveness in children aged 5-14 years 71](#_Toc497227751)

[Figure 52 Total breastfeeding (dose response) short vs. never and risk of bronchial hyper-responsiveness in children aged 5-14 years 72](#_Toc497227752)

[Figure 53 Total breastfeeding (dose response) medium vs. never and risk of bronchial hyper-responsiveness in children aged 5-14 years 72](#_Toc497227753)

[Figure 54 Total breastfeeding (dose response) long vs. never and risk of bronchial hyper-responsiveness in children aged 5-14 years 72](#_Toc497227754)

[Figure 55 Total Breastfeeding ≥1-2 months vs. <1-2 months and risk of bronchial hyper-responsiveness in children aged 15+ years 73](#_Toc497227755)

[**Figure 25 Total breastfeeding and FEV1 (ml) in children aged 5-14 years** 74](#_Toc497227756)

[**Figure 26 Total breastfeeding and PEF (ml/sec) in children aged 5-14 years** 74](#_Toc497227757)

[**Figure 27 Total breastfeeding and FEV1% predicted in children aged 5-14 years** 75](#_Toc497227758)

[**Figure 28 Total breastfeeding and FVC (ml) in children aged 5-14 years** 76](#_Toc497227759)

[Figure 56 Risk of bias in studies of exclusive breastfeeding and wheeze 98](#_Toc497227760)

[Figure 57 Exclusive breast feeding ≥0-2 months vs. <0-2 months and risk of wheeze in children aged 0-4 years 100](#_Toc497227761)

[Figure 58 Exclusive breast feeding ≥3-4 months vs. <3-4 months and risk of wheeze in children aged 0-4 years 101](#_Toc497227762)

[Figure 59 Risk of publication bias in studies of exclusive breast feeding ≥3-4 months vs. <3-4 months and risk of wheeze in children aged 0-4 years 101](#_Toc497227763)

[Figure 60 Exclusive breast feeding ≥5 months vs. <5 months and risk of wheeze in children aged 0-4 years 102](#_Toc497227764)

[Figure 61 Exclusive breast feeding ≥0-2 months vs. <0-2 months and risk of wheeze in children aged 5-14 years 105](#_Toc497227765)

[Figure 62 Exclusive breast feeding ≥3-4 months vs. <3-4 months and risk of wheeze in children aged 5-14 years 106](#_Toc497227766)

[Figure 63 Exclusive breast feeding ≥5 months vs. <5 months and risk of wheeze in children aged5-14 years 106](#_Toc497227767)

[Figure 64 Exclusive breast feeding ≥0-2 months vs. <0-2 months and risk of wheeze in children aged 15+ years 107](#_Toc497227768)

[Figure 65 Exclusive breastfeeding per month and risk of wheeze in children aged 0-4 years 107](#_Toc497227769)

[Figure 66 Exclusive breast feeding ≥0-2 months vs. <0-2 months and risk of recurrent wheeze in children aged 0-4 years 108](#_Toc497227770)

[Figure 67 Exclusive breast feeding ≥3-4 months vs. <3-4 months and risk of recurrent wheeze in children aged 0-4 years 109](#_Toc497227771)

[Figure 68 Risk of publication bias in studies of exclusive breast feeding ≥3-4 months vs. <3-4 months and risk of wheeze in children aged 0-4 years 110](#_Toc497227772)

[Figure 69 Exclusive breast feeding ≥5 months vs. <5 months and risk of recurrent wheeze in children aged 0-4 years 110](#_Toc497227773)

[Figure 70 Exclusive breast feeding ≥0-2 months vs. <0-2 months and risk of recurrent wheeze in children aged 5-14 years 113](#_Toc497227774)

[Figure 71 Exclusive breast feeding ≥3-4 months vs. <3-4 months and risk of recurrent wheeze in children aged 5-14 years 114](#_Toc497227775)

[Figure 72 Risk of publication bias of studies investigating exclusive breast feeding ≥3-4 months vs. <3-4 months and risk of recurrent wheeze in children aged 5-14 years 115](#_Toc497227776)

[Figure 73 Exclusive breastfeeding ≥5 months vs. <5 months and risk of recurrent wheeze in children aged 5-14 years 115](#_Toc497227777)

[Figure 74 Exclusive breast feeding ≥0-2 months vs. <0-2 months and risk of recurrent wheeze in children aged 15+ years 118](#_Toc497227778)

[Figure 75 Exclusive breast feeding ≥3-4 months vs. <3-4 months and risk of recurrent wheeze in children aged 15+ years 118](#_Toc497227779)

[Figure 76 Exclusive breast feeding ≥5 months vs. <5 months and risk of recurrent wheeze in children aged15+ years 119](#_Toc497227780)

[Figure 77 Exclusive breast feeding ≥0-2 months vs. <0-2 months and risk of atopic wheeze in children aged 0-4 years 119](#_Toc497227781)

[Figure 78 Exclusive breast feeding ≥3-4 months vs. <3-4 months and risk of atopic wheeze in children aged 0-4 years 120](#_Toc497227782)

[Figure 79 Exclusive breast feeding ≥0-2 months vs. <0-2 months and risk of atopic wheeze in children aged 5-14 years 120](#_Toc497227783)

[Figure 80 Exclusive breast feeding ≥3-4 months vs. <3-4 months and risk of atopic wheeze in children aged 5-14 years 121](#_Toc497227784)

[Figure 81 Exclusive breastfeeding per month and risk of recurrent wheeze in children aged 0-4 years 121](#_Toc497227785)

[Figure 82 Exclusive breastfeeding per month and risk of recurrent wheeze in children aged 5-14 years 122](#_Toc497227786)

[Figure 83 Exclusive breastfeeding ≥0-2 months vs. <0-2 months and risk of bronchial hyper-responsiveness in children aged 5-14 years 122](#_Toc497227787)

[Figure 84 Exclusive breastfeeding ≥3-4 months vs. <3-4 months and risk of bronchial hyper-responsiveness in children aged 5-14 years 123](#_Toc497227788)

[**Figure 84 Exclusive breastfeeding and FEV1/FVC% in children aged 5-14 years** 124](#_Toc497227789)

[Figure 85 Risk of bias in studies of solid food introduction and wheeze 134](#_Toc497227790)

[Figure 86 Solid Food Introduction ≥3-4 months vs. <3-4 months and risk of wheeze in children aged 0-4 years 135](#_Toc497227791)

[Figure 87 Solid Food Introduction ≥3-4 months vs. <3-4 months and risk of wheeze in children aged 5-14 years 136](#_Toc497227792)

[Figure 88 Solid Food Introduction ≥3-4 months vs. <3-4 months and risk of recurrent wheeze in children aged 0-4 years 136](#_Toc497227793)

[Figure 89 Solid Food Introduction ≥3-4 months vs. <3-4 months and risk of recurrent wheeze in children aged 5-14 years 137](#_Toc497227794)

# List of Tables

[**Table 1 Characteristics of included studies evaluating total breastfeeding and wheeze** 15](#_Toc497227795)

[Table 2 Subgroup and stratified analysis of TBF ever and risk of wheeze in children aged 0-4 years 33](#_Toc497227796)

[Table 3 Subgroup Analysis of risk of wheeze and total breastfeeding ≥5-7 months vs. <5-7 months in children aged 0-4 years 37](#_Toc497227797)

[Table 4 Subgroup Analysis of risk of wheeze and total breastfeeding never vs. ever in children aged 5-14 years 41](#_Toc497227798)

[Table 5 Subgroup Analysis of risk of recurrent wheeze and total breastfeeding ever vs. never (or ever) months in children aged 0-4 years 49](#_Toc497227799)

[Table 6 Subgroup Analysis of risk of recurrent wheeze and total breastfeeding ≥5-7 months vs. <5-7 months in children aged 0-4 years 53](#_Toc497227800)

[Table 7 Subgroup Analysis of risk of recurrent wheeze and total breastfeeding ever vs. never months in children aged 5-14 years 57](#_Toc497227801)

[Table 8 Subgroup Analysis of risk of recurrent wheeze and total breastfeeding ≥3-4 months vs. <3-4 months in children aged 5-14 years 62](#_Toc497227802)

[Table 9 Subgroup Analysis of risk of recurrent wheeze and total breastfeeding ≥5-7 months vs. <5-7 months in children aged 5-14 years 65](#_Toc497227803)

[Table 10 Subgroup Analysis of risk of recurrent wheeze and total breastfeeding ever vs. never in children aged 15+ years 68](#_Toc497227804)

[Table 11 Studies investigating the association between total breastfeeding and wheeze which were not eligible for meta-analysis 78](#_Toc497227805)

[**Table 12 Studies investigating the association between total breastfeeding and lung function which were not eligible for meta-analysis** 82](#_Toc497227806)

[Table 13 Characteristics of included studies evaluating exclusive breastfeeding and wheeze 87](#_Toc497227807)

[Table 14 Subgroup Analyses of risk of wheeze and exclusive breastfeeding ≥0-2 months vs. <0-2 months in children aged 0-4 years 103](#_Toc497227808)

[Table 15 Subgroup Analyses of risk of wheeze and exclusive breastfeeding ≥3-4 months vs. <3-4 months in children aged 0-4 years 104](#_Toc497227809)

[Table 16 Subgroup Analyses of risk of recurrent wheeze and exclusive breastfeeding ≥0-2 months vs. <0-2 months in children aged 0-4 years 111](#_Toc497227810)

[Table 17 Subgroup Analyses of risk of recurrent wheeze and exclusive breastfeeding ≥3-4 months vs. <3-4 months in children aged 0-4 years 112](#_Toc497227811)

[Table 18 Subgroup Analyses of risk of recurrent wheeze and exclusive breastfeeding ≥0-2 months vs. <0-2 months in children aged 5-14 years 116](#_Toc497227812)

[Table 19 Subgroup Analyses of risk of recurrent wheeze and exclusive breastfeeding ≥3-4 months vs. <3-4 months in children aged 5-14 years 117](#_Toc497227813)

[Table 20 Studies investigating the association between exclusive breastfeeding and wheeze which were not eligible for meta-analysis 125](#_Toc497227814)

[Table 21 Studies investigating the association between exclusive breastfeeding and BHR or lung function which were not eligible for meta-analysis 127](#_Toc497227815)

[Table 21 Characteristics of included studies evaluating solid food introduction and wheeze 131](#_Toc497227816)

[Table 22 Subgroup Analyses of risk of wheeze and solid food introduction ≥3-4 months vs. <3-4 months in children aged 5-14 years 138](#_Toc497227817)

[Table 23 Studies investigating the association between solid food introduction and wheeze which were not eligible for meta-analysis 139](#_Toc497227818)

# Total breastfeeding and wheeze

## Overall characteristics of studies, risk of bias and summary of results

Table 1 describes the main characteristics of the studies that assessed total breastfeeding duration (TBF) in relation to wheeze risk. There were a total of 105 studies of which one was a cluster randomised controlled trial, 68 were prospective cohort studies (2 of which were parental studies to a cross-sectional (CS) and to a nested case-control study), 3 retrospective cohorts, 10 case-control, 2 nested case-control, and 19 cross-sectional studies. The majority of studies (n=57) are from Europe – others are from the Asia-Pacific region (n=18) and North America (n=17), 5 from South America, 3 from Africa, 1 from the Middle East (Iraq), and 1 has an unknown provenance. There are also 2 studies from the International Study of Allergies and Asthma in Childhood (ISAAC), which included multiple countries.

Overall, valid data on TBF duration in the first 2 years of life and wheeze risk were available from over 463,000 subjects. Information on wheeze was obtained solely from parental or self-reported in 25 studies, through Dr-diagnosis in 31 studies, and from the ISAAC questionnaire in 16 studies. A further 4 studies use spirometry alone. Another 27 studies used a combination of self /parental report, Doctor diagnosis, and/ or objective measures (e.g. bronchial hyper-responsiveness (BHR)). One study used an unclear method for defining wheeze.

With regards to time of outcome diagnosis, 34 studies explored the association between TBF duration and wheeze at age 0-4 years, 41 at age 5-14 years, and 14 at age 15 years or beyond. A further 15 studies investigated the association between TBF duration and wheeze at various time points between the ages of 1 and 22 years. To ascertain exposure to TBF, 64 studies used a questionnaire method, 13 used an interview, 3 used medical records, 2 used a diary, and 1 used a food frequency questionnaire (FFQ). A further 18 studies used a combination of 2 or more of these methods, whilst 3 studies had no information available on the method used.

Risk of bias was assessed using the NICE Methodological checklists for cohort and case-control studies. Figure 1 illustrates the distribution of bias across the five main methodological areas of the studies. At least half of the studies were considered to have a low risk of assessment, selection and confounding bias, with over a third of studies had a low risk of overall bias. Over a third of the studies had a high risk of overall bias, mainly due to lack of controlling for potential confounders. Risk of conflict of interest was generally assessed as low.

Where data were available, five levels of comparison were used to assess the risk of wheeze according to TBF duration, namely ‘ever vs. never’, ‘≥1-2 months vs. <1-2 months’, ‘≥3-4 months vs. <3-4 months’, ‘≥5-7 months vs. <5-7 months’, and ‘≥8-12 months vs. <8-12 months’.

*Main findings*

In the single intervention trial, there was no evidence of a relationship between breastfeeding promotion and risk of wheeze at age 1 year or 6.5 years. For observational studies, across in children aged 0-4 years, we found no consistent evidence for an association between TBF and wheeze or recurrent wheeze. There was weak evidence for an association in dose-response analysis of four studies for wheeze (Figures 4 to 6) and three studies for recurrent wheeze (Figures 28 to 30). In other analyses there was either extreme heterogeneity, or no consistent association between TBF duration and wheeze or recurrent wheeze. Heterogeneity could not always be explained, but in some cases was likely due to differences in adjustment for potential confounders. In some sensitivity analyses unadjusted data showed greater evidence of association than adjusted data.

In children aged 5-14 years old, we found limited evidence from observational studies for an association between TBF and wheeze or recurrent wheeze. Meta-analysis of data for ever vs. never breastfed showed reduced odds of wheeze, but with high heterogeneity and evidence of publication bias; inconclusive findings from dose/response analysis, and no association seen with other durations of TBF. Meta-analysis of data for ever vs. never breastfed showed reduced odds of recurrent wheeze, with moderate heterogeneity and evidence of publication bias; but supportive findings from dose-response analysis, and significant or borderline significant associations seen with other durations of TBF (3 to 4 months, 5 to 7 months, over 18 months). The evidence for an association between TBF and recurrent wheeze at age 5-14 was graded as VERY LOW (-1 publication bias).

In people aged 15 years or older, we found no consistent evidence for an association between TBF and risk of wheeze or recurrent wheeze. Data were sparse for analyses of wheeze. For analyses of recurrent wheeze, TBF 1 to 2 months or longer was associated with increased risk of recurrent wheeze in one prospective and one retrospective study (Figure 47); TBF 5 to 7 months or longer was associated with decreased risk of recurrent wheeze in one prospective study at low risk of bias (Figure 49); and analyses of TBF ever vs. never, or TBF 3 to 4 months or longer showed no significant association with recurrent wheeze.

We found no evidence for an association between TBF and atopic wheeze, or between TBF and wheeze when TBF was reported as a continuous measure (e.g. average number of months breastfed in those with and without a history of wheeze).

We found no evidence for an association between TBF and bronchial hyper-reactivity (BHR) in children aged 5 to 14 years, and no consistent evidence for an association between TBF and measures of lung function. Eight studies investigated the association between lung function outcomes and TBF in over 37,800 subjects aged 10 to 51 years old, summarised in Table 12. Outcomes reported included forced expiratory volume in 1 second (FEV_1_), FEV_1_ as percentage of predicted value (FEV_1_%predicted), peak expiratory flow rate (PEFR), forced expiratory mid flow (FEF_50_), FEV_1_ decline, airflow obstruction (FEV_1_/FVC<0.80), FEV_1_/FVC as percentage of predicted value, and post-bronchodilator measures of FEV_1_, FVC and FEV_1_/FVC. One cross-sectional study reported a positive association between FEV_1_% and TBF ever vs. never. A prospective cohort reported a positive association between TBF and FEF_50_, whilst two prospective cohort studies reported a positive association between TBF and FEV_1_. Two studies reported an association between TBF ever and FEV_1_/FVC% and FVC. However a number of non-significant associations reported, and no measure of lung function was consistently associated with TBF across different studies.

A total of 36 studies with data on wheeze and TBF exposure were identified which did not report data in a way that could be included in meta-analyses (Table 11). There were 25 prospective cohorts, 1 retrospective cohort, 1 case-control, 1 a nested case-control, and 8 cross-sectional studies. Seven prospective cohort studies reported associations between TBF and reduced wheeze (n=1) or recurrent wheeze (n=6), and 2 cross-sectional studies reported associations between TBF and reduced recurrent wheeze. Twenty five studies showed no evidence of an association between TBF and wheeze outcomes. Four studies reported borderline associations (P=0.05).

Overall, TBF analyses were marked by statistical heterogeneity, evidence of publication bias in 3 analyses (ever vs. never and wheeze at age 5-14; ever vs. never and recurrent wheeze at age 5-14; TBF for at least 3 to 4 months and recurrent wheeze at age 5-14) – suggesting that the relationship reported between TBF and wheeze/recurrent wheeze may be influenced by publication of studies showing a protective association, and non-publication of studies finding TBF is not associated with, or is associated with increased risk of wheeze/recurrent wheeze.

*Conclusion*

We found VERY LOW (-1 publication bias) evidence that longer TBF is associated with reduced risk of recurrent wheeze at age 5-14 years, and no consistent evidence for an association between TBF and other wheeze or lung function outcomes.

**Table 1 Characteristics of included studies evaluating total breastfeeding and wheeze**

| **First Author & Publication Year** | **Design** | **N** | **Exposure assessment** | **Method of outcome assessment** | **Age at outcome (years)** | **Country** | **Population characteristics** |
| --- | --- | --- | --- | --- | --- | --- | --- |
| Kramer, 2001 ([1](#_ENREF_1)); Kramer, 2007 ([2](#_ENREF_2)) | 8865/8181 | Cluster RCT | Belarus | - | SPT-Aero | 6.5 | Breastfeeding promotion program based on the WHO/UNICEF baby friendly hospital initiative, versus standard local breastfeeding policies |
| Wright, 2002 ([3](#_ENREF_3)) | PC | 499 | Q/I | Parent reported wheeze | 0-1 | USA | Part of a metropolitan Boston prospective birth cohort study of infants born between 1994-1996 with family history of asthma or recruited from a Boston hospital |
| Al-Kubaisy, 2005 ([4](#_ENREF_4)) | CS | 2262 | Q | DD plus ISAAC | 12 | Iraq | Primary school urban and rural children |
| Hesselmar, 2010 ([5](#_ENREF_5)) | PC | 184 | I | DD asthma (>=3 episodes of wheeze) | 1.5 | Sweden | ALLERGYFLORA study. Population based study of babies selected from antenatal clinics between 1998 and 2003 - mainly high risk of allergic disease |
| Elliott, 2008; Granell, 2012; Sherriff, 2001  ([6-8](#_ENREF_6)) | PC | 9100 | Q | DD asthma PLUS current wheeze; Parent reported wheeze; BHR: (metacholine PC20) | 3, 3.5, 7, 7.5 | UK | ALSPAC study. Population based cohort of children born 1991-1992 |
|  |  |  |  |  |  |  |  |
|  |  |  |  |  |  |  |  |
| Kull, 2002 ([9](#_ENREF_9)) | PC | 3790 | Q | Self-reported wheeze; >=3 episodes of wheeze OR inhaled corticosteroids | 2 | Sweden | BAMSE study. Population based cohort of children born between 1994-1996 |
| Lewis, 1996 (BCS58) ([10](#_ENREF_10)) | PC | 9820 | I | Parent reported current wheeze | 16 | UK | British Cohort Study: Infants born in England, Wales, and Scotland England, Scotland and Wales in 1958 |
| Lewis, 1995; Lewis, 1996 (BCS70) ([10](#_ENREF_10), [11](#_ENREF_11)) | PC | 12835 | Q, I | Parent reported wheeze ever/ current wheeze | 5, 16 | UK | British Cohort Study: sample of all infants born in 1970 in Britain |
|  |  |  |  |  |  |  |  |
| Burr, 1989; Burr, 1993; Burr, 1993 (b) ([12-14](#_ENREF_12)) | PC | 483 | D, Q | Parent reported wheeze; Wheeze ever, DD | 1, 6.7, 7 | UK | Infants with family history of allergic diseases in South Wales |
|  |  |  |  |  |  |  |  |
|  |  |  |  |  |  |  |  |
| Alper, 2006 ([15](#_ENREF_15)) | CS | 858 | Q | Parent reported wheeze classified using Martinez criteria | 7 | Turkey | 7 years old children randomly selected from seven primary schools in Bursa in 1999 |
| Businco, 1987 ([16](#_ENREF_16)) | PC | 244 | I | DD asthma (>=3 episodes of wheeze) | 8 | Italy | Infants of atopic parents recruited from hospital and born in 1985-1988 |
| Camara, 2003 ([17](#_ENREF_17)) | CC | 91 | Q | DD (wheezing that required therapy with inhaled β2-agonists as judged by the attending physician) | 12 | Brazil | Cases and control were children who sought ED care |
| Midodzi, 2010 ([18](#_ENREF_18)) | PC | 8499 | Q | DD | 5 | Canada | Canadian Early Childhood Development Cohort Study- part of NLSCY. Longitudinal surveys of children representative of the Canadian population |
| Taylor, 1983 ([19](#_ENREF_19)) | PC | 12608 | I | Parent reported wheeze; DD | 5 | UK | CHES study. Population based cohort of children born in England, Scotland, and Wales in 1970 |
| Mihrshahi, 2007 ([20](#_ENREF_20)) | PC | 516 | I | DD PLUS current wheeze | 5 | Australia | CAPS study. Infants born in 1997-1999 with family history of asthma or wheezing |
| Simon, 2008 ([21](#_ENREF_21)) | PC | 372 | R/I | Transient wheezing: wheezing in the last 12 months at ages of 1,2 and/or 4 years but not at age of 6 years | 6 | USA | CAS study. Middle class mother-infant pairs enrolled in a health maintenance organisation in 1987-89 |
| Larsson, 2008 ([22](#_ENREF_22)) | PC | 4779 | Q | DD | 9 | Sweden | DBH study. Preschool children aged 1–6 years surveyed in 2000 and 2005. |
| Devereux, 2006 ([23](#_ENREF_23)) | PC | 1704 | FFQ | ISAAC | 5 | Scotland, UK | Population based birth cohort of infants born in 1998 |
| Nwaru, 2013 ([24](#_ENREF_24)) | PC | 3675 | D | DD plus ISAAC; DD plus ISAAC (+/- sIgE) | 5 | Finland | DIPP study. Infants at high risk (HLA) for TIDM born between 1996-2004 invited to the allergy study between 1998 and 2000 |
| Wilson, 1998 ([25](#_ENREF_25)) | PC | 545 | Q | Self-reported wheeze; DD | 7 | UK | Dundee infant feeding study. Population based cohort of infants born between 1983-1986 |
| Ehrlich, 1996 ([26](#_ENREF_26)) | CS | 620 | Q/I | ISAAC | 9 | South Africa | Second year elementary school (7- 8 years) children. All black |
| Visser, 2010 ([27](#_ENREF_27)) | CS | 1115 | Q | Parent reported wheeze ever; >=3 episodes of wheeze | 1 | Netherlands | EISL study. One-year old infants from urban and rural primary care health clinics born in 2004-2006 |
| Munro, 2011 ([28](#_ENREF_28)) | PC | 700 | Q | Parent reported wheeze | 1 | UK | EuroPrevall-UK. Population based birth cohort of infants born in 2008 |
| Farooqi, 1998 ([29](#_ENREF_29)) | PC | 1453 | R | DD (recurrent episodes of wheeze after the age of two years) | 16 | UK | Representative sample of general practice both in 1975-84 |
| Friday, 2000 ([30](#_ENREF_30)) | RC | 94 | NA | Physician assessed asthma | 10? | no data | Unclear |
| Fredriksson, 2007 ([31](#_ENREF_31)) | PC | 1933 | Q | ATS questionnaire: wheezing apart from colds or wheezing most days or nights during the past year; DD | 15 | Finland | Population-based study of children born between 1984 and 1989 |
| Tanaka, 2009([32](#_ENREF_32)) | CS | 1957 | Q | ISAAC | 3 | Japan | Fukuoka Child Health Study. All 3-year old children who had the examination at public public health centers in Fukuoka city |
| van der Voort, 2012 ([33](#_ENREF_33)) | PC | 5368 | Q | ISAAC | 1, 4 | Netherlands | Generation R study. Population-based multicultural birth cohort of infants born between 2002 and 2009 |
| Gruskay 1982 ([34](#_ENREF_34)) | PC | 328 FH+/ 580 FH- | NA/Q | Physician assessment (recurrent wheezing) | 3, 5, 15 | USA | Children born in 1961-1966 in a private pediatric practice |
| Guida, 2009 ([35](#_ENREF_35)) | PC | 3041 | Q | Parent reported wheeze | 1 | France | Population based birth cohort of infants born in 2003 |
| Gustafsson, 1999 ([36](#_ENREF_36)) | PC | 94 | Q | >=3 episodes of physician diagnosed wheezing | 8 | Sweden | Children with atopic dermatitis attending allergic clinic or referred by child welfare clinics |
| Halken, 1991([37](#_ENREF_37)) | PC | 276 | Q | >=2 episodes of physician diagnosed wheeze | 1.5 | Denmark | Population based birth cohort of children born in 1985 |
| Han, 2009 ([38](#_ENREF_38)) | CS | 21371 | Q | ISAAC | 15 | Taiwan | Elementary and middle school children aged 6-15 years old in 2004 |
| Puig, 2010 ([39](#_ENREF_39)) | PC | 368 | Q | DD | 6 | Spain | Part of AMICS. Population based cohort of infants born in 1996-1998 in Barcelona |
| Soto-Ramirez, 2013 ([40](#_ENREF_40)) | PC | 2833 | Q | Parent reported current wheeze | 1 | USA | Population based birth cohort selected from nationally distributed consumer opinion panel of 500 000 household between 2005-2007 |
| Oddy, 2003 ([41](#_ENREF_41)) | PC | 243 | Q | Parent reported wheeze ever | 1 | USA | Birth cohort of infants participating in the Infant Immune Study in Tucson, Arizona. |
| Infante-Rivard, 1993([42](#_ENREF_42)) | CC | 914 | I | DD | 3.5 | Canada | Cases were 3- and 4-year-old children with a first-time diagnosis of asthma made by a paediatrician 1988-90. Age and area matched controls chosen from computerized family allowance files for the target region. |
| Alm, 2008; Goksor, 2009  ([43](#_ENREF_43), [44](#_ENREF_44)) | PC | 4987 | Q, Q/FFQ | Wheeze ever; >=3 episodes of wheeze in past year | 1.4, 4.5 | Sweden | Infants of Western Sweden. Population birth cohort of infants born in 2003 |
|  |  |  |  |  |  |  |  |
| Morales, 2012 ([45](#_ENREF_45)) | PC | 467 | Q/I | Parent reported wheeze | 1 | Spain | INMA project. Population based birth cohort of infants born 2004-2006 |
| Nagel, 2009 ([46](#_ENREF_46)) | CS | 31579 | Q | Parent reported current wheeze; Parent reported asthma (+/- SPT); Spirometry; BHR:hypertonic saline PC1 | 12 | Worldwide | ISAAC Phase 2. Schoolchildren aged 8–12 years from 27 centres in 21 affluent and nonaffluent countries |
| Björkstén, 2011 ([47](#_ENREF_47)) | CS | 103716 | Q | ISAAC | 7 | Worldwide | ISAAC Phase 3: Schoolchildren aged 6-7 years from different countries and geographic regions |
| Awasthi, 2004 ([48](#_ENREF_48)) | CS | 2471 | Q | ISAAC | 7 | India | ISAAC Phase 3-India: Schoolchildren aged 6-7 and 13-14 years old from India |
| Karmaus, 2008 ([49](#_ENREF_49)) | PC | 1336 | Q | ISAAC PLUS DD asthma | 10 | UK | Isle of Wight Prevention Study. Population based birth cohort of infants born in semi-rural areas between 1989 and 1990 |
| Juca, 2012 ([50](#_ENREF_50)) | CC | 590 | Q | ISAAC | 14 | Brazil | Adolescents age 13-14 years old in Mato Grosso State, Brazil |
| Karino, 2008 ([51](#_ENREF_51)) | CS | 9615 | Q | Self-reported asthma | 18 | Japan | University freshmen students aged 18–19 years enrolled from 2003 through 2005. |
| Karunasekera, 2001 ([52](#_ENREF_52)) | CC | 582 | Q | Physician assessment | 10 | Sri Lanka | Hospital-based cases aged 1-10 years old with age matched controls from inpatient clinics |
| Kaufman, 1976 ([53](#_ENREF_53)) | PC | 94 | NA | DD | 2 | USA | Birth cohort of infants from allergic mothers |
| Kemeny, 1991([54](#_ENREF_54)) | PC | 180 | NA | >=2 episodes of wheeze | 1 | UK | Population based birth cohort of infants born at Dulwich and King’s College Hospitals in London |
| Klinnert, 2001([55](#_ENREF_55)) | PC | 145 | Q | DD | 8 | USA | Birth cohort of infants at increased risk for atopy born between 1985 and 1987 |
| Snijders, 2007; Snijders, 2008 ([56](#_ENREF_56), [57](#_ENREF_57)) | PC | 2505 | Q | >=4 episodes of wheeze | 2 | Netherlands | KOALA study. Population based birth cohort of infants born between 2000-2002 (consisting of cohorts with conventional and alternative lifestyle) |
|  |  |  |  |  |  |  |  |
| Galbally, 2013 ([58](#_ENREF_58)) | PC | 4507 | I | Parent reported wheezing >=4 nights per week | 1 | Australia | Longitudinal Study of Australian Children. Population based study of infants born between March 2003 and February 2004 and were enrolled in the Australian Medicare database |
| Marini, 1996 ([59](#_ENREF_59)) | PC | Unclear | Q | Physician assessment (>=3 episodes of wheeze) | 3 | Italy | Infants with family history of allergy whose mother were proposed to participate in an allergy prevention program |
| Martel, 2008 ([60](#_ENREF_60)) | NCC | 1578 | Q | DD PLUS asthma medication | <10 | Canada | Data originating from 3 interlinked administrative health databases on children health in the first 10 years of life |
| Burgess, 2006 ([61](#_ENREF_61)) | PC | 4964 | Q | Parent reported asthma | 14 | Australia | Mater-University of Queensland Study of Pregnancy. Population based birth cohort of infants born 1981-1984 |
| Mavale-Manuel, 2003 ([62](#_ENREF_62)) | CC | 199 | Q/I | DD PLUS asthma medication | 8 | Mozambique | Children aged between 18 months and 8 years attending pediatrics clinic with history of asthma with age-match controls attending the clinic immediately after selection of the index case |
| McConnochie, 1986([63](#_ENREF_63)) | RC | 223 | R/I | ATS guideline: wheezing with and without colds or most days or nights; DD (ATS guideline) | 8 | USA | Historical cohort with subjects were drawn from the patient population of a five-paediatrician group practice in a suburb of Rochester, New York |
| Miskelly, 1988 ([64](#_ENREF_64)) | PC | 482 | D | Parent reported wheeze | 1 | UK | Infants from antenatal clinics with family history of allergy randomised into a dietary intervention program |
| Miyake, 2003 ([65](#_ENREF_65)) | CS | 6845 | Q | ISAAC | 15 | Japan | 12-15 years old children from all public junior high schools in Suita, Japan. |
| Morgan, 2004 ([66](#_ENREF_66)) | PC | 1600 | I | DD | 1.5 | U.K. | Infants from five prospective randomised dietary trials conducted in the UK 1993-1997. Two term infant trials; one LBW infant trial; two preterm infant trials. |
| Mann, 1992 ([67](#_ENREF_67)) | PC | 2139 | Q | Self-reported wheeze; Spirometry | 36 | UK | MRC National Survey of Health and Development. Birth cohort of infants to wives of non-manual and agricultural workers, and one in four of all single, legitimate infants to wives of manual workers in England, Wales, or Scotland |
| Muiño, 2008 ([68](#_ENREF_68)) | PC | 897 | Q | Parent reported current wheeze; Persistent wheeze: parent reported wheeze at 1, 4 and 8-10 year assessments; Early transient wheeze: parent reported | 12 | Brazil | Population based cohort of infants born in 1993 |
| Bergmann, 2000 ([69](#_ENREF_69)) | PC | 1314 | Q/I | Physician assessment | 6 | Germany | MAS study. Atopic risk enriched cohort of infants born in 1990 in 5 German cities |
| Rust, 2001([70](#_ENREF_70)) | CS | 6783 | I | DD; DD asthma ever | <6 | USA | NHANES III survey. Children ages 2 months to 5 years from noninstitutionalized U.S. population |
| Evenhouse, 2005 ([71](#_ENREF_71)) | CS | 16903 | Q | Unclear | 12-18 | USA | National Longitudinal Study of Adolescent Health (Add Health). Nationally representative samples of adolescents from 80 school districts, since 1994 |
| Dell, 2001; Midodzi, 2008 ([72](#_ENREF_72), [73](#_ENREF_73)) | PC; CS | 2711 | NA; Q | >=2 episodes of wheeze; Preschool wheeze: <5 years but not beyond 6 years; Parent reported wheeze; DD | 2,9 | Canada | National Longitudinal Survey of Children and Youth (NLSCY). First of longitudinal surveys of 12-24 months old children representative of the Canadian population |
|  |  |  |  |  |  |  |  |
| Milner, 2004 ([74](#_ENREF_74)) | PC | 8071 | Q | DD | 3 | USA | National Maternal and Infant Health Survey and Longitudinal Follow Up. Representative US population born in 1988. Black, low socioeconomic status, and premature infants intentionally overrepresented |
| Silvers, 2009; Silvers, 2011 ([75](#_ENREF_75), [76](#_ENREF_76)) | PC | 987 | Q | DD PLUS current wheeze; Parent reported wheeze | 1, 5 | New Zealand | New Zealand Asthma and Allergy Cohort Study. Population based birth cohort of infants born between 1997 and 2001 |
|  |  |  |  |  |  |  |  |
| Ronmark, 1999 ([77](#_ENREF_77)) | NCC | 258 | Q | DD plus ISAAC; Physician assessment (+/-sensitisation) | 8 | Sweden | Obstructive Lung Disease in Northern Sweden Study. 7-8 years old children enrolled in school in 1996 in northern Sweden (born 1988-1989) ([77](#_ENREF_77)) |
| Oliveti, 1995 ([78](#_ENREF_78)) | CC | 262 | Q | DD PLUS asthma medication | 9 | USA | Cases and age matched controls were identified using rosters of patients followed during the previous year. he majority of children from each group were insured by Medicaid (low income) |
| Miyake, 2008 ([79](#_ENREF_79)) | PC | 763 | Q | ISAAC | 2 | Japan | OMCHS study. Population based birth cohort of infants born in 2002-2003 |
| da Costa Lima, 2003; Menezes, ([80](#_ENREF_80), [81](#_ENREF_81)) | PC | 4297 | I, Q/I | ISAAC | 18, 22 | Brazil | Pelotas Birth Cohort. Population based birth cohort of infants born in 1982 in the city of Pelotas |
|  |  |  |  |  |  |  |  |
| Perez Tarazona, 2010 ([82](#_ENREF_82)) | PC | 620 | Q | Parent reported wheeze | 1 | Spain | Population based birth cohort of infants born in 2007-2008 in Valencia |
| Caudri, 2013; Scholtens, 2009 ([83](#_ENREF_83), [84](#_ENREF_84)) | PC | 3115 | Q | Current wheeze; Parent reported wheeze; Parent reported wheeze ever (+/- sIgE); ISAAC; BHR: metacholine PC20 | 8 | Netherlands | PIAMA: population-based born in 1996-1997 (normal risk of disease) |
|  |  |  |  |  |  |  |  |
| Kerr, 1981([85](#_ENREF_85)) | PC | 269 | I | Parent reported wheeze | 0.5 | New Zealand | Birth cohort hospital based, born 1977-1978 (normal risk of disease) |
| Porro, 1993 ([86](#_ENREF_86)) | CC | 465 | Q | Parent reported wheeze | <1.6 | Italy | Hospital based study with matched controls (normal risk of disease) |
| Kurt, 2008([87](#_ENREF_87)) | CS | 25843 | Q | Current wheeze; Parent reported current wheeze | 15 | Turkey | Prevalence and Risk Factors of Allergies in Turkey (PARFAIT): population representative sample of children aged 9-15 years old (normal risk of disease0 |
| Schonberger, 2005 ([88](#_ENREF_88)) | PC | 443 | D/Q | ISAAC | 2 | Netherlands | PREVASC study: cohort born in 1997-2000 with family history of asthma (high risk of disease) |
| Hagendorens, 2005 ([89](#_ENREF_89)) | PC | 693 | Q | Parent reported current wheeze | 1 | Belgium | PIPO study: recruited from university service, born 1997-2001 (normal risk of disease) |
| Alho, 1990 ([90](#_ENREF_90)) | PC | 2130 | Q | DD Wheeze | 2 | Finland | Birth cohort, population representative sample born 1985-1986 (normal risk of disease) |
| Rhodes, 2001([91](#_ENREF_91)) | PC | 63 | Q | Current wheeze AND BHR | 22 | UK | Hospital based, born 1976-1977, family history of allergy (high risk of disease) |
| Rona, 2005 ([92](#_ENREF_92)) | RC | 1213 | R | ECRHS questionnaire; ECRHS questionnaire AND SPT AND BHR; BHR: metacholine PC_20_ | 27 | Chile | Infants born in a hospital in 1974-1978 (normal risk of disease) |
| Rosas Vargas 2002 ([93](#_ENREF_93)) | CC | 148 | Q | DD | 3 | Mexico | Hospital-based study, cases born in 2000 (normal risk of disease) |
| Rothenbacher, 2005([94](#_ENREF_94)) | PC | 803 | Q/I | DD | 2 | Germany | Recruited from university service, born in 2000-2001 (normal risk of disease) |
| Rusconi, 1999; Rusconi, 2005 ([95](#_ENREF_95), [96](#_ENREF_96)) | CS | 16933 | Q | Parent reported persistent wheezing: >=1 in first 2 years, and in past 12 months; Transient early wheeze: wheeze in first 2 years but not past 12 months; ISAAC | 7 | Italy | SIDRIA survey of a representative sample of children aged 6-7 years old |
| Saarinen, 1995 ([97](#_ENREF_97)) | PC | 150 | R | Physician assessment | 5, 17 | Finland | Recruited from hospital and born in 1975 (normal risk of disease) |
| Selcuk, 1997 ([98](#_ENREF_98)) | CS | 5412 | Q | Parent reported wheeze ever; Parent reported current asthma | 12 | Turkey | Children 7-12 ys of 18 primary schools (normal risk of disease) |
| Sunyer, 2006 ([99](#_ENREF_99)) | PC | 462 | Q | DD | 6.5 | Spain | Population representative sample born in 1997-1998 (normal risk of disease) |
| Sunyer, 2001([100](#_ENREF_100)) | PC | 596 | Q | ISAAC | 4 | Tanzania | Cohort born in 1995-1996 in urban area (normal risk of disease) |
| Hide, 1981([101](#_ENREF_101)) | PC | 843 | D/Q | Wheeze ever; Parent reported wheeze | 1 | UK | The Isle of Wight Prevention study: born in 1977-1978 (normal risk of disease) |
| Takemura, 2002 ([102](#_ENREF_102)) | CS | 23828 | Q | ATS questionnaire: DD asthma PLUS >=2 episodes of wheeze | 15 | Japan | The Tokorozawa Childhood Asthma and Pollinosis Study: Representative sample of children in public elementary schools (normal risk of disease) |
| Tian, 2009 ([103](#_ENREF_103)) | PC | 472 | I | Physician assessment | 2 | China | Infants from urban areas born in 2004-2006 (normal risk of disease) |
| Yamamoto, 2011([104](#_ENREF_104)) | PC | 1344 | Q | ISAAC | 3 | Japan | Tokyo Children's Health Illness and Development study (T-CHILD) |
| Wright, 1989; Wright, 1995 ([105](#_ENREF_105), [106](#_ENREF_106)) | PC | 988 | Q/I | DD; >=4 episodes of wheeze in past year | 1, 6 | USA | Tuscon Children's Respiratory Study: Healthy newborn infants recruited from local health maintenance organisation born in 1980-1984 (normal risk of disease) |
|  |  |  |  |  |  |  |  |
| Van Beijstervelft, 2008 ([107](#_ENREF_107)) | PC | 24018 | Q | DD | 5 | The Netherlands | Netherlands Twin Register: born in 1987-2000 (normal risk of disease) |
| Wang, 2006 ([108](#_ENREF_108)) | CS | 8733 | Q | ISAAC | 10 | China | Population representative sample of children in elementary schools and nurseries (normal risk of disease) |
| Oddy, 1999; Oddy, 2003; Oddy, 2004  ([109-111](#_ENREF_109)) | PC, NCC | 2456 | D/I, Q, D/Q | DD; DD PLUS current wheeze; Parent reported current wheeze; DD asthma PLUS >=3 episodes of wheeze | 1, 6, 8 | Australia | Western Australia Pregnancy Cohort: Recruited from antenatal clinics born in 1989-1992 (normal risk of disease) |
|  |  |  |  |  |  |  |  |
|  |  |  |  |  |  |  |  |
| Wickens, 2001 ([112](#_ENREF_112)) | CC | 474 | I | ISAAC | 6.5 | New Zealand | Population-based study (normal risk of disease) |
| Zhu, 2012 ([113](#_ENREF_113)) | CC | 542 | Q | DD | 14 | China | Population-based study (normal risk of disease) |
| Zutavern, 2004 ([114](#_ENREF_114)) | PC | 606 | I | Parent reported current wheeze | 2, 5.5 | UK | Cohort recruited from general practices and born in 1993-1995 (normal risk of disease) |
| Ogbuanu, 2009 ([115](#_ENREF_115)); Soto-Ramırez, 2012 ([116](#_ENREF_116)) | PC | 1033 | NA, Q | Spirometry | 18 | UK | Isle of Wight Prevention Study. Population based birth cohort of infants born in semi-rural areas between 1989 and 1990 |
| Dogaru, 2012 ([117](#_ENREF_117)) | PC | 1458 | Q | Spirometry | 12 | UK | Population based sample of children of white and south Asian ethnic origin born between 1993 and 1997, part of the Leicestershire cohorts |
| Tennant, 2008 ([118](#_ENREF_118)) | PC | 392 | Q | Spirometry | 50 | UK | Newcastle Thousand Families Study. Population based sample of subject born in 1947 who were either traced through the National Health Service Central Register or contacted the study team in response to media publicity in the mid 1990 |
| Suwanpromma, 2012 ([119](#_ENREF_119)) | CS | 215 | Q | Spirometry; BHR:hypertonic saline PC15 | 18 | Thailand | Schoolchildren aged 6-18 years (normal risk of disease) |
| Eneli, 2006 ([120](#_ENREF_120)) | PC | 536 | Q | BHR:hypertonic saline PC15 | 10 | Germany | The Child Health and Environment Cohort Study: community based in urban area, born 1994 (normal risk of disease) |
| Tennant, 2010 ([121](#_ENREF_121)) | PC | 122 | Q | Spirometry | 14, 51 | UK | The Newcastle Thousand Families Study: born in 1947 (normal risk of disease) |

Figure 1 Risk of bias in studies of total breastfeeding and wheeze

## Total Breastfeeding and wheeze

The single intervention trial of a breastfeeding promotion intervention was rated as having a low risk of bias on all domains, and a low risk of conflict of interest. Kramer found no significant difference in odds of recurrent wheeze at age 1 year – cluster adjusted odds ratio 0.7 (95% CI 0.29, 1.70); or at age 6.5 years – cluster adjusted odds ratio 1.2 (95% CI 0.7, 1.9). There was also no significant difference in odds of wheeze ever OR 1.1 (0.6, 1.8) or wheeze in the past 12 months OR 1.0 (0.7, 1.6) at 6.5 years. All other evidence was derived from observational studies.

### Age at outcome measurement 0-4

#### Ever vs. never

Figure 2 shows the outcomes of 14 eligible observational studies evaluating TBF ever vs never and risk of wheeze. The data were not pooled due to extreme heterogeneity across studies (I^2^=83.3%). Thirteen studies were prospective cohorts and one was a cross-sectional study. All studies had low or unclear risk of bias in most domains. There was no evidence of publication bias and the reason for extreme statistical heterogeneity was not clear (Figure 3). Adjusted data showed no relationship, whereas unadjusted data showed reduced wheeze with TBF. There was a significant subgroup difference in the relationship between TBF ever and wheeze, between infants with a family history of wheeze or allergic disorder, and more representative populations, with a significant association and reduced heterogeneity in the high risk infants (Table 1).

In dose-response analysis there was some evidence that increasing duration of TBF is associated with a greater reduction in risk of wheeze, in this age group (Figure 4-Figure 6). This analysis was dominated by one study Elliott, which reported appropriately adjusted OR for different durations of TBF in relation to wheeze in the first 3 years. In the same cohort there was no relationship between TBF and wheeze at older ages.

Figure 2 Total breastfeeding ever vs. never and risk of wheeze in children aged 0-4 years


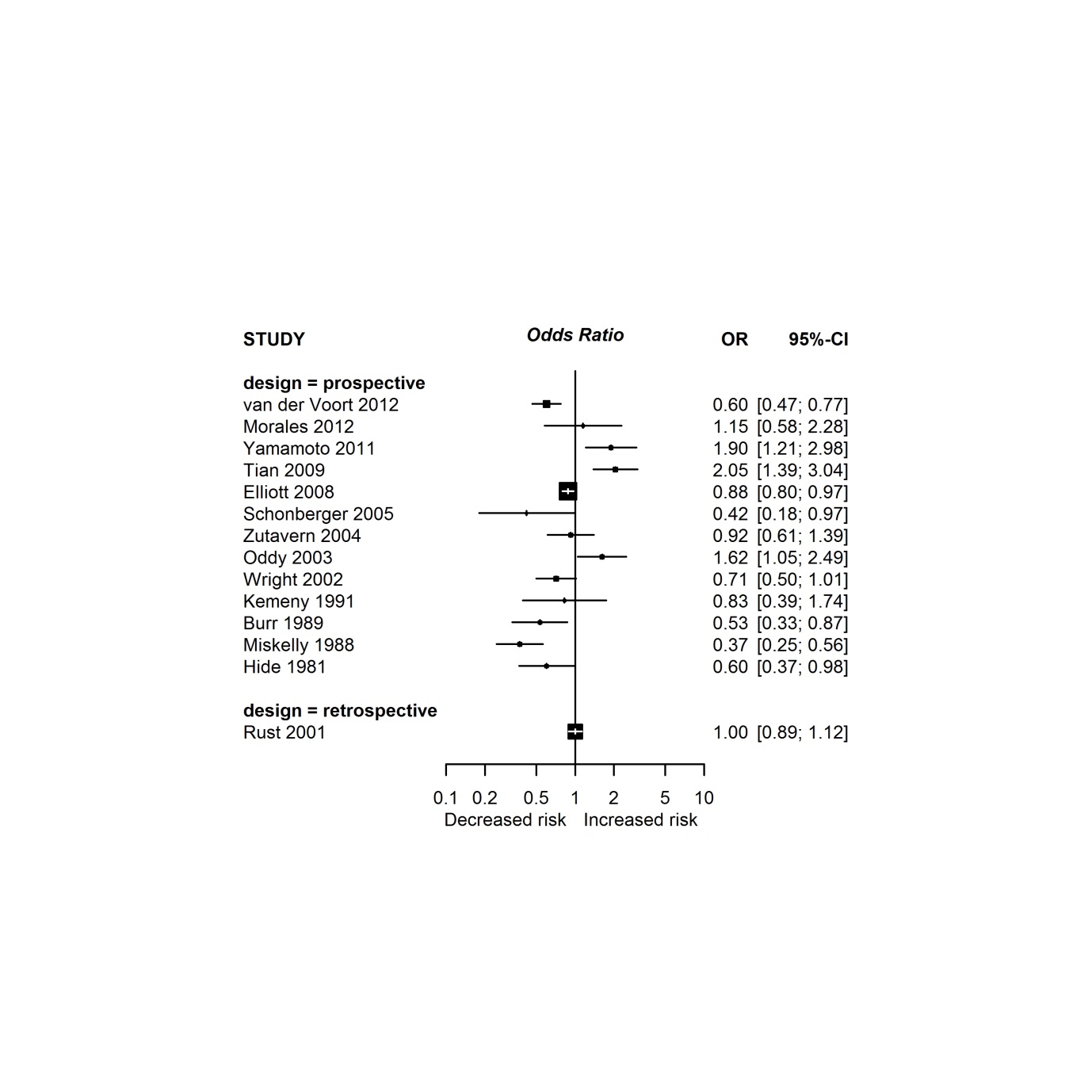


Figure 3 Risk of publication bias in studies investigating total breastfeeding ever vs. never and risk of wheeze in children aged 0-4 years


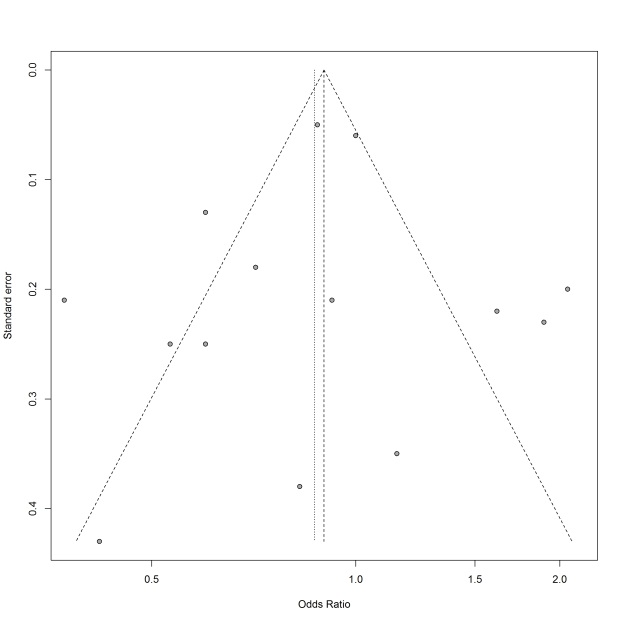


Egger’s test p-value =0.736

Table 2 Subgroup and stratified analysis of TBF ever and risk of wheeze in children aged 0-4 years

|  | **Number of studies** | **OR [95% CI]** | **I^2^ (%)** | **P-value for between groups difference** |
| --- | --- | --- | --- | --- |
| **Overall (if adjusted NA, unadjusted used)** | 14 | 0.87 [0.71; 1.06] | 83.3 |  |
| **Adjusted** | 7 | 0.98 [0.72; 1.35] | 76.8 | Not tested |
| **Unadjusted** | 12 | 0.79 [0.63; 0.99] | 87.7 |  |
| Study Design – Prospective | 13 | 0.85 [0.66; 1.10] | 83.6 | 0.26 |
| Study Design – Retrospective | 1 | 1.00 [0.89; 1.12] | -- |  |
| Risk of disease – High | 4 | 0.51 [0.37; 0.72] | 48.7 | < 0.001 |
| Risk of disease – Normal | 10 | 1.04 [0.85; 1.28] | 81.7 |  |
| Risk of bias – Low | 6 | 0.75 [0.54; 1.05] | 74.8 | 0.25 |
| Risk of bias – High/Unclear | 8 | 0.96 [0.74; 1.25] | 86.2 |  |

Figure 4 Total breastfeeding (dose response) short vs. never and risk of wheeze in children aged 0-4 years


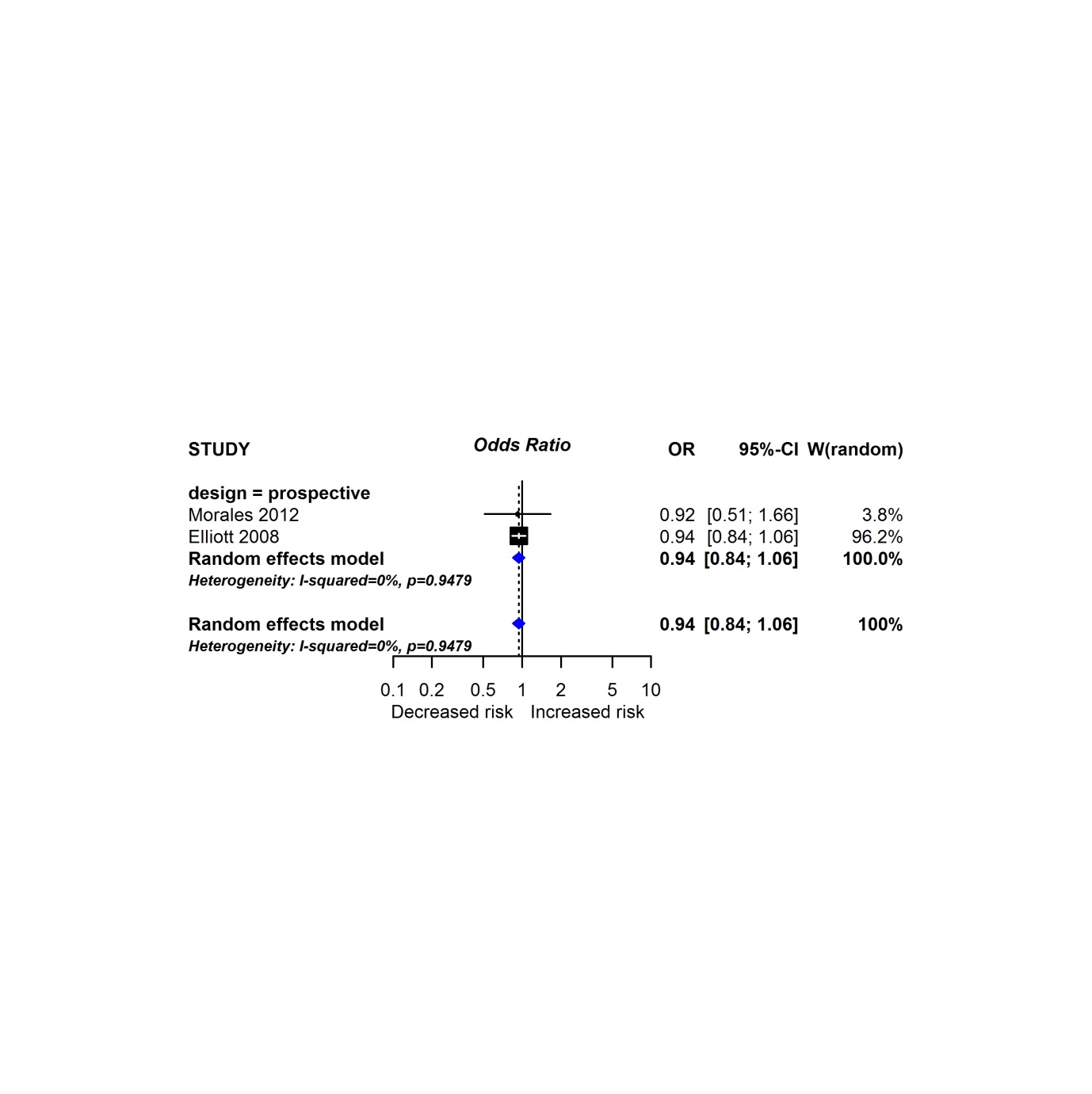


Figure 5 Total breastfeeding (dose response) medium vs. never and risk of wheeze in children aged 0-4 years


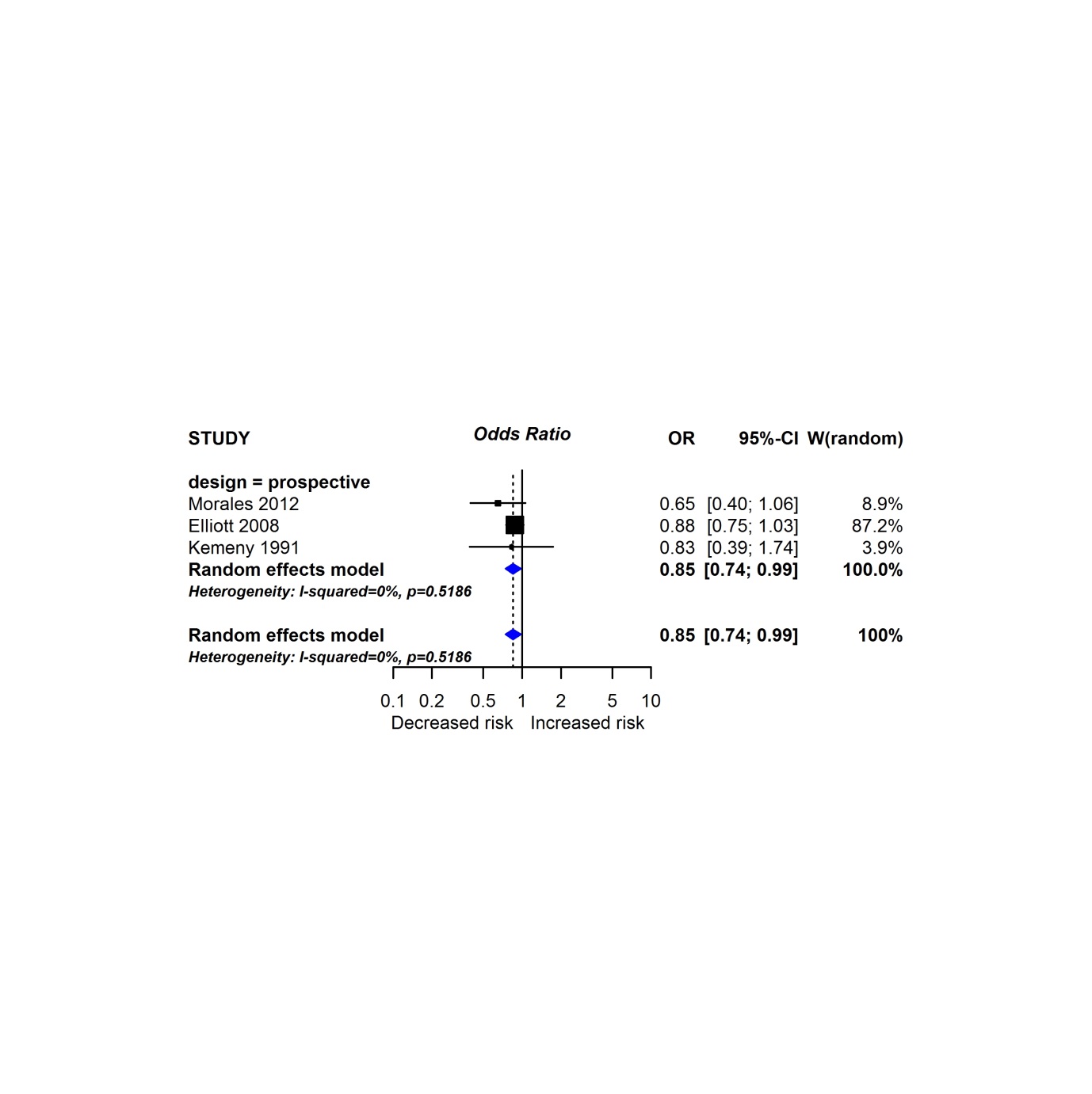


Figure 6 Total breastfeeding (dose response) long vs. never and risk of wheeze in children aged 0-4 years


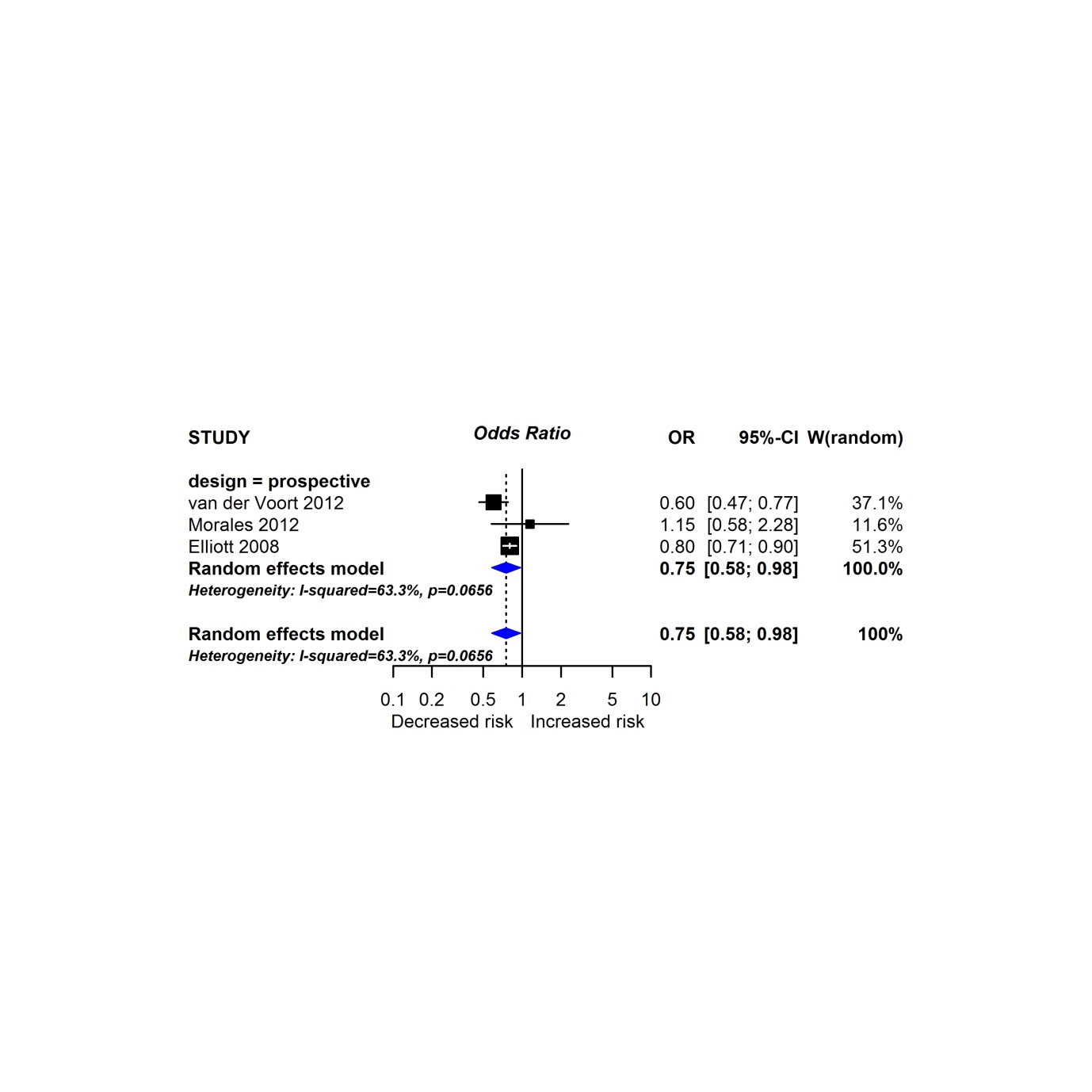


#### 1-2 Months

Figure 7 shows the pooled estimates of three studies which assessed risk of wheeze and TBF for ≥1-2 months vs. <1-2 months, suggesting no association, with very high heterogeneity between studies (I^2^=77.7%). The study of Oddy and Dell provided adjusted odds ratios, whilst the study of Kaufman provided unadjusted estimates, and therefore carried a high risk of bias.

Figure 7 Total breastfeeding for ≥1-2 months vs. <1-2 months and risk of wheeze in children aged 0-4 years


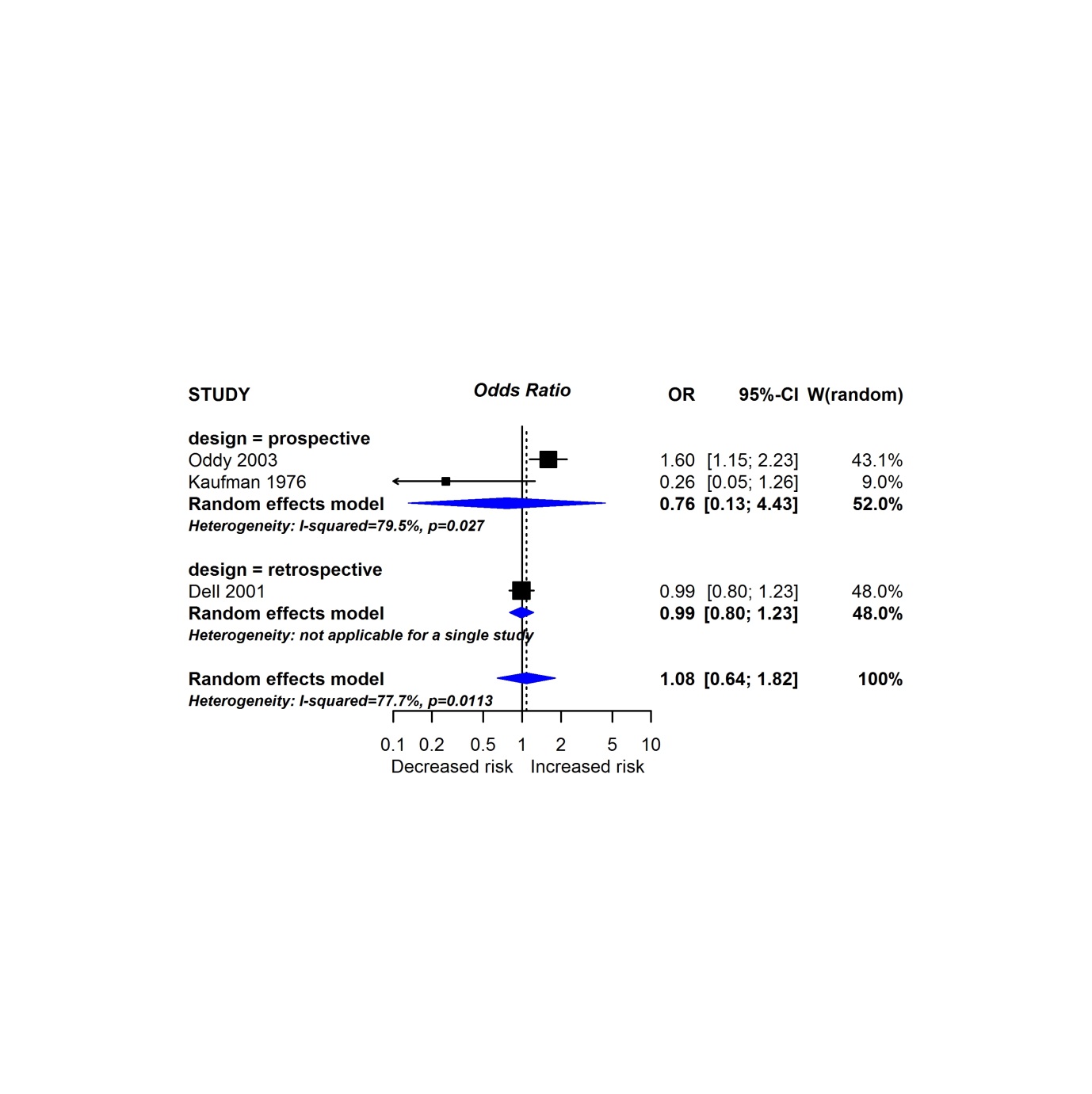


#### 3-4 Months

Five studies (all prospective cohorts) reported relevant data, which could not be combined in a meta-analysis due to extreme heterogeneity (I^2^=81%; Figure 8). With the exception of the Oddy study, all the studies showed a tendency for a lower risk of wheeze if TBF lasted for at least 3-4 months when compared to less than this cut-off. The studies of Perez-Tarazona and Alho provided unadjusted estimates and therefore carried a high risk of bias. The other three studies had a low overall risk of bias and the source of heterogeneity is not clear.

Figure 8 Total breastfeeding for ≥3-4 months vs. <3-4 months and risk of wheeze in children aged 0-4 years


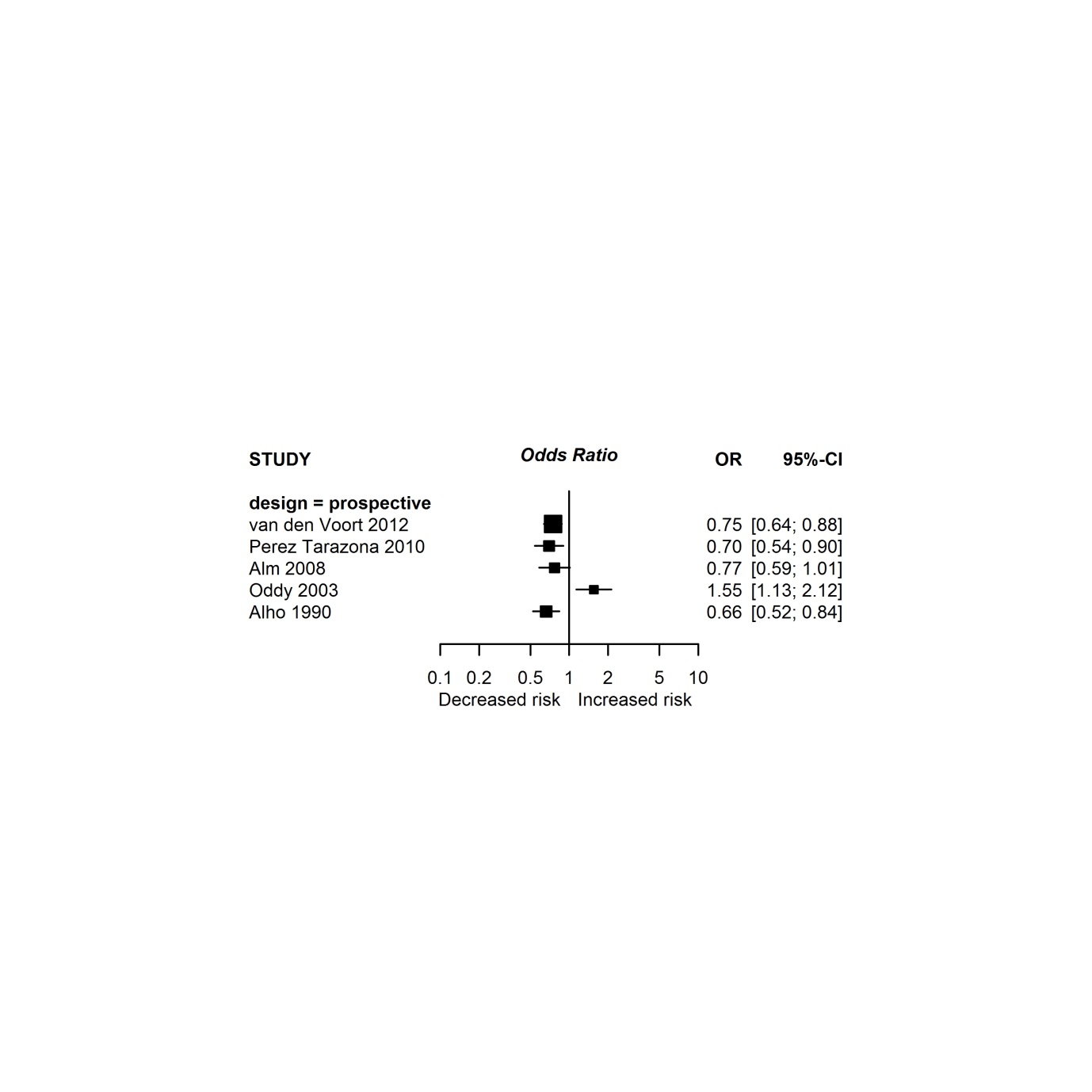


#### 5-7 Months

Nine observational studies examined the association between TBF for ≥5-7 months vs. <5-7 months and risk of wheeze in children aged 0-4 years (Figure 9). There was no association between TBF and risk of wheeze in this analysis, but with high heterogeneity across studies (I^2^=72.3%). Subgroup analyses (Table 3) show no important subgroup differences, and outcomes are similar in unadjusted and adjusted analyses. Heterogeneity remains high in all analyses, and we were unable to explain the heterogeneity in outcomes between studies.

Figure 9 Total breastfeeding for ≥5-7 months vs. <5-7 months and risk of wheeze in children aged 0-4 years


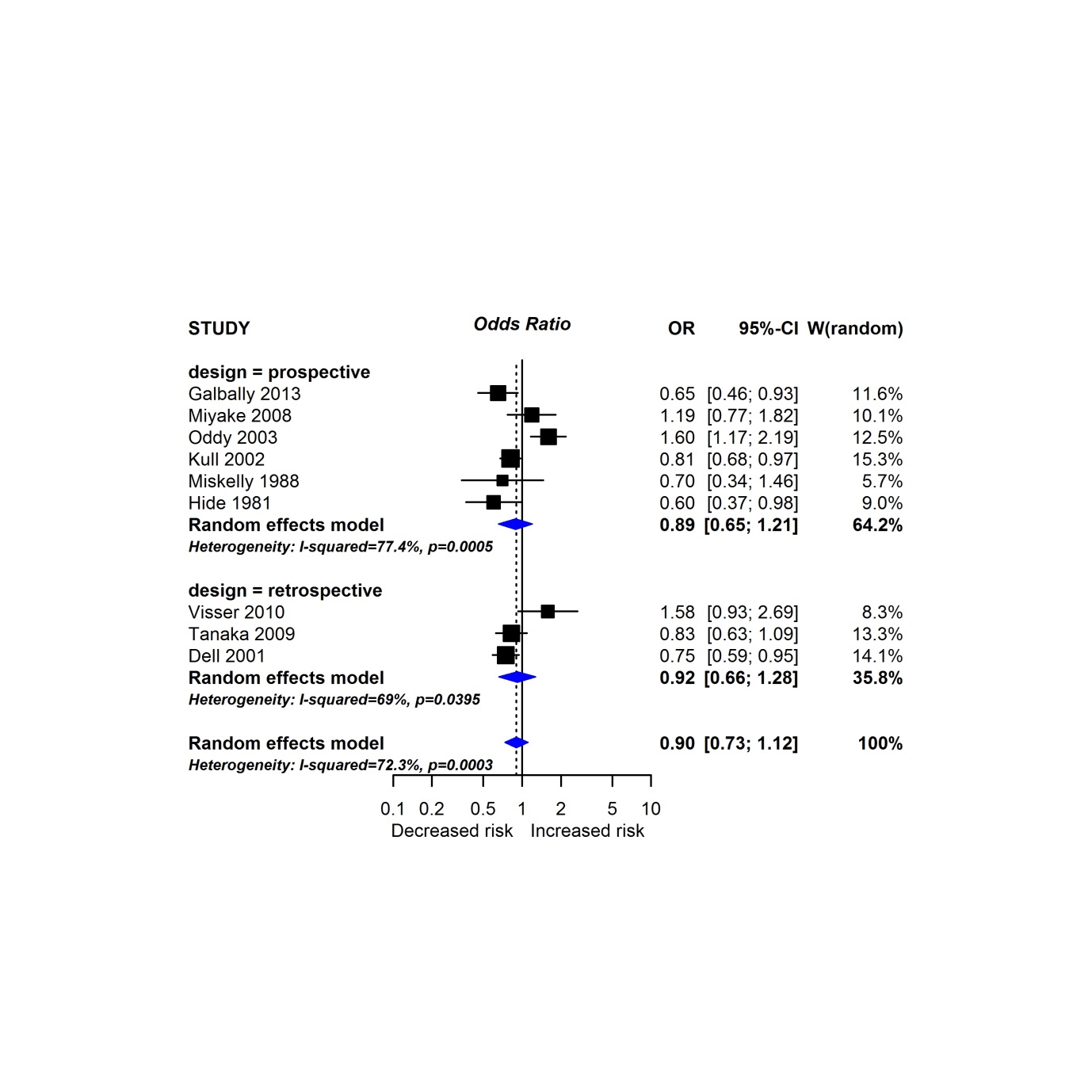


Table 3 Subgroup Analysis of risk of wheeze and total breastfeeding ≥5-7 months vs. <5-7 months in children aged 0-4 years

|  | **Number of studies** | **OR [95% CI]** | **I^2^ (%)** | **P-value for between groups difference** |
| --- | --- | --- | --- | --- |
| **Overall (if adjusted NA, unadjusted used)** | 9 | 0.91 [0.73; 1.12] | 72.3 |  |
| **Adjusted** | 7 | 0.96 [0.76; 1.22] | 77.1 | Not tested |
| **Unadjusted** | 8 | 0.89 [0.63; 1.27] | 89.3 |  |
| Study Design – Prospective | 6 | 0.89 [0.65; 1.21] | 77.4 | 0.89 |
| Study Design – Retrospective | 3 | 0.92 [0.66; 1.28] | 69 |  |
| Risk of disease – High | 1 | 0.71 [0.34; 1.46] | -- | 0.49 |
| Risk of disease – Normal | 8 | 0.92 [0.73; 1.15] | 75.5 |  |
| Risk of bias – Low | 3 | 0.94 [0.59; 1.51] | 88.6 | 0.78 |
| Risk of bias – High/Unclear | 6 | 0.88 [0.69; 1.12] | 54.3 |  |

#### 8-12 Months

Two prospective cohort studies had eligible data that could be pooled to calculate OR for wheeze in infants with TBF for ≥8-12 months vs. <8-12 months and are shown in Figure 10. Data could not be pooled due to extreme statistical heterogeneity (I^2^=93.1%). The overall risk of bias in the Oddy study was assessed as low and that of Dell unclear.

Figure 10 Total breastfeeding for ≥8-12 months vs. <8-12 months and risk of wheeze in children aged 0-4 years

**
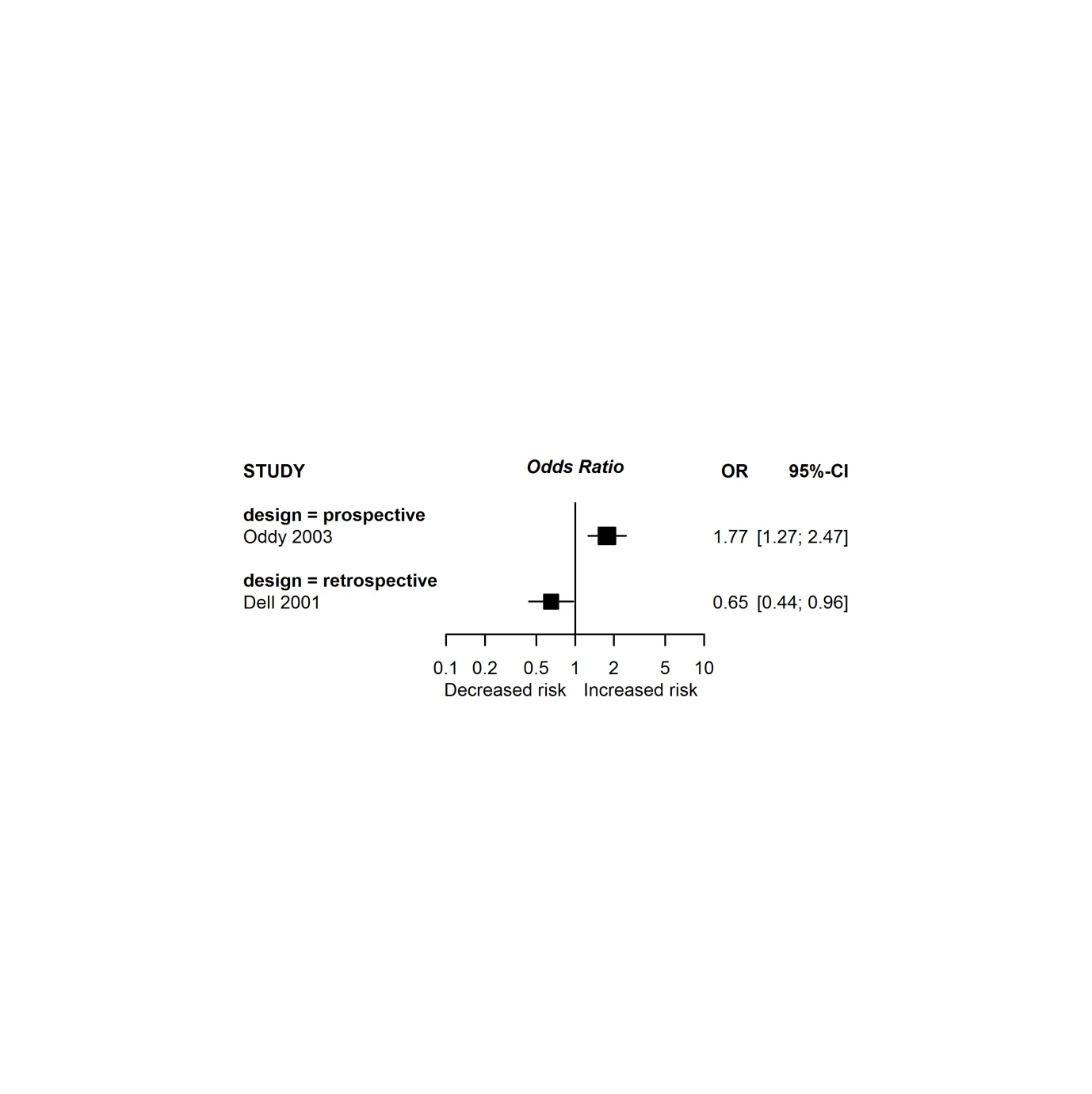
**

### Age at outcome measurement 5-14

#### Ever vs. never

Figure 11 illustrates the pooled OR from 12 studies for risk of wheeze in children aged 5-14 years who ever initiated TBF vs. those who never did, suggesting an association between ever BF and reduced risk of wheeze. The statistical heterogeneity between studies was high (I^2^=56.9%). Five studies were prospective cohorts, two of which had an overall low risk of bias, one had an unclear risk of bias due to unclear selection method, and two had unadjusted estimates (Wilson and Lewis), which carried a high risk of confounding. There were 7 case-control studies, five of which were considered to have a low or unclear overall risk of bias, whilst the studies of Nagel (affluent and non-affluent countries) and Miyake were considered to have a high risk of bias due to the selection of participants.

Figure 11 Total breastfeeding ever vs. never and risk of wheeze in children aged 5-14 year

**
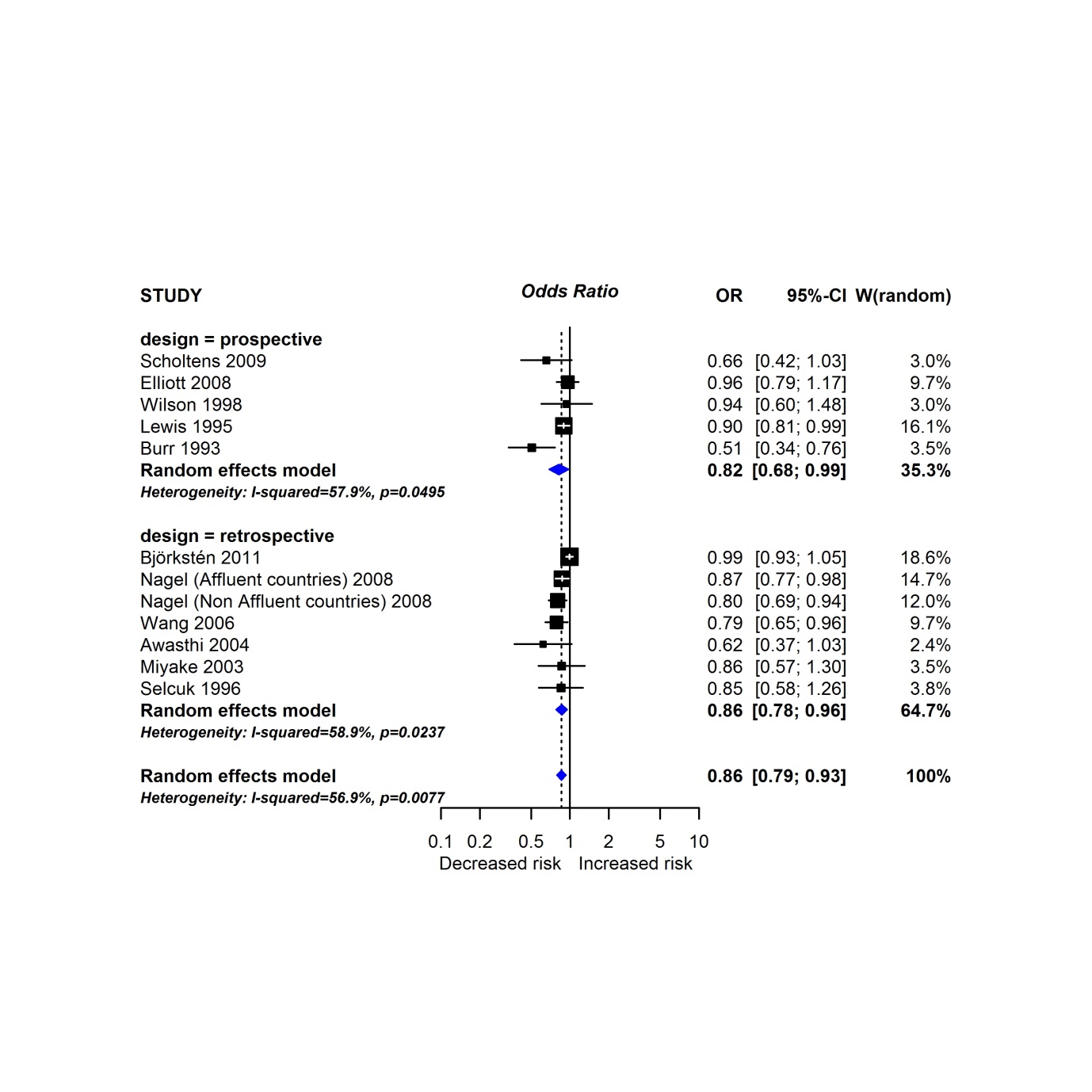
**

A Funnel plot to explore publication bias is shown in Figure 12. The plot is not symmetrical, and the Egger’s test for asymmetry reaches statistical significance suggesting evidence of publication bias.

Subgroup and stratified analyses (Table 4) did not show clear subgroup differences, other than a difference for risk of disease based on one study (Burr 1993); and findings were similar in adjusted and unadjusted analyses.

Dose response analysis (Figure 13-Figure 15) did not show evidence of a dose response effect of increasing durations of TBF compared with never TBF on risk of wheeze.

Figure 12 Risk of publication bias in studies investigating breastfeeding ever vs. never and risk of wheeze in children aged 5-14 years


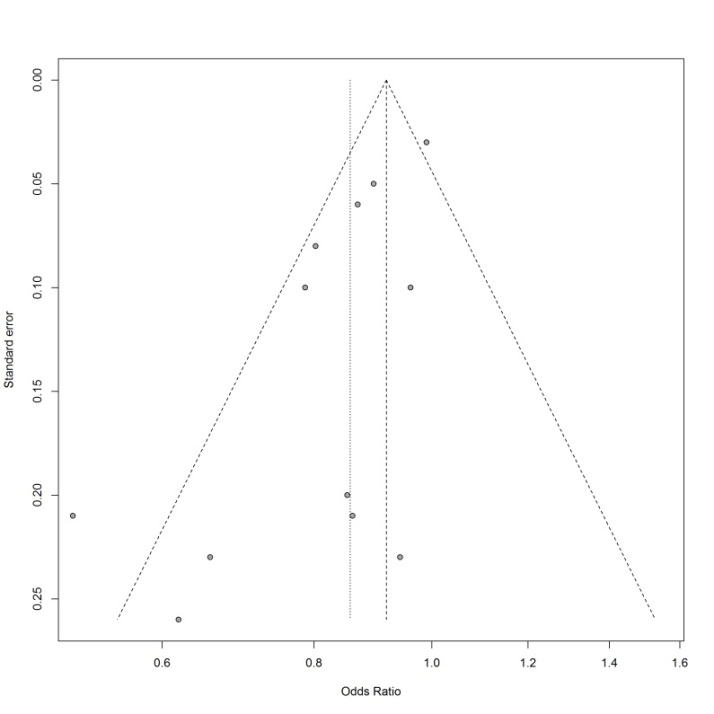


Egger’s test p-value = 0.007

Table 4 Subgroup Analysis of risk of wheeze and total breastfeeding never vs. ever in children aged 5-14 years

|  | **Number of studies** | **OR [95% CI]** | **I^2^ (%)** | **P-value for between groups difference** |
| --- | --- | --- | --- | --- |
| **Overall (if adjusted NA, unadjusted value used)** | 12 | 0.86 [0.79; 0.93] | 56.9 |  |
| **Adjusted** | 10 | 0.88 [0.82; 0.95] | 48.3 | Not tested |
| **Unadjusted** | 9 | 0.84 [0.77; 0.91] | 33.9 |  |
| Study Design – Prospective | 5 | 0.82 [0.69; 0.99] | 57.9 | 0.65 |
| Study Design – Retrospective | 7 | 0.86 [0.78; 0.96] | 58.9 |  |
| Risk of disease – High | 1 | 0.51 [0.34; 0.77] | -- | 0.01 |
| Risk of disease – Normal | 11 | 0.88 [0.82; 0.95] | 42.6 |  |
| Risk of bias – Low | 4 | 0.76 [0.55; 1.05] | 77.5 | 0.43 |
| Risk of bias – High/Unclear | 8 | 0.87 [0.82; 0.92] | 0.0 |  |

Figure 13 Total breastfeeding (dose response) short vs. never and risk of wheeze in children aged 5-14 years


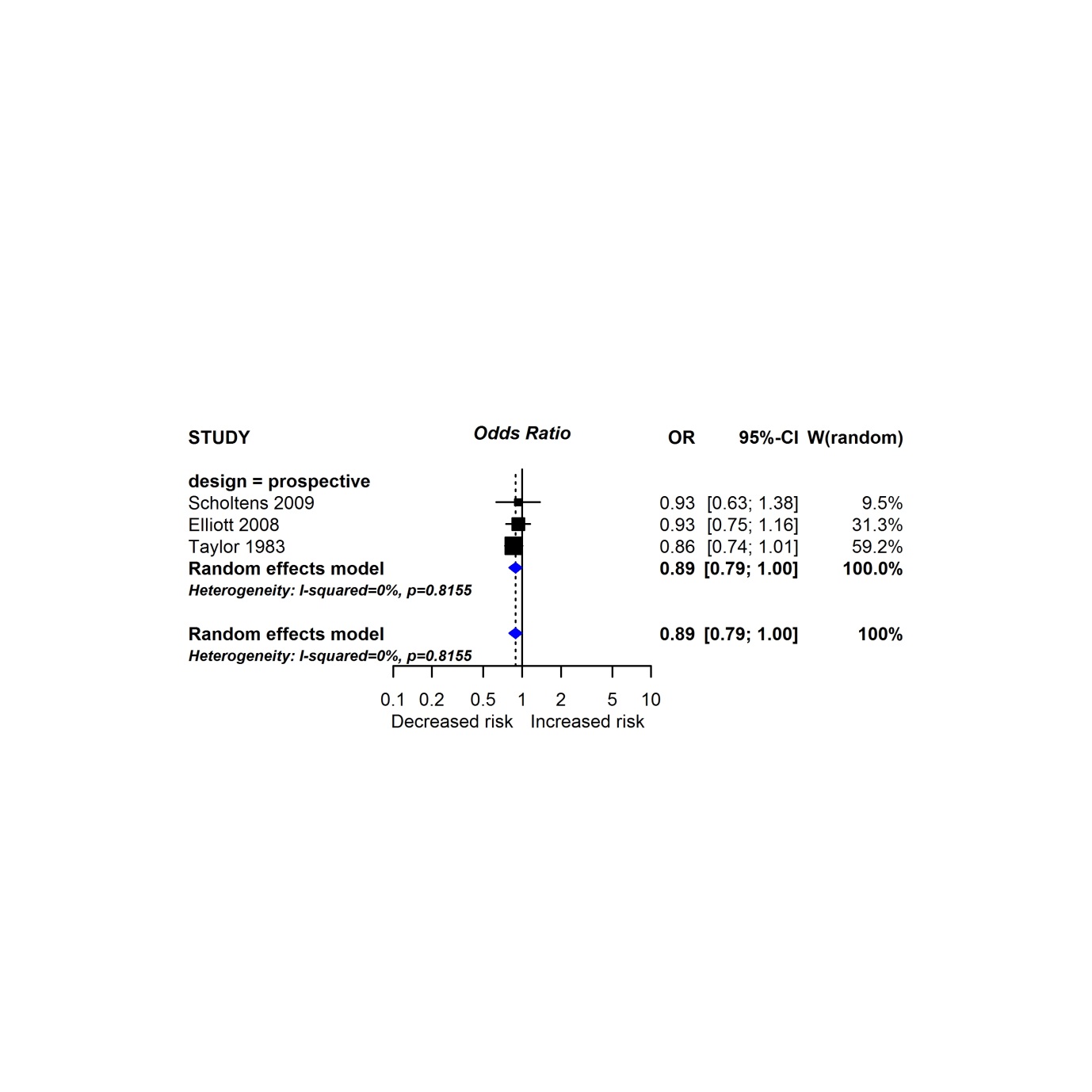


Figure 14 Total breastfeeding (dose response) medium vs. never and risk of wheeze in children aged 5-14years


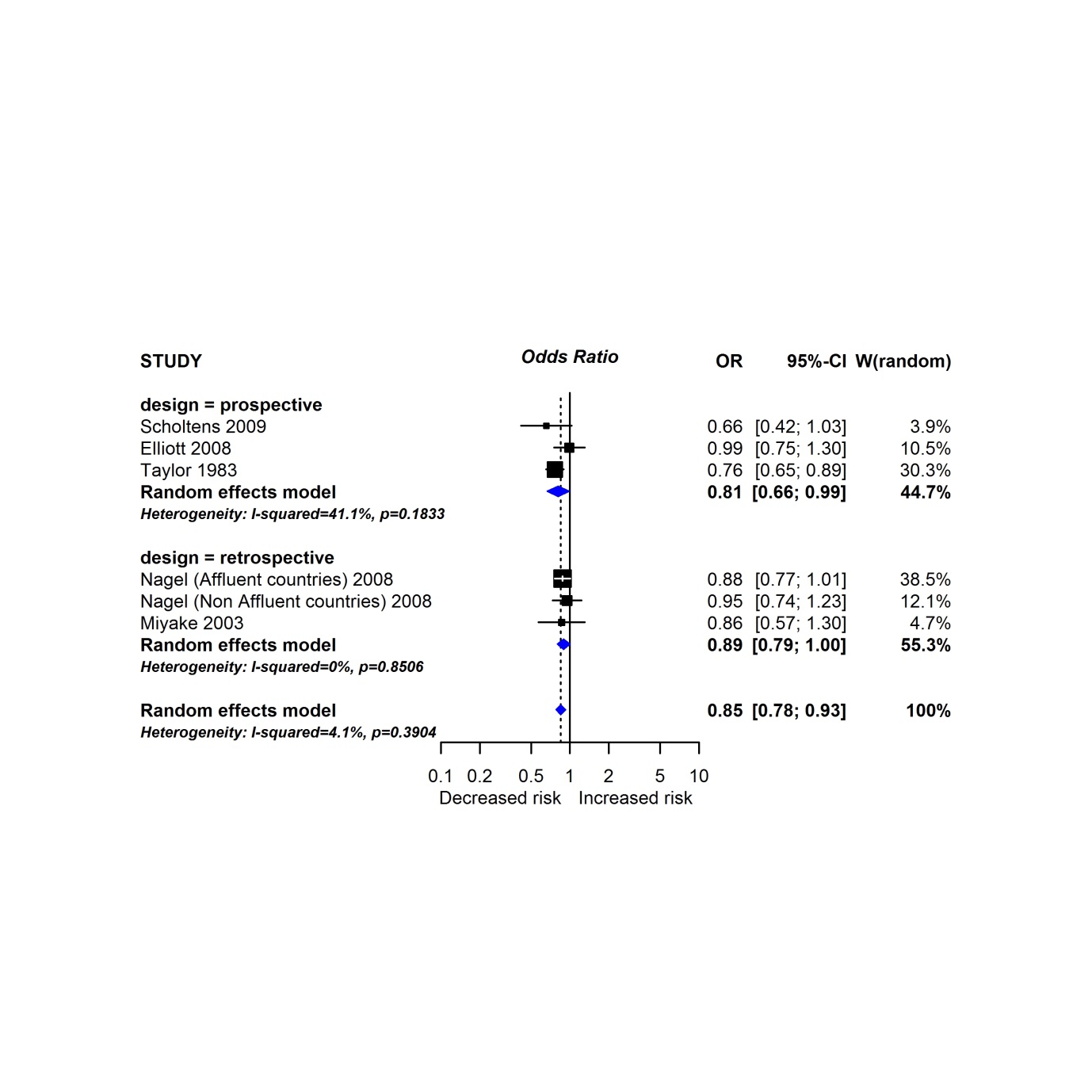


Figure 15 Total breastfeeding (dose response) long vs. never and risk of wheeze in children aged 5-14 years


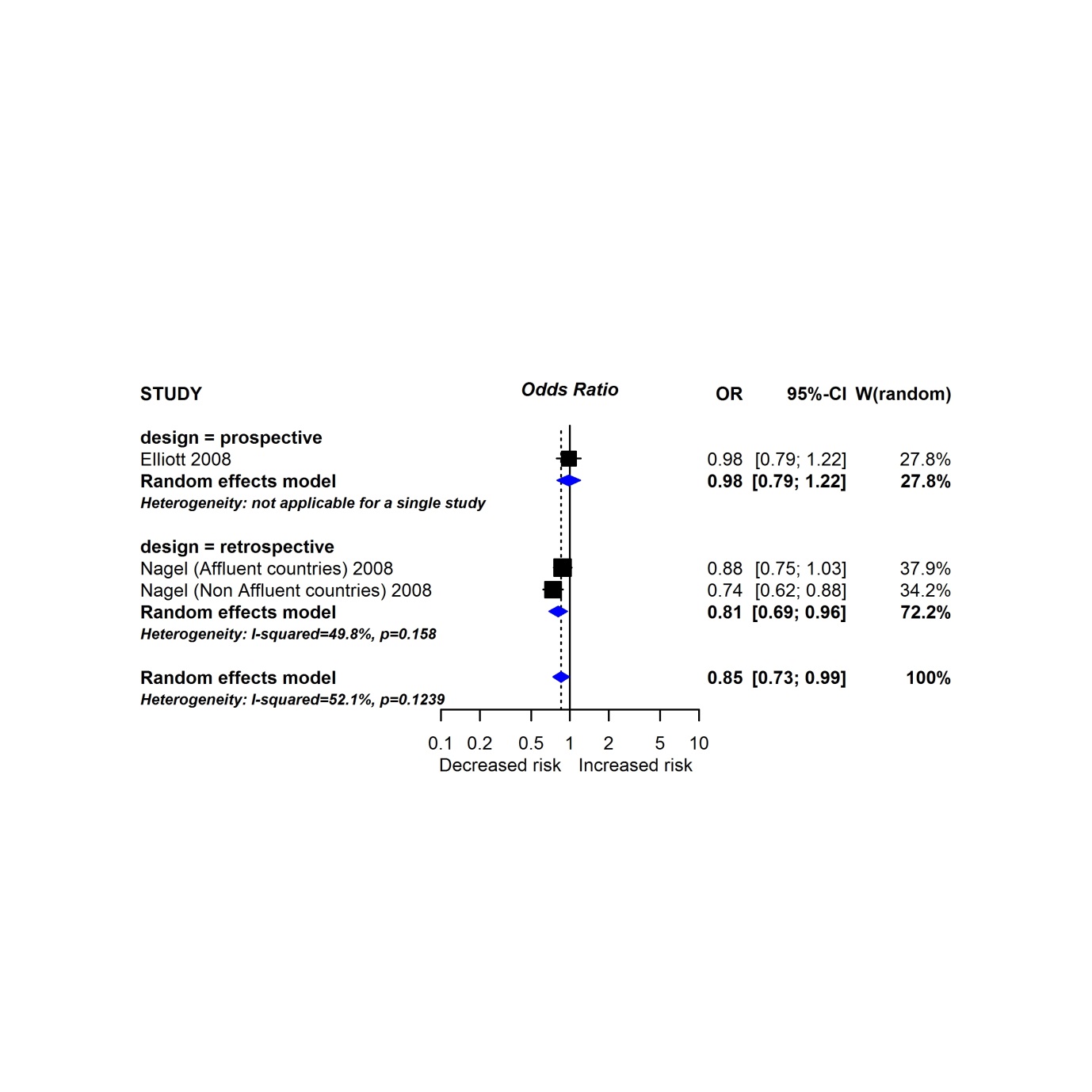


#### 1-2 Months

A cohort study of 897 infants born in Brazil showed no evidence of association between TBF for ≥1-2 months vs. <1-2 months in children aged 5-14 year (Figure 16). The study had an overall high risk of bias due to not controlling for relevant potential confounders.

Figure 16 Total breastfeeding for ≥1-2 months vs. <1-2 months and risk of wheeze in children aged 5-14 years


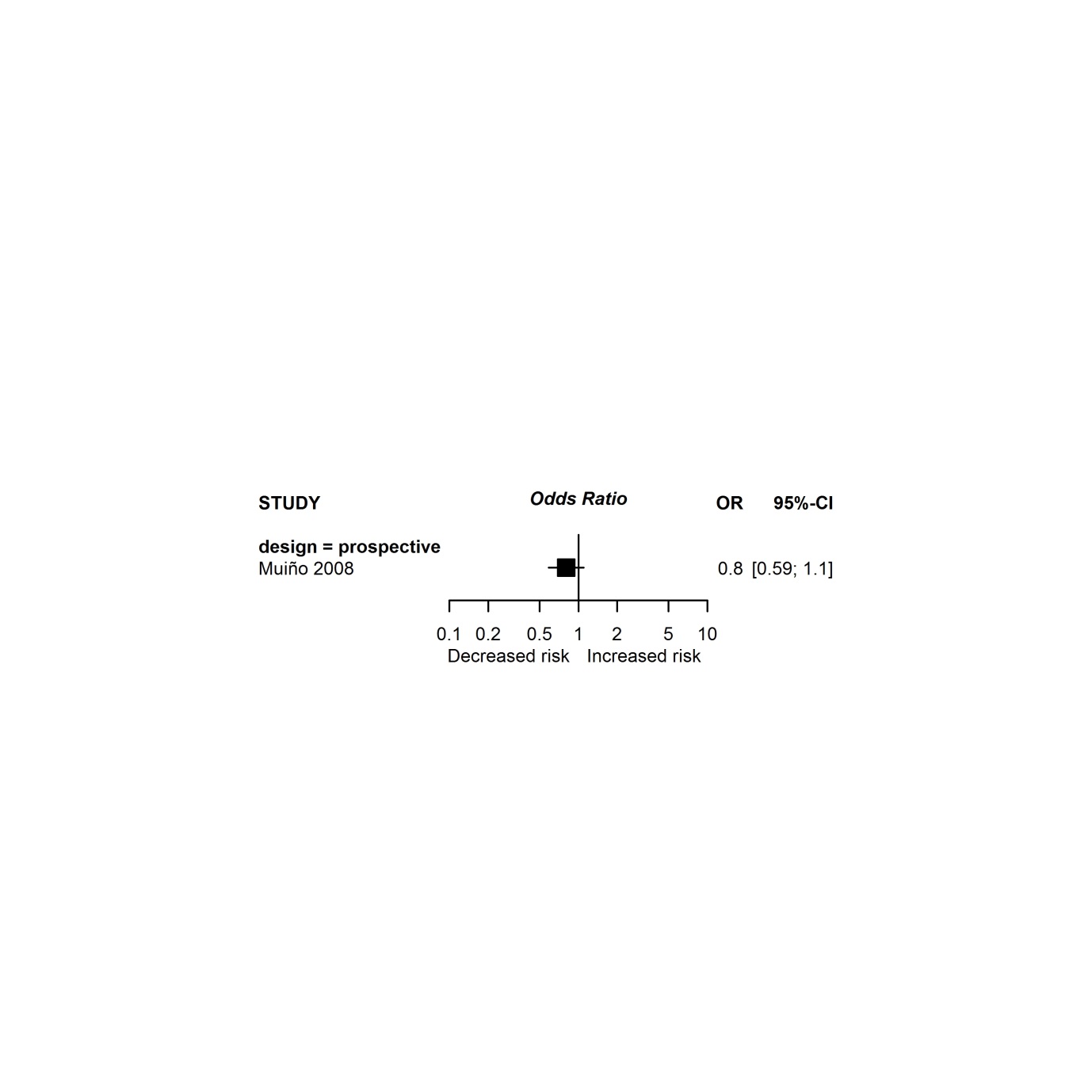


#### 3-4 Months

Two prospective cohort studies and one case-control study had eligible data to calculate a combined OR of wheeze in infants who had TBF for ≥3-4 months vs. <3-4 months at age 5-14 years, showing no clear evidence of an effect (Figure 17). There was high statistical heterogeneity between studies (I^2^=66.8%). Two studies had an unclear risk of bias and the study of Oddy had a low risk of bias.

Figure 17 Total breastfeeding for ≥3-4 months vs. <3-4 months and risk of wheeze in children aged 5-14 years


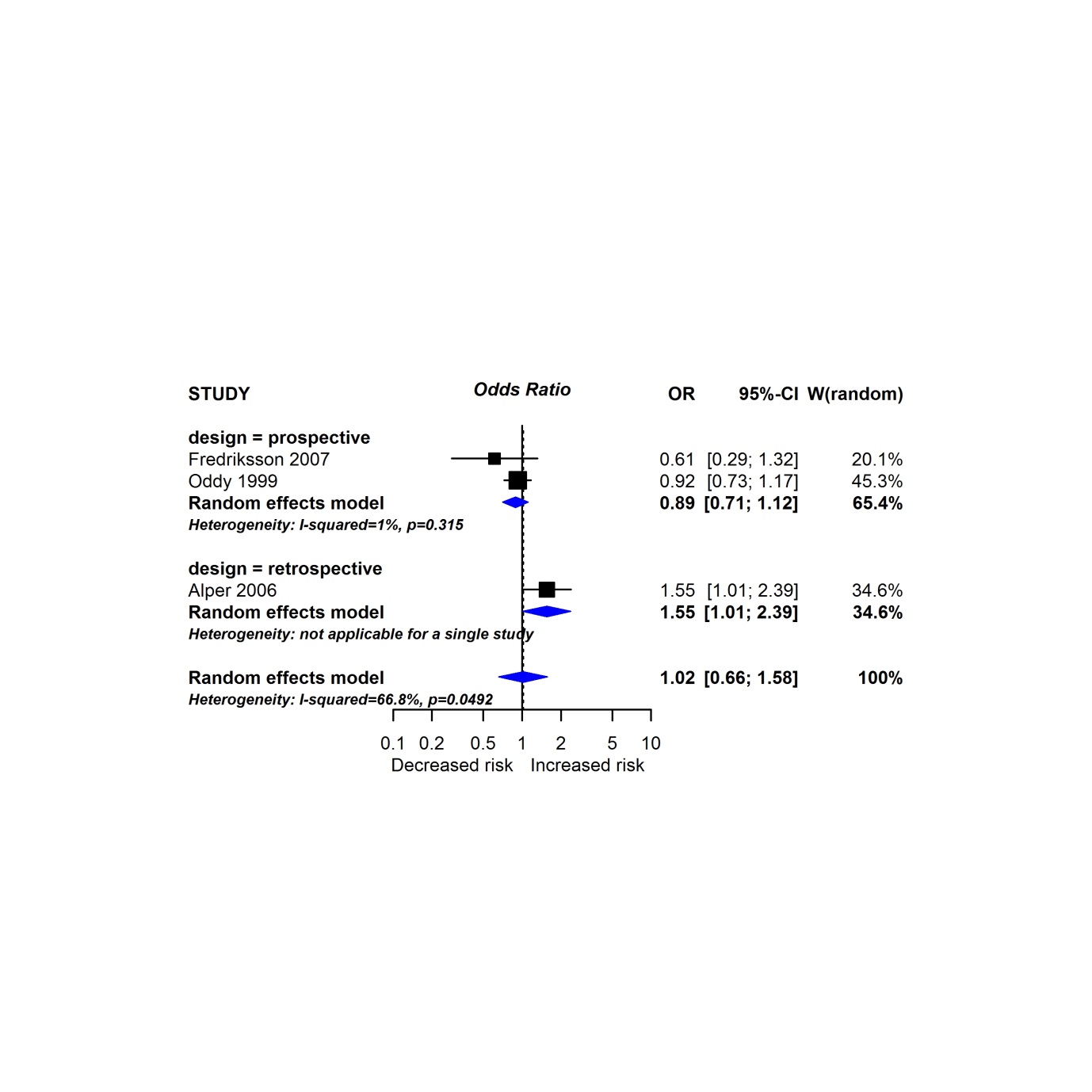


#### 5-7 Months

A case-control study and a prospective cohort had data eligible for meta-analysis, and their combined effect showed no evidence of association between TBF ≥5-7 months vs. <5-7 months and risk of wheeze in children aged 5-14 years (Figure 18). The study of Camara had a high risk of overall bias as it provided unadjusted data. There was no heterogeneity between studies (I^2^=0.0%).

Figure 18 Total breastfeeding for ≥5-7 months vs. <5-7 months and risk of wheeze in children aged 5-14 years


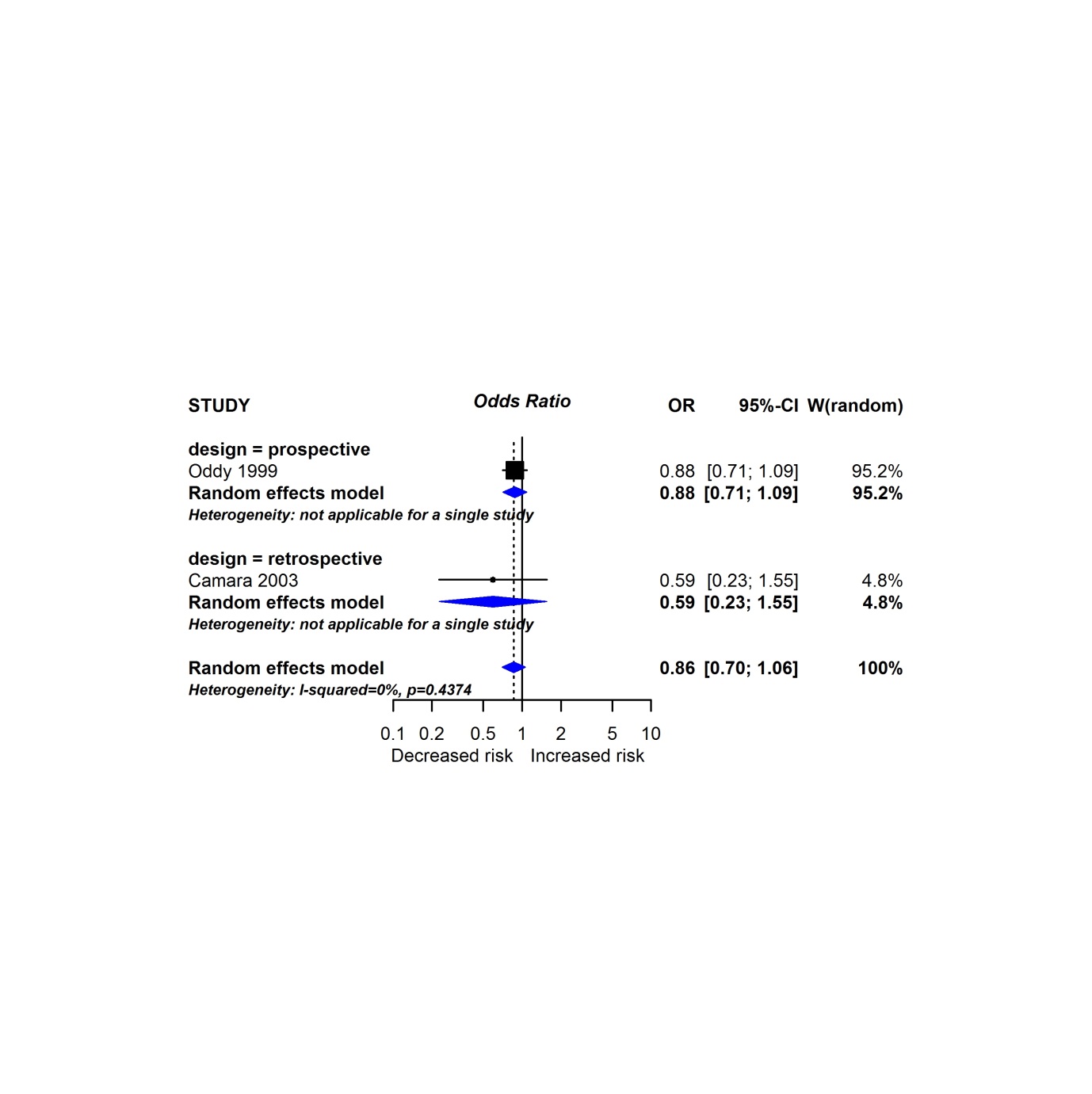


### Age at outcome measurement 15+

#### Ever vs. never

One case-control study reported risk of wheeze at age 15 or beyond in infants who received any TBF compared to those who never did. The data are adjusted, the study had a low overall risk of bias, and they show a reduced risk of wheeze (Figure 19).

Figure 19 Total breastfeeding ever vs. never and risk of wheeze in children aged 15+ years


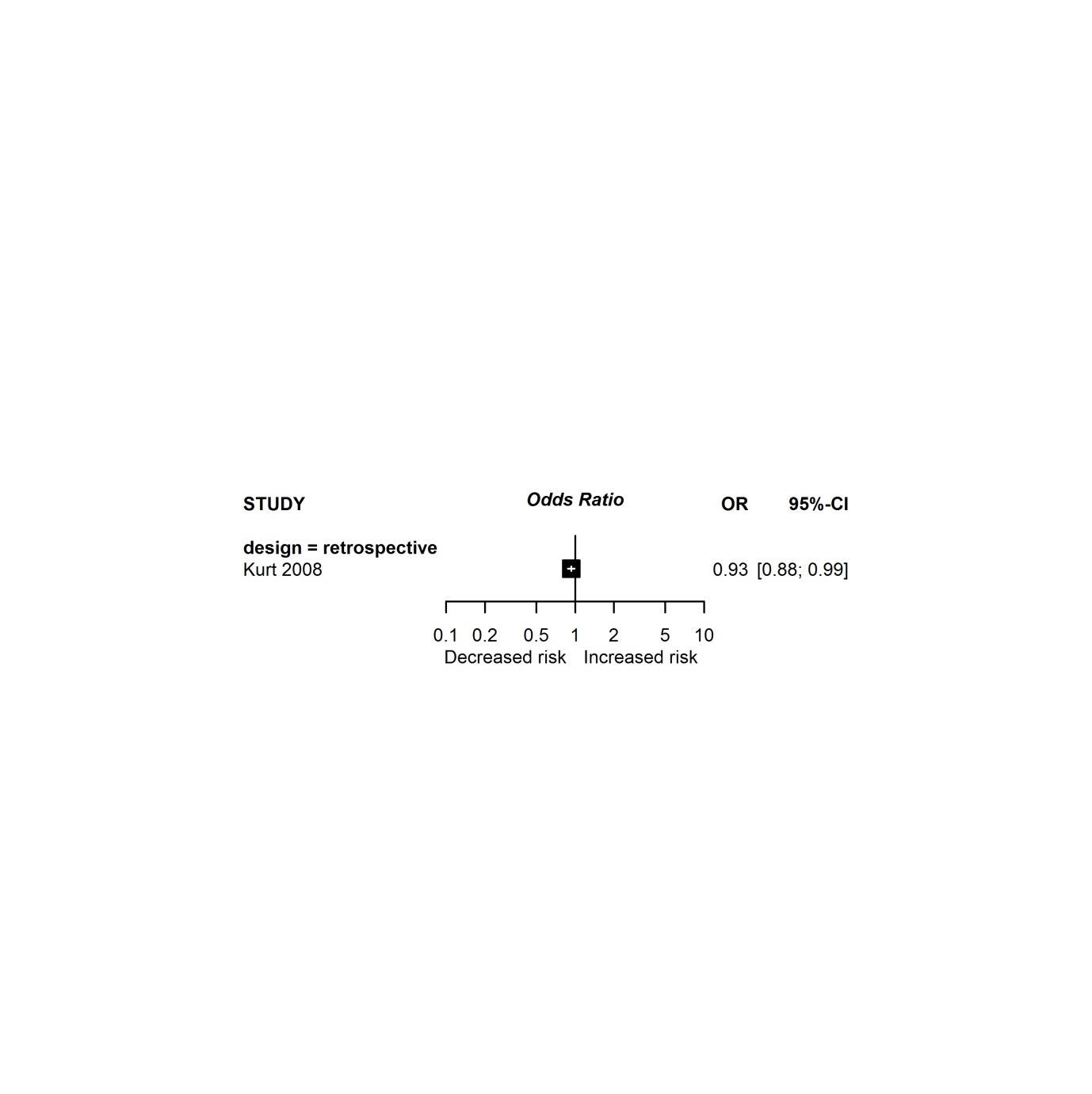


#### 1-2 Months

Figure 20 illustrates the combined effect of two eligible studies, a retrospective and a prospective cohort, showing no clear evidence of an association with risk of wheeze in children at age 15 or beyond who received TBF for ≥1-2 months vs. <1-2 months. The studies had moderate statistical heterogeneity between them (I^2^=35.3%) and had low or unclear overall risk of bias.

Figure 20 Total breastfeeding for ≥1-2 months vs. <1-2 months and risk of wheeze in children aged 15+ years


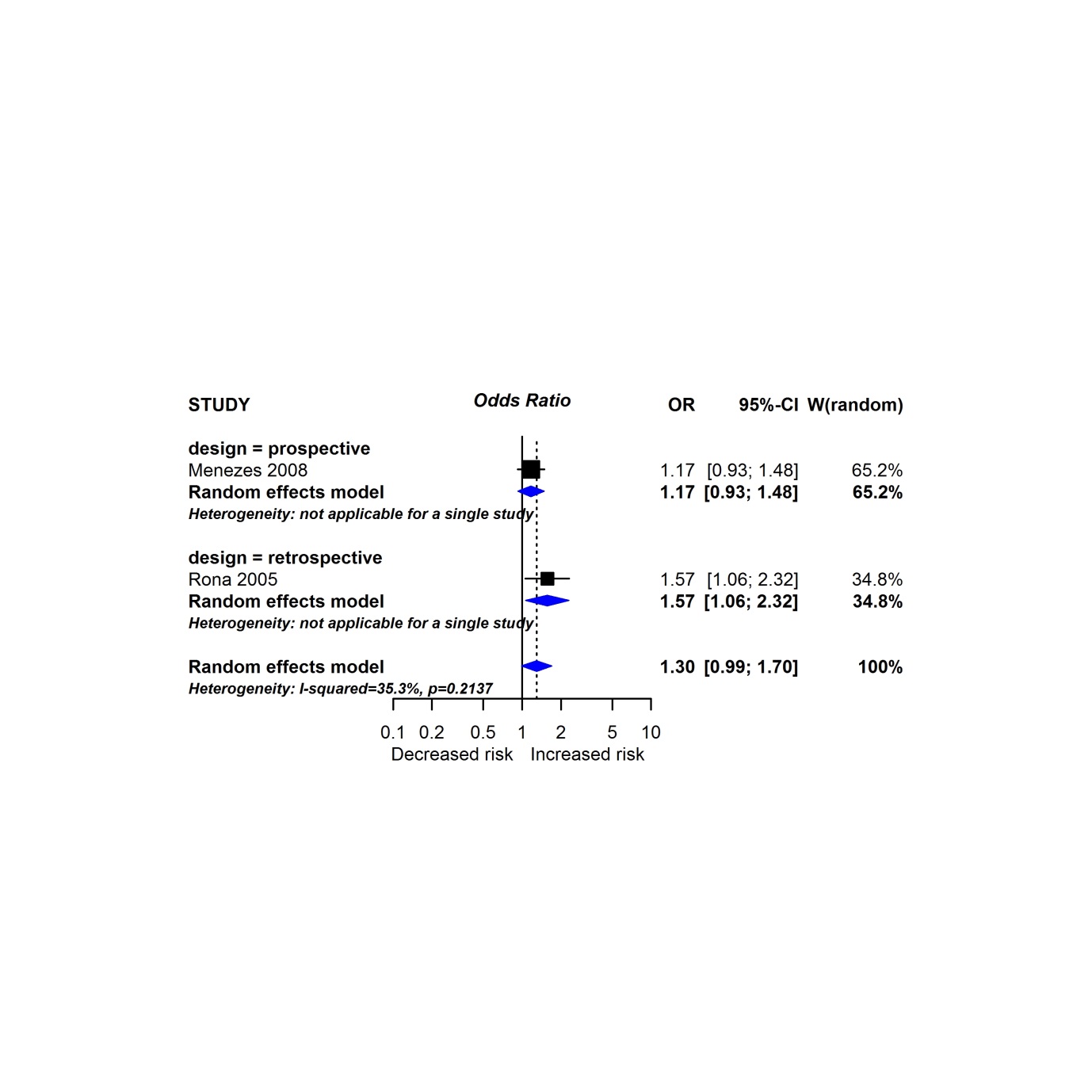


#### 3-4 Months

One cross-sectional study reported the risk of wheeze at age 15 or beyond, in infants who had TBF for ≥3-4 months vs. <3-4 months, showing significantly reduced odds of wheeze with longer TBF (Figure 21). The study had a low overall risk of bias.

Figure 21 Total breastfeeding for ≥3-4 months vs. <3-4 months and risk of wheeze in children aged 15+ years


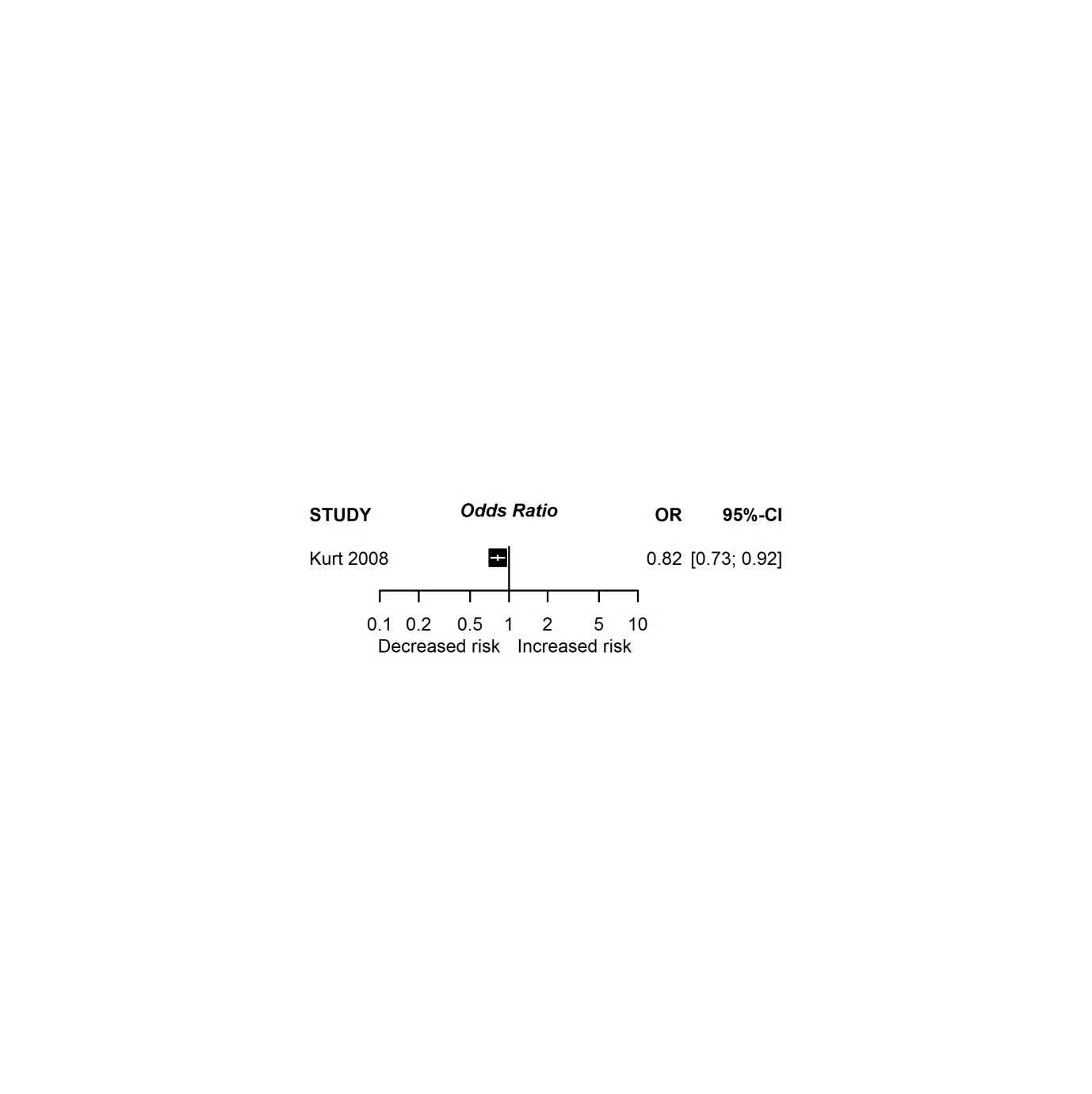


## Total Breastfeeding and atopic wheeze

### Age at outcome measurement 5-14

One study reported atopic wheeze at age 5-14 in relation to TBF. The findings are shown in Figure 22, and show no association. It was also possible to assess does response using this study, and there was no evidence of a dose-response relationship (Figure 23-Figure 24).

Figure 22 Total breastfeeding (dose response) ever vs. never and risk of atopic wheeze in children aged 5-14 years


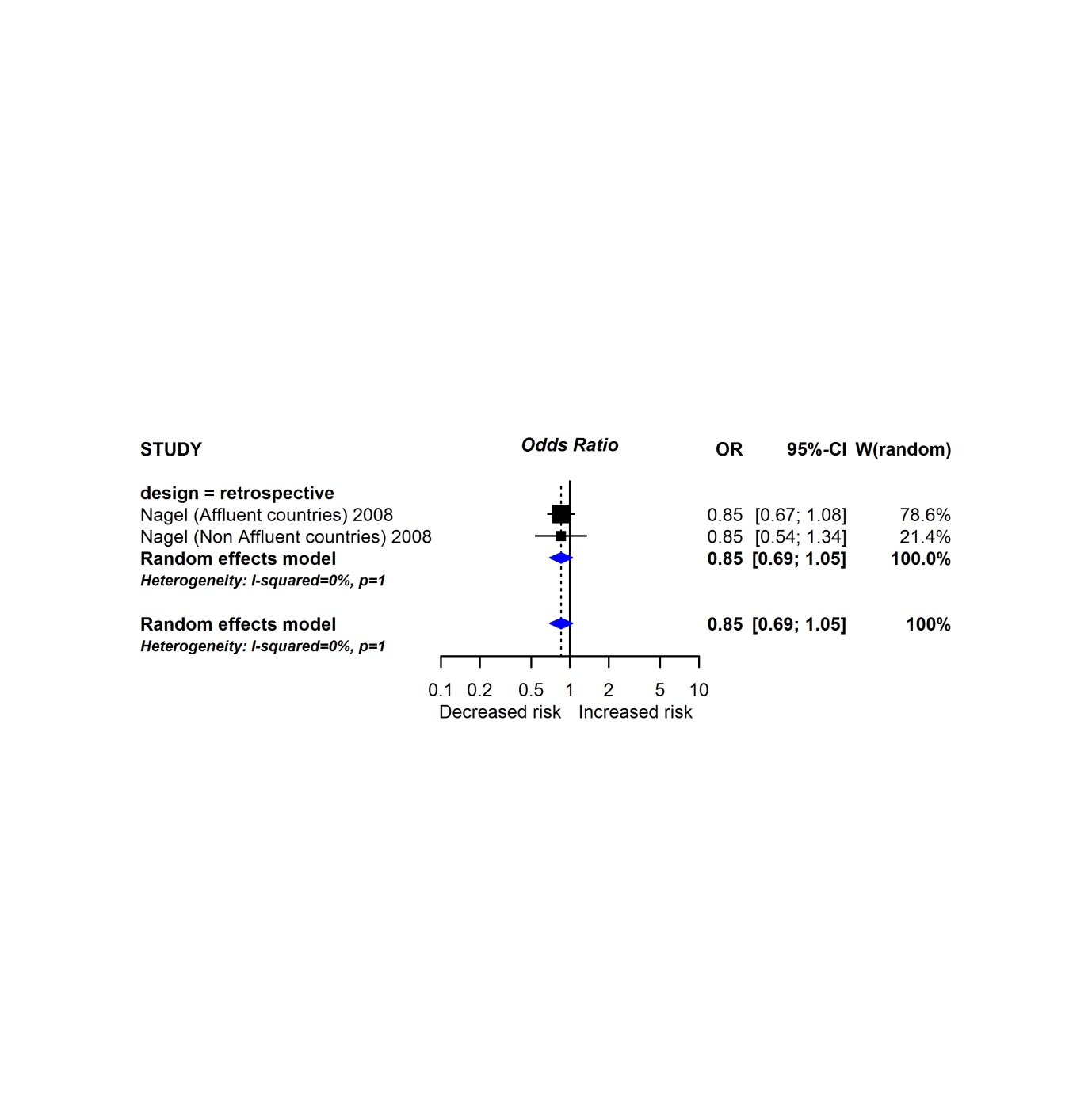


Figure 23 Total breastfeeding (dose response) medium vs. never and risk of atopic wheeze in children aged 5-14 years


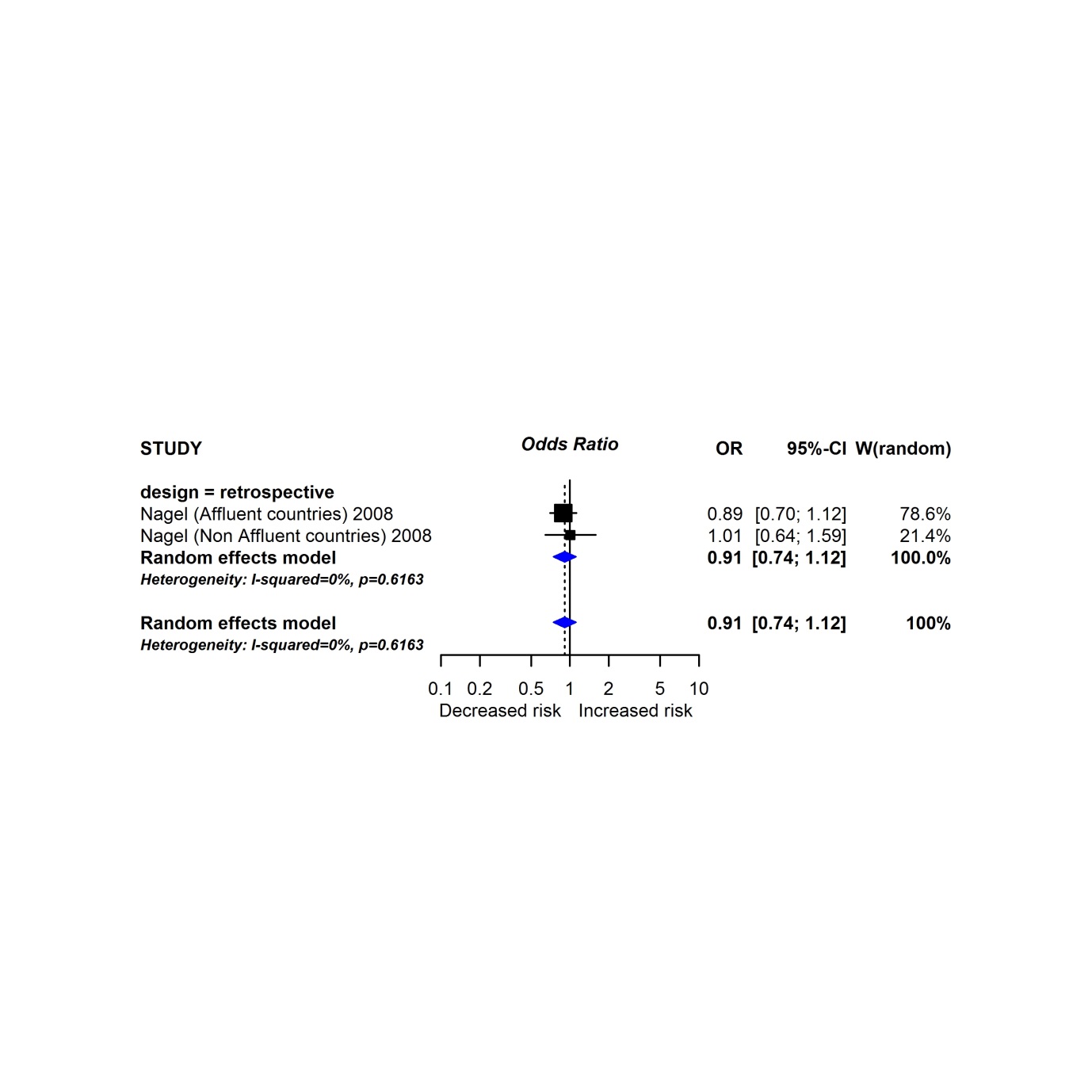


Figure 24 Total breastfeeding (dose response) long vs. never and risk of atopic wheeze in children aged 5-14 years


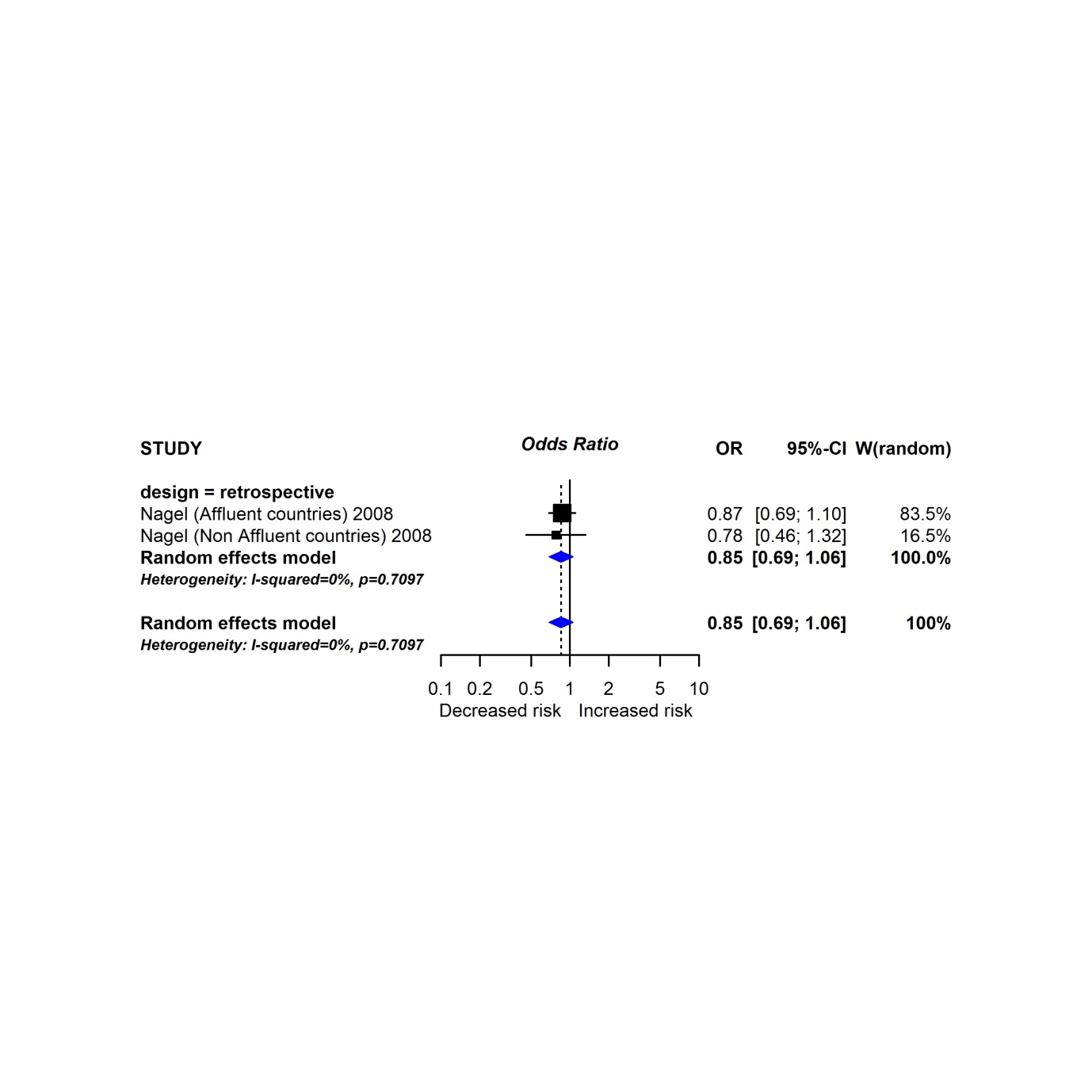


## Total Breastfeeding per month and Wheeze

### Age at outcome measurement 5-14

Two studies reported data for TBF exposure per month and risk of wheeze at age 5-14, and are shown in Figure 25. They show no significant association, with high statistical heterogeneity. Both studies are prospective cohort studies in a normal risk population, with the study of Silvers having a low risk of bias in all domains, whilst the study of Munro carried an unclear risk due to unclear selection bias, which may contribute to explain the high heterogeneity between studies.

Figure 25 Total breastfeeding per month and risk of wheeze in children aged 5-14 years


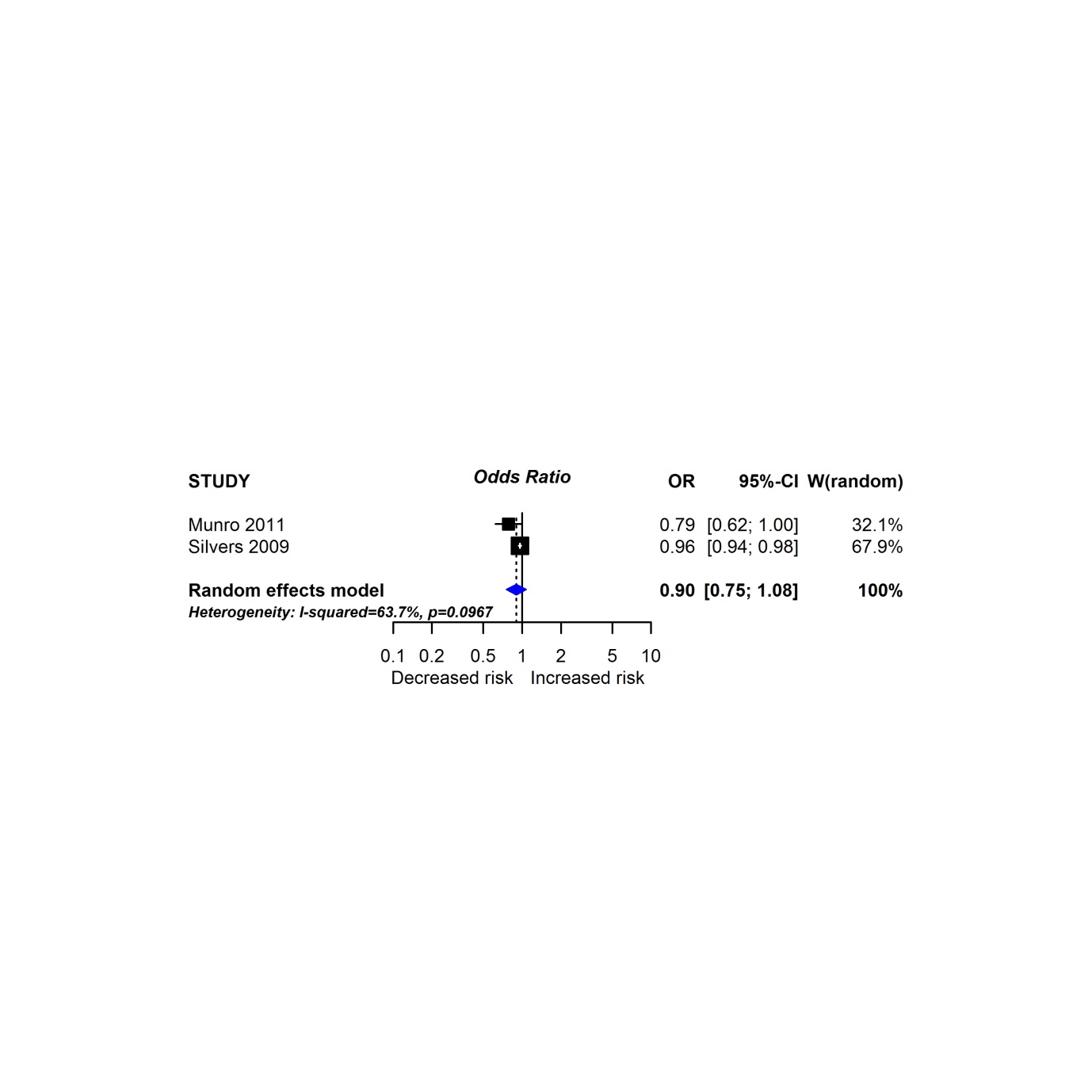


## Total Breastfeeding and Recurrent Wheeze

### Age at outcome measurement 0-4

#### Ever vs. never

Eleven observational studies had data that could be used to calculate combined OR of risk of recurrent wheeze in children 0-4 years old who were exposed to ever vs. never TBF, showing a negative association between this exposure and risk of wheeze. There were 9 prospective cohorts and 2 case-control studies. The study of Businco had a high overall risk of bias due to lack of adjustment for relevant potential confounders. The two case-control studies had unclear risk of bias, three prospective cohorts had unclear risk of bias, and 5 had a low risk of overall bias. These differences in overall risk might partly explain the very high heterogeneity observed between studies (I^2^=80.7%). For this reason data were not pooled in meta-analysis (Figure 26). There was no visual or statistical evidence of publication bias as illustrated in Figure 27. The subgroup analysis showed no difference between the various groups compared, with extreme heterogeneity in most analyses (Table 5). Dose response analysis (Figure 28-Figure 30) showed some evidence of a dose response, although only a small proportion of studies could be included in dose response analysis due to available data.

Figure 26 Total breastfeeding ever vs. never and risk of recurrent wheeze in children aged 0-4 years

**
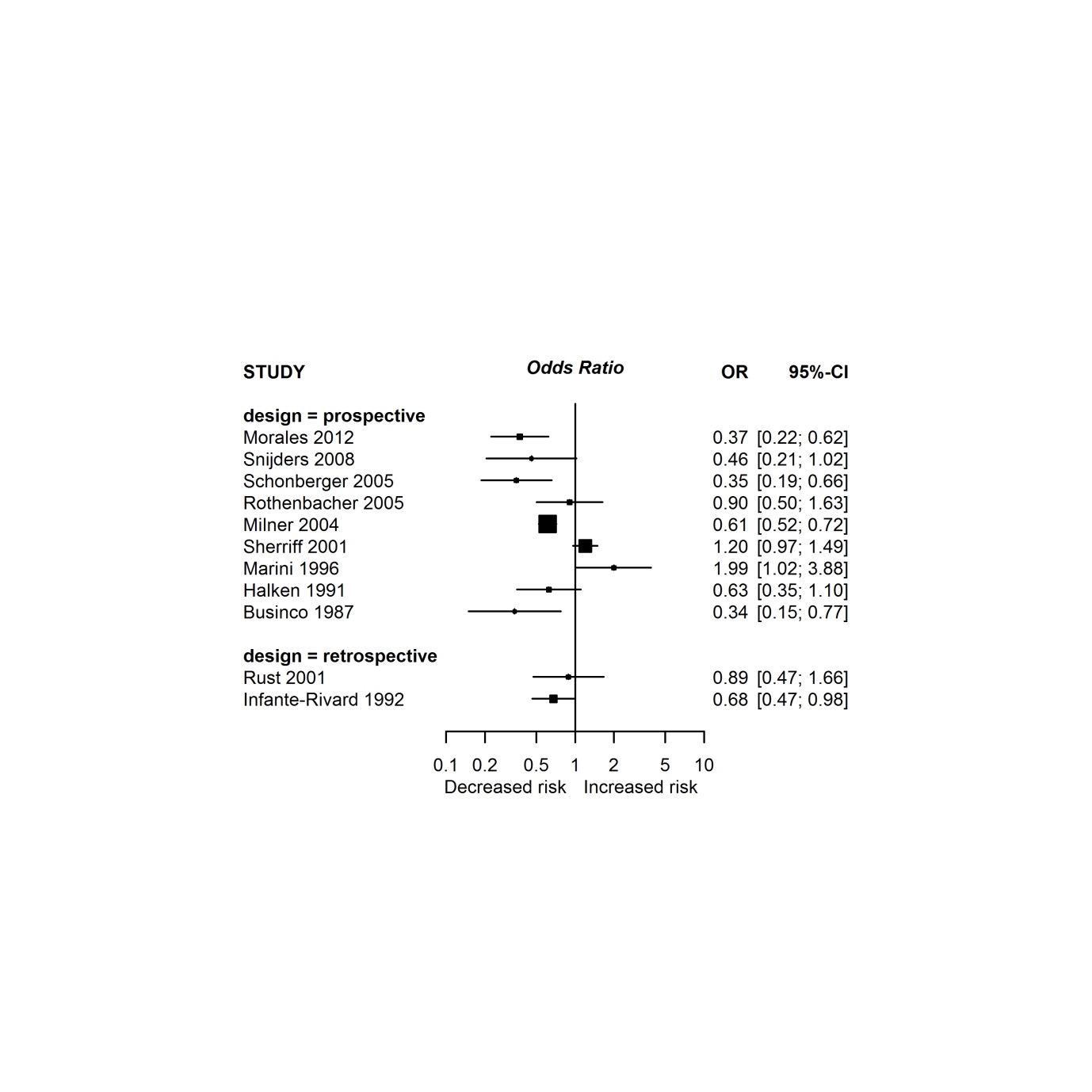
**

Figure 27 Risk of publication bias in studies investigating total breastfeeding ever vs. never and risk of recurrent wheeze in children aged 0-4 years


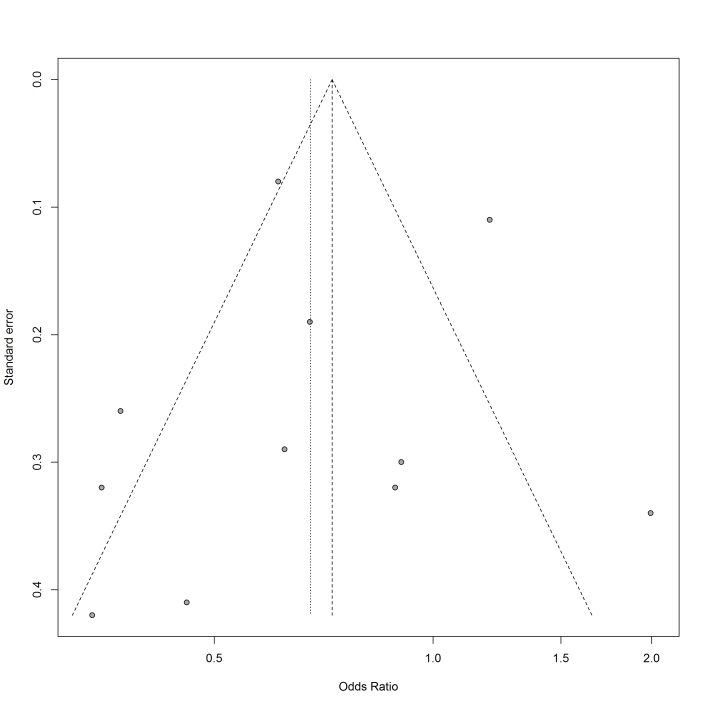


Egger’s test p-value = 0.663

Table 5 Subgroup Analysis of risk of recurrent wheeze and total breastfeeding ever vs. never (or ever) months in children aged 0-4 years

|  | **Number of studies** | **OR [95% CI]** | **I^2^ (%)** | **P-value for between groups difference** |
| --- | --- | --- | --- | --- |
| **Overall (if adjusted NA, unadjusted value used)** | 11 | 0.68 [0.51; 0.91] | 80.7 |  |
| **Adjusted** | 7 | 0.78 [0.53; 1.15] | 78.0 | Not tested |
| **Unadjusted** | 9 | 0.63 [0.50; 0.79] | 77.1 |  |
| Study Design – Prospective | 9 | 0.66 [0.46; 0.94] | 84.4 | 0.68 |
| Study Design – Retrospective | 2 | 0.73 [0.53; 1.00] | 0.0 |  |
| Risk of disease – High | 3 | 0.62 [0.19; 2.03] | 88.2 | 0.86 |
| Risk of disease – Normal | 8 | 0.69 [0.52; 0.94] | 79.6 |  |
| Risk of bias – Low | 5 | 0.68 [0.41; 1.12] | 79.4 | 0.96 |
| Risk of bias – High/Unclear | 6 | 0.67 [0.47; 0.96] | 73.9 |  |

Figure 28 Total breastfeeding short vs. never term and risk of recurrent wheeze in children aged 0-4 years


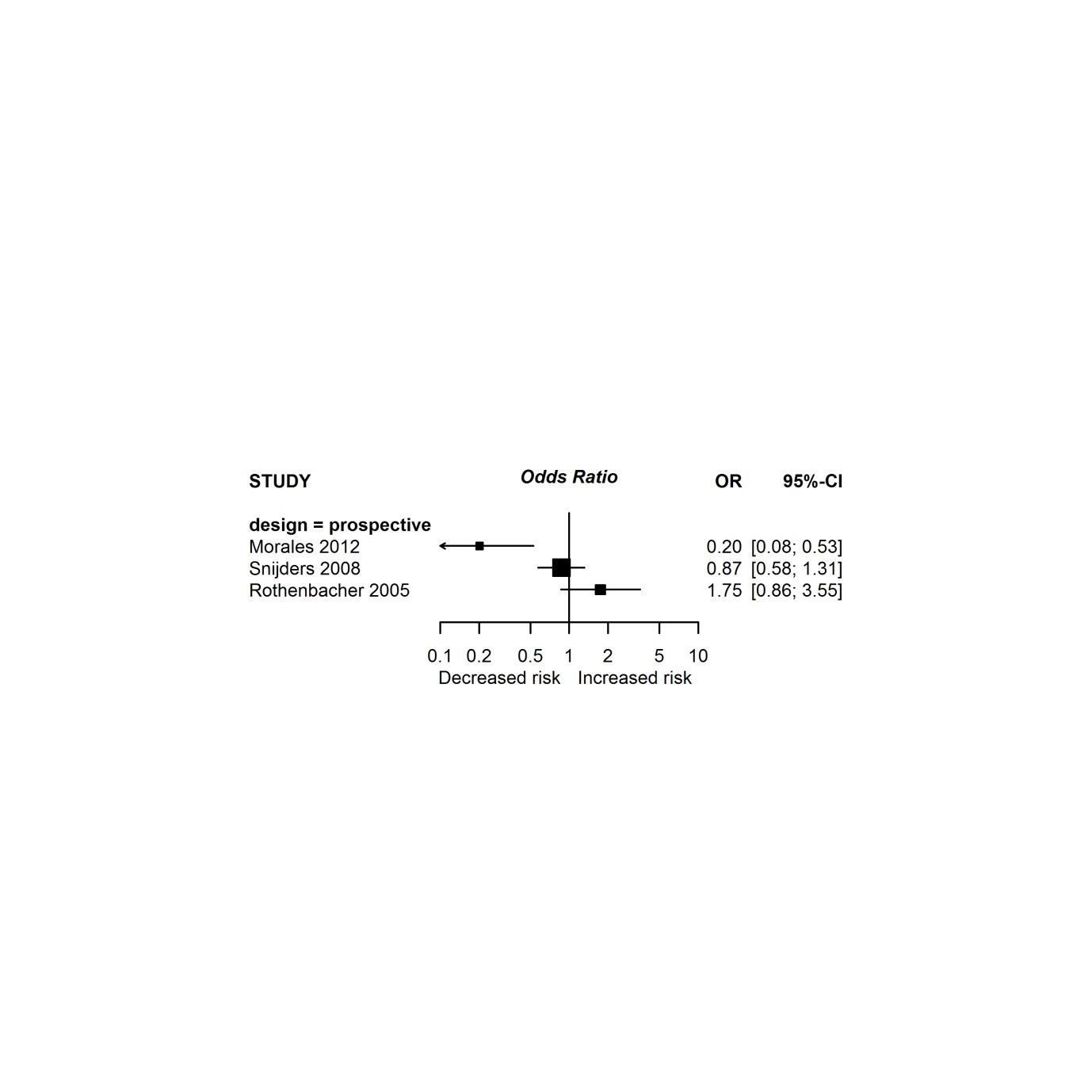


Figure 29 Total breastfeeding medium vs. never term and risk of recurrent wheeze in children aged 0-4 years


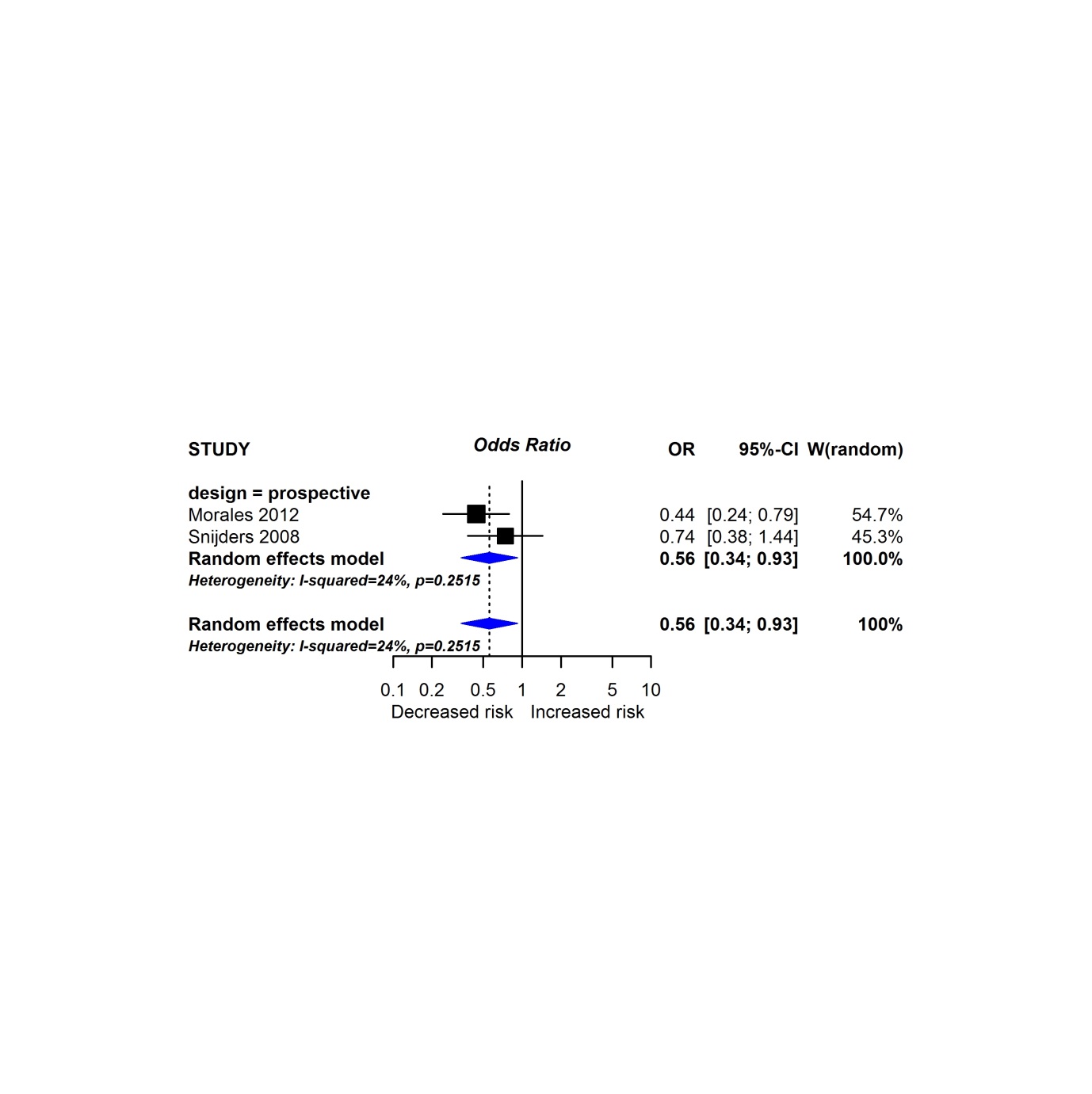


Figure 30Total breastfeeding long vs. never term and risk of recurrent wheeze in children aged 0-4 years

**
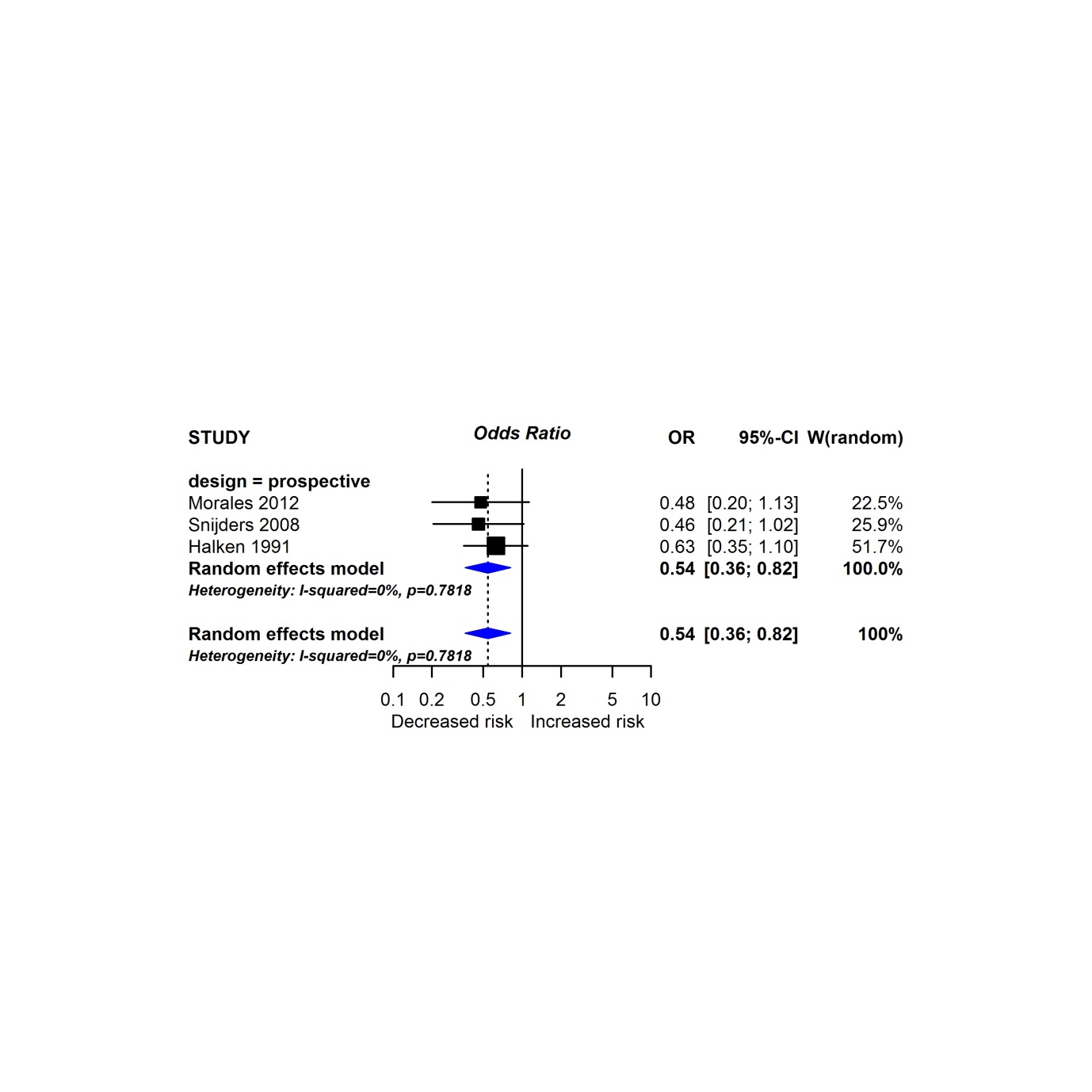
**

#### 1-2 Months

Two prospective cohort studies had outcome data suitable for meta-analysis, showing no evidence of association between TBF for ≥1-2 months vs. <1-2 months and risk of recurrent wheeze in children aged 0-4 years. The study of Halken had an overall low risk of bias whilst the study of Dell had an unclear overall risk bias. There was evidence of high heterogeneity between the studies (I^2^=54.4%)

Figure 31 Total breastfeeding for ≥1-2 months vs. <1-2 months and risk of recurrent wheeze in children aged 0-4 years

**
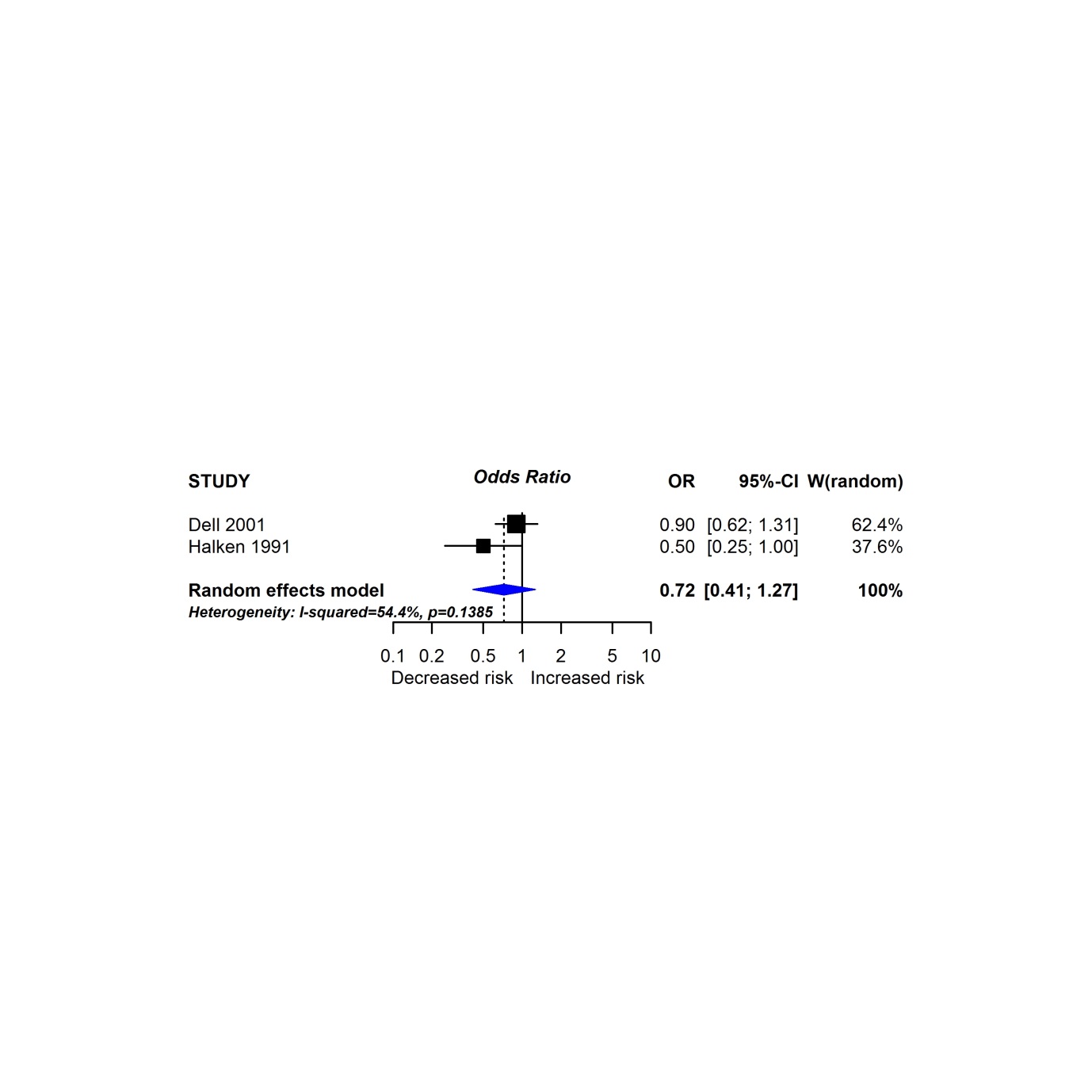
**

#### 3-4 Months

Three observational studies had data eligible for meta-analysis, however they could not be pooled due to extreme heterogeneity (Figure 32). The studies of Alm and Rothenbacher were prospective cohorts and one (Rosas-Vargas) was a case-control study. The latter had a high risk of overall bias due to lack of adjustment for potential confounders. The other two studies had a low overall risk of bias.

Figure 32 Total breastfeeding for ≥3-4 months vs. <3-4 months and risk of recurrent wheeze in children aged 0-4 years


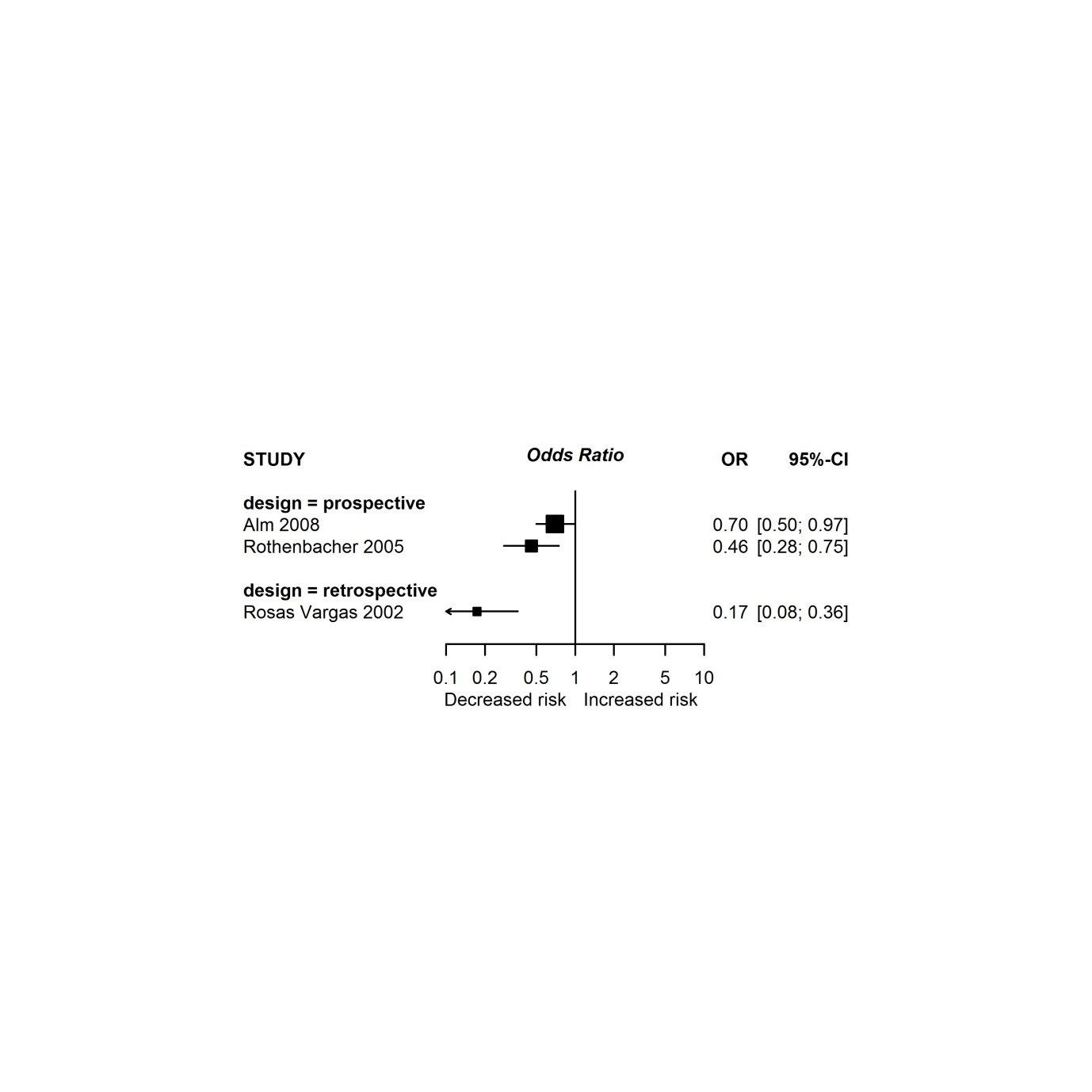


#### 5-7 Months

Three prospective cohort studies (Miyake, Kull, Gustaffson) and three case-control studies (Visser, Tanaka, Dell) reported data that could be pooled to calculate the OR for wheeze in children aged 0-4 years who were exposed to TBF for ≥5-7 months vs. <5-7 months. As shown in Figure 33, their combined effect suggests no difference in risk of wheeze (OR 0.79; 95% CI 0.61, 1.04), with moderate heterogeneity between studies (I^2^=39.7%). In subgroup and stratified analyses (Table 6), there was evidence for a relationship between TBF ≥5-7 months and reduced risk of recurrent wheeze at age 0-4 in prospective studies, and studies reporting adjusted data and low risk of bias, but not in retrospective or unadjusted studies or studies at high/unclear risk of bias.

Figure 33 Total breastfeeding for ≥5-7 months vs. <5-7 months and risk of recurrent wheeze in children aged 0-4 years


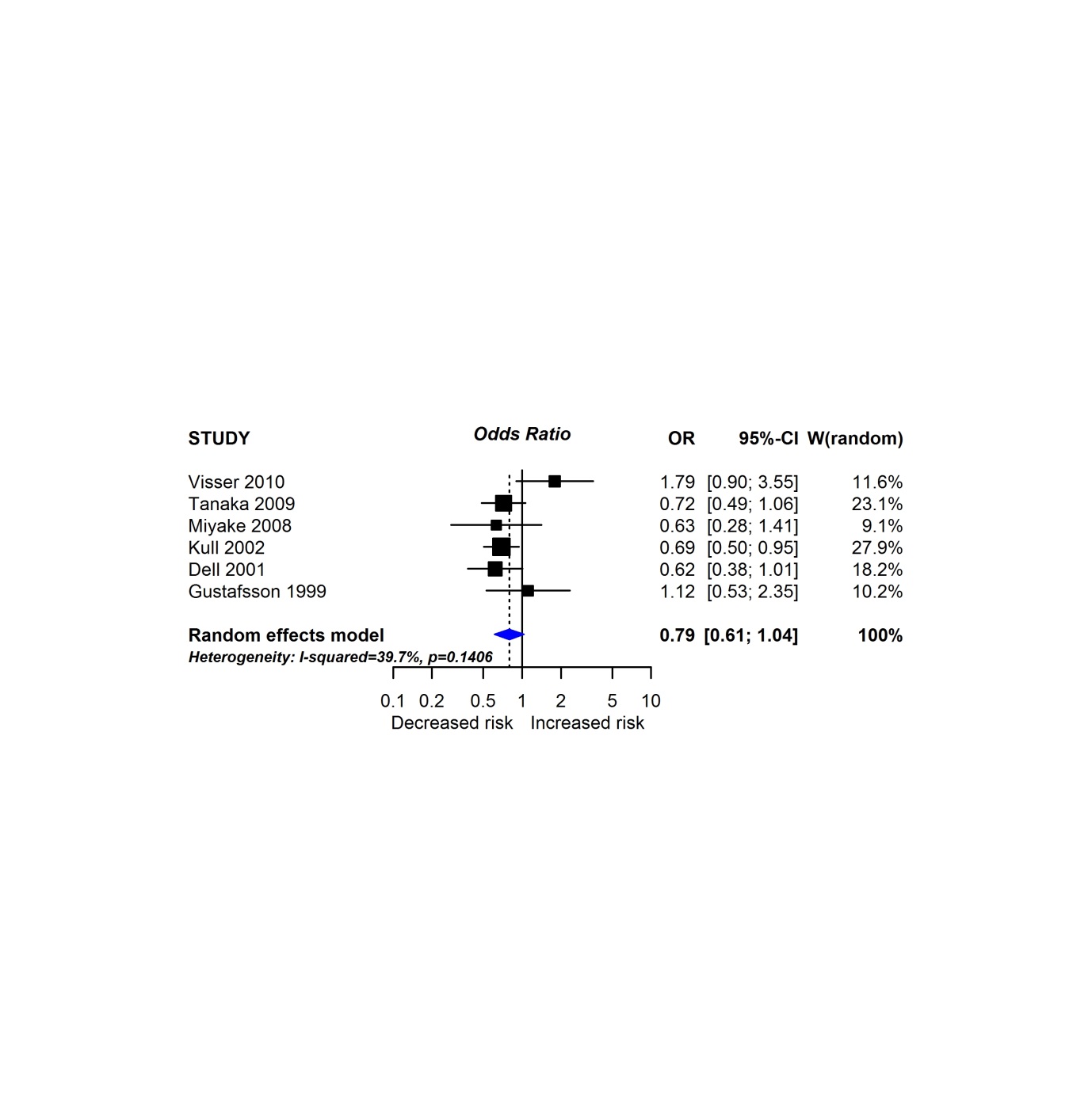


Table 6 Subgroup Analysis of risk of recurrent wheeze and total breastfeeding ≥5-7 months vs. <5-7 months in children aged 0-4 years

|  | **Number of studies** | **OR [95% CI]** | **I^2^ (%)** | **P-value for between groups difference** |
| --- | --- | --- | --- | --- |
| **Overall (if adjusted NA, unadjusted value used)** | 6 | 0.79 [0.61; 1.04] | 39.7 |  |
| **Adjusted** | 4 | 0.68 [0.55; 0.84] | 0.0 | Not tesed |
| **Unadjusted** | 5 | 0.80 [0.56; 1.16] | 60.6 |  |
| Study Design – Prospective | 3 | 0.73 [0.56; 0.96] | 0.0 | 0.56 |
| Study Design – Retrospective | 3 | 0.87 [0.51; 1.49] | 69.7 |  |
| Risk of disease – High | -  6 | -  0.79 [0.61; 1.04] | -  39.7 | - |
| Risk of disease – Normal |  |  |  |  |
| Risk of bias – Low | 1 | 0.69 [0.51; 0.95] | -- | 0.39 |
| Risk of bias – High/Unclear | 5 | 0.85 [0.59; 1.23] | 48.1 |  |

#### 8-12 Months

One case-control study reported risk of recurrent wheeze in children breastfeed ≥8-12 months vs. <8-12 months. The study had an overall unclear risk of bias and suggested no significant difference in OR of disease (Figure 34).

Figure 34 Total breastfeeding for ≥8-12 months vs. <8-12 months and risk of recurrent wheeze in children aged 0-4 years


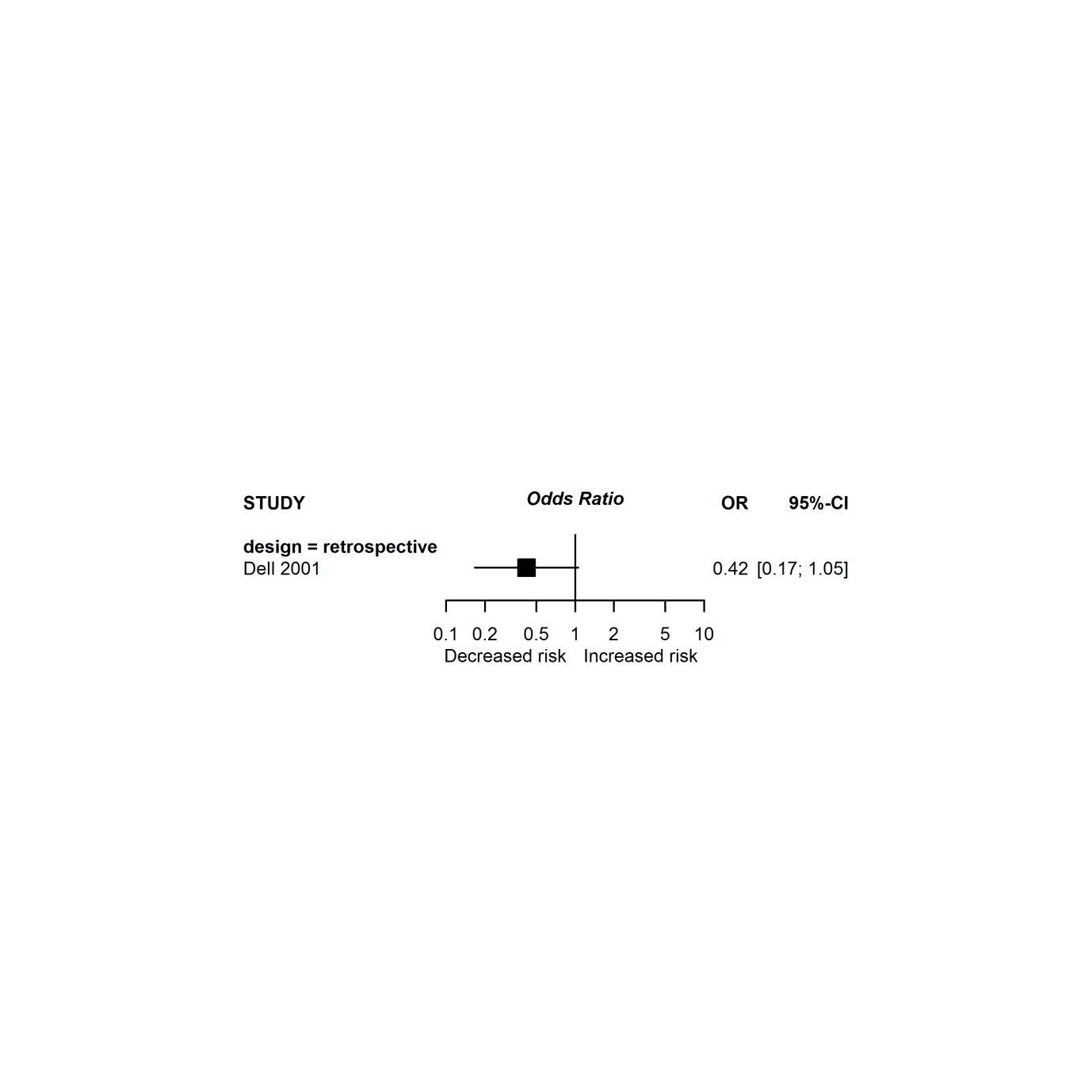


Subgroup analyses to investigate differences in risk of recurrent wheeze and TBF ≥5-7 months vs. <5-7 months in children aged 0-4 years, showed no statistically significant differences (Table 7).

### Age at outcome measurement 5-14

#### Risk of recurrent wheeze at age 5-14, in relation to TBF Ever vs. never

Twenty five observational studies reported the association between recurrent wheeze at age 5-14 and TBF ever vs. never. Most studies reported ORs below 1, with an overall effect size suggestive of a negative association between TBF and disease (OR 0.88; 95% CI 0.82, 0.94) and moderate heterogeneity across studies (I^2^=36.5%) (Figure 35). Thirteen studies were prospective cohorts (9 of which had a low overall risk of bias, 3 had high risk, and 1 unclear), 1 retrospective cohort study (with a high overall risk of bias due to confounding not being accounted for in the analyses), 4 case-control studies (of which 3 had a high overall risk of bias and one had an unclear overall risk of bias), 1 nested case-control study (with high risk of bias due to selection bias), and six cross-sectional studies (of which 3 were considered to have a high overall risk of bias mainly due to selection or confounding bias, 2 had an unclear risk of bias and 1 study had a low risk of bias). A funnel plot was used to examine the likelihood of publication bias (Figure 36), which showed significant asymmetry, confirmed by a statistically significant Egger’s test P=0.01. Dose response analysis (Figure 37-Figure 39) suggests an association between increasing duration of TBF and greater protection against recurrent wheeze at age 5-14.

Figure 35 Total Breastfeeding ever vs. never and risk of recurrent wheeze in children aged 5-14 years

**
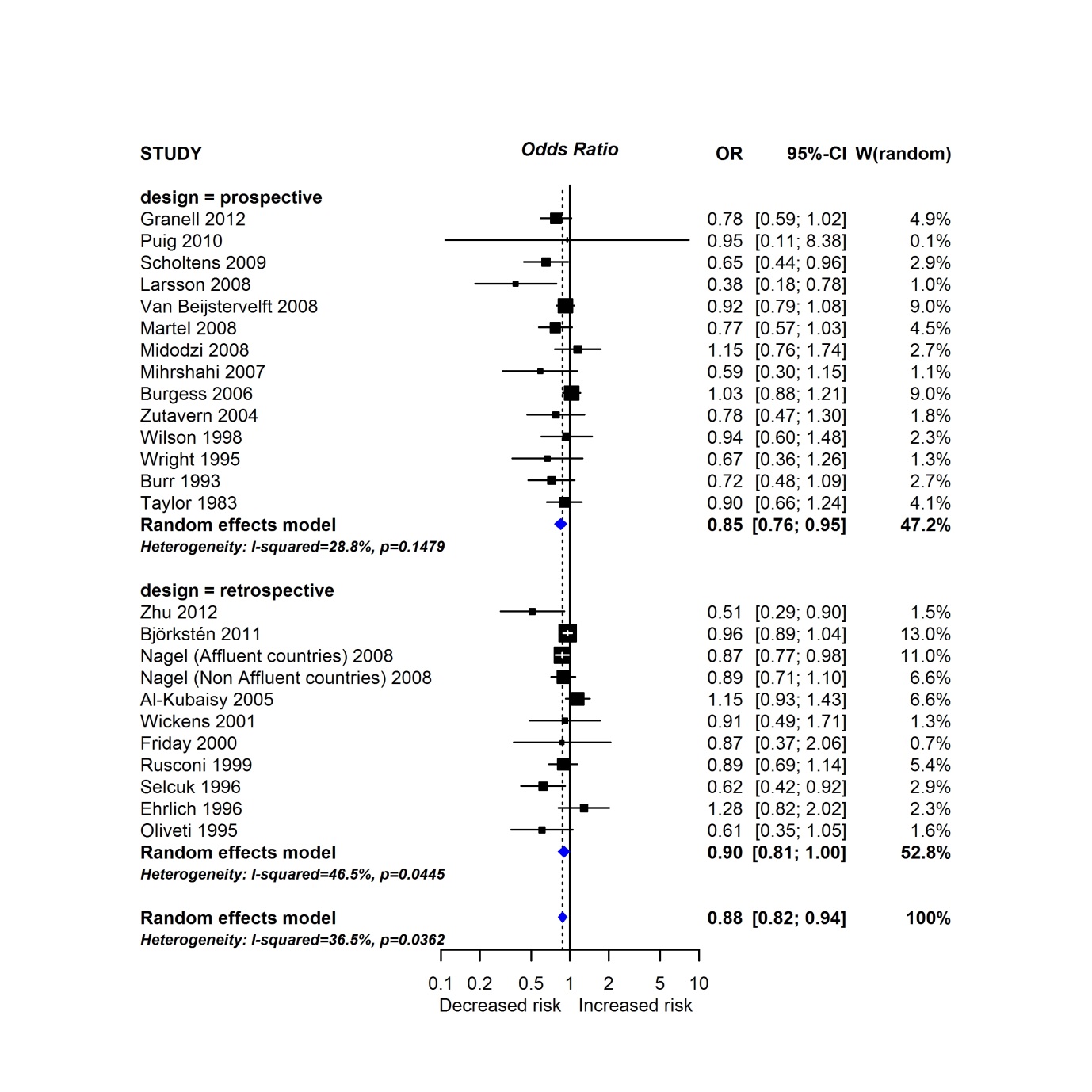
**

Figure 36 Risk of publication bias in studies investigating total breastfeeding ever vs. never and risk of recurrent wheeze in children aged 5-14 years


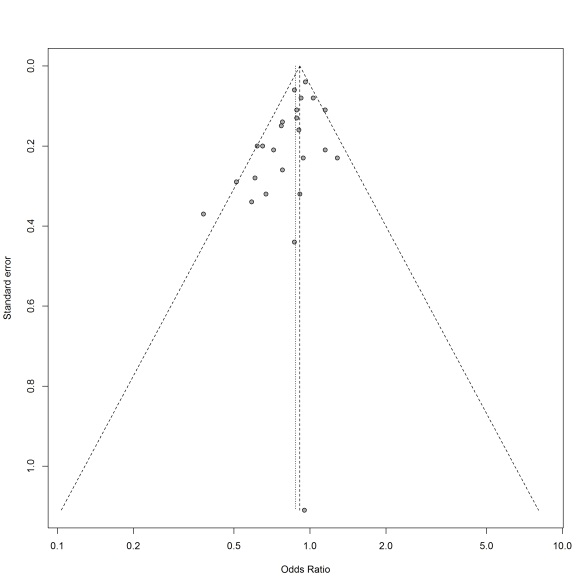


Egger’s test p-value = 0.012

Table 7 Subgroup Analysis of risk of recurrent wheeze and total breastfeeding ever vs. never months in children aged 5-14 years

|  | **Number of studies** | **OR [95% CI]** | **I^2^ (%)** | **P-value for between groups difference** |
| --- | --- | --- | --- | --- |
| **Overall (if adjusted NA, unadjusted value used)** | 25 | 0.88 [0.82; 0.95] | 36.5 |  |
| **Adjusted** | 15 | 0.82 [0.74; 0.91] | 44.9 | Not tested |
| **Unadjusted** | 22 | 0.83 [0.75; 0.91] | 57.4 |  |
| Study Design – Prospective | 14 | 0.85 [0.76; 0.95] | 28.8 | 0.44 |
| Study Design – Retrospective | 11 | 0.90 [0.81; 1.00] | 46.5 |  |
| Risk of disease – High | 3 | 0.70 [0.51; 0.97] | 0.0 | 0.18 |
| Risk of disease – Normal | 22 | 0.89 [0.82; 0.96] | 39.7 |  |
| Risk of bias – Low | 10 | 0.80 [0.68; 0.93] | 55.9 | 0.16 |
| Risk of bias – High/Unclear | 15 | 0.90 [0.83; 0.98] | 18.7 |  |

Figure 37 Total breastfeeding (dose response) short vs. never term and risk of recurrent wheeze in children aged 5-14 years


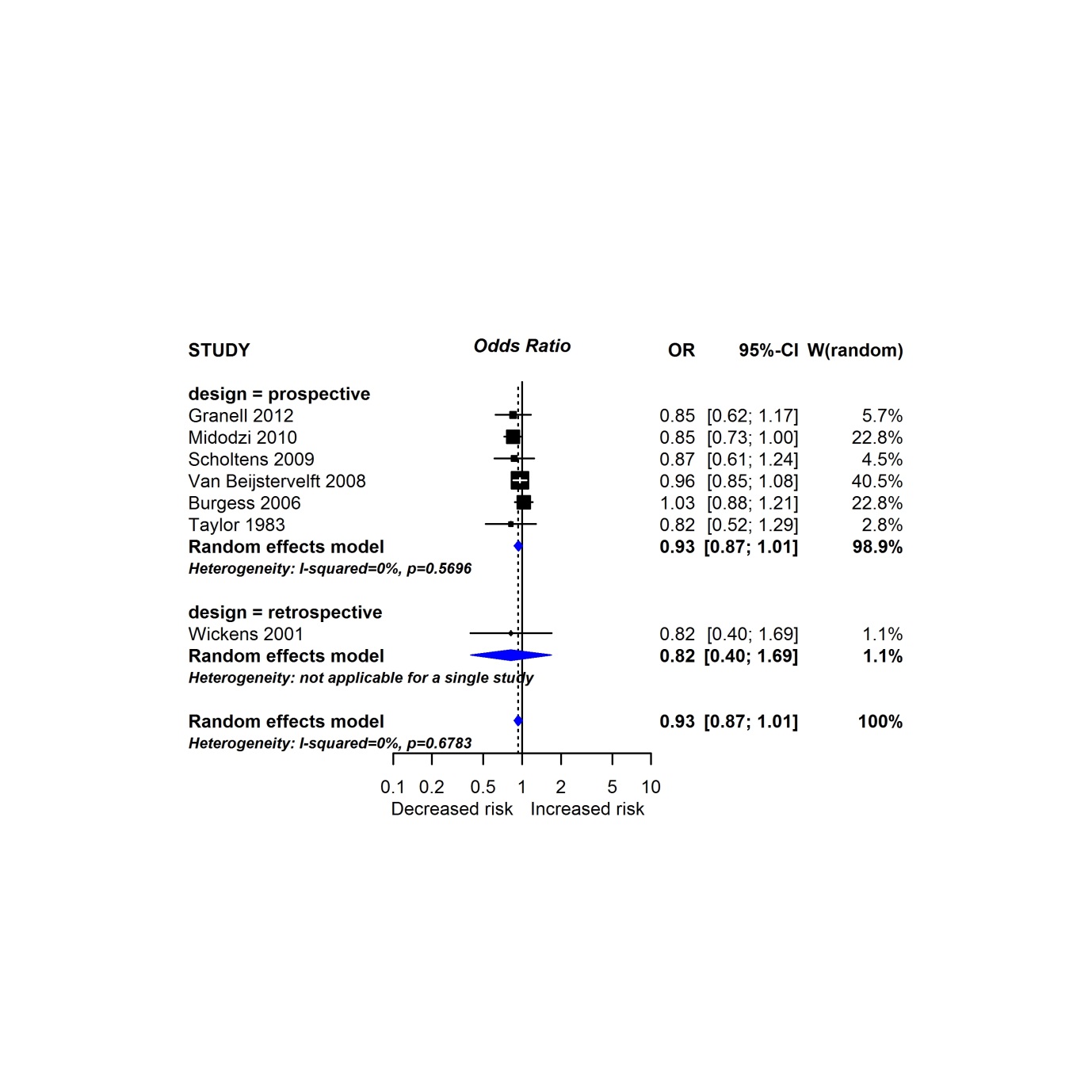


Figure 38 Total breastfeeding (dose response) medium vs. never term and risk of recurrent wheeze in children aged 5-14 years


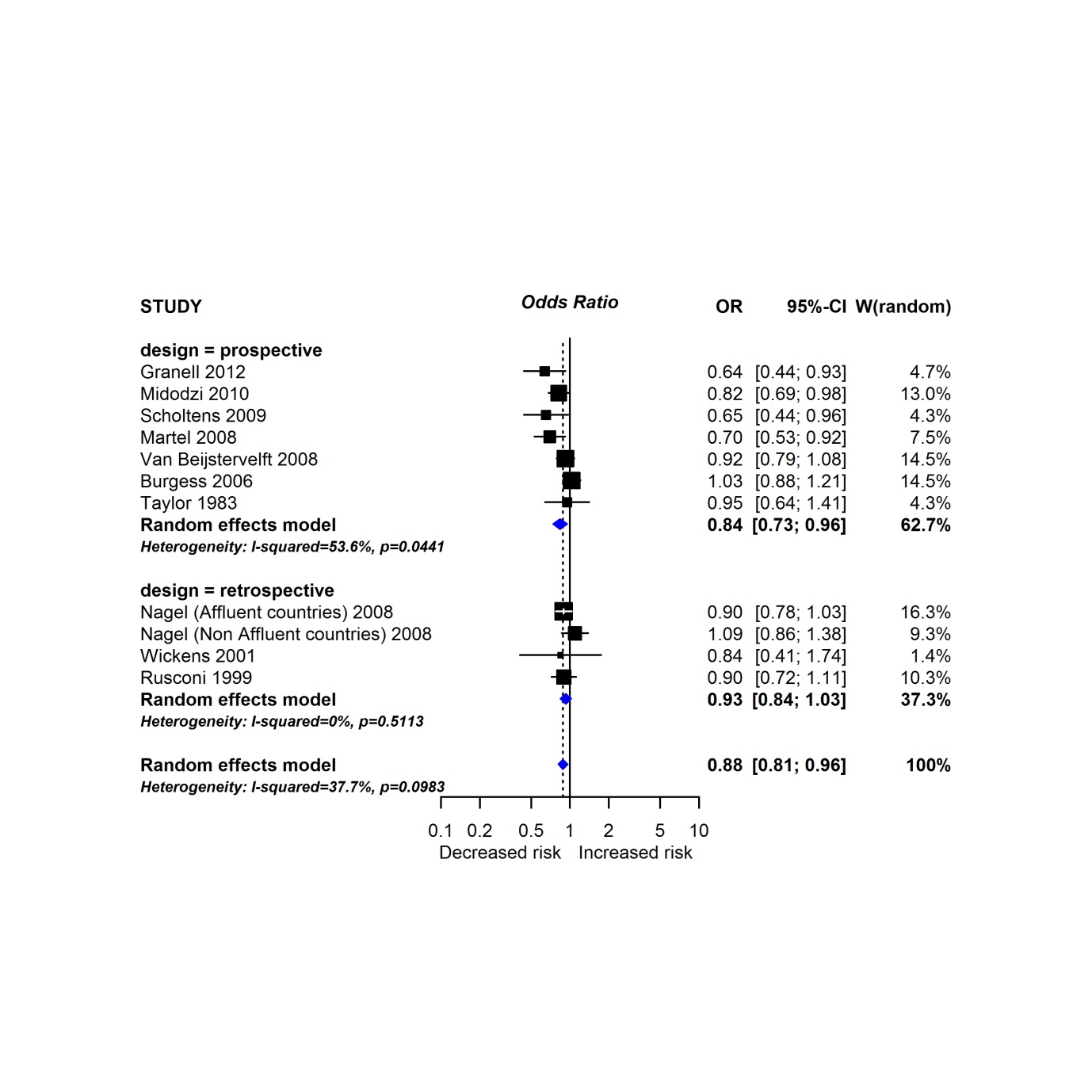


Figure 39 Total breastfeeding (dose response) long vs. never term and risk of recurrent wheeze in children aged 5-14 years


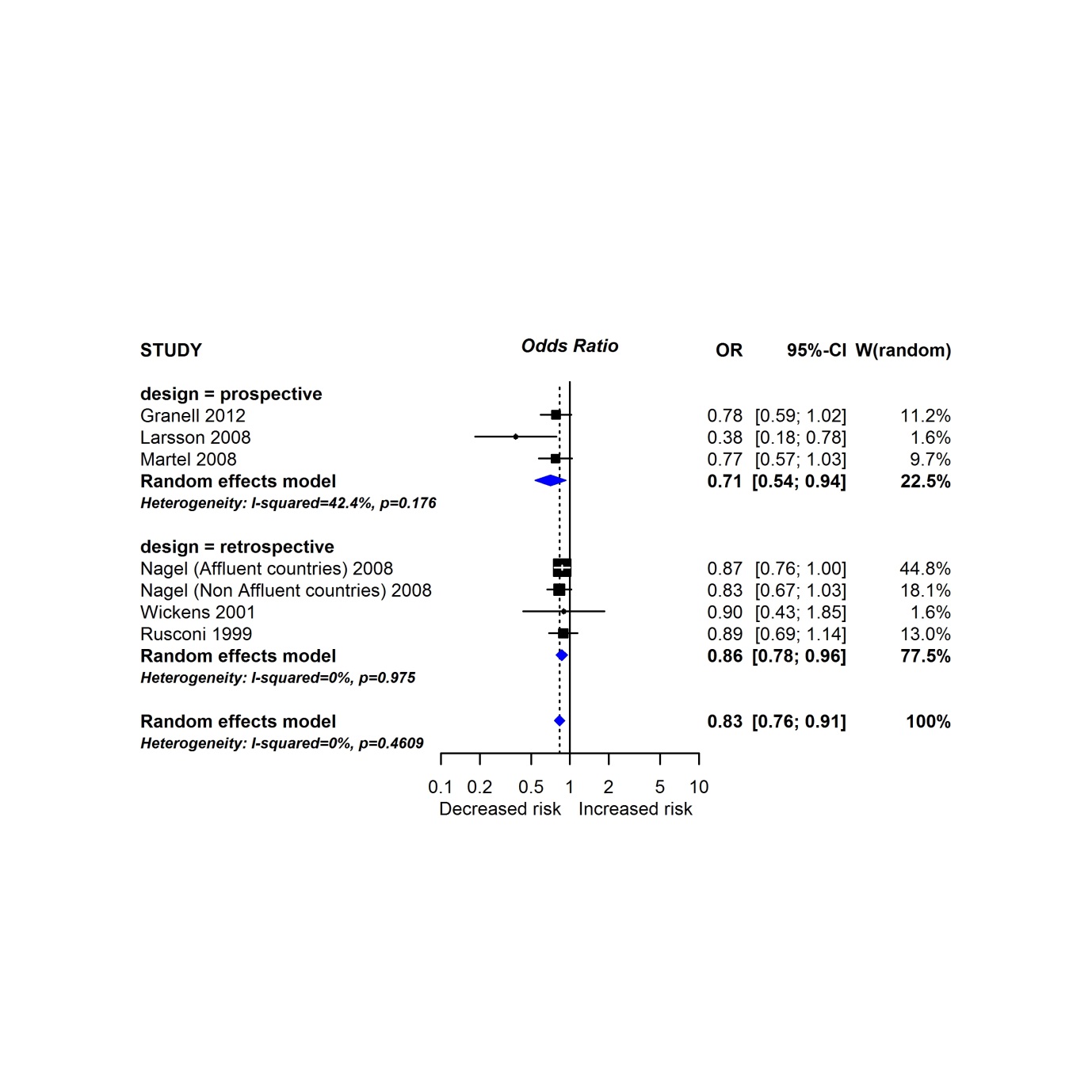


#### 1-2 Months

Two observational studies reported risk of recurrent wheeze in infants with TBF for ≥1-2 months vs. <1-2 months, and could not be pooled due to extreme heterogeneity (I^2^>80%). The study of Muino had an overall high risk of bias due to lack of adjustment for potential confounders, whilst the study of Sears reported adjusted data and had an unclear overall risk of bias (Figure 40).

Figure 40 Total breastfeeding for ≥1-2 months vs. <1-2 months and risk of recurrent wheeze in children aged 5-14 years


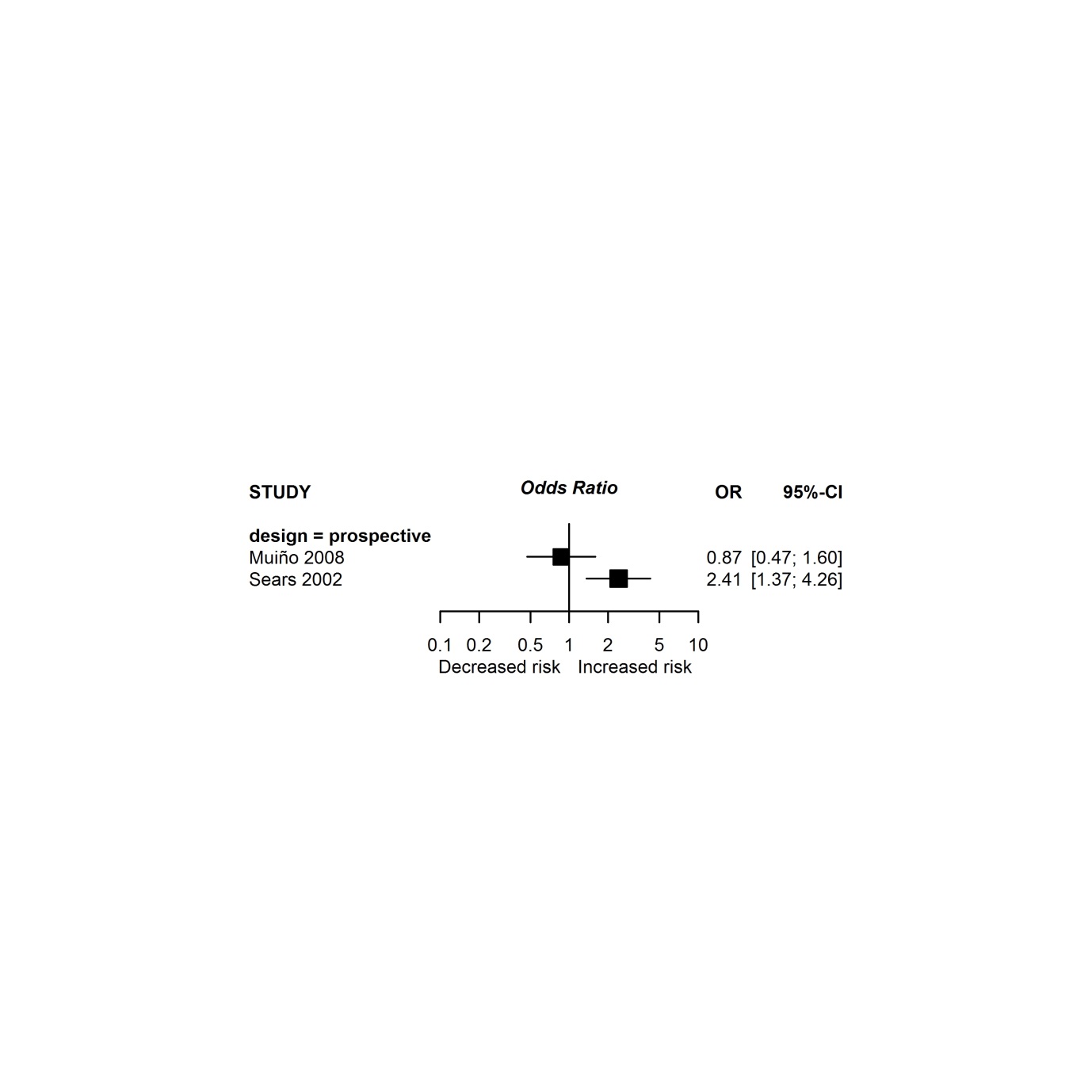


#### 3-4 Months

Figure 41 shows the pooled OR from ten observational studies that reported risk of recurrent wheeze at age 5-14 in infants who had TBF for ≥3-4 months vs. <3-4 months, showing a statistically significant association with reduced risk of wheeze (OR 0.76; 95% CI 0.67, 0.87), and moderate heterogeneity across studies (I^2^=33.7%). Six studies were prospective cohorts (4 of which had a low overall risk of bias, 1 had a high risk of bias due to lack of adjustment for potential confounders, and 1 had an unclear overall risk of bias), 2 were retrospective cohorts (1 with a high risk of bias due to lack of adjustment for confounders and 1 was considered to carry a low overall risk of bias), 1 nested case-control study (with unclear overall risk of bias) and one cross-sectional (with unclear overall risk of bias). Egger’s test showed that there was statistical evidence of publication bias (P=0.01) (Figure 42). Subgroup and stratified analyses (Table 8) did not show important group differences, and findings were similar in adjusted and unadjusted analyses.

Figure 41 Total breastfeeding for ≥3-4 months vs. <3-4 months and risk of recurrent wheeze in children aged 5-14 years


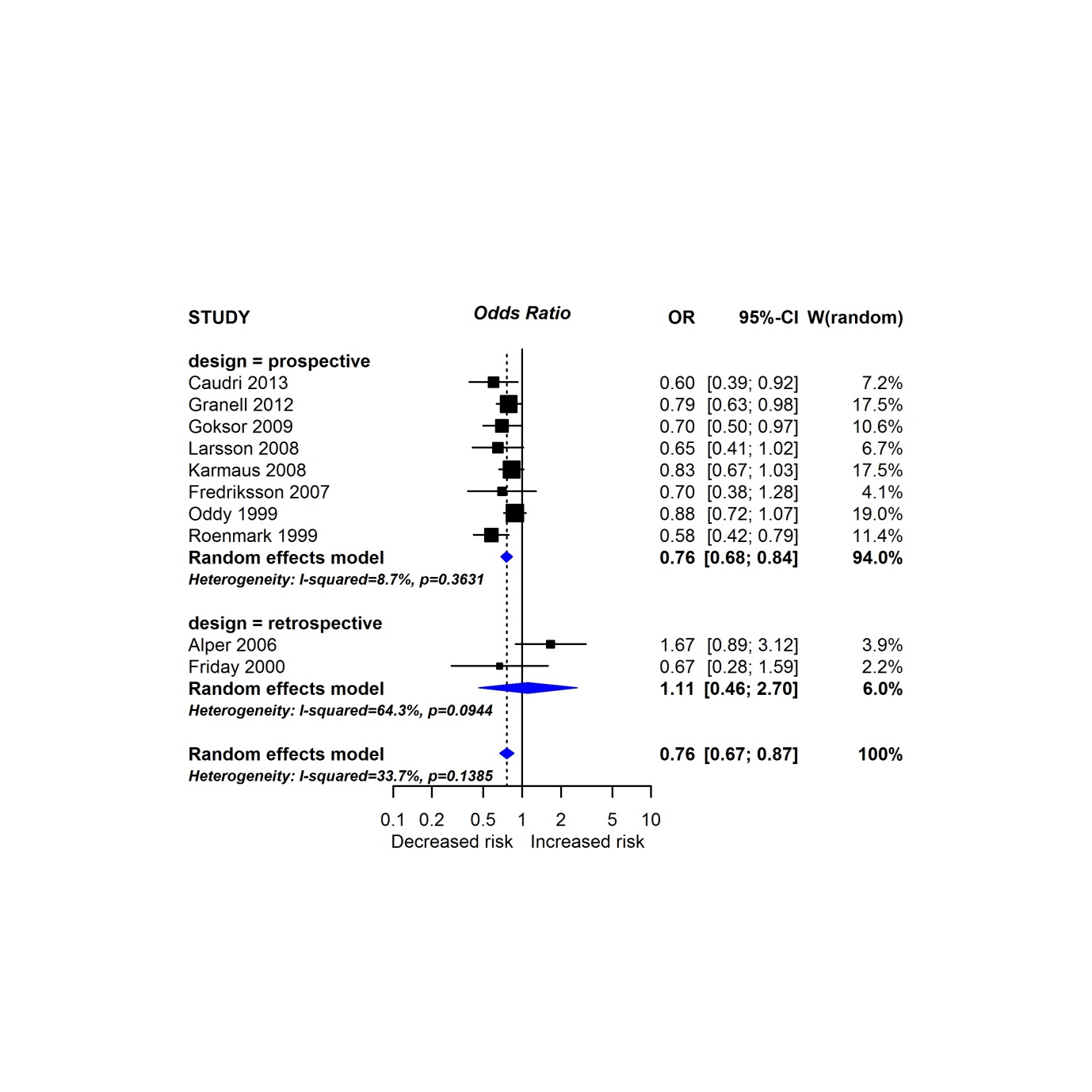


Figure 42 Risk of publication bias in studies investigating total breast feeding and recurrent wheeze ≥3-4 months vs. <3-4 months in children aged 5-14 years


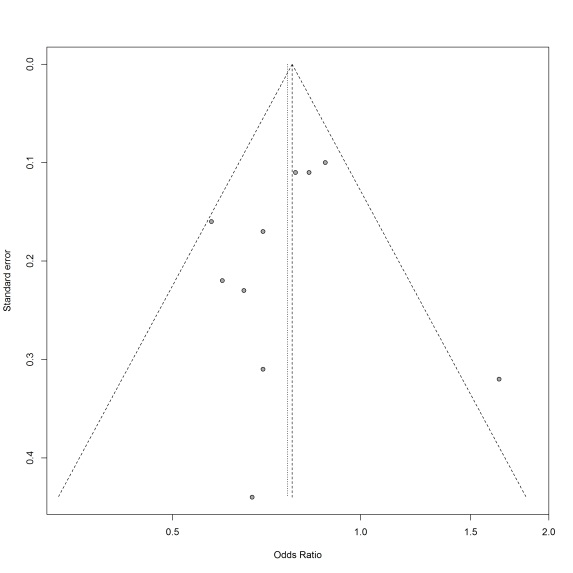


Egger’s test p-value = 0.012

Table 8 Subgroup Analysis of risk of recurrent wheeze and total breastfeeding ≥3-4 months vs. <3-4 months in children aged 5-14 years

|  | **Number of studies** | **OR [95% CI]** | **I^2^ (%)** | **P-value for between groups difference** |
| --- | --- | --- | --- | --- |
| **Overall (if adjusted NA, unadjusted value used)** | 10 | 0.76 [0.67; 0.87] | 33.7 |  |
| **Adjusted** | 5 | 0.74 [0.56; 0.96] | 48.0 | Not tested |
| **Unadjusted** | 7 | 0.67 [0.58; 0.77] | 0.0 |  |
| Study Design – Prospective | 8 | 0.76 [0.68; 0.84] | 8.7 | 0.40 |
| Study Design – Retrospective | 2 | 1.11 [0.46; 2.70] | 64.3 |  |
| Risk of disease – High | 1 | 0.67 [0.28; 1.59] | -- | 0.77 |
| Risk of disease – Normal | 9 | 0.76 [0.67; 0.88] | 40.5 |  |
| Risk of bias – Low | 5 | 0.80 [0.72; 0.90] | 0.0 | 0.74 |
| Risk of bias – High/Unclear | 5 | 0.76 [0.55; 1.05] | 54.6 |  |

#### 5-7 Months

Nine observational studies reported the risk of recurrent wheeze in infants who had TBF for ≥5-7 months vs. <5-7 months that was eligible to calculate the overall OR (Figure 43), showing a reduced overall risk of disease (OR 0.76; 95% CI 0.62, 0.92). There was no evidence of heterogeneity across studies (I^2^=0.0%). Four studies were prospective cohorts (of which 1 had a high overall risk of bias mainly due to selection bias, 1 had unclear risk of bias, and 2 had a low overall risk of bias), 1 retrospective cohort (carrying a high risk of overall bias due to lack of adjustment for potential confounders), 2 case-control (one of which lacked adjustment for potential confounders, therefore it carries a high risk of bias, and the other had unclear risk of bias) and 2 cross-sectional studies (both of which did not include adjustment for potential confounders, therefore carrying a high overall risk of bias). Funnel plot showed some asymmetry, but Egger’s test did not confirm any clear evidence of publication bias (Figure 44). Subgroup and stratified analyses (Table 9) did not show important group differences, and findings were similar in adjusted and unadjusted analyses.

Figure 43 Total breastfeeding for ≥5-7 months vs. <5-7 months and risk of recurrent wheeze in children aged 5-14 years


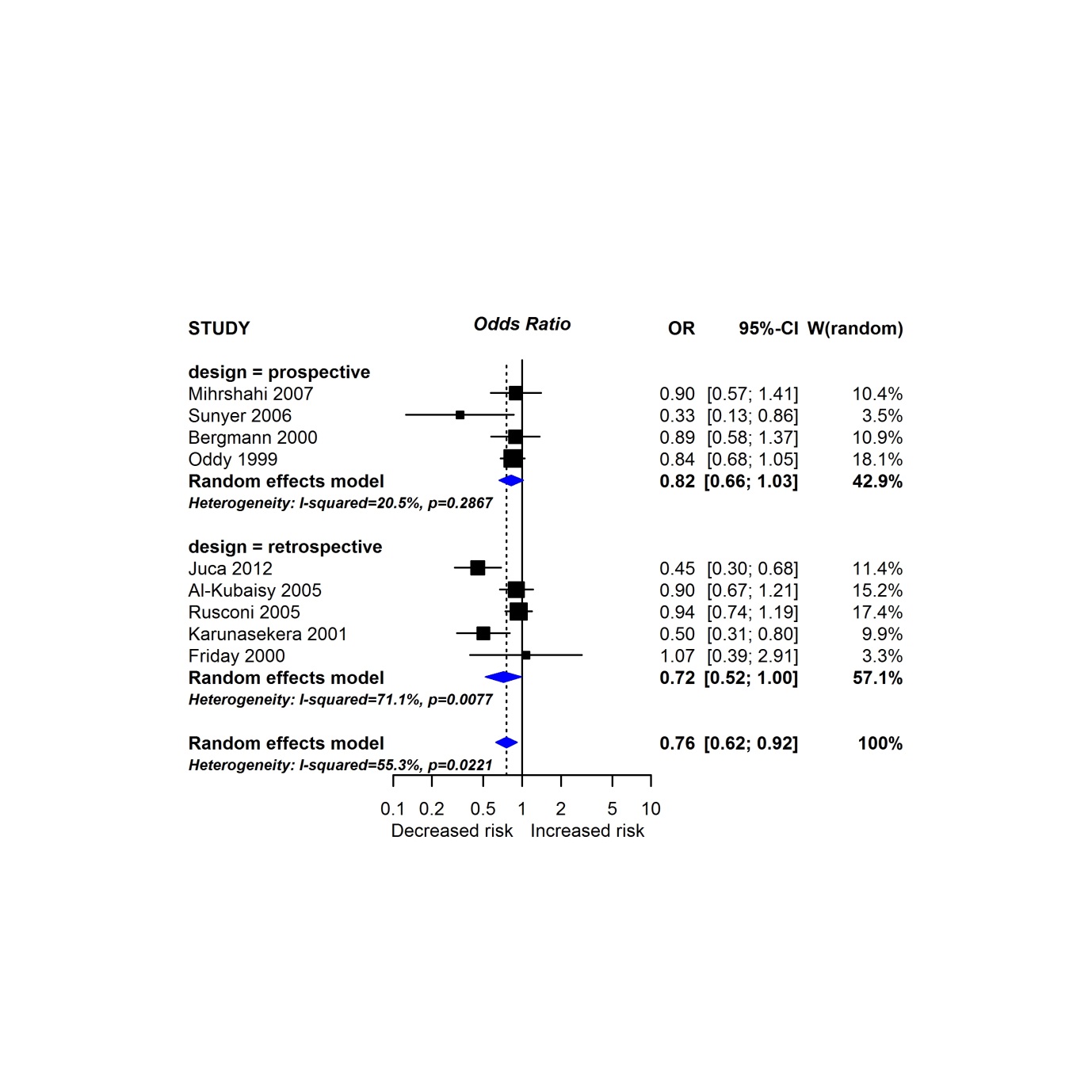


Figure 44 Risk of publication bias in studies investigating total breast feeding and recurrent wheeze ≥5-7 months vs. <5-7 months in children aged 5-14 years


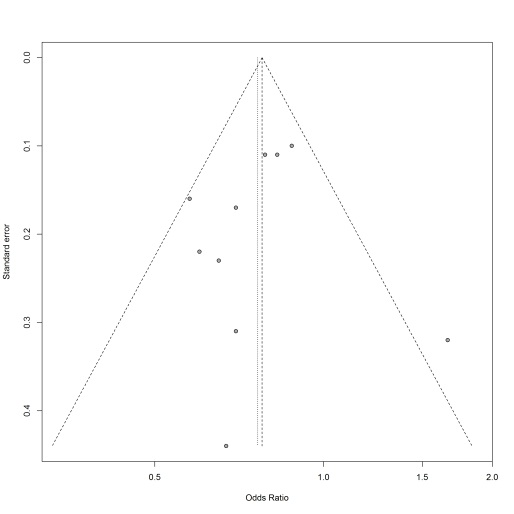


Egger’s test p-value = 0.637

Table 9 Subgroup Analysis of risk of recurrent wheeze and total breastfeeding ≥5-7 months vs. <5-7 months in children aged 5-14 years

|  | **Number of studies** | **OR [95% CI]** | **I^2^ (%)** | **P-value for between groups difference** |
| --- | --- | --- | --- | --- |
| **Overall (if adjusted NA, unadjusted value used)** | 9 | 0.76 [0.62; 0.92] | 55.3 |  |
| **Adjusted** | 5 | 0.74 [0.56; 0.96] | 48.0 | Not tested |
| **Unadjusted** | 6 | 0.72 [0.54; 0.97] | 71.1 |  |
| Study Design – Prospective | 4 | 0.82 [0.66; 1.03] | 20.5 | 0.50 |
| Study Design – Retrospective | 5 | 0.72 [0.52; 1.00] | 71.1 |  |
| Risk of disease – High | 2 | 0.92 [0.61; 1.39] | 0.0 | 0.32 |
| Risk of disease – Normal | 7 | 0.73 [0.58; 0.91] | 65.3 |  |
| Risk of bias – Low | 3 | 0.78 [0.55; 1.10] | 45.6 | 0.87 |
| Risk of bias – High/Unclear | 6 | 0.75 [0.57; 0.98] | 64.6 |  |

#### 18+ Months

One case-control study reported the risk of recurrent wheeze in children aged 5-14 years old if they were exposed to TBF for ≥18+ months vs. <18+ months, showing no indication of effect (Figure 45). The study did not adjust for potential confounders and therefore carried an overall high risk of bias.

Figure 45 Total breastfeeding for ≥18+ months vs. <18+ months and risk of recurrent wheeze in children aged 5-14 years


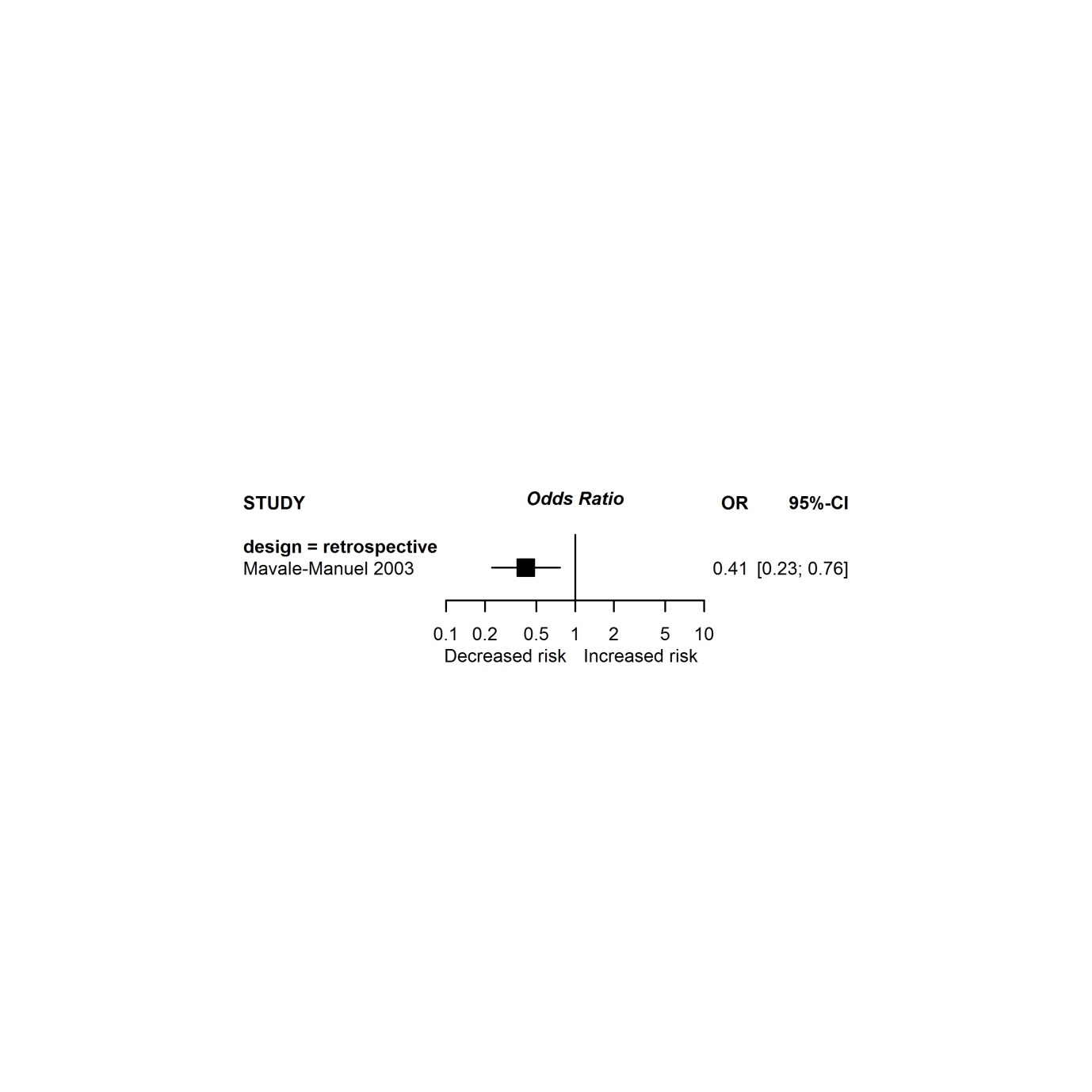


### Age at outcome measurement 15+

#### Ever vs. never

Six observational studies reported risk of recurrent wheeze in children over 15 years old if they were exposed to any TBF, showing no indication of an effect (OR 0.99; 95% CI 0.89, 1.11) (Figure 46). Three studies were prospective cohorts, all of which had an overall high risk of bias due to selection bias or lack of adjustment for relevant potential confounders. The other three studies were of cross-sectional design, one of which had a high risk of overall bias (due to selection bias), and the other two had a low or unclear overall risk of bias. The presence of high risk of bias in these studies might partly explain the high heterogeneity observed across studies (I^2^=73.2%). Subgroup and stratified analyses (Table 10) did not show important group differences, and findings were similar in adjusted and unadjusted analyses. There were insufficient data for meaningful dose response analysis for this comparison.

Figure 46 Total breastfeeding ever vs. never and risk of recurrent wheeze in children aged 15+ years


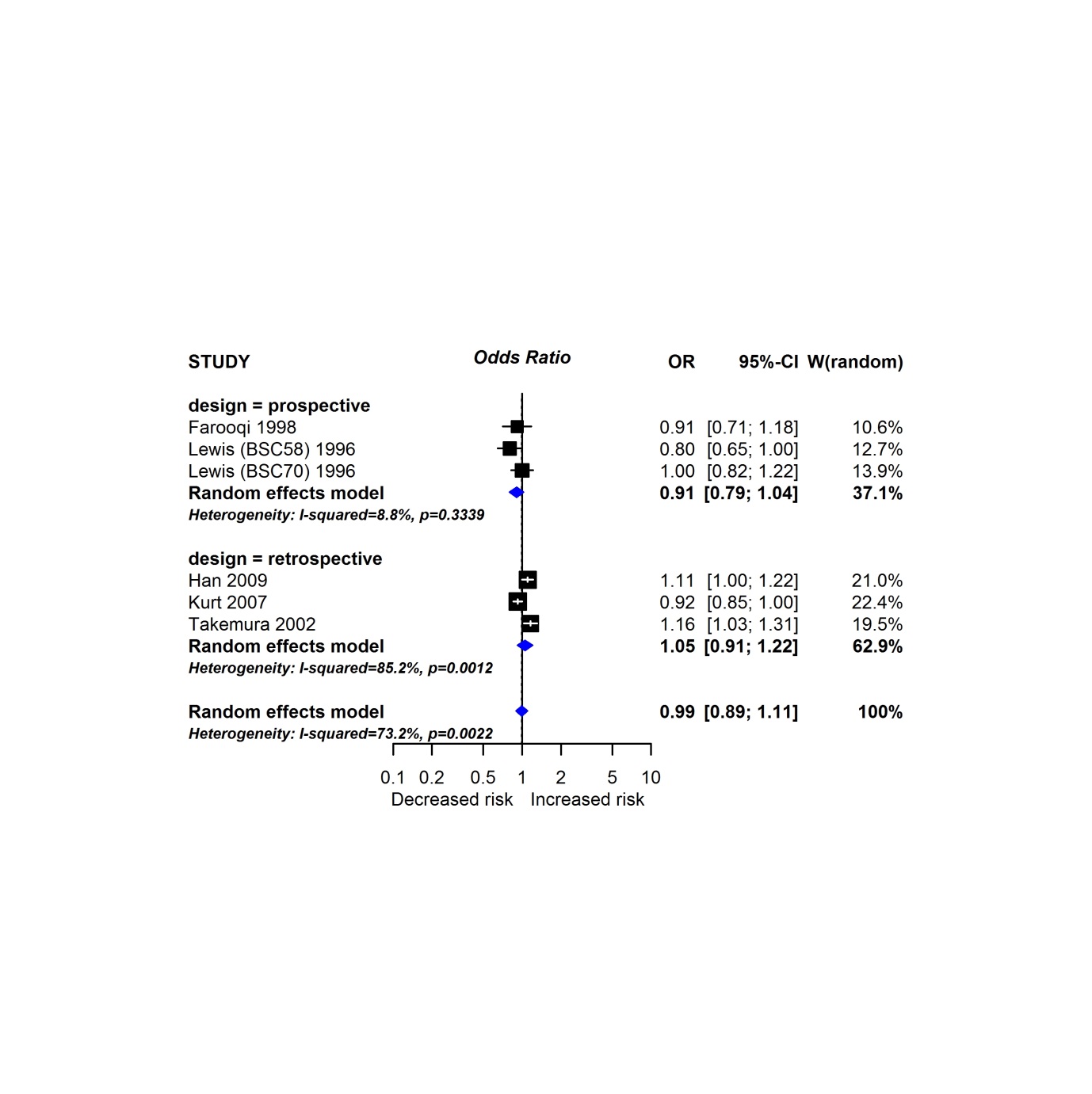


Table 10 Subgroup Analysis of risk of recurrent wheeze and total breastfeeding ever vs. never in children aged 15+ years

|  | **Number of studies** | **OR [95% CI]** | **I^2^ (%)** | **P-value for between groups difference** |
| --- | --- | --- | --- | --- |
| **Overall (if adjusted NA, unadjusted value used)** | 6 | 1.00 [0.89; 1.11] | 73.2 |  |
| **Adjusted** | 2 | 1.01 [0.85; 1.20] | 87.3 | Not tested |
| **Unadjusted** | 5 | 1.01 [0.90; 1.14] | 62.1 |  |
| Study Design – Prospective | 3 | 0.91 [0.79; 1.04] | 8.8 | 0.14 |
| Study Design – Retrospective | 3 | 1.05 [0.91; 1.22] | 85.2 |  |
| Risk of disease – High | -  6 | -  1.00 [0.89; 1.11] | -  73.2 | - |
| Risk of disease – Normal |  |  |  |  |
| Risk of bias – Low | 1 | 0.92 [0.85; 1.00] | -- | 0.18 |
| Risk of bias – High/Unclear | 5 | 1.02 [0.90; 1.15] | 63.8 |  |
| Clear definition of breastfeeding duration | 4 | 0.92 [0.86; 0.99] | 0.0 | 0.14 |
| Unclear definition of breastfeeding duration | 2 | 1.05 [0.91; 1.18] | 68.6 |  |

#### 1-2 Months

Two retrospective cohort reported the risk of recurrent wheeze in infants exposed to TBF for ≥1-2 months vs. <1-2 months. In both studies, the data were adjusted, and the studies had an unclear overall risk of bias. Both studies showed an increased risk of recurrent wheeze in children aged 15 or more who received TBF for≥1-2 months vs. <1-2 months, and this was confirmed in their combined effect estimate (OR 1.71; 95% CI 1.35, 2.16). There was no heterogeneity between studies (Figure 47).

Figure 47 Total breastfeeding for ≥1-2 months vs. <1-2 months and risk of recurrent wheeze in children aged 15+ years


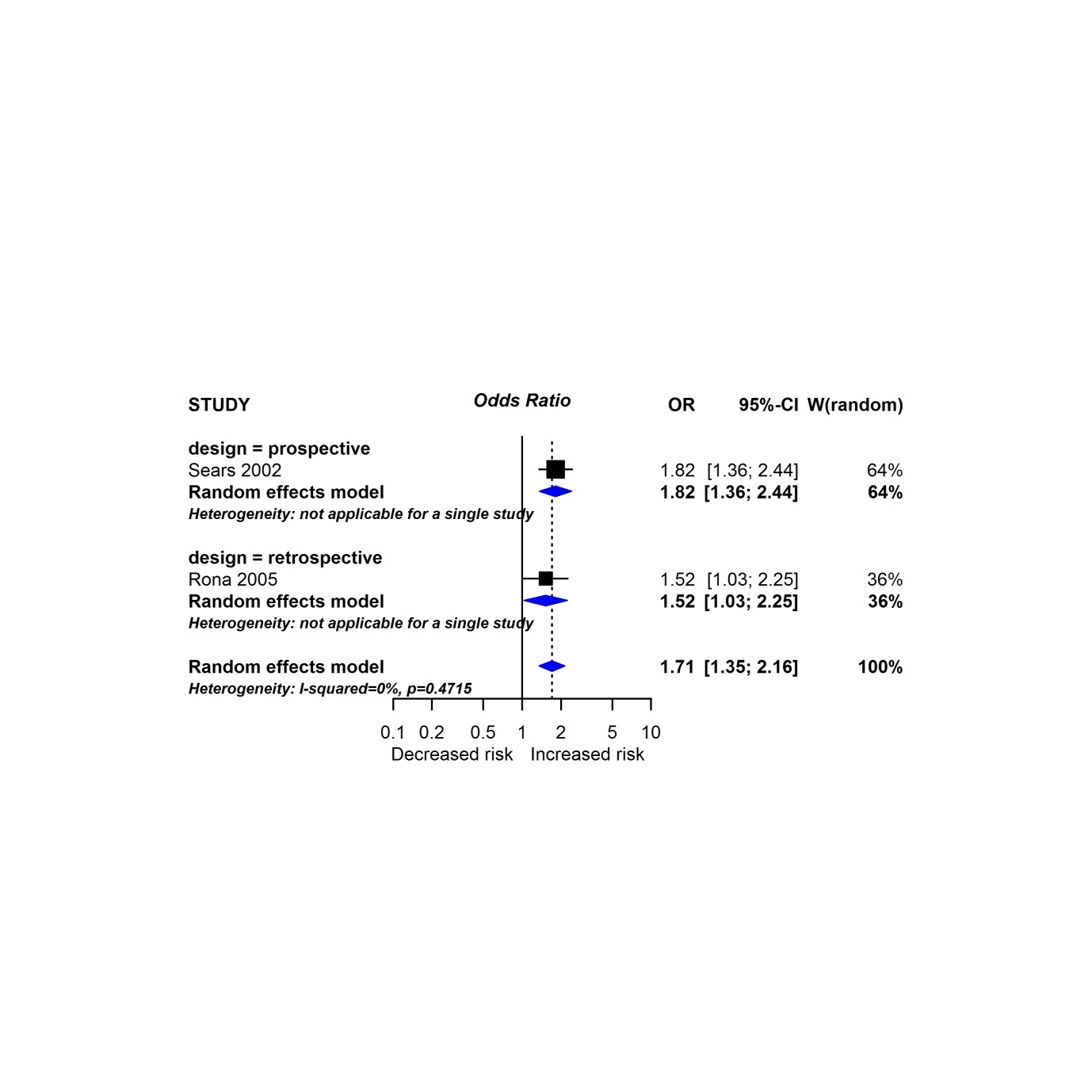


#### 3-4 Months

One prospective cohort study reported OR for recurrent wheeze in infants breastfed for ≥3-4 months vs. <3-4 months, and is shown in Figure 48. The data are unadjusted and had a high risk of selection bias, and therefore carry a high overall risk of bias, and show no significant difference in OR for recurrent wheeze in relation to breastfeeding status.

Figure 48 Total breastfeeding for ≥3-4 months vs. <3-4 months and risk of recurrent wheeze in children aged 15+ years

**
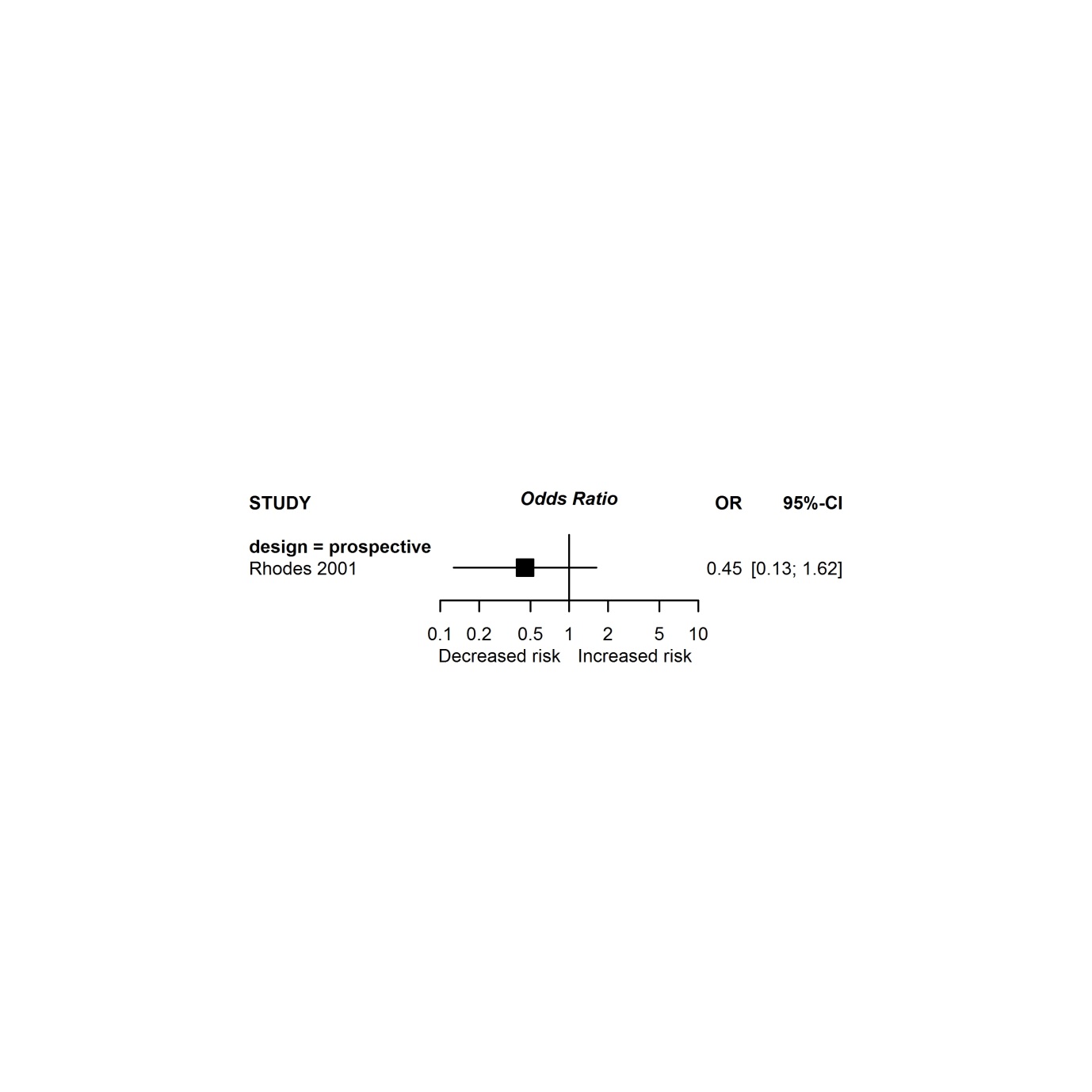
**

#### 5-7 Months

One cross-sectional study reported OR for recurrent wheeze in infants breastfed for ≥5-7 months vs. <5-7 months, and is shown in Figure 49. The data are adjusted, and carry a low overall risk of bias, and show a statistically significant difference in the OR for recurrent wheeze in relation to breastfeeding status (OR 0.79; 95% CI 0.70, 0.88).

Figure 49 Total breastfeeding for ≥5-7 months vs. <5-7 months and risk of recurrent wheeze in children aged 15+ years


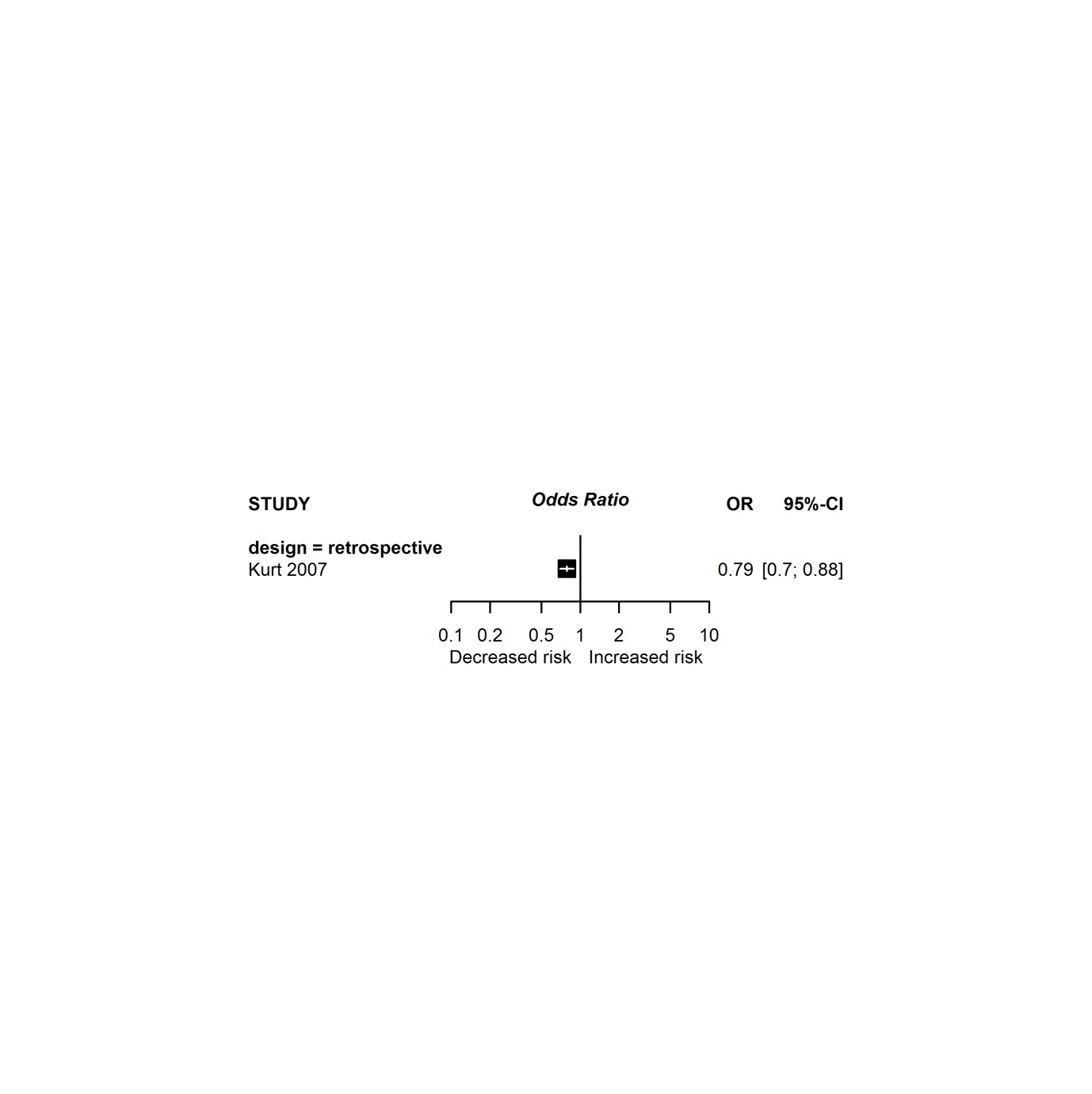


#### Total breastfeeding and bronchial hyper-responsiveness (BHR)

#### Age at outcome 5-14 years

#### Ever vs. never

Five observational studies reported ORs that could be pooled to assess the overall risk of BHR in children aged 5-14 years old if they were exposed to ever vs. never TBF. There was no suggestion of an association with BHR in the exposed group (Figure 50). Three studies were prospective cohorts and carry low or unclear overall risk of bias, and there were two cross-sectional studies with selection bias, and therefore carried an overall high risk of bias. Dose response analysis shows no evidence that prolonged TBF is associated with altered BHR (Figure 51-Figure 54).

Figure 50 Total Breastfeeding any duration vs. never and risk of bronchial hyper-responsiveness in children aged 5-14 years


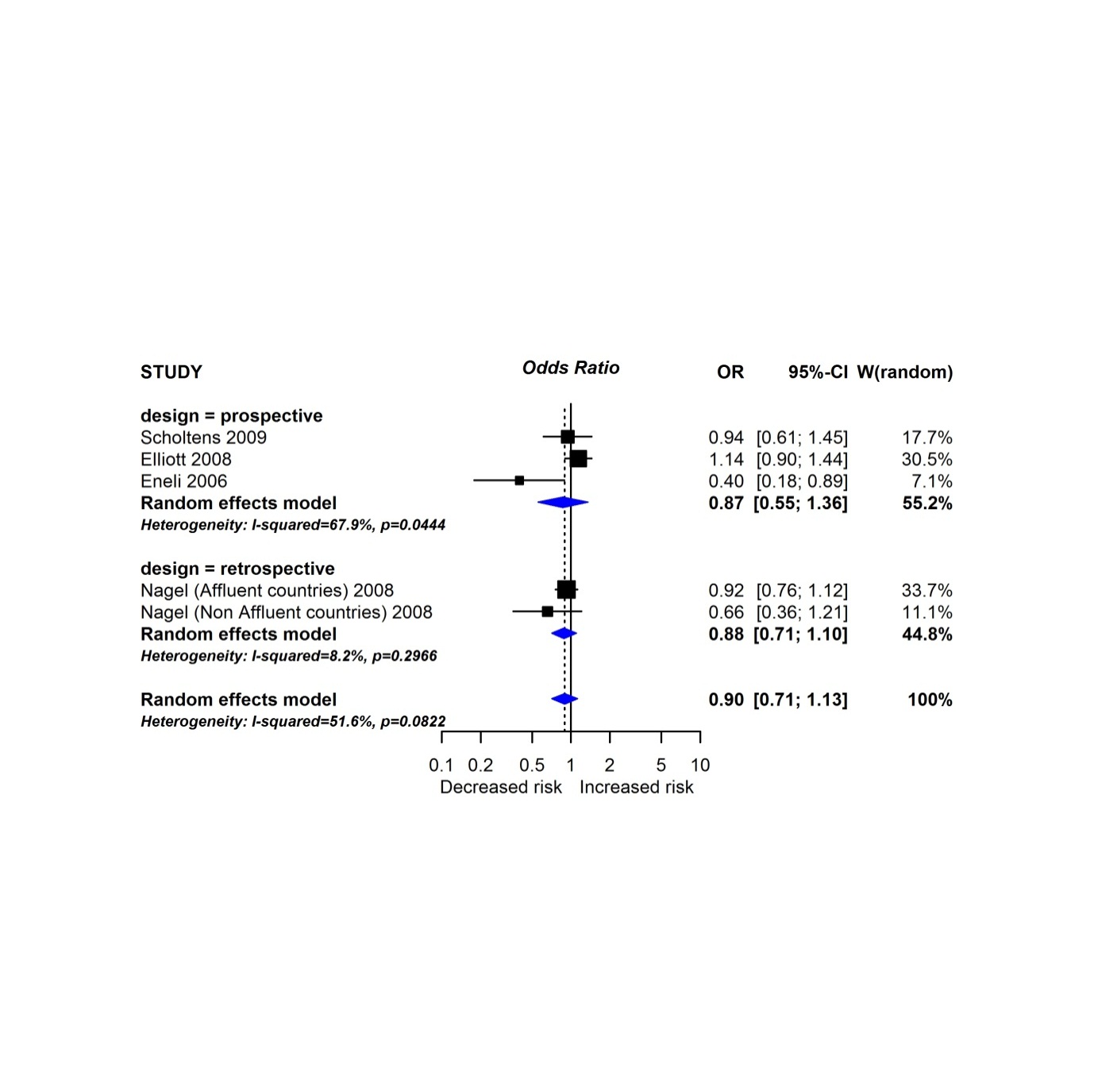


Figure 51 Total breastfeeding (dose response) never vs. ever and risk of bronchial hyper-responsiveness in children aged 5-14 years


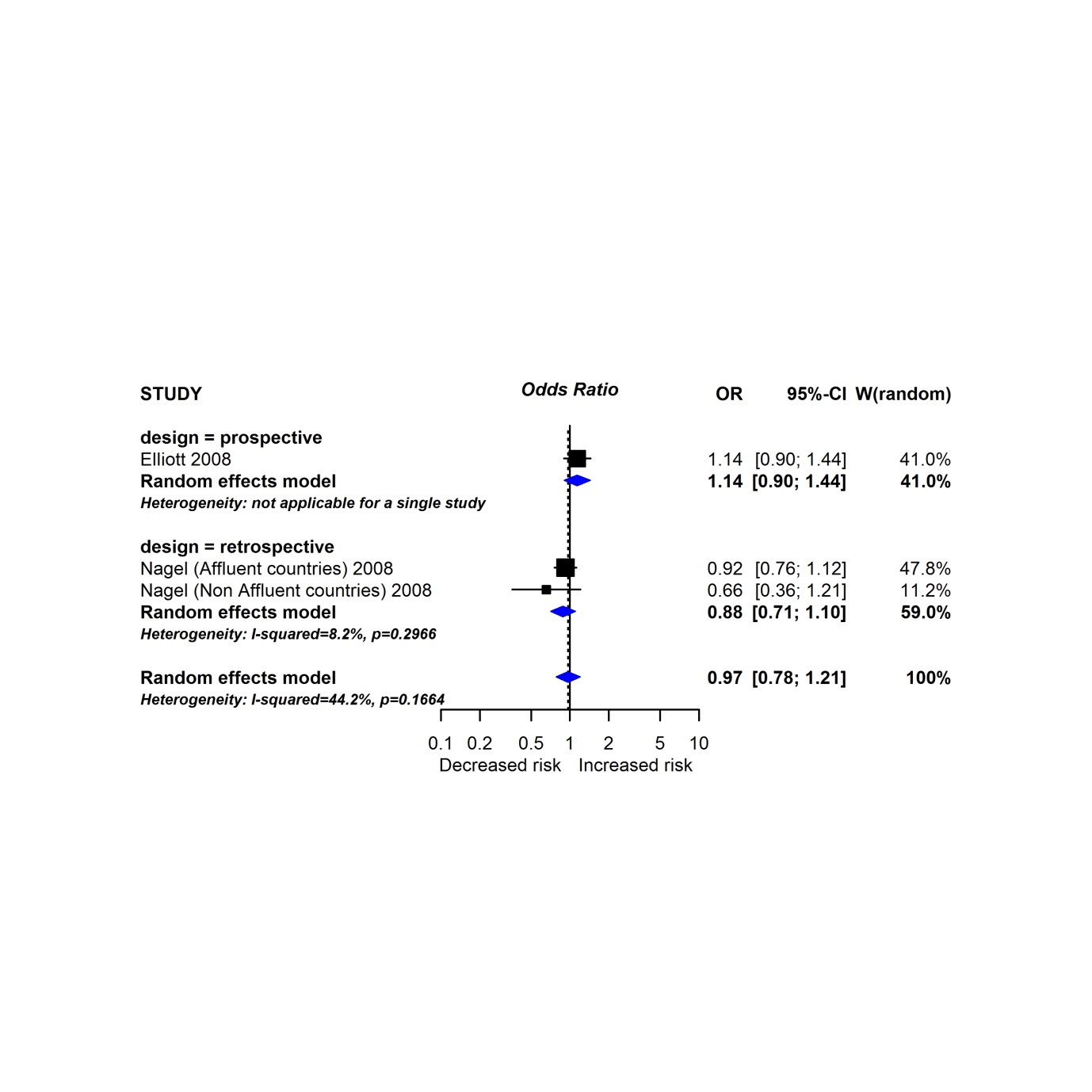


Figure 52 Total breastfeeding (dose response) short vs. never and risk of bronchial hyper-responsiveness in children aged 5-14 years


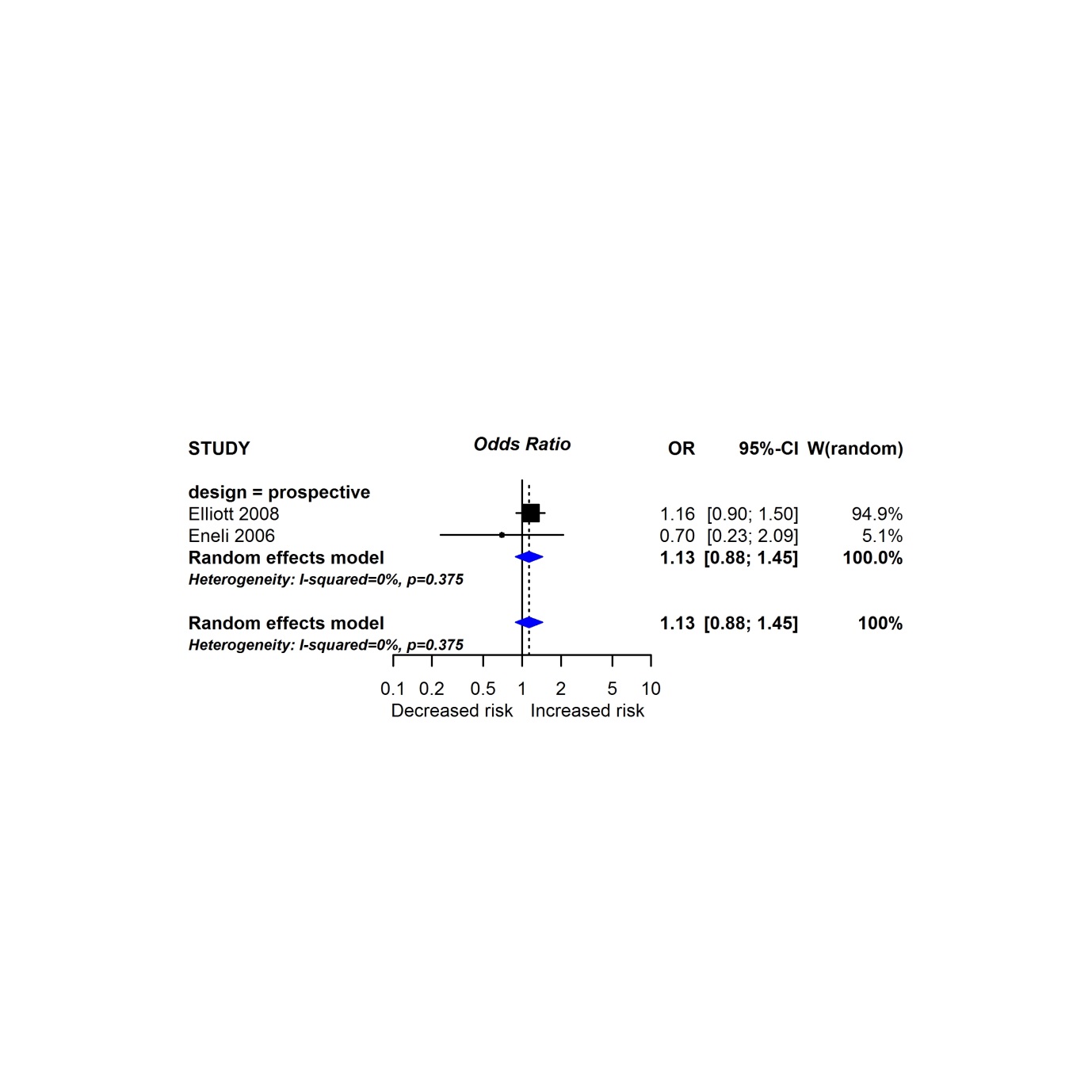


Figure 53 Total breastfeeding (dose response) medium vs. never and risk of bronchial hyper-responsiveness in children aged 5-14 years


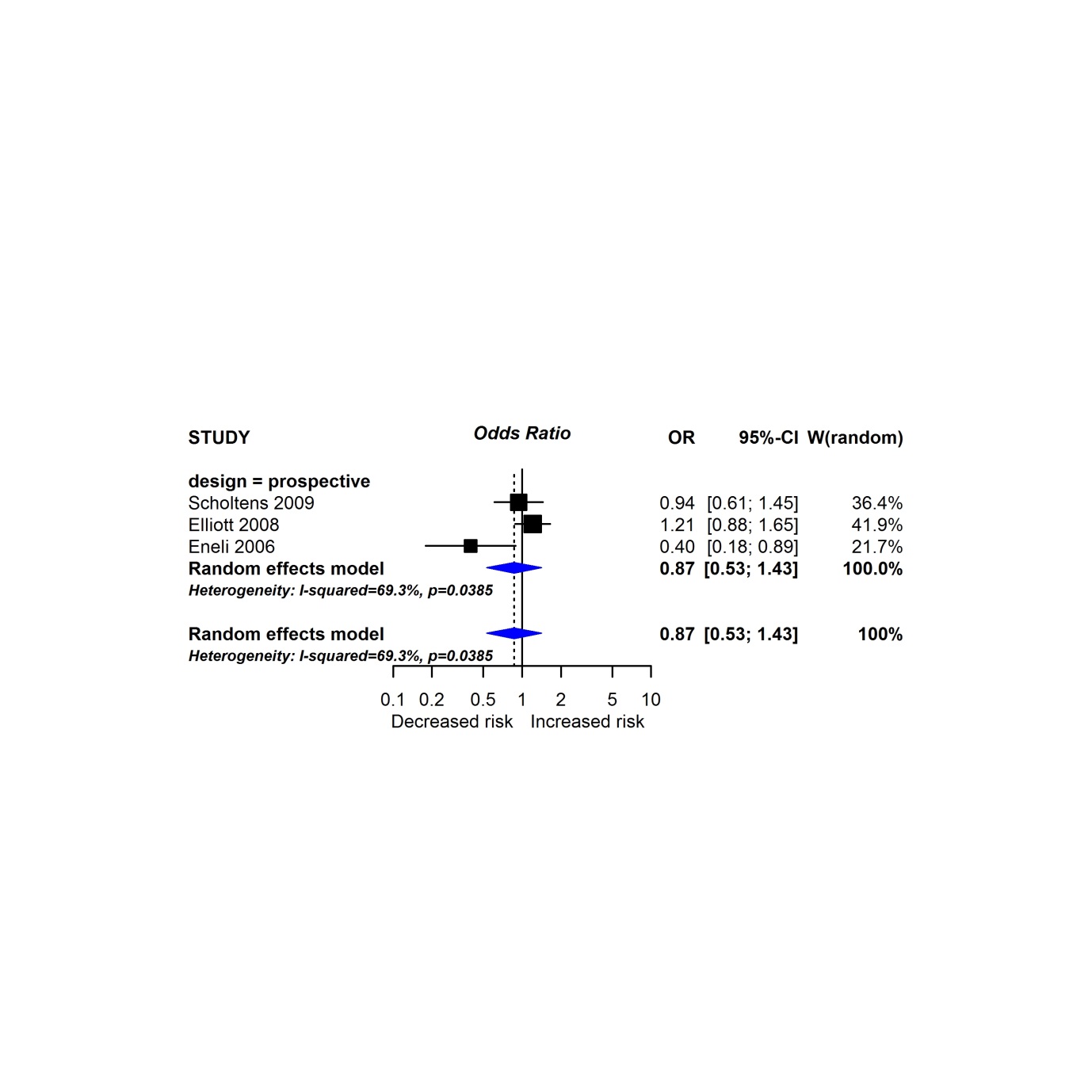


Figure 54 Total breastfeeding (dose response) long vs. never and risk of bronchial hyper-responsiveness in children aged 5-14 years


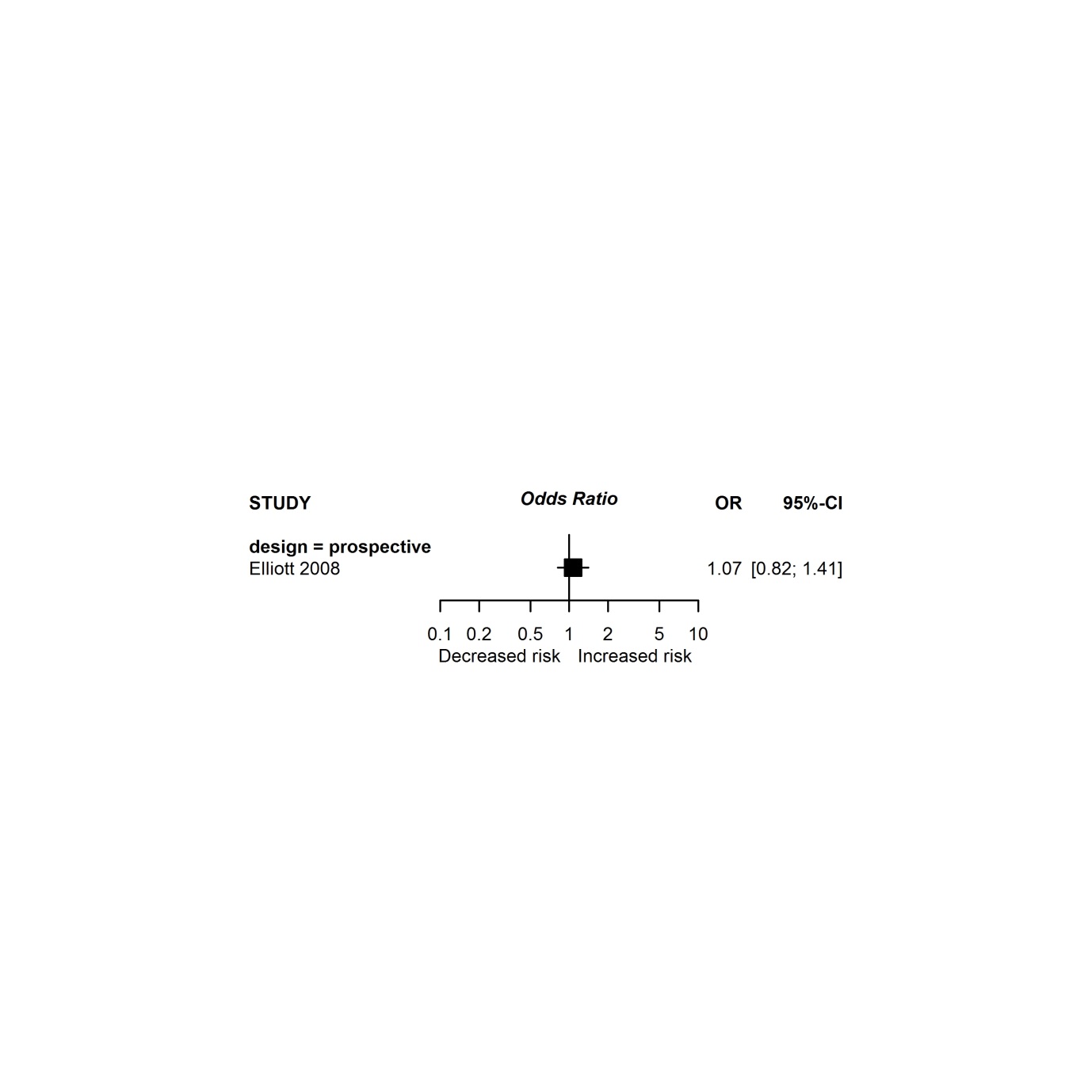


#### Age at outcome 15+ years

#### 1-2 months

One retrospective cohort study reported risk of BHR in young adults according to their exposure to ≥1-2 months vs. <1-2 months of TBF (Figure 55). They show no suggestion of an association, and the study had unclear overall risk of bias.

Figure 55 Total Breastfeeding ≥1-2 months vs. <1-2 months and risk of bronchial hyper-responsiveness in children aged 15+ years


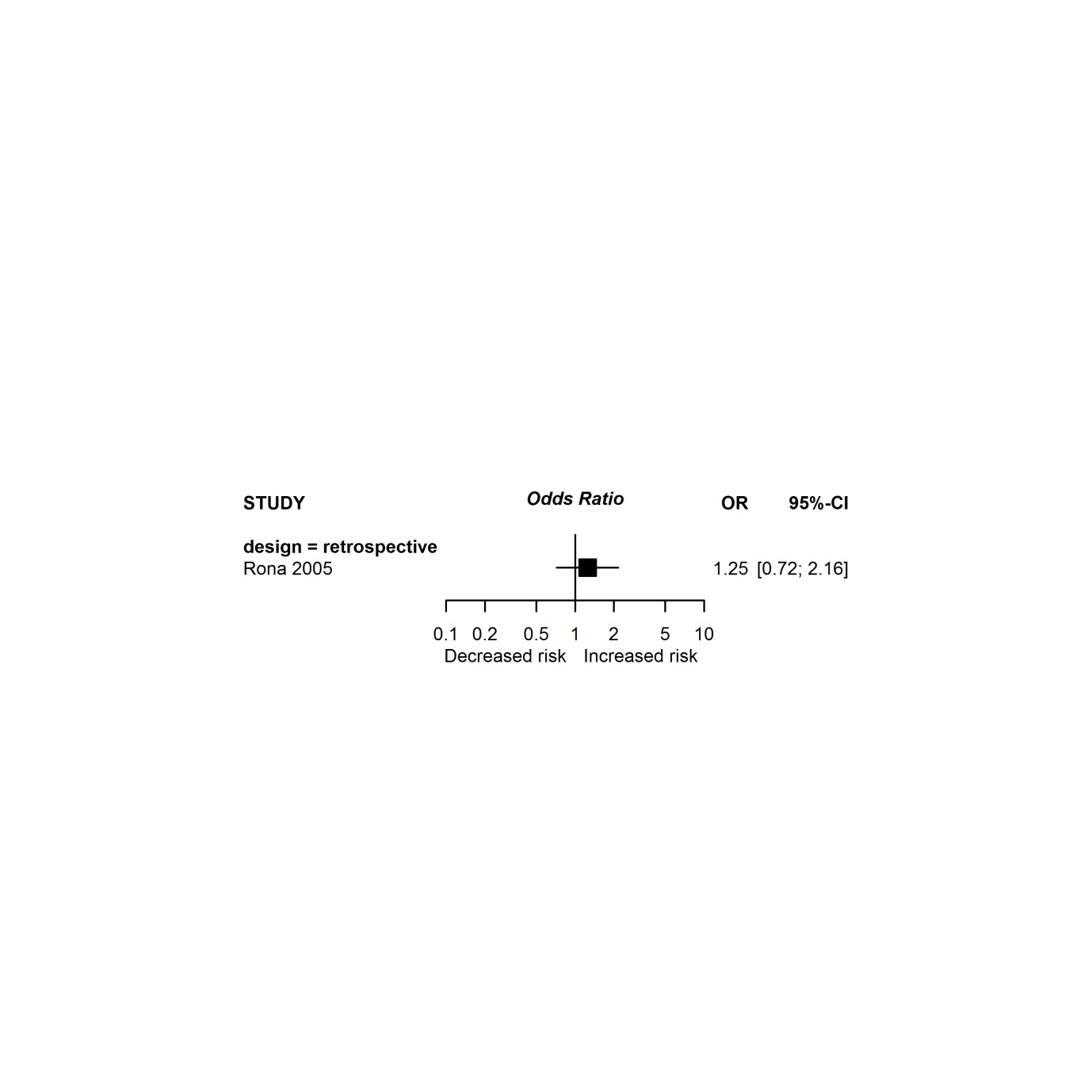


- 1. **Data for TBF duration and lung function in children aged 5-14 years**

### Outcome: FEV1(ml)

Two prospective cohorts had results based on analysis with linear regression and adjusted mean differences: (1) Ogbuanu comparing breastfeeding never (reference) versus ≥ 4 months at 10 years of age; (2) Dogaru breastfeeding never (reference) versus ≥ 6 months at 12.2 years of age.

**Figure 25 Total breastfeeding and FEV1 (ml) in children aged 5-14 years**


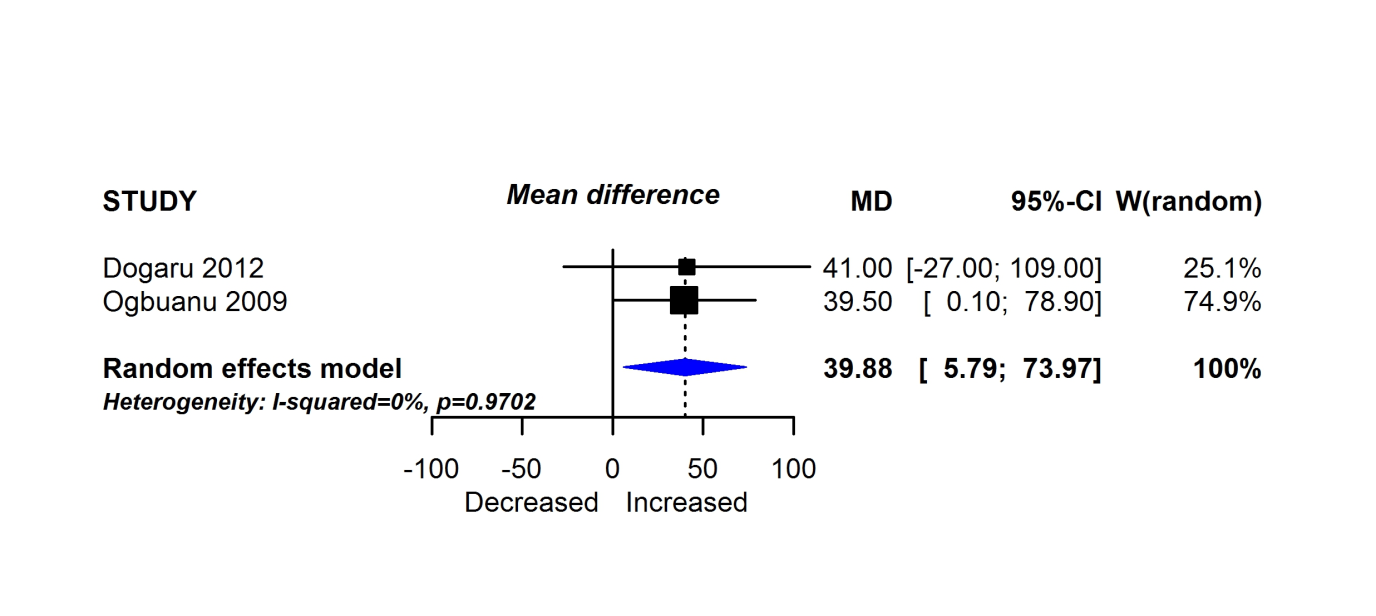


### Outcome: PEF (ml/sec)

Two prospective cohorts had results with adjusted mean differences based on linear regression: (1) Ogbuanu comparing breastfeeding never (reference) versus ≥ 4 months at 10 years of age; (2) Dogaru breastfeeding never (reference) versus ≥ 6 months at 12.2 years of age.

**Figure 26 Total breastfeeding and PEF (ml/sec) in children aged 5-14 years**


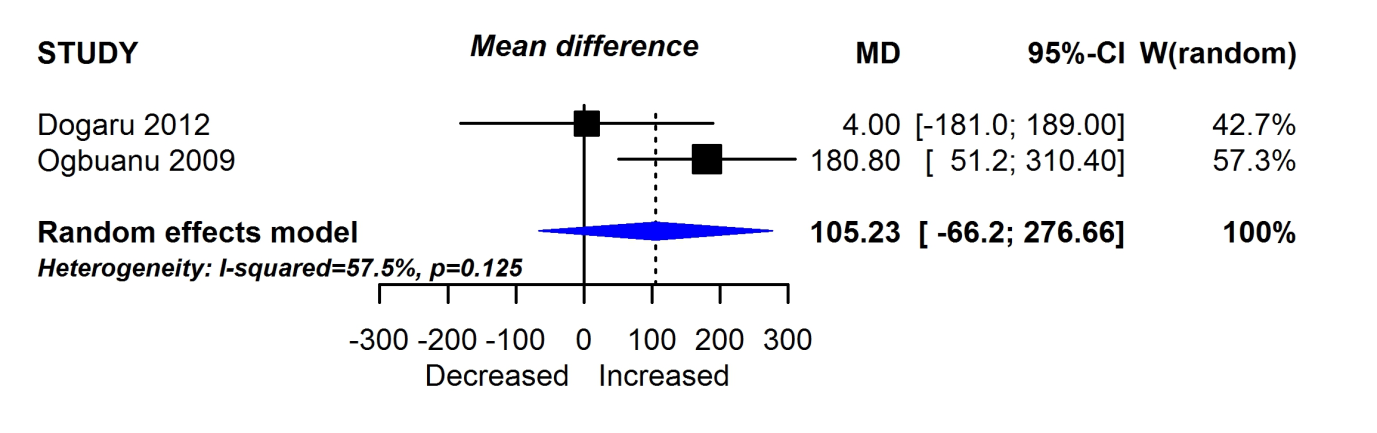


### Outcome: FEV1% predicted

One large multicentre cross-sectional study, Nagel presented 2 adjusted estimates of mean ratio (MR) comparing breastfeeding never (reference) versus < 6 months, separately for affluent and non-affluent countries, based on ISAAC methodology, in children aged 8-12 years of age.

**Figure 27 Total breastfeeding and FEV1% predicted in children aged 5-14 years**


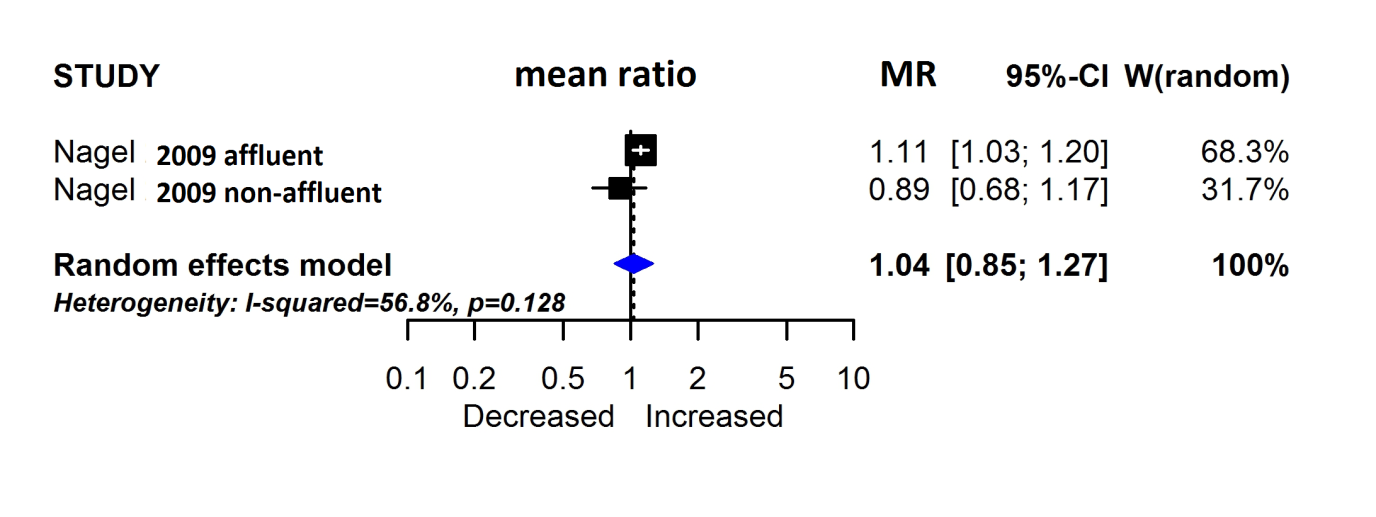


### Outcome: FVC (ml)

Two prospective cohorts had results with adjusted mean differences based on linear regression: (1) Ogbuanu comparing breastfeeding never (reference) versus ≥ 4 months at 10 years of age; (2) Dogaru breastfeeding never (reference) versus ≥ 6 months at 12.2 years of age.

**Figure 28 Total breastfeeding and FVC (ml) in children aged 5-14 years**


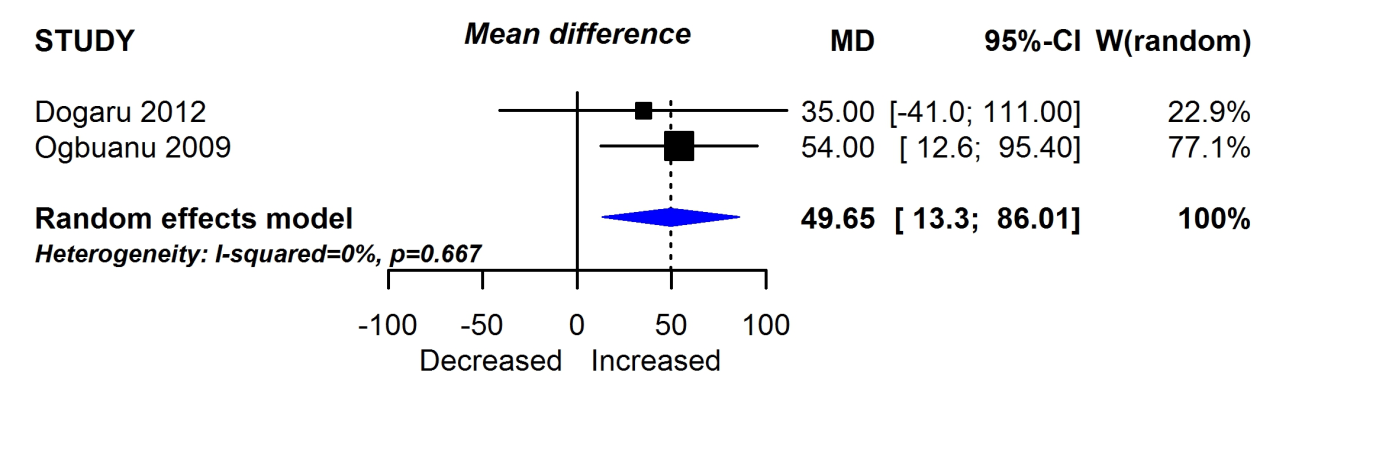


### Data for TBF duration and wheeze, BHR or lung function not included in meta-analysis

A total of 36 studies with data on wheeze and TBF exposure, not eligible for meta-analyses were identified (Table 11). Wheeze was measured between age 1 year and 36 years. There were 25 prospective cohorts, 1 retrospective cohort, 1 case-control, 1 a nested case-control, and 8 cross-sectional studies. 7 prospective cohort studies showed a negative association between wheeze (n=1) or recurrent wheeze (n=6) and TBF, whilst 2 cross-sectional studies showed a negative association between recurrent wheeze and TBF. Twenty one studies showed no evidence of an association between wheeze outcomes and TBF. Four studies showed a borderline association (P=0.05) and 2 studies had no data available to estimate size effect.

Data on the association between lung function outcomes and TBF from seven studies that could not be meta-analysed are summarised in Table 12. When P value was not reported in the paper, it was calculated (Altman, D. G. and J. M. Bland (2011). "How to obtain the P value from a confidence interval." BMJ **343**: d2304). Two studies were cross-sectional and five were prospective cohorts. Outcomes studied included forced expiratory volume in 1 second (FEV_1_), forced expiratory mid flow (FEF_50_), airflow obstruction (FEV_1_/FVC<0.80), FEV_1_/FVC as percentage of predicted value, FVC and BHR. Overall of 7 studies, 3 found a positive and statistically significant association between longer duration of TBF and at least one measure of lung function; however findings were not consistent between studies, and between meta-analyses and studies not included in meta-analysis.

Table 11 Studies investigating the association between total breastfeeding and wheeze which were not eligible for meta-analysis

| **First Author and year of publication** | **Design** | **Outcome** | **Age** | **N/n** | **TBF duration (continuous or categorical in months)** | **Measure of association** | **Effect** | **P-value** |
| --- | --- | --- | --- | --- | --- | --- | --- | --- |
| Munro, 2011 ([28](#_ENREF_28)) | PC | wheeze | 1 | 700 | continuous per week | OR (95%CI) average | 0.94  (0.97, 1.00) | <0.05 |
| Soto-Ramirez, 2013  ([40](#_ENREF_40)) | PC | wheeze | 1 | 2833 | continuous | Adjusted RR average | 1.26  (1.08, 1.47) | <0.05 |
| Kerr, 1981 ([85](#_ENREF_85)) | PC | wheeze | 0.5 | 269 | 0+ | average |  | NS |
| Hagendorens, 2005 ([89](#_ENREF_89)) | PC | wheeze | 1 | 693 | 0+ |  |  | NS |
| Sunyer, 2001 ([100](#_ENREF_100)) | PC | wheeze | 4 | 596 | 0+ |  |  | NS |
| Porro, 1993 ([86](#_ENREF_86)) | CC | wheeze | <1.6 | 465/266 | >1 |  |  | NS |
| Wright, 1989 ([105](#_ENREF_105)) | PC | wheeze | 1 | 949 | >1 |  |  | NS |
| Morgan, 2004 ([66](#_ENREF_66)) | PC | wheeze, recurrent wheeze | 1.5 | ? | >3 |  |  | NS |
| Devereux, 2006 ([23](#_ENREF_23)) | PC | wheeze | 5 | 1704 | 0+ | Adjusted OR |  | NS |
| McConnochie, 1986 ([63](#_ENREF_63)) | RC | wheeze | 8 | 223/29 | 0+ | Adjusted OR | 2.1 | 0.05 |
| Mann, 1992 ([67](#_ENREF_67)) | PC | wheeze | 36 | 2139 | 0+ |  |  | NS |
| da Costa Lima, 2003 ([81](#_ENREF_81)) | PC | Recurrent wheeze | 18 | 2247 | 0+, 3+, 6+, 9+, 12+ | Adjusted PR (95%CI) | Increased recurrent wheeze with longer TBF | NS for 0+, 3+, 6+;  P<0.05 for 9+, 12+ |
| Burr, 1993 ([13](#_ENREF_13)) | PC | wheeze-atopic | 7 | 453 | continuous |  |  | NS |
| Silvers, 2009 ([76](#_ENREF_76)) | PC | recurrent wheeze | 1, 5 | 987 | continuous | Adjusted OR (95%CI) | 0.93  (0.89-0.97) | <0.05 |
| Silvers, 2012 ([75](#_ENREF_75)) | PC | recurrent wheeze | 5 | 984 | continuous | Adjusted OR (95%CI) average | 0.99 (0.96-1.0) | NS |
| Hesselmar 2010 ([5](#_ENREF_5)) | PC | recurrent wheeze | 1.5 | 184 | continuous | Median (IQR) |  | - |
| Oddy, 2004 ([110](#_ENREF_110)) | NCC | recurrent wheeze | 8 | 335/166 | continuous | Adjusted OR average |  | NS |
| Klinnert, 2001 ([55](#_ENREF_55)) | PC | recurrent wheeze | 8 | unclear | continuous | average |  | NS |
| Awasthi, 2004 ([48](#_ENREF_48)) | CS | recurrent wheeze | 7 | 2471 | 0+ | Adjusted OR |  | NS |
| McConnochie, 1986 ([63](#_ENREF_63)) | RC | recurrent wheeze | 8 | 223/15 | 0+ | RR | 2.6 | 0.05 |
| Midodzi, 2010 ([18](#_ENREF_18)) | PC | recurrent wheeze | 5 | 8499/888 | 0+ | Adjusted HR (95%CI) | 0.8 (0.7-0.97) | <0.05 |
| Saarinen, 1995 ([97](#_ENREF_97)) | PC | recurrent wheeze | 5, 17 | unclear | >1 | OR | <1 | <0.05 |
| Nwaru, 2013 ([24](#_ENREF_24)) | PC | recurrent wheeze | 5 | 3142 | >5 | Adjusted HR (95%CI) | 1.9 (1.2-3.0) | <0.05 |
| Nwaru, 2013 ([24](#_ENREF_24)) | PC | atopic wheeze | 5 | 3675 | >5, >9 | Adjusted HR |  | NS |
| Evenhouse, 2005 ([71](#_ENREF_71)) | CS | recurrent wheeze | 12-18 | 16903 | 0+, continuous | Adjusted OR |  | NS |
| Karino, 2008 ([51](#_ENREF_51)) | CS | recurrent wheeze | 18 | 9615 | 0+, >1, >3, >6 |  |  | NS |

**Table 12 Studies investigating the association between total breastfeeding and lung function which were not eligible for meta-analysis**

| **First Author and year of publication** | **Outcome** | **Exposure variable** | **Comparison**  **(reference)** | **Age at outcome** | **Statistics and results** | | |
| --- | --- | --- | --- | --- | --- | --- | --- |
|  |  |  |  |  | **Analysis** | **Result**  **95% C.I.** | **P value** |
| Soto-Ramırez 2012 (114) | FEV1(ml) | continuous | ml/week | 10-18 | linear mixed model, adjusted | 1.21 (0.07; 2.35) | 0.03 |
| Tennant 2008 (116) | FEV1(ml) | categorical | <4mo (ref.) vs ≥ 4mo | 50 | linear regression, adjusted | -148 (-269; -027)**^2^** | 0.02 |
| Tennant 2010 (119) | FEV1(ml) | categorical | <1mo (ref.) vs ≥1mo | 14 | linear regression, adjusted | 115 (13; 218) | 0.03 |
| Ogbuanu 2009 (113) | FEV1/FVC% | categorical | 0 (ref) vs ≥4mo | 10 | linear regression, adjusted | -0.4 (-1.44; 0.64) | 0.45 |
| Soto-Ramırez 2012 (114) | FEV1/FVC% | continuous | % increase per week | 10-18 | linear mixed model, adjusted | -0.001 (-0.02; 0.02) | 0.92 |
| Dogaru 2012 (115) | FEF50 (L/s) | categorical | 0 (ref) vs ≤3mo | 12.2 | linear regression, adjusted | 0.04 (0.06; 0.14) | **0.05*** |
| Dogaru 2012 (115) | FEF50 (L/s) | categorical | 0 (ref) vs 4-6mo | 12.2 | linear regression, adjusted | 0.13 (0.01; 0.26) | **0.05** |
| Dogaru 2012 (115) | FEF50 (L/s) | categorical | 0 (ref) vs >6mo | 12.2 | linear regression, adjusted | 0.12 (0.01; 0.22) | **0.03** |
| Suwanpromma 2012 (117) | FEV1/FCV<0.8 | categorical | <3mo (ref) vs ≥3mo | 6-18 | Odds ratio, model, adjusted | **0.87 (0.59; 2.24)** | **<0.05** |
| Soto-Ramırez 2012 (114) | FVC (ml) | continuous | mL/week | 10-18 | linear mixed model, adjusted | 1.48 (0.30; 2.66) | 0.01 |
| Guilbert 2007 (176) | FVC (ml) | categorical | <1mo (ref) vs >4mo | 11-16 | random-effects model, adjusted | 103 (24.6; 181.4)**^8^** | **0.01** |
| Rona 2005 (90) | Bronchial hyper-responsiveness | categorical | ≥1-2mo vs <1-2mo | ≥15 | polytomous logistic regression, adjusted | 1.25 (0.72-2.16) | **0.43** |

*Note that there may be a data entry error in this paper, since the mean is not within the 95% confidence interval. It is possible that the correct finding is 0.04 (-0.06, 0.14) which is not statistically significant.

### Conclusion on association between TBF duration and lung function and bronchial hyper-responsiveness

The majority of the studies (**~**70%) reported at least one result indicating improvement in lung function, and the majority of these study estimates (around 70%) were statistically significant. However, findings were not consistent between studies or across closely related measures of lung function. Therefore we assessed the body of evidence as being inconclusive.

# Exclusive breastfeeding and wheeze

## Overall characteristics of studies, risk of bias and summary of results

Table 13 describes the main characteristics of the studies that assessed exclusive breastfeeding duration (EBF) in relation to wheeze risk. 2 systematic reviews reported combined risk of exposure to EBF and risk of wheeze. A total of 62 observational studies reported the association between EBF and wheeze. Of the observational studies, 42 were prospective cohort studies, 5 case-control studies, 3 nested case-control studies and 12 cross-sectional studies. The majority of studies (n=27) are from Europe – others are from the Asia-Pacific region (n=14) and North America (n=8), 7 from South America, 2 from South Asia and 2 from the Middle East. There are also 2 studies, which included multiple countries.

Overall, valid data on EBF duration in the first 2 years of life and wheeze risk were available from over 220,000 subjects. Information on wheeze was obtained solely from parental or self-reported in 13 studies, through Dr-diagnosis in 12 studies, and from the ISAAC questionnaire in 14 studies. One other study used spirometry alone. Another 18 studies used a combination of self /parental report, Doctor diagnosis, and/ or objective measures (e.g. bronchial hyper-responsiveness (BHR)). One study used the Canadian Asthma Consensus Guidelines. Three studies used an unclear method for defining wheeze.

With regards to time of outcome diagnosis, 25 studies explored the association between EBF duration and wheeze at age 0-4 years, 18 at age 5-14 years, and 2 at age 15 years or beyond. A further 15 studies investigated the association between EBF duration and wheeze at various time points between the ages of 1 and 22 years. Two studies examined children at a range of ages (8-18, 6-15). To ascertain exposure to EBF, 31 studies used a questionnaire method, 14 used an interview and 1 used a diary. A further 11 studies used a combination of 2 or more of these methods, whilst 5 studies had no information available on the method used.

Risk of bias in the intervention trials was low in the study of Kramer, and unclear due to unclear selection bias in the study of Kajosaari. Risk of bias in observational studies was assessed using the NICE Methodological checklists for cohort and case-control studies. Figure 56 illustrates the distribution of bias across the five main methodological areas of the observational studies. Nearly 40% of studies were considered to have an overall high risk of bias, mainly due to lack of controlling for potential confounders and selection bias issues. Risk of conflict of interest was generally assessed as low.

Where data were available, five levels of comparison were used to assess the risk of wheeze according to TBF duration, namely ‘ever vs. never’, ‘≥0-2 months vs. <0-2 months’, ‘≥3-4 months vs. <3-4 months’, ‘≥5-7 months vs. <5-7 months’, and ‘≥8-12 months vs. <8-12 months’.

*Main findings*

In children aged 0-4 years we found no consistent evidence for an association between EBF duration and risk of wheeze or recurrent wheeze. EBF ≥0-2 months was associated with reduced wheeze at age 0-4, but not recurrent wheeze. There was high heterogeneity in the analysis of wheeze, and subgroup analysis found a significant association in studies at low risk of bias, with no heterogeneity. EBF ≥3-4 months was not associated with wheeze or recurrent wheeze at age 0-4, but there was significant statistical heterogeneity in both analyses. EBF ≥5 months was associated with reduced wheeze in one prospective study, but not in one retrospective study, and there was no association with recurrent wheeze.

In children aged 5-14 we found no consistent evidence for an association between EBF duration and risk of wheeze or recurrent wheeze. EBF ≥0-2 months was associated with reduced wheeze in meta-analysis of two prospective studies, but not in two retrospective studies; and there was no association with recurrent wheeze. EBF ≥3-4 months was not associated with wheeze or recurrent wheeze, and there was extreme statistical heterogeneity and publication bias in the analysis of recurrent wheeze. EBF ≥5 months was associated with reduced wheeze in one prospective study and one retrospective study, but with borderline statistical significance; and there was extreme heterogeneity in the analysis of EBF ≥5 months and recurrent wheeze.

In people aged 15 years and over we found no evidence for an association between EBF and wheeze or recurrent wheeze.

When EBF was analysed as a ‘per month’ exposure, a significantly lower risk of wheeze was observed in children aged 0-4 years old, albeit with high statistical heterogeneity; and there was significantly lower risk of recurrent wheeze at both 0-4 years and 5-14 years, with low heterogeneity.

We found no consistent evidence for a relationship between EBF and atopic wheeze, with one study reporting reduced atopic wheeze at age 0-4 with EBF ≥0-2 months, but no relationship with EBF ≥3-4 months; no evidence for a relationship between EBF ≥0-2 months and atopic wheeze at age 5-14 years; and one study reporting reduced risk of atopic wheeze at age 5-14 years with EBF ≥3-4 months.

Two studies reported decreased FEV1/FVC% associated with EBF ≥1 month, and meta-analysis was statistically significant with no heterogeneity. Other studies which could not be included in meta-analysis showed no consistent evidence for an association between EBF and measures of lung function.

Overall 18 studies reported data which could not be included in meta-analysis - 16 prospective cohort studies and 2 cross-sectional studies. These studies reported no consistent evidence for an association between EBF and wheeze.

*Conclusion*

We found no consistent evidence for an association between EBF and risk of wheeze, recurrent wheeze, atopic wheeze, BHR or measures of lung function.

Table 13 Characteristics of included studies evaluating exclusive breastfeeding and wheeze

| **First Author & Publication Year** | **Design** | **N** | **Exposure assessment** | **Method of outcome assessment** | **Age at outcome (years)** | **Country** | **Population characteristics** |
| --- | --- | --- | --- | --- | --- | --- | --- |
| Brew, 2011 ([122](#_ENREF_122)) | SR/PC/CC/C-S | 417,880 | Q | ‘Current wheezing illness’, defined in various ways, but usually including wheezing in last 12 months | 5-18 | | 31 studies included in the SR  (180 – 168,330) participants and EBF for 3-4 months vs. less than 3-4 months |
| Kramer, 2012 ([123](#_ENREF_123)) | SR /PC nested in RCTs | 3,993 with wheeze data | Q | Self-reported symptoms, objective markers | 5-7 | | Lactating mothers and their healthy, term, singleton infants exclusively or predominantly breast fed for at least 3 months |
| Kajosaari, 1991[1] | PC | 135 | - | DD asthma | 1, 5 | Finland | Solid food introduction at 6 months versus 3 months, in exclusively breastfed infants |
| de Vries, 2010 ([124](#_ENREF_124)) | PC | 4860 | Q | Parent reported wheeze | 0.5 | Netherlands | ABCD study. Population based study of babies born between 2003 and 2004 |
| Garcia-Marcos, 2010 ([125](#_ENREF_125)) | CS | 28687 | Q | ISAAC ; >= 3 episodes of wheeze in the first 2 years | 1 | Spain, Netherlands and Latin America | EISL study. One-year old infants from urban and rural primary care health clinics born in 2004-2006 |
| Hesselmar, 2010 ([5](#_ENREF_5)) | PC | 184 | I | DD asthma : >=3 episodes of wheeze | 1.5 | Sweden | ALLERGYFLORA study. Population based study of babies selected from antenatal clinics between 1998 and 2003 - mainly high risk of allergic disease |
| Elliott, 2008 ([6](#_ENREF_6)) | PC | 8191 | Q | Current wheeze; Wheeze ever; BHR: metacholine PC20 | 3, 7.5 | UK | ALSPAC study. Population based cohort of children born 1991-1992. |
| Kull, 2002; Kull, 2004; Kull, 2010  ([9](#_ENREF_9), [126](#_ENREF_126), [127](#_ENREF_127)) | PC | 3825 | Q | >=4 episodes of wheeze OR inhaled corticosteroids (+/- sIgE); >=3 episodes of wheeze OR inhaled corticosteroids (+/- sIgE); Self reported wheeze; >=3 episodes of wheeze OR inhaled corticosteroids; >=3 episodes of wheeze PLUS inhaled glucocorticoids or cough/wheeze at excitement/stress; Spirometry | 2, 4, 8 | Sweden | BAMSE study. Population based cohort of children born between 1994-1996 |
|  |  |  |  |  |  |  |  |
|  |  |  |  |  |  |  |  |
| Besednjak-Kocijancic, 2010 ([128](#_ENREF_128)) | PC | 408 | NA | Unclear | 1, 5 | Slovenia | Infants with a positive history of parental allergy |
| Bacopoulou, 2009 ([129](#_ENREF_129)) | PC | 6643 | Q | DD | 7 | Greece | Population based sample of neonates born in 1983 |
| Cano Garcinuno, 2003 ([130](#_ENREF_130)) | CS? Or PC | 234 | Q | DD | 3 | Spain | Children born in 1998-2002 attending primary health center |
| Salam, 2003 ([131](#_ENREF_131)) | NCC | 691 | I | DD PLUS current wheeze; DD | 8-18 | USA | Cases and controls selected within the CHS study, a population based study of children recruited from public school classrooms from grades 4, 7, and 10 in 12 communities in southern California. |
| Mihrshahi, 2007 ([20](#_ENREF_20)) | PC | 516 | I | DD PLUS current wheeze | 5 | Australia | CAPS study. Infants born in 1997-1999 with family history of asthma or wheezing |
| Simon, 2008 ([21](#_ENREF_21)) | PC | 372 | R/I | Transient wheezing: wheezing in the last 12 months at ages of 1,2 and/or 4 years but not at age of 6 years. vs never wheeze | 6 | USA | CAS study. Middle class mother-infant pairs enrolled in a health maintenance organisation in 1987-89 |
| Fergusson, 1983; Horwood, 1995  ([132](#_ENREF_132), [133](#_ENREF_133)) | PC | 1110 | R/I | DD asthma (>=2 episodes of wheeze) | 4, 6 | New Zealand | Christchurch Child Development Study. Population based cohort of infants born in 1977 in the Christchurch urban region |
|  |  |  |  |  |  |  |  |
| Giwercman, 2010 ([134](#_ENREF_134)) | PC | 313 | I | Parent reported wheeze | 2 | Denmark | COPSAC study. Infants of mothers with a history of doctor-diagnosed asthma, recruited from August 1998 to December 2001. |
| Linneberg, 2006 ([135](#_ENREF_135)) | PC | 34793 | Q/I | Parent reported wheeze | 1.5 | Denmark | DNBC. Population based birth cohort of children born between 1997-2002 |
| Erkkola, 2012; Nwaru, 2013  ([24](#_ENREF_24), [136](#_ENREF_136)) | PC | 3675 | D | DD plus ISAAC (+/-) sIgE | 5 | Finland | DIPP study. Infants at high risk (HLA) for TIDM born between 1996-2004 invited to the allergy study between 1998 and 2000 |
|  |  |  |  |  |  |  |  |
| Wilson, 1998 ([25](#_ENREF_25)) | PC | 545 | Q | Self-reported wheeze; DD | 7 | UK | Dundee infant feeding study. Population based cohort of infants born between 1983-1986 |
| Mandhane, 2007; Sears, 2002  ([137](#_ENREF_137)) ([138](#_ENREF_138)) | PC | 1037 | R/I | DD PLUS current wheeze; Current wheeze PLUS airway hyperresponsiveness; Spirometry; BHR: metacholine PC20 | 9, 26 | New Zealand | Dunedin Multidisciplinary Health and Development Research Study. Population based cohort of infants born between 1972-1973 |
|  |  |  |  |  |  |  |  |
| Hetzner, 2009 ([139](#_ENREF_139)) | PC | 7900 | I | DD | 2 | USA | Early Child Longitudinal Study Birth Cohort. Nationally representative sample of children born in the United States during 2001 |
| Castro-Rodriguez, 2010 ([140](#_ENREF_140)) | CS | 1409 | Q | ISAAC | 1.4 | Spain | EISL study. One-year old infants from urban and rural primary care health clinics born in 2004-2006 |
| Chong Neto, 2007 ([141](#_ENREF_141)) | CS | 3003 | Q | Parent reported wheeze ever | 1 | Brazil | EISL study. One-year old infants from urban and rural primary care health clinics born in 2004-2006 |
| Munro, 2011 ([28](#_ENREF_28)) | PC | 700 | Q | Parent reported wheeze | 1, 2 | UK | EuroPrevall-UK. Population based birth cohort of infants born in 2008 |
| Tanaka, 2009 ([32](#_ENREF_32)) | CS | 1957 | Q | ISAAC | 3 | Japan | Fukuoka Child Health Study. All 3-year old children who had the examination at public public health centers in Fukuoka city |
| van der Voort, 2012 ([33](#_ENREF_33)) | PC | 5368 | Q | ISAAC | 1, 4 | Netherlands | Generation R study. Population-based multicultural birth cohort of infants born between 2002 and 2009 |
| Ehlayel, 2008 ([142](#_ENREF_142)) | CS | 1278 | Q | ISAAC; Parent reported recurrent wheezing/asthma | 5 | Qatar | Children 0-5 years old attending primary healthcare centers for routine immunisation |
| Huang, 2013 ([143](#_ENREF_143)) | PC | 684 | Q | ISAAC | 2 | China | Mother-infant pairs registered in Putuo District, Changzheng Town Community Health Service Center Child Health Clinic within the period from January to December, 2008 |
| Nagel, 2009 ([46](#_ENREF_46)) | CS | 31579 | Q | Parent reported current wheeze/asthma; Parent reported current wheeze (+/-SPT) | 12 | International - Affluent countries (China, Germany, Greece, Iceland, Italy, Netherlands, New Zealand, Norway, Spain); International - Nonaffluent countries (Albania, Brazil, China, Ecuador, Georgia, Ghana, India, Latvia, West Bank, Turkey) | ISAAC Phase 2. Schoolchildren aged 8–12 years from 27 centres in 21 affluent and nonaffluent countries |
| Arshad, 1992; Kurukulaaratchy, 2004; Kurukulaaratchy, 2006  ([144-146](#_ENREF_144)) | PC | 1167 | D/Q | DD asthma (>=3 episodes of wheeze); Parent reported wheeze; >=2 episodes of wheeze between age <4 and 10 years | 1, 10 | UK | Isle of Wight Prevention Study. Population based birth cohort of infants born in semi-rural areas between 1989 and 1990 |
|  |  |  |  |  |  |  |  |
|  |  |  |  |  |  |  |  |
| Juca ([147](#_ENREF_147)) | CC | 590 | Q | ISAAC | 14 | Brazil |  |
| Kemeny, 1991 ([54](#_ENREF_54)) | PC | 180 | NA | >=2 episodes of wheeze | 1 | UK | Population based birth cohort of infants born at Dulwich and King’s College Hospitals in London |
| Liu, 2012 ([148](#_ENREF_148)) | CS | 8733 | Q | ISAAC; ATS guideline | 8 | China | Sample of children from kindergarden and elementary schools in Shenyang |
| Mai, 2007 ([149](#_ENREF_149)) | NCC | 723 | Q | Canadian Asthma Consensus Guidelines: symptoms plus variable airway obstruction | 10 | Canada | Cases and controls selected from records of the Manitoba Health Services Insurance Plan and all were born 1995 |
| Majeed, 2008 ([150](#_ENREF_150)) | CC | 398 | Q/I | >=3 episodes of wheeze in past year | 3 | Pakistan | Cases are 1-12 years old children with wheeze/asthma admitted to paediatrics wards. Controls are age matched children and adolescents attending immediately after selection of the index case |
| Marini, 1996 ([59](#_ENREF_59)) | PC | Unclear | Q | >=3 episodes of wheeze | 1 | Italy | Infants with family history of allergy whose mother were proposed to participate in an allergy prevention program |
| Midwinter, 1987 ([151](#_ENREF_151)) | PC | 453 | NA | DD asthma | 5 | UK | Children born to parents with a family history of atopy in 1979-1981 |
| Miyake, 2003 ([65](#_ENREF_65)) | CS | 6845 | Q | ISAAC | 15 | Japan | 12-15 years old children from all public junior high schools in Suita, Japan. |
| Silvers, 2009; Silvers, 2012  ([75](#_ENREF_75), [76](#_ENREF_76)) | PC | 987 | Q | DD PLUS current wheeze; Parent reported wheeze | 1, 5 | New Zealand | New Zealand Asthma and Allergy Cohort Study. Population based birth cohort of infants born between 1997 and 2001 |
|  |  |  |  |  |  |  |  |
| Nielsen, 2013 ([152](#_ENREF_152)) | PC | 5429 | Q | Unclear | 0.5 | Denmark | Population based birth cohort of infants born in 1995 |
| Miyake, 2008 ([79](#_ENREF_79)) | PC | 763 | Q | ISAAC | 2 | Japan | OMCHS study. Population based birth cohort of infants born in 2002-2003 |
| da Costa Lima, 2003([81](#_ENREF_81)) | PC | 2247 | I | ISAAC | 18 | Brazil | Pelotas Birth Cohort. Population based birth cohort of infants born in 1982 in the city of Pelotas |
| Pesonen, 2006 ([153](#_ENREF_153)) | PC | 164 | I | DD OR >=2 episodes of wheezing | 5, 20 | Finland | Population based birth cohort of infants born in 1981 |
| van Merode, 2007 ([154](#_ENREF_154)) | PC | 222 | Q | ISAAC | 1 | Holland | PREVASC study: cohort born in 2005 with family history of asthma (high risk of disease) |
| Prietsch, 2006 ([155](#_ENREF_155)) | CS | 685 | Q | Unclear | 13 | Brazil | Population representative sample of infants aged 12-15 months (normal risk of disease) |
| Kramer, 2003; 2009; 2009 (b)  ([156-158](#_ENREF_156)) | PC | 2951 | I | ISAAC | 1, 6.5 | Belarus | PROBIT study: recruited in hospitals, born 1996-1997 (normal risk of disease) |
|  |  |  |  |  |  |  |  |
|  |  |  |  |  |  |  |  |
| Ratageri, 2000 ([159](#_ENREF_159)) | CC | 180 | NA | DD asthma using International Paediatric Consensus Group criteria | 9 | India | Hospital-based study (normal risk of disease) |
| Rothenbacher, 2005 ([94](#_ENREF_94)) | PC | 803 | Q/I | DD | 2 | Germany | Recruited from university service, born in 2000-2001 (normal risk of disease) |
| Rullo, 2007; Rullo, 2009; Rullo, 2009 (b); Rullo, 2010  ([160-163](#_ENREF_160)) | PC | 101 | Q/I | >=3 episodes of wheeze in past year; Persistent wheeze: ever wheezing treated with inhaled corticosteroids and beta-2 agonists in the past year | 1.5, 2.5, 4, 5 | Brazil | Recruited from hospital (high risk of asthma) |
|  |  |  |  |  |  |  |  |
|  |  |  |  |  |  |  |  |
|  |  |  |  |  |  |  |  |
| Rylander, 1993 ([164](#_ENREF_164)) | CC | 550 | I | Physician assessment | 4 | Sweden | Cases from a health service and control from population (normal risk of disease) |
| Siltanen,2003 ([165](#_ENREF_165)) | PC | 285 | Q | DD PLUS current wheeze; >=3 episodes of wheezing; | 4 | Finland | Infants recruited from maternal hospital born in 1994-1995 (normal risk of disease) |
| Salem, 2002 ([166](#_ENREF_166)) | CS | 424 | Q | Wheeze ever | 5 | Iraq | Population representative sample of children aged 0.16-2 years old (normal risk of disease) |
| Silva, 2005 ([167](#_ENREF_167)) | PC | 73 | Q | Physician assessment (>=3 episodes of wheeze) | 4 | Brazil | Recruited from health services in urban area, born in 1998 (high risk of disease) |
| Strassburger, 2010 ([168](#_ENREF_168)) | PC | 343 | I | ISAAC | 3.5 | Brazil | Recruited from hospital, born 2001-2002 (normal risk of disease) |
| Mai, 2008 ([169](#_ENREF_169)) | NCC | 723 | Q | Physician assessment | 10 | Canada | Study of Asthma, Genes and the Environment (SAGE) project: population-based study with children born in 1995 (normal risk of disease) |
| Matheson, 2007 ([170](#_ENREF_170)) | PC | 5729 | Q | Self-reported current asthma | 7, 32 | Tasmania | Tasmanian ASTHMA Study: population based born in 1961 (high risk/low risk of disease) |
| Takemura, 2001 ([102](#_ENREF_102)) | CS | 23828 | Q | >=2 episodes of wheeze PLUS DD asthma | 6-15 | Japan | The Tokorozawa Childhood Asthma and Pollinosis Study: Representative sample of children in public elementary schools (normal risk of disease) |
| Wright, 2001 ([171](#_ENREF_171)) | PC | 1043 | Q/I | DD PLUS wheeze >=2 times between 6 and 13 years | 13 | USA | Tuscon Children's Respiratory Study: Recruited from local health maintenance organisation born in 1980-1984 (normal risk of disease) |
| Van Asperen, 1983 ([172](#_ENREF_172)) | PC | 79 | I | Parent reported wheeze | 1 | Australia | Cohort recruited from medical service, born in 1980-1981 with family history of atopy (high risk of disease) |
| Watson,2013 ([173](#_ENREF_173)) | PC | 369 | Q/I | ISAAC | 1.5 | New Zealand | Recruited from Polynesian women, non-random sample (high risk of disease) |
| Oddy, 1999; Oddy, 2002; Oddy, 2003; Oddy, 2004  ([109](#_ENREF_109), [111](#_ENREF_111), [174](#_ENREF_174), [175](#_ENREF_175)) | PC | 2456 | D/I | DD PLUS wheeze in the past 12 months; Parent reported current wheeze; DD asthma PLUS >=3 episodes of wheeze | 1, 6 | Australia | Western Australia Pregnancy Cohort: Recruited from antenatal clinics born in 1989-1992 (high risk/low risk of disease) |
|  |  |  |  |  |  |  |  |
|  |  |  |  |  |  |  |  |
|  |  |  |  |  |  |  |  |
| Whu, 2007 ([176](#_ENREF_176)) | CC | 261 | I | DD PLUS asthma medication | 4 | USA | Hospital-based study cases born in 2000 (normal risk of disease) |
| Zutavern 2004 ([114](#_ENREF_114)) | PC | 606 | I | Parent reported current wheeze | 2, 5.5 | UK | Cohort recruited from general practices and born in 1993-1995 (normal risk of disease) |
| Poysa, 1992 ([177](#_ENREF_177)) | PC | 68 | NA | BHR: metacholine PC20 | 10 | Finland | High risk of disease, born 1979-1980 |
| Guilbert, 2007 ([178](#_ENREF_178)) | PC | 679 | I | Spirometry | 13 | USA | Tuscon Children's Respiratory Study: A population-based cohort of healthy infants born in 1980-1984 (normal risk of disease) |

Figure 56 Risk of bias in studies of exclusive breastfeeding and wheeze

## Exclusive Breastfeeding and Wheeze

### Systematic reviews

Two systematic reviews investigated overall association between exclusive breastfeeding and wheeze. Brew and colleague found no evidence that EBF for more than 3-4 months vs. less was associated with wheeze (OR 0.96 [0.86, 1.06]) (I^2^=54.2%). Kramer reported no association between EBF for 6 or more months’ vs 3-4 months and having at least 2 episodes of wheezing in the first 12 months RR 0.79 (0.49, 1.28; I^2^=0%). No intervention trials were identified, other than the cluster RCT of Kramer which is included in the ‘TBF’ section of this report.

### Age at outcome measurement 0-4

#### 0-2 months

Six prospective cohort studies reported data on risk of wheeze that could be pooled to estimate the effect of exposure to EBF for ≥0-2 months vs. <0-2 months in children aged 0-4 years, suggesting a protective effect of EBF on wheeze in the exposed group (OR 0.82; 95% CI 0.67, 0.99) (Figure 57). There was very high heterogeneity between studies (I^2^=74.3%). Two of the studies had a low overall risk of bias, whilst three had an unclear overall risk, and the study of Van Asperen lacked controlling for potential confounders, so carries an overall high risk of bias.

Subgroup analyses for EBF for ≥0-2 months vs. <0-2 months and risk of wheeze showed a statistically significant difference between studies at low risk of overall bias and high/unknown risk (P=0.01) (Table 14).

Figure 57 Exclusive breast feeding ≥0-2 months vs. <0-2 months and risk of wheeze in children aged 0-4 years


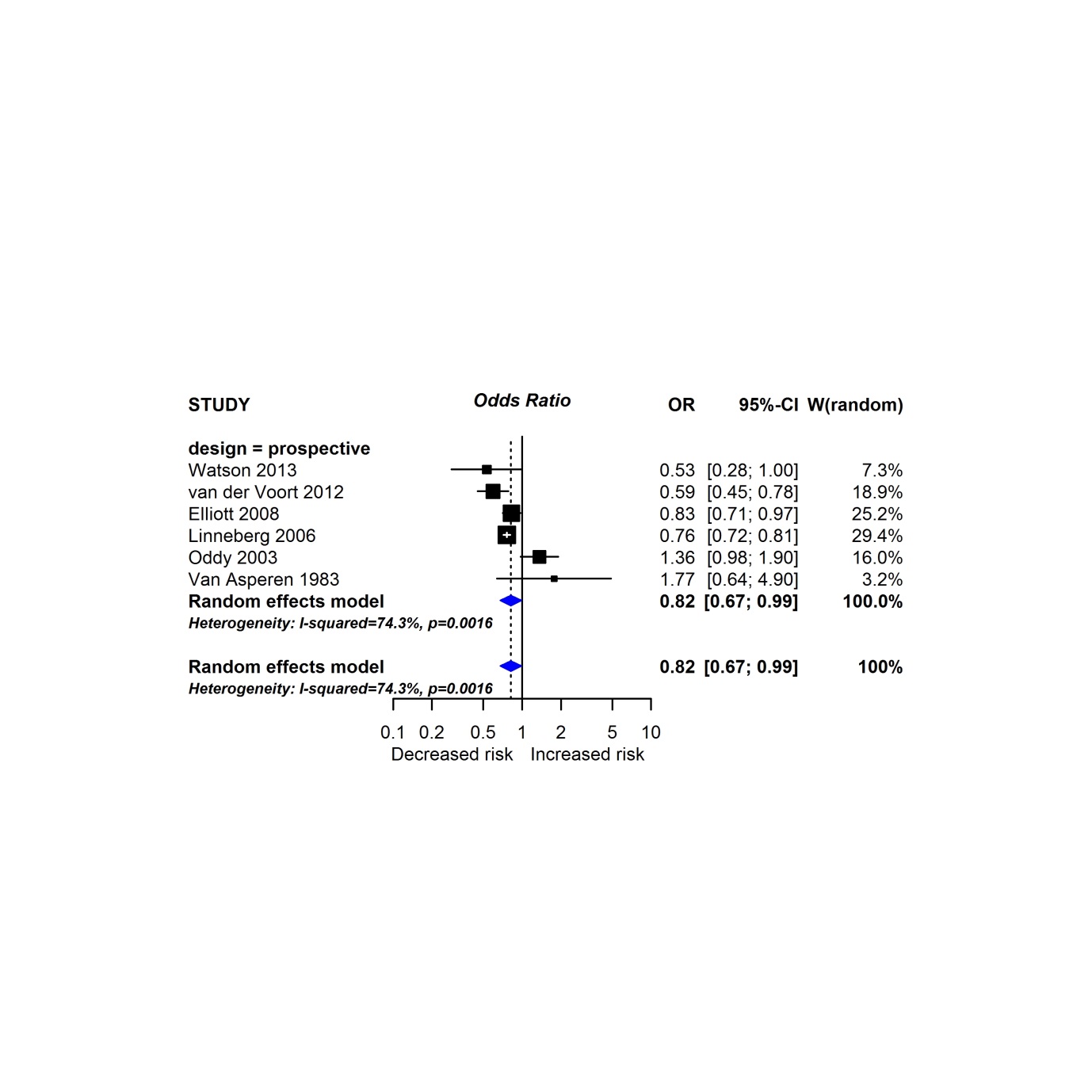


#### 3-4 months

Nine observational studies reported data on risk of wheeze that could be pooled to estimate the effect of exposure to EBF for ≥3-4 months vs. <3-4 months in children aged 0-4 years, suggesting no evidence of an effect of EBF on wheeze in the exposed group (Figure 58). There was very high heterogeneity between studies (I^2^=73.2%). Six studies were prospective cohorts (of which 2 had an overall high risk of bias due to not accounting for potential confounders), two were cross sectional (both carry a high risk of overall bias due to selection bias), and one is a case-control study (which did not account for confounding and therefore carries an overall high risk of bias). A Funnel plot was used to investigate publication bias, showing a slight asymmetry but no statistical evidence of such a bias (Figure 59).

Prolonged exposure to EBF for ≥3-4 months vs. <3-4 months showed no difference in the subgroup analyses (Table 15).

Figure 58 Exclusive breast feeding ≥3-4 months vs. <3-4 months and risk of wheeze in children aged 0-4 years


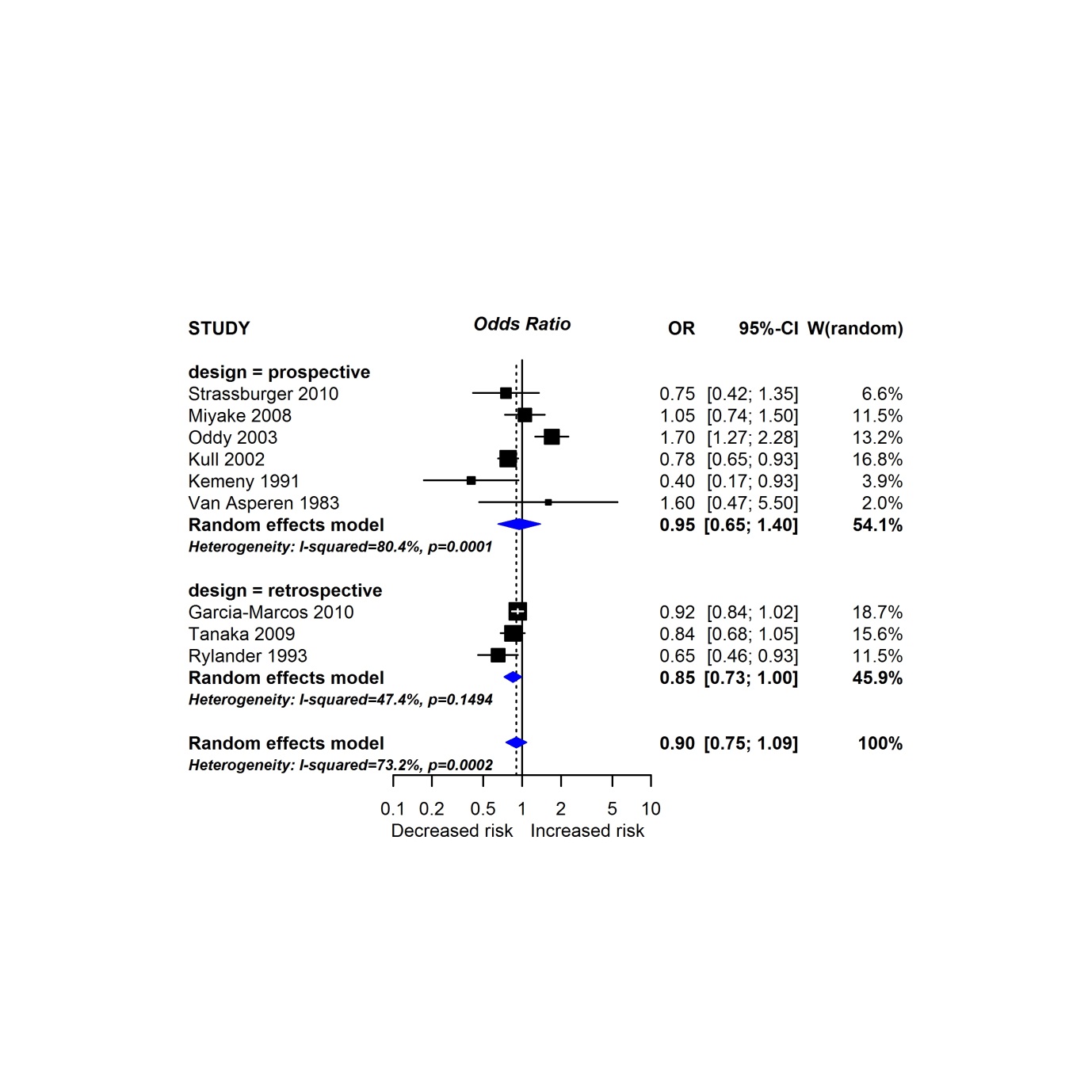


Figure 59 Risk of publication bias in studies of exclusive breast feeding ≥3-4 months vs. <3-4 months and risk of wheeze in children aged 0-4 years


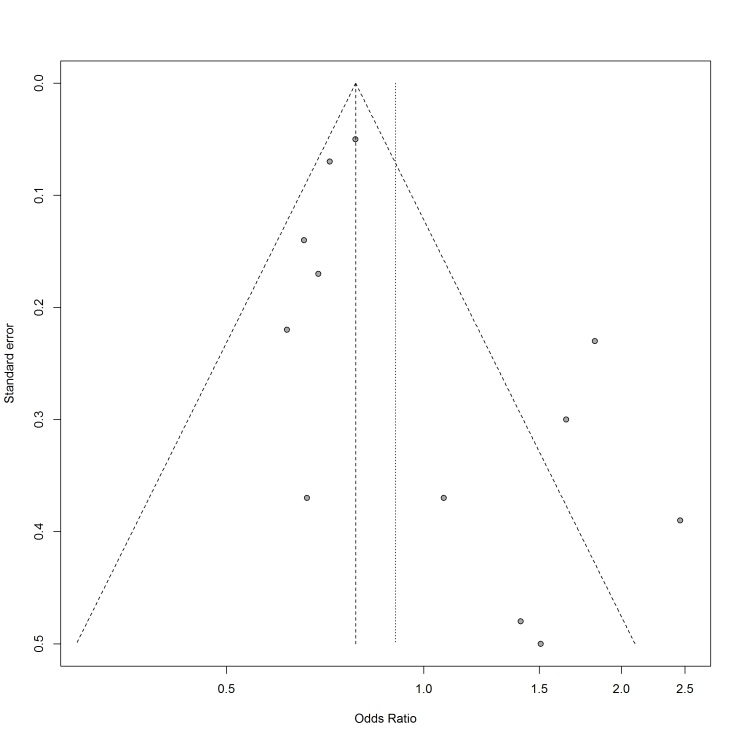


Egger’s test p-value = 0.141

#### 5+ months

Two observational studies, a prospective cohort and a cross-sectional study reported data on risk of wheeze that could be pooled to estimate the effect of exposure to EBF for ≥5 months vs. <5 months in children aged 0-4 years (Figure 60). There was extremely high heterogeneity between the two studies (I^2^=86.6%), possibly explained by the overall high risk of bias of the retrospective Salem study (mainly due to lack of adjustment for potential confounders) and the unclear overall risk of bias in the prospective Oddy study.

Figure 60 Exclusive breast feeding ≥5 months vs. <5 months and risk of wheeze in children aged 0-4 years


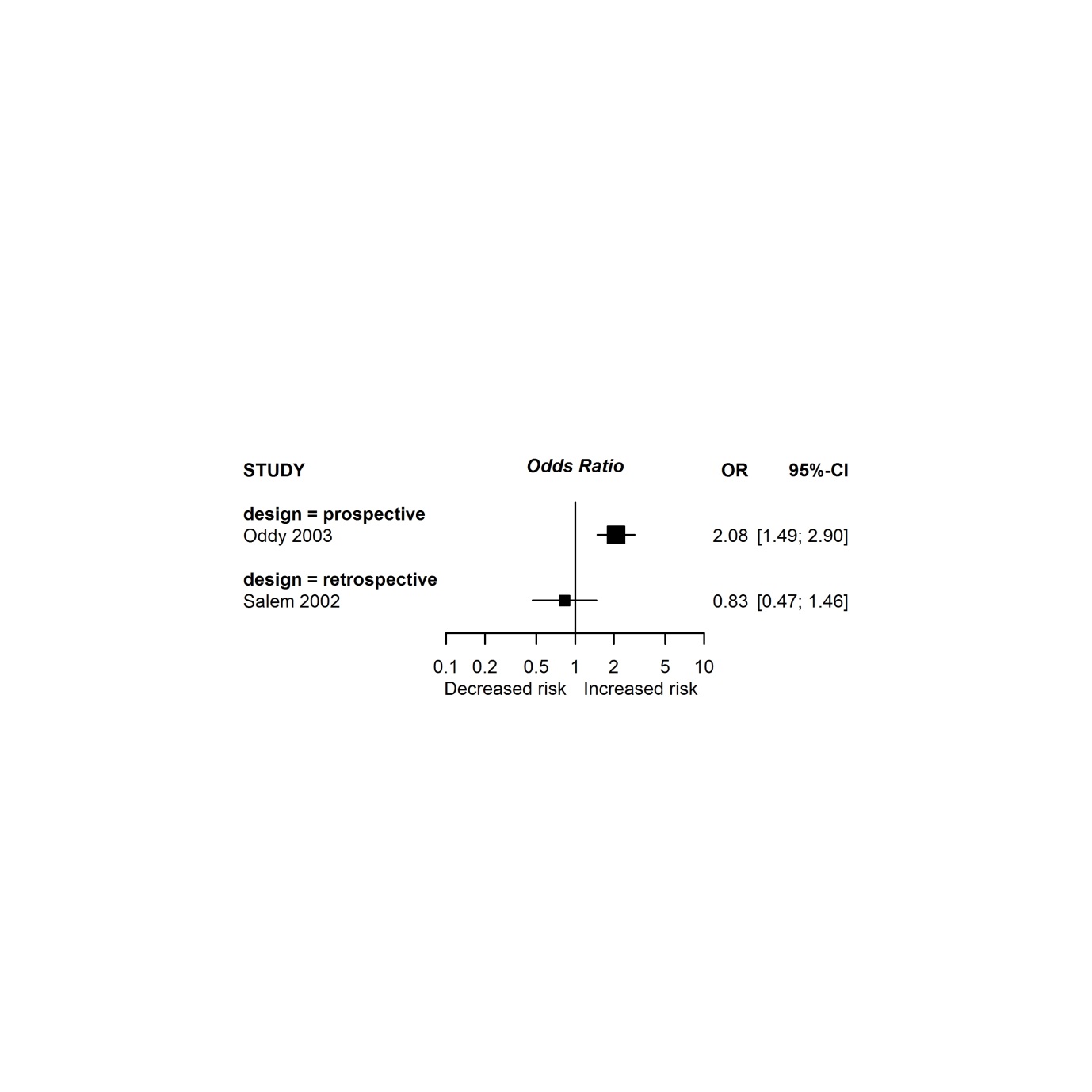


Table 14 Subgroup Analyses of risk of wheeze and exclusive breastfeeding ≥0-2 months vs. <0-2 months in children aged 0-4 years

|  | **Number of studies** | **OR [95% CI]** | **I^2^ (%)** | **P-value for between groups difference** |
| --- | --- | --- | --- | --- |
| **Overall (if adjusted NA, unadjusted value used)** | 6 | 0.82 [0.67; 0.99] | 74.3 |  |
| **Adjusted** | 4 | 0.85 [0.69; 1.05] | 77.4 | Not tested |
| **Unadjusted** | 5 | 0.88 [0.52; 1.48] | 93.6 |  |
| Study Design – Prospective | 6 | 0.82 [0.67; 0.99] | 74.3 | -- |
| Study Design – Retrospective | -- | -- | -- |  |
| Risk of disease – High | 2 | 0.91 [0.28; 2.91] | 74.1 | 0.87 |
| Risk of disease – Normal | 4 | 0.82 [0.67; 1.00] | 80.7 |  |
| Risk of bias – Low | 2 | 0.58 [0.45; 0.75] | 0.0 | 0.01 |
| Risk of bias – High/Unclear | 4 | 0.92 [0.74; 1.16] | 79.0 |  |
| Clear definition of breastfeeding duration | 4 | 0.73 [0.54; 0.99] | 62.8 | 0.34 |
| Unclear definition of breastfeeding duration | 2 | 1.00 [0.57; 1.76] | 91.1 |  |

Table 15 Subgroup Analyses of risk of wheeze and exclusive breastfeeding ≥3-4 months vs. <3-4 months in children aged 0-4 years

|  | **Number of studies** | **OR [95% CI]** | **I^2^ (%)** | **P-value for between groups difference** |
| --- | --- | --- | --- | --- |
| **Overall (if adjusted NA, unadjusted value used)** | 9 | 0.90 [0.75; 1.09] | 73.2 | 0.28 |
| **Adjusted** | 6 | 0.97 [0.80; 1.17] | 77.1 |  |
| **Unadjusted** | 8 | 0.89 [0.65; 1.2157] | 84.5 |  |
| Study Design – Prospective | 9 | 0.95 [0.65; 1.40] | 80.4 | 0.60 |
| Study Design – Retrospective | 3 | 0.85 [0.73; 1.00] | 47.4 |  |
| Risk of disease – High | 1 | 1.6000 [0.4654; 5.5002] |  | 0.36 |
| Risk of disease – Normal | 8 | 0.8917 [0.7380; 1.0773] | 75.9 |  |
| Risk of bias – Low | 4 | 0.8468 [0.7022; 1.0213] | 12.5 | 0.74 |
| Risk of bias – High/Unclear | 5 | 0.8980 [0.6724; 1.1993] | 83.8 |  |

### Age at outcome measurement 5-14

#### 0-2 months

Four observational studies reported data that could be pooled to estimate risk of wheeze with duration of EBF ≥0-2 months vs. <0-2 months, suggesting a reduced risk of disease (OR 0.84; 95% CI 0.75, 0.92; [Figure 61]). The analysis showed no statistical heterogeneity between studies (I^2^=0.0%). Two studies were prospective cohorts, the study of Erkkola had an overall high risk of bias, mainly due to lack of adjustment for potential confounders, and the study of Elliot had an unclear risk of bias. The other two studies were of cross-sectional design and used the ISAAC questionnaire. Both had an overall high risk of bias, due to selection bias.

Figure 61 Exclusive breast feeding ≥0-2 months vs. <0-2 months and risk of wheeze in children aged 5-14 years


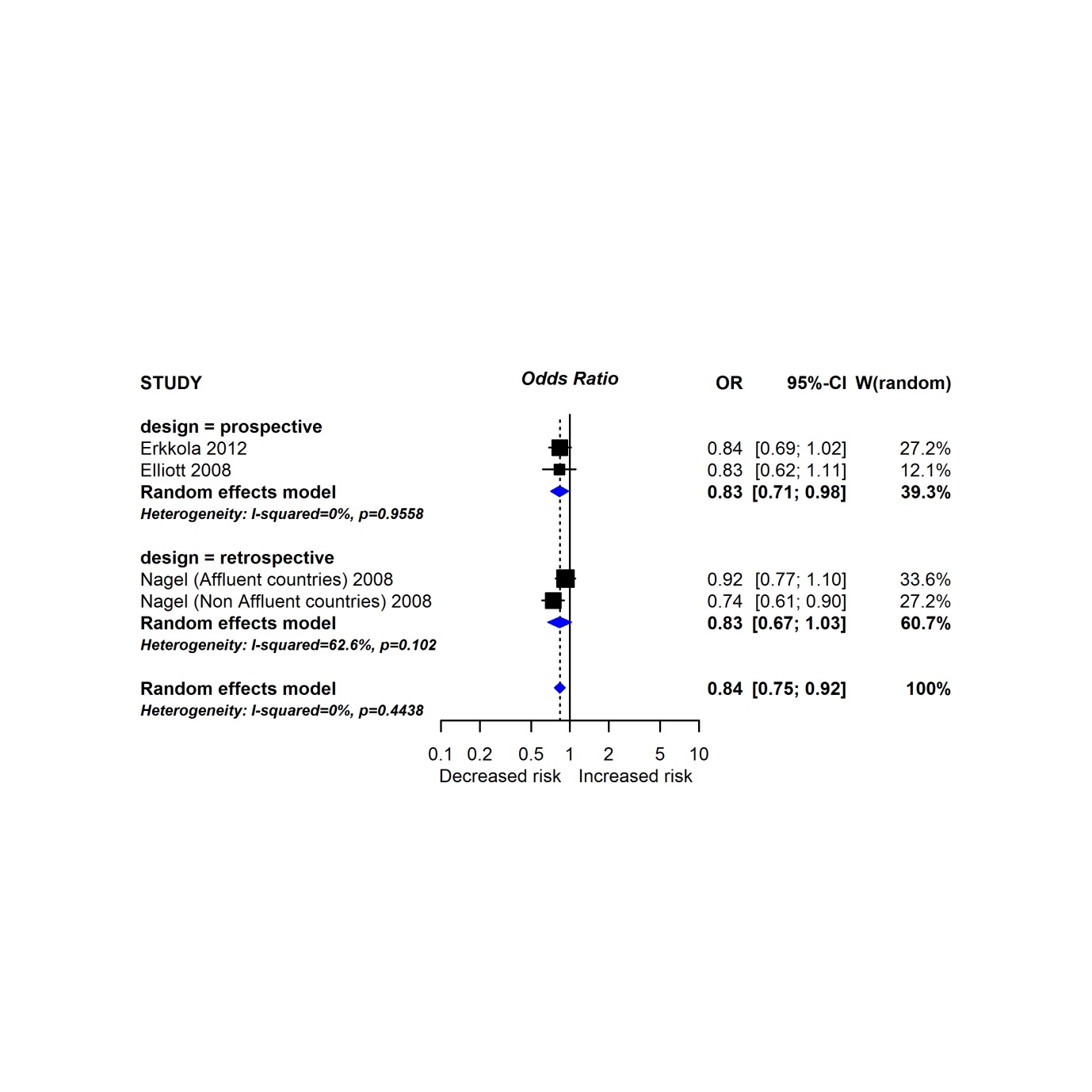


#### 3-4 months

Four observational studies reported data that could be pooled to estimate risk of wheeze with exposure to EBF ≥3-4 months vs. <3-4 months. Although all studies showed an OR below 1.0, there was no evidence of an association in the meta-analysis (Figure 62). Three of the studies were prospective cohorts (one of which had a high risk of bias due to confounding), and one was cross-sectional (with high risk of overall bias due to confounding).

Figure 62 Exclusive breast feeding ≥3-4 months vs. <3-4 months and risk of wheeze in children aged 5-14 years


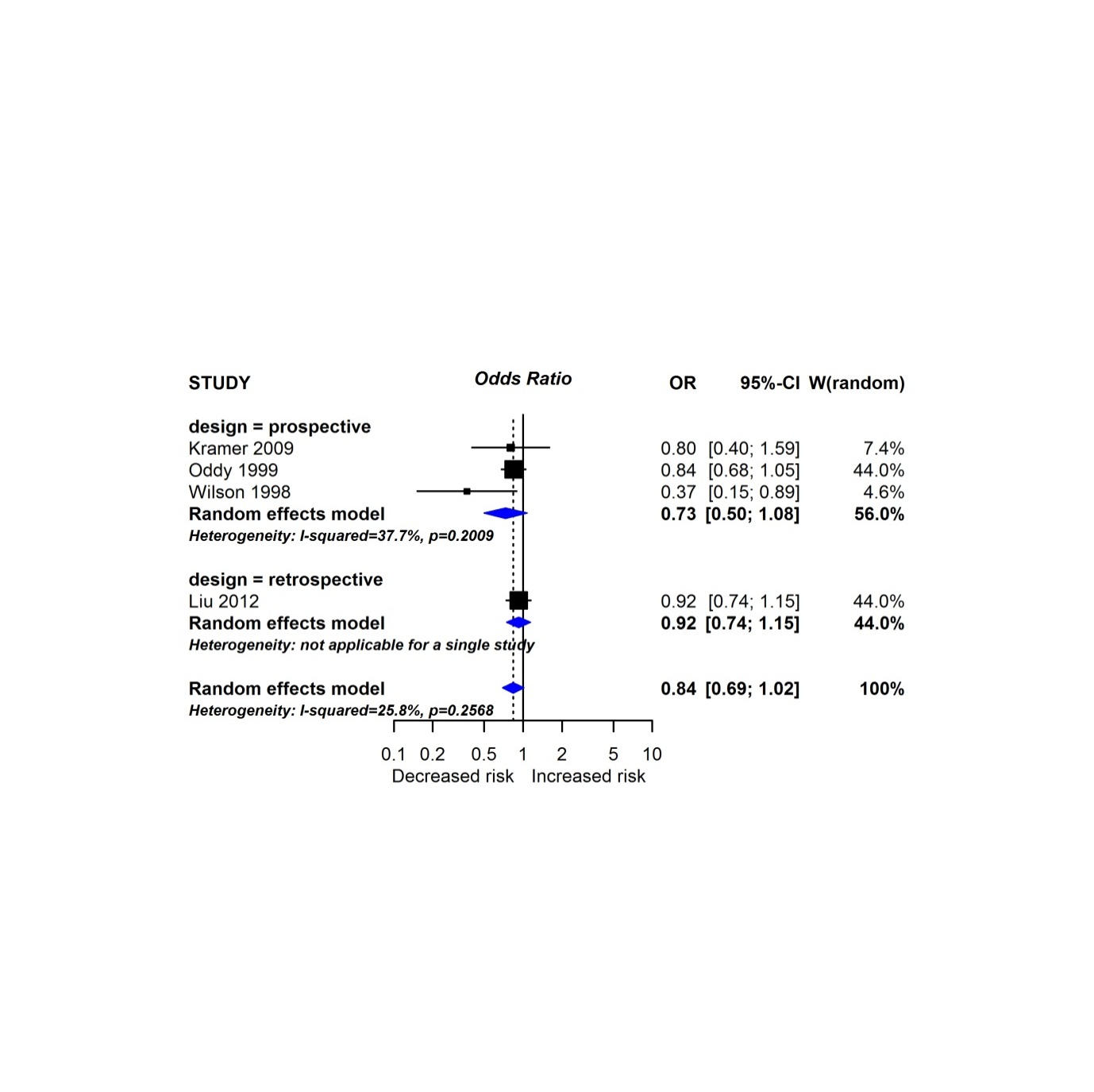


#### 5+ months

Data were available from one cross-sectional study and one prospective study to examine the pooled effect of EBF for ≥5 months vs. <5 months and risk of wheeze in children aged 5-14 years old, showing a reduced risk of disease in the exposed group (OR 0.76; 95% CI 0.63, 0.92). The studies had no heterogeneity (I^2^=0.0%) (Figure 63).

Figure 63 Exclusive breast feeding ≥5 months vs. <5 months and risk of wheeze in children aged5-14 years


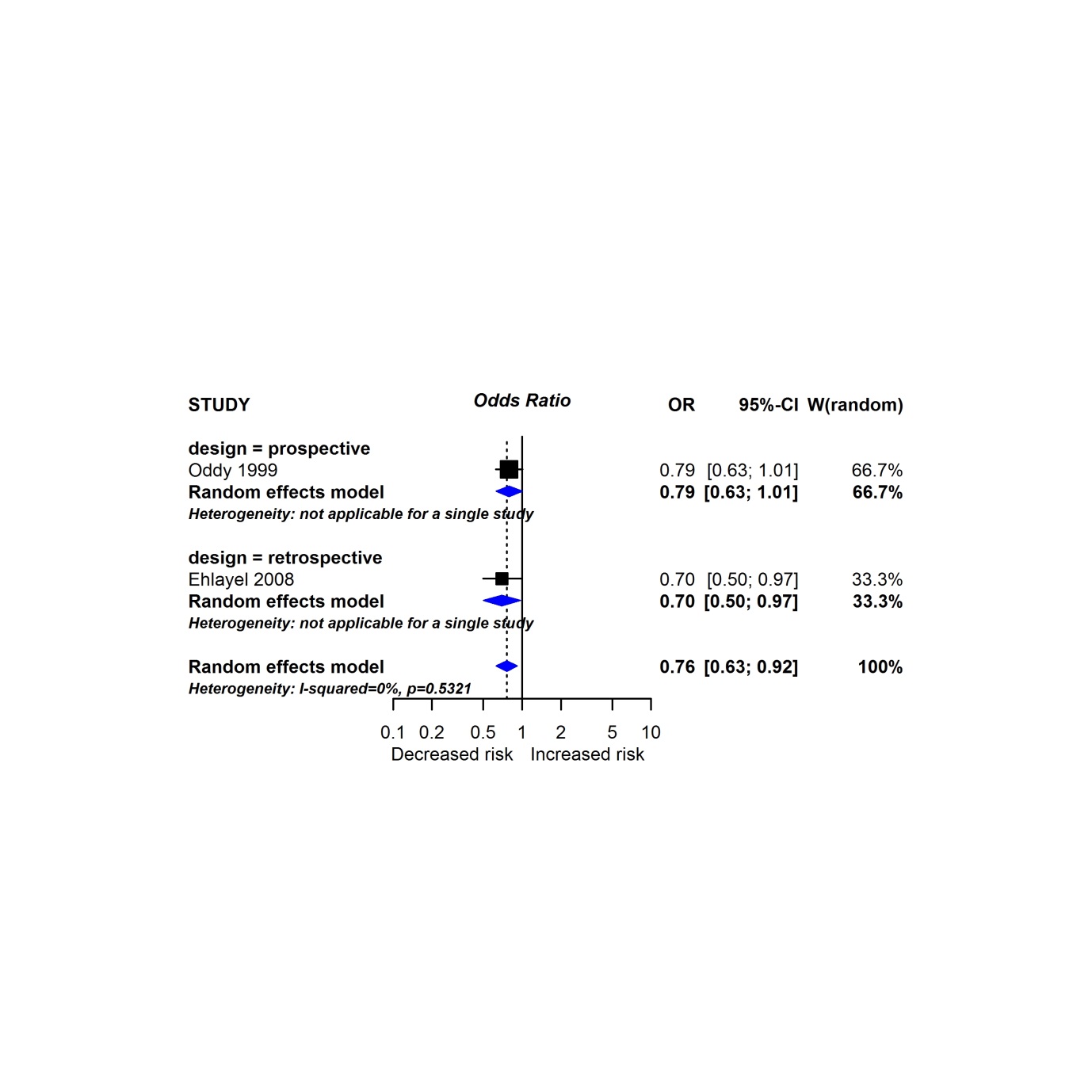


### Age at outcome measurement 15+

#### 0-2 months

One cross-sectional study reported risk of wheeze in children aged over 15 years old who were exposed to EBF for ≥0-2 months vs. <0-2 months (Figure 64). The study had high risk of bias due to lack of adjustment for potential confounders, and showed no association between exposure and disease.

Figure 64 Exclusive breast feeding ≥0-2 months vs. <0-2 months and risk of wheeze in children aged 15+ years


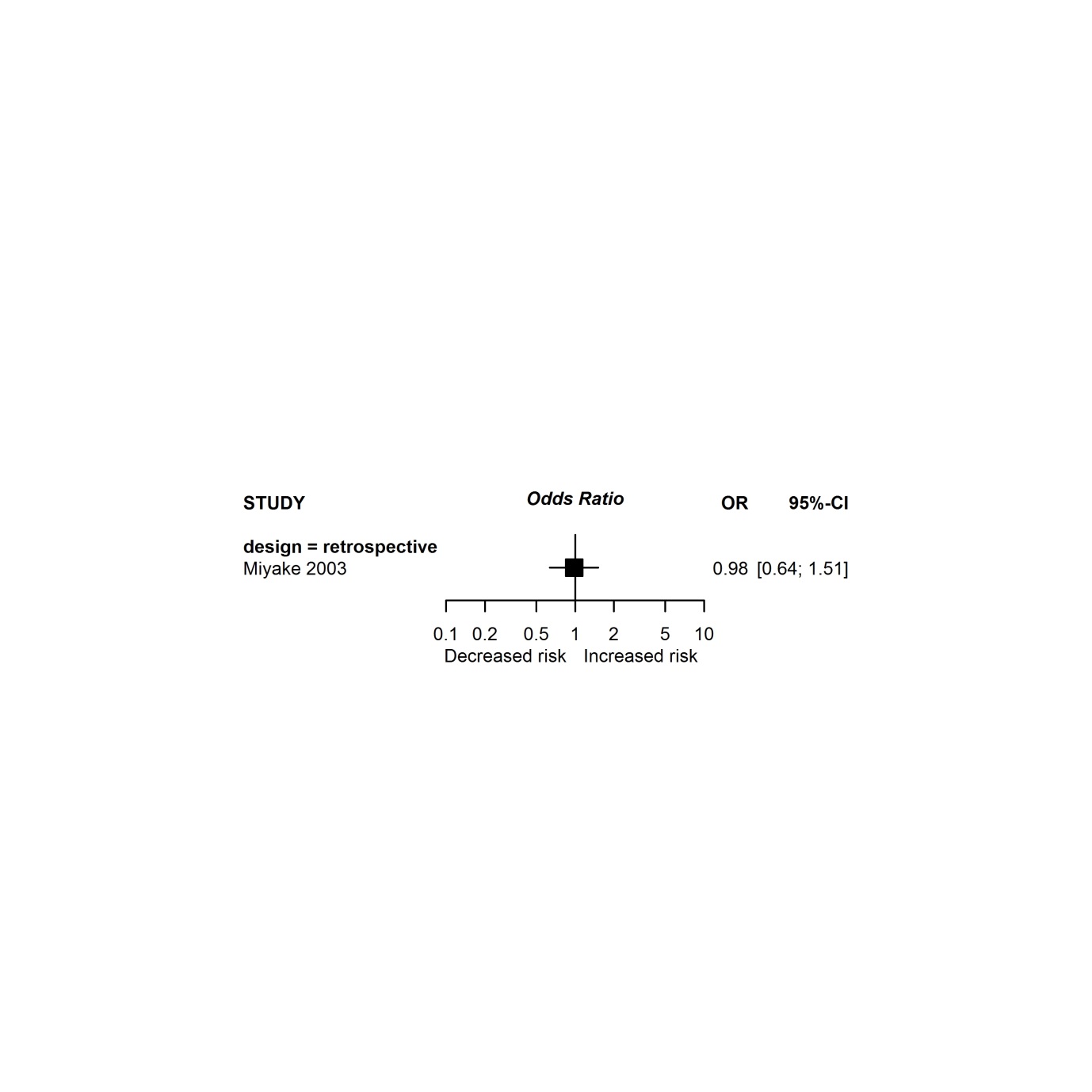


## Exclusive Breastfeeding per month and Wheeze

### Age at outcome measurement 0-4

Four observational studies reported associations between EBF per month and risk of wheeze in children aged 0-4 (Figure 65). The overall combined effect suggests a lower risk of disease in the exposed group (OR 0.92; 95% CI 0.87, 0.97). The analysis showed high statistical heterogeneity across studies (I^2^=68.8%).

Figure 65 Exclusive breastfeeding per month and risk of wheeze in children aged 0-4 years


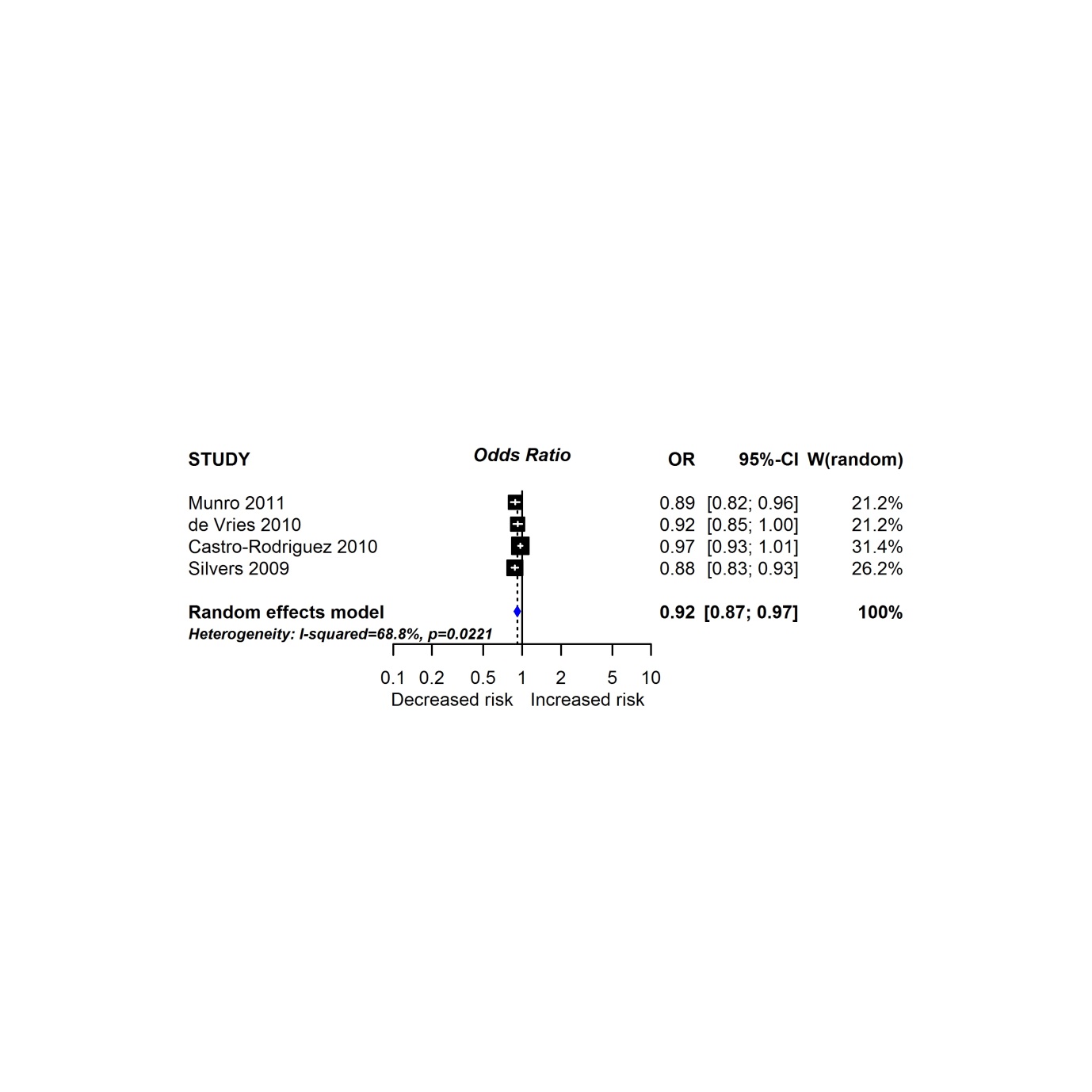


## Exclusive Breastfeeding and Recurrent Wheeze

### Age at outcome measurement 0-4

#### 0-2 months

Five observational studies (six analyses) reported risk of recurrent wheeze with exposure to EBF for ≥0-2 months vs. <0-2 months in children aged 0-4 years old (Figure 66). There was no clear evidence of a significant effect and there was very high heterogeneity between studies (I^2^=69.4%).

Subgroup analyses of risk of recurrent wheeze and exclusive breastfeeding ≥0-2 months vs. <0-2 months in children aged 0-4 years, showed statistically significant differences between children at high and low risk of disease (P<0.01) and between risk of bias (P=0.01) (Table 16). Studies at low risk of bias found no association between EBF and recurrent wheeze, with no statistical heterogeneity.

Figure 66 Exclusive breast feeding ≥0-2 months vs. <0-2 months and risk of recurrent wheeze in children aged 0-4 years


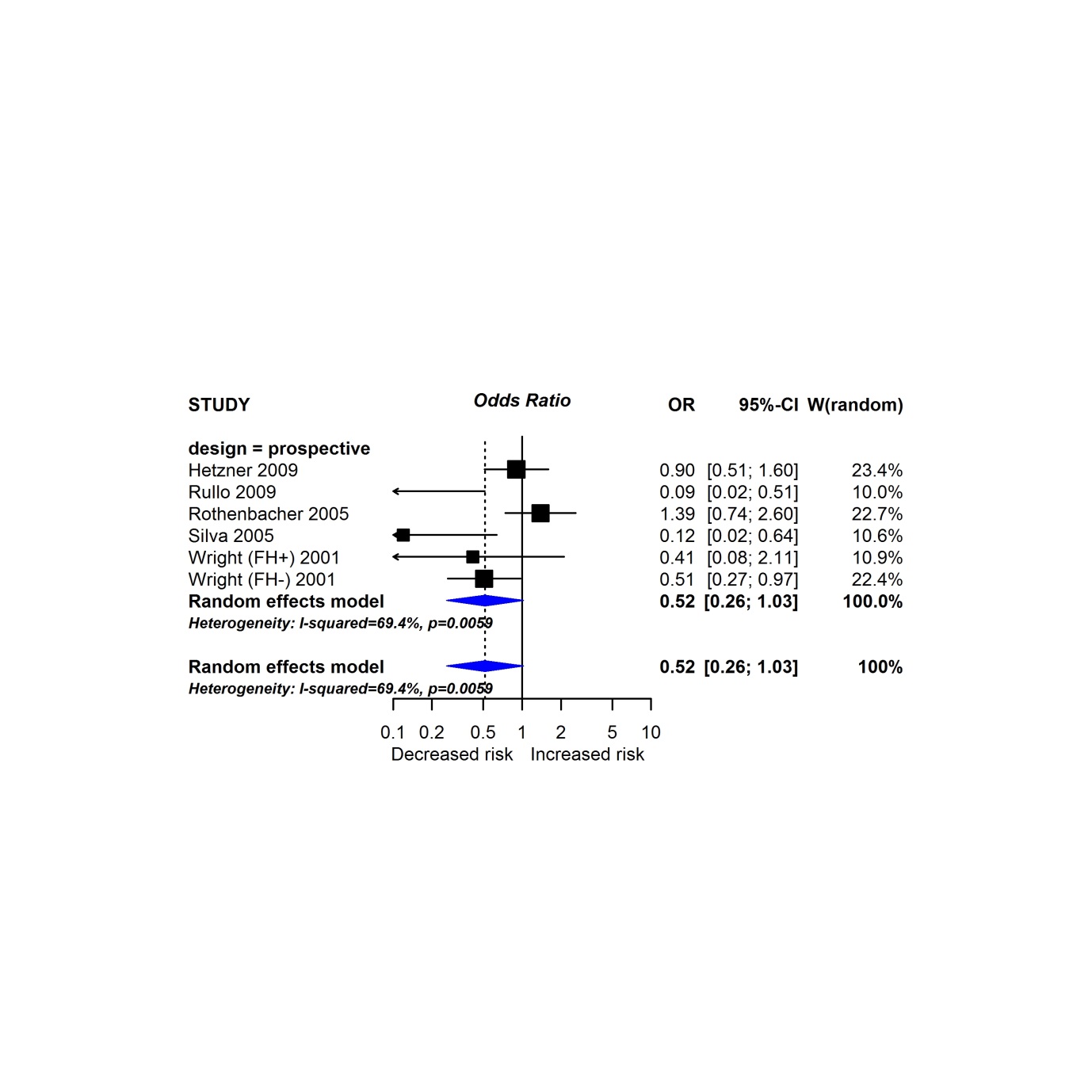


#### 3-4 months

Thirteen observational studies reported data eligible to calculate combined OR of wheeze in children aged 0-4 comparing EBF ≥3-4 months vs. <3-4 months. There was no evidence of an overall effect (Figure 67) and the heterogeneity was very high (I^2^=68.7%), which might have been partly influenced by the different outcome measurements used across studies. Nine studies were prospective cohorts (six of which had high risk of overall bias, mainly due to confounding or selection bias), 2 were cross-sectional studies (both of which had a high risk of overall bias) and one was a case-control study (which also had a high risk of overall bias, due to lack of adjustment for potential confounders). There was no evidence of publication bias (Figure 68).

Subgroup analyses of risk of recurrent wheeze and exclusive breastfeeding ≥3-4 months vs. <3-4 months in children aged 0-4 years showed no statistically significant differences between groups (Table 17).

Figure 67 Exclusive breast feeding ≥3-4 months vs. <3-4 months and risk of recurrent wheeze in children aged 0-4 years


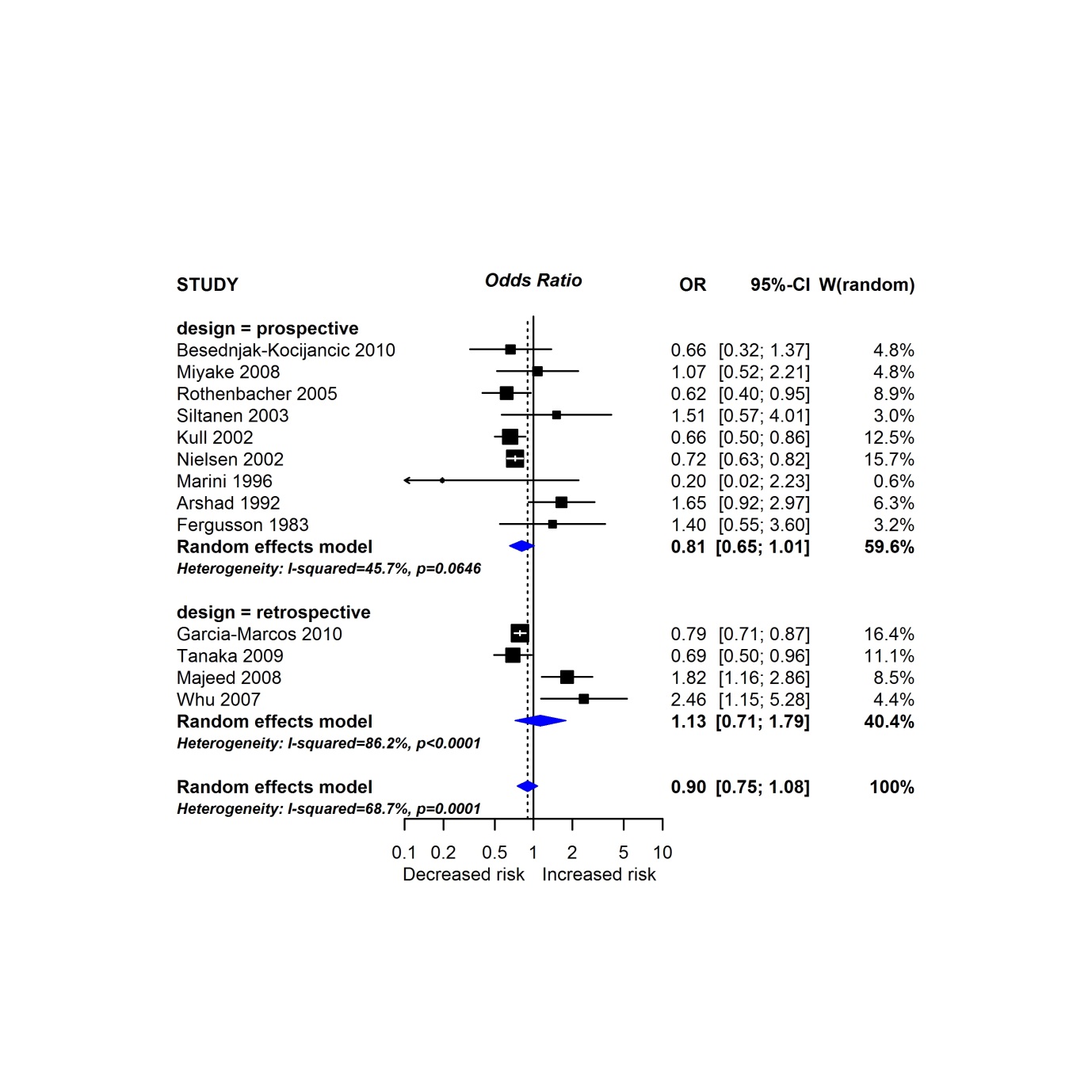


Figure 68 Risk of publication bias in studies of exclusive breast feeding ≥3-4 months vs. <3-4 months and risk of wheeze in children aged 0-4 years


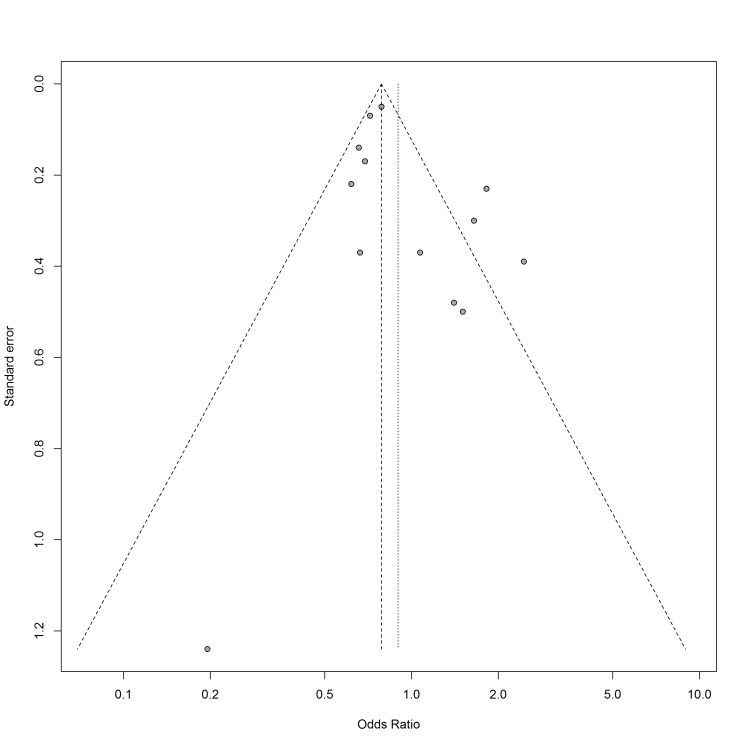


Egger’s test p-value = 0.159

#### 5+ months

Three observational studies reported data on risk recurrent wheeze with EBF ≥5 months vs. <5 months in children aged 0-4 years old. There was no evidence of an effect in the combined analysis (Figure 69), and the studies had high heterogeneity across studies (I^2^=61.7%).

Figure 69 Exclusive breast feeding ≥5 months vs. <5 months and risk of recurrent wheeze in children aged 0-4 years

**
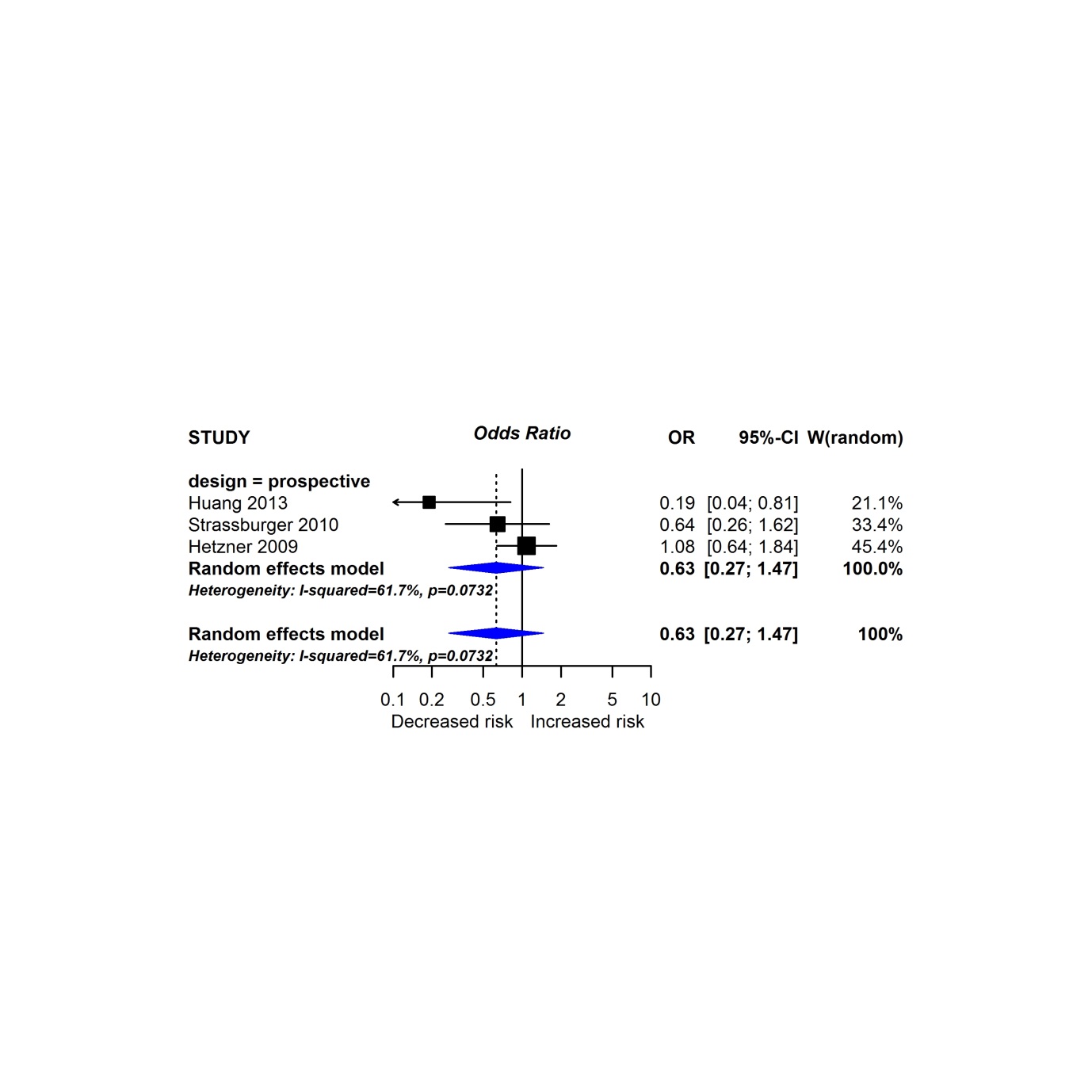
**

Table 16 Subgroup Analyses of risk of recurrent wheeze and exclusive breastfeeding ≥0-2 months vs. <0-2 months in children aged 0-4 years

|  | **Number of studies** | **OR [95% CI]** | **I^2^ (%)** | **P-value for between groups difference** |
| --- | --- | --- | --- | --- |
| **Overall (if adjusted NA, unadjusted value used)** | 6 | 0.52 [0.26; 1.03] | 69.4 |  |
| **Adjusted** | 3 | 0.25 [0.05; 1.37] | 80.3 | Not tested |
| **Unadjusted** | 5 | 0.43 [0.18; 1.00] | 73.7 |  |
| Risk of disease – High | 3 | 0.17 [0.07; 0.45] | 0.0 | <0.01 |
| Risk of disease – Normal | 3 | 0.87 [0.50 1.50] | 58.8 |  |
| Risk of bias – Low | 2 | 1.10 [0.72; 1.67] | 0.0 | 0.01 |
| Risk of bias – High/Unclear | 4 | 0.28 [0.12; 0.66] | 42.3 |  |

Table 17 Subgroup Analyses of risk of recurrent wheeze and exclusive breastfeeding ≥3-4 months vs. <3-4 months in children aged 0-4 years

|  | **Number of studies** | **OR [95% CI]** | **I^2^ (%)** | **P-value for between groups difference** |
| --- | --- | --- | --- | --- |
| **Overall (if adjusted NA, unadjusted value used)** | 13 | 0.90 [0.75; 1.08] | 68.7 |  |
| **Adjusted** | 9 | 0.79 [0.69; 0.92] | 63.0 | Not tested |
| **Unadjusted** | 12 | 0.85 [0.67; 1.08] | 69.2 |  |
| Study Design – Prospective | 9 | 0.81 [0.65; 1.01] | 45.7 | 0.20 |
| Study Design – Retrospective | 4 | 1.13 [0.71; 1.79] | 86.2 |  |
| Risk of disease – High | 2 | 0.60 [0.30; 1.20] | 0.0 | 0.24 |
| Risk of disease – Normal | 11 | 0.92 [0.76; 1.12] | 72.9 |  |
| Risk of bias – Low | 3 | 0.83 [0.50; 1.37] | 76.5 | 0.65 |
| Risk of bias – High/Unclear | 10 | 0.94 [0.76; 1.17] | 69.4 |  |

### Age at outcome measurement 5-14

#### 0-2 months

Eight observational studies reported data that could be pooled to assess the overall risk of recurrent wheeze in children aged 5-14 if with EBF ≥0-2 months vs. <0-2 months, and showed no evidence of an association with disease, but high heterogeneity between prospective studies was observed (I^2^=57.4%) (Figure 70).

Subgroup analyses of risk of recurrent wheeze and EBF for ≥0-2 months vs. <0-2 months (Table 18) in children aged 5-14 years, showed no evidence of statistically significant differences between groups.

Figure 70 Exclusive breast feeding ≥0-2 months vs. <0-2 months and risk of recurrent wheeze in children aged 5-14 years


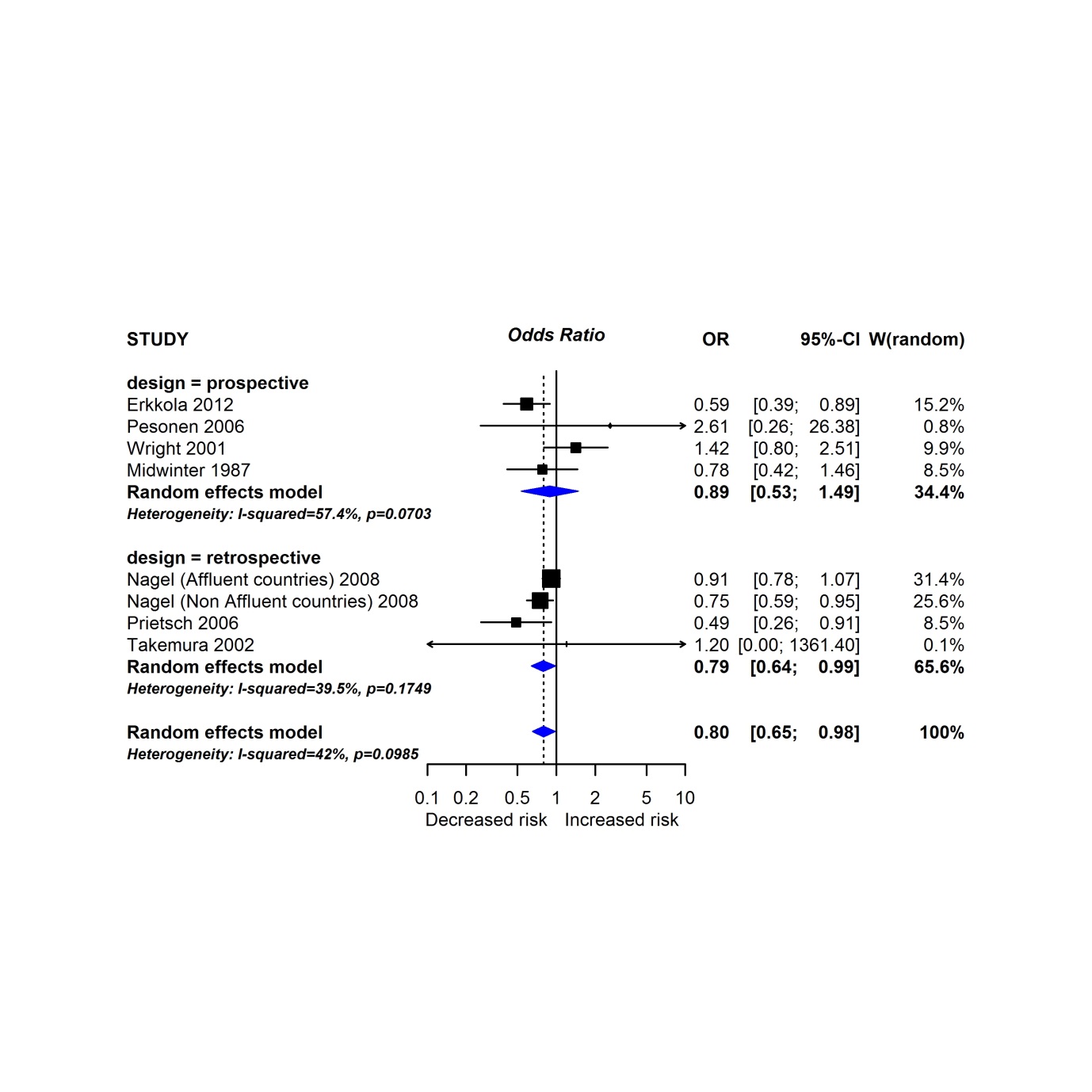


#### 3-4 months

Fifteen observational studies reported data eligible to calculated pooled effect of EBF ≥3-4 months vs. <3-4 months on recurrent wheeze in children aged 5-14 years. Data could not be pooled due to extreme statistical heterogeneity (Figure 71). Ten of them were prospective cohorts, two were nested case-control studies, 1 was a case-control study and 2 were cross-sectional studies. Most studies had low or unclear risk of bias, with the exceptions of three studies in which confounding was not accounted for and therefore they carry an overall high risk of bias. A funnel plot was used to explore publication bias (Figure 71). There was asymmetry across studies indicating risk of publication bias, which was confirmed by the Egger’s test (P=0.01).

Subgroup analyses of risk of recurrent wheeze and EBF for ≥3-4 months vs. <3-4 months (Table 19) showed no evidence of statistically significant differences between groups.

Figure 71 Exclusive breast feeding ≥3-4 months vs. <3-4 months and risk of recurrent wheeze in children aged 5-14 years


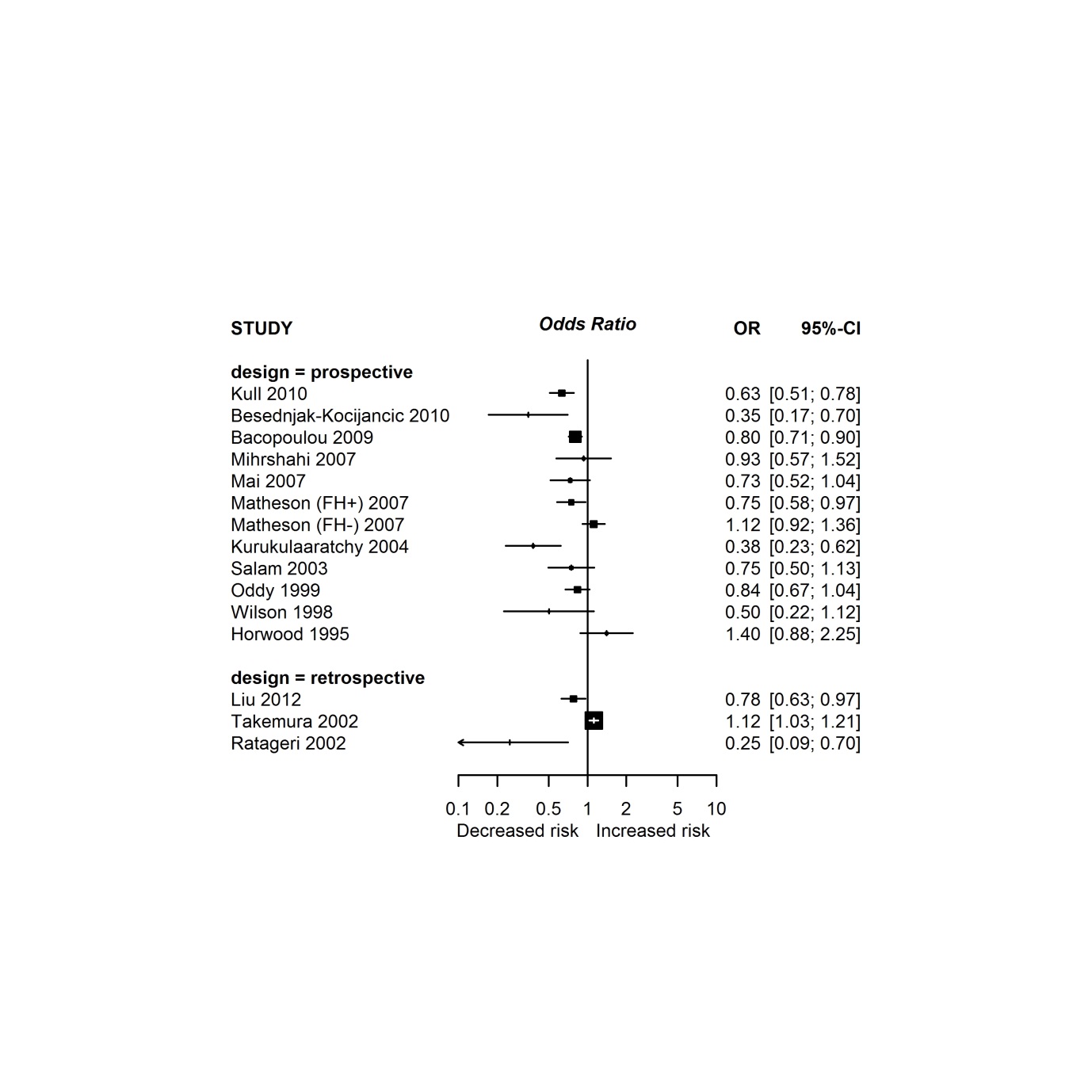


Figure 72 Risk of publication bias of studies investigating exclusive breast feeding ≥3-4 months vs. <3-4 months and risk of recurrent wheeze in children aged 5-14 years


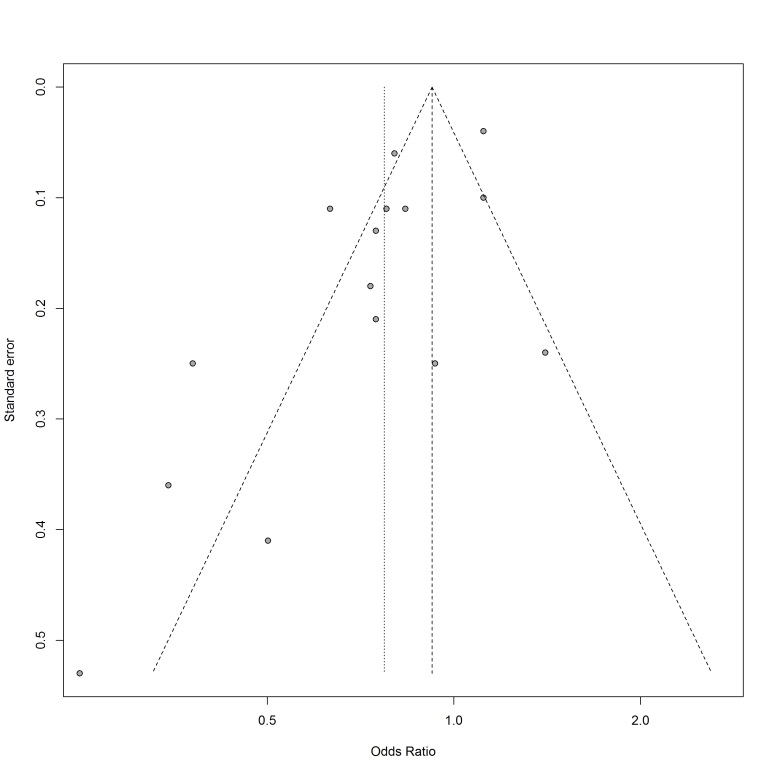


Egger’s test p-value = 0.01

#### 5+ months

Four observational studies reported data that was eligible to calculate pooled estimates of risk of recurrent wheeze in children aged 5-14 years old and exposure to EBF for 5 or more months, showing no evidence of an association (Figure 73) and an extremely high heterogeneity between studies (I^2^=86.9%).

Figure 73 Exclusive breastfeeding ≥5 months vs. <5 months and risk of recurrent wheeze in children aged 5-14 years


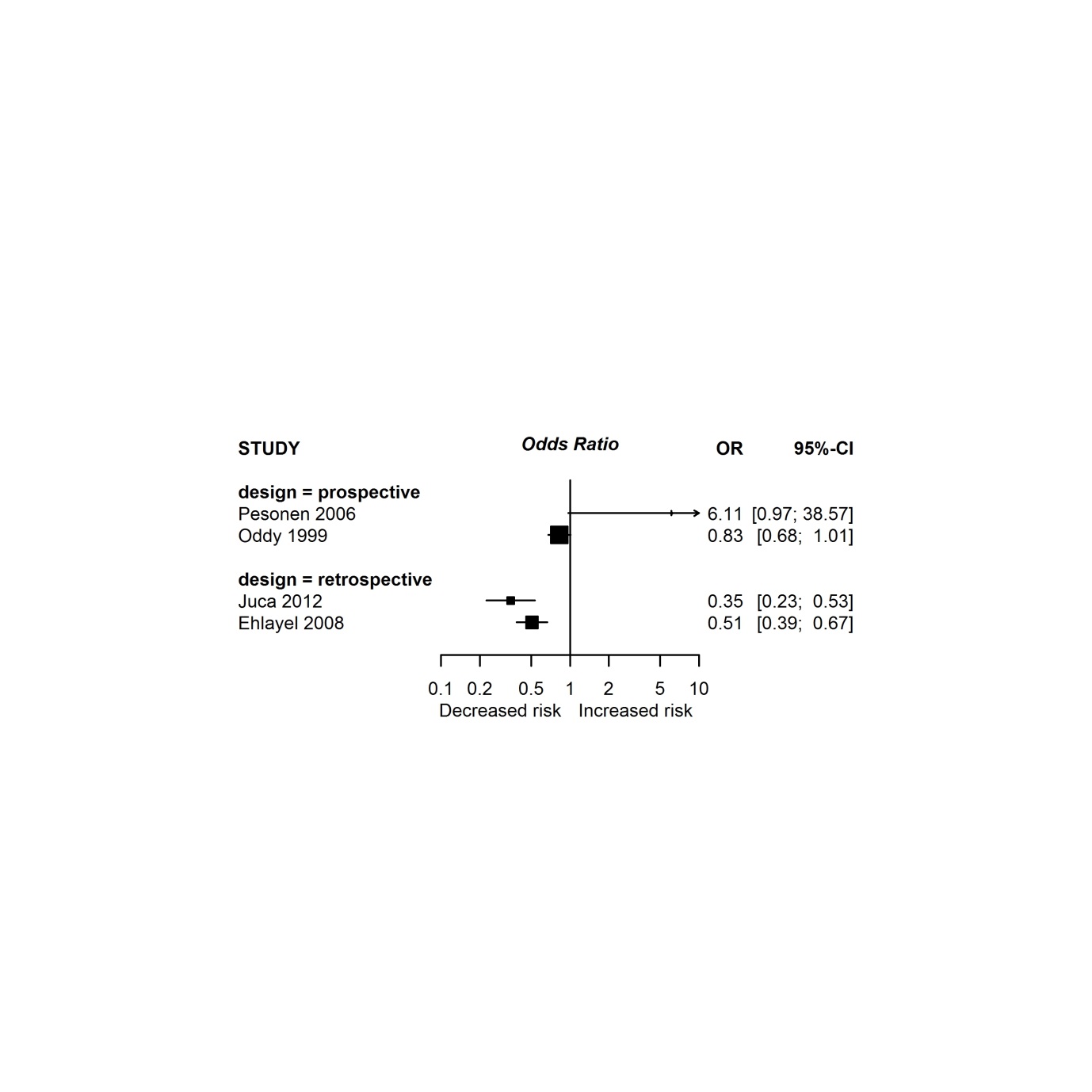


Table 18 Subgroup Analyses of risk of recurrent wheeze and exclusive breastfeeding ≥0-2 months vs. <0-2 months in children aged 5-14 years

|  | **Number of studies** | **OR [95% CI]** | **I^2^ (%)** | **P-value for between groups difference** |
| --- | --- | --- | --- | --- |
| **Overall (if adjusted NA, unadjusted value used)** | 9 | 0.91 [0.69; 1.19] | 67.9 |  |
| **Adjusted** | 5 | 0.93 [0.64; 1.35] | 77.2 | Not tested |
| **Unadjusted** | 9 | 0.96 [0.75; 1.23] | 81.9 |  |
| Study Design – Prospective | 5 | 1.16 [0.63; 2.16] | 78.1 | 0.26 |
| Study Design – Retrospective | 4 | 0.79 [0.64; 0.99] | 39.5 |  |
| Risk of disease – High | 1 | 0.78 [0.42; 1.46] | -- | 0.63 |
| Risk of disease – Normal | 8 | 0.93 [0.68; 1.25] | 71.8 |  |
| Risk of bias – Low | 1 | 2.61 [0.26; 26.38] | -- | 0.37 |
| Risk of bias – High/Unclear | 8 | 0.89 [0.68; 1.18] | 70.9 |  |

Table 19 Subgroup Analyses of risk of recurrent wheeze and exclusive breastfeeding ≥3-4 months vs. <3-4 months in children aged 5-14 years

|  | **Number of studies** | **OR [95% CI]** | **I^2^ (%)** | **P-value for between groups difference** |
| --- | --- | --- | --- | --- |
| **Overall (if adjusted NA, unadjusted value used)** | 15 | 0.77 [0.66; 0.91] | 83.2 |  |
| **Adjusted** | 9 | 0.79 [0.69; 0.92] | 63.0 | Not tested |
| **Unadjusted** | 11 | 0.77 [0.63; 0.95] | 83.5 |  |
| Study Design – Prospective | 12 | 0.76 [0.65; 0.90] | 71.0 | 0.83 |
| Study Design – Retrospective | 3 | 0.80 [0.53; 1.22] | 88.2 |  |
| Risk of disease – High | 3 | 0.68 [0.44; 1.04] | 62.1 | 0.51 |
| Risk of disease – Normal | 12 | 0.79 [0.66; 0.94] | 85.0 |  |
| Risk of bias – Low | 5 | 0.76 [0.55; 1.05] | 78.3 | 0.87 |
| Risk of bias – High/Unclear | 10 | 0.79 [0.65; 0.95] | 83.1 |  |

### Age at outcome measurement 15+

#### 0-2 months

One prospective cohort study reported data for risk of recurrent wheeze in children aged 15+ and feeding ≥0-2 months vs. <0-2 months, showing no evidence of an association (Figure 74).

Figure 74 Exclusive breast feeding ≥0-2 months vs. <0-2 months and risk of recurrent wheeze in children aged 15+ years


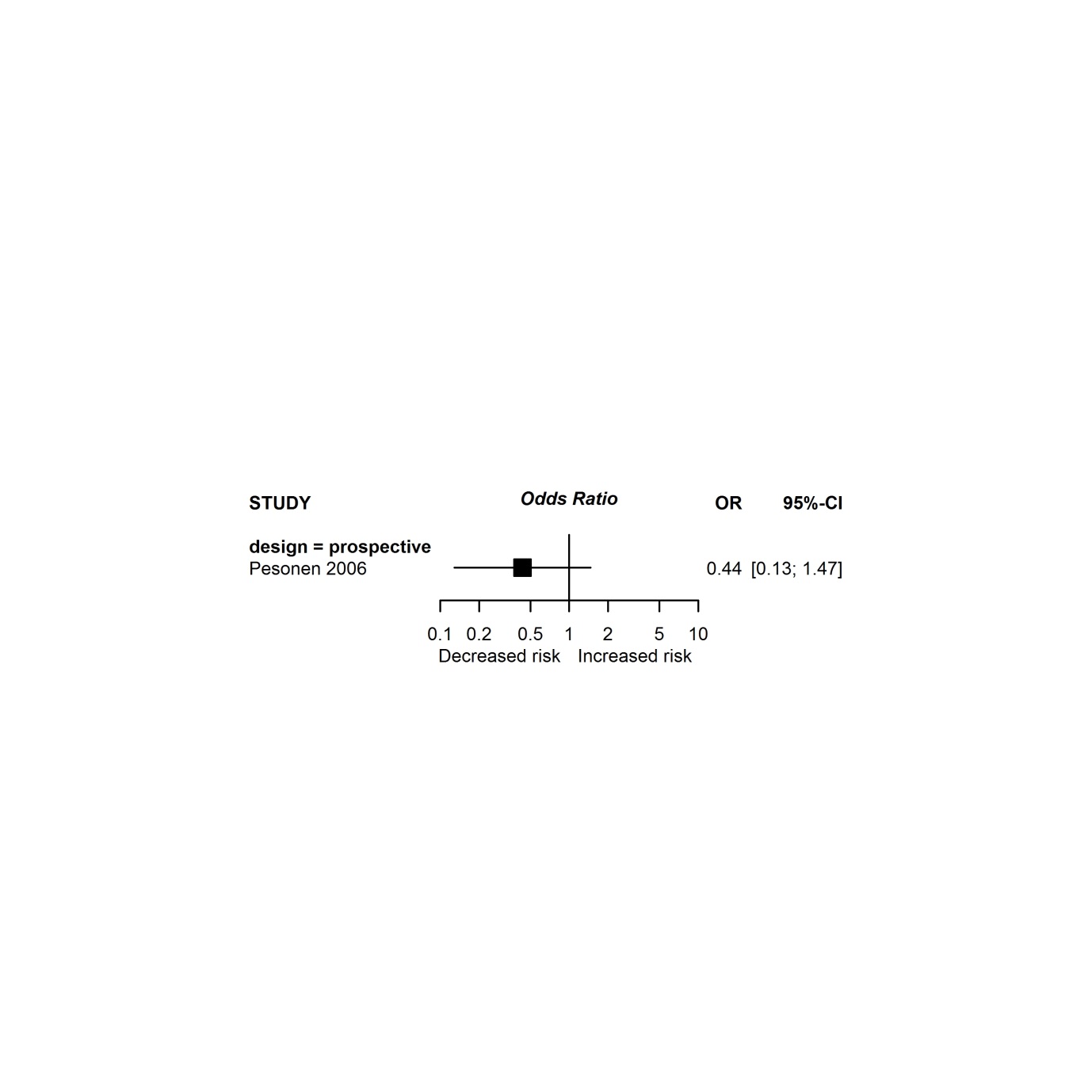


#### 3-4 months

The prospective cohort study of Matheson reported ORs that were eligible to calculate pooled ORs or recurrent wheeze, showing no evidence of an association with the disease in the exposed group. The study had an overall low risk of bias (Figure 75).

Figure 75 Exclusive breast feeding ≥3-4 months vs. <3-4 months and risk of recurrent wheeze in children aged 15+ years


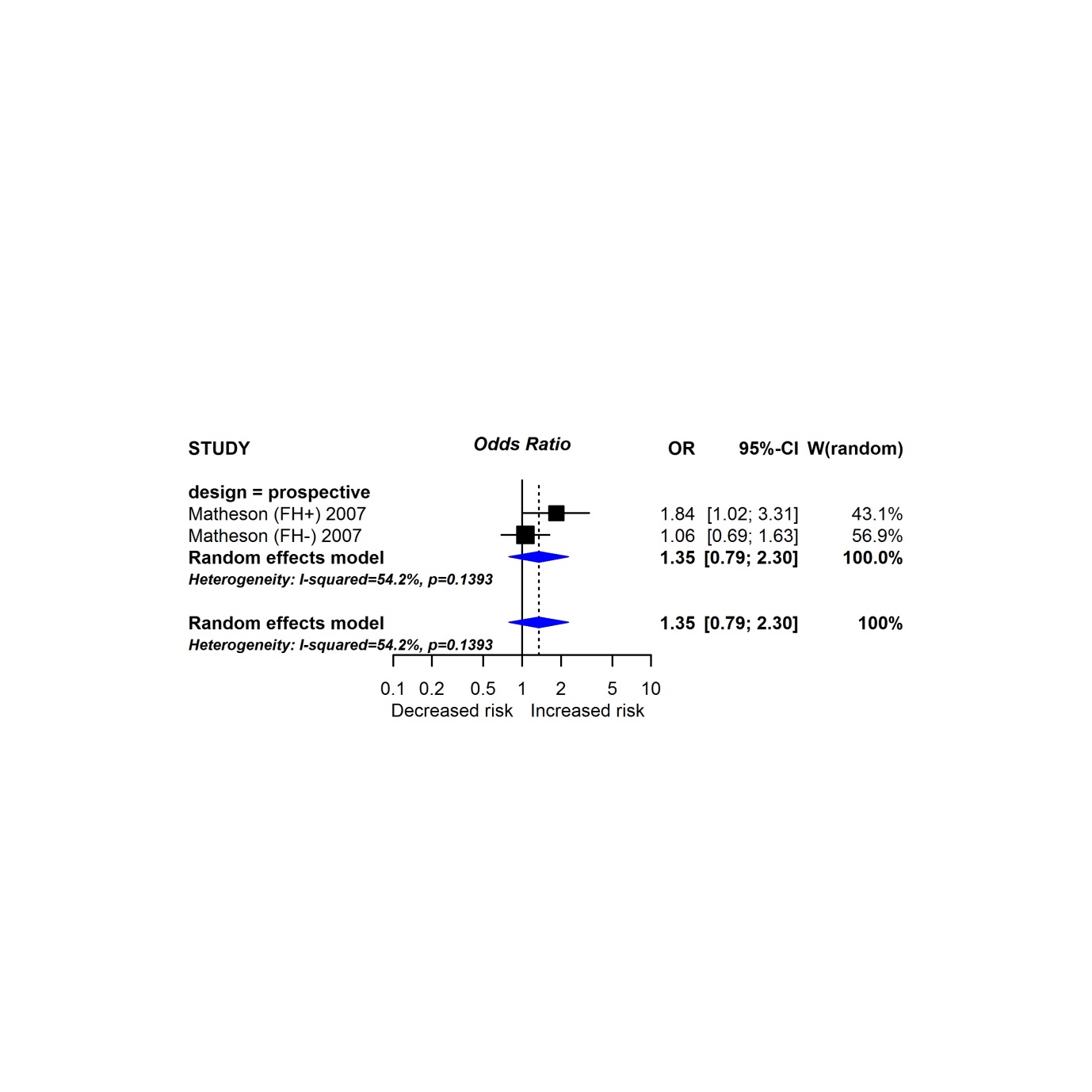


#### 5+ months

The study of Pesonen had reported data on risk of recurrent wheeze in children aged 15+ who were exposed to EBF for ≥5 months vs. <5 months, showing no indication of an effect (Figure 76).

Figure 76 Exclusive breast feeding ≥5 months vs. <5 months and risk of recurrent wheeze in children aged15+ years


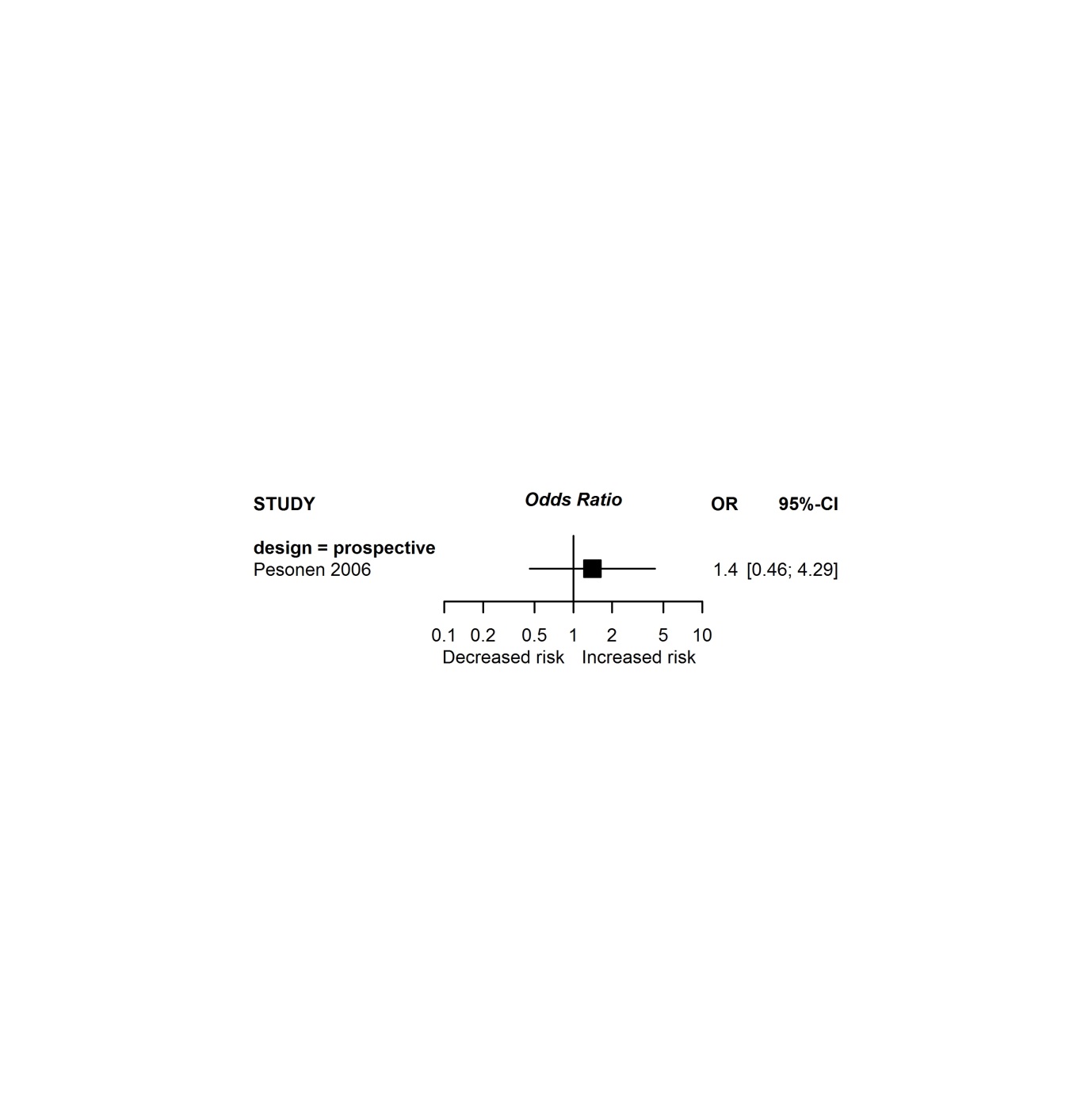


## Exclusive Breastfeeding and Atopic Wheeze

### Age at outcome measurement 0-4

#### 0-2 months

The prospective cohort study of Kull (Figure 77) showed a lower risk of atopic wheeze in children aged 0-4 who were exposed to EBF for feeding ≥0-2 months vs. <0-2 months (OR 0.45; 95% CI 0.21, 0.97). The study carried a low risk of bias across all the domains studied.

Figure 77 Exclusive breast feeding ≥0-2 months vs. <0-2 months and risk of atopic wheeze in children aged 0-4 years


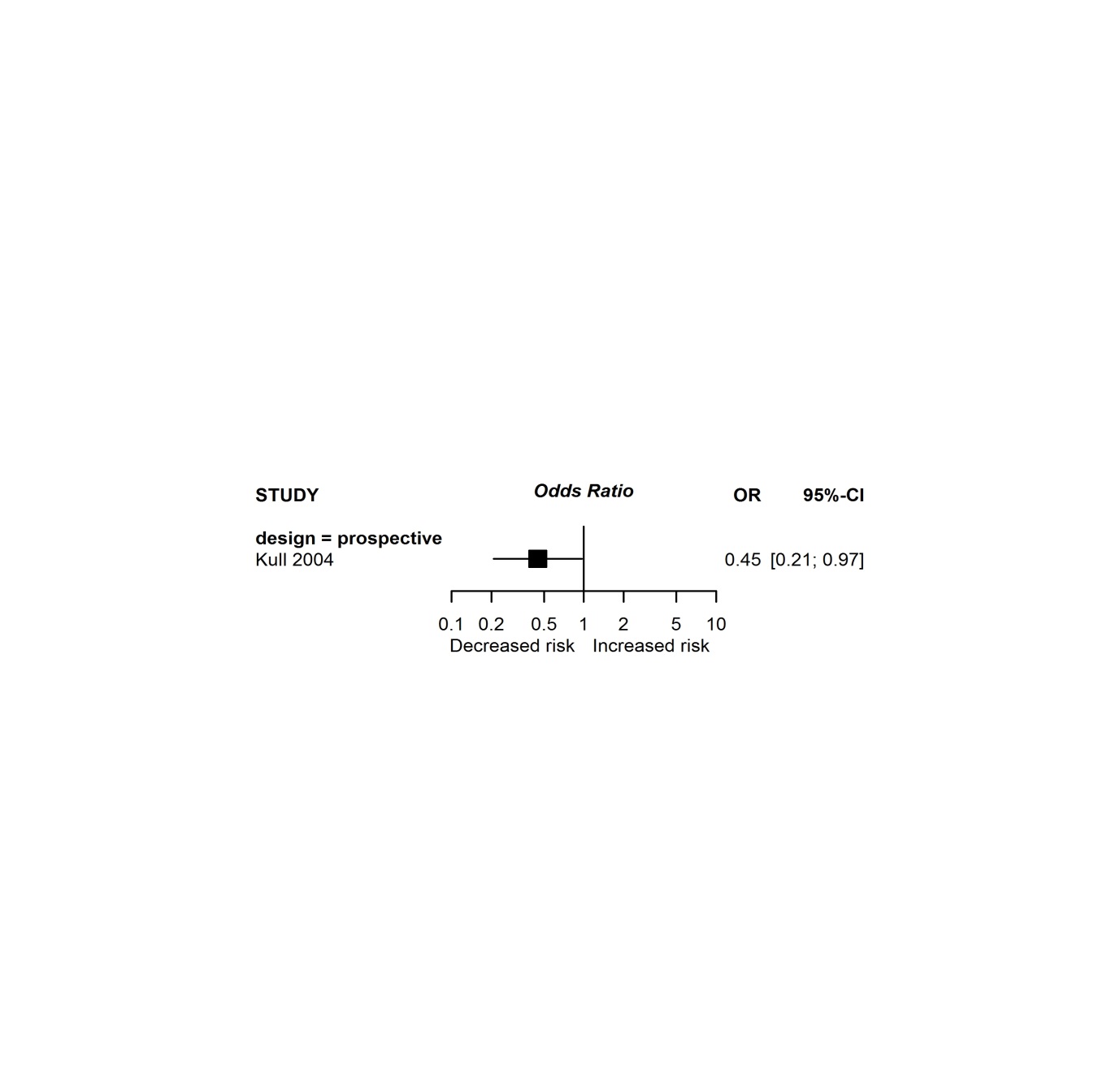


#### 3-4 months

The prospective cohort study of Kull (Figure 78) showed no evidence of a protective effect of prolonged EBF for ≥3-4 months vs. <3-4 months on atopic wheeze in children aged 0-4. The study carried a low risk of bias across all the domains studied.

Figure 78 Exclusive breast feeding ≥3-4 months vs. <3-4 months and risk of atopic wheeze in children aged 0-4 years


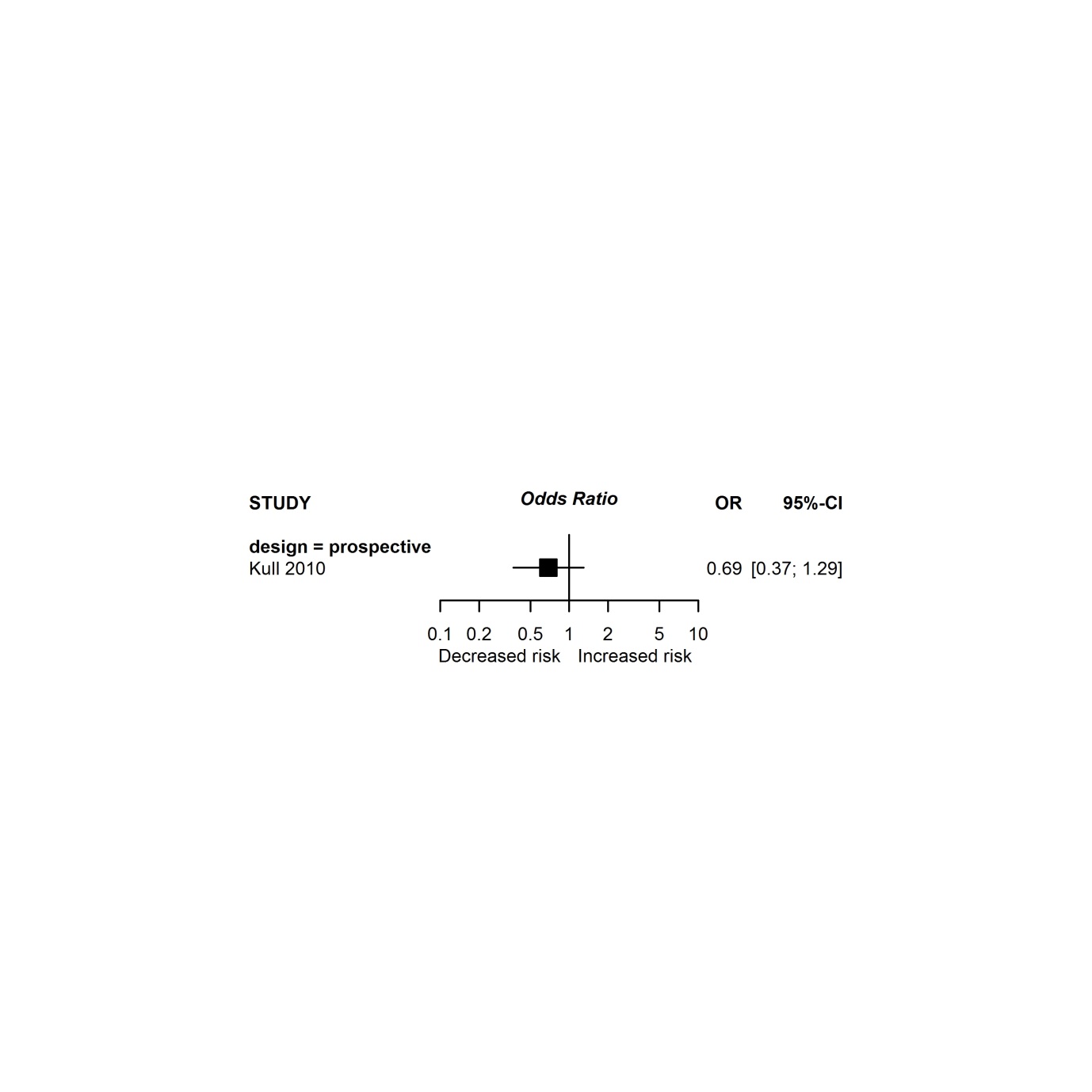


### Age at outcome measurement 5-14

#### 0-2 months

The cross-sectional study of Nagel investigated the risk of atopic wheeze at age 5-14 if EBF was ≥0-2 months vs. <0-2 months and reported no association with disease (Figure 79)

Figure 79 Exclusive breast feeding ≥0-2 months vs. <0-2 months and risk of atopic wheeze in children aged 5-14 years


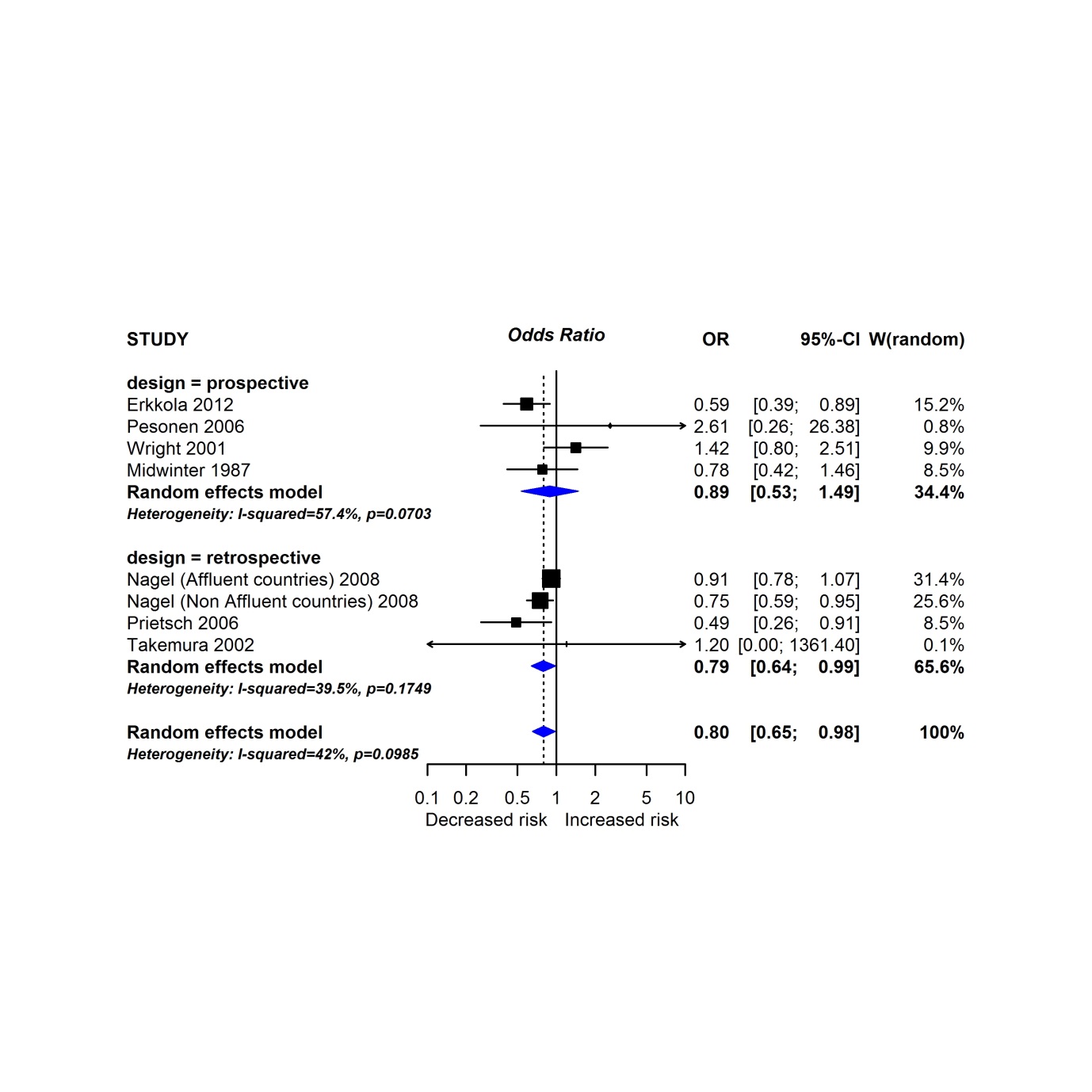


#### 3-4 months

The prospective cohort study of Kull (Figure 80) showed a reduced risk of atopic wheeze in children aged 5-14 years who were EBF for ≥3-4 months vs. <3-4 months (OR 0.58; 95% CO 0.36, 0.93). The study carried a low risk of bias across all the domains studied.

Figure 80 Exclusive breast feeding ≥3-4 months vs. <3-4 months and risk of atopic wheeze in children aged 5-14 years


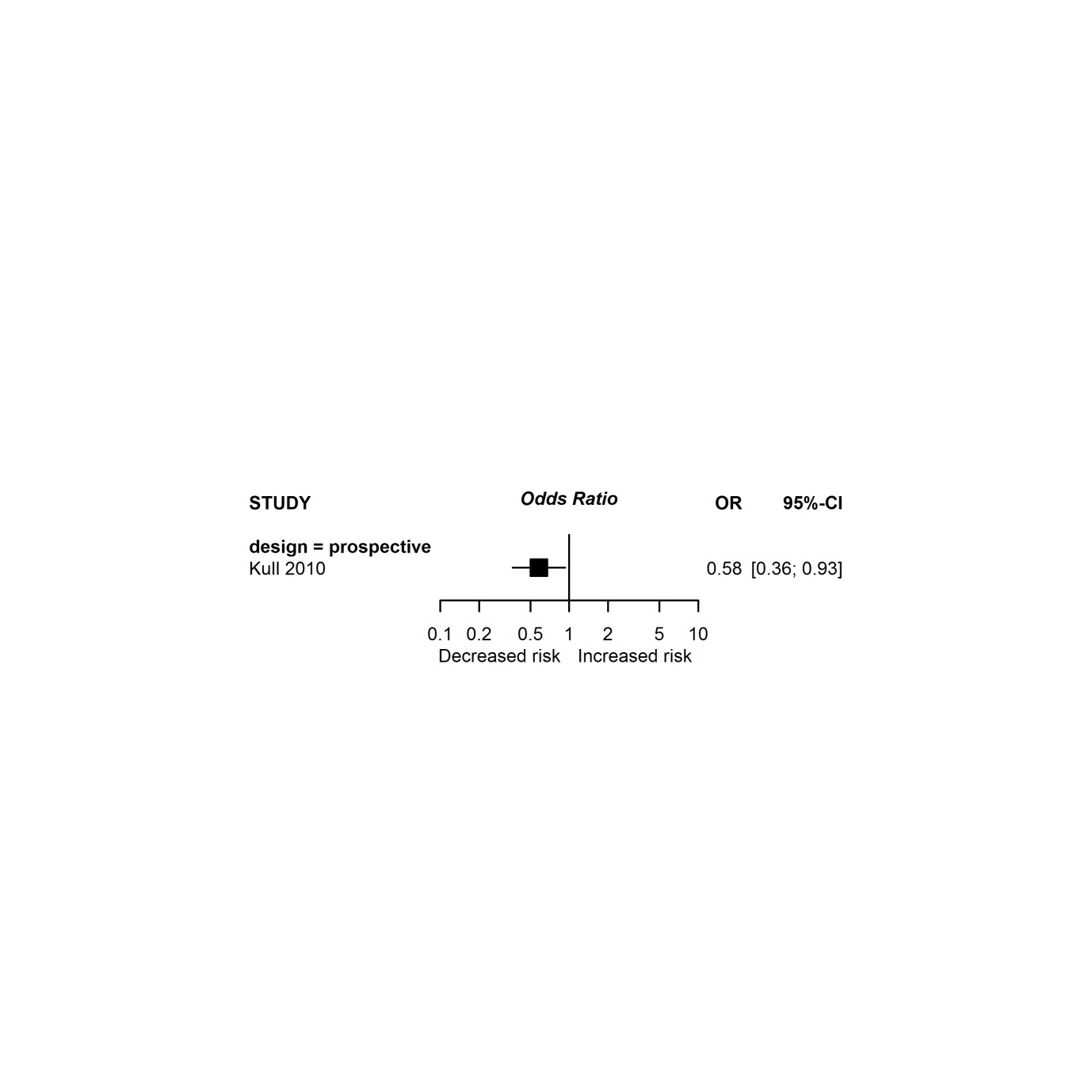


## Exclusive Breastfeeding per month and recurrent wheeze

### Age at outcome measurement 0-4

Two prospective cohort studies reported data eligible to calculate pooled effect estimates of risk of recurrent wheeze and exposure to EBF per month (Figure 81), suggesting a protective effect against disease in the exposed children between 0-4 years (OR 0.83; 0.76, 0.91). There was no evidence of heterogeneity between the two studies (I^2^=0.0%). The study of Silvers had a low overall risk of bias, and the study of Munro had an unclear overall risk of bias.

Figure 81 Exclusive breastfeeding per month and risk of recurrent wheeze in children aged 0-4 years


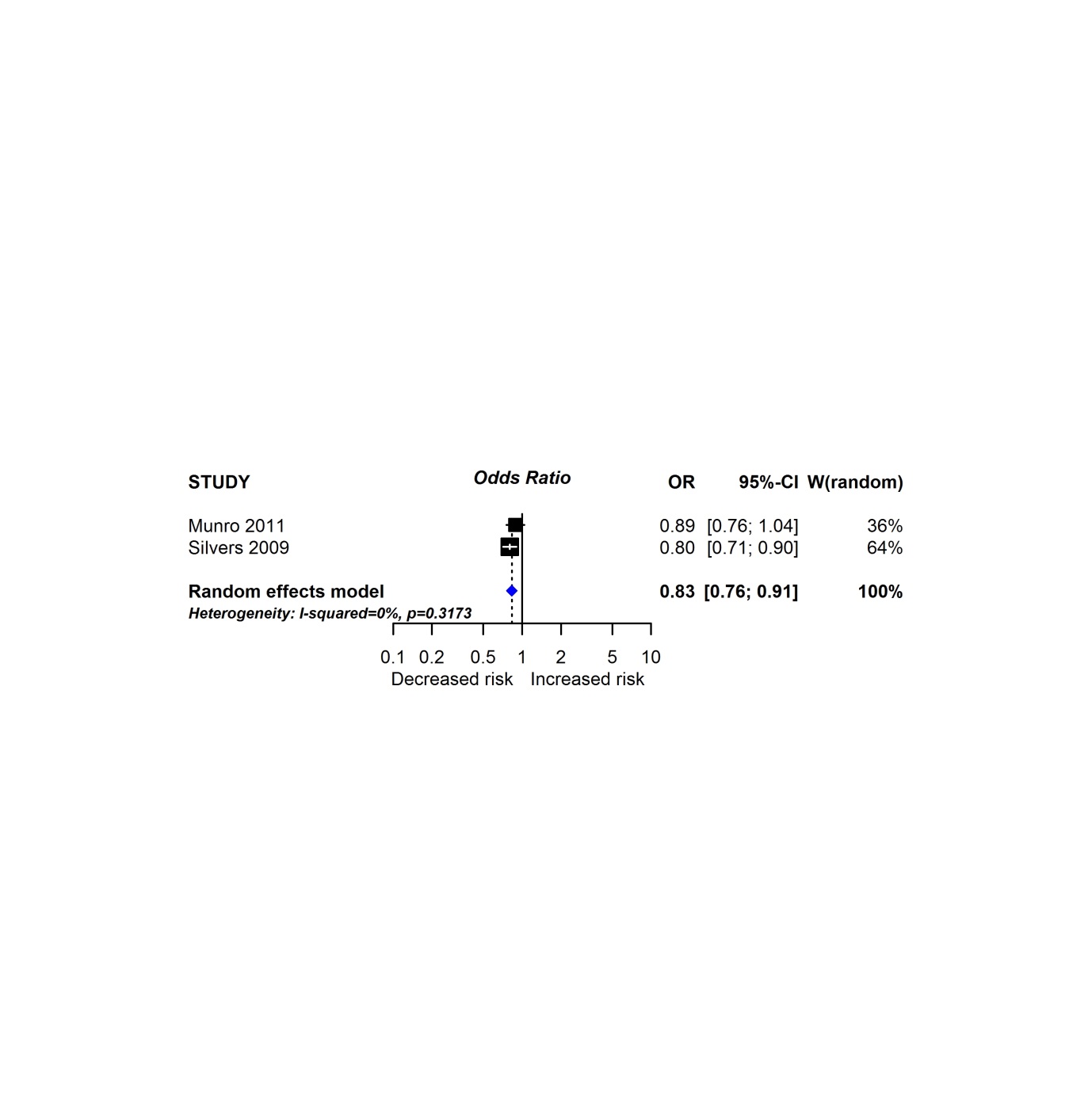


### Age at outcome measurement 5-14

Two prospective cohort studies reported data eligible to calculate pooled effect estimates of risk of recurrent wheeze and exposure to EBF per month (Figure 82), suggesting a protective effect against disease in the exposed children between 5-14 years (OR 0.94; 0.90, 0.99). There was no evidence of heterogeneity between the two studies (I^2^=0.0%). The study of Silvers had a low overall risk of bias, and the study of Oddy had an unclear overall risk of bias.

Figure 82 Exclusive breastfeeding per month and risk of recurrent wheeze in children aged 5-14 years

**
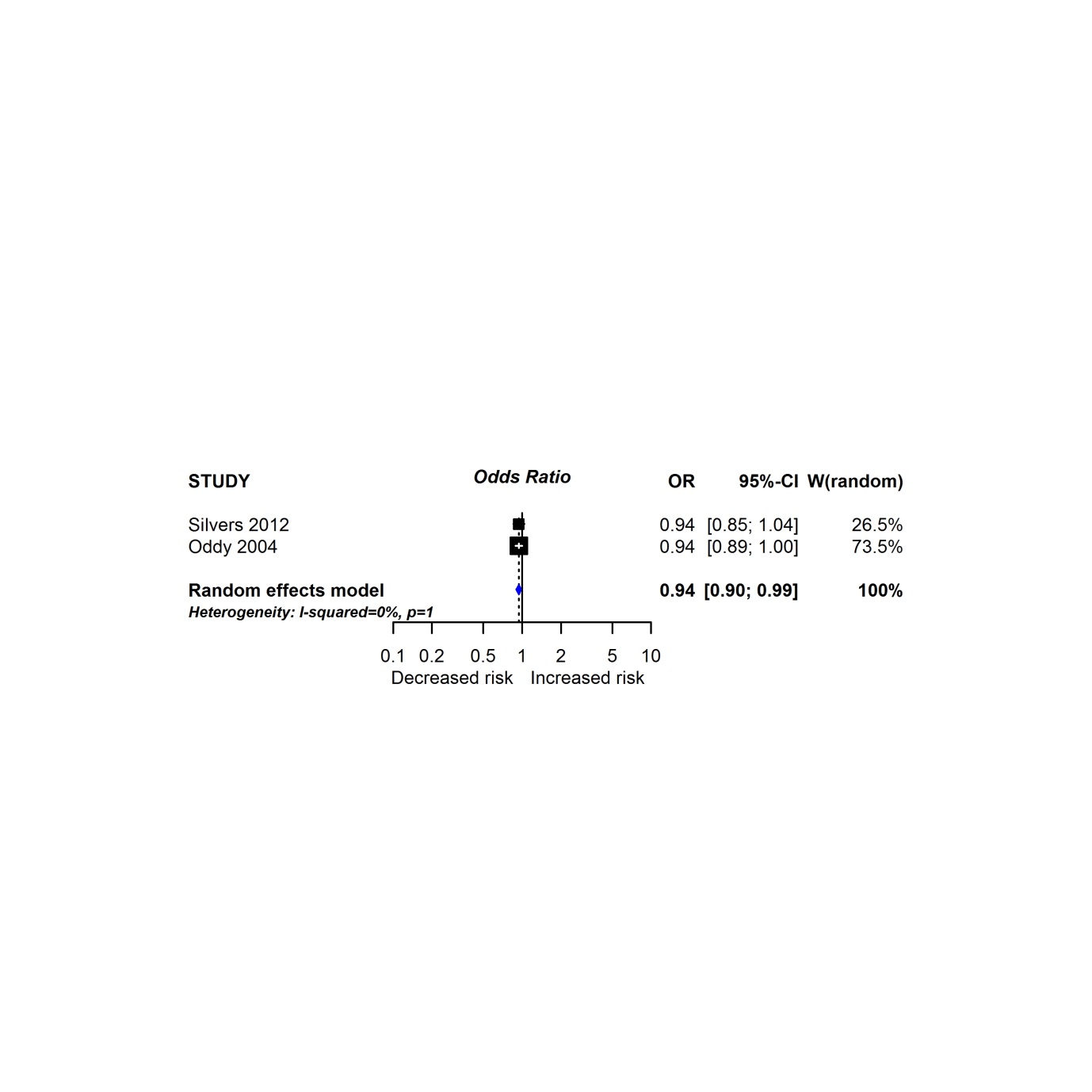
**

#### Exclusive breastfeeding and BHR

#### Age at outcome 5-14 years

#### 0-2 Months

The prospective study of Elliot reported risk of BHR in children aged 5-14 who were exposed to EBF for ≥0-2 months vs. <0-2 months, showing no evidence of an association (Figure 83). The study had an unclear overall risk of bias, due to unclear risk of selection bias.

Figure 83 Exclusive breastfeeding ≥0-2 months vs. <0-2 months and risk of bronchial hyper-responsiveness in children aged 5-14 years


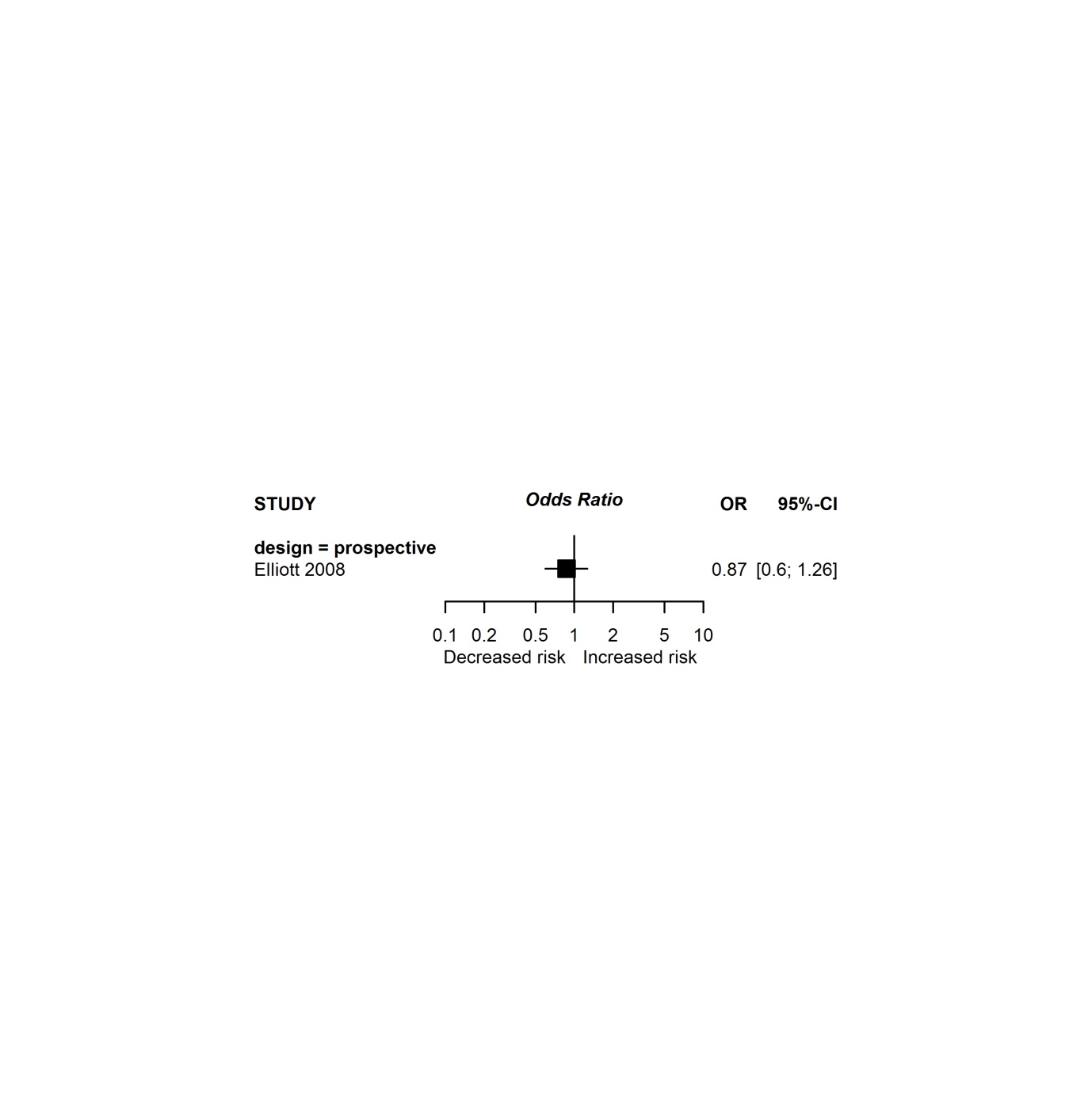


#### 3-4 Months

The prospective study of Poysa reported risk of BHR in children aged 5-14 who were exposed to EBF for ≥3-4 months vs. <3-4 months, showing no evidence of an association (Figure 84). The study had a high overall risk of bias, due to risk of selection and confounding biases.

Figure 84 Exclusive breastfeeding ≥3-4 months vs. <3-4 months and risk of bronchial hyper-responsiveness in children aged 5-14 years


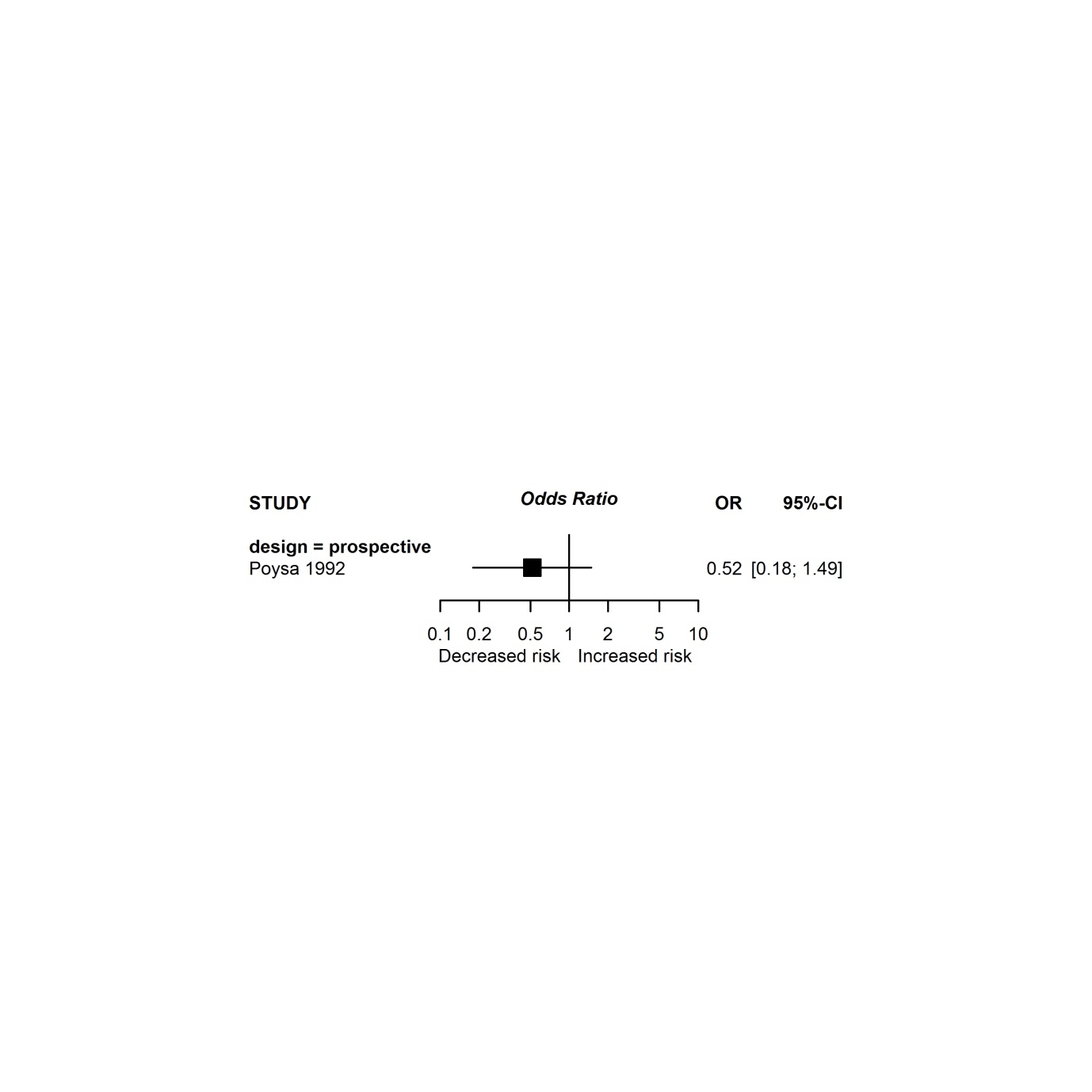


## Data for EBF duration and lung function (FEV1/FVC%)

Two prospective cohorts had results with mean differences: (1) Mandhane, 2007, comparing breastfeeding < 1 month (reference) versus ≥ 1 months at 9 years of age, with unadjusted estimate; (2) Guilbert, 2007, breastfeeding < 1 month (reference) versus > 4 months between 11-16 years of age, with adjusted estimate.

**Figure 84 Exclusive breastfeeding and FEV1/FVC% in children aged 5-14 years**


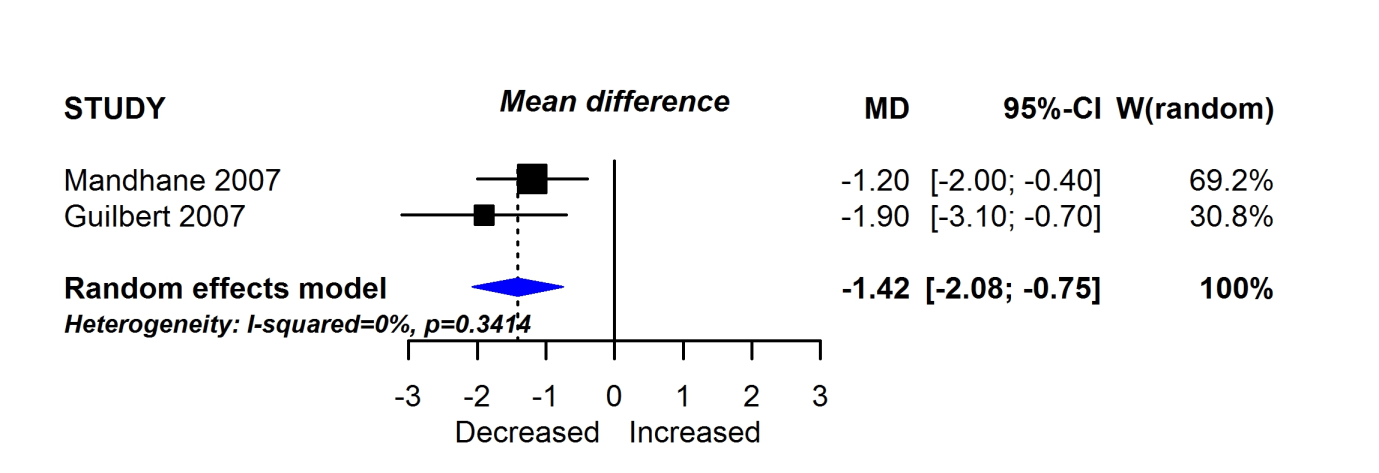


- 1. **Data for EBF duration and Wheeze, BHR or lung function that were not suitable for meta-analysis**

Table 20 summarises the studies on wheeze, BHR or lung function for which data could not be meta-analysed. Meta-analysis was not possible due to the way the data were reported. In total there were 18 reports, of which 16 were prospective cohort studies and 2 cross-sectional studies.

From 9 studies reporting wheeze, and 10 reporting recurrent wheeze, just 2 reported a statistically significant association between increased EBF and reduced wheeze and 2 studies reported a significant association between increased EBF and increased wheeze.

There were 2 studies for BHR, both reporting no significant association between EBF and BHR. There were 14 measures reported from 3 prospective studies of lung function, in individuals aged 8-26 years old. Of these 14 measures, 2 were significantly associated with increased EBF, 2 with reduced EBF and 10 were not associated with EBF.

Table 20 Studies investigating the association between exclusive breastfeeding and wheeze which were not eligible for meta-analysis

| **First Author and year of publication** | **Design** | **Outcome** | **Age** | **N/n** | **EBF duration (continuous or categorical in months)** | **Measure of association** | **Effect** | **P-value** |
| --- | --- | --- | --- | --- | --- | --- | --- | --- |
| Giwercman, 2010 ([134](#_ENREF_134)) | PC | wheeze | 2 | 313 | 0+ | Adjusted HR (95%CI) | 0.67 (0.48-0.96) | <0.05 |
| Zutavern 2004 ([114](#_ENREF_114)) | PC | wheeze, recurrent wheeze | 2, 5.5 | 606 | >2 | Adjusted OR |  | NS |
| Cano Garcinuno ([130](#_ENREF_130)) (2003) | CS | wheeze | 3 | 234 | >3 | Adjusted HR (95%CI) | 0.83 (0.42-1.64) | 0.60 |
| Chong Neto, 2007 ([141](#_ENREF_141)) | CS | wheeze | 1 | 3003/1364 | >4, >6 | Adjusted PR |  | NS |
| Kramer, 2003 ([156](#_ENREF_156)) | PC | wheeze | 1 | 3483/8 | >6 | Adjusted OR (95%CI) | 1.49 (0.66-3.36) | 0.34 |
| da Costa Lima, 2003 ([81](#_ENREF_81)) | PC | wheeze | 18 | 2247 | 0+ | Adjusted PR (95%CI) | 1.22 (1-1.5) | 0.054 |
| Kurukulaaratchy, 2006 ([145](#_ENREF_145)) | PC | wheeze-atopic | 10 | unclear | >3 | Adjusted OR (95%CI) | 0.09 (0.01-0.93) | 0.04 |
| Kurukulaaratchy, 2006 ([145](#_ENREF_145)) | PC | wheeze-non atopic | 10 | 492/178 | >3 | Adjusted OR |  | NS |
| Rullo, 2007 ([163](#_ENREF_163)) | PC | recurrent wheeze | 1.5, 5 | 101 | >6 | Adjusted OR |  | NS |
| Hesselmar 2010 ([5](#_ENREF_5)) | PC | recurrent wheeze | 1.5 | 184 | continuous |  |  | NS |
| Nwaru, 2013 ([24](#_ENREF_24)) | PC | recurrent wheeze | 5 | 3675 | 0+, >3 | Adjusted HR |  | NS |
| Mandhane, 2007 ([138](#_ENREF_138)) | PC | recurrent wheeze | 9 | 714/76 | >1 | Adjusted OR | 1.22 | 0.04 |
| Pesonen, 2006 ([153](#_ENREF_153)) | PC | recurrent wheeze | 5 | 160/1 | >2 | Prevalence | 3.5% Vs 0% | - |
| Kramer, 2009 ([157](#_ENREF_157)) | PC | recurrent wheeze | 6.5 | 2951/44 | >6 | Adjusted OR (95%CI) | 1.2 (0.6-2.4) | 0.62 |

Table 21 Studies investigating the association between exclusive breastfeeding and BHR or lung function which were not eligible for meta-analysis

| **First Author and year of publication** | **Design** | | **Outcome** | | **Age** | | | **N/n** | | **EBF duration (continuous or categorical in months)** | | **Measure of association** | | | | **Effect** | **P-value** |
| --- | --- | --- | --- | --- | --- | --- | --- | --- | --- | --- | --- | --- | --- | --- | --- | --- | --- |
| Elliot  2008 (4) | PC | Bronchial hyper-responsiveness | | | | 5-14 | | | 3295 | | ≥0-2mo vs <0-2mo | | logistic regression, adjusted | | 0.87 (0.60-1.26) | | 0.46 |
| Poysa  1992 (175) | PC | Bronchial hyper-responsiveness | | | | 5-14 | | | 119 | | ≥3-4mo vs <3-4mo | | Compared proportions, unadjusted | | 0.52 (0.18-1.49) | | 0.12 |
| Kull, 2010 ([127](#_ENREF_127)) | PC | | FEV1(ml) | | 8 | | | 1838 | | >4 | | Adjusted Mean difference (95%CI) | | | | 17.4 (-5.7, 40.5) | NS |
|  |  |  | PEF(L) | | 8 | | | 2168 | | >4 | | Adjusted Mean difference (95%CI) | | | | 4.5 (0.68, 8.29) | 0.02 |
| Mandhane, 2007 ([138](#_ENREF_138)) | PC | | FEV1/FVC% | | 26 | | | 714 | | >1 | | Mean difference (95%CI), unadjusted | | | | -0.1 (-0.93; 0.73) | 0.83 |
|  |  |  |  |  | 9 | | | 714 | | >1 | | Mean difference (95%CI), unadjusted | | | | -1.2 (-2.00; -0.40) | 0.004 |
| Guilbert, 2007 ([178](#_ENREF_178)) | PC | | FEF25–75(ml/s) | | 13 | | | 679 | | 2-4 vs >1 | | Adjusted Mean difference (95%CI) | | | | -2.9 (-132.3-126.5) | 0.9 |
| Guilbert, 2007 (176) | PC | | FEF25–75(ml/s) | | 13 | | | 679 | | >4 vs <1 | | Adjusted Mean difference (95%CI) | | | | -124 (-267-19) | 0.009 |
| Guilbert, 2007 (176) | PC | | FEF25–75/ FVC% | | 13 | | | 679 | | 2-4 vs >1 | | Adjusted Mean difference (95%CI) | | | | -2.7 (-6.8-1.5) | 0.02 |
| Guilbert, 2007 (176) | PC | | FEF25–75/ FVC% | | 13 | | | 679 | | >4 vs <1 | | Adjusted Mean difference (95%CI) | | | | -7.6 (-12.1;-3.1) | 0.001 |
| Guilbert, 2007 (176) | PC | | FEV1(ml) | | 13 | | | 679 | | 2-4 vs >1 | | Adjusted Mean difference (95%CI) | | | | 27 (-33.8-87.8) | 0.1 |
| Guilbert, 2007 (176) | PC | | FEV1(ml) | | 13 | | | 679 | | >4 vs <1 | | Adjusted Mean difference (95%CI) | | | | 20 (-38 .8-78.8) | 0.5 |
| Guilbert, 2007 (176) | PC | | FEV1/FVC% | | 13 | | | 679 | | 2-4 vs >1 | | Adjusted Mean difference (95%CI) | | | | -0.7 (-1.9, -0.5) | 0.2 |
| Guilbert, 2007 (176) | PC | | FEV1/FVC% | | 13 | | | 679 | | >4 vs <1 | | Adjusted Mean difference (95%CI) | | | | -1.9 (-3.1; -0.7) | 0.004 |
| Guilbert, 2007 (176) | PC | | FVC(ml) | 13 | | | 679 | | | 2-4 vs >1 | | Adjusted Mean difference (95%CI) | | 43 (-84.4, 170.4) | | | 0.2 |
| Guilbert, 2007 (176) | PC | | FVC(ml) | 13 | | | 679 | | | >4 vs <1 | | Adjusted Mean difference (95%CI) | | 103 (24.6, 181.4) | | | 0.01 |
| Elliot  2008 (4) | PC | Bronchial hyper-responsiveness | | | | 5-14 | | | 3295 | | ≥0-2mo vs <0-2mo | | logistic regression, adjusted | | 0.87 (0.60-1.26) | | 0.46 |
| Poysa  1992 (175) | PC | Bronchial hyper-responsiveness | | | | 5-14 | | | 119 | | ≥3-4mo vs <3-4mo | | Compared proportions, unadjusted | | 0.52 (0.18-1.49) | | 0.12 |

# Solid Food Introduction and Wheeze

## Overall characteristics of studies, risk of bias and summary of results

Table 21 describes the main characteristics of the studies that assessed solid food introduction (SFI) in relation to wheeze risk. A total of 15 observational studies, and no intervention studies, reported the association between SFI and wheeze. Of these, 13 were prospective cohort studies and 2 were case-control studies. The majority of studies (n=9) are from Europe – others are from the Asia-Pacific region (n=4), North America (n=1), 1 from South Asia.

Overall, valid data on SFI duration in the first 2 years of life and wheeze risk were available from over 14,000 subjects. Information on wheeze was obtained solely from parental or self-reported in 3 studies and through Dr-diagnosis in 7 studies. Another 4 studies used a combination of self /parental report, Doctor diagnosis, and/ or objective measures (e.g. bronchial hyper-responsiveness (BHR)). One study used an unclear method for defining wheeze.

With regards to time of outcome diagnosis, 8 studies explored the association between SFI duration and wheeze at age 0-4 years and 4 at age 5-14 years. No studies investigated outcomes at age 15 years or beyond. A further three studies investigated the association between SFI duration and wheeze at various time points between the ages of 1 and 10 years. To ascertain exposure to SFI, 4 studies used a questionnaire method, 6 used an interview and 1 used a food frequency questionnaire (FFQ). A further 4 studies used a combination of 2 or more of these methods.

Risk of bias was assessed using the NICE Methodological checklists for cohort and case-control studies. Figure 84 illustrates the distribution of bias across the five main methodological areas of the studies. Over 40% of the studies had a high risk of overall bias, mainly due to confounding bias. The majority of the studies were considered to have a low risk of conflict of interest bias. Overall, there was no suggestion of an effect of SFI on wheeze. None of the meta-analyses that could be carried out showed an effect of SFI on wheeze. There were additionally three studies that could not be meta-analysed and showed no association with wheeze.

Table 21 Characteristics of included studies evaluating solid food introduction and wheeze

| **First Author & Publication Year** | **Design** | **N** | **Exposure assessment** | **Method of outcome assessment** | **Age at outcome (years)** | **Country** | **Population characteristics** |
| --- | --- | --- | --- | --- | --- | --- | --- |
| Hesselmar, 2010 ([5](#_ENREF_5)) | PC | 184 | I | DD asthma (>=3 episodes of wheeze) | 1.5 | Sweden | ALLERGYFLORA study. Population based study of babies selected from antenatal clinics between 1998 and 2003 - mainly high risk of allergic disease |
| Mihrshahi, 2007 ([20](#_ENREF_20)) | PC | 516 | I | DD PLUS current wheeze | 5 | Australia | CAPS study. Infants born in 1997-1999 with family history of asthma or wheezing |
| Fergusson, 1983([132](#_ENREF_132)) | PC | 1110 | R/I | DD asthma (>=2 episodes of wheeze) | 4 | New Zealand | Christchurch Child Development Study. Population based cohort of infants born in 1977 in the Christchurch urban region |
| Larsson, 2008 ([22](#_ENREF_22)) | PC | 4779 | Q | DD | 9 | Sweden | DBH study. Preschool children aged 1–6 years surveyed in 2000 and 2005. |
| Forsyth, 1993; Wilson, 1998  ([25](#_ENREF_25), [179](#_ENREF_179)) | PC | 545 | Q, D/I | Self-reported wheeze; DD; Physician assessment | 2, 7.3 | UK | Dundee infant feeding study. Population based cohort of infants born between 1983-1986 |
|  |  |  |  |  |  |  |  |
| Karunasekera, 2001 ([52](#_ENREF_52)) | CC | 600 | Q | Physician assessment | 10 | Sri Lanka | Hospital-based cases aged 1-10 years old with age matched controls from inpatient clinics |
| Snijders, 2008 ([56](#_ENREF_56)) | PC | 1894 | Q | >=4 episodes of wheeze | 2.0 | Netherlands | KOALA study. Population based birth cohort of infants born between 2000-2002 (consisting of cohorts with conventional and alternative lifestyle) |
| Zutavern, 2008 ([180](#_ENREF_180)) | PC | 2073 | FFQ | Parent reported current wheeze OR asthma medication; DD | 6 | Germany | LISA study. Population based birth cohort of infants born between November 1997 and January 1999 at selected maternity hospitals in 4 German cities |
| Majeed, 2008 ([150](#_ENREF_150)) | CC | 398 | Q/I | DD asthma AND >=2 episodes of wheeze | 3 | Pakistan | Cases are 1-12 years old children with wheeze/asthma admitted to paediatrics wards. Controls are age matched children and adolescents attending immediately after selection of the index case |
| Marini, 1996 ([59](#_ENREF_59)) | PC | Unclear | Q | Physician assessment (>=3 episodes of wheeze) | 3 | Italy | Infants with family history of allergy whose mother were proposed to participate in an allergy prevention program |
| Morgan, 2004 ([66](#_ENREF_66)) | PC | 144 | I | DD | 1.5 | U.K. | Infants from five prospective randomised dietary trials conducted in the UK between 1993 and 1997. Two trials involved term appropriate for gestational age (AGA) infants, one trial involved term small for gestational age (SGA) infants (birth weight 10th centile for gestational age and sex), and two trials involved preterm infants (37 weeks gestation, birth weight 2000g). |
| Hide, 1981; Kurukulaaratchy, 2004  ([101](#_ENREF_101), [146](#_ENREF_146)) | PC | 843 | D/Q, Q | Parent reported wheeze; >=2 episodes of wheeze between age <4 and 10 years; Late onset wheeze: onset >4 years of age and still wheezed at 10 years | 1, 10 | UK | The Isle of Wight Prevention study: born in 1977-1978 (normal risk of disease) |
|  |  |  |  |  |  |  |  |
| Van Asperen, 1983 ([172](#_ENREF_172)) | PC | 79 | I | Parent reported wheeze | 1.3 | Australia | Cohort recruited from medical service, born in 1980-1981 with family history of atopy (high risk of disease) |
| Joseph, 2012 ([181](#_ENREF_181)) | PC | 594 | I | Unclear | 3 | USA | WHEALS STUDY: Recruited from hospital prenatal care and born in 2005 (normal risk of disease) |
| Zutavern 2004 ([114](#_ENREF_114)) | PC | 606 | I | Parent reported current wheeze | 2, 5.5 | UK | Cohort recruited from general practices and born in 1993-1995 (normal risk of disease) |

Figure 85 Risk of bias in studies of solid food introduction and wheeze

## Solid Food Introduction and Wheeze

### Age at outcome measurement 0-4

#### 3-4 months

Two prospective cohort studies reported data that could be pooled to estimate the combined effect of SFI on wheeze at age 0-4 years, showing no suggestion of a protective effect (Figure 86). The study of Hide had a low overall risk of bias, whilst the study of Van Asperen had a high overall risk of bias (due to confounding bias), which might contribute to explain the high heterogeneity observed between studies (I^2^=58.7%)

Figure 86 Solid Food Introduction ≥3-4 months vs. <3-4 months and risk of wheeze in children aged 0-4 years


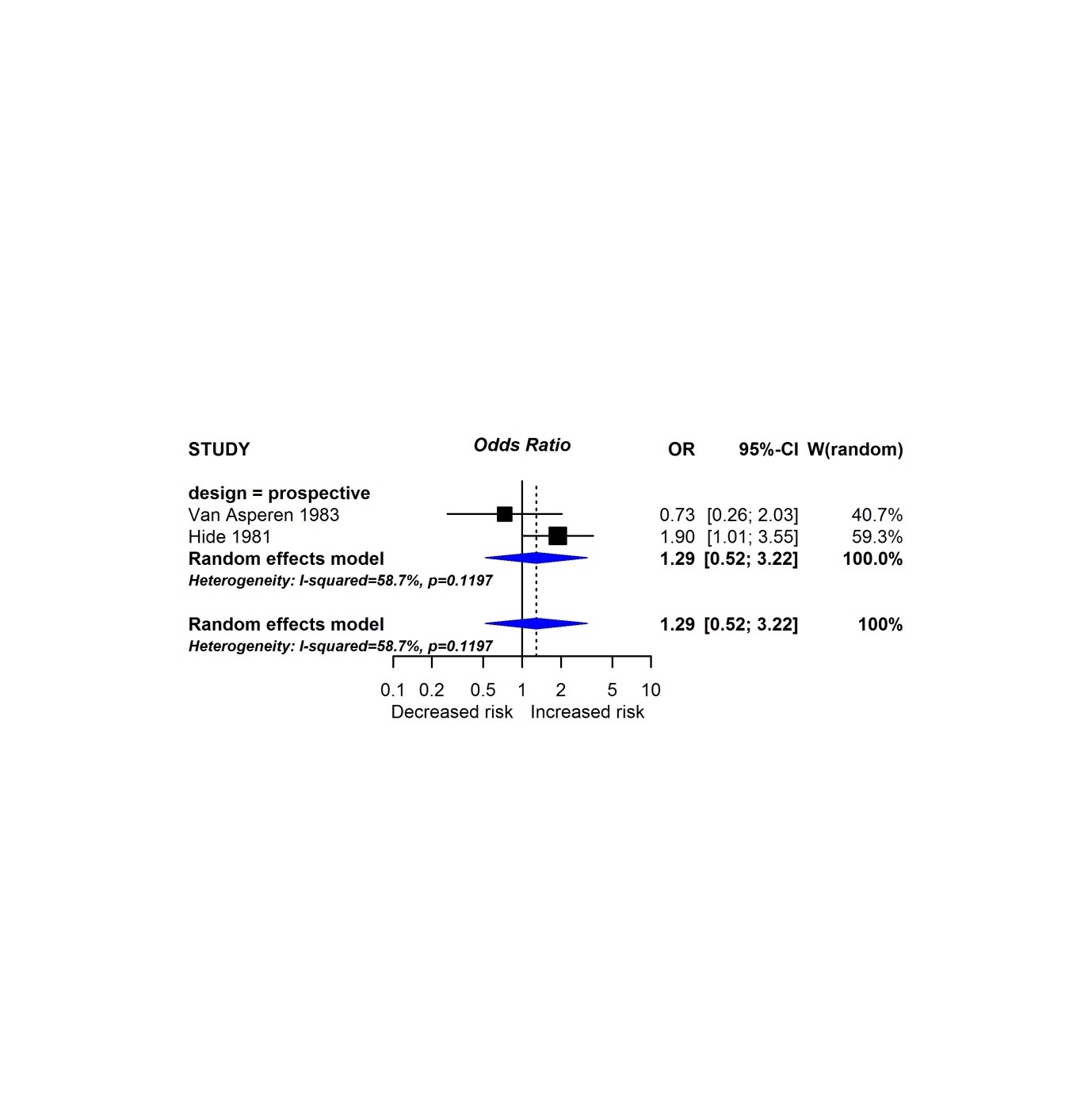


### Age at outcome measurement 5-14

#### 3-4 months

Two prospective cohort studies reported data that could be pooled to estimate the combined effect of SFI at ≥3-4 months vs. <3-4 months, on wheeze at age 5-14 years, showing no suggestion of a protective effect (Figure 87). The study of Zutavern had a low overall risk of bias, whilst the study of Wilson carried a had a high overall risk of bias (due to confounding bias), which might contribute to explain the high heterogeneity observed between studies (I^2^=60.0%)

Figure 87 Solid Food Introduction ≥3-4 months vs. <3-4 months and risk of wheeze in children aged 5-14 years


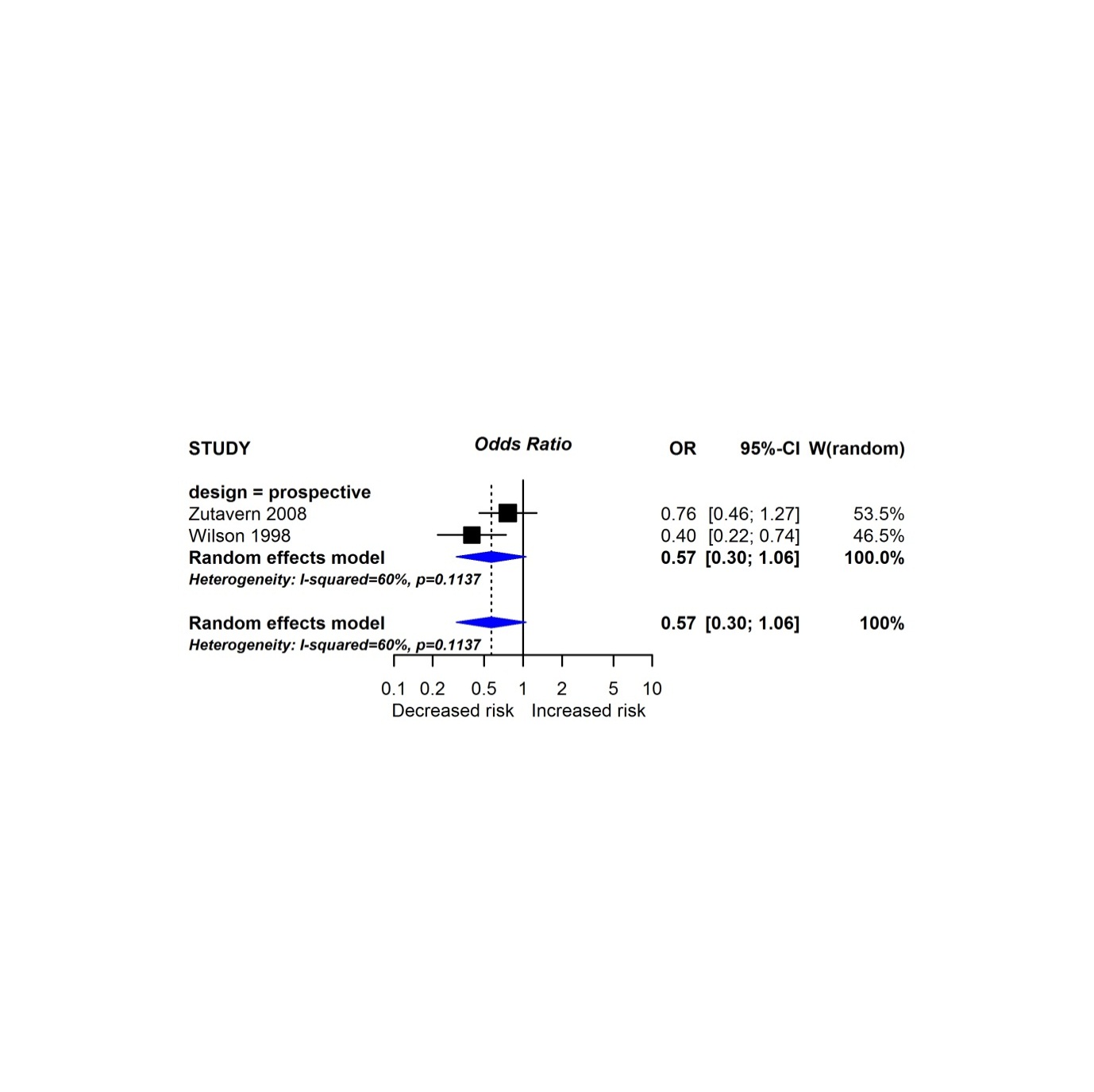


## Solid Food Introduction and Recurrent Wheeze

### Age at outcome measurement 0-4

#### 3-4 months

Three prospective cohort studies and one case-control study reported data that could be pooled to estimate overall risk of recurrent wheeze at age 0-4 years comparing SFI at ≥3-4 months vs. <3-4 months. There was no evidence of an effect (Figure 88). Two studies (Majeed and Ferguson) had a high risk of overall bias (due to confounding bias), one had a low risk and one was unclear. These variations might explain the high heterogeneity observed between studies (I^2^=87.3%)

Figure 88 Solid Food Introduction ≥3-4 months vs. <3-4 months and risk of recurrent wheeze in children aged 0-4 years


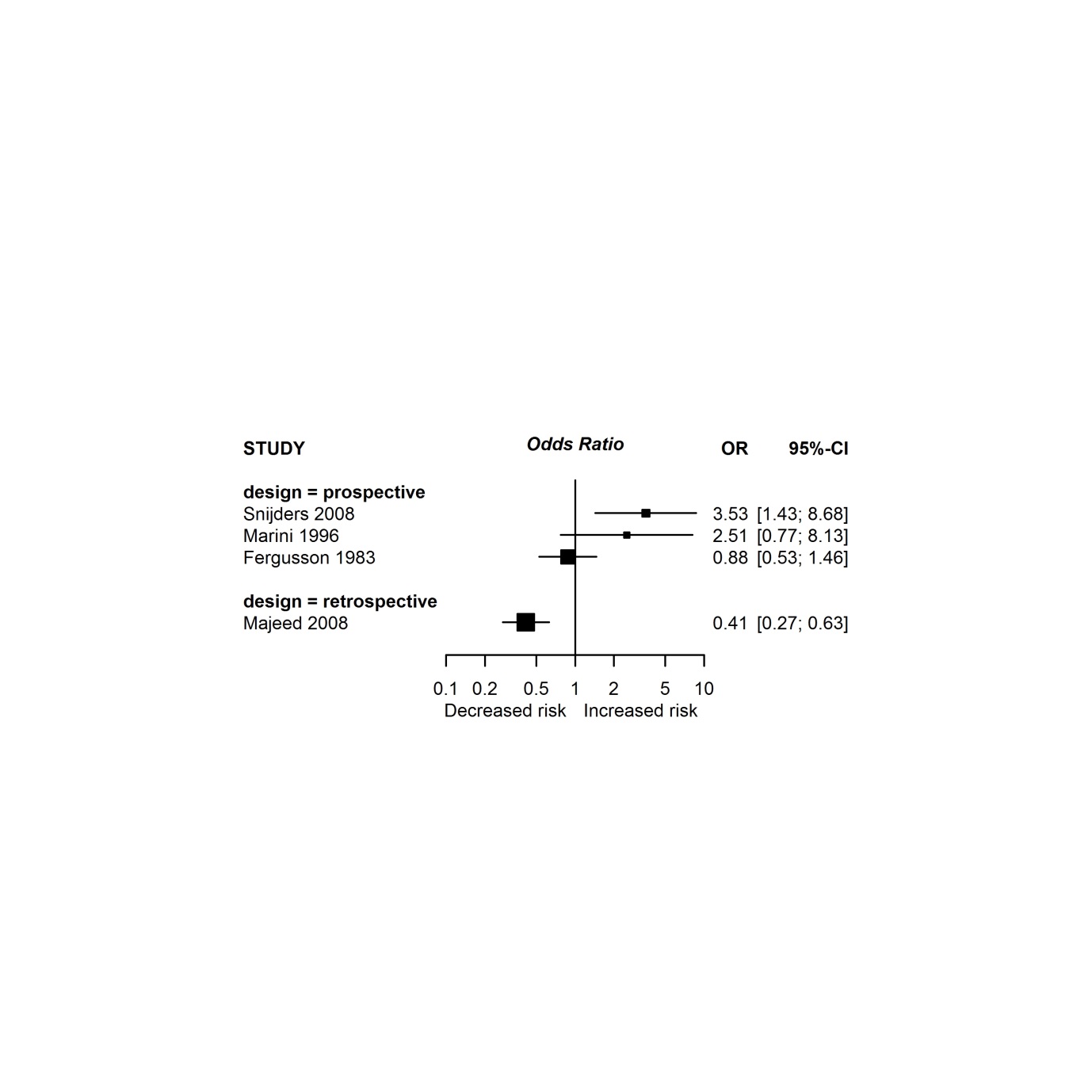


### Age at outcome measurement 5-14

#### 3-4 months

Five prospective cohort studies and one case-control study reported data that could be pooled to estimate overall risk of recurrent wheeze at age 5-14 years comparing SFI at ≥3-4 months vs. <3-4 months. There was no evidence of an effect (Figure 89). With the exception of the study of Wilson (which had a high risk of overall bias due to confounding bias), all prospective cohorts had a low risk of overall bias. The study of Karunasekera (case-control) had an unclear overall risk of bias. There was moderate heterogeneity observed between studies (I^2^=45.8%)

Figure 89 Solid Food Introduction ≥3-4 months vs. <3-4 months and risk of recurrent wheeze in children aged 5-14 years


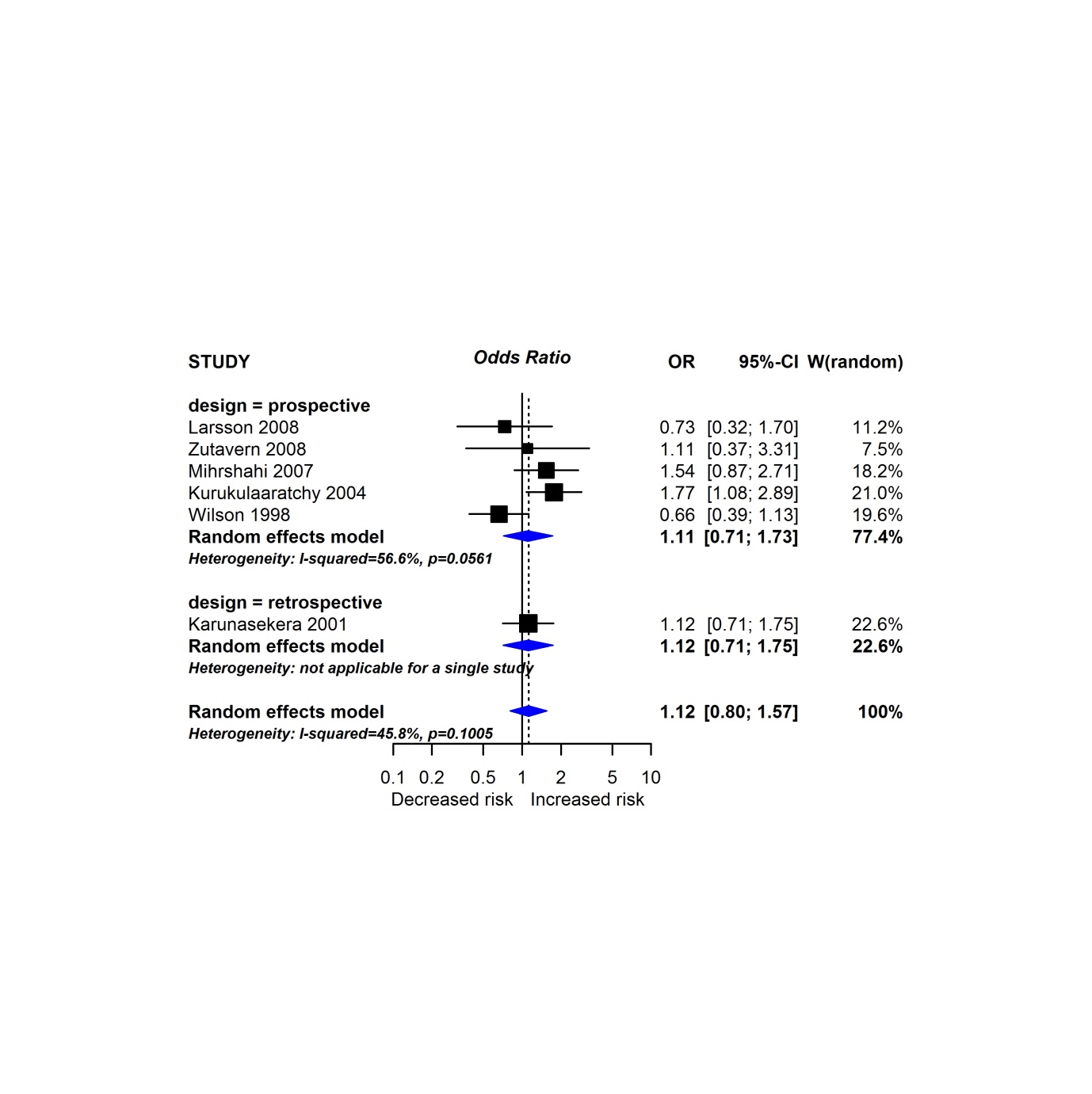


Subgroup analyses of risk of wheeze and solid food introduction ≥3-4 months vs. <3-4 months in children aged 5-14 years showed no indication of statistically significant differences between the various groups examined (Table 22). There were additionally three studies that could not be meta-analysed and showed no association with wheeze (Table 23).

Table 22 Subgroup Analyses of risk of wheeze and solid food introduction ≥3-4 months vs. <3-4 months in children aged 5-14 years

|  | **Number of studies** | **OR [95% CI]** | **I^2^ (%)** | **P-value for between groups difference** |
| --- | --- | --- | --- | --- |
| **Overall (if adjusted NA, unadjusted value used)** | 6 | 1.12 [0.80; 1.57] | 45.8 |  |
| **Adjusted** | 2 | 1.43 [0.87; 2.38] | 0.0 | Not tested |
| **Unadjusted** | 5 | 1.11 [0.77; 1.60] | 54.7 |  |
| Study Design – Prospective | 5 | 1.11 [0.72; 1.73] | 56.6 | 0.99 |
| Study Design – Retrospective | 1 | 1.12 [0.71; 1.75] | -- |  |
| Risk of disease – High | 1 | 1.54 [0.87; 2.71] | -- | 0.27 |
| Risk of disease – Normal | 5 | 1.04 [0.71; 1.54] | 49.8 |  |
| Risk of bias – Low | 3 | 1.20 [0.77; 1.86] | 3.0 | 0.82 |
| Risk of bias – High/Unclear | 3 | 1.10 [0.67; 1.88] | 71.8 |  |
| Clear definition of breastfeeding duration | 5 | 1.11 [0.72; 1.73] | 56.6 | 0.99 |
| Unclear definition of breastfeeding duration | 1 | 1.12 [0.71; 1.75] | -- |  |

Table 23 Studies investigating the association between solid food introduction and wheeze which were not eligible for meta-analysis

| **First Author and year of publication** | **Design** | **Outcome** | **Age** | **N/n** | **Age at SF introduction (continuous or categorical in months)** | **Average age at SF introduction** | **SF introduction Unaffected** | | **SF introduction in Affected** | | **P-value** |
| --- | --- | --- | --- | --- | --- | --- | --- | --- | --- | --- | --- |
| Hesselmar 2010 ([5](#_ENREF_5)) | PC | recurrent wheeze | 1.5 | 184 | continuous | Median (IQR) | 4 | 4 | 4.2 | 4 | NS |
| Joseph 2012 ([181](#_ENREF_181)) | PC | wheeze | 3 | 594 | >4 |  |  |  |  |  | NS |
| Forsyth, 1993 ([179](#_ENREF_179)) | PC | wheeze | 2 | 455 | >2 |  |  |  |  |  | NS |

# Conclusion

This systematic review on breastfeeding and solid food introduction did not find strong evidence of a relationship between these exposures and the outcomes of wheeze, recurrent wheeze, atopic wheeze, BHR or lung function. There were asymmetrical Funnel plots with evidence of publication bias in several meta-analyses of observational studies for TBF and EBF and wheezing, with small negative studies potentially missing. For this reason the only consistent finding, that TBF is associated with reduced risk of recurrent wheeze at age 5-14 years, was downgraded to VERY LOW certainty evidence, due to risk of publication bias. These findings highlight the importance of rigorous assessment of bias, including publication bias in the synthesis of existing scientific literature in this area, since previous reviews have not identified publication bias as an issue with this body of literature. The single intervention trial in this area found reduced risk of recurrent wheezing at age 1 year in infants born in centres randomised to a breastfeeding promotion intervention, but the outcome was not statistically significant OR 0.70 (0.29, 1.70). In the same trial there was no evidence for a difference in recurrent wheeze (‘asthma ever’) at age 6.5 years OR 1.2 (0.7, 1.9). Thus the intervention trial neither excludes nor supports an effect on wheezing.

Overall, the data for TBF and other outcomes, and for EBF and SF timing and all outcomes, showed significant heterogeneity in findings and rarely excluded the possibility of biological relationships. Thus we cannot confidently exclude the possibility that breastfeeding and solid introduction are related to risk of wheeze; however the currently available data do not support a relationship, aside from the VERY LOW certainty evidence that longer TBF is associated with reduced risk of recurrent wheeze at age 5-14 years.

We did not identify recent systematic reviews that met our requirements for inclusion in the review (i.e. R-AMSTAR score ≥32). There are however 3 recent important systematic reviews in this area which are relevant to discuss. The study of Dick ([182](#_ENREF_182)) did not undertake meta-analysis, and no firm conclusions were made regarding the relationship between breastfeeding duration and risk of asthma at age ≤ 9 years. The systematic review of Waidyatillake ([183](#_ENREF_183)) assessed the relationship between breastfeeding duration and lung function. Consistent with our approach and findings, Waidyatillake et al did not undertake meta-analysis, but identified the same studies as we did, and found evidence that increased duration of TBF and EBF is associated with increased measures of lung function – especially FVC but also FEV1. We found evidence for a positive relationship between breastfeeding duration and lung function on at least one measure reported, for 5 of 7 studies reporting TBF, and 2 of 3 studies reporting EBF, with strongest evidence for FVC but also positive findings for measures of airway obstruction. However, findings were not consistent, and for two studies of EBF we found significant associations between longer duration and poorer lung function. We additionally studied bronchial hyper-responsiveness (BHR) where we were able to undertake limited meta-analysis and found no evidence of a relationship between TBF or EBF duration and BHR. The third relevant systematic review is the study of Dogaru, whose authors reported a strong association between increased breastfeeding duration and reduced risk of asthma, especially at age 0-2 years ([184](#_ENREF_184)). Their approach of combining all durations of breastfeeding and creating a single pooled estimate for ‘more’ versus ‘less’ could be criticised on the basis that the reference group in some studies has significant overlap with the exposure group in other studies within the same meta-analysis. This may also lead to an exaggeration of any biases inherent in the included dataset. Our new finding of probable publication bias in this area, which has not been reported before, weakens the evidence base that prolonged breastfeeding duration reduces wheeze or asthma risk.

In summary we have found VERY LOW evidence that longer total duration of breastfeeding may be associated with reduced risk of recurrent wheeze at age 5-14 years. We have not found evidence for associations between duration of breastfeeding, or timing of solid food introduction, and other wheeze or lung function outcomes. The finding of probable publication bias means that these findings need to be interpreted with caution. We have identified a need for further investigation in this area – especially the study of mechanisms through which human milk feeding might promote lung growth in the developing infant, since we did not find evidence for an association between these exposures and allergic sensitisation; so if the association found here is real, then other mechanisms must be explored ([183](#_ENREF_183)). A number of hypotheses have been proposed through which breastfeeding might promote lung growth – including reduced respiratory tract infections, reduced weight gain, altered respiratory dynamics during breastfeeding, increased exposure to breast milk derived growth factors, and as a secondary consequence of reduced asthma prevalence. Further work is needed to establish whether breastfeeding influences lung function in the developing infant, and whether the association seen here with recurrent wheezing from age 5-14 years can be confirmed.

# References

1. Kramer MS, Chalmers B, Hodnett ED, Sevkovskaya Z, Dzikovich I, Shapiro S, et al. Promotion of Breastfeeding Intervention Trial (PROBIT): a randomized trial in the Republic of Belarus. JAMA. 2001;285(4):413-20.

2. Kramer MS, Matush L, Vanilovich I, Platt R, Bogdanovich N, Sevkovskaya Z, et al. Effect of prolonged and exclusive breast feeding on risk of allergy and asthma: cluster randomised trial. Bmj. 2007;335(7624):815.

3. Wright RJ, Cohen S, Carey V, Weiss ST, Gold DR. Parental stress as a predictor of wheezing in infancy: a prospective birth-cohort study. American Journal of Respiratory & Critical Care Medicine. 2002;165(3):358-65.

4. Al-Kubaisy W, Ali SH, Al-Thamiri D. Risk factors for asthma among primary school children in Baghdad, Iraq. Saudi Medical Journal. 2005;26(3):460-6.

5. Hesselmar B, Saalman R, Rudin A, Adlerberth I, Wold A. Early fish introduction is associated with less eczema, but not sensitization, in infants. Acta Paediatrica. 2010;99(12):1861-7.

6. Elliott L, Henderson J, Northstone K, Chiu GY, Dunson D, London SJ. Prospective study of breast-feeding in relation to wheeze, atopy, and bronchial hyperresponsiveness in the Avon Longitudinal Study of Parents and Children (ALSPAC). Journal of Allergy & Clinical Immunology. 2008;122(1):49-54, .e1-3.

7. Granell R, Sterne JAC, Henderson J. Associations of Different Phenotypes of Wheezing Illness in Early Childhood with Environmental Variables Implicated in the Aetiology of Asthma. PLoS ONE. 2012;7(10).

8. Sherriff A, Peters TJ, Henderson J, Strachan D, Team AS. Risk factor associations with wheezing patterns in children followed longitudinally from birth to 3 1/2 years. International Journal of Epidemiology. 2001;30(6):1473-84.

9. Kull I, Wickman M, Lilja G, Nordvall SL, Pershagen G. Breast feeding and allergic diseases in infants-a prospective birth cohort study. Archives of Disease in Childhood. 2002;87(6):478-81.

10. Lewis S, Butland B, Strachan D, Bynner J, Richards D, Butler N, et al. Study of the aetiology of wheezing illness at age 16 in two national British birth cohorts. Thorax. 1996;51(7):670-6.

11. Lewis S, Richards D, Bynner J, Butler N, Britton J. PROSPECTIVE-STUDY OF RISK-FACTORS FOR EARLY AND PERSISTENT WHEEZING IN CHILDHOOD. European Respiratory Journal. 1995;8(3):349-56.

12. Burr ML, Miskelly FG, Butland BK, Merrett TG, Vaughan-Williams E. Environmental factors and symptoms in infants at high risk of allergy. Journal of Epidemiology & Community Health. 1989;43(2):125-32.

13. Burr ML, Limb ES, Maguire MJ, Amarah L, Eldridge BA, Layzell JCM, et al. INFANT-FEEDING, WHEEZING, AND ALLERGY - A PROSPECTIVE-STUDY. Archives of Disease in Childhood. 1993;68(6):724-8.

14. Burr ML, Limb ES, Maguire MJ, Amarah L, Eldridge BA, Layzell JC, et al. Infant feeding, wheezing, and allergy: a prospective study. Archives of Disease in Childhood. 1993;68(6):724-8.

15. Alper Z, Sapan N, Ercan I, Canitez Y, Bilgel N. Risk factors for wheezing in primary school children in Bursa, Turkey. American Journal of Rhinology. 2006;20(1):53-63.

16. Businco L, Cantani A, Meglio P, Bruno G. Prevention of atopy: Results of a long-term (7 months to 8 years) follow-up. Annals of Allergy. 1987;59(5 PART II):183-6.

17. Camara AA, Silva JM, Ferriani VPL, Tobias KRC, Macedo IS, Padovani MA, et al. Risk factors for wheezing in a subtropical environment: Role of respiratory viruses and allergen sensitization. Journal of Allergy and Clinical Immunology. 2004;113(3):551-7.

18. Midodzi WK, Rowe BH, Majaesic CM, Saunders LD, Senthilselvan A. Early life factors associated with incidence of physician-diagnosed asthma in preschool children: results from the Canadian Early Childhood Development cohort study. Journal of Asthma. 2010;47(1):7-13.

19. Taylor B, Wadsworth J, Golding J, Butler N. Breast feeding, eczema, asthma, and hayfever. Journal of Epidemiology and Community Health. 1983;37:95-9.

20. Mihrshahi S, Ampon R, Webb K, Almqvist C, Kemp AS, Hector D, et al. The association between infant feeding practices and subsequent atopy among children with a family history of asthma. Clinical & Experimental Allergy. 2007;37(5):671-9.

21. Simon MR, Havstad SL, Wegienka GR, Ownby DR, Johnson CC. Risk factors associated with transient wheezing in young children. Allergy & Asthma Proceedings. 2008;29(2):161-5.

22. Larsson M, Hagerhed-Engman L, Sigsgaard T, Janson S, Sundell J, Bornehag CG. Incidence rates of asthma, rhinitis and eczema symptoms and influential factors in young children in Sweden. Acta Paediatrica. 2008;97(9):1210-5.

23. Devereux G, Turner SW, Craig LC, McNeill G, Martindale S, Harbour PJ, et al. Low maternal vitamin E intake during pregnancy is associated with asthma in 5-year-old children. American Journal of Respiratory & Critical Care Medicine. 2006;174(5):499-507.

24. Nwaru BI, Takkinen HM, Niemela O, Kaila M, Erkkola M, Ahonen S, et al. Timing of infant feeding in relation to childhood asthma and allergic diseases. Journal of Allergy and Clinical Immunology. 2013;131(1):78-86.

25. Wilson AC, Forsyth JS, Greene SA, Irvine L, Hau C, Howie PW. Relation of infant diet to childhood health: seven year follow up of cohort of children in Dundee infant feeding study. Bmj. 1998;316(7124):21-5.

26. Ehrlich RI, Du Toit D, Jordaan E, Zwarenstein M, Potter P, Volmink JA, et al. Risk factors for childhood asthma and wheezing. Importance of maternal and household smoking. American Journal of Respiratory & Critical Care Medicine. 1996;154(3 Pt 1):681-8.

27. Visser CAN, Garcia-Marcos L, Eggink J, Brand PLP. Prevalence and Risk Factors of Wheeze in Dutch Infants in Their First Year of Life. Pediatric Pulmonology. 2010;45(2):149-56.

28. Munro A, Grimshaw K, Oliver E, Foote K, Roberts G. Risk factors associated with wheezing in the first 2 years of life: Europrevall cohort. Clinical and Experimental Allergy. 2011;41 (12):1856.

29. Farooqi IS, Hopkin JM. Early childhood infection and atopic disorder. Thorax. 1998;53(11):927-32.

30. Friday GA, Jr., Smith J. Breast-feeding and atopic dermatitis. Pediatric Asthma, Allergy and Immunology. 2000;14(3):205-9.

31. Fredriksson P, Jaakkola N, Jaakkola JJ. Breastfeeding and childhood asthma: a six-year population-based cohort study. BMC Pediatrics. 2007;7:39.

32. Tanaka K, Miyake Y, Sasaki S. Association between breastfeeding and allergic disorders in Japanese children. International Journal of Tuberculosis and Lung Disease. 2010;14(4):513-8.

33. Sonnenschein-van der Voort AM, Jaddoe VW, van der Valk RJ, Willemsen SP, Hofman A, Moll HA, et al. Duration and exclusiveness of breastfeeding and childhood asthma-related symptoms. European Respiratory Journal. 2012;39(1):81-9.

34. Gruskay FL. Comparison of breast, cow, and soy feedings in the prevention of onset of allergic disease: a 15-year prospective study. Clinical Pediatrics. 1982;21(8):486-91.

35. Guida F, Clarisse B, Momas I. Risk factors of wheezing onset during infancy in the Paris birth cohort: Benefits of the cox model. Allergy: European Journal of Allergy and Clinical Immunology. 2009;64:202.

36. Gustafsson D, Sjoberg O, Foucard T. Development of allergies and asthma in infants and young children with atopic dermatitis--a prospective follow-up to 7 years of age. Allergy. 2000;55(3):240-5.

37. Halken S, Host A, Husby S, Hansen LG, Osterballe O, Nyboe J. Recurrent wheezing in relation to environmental risk factors in infancy. A prospective study of 276 infants. Allergy. 1991;46(7):507-14.

38. 6th International Conference on Environmental Health Science. Toxicology and Environmental Health Sciences Conference: 6th International Conference on Environmental Health Science. 2013;5(pp S80).

39. Puig C, Friguls B, Gomez M, Garcia-Algar O, Sunyer J, Vall O. Relationship between lower respiratory tract infections in the first year of life and the development of asthma and wheezing in children. [Spanish] Relacion entre las infecciones respiratorias de vias bajas durante el primer ano de vida y el desarrollo de asma y sibilancias en ninos. Archivos de Bronconeumologia. 2010;46(10):514-21.

40. Soto-Ramirez N, Karmaus W, Zhang H, Davis S, Agarwal S, Albergottie A. Modes of infant feeding and the occurrence of coughing/wheezing in the first year of life. Journal of Human Lactation. 2013;29(1):71-80.

41. Oddy WH, Halonen M, Martinez FD, Lohman IC, Stern DA, Kurzius-Spencer M, et al. TGF-beta in human milk is associated with wheeze in infancy. Journal of Allergy & Clinical Immunology. 2003;112(4):723-8.

42. Erratum: Allergies in high-risk schoolchildren after early intervention with cow's milk protein hydrolysates: 10-year results from the German Infant Nutritional Intervention [GINI] study (Journal of Allergy and Clinical Immunology (2013) 131 (1565-1573)). Journal of Allergy and Clinical Immunology. 2013;132(3):773.

43. Goksor E, Alm B, Thengilsdottir H, Erdes L, Mollborg P, Pettersson R, et al. Neonatal antibiotic treatment is a risk factor for multiple trigger wheeze at age 41/2 years. Pediatric Allergy and Immunology. 2009;20:17.

44. Alm B, Erdes L, Mollborg P, Pettersson R, Norvenius SG, Aberg N, et al. Neonatal antibiotic treatment is a risk factor for early wheezing. Pediatrics. 2008;121(4):697-702.

45. Morales E, Garcia-Esteban R, Guxens M, Guerra S, Mendez M, Molto-Puigmarti C, et al. Effects of prolonged breastfeeding and colostrum fatty acids on allergic manifestations and infections in infancy. Clinical and Experimental Allergy. 2012;42(6):918-28.

46. Nagel G, Buchele G, Weinmayr G, Bjorksten B, Chen YZ, Wang H, et al. Effect of breastfeeding on asthma, lung function and bronchial hyperreactivity in ISAAC Phase II. European Respiratory Journal. 2009;33(5):993-1002.

47. Innes Asher M, Robertson C, Ait-Khaled N, Anderson HR, Beasley R, Bjorksten B, et al. Global analysis of breast feeding and risk of symptoms of asthma, rhinoconjunctivitis and eczema in 6-7 year old children: ISAAC Phase Three. Allergologia et Immunopathologia. 2011;39(6):318-25.

48. Awasthi S, Kalra E, Roy S, Awasthi S. Prevalence and risk factors of asthma and wheeze in school-going children in Lucknow, North India. Indian Pediatrics. 2004;41(12):1205-10.

49. Karmaus W, Dobai AL, Ogbuanu I, Arshard SH, Matthews S, Ewart S. Long-term effects of breastfeeding, maternal smoking during pregnancy, and recurrent lower respiratory tract infections on asthma in children. Journal of Asthma. 2008;45(8):688-95.

50. Juca SC, Takano OA, Moraes LS, Guimaraes LV. [Asthma prevalence and risk factors in adolescents 13 to 14 years of age in Cuiaba, Mato Grosso State, Brazil]. Cadernos de Saude Publica. 2012;28(4):689-97.

51. Karino S, Okuda T, Uehara Y, Toyo-oka T. Breastfeeding and prevalence of allergic diseases in Japanese university students. Annals of Allergy Asthma & Immunology. 2008;101(2):153-9.

52. Karunasekera KA, Jayasinghe JA, Alwis LW. Risk factors of childhood asthma: a Sri Lankan study. Journal of Tropical Pediatrics. 2001;47(3):142-5.

53. Kaufman HS, Frick OL. The development of allergy in infants of allergic parents: a prospective study concerning the role of heredity. Annals of Allergy. 1976;37(6):410-5.

54. Kemeny DM, Price JF, Richardson V, Richards D, Lessof MH. THE IGE AND IGG SUBCLASS ANTIBODY-RESPONSE TO FOODS IN BABIES DURING THE 1ST YEAR OF LIFE AND THEIR RELATIONSHIP TO FEEDING REGIMEN AND THE DEVELOPMENT OF FOOD ALLERGY. Journal of Allergy and Clinical Immunology. 1991;87(5):920-9.

55. Klinnert MD, Nelson HS, Price MR, Adinoff AD, Leung DYM, Mrazek DA. Onset and persistence of childhood asthma: Predictors from infancy. Pediatrics. 2001;108(4):art. no.-e69.

56. Snijders BE, Thijs C, van Ree R, van den Brandt PA. Age at first introduction of cow milk products and other food products in relation to infant atopic manifestations in the first 2 years of life: the KOALA Birth Cohort Study. Pediatrics. 2008;122(1):e115-22.

57. Snijders BE, Thijs C, Dagnelie PC, Stelma FF, Mommers M, Kummeling I, et al. Breast-feeding duration and infant atopic manifestations, by maternal allergic status, in the first 2 years of life (KOALA study). Journal of Pediatrics. 2007;151(4):347-51, 51.e1-2.

58. Galbally M, Lewis AJ, McEgan K, Scalzo K, Islam FA. Breastfeeding and infant sleep patterns: an Australian population study. Journal of Paediatrics & Child Health. 2013;49(2):E147-52.

59. Marini A, Agosti M, Motta G, Mosca F. Effects of a dietary and environmental prevention programme on the incidence of allergic symptoms in high atopic risk infants: three years' follow-up. Acta Paediatrica Supplement. 1996;414:1-21.

60. Martel MJ, Rey E, Malo JL, Perreault S, Beauchesne MF, Forget A, et al. Determinants of the incidence of childhood asthma: a two-stage case-control study. American Journal of Epidemiology. 2009;169(2):195-205.

61. Burgess SW, Dakin CJ, O'Callaghan MJ. Breastfeeding does not increase the risk of asthma at 14 years. Pediatrics. 2006;117(4):e787-92.

62. Mavale-Manuel S, Alexandre F, Duarte N, Albuquerque O, Scheinmann P, Poisson-Salomon AS, et al. Risk factors for asthma among children in Maputo (Mozambique). Allergy. 2004;59(4):388-93.

63. McConnochie KM, Roghmann KJ. Breast feeding and maternal smoking as predictors of wheezing in children age 6 to 10 years. Pediatric Pulmonology. 1986;2(5):260-8.

64. Miskelly FG, Burr ML, Vaughan-Williams E, Fehily AM, Butland BK, Merret TG. Infant feeding and allergy. Archives of Disease in Childhood. 1988;63(4):388-93.

65. Miyake Y, Yura A, Iki M. Breastfeeding and the prevalence of symptoms of allergic disorders in Japanese adolescents. Clinical and Experimental Allergy. 2003;33(3):312-6.

66. Morgan JB, Lucas A, Fewtrell MS. Does weaning influence growth and health up to 18 months? Archives of Disease in Childhood. 2004;89(8):728-33.

67. Mann SL, Wadsworth MEJ, Colley JRT. ACCUMULATION OF FACTORS INFLUENCING RESPIRATORY ILLNESS IN MEMBERS OF A NATIONAL BIRTH COHORT AND THEIR OFFSPRING. Journal of Epidemiology and Community Health. 1992;46(3):286-92.

68. Muiño A, Menezes AMB, Reichert FF, Duquia RP, Chatkin M. Padrões de sibilância respiratória do nascimento até o início da adolescência: coorte de Pelotas (RS) Brasil, 1993-2004Wheezing phenotypes from birth to adolescence: a cohort study in Pelotas, Brazil, 1993-2004. J Bras Pneumol. 2008;34(6):347-55.

69. Bergmann RL, Edenharter G, Bergmann KE, Lau S, Wahn U. Socioeconomic status is a risk factor for allergy in parents but not in their children. Clinical & Experimental Allergy. 2000;30(12):1740-5.

70. Rust GS, Thompson CJ, Minor P, Davis-Mitchell W, Holloway K, Murray V. Does breastfeeding protect children from asthma? Analysis of NHANES III survey data. Journal of the National Medical Association. 2001;93(4):139-48.

71. Evenhouse E, Reilly S. Improved estimates of the benefits of breastfeeding using sibling comparisons to reduce selection bias. Health Services Research. 2005;40(6):1781-802.

72. Midodzi WK, Rowe BH, Majaesic CM, Saunders LD, Senthilselvan A. Predictors for wheezing phenotypes in the first decade of life. Respirology. 2008;13(4):537-45.

73. Dell S, To T. Breastfeeding and asthma in young children: findings from a population-based study. Archives of Pediatrics & Adolescent Medicine. 2001;155(11):1261-5.

74. Milner JD, Stein DM, McCarter R, Moon RY. Early infant multivitamin supplementation is associated with increased risk for food allergy and asthma. Pediatrics. 2004;114(1):27-32.

75. Silvers KM, Frampton CM, Wickens K, Pattemore PK, Ingham T, Fishwick D, et al. Breastfeeding protects against current asthma up to 6 years of age. Journal of Pediatrics. 2012;160(6):991-6.e1.

76. Silvers KM, Frampton CM, Wickens K, Epton MJ, Pattemore PK, Ingham T, et al. Breastfeeding protects against adverse respiratory outcomes at 15 months of age. Maternal & Child Nutrition. 2009;5(3):243-50.

77. Ronmark E, Jonsson E, Platts-Mills T, Lundback B. Different pattern of risk factors for atopic and nonatopic asthma among children--report from the Obstructive Lung Disease in Northern Sweden Study. Allergy. 1999;54(9):926-35.

78. Oliveti JF, Kercsmar CM, Redline S. Pre- and perinatal risk factors for asthma in inner city African-American children. American Journal of Epidemiology. 1996;143(6):570-7.

79. Miyake Y, Tanaka K, Sasaki S, Kiyohara C, Ohya Y, Fukushima W, et al. Breastfeeding and the risk of wheeze and asthma in Japanese infants: the Osaka Maternal and Child Health Study. Pediatric Allergy & Immunology. 2008;19(6):490-6.

80. Menezes AM, Lima RC, Minten GC, Hallal PC, Victora CG, Horta BL, et al. [Prevalence of wheezing in the chest among adults from the 1982 Pelotas birth cohort, Southern Brazil]. Revista de Saude Publica. 2008;42 Suppl 2:101-7.

81. da Costa Lima R, Victora CG, Menezes AM, Barros FC. Do risk factors for childhood infections and malnutrition protect against asthma? A study of Brazilian male adolescents. American Journal of Public Health. 2003;93(11):1858-64.

82. Perez Tarazona S, Alfonso Diego J, Amat Madramany A, Chofre Escrihuela L, Lucas Saez E, Bou Monterde R. [Incidence of wheezing and associated risk factors in the first 6 months of life of a cohort in Valencia (Spain)]. Anales de Pediatria. 2010;72(1):19-29.

83. Scholtens S, Wijga AH, Brunekreef B, Kerkhof M, Hoekstra MO, Gerritsen J, et al. Breast feeding, parental allergy and asthma in children followed for 8 years. The PIAMA birth cohort study. Thorax. 2009;64(7):604-9.

84. Caudri D, Savenije O, Smit HA, Postma DS, Koppelman G, Wijga A, et al. The relation between perinatal factors and phenotypes of wheeze in the first 8 years of life. American Journal of Respiratory and Critical Care Medicine. 2010;181 (1 MeetingAbstracts).

85. Kerr AA. Lower respiratory tract illness in Polynesian infants. New Zealand Medical Journal. 1981;93(684):333-5.

86. Porro E, Indinnimeo L, Antognoni G, Midulla F, Criscione S. Early wheezing and breast feeding. Journal of Asthma. 1993;30(1):23-8.

87. Kurt S, Kisacik B, Kaplan Y, Yildirim B, Etikan I, Karaer H. Obesity and carpal tunnel syndrome: Is there a causal relationship? European Neurology. 2008;59(5):253-7.

88. Schonberger HJ, Dompeling E, Knottnerus JA, Maas T, Muris JW, van Weel C, et al. The PREVASC study: the clinical effect of a multifaceted educational intervention to prevent childhood asthma. European Respiratory Journal. 2005;25(4):660-70.

89. Hagendorens MM, Bridts CH, Lauwers K, van Nuijs S, Ebo DG, Vellinga A, et al. Perinatal risk factors for sensitization, atopic dermatitis and wheezing during the first year of life (PIPO study). Clinical and Experimental Allergy. 2005;35(6):733-40.

90. Alho OP, Koivu M, Sorri M, Rantakallio P. Risk factors for recurrent acute otitis media and respiratory infection in infancy. International Journal of Pediatric Otorhinolaryngology. 1990;19(2):151-61.

91. Rhodes HL, Sporik R, Thomas P, Holgate ST, Cogswell JJ. Early life risk factors for adult asthma: a birth cohort study of subjects at risk. Journal of Allergy & Clinical Immunology. 2001;108(5):720-5.

92. Rona RJ, Smeeton NC, Bustos P, Amigo H, Diaz PV. The early origins hypothesis with an emphasis on growth rate in the first year of life and asthma: a prospective study in Chile. Thorax. 2005;60(7):549-54.

93. Rosas Vargas MA, Gonzalez Reyes M, del Rio Navarro BE, Avila Castanon L, Velazquez Armenta Y, Sienra Monge JJ. [Allergen sensitization and asthma in children from 1 to 3 years of age]. Revista Alergia Mexico. 2002;49(6):171-5.

94. Rothenbacher D, Weyermann M, Beermann C, Brenner H. Breastfeeding, soluble CD14 concentration in breast milk and risk of atopic dermatitis and asthma in early childhood: birth cohort study. Clinical & Experimental Allergy. 2005;35(8):1014-21.

95. Rusconi F, Galassi C, Bellasio M, Piffer S, Lombardi E, Bonci E, et al. [Risk factors in the pre-, perinatal and early life (first year) for wheezing in young children]. Epidemiologia e Prevenzione. 2005;29(2 Suppl):47-51.

96. Rusconi F, Galassi C, Corbo GM, Forastiere F, Biggeri A, Ciccone G, et al. Risk factors for early, persistent, and late-onset wheezing in young children. SIDRIA Collaborative Group. American Journal of Respiratory & Critical Care Medicine. 1999;160(5 Pt 1):1617-22.

97. Saarinen UM, Kajosaari M. Breastfeeding as prophylaxis against atopic disease: prospective follow-up study until 17 years old. Lancet. 1995;346(8982):1065-9.

98. Selcuk ZT, Caglar T, Enunlu T, Topal T. The prevalence of allergic diseases in primary school children in Edirne, Turkey. Clinical & Experimental Allergy. 1997;27(3):262-9.

99. Sunyer J, Torrent M, Garcia-Esteban R, Ribas-Fito N, Carrizo D, Romieu I, et al. Early exposure to dichlorodiphenyldichloroethylene, breastfeeding and asthma at age six. Clinical & Experimental Allergy. 2006;36(10):1236-41.

100. Sunyer J, Mendendez C, Ventura PJ, Aponte JJ, Schellenberg D, Kahigwa E, et al. Prenatal risk factors of wheezing at the age of four years in Tanzania. Thorax. 2001;56(4):290-5.

101. Hide DW, Guyer BM. Clinical manifestations of allergy related to breast and cows' milk feeding. Archives of Disease in Childhood. 1981;56(3):172-5.

102. Takemura Y, Sakurai Y, Honjo S, Kusakari A, Hara T, Gibo M, et al. Relation between breastfeeding and the prevalence of asthma : the Tokorozawa Childhood Asthma and Pollinosis Study. American Journal of Epidemiology. 2001;154(2):115-9.

103. Tian M, Zhao DY, Wen GY, Shi SY. [The correlation factor about respiratory syncytial virus bronchiolitis and post-bronchiolitis wheezing in infant]. Chinese Journal of Experimental & Clinical Virology. 2009;23(5):371-4.

104. Yamamoto K, Shoda T, Futamura M, Narita M, Sakamoto N, Aizawa S, et al. Associated factors of wheezing at 3 years of age in Japan: Prospective birth cohort study. Allergy: European Journal of Allergy and Clinical Immunology. 2011;66:549-50.

105. Wright AL, Holberg CJ, Martinez FD, Morgan WJ, Taussig LM. Breast feeding and lower respiratory tract illness in the first year of life. Group Health Medical Associates. Bmj. 1989;299(6705):946-9.

106. Wright AL, Holberg CJ, Taussig LM, Martinez FD. Relationship of infant feeding to recurrent wheezing at age 6 years. Archives of Pediatrics & Adolescent Medicine. 1995;149(7):758-63.

107. van Beijsterveldt TC, Boomsma DI. An exploration of gene-environment interaction and asthma in a large sample of 5-year-old Dutch twins. Twin Research & Human Genetics: the Official Journal of the International Society for Twin Studies. 2008;11(2):143-9.

108. Wang HY, Chen YZ, Ma Y, Wong GW, Lai CK, Zhong NS. [Disparity of asthma prevalence in Chinese schoolchildren is due to differences in lifestyle factors]. Zhonghua Erke Zazhi. 2006;44(1):41-5.

109. Oddy WH, Sly PD, de Klerk NH, Landau LI, Kendall GE, Holt PG, et al. Breast feeding and respiratory morbidity in infancy: a birth cohort study. Archives of Disease in Childhood. 2003;88(3):224-8.

110. Oddy WH, de Klerk NH, Kendall GE, Mihrshahi S, Peat JK. Ratio of omega-6 to omega-3 fatty acids and childhood asthma. Journal of Asthma. 2004;41(3):319-26.

111. Oddy WH, Holt PG, Sly PD, Read AW, Landau LI, Stanley FJ, et al. Association between breast feeding and asthma in 6 year old children: findings of a prospective birth cohort study. Bmj. 1999;319(7213):815-9.

112. Wickens K, Crane J, Kemp T, Lewis S, D'Souza W, Sawyer G, et al. A case-control study of risk factors for asthma in New Zealand children. Aust N Z J Public Health. 2001;25(1):44-9.

113. Zhu CH, Liu JX, Zhao XH. [Risk factors of asthma among children aged 0 - 14 in Suzhou city]. Chung-Hua Yu Fang i Hsueh Tsa Chih [Chinese Journal of Preventive Medicine]. 2012;46(5):456-9.

114. Zutavern A, von Mutius E, Harris J, Mills P, Moffatt S, White C, et al. The introduction of solids in relation to asthma and eczema. Archives of Disease in Childhood. 2004;89(4):303-8.

115. Ogbuanu IU, Karmaus W, Arshad SH, Kurukulaaratchy RJ, Ewart S. Effect of breastfeeding duration on lung function at age 10 years: a prospective birth cohort study. Thorax. 2009;64(1):62-6.

116. Soto-Ramirez N, Alexander M, Karmaus W, Yousefi M, Zhang H, Kurukulaaratchy RJ, et al. Breastfeeding is associated with increased lung function at 18 years of age: a cohort study. European Respiratory Journal. 2012;39(4):985-91.

117. Dogaru CM, Strippoli MP, Spycher BD, Frey U, Beardsmore CS, Silverman M, et al. Breastfeeding and lung function at school age: does maternal asthma modify the effect? American Journal of Respiratory & Critical Care Medicine. 2012;185(8):874-80.

118. Tennant PW, Gibson GJ, Pearce MS. Lifecourse predictors of adult respiratory function: results from the Newcastle Thousand Families Study. Thorax. 2008;63(9):823-30.

119. Suwanpromma S, Boonlarbtaweechoke C, Udomsubpayakul U, Preutthipan A. Spirometric airflow obstruction in Bangkok school children: prevalence and risk factors. Journal of the Medical Association of Thailand. 2012;95(11):1411-7.

120. Eneli IU, Karmaus WK, Davis S, Kuehr J. Airway hyperresponsiveness and body mass index: the Child Health and Environment Cohort Study in Hesse, Germany. Pediatric Pulmonology. 2006;41(6):530-7.

121. Tennant PW, Gibson GJ, Parker L, Pearce MS. Childhood respiratory illness and lung function at ages 14 and 50 years: childhood respiratory illness and lung function. Chest. 2010;137(1):146-55.

122. Brew BK, Allen CW, Toelle BG, Marks GB. Systematic review and meta-analysis investigating breast feeding and childhood wheezing illness. Paediatric and Perinatal Epidemiology. 2011;25(6):507-18.

123. Kramer Michael S, Kakuma R. Optimal duration of exclusive breastfeeding. Cochrane Database of Systematic Reviews [Internet]. 2012; (8). Available from: <http://onlinelibrary.wiley.com/doi/10.1002/14651858.CD003517.pub2/abstract>.

124. De Vries A, Reynolds RM, Seckl JR, Van Der Wal M, Bonsel GJ, Vrijkotte TG. Increased maternal BMI is associated with infant wheezing in early life. Brain, Behavior, and Immunity. 2010;24:S20.

125. Garcia-Marcos L, Mallol J, Sole D, Brand PL, Group ES. International study of wheezing in infants: risk factors in affluent and non-affluent countries during the first year of life. Pediatric Allergy & Immunology. 2010;21(5):878-88.

126. Kull I, Almqvist C, Lilja G, Pershagen G, Wickman M. Breast-feeding reduces the risk of asthma during the first 4 years of life. Journal of Allergy & Clinical Immunology. 2004;114(4):755-60.

127. Kull I, Melen E, Alm J, Hallberg J, Svartengren M, van Hage M, et al. Breast-feeding in relation to asthma, lung function, and sensitization in young schoolchildren. Journal of Allergy & Clinical Immunology. 2010;125(5):1013-9.

128. Besednjak-Kocijancic L. Is longer exclusive breastfeeding associated with lover prevalence of asthma, atopic dermatitis and atopic sensitisation in 1 and 5-year-old Slovene children? Allergy: European Journal of Allergy and Clinical Immunology. 2010;65:311-2.

129. Bacopoulou F, Veltsista A, Vassi I, Gika A, Lekea V, Priftis K, et al. Can we be optimistic about asthma in childhood? A Greek cohort study. Journal of Asthma. 2009;46(2):171-4.

130. Cano Garcinuno A, Perez Garcia I, Garcia Puertas J, Casas Rodriguez P. [Tobacco, infant feeding, and wheezing in the first three years of life]. Anales de Pediatria. 2003;59(6):541-7.

131. Salam MT, Li YF, Langholz B, Gilliland FD, Children's Health S. Early-life environmental risk factors for asthma: findings from the Children's Health Study. Environmental Health Perspectives. 2004;112(6):760-5.

132. Fergusson DM, Horwood LJ, Shannon FT. Asthma and infant diet. Archives of Disease in Childhood. 1983;58(1):48-51.

133. Horwood LJ, Fergusson DM, Shannon FT. Social and familial factors in the development of early childhood asthma. Pediatrics. 1985;75(5):859-68.

134. Giwercman C, Halkjaer LB, Jensen SM, Bonnelykke K, Lauritzen L, Bisgaard H. Increased risk of eczema but reduced risk of early wheezy disorder from exclusive breast-feeding in high-risk infants. Journal of Allergy & Clinical Immunology. 2010;125(4):866-71.

135. Linneberg A, Simonsen JB, Petersen J, Stensballe LG, Benn CS. Differential effects of risk factors on infant wheeze and atopic dermatitis emphasize a different etiology. Journal of Allergy & Clinical Immunology. 2006;117(1):184-9.

136. Erkkola M, Nwaru BI, Kaila M, Kronberg-Kippila C, Ilonen J, Simell O, et al. Risk of asthma and allergic outcomes in the offspring in relation to maternal food consumption during pregnancy: a Finnish birth cohort study. Pediatric Allergy & Immunology. 2012;23(2):186-94.

137. Sears MR, Greene JM, Willan AR, Taylor DR, Flannery EM, Cowan JO, et al. Long-term relation between breastfeeding and development of atopy and asthma in children and young adults: a longitudinal study. Lancet. 2002;360(9337):901-7.

138. Mandhane PJ, Greene JM, Sears MR. Interactions between breast-feeding, specific parental atopy, and sex on development of asthma and atopy. Journal of Allergy & Clinical Immunology. 2007;119(6):1359-66.

139. Hetzner NMP, Razza RA, Malone LM, Brooks-Gunn J. Associations among feeding behaviors during infancy and child illness at two years. Maternal and Child Health Journal. 2009;13(6):795-805.

140. Castro-Rodriguez JA, Garcia-Marcos L, Sanchez-Solis M, Perez-Fernandez V, Martinez-Torres A, Mallol J. Olive oil during pregnancy is associated with reduced wheezing during the first year of life of the offspring. Pediatric Pulmonology. 2010;45(4):395-402.

141. Chong Neto HJ, Rosário NA, Solé D, Mallol J. Prevalência de sibilância recorrente em lactentes

Prevalence of recurrent wheezing in infants. J Pediatr (Rio J). 2007;83(4):357-62.

142. Ehlayel MS, Bener A. Duration of breast-feeding and the risk of childhood allergic diseases in a developing country. Allergy & Asthma Proceedings. 2008;29(4):386-91.

143. Huang H, Zhang FY, Hang JQ, Zhu J, Wang R, Chen PF, et al. [Cohort study of 684 pairs of mother-and-child allergic diseases]. Zhonghua Erke Zazhi. 2013;51(3):168-71.

144. Arshad SH, Hide DW. Effect of environmental factors on the development of allergic disorders in infancy. Journal of Allergy & Clinical Immunology. 1992;90(2):235-41.

145. Kurukulaaratchy RJ, Matthews S, Arshad SH. Relationship between childhood atopy and wheeze: what mediates wheezing in atopic phenotypes? Annals of Allergy, Asthma, & Immunology. 2006;97(1):84-91.

146. Kurukulaaratchy RJ, Matthews S, Arshad SH. Does environment mediate earlier onset of the persistent childhood asthma phenotype? Pediatrics. 2004;113(2):345-50.

147. Macedo CLD, Ferreira MC, Naujorks AA, Tercziany A, Costa FJB, David HCd, et al. Aleitamento materno e diabetes mellitus do tipo 1

Breast feeding and type 1 diabetes mellitus. Arq Bras Endocrinol Metabol. 1999;43(5):360-5.

148. Liu JR, Xu BP, Li HM, Sun JH, Tian BL, Zhao SY, et al. [Clinical analysis of 20 cases with Streptococcus pneumoniae necrotizing pneumonia in China]. Zhonghua Erke Zazhi. 2012;50(6):431-4.

149. Mai XM, Becker AB, Sellers EAC, Liem JJ, Kozyrskyj AL. The relationship of breast-feeding, overweight, and asthma in preadolescents. Journal of Allergy and Clinical Immunology. 2007;120(3):551-6.

150. Majeed R, Rajar UD, Shaikh N, Majeed F, Arain AA. Risk factors associated with childhood asthma. Jcpsp, Journal of the College of Physicians & Surgeons - Pakistan. 2008;18(5):299-302.

151. Midwinter RE, Moore WJ, Soothill JF, Turner MW, Colley JR. Infant feeding and atopy. Lancet. 1982;1(8267):339.

152. Nielsen AM, Rasmussen S, Christoffersen MN. [Morbidity of Danish infants during their first months of life. Incidence and risk factors]. Ugeskrift for Laeger. 2002;164(48):5644-8.

153. Pesonen M, Kallio MJ, Ranki A, Siimes MA. Prolonged exclusive breastfeeding is associated with increased atopic dermatitis: a prospective follow-up study of unselected healthy newborns from birth to age 20 years. Clinical & Experimental Allergy. 2006;36(8):1011-8.

154. van Merode T, Maas T, Twellaar M, Kester A, van Schayck CP. Gender-specific differences in the prevention of asthma-like symptoms in high-risk infants. Pediatric Allergy & Immunology. 2007;18(3):196-200.

155. Prietsch SO, Fischer GB, Cesar JA, Cervo PV, Sangaletti LL, Wietzycoski CR, et al. [Risk factors for recurrent wheezing in children under 13 years old in the South of Brazil]. Pan American Journal of Public Health. 2006;20(5):331-7.

156. Kramer MS, Guo T, Platt RW, Sevkovskaya Z, Dzikovich I, Collet JP, et al. Infant growth and health outcomes associated with 3 compared with 6 mo of exclusive breastfeeding. American Journal of Clinical Nutrition. 2003;78(2):291-5.

157. Kramer MS, Matush L, Bogdanovich N, Aboud F, Mazer B, Fombonne E, et al. Health and development outcomes in 6.5-y-old children breastfed exclusively for 3 or 6 mo. American Journal of Clinical Nutrition. 2009;90(4):1070-4.

158. Kramer MS, Matush L, Bogdanovich N, Dahhou M, Platt RW, Mazer B. The low prevalence of allergic disease in Eastern Europe: are risk factors consistent with the hygiene hypothesis? Clinical & Experimental Allergy. 2009;39(5):708-16.

159. Ratageri VH, Kabra SK, Dwivedi SN, Seth V. Factors associated with severe asthma. Indian Pediatrics. 2000;37(10):1072-82.

160. Rullo VE, Arruda LK, Cardoso MR, Valente V, Zampolo AS, Nobrega F, et al. Respiratory infection, exposure to mouse allergen and breastfeeding: role in recurrent wheezing in early life. International Archives of Allergy & Immunology. 2009;150(2):172-8.

161. Rullo V, Arruda K, Valente V, Zampolo A, Cardoso M, Nobrega F, et al. Allergen and endotoxin exposure, infection, and breastfeeding in early life, and recurrent wheezing in children: 48-month follow-up cohort study. Allergy: European Journal of Allergy and Clinical Immunology. 2009;64:22.

162. Rullo VEV, Arruda LK, Valente V, Zampolo AS, Cardoso MR, Nobreg FJ, et al. Allergen and endotoxin exposure, infection, and breastfeeding in early life, and persistent wheezing in children: 60-month follow-up of a cohort study. Journal of Allergy and Clinical Immunology. 2010;1):AB56.

163. Rider NL, Morton D, Strauss KA. Allergen and endotoxin exposure, infection, and breastfeeding in early life, and recurrent wheezing in infants: 18-month follow-up of a cohort study. Journal of Allergy and Clinical Immunology. 2007;119(1):S70-S1.

164. Rylander E, Pershagen G, Eriksson M, Nordvall L. Parental smoking and other risk factors for wheezing bronchitis in children. European Journal of Epidemiology. 1993;9(5):517-26.

165. Siltanen M, Kajosaari M, Poussa T, Saarinen KM, Savilahti E. A dual long-term effect of breastfeeding on atopy in relation to heredity in children at 4 years of age. Allergy. 2003;58(6):524-30.

166. Salem MB, Al-Sadoon IO, Hassan MK. Prevalence of wheeze among preschool children in Basra governonate, southern Iraq. Eastern Mediterranean Health Journal. 2002;8(4-5):503-8.

167. Silva JM, Camara AA, Tobias KRC, Macedo IS, Cardoso MRA, Arruda E, et al. A prospective study of wheezing in young children: The independent effects of cockroach exposure, breast-feeding and allergic sensitization. Pediatric Allergy and Immunology. 2005;16(5):393-401.

168. Strassburger SZ, Vitolo MR, Bortolini GA, Pitrez PM, Jones MH, Stein RT. Nutritional errors in the first months of life and their association with asthma and atopy in preschool children. Jornal de Pediatria. 2010;86(5):391-9.

169. Mai XM, Becker AB, Liem JJ, Kozyrskyj AL. Fast food consumption counters the protective effect of breastfeeding on asthma in children? Clinical & Experimental Allergy. 2009;39(4):556-61.

170. Matheson MC, Erbas B, Balasuriya A, Jenkins MA, Wharton CL, Tang ML, et al. Breast-feeding and atopic disease: a cohort study from childhood to middle age. Journal of Allergy & Clinical Immunology. 2007;120(5):1051-7.

171. Wright AL, Holberg CJ, Taussig LM, Martinez FD. Factors influencing the relation of infant feeding to asthma and recurrent wheeze in childhood. Thorax. 2001;56(3):192-7.

172. Van Asperen PP, Kemp AS, Mellis CM. Relationship of diet in the development of atopy in infancy. Clinical Allergy. 1984;14(6):525-32.

173. Watson PE, McDonald BW. Subcutaneous body fat in pregnant new zealand women: association with wheeze in their infants at 18months. Maternal & Child Health Journal. 2013;17(5):959-67.

174. Oddy WH, Peat JK, de Klerk NH. Maternal asthma, infant feeding, and the risk of asthma in childhood. Journal of Allergy & Clinical Immunology. 2002;110(1):65-7.

175. Oddy WH, Sherriff JL, de Klerk NH, Kendall GE, Sly PD, Beilin LJ, et al. The relation of breastfeeding and body mass index to asthma and atopy in children: a prospective cohort study to age 6 years. American Journal of Public Health. 2004;94(9):1531-7.

176. Whu R, Cirilo G, Wong J, Finkel ML, Mendez HA, Leggiadro RJ. Risk factors for pediatric asthma in the South Bronx. Journal of Asthma. 2007;44(10):855-9.

177. Poysa L, Pulkkinen A, Korppi M, Remes K, Juntunen-Backman K. Diet in infancy and bronchial hyperreactivity later in childhood. Pediatric Pulmonology. 1992;13(4):215-21.

178. Guilbert TW, Stern DA, Morgan WJ, Martinez FD, Wright AL. Effect of breastfeeding on lung function in childhood and modulation by maternal asthma and atopy. American Journal of Respiratory & Critical Care Medicine. 2007;176(9):843-8.

179. Forsyth JS, Ogston SA, Clark A, Florey CD, Howie PW. Relation between early introduction of solid food to infants and their weight and illnesses during the first two years of life. Bmj. 1993;306(6892):1572-6.

180. Zutavern A, Brockow I, Schaaf B, von Berg A, Diez U, Borte M, et al. Timing of solid food introduction in relation to eczema, asthma, allergic rhinitis, and food and inhalant sensitization at the age of 6 years: results from the prospective birth cohort study LISA. Pediatrics. 2008;121(1):e44-52.

181. Joseph CL, Ownby DR, Havstad SL, Woodcroft KJ, Wegienka G, MacKechnie H, et al. Early complementary feeding and risk of food sensitization in a birth cohort. Journal of Allergy & Clinical Immunology. 2011;127(5):1203-10.e5.

182. Dick S, Friend A, Dynes K, AlKandari F, Doust E, Cowie H, et al. A systematic review of associations between environmental exposures and development of asthma in children aged up to 9 years. BMJ Open. 2014;4(11).

183. Waidyatillake NT, Allen KJ, Lodge CJ, Dharmage SC, Abramson MJ, Simpson JA, et al. The impact of breastfeeding on lung development and function: a systematic review. Expert Review of Clinical Immunology. 2013;9(12):1253-65.

184. Dogaru CM, Nyffenegger D, Pescatore AM, Spycher BD, Kuehni CE. Breastfeeding and childhood asthma: systematic review and meta-analysis. Am J Epidemiol. 2014;179(10):1153-67.
