# Supplementary material for: Diet during pregnancy and infancy and risk of allergic or autoimmune disease: A systematic review and meta-analysis
Source: PLoS Med. 2018 Feb 28;15(2):e1002507. doi: 10.1371/journal.pmed.1002507 (PMC5830033; doi:10.1371/journal.pmed.1002507)
Supplement: S2 Data — (ZIP) [file pmed.1002507.s007.zip › Review_C_reports/MATERNAL ALLERGENIC FOOD AVOIDANCE.docx]

**MATERNAL ALLERGENIC FOOD AVOIDANCE AND RISK OF ALLERGIC OR AUTO-IMMUNE DISEASES IN OFFSPRING**

Robert J Boyle^1^, Vanessa Garcia-Larsen^2^, Despo Ierodiakonou^3^, Jo Leonardi-Bee^4^, Tim Reeves^5^, Jennifer Chivinge^6^, Zoe Robinson^6^, Natalie Geoghegan^6^, Katharine Jarrold^6^, Andrew Logan^6^, Annabel Groome^6^ , Evangelia Andreou^7^, Nara Tagiyeva-Milne^8^, Ulugbek Nurmatov^9^, Sergio Cunha^10^

^1^ Clinical Senior Lecturer, Section of Paediatrics ^2^ Post-Doctoral Research Associate, Respiratory Epidemiology and Public Health, National Heart and Lung Institute ^3^ Post-Doctoral Research Associate, Departments of Paediatric and Respiratory Epidemiology and Public Health Group, all at Imperial College London

^4^Associate Professor of Community Health Sciences, University of Nottingham

^5^ Research Support Librarian, Faculty of Medicine, Imperial College London

^6^ Undergraduate medical students, Imperial College London

^7^Research Associate, Imperial Consultants

^8^Research Fellow, University of Aberdeen

^9^Research Fellow, University of Edinburgh

^10^Research Associate, Respiratory Epidemiology and Public Health, National Heart and Lung Institute, Imperial College London

Imperial College London

Table of Contents

[List of Figures 3](#_Toc485208380)

[1. Maternal AFA and risk of allergic/ autoimmune outcomes – summary of interventions and findings 5](#_Toc485208381)

[2. Maternal AFA and risk of AD 13](#_Toc485208382)

[3. Maternal AFA and risk of allergic rhinitis +/- conjunctivitis 17](#_Toc485208383)

[4. Maternal AFA and risk of food allergy 20](#_Toc485208384)

[5 Maternal AFA and risk of allergic sensitisation 24](#_Toc485208385)

[6. Maternal AFA and lung function, wheeze or asthma 30](#_Toc485208386)

[7. Maternal AFA and risk of TIDM 35](#_Toc485208387)

[8. Conclusions 36](#_Toc485208388)

[References 38](#_Toc485208389)

# List of Figures

[Figure 1 Risk of bias in studies of maternal AFA and allergic or auto-immune diseases 12](#_Toc427749848)

[Figure 2 Risk of bias in studies of maternal AFA and AD risk 14](#_Toc427749849)

[Figure 3 Maternal AFA and risk of AD at age ≤ 4 yrs - RCT 14](#_Toc427749850)

[Figure 4 Maternal AFA and risk of AD at age ≤ 4 yrs - CCT 16](#_Toc427749851)

[Figure 5 Maternal AFA and risk of AD at age 5-14 yrs - RCT 16](#_Toc427749852)

[Figure 6 Maternal AFA and risk of ‘atopic’ AD at age 5-14 yrs - RCT 16](#_Toc427749853)

[Figure 7 Maternal AFA and risk of AD at age 5-14 yrs - CCT 16](#_Toc427749854)

[Figure 8 Maternal AFA and risk of AD at age ≥15 yrs - RCT 16](#_Toc427749855)

[Figure 9 Risk of bias in studies on maternal AFA and AR risk 18](#_Toc427749856)

[Figure 10 Maternal AFA and risk of AR at age ≤4 yrs - RCT 18](#_Toc427749857)

[Figure 11 Maternal AFA and risk of AR at age ≤4 yrs - CCT 18](#_Toc427749858)

[Figure 12 Maternal AFA and risk of AR at age 5-14 yrs - RCT 19](#_Toc427749859)

[Figure 13 Maternal AFA and risk of ‘atopic’ AR at age 5-14 yrs - RCT 19](#_Toc427749860)

[Figure 14 Maternal AFA and risk of AR at age 5-14 yrs - CCT 19](#_Toc427749861)

[Figure 15 Risk of bias of intervention studies of maternal AFA and FA risk 21](#_Toc427749862)

[Figure 16 Maternal AFA and risk of food allergy (any) at age ≤ 4 yrs - RCT 21](#_Toc427749863)

[Figure 17 Maternal AFA and risk of food allergy (any) at age ≤ 4 yrs - CCT 21](#_Toc427749864)

[Figure 18 Maternal AFA and risk of food allergy (any) at age 5-14 yrs - RCT 22](#_Toc427749865)

[Figure 19 Maternal AFA and risk of food allergy (any) at age 5-14 yrs - CCT 22](#_Toc427749866)

[Figure 20 Maternal AFA and risk of CMA at age ≤ 4 yrs - RCT 22](#_Toc427749867)

[Figure 21 Maternal AFA and risk of CMA at age ≤ 4 yrs - CCT 22](#_Toc427749868)

[Figure 22 Maternal AFA and risk of CMA at age 5-14 yrs - CCT 22](#_Toc427749869)

[Figure 23 Maternal AFA and risk of Egg allergy at age ≤ 4 yrs - RCT 23](#_Toc427749870)

[Figure 24 Maternal AFA and risk of Egg allergy at age ≤ 4 yrs - CCT 23](#_Toc427749871)

[Figure 25 Maternal AFA and risk of Egg allergy at age 5-14 yrs - RCT 23](#_Toc427749872)

[Figure 26 Maternal AFA and risk of Egg allergy at age 5-14 yrs - CCT 23](#_Toc427749873)

[Figure 27 Risk of bias of intervention studies of maternal AFA and AS 26](#_Toc427749874)

[Figure 28 AFA and risk of allergic sensitisation (any) - RCT 27](#_Toc427749875)

[Figure 29 AFA and risk of allergic sensitisation (any) - CCT 27](#_Toc427749876)

[Figure 30 AFA and risk of allergic sensitisation (aero) - RCT 27](#_Toc427749877)

[Figure 31 AFA and risk of allergic sensitisation (aero) - CCT 27](#_Toc427749878)

[Figure 32 AFA and risk of allergic sensitisation (food) - RCT 28](#_Toc427749879)

[Figure 33 AFA and risk of allergic sensitisation (food) - CCT 28](#_Toc427749880)

[Figure 34 AFA and risk of allergic sensitisation (CM) - RCT 28](#_Toc427749881)

[Figure 35 AFA and risk of allergic sensitisation (CM) - CCT 28](#_Toc427749882)

[Figure 36 AFA and risk of allergic sensitisation (Egg) - RCT 29](#_Toc427749883)

[Figure 37 AFA and risk of allergic sensitisation (Egg) - CCT 29](#_Toc427749884)

[Figure 38 AFA and risk of allergic sensitisation (Peanut) - RCT 29](#_Toc427749885)

[Figure 39 AFA and risk of allergic sensitisation (Peanut) - CCT 29](#_Toc427749886)

[Figure 40 Risk of bias in studies of maternal AFA and lung function, wheeze/asthma 31](#_Toc427749887)

[Figure 41 Maternal AFA and risk of wheeze at ≤ 4 yrs - CCT 32](#_Toc427749888)

[Figure 42 Maternal AFA and risk of recurrent wheeze at ≤ 4 yrs - RCT 32](#_Toc427749889)

[Figure 43 Maternal AFA and risk of recurrent wheeze at ≤ 4 yrs - CCT 32](#_Toc427749890)

[Figure 44 Maternal AFA and risk of wheeze at 5-14 yrs - RCT 33](#_Toc427749891)

[Figure 45 Maternal AFA and risk of recurrent wheeze at 5-14 yrs - RCT 33](#_Toc427749892)

[Figure 46 Maternal AFA and risk of recurrent wheeze at age 5-14 yrs – CCT 33](#_Toc427749893)

[Figure 47 Maternal AFA and risk of recurrent wheeze at ≥15 yrs - RCT 33](#_Toc427749894)

[Figure 48 Maternal AFA and risk of BHR at 5-14 - RCT 34](#_Toc427749895)

[Figure 49 Multi-faceted interventions and FEV_1_ as % predicted 34](#_Toc427749896)

[Figure 50 Risk of bias in studies on maternal AFA and TIDM 35](#_Toc427749897)

# Maternal AFA and risk of allergic/ autoimmune outcomes – summary of interventions and findings

In this analysis we included studies of any type of allergenic food avoidance (AFA) in mothers – during any part of pregnancy, lactation or both periods of time. We did not include studies where maternal diet was modified during lactation for management of established allergic disease in the infant. We included studies of multifaceted interventions, if maternal AFA was part of the intervention – and where appropriate undertook subgroup analysis of single versus multifaceted interventions. We defined allergenic foods as cow’s milk, soya, egg, peanut, tree nuts, wheat, fish, seafood, which are the ‘major food allergens’ as defined by the US Food Allergen Labelling and consumer Protection Act of 2004. We planned to undertake subgroup analyses for meta-analyses which included >5 studies, which was possible only for AD ≤4 years. We planned to assess publication bias using Funnel plots and Egger’s test where there were ≥10 studies in a meta-analysis, although no such meta-analysis was undertaken.

In total we identified 1 high quality recent systematic review which included 5 trials (925 participants), and we identified 12 original trials (9 RCT with ~1500 participants, 3 CCT with ~330 participants) investigating the effect of maternal AFA on allergic or autoimmune outcomes.

*Interventions used:*

Two studies intervened in pregnancy only, 6 during lactation and 4 during both periods of time. Seven studies were single intervention (AFA), 5 were multifaceted (typically both maternal and infant dietary interventions, with environmental control measures to reduce allergen/ irritant exposure). The foods excluded were: milk alone (2), milk and egg (3), egg alone (1) or multiple other food groups (6), the latter usually including milk and egg as well.

*Populations and outcomes assessed:*

Seven studies were undertaken in Europe, 1 in Australia, 2 in Asia and 2 in North America. All study populations were at high allergic disease risk based on family history, but populations were not at increased risk of autoimmune outcomes. Outcomes assessed were wheeze/lung function (6), allergic rhinitis (AR; 6), eczema (AD; 11), food allergy (FA; 5), allergic sensitisation (AS; 10) and type 1 diabetes (TIDM; 2). Age at outcome assessment ranged between 3 months and 18 years.

*Overall findings:*

Overall risk of bias was high or unclear in all studies, mainly due to risk of selection bias, based on inadequate randomisation or treatment allocation procedures – seen most clearly in the 3 CCTs where the process used for treatment allocation was judged likely to lead to imbalanced treatment groups. Risk of assessment bias was also unclear in the majority of cases, and risk of conflict of interest was judged as low in most studies.

Data were sparse for all outcomes, especially TIDM, food allergy and allergic rhinitis. No studies evaluated participants at ‘normal risk’ of allergic outcomes.

**Overall there is no evidence that maternal allergenic food avoidance reduces risk of allergic of autoimmune outcomes in the offspring.**

**Table 1 Characteristics of included studies evaluating maternal AFA and allergic outcomes**

| **Study** | **Design** | **N Intervention/ Control** | **Country** | **Excluded food** | **Intervention** | **Disease risk** | **Age at outcome (years)** | **Outcomes reported** |
| --- | --- | --- | --- | --- | --- | --- | --- | --- |
| **Kramer (**[**1**](#_ENREF_1)**)** | SR | 5 trials (925) | - |  | Pregnant/lactating women | high |  | Eczema, Allergic sensitisation, Wheeze, Allergic rhinitis |
| Falth-Magnusson, 1987 ([2](#_ENREF_2))  Falth-Magnusson 1992 ([3](#_ENREF_3))  Ludvigsson, 2003 ([4](#_ENREF_4)) | RCT | 108/ 104 | Sweden | Cow's milk and egg | **Pregnant women**. Cow's milk and egg exclusion from 28 weeks gestation to delivery. | high | 1.5, 5 | Allergic rhinitis (physician assessment), Asthma (≥3 episodes of wheeze), Eczema (Hanifin and Lobitz), Allergic sensitisation (SPT), Food allergy (history), Total IgE, TIDM (serology) |
| Hattevig, 1990 ([5](#_ENREF_5))  Paronen, 2000([6](#_ENREF_6))  Hattevig, 1999 ([7](#_ENREF_7))  Hattevig, 1989 ([8](#_ENREF_8))  Sigurs, 1992 ([9](#_ENREF_9)) | CCT | 54/ 67 | Sweden | Cow's milk, fish and egg | **Lactating women**. Cow's milk, egg and fish exclusion during first 3 months post-partum. | high | 0.25, 1.5, 4, 10 | Allergic sensitisation TIDM (serology), Eczema, Asthma, Wheeze, Recurrent wheeze Allergic rhinitis, Food allergy (history), Total IgE |
| Herrmann, 1996 ([10](#_ENREF_10)) | CCT | 50/ 50 | Germany | Cow's milk and egg | **Pregnant/lactating women**. Cow's milk and egg exclusion from 28 weeks gestation through lactation, versus 1 litre cow's milk and 1 egg per day. | high | 1 | Eczema (DD), Allergic sensitisation (sIgE) |
| Jirapinyo, 2013 ([11](#_ENREF_11)) | RCT | 30/ 32 | Thailand | Cow's milk | **Lactating women**. Cow's milk exclusion during lactation up to 4 months postpartum. | high | <0.5 | Eczema (method unclear) |
| Kilburn, 1998 ([12](#_ENREF_12)) | CCT | 15/ 96 | UK | ‘Allergenic foods’ | **Lactating women**. Milk, egg, fish and nuts exclusion throughout lactation. | high | 1.5 | Eczema (Hanifin and Rajka), Allergic sensitisation (SPT) |
| Lovegrove, 1994 ([13](#_ENREF_13)) | RCT | 12/ 14 | UK | Cow’s milk | **Pregnant/lactating women**. CM exclusion during pregancy and lactation with hydrolysed milk if necessary. BF encouraged for 6 months and eHF if needed. | high | 1 | Eczema (DD) |
| Metcalfe, 2016{Metcalfe, 2016 #832} | RCT | 40, 44, 36 | Australia | Egg | **Lactating women.** First 6 weeks of lactation. Randomised to high-egg diet (>4 eggs per week), low-egg diet (1-3 eggs per week) and egg-free diet | high | 0.33 | Allergic sensitisation (sIgE-egg) |
| Shao, 2006 ([14](#_ENREF_14)) | RCT | 23/ 23 | China | ‘Allergenic foods’ | **Lactating women**.  eBF encouraged for 4 months, allergenic food exclusion during lactation, delayed solid (4 months) and allergenic (6-12 months) food, pHF if necessary. | high | 1.5 | Eczema (Wolkerstorfer score), Allergic Sensitisation (SPT) |
| Becker, 2004 ([15](#_ENREF_15)); Chan-Yeung, 2000 ([16](#_ENREF_16)); Chan-Yeung 2005 ([17](#_ENREF_17)); Wong, 2013 ([18](#_ENREF_18))Protudjer, 2011([19](#_ENREF_19)); Carlsten 2013([20](#_ENREF_20))  **CAPPS Study** | RCT | 281/ 268 | Canada | Nuts, seafood | **Pregnant/lactating women**. BF encouraged for 4 months, allergenic food exclusion during pregnancy/lactation, delayed solid (6 months) and allergenic (12 months) food, whey pHF if necessary, environmental control. | high | 1, 7, 15 | Allergic Sensitisation (SPT), Allergic Rhinitis (DD), Wheeze (ISAAC and modified ECRHS), Eczema (DD), bronchial hyper-responsiveness (PC20 <7.8mg/ml), Lung function (FEV1) |
| Hide, 1994 ([21](#_ENREF_21))  Hide, 1996([22](#_ENREF_22))  Arshad, 1992 ([23](#_ENREF_23))  Arshad, 2003 ([24](#_ENREF_24))  Arshad, 2007 ([25](#_ENREF_25))  Scott, 2012 ([26](#_ENREF_26))  **Isle of Wight Study** | RCT | 71/ 68 | UK | Cow’s milk, egg, fish and nuts | **Lactating women.** Allergenic food exclusion during lactation, delayed allergenic (9-11 months) food, soya hydrolysate if necessary, environmental control. | high | 1, 2,4,8,18 | Recurrent wheeze (>=3 episodes), BHR (PC20<8mg/ml), Eczema (Physician assessment), Food allergy (Open food challenge ; Physician assessment), Allergic sensitisation (SPT, total IgE), Allergic rhinitis (physician assessment), Total IgE |
| Zeiger, 1992  ([27](#_ENREF_27))  Zeiger 1989 ([28](#_ENREF_28)), 1994 ([29](#_ENREF_29)) | RCT | 103/ 185 [seen at 4 months] | USA | Cow’s milk, egg, peanut; and partial soya and wheat avoidance | **Pregnant/lactating women**. Allergenic food exclusion during pregnancy/lactation, delayed allergenic (1-3 years) food, casein eHF if necessary. | high | 1, 4, 7 | Eczema (Hanifin and Rajka Criteria), Allergic Rhinoconjunctivitis (DD), Food Allergy - Any (DD), Wheeze ( ≥2 physician diagnosed episodes), Allergic Sensitisation (SPT) |
| Lilja, 1989 ([30](#_ENREF_30)) | RCT | 84/ 87 | Sweden | Cow’s milk, egg | **Pregnant women.** Milk and Egg exclusion during third trimester, versus 1 litre milk and 1 egg per day. | High | 1.5 | Asthma (physician assessment), Allergic sensitisation (SPT), Allergic rhinitis (physician assessment), Eczema (physician assessment), Food allergy (history) |

BF breastfeeding; eBF exclusive breastfeeding; pHF partially hydrolysed formula; eHF extensively hydrolysed formula; CM cow’s milk; RCT randomised clinical trial, SPT skin prick test, BHR bronchial hyperresponsiveness, FE_V1_ forced expiratory volume in one second; Physician assessment refers to assessment by a study physician , DD refers to community diagnosis.

Figure 1 Risk of bias in studies of maternal AFA and allergic or auto-immune diseases

# Maternal AFA and risk of AD

One systematic review and 11 original trials (8 RCT, 3 CCT; total 1700 participants) were included in this analysis. Table 2 summarises findings from the recent high quality systematic review of Kramer, which excluded multi-faceted studies and CCTs, but did not exclude studies where AFA was practiced in the context of infant allergic disease. Kramer identified 2 studies of AFA in pregnancy and 1 study of AFA in lactation. Meta-analysis did not show any evidence for a protective effect.

Figure 2 summarises the risk of bias for original studies of maternal AFA and AD. Overall risk of bias was high or unclear in all studies, mainly due to risk of selection bias, based on inadequate randomisation or treatment allocation procedures – seen most clearly in the 3 CCTs where the process used for treatment allocation was judged likely to lead to imbalanced treatment groups. Risk of assessment bias was also unclear in the majority of cases, and risk of conflict of interest was judged as low in most studies.

Meta-analysis of data from the original studies did not show a significant association between maternal AFA and AD risk – albeit with high heterogeneity in both the RCT (Figures 3 and 5) and CCT (Figure 4) analyses. A single small RCT (Arshad) found evidence that maternal AFA, as part of a multi-faceted intervention study, reduces risk of AD associated with positive SPT at the age of 8 (Figure 6). Other small single study analyses were not statistically significant (Figures 7 and 8). Subgroup analysis of RCT evidence for prevention of AD at age ≤4 years showed evidence for a subgroup difference according to timing of intervention – with AFA during lactation reducing AD risk, but AFA during pregnancy having no effect (Table 3). The effect of AFA during lactation on AD risk was not supported by the CCT evidence from 2 trials with a similar number of participants (Figure 4).

The CCT of Kilburn did not report data for meta-analysis, but did report a trend towards increased AD in the AFA group, which was not statistically significant. In the CCT of Hattevig AD was significantly reduced at age 4, but not at other ages (Figures 4 and 7). In the RCT of Arshad AD was significantly reduced at age 2, but not at other ages (Figures 3, 5 and 8).

**Overall we found no consistent evidence that maternal AFA impacts on child AD risk.**

Figure 2 Risk of bias in studies of maternal AFA and AD risk

Table 2 Systematic review evidence for AFA and AD risk

| **Study** | **Outcome measure** | **Intervention timing** | **No. participants (studies)** | **Outcome (95% CI)** | I^2^ |
| --- | --- | --- | --- | --- | --- |
| **Kramer** ([1](#_ENREF_1)) | AD at ≤18 months | Pregnancy | 334 (2) | RR 1.01 [0.57, 1.79] | 48% |
| **Kramer** ([1](#_ENREF_1)) | AD at ≤18 months | Lactation | 26 [1] | RR 0.73 [0.32, 1.64] | - |

Figure 3 Maternal AFA and risk of AD at age ≤ 4 yrs - RCT


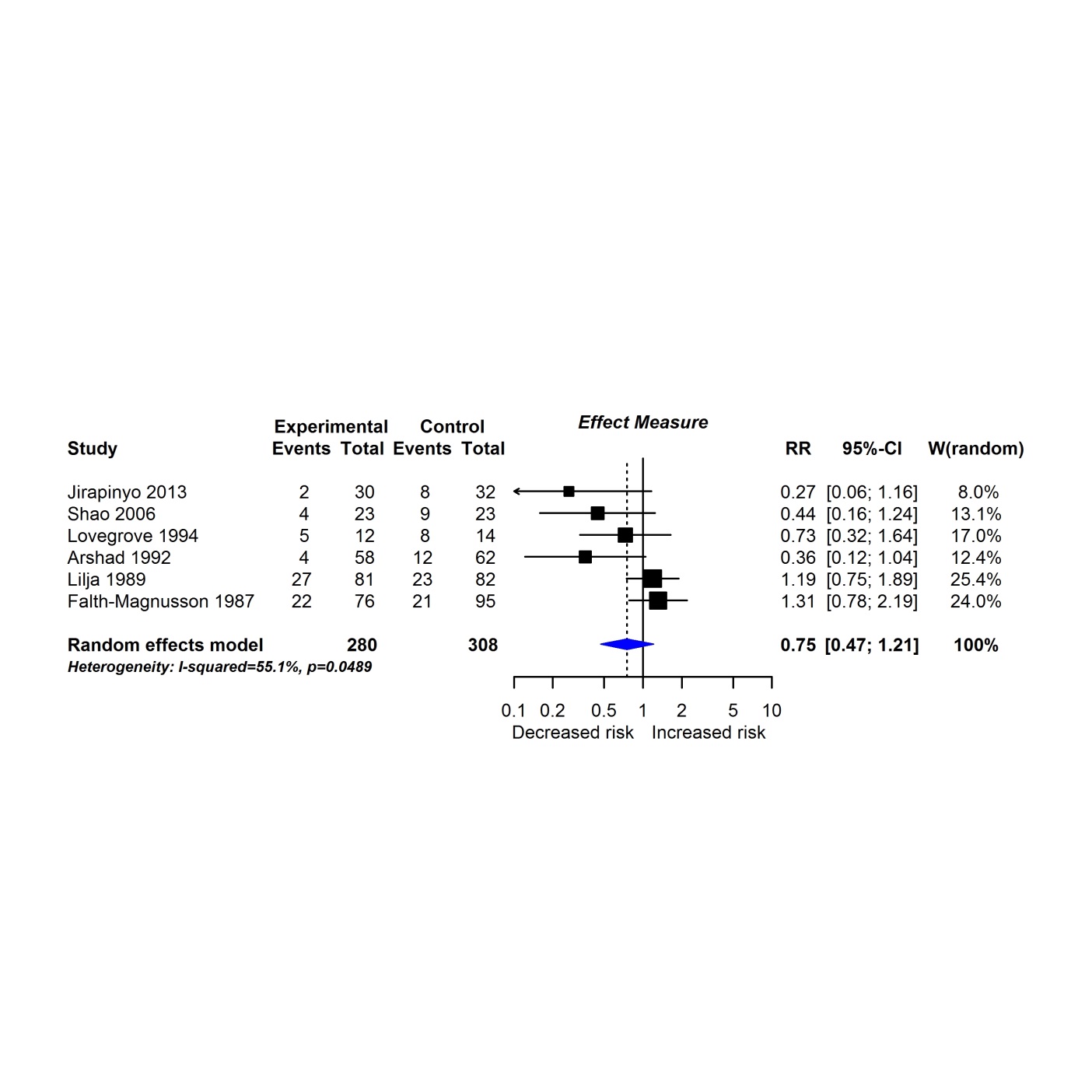


Table 3 Subgroup analysis of maternal AFA and AD risk at age ≤4 years

|  | | **Number of studies** | **RR [95% CI]** | **I^2^ (%)** | **P-value for between groups difference** |
| --- | --- | --- | --- | --- | --- |
| Intervention – pregnancy only  Intervention – lactation +/- pregnancy | 2  4 | | 1.24 [0.88-1.75]  0.48 [0.29-0.81] | 0  0 | 0.003 |
| Intervention – multifaceted including AFA  Intervention – AFA alone | 3  3 | | 0.53 [0.30-0.91]  1.04 [0.60-1.80] | 0  52 | 0.09 |
| Overall risk of bias – Low  Overall risk of bias – High/Unclear | -  4 | | -  0.75 [0.47-1.21] | -  55 | - |
| Conflict of interest bias – Low  Conflict of interest bias – High/Unclear | 2  4 | | 0.66 [0.15-2.84]  0.70 [0.38-1.31] | 73  57 | 0.94 |

Figure 4 Maternal AFA and risk of AD at age ≤ 4 yrs - CCT


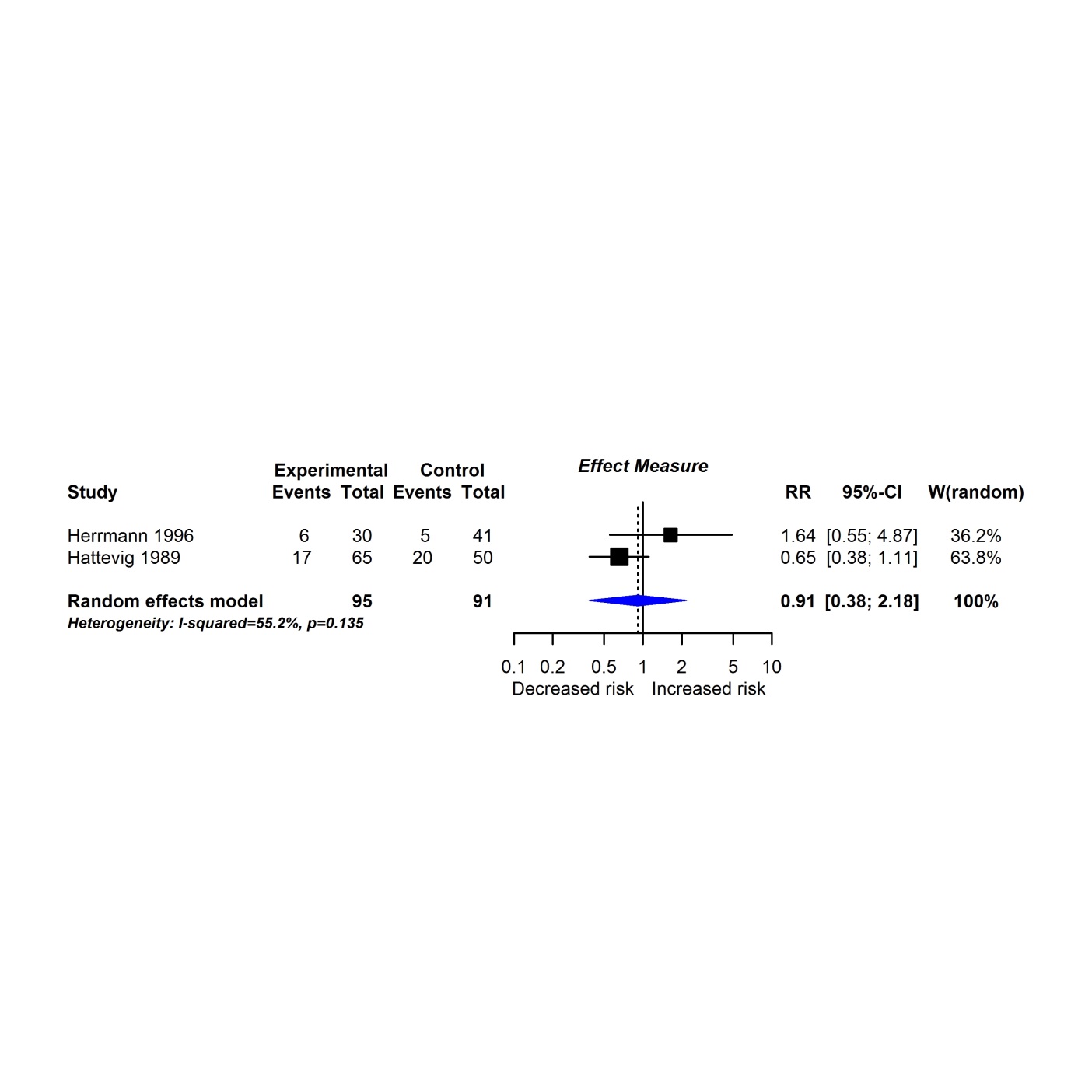


Figure 5 Maternal AFA and risk of AD at age 5-14 yrs - RCT


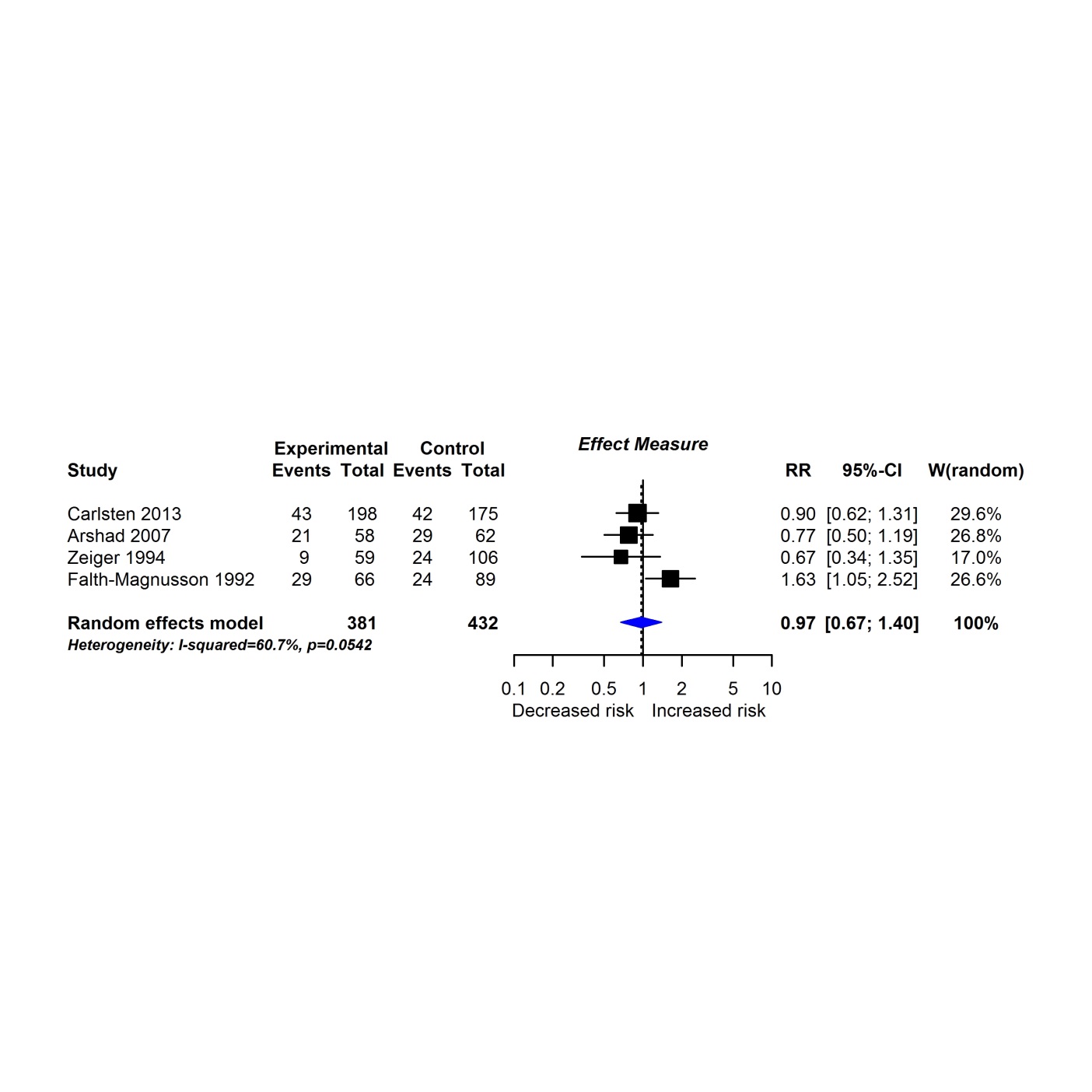


Figure 6 Maternal AFA and risk of ‘atopic’ AD at age 5-14 yrs - RCT


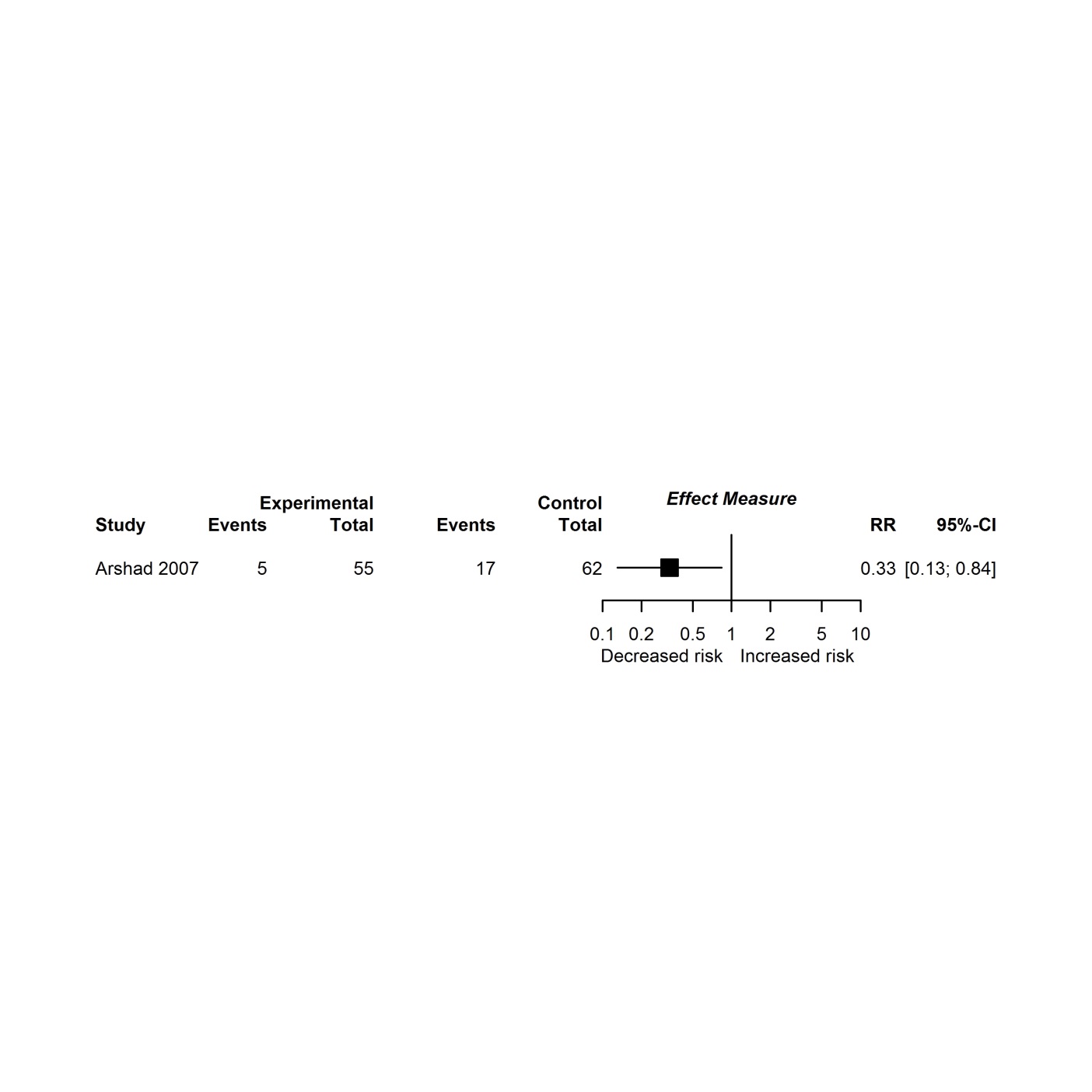


Figure 7 Maternal AFA and risk of AD at age 5-14 yrs - CCT


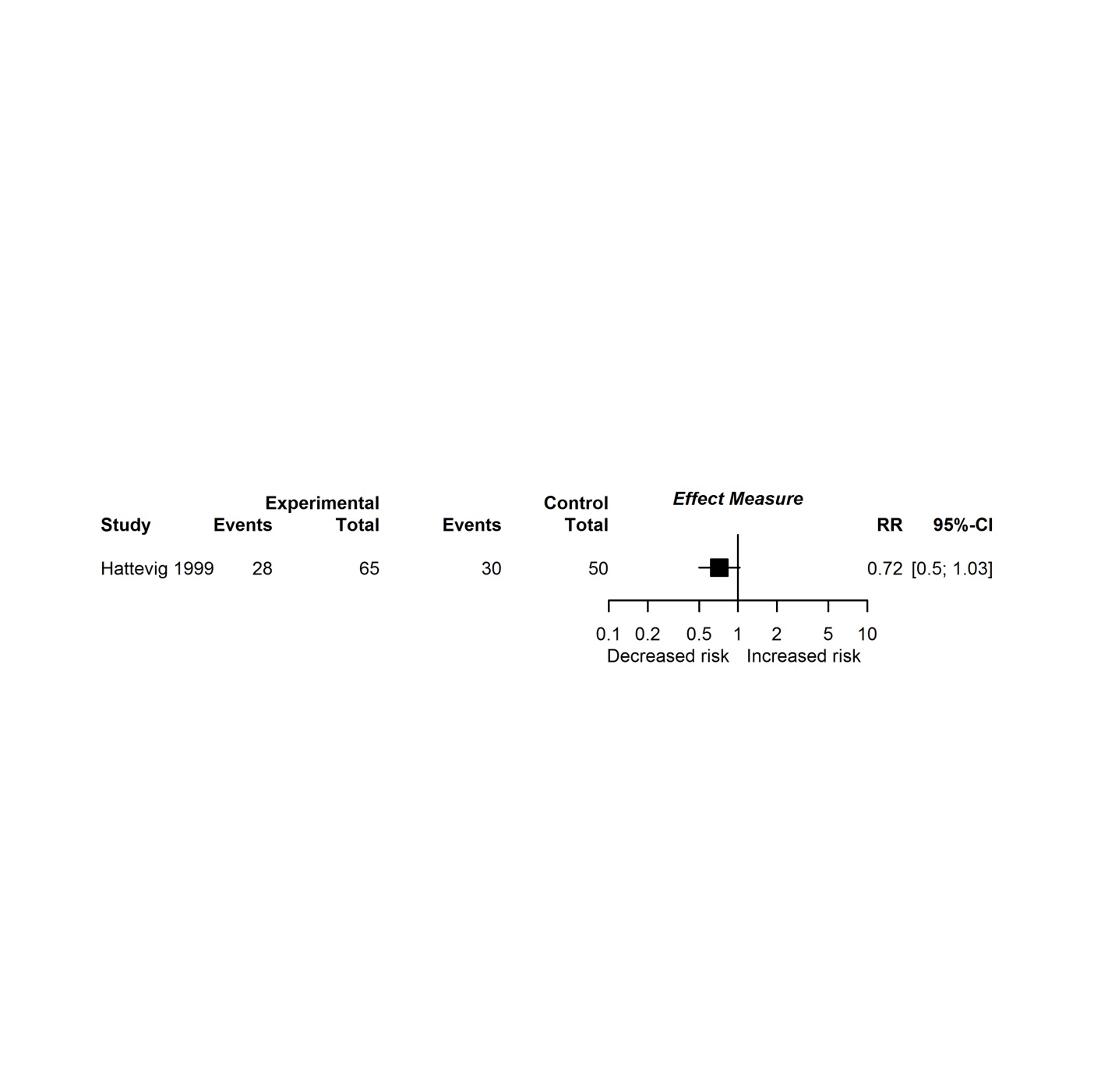


Figure 8 Maternal AFA and risk of AD at age ≥15 yrs - RCT


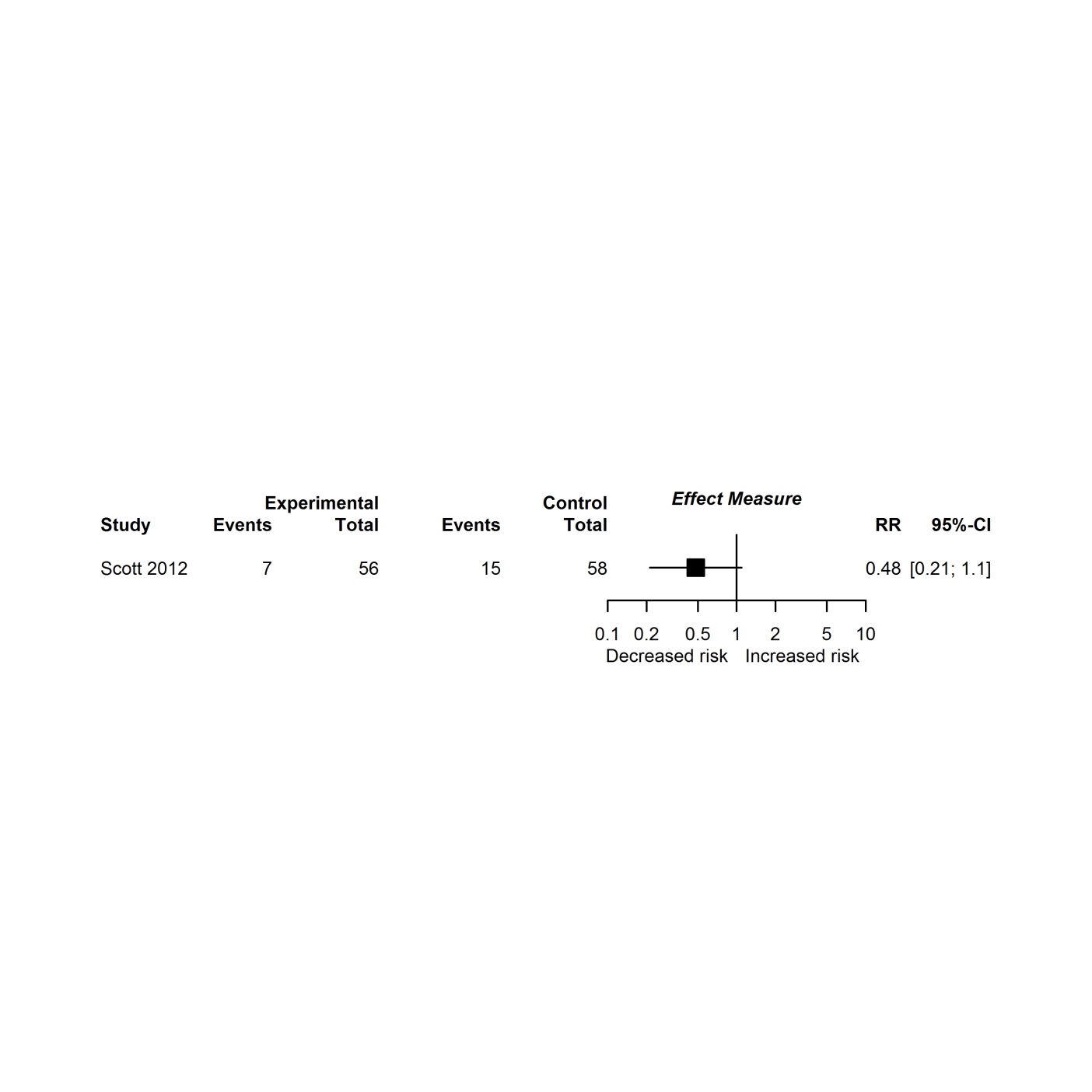


# Maternal AFA and risk of allergic rhinitis +/- conjunctivitis

One systematic review and 6 original trials (1400 participants) were included in this analysis.

The recent high quality systematic review of Kramer identified 1 study (163 participants) of AFA in pregnancy from which a RR could not be estimated due to low event numbers.

Figure 9 summarises the risk of bias for original studies of maternal AFA and AR. Overall risk of bias was high or unclear in 4 of 5 studies, mainly due to risk of selection bias, based on inadequate randomisation or treatment allocation procedures. Risk of assessment bias was also unclear in the majority of cases, and risk of conflict of interest was judged as low in most studies.

Meta-analysis of data from the original studies showed a borderline significant association between maternal AFA and AR risk, with low heterogeneity in the RCT analysis (Figure 10) and just 1 study in the CCT analysis (Figure 11) analysis. The RCT analysis was dominated by a single multifaceted intervention trial (Chan-Yeung) which is described further in the multifaceted intervention report. The AFA-only trials (Lilja, Falth-Magnusson and Sigurs) included in these analyses did not show evidence of an impact on AR at age ≤4 years. Analysis at age 5-14 showed no evidence of an effect (Figure 12), although a single small RCT (Arshad; Figure 13) found reduced ‘atopic’ AR at aged 5-14. This was not seen in a similar sized CCT evaluating AR at the same age (Figure 14).

The multifaceted RCT of Zeiger also reported AR at age 4 in graphical form, where no significant difference was seen in this outcome between treatment groups. The CCT of Hattevig/Sigurs and the RCT of Arshad/Hide reported AR at other timepoints and/or using other methods in addition to those shown in meta-analysis – with broadly similar findings. We did not identify any studies reporting allergic conjunctivitis as an outcome.

**Overall we found no consistent evidence that maternal AFA impacts on child AR risk.**

Figure 9 Risk of bias in studies on maternal AFA and AR risk

Figure 10 Maternal AFA and risk of AR at age ≤4 yrs - RCT


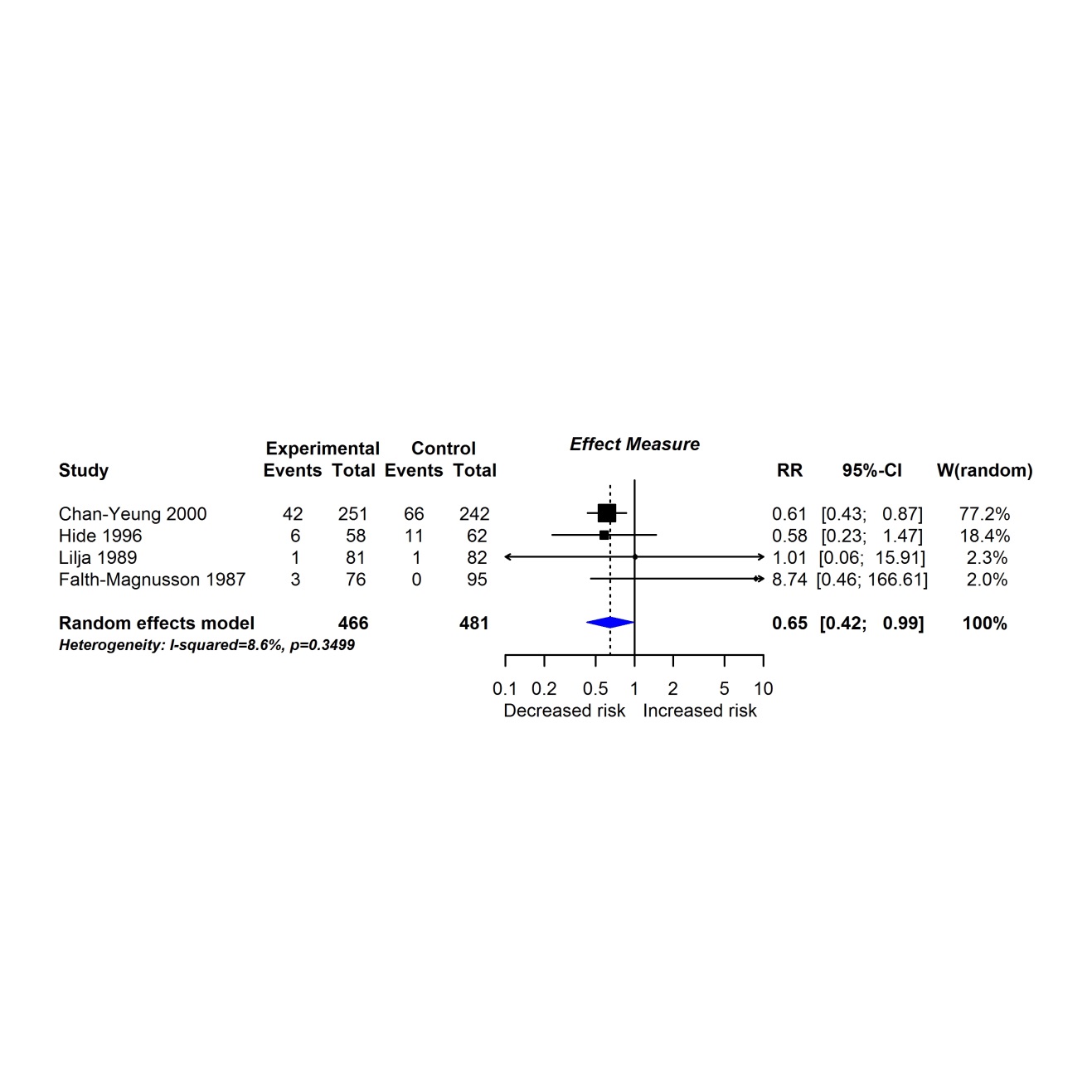


Figure 11 Maternal AFA and risk of AR at age ≤4 yrs - CCT


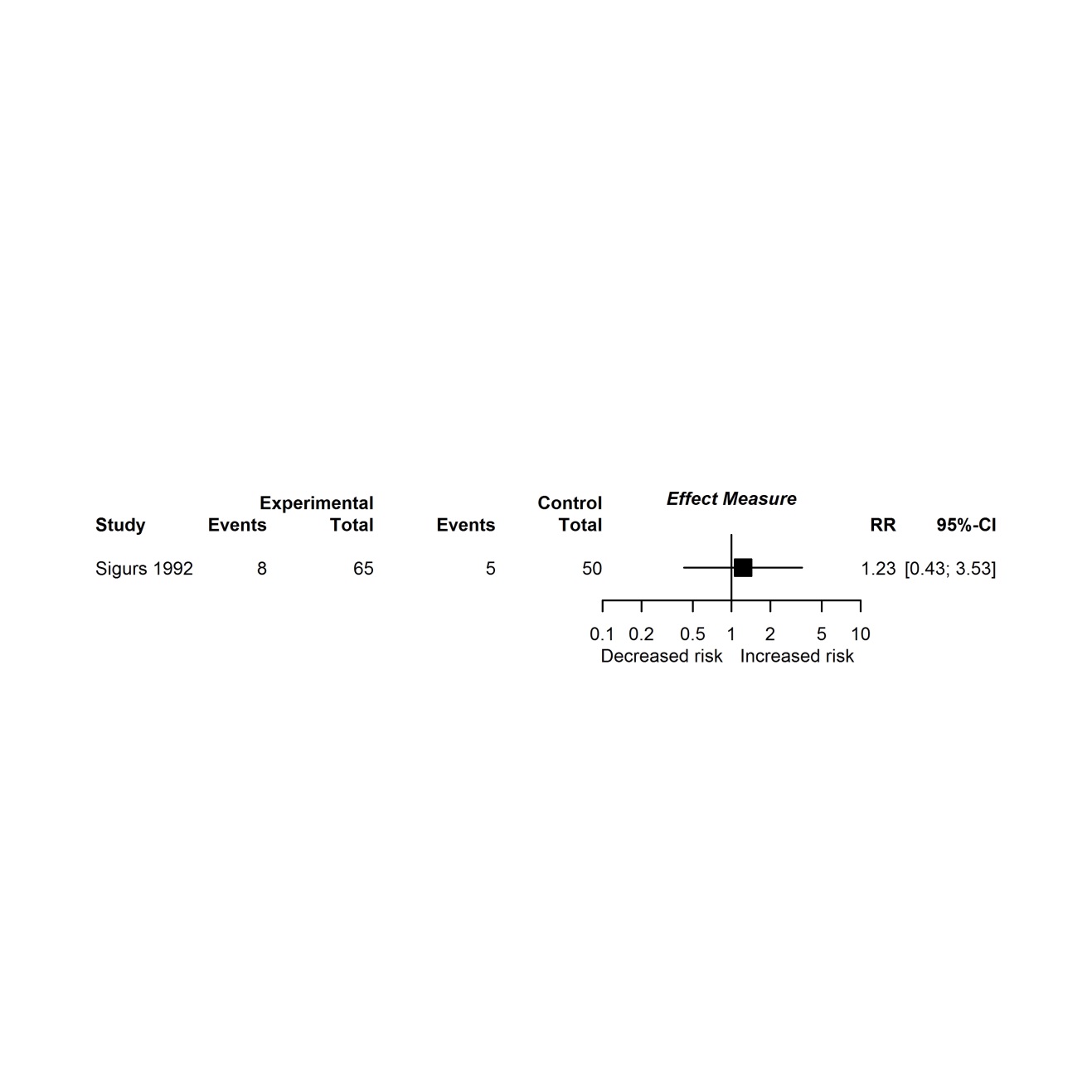


Figure 12 Maternal AFA and risk of AR at age 5-14 yrs - RCT


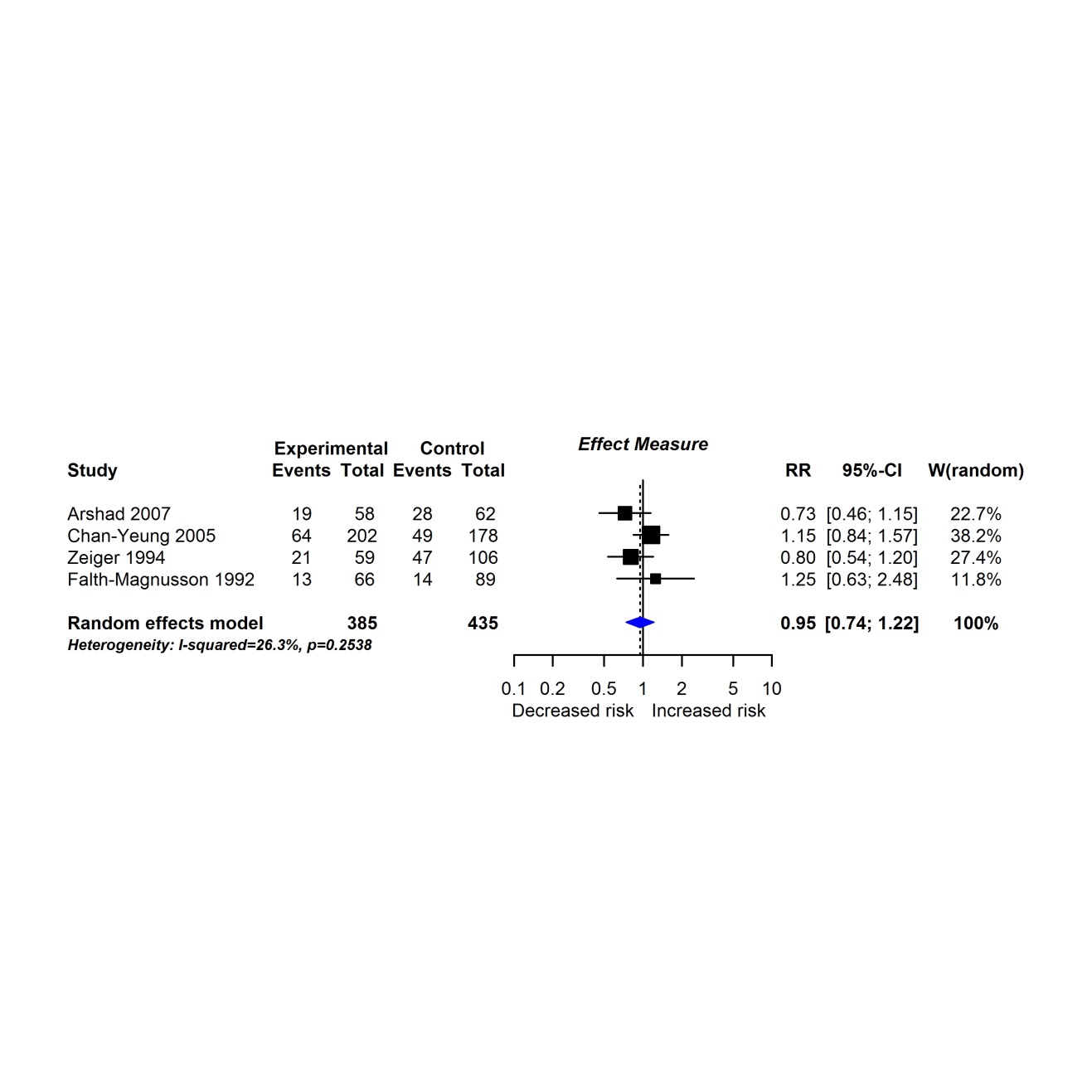


Figure 13 Maternal AFA and risk of ‘atopic’ AR at age 5-14 yrs - RCT


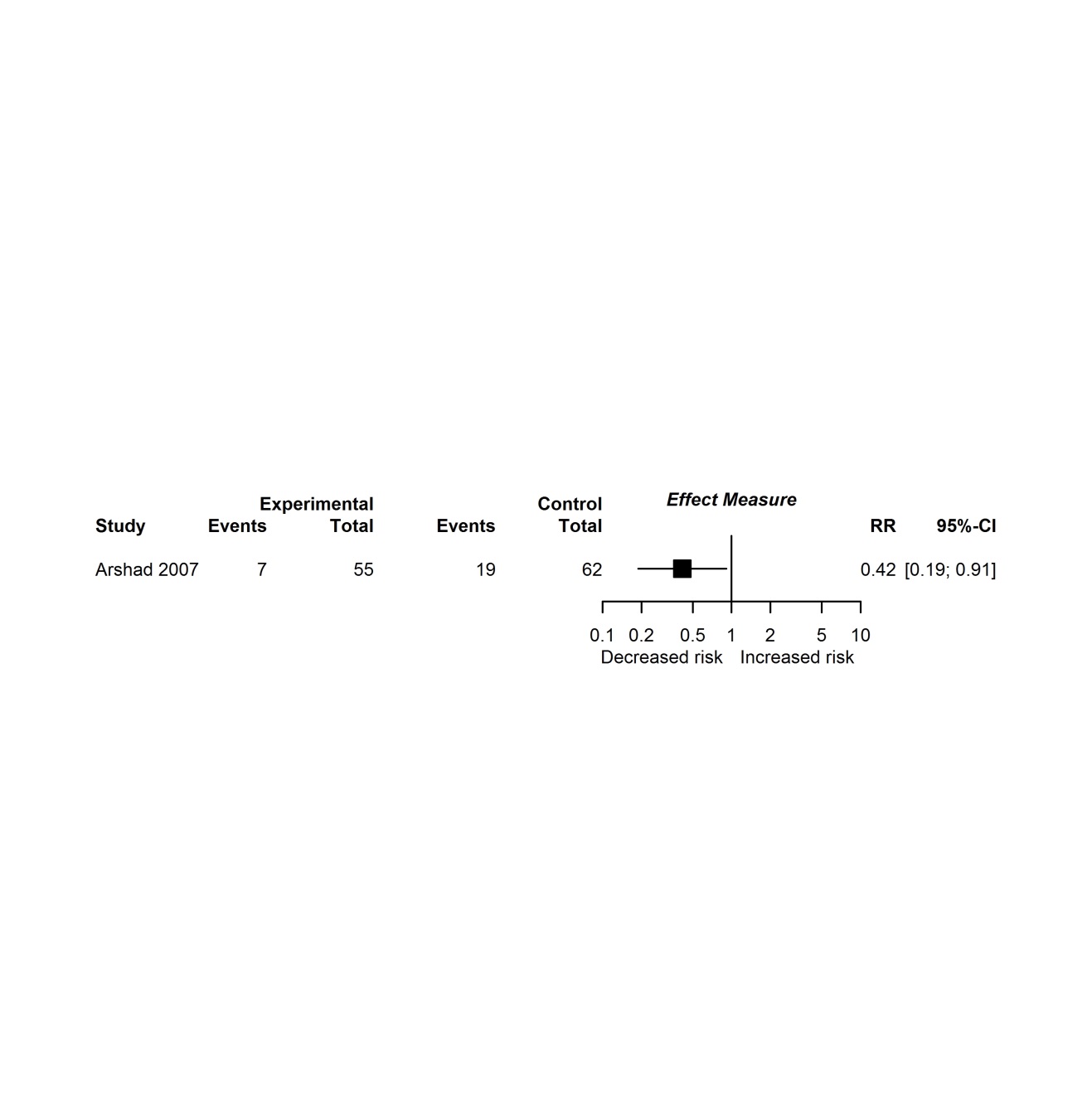


Figure 14 Maternal AFA and risk of AR at age 5-14 yrs - CCT


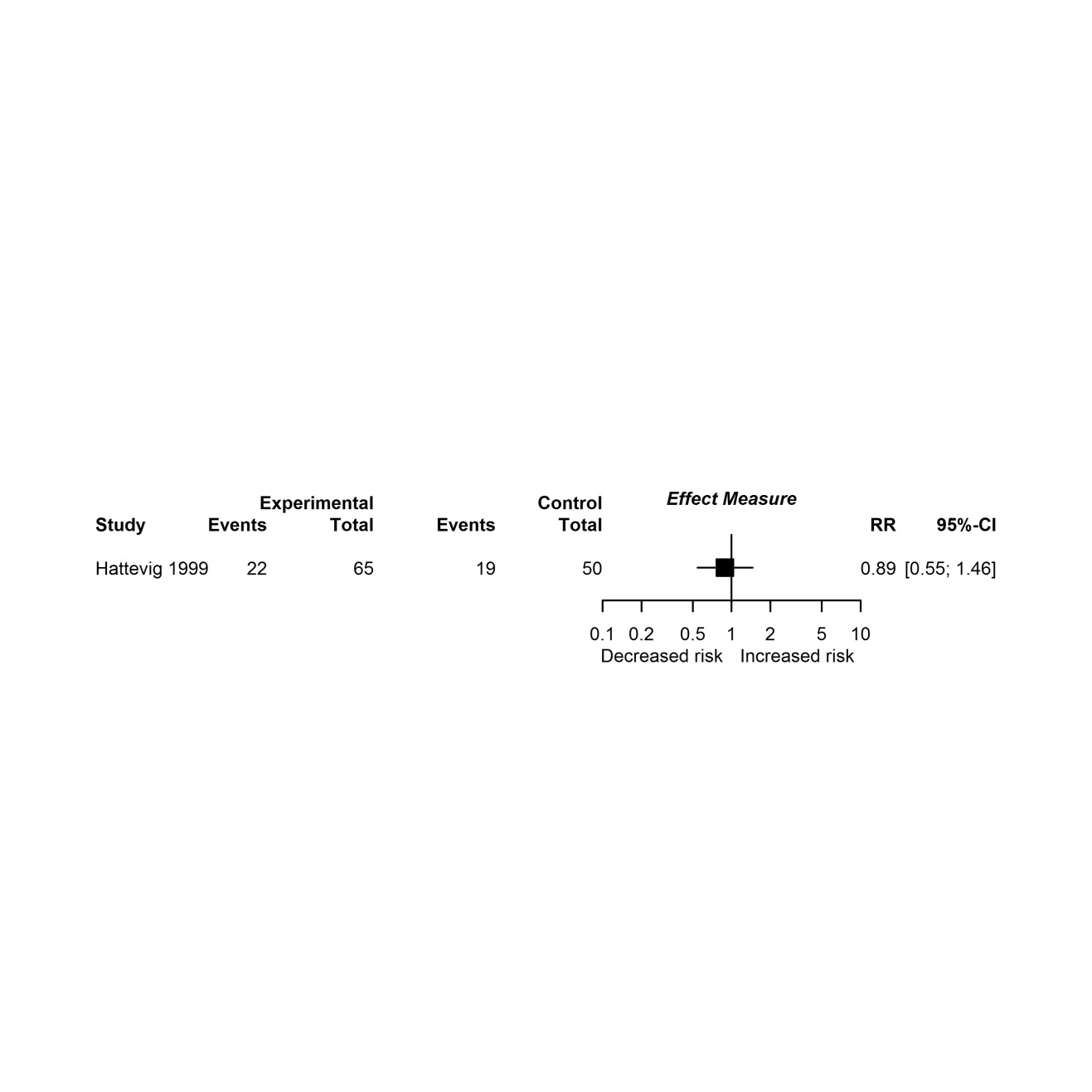


# Maternal AFA and risk of food allergy

No systematic review and 5 original trials (800 participants) were included in this analysis.

Figure 15 summarises the risk of bias for original studies of maternal AFA and FA. Overall risk of bias was high or unclear in all 5 studies, mainly due to risk of selection bias, based on inadequate randomisation or treatment allocation procedures. Risk of conflict of interest was judged as low in most studies.

Meta-analysis of data from the original studies showed no significant association between maternal AFA and FA risk, with high heterogeneity in the RCT analysis (Figure 16) and just 1 study in the CCT analysis (Figure 17) analysis. The heterogeneity in the RCT analysis may be explained by combination of a multifaceted intervention trial (Arshad) with a AFA-only intervention trial (Falth-Magnusson). At age 5-14 there was also no significant effect, again with high heterogeneity which may be explained by combination of 2 multifaceted with 1 AFA-only study (Figure 18). Other analyses, including analysis of cow’s milk and egg allergy were limited by small study and participant numbers and wide confidence intervals, but showed no evidence of an effect of maternal AFA on child food allergy (Figures 19 to 26).

The multifaceted RCT of Zeiger also reported FA at age 4 in graphical form, where no significant difference was seen in current FA, but a significant reduction in FA ever was seen in the intervention group (P<0.01). The multifaceted RCT of Arshad/Hide reported no significant difference in physician diagnosed food allergy at age 2.

**Overall we found no consistent evidence that maternal AFA impacts on child FA risk.**

Figure 15 Risk of bias of intervention studies of maternal AFA and FA risk

Figure 16 Maternal AFA and risk of food allergy (any) at age ≤ 4 yrs - RCT


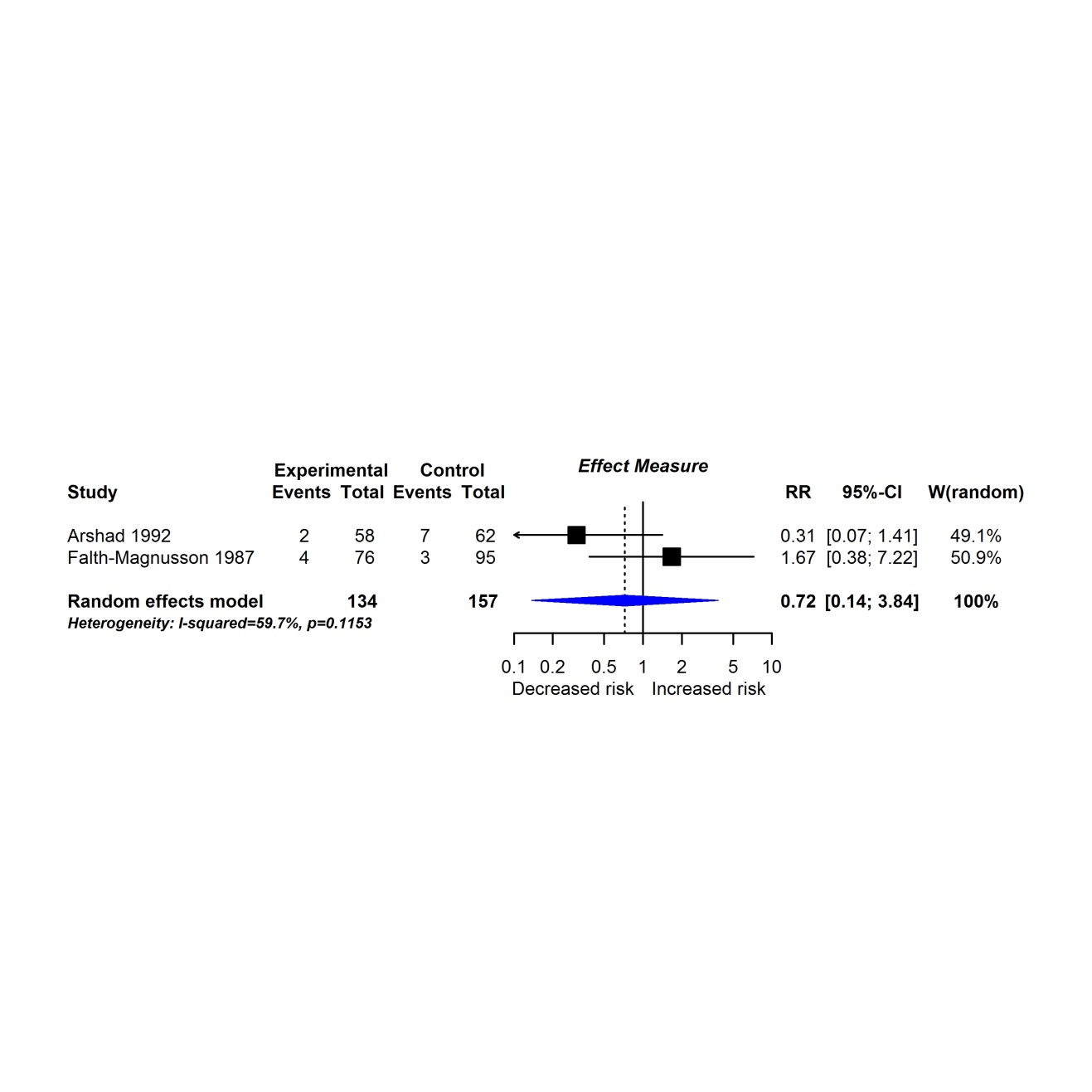


Figure 17 Maternal AFA and risk of food allergy (any) at age ≤ 4 yrs - CCT


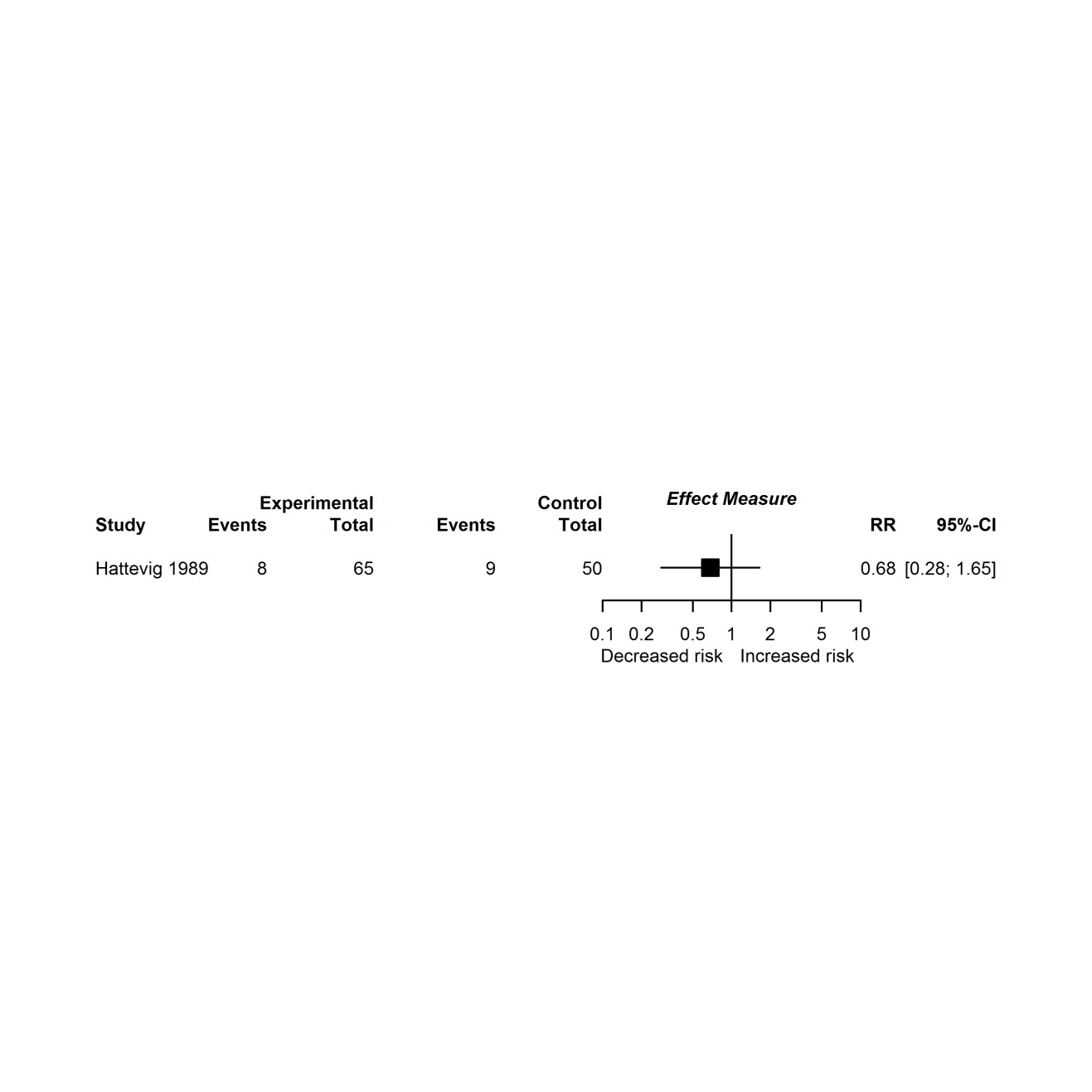


Figure 18 Maternal AFA and risk of food allergy (any) at age 5-14 yrs - RCT


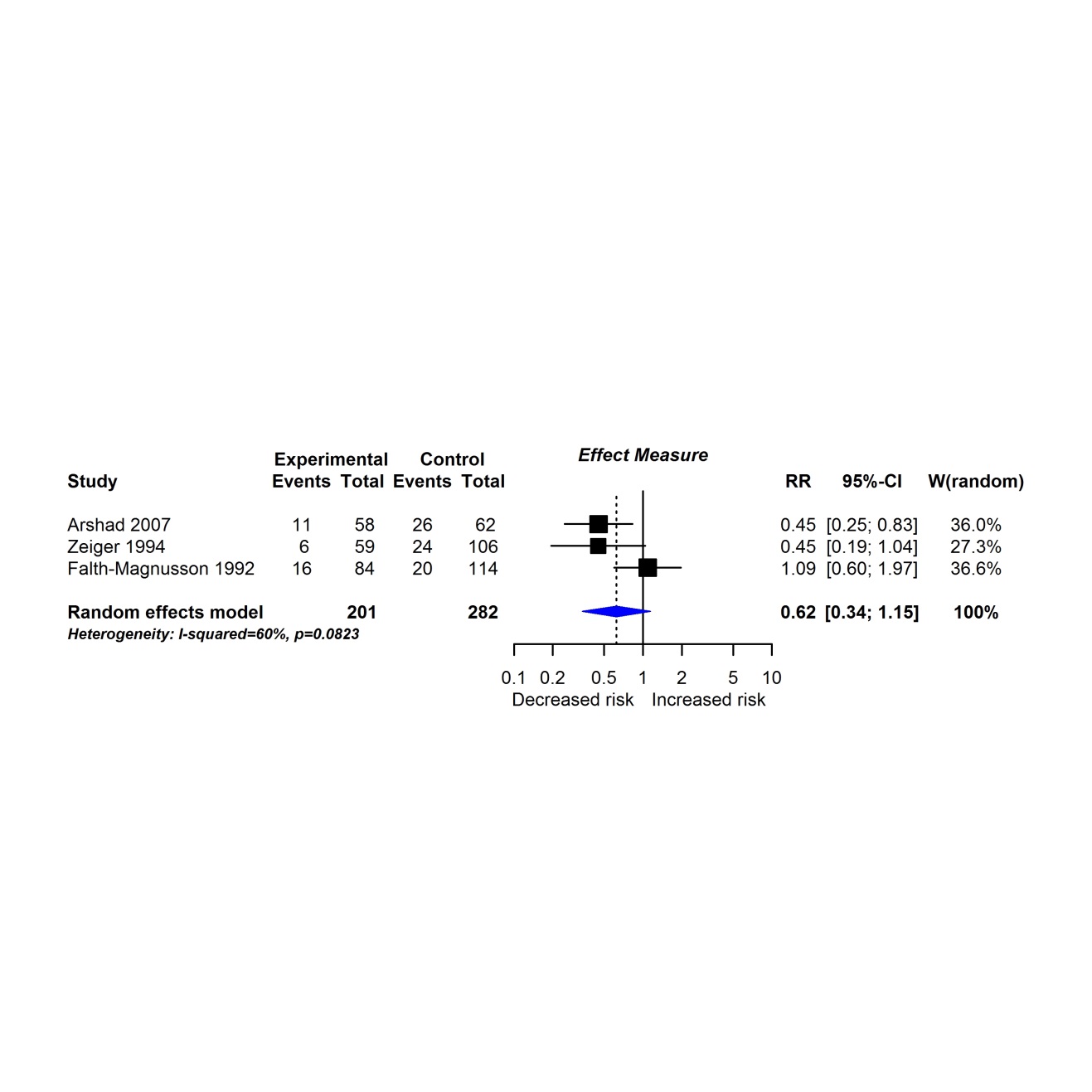


Figure 19 Maternal AFA and risk of food allergy (any) at age 5-14 yrs - CCT


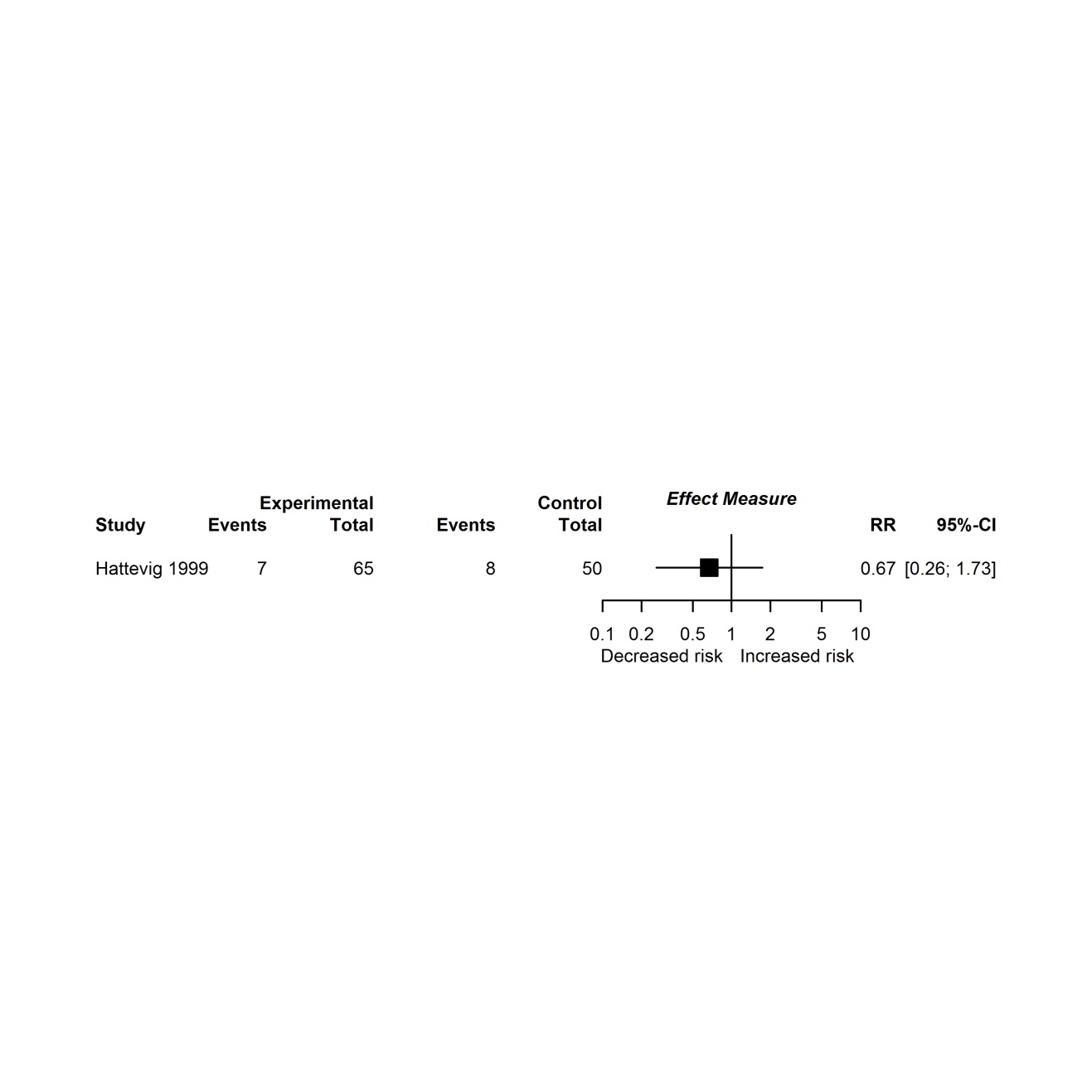


Figure 20 Maternal AFA and risk of CMA at age ≤ 4 yrs - RCT


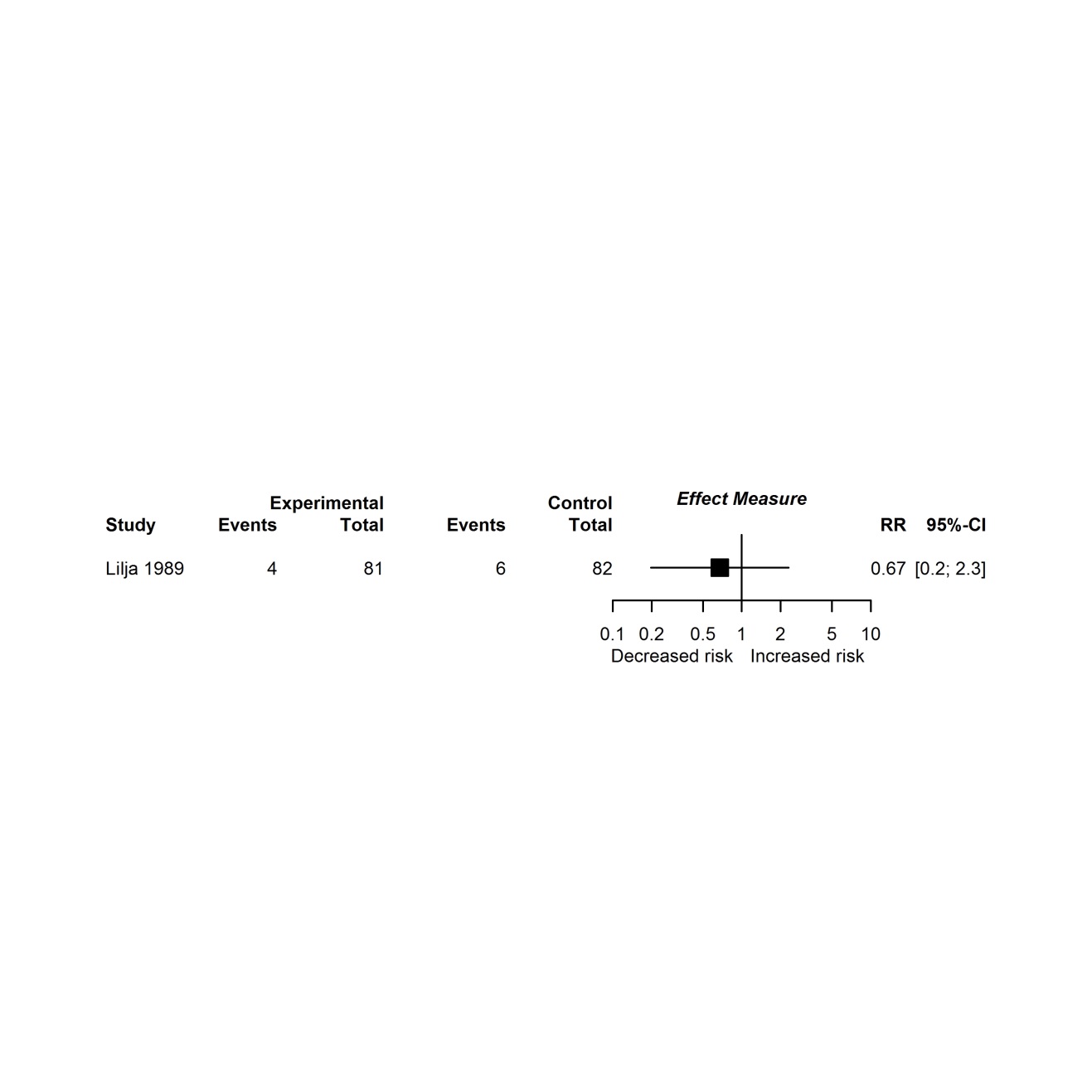


Figure 21 Maternal AFA and risk of CMA at age ≤ 4 yrs - CCT


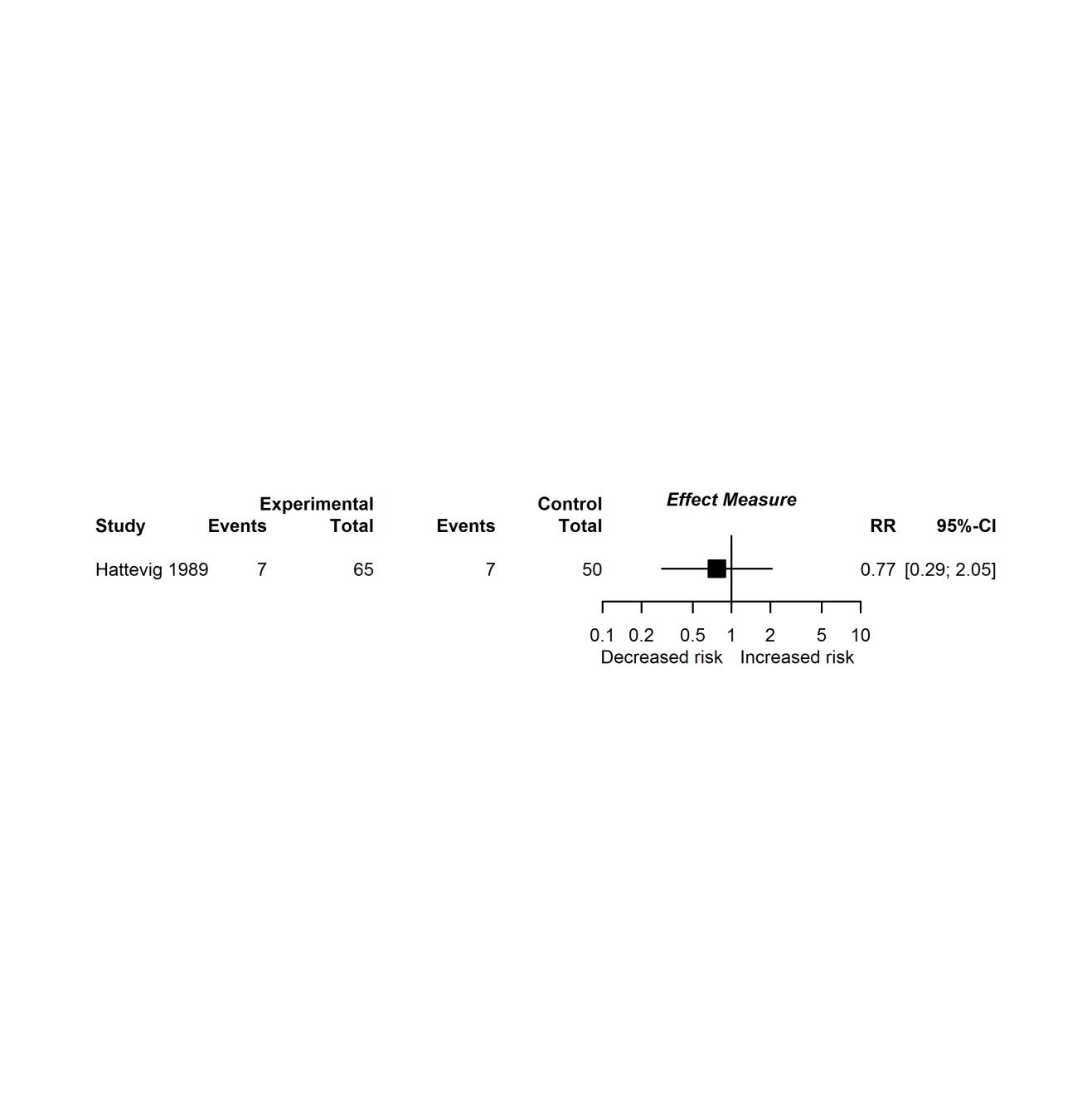


Figure 22 Maternal AFA and risk of CMA at age 5-14 yrs - CCT


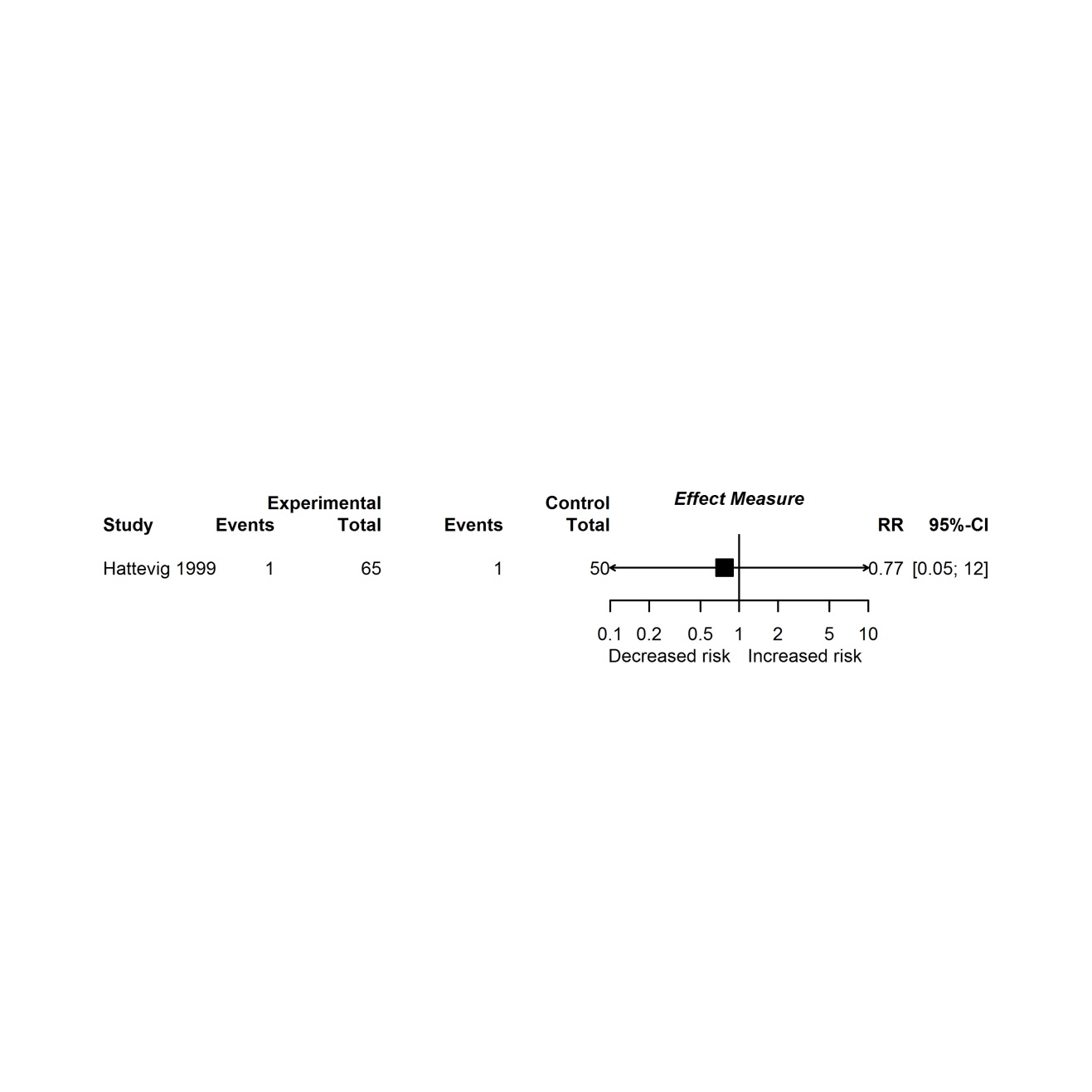


Figure 23 Maternal AFA and risk of Egg allergy at age ≤ 4 yrs - RCT


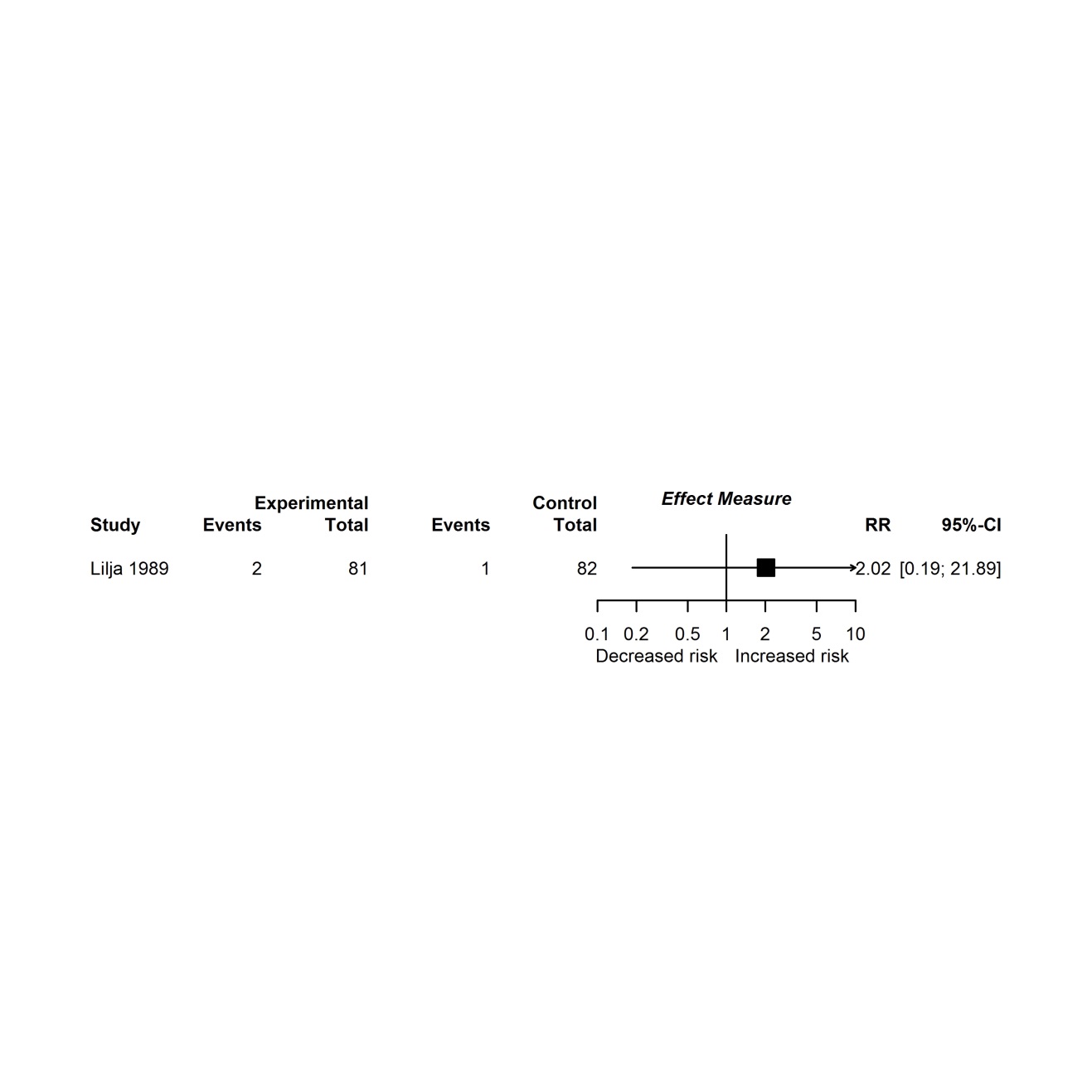


Figure 24 Maternal AFA and risk of Egg allergy at age ≤ 4 yrs - CCT


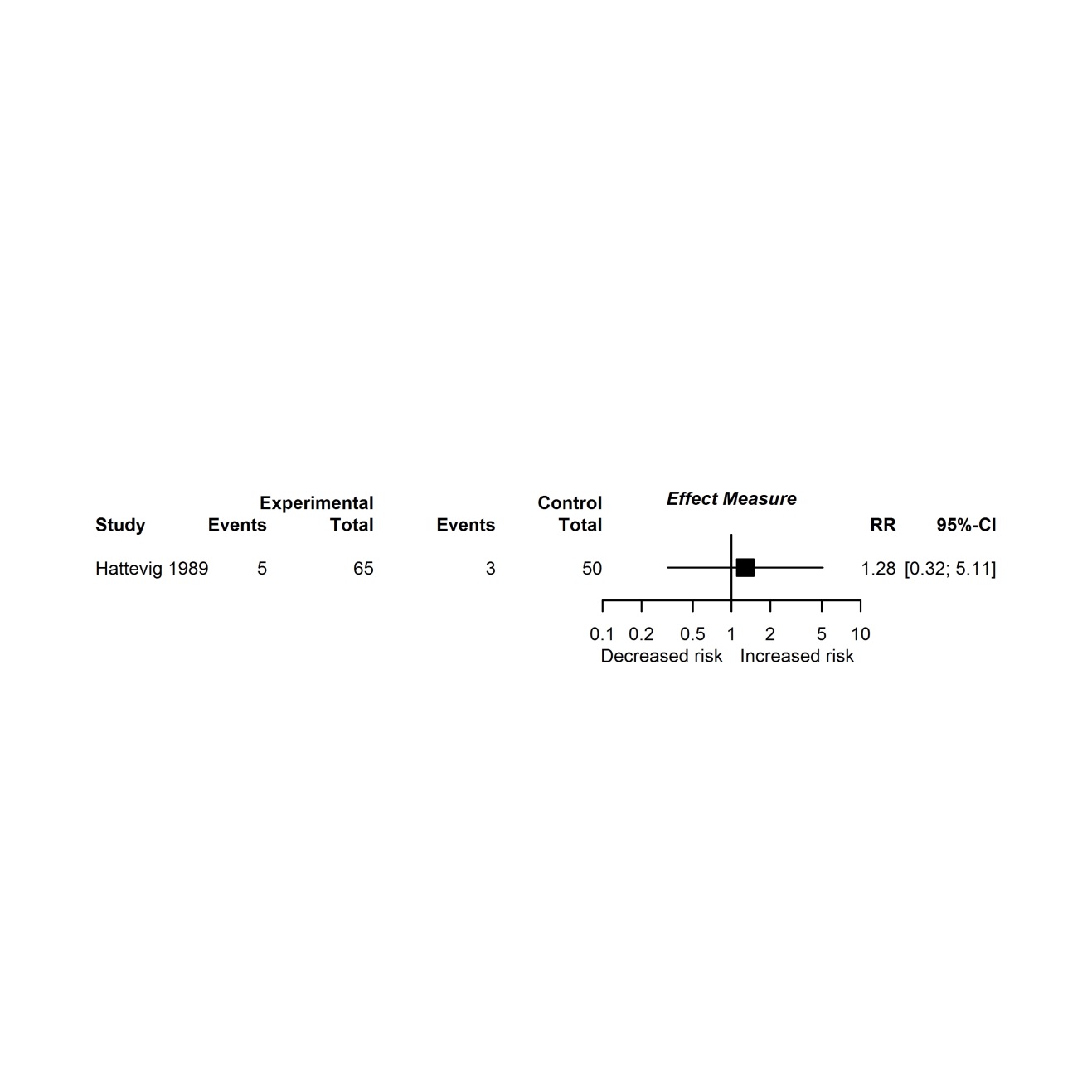


Figure 25 Maternal AFA and risk of Egg allergy at age 5-14 yrs - RCT


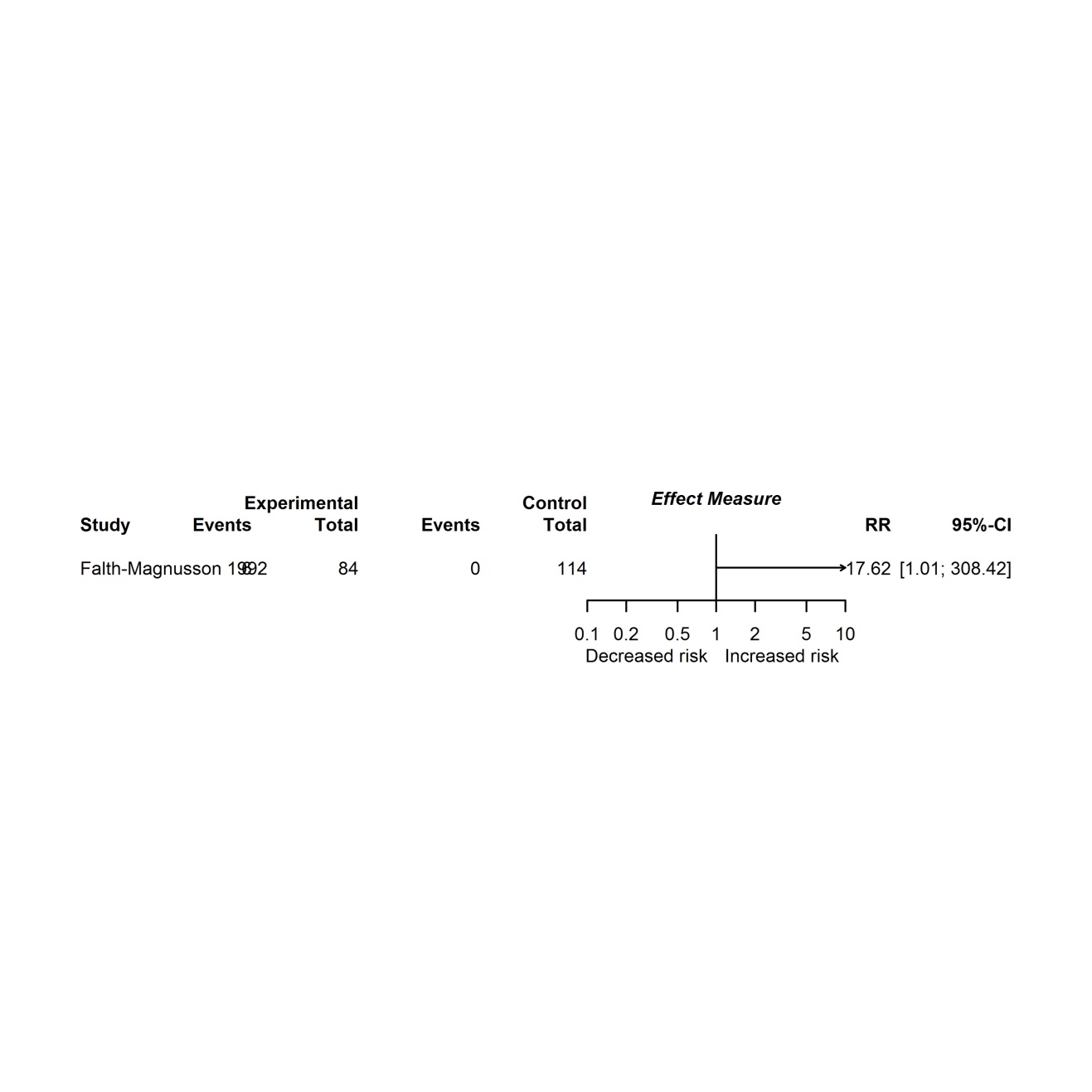


Figure 26 Maternal AFA and risk of Egg allergy at age 5-14 yrs - CCT


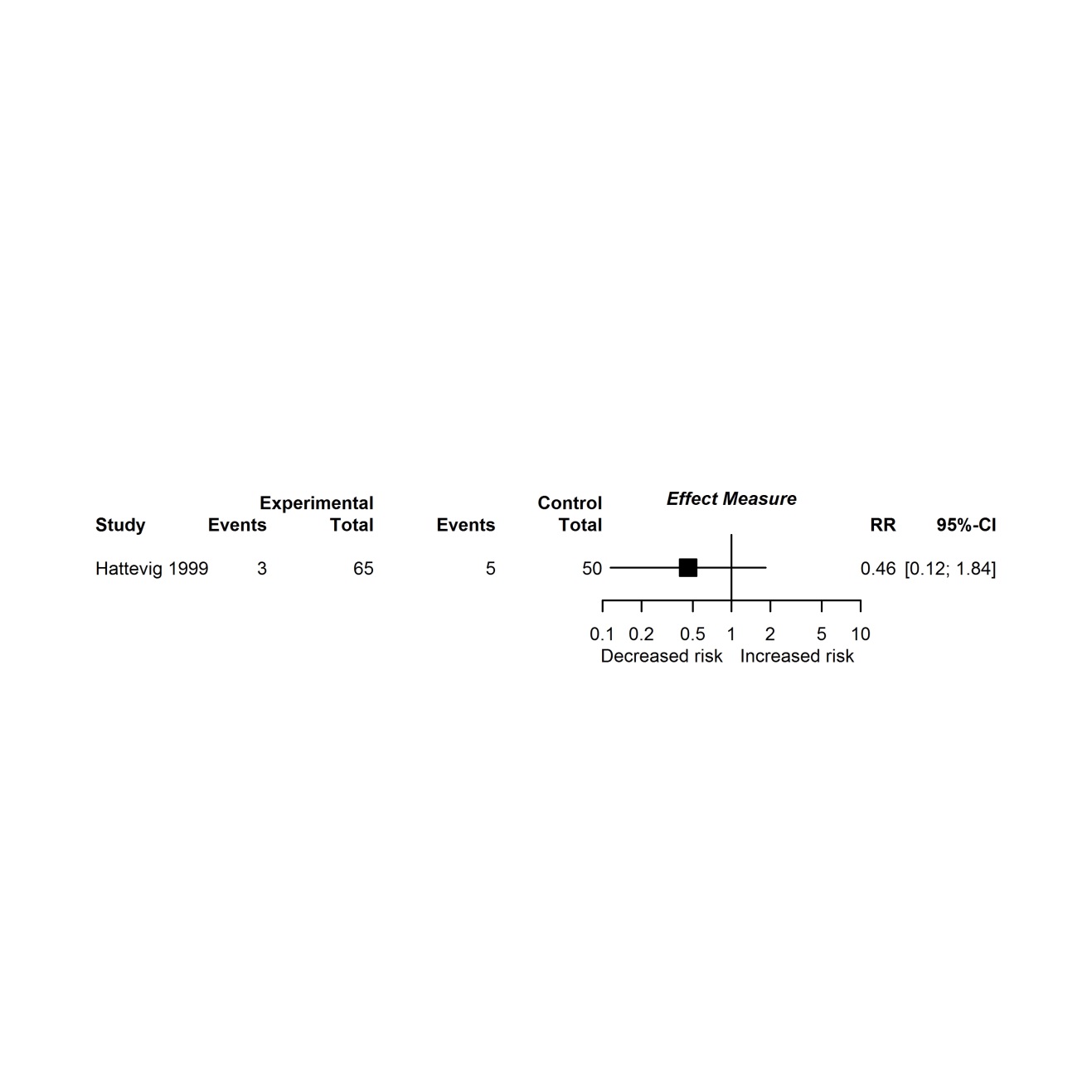


# Maternal AFA and risk of allergic sensitisation

One systematic review and 10 original trials (1700 participants) were included in this analysis. The recent high quality systematic review of Kramer identified 2 studies (340 participants) of AFA in pregnancy and 1 study (497 participants) of AFA during lactation which examined allergic sensitisation. Data are summarised in Table 4 and do not show consistent evidence for an effect of maternal AFA on child AS to egg, milk or peanut.

Figure 27 summarises the risk of bias for original studies of maternal AFA and AS. Overall risk of bias was high or unclear in most studies, mainly due to risk of selection bias, based on inadequate randomisation or treatment allocation procedures. Risk of assessment bias and risk of conflict of interest were judged as low in most studies.

Meta-analysis of data from the original studies showed no evidence for an effect of maternal AFA on AS to any allergen (Figures 28 and 29). RCT but not CCT evidence supported a reduction in AS to aeroallergens with maternal AFA (Figures 30 and 31) however all RCTs included in this analysis were multifaceted intervention studies, which included measures to reduce aeroallergen exposure in mother and infant. There was some evidence that maternal AFA may reduce AS to ‘any food’ in the child, from RCT (Figure 32) but not CCT (Figure 33) studies. However, larger numbers of studies and participants could be included in analysis of specific AS to individual foods, since some studies reported AS to individual foods but not a summed ‘any food’ outcome. Here there was no evidence that maternal AFA influences AS to cow’s milk (Figures 34 and 35), egg (Figures 36 and 37) or peanut (Figures 38 and 39).

*Data that could not be included in meta-analysis*

The studies of Falth-Magnusson, Hattevig and Arshad/Hide all reported total IgE, and found no difference between active and control groups although data could not be meta-analysed. The CCT of Kilburn reported SPT to aeroallergens at 1.5 years and stated there was a non-significant trend to increased sensitisation in the control group. The RCT of Lilja reported SPT to milk and egg components at 1.5 years and stated there was no significant difference between groups in any outcome. The RCT of Metcalfe reported specific IgE to egg at 4 months was detectable in 1 infant from the egg-free group, 1 infant from the high-egg group and two infants from the low-egg group. Denominators were not presented for these data, but they do not suggest a significant difference between groups. Several of the studies included in meta-analyses reported AS outcomes at other times and/or using other methods, with similar findings to those presented in the analysis Figures.

**Overall we found no consistent evidence that maternal AFA impacts on child AS risk.**

Figure 27 Risk of bias of intervention studies of maternal AFA and AS

Table 4 Systematic review evidence for AFA and AS risk

| **Study** | **Outcome measure** | **Intervention/ outcome timing** | **No. participants (studies)** | **Outcome (95% CI)** | I^2^ |
| --- | --- | --- | --- | --- | --- |
| **Kramer** ([1](#_ENREF_1)) | Egg SPT | **Pregnancy**  6 months | 340 (2) | RR 0.58 [0.32,1.05] | 0% |
|  |  | 18 months | 335 (2) | RR 0.95 [0.52,1.74] | 0% |
|  | Milk SPT | 6 months | 340 (2) | RR 1.15 [0.29, 4.51] | 0% |
|  |  | 18 months | 335 (2) | RR 0.86 [0.16, 4.59] | 13% |
| **Kramer** ([1](#_ENREF_1)) | Egg SPT | **Lactation**  1 year | 497 (1) | RR 1.31 [0.88, 1.94] | - |
|  |  | 2 years | 473 (1) | **RR 1.91 [1.03, 3.53]** | - |
|  |  | 7 years | 354 (1) | RR 0.15 [0.02, 1.25] | - |
|  | Milk SPT | 1 year | 497 (1) | RR 1.43 [0.68, 3.01] | - |
|  |  | 2 years | 473 (1) | RR 4.30 [0.94, 19.67] | - |
|  |  | 7 years | 354 (1) | RR 0.91 [0.13, 6.41] | - |
|  | Peanut SPT | 1 year | 497 (1) | RR 1.41 [0.76, 2.60] | - |
|  |  | 2 years | 473 (1) | RR 1.00 [0.56, 1.80] | - |
|  |  | 7 years | 354 (1) | RR 1.60 [0.81, 3.15] | - |

Figure 28 AFA and risk of allergic sensitisation (any) - RCT


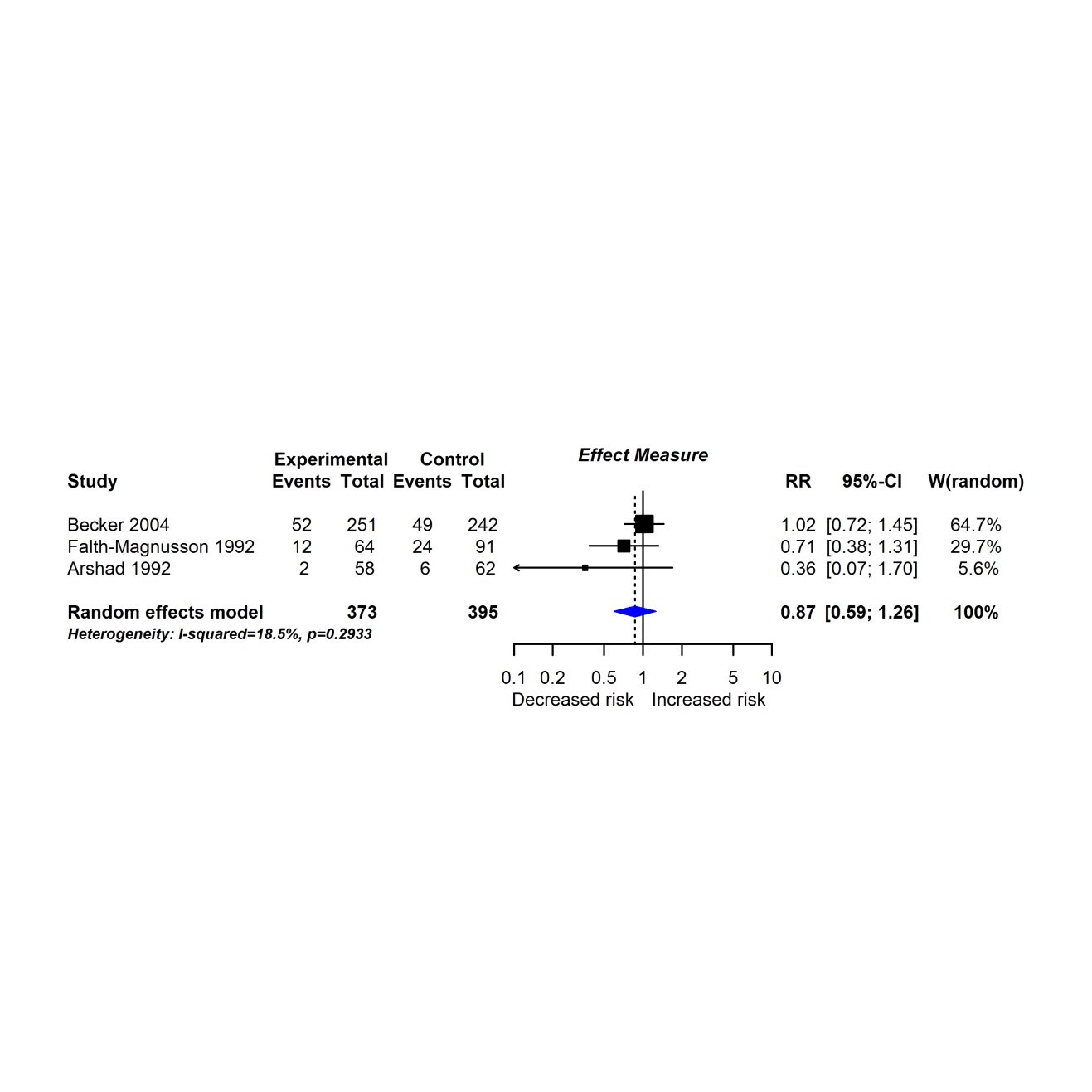


Figure 29 AFA and risk of allergic sensitisation (any) - CCT


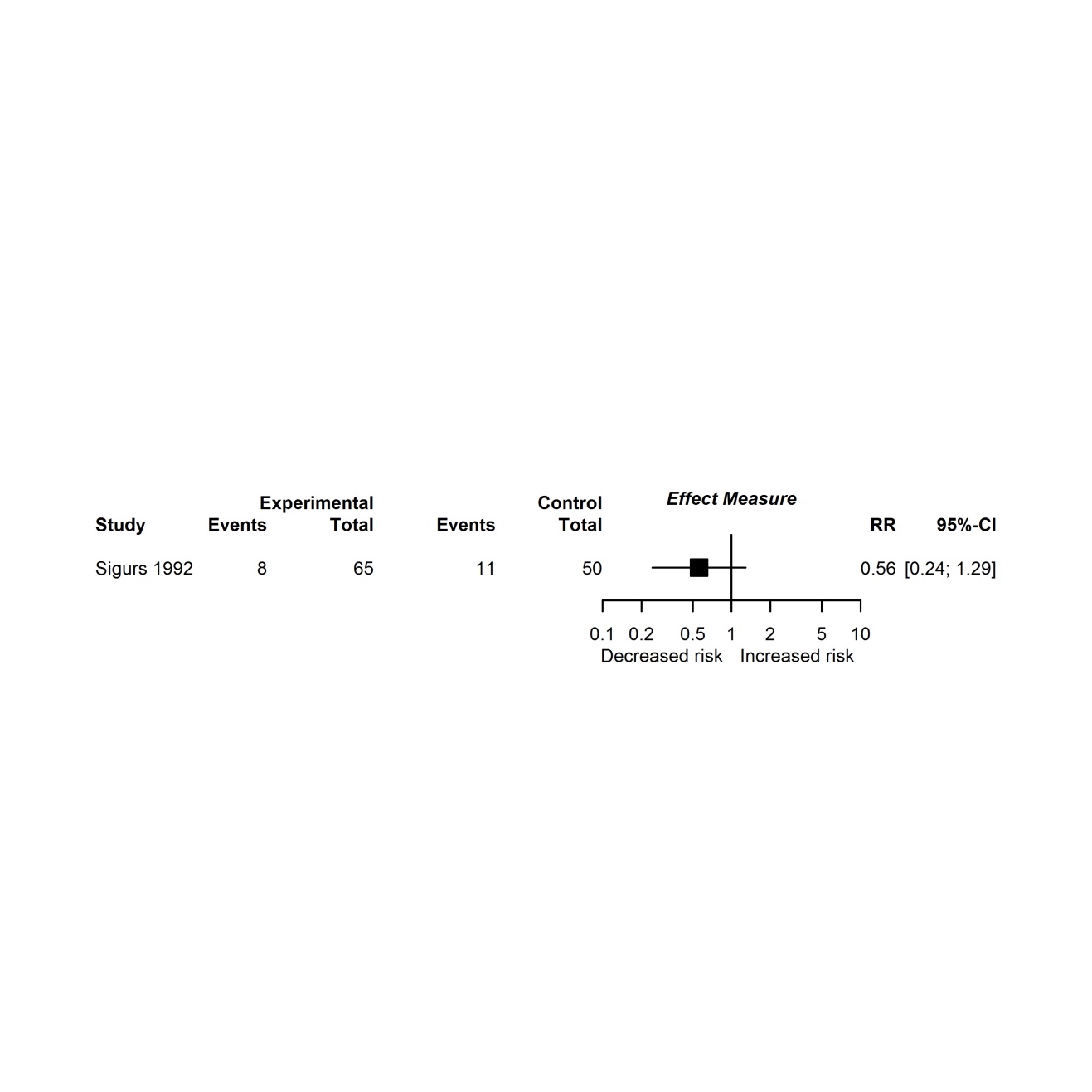


Figure 30 AFA and risk of allergic sensitisation (aero) - RCT


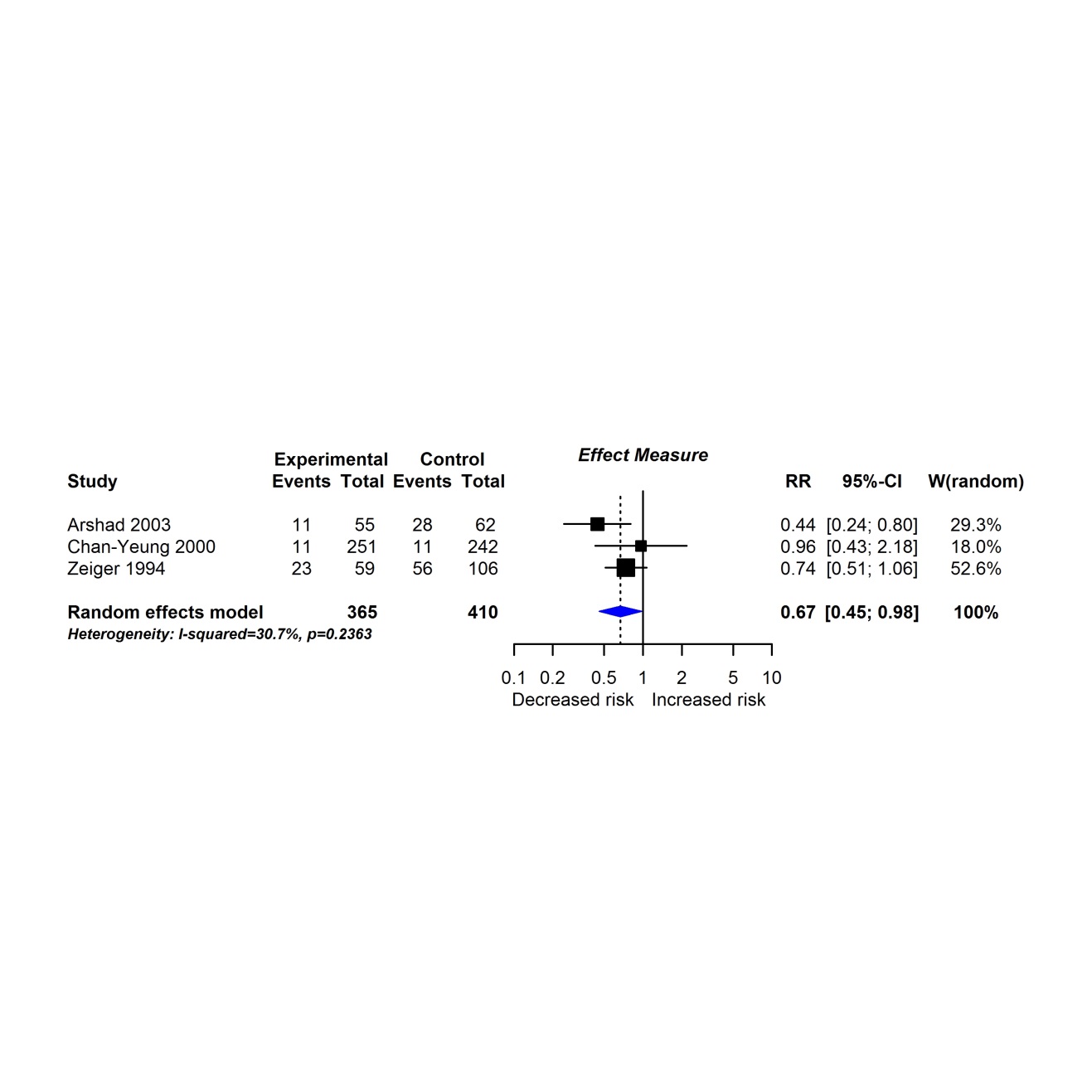


Figure 31 AFA and risk of allergic sensitisation (aero) - CCT


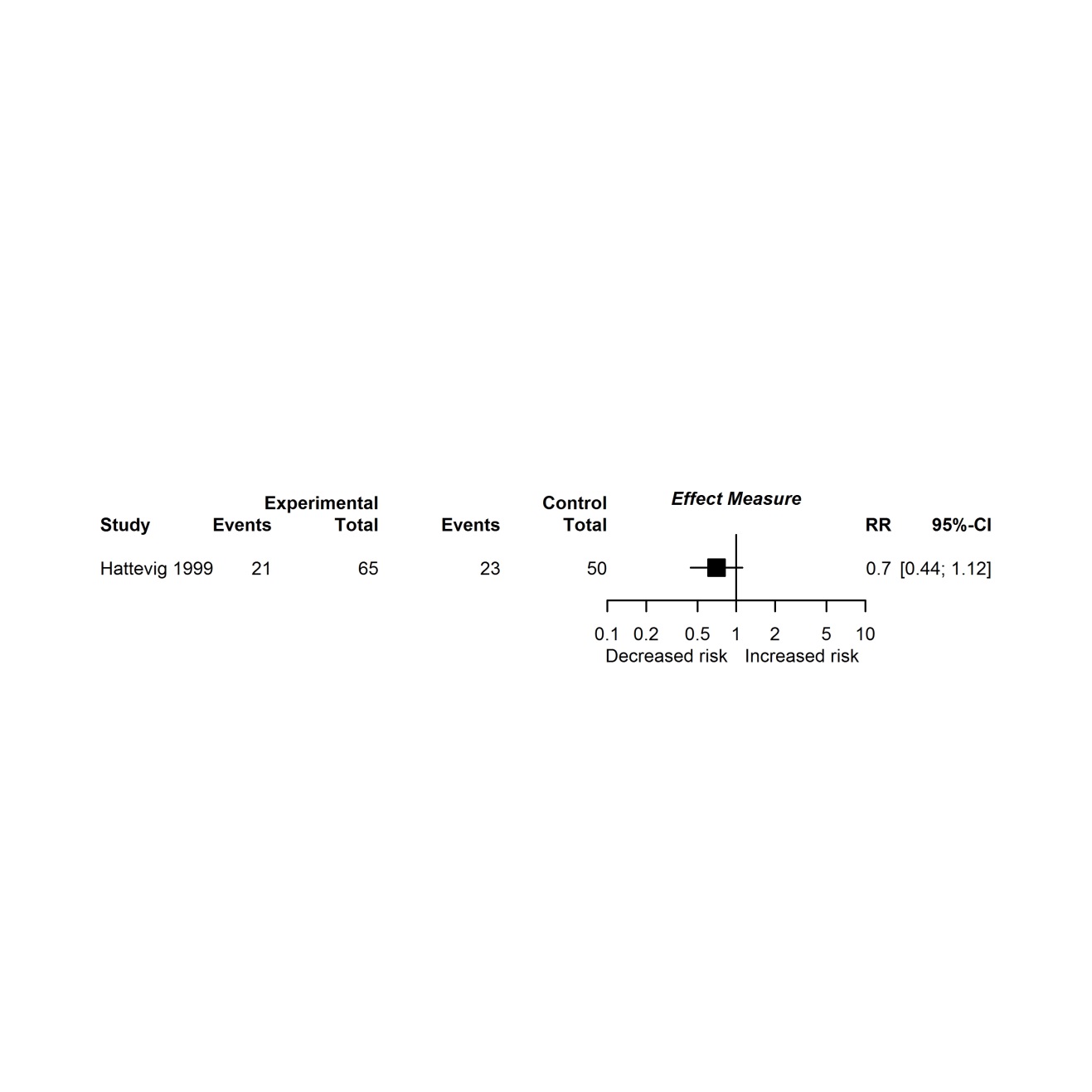


Figure 32 AFA and risk of allergic sensitisation (food) - RCT


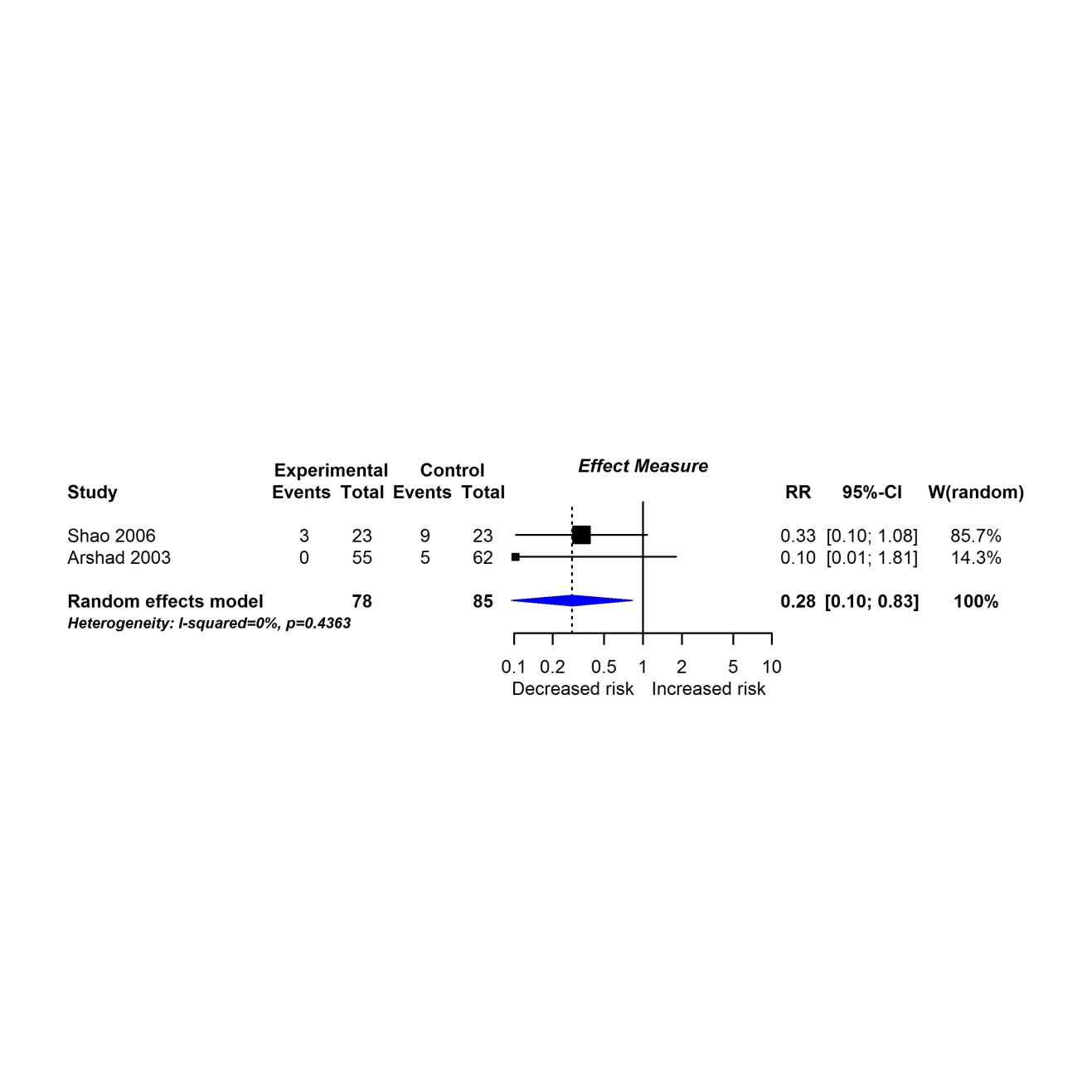


Figure 33 AFA and risk of allergic sensitisation (food) - CCT


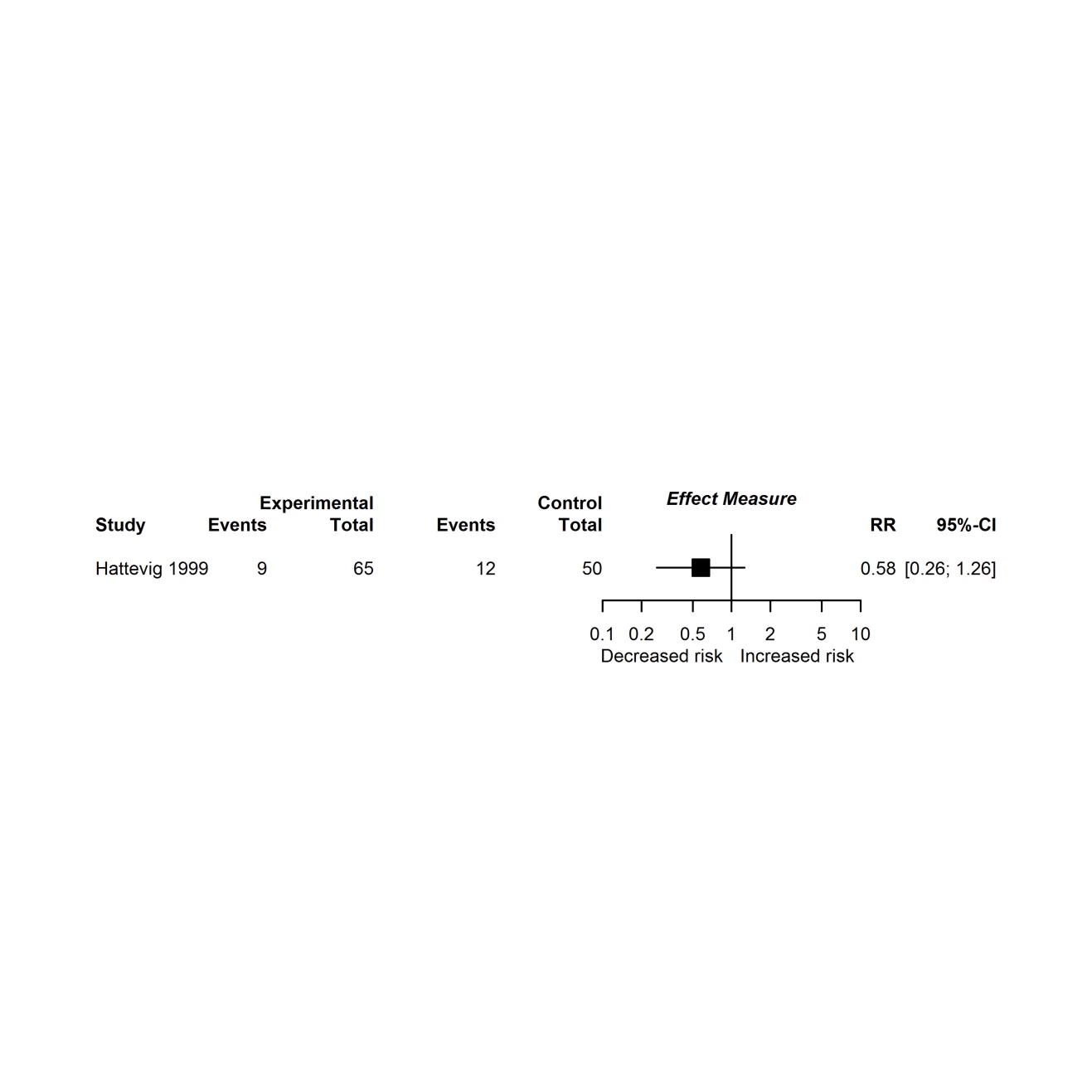


Figure 34 AFA and risk of allergic sensitisation (CM) - RCT


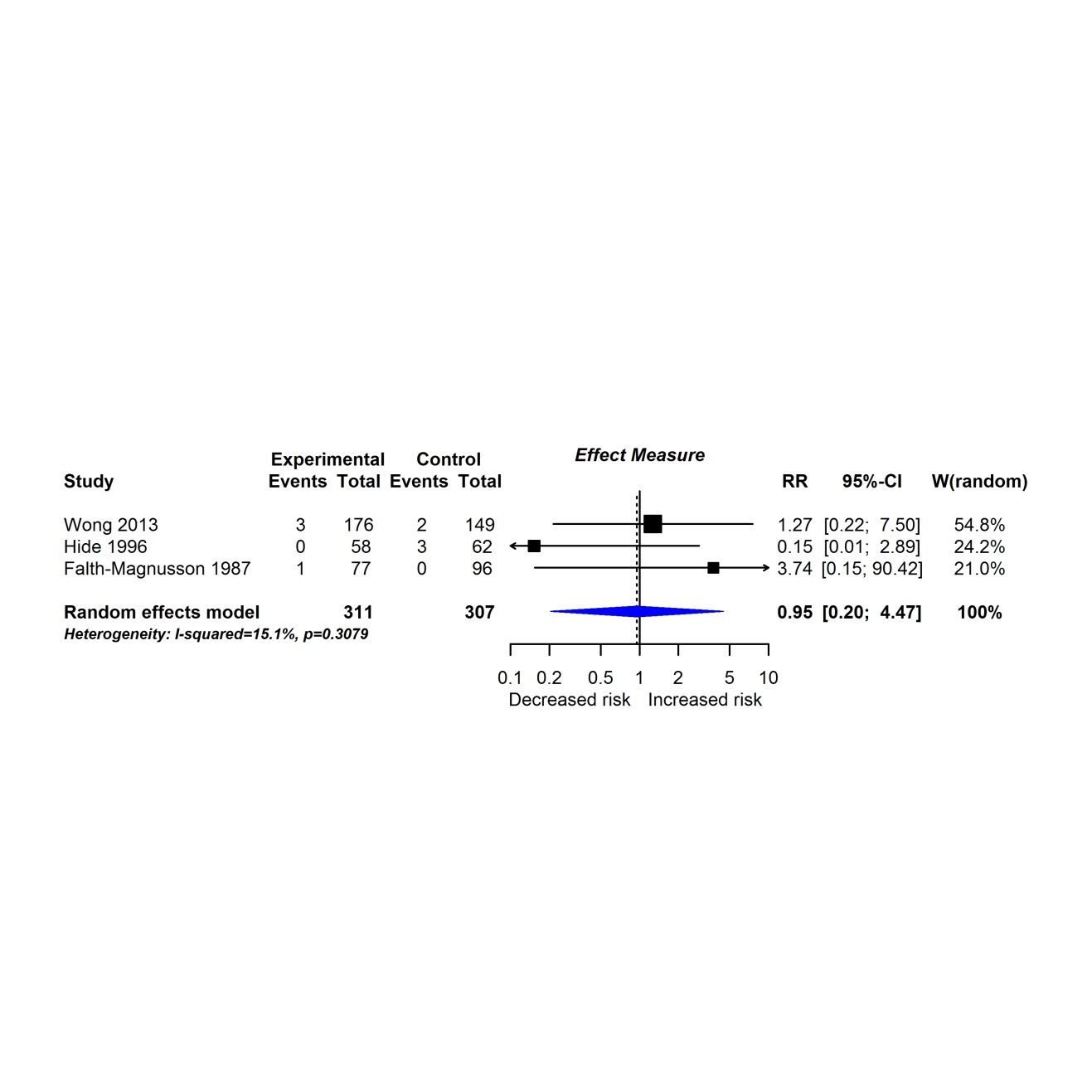


Figure 35 AFA and risk of allergic sensitisation (CM) - CCT


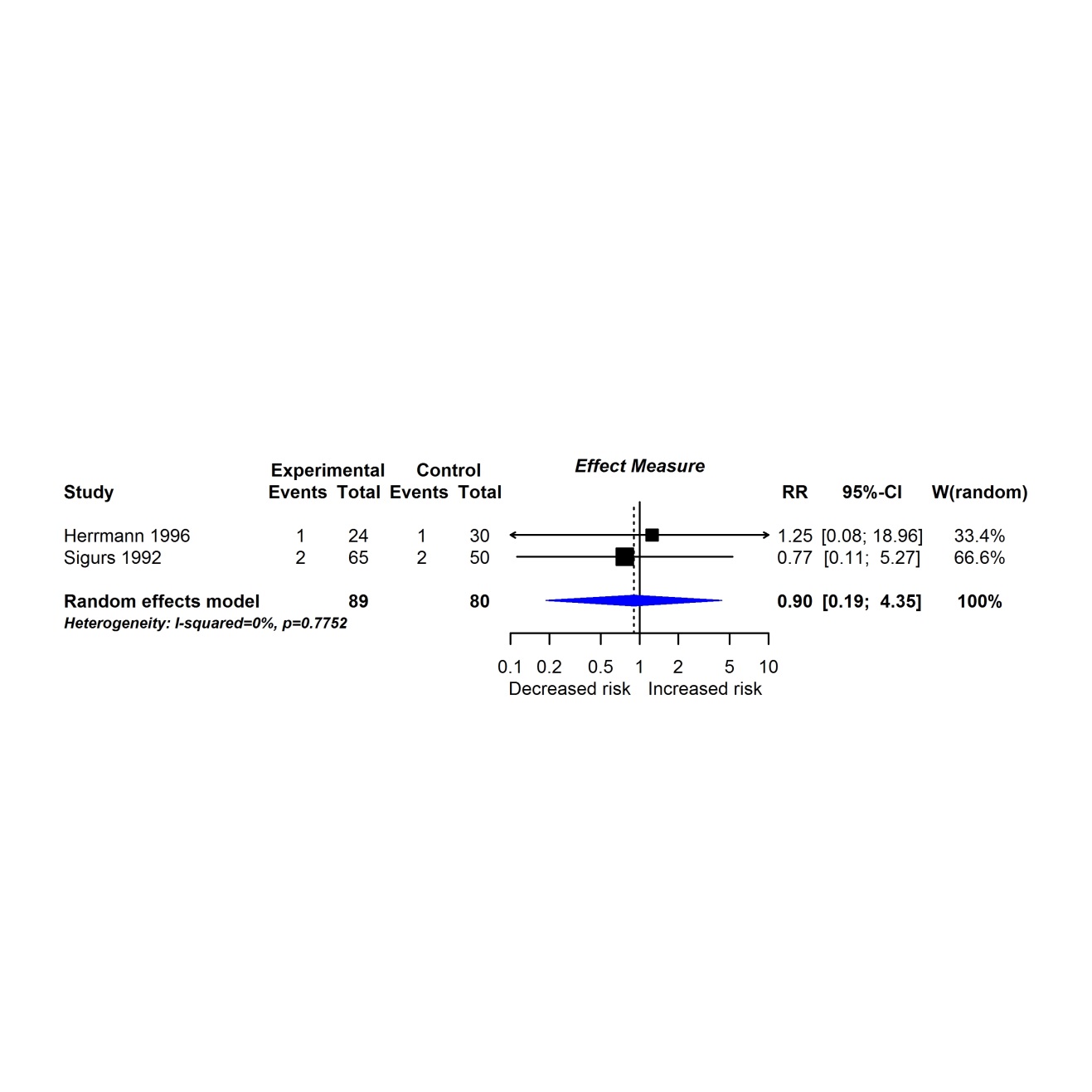


Figure 36 AFA and risk of allergic sensitisation (Egg) - RCT


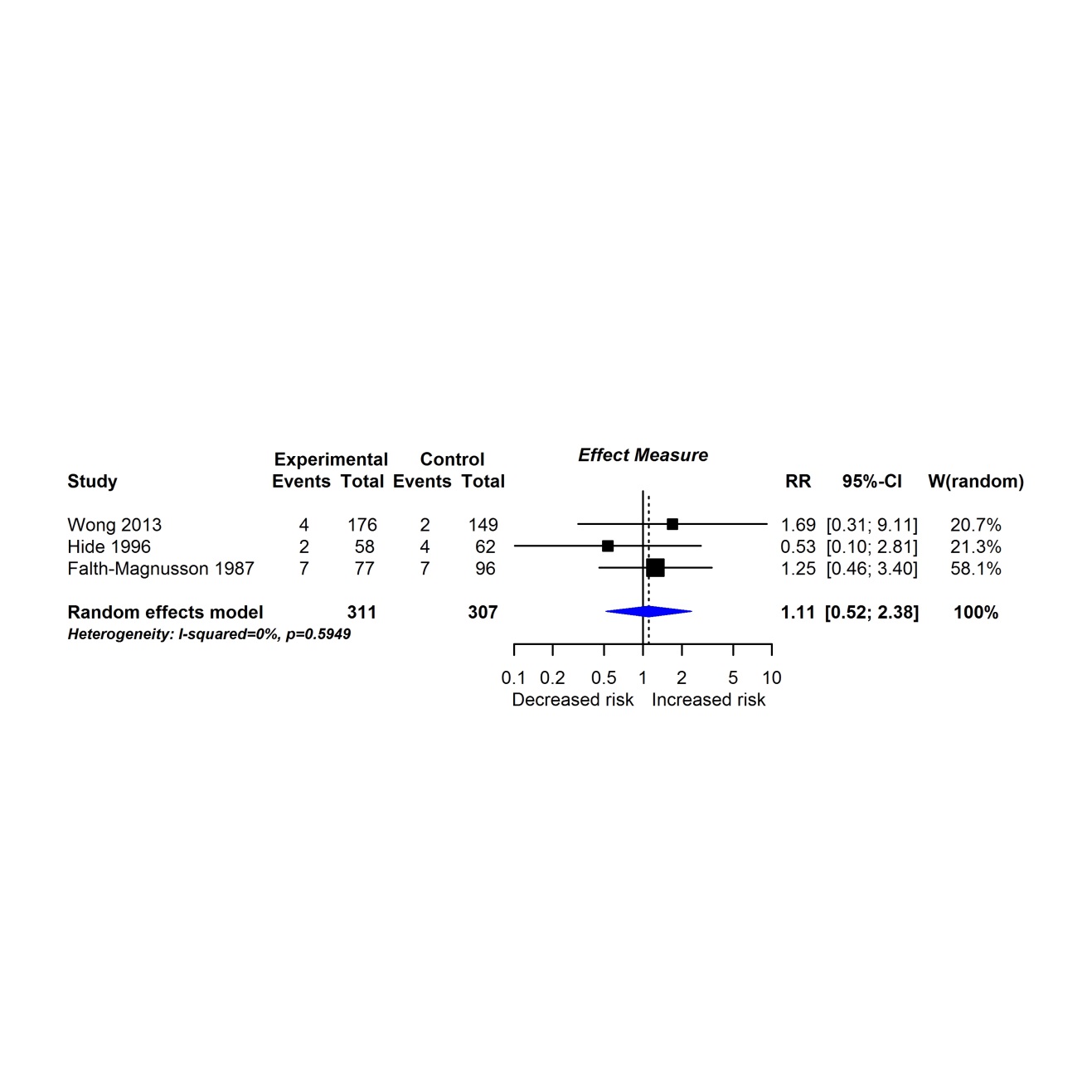


Figure 37 AFA and risk of allergic sensitisation (Egg) - CCT


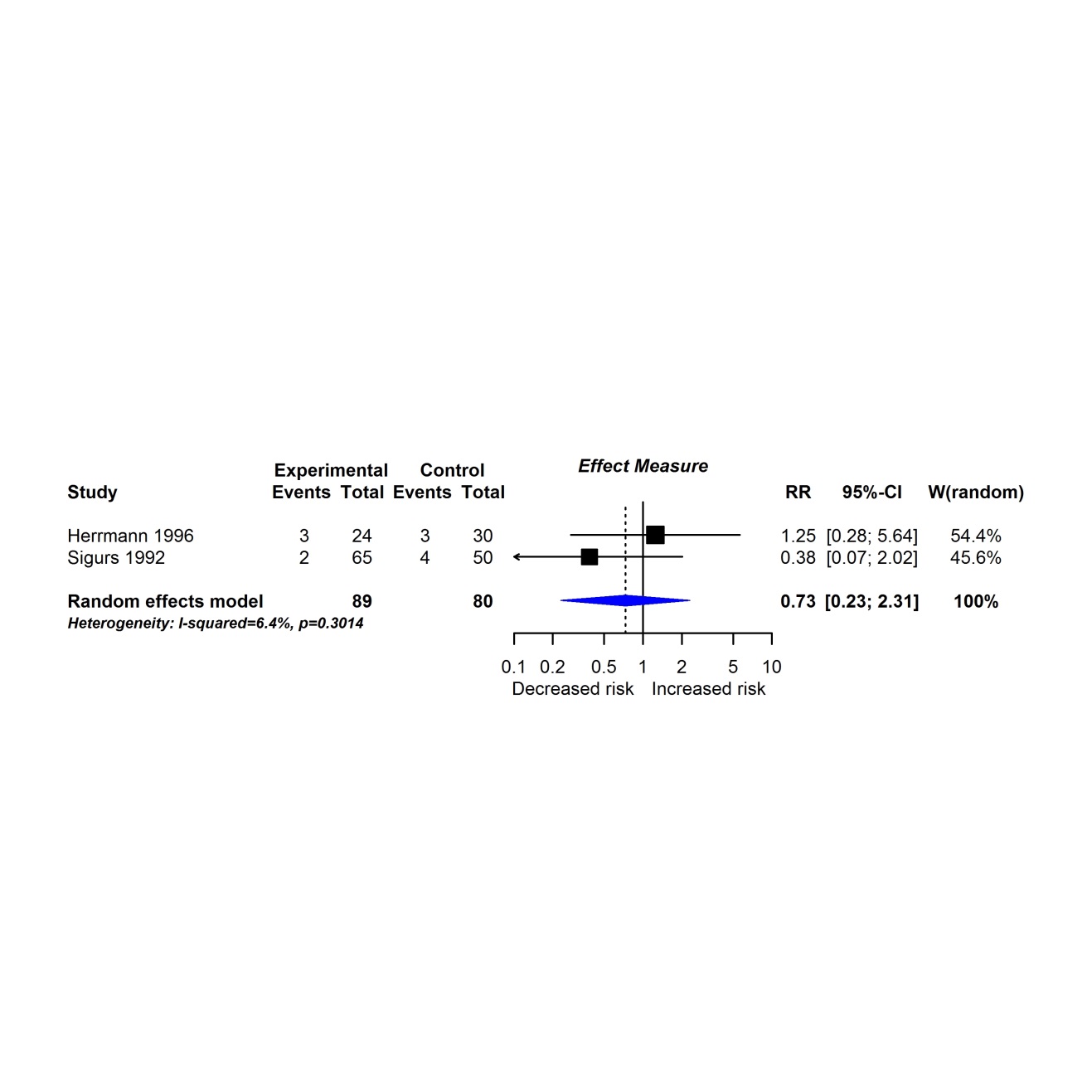


Figure 38 AFA and risk of allergic sensitisation (Peanut) - RCT


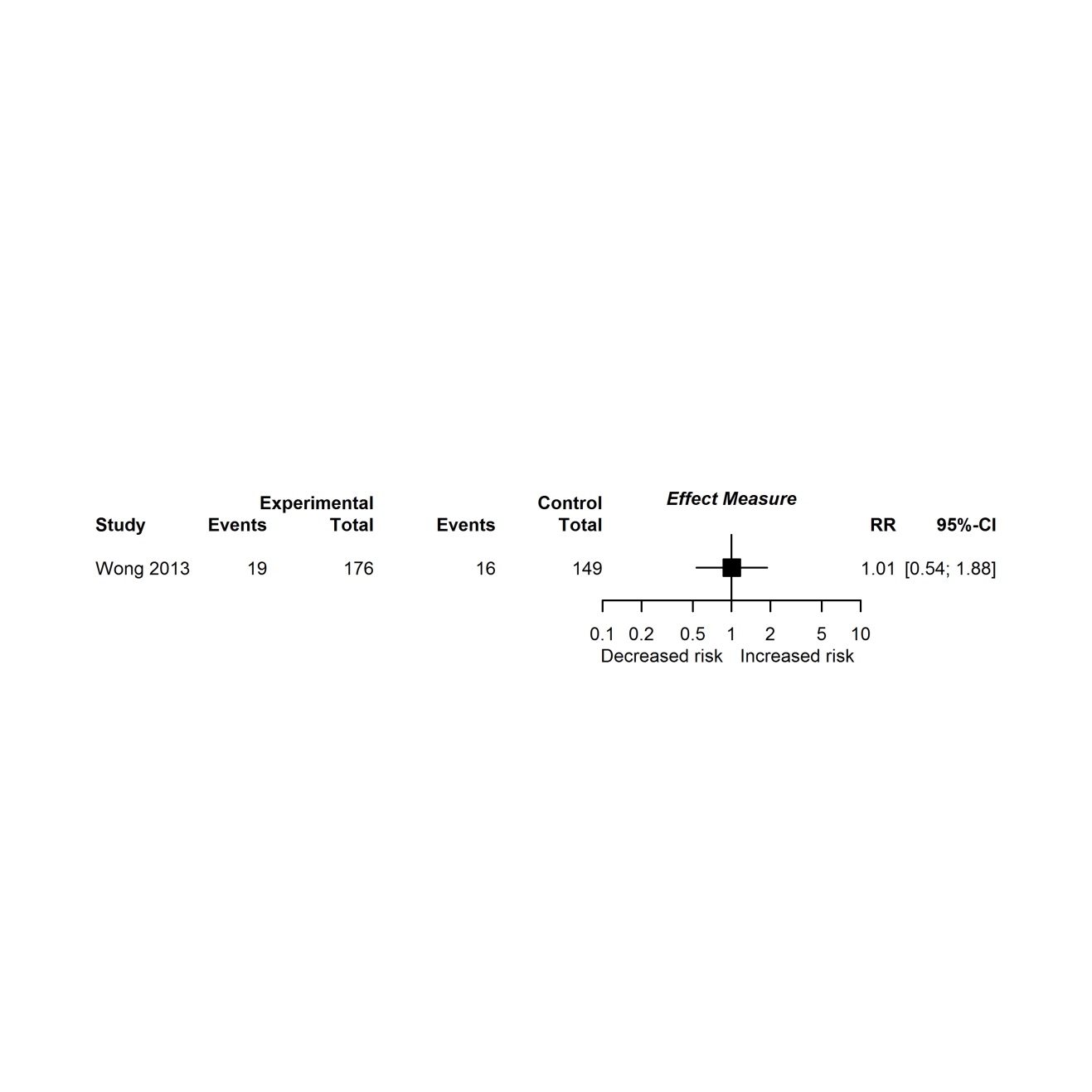


Figure 39 AFA and risk of allergic sensitisation (Peanut) - CCT


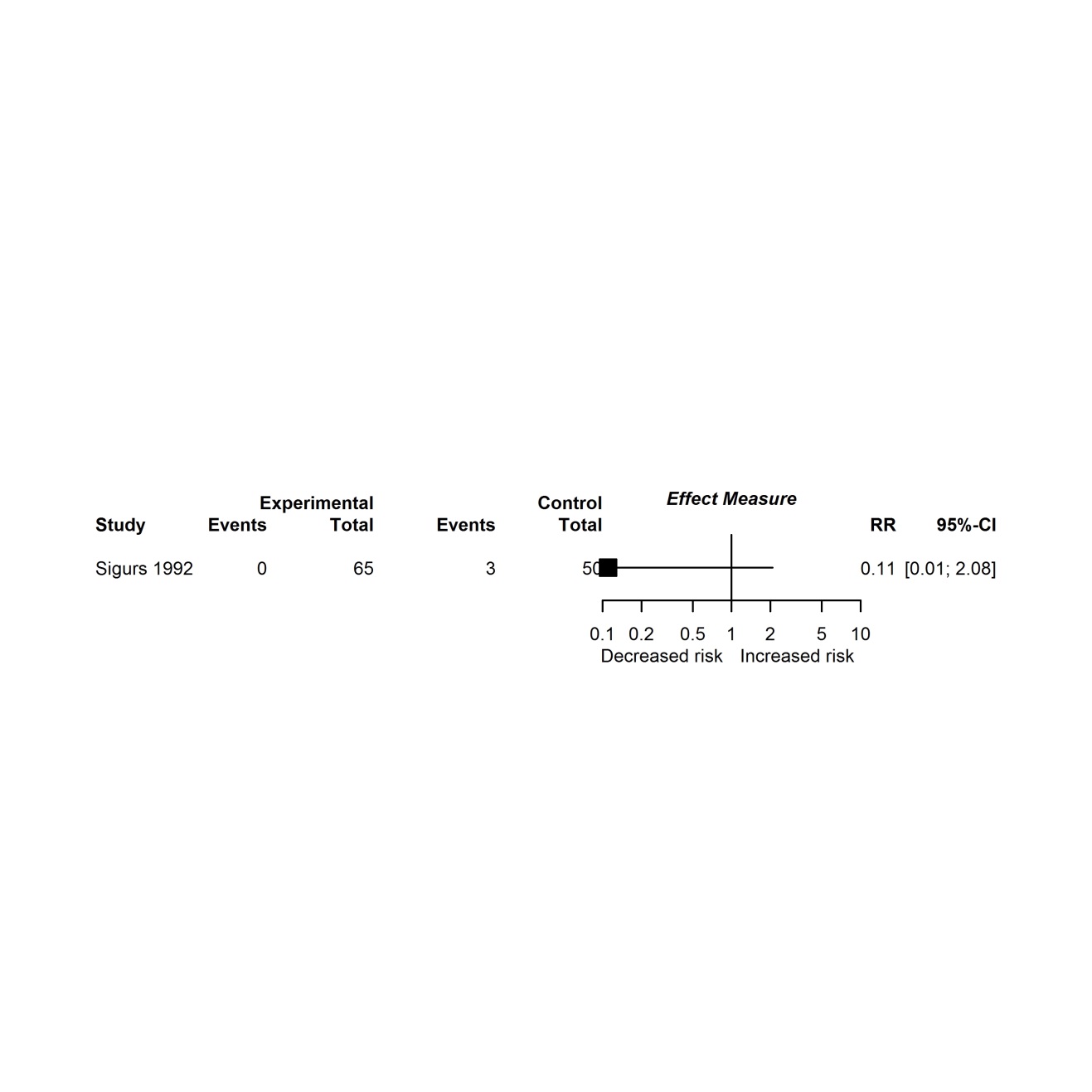


# Maternal AFA and lung function, wheeze or asthma

One systematic review and 6 original trials (1400 participants) were included in this analysis. The recent high quality systematic review of Kramer identified 2 studies (334 participants) of AFA in pregnancy and wheeze. Data are summarised in Table 5 and do not show any evidence for an effect of maternal AFA on child wheeze, with no statistical heterogeneity.

Figure 40 summarises the risk of bias for original studies of maternal AFA and AS. Overall risk of bias was high or unclear in most studies, mainly due to risk of selection bias, based on inadequate randomisation or treatment allocation procedures. Risk of assessment bias and risk of conflict of interest were judged as low in most studies.

Meta-analysis of data from the original studies showed no evidence for an effect of maternal AFA on wheeze or recurrent wheeze at age ≤4 (Figures 41-43). Two multifaceted RCTs found significantly reduced risk of wheeze at age 5-14, with no statistical heterogeneity (Figure 44). However a larger number of participants and studies reported recurrent wheeze at this age, where no evidence for an effect of maternal AFA was seen albeit with high heterogeneity (Figures 45-46). The statistical heterogeneity seen in this analysis may be explained by the difference between the multifaceted intervention trials (RR generally <1) and the AFA-only trial (RR>1). A single RCT found no evidence of an effect on asthma at age 18 (Figure 47). Two multifaceted RCTs found no evidence for an effect on bronchial hyper-responsiveness (Figure 48), nor in one of them on FEV1 (Figure 49).

*Data that could not be included in meta-analysis*

Zeiger reported current wheeze and wheeze ever at aged 4 in graphical form, with similar proportions in each treatment group and no significant difference found. The studies of Becker, Hattevig, and Arshad/Hide also reported wheeze at other times and/or using other methods, with similar findings to those presented in the analysis Figures.

**Overall we found no consistent evidence that maternal AFA impacts on child wheezing risk or lung function.**

Figure 40 Risk of bias in studies of maternal AFA and lung function, wheeze/asthma

Table 5 Systematic review evidence for AFA and wheeze risk

| **Study** | **Outcome measure** | **Intervention timing** | **No. participants (studies)** | **Outcome (95% CI)** | I^2^ |
| --- | --- | --- | --- | --- | --- |
| **Kramer** ([1](#_ENREF_1)) | Wheeze at ≤18 months | Pregnancy | 334 (2) | RR 2.22 [0.39, 12.67] | 0% |

Figure 41 Maternal AFA and risk of wheeze at ≤ 4 yrs - CCT


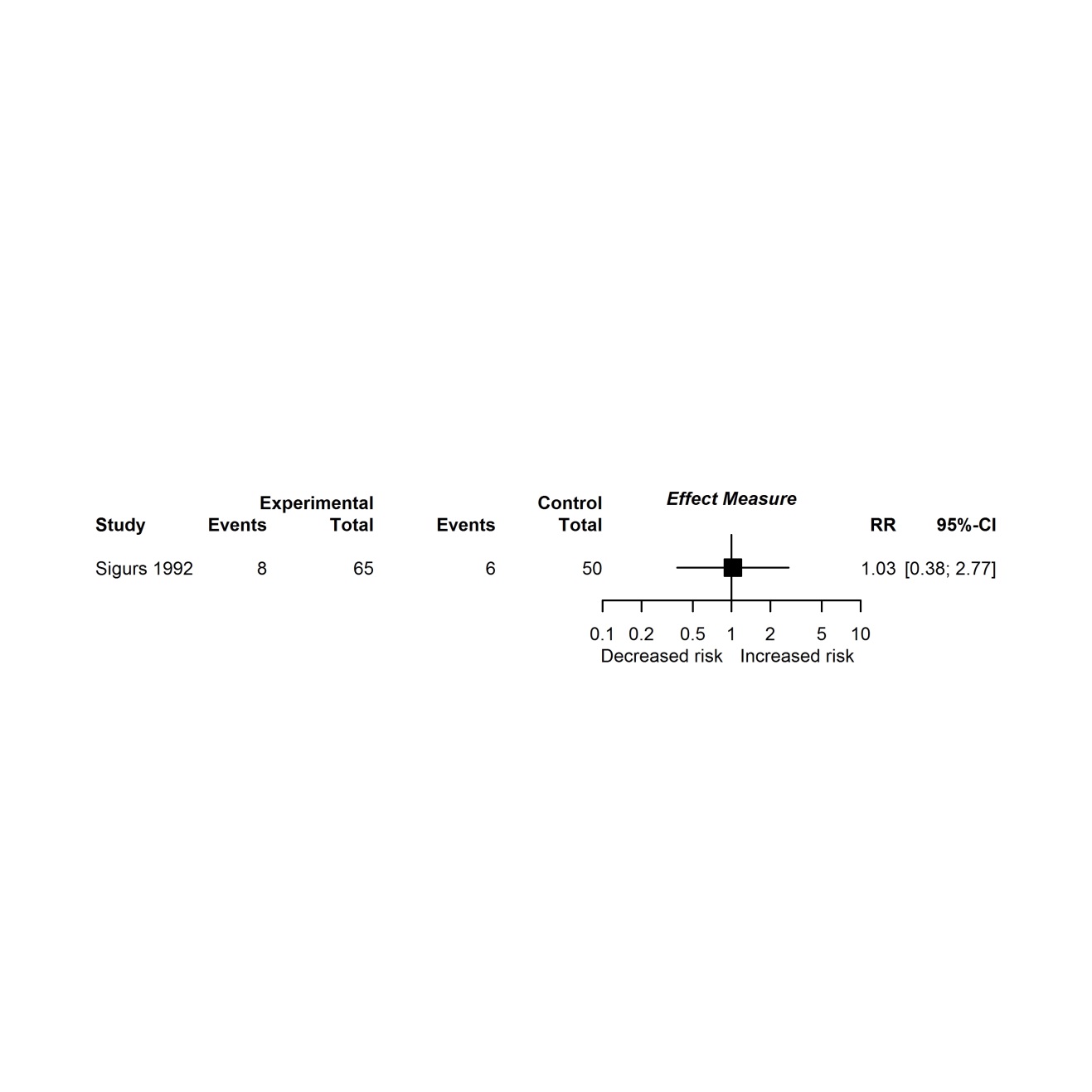


Figure 42 Maternal AFA and risk of recurrent wheeze at ≤ 4 yrs - RCT


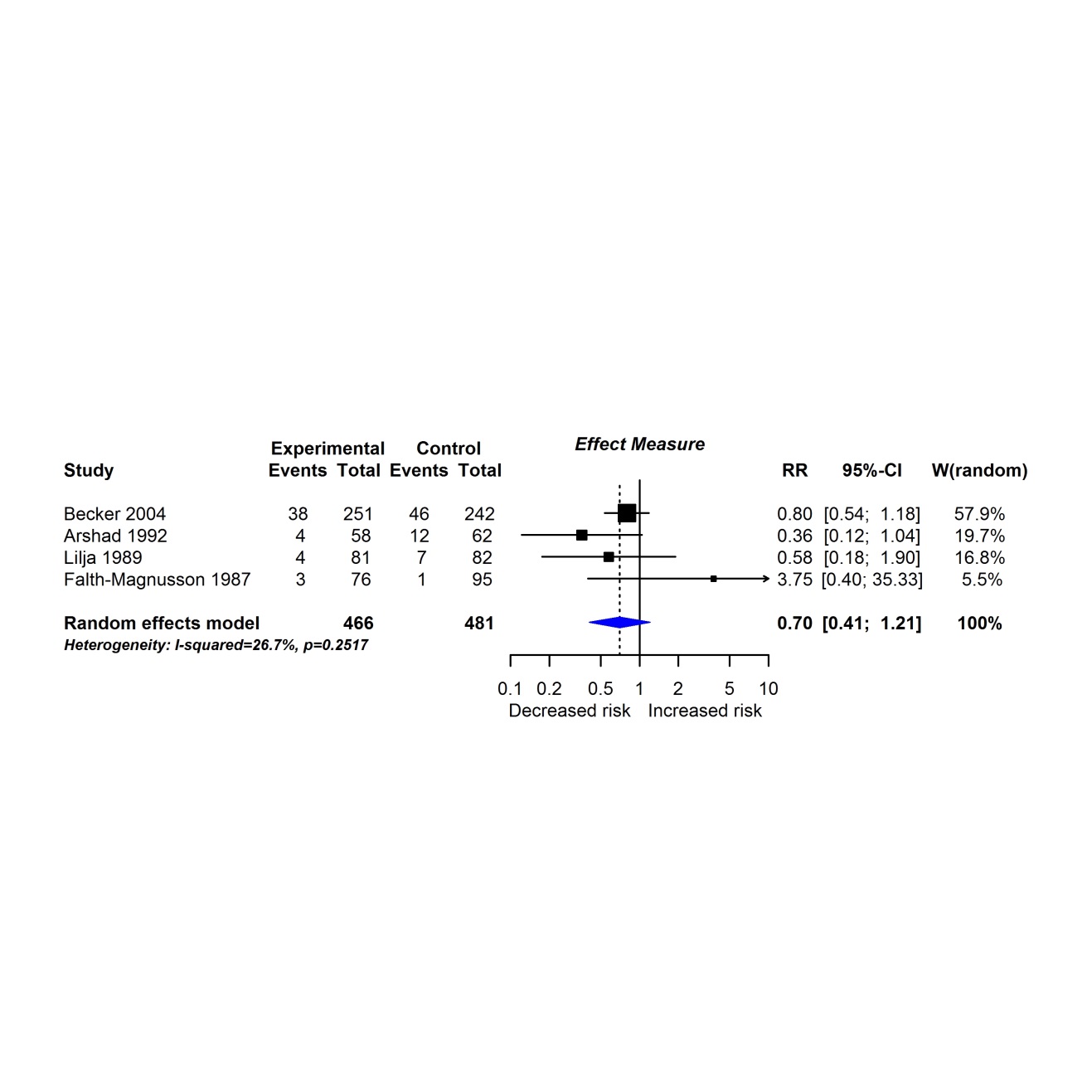


Figure 43 Maternal AFA and risk of recurrent wheeze at ≤ 4 yrs - CCT


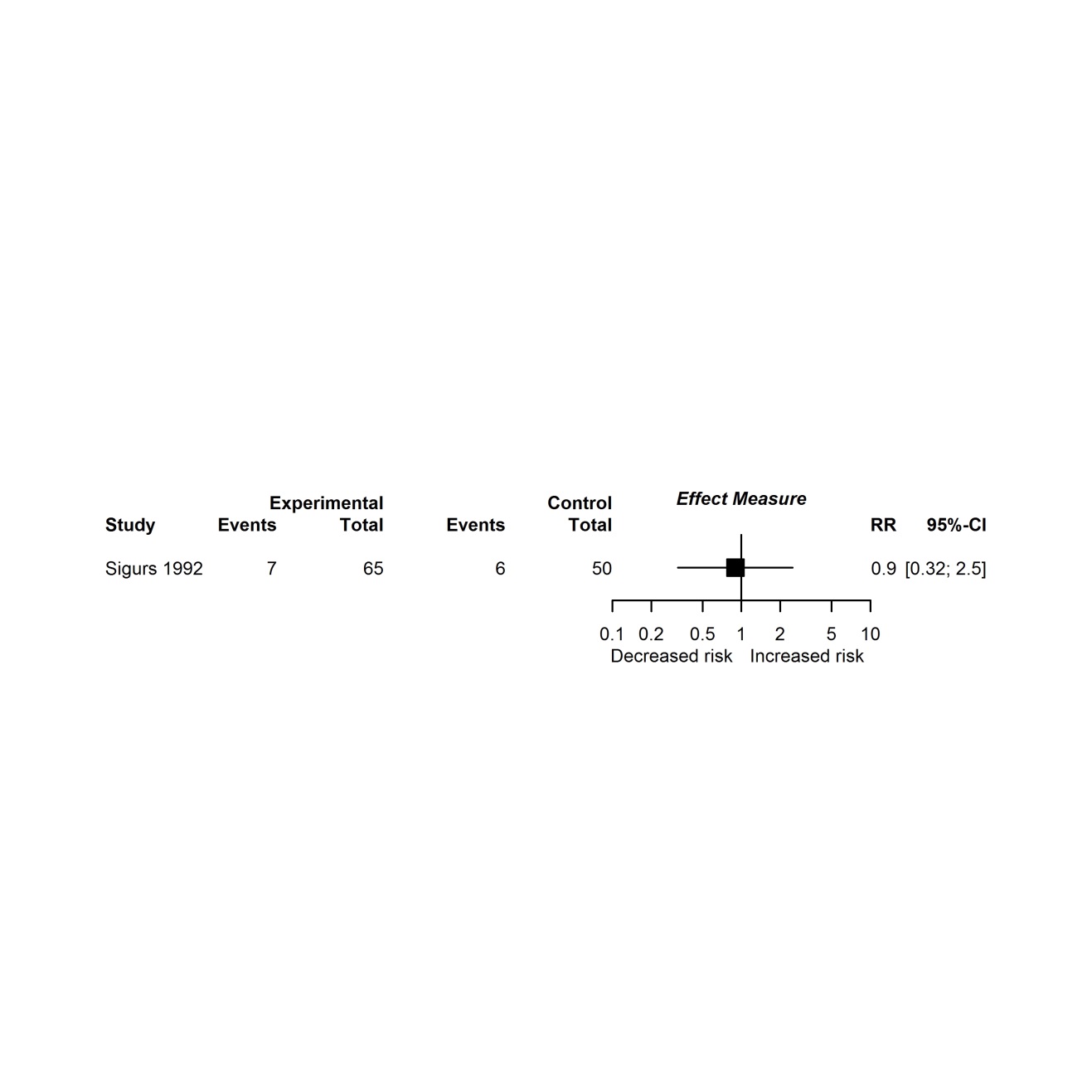


Figure 44 Maternal AFA and risk of wheeze at 5-14 yrs - RCT


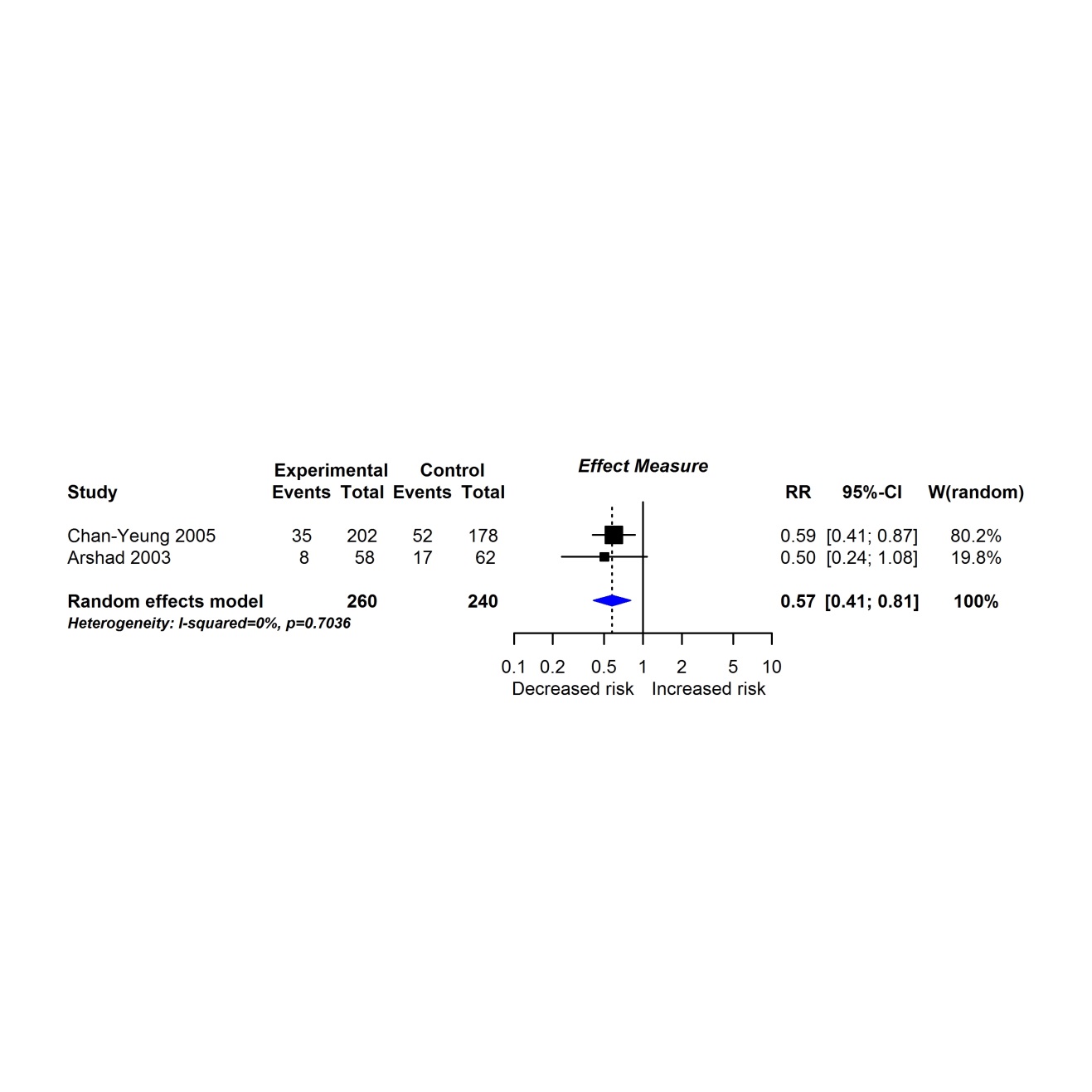


Figure 45 Maternal AFA and risk of recurrent wheeze at 5-14 yrs - RCT


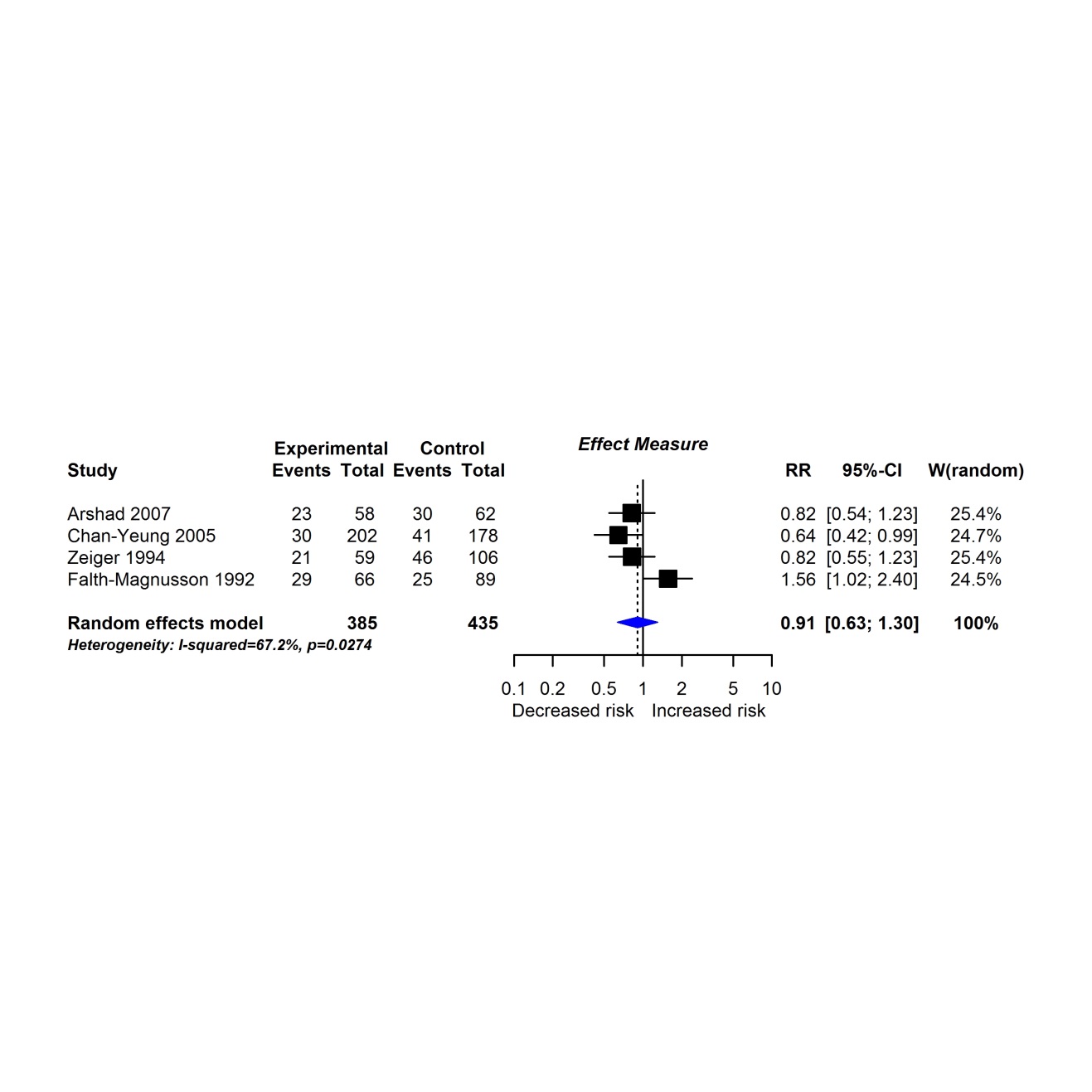


Figure 46 Maternal AFA and risk of recurrent wheeze at age 5-14 yrs – CCT


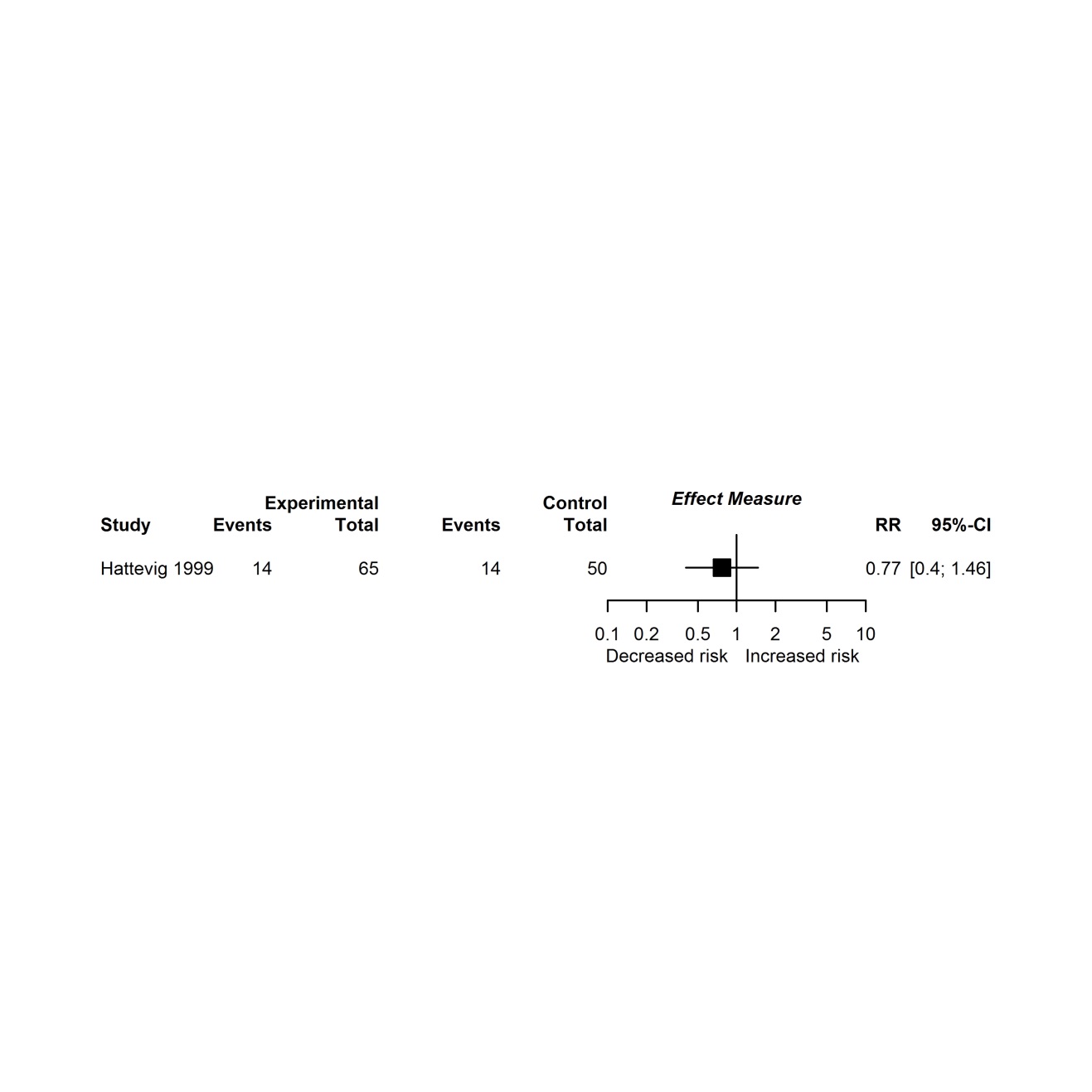


Figure 47 Maternal AFA and risk of recurrent wheeze at ≥15 yrs - RCT


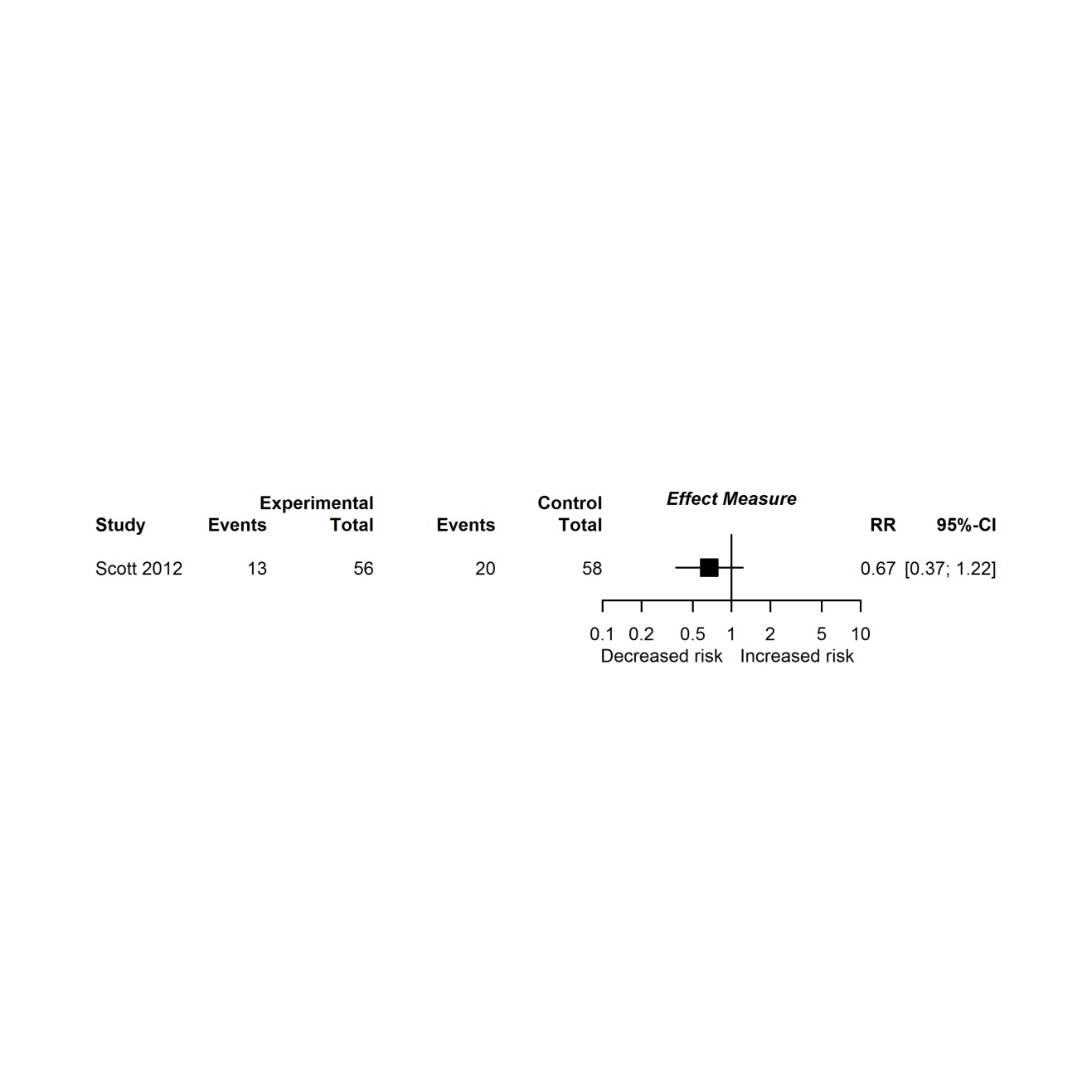


Figure 48 Maternal AFA and risk of BHR at 5-14 - RCT


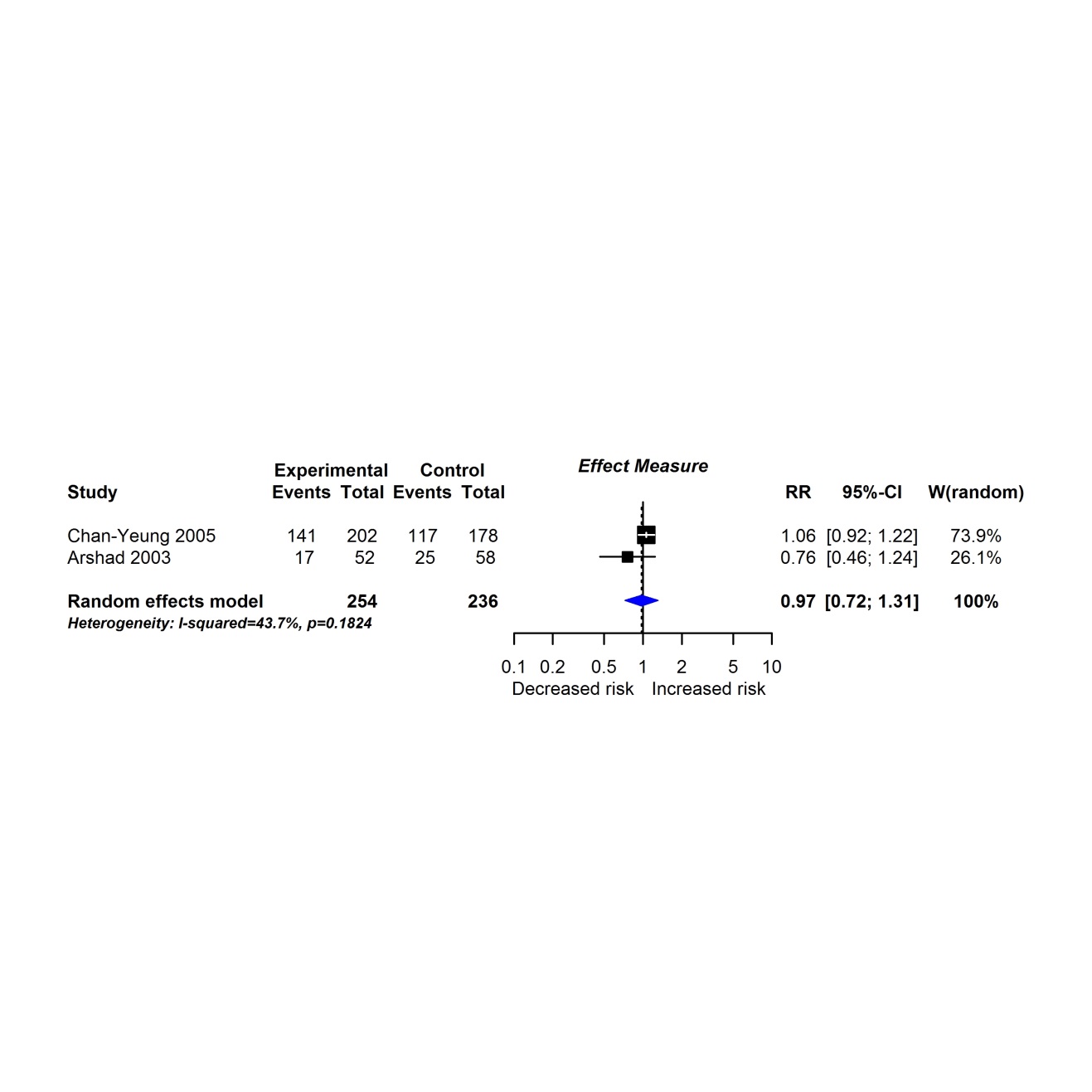


Figure 49 Multi-faceted interventions and FEV_1_ as % predicted


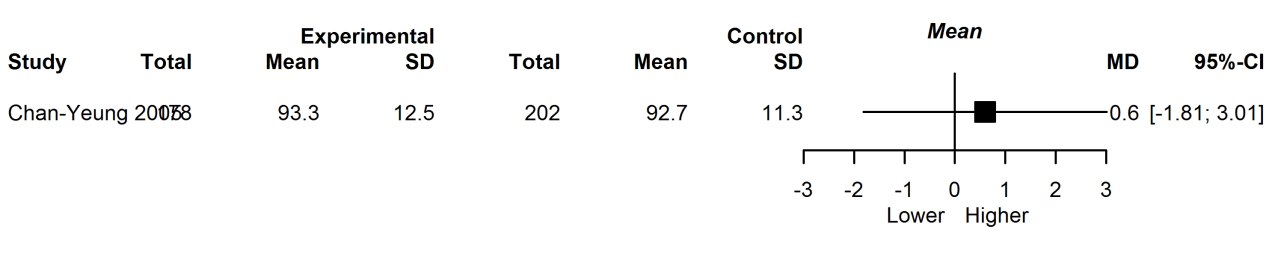


# Maternal AFA and risk of TIDM

No systematic review, and 2 original trials (340 participants) reported serological measures of TIDM – with mixed risk of bias (Figure 50). These data were not reported in a way that they could be used in meta-analysis. The CCT of Paronen assessed human IgG anti-insulin antibodies at 3 months, 1, 2 and 4 years. They reported a significantly raised level of insulin-IgG at age 2 in the AFA group, but this was not seen at age 3 months, 1 and 4 years, with no significant differences at these other timepoints. The RCT of Falth-Magnusson assessed multiple TIDM associated antibodies (IA2A, GADA and IAA) using a radioligand binding assay at 6 weeks, 6 months, 18 months and 5 years, and reported no significant difference between groups in autoantibody levels.

**Overall we found no evidence that maternal AFA influences TIDM risk. We did not identify any intervention trials of AFA and other autoimmune diseases.**

Figure 50 Risk of bias in studies on maternal AFA and TIDM

# Conclusions

In this review we found no evidence to support a relationship between maternal AFA during pregnancy and/or lactation, and allergic or autoimmune outcomes in offspring. In general data were sparse for all outcomes reported, such that clinically meaningful effects cannot be confidently excluded. However the available data do not suggest a relationship. Data were absent for autoimmune diseases other than TIDM, and the included trials all carried high or unclear overall risk of bias, mainly related to randomisation and treatment allocation procedures. It is also worth noting that all included trials studied infants at high risk of allergic (but not autoimmune) outcomes due to family history of allergic disease.

In our overview of recent high quality systematic reviews, we identified a 2012 Cochrane review of maternal AFA and allergic outcomes ([31](#_ENREF_31)). Kramer’s review differed from ours in some important respects. Kramer excluded multi-faceted studies and CCTs, but did not exclude studies where AFA was practiced in the context of infant allergic disease. In our review we included multifaceted studies, but interpreted their findings cautiously if not supported by similar findings from AFA-only trials. We included CCTs and indeed observational studies (summarised in a separate report), but our focus was on primary prevention so we did not include studies of maternal AFA for established allergic disease in the infant. For these reasons Kramer identified fewer studies and participants than we did - 5 trials including 925 participants. However findings are similar, with quite sparse data, high or unclear risk of bias in most studies, but overall no evidence to support the hypothesis that maternal AFA can influence allergic outcomes in childhood. In the 2017 update of our systematic review, we did not identify any further high quality systematic reviews of maternal allergenic food avoidance intervention studies and allergic or autoimmune outcomes.

**These data do not support any specific dietary recommendations for pregnant or lactating women with regard to allergenic food intake.**

Some recent studies suggest that allergenic food exposure from environmental sources may be an important determinant of food allergy, so that total household consumption of allergenic foods may be more important than maternal consumption ([32](#_ENREF_32))([33](#_ENREF_33)). Other data suggest that there may be an important gene-environment interaction in the relationship between environmental allergenic food exposure and food allergy ([34](#_ENREF_34)). Future work might explore the association between whole household allergenic food exposure, environmental allergenic food protein levels, and infant allergic outcomes in the context of specific predisposing genotypes.

# References

1. Kramer MS, Kakuma R. Maternal dietary antigen avoidance during pregnancy or lactation, or both, for preventing or treating atopic disease in the child. Cochrane Database of Systematic Reviews. 2012;9:CD000133.

2. Falth-Magnusson K, Kjellman NI. Development of atopic disease in babies whose mothers were receiving exclusion diet during pregnancy--a randomized study. Journal of Allergy & Clinical Immunology. 1987;80(6):868-75.

3. Falth-Magnusson K, Kjellman NI. Allergy prevention by maternal elimination diet during late pregnancy--a 5-year follow-up of a randomized study. Journal of Allergy & Clinical Immunology. 1992;89(3):709-13.

4. Ludvigsson J. Cow-milk-free diet during last trimester of pregnancy does not influence diabetes-related autoantibodies in nondiabetic children. Annals of the New York Academy of Sciences. 2003;1005:275-8.

5. Hattevig G, Kjellman B, Sigurs N, Grodzinsky E, Hed J, Bjorksten B. The effect of maternal avoidance of eggs, cow's milk, and fish during lactation on the development of IgE, IgG, and IgA antibodies in infants. Journal of Allergy & Clinical Immunology. 1990;85(1 Pt 1):108-15.

6. Paronen J, Bjorksten B, Hattevig G, Akerblom HK, Vaarala O. Effect of maternal diet during lactation on development of bovine insulin-binding antibodies in children at risk for allergy. Journal of Allergy & Clinical Immunology. 2000;106(2):302-6.

7. Hattevig G, Sigurs N, Kjellman B. Effects of maternal dietary avoidance during lactation on allergy in children at 10 years of age. Acta Paediatrica. 1999;88(1):7-12.

8. Hattevig G, Kjellman B, Sigurs N, Bjorksten B, Kjellman NI. Effect of maternal avoidance of eggs, cow's milk and fish during lactation upon allergic manifestations in infants. Clin Exp Allergy. 1989;19(1):27-32.

9. Sigurs N, Hattevig G, Kjellman B. Maternal avoidance of eggs, cow's milk, and fish during lactation: effect on allergic manifestations, skin-prick tests, and specific IgE antibodies in children at age 4 years. Pediatrics. 1992;89(4 Pt 2):735-9.

10. Herrmann ME, Dannemann A, Gruters A, Radisch B, Dudenhausen JW, Bergmann R, et al. Prospective study of the atopy preventive effect of maternal avoidance of milk and eggs during pregnancy and lactation. European Journal of Pediatrics. 1996;155(9):770-4.

11. Jirapinyo P, Densupsoontorn N, Kangwanpornsiri C, Limlikhit T. Lower prevalence of a topic dermatitis in breast-fed infants whose allergic mothers restrict dairy products. Journal of the Medical Association of Thailand. 2013;96(2):192-5.

12. Kilburn SA, Pollard C, Bevin S, Hourihane JO, Warner JO, Dean T. Allergens in mother's milk: Tolerisation or sensitization. Nutrition Research. 1998;18(8):1351-61.

13. Lovegrove JA, Hampton SM, Morgan JB. The immunological and long-term atopic outcome of infants born to women following a milk-free diet during late pregnancy and lactation: a pilot study. British Journal of Nutrition. 1994;71(2):223-38.

14. Shao J, Sheng J, Dong W, Li YZ, Yu SC. [Effects of feeding intervention on development of eczema in atopy high-risk infants: an 18-month follow-up study]. Zhonghua Erke Zazhi. 2006;44(9):684-7.

15. Becker A, Watson W, Ferguson A, Dimich-Ward H, Chan-Yeung M. The Canadian asthma primary prevention study: outcomes at 2 years of age. Journal of Allergy & Clinical Immunology. 2004;113(4):650-6.

16. Chan-Yeung M, Manfreda J, Dimich-Ward H, Ferguson A, Watson W, Becker A. A randomized controlled study on the effectiveness of a multifaceted intervention program in the primary prevention of asthma in high-risk infants. Archives of Pediatrics & Adolescent Medicine. 2000;154(7):657-63.

17. Chan-Yeung M, Ferguson A, Watson W, Dimich-Ward H, Rousseau R, Lilley M, et al. The Canadian Childhood Asthma Primary Prevention Study: outcomes at 7 years of age. Journal of Allergy & Clinical Immunology. 2005;116(1):49-55.

18. Wong T, Chan-Yeung M, Rousseau R, Dybuncio A, Kozyrskyj AL, Ramsey C, et al. Delayed introduction of food and effect on incidence of food allergy in a population at high risk for atopy: The Canadian asthma primary prevention study (CAPPS). Journal of Allergy and Clinical Immunology. 2013;1):AB96.

19. Protudjer JLP, Robertson L, Kozyrskyj A, Ramsey CD, Chan-Yeung M, Becker AB. Non-atopic asthma and atopic asthma are associated with reduced pulmonary function by age 7 years. American Journal of Respiratory and Critical Care Medicine. 2011;183 (1 MeetingAbstracts).

20. Carlsten C, Dimich-Ward H, Ferguson A, Watson W, Rousseau R, Dybuncio A, et al. Atopic dermatitis in a high-risk cohort: Natural history, associated allergic outcomes, and risk factors. Annals of Allergy, Asthma and Immunology. 2013;110(1):24-8.

21. Hide DW. The Isle of Wight study, an approach to allergy prevention. Pediatric Allergy & Immunology. 1994;5(6 Suppl):61-4.

22. Hide DW, Matthews S, Tariq S, Arshad SH. Allergen avoidance in infancy and allergy at 4 years of age. Allergy. 1996;51(2):89-93.

23. Arshad SH, Matthews S, Gant C, Hide DW. Effect of allergen avoidance on development of allergic disorders in infancy. Lancet. 1992;339(8808):1493-7.

24. Arshad SH, Bateman B, Matthews SM. Primary prevention of asthma and atopy during childhood by allergen avoidance in infancy: a randomised controlled study. Thorax. 2003;58(6):489-93.

25. Arshad SH, Bateman B, Sadeghnejad A, Gant C, Matthews SM. Prevention of allergic disease during childhood by allergen avoidance: the Isle of Wight prevention study. Journal of Allergy & Clinical Immunology. 2007;119(2):307-13.

26. Scott M, Roberts G, Kurukulaaratchy RJ, Matthews S, Nove A, Arshad SH. Multifaceted allergen avoidance during infancy reduces asthma during childhood with the effect persisting until age 18 years. Thorax. 2012;67(12):1046-51.

27. Zeiger RS, Heller S, Mellon MH, Halsey JF, Hamburger RN, Sampson HA. Genetic and environmental factors affecting the development of atopy through age 4 in children of atopic parents: A prospective randomized study of food allergen avoidance. Pediatric Allergy and Immunology. 1992;3(3):110-27.

28. Zeiger RS, Heller S, Mellon MH, Forsythe AB, O'Connor RD, Hamburger RN, et al. Effect of combined maternal and infant food-allergen avoidance on development of atopy in early infancy: A randomized study. Journal of Allergy and Clinical Immunology. 1989;84(1):72-89.

29. Zeiger RS. Dietary manipulations in infants and their mothers and the natural course of atopic disease. Pediatric Allergy & Immunology. 1994;5(6 Suppl):33-43.

30. Lilja G, Dannaeus A, Foucard T, Graff-Lonnevig V, Johansson SG, Oman H. Effects of maternal diet during late pregnancy and lactation on the development of atopic diseases in infants up to 18 months of age--in-vivo results. Clinical & Experimental Allergy. 1989;19(4):473-9.

31. Kramer Michael S, Kakuma R. Maternal dietary antigen avoidance during pregnancy or lactation, or both, for preventing or treating atopic disease in the child. Cochrane Database of Systematic Reviews [Internet]. 2012; (9).

32. Fox AT, Sasieni P, du Toit G, Syed H, Lack G. Household peanut consumption as a risk factor for the development of peanut allergy. Journal of Allergy and Clinical Immunology. 2009;123(2):417-23.

33. Brough H, Penagos M, Stephens A, Turcanu V, Grieve A, Lack G. Household peanut consumption over 6 months is positively correlated with measurable peanut protein in an infant's home environment. Allergy: European Journal of Allergy and Clinical Immunology. 2010;65:31-2.

34. Brough HA, Simpson A, Makinson K, Hankinson J, Brown S, Douiri A, et al. Peanut allergy: Effect of environmental peanut exposure in children with filaggrin loss-of-function mutations. Journal of Allergy and Clinical Immunology. 2014;134(4):867-75.e1.
