# Supplementary material for: Diet during pregnancy and infancy and risk of allergic or autoimmune disease: A systematic review and meta-analysis
Source: PLoS Med. 2018 Feb 28;15(2):e1002507. doi: 10.1371/journal.pmed.1002507 (PMC5830033; doi:10.1371/journal.pmed.1002507)
Supplement: S2 Data — (ZIP) [file pmed.1002507.s007.zip › Review_C_reports/MULTIFACETED INTERVENTIONS.docx]

**MULTI-FACETED INTERVENTIONS IN PREGNANCY/ LACTATION/ INFANCY AND RISK OF ALLERGIC SENSITISATION OR ALLERGIC DISEASE**

Robert J Boyle^1^, Vanessa Garcia-Larsen^2^, Despo Ierodiakonou^3^, Jo Leonardi-Bee^4^, Tim Reeves^5^, Jennifer Chivinge^6^, Zoe Robinson^6^, Natalie Geoghegan^6^, Katharine Jarrold^6^, Andrew Logan^6^, Annabel Groome^6^ , Evangelia Andreou^7^, Nara Tagiyeva-Milne^8^, Ulugbek Nurmatov^9^, Sergio Cunha^10^

^1^ Clinical Senior Lecturer, Section of Paediatrics ^2^ Post-Doctoral Research Associate, Respiratory Epidemiology and Public Health, National Heart and Lung Institute ^3^ Post-Doctoral Research Associate, Departments of Paediatric and Respiratory Epidemiology and Public Health Group, all at Imperial College London

^4^Associate Professor of Community Health Sciences, University of Nottingham

^5^ Research Support Librarian, Faculty of Medicine, Imperial College London

^6^ Undergraduate medical students, Imperial College London

^7^Research Associate, Imperial Consultants

^8^Research Fellow, University of Aberdeen

^9^Research Fellow, University of Edinburgh

^10^Research Associate, Respiratory Epidemiology and Public Health, National Heart and Lung Institute, Imperial College London

Imperial College London

Table of Contents

[List of Figures 3](#_Toc427181441)

[1. Multi-faceted interventions and risk of allergic or auto-immune diseases – summary of interventions and findings 4](#_Toc427181442)

[2. Multi-faceted interventions and risk of eczema 11](#_Toc427181443)

[3. Multi-faceted interventions and risk of allergic rhinitis or conjunctivitis 14](#_Toc427181444)

[4. Multi-faceted interventions and risk of food allergy 17](#_Toc427181445)

[5 Multi-faceted interventions and risk of allergic sensitisation 19](#_Toc427181446)

[6. Multi-faceted interventions and lung function, bronchial hyper-responsiveness and risk of wheeze 23](#_Toc427181447)

[General Conclusions 26](#_Toc427181448)

[References 28](#_Toc427181449)

# List of Figures

[Figure 1 Risk of bias in trials of multi-faceted intervention studies 10](#_Toc423021976)

[Figure 2 Risk of bias in multi-faceted interventions and risk of AD 12](#_Toc423021977)

[Figure 3 Multi-faceted interventions and risk of AD at age ≤ 4 years 12](#_Toc423021978)

[Figure 4 Multi-faceted interventions and risk of AD at age > 5-14 years 12](#_Toc423021979)

[Figure 5 Risk of bias in multi-faceted interventions and risk of ARC 15](#_Toc423021980)

[Figure 6 Multi-faceted interventions and risk of ARC at age ≤ 4 years 15](#_Toc423021981)

[Figure 7 Multi-faceted interventions and risk of ARC at age 5-14 years 15](#_Toc423021982)

[Figure 8 Multi-faceted interventions and risk of ARC at age 5-14 years 16](#_Toc423021983)

[Figure 9 Multi -faceted interventions and risk of atopic ARC at age 5-14 years 16](#_Toc423021984)

[Figure 10 Risk of bias of multi-faceted interventions and risk of food allergy 18](#_Toc423021985)

[Figure 11 Multi-faceted interventions and risk of food and risk of food allergy at age ≤ 4 years 18](#_Toc423021986)

[Figure 12 Multi-faceted interventions and risk of food allergy at age 5-14 years 18](#_Toc423021987)

[Figure 13 Risk of bias of multi-faceted interventions and risk of allergic sensitisation 20](#_Toc423021988)

[Figure 14 Multi-faceted interventions and risk of AS to any allergen 20](#_Toc423021989)

[Figure 15 Multi-faceted interventions and risk of AS to aeroallergens 20](#_Toc423021990)

[Figure 16 Multi-faceted interventions and risk of AS to any food 21](#_Toc423021991)

[Figure 17 Multi-faceted interventions and risk of AS to cow’s milk 21](#_Toc423021992)

[Figure 18 Multi-faceted interventions and risk of AS to egg 21](#_Toc423021993)

[Figure 19 Multi-faceted interventions and risk of AS to peanut 21](#_Toc423021994)

[Figure 20 Multi-faceted interventions and Total IgE 22](#_Toc423021995)

[Figure 21 Risk of bias in multi-faceted studies and risk of wheeze or asthma 24](#_Toc423021996)

[Figure 22 Multi-faceted interventions and FEV_1_ as % predicted 24](#_Toc423021997)

[Figure 23 Multi-faceted interventions and risk of bronchial hyper-responsiveness 24](#_Toc423021998)

[Figure 24 Multi-faceted interventions and risk of wheeze at age ≤ 4 years 25](#_Toc423021999)

[Figure 25 Multi-faceted interventions and risk of recurrent wheeze at ≤ 4 years 25](#_Toc423022000)

[Figure 26 Multi-faceted interventions and risk of wheeze at age 5-14 years 25](#_Toc423022001)

[Figure 27 Multi-faceted interventions and risk of recurrent wheeze at age 5-14 years 25](#_Toc423022002)

# Multi-faceted interventions and risk of allergic or auto-immune diseases – summary of interventions and findings

In this analysis we included studies of multi-faceted interventions, defined as an intervention which included more than one category of dietary intervention that was eligible for inclusion in the overall FSA systematic review project. Many such studies included non-dietary components to the intervention – environmental control measures designed to reduce exposure to airborne inhalants/ irritants. We undertook subgroup/stratified analyses for meta-analyses which included >5 studies, in this case only eczema (AD) at age ≤4 years. We planned to assess publication bias where there were ≥10 studies in a meta-analysis but no meta-analysis had sufficient studies included. In total we identified 9 RCT investigating the effect of multifaceted interventions during pregnancy/lactation/infancy on allergic outcomes.

*Interventions used*

Six of the 9 trials used an intervention which included promotion of prolonged breastfeeding, avoidance of at least some allergenic foods for the mother and/or infant, and delayed introduction of solid foods to the infant. Five of these trials also included environmental control measures, and 4 used an alternative formula when infant formula milk was introduced - soya in the study of **Matthew** **(**[**1**](#_ENREF_1)**)**, pHF in the **Canadian asthma primary prevention study (CAPPS)** of Becker **(**[**2-4**](#_ENREF_2)**)**, and the study of **Shao** **(**[**5**](#_ENREF_5)**)**, and eHF in the **PREVASC study** of Schonberger **(**[**6**](#_ENREF_6)**).** The **SPACE** study of Halmerbauer **(**[**7**](#_ENREF_7)**,** [**8**](#_ENREF_8)**)**, and the study of **Poysa** **(**[**9-11**](#_ENREF_9)**)** did not use special formula milk.

The other three studies used more limited interventions – delayed allergenic food exposure plus extensively hydrolysed formula in the studies of **Lovegrove** **(**[**12**](#_ENREF_12)**)** and **Zeiger** **(**[**13**](#_ENREF_13)**)**; plus soya hydrolysate formula and environmental control in the **Isle of Wight study** of Hide **(**[**14**](#_ENREF_14)**,** [**15**](#_ENREF_15)**)**. Environmental control measures were aimed at reducing house dust mite allergen exposure, pet exposure and/or tobacco smoke exposure.

*Populations and Outcomes assessed*

Outcomes studied were allergic sensitisation, total IgE, food allergy, AD, allergic rhinitis, wheeze/asthma and lung function – forced expiratory volume in 1 second (FEV1), bronchial hyper-responsiveness (BHR). Overall approximately 2,200 infants were randomised, 1,100 to an intervention arm. All studies were in infants at high risk of allergic disease, six studies were carried out in Europe, one in Asia-Pacific and two in North America. The definitions of study outcomes varied across studies but in general studies used accepted criteria for outcome assessment.

*Overall findings*

Overall the quality of evidence was mixed. There was an unclear risk of bias in most studies, largely due to insufficient details about the randomisation process (Selection bias; Figure 1), and this may be partly explained by many of the included studies having taken place prior to CONSORT (1996) clinical trial guidance and/or ICMJE (2005) clinical trial registration requirements were developed. Individual meta-analyses were usually limited by small numbers of studies and participants leading to imprecision in estimates of treatment effect. Overall we found no clear evidence that multifaceted interventions prevent AD, allergic sensitisation or food allergy, although analyses were limited by small study numbers and wide confidence intervals – such that further work will be needed in order to confidently exclude a beneficial effect. All studies were undertaken in populations at high risk of allergic disease, so conclusions only apply to this population.

We did find evidence for reduced allergic rhinitis at age ≤4 years based on meta-analysis of 2 studies (RR=0.61; 95% CI 0.44 to 0.84) with no statistical heterogeneity. However a further study which couldn’t be included in this meta-analysis found no significant difference in allergic rhinitis. Both studies included house dust mite avoidance measures, delayed allergenic food introduction to the infant, and use of a hydrolysed formula if necessary as part of the intervention. The intervention in the study which found no significant difference, was limited to allergenic food exclusion in mother and infant. The allergic rhinitis meta-analysis was dominated by the CAPPS study, which also included avoidance of nursery/daycare until age 1, avoidance of environmental tobacco smoke exposure, and pet avoidance as part of the intervention. Given that environmental tobacco smoke is a major trigger of respiratory symptoms, it is unclear whether the dietary components of the intervention were relevant. We did not find any evidence that multifaceted interventions reduce allergic rhinitis at age 5-14, although in one small study atopic AR (here AR associated with allergic sensitisation measured by SPT) was reduced.

We found evidence that multifaceted interventions reduce the risk of wheeze (RR 0.57 95%CI 0.41, 0.81) or recurrent wheeze/asthma at age 5-14 (RR 0.77 95%CI 0.61, 0.97), with no statistical heterogeneity. However no such effects were seen at age 0-4 years, and no effect on lung function.

*Conclusion*

These data suggest that in infants at high risk of allergic disease, multifaceted interventions which include environmental control measures, may reduce the risk of AR at age ≤ 4 years, and recurrent wheeze in school-age children and adolescents. Evidence is inconsistent, and effect estimates are imprecise due to sparse data, so further work is needed to confirm these findings.

**Multifaceted interventions prevent AR at age ≤4.**

**GRADE of evidence: LOW (-1 indirectness of intervention; -1 imprecision due to sparse data, inconsistent with data at age 5 to 14).**

**Multifaceted interventions prevent wheeze/recurrent wheeze at age 5-14.**

**GRADE of evidence: LOW (-1 indirectness of intervention; -1 imprecision due to sparse data, inconsistent with data at age ≤4).**

**Table 1 Characteristics of included studies**

| **Study** | **Design** | **N Int/ Control** | **Country** | **Intervention** | **Disease risk** | **Age (yrs)** | **Outcomes reported** |
| --- | --- | --- | --- | --- | --- | --- | --- |
| Becker, 2004 ([2](#_ENREF_2)); Chan-Yeung, 2000 ([16](#_ENREF_16)); Chan-Yeung 2005 ([3](#_ENREF_3)); Wong, 2013 ([17](#_ENREF_17))Protudjer, 2011([18](#_ENREF_18)); Carlsten 2013([4](#_ENREF_4))  **CAPPS Study** | RCT | 281/ 268 | Canada | BF encouraged for 4 months, allergenic food exclusion during pregnancy/lactation, delayed solid (6 months) and allergenic (12 months) food, whey pHF if necessary, environmental control. | High | 1, 7, 15 | Allergic Sensitisation (SPT), Allergic Rhinitis (DD), Wheeze (ISAAC and modified ECRHS), AD (DD), bronchial hyper-responsiveness (PC20 <7.8mg/ml), Lung function (FEV1) |
| Lovegrove, 1994 ([12](#_ENREF_12)) | RCT | 12/ 14 | UK | CM exclusion during pregancy and lactation with hydrolysed milk if necessary. BF encouraged for 6 months and eHF if needed. | High | 1 | AD (DD) |
| Matthew, 1977 ([1](#_ENREF_1)) | RCT | 27/ 35 | UK | BF encouraged for 6 months, delayed solid food (3 months) and allergenic food (6 months), soy milk if necessary, environmental control. | High | 1 | AD (Physician assessment), Allergic sensitisation, Total IgE |
| Poysa, 1991 ([9](#_ENREF_9))  Poysa 1989 ([10](#_ENREF_10))  Kuikka 1985 ([11](#_ENREF_11)) | RCT | Unclear - outcome reported in 35/ 33 | Finland | eBF encouraged for 3 months, solid food and cow's milk formula after 3 months plus environmental control. | High | 5, 10 | Asthma (>=3 episodes of wheeze), Allergic rhinitis/ conjunctivitis (Physician assessment), Allergic sensitisation (SPT, total IgE), Food allergy (oral food challenge), AD (Hanifin and Rajka) |
| Shao, 2006 ([5](#_ENREF_5)) | RCT | 23/ 23 | China | eBF encouraged for 4 months, allergenic food exclusion during lactation, delayed solid (4 months) and allergenic (6-12 months) food, pHF if necessary. | High | 1.5 | AD (Wolkerstorfer score), Allergic Sensitisation (SPT) |
| Zeiger, 1992  ([13](#_ENREF_13))  Zeiger 1989 ([19](#_ENREF_19)), 1994 ([20](#_ENREF_20)) | RCT | 103/ 185 [seen at 4 months] | USA | Allergenic food exclusion during pregnancy/lactation, delayed allergenic (1-3 years) food, casein eHF if necessary. | High | 1, 4, 7 | AD (Hanifin and Rajka Criteria), Allergic Rhinoconjunctivitis (DD), Food Allergy - Any (DD), Wheeze ( ≥2 physician diagnosed episodes), Allergic Sensitisation (SPT) |
| Hide, 1994 ([14](#_ENREF_14))  Hide, 1996([21](#_ENREF_21))  Arshad, 1992 ([15](#_ENREF_15))  Arshad, 2003 ([22](#_ENREF_22))  Arshad, 2007 ([23](#_ENREF_23))  Scott, 2012 ([24](#_ENREF_24))  **Isle of Wight Study** | RCT | 71/ 68 | UK | Allergenic food exclusion during lactation, delayed allergenic (9-11 months) food, soya hydrolysate if necessary, environmental control. | High | 1, 2,4,18 | Recurrent wheeze (>=3 episodes), BHR (PC20<8mg/ml), AD (Physician assessment), Food allergy (Open food challenge ; Physician assessment), Allergic sensitisation (SPT, total IgE), Allergic rhinitis (physician assessment), Total IgE |
| Halmerbauer, 2002  Halmerbauer, 2003 ([7](#_ENREF_7), [8](#_ENREF_8))  **SPACE Study** | RCT | 349/ 347 | UK, Germany, Austria | eBF encouraged beyond 3 months, delayed solid (6 months) and allergenic (1-3 years) food, environmental control. | High | 1 | AD (DD), Allergic sensitisation (SPT, sIgE), Wheeze (Parent reported wheeze ever), Asthma (>=3 episodes of wheeze), Food allergy (DD) |
| Schonberger, 2005  ([6](#_ENREF_6))  **PREVASC study** | RCT | 212/ 208 | Netherlands | BF encouraged to ≥6 months, delayed solid and allergenic (6 months) food, eHF if formula introduced, environmental control. | High | 2 | AD (ICHPPC), Wheeze (Dutch Guideline ‘‘Asthma in Children’’ and ISAAC), Allergic Sensitisation (sIgE) |

BF breastfeeding; eBF exclusive breastfeeding; pHF partially hydrolysed formula; eHF extensively hydrolysed formula; CM cow’s milk; RCT randomised clinical trial, SPT skin prick test, BHR bronchial hyperresponsiveness, FE_V1_ forced expiratory volume in one second; Physician assessment refers to assessment by a study physician , DD refers to community diagnosis.

Figure 1 Risk of bias in trials of multi-faceted intervention studies

# Multi-faceted interventions and risk of AD

All 9 trials (2200 participants) reported AD (AD) as an outcome. Age at time of outcome measurement ranged between 1 and 7 years. In 4 studies diagnosis was made by a doctor – in 3 cases at a scheduled study visit by a study physician; 2 studies used Hanifin & Rajka and others used either unclear or other methods of outcome assessment. The risk of bias was unclear in most studies, largely due to unclear selection bias (Figure 2).

Multi-faceted interventions did not significantly reduce AD risk at age ≤4, 5-14 or ≥15 years (Figures 3-5). In one small study (Isle of Wight) there was evidence for reduced atopic AD (i.e. AD associated with positive skin prick test) RR 0.3 95% CI 0.13, 0.84 (Figure 6). Subgroup analysis was undertaken for AD at ≤4 by risk of Conflict of Interest bias, and by inclusion of environmental control (house dust mite, tobacco smoke, pet exposure reduction methods) within the intervention. This showed no significant subgroup differences, as summarised below.

Environmental control 4 studies RR 0.72 (0.42, 1.22; I^2^=69%); no environmental control 2 studies RR 0.60 (0.32, 1.14; I^2^=0%) – test for subgroup difference P=0.68.

Low conflict of interest risk 1 study RR 0.86 (0.58, 1.29); unclear/high conflict of interest risk 5 studies RR 0.59 (0.32, 1.10; I^2^=66%) – test for subgroup difference P=0.32.

*Data that could not be included in meta-analysis*

In the Isle of Wight Study 2-year outcomes were also reported, which could not be included in meta-analysis. Here there were increased odds for AD in control compared with intervention group – OR 9.5 95%CI 1.8, 50.1 ([14](#_ENREF_14)). The study by Zeiger ([13](#_ENREF_13)) reported AD in graphical form in 225 participants, so could not be included in meta-analysis. The graph suggests that ~20% in the control, and ~14% in the active group developed AD by 4 years, which the authors describe at not statistically significant.

**Overall we found no evidence that multifaceted interventions reduce risk of AD, in high risk infants. However data were sparse and confidence intervals were wide, such that clinically significant beneficial effects cannot be confidently excluded.**

Figure 2 Risk of bias in multi-faceted interventions and risk of AD

Figure 3 Multi-faceted interventions and risk of AD at age ≤ 4 years


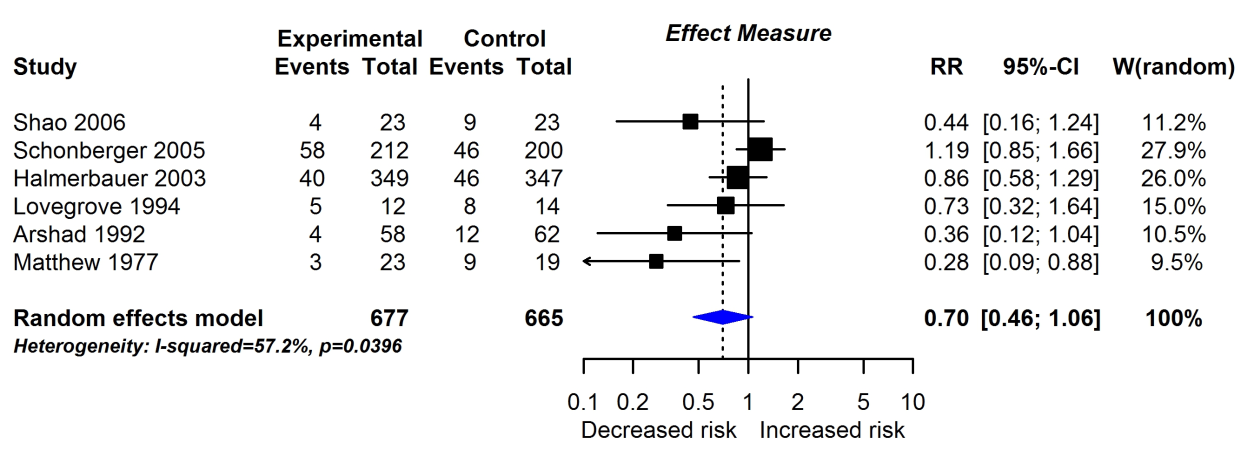


Figure 4 Multi-faceted interventions and risk of AD at age ≥ 5-14 years


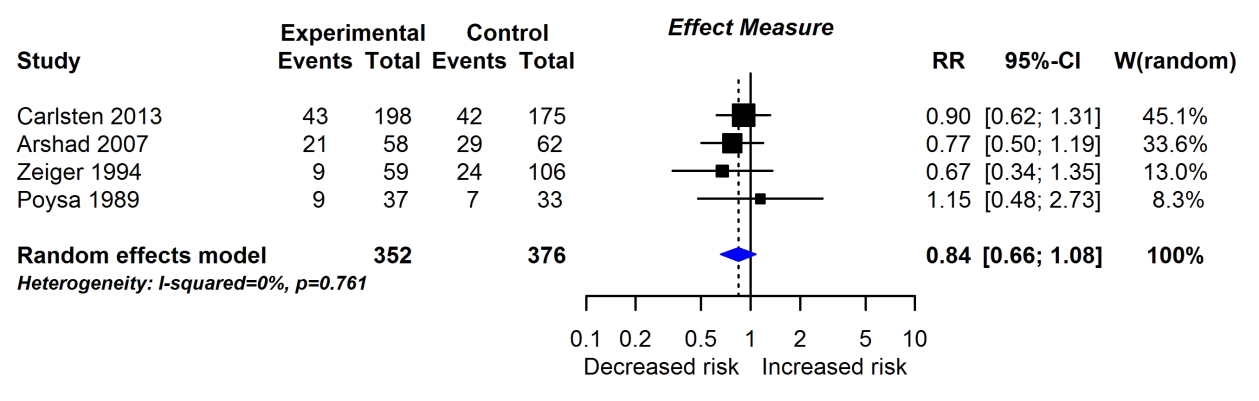


Figure 5 Multi-faceted interventions and risk of AD at age ≥ 15 years


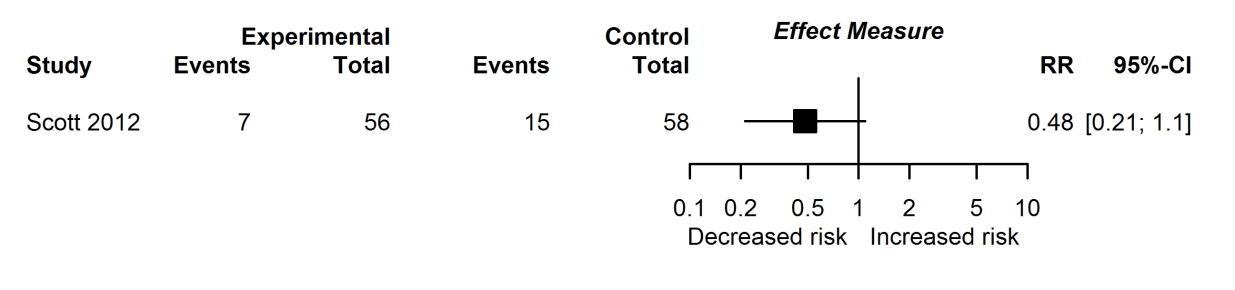


**Figure 6 Multi-faceted interventions and risk of atopic AD at age 5-14 years**


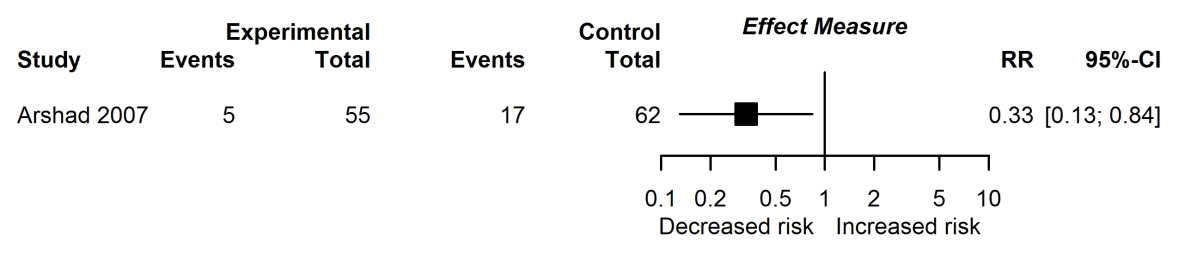


# Multi-faceted interventions and risk of allergic rhinitis or conjunctivitis

Four trials (900 participants) reported allergic rhinitis (AR) and/or allergic conjunctivitis (AC) as an outcome. Age at time of outcome measurement ranged between 3 and 10 years. Diagnosis was made by a study physician in two studies, community doctor diagnosis in two studies. The risk of bias was unclear in most studies, largely due to unclear selection bias due to poorly reported methods of randomisation and treatment allocation (Figure 5).

At age ≤4 years two studies were suitable for meta-analysis which showed reduced risk of AR with multifaceted interventions (RR 0.61 95% CI 0.44, 0.84; 2 studies; 613 participants; Figure 6). There was no statistical heterogeneity. The study by Zeiger ([13](#_ENREF_13)) reported AR in graphical form in a further 225 participants, so could not be included in meta-analysis. There was no significant difference between groups, with ~30% in both the intervention and the control groups developing AR by 4 years.

We found no evidence that multi-faceted interventions reduce AR or allergic conjunctivitis (AC) risk at age 5-14 years (Figure 7-8) but in one small study there was reduced atopic AR (i.e. AR associated with a positive SPT) in the intervention group at 8 years (Figure 9)

**Overall we found LOW level evidence that multifaceted interventions reduce risk of AR at age ≤ 4 years (-1 indirect intervention; -1 imprecision). We found no evidence for an effect on AC, or on AR at age 5-14.**

Figure 5 Risk of bias in multi-faceted interventions and risk of AR

Figure 6 Multi-faceted interventions and risk of AR at age ≤ 4 years


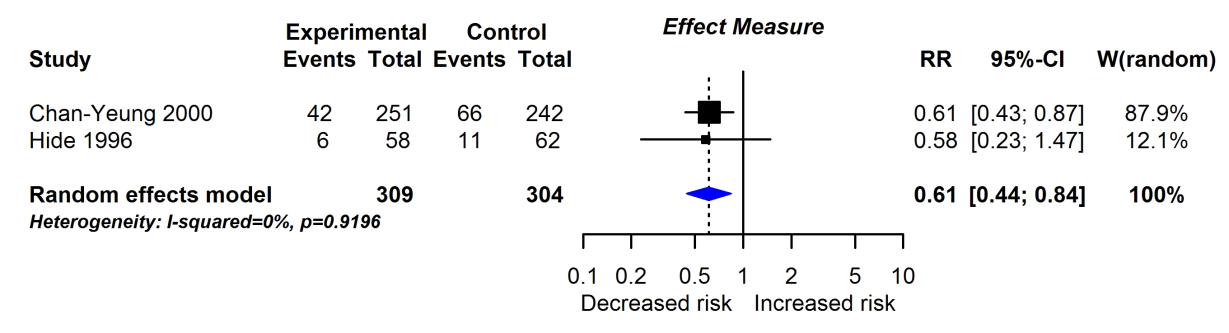


Figure 7 Multi-faceted interventions and risk of AR at age 5-14 years


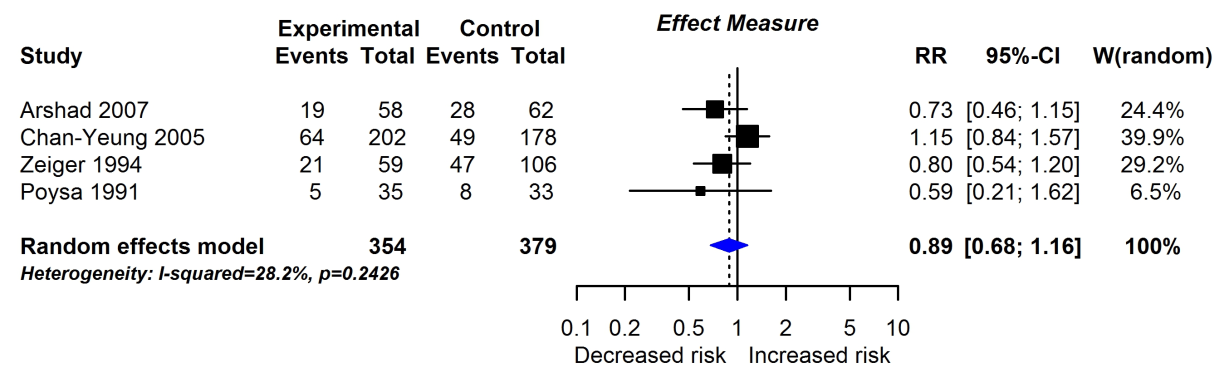


Figure 8 Multi-faceted interventions and risk of AC at age 5-14 years


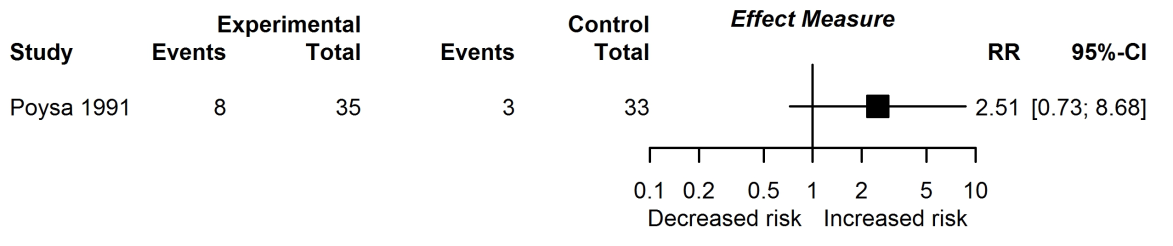


**Figure 9 Multi -faceted interventions and risk of atopic AR at age 5-14 years**


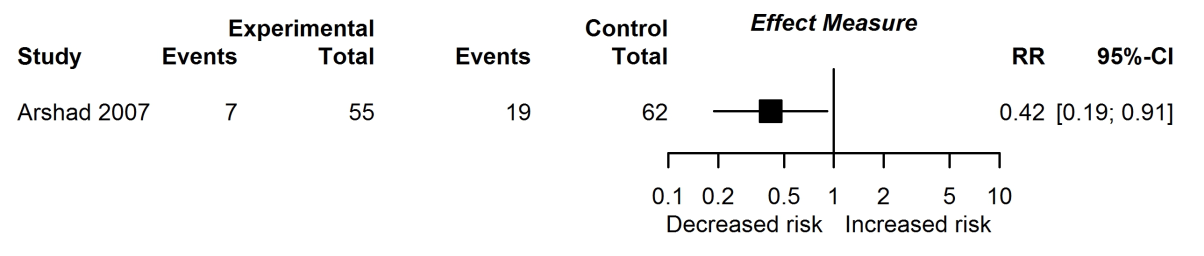


# Multi-faceted interventions and risk of food allergy

Four trials (1000 participants) reported food allergy as an outcome. Age at time of outcome measurement ranged between 1 and 10 years. Diagnosis was made by doctor’s diagnosis in two studies, oral food challenge in two studies. The risk of bias was unclear in most studies, largely due to unclear selection bias (Figure 10).

At age ≤4 years two studies were suitable for meta-analysis which showed no significant difference in food allergy risk, with high statistical heterogeneity (Figure 11). At age 5-14 three studies contributed to meta-analysis which again showed no significant difference in food allergy risk, with moderate statistical heterogeneity (Figure 12).

*Data that could not be included in meta-analyses*

The study of Arshad and Hide (Isle of Wight) found no significant difference in doctor diagnosed food allergy by age 2 – OR 1.25 (95% CI 0.27, 5.79). The study by Zeiger ([13](#_ENREF_13)) reported food allergy in graphical form in a further 225 participants at age 4, which could not be included in meta-analysis. They found reduced food allergy to any food in the intervention group when using lifetime prevalence, but no significant difference when using 12-month prevalence at age 4.

**Overall we found no evidence that multifaceted interventions reduce risk of food allergy. However data were sparse and confidence intervals were wide, such that clinically significant beneficial effects cannot be confidently excluded.**

Figure 10 Risk of bias of multi-faceted interventions and risk of food allergy

Figure 11 Multi-faceted interventions and risk of food and risk of food allergy at age ≤ 4 years


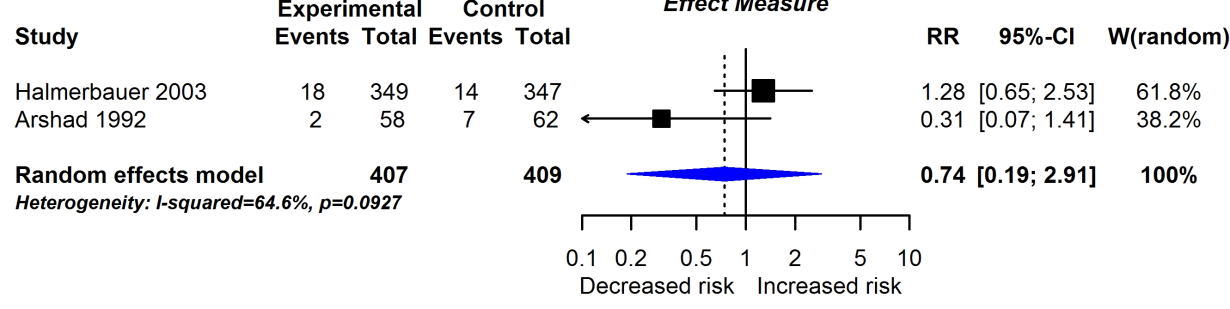


Figure 12 Multi-faceted interventions and risk of food allergy at age 5-14 years


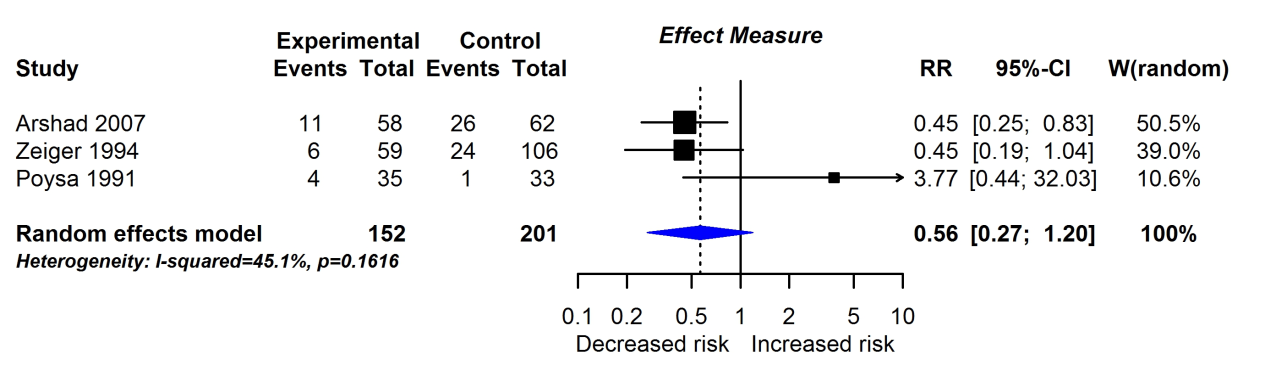


# Multi-faceted interventions and risk of allergic sensitisation

Eight trials (2100 participants) reported allergic sensitisation (AS) as an outcome. Age at time of outcome measurement ranged between 1 and 18 years. Diagnosis was made using SPT in seven studies, and sIgE in one study. Two studies reported total IgE. The risk of bias was unclear in most studies, largely due to unclear selection bias (Figure 13). For meta-analysis of specific allergic sensitisation SPT and sIgE were combined, and all age groups were combined – consistent with the approach taken in the other reports in this systematic review project.

We found no evidence for an effect of multi-faceted interventions on AS to any allergen (Figure 14), aeroallergens (Figure 15), and no consistent effect for food allergens (Figures 16-19). Zeiger separately reported no significant difference in AS to cow’s milk, egg, peanut, any food or any aeroallergen at age 4, but no data were shown that could be included in meta-analysis. Chan-Yeung separately reported no significant difference in AS to any allergen at age 2 and age 7 in the same study as Wong 2013 (CAPPS). Scott separately reported no significant difference in AS to any food allergen, in a later follow up of the study of Arshad 2003 (Figure 16; RR 1.29 95% CI 0.54, 3.08).

There was evidence from one study that multifaceted interventions may be associated with significantly increased total IgE compared to the control group (Figure 20), however a separate study (Matthew 1977) found no such difference. Matthew found no significant difference in total IgE between intervention and control group at one year of age – mean IgE level 6.0 (sd 3.4; n=16) intervention, 7.6 (sd 4.4; n=11) control P=0.30.

**Overall we found no evidence that multifaceted interventions reduce risk of AS.**

Figure 13 Risk of bias of multi-faceted interventions and risk of allergic sensitisation

Figure 14 Multi-faceted interventions and risk of AS to any allergen


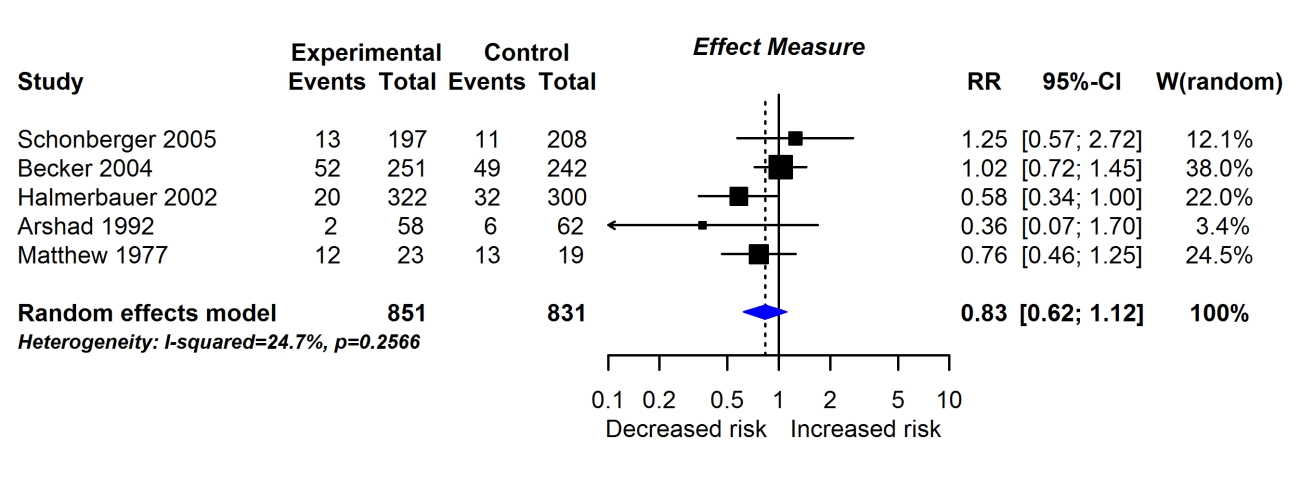


Figure 15 Multi-faceted interventions and risk of AS to aeroallergens


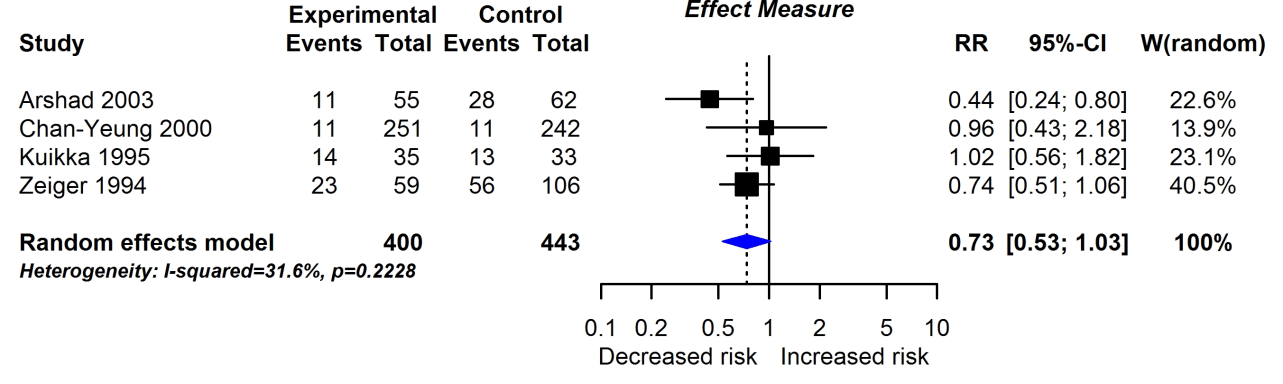


Figure 16 Multi-faceted interventions and risk of AS to any food


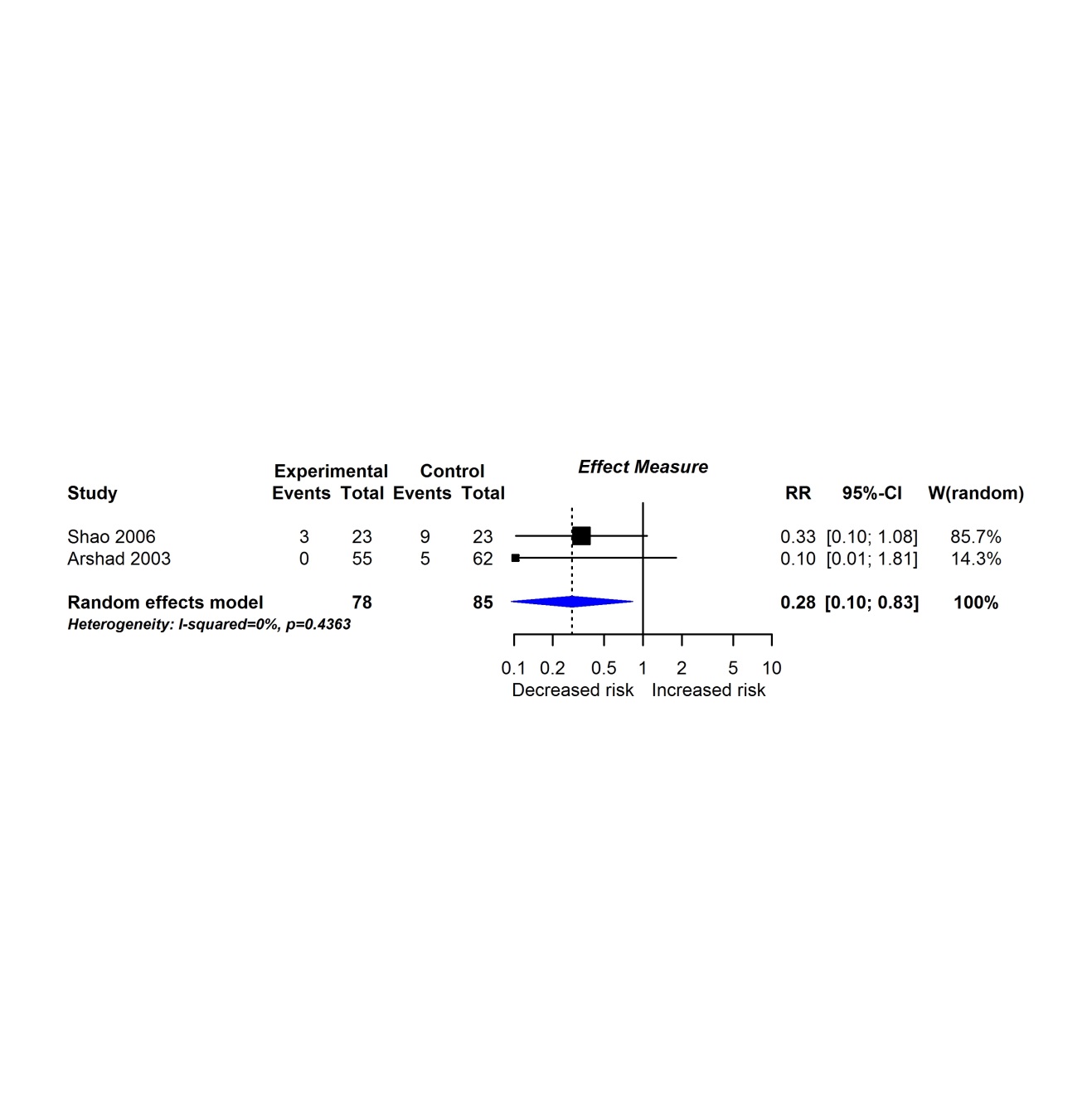


Figure 17 Multi-faceted interventions and risk of AS to cow’s milk


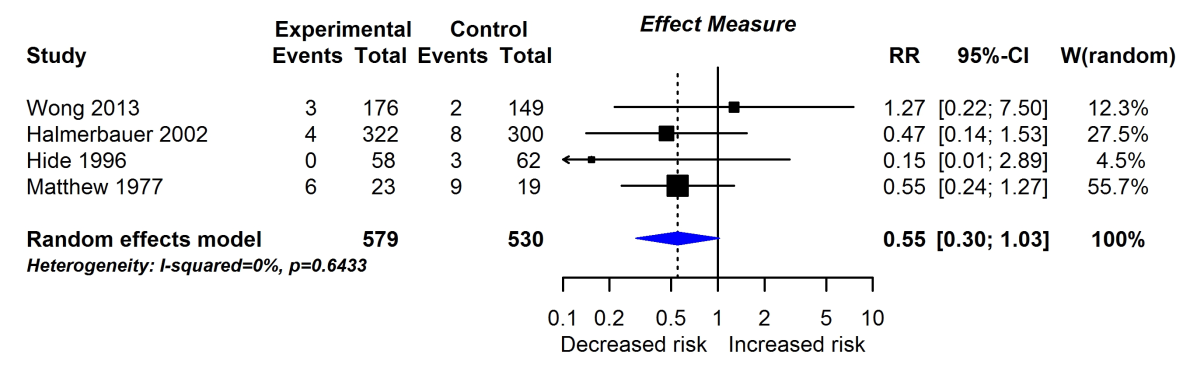


Figure 18 Multi-faceted interventions and risk of AS to egg


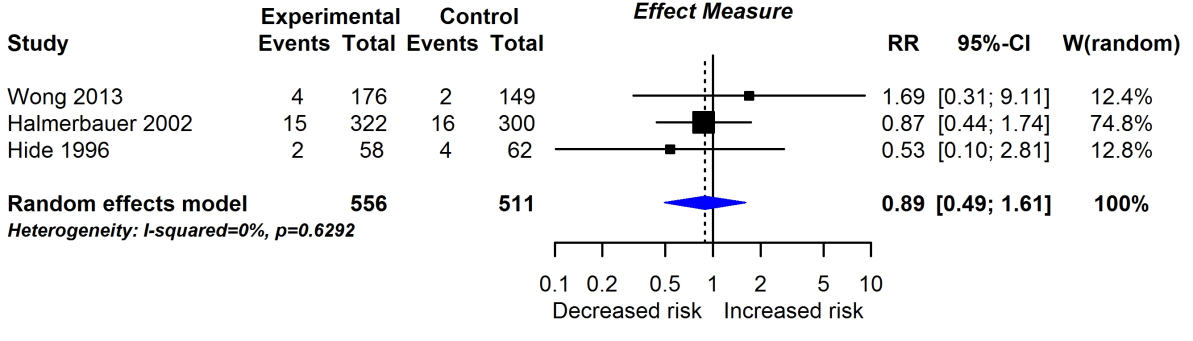


Figure 19 Multi-faceted interventions and risk of AS to peanut


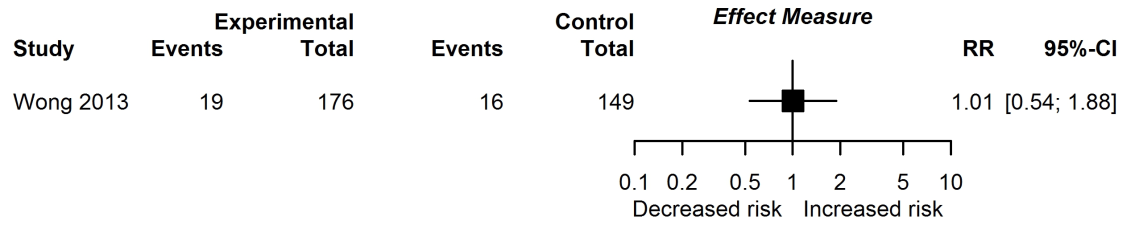


**Figure 20 Multi-faceted interventions and Total IgE**

**
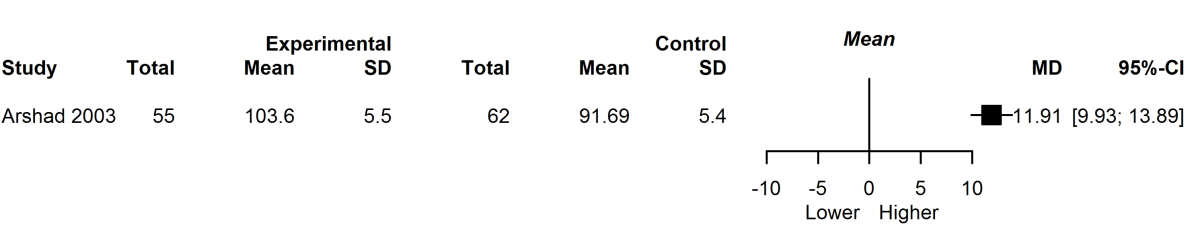
**

# Multi-faceted interventions and lung function, bronchial hyper-responsiveness and risk of wheeze

Six trials (1900 participants) reported wheeze as an outcome, and in two cases this included lung function or bronchial hyper-responsiveness. Age at time of outcome measurement ranged between 1 and 18 years. Studies mainly reported recurrent wheeze, using parent report. The risk of bias was unclear in most studies, largely due to unclear selection bias (Figure 21).

We found no evidence for an effect of multi-faceted interventions on lung function or bronchial hyper-responsiveness at the age of 5-14 years (Figures 22-23), and no evidence for an effect on wheeze or recurrent wheeze at age 0-4 (Figures 24-25).

We found evidence that multifaceted interventions reduce risk of wheeze (RR 0.57 95% CI 0.41, 0.81) or recurrent wheeze (RR 0.77 95% CI 0.61, 0.97) at age 5-14 (Figures 26-27), with no statistical heterogeneity. One of the four studies which contributed to this analysis also reported asthma at age 17 and found reduced asthma (OR 0.28 95% CI 0.09, 0.86) and reduced persistent asthma (OR 0.21 95% CI 0.05, 0.90) at this age. All these 4 studies included delayed introduction of allergenic foods into the infant diet as part of the intervention. Three of the studies also included maternal allergenic food exclusion during pregnancy/lactation, and three of the studies included environmental control measures such as house dust mite, pet and tobacco smoke avoidance.

**Overall we found LOW level evidence (-1 indirectness of intervention; -1 imprecision) that multifaceted interventions reduce risk of wheeze/recurrent wheeze at age 5-14 years, in high risk children. We found no evidence for an effect on lung function, or on wheeze at age ≤ 4 years.**

Figure 21 Risk of bias in multi-faceted studies and risk of wheeze or asthma

Figure 22 Multi-faceted interventions and FEV_1_ as % predicted at age 5-14 years


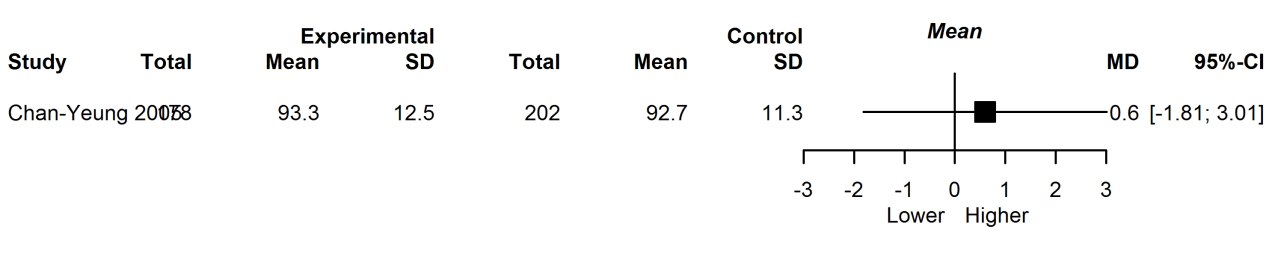


Figure 23 Multi-faceted interventions and risk of BHR at age 5-14 years


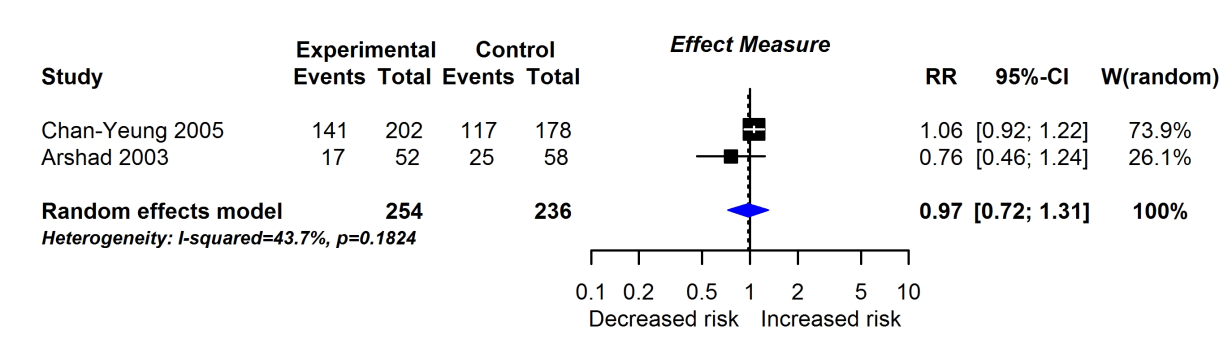


Figure 24 Multi-faceted interventions and risk of wheeze at age ≤ 4 years


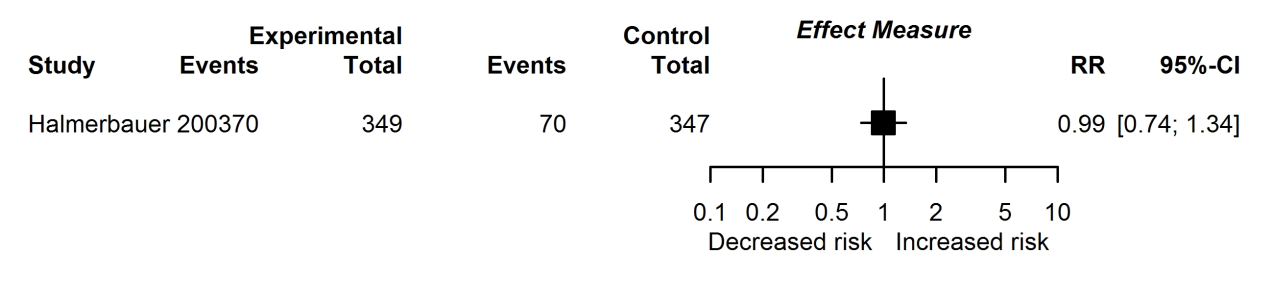


Figure 25 Multi-faceted interventions and risk of recurrent wheeze at ≤ 4 years


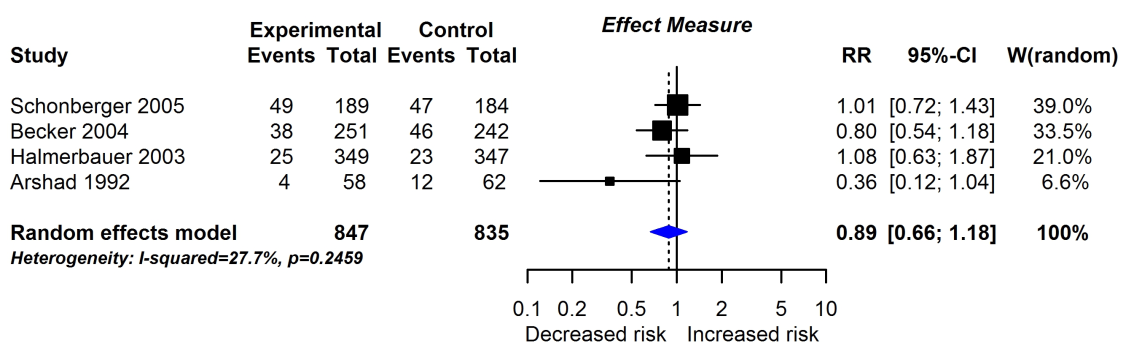


Figure 26 Multi-faceted interventions and risk of wheeze at age 5-14 years


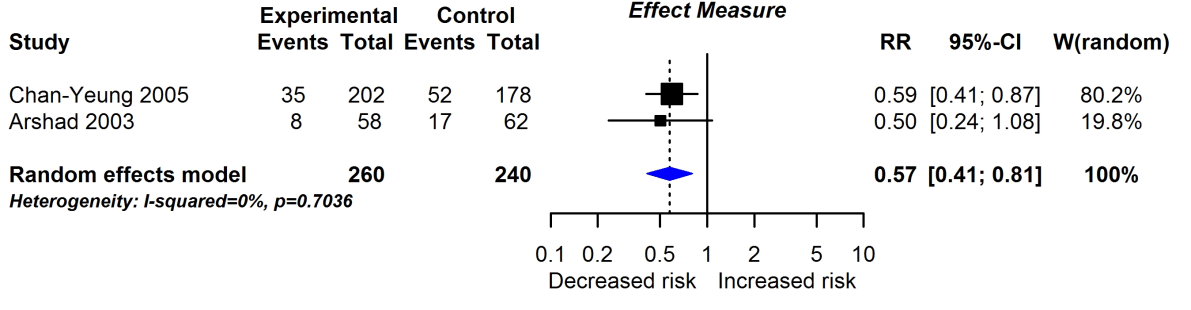


Figure 27 Multi-faceted interventions and risk of recurrent wheeze at age 5-14 years


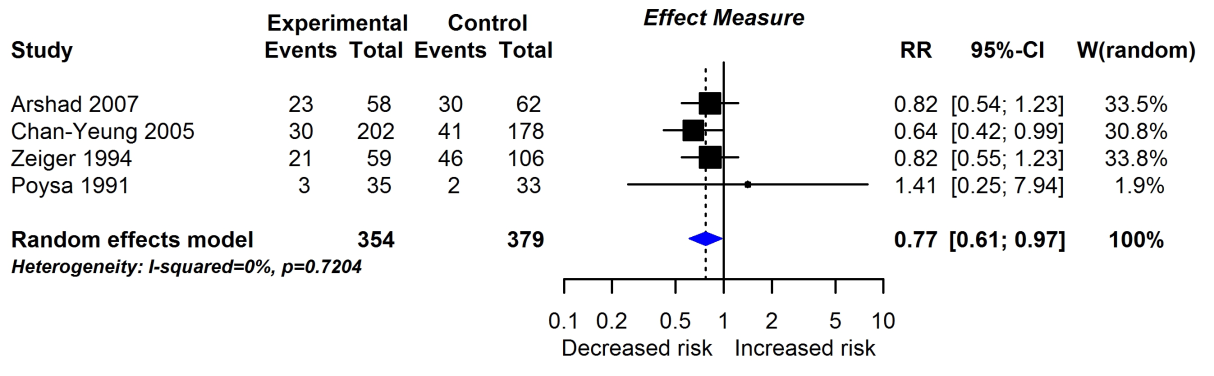


# General Conclusions

In this systematic review of multi-faceted interventions during pregnancy/lactation/infancy and risk of allergic outcomes, we found evidence that such interventions may reduce the risk of AR at age ≤ 4 years, and wheeze and recurrent wheeze at the age of 5-14 and 15+. We found no evidence for such effects at different ages, nor any effect on lung function. We found no evidence that multifaceted interventions influence other allergic outcomes, and we found no data for autoimmune outcomes. The findings are based on relatively small numbers of studies and participants, with unclear risk of bias due to uncertainty around methods of randomisation and treatment allocation. This is partly because some of the studies are relatively old and were therefore undertaken prior to guidance for reporting and registering of clinical trials in the form of CONSORT and ICMJE statements.

For AR at age ≤ 4 years there was some inconsistency (with the study of Zeiger finding no effect, but the 2 studies in meta-analysis finding an effect), and serious imprecision due to sparse data. The 2 studies included in meta-analysis used house dust mite avoidance measures, delayed allergenic food introduction to the infant, and use of a hydrolysed formula if necessary as part of the intervention. The Zeiger study, which found no significant difference, was limited to allergenic food exclusion in mother and infant. The CAPPS study, which dominated the positive meta-analysis, included avoidance of nursery/daycare until age 1, avoidance of environmental tobacco smoke exposure, and pet avoidance as part of the intervention. Given that environmental tobacco smoke is a major trigger of respiratory symptoms, it is unclear whether the dietary components of the intervention were relevant.

For analyses of wheeze/recurrent wheeze at age 5-14, there was no statistical heterogeneity, but there was imprecision due to sparse data. The included studies had diverse multifaceted interventions. However all studies that reported recurrent wheeze as an outcome measure included delayed introduction of allergenic foods into the infant diet as part of the intervention. Three of the studies also included maternal allergenic food exclusion during pregnancy/lactation, and three of the studies included environmental control measures such as house dust mite, pet and tobacco smoke avoidance.

Systematic reviews of multi-faceted interventions for preventing allergic outcomes have not previously been undertaken, to our knowledge, and none was identified in our search for recent systematic reviews. However the systematic review of Dick 2014 ([25](#_ENREF_25)) did assess a wide variety of environmental exposures in relation to childhood asthma. Dick found consistent evidence that tobacco smoke exposure, mould and air pollution are associated with increased risk for childhood asthma, and less consistent evidence that pet exposure is associated with increased asthma risk. Some of the house dust mite avoidance measures used in these multifaceted intervention studies may have also impacted on mould and/or pollutant exposure due to improved ventilation and cleaning of the indoor environment, so this should be considered as a possible mechanism of effect.

In our systematic review of breastfeeding and allergic/autoimmune outcomes (Review A) we found no consistent evidence for an association between increased exclusive breastfeeding duration and reduced risk of asthma, and Dick reported inconsistent evidence supporting the same association. Dick found evidence that exposure to house dust mite in isolation, is not associated with asthma risk. The multifaceted interventions also may have reduced wheeze/recurrent wheeze at age 5-14 and 15+ through the advice to increase breastfeeding / exclusive breastfeeding duration included in some intervention packages, but there is no direct evidence to support this possibility.

**Overall these data suggest that multifaceted interventions, which include tobacco smoke avoidance, pet avoidance and increased duration of breastfeeding, and possibly allergenic food exclusion from the infant +/- maternal diet, improved home ventilation and cleaning, may reduce the risk of AR at age ≤ 4, and wheeze in older children and adolescents at high risk of allergic disease due to a family history.**

# References

1. Matthew DJ, Taylor B, Norman AP, Turner MW. Prevention of AD. Lancet. 1977;1(8007):321-4.

2. Becker A, Watson W, Ferguson A, Dimich-Ward H, Chan-Yeung M. The Canadian asthma primary prevention study: outcomes at 2 years of age. Journal of Allergy & Clinical Immunology. 2004;113(4):650-6.

3. Chan-Yeung M, Ferguson A, Watson W, Dimich-Ward H, Rousseau R, Lilley M, et al. The Canadian Childhood Asthma Primary Prevention Study: outcomes at 7 years of age. Journal of Allergy & Clinical Immunology. 2005;116(1):49-55.

4. Carlsten C, Dimich-Ward H, Ferguson A, Watson W, Rousseau R, Dybuncio A, et al. Atopic dermatitis in a high-risk cohort: Natural history, associated allergic outcomes, and risk factors. Annals of Allergy, Asthma and Immunology. 2013;110(1):24-8.

5. Shao J, Sheng J, Dong W, Li YZ, Yu SC. [Effects of feeding intervention on development of AD in atopy high-risk infants: an 18-month follow-up study]. Zhonghua Erke Zazhi. 2006;44(9):684-7.

6. Schonberger HJAM, Dompeling E, Knottnerus JA, Maas T, Muris JWM, van Weel C, et al. The PREVASC study: The clinical effect of a multifaceted educational intervention to prevent childhood asthma. European Respiratory Journal. 2005;25(4):660-70.

7. Halmerbauer G, Gartner C, Schierl M, Arshad H, Dean T, Koller DY, et al. Study on the Prevention of Allergy in Children in Europe (SPACE): Allergic sensitization at 1 year of age in a controlled trial of allergen avoidance from birth. Pediatric Allergy and Immunology. 2003;14(1):10-7.

8. Halmerbauer G, Gartner C, Schierl M, Arshad H, Dean T, Koller DY, et al. Study on the Prevention of Allergy in Children in Europe (SPACE): Allergic sensitization in children at 1 year of age in a controlled trial of allergen avoidance from birth. Pediatric Allergy and Immunology, Supplement. 2002;13(15):47-54.

9. Poysa L, Korppi M, Remes K, Juntunen-Backman K. Atopy in childhood and diet in infancy. A nine-year follow-up study. I. Clinical manifestations. Allergy Proceedings. 1991;12(2):107-11.

10. Poysa L, Remes K, Korppi M, Juntunen-Backman K. Atopy in children with and without a family history of atopy. I. Clinical manifestations, with special reference to diet in infancy. Acta Paediatrica Scandinavica. 1989;78(6):896-901.

11. Kuikka L, Korppi M, Remes K. Atopy and skin reactivity at school age in children followed up from birth, with special reference to atopy prevention in infancy and atopic findings at preschool age. Allergy Proceedings. 1995;16(6):313-7.

12. Lovegrove JA, Hampton SM, Morgan JB. The immunological and long-term atopic outcome of infants born to women following a milk-free diet during late pregnancy and lactation: a pilot study. British Journal of Nutrition. 1994;71(2):223-38.

13. Zeiger RS, Heller S, Mellon MH, Halsey JF, Hamburger RN, Sampson HA. Genetic and environmental factors affecting the development of atopy through age 4 in children of atopic parents: A prospective randomized study of food allergen avoidance. Pediatric Allergy and Immunology. 1992;3(3):110-27.

14. Hide DW. The Isle of Wight study, an approach to allergy prevention. Pediatric Allergy & Immunology. 1994;5(6 Suppl):61-4.

15. Arshad SH, Matthews S, Gant C, Hide DW. Effect of allergen avoidance on development of allergic disorders in infancy. Lancet. 1992;339(8808):1493-7.

16. Chan-Yeung M, Manfreda J, Dimich-Ward H, Ferguson A, Watson W, Becker A. A randomized controlled study on the effectiveness of a multifaceted intervention program in the primary prevention of asthma in high-risk infants. Archives of Pediatrics & Adolescent Medicine. 2000;154(7):657-63.

17. Wong T, Chan-Yeung M, Rousseau R, Dybuncio A, Kozyrskyj AL, Ramsey C, et al. Delayed introduction of food and effect on incidence of food allergy in a population at high risk for atopy: The Canadian asthma primary prevention study (CAPPS). Journal of Allergy and Clinical Immunology. 2013;1):AB96.

18. Protudjer JLP, Robertson L, Kozyrskyj A, Ramsey CD, Chan-Yeung M, Becker AB. Non-atopic asthma and atopic asthma are associated with reduced pulmonary function by age 7 years. American Journal of Respiratory and Critical Care Medicine. 2011;183 (1 MeetingAbstracts).

19. Zeiger RS, Heller S, Mellon MH, Forsythe AB, O'Connor RD, Hamburger RN, et al. Effect of combined maternal and infant food-allergen avoidance on development of atopy in early infancy: A randomized study. Journal of Allergy and Clinical Immunology. 1989;84(1):72-89.

20. Zeiger RS. Dietary manipulations in infants and their mothers and the natural course of atopic disease. Pediatric Allergy & Immunology. 1994;5(6 Suppl):33-43.

21. Hide DW, Matthews S, Tariq S, Arshad SH. Allergen avoidance in infancy and allergy at 4 years of age. Allergy. 1996;51(2):89-93.

22. Arshad SH, Bateman B, Matthews SM. Primary prevention of asthma and atopy during childhood by allergen avoidance in infancy: a randomised controlled study. Thorax. 2003;58(6):489-93.

23. Arshad SH, Bateman B, Sadeghnejad A, Gant C, Matthews SM. Prevention of allergic disease during childhood by allergen avoidance: the Isle of Wight prevention study. Journal of Allergy & Clinical Immunology. 2007;119(2):307-13.

24. Scott M, Roberts G, Kurukulaaratchy RJ, Matthews S, Nove A, Arshad SH. Multifaceted allergen avoidance during infancy reduces asthma during childhood with the effect persisting until age 18 years. Thorax. 2012;67(12):1046-51.

25. Dick S, Friend A, Dynes K, AlKandari F, Doust E, Cowie H, et al. A systematic review of associations between environmental exposures and development of asthma in children aged up to 9 years. BMJ Open. 2014;4(11).
