# Supplementary material for: Diet during pregnancy and infancy and risk of allergic or autoimmune disease: A systematic review and meta-analysis
Source: PLoS Med. 2018 Feb 28;15(2):e1002507. doi: 10.1371/journal.pmed.1002507 (PMC5830033; doi:10.1371/journal.pmed.1002507)
Supplement: S2 Data — (ZIP) [file pmed.1002507.s007.zip › Review_C_reports/PREBIOTICS.docx]

**PREBIOTIC SUPPLEMENTATION IN INFANTS AND RISK OF ALLERGIC SENSITISATION OR DISEASE**

Robert J Boyle^1^, Vanessa Garcia-Larsen^2^, Despo Ierodiakonou^3^, Jo Leonardi-Bee^4^, Tim Reeves^5^, Jennifer Chivinge^6^, Zoe Robinson^6^, Natalie Geoghegan^6^, Katharine Jarrold^6^, Andrew Logan^6^, Annabel Groome^6^ , Evangelia Andreou^7^, Nara Tagiyeva-Milne^8^, Ulugbek Nurmatov^9^, Sergio Cunha^10^

^1^ Clinical Senior Lecturer, Section of Paediatrics ^2^ Post-Doctoral Research Associate, Respiratory Epidemiology and Public Health, National Heart and Lung Institute ^3^ Post-Doctoral Research Associate, Departments of Paediatric and Respiratory Epidemiology and Public Health Group, all at Imperial College London

^4^Associate Professor of Community Health Sciences, University of Nottingham

^5^ Research Support Librarian, Faculty of Medicine, Imperial College London

^6^ Undergraduate medical students, Imperial College London

^7^Research Associate, Imperial Consultants

^8^Research Fellow, University of Aberdeen

^9^Research Fellow, University of Edinburgh

^10^Research Associate, Respiratory Epidemiology and Public Health, National Heart and Lung Institute, Imperial College London**Table of Contents**

[1. Prebiotics and risk of allergic outcomes - summary of interventions and findings 3](#_Toc423210691)

[2. Prebiotics and risk of allergic sensitisation 9](#_Toc423210692)

[3. Prebiotics and risk of food allergy 12](#_Toc423210693)

[4. Prebiotics and risk of eczema 14](#_Toc423210694)

[5. Prebiotics and risk of wheeze 20](#_Toc423210695)

[6. Prebiotics and risk of allergic rhinitis 24](#_Toc423210696)

[Conclusions 26](#_Toc423210697)

[References 28](#_Toc423210698)

**List of Figures**

Figure 1 Risk of bias in intervention studies of prebiotics and allergic sensitisation 10

Figure 2 Prebiotics and allergic sensitisation to any allergen 10

Figure 3 Prebiotics and allergic sensitisation to any aeroallergen 10

Figure 4 Prebiotics and allergic sensitisation to any food 11

Figure 5 Prebiotics and allergic sensitisation to cow’s milk 11

Figure 6 Prebiotics and allergic sensitisation to egg 11

Figure 7 Risk of bias in intervention studies of prebiotics and food allergy 13

Figure 8 Prebiotics for preventing Food Allergy 13

Figure 9 Prebiotics for preventing Cow’s Milk Allergy 13

Figure 10 Risk of bias in intervention studies of prebiotics and eczema 16

Figure 11 Prebiotics for preventing eczema at age ≤4 years 18

Figure 12 Prebiotics for preventing eczema at age 5-14 years 18

Figure 13 Risk of bias in intervention studies of prebiotics and wheeze 22

Figure 14 Prebiotics for preventing recurrent wheeze at age ≤ 4 years 23

Figure 15 Prebiotics for preventing recurrent wheeze at age 5-14 years 23

Figure 16 Risk of bias in intervention studies of prebiotics and risk of allergic rhinitis 25

Figure 17 Prebiotics for preventing allergic rhinitis at age 5-14 years 25

# Prebiotics and risk of allergic outcomes - summary of interventions and findings

Prebiotics are defined as selectively fermented ingredients (typically oligosaccharides), that allow specific changes in the composition and/or activity of the gastrointestinal microflora, and thereby confer a health benefit. Synbiotics are a combination of probiotic and prebiotic administered to the same individual. In this analysis we included studies of any oligosaccharide, given alone (=prebiotic) or in combination with a probiotic (=synbiotic) or other co-intervention. We planned to undertake subgroup/stratified analyses for meta-analyses which contained a total of >5 studies, and where appropriate to include probiotic/synbiotic intervention as a subgroup. We planned to assess publication bias using Funnel plots and Egger’s test where there were ≥10 studies in a meta-analysis. In total we identified 10 trials with over 4000 participants investigating the effect of 9 different prebiotic interventions during infancy on allergic outcomes (Table 1). We identified 1 recent high-quality systematic review which included 4 of these studies, in our July 2013 search for existing systematic reviews. We did not identify any further systematic reviews in our updated search on 26^th^ February 2017. Nine of the original trials studied supplementation of infant formula, and one studied direct supplementation of mothers and infants. Four trials studied variations of the same prebiotic (Immunofortis). Six trials investigated prebiotic, and 4 synbiotic.

*Interventions used*

The **Moro** study ([1](#_ENREF_1)) used extensively hydrolysed cow’s milk whey protein formula [Aptamil HA] supplemented with 8g/L galactooligosaccharides (GOS)/ fructooligosaccharides (FOS) per 100ml [a product called Immunofortis, Nutricia, Netherlands] for six months. The **MIPS-1** study ([2](#_ENREF_2)) used cow’s milk formula supplemented with Immunofortis plus specific acidic oligosaccharides (AOS) at 8g/L (6.8g/L neutral and 1.2g/L AOS). The study of **Van der Aa** ([3](#_ENREF_3)) used an extensively hydrolyzed whey-based formula (Nutrilon Pepti ; Nutricia, Netherlands) with synbiotics for 12 weeks. The synbiotic contained Immunofortis at 8g/L, and *Bifidobacterium breve* M-6V (Morinaga Milk Co, Ltd., Tokyo, Japan) 1.3 x 10^9^ colony forming units (cfu)/100 ml. The study of **Kukkonen** ([4](#_ENREF_4)) used a probiotic during the last 2-4 weeks of pregnancy - *Lactobacillus rhamnosus* GG(ATCC 3103), 10^10^ cfu/day; *L rhamnosus* LC705 (DSM 7061), 10^10^ cfu/day; *Bifidobacterium breve* Bb99(DSM 13692), 4 x 10^8^ cfu/day; and *Propionibacterium freudenreichii* ssp. shermanii JS(DSM 7076), 4 x 10^9^ cfu/day (Valio, Helsinki, Finland). Infants then received the same probiotics with 20 drops of syrup containing 0.8 g GOS prebiotic daily for 6 months. The study of **Roze** ([5](#_ENREF_5)) used infant formula containing a synbiotic - *Lactobacillus rhamnosus* LCS-742 and *Bifidobacterium longum* subsp infantis M63, and a 96% GOS/ 4% FOS prebiotic. The formula was also enriched with bovine α-lactalbumin, using whey protein concentrate, and was fed to infants for the first 6 months. The study of **Ziegler** ([6](#_ENREF_6)) used infant formula containing prebiotics polydextrose (PDX) and GOS at 50:50 ratio 4g/L (n=74) or PDX, GOS and lactulose at 50:33:17 ratio 8g/L (n=76), from 14 days to 120 days age. The study of **Boyle** ([11](#_ENREF_11)**)** used partially hydrolysed whey-based infant formula containing Immunofortis in infants up to 6 months age. The study of **Ivakhnenko (**[**7**](#_ENREF_7)**)** used a standard infant formula supplemented with scGOS/lcFOS 9:1 at 8g/L for 2 months. The study of **Sierra** ([8](#_ENREF_8)) used a standard formula supplemented with GOS 0.44g/dl initially and then 0.5g/dl when switched to follow on formula (6 months age) – infants received study formula until 1 year age. The study of Chien ([9](#_ENREF_9)) used a standard infant formula, or a formula supplemented with either scGOS/lcFOS (0.8g/100ml) and B. breve M-16V (7.5x108CFU/100ml), or scGOS/lcFOS (0.8g/100ml) alone – from birth until age 4 months.

*Populations and Outcomes assessed*

The outcomes allergic sensitisation, eczema, wheeze, and allergic rhinitis were reported. No studies were identified reporting allergic conjunctivitis, food allergy or autoimmune outcomes. Overall 4000 infants were randomised. In four studies infants were at high risk of allergic outcomes, in seven studies the population studied was unrepresentative of the general population, due to early full formula feeding (6) or established eczema (one). Six studies were carried out in Europe, one in North America, two in Asia and one in Asia/Europe/Australasia. Definitions used to assess the same outcome varied across studies, but most studies used recognised assessment tools to define the outcomes of interest.

*Overall findings*

All studies had a high or unclear risk of conflict of interest due to direct or unclear industry involvement in the trial, in the form of possible study sponsorship, employment of study authors, and/or consulting fees paid to study authors. 20-50% of studies in each analysis had a high risk of attrition bias due to loss to follow up of over 30% of participants prior to outcome assessment. Assessment of publication bias was not undertaken due to small numbers of studies included in each meta-analysis.

**Overall there was no evidence that prebiotics reduce risk of wheezing, allergic sensitisation, AD, food allergy or allergic rhinitis.**

**Table 1 Characteristics of included studies evaluating prebiotic supplementation and allergic outcomes**

| First Author & Publication Year | N Intervention/ Control | Study Design | Country | Population Studied | Disease risk | Intervention | Age (yrs) | Outcomes Reported |
| --- | --- | --- | --- | --- | --- | --- | --- | --- |
| Osborn 2013 ([10](#_ENREF_10)) | 4 RCT (1428 participants) | SR | - | Infants in the first six months of life without clinical evidence of allergy. | All | Prebiotics added to human milk or infant formula, compared to control or a different prebiotic | All | Asthma/wheeze, , eczema, rhinitis  (no data), food allergy (no data) |
| van Hoffen 2009 ([11](#_ENREF_11)) Moro 2006 ([1](#_ENREF_1)), Arslanoglu 2008 ([12](#_ENREF_12)) Arslanoglu 2012 ([13](#_ENREF_13)) | 129/ 130 | RCT | Italy | Formula introduced <2 weeks; full formula feeds <6 weeks (excluded post-randomisation, if BF continued beyond 6 weeks) | high | **Prebiotic**  Immunofortis [GOS/FOS]  until 6 months | 0.5, 2, 5 | Allergic sensitisation (sIgE to CM; total IgE), Eczema (Harrigan and Rabinowitz), Wheeze (≥3 episodes), Allergic rhinoconjunctivitis (doctor-diagnosed nasal and ocular symptoms not related to infection) |
| Boyle 2015 ([14](#_ENREF_14)) Boyle 2016 ([15](#_ENREF_15))  PATCH Study | 432/431 | RCT | Australia, Singapore, England and Ireland | Term infants with ≥one parent with allergic disease, and formula introduction <18 weeks. | high | **Prebiotic**  Immunofortis [GOS/FOS] + AOS until 6 months. Co-intervention with partially hydrolysed formula | 1 | Eczema (Hanifin and Rajka) |
| Gruber 2010 ([2](#_ENREF_2))  Gruber, 2015 ([16](#_ENREF_16))  MIPS-1 Study | 414/ 416 | RCT | NetherlandsAustria Switzerland Italy  Germany | Full formula feeds <8 weeks | low | **Prebiotic**  Immunofortis [GOS/FOS] + AOS | 1 | Allergic sensitisation (sIgE to CM or egg; total IgE), Eczema (UK Working Party criteria) |
| Ivakhnenko 2013 ([7](#_ENREF_7)) | 80/ 80 | RCT | Ukraine | Fully formula fed – no breastfeeding at all | normal | **Prebiotic**  scGOS/lcFOS 9:1 at 8g/L until 2 months. | 1.5 | Eczema (Harrigan and Rabinowitz), Food allergy (unclear) |
| Sierra 2015 ([8](#_ENREF_8)) | 188/ 177 | RCT | Spain | Infants aged <2 months, exclusively formula fed for ≥15 days | normal | **Prebiotic**  Nutradefense. GOS 0.44-0.5g/dl formula until 1 year age | 1 | Eczema (physician assessment), Wheeze (physician assessment), Allergic sensitisation (SPT), Food allergy (unclear) |
| Ziegler 2007 ([6](#_ENREF_6)) | 150/ 76 | RCT | USA | Full formula feeds <2 weeks | normal | **Prebiotic**  GOS/Polydextrose +/-lactulose | 0.3 | Eczema (unclear) |
| Chien, 2016 ([9](#_ENREF_9)) | Unclear – outcome reported in 45 (synbiotic), 39 (prebiotic), 45 (control) | RCT | Singapore | Mixed fed infants born by elective Caesarean. Intervention included in formula milk from birth until 4 months. | unclear | **Prebiotic & Synbiotic** scGOS/lcFOS (0.8g/100ml) and B. breve M-16V (7.5x108CFU/100ml), or scGOS/lcFOS (0.8g/100ml), or control formula | 0.4 | Eczema (unclear) |
| Van der Aa 2010 ([3](#_ENREF_3)) | 46/ 44 | RCT | Netherlands | Infants who already had AD, with SCORAD >15 | high | **Synbiotic**  Immunofortis [GOS/FOS] + B. breve | 1 | Allergic sensitisation (Total IgE), Wheeze (≥3 episodes + interval symptoms) |
| Kuitunen 2009 ([17](#_ENREF_17)) Kukkonen 2007 ([4](#_ENREF_4))  Kukkonen 2011 ([18](#_ENREF_18)) | 610/ 613 | RCT | Finland | Representative population | high | **Synbiotic**  GOS + B. breve + L. rhamnosus. Cointervention with probiotic during pregnancy. | 2, 5 | Allergic sensitisation (+ve SPT or sIgE; total IgE), Eczema (Hanifin and Rajka), Wheeze (≥2 episodes + interval symptoms), Allergic rhinoconjunctivitis (symptoms + sensitisation) |
| Roze 2012 ([5](#_ENREF_5)) | 48/ 49 | RCT | France | Full formula feeds until randomised (up to day 3) | normal | **Synbiotic**  GOS/FOS + L.rhamnosus + B.infantis | 0.5 | Eczema (UK Working Party criteria) |

# Prebiotics and risk of allergic sensitisation

Five intervention studies examining the effect of prebiotic supplementation on allergic sensitisation were eligible for inclusion. In total over 3000 participants were randomised. Studies reported total IgE, and allergic sensitisation measured using specific IgE (sIgE) or skin prick testing (SPT). Four studies were carried out in high risk populations, and 4 studies were in very specific populations with either early formula feeding or established eczema. Risk of disease (outcome) was assessed between 6 months and 5 years age. Three of the studies were at high risk of attrition bias, and all studies were considered to have a high risk of conflict of interest (Figure 1). The outcomes are shown in Figures 2 to 6, and show no evidence for an effect on AS to any allergen, aeroallergen, food, or cow’s milk or egg specifically.

*Data that could not be included in meta-analyses*

In the study of Kukkonen there was no significant reduction in odds of ‘any allergic sensitisation’ with synbiotics, using a combination of SPT and sIgE to common allergens in the first 2 years of life – OR 0.86 (95%CI 0.65 – 1.14; p=0.29) – SPT data shown in Figure 2 - and no effect on total IgE at this age – mean 25.7 ku/L synbiotic (95%CI 22.2 – 29.8), 27.2 control (23.1– 32.0) ([4](#_ENREF_4)). At age 5 the same authors found no reduction in ‘any allergic sensitisation’ again using a combination of SPT and sIgE – OR 1.0 (95% CI 0.77 – 1.33; p=0.99). At age 5 they also examined SPT and sIgE to common allergens, SPT to foods separately and total IgE, and found no significant effect of synbiotics ([17](#_ENREF_17), [18](#_ENREF_18)). In the study of Moro there was reduced sIgE to cow’s milk (median 0.75 versus 1.04 U/ml; p<0.05) and reduced total IgE (median 4 versus 10 kU/L; p<0.01) at 6 months in the prebiotic group ([11](#_ENREF_11)), however, separate publications from the same study reported no significant difference in these outcomes at 6 months age ([19](#_ENREF_19), [20](#_ENREF_20)). In the MIPS-1 study there was no significant effect of prebiotics on total IgE at 1 year – mean 13 ku/L (95%CI 11 – 15.4) prebiotic, 11.9 (10.2 – 13.8) control (p=0.43) ([21](#_ENREF_21)). In the study of Van der Aa there was a non-significant reduction in total IgE at 1 year in the synbiotic (median 20.4 range 2.9, 628.0) versus control group (median 47.7 range 3.7, 1529; p=0.13) ([3](#_ENREF_3)). In the study of Boyle median total and specific IgE levels in a subgroup randomised before 4 weeks did not differ between groups. Total IgE 8.6 active, 9.0 control P=0.51; milk IgE 0.03 active 0.03 control P=0.46; egg IgE 0.0 active 0.01 control P=0.15

**Overall we found no evidence that prebiotics reduce risk of allergic sensitisation.**

Figure 1 Risk of bias in intervention studies of prebiotics and allergic sensitisation

Figure 2 Prebiotics and allergic sensitisation to any allergen


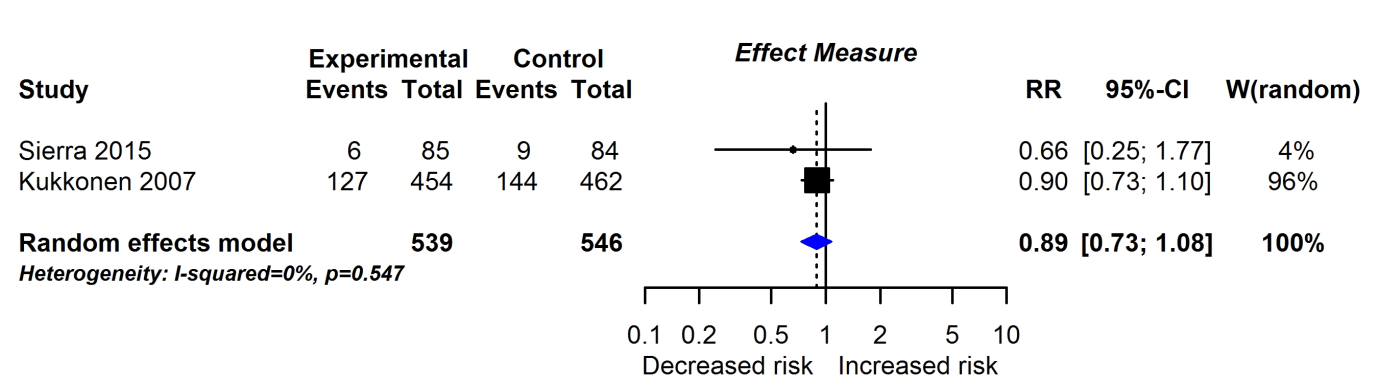


Figure 3 Prebiotics and allergic sensitisation to any aeroallergen


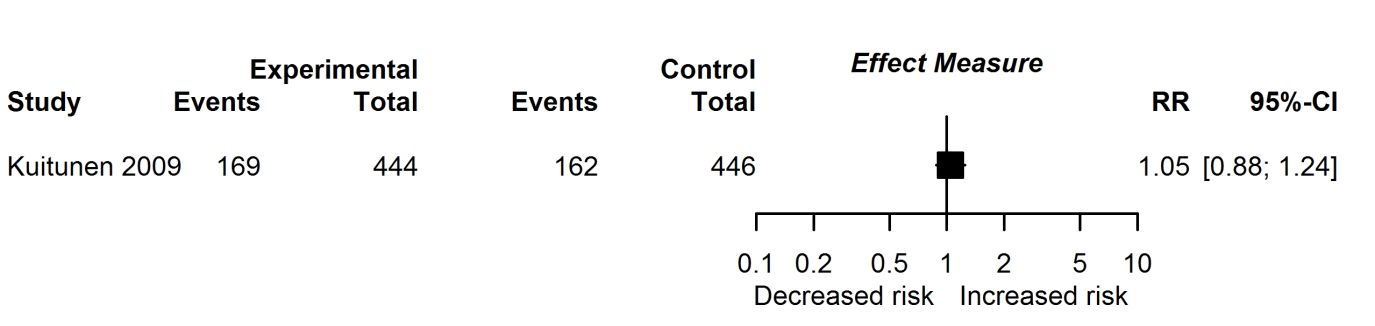


Figure 4 Prebiotics and allergic sensitisation to any food


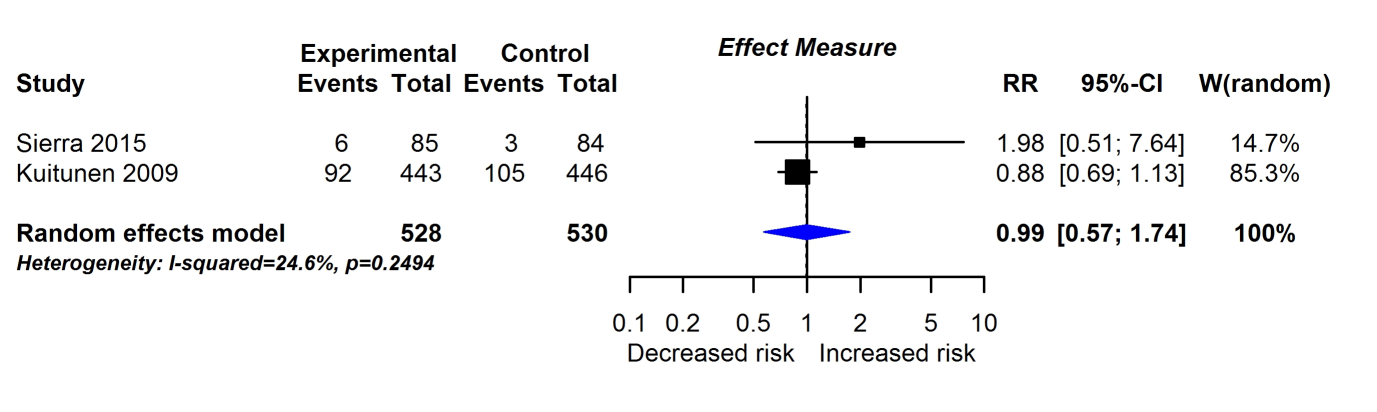


Figure 5 Prebiotics and allergic sensitisation to cow’s milk


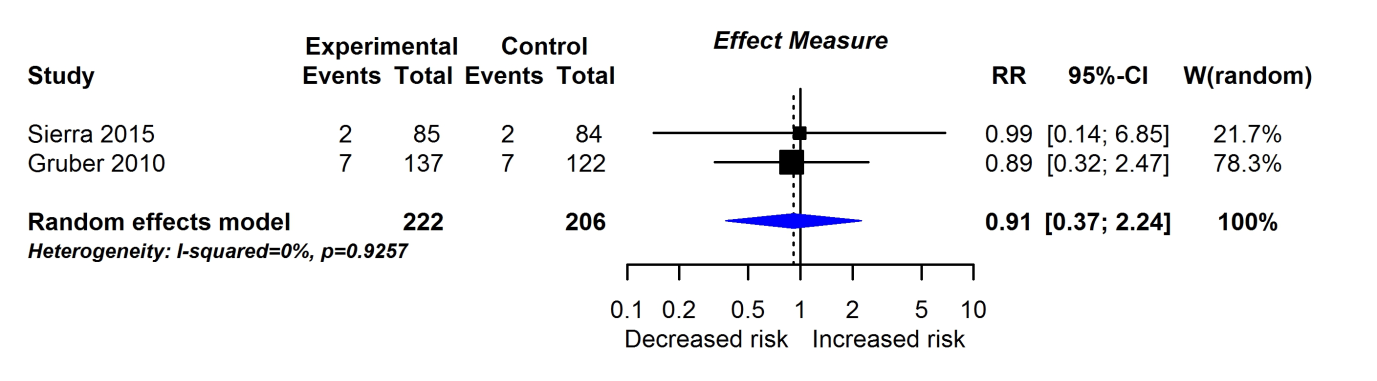


Figure 6 Prebiotics and allergic sensitisation to egg


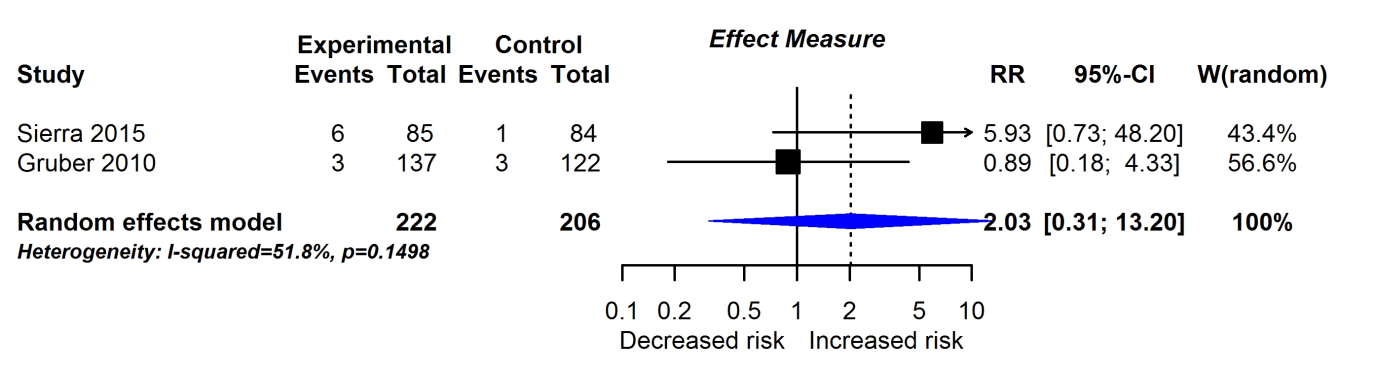


# Prebiotics and risk of food allergy

Three intervention studies investigated the effect of prebiotic supplementation on risk of food allergy. In total over 1300 participants were randomised to prebiotics or control treatment. The investigators studied infants at normal or low risk of disease. Food allergy (outcome) was assessed between 1 and 5 years of age. Studies’ outcome assessment methods were either doctor diagnosis or not reported. All studies were considered to be at high or unclear risk of attrition bias, and either high or unclear risk of conflict of interest (Figure 7).

One study reported data that could be presented in a forest plot, and showed weak evidence of reduced food allergy and cow’s milk allergy, of borderline statistical significance (Figures 8 and 9). In contrast in the study of Sierra no difference was reported in food allergy between prebiotic and control groups, but numerical data were only presented for a combined outcome of wheeze/ eczema/ food allergy, and not for food allergy as an outcome alone. In the study of Gruber no significant difference was reported between groups in food allergy – data were not presented in a form that could be included in meta-analysis.

**Overall we found no evidence that prebiotics can prevent food allergy, but data were sparse and further work is needed to confirm whether or not prebiotics can prevent food allergy.**

Figure 7 Risk of bias in intervention studies of prebiotics and food allergy

Figure 8 Prebiotics for preventing Food Allergy


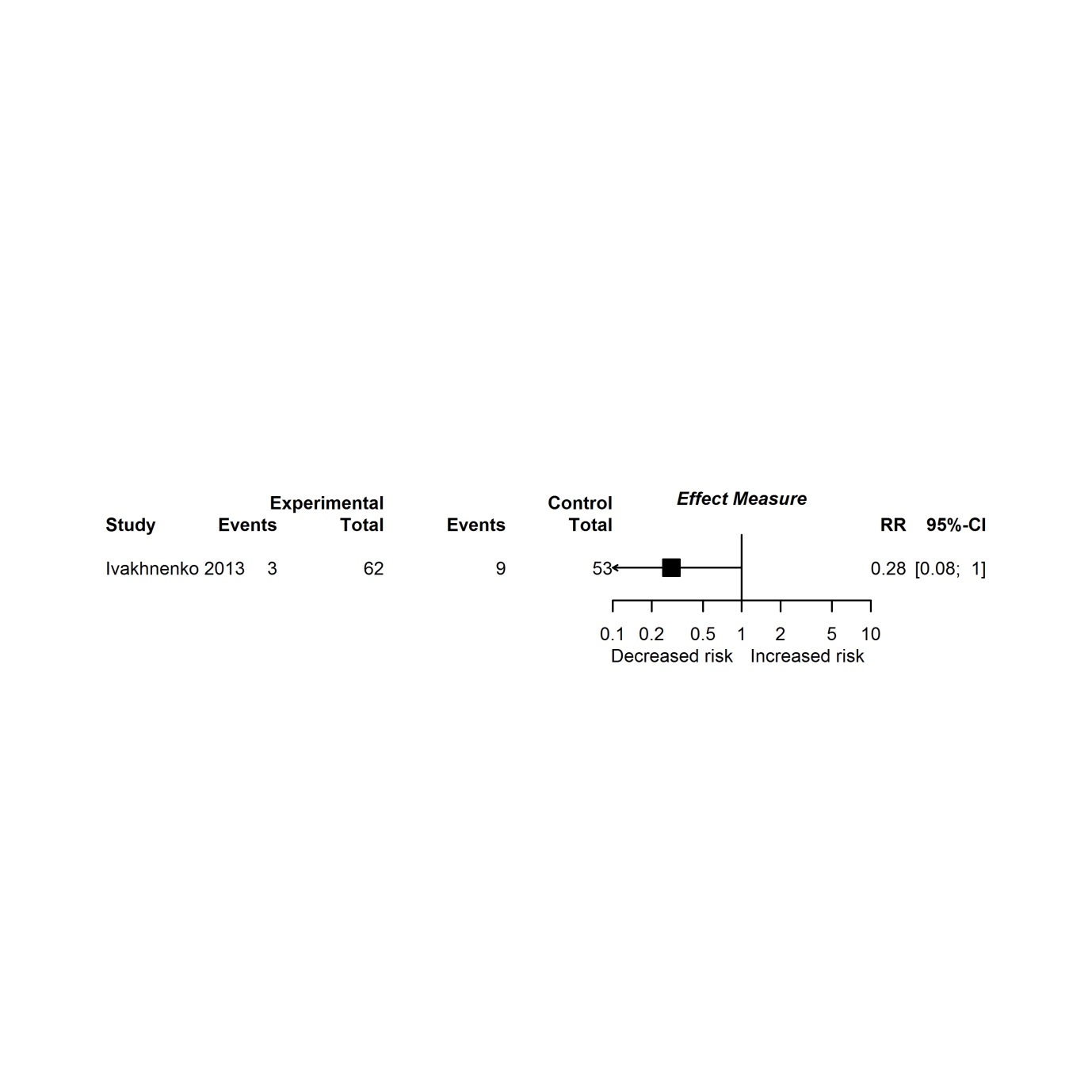


Figure 9 Prebiotics for preventing Cow’s Milk Allergy


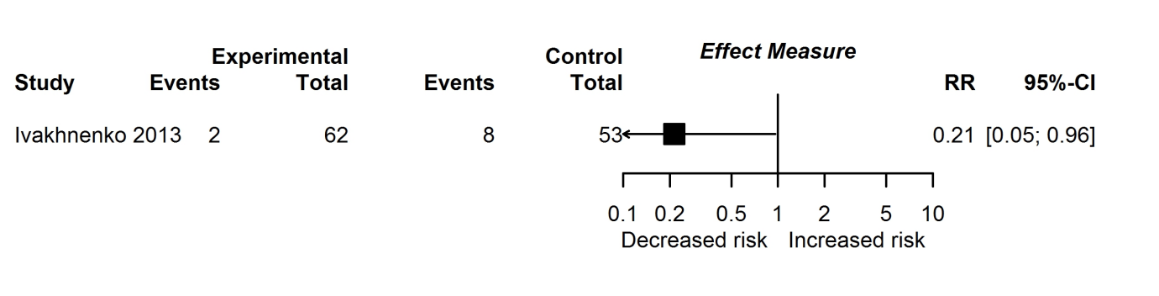


# Prebiotics and risk of eczema

One systematic review and 9 original intervention studies investigated the effect of prebiotic supplementation on risk of eczema. The systematic review identified 4 studies, with overall significantly reduced AD with moderate statistical heterogeneity (Table 2). The benefit was seen most clearly in a single small study of high risk infants using a hydrolysed formula with vs without added prebiotic (Moro/Arslanoglu).

In total over 3800 participants were randomised in the original studies. Three studied infants at high risk of disease, four in infants at normal risk, one on infants with low disease risk and in one study disease risk was unclear. Risk of eczema (outcome) was assessed between 3 months and 5 years of age. Studies used one of four different outcome assessment methods. All studies were considered to be at high or unclear risk of conflict of interest, and one-third had a high risk of attrition bias (Figure 10).

Seven original studies were eligible for meta-analysis of eczema at age ≤4, and two at age >4. Meta-analysis showed evidence for a reduction in eczema risk in children at age ≤4 with borderline statistical significance, but not at age 5-14 (Figures 11 and 12). The heterogeneity in both meta-analyses was high. Subgroup analysis for eczema at age ≤4 showed no subgroup differences.

The study of Moro/Arslanoglu also reported eczema at the earlier time-point of 6 months, where this was significantly reduced in the prebiotic group - 10/102 (9.8%) prebiotic, 24/104 (23.1%) control (p=0.01) ([1](#_ENREF_1)). The study of Kukkonen also reported atopic eczema (i.e. eczema plus positive SPT), where synbiotics reduced atopic eczema at 2 years – OR 0.66 (95%CI 0.46 – 0.95; p=0.02) but not at 5 years – OR 0.94 (0.70 – 1.28; p=0.71) ([4](#_ENREF_4), [17](#_ENREF_17)). In the study of Sierra no difference was reported in eczema between prebiotic and control groups, but data were only presented for a combined outcome of wheeze/ eczema/ food allergy, and not for eczema outcomes alone. In the study of Chien 3 participants in the synbiotic group, 9 in the prebiotic and 10 in the control group developed eczema by 3 months. The difference between synbiotic and other groups, adjusted for family history, was reported as statistically significant P<0.05. In the study of Gruber no significant difference was reported between groups in eczema at 5 years – data were not presented in a form that could be included in meta-analysis. In the study of Boyle data were also reported for cumulative incidence of eczema at 18 months, where there was no significant difference between groups RR 1.09 (0.89, 1.34); the authors also reported no difference between groups in time to first onset of eczema (P=0.81).

Evidence was downgraded to no evidence, due to concerns about study quality (-1, due to risk of bias and risk of conflict of interest in included studies); imprecision (-1, wide confidence intervals, borderline statistical significance); indirectness (-1, of study populations); and inconsistency (-1, high statistical heterogeneity) in analyses of prebiotics and AD.

**So we conclude that there is no evidence that prebiotics prevent AD, but high quality studies at low risk of bias or conflict of interest are lacking.**

Figure 10 Risk of bias in intervention studies of prebiotics and eczema

Table 2 Findings from the previous systematic review

| **Study** | **Outcome measure** | **No. participants (studies)** | **Outcome (95% CI)** | **I^2^** |
| --- | --- | --- | --- | --- |
| **Osborn** ([10](#_ENREF_10)) | AD at < 2 years* | 1220 (4) | **RR 0.68 [0.48, 0.97]** | 34% |
|  | AD at < 2 years  (high risk infants) | 134 (1) | **RR 0.49 [0.24, 1.00]** | - |
|  | AD at < 2 years  (normal/low risk infants) | 1086 (3) | RR 0.76 [0.51, 1.14] | 42% |
|  | AD at < 2 years  (fed human milk) | 92 (1) | RR 1.05 [0.41, 2.65] | - |
|  | AD at < 2 years  (fed mainly standard formula) | 994 (2) | RR 0.71 [0.45, 1.11] | 65% |
|  | AD at < 2 years  (fed mainly hydrolysed formula) | 134 (1) | **RR 0.49 [0.24, 1.00]** | - |

* the authors also analysed eczema according to type of prebiotic used, and found evidence that GOS/FOS (9:1) 6.8 grams/ and acidic OS 1.2grams/L reduced the incidence of eczema (1 trial, 830 participants, RR 0.58 95%CI 0.35, 0.97); GOS/FOS (9:1) 8grams/L was also associated with reduced eczema (1 trial, 134 participants, RR 0.49 95% CI 0.24, 1.00) whereas other prebiotics namely polydextrose and GOS 4g/L, polydextrose, GOS and lactulose 8grams/L and GOS/FOS and acidic OS (4:1) 1.5 grams/kg/day were not associated with a significant effect when analysed alone.

Figure 11 Prebiotics for preventing eczema at age ≤4 years


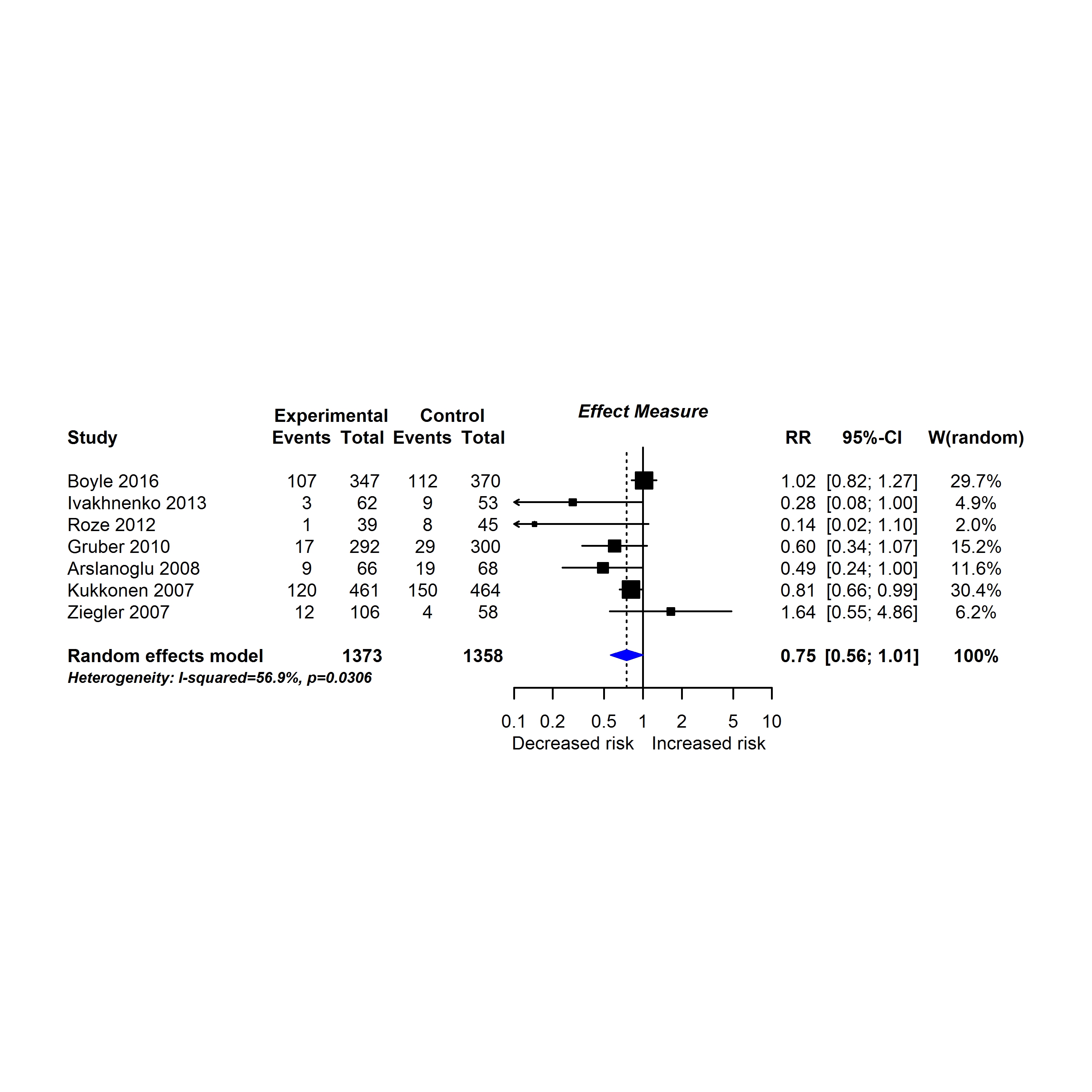


Figure 12 Prebiotics for preventing eczema at age 5-14 years


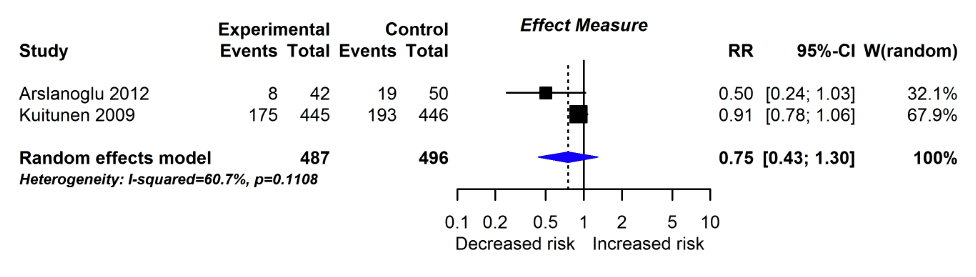


.

**Table 3 Subgroup analysis for prebiotics and risk of AD at age <4 years**

|  | | **Number of studies** | **RR [95% CI]** | **I^2^ (%)** | **P-value for between groups difference** |
| --- | --- | --- | --- | --- | --- |
| Risk of disease – High  Risk of disease – Normal/Low | 3  4 | | 0.84 [0.64-1.10]  0.56 [0.24-1.26] | 60  54 | 0.35 |
| Intervention – prebiotic  Intervention – synbiotic | 5  2 | | 0.74 [0.44-1.22]  0.46 [0.09-2.27] | 67  64 | 0.59 |
| Overall risk of bias – Low  Overall risk of bias – High/Unclear | 2  5 | | 0.50 [0.08-3.24]  0.69 [0.48-0.98] | 72  40 | 0.74 |
| Conflict of interest bias – Low  Conflict of interest bias – High/Unclear | 0  7 | | -  0.75 [0.56-1.01] | --  57 | - |

# Prebiotics and risk of wheeze

One systematic review including 2 trials, and five original intervention trials investigated the effect of prebiotics on risk of wheezing or asthma. The systematic review found reduced wheezing in a subgroup analysis which included one study (Moro/Arslonagu) of high risk infants, but did not find an overall reduction in wheezing in the main analysis (Table 4).

In the original studies a combined total of over 2700 children were allocated to prebiotics or control treatment. All original studies were carried out in Europe, and in three studies children were at high risk of disease. Outcomes were assessed at 1 to 5 years, using different definitions of wheeze/asthma.

All studies had a high risk of conflict of interest, and 20% had a high risk of attrition bias (Figure 12). Three studies reported data eligible for meta-analysis, one in children below the age of 4, one over this age, and one at both age groups. In younger children (below 4 years old, Figure 13) there was evidence of a lower risk of recurrent wheeze if infants were fed with prebiotics (RR 0.39; 95% CI 0.20 – 0.77) in two studies with no statistical heterogeneity (I^2^=0%). However this was not evident in older children (RR 0.90; 95% CI 0.66 – 1.23; figure 14) nor in data that could not be included in meta-analysis (discussed below). Both studies included in Figure 13 were in selected populations – one in infants who already had AD, one in infants fully formula fed prior to 6 weeks age. Both were at high risk of conflict of interest, and one at high overall risk of bias. This evidence was downgraded for study quality, inconsistency, imprecision and indirectness and is therefore not supportive of a relationship between prebiotic supplementation and reduced risk of recurrent wheezing.

*Data that could not be included in meta-analyses*

Analysis of a subgroup from the study of Kukkonen and colleagues found doctor diagnosis of asthma at age 5 in 4/64 (6.3%) children from the synbiotic group, and 4/67 (6.0%) control (p=1.0) ([18](#_ENREF_18)), and no difference between groups in atopic asthma (ie asthma associated with positive SPT or sIgE) – OR 1.09 (95%CI 0.69 – 1.71; p=0.39). In the study of Van der Aa, there was a trend to reduced prevalence of ‘wheezing without colds’ (p=0.06) and significantly reduced ‘wheezing +/- noisy breathing’ (p<0.01) in the first year in synbiotic treated infants ([3](#_ENREF_3)). In the study of Sierra no difference was reported in wheeze between prebiotic and control groups, but data were only presented for a combined outcome of wheeze/ eczema/ food allergy, and not for wheeze outcomes alone – so data from this study could not be included in meta-analysis. In the study of Gruber no significant difference was reported between groups in doctor diagnosed asthma – data were not presented in a form that could be included in meta-analysis.

**Overall we found no evidence that prebiotics reduce risk of wheezing or recurrent wheezing.**

Figure 13 Risk of bias in intervention studies of prebiotics and wheeze

Table 4 Findings from the previous systematic review

| **Study** | **Outcome measure** | **No. participants (studies)** | **Outcome (95% CI)** | **I^2^** |
| --- | --- | --- | --- | --- |
| **Osborn** ([10](#_ENREF_10)) | Wheeze at < 2 years* | 226 (2) | RR 0.70 (0.41, 1.19) | 34% |
|  | Wheeze at < 2 years  (high risk infants) | 134(1) | **RR 0.37 (0.14, 0.96)** | - |
|  | Wheeze at < 2 years  (normal/low risk infants) | 92 (1) | RR 1.07 (0.56, 2.06) | 42% |
|  | Wheeze at < 2 years  (fed human milk) | 92 (1) | RR 1.07 (0.56, 2.06) | - |
|  | Wheeze at < 2 years  (fed mainly standard formula) | 0 (0) | - | 65% |
|  | Wheeze at < 2 years  (fed mainly hydrolysed formula) | 134 (1) | **RR 0.37 (0.14, 0.96)** | - |

* the authors also analysed wheeze according to specific prebiotic used, and found no evidence that any specific prebiotic is effective at reducing incidence of infant wheeze.

Figure 14 Prebiotics for preventing recurrent wheeze at age ≤ 4 years


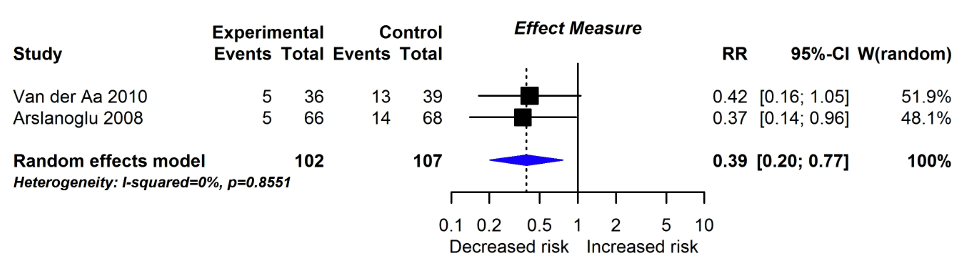


Figure 15 Prebiotics for preventing recurrent wheeze at age 5-14 years


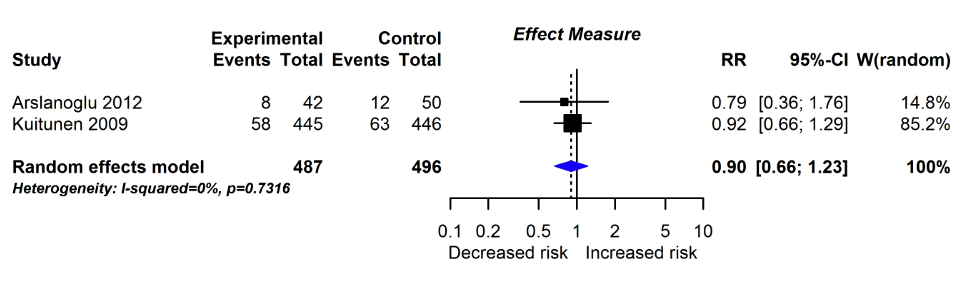


# Prebiotics and risk of allergic rhinitis

Three intervention trials assessed the effect of prebiotic supplementation on risk of allergic rhinitis. All were European studies, two in high risk populations and one in a low risk population. In total, over 1100 infants were allocated to prebiotics, and 1100 to a control treatment. The interventions lasted for 6 months, and the outcome was measured at age 5 years. The risk of conflict of interest was high in all studies, and one study had a high risk of attrition bias (Figure 15). The combined effect of prebiotic supplementation on risk of allergic rhinitis from two studies is shown in Figure 16. There was no evidence that prebiotics reduce the risk of allergic rhinitis (RR 0.71; 95% CI 0.2, 2.34), with high statistical heterogeneity. In the study of Gruber no significant difference was reported between groups in doctor diagnosed hayfever – data were not presented in a form that could be included in meta-analysis.

**Overall we found no evidence that prebiotics influence risk of allergic rhinitis, in high risk infants.**

Figure 16 Risk of bias in intervention studies of prebiotics and risk of allergic rhinitis

Figure 17 Prebiotics for preventing allergic rhinitis at age 5-14 years


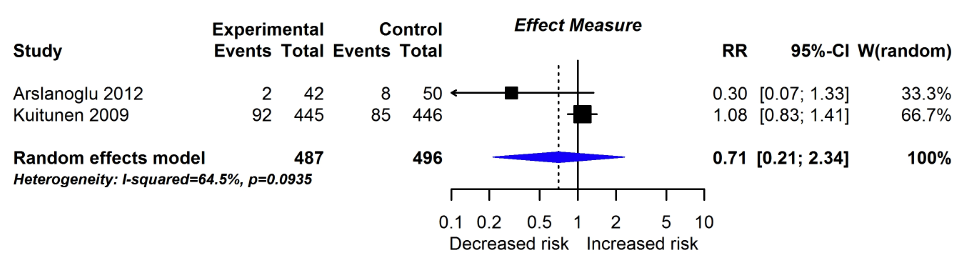


# Conclusions

In this systematic review of prebiotic supplementation for reducing risk of allergic outcomes, we found no evidence that prebiotics prevent wheeze, allergic sensitisation, allergic rhinitis or food allergy. Most studies identified had high risk of conflict of interest due to direct industry involvement in the trials, and a significant number of studies had high risk of attrition bias.

Our findings are similar to those of Osborn and colleagues ([22](#_ENREF_22)), who conducted a Cochrane systematic review of prebiotics for preventing allergic outcomes which was included in our overview of recent systematic reviews in summer 2013. They included one trial in premature infants, which we excluded based on our study protocol which excludes populations which clearly represent <5% of the UK population, and they did not include some of the other publications identified in our systematic review. Osborn et al found that prebiotic supplementation reduced eczema risk in the first 2 years (RR 0.68; 95% CI 0.48 – 0.97) with moderate statistical heterogeneity (I^2^ = 34%). In our analysis, which excluded one of the trials in Osborn’s analysis, included two that were excluded by Osborn (Roze 2012; Kukkonen 2007) and included further trials published since Osborn’s search, this effect was of borderline statistical significance at age 0-4 and not seen at all at age 5-14. Heterogeneity in our analysis was higher (I^2^ = 57%), and all studies contributing to AD meta-analysis were considered to be at high risk of conflict of interest. We therefore judge that there is no evidence that prebiotics reduce AD risk.

Osborn also found a protective effect for wheeze in the first 2 years in infants at high risk of atopy based on one study (RR 0.37, 95% CI 0.14 – 0.96), but did not find a protective effect in all infants (2 studies; RR 0.70; 95%CI 0.41 – 1.19; I^2^=70%). They concluded that further research is needed and the results should be interpreted with caution, due to the high risk of bias – citing attrition bias and possible publication bias – they identified several prebiotic trials where allergic outcomes may have been captured but had not yet been reported. We found a reduced risk of recurrent wheeze at aged 0-4 years in one analysis, based on two small studies (the same one as Osborn identified, and one extra). We did not however find an effect at age 5-14 years, and we downgraded this to no evidence due to inconsistency with other studies that could not be included in the meta-analysis, study quality, indirectness due to the specific study populations, and imprecision. Our updated search on 26^th^ February 2017 identified a further 2 systematic reviews of prebiotics or synbiotics for prevention of allergic outcomes. R-AMSTAR scoring identified no further high quality reviews. Dang 2013 ([23](#_ENREF_23)) identified 18 trials of prebiotics, probiotics or synbiotics for preventing eczema (our review identified 29 such trials). They did not find any effect of prebiotics on AD risk or risk of allergic sensitisation, and did not assess later timepoints or other outcomes. De Moura 2013 ([24](#_ENREF_24)) - included both treatment and prevention studies in the same analysis which may not be appropriate – and failed to identify several of the studies included in our review.

Taken together, the results of the current systematic review do not provide evidence to support the use of prebiotics for preventing allergic sensitisation or disease. The findings have largely arisen from industry-sponsored studies undertaken in populations of infants with very limited or no breastfeeding, and often with significant post-randomisation losses/exclusions. Given the very high levels of prebiotics naturally present in human breast milk, the role of prebiotics in infant nutrition is likely to be as an additive to formula milk in formula-fed infants, rather than as a supplement for all infants, but an allergy-prevention effect remains to be proven.

**Conclusions:**

**Current evidence does not support an effect for prebiotics in reducing risk of wheeze, allergic sensitisation, AD, food allergy or allergic rhinitis.**

# References

1. Moro G, Arslanoglu S, Stahl B, Jelinek J, Wahn U, Boehm G. A mixture of prebiotic oligosaccharides reduces the incidence of atopic dermatitis during the first six months of age. Archives of Disease in Childhood. 2006;91(10):814-9.

2. Gruber C, Van Stuijvenberg M, Mosca F, Moro G, Chirico G, Braegger CP, et al. Reduced occurrence of early atopic dermatitis because of immunoactive prebiotics among low-atopy-risk infants. Journal of Allergy and Clinical Immunology. 2010;126(4):791-7.

3. Van Der Aa L, Heymans H, Van Aalderen W, Sillevis Smitt H, Nauta A, Knippels L, et al. Specific synbiotic mixture prevents asthma-like symptoms in infants with atopic dermatitis. Allergy: European Journal of Allergy and Clinical Immunology. 2010;65:313.

4. Kukkonen K, Savilahti E, Haahtela T, Juntunen-Backman K, Korpela R, Poussa T, et al. Probiotics and prebiotic galacto-oligosaccharides in the prevention of allergic diseases: a randomized, double-blind, placebo-controlled trial. Journal of Allergy & Clinical Immunology. 2007;119(1):192-8.

5. Roze JC, Barbarot S, Butel MJ, Kapel N, Waligora-Dupriet AJ, De Montgolfier I, et al. An alpha-lactalbumin-enriched and symbiotic-supplemented v. a standard infant formula: A multicentre, double-blind, randomised trial. British Journal of Nutrition. 2012;107(11):1616-22.

6. Ziegler E, Vanderhoof JA, Petschow B, Mitmesser SH, Stolz SI, Harris CL, et al. Term infants fed formula supplemented with selected blends of prebiotics grow normally and have soft stools similar to those reported for breast-fed infants. Journal of Pediatric Gastroenterology & Nutrition. 2007;44(3):359-64.

7. Ivakhnenko OS, Nyankovskyy SL. Effect of the specific infant formula mixture of oligosaccharides on local immunity and development of allergic and infectious disease in young children: Randomized study. Pediatria Polska. 2013;88(5):398-404.

8. Sierra C, Bernal MJ, Blasco J, Martinez R, Dalmau J, Ortuno I, et al. Prebiotic effect during the first year of life in healthy infants fed formula containing GOS as the only prebiotic: a multicentre, randomised, double-blind and placebo-controlled trial. European Journal of Nutrition. 2015;54(1):89-99.

9. Chien CM, Anne GEN, Chin CW, Rao R, Charmaine C, Lay C, et al. A synbiotic mixture of scGOS/lcFOS and Bifidobacterium breve M-16V is able to restore the delayed colonization of bifidobacterium observed in C-section Delivered Infants. Journal of pediatric gastroenterology and nutrition [Internet]. 2016; 62:[681 p.]. Available from: <http://onlinelibrary.wiley.com/o/cochrane/clcentral/articles/821/CN-01167821/frame.html>.

10. Osborn DA, Sinn JK. Prebiotics in infants for prevention of allergy. The Cochrane database of systematic reviews. 2013;3:CD006474.

11. van Hoffen E, Ruiter B, Faber J, M'Rabet L, Knol EF, Stahl B, et al. A specific mixture of short-chain galacto-oligosaccharides and long-chain fructo-oligosaccharides induces a beneficial immunoglobulin profile in infants at high risk for allergy. Allergy. 2009;64(3):484-7.

12. Arslanoglu S, Moro GE, Schmitt J, Tandoi L, Rizzardi S, Boehm G. Early dietary intervention with a mixture of prebiotic oligosaccharides reduces the incidence of allergic manifestations and infections during the first two years of life. Journal of Nutrition. 2008;138(6):1091-5.

13. Arslanoglu S, Moro GE, Boehm G, Wienz F, Stahl B, Bertino E. Early neutral prebiotic oligosaccharide supplementation reduces the incidence of some allergic manifestations in the first 5 years of life. Journal of Biological Regulators & Homeostatic Agents. 2012;26(3 Suppl):49-59.

14. Boyle RJ, Brown N, Chiang WC, Chien CM, Gold M, Hourihane J, et al. Partially hydrolysed prebiotic supplemented whey formula for the prevention of allergic manifestations in high risk infants: a multicentre double blind randomised controlled trial. Clin Trans Allergy. 2015;5(Suppl 3):P30.

15. Boyle RJ, Tang MLK, Chiang WC, Chua MC, Ismail I, Nauta A, et al. Prebiotic-supplemented partially hydrolysed cow's milk formula for the prevention of eczema in high-risk infants: a randomized controlled trial. Allergy. 2016;71(5):701-10.

16. Grüber C, Stuivenberg M, Mosca F, Moro G, Chirico G, Braegger CP, et al. Immunoactive prebiotics transiently prevent occurrence of early atopic dermatitis among low-atopy-risk infants. The Journal of allergy and clinical immunology [Internet]. 2015; 136(6):[1696-8.e1 pp.]. Available from: <http://onlinelibrary.wiley.com/o/cochrane/clcentral/articles/673/CN-01169673/frame.html>.

17. Kuitunen M, Kukkonen K, Juntunen-Backman K, Korpela R, Poussa T, Tuure T, et al. Probiotics prevent IgE-associated allergy until age 5 years in cesarean-delivered children but not in the total cohort. Journal of Allergy & Clinical Immunology. 2009;123(2):335-41.

18. Kukkonen AK, Kuitunen M, Savilahti E, Pelkonen A, Malmberg P, Makela M. Airway inflammation in probiotic-treated children at 5 years. Pediatric Allergy & Immunology. 2011;22(2):249-51.

19. Knipping K, Orsi A, Boehm G, Castoldi F, Garssen J, Gianni M, et al. Explorative evaluation of microbial and immune biomarkers in infants at risk for allergies fed an intact cow's-milk formula-Containing specific nondigestible carbohydrates. Journal of Pediatric Gastroenterology and Nutrition. 2011;52:E90.

20. Nauta AJ, Arsnalognu S, Boehm G, Moro G, Faber J, Knol E, et al. A specific mixture of short-chain galacto-oligosaccharides and long-chain fructo-oligosaccharides induced an anti-allergic Ig profile in infants at risk for allergy. Proceedings of the Nutrition Society. 2008;67(OCE):E82.

21. Gruber C, van Stuijvenberg M, Mosca F, Moro G, Chirico G, Braegger CP, et al. Reduced occurrence of early atopic dermatitis because of immunoactive prebiotics among low-atopy-risk infants. Journal of Allergy & Clinical Immunology. 2010;126(4):791-7.

22. Osborn DA, Sinn JK. Prebiotics in infants for prevention of allergy. Cochrane Database of Systematic Reviews. 2013;3:CD006474.

23. Dang D, Zhou W, Lun ZJ, Mu X, Wang DX, Wu H. Meta-analysis of probiotics and/or prebiotics for the prevention of eczema. Journal of International Medical Research. 2013;41(5):1426-36.

24. de Moura PN, Rosario Filho NA. The use of prebiotics during the first year of life for atopy prevention and treatment. Immunity, Inflammation and Disease. 2013;1(1):63-9.
