# Supplementary material for: Diet during pregnancy and infancy and risk of allergic or autoimmune disease: A systematic review and meta-analysis
Source: PLoS Med. 2018 Feb 28;15(2):e1002507. doi: 10.1371/journal.pmed.1002507 (PMC5830033; doi:10.1371/journal.pmed.1002507)
Supplement: S2 Data — (ZIP) [file pmed.1002507.s007.zip › Review_C_reports/PROBIOTICS.docx]

**PROBIOTIC SUPPLEMENTATION IN PREGNANCY, LACTATION AND/OR INFANCY, AND RISK OF ALLERGIC SENSITISATION OR ALLERGIC DISEASE**

Robert J Boyle^1^, Vanessa Garcia-Larsen^2^, Despo Ierodiakonou^3^, Jo Leonardi-Bee^4^, Tim Reeves^5^, Jennifer Chivinge^6^, Zoe Robinson^6^, Natalie Geoghegan^6^, Katharine Jarrold^6^, Andrew Logan^6^, Annabel Groome^6^ , Evangelia Andreou^7^, Nara Tagiyeva-Milne^8^, Ulugbek Nurmatov^9^, Sergio Cunha^10^

^1^ Clinical Senior Lecturer, Section of Paediatrics ^2^ Post-Doctoral Research Associate, Respiratory Epidemiology and Public Health, National Heart and Lung Institute ^3^ Post-Doctoral Research Associate, Departments of Paediatric and Respiratory Epidemiology and Public Health Group, all at Imperial College London

^4^Associate Professor of Community Health Sciences, University of Nottingham

^5^ Research Support Librarian, Faculty of Medicine, Imperial College London

^6^ Undergraduate medical students, Imperial College London

^7^Research Associate, Imperial Consultants

^8^Research Fellow, University of Aberdeen

^9^Research Fellow, University of Edinburgh

^10^Research Associate, Respiratory Epidemiology and Public Health, National Heart and Lung Institute, Imperial College London

Imperial College London

**Table of Contents**

[List of Figures 3](#_Toc423291416)

[1. Probiotics and risk of allergic outcomes – summary of interventions and findings 5](#_Toc423291417)

[2. Probiotics and allergic sensitisation 20](#_Toc423291418)

[3. Probiotics and risk of food allergy 34](#_Toc423291419)

[4. Probiotics and risk of AD 38](#_Toc423291420)

[5. Probiotics and risk of allergic rhinitis 48](#_Toc423291421)

[6. Probiotics and risk of wheeze or asthma 53](#_Toc423291422)

[Conclusions 65](#_Toc423291423)

[References 68](#_Toc423291424)

#

# List of Figures

[Figure 1 Risk of bias in intervention studies of probiotics and allergic sensitisation 22](#_Toc497234488)

[Figure 2 Probiotics and risk of AS to any allergen 23](#_Toc497234489)

[Figure 3 Risk of publication bias: probiotics and AS to any allergen 25](#_Toc497234490)

[Figure 4 Probiotics and risk of AS to any aeroallergen 26](#_Toc497234491)

[Figure 5 Probiotics and risk of AS to any food 26](#_Toc497234492)

[Figure 6 Risk of publication bias: probiotics and AS to any food allergen 29](#_Toc497234493)

[Figure 7 Probiotics and risk of AS to CM 30](#_Toc497234494)

[Figure 8 Probiotics and risk of AS to egg 30](#_Toc497234495)

[Figure 9 Probiotics and risk of AS to peanut 30](#_Toc497234496)

[Figure 10 Probiotics and total IgE 33](#_Toc497234497)

[Figure 11 Risk of bias in intervention studies of probiotics and food allergy 35](#_Toc497234498)

[Figure 12 Probiotics and risk of any food allergy at age ≤ 4 years old 35](#_Toc497234499)

[Figure 13 Probiotics and risk of any food allergy at age 5-4 years old 35](#_Toc497234500)

[Figure 14 Probiotics and risk of CMA at age ≤ 4 years old 37](#_Toc497234501)

[Figure 15 Risk of bias in intervention studies of probiotics and AD 40](#_Toc497234502)

[Figure 16 Probiotics and risk of AD at age ≤ 4 years - RCT 41](#_Toc497234503)

[Figure 17 Probiotics and risk of AD at age ≤ 4 years - CCT 41](#_Toc497234504)

[Figure 18 Risk of publication bias: probiotics and AD at age ≤4 43](#_Toc497234505)

[Figure 19 Probiotics and risk of atopic AD at age ≤ 4 years 44](#_Toc497234506)

[Figure 20 Risk of publication bias: probiotics and atopic AD at age ≤4 44](#_Toc497234507)

[Figure 21 Probiotics and risk of AD at age 5-14 years 46](#_Toc497234508)

[Figure 22 Probiotics and risk of atopic AD at age 5-14 years 46](#_Toc497234509)

[Figure 23 Risk of publication bias: probiotics and AD at age 5 to 14 years 46](#_Toc497234510)

[Figure 24 Risk of bias in intervention studies of probiotics and allergic rhinitis 49](#_Toc497234511)

[Figure 25 Probiotics and risk of allergic rhinitis at ≤ 4 years 49](#_Toc497234512)

[Figure 26 Probiotics and risk of allergic rhinitis at age 5-14 years 50](#_Toc497234513)

[Figure 27 Risk of publication bias: probiotics and AR at age 5 to 14 years 50](#_Toc497234514)

[Figure 28 Risk of bias in intervention studies of probiotics and wheeze 55](#_Toc497234515)

[Figure 29 Probiotics and risk of any wheeze at age ≤ 4 years – RCT 56](#_Toc497234516)

[Figure 30 Risk of publication bias: probiotics and wheeze at age ≤ 4 years 56](#_Toc497234517)

[Figure 31 Probiotics and risk of any wheeze at age ≤ 4 years - CCT 57](#_Toc497234518)

[Figure 32 Probiotics and risk of recurrent wheeze at age ≤ 4 years 57](#_Toc497234519)

[Figure 33 Probiotics and risk of any wheeze at age 5-14 years 60](#_Toc497234520)

[Figure 34 Risk of publication bias: probiotics and wheeze at age 5-14 years 60](#_Toc497234521)

[Figure 35 Probiotics and risk of recurrent wheeze at age 5-14 years 61](#_Toc497234522)

[Figure 36 Probiotics and lung function at age 5-14 (FEV1<80% predicted) 64](#_Toc497234523)

[Figure 37 Probiotics and lung function at age 5-14 (FEV1 reversibility >12%) 64](#_Toc497234524)

# Probiotics and risk of allergic outcomes – summary of interventions and findings

Probiotics are traditionally defined as live microorganisms which, when administered in adequate amounts, confer a health benefit on the host. Synbiotics are defined as a combination of probiotic and prebiotic administered to the same individual. In this analysis we included studies of any microbial intervention, whether live or heat-killed, given either alone (=probiotic) or in combination with a prebiotic (=synbiotic). We included heat-killed microbes for two reasons. First observational studies exploring the hygiene hypothesis have found relationships between both live microbial exposures, and non-live microbial components, and allergic outcomes. Second there is a lack of good understanding of the mechanisms through which probiotics might prevent allergic outcomes, and while microbes may need to be live for some health indications of probiotics, it is unclear whether this is the case when they are used for the prevention of allergic outcomes. We planned to undertake subgroup/stratified analyses for meta-analyses which included >5 studies, and where relevant to include probiotic/synbiotic intervention as a subgroup. We planned to assess publication bias using Funnel plots and Egger’s test where there were ≥10 studies in a meta-analysis. Due to the significant number of studies and participants included in intervention trials of reasonable quality, we did not analyse the very small number of observational studies which we identified reporting probiotic intake in relation to allergic outcomes, in keeping with the hierarchical approach outlined in the review protocols. In total we identified two high quality systematic reviews in our July 2013 literature search – data were extracted from these reviews, and included in relevant sections of this report. In our updated search on 26^th^ February 2017, we identified three further high quality systematic reviews – the findings of these reviews are referred to in the Conclusions section of this report. In total we identified 28 original trials (27 RCT, 1 CCT) investigating the effect of 30 different probiotic interventions during infancy and/or pregnancy on allergic outcomes.

*Interventions used*

Characteristics of studies are shown in Table 1, and interventions used are summarised in Table 2. Below is a brief description of the intervention for each original study:

The **Abrahamsson** study ([1](#_ENREF_1)) used 1 x 10^8^ CFU/day freeze-dried *Lactobacillus reuteri* (strain American Type Culture Collection 55730; BioGaia AB, Stockholm, Sweden), suspended in three-quarters refined coconut oil and one-quarter refined peanut oil containing cryo-protective components. This was given to pregnant mothers from week 36 until delivery, and then to the infants until 12 months of age. The **Allen** study ([2](#_ENREF_2)) administered 2 strains of lactobacilli and 2 strains of bifidobacteria to pregnant mothers from week 36 and to their offspring until six months of age. The study by **Boyle** and colleagues ([3](#_ENREF_3)) used 1.8 x 10^10^ colony forming units (cfu)/day of *L. rhamnosus* GG (LGG; American Type Culture Collection 53103; Dicofarm, Italy) each morning from 36 weeks gestation until delivery. The Cabana study used 10^10^ cfu/day of LGG to infants for the first 6 months ([4](#_ENREF_4)). The Chien study used B. breve M-16V (7.5x10^8^CFU/100ml) for the first 4 months to infants, within a formula milk, with added prebiotic ([5](#_ENREF_5)). The **De Leon** study ([6](#_ENREF_6)) used lactobacillus/bifidobacterium strains given daily for 4 months to infants or their breastfeeding mothers. The **Dotterud** study ([7](#_ENREF_7)) used 250 mL probiotic low fat fermented milk, for 4 months, from 36 weeks prenatally to 3 months of age while breastfeeding (i.e. mother only). The probiotic milk, Biola (Tine BA, Oslo, Norway), contained LGG, *Bifidobacterium animalis* subsp. lactis Bb-12 (Bb-12) and *L. acidophilus* La-5 (La-5), equalling 5 x 10^10^ cfu of LGG and Bb-12, and 5 x 10^9^ of La-5 per day for its entire shelf life. The **Enomoto** study ([8](#_ENREF_8)) used 5 x 10^9^ cfu *B. longum* BB536 (ATCC BAA-999) and 5 x 10^9^ cfu *B. breve* M-16V given to pregnant women from 36 weeks gestation then for 6 months to the infant. The **Huurre** study ([9](#_ENREF_9)) used 1 x 10^10^ cfu/day each of LGG (ATCC 53103, Valio Ltd, Helsinki, Finland) and *B. lactis* Bb12 (Chr. Hansen, Horsholm, Denmark). The **Kalliomaki** study ([10](#_ENREF_10)) used 2 x 10^10^ cfu of LGG (Valio Ltd; Helsinki, Finland) daily for 2–4 weeks before expected delivery and 6 months after birth (to mothers if breastfeeding, or to infants if not). The **Kim** study ([11](#_ENREF_11)) used a mixture of *B. bifidum* BGN4 (1.6 x 10^9^ cfu), *B. lactis* AD011 (1.6 x 10^9^ cfu), and *L. acidophilus* AD031 (1.6 x 10^9^ cfu) (Bifido Inc., Hongchungun, Korea) daily from 8 weeks before expected delivery to 3 months after delivery to mother, and from 4 to 6 months to infant. The **Kopp** study ([12](#_ENREF_12)) used 2 capsules containing 5 x 10^9^ cfu/day of LGG (American type culture collection 53103) by Infectopharm, Heppenheim, Germany. Mothers consumed this from 2-4 weeks before delivery until 6 months after birth. The **Kukkonen** study ([13](#_ENREF_13)) used a probiotic during the last 2-4 weeks of pregnancy – LGG (ATCC 3103), 10^10^ cfu/day; *L.rhamnosus* LC705 (DSM 7061), 1010 cfu/day; *B. breve* Bb99(DSM 13692), 4 x 10^8^ cfu/day; and *Propionibacterium freudenreichii* ssp. shermanii JS(DSM 7076), 4 x 10^9^ cfu/day (Valio, Helsinki, Finland). Infants then received the same probiotics with 20 drops of syrup containing 0.8 g GOS prebiotic daily for 6 months. The **Lau** study used heat-killed *Escherichia coli* Symbio DSM 17252 and *E faecalis* Symbio DSM 16440 4.5-13.5 x 10^7^ cfu/day to the infant only, from 5 weeks age to 7 months age. The **Lodinová-Žádníková** study ([14](#_ENREF_14)) gave 0.8 x 10^9^ cfu *E. coli* 3 times per weeks to infants from <48 hours age to 4 weeks (Dyntec. Co. (Terezín, Czech Republic)). The Lundelin study used L. rhamnosus GG, B. lactis, L. paracasei ST11, B.longum BL999 for mothers and infants ([15](#_ENREF_15)). In the study of **Morisset** and colleagues ([16](#_ENREF_16)), participants were fed a fermented formula without live bacteria (FWLB), given from birth -or weaning- to 12 month of age. During breastfeeding, mothers ingested the same formula as their child. In the study of **Niers** ([17](#_ENREF_17)), pregnant mothers during the last 6 weeks of pregnancy and their infants for 12 months, were fed 1 x 10^9^ cfu each of *B. bifidum* W23, B. lactis W52 and *Lactococcus lactis* W58 (Ecologic Panda, Winclove Bio Industries B.V. In the **Ou** study ([18](#_ENREF_18)), the authors used LGG (ATCC 53103; 1 x 10^10^ cfu/day; Valio Ltd.) beginning from 24 weeks gestation (second trimester) of pregnancy until delivery. After delivery, LGG was administered exclusively to breastfeeding mothers or to non-breastfeeding neonates, where it was mixed with water and given by spoon for 6 months. In the **Prescott** study ([19](#_ENREF_19)), infants were fed 3 x 10^9^ cfu/day *L. acidophilus* LAVRI-A1 in maltodextrin (Probiomics, Sydney, Australia) for six months. In the **Rautava 2006** study ([20](#_ENREF_20)), authors used an infant formula (Enfamil, Mead Johnson Nutritionals, Evansville, IN) supplemented with 1 x 10^10^ cfu of both LGG and *B. lactis* Bb-12 (Chr. Hansen, Hoersholm, Denmark), given to infants daily until 12 months of age. The same group (**Rautava 2012**) investigated the effect of probiotics in a separate intervention ([21](#_ENREF_21)), using *L. rhamnosus* LPR (CGMCC 1.3724) and *B. longum* BL999 (ATCC: BAA-999) [Nestle S.A.] at 1 x 10^9^ cfu, each given daily as a sachet diluted in a glass of water. The study of **Roze** ([22](#_ENREF_22)) used infant formula containing a synbiotic - *L. rhamnosus* LCS-742 and *B. longum* subsp infantis M63, and a 96% galactooligosaccharides (GOS)/ 4% fructooligosaccharides (FOS) prebiotic. The formula was also enriched with bovine a-lactalbumin, using whey protein concentrate, and was fed to infants for the first 6 months. The study of **Scalabrin** ([23](#_ENREF_23)) used Nutramigen plus LGG at 10^8^ cfu per gram of formula powder, given to infants for 1 year. In the study of **Soh** ([24](#_ENREF_24)), the authors used a cow’s milk-based infant formula supplemented with *B. longum* BL999 (BB536, Morinaga, Japan) 1 x 10^7^ cfu/g and *L. rhamnosus* LPR (CGMCC 1.3724) 2 x 10 ^7^ cfu/g, initiated within 12 hours of life for the first six months. The study of **Van der Aa** ([25](#_ENREF_25)) used an extensively hydrolyzed whey-based formula (Nutrilon Pepti; Nutricia, Netherlands) with synbiotics for 12 weeks. The synbiotic contained Immunofortis at 8g/L, and *B. breve* M-6V (Morinaga Milk Co, Ltd., Tokyo, Japan) 1.3 x 10^9^ cfu/100 ml. The study of **West** ([26](#_ENREF_26)) combined a daily intake of cereals with 1 x 10^8^ cfu of strain LF19 per serving (Semper AB, Stockholm, Sweden) during weaning, from 4 to 13 months of age. The study of **Wickens** ([27](#_ENREF_27)) used 6 x 10^9^ cfu/day *L rhamnosus* HN001 or 9 x 10^9^ cfu/day *B animalis* subsp lactis HN019, delivered in a freeze dried capsule (Fonterra Cooperative Group, Auckland, New Zealand). Capsule powder was given to the infant either undiluted or mixed with water, breast milk, or formula and given with a teaspoon. Mothers took the capsules from 35 weeks gestation until the end of the breastfeeding period or up to 6 months post-partum, whilst infants took it from 2-16 days of birth until 2 years of age.

*Populations and Outcomes assessed*

Outcomes studied were allergic sensitisation (total IgE, specific IgE and skin prick test (SPT)), food allergy, AD, allergic rhinitis, and wheeze/recurrent wheeze. Overall 6000 infants were randomly or non-randomly allocated to an intervention arm. Nineteen studies were in infants at high risk of allergic disease. 17 studies were carried out in Europe, 9 in Asia-Pacific and 2 in the USA. The definitions of diseases varied across studies but with few exceptions,studies used commonly accepted criteria for these definitions.

*Overall findings*

Overall the quality of evidence was moderate - there was a low or unclear risk of bias in most studies, although approximately a quarter of studies were considered to be at high risk of conflict of interest bias due to direct industry involvement. Overall we found MODERATE level evidence (-1 inconsistency) that probiotics can prevent atopic AD ie AD with associated positive skin prick test or specific IgE test (11 studies, 12 interventions; 3000 participants RR 0.78; 95% CI 0.65 to 0.92; I^2^=0%). We found MODERATE level evidence (-1 inconsistency) that probiotics can prevent *all* AD (19 studies, 21 interventions; 4700 participants; RR 0.78; 95% CI 0.68 to 0.90; I^2^=61%) in children ≤ 4 years old. The majority of the evidence in these positive meta-analyses came from studies of high risk infants i.e. those with a positive family history of allergic disease (10 of 12 interventions for atopic AD; 17 of 21 interventions for *all* AD).

We found LOW level evidence (-1 indirect outcome; -1 imprecision) that probiotics can prevent allergic sensitisation to cow’s milk, but no evidence that probiotics or synbiotics prevent allergic sensitisation to other allergens or prevent clinical cow’s milk allergy.

We found no evidence for persistence of the effect on atopic AD or *all* AD beyond age 4 years, and no evidence that probiotics prevent other allergic outcomes.

**Table 1 Characteristics of included studies evaluating probiotic supplementation and allergic outcomes**

| Study | N Intervention/ Control | Study Design | Country | Intervention & Population studied | Disease risk | Age at outcome (years) | Outcomes reported |
| --- | --- | --- | --- | --- | --- | --- | --- |
| Pelucchi ([28](#_ENREF_28)) | 14 RCT (1417 participants) | SR | - | Pregnant or lactating women or their infants; any probiotic(s) versus no probiotic | all | Up to age 12 | AD, IgE-associated AD |
| Tang ([29](#_ENREF_29)) | 11 RCT (1007 participants) | SR | - | Pregnant or lactating women or their infants; probiotic versus no probiotic | high | All | AD, asthma, allergic sensitisation to any food |
| Abrahamsson 2007 ([1](#_ENREF_1))  Abrahamsson 2013 ([30](#_ENREF_30)) | 117/ 115 | RCT | Sweden | Probiotic  Pregnant women (week 36) and infants (for 12 months) | high | 2, 7 | AD (Seymour), Wheeze (single, or ≥2 episodes), ARC (watery discharge ≥2 times with same allergen), Allergic sensitisation (SPT common allergens) |
| Allen 2012 ([2](#_ENREF_2))  Allen 2014 ([31](#_ENREF_31)) | 220/ 234 | RCT | UK | Probiotic  Pregnant women (week 36) and infants (for 6 months) | high | 2 | AD (DD), Allergic sensitisation (SPT common allergens, CM, Egg), Wheeze (unclear), ARC (unclear), Food Allergy (parent report) |
| Boyle 2011 ([3](#_ENREF_3)) | 125/ 125 | RCT | Australia | Probiotic  Pregnant women (week 36) | high | 1 | AD (UK Working party criteria), Wheeze (wheeze + loose API), Allergic sensitisation (SPT common allergens) |
| Cabana, 2015 ([4](#_ENREF_4)) | 93/ 92 | RCT | USA | Probiotic  Infants (for 6 months) | high | 2 | Wheeze (unclear); AD (unclear) |
| Chien, 2016 ([5](#_ENREF_5)) | Unclear – outcome reported in 45 (synbiotic), 39 (prebiotic), 45 (control) | RCT | Singapore | Prebiotic & Synbiotic scGOS/lcFOS (0.8g/100ml) and B. breve M-16V (7.5x108CFU/100ml), or scGOS/lcFOS (0.8g/100ml), or control formula from birth to 4 months, in mixed fed infants born by elective Caesarean | unclear | 0.4 | AD (unclear) |
| De Leon 2007 ([6](#_ENREF_6)) Simon 2007 ([32](#_ENREF_32)) | Total = 33 | RCT | Philippines | Probiotic for 4 months to infants or to their breastfeeding mothers | high | 0.5 | AD (unclear)  Allergic sensitisation (Total IgE) |
| Dotterud 2010 ([7](#_ENREF_7))  Simpson 2015 ([33](#_ENREF_33)) | 211/ 204 | RCT | Norway | Probiotic  Mothers only (36 weeks gestation to 3 months after birth) | normal | 2 | AD (UK Working party criteria), Wheeze (≥3 episodes + ICS), ARC (DD), Allergic sensitisation (SPT common allergens) |
| Enomoto 2014 ([8](#_ENREF_8)) | 130/36 | CCT | Japan | Probiotic  Pregnant women (week 36) and infants (for 6 months) | normal | 1.5 | AD (Hanifin and Rajka), Wheeze (physician assessment), ARC (physician assessment) |
| Huurre 2008 ([9](#_ENREF_9)) | 72/ 68 | RCT | Finland | Probiotic  Infants (for 6 months) | high | 1 | AD (Hanifin and Rajka),  Allergic sensitisation (SPT common allergens) |
| Kalliomaki 2001 ([34](#_ENREF_34)) Kalliomaki 2003 ([10](#_ENREF_10)) Kalliomaki 2007 ([35](#_ENREF_35))  Rautava 2002 ([36](#_ENREF_36)) | 77/ 82 | RCT | Finland | Mothers and infants; from 2–4 weeks before expected delivery and 6 months of age | high | 2 | AD (relapsing itchy lesions with typical location), Wheeze (symptoms + ICS), ARC (symptoms with allergen exposure), Food Allergy (CMA by DBPCFC), Allergic sensitisation (Total IgE and SPT/sIgE to common allergens) |
| Kim 2010 ([11](#_ENREF_11)) | 57/ 55 | RCT | Korea | Probiotic, pregnant women and infants from 4 to 6 months | high | 1 | AD (Hanifin and Rajka),  Allergic sensitisation (Total IgE and sIgE to common allergens) |
| Kopp 2008 ([12](#_ENREF_12)) | 54/ 51 | RCT | Germany | Probiotic  Mothers (2-4 weeks before birth until 3 months post birth) and infants (months 4-6) | high | 2 | AD (UK Working party criteria), Wheeze (≥5 episodes),  Allergic sensitisation (Total IgE and sIgE to common allergens) |
| Kukkonen 2007 ([13](#_ENREF_13)) Kuitunen 2009 ([37](#_ENREF_37)) Kukkonen 2011([38](#_ENREF_38)) | 610/ 613 | RCT | Finland | Synbiotic  Pregnant women (2-4 weeks before delivery) and infants up to 6 months | high | 2, 5 | AD (UK Working party criteria), Wheeze (≥2 episodes + interval symptoms), ARC (symptoms + sensitisation), Allergic sensitisation (Total IgE and SPT/sIgE to common allergens) |
| Lau 2012 ([39](#_ENREF_39)) | 303/ 303 | RCT | Germany | Probiotic  Infant from 5 weeks up to 7 months age | high | 2 | AD (Hanifin and Rajka), Allergic sensitisation (Total IgE and sIgE) |
| Lodinová-Žádníková 2010 ([40](#_ENREF_40)) | 56/ 57 | RCT | Czech Republic | Probiotic  Infants - birth to age 4 weeks | high | 1 | AD (unclear), Food allergy (unclear), Wheeze (unclear)  Allergic sensitisation (Total IgE and sIgE to common allergens) |
| Lundelin, 2016 ([15](#_ENREF_15))  Luoto, 2014 ([41](#_ENREF_41)) | 31, 31, 32 | RCT | Finland | Probiotic  Pregnant women and infants | normal | 1 | Wheeze (ISAAC); ARC ISAAC); AD (ISAAC); FA (unclear) |
| Morisset 2008 ([16](#_ENREF_16)) | 59/ 56 | RCT | France | Probiotic  Infant – from birth or weaning, to age 1 year | high | 1 | Food allergy (CMA by physician assessment)  Allergic sensitisation (sIgE to cow’s milk) |
| Niers 2009 ([17](#_ENREF_17))  Gorissen, 2014 ([42](#_ENREF_42)) | 78/ 78 | RCT | Netherlands | Probiotic  Pregnant women during the last 6 weeks of pregnancy and their infants until age 1 year | high | 2 | AD (modified ECRHS)  Allergic sensitisation (Total IgE and SPT/sIgE to common allergens)  AR (ISAAC)  Wheeze (physician assessment),  Food allergy (physician assessment), lung function (FEV1) |
| Ou 2012 ([18](#_ENREF_18)) | 95/ 96 | RCT | Taiwan | Probiotic  Pregnant women (third trimester) and then breastfeeding mothers, or directly to infants, until age 6 months | high | 3 | AD (ISAAC), Wheeze (ISAAC), ARC (ISAAC), Allergic sensitisation (sIgE to common allergens) |
| Taylor 2007 ([43](#_ENREF_43)), Prescott 2008 ([44](#_ENREF_44)) Jensen 2012 (termed Taylor 2012 in some meta-analyses) ([45](#_ENREF_45)) | 115/ 111 | RCT | Australia | Probiotic, infants until 6 months | high | 1, 2.5, 5 | AD (DD), Food allergy (any food - physician assessment), Wheeze (DD), ARC (symptoms + sensitisation), Allergic sensitisation (SPT common allergens) |
| Rautava 2006 ([20](#_ENREF_20)) | 38/ 43 | RCT | Finland | Probiotic  Infant formula; infants until age 1 year | normal | 1 | AD (Hanifin and Rajka), Food allergy (CMA by DBPCFC)  Allergic sensitisation (SPT food allergens) |
| Rautava 2012 ([21](#_ENREF_21)) | 82/ 78 | RCT | Finland | Probiotic  Infants fed daily until age 1 year | high | 2 | AD (Hanifin and Rajka),  Allergic sensitisation (SPT common allergens) |
| Roze 2012 ([22](#_ENREF_22)) | 48/ 49 | RCT | France | Synbiotic  Infants (first 6 months) | normal | 0.5 | AD (UK Working party criteria) |
| Scalabrin 2009 ([46](#_ENREF_46))  Scalabrin 2014 ([47](#_ENREF_47))  Scalabrin 2017 ([48](#_ENREF_48)) | 95/ 95 | RCT | USA | Probiotic  Infants (first 12 months) | normal | 0.4, 5 | Allergic sensitisation (sIgE to common allergens), AD (unclear), Wheeze (unclear), ARC (unclear), Food Allergy (unclear) |
| Soh 2009 ([49](#_ENREF_49))  Loo 2014 ([50](#_ENREF_50)) | 127/ 126 | RCT | Singapore | Probiotic  Cow’s milk-based infant formula supplemented with Probiotic; infants up to 6 months of age | high | 1 | AD (Seymour),  Allergic sensitisation (Total IgE and SPT/sIgE to common allergens)  Food allergy (physician assessment), AR (physician assessment) |
| Van der Aa 2010 ([51](#_ENREF_51)) | 46/ 44 | RCT | Netherlands | Synbiotic  Whey-based formula combined with Synbiotic, for 3 months | high | 1 | Wheeze (≥3 episodes),  Allergic sensitisation (Total IgE) |
| West 2009 ([26](#_ENREF_26))  West 2013 ([52](#_ENREF_52)) | 89/ 90 | RCT | Sweden | Probiotic  To infant from 4 until 13 months | normal | 1, 8-9 | AD (itchy rash with typical distribution, or DD), Wheeze (DD), ARC (DD), Allergic sensitisation (Total IgE and sIgE to common allergens) Food allergy (physician assessment) |
| Wickens 2008 ([27](#_ENREF_27))  Wickens 2012 ([53](#_ENREF_53))  Wickens 2013 ([54](#_ENREF_54)) | 341/ 171 | RCT | New Zealand | Probiotic  To pregnant women (35 weeks gestation to end up of BF or 6 months post-partum) and infants from 2-16 days of birth to 2 years | high | 2, 4, 6 | AD (UK Working party criteria), Wheeze (ISAAC), ARC (ISAAC), Allergic sensitisation (SPT common allergens) |

Seymour criteria and UK working party criteria are both modifications of the Hanifin and Rajka criteria. AR: Allergic Rhinitis. ARC: Allergic Rhinoconjunctivis. DD doctor’s diagnosis; SPT skin prick test; sIgE specific IgE; RCT randomised controlled trial; CCT controlled clinical trial; ISAAC International Study of Asthma and Allergies in Childhood; ICS inhaled corticosteroids; ECRHS European Community Respiratory Health Survey; API Asthma Predictive Index

Table 2 Probiotic(s) and daily dose used in intervention trials of probiotics/synbiotics for primary prevention of allergic outcomes

| First Author & Publication Year | Intervention |
| --- | --- |
| Abrahamsson 2007 ([1](#_ENREF_1)) | 1 x 10^8^ cfu/day freeze-dried L reuteri (strain American Type Culture Collection 55730) |
| Allen 2012 ([2](#_ENREF_2)) | 6.5 x 10^9^ cfu/day L. salivarius CUL61, 1.25 x 10^9^ cfu/day L. paracasei CUL08, 1.25 x 10^9^ cfu/day B. animalis subsp. Lactis CUL34, 1.25 x 10^9^ cfu/day B. bifidum CUL20 |
| Boyle 2011 ([3](#_ENREF_3)) | 1.8 x 10^10^ cfu/day L. rhamnosus strain GG (ATCC 53103) |
| Dotterud 2010 ([7](#_ENREF_7)) | 250 mL probiotic milk with L. rhamnosus strain GG (ATCC 53103), B. animalis subsp. lactis Bb-12 and L. acidophilus La-5 |
| Cabana, 2015 ([4](#_ENREF_4)) | Lactobacillus GG from birth to 6 months to infants at 10^10^ cfu/day |
| Chien, 2016 ([5](#_ENREF_5)) | Infant formula supplemented with scGOS/lcFOS (0.8g/100ml) and B. breve M-16V (7.5x108CFU/100ml), or formula with scGOS/lcFOS (0.8g/100ml), or control formula from birth until 4 months |
| Enomoto 2014 ([8](#_ENREF_8)) | 5 x 10^9^ cfu B. longum BB536 (ATCC BAA-999) and 5 x 10^9^ cfu B. breve M-16V |
| Huurre 2008 ([9](#_ENREF_9)) | 1 x 10^10^ cfu/day each of L. rhamnosus strain GG (ATCC 53103) and B. lactis Bb12 |
| Kalliomaki 2001 ([10](#_ENREF_10), [34-36](#_ENREF_34)) | 2 x 10^10^ cfu L. rhamnosus strain GG (ATCC 53103) |
| Kim 2010 ([11](#_ENREF_11)) | B. bifidum BGN4 [1.6 x 10 ^9^ cfu], B. lactis AD011 (1.6 x 10 ^9^ cfu), and L. acidophilus AD031 (1.6 x 10^9^ cfu) |
| Kopp 2008 ([12](#_ENREF_12)) | 2 capsules containing 5 x 10 ^9^ cfu/day L. rhamnosus strain GG (ATCC 53103) |
| Kuitunen 2009 ([37](#_ENREF_37)) | 5 x 10^9^ cfu L. rhamnosus strain GG (ATCC 53103), 5 x 10^9^ cfu L rhamnosus LC705 (DSM 7061), 2 x 10^8^ cfu B. breve Bb99(DSM 13692), 2 x 10^9^ cfu Propionibacterium freudenreichii ssp. shermanii JS(DSM 7076). Infants received 1 capsule with 20 drops of syrup containing 0.8 g GOS daily for 6 months. |
| Lau 2012 ([39](#_ENREF_39)) | Heat-killed E coli Symbio DSM 17252 and E faecalis Symbio DSM 16440 4.5-13.5 x 10^7^ cfu/day |
| Lodinová-Žádníková 2010 ([40](#_ENREF_40)) | 0.8 x 10^9^ cfu E. coli 3 times weekly to infant, from <48 hours age to 4 weeks |
| Lundelin, 2016 ([15](#_ENREF_15))  Luoto, 2014 ([41](#_ENREF_41)) | L. rhamnosus GG, B. lactis, L. paracasei ST11, B.longum BL999 given to pregnant women and their infants |
| Morisset 2008 ([16](#_ENREF_16)) | Fermented formula without live bacteria (FWLB) |
| Niers 2009 ([17](#_ENREF_17)) | 1 x 10^9^ cfu each of B. bifidum W23, B. lactis W52 and Lc. lactis W58 |
| Ou 2012 ([18](#_ENREF_18)) | L. rhamnosus strain GG (ATCC 53103) 1 x 10^10^ cfu/day |
| Rautava 2006 ([20](#_ENREF_20)) | Infant formula supplemented with 1 x 10^10^ cfu L. rhamnosus strain GG (ATCC 53103) and B. lactis Bb-12 |
| Rautava 2012 ([21](#_ENREF_21)) | L. rhamnosus LPR (CGMCC 1.3724) and B. longum BL999 (ATCC: BAA-999) at 1 x 10^9^ cfu each daily |
| Roze 2012 ([22](#_ENREF_22)) | Extensively hydrolysed casein based formula, plus L. rhamnosus strain GG (ATCC 53103) at 10e8 cfu per gram of formula powder |
| Scalabrin 2009 ([46](#_ENREF_46)) | Extensively hydrolysed casein based formula, plus L. rhamnosus strain GG (ATCC 53103) at 10e8 cfu per gram of formula powder |
| Simon 2007 ([32](#_ENREF_32)) | L./B. strains |
| Soh 2009 ([49](#_ENREF_49)) | At least 60 mL (9.26 g) a day of cow’s milk-based infant formula supplemented with [B. longum BL999 (BB536) 1 x 10^7^ cfu/g and L. rhamnosus LPR (CGMCC 1.3724) 2 x 10^7^ cfu/g |
| Prescott 2008 ([44](#_ENREF_44)) | 3 x 10^9^ cfu/day L acidophilus LAVRI-A1 |
| Van der Aa 2010 ([51](#_ENREF_51)) | Extensively hydrolyzed whey-based formula with synbiotics [B. breve M-6V (1.3 x 10^9^ cfu/100 ml and 90% scGOS / 10% lcFOS (Immunofortis ), 0.8 g/100 ml] |
| West 2009 ([26](#_ENREF_26)) | 1 x 10^8^ cfu/serving L.paracasei LF19 per serving fed to infant from 4 to 13 months age |
| Wickens 2008 ([27](#_ENREF_27)) | 6 x 10 ^9^ cfu/day L rhamnosus HN001 or 9 x 10 ^9^ cfu/day B animalis subsp lactis HN019 |

# Probiotics and allergic sensitisation

Twenty-four studies reported the effect of probiotics on allergic sensitisation in over 5000 participants. Studies reported skin prick test (SPT) or specific IgE to common allergens, SPT to food allergens, and total IgE. Children’s age at time of outcome measurement ranged between 4 months and 7 years old – as in other parts of this project, allergic sensitisation data were combined for all ages, due to limited data available when age-groups were analysed separately. Assessment bias was low in 85% of studies, and over half had a low risk of selection or attrition bias. Approximately a quarter of studies were considered to have a high risk of conflict of interest (Figure 1).

Fourteen studies (16 interventions) reported AS to any allergen. There was no evidence that probiotics reduce the prevalence of AS to any one of a panel of common allergens (Figure 2), with low statistical heterogeneity. Subgroup analyses showed no evidence of different treatment efficacy according to features of the intervention, population or risk of bias (Table 4). We found no evidence of publication bias (Figure 3). Similarly analysis of AS to aeroallergen or any food (Figures 4 and 5) did not show any evidence of a treatment effect, and subgroup analyses showed no important subgroup differences (Tables 5 and 6). We found no evidence for publication bias in the analysis of ‘sensitisation to any food’ as an outcome (Figure 6).

Analysis of allergic sensitisation to specific foods showed some evidence that sensitisation to cow’s milk may be reduced by probiotics (Figure 7) but not egg or peanut (Figures 8 and 9). Subgroup analyses for milk and egg allergy showed no evidence of different efficacy according to features of the intervention, population or risk of bias (Tables 7 and 8). It is worth noting that 7 of 8 studies included in meta-analysis of cow’s milk sensitisation were considered to be at high/unclear overall risk of bias, and 5 of 8 at high/unclear risk of conflict of interest.

Four studies (~2000 participants) which reported geometric mean total IgE could not be pooled due to extreme statistical heterogeneity, but the studies tended to show reduced total IgE in the probiotic compared to control group (Figure 10). However ten further studies (also ~2000 participants) which couldn’t be presented on a forest plot due to the nature of outcome data reported, all showed no significant difference in total IgE between probiotic and control group ([11](#_ENREF_11), [12](#_ENREF_12), [17](#_ENREF_17), [18](#_ENREF_18), [26](#_ENREF_26), [32](#_ENREF_32), [39](#_ENREF_39), [46](#_ENREF_46), [51](#_ENREF_51), [54](#_ENREF_54)).

Four studies reported some data which could not be included in meta-analyses. Huurre and colleagues ([9](#_ENREF_9)) found no effect of probiotic supplementation with L.rhamnosus on risk of positive SPT to any food at age 1 year (OR 0.92; 95% CI 0.45, 1.9; p=0.83). Rautava (2006) found no statistically significant differences in the percentage of children having a positive SPT to any food (p=0.84) ([20](#_ENREF_20)). Scalabrin and colleagues ([46](#_ENREF_46)) found no effect on sensitisation to cow’s milk using sIgE (p=0.63). Finally Kalliomaki and colleagues ([10](#_ENREF_10)) reported allergic sensitisation at 2 years (shown in Figure 2), but also reported no effect on AS-any at ages 4 and 7 ([35](#_ENREF_35)). Gorrisen ([42](#_ENREF_42)) reported outcomes from the trial of Niers ([17](#_ENREF_17)) at 6 years, and found no difference between groups in total IgE, specific IgE or skin prick test response to common allergens. Simpson reported outcomes from the trial of Dotterud at 6 years, and found no difference between groups in specific IgE or skin prick test response to common allergens.

**Overall we found LOW (-1 indirect outcome; -1 imprecision due to low event numbers and borderline statistical significance) evidence that probiotics reduce allergic sensitisation to cow’s milk, but no evidence that probiotics or synbiotics reduce allergic sensitisation to other allergens.**

Figure 1 Risk of bias in intervention studies of probiotics and allergic sensitisation

Table 3 Findings from a high quality systematic review identified in the 07/13 search

| **Study** | **Outcome measure** | **No. participants (studies)** | **Outcome (95% CI)** | **I^2^** |
| --- | --- | --- | --- | --- |
| **Tang** ([29](#_ENREF_29)) | AS-Food | 1826 (7) | **RR 0.95 [0.80, 1.12]** | 19 |

Figure 2 Probiotics and risk of AS to any allergen


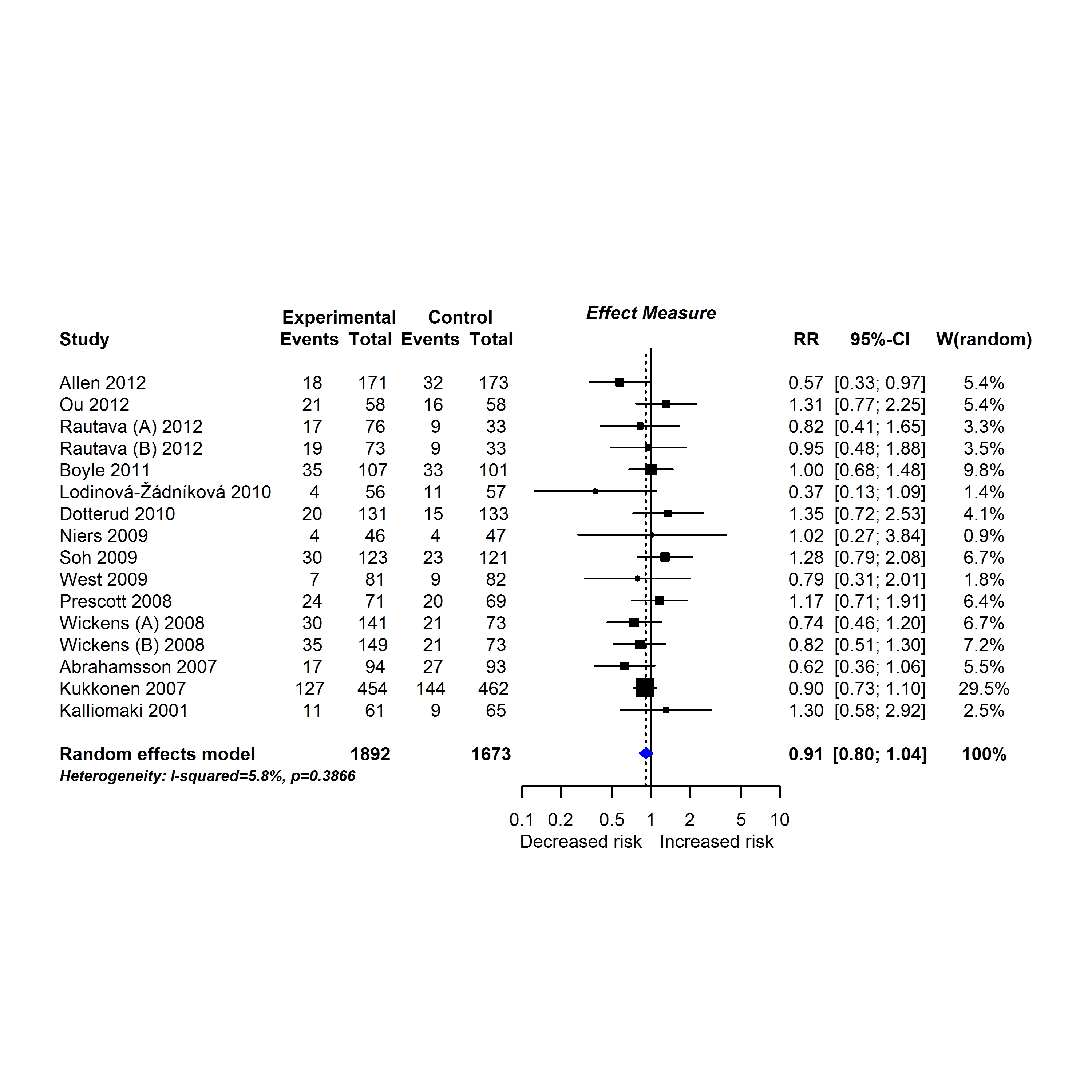


Table 4 Subgroup analyses of effect of probiotics on risk of allergic sensitisation to common allergen

|  | **Number of studies** | **RR [95% CI]** | **I^2^ (%)** | **P for between groups difference** |
| --- | --- | --- | --- | --- |
| Risk of disease – High  Risk of disease – Normal | 14  2 | 0.90 [0.76; 1.07]  0.94 [0.45; 1.94] | 16  0 | 0.92 |
| Method of assessment – sIgE  Method of assessment – SPT | 3  13 | 0.82 [0.39; 1.69]  0.90 [0.79; 1.02] | 56  0 | 0.80 |
| Type of Intervention – Probiotic  Type of Intervention – Synbiotic | 15  1 | 0.91 [0.77; 1.06]  0.90 [0.73; 1.10] | 4  -- | 0.95 |
| Type of Probiotic – L.rhamnosus  Type of Probiotic – Other | 9  7 | 0.96 [0.83; 1.10]  0.77 [0.60; 0.99] | 0  14 | 0.15 |
| Timing – Postnatal only  Timing – Pre +/-Post-natal | 4  12 | 0.98 [0.64; 1.50]  0.88 [0.77; 1.00] | 38  0 | 0.62 |
| *Postnatal Administration – Infant only  *Postnatal Administration – Other | 8  7 | 0.85 [0.67; 1.07]  0.97 [0.78; 1.21] | 33  0 | 0.65 |
| Overall risk of bias – High/Unclear  Overall risk of bias – Low | 10  6 | 0.90 [0.73; 1.11]  0.90 [0.72; 1.11] | 29  0 | 0.96 |
| Conflict of interest – High/Unclear risk  Conflict of interest – Low risk | 8  8 | 0.90 [0.71; 1.14]  0.91[0.74; 1.11] | 35  0 | 0.93 |

*no postnatal administration in study of Boyle, 2011 – hence only 15 interventions included in this analysis

Figure 3 Risk of publication bias: probiotics and AS to any allergen


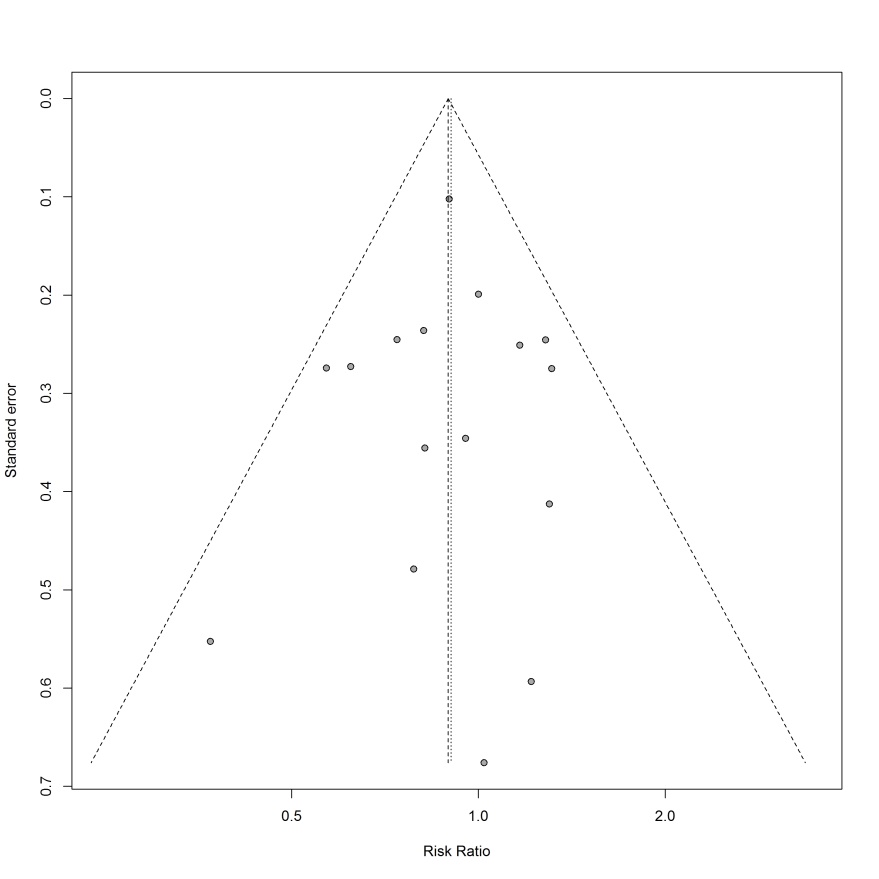


Egger’s test p = 0.84

Figure 4 Probiotics and risk of AS to any aeroallergen


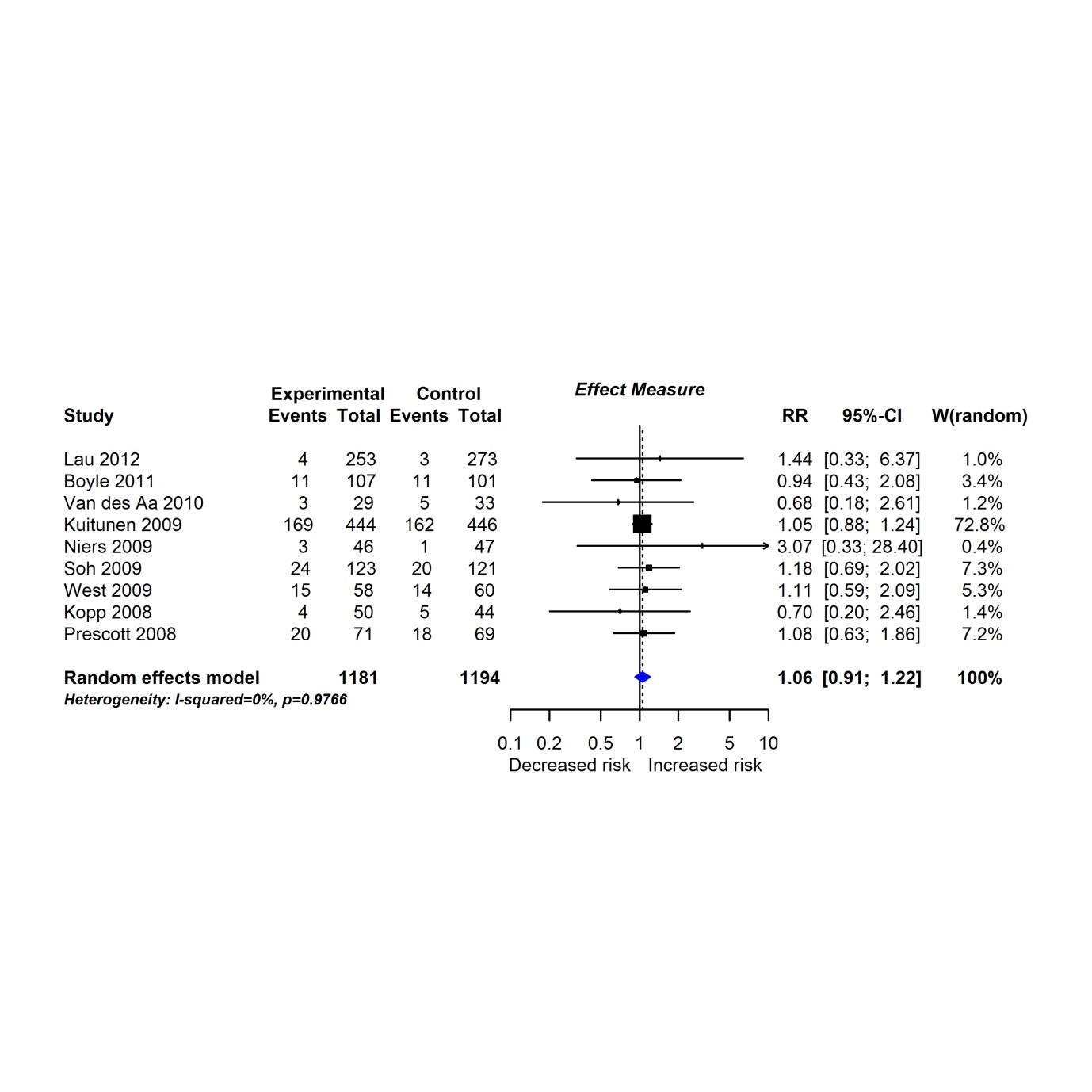


Figure 5 Probiotics and risk of AS to any food


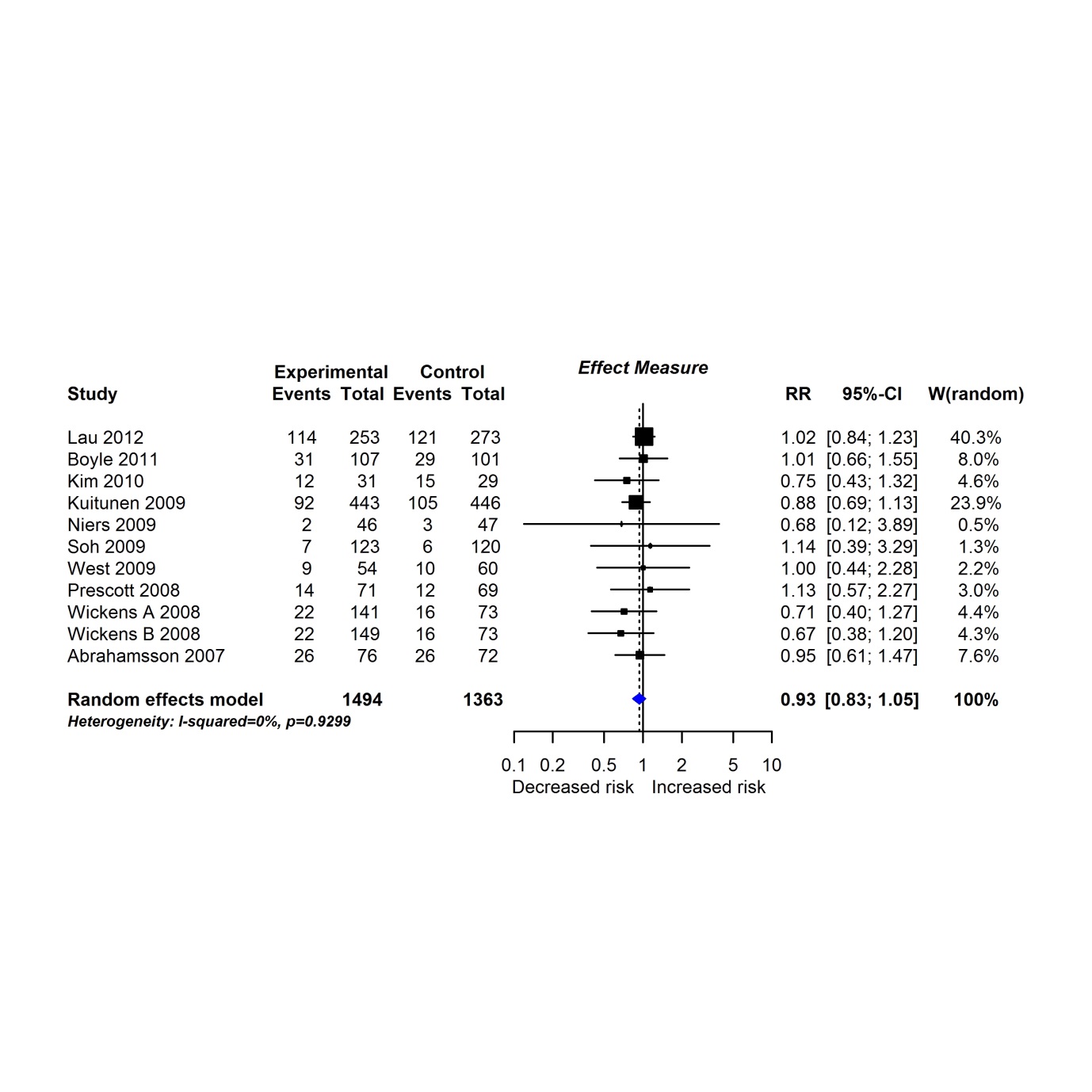


Table 5 Subgroup analyses of effect of probiotics on risk of allergic sensitisation to aeroallergen

|  | **Number of studies** | **RR [95% CI]** | **I^2^ (%)** | **P for between groups difference** |
| --- | --- | --- | --- | --- |
| Risk of disease – High  Risk of disease – Normal | 8  1 | 1.06 [0.91-1.22]  1.11 [0.59-2.09] | 0  - | 0.88 |
| Method of assessment – sIgE  Method of assessment – SPT | 4  5 | 1.00 [0.61; 1.63]  1.12 [0.80; 1.57] | 0  0 | 0.70 |
| Type of Intervention – Probiotic  Type of Intervention – Synbiotic | 8  1 | 1.10 [0.83; 1.46]  0.68 [0.18; 2.61] | 0  -- | 0.50 |
| Type of Probiotic – L.rhamnosus  Type of Probiotic – Other | 5  4 | 1.05 [0.90-1.23]  1.10 [0.69-1.75] | 0  0 | 0.86 |
| Timing – Postnatal only  Timing – Pre +/-Post-natal | 5  4 | 1.11 [0.81-1.51]  1.04 [0.88-1.23] | 0  0 | 0.74 |
| *Postnatal Administration – Infant only  *Postnatal Administration – Other | 7  1 | 1.07 [0.92-1.24]  0.70 [0.20-2.46] | 0  - | 0.78 |
| Overall risk of bias – High/Unclear  Overall risk of bias – Low | 6  3 | 1.06 [0.91-1.24]  0.95 [0.51-1.74] | 0  0 | 0.71 |
| Conflict of interest – High/Unclear risk  Conflict of interest – Low risk | 3  6 | 0.95 [0.51-1.74]  1.06 [0.91-1.24] | 0  0 | 0.71 |

*no postnatal administration in study of Boyle, 2011 – hence only 8 interventions included in this analysis

Table 6 Subgroup analyses of effect of probiotics on risk of allergic sensitisation to any food

|  | **Number of studies** | **RR [95% CI]** | **I^2^ (%)** | **P for between groups difference** |
| --- | --- | --- | --- | --- |
| Risk of disease – High  Risk of disease – Normal | 10  1 | 0.93 [0.83-1.05]  1.00 [0.44-2.27] | 0  - | 0.87 |
| Method of assessment – sIgE  Method of assessment – SPT | 4  7 | 0.98 [0.83; 1.15]  0.88 [0.68; 1.14] | 0  0 | 0.49 |
| Type of Intervention – Probiotic  Type of Intervention – Synbiotic | 11  0 | 0.95 [0.83; 1.09]  -- | 0  -- | -- |
| Type of Probiotic – L.rhamnosus  Type of Probiotic – Other | 5  6 | 0.90 [0.74-1.09]  0.96 [0.82-1.12] | 0  0 | 0.61 |
| Timing – Postnatal only  Timing – Pre +/-Post-natal | 4  7 | 1.02 [0.86-1.22]  0.86 [0.73-1.01] | 0  0 | 0.15 |
| *Postnatal Administration – Infant only  *Postnatal Administration – Other | 7  3 | 0.97 [0.85-1.11]  0.71 [0.51-0.99] | 0  - | 0.22 |
| Overall risk of bias – High/Unclear  Overall risk of bias – Low | 7  4 | 0.90 [0.75-1.09]  0.96 [0.82-1.12] | 0  0 | 0.64 |
| Conflict of interest – High/Unclear risk  Conflict of interest – Low risk | 6  5 | 0.95 [0.83-1.09]  0.88 [0.68-1.14] | 0  0 | 0.62 |

*no postnatal administration in study of Boyle, 2011 – hence only 10 interventions included in this analysis

Figure 6 Risk of publication bias: probiotics and AS to any food allergen


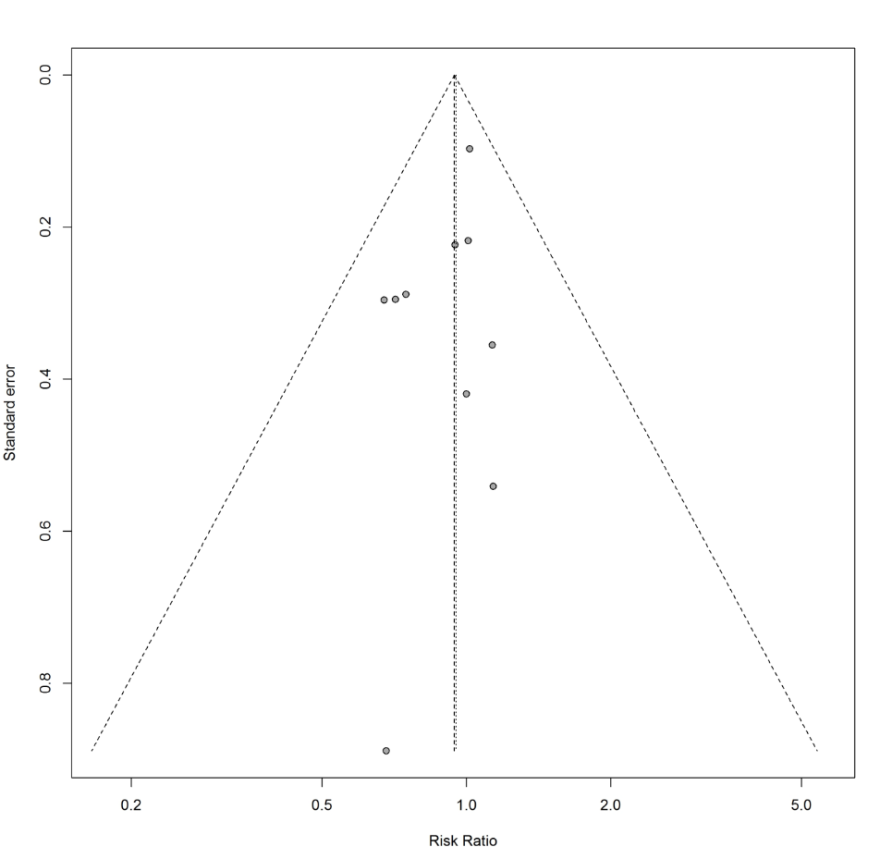


Egger’s test p = 0.22

Figure 7 Probiotics and risk of AS to CM


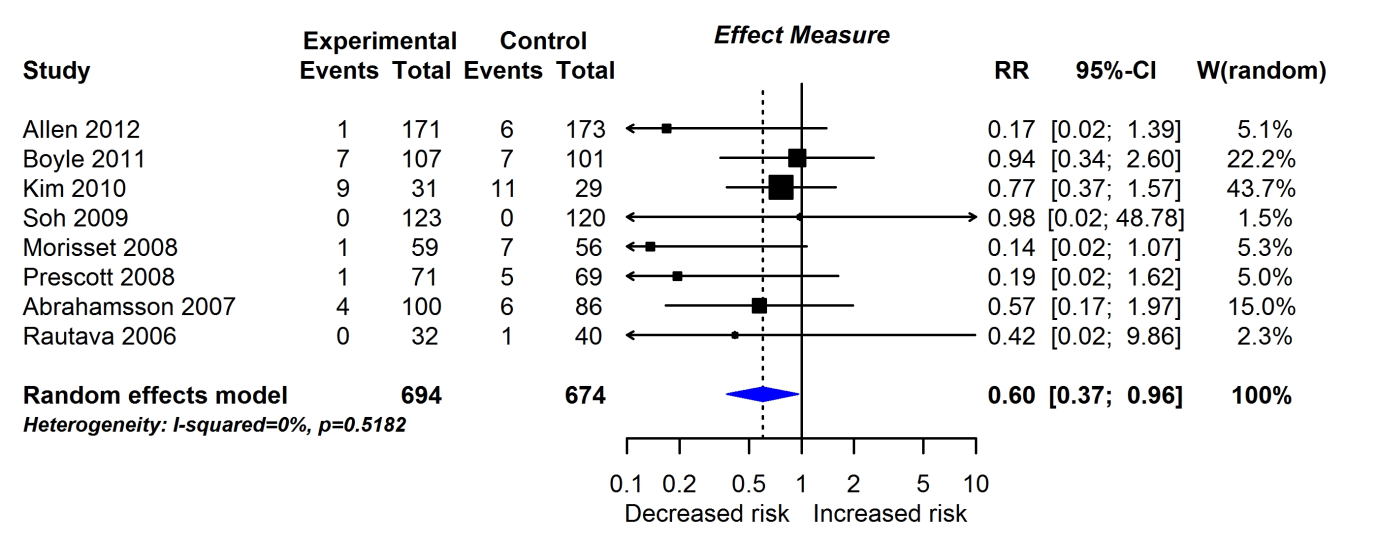


Figure 8 Probiotics and risk of AS to egg


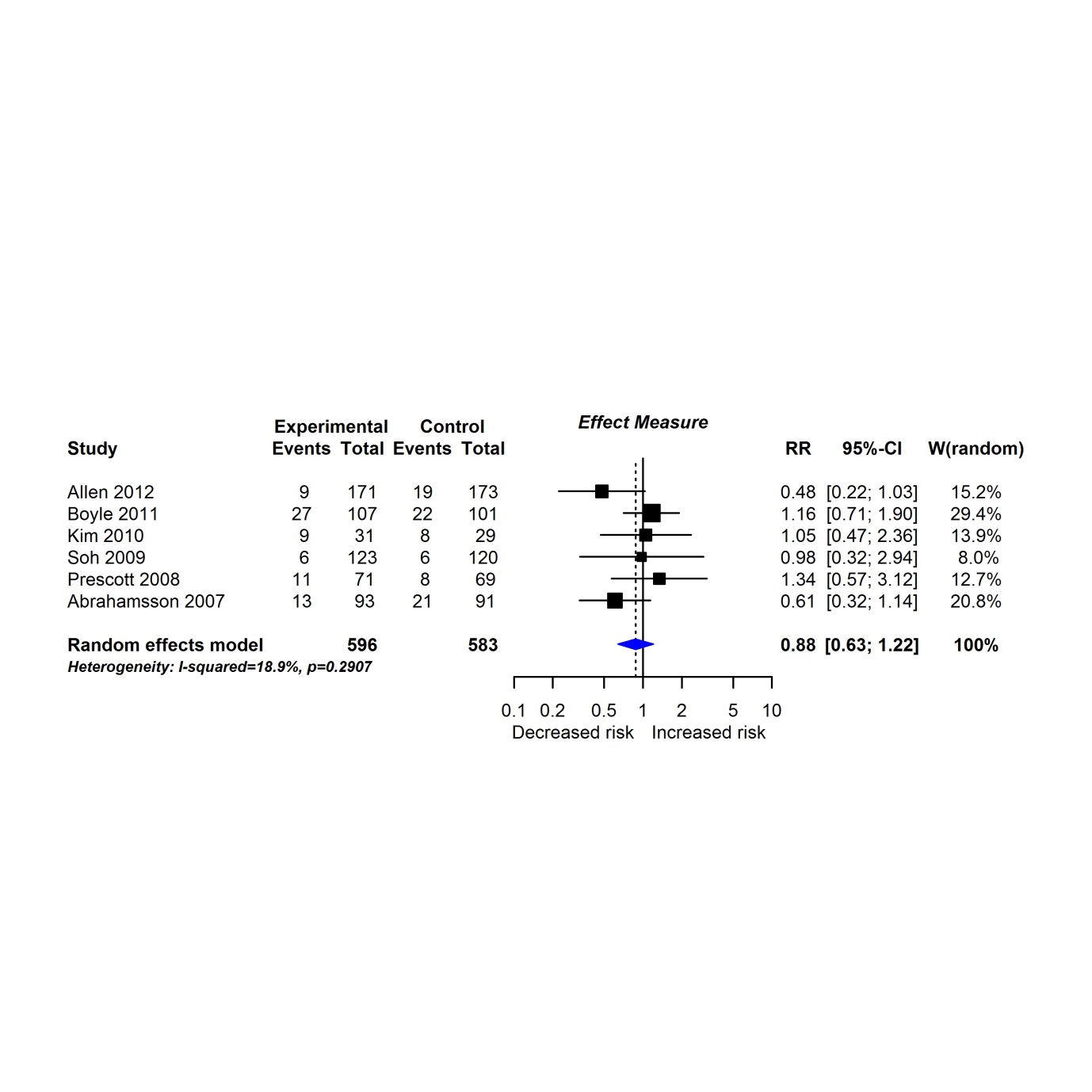


Figure 9 Probiotics and risk of AS to peanut


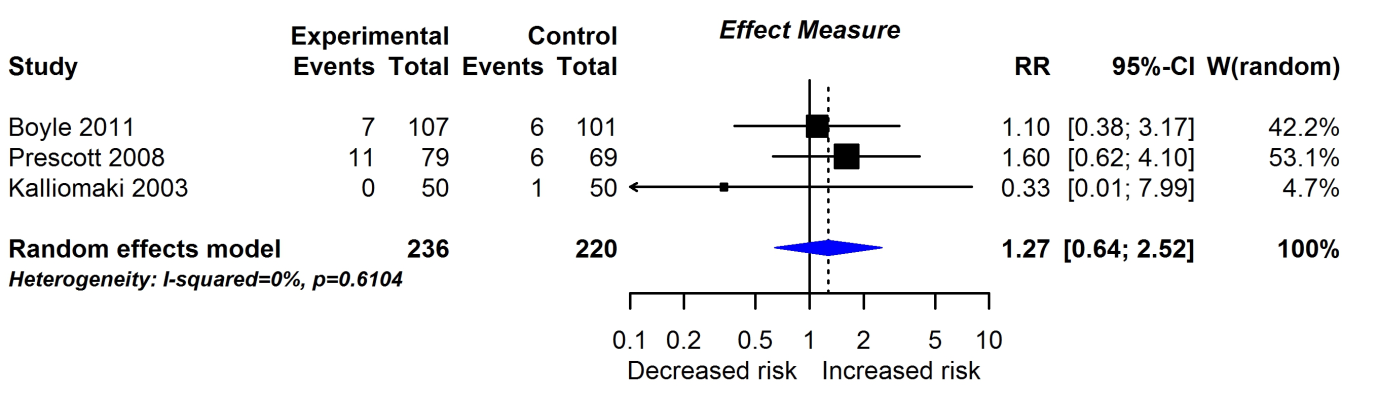


Table 7 Subgroup analyses of effect of probiotics on risk of allergic sensitisation to cow’s milk

|  | **Number of studies** | **RR [95% CI]** | **I^2^ (%)** | **P for between groups difference** |
| --- | --- | --- | --- | --- |
| Risk of disease – High  Risk of disease – Normal | 7  1 | 0.60 [0.36; 0.98]  0.42 [0.02; 9.86] | 2  -- | 0.82 |
| Method of assessment – sIgE  Method of assessment – SPT | 2  6 | 0.41 [0.07; 2.33]  0.57 [0.29; 1.10] | 63  0 | 0.74 |
| Type of Intervention – Probiotic  Type of Intervention – Synbiotic | 8  0 | 0.60 [0.37; 0.96]  -- | 0  -- | -- |
| ¶Type of Probiotic – L.rhamnosus  ¶Type of Probiotic – Other | 3  4 | 0.88 [0.35; 2.24]  0.56 [0.30; 1.04] | 0  6 | 0.26 |
| Timing – Postnatal only  Timing – Pre +/-Post-natal | 4  4 | 0.23 [0.06; 0.81]  0.70 [0.42; 1.17] | 0  0 | 0.11 |
| *Postnatal Administration – Infant only  *Postnatal Administration – Other | 5  2 | 0.38 [0.16; 0.93]  0.41 [0.07; 2.33] | 0  63 | 0.40 |
| Overall risk of bias – High/Unclear  Overall risk of bias – Low | 7  1 | 0.53 [0.31; 0.90]  0.94 [0.34; 2.56] | 0  -- | 0.32 |
| Conflict of interest – High/Unclear risk  Conflict of interest – Low risk | 5  3 | 0.55 [0.29; 1.01]  0.68 [0.28; 1.63] | 6  0 | 0.69 |

¶the study of Morisset, 2008 did not name the probiotic given, hence only 7 interventions included in this analysis

*no postnatal administration in study of Boyle, 2011 – hence only 7 interventions included in this analysis

Table 8 Subgroup analyses of effect of probiotics on risk of allergic sensitisation to egg

|  | **Number of studies** | **RR [95% CI]** | **I^2^ (%)** | **P for between groups difference** |
| --- | --- | --- | --- | --- |
| Risk of disease – High  Risk of disease – Normal | 6  0 | 0.88 [0.63; 1.22]  -- | 19  -- | -- |
| Method of assessment – sIgE  Method of assessment – SPT | 1  5 | 1.05 [0.47; 2.36]  0.85 [0.57; 1.25] | 0  32 | 0.65 |
| Type of Intervention – Probiotic  Type of Intervention – Synbiotic | 6  0 | 0.88 [0.63; 1.22]  -- | 19  -- | -- |
| Type of Probiotic – L.rhamnosus  Type of Probiotic – Other | 2  4 | 1.13 [0.72; 1.77]  0.77 [0.49; 1.20] | 0  29 | 0.23 |
| Timing – Postnatal only  Timing – Pre +/-Post-natal | 2  4 | 1.19 [0.61; 2.33]  0.80 [0.52; 1.22] | 0  41 | 0.33 |
| *Postnatal Administration – Infant only  *Postnatal Administration – Other | 4  1 | 0.73 [0.47; 1.14]  1.05 [0.47; 2.36] | 19  -- | 0.37 |
| Overall risk of bias – High/Unclear  Overall risk of bias – Low | 5  1 | 0.78 [0.53; 1.13]  1.16 [0.71; 1.90] | 10  -- | 0.21 |
| Conflict of interest – High/Unclear risk  Conflict of interest – Low risk | 4  2 | 0.67 [0.46; 0.99]  1.21 [0.79; 1.85] | 0  0 | 0.06 |

*no postnatal administration in study of Boyle, 2011 – hence only 6 interventions included in this analysis

Figure 10 Probiotics and total IgE


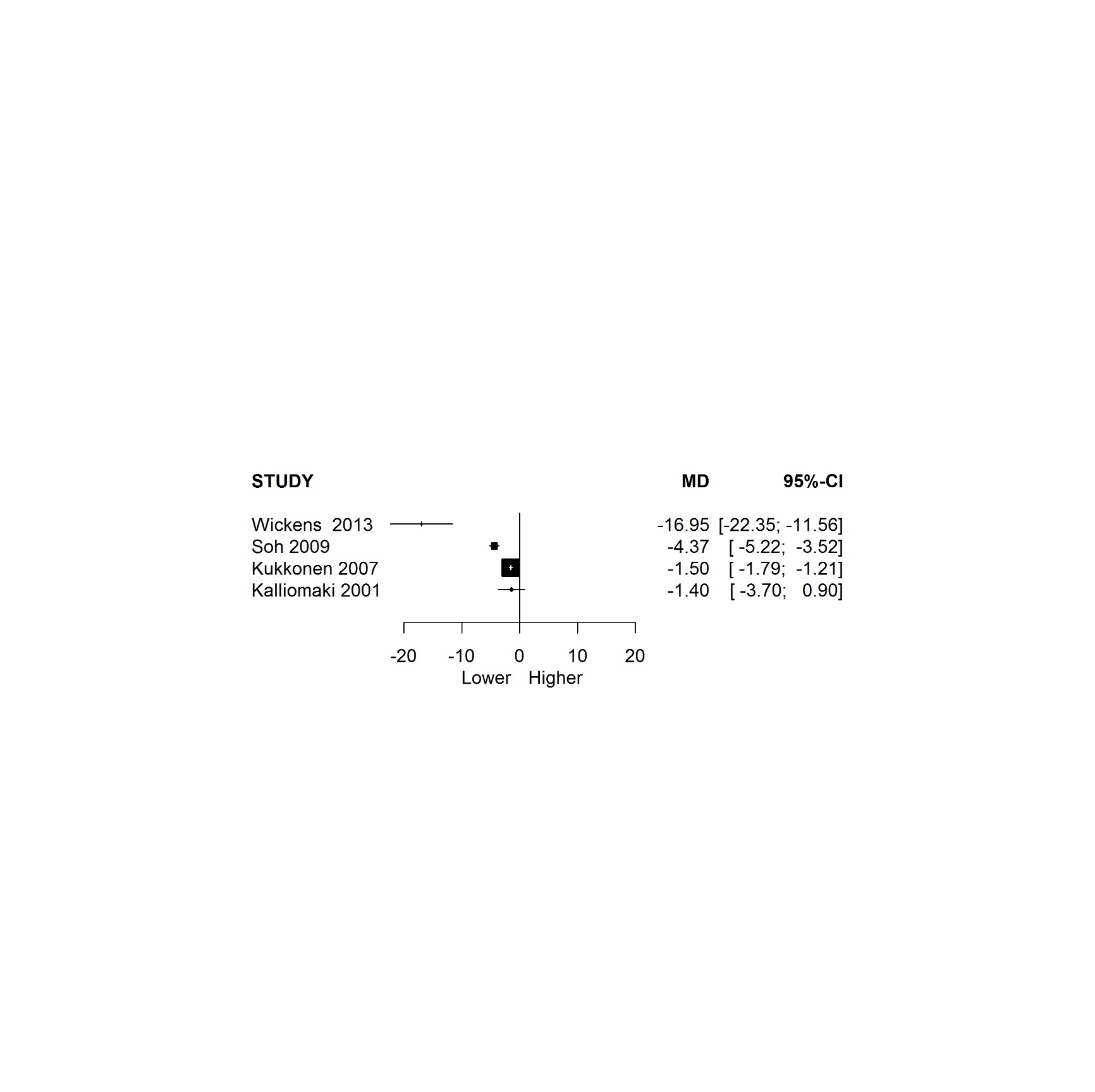


# Probiotics and risk of food allergy

Eleven intervention trials (1167 participants, 580 randomised to probiotics) assessed the effect of supplementation with probiotics on risk of developing food allergy. Interventions lasted 6-12 months postnatally. One study was carried out in Australia, one in the USA and 9 in Europe. Four studies had a normal disease risk, whilst the other 7 were carried out in high risk infants. Children’s age at the time of outcome measurement ranged between 1 and 6 years. Three studies measured allergy to cow’s milk, using either DBPCFC (Kalliomaki and Rautava), or physician assessment (Morisset). The other studies measured food allergy to any food, mainly using a physician’s assessment, relying on a combination of a suggestive history and positive allergy test.

Risk of bias was generally unclear (Figure 11). There was a low risk of assessment and attrition bias in over 60% of studies. There was a high risk of conflict of interest in almost 30% of studies.

We found no evidence that probiotic supplementation reduces the risk of any food allergy at age ≤4 years or age 5 to 14 years (Figures 12 and 13), and no evidence that probiotics reduce the risk of having cow’s milk allergy at age ≤4 years (Figure 14). Subgroup analysis showed no evidence that specific subgroups have different outcome with respect to any food allergy at age 5 to 14 years (Table 9).

The data from Prescott ([19](#_ENREF_19)) at age 2.5 years old could not be combined with other studies in meta-analysis. They reported no effect of probiotics on odds of food allergy (OR 0.83; 95% CI 0.33 – 2.20). In an analysis including data from Luoto, together with other probiotic intervention studies, Lundelin ([15](#_ENREF_15)) found no significant difference in food allergy between probiotic and placebo groups.

**Overall we found no evidence that probiotics influence risk of food allergy.**

Figure 11 Risk of bias in intervention studies of probiotics and food allergy

Figure 12 Probiotics and risk of any food allergy at age ≤ 4 years old


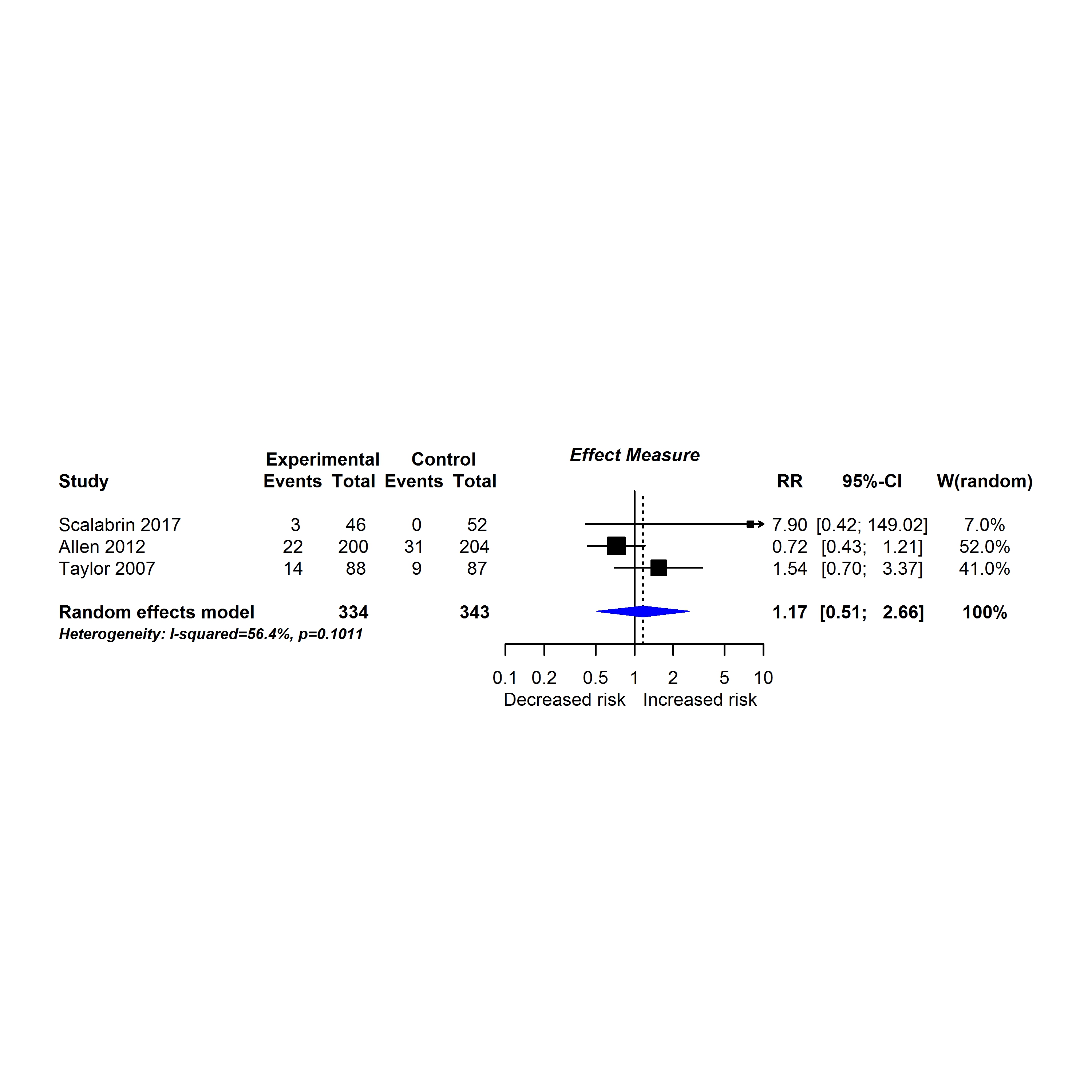


Figure 13 Probiotics and risk of any food allergy at age 5-4 years old


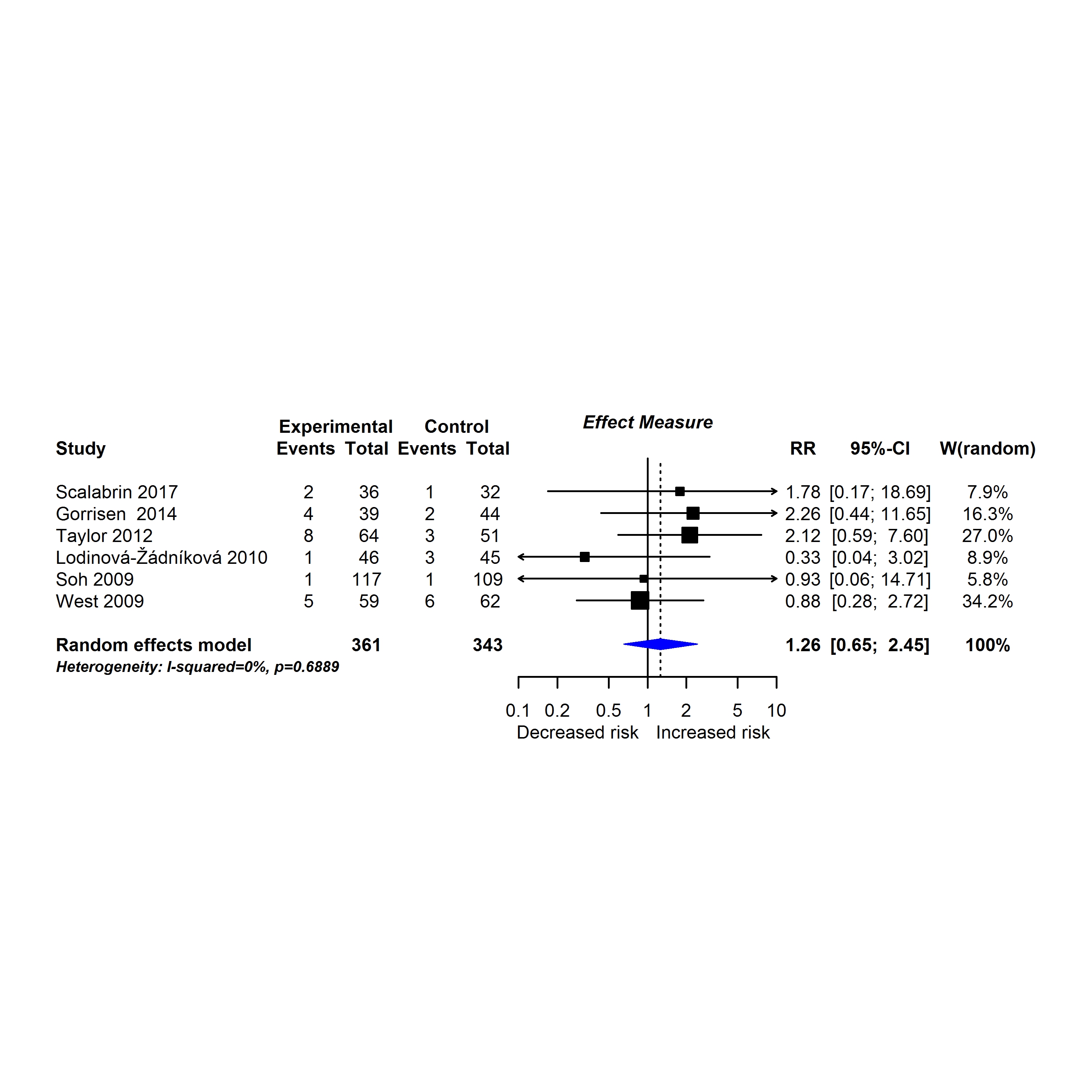


Table 9 Subgroup analyses of effect of probiotics on risk of any food allergy at age 5 to 14 years

|  | **Number of studies** | **RR [95% CI]** | **I^2^ (%)** | **P for between groups difference** |
| --- | --- | --- | --- | --- |
| Risk of disease – High  Risk of disease – Normal | 4  2 | 1.49 [0.63; 3.57]  1.00 [0.36; 2.77] | 0  0 | 0.56 |
| Type of Intervention – Probiotic  Type of Intervention – Synbiotic | 6  0 | 1.26 [0.65; 2.45]  -- | 0  -- | -- |
| Type of Probiotic – L.rhamnosus  Type of Probiotic – Other | 3  3 | 0.99 [0.38; 2.58]  1.52 [0.55; 4.18] | 0  14 | 0.55 |
| Timing – Postnatal only  Timing – Pre +/-Post-natal | 5  1 | 1.13 [0.55; 2.32]  2.26 [0.44; 11.65] | 0  -- | 0.45 |
| Postnatal Administration – Infant only  Postnatal Administration – Other | 5  1 | 1.13 [0.55; 2.32]  2.26 [0.44; 11.65] | 0  -- | 0.45 |
| Overall risk of bias – High/Unclear  Overall risk of bias – Low | 6  0 | 1.26 [0.65; 2.45]  -- | 0  -- | -- |
| Conflict of interest – High/Unclear risk  Conflict of interest – Low risk | 4  2 | 1.21 [0.42; 3.51]  1.30 [0.55; 3.08] | 0  4 | 0.93 |

Figure 14 Probiotics and risk of CMA at age ≤ 4 years old


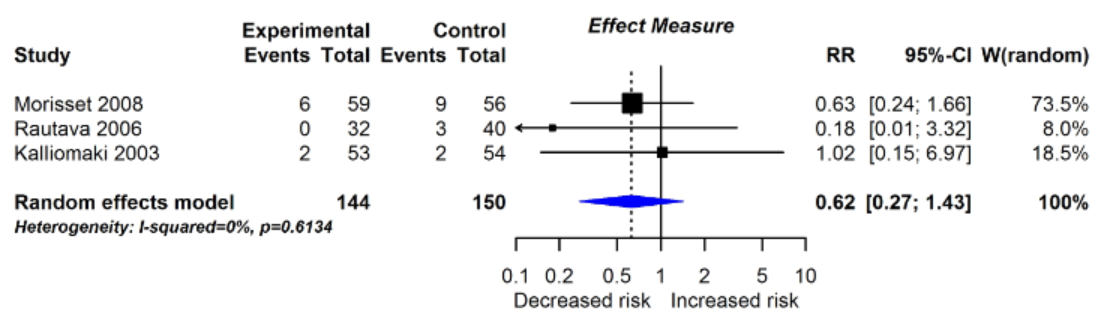


#

# Probiotics and risk of AD

One systematic review (13 trials, 3000 participants) evaluated AD ([28](#_ENREF_28)). They reported significantly reduced AD and atopic AD, with low statistical heterogeneity, and did not identify important subgroup differences.

Twenty-six original intervention trials (25 RCT; 1 CCT) evaluated over 6000 participants allocated to probiotic or control treatment with AD as an outcome. Sixteen studies were carried out in Europe, 2 in USA and 8 in Asia-Pacific. Seven studies were carried out in infants with normal risk of AD, 18 in infants at high risk of AD and in one study this was unclear. Age at time of outcome measurement ranged between 4 months and 7 years. Fourteen studies used Hanifin & Rajka or modifications of this method for outcome assessment, 5 used doctor diagnosis of AD and others used different assessment methods or (for 5 studies) method of outcome assessment was unclear. The overall risk of bias was low or unclear in over 80% of studies based on assessment, selection and attrition bias, however over 30% of studies had a high risk of conflict of interest due to direct industry involvement in the trial (Figure 15).

Probiotics reduced AD risk at age ≤4 years (Figure 16 – RCT) with high statistical heterogeneity (I^2^=61%). In the CCT of Enomoto ([8](#_ENREF_8)) there was significantly reduced AD risk at age 18 months in probiotic versus control group (Figure 17), despite a significant imbalance at randomisation with increased risk factors for allergic disease in the probiotic group. There was no evidence of publication bias in these two meta-analyses (Figure 18; Egger’s test p=0.12). Subgroup analyses showed no clear evidence that one subgroup had different efficacy to another, however there was some evidence that postnatal administration to mother during lactation is more effective than infant supplementation alone during the postnatal period (p=0.016) – high statistical heterogeneity remained in this subgroup analysis. Probiotics also reduced risk of atopic AD at age ≤4 years (Figure 19), with no statistical heterogeneity (I^2^=0%) and no evidence of publication bias (Figure 20). Subgroup analyses did not identify any important subgroup differences (Table 11).

We found no evidence that probiotics reduce AD or atopic AD risk at age 5-14 years (Figures 21 and 22) with moderate and no statistical heterogeneity respectively, and no evidence for important subgroup differences in analysis of all AD (Table 12) or publication bias (Figure 23).

A further five studies were not eligible for meta-analysis. Huurre ([9](#_ENREF_9)) reported reduced AD, but this was not statistically significant (9.7% probiotic, 17.6% control; p=0.13). De Leon ([6](#_ENREF_6), [32](#_ENREF_32)) found no difference at age 6 months (45% probiotic, 56% control). Scalabrin reported no difference in AD by age 5, but did not present numerical data. In an analysis including data from Luoto, together with other probiotic intervention studies, Lundelin found no significant difference in food allergy between probiotic and placebo groups. In the study of Chien 3 participants in the synbiotic group, 9 in the prebiotic and 10 in the control group developed eczema by 3 months. The difference between synbiotic and other groups, adjusted for family history, was reported as statistically significant P<0.05.

**Overall we found MODERATE level evidence (-1 inconsistency between age ≤4 and age 5-14 findings) that probiotics can prevent atopic AD and MODERATE level evidence (-1 inconsistency between studies i.e. high statistical heterogeneity) that probiotics can prevent *all* AD in children ≤ 4 years old. The majority of the evidence in these positive meta-analyses came from studies of high risk infants i.e. those with a positive family history of allergic disease. So although in subgroup analyses we found no evidence that outcomes differed according to disease risk, our view is that these conclusions should only be seen as relevant for infants at high risk of AD.**

Figure 15 Risk of bias in intervention studies of probiotics and AD

Table 10 Findings from the previous high quality systematic review found in 07/13 search

| **Study** | **Outcome measure** | **No. participants (studies)** | **Outcome (95% CI)** | **I^2^** |
| --- | --- | --- | --- | --- |
| **Pelucci** ([28](#_ENREF_28))* | AD | 3092 (13) | **RR 0.79 [0.71, 0.88]** | 24 |
|  | IgE-associated AD | 2711 (10) | **RR 0.80 [0.66, 0.96]** | 32 |
|  | AD  (high risk infants) | (12) | **RR 0.80 [0.70, 0.91]** | 24 |
|  | AD  (normal/low risk infants) | (2) | RR 0.35 [0.06, 2.01] | 49 |
|  | AD  (Hanifin and Rajka criteria) | (6) | RR 0.80 [0.61, 1.06] | 49 |
|  | AD  (UK working party criteria) | (5) | **RR 0.78 [0.67, 0.90]** | 0 |
|  | AD (parent reported) | (2) | RR 0.70 [0.47, 1.04] | 26 |
|  | AD at < 2 years | (6) | **RR 0.78 [0.65, 0.93]** | 40 |
|  | AD at ≥ 2 years | (7) | **RR 0.79 [0.65, 0.95]** | 1 |

* The authors also undertook meta-regression to examine the effect of pre/postnatal/combined treatment, treatment of mother/child/both, duration of treatment, treatment dose, single versus multiple probiotic treatment, location of trial and apparent conflict of interest and found no significant effect for any of these factors.

Figure 16 Probiotics and risk of AD at age ≤ 4 years - RCT


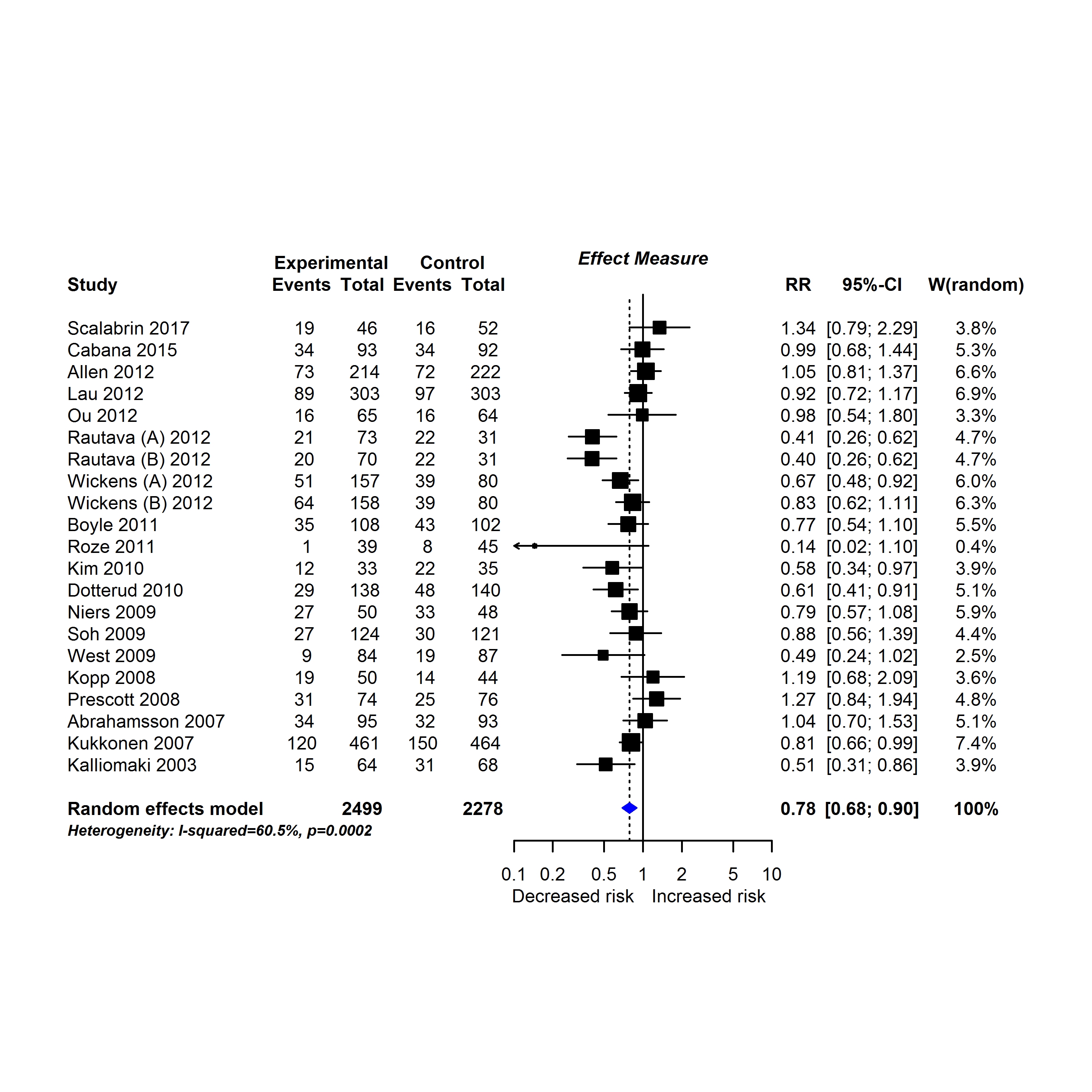


**Figure 17 Probiotics and risk of AD at age ≤ 4 years - CCT**


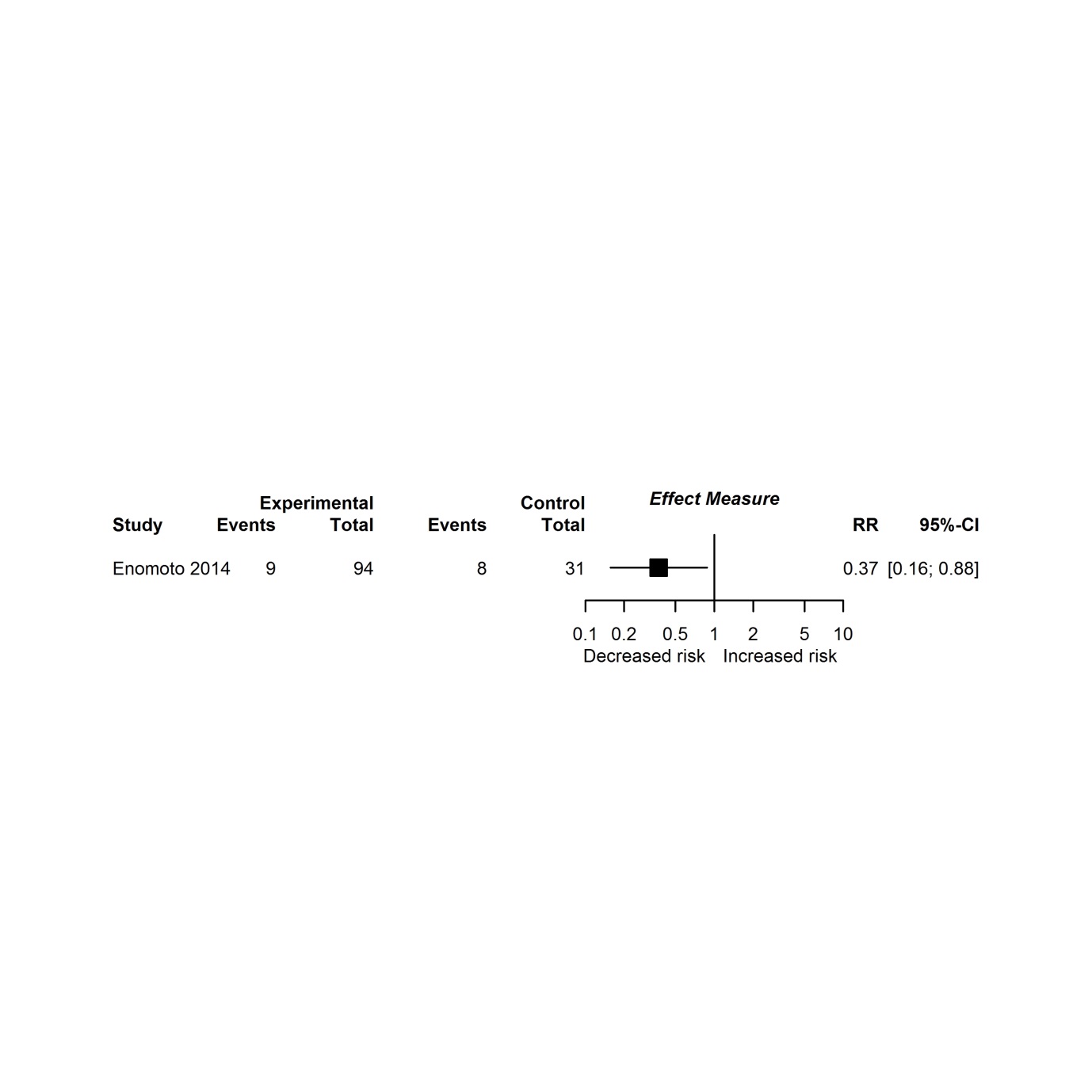


Table 10 Subgroup analyses of probiotics and risk of AD at age ≤ 4 years

| **Subgroups compared** | **Number of studies** | **RR [95% CI]** | **I^2^ (%)** | **P for between groups difference** |
| --- | --- | --- | --- | --- |
| Type of Probiotic – L.rhamnosus  Type of Probiotic – Other | 13  8 | 0.75 [0.62-0.89]  0.84 [0.68-1.03] | 55  67 | 0.42 |
| Type of Intervention – Probiotic  Type of Intervention – Synbiotic | 19  2 | 0.79 [0.68; 0.91]  0.46 [0.09; 2.27] | 62  64 | 0.51 |
| Timing – Postnatal only  Timing – Pre +/-Post-natal | 7  14 | 0.95 [0.76-1.20]  0.73 [0.62-0.86] | 42  62 | 0.06 |
| *Postnatal Administration – Infant only  *Postnatal Administration – Other | 11  9 | 0.93 [0.81-1.06]  0.64 [0.51-0.80] | 31  59 | 0.016 |
| Risk of disease – High  Risk of disease – Normal | 17  4 | 0.80 [0.69-0.92]  0.55 [0.38-0.80] | 61  69 | 0.58 |
| Overall risk of bias – High/Unclear  Overall risk of bias – Low | 12  9 | 0.88 [0.76-1.02]  0.67 [0.53-0.85] | 43  69 | 0.06 |
| Conflict of interest – High/Unclear risk  Conflict of interest – Low risk | 14  7 | 0.86 [0.76-0.98]  0.66 [0.49-0.88] | 37  74 | 0.10 |

*no postnatal administration in study of Boyle, 2011 – hence only 18 interventions included in this analysis

Figure 18 Risk of publication bias: probiotics and AD at age ≤4


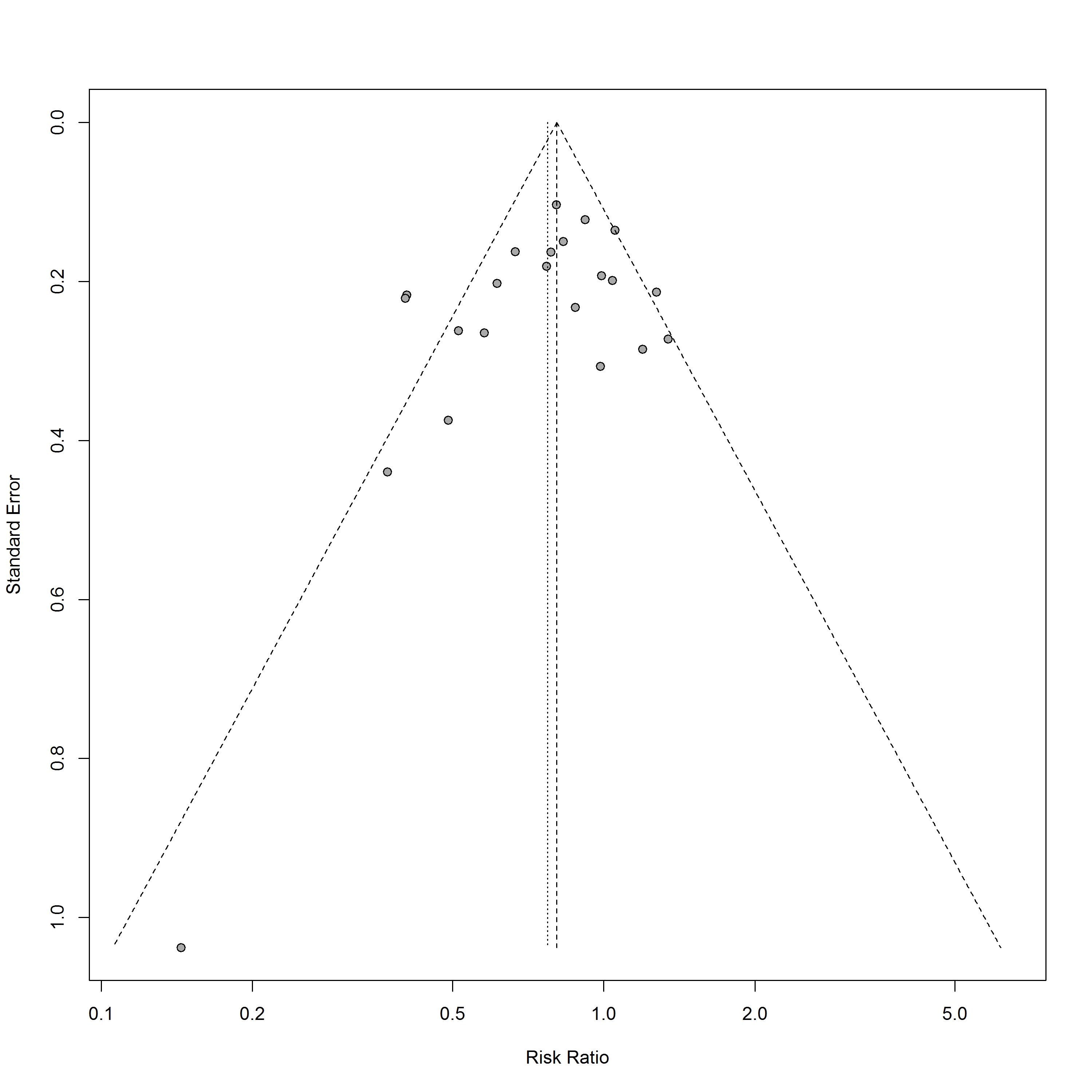


Egger’s test p = 0.12

**Figure 19 Probiotics and risk of atopic AD at age ≤ 4 years**


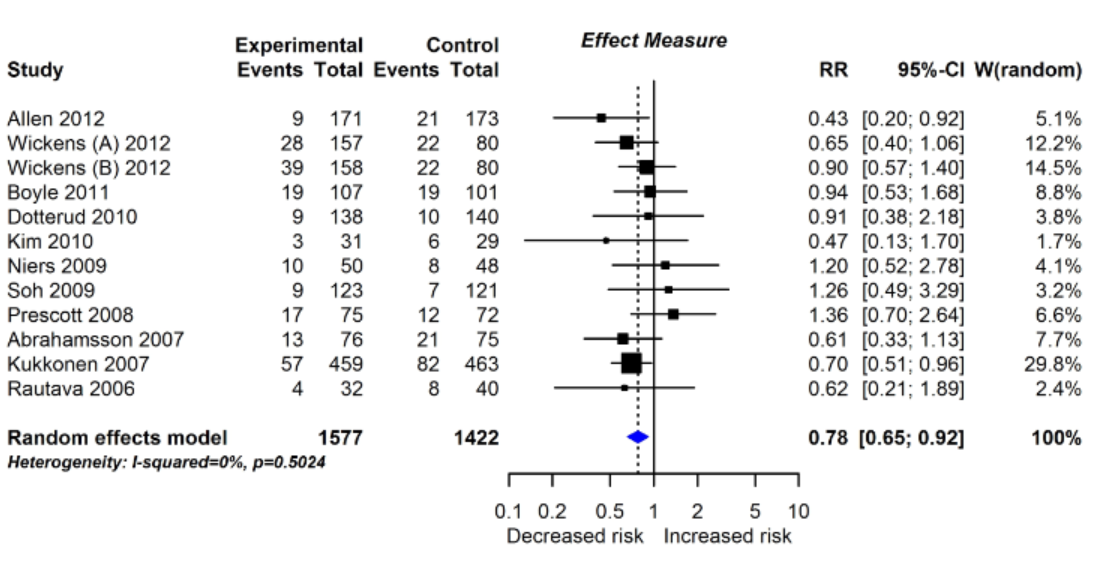


Figure 20 Risk of publication bias: probiotics and atopic AD at age ≤4


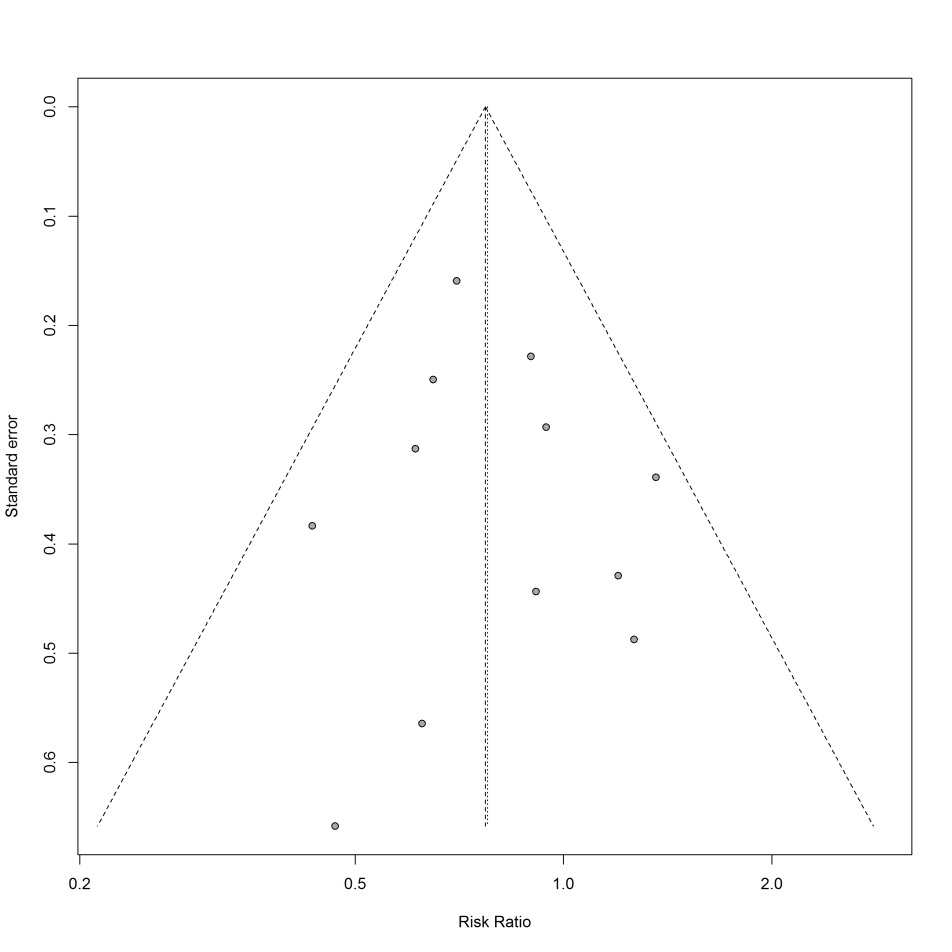


Egger’s test p = 0.70

Table 11 Subgroup analyses of probiotics and risk of atopic AD at age ≤ 4 years

| **Subgroups compared** | **Number of studies** | **RR [95% CI]** | **I^2^ (%)** | **P for between groups difference** |
| --- | --- | --- | --- | --- |
| Intervention – Probiotics  Intervention – Synbiotics | 11  1 | 0.81 [0.66-0.99]  0.70 [0.51-0.96] | 0  - | 0.45 |
| Type of Probiotic – L.rhamnosus  Type of Probiotic – Other | 6  6 | 0.75 [0.60-0.94]  0.80 [0.56-1.13] | 0  34 | 0.38 |
| Timing – Postnatal only  Timing – Pre +/-Post-natal | 3  9 | 1.15 [0.70-1.87]  0.73 [0.61-0.88] | 0  0 | 0.10 |
| *Postnatal Administration – Infant only  *Postnatal Administration – Other | 7  4 | 0.78 [0.58-1.05]  0.77 [0.57-1.04] | 27  0 | 0.93 |
| Risk of disease – High  Risk of disease – Normal | 10  2 | 0.78 [0.64-0.95]  0.79 [0.40-1.57] | 10  0 | 0.97 |
| Overall risk of bias – Low  Overall risk of bias – High/Unclear | 4  8 | 0.79 [0.60-1.05]  0.78 [0.60-1.01] | 0  16 | 0.96 |
| Conflict of interest – High/Unclear risk  Conflict of interest – Low risk | 7  5 | 0.71 [0.57-0.90]  0.87 [0.67-1.12] | 0  0 | 0.27 |

*no postnatal administration in the study of Boyle, 2011 – hence only 11 interventions included in this analysis

Figure 21 Probiotics and risk of AD at age 5-14 years


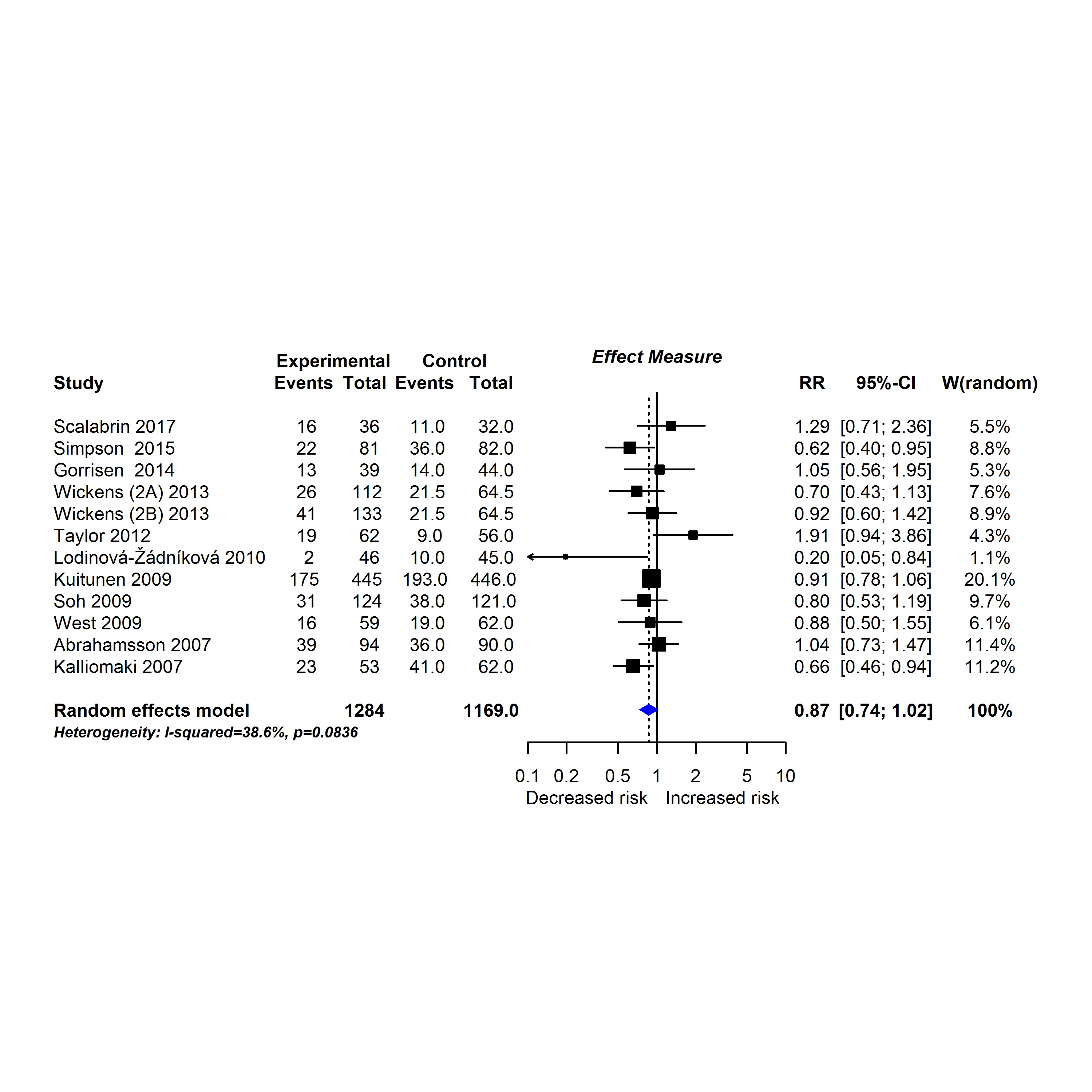


Figure 22 Probiotics and risk of atopic AD at age 5-14 years


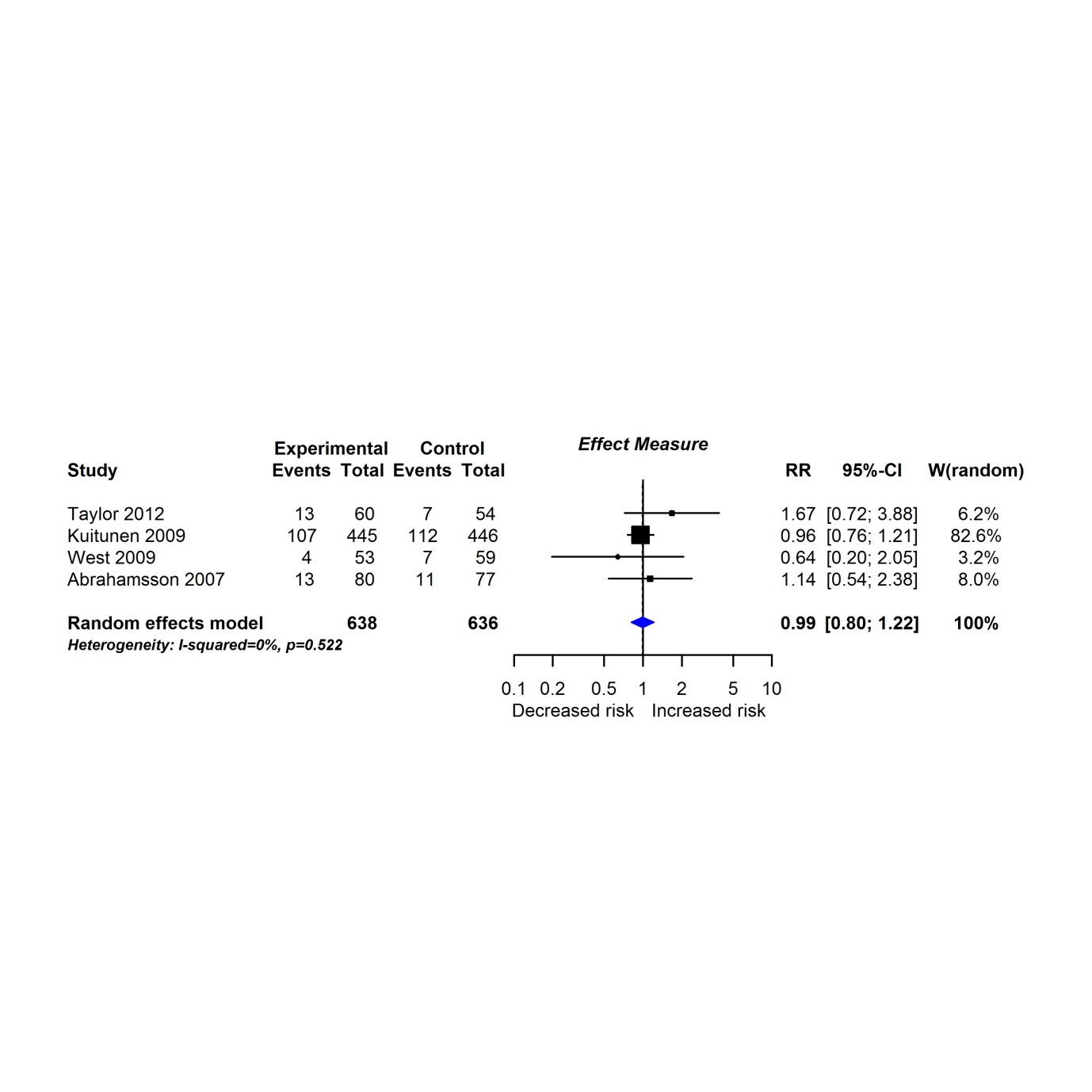


**Figure 23 Risk of publication bias: probiotics and AD at age 5 to 14 years**


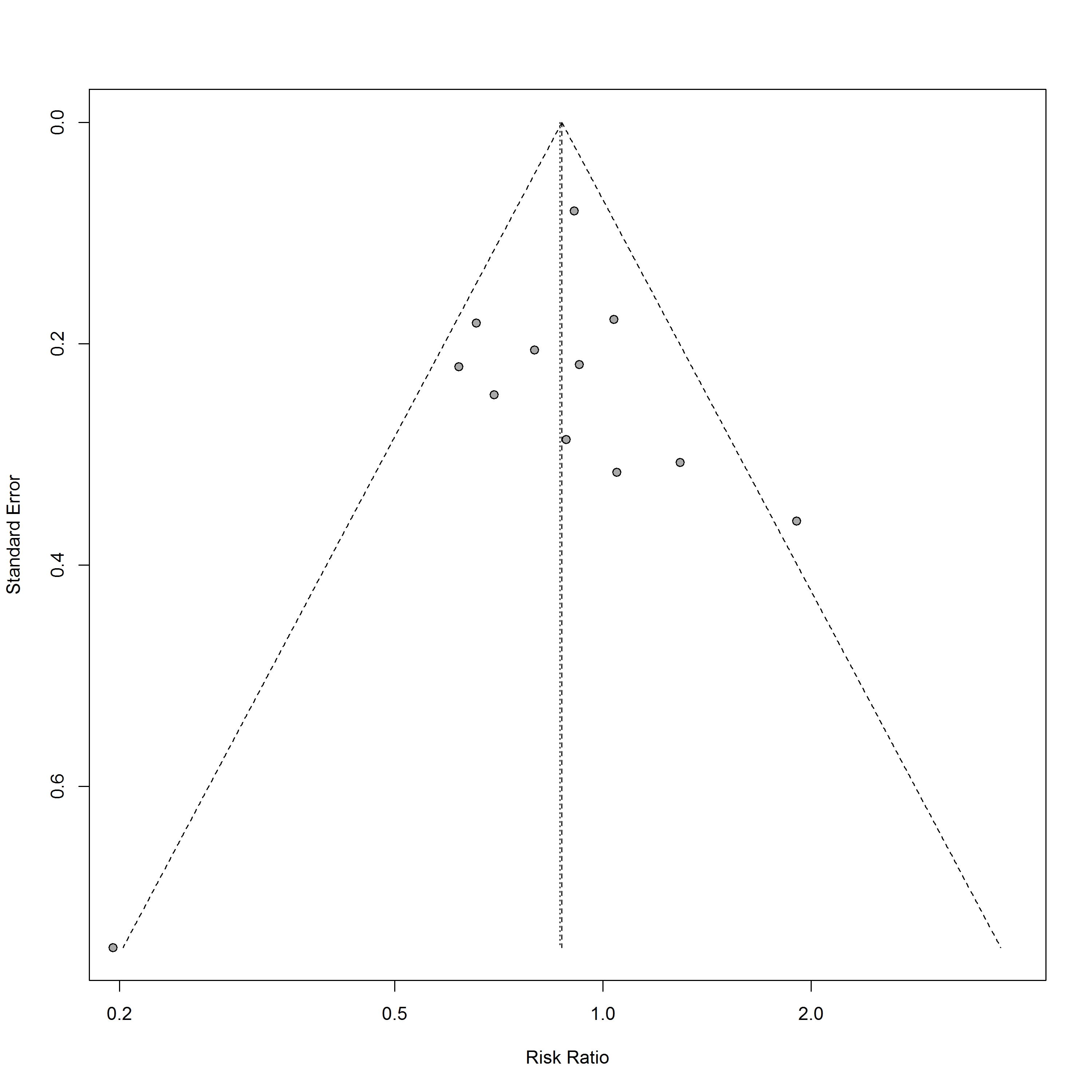


Egger’s test p = 0.73

Table 12 Subgroup analyses of probiotics and risk of AD at age 5-14 years

| **Subgroups compared** | **Number of studies** | **RR [95% CI]** | **I^2^ (%)** | **P for between groups difference** |
| --- | --- | --- | --- | --- |
| Type of Probiotic – L.rhamnosus  Type of Probiotic – Other | 7  5 | 0.82 [0.70-0.95]  1.02 [0.70-1.47] | 19  51 | 0.28 |
| Type of Intervention – Probiotic  Type of Intervention – Synbiotic | 11  1 | 0.86 [0.70; 1.05]  0.91 [0.78; 1.06] | 43  - | 0.67 |
| Timing – Postnatal only  Timing – Pre +/-Post-natal | 5  7 | 0.96 [0.61-1.52]  0.84 [0.73-0.97] | 60  18 | 0.58 |
| Postnatal Administration – Infant only  Postnatal Administration – Other | 7  5 | 0.97 [0.77-1.21]  0.74 [0.61-0.90] | 43  0 | 0.08 |
| Risk of disease – High  Risk of disease – Normal | 10  2 | 0.87 [0.74-1.03]  0.87 [0.42-1.78] | 35  74 | 0.98 |
| Overall risk of bias – High/Unclear  Overall risk of bias – Low | 10  2 | 0.88 [0.73-1.06]  0.82 [0.59-1.12] | 47  0 | 0.69 |
| Conflict of interest – High/Unclear risk  Conflict of interest – Low risk | 8  4 | 0.84 [0.69-1.01]  0.96 [0.67-1.37] | 43  45 | 0.51 |

# Probiotics and risk of allergic rhinitis or conjunctivitis

Fourteen intervention trials (13 RCT; 1 CCT – in total 15 interventions; 4450 participants) reported allergic rhinitis (AR) or allergic rhinoconjunctivitis (ARC) – here analysed together as ‘allergic rhinitis’. No studies reported allergic conjunctivitis as an outcome. Eight studies were carried out in Europe, one in USA and five Asia-Pacific. Five studies were carried out in infants with normal risk of allergic disease, whilst the other nine were in children at high risk. Age at outcome assessment varied between 1 and 9 years. Two studies defined allergic rhinitis according to specific symptoms, 4 were based on previous Dr-diagnosis or physician assessment during the trial, 2 studies combined symptoms and having 1 or more positive SPTs, and four studies based the diagnosis on ISAAC questionnaires; two studies didn’t report the method of outcome assessment. Risk of bias was similar to other analyses, with a low overall risk of bias in <10%, unclear in the majority, and high in over 35%. (Figure 24).

There was no evidence that probiotics reduce risk of AR at age ≤4 (Figure 25) with low statistical heterogeneity. There was also no evidence for an effect at age 5-14 (Figure 26) with no statistical heterogeneity, and no evidence of publication bias (Figure 27). Subgroup analyses did not identify a subgroup with different outcomes (Tables 13 and 14).

Enomoto ([8](#_ENREF_8)) reported no cases of ARC in either active (n=94) or control (n=31) group at 18 months age. In an analysis including data from Luoto, together with other probiotic intervention studies, Lundelin found no significant difference in allergic rhinitis between probiotic and placebo groups.

**Overall we found no evidence that probiotics influence risk of allergic rhinitis, and data were lacking for allergic conjunctivitis.**

Figure 24 Risk of bias in intervention studies of probiotics and allergic rhinitis

Figure 25 Probiotics and risk of allergic rhinitis at ≤ 4 years


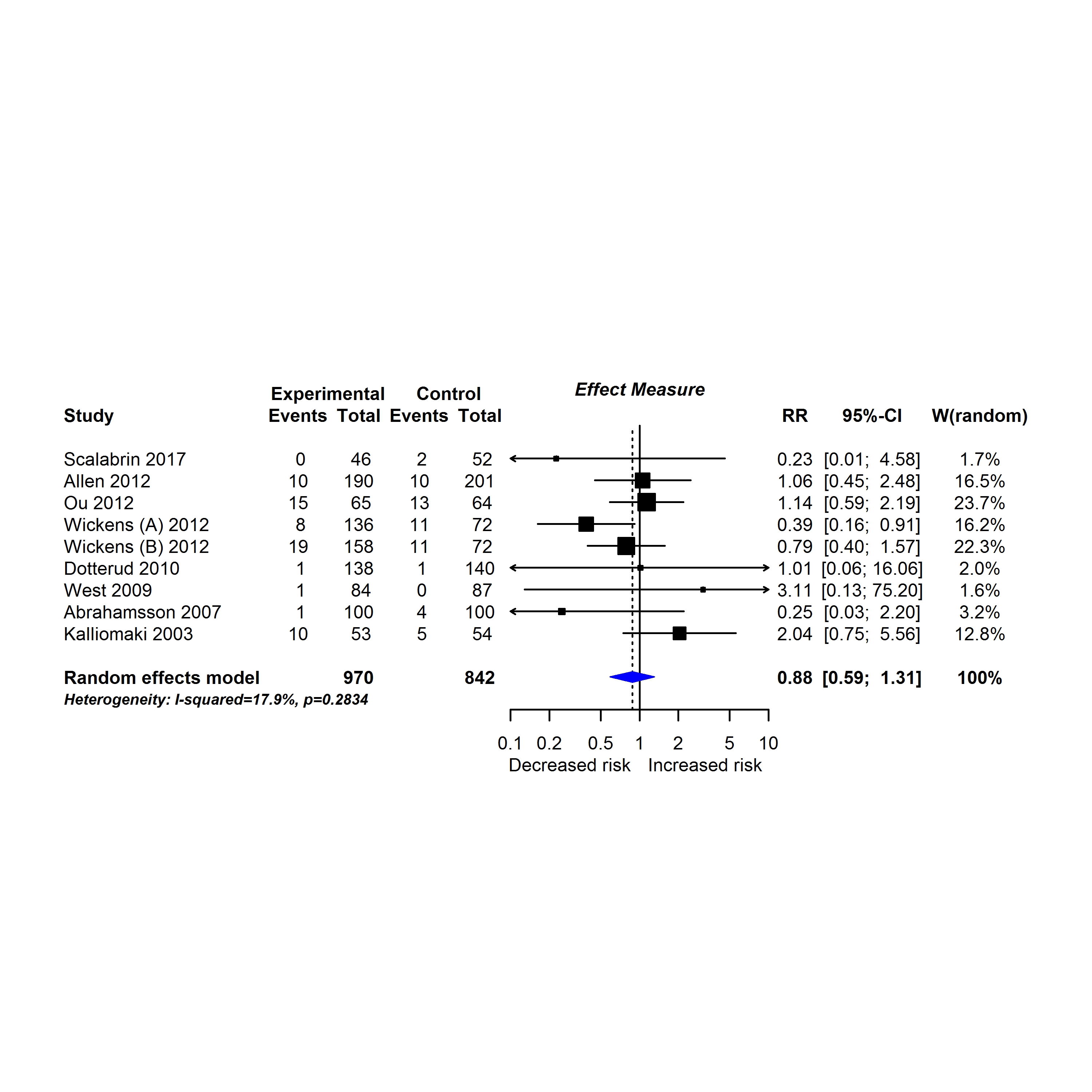


Figure 26 Probiotics and risk of allergic rhinitis at age 5-14 years


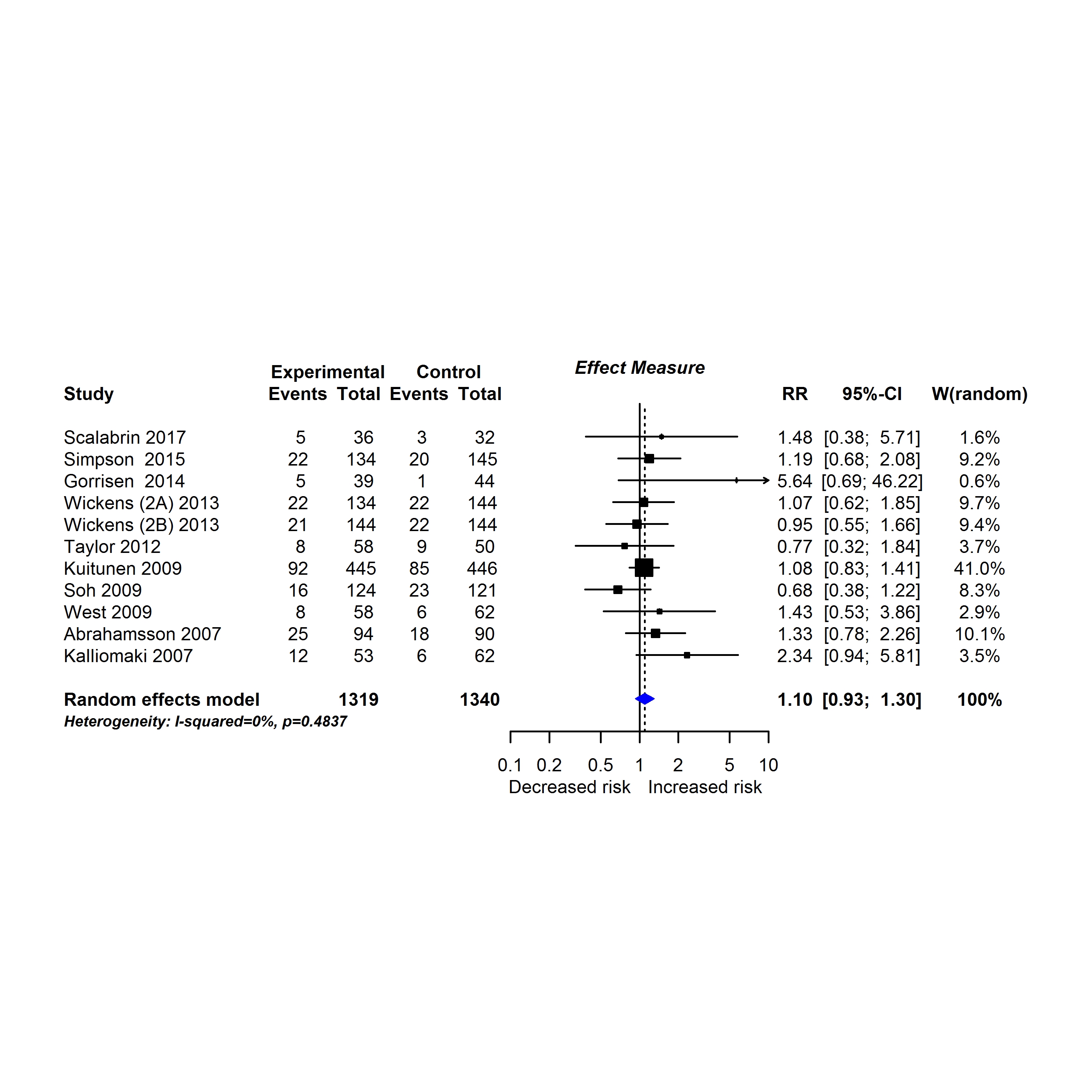


**Figure 27 Risk of publication bias: probiotics and AR at age 5 to 14 years**


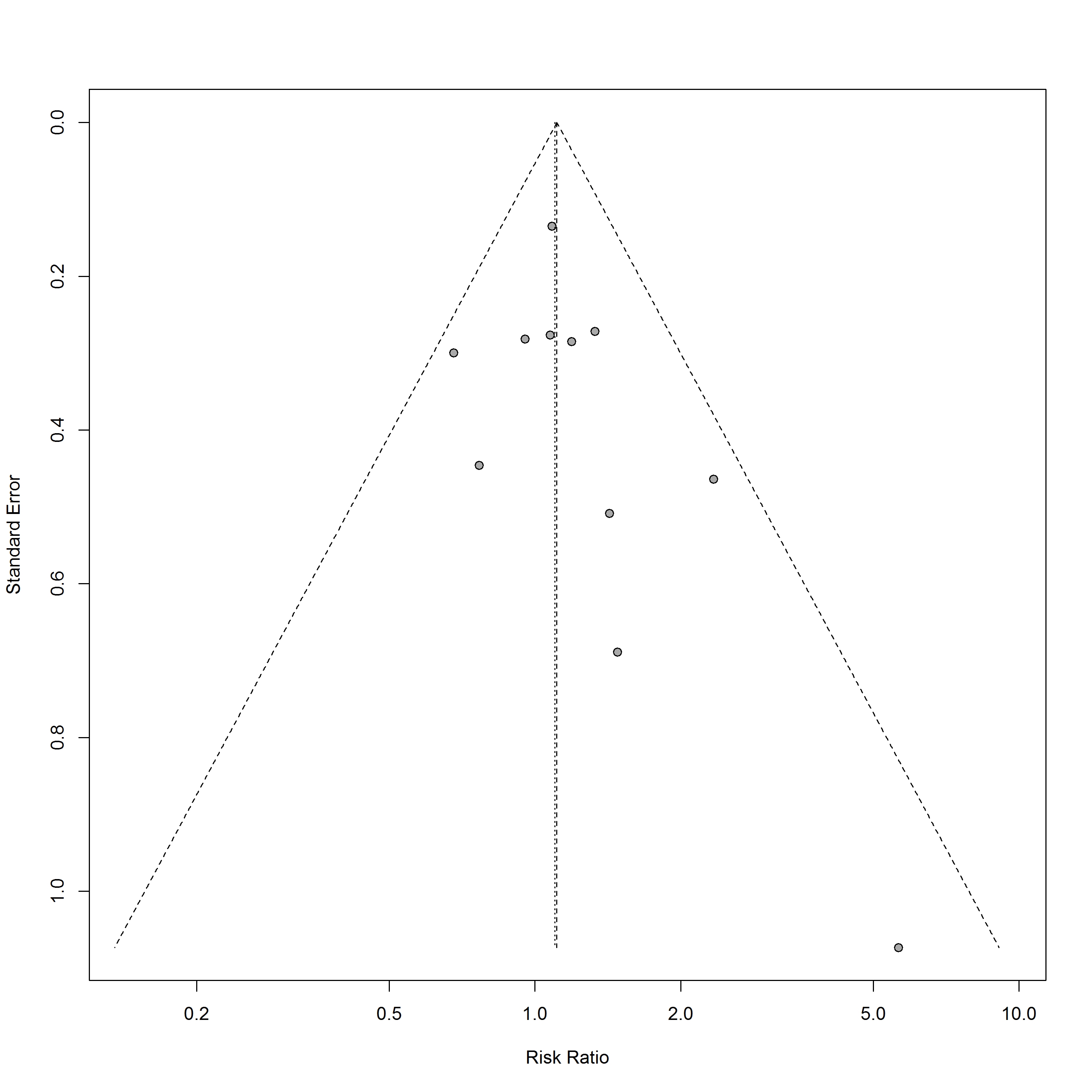


Egger’s test p = 0.22

Table 13 Subgroup analyses of probiotics and risk of allergic rhinitis at age ≤ 4 years

| **Subgroups compared** | **Number of studies** | **RR [95% CI]** | **I^2^ (%)** | **P for between groups difference** |
| --- | --- | --- | --- | --- |
| Type of Intervention – Probiotic  Type of Intervention – Synbiotic | 9  0 | 0.81[0.59; 1.31]  -- | 18 | -- |
| Type of Probiotic – L.rhamnosus  Type of Probiotic – Other | 6  3 | 0.86 [0.30-2.42]  0.82 [0.49-1.38] | 47  0 | 0.94 |
| Timing – Postnatal only  Timing – Pre +/-Post-natal | 2  6 | 0.79 [0.06-10.38]  0.80 [0.44-1.45] | 27  47 | 0.99 |
| Postnatal Administration – Infant only  Postnatal Administration – Other | 4  5 | 0.86 [0.41-1.82]  0.83 [0.39-1.74] | 0  51 | 0.94 |
| Risk of disease – High  Risk of disease – Normal | 6  3 | 0.87 [0.55-1.40]  0.86 [0.16-4.79] | 40  0 | 0.99 |
| Overall risk of bias – Low  Overall risk of bias – High/Unclear | 2  7 | 0.58 [0.29-1.16]  1.15 [0.74-1.78] | 38  0 | 0.10 |
| Conflict of interest – High/Unclear risk  Conflict of interest – Low risk | 6  3 | 1.13 [0.73-1.76]  0.62 [0.32-1.21] | 0  23 | 0.15 |

Table 14 Subgroup analyses of probiotics and risk of allergic rhinitis at age 5-14 years

| **Subgroups compared** | **Number of studies** | **RR [95% CI]** | **I^2^ (%)** | **P for between groups difference** |
| --- | --- | --- | --- | --- |
| Type of Intervention – Probiotic  Type of Intervention – Synbiotic | 10  1 | 1.11 [0.89; 1.40]  1.08 [0.83; 1.41] | 12  -- | 0.88 |
| Type of Probiotic – L.rhamnosus  Type of Probiotic – Other | 7  4 | 1.10 [0.90; 1.33]  1.12 [0.74; 1.68] | 0  20 | 0.94 |
| Timing – Postnatal only  Timing – Pre +/-Post-natal | 4  7 | 0.86 [0.56; 1.30]  1.16 [0.96; 1.39] | 0  0 | 0.19 |
| Postnatal Administration – Infant only  Postnatal Administration – Other | 6  5 | 1.06 [0.86; 1.30]  1.23 [0.87; 1.74] | 0  20 | 0.45 |
| Risk of disease – High  Risk of disease – Normal | 8  3 | 1.08 [0.86; 1.36]  1.27 [0.80; 2.00] | 22  0 | 0.54 |
| Overall risk of bias – Low  Overall risk of bias – High/Unclear | 2  9 | 1.01 [0.69; 1.49]  1.14 [0.91; 1.42] | 0  13 | 0.62 |
| Conflict of interest – High/Unclear risk  Conflict of interest – Low risk | 7  4 | 1.17 [0.89; 1.54]  1.01 [0.72; 1.41] | 27  0 | 0.50 |

# Probiotics and risk of wheeze

One systematic review was identified in our July 2013 search. The study of Tang ([29](#_ENREF_29)) included 5 trials and over 1500 participants, and concluded that probiotics do not reduce risk of wheezing prior to age 5 years (Table 15).

In our own systematic review we identified 18 intervention studies (17 RCT; 1 CCT; in total 19 interventions; 4950 participants) investigating the effect of probiotic supplementation on risk of wheeze. Five studies were from Asia-Pacific countries, two from USA, eleven from Europe. Age at time of outcome assessment ranged between 1 and 9 years old. In four studies the definition used for asthma was prior doctor diagnosis or study physician assessment, in 7 asthma was defined according to whether the child had had from 1 to 3 episodes of wheeze, accompanied sometimes by other symptoms; three studies used the ISAAC definition of wheeze, and in four studies the definition was unclear. Overall risk of bias was low in just over 20%, and unclear in over half of studies. Over one third of studies were considered to be at high risk of conflict of interest due to direct industry involvement in the trial (Figure 28).

We found no evidence that probiotics reduce risk of wheeze or recurrent wheeze at age ≤4 years (Figure 29 – RCT; Figure 31 – CCT; Figure 32– recurrent wheeze) or at 5-14 years (Figures 33 and 35), with low/moderate and no statistical heterogeneity respectively, and no evidence of publication bias for the analysis of wheeze at age ≤4 years or age 5-14 years (Figures 30 and 34).

Subgroup analyses (Table 16-19) demonstrated that the single synbiotic trial showed a positive effect, whereas the trials of probiotics without prebiotic showed no effect on wheeze at age ≤4 (test for subgroup difference p=0.02). The synbiotic trial also differed from other trials in the inclusion criteria of eczema and SCORAD >15 at enrolment. No other consistent subgroup differences were identified.

In an analysis including data from Luoto, together with other probiotic intervention studies, Lundelin found no significant difference in asthma between probiotic and placebo groups - OR 0.55 (0.24, 1.25) P=0.15.

Four studies reported measures of lung function. Abrahamsson reported no difference in forced expiratory volume in 1 second (FEV1) as % predicted at age 7 (Figure 36), and meta-analysis of 3 studies showed no difference in FEV1 reversibility after administration of bronchodilator (Figure 37). Abrahamsson and Wickens both reported FEV1 as % of forced vital capacity (FVC) – in Abrahamsson the median FEV1 as % of FVC was 87 (IQR 83.5, 95) in the intervention group, 88.5 (84.4, 96) in the control group. In the study of Wickens neither probiotic group showed a significant difference in FEV1 as % of FVC – mean difference compared with control group was -1.5% (95% CI -3.7, 0.8) for L. rhamnosus HN001, and -1.2 (-3.4, 0.9) for B. animalis HN019. Gorissen reported ‘all spirometry measures were essentially equal in both groups’, without presenting specific data.

**Overall we found no evidence that probiotics influence risk of wheeze, recurrent wheeze or lung function.**

Figure 28 Risk of bias in intervention studies of probiotics and wheeze

Table 15 Findings from the previous high quality systematic review found in 7/13 search

| **Study** | **Outcome measure** | **No. participants (studies)** | **Outcome (95% CI)** | **I^2^** |
| --- | --- | --- | --- | --- |
| **Tang** ([29](#_ENREF_29)) | Wheeze (under 5 years) | 1536 (5) | **RR 0.93 [0.71, 1.21]** | 0 |

Figure 29 Probiotics and risk of any wheeze at age ≤ 4 years – RCT


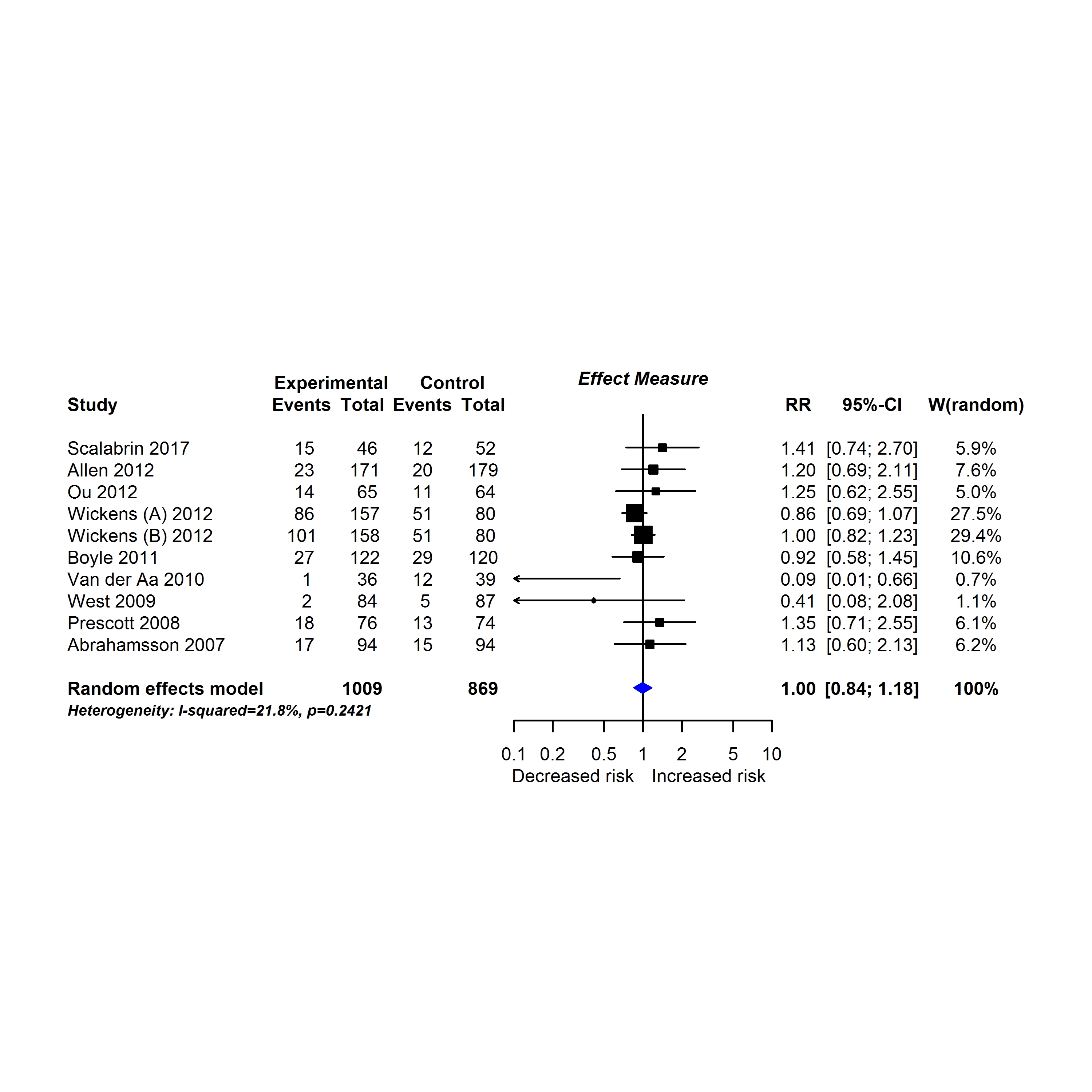


**Figure 30 Risk of publication bias: probiotics and wheeze at age ≤ 4 years**


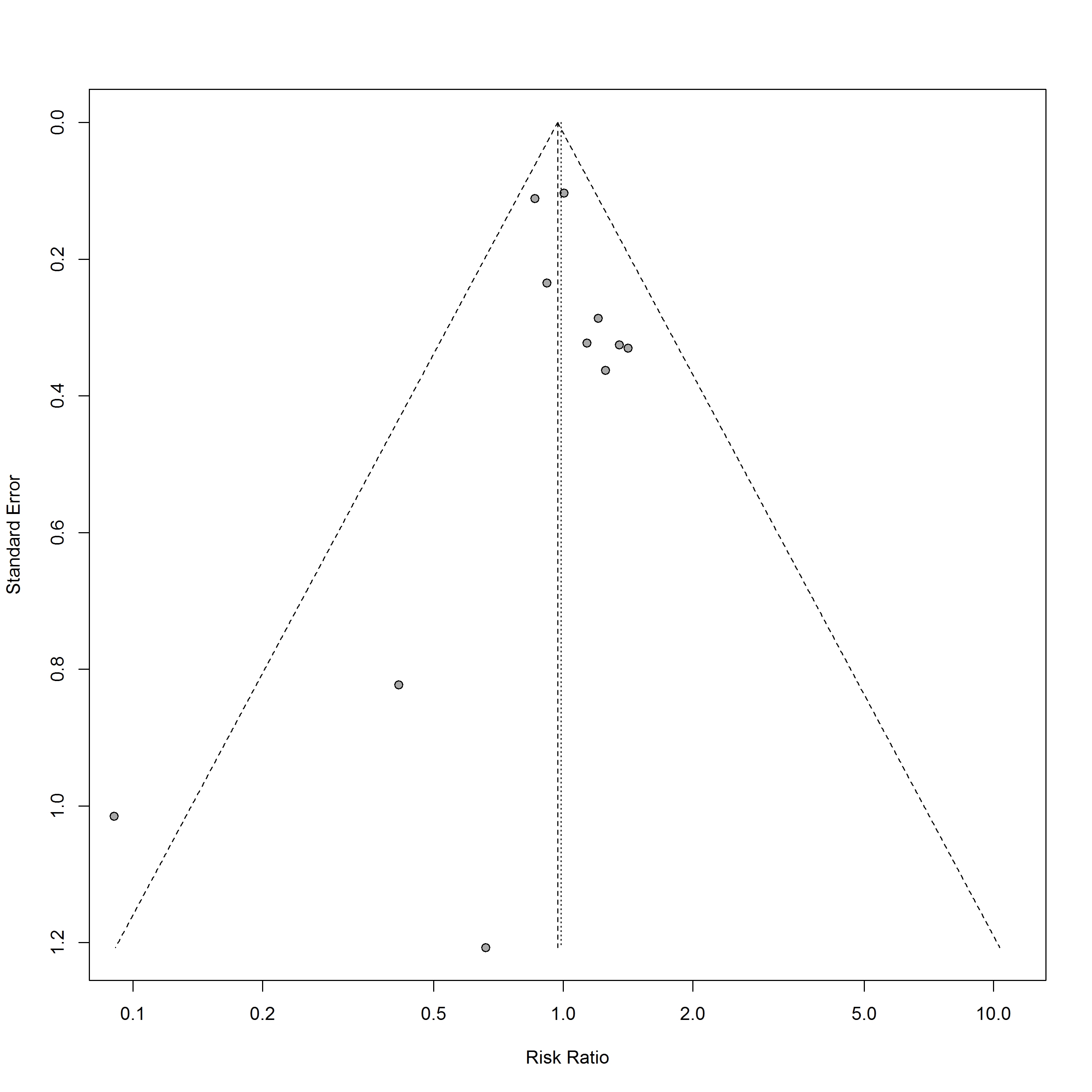


Egger’s test p = 0.73

Figure 31 Probiotics and risk of any wheeze at age ≤ 4 years - CCT


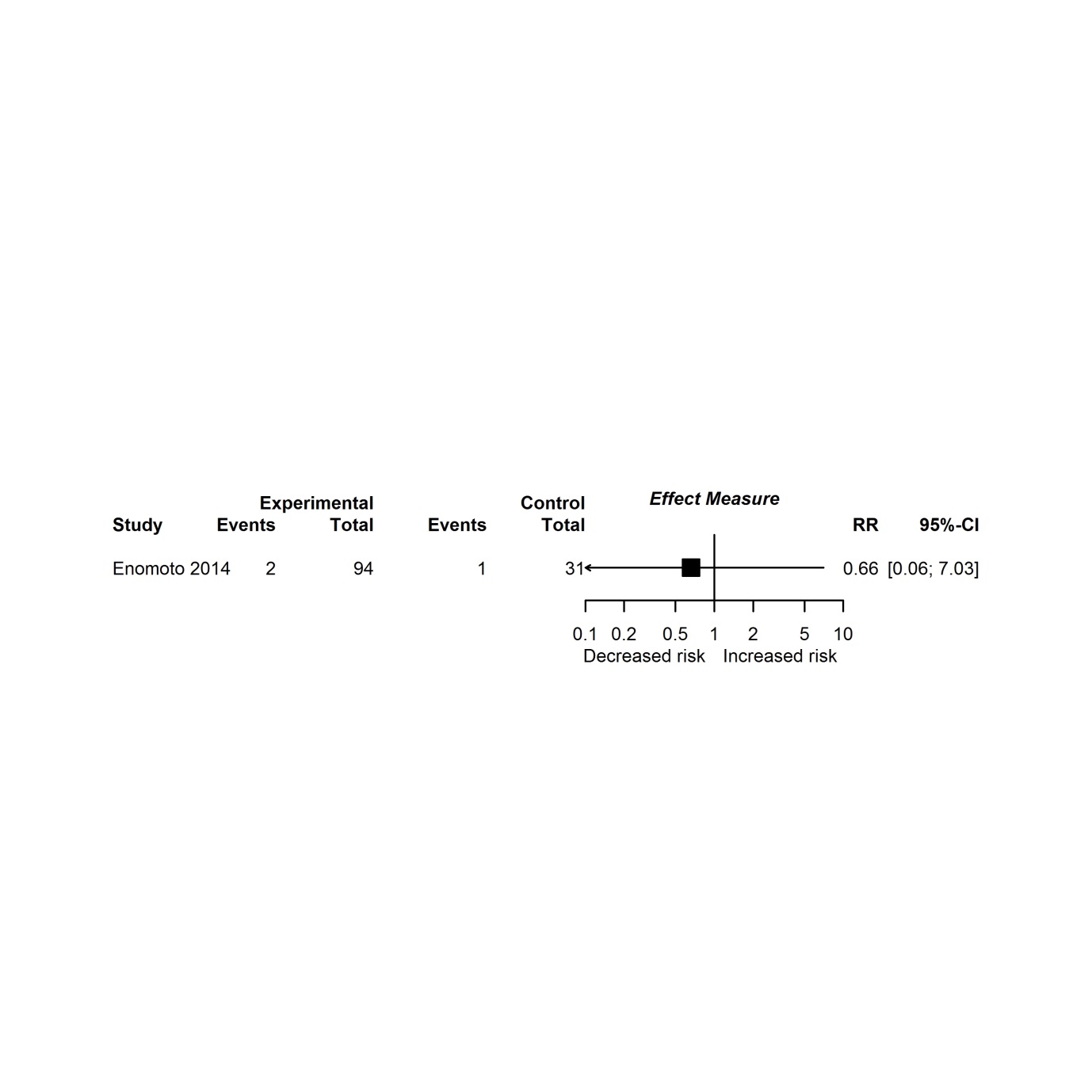


Figure 32 Probiotics and risk of recurrent wheeze at age ≤ 4 years


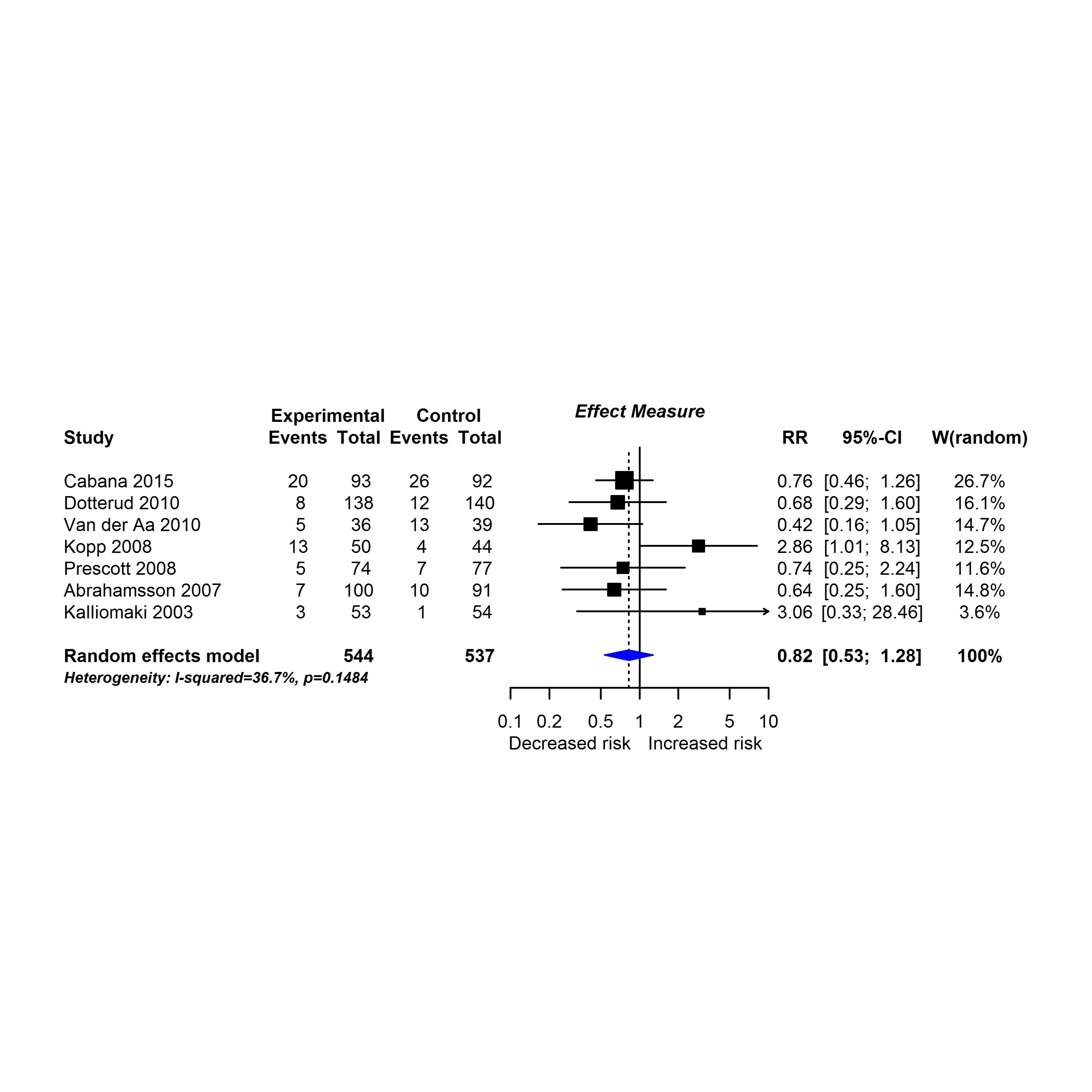


Table 16 Subgroup analyses of probiotics and risk of wheeze at age ≤ 4 years

| **Subgroups compared** | **Number of studies** | **RR [95% CI]** | **I^2^ (%)** | **P for between groups difference** |
| --- | --- | --- | --- | --- |
| Intervention – Probiotics  Intervention – Synbiotics | 9  1 | 0.98 [0.87; 1.12]  0.09 [0.01; 0.66] | 0  - | 0.02 |
| Type of Probiotic – L.rhamnosus  Type of Probiotic – Other | 5  5 | 0.92 [0.76; 1.10]  1.05 [0.76; 1.46] | 0  43 | 0.47 |
| Timing – Postnatal only  Timing – Pre +/-Post-natal | 4  6 | 0.77 [0.31; 1.89]  0.96 [0.84; 1.10] | 68  0 | 0.62 |
| *Postnatal Administration – Infant only  *Postnatal Administration – Other | 6  3 | 1.06 [0.68; 1.63]  0.95 [0.82; 1.09] | 45  0 | 0.88 |
| Risk of disease – High  Risk of disease – Normal | 8  2 | 0.98 [0.83; 1.17]  0.96 [0.31; 2.98] | 24  49 | 0.97 |
| Overall risk of bias – High/Unclear  Overall risk of bias – Low | 6  4 | 1.22 [0.92; 1.61]  0.89 [0.69; 1.16] | 0  56 | 0.11 |
| Conflict of interest – High/Unclear risk  Conflict of interest – Low risk | 5  5 | 1.11 [0.71; 1.72]  0.94 [0.82; 1.08] | 45  0 | 0.50 |

*no postnatal administration in the study of Boyle, 2011 – hence only 7 interventions included in this analysis

Table 17 Subgroup analyses of probiotics and risk of recurrent wheeze at age ≤ 4 years

| **Subgroups compared** | **Number of studies** | **RR [95% CI]** | **I^2^ (%)** | **P for between groups difference** |
| --- | --- | --- | --- | --- |
| Intervention – Probiotics  Intervention – Synbiotics | 6  1 | 0.92 [0.58-1.46]  0.42 [0.17-1.05] | 32  - | 0.13 |
| Type of Probiotic – L.rhamnosus  Type of Probiotic – Other | 4  3 | 1.14 [0.56-2.33]  0.57 [0.32-1.00] | 56  0 | 0.13 |
| Timing – Postnatal only  Timing – Pre +/-Post-natal | 3  4 | 0.67 [0.45-1.02]  1.16 [0.51-2.65] | 0  54 | 0.25 |
| Postnatal Administration – Infant only  Postnatal Administration – Other | 4  3 | 0.67 [0.46-0.97]  1.55 [0.50-4.76] | 0  60 | 0.16 |
| Risk of disease – High  Risk of disease – Normal | 6  1 | 0.87 [0.51-1.49]  0.68 [0.29-1.60] | 46  0 | 0.63 |
| Overall risk of bias – Low  Overall risk of bias – High/Unclear | 2  5 | 1.07 [0.16-7.13]  0.75 [0.52-1.08] | 86  0 | 0.71 |
| Conflict of interest – High/Unclear  Conflict of interest – Low | 6  1 | 0.85 [0.51-1.42]  0.74 [0.25-2.24] | 47  - | 0.83 |

Figure 33 Probiotics and risk of any wheeze at age 5-14 years
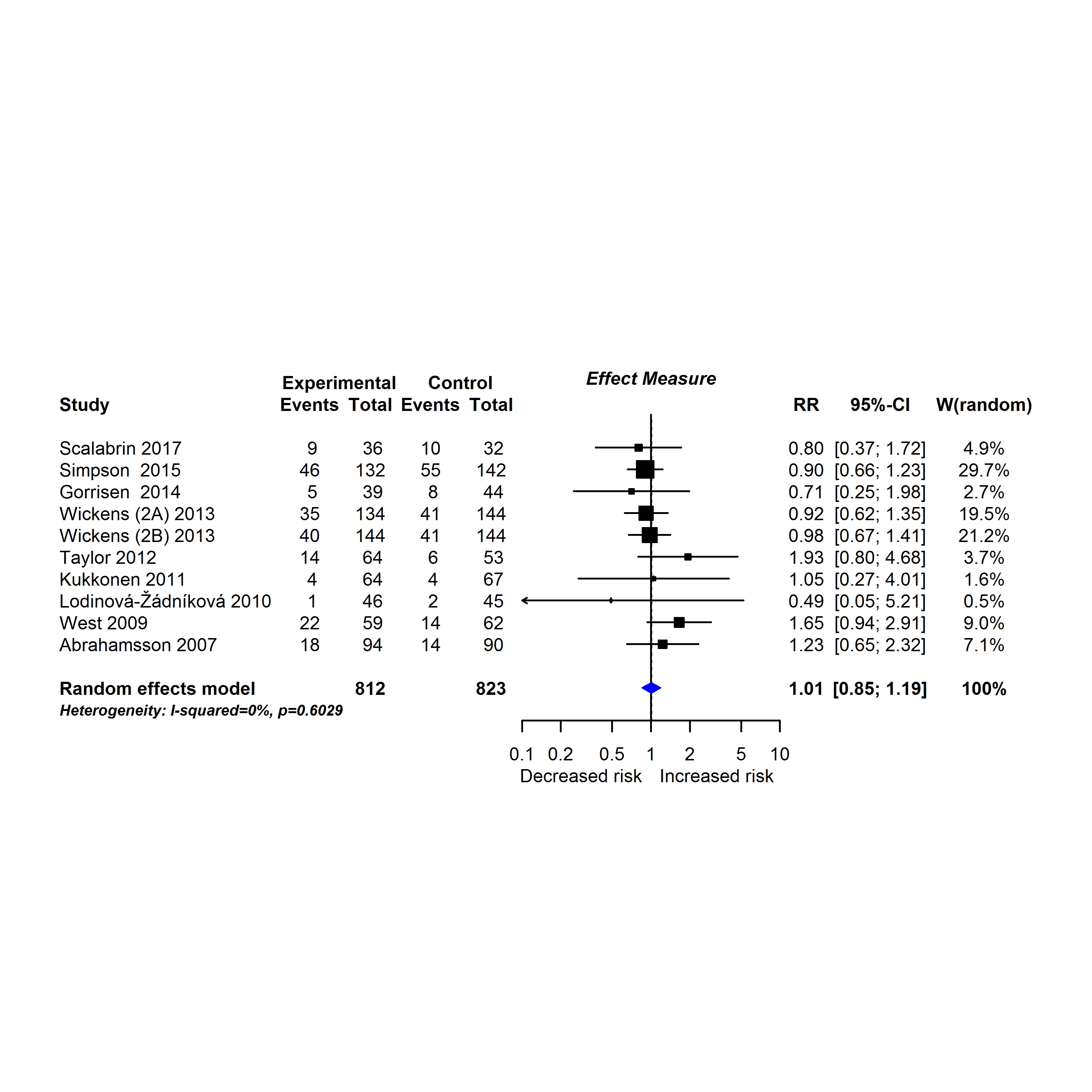


Figure 34 Risk of publication bias: probiotics and wheeze at age 5-14 years


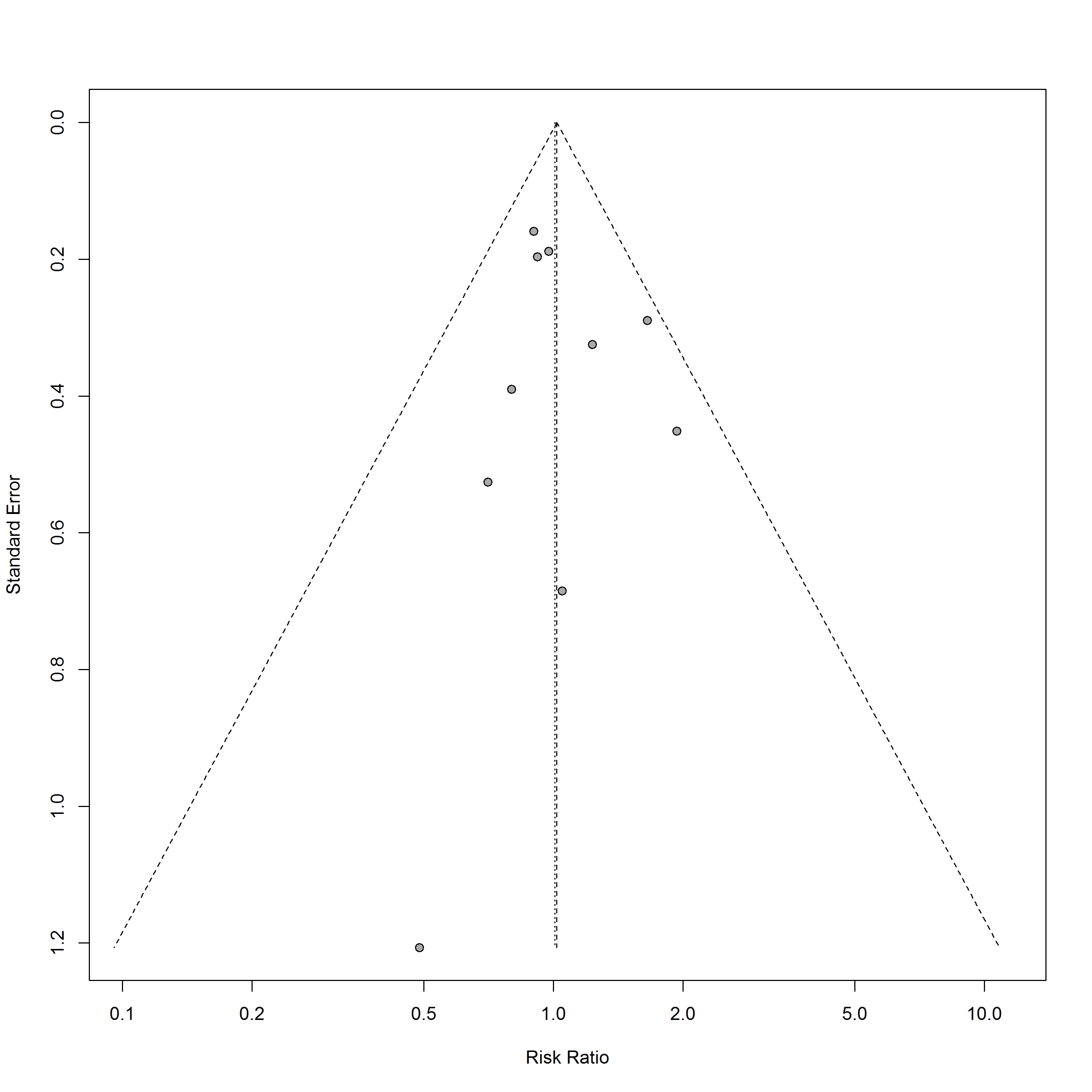


Egger’s test p = 0.68

Figure 35 Probiotics and risk of recurrent wheeze at age 5-14 years


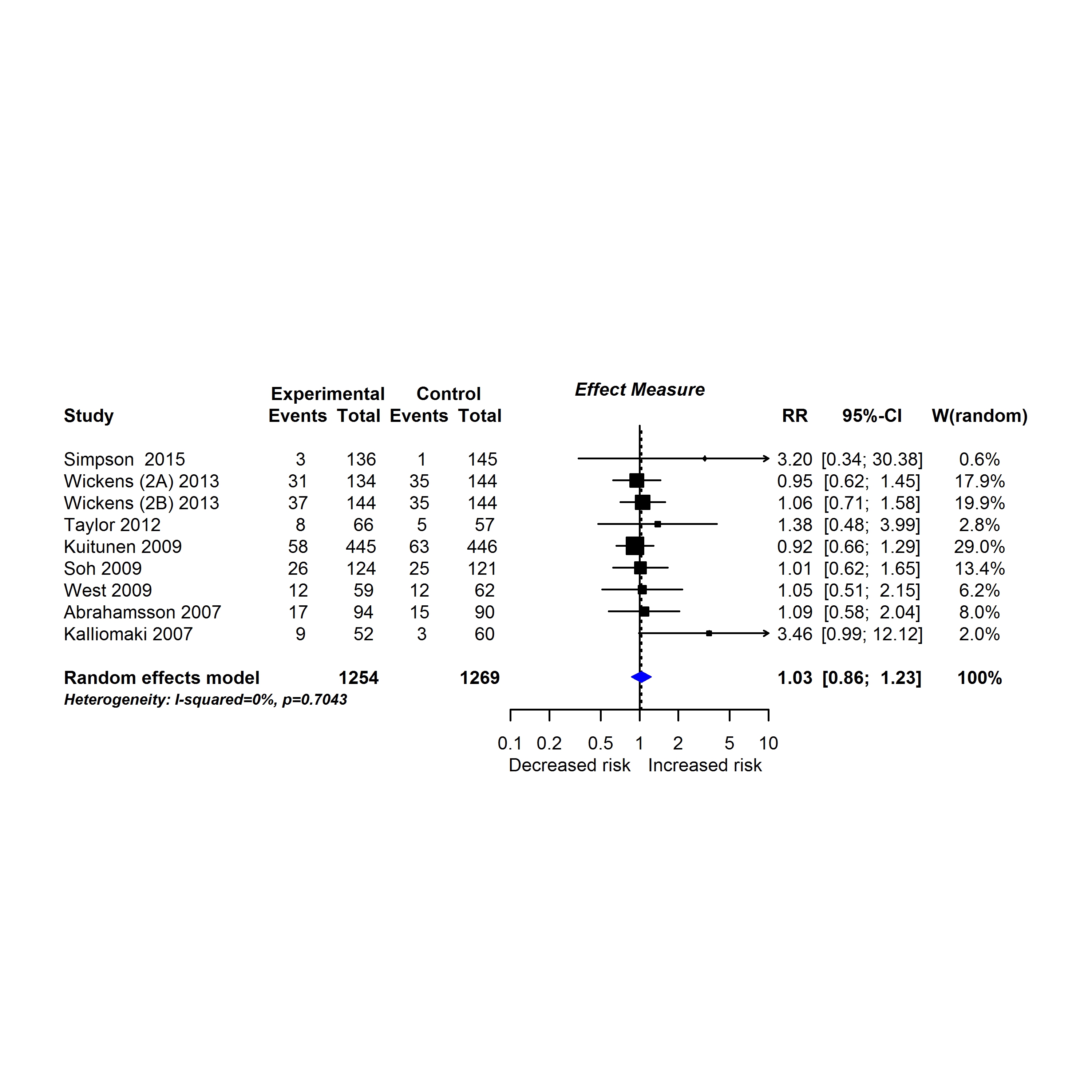
Table 18 Subgroup analyses of probiotics and risk of wheeze at age 5-14 years

| **Subgroups compared** | **Number of studies** | **RR [95% CI]** | **I^2^ (%)** | **P for between groups difference** |
| --- | --- | --- | --- | --- |
| Intervention – Probiotics  Intervention – Synbiotics | 9  1 | 1.01 [0.85-1.19]  1.05 [0.27-4.01] | 0  - | 0.95 |
| Type of Probiotic – L.rhamnosus  Type of Probiotic – Other | 5  5 | 0.97 [0.79-1.21]  1.06 [0.80-1.41] | 0  0 | 0.66 |
| Timing – Postnatal only  Timing – Pre +/-Post-natal | 4  6 | 1.33 [0.84-2.11]  0.94 [0.78-1.14] | 17  0 | 0.18 |
| Postnatal Administration – Infant only  Postnatal Administration – Other | 6  4 | 1.30 [0.94-1.80]  0.92 [0.75-1.12] | 0  0 | 0.08 |
| Risk of disease – High  Risk of disease – Normal | 8  2 | 1.08 [0.87-1.33]  0.88 [0.66-1.18] | 0  0 | 0.28 |
| Overall risk of bias – Low  Overall risk of bias – High/Unclear | 2  8 | 0.95 [0.73-1.24]  1.05 [0.84-1.31] | 0  0 | 0.56 |
| Conflict of interest – High/Unclear  Conflict of interest – Low | 6  4 | 0.92 [0.72-1.18]  1.15 [0.84-1.57] | 0  38 | 0.29 |

Table 19 Subgroup analyses of probiotics and risk of recurrent wheeze at age 5-14 years

| **Subgroups compared** | **Number of studies** | **RR [95% CI]** | **I^2^ (%)** | **P for between groups difference** |
| --- | --- | --- | --- | --- |
| Intervention – Probiotics  Intervention – Synbiotics | 8  1 | 1.08 [0.87-1.34]  0.92 [0.66-1.29] | 0  - | 0.43 |
| Type of Probiotic – L.rhamnosus  Type of Probiotic – Other | 6  3 | 1.01 [0.81-1.26]  1.09 [0.79-1.51] | 2  0 | 0.70 |
| Timing – Postnatal only  Timing – Pre +/-Post-natal | 3  6 | 1.07 [0.73-1.55]  1.03 [0.83-1.27] | 0  4 | 0.87 |
| Postnatal Administration – Infant only  Postnatal Administration – Other | 5  4 | 1.00 [0.79-1.26]  1.18 [0.78-1.78] | 0  35 | 0.50 |
| Risk of disease – High  Risk of disease – Normal | 8  1 | 1.03 [0.86-1.23]  3.20 [0.34-30.38] | 0  - | 0.32 |
| Overall risk of bias – Low  Overall risk of bias – High/Unclear | 2  7 | 1.00 [0.75-1.35]  1.05 [0.84-1.32] | 0  0 | 0.82 |
| Conflict of interest – High/Unclear  Conflict of interest – Low | 5  4 | 1.08 [0.79-1.47]  1.03 [0.79-1.34] | 21  0 | 0.83 |

Figure 36 Probiotics and lung function at age 5-14 (FEV1<80% predicted)


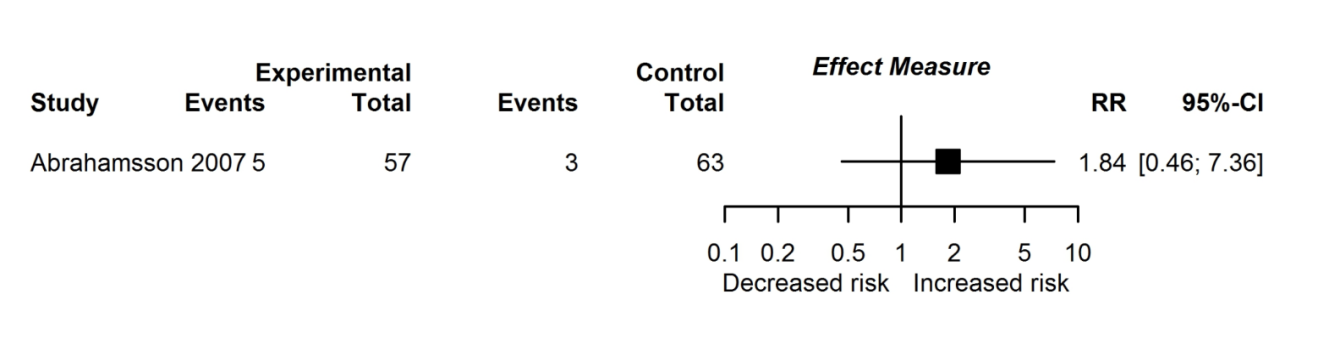


Figure 37 Probiotics and lung function at age 5-14 (FEV1 reversibility >12%)


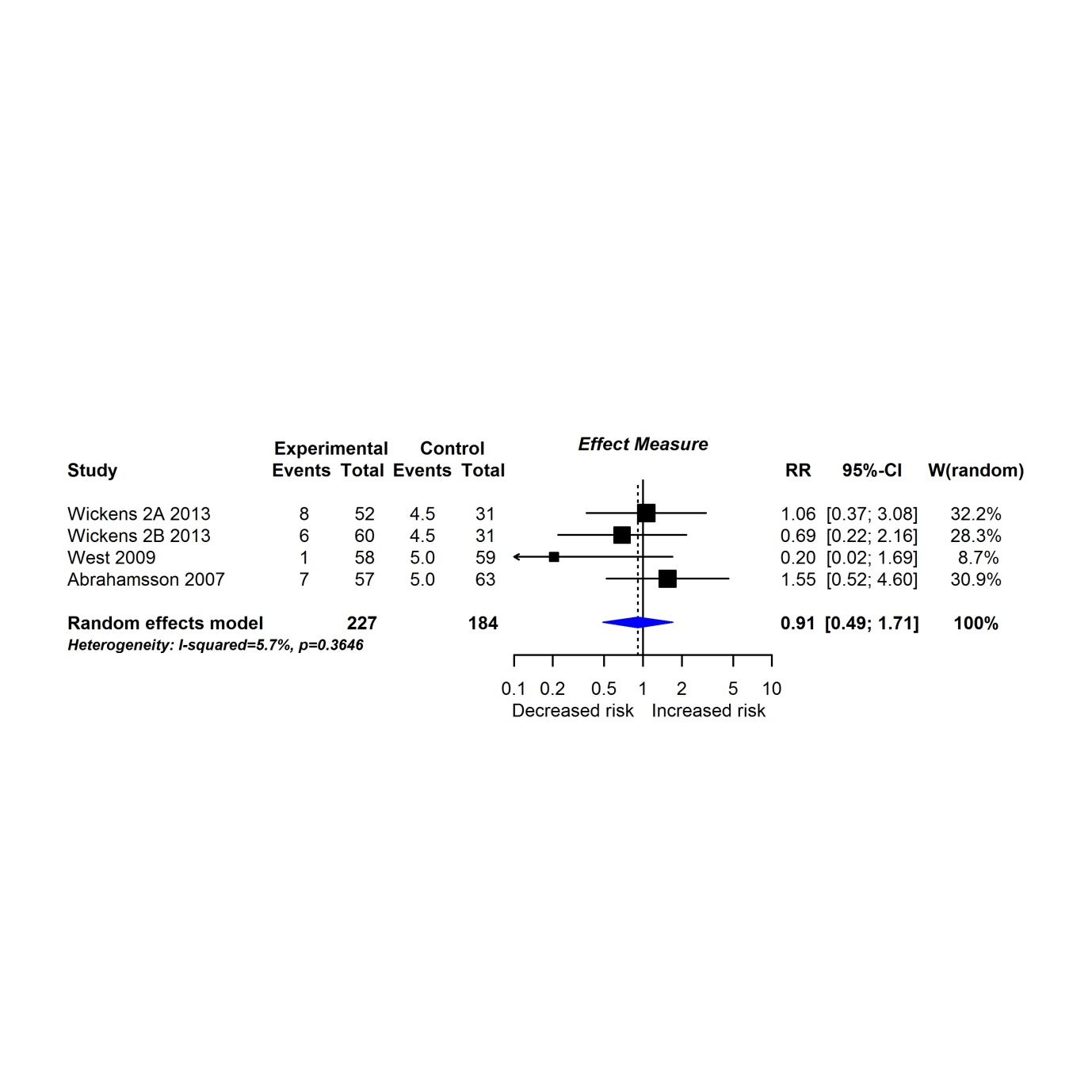


# Conclusions

In this systematic review of probiotic supplementation during pregnancy/lactation/infancy, (either as a probiotic alone or as part of a symbiotic) and risk of allergic outcomes, we found evidence that probiotics reduce the risk of AD or atopic AD, and allergic sensitisation to cow’s milk. The positive findings for AD/atopic AD were not supported in analyses of longer term follow up data from a smaller number of trials at age 5-14. The positive finding for allergic sensitisation to cow’s milk was not supported by data for allergic sensitisation to other allergens, nor by data for clinical cow’s milk allergy. For the outcomes allergic rhinitis, food allergy and wheeze, there was no evidence that probiotics impact on disease risk. For allergic conjunctivitis and autoimmune disease, data were lacking.

In the analysis of AD, there was high statistical heterogeneity. Subgroup analysis identified that postnatal supplementation of mother during lactation may be important for a beneficial effect, and the data were predominantly from studies of infants at high risk of AD due to family history of allergic disease. In the analysis of atopic AD, there was no statistical heterogeneity. In the analysis of allergic sensitisation to cow’s milk there was no statistical heterogeneity, but the number of events was low, and this led to high imprecision and borderline statistical significance.

In the systematic review of Pelucchi ([28](#_ENREF_28)), which we included in our overview of systematic reviews in 2013, the authors also found reduced AD risk in children supplemented with probiotics (RR 0.79; 95%CI 0.71 – 0.88), with less statistical heterogeneity than we found (I^2^=24%). For atopic AD they found RR 0.80 (95% CI 0.66 – 0.96), this time with more statistical heterogeneity than we found (I^2^=31.5%). Also included in our overview Tang found no effect of probiotic supplementation on risk of allergic sensitisation to food in children (RR 0.95; 95% CI 0.80 – 1.12).

Our updated search on 26^th^ February 2017 identified several new systematic reviews of probiotics or synbiotics for prevention of allergic outcomes. R-AMSTAR scoring identified 3 high quality reviews (Dick; Elazab; Azad) and a recent international guideline and systematic review (Fiocchi), which together found the following:

1. Fiocchi 2015 ([55](#_ENREF_55)) in a World Allergy Organization systematic review and position paper have recommended the use of probiotics for preventing AD in high risk pregnancies/infants – for pregnant and lactating women, and directly to their infant. This is based on very low grade evidence.
2. The review of Dick 2014 ([56](#_ENREF_56)) covered a wide range of dietary influences on the development of childhood asthma. They cited the negative finding of Kukkonen 2011, but did not identify the other studies included in our systematic review.
3. Elazab 2013 ([57](#_ENREF_57)) found no evidence that probiotics prevent wheeze, but did find evidence for a protective effect on specific allergic sensitisation with borderline statistical significance. They also found evidence for reduced total IgE, but not in the context of primary prevention. The finding for specific sensitisation was statistically significant for studies which included a prenatal component to the intervention.. We did not find clear evidence for a subgroup difference in allergic sensitisation according to timing of probiotic intervention. Taken together our findings and the findings of Elazab suggest that a small effect (<20% risk reduction) of probiotics on risk of allergic sensitisation cannot be confidently excluded at this stage, and further research is warranted if a small reduction in incidence of allergic sensitisation is considered an important intervention goal.
4. Azad and colleagues published a systematic review on probiotic supplementation and risk of wheeze, asthma or lower tract respiratory infections subsequent to our overview of systematic reviews and found no evidence of a protective effect of probiotics on this outcome (risk ratio 0.97; 95% CI 0.87 – 1.09; I^2^=0%), nor on doctor diagnosed asthma (risk ratio 0.99; 95% CI 0.81 – 1.21; I^2^=0%) ([58](#_ENREF_58)). Azad 2013 found no evidence that probiotics prevent asthma (RR 0.99 95% CI 0.81, 1.21 I^2^=0) or wheeze.

Two further systematic reviews had R-AMSTAR scores below 30, but were nevertheless reasonably comprehensive, and came to similar conclusions to ours. A systematic review by Mansfield identified 27 publications corresponding to 16 trials, and reported a protective effect of probiotic exposure for AD (RR 0.74; CI 0.67 – 0.82) ([59](#_ENREF_59)). Dang 2013 ([60](#_ENREF_60)) identified 18 trials of prebiotics, probiotics or synbiotics for preventing AD. We identified 31 trials (29 with AD as an outcome measure). Dang reported similar conclusions to us – probiotics/synbiotics reduce AD at age <2. They did not review later outcomes, and found no effect on allergic sensitisation.

Overall, our systematic review on probiotics and allergic outcomes provides evidence of a protective effect of these supplements against AD and atopic AD in high risk children ≤ 4 years old, but not beyond this age. Our data are consistent with other recent systematic reviews which did not include all the studies identified in our review.

**Conclusion:**

Probiotics/synbiotics reduce risk of AD in the first 4 years: grade of evidence MODERATE (-1 inconsistency i.e. statistical heterogeneity)

Probiotics/synbiotics reduce risk of atopic (IgE-associated) AD in the first 4 years: grade of evidence MODERATE (-1 inconsistency with age 5-14 outcome).

Probiotics may reduce risk of allergic sensitisation to cow’s milk: grade of evidence LOW (-1 indirectness; -1 imprecision).

Further research is required in order to confirm whether probiotics reduce risk of allergic sensitisation.

# References

1. Abrahamsson TR, Jakobsson T, Bottcher MF, Fredrikson M, Jenmalm MC, Bjorksten B, et al. Probiotics in prevention of IgE-associated eczema: a double-blind, randomized, placebo-controlled trial. Journal of Allergy & Clinical Immunology. 2007;119(5):1174-80.

2. Allen SJ, Jordan S, Storey M, Thornton CA, Gravenor MB, Garaiova I, et al. Probiotics and atopic ECZEMA: A double-blind randomised controlled trial. Archives of Disease in Childhood. 2012;97:A2.

3. Boyle RJ, Ismail IH, Kivivuori S, Licciardi PV, Robins-Browne RM, Mah LJ, et al. Lactobacillus GG treatment during pregnancy for the prevention of eczema: a randomized controlled trial. Allergy. 2011;66(4):509-16.

4. Cabana M, McKean M, Caughey A, Leong R, Wong A, Hilton J, et al. A randomized controlled trial of early probiotic supplementation to prevent early markers of asthma for high-risk infants. European Respiratory Journal ( varpagings) [Internet]. 2015; 46(no pagination). Available from: <http://erj.ersjournals.com/content/46/suppl_59/OA4770>.

5. Chien CM, Anne GEN, Chin CW, Rao R, Charmaine C, Lay C, et al. A synbiotic mixture of scGOS/lcFOS and Bifidobacterium breve M-16V is able to restore the delayed colonization of bifidobacterium observed in C-section Delivered Infants. Journal of pediatric gastroenterology and nutrition [Internet]. 2016; 62:[681 p.]. Available from: <http://onlinelibrary.wiley.com/o/cochrane/clcentral/articles/821/CN-01167821/frame.html>.

6. De Leon J, Sumpaico MW, Recto MT, Tan RA. A preliminary study on the role of probiotics (lactobacillus acidophilus/bifidobacterium) in the prevention of atopic dermatitis in high-risk infants (0-2 weeks old): A randomized placebo-controlled trial. Annals of Allergy Asthma & Immunology. 2007;98(1):A84-A.

7. Dotterud CK, Storro O, Johnsen R, Oien T. Probiotics in pregnant women to prevent allergic disease: a randomized, double-blind trial. British Journal of Dermatology. 2010;163(3):616-23.

8. Enomoto T, Sowa M, Nishimori K, Shimazu S, Yoshida A, Yamada K, et al. Effects of bifidobacterial supplementation to pregnant women and infants in the prevention of allergy development in infants and on fecal microbiota. Allergology International. 2014;63(4):575-85.

9. Huurre A, Laitinen K, Rautava S, Korkeamaki M, Isolauri E. Impact of maternal atopy and probiotic supplementation during pregnancy on infant sensitization: a double-blind placebo-controlled study. Clinical & Experimental Allergy. 2008;38(8):1342-8.

10. Kalliomaki M, Salminen S, Poussa T, Arvilommi H, Isolauri E. Probiotics and prevention of atopic disease: 4-year follow-up of a randomised placebo-controlled trial. Lancet. 2003;361(9372):1869-71.

11. Kim JY, Kwon JH, Ahn SH, Lee SI, Han YS, Choi YO, et al. Effect of probiotic mix (Bifidobacterium bifidum, Bifidobacterium lactis, Lactobacillus acidophilus) in the primary prevention of eczema: a double-blind, randomized, placebo-controlled trial. Pediatric Allergy & Immunology. 2010;21(2 Pt 2):e386-93.

12. Kopp MV, Hennemuth I, Heinzmann A, Urbanek R. Randomized, double-blind, placebo-controlled trial of probiotics for primary prevention: no clinical effects of Lactobacillus GG supplementation. Pediatrics. 2008;121(4):e850-6.

13. Kukkonen K, Savilahti E, Haahtela T, Juntunen-Backman K, Korpela R, Poussa T, et al. Probiotics and prebiotic galacto-oligosaccharides in the prevention of allergic diseases: a randomized, double-blind, placebo-controlled trial. Journal of Allergy & Clinical Immunology. 2007;119(1):192-8.

14. Lodinova-Zadnikova R, Prokesova L, Kocourkova I, Hrdy J, Zizka J. Prevention of allergy in infants of allergic mothers by probiotic escherichia coli. International Archives of Allergy and Immunology. 2010;153(2):201-6.

15. Lundelin K, Poussa T, Salminen S, Isolauri E. Long-term safety and efficacy of perinatal probiotic intervention: Evidence from a follow-up study of four randomized, double-blind, placebo-controlled trials. Pediatric allergy and immunology : official publication of the European Society of Pediatric Allergy and Immunology. 2017;28(2):170-5.

16. Morisset M, Soulaines P, Aubert-Jacquin C, Codreanu F, Maamri N, Hatahet R. Double blind test of a fermented infantile formula in cow's milk allergy prevention [Abstract]. Journal of Allergy and Clinical Immunology [Internet]. 2008; 21(2 Suppl 1):[S244 [941] p.]. Available from: <http://onlinelibrary.wiley.com/o/cochrane/clcentral/articles/516/CN-00679516/frame.html>.

17. Niers L, Martin R, Rijkers G, Sengers F, Timmerman H, van Uden N, et al. The effects of selected probiotic strains on the development of eczema (the PandA study). Allergy. 2009;64(9):1349-58.

18. Ou CY, Kuo HC, Wang L, Hsu TY, Chuang H, Liu CA, et al. Prenatal and postnatal probiotics reduces maternal but not childhood allergic diseases: a randomized, double-blind, placebo-controlled trial. Clinical & Experimental Allergy. 2012;42(9):1386-96.

19. Prescott SL, Wiltschut J, Taylor A, Westcott L, Jung W, Currie H, et al. Early markers of allergic disease in a primary prevention study using probiotics: 2.5-year follow-up phase. Allergy. 2008;63(11):1481-90.

20. Rautava S, Arvilommi H, Isolauri E. Specific probiotics in enhancing maturation of IgA responses in formula-fed infants. Pediatric Research. 2006;60(2):221-4.

21. Rautava S, Kainonen E, Salminen S, Isolauri E. Maternal probiotic supplementation during pregnancy and breast-feeding reduces the risk of eczema in the infant. Journal of Allergy & Clinical Immunology. 2012;130(6):1355-60.

22. Roze JC, Barbarot S, Butel MJ, Kapel N, Waligora-Dupriet AJ, De Montgolfier I, et al. An alpha-lactalbumin-enriched and symbiotic-supplemented v. a standard infant formula: A multicentre, double-blind, randomised trial. British Journal of Nutrition. 2012;107(11):1616-22.

23. Scalabrin D, Berseth C, Marunycz J, Mitmesser S. Long term safety, growth, and health effects of lactobacillus GG (LGG) added to partially and extensively hydrolyzed infant formulas. Allergy: European Journal of Allergy and Clinical Immunology. 2009;64:447.

24. Soh SE, Aw M, Gerez I, Chong YS, Rauff M, Ng YPM, et al. Probiotic supplementation in the first 6 months of life in at risk Asian infants - Effects on eczema and atopic sensitization at the age of 1 year. Clinical and Experimental Allergy. 2009;39(4):571-8.

25. van der Aa LB, Heymans HS, van Aalderen WM, Sillevis Smitt JH, Knol J, Ben Amor K, et al. Effect of a new synbiotic mixture on atopic dermatitis in infants: a randomized-controlled trial. Clinical & Experimental Allergy. 2010;40(5):795-804.

26. West CE, Hammarstrom ML, Hernell O. Probiotics during weaning reduce the incidence of eczema. Pediatric Allergy & Immunology. 2009;20(5):430-7.

27. Wickens K, Black PN, Stanley TV, Mitchell E, Fitzharris P, Tannock GW, et al. A differential effect of 2 probiotics in the prevention of eczema and atopy: a double-blind, randomized, placebo-controlled trial. Journal of Allergy & Clinical Immunology. 2008;122(4):788-94.

28. Pelucchi C, Chatenoud L, Turati F, Galeone C, Moja L, Bach JF, et al. Probiotics supplementation during pregnancy or infancy for the prevention of atopic dermatitis: a meta-analysis. Epidemiology. 2012;23(3):402-14.

29. Tang LJ, Chen J, Shen Y. [Meta-analysis of probiotics preventing allergic diseases in infants]. Zhonghua Erke Zazhi. 2012;50(7):504-9.

30. Abrahamsson TR, Jakobsson T, Bjorksten B, Oldaeus G, Jenmalm MC. No effect of probiotics on respiratory allergies: a seven-year follow-up of a randomized controlled trial in infancy. Pediatric Allergy & Immunology. 2013;24(6):556-61.

31. Allen SJ, Jordan S, Storey M, Thornton CA, Gravenor MB, Garaiova I, et al. Probiotics in the prevention of eczema: a randomised controlled trial. Archives of Disease in Childhood. 2014;99(11):1014-9.

32. Simon AL, Sumpaico MW, Recto MT, Castor MR, Tan RA. The effects of probiotics on total ige levels of infants at risk for the development of atopic disease: A randomized triple blind placebo controlled clinical trial. Annals of Allergy Asthma & Immunology. 2007;98(1):A94-A5.

33. Simpson MR, Dotterud CK, Storro O, Johnsen R, Oien T. Perinatal probiotic supplementation in the prevention of allergy related disease: 6 year follow up of a randomised controlled trial. BMC Dermatology. 2015;15(1):no pagination.

34. Kalliomaki M, Salminen S, Arvilommi H, Kero P, Koskinen P, Isolauri E. Probiotics in primary prevention of atopic disease: a randomised placebo-controlled trial. Lancet. 2001;357(9262):1076-9.

35. Kalliomaki M, Salminen S, Poussa T, Isolauri E. Probiotics during the first 7 years of life: a cumulative risk reduction of eczema in a randomized, placebo-controlled trial. Journal of Allergy & Clinical Immunology. 2007;119(4):1019-21.

36. Rautava S, Kalliomäki M, Isolauri E. Probiotics during pregnancy and breast-feeding might confer immunomodulatory protection against atopic disease in the infant. The Journal of allergy and clinical immunology [Internet]. 2002; 109(1):[119-21 pp.]. Available from: <http://onlinelibrary.wiley.com/o/cochrane/clcentral/articles/174/CN-00377174/frame.html>

37. Kuitunen M, Kukkonen K, Juntunen-Backman K, Korpela R, Poussa T, Tuure T, et al. Probiotics prevent IgE-associated allergy until age 5 years in cesarean-delivered children but not in the total cohort. Journal of Allergy & Clinical Immunology. 2009;123(2):335-41.

38. Kukkonen AK, Kuitunen M, Savilahti E, Pelkonen A, Malmberg P, Makela M. Airway inflammation in probiotic-treated children at 5 years. Pediatric Allergy & Immunology. 2011;22(2):249-51.

39. Lau S, Gerhold K, Zimmermann K, Ockeloen CW, Rossberg S, Wagner P, et al. Oral application of bacterial lysate in infancy decreases the risk of atopic dermatitis in children with 1 atopic parent in a randomized, placebo-controlled trial. Journal of Allergy & Clinical Immunology. 2012;129(4):1040-7.

40. Lodinová-Zádníková R, Prokesová L, Kocourková I, Hrdý J, Zizka J. Prevention of allergy in infants of allergic mothers by probiotic Escherichia coli. International archives of allergy and immunology [Internet]. 2010; 153(2):[201-6 pp.]. Available from: <http://onlinelibrary.wiley.com/o/cochrane/clcentral/articles/180/CN-00762180/frame.html>

41. Luoto R, Kinnunen TI, Aittasalo M, Kolu P, Raitanen J, Ojala K, et al. Primary prevention of gestational diabetes mellitus and large-for-gestational-age newborns by lifestyle counseling: a cluster-randomized controlled trial. Plos medicine [Internet]. 2017; 8(5):[e1001036 p.]. Available from: <http://onlinelibrary.wiley.com/o/cochrane/clcentral/articles/060/CN-01261060/frame.html>.

42. Gorissen DM, Rutten NB, Oostermeijer CM, Niers LE, Hoekstra MO, Rijkers GT, et al. Preventive effects of selected probiotic strains on the development of asthma and allergic rhinitis in childhood. The Panda study. Clin Exp Allergy. 2014;44:1431-33.

43. Taylor AL, Dunstan JA, Prescott SL. Probiotic supplementation for the first 6 months of life fails to reduce the risk of atopic dermatitis and increases the risk of allergen sensitization in high-risk children: a randomized controlled trial. Journal of Allergy & Clinical Immunology. 2007;119(1):184-91.

44. Taylor SN, Wagner CL, Hollis BW. Vitamin D supplementation during lactation to support infant and mother. Journal of the American College of Nutrition. 2008;27(6):690-701.

45. Jensen MP, Meldrum S, Taylor AL, Dunstan JA, Prescott SL. Early probiotic supplementation for allergy prevention: long-term outcomes. Journal of Allergy & Clinical Immunology. 2012;130(5):1209-11.e5.

46. Scalabrin DM, Johnston WH, Hoffman DR, P'Pool VL, Harris CL, Mitmesser SH. Growth and tolerance of healthy term infants receiving hydrolyzed infant formulas supplemented with Lactobacillus rhamnosus GG: randomized, double-blind, controlled trial. Clinical Pediatrics. 2009;48(7):734-44.

47. Scalabrin DMF, Harris C, Strong PV, Liu B, Berseth CL. Infant supplementation with Lactobacillus rhamnosus GG (LGG) and long-term growth and health: A 5-year follow-up. Allergy: European Journal of Allergy and Clinical Immunology. 2014;69:196-7.

48. Scalabrin D, Harris C, Johnston W, Berseth C. Long-term safety assessment in children who received hydrolyzed protein formulas with Lactobacillus rhamnosus GG: a 5-year follow-up. European Journal of Pediatrics. 2017;176(2):217-24.

49. Soh SE, Aw M, Gerez I, Chong YS, Rauff M, Ng YP, et al. Probiotic supplementation in the first 6 months of life in at risk Asian infants--effects on eczema and atopic sensitization at the age of 1 year. Clinical & Experimental Allergy. 2009;39(4):571-8.

50. Loo EX, Llanora GV, Lu Q, Aw MM, Lee BW, Shek LP. Supplementation with probiotics in the first 6 months of life did not protect against eczema and allergy in at-risk Asian infants: a 5-year follow-up. International Archives of Allergy & Immunology. 2014;163(1):25-8.

51. Van Der Aa L, Heymans H, Van Aalderen W, Sillevis Smitt H, Nauta A, Knippels L, et al. Specific synbiotic mixture prevents asthma-like symptoms in infants with atopic dermatitis. Allergy: European Journal of Allergy and Clinical Immunology. 2010;65:313.

52. West CE, Hammarstrom ML, Hernell O. Probiotics in primary prevention of allergic disease--follow-up at 8-9 years of age. Allergy. 2013;68(8):1015-20.

53. Wickens K, Black P, Stanley TV, Mitchell E, Barthow C, Fitzharris P, et al. A protective effect of Lactobacillus rhamnosus HN001 against eczema in the first 2 years of life persists to age 4 years. Clinical & Experimental Allergy. 2012;42(7):1071-9.

54. Wickens K, Stanley TV, Mitchell EA, Barthow C, Fitzharris P, Purdie G, et al. Early supplementation with Lactobacillus rhamnosus HN001 reduces eczema prevalence to 6 years: does it also reduce atopic sensitization? Clinical & Experimental Allergy. 2013;43(9):1048-57.

55. Fiocchi A, Pawankar R, Cuello-Garcia C, Ahn K, Al-Hammadi S, Agarwal A, et al. World Allergy Organization-McMaster University Guidelines for Allergic Disease Prevention (GLAD-P): Probiotics. World Allergy Organization Journal. 2015;8(1):4.

56. Dick S, Friend A, Dynes K, AlKandari F, Doust E, Cowie H, et al. A systematic review of associations between environmental exposures and development of asthma in children aged up to 9 years. BMJ Open. 2014;4(11).

57. Elazab N, Mendy A, Gasana J, Vieira ER, Quizon A, Forno E. Probiotic administration in early life, atopy, and asthma: a meta-analysis of clinical trials. Pediatrics. 2013;132(3):e666-76.

58. Azad MB, Coneys JG, Kozyrskyj AL, Field CJ, Ramsey CD, Becker AB, et al. Probiotic supplementation during pregnancy or infancy for the prevention of asthma and wheeze: systematic review and meta-analysis. Bmj. 2013;347:f6471.

59. Mansfield JA, Bergin SW, Cooper JR, Olsen CH. Comparative probiotic strain efficacy in the prevention of eczema in infants and children: a systematic review and meta-analysis. Military medicine. 2014;179(6):580-92.

60. Dang D, Zhou W, Lun ZJ, Mu X, Wang DX, Wu H. Meta-analysis of probiotics and/or prebiotics for the prevention of eczema. Journal of International Medical Research. 2013;41(5):1426-36.
