# Supplementary material for: Diet during pregnancy and infancy and risk of allergic or autoimmune disease: A systematic review and meta-analysis
Source: PLoS Med. 2018 Feb 28;15(2):e1002507. doi: 10.1371/journal.pmed.1002507 (PMC5830033; doi:10.1371/journal.pmed.1002507)
Supplement: S2 Data — (ZIP) [file pmed.1002507.s007.zip › Review_C_reports/PUFA.docx]

**POLYUNSATURATED FATTY ACID SUPPLEMENTATION IN PREGNANCY/ LACTATION/ INFANCY, AND RISK OF ALLERGIC SENSITISATION OR ALLERGIC DISEASE**

Robert J Boyle^1^, Vanessa Garcia-Larsen^2^, Despo Ierodiakonou^3^, Jo Leonardi-Bee^4^, Tim Reeves^5^, Jennifer Chivinge^6^, Zoe Robinson^6^, Natalie Geoghegan^6^, Katharine Jarrold^6^, Andrew Logan^6^, Annabel Groome^6^ , Evangelia Andreou^7^, Nara Tagiyeva-Milne^8^, Ulugbek Nurmatov^9^, Sergio Cunha^10^

^1^ Clinical Senior Lecturer, Section of Paediatrics ^2^ Post-Doctoral Research Associate, Respiratory Epidemiology and Public Health, National Heart and Lung Institute ^3^ Post-Doctoral Research Associate, Departments of Paediatric and Respiratory Epidemiology and Public Health Group, all at Imperial College London

^4^Associate Professor of Community Health Sciences, University of Nottingham

^5^ Research Support Librarian, Faculty of Medicine, Imperial College London

^6^ Undergraduate medical students, Imperial College London

^7^Research Associate, Imperial Consultants

^8^Research Fellow, University of Aberdeen

^9^Research Fellow, University of Edinburgh

^10^Research Associate, Respiratory Epidemiology and Public Health, National Heart and Lung Institute, Imperial College London

Imperial College London

Table of Contents

[List of Figures 3](#_Toc427132456)

[1. PUFA supplementation and allergic outcomes – summary of interventions and findings 4](#_Toc427132457)

[2. PUFA supplementation and risk of atopic dermatitis 12](#_Toc427132458)

[3. PUFA supplementation and risk of allergic rhino-conjunctivitis 19](#_Toc427132459)

[4. PUFA supplementation and risk of food allergy 21](#_Toc427132460)

[5. PUFA supplementation and risk of allergic sensitisation 24](#_Toc427132461)

[6. PUFA supplementation and lung function/ wheeze 31](#_Toc427132462)

[General conclusions 39](#_Toc427132463)

[References 41](#_Toc427132464)

# List of Figures

[**Figure 1 Risk of bias in intervention studies of PUFAs** 11](#_Toc497234166)

[Figure 2 Risk of bias in intervention studies of PUFAs and AD 14](#_Toc497234167)

[Figure 3 Omega 3 fatty acids and risk of AD at age ≤ 4 15](#_Toc497234168)

[Figure 4 Omega 3 fatty acids and risk of AD at age 5-14 17](#_Toc497234169)

[Figure 5 Omega 3 fatty acids and risk of ‘atopic’ AD at age ≤ 4 17](#_Toc497234170)

[Figure 6 Omega 6 fatty acids and AD at age ≤ 4 17](#_Toc497234171)

[Figure 7 Risk of bias in trials of PUFAs for preventing AR 20](#_Toc497234172)

[Figure 8 Omega 3 fatty acids and risk of AR at age ≤ 4 20](#_Toc497234173)

[Figure 9 Omega 3 fatty acids and risk of AR at age 5-14 20](#_Toc497234174)

[Figure 10 Omega 3 fatty acids and risk of AR at age ≥15 21](#_Toc497234175)

[Figure 11 Omega 3 fatty acids and risk of atopic AR at age 5-14 21](#_Toc497234176)

[Figure 12 Risk of bias of intervention studies on PUFAs and risk of food allergy 23](#_Toc497234177)

[Figure 13 Omega 3 and risk of food allergy at age ≤ 4 23](#_Toc497234178)

[Figure 14 Risk of bias of intervention studies on PUFAs and risk of allergic sensitisation 26](#_Toc497234179)

[Figure 15 Omega 3 fatty acids and risk of AS to any allergen 26](#_Toc497234180)

[Figure 16 Omega 3 fatty acids and risk of AS to any aeroallergen 28](#_Toc497234181)

[Figure 17 Omega 3 fatty acids and risk of AS to food 28](#_Toc497234182)

[Figure 18 Omega 3 fatty acids and risk of AS to egg 28](#_Toc497234183)

[Figure 19 Omega 3 fatty acids and risk of AS to peanut 30](#_Toc497234184)

[Figure 20 Omega 6 fatty acids and risk of AS to food 30](#_Toc497234185)

[Figure 21 Omega 3 fatty acids and risk of AS to cow’s milk 30](#_Toc497234186)

[Figure 22 Omega 6 fatty acids and risk of AS to egg 30](#_Toc497234187)

[Figure 23 Risk of bias in intervention studies on PUFAs and risk of wheeze 34](#_Toc497234188)

[Figure 24 Omega 3 fatty acids and risk of wheeze at age ≤ 4 35](#_Toc497234189)

[Figure 25 Omega 3 fatty acids and risk of wheeze at age 5-14 37](#_Toc497234190)

[Figure 26 Omega 3 fatty acids and risk of atopic wheeze at age ≤ 4 37](#_Toc497234191)

[Figure 27 Omega 3 fatty acids and risk of atopic wheeze at age 5-14 37](#_Toc497234192)

[Figure 28 Omega 3 fatty acids and risk of recurrent wheeze at age ≤ 4 37](#_Toc497234193)

[Figure 29 Omega 3 fatty acids and risk of recurrent wheeze at age 5-14 37](#_Toc497234194)

# PUFA supplementation and allergic outcomes – summary of interventions and findings

In this analysis we included studies of any type of polyunsaturated fatty acid (PUFA) supplementation, whether given as n-3, n-6 or combined interventions, as part of an infant formula, as a supplement, as part of a fish oil or dietary fish supplement during pregnancy, lactation and/or infancy. A formal definition of PUFA is given in the Glossary section of the summary document. We planned to undertake subgroup/stratified analyses for meta-analyses which included >5 studies, and where relevant to include specific fatty acid intervention as a subgroup. We planned to assess publication bias using Funnel plots and Egger’s test where there were ≥10 studies in a meta-analysis. In total we identified 1 high quality recent systematic review in our July 2013 search, and no further high quality systematic reviews in our search update on 26^th^ February 2017. We found 19 original trials investigating the effect of (predominantly) omega 3 fatty acids (n-3), omega 6 fatty acids (n-6), or both, during infancy and/or pregnancy on allergic outcomes. We did not identify any studies reporting autoimmune outcomes.

*Interventions used:*

The **Berman** study ([1](#_ENREF_1)) used DHA (Ω -3) or EPA (Ω -3) rich fish oil supplementation prenatally, compared with soy oil. The **Bisgaard** study ([2](#_ENREF_2)) used fish oil containing 2.4grams per day Ω -3 LCPUFA daily, or olive oil to pregnant women 24 weeks gestation until 1 week post-partum. The **Birch** study ([3](#_ENREF_3)) used supplementation of infant formula with docosahexanoic acid (DHA; n-3) and arachidonic acid (AA; n-6) at concentrations of 0.32%-0.36%/0.64%-0.72% of total fatty acids, respectively. This was given to infants during their first year of life. The **Lucas** study ([4](#_ENREF_4)) gave AA (0.30%) and DHA (0.32%) supplemented to formula. This was given to infants from birth to 6 months. The **van Gool** study ([5](#_ENREF_5)) used Borage oil (containing 100 mg of gamma linoleic acid; GLA). This was given to infants, daily from 1-2 weeks of life to 6 months age. The **Harslof** study ([6](#_ENREF_6)) used fish oil containing 1.2grams Ω-3 PUFA daily, or sunflower oil to infants from 9 to 18 months. The **Kitz study** ([7](#_ENREF_7)) gave GLA at a dose of 100mg/day supplemented to mothers during lactation, or as infant formula (160mg/day) to the infants in their first 5 months of life. The **Linnamaa** study ([8](#_ENREF_8)) used blackcurrant seed oil. This was given in a dose of 3g/day to pregnant women and to infants exclusively breastfeeding in a dose of 1ml/day until age 2 years. The **Mihrshahi** study ([9](#_ENREF_9)) used fish oil (500mg, equivalent to 184mg n-3) daily from 6 months to 5 years, also given in infant formula if that was introduced before 6 months. The family of the participant infant was given canola oil (high in n-3 PUFA) for use in food. There were follow-up times at 3 and 5 years of age([10](#_ENREF_10), [11](#_ENREF_11)). The **Damsgaard** study ([12](#_ENREF_12)) administered fish oil at a dose of 3.4mls/day (equivalent to 571mg eicosapentaenoic acid; EPA, and 381mg DHA) to infants from 9 to 12 months. The **Palmer** study ([13](#_ENREF_13)) used fish oil capsules containing 800mg DHA, 100mg EPA daily. This was given to pregnant women from 21 weeks gestation to delivery. The **Dunstan** study ([14](#_ENREF_14)) used 4g of fish oil 4g (containing 2.07g DHA, 1.02g EPA) to pregnant women, daily from 20 weeks gestation to delivery. The **D’Vaz** study ([15](#_ENREF_15)) used fish oil (containing 280mg DHA and 110mg EPA). This was given to infants from birth to 6 months of age. The **Furuhjelm** study ([16](#_ENREF_16)) used a daily dose of 1.6 g EPA, 1.1 g DHA, as fish oil. This was given to pregnant women from 25 weeks gestation through to the end of lactation. Outcomes were studied at age 1 ([16](#_ENREF_16)) and 2 ([17](#_ENREF_17)). The **Lauritzen** study ([18](#_ENREF_18)) used 4.5 g fish oil daily (equivalent to 1.5 g/d of n-3) to lactating mothers from week 1-2 to 4 months of breastfeeding. The **Olsen** study ([19](#_ENREF_19)) used 4g/day of fish oil (equivalent to 1.28g EPA, and 920mg DHA). This was given to pregnant women from 30 weeks of gestation to delivery. The **Dotterud** study ([20](#_ENREF_20)) used oily fish twice a week and 5ml cod liver oil (1.2 g n-3) daily throughout pregnancy and to infants from 4–6 weeks. It then included a recommendation to give infants oily fish twice a week from the age of 6 months. The **Imhoff-Kunsch** study ([21](#_ENREF_21)) used 400 mg DHA daily. This was given to pregnant women from 20 weeks of gestation to delivery. The **Noakes** study ([22](#_ENREF_22)) used 2 portions (150g each) per week of farmed salmon (equivalent to 163mg EPA, 331mg DHA per day). This was given to pregnant women from 20 weeks of gestation to delivery.

*Populations and Outcomes assessed:*

Outcomes studied were allergic sensitisation (total IgE and skin prick test or specific IgE), food allergy, eczema, allergic rhinitis, wheeze, and lung function (forced expiratory volume in 1 second, FEV_1_). Overall 14,479 infants were included. Eight studies were in infants at high risk of allergic diseases. Eleven studies were carried out in Europe, one in Latin America, three in the USA, and four in Australia. The definitions of diseases varied across studies.

*Overall findings*

Overall the quality of evidence was moderate - there was a low or unclear risk of bias in most studies. Just over 20% of studies were considered to have a high risk of overall bias. Overall we found some evidence that n-6 supplementation can prevent atopic dermatitis (RR 0.76; 95% CI 0.59 to 0.98) in children ≤ 4 years old, although this was considered unreliable because it was based on studies at high risk of bias or conflict of interest, and was dominated by one study (Linnamaa) which found no such effect at a later timepoint. We found evidence from one study that n-3 fatty acid supplements during late pregnancy reduce asthma risk at age 16 compared to olive oil, but not compared to no oil supplement during late pregnancy. In one sensitivity analysis we found evidence that n-3 fatty acid supplementation during pregnancy may reduce risk of allergic rhinitis (AR) at age 5-14 years, however this effect was not seen at age ≤ 4 or ≥15 years, was not seen for the outcome atopic AR, and was not seen for infant supplementation.

We found MODERATE level evidence that n-3 fatty acids reduce the risk of allergic sensitisation to egg (RR 0.69; 95% CI 0.53 to 0.90), although one of the studies contributing to this analysis found no significant effect at later follow up, and no such effect was found for allergic sensitisation to other allergens or ‘any allergen’ or food allergy. This finding was derived from studies of fish oil supplementation during pregnancy +/- lactation, which were all undertaken in infants at high risk of allergic outcomes based on family history. Subgroup analysis found evidence that the effect was related to n-3 fatty acid supplementation initiated during pregnancy (RR 0.55 95% CI 0.40 to 0.76; test for subgroup difference P=0.03), and a similar finding was seen for n-3 fatty acid supplementation initiated during pregnancy, and allergic sensitisation to peanut (RR 0.62 95% CI 0.40 to 0.96). There was no evidence for any other protective effects on the rest of the outcomes studied, but data were sparse and inconclusive for n-3 supplementation and risk of food allergy, and for n-6 supplementation and all allergic outcomes. We did not find any studies reporting the effects of PUFA supplementation on autoimmune outcomes.

**Overall we found MODERATE level evidence (-1 indirectness of outcome measure) that n-3 supplementation using fish oil during pregnancy +/- lactation, reduces allergic sensitisation to egg in high risk infants. No effect was seen for allergic sensitisation to other allergens, but the data for allergic sensitisation to peanut were consistent with those for allergic sensitisation to egg. We did not find evidence that fatty acid supplementation during pregnancy, lactation or infancy has an impact on risk of other allergic outcomes.**

**Table 1 Characteristics of included studies evaluating supplementation of long chain fatty acids and allergic outcomes**

| **Study** | **Design** | **N**  **Int/ Ctrl** | **Country** | **Fatty acid class** | **Intervention** | **Disease risk** | **Age** | **Outcomes reported** |
| --- | --- | --- | --- | --- | --- | --- | --- | --- |
| Klemens ([23](#_ENREF_23)) | SR | 5 RCT  (949) | - | Ω-3 | **Pregnant/lactating women**. Supplementation of pregnant or lactating women with n-3 PUFA, versus olive oil (3), placebo not described (1) or soya oil (1) | - | - | Wheeze, Eczema, Food allergy, Allergic sensitisation |
| Birch, 2010([3](#_ENREF_3))  Foiles, 2015 ([24](#_ENREF_24)) | RCT | 88/ 90 | USA | Ω-3 and Ω-6 | **Infants**. DHA (n-3) and Arachidonic acid (n-6) supplemented formula for first year as 0.32%-0.36%/0.64%-0.72% of total fatty acids, respectively. | normal | 3 | Wheeze (DD), Eczema (DD) |
| Lucas, 1999([4](#_ENREF_4)) | RCT | 154/ 155 | UK | Ω-3 and Ω-6 | **Infants**. 0.3% arachidonic acid (n-6), 0.32% DHA (n-3) supplemented formula from birth to 6 months. | normal | 0.75 | Eczema (parent report), Wheeze/Asthma (parent report; DD) |
| van Gool, 2003([5](#_ENREF_5)) | RCT | 61/ 60 | The Netherlands | Ω-6 | **Infants**. EFATOP trial. Borage oil (100 mg GLA) daily from 1-2 weeks to 6 months age, or sunflower oil. | high | 1 | Eczema (UKWP), Allergic sensitisation (sIgE), Total IgE |
| Kitz, 2006([7](#_ENREF_7)) | RCT | 55/ 76 | Germany | Ω-6 | **Lactating women or their infants**. 100mg/day GLA supplementation of mothers during lactation, or infant formula (160mg/day) in first 5 months. | high | 1 | Eczema (Hanifin), Total IgE |
| Linnamaa, 2010([8](#_ENREF_8)) | RCT | 151/ 162 | Finland | Ω-6 | **Pregnancy/lactating women**. 3g/day blackcurrant seed oil (essential fatty acid) during pregnancy and exclusive breastfeeding, 1ml/day to infant to age 2, or olive oil. | normal | 1 | Eczema (H&R), Allergic sensitisation (SPT), Total IgE |
| Mihrshahi, 2003([9](#_ENREF_9))  Peat, 2004([10](#_ENREF_10)) Marks, 2006([11](#_ENREF_11)) | RCT | 312/ 304 | Australia | Ω-3 | **Infants**. CAPS trial. 500mg fish oil (=184mg n-3 PUFA) daily from 6 months, or in formula if introduced before then, plus canola oil (high in n-3 PUFA) for family, plus environmental control, until 5 years age. | high | 1.5, 3, 5 | Asthma (ISAAC/ parent report), FEV_1_, Eczema (ISAAC-UKWP), Allergic Rhinitis (ISAAC), Allergic sensitisation (SPT), Total IgE |
| Damsgaard, 2007([12](#_ENREF_12)) | RCT | 45/ 49 | Denmark | Ω-3 | **Infants**. Fish oil ~3.4mls/day (571mg EPA, 381mg DHA) from 9 to 12 months. | normal | 1 | Total IgE |
| Palmer, 2012  & 2013 ([13](#_ENREF_13), [25](#_ENREF_25))  Best, 2015 & 2016 ([26](#_ENREF_26), [27](#_ENREF_27)) | RCT | 368/ 338 | Australia | Ω-3 | **Pregnant women**. DOMINO trial. Fish oil capsules with 800mg DHA, 100mg EPA daily from 21 weeks gestation to delivery, or vegetable oil. | high | 1, 3 | Eczema (Itchy rash on facial, flexural or extensor surface), Asthma (≥3 episodes of wheeze + SPT), Food allergy (parent report + SPT), Allergic rhinitis (history + SPT), Allergic sensitisation (SPT) |
| Dunstan, 2003([14](#_ENREF_14)) | RCT | 52/ 46 | Australia | Ω-3 | **Pregnant women**. Fish oil 4g daily from 20 weeks gestation to delivery, containing 2.07g DHA, 1.02g EPA, from 20 week of gestation until delivery - or olive oil. | high | 1 | Food allergy (history), Asthma (≥2 episodes of wheeze), Eczema (DD), Allergic sensitisation (SPT) |
| D'Vaz, 2012([15](#_ENREF_15)) | RCT | 218/ 202 | Australia | Ω-3 | **Infants**. Daily fish oil with 280mg DHA and 110mg EPA (changed to 250/60 part way through trial) from birth to 6 months, or 650mg olive oil. | high | 1 | Eczema (´typical skin lesions´), Allergic sensitisation (SPT), Food Allergy (parent report + SPT), Wheeze (unclear) |
| Furuhjelm, 2009([16](#_ENREF_16))  Furuhjelm, 2011([17](#_ENREF_17)) | RCT | 70/ 75 | Sweden | Ω-3 | **Pregnant/lactating women**. Daily fish oil (1.6 g EPA, 1.1 g DHA) from 25 weeks gestation to end of lactation (or through 3.5 months of breastfeeding), or soy oil. | high | 1, 2 | Allergic sensitisation (SPT and sIgE), Food allergy (parent report), Allergic rhinitis (seasonal symptoms), Asthma (DD), Eczema (recurrent typical itchy rash) |
| Lauritzen, 2005([18](#_ENREF_18)) | RCT | 62/ 60 | Denmark | Ω-3 | **Lactation**. 4.5 g fish oil daily (=~1.5 g/d of n-3 LCPUFA) from 1-2 week to 4 months, or olive oil. | normal | 2.5 | Wheeze (parent report), Food allergy (parent report), Eczema (DD),Total IgE |
| Olsen, 2008([19](#_ENREF_19))  Hansen, 2017 ([28](#_ENREF_28)) | RCT | 266/ 136 | Denmark | Ω-3 | **Pregnant women**. Fish oil 4g/day (=1.28g EPA, 920mg DHA) from 30 weeks gestation to delivery. | normal | 16 | Asthma (DD) |
| Dotterud, 2013([20](#_ENREF_20)) | CCT | 2860/ 5743 | Norway | Ω-3 | **Pregnancy and infant**. PACT trial. Oily fish twice a week and 5ml cod liver oil (1.2 g n-3 PUFA) daily throughout pregnancy and to infant from 4–6 weeks with oily fish twice a week from 6 months. | normal | 2 | Wheeze/Asthma (parent report; DD), Eczema (Parent report) |
| Imhoff-Kunsch, 2011([21](#_ENREF_21)) | RCT | 547/ 547 | Mexico | Ω-3 | **Pregnant women**. 400 mg DHA daily from ~20 weeks gestation to delivery, or corn/soy oil. | normal | 0.5 | Wheeze (parent report) |
| Noakes, 2012([22](#_ENREF_22)) | RCT | 62/ 61 | UK | Ω-3 | **Pregnant women**. SiPS trial. 2 x 150g portions/week of farmed salmon (=~163mg EPA, 331mg DHA per day) from 20 weeks gestation to delivery. | high | 0.5 | Wheeze (study assessment), Eczema (Physician assessment), Allergic sensitisation (SPT) |
| Berman, 2015 ([1](#_ENREF_1)) | RCT | Unclear – total 114 | USA | **Fatty acids**  Ω-3 | DHA (Ω -3) or EPA (Ω -3) rich fish oil prenatal supplementation, or soy oil | Unclear | 3 | Wheeze (parent report); Eczema (parent report) |
| Bisgaard, 2016 ([2](#_ENREF_2)) | RCT | 365/371 | Denmark | **Fatty acids**  Ω-3 | **COPSAC trial.** Fish oil (2.4g/d Ω -3 LCPUFA), or olive oil to pregnant women 24 weeks gestation until 1 week post-partum | Normal | 5 | Wheeze (physician assessment); Eczema (Hanifin and Rajka); AS (SPT, sIgE); ARC (DD);  Lung function (FEV1) |
| Harslof, 2014 ([6](#_ENREF_6)) | RCT | 75/ 79 | Denmark | **Fatty acids**  Ω-3 | Fish oil (1.2g/d Ω-3 PUFA), or sunflower oil to infants from 9 to 18 months | Normal | 1.5 | AS (total IgE) |

SR systematic review; RCT randomised controlled trial; DHA docosahexanoic acid; EPA eicosapentaenoic acid; GLA gamma linolenic acid; LCPUFA long chain polyunsaturated fatty acid; SPT skin prick test; FEV_1_ forced expiratory volume in one second; Physician assessment refers to assessment by a study physician , DD ‘doctor diagnosis’, which refers to community diagnosis; H&R Hanifin and Rajka criteria.

**Figure 1 Risk of bias in intervention studies of PUFAs**

# PUFA supplementation and risk of atopic dermatitis

One systematic review (Table 2) and sixteen intervention trials (12,465 participants, at least 4,878 randomised to intervention) investigated the effect of n-3 or n-6 fatty acids on dermatitis. Ten studies were carried out in Europe and 5 in Australia. Seven studies were carried out in infants at high risk of atopic dermatitis (AD). One study used assessment of medical records by study nurse to define outcome, three studies used parental reported eczema, five studies used either the UK Working Party or Hanifin & Rajka criteria, one study used the ISAAC definition, three studies used typical skin lesions or rash (alone or combined with sIgE or SPT), and three studies used doctor diagnosed eczema. Children’s ages at time of outcome measurement ranged between 6 months and 5 years of age. Assessment bias was low in two-thirds of studies, whilst 60% had a low risk of selection bias and 60% had low risk of attrition bias. Less than 20% of the studies were considered to have a high risk of conflict of interest due to direct industry funding of the trial or relevant industry connections of participating of authors (Figure 2).

The systematic review of 3 studies using n-3 fatty acid supplements during pregnancy/lactation found no evidence of an effect on AD. Twelve original studies reported data that could be included in meta-analysis. There was no evidence for an effect of n-3 fatty acids on AD risk in children ≤ 4 years (Figure 3; RR 1.08, 95% CI 0.82 to 1.42), at age 5-14 (Figure 4), or on atopic-AD (Figure 5; 3 studies; RR 0.69; 95% CI 0.42 – 1.14). The study of Palmer reported outcomes at 1 year (included in Figure 5) and at 3 years. At 3 years the authors used random imputation of missing outcome data, and found no significant difference in atopic AD in the supplemented group (RR 0.73 95% CI 0.51, 1.03). The study of Berman found increased eczema associated with n-3 fatty acid supplementation during pregnancy, but caused moderate heterogeneity in the meta-analysis (Figure 3). The reason why this small trial had different results to others was not clear, since the trial was reported in abstract form only. The CCT of Dotterud reported no difference in AD at age ≤ 4 – RR 0.95 (95%CI 0.83, 1.09).

There was evidence of a protective effect of n-6 fatty acids on AD in children age ≤ 4 years (Figure 6; 3 studies; RR 0.56; 95% CI 0.59 – 0.98) with no heterogeneity, based on 3 trials. Linnamaa found a significant reduction in AD at age 1 (shown in Figure 6) but no significant difference at age 2, and was at high risk of bias due to high attrition. The study of van Gool was at high risk of conflict of interest; the study of Kitz, which showed no evidence of an effect, at unclear or low risk of bias on all domains including conflict of interest. Thus the GRADE of evidence for n-6 fatty acid supplementation and AD was downgraded (-2 study quality; -1 inconsistency; -1 sparse/imprecise data) to no evidence.

Figure 2 Risk of bias in intervention studies of PUFAs and AD

Table 2 Findings from the previous systematic review

| **Study** | **Outcome measure** | **No. participants (studies)** | **Outcome (95% CI)** | **I^2^** |
| --- | --- | --- | --- | --- |
| **Klemens** ([23](#_ENREF_23)) | Eczema | 262 (3) | OR 0.92 [0.49, 1.75] | 70% |

Meta-analysis of 2 studies (482 participants) where supplementation was initiated during pregnancy rather than during lactation showed similar results to supplementation during pregnancy and/or lactation overall.

Figure 3 Omega 3 fatty acids and risk of AD at age ≤ 4


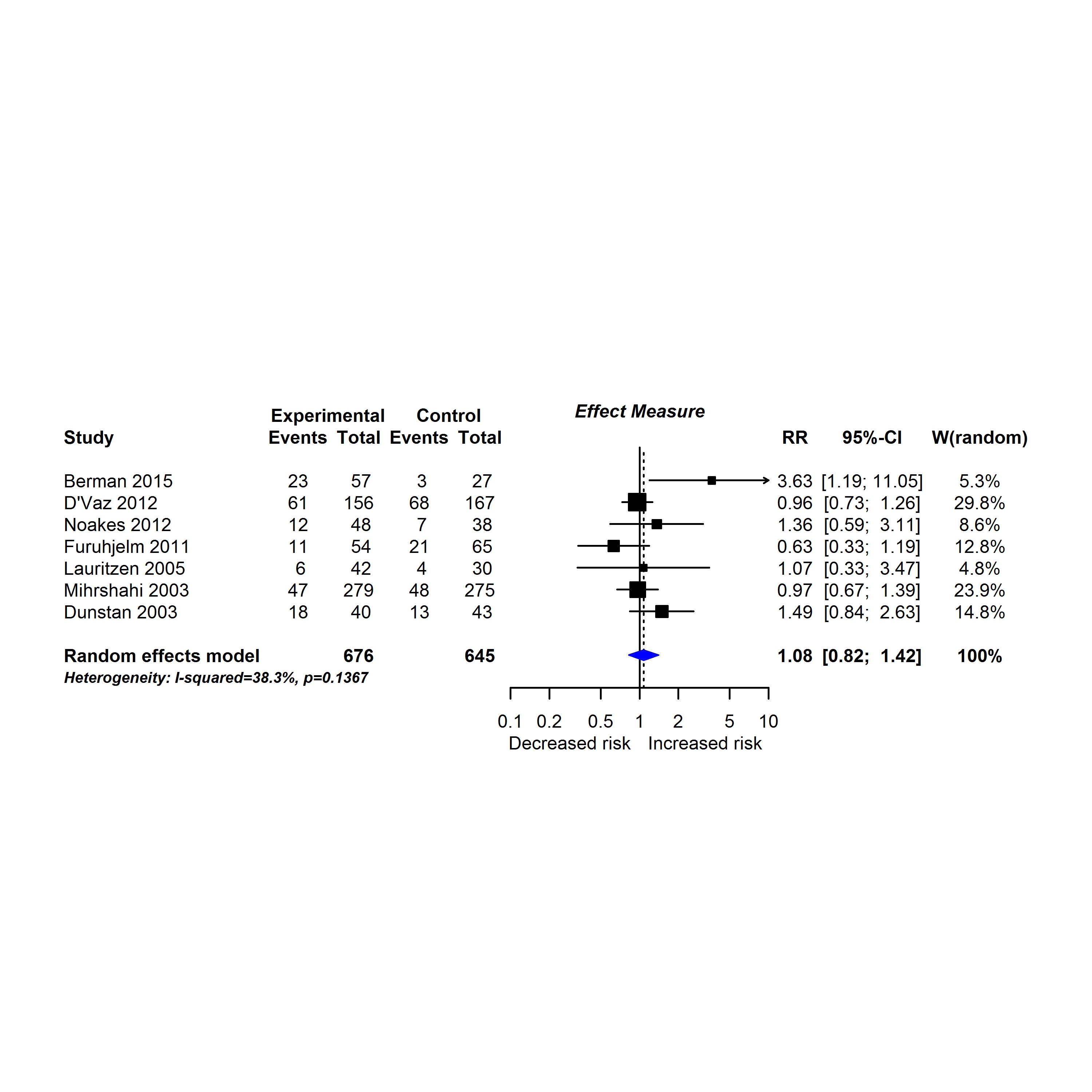


Table 3 Subgroup analysis of n-3 fatty acids and eczema risk in children aged 0-4 years

|  | | **Number of studies** | **RR [95% CI]** | **I^2^ (%)** | **P-value for between groups difference** |
| --- | --- | --- | --- | --- | --- |
| Intervention – prenatal +/- postnatal  Intervention – postnatal only | 5  2 | | 1.28 [0.75-2.17]  0.96 [0.77-1.19] | 53  0 | 0.43 |
| Risk of disease – High  Risk of disease – Normal/Low | 6  1 | | 1.09 [0.81-1.47]  1.07 [0.33-3.47] | 49  - | 0.43 |
| Overall risk of bias – Low  Overall risk of bias – High/Unclear | 3  4 | | 0.98 [0.65-1.49]  1.31 [0.77-2.23] | 49  49 | 0.83 |
| Conflict of interest bias – Low  Conflict of interest bias – High/Unclear | 5  2 | | 1.03 [0.85-1.26]  1.43 [0.25-8.29] | 0  87 | 0.68 |

Figure 4 Omega 3 fatty acids and risk of AD at age 5-14


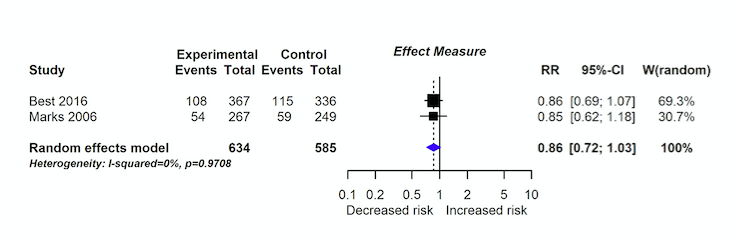


Figure 5 Omega 3 fatty acids and risk of ‘atopic’ AD at age ≤ 4


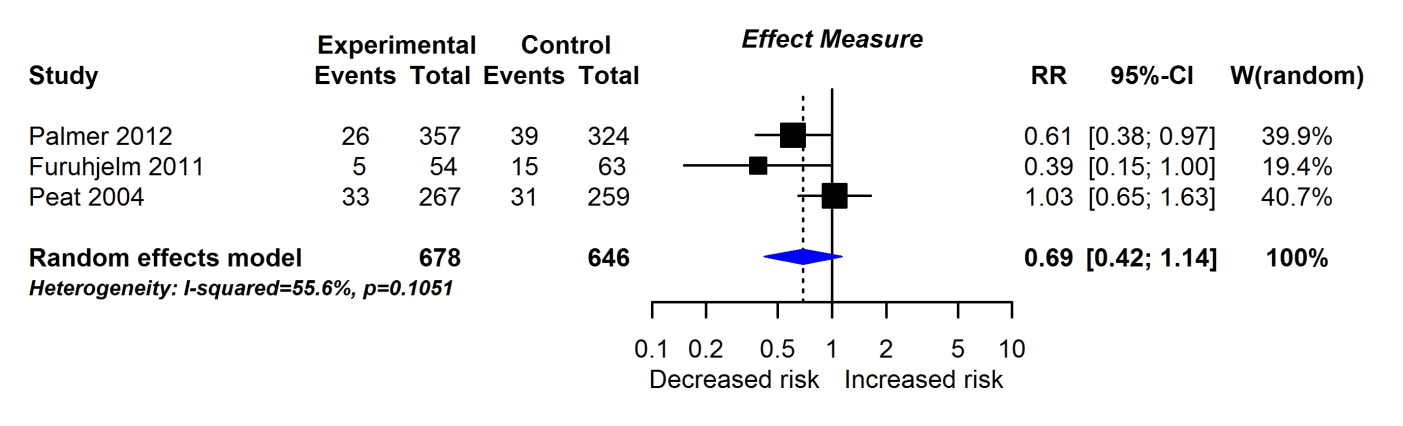


Figure 6 Omega 6 fatty acids and AD at age ≤ 4


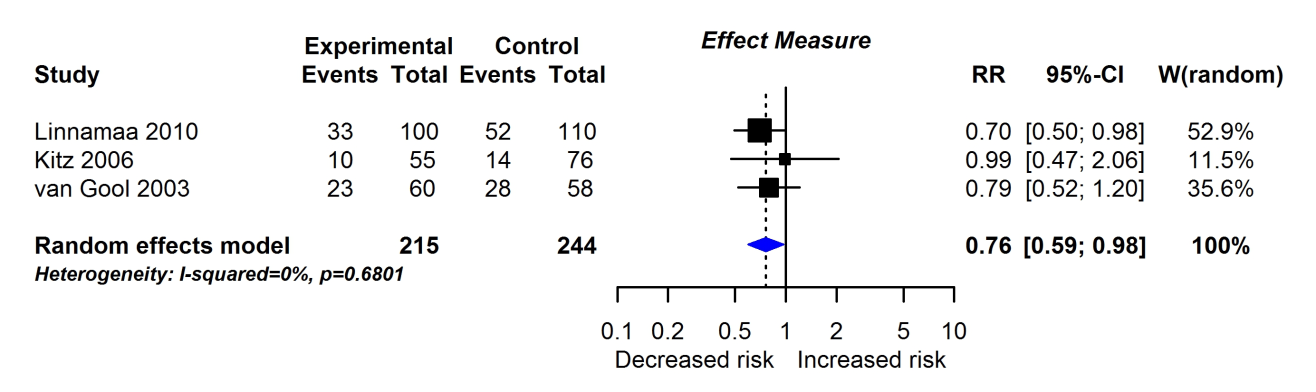


*PUFA supplementation and risk of atopic dermatitis – data that could not be included in meta-analyses*

Two studies assessed an intervention which combined an n-3 (DHA) and an n-6 (Arachidonic acid) in the intervention. In both cases the intervention was as a supplementary formula milk introduced very early (<1 week) into the infant diet, and both studies were considered at high risk of conflict of interest due to direct industry involvement. The study of Birch, considered at high risk of bias due to 50% loss to follow up, found no difference in AD risk between active and control groups (RR 0.52, 95%CI 0.24, 1.12). Follow up of the same study by Foiles reported reduced 'skin allergic disease' in fatty acid versus control group (P=0.05), but the outcome definition included contact dermatitis and urticaria as well as eczema. The study of Lucas, considered at unclear overall risk of bias due to unclear assessment bias, also found no difference (OR 1.20, 95%CI 0.70, 2.10). The study of Bisgaard, considered at low overall risk of bias, due to low assessment, selection and attrition bias, furthermore found no difference (HR 1.10 95%CI 0.83, 1.44).

**Overall we found no evidence that n-3 or n-6 supplementation reduces risk of AD. For n-3 supplementation the data make clinically meaningful effects unlikely. For n-6 supplementation the data are sparse and more work is needed to assess any possible role.**

# PUFA supplementation and risk of allergic rhinitis/rhinoconjunctivitis (AR)

Six intervention studies (2914 participants, 1469 randomised to the intervention arm) investigated the effect of n-3 fatty acids and risk of allergic rhino-conjunctivitis. One study was carried out in Australia, one in USA and two in Europe. Two studies recruited infants at high risk of atopic disease, and two a normal risk population. One study used the ISAAC definition of outcome, one used Hanifin and Rajka, one used doctor diagnosis and one used seasonal symptoms, combined with sIgE or SPT. The children’s age at the time of outcome measure ranged between 2 and 5 years old. Four studies showed a low risk of bias, one had a high risk of attrition bias. One study had a high risk of conflict of interest (Figure 7). Forest plots showing data from five studies show no overall evidence for an effect on RC at age ≤ 4, age 5-14 or age ≥15 (Figures 8 to 11). High heterogeneity is seen in the analysis of AR at age 5-14 (Figure 9), and this is accounted for by the null findings of Marks, where infants were supplemented, and a reduction in AR in the studies of Best and Bisgaard (sensitivity analysis RR 0.75 95%CI 0.60, 0.93) where pregnant women were supplemented. Data from one study at high risk of bias and high risk of conflict of interest could not be included in a forest plot – Foiles reported that this outcome AR was recorded, but did not report outcome data for AR. They did report a composite outcome ‘any allergic disease’, which included AR and was reduced in the fatty acid supplemented group (P=0.04).

**Overall we found no consistent evidence that n-3 supplementation reduces risk of allergic rhinitis. In one sensitivity analysis we found evidence that omega 3 fatty acid supplementation during pregnancy may reduce risk of AR at age 5-14 years, however this effect was not seen at age ≤ 4 or ≥15 years, was not seen for the outcome atopic AR, and was not seen for infant supplementation. We found no studies reporting data for allergic conjunctivitis.**

Figure 7 Risk of bias in trials of PUFAs for preventing AR

Figure 8 Omega 3 fatty acids and risk of AR at age ≤ 4


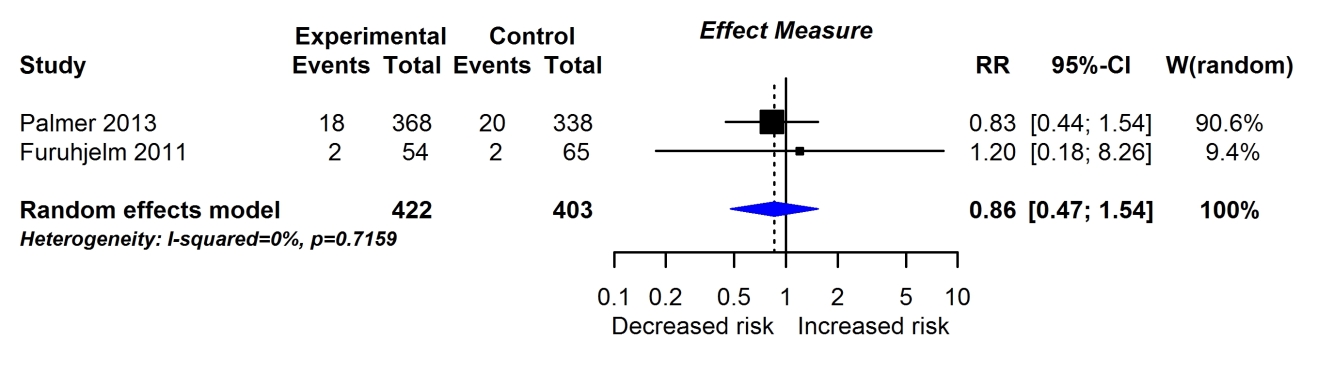


Figure 9 Omega 3 fatty acids and risk of AR at age 5-14


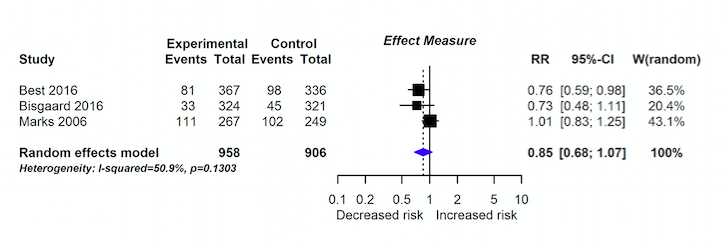


Figure 10 Omega 3 fatty acids and risk of AR at age ≥15


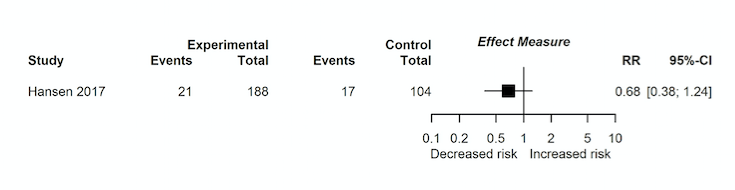


#

Figure 11 Omega 3 fatty acids and risk of atopic AR at age 5-14


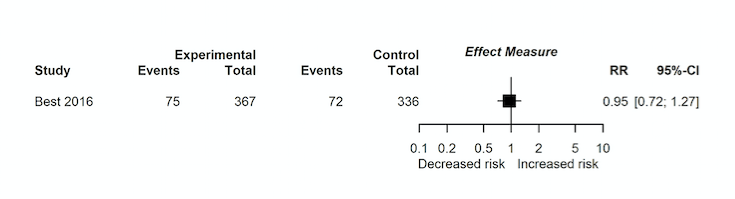


# PUFA supplementation and risk of food allergy

One systematic review and six intervention studies (1,669 participants, 858 randomised to the intervention arm) investigated the effect of n-3 fatty acid supplementation and risk of food allergy. Three studies were carried out in Australia, two in Europe and one in USA. All but one study was carried out in infants at high risk of food allergy. Two studies used parent reported food allergy as outcome definition, three used food allergy history plus SPT, one used doctor diagnosis. Children’s age at time of outcome measurement ranged between 1 and 4 years old. Risk of bias was low in 50% (Figure 12). Risk of conflict of interest was low in most studies (Figure 12).

The systematic review of 3 studies using n-3 fatty acid supplementation during pregnancy and/or lactation found no evidence that this influenced risk of food allergy (Table 4).

Five of 6 studies were eligible for meta-analysis (Figure 13). There was no evidence that n-3 fatty acid supplementation alters food allergy risk (RR 0.76; 95% CI 0.52, 1.11), with no statistical heterogeneity between studies.

The study of Palmer reported outcomes at 1 year (included in Figure 13) and at 3 years. At 3 years the authors used random imputation of missing outcome data, and found no significant difference in food allergy in the supplemented group (RR 1.20 95% CI 0.60, 2.41). The study of Foiles was considered at a high risk of bias due to high attrition bias. The study recorded food allergy outcomes, but did not report results for food allergy separately, other than as part of ‘any allergic disease’, where there was reduced incidence in the PUFA supplemented group (P = 0.04).

**Overall we found no evidence that n-3 supplementation reduces risk of food allergy, although data were sparse and further work is needed since significant effects cannot be confidently excluded based on the available data.**

Figure 12 Risk of bias of intervention studies on PUFAs and risk of food allergy

Table 4 Findings from the previous systematic review

| **Study** | **Outcome measure** | **No. participants (studies)** | **Outcome (95% CI)** | I^2^ |
| --- | --- | --- | --- | --- |
| **Klemens** ([23](#_ENREF_23)) | Food allergy* | 264 (3) | OR 0.46 [0.16, 1.38] | 33% |

*Meta-analysis of 2 studies (200 participants) where supplementation was initiated during pregnancy rather than during lactation showed OR 0.34, 95%CI 0.10, 1.15.

Figure 13 Omega 3 and risk of food allergy at age ≤ 4


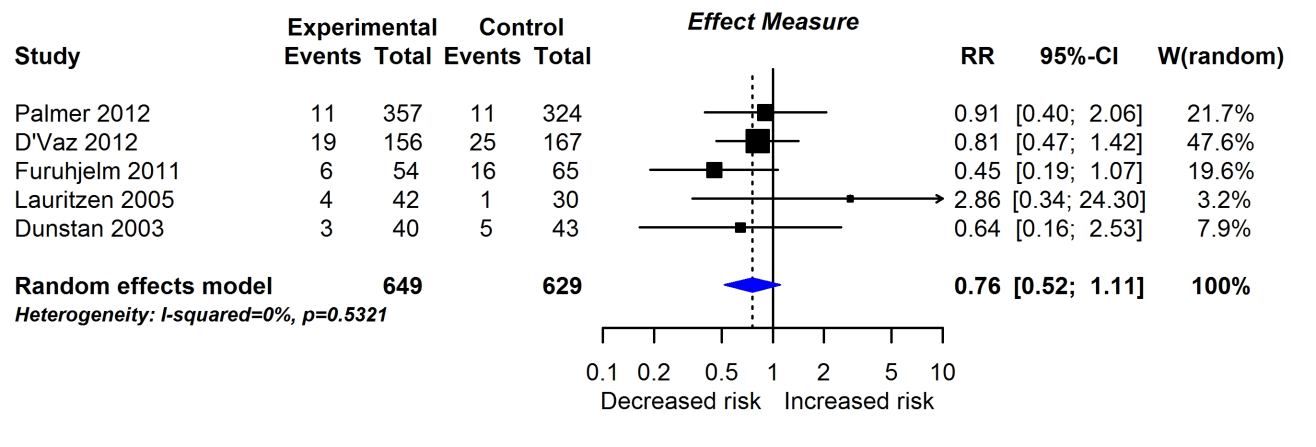


# PUFA supplementation and risk of allergic sensitisation

One systematic review (Table 5) and 14 intervention studies (4,131 participants, 2,162 randomised to the intervention arm) investigated the effect of fatty acids on allergic sensitisation (Table 6). Two original studies used n-6 fatty acids, and 12 used n-3 fatty acids. Ten studies were carried out in Europe and four in Australia. Eight studies were carried out in children at high risk of having allergic sensitisation. The age of the participants at time of outcome measurement ranged from 6 months to 24 years. The risk of bias was high in one third of studies, due to attrition bias (Figure 14).

The systematic review of 3 studies using n-3 fatty acid supplementation during pregnancy and/or lactation found evidence that this reduced the risk of egg sensitisation (Table 3).

Figures 15 to 22 show analyses of original studies assessing the effect of fatty acid supplementation on allergic sensitisation at any age. We did not find evidence of an effect of n-3 fatty acids on AS to any allergen or aeroallergen. There was however reduced AS to egg with n-3 supplementation (Figure 18; RR 0.71; 95% CI 0.56, 0.91), with low statistical heterogeneity. This was seen most clearly in the studies with a prenatal component to treatment (Table 5). Although the same effect was not seen for other foods individually when all studies were pooled, the same effect was seen for AS to peanut with *prenatal* n-3 supplementation (RR 0.62 95% CI 0.40, 0.96), with no heterogeneity. No effect was seen for n-6 fatty acids, or in the 2 studies reporting AS to any food. The study of Palmer reported reduced AS to egg at 1 year (included in Figure 18), but at 3 years there was no significant difference in egg sensitisation (RR 0.65; 95% CI 0.30, 1.41) or indeed any other allergic sensitisation, and at 6 years there was no difference in egg sensitisation (RR 1.12; 95%CI 0.35, 3.63) or any allergic sensitisation (Figure 15).

Eight studies assessed total IgE level as an outcome. In all studies there was no significant difference in total IgE between intervention and control groups. Marks reported the ratio of mean IgE at age 5 to be 0.86 (95% CI 0.64, 1.16). Damsgaard reported median total IgE 16μg/L (IQR 2, 475) in the supplemented group, 12 (2, 128) in the control group. Van Gool reported geometric mean total IgE 9.31 kU/L (sd 4.45) in the intervention group, 6.87 (3.56 in the control group). Kitz reported no significant difference (data not shown). Linnamaa reported mean 20.5 (sd 37.2) intervention, 14.8 (17.5) control. Lauritzen reported similar average total IgE in intervention (8.9) and control (8.8) groups. Harslof reported that Total IgE did not differ between intervention and control groups (median fish oil 15.5 (IQR 3.3, 30.8); soya oil 7.5 (2.9, 29.7). Hansen also reported no difference in total IgE between groups (median 36 (IQR 15,84) fish oil; 38 (12,90) olive oil.

**Overall we found MODERATE level evidence (-1 indirectness of outcome measure) that n-3 supplementation using fish oil during pregnancy +/- lactation, reduces allergic sensitisation to egg in high risk infants. No clear effect was seen for allergic sensitisation to other allergens, but the data for allergic sensitisation to peanut were consistent with those for allergic sensitisation to egg.**

Figure 14 Risk of bias of intervention studies on PUFAs and risk of allergic sensitisation

Table 5 Findings from the previous systematic review

| **Study** | **Outcome measure** | **No. participants (studies)** | **Outcome (95% CI)** | **I^2^** |
| --- | --- | --- | --- | --- |
| **Klemens** ([23](#_ENREF_23)) | SPT to egg at 1 yr | 187 (2) | OR 0.33 [0.16, 0.70] | 0% |

Figure 15 Omega 3 fatty acids and risk of AS to any allergen

**
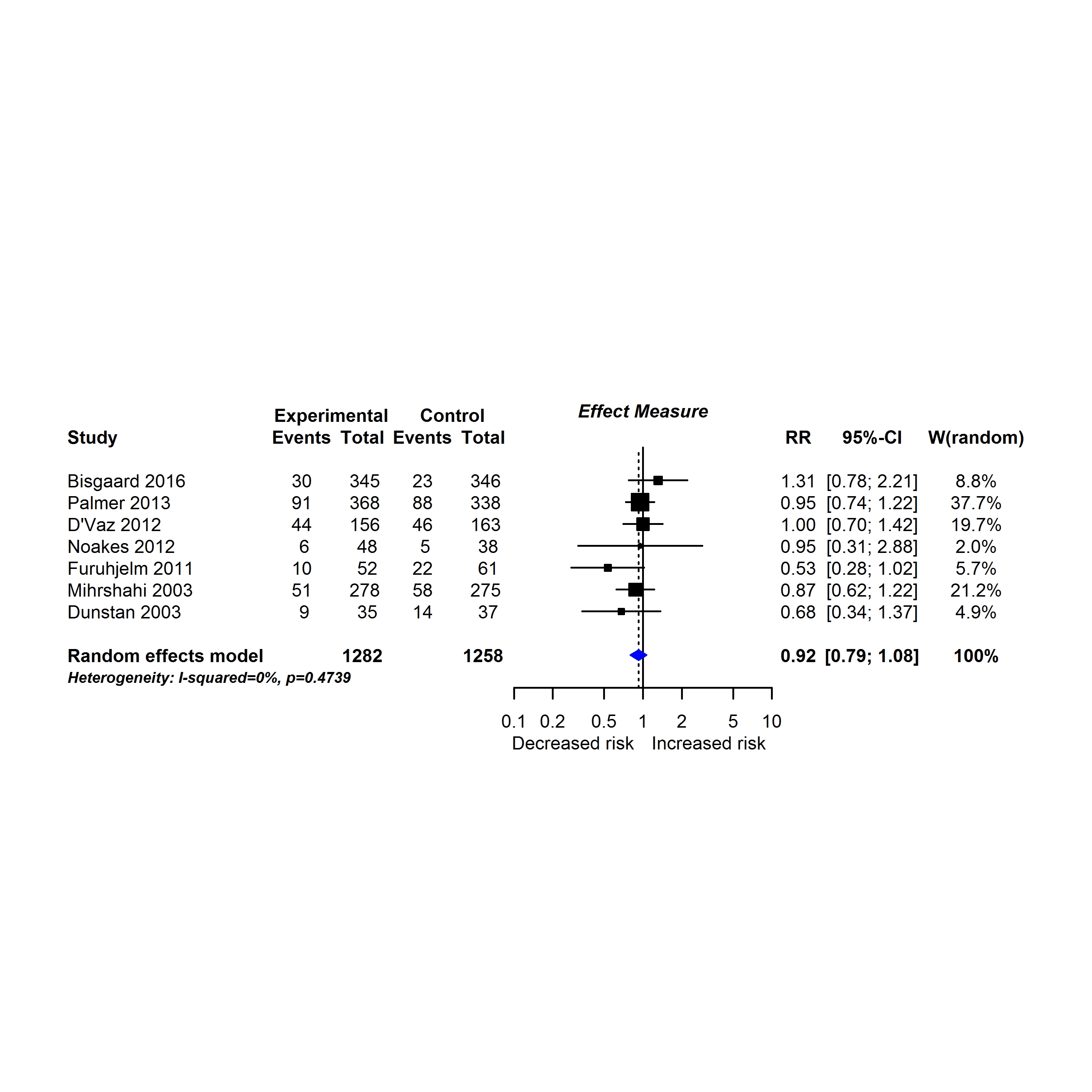
**

Data shown from Bisgaard are for SPT. The same study reported similar findings for sIgE – RR 1.65 (95% CI 0.95, 2.86).

Table 6 Subgroup analysis of n-3 fatty acids and allergic sensitisation to any allergen in children

|  | | **Number of studies** | **RR [95% CI]** | **I^2^ (%)** | **P-value for between groups difference** |
| --- | --- | --- | --- | --- | --- |
| Intervention – postnatal only  Intervention – prenatal +/- postnatal | 2  5 | | 0.93 [0.73-1.19]  0.89 [0.68-1.17] | 0  24 | 0.83 |
| Risk of disease – High  Risk of disease – Normal/Low | 6  1 | | 0.85 [0.69-1.06]  1.34 [0.76-2.35] | 0  -- | 0.15 |
| Overall risk of bias – Low  Overall risk of bias – High/Unclear | 5  2 | | 0.88 [0.71-1.11]  0.99 [0.63-1.56] | 31  0 | 0.66 |
| Conflict of interest bias – Low  Conflict of interest bias – High/Unclear | 6  1 | | 0.95 [0.81-1.12]  0.53 [0.28-1.02] | 0  -- | 0.09 |

Figure 16 Omega 3 fatty acids and risk of AS to any aeroallergen


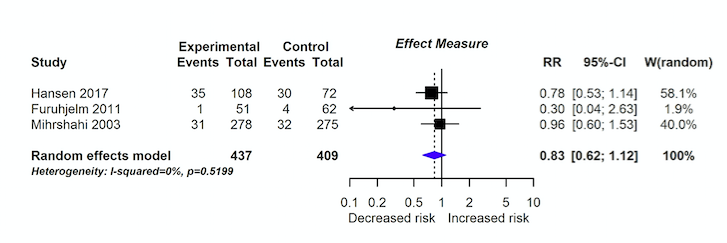


Figure 17 Omega 3 fatty acids and risk of AS to food


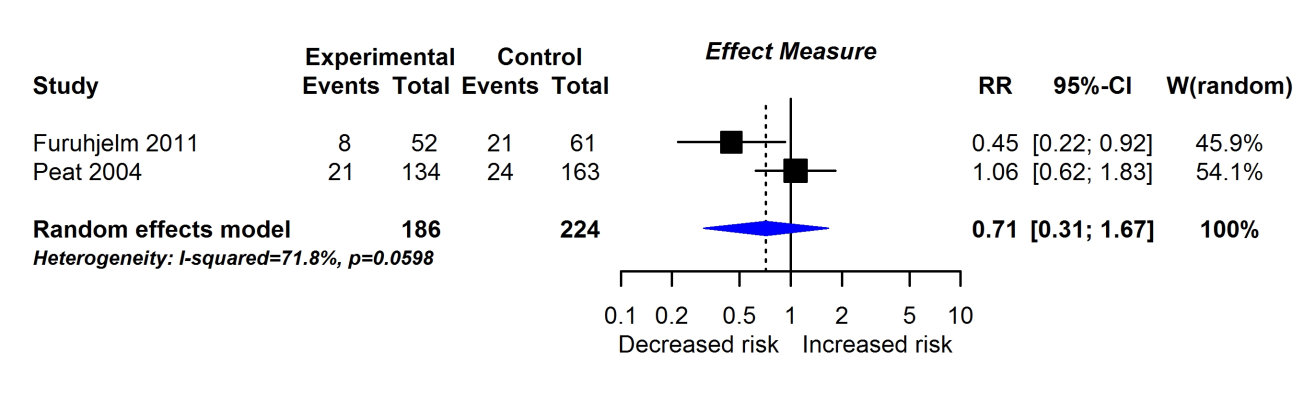


Figure 18 Omega 3 fatty acids and risk of AS to egg


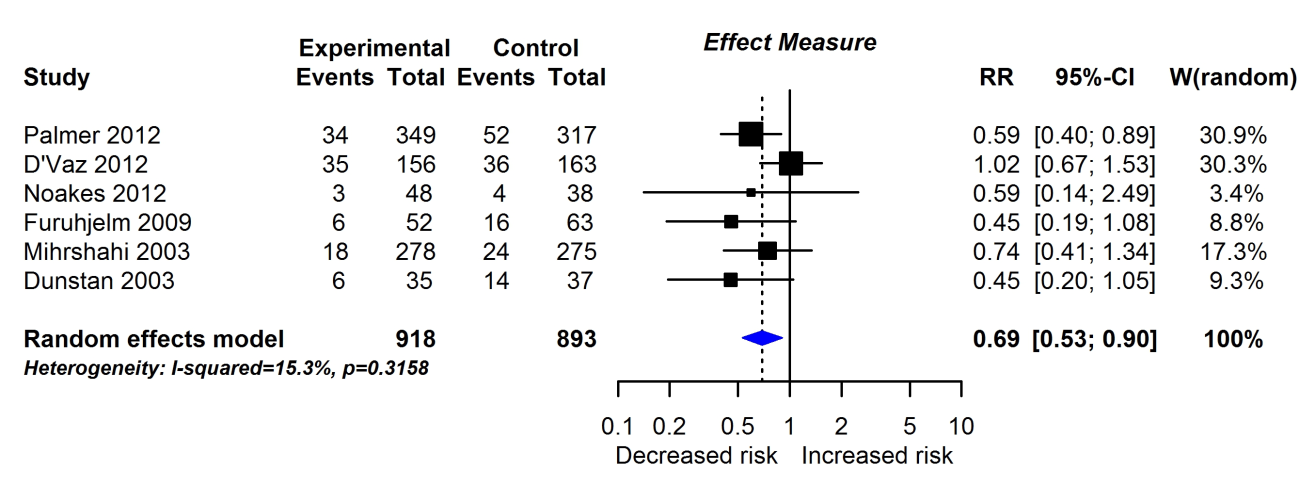


**Table 7 Subgroup analysis of n-3 fatty acids and risk of AS to egg**

|  | | **Number of studies** | **RR [95% CI]** | **I^2^ (%)** | **P-value for between groups difference** |
| --- | --- | --- | --- | --- | --- |
| Intervention – prenatal +/- postnatal  Intervention – postnatal only | 4  2 | | 0.55 [0.40-0.76]  0.92 [0.65-1.28] | 0  0 | 0.03 |
| Risk of disease – High  Risk of disease – Normal/Low | 6  0 | | 0.69 [0.53-0.90]  - | 15  - | - |
| Overall risk of bias – Low  Overall risk of bias – High/Unclear | 4  2 | | 0.59 [0.44-0.79]  0.98 [0.66-1.45] | 0  0 | 0.044 |
| Conflict of interest bias – Low  Conflict of interest bias – High/Unclear | 4  2 | | 0.80 [0.58-1.12]  0.57 [0.39-0.82] | 8.8  0 | 0.17 |

Figure 19 Omega 3 fatty acids and risk of AS to peanut


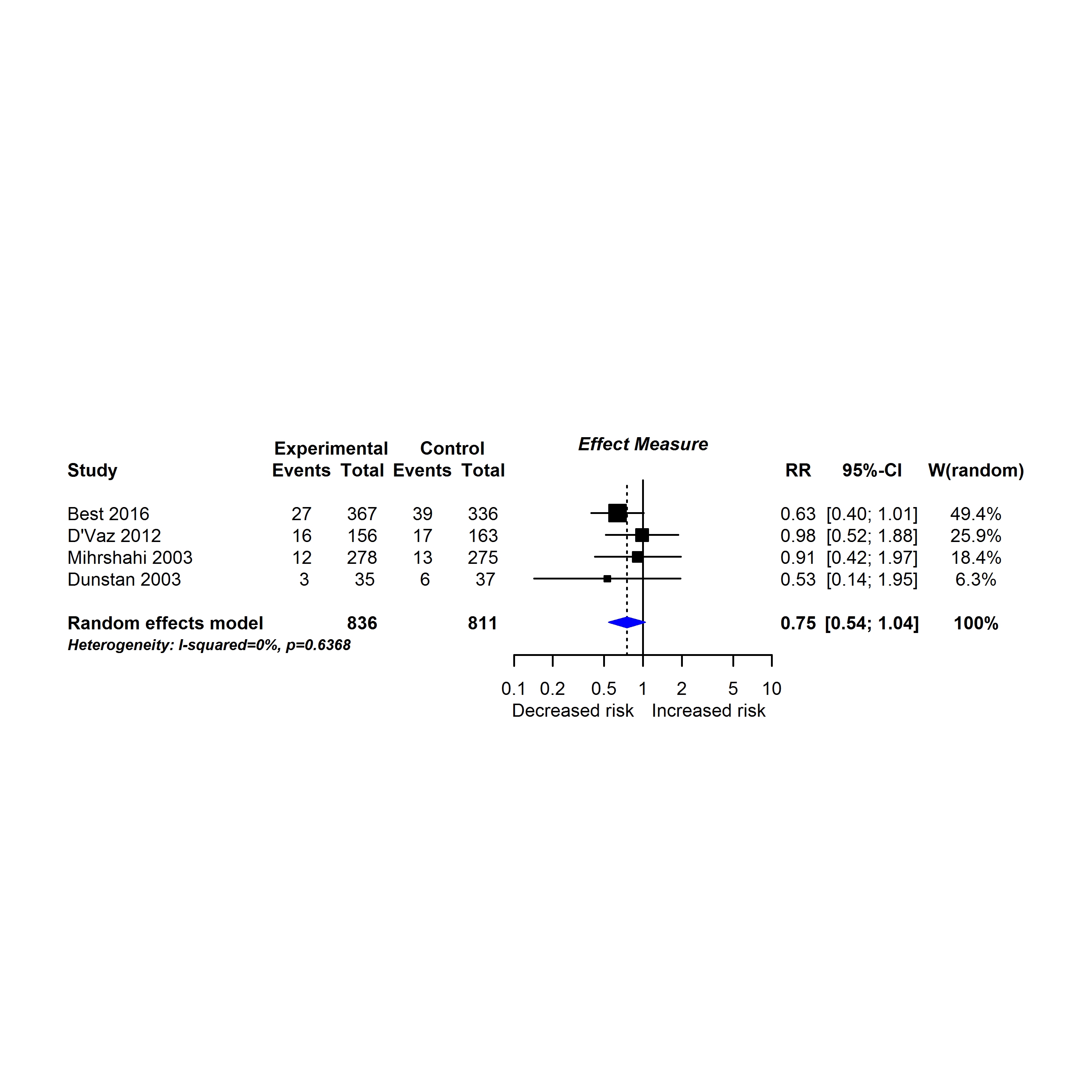


Figure 20 Omega 6 fatty acids and risk of AS to food


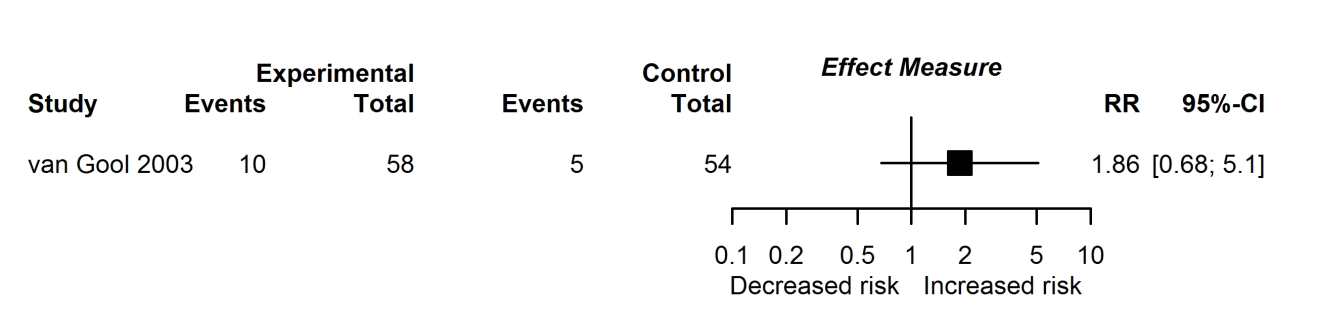


Figure 21 Omega 3 fatty acids and risk of AS to cow’s milk


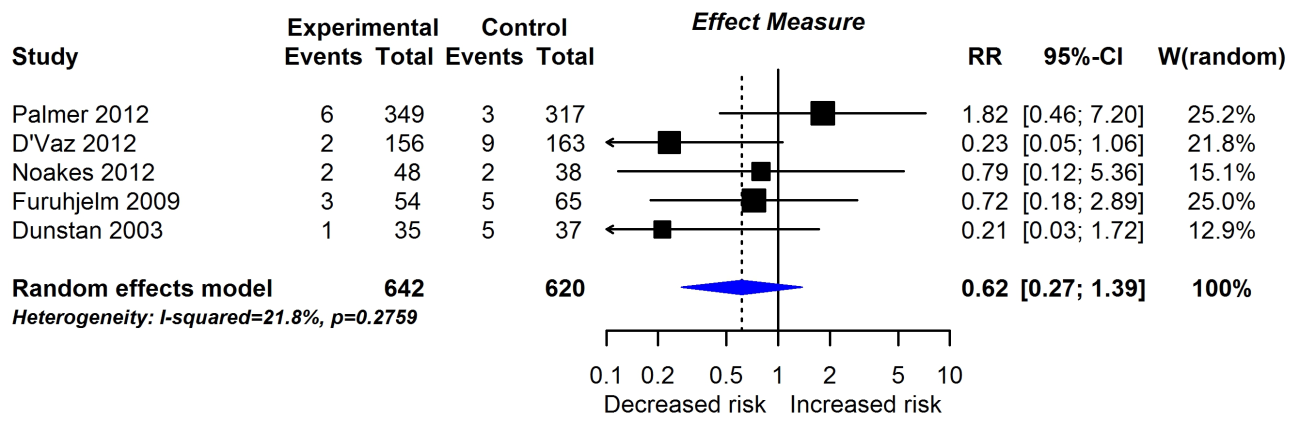


Figure 22 Omega 6 fatty acids and risk of AS to egg


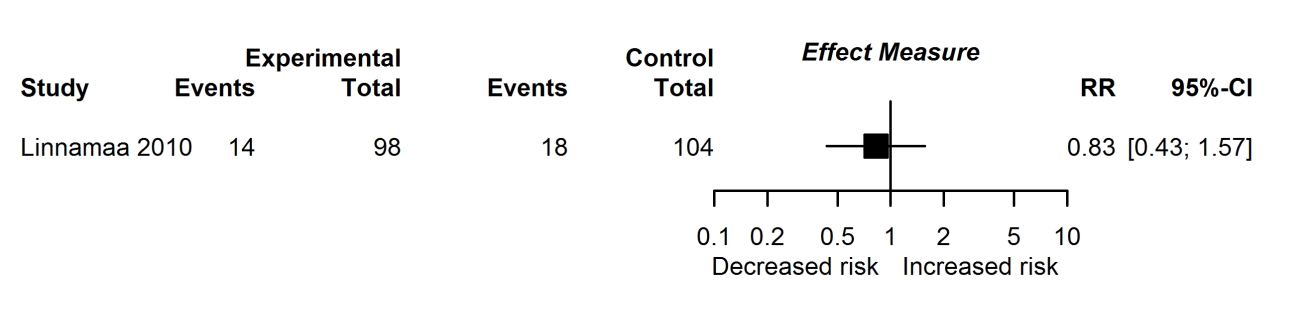


# PUFA supplementation and wheeze

One systematic review and thirteen intervention studies (13,666 participants, 5,771 randomised to the intervention arm) investigated the effect of fatty acids on wheeze +/- lung function (Table 8). Six studies were from Europe; four were from Australia, and four from the USA. Two studies used a combination of n-3 and n-6 fatty acids, and all other studies used n-3. Six studies were carried out in infants with high risk of disease. Age at time of outcome measurement ranged from 6 months to 24 years old. Nine studies used parental report of Dr diagnosed asthma or wheeze, one study used an unclear method to define the outcome, one study used current wheeze as definition, whilst another study used number of episodes of wheeze, and one study defined wheeze according to the ISAAC questionnaire. The risk of bias was low for just under half of studies (Figure 23). Slightly under 30% of studies had a high risk of bias, most commonly due to attrition bias, and 14% were considered to have a high risk of conflict of interest.

The systematic review of three trials of n-3 fatty acid supplementation during pregnancy/lactation, found no effect of this intervention on risk of wheeze.

Eight original studies were eligible to combine in a meta-analysis to examine the effect of n-3 fatty acids on risk of wheeze in children aged ≤ 4 years old (Figure 24) but there was no evidence of an effect (RR 0.95; 95% CI 0.79 – 1.14; I^2^=13%). Subgroup analyses did not identify important subgroup differences. The CCT of Dotterud reported no difference in wheeze at age ≤ 4 – RR 0.94 (95%CI 0.85, 1.05). Smaller numbers of studies contributed to non-significant analyses of wheeze at age 5-14, atopic wheeze at age ≤ 4 years and age 5-14 years (Figures 25 to 27).

Four studies evaluated the effect of n-3 supplementation on recurrent wheeze in children aged ≤ 4 years old (Figure 28). There was no evidence of an effect in meta-analysis of 3 RCTs (RR 1.06; 95% CI 0.69 – 1.63; I^2^=8%). In the CCT of Dotterud there was reduced recurrent wheeze at age ≤ 4 – RR 0.73 (95%CI 0.57, 0.94).It should be noted that the study of Dotterud included a smoking cessation programme as part of the intervention, which was successful, and this may have contributed to any positive effect on risk of wheeze/recurrent wheeze. Three studies evaluated the effect of n-3 supplementation on recurrent wheeze in children aged 5-14 years (Figure 29). There was no evidence of an effect (RR 0.92; 95% CI 0.69 – 1.22; I^2^=59.7%). The high statistical heterogeneity was caused by the study of Bisgaard, and could not be easily explained.

Marks reported both wheeze using ISAAC, and recurrent wheeze using ISAAC PLUS either DD wheeze or >12% FEV1 increase after bronchodilator at the age of 5 and found no effect (Figure 25 and 29).

Two studies assessed an intervention with combined omega 3 (DHA) and omega 6 (Arachidonic acid) supplements. In both cases the intervention was as a supplementary formula milk introduced very early (<1 week) into the infant diet, and both studies were considered at high risk of conflict of interest due to direct industry involvement. The study of Birch, considered at high risk of bias due to 50% loss to follow up, found reduced odds of recurrent wheeze by age 3 in those receiving n-6 supplements OR 0.31 (95%CI 0.11-0.97). The study of Lucas, considered at unclear overall risk of bias due to unclear assessment bias, found no significant difference for parent reported wheeze OR 1.10 (95%CI 0.60-1.80) or for doctor diagnosed asthma; OR 0.80 (95%CI 0.30- 2.50) at 9 months.

The study of Berman found no difference between intervention and control groups for childhood respiratory outcomes, including wheeze – no data were reported that could be included in meta-analysis.

Olsen reported significantly reduced recurrent wheeze at 16 years, when compared with olive oil, with small numbers of cases identified (RR 0.13, 95% CI 0.03, 0.60). However a third randomised group in this study, who received no oil supplementation at all, had similar outcomes to the fish oil group (0/ 129 cases of allergic asthma) suggesting that the outcomes in this study may have been due to an adverse effect of olive oil rather than a beneficial effect of fish oil. A later follow up of the same cohort by Hansen at 18 years of age found there was no significant difference for recurrent wheeze when comparing intervention and control groups (RR 0.79, 95% CI 0.55, 1.13).

This study also found that forced expiratory volume in one second (FEV_1_) did not differ between groups at later follow up (mean 99.5 (SD 10.8) fish oil; 100.0 (10.0) olive oil). Bisgaard also found no difference between groups in FEV_1_ in an assessment of 536 participants at age 5 years - mean difference 0 litres per minute 95% CI -0.02 to +0.03 P=0.70.

One other intervention study from Australia (616 participants, 312 randomised to the intervention arm) investigated the association between n-3 fatty acids and FEV_1_ in infants at high risk of disease. Age at time of outcome measurement was 5 years old. The study had low risk of selection and assessment bias but this analysis had high risk of attrition bias. The authors reported mean FEV_1_1.07 litres in active, 1.07 in control group - mean difference 0.0 litres (-0.03, 0.03). P=0.90.

**Overall we found no consistent evidence that n-3 or n-6 fatty acid supplementation reduces risk of wheezing.**

Figure 23 Risk of bias in intervention studies on PUFAs and risk of wheeze

Table 8 Findings from the previous systematic review

| **Study** | **Outcome measure** | **No. participants (studies) contributing data** | **Outcome (95% CI)** | **I2** |
| --- | --- | --- | --- | --- |
| **Klemens** ([23](#_ENREF_23)) | Wheezing | 546 (3) | OR 0.58 [0.30, 1.13] | 56% |

*Meta-analysis of 2 studies (482 participants) where supplementation was initiated during pregnancy rather than during lactation showed reduced wheeze (OR 0.35, 95%CI 0.15, 0.79). However wheeze assessment was at 12 months and 16 years in these 2 studies, so meta-analysis may not be appropriate. The third study included above assessed wheeze at 30 months.

Figure 24 Omega 3 fatty acids and risk of wheeze at age ≤ 4


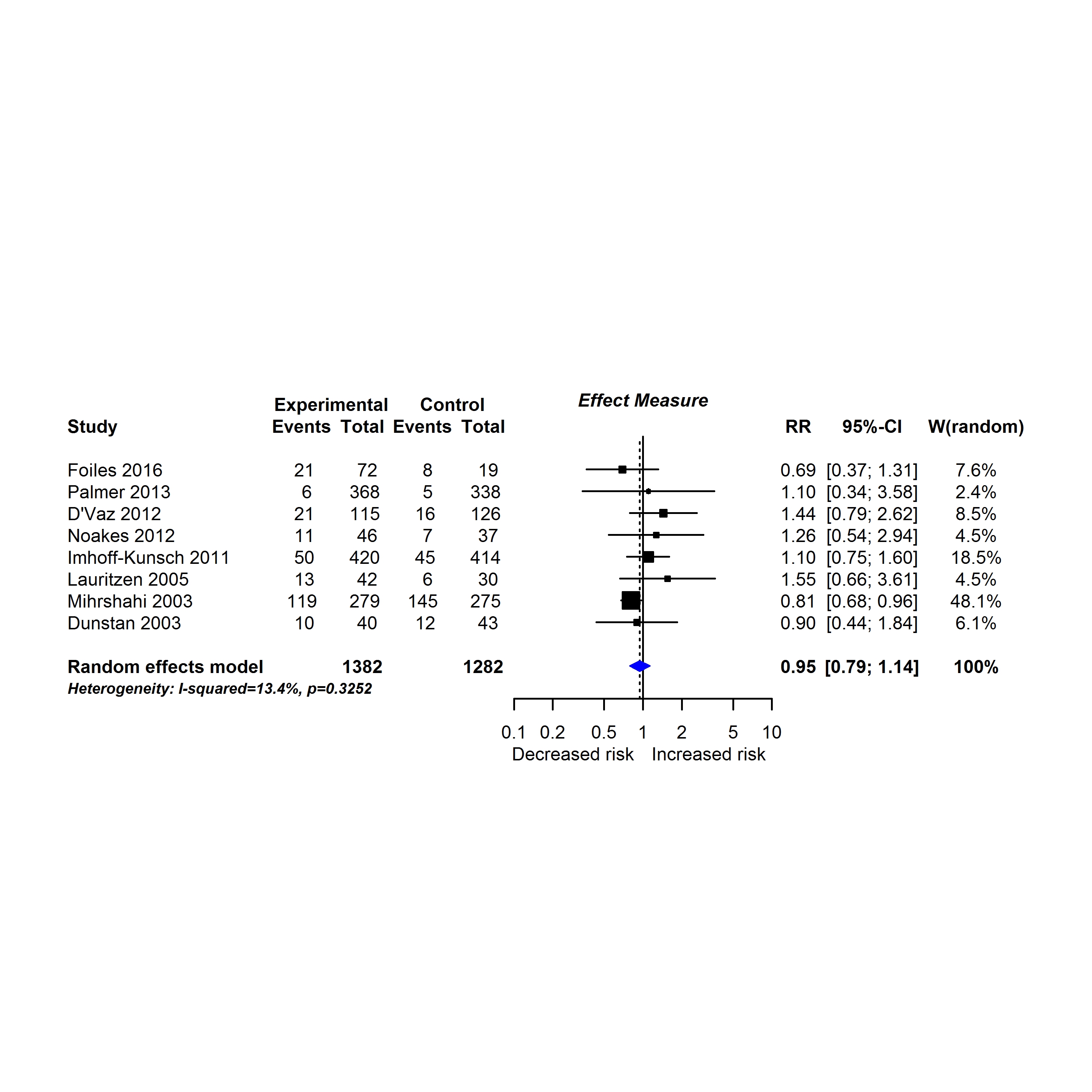


Table 9 Subgroup analysis of n-3 fatty acids and wheeze at age ≤ 4

|  | | **Number of studies** | **RR [95% CI]** | **I^2^ (%)** | **P-value for between groups difference** |
| --- | --- | --- | --- | --- | --- |
| Intervention – prenatal +/- postnatal  Intervention – postnatal only | 5  3 | | 1.12 [0.84-1.49]  0.89 [0.63-1.26] | 0  46 | 0.31 |
| Risk of disease – High  Risk of disease – Normal/Low | 5  3 | | 0.91 [0.73-1.12]  1.03 [0.71-1.48] | 9  20 | 0.57 |
| Overall risk of bias – Low  Overall risk of bias – High/Unclear | 4  4 | | 0.86 [0.74-1.00]  1.14 [0.78-1.69] | 0  16 | 0.18 |
| Conflict of interest bias – Low  Conflict of interest bias – High/Unclear | 7  1 | | 0.99 [0.80-1.21]  0.69 [0.37-1.31] | 19  - | 0.30 |

Figure 25 Omega 3 fatty acids and risk of wheeze at age 5-14


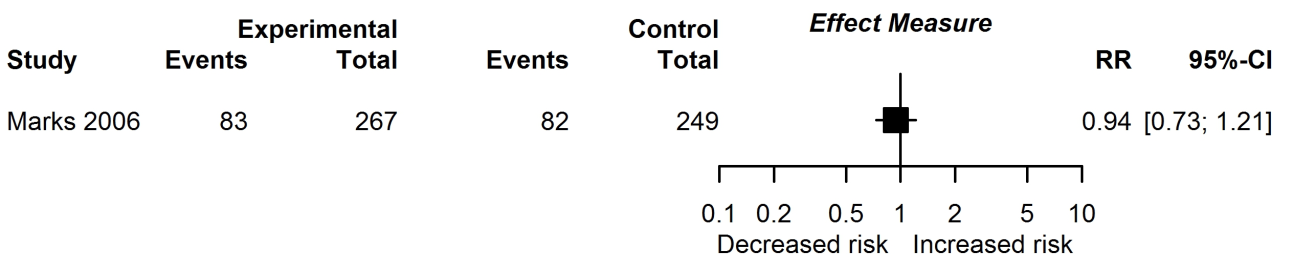


Figure 26 Omega 3 fatty acids and risk of atopic wheeze at age ≤ 4


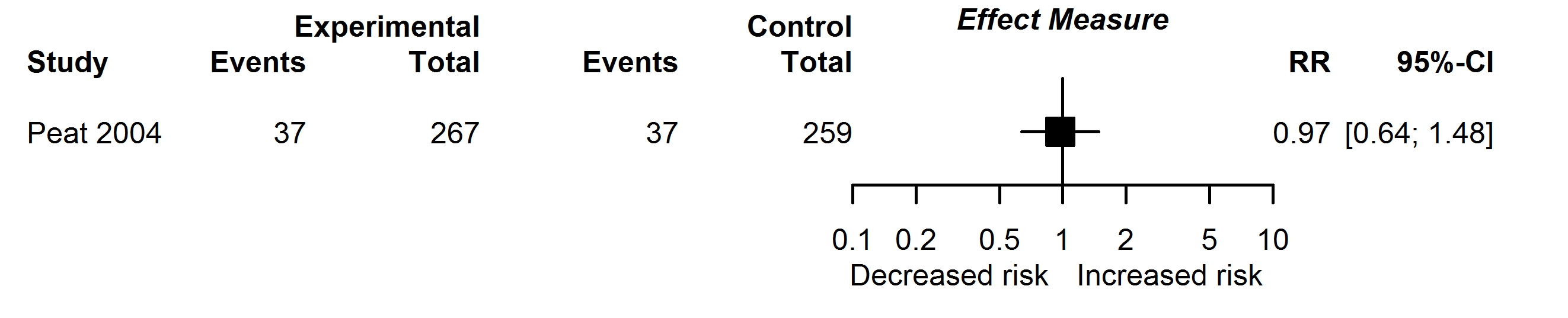


Figure 27 Omega 3 fatty acids and risk of atopic wheeze at age 5-14


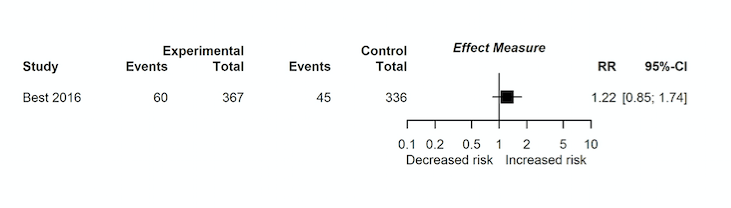


Figure 28 Omega 3 fatty acids and risk of recurrent wheeze at age ≤ 4


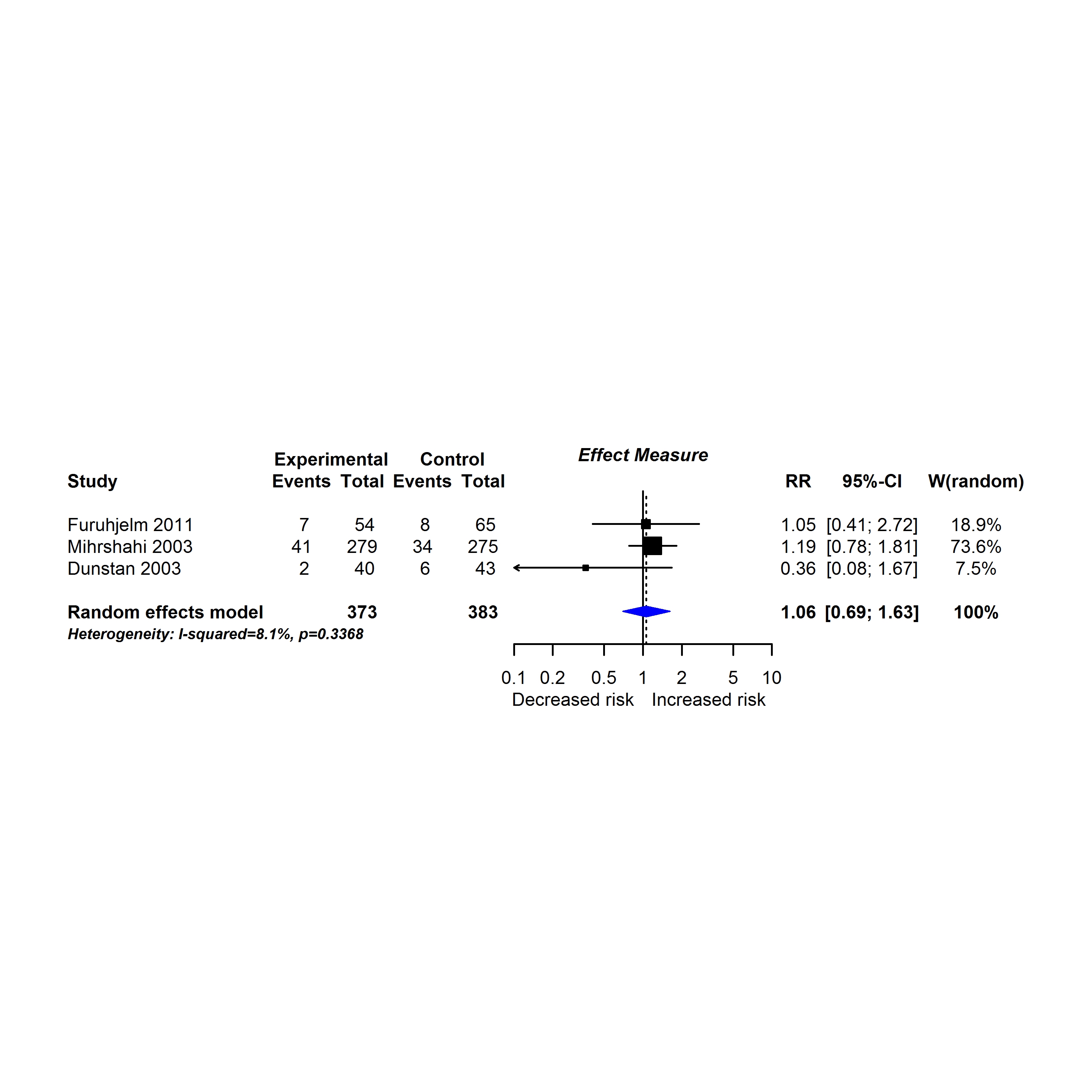


Figure 29 Omega 3 fatty acids and risk of recurrent wheeze at age 5-14


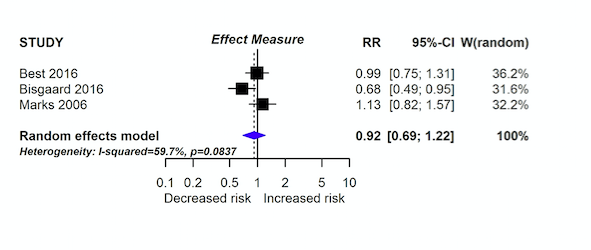


# General conclusions

A systematic review of this area by Sheikh and colleagues ([29](#_ENREF_29)) published in 2009 found no evidence that fatty acid supplements reduce risk of allergic disease, but with wide confidence intervals suggesting that further trials were needed. They identified 6 relevant trials, which indicated the review was in need of updating.

A more recent systematic review from 2011 included 5 trials, restricted to omega 3 fatty acid supplementation during pregnancy/lactation, and has been incorporated in our report ([23](#_ENREF_23)). The authors found evidence that n-3 fatty acid supplementation during pregnancy/lactation may reduce allergic sensitisation to egg, but not other allergic outcomes (wheeze, AD and food allergy).

There have been several subsequent systematic reviews, narrative reviews and overviews of this area by Best ([30](#_ENREF_30)), Dick ([31](#_ENREF_31)), Koletzko ([32](#_ENREF_32)), Netting ([33](#_ENREF_33)), Yang ([34](#_ENREF_34)), Girdhar ([35](#_ENREF_35)). These reports have all included small numbers of fatty acid supplementation trials, with limited or no new meta-analysis undertaken. The authors have generally reported positive findings in relation to allergic outcomes. One of these reviews (Dick) met our quality criteria of R-AMSTAR score over 32. This review identified one cohort study of fish consumption, and one of the intervention trials included in our review (Dunstan) which reported no association between fish oil supplementation during pregnancy and risk of wheezing.

The Cochrane review of Gunaratne differed from our systematic review in that they excluded the CCT of Dotterud 2013, and limited their review to maternal supplementation, with exclusion of infant supplementation studies which didn’t also supplement mothers ([36](#_ENREF_36)). Their findings and conclusions are overall similar to ours – that there is some evidence for a reduction in allergic sensitisation to egg for omega 3 supplementation during pregnancy/lactation. They stratified analyses by age at outcome, which led to some mixed positive and null findings for eczema and food allergy outcomes. But overall they concluded that omega 3 supplements during pregnancy or lactation showed little benefit in the reduction of allergic disease in childhood. Their positive finding in one age stratum for medically diagnosed eczema (but not for other age strata, nor for parent-reported eczema) has led to a national recommendation to use omega 3 fatty acid supplements for eczema prevention <https://www.allergy.org.au/images/pcc/ASCIA_Guidelines_infant_feeding_and_allergy_prevention.pdf> .

In our more recent and comprehensive review we identified 19 trials, some of which were published subsequent to the reviews of Sheikh, Klemens and Gunaratne. We found evidence that maternal n-3 fatty acid supplementation may reduce allergic sensitisation to egg and peanut, the commonest food allergens in young children, but no evidence for other allergic sensitisation either to foods or aeroallergens or to ‘any allergen’. We found no evidence of a reduction in food allergy, but this needs to be further explored because data were sparse with wide confidence intervals. One study of n-3 fatty acid supplementation during pregnancy found reduced asthma at age 16 when compared with olive oil supplementation during pregnancy, but no reduction when compared with no oil supplementation during pregnancy. Finally one analysis of n-6 supplementation during pregnancy/lactation, based mainly on studies at high risk of attrition bias or conflict of interest bias, found reduced eczema at ≤4 years. However, the study of Linnamaa which dominated this analysis found reduced eczema at age 1 (included in the meta-analysis), but no reduction at age 2.

Taken together these data do not provide conclusive evidence that fatty acid supplementation during pregnancy, lactation or infancy has an impact on childhood allergic disease risk. However further work is needed, especially for food allergy, given the positive findings for allergic sensitisation to food.

**Overall we found MODERATE level evidence (-1 inconsistency; -1 indirectness of outcome measure) that n-3 supplementation using fish oil during pregnancy +/- lactation, reduces allergic sensitisation to egg in high risk infants. Data for peanut sensitisation were consistent with those for egg, but no effect was seen for allergic sensitisation to other allergens, nor for the outcomes AD, wheeze, food allergy or AR.**

# References

1. Berman D, Limb R, Somers E, Clinton C, Romero V, Mozurkewich E. Prenatal omega-3 supplementation and risk of eczema among offspring at age 36 months: Long-term follow-up of the mothers, omega-3, & mental health trial. American journal of obstetrics and gynecology [Internet]. 2015 B]; 212(1 suppl. 1):[S162 p.]. Available from: <http://onlinelibrary.wiley.com/o/cochrane/clcentral/articles/834/CN-01049834/frame.html>.

2. Bisgaard H, Stokholm J, Chawes BL, Vissing NH, Bjarnadottir E, Schoos AMM, et al. Fish oil-derived fatty acids in pregnancy and wheeze and asthma in offspring. New England Journal of Medicine. 2016;375(26):2530-9.

3. Birch EE, Khoury JC, Berseth CL, Castaneda YS, Couch JM, Bean J, et al. The Impact of Early Nutrition on Incidence of Allergic Manifestations and Common Respiratory Illnesses in Children. Journal of Pediatrics. 2010;156(6):902-U68.

4. Lucas A, Stafford M, Morley R, Abbott R, Stephenson T, MacFadyen U, et al. Efficacy and safety of long-chain polyunsaturated fatty acid supplementation of infant-formula milk: a randomised trial. Lancet. 1999;354(9194):1948-54.

5. van Gool CJ, Thijs C, Henquet CJ, van Houwelingen AC, Dagnelie PC, Schrander J, et al. Gamma-linolenic acid supplementation for prophylaxis of atopic dermatitis--a randomized controlled trial in infants at high familial risk. American Journal of Clinical Nutrition. 2003;77(4):943-51.

6. Harslof LBS, Damsgaard CT, Andersen AD, Aakjaer DL, Michaelsen KF, Hellgren LI, et al. Reduced ex vivo stimulated IL-6 response in infants randomized to fish oil from 9 to 18 months, especially among PPARG2 and COX2 wild types. Prostaglandins Leukotrienes and Essential Fatty Acids. 2015;94:21-7.

7. Kitz R, Rose MA, Schonborn H, Zielen S, Bohles HJ. Impact of early dietary gamma-linolenic acid supplementation on atopic eczema in infancy. Pediatric Allergy & Immunology. 2006;17(2):112-7.

8. Linnamaa P, Savolainen J, Koulu L, Tuomasjukka S, Kallio H, Yang B, et al. Blackcurrant seed oil for prevention of atopic dermatitis in newborns: a randomized, double-blind, placebo-controlled trial. Clinical & Experimental Allergy. 2010;40(8):1247-55.

9. Mihrshahi S, Peat JK, Marks GB, Mellis CM, Tovey ER, Webb K. Eighteen-month outcomes of house dust mite avoidance and dietary fatty acid modification in the Childhood Asthma Prevention Study (CAPS) J Allergy Clin Immunol. 2003 Apr;111(4):735. Journal of Allergy and Clinical Immunology [Internet]. 2003; 111(1):[162-8 pp.]. Available from: <http://onlinelibrary.wiley.com/o/cochrane/clcentral/articles/174/CN-00676174/frame.html>

10. Peat JK, Mihrshahi S, Kemp AS, Marks GB, Tovey ER, Webb K, et al. Three-year outcomes of dietary fatty acid modification and house dust mite reduction in the Childhood Asthma Prevention Study. Journal of Allergy & Clinical Immunology. 2004;114(4):807-13.

11. Marks GB, Mihrshahi S, Kemp AS, Tovey ER, Webb K, Almqvist C, et al. Prevention of asthma during the first 5 years of life: a randomized controlled trial. Journal of Allergy & Clinical Immunology. 2006;118(1):53-61.

12. Damsgaard CT, Lauritzen L, Kjaer TMR, Holm PMI, Fruekilde MB, Michaelsen KF, et al. Fish oil supplementation modulates immune function in healthy infants. Journal of Nutrition. 2007;137(4):1031-6.

13. Palmer DJ, Sullivan T, Gold MS, Prescott SL, Heddle R, Gibson RA, et al. Effect of n-3 long chain polyunsaturated fatty acid supplementation in pregnancy on infants' allergies in first year of life: randomised controlled trial. Bmj. 2012;344:e184.

14. Dunstan JA, Mori TA, Barden A, Beilin LJ, Taylor AL, Holt PG, et al. Fish oil supplementation in pregnancy modifies neonatal allergen-specific immune responses and clinical outcomes in infants at high risk of atopy: a randomized, controlled trial. Journal of Allergy & Clinical Immunology. 2003;112(6):1178-84.

15. D'Vaz N, Meldrum SJ, Dunstan JA, Martino D, McCarthy S, Metcalfe J. Postnatal fish oil supplementation in high-risk infants to prevent allergy: Randomized controlled trial. Pediatrics [Internet]. 2012; 130(4):[674-82 pp.]. Available from: <http://onlinelibrary.wiley.com/o/cochrane/clcentral/articles/209/CN-00837209/frame.html>

16. Furuhjelm C, Warstedt K, Larsson J, Fredriksson M, Bottcher MF, Falth-Magnusson K, et al. Fish oil supplementation in pregnancy and lactation may decrease the risk of infant allergy. Acta Paediatrica. 2009;98(9):1461-7.

17. Furuhjelm C, Warstedt K, Fagerås M, Fälth-Magnusson K, Larsson J, Fredriksson M, et al. Allergic disease in infants up to 2 years of age in relation to plasma omega-3 fatty acids and maternal fish oil supplementation in pregnancy and lactation. Pediatric allergy and immunology : official publication of the European Society of Pediatric Allergy and Immunology [Internet]. 2011; 22(5):[505-14 pp.]. Available from: <http://onlinelibrary.wiley.com/o/cochrane/clcentral/articles/572/CN-00812572/frame.html>

18. Lauritzen L, Kjaer TM, Fruekilde MB, Michaelsen KF, Frokiaer H. Fish oil supplementation of lactating mothers affects cytokine production in 2 1/2-year-old children. Lipids. 2005;40(7):669-76.

19. Olsen SF, Osterdal ML, Salvig JD, Mortensen LM, Rytter D, Secher NJ, et al. Fish oil intake compared with olive oil intake in late pregnancy and asthma in the offspring: 16 y of registry-based follow-up from a randomized controlled trial. American Journal of Clinical Nutrition. 2008;88(1):167-75.

20. Dotterud CK, Storro O, Simpson MR, Johnsen R, Oien T. The impact of pre- and postnatal exposures on allergy related diseases in childhood: a controlled multicentre intervention study in primary health care. BMC Public Health. 2013;13:123.

21. Imhoff-Kunsch B, Stein AD, Martorell R, Parra-Cabrera S, Romieu I, Ramakrishnan U. Prenatal docosahexaenoic acid supplementation and infant morbidity: randomized controlled trial. Pediatrics. 2011;128(3):e505-12.

22. Noakes PS, Vlachava M, Kremmyda LS, Diaper ND, Miles EA, Erlewyn-Lajeunesse M. Increased intake of oily fish in pregnancy: Effects on neonatal immune responses and on clinical outcomes in infants at 6 mo. American journal of clinical nutrition [Internet]. 2012; 95(2):[395-404 pp.]. Available from: <http://onlinelibrary.wiley.com/o/cochrane/clcentral/articles/023/CN-00834023/frame.html>

23. Klemens CM, Berman DR, Mozurkewich EL. The effect of perinatal omega-3 fatty acid supplementation on inflammatory markers and allergic diseases: a systematic review. BJOG: An International Journal of Obstetrics & Gynaecology. 2011;118(8):916-25.

24. Foiles AM, Kerling EH, Wick JA, Scalabrin DMF, Colombo J, Carlson SE. Formula with long-chain polyunsaturated fatty acids reduces incidence of allergy in early childhood. Pediatric Allergy and Immunology. 2016;27(2):156-61.

25. Palmer DJ, Sullivan T, Gold MS, Prescott SL, Heddle R, Gibson RA, et al. Randomized controlled trial of fish oil supplementation in pregnancy on childhood allergies. Allergy. 2013;68(11):1370-6.

26. Best K, Sullivan T, Gold M, Kennedy D, Martin J, Palmer D. Six-year follow up of children at high hereditary risk of allergy, born to mothers supplemented with docosahexaenoic acid (DHA) in the domino trial. Journal of Paediatrics and Child Health [Internet]. 2015; 51:[58 p.]. Available from: <http://onlinelibrary.wiley.com/o/cochrane/clcentral/articles/001/CN-01073001/frame.html>.

27. Best KP, Sullivan T, Palmer D, Gold M, Kennedy DJ, Martin J, et al. Prenatal fish oil supplementation and allergy: 6-Year follow-up of a randomized controlled trial. Pediatrics [Internet]. 2016; 137(6) (no pagination). Available from: <http://onlinelibrary.wiley.com/o/cochrane/clcentral/articles/874/CN-01159874/frame.html>

28. Hansen S, Strom M, Maslova E, Dahl R, Hoffmann HJ, Rytter D, et al. Fish oil supplementation during pregnancy and allergic respiratory disease in the adult offspring. Journal of Allergy and Clinical Immunology. 2017;139(1):104.

29. Anandan C, Nurmatov U, Sheikh A. Omega 3 and 6 oils for primary prevention of allergic disease: systematic review and meta-analysis. Allergy. 2009;64(6):840-8.

30. Best K, Makrides M, Gold M. Effect of maternal dietary long chain polyunsaturated fatty acid intake during pregnancy on clinical outcomes of allergic disease in the offspring: A systematic review. Internal Medicine Journal. 2014;44:2.

31. Dick S, Friend A, Dynes K, AlKandari F, Doust E, Cowie H, et al. A systematic review of associations between environmental exposures and development of asthma in children aged up to 9 years. BMJ Open. 2014;4(11).

32. Koletzko B, Boey CC, Campoy C, Carlson SE, Chang N, Guillermo-Tuazon MA, et al. Current information and Asian perspectives on long-chain polyunsaturated fatty acids in pregnancy, lactation, and infancy: systematic review and practice recommendations from an early nutrition academy workshop. Annals of Nutrition & Metabolism. 2014;65(1):49-80.

33. Netting MJ, Middleton PF, Makrides M. Does maternal diet during pregnancy and lactation affect outcomes in offspring? A systematic review of food-based approaches. Nutrition. 2014;30(11-12):1225-41.

34. Yang H, Xun P, He K. Fish and fish oil intake in relation to risk of asthma: A systematic review and meta-analysis. PLoS ONE. 2013;8(11).

35. Girdhar M, Ciaccio CE. Does omega-3 fatty acid supplementation during pregnancy prevent childhood atopic disease? Journal of Allergy and Clinical Immunology. 2014;1):AB124.

36. Gunaratne Anoja W, Makrides M, Collins Carmel T. Maternal prenatal and/or postnatal n-3 long chain polyunsaturated fatty acids (LCPUFA) supplementation for preventing allergies in early childhood. Cochrane Database of Systematic Reviews [Internet]. 2015; (7). Available from: <http://onlinelibrary.wiley.com/doi/10.1002/14651858.CD010085.pub2/abstract>

<http://onlinelibrary.wiley.com/store/10.1002/14651858.CD010085.pub2/asset/CD010085.pdf?v=1&t=izmdufvr&s=d91fd0ca9e59df1bc8a24441a8eb5fd93751b107>.
