# Supplementary material for: Diet during pregnancy and infancy and risk of allergic or autoimmune disease: A systematic review and meta-analysis
Source: PLoS Med. 2018 Feb 28;15(2):e1002507. doi: 10.1371/journal.pmed.1002507 (PMC5830033; doi:10.1371/journal.pmed.1002507)
Supplement: S2 Data — (ZIP) [file pmed.1002507.s007.zip › Review_C_reports/FATS & FATTY ACIDS.doc]

**dietary intake of sources of fatty acids IN INFANTS AND MOTHERS, AND RISK OF ALLERGIC AND AUTOIMMUNE DISEASES**

Vanessa Garcia-Larsen^1^, Robert J Boyle^2^, Despo Ierodiakonou^3^, Jo Leonardi-Bee^4^, Tim Reeves^5^, Jennifer Chivinge^6^, Zoe Robinson^6^, Natalie Geoghegan^6^, Katharine Jarrold^6^, Andrew Logan^6^, Annabel Groome^6^ , Evangelia Andreou^7^, Nara Tagiyeva-Milne^8^, Ulugbek Nurmatov^9^, Sergio Cunha^7^

^1^ Post-Doctoral Research Associate, Respiratory Epidemiology and Public Health, National Heart and Lung Institute; ^2^Clinical Senior Lecturer, Section of Paediatrics; ^3^ Post-Doctoral Research Associate, Departments of Paediatric and Respiratory Epidemiology and Public Health Group, all at Imperial College London

^4^Associate Professor of Community Health Sciences, University of Nottingham

^5^ Research Support Librarian, Faculty of Medicine, Imperial College London

^6^ Undergraduate medical students, Imperial College London

^7^Research Associate, Imperial Consultants

^8^Research Fellow, University of Aberdeen

^9^Research Fellow, University of Edinburgh

**Table of contents**

List of figures	3

List of tables	5

1.	Executive summary of findings on Infant and maternal intake of sources of fatty acids and risk of allergic or autoimmune outcomes	6

1.1.	Context of this report	6

1.2.	Studies identified	6

1.3.	Outcomes evaluated	6

1.4.	Dietary exposures studied	6

1.5.	Dietary assessment	7

1.6.	Presentation of results	7

1.7.	Risk of bias assessment	8

1.8.	Key findings	9

2.	Infant or maternal dietary intake of fats and risk of atopic dermatitis	22

2.1.	Studies that investigated the association between infant or maternal intake of fats and risk of AD which were not eligible for meta-analysis	25

3.	Infant or maternal dietary intake of fats and risk of food allergy	36

3.1.	Studies that investigated the association between infant or maternal intake of fats and risk of food allergy which were not eligible for meta-analysis	36

4.	Infant or maternal dietary intake of fats and risk of wheeze	38

4.1.	Studies that investigated the association between wheeze and infant or maternal intake of fats which were not eligible for meta-analysis	40

5.	Infant or maternal dietary intake of fats and lung function	48

6.	Infant or maternal dietary intake of fats and risk of allergic rhino-conjunctivitis	50

6.1.	Studies that investigated the association between infant or maternal intake of dietary sources of fat and risk of RC which could not be included in the meta-analyses	51

7.	Infant or maternal dietary intake of fats and risk of allergic sensitisation	56

7.1.	Studies that investigated the association between infant or maternal intake of fats and risk of allergic sensitisation which could not be included in the meta-analyses	60

8.	Infant or maternal dietary intake of fats and risk of type 1 diabetes mellitus	68

8.1.	Studies that investigated the association between infant or maternal intake of dietary fats and risk of T1DM which were not eligible for meta-analysis	68

9.	Infant or maternal dietary intake of fats and risk of juvenile idiopathic arthritis	72

General conclusions	74

References	75

# List of figures

Figure 1 Risk of Bias in studies of fat intake and atopic dermatitis	22

Figure 2 Maternal butter intake (highest vs. lowest) and risk of AD in children at age 0-4 years old	23

Figure 3 Maternal intake of marine sources of fatty acids intake (highest vs. lowest) and risk of atopic dermatitis (eczema) in children at age 0-4 years old	23

Figure 4 Maternal intake of marine sources of fatty acids intake (often vs. rare) and risk of atopic dermatitis (eczema) in children at age 0-4 years old (odds ratio)	24

Figure 5 Maternal fish oil supplementation (often vs. rare) and risk of atopic dermatitis (eczema) in children at age 0-4 years old (odds ratio)	24

Figure 6 Introduction of fats in the infant’s diet (> 8 months vs earlier) and risk of AD at age 0-4 years old	25

Figure 7 Risk of Bias in studies of fat intake and food allergy	36

Figure 8 Risk of Bias in studies of fat intake and wheeze	38

Figure 9 Maternal intake of fish or seafood (highest vs lowest) and risk of wheeze in children at age 0-4 years old	39

Figure 10 Maternal intake of fish (often vs rare-never) and risk of recurrent wheeze in children at age 5-14 years old	39

Figure 11 Infant intake of fish (often vs rare) and risk of recurrent wheeze at age 0-4 years old	40

Figure 12 Risk of Bias in studies of fat intake and Rhino-conjunctivitis	50

Figure 13 Infant fish intake (often vs. rare) and risk of and rhino-conjunctivitis in children 0 to 4 years old (odds ratio)	51

Figure 14 Risk of bias in studies of fat intake and risk of allergic sensitisation at any age	56

Figure 15 Maternal fat intake (highest vs. lowest) and risk of allergic sensitisation to aeroallergens in children at any age	57

Figure 16 Maternal fish or omega 3 fatty acid intake (often vs. rare) and risk of allergic sensitisation to aeroallergens in children at any age	57

Figure 17 Maternal fish or omega-3 intake (highest vs. lowest) and risk of specific sensitisation to aeroallergen in children at any age	57

Figure 18 Maternal fish intake (often vs. rare) and risk of sensitisation to food allergens in children at any age	58

Figure 19 Maternal fish omega-3 intake (highest vs. lowest) and risk of sensitisation to food allergens in children (odds ratio)	59

Figure 20 Maternal fat intake (highest vs lowest) and risk of allergenic sensitisation to any food in children (odds ratio)	59

Figure 21 Maternal fish or omega 3 intake (highest vs lowest) and risk of allergenic sensitisation to any food in children (odds ratio)	59

Figure 22 Risk of bias in studies of fat intake and risk of allergic sensitisation at any age	68

Figure 24 Risk of Bias in studies of fat intake and juvenile idiopathic arthritis	72

**L**ist of tables

Table 1 Characteristics of included studies evaluating infant or maternal fat intake and allergic outcomes	10

Table 2 Characteristics of included studies evaluating infant or maternal fat intake and autoimmune outcomes	18

Table 3 Dietary instrument used to ascertain infant or maternal intake of dietary sources of fats	20

Table 4 Studies investigating the association between fat intake and atopic dermatitis which were not eligible for meta-analysis	27

Table 5 Studies investigating the association between intake of dietary sources of fat and food allergy which were not eligible for meta-analysis	37

Table 6 Studies investigating the association between fat intake and wheeze which were not eligible for meta-analysis	42

Table 7 Studies investigating the association between fat intake and lung function which were not eligible for meta-analysis	49

Table 8 Studies investigating the association between fat intake and rhino-conjunctivitis which were not eligible for meta-analysis	52

Table 9 Studies investigating the association between fat intake and allergic sensitization which were not eligible for meta-analysis	61

Table 10 Studies investigating the association between fat intake and type 1 diabetes mellitus which were not eligible for meta-analysis	70

Table 11 Studies investigating the association between fat intake (dietary exposure) and Juvenile idiopathic arthritis (outcome) which were not eligible for meta-analysis	73

# 1. Executive summary of findings on infant and maternal intake of sources of fatty acids and risk of allergic or autoimmune outcomes

# 1.1. Context of this report

This report is one of a series of systematic review reports included in Review C Part II, which focusses on evidence on dietary exposures during pregnancy, lactation and or infancy for reducing risk of allergic or autoimmune outcomes.. This report focuses on infant and maternal intake of dietary sources fats. It includes dietary fats derived from vegetable or animal sources, as well as specific foods rich in fat. Dietary supplementation with specific fatty acids is also included. As fish are a known source of essential omega 3 fatty acids, these are included in this report. Lean fish is also included as although its content of omega 3 fatty acids is much lower than that found in oily fish, they contain modest amounts of this macro-nutrient. Whenever possible, this report specifies the type of dietary source of fat studied, although in some cases this remained unspecified (e.g. ‘fish’).

# 1.2. Studies identified

A total of 26 studies investigated intake of fats and risk of allergic or autoimmune diseases. Thirty four studies investigated the association between infant or maternal intake of dietary sources of fats, and risk of allergic diseases. Amongst these, there were 20 were prospective cohort studies (PC), 1 nested case-control (NCC), 1 retrospective cohort (RC), 3 case-control (CC) and 1 cross-sectional study.

# 1.3. Outcomes evaluated

Outcomes studied included atopic dermatitis (AD) (n=13), food allergy (FA) (n=3), wheeze (n=13), lung function (LF) (n=1), allergic sensitisation (AS) (n=6), type 1 diabetes mellitus (T1DM) (n=3) and juvenile idiopathic arthritis (JIA) (n=1).

# 1.4. Dietary exposures studied

Studies showed a high degree of variation in the types of dietary fats studied. When studies had data not eligible for meta-analysis, these were summarised in a descriptive table. This included the reported effect sizes for each relevant dietary exposure included in the study and their effect size (if available). The main nutrient exposures included fat intake (in general), as well as specific macro-nutrients omega 3, omega 6, poly-unsaturated fatty acids (PUFAs), saturated fatty acids, mono-unsaturated fatty acids (MUFA), α-linolenic acid, linoleic acid, docosahexaenoic acid (DHA), eicosapentaenoic acid (EPA). Foods rich in fat included those of animal origin e.g. butter, as well those derived from vegetables e.g. margarine and oils. Several studies also included fish (any, unspecified, oily, lean, or both).

# 1.5. Dietary assessment

Table 3 summarises the types dietary instruments used to ascertain intake of fats. Most of the studies (n=32) used a form of questionnaire (specific dietary questionnaire), or as part of a general questionnaire). Other forms of dietary assessment included interviews and hospital records.

# 1.6. Presentation of results

We created a summary Table of Study Characteristics with key study features, presented separately for allergic (Table 1) and autoimmune outcomes (Table 2). There are seven main sections in this report that summarise the main findings for each of the following outcomes: atopic dermatitis (AD), food allergy (FA), lung function (LF), wheeze, rhino-conjunctivitis (RC), allergic sensitisation (AS), type I diabetes mellitus (T1DM), and juvenile idiopathic arthritis (JIA). We examined each of the studies for their eligibility to be meta-analysed. If two or more studies had data that could be combined, a meta-analysis was included in the corresponding section. If the heterogeneity across studies ≥ 80% then the overall effect of the studies is not included but that of each individual study is presented. As most of the studies included in this report had data that could not be combined in a meta-analysis, a table of main characteristics and main findings is provided at the end of each section. The tables provide specific details on the dietary intakes compared for each nutritional exposure as these varied between, and sometimes within study.

# 1.7. Risk of bias assessment

The risk of bias in included cohort and case control studies was assessed using a modified version of the National Institute for Clinical Excellence (NICE) methodological checklist for cohort and case-control studies, respectively (1). Key domains were:

- Selection Bias (low if cases and controls were selected from similar populations, if the participation rate was ≥80%, or <80% but investigators explored and adjusted for characteristic differences between participants and non-participants);
- Assessment Bias (low if validated and reliable tools were used to assess exposure and/or outcome), and;
- Confounding Bias (low if most likely confounders are identified and taken into account in study design and analysis).

Observational studies were considered at low overall risk of bias where the risk of bias was judged to be low for all 3 key domains for selection, assessment and confounding bias. For assessment of Confounding Bias, factors that we expected to be adjusted for within studies of allergic outcomes were: siblings (parity or birth order or family size); gender; age at outcome assessment; disease risk based on family history; maternal or household smoking (asthma/wheeze outcomes); maternal age; maternal education or socioeconomic status; mode of delivery. For studies on autoimmune outcomes we expected matching and/or adjusting for gender, age, address, socioeconomic status, smoking and disease risk. We also assessed possible Conflict of Interest, this being judged as low where there was no evidence of industry involvement in study design, analysis, interpretation or publication, and no evidence that study authors received remuneration from relevant industry partners for other activities. Given the nature of the studies included in this report (observational studies on reported dietary intake of foods or food groups), the risk of conflict of interest was judged as low in all cases. Therefore, conflict of interest bias is not systematically shown through this report. A Risk of Bias Figure is presented in each outcome section, reported for all studies contributing data, whether included in meta-analysis or reported narratively.

# 1.8. Key findings

i. **Overall risk of bias was low or unclear for the majority of the outcomes assessed.**

The overall bias was considered to be low in the majority of the studies on atopic dermatitis, food allergy, wheeze, allergic sensitisation, and for autoimmune diseases. In several studies it was unclear whether adjustment for potential confounders had been made, which was the main factor influencing the ‘unclear’ assessment of this domain.

ii. **Large variations in the dietary exposures studied and their comparison levels limited the possibility of meta-analyses**

Although most studies used a questionnaire to ascertain dietary intake of the infant or the mother, there were large variations in the types of nutritional exposures assessed and the levels of intake compared. This means that opportunities for meta-analysis were very limited in this report. We present most data in narrative tables, and have detailed the effect estimates for each relevant exposure and indicated the levels of comparisons used for each effect estimate.

iii. **There is no consistent evidence that maternal or infant fat intake influences risk of allergic outcomes or autoimmune diseases**

We found inconsistent evidence regarding infant intake of fish and risk of AD and RC, and no evidence that other measures of infant fat intake, or maternal fat intake are associated with allergic outcomes or autoimmune diseases.

**Overall we found no evidence of associations with infant or maternal intake of fats, fatty acids or fish, and risk of allergic or autoimmune diseases.**

Table 1 Characteristics of included studies evaluating infant or maternal fat intake and allergic outcomes

| **Study** | **Design** | **N** | **Country** | **Type of dietary fat measured** | **Exposure assessment** | **Outcomes** |  | **Age at outcome (years)** | **Population characteristics** |
| --- | --- | --- | --- | --- | --- | --- | --- | --- | --- |
| Willers, 2007 (2) | PC | 1212 | UK | Maternal fat intake from different sources | Q | AD (ISAAC ), Lung function (Spirometry), AS-any by SPT, Wheeze (DD), Recurrent wheeze (DD asthma) |  | 5 | **Aberdeen birth cohort**: Population based birth cohort with pregnant women recruited 1997-99 while attending a hospital antenatal clinic at ~12weeks gestation |
| Kull, 2006; (3) Magnusson, 2013 (4) | PC | 3230 | Sweden | Infant fish intake | Q | AD (DD), AR (DD) AS-any by sIgE, Recurrent wheeze (≥ 3 episodes in past year) |  | 4, 12 | **BAMSE**: Prospective birth cohort of newborns in a predefined area of Stockholm, Sweden between 1994 and 1997 |
| Nwaru, 2012; (5)  Nwaru, 2011; (6); Nwaru 2010 (7); Niinisto, 2012; (8) Lumia, 2011; (9) Erkkola, 2012; (10) | PC | 3730 | Finland | Maternal fish and margarine intake; infant fish intake | Q | AD (ISAAC), Wheeze (ISAAC), Recurrent wheeze (DD), AS (sIgE) |  | 5, 0.5, <10 | **DIPP**: Prospective birth cohort of children at high risk of TIDM (HLA genotype conferred susceptibility) born between 1997 and 2004 in Oulu and Tampere University Hospital Finland |
| Leermakers, 2013; (11) De Jong, 2012 (12) | PC | 7210 | The Netherlands | Maternal fish intake | Q | AD (ISAAC), Wheeze (ISAAC) |  | 4 | **GENERATION R**: Population based birth cohort , with pregnant women recruited < 25 weeks gestation in Rotterdam, The Netherlands |
| Hoppu, 2000 (13) | PC | 114 | Finland | Maternal fat intake | R | AS-any by SPT |  | 1 | Birth cohort of infants of breastfeeding mothers (for at least 3 months) with a positive family history of atopic disease |
| Alm, 2009; (14) Goksor, 2011; (15) Alm, 2012;(16) | PC | 4941 | Sweden | Infant fish intake; infant fat intake | Q | Parent reported AD; DD Food Allergy |  | 1, 4.5 | **Infants of Western Sweden**: Population based birth cohort of infants born in the region in 2003 |
| Jedrychowski, 2008 (17); 2011 (18) | PC | 469 | USA and Poland | Maternal fish intake | Q | AD - physician assessment; wheeze (parent reported) |  | 1, 2 | Prospective birth cohort of infants from non-smoking healthy mothers who gave birth at 29 - 43 weeks of gestation between 2001 and 2004 |
| Laitinen, 2005 (19) | PC | 95 | Finland | Infant fat intake | D | AD (physician assessment), FA-cow’s milk |  | 0.5, 1 | Children with a family history of AD (mother, father and/or older sibling with AD, AR or asthma), who participated in a prospective allergy prevention study (probiotic intervention trial) |
| Fitzsimon, 2007 (20) | PC | 631 | Ireland | Maternal fat intake | Q | Recurrent wheeze (physician assessment of asthma) |  | 3 | **LIFE-WAYS**: cohort of children born in 2002 whose mother had completed FFQ during pregnancy were followed up from birth through general practice records |
| Sausenthaler, 2007 (21) | PC | 2540 | Germany | Maternal fat intake; maternal fish intake | Q | AD (DD; Parent reported), AS – any, food, aeroallergen by sIgE |  | 2 | **LISA**: Population based cohort study of newborns recruited between 1997 and1999 from 4 German cities: Munich, Leipzig, Wesel, and Bad Honnef. |
| Romieu, 2007; (22) Chatzi, 2008 (23) | PC | 468 | Spain | Maternal fish intake | I/Q, Q | Recurrent wheeze (parent reported), atopic wheeze (parent reported wheeze plus positive SPT), AS-aeroallergen by sIgE/SPT |  | 4, 6, 6.5 | **Menorca birth cohort**: Population based birth cohort with women recruited from antenatal care at all general practices in Menorca between 1997-1998 |
| Miyake, 2009 (24) Miyake, 2010 (25) | PC | 771 | Japan | Maternal fat intake, Maternal fish intake | Q | AD (DD, ISAAC), Wheeze (ISAAC) |  | 0.33, 2 | **OMCHS**: Population birth cohort with pregnant women between the 5-39th week of pregnancy recruited from a university hospital and three obstetric hospitals in municipality of Osaka between 2001 and 2003 |
| Oien, 2010 (26) | PC | 3067 | Norway | Maternal fish/ fish oil intake | Q | AD (ISAAC), recurrent wheeze (DD asthma) |  |  | **PACT**: A controlled primary intervention study on allergic diseases conducted in the central part of Norway in the city of Trondheim. Inclusion in the control cohort began in September 2000, and the interventional programme started in a separate cohort in July 2002 |
| Roduit, 2012 (27) | PC | 1041 | Austria, Finland, France, Germany, and Switzerland | Infant fat source introduction | D | AD (DD) |  | 1, 4 | **PASTURE**: Population based birth cohort with women recruited in third trimester of pregnancy from rural areas in 5 European countries (Austria, Finland, France, Germany, and Switzerland) and divided into 2 groups: those who lived or worked on family run farms and those not living on a farm from the same area |
| Willers, 2008 (28) | PC | 2811 | The Netherlands | Maternal fish intake | Q | Wheeze (ISAAC), recurrent wheeze (DD asthma) |  | 8 | **PIAMA**: Population based birth cohort of children born in 1996/97 after prenatal recruitment through prenatal clinics in the northern, middle and south western part of the Netherlands. The children were allocated to an intervention study or to a natural history study depending on their family risk for allergy |
| Narita, 2011 (29) | PC | 1344 | Japan | Maternal fish intake | Q | AD (ISAAC), wheeze (ISAAC) |  | 3 | **T-CHILD**: Population based birth cohort of Japanese mother-infant pairs with women recruited ~ second trimester in Tokyo |
| Wang, 2007 (30) | PC | 1760 | Taiwan | Maternal seafood intake/ fish oil supplementation | Q | AD (DD) |  | 0.5 | **Pilot study of Taiwan National Birth Cohort Study**: All postpartum women and new-borns from 29 towns were recruited with the multistage stratified systematic sampling design to obtain the representative samples from the national birth registration data in 2003 |
| Oliver, 2010; (31) Grimshaw, 2012 (32) | NCC | 123  (41 cases) | UK | Infant fat intake, maternal fish oil supplement | D, Q | FA-any (DBPCFC) |  | 1, 2 | **EuroPrevall** (UK birth cohort): cases were infants with food allergy, each matched to two controls. |
| Calvani, 2006 (FH+),Calvani, 2006 (FH-); (33) | RC | 988 | Italy | Maternal fat intake | Q | AS-aero-allergy, food, cow’s milk, egg by SPT |  | 5 | **APAL**: RC study of children attending outpatients allergy clinic in Rome between 2001-2002 |
| Salam, 2005 (34) | CC | 691  (279 cases) | USA | Maternal fish intake | I | Recurrent wheeze (DD asthma) |  | 9-16 | Children's Health Study: cases and controls selected from school-children who participated in a population-based study: USA |
| Castro-Rodriguez, 2010 (35) | CS | 1409  (594 cases) | Spain | Maternal fat intake | Q | Wheeze (ISAAC) |  | 1.4 | **EISL**: Spanish population attending primary healthcare clinics, cases with wheeze in first year of life and controls without: Spain |

PC Prospective cohort study; RC retrospective cohort study; Q questionnaire; AD atopic dermatitis; DBPCFC double blind placebo control food challenge; ISAAC International Study of Allergy and Asthma in Children; AS allergic sensitisation; SPT skin prick test, sIgE specific IgE; DD Doctor diagnosis (community); Physician assessment is assessment by study physician; AR allergic rhinitis, FA food allergy; CS cross-sectional study; NCC nested case-control study; FH+ having a family history of disease; FH- no family history of disease

Table 2 Characteristics of included studies evaluating infant or maternal fat intake and autoimmune outcomes

| **Study** | | **Design** | | **N** | | | **Country** | | **Type of dietary fat measured** | **Exposure assessment** | | **Outcomes** | |  | | **Age at outcome (years)** | | **Population characteristics** | | |
| --- | --- | --- | --- | --- | --- | --- | --- | --- | --- | --- | --- | --- | --- | --- | --- | --- | --- | --- | --- | --- |
| Fronczak, 2003; (36) Lamb 2008 (37) | | PC | | 642  (27 cases) | | | America, Australia | | Maternal fat intake | Q | | TIDM (serology) | |  | | 4 | | **DAISY**: Prospective birth cohort of children at increased risk for T1DM (relative with T1DM via registries and hospital records) recruired from 1993 to 2004 in Denver, Colorado US were screened for human leukocyte antigen (HLA) genotype associated with celiac disease and TIDM | | |
| Brekke, 2010 (38) | | PC | | 4005  (191 cases) | | | Sweden | | Maternal fish intake | Q | | TIDM (serology) | |  | | 5 | | **ABIS**: Population based birth cohort of children born in Southeast Sweden between 1997 and 1999 | | |
| Nwaru, 2012; (5)  Nwaru, 2011; (6) Niinisto, 2012; (8) Lumia, 2011; (9) Erkkola, 2012; (10) Virtanen, 2011 (39) | | PC | | 3730 | | Finland | | | Maternal fish and margarine intake; infant fish intake | | Q | | TIDM (serology) | |  | | 5, 0.5, <10 | | **DIPP**: Prospective birth cohort of children at high risk of TIDM (HLA genotype conferred susceptibility) born between 1997 and 2004 in Oulu and Tampere University Hospital Finland |  |
| Stene, 2000; (40) | CC | | 1131  (84 cases) | | Norway | | | Maternal fish oil supplement | | Q | | TIDM (medical diagnosis) | |  | | <15 | | Cases were T1DM patients in Vest-Agder & on the National Childhood Diabetes Register 1982-98 and controls were selected randomly from the population register for the same area and period | | |
| Stene, 2003 (41), Stene, 2008 (42) | CC | | 2213  (545 cases) | | Norway | | | Infant oil supplement, maternal fish oil supplement | | Q | | TIDM (medical diagnosis) | |  | | 8.8, 10 | | **Norwegian Childhood Diabetes Study** Group: cases were all children on the diabetes registry diagnosed 1997-2000 and controls were matched from the national population registry. | | |
| Ellis, 2012 (43) | CC | | 655  (246 cases) | | Australia | | | Maternal fish oil supplement | | Q | | JIA (ILAR criteria) | |  | | 0-18 | | **CLARITY**: cases were recruited during a clinic visit to Royal Children's Hospital, with diagnosed JIA using ILAR criteria: controls were patients in for elective surgery, also at the Royal Children's Hospital Day Surgery Unit | | |

PC Prospective cohort study; Q questionnaire; CC case-control study; SPT skin prick test, sIgE specific IgE; DD Doctor diagnosis (community); Physician assessment is assessment by study physician; AR allergic rhinitis, FA food allergy, T1DM Type 1 diabetes mellitus, RC retrospective cohort, JIA juvenile idiopathic arthritis; ILAR International League of Associations for Rheumatology

Table 3 Dietary instrument used to ascertain infant or maternal intake of dietary sources of fats

| First author, year | Dietary assessment used |
| --- | --- |
| Alm, 2011 | Specific question on fish intake in first year of life, as part of main questionnaire on risk factors for respiratory diseases |
| Brekke, 2010 | 22-item FFQ |
| Calvani, 2006 | Standardised self-administered questionnaire |
| Castro-Rodriguez, 2010 | Environmental questionnaire which included type of oil used for cooking or in dressing salads |
| Chatzi, 2008 | Spanish version of the validated EPIC-Norfolk FFQ |
| Ellis, 2012 | General questionnaire with dietary questions |
| Erkkola, 2012 | Retrospective diary records of foods eaten during the month preceding delivery (8^th^ month of pregnancy) |
| Fitzimon, 2007 | ‘Hospital records’ |
| Fronczak, 2003 | Willett FFQ regarding third trimester diet of mother, administered at 2-3 months post-delivery |
| Goksor, 2011 | Questions based on the ISAAC questionnaire for Sweden |
| Grimshaw, 2012 | Questionnaire data from the general EuroPrevall questionnaire (fish oil use during pregnancy) |
| Hoppu, 2010 | Four-consecutive-day food record with household measures |
| Jedrychowski, 2008 | Interviews with a validated FFQ |
| Kull, 2006 | Questionnaire about frequency of fish intake by the infant and age (months) of fish introduction |
| Lamb, 2008 | Willet FFQ |
| Laitinen, 2005 | Four day food diaries completed by parents or personnel at day-care |
| Leermakers, 2013 | Modified version of a semi-quantitative FFQ |
| Lumia, 2011 | Validated, self-administered, semi-quantitative 181-item FFQ |
| Magnusson, 2013 | General parental questionnaire which included questions on other risk factors |
| Miyake, 2009 | Self-administered DHQ that assessed dietary habits during the preceding month (150 foods) |
| Narita, 2011 | Dietary history questionnaire |
| Niinisto, 2012 | FFQ |
| Nwaru, 2010, 2011, and 2012 | 181-item FFQ |
| Oien, 2009 | Questionnaire administered to the mother 1 year after delivery |
| Oliver, 2010 | Food diaries with qualitative and quantitative data completed and returned every 4 weeks in the first year of life |
| Roduit, 2012 | Dietary intake reported by parents in monthly diaries between the 3rd and 12th month of life |
| Romieu, 2007 | Interviewer-administered FFQ at 3 months after delivery |
| Salem, 2005 | Telephone interviews with mothers/guardians |
| Sausenthaler, 2007 | Semi-quantitative FFQ capturing intake in the last 4 weeks of pregnancy |
| Stene, 2000 and 2003 | Parental questionnaire, which included questions on fat intake and duration of exclusive breastfeeding |
| Wang, 2007 | Questions on dietary intake included in environmental questionnaire |
| Willers, 2007 | Semi-quantitative FFQ (Version 5.4 of the Scottish Collaborative Group FFQ) mailed at 32 weeks gestation (150 items divided into 20 food groups |

EPIC= The European Prospective Investigation into Cancer Study

FFQ= Food frequency questionnaire

ISAAC=International Study of Allergy and Asthma in Children

DHQ= Dietary history questionnaire

# 2. Infant or maternal dietary intake of fats and risk of atopic dermatitis

Sixteen studies including over 26,000 children investigated the association between infant or maternal intake of fats and risk of AD. The quality assessment of studies on AD and intake of various types of fats is summarised in Figure 1. Overall, 70% of the studies were considered to be of low risk of bias. The assessment and confounding biases were judged to be low in over 80% of the studies. This was largely driven by the methodological quality of the prospective cohort studies that examined the association between this outcome and the exposure of interest.

Figure 1 Risk of Bias in studies of fat intake and atopic dermatitis

Figures 2 to 5 illustrate the combined effect sizes of cohort studies that had comparable estimates of maternal intake of fats and risk of AD in the offspring. The studies of **Miyake (2009)** and **Sausenthaler (2007)** showed no evidence of an association between maternal intake of butter during pregnancy and risk of this outcome in their children (Figure 2). Both studies had a similar effect size, and there was very low heterogeneity. This was likely to be a reflection of the similar methods used in both studies to ascertain dietary intake, and the time of outcome assessment (2 years old).

Figure 2 Maternal butter intake (highest vs. lowest) and risk of AD in children at age 0-4 years old

Figure 3 shows the combined effect estimates of association between maternal intake of marine sources of fat (omega 3, seafood or fish) and risk of AD. The prospective cohort studies of **Jedrychowski (2011)**, and **Sausenthaler (2007)** showed a statistically significant negative association, whilst **Miyake**’s findings **(2009)** were in the same direction but did not reach statistical significance. The overall effect of these three studies showed a 30% lower risk of AD (95% CI 0.56, 0.89) in the child of mothers who ate the highest vs lowest consumption categories of omega 3 fatty acids during pregnancy. There was no evidence of heterogeneity (I^2^=0%) across studies.

Figure 3 Maternal intake of marine sources of fatty acids intake (highest vs. lowest) and risk of atopic dermatitis (eczema) in children at age 0-4 years old

Three prospective cohort studies (**Leermakers (2013)**, **Oien (2009)** and **Wang (2007))** had comparable data on maternal fish intake and risk of AD in the offspring (often vs rare) (Figure 4). There was no evidence of an association with AD in any of the studies or in the combined effect size. There was no heterogeneity across studies (I^2^=0%). Similarly, the studies of **Oien (2009)** and **Wang (2007)** showed no evidence of association between maternal supplementation with fish oils during pregnancy and risk of AD in the offspring (Figure 5).

Figure 4 Maternal intake of marine sources of fatty acids intake (often vs. rare) and risk of atopic dermatitis (eczema) in children at age 0-4 years old (odds ratio)

Figure 5 Maternal fish oil supplementation (often vs. rare) and risk of atopic dermatitis (eczema) in children at age 0-4 years old (odds ratio)

Figure 6 shows the association between introduction of fats in the infant’s diet and risk of AD at age 0-4. The prospective studies of Roduit (2012) and Alm (2009) reported no evidence of association between introduction of any type of fat spread for bread (Alm) or butter (Roduit) after month 8 of life compared to earlier, and AD.

Figure 6 Introduction of fats in the infant’s diet (> 8 months vs earlier) and risk of AD at age 0-4 years old

# 2.1. Studies that investigated the association between infant or maternal intake of fats and risk of AD which were not eligible for meta-analysis

Table 4 summarises the findings of observational studies investigating intake of fats and risk of AD in childhood. Six studies investigated infant intake, whereas eight had data on maternal intake during pregnancy or lactation. The BAMSE prospective cohort study of **Kull (2006)** investigated risk of AD at age 4 in Swedish children according to their fish intake during the first year of life. The study showed a negative association between fish intake and risk of AD at age 4 in the entire sample of children studied (OR 0.69; 95% CI 0.57-0.84), and this association remained statistically significant when children with early onset eczema were excluded from analysis to account for potential reverse causation. The same cohort study followed up these children to age 12 years old (**Magnussonn, 2013**) and found that a higher intake of fish at age 1 was associated with a lower risk of AD when age 12. . The prospective study of **Alm (2009)** reported no association between level of fish intake in infancy and AD, although they did find an association between timing of fish introduction in infancy and AD (discussed in review B report). The rest of the studies on infant intake, showed no evidence that other dietary sources of fats (e.g. margarine, lean fish, and cod liver oil) were associated with AD in children, regardless of the disease risk level studied.

The study of **Oien (2009)** showed that a higher intake of oily fish (any) in pregnancy women was negatively associated with risk of AD at age 2 (OR 0.65; 95% CI 0.45-0.94) and the association was strongest for oily fish. However this was not a consistent finding across studies.

Amongst the studies examining maternal intake of dietary sources and risk of AD in the offspring, there was little evidence of a consistent effect. The study of **Sausenthaler (2007)** showed that intake during the final 4 weeks of pregnancy of margarine and butter, but not deep frying vegetable fat, was associated with higher risk of AD at age 2 years in their offspring. However, these findings were not confirmed in two other studies who found no evidence of an association between intake of butter (**Nwaru 2012**) or margarine (**Willers 2007**) and risk of AD.

**Conclusion**

We found no evidence that fatty acid or fat intake during pregnancy, lactation or infancy is associated with risk of AD in offspring.

Table 4 Studies investigating the association between fat intake and atopic dermatitis which were not eligible for meta-analysis

| **First Author and year of publication** | **Design** | **Outcome** | **Age** | **N/n** | **Dietary exposure and comparison level** | **Measure of association** | **Effect (bold indicates p<0.05)** |
| --- | --- | --- | --- | --- | --- | --- | --- |
|  |  |  |  |  | **INFANT INTAKE** |  |  |
| Alm, 2009 (14) | PC | AD | 1 | 4941 | Fish intake in the first year of life (a few times a year vs ≥3 times per week) | OR | **1.32 (0.86-2.02)** |
|  |  |  |  | 2536/358 | **Fish intake in the child's diet at 12 months of age** (≥2-3 times per month vs ≤ once month)  Children who have eczema at age 1 excluded | OR | **0.76 (0.60-0.98)** |
| Kull, 2006 (3) | PC | AD | 4 | 2896/551 | All children | OR | **0.69 (0.57-0.84)** |
| Magnusson, 2013 (4) | PC | AD | 12 | 2456 | Fish intake in first year of life ( ≥2-3 times per month vs ≤ once a month) | OR | **0.61 (0.53-0.70)** |
| Laitinen, 2005 (19) |  |  | 0.5 | 60 | **Fat intake during the first year of life:**  Grams of fat intake/ mega joule per day energy intake | OR | 1.06 (0.99-1.14) |
|  |  |  | 0.5 | 60 | Percentage of energy intake as fat | OR | NS |
|  | PC | AD | 1 | 95 | Grams of fat intake/ mega joule per day energy intake | OR | NS |
| Roduit, 2012 (27)  Roduit, 2012 (25) |  |  |  |  | **Food introduced in the first year of life vs none in the first year:**  Margarine (high risk infants) | OR | 1.35 (0.75-2.45) |
|  | PC | AD | 4 | 1041 | Butter (high risk infants) | OR | 0.92 (0.53-1.59) |
|  |  |  |  |  | Margarine (low risk infants) | OR | 1.61 (0.62-4.21) |
|  |  |  |  |  | **Food introduced in the first year of life vs none in the first year:**  Butter (low risk infants) | OR | 1.15 (0.55-2.42) |
|  |  |  |  |  | Margarine (any risk infants) | OR | 1.45 (0.86-2.44) |
|  |  |  | 4 | 1041 | Butter (any risk infants) | OR | 1.00 (0.65-1.55) |
|  | PC | AD | 4 | 912 | Margarine introduced into diet>12 months vs 3 to 12 months | OR | 1.45 (0.86-2.44) |
|  |  |  | 4 | 912 | Butter introduced into diet>12 months vs 3 to 12 months | OR | 1.00 (0.65-1.55) |
|  |  |  | 1 | 912 | Margarine introduced into diet>12 months vs 3 to 12 months (single point measurement) | OR | 1.32 (0.88-1.97) |
|  |  |  | 1 | 912 | Butter introduced into diet >12 months vs 3-12 months (single point measurement) | OR | 1.28 (0.86-1.91) |
|  |  |  |  |  | **MATERNAL INTAKE** |  |  |
| Leermakers, 2013 (11) | PC | AD | 4 | 2796 | **Maternal intake in first trimester of pregnancy (intake indicated below vs no intake):**  Total fish 140-209g/week |  | 0.96 (0.72-1.28) |
|  |  |  |  |  | Total fish 1-69g/week |  | 1.03 (0.85-1.25) |
|  |  |  |  |  | Total fish 70-139g/week |  | 1.06 (0.88-1.27) |
|  |  |  |  |  | Lean fish 35-69 g/week |  | 1.04 (0.89-1.22) |
|  |  |  |  |  | Lean fish >70 g/week |  | 0.99 (0.79-1.24) |
|  |  |  |  |  | Lean fish 1-34g/week |  | 1.06 (0.88-1.27) |
|  |  |  |  |  | Fatty fish 1-34g/week |  | 1.17 (0.99-1.38) |
|  |  |  |  |  | Fatty fish 35-69 g/week |  | 1.17 (0.99-1.38) |
|  |  |  |  |  | Fatty fish >70 g/week |  | 1.06 (0.88-1.27) |
|  |  |  |  |  | Shellfish 1-13 g/week |  | **1.18 (1.02-1.37)** |
|  |  |  |  |  | Shellfish >14 g/week |  | 1.02 (0.86-1.21) |
| Miyake (Saito), 2010 (25) | PC | AD | 0.33 | 771 | **Maternal intake during pregnancy (in the previous month) (highest vs lowest quartile):**  Saturated fat | OR | 0.95 (0.43-2.09) |
|  |  |  |  |  | Cholesterol | OR | 0.96 (0.43-2.12) |
|  |  |  |  |  | Arachidonic acid | OR | 0.81 (0.37-1.79) |
|  |  |  |  |  | Linoleic acid | OR | 1.04 (0.43-2.49) |
|  |  |  |  |  | n6 fatty acids | OR | 1.14 (0.46-2.81) |
|  |  |  |  |  | MUFA | OR | 1.48 (0.59-3.73) |
|  |  |  |  |  | Total fat | OR | 1.38 (0.57-3.33) |
|  |  |  |  |  | Fish | OR | 1.15 (0.50-2.62) |
|  |  |  |  |  | α-linolenic acid | OR | 0.76 (0.31-1.87) |
|  |  |  |  |  | Omega 3/omega 6 fatty acids | OR | 1.17 (0.52-2.62) |
|  |  |  |  |  | DHA | OR | 1.43 (0.67-3.07) |
|  |  |  |  |  | Omega 3 fatty acids | OR | 1.45 (0.64-3.31) |
|  |  |  |  |  | EPA | OR | 1.84 (0.82-4.15) |
| Miyake, 2009 (24) | PC | AD | 2 | 763 | **Maternal intake during pregnancy (in the previous month) (lowest vs highest quartile):**  Cholesterol | OR | 0.71 (0.40-1.27) |
|  |  |  |  |  | Arachidonic acid | OR | 0.69 (0.37-1.27) |
|  |  |  |  |  | MUFA | OR | 1.32 (0.70-2.49) |
|  |  |  |  |  | Linoleic acid | OR | **2.11 (1.05-4.26)** |
|  |  |  |  |  | Omega 6 fatty acids | OR | **2.25 (1.12-4.54)** |
|  |  |  |  |  | Total fat | OR | 1.49 (0.78-2.83) |
|  |  |  |  |  | α-linolenic acid | OR | 1.79 (0.92-3.50) |
|  |  |  |  |  | DHA | OR | 0.86 (0.32-2.28) |
|  |  |  |  |  | EPA | OR | 0.98 (0.38-2.50) |
|  |  |  |  |  | Omega 3/omega 6 fatty acids | OR | 1.32 (0.64-2.71) |
|  |  |  |  |  | Omega 3 fatty acids | OR | 1.74 (0.81-3.73) |
|  |  |  |  |  | **Maternal consumption of fish during pregnancy: (Lowest vs highest quartile):**  Fish |  | NS |
| Narita, 2011 (29) | PC | AD | 3 | 1344 | Omega 3/omega 6 fatty acids |  | NS |
| Nwaru, 2012 (5) | PC | AD | 5 | 2441 | **Maternal intake during the 8^th^ month of pregnancy (second/third tertile of intake vs lowest):**  Consumption of margarines (no vs. yes) | OR | 0.92 (0.78-1.09) |
|  |  |  |  |  | Butter and butter spreads | OR | 1.06 (0.85-1.32) |
|  |  |  |  |  | Saturated fatty acid | OR | 1.01 (0.81-1.25) |
|  |  |  |  |  | MUFA | OR | 1.11 (0.90-1.37) |
|  |  |  |  |  | γ-linoleic fatty acids | OR | 1.10 (0.89-1.35) |
|  |  |  |  |  | Conjugated linoleic fatty acids | OR | 1.08 (0.87-1.33) |
|  |  |  |  |  | Omega 6 fatty acids | OR | 1.03 (0.83-1.28) |
|  |  |  |  |  | Linoleic fatty acid | OR | 1.02 (0.83-1.26) |
|  |  |  |  |  | Arachidonic acid | OR | 0.98 (0.79-1.21) |
|  |  |  |  |  | Oils | OR | 1.10 (0.88-1.36) |
|  |  |  |  |  | ‘Industrial fats’ (unclear term) | OR | 1.06 (0.85-1.32) |
|  |  |  |  |  | Trans-fatty acid | OR | 1.19 (0.96-1.47) |
|  |  |  |  |  | Total fatty acid | OR | 1.19 (0.96-1.47) |
|  |  |  |  |  | α-linoleic fatty acids | OR | 0.95 (0.77-1.18) |
|  |  |  |  |  | PUFA | OR | 1.06 (0.85-1.32) |
|  |  |  |  |  | **Maternal intake during the 8^th^ month of pregnancy(second/third tertile of intake vs lowest):**  Omega 3 fatty acids from plants | OR | 1.03 (0.84-1.26) |
|  |  |  |  |  | Omega 3 fatty acids | OR | 0.97 (0.79-1.20) |
|  |  |  |  |  | EPA | OR | 0.93 (0.75-1.16) |
|  |  |  |  |  | Omega 3 fatty acids from fish | OR | 0.86 (0.70-1.06) |
| Nwaru, 2012 (5) | PC | AD | 5 | 2441 | DHA | OR | 0.85 (0.68-1.05) |
| Oien, 2010 (25)  Oien, 2010 (26) | PC  PC | AD    AD | 2  2 | 3055 | **Maternal intake of the following sources of fish up to the first year of life**  Any fish (≥4 times/week vs never or < 1x/week ) | OR | **0.65 (0.45-0.94)** |
|  |  |  |  | 3055 | Cod liver oil (≥4 times/week vs never ) | OR | 0.89 (0.59-1.34) |
|  |  |  |  | 3055 | Cod liver oil (≤3 times/week vs never) | OR | 1.11 (0.68-1.81) |
|  |  |  |  | 3050 | Lean fish e.g. cod and coalfish (≥1 per week vs never or < once per week ) | OR | 0.67 (0.42-1.08) |
|  |  |  |  | 3050 | Oily fish e.g. redfish, halibut, salmon, trout herring and mackerel (≥ 1 per week vs never or < once per week) | OR | **0.21 (0.05-0.86)** |
|  |  |  |  | 2427 | **Maternal consumption during the last 4 weeks of pregnancy (high vs low tertile):**  Margarine | OR | **1.49 (1.09-2.04)** |
|  |  |  |  | 2465 | Deep frying vegetable fat | OR | 1.10 (0.86-1.41) |
| Sausenthaler, 2007 (21) | PC | AD | 2 | 2492 | Butter | OR | **1.48 (1.15-1.91)** |
|  |  |  |  |  | **Maternal diet during pregnancy:**  Fish intake ≥1/week vs never | OR | **0.57 (0.35-0.92)** |
|  |  |  |  |  | Fat from dairy products (low vs high) |  | NS |
| Willers, 2007 (2) | PC | AD | 5 | 1212 | Margarine/low fat spread vs Butter spread |  | NS |

# 3. Infant or maternal dietary intake of fats and risk of food allergy

Five studies which included over 5,500 children investigated the association between infant or maternal intake of fats and risk of FA. The quality assessment of observational studies examining the association between infant or maternal intake of fats and risk of food allergy is illustrated in Figure 7. Most of the studies were judged to have low or unclear risk of bias across the three domains investigated.

Figure 7 Risk of Bias in studies of fat intake and food allergy

# 3.1. Studies that investigated the association between infant or maternal intake of fats and risk of food allergy which were not eligible for meta-analysis

Table 5 summarises the findings of three observational studies on infant intake, and two on maternal intake of fats and risk of FA in the offspring, none of which showed evidence of an association between dietary fats and this outcome.

**Conclusion**

We found no evidence to suggest that infant or maternal intake of dietary fats influences the risk of FA in children.

Table 5 Studies investigating the association between intake of dietary sources of fat and food allergy which were not eligible for meta-analysis

| **First Author and year of publication** | **Design** | **Outcome** | **Age** | **N/n** | **Dietary exposure and comparison level** | **Measure of association** | **Effect (bold indicates p<0.05)** |
| --- | --- | --- | --- | --- | --- | --- | --- |
|  |  |  |  |  | **INFANT INTAKE** |  |  |
| Goksor, 2011 (15) | PC | FA any food | 4.5 | 4496 | Frequency of fish consumption, assessed at 12 months age (unclear level of comparison) | Unclear | NS |
| Laitinen, 2005 (19) | PC | FA any food | 1 | 95 | Infant fat intake ( % of energy intake [MJ/day] ) | Mean | (28% vs 33% in healthy vs allergic) NS |
| Oliver, 2010 (32) | NCC | FA any food | 1 | 93/31 | Dietary fat intake during the first year of life (unclear level of comparison) | OR | NS |
|  |  |  |  |  | **MATERNAL INTAKE** |  |  |
| Nwaru, 2010 (5) | PC | FA any food | 5 | 931 | Maternal n-3 PUFA intake at 8^th^ month of pregnancy lowest tertile vs highest 2 tertiles | OR | 0.73 (0.33-1.61) |
|  |  |  |  |  | Fish oils supplement during pregnancy (no vs yes) | OR | NS |
| Grimshaw, 2012 (31) | NCC | FA any food | 2 | 123/41 | Fish oils supplement during lactation (no vs yes) | OR | NS |

# 4. Infant or maternal dietary intake of fats and risk of wheeze

Fifteen studies which included over 27,000 children examined the association between infant or maternal intake of fats and risk of wheeze. The overall risk of bias was low for the majority of studies. The assessment of outcome and exposure were clearly described, and almost all studies reported adjusted data (Figure 8).

Figure 8 Risk of Bias in studies of fat intake and wheeze

Two birth cohorts from Japan (**Miyake 2009**; healthy, non-smoking mothers) and Poland (**Jedrychowski 2008**, population-based) had comparable data on maternal intake of marine sources of fat and risk of wheeze in the offspring (Figure 9). The study of Miyake compared highest vs lowest quartile of intake of seafood, whilst the study of Jedrychowski compared the effect of maternal intake of 150g of fish per week vs less than this cut-off. The combined effect estimates showed a small but statistically significantly lower risk of wheeze in children at age 2 from mothers whose fish intake was in the highest level of comparison (OR 0.97; 95% CI 0.95, 0.99). There was no heterogeneity between studies.

Another two prospective studies (**Chatzi 2008**, and **Willers 2008**) and one retrospective study (**Salam, 2005**) had comparable data to on maternal intake of fish and risk of childhood wheeze (Figure 10). The study of Chatzi compared a maternal reported intake of fish of at least 2.5 times per week vs < 2.5 times per week; in the study of Willers the authors compared daily or regular intake of fish during pregnancy vs rare consumption or never. The study of Salam compared at least monthly intake of fish during pregnancy vs never or rare. There was no evidence of an association between this outcome and the dietary exposures. There was a high level of heterogeneity (I^2^=65.8%), driven mostly by the heterogeneity observed between the two prospective studies.

Figure 9 Maternal intake of fish or seafood (highest vs lowest) and risk of wheeze in children at age 0-4 years old

Figure 10 Maternal intake of fish (often vs rare-never) and risk of recurrent wheeze in children at age 5-14 years old

Two studies (**Oien 2009**, and **Kull 2006**) investigated infant intake of any type of fish in the first year of life and risk of recurrent wheeze. The study of Oien compared intake once a week vs never, and the study of Kull compared an intake of 2-3 times per month vs ≤ once per month. The pooled effect estimate showed an overall negative association between higher intake of fish and risk of recurrent wheeze at age 0-4 years old (OR 0.74; 95% CI 0.60, 0.92) (Figure 11), with no heterogeneity between studies (I^2^=0%). Data from Kull which excluded infants with early onset eczema, in order to account for possible reverse causation, did not show an association between fish intake and recurrent wheeze (Table 6).

Figure 11 Infant intake of fish (often vs rare) and risk of recurrent wheeze at age 0-4 years old

# 4.1. Studies that investigated the association between wheeze and infant or maternal intake of fats which were not eligible for meta-analysis

Three prospective cohort studies investigated the association between infant intake of fish-related foods and risk of wheeze. The study of **Kull (2006)** and **Oien (2006)** measured the outcome at age 4 and 2 years old, respectively, whilst the study of **Magnusson (2013)**, measured the outcome at age 12. There was no evidence of an association between intake of specific types of fish or fatty acids in the first year of life and risk of wheeze later in childhood in these 3 studies (Table 6).

With regards to maternal intake, the studies of **Leermakers (2013)**, **Lumia (2011)**, and **Narita (2011)** found no association between maternal intake of fish observed in older children (5-14 years old) when the outcome was studied in children aged 4, 5, and 3 years old, respectively. **Miyake (2009)** reported a lower risk of wheeze in children at age 2 whose mothers had a higher intake of α-linolenic acid and DHA. The study of **Nwaru (2012)** however, did not confirm an association between these nutrients and risk of wheeze in childhood.

**Conclusion**

**We found no consistent evidence to suggest that a high infant or maternal intake of fish, seafood or specific fatty acids is associated with risk of wheeze or recurrent wheeze in children aged 0-4 or 5-14 years old. We also found no consistent evidence that dietary sources of fats other than those found in fish influence the risk of wheeze in childhood.**

Table 6 Studies investigating the association between fat intake and wheeze which were not eligible for meta-analysis

| **First Author and year of publication** | **Design** | **Outcome** | **Age** | | | **N/n** | **Dietary exposure and comparison level** | | | **Measure of association** | **Effect (bold indicates p<0.05)** | | |
| --- | --- | --- | --- | --- | --- | --- | --- | --- | --- | --- | --- | --- | --- |
|  |  |  |  | | |  | **INFANT INTAKE** | | |  |  | | |
| Kull, 2006 (3) | PC | Rec wheeze | 4 | | | 3926 | **Infant’s fish intake in first 12 months of life ≥2-3 times/month vs ≤1/month):**  Children with eczema in first year excluded | | | OR | 0.76 (0.52-1.11) | | |
| Magnusson, 2013 (4) | PC | Rec wheeze | 12 | | | 2456 | **Fish intake in 1^st^ year of life:**  ≥ 2-3 time/month vs ≤ once per month | | | OR | **0.71 (0.58-0.87)** | | |
|  |  |  |  | | | 3067 | **Infant intake of cod liver oil up to 1 year of age:**  ≤3 times per week vs never | | | OR | 0.84 (0.55-1.29) | | |
|  |  |  |  | | | 3067 | ≥4 times per week vs never | | | OR | 0.90 (0.64-1.26) | | |
| Oien, 2010 (26) | PC | Rec wheeze | 2 | | | 2220 | **Lean fish**  ≥1 times per week vs never or < once per week | | | OR | 0.76 (0.51-1.13) | | |
|  |  |  | 2 | | | 1681 | **Oily fish (redfish, halibut, salmon, trout herring and mackerel)**  ≥1 times per week vs never or < once per week | | | OR | 1.06 (0.58-1.93) | | |
|  |  |  |  | | |  | **MATERNAL INTAKE** | | |  |  | | |
| Castro-Rodriguez, 2010 (35) | RC | Wheeze | 1.4 | | | 1409 | Maternal intake of olive oil for salads (yes vs no) | | | OR | **0.57 (0.41-0.80)** | | |
|  |  |  |  | | |  | **Maternal intake during pregnancy ( comparison levels in quarters, with mid-half (median) values as reference i.e. 149g to 315g) :**  Dietary sources of fat: 31-57.3 vs <31 | | | OR | 0.92 (0.71-1.18) | | |
|  |  |  |  | | |  | Dietary sources of fat: 31-57.3 vs 57.3-264.8 | | | OR | 0.86 (0.66-1.12) | | |
| Erkkola, 2012 (10) | PC | Rec wheeze | 5 | | | 2441 | Fish: 10.9-33.27 vs <10.9 | | | OR | 0.94 (0.74-1.20) | | |
|  |  |  |  | | |  | Fish: 10.9-33.3 vs 33.3-254.8 | | | OR | 0.99 (0.78-1.25) | | |
|  |  |  |  | | |  | Fish: 10.9-33.27 vs <10.9 | | | HR | 1.15 (0.70-1.89) | | |
|  |  |  |  | | |  | Fish: 10.9-33.27 vs 33.28-254.8 | | | HR | 1.09 (0.67-1.78) | | |
|  |  |  |  | | |  | Dietary sources of fat: 31-57.3 vs <31 | | | HR | 0.75 (0.46-1.21) | | |
| Fitzsimon, 2007 (20) | PC | Rec wheeze | 3 | | | 631 | **Added or spreadable fats considered included butter, margarine and other spreads, salad dressings and mayonnaise:**  Q1 vs Q3 | | | OR | 0.62 (0.24-1.60) | | |
|  |  |  |  | | |  | Q1 vs Q2 | | | OR | 1.90 (0.98-3.70) | | |
|  |  |  |  | | |  | **Maternal intake during 1^st^ trimester of pregnancy (each of the following categories vs no fish):**  Total fish 140-209g/week | | | OR | 0.86 (0.70-1.06) | | |
|  |  |  |  | | |  | Total fish 70-139g/week | | | OR | 0.91 (0.77-1.08) | | |
|  |  |  |  | | |  | Total fish 1-69g/week | | | OR | 0.92 (0.78-1.09) | | |
|  |  |  |  | | |  | Lean fish 35-69 g/week | | | OR | 0.86 (0.73-1.02) | | |
|  |  |  |  | | |  | Lean fish 1-34g/week | | | OR | 1.02 (0.87-1.19) | | |
| Leermakers, 2013 (11) | PC | Wheeze | 4 | | | 2796 | Fatty fish 35-69 g/week | | | OR | 0.91 (0.77-1.08) | | |
|  |  |  |  | | |  | Fatty fish 1-34g/week | | | OR | 1.02 (0.87-1.19) | | |
|  |  |  |  | | |  | Shellfish 1-13 g/week | | | OR | 1.20 (1.03-1.40) | | |
|  |  |  |  | | |  | Lean fish >70 g/week | | | OR | 0.99 (0.80-1.23) | | |
|  |  |  |  | | |  | Fatty fish >70 g/week | | | OR | 0.94 (0.77-1.15) | | |
|  |  |  |  | | |  | Shellfish >14 g/week | | | OR | 1.06 (0.92-1.22) | | |
| Lumia, 2011 (9) | PC | Rec wheeze | 5 | | | 2679 | **Dietary intakes during pregnancy (highest quartile vs mid-half of intake):**  MUFA | | | HR | 0.71 (0.46-1.10) | | |
|  |  |  |  | | |  | Omega 6 | | | HR | 0.75 (0.47-1.20) | | |
|  |  |  |  | | |  | Oils | | | HR | 1.26 (0.82-1.94) | | |
|  |  |  |  | | |  | **Dietary intakes during pregnancy (highest quartile vs mid-half of intake):**  Fish | | | HR | 1.06 (0.68-1.65) | | |
| Lumia, 2011 (9) | PC | Rec wheeze | 5 | | | 2679 | PUFA | | | HR | 0.84 (0.53-1.32) | | |
|  |  |  |  | | |  | **Maternal intake pregnancy (highest vs lowest quartile):**  Cholesterol | | | OR | 0.85 (0.49-1.47) | | |
|  |  |  |  | | |  | Arachidonic acid | | | OR | 0.77 (0.43-1.37) | | |
|  |  |  |  | | |  | Linoleic acid | | | OR | 0.79 (0.42-1.50) | | |
| Miyake, 2009 (24) | PC | Wheeze | 2 | | | 763 | MUFA | | | OR | 0.79 (0.43-1.45) | | |
|  |  |  |  | | |  | Omega 6 | | | OR | 0.80 (0.42-1.54) | | |
|  |  |  |  | | |  | Total fat | | | OR | 0.80 (0.45-1.43) | | |
|  |  |  |  | | |  | α-linolenic acid | | | OR | **0.52 (0.28-0.97)** | | |
|  |  |  |  | | |  | DHA | | | OR | **0.37 (0.15-0.91)** | | |
|  |  |  |  | | |  | Omega 3 | | | OR | 0.53 (0.26-1.08) | | |
|  |  |  |  | | |  | EPA | | | OR | 0.76 (0.32-1.80) | | |
|  |  |  |  | | |  | Omega 3/Omega 6 fatty acids | | | OR | 0.81 (0.42-1.55) | | |
| Narita, 2011 (29) | PC | Wheeze | 3 | | | 1344 | **Maternal consumption of fish during pregnancy (highest vs lowest quartile [specific amounts not reported]):**  Fish, omega 3 | | |  | NS | | |
|  |  |  |  | | |  | **Maternal intake at 8^th^ month of pregnancy (Second/ third quartile of intake vs lowest)**  Saturated fatty | | | HR | 1.01 (0.79-1.30) | | |
|  |  |  |  | | |  | Butter and butter spreads | | | HR | 0.92 (0.71-1.19) | | |
|  |  |  |  | | |  | Arachidonic acid | | | HR | 1.05 (0.81-1.36) | | |
|  |  |  |  | | |  | Linoleic fatty acid | | | HR | 0.99 (0.77-1.27) | | |
| Nwaru, 2012 (5) | PC | Wheeze | 5 | | | 2441 | Omega 6 fatty acids | | | HR | 0.99 (0.77-1.27) | | |
|  |  |  |  | | |  | Conjugated linoleic fatty acids | | | HR | 0.84 (0.66-1.07) | | |
|  |  |  |  | | |  | γ-linoleic fatty acids | | | HR | 0.83 (0.65-1.06) | | |
|  |  |  |  | | |  | Oils | | | HR | 1.14 (0.88-1.47) | | |
|  |  |  |  | | |  | α-linoleic fatty acids | | | HR | 1.15 (0.90-1.47) | | |
|  |  |  |  | | |  | PUFA | | | HR | 1.06 (0.83-1.37) | | |
|  |  |  |  | | |  | Omega 3 fatty acids from plant sources | | | HR | 1.06 (0.84-1.35) | | |
|  |  |  |  | | |  | Omega 3 fatty acids | | | HR | 1.02 (0.80-1.30) | | |
|  |  |  |  | | |  | EPA | | | HR | 0.99 (0.83-1.19) | | |
|  |  |  |  | | |  | DHA | | | HR | 0.94 (0.74-1.21) | | |
|  |  |  |  | | |  | Omega 3 fatty acids from fish | | | HR | 0.93 (0.72-1.18) | | |
| Oien, 2009 (26) | PC | Rec wheeze | 2 | | | 3018 | **Maternal intake of cod liver oil during pregnancy**  ≤ 3 times per week vs never | | | OR | 0.97 (0.63-1.50) | | |
|  |  |  |  | |  | | | **Maternal consumption during pregnancy**  Oily fish (less then monthly (0.25 times per week vs never) | OR | | | 1.01 (0.54-1.89) |  |
|  |  |  |  | |  | | | ‘Fish stick’ [source of trans-fats] (less then monthly (0.25 times per week) vs never) | OR | | | 1.15 (0.66-2.01) |  |
| Salam, 2005 (34) | CC | Rec wheeze | 9-16 | | 691/  279 | | | ‘Fish stick’ (at least monthly i.e. 0.25 times per week vs never) | OR | | | **2.04 (1.19-3.51)** |  |
| Willers, 2007 (2) | PC | Wheeze  Rec wheeze | 5 | 1212 | | | **Maternal diet during pregnancy:**  Margarine (low fat spread vs butter spread)  Fat from dairy (low vs high) | | | OR | NS | | |

# 5. Infant or maternal dietary intake of fats and lung function

The prospective study of **Willers (2007)** investigated the association between maternal intake of various dietary sources of fat and lung function (assessed using spirometry) in a subsample of 510 children aged 5 years old from the Aberdeen prospective cohort (Table 7). The study was considered to have a low risk of bias across all domains. The authors reported no association between any of the dietary exposures and risk of wheeze (effect estimates not shown).

Table 7 Studies investigating the association between fat intake and lung function which were not eligible for meta-analysis

| **First Author and year of publication** | **Design** | **Outcome** | **Age** | **N/n** | **Dietary exposure and comparison level** | **Measure of association** | **Effect (bold indicates p<0.05)** |
| --- | --- | --- | --- | --- | --- | --- | --- |
| Willers, 2007 (2) | PC | LF | 5 | 510 | **Maternal diet during pregnancy:**  Margarine/low fat spread vs Butter spread |  | NS |
|  |  |  |  |  | Fat from dairy products (low vs high) |  | NS |
|  |  |  |  |  | Fish (low vs high) |  | NS |
|  |  |  |  |  | Fatty fish (low vs high) |  | NS |

# 6. Infant or maternal dietary intake of fats and risk of allergic rhino-conjunctivitis

Six studies including over 10,000 children investigated the association between infant or maternal intake of fats and risk of RC. All the studies had a low risk of confounding bias, and over 80% had a low risk of selection bias. Nearly half of the studies were considered to have an overall high risk of bias due selection or assessment methods (Figure 12).

Figure 12 Risk of Bias in studies of fat intake and Rhino-conjunctivitis

The prospective cohort studies of **Alm (2012)** and **Kull (2006)**, both on Swedish children, had data eligible for meta-analysis on infant fish intake and risk of RC at age 4 years old (Figure 13). Both studies reported a reduced risk of disease in children who ate fish at least once a month (**Alm, 2012**) or 2-3 times per month (**Kull, 2006**) compared with children who ate fish less frequently. The overall effect size showed a 49% reduced risk of having RC if infants consumed fish more regularly in the first year of life. There was moderate statistical heterogeneity (I^2^=32.4%). In the study of Alm, fish consumption was not statistically significantly associated with RC risk in the multivariate model – data shown in Figure 13 are for univariate analysis. In the study of Kull the relationship persisted in multivariate analysis excluding infants with early onset disease to account for potential reverse causation. Adjusted OR for rhinitis at age 1-12 in infants with regular (≥2-3 times per month) versus irregular (≤1 time per month) fish intake at age 1 were 0.74 (95% CI 0.60, 0.93) for prevalent rhinitis and 0.78 (95% CI 0.63, 0.95) for incident rhinitis.

Figure 13 Infant fish intake (often vs. rare) and risk of and rhino-conjunctivitis in children 0 to 4 years old (odds ratio)

# 6.1. Studies that investigated the association between infant or maternal intake of dietary sources of fat and risk of RC which could not be included in the meta-analyses

Table 8 summarises the main findings of studies that investigated infant or maternal intake of dietary sources of fats which were not eligible for meta-analysis. The prospective cohort study of **Kull (2006)** and **Magnusson (2013**) showed that a more frequent intake of fish during the first year of life was associated with a reduced risk of RC later in childhood. A further three studies investigated maternal intake of various types of fats in pregnancy in relation to RC but none of the exposures studied showed evidence of an association with RC later in childhood.

**Conclusion**

**We found no consistent evidence for an association between fat intake in infants or lactating/pregnant women and risk of RC in the offspring.**

Table 8 Studies investigating the association between fat intake and rhino-conjunctivitis which were not eligible for meta-analysis

| **First Author and year of publication** | **Design** | **Outcome** | **Age** | **N/n** | **Dietary exposure and comparison level** | **Measure of association** | **Effect (bold indicates p<0.05)** |
| --- | --- | --- | --- | --- | --- | --- | --- |
|  |  |  |  |  | **INFANT INTAKE** |  |  |
|  |  |  |  |  | Fish intake in the child’s first 12 months of life (≥2-3 time/month vs ≤ once per month)  Atopic children without sensitisation | OR | 0.55 (0.30-1.01) |
|  |  |  |  |  | High risk infants | OR | **0.62 (0.41-0.94)** |
|  |  |  |  |  | Normal risk infants | OR | **0.56 (0.33-0.95)** |
| Kull, 2006 (3) | PC | RC | 4 | 3230 | Non-atopic infants without sensitisation | OR | **0.58 (0.35-0.96)** |
| Magnusson, 2013 (4) | PC | RC | 12 | 2456 | Fish intake in first year of life (≥2-3 time/month vs ≤ once per month) | OR | **0.68 (0.59-0.79)** |
|  |  |  |  |  | **MATERNAL INTAKE** |  |  |
|  |  |  |  |  | **Maternal intake during pregnancy:**  Dietary fat (31-57.3 vs <31 g/d) | HR | 0.88 (0.63-1.22) |
| Erkkola, 2012 (10) | PC | RC | 5 | 2441 | Dietary fat (31-57.3 vs 57.3-264.8 g/d) | HR | 1.14 (0.80-1.62) |
|  |  |  |  |  | Fish (10.9-33.27 vs <10.9 g/d) | HR | 1.03 (0.75-1.41) |
|  |  |  |  |  | Fish (10.9-33.27 vs 33.28-254.8 g/d) | HR | 0.92 (0.66-1.28) |
|  |  |  |  |  | **Maternal intake during the 8^th^ month of pregnancy** (Second/third quartile of intake vs lowest quartile of intake)  Saturated fatty acid | HR | 0.94 (0.77-1.16) |
|  |  |  |  |  | Butter and butter spreads | HR | 0.93 (0.70-1.24) |
|  |  |  |  |  | Omega 6 fatty acids | HR | 1.18 (0.90-1.54) |
|  |  |  |  |  | Arachidonic acid | HR | 1.16 (0.88-1.54) |
| Nwaru, 2012 (5) | PC | RC | 5 | 2441 | Linoleic acid | HR | 1.16 (0.91-1.49) |
|  |  |  |  |  | **Maternal intake during the 8^th^ month of pregnancy** (Second/third quartile of intake vs lowest quartile of intake):  MUFA | HR | 1.12 (0.85-1.48) |
|  |  |  |  |  | Conjugated linoleic acids | HR | 1.02 (0.79-1.32) |
|  |  |  |  |  | γ-linoleic acid | HR | 0.93 (0.72-1.21) |
|  |  |  |  |  | ‘Industrial fats’ | HR | 1.32 (1.00-1.73) |
| Nwaru, 2012 (5) | PC | RC | 5 | 2441 | Oils | HR | 1.16 (0.89-1.51) |
|  |  |  |  |  | Total fatty acid | HR | 1.09 (0.83-1.42) |
|  |  |  |  |  | Trans-fatty acid | HR | 1.05 (0.80-1.39) |
|  |  |  |  |  | α-linoleic acids | HR | 0.99 (0.78-1.26) |
|  |  |  |  |  | PUFA | HR | 1.22 (0.93-1.59) |
|  |  |  |  |  | Omega 3 fatty acids | HR | 1.12 (0.87-1.45) |
|  |  |  |  |  | DHA | HR | 1.06 (0.82-1.38) |
|  |  |  |  |  | **Maternal intake during the 8^th^ month of pregnancy** (Second/third quartile of intake vs lowest quartile of intake):  Omega 3 fatty acids from plants | HR | 1.03 (0.80-1.33) |
| Nwaru, 2012 (5) | PC | RC | 5 | 2441 | EPA | HR | 1.01 (0.78-1.32) |
|  |  |  |  |  | Omega 3 fatty acids from fish | HR | 1.01 (0.78-1.31) |
|  |  |  |  |  | Margarine (no vs yes) | HR | 0.89 (0.71-1.11) |
|  |  |  |  |  | **Maternal intake during pregnancy**  Butter spread vs margarine | OR | NS |
| Willers, 2007 (2) | PC | RC | 5 | 1212 | Oily fish (≥1/week vs never) | OR | 0.28 (0.07-1.19) |

# 7. Infant or maternal dietary intake of fats and risk of allergic sensitisation

Nine studies including over 10,000 children investigated the association between infant or maternal intake of fats and risk of RC. Over 70% of the studies were considered to have a low risk of assessment bias, and all but one study were considered to have a low risk of confounding bias. Some studies had an unclear method of assessment or selection bias, which resulted in over half of the studies having an overall risk of bias judged to be unclear (Figure 14).

Figure 14 Risk of bias in studies of fat intake and risk of allergic sensitisation at any age

Two prospective cohort studies (**Nwaru, 2010**; and **Sausenthaler, 2007**) had comparable data to estimate the combined effect size of maternal intake of omega 6 fatty acids, and butter, respectively, and risk of allergic sensitisation to aero-allergens in children (Figure 15). Neither study showed evidence of an association. The overall effect confirmed the lack of association. There was no heterogeneity between studies (I^2^=0%). The studies of **Nwaru** and **Sausenthaler** also had comparable data to examine the association between frequent maternal intake of omega 3 fatty acids (**Nwaru, 2010**) and fish (**Sausenthaler, 2007**) compared with rare, and risk of sensitisation to aero-allergens in children (Figure 16). The combined effect estimate showed no evidence of an association. There was no heterogeneity between studies (I^2^=0%). When the highest intakes (highest quartiles) were compared to the lowest in these two studies, the lack of association between fish or omega 3 fatty acids and risk of AS was confirmed (I^2^=0%) (Figure 17).

Figure 15 Maternal fat intake (highest vs. lowest) and risk of allergic sensitisation to aeroallergens in children at any age

Figure 16 Maternal fish or omega 3 fatty acid intake (often vs. rare) and risk of allergic sensitisation to aeroallergens in children at any age

Figure 17 Maternal fish or omega-3 intake (highest vs. lowest) and risk of specific sensitisation to aeroallergen in children at any age

We identified two prospective cohort studies (**Nwaru, 2010; and Sausenthaler, 2007**) and one retrospective cohort study (**Calvani, 2006**) which had data that could be combined to estimate the overall effect of maternal fat intake and risk sensitisation to food allergens in the child. In the study of **Calvani (2006)**, the authors separately examined the risk of food allergy in the child according to whether the mother was allergic or not. There was no evidence of an association between maternal intake of fish and risk of this outcome in children at age 5 years old (Figure 18).

Figure 18 Maternal fish intake (often vs. rare) and risk of sensitisation to food allergens in children at any age

Similarly, there was no evidence of an association between maternal intake of omega 3 fatty acids (**Nwaru, 2010**) or fish (**Sausenthaler, 2007**) and risk of sensitisation to food allergens in the offspring (Figure 19). The level of heterogeneity was very high (I^2^=75%). Both studies were birth cohorts, and they used a similar diagnostic tool (IgE concentrations assayed using CAP and considering a response as positive if>0.35kU/l). However, there were some differences in the participants; the Finnish infants in the study of **Nwaru** were all at high risk of type 1 diabetes, whilst the **Sausenthaler** study was a population-based cohort study of German children, and it specifically excluded any children with existing chronic diseases. The time at outcome measurement was also different (2 and 5 years old, in the Nwaru and Sausenthaler studies, respectively). Maternal intake of varying frequencies of fat or fish intake in these two studies in relation to food sensitisation showed no evidence of an association (Figures 20 and 21).

Figure 19 Maternal fish omega-3 intake (highest vs. lowest) and risk of sensitisation to food allergens in children (odds ratio)

Figure 20 Maternal fat intake (highest vs lowest) and risk of allergenic sensitisation to any food in children (odds ratio)

Figure 21 Maternal fish or omega 3 intake (highest vs lowest) and risk of allergenic sensitisation to any food in children (odds ratio)

# 7.1. Studies that investigated the association between infant or maternal intake of fats and risk of allergic sensitisation which could not be included in the meta-analyses

We identified one study that investigated infant intake of fish in the first year of life and risk of having specific IgE (sIgE) to any allergens (**Kull, 2006**). The authors reported that in normal/low risk infants there was an association between frequent intake of fish and reduced sensitisation to any allergen (AS-Any) at age 4 (OR 0.52; 95% CI 0.36-0.76) (Table 9).

With regards to maternal intake (Table 9), nine studies investigated the association of dietary sources of fat or fish-related products with sensitisation to various allergens. Overall, there were few studies that found a statistically significant association. The study of **Calvani (2006)** reported that in children of mothers who were not allergic, fish intake was associated with reduced AS-CM or AS-Egg at age 5 years. However, **Nwaru (2010)** found no evidence of an association between a higher intake of fish (or related foods) and risk of sIgE to egg in children at the same age. **Nwaru (2010**) reported that a higher intake of omega 6 fatty acids in late pregnancy was associated with reduced risk of being sensitised to aero-allergens (OR 0.60; 95% CI 0.39, 0.93). Several other studies showed no association between omega 6, omega 3 or any other type of PUFAs and allergic sensitisation.

**Conclusion**

**We found no evidence that maternal or infant intake of fish, fat or fatty acids is associated with risk of allergic sensitisation.**

Table 9 Studies investigating the association between fat intake and allergic sensitization which were not eligible for meta-analysis

| **First Author and year of publication** | **Design** | **Outcome** | **Age** | **N/n** | **Dietary exposure and comparison level** | **Measure of association** | **Effect (bold indicates p<0.05)** |
| --- | --- | --- | --- | --- | --- | --- | --- |
|  |  |  |  |  | **INFANT INTAKE** |  |  |
|  |  |  |  |  | **Infant intake of fish in the first 12 months of life (**≥2-3 times per month vs ≤ once per month):  High risk infants | OR | 1.14 (0.76-1.72) |
| Kull, 2006 (3) | PC | sIgE any | 4 | 3230 | Low risk infants | OR | **0.52 (0.36-0.76)** |
|  |  |  |  |  | **MATERNAL INTAKE** |  |  |
|  |  | SPT aero |  |  | **Maternal intake during pregnancy (in children with family history of allergies [FH+]):**  Margarine (≥ 2-3 times/week vs ≤1 month) | OR | 0.81 (0.60-1.10) |
|  |  | SPT aero |  |  | Margarine (once a week vs ≤1 month) | OR | 0.39 (0.10-1.48) |
|  |  | SPT any food |  |  | Margarine (once a week vs ≤1 month) | OR | 0.26 (0.03-2.54) |
| Calvani, 2006 (FH+) (33) | RC | SPT any food | 5 | 988 | Butter (once a week vs ≤1 month) | OR | 0.49 (0.17-1.43) |
|  |  | SPT cow’s milk |  |  | **Maternal intake during pregnancy (once a week vs ≤1 month) (in children with no family history of allergies [FH-]) :**  Fish | OR | **0.15 (0.04-0.59)** |
|  |  | SPT egg |  |  | Fish | OR | **0.26 (0.09-0.76)** |
|  |  | SPT any food |  |  | Butter | OR | 0.91 (0.37-2.25) |
|  |  | SPT aero |  |  | Margarine | OR | 1.28 (0.53-3.07) |
|  |  | SPT any food |  |  | Margarine | OR | 1.63 (0.39-6.87) |
|  |  | SPT aero |  |  | Butter | OR | **1.73 (1.00-2.99)** |
| Calvani, 2006 (FH-) (33) | RC | SPT aero | 5 | 988 | Margarine (≥ 2-3 times/week vs ≤1 month) | OR | 0.92 (0.66-1.28) |
|  |  |  |  |  | **Maternal intake during pregnancy:**  Butter (once a week vs ≤1 month; allergic mother) | OR | **0.27 (0.10-0.73)** |
|  |  |  |  |  | Fish (≥ 2-3 times/week vs ≤1 month; allergic mother) | OR | 0.74 (0.23-2.37) |
|  |  |  |  |  | Fish (once a week vs ≤1 month; allergic mother) | OR | 0.89 (0.30-2.60) |
| Calvani, 2006 (33) | RC | SPT aero | 5 | 988 | Fish (≥ 2-3 times/week vs ≤1 month; non-allergic mother) | OR | 0.55 (0.28-1.08) |
|  |  |  |  |  | Fish (once a week vs ≤1 month; non allergic mother) | OR | 0.70 (0.38-1.30) |
|  |  |  |  |  | **Maternal intake during pregnancy:**  Fish (≥ 2-3 times/week vs ≤1 month; allergic mother) | OR | 1.13 (0.31-4.10) |
|  |  |  |  |  | Fish (once a week vs ≤1 month; allergic mother) | OR | 1.15 (0.38-3.47) |
|  |  |  |  |  | Fish (once a week vs ≤1 month; non-allergic mother) | OR | **0.22 (0.09-0.55)** |
|  |  |  |  |  | Fish (≥ 2-3 times/week vs ≤1 month; non-allergic mother) | OR | **0.23 (0.08-0.69)** |
| Calvani, 2006 (33) | RC | SPT any food | 5 | 988 | Fish (once a week vs ≤1 month; all) | OR | **0.34 (0.15-0.75)** |
| Chatzi, 2008 (23) | PC | SPT aero | 6.5 | 468 | **Maternal intake of fish during pregnancy** (>2.5 times per week vs ≤ 2.5 per week) | % | NS |
| Hoppu, 2000 (13) | PC | SPT any food | 1 | 114 | **Maternal intake of saturated fats during breastfeeding** (mean % energy from saturated fat) | OR | 1.15 (0.98-1.35) |
|  |  | sIgE aero | 5 | 931 | **Maternal intake at 8^th^ month of pregnancy**  Saturated fat (mean daily intake in grams) | OR | 0.83 (0.35-1.99) |
| Nwaru, 2010 (7) | PC | sIgE aero | 5 | 931 | Margarine (mean daily intake in grams) | OR | 0.96 (0.79-1.17) |
|  |  |  |  |  | **Maternal intake at 8^th^ month of pregnancy:**  Omega 6 (2^nd^ and 3^rd^ quartile vs 1^st^) | OR | **0.60 (0.39-0.93)** |
|  |  |  |  |  | Oils (mean daily intake in grams) | OR | 0.97 (0.63-1.49) |
|  |  |  |  |  | Fat (mean daily intake in grams) | OR | 1.12 (0.32-3.93) |
|  |  |  |  |  | Fish (mean daily intake in grams) | OR | 1.02 (0.93-1.12) |
|  |  |  |  |  | Omega 3 4^th^ quartile vs 2^nd^ and 3^rd^ ) | OR | 0.68 (0.44-1.05) |
|  |  |  |  |  | PUFA (1^st^ quartile vs 2^nd^ and 3^rd^ ) | OR | 0.63 (0.41-0.97) |
| Nwaru, 2010 (7) | PC | sIgE aero | 5 | 931 | PUFA (4^th^ quartile vs 2^nd^ and 3^rd^ ) | OR | 0.33 (0.06-1.82) |
|  |  |  |  |  | Saturated fats (mean daily intake in grams) | OR | 0.91 (0.34-2.43) |
|  |  |  |  |  | Omega 6 (2^nd^ and 3^rd^ quartile vs 1^st^ ) | OR | 0.78 (0.49-1.25) |
|  |  |  |  |  | Fat (mean daily intake in grams) | OR | 0.68 (0.17-2.76) |
|  |  |  |  |  | Oils (mean daily intake in grams) | OR | 0.83 (0.51-1.36) |
|  |  | sIgE any food | 5 | 931 | PUFA (2^nd^ and 3^rd^ quartile vs 1^st^ ) | OR | 1.03 (0.65-1.62) |
|  |  |  |  |  | **Maternal intake at 8th month of pregnancy:**  Margarine (mean daily intake in grams) | OR | 0.95 (0.75-1.20) |
| Nwaru, 2010 (7) | PC | SPT any food | 5 | 931 | Fish (mean daily intake in grams) | OR | 0.99 (0.90-1.09) |
|  |  |  |  |  | Omega 3 (4^th^ quartile vs 2^nd^ and 3^rd^ ) | OR | 0.80 (0.49-1.31) |
|  |  |  |  |  | **Maternal intake during breastfeeding (g/day increase in total intake during 2nd month lactation):**  Saturated fatty acid | OR | 1.14 (0.62-2.08) |
|  |  |  |  |  | Margarine and low fat spread | OR | 0.96 (0.70-1.31) |
|  |  |  |  |  | Omega 6 | OR | 0.92 (0.60-1.41) |
| Nwaru, 2011 (6) | PC | sIgE cow’s milk | 5 | 652 | Oil consumption | OR | 1.15 (0.86-1.54) |
|  |  |  |  |  | Fish and fish product | OR | 0.80 (0.57-1.12) |
|  |  |  |  |  | Total PUFA | OR | 0.96 (0.62-1.49) |
|  |  |  |  |  | Omega 3 | OR | 1.09 (0.71-1.68) |
|  |  |  |  |  | Omega 3 from vegetables | OR | 1.21 (0.81-1.81) |
|  |  |  |  |  | **Maternal intake during breastfeeding (g/day increase in total intake during 2^nd^ month lactation):**  Saturated fatty acid | OR | 1.71 (0.89-3.27) |
|  |  |  |  |  | Margarine and low fat spread | OR | 0.80 (0.51-1.26) |
|  |  |  |  |  | Omega 6 | OR | 0.88 (0.51-1.53) |
| Nwaru, 2011 (6) | PC | sIgE egg | 5 | 652 | Oils | OR | 0.92 (0.62-1.36) |
|  |  |  |  |  | Fish and fish products | OR | 0.97 (0.69-1.37) |
|  |  |  |  |  | Omega 3 | OR | 0.76 (0.47-1.24) |
|  |  |  |  |  | PUFA | OR | 0.84 (0.47-1.49) |
|  |  |  |  |  | Omega 3 from fish | OR | 0.84 (0.53-1.33) |
|  |  |  | 4 | 333 | Maternal fish consumption during all pregnancy | OR | 0.93 (0.59-1.47) |
| Romieu, 2007 (22) | PC | allergic sensitization | 6 | 399 | Maternal fish consumption during all pregnancy | OR | 0.74 (0.50-1.09) |
|  |  |  | 2 | 2068 | **Maternal consumption during the last 4 weeks of pregnancy (highest vs lowest tertile) (variation in N refers to individuals with data in the multi-variate analysis)**  Margarine | OR | 0.93 (0.50-1.73) |
|  |  | sIgE aero | 2 | 2117 | Vegetable oils | OR | 0.89 (0.52-1.51) |
|  |  |  | 2 | 2091 | Deep frying vegetable fat | OR | 1.61 (1.02-2.54) |
| Sausenthaler, 2007 (21) | PC |  | 2 | 2069 | Margarine | OR | 0.85 (0.57-1.27) |
|  |  |  | 2 | 2118 | Vegetable oils | OR | 0.88 (0.62-1.25) |
|  |  |  | 2 | 2092 | Deep frying vegetable fat | OR | 1.25 (0.92-1.70) |
|  |  |  | 2 | 2076 | Margarine | OR | 0.80 (0.50-1.27) |
|  |  |  | 2 | 2125 | Vegetable oils | OR | 0.91 (0.62-1.34) |
|  |  | sIgE any food | 2 | 2099 | Deep frying vegetable oil | OR | 1.12 (0.79-1.58) |
|  |  |  |  |  | **Maternal diet during pregnancy:**  Margarine/low fat spread vs Butter spread |  | NS |
|  |  |  |  |  | Fat from dairy (low vs high) |  | NS |
| Willers, 2007 (2) | PC | SPT any | 5 | 1212 | Fish |  | NS |
|  |  |  |  |  | Oily fish |  | NS |

# 8. Infant or maternal dietary intake of fats and risk of type 1 diabetes mellitus

Six studies including over 9,000 children examined the association between infant or maternal intake of fats and risk of T1DM. The majority of the studies had a low or unclear bias in most domains (Figure 22).

Figure 22 Risk of bias in studies of fat intake and risk of allergic sensitisation at any age

# 8.1. Studies that investigated the association between infant or maternal intake of dietary fats and risk of T1DM which were not eligible for meta-analysis

We identified one study (**Stene, 2003**) reporting the association between dietary intake of cod liver oil supplement in the first year of life and risk of T1DM later in childhood (Table 10). The authors reported that a more frequent intake of cod liver oil supplement in the first year was associated with a lower risk of disease in children. With regards to maternal intake (Table 10), five prospective cohort studies investigated the association between intake of various types of fats or oils and risk of T1DM in the offspring. In the study of **Niinisto (2012)** the authors reported that maternal consumption of vegetable oils during lactation was associated with a higher risk of T1DM later in childhood, whilst **Virtanen (2011, [2])** reported that maternal intake of butter was associated with a lower risk of disease of T1DM in children at age 8 years old. Two other studies reported no association between maternal intake of various types of fatty acids (**Fronczak 2003**) or fish (**Lamb 2008**) and risk of T1DM.

**Conclusion**

**We found VERY LOW evidence from one case-control study suggesting that intake of cod liver oil in the first year of life (after month 7) is associated with reduced T1DM later in childhood. Maternal intake of vegetable oils during lactation was associated with an increased risk of T1DM, and intake of butter during pregnancy was associated with a reduced risk of T1DM. Overall, we found no conclusive evidence that maternal intake of fats influences the risk of T1DM in childhood.**

Table 10 Studies investigating the association between fat intake and type 1 diabetes mellitus which were not eligible for meta-analysis

| **First Author and year of publication** | **Design** | **Outcome** | **Age** | **N/n** | **Dietary exposure and comparison level** | **Measure of association** | **Effect** |
| --- | --- | --- | --- | --- | --- | --- | --- |
|  |  |  |  |  | **INFANT INTAKE** |  |  |
|  |  |  |  |  | **Infant intake of cod liver oil supplement in 1^st^ year of life:**  Starting at age 7-12 months (Yes vs no) | OR | **0.55 (0.32-0.96)** |
| Stene, 2003 (41) | CC | TIDM | 8.8 | 2213/545 | Starting at age 0-6 months | OR | 0.80 (0.60-1.06) |
|  |  |  |  |  | 1-4 times per week vs No | OR | 0.81 (0.55-1.19) |
|  |  |  |  |  | ≥5 times per week vs No | OR | **0.74 (0.55-0.99)** |
|  |  |  |  |  | **MATERNAL INTAKE** |  |  |
| Brekke, 2010 (38) | PC | DM |  |  | Maternal intake in the 3^rd^ trimester of pregnancy:  Fish (open sea) (1-2 times per week vs <1 per week) | OR | 0.76 (0.5-1.17) |
|  |  |  |  |  | **Maternal intake during pregnancy**:  Linoleic acid (7.92-11.78g/day vs <7.92g/day ) | HR | 2.01 (0.55-7.37) |
|  |  |  |  |  | Omega 6 (g/day, continuous) | HR | 0.88 (0.50-1.56) |
| Fronczak, 2003 (36) | PC | DM | 4 | 222/16 | Omega 3 (g/day, continuous) | HR | 1.16 (0.75-1.80) |
|  |  |  |  |  | Omega 3 (highest vs. lowest) | OR | 0.64 (0.24-1.70) |
|  |  |  |  |  | Omega 6 (highest vs. lowest) | OR | 1.04 (0.26-4.19) |
| Lamb 2008 (37) | PC | TIDM | 15 | 642/27 | **Maternal intake in the last trimester of pregnancy**  Fish (No. servings) | HR | 0.90 (0.54-1.51) |
| Niinisto, 2012 (8) | PC | TIDM | 9.7 | 2939/143 | **Maternal intake of vegetable oils during lactation**  (Yes vs no) | HR | **1.22 (1.03-1.44)** |
| Virtanen, 2011 (39) | PC | TIDM | <10 | 3730/165 | Maternal intake of butter during pregnancy | Change in risk per 2-fold increase in energy-adjusted consumption | **0.83 (0.70-0.98)** |

# 9. Infant or maternal dietary intake of fats and risk of juvenile idiopathic arthritis

We identified one study that investigated the association between maternal intake of fats and risk of JIA, which was considered to have an overall unclear bias due to unclear assessment and selection of participants (Figure 24).

Figure 23 Risk of Bias in studies of fat intake and juvenile idiopathic arthritis

The CLARITY - ChiLdhood Arthritis Risk factor Identification sTudY (**Ellis, 2012**) is a case-control study established in Australia with the aim of identifying genomic and environmental disease risk factors in children diagnosed with juvenile idiopathic arthritis (JIA). Cases (n=262) were children ≤18 years old with a diagnosis of JIA, who were matched to healthy children. As part of the early life risk factors investigated, the author reported that maternal fish oil supplementation during pregnancy was not related to the risk of having JIA later in childhood (Table 11).

**Conclusion**

**We found no evidence to suggest that maternal intake of dietary sources of fat modulates the risk of JIA in childhood.**

Table 11 Studies investigating the association between fat intake (dietary exposure) and Juvenile idiopathic arthritis (outcome) which were not eligible for meta-analysis

| **First Author and year of publication** | **Design** | **Outcome** | **Age** | **N/n** | **Dietary exposure and comparison level** | **Measure of association** | **Effect** |
| --- | --- | --- | --- | --- | --- | --- | --- |
| Ellis, 2012 (43) | RC | JIA |  | 262/458 | Maternal fish oil supplement intake (often vs. rare) and risk of juvenile idiopathic arthritis in children | OR | 0.7 (0.15-3.35) |

# General conclusions

In this report of observational studies on infant or maternal intake of dietary sources of fats and risk of allergic and selected autoimmune diseases, we found some evidence to suggest that intake of fish, particularly oily/fatty, in the first year of life reduces the risk of AD in early and late childhood. Consumption of other sources of dietary fats during infancy did not appear to be associated with risk of AD. We found inconsistent evidence suggesting that a more frequent intake of fish during the first year of life is associated with a reduced risk of RC later in childhood and that maternal fish or omega 3 intakes could reduce the risk of wheeze and AD early in childhood.

With regards to wheeze, we found evidence of a 3% reduction in risk in infants with a higher intake of fish, but this was not consistent. Our review also shows no evidence to suggest that infant or maternal intake of dietary fats influences the risk of food allergy or that is related to lung function in children.

We found very little evidence to suggesting that a higher intake of fish in normal/low risk infants is associated with a lower risk of allergic sensitisation. Maternal intake of omega 3 fatty acids does not appear to influece the risk of sensitisation in childhood. Maternal intake of other dietary sources of fats showed limited and contradictory evidence on the direction in which fat intake could modulate the risk of sensitisation.

With regards to autoimmune diseases, we found limited evidence suggesting that intake of cod liver oil in the first year of life (after month 7) might reduce the risk of T1DM later in childhood. Maternal intake of butter during pregnancy was associated with a reduced risk of disease. But this evidence was too limited to suggest that maternal intake of fats during pregnancy is involved in the modulation of risk of childhood T1DM. Similarly, we found no evidence to suggest that maternal intake of dietary sources of fat modulates the risk of JIA in childhood.

Overall, we found no consistent evidence to suggest that maternal or infant intake of fats might contribute to reduce the risk of allergic or autoimmune outcomes in early childhood.

# References

1.	(NICE) NIfCaHE. The guidelines manual methodology checklist. 2012.

2.	Willers SM, Devereux G, Craig LC, McNeill G, Wijga AH, Abou El-Magd W, et al. Maternal food consumption during pregnancy and asthma, respiratory and atopic symptoms in 5-year-old children. Thorax. 2007;62(9):773-9.

3.	Kull I, Bergstrom A, Lilja G, Pershagen G, Wickman M. Fish consumption during the first year of life and development of allergic diseases during childhood. Allergy. 2006;61(8):1009-15.

4.	Magnusson J, Kull I, Rosenlund H, Hakansson N, Wolk A, Melen E, et al. Fish consumption in infancy and development of allergic disease up to age 12 y. American Journal of Clinical Nutrition. 2013;97(6):1324-30.

5.	Nwaru BI, Erkkola M, Lumia M, Kronberg-Kippila C, Ahonen S, Kaila M, et al. Maternal intake of fatty acids during pregnancy and allergies in the offspring. British Journal of Nutrition. 2012;108(4):720-32.

6.	Nwaru BI, Erkkola M, Ahonen S, Kaila M, Lumia M, Prasad M, et al. Maternal diet during lactation and allergic sensitization in the offspring at age of 5. Pediatric Allergy & Immunology. 2011;22(3):334-41.

7.	Nwaru BI, Ahonen S, Kaila M, Erkkola M, Haapala AM, Kronberg-Kippila C, et al. Maternal diet during pregnancy and allergic sensitization in the offspring by 5 yrs of age: a prospective cohort study. Pediatric Allergy & Immunology. 2010;21(1 Pt 1):29-37.

8.	Niinisto S, Takkinen HM, Uusitalo L, Nevalainen J, Kenward M, Lumia M, et al. Maternal fatty acid composition of diet during lactation and risk of preclinical and clinical type 1 diabetes. Diabetologia. 2012;55:S141-S2.

9.	Lumia M, Luukkainen P, Tapanainen H, Kaila M, Erkkola M, Uusitalo L, et al. Dietary fatty acid composition during pregnancy and the risk of asthma in the offspring. Pediatric Allergy & Immunology. 2011;22(8):827-35.

10.	Erkkola M, Nwaru BI, Kaila M, Kronberg-Kippila C, Ilonen J, Simell O, et al. Risk of asthma and allergic outcomes in the offspring in relation to maternal food consumption during pregnancy: a Finnish birth cohort study. Pediatric Allergy & Immunology. 2012;23(2):186-94.

11.	Leermakers ET, Sonnenschein-van der Voort AM, Heppe DH, de Jongste JC, Moll HA, Franco OH, et al. Maternal fish consumption during pregnancy and risks of wheezing and eczema in childhood: the Generation R Study. European Journal of Clinical Nutrition. 2013;67(4):353-9.

12.	Kiefte-de Jong JC, de Vries JH, Franco OH, Jaddoe VW, Hofman A, Raat H, et al. Fish consumption in infancy and asthma-like symptoms at preschool age. Pediatrics. 2012;130(6):1060-8.

13.	Hoppu U, Kalliomaki M, Isolauri E. Maternal diet rich in saturated fat during breastfeeding is associated with atopic sensitization of the infant. European Journal of Clinical Nutrition. 2000;54(9):702-5.

14.	Alm B, null, Thengilsdottir H, Erdes L, Mollborg P, Pettersson R, et al. Early introduction of fish decreases the risk of preschool wheezing disorder. Allergy: European Journal of Allergy and Clinical Immunology. 2009;64:64-5.

15.	Goksor E, Alm B, Thengilsdottir H, Mollborg P, Pettersson R, Erdes L, et al. Characteristics and risk factors for doctor-diagnosed food allergy at preschool age. Allergy: European Journal of Allergy and Clinical Immunology. 2011;66:219.

16.	Alm B, Goksor E, Thengilsdottir H, Pettersson R, Mollborg P, Norvenius G, et al. Early protective and risk factors for allergic rhinitis at age 41/2 yr. Pediatric Allergy & Immunology. 2011;22(4):398-404.

17.	Jedrychowski W, Flak E, Mroz E, Pac A, Jacek R, Sochacka-Tatara E, et al. Modulating effects of maternal fish consumption on the occurrence of respiratory symptoms in early infancy attributed to prenatal exposure to fine particles. Annals of Nutrition & Metabolism. 2008;52(1):8-16.

18.	Jedrychowski W, Perera F, Maugeri U, Mrozek-Budzyn D, Miller RL, Flak E, et al. Effects of prenatal and perinatal exposure to fine air pollutants and maternal fish consumption on the occurrence of infantile eczema. International Archives of Allergy & Immunology. 2011;155(3):275-81.

19.	Laitinen K, Kalliomaki M, Poussa T, Lagstrom H, Isolauri E. Evaluation of diet and growth in children with and without atopic eczema: follow-up study from birth to 4 years. British Journal of Nutrition. 2005;94(4):565-74.

20.	Fitzsimon N, Fallon U, O'Mahony D, Loftus BG, Bury G, Murphy AW, et al. Mothers' dietary patterns during pregnancy and risk of asthma symptoms in children at 3 years. Irish Medical Journal. 2007;100(8):suppl 27-32.

21.	Sausenthaler S, Koletzko S, Schaaf B, Lehmann I, Borte M, Herbarth O, et al. Maternal diet during pregnancy in relation to eczema and allergic sensitization in the offspring at 2 y of age. American Journal of Clinical Nutrition. 2007;85(2):530-7.

22.	Romieu I, Torrent M, Garcia-Esteban R, Ferrer C, Ribas-Fito N, Anto JM, et al. Maternal fish intake during pregnancy and atopy and asthma in infancy. Clinical & Experimental Allergy. 2007;37(4):518-25.

23.	Chatzi L, Torrent M, Romieu I, Garcia-Esteban R, Ferrer C, Vioque J, et al. Mediterranean diet in pregnancy is protective for wheeze and atopy in childhood. Thorax. 2008;63(6):507-13.

24.	Miyake Y, Sasaki S, Tanaka K, Ohfuji S, Hirota Y. Maternal fat consumption during pregnancy and risk of wheeze and eczema in Japanese infants aged 16-24 months: the Osaka Maternal and Child Health Study. Thorax. 2009;64(9):815-21.

25.	Saito K, Yokoyama T, Miyake Y, Sasaki S, Tanaka K, Ohya Y, et al. Maternal meat and fat consumption during pregnancy and suspected atopic eczema in Japanese infants aged 3-4 months: the Osaka Maternal and Child Health Study. Pediatric Allergy & Immunology. 2010;21(1 Pt 1):38-46.

26.	Oien T, Storro O, Johnsen R. Do early intake of fish and fish oil protect against eczema and doctor-diagnosed asthma at 2 years of age? A cohort study. Journal of Epidemiology & Community Health. 2010;64(2):124-9.

27.	Roduit C, Frei R, Loss G, Buchele G, Weber J, Depner M, et al. Development of atopic dermatitis according to age of onset and association with early-life exposures. Journal of Allergy & Clinical Immunology. 2012;130(1):130-6.e5.

28.	Willers SM, Wijga AH, Brunekreef B, Kerkhof M, Gerritsen J, Hoekstra MO, et al. Maternal food consumption during pregnancy and the longitudinal development of childhood asthma. American Journal of Respiratory & Critical Care Medicine. 2008;178(2):124-31.

29.	Narita M, Qiu D, Hamaguchi M, Doi M, Futamura M, Sakamoto N, et al. Maternal diet during pregnancy and wheeze and eczema in infants; the Japanese birth cohort (T-CHILD) study. Journal of Allergy and Clinical Immunology. 2011;1):AB174.

30.	Wang IJ, Guo YL, Weng HJ, Hsieh WS, Chuang YL, Lin SJ, et al. Environmental risk factors for early infantile atopic dermatitis. Pediatric Allergy & Immunology. 2007;18(5):441-7.

31.	Grimshaw KEC, Oliver EM, Kemp T, Mills ENC, Beyer K, Foote K, et al. Maternal dietary intake and subsequent allergy development. Journal of Allergy and Clinical Immunology. 2012;1):AB173.

32.	Oliver E, Grimshaw K, Scally K, Garland J, Gudgeon L, Foote K, et al. Does nutritional intake in infancy effect later development of food allergy? Allergy: European Journal of Allergy and Clinical Immunology. 2010;65:313.

33.	Calvani M, Alessandri C, Sopo SM, Panetta V, Pingitore G, Tripodi S, et al. Consumption of fish, butter and margarine during pregnancy and development of allergic sensitizations in the offspring: role of maternal atopy. Pediatric Allergy and Immunology. 2006;17(2):94-102.

34.	Salam MT, Li YF, Langholz B, Gilliland FD. Maternal fish consumption during pregnancy and risk of early childhood asthma. Journal of Asthma. 2005;42(6):513-8.

35.	Castro-Rodriguez JA, Garcia-Marcos L, Sanchez-Solis M, Perez-Fernandez V, Martinez-Torres A, Mallol J. Olive oil during pregnancy is associated with reduced wheezing during the first year of life of the offspring. Pediatric Pulmonology. 2010;45(4):395-402.

36.	Fronczak CM, Baron AE, Chase HP, Ross C, Brady HL, Hoffman M, et al. In utero dietary exposures and risk of islet autoimmunity in children. Diabetes Care. 2003;26(12):3237-42.

37.	Lamb MM, Myers MA, Barriga K, Zimmet PZ, Rewers M, Norris JM. Maternal diet during pregnancy and islet autoimmunity in offspring. Pediatric Diabetes. 2008;9(2):135-41.

38.	Brekke HK, Ludvigsson J. Daily vegetable intake during pregnancy negatively associated to islet autoimmunity in the offspring--the ABIS study. Pediatric Diabetes. 2010;11(4):244-50.

39.	Virtanen SM, Uusitalo L, Kenward MG, Nevalainen J, Uusitalo U, Kronberg-Kippilad C, et al. Maternal food consumption during pregnancy and risk of advanced beta-cell autoimmunity in the offspring. Pediatric Diabetes. 2011;12(2):95-9.

40.	Stene LC, Ulriksen J, Magnus P, Joner G. Use of cod liver oil during pregnancy associated with lower risk of Type I diabetes in the offspring.[Erratum appears in Diabetologia 2000 Nov;43(11):1451]. Diabetologia. 2000;43(9):1093-8.

41.	Stene LC, Joner G, Norwegian Childhood Diabetes Study G. Use of cod liver oil during the first year of life is associated with lower risk of childhood-onset type 1 diabetes: a large, population-based, case-control study. American Journal of Clinical Nutrition. 2003;78(6):1128-34.

42.	Stene LC, Thorsby PM, Berg JP, Ronningen KS, Joner G, Aabech H, et al. Peroxisome proliferator-activated receptor-2 Pro12Ala polymorphism, cod liver oil and risk of type 1 diabetes. Pediatric Diabetes. 2008;9(1):40-5.

43.	Ellis JA, Ponsonby AL, Pezic A, Chavez RA, Allen RC, Akikusa JD, et al. CLARITY - ChiLdhood Arthritis Risk factor Identification sTudY. Pediatric Rheumatology Online Journal. 2012;10(1):37.
