# Supplementary material for: Diet during pregnancy and infancy and risk of allergic or autoimmune disease: A systematic review and meta-analysis
Source: PLoS Med. 2018 Feb 28;15(2):e1002507. doi: 10.1371/journal.pmed.1002507 (PMC5830033; doi:10.1371/journal.pmed.1002507)
Supplement: S2 Data — (ZIP) [file pmed.1002507.s007.zip › Review_C_reports/FRUITS & VEGETABLES.doc]

**INFANT AND MATERNAL FRUIT AND VEGETABLE INTAKE, AND RISK OF ALLERGIC AND AUTOIMMUNE DISEASES**

Vanessa Garcia-Larsen^1^, Robert J Boyle^2^, Despo Ierodiakonou^1,3^, Jo Leonardi-Bee^4^, Tim Reeves^5^, Jennifer Chivinge^6^, Zoe Robinson^6^, Natalie Geoghegan^6^, Katharine Jarrold^6^, Andrew Logan^6^, Annabel Groome^6^ , Evangelia Andreou^7^, Nara Tagiyeva-Milne^8^, Ulugbek Nurmatov^9^, Sergio Cunha^7^

1 Post-Doctoral Research Associate, Respiratory Epidemiology, Occupational Medicine, and Public Health, National Heart and Lung Institute Imperial College London

2 Clinical Senior Lecturer, Department of Paediatrics, Imperial College London

3 Post-Doctoral Research Associate, Department of Paediatrics, Imperial College London

4 Associate Professor of Community Health Sciences, University of Nottingham

5 Research Support Librarian, Faculty of Medicine, Imperial College London

6 Undergraduate medical students, Imperial College London

7 Research Associate, Imperial Consultants

8 Research Fellow, University of Aberdeen

9 Research Fellow, University of Edinburgh

Table of Contents

List of figures	4

List of tables	5

1.	Executive summary of findings on infant and maternal intake of fruits and vegetables and risk of allergic and autoimmune diseases	6

2.	Infant or maternal dietary intake of fruit and vegetables and risk of atopic dermatitis (AD)	25

2.1.	Infant Intake of fruit and vegetable and AD risk	25

2.2.	Maternal Intake of fruits and vegetables and risk of AD in childhood	26

2.3.	Studies on infant or maternal intake of fruits and vegetables that were not suitable for meta-analysis	27

3.	Infant or maternal dietary intake of fruit and vegetable and risk of food allergy	33

3.1.	Maternal Intake of fruit and vegetables and risk of childhood food allergy	33

3.2.	Studies on infant or maternal intake of fruits and vegetables that were not suitable for meta-analysis	34

4.	Infant or maternal dietary intake of fruit and vegetables and risk of poor lung function	38

4.1.	Lung function	38

5.	Infant or maternal dietary intake of fruit and vegetables and risk of wheeze	40

5.1.	Infant Intake of vegetables and fruits and risk of wheeze	40

5.2.	Maternal Intake of fruits and vegetables and risk of wheeze	41

5.3.	Studies on infant or maternal intake of fruits and vegetables that were not suitable for meta-analysis	42

6.	Infant or maternal dietary intake of fruit and vegetables and risk of allergic rhino-conjunctivitis (RC)	54

7.	Infant or maternal dietary intake of fruits and vegetables and risk of allergic sensitisation	60

7.1.	Studies on infant or maternal intake of fruits and vegetables that were not suitable for meta-analysis	62

8.	Infant or maternal dietary intake of fruits and vegetables and risk of autoimmune diseases	74

8.1.	Type 1 diabetes mellitus (TIDM)	74

8.2.	Infant or maternal dietary intake of fruit and vegetable and risk of inflammatory bowel disease (IBD)	79

Conclusions	81

References	82

# List of figures

Figure 1 Risk of bias in studies of fruit and vegetable intake and risk of atopic dermatitis	25

Figure 2 Introduction of fruit at age >4 months vs 0-4 months and risk of atopic dermatitis (odds ratio) in children aged 0-4 years old	26

Figure 3 Introduction of fruit at age > 4 months vs 0-4 months and risk of atopic dermatitis (odds ratio) in children aged 5-14 years old	26

Figure 5 Maternal fruit intake (highest vs. lowest) and risk of atopic dermatitis (odds ratio) in children aged 0-4 years old	27

Figure 6 Risk of Bias in studies of fruit and vegetable intake and food allergy	33

Figure 7 Maternal peanut avoidance during pregnancy (often vs. rare) and risk of food allergy to peanut (odds ratio) in children (at any age)	34

Figure 8 Maternal peanut avoidance during pregnancy (often vs. rare) and risk of food allergy to peanut (odds ratio) in children aged 0 to 4 years old	34

Figure 10 Risk of bias in studies of fruit and vegetables intake and wheeze	40

Figure 11 Introduction of fruit in months 0 to 4 and risk of wheeze (odds ratio) in children aged 0 to 4 years old	41

Figure 12 Introduction of fruit in month ≥4 and risk of recurrent wheeze (odds ratio) in children aged 0 to 4 years old	41

Figure 13 Maternal fruit intake (often vs rare) and risk of wheeze (odds ratio) in children aged 5 to 14 years old	42

Figure 16 Risk of Bias in studies of fruit and vegetables intake and rhino-conjunctivitis	54

Figure 17 Risk of Bias in studies of fruits and vegetables intake and allergic sensitisation	60

Figure 18 Introduction of vegetables months 0 to4 and risk of specific sensitisation to aero-allergens in children (any age) (odds ratio)	61

Figure 19 Introduction of fruit months 0 to4 and risk of specific sensitisation to aero-allergens in children (any age) (odds ratio)	61

Figure 20 Maternal peanut intake during pregnancy or lactation (often vs. rare) and risk of specific sensitisation to peanut in children (any age) (odds ratio)	61

Figure 21 Maternal fruit intake (highest vs. lowest) and risk of specific sensitisation to aero-allergens in children (any age) (odds ratio)	62

Figure 22 Risk of Bias in studies of fruit and vegetable intake and type I diabetes mellitus	74

Figure 23 Infant fruit introduction in months 0 to 4 and risk of type I diabetes mellitus in children	75

Figure 24 Risk of Bias in studies of fruit and vegetable intake and IBD	79

# List of tables

Table 1 Characteristics of included studies evaluating fruit and vegetable supplementation and allergic outcomes	9

Table 2 Characteristics of included studies evaluating fruit and vegetable supplementation and autoimmune outcomes	22

Table 3 Studies investigating the association between fruit and vegetable and atopic dermatitis which were not eligible for meta-analysis	28

Table 4 Studies investigating the association between fruit and vegetable and any food allergy which were not eligible for meta-analysis	35

Table 5 Studies investigating the association between fruit and vegetable (dietary exposure) and lung function (outcome) which were not eligible for meta-analysis	39

Table 6 Studies investigating the association between fruit and vegetable and wheeze which were not eligible for meta-analysis	44

Table 7 Studies investigating the association between fruit and vegetable and rhino-conjunctivitis which were not eligible for meta-analysis	55

Table 8 Studies investigating the association between fruit and vegetable and allergic sensitization which were not eligible for meta-analysis	63

Table 9 Studies investigating the association between fruit and vegetable and type 1 diabetes mellitus which were not eligible for meta-analysis	76

Table 10 Studies investigating the association between fruit and vegetable (dietary exposure) and IBD (outcome) which were not eligible for meta-analysis	80

# 1. Executive summary of findings on infant and maternal intake of fruits and vegetables and risk of allergic and autoimmune diseases

1.1. **Context of this report**

This report is one of a series of systematic review reports included in Review C Part II, which focusses on evidence on dietary exposures during pregnancy, lactation and or infancy for reducing risk of allergic or autoimmune outcomes. It describes the current evidence from observational studies on infant or maternal intake of fruits and vegetables and risk of allergic or autoimmune diseases. There was a high degree of variation in the type of dietary exposures studied, which limited the possibilities for meta-analyses. Within each outcome section, a narrative table is provided, which describes the main findings and effect sizes (when available) for each study.

1.2. **Studies identified**

A total of 35 observational studies reported the association between infant and/or maternal intake of fruit and vegetable and allergic (n=28) or autoimmune (n=7) diseases. Of these studies, 27 were prospective cohort studies, 6 case-controls, and 2 nested case control studies. The majority of studies (n=24) are from Europe – others are from North America (n=5), Asia Pacific (n=5), and Latin America (n=1).

1.3. **Outcomes evaluated**

Nineteen studies investigated the association between risk of atopic dermatitis (AD) and fruit or vegetable intake, nine studies investigated food allergy (FA), 1 study investigated lung function (LF), 19 studied wheeze, seven studied rhino-conjunctivitis (RC), 15 studied allergic sensitisation, nine studied type 1 diabetes mellitus (T1DM) and one investigated inflammatory bowel disease (IBD).

1.4. **Dietary exposures studied**

Dietary exposures for fruits and vegetables varied greatly across studies. In some studies, only a single question was used to ascertain habit of consumption, often as part of other risk factors investigated in the study. In others, there was a specific questionnaire used to ascertain intake of several individual and/or grouped fruits and vegetables. Our approach to meta-analysis considered the classification of foods of the same family if possible (e.g. ‘apples’, ‘any fruit’). If studies used only a generic question to ascertain fruit or vegetable intake (e.g. ‘does your baby eat any fruit’) this variable was included in the meta-analysis along with other studies where only a single fruit was assessed, provided that the outcomes were comparable.

1.5. **Presentation of results**

We created a summary Table of Study Characteristics with key study features, presented separately for allergic (Table 1) and autoimmune outcomes (Table 2). There are seven main sections in this report that summarise the main findings for each of the following outcomes: atopic dermatitis (AD), food allergy (FA), lung function (LF), wheeze, rhino-conjunctivitis (RC), allergic sensitisation (AS), type I diabetes mellitus (T1DM), and inflammatory bowel disease (IBD).We examined each of the studies for their eligibility to be meta-analysed. If two or more studies had data that could be combined, a meta-analysis was included in the corresponding section. If the heterogeneity across studies ≥ 80% then the overall effect of the studies is not included but that of each individual study is presented. As most of the studies included in this report had data that could not be combined in a meta-analysis, a table of main characteristics and main findings is provided at the end of each section. The tables provide specific details on the intakes compared for each dietary exposure as these varied between, and sometimes within study.

1.6. **Risk of bias assessment**

The risk of bias in included cohort and case control studies was assessed using a modified version of the National Institute for Clinical Excellence (NICE) methodological checklist for cohort and case-control studies, respectively . Key domains were:

- Selection Bias (low if cases and controls were selected from similar populations, if the participation rate was ≥80%, or <80% but investigators explored and adjusted for characteristic differences between participants and non-participants);
- Assessment Bias (low if validated and reliable tools were used to assess exposure and/or outcome), and;
- Confounding Bias (low if most likely confounders are identified and taken into account in study design and analysis).

Observational studies were considered at low overall risk of bias where the risk of bias was judged to be low for all 3 key domains for selection, assessment and confounding bias. For assessment of Confounding Bias, factors that we expected to be adjusted for within studies of allergic outcomes were: siblings (parity or birth order or family size); gender; age at outcome assessment; disease risk based on family history; maternal or household smoking (asthma/wheeze outcomes); maternal age; maternal education or socioeconomic status; mode of delivery. For studies on autoimmune outcomes we expected matching and/or adjusting for gender, age, address, socioeconomic status, smoking and disease risk. We also assessed possible Conflict of Interest, this being judged as low where there was no evidence of industry involvement in study design, analysis, interpretation or publication, and no evidence that study authors received remuneration from relevant industry partners for other activities. Given the nature of the studies included in this report (observational studies on reported dietary intake of foods or food groups), the conflict of interest was low in all cases. Therefore, we only included the first three domains, and the overall bias. These domains were assessed for all studies and included in the Risk of Bias Figure regardless of whether the studies were eligible for meta-analysis or for narrative. A Risk of Bias Figure is presented in each outcome section.

1.7. **Key findings**

i. **Overall risk of bias was low or unclear for the majority of the outcomes assessed**

The overall risk of bias was considered to be low in the majority of included studies. In several studies it was unclear whether adjustment for potential confounders had been made, leading to an ‘unclear’ risk of bias.

ii. **Classification of dietary exposures and varying comparison levels of intake reduced the possibilities of meta-analyses**

Very few studies had comparable information which could be meta-analysed. The main reason for this was the large variations in the ways of reporting associations between the relevant dietary exposures and risk of allergic or autoimmune diseases. In general, the few available meta-analyses showed low statistical heterogeneity.

iii. **There was no evidence of an effect of fruits or vegetables on studied outcomes**

Overall, we found no consistent evidence to suggest that intake of fruits and vegetables early in life influences risk of other allergic or autoimmune diseases.

Table 1 Characteristics of included studies evaluating fruit and vegetable supplementation and allergic outcomes

| **Study** | **Design** | | **N/n** | | | **Country** | | | **Type of fruit and vegetable studied** | | | **Exposure assessment** | | | **Outcomes** | | | **Age at outcome (years)** | | | **Population characteristics** |  |
| --- | --- | --- | --- | --- | --- | --- | --- | --- | --- | --- | --- | --- | --- | --- | --- | --- | --- | --- | --- | --- | --- | --- |
| Martindale, 2005; Willers, 2007 | PC | 1300 | | | UK, Netherlands | | | Fruits; citrus fruits; fruit juice; vegetables; green leafy vegetables; apples | | | Q | | | AD: Parent reported and/or UK Working Party Criteria; LF: Spirometry; RC and wheeze: Parent reported (ISAAC Q); SPT any; rec wheeze: DD asthma | | | 2, 5 | | | **Aberdeen birth cohort**: Population based birth cohort with pregnant women recruited 1997-99 while attending a hospital antenatal clinic at ~12weeks gestation | | |
| Hesselmar, 2010 | PC | 184 | | Sweden | | | Fruits | | | I/Q | | | AD: Physician assessment, and full-filling Williams’ criteria; FA cm: Physician assessment plus Open Food Challenge; sIgE any | | | 0.5; 1.5 | | | **ALLERGYFLORA:** Birth cohort in Sweden enriched with children with family history of allergies | | |  |
| Lack, 2003 | PC; NCC | 11352 | | UK | | | Allergenic food avoidance (peanuts/nuts); allergenic food avoidance (soybean meat); peanut | | | Q | | | FA peanut: History of reaction to peanut (without food challenge), DBPCFC alone or plus DD | | | 5.5, 6 | | | **ALSPAC**: The study enrolled pregnant women living in the Avon Health Authority are, UK, expected to delivery between 1991-92. Cases and controls, who did and did not develop food allergy respectively, were not matched. | | |  |
| Binkley, 2011 | CC | 1413/1300 | | Canada | | | Allergenic food avoidance (peanut) | | | Q | | | FA peanut DD | | | 5to14, <18 | | | Cases & controls were anaphylaxis registry's previous survey respondents, all having had previous anaphylactic food allergy reactions, although only cases to peanuts: Canada. | | |  |
| Fergusson, 1990 | PC | 1067 | | New Zealand | | | Fruits; vegetables | | | R/D | | | AD Physician assessment | | | 10 | | | **CHRISTCHURCH CHILD DEVELOPMENT STUDY:** A cohort of children born in the Christchurch urban region New Zealand during mid1977 | | |  |
| Maslova, 2012 | PC | 4501 | | Denmark | | | Peanut and pistachio; nuts (tree nuts) | | | Q | | | RC:DD plus medication records; wheeze: DD; Rec wheeze: parent reported >3 episodes of wheeze; Rec wheeze: DD asthma plus medication records | | | 1.5, 7 | | | **DNBC**: Population based birth cohort with pregnant women recruited between 1996 and 2002 at ~12weeks gestation in Denmark | | |  |
| DesRoches, 2010 | CC | 401/202 | | Canada | | | Allergenic food avoidance (nut); allergenic food avoidance (soya); allergenic food avoidance (peanut) | | | Q | | | FA peanut :History of a clinical reaction within 60 minutes of exposure to peanuts, combined with positive IgE and/or SPT to peanut | | | <1.5 | | | Cases and controls were recruited from the Paediatric University Centre between 1998-2004: Canada | | |  |
| Maijaliisa, 2011; Nwaru, 2010; Nwaru, 2011; Nwaru, 2013; Erkkola, 2012; Nwaru, 2010b; | PC | 5619/237 | | Finland | | | malaceous fruits; green leafy vegetables; berries; citrus fruits; fruit juice; fruits; vegetables and roots; nuts and pulses; potatoes; fruits and berries; root vegetables; fruits and vegetables; vegetables; cabbage; carrots | | | Q | | | AD, wheeze, rec wheeze, and RC: Parent reported (ISAAC Q); sIgE-aero; sIgE cm; sIgE to egg; sIgE to food; wheeze; rec wheeze (atopic): DD asthma plus positive sIgE; rec wheeze: DD asthma plus medication and/or current symptoms | | | 0.5, 5, <10 | | | **DIPP:** Prospective birth cohort of children at high risk of TIDM (HLA genotype conferred susceptibility) born between 1997 and 2004 in Oulu and Tampere University Hospital Finland | | |  |
| Fox, 2009 | CC | 293/133 | | UK | | | peanut | | | Q | | | FA to peanut: DD DBPCFC | | | <4 | | | Cases and controls from specialist food allergy clinics, with cases sensitised to peanut and controls sensitised to egg but not peanut: UK. | | |  |
| Venter, 2009; Dean, 2007 | PC | 937 | | UK | | | allergenic food avoidance (peanut) | | | Q | | | SPT peanut | | | 2 | | | **The Isle of Wight cohort:** Population based birth cohort recruited through antenatal clinics and included all babies born on the Isle of Wight UK between 2001 and 2002 | | |  |
| Fitzsimon, 2007 | PC | 631 | | Ireland | | | fruits and vegetables | | | Q | | | Rec wheeze: Physician assessment asthma | | | 3 | | | **LIFE-WAYS**: cohort of children born in 2002 whose mother had completed FFQ during pregnancy were followed up from birth through general practice records | | |  |
| Zutavern, 2006; Zutavern, 2008; Sausenthaler, 2007 | PC | 2540 | | Germany | | | fruits; vegetables; apples; bananas; strawberries; citrus fruits; fruit juice; cabbage; salad; celery; sweet peppers; spinach; nuts; carrots; seeds; tomatoes | | | Q | | | AD:DD; Parent reported; sIgE to food; sIgE aero; sIgE any | | | 2, 6 | | | **LISA:** Population based cohort study of newborns recruited between 1997 and1999 from 4 German cities: Munich, Leipzig, Wesel, and Bad Honnef. | | |  |
| Marini, 1996 | PC | 68 | | Italy | | | citrus fruits | | | Q | | | AD: Physician assessment plus parent reported; RC: Physician assessment plus parent reported; Recurrent wheeze: Physician assessment > 3 episodes of wheeze | | | 1, 3 | | | Infants with family history of allergy born in maternity wards of 3 hospitals from 1989 whose mothers were refused to participate in an allergy prevention intervention program | | |  |
| Chatzi, 2008 | PC | 468 | | Spain | | | fruits; legumes; nuts; vegetables | | | Q | | | SPT-aero; Atopic wheeze: Parent reported wheeze plus positive SPT; rec wheeze: parent reported ≥1 episodes of wheeze | | | 6.5 | | | **Menorca birth cohort:** Population based birth cohort with women recruited from antenatal care at all general practices in Menorca between 1997-1998 | | |  |
| Miskelly, 1988 | PC | 482 | | UK | | | fruits | | | D | | | AD: Physician assessment and parent reported; wheeze :Parent reported wheeze | | | 1 | | | Infant recruited through two antenatal clinics in South Wales born to mothers with positive allergy history in at least one member of family, whose mothers were asked to participate in allergy preventive program | | |  |
| Morgan, 2004 | PC | 257 | | UK | | | fruits; vegetables | | | I | | | AD: Physician assessment and parent reported | | | 1 | | | Healthy preterm births (<37 weeks gestational) from 3 hospitals in southeast England | | |  |
| Miyake, 2010 | PC | 763 | | Japan | | | fruits; apples; citus fruits; vegetables; green and yellow vegetables;vegetables other | | | Q | | | AD and wheeze: Parent reported (ISAAC Q) | | | 2 | | | **OMCHS:** Population birth cohort recruited during pregnancy from a university hospital and three obstetric hospitals in municipalities of Osaka 2001 - 2003 | | |  |
| Oien, 2009 | PC | 3050 | | Norway | | | vegetables | | | Q | | | AD: Parent reported (ISAAC Q); rec wheeze: DD asthma | | | 2 | | | **PACT:** Trondheim, Norway. Inclusion in the control cohort began in September 2000, and the interventional programme started in a separate cohort in July 2002 | | |  |
| Roduit, 2012 | PC | 1041 | | Austria, Finland, France, Germany, and Switzerland | | | fruits and vegetables | | | D | | | AD:DD | | | 1, 4 | | | **PASTURE:** Population based birth cohort recruited in third trimester of pregnancy from rural areas in 5 European countries from farming and non-farming families | | |  |
| Willers, 2008 | PC | 2828 | | The Netherlands | | | nuts; fruits; vegetables | | | Q | | | Total IgE; sIgE-aero; sIgE to food; wheeze: Parent reported (ISAAC Q) wheeze; Rec wheeze: DD asthma plus medication and/or current symptoms | | | 8 | | | **PIAMA:** Population based birth cohort recruited prenatally Netherlands. The children were allocated to an intervention study or to a natural history study depending on their family risk for allergy | | |  |
| Sariachvili, 2010 | NCC | 557/252 | | Belgium | | | fruits; fruit juice; vegetables | | | Q | | | AD: Parent reported  (ISAAC Q) | | | 4 | | | **PIPO Cohort:** cases and controls with data regarding development of eczema and timing of introduction of solid foods were identified from this PC: Belgium. | | |  |
| Litonjua, 2006 | PC | 1290 | | USA | | |  | | | Q | | | AD:DD; wheeze: parent reported wheeze; rec wheeze: parent reported wheeze in at least one questionnaire (1,2,3 years old) | | | 2 | | | **Project Viva:** Population based birth cohort with pregnant women at <22 weeks of gestation recruited from 8 obstetric offices of a large multispecialty suburban/urban group practice in eastern Massachusetts US between 1999 and 2002 | | |  |
| Dunlop, 2006 | PC | 1326 | | Slovakia | | | citrus fruits | | | Q | | | AD: Physician assessment | | | 1 | | | **Slovak birth cohort:** The 1st 250 pregnant women delivering at maternity hospitals in the selected study sites were recruited between 1997 and 1999, | | |  |
| Strassburger, 2010 | PC | 338 | | Brazil | | | salty pureed food; fruit juice | | | R | | | SPT aero; Wheeze: Parent reported wheeze | | | 3.5 | | | Birth cohort study nested in a dietary intervention randomized field trial in the city of São Leopoldo, southern Brazil in 2002 | | |  |
| Kemp, 2011 | PC | 310 | | Tasmania | | | allergenic food avoidance (peanut) | | | Q | | | sIgE to peanut | | | 16 | | | **THIS:** cohort of infants at high risk for sudden infant death syndrome born in 1988-1989 were identified in the northern region of Tasmania through school records | | |  |
| Andreasyan, 2007; | PC | 498 | | Australia | | | fruit syrup; fruit juice (orange) | | | Q | | | AD, wheeze and RC: Parent reported (ISAAC Q); SPT any | | | 9 | | | **CARHS:** Some of the participants in the THIS cohort of infants at high risk for sudden infant death syndrome born in 1988-1989 were identified in the northern region of Tasmania through school records | | |  |
| Sicherer, 2010 | PC | 503 | | USA | | | Peanut | | | Q | | | sIgE to peanut | | | 1 | | | **The Consortium of Food Allergy Research** enrolled infants at 3 to 15 months of age with likely egg or milk allergy but without previously known peanut allergy | | |  |
| Zutavern, 2004; | PC | 606 | | UK | | | vegetables; fruits | | | Q | | | AD:DD; SPT aero; wheeze: Parent reported wheeze; rec wheeze: Parent reported asthma | | | 2, 5.5 | | | Population based birth cohort of newly pregnant women who presented at one of three general practices in Ashford, Kent UK between 1993 and 1995 | | |  |

PC Prospective cohort study; RC retrospective cohort study; Q questionnaire; AD atopic dermatitis; DBPCFC double blind placebo control food challenge; ISAAC International Study of Allergy and Asthma in Children; AS allergic sensitisation; SPT skin prick test, sIgE specific IgE; DD Doctor diagnosis (community); Physician assessment is assessment by study physician; AR allergic rhinitis, FA food allergy; rec wheeze recurrent wheeze; CS cross-sectional study; NCC nested case-control study

Table 2 Characteristics of included studies evaluating fruit and vegetable supplementation and autoimmune outcomes

| **Study** | **Design** | **N/n** | **Country** | **Type of fruit and vegetable studied** | **Exposure assessment** | **Outcomes** | **Age at outcome (years)** | **Population characteristics** |
| --- | --- | --- | --- | --- | --- | --- | --- | --- |
| Brekke, 2010 | PC | 4005/191 | Sweden | potatoes and root vegetables; vegetables | Q | TIDM: Islet autoimmunity (GADA and/or IA2A and/or IAA) | 5 | **ABIS:** Population based birth cohort of children born in Southeast Sweden between 1997 and 1999 |
| Baron, 2005 | CC | 444/ 222 | France | Vegetables | I | IBD-CR:DD; IBD-UC:DD | <17 | Cases with Crohn's disease were identified from the **EPIMAD** registry with matched controls from the same area identified by random digit dialling: France; Cases with ulcerative colitis were identified from the EPIMAD registry with matched controls from the same area |
| Norris, 1996; Frederikson, 2012; Lamb 2008 | NCC; PC | 1698/49 | USA; Australia | fruits; vegetables; potatoes; root vegetables | Q/I | TIDM: Islet autoimmunity (GADA and/or IA2A and/or IAA) | <7; 15; unclear | **DAISY:** Prospective birth cohort of children at increased risk of T1DM. Cases & their non-diabetic siblings, as controls, taken from diabetes care clinics & Colorado IDDM registry; recruited from 1993 to 2001 |
| Visalli, 2003 | CC | 900/ 150 | Italy | fruits; vegetables | Q | TIDM:DD WHO criteria | 12 | **EURODIAB Italy**: Cases with T1DM selected from within the EURODIAB ACE study, born 1977-89, with controls selected from school records for the same period |
| Savilahti, 2009 | NCC | 6209/45 | Finland | vegetables | R/D | TIDM:DD | 11.5 | Cases and controls taken from the NHI database, Finland |
| Strotmeyer, 2004; | CC | 688/ 247 | China | vegetables; fruits | Q | TIDM: DD WHO criteria | 9.7 | **DiaMond:** WHO Multinational Project; cases selected from T1DM incidence registries 1985-98 and matched controls from local population |
| Virtanen, 2011; Virtanen, 2006; | PC | 6069 | Finland | Vegetables | Q | TIDM: DD WHO criteria | <10 | A prospective birth cohort infants with HLA-conferred susceptibility to type 1 diabetes was recruited between 1996 and 2004 |

PC Prospective cohort study; Q questionnaire; CC case-control study; SPT skin prick test, sIgE specific IgE; DD Doctor diagnosis (community); Physician assessment is assessment by study physician; AR allergic rhinitis, FA food allergy, T1DM type 1 diabetes mellitus, RC retrospective cohort, JIA juvenile idiopathic arthritis; ILAR International League of Associations for Rheumatology; HLA human leukocyte antigen serotype

# 2. Infant or maternal dietary intake of fruit and vegetables and risk of atopic dermatitis (AD)

Nineteen studies investigated intake of fruits or vegetables during infancy or pregnancy in relation to AD. The majority showed a low risk of bias, which was reflected in the low risk of assessment and selection bias of the prospective cohort studies. A few studies had a high risk of bias due to the absence of adjustment for confounding (Figure 1).

Figure 1 Risk of bias in studies of fruit and vegetable intake and risk of atopic dermatitis

# 2.1.  Infant Intake of fruit and vegetable and AD risk

Three prospective cohort studies with comparable data investigated fruit introduction between 0 and 4 months, and risk of AD at age 0-4 years old (). The study of **Sariachvilli (2010)** and **Zutavern (2006)** examined the association between fruit introduction and risk of AD, and the study of **Marini (2006)** studied citrus fruit introduction. **Andreasyan** (**2007)** investigated fruit or fruit syrup introduction and **Zutavern** (**2004**) investigated fruit introduction, and risk of AD at age 5-14 years (Figure 3). Neither meta-analysis showed evidence of association nor evidence of statistical heterogeneity (I^2^=0%).

Figure 2 Introduction of fruit at age >4 months vs 0-4 months and risk of atopic dermatitis (odds ratio) in children aged 0-4 years old

Figure 3 Introduction of fruit at age > 4 months vs 0-4 months and risk of atopic dermatitis (odds ratio) in children aged 5-14 years old

# 2.2. Maternal Intake of fruits and vegetables and risk of AD in childhood

Maternal dietary intake of fruits or vegetables and risk of AD in childhood was investigated in three prospective cohort studies with comparable data for meta-analysis. Intake of fruit during pregnancy was unrelated to AD in children at age 0-4 years (Figure 4). The studies of **Miyake** (**2010**; any fruits), **Martindale** (**2005**; any fruits) and **Sausenthaler** (**2007**; apples) had high heterogeneity. This may be explained by differences in the populations and dietary habits of the study populations, and/or the method of assessing fruit intake. The LISA study **(Sausenthaler)** was based on German children, whereas the Osaka cohort (OMCHS, **Miyake)** recruited Japanese children, and the Aberdeen cohort was based on a Scottish population (**Martindale**). Dietary intake of fruits was analysed using different, but comparable approaches.

Figure 4 Maternal fruit intake (highest vs. lowest) and risk of atopic dermatitis (odds ratio) in children aged 0-4 years old

# 2.3. Studies on infant or maternal intake of fruits and vegetables that were not suitable for meta-analysis

The main results from observational studies that could not be included in meta-analysis are shown in Table 3. All the studies were prospective cohorts, with the exception of **Sariachvili (2010)**, which was a nested case-control study. There was no evidence of an association between fruit and vegetable intake or introduction and AD. There was some evidence of an association in three studies on infant intake. The study of **Sariachvili (2010)** which reported a higher risk of AD in infants who were introduced to fruit juice after 4 months of age in their diet, compared to those who started drinking it earlier. The study of **Nwaru (2010)** reported that a higher intake of berries was statistically negatively associated with risk of AD. There was some evidence on maternal intake of green and yellow vegetables, and citrus fruit reducing risk of AD in one study (**Miyake 2010**) but this was not confirmed in other studies.

**We found no consistent evidence to suggest that infant or maternal intake of fruits and vegetables might influence the risk of AD.**

Table 3 Studies investigating the association between fruit and vegetable and atopic dermatitis which were not eligible for meta-analysis

| **First Author and year of publication** | **Design** | **Outcome** | **Age** | **N/n** | **Dietary exposure and comparison level** | **Measure of association** | | **Effect (bold indicates p-value<0.05)** | |  | |
| --- | --- | --- | --- | --- | --- | --- | --- | --- | --- | --- | --- |
|  |  |  |  |  | **INFANT INTAKE** |  | |  | | |  |
| Fergusson, 1990 | PC | AD | 10 | 1067 | Fruit and vegetable introduction (>4 months vs. ≤ 4months) | HR | | NS | | |  |
|  |  |  |  |  | Fruits and berries >4 months vs. <3.5 and 3.5-4 months |  | | NS | | |  |
| Nwaru, 2013 | PC | AD | 0.5 | 3109 | Fruits and berries (continuous intake) | Reduced AD | | **P=0.03** | | |  |
|  |  |  |  |  | Root vegetables/ >4 months vs. <3.2 and 3.2-4 months |  | | NS | | |  |
|  |  |  |  | 1041 | **Fruit and vegetable intake in the first year of life**  Intake before 6 months (high risk infants) | OR | | 1.92 (0.94-3.92) | |  | |
| Roduit, 2012 | PC | AD | 4 |  | Intake before 6 months (low risk infants) | OR | | 1.69 (0.74-3.91) | | | |
|  |  |  |  | 912 | Fruits and vegetables introduced into diet >6 months vs. <6 months | OR | | 1.23 (0.80-1.91) | |  | |
| Andreasyan, 2007 | PC | AD | 9 | 495 | Fruit juice (orange) consumption at >1 month vs. ≤ 1 month | RR | | 1.49 (0.42-5.35) | |  | |
| Hesselmar, 2010 | PC | AD | 1.5 | 184 | Fruits introduced to the diet (age in months compared as continuous variable) |  | | NS | |  | |
|  |  | Parental reported AD | 2 | 2,540 | Fruits at 5-6 months vs. 0-4 months | OR | | 1.02 (0.77-1.35) | |  | |
|  |  |  |  |  | Fruits >6 months vs. 0-4 months | OR | | 1.03 (0.74-1.44) | |  | |
| Zutavern, 2006 | PC |  |  |  | Vegetables at 5-6 months vs. 0-4 months | OR | | 1.02 (0.78-1.34) | |  | |
|  |  |  |  |  | Vegetables>6 months vs. 0-4 months | OR | | 1.22 (0.88-1.69) | |  | |
|  |  |  |  |  | Fruits at 5-6 months vs. 0-4 months | OR | | 1.14 (0.85-1.53) | |  | |
|  |  | DD AD | 2 | 2,540 | Vegetables at 5-6 months vs. 0-4 months | OR | | 1.21 (0.90-1.62) | |  | |
| Zutavern, 2004 | PC | AD | 5.5 | 642 | Introduction of vegetables >4 months vs earlier | OR | | 0.83 (0.53-1.30) | |  | |
| Miskelly, 1988 | PC (of RCT) | AD | 1 | 482 | Fruits/ early introduction (continuous comparison scale (months) |  | | NS | |  | |
| Morgan, 2004 | PC | AD | 1 | 257 | Introduction of fruits or vegetables (weeks) |  | | NS | |  | |
| Oien, 2009 | PC | AD | 2 | 3050 | Infant intake of vegetables during 1st year / ≤1x/week vs. 2 to 5 times | OR | | 0.91 (0.46-1.80) | |  | |
| Sariachvili, 2010 | NCC | AD | 4 | 557/252 | **Infant food introduction (**>4 months vs. ≤4 months)  Fruit juice | OR | | **1.56 (1.08-2.27)** | |  | |
|  |  |  |  |  | Vegetables | OR | | 1.39 (0.93-2.06) | |  | |
| Dunlop, 2006 | PC | AD | 1 | 1326 | Introduction of citrus fruit in months 8 and beyond | OR | | 0.90 (0.55-1.48) | |  | |
|  |  |  |  |  | **MATERNAL INTAKE** |  | |  | |  | |
| Oien, 2009 | PC | AD | 2 | 3013 | Vegetables/maternal intake of vegetables during pregnancy (2-5 times per week vs ≤once a week) | OR | | 0.76 (0.54-1.06) | |  | |
| Maijaliisa, 2011 | PC | AD | 5 | 2441 | **Maternal intake of malaceous fruits during 8^th^ month pregnancy** (not low consumption vs. low consumption)  Apple  Green leafy vegetables |  | NS  NS | |  | | |
| Willers, 2007 |  |  |  |  | **Maternal intake during pregnancy** (highest vs lowest tertile)  Any fruits |  | NS | |  | | |
|  | PC | AD | 5 | 1212 | Any vegetables |  | | NS | |  | |
| Martindale, 2005 | PC | AD | 2 | 1300 | **Maternal intake during pregnancy** (>1 portion per day vs ≤ 1 portion per day)  Fruit juice | OR | | NS | |  | |
|  |  |  |  |  | Any vegetables | OR | | NS | |  | |
| Sausenthaler, 2007 | PC | AD | 2 | 2473 | **Maternal consumption during the last 4 weeks of pregnancy** (highest vs lowest tertile)  Bananas | OR | | 1.03 (0.77-1.38) | |  | |
|  |  |  |  |  | Strawberries | OR | | 1.02 (0.77-1.35) | |  | |
|  |  |  |  |  | Citrus fruits | OR | | 1.03 (0.79-1.35) | |  | |
|  |  |  |  |  | Fruit juice | OR | | 1.18 (0.90-1.54) | |  | |
|  |  | | | | Salad | OR | | 0.92 (0.69-1.22) | |  | |
|  |  |  |  |  | Celery | OR | | 0.94 (0.67-1.31) | |  | |
|  |  |  |  |  | Sweet pepper | OR | | 0.97 (0.74-1.27) | |  | |
|  |  |  |  |  | Spinach | OR | | 1.26 (0.99-1.61) | |  | |
|  |  |  |  |  | Raw carrots | OR | | 1.12 (0.86-1.46) | |  | |
|  |  |  |  |  | Seeds | OR | | 1.24 (0.94-1.64) | |  | |
|  |  |  |  |  | Tomato | OR | | 0.83 (0.63-1.10) | |  | |
| Miyake, 2010 | PC | AD | 2 | 763 | **Maternal intake during pregnancy** (highest vs lowest tertile)  Any vegetables | OR | | 0.85 (0.49-1.46) | |  | |
|  |  |  |  |  | Green and yellow vegetables | OR | | **0.41 (0.24-0.71)** | |  | |
|  |  |  |  |  | Apple | OR | | 0.87 (0.50-1.52) | |  | |
|  |  |  |  |  | Citrus fruits | OR | | **0.53 (0.30-0.93)** | |  | |
| Litonjua, 2006 | PC | AD | 2 | 1290 | **Maternal intake at 26-28 weeks of pregnancy**( highest quartile intake vs lowest)  Any fruit  Any vegetable |  | | NS  NS | |  | |
| Maijaliisa, 2011 | PC | AD | 5 | 2441 | **Maternal intake at 8^th^ month of pregnancy** (not low consumption vs. low consumption)  Apple  Green/leafy vegetables |  | | NS  NS | |  | |

# 3. Infant or maternal dietary intake of fruit and vegetable and risk of food allergy

Nine observational studies (5 prospective cohorts, and 4 case-control studies) investigated the association between intake of fruits and vegetables and risk of food allergy. Over one third of studies were assessed as at high risk of bias. This was mostly due to unadjusted or poorly reported adjusting leading to a risk of confounding bias. Assessment bias was considered to be of low risk for over 90% of the studies (Figure 5).

Figure 5 Risk of Bias in studies of fruit and vegetable intake and food allergy

# 3.1. Maternal Intake of fruit and vegetables and risk of childhood food allergy

Three prospective cohort studies with data eligible for meta-analysis investigated the association between maternal peanut avoidance and risk of peanut allergy in children at any age, between 0-4 years old, or later in childhood (5-14 years old). The pooled estimates of three prospective cohorts examining the association between maternal peanut avoidance during pregnancy and risk of peanut allergy in children showed no indication of association (Figure 6). The studies compared eating peanuts ever vs never (Kemp 2011), ≥2 times per week vs ≤ twice per week, (Sicherer 2010), and no vs yes (Dean 2007). There was no evidence of heterogeneity in these studies (I^2^=0%). The retrospective studies of DesRoches (2010) and Fox (2009) showed no association between maternal peanut avoidance in pregnancy and risk of peanut allergy in children at age 0-4 years old (Figure 7). The study of DesRoches (2010) investigated peanut avoidance as a binary exposure (no vs yes) and the study of Fox (2009) compared an intake of >15g per week vs none per week. There was very high heterogeneity in these studies (I^2^=72.1%) probably due to the variability in the assessment of peanut consumption in these two studies.

Figure 6 Maternal peanut avoidance during pregnancy (often vs. rare) and risk of food allergy to peanut (odds ratio) in children (at any age)

Figure 7 Maternal peanut avoidance during pregnancy (often vs. rare) and risk of food allergy to peanut (odds ratio) in children aged 0 to 4 years old

# 3.2. Studies on infant or maternal intake of fruits and vegetables that were not suitable for meta-analysis

Table 4 summarises the findings of six observational studies that investigated the association between fruits and vegetable intake and risk of food allergy. **Des Roches 2010** reported increased peanut allergy in infants of mothers who consumed peanut and soya during pregnancy or lactation. This association (for peanut) was not supported by a second study (**Fox 2009)**.

**Overall we found no evidence for association between fruit and vegetable intake and food allergy.**

Table 4 Studies investigating the association between fruit and vegetable and any food allergy which were not eligible for meta-analysis

| **First Author and year of publication** | **Design** | **Outcome** | **Age** | **N/n** | **Dietary exposure and comparison level** | **Measure of association** | **Effect (bold indicates p-value <0.05)** |
| --- | --- | --- | --- | --- | --- | --- | --- |
|  |  |  |  |  | **INFANT INTAKE** |  | |
| Hesselmar, 2010 | PC | Any food allergy | 1.5 | 184 | Fruit introduction (in months) | OR NS | |
|  |  | Food allergy to cow’s milk | 0.5 | 184 | Fruit introduction (in months) | OR NS | |
|  |  |  |  |  | **MATERNAL INTAKE** |  |  |
| Lack, 2003 | PC | Food allergy to peanut | 5.5 | 11352 | **Maternal avoidance during pregnancy:**  Nuts (including peanuts) during pregnancy yes vs. no | OR | 0.98 (0.53-1.81) |
|  |  |  |  |  | Soybean meat avoidance (intake vs no intake) | OR | 1.09 (0.39-3.03) |
|  |  |  |  |  | Maternal fruit intake (highest vs. lowest) | OR | 0.33 (0.10-1.07) |
| Sausenthaler, 2007 | PC | Food allergy (any) | 2 | 2473 | Maternal vegetable intake (highest vs. lowest) | OR | 0.81 (0.50-1.32) |
| Binkley, 2011 | CC | Food allergy to peanut | 18 | 1413/1300 | Peanut avoidance in pregnancy ( reduced intake vs no intake) | OR | 0.78 (0.43-1.40) |
| DesRoches, 2010 | CC | Food allergy to peanut or soy | 1.5 | 202/201 | Maternal avoidance  Peanut avoidance during pregnancy (no vs yes) | OR | **4.22 (1.57-11.30)** |
|  |  |  |  | 202/201 | Soya avoidance during pregnancy (no vs yes) | OR | **1.88 (1.20-2.94)** |
|  |  |  |  | 202/201 | Maternal avoidance  Peanut avoidance during lactation (no vs yes) | OR | **2.28 (1.31-3.97)** |
|  |  |  |  | 202/201 | Soya avoidance during lactation (no vs yes) | OR | **1.81 (1.08-3.03)** |
| Fox, 2009 (15)  Fox, 2009 | CC  CC | Food allergy to peanut  Food allergy to peanut | <4  <4 | 293/133  293/133 | **Maternal peanut intake during pregnancy/lactation:**  Lactation (≥15g per week vs no intake) | OR | 0.63 (0.15-2.70) |
|  |  |  |  |  | Lactation ( 3.75-15 g/week vs no intake) | OR | 0.70 (0.27-1.80) |
|  |  |  |  |  | Lactation ( 0.1-3.75 g/week vs no intake) | OR | 0.80 (0.34-1.90) |
|  |  |  |  |  | Pregnancy ( 3.75-15 g/week vs no intake) | OR | 1.00 (0.42-2.40) |
|  |  |  |  |  | Pregnancy ( 0.1-3.75 g/week vs no intake) | OR | 2.11 (0.93-4.80) |
|  |  |  |  |  | Pregnancy (≥15g per week vs no intake) | OR | 2.25 (0.70-7.10) |

# 4. Infant or maternal dietary intake of fruit and vegetables and risk of poor lung function

# 4.1. Lung function

One prospective cohort study was identified, which investigated intake of various types of fruits and vegetables in relation to lung function. The study was considered to have a low overall risk of bias. There was no evidence of an association between maternal intake of specific fruits and vegetable groups during pregnancy and lung function measured in the offspring when 5 years old (Table 5).

Table 5 Studies investigating the association between fruit and vegetable (dietary exposure) and lung function (outcome) which were not eligible for meta-analysis

| **First Author and year of publication** | **Design** | **Outcome** | **Age** | **N/n** | **Dietary exposure and comparison level** | **Measure of association** | **Effect** |
| --- | --- | --- | --- | --- | --- | --- | --- |
|  |  |  |  |  | **MATERNAL INTAKE** |  |  |
| Willers, 2007 | PC | LF | 5 | 510 | **Maternal intake during pregnancy (high vs low tertiles):**  Fruits |  | NS |
|  |  |  |  |  | Citrus fruits |  | NS |
|  |  |  |  |  | Fruit juice |  | NS |
|  |  |  |  |  | Vegetables |  | NS |
|  |  |  |  |  | Green leafy vegetables |  | NS |

# 5. Infant or maternal dietary intake of fruit and vegetables and risk of wheeze

Nineteen observational studies were identified which investigated the relation between infant or maternal intake of fruits and vegetables and risk of wheeze or recurrent wheeze. Over half of the studies were considered to be of low risk of overall bias, with most of them having a low risk of selection or confounding bias (Figure 8).

Figure 8 Risk of bias in studies of fruit and vegetables intake and wheeze

# 5.1. Infant intake of vegetables and fruits and risk of wheeze

Meta-analysis was possible for two prospective studies reporting the association between introduction of fresh fruit in the infant diet between 0-4 months of age and risk of wheeze (). The study of Strassburger (2010) investigated ‘introduction of fruit juice’ and risk of wheeze at age 3.5 years, whereas the study of Zutavern (2004) investigated ‘introduction of fruit’ and risk of wheeze at age 2 years. The pooled effect estimates showed no evidence of a relationship between early fruit intake and this outcome, and there was no statistical heterogeneity between studies (I^2^=0%). Similarly, there was no evidence of an association between introduction of fruit juice (Strassburger 2010) and introduction of citrus fruit (Marini 1996) and recurrent wheeze, as shown in Figure 10. There was moderate heterogeneity between these studies (I^2^=40.6%).

Figure 9 Introduction of fruit in months 0 to 4 and risk of wheeze (odds ratio) in children aged 0 to 4 years old

Figure 10 Introduction of fruit in month ≥4 and risk of recurrent wheeze (odds ratio) in children aged 0 to 4 years old

# 5.2. Maternal intake of fruits and vegetables and risk of wheeze

shows the pooled effect estimates of the two prospective studies of Willers, one from 2007 (apple intake) and one from 2008 (any fruit intake) and risk of wheeze at age 5 years and 8 years, respectively. The meta-analysis showed no evidence of association between this outcome and fruit intake.

Figure 11 Maternal fruit intake (often vs rare) and risk of wheeze (odds ratio) in children aged 5 to 14 years old

# 5.3. Studies on infant or maternal intake of fruits and vegetables that were not suitable for meta-analysis

Table 6 summarises the results of 15 studies with data on dietary intake of fruits or vegetables during pregnancy or infancy and its association with risk of wheeze or recurrent wheeze. These studies were considered not suitable for meta-analysis due to the large variations in the comparison levels used to describe dietary intake and its relation to disease. The age at time of outcome measurement ranged between 1 year and 9 years old.

There was some evidence of an association between higher intake of fruits or vegetables during pregnancy and reduced wheeze in children at age 5 years. The study of **Maijaliisa** (**2011**) showed that higher intakes of green/leafy vegetables and of malaceous fruits in the last month of pregnancy were negatively associated with disease risk. In the study of **Erkkola** (**2012)** and colleagues there was evidence suggesting that pregnant mothers with a higher intake of various types of fruits (including malaceous and berry juice), as well as of several groups of vegetables, was related to a lower risk of wheeze in the offspring at age 5. In The Danish prospective cohort study of **Maslova** **(2012)** the authors reported that a lower intake of peanuts (avoidance) during the second trimester of gestation was associated with a reduced risk of recurrent wheeze in the offspring, both at age 18 months and at 7 years old. There was no evidence from other studies that infant intake of fruits or vegetables to suggest an association between fruit or vegetable intake and risk of wheeze or recurrent wheeze, nor there was evidence that early introduction of these foods was related to the outcomes of interest.

**Overall, we found no consistent evidence to suggest that intake of fruits or vegetables in infancy, pregnancy or lactation influences the risk of wheeze.**

Table 6 Studies investigating the association between fruit and vegetable and wheeze which were not eligible for meta-analysis

| **First Author and year of publication** | **Design** | **Outcome** | **Age** | **N/n** | | **Dietary exposure and comparison level** | **Measure of association** | | **Effect (bold indicates p-value <0.05)** | |
| --- | --- | --- | --- | --- | --- | --- | --- | --- | --- | --- |
|  |  |  |  |  | | **INFANT INTAKE** |  | |  | |
| Strassburger, 2010 | PC | Wheeze | 3.5 | 338 | | **Infant introduction (≥4 months vs. <4 months)**  Salty pureed food | OR | | 0.60 (0.25-1.39) | |
|  |  | Rec wheeze |  |  |  | Salty pureed food | OR | | 0.45 (0.12-1.61) | |
| Andreasyan, 2007 | PC | Wheeze | 9 | 497 | | Infant fruit juice (orange) introduction >1 month vs. ≤ 1 month | RR | | 1.33 (0.49-3.66) | |
| Miskelly, 1988 | PC | Wheeze | 1 | 482 | | Fruits intake (continuous months) | OR | | NS | |
| Hesselmar, 2010 | PC | Rec wheeze | 1.5 | 184 | | Fruits/age in months when introduced | OR | | NS | |
| Oien, 2009 (35) | PC | Rec wheeze | 2 | 2220 | | Infant intake of vegetables during 1st year (2 to 5 times per week vs ≤1/week ) | OR | | NS | |
|  |  |  |  |  | | Fruits and berries/ intake >4 months vs. <3.5 and 3.5-4 months | HR | | NS | |
|  |  |  |  |  | | Root vegetables//>4 months vs. <3.2 and 3.2-4 months | HR | | NS | |
| Nwaru, 2013 | PC | Rec wheeze | 0.5 | 3109 | | Fruits and berries// >4 months vs. <3.5 and 3.5-4 months | HR | | NS | |
|  |  |  |  |  | | Root vegetables/ >4 months vs. <3.2 and 3.2-4 months | HR | | **0.65 (0.51-0.82)** | |
| Maijaliisa, 2011 | PC | Wheeze | 5 | 2441 | | Malaceous fruit (not low vs. low consumption) | OR | | **0.69 (0.54-0.88)** | |
|  |  |  |  |  | | **MATERNAL INTAKE** |  | |  | |
| Erkkola, 2012 | PC | Wheeze | 5 | 2441 | | **Intake during pregnancy:**  Fruits/ 326.2-793.9 g/d vs. <326.2 g/d | OR | | 1.19 (0.93-1.53) | |
|  |  |  |  |  |  | **Intake during pregnancy:**  Malaceous fruits/17.1-97.5 g/d quarter vs. <17.7 g/d | OR | | **0.74 (0.58-0.94)** | |
|  |  |  |  |  |  | Fruits and vegetables/ 49.5-138.7g/d vs. <49.5 g/d | OR | | 0.89 (0.70-1.14) | |
|  |  |  |  |  |  | Berries / 6.74-32.7 g/d vs. <6.74 g/d | OR | | 1.11 (0.87-1.42) | |
|  |  |  |  |  |  | Citrus fruits / 7.04-71.8 g/d vs. <7.04 g/d | OR | | 0.87 (0.69-1.10) | |
|  |  |  |  |  | | Fruit and berry juices / 5.67-145.7 g/d vs. <5.67 g/d | OR | | 1.03 (0.81-1.31) | |
|  |  |  |  |  | | Malaceous fruit / 17.1-97.5 g/d vs. 97.6-935 g/d | OR | | 1.14 (0.90-1.45) | |
|  |  |  |  |  | | Berries 6.74-32.7 g/d vs. 32.7-611 g/d | OR | | 1.12 (0.88-1.42) | |
|  |  |  |  |  | | Citrus fruit / 7.04-71.8 g/d vs. 71.8-778.8 g/d | OR | | 1.00 (0.79-1.27) | |
|  |  |  |  |  | | Fruit and berry juice / 5.67-145.7 g/d vs. 145.7-1700 s g/d | OR | | **1.27 (1.00-1.61)** | |
|  |  |  |  |  | | Vegetables / 149.1-315.1 g/d vs. <149.1 g/d | OR | | **0.76 (0.60-0.97)** | |
| Erkkola, 2012 |  | Wheeze | 5 | 2441 | | Leafy vegetable / 9.6-36.1 g/d vs. <9.6 g/d | OR | | **0.68 (0.53-0.86)** | |
|  |  |  |  |  | | Root vegetables and potatoes/ 117.8-207.5 g/d vs. <117.8 g/d | OR | | **0.78 (0.61-0.99)** | |
|  |  |  |  |  |  | Green leafy vegetables/ 9.6-36.1 g/d vs. 36.1-260.7 g/d | OR | | 1.05 (0.82-1.34) | |
|  |  |  |  |  |  | Root vegetables and potatoes/ 49.5-138.7 g/d vs. 207.5-788.5 g/d | OR | | 1.14 (0.88-1.47) | |
|  |  |  | | | | Fruit and berry juices/ 5.67-145.7 g/d vs. 145.7-1700 g/d | HR | | 0.71 (0.45-1.13) | |
|  |  |  |  |  |  | Root vegetables and potatoes/ 117.8-207.5 g/d vs. <117.8 g/d | HR | | 0.93 (0.58-1.50) | |
|  |  |  |  |  |  | Green leafy vegetables/ 9.6-36.1 g/d vs. <9.6 g/d | HR | | 0.96 (0.59-1.56) | |
|  |  |  |  |  |  | Root vegetables and potatoes/49.5-138.7 g/d vs. 207.5-788.5 g/d | HR | | 1.52 (0.88-2.64) | |
|  |  |  |  |  |  | Berries/ 6.74-32.7 g/d  vs. <6.74 g/d | HR | | 0.93 (0.53-1.61) | |
|  |  |  |  |  |  | Citrus fruits/ 7.04-71.8 g/d vs. <7.04 g/d | HR | | 0.77 (0.48-1.23) | |
|  |  |  |  |  | | Fruit and berry juices/ 5.67-145.7 g/d vs. <5.67 g/d | HR | | 0.93 (0.57-1.49) | |
|  |  |  |  |  | | Malaceous fruits/ 17.1-97.5 g/d vs. 97.6-935 g/d | HR | | 1.12 (0.68-1.85) | |
|  |  |  |  |  |  | Berries/6.74-32.7 g/d vs. 32.7-611 g/d | HR | | 1.56 (0.96-2.54) | |
| Erkkola, 2012 (18) | PC | Rec wheeze | 5 | 2441 | | Citrus fruits/ 7.04-71.8 g/d vs. 71.8-778.8 g/d | HR | | 0.77 (0.47-1.27) | |
|  |  |  |  | |  | **Maternal intake during pregnancy (high vs low tertiles)**  Green leafy vegetables | OR | NS | |  |
|  |  |  |  | |  | Citrus fruits | OR | NS | |  |
| Willers, 2007 | PC | Rec Wheeze | 5 | | 1212 | Fruit juice | OR | NS | |  |
|  |  |  |  | |  | Vegetables | OR | NS | |  |
|  |  |  |  | |  | Green leafy vegetables | OR | NS | |  |
|  |  |  |  | |  | Vegetables | OR | NS | |  |
| Martindale, 2005 | PC | Wheeze | 2 | | 1300 | Fruit juice intake during pregnancy / >1 portion per day vs ≤1 portion per day | OR | NS | |  |
|  |  |  |  | |  | **Maternal intake during pregnancy (highest vs lowest quartile)**  Apple | OR | NS | |  |
| Miyake, 2010 | PC | Wheeze | 2 | | 763 | Citrus fruits | OR | NS | |  |
| Litonjua, 2006 | PC | Wheeze | 2 | | 1290 | Fruits/ maternal diet / lowest vs. highest quartiles | OR | NS | |  |
|  |  |  |  | | 38223 | **Maternal intake of peanut 21-25^th^ week of gestation**  ≥once per week vs 0 times per week | OR | NS | |  |
|  |  |  |  | | 38279 | 2-3 times per month vs 0 times per week | OR | NS | |  |
|  |  |  |  | | 13313 | . ≥ once per week vs 0 per week | OR | NS | |  |
|  |  |  |  | | 13415 | ≥ once per month vs 0 times per month | OR | NS | |  |
| Maslova, 2012 | PC | Wheeze | 1.5 | | 38223 | ≥once per week 0 times per week | OR | NS | |  |
| Martindale, 2005 | PC | Wheeze | 2 | | 1300 | Vegetable maternal intake in pregnancy / ≤1 portion per day vs. >1 portion per day | OR | NS | |  |
| Miyake, 2010 | PC | Wheeze | 2 | | 763 | Maternal intake during pregnancy (highest vs lowest quartiles)  Green and yellow vegetables | OR | NS | |  |
|  |  |  |  | |  | **Maternal intake during pregnancy**  Nuts (daily vs rarely) | OR | NS | |  |
| Willers, 2008 | PC | Wheeze | 8 | | 2806 | Nuts (regularly vs rarely) | OR | NS | |  |
| Litonjua, 2006 | PC | Wheeze | 2 | | 1290 | **Maternal intake in the first trimester of gestation (highest vs lowest quartiles)**  Vegetables | OR | NS | |  |
|  |  |  |  | |  | Fruits | OR | NS | |  |
| Oien, 2009 | PC | Rec wheeze | 2 | | 2220 | Vegetables/Maternal consumption during pregnancy / ≤ once/week vs. almost daily | OR | 0.98 (0.86-1.04) | |  |
| Fitzsimon, 2007 | PC | Rec wheeze | 3 | | 631 | Grouped fruits and vegetables (highest vs. lowest quartiles) | OR | NS | |  |
| Maijaliisa, 2011 | PC | Rec wheeze | 5 | | 2441 | **Maternal intake during pregnancy**  Fruit / 326.2-793.9 g/d vs. <326.2 g/d | HR | NS | |  |
|  |  |  |  | |  | Malaceous fruit 17.1-97.5 g/d vs. <17.7 g/d | OR | NS | |  |
|  |  |  |  | |  | Green leafy vegetable/ Not low vs. low consumption | OR | NS | |  |
| Erkkola, 2012 | PC | Wheeze | 5 | | 2441 | **Maternal intake during pregnancy**  Vegetable / 149.1-315.1 g/d  vs. <149.1 g/d | HR | NS | |  |
|  |  |  |  | |  | Green leafy vegetables/ 9.6-36.1 g/d vs. 36.1-260.7 g/d | HR | NS | |  |
|  |  | Rec wheeze | 6.5 | | 468 | **Maternal intake during pregnancy:**  Fruit >14 servings per week vs ≤14 servings per week | OR | NS | |  |
|  |  |  |  | |  | Legumes >1 serving per week vs ≤1 servings per week | OR | NS | |  |
| Chatzi, 2008 | PC |  |  | |  | Nut >1 serving per week vs ≤1 serving per week | OR | NS | |  |
|  |  | Wheeze | 6.5 | | 468 | Fruit intake >14 servings per week vs ≤14 servings per week | OR | NS | |  |
|  |  |  |  | |  | Vegetable >8 servings per week vs ≤8 servings per week | OR | NS | |  |
|  |  |  |  | |  | Legume >1 servings per week vs ≤1 servings per week. | OR | NS | |  |
|  |  |  |  | |  | Nut >1 servings per week vs ≤1 serving per week | OR | NS | |  |

# 6. Infant or maternal dietary intake of fruit and vegetables and risk of allergic rhino-conjunctivitis (RC)

Seven prospective cohorts investigated the association between infant or maternal dietary intake of fruits and vegetables and risk of RC in childhood. None of the studies had comparable data eligible for a meta-analysis. All studies had low selection bias, and the majority had low risk of confounding bias. Due to the proportion of studies with high risk in assessment bias, half of them were considered to have an overall high risk of bias (Figure 12).

Figure 12 Risk of Bias in studies of fruit and vegetables intake and rhino-conjunctivitis

The summary results of the studies that assessed the association between infant or maternal dietary intake of fruits and vegetables is presented in Table 7. The studies of **Nwaru (2013)**, **Marini (2006)** and **Andreasyan (2007)** investigated the association between early exposure to fruits (0-4 months age) and risk of RC, showing no evidence of association. Four studies investigated the association between maternal intake of fruits or vegetables at various stages of pregnancy, with risk of RC. Only one study found a evidence of an association between maternal intake of vegetables and reduced RC in children at age 7 years (**Maslova 2012).** They also reported reduced RC in the offspring of mothers who consumed peanuts 2-3 times per month vs less frequently. There was no evidence from other studies to suggest an association between intake of fruits or vegetables and risk of RC. We found no consistent evidence to suggest that maternal or infant fruit or vegetable intake influences risk of RC in childhood.

Table 7 Studies investigating the association between fruit and vegetable and rhino-conjunctivitis which were not eligible for meta-analysis

| **First Author and year of publication** | **Design** | **Outcome** | **Age** | **N/n** | **Dietary exposure and comparison level** | **Measure of association** | **Effect (bold indicates p-value <0.05)** |
| --- | --- | --- | --- | --- | --- | --- | --- |
|  |  |  |  |  | **INFANT INTAKE** |  |  |
| Nwaru, 2013 | PC | RC | 0.5 | 3109 | Fruits and berries introduction / >4 months vs. <3.5 and 3.5-4 months old | HR | NS |
|  |  |  |  |  | Root vegetables introduction / >4 months vs. <3.2 and 3.2-4 months old | HR | NS |
|  |  |  |  |  | Fruit juice (orange) introduction / >1 vs. ≤1 month old | RR | 3.45 (0.52-22.83) |
| Andreasyan, 2007 | PC | RC | 9 | 497 | Introduction of fruit >1 vs. ≤1 month old | RR | 0.84 (0.56-1.27) |
| Marini, 1996 | PC | RC | <4 |  | Introduction of fruit at age 0-4 months | OR | 1.12 (0.83-1.50) |
|  |  |  |  |  | **MATERNAL INTAKE** |  |  |
|  |  |  |  |  | **Fruit and vegetable intake during pregnancy**  Fruit and vegetable/ 49.5-138.7 g/d vs. <49.5 g/d | HR | 1.15 (0.82-1.61) |
|  |  |  |  |  | Malaceous fruit / 17.1-97.5 g/d vs. <17.7 g/d | HR | 0.85 (0.62-1.16) |
| Erkkola 2012 | PC | RC | 5 | 2441 | Malaceous / 17.1-97.5 g/d vs. 97.6-935 g/d | HR | 1.14 (0.83-1.57) |
|  |  |  |  |  | Berries / 6.74-32.7 g/d vs. <6.74 g/d | HR | 0.91 (0.66-1.26) |
|  |  |  |  |  | Berries / 6.74-32.7 g/d vs. 32.7-611 g/d | HR | 1.14 (0.83-1.57) |
| Erkkola 2012 | PC | RC | 5 | 2441 | Citrus fruit / 7.04-71.8 g/d vs. <7.04 g/d | HR | 0.88 (0.65-1.21) |
|  |  |  |  |  | Citrus fruit / 7.04-71.8 g/d vs. 71.8-778.8 g/d | HR | 1.03 (0.75-1.42) |
|  |  |  |  |  | Fruit and berry juices / 5.67-145.7 g/d vs. <5.67 g/d | HR | 1.04 (0.75-1.44) |
|  |  |  |  |  | Fruit and berry juices / 5.67-145.7 g/d vs. 145.7-1700 g/d | HR | 1.40 (1.03-1.90) |
|  |  |  |  |  | Fruits/ 326.2-793.9 g/d vs. >793.9-3908 g/d | HR | 0.95 (0.67-1.34) |
| Erkkola, 2012 | PC | RC | 5 | 2441 | Vegetable intake / 149.1-315.1 g/d g/d vs. <149.1 g/d | HR | 1.06 (0.75-1.51) |
|  |  |  |  |  | Leafy vegetable / 9.6-36.1 quarter vs. <9.6 g/d | HR | 1.15 (0.81-1.63) |
|  |  |  |  |  | Leafy vegetable / 9.6-36.1 g/d vs. 36.1-260.7 g/d | HR | 1.06 (0.78-1.44) |
|  |  |  |  |  | Root vegetable and potato/ 117.8-207.5 g/d vs. <117.8 g/d | HR | 0.88 (0.64-1.20) |
|  |  |  |  |  | Root vegetable and potato / 117.8-207.5 g/d vs. 207.5-788.5 g/d | HR | 0.97 (0.69-1.37) |
|  |  |  |  |  | **Maternal intake during 8^th^ month pregnancy / Not low consumption vs. low consumption**  Malaceous fruit intake | OR | NS |
| Maijaliisa, 2011 | PC | RC | 5 | 2441 | Green leafy vegetable | OR | NS |
| Maslova, 2012 (1.5 years) | PC | RC | 7  1.5  7  1.5 | 13313 | **Maternal intake of peanut during 21-25^th^ week of gestation**  Peanut ≥1 per week vs 0 per week | OR | 0.78 (0.56-1.08) |
|  |  |  |  | 44956 | Peanut 2-3 times per month vs 0 per month | OR | **0.83 (0.72-0.96)** |
|  |  |  |  | 38223 | Peanut ≥1 per week vs 0 per week | OR | 0.92 (0.77-1.10) |
|  |  |  |  | 13415 | Peanut once a month vs 0 per month | OR | 0.95 (0.84-1.08) |
|  |  |  |  | 38279 | Peanut 2-3 times per month vs 0 per month | OR | 0.99 (0.86-1.14) |
| Maslova, 2012 (12) (7 years) | PC | RC | 7 |  | Maternal vegetable intake (often vs. rare) | OR | **0.84 (0.75-0.95)** |
| Willers, 2007 | PC | RC | 5 | 1212 | **Intake during pregnancy**(high vs lowest tertile)  Vegetable | OR | NS |
|  |  |  |  |  | Green leafy vegetables | OR | NS |
|  |  |  |  |  | Fruit | OR | NS |
|  |  |  |  |  | Citrus fruit | OR | NS |

# 7. Infant or maternal dietary intake of fruits and vegetables and risk of allergic sensitisation

Fifteen studies investigated the association between infant or maternal intake of fruits and vegetables and risk of allergic sensitisation (positive skin prick test [SPT] or sensitisation to a specific allergen [sIgE]). Overall, the studies were considered to have a low or unclear risk of bias. Most of the studies had a low risk of assessment and confounding bias, with nearly 40% of the studies having an unclear method of sample selection (Figure 13).

Figure 13 Risk of Bias in studies of fruits and vegetables intake and allergic sensitisation

Few prospective cohort studies had comparable data to estimate pooled size effects of fruit or vegetable intake on allergic sensitisation. Early introduction of vegetables or fruits was unrelated to the risk of having specific sensitisation to aero-allergens in children. The studies of **Nwaru (2010)** and **Zutavern (2004)** showed no evidence of an effect of introduction of cabbage or any vegetables, respectively, with the meta-analysis having no heterogeneity (Figure 14, I^2^=0%). The studies of **Strassburger (2010)** and **Zutavern (2004)** showed no evidence of an association between early introduction of fruit juice or fruit, respectively, and specific sensitisation to aero-allergens (Figure 15). The meta-analysis had moderate heterogeneity (I^2^=45.8%). Maternal intake of peanuts during pregnancy (**Kemp 2011**; **Dean 2007**) or during lactation (**Sicherer 2010**) was unrelated to risk of specific aero-allergen sensitisation in childhood (Figure 16). Maternal intake of any fruit (**Nwaru 2010**) or of apple (**Sausenthaler 2007**) was not associated with risk of specific sensitisation in childhood (Figure 17).

Figure 14 Introduction of vegetables months 0 to4 and risk of specific sensitisation to aero-allergens in children (any age) (odds ratio)

Figure 15 Introduction of fruit months 0 to4 and risk of specific sensitisation to aero-allergens in children (any age) (odds ratio)

Figure 16 Maternal peanut intake during pregnancy or lactation (often vs. rare) and risk of specific sensitisation to peanut in children (any age) (odds ratio)

Figure 17 Maternal fruit intake (highest vs. lowest) and risk of specific sensitisation to aero-allergens in children (any age) (odds ratio)

# 7.1. Studies on infant or maternal intake of fruits and vegetables that were not suitable for meta-analysis

Findings from studies that had narrative or descriptive information on the association between infant or maternal intake of fruits and vegetables and risk of allergic sensitisation are summarised in Table 8. The studies of **Nwaru (2010)** and **Sausenthaler (2007)** reported increased maternal citrus fruit intake during pregnancy was associated with increased sensitisation to aero-allergens. The study of **Willers (2007)** found no association between citrus fruit intake in pregnancy and allergic sensitisation in offspring. The study of **Kemp (2011)** found that consuming peanuts during pregnancy was related to a lower risk of sensitisation to peanut in children at age 16 years old, but only in those with no parental history of asthma. The study of **Sicherer (2010)** reported that a reduced intake of peanuts during late stages of pregnancy was associated with a higher risk of sensitisation to this food in high risk infants aged 1 year. Other studies found no association between maternal peanut intake and AS-Peanut.

**We found no consistent evidence to suggest that intake of fruits or vegetables in infancy or pregnancy influences the risk of allergic sensitisation later in life.**

Table 8 Studies investigating the association between fruit and vegetable and allergic sensitization which were not eligible for meta-analysis

| **First Author and year of publication** | **Design** | **Outcome** | **Age** | | **N/n** | | **Dietary exposure and comparison**  **level** | **Measure of association** | | **Effect (bold indicates p-value <0.05)** | |  |
| --- | --- | --- | --- | --- | --- | --- | --- | --- | --- | --- | --- | --- |
|  |  |  |  | |  | | **INFANT INTAKE** |  | |  | |  |
| Strassburger, 2010 | PC | Aero | 3.5 | | 321 | | Time of introduction of salty pureed food [mostly vegetables] / ≥4 months vs. <4 months | OR | | 1.11 (0.65-1.89) | |  |
| Nwaru, 2010 | PC | sIgE-aero | 5 | | 994 | | **Introduction of vegetables:**  Cabbage/. 4.1-5.2 months vs <4.1 months | OR | | 1.00 (0.64-1.57) | |  |
|  |  |  |  |  |  |  | Fruits and berries/ 3.46-4 months vs < 3.46 months | OR | | 1.09 (0.70-1.69) | |  |
|  |  |  |  |  |  |  | Fruits and berries/ >4 months vs < 3.46 months | OR | | 1.54 (0.97-2.45) | |  |
|  |  |  |  |  |  |  | Carrots/ 3. 5-4 months vs <3.5 months | OR | | 1.02 (0.66-1.57) | |  |
|  |  |  |  |  |  |  | Potatoes/ 3.1-4 months vs <3.1 months | OR | | 1.30 (0.87-1.95) | |  |
|  |  |  |  |  |  |  | Carrots/ >4 months vs <3.5 months | OR | | 1.37 (0.86-2.18) | |  |
|  |  |  |  |  |  |  | Potatoes/>4 months vs <3.1 months | OR | | **1.93 (1.20-3.11)** | |  |
| Hesselmar, 2010 | PC | sIgE-any | 1.5 | | 169 | | Fruits/age in months when foods were introduced in months (continuous) | OR | | NS | |  |
| Nwaru, 2010 | PC | sIgE cow’s milk | 5 | | 994 | | **Introduction during first month vs later:**  Cabbage, fruits and berries, Carrots, potatoes | OR | | NS | |  |
| Nwaru, 2010 | PC | sIgE to egg | 5 | | 994 | | **Introduction to infant diet in the first month vs. 2nd and 3rd months**  Cabbage, fruits and berries, carrots, potatoes | OR | | NS | |  |
| Nwaru, 2010 | PC | sIgE to egg | 5 | | 994 | | **Introduction to infant diet in the first month vs. 2^nd^ and 3^rd^ months**  Cabbage/ 4.1-5.2 months vs <4.1 months | OR | | 1.00 (0.61-1.65) | |  |
|  |  |  |  |  |  |  | Fruits and berries / 3.5-4 months vs < 3.5 months | OR | | 1.30 (0.78-2.16) | |  |
|  |  |  |  |  |  |  | Fruits and berries/ >4 months vs < 3.5 months | OR | | **1.70 (1.00-2.90)** | |  |
|  |  |  |  |  |  |  | Carrots/ 3.5-4 months vs <3.5 months | OR | | 1.15 (0.70-1.90) | |  |
|  |  |  |  |  |  |  | Potatoes/ 3.1-4 months vs <3.1 months | OR | | 1.50 (0.93-2.42) | |  |
|  |  |  |  |  |  |  | Carrots/ >4 months vs <3.5 months | OR | | 1.66 (0.97-2.83) | |  |
|  |  |  |  |  |  |  | Potatoes/>4 months vs <3.1 months | OR | | **2.56 (1.49-4.39)** | |  |
| Zutavern, 2008 | PC | sIgE to food | 6 | | 2073 | | Fruits or vegetables introduced into infant’s diet by 4 months vs. 4-6 months | OR | | NS | | |
| Andreasyan, 2007 | PC | SPT any | 9 | 498 | | | Fruit juice (orange)/Introduction at 1 month. / >1 month vs. ≤1 months | RR | | 1.16 (0.58-2.34) | | |
|  |  |  |  | |  | | **MATERNAL INTAKE** |  | |  | |  |
|  |  |  |  | |  | | **Consumption by the mother during the 8^th^ month of pregnancy: (continuous):**  Nuts and pulses | OR | | 1.10 (0.95-1.28) | |  |
|  |  | sIgE-aero | 5 | | 931 | | Root vegetables | OR | | 0.92 (0.63-1.35) | |  |
|  |  |  |  | |  | | Citrus fruit | OR | | 1.14 (1.04-1.25) | |  |
| Nwaru, 2010 | PC |  |  | |  | | Fruit juice/syrup | OR | | 0.98 (0.91-1.06) | |  |
|  |  |  |  | |  | | Apple | OR | | 0.97 (0.83-1.13) | |  |
|  |  | sIgE food | 5 | | 931 | | Berries | OR | | 1.07 (0.92-1.25) | |  |
|  |  |  |  | |  | | Citrus fruit | OR | | 1.00 (0.92-1.09) | |  |
|  |  |  |  | |  | | **Consumption by the mother during the 8th month of pregnancy: (continuous):**  Fruit juice/syrup | OR | | 0.99 (0.91-1.08) | |  |
| Nwaru, 2010 | PC | sIgE food | 5 | | 931 | | Nuts | OR | | 1.11 (0.93-1.33) | |  |
|  |  |  |  | |  | | Root vegetables | OR | | 0.73 (0.43-1.23) | |  |
| Sausenthaler, 2007 | PC | sIgE aero | 2 | | 2102 | | **Maternal consumption during the last 4 weeks of pregnancy /High intake vs low:**  Bananas | OR | | 1.10 (0.63-1.93) | |  |
|  |  |  |  |  |  |  | Strawberries | OR | | 1.46 (0.86-2.47) | |  |
|  |  |  |  |  |  |  | Citrus fruits | OR | | **1.72 (1.01-2.92)** | |  |
|  |  |  |  | |  | | Salad | OR | | 0.92 (0.52-1.62) | |  |
|  |  |  |  |  |  |  | Fruit juice | OR | | 0.78 (0.47-1.30) | |  |
|  |  |  |  |  |  |  | Spinach | OR | | 1.18 (0.73-1.91) | |  |
|  |  |  |  |  |  |  | Celery | OR | | 1.39 (0.75-2.58) | |  |
|  |  |  |  |  |  |  | Sweet pepper | OR | | **2.16 (1.20-3.90)** | |  |
|  |  |  |  |  |  |  | Nuts | OR | | 0.84 (0.46-1.53) | |  |
|  |  |  |  |  |  |  | **Maternal consumption during the last 4 weeks of pregnancy/ High maternal intake vs low**  Carrots | OR | | 0.77 (0.46-1.28) | |  |
|  |  |  |  |  |  |  | Seeds | OR | | 0.75 (0.42-1.33) | |  |
|  |  |  |  |  |  |  | Tomatoes | OR | | 1.05 (0.62-1.77) | |  |
| Sausenthaler, 2007 | PC | sIgE any | 2 | | 2103 | | Bananas | OR | | 1.08 (0.75-1.55) | |  |
|  |  |  |  |  |  |  | Strawberries | OR | | 1.06 (0.74-1.51) | |  |
|  |  |  |  |  |  |  | Citrus fruits | OR | | **1.82 (1.29-2.56)** | |  |
|  |  |  |  |  |  |  | Fruit juice | OR | | 1.03 (0.73-1.46) | |  |
|  |  |  |  |  |  |  | Spinach | OR | | 0.97 (0.71-1.32) | |  |
|  |  |  |  |  |  |  | Salad | OR | | 1.09 (0.76-1.57) | |  |
|  |  |  |  |  |  |  | Sweet pepper | OR | | **1.45 (1.02-2.06)** | |  |
|  |  |  |  |  |  |  | **Maternal consumption during the last 4 weeks of pregnancy/ High maternal intake vs low**  Celery | OR | | **1.61 (1.08-2.41)** | |  |
|  |  |  |  |  |  |  | Nuts | OR | | 0.92 (0.63-1.34) | |  |
|  |  |  |  |  |  |  | Carrots | OR | | 0.85 (0.61-1.18) | |  |
|  |  |  |  |  |  |  | Seeds | OR | | 0.78 (0.53-1.14) | |  |
| Sausenthaler, 2007 (21) | PC | sIgE any | 2 | | 2103 | | Tomatoes | OR | | 0.81 (0.57-1.16) | |  |
| Nwaru, 2011 | PC | sIgE to cow’s milk | 5 | | 652 | | **Maternal intake during breastfeeding/ g/day increase in total intake during 2^nd^ month lactation:**  Berries | OR | | 1.12 (0.86-1.46) | |  |
|  |  |  |  |  |  |  | Nuts and pulses | OR | | 1.02 (0.77-1.36) | |  |
|  |  |  |  |  |  |  | Potatoes | OR | | 0.77 (0.54-1.10) | |  |
|  |  | sIgE to egg | 5 | | 652 | | Butter/butter | OR | | 1.05 (0.74-1.50) | |  |
| Nwaru, 2010 | PC | Allergic Sensitization | 5 | | 931 | | **Maternal intake during the 8^th^ month of pregnancy (mean daily consumption [grams]):**  Malaceous fruits | OR | 0.97 (0.83-1.13) | |  | |
|  |  |  |  |  |  |  | Berries | OR | 1.07 (0.92-1.25) | |  | |
|  |  |  |  |  |  |  | Citrus fruits | OR | 1.00 (0.92-1.09) | |  | |
|  |  |  |  |  |  |  | Fruit juice | OR | 0.99 (0.91-1.08) | |  | |
|  |  |  |  | |  | | Nuts and pulses | OR | 1.11 (0.93-1.33) | |  | |
|  |  |  |  |  |  |  | Potatoes | OR | 0.73 (0.43-1.23) | |  | |
|  |  |  |  | |  | | Fruits | OR | 0.97 (0.76-1.23) | |  | |
|  |  |  |  | |  | | Vegetables and roots | OR | 1.28 (0.82-1.99) | |  | |
| Sausenthaler, 2007 | PC | sIgE food | 2 | | 2110 | | **Maternal consumption during the last 4 weeks of pregnancy/ high vs low maternal intake:**  Spinach | OR | 0.82 (0.57-1.17) | |  | |
|  |  |  |  |  |  |  | Salad | OR | 1.14 (0.76-1.72) | |  | |
|  |  |  |  |  |  |  | Sweet pepper | OR | 1.16 (0.80-1.69) | |  | |
|  |  |  |  |  |  |  | Celery | OR | **1.85 (1.18-2.89)** | |  | |
|  |  |  |  |  |  |  | Nuts | OR | 1.10 (0.72-1.67) | |  | |
|  |  |  |  | | |  | Carrots | OR | 1.02 (0.70-1.49) | |  | |
|  |  |  |  |  |  |  | Seeds | OR | 0.72 (0.46-1.12) | |  | |
|  |  |  |  |  |  |  | **Maternal consumption during the last 4 weeks of pregnancy/ high vs low maternal intake:**  Tomatoes | OR | 0.74 (0.49-1.11) | |  | |
|  |  |  |  | | |  | Bananas | OR | 1.14 (0.76-1.72) | |  | |
| Sausenthaler, 2007 | PC | sIgE to food | 2 | | | 2110 | Strawberries | OR | 0.90 (0.60-1.34) | |  | |
|  |  |  |  | | |  | Citrus fruits | OR | **1.73 (1.18-2.53)** | |  | |
|  |  |  |  | | |  | Fruit juice | OR | 1.12 (0.76-1.65) | |  | |
| Kemp, 2011 | PC | sIgE to peanut | 16 | 310 | | | **Maternal peanut avoidance /Intake in 3^rd^ trimester vs./ never in a month:**  Ever eaten vs never (no family history of asthma) | OR | **0.18 (0.04-0.78)** | |  | |
|  |  |  |  |  |  |  | Ever eaten vs never (parental history of asthma) | OR | NS | |  | |
| Sicherer, 2010 | PC | sIgE to peanut | 1 | 503 | | | **Maternal intake during pregnancy:**  Peanut/ ≥2 times per week vs <2 times per week | OR | **2.93 (1.76-4.88)** | |  | |
| Chatzi, 2008 | PC | SPT-aero | 6.5 | 468 | | | **Maternal intake during pregnancy:**  Fruits >14 servings per week vs ≤14 servings per week | OR | NS | |  | |
|  |  |  |  |  |  |  | Legumes >1 servings per week vs ≤1 servings per week | OR | NS | |  | |
|  |  |  |  |  |  |  | Nuts >1servings per week vs ≤1 servings per week | OR | NS | |  | |
| Willers, 2007 | PC | SPT any | 5 | 1212 | | | **Maternal intake during pregnancy (high vs low intakes):**  Fruit | OR | NS | |  | |
|  |  |  |  |  |  |  | Citrus fruits | OR | NS | |  | |
|  |  |  |  |  |  |  | Fruit juice | OR | NS | |  | |
|  |  |  |  |  |  |  | Vegetables | OR | NS | |  | |
|  |  |  |  |  |  |  | Green leafy vegetables | OR | NS | |  | |
| Venter, 2009 | PC | SPT to peanut | 2 | 937 | | | Peanut avoidance during pregnancy (yes/no [unclear]) |  | NS | |  | |
| Willers, 2008 | PC | Total-IgE  sIgE to food  sIge aero | 8 | 1657 | | | Nut consumption during pregnancy (frequency in the last month of reporting data) | OR | NS | |  | |

# 8. Infant or maternal dietary intake of fruits and vegetables and risk of autoimmune diseases

# 8.1.  Type 1 diabetes mellitus (TIDM)

Nine observational studies (7 prospective cohorts and 2 case-control studies) investigated the association between dietary intake of fruits or vegetables during infancy or pregnancy and risk of type I diabetes mellitus. Overall, studies were considered to be of low or unclear risk of bias, with one study having high risk of bias due to lack of control for confounding (Figure 18).

Figure 18 Risk of Bias in studies of fruit and vegetable intake and type I diabetes mellitus

Two prospective cohorts and a case-control study which examined the association between fruit introduction and risk of T1DM had comparable data suitable to be pooled in a meta-analysis. The pooled effect of the prospective studies showed a lower risk of TIDM in children who started eating fruits between 0-4 months of age compared with those who started later. This meta-analysis had no heterogeneity (I^2^=0%). The effect size was attenuated and lost statistical significance when the three studies were pooled, and the heterogeneity increased slightly but remained low (I^2^=25.8%) (Figure 19).

Figure 19 Infant fruit introduction in months 0 to 4 and risk of type I diabetes mellitus in children

Table 9 summarises the findings of studies that could not be meta-analysed. The study of Virtanen found that early introduction of root vegetables (including potatoes), was associated with increased risk of T1DM later in childhood. These findings were not supported by other studies. The finding in the meta-analysis of 2 prospective studies that early fruit introduction is associated with reduced TIDM was also not supported by other studies which could not be included in the same meta-analysis.

**Overall, we found no consistent evidence that infant or maternal intake of other fruits or vegetables influences the risk of T1DM.**

Table 9 Studies investigating the association between fruit and vegetable and type 1 diabetes mellitus which were not eligible for meta-analysis

| **First Author and year of publication** | **Design** | **Outcome** | **Age** | **N/n** | **Dietary exposure and comparison level** | **Measure of association** | **Effect (bold indicates p-value <0.05)** |
| --- | --- | --- | --- | --- | --- | --- | --- |
|  |  |  |  |  | **INFANT INTAKE** |  |  |
| Visalli, 2003 | CC | TIDM | 12 | 900/150 | Vegetables/age at introduction / ≥3 months vs. <3 months | OR | 1.32 (0.16-10.86) |
| Strotmeyer, 2004 | CC | TIDM | 9.7 | 688/247 | **Fruit introduction first 12months**  >7.0-12 months vs. 7-12 months | OR | NS |
|  |  |  |  |  | >4–6 months vs. 4-6 months | OR | NS |
|  |  |  |  |  | >1–3 months vs. 1-3months months | OR | NS |
|  |  |  |  |  | **Vegetable introduction first 12 months**  5-7 months vs. <5-7 months | OR | **0.66 (0.45-0.98)** |
|  |  |  |  |  | >7.0-12 months vs. 7-12 months | OR | NS |
| Virtanen, 2006 | PC | TIDM | <10 | 2435/54 | Fruits and berries introduction / 0-3.5 months vs. 4+ months | HR | **0.50 (0.25-0.97)** |
|  |  |  |  | 2435/53 | Root vegetable introduction / 0-3 months vs. 4+ months | HR | 0.96 (0.53-1.75) |
| Virtanen, 2011 | PC | TIDM | <10 | 5619/237 | Fruits and berries introduction / 3.5-4 months vs. 4+ months | HR | 0.75 (0.54-1.04) |
|  |  |  |  |  | Root vegetables introduction / 3-4 months vs. 4+ months | HR | **1.79 (1.22-2.62)** |
|  |  |  |  |  | Root vegetables introduction / 0-3 months vs. 4+ months | HR | **1.75 (1.11-2.75**) |
| Brekke, 2010 | PC | TIDM | 5 | 4005/191 | Infant vegetable intake (often vs. rare) | OR | 0.77 (0.41-1.44) |
| Savilahti, 2009 | PC | T1DM |  |  | Infant vegetable introduction in months 0-4 | OR | 1.73 (0.84-3.58) |
| Norris, 1996 | PC | TIDM |  |  | Fruit and vegetable introduction months 5-7 vs. <5-7 months | OR | 0.73 (0.28-1.95) |
|  |  |  |  |  | **MATERNAL INTAKE** |  |  |
| Brekke, 2010 | PC | TIDM | 5 | 4005/191 | Maternal intake of potatoes and root vegetables during pregnancy/ 3-5 times/week vs. 1-2 times/week | OR | 0.75 (0.44-1.29) |
| Virtanen, 2011 | PC | TIDM | <10 | 3730/165 | **Maternal intake during pregnancy / Change in risk per 2-fold increase in energy-adjusted consumption of the specific food:**  Potatoes and root vegetables | HR | 1.20 (0.92-1.56) |
|  |  |  |  |  | Fruit | HR | 1.02 (0.88-1.18) |
|  |  |  |  |  | Vegetables | HR | 0.92 (0.74-1.14) |
| Lamb, 2008 | PC | TIDM | 15 | 642/27 | **Food intake during last trimester of pregnancy (continuous; number servings per month):**  Fruits | HR | 0.86 (0.52-1.42) |
|  |  |  |  |  | Potatoes | HR | **0.49 (0.28-0.87)** |
|  |  |  |  |  | Vegetables | HR | 0.61 (0.34-1.09) |
|  |  |  |  |  | Other root vegetables | HR | 1.04 (0.72-1.51) |

# 8.2.  Infant or maternal dietary intake of fruit and vegetable and risk of inflammatory bowel disease (IBD)

One case-control study was identified, which investigated the risk of IBD according to the timing of vegetable introduction. The study of **Baron (2005)** included as cases, children aged <17 who were diagnosed with Crohn’s disease and who were matched to healthy controls. The study was considered to have unclear overall bias as there was no clear description of the assessment of outcomes (Figure 20). There was difference in the risk of IBD according to the months of vegetable introduction (Table 10).

Figure 20 Risk of Bias in studies of fruit and vegetable intake and IBD

Table 10 Studies investigating the association between fruit and vegetable (dietary exposure) and IBD (outcome) which were not eligible for meta-analysis

| **First Author and year of publication** | **Design** | **Outcome** | **Age** | **N/n** | **Dietary exposure and comparison level** | **Measure of association** | **Effect** |  |
| --- | --- | --- | --- | --- | --- | --- | --- | --- |
| Baron, 2005 | CC | IBD-CR | <17 | 444/222 | Vegetables introduction in months (continuous) | OR | NS |  |
|  |  | IBD-UC |  |  | Vegetable introduction in months (continuous) | OR | NS |  |

# Conclusions

Overall, this report on dietary intake of fruits and vegetables and risk of allergic and autoimmune diseases shows that although there are a significant number of well designed (low bias) observational studies, the evidence base for these foods is hindered by the lack of comparable data between studies. The few studies that could be meta-analysed contributed just a small part of the overall picture of no consistent evidence that fruit or vegetable intake or introduction during infancy, pregnancy or lactation are associated with allergic or autoimmune outcomes.

From the point of view of epidemiological research, these findings highlight the need to improve the homogenisation of epidemiological tools to assess dietary exposure in population-based studies.

**Overall we found no evidence to suggest that infant or maternal intake of fruits or vegetables influences the risk of allergic or autoimmune diseases.**

# References

1.	(NICE) NIfCaHE. The guidelines manual methodology checklist. 2012.

2.	Martindale S, McNeill G, Devereux G, Campbell D, Russell G, Seaton A. Antioxidant intake in pregnancy in relation to wheeze and eczema in the first two years of life. American Journal of Respiratory & Critical Care Medicine. 2005;171(2):121-8.

3.	Willers SM, Devereux G, Craig LC, McNeill G, Wijga AH, Abou El-Magd W, et al. Maternal food consumption during pregnancy and asthma, respiratory and atopic symptoms in 5-year-old children. Thorax. 2007;62(9):773-9.

4.	Hesselmar B, Saalman R, Rudin A, Adlerberth I, Wold A. Early fish introduction is associated with less eczema, but not sensitization, in infants. Acta Paediatrica. 2010;99(12):1861-7.

5.	Lack G, Fox D, Northstone K, Golding J, Avon Longitudinal Study of P, Children Study T. Factors associated with the development of peanut allergy in childhood. New England Journal of Medicine. 2003;348(11):977-85.

6.	Binkley KE, Leaver C, Ray JG. Antenatal risk factors for peanut allergy in children. Allergy, Asthma, & Clinical Immunology : Official Journal of the Canadian Society of Allergy & Clinical Immunology. 2011;7:17.

7.	Fergusson DM, Horwood LJ, Shannon FT. Early solid feeding and recurrent childhood eczema: a 10-year longitudinal study. Pediatrics. 1990;86(4):541-6.

8.	Maslova E, Granstrom C, Hansen S, Petersen SB, Strom M, Willett WC, et al. Peanut and tree nut consumption during pregnancy and allergic disease in children-should mothers decrease their intake? Longitudinal evidence from the Danish National Birth Cohort. Journal of Allergy & Clinical Immunology. 2012;130(3):724-32.

9.	DesRoches A, Infante-Rivard C, Paradis L, Paradis J, Haddad E. Peanut allergy: is maternal transmission of antigens during pregnancy and breastfeeding a risk factor? Journal of Investigational Allergology & Clinical Immunology. 2010;20(4):289-94.

10.	Maijaliisa E, Bright IN, Minna K, Kronberg-Kippila C, Jorma I, Olli S, et al. Maternal food consumption during pregnancy and risk of asthma and allergic outcomes in the offspring. Annals of Nutrition and Metabolism. 2011;58:369.

11.	Nwaru BI, Ahonen S, Kaila M, Erkkola M, Haapala AM, Kronberg-Kippila C, et al. Maternal diet during pregnancy and allergic sensitization in the offspring by 5 yrs of age: a prospective cohort study. Pediatric Allergy & Immunology. 2010;21(1 Pt 1):29-37.

12.	Nwaru BI, Erkkola M, Ahonen S, Kaila M, Lumia M, Prasad M, et al. Maternal diet during lactation and allergic sensitization in the offspring at age of 5. Pediatric Allergy & Immunology. 2011;22(3):334-41.

13.	Nwaru BI, Takkinen HM, Niemela O, Kaila M, Erkkola M, Ahonen S, et al. Timing of infant feeding in relation to childhood asthma and allergic diseases. Journal of Allergy and Clinical Immunology. 2013;131(1):78-86.

14.	Erkkola M, Nwaru BI, Kaila M, Kronberg-Kippila C, Ilonen J, Simell O, et al. Risk of asthma and allergic outcomes in the offspring in relation to maternal food consumption during pregnancy: a Finnish birth cohort study. Pediatric Allergy & Immunology. 2012;23(2):186-94.

15.	Nwaru BI, Erkkola M, Ahonen S, Kaila M, Haapala AM, Kronberg-Kippila C, et al. Age at the introduction of solid foods during the first year and allergic sensitization at age 5 years. Pediatrics. 2010;125(1):50-9.

16.	Fox AT, Sasieni P, du Toit G, Syed H, Lack G. Household peanut consumption as a risk factor for the development of peanut allergy. Journal of Allergy and Clinical Immunology. 2009;123(2):417-23.

17.	Venter C, Pereira B, Voigt K, Grundy J, Clayton CB, Higgins B, et al. Factors associated with maternal dietary intake, feeding and weaning practices, and the development of food hypersensitivity in the infant. Pediatric Allergy and Immunology. 2009;20(4):320-7.

18.	Dean T, Venter C, Pereira B, Grundy J, Clayton CB, Higgins B. Government advice on peanut avoidance during pregnancy--is it followed correctly and what is the impact on sensitization? Journal of Human Nutrition & Dietetics. 2007;20(2):95-9.

19.	Fitzsimon N, Fallon U, O'Mahony D, Loftus BG, Bury G, Murphy AW, et al. Mothers' dietary patterns during pregnancy and risk of asthma symptoms in children at 3 years. Irish Medical Journal. 2007;100(8):suppl 27-32.

20.	Zutavern A, Brockow I, Schaaf B, Bolte G, von Berg A, Diez U, et al. Timing of solid food introduction in relation to atopic dermatitis and atopic sensitization: results from a prospective birth cohort study. Pediatrics. 2006;117(2):401-11.

21.	Zutavern A, Brockow I, Schaaf B, von Berg A, Diez U, Borte M, et al. Timing of solid food introduction in relation to eczema, asthma, allergic rhinitis, and food and inhalant sensitization at the age of 6 years: results from the prospective birth cohort study LISA. Pediatrics. 2008;121(1):e44-52.

22.	Sausenthaler S, Koletzko S, Schaaf B, Lehmann I, Borte M, Herbarth O, et al. Maternal diet during pregnancy in relation to eczema and allergic sensitization in the offspring at 2 y of age. American Journal of Clinical Nutrition. 2007;85(2):530-7.

23.	Marini A, Agosti M, Motta G, Mosca F. Effects of a dietary and environmental prevention programme on the incidence of allergic symptoms in high atopic risk infants: three years' follow-up. Acta Paediatrica Supplement. 1996;414:1-21.

24.	Chatzi L, Torrent M, Romieu I, Garcia-Esteban R, Ferrer C, Vioque J, et al. Mediterranean diet in pregnancy is protective for wheeze and atopy in childhood. Thorax. 2008;63(6):507-13.

25.	Miskelly FG, Burr ML, Vaughan-Williams E, Fehily AM, Butland BK, Merret TG. Infant feeding and allergy. Archives of Disease in Childhood. 1988;63(4):388-93.

26.	Morgan J, Williams P, Norris F, Williams CM, Larkin M, Hampton S. Eczema and early solid feeding in preterm infants. Archives of Disease in Childhood. 2004;89(4):309-14.

27.	Miyake Y, Sasaki S, Tanaka K, Hirota Y. Consumption of vegetables, fruit, and antioxidants during pregnancy and wheeze and eczema in infants. Allergy. 2010;65(6):758-65.

28.	Oien T, Storro O, Johnsen R. Do early intake of fish and fish oil protect against eczema and doctor-diagnosed asthma at 2 years of age? A cohort study. Journal of Epidemiology & Community Health. 2010;64(2):124-9.

29.	Roduit C, Frei R, Loss G, Buchele G, Weber J, Depner M, et al. Development of atopic dermatitis according to age of onset and association with early-life exposures. Journal of Allergy & Clinical Immunology. 2012;130(1):130-6.e5.

30.	Willers SM, Wijga AH, Brunekreef B, Kerkhof M, Gerritsen J, Hoekstra MO, et al. Maternal food consumption during pregnancy and the longitudinal development of childhood asthma. American Journal of Respiratory & Critical Care Medicine. 2008;178(2):124-31.

31.	Sariachvili M, Droste J, Dom S, Wieringa M, Hagendorens M, Stevens W, et al. Early exposure to solid foods and the development of eczema in children up to 4 years of age. Pediatric Allergy & Immunology. 2010;21(1 Pt 1):74-81.

32.	Litonjua AA, Rifas-Shiman SL, Ly NP, Tantisira KG, Rich-Edwards JW, Camargo CA, Jr., et al. Maternal antioxidant intake in pregnancy and wheezing illnesses in children at 2 y of age. American Journal of Clinical Nutrition. 2006;84(4):903-11.

33.	Dunlop AL, Reichrtova E, Palcovicova L, Ciznar P, Adamcakova-Dodd A, Smith SJ, et al. Environmental and dietary risk factors for infantile atopic eczema among a Slovak birth cohort. Pediatric Allergy & Immunology. 2006;17(2):103-11.

34.	Strassburger SZ, Vitolo MR, Bortolini GA, Pitrez PM, Jones MH, Stein RT. Nutritional errors in the first months of life and their association with asthma and atopy in preschool children. Jornal de Pediatria. 2010;86(5):391-9.

35.	Kemp AS, Ponsonby AL, Dwyer T, Cochrane JA, Pezic A, Jones G. Maternal antenatal peanut consumption and peanut and rye sensitization in the offspring at adolescence. Clinical & Experimental Allergy. 2011;41(2):224-31.

36.	Andreasyan K, Ponsonby AL, Dwyer T, Dear K, Cochrane J. Infant feeding and childhood atopy: does early introduction of non-milk fluids matter? Pediatric Allergy & Immunology. 2007;18(3):250-7.

37.	Sicherer SH, Wood RA, Stablein D, Lindblad R, Burks AW, Liu AH, et al. Maternal consumption of peanut during pregnancy is associated with peanut sensitization in atopic infants. Journal of Allergy & Clinical Immunology. 2010;126(6):1191-7.

38.	Zutavern A, von Mutius E, Harris J, Mills P, Moffatt S, White C, et al. The introduction of solids in relation to asthma and eczema. Archives of Disease in Childhood. 2004;89(4):303-8.

39.	Brekke HK, Ludvigsson J. Daily vegetable intake during pregnancy negatively associated to islet autoimmunity in the offspring--the ABIS study. Pediatric Diabetes. 2010;11(4):244-50.

40.	Baron S, Turck D, Leplat C, Merle V, Gower-Rousseau C, Marti R, et al. Environmental risk factors in paediatric inflammatory bowel diseases: a population based case control study. Gut. 2005;54(3):357-63.

41.	Norris JM, Beaty B, Klingensmith G, Yu L, Hoffman M, Chase HP, et al. Lack of association between early exposure to cow's milk protein and beta-cell autoimmunity. Diabetes Autoimmunity Study in the Young (DAISY). Jama. 1996;276(8):609-14.

42.	Frederiksen B, Kroehl M, Lamb M, Seifert J, Barriga K, Rewers M, et al. Infant exposures and development of type 1 diabetes-the diabetes autoimmunity study in the young (DAISY). Diabetes. 2012;61:A352.

43.	Lamb MM, Myers MA, Barriga K, Zimmet PZ, Rewers M, Norris JM. Maternal diet during pregnancy and islet autoimmunity in offspring. Pediatric Diabetes. 2008;9(2):135-41.

44.	Visalli N, Sebastiani L, Adorisio E, Conte A, De Cicco AL, D'Elia R, et al. Environmental risk factors for type 1 diabetes in Rome and province. Archives of Disease in Childhood. 2003;88(8):695-8.

45.	Savilahti E, Saarinen KM. Early infant feeding and type 1 diabetes. European Journal of Nutrition. 2009;48(4):243-9.

46.	Strotmeyer ES, Yang Z, LaPorte RE, Chang YF, Steenkiste AR, Pietropaolo M, et al. Infant diet and type 1 diabetes in China. Diabetes Research & Clinical Practice. 2004;65(3):283-92.

47.	Virtanen SM, Takkinen HM, Nevalainen J, Kronberg-Kippila C, Salmenhaara M, Uusitalo L, et al. Early introduction of root vegetables in infancy associated with advanced beta-cell autoimmunity in young children with human leukocyte antigen-conferred susceptibility to Type 1 diabetes. Diabetic Medicine. 2011;28(8):965-71.

48.	Virtanen SM, Kenward MG, Erkkola M, Kautiainen S, Kronberg-Kippila C, Hakulinen T, et al. Age at introduction of new foods and advanced beta cell autoimmunity in young children with HLA-conferred susceptibility to type 1 diabetes. Diabetologia. 2006;49(7):1512-21.
