# Supplementary material for: Diet during pregnancy and infancy and risk of allergic or autoimmune disease: A systematic review and meta-analysis
Source: PLoS Med. 2018 Feb 28;15(2):e1002507. doi: 10.1371/journal.pmed.1002507 (PMC5830033; doi:10.1371/journal.pmed.1002507)
Supplement: S2 Data — (ZIP) [file pmed.1002507.s007.zip › Review_C_reports/OTHER FOODS.doc]

**OTHER DIETARY EXPOSURES IN INFANTS AND MOTHERS, AND RISK OF ALLERGIC AND AUTOIMMUNE DISEASES**

Vanessa Garcia-Larsen^1^, Robert J Boyle^2^, Despo Ierodiakonou^1,3^, Jo Leonardi-Bee^4^, Tim Reeves^5^, Jennifer Chivinge^6^, Zoe Robinson^6^, Natalie Geoghegan^6^, Katharine Jarrold^6^, Andrew Logan^6^, Annabel Groome^6^ , Evangelia Andreou^7^, Nara Tagiyeva-Milne^8^, Ulugbek Nurmatov^9^, Sergio Cunha^7^

1 Post-Doctoral Research Associate, Respiratory Epidemiology, Occupational Medicine, and Public Health, National Heart and Lung Institute Imperial College London

2 Clinical Senior Lecturer, Department of Paediatrics, Imperial College London

3 Post-Doctoral Research Associate, Department of Paediatrics, Imperial College London

4 Associate Professor of Community Health Sciences, University of Nottingham

5 Research Support Librarian, Faculty of Medicine, Imperial College London

6 Undergraduate medical students, Imperial College London

7 Research Associate, Imperial Consultants

8 Research Fellow, University of Aberdeen

9 Research Fellow, University of Edinburgh

**Contents**

List of figures	4

List of tables	5

1.	Executive summary of studies and findings: Maternal and infant intake of other foods and risk of allergic and autoimmune diseases – Observational studies	6

1.1.	Context of this report	6

1.2.	Studies and outcomes identified	6

1.3.	Outcomes evaluated	6

1.4.	Dietary exposures studied	7

1.5.	Dietary assessment and comparators	7

1.6.	Presentation of findings	7

1.7.	Risk of bias assessment	8

1.8.	Key findings	9

1.9.	Conclusions	10

2.	Other foods and risk of atopic dermatitis	38

2.1.	Overall risk of bias between other foods and atopic dermatitis	38

2.2.	Introduction of meat into infant’s diet and risk of atopic dermatitis	38

2.3.	Dairy intake (maternal or infant) and risk of atopic dermatitis	39

2.4.	Studies not eligible for meta-analysis	39

3.	Other foods and risk of food allergy	51

3.1.	Overall risk of bias between other foods and food allergy	51

4.	Other foods and lung function	54

4.1.	Risk of Bias in studies of other food exposures and lung function	54

5.	Other foods and risk of wheeze	57

5.1.	Risk of bias in studies investigating the association between other foods and risk of wheeze	57

5.2.	Studies investigating the association between wheeze and other foods which were not eligible for meta-analysis	58

6.	Other foods and risk of rhino-conjunctivitis	70

6.1.	Overall risk of bias between other foods and rhino-conjunctivitis	70

6.2.	Studies not meta-analysed that examined risk of rhino-conjunctivitis and intake of various foods or nutrients	70

7.	Other foods and risk of allergic sensitization	75

7.1.	Overall risk of bias between other foods and allergic sensitisation	75

7.2.	Introduction of meat and risk of aero-allergen sensitisation	75

7.3.	Studies that were not meta-analysed and that investigated the association between other foods and risk of allergic sensitisation	76

8.	Other foods and risk of type 1 diabetes mellitus	84

8.1.	Overall risk of bias between other foods and type 1 diabetes mellitus	84

8.1. Studies not meta-analysed that explored the association between food intake and T1DM	84

9.	Other foods and risk of juvenile idiopathic arthritis (JIA)	89

9.1.	Overall risk of bias between other foods and juvenile idiopathic arthritis	89

10.	Other foods and risk of inflammatory bowel disease	91

10.1.	Overall risk of bias in studies on other foods and inflammatory bowel disease	91

Conclusions	93

References	94

# List of figures

Figure 1 Risk of Bias in studies of other food intake and atopic dermatitis	38

Figure 2 Introduction of meat in months 0 to 4 and risk of atopic dermatitis in children aged 0 to 4 years old	39

Figure 3 Maternal intake of yoghurt (highest vs. lowest) and risk of atopic dermatitis in children aged 0 to 4 years old	39

Figure 4 Risk of Bias in studies of other foods intake and food allergy	51

Figure 5 Risk of bias in studies of other foods intake and lung function	54

Figure 6 Risk of Bias in studies of other foods intake and wheeze	57

Figure 7 Risk of Bias in studies of other foods intake and rhino-conjunctivitis	70

Figure 8 Risk of Bias in studies of other foods intake and allergic sensitisation	75

Figure 9 Introduction of meat in months 5 to 7 and risk of sensitisation to aero-allergens (odds ratio) in children	76

Figure 10 Risk of Bias in studies of other foods intake and type 1 diabetes mellitus	84

Figure 11 Risk of Bias in studies of other foods intake and juvenile idiopathic arthritis	89

Figure 12 Risk of Bias in studies on other foods intake and inflammatory bowel disease	91

# List of tables

Table 1 Characteristics of included studies on allergic outcomes and infant or maternal intake of other foods	11

Table 2 Characteristics of included studies on autoimmune diseases and infant or maternal intake	27

Table 3 Dietary instrument used to ascertain infant or maternal intake of other foods	34

Table 4 Studies investigating the association between other foods and atopic dermatitis which were not eligible for meta-analysis	41

Table 5 Studies investigating the association between other foods and food allergy which were not eligible for meta-analysis	53

Table 6 Studies investigating the association between other foods (dietary exposure) and lung function (outcome) which were not eligible for meta-analysis	55

Table 7 Studies investigating the association between other foods and wheeze which were not eligible for meta-analysis	60

Table 8 Studies investigating the association between other foods and rhino-conjunctivitis which were not eligible for meta-analysis	72

Table 9 Studies investigating the association between other foods and allergic sensitization which were not eligible for meta-analysis	78

Table 10 Studies investigating the association between other foods and type 1 diabetes mellitus which were not eligible for meta-analysis	86

Table 11 Studies investigating the association between other foods and juvenile idiopathic arthritis which were not eligible for meta-analysis	90

Table 12 Studies investigating the association between other food and irritable bowel disease which were not eligible for meta-analysis	92

# 1. Executive summary of studies and findings: Maternal and infant intake of other foods and risk of allergic and autoimmune diseases – Observational studies

# 1.1.  Context of this report

This report is one of a series of systematic review reports included in Review C Part II, which focusses on evidence on dietary exposures during pregnancy, lactation and or infancy for reducing risk of allergic or autoimmune outcomes. This report focuses on dietary exposures that were considered inappropriate for inclusion in the themes of the other three reports, namely: ‘Fruits and vegetables’, ‘Vitamins and Minerals’, or ‘Fats’. The report includes food introduction in the infant’s diet if these foods are not considered as allergenic, in which case they will be added to the corresponding report in Review B (Introduction of allergenic food and risk of allergic or autoimmune diseases). This report also includes consumption of dairy products, and therefore observational studies that included dairy foods rich in probiotics are also included here. A separate report covers all intervention studies on pre- and probiotics.

# 1.2. Studies and outcomes identified

We identified a total of 47 observational studies that investigated the association between infant or maternal intake of other foods and allergic or autoimmune diseases. Of these, 27 studies were prospective cohorts (PC), 4 were nested case-controls (NCC), 12 were case-controls (CC), and 4 cross-sectional studies (CS).

# 1.3. Outcomes evaluated

22 studies included atopic dermatitis (AD) as outcome; 2 studied food allergy (FA); 2 lung function (LF); 21 wheeze; 7 rhino-conjunctivitis (RC); 10 allergic sensitisation (AS); 10 type 1 diabetes mellitus (T1DM); one studied juvenile idiopathic arthritis (JIA), and one studied inflammatory bowel disease (IBD). The characteristics of included studies that investigated allergic outcomes are summarised in Table 1, whilst those of studies that investigated autoimmune outcomes are presented in Table 2.

# 1.4. Dietary exposures studied

The majority of the studies included in this report examined the association between several foods and outcomes of interest. Dietary exposures that did not fit the characteristics of those included in the other three reports are included here. Alcoholic and non-alcoholic beverages such as alcohol, coffee, tea, and sterilised water were included in addition to individual foods, food groups, dietary patterns and dietary micro/macronutrients. Fats, fatty acids and specific foods rich in fatty acids are not included as they are covered in a separate report.

Table 3 summarises the types of approaches used by each study to ascertain dietary intake. The majority of the studies used some form of questionnaire, although it was not always possible to confirm if the questionnaires were a) designed for the specific study, and b) whether the questionnaire had been validated or piloted in any form.

# 1.5. Dietary assessment and comparators

Dietary assessment was performed via questionnaire in all the studies included in this report. Food intake was often analysed by deriving dietary patterns, though the definitions and methodological approaches to derive those patterns varied across studies. In some cases dietary patterns were derived using Principal Component Analysis (PCA), whereas in other studies the use of term ‘pattern’ only meant grouping certain foods according to their similarities e.g. Mediterranean foods. The intakes compared and their cut-offs varied greatly between studies, which made meta-analyses only rarely possible. We have illustrated the main comparison levels used in the studies in the tables within each section.

# 1.6. Presentation of findings

We created a summary Table of Study Characteristics with key study features separating them by allergic (Table 1) and autoimmune (Table 2) outcomes. There are seven main sections that summarise the findings for each outcome: atopic dermatitis (AD), food allergy (FA), lung function (LF), wheeze, rhino-conjunctivitis (RC), allergic sensitisation (AS), type I diabetes mellitus (T1DM), inflammatory bowel disease (IBD), and juvenile idiopathic arthritis (JIA). As most of the studies included in this report had data that could not be combined in a meta-analysis, a narrative table summarising study findings is provided at the end of each section.

# 1.7. Risk of bias assessment

The risk of bias in included cohort and case control studies was assessed using a modified version of the National Institute for Clinical Excellence (NICE) methodological checklist for cohort and case-control studies, respectively . Key domains were:

- Selection Bias (low if cases and controls were selected from similar populations, if the participation rate was ≥80%, or <80% but investigators explored and adjusted for differences between the characteristics of participants and non-participants);
- Assessment Bias (low if validated and reliable tools were used to assess exposure and outcome), and;
- Confounding Bias (low if most likely confounders are identified and taken into account in study design and analysis).

Observational studies were considered at low overall risk of bias where the risk of bias was judged to be low for all 3 key domains for selection, assessment and confounding bias. For assessment of Confounding Bias, factors that we expected to be adjusted for within studies of allergic outcomes were: siblings (parity or birth order or family size); gender; age at outcome assessment; disease risk based on family history; maternal or household smoking (asthma/wheeze outcomes); maternal age; maternal education or socioeconomic status; mode of delivery. For studies on autoimmune outcomes we expected matching and/or adjusting for gender, age, address, socioeconomic status, smoking and disease risk. We also assessed possible Conflict of Interest, this being judged as low where there was no evidence of industry involvement in study design, analysis, interpretation or publication, and no evidence that study authors received remuneration from relevant industry partners for other activities. Risk of conflict of interest bias was low in all studies presented in this report, so conflict of interest bias was not included in the risk of bias summary figures. A Risk of Bias Figure is presented in each outcome section, which summarises the risk of bias in all included studies reporting that outcome, whether presented in meta-analysis forest plots, in the narrative table or both.

# 1.8. Key findings

i. **Overall risk of bias was low or unclear for the majority of the outcomes assessed.**

The overall bias was considered to be low in the majority of the studies on atopic dermatitis, food allergy, wheeze, allergic sensitisation, and for autoimmune diseases. In several studies it was unclear whether adjustment for potential confounders had been made, which was the main factor influencing the ‘unclear’ assessment in this domain for some studies.

ii. **There was limited opportunity to meta-analyse results**

The large variations observed in the way data were reported made meta-analysis possible for only a few dietary exposures relevant to atopic dermatitis, rhino-conjunctivitis and Type I Diabetes Mellitus. Each section includes a table that summarises the main characteristics and findings of those studies that were not eligible for meta-analysis, and this represents the majority of the evidence in all sections of the report.

- **There was very little evidence suggesting that any of the foods included in this report were associated with allergic or autoimmune diseases.**

Allergic outcomes

- One prospective cohort study reported that maternal intake of yoghurt during pregnancy is associated with reduced rhino-conjunctivitis and reduced AD at age 3 years.
- One cross-sectional study reported that intake of farm milk during infancy is associated with reduced RC and reduced wheeze in childhood.
- One cross-sectional study reported that infant farm milk intake in the first year of life is associated with reduced AS-Aero as measured by specific IgE antibody testing.
- One study reported that a Mediterranean diet during pregnancy is associated with reduced AS-Aero at age 6.5, as measured by specific IgE blood testing

Autoimmune outcomes

- One case control study with a high risk of assessment bias, reported that consumption of nitrosamine-rich foods during pregnancy is associated with TIDM.

We found no evidence for associations between other maternal or infant dietary exposures and allergic or autoimmune outcomes.

# 1.9. Conclusions

Overall we found no consistent evidence for associations between maternal or infant diet and allergic or autoimmune outcomes in this report. Some single studies reported significant associations, but these require replication.

Table 1 Characteristics of included studies on allergic outcomes and infant or maternal intake of other foods

| **Study** | **Design** | | **N/N cases** | **Country** | | **Type of food or nutrient measured** | | **Exposure assessment** | | | **Outcomes** | | **Age at outcome (years)** | | | **Population characteristics** |
| --- | --- | --- | --- | --- | --- | --- | --- | --- | --- | --- | --- | --- | --- | --- | --- | --- |
| Willers, 2007 | PC | | 1212 | Netherlands | | Whole grain products | | | Q | AD, RC, and wheeze: Parent reported (ISAAC Q); LF: Spirometry; SPT any; Rec wheeze: DD asthma | | 5 | | | **Aberdeen birth cohort**: Population based birth cohort with pregnant women recruited 1997-99 while attending a hospital antenatal clinic at ~12weeks gestation | |
| Shaheen, 2009 | PC | | 9516 | UK | | Dietary Patterns: 'Traditional'; 'Health Conscious'; 'Processed'; 'Confectionery'; 'Vegetarian'; 'Processed'; 'Vegetarian' | | | Q | Rec wheeze: Parent reported persistent wheeze; DD asthma; AD: Parent reported; LF-BHR slope: Spirometry; LF-FEV_1_: RC: Parent reported; SPT aero; Total IgE | | 0.5, 2.5, 3.5, 7, 7.5 | | | **ASLPAC study**: Population based birth cohort study, of mother-child pair recruited in 1991-1992 | |
| Fergusson, 1990 | PC | | 1067 | New Zealand | | Cereal, meat | | | R/D/Q | AD: Physician assessment; AD: DD , duration of > 3years and use of regular medication | | 10 | | | **CHRISTCHURCH CHILD DEVELOPMENT STUDY:** A cohort of children born in the Christchurch urban region New Zealand during 1977 | |
| Bisgaard, 2009 | PC | | 354 | Denmark | | Alcohol | | | Q | AD: Physician assessment | | 3 | | | **COPSAC study**:  Healthy newborns of mothers with a history of doctor-diagnosed asthma recruited in Copenhagen, Denmark between 1998 and 2001 | |
| Linneberg, 2004; Maslova, 2013 | PC | | 60466 | Denmark | | alcohol; artificially-sweetened non-carbonated soft drinks | | | I, Q | AD: Parent reported + DD; RC: DD + Medication registry ; Rec wheeze: DD asthma | | 1.5, 7 | | | **DNBC study**: Population based birth cohort recruited 1996-2002 at ~12weeks gestation in Denmark | |
| Nwaru, 2010/11/13 Virtanen, 2010  Maijaliisa, 2011 Niinisto, 2012 Lumia, 2011 Erkkola, 2012 Uusitalo 2008 | PC | | 5619/237 | Finland | | Various cereals, dairy products, meat, egg, alcohol, rice, tea, coffee, chocolate, sweets | | | Q | AD: DD, Parent reported ISAAC; RC: Parent reported (ISAAC); sIgE aero; sIgE cm; sIgE egg; sIgE food; Rec atopic wheeze: DD asthma + positive IgE; Rec wheeze: DD asthma; Wheeze; Rec wheeze: Parent reported (ISAAC Q) asthma | | 0.5, 5 | | | **DIPP study**: Prospective birth cohort of children at high risk of TIDM (HLA genotype conferred susceptibility) born between 1997 and 2004 in Oulu and Tampere University Hospital Finland | |
| Alm, 2009 | PC | | 4941 | Sweden | | Dietary Pattern-Vegetarianism in the family, gluten, type of fat on bread, lactic acid (yoghurt ,fermented) vegetables, alcohol | | | Q | AD: Parent reported | | 1 | | | **Infants of Western Sweden**: Population based birth cohort of infants born in the region in 2003 | |
| Laitinen, 2005 | PC | | 60, 95 | Finland | | Dietary carbohydrates; dietary energy; dietary protein | | | D | AD: Physician assessment; FA cow’s milk: Physician assessment | | 0.5, 1 | | | Children with a family history of AD (mother, father and/or older sibling with AD, AR or asthma), who participated in a prospective allergy prevention study (probiotic intervention trial) | |
| Zutavern, 2006; Zutavern, 2008; Sausenthaler, 2007 | PC | | 2532 | Germany | | Cereal, milk, cheese, yoghurt | | | Q | AD: DD; Parent reported; sIgE food; sIgE aero; sIgE any | | 2, 6 | | | **LISA study**: Population based cohort study of newborns recruited between 1997 and1999 from 4 German cities: Munich, Leipzig, Wesel, and Bad Honnef. | |
| Marini, 1996 | PC | | 68 | Italy | | Meat | | | Q | AD: Physician assessment + parent reported; RC: Physician assessment + parent reported; Rec wheeze: Physician assessment > 3 episodes of wheeze | | 1, 3 | | | Infants with family history of allergy born in maternity wards of 3 hospitals from 1989 whose mothers were refused to participate in an allergy prevention intervention program | |
| Chatzi, 2008 | PC | | 468 | Spain | | Dietary Pattern-adherence to Mediterranean diet; cereal; dairy; meat; white meat | | | Q | Rec wheeze: parent reported ≥1 episodes of wheeze; Atopic wheeze: Parent reported wheeze + positive SPT; SPT aero | | 6.5 | | | **Menorca birth cohort study**: Population based birth cohort with women recruited from antenatal care at all general practices in Menorca between 1997-1998 | |
| Miskelly, 1988 | PC | | 482 | UK | | Meat; meat other than beef | | | D | AD: Physician assessment and parent reported; wheeze: parent reported | | 1 | | | Infant recruited through two antenatal clinics in South Wales born to mothers with positive allergy history in at least one member of family, whose mothers were asked to participate in allergy preventive program | |
| Morgan, 2004 | PC | | 257 | UK | | Cereal; rice; rusks; desserts; meat; meat with vegetables | | | I | AD: Physician assessment and parent reported | | 1 | | | Healthy preterm birth (<37 weeks gestational)were recruited from a cross section of socioeconomic groups in southeast England using the Royal Surrey County Hospital (Guildford), St Peter’s Hospital (Chertsey), and Frimley Park Hospital (Frimley) | |
| Bertelsen, 2013 | PC | | 54740 | Norway | | Milk or yogurt products rich in probiotics | | | Q | AD: Parent reported; Rec wheeze: Parent reported asthma | | 0.5, 3 | | | **MoBa study**: Population birth cohort with pregnant women recruited at ~17 weeks of pregnancy with children born between 2000 and 2005 in Denmark | |
| Miyake, 2011; Miyake, 2010a; Miyake, 2010b Miyake, 2009; Satio, 2010 | PC | | 771 | Japan | | Dietary patterns derived from Factor Analysis; dairy; cheese; milk; egg; meat; yoghurt | | | Q | AD and wheeze: Parent reported (ISAAC Q) wheeze; AD: DD | | 2, 0.33, 2 | | | **OMCHS study**: Population birth cohort with pregnant women between the 5-39th week of pregnancy recruited from a university hospital and three obstetric hospitals in municipality of Osaka between 2001 and 2003 | |
| Roduit, 2012 | PC | | 1041 | Austria, Finland, France, Germany, and Switzerland | | Cereal; bread; chocolate; cake; Farm milk' - either boiled or not boiled; meat | | | D | AD: DD | | 1, 4 | | | **PASTURE study**:  Population based birth cohort with women recruited in third trimester of pregnancy from rural areas in 5 European countries (Austria, Finland, France, Germany, and Switzerland) and were divided into 2 group: those who lived or worked on family run farms and those not living on a farm of the same area | |
| Willers, 2008 | PC | | 2818 | The Netherlands | | milk; egg | | | Q | Wheeze and rec wheeze: Parent reported (ISAAC Q) wheeze; Rec wheeze: DD asthma + medication and/or current symptoms | | 8 | | | **PIAMA study**: Population based birth cohort of children born in 1996/97 after prenatal recruitment through prenatal clinics in the northern, middle and southwestern part of the Netherlands. The children were allocated to an intervention study or to a natural history study depending on their family risk for allergy | |
| Lange, 2010 | PC | | 1376 | USA | | Dietary Pattern derived from Principal Component Analysis -Alternate Healthy Eating Index for pregnancy (AHEI-P); Adherence to intake of Mediterranean foods | | | Q | DD asthma; rec wheeze: Parent reported wheeze in at least one questionnaire (1,2,3 years old), AD: DD | | 3 | | | **Project Viva study**:  Population based birth cohort with pregnant women at <22 weeks of gestation recruited from 8 obstetric offices of a large multispecialty suburban/urban group practice in eastern Massachusetts US between 1999 and 2002 | |
| Andreasyan, 2007 | PC | | 498 | Australia | | Honey | | | Q | AD, RC and wheeze: Parent reported (ISAAC Q); SPT any | | 9 | | | **CARHS study**: Some of the participants in an existing cohort (‘THIS’) of infants at high risk for sudden infant death syndrome born in 1988-1989 were identified in the northern region of Tasmania through school records | |
| Narita, 2011; Ohya, 2011 | PC | | 1463 | Japan | | dietary energy | | | Q | AD, and wheeze: Parent reported (ISAAC Q) | | 3 | | | **T-CHILD study**: Population based birth cohort of Japanese mother-infant pairs with women recruited ~ second trimester in Tokyo | |
| Wang, 2007 | PC | | 1760 | Taiwan | | Ginseng | | | Q | AD: DD | | 0.5 | | | Pilot study of Taiwan National Birth Cohort Study: All postpartum women and newborns from these 29 towns were recruited with the multistage stratified systematic sampling design to obtain the representative samples from the national birth registration data in 2003 | |
| Harris, 2001; Zutavern, 2004 | PC | | 604, 622 | UK | | Rice; cereal; meat | | | Q | AD: DD; AD: Visible AD on examination; SPT aero; rec wheeze: Parent reported wheeze; rec wheeze: parent reported | | 2, 5.5 | | | Po Population based birth cohort of newly pregnant women who presented at one of three general practices in Ashford, Kent UK between 1993 and 1995; Population based birth cohort of infants from all pregnant women presenting in three general practices in Ashford, Kent UK between 1993 and 1995 | |
| Dubakiene, 2012 (41) | | PC 128 | | | Lithuania Maternal allergenic food avoidance [more than one food group]: milk and egg | | Q SPT 1 | | | | | | | The Lithuanian birth cohort “Alergemol” (n=1158) was established as a part of the multi-centric “EuroPrevall” birth cohort that recruited over 12000 newborns in 9 European countries. The “EuroPrevall” cohort was established in 2005–2009 using a standardised approach across 9 European countries | | |
| Oliver, 2010; | NCC | | 117/39 | UK | | Dietary carbohydrates; Wheat; dietary energy; dietary protein | | | D | FA any: DD with DBPCFC | | 1 | | | This case-control study was nested in the **EuroPrevall study** (UK birth cohort): cases were infants with food allergy, each matched to two controls. | |
| Sariachvili, 2010 | NCC | | 557/252 | Belgium | | Cereal, meat | | | Q | AD: Parent reported (ISAAC Q) | | 4 | | | **PIPO Cohort study**: cases and controls with data regarding development of eczema and timing of introduction of solid foods were identified from this prospective cohort: Belgium. | |
| Butiene, 2011 | NCC | | 43 | Lithuania | | Maternal allergenic food avoidance [more than one food group]: milk and egg | | | I | SPT/sIgE milk or egg | | 1 | | | Children in Lithuanian birth cohort study, gestational age at least 34 weeks and APGAR score ≥7 at 5 min; 51.3% boys; <1year; sensitised to food allergens and their age-matched controls | |
| Dai, 1993 | CC | | 70 | China | | Maternal allergenic food avoidance [more than one food group]: any | | | Q | Rec wheeze (unclear) | | 0.5 | | | Children from the community and they had to reside there the last year | |
| Oliveti, 1995 | CC | | 262/131 | USA | | Alcohol | | | R | Rec wheeze; DD asthma | | 4-9 | | | Cases & controls were identified from rosters of patients followed up at the Rainbow Babies & Children's Hospital continuity care clinic, Ohio. | |
| Lopez Campos, 2001 | CC | | 75/58 | Mexico | | chocolate; mango | | | Q | Rec wheeze: DD asthma | | 6-10 | | | Asthmatic patients were recruited from Allergy clinics and control patients from familial medicine clinics of Hospital de Especialidades, Mexico. | |
| de Batlle, 2008 | CS | | 1476/402 | Mexico | | Dietary pattern intake in pregnant mothers | | | Q | Wheeze: parent reported wheeze; Rec wheeze: parent reported asthma; RC: parent reported | | 6-7 | | | Participants were recruited from a random sample of children in primary school in the Mexicali province, Mexico. | |
| Dela Bianca, 2012 | CS | | 467/467 | Brazil | | Processed food | | | Q | Rec wheeze: parent reported ≥1 episode of wheezing in the past year | | 1 | | | Participants were infants aged from 12 to 15 months who attended 9 selected health centres for routine immunisations. | |
| Riedler, 2001 | CS | | 812/unclear | Austria, Germany, and Switzerland | | Farm milk/environment | | | I | AD: DD; RC: parent reported; sIgE aero; Rec wheeze: DD asthma; asthma ≥1 episode of wheezing in the past year | | 9 | | | **ALEX STUDY TEAM**: cases were children of farming families and controls of non-farming families from the study schools: Austria, Germany, & Switzerland | |
| Castro-Rodriguez, 2010 Binkley, 2011 | CS | | 1409/594 | Spain | | Mediterranean diet; industrial food | | | Q | Wheeze: Parent reported (ISAAC Q) wheeze | | 1.4 | | | **EISL study**: Spanish population attending primary healthcare clinics, cases with wheeze in first year of life and controls without: Spain | |

PC Prospective cohort study; Q questionnaire; AD atopic dermatitis; ISAAC International Study of Allergy and Asthma in Children; AS allergic sensitisation; SPT skin prick test, sIgE specific IgE; DD Doctor diagnosis (community); Physician assessment is assessment by study physician; AR allergic rhinitis, FA food allergy; CS cross-sectional study

Table 2 Characteristics of included studies on autoimmune diseases and infant or maternal intake

| **Study** | **Design** | **N/N cases** | **Country** | **Type of dietary fat measured** | **Exposure assessment** | | | **Outcomes** | | **Age at outcome (years)** | | **Population characteristics** |
| --- | --- | --- | --- | --- | --- | --- | --- | --- | --- | --- | --- | --- |
| Wahlberg, 2006; Brekke, 2010; | PC | 9414/44 | Sweden | Coffee | | Q | CD: DD + IgA-tTG +symptoms; TIDM: Islet autoimmunity (GADA and/or IA2A) | | 8.4, 2, 5 | | **ABIS study**: Population based birth cohort of children born in Southeast Sweden between 1997 and 1999 | |
| Wahlberg, 2006 | PC | 8715 | Sweden | Maternal allergenic food avoidance [more than one food group]: all dairy | | Q | TIDM: Islet autoimmunity (GADA and/or IA2A) | | 2.5 | | Born between Oct 1997 and Oct 1999 in South East Sweden. Representative of general Swedish population | |
| Nwaru, 2013; Virtanen, 2011; Nwaru, 2010; Virtanen, 2011/10; Virtanen, 2006; Nwaru, 2011; Niinisto, 2012;  Lumia, 2011; Erkkola, 2012; Uusitalo, 2008; Nwaru, 2010  2011 | PC | 561 | Finland  Finland | Wheat , Rye, Oats, Barley, Other cereals (maize, rice, millet, and buckwheat); cereal; Other cereals (maize, rice, millet, and buckwheat); milk; milk and dairy; cheese; meat; egg; alcohol; rice; tea; coffee; chocolate; chocolate and  sweets; dairy | | Q | TIDM: Islet autoimmunity (ICA plus GADA and/or IA2A and/or IAA) | | 0.5, 5 | | **DIPP study**: Prospective birth cohort of children at high risk of TIDM (HLA genotype conferred susceptibility) born between 1997 and 2004 in Oulu and Tampere University    Hospital Finland | |
| Lamb, 2008  Lamb, 2013;  Norris, 1996; | PC, NCC | 1698/49 | USA, UK, Australia | rice; oats; wheat, rye, oats, cereal, meat | | I, Q | CD: positive IgA-tTG on 2 consecutive visits or a positive small bowel biopsy after only a single tTG-positive visit. Secondary only those children who had a biopsy positive, as defined by Marsh score; TIDM: DD; TIDM: Islet autoimmunity (GADA and/or IA2A and/or IAA) | | 4; <5; <7; 9; <10; 15 ; unclear | | **DAISY study:** Prospective birth cohort of children at increased risk for T1DM (relative with T1DM via registries and hospital records) recruited from 1993 to 2004 in Denver, Colorado US were screened for human leukocyte antigen (HLA) genotype associated with celiac disease and TIDM; DAISY: cases & their non-diabetic siblings, as controls, taken from Barbara Davies Centre Colorado, other diabetes care clinics & Colorado IDDM registry. | |
| Harsunen, 2012 | NCC | 33/33 | Germany | Dietary energy intake | | D | TIDM | | <18 | | **BABYDIET study**: cases were study children who developed islet autoantibodies and T1DM during BABYDIET study follow-up and controls were children from the same original study cohort who did not: Germany. | |
| Baron, 2005 | CC | 444/222 | France | Flour, meat | | I | IBD-CR: DD; IBD-UC: DD | | <17 | | Cases with Crohn's disease were identified from the EPIMAD registry with matched controls from the same area identified by random digit dialling: France. | |
| Virtanen, 1994 | CC | 1136/600 | Finland | Coffee | | Q | TIDM: DD | | ≤14 | | **Childhood Diabetes in Finland**: cases were newly diagnosed T1DM with matched controls selected from the general population | |
| Ellis, 2012 | CC | 655/246 | Australia | Alcohol; coffee | | Q | JIA: DD ILAR criteria | | 0-18 | | **CLARITY study**: cases were recruited during a clinic visit to Royal Children's Hospital, with diagnosed JIA using ILAR criteria: controls were patients in for elective surgery, also at the Royal Children's Hospital Day Surgery Unit: Australia | |
| Visalli, 2003 | CC | 900/150 | Italy | meat; tea; coffee | | Q | TIDM: DD WHO criteria | | 12 | | **EURODIAB Italy** **study:** Cases with T1DM selected from within the EURODIAB ACE study, born 1977-89, with controls selected from school records for the same period | |
| Majeed, 2011 | CC | 395/96 | Iraq | Coffee; tea | | Q | TIDM: DD | | 1.5-17 | | Cases were T1DM patients admitted to hospitals or primary health centres in Basrah and controls were attendees to outpatients of the same institutions for non-diabetic complaints: Iraq | |
| Rosenbauer, 2007/2008 2007 | CC | 2631/760 | Germany | coffee | | Q | TIDM: DD | | <5 | | German study of newly diagnosed T1DM cases selected from a hospital-based surveillance system ESPED and controls from local registration office records. | |
| Sipetic, 2003 | CC | 315/105 | Serbia | Alcohol; coca cola, nitrosamine-rich foods, coffee | | Q | TIDM: maternal report | | 1-16 | | Cases and controls were children admitted to hospital due to allergic conditions 1994-97, Serbia | |
| Sipetic, 2005 | CC | 315/105 | Serbia | Alcohol; coca cola; nitrosamines; tea; coffee | | Q | TIDM: DD WHO criteria | | ≤16 | | Cases were children hospitalized with new diagnosis of T1DM in Belgrade, with controls selected from a population of children treated for skin disease as outpatients: Serbia | |
| Strotmeyer, 2004 | CC | 690/247 | China | steamed bread; rice; noodles; meat | | Q | TIDM: DD WHO criteria | | 9.7 | | **DiaMond study**:  WHO Multinational Project for Childhood Diabetes (cases selected from T1DM incidence registries 1985-98 and matched controls from locally resident populations (>95%). | |

PC Prospective cohort study; Q questionnaire; CC case-control study; SPT skin prick test, sIgE specific IgE; DD Doctor diagnosis (community); Physician assessment is assessment by study physician; AR allergic rhinitis, FA food allergy, T1DM Type 1 Diabetes Mellitus, RC retrospective cohort, JIA juvenile idiopathic arthritis; ILAR International League of Associations for Rheumatology

Table 3 Dietary instrument used to ascertain infant or maternal intake of other foods

| First author, year | Dietary assessment used |
| --- | --- |
| Alm, 2009 | Questions on maternal diet and timing of food introduction and allergic diseases. Food questions asked at 6 and 12 months |
| Andreasyan, 2007 | Parental questionnaire including the ISAAC respiratory and dietary questions |
| Baron, 2005 | Trained researchers interviewed child and mother |
| Bertelsen, 2013 | FFQ |
| Binkley, 2011 | Additional questions on diet added to anaphylaxis survey questionnaire |
| Bisgaard, 2009 | Information collected during personal interviews at the clinical research unit during pregnancy by doctors with paediatric training |
| Brekke, 2010 | 22-item FFQ |
| Butiene, 2011 | Self-administered parental questionnaire. Follow-up with telephone interview if there were missing data |
| Castro-Rodriguez, 2010 | Environmental questionnaire which included food consumption of oil type used for cooking or dressing salads |
| Chatzi, 2008 | Spanish version of the validated EPIC-Norfolk FFQ |
| De Batlle, 2008 | Validated and adapted FFQ with 70 food items asking about the diet of the children in the last 12 months |
| Dubakiene, 2012 | Data collected pre- and post-natal. Questionnaire with questions on food intake and nutritional supplement as well as other relevant risk factors and co-morbidities |
| Ellis, 2012 | General questionnaire with dietary questions |
| Erkkola, 2012 | Retrospective diary records of foods eaten during the month preceding delivery (8^th^ month of pregnancy) |
| Fergusson, 1990 | Child and maternal records kept at Plunket Community Nursery were reviewed to obtain estimates of the types of  solid food that the child was given in first 4 months of life |
| Harris, 2001 | Questionnaire - Trained nurses visited mothers and asked about breastfeeding and food introduction practices |
| Harsunen, 2012 | 3-day dietary records (after termination of breastfeeding) collected every 3 months |
| Lamb, 2008 and 2013 | Willet FFQ (146 food items) |
| Laitinen, 2005 | Four day food diaries completed by parents or personnel at day-care |
| Lange 2010 | Self-administered 166-item semi-quantitative FFQ, answered twice, at 1^st^ and 3^rd^ trimester of pregnancy |
| Leermakers, 2013 | Modified version of a semi-quantitative FFQ |
| Linneberg, 2004 | Interview at 12 and 30 weeks of gestation, during which the mother answered a question on alcohol intake habits |
| Long, 1986 | Unclear |
| Lopez-Campos, 2001 | Questionnaire |
| Lumia, 2011 | Validated, self-administered, semi-quantitative 181-item FFQ |
| Maslova, 2013 | A validated 360-itemsemi-quantitative FFQ completed around gestation week 25 and covered intake during the previous four weeks |
| Maijaliisa 2011 | Validated FFQ enquiring about maternal diet in 8^th^ month pregnancy |
| Majeed, 2011 | Specially designed study questionnaire for the study, which included questions on maternal habits, including maternal coffee/tea consumption habits |
| Marini, 1996 | Parental questionnaire |
| Miyake, 2009, 2010, and 2011; and Saito 2010 | Self-administered DHQ that assessed dietary habits during the preceding month (150 foods); dietary patterns derived with PCA |
| Miskelly, 1988 | Feeding diaries were completed for the babies, dietary information being supplemented by notes made by the dietician |
| Morgan, 2004 | FFQ administered by the study midwives to the mother on enrolment and at four monthly intervals to 12 months post-term |
| Narita, 2011 | Dietary history questionnaire administered in the 3^rd^ trimester of pregnancy |
| Niinisto, 2012 | Validated FFQ capturing maternal diet during the 3^rd^ month of lactation |
| Norris 1996 | Infant diet questionnaire prior to auto-antibody knowledge. List of milks commonly given to infant in the first 15 months |
| Nwaru, 2010, 2011, and 2012 | Validated 181-item FFQ |
| Nwaru 2013 | Repeated age-specific dietary questionnaires at the ages of 3, 6, and 12 months and a follow-up “age at introduction of new foods-form” |
| Ohya 2011 | Self-administered DHQ |
| Oliver, 2010 | Food diaries with qualitative and quantitative data completed and returned every 4 weeks in the first year of life |
| Oliveti, 1995 | Medical obstetric records had data on maternal alcohol intake as part of other variables registered during pregnancy |
| Riedler, 2001 | Interview with parents to ascertain children’s exposure to stables and farm and pet animals; duration of breastfeeding; timing of consumption |
| Roduit, 2012 | Dietary intake reported by parents in monthly diaries between the 3rd and 12th month of life |
| Rosenbauer, 2007 and 2008 | Self-administered parental questionnaire. Follow-up done with telephone interview if data missing |
| Sariachvili, 2010 | When children were 1 year old, parents provided information on introduction of specific food item in weeks by answering the following question ‘How old (in weeks) was your child when you gave him/her the following food items to eat or drink?’ |
| Sausenthaler, 2007 | Semi-quantitative FFQ capturing intake in the last 4 weeks of pregnancy |
| Shaheen, 2009 | FFQ to assess maternal intake of 110 foods (43 groups) at 32 weeks of gestation; dietary patterns derived with PCA |
| Sipetic, 2003 and 2005 | A main questionnaire was used, which covered a variety of parental and neonatal factors including diet |
| Strotmeyer, 2004 | Retrospective questionnaire (yes/no), completed by primary caregiver for the age ranges of <1, 1–3, 4–6, and 7–12 months |
| Visalli, 2003 | Parent-completed environmental questionnaire |
| Virtanen, 1994; 2006; 2010, and 2011; Uusitalo, 2008 | A validated 181 food item FFQ |
| Wang, 2007 | Questions on dietary intake included in environmental questionnaire |
| Wahlberg, 2006 | Parental questionnaire at birth, 1 and 2.5 years |
| Willers, 2007 | Semi-quantitative FFQ (Version 5.4 of the Scottish Collaborative Group FFQ) mailed at 32 weeks gestation (150 items divided into 20 food groups |
| Willers, 2008 | The pregnancy questionnaire contained a question on frequency of consumption: ‘How often did you consume vegetables, fresh fruit, fish, egg, milk, milk products, nuts, and nut products such as peanut butter during the last month?’ |
| Zutavern, 2006 and 2008 | A 48 food item questionnaire on timing of food introduction was asked to parents when babies were 6 months old |
| Zutavern, 2004 | Information on dietary intake was obtained through a question included in the 1^st^ year questionnaire: “When did you start feeding your son/ daughter the following foods?”(9 items) |

EPIC= The European Prospective Investigation into Cancer Study

FFQ= Food frequency questionnaire

ISAAC=International Study of Allergy and Asthma in Children

PCA= Principal component analysis

DHQ= Diet history questionnaire

# 2. Other foods and risk of atopic dermatitis

# 2.1. Overall risk of bias between other foods and atopic dermatitis

The overall risk of bias in the studies investigating the association between atopic dermatitis and various foods was considered to be low. Most studies had low risk of bias in all the domains evaluated. Just over 10% of studies had a high risk of confounding bias due to the lack of controlling for potential confounders (Figure 1).

Figure 1 Risk of Bias in studies of other food intake and atopic dermatitis

# 2.2. Introduction of meat into infant’s diet and risk of atopic dermatitis

The studies of **Sariachvili** (**2010**) and **Zutavern** (**2006**) had comparable data to examine the pooled effect estimate of timing of meat introduction and risk of atopic dermatitis in children aged 0-4 years old (Figure 2). The generic term ‘meat’ was used when assessing exposure. There was no evidence of an association between this outcome and meat introduction.. Differences in exposure assessment may account for the high statistical heterogeneity seen.

Figure 2 Introduction of meat in months 0 to 4 and risk of atopic dermatitis in children aged 0 to 4 years old

# 2.3. Dairy intake (maternal) and risk of atopic dermatitis

Figure 3 shows the pooled estimates from two prospective cohort studies (**Miyake 2010** and **Sausenthaler 2007**) that examined the association between maternal intake of yoghurt during pregnancy and risk of atopic dermatitis in children at age 0-4 years old. For both dietary exposures there was no evidence of an association with this outcome. There was no heterogeneity between studies (I^2^=0.0%)

Figure 3 Maternal intake of yoghurt (highest vs. lowest) and risk of atopic dermatitis in children aged 0 to 4 years old

# 2.4. Studies not eligible for meta-analysis

Table 4 summarises the main characteristics and findings of the studies examining the association between various types of dietary exposures and risk of atopic dermatitis. The main dietary exposures studied included intake of confectionary products (e.g. chocolate, cakes, and desserts) (**Roduilt 2012; Morgan 2004; Maijaliisa 2011; Andreasyan 2007**). A further three studies used different methods to characterise dietary patterns i.e. Healthy Index (**Lange 2010)** or Principal Component Analysis (**Shaheen 2009; Miyake 2011**). Infant exposure to farm milk (unclear subtypes but assumed to be unpasteurised) was included in the studies of **Roduit (2012)** and **Riedler** **(2001)**. Six studies investigated meat **(Roduilt 2012; Ferguson 1990; Zutavern 2006; Zutavern 2004; Miskelly 1998; Morgan 2004)**. Two studies investigated macro-nutrients or total energy intake **(Laitinen 2005, Ohya 2011),** and three studies investigated maternal intake of alcohol **(Linneberg 2004; Alm 2009; Bisgaard 2009).** One study investigated timing of introduction of rice in the infant **(Harris, 2001).** One study **(Bertelsen, 2013)** investigated maternal intake of probiotics and showed probiotic intake was associated with reduced AD in the offspring at age 6 months. Overall, there was no evidence to suggest that dietary intake of any of these (by the mother or the infant) could influence risk of AD.

**Conclusion**

**The finding that intake of probiotic-rich milk products during pregnancy is associated with reduced AD requires replication. Overall we found no consistent evidence that other infant or maternal dietary exposures or dietary patterns are related to AD in the child.**

Table 4 Studies investigating the association between other foods and atopic dermatitis which were not eligible for meta-analysis

| **First Author and year of publication** | **Design** | **Outcome** | **Age** | **N/n** | **Dietary exposure and comparison level** | **Measure of association** | **Effect (bold indicates p-value <0.05)** |
| --- | --- | --- | --- | --- | --- | --- | --- |
|  |  |  |  |  | **INFANT INTAKE** |  |  |
| Laitinen, 2005 | PC | AD |  | 60 | **Total infant daily energy intake (various measurements) (continuous):**  Total energy intake (mega joules) |  | NS |
|  |  |  | 0.5 | 60 | % of energy intake as carbohydrate |  | NS |
|  |  |  | 1 | 95 | Total energy intake (mega joules) |  | NS |
|  |  |  | 0.5 | 60 | Energy intake (as kilo joule per day) |  | NS |
|  |  |  | 0.5 | 60 | Energy intake (as kilo joule/ kg body weight) |  | NS |
|  |  |  | 1 | 95 | Kilo joule per day energy intake |  | NS |
|  |  |  | 0.5 | 60 | Protein intake (continuous) |  | NS |
|  |  |  | 0.5 | 60 | % of energy intake as protein (continuous) |  | NS |
|  |  |  | 1 | 95 | Protein intake (g) as mega joule per day energy intake (continuous) |  | NS |
| Roduit, 2012 | PC | AD |  |  | **Intake in infancy (none vs introduction in the 1^st^ year of life):**  Chocolate | OR | 1.49 (0.85-2.61) |
|  |  |  |  |  | Cake (None vs yes <8 Months) | OR | 1.33 (0.66-2.68) |
|  |  |  | 4 | 483 | Chocolate/ introduced into diet >12 months vs. 3 to 12 months | OR | 1.37 (0.87-2.16) |
|  |  |  | 4 | 912 | Cake/ introduced into diet >8 months vs. 3 to 8 months | OR | 1.33 (0.83-2.15) |
|  |  |  | 1 | 912 | Chocolate/ introduced into diet >12 months vs. 3 to 12 months | OR | **1.52 (1.02-2.25)** |
|  |  |  | 4 | 912 | Cake/ introduced into diet >8 months vs. 3 to 8 months | OR | 1.43 (0.98-2.08) |
|  |  |  | 1 | 912 | Cake introduced into diet / >8 months vs. 3 to 8 months | OR | 1.39 (0.93-2.06) |
| Morgan, 2004 | PC | AD | 1 | 257 | Desserts/time of introduction (continuous) | OR | NS |
| Andreasyan, 2007 | PC | AD | 9 | 699 | Introduction of confectionery in months 0 to 4 vs later | OR | 2.27 (0.61-8.44) |
|  |  |  |  |  | Meat/ no intake in the first 8 months vs. <8 months (high risk infants) | OR | 1.23 (0.56-2.70) |
|  |  |  |  | 1,041 | Meat/ no intake in the first 8 months vs. <8 months (low risk infants) | OR | 0.99 (0.41-2.37) |
| Roduit, 2012 | PC | AD | 4 | 483 | Meat/ introduced into diet >8 months vs. 3-8 months (onset after 1^st^ year of life) | OR | 1.15 (0.65-2.03) |
|  |  |  | 1 | 912 | Meat/ introduced into diet >8 months vs. 3-8 months | OR | 1.41 (0.93-2.14) |
| Fergusson, 1990 | PC | AD | .33-10 | 1,067 | Meat/introduced >4 months vs. ≤4 months | OR | NS |
| Nwaru, 2013 | PC | AD | 0-0.5 | 3,109 | Meat introduction (median month of introduction in those with AD vs those with no AD) | OR | P-value **<0.001** |
| Nwaru, 2013 | PC | AD | 0-0.5 | 2,951 | Meat introduction (median month in those with AD vs non AD) infants with parental history of allergy | OR | NS |
|  |  |  |  |  | Meat/ 0-4 months vs. >6 months | OR | 1.20 (0.79-1.83) |
| Zutavern, 2006 | PC | AD | 0-2 | 2,532 | Meat/ 0-4 months vs. 5-6 months | OR | 1.41 (0.88-2.25) |
| Zutavern, 2004 | PC | AD | 5 ½ | 642 | Introduction of meat in months 5 to 7 vs earlier | OR | 1.08 (0.78-1.51) |
|  |  |  |  |  | Meat/early months introduction vs later (continuous) | OR | NS |
| Miskelly, 1988 | PC | AD | 1 | 482 | Meat other than beef/ early months introduction vs later (continuous) | OR | NS |
|  |  |  |  |  | Meat/number of weeks from term meat introduced (continuous) | OR | NS |
| Morgan, 2004 | PC | AD | 1 | 257 | Meat with vegetables/number of weeks from term meat with vegetables introduced (continuous) | OR | NS |
| Marini, 1996 | PC | AD | 3 | 80 | Introduction of meat in months 5 to 7 vs earlier | OR | **0.18 (0.04-0.88)** |
|  |  |  |  |  | Lactic acid (yoghurt, fermented vegetables, etc consumption first year / more than 3 times/ week vs. ≤3 times/per week | OR | 1.14 (0.96-1.34) |
|  |  |  |  |  | Consumption of dairy in infants (often vs. Rare) | HR | 0.94 (0.67-1.31) |
| Alm, 2009 | PC | AD | 1 | 4941 | Consumption of fermented food (rare vs. often) | **OR** | **2.86 (2.21-3.69)** |
| Roduit, 2012 | PC | AD | 4 | 1,041 | ‘Farm milk' (either boiled or not boiled) none in 1^st^ year life vs. farm milk in infant diet | OR | 1.50 (0.78-2.89) |
| Riedler, 2001 | CS | AD | 9 | 812 | Exposed to farm milk in the 1^st^ year of life vs neither farm milk or stable exposure | OR | NS |
|  |  |  | 1.5-2 | 763 | **Infant intake of dairy foods** (categorized at quartile points based on the distribution in 763 subject) / lowest vs. highest quartile  Cheese | OR | 0.67 (0.38-1.16) |
| Miyake, 2010 | PC | AD | 1.5-2 | 763 | Milk | OR | 1.19 (0.69-2.06) |
| Harris, 2001 (39) | PC | AD | 2 | 624 | Introduction of rice in infant’s diet >12 months vs earlier | OR | 0.85 (0.17-1.18) |
|  |  |  |  |  | **MATERNAL INTAKE** |  |  |
|  |  |  |  |  | **Maternal alcohol intake in late (30 weeks of gestation) Beer/wine/spirits:**  No drinks per week/tiles vs. 0.5 drinks per week/tiles | HR | 0.98 (0.82-1.17) |
|  |  |  | 0.1-1.5 | 10,218 | No drinks per week vs. 2-3.5 drinks per week | HR | 1.00 (0.83-1.21) |
| Linneberg, 2004 | PC | AD | 0.1-1.5 | 10,218 | No drinks per week vs. 1-1.5 drinks per week | HR | 1.08 (0.93-1.26) |
|  |  |  |  |  | **Maternal alcohol intake in late pregnancy (30 weeks of gestation) Beer/wine/spirits:**  No drinks per week vs. ≥4 drinks per week | HR | 1.06 (0.72-1.57) |
|  |  |  |  |  | No drinks per week vs. 2-3.5 drinks per week | HR | 1.09 (0.87-1.37) |
| Linneberg, 2004 | PC | AD | 0.1-1.5 | 11,357 | No drinks per week vs. 1-1.5 drinks per week | HR | **1.32 (1.10-1.59)** |
|  |  |  |  |  | **Maternal alcohol intake in early pregnancy (12 weeks gestation) – Beer/wine/spirits:**  No drinks per week vs. 1-1.5 drinks per week | HR | 0.90 (0.80-1.01) |
|  |  |  |  |  | No drinks per week vs. 0.5 drinks per week | HR | 0.94 (0.83-1.06) |
| Linneberg, 2004 | PC | AD | 0.1-1.5 | 24,341 | No drinks per week vs. 2-3.5 drinks per week | HR | 1.02 (0.89-1.17) |
|  |  |  |  |  | Dietary pattern-vegetarianism in family/ descriptive (Yes vs. No) | OR | NS |
|  |  |  |  |  | Alcohol intake during pregnancy throughout lactation until 6 months of age (Yes vs. No) | OR | NS |
| Alm, 2009 | PC | AD | 1 | 4,941 | Alcohol intake during lactation - 6 months of age (Yes vs. No) | OR | NS |
| Willers, 2007 | PC | AD | 5 | 1,212 | Whole grain products/maternal diet during pregnancy. / low vs. High tiles | OR | NS |
| Maijaliisa, 2011 | PC | AD | 5 | 2,441 | Chocolate/not low consumption vs. Low consumption | OR | NS |
| Lange, 2010 | PC | AD | 3 | 1,376 | **Dietary pattern alternate healthy eating index for pregnancy (AHEI-P)/score comprised of 9 components contributing 10 possible points each:**  Vegetables, fruit, ratio of white to red meat, fibre, trans fat, ratio of poly- to saturated fatty acids, folate, calcium, and iron from foods | OR | 0.94 (0.82-1.08) |
|  |  |  |  |  | Adherence to Mediterranean foods/ dairy, fish, fruit, legumes, nuts, fats, vegetables and whole grains (alcohol intake excluded) / | OR | 1.00 (0.94-1.06) |
|  |  |  |  |  | **Maternal dietary pattern derived from principal component analysis (PCA**)  'Prudent' pattern i.e. Fruits, tomatoes, cabbages, green leafy vegetables, poultry, and fish | OR | 0.95 (0.83-1.09) |
|  |  |  |  |  | ‘Processed’ i.e. red meat, processed meat, refined grains, snacks, sweets, desserts, French fries, pizza | OR | 1.06 (0.92-1.22) |
|  |  |  | 7.5 | 7,693 | **Maternal dietary patterns (effect estimates based on per-SD increase in dietary pattern score)**  ‘Processed' | OR | 0.96 (0.88-1.05) |
|  |  |  | 2.5 | 9,516 | 'Processed' | OR | 0.97 (0.91-1.03) |
| Shaheen, 2009 (6) | PC | AD | 7.5 | 7,693 | ‘Traditional' | OR | 0.99 (0.93-1.05) |
|  |  |  |  | 9,516 | **Maternal dietary patterns (effect estimates based on per-SD increase in dietary pattern score)**  ‘Vegetarian' | OR | 0.99 (0.94-1.04) |
|  |  |  | 2.5 | 9,516 | ‘Traditional' | OR | 1.00 (0.95-1.05) |
|  |  |  | 7.5 | 7,693 | 'Vegetarian' | OR | 1.01 (0.94-1.08) |
|  |  |  | 2.5 | 9,516 | ‘Confectionery' | OR | 1.03 (0.98-1.08) |
|  |  |  |  | 7,693 | ‘Confectionery' | OR | 1.03 (0.96-1.11) |
|  |  |  | 7.5 | 7,693 | 'health conscious' | OR | 1.04 (0.96-1.13) |
| Shaheen, 2009 | PC | AD | 2.5 | 9,516 | 'health conscious' | OR | **1.06 (1.00-1.12)** |
|  |  |  | 1.3 - 2 | 763 | **Dietary pattern derived from factor analysis**/maternal dietary habits based on intake of 150 foods in the last month / scores for each dietary pattern categorised in quartiles vs. Lowest quartile (1) scores for each dietary pattern categorised in quartiles  ‘Western’ | OR | 0.70 (0.41-1.20) |
|  |  |  | 1.3 - 2 | 763 | ‘Health conscious’ | OR | 1.09 (0.64-1.85) |
| Miyake, 2011 | PC | AD | 1.3 - 2 | 763 | ‘Japanese’ | OR | 1.11 (0.64-1.94) |
| Ohya, 2011 | PC | AD | 1.5 | 1,463 | Dietary energy (continuous) | OR | NS |
|  |  |  | 3-4 months | 771 | **Maternal intake during pregnancy** / highest vs lowest quartile  Meat | OR | **2.59 (1.15-6.17)** |
| Saito, 2010 | PC | AD | 3-4 months | 771 | Dairy | OR | 1.84 (0.82-4.27) |
| Sausenthaler, 2007 | PC | AD | 2 | 2,473 | **Maternal intake** of cheese during the last 4 months of pregnancy (≥4 times/week vs <2 times per week or never) | OR | 0.87 (0.67-1.13) |
| Narita, 2011 | PC | AD | 3 | 1,344 | **Maternal intake** of dairy during pregnancy (highest vs lowest quartile) | OR | NS |
| Bertelsen, 2013 | PC | AD | 0.5 | 40,614 | Maternal intake of milk or yogurt rich in probiotics (yes vs no) | RR | **0.94 (0.89-0.99)** |
| Bisgaard, 2009 | PC | AD | 0-4 |  | Maternal intake of alcohol (often vs. Rare) | OR | 1.88 (0.94-3.75) |
| Wang, 2007 | PC | AD | 0-4 |  | Maternal intake of tea (often vs. rare) | OR | 0.59 (0.08-4.42) |

# 3. Other foods and risk of food allergy

# 3.1. Overall risk of bias between other foods and food allergy

Three studies were identified in this part of the report. All studies were considered to have a low risk of assessment bias, whilst two of them had an unclear description of the selection of participants. Therefore the overall bias was considered to be low or unclear for the majority (Figure 4).

**Figure 4 Risk of Bias in studies of other foods intake and food allergy**

We found no data eligible for meta-analysis. Table 5 summarises the characteristics of studies with data that could not be meta-analysed. Two prospective cohort studies (**Laitinen 2005; Sausenthaler 2007**), one nested case-control study (**Oliver 2010**), investigated the association between infant intake of macro-nutrients (daily estimates) and food allergy, showing no evidence of an effect. The study of Sausenthaler also examined infant intake of dairy products and reported a statistically significant negative association with risk of food allergy. One case-control study (**Binkley, 2011**) investigated maternal avoidance of peanut intake in relation to food allergy to peanut in the offspring, but reported no association between outcome and exposure. We found no evidence of an association between risk of food allergy and dietary exposures relevant to this report.

**Conclusion**

**We found no evidence that intake of proteins, carbohydrates or total energy is related to risk of food allergy. The finding of a single study reporting an association between dairy intake and reduced food allergy requires replication.**

Table 5 Studies investigating the association between other foods and food allergy which were not eligible for meta-analysis

| **First Author and year of publication** | **Design** | **Outcome** | **Age** | **N/n** | **Dietary exposure and comparison level** | **Measure of association** | **Effect (bold indicates p-value <0.05)** |
| --- | --- | --- | --- | --- | --- | --- | --- |
|  |  |  |  |  | Infant intake of Energy (continuous) - Total energy intake kJ/day |  | NS |
| Laitinen, 2005 | PC | FA | 1 | 95 | Infant intake of Protein (continuous) - % of energy intake as Protein |  | NS |
| Sausenhaler, 2007 (22) | PC | FA | Any |  | Maternal intake of dairy (often vs. rare) | OR | **0.4 (0.22-0.72)** |
|  |  |  | 1 | 93 | Dietary carbohydrate intake during the first year of life |  | NS |
|  |  |  | 1 | 93 | Dietary energy intake during the first year of life |  | NS |
| Oliver, 2010 | NCC | FA any | 1 | 93 | Dietary protein intake during the first year of life |  | NS |

# 4. Other foods and lung function

# 4.1. Risk of Bias in studies of other food exposures and lung function

The prospective studies of **Shaheen (2009)** and **Willers (2007)** investigated the associations between relevant dietary exposures and measures of lung function. Both studies were considered to have a low overall risk of bias (Figure 5).

Figure 5 Risk of bias in studies of other foods intake and lung function

The study of **Shaheen (2009)** investigated the association between maternal dietary patterns and risk of bronchial hyper-responsiveness (BHR) in children from the ALSPAC cohort study at age 8-9 years old. The authors identified five types of patterns derived from PCA and there was no evidence that any of these patterns influenced the risk of BHR in the infant. There was also no association with Forced expiratory volume in 1 second (FEV_1_). The authors reported weak evidence for a relationship between ‘processed’ foods type dietary pattern and reduced forced vital capacity (FVC). The study of **Willers (2007)** used a sample of children from the Aberdeen cohort study, a population-based birth cohort with pregnant women recruited from antenatal hospital clinics. They found no association between maternal cereal intake and measures of lung function in their children (Table 6).

**Conclusion**

Overall we found no consistent evidence to suggest that dietary patterns during pregnancy influence BHR or lung function in children.

Table 6 Studies investigating the association between other foods (dietary exposure) and lung function (outcome) which were not eligible for meta-analysis

| **First Author and year of publication** | **Design** | **Outcome** | **Age** | **N/n** | **Dietary exposure and comparison level** | **Measure of association** | **Effect (bold indicates p>0.05)** |
| --- | --- | --- | --- | --- | --- | --- | --- |
|  |  |  |  |  | **Maternal dietary patterns** (per SD increase in score; GM ratio) 'Confectionery' |  | NS |
|  |  |  |  |  | 'Health Conscious' |  | NS |
|  |  |  |  |  | 'Processed’ |  | NS |
|  |  |  |  |  | 'Traditional' |  | NS |
| Shaheen, 2009 | PC | LF-BHR slope | 8-9 | 4,113 | 'Vegetarian' |  | NS |
|  |  |  |  |  | **Maternal dietary pattern (Difference per SD of dietary pattern score)**  'Health Conscious' |  | NS |
|  |  | LF-FEV_1_ | 8-9 | 6,192 | 'Confectionery' |  | NS |
|  |  |  |  |  | **Maternal dietary pattern (Difference per SD of dietary pattern score)**  'Processed' |  | NS |
|  |  |  |  |  | 'Traditional' |  | NS |
| Shaheen, 2009 | PC | LF-FEV_1_(SDS) | 8-9y | 6,192 | 'Vegetarian' |  | NS |
|  |  |  |  |  | 'Confectionery' |  | NS |
|  |  |  |  |  | 'Health conscious’ |  | NS |
|  |  |  |  |  | **'Processed'** |  | P-value= 0.02 |
|  |  |  |  |  | 'Traditional' |  | NS |
|  |  | LF-FVC (SDS) | 8-9 | 6,285 | 'Vegetarian' |  | NS |
| Willers, 2007 | PC | LF | 5 | 510 | Whole grain products/ Low vs. high quintile |  | NS |

# 5. Other foods and risk of wheeze

# 5.1. Risk of bias in studies investigating the association between other foods and risk of wheeze

Twenty two studies investigated the relationship between maternal or infant intake of various foods and risk of wheeze. Most of the studies were considered to have a low risk of assessment, selection and confounding bias. Therefore the majority of the studies had an overall risk of bias judged as low. This was driven largely by population-based prospective cohorts. Approximately 15% of the studies were considered to have a high risk of assessment, and less than 10% were considered to have a high risk of bias due to lack of controlling for confounders (Figure 6).

Figure 6 Risk of Bias in studies of other foods intake and wheeze

# 5.2. Studies investigating the association between wheeze and other foods which were not eligible for meta-analysis

Despite of the number of studies investigating the association between infant or maternal intake and risk of wheeze in the offspring (Table 7), we did not identify any suitable studies that could have comparable data to pool estimates.

The main dietary exposures studied were maternal dietary patterns during pregnancy, maternal or infant intake of dairy products and intake of confectionary products. With regards to dietary patterns, these used different methodological approaches to define them. Some used a Healthy Index and adherence to Mediterranean diet (score) (**Lange 2010; de Batlle 2008; Castro-Rodriguez 2010; Chatzi 2008**), whilst the studies of **Shaheen (2009)** and **Miyake (2011)** derived dietary patterns from PCA using maternal reported intake of foods during pregnancy. The study of **Chatzi** reported a reduced risk of atopic wheeze (OR 0.55, 95% CI 0.31, 0.97) in children whose mothers had a high adherence to a Mediterranean diet compared with those with a lower score, but no association with other outcomes. The study of **Miyake (2011)** found that in Japanese women, a ‘Western’ dietary pattern was associated with a reduced risk of childhood wheeze (OR 0.59; 95% CI 0.36-0.98). The study of **Shaheen (2009)** identified five distinctive maternal dietary patterns, and found no evidence that any of these influenced the risk of childhood asthma at age 6 months, 3.5 years or 7 years old. Similarly, the study of **Lange (2010)** used three approaches to derive patterns of intake in pregnant mothers: a healthy eating index, adherence to Mediterranean diet, and PCA but none of these patterns showed evidence of an association between maternal intake of specific patterns and risk of childhood wheeze.

The study of **Andreasyan (2007)** showed that introduction of sterilised water in the first month was associated with a reduced risk of asthma at age 8 years (RR 0.64; 95% CI 0.45-0.88). This association remained after adjusting for several potential confounders including maternal and infant factors and style of bottle cleaning. However, the association was no longer statistically significant after adding low maternal educational level as potential confounder (RR 1.50; 95%CI 0.95–2.36).

Maternal intake of dairy products was investigated by several studies (**Bertelsen 2013; Erkkola 2012; Narita 2011; Willers 2007; Miyake 2010)** but there was no evidence of an association with wheeze. The study of **Riedler (2001)** examined infant intake in first year of life of farm milk (ie unpasteurised milk) with or without additional exposure to being in stables, and they found that infants who only consumed farm milk alone or that in addition to this intake they were in contact with stables, had a lower risk of wheeze or (for farm milk plus stables exposure only) recurrent wheeze. The study of **Bertelsen 2013** reported no association between maternal intake of milk or yogurt rich in probiotics during pregnancy and recurrent wheeze in offspring in the first 3 years.

With regards to maternal or infant intake of confectionary products, (e.g. sugary foods, chocolates, artificially sweetened products) the study of **Maijaliisa (2011)** found an association between infant intake of chocolate and reduced childhood wheeze (OR 0.74; 95% CI 0.59-0.72) but not recurrent wheeze. These findings were confirmed in the study of **Lopez-Campos (2001)** who reported a statistically significant association between infant intake of chocolate and wheeze. Maternal intake of chocolate was associated with reduced risk of wheeze in the study of **Erkkola (2012)** (OR 0.74; 95% CI 0.57-0.94). In the study of **Maslova (2013)** the authors found that pregnant mothers who had a higher intake of artificially-sweetened soft drinks were more likely to have a child with asthma. The study of **Long (1986)** reported that children who wheezed were more likely to have mothers who avoided peanuts (p<0.05)

**Conclusion**

**One cross-sectional study that intake of farm milk in the first year of life is associated with a lower risk of wheeze, but this requires replication. Overall we found no consistent evidence of association between the dietary exposures included in this report, and wheezing or recurrent wheezing.**

Table 7 Studies investigating the association between other foods and wheeze which were not eligible for meta-analysis

| **First Author and year of publication** | **Design** | **Outcome** | **Age** | **N/n** | **Dietary exposure and comparison level** | **Measure of association** | **Effect (bold indicates p-value <0.05)** |
| --- | --- | --- | --- | --- | --- | --- | --- |
|  |  |  |  |  | **INFANT INTAKE** |  |  |
|  |  |  |  |  | **Introduction in 1^st^ month yes/no**  Sterilised water intake | OR | **0.64 (0.45-0.88)** |
| Andreasyan, 2007 (35) | PC | Wheeze | 9 | 699 | Honey | RR | 1.12 (0.61-2.06) |
|  |  | Wheeze |  |  | Chocolate (not low consumption vs. low consumption) | OR | **0.74 (0.59-0.92)** |
| Maijaliisa, 2011 | PC | Rec wheeze | 5 | 2,441 | Chocolate (not low consumption vs. low consumption) |  | NS |
| Lopez Campos, 2001 | CC | Rec wheeze | 6-10 | 75 | Chocolate during first year of life (yes/no) |  | **P-value<0.05** |
|  |  | Rec wheeze |  |  | **Rural exposure in 1^st^ year of life** (neither farm milk nor stable exposure as reference) **:**  Exposed to farm milk and stables | OR | **0.14 (0.04-0.48)** |
|  |  | Wheeze |  |  | Exposed to farm milk and stables | OR | **0.17 (0.06-0.45)** |
|  |  | Wheeze |  |  | Farm milk but not stables | OR | **0.43 (0.20-0.92)** |
| Riedler, 2001 | C-S | Rec wheeze | 9 | 812 | Farm milk but not stables | OR | 0.48 (0.21-1.10) |
| Lopez Campos, 2001 | CC | Rec wheeze | 6-10 | 75 | ‘Industrial milk’ from birth to 1^st^ year of life | OR | NS |
|  |  |  |  |  | **Infant intake (earlier vs. later months**):  Meat |  | NS |
| Miskelly, 1988 | PC | Wheeze | 0-1 | 482 | Meat other than beef |  | NS |
|  |  |  |  |  | **MATERNAL INTAKE** |  |  |
| Lange, 2010 | PC | DD asthma | 3 | 1,376 | **Alternate Healthy Eating Index for pregnancy (AHEI-P)/score comprised of 9 components contributing 10 possible points each:**  Vegetables, fruit, ratio of white to red meat, fibre, trans fat, ratio of poly- to saturated fatty acids, folate, calcium, and iron from foods | OR | 1.07 (0.88-1.30) |
|  |  | Rec wheeze |  |  | **Alternate Healthy Eating Index for pregnancy (AHEI-P)**  Vegetables, fruit, ratio of white to red meat, fibre, trans fat, ratio of poly- to saturated fatty acids, folate, calcium, and iron from foods | OR | 1.07 (0.88-1.30) |
|  |  | Rec wheeze |  |  | **Adherence to intake of traditional Mediterranean foods**  Dairy, fish, fruit, legumes, nuts, fats, vegetables and whole grains (alcohol intake excluded from score due to pregnant status of sample studied) | OR | 0.98 (0.89-1.08) |
| Lange, 2010 | PC | DD asthma | 3 | 1,376 | **Adherence to intake of traditional Mediterranean foods**  Dairy, fish, fruit, legumes, nuts, fats, vegetables and whole grains | OR | 1.01 (0.94-1.09) |
|  |  | Rec wheeze |  |  | **Maternal dietary pattern derived from Principal Component Analysis (PCA):**  Pattern comprised of red meat, processed meat, refined grains, snacks, sweets, desserts, French fries, pizza | OR | 0.98 (0.81-1.19) |
|  |  | Rec wheeze |  |  | 'Prudent' pattern i.e. fruits, tomatoes, cabbages, green leafy vegetables, poultry, and fish | OR | 1.02 (0.83-1.26) |
|  |  | DD asthma |  |  | 'Prudent' pattern i.e. fruits, tomatoes, cabbages, green leafy vegetables, poultry, and fish / | OR | 1.08 (0.93-1.26) |
| Lange, 2010 | PC | DD asthma | 3 | 1,376 | Pattern comprised of red meat, processed meat, refined grains, snacks, sweets, desserts, French fries, pizza | OR | 1.08 (0.93-1.26) |
| Castro-Rodriguez, 2010 | C-S | Rec wheeze | 1.4 | 1,409 | Mediterranean diet during pregnancy-Mediterranean diet score (22 - point scale) | OR | 0.96 (0.84-1.10) |
| Chatzi, 2008 | PC | Atopic wheeze | 6.5 | 468 | Maternal adherence to Mediterranean diet (MD) during pregnancy / high MD quality/tiles vs. low MD quality/tiles ( score=4-7 high MD quality vs ≤3, low MD quality) | OR | **0.55 (0.31-0.97)** |
|  |  |  |  |  | **Maternal intake during pregnancy/ Dietary patterns derived from PCA**/ Highest vs lowest quartiles of scores for each dietary pattern  ‘Western’ | OR | **0.59 (0.36-0.98)** |
|  |  |  |  |  | ‘Healthy’ | OR | 0.68 (0.40-1.15) |
| Miyake, 2011 | PC | Wheeze | 1.3 - 2 | 763 | ‘Japanese’ | OR | 1.41 (0.85-2.35) |
|  |  |  |  |  | **Maternal dietary patterns during pregnancy (estimates of risk per SD of dietary pattern score):**  'Traditional' | OR | 0.96 (0.85-1.08) |
|  |  | Rec wheeze | 0.5 | 8,886 | 'Health Conscious' | OR | 1.00 (0.86-1.16) |
|  |  |  |  |  | 'Processed' | OR | 1.00 (0.88-1.13) |
|  |  |  |  |  | 'Confectionery' | OR | 1.02 (0.90-1.16) |
| Shaheen, 2009 | PC |  |  |  | 'Vegetarian' | OR | 1.06 (0.97-1.16) |
|  |  |  |  |  | **Maternal dietary patterns during pregnancy (estimates of risk per SD of dietary pattern score):**  ‘Health conscious’ | OR | 0.96 (0.88-1.05) |
|  |  |  |  |  | ‘Vegetarian’ | OR | 0.97 (0.90-1.04) |
|  |  |  |  |  | 'Confectionery' | OR | 0.98 (0.91-1.06) |
|  |  |  |  |  | 'Traditional' | OR | 1.00 (0.93-1.07) |
| Shaheen, 2009 | PC | Wheeze | 3.5 | 8,886 | 'Processed' | OR | 1.02 (0.95-1.10) |
|  |  |  |  | 7,707 | 'Processed' | OR | 0.92 (0.84-1.01) |
|  |  |  |  | 7,625 | 'Health Conscious' | OR | 0.95 (0.87-1.04) |
|  |  |  |  | 7,625 | 'Traditional' | OR | 0.96 (0.89-1.04) |
|  |  |  |  | 7,625 | 'Processed' | OR | 0.98 (0.90-1.07) |
|  |  |  |  | 7,625 | 'Confectionery' | OR | 1.00 (0.93-1.08) |
| Shaheen, 2009 | PC | Asthma | 7.5 | 7,707 | 'Health Conscious' | OR | 1.00 (0.90-1.11) |
|  |  |  |  | 7,707 | 'Traditional' | OR | 1.00 (0.93-1.08) |
|  |  |  |  | 7,707 | 'Confectionery' | OR | 1.02 (0.93-1.12) |
|  |  |  |  | 7,625 | 'Vegetarian' | OR | 1.02 (0.95-1.09) |
|  |  |  |  | 7,707 | 'Vegetarian' | OR | 1.02 (0.95-1.10) |
| Narita, 2011 | PC | Wheeze | 3 | 1,344 | Maternal consumption of dairy products during pregnancy (Lowest vs. highest quartile) |  | NS |
|  |  |  |  |  | **Maternal intake of various foods during pregnancy** / in quarters ( mid-half values as reference; cut-off values g/d):  Chocolate (7.14-26.7 vs. <7.14g/d) | OR | **0.74 (0.57-0.94)** |
|  |  |  |  |  | Cereal (155.0-246.2 vs. <155.0 g/d) | OR | 0.94 (0.72-1.23) |
|  |  |  |  |  | Meat (119.2-197.7 vs. <119.2 g/d) | OR | 1.06 (0.83-1.36) |
| Erkkola, 2012, | PC | Wheeze | 5 | 2,441 | Fermented milk (100.0-309.6 vs. <100.0 g/d) | OR | 0.92 (0.72-1.16) |
|  |  |  |  |  | Cheese (30.3-85.8 vs. <30.3 g/d) | OR | 1.16 (0.91-1.49) |
|  |  |  |  |  | Milk (214.0-766.4 mL/d vs. <214.0 mL/d) | OR | 1.04 (0.82-1.32) |
|  |  |  |  |  | Probiotic (highest vs. lowest) | HR | 1.38 (0.81-2.34) |
|  |  |  |  |  | Probiotic (highest vs. lowest) | OR | 0.81 (0.64-1.03) |
|  |  |  |  |  | Dairy (highest vs. lowest) | OR | 1.12 ( 0.88-1.41) |
|  |  |  |  |  | **Maternal intake of various foods during pregnancy / in quarters ( mid-half values as reference; cut-off values g/d):**  Chocolate (7.14-26.7 vs. <7.14g/d) | HR | 0.73 (0.45-1.18) |
|  |  |  |  |  | Fermented milk (100.0-309.6 vs. <100.0 g/d) | HR | 0.83 (0.52-1.32) |
|  |  |  |  |  | Cereal (155.0-246.2 vs. <155.0 g/d) | HR | 0.62 (0.35-1.08) |
| Erkkola, 2012 | PC | Rec wheeze | 5 | 2,441 | Meat (119.2-197.7 vs. <119.2 g/d) | HR | 0.80 (0.46-1.39) |
|  |  |  |  |  | Cheese (30.3-85.8 vs. <30.3 g/d) | HR | 1.61 (0.95-2.73) |
|  |  |  |  |  | Milk (214.0-766.4 mL/d vs. <214.0 mL/d) | HR | 0.98 (0.60-1.61) |
|  |  |  |  |  |  |  |  |
|  |  |  |  |  | Confectionery/ sweet drinks (highest vs. lowest) | OR | 1.00 (0.66-1.82) |
| Dai, 1993 | CC | Rec wheeze | 0.5 | 70 | Maternal allergenic food avoidance (any) (no vs yes) |  | **P-value <0.001** |
|  |  |  |  |  | **Maternal intake during pregnancy** (highest quartile vs mid-half intake) :  Meat (>156.4 vs. 88.49–156.44) | HR | 1.06 (0.67-1.67) |
| Lumia, 2011 | PC | Rec wheeze | 5 | 2,679 | Milk and milk products (>1099.8 vs. 546.62–1099.8) | HR | 0.73 (0.44-1.21) |
|  |  |  | 1.5-7 |  | **Maternal intake of artificially-sweetened carbonated soft drinks:**  None vs. any servings (per week) | OR | 0.98 (0.84-1.14) |
|  |  |  | 1.5-7 |  | None vs. <1servings per week | OR | 1.01 (0.86-1.19) |
|  |  |  | 1.5 |  | None vs. any servings (weekly) | OR | 1.06 (0.98-1.15) |
|  |  |  | 1.5 |  | None vs. ≥ 1 servings per day | OR | 1.10 (0.95-1.27) |
| Maslova, 2013 | PC | Rec wheeze | 1.5-7 |  | **Maternal intake of artificially-sweetened carbonated soft drinks:**  any (weekly) vs none | OR | 1.13 (0.96-1.33) |
|  |  |  | 1.5 |  | <1 serving per week vs none | OR | 1.17 (1.08-1.27) |
|  |  |  | 1.5-7 |  | <1 serving per week vs none | OR | 1.20 (0.97-1.48) |
|  |  |  | 1.5 |  | Any (weekly) vs none | OR | **1.21 (1.12-1.31)** |
|  |  |  | 1.5-7 | 60,466 | ≥ 1 serving per day vs none | OR | **1.30 (1.02-1.66)** |
|  |  |  |  |  | **Maternal intake (highest vs lowest quartile):**  Cheese | OR | 0.51 (0.31-0.85) |
|  |  |  | 1.5-2 |  | Milk | OR | **0.50 (0.29-0.87)** |
|  |  |  |  |  | Yoghurt | OR | 1.26 (0.74-2.16) |
| Miyake, 2010 | PC | Wheeze | 0-4 | 763 | Dairy (highest vs. lowest) | OR | **0.45 (0.26-0.79)** |
| Bertelsen, 2013 | PC | Rec wheeze | <3 | 54,740 | **Maternal intake of milk-based products** containing probiotic lactobacilli during pregnancy / yes vs. no |  | NS |
|  |  | Rec wheeze |  |  | Meat/ Introduction after 5.5 months vs. <5.1; or between 5.1-5.5 months vs less | HR | NS |
| Nwaru, 2013 | PC | Rec atopic wheeze | 0-0.5 | 3,109 | Meat/ Introduction after 5.5 months vs. <5.1; or between 5.1-5.5 months vs less | HR | NS |
| Oliveti, 1995 | PC | Rec wheeze | 9 | 262 | Maternal intake of alcohol (often vs. rare) | OR | 2.28 (0.91-5.68) |
|  |  | Wheeze |  |  | **Maternal intake pregnancy**  Whole grain products |  | NS |
|  |  |  |  |  | Whole grain products |  | NS |
|  |  |  |  |  | Dairy (often vs. rare) | OR | 0.88 (0.71-1.09) |
| Willers, 2008 | PC | Rec wheeze | 5 | 1,212 | Egg (often vs. rare) | OR | 0.96 (0.82-1.12) |

# 6. Other foods and risk of rhino-conjunctivitis

# 6.1. Overall risk of bias between other foods and rhino-conjunctivitis

Eleven studies investigated the association between various foods and risk of rhino-conjunctivitis. Over 50% of these were judged to have a low or unclear overall risk of bias. The majority of the studies had a low risk of bias due to lack of confounding, whilst over 40% were considered to have a high risk of bias due to risk of selection or assessment bias (Figure 7).

Figure 7 Risk of Bias in studies of other foods intake and rhino-conjunctivitis

# 6.2. Studies not meta-analysed that examined risk of rhino-conjunctivitis and intake of various foods or nutrients

Across the nine studies that investigated the association between other foods and risk of rhino-conjunctivitis, there was little evidence for any associations. Two studies investigated dietary patterns. The study of **Shaheen (2009)** investigated maternal dietary patterns during pregnancy derived from PCA, whilst **de Batlle (2008)** derived a Mediterranean score adherence in children and examined their association with symptoms of rhino-conjunctivitis. Neither study found evidence of a relationship between dietary patterns and risk. The study of **Bertelsen (2013)** reported that regular intake of probiotics during pregnancy was associated with lower risk of RC in children (OR 0.87; 95% CI 0.77-0.98). **Riedler (2001)** found that intake of farm milk (with or without spending time in the stables) in the first year was associated with a lower risk of RC ever and current RC at age 9 years old. Other dietary exposures studied included several single foods, for which no evidence of association was reported.

**Conclusion**

**We found no evidence that specific dietary patterns consumed during pregnancy or childhood are related to risk of rhino-conjunctivitis. One prospective cohort study reported maternal intake of probiotics during pregnancy was associated with reduced rhino-conjunctivitis at age 3 years, but this requires replication. One cross-sectional study reported intake of farm milk during infancy is associated with reduced RC in childhood, but this also requires replication.**

Table 8 Studies investigating the association between other foods and rhino-conjunctivitis which were not eligible for meta-analysis

| **First Author and year of publication** | **Design** | **Outcome** | **Age** | **N/n** | **Dietary exposure and comparison level** | **Measure of association** | **Effect (bold indicates p-value <0.05)** |
| --- | --- | --- | --- | --- | --- | --- | --- |
|  |  |  |  |  | **INFANT INTAKE** |  |  |
| Andreasyan, 2007 | PC | RC | 9 | 711 | **Introduction of non-milk fluids >1 month vs <1 month**  Fruit syrup | OR | 3.45 (0.52-22.83) |
|  |  |  |  |  | Sterilised water | OR | 1.08 (0.77-1.51) |
|  |  |  |  |  | Honey (by 1^st^ month vs later) | OR | 1.31 (0.65-2.64) |
| Marini, 1996 | PC | RC | 3 | 80 | Introduction of meat in months 5 to 7 vs earlier | OR | 1.43 (0.84-2.43) |
| Riedler, 2001 | CS | RC | 9 | 812 | **Exposure to rural environment in the 1^st^ year:**  Exposed to farm milk and stables | OR | **0.20 (0.08-0.50)** |
|  |  |  |  |  | Exposed to farm milk but not stables vs neither | OR | **0.24 (0.10-0.56)** |
|  |  |  |  |  |  |  |  |
|  |  |  |  |  |  |  |  |
|  |  |  |  |  |  |  |  |
|  |  |  |  |  |  |  |  |
|  |  |  |  |  |  |  |  |
|  |  |  |  |  | **MATERNAL INTAKE** |  |  |
|  |  |  |  |  | **Maternal dietary intake of various foods (median intake g/d as reference)**  Chocolate (7.14-26.7 vs. <7.14 ) | HR | 0.85 (0.61-1.17) |
|  |  |  |  |  | Cereal (155.0-246.2 vs. <155.0) | HR | 0.88 (0.62-1.24) |
|  |  |  |  |  | Meat (119.2-197.7 vs. <119.2) | HR | 1.18 (0.84-1.65) |
| Erkkola, 2012 | PC | RC | 5 | 2,441 | Fermented milk products (100.0-309.6 vs. <100.0 mL) | HR | 1.00 (0.72-1.38) |
|  |  |  |  |  | Cheese (30.3-85.8 vs. <30.3) | HR | 1.27 (0.89-1.79) |
|  |  |  |  |  | Cheese (85.8-380.9 vs. 30.3-85.8) | HR | 1.17 (0.86-1.60) |
|  |  |  |  |  | Milk (214.0-766.4 vs. <214.0) | HR | 1.09 (0.80-1.48) |
| Maijaliisa, 2011 | PC | RC | 5 | 2,441 | Maternal intake of chocolate/ Not low consumption vs. low consumption | OR | NS |
|  |  |  | 1.5-7 |  | **Maternal intake of artificially-sweetened carbonated soft drinks** during pregnancy Weekly vs none | OR | 1.15 (0.98-1.35) |
|  |  |  | 1.5 |  | Weekly vs none | OR | 0.86 (0.71-1.04) |
|  |  |  | 1.5 |  | <1 serving per week vs none | OR | 0.96 (0.76-1.21) |
| Maslova, 2013 | PC | RC | 1.5-7 | 60,466 | <1 serving per week vs none | OR | 0.97 (0.81-1.16) |
|  |  |  |  |  | **Maternal dietary intake (estimated as dietary patterns derived with PCA) -'risk estimates per SD of dietary pattern score)**  ‘Traditional' | OR | 1.04 (0.96-1.13) |
|  |  |  |  |  | 'Vegetarian' | OR | 0.97 (0.89-1.06) |
| Shaheen, 2009 (2) | PC | RC | 7.5 | 7,674 | 'Health Conscious' | OR | 1.00 (0.90-1.11) |
|  |  |  |  |  | 'Confectionery' | OR | 1.01 (0.92-1.11) |
|  |  |  |  |  | 'Processed' | OR | 1.04 (0.96-1.13) |
| Willers, 2007 | PC | RC | 5 | 1,212 | Maternal intake of whole grain products |  | NS |
| Bertelsen, 2013 | PC | RC | 3 | 40,614 | Maternal intake of probiotic (often vs. rare) | OR | **0.87 (0.77-0.98)** |

# 7. Other foods and risk of allergic sensitization

# 7.1. Overall risk of bias between other foods and allergic sensitisation

We identified ten studies that investigated the association between other foods and risk of allergic sensitisation. Over 60% of the studies were judged to have a low risk of bias, whilst a third of the studies had an unclear risk of selection bias (Figure 8).

Figure 8 Risk of Bias in studies of other foods intake and allergic sensitisation

# 7.2. Introduction of meat and risk of aero-allergen sensitisation

The prospective cohort studies of **Nwaru (2010)** and **Zutavern (2004)** had comparable data to pool estimates of risk of rhino-conjunctivitis and meat introduction at months 5-7 (Figure 9). There was no evidence of an association with this exposure and outcome, with no statistical heterogeneity between studies (I^2^=0%)

Figure 9 Introduction of meat in months 5 to 7 and risk of sensitisation to aero-allergens (odds ratio) in children

# 7.3. Studies that were not meta-analysed and that investigated the association between other foods and risk of allergic sensitisation

Amongst the studies identified (Table 9) the dietary exposures investigated included single foods (dairy, confectionary, meat, and cereal intake). Two studies included maternal food avoidance during pregnancy **(Butiene 2001; Dubakiene 2012**), and two studies (**Shaheen 2009; Chatzi 2008)** studied dietary patterns during pregnancy. The study of **Chatzi (2008)** found that children of mothers who had a higher adherence to a Mediterranean diet (MD) during pregnancy were less likely to be sensitised to aero-allergens at age 6.5 years old (OR 0.55; 95% CI 0.31–0.97). One further study investigated intake of farm milk in the first year (**Riedler 2001).** The studies of **Nwaru (2010)**, **Sausenthaler (2007)**, and **Chatzi (2008)** also investigated maternal intake of dairy products, but none of these found evidence of an association with outcomes of sensitisation in the offspring. While **Nwaru (2010)** found evidence from one analysis of meat introduction and AS-Egg that early meat introduction may decrease AS, other measures of AS and other comparisons related to timing or amount of infant meat intake from this study and others did not support an association.

**Conclusion**

**We found no consistent evidence that any of these exposures are associated with risk of AS.**

Table 9 Studies investigating the association between other foods and allergic sensitization which were not eligible for meta-analysis

| **First Author and year of publication** | **Design** | **Outcome** | **Age** | **N/n** | **Dietary exposure and comparison level** | **Measure of association** | **Effect (bold indicates p-value <0.05)** |
| --- | --- | --- | --- | --- | --- | --- | --- |
|  |  |  |  |  | **INFANT INTAKE** |  |  |
|  |  |  |  |  | **Introduction of non-milk fluids** (After 1^st^ month vs earlier)  Fruit syrup | OR | 1.16 (0.58-2.34) |
| Andreasyan, 2007 | PC | SPT to any | 9 | 699 | Sterilised water | OR | 1.01 (0.81-1.27) |
|  |  |  |  |  | Honey (By 1^st^ month vs later) | OR | 1.22 (0.80-1.86) |
| Zutavern, 2008 | PC | sIgE food | 6 | 2073 | Infant meat introduction by 4 months vs. 4-6 months | OR | NS |
|  |  | sIgE aero | 9 | 812 | **Exposure to farm milk AND stable in the 1^st^ year:** Exposed to farm milk and stables vs. Neither farm milk nor stable exposure | OR | **0.32 (0.17-0.62)** |
| Riedler, 2001 | CS | sIgE aero | 9 | 812 | Exposed to farm milk but not stables vs. Neither farm milk nor stable exposure | OR | **0.43 (0.24-0.77)** |
|  |  | sIgE aero |  |  | **Introduction to infant’s diet:**  Meat (5.1-5.5 months vs. <5.1 months) | OR | 0.80 (0.48-1.33) |
| Nwaru, 2010 | PC | sIgE cm |  |  | Meat (third tertile vs. second tertile) |  | NS |
|  |  | sIgE egg |  |  | Meat (third tertile vs. first tertile) | Increased sensitisation | **P=0.029** |
|  |  | sIgE food | 5 | 994 | Meat (5.1-5.5 months vs. <5.1 months) | OR | 0.82 (0.45-1.48) |
| Chatzi, 2008 | (C-S/ PC) | SPT aero | 6.5 | 468 | **Child’s adherence to Mediterranean diet (MD) at age 6.5 years** (high MD quality vs. low MD quality ; where score≥7 high, 4-6 medium, and <4 low MD quality)  High MD score vs. low | OR | 0.49 (0.18–1.32) |
|  |  |  |  |  | Medium MD score vs. low | OR | 0.58 (0.23–1.45) |
|  |  |  |  |  |  |  |  |
|  |  |  |  |  |  |  |  |
|  |  |  |  |  |  |  |  |
|  |  |  |  |  | **MATERNAL INTAKE** |  |  |
| Chatzi, 2008 | PC | SPT aero | 6.5 | 468 | **Maternal dietary patterns during pregnancy:**  Adherence to Mediterranean diet (MD) (high MD quality vs. low MD quality ; where score≥7 high, 4-6 medium, and <4 low MD quality)  High MD score vs. low | OR | **0.55 (0.31–0.97)** |
|  |  |  |  |  | **Maternal intake as dietary patterns/ (odds per SD of dietary pattern score):**  ‘Processed' | OR | 0.93 (0.86-1.01) |
| Shaheen, 2009 | PC | SPT aero | 7 | 6085 | 'Health Conscious’ | OR | 0.95 (0.87-1.04) |
|  |  |  |  |  | 'Traditional' | OR | 0.98 (0.91-1.05) |
|  |  |  |  |  | 'Vegetarian' | OR | 1.02 (0.95-1.09) |
|  |  |  |  |  | 'Confectionery' | OR | **1.07 (1.00-1.15)** |
|  |  |  |  |  | **Maternal intake during pregnancy (median servings per week):**  Cereal (>11.5 vs. ≤ 11.5) | Prevalence (%) | NS |
| Chatzi, 2008 | PC | SPT aero | 6.5 | 468 | Meat (≥3.25 vs. ≤3.25) |  | NS |
|  |  |  |  |  | White meat (≥2.5 vs. ≤2.5) |  | NS |
|  |  |  |  |  | Dairy (. >23 Median vs ≤23) |  | NS |
|  |  | sIgE aero | 5 | 931 | **Maternal intake at 8^th^ month pregnancy** (continuous g/d):  Cereal | OR | 0.94 (0.53-1.66) |
|  |  | sIgE food | 5 | 931 | Cereal | OR | 1.26 (0.65-2.43) |
| Nwaru, 2010 | PC | sIgE aero | 5 | 931 | Cheese | OR | 1.05 (0.85-1.29) |
|  |  | sIgE food | 5 | 931 | Cheese | OR | 0.96 (0.77-1.19) |
|  |  | sIgE aero | 5 | 931 | Milk | OR | 0.91 (0.74-1.12) |
|  |  | sIgE food | 5 | 931 | Milk | OR | 0.95 (0.75-1.20) |
|  |  | sIgE aero |  | 2102 | **Maternal intake last 4 months of pregnancy (highest vs lowest tertile):**  Cheese | OR | 0.93 (0.57-1.53) |
|  |  | sIgE any |  | 2103 | Cheese | OR | 0.99 (0.72-1.36) |
| Sausenthaler, 2007 | PC | sIgE food |  | 2110 | Cheese | OR | 0.97 (0.68-1.39) |
|  |  | sIgE any |  |  | Yoghurt | OR | 0.81 (0.59-1.10) |
|  |  | sIgE food |  |  | Yoghurt | OR | 0.89 (0.62-1.27) |
|  |  | sIgE aero | 2 | 2110 | Yoghurt | OR | 0.69 (0.43-1.12) |
|  |  | sIgE aero |  |  | **Maternal intake last 4 months of pregnancy** (highest vs lowest tertile):  Milk | OR | 0.95 (0.58-1.57) |
| Sausenthaler, 2007 | PC | sIgE any |  |  | Milk | OR | 0.93 (0.67-1.28) |
|  |  | sIgE food |  |  | Milk | OR | 0.95 (0.66-1.37) |
|  |  | sIgE any |  |  | Egg | OR | 0.91 (0.56-1.28) |
|  |  | sIgE food |  |  | Egg | OR | 0.93 (0.63-1.38) |
|  |  | sIgE aero | 2 | 2110 | Egg | OR | 0.90 (0.53-1.53) |
| Willers, 2007 | PC | SPT any | 5 | 1212 | Maternal intake of whole grain products | OR | NS |
| Dubakiene, 2012 | PC | SPT food | 1 | 128 | Maternal avoidance of milk and egg products during pregnancy and lactation | OR | NS |
| Butiene, 2011 | NCC | SPT/sIgE milk or egg | 1 | 43 | Maternal allergenic food avoidance during pregnancy / milk and egg products | OR | NS |

# 8. Other foods and risk of type 1 diabetes mellitus

# 8.1. Overall risk of bias between other foods and type 1 diabetes mellitus

We identified fifteen studies that investigated the association between other foods and risk of T1DM. The majority of these studies were considered to have a low or unclear overall risk of bias (Figure 10).

Figure 10 Risk of Bias in studies of other foods intake and type 1 diabetes mellitus

# 8.2.  Studies not meta-analysed that explored the association between food intake and T1DM

The most commonly studied dietary exposure was coffee (six observational studies). Other foods included maternal intake of dairy products, eggs, meat, cereals (containing or not gluten), tea and alcohol intake (Table 10). For most of these exposures, there was no consistent evidence of association with T1DM in the offspring. The abstract publication of Ninisto (2012) reported an association between higher intake of fresh milk by mothers during lactation, and reduced T1DM in childhood (OR 0.49; 95% CI 0.28-0.87). However this was in the context of multiple dietary exposure assessments, and the association was only seen for serological diabetes in the first 3 years, in a study which also assessed clinical TIDM and outcomes up to 9 years. It is unclear from this abstract publication how robust this finding was due to variations in the timing and nature of the outcome assessment. The study of Sipetic (2003) found increased maternal intake of tea, coffee, alcohol and coca-cola during pregnancy was associated with T1DM in children in univariate analysis, but this was not confirmed in multivariate analysis, nor in the separate study of Virtanen (2011). There was evidence from one study (Sipetic 2003) that maternal intake of foods rich in nitrosamines was associated with increased T1DM in childhood, an association which persisted in adjusted analysis.

**Conclusion**

**We found evidence from just one case control study with a high risk of assessment bias, that consumption of nitrosamine-rich foods during pregnancy is associated with TIDM. We found no evidence to suggest that other infant or maternal dietary exposures are associated with T1DM, and the finding for nitrosamine-rich foods requires replication.**

Table 10 Studies investigating the association between other foods and type 1 diabetes mellitus which were not eligible for meta-analysis

| **First Author and year of publication** | **Design** | **Outcome** | **Age** | **N/n** | **Dietary exposure and comparison level** | **Measure of association** | **Effect (bold indicates p-value <0.05)** |
| --- | --- | --- | --- | --- | --- | --- | --- |
|  |  |  |  |  | **INFANT INTAKE** |  |  |
|  |  |  |  |  | Milk (1-2dL/day vs no milk) | OR | 1.05 (0.71-1.58) |
| Brekke, 2010 | PC | TIDM | 5 | 4,005 | Egg (1-2 times per week vs <1 per week) | OR | 1.19 (0.82-1.72) |
| Norris, 1996 | PC | TIDM | <7 | 171 | Introduction of meat 5 to 7 months vs. earlier | OR | 4.66 (0.92-23.73) |
| Visalli, 2003 | PC | TIDM | 12 | 900 | Introduction of meat ≥3 months vs <3 months | OR | 0.57 (0.06-5.49) |
| Harsunen, 2012 | NCC | TIDM | <18 | 33/33 | Infant intake of soluble fibre in the diet in the first year of life , adjusted for total energy intake | HR | 0.68 (0.23-1.97) |
| Strotmeyer, 2004 | CC | TIDM | 9.7 | 688 | **Introduction of meat to infant’s diet**:  >4–6 months vs. 4-6 months; or >7-12 vs 7-12 | OR | NS |
|  |  |  |  |  |  |  |  |
|  |  |  |  |  | **MATERNAL INTAKE** |  |  |
| Majeed, 2011 | PC | T1DM | 17 | 395 | Maternal consumption of tea during pregnancy (often vs no intake) | OR | **4.66 (1.36-16.05)** |
|  |  |  |  |  | **Maternal intake in the last trimester (No servings per month)**:  Gluten-containing foods | HR | 0.89 (0.50-1.58) |
|  |  |  |  |  | Non-gluten cereal grains | HR | 0.98 (0.64-1.51) |
|  |  |  |  |  | Meat | HR | 0.91 (0.54-1.52) |
| Lamb 2008 | PC | TIDM | 15 | 642/27 | Cow's milk products | HR | 1.18 (0.74-1.87) |
| Niinisto, 2012 | PC | TIDM | 10 | 2939 | Maternal diet during the 3^rd^ month of lactation / High maternal consumption of fresh milk during lactation or intermediate intake vs. low maternal consumption of fresh milk during lactation | HR | **0.49 (0.28-0.87)** |
|  |  |  |  |  | **Maternal intake in pregnancy (yes vs. no):** Alcohol |  | NS |
|  |  |  |  |  | Coca-Cola |  | NS |
|  |  |  |  |  | Tea, Coffee |  | NS |
| Sipetic, 2005 | CC | TIDM | 16 | 315 | Nitrosamine-rich foods |  | **4.33 (1.95–9.61)** |
| Visalli, 2003 | PC | T1DM | 12 | 900 | Tea intake during pregnancy (yes vs. no) | OR | 0.66 (0.4-1.08) |
| Uusitalo, 2008 | PC | TIDM | <10 | 3730/165 | Maternal milk intake during pregnancy (change in risk per 2-fold increase in intake) | HR | 1.03 (0.82-1.30) |
| Virtanen, 1994 | CC | TIDM | 9 | 1136 | Maternal coffee intake during pregnancy (high intake vs low; cups per day) |  | NS |
| Virtanen, 2011 | PC | TIDM | <10 | 3730/165 | **Maternal intake of during pregnancy:**  Coffee (highest vs. lowest) | OR | **0.59 (0.37-0.96)** |
|  |  |  |  |  | Tea (highest vs. lowest) | OR | 0.74 (0.45-1.21) |
|  |  |  |  |  | Wheat (Continuous exposure) | HR | 1.04 (0.79-1.37) |
|  |  |  |  |  | Cereal | HR | 1.09 (0.72-1.65) |
|  |  |  |  | 3730 | Rice | HR | 1.11 (0.93-1.32) |
|  |  |  |  |  | Chocolate and sweets | HR | 1.00 (0.89-1.12) |
| Virtanen, 2011 | PC | TIDM |  |  | Egg | HR | 0.94 (0.74-1.20) |
|  |  |  |  |  | Meat | HR | 1.12 (0.87-1.44) |
|  |  |  | <10 | 3730/165 | Milk and dairy | HR | 0.92 (0.72-1.17) |
| Wahlberg, 2006 | PC | T1DM | 2.5 | 8,715 | Maternal food avoidance of milk | OR | NS |

# 9. Other foods and risk of juvenile idiopathic arthritis (JIA)

# 9.1. Overall risk of bias between other foods and juvenile idiopathic arthritis

We identified one case-control study examining the association between maternal intake of alcohol and risk of JIA. The CLARITY - ChiLdhood Arthritis Risk factor Identification sTudY- is an Australian study aimed at identifying genomic and environmental disease risk factors for JIA. Within this study, the authors retrospectively examined alcohol intake in mothers during pregnancy and risk of JIA before age of 18 years old. The study was considered to have an unclear overall risk (Figure 15). The study found no evidence to suggest that maternal alcohol intake was a risk factor for JIA (Table 9).

Figure 11 Risk of Bias in studies of other foods intake and juvenile idiopathic arthritis

Table 11 Studies investigating the association between other foods and juvenile idiopathic arthritis which were not eligible for meta-analysis

| **First Author and year of publication** | **Design** | **Outcome** | **Age** | **N/n** | **Dietary exposure and comparison level** | **Measure of association** | **Effect** |
| --- | --- | --- | --- | --- | --- | --- | --- |
| Ellis, 2012 | RC | JIC | Any |  | Maternal intake of alcohol (often vs. rare) | OR | 0.66 (0.36-1.21) |

# 10. Other foods and risk of inflammatory bowel disease

# 10.1. Overall risk of bias in studies on other foods and inflammatory bowel disease

We identified a case-control study investigating the association between earlier introduction of meat and risk of IBD in children. The study had a low risk of selection and confounding bias, but had unclear assessment bias, which influenced the overall bias as being considered unclear (Figure 16). There was no evidence of an association between meat introduction and risk of disease (Table 10).

Figure 12 Risk of Bias in studies on other foods intake and inflammatory bowel disease

Table 12 Studies investigating the association between other food and irritable bowel disease which were not eligible for meta-analysis

| **First Author and year of publication** | **Design** | **Outcome** | **Age** | **N/n** | **Dietary exposure and comparison level** | **Measure of association** | **Effect** |
| --- | --- | --- | --- | --- | --- | --- | --- |
|  |  | IBD-CR | <17 | 444 | Meat introduction months (continuous) | OR | NS |
| Baron, 2005 | CC | IBD-UC | <17 | 444 | Meat introduction months (continuous) | OR | NS |

# Conclusions

In this report, intake of several foods during pregnancy, or in the first year of life was investigated in relation to the risk of allergic and autoimmune diseases. Exposures were dietary pattern, maternal allergenic food avoidance, alcohol, tea or coffee intake, and maternal or infant meat, cereal, milk or egg intake. We found no consistent evidence to suggest that maternal dietary habits during pregnancy or lactation, or infant diet, influence the risk of any of the outcomes considered in this report. Our findings are consistent with those reported recently by Lv *at al* who found no evidence for a relationship between maternal dietary patterns and risk of allergic diseases in the offspring . The weak evidence generated from single case-control studies, studies at high risk of bias, and inconsistent datasets requires confirmation in replication studies before it can be accepted as evidence for relationships between other dietary exposures and allergic or autoimmune outcomes.

# References

1.	(NICE) NIfHaCE. The guidelines manual methodology checklist. 2012

2.	Willers SM, Devereux G, Craig LC, McNeill G, Wijga AH, Abou El-Magd W, et al. Maternal food consumption during pregnancy and asthma, respiratory and atopic symptoms in 5-year-old children. Thorax. 2007;62(9):773-9.

3.	Shaheen SO, Northstone K, Newson RB, Emmett PM, Sherriff A, Henderson AJ. Dietary patterns in pregnancy and respiratory and atopic outcomes in childhood. Thorax. 2009;64(5):411-7.

4.	Fergusson DM, Horwood LJ, Shannon FT. Early solid feeding and recurrent childhood eczema: a 10-year longitudinal study. Pediatrics. 1990;86(4):541-6.

5.	Bisgaard H, Halkjaer LB, Hinge R, Giwercman C, Palmer C, Silveira L, et al. Risk analysis of early childhood eczema. Journal of Allergy & Clinical Immunology. 2009;123(6):1355-60.e5.

6.	Linneberg A, Petersen J, Gronbaek M, Benn CS. Alcohol during pregnancy and atopic dermatitis in the offspring. Clinical & Experimental Allergy. 2004;34(11):1678-83.

7.	Maslova E, Strom M, Olsen SF, Halldorsson TI. Consumption of Artificially-Sweetened Soft Drinks in Pregnancy and Risk of Child Asthma and Allergic Rhinitis. PLoS ONE. 2013;8(2).

8.	Nwaru BI, Takkinen HM, Niemela O, Kaila M, Erkkola M, Ahonen S, et al. Timing of infant feeding in relation to childhood asthma and allergic diseases. Journal of Allergy and Clinical Immunology. 2013;131(1):78-86.

9.	Nwaru BI, Erkkola M, Ahonen S, Kaila M, Haapala AM, Kronberg-Kippila C, et al. Age at the introduction of solid foods during the first year and allergic sensitization at age 5 years. Pediatrics. 2010;125(1):50-9.

10.	Nwaru BI, Ahonen S, Kaila M, Erkkola M, Haapala AM, Kronberg-Kippila C, et al. Maternal diet during pregnancy and allergic sensitization in the offspring by 5 yrs of age: a prospective cohort study. Pediatric Allergy & Immunology. 2010;21(1 Pt 1):29-37.

11.	Nwaru BI, Erkkola M, Ahonen S, Kaila M, Lumia M, Prasad M, et al. Maternal diet during lactation and allergic sensitization in the offspring at age of 5. Pediatric Allergy & Immunology. 2011;22(3):334-41.

12.	Virtanen SM, Kaila M, Pekkanen J, Kenward MG, Uusitalo U, Pietinen P, et al. Early introduction of oats associated with decreased risk of persistent asthma and early introduction of fish with decreased risk of allergic rhinitis. British Journal of Nutrition. 2010;103(2):266-73.

13.	Maijaliisa E, Bright IN, Minna K, Kronberg-Kippila C, Jorma I, Olli S, et al. Maternal food consumption during pregnancy and risk of asthma and allergic outcomes in the offspring. Annals of Nutrition and Metabolism. 2011;58:369.

14.	Niinisto S, Takkinen HM, Uusitalo L, Nevalainen J, Kenward M, Lumia M, et al. Maternal fatty acid composition of diet during lactation and risk of preclinical and clinical type 1 diabetes. Diabetologia. 2012;55:S141-S2.

15.	Lumia M, Luukkainen P, Tapanainen H, Kaila M, Erkkola M, Uusitalo L, et al. Dietary fatty acid composition during pregnancy and the risk of asthma in the offspring. Pediatric Allergy & Immunology. 2011;22(8):827-35.

16.	Erkkola M, Nwaru BI, Kaila M, Kronberg-Kippila C, Ilonen J, Simell O, et al. Risk of asthma and allergic outcomes in the offspring in relation to maternal food consumption during pregnancy: a Finnish birth cohort study. Pediatric Allergy & Immunology. 2012;23(2):186-94.

17.	Uusitalo L, Kenward MG, Virtanen SM, Uusitalo U, Nevalainen J, Niinisto S, et al. Intake of antioxidant vitamins and trace elements during pregnancy and risk of advanced beta cell autoimmunity in the child. American Journal of Clinical Nutrition. 2008;88(2):458-64.

18.	Alm B, Aberg N, Erdes L, Mollborg P, Pettersson R, Norvenius SG, et al. Early introduction of fish decreases the risk of eczema in infants. Archives of Disease in Childhood. 2009;94(1):11-5.

19.	Laitinen K, Kalliomaki M, Poussa T, Lagstrom H, Isolauri E. Evaluation of diet and growth in children with and without atopic eczema: follow-up study from birth to 4 years. British Journal of Nutrition. 2005;94(4):565-74.

20.	Zutavern A, Brockow I, Schaaf B, Bolte G, von Berg A, Diez U, et al. Timing of solid food introduction in relation to atopic dermatitis and atopic sensitization: results from a prospective birth cohort study. Pediatrics. 2006;117(2):401-11.

21.	Zutavern A, Brockow I, Schaaf B, von Berg A, Diez U, Borte M, et al. Timing of solid food introduction in relation to eczema, asthma, allergic rhinitis, and food and inhalant sensitization at the age of 6 years: results from the prospective birth cohort study LISA. Pediatrics. 2008;121(1):e44-52.

22.	Sausenthaler S, Koletzko S, Schaaf B, Lehmann I, Borte M, Herbarth O, et al. Maternal diet during pregnancy in relation to eczema and allergic sensitization in the offspring at 2 y of age. American Journal of Clinical Nutrition. 2007;85(2):530-7.

23.	Marini A, Agosti M, Motta G, Mosca F. Effects of a dietary and environmental prevention programme on the incidence of allergic symptoms in high atopic risk infants: three years' follow-up. Acta Paediatrica Supplement. 1996;414:1-21.

24.	Chatzi L, Torrent M, Romieu I, Garcia-Esteban R, Ferrer C, Vioque J, et al. Mediterranean diet in pregnancy is protective for wheeze and atopy in childhood. Thorax. 2008;63(6):507-13.

25.	Miskelly FG, Burr ML, Vaughan-Williams E, Fehily AM, Butland BK, Merret TG. Infant feeding and allergy. Archives of Disease in Childhood. 1988;63(4):388-93.

26.	Morgan J, Williams P, Norris F, Williams CM, Larkin M, Hampton S. Eczema and early solid feeding in preterm infants. Archives of Disease in Childhood. 2004;89(4):309-14.

27.	Bertelsen RJ, Brantsaeter AL, Haugen M, Meltzer HM, London SJ. Maternal probiotic intake and respiratory and allergy outcomes in early childhood. Journal of Allergy and Clinical Immunology. 2013;1):AB129.

28.	Miyake Y, Okubo H, Sasaki S, Tanaka K, Hirota Y. Maternal dietary patterns during pregnancy and risk of wheeze and eczema in Japanese infants aged 16-24 months: the Osaka Maternal and Child Health Study. Pediatric Allergy & Immunology. 2011;22(7):734-41.

29.	Miyake Y, Sasaki S, Tanaka K, Hirota Y. Dairy food, calcium and vitamin D intake in pregnancy, and wheeze and eczema in infants. European Respiratory Journal. 2010;35(6):1228-34.

30.	Miyake Y, Sasaki S, Tanaka K, Ohfuji S, Hirota Y. Maternal fat consumption during pregnancy and risk of wheeze and eczema in Japanese infants aged 16-24 months: the Osaka Maternal and Child Health Study. Thorax. 2009;64(9):815-21.

31.	Saito K, Yokoyama T, Miyake Y, Sasaki S, Tanaka K, Ohya Y, et al. Maternal meat and fat consumption during pregnancy and suspected atopic eczema in Japanese infants aged 3-4 months: the Osaka Maternal and Child Health Study. Pediatric Allergy & Immunology. 2010;21(1 Pt 1):38-46.

32.	Roduit C, Frei R, Loss G, Buchele G, Weber J, Depner M, et al. Development of atopic dermatitis according to age of onset and association with early-life exposures. Journal of Allergy & Clinical Immunology. 2012;130(1):130-6.e5.

33.	Willers SM, Wijga AH, Brunekreef B, Kerkhof M, Gerritsen J, Hoekstra MO, et al. Maternal food consumption during pregnancy and the longitudinal development of childhood asthma. American Journal of Respiratory & Critical Care Medicine. 2008;178(2):124-31.

34.	Lange NE, Rifas-Shiman SL, Camargo CA, Jr., Gold DR, Gillman MW, Litonjua AA. Maternal dietary pattern during pregnancy is not associated with recurrent wheeze in children. Journal of Allergy & Clinical Immunology. 2010;126(2):250-5, 5.e1-4.

35.	Andreasyan K, Ponsonby AL, Dwyer T, Dear K, Cochrane J. Infant feeding and childhood atopy: does early introduction of non-milk fluids matter? Pediatric Allergy & Immunology. 2007;18(3):250-7.

36.	Narita M, Qiu D, Hamaguchi M, Doi M, Futamura M, Sakamoto N, et al. Maternal diet during pregnancy and wheeze and eczema in infants; the Japanese birth cohort (T-CHILD) study. Journal of Allergy and Clinical Immunology. 2011;1):AB174.

37.	Ohya Y, Futamura M, Saito H, Sakamoto N, Aizawa S, Kitagawa M. Maternal retinol intake during pregnancy was a risk factor of offspring's atopic eczema at 18 months. Allergy: European Journal of Allergy and Clinical Immunology. 2010;65:310.

38.	Wang IJ, Guo YL, Weng HJ, Hsieh WS, Chuang YL, Lin SJ, et al. Environmental risk factors for early infantile atopic dermatitis. Pediatric Allergy & Immunology. 2007;18(5):441-7.

39.	Harris JM, Cullinan P, Williams HC, Mills P, Moffat S, White C, et al. Environmental associations with eczema in early life. British Journal of Dermatology. 2001;144(4):795-802.

40.	Zutavern A, von Mutius E, Harris J, Mills P, Moffatt S, White C, et al. The introduction of solids in relation to asthma and eczema. Archives of Disease in Childhood. 2004;89(4):303-8.

41.	Oliver E, Grimshaw K, Scally K, Garland J, Gudgeon L, Foote K, et al. Does nutritional intake in infancy effect later development of food allergy? Allergy: European Journal of Allergy and Clinical Immunology. 2010;65:313. 42.	Sariachvili M, Droste J, Dom S, Wieringa M, Hagendorens M, Stevens W, et al. Early exposure to solid foods and the development of eczema in children up to 4 years of age. Pediatric Allergy & Immunology. 2010;21(1 Pt 1):74-81.

43.	Butiene I, Dubakiene R, Vaicekauskaite D, Drasutiene G. Prevalence of food sensitisation in young children from EuroPrevall Lithuanian birth cohort. Allergy: European Journal of Allergy and Clinical Immunology. 2011;66:233.

44.	Dai L. [An analysis of the risk factors of child bronchial asthma]. Chung-Hua Liu Hsing Ping Hsueh Tsa Chih Chinese Journal of Epidemiology. 1993;14(3):131-5.

45.	Oliveti JF, Kercsmar CM, Redline S. Pre- and perinatal risk factors for asthma in inner city African-American children. American Journal of Epidemiology. 1996;143(6):570-7.

46.	Lopez Campos C, Munoz Hernandez MA, Lopez Campos JE, Carrillo Lucero J, Rincon Castaneda CB, Martinez Cairo Cueto S. [Asthma risk factors]. Revista Alergia Mexico. 2001;48(4):103-6.

47.	de Batlle J, Garcia-Aymerich J, Barraza-Villarreal A, Anto JM, Romieu I. Mediterranean diet is associated with reduced asthma and rhinitis in Mexican children. Allergy. 2008;63(10):1310-6.

48.	Dela Bianca A, Wandalsen G, Mallol J, Sole D. Risk factors for wheezing disorders in infants in the first year of life living in Sao Paulo, Brazil. Journal of Tropical Pediatrics. 2012;58(6):501-4.

49.	Riedler J, Braun-Fahrlander C, Eder W, Schreuer M, Waser M, Maisch S, et al. Exposure to farming in early life and development of asthma and allergy: a cross-sectional survey. Lancet. 2001;358(9288):1129-33.

50.	Castro-Rodriguez JA, Garcia-Marcos L, Sanchez-Solis M, Perez-Fernandez V, Martinez-Torres A, Mallol J. Olive oil during pregnancy is associated with reduced wheezing during the first year of life of the offspring. Pediatric Pulmonology. 2010;45(4):395-402.

51.	Binkley KE, Leaver C, Ray JG. Antenatal risk factors for peanut allergy in children. Allergy, Asthma, & Clinical Immunology : Official Journal of the Canadian Society of Allergy & Clinical Immunology. 2011;7:17.

52.	Wahlberg J, Fredriksson J, Nikolic E, Vaarala O, Ludvigsson J, Group AB-S. Environmental factors related to the induction of beta-cell autoantibodies in 1-yr-old healthy children. Pediatric Diabetes. 2005;6(4):199-205.

53.	Brekke HK, Ludvigsson J. Daily vegetable intake during pregnancy negatively associated to islet autoimmunity in the offspring--the ABIS study. Pediatric Diabetes. 2010;11(4):244-50.

54.	Wahlberg J, Vaarala O, Ludvigsson J, group AB-s. Dietary risk factors for the emergence of type 1 diabetes-related autoantibodies in 21/2 year-old Swedish children. British Journal of Nutrition. 2006;95(3):603-8.

55.	Virtanen SM, Uusitalo L, Kenward MG, Nevalainen J, Uusitalo U, Kronberg-Kippilad C, et al. Maternal food consumption during pregnancy and risk of advanced beta-cell autoimmunity in the offspring. Pediatric Diabetes. 2011;12(2):95-9.

56.	Virtanen SM, Takkinen HM, Nevalainen J, Kronberg-Kippila C, Salmenhaara M, Uusitalo L, et al. Early introduction of roots in infancy associated with advanced beta cell autoimmunity in young children with HLA-conferred susceptibility to type 1 diabetes. Diabetologia. 2010;53:S64.

57.	Virtanen SM, Kenward MG, Erkkola M, Kautiainen S, Kronberg-Kippila C, Hakulinen T, et al. Age at introduction of new foods and advanced beta cell autoimmunity in young children with HLA-conferred susceptibility to type 1 diabetes. Diabetologia. 2006;49(7):1512-21.

58.	Lamb MM, Myers MA, Barriga K, Zimmet PZ, Rewers M, Norris JM. Maternal diet during pregnancy and islet autoimmunity in offspring. Pediatric Diabetes. 2008;9(2):135-41.

59.	Lamb MM, Simpson MD, Seifert J, Scott FW, Rewers M, Norris JM. The association between IgG4 antibodies to dietary factors, islet autoimmunity and type 1 diabetes: the Diabetes Autoimmunity Study in the Young. PLoS ONE [Electronic Resource]. 2013;8(2):e57936.

60.	Norris JM, Beaty B, Klingensmith G, Yu L, Hoffman M, Chase HP, et al. Lack of association between early exposure to cow's milk protein and beta-cell autoimmunity. Diabetes Autoimmunity Study in the Young (DAISY). JAMA. 1996;276(8):609-14.

61.	Harsunen MH, Hummel S, Beyerlein A, Joslowski G, Von Meyer A, Pfluger M, et al. Short chain fatty acids and neutrophils in the pathogenesis of type 1 diabetes. Diabetologia. 2012;55:S141.

62.	Baron S, Turck D, Leplat C, Merle V, Gower-Rousseau C, Marti R, et al. Environmental risk factors in paediatric inflammatory bowel diseases: a population based case control study. Gut. 2005;54(3):357-63.

63.	Virtanen SM, Rasanen L, Aro A, Ylonen K, Lounamaa R, Akerblom HK, et al. Is children's or parents' coffee or tea consumption associated with the risk for type 1 diabetes mellitus in children? European Journal of Clinical Nutrition. 1994;48(4):279-85.

64.	Ellis JA, Ponsonby AL, Pezic A, Chavez RA, Allen RC, Akikusa JD, et al. CLARITY - ChiLdhood Arthritis Risk factor Identification sTudY. Pediatric Rheumatology Online Journal. 2012;10(1):37.

65.	Visalli N, Sebastiani L, Adorisio E, Conte A, De Cicco AL, D'Elia R, et al. Environmental risk factors for type 1 diabetes in Rome and province. Archives of Disease in Childhood. 2003;88(8):695-8.

66.	Majeed AA, Mea, Hassan K. Risk Factors for Type 1 Diabetes Mellitus among Children and Adolescents in Basrah. Oman Medical Journal. 2011;26(3):189-95.

67.	Rosenbauer J, Herzig P, Kaiser P, Giani G. Early nutrition and risk of Type 1 diabetes mellitus--a nationwide case-control study in preschool children. Experimental & Clinical Endocrinology & Diabetes. 2007;115(8):502-8.

68.	Rosenbauer J, Herzig P, Giani G. Early infant feeding and risk of type 1 diabetes mellitus-a nationwide population-based case-control study in pre-school children. Diabetes/Metabolism Research Reviews. 2008;24(3):211-22.

69.	Sipetic S, Vlajinac H, Kocev N, Radmanovic S. Characteristics and habits of parents of children with insulin-dependent diabetes mellitus. [Croatian]

Neke karakteristike i navike roditelja dece sa insulin-zavisnim dijabetes melitusom. Srpski arhiv za celokupno lekarstvo. 2003;131(5-6):238-43.

70.	Sipetic SB, Vlajinac HD, Kocev NI, Marinkovic JM, Radmanovic SZ, Bjekic MD. The Belgrade childhood diabetes study: a multivariate analysis of risk determinants for diabetes. European Journal of Public Health. 2005;15(2):117-22.

71.	Strotmeyer ES, Yang Z, LaPorte RE, Chang YF, Steenkiste AR, Pietropaolo M, et al. Infant diet and type 1 diabetes in China. Diabetes Research & Clinical Practice. 2004;65(3):283-92.

72.	Dubakiene R, Rudzeviciene O, Butiene I, Sezaite I, Petronyte M, Vaicekauskaite D, et al. Studies on early allergic sensitization in the Lithuanian birth cohort. Thescientificworldjournal. 2012;2012:909524.

73.	Lv N, Xiao L, Ma J. Dietary pattern and asthma: a systematic review and meta-analysis. Journal of asthma and allergy. 2014;7:105-21.
