# Supplementary material for: Diet during pregnancy and infancy and risk of allergic or autoimmune disease: A systematic review and meta-analysis
Source: PLoS Med. 2018 Feb 28;15(2):e1002507. doi: 10.1371/journal.pmed.1002507 (PMC5830033; doi:10.1371/journal.pmed.1002507)
Supplement: S2 Data — (ZIP) [file pmed.1002507.s007.zip › Review_C_reports/VITAMINS & MINERALS.doc]

**MATERNAL AND INFANT INTAKE OF VITAMINS AND MINERALS, AND RISK OF ALLERGIC AND AUTOIMMUNE DISEASES – Observational and intervention studies**

Vanessa Garcia-Larsen^1^, Robert J Boyle^2^, Despo Ierodiakonou^1,3^, Jo Leonardi-Bee^4^, Tim Reeves^5^, Jennifer Chivinge^6^, Zoe Robinson^6^, Natalie Geoghegan^6^, Katharine Jarrold^6^, Andrew Logan^6^, Annabel Groome^6^ , Evangelia Andreou^7^, Nara Tagiyeva-Milne^8^, Ulugbek Nurmatov^9^, Sergio Cunha^7^

1 Post-Doctoral Research Associate, Respiratory Epidemiology, Occupational Medicine, and Public Health, National Heart and Lung Institute Imperial College London

2 Clinical Senior Lecturer, Department of Paediatrics, Imperial College London

3 Post-Doctoral Research Associate, Department of Paediatrics, Imperial College London

4 Associate Professor of Community Health Sciences, University of Nottingham

5 Research Support Librarian, Faculty of Medicine, Imperial College London

6 Undergraduate medical students, Imperial College London

7 Research Associate, Imperial Consultants

8 Research Fellow, University of Aberdeen

9 Research Fellow, University of Edinburgh

**Table of contents**

List of figures	4

List of Tables	7

1.	Executive summary of studies and findings: Maternal and infant intake of vitamins and minerals and risk of allergic and autoimmune diseases – Observational and intervention studies	8

1.1.	Context of this report	8

1.2.	Studies identified	8

1.3.	Populations	8

1.4.	Dietary assessment and comparators used	8

1.5.	Outcomes evaluated	9

1.6.	Presentation of results	9

1.7.	Risk of bias assessment	9

1.8.	Key findings	10

2.	Infant or maternal dietary intake of vitamins and minerals and risk of atopic dermatitis (AD)	33

2.1.	Intervention studies eligible for meta-analysis	34

2.2.	Observational studies eligible for meta-analysis	35

2.3.	Studies on vitamin and mineral intake and risk of AD which were not eligible for meta-analysis	42

3.	Infant or maternal dietary intake of vitamins and minerals and risk of food allergy	52

3.1.	Intervention studies eligible for meta-analysis	53

3.2.	Observational studies eligible for meta-analysis	53

3.3.	Studies on vitamin and mineral intake and risk of food allergy which were not eligible for meta-analysis	53

4.	Infant or maternal dietary intake of vitamins and minerals and lung function	60

4.1.	Intervention studies eligible for meta-analysis	61

4.2.	Observational studies with data eligible for meta-analysis	61

4.3.	Studies on vitamin and mineral intake and lung function which were not eligible for meta-analysis	61

5.	Infant or maternal dietary intake of vitamins and minerals and risk of wheeze	68

5.1. Intervention studies	69

5.2.	Observational studies with data eligible for meta-analysis	72

5.3.	Studies on vitamin and mineral intake and wheeze which were not eligible for meta-analysis	77

6.	Infant or maternal dietary intake of vitamins and minerals and risk of allergic rhino-conjunctivitis	91

6.1. Intervention studies with data eligible for meta-analysis	92

6.2.	Observational studies with data eligible for meta-analysis	92

6.3.	Studies on vitamin and mineral intake and rhino-conjunctivitis which were not eligible for meta-analysis	92

7.	Infant or maternal dietary intake of vitamins and minerals and risk of allergic sensitisation (AS)	99

7.1. Intervention studies	100

7.2.	Observational studies with data eligible for meta-analysis	102

7.3.	Studies on vitamin and mineral intake and allergic sensitisation which were not eligible for meta-analysis	102

8.	Infant or maternal dietary intake of vitamins and minerals and risk of type 1 diabetes mellitus (T1DM)	114

8.1. Intervention studies with data eligible for meta-analysis	114

8.2.	Observational studies with data eligible for meta-analysis	114

8.3.	Studies on vitamin and mineral intake and T1DM which were not eligible for meta-analysis	117

9.	Infant or maternal dietary intake of vitamins and minerals and risk of inflammatory bowel disease (IBD)	121

10.	Infant or maternal dietary intake of vitamins and minerals and risk juvenile idiopathic arthritis (JIA)	123

Conclusion	125

References	128

# List of figures

Figure 1 Risk of Bias in observational studies of vitamin and mineral intake and AD	33

Figure 2 Risk of Bias in intervention trials of vitamin and mineral intake and AD	33

Figure 3 Maternal vitamin interventions (by type) and risk of AD in children aged 0-4 years	35

Figure 4 Maternal vitamin interventions (by type) and risk of AD in children aged 5-14 years	35

Figure 5 Maternal folic acid supplementation (often vs. rare) and AD at 0 – 4 years	36

Figure 6 Maternal intake of folic acid supplement (often vs. rare) and AD at any age	36

Figure 7 Maternal intake of calcium (highest vs. lowest) and AD at 0 – 4 years	37

Figure 8 Maternal intake of zinc (highest vs lowest) and risk of AD in children aged 0-4 years	37

Figure 9 Maternal intake of zinc (highest vs lowest) and risk of AD in children aged 0-5 years	37

Figure 10 Maternal intake of vitamin A (highest vs lowest) and risk of AD in children (at any age)	39

Figure 11 Maternal intake of vitamin C (highest vs lowest) and risk of AD in children aged 0-4 years	39

Figure 12 Maternal intake of vitamin D (highest vs lowest) and risk of AD in children aged 0-4 years	39

Figure 13 Maternal intake of vitamin D (highest vs lowest) and risk of AD in children aged 0-5 years	40

Figure 14 Maternal intake of vitamin D supplement (often vs rare) and risk of AD in children aged 0-4 years	40

Figure 15 Maternal plasma levels of vitamin D (highest vs lowest) and risk of AD in children (at any age)	40

Figure 16 Maternal intake of vitamin D (highest vs lowest) and risk of AD in children (at any age)	41

Figure 17 Maternal intake of vitamin E (highest vs lowest) and risk of AD in children aged 0-4 years	41

Figure 18 Infant plasma vitamin D (highest vs. lowest) and risk of AD in children aged 5– 14 years	41

Figure 19 Infant plasma vitamin D (highest vs. lowest)and atopic dermatitis in children aged 0-4 years	42

Figure 20 Infant vitamin A intake (highest vs lowest) and risk of AD in children aged 0-4 years	42

Figure 21 Risk of Bias in observational studies of vitamin and mineral intake and food allergy	52

Figure 22 Risk of Bias in intervention trials of vitamins/minerals and food allergy	52

Figure 23 Interventions with vitamins (by type) and risk of food allergy in children aged 0-4 years	53

Figure 24 Risk of Bias in observational studies of vitamin and mineral intake and lung function	60

Figure 25 Risk of Bias in intervention trials of vitamins/ minerals and lung function	60

Figure 26 Maternal plasma vitamin D (mean difference) and lung function (FEV_1_; standard deviations) in children at age 5-14 years	61

Figure 27 Risk of Bias in observational studies of vitamin and mineral intake and wheeze	68

Figure 28 Risk of Bias in intervention trials of vitamins and minerals and wheeze	68

Figure 29 Vitamin interventions (by type) and risk of wheeze in children aged 0-4 years	69

Figure 30 Vitamin interventions (by type) and risk of recurrent wheeze in children aged 0-4 years	70

Figure 31 Vitamin interventions (by type) and risk of wheeze in children aged 5-14 years	70

Figure 32 Maternal copper intake (highest vs. lowest) and risk of wheeze in children aged 0 – 4 years	72

Figure 33 Maternal zinc intake (highest vs. lowest) and risk of wheeze in children at age 0-4 years	72

Figure 34 Maternal plasma vitamin D (highest vs. lowest) and risk of recurrent wheeze in children aged 5 – 14 years	73

Figure 35 Maternal plasma vitamin D (per-unit increase) and risk of recurrent wheeze in children at age 5-14 years	73

Figure 36 Maternal vitamin D intake (often vs. rare) and risk of wheeze in children aged 5 –14 years	73

Figure 37 Maternal vitamin D intake (often vs. rare) and risk of recurrent wheeze in children aged 5 –14 years	74

Figure 38 Maternal vitamin D intake (highest vs lowest) and risk of recurrent wheeze in children (at any age)	74

Figure 39 Cord blood vitamin D levels (highest vs. lowest) and risk of recurrent wheeze in children	74

Figure 40 Maternal folic acid supplement use (often vs. rare) and risk of wheeze in children aged 0 – 4 years	75

Figure 41 Maternal folic acid supplement use (highest vs lowest) and risk of wheeze in children aged 0-4 years	75

Figure 42 Maternal folic acid supplement use (often vs. rare) and risk of recurrent wheeze in children aged 5-14 years	75

Figure 43 Maternal vitamin C intake (highest vs lowest) and risk of wheeze in children aged 0-4 years	76

Figure 44 Maternal vitamin E intake (highest vs lowest) and risk of wheeze in children aged 0-5 years	76

Figure 45 Maternal vitamin A intake (highest vs lowest) and risk of wheeze in children aged 0-4 years	76

Figure 46 Risk of bias in studies of vitamin and mineral intake and rhino-conjunctivitis	91

Figure 47 Risk of bias in intervention studies of vitamin and mineral intake and rhino-conjunctivitis	92

Figure 48 Risk of Bias in observational studies of vitamin and mineral intake and allergic sensitisation	99

Figure 49 Risk of Bias in intervention trials of vitamins/minerals and allergic sensitisation	100

Figure 50 Vitamin supplementation during pregnancy/infancy and risk of AS	101

Figure 51 Vitamin supplementation during pregnancy/infancy and risk of AS to aero allergens	101

Figure 52 Risk of Bias in studies of vitamin and mineral intake and type I diabetes mellitus	114

Figure 53 Infant vitamin D supplementation (often vs. rare) and risk of type 1 diabetes mellitus in children	115

Figure 54 Maternal vitamin D intake (highest vs. lowest) and risk of type 1 diabetes mellitus in children at (any age)	116

Figure 55 Maternal vitamin D supplement use (often vs. rare) and risk of type 1 diabetes mellitus in children (at any age)	116

Figure 56 Maternal plasma vitamin D (mean difference) and risk of T1DM in children (at any age)	116

Figure 57 Risk of bias in study of vitamin and mineral intake and IBD	121

Figure 58 Risk of Bias in studies of vitamin and mineral intake and JIA	123

# List of Tables

Table 1 Characteristics of included studies evaluating vitamin and mineral supplementation or dietary intake and allergic diseases	12

Table 2 Characteristics of included studies evaluating vitamin and mineral supplementation and autoimmune diseases	28

Table 3 Observational studies investigating the association between vitamins and minerals and atopic dermatitis which were not eligible for meta-analysis	44

Table 4 Studies investigating the association between vitamins and minerals and food allergy which were not eligible for meta-analysis	55

Table 5 Studies investigating the association between vitamins and minerals and lung function which were not eligible for meta-analysis	64

Table 6 Observational studies investigating the association between vitamins and minerals and wheeze which were not eligible for meta-analysis	79

Table 7 Observational studies investigating the association between vitamins and minerals and rhino-conjunctivitis which were not eligible for meta-analysis	94

Table 8 Studies investigating the association between vitamins and minerals and allergic sensitization which were not eligible for meta-analysis	103

Table 9 Studies investigating the association between vitamins and minerals and type I diabetes mellitus which were not eligible for meta-analysis	118

Table 10 Studies investigating the association between vitamins and minerals and inflammatory bowel disease which were not eligible for meta-analysis	122

Table 11 Studies investigating the association between vitamins and minerals and juvenile idiopathic which were not eligible for meta-analysis	124

# 1. Executive summary of studies and findings: Maternal and infant intake of vitamins and minerals and risk of allergic and autoimmune diseases – Observational and intervention studies

# 1.1. Context of this report

This report is one of a series of systematic review reports included in Review C Part II, which focusses on evidence on dietary exposures during pregnancy, lactation and or infancy for reducing risk of allergic or autoimmune outcomes. This report focuses on infant and maternal intake of vitamins and minerals. It includes all measures of vitamin and mineral intake, including those derived from food frequency questionnaires, records of direct vitamin supplementation, and for vitamin D for plasma/serum measurements of vitamin D level. Whenever possible, this report specifies the precise vitamin or mineral measured or used for supplementation, if such information is available from the original report.

# 1.2. Studies identified

We identified a total of 61 studies investigating the association between vitamins or minerals and allergic or autoimmune diseases. Of these, there were 11 RCT of vitamin supplementation in pregnant women (9) or infants (2), and 50 observational studies. Amongst these, 32 were prospective cohorts (PC), 4 were nested case-control studies (NCC), and 14 were case-control studies (CC).

# 1.3. Populations

Amongst the intervention studies, four were carried out in Europe, two in the US, two in Asia, one in Australasia and two in Africa. The majority of observational studies were carried out in European populations (n=30). The rest are from North America (n=8), Asia Pacific region (n=10), and the Middle East (n=2).

# 1.4. Dietary assessment and comparators used

The majority of the studies used a questionnaire to ascertain dietary intake of vitamins and minerals. Other studies used sera to analyse levels of vitamin D, 1 study used medical records or a combination of the above approaches. It was unclear if all studies using a dietary questionnaire had validated or piloted the questionnaire, or if it was adapted to reflect the research question being addressed in the study.

# 1.5.  Outcomes evaluated

Amongst the intervention studies, the outcomes investigated included wheeze (n=10), allergic sensitisation (n=6), atopic dermatitis (AD) (n=6), food allergy (n=2) and lung function (n=3). Amongst the observational studies, the outcomes studied included AD (n=18), food allergy (FA) (n=10), lung function (LF) (n=4), wheeze (n=28), rhino conjunctivitis (RC) (n=4), allergic sensitisation (AS) (n=12), type 1 diabetes mellitus (T1DM) (n=15), inflammatory bowel disease (IBD) (n=1), and juvenile idiopathic arthritis (JIA) (n=1).

# 1.6. Presentation of results

We created summary Tables of Study Characteristics with key study features (Table 1 allergic outcomes; Table 2 autoimmune outcomes). As most of the observational studies had data that could not be combined in a meta-analysis, a table of main characteristics and main findings for observational studies is also provided at the end of each section of this report.

# 1.7. Risk of bias assessment

Overall risk of bias for the intervention studies was high in 45% of studies, due to high attrition bias; no intervention study had a high risk of conflict of interest. The risk of bias in included cohort and case control studies was assessed using a modified version of the National Institute for Clinical Excellence (NICE) methodological checklist for cohort and case-control studies, respectively . Key domains were:

•	Selection Bias (low if cases and controls were selected from similar populations, if the participation rate was ≥80%, or <80% but investigators explored and adjusted for characteristic differences between participants and non-participants);

•	Assessment Bias (low if validated and reliable tools were used to assess exposure and/or outcome), and;

•	Confounding Bias (low if most likely confounders are identified and taken into account in study design and analysis).

Observational studies were considered at low overall risk of bias where the risk of bias was judged to be low for all 3 key domains for selection, assessment and confounding bias. For assessment of Confounding Bias, factors that we expected to be adjusted for within studies of allergic outcomes were: siblings (parity or birth order or family size); gender; age at outcome assessment; disease risk based on family history; maternal or household smoking (asthma/wheeze outcomes); maternal age; maternal education or socioeconomic status; mode of delivery. For studies on autoimmune outcomes we expected matching and/or adjusting for gender, age, address, socioeconomic status, smoking and disease risk. We also assessed possible Conflict of Interest, this being judged as low where there was no evidence of industry involvement in study design, analysis, interpretation or publication, and no evidence that study authors received remuneration from relevant industry partners for other activities. Given the nature of the studies included in this report (observational studies on reported dietary intake of foods or food groups), the risk of conflict of interest was judged as low in all cases. Therefore, conflict of interest bias is not systematically shown through this report. A Risk of Bias Figure is presented in each outcome section, reported for all studies contributing data, whether included in meta-analysis or reported narratively.

# 1.8. Key findings

i. **Overall risk of bias was low or unclear for the majority of the outcomes assessed.**

The overall bias was considered to be low in the majority of the studies on atopic dermatitis, food allergy, wheeze, allergic sensitisation, and for autoimmune diseases. In some observational studies it was unclear whether adjustment for potential confounders had been done, which led to a judgement of ‘unclear’ overall risk of bias. In 5 of the 11 intervention trials identified in this review attrition bias was high; no intervention study had a high risk of assessment or selection bias, nor a high risk of conflict of interest.

ii. **The large variations in the methods of dietary assessment prevented us from combining the effect estimates reported in the majority of the studies**.

Despite the number of studies included in this report, meta-analysis of effect estimates was seldom possible due to the lack of comparability of dietary estimates for observational studies, and sparse data/mixed outcome assessments in the intervention trials. We summarised any evidence that could not be included in meta-analysis, in a narrative table for each study outcome.

iii. **There was no consistent evidence to suggest that maternal or infant intake of specific vitamins or minerals might be protective against allergic outcomes or autoimmune diseases**

The main dietary exposures studied were antioxidant vitamins A, C, E and their precursors; and vitamin D and folic acid.

**Overall, we found no consistent evidence from observational or intervention studies to suggest that maternal or infant intake of vitamins or minerals influences the risk of AD, AR, FA, AS, TIDM or JIA.**

**From the intervention trials we found LOW (-1 indirectness of study population; -1 imprecision) evidence that vitamin supplementation during pregnancy increases lung function in offspring; but no evidence that vitamin supplementation during pregnancy reduces risk of wheezing in offspring.**

Table 1 Characteristics of included studies evaluating vitamin and mineral supplementation or dietary intake and allergic diseases

| **Study** | **Design** | **No. participants**  **(Intervention/Ctrl)** | **Country** | **Population characteristics** | **Intervention/ Exposure** | **Intervention/ Exposure details** | **Age at outcome (years)** | **Outcomes and Method of Assessment** |
| --- | --- | --- | --- | --- | --- | --- | --- | --- |
| **INTERVENTION STUDIES** | | | | | | | | |
| **Aage, 2015** | RCT | 2145/ 2200 | Guinea-Bissau | Neonates at time of BCG vaccination. Population at high risk of vitamin A deficiency. | **Vitamin A** | Neonates supplemented with Vitamin A 50,000 IU plus 10IU vitamin E, or 10IU vitamin E alone at time of BCG vaccination. | **10** | AS (SPT); Wheeze (ISAAC); Eczema (ISAAC) |
| **Chawes, 2016** | RCT | 315/308 | Denmark | **COPSAC**. Pregnant women 24 weeks gestation to 1 week post-partum. | **Vitamin D** | Vitamin D3 (2400 IU/d) or placebo from 24 weeks gestation to 1 week post-partum. All women also took 400 IU/day of vitamin D3. | **3** | Wheeze (physician assessment); Eczema (Hanifin and Rajka); AS (SPT, sIgE) |
| **Checkley, 2010**  **Checkley, 2011** | cluster RCT | 803, 885, 771 | Nepal | **NIPPS 2 study.** Married women at high risk of vitamin A deficiency. | **Vitamin A or Beta Carotene** | Vitamin A (23,300 IU) or Beta Carotene (42mg) versus peanut oil placebo weekly from preconception through lactation | **11** | Wheeze (ISAAC, DD); LF (FEV_1_; FVC; PEF) |
|  |  |  |  |  |  |  |  |  |
| **Study** | **Design** | **No. participants**  **(Intervention/Ctrl)** | **Country** | **Population characteristics** | **Intervention/ Exposure** | **Intervention/ Exposure details** | **Age at outcome (years)** | **Outcomes and Method of Assessment** |
| **Czeizel, 1994 ; Dobo, 1998** | RCT | 2090/2032 | Hungary | **Hungarian Optimal Family Planning Programme**. Women who wanted to become pregnant. | **Multivitamin** | Multivitamins from preconception to second missed period. | **1, 6** | Wheeze (physician assessment); Recurrent wheeze (physician assessment); Eczema (physician assessment); FA (unclear) |
| **Devakumar, 2015** | RCT | 600/600 | Nepal | Pregnant women in the second and third trimester. Population at risk of micronutrient deficiencies. | **Multivitamin** | Micronutrient daily supplement (800 g Vit A, 10mg Vit E, 5 g Vit D, 1.4mg Vit B1, 1.4mg Vit B2, 18mg Niacin, 1.9mg Vit B6, 2.6 g Vit B12, 400 g Folic acid, 70mg Vit C, 30mg Iron, 15mg Zinc, 2mg Copper, Selenium 65 g, Iodine 150 g) versus iron 60mg and folic acid 400 g, to pregnant women. | **8** | Lung function (FEV1, FVC); Wheeze (ISAAC); AR (ISAAC) |
| **Grant, 2016** | RCT | 87, 86, 87 | New Zealand | Pregnant women from 27 weeks gestation to birth and infants birth to 6 months | **Vitamin D** | Vitamin D daily from 27 weeks gestation to birth (pregnant women) and birth to 6 months (infants) at 1000 IU/ 400 IU, or 2000 IU/ 800 IU, versus placebo | **1.5** | Wheeze (DD); AS (SPT, sIgE) |
| **Study** | **Design** | **No. participants**  **(Intervention/Ctrl)** | **Country** | **Population characteristics** | **Intervention/ Exposure** | **Intervention/ Exposure details** | **Age at outcome (years)** | **Outcomes and Method of Assessment** |
| **Greenough, 2010** | RCT | 1199/1205 | UK | **Vitamins in Pre-eclampsia trial.** Women with clinical risk factors for pre-eclampsia. | **Vitamin C + E** | Vitamin C (100mg) and Vitamin E (400IU) from 14-21 weeks to delivery. | **1** | Wheeze (parent-reported); Recurrent wheeze (parent-reported); Eczema (parent-reported) |
| **Goldring, 2013** | RCT | 120/60 | UK | Pregnant women from 27 weeks. Ethnically stratified as Asian, Middle Eastern, Black, White. | **Vitamin D** | Vitamin D 200,000 IU bolus at 27 weeks or 800IU daily from 27 weeks to delivery, versus no treatment | **3** | Wheeze (ISAAC); Recurrent wheeze (≥episodes); AR (ISAAC); AS (SPT); Eczema (ISAAC); FA (physician assessment); total IgE |
| **Kiraly, 2013** | RCT | 227/235 | Guinea-Bissau | Infants with no history of measles or vitamin A supplementation. Population at risk of vitamin A deficiency. | **Vitamin A** | Vitamin A 100,000-200,000 IU at 6-9 months age, versus no supplement, | **7** | AS (SPT) |
|  |  |  |  |  |  |  |  |  |
| **Study** | **Design** | **No. participants**  **(Intervention/Ctrl)** | **Country** | **Population characteristics** | **Intervention/ Exposure** | **Intervention/ Exposure details** | **Age at outcome (years)** | **Outcomes and Method of Assessment** |
| **Litonjua, 2016** | RCT | Unclear – 881 total | USA | Pregnant women from 10-18 weeks gestation to delivery. | **Vitamin D** | Vitamin D (4000 IU/d) versus placebo from 10-18 weeks gestation to delivery. All women also received 400 IU/d vitamin D as part of a multivitamin supplement. | **3** | Wheeze (physician assessment); Eczema (DD); AS (total IgE, sIgE) |
| **McEvoy, 2014** | RCT | 89/90 | USA | Women aged ≥15 years old who reported being current smokers (≥1 cigarette per day) randomised at 22 weeks of gestation. | **Vitamin C** | Vitamin C 500mg daily from ≤22 weeks gestation to delivery versus corn starch. | **1** | Wheeze (unclear); LF (spirometry) |

| **Study** | **Design** | **No. participants**  **(Case/Ctrl)** | **Country** | **Population characteristics** | **Exposure** | | | **Method of Exposure Assessment** | **Age (years)** | **Outcomes and Method of Assessment** |
| --- | --- | --- | --- | --- | --- | --- | --- | --- | --- | --- |
|  |  |  |  | **OBSERVATIONAL STUDIES** | | | | |  |  |
| Martindale, 2005; Devereux, 2006; Devereux, 2007 | PC | 1704 | UK | **Aberdeen birth cohort:** Population based birth cohort with pregnant women recruited 1997-99 while attending a hospital antenatal clinic at ~12weeks gestation | | Maternal vitamin intake and mineral intake | Q | | 2,5 | AD: Parent reported (ISAAC Q); UK Working Party Criteria; LF: Spirometry; RC: Parent reported (ISAAC Q); SPT any; wheeze: Parent reported (ISAAC Q) wheeze |
| Wills, 2013; Granell, 2008 | PC | 5368 | UK | **ALSPAC:** Population based birth cohort study, of mother-child pair recruited in 1991-1992 | | Maternal plasma vitamin and vitamin supplement [any] | S, Q | | 7.5, 8, 8.7 | AD: Parent reported; LF-BHR, -FEV_1_(SDS): Spirometry; RC: Parent reported; SPT aero; Wheeze: Parent reported wheeze; Rec wheeze: DD asthma |
| Back, 2009 | PC | 123 | Sweden | The first 206 babies born in 1998 recruited at University Hospital of Umea. All children were prescribed vitamin A&D supplements from 6 weeks to 24 months of age | | Infant vitamin intake [any] | Q | | 6 | AD: Parent reported (ISAAC Q); wheeze and rec wheeze: Parent reported (ISAAC Q) |
| Liu, 2011 | PC | 649 | USA | **Boston Birth Cohort**: Mother-infant pairs recruited at birth at Boston Medical Centre | | Vitamin D cord blood | S | | 2 | sIgE cow’s milk; sIgE egg; sIgE food; sIgE peanut |
| Maslova, 2013 | PC | 28758 | Denmark | **DNBC**: Population based birth cohort with pregnant women recruited between 1996 and 2002 at ~12weeks gestation | | Maternal vitamin intake | Q | | 1.5, 7 | RC:DD; rec wheeze :DD asthma; rec wheeze: DD plus ≥1 episode of wheeze in the last year; rec wheeze :Admission registry - asthma |
| Erkkola, 2009; Nwaru, 2010/11/11; Marjamaki, 2010; Uusitalo, 2008 | PC; RC | 3730 | Finland | **DIPP:** Prospective birth cohort of children at high risk of TIDM (HLA genotype conferred susceptibility) born between 1997 and 2004 in Oulu and Tampere University Hospital | | Maternal vitamin D | Q | | 5, <10 | AD: Parent reported (ISAAC Q); sIgE aero; sIgE cow’s milk; sIgE egg; sIgE food; Rec wheeze :DD asthma plus medication and/or current symptoms |
| Baiz, 2013 | PC | 239 | France | **EDEN:** Population based birth cohort with pregnant women recruited from prenatal clinics in Nancy and Poitiers,  < 24th weeks of gestation in 2003 | | Vitamin D cord blood | S | | 1, 2, 3, 5 | AD:DD; RC: Parent reported (ISAAC Q); rec wheeze :DD asthma plus medication and/or current symptoms |
| Gale, 2008 | PC | 440 | UK | Population based birth cohort with pregnant women recruited on their first visit to midwives' antenatal booking clinic in 1991 | | Maternal plasma Vitamin D | S | | 0.75, 9 | AD:UK Working Party Criteria; Visible AD on examination; Parent reported; Rec wheeze: Parent reported asthma |
| Whitrow, 2009 | PC | 490 | Australia | **Generation 1**: Population based birth cohort of women and their children recruited in the first 16 weeks of pregnancy between 1998 and 2000 from 4 antenatal clinics in Adelaide | | Maternal vitamin intake | Q | | 3.5, 5.5 | Rec wheeze :DD asthma |
| de Jong, 2012 | PC | 8742 | The Netherlands | **GENERATION R**: Population based birth cohort, with pregnant women recruited < 25 weeks gestation in Rotterdam | | Maternal vitamin supplement [any] | Q | | 4 | AD: Parent reported (ISAAC Q); wheeze: Parent reported (ISAAC Q) wheeze |
| Hoppu, 2000 | PC | 115 | Finland | Birth cohort of infants of breastfeeding mothers (for at least 3 months) with a positive family history of atopic disease | | Maternal vitamin intake | R | | 1 | SPT any |
| Rothers, 2011 | PC | 208 | US | **IIS:** prospective birth cohort study of healthy children born to pregnant women who planned to obtain care for their new-borns from collaborating paediatricians | | Vitamin D cord blood | S | | 1, 2, 3, 5 | RC:DD; sIgE aero: SPT aero: Total IgE; rec wheeze: DD asthma |
| Alm, 2009 | PC | 4941 | Sweden | Infants of Western Sweden: Population based birth cohort of infants born in the region in 2003 | | Infant vitamin intake [any] | Q | | 1 | AD: Parent reported |
| Morales, 2012 | PC | 1724 | Spain | **INMA Project**: Population based birth cohort study with pregnant women attending their first routine specialized antenatal care visit in 4 study areas: Menorca (1997-98), Valencia (2003-05), Sabadell (2004-06), Gipuzkoa (2006-08) | | Maternal plasma Vitamin D | S | | 1, 4, 5 | Wheeze: parent reported wheeze; rec wheeze: DD asthma plus medication and/or current symptoms |
| Laitinen, 2005 | PC | 95 | Finland | Children with a family history of AD (mother, father and/or older sibling with AD, AR or asthma), who participated in a prospective allergy prevention study (probiotic intervention trial) | | Infant mineral intake and vitamin intake [any] | D | | 0.5, 1 | AD: Physician assessment; FA cow’s milk: Physician assessment |
| Mommers, 2009; Magdelijn, 2011; Cremers, 2011 | PC | 2465 | The Nether-lands | **KOALA**: Population based birth cohort with healthy pregnant women recruited in week 10 -14 of their pregnancy from an ongoing PC study on pregnancy-related pelvic girdle pain and through posters in organic food shops, anthroposophical, physician offices, and midwives. | | Maternal plasma vitamin D and vitamin supplement [any] | S, Q | | 2, 5, 6.5 , 7 | AD:UK Working Party Criteria; Parent reported (ISAAC Q); LF-FEV_1_ (SDS):Spirometry; FEV_1_%predictedy; FVC% predicted ; sIgE any: Total IgE: Wheeze: parent reported (ISAAC Q) wheeze; rec wheeze :DD asthma plus medication |
| Weisse, 2012 | PC | 272 | Germany | **LINA**: Population based birth cohort with mother–child pairs recruited between 2006 and 2008 in Leipzig | | Maternal plasma Vitamin D and Vitamin D cord blood | S | | 1, 2 | AD:DD; Parent reported; FA any: DD; Total IgE; sIgE |
| Milner, 2004 | PC | 8073 | USA | **NMIH:** survey of mothers who gave birth in 1988 with a follow-up survey (LF), conducted in 1991. Blacks, individuals with low socioeconomic status, and premature infants were intentionally overrepresented in the survey sample | | Infant vitamin intake [any] | Q | | 3 | FA any: DD; Rec wheeze: DD asthma |
| Carmargo, 2010 | PC | 823 | New Zealand | **The New Zealand Asthma and Allergy Cohort study:** Population based birth cohort with pregnant women recruited at maternity care centres in Wellington and Christchurch 1997-2001 | | Vitamin D cord blood | S | | 1.25, 3, 5 | Wheeze: Parent reported wheeze; rec wheeze :DD asthma plus medication and/or current symptoms |
| Hypponen, 2004;  Hypponen, 2001 | PC | 10366/81 | Finland | **Northern Finland Birth Cohort:** Population based birth cohort with women recruited between the 24th and 28th week of gestation in Oulu and Lapland in 1995-1966 | | Infant vitamin intake [any] | Q | | 31, 2 | SPT aero; rec wheeze: self-reported asthma plus medication and/or symptoms |
| Magnus, 2013 Haberg, 2009 | NCC, PC | 32077 | Norway | Cases with asthma & non asthmatic controls were recruited from the participants of the Norwegian Mother and Child Cohort Study. | | Maternal plasma vitamin d and vitamin supplement [any] | S, R | | 1.5, 3 | Rec wheeze :DD or asthma medication; wheeze: Parent reported |
| Miyake, 2010; Miyake, 2010; Miyake, 2011 | PC | 763 | Japan | **OMCHS:** Population birth cohort with pregnant women between the 5-39th week of pregnancy recruited from a university hospital and three obstetric hospitals in municipalities of Osaka between 2001 and 2003 | | Maternal mineral and vitamin intake | Q | | 2 | AD: Parent reported (ISAAC Q); wheeze: Parent reported (ISAAC Q) |
| Bekkers, 2012 | PC | 3786 | The Nether-lands | **PIAMA:** Population based birth cohort of children born in 1996/97 recruited prenatally, Netherlands. The children were allocated to an intervention study or natural history study depending on their family risk for allergy | | Maternal vitamin supplement [any] and Vitamin D cord blood | Q | | 2, 8 | AD; LF-BHR: Spirometry; sIgE any: wheeze: Parent reported (ISAAC Q) wheeze; rec wheeze :DD asthma plus medication and/or current symptoms; rec wheeze :DD asthma plus ≥4 episodes of wheeze |
| Litonjua, 2006; Carmargo, 2007 | PC | 1290 | USA | **Project Viva:** Population based birth cohort with pregnant women at <22 weeks of gestation recruited from 8 obstetric offices of a large multispecialty suburban/urban group practice in eastern Massachusetts between 1999 and 2002 | | Maternal vitamin and mineral intake; infant vitamin and vitamin supplement [any] | Q | | 2, 3 | AD:DD; wheeze: Parent reported wheeze; rec wheeze: Parent reported in at least one questionnaire (1,2,3 years old); rec wheeze: parent reported ≥2 episodes of wheeze plus medication and/or current symptoms |
| Pike, 2012 | PC | 739 | UK | **SWS**: Population birth cohort with 20-34 year old women recruited pre-conception from general practitioners in Southampton between 1998 and 2002 and subsequently become pregnant | | Maternal plasma vitamin d and vitamin intake | Q | | 1, 3, 6 | LF-BHR slope, LF-FEV_1_ and LF-FVC (SD):Spirometry; SPT any; wheeze: Parent reported (ISAAC Q) wheeze; rec wheeze: DD asthma |
| Narita, 2011; Ohya, 2011 Ohya, 2011 | PC | 1463 | Japan | **T-CHILD:** Population based birth cohort of Japanese mother-infant pairs with women recruited ~ second trimester in Tokyo | | Maternal vitamin and mineral intake | Q | | 0.6, 1.5, 3 | AD: Parent reported (ISAAC Q); wheeze: Parent reported (ISAAC Q) wheeze; rec wheeze: DD asthma; wheeze: Parent reported (ISAAC Q) |
| West, 2012 | PC | 319 | Australia | Mother-infant pairs from a pregnancy cohort, recruited in Perth, Western Australia from 2005 to 2008. Pregnant women with a family history of allergic diseases recruited to participate in a postnatal infant dietary intervention study | | Maternal mineral and vitamin intake | Q | | 1 | AD:DD; FA any: History of reaction to food plus positive SPT-food; SPT any; wheeze: DD |
| Wang, 2007 | PC | 1760 | Taiwan | Pilot study of Taiwan National Birth Cohort Study: representative samples recruited postnatally using the national birth registration data in 2003 | | Maternal mineral supplement | Q | | 0.5 | AD:DD |
| Jones, 2012 | RC | 231 | Western Australia | Children with family history of atopy derived from a larger birth cohort in 2002-2009 | | Maternal vitamin supplement [any] and Vit D cord blood | Q, S | | 1 | AD :DD or evidence of typical skin lesions ; FA any: History of reaction to food plus positive SPT-food; SPT any rec wheeze: unclear |
| Oliver, 2010; Grimshaw, 2012 | NCC | 123/41 | UK | **EuroPrevall** (UK birth cohort): cases were infants with food allergy, each matched to two controls | | Infant mineral intake | D, Q | | 1, 2 | FA any :DD with DBPCFC |
| Allen, 2013 | NCC | 2758/240 | Australia | **HealthNUTS:** Cases & controls recruited from population-based sampling from governmental immunisation clinics in Melbourne | | Infant plasma Vitamin D | S, Q | | 1 | AD:DD; FA any, egg, peanut: :DD with OFCs |
| Binkley, 2011 | CC | 1413/1300 | Canada | Cases & controls were anaphylaxis registry's previous survey respondents, all having had previous anaphylactic food allergy reactions, although only cases to peanuts | | Maternal vitamin supplement [any] | Q | | <18 | FA peanut: DD |
| Mullins, 2012 | CC | 115/115 | Australia | Cases were peanut allergic patients born in Australian Capital Territory and population matched-controls: Australia | | Infant plasma vitamin D | S | | 6 | FA peanut: History of acute systemic allergic reaction within 2 hours of known food exposure, combined with a SPT to the relevant food. |

PC Prospective cohort study; Q questionnaire; AD atopic dermatitis; ISAAC International Study of Allergy and Asthma in Children; AS allergic sensitisation; SPT skin prick test, sIgE specific IgE; DD Doctor diagnosis (community); Physician assessment is assessment by study physician; AR allergic rhinitis, FA food allergy; LF lung function; FEV1 forced expiratory volume in 1 second; BHR bronchial hyper-responsiveness; PEF peak expiratory flow; CS cross-sectional study; DBPCFC double blind placebo-controlled food challenge; OFCs oral food challenges; SD standard deviation

Table 2 Characteristics of included studies evaluating vitamin and mineral supplementation and autoimmune diseases

| **Study** | **Design** | **No. participants** | **Country** | **Population characteristics** | **Intervention/ Exposure** | **Exposure assessment** | **Age (yrs)** | **Outcomes** |
| --- | --- | --- | --- | --- | --- | --- | --- | --- |
| Brekke, 2007 | PC | 8694 | Sweden | **ABIS:** Population based birth cohort of children born in Southeast Sweden between 1997 and 1999 | Infant vitamin intake [any] and maternal vitamin supplement [any] | Q | 1, 2.5+17 | TIDM: Islet autoimmunity |
| Fronczak, 2003 ; Simpson, 2011 | PC | 222, 222/16 | USA | **DAISY**: Prospective birth cohort of children at increased risk for T1DM recruited 1993 to 2004 in Denver, Colorado | Maternal vitamin intake; infant plasma vitamin D | Q | 4, 9 | TIDM: Islet autoimmunity (GADA and/or IA2A and/or IAA) |
| Hypponen, 2004  Hypponen, 2001 | PC | 10366/81 | Finland | **Northern Finland Birth Cohort:** Population based birth cohort with women recruited between the 24th and 28th week of gestation in Oulu and Lapland in 1995-1966 | Infant vitamin intake [any] | Q | 31, 2 | TIDM:DD |
| Sørensen, 2012 | NCC | 328/109 | Norway | Cases and controls selected from a population birth cohort linked to Norwegian Childhood Diabetes Registry | Maternal plasma Vitamin D | S | <15 | TIDM:DD |
| Miettinen, 2012 | NCC | 686/343 | Finland | All selected from the Finnish Maternity Cohort, with cases identified from the Finnish Diabetes Register | Maternal plasma Vitamin D | S | 3.4 | TIDM:DD |
| Ahadi, 2011 | CC | 202/101 | Iran | Cases were diagnoses of T1DM referred to Children's Medical Centre Hospital and matched controls | Infant vitamin intake [any] | Q | 6.7 | TIDM: DD |
| Ashraf, 2010 | CC | 195 | USA | Cases and controls selected from electronic medical records | Infant mineral intake | Q | 10 | TIDM: DD |
| Ellis, 2012 | CC | 655/246 | Australia | **CLARITY:** cases were recruited during a clinic visit to Royal Children's Hospital, with diagnosed JIA using ILAR criteria: controls were patients in for elective surgery, also at the Royal Children's Hospital Day Surgery Unit | Maternal iron, multivitamin, folate, calcium and vitamin d supplement | Q | 18 | JIA:DD ILAR criteria |
| Bener, 2009 | CC | 340/170 | Qatar | Cases were insulin dependent or had a venous blood glucose >6.7mmol/L on 2 occasions with matched healthy controls selected from the community | Infant vitamin intake [any] | I/Q | <16 | TIDM: DD |
| EURODIAB substudy 2 study group, 1999 | CC | 2934/746 | Luxembourg, N. Ireland, Romania, Lithuania, Bulgaria and Australia | **EURODIAB**: cases were <15years at T1DM diagnosis and matched with population-based controls. | Infant vitamin intake [any] | I/Q | <15 | TIDM:DD; TIDM :DD WHO criteria |
| Visalli, 2003 | CC | 900/150 | Italy | **EURODIAB Italy**: Cases with T1DM selected from within the EURODIAB ACE study, born 1977-89, with controls selected from school records for the same period | Infant vitamin intake [any] | Q | 12 | TIDM :DD WHO criteria |
| EURODIAB substudy 2 study group, 1999 Europe | CC | 2934/746 | Europe - multicentre | **EURODIAB:** cases were <15years at T1DM diagnosis and matched with population-based controls. | Infant vitamin intake [any] | I/Q | <15 | TIDM:DD |
| Malcova, 2005 | CC | 2334/868 | Czech Republic | Cases were identified from the Czech Childhood Diabetes Register, with unrelated aged-match controls selected from among the schoolmates of cases | Infant vitamin intake [any] | Q | 7 | TIDM:DD |
| Stene, 2003 | CC | 2213/545 | Norway | **Norwegian Childhood Diabetes Study Group**: cases were all children on the diabetes registry diagnosed 1997-2000 and controls were matched from the national population registry | Infant vitamin intake [any] and maternal vitamin supplement [any] | Q | 8.8 | TIDM : DD WHO criteria |
| Stene, 2000 | CC | 1131/84 | Norway | Cases were T1DM patients in Vest-Agder & on the National Childhood Diabetes Register 1982-98 and controls were selected randomly from the population register for the same age and period | Maternal vitamin supplement [any] | Q | <15 | TIDM:DD |
| Svensson, 2005 | CC | 1152/475 | Denmark | Cases identified from the Danish National Register of incident cases diagnosed 1996-99 and matched controls from the Danish Population Register | Infant vitamin intake [any] | Q | 8.4 | TIDM:DD |
| Tenconi, 2007 | CC | 429/131 | Italy | Cases identified from T1DM population registry 1988-2000 and matched controls selected from hospitalised patients, not affected by metabolic disease or cancer | Maternal vitamin supplement [any] | Q | 15.5 | TIDM:DD |
| Gilat, 1987 | CC | 504/167 | 9 countries: USA, Canada, UK, Sweden, Denmark, Holland, France, Italy, Israel | **The International IBD Study Group**: cases were patients with proven Crohn's Disease in 14 centres across 9 countries with 2 controls per case, one with a different minor GI disease and the other with minor non-GI disease taken from hospitals or clinics. | Maternal vitamin supplement [any] | Q | <25 | IBD-CR:DD; IBD-UC:DD |

PC Prospective cohort study; Q questionnaire; NCC nested case-control study; CC case-control study; SPT skin prick test; sIgE specific IgE; DD Doctor diagnosis (community); Physician assessment is assessment by study physician; AR allergic rhinitis; FA food allergy; T1DM type 1 diabetes mellitus; RC retrospective cohort; JIA juvenile idiopathic arthritis; ILAR International League of Associations for Rheumatology; OBD inflammatory bowel disease; UC ulcerative colitis; CR Crohn’s disease; GI gastro-intestinal

# 2. Infant or maternal dietary intake of vitamins and minerals and risk of atopic dermatitis (AD)

Nineteen observational studies and 6 intervention trials investigated the association between vitamin or mineral intake and risk of AD in childhood. The risk of bias in studies on vitamin and mineral intake during infancy or pregnancy in relation to atopic dermatitis is illustrated in Figure 1. This is low in most observational studies, but high in two intervention trials due to high loss to follow up (Figure 2).

Figure 1 Risk of Bias in observational studies of vitamin and mineral intake and AD

Figure 2 Risk of Bias in intervention trials of vitamin and mineral intake and AD

 Studies which investigated AD as an outcome included vitamin intake/supplementation (A, D, C, E, folic acid), minerals copper and zinc, and selenium and magnesium.

# 2.1. Intervention studies eligible for meta-analysis

We identified five intervention studies using vitamin supplements during pregnancy: multi-vitamins (Czeizel 1994), vitamins C and E (Greenough 2010) or vitamin D (Goldring 2013; Chawes 2016 and Litonjua 2016), which had comparable data for meta-analysis (Figure 3). The study of Czeizel reported an increased risk of childhood AD in children of mothers who received the multi-vitamin supplement, in a population with a very low rate of eczema. The other four intervention studies showed no effect on AD, and the pooled estimate for the three studies of vitamin D supplementation showed no evidence of association. There was no heterogeneity between studies of vitamin D supplementation (I^2^=0%). In the study of Czeizel, vitamins were given periconceptually rather than during the second/third trimesters of pregnancy, as in the other four intervention studies. Dobo 1998 reported a substudy of 400 participants from Czeizel’s trial, and found no significant difference in AD - RR 1.53 (0.91, 2.59) at age 2, and RR 1.05 (0.48, 2.31) at age 6 for the infants of supplemented women. Data from Czeizel’s trial shown in Figure 3 are for AD at age 1 on the complete study dataset.

One intervention study supplemented infants with vitamins A and E at BCG vaccination (Aage 2015); no significant reduction in risk of AD in children at age 10 was observed (Figure 4).

Figure 3 Maternal vitamin interventions (by type) and risk of AD in children aged 0-4 years

Figure 4 Maternal vitamin interventions (by type) and risk of AD in children aged 5-14 years

# 2.2. Observational studies eligible for meta-analysis

Thirteen studies contributed to one or more meta-analyses of observational study data. Two prospective cohort studies with data eligible for meta-analysis (De Jong 2012; Magdeljins 2011), showed no association between maternal folic acid supplementation during pregnancy and risk of AD at age 0-4 years. There was no heterogeneity in these studies (Figure 5; I^2^=0%). When the risk of AD was studied in children at any age in a *post hoc* analysis, the combined effect sizes of these two studies and that of Bekkers (2012) also showed no evidence of association with AD (Figure 6).

Figure 5 Maternal folic acid supplementation (often vs. rare) and AD at 0 – 4 years

Figure 6 Maternal intake of folic acid supplement (often vs. rare) and AD at any age

Studies with comparable data on the association between maternal intake of calcium and zinc and risk of AD in children aged 0-4 years are shown in Figures 7 to 9. There was no evidence of association in either meta-analysis. The meta-analysis on calcium intake showed high heterogeneity between studies (I^2^=59.4%; Figure 7). Although both studies were carried out in Japanese populations of normal risk infants, they used a different approach to obtain information on the exposure to dietary intake of sources of calcium, which might explain the heterogeneity observed. We did a post-hoc meta-analysis which enabled data from the study of Devereux at age 5 years to be included within a meta-analysis alongside data for 0-4 years of age (Figure 9) which showed extreme statistical heterogeneity.

**Figure 7 Maternal intake of calcium (highest vs. lowest) and AD at 0 – 4 years**

Figure 8 Maternal intake of zinc (highest vs lowest) and risk of AD in children aged 0-4 years

Figure 9 Maternal intake of zinc (highest vs lowest) and risk of AD in children aged 0-5 years

With regards to vitamin A, the studies of **West (2012)** and **Miyake (2010)** had comparable data which was meta-analysed (Figure 10). The combined effect size showed a 43% reduced risk of AD in children whose mothers had a higher intake of vitamin A. There was no heterogeneity between studies (I^2^=0%). A large study which could not be included in this meta-analysis (**Nwaru 2011**) failed to confirm this association. Maternal intake of vitamin C on the other hand, was unrelated to risk of AD in children at age 0-4 years (Figure 11).

With regards to maternal intake of vitamin D, highest vs lowest intakes showed no evidence of association with AD risk at age 0-4 years (Figure 12) or at age 0-5 years (Figure 13). A reduced risk of AD was observed in children of mothers who consumed vitamin D supplements often (monthly) vs those who rarely consumed them (OR 0.57; 95% CI 0.37, 0.88) (Figure 14), with no evidence of heterogeneity between studies (I^2^=0%). Reports of other measures of vitamin D intake and status failed to confirm this association. Maternal plasma levels of vitamin D (Figure 15) or maternal intake of vitamin D were not related to AD risk at any age (Figure 16). Maternal intake of vitamin E was unrelated to risk of AD in children (Figure 17).

In infants plasma Vitamin D levels (Figure 18; Figure 19) were unrelated to risk of AD in childhood. Similarly, there was no evidence of association between infant intake of vitamin A and risk of AD (Figure 20). The studies of **Wills** (**2013**) and **Mommers** (**2009**) assessed the association between plasma levels of vitamin D in infants and risk of AD (Figure 18). There was no evidence of a relationship with this outcome. The studies showed high heterogeneity (I^2^=66.6%). Both studies included populations from the UK with normal risk of disease, but they used different outcome definitions, which might partly explain the heterogeneity. The study of Wills used parental reported AD (subjective measure), whilst the study of Mommers also used this measure and clinical examination. Similarly, the meta-analysis on vitamin A intake showed no evidence of association between this exposure and risk of AD. There was very high heterogeneity between studies (I^2^=72.2%), which could be partly explained by the different methods used to ascertain consumption of vitamin A.

Figure 10 Maternal intake of vitamin A (highest vs lowest) and risk of AD in children (at any age)

Figure 11 Maternal intake of vitamin C (highest vs lowest) and risk of AD in children aged 0-4 years

Figure 12 Maternal intake of vitamin D (highest vs lowest) and risk of AD in children aged 0-4 years

Figure 13 Maternal intake of vitamin D (highest vs lowest) and risk of AD in children aged 0-5 years

Figure 14 Maternal intake of vitamin D supplement (often vs rare) and risk of AD in children aged 0-4 years

Figure 15 Maternal plasma levels of vitamin D (highest vs lowest) and risk of AD in children (at any age)

Figure 16 Maternal intake of vitamin D (highest vs lowest) and risk of AD in children (at any age)

Figure 17 Maternal intake of vitamin E (highest vs lowest) and risk of AD in children aged 0-4 years

Figure 18 Infant plasma vitamin D (highest vs. lowest) and risk of AD in children aged 5– 14 years

Figure 19 Infant plasma vitamin D (highest vs. lowest)and atopic dermatitis in children aged 0-4 years

**Figure 20 Infant vitamin A intake (highest vs lowest) and risk of AD in children aged 0-4 years**

# 2.3.  Studies on vitamin and mineral intake and risk of AD which were not eligible for meta-analysis

We identified 6 further studies that examined the association between AD and vitamins or minerals and had no data eligible for meta-analysis, and other relevant data from some of the studies included in meta-analysis (Table 3).

Two prospective cohort studies (**Baiz, 2013**; and **Jones, 2012**) investigated the association between vitamin D levels in cord blood and risk of AD. The study of Jones showed a negative association between cord levels of vitamin D and risk of AD in infants aged 1 year, whereas the study of **Baiz**, reported a negative association at age 2, 3 and 5 years. There was no evidence of infant vitamin D intake having an association with AD as examined by the studies of **Back (2009)** and **Allen (2013**). Another six studies investigated various levels of maternal vitamin D intake and risk of AD. The studies of **Camargo** (**2007**) and **Erkkola** (**2009**) found no evidence of association at age 3 or 5, respectively. The study of **Miyake** (**2011)** found a lower risk of AD in children aged 2 whose mothers had an intake of vitamin D ≥172.4 µg/d. These authors also reported some, but not consistent, evidence of a negative association at age 18 months (**Miyake 2010**). Maternal serum levels of vitamin D were unrelated to risk of AD in infants at age 1 or 2 years (**Weisse, 2012**) or later in childhood at age 7.5 years (**Wills, 2013**).

The study of **Miyake** (**2010**) also reported a negative association between β-carotene and risk of AD in children at age 1.5 years. There was no other evidence of associations between vitamin intake (maternal or infant) and risk of AD. We found two studies (**Laitinen, 2005**; and **Nwaru, 2011**) reporting a negative association between AD and calcium and magnesium intake in children, respectively. The study of **Laitinen** showed a negative association at 6 months of age, whilst the study of **Nwaru** examined this association in children aged 5.

**Overall, we found no consistent evidence for an association between vitamin or mineral intake during pregnancy, lactation or infancy and risk of AD.**

Table 3 Observational studies investigating the association between vitamins and minerals and atopic dermatitis which were not eligible for meta-analysis

| **First Author and year of publication** | **Design** | **Age** | **N/n** | **Dietary exposure and comparison level** | **Measure of association** | | **Effect (bold indicates p-value <0.05)** |
| --- | --- | --- | --- | --- | --- | --- | --- |
|  |  |  |  | **INFANT INTAKE** |  | |  |
| Alm, 2009 | PC | 1 | 4,941 | Infant supplementation 1st year with vitamin D/ yes vs. no | OR | | NS |
| Back, 2009 | PC | 6 | 123 | Infant vitamin D intake (estimated >13.1mcg/kg versus ≤13.1mcg/kg) | OR | | **3.63 (1.49-8.87)** |
|  |  | 1 |  | Vitamin D Cord Blood/ ng/Ml (continuous) | OR | | 0.84 (0.71-1.00) |
|  |  | 2 |  | Vitamin D Cord Blood/ ng/Ml (continuous) | OR | | **0.82 (0.71-0.95)** |
|  |  | 3 |  | Vitamin D Cord Blood/ ng/Ml (continuous) | OR | | **0.82 (0.69-0.97)** |
| Baiz, 2014 | PC | 5 | 239 | Vitamin D Cord Blood/ ng/Ml (continuous) | OR | | **0.75 (0.64-0.88)** |
|  |  |  | 108 | Infant energy-adjusted calcium intake (mg/MJ/day) | OR | | **0.96 (0.94-0.99)** |
| Laitinen, 2005 | PC | 4 | 88 | Zinc, Iron, calcium, vitamins A, C, D, E | OR | | NS |
|  |  |  |  | **MATERNAL INTAKE** |  | |  |
| Bekkers, 2012 | PC | 1-8 years | 3,786 | Supplementation during pregnancy (no vs yes)  Folic acid, vitamins complex B, multivitamins | OR | | NS |
|  |  |  |  | Maternal vitamin D intake in 1^st^ and 2^nd^ trimesters (Harvard nutrient composition database and mean FFQ score) / Quartile 2 (513, 446-562 IU) vs Quartile 1 (356, 60-445 IU) IU | OR | | 0.88 (0.61-1.27) |
| Carmargo, 2007 | PC | 3 | 1,194 | / Quartile 3 (603, 563-658) IU vs Quartile 1 (356, 60-445 IU) IU | OR | | 0.94 (0.64-1.37) |
| Devereux, 2006 | PC | 5 | 1,704 | Maternal and children’s dietary and supplement intakes summated / highest (Q5) quintile vs lowest (Q1)  Zinc, copper , iron, magnesium, B-carotene, and vitamin C | OR | | NS |
|  |  |  |  | Daily vitamin D intake during pregnancy (8th month)/ 4^th^ quartile (>0.54) vs 2^nd^ & 3^rd^ quartiles [µg/MJ] (0.31-0.54) | OR | | 0.92 (0.71-1.20) |
| Erkkola, 2009 | PC | 5 | 1,669 | Daily vitamin D intake during pregnancy (8th month)/ 2^nd^ & 3^rd^ quartiles [µg/MJ] (0.31-0.54) vs. 1^st^ quartile (<0.31) | OR | | 1.07 (0.82-1.39) |
|  |  | 9 | 178 | Serum maternal vitamin D during late pregnancy (28-42 weeks, nmol/L):  Q3 (50-75) vs. Q1 (<30) | OR | | 0.47 (0.08-2.68) |
|  |  | 9 | 178 | Q4 (>75) vs. Q1 (<30) | OR | | 1.89 (0.51-6.99) |
| Gale, 2008 | PC | 9 | 178 | Q2 (30-50) vs. Q1 (<30) | OR | | 0.71 (0.15-3.39) |
|  |  | 0.75 | 440 | Q2 (30-50) vs. Q1 (<30) | OR | | 1.11 (0.43-2.84) |
|  |  | 0.75 | 440 | Q3 (50-75) vs. Q1 (<30) | OR | | 1.75 (0.73-4.17) |
| Gale, 2008 | PC | 0.75 | 440 | Q4 (<75) vs. Q1 (<30) | OR | | 1.62 (0.67-3.89) |
| Jones, 2012 | RC | 1 | 231 | Cord blood 25(OH)D3 highest versus lowest tertile | OR | | 0.86 (0.74-1.00) |
| Litonjua, 2006 | PC | 2 | 1,290 | **Maternal diet (FFQ) validated for use during pregnancy/ lowest vs. highest quartiles** :  Multivitamins, copper, zinc, α- and β- carotene, β-crytoxanthin, lutein and zeaxanthin, lycopene, vitamins C, E and folic acid | OR | | NS |
|  |  |  |  | Total maternal intake (diet +supplements) in pregnancy/ lowest vs. highest pentile  Vitamin-C | OR | | 1.30 (0.66-2.56) |
| Martindale, 2005 | PC | 1-2y | 1,300 | Maternal intake vitamin E (diet and supplement) | OR | | NS |
|  |  |  |  | **Maternal intake during pregnancy highest vs lowest quartile**  Zinc | OR | | 1.43 (0.81-2.52) |
|  |  |  |  | β-carotene | OR | | **0.52 (0.30-0.89)** |
|  |  |  |  | α-carotene | OR | | 0.71 (0.41-1.23) |
|  |  |  |  | Vitamin C | OR | | 0.67 (0.38-1.18) |
| Miyake, 2010 | PC | 1.5-2 | 763 | Vitamin E | | OR | 0.59 (0.34-1.02) |
|  |  |  |  | **Maternal vitamin D intake from food during pregnancy** (quartiles; 5µg/d ):  Q3 (6.4) vs Q 1 (3.5) | OR | | 0.59 (0.34-1.02) |
|  |  |  |  | Q2 (5.1) vs Q1 (3.5) | OR | | 0.63 (0.36-1.09) |
|  |  |  |  | ≥4.31 (25^th^ percentile) vs <4.31 | OR | | **0.63 (0.41-0.98)** |
| Miyake, 2010 | PC | 1.6 | 763 | Q4 (9.1) vs Q1 (3.5) | OR | | 0.67 (0.40-1.13) |
|  |  |  |  | **Maternal intake during pregnancy (highest vs lowest quartile)**:  Vitamin B2 supplement | OR | | 0.72 (0.35-1.49) |
|  |  |  |  | Vitamin B12 supplement | OR | | 0.85 (0.39-1.84) |
| Miyake, 2011 | PC | 2 | 763 | Vitamin B6 supplement | OR | | 1.06 (0.52-2.15) |
|  |  |  |  | **Maternal food consumption during the eighth month of pregnancy (unclear levels of comparison)**  Copper | OR | | 0.94 (0.78-1.14) |
|  |  |  |  | Magnesium | OR | | **0.78 (0.63-0.97)** |
|  |  |  |  | Selenium | OR | | 0.99 (0.81-1.21) |
| Nwaru, 2011 | PC | 5 | 2,441 | Zinc | OR | | 0.92 (0.74-1.15) |
|  |  |  |  | Folate | OR | | 0.91 (0.76-1.09) |
|  |  |  |  | Vitamin A | OR | | **1.12 (1.00-1.26)** |
|  |  |  |  | Vitamin B2 | OR | | 0.87 (0.73-1.03) |
|  |  |  |  | Vitamin C | OR | | 0.91 (0.80-1.03) |
|  |  |  |  | Vitamin E | OR | | 1.00 (0.84-1.19) |
| Ohya, 2011 | PC | 1.5, 3 | 1,463 | **Maternal intake during pregnancy (measure of comparison unclear)**  Folic acid, carotene, vitamin A, retinol | OR | | NS |
| Weisse, 2012 | PC | 1, 2 | 272 | Maternal serum 25(OH)D levels at 34 weeks gestation and disease risk at age 1 and 2 years | OR | | NS |
|  |  |  | 316 | **Maternal intake in pregnancy / highest vs lowest**  Zinc | OR | | 1.73 (0.80-3.73) |
|  |  |  | 304 | Vitamin C | OR | | 0.72 (0.33-1.57) |
| West, 2012 | PC | 1 | 308 | Vitamin E | OR | | 1.42 (0.68-2.98) |
|  |  |  |  | **Maternal serum vitamin D during pregnancy / (quintiles; nml/L)**  Q3 (52-67) vs Q1 (min -38) | OR | | 0.91 (0.70-1.18) |
|  |  |  |  | Q4 (67-89) vs Q1 (min -38) | OR | | 0.97 (0.75-1.26) |
| Wills, 2013 | PC | 7.5 | 4,686 | Q2 (38-52) vs Q 1 (min -38) | OR | | 1.04 (0.81-1.34) |
| Wang, 2007 | PC | 0.5 | 1,760 | Maternal calcium supplementation (maternal intake during pregnancy vs no intake during pregnancy) | OR | | 1.22 (0.83-1.81) |

# 3. Infant or maternal dietary intake of vitamins and minerals and risk of food allergy

Thirteen studies investigated the association between vitamin and mineral intake and risk of FA. Figure 21 illustrates the risk of bias in studies of vitamin and mineral intake in relation to food allergy. Nearly a third of observational studies were considered to be of high overall risk of bias, and none were at low risk of bias, due to the lack of control for potential confounding. The two intervention trials were at low and unclear risk of bias respectively (Figure 22).

Figure 21 Risk of Bias in observational studies of vitamin and mineral intake and food allergy

Figure 22 Risk of Bias in intervention trials of vitamins/minerals and food allergy

# 3.1. Intervention studies eligible for meta-analysis

We identified two intervention studies (**Czeizel 1994** and **Goldring 2013**) on multivitamins and vitamin D, respectively. There was no evidence of an association with food allergy in either study, nor was there evidence of a reduced risk of disease when the two effect sizes were combined (Figure 23). There was no heterogeneity between studies (I^2^=0%). Goldring also measured food allergy from primary care record of food allergy diagnosis (study physician assessment shown in plot) and found RR 0.44 (0.07, 3.00) in that analysis.

Figure 23 Interventions with vitamins (by type) and risk of food allergy in children aged 0-4 years

# 3.2. Observational studies eligible for meta-analysis

We identified no observational studies with data eligible for meta-analysis.

# 3.3.  Studies on vitamin and mineral intake and risk of food allergy which were not eligible for meta-analysis

We identified 11 observational studies that investigated the association between food allergy and vitamins or minerals (Table 4). The exposures studied included vitamin D (infant/maternal intake, serum and cord blood levels), maternal folic acid or multivitamin supplementation, as well vitamins A, B, C, E, and minerals zinc, calcium, iron, selenium and sodium.

The nested case-control study of **Allen** (**2013**) showed that higher serum levels of vitamin D in infants was associated with a lower risk of having any food allergy at age 1 year. However, maternal vitamin D was unrelated to the risk of food allergy in this study. In the study of **Weisse (2012**), maternal and cord blood vitamin D was associated with an increased risk of food allergy within the first 2 years. Further, higher maternal vitamin D was associated with a higher risk for sensitization to food allergens at age 2 years.

The pilot case-control study of **Mullins** (**2012**) in 115 Australian children showed a non-linear relationship between serum levels of vitamin D and risk of peanut allergy: compared with the reference group (50-74.9 nmol/L), levels of 75 to 99.9 nmol/L were associated with lower risk of peanut allergy (P = 0.02). This effect was not observed at levels ≥100 nmol/L, and the risk of peanut allergy at levels less than 50 nmol/L was not significantly different from the reference group.

In the cohort study of **West** (**2012**), a higher intake of copper assessed prospectively by a food frequency questionnaire in 420 pregnant women, was associated with a reduced risk of food allergy at age 1 year. However, this was not the case for all comparison levels, and the trend was not statistically significant.

In the cohort study of **Milner** (**2004**) the authors reported that early vitamin supplementation was associated with increased risk of food allergies in exclusively formula-fed children. Vitamin supplementation at 3 years of age was associated with increased risk of food allergies, but not asthma, in both breastfed (OR: 1.62; 95% CI: 1.19-2.21) and exclusively formula-fed infants (OR: 1.39; 95% CI: 1.03-1.88).

**Overall, we found no consistent evidence to suggest that infant or maternal dietary intake or supplementation with vitamins or minerals influences the risk of food allergies.**

Table 4 Studies investigating the association between vitamins and minerals and food allergy which were not eligible for meta-analysis

| **First Author and year of publication** | **Design** | **Outcome** | **Age** | **N/n** | **Dietary exposure and**  **comparison level** | **Measure of association** | **Effect (bold indicates p<0.05)** |
| --- | --- | --- | --- | --- | --- | --- | --- |
|  |  |  |  |  | **INFANT INTAKE OR CORD BLOOD LEVELS** |  |  |
|  |  | Cow’s milk allergy | 1 | 95 | **Infant intake**  Iron, vitamins C, D, and E | OR | NS |
| Laitinen, 2005 | PC | Food allergy | 1 | 95 | Calcium | OR | NS |
| Milner, 2004 | PC | Food allergy | 0-4 | 8285 | Infant multi-vitamin intake | OR | **0.77 (0.62-0.96)** |
|  |  |  | 5-7 | 8285 | Infant multi-vitamin intake |  | **0.75 (0.62-0.91)** |
| Liu, 2011 | PC | Food allergy | 0-4 | 649 | Cord blood vitamin D intake and risk of food allergy (highest vs. lowest) | OR | 1.19 (0.37-3.84) |
|  |  | Cow’s milk allergy | 0-4 | 649 | Cord blood vitamin D (highest vs. lowest) | OR | 0.76 (0.49-1.16) |
|  |  | Egg allergy | 0-4 | 649 | Cord blood vitamin D (highest vs. lowest) | OR | 1.01 (0.81-1.28) |
|  |  |  |  |  | Infant plasma vitamin D intake and risk of food allergy (highest vs. lowest) | OR | 0.78 (0.31-1.96) |
| Allen, 2013 | PC | Food allergy | 1 | 361 | Vitamin D infant serum levels / 25(OH)D3 >50 vs. 25(OH)D3 ≤50 nmol/L nMol/L | OR | **0.29 (0.11-0.80)** |
| Oliver, 2010 | NCC | Food Allergy | 1 | 93 | **Infant dietary intake:**  Calcium, iron, selenium, sodium, zinc, vitamin B, vitamins A, D, and E. | OR | NS |
| Mullins, 2012 | CC | Peanut Allergy | 6 | 115 | Vitamin D infant serum levels / 50-74.9 nmol/l vs. <50 nmol/l | OR | 1.54 (0.77-3.08) |
|  |  |  |  |  | Vitamin D infant serum levels / 75-99.9 nmol/l vs/ 50-74.9 nmol/l | OR | **0.37 (0.16-0.83)** |
|  |  |  |  |  | **MATERNAL INTAKE** |  |  |
| West, 2012 | PC | Food allergy | 1 | 317 | Maternal intake (highest vs. lowest) Copper | OR | **0.38 (0.15-0.95)** |
|  |  |  |  |  | Vitamin A |  | 0.38 (0.11-1.28) |
|  |  |  |  |  | Zinc |  | 0.52 (0.16-1.73) |
|  |  |  |  |  | Vitamin C |  | 0.46 (0.16-1.36) |
|  |  |  |  |  | Vitamin E |  | 0.57 (0.19-1.72) |
|  |  |  |  |  | **STUDIES EXAMINING MATERNAL AND INFANT LEVELS** |  |  |
|  |  |  | 1 | 272 | Infant plasma vitamin D intake and risk of food allergy (highest vs. lowest) | OR | **1.92 (1.09-3.38)** |
| Weisse, 2013 | PC | Food allergy | 1 | 272 | Cord blood vitamin D intake and risk of food allergy (highest vs. lowest) | OR | 1.7 (0.93-3.12) |
|  |  |  | 1 | 272 | Maternal serum 25(OH)D at 34 weeks (quartiles) | OR | 1.27 (0.67-2.40) |
|  |  |  | 2 | 272 | Maternal serum 25(OH)D at 34 weeks (quartiles) | OR | **3.66 (1.36-9.87)** |
|  |  |  | 1 | 272 | Cord blood 25(OH)D levels ( quartiles) | OR | 0.92 (0.46-1.85) |
|  |  |  | 2 | 272 | Cord blood 25(OH) D (quartiles) | OR | **4.65 (1.49-14.48)** |
| Allen, 2013 | PC | Food allergy | 1 | 361 | Maternal multivitamin supplement intake and risk of food allergy (often vs. rare) | OR | 0.9 (0.62-1.3) |
| Jones, 2012 | RC | Food Allergy | 1 | 146 | Maternal vitamin D supplement during pregnancy (analysed in increments of 50 IU) | OR | 1.08 (0.98-1.19) |
|  |  |  |  | 231 | Cord blood 25(OH)D3 in nmol/L (continuous) | OR | NS |
| Allen, 2013 | NCC | Food Allergy | 1 | 2758 | Additional vitamin D in pregnancy/ yes vs. no multivitamins, no additional vitamin D in pregnancy | OR | 0.67 (0.35-1.28) |
| Grimshaw, 2012 | CC | Food Allergy | 2 | 123 | Folic acid, multivitamin, vitamin D supplement during lactation / No vs yes | OR | NS |
| Binkley, 2011 | CC | Peanut Allergy | 1 | 1413 | Folic acid supplementation before conception / - no vs. yes | OR | **1.48 (1.00-2.20)** |
|  |  |  |  | 1413 | Folic acid supplement initiation during pregnancy / no vs. yes | OR | 0.78 (0.27-2.24) |

# 4. Infant or maternal dietary intake of vitamins and minerals and lung function

Eight observational studies and three intervention trials investigated the association between various lung function outcomes and maternal or infant intake of vitamins or minerals. Although all studies were considered to have a low risk of assessment bias, the overall bias of all included observational studies was judged to be high, due to high bias observed in the selection of participants, and to lack of adjustment for confounders in a third of studies (Figure 24); two of the intervention trials were at high risk of bias due to high loss to follow up, the other at low risk of bias (Figure 25).

Figure 24 Risk of Bias in observational studies of vitamin and mineral intake and lung function

Figure 25 Risk of Bias in intervention trials of vitamins/ minerals and lung function

# 4.1.  Intervention studies eligible for meta-analysis

We identified no intervention studies with data eligible for meta-analysis

# 4.2. Observational studies with data eligible for meta-analysis

We identified two studies which investigated the association between plasma levels of vitamin D in pregnant mothers (Figure 26) and changes in forced expiratory volume in 1 second (FEV_1_). There was no evidence that maternal plasma vitamin D influences FEV_1_ in children aged 5-15 years.

Figure 26 Maternal plasma vitamin D (mean difference) and lung function (FEV_1_; standard deviations) in children at age 5-14 years

# 4.3. Studies on vitamin and mineral intake and lung function which were not eligible for meta-analysis

Three intervention studies reported data on lung function, which could not be included in meta-analyses. In the cluster RCT study of **Checkley (2010)** children whose mothers had received vitamin A had a forced expiratory volume in 1 second (FEV_1_) and a forced vital capacity (FVC) that were significantly higher than those of children whose mothers had not received vitamin A supplementation, after adjustment for various potential confounders Children whose mothers had received β-carotene had adjusted FEV_1_ and FVC values that were similar to those of children whose mothers had received placebo, and in neither group was there any difference in FEV1:FVC ratio or peak expiratory flow rate. **McEvoy (2014)** found significantly increased lung function (ratio of time to peak tidal expiratory flow to expiratory time; and passive respiratory compliance per kilogram) in infants born to mothers in the intervention compared with the control group in the first 72 hours of life, but no significant difference at age 1 year. The positive findings regarding lung function from the study of Checkley were not replicated in a similar trial undertaken in Nepal using a multivitamin supplement which included a lower vitamin A dose than the trial of Checkley. **Devakumar (2015)** reported no significant difference in lung function between the children of women supplemented with multivitamins during pregnancy and a control group - FEV1 mean 1.2L SD0.2 intervention, 1.21 SD0.2 control; FVC mean 1.37L SD0.2 intervention, 1.38 SD0.2 control. We concluded that the Checkley trial findings for vitamin A were not reproduced in the trial of Devakumar using a lower vitamin A dose, were not reproduced using beta-carotene in the trial of Checkley, and were not supported by clinical evidence of differences in respiratory outcomes (see wheeze section). This finding was downgraded to no evidence due to inconsistency (-2), indirectness of the study population (-1) and imprecision (-1).

Observational studies reported inconsistent evidence for relationships between lung function and vitamin D (maternal serum or supplement intake), maternal folic acid supplementation, zinc intake (maternal and infant, including supplement use) and multivitamin use (Table 5).

In the cohort study of **Wills** (**2013**) which was carried out in a population residing in south west England, there was no evidence to suggest that maternal vitamin D blood level in pregnancy was associated with altered lung function or bronchial hyper-responsiveness measurements in the offspring measured at age 8.7 years old (n=3,728). These findings remained after adjustment for season of measurement and for other potential confounders. There was also no evidence that these relationships followed a non-linear form, and no evidence that either deficient or high concentrations of maternal vitamin D were associated with atopic or respiratory outcomes. Two other smaller studies also reported no association between maternal blood levels of vitamin D and measures of lung function in the offspring. The study of **Pike (2012)**, which included 800 pregnant mothers from Southampton, showed no significant associations between maternal late-pregnancy vitamin D status and lung function in their offspring at age 6 years. In the KOALA Birth study (**Cremers, 2011**) the authors found no association between vitamin D levels, vitamin D supplementation in childhood or recommended vitamin D dosage of ≥10 μg/day during pregnancy and lung function in children aged 6-7 years.

With regards to folic acid use (or plasma levels) in pregnant mothers, the studies of **Bekkers (2012**) and **Magdelijns (2011)** showed no evidence of an association between this exposure and measures of lung function at age 8 or 7, respectively.

**Overall we found no evidence that vitamin or mineral supplementation or intake to mothers or infants influences child lung function from observational studies. We found no consistent evidence from the intervention studies that vitamin A or other vitamin supplementation during pregnancy influences lung function during childhood.**

Table 5 Studies investigating the association between vitamins and minerals and lung function which were not eligible for meta-analysis

| **First Author and year of publication** | **Design** | **Outcome** | **Age** | **N/n** | **Dietary exposure and comparison level** | **Measure of association** | **Effect (bold indicates p<0.05)** |
| --- | --- | --- | --- | --- | --- | --- | --- |
|  |  |  |  |  | **MATERNAL INTAKE OR BLOOD LEVELS** |  |  |
| Checkley, 2010 | cluster RCT | FEV1 (ml) | 11 | 591/506 | Maternal supplementation of vitamin A 23,300 IU (7000μg retinol equivalents) weekly from pre-conception through lactation versus peanut oil placebo | MD | **46 mls (6, 86)** |
|  |  | FVC (ml) |  |  | Maternal supplementation of vitamin A 23,300 IU (7000μg retinol equivalents) weekly from pre-conception through lactation versus peanut oil placebo | MD | **46 mls (8, 84)** |
|  |  | FEV1 (ml) |  |  | Maternal supplementation beta-carotene 42mg (7000μg retinol equivalents) weekly from pre-conception through lactation versus peanut oil placebo | MD | 14 mls (-24, 54) |
|  |  | FVC (ml) |  |  | Maternal supplementation beta-carotene 42mg (7000μg retinol equivalents) weekly from pre-conception through lactation versus peanut oil placebo | MD | 17 mls (-21, 55) |
| McEvoy, 2014 | RCT | Time to peak tidal expiratory flow: expiratory time ratio(TPTEF:TE) | 0 | 89/90 | Vitamin C supplementation (500mg/d) to pregnant, smoker women compared to placebo | MD | **0.04 (0.01 – 0.06)** |
|  |  |  | 1 |  |  | MD | NS |
|  |  | Passive respiratory system compliance (Crs/kg) | 0 |  |  | MD | **0.11 (0.02 – 0.20)** |
|  |  |  | 1 |  |  | MD | NS |
| Bekkers, 2012 | PC | LF-BHR | 8 | 3,786 | Maternal vitamin or mineral supplements during pregnancy, including folic acid, pre-natal vitamins (multivitamin supplements especially for pregnant females)/ no use vs. yes  Folic acid, multivitamins | OR | NS |
| Cremers, 2011 |  | LF-FEV_1_ and FVC |  |  | Maternal plasma vitamin D at 36 weeks gestation / Quintile 1 (21.5, 12.0-28.2) nmol/l vs. upper quintiles | β-coeff | NS |
|  | PC |  | 6.5 | 436 | Maternal vitamin D intake from supplements / no use vs variable amounts at 1^st^, 2^nd^ or 3^rd^ trimester | β-coeff | NS |
| Devereux, 2006 | PC | LF | 5 | 1,704 | Maternal and children’s dietary and supplement intakes of zinc were summated and energy adjusted/lowest (Q1) vs. highest (Q5) quintiles | OR | NS |
| Magdelijns, 2011 | PC | LF-FEV_1_% and FVC% predicted | 7 | 1,902 | Maternal plasma vitamin D at 36 weeks gestation / lowest vs upper quintiles | β-coeff | NS |
|  |  | LF-BHR slope | 6 | 216 | Vitamin D intake during pregnancy (average total daily mcg/kg) | β-coeff | NS |
| Pike, 2012 | PC | LF-FEV_1_ or FVC (L) | 6 | 739 | Vitamin D intake or plasma levels ,during pregnancy (average total daily mcg/kg), serum levels measured as 10 nmol/litre change | β-coeff | NS |
|  |  | Spirometry | 8.7 | 3,728 | Maternal serum vitamin D (nmol/L) in pregnancy: Higher quintiles vs lowest (Q1 min -38):  Quintile 5 (89-max) | β-coeff | NS |
|  |  |  |  |  | Quintile 4 (67-89) | OR | **0.71 (0.53-0.96)** |
|  |  |  |  |  | Quintile 3 (52-67) | OR | 0.79 (0.58-1.07) |
| Wills, 2013 | PC | LF-BHR | 8.7 | 2,409 | Quintile 2 (38-52) | OR | 0.87 (0.65-1.17) |
|  |  |  |  |  | Quintile 2 (38-52) | β-coeff | NS |
|  |  |  |  |  | Quintile 3 (52-67) | β-coeff | NS |
|  |  | LF-FEV_1_ | 8.7 | 3728 | Quintile 4 (67-89) | β-coeff | NS |
|  |  |  |  |  | Quintile 2 (38-52) | β-coeff | NS |
|  |  |  |  |  | Quintile 3 (52-67) | β-coeff | NS |
| Wills, 2013 | PC | LF-FVC | 8.7 | 3784 | Quintile 4 (67-89) | β-coeff | NS |

# 5. Infant or maternal dietary intake of vitamins and minerals and risk of wheeze

Forty studies investigated the association between intake of vitamins or minerals and risk of wheeze in childhood. Most observational studies were judged to be of low risk in all the domains assessed (Figure 27). Four of the ten intervention trials were at high risk of bias due to high loss to follow up (Figure 28).

Figure 27 Risk of Bias in observational studies of vitamin and mineral intake and wheeze

Figure 28 Risk of Bias in intervention trials of vitamins and minerals and wheeze

# 5.1. Intervention studies

We identified ten intervention studies. Five studies could be included in meta-analysis for the effect of vitamins on risk of wheeze at age 0-4 (Figure 29). Supplementation with vitamin D (**Goldring 2013 and Grant 2016**), vitamin C and E (**Greenough 2010 and McEvoy 2014**) or multivitamins (**Cziezel 1994**) did not demonstrate a significant effect on wheeze at age 0-4. Data from four studies (**Chawes 2016; Goldring 2013; Grant 2016; Litonjua 2016**) found no significant effect of vitamin D on risk of recurrent wheeze at age 0-4 (Figure 30). Goldring also measured current wheeze (wheeze ever is shown in Figure 29) with RR 0.98 (0.46, 2.13), and wheeze plus positive Asthma Predictive Index RR 1.00 (0.44, 2.30); and recurrent wheeze using primary healthcare record (study physician assessment is shown in Figure 30) with RR 1.48 (0.44, 5.00). Overall there was no evidence that vitamins (separately or combined) influenced the risk of wheeze outcomes at age 0-4 years in offspring. The heterogeneity across studies was moderate for both wheeze and recurrent wheeze.

Two intervention trials reported wheeze outcomes at age 5-14 years (**Checkley 2011 and Aage 2015**). No effect of maternal vitamin A supplementation on wheeze risk in offspring at age 5-14 was demonstrated (Figure 31). Checkley separately reported RR 1.37 (0.77, 2.45) with vitamin A, and RR 1.47 (0.83, 2.61) with β-carotene for current wheeze, and RR 2.66 (0.54, 13.14) with vitamin A, and RR 1.32 (0.22, 7.89) with β-carotene for recurrent wheeze.

Figure 29 Vitamin interventions (by type) and risk of wheeze in children aged 0-4 years

Figure 30 Vitamin interventions (by type) and risk of recurrent wheeze in children aged 0-4 years

Figure 31 Vitamin interventions (by type) and risk of wheeze in children aged 5-14 years

# 5.2. Observational studies with data eligible for meta-analysis

For the dietary exposures copper, zinc, folic acid, and vitamins D, C, E, and A, there were studies with comparable data for a meta-analysis. These are presented in Figures 32 to 45. With few exceptions, most of the studies showed no evidence to suggest that maternal intake, or blood/cord levels in the case of vitamin D, influence the risk of wheeze in early or later childhood. Figure 33 shows the combined effect sizes of three studies (**West 2012; Miyake 2010** and **Litonjua 2006**) that investigated the association between maternal intake of zinc and risk of wheeze in children aged 0-4 years. A higher intake of zinc was associated with a 40% reduced risk of wheeze (OR 0.60; 95% CI 0.45, 0.81). There was no heterogeneity across studies (I^2^=0%).

Figure 44 shows the effect sizes of the studies of **Devereux (2006)** and **Litonjua (2006),** both of which reported a reduced risk of wheeze in children of mothers who had a higher intake of vitamin E. The overall effect showed a 53% reduction in the risk of wheeze (OR 0.47; 95% CI 0.30, 0.74). There was no evidence of heterogeneity between studies (I^2^=0%).

Figure 32 Maternal copper intake (highest vs. lowest) and risk of wheeze in children aged 0 – 4 years

Figure 33 Maternal zinc intake (highest vs. lowest) and risk of wheeze in children at age 0-4 years

Figure 34 Maternal plasma vitamin D (highest vs. lowest) and risk of recurrent wheeze in children aged 5 – 14 years

Figure 35 Maternal plasma vitamin D (per-unit increase) and risk of recurrent wheeze in children at age 5-14 years

Figure 36 Maternal vitamin D intake (often vs. rare) and risk of wheeze in children aged 5 –14 years

Figure 37 Maternal vitamin D intake (often vs. rare) and risk of recurrent wheeze in children aged 5 –14 years

Figure 38 Maternal vitamin D intake (highest vs lowest) and risk of recurrent wheeze in children (at any age)

Figure 39 Cord blood vitamin D levels (highest vs. lowest) and risk of recurrent wheeze in children

Figure 40 Maternal folic acid supplement use (often vs. rare) and risk of wheeze in children aged 0 – 4 years

Figure 41 Maternal folic acid supplement use (highest vs lowest) and risk of wheeze in children aged 0-4 years

Figure 42 Maternal folic acid supplement use (often vs. rare) and risk of recurrent wheeze in children aged 5-14 years

Figure 43 Maternal vitamin C intake (highest vs lowest) and risk of wheeze in children aged 0-4 years

Figure 44 Maternal vitamin E intake (highest vs lowest) and risk of wheeze in children aged 0-5 years

Figure 45 Maternal vitamin A intake (highest vs lowest) and risk of wheeze in children aged 0-4 years

# 5.3. Studies on vitamin and mineral intake and wheeze which were not eligible for meta-analysis

One intervention study reported findings that could not be included in meta-analysis. **Devakumar 2015** reported no significant difference in prevalence of wheeze or recurrent wheeze between children of women allocated multivitamins during pregnancy, and a control group – numerical data were not reported. The studies that reported data which could not be included in meta-analysis are summarised in Table 6. In observational studies Vitamin D (intake/blood) and folic acid supplementation were the most commonly studied exposures. Others included antioxidant vitamins and minerals, mostly self-reported.

With regards to vitamin D, there was no consistent evidence that maternal intake was related to a lower risk of wheeze in childhood. The studies of **Pike (2012**) and **Back (2009)** did not find an association between maternal (Pike) or infant (Back) intake and risk of wheeze or recurrent wheeze at age 6 years. The study of **Baiz (2013)** did not find an association between concentrations of vitamin D in cord blood and risk of recurrent wheeze at age 5 years. Similarly, **Wills (2013**) reported no association between maternal serum levels of vitamin D and risk of wheeze at age 7 years.

On the contrary, the study of **Devereux (2007)** reported a lower risk of wheeze in children at age 5 in pregnant mothers who had a high intake of vitamin D (when comparing highest vs lowest quintiles); the study of **Miyake (2010)** also found that a higher maternal intake of vitamin D was negatively associated with risk of wheeze at age 2. The study of **Camargo (2010)** found reduced recurrent wheeze with increased maternal vitamin D intake during pregnancy; the same authors found a similar relationship for cord-blood vitamin D levels, but only for some measures of wheezing and not for others. The studies of **Miyake 2010**, **Litonjua 2006** and the Seaton cohort (Martindale/ Devereux) all reported significantly reduced wheeze with higher estimated maternal vitamin E intake during pregnancy. However, the study of **Nwaru 2011** failed to confirm this association, and had a similar number of participants as the studies combined in meta-analysis in Figure 39. Similarly, **Nwaru 2011** failed to confirm the association between zinc intake and reduced risk of wheezing seen in Figure 31, despite including more participants in analysis than the combined studies in Figure 31.

**Overall we found no consistent evidence from intervention studies to suggest that maternal vitamin supplementation during pregnancy may reduce risk of wheeze in childhood, and no consistent evidence from observational studies for a relationship between vitamin or mineral intake during pregnancy, lactation or infancy and risk of wheeze.**

Table 6 Observational studies investigating the association between vitamins and minerals and wheeze which were not eligible for meta-analysis

| **First Author and year of publication** | **Design** | **Outcome** | **Age** | **N/n** | **Dietary exposure and comparison level** | **Measure of association** | **Effect (bold indicates p-value <0.05)** | |
| --- | --- | --- | --- | --- | --- | --- | --- | --- |
|  |  |  |  |  | **INFANT OR CORD BLOOD** |  |  | |
|  | PC | Asthma-Wheeze | 6 | 123 | Infant vitamin D intake (continuous) | OR | 1.06 (0.44-2.57) | |
| Back, 2009 |  | Rec wheeze |  |  |  | OR | 3.16 (0.61-16.4) | |
| Hypponen, 2004 | PC | Rec wheeze | 31 | 6722 | Infant vitamin D supplementation in the first year of life – daily dose/ 2000 IU vs < 2000 IU | OR | 3.91 (0.53 -29.9) |  |
|  |  | Rec wheeze | 5 | 239 | Vitamin D in cord blood (continuous scale) | OR | 1.15 (0.78-1.69) |  |
| Baiz, 2013 | PC | Asthma-Wheeze | 5 | 239 | Vitamin D in cord blood (continuous scale) | OR | 1.07 (0.79-1.45) |  |
| Jones, 2012 | RC | Asthma-Wheeze | 1 | 231 | Cord blood 25(OH)D3 in nmol/L (continuous) | OR | NS |  |
|  |  |  |  |  | **Infant vitamin supplementation (highest vs lowest quartiles):**  α-carotene | OR | 0.92 (0.63-1.34) |  |
|  |  |  |  |  | b-carotene | OR | 0.98 (0.65-1.47) |  |
| Litonjua, 2006 | PC | Wheeze | 2 | 1290 | b-cryptoxanthin | OR | 0.97 (0.67-1.41) |  |
|  |  |  |  |  | Lutein and zeaxanthin | OR | 0.84 (0.56-1.25) |  |
|  |  |  |  |  | Lycopene | OR | 0.96 (0.65-1.42) |  |
|  |  |  |  |  | Vitamin C | OR | 0.79 (0.54-1.15) |  |
| Litonjua, 2006 | PC | Wheeze | 2 | 1290 | Vitamin E | OR | 0.70 (0.48-1.03) |  |
|  |  |  |  |  | **MATERNAL INTAKE OR BLOOD LEVELS** |  |  |  |
|  |  |  |  |  | **Maternal vitamin D intake in 1^st^ and 2^nd^ trimesters**:  Q2 (513, 446-562 IU) vs Q1 (356, 60-445 IU) | OR | **0.47 (0.28-0.77)** |  |
|  |  |  |  |  | Q3 (603, 563-658) vs Q 1 (356, 60-445 IU) | OR | **0.54 (0.33-0.89)** |  |
|  |  |  |  |  | Q4 (724, 659-1145 UI) vs Q1 (356, 60-445 UI | OR | **0.38 (0.22, 0.65)** |  |
| Camargo, 2007 | PC | Rec wheeze | 3 | 1194 | Vitamin D intake from food only (per 100IU/d) | OR | **0.80 (0.66-0.97)** |  |
|  |  |  |  |  | Total vitamin D intake | OR | **0.81 (0.71-0.93)** |  |
|  |  |  |  |  | Vitamin D intake from supplements only per 100IU/d; (continuous) | OR | **0.82 (0.71-0.95)** |  |
|  |  | Rec wheeze |  | 823  823 | Vitamin D cord blood/ ≥75 nmol/L vs. 25-74.9 nmol/L | OR | 0.84 (0.55-1.29) |  |
| Camargo, 2010 (37) | PC | Wheeze | 1 |  | Cord blood 25(OH)D in nmol/L (per ten nmol/L increase) | OR | 0.98 (0.93, 1.02) |  |
| Camargo, 2010 | PC | Wheeze | 3 |  | Cord blood 25(OH)D in nmol/L (per ten nmol/L increase) | OR | **0.96 (0.91, 1.00)** |  |
|  |  | Wheeze | 5 |  | Cord blood 25(OH)D in nmol/L (per ten nmol/L increase) | OR | **0.95 (0.91, 0.99)** |  |
|  |  | Incident asthma | 5 |  | Cord blood 25(OH)D in nmol/L (per ten nmol/L increase) | OR | 1.03 (0.97, 1.10) |  |
|  |  |  |  |  | **Maternal vitamin D intake in 3^rd^ trimester** (median, range):  Q5 (275, 189-751 IU/d) vs Q 1 (77, 46-92 IU/d) | OR | **0.45 (0.21-0.97)** |  |
| Devereux, 2007 | PC | Wheeze | 5 | 1120 | Q 4 (157, 142-182 IU/d) vs Q1 (77, 46-92 IU/d) | OR | 0.65 (0.38-1.11) |  |
|  |  |  |  |  | Q3 (128, 117-139 IU/d) vs Q1 (77, 46-92 IU/d) | OR | 0.77 (0.47-1.27) |  |
|  |  |  |  |  | Q2 (104, 94-115 IU/d) vs Q1 (77, 46-92 IU/d) | OR | 0.84 (0.52-1.37) |  |
| Devereux, 2007 | PC | Rec wheeze | 5 | 1120 | **Maternal vitamin D intake in 3^rd^ trimester:**  Q5 (157, 142-182 IU/d) vs Q1 (77, 46-92 IU/d) | OR | 0.51 (0.22-1.20) |  |
|  |  |  |  |  | Q3 (128, 117-139 IU/d) vs Q 1 (77, 46-92 IU/d) | OR | 0.75 (0.35-1.61) |  |
| Devereux, 2007 | PC | Rec wheeze | 5 | 1120 | Q2 (104, 94-115 IU/d) vs Q 1 (77, 46-92 IU/d) | OR | 0.91 (0.44-1.88) |  |
| Erkkola, 2009 | PC | Rec wheeze | 5 | 1669 | **Maternal vitamin D intake from food in pregnancy (8th month)**  2^nd^ & 3^rd^ Quartiles (0.31-0.54 µg/MJ) vs. 1^st^ Quartile (<0.31 µg/MJ) | HR | 1.64 (1.02-2.64) |  |
|  |  |  |  |  | Vitamin D intake from supplements in pregnancy (8th month)/no use vs. use | HR | 0.99 (0.62-1.57) |  |
| Gale, 2008 | PC | Rec wheeze | 9 | 178 | Maternal vitamin D level in serum [ nmol/L] in late pregnancy (28-42 weeks) :  Q2 (30-50) vs Q 1 (<30) | OR | NS |  |
|  |  |  |  |  | Q3 (51-75) vs Q1 (<30) | OR | NS |  |
| Magnus, 2013 | NCC | Rec wheeze | 3 | 1684/489 | Plasma vitamin D at 18 weeks gestation | OR | 0.96 (0.84-1.10) |  |
|  |  |  |  |  | **Maternal vitamin D intake** (quintiles[IQR]) from supplements during pregnancy (µg/d)  Q3 (11.7, 9.9-12.3) vs Q1 (5.5, 1.8-7.1) | RR | 0.78 (0.55-1.10) |  |
| Maslova, 2013 | PC | Rec wheeze | 7 | 28767 | Q2 (8.2, 7.3-9.4) vs Q1 (5.5, 1.8-7.1) | RR | 0.92 (0.78-1.08) |  |
|  |  |  | 7 | 28767 | Q4 (13.0, 12.4-14.0) vs Q1 (5.5, 1.8-7.1) | RR | 0.92 (0.78-1.08) |  |
|  |  |  | 7 | 28767 | Q5 (16.5, 14.2-27.4) vs Q 1 (5.5, 1.8-7.1) | RR | 0.92 (0.78-1.08) |  |
| Maslova, 2013 | PC | Rec wheeze | 1.5 | 33425 | Q3 (11.7, 9.9-12.3) vs Q1 (5.5, 1.8-7.1) | RR | 0.95 (0.84-1.08) |  |
|  |  |  |  |  | **Vitamin D intake from food during pregnancy** / ≥4.309 µcg /d vs <4.309 (25th percentile) mcg/d | OR | **0.64 (0.42-0.97)** |  |
|  |  |  |  |  | Vitamin D intake / Q4 (9.1) µcg /d vs Q1 (3.5) µcg/d | OR | NS |  |
|  |  |  |  |  | Vitamin D intake / Q3 (6.4) µcg/d vs Q1 (3.5) µcg/d | OR | NS |  |
| Miyake, 2010 | PC | Wheeze | 1.6 | 763 | Vitamin D intake / Q4 vs Q1 | OR | 0.69 (0.42-1.14) |  |
| Morales, 2012 | PC | Wheeze | 4 | 1223 | Maternal plasma 25(OH)D3 at ~12.5 weeks gestation:  Q3 (29.2-37ng/ml) vs Q1 (<21.9ng/ml) | OR | 0.82 (0.54-1.25) |  |
|  |  |  | 4 | 1223 | Q2 (21.9-29.1ng/ml) vs Q1 (<21.9ng/ml) | OR | 0.89 (0.59-1.34) |  |
| Morales, 2012 | PC | Wheeze | 1 | 1724 | Q4 (>37 ng/ml) vs Q1 (<21.9ng/ml) | OR | 0.94 (0.62-1.43) |  |
|  |  |  |  |  | Maternal plasma 25(OH)D3 at ~12.5 weeks gestation / Quartile 3 (29.2-37ng/ml) vs Q1 (<21.9ng/ml) | OR | 0.81 (0.55-1.20) |  |
| Morales, 2012 | PC | Rec wheeze | 5 | 1233 | Vitamin D plasma/Maternal plasma 25(OH)D3 at ~12.5 weeks gestation / Q2 (21.9-29.1ng/ml) vs Q1 (<21.9ng/ml) | OR | 0.88 (0.60-1.29) |  |
|  |  |  |  |  | Maternal vitamin D intake during pregnancy/ highest vs lowest quartile | RR | 1.23 (0.65-2.33) | |
| Pike, 2012 | PC | Rec wheeze | 6 | 856 | Maternal serum 25(OH)-D status at 34 weeks pregnancy /(continuous scale in nmol/L) | RR | NS | |
|  |  |  |  |  | **Vitamin D cord blood [nml/L]:**  50-74.9 vs. < 50 | OR | 2.00 (0.63-6.40) |  |
| Rothers, 2011 | PC | Rec wheeze | 5 | 194 | 75-99.9 vs 50-74.9 | OR | 1.10 (0.39-3.10) |  |
|  |  |  |  |  | **Maternal serum [nmol/L] vitamin D in pregnancy:**  Quintile 3 (52-67) vs Quintile1 (min -38) | OR | 0.99 (0.73-1.35) |  |
|  |  | Wheeze | 7.5 | 4,696 | Quintile 2 (38-52) vs Quintile1 (min -38) | OR | 1.03 (0.75-1.41) |  |
| Wills, 2013 | PC |  |  |  | Maternal serum vitamin D in pregnancy [quintiles; nmol/L]  Q 4 (67-89) vs Q1 (min -38) | OR | 0.92 (0.69-1.23) |  |
|  |  |  |  |  | Q3 (52-67) vs Q1 (min -38) | OR | 1.00 (0.75-1.34) |  |
|  |  | Rec wheeze | 7.5 | 4,648 | Q2 (38-52) vs Q1 (min -38) | OR | 1.02 (0.77-1.36) |  |
|  |  |  |  |  | **Maternal intake in pregnancy (highest vs lowest quartiles):**  Multivitamins, α-carotene, β-carotene, β-cryptoxanthin, lutein and zeaxanthin, lycopene |  | NS |  |
|  |  |  |  |  | Zinc | OR | **0.49 (0.28-0.87)** |  |
| Litonjua, 2006 | PC | Rec wheeze | 2 | 1,290 | Vitamin E | OR | **0.49 (0.27-0.90)** |  |
| Magdelijns, 2011 | PC | Wheeze | 7 | 1,902 | Folic acid supplementation before or during pregnancy, alone or with other supplements use vs no use | OR | 0.99 (0.8-1.23) |  |
| Bekkers, 2012 | PC | Rec wheeze | 8 | 3,786 | Folic acid supplementation during pregnancy (yes vs no) | Prevalence ratio | 1.03 (0.96- 1.16) |  |
|  |  |  |  |  | **Vitamin and mineral intake** (highest vs lowest pentile)  Total maternal vitamin C intake (diet and supplements) | OR | **3.00 (1.47-6.12)** |  |
| Martindale, 2005 | PC | Wheeze | 1-2 | 1,300 | Total maternal vitamin E intake (diet and supplements) | OR | 0.53 (0.27-1.01) |  |
| Martindale, 2005 | PC | Wheeze | 1-2 | 1,300 | Copper, magnesium, manganese, zinc, selenium, b-carotene | OR | NS |  |
| Milner, 2004 | PC | Rec wheeze | 0-3 | 8,073 | Vitamin/ mineral drops given to infants at least 3 days a week / intake in the first 3 months vs no intake | OR | 0.89 (0.77-10.2) |  |
|  |  |  |  |  | **Maternal intake during pregnancy** (highest vs lowest quartiles of intake[energy adjusted]):  β-carotene or α-carotene, vitamin C | OR | NS |  |
| Miyake, 2010 | PC | Wheeze | 1.6 | 763 | Vitamin D | OR | **0.48 (0.29-0.80)** |  |
|  |  |  |  |  | Vitamin E intake | OR | **0.54 (0.32-0.90)** |  |
| Miyake, 2011 | PC | Wheeze | 2 | 763 | Vitamins B6, B12, Folate, B2, supplementation during pregnancy (FFQ) / highest [median 1.2mg per day] vs lowest quartile [median 0.7 mg per day] | OR | NS |  |
| Miyake, 2010 | PC | Rec wheeze | 2 | 763 | Maternal calcium intake (highest vs. lowest) | OR | **0.57 (0.33-0.99)** |  |
| Narita, 2011 | PC | Wheeze | 0.6, 3 | 1,344 | Maternal consumption of calcium or vitamin D during pregnancy / highest quartile vs lowest | OR | NS |  |
|  |  |  |  |  | **Maternal food consumption during the eighth month of pregnancy** (continuous):  Copper | HR | 1.00 (0.71-1.41) |  |
|  |  |  |  |  | Magnesium | HR | 0.91 (0.59-1.41) |  |
|  |  |  |  |  | Selenium | HR | 1.37 (0.89-2.11) |  |
| Nwaru, 2011 | PC | Rec wheeze | 5 | 2,441 | Zinc | HR | 1.15 (0.77-1.72) |  |
|  |  |  |  |  | Folate | HR | 1.04 (0.71-1.52) |  |
|  |  |  |  |  | Vitamin A | HR | 1.20 (0.92-1.56) |  |
|  |  |  |  |  | Vitamin B2 | HR | 1.07 (0.82-1.40) |  |
|  |  |  |  |  | Vitamin C | HR | 0.92 (0.69-1.22) |  |
|  |  |  |  |  | Vitamin E | HR | 1.02 (0.71-1.47) |  |
| Ohya, 2011 | PC | Rec wheeze | 1.5, 3 | 1463 | Iron and folic acid (unclear measure of comparison) / |  | NS |  |
|  |  |  |  | 318 | Maternal intake during pregnancy (highest vs lowest quartile)  Zinc, vitamin A, vitamin E | OR | NS |  |
| West, 2012 | PC | Wheeze | 1 | 306 | Vitamin C supplementation (yes/no) | OR | **0.40 (0.16-0.98)** |  |
|  |  |  | 3.5 | 490 | Folic acid [Maternal intake during pregnancy] <16 weeks gestation: <100 µg/d vs. 100 µg/d | RR | 1.15 (0.82-1.61) |  |
|  |  |  | 3.5 | 490 | µg /day at 30-34 weeks gestation [late pregnancy] vs. early pregnancy | RR | 1.32 (1.14-1.53) |  |
| Whitrow, 2009 | PC | Rec wheeze | 5.5 | 423 | [Maternal intake at 30-34 weeks gestation] µg /day at 30-34 weeks gestation [early vs. late pregnancy] | RR | 1.38 (1.06-1.79) |  |

# 6. Infant or maternal dietary intake of vitamins and minerals and risk of allergic rhino-conjunctivitis

Eleven observational studies investigated the association between allergic rhino-conjunctivitis in relation to intake of vitamins or minerals. The assessment of risk of bias in these studies showed that all studies included some form of adjustment for confounders. Several studies were considered to have a high risk of assessment or selection bias which contributed to over two thirds of them to be considered to have a high overall risk of bias (Figure 46). Of the two intervention trials identified, one had high attrition bias (Figure 47).

Figure 46 Risk of bias in studies of vitamin and mineral intake and rhino-conjunctivitis

Figure 47 Risk of bias in intervention studies of vitamin and mineral intake and rhino-conjunctivitis

# 6.1. Intervention studies with data eligible for meta-analysis

We found no intervention studies with data eligible for meta-analysis

# 6.2. Observational studies with data eligible for meta-analysis

We found no observational studies with data eligible for meta-analysis

# 6.3. Studies on vitamin and mineral intake and rhino-conjunctivitis which were not eligible for meta-analysis

The main findings of the studies which investigated the association between the relevant exposures and rhino-conjunctivitis are summarised in Table 7. The majority of the studies investigated varying vitamin D intake or serum levels, with one observational study investigating micro-nutrients (**Nwaru 2011**).

The RCT of **Goldring (2013)** examined the effect of vitamin D supplementation during pregnancy and the risk of RC at age 3 years. They found no evidence of an effect. The RCT of **Devakumar (2015)** reported no significant difference in prevalence of RC between children of women allocated multivitamins during pregnancy, and a control group – numerical data were not reported.

The observational studies of **Baiz (2013)**, **Maslova (2013)**, **Wills (2013)** and **Rother (2011)** showed no evidence of an association between Vitamin D and risk of RC assessed at ages 3 to 7 years of age. The study of **Hypponen (2004)** found no association between infant vitamin D supplementation and risk of adult RC (age 31 years old). In the cohort study of **Erkkola (2009)**, the authors found a negative association between maternal dietary intake of vitamin D in the last trimester and risk of RC at age 5 years. This association was not observed when vitamin D was combined with intake from supplements. There were no other significant associations in relation to other vitamins or minerals and RC.

**Overall, we found no evidence to suggest that infant or maternal intake of vitamins or minerals influences the risk of RC.**

Table 7 Observational studies investigating the association between vitamins and minerals and rhino-conjunctivitis which were not eligible for meta-analysis

| **First Author and year of publication** | **Design** | **Outcome** | **Age** | **N/n** | **Dietary exposure and comparison level** | **Measure of association** | **Effect (bold indicates p-value <0.05)** |
| --- | --- | --- | --- | --- | --- | --- | --- |
|  |  |  |  |  | **INFANT INTAKE AND CORD BLOOD** |  |  |
|  |  |  |  |  | Plasma level of 25(OH)D measured in cord blood specimens (nml/L)  50-74.9 vs. < 50 | OR | 0.91 (0.36-2.31) |
| Rothers, 2011 | PC | RC | 5 | 192 | 75-99.9 vs 50-74.9 | OR | 0.60 (0.20-1.80) |
| Back, 2009 | PC | RC | 5-14 | 123 | Infant vitamin intake (highest vs. lowest) | OR | 1.67 (0.6-4.61) |
| Baiz, 2013 | PC | RC | 5 | 239 | Cord blood vitamin D (infant-1st year-blood) in ng/ml (continuous) | OR | 0.99 (0.71-1.38) |
| Hypponen, 2004 | PC | RC | 31 | 6722 | Infant vitamin D supplementation in the first year of life – daily dose / 2000 IU vs <2000 IU | OR | 1.34 (0.71-2.53) |
|  |  |  |  |  | **MATERNAL INTAKE OR BLOOD LEVELS** |  |  |
| Goldring, 2013 | RCT | RC | 3 | 120/60 | Pregnant women. Single oral bolus of 200,000 IU vitamin D (cholecalciferol) at 27 weeks gestation, or daily 800IU vitamin D (ergocalciferol) from 27 weeks to delivery, versus no treatment | RR | 0.76 (0.31, 1.85) |
| Rothers, 2011 | PC | RC | 5 | 192 | Maternal vitamin D supplements and risk of rhino-conjunctivitis (high vs. lowest) | OR | 2.41 (0.79-7.37) |
| Devereux, 2006 | PC | RC | 5 | 1704 | Maternal and children’s dietary and supplement intakes summated to give total nutrient intake [lowest vs. highest quintile]: Copper, Iron, magnesium, b-carotene, vitamin C | OR | NS |
| Erkkola, 2009 | PC | RC | 5 | 1669 | Vitamin D intake in pregnancy (8^th^ month) in µg/MJ:  from food (diet) | HR | **0.85 (0.75-0.96)** |
|  |  |  |  |  | Maternal diet and supplements | HR | 0.90 (0.79-1.02) |
| Erkkola, 2009 | PC | RC | 5 | 1,669 | Q4 (>0.54) vs Q2 and Q3 (0.31-0.54) | HR | 0.98 (0.70-1.37) |
|  |  |  |  |  | Q2 and Q3 (0.31-0.54) vs. Q1 (<0.3) | HR | 1.53 (1.14-2.06) |
|  |  |  |  |  | Maternal vitamin D intake in pregnancy from food (in quintiles; µg/d)  Q 3 (11.7, 9.9-12.3) vs Q1 (5.5, 1.8-7.1) | RR | 0.90 (0.76-1.07) |
|  |  |  |  |  | Q2 (8.2, 7.3-9.4) vs Q1 (5.5, 1.8-7.1) | RR | 0.91 (0.77-1.07) |
| Maslova, 2013 | PC | RC | 7 | 28,545 | Q 4 (13.0, 12.4-14.0) vs Q 1 (5.5, 1.8-7.1) | RR | 1.05 (0.90-1.23) |
|  |  |  |  |  | **Maternal vitamin D intake in pregnancy from supplements (in quintiles; µg/d)**  Q3 (11.7, 9.9-12.3) vs Q1 (5.5, 1.8-7.1) | RR | 0.96 (0.73-1.27) |
|  |  |  |  |  | Q 4 (13.0, 12.4-14.0) vs Q1 (5.5, 1.8-7.1) | RR | 1.06 (0.89-1.26) |
|  |  |  |  |  | Q 2 (8.2, 7.3-9.4) vs Q1 (5.5, 1.8-7.1) | RR | 1.12 (0.94-1.33) |
| Maslova, 2013 | PC | RC | 7 | 28,545 | **Total maternal vitamin D intake during pregnancy (in quintiles; µg/d)**  Q5 (16.5, 14.2-27.4) vs Q1 (5.5, 1.8-7.1) | RR | 1.00 (0.84-1.19) |
|  |  |  |  |  | Q 3 (11.7, 9.9-12.3) vs Q 1 (5.5, 1.8-7.1) | RR | 1.01 (0.86-1.19) |
|  |  |  |  |  | Q 4 (13.0, 12.4-14.0) vs Q1 (5.5, 1.8-7.1) | RR | 1.02 (0.86-1.21) |
|  |  |  |  |  | Q2 (8.2, 7.3-9.4) vs Q 1 (5.5, 1.8-7.1) | RR | 1.05 (0.88-1.25) |
|  |  |  |  |  | **Maternal serum vitamin D in pregnancy (quintiles; nmol/L)/**  Q4 (67-89) vs Q1 (min -38) | OR | 0.95 (0.67-1.35) |
| Wills, 2013 | PC | RC | 7.5 | 4,673 | Q 2 (38-52) vs Q1 (min -38) | OR | 1.10 (0.79-1.54) |
|  |  |  |  |  | Q 3 (52-67) vs Q1 (min -38) | OR | 1.23 (0.89-1.70) |
| Nwaru, 2011 | PC | RC | 5 | 2,441 | **Maternal food consumption during the eighth month of pregnancy**  Copper, magnesium, selenium, zinc, folate, Vitamins A, B2, C, E | HR | NS |

# 7. Infant or maternal dietary intake of vitamins and minerals and risk of allergic sensitisation (AS)

Eighteen studies investigated the association between intake of vitamins or minerals and risk of AS in childhood. Overall, the observational studies were almost all considered to have a low or unclear risk of bias. Most of the studies included some form of adjustment for confounders and therefore were considered to be of low risk in this domain. Some studies had unclear information about the methods of sample selection and assessment, therefore over half of the studies were considered to have an unclear overall risk of bias (Figure 48); of the six intervention trials, two had a high risk of bias due to high loss to follow up (Figure 49).

Figure 48 Risk of Bias in observational studies of vitamin and mineral intake and allergic sensitisation

Figure 49 Risk of Bias in intervention trials of vitamins/minerals and allergic sensitisation

# 7.1. Intervention studies

Six intervention studies with data eligible for meta-analysis were identified. These studies demonstrated no significant effect of vitamin supplementation during pregnancy/infancy and risk of AS (**Aage 2015; Chawes 2016; Litonjua 2016**) (Figure 50) and specifically AS to aero allergens (**Kiraly 2013; Grant 2016; Goldring 2013**) (Figure 51).

**Figure 50 Vitamin supplementation during pregnancy/infancy and risk of AS**

Figure 51 Vitamin supplementation during pregnancy/infancy and risk of AS to aero allergens

# 7.2. Observational studies with data eligible for meta-analysis

We found no observational studies with data eligible for meta-analysis

# 7.3. Studies on vitamin and mineral intake and allergic sensitisation which were not eligible for meta-analysis

The main findings of observational studies investigating the relationship between vitamins or minerals with AS, are presented in Table 8. The outcomes studied included specific and total IgE levels, as well as SPT. Maternal vitamin D and folic acid intake or blood levels were the commonest dietary exposures studied.

The cohort study of **Rothers (2011)** in the Tucson Infant Immune Study found that both low and high levels of cord blood vitamin D were associated with increased aeroallergen sensitization. Relative to the reference group (50-74.9 nmol/L), both low (<50 nmol/L) and high (≥ 100 nmol/L) levels were associated with increased total IgE, and detectable inhalant allergen-specific IgE at age 5 years. High cord blood levels of vitamin D were also associated with increased SPT positivity in these children. In the LINA cohort study (Lifestyle and environmental factors and their Influence on Newborns Allergy risk), **Weisse** and colleagues **(2012)** found that higher maternal blood levels of vitamin D were associated with a higher risk for sensitization against food allergens at age 2 years. The studies of **Nwaru (2010)**, **Hypponen (2004)**, **Jones (2012)** and **Pike (2012)** on maternal intake of vitamin D showed no evidence of an association with outcomes of AS.

**Overall, we found no consistent evidence that minerals or vitamins are associated with risk of allergic sensitisation. We found conflicting evidence on the role of vitamin D on allergic sensitisation, which is insufficient to draw conclusions.**

Table 8 Studies investigating the association between vitamins and minerals and allergic sensitization which were not eligible for meta-analysis

| **First Author and year of publication** | **Design** | **Outcome** | **Age** | **N/n** | **Dietary exposure and comparison level** | **Measure of association** | **Effect (bold indicates p-value <0.05)** |
| --- | --- | --- | --- | --- | --- | --- | --- |
|  |  |  |  |  | INFANT INTAKE OR CORD BLOOD LEVELS |  |  |
|  |  | SPT aero | 31 | 4,400 | Infant vitamin D supplementation in 1^st^ year of life 2000 IU <2000 IU | OR | 1.02 (0.45-2.29) |
|  |  | AS any | 31 | 4,400 | Infant vitamin D intake (highest vs. lowest) | OR | **1.28 (1.06-1.56)** |
| Hypponen, 2004 | PC | AS any | 31 | 4,400 | Infant vitamin D intake (often vs. rare) | OR | 1.04 (0.75-1.45) |
| Jones, 2012 | RC | AS | 1 | 217 | Cord blood 25(OH)D3 in nmol/L (continuous) |  | 1.00 (0.99-1.01) |
| Liu, 2011 | PC | AS peanut | Any | 123 | Cord blood vitamin D (highest vs. lowest) | OR | 0.55 (0.15-2.02) |
|  |  |  |  |  |  |  |  |
|  |  |  |  |  | **MATERNAL INTAKE OR BLOOD LEVELS** |  |  |
|  |  |  |  |  | Total energy-adjusted daily maternal vitamin D intake during pregnancy / Q3 vs Q1 | RR | **1.39 (1.15-1.67)** |
|  |  | SPT any | 1 | 635 | Maternal serum 25(OH)D status at 34 weeks pregnancy in nmol/L (continuous) | RR | 0.96 (0.89-1.03) |
|  |  |  |  |  | Average total daily vitamin D intake during pregnancy in mcg/day (continuous) | RR | 0.33 (0.10-1.07) |
| Pike, 2012 |  |  |  |  | Total energy-adjusted daily maternal vitamin D intake during pregnancy /Q3 vs Q1 | RR | 0.69 (0.43-1.11) |
|  |  |  |  |  | Total energy-adjusted daily maternal vitamin D intake during pregnancy / Q2 vs Q1 | RR | 0.86 (0.53-1.40) |
|  |  | SPT any | 6 | 635 | Average maternal daily vitamin D intake from FOOD during pregnancy in mcg/day (continuous) | RR | 1.03 (0.95-1.12) |
|  |  | AS | 3 | 635 | Maternal plasma 25(OH)D (pregnancy) | RR | 0.99 (0.94-1.04) |
|  | PC | AS | 6 | 635 | Maternal plasma 25(OH)D (pregnancy) | RR | 0.99 (0.94-1.04) |
|  |  |  | 2 | 207 | Plasma level of 25(OH)D measured in cord blood specimens [nmol/L]:  50-74.9 vs. < 50 | OR | 0.63 (0.19-2.03) |
|  |  |  | 5 | 207 | 50-74.9 vs. < 50 | OR | 0.50 (0.19-1.30) |
|  |  |  | 3 | 207 | 50-74.9 vs. < 50 | OR | 0.38 (0.14-1.07) |
| Rothers, 2011 (28) | PC | sIgE aero | 5 | 208 | 50-74.9 vs. < 50 | OR | **0.36 (0.15-0.84)** |
|  |  |  | 1 | 207 | 50-74.9 vs. < 50 | OR | 0.26 (0.05-1.50) |
|  |  |  | 2 | 207 | 75-99.9 vs 50-74.9 | OR | 0.50 (0.10-2.50) |
|  |  |  | 1 | 207 | 75-99.9 vs 50-74.9 | OR | 0.90 (0.08-10.2) |
|  |  |  | 3 | 207 | 50-74.9 vs 75-99.9 | OR | 1.90 (0.64-5.6) |
|  |  |  | 5 | 208 | Plasma level of 25(OH)D measured in cord blood specimens [nmol/L]:  75-99.9 vs 50-74.9 | OR | 2.10 (0.94-4.7) |
|  |  |  | 5 | 207 | ≥ 100 vs 50-74.9 | OR | 2.80 (0.75-10.5) |
|  |  |  | 5 | 207 | 75-99.9 vs 50-74.9 | OR | **3.00 (1.13-8.0)** |
| Rothers, 2011 (28) | PC | sIgE aero | 2 | 207 | ≥ 100 vs 50-74.9 | OR | **4.70 (1.33-16.6)** |
|  |  |  | 1 | 207 | ≥ 100 vs 50-74.9 | OR | **2.02 (1.23-3.32)** |
|  |  |  | 5 | 208 | ≥100 vs 50-74.9 | OR | 0.86 (0.61-1.21) |
|  |  |  | 5 | 172 | 50-74.9 vs. < 50 | OR | 0.83 (0.37-1.88) |
|  |  | SPT aero | 5 | 172 | Plasma level of 25(OH)D measured in cord blood specimens [nmol/L]/  75-99.9 vs 50-74 | OR | 2.00 (0.89-4.50) |
|  |  | Total-IgE | 5 | 207 | 50-74.9 vs. < 50 | β-coeff | NS |
| Rothers, 2011 | PC | Total-IgE | 5 | 207 | 75-99.9 vs 50-74 | β-coeff | NS |
|  |  |  | 2 | 191 | Maternal serum 25(OH)D levels at 34 weeks gestation (unclear if continuous or categorical) | OR | 0.97 (0.69-1.36) |
| Weisse, 2012 | PC |  | 1 | 184 | Maternal serum 25(OH)D levels at 34 weeks gestation (unclear if continuous or categorical) | OR | 1.06 (0.76-1.47) |
|  |  |  | 1 | 184 | Cord blood 25(OH)D levels (unclear if continuous or categorical) | OR | 1.07 (0.74-1.54) |
|  |  | Total-IgE | 2 | 191 | Cord blood 25(OH)D levels (unclear if continuous or categorical) | OR | 1.07 (0.72-1.58) |
|  |  | sIgE any | 1 | 184 | Maternal serum 25(OH)D levels at 34 weeks gestation | OR | 0.90 (0.61-1.32) |
|  |  |  | 2 | 191 | Maternal serum 25(OH)D levels at 34 weeks gestation | OR | **1.59 (1.03-2.45)** |
|  |  |  | 1 | 184 | Cord blood 25(OH)D levels | OR | 0.76 (0.49-1.18) |
| Weisse, 2012 | PC | sIgE any | 2 | 191 | Cord blood 25(OH)D levels | OR | 1.60 (1.00-2.57) |
| Jones, 2012 | RC | AS | 1 | 146 | Maternal vitamin D intake (supplementation during pregnancy) in IU (continuous) | OR | 0.98 (0.90-1.07) |
| Goldring, 2013 | RCT | SPT any  Total IgE | 3 | 60/60 | Pregnant women. Single oral bolus of 200,000 IU vitamin D (cholecalciferol) at 27 weeks of gestation, or daily 800IU vitamin D (ergocalciferol) from 27 weeks to delivery, or a combination of both, versus no treatment. | OR | NS |
| Bekkers, 2012 | PC | sIgE any | 8 | 3786 | Maternal intake of specific vitamin or mineral supplements during pregnancy, including folic acid, multivitamin and vitamin B complex supplements (yes vs no)  Folic acid, multivitamins, vitamins B | OR | NS |
|  |  |  |  |  | Multivitamins | OR | NS |
|  |  |  | 8 | 5368 | Mothers dietary folate intake at 32 weeks gestation (yes vs no) | OR | 0.98 (0.87-1.10) |
| Granell, 2008 | PC | AS-Any | 8 | 5368 | Maternal folic acid supplementation (highest vs. lowest) | OR | **1.65 (1.07-2.54)** |
| Hoppu, 2000 | PC | AS | Any |  | Maternal vitamin C intake (highest vs. lowest) | OR | 0.84 (0.54 - 1.3) |
| Magdelijns, 2011 | PC | Total-IgE | 2 | 2465 | Folic acid supplementation before or during pregnancy (questionnaire completed in weeks 14 and 34 of gestation) (use vs no use) | OR | 0.72 (0.46-1.12) |
|  |  |  |  |  | Maternal consumption during t8^th^ month pregnancy (continuous):  Copper mg/d | OR | 1.12 (0.42-3.01) |
|  |  |  |  |  | Zinc mg/d | OR | 0.53 (0.17-1.63) |
| Nwaru, 2010 | PC | AS | 5 | 931 | Vitamin C mg/d | OR | 1.25 (0.89-1.76) |
|  |  |  |  |  | Vitamin D µg/day | OR | 0.76 (0.49-1.17) |
|  |  |  |  |  | **Maternal consumption during t8th month pregnancy (continuous):**  Vitamin E mg /d | OR | 1.97 (0.91-4.26) |
| Nwaru, 2010 | PC | AS | 5 | 931 | Iron - mg/day | OR | 1.37 (0.54-3.49) |
| Devereux, 2006 | PC | SPT any | 5 | 1704 | Maternal and children’s dietary and supplement intakes were summated to give total nutrient intake (highest vs lowest quintile)  Copper, Iron, magnesium, vitamin C | OR | NS |
| Nwaru, 2010 | PC | AS | 5 | 931 | Maternal intake of vitamin A -µ/day | OR | 0.22 (0.04-1.33) |
| Nwaru, 2011 | PC | sIgE to cow’s milk | 5 | 652 | Maternal intake during 2^nd^ and 3^rd^ month of breastfeeding (g/day increase in intake)  Copper | OR | 1.20 (0.82-1.75) |
|  |  |  |  |  | **Maternal intake during 2nd and 3rd month of breastfeeding (g/day increase in intake)**  Selenium | OR | 1.05 (0.68-1.62) |
| Nwaru, 2011 | PC | sIgE to cow’s milk | 5 | 652 | Zinc | OR | 1.05 (0.73-1.51) |
|  |  |  |  |  | Vitamin C | OR | 1.11 (0.84-1.47) |
|  |  |  |  |  | Vitamin E | OR | 0.76 (0.44-1.30) |
|  |  |  |  |  | Vitamin D | OR | 1.05 (0.76-1.45) |
| Nwaru, 2011 | PC | sIg E to egg | 5 | 652 | **Maternal intake during 2^nd^ and 3^rd^ month of breastfeeding (g/day):**  Copper | OR | 0.87 (0.54-1.40) |
|  |  |  |  |  | Selenium | OR | 1.07 (0.68-1.68) |
|  |  |  |  |  | **Maternal intake during 2nd and 3rd month of breastfeeding (g/day):**  Zinc | OR | 0.93 (0.63-1.38) |
|  |  |  |  |  | Vitamin C | OR | 1.08 (0.82-1.42) |
| Nwaru, 2011 | PC | sIg E to egg | 5 | 652 | Vitamin E | OR | 0.81 (0.43-1.52) |
|  |  |  |  |  | Vitamin D | OR | 0.87 (0.61-1.25) |
|  |  |  |  |  | Iron | OR | 1.17 (0.88-1.55) |
|  |  |  |  |  | **Maternal intake 8^th^ month of pregnancy / mean daily consumption (continuous)**  Copper | OR | 1.21 (0.40-3.69) |
| Nwaru, 2010 | PC | AS | 5 | 931 | Zinc | OR | 0.92 (0.25-3.37) |
|  |  |  |  |  | Vitamin C | OR | 0.83 (0.56-1.22) |
|  |  |  |  |  | Vitamin D | OR | **0.56 (0.34-0.91)** |
|  |  |  |  |  | **Maternal intake 8th month of pregnancy / mean daily consumption (continuous)**  Vitamin E | OR | 0.62 (0.25-1.52) |
| Nwaru, 2010 | PC | AS | 5 | 931 | Iron | OR | 1.47 (0.51-4.23) |
|  |  |  |  |  | Vitamin A | OR | 0.84 (0.56-1.27) |
|  |  |  |  | 307 | **Maternal vitamin intake during pregnancy:**  Vitamin C | OR | 0.76 (0.35-1.67) |
| West, 2012 | PC | SPT any | 1 | 311 | Vitamin E | OR | 0.64 (0.29-1.41) |
|  |  | AS aero | any |  | Vitamin D | OR | 1.54 (1.02-2.28) |
| West, 2012 (60) | PC | SPT aero | 7.5 | 3652 | **Maternal serum vitamin D in pregnancy (quintiles; nmol/L)**  Q4 (67-89) vs Q1 (min -38) | OR | 0.92 (0.71-1.19) |
|  |  |  |  |  | Q3 (52-67) vs Q1 (min -38) | OR | 0.95 (0.73-1.24) |
|  |  | SPT aero | 7.5 | 3652 | Q2 (38-52 vs Q1 (min -38) | OR | 0.96 (0.74-1.25) |

# 8. Infant or maternal dietary intake of vitamins and minerals and risk of type 1 diabetes mellitus (T1DM)

Nineteen observational studies and no intervention trials investigated the association between vitamin or mineral intake and risk of T1DM in childhood. These studies were considered to have a low risk of bias across most domains. Over 60% of the studies had a low overall risk of bias, due to appropriate account for assessment, sample selection and confounding (Figure 52).

Figure 52 Risk of Bias in studies of vitamin and mineral intake and type I diabetes mellitus

# 8.1. Intervention studies with data eligible for meta-analysis

We identified no intervention studies for vitamin/mineral supplementation and TIDM.

# 8.2. Observational studies with data eligible for meta-analysis

Data from nine observational studies examining the association between T1DM and vitamin D were suitable for meta-analysis.

Seven case control studies and two prospective cohort studies (Brekke 2007, and Hypponen 2001) investigated the relationship of infant dietary intake of vitamin D and T1DM (Figure 53). The study of Bekker (2007) showed that use of supplements containing vitamin D, amongst other nutrients, during pregnancy was associated with reduced diabetes-related autoimmunity at 1 year of age, but not at 2.5 years old. The prospective cohort study of Hypponen (2001) showed that infant vitamin D supplementation was associated with a decreased frequency of T1DM (at age 30 years) when adjusted for neonatal, anthropometric, and social characteristics (rate ratio [RR] for regular vs no supplementation 0.12, 95% CI 0.03-0.51, and irregular vs no supplementation 0.16, 0.04-0.74). Children who regularly took the recommended dose of vitamin D (2000 IU daily) also had a reduced risk of T1DM compared with those who regularly received less than the recommended amount. The combined effect of these prospective cohorts was not statistically significant; the pooled effect of the seven retrospective studies also failed to reach statistical significance. Both analyses had significant statistical heterogeneity. When retrospective and prospective study data were pooled, which may not be appropriate due to differences in study design, the pooled association was statistically significant, but with high heterogeneity across studies (I^2^=62.4%). The evidence for an association between infant vitamin D supplementation and risk of TIDM was downgraded -1 for inconsistency and -1 for imprecision leading to no conclusive evidence of an association.

Figure 53 Infant vitamin D supplementation (often vs. rare) and risk of type 1 diabetes mellitus in children

In the DAISY cohort study, Fronczak (2003) showed that maternal intake of vitamin D from diet was significantly associated with a decreased risk of islet auto-immunity appearance in offspring, independent of related factors such as family history of T1DM, presence of gestational diabetes mellitus, and ethnicity. The study of Stene (2003) found no evidence of an association between vitamin D intake and risk of T1DM. There was no evidence to suggest that maternal supplement use or plasma levels of vitamin D is associated with risk of T1DM in the offspring (Figures 53 to 56).

Figure 54 Maternal vitamin D intake (highest vs. lowest) and risk of type 1 diabetes mellitus in children at (any age)

Figure 55 Maternal vitamin D supplement use (often vs. rare) and risk of type 1 diabetes mellitus in children (at any age)

Figure 56 Maternal plasma vitamin D (mean difference) and risk of T1DM in children (at any age)

# 8.3. Studies on vitamin and mineral intake and T1DM which were not eligible for meta-analysis

We identified seven studies which investigated the association between various minerals and vitamins with outcomes of T1DM (Table 9). Most of the studies investigated vitamin D. The study of **Mettienen (2012)** measured serum levels of vitamin D in the first trimester of pregnancy and reported no association with risk of T1DM at age 3 years. The case-control study of **Sorensen (2012)** nested in a cohort of 29,000 Norwegian women measured serum levels of vitamin D in late pregnancy of 109 mothers with a child diagnosed with T1DM (before 15 years old) and 209 controls. Dividing the levels of maternal vitamin D into quartiles, there was a trend (although not completely linear) toward a higher risk of T1DM with lower levels of vitamin D during pregnancy. The odds of T1DM were more than twofold higher for the offspring of women with the lowest levels of vitamin D compared with the offspring of those with levels above the upper quartile. No other associations of relevance were observed.

**Overall, we found no consistent evidence to suggest that infant or maternal intake of vitamins or minerals influences the risk of T1DM in the offspring.**

Table 9 Studies investigating the association between vitamins and minerals and type I diabetes mellitus which were not eligible for meta-analysis

| **First Author and year of publication** | **Design** | **Outcome** | **Age** | **N/n** | **Dietary exposure and**  **comparison level** | **Measure of association** | **Effect (bold indicates p-value <0.05)** |
| --- | --- | --- | --- | --- | --- | --- | --- |
|  |  |  |  |  | **INFANT INTAKE OR BLOOD LEVEL** |  |  |
| Simpson, 2011 | NCC | DM | 9 | 128/30 | Infant serum vitamin D level | HR | 1.16 (0.70-1.93) |
|  |  |  |  |  | **MATERNAL INTAKE OR BLOOD LEVEL** |  |  |
|  |  |  |  |  | **Dietary intake during pregnancy** [HR is change in risk per 2-fold increase in intake, from foods plus supplements]:  Manganese | HR | 1.03 (0.76-1.40) |
|  |  |  |  |  | Selenium | HR | 1.11 (0.75-1.64) |
|  |  |  |  |  | Vitamin C | HR | 0.96 (0.79-1.17) |
| Uusitalo, 2008 | PC | TIDM | 9 | 3730 | Vitamin E | HR | 1.06 (0.75-1.49) |
|  |  |  |  |  | **VIT D/Maternal intake in 8^th^ month of pregnancy** [quartiles] : |  |  |
|  |  |  |  |  | From food  Q4 vs. Q1 | HR | 1.17 (0.73-1.90) |
|  |  | Advanced β-cell T1DM |  |  | Q3 vs. Q 1 | HR | 1.12 (0.69-1.82) |
|  |  |  |  |  | Q2 vs. Q1 | HR | 0.74 (0.44-1.25) |
| Marjamaki, 2010 |  |  |  |  | Total vitamin D intake (food and supplements) | HR | 1.26 (0.92-1.73) |
|  |  |  |  |  | Vitamin D from supplements | HR | 1.05 (0.95-1.16) |
|  | PC | Clinical T1DM alone | 1-8y | 3723 | Q4 vs. Q1 | HR | 0.77 (0.35-1.72) |
|  |  |  |  |  | Q3 vs. Q1 | HR | 0.84 (0.38-1.83) |
|  |  |  |  |  | Q2 vs. Q1 | HR | 1.00 (0.48-2.11) |
|  |  |  |  |  | Total vitamin D intake (food and supplements) | HR | 1.08 (0.65-1.79) |
|  |  |  |  |  | Vitamin D from supplements | HR | 1.09 (0.99-1.20) |
| Miettinen, 2012 | NCC | TIDM | 3.4 | 686 | Maternal plasma 25(OH)D levels in 1^st^ trimester of pregnancy | OR | NS |
| Ashraf, 2010 | CC | TIDM | 1-10 | 195/128 | Specific nutrient exposures (mother or child) | OR per SD [=540mg over 4 months] increase in exposure | NS |
| Stene, 2003 | CC | TIDM | <15 | 1058 | Yes vs no vitamin D supplementation during 1^st^ year of life | OR | 0.82 (0.47 -1.42) |

# 9. Infant or maternal dietary intake of vitamins and minerals and risk of inflammatory bowel disease (IBD)

A single case control study reported infant or maternal intake of vitamins and minerals and risk of inflammatory bowel disease: the international multi-centric case-control study of Gilat (1987) investigated the association between risk of IBD in cases with Crohn’s Disease (CD) or ulcerative colitis (UC) and use of maternal multivitamin supplements, compared to controls. The assessment of bias risk was considered unclear overall, due to lack of information on assessment and selection bias (Figure 57).

Mothers of patients with UC and CD took vitamin, mineral, and iron preparations during pregnancy significantly less frequently than mothers of controls. The authors reported that multivitamin supplementation during pregnancy was negatively associated with risk of IBD in children. We did not consider data from a single case-control study to be sufficient quality or quantity to be classed as evidence for association, and further work is needed to test the hypothesis that vitamin supplementation during pregnancy influences risk of IBD.

Overall we found no consistent evidence that maternal vitamin supplementation during pregnancy is associated with reduced UC and Crohn’s disease.

Figure 57 Risk of bias in study of vitamin and mineral intake and IBD

Table 10 Studies investigating the association between vitamins and minerals and inflammatory bowel disease which were not eligible for meta-analysis

| **First Author and year of publication** | **Design** | **Outcome** | **Age** | **N/n** | **Dietary exposure and comparison level** | **Measure of association** | **Association (bold indicates p-value <0.05)** |
| --- | --- | --- | --- | --- | --- | --- | --- |
| Gilat,1987 | CC | IBD | Any | 167/ 337 | Maternal multivitamin supplementation and risk of inflammatory bowel disease- CD) in children (often vs. rare) | OR | **0.46 (0.26-0.81)** |
|  |  |  |  | 128/ 251 | Maternal multivitamin supplementation and risk of irritable bowel disease-UC in children (often vs. rare) | OR | **0.49 (0.25-0.99)** |

# 10. Infant or maternal dietary intake of vitamins and minerals and risk juvenile idiopathic arthritis (JIA)

The Childhood Arthritis Risk factor Identification study (CLARITY) was set up in Australia to investigate genetic and environmental risk factors for JIA (Ellis 2012) in children <18 years old. In this case-control study, the authors report some of the risk factors found in the first three years of the study. The overall risk of bias was considered to be unclear (Figure 58).

Figure 58 Risk of Bias in studies of vitamin and mineral intake and JIA

Amongst the early life risk factors investigated, use of vitamin D during pregnancy was lower in case mothers (less frequent); however, these associations were not significant after adjusting for potential confounders (Table 11). There was also no evidence of difference in risk of JIA by use of maternal supplementation of folic acid, multivitamins, or calcium.

| **First Author and year of publication** | **Design** | **Outcome** | **Age** | **N/n** | **Dietary exposure and comparison level** | **Measure of association** | **Effect (bold indicates p-value <0.05)** |
| --- | --- | --- | --- | --- | --- | --- | --- |
|  |  |  |  |  | Maternal supplementation of:  Iron (often vs. rare) | OR | 0.82 (0.59-1.14) |
|  |  |  |  |  | Folic acid (often vs. rare) | OR | 0.82 (0.59-1.14) |
|  |  |  |  |  | Multivitamin (often vs. rare) | OR | 0.76 (0.52-1.1) |
|  |  |  |  |  | Calcium (often vs. rare) | OR | 0.61 (0.36- 1.04) |
| Ellis, 2012 | CC | JIA | <18 years | 262/458 | Vitamin D (often vs. rare) | OR | 0.26 (0.03-2.11) |

Table 11 Studies investigating the association between vitamins and minerals and juvenile idiopathic which were not eligible for meta-analysis

# Conclusion

In this systematic review of maternal and infant intake of vitamins and minerals, we found some evidence to support associations with respiratory outcomes from intervention trials, but no consistent evidence to suggest that these nutrients are related to allergic or autoimmune diseases in observational studies.

In one intervention trials there was an association between maternal vitamin A supplementation during pregnancy and increased childhood lung function, however this was not consistent with findings in another similar trial, in the same trial using another form of vitamin A (beta-carotene), or with clinical outcomes. Trials of vitamin D and risk of recurrent wheeze showed unexplained,, high heterogeneity, but overall showed no evidence of an effect.The large variations in the intake levels and the measures used to evaluate them in comparisons limited our ability to carry out meta-analyses in observational studies. However, the majority of the findings suggested that there was no association between vitamins and minerals with allergic and autoimmune outcomes.

The most common nutrients studied were vitamin D and folic acid, as well as vitamins C, E, and A, and minerals zinc and copper. With regards to vitamin D, several studies suggested that a higher intake or higher concentrations in maternal or cord blood were related to a reduced risk of atopic dermatitis, wheeze, and rhino conjunctivitis, whereas others found a positive association between increasing intake/levels of vitamin D and risk of disease. These conflicting findings may partly be explained by the various study designs and exposure and outcome assessment strategies employed in the epidemiologic studies conducted to date. In observational studies where meta-analysis was possible, we found no evidence that maternal intake of vitamin D supplements or vitamin A intake was associated with allergic or autoimmune outcomes.

We found some evidence from observational studies that infant dietary intake of vitamin D supplements was associated with a reduced risk of T1DM later in childhood, however this was downgraded for inconsistency and imprecision to no consistent evidence. Maternal dietary intake or blood levels of this vitamin were unrelated to TIDM risk. The results of our meta-analysis are in agreement with those reported in the systematic review by **Dong** *et al* (**2013**) both with regards to the lack of association between maternal intake of vitamin D and risk of T1DM. Dong reported an association between infant vitamin D supplementation/intake and reduced TIDM . A more extensive systematic review by **Harvey** *et al* **(2014)** concluded that there was no evidence to support a relationship between maternal 25(OH)D status and asthma, atopy or T1DM in children, consistent with our conclusions . The authors also highlighted that the current evidence is limited by the observational nature of the majority of the relevant studies. We found VERY LOW (-1 imprecision) evidence from a single case control study that maternal multivitamin supplementation during pregnancy is associated with reduced inflammatory bowel disease in offspring, and no evidence to suggest that vitamin or minerals influence risk of JIA.

We found no evidence to suggest that different levels of intake of folic acid can modulate risk of allergic or autoimmune diseases. This observation was confirmed in studies where the timing of exposure studied differed. We were able to combine results from 4 studies investigating the association between maternal intake of folic acid and risk of wheeze, but there was no evidence of a relationship. One study (**Granell, 2008**) suggested that a higher intake during pregnancy was related to a higher risk of AS in the offspring, although several other studies found no indication of an association between folic acid consumption during pregnancy and AS. From a clinical point of view, the role of folic acid in protecting an adequate neural tube development in the foetus makes it a commonly used maternal supplement (before and after gestation). We found that studies investigated different timings of exposure throughout the pregnancy, as well as varying doses and frequency of supplementation. Our results are in agreement with those reported recently by **Brown** *et al* , which showed no evidence suggesting an association between folate consumption by the mother or the infant, and risk of allergic diseases.

Due to the observational nature of the majority of studies included in this review, dietary questionnaires were the main source to ascertain dietary intake of vitamins and minerals. The use of such instruments allows estimates of a wide range of nutrients to be derived, which was often the way in which the dietary intake of vitamins and minerals were reported in these studies. Although there were some studies finding statistically significant associations between some vitamins (e.g. vitamin C and respiratory outcomes), within the same study there were multiple comparisons being carried out. We found that none of these studies formally tested or controlled for multiple comparisons, which weakens even more any isolated statistically significant findings.

**Overall, we found no consistent evidence from observational or intervention studies to suggest that maternal or infant intake of vitamins and minerals influences the risk of AD, AR, FA, AS, TIDM or JIA.**

# References

1.	(NICE) NIfCaHE. The guidelines manual methodology checklist. 2012.

2.	Aage S, Kiraly N, Da Costa K, Byberg S, Bjerregaard-Andersen M, Fisker AB, et al. Neonatal vitamin A supplementation associated with increased atopy in girls. Allergy: European Journal of Allergy and Clinical Immunology. 2015;70(8):985-94.

3.	Chawes BL, Bonnelykke K, Stokholm J, Vissing NH, Bjarnadottir E, Schoos AMM, et al. Effect of Vitamin D3 supplementation during pregnancy on risk of persistent wheeze in the offspring: A randomized clinical trial. JAMA - Journal of the American Medical Association. 2016;315(4):353-61.

4.	Checkley W, West KP, Wise RA, Baldwin MR, Wu L, LeClerq SC, et al. Maternal Vitamin A Supplementation and Lung Function in Offspring. New England Journal of Medicine. 2010;362(19):1784-94.

5.	Checkley W, West Jr KP, Wise RA, Wu L, LeClerq SC, Khatry S, et al. Supplementation with vitamin A early in life and subsequent risk of asthma. European Respiratory Journal. 2011;38(6):1310-9.

6.	Czeizel AE, Dobo M. Postnatal somatic and mental development after periconceptional multivitamin supplementation. Archives of Disease in Childhood. 1994;70(3):229-33.

7.	Dobo M, Czeizel AE. Long-term somatic and mental development of children after periconceptional multivitamin supplementation. European Journal of Pediatrics. 1998;157(9):719-23.

8.	Devakumar D, Stocks J, Ayres JG, Kirkby J, Yadav SK, Saville NM, et al. Effects of antenatal multiple micronutrient supplementation on lung function in mid-childhood: Follow-up of a double-blind randomised controlled trial in Nepal. European Respiratory Journal. 2015;45(6):1566-75.

9.	Grant CC, Crane J, Mitchell EA, Sinclair J, Stewart A, Milne T, et al. Vitamin D supplementation during pregnancy and infancy reduces aeroallergen sensitization: a randomized controlled trial. Allergy: European Journal of Allergy and Clinical Immunology. 2016;71(9):1325-34.

10.	Greenough A, Shaheen SO, Shennan A, Seed PT, Poston L. Respiratory outcomes in early childhood following antenatal vitamin C and E supplementation. Thorax. 2010;65(11):998-1003.

11.	Goldring ST, Griffiths CJ, Martineau AR, Robinson S, Yu C, Poulton S, et al. Prenatal vitamin d supplementation and child respiratory health: a randomised controlled trial. PLoS ONE [Electronic Resource]. 2013;8(6):e66627.

12.	Kiraly N, Balde A, Lisse IM, Eriksen HB, Aaby P, Benn CS. Vitamin A supplementation and risk of atopy: long-term follow-up of a randomized trial of vitamin A supplementation at six and nine months of age. BMC pediatrics. 2013;13:190.

13.	Litonjua AA, Carey VJ, Laranjo N, Harshfield BJ, McElrath TF, O'Connor GT, et al. Effect of prenatal supplementation with Vitamin D on asthma or recurrent wheezing in offspring by age 3 years: The VDAART randomized clinical trial. JAMA - Journal of the American Medical Association. 2016;315(4):362-70.

14.	McEvoy CT, Schilling D, Clay N, Jackson K, Go MD, Spitale P, et al. Vitamin C supplementation for pregnant smoking women and pulmonary function in their newborn infants: a randomized clinical trial. JAMA. 2014;311(20):2074-82.

15.	Martindale S, McNeill G, Devereux G, Campbell D, Russell G, Seaton A. Antioxidant intake in pregnancy in relation to wheeze and eczema in the first two years of life. American Journal of Respiratory & Critical Care Medicine. 2005;171(2):121-8.

16.	Devereux G, Turner SW, Craig LC, McNeill G, Martindale S, Harbour PJ, et al. Low maternal vitamin E intake during pregnancy is associated with asthma in 5-year-old children. American Journal of Respiratory & Critical Care Medicine. 2006;174(5):499-507.

17.	Devereux G, Litonjua AA, Turner SW, Craig LC, McNeill G, Martindale S, et al. Maternal vitamin D intake during pregnancy and early childhood wheezing. American Journal of Clinical Nutrition. 2007;85(3):853-9.

18.	Wills AK, Shaheen SO, Granell R, Henderson AJ, Fraser WD, Lawlor DA. Maternal 25-hydroxyvitamin D and its association with childhood atopic outcomes and lung function. Clinical and experimental allergy : journal of the British Society for Allergy and Clinical Immunology. 2013;43(10):1180-8.

19.	Granell R, Heron J, Lewis S, Davey Smith G, Sterne JA, Henderson J. The association between mother and child MTHFR C677T polymorphisms, dietary folate intake and childhood atopy in a population-based, longitudinal birth cohort.[Erratum appears in Clin Exp Allergy. 2008 Apr;38(4):699 Note: Smith, G D [corrected to Davey Smith, G]]. Clinical & Experimental Allergy. 2008;38(2):320-8.

20.	Back O, Blomquist HK, Hernell O, Stenberg B. Does vitamin D intake during infancy promote the development of atopic allergy? Acta dermato-venereologica. 2009;89(1):28-32.

21.	Liu X, Wang G, Hong X, Wang D, Tsai HJ, Zhang S, et al. Gene-vitamin D interactions on food sensitization: a prospective birth cohort study. Allergy. 2011;66(11):1442-8.

22.	Maslova E, Hansen S, Strom M, Jensen CB, Thorne-Lyman AL, Cohen A, et al. Maternal 25(OH) vitamin D prediction scores in pregnancy and child asthma and allergic rhinitis: a prospective study from the Danish National Birth Cohort. Acta Obstetricia Et Gynecologica Scandinavica. 2013;92:24-5.

23.	Erkkola M, Kaila M, Nwaru BI, Kronberg-Kippila C, Ahonen S, Nevalainen J, et al. Maternal vitamin D intake during pregnancy is inversely associated with asthma and allergic rhinitis in 5-year-old children. Clinical & Experimental Allergy. 2009;39(6):875-82.

24.	Nwaru BI, Erkkola M, Ahonen S, Kaila M, Kronberg-Kippila C, Ilonen J, et al. Intake of antioxidants during pregnancy and the risk of allergies and asthma in the offspring. European Journal of Clinical Nutrition. 2011;65(8):937-43.

25.	Nwaru BI, Erkkola M, Ahonen S, Kaila M, Lumia M, Prasad M, et al. Maternal diet during lactation and allergic sensitization in the offspring at age of 5. Pediatric Allergy & Immunology. 2011;22(3):334-41.

26.	Nwaru BI, Ahonen S, Kaila M, Erkkola M, Haapala AM, Kronberg-Kippila C, et al. Maternal diet during pregnancy and allergic sensitization in the offspring by 5 yrs of age: a prospective cohort study. Pediatric Allergy & Immunology. 2010;21(1 Pt 1):29-37.

27.	Marjamaki L, Niinisto S, Kenward MG, Uusitalo L, Uusitalo U, Ovaskainen ML, et al. Maternal intake of vitamin D during pregnancy and risk of advanced beta cell autoimmunity and type 1 diabetes in offspring. Diabetologia. 2010;53(8):1599-607.

28.	Uusitalo L, Kenward MG, Virtanen SM, Uusitalo U, Nevalainen J, Niinisto S, et al. Intake of antioxidant vitamins and trace elements during pregnancy and risk of advanced beta cell autoimmunity in the child. American Journal of Clinical Nutrition. 2008;88(2):458-64.

29.	Baiz N, Dargent-Molina P, Wark JD, Souberbielle JC, Annesi-Maesano I, Group EM-CCS. Cord serum 25-hydroxyvitamin D and risk of early childhood transient wheezing and atopic dermatitis. The Journal of allergy and clinical immunology. 2014;133(1):147-53.

30.	Gale CR, Robinson SM, Harvey NC, Javaid MK, Jiang B, Martyn CN, et al. Maternal vitamin D status during pregnancy and child outcomes. European Journal of Clinical Nutrition. 2008;62(1):68-77.

31.	Whitrow MJ, Moore VM, Rumbold AR, Davies MJ. Effect of supplemental folic acid in pregnancy on childhood asthma: a prospective birth cohort study. American Journal of Epidemiology. 2009;170(12):1486-93.

32.	Kiefte-de Jong JC, Timmermans S, Jaddoe VW, Hofman A, Tiemeier H, Steegers EA, et al. High circulating folate and vitamin B-12 concentrations in women during pregnancy are associated with increased prevalence of atopic dermatitis in their offspring. Journal of Nutrition. 2012;142(4):731-8.

33.	Hoppu U, Kalliomaki M, Isolauri E. Maternal diet rich in saturated fat during breastfeeding is associated with atopic sensitization of the infant. European Journal of Clinical Nutrition. 2000;54(9):702-5.

34.	Rothers J, Wright AL, Stern DA, Halonen M, Camargo CA, Jr. Cord blood 25-hydroxyvitamin D levels are associated with aeroallergen sensitization in children from Tucson, Arizona. Journal of Allergy & Clinical Immunology. 2011;128(5):1093-9.e1-5.

35.	Alm B, Aberg N, Erdes L, Mollborg P, Pettersson R, Norvenius SG, et al. Early introduction of fish decreases the risk of eczema in infants. Archives of Disease in Childhood. 2009;94(1):11-5.

36.	Morales E, Romieu I, Guerra S, Ballester F, Rebagliato M, Vioque J, et al. Maternal vitamin D status in pregnancy and risk of lower respiratory tract infections, wheezing, and asthma in offspring. Epidemiology. 2012;23(1):64-71.

37.	Laitinen K, Kalliomaki M, Poussa T, Lagstrom H, Isolauri E. Evaluation of diet and growth in children with and without atopic eczema: follow-up study from birth to 4 years. British Journal of Nutrition. 2005;94(4):565-74.

38.	Mommers M, Bronsveld A, Van Ree R, Jansen E, Thijs C. Vitamin D status during pregnancy and early life and atopic outcomes in childhood. Allergy: European Journal of Allergy and Clinical Immunology. 2009;64:21.

39.	Magdelijns FJ, Mommers M, Penders J, Smits L, Thijs C. Folic acid use in pregnancy and the development of atopy, asthma, and lung function in childhood. Pediatrics. 2011;128(1):e135-44.

40.	Cremers E, Thijs C, Penders J, Jansen E, Mommers M. Maternal and child's vitamin D supplement use and vitamin D level in relation to childhood lung function: the KOALA Birth Cohort Study. Thorax. 2011;66(6):474-80.

41.	Weisse K, Winkler S, Hirche F, Herberth G, Hinz D, Bauer M, et al. Maternal and newborn vitamin D status and its impact on food allergy development in the German LINA cohort study. Allergy. 2013;68(2):220-8.

42.	Milner JD, Stein DM, McCarter R, Moon RY. Early infant multivitamin supplementation is associated with increased risk for food allergy and asthma. Pediatrics. 2004;114(1):27-32.

43.	Camargo CA, Jr., Ingham T, Wickens K, Thadhani R, Silvers KM, Epton MJ, et al. Cord-blood 25-hydroxyvitamin D levels and risk of respiratory infection, wheezing, and asthma. Pediatrics. 2011;127(1):e180-7.

44.	Hypponen E, Sovio U, Wjst M, Patel S, Pekkanen J, Hartikainen AL, et al. Infant vitamin d supplementation and allergic conditions in adulthood: northern Finland birth cohort 1966. Annals of the New York Academy of Sciences. 2004;1037:84-95.

45.	Hypponen E, Laara E, Reunanen A, Jarvelin MR, Virtanen SM. Intake of vitamin D and risk of type 1 diabetes: a birth-cohort study. Lancet. 2001;358(9292):1500-3.

46.	Magnus MC, Nafstad P, Stene LC, Haberg SE, London SJ, Stigum H, et al. Maternal vitamin D status during pregnancy and asthma in the offspring among participants in the Norwegian Mother and Child Cohort Study. Journal of Allergy and Clinical Immunology. 2013;1):AB128.

47.	Haberg SE, London SJ, Stigum H, Nafstad P, Nystad W. Folic acid supplements in pregnancy and early childhood respiratory health.[Erratum appears in Arch Dis Child. 2009 Jun;94(6):485]. Archives of Disease in Childhood. 2009;94(3):180-4.

48.	Miyake Y, Sasaki S, Tanaka K, Hirota Y. Consumption of vegetables, fruit, and antioxidants during pregnancy and wheeze and eczema in infants. Allergy. 2010;65(6):758-65.

49.	Miyake Y, Sasaki S, Tanaka K, Hirota Y. Dairy food, calcium and vitamin D intake in pregnancy, and wheeze and eczema in infants. European Respiratory Journal. 2010;35(6):1228-34.

50.	Miyake Y, Sasaki S, Tanaka K, Hirota Y. Maternal B vitamin intake during pregnancy and wheeze and eczema in Japanese infants aged 16-24 months: the Osaka Maternal and Child Health Study. Pediatric Allergy & Immunology. 2011;22(1 Pt 1):69-74.

51.	Bekkers MB, Elstgeest LE, Scholtens S, Haveman-Nies A, de Jongste JC, Kerkhof M, et al. Maternal use of folic acid supplements during pregnancy, and childhood respiratory health and atopy. European Respiratory Journal. 2012;39(6):1468-74.

52.	Litonjua AA, Rifas-Shiman SL, Ly NP, Tantisira KG, Rich-Edwards JW, Camargo CA, Jr., et al. Maternal antioxidant intake in pregnancy and wheezing illnesses in children at 2 y of age. American Journal of Clinical Nutrition. 2006;84(4):903-11.

53.	Camargo CA, Jr., Rifas-Shiman SL, Litonjua AA, Rich-Edwards JW, Weiss ST, Gold DR, et al. Maternal intake of vitamin D during pregnancy and risk of recurrent wheeze in children at 3 y of age. American Journal of Clinical Nutrition. 2007;85(3):788-95.

54.	Pike KC, Inskip HM, Robinson S, Lucas JS, Cooper C, Harvey NC, et al. Maternal late-pregnancy serum 25-hydroxyvitamin D in relation to childhood wheeze and atopic outcomes. Thorax. 2012;67(11):950-6.

55.	Narita M, Qiu D, Hamaguchi M, Doi M, Futamura M, Sakamoto N, et al. Maternal diet during pregnancy and wheeze and eczema in infants; the Japanese birth cohort (T-CHILD) study. Journal of Allergy and Clinical Immunology. 2011;1):AB174.

56.	Ohya Y, Yonemoto J, Ogata T, Sone H, Sakamoto N, Qiu D, et al. Influence of environmental chemicals and drugs taken before and during pregnancy on onset of childhood asthma and eczema. Allergy: European Journal of Allergy and Clinical Immunology. 2011;66:554.

57.	West CE, Dunstan J, McCarthy S, Metcalfe J, D'Vaz N, Meldrum S, et al. Associations between maternal antioxidant intakes in pregnancy and infant allergic outcomes. Nutrients. 2012;4(11):1747-58.

58.	Wang IJ, Guo YL, Weng HJ, Hsieh WS, Chuang YL, Lin SJ, et al. Environmental risk factors for early infantile atopic dermatitis. Pediatric Allergy & Immunology. 2007;18(5):441-7.

59.	Jones AP, Palmer D, Zhang G, Prescott SL. Cord blood 25-hydroxyvitamin D3 and allergic disease during infancy. Pediatrics. 2012;130(5):e1128-35.

60.	Oliver E, Grimshaw K, Scally K, Garland J, Gudgeon L, Foote K, et al. Does nutritional intake in infancy effect later development of food allergy? Allergy: European Journal of Allergy and Clinical Immunology. 2010;65:313.

61.	Grimshaw KEC, Oliver EM, Kemp T, Mills ENC, Beyer K, Foote K, et al. Maternal dietary intake and subsequent allergy development. Journal of Allergy and Clinical Immunology. 2012;1):AB173.

62.	Allen KJ, Koplin JJ, Ponsonby AL, Gurrin LC, Wake M, Vuillermin P, et al. Vitamin D insufficiency is associated with challenge-proven food allergy in infants. Journal of Allergy and Clinical Immunology. 2013;131(4):1109-16.e6.

63.	Binkley KE, Leaver C, Ray JG. Antenatal risk factors for peanut allergy in children. Allergy, Asthma, & Clinical Immunology : Official Journal of the Canadian Society of Allergy & Clinical Immunology. 2011;7:17.

64.	Mullins RJ, Clark S, Wiley V, Eyles D, Camargo Jr CA. Neonatal vitamin D status and childhood peanut allergy: A pilot study. Annals of Allergy, Asthma and Immunology. 2012;109(5):324-8.

65.	Brekke HK, Ludvigsson J. Vitamin D supplementation and diabetes-related autoimmunity in the ABIS study. Pediatric Diabetes. 2007;8(1):11-4.

66.	Fronczak CM, Baron AE, Chase HP, Ross C, Brady HL, Hoffman M, et al. In utero dietary exposures and risk of islet autoimmunity in children. Diabetes Care. 2003;26(12):3237-42.

67.	Simpson M, Brady H, Yin X, Seifert J, Barriga K, Hoffman M, et al. No association of vitamin D intake or 25-hydroxyvitamin D levels in childhood with risk of islet autoimmunity and type 1 diabetes: the Diabetes Autoimmunity Study in the Young (DAISY). Diabetologia. 2011;54(11):2779-88.

68.	Sorensen IM, Joner G, Jenum PA, Eskild A, Torjesen PA, Stene LC. Maternal serum levels of 25-hydroxy-vitamin D during pregnancy and risk of type 1 diabetes in the offspring. Diabetes. 2012;61(1):175-8.

69.	Miettinen ME, Reinert L, Kinnunen L, Harjutsalo V, Koskela P, Surcel HM, et al. Serum 25-hydroxyvitamin D level during early pregnancy and type 1 diabetes risk in the offspring. Diabetologia. 2012;55(5):1291-4.

70.	Ahadi M, Tabatabaeiyan M, Moazzami K. Association between environmental factors and risk of type 1 diabetes - a case-control study. Endokrynologia Polska. 2011;62(2):134-7.

71.	Ashraf AP, Eason NB, Kabagambe EK, Haritha J, Meleth S, McCormick KL. Dietary iron intake in the first 4 months of infancy and the development of type 1 diabetes: A pilot study. Diabetology and Metabolic Syndrome. 2010;2(1).

72.	Ellis JA, Ponsonby AL, Pezic A, Chavez RA, Allen RC, Akikusa JD, et al. CLARITY - ChiLdhood Arthritis Risk factor Identification sTudY. Pediatric Rheumatology Online Journal. 2012;10(1):37.

73.	Bener A, Alsaied A, Al-Ali M, Al-Kubaisi A, Basha B, Abraham A, et al. High prevalence of vitamin D deficiency in type 1 diabetes mellitus and healthy children. Acta Diabetologica. 2009;46(3):183-9.

74.	Anonymous. Vitamin D supplement in early childhood and risk for Type I (insulin-dependent) diabetes mellitus. The EURODIAB Substudy 2 Study Group. Diabetologia. 1999;42(1):51-4.

75.	Visalli N, Sebastiani L, Adorisio E, Conte A, De Cicco AL, D'Elia R, et al. Environmental risk factors for type 1 diabetes in Rome and province. Archives of Disease in Childhood. 2003;88(8):695-8.

76.	Malcova H, Sumnik Z, Drevinek P, Venhacova J, Lebl J, Cinek O. Absence of breast-feeding is associated with the risk of type 1 diabetes: a case-control study in a population with rapidly increasing incidence. European Journal of Pediatrics. 2006;165(2):114-9.

77.	Stene LC, Joner G, Norwegian Childhood Diabetes Study G. Use of cod liver oil during the first year of life is associated with lower risk of childhood-onset type 1 diabetes: a large, population-based, case-control study. American Journal of Clinical Nutrition. 2003;78(6):1128-34.

78.	Stene LC, Ulriksen J, Magnus P, Joner G. Use of cod liver oil during pregnancy associated with lower risk of Type I diabetes in the offspring.[Erratum appears in Diabetologia 2000 Nov;43(11):1451]. Diabetologia. 2000;43(9):1093-8.

79.	Svensson J, Carstensen B, Mortensen HB, Borch-Johnsen K, Danish Study Group of Childhood D. Early childhood risk factors associated with type 1 diabetes--is gender important? European Journal of Epidemiology. 2005;20(5):429-34.

80.	Tenconi MT, Devoti G, Comelli M, Pinon M, Capocchiano A, Calcaterra V, et al. Major childhood infectious diseases and other determinants associated with type 1 diabetes: a case-control study. Acta Diabetologica. 2007;44(1):14-9.

81.	Gilat T, Hacohen D, Lilos P, Langman MJ. Childhood factors in ulcerative colitis and Crohn's disease. An international cooperative study. Scandinavian Journal of Gastroenterology. 1987;22(8):1009-24.

82.	Dong JY, Zhang WG, Chen JJ, Zhang ZL, Han SF, Qin LQ. Vitamin D intake and risk of type 1 diabetes: a meta-analysis of observational studies. Nutrients. 2013;5(9):3551-62.

83.	Harvey NC, Holroyd C, Ntani G, Javaid K, Cooper P, Moon R, et al. Vitamin D supplementation in pregnancy: a systematic review. Health technology assessment. 2014;18(45):1-190.

84.	Brown SB, Reeves KW, Bertone-Johnson ER. Maternal folate exposure in pregnancy and childhood asthma and allergy: a systematic review. Nutrition reviews. 2014;72(1):55-64.
